# Creatinine assay interferences compromises MELD accuracy and may bias liver allocation

**DOI:** 10.1038/s41467-026-75011-x

**Published:** 2026-07-23

**Authors:** Eda Kaya, Christin Quast, Maria Stepanova, Jasmin Weninger, Oliver Goetze, Antonios Katsounas, Abdurrahman Coskun, Martina Bröcker-Preuß, Zobair M. Younossi, Jan-Peter Sowa, Caroline Stobe, Mustafa Kemal Özcürümez, Ali Canbay

**Affiliations:** 1 Department of Medicine, Knappschaft Kliniken University Hospital Bochum, Ruhr University Bochum, Bochum, Germany; 2 Global NASH/MASH Council, Center for Outcomes Research in Liver Disease, Washington, DC USA; 3 Global Center for Liver Outcomes & Policy Research, Georgetown University School of Medicine, Washington, DC USA; 4 Department of Biochemistry, School of Medicine, Acibadem Mehmet Ali Aydinlar University, Istanbul, Türkiye; 5 Calibration Laboratory 1, Reference Institute for Bioanalytics, Cologne, Germany

**Keywords:** Liver diseases, Prognostic markers, Hepatology

## Abstract

The Model for End-Stage Liver Disease (MELD) score is widely used to prioritize patients for liver transplantation and to estimate short-term mortality in end-stage liver disease. Inaccuracies in serum creatinine measurements, particularly interference from bilirubin, both key components of the MELD, may influence clinical decision-making. However, standardized approaches to address such analytical bias are lacking. Here we show that bilirubin-related interference leads to clinically relevant MELD distortions and associated outcomes. We developed a correction model using controlled in vitro matrices and validated it against representative patient samples. The model was applied to a large cohort from registry data and a separate clinical population. After correction, clinically meaningful score shifts occurred in a substantial proportion of patients, with lower corrected scores associated with altered transplantation probability and mortality estimates. These findings highlight the importance of harmonized, interference-resistant creatinine assays to improve fairness and accuracy in liver transplant allocation.

## Abstract (web summary)

The MELD score is a key tool to assess severity of liver disease. Here the authors show that assay-related inaccuracies in serum creatinine measurements can significantly alter MELD scores in 8−14 % of patients, potentially influencing mortality risk assessment and liver transplant prioritization.

## Introduction

The Model for End Stage Liver Disease (MELD) score was developed as a prognostic tool for patients with end-stage liver disease (ESLD), which is currently used as the core system in decision-making processes in liver transplantation since 2002^1^. Over the years, MELD has been continuously updated to reflect accumulating liver transplantation data. Various parameters, including sodium, albumin, and sex, have been added, along with mathematical adjustments to enhance the accuracy of the model^2–4^. In the United States (US), the current transplant policy has evolved in response to these adaptations, recently incorporating MELD 3.0^5^. Despite the development of optimized MELD scores, liver transplantation strategies in most regions worldwide continue to rely on the original MELD score. In Europe, the original MELD score remains the backbone for liver transplant allocation and is applied without any of the proposed updates^6–8^. Though the original MELD was derived from U.S. patient data from 1991 to 1995, which may not reflect the current clinical and demographic characteristics of the Eurotransplant region. In 2021, based on data from the Eurotransplant region, an optimized MELD score called reMELD-Na was introduced^9^. In their study, Goudsmit et al. proposed a MELD score tailored to the Eurotransplant population, addressing both general limitations of the original MELD model and specific considerations relevant to the Eurotransplant setting. In addition, methodological assumptions such as fixed lower boundaries at 0.7 or 1 mg/dL, a creatinine cap at 4, 3, or 2.5 mg/dL without objective justification, and the lack of adjustment for analytical interference (e.g., between bilirubin and creatinine) may affect score performance. To minimize these limitations, the authors reweighted coefficients and adjusted score boundaries^9^.

Indeed, creatinine measurement is influenced by various interfering factors, including glucose, protein, cephalosporins, and bilirubin. Importantly, the MELD score incorporates both bilirubin and creatinine, which can interfere with each other, leading to falsely measured creatinine levels depending on bilirubin concentration and used creatinine measurement methodology^10–12^. For creatinine measurement, the Jaffe method and an enzymatic assay are the most commonly used methodologies. Modern Jaffe assays determine creatinine levels in serum samples based on the rate of change in spectrophotometric absorption^13^. However, bilirubin can interfere with this method due to its chromogenic effect. In contrast, the enzymatic assay is less affected by bilirubin interference, as it relies on a more specific reaction^12^. Nonetheless, despite the uncertainties due to many known and unknown interferents Jaffe method is still a widely used method to measure creatinine^14^.

Above mentioned interference affects the MELD score, ultimately impacting liver allocation due to inaccurately measured creatinine values. Moreover, as bilirubin levels increase, the variability in creatinine measurements becomes more pronounced, depending on the methodology used, leading to inconsistencies in the MELD score^15,16^. The initial MELD score studies did not include a patient population with high bilirubin values, where interference becomes more pronounced. Additionally, the authors did not specify the methodology used for creatinine measurement^1,17^. As a result, the initial formula was not influenced by high bilirubin levels, which introduces a preselection bias. Therefore, the use of the current MELD score may be limited when selecting patients with higher bilirubin levels, complicating its use in real-world data and clinical settings.

The primary aim of our study was to develop a reference model based on validated experimental data for both the Jaffe assay and the enzymatic assay, to approximate true serum creatinine levels and reduce method- and bilirubin interference-related bias. The secondary objective was to assess the impact of this correction on variants of the MELD score, specifically by analyzing score shifts using data from liver cirrhosis patients in tertiary care centres and liver allocation data from the Scientific Registry of Transplant Recipients (SRTR)^18^. These effects were evaluated by identifying the proportion of MELD scores misclassified relative to clinically relevant thresholds, potentially altering patient outcomes. Additionally, we aimed to assess the impact of these analytical biases on the prognostic performance of MELD scores, potentially contributing to inequities in organ allocation among liver transplant candidates.

## Results

### Reference model to estimate true creatinine values independent from bilirubin interference reduces detection method dependent bias

**Fig. 1:**
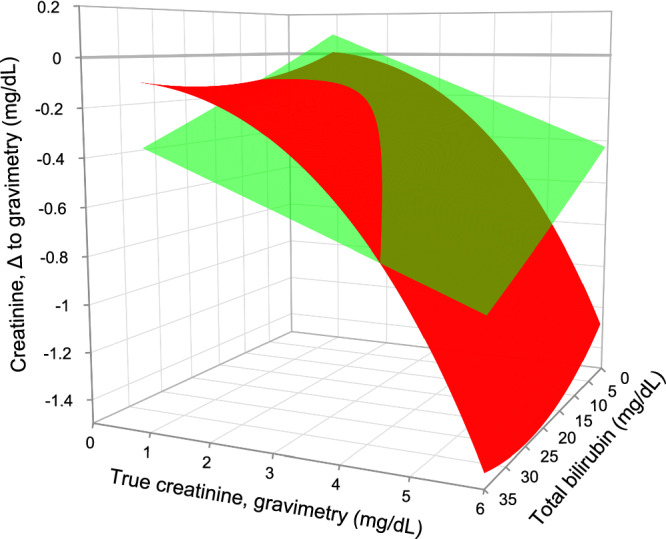
Three-dimensional visualization of correction models for Jaffe (CreJ, red surface) and enzymatic (CreE, green surface) creatinine assays in the presence of bilirubin interference. The *x*-axis indicates true creatinine values determined gravimetrically (mg/dL), the *y*-axis represents total bilirubin concentrations (mg/dL). The *z*-axis represents the correction applied to the measured creatinine values, as calculated by Eq. (1) for CreJ (red surface) and Eq. (2) for CreE (green surface), i.e., true creatinine (gravimetry)—measured creatinine (Jaffe or enzymatic). Negative Δ values indicate overestimation by the assay, meaning that correction lowers the reported value. These correction models account for the concentration-dependent differences between the two assays, highlighting the varying degrees of interference caused by bilirubin. Source data are provided as a Source Data file (F1 tb cre experimental array raw.csv contained within MELD_public_data_package).

Sample arrays containing gravimetrically determined masses of creatinine and bilirubin in a serum-free matrix were created in vitro, resulting in 396 creatinine/bilirubin concentration combinations per array. These arrays were analyzed using both creatinine assessments by Jaffe methodology (CreJ) and enzymatic creatinine measurements (CreE). The modeled correction to reduce assay deviation from true creatinine values for CreJ and CreE (given in detail as Eqs. (1) and (2) in the “Methods” section), illustrated in Fig. 1, revealed significant differences between the two assays. As these differences are concentration-dependent even in the absence of bilirubin, both method-specific components and the interferent contribute to varying degrees to the results. The reference models for CreJ and CreE intersect along a curve in the modelled surface space where both methods yield identical adjustments.

Based on the spatial relationship between the two deviation surfaces, 60.2% of all possible creatinine–bilirubin constellations CreJ, overestimates creatinine compared to CreE, assuming a uniform distribution of concentration pairs. In the remaining 39.8%, CreE yields higher deviations from the true creatinine value. This indicates a systematic tendency of CreJ to overestimate creatinine to a greater extent than CreE under most conditions.

To verify that the model-based expectations from the artificial matrix experiments could serve as a reference function, we validated them against true serum creatinine values from patient samples (*n* = 32). These values were determined with gas chromatography–isotope dilution mass spectrometry (GC-IDMS) as independent reference method to exclude potential serum matrix effects. The true creatinine concentrations of these samples ranged from 0.63 to 4.26 mg/dL; corresponding total bilirubin levels ranged from 0.107 to 28.5 mg/dL.

Next, weighted Deming regression was used to estimate systematic and proportional biases by comparing CreE and CreJ results with GC-IDMS measurements, before and after applying the reference models. CreJ overestimated true serum creatinine by 7.19%, with an additional systematic bias of +0.06 mg/dL. Applying Eq. (1) reduced the proportional bias to 0.26%, while the systematic bias remained nearly unchanged at +0.07 mg/dL.

Analytical precision was assessed using two metrics: the 95% central range of deviations and the standard deviation of creatinine measurements from their true values. Applying the reference model reduced imprecision by 10.2% in both metrics, indicating improved consistency and reduced variability for CreJ measurements.

Serum creatinine measured by CreE exhibited a noticeably lower proportional error of 0.89%, along with a systematic negative deviation of −0.081 mg/dL, even without correction. After applying Eq. (2), the proportional bias was further reduced to 0.004%, while the absolute systematic negative bias slightly increased to −0.11 mg/dL.

In line with these findings, direct comparison of both methods indicated that CreJ yielded results 6.2% higher than CreE, with an additional systematic bias of 0.15 mg/dL. After applying Eq. (1) to CreJ, the remaining proportional error relative to CreE was reduced to 0.3%, confirming a substantial reduction in method-related discrepancy.

In summary, the method- and interference-dependent corrections defined by the experimentally developed equations were confirmed by GC-IDMS as an independent reference method in patient samples representing a true biological matrix. CreE remained in good agreement with GC-IDMS results, with and without correction. In contrast, applying the correction equation to CreJ resulted in substantial adjustments. Therefore, the impact of applying the reference model to CreJ-derived creatinine values on MELD and its derivatives was further investigated.

### Effects of the reference model on creatinine values

The ESLD dataset comprises 20,359 sample-level observations from 1375 patients. Detailed demographic and clinical information are shown in Table 1; information on transplant list status in Supplementary Table 1), total bilirubin values ranged from 0.07 to 49.3 mg/dL and were significantly higher in deceased compared to surviving patients (*p* < 0.0001), with median (5–95% percentile) levels of 1.5 mg/dL (0.32–13.6 mg/dL) vs. 1.0 mg/dL (0.29–10.4 mg/dL). In the SRTR cohort (*N* = 251,637; Table 1), including only patients from centers with confirmed use of the Jaffe assay for creatinine, total bilirubin levels were between 0.1 mg/dL and 91.6 mg/dL, with a median of 2.4 mg/dL (95% CI: 0.6–18.4 mg/dL). Bilirubin concentrations were again significantly higher in deceased patients (2.7 mg/dL; 0.7–24.0 mg/dL) compared to survivors (2.4 mg/dL; 0.6–17.1 mg/dL; *p* < 0.0001).

**Table 1:** The general characteristics of the study populations. Categories are not mutually exclusive; multiple conditions can apply

Parameter	Cohort 1, ESLD	Cohort 2, SRTR
Total patients, *N* (%)	1375 (100)	20,444 (100)
Sex, male, *N* (%)	759 (55.2)	12,606 (61.7)
Age, years, median (IQR)	63 (53–71)	55 (48–61)
Death count, *N* (%)	233 (16.9)	2740 (13.4)
MELD scores calculated, *N*	20,359	251,637
Creatinine (Jaffé), mg/dL, median (IQR)	0.94 (0.71–1.34)	1.16 (0.83–1.85)
Total bilirubin, mg/dL, median (IQR)	1.10 (0.58–2.54)	3.4 (1.7–8.4)^a^
INR, ratio, median (IQR)	1.27 (1.10–1.52)	1.6 (1.3–2.2)
Sodium, mmol/L, median (IQR)	138 (135–141)	137 (133–140)
Albumin, g/dL, median (IQR)	3.07 (2.54–3.65)	3.0 (2.6–3.5)
Creatinine capping due to dialysis, *N* (%)	83 (6.0)	2064 (10.1)
MELD, median (IQR)	12 (9–17)	20 (14–30)
MELD-Na, median (IQR)	14 (9–19)	23 (15–31)
reMELD-Na, median (IQR)	12 (8–17)	19 (14–26)
MELD 3.0, median (IQR)	15 (11–21)	23 (16–31)
ALD, *N* (%)	369 (26.8)	5059 (27.4)
MASH, *N* (%)	297 (21.6)	3961 (21.4)
Hepatitis B, *N* (%)	55 (4.0)	367 (2.0)
Hepatitis C, *N* (%)	89 (6.5)	4,115 (22.3)
ALD + Hepatitis C, *N* (%)	15 (1.1)	954 (5.1)
Autoimmune hepatitis, *N* (%)	68 (4.9)	723 (3.9)
PBC, *N* (%)	130 (9.5)	781 (4.2)
Other causes of liver cirrhosis *N* (%)	482 (35.1)	2531 (13.7)

*ALD* alcoholic liver disease, *INR* international normalized ratio, *IQR* interquartile range, *MASH* metabolic dysfunction associated steatohepatitis, *PBC* primary biliary cirrhosis.

^a^last value reported per patient.

The characteristics of serum creatinine values with and without correction (Table 2) illustrate the impact of bilirubin interference and method-specific measurement bias for both cohorts. The decrease in the corrected creatinine values was more pronounced in deceased patients. Compared to our experimental data, Eq. (1) achieved a further reduction in standard deviations among both surviving (−18.0%) and deceased patients (−12.2%). For creatinine concentrations, comparisons between surviving and deceased patients, both with and without corrections by the reference model, yielded *p*-values < 0.0001. Table 2 also presents the changes in the proportion of truncated creatinine values following application of different cut-off thresholds defined by the various MELD models. Significant differences were observed between deceased and surviving patients, both before and after applying the reference model. However, within each group (deceased and surviving), no statistically significant differences were found between values before and after correction.

**Table 2:** Impact of reference model on serum-creatinine values and truncation rules by score variants in ESLD and SRTR patients

Serum-creatinine characteristics in samples from patients with ESLD	Overall samples 20,359 (100%)		Samples, patients alive 15,273 (75%)		Samples, patients deceased within 90 days after MELD calculation 5086 (25%)	
	Application of reference model					
	Before	After	Before	After	Before	After
Mean (SD)^a^	1.18 (0.91)	1.15 (0.77)	1.12 (0.88)	1.09 (0.73)	1.37 (0.98)	1.32 (0.86)
Median (2.5–97.5%)^a^	0.94 (0.43–3.36)	0.94 (0.42–3.13)	0.91 (0.42–3.00)	0.91 (0.41–2.83)	1.08 (0.44–3.97)	1.07 (0.43–3.65)
Boundaries, *n* (%)^b^						
<0.7 mg/dL	4749 (23.3)	4834 (23.7)	3767 (24.7)	3816 (25.0)	982 (19.3)	1018 (20.0)
<1.0 mg/dL	11,146 (54.7)	11,308 (55.5)	8877 (58.1)	8992 (58.9)	2269 (44.6)	2316 (45.5)
1.0–2.5 mg/dL	8126 (39.9)	8100 (39.8)	5811 (38.0)	5758 (37.7)	2315 (45.5)	2342 (46.0)
> 2.5 mg/dL	1087 (5.3)	951 (4.7)	585 (3.8)	523 (3.4)	502 (9.9)	428 (8.4)
> 3.0 mg/dL	670 (3.3)	575 (2.8)	382 (2.5)	337 (2.2)	288 (5.7)	238 (4.7)
> 4.0 mg/dL	321 (1.6)	254 (1.2)	197 (1.3)	164 (1.1)	124 (2.4)	90 (1.8)

Serum-creatinine characteristics in samples from SRTR patients	Overall samples 251,637 (100%)		Samples, patients alive 211,811 (84%)		Samples, patients deceased within 90 days after MELD calculation 39,826 (16%)	
	Application of reference model					
	Before	After	Before	After	Before	After
Mean (SD)^a^	1.66 (1.44)	1.54 (1.18)	1.62 (1.43)	1.51 (1.17)	1.90 (1.49)	1.75 (1.24)
Median (2.5–97.5%) ^a^	1.16 (0.83–1.85)	1.12 (0.81–1.77)	1.11 (0.81–1.78)	1.1 (0.81–1.7)	1.36 (0.90–2.39)	1.30 (0.90–2.23)
Boundaries, *n* (%)^b^						
<0.7 mg/dL	28,196 (11.2)	37,699 (15.0)	23,634 (11.2)	31,430 (14.8)	4562 (11.5)	6269 (15.7)
<1.0 mg/dL	102,593 (40.8)	112,694 (44.8)	86,586 (40.9)	94,877 (44.8)	16,007 (40.2)	17,817 (44.7)
1.0–2.5 mg/dL	114,720 (45.6)	106,420 (42.3)	97,007 (45.8)	90,182 (42.6)	17,713 (44.5)	16,238 (40.8)
> 2.5 mg/dL	34,324 (13.6)	32,523 (12.9)	28,218 (13.3)	26,752 (12.6)	6106 (15.3)	5771 (14.5)
> 3.0 mg/dL	27,473 (10.9)	25,715 (10.2)	22,542 (10.6)	21,129 (10.0)	4931 (12.4)	4586 (11.5)
> 4.0 mg/dL	18,904 (7.5)	16,157 (6.4)	15,581 (7.4)	13,356 (6.3)	3323 (8.3)	2801 (7.0)

^a^Comparison of creatinine values alive versus dead and before versus after: *p* < 0.00001.

^b^Comparison of categorial distribution defined by boundaries before and after application of reference model: *p* > 0.05.

Comparison of categorial distribution defined by boundaries between patients alive and dead: *p* < 0.00001.

### Creatinine correction by the reference models potentially affects up to 50% of MELD scores in data of two patient cohorts

**Fig. 2:**
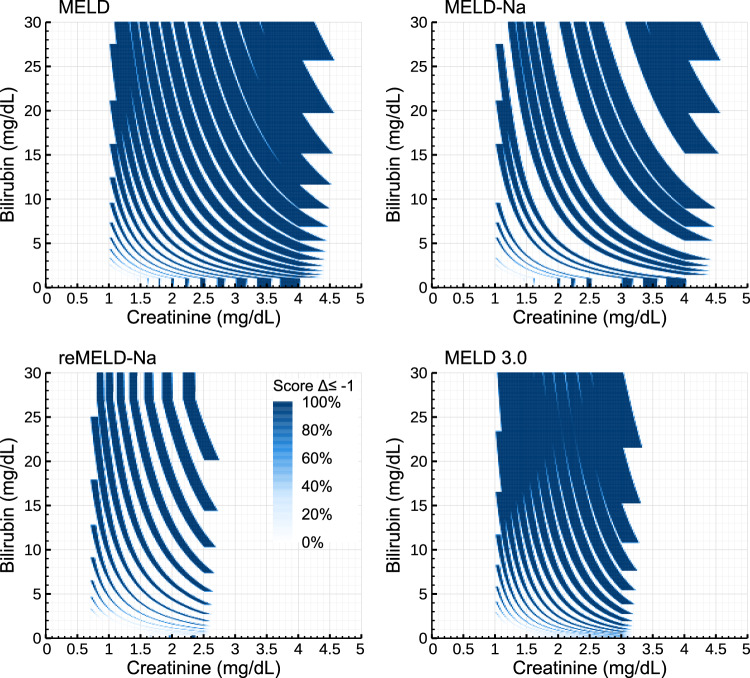
Distribution patterns of score corrections by the proposed correction model, assuming a uniform distribution of creatinine and bilirubin. The heatmaps illustrate the effects of creatinine–bilirubin interactions at varying concentrations, as well as the impact of boundary and rounding effects during score calculation. Blue colours represent reductions, red colours increase of the score due to the modelled creatinine correction. Upper left panel: classical MELD; upper right panel: MELD-Na; lower left panel: reMELD-Na; lower right panel: MELD 3.0. Colour codes for all panels are shown in the lower left panel. Source data are provided as a Source Data file (F2 simulated heatmap repository.csv contained within MELD_public_data_package).

Figure 2 illustrates the expected changes in variants of the MELD score due to creatinine correction across the modeled range of creatinine and bilirubin values, based on a hypothetical uniform distribution of both analytes. Each score variant exhibits a characteristic pattern determined by its specific calculation method, including rounding effects and boundary constraints. These patterns highlight the complex relationship between creatinine correction and score deviations, which must be addressed in clinical practice. Assuming a uniform distribution of creatinine and bilirubin, corrections leading to a decrease of at least one point (Δ ≤ −1) occur in 50.4, 39.2, 33.7, and 16.7% of modeled concentration pairs for MELD, MELD-Na, MELD 3.0, and reMELD-Na, respectively. The graduation between the score variants results mainly from the truncation of high creatinine values and to a lesser extent from the model-specific interaction terms between creatinine and sodium. Given the varying weightings of bilirubin and creatinine, and the inclusion of creatinine as a model-specific interaction term, discordant shifts between the four score variants are expected. Supplementary Fig. 1 displays a Venn diagram illustrating the distribution of score deviations across 8614 complete cases (ESLD), i.e., based on MELD 3.0, with complete data for all four models and both cohorts. In most cases (*n* = 6560; 76.2%), no deviation occurred in any score. Deviations in at least one score were observed in 2054 cases (23.8%), with all four scores deviating in 155 cases (1.8%). Among the deviating cases, the highest overlap was between MELD and MELD-Na (*n* = 804; 39.1%), while concurrent deviations between reMELD-Na and MELD 3.0 occurred only in the subgroup with deviations in all four scores (*n* = 155; 1.8%). ReMELD-Na most frequently showed isolated changes—i.e., deviations not shared with the other scores—in 721 cases (35.1%), including 431 reductions of −1, 6 scores reduced by −2 points, and 290 increasing by +1 point. Similar concordances/discordances were observed in the SRTR group.

**Fig. 3:**
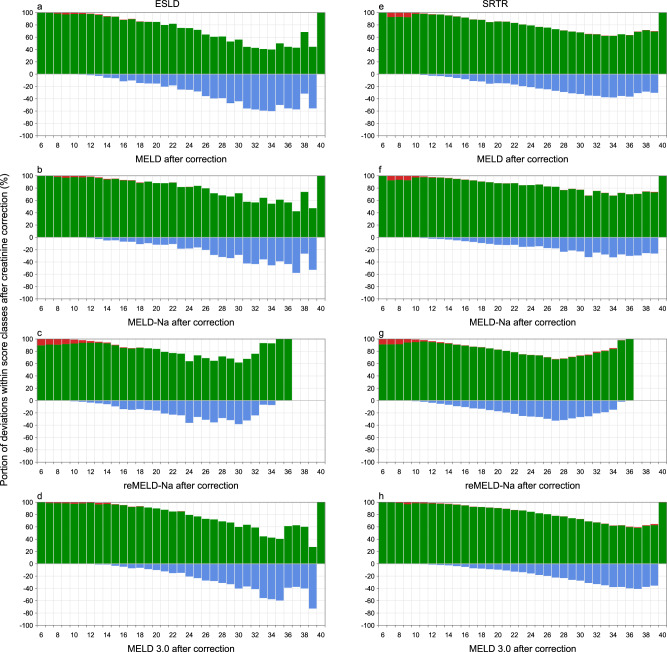
Distribution of changes across original score classes after creatinine correction by score variant. Stacked bar plots show the proportion of datasets with unchanged scores (green) or changes following modelled creatinine correction: Δ = +2 (violet), Δ = +1 (red), Δ = −1 (light blue), and Δ = −2 (dark blue), stratified by original score class. **a**–**d** left column, ESLD (*N* = 20,359); **e**–**h** right column, SRTR (*N* = 251,637), **a**/**e**: MELD, **b**/**f**: MELD-Na, **c**/**g**: reMELD-Na, **d**/**h**: MELD 3.0. Score changes of +2 points were extremely rare (each ≤0.1% within any score class in both cohorts) and therefore are not visually apparent in most bars; the exact distribution by score class and score variant is reported in Supplementary Tables 2 (ESLD End stage liver disease cohort) and 3 (SRTR Scientific registry of transplant recipients cohort). Any positive deviations (Δ +1/2) occurred in 0.9, 0.9, 4.1, and 0.9% for the ESLD group and 14.9, 13.3, 16.9, and 11.6% for the SRTR group for MELD, MELD-Na, ReMELD-Na, and MELD 3.0, respectively. Any negative deviations (Δ −1/2) were present in 7.9, 6.9, 7.4, and 8.6% for the ESLD group and 0.8, 0.8, 1.4, and 0.3% for the SRTR group for MELD, MELD-Na, ReMELD-Na, and MELD 3.0, respectively. Source data are provided as a Source Data file (Esld master long.csv and Esld F3 score shift aggregate.csv contained within MELD_public_data_package) and Supplementary Tables 2 and 3.

Figure 3 (with Supplementary Tables 2 and 3) illustrates the extent and proportion of deviations observed for recalculated score variants after applying the correction (Eq. (1)) to historical creatinine values in both the ESLD and the SRTR cohorts. Decreasing scores due to creatinine corrections occurred at baseline scores from 11 to 12 points upward in the ESLD and SRTR cohorts, respectively. Score deviations were most frequent above 30 score points, except for reMELD-Na, which showed peak proportion at 27 points. In contrast, increased scores occurred only below 20 points, affecting less than 1% of cases across all score variants. An exception was reMELD-Na, where they accounted for 4.0% of all shifts and thus contributed to its higher overall deviation rate. Score deviations due to the correction ranged from +2 to −2 in both cohorts. In the ESLD data, the proportion of deviating scores was 7.9%, 8.8%, 9.5%, and 12.0% for MELD-Na, MELD, MELD 3.0, and reMELD-Na, respectively. In the SRTR cohort, the same order was observed with slightly higher proportions, i.e., 10.9, 12.1, 11.7, and 13.8%, respectively.

### Correction-related MELD score decreases were associated with longer survival times among deceased patients

Patients with score deviations ≤ −1 were stratified by survival status (Table 3, overall prevalence), firstly without restricting the time interval between score assessment and death. In both cohorts and across all four score variants, the proportion of deceased individuals was significantly higher among those deviating ≤ −1 points. The odds of death associated with a shift of ≤ −1 points were higher in the SRTR cohort (OR: 5.4–6.3) than in the ESLD cohort (OR: 1.3–2.3). In the subgroup of deceased patients, we compared survival indicators between patients with no score deviation and those with a decrease of ≤ –1 points across the ESLD and SRTR cohorts (Table 3, 30-day and 90-day survival probabilities).

**Table 3:** Score deviations (Δ) of ≤ −1 stratified by outcome (alive/dead within 90 days after MELD calculation); subanalysis of 30- and 90-days survival indicators in deceased ESLD patients only. Data are presented as point estimates with 95% confidence intervals in parentheses

		ESLD			
Metrics		MELD (*N* = 20,359)	MELD-Na (*N* = 19,213)	reMELD-Na (*N* = 19,213)	MELD 3.0 (*N* = 8614)
Overall prevalence of score Δ ≤ −1, %	Alive	6.2 (5.8–6.6)	5.7 (5.4–6.1)	6.9 (6.5–7.3)	6.7 (6.2–7.4)
	Deceased	12.8 (11.9–13.8)	10.7 (9.9–11.6)	9.0 (8.2–9.8)	14.5 (13.0–16.1)
Odds ratio		2.222 (1.997–2.472)	1.978 (1.757–2.225)	1.330 (1.178–1.501)	2.346 (1.999–2.750)
30 days survival probability	Score Δ ≤ −1	0.677 (0.608–0.754)	0.672 (0.597–0.755)	0.714 (0.638–0.800)	0.659 (0.569–0.764)
	Score Δ = 0	0.463 (0.357–0.599)	0.524 (0.427–0.642)	0.412 (0.318–0.536)	0.590 (0.504–0.692)
90 days survival probability	Score Δ ≤ −1	0.506 (0.434–0.591)	0.496 (0.419–0.588)	0.555 (0.472–0.652)	0.495 (0.402–0.609)
	Score Δ = 0	0.388 (0.287–0.524)	0.440 (0.346–0.561)	0.300 (0.215–0.419)	0.467 (0.380–0.573)
90 days restricted mean survival time (RMST90)	Score Δ ≤ −1	58.2 (52.7–63.8)	57.5 (51.4–63.6)	61.9 (55.6–68.3)	56.6 (49.2–64.0)
	Score Δ = 0	44.3 (34.9–53.7)	49.1 (40.8–57.4)	38.2 (30.1–46.3)	52.2 (44.9–59.6)
RMST90-ratio	Δ ≤ −1/Δ = 0	1.32 (1.04–1.66)	1.17 (0.96–1.43)	1.62 (1.28–2.05)	1.08 (0.89–1.31)
90 days hazard ratio	Δ ≤ −1 vs. Δ = 0	0.754 (0.540–1.052)	0.884 (0.642–1.218)	0.548 (0.395–0.760)	0.880 (0.639–1.211)

		SRTR			
Metrics		MELD (*N* = 251,637)	MELD-Na (*N* = 251,637)	reMELD-Na (*N* = 251,637)	MELD 3.0 (*N* = 251,637)
Overall prevalence of score Δ ≤ −1, %	Alive	11.1 (11.0–11.3)	9.9 (9.8–10.0)	12.1 (12.0–12.2)	11.0 (10.8–11.1)
	Deceased	40.3 (38.4–42.1)	38.2 (36.3–40.1)	46.4 (44.5–48.3)	41.6 (39.7–43.4)
Odds ratio		5.373 (4.970–5.809)	5.615 (5.190–6.076)	6.282 (5.818–6.783)	5.775 (5.343–6.241)
30 days survival probability	Score Δ ≤ −1	0.760 (0.737–0.783)	0.760 (0.737–0.783)	0.760 (0.738–0.784)	0.762 (0.739–0.785)
	Score Δ = 0	0.755 (0.733–0.778)	0.755 (0.733–0.778)	0.754 (0.732–0.778)	0.755 (0.733–0.779)
90 days survival probability	Score Δ ≤ −1	0.630 (0.604–0.656)	0.630 (0.604–0.656)	0.631 (0.606–0.658)	0.632 (0.607–0.659)
	Score Δ = 0	0.610 (0.585–0.637)	0.610 (0.585–0.637)	0.612 (0.587–0.639)	0.612 (0.586–0.638)
90 days restricted mean survival time (RMST90)	Score Δ ≤ −1	66.9 (65.1–68.7)	66.9 (65.1–68.7)	66.9 (65.1–68.8)	67.0 (65.2–68.9)
	Score Δ = 0	66.1 (64.3–67.9)	66.1 (64.3–67.9)	66.1 (64.3–67.9)	66.1 (64.3–67.9)
RMST90-ratio	Δ ≤ −1/Δ = 0	1.01 (0.97–1.05)	1.01 (0.97–1.05)	1.01 (0.97–1.05)	1.01 (0.98–1.05)
90 days hazard ratio	Δ ≤ −1 vs. Δ =	0.943 (0.834–1.066)	0.943 (0.834–1.066)	0.944 (0.834–1.067)	0.939 (0.830–1.061)

Source data are provided as a Source Data file (Esld master long.csv and Esld T4 score deviation outcome table.csv contained within MELD_public_data_package).

**Fig. 4:**
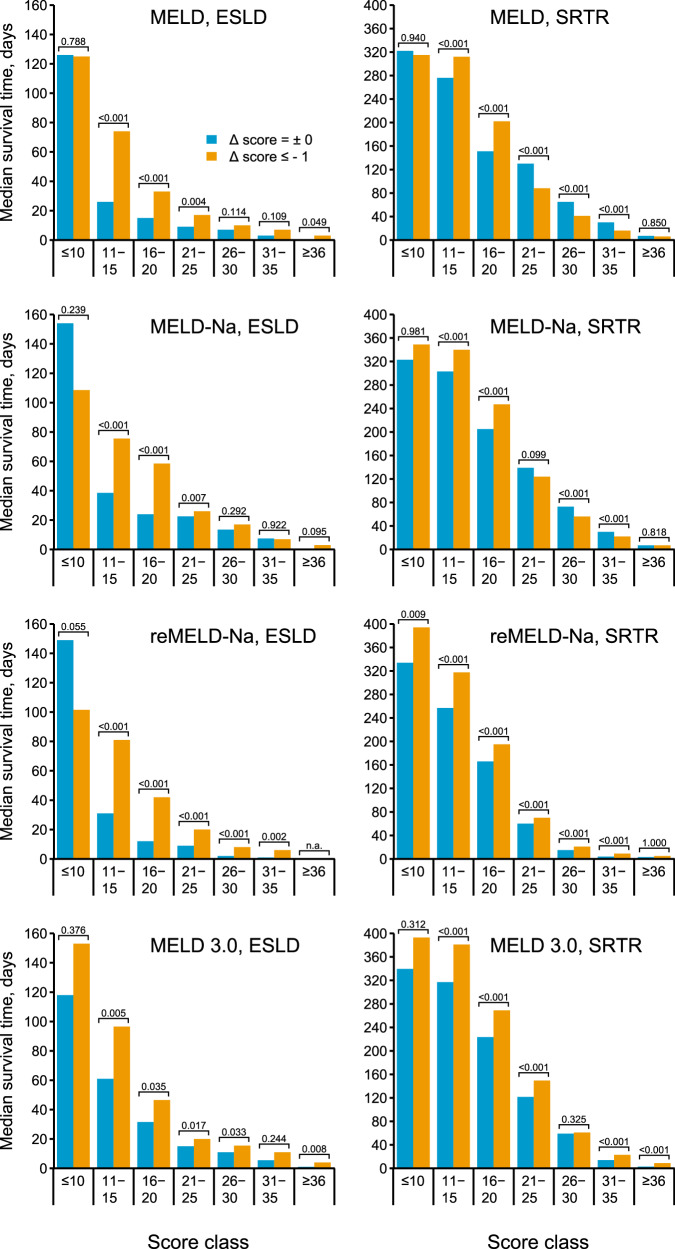
Effect of score variants stability on median survival across score classes. Median retrospective two-years survival times are compared between deceased patients stratified by score class (*x-*axis) and post-correction score stability. Blue bars represent patients with unchanged scores (Δ = 0), and orange bars represent those which had at least one score decrease of Δ ≤ −1 following modelled creatinine correction. ESLD End stage liver disease cohort, SRTR Scientific registry of transplant recipients cohort. *P*-values displayed above the brackets indicate within-score class comparisons (two-sided Wilcoxon rank sum test). Source data are provided as a Source Data file (Esld master long.csv and Esld F4 survival stats.csv contained within MELD_public_data_package).

Figure 4 illustrates the interrelation between score classes and median survival times in deceased patients within the last two years before death, comparing median survival between those deviating ≤ −1 points and those without any changes. In both cohorts, patients with decreased scores (Δ ≤ –1), indicating creatinine overestimation in the uncorrected score, consistently presented significantly longer median survival compared to patients with unchanged scores (Δ = 0), particularly within intermediate MELD ranges. Exceptions were rare and most evident for MELD-Na in the SRTR cohort.

### Corrected MELD scores affected observed survival and incidence of events on transplant waitlist

**Fig. 5:**
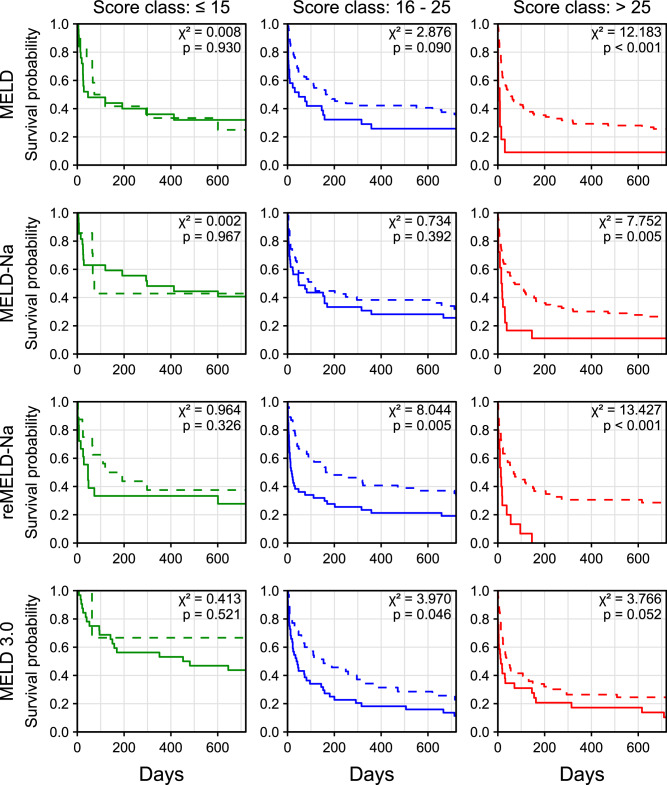
Survival analysis comparing score variants deviations following creatinine correction in ESLD patients. Two-year survival rate of deceased patients of the ESLD (End-stage liver disease) cohort, stratified by score class (green lines: ≤ 15; blue lines: 16 to 25; red lines: ≥ 25) and score stability after creatinine correction, shown as Kaplan–Meier survival curve. In each panel, patients with no change in the respective MELD-based score (Δ = 0; full lines) are compared to those with at least one decrease of Δ ≤ −1 (dashed lines). Log-rank p-values of all comparisons are given in Supplementary Table 4. (Esld master long.csv and Esld F5 stratified survival stats.csv contained within MELD_public_data_package).

Two-year survival rates were calculated for the ESLD cohort, stratified by MELD classes (≤ 15; 16–25; > 25; Fig. 5) and score stability after creatinine correction to generate Kaplan–Meier curves. Log-Rank tests (Supplementary Table 4) revealed significantly lower mortality hazard in class > 25 for MELD, MELD-Na, and reMELD-Na, but not for MELD 3.0 (*p* = 0.052).

**Fig. 6:**
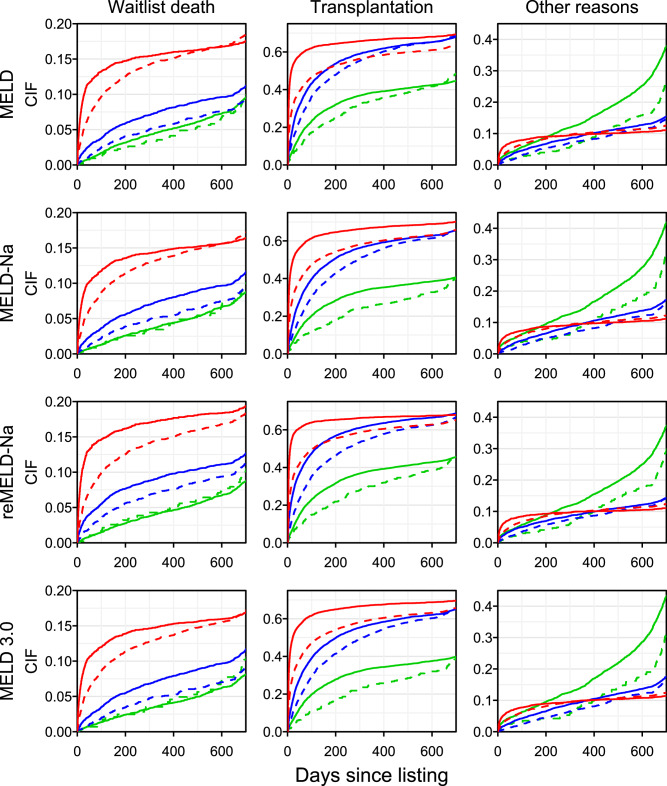
Competing risk analysis comparing score variants deviations following creatinine correction in SRTR Scientific Registry of Transplant Recipients, patients. CIF Cumulative incidence functions are stratified by causes of removal from waitlist: death on waitlist (left column), transplantation (middle column), and other causes for removal (right column), and further grouped by score classes. Green lines: MELD ≤ 15 blue lines: MELD 16–25; red lines: MELD > 25. In each panel, patients with no change (Δ = 0; full lines) are compared to those with at least one decrease of Δ ≤ −1 (dashed lines). Detailed statistical analysis for competing risks is provided in Supplementary Table 5a (Gray’s test for competing risks of removal from waitlist) and Supplementary Table 6a (Fine–Gray subdistribution hazard for waitlist death).

For the SRTR cohort, cumulative incidence functions were calculated to illustrate death on waitlist, transplantation and other causes for removal as competing risks up to two years after score assessment (Fig. 6). Competing risks in this group, stratified by MELD class and score stability after application of the creatinine reference model were assessed by Gray’s Test (Supplementary Table 5a), indicating significant differences associated with correction-related score decreases for waitlist death, transplant, and other causes of removal depending on score variant and class. Fine–Gray subdistribution hazard ratio was calculated for waitlist death (Supplementary Table 6a), resulting in a significantly lower subdistribution hazard ratio for patients with MELD correction of Δ ≤ −1 in the intermediate classes (16–25) of MELD, MELD-Na, and MELD 3.0.

For SRTR, competing-risk analyses was separately conducted in the 2023–2025 subgroup to reflect the contemporary allocation and transplantation practice, yielding consistent results. A lower subdistribution hazard of transplantation in the Δ ≤ −1 group was identified by Gray’s test and Fine–Gray models across MELD, MELD-Na, and MELD 3.0 strata (e.g., MELD > 25: SHR 0.78 [0.69–0.89], *p* = 0.0003; MELD 3.0 ≤ 15: SHR 0.55 [0.42–0.73], *p* = 0.0001; Supplementary Table 6b), reinforcing that assay-related MELD inflation translates into clinically relevant differences in transplant access. By contrast, effects on waitlist death or other removals were less consistently detectable, likely reflecting reduced event rates and increasing pre-emption by transplantation, with significance limited to selected strata (Supplementary Table 6b). Contemporary competing-risk patterns are illustrated in Supplementary Fig. 2, updating Fig. 6, with corresponding Gray/Fine–Gray and horizon-specific results provided in Supplementary Tables 5b and 6b.

Horizon-specific analyses at 90 and 365 days since listing confirmed that creatinine correction predominantly affected transplantation outcomes. Across both time horizons, the Δ≤−1 group consistently showed a lower probability and hazard of transplantation compared with Δ = 0 across MELD variants and score classes (Supplementary Tables 7a/b and 8a/b).

## Discussion

In this study, we systematically characterized the analytical bias in serum creatinine measurements used for MELD score calculation, focusing on method-related variation and bilirubin-dependent interference. We examined the impact of a correction algorithm compensating this interference across four variants of the MELD score. The Jaffe method, which tended to overestimate creatinine concentrations, was shown to artificially increase score variants by up to 2 points. Correction of the creatinine value resulted in a reduced MELD significantly more often in deceased patients in two independent cohorts. Those deceased patients whose MELD was reduced by the correction had longer survival times than those with unaffected MELD scores. Score correction was also independently associated with waitlist mortality and waitlist removal due to transplantation over the whole time frame analysed, including the contemporary 2023–2025 subgroup. These findings imply a clinical impact of the proposed correction of creatinine measurements for score variants, assessment of ESLD severity, and potentially improved fairness in liver transplant allocation.

Our experimental in vitro approach and the subsequent validation in selected patient samples demonstrated that creatinine test accuracy depends significantly on bilirubin concentration, with a markedly greater interference effect observed in the Jaffe method. Across all bilirubin levels, the Jaffe assay consistently produced higher creatinine values—a finding that aligns with previous studies^15,16,19,20^. For instance, Kaiser et al. compared enzymatic and Jaffe-based creatinine measurements using 60 residual serum samples from patients with ESLD, referencing GC-IDMS as the gold standard^21^. To our knowledge, no other study applied GC-IDMS on samples from patients on waitlists for liver transplantation. Samples with creatinine levels below 1 mg/dL were excluded due to the MELD score’s boundary rules. Among the remaining 28 samples, 25 showed measurable differences in creatinine, leading to MELD score deviations of up to 4 points. Given the greater clinical relevance of creatinine levels ≥1 mg/dL and the rising burden of renal impairment among liver transplant candidates ^22^, this analytical bias could become increasingly consequential. In a larger cohort of 1013 samples from 445 liver transplant candidates, MELD scores based on Jaffe-derived creatinine values were up to 4 points higher than those calculated using enzymatic assays; the latter showed a maximum variation of only 2 points^19^. Notably, higher MELD scores occurred in 16.1% of Jaffe-based assessments, compared to just 1.5% when the enzymatic method was used. This discrepancy widened further in patients with MELD scores >20, where 32.9% of Jaffe-based scores were elevated versus 2.6% for enzymatic measurements. Although these studies considered potential confounders—including glucose, albumin, bilirubin, and medications—none could fully account for the observed differences, likely due to the complex biochemical milieu in ESLD serum. Our findings confirm that enzymatic creatinine assays align more closely with true values and are substantially less susceptible to interference from elevated bilirubin.

Inconsistencies in creatinine measurement between centers—largely due to differences in analytical methodologies—pose a significant threat to the fairness of liver allocation^23^. This concern has been recognized since the early adoption of the MELD score and underscores the need for standardized laboratory procedures to ensure objective and equitable assessments^24^. The clinical chemistry analyzer employed in our study, introduced in 2006, is widely used in diagnostic laboratories globally and thus reflects the commonly applied methodology for MELD score calculations. Importantly, creatinine is not the only MELD component subject to inter-laboratory variation. Lisman et al. identified an average MELD score discrepancy of 4.8 points across seven European centers, primarily attributed to differences in INR measurements. However, the small sample size in that study limits the generalizability of its findings^25^. Despite longstanding awareness of the inconsistencies in creatinine assessment, the Jaffe method remains prevalent: 68% of the 1094 medical laboratories in Germany (2025) and 61.6% of the 6137 laboratories in the US (2024) participating in external quality assurance programs still rely on this approach. A key limitation is that interference testing by manufacturers is typically performed on healthy serum samples spiked with bilirubin, which does not adequately replicate the complex biochemical environment in patients with ESLD^19,20^. Consequently, the real-world impact of bilirubin interference is likely underestimated. In response, our study employed an experimental approach to correct for bilirubin interference, systematically covering the entire spectrum of bilirubin and creatinine level combinations. This enabled the development of a correction formula applicable across the full clinical spectrum, aiming to more accurately reflect true creatinine concentrations and improve MELD score reliability. For confirmation in patient samples, we deliberately included a broad range encompassing diverse bilirubin and creatinine levels from two distinct cohorts (ESLD and SRTR).

Laboratory method-dependent variations and risk of potential inaccuracies in creatinine values are aggravated by elevated bilirubin levels^15^. Bilirubin concentration-dependent effects have been reported previously with varying magnitudes and differing directions of interference^15,16,23^. This issue is particularly critical for patients with liver cirrhosis, where hyperbilirubinemia is both common and a marker of disease severity^26^. As a result, patients with more advanced disease stages are disproportionately affected by analytical inconsistencies, complicating decisions on organ allocation. In our study, we observed a parallel rise in creatinine values with increasing bilirubin concentrations, specifically in the Jaffe method. A methodological strength of our approach lies in the use of a defined matrix in which creatinine was dissolved and systematically spiked with bilirubin^10,27–30^. This design avoids confounding effects of endogenous serum components, which complicate traditional interference studies. Our findings are in part supported by Dissanayake et al., who similarly employed an artificial matrix using phosphate-buffered saline with spiked creatinine and bilirubin^11^. They reported that at creatinine concentrations up to 2 mg/dL, increasing bilirubin led to underestimation of creatinine, while concentrations above 2 mg/dL were increasingly overestimated. In contrast, the enzymatic assay showed only minimal underestimation, which the authors attributed to a negative interference effect of bilirubin conversion to biliverdin. At higher creatinine levels, the shift toward positive interference was thought to reflect unconverted bilirubin exerting its spectral effect. The differences between their findings and ours may reflect variability in assay manufacturers, interference mitigation strategies, and analyzer platforms, which can significantly influence results even within the same methodological category. Thus, any mathematical correction should be assay-specific to ensure accurate clinical interpretation. A further advantage of our study is the integration of these findings into a mathematical model that was independently validated using patient samples analyzed via GC-IDMS, the gold standard for creatinine measurement. This allowed us to confirm the model’s applicability in real-world clinical conditions. Notably, the resulting algorithm is both robust and easy to apply.

The clinical consequences of measurement variability become particularly evident when examining its direct impact on MELD scores and patient outcomes. The overestimation of creatinine may lead to patients being prioritized for transplantation based on inaccurate estimates of disease severity, potentially disadvantaging others whose values more accurately reflect their condition. Such variability undermines the principle of equitable organ allocation, which stipulates that patients with comparable clinical needs should have equal access to transplantation. In our study, deceased patients consistently showed a higher likelihood of MELD deviations of Δ ≤ −1 compared with survivors in both the ESLD and SRTR cohorts, with higher odds ratios in SRTR. The increased frequency of corrections likely reflects the higher bilirubin levels commonly seen in deceased patients, as well as the overall higher bilirubin values in SRTR compared with ESLD. As a result, the particularly vulnerable group of patients on the transplant waitlist is disproportionately affected by analytical discrepancies and the resulting inequities. MELD score ranges also dictate the frequency of recertification, with higher scores requiring more frequent medical evaluations (i.e., MELD scores 16–20 every 30 days; 10−15 every 90 days). In a hypothetical allocation scenario where all other factors are equal, a patient with an inaccurately elevated MELD score could be prioritized over another candidate. While such cases may be rare, they highlight the potential consequences of even small inaccuracies in MELD scoring. Importantly, these deviations were not random. Higher MELD categories, elevated creatinine or bilirubin levels, or both were associated with a greater likelihood of correction-induced changes. Thus, deviations depending on the assay applied occur particularly in patients with an already elevated baseline mortality risk, making fair and accurate transplantation decisions even more critical. This raises the key question: does a MELD shift of Δ ≤ −1 represent a clinically meaningful difference, or does it instead improve the accuracy of survival prediction?

To address this, we conducted a targeted analysis of the subgroup of deceased patients. Both median overall survival times and 90-day restricted mean survival times were longer among deceased ESLD and SRTR patients who experienced a MELD score reduction of Δ ≤ −1 following creatinine correction, compared to those whose scores remained unchanged. This result indicates that initial MELD values were inflated by falsely elevated creatinine measurements, leading to artificially higher scores. Adjusting creatinine toward its true value could provide a more accurate reflection of disease severity and prognosis, as the down-corrected MELD scores were associated with longer survival times. In Cohort 1, patients with Δ ≤ −1 deviations had significantly longer survival times compared to those in Cohort 2, which could be explained by clinical differences between these groups. Cohort 1 included patients with ESLD not necessarily listed for liver transplantation (2% of the cohort). Thus, Cohort 1 represented a comparatively healthier population than Cohort 2. Supporting this interpretation, laboratory markers of disease severity, as well as MELD scores, were significantly higher in Cohort 2. Conversely, 90-day mortality was higher in Cohort 2 than in Cohort 1, before and after creatinine correction. Additionally, this difference may be reinforced by systematic differences in waitlist removal reasons, which introduce competing risks and informative censoring in SRTR. In the SRTR subgroup of Jaffe-confirmed centers, >50% of candidates were removed due to transplantation (deceased or living donor; *n* = 10,778 and *n* = 794, respectively), with additional removals for other reasons (*n* = 5263). Thus, in SRTR, competing risks (as detailed in Supplementary Table 6a) for death had to be taken into account in the analysis. In contrast, survival analyses in ESLD focused on overall survival independent of waitlist events (1 death among 5 on waitlist). The combined results from corrected biochemical analyses and survival data indicate that method-dependent differences can distort the predictive validity of the MELD score over a longer observation period as well as for 90-day and 1-year survival times. This underscores the urgent need to adjust for analytical biases or harmonize diagnostic methodologies to ensure equity in liver allocation. Among the evaluated score variants, reMELD-Na was most susceptible to analytical bias and showed the poorest concordance with MELD 3.0. By contrast, MELD 3.0 was the most robust, displaying minimal impact on survival prediction and closer alignment with corrected scores.

Liver allocation worldwide is based on the “sickest first” principle, operationalized through the MELD score since 2002. While this system has introduced much-needed objectivity, it is not free from limitations and biases. One well-documented source of bias involves sex-related disparities: women are 8.6% more likely to die while awaiting transplantation and 14.4% less likely to receive a liver than men^31^. A major contributor is the creatinine component of MELD^32,33^, which does not adjust for naturally lower creatinine levels in women. As a result, women often receive MELD scores that underestimate disease severity by 1.0 to 2.4 points, a gap that became more pronounced with the introduction of Na-MELD^21^. In response, MELD 3.0 incorporates a 1.3-point adjustment for female sex, aiming to reduce these disparities and improve fairness in organ allocation^2^. In our study, score corrections of Δ ≤ −1 in the non-deceased population occurred more frequently in men than in women (Supplementary Table 9), likely reflecting physiologically higher creatinine levels in men and indicating a potential sex related bias. This trend was absent among deceased patients, possibly due to more advanced disease states influenced by additional pathological factors. Whether the proposed creatinine correction could further enhance fairness in score variants that do not account for sex will require more detailed future analyses.

Among the three components of the MELD score, creatinine has emerged as the most influential variable. A recent analysis showed that patients whose MELD scores were primarily driven by elevated creatinine had the poorest outcomes^34^. This highlights the importance of accurate creatinine measurement for fair prioritization on the transplant waiting list. At the same time, the demographic and clinical profile of transplant candidates has shifted considerably. The waiting list population is now older, hepatitis C has declined as the leading indication for transplantation, and cases of alcoholic liver disease and metabolic dysfunction–associated steatotic liver disease have risen sharply^35,36^. Additionally, the prevalence of chronic kidney disease has doubled^22^. These trends point to a progressively sicker patient population with increasing renal burden, further amplifying the need for precise assessment of kidney function in liver transplant candidates. Despite the central role of creatinine in the MELD score, there is currently no requirement to report the method used for creatinine measurement when submitting MELD scores to transplant allocation authorities. Furthermore, none of the original MELD publications or their derivatives specify the creatinine assay used during score development. This omission suggests that potential methodological biases in creatinine measurement were not fully considered, despite their now-demonstrated clinical impact.

In our study, reMELD-Na was the most affected variant among the evaluated MELD scores, showing the largest deviations in survival indicators within the ESLD group and limited concordance with other variants. This discordance may reflect its design for the Eurotransplant region, which applies lower creatinine capping values than other MELD-based scores. By contrast, MELD 3.0 includes additional variables such as sex and albumin and uses different capping thresholds, factors that may have reduced the impact of creatinine correction in our analysis. To our knowledge, only one prior study has assessed the diagnostic performance of reMELD-Na relative to other score variants, reflecting its recent introduction. However, this study was restricted to a 90-day survival analysis and evaluated only the reMELD score without the sodium component^37^, thus limiting its applicability to our findings. Although reMELD-Na applies the lowest boundary for creatinine, we did not observe a clear impact on the proportion of scores affected by a deviation of Δ ≤ −1 in our study. This suggests that underlying methodology for creatinine measurement and its integration into the scoring formula may play a more decisive role in score variability and its clinical implications than boundary limits.

The findings of this study should be considered in light of both its strengths and limitations. One of the main strengths lies in the development of an experimentally derived and independently validated correction formula, specifically tailored to individual combinations of creatinine and bilirubin concentrations. This model covers the full clinically relevant spectrum of values for the Jaffe method, offering a practical solution to mitigate bilirubin-related interference. Although alternative strategies exist—such as sample dilution, rate blanking, or deproteinization—these approaches are either labor-intensive, require manual handling, or are not routinely feasible in clinical laboratories^38,39^. Another strength is the scope of data utilized for model development and evaluation, which comprise datasets containing over 270,000 MELD score observations. The data from a large tertiary care center in Germany encompass a broad and heterogeneous cohort of patients with ESLD. The inclusion of an external validation branch with a subset of the SRTR, which represents the entire population of liver transplant candidates in the US since 1990, further strengthens the generalizability and population-independent relevance of our findings. However, several limitations should be acknowledged. First, the experimental design did not allow for a complete isolation of bilirubin interference from other potential method-specific sources of variability in creatinine measurement. Second, the primary aim of this study was to demonstrate that specific creatinine assay types can systematically disadvantage patients within MELD-based allocation systems. The proposed correction formula addresses this issue in our setting, though may not be directly generalizable to all laboratories. Additionally, we did not assess possible inter-laboratory variation in the measurement of other MELD-relevant analytes such as INR, bilirubin, or albumin—some of which are known to exhibit even greater variability than creatinine^40^. Finally, while we applied our correction model to the SRTR cohort, we cannot guarantee that all participating centers used the same Jaffe assay protocol. This lack of standardization may limit the consistency and applicability of our correction across this dataset.

In conclusion, our findings underscore the critical need for methodological standardization in creatinine measurement to ensure fairness in liver transplant allocation. We developed a correction model for creatinine–bilirubin interference based on gravimetrically prepared in vitro concentrations and validated it in representative patient samples. Applying this model to two large clinical cohorts demonstrated that MELD scores can vary systematically depending on the creatinine assay used and its susceptibility to bilirubin interference. In particular, the Jaffe method tended to overestimate creatinine, which may result in inequitable prioritization of patients compared with those whose scores are calculated using more accurate enzymatic assays. These inconsistencies not only challenge the objectivity of the MELD allocation system but also raise concerns about fairness in the distribution of scarce donor organs. To address these disparities, transplant authorities and policymakers should consider implementing standardized, interference-resistant creatinine assays across all transplantation centres. Meanwhile, revising allocation protocols to account for methodological differences in MELD calculations could further enhance the fairness and accuracy of organ distribution systems.

## Methods

### Ethics statement

This study was conducted in accordance with the Declaration of Helsinki (2024) and complied all relevant ethical regulations. Ethical approval was obtained from the institutional review board of Ruhr University Bochum (Register Number: 23-7840). The Inova Health Systems Institutional Review Board classified the study as non-human subject research. Both boards waived the need for informed consent.

### Study design and setting

We conducted a mixed-method study integrating experimental in vitro model development and validation in patient samples with a retrospective comparative analysis of clinical implications. The retrospective analysis employed two cohorts from two distinct data sources to evaluate the impact of creatinine correction on MELD scores. The general characteristics of both cohorts investigated are summarized in Table 1 further details are given below under general information on patient cohorts.

### Experimental development of a reference model to approximate true creatinine values

A two-stage approach was designed to create a model that can compensate for both method-dependent and interference-dependent inaccuracies. The procedure was divided into a development phase and a subsequent validation phase.

#### Development phase

Target creatinine concentrations were prepared by gravimetric dilution of a certified reference material (Sigma-Aldrich Chemie GmbH, Germany; product code PHR1462) in a serum-free matrix. The concentration range covered 0 to 5 mg/dL in 0.5 mg/dL increments. From each of these creatinine concentrations, a series of aliquots was prepared. Bilirubin (Bilirubin-Ditaurat, Calbiochem-Sigma-Aldrich Chemie GmbH, Germany; product code 201102) was also quantified gravimetrically and added in increasing concentrations to each of these series to cover a total bilirubin range from 0 to 35 mg/dL in 1 mg/dL increments. This approach creates an array of 396 concentration-combinations in which a true creatinine value is assigned to each possible creatinine-bilirubin combination. Sample arrays were repeatedly prepared on different days under identical conditions (*n* = 3, duplicate measurements). Complete sets of sample arrays were analyzed within the same day using two different creatinine assay applications, Creatinine Jaffe Gen.2 (CreJ) and Creatinine plus ver.2 (CreE) on a cobas® c 501 modules integrated into a cobas 6000 clinical chemistry analyzer system. Total bilirubin concentrations were determined using the Bilirubin Total Gen.3 assay on a uniform analyzer platform, spanning a measurement range of 0.146 to 38.0 mg/dL. Bilirubin levels exceeding this range were diluted according to the manufacturer’s instructions. All assays and analyzers have been purchased from Roche Diagnostics, Mannheim, Germany.

Results from all sample arrays and gravimetrically defined reference concentrations were incorporated into a second-degree polynomial regression model, which included creatinine, total bilirubin, and their interaction term as independent variables. Solving the output of the nonlinear regression model for the dependent variable will be defined as reference function since true values are deduced from a certified reference material traceable to gravimetry. Independent reference functions were computed for CreJ and CreE. 3D-surface plots are rendered to visualize the observed dependencies (Fig. 1).

The resulting second-degree polynomials to reduce assay deviation from true creatinine values are presented below as Eqs. (1) and (2),

1 $$\hat{{{\rm{CreJ}}}}\,=	0.0115+\left(-0.0120\right)\cdot {{{\rm{TB}}}}_{{{\rm{M}}}}+0.00025\cdot {{{{\rm{TB}}}}_{{{\rm{M}}}}}^{2} \\ 	+1.0390\cdot {{{\rm{CreJ}}}}_{{{\rm{M}}}}+\left(-0.0294\right)\cdot {{{{\rm{CreJ}}}}_{{{\rm{M}}}}}^{2}$$

2 $$\hat{{{\rm{CreE}}}}=	 0.1945+\left(-0.0130\right)\cdot {{{\rm{TB}}}}_{{{\rm{M}}}}+0.00003\cdot {{{{\rm{TB}}}}_{{{\rm{M}}}}}^{2} \\ 	+0.9076\cdot {{{\rm{CreE}}}}_{{{\rm{M}}}}+\left(-0.0003\right)\cdot {{{{\rm{CreE}}}}_{{{\rm{M}}}}}^{2}$$

where

$\hat{{{\rm{CreJ}}}}$ and $\hat{{{\rm{CreE}}}}$ are the estimated true creatinine values,

${{{\rm{TB}}}}_{{{\rm{M}}}}$ is the measured total bilirubin value, and

${{{\rm{CrJ}}}}_{{{\rm{M}}}}$ and ${{{\rm{CrE}}}}_{{{\rm{M}}}}$ indicate the measured creatinine value.

#### Validation phase

The validity of the model was confirmed by independent sample aliquots (*n* = 32) from routine diagnostics that representatively covered the 3D-surface plots of the reference function. All aliquots were reanalysed by both CreJ and CreE. One blinded aliquot of each sample was transferred to the German Reference Institute for Bioanalytics and reanalyzed using GC-IDMS, the internationally recognized reference method. The analysis was carried out according to the standards defined by the Joint Committee for Traceability in Laboratory Medicine. These results were regarded as true creatinine values to be compared with the values determined by the reference model.

### General information on patient cohorts for retrospective validation and MELD assessment

Cohort 1 consisted of adult inpatients and outpatients with ESLD recruited at a single tertiary care university hospital in Germany. This center functions as a major referral center for liver disease and transplantation. Data were retrospectively extracted from structured electronic health records between May 2006 and April 2025. The pseudonymized dataset included laboratory findings, diagnoses encoded by the International Classification of Diseases (ICD), and the German Operation and Procedure Code (OPS), the official classification system for surgical procedures, medical interventions, and measures.

Cohort 2 comprised data from the SRTR. The SRTR data system includes data on all donors, wait-listed candidates, and transplant recipients in the U.S., submitted by the members of the Organ Procurement and Transplantation Network (OPTN). The Health Resources and Services Administration, U.S. Department of Health and Human Services, provides oversight to the activities of the OPTN and SRTR contractors. Cohort 2 includes patient records from 1990 to 2022, comprising laboratory and clinical data for retrospective calculation of MELD scores. Since MELD was not implemented for liver allocation before 2002, these retrospectively derived scores were not used in any analyses related to allocation decisions. Twelve centres of the OPTN met the predefined inclusion criteria, contributing a total of 20,444 patient records. Creatinine measurement methods were available from 42 centers, of which 8 used the Jaffe method. An additional 21 centers were contacted directly, yielding a 29% response rate and identifying 4 further centers using the Jaffe method. In total, 12 transplant centers were included in the analysis, spanning 9 OPTN regions. For an additional subgroup analysis reflecting current allocation and transplantation practice, we analyzed a cohort restricted to data from 2023 to 2025. All eligibility criteria, definitions, and analytical procedures were identical to those used in the primary analysis.

Cohort 1 served as an initial reference setting, where all measurements were performed on the same analyzer system, consistent with the approach used to develop the reference functions. This ensured methodological consistency before extending the correction model to Cohort 2, where creatinine analyzes was more heterogeneous, regarding assays and analyzer systems.

### Inclusion criteria

Inclusion of adult patients (age ≥ 18 years) in cohort 1 required the presence of liver cirrhosis (ICD: K74), as well as complete demographic (age, sex) and laboratory data necessary for calculation of all four variants of the MELD score, including total bilirubin, serum creatinine, and the international normalized ratio (INR) from citrated plasma, all measured on the same date. Subsets for MELD-Na, reMELD-Na, and MELD 3.0 calculations included only cases with additional serum sodium and albumin measurements. Hemodialysis periods were retrieved using OPS codes 8–85a, 8–853, 8–854, and 8–855, requiring at least two dialysis treatments within the preceding week. Sex is presented and analyzed as documented in clinical records. Sex or gender were not considered in the study design (post-hoc analysis in Supplementary Table 9 by two-sided *χ*² tests).

A survey was conducted among transplant centers reporting patient data to the SRTR to determine the creatinine assay used by their laboratories. Only adult patients from liver transplantation centers that confirmed the use of the Jaffe assay were included in cohort 2. For individuals in cohort 2 who were no longer on the waitlist at the study cutoff (December 2022), the possible outcomes were: transplantation, death, removal due to clinical deterioration (too sick for transplant) or improvement (no longer eligible), refusal, or transfer to another center. For patients who underwent retransplantation, only the most recent transplant was considered in the mortality analysis. Patients without a documented date of death were assumed to be alive at the study cutoff. Depending on the analyses, survival was analysed at 30-days, 90-days, within 2 years, or without time restriction (see results for details). For contemporary validation, additional horizon-specific analyses at 90 and 365 days were performed in an SRTR subgroup restricted to data from 2023 to 2025.

### Exclusion criteria

Patients of both cohorts suffering from primary sclerosing cholangitis (ICD-10: K83.0), biliary atresia (ICD-10: Q44.2), benign and malignant liver tumors (ICD-10: D18.03, C22.-), cystic fibrosis (ICD-10: E84.-), and primary hyperoxaluria (ICD-10: E74.8) were excluded from the study due to exceptional MELD-related allocation criteria under the German guideline for waiting list management and organ allocation for liver transplantation^41–43^.

### Statistical analysis

A second-degree polynomial regression was used to model the predicted true creatinine values as a function of creatinine and bilirubin as predictors. The model included all quadratic terms and their interaction to capture curvature in the surface response. Proportional and systematic biases were estimated using weighted Deming regression analyses, accounting for the differing heteroscedastic measurement errors of CreJ, CreE, and GC-IDMS. Comparisons of continuous laboratory values—either between subgroups or before and after applying the reference model—were performed using one-sample and two-sample Wilcoxon tests. Unpaired Wilcoxon tests were applied for between-group comparisons, while paired tests were used for before–after comparisons. Categorical variables were compared using chi-squared tests. Non-parametric methods were selected due to the non-normal distribution of all continuous variables. MELD, MELD-Na, reMELD-Na, and MELD 3.0 were calculated using published formulas^1,2,4,9^ with variable-specific boundary conditions as defined in Table 4.

**Table 4:** MELD score variants, their corresponding formulas, and parameter boundaries

Score variant	Formula	Parameter Boundaries
MELD^1^	*0.957 × ln(Cr)* + *0.378 × ln(bilirubin)* + *1.120 × ln(INR)* + *0.643*	*if* < *1:Cr* = *1 mg/dL* *Bilirubin* = *1 mg/dL* *INR* = *1* *if* > *4:Cr* = *4 mg/dL* *if hemodialysis:* *Cr* = *4 mg/dL*
Na-MELD, if MELD > 11^3^	*MELD(i)* + *1.32 × (137 – Na) – [0.033 × MELD(i) × (137 – Na)]*	*if* < *125:Na* = *125 mmol/L* *if* > *137:Na* = *137 mmol/L*
MELD 3.0^2^	*1.33 × (Female)* + *4.56 × ln(bilirubin)* + *0.82 × (137 - Na) – 0.24 × (137 - Na) × ln(bilirubin)* + *9.09 × ln(INR)* + *11.14 × ln(Cr)* + *1.85 × (3.5 – albumin) – 1.83 × (3.5 – albumin) × ln(Cr)* + *6*	*if* < *1:Cr* = *1 mg/dL* *Bilirubin* = *1 mg/dL* *INR* = *1* *if* > *3:Cr* = *3 mg/dL* *if* < *125:Na* = *125 mmol/L* *if* > *137:Na* = *137 mmol/L* *if* < *1.5:Albumin* = *1.5 g/dL* *if* > *3.5:Albumin* = *3.5 g/dL*
re-MELD-Na^9^	*9.025 × ln(Cr)* + *2.969 × ln(bilirubin)* + *9.518 × ln(INR) – 0.392 × (139-Na) – 0.351 × ln(139-Na) × ln(Cr)*	*if* < *0.7:Cr* = *0.7 mg/dL* *if* > *2.5:Cr* = *2.5 mg/dL* *if hemodialysis:* *Cr* = *2.5 mg/dL* *if* < *0.1:INR* = *0.1* *if* > *2.6:INR* = *2.6* *if* < *0.3:Bilirubin* = *0.3 mg/dL* *if* > *27:Bilirubin* = *27 mg/dL* *if* < *121:Na* = *121 mmol/L* *if* > *138:Na* = *138 mmol/L*

*Cr* Creatinine, *INR* international normalized ratio, *Na* sodium.

The prognostic implications of MELD score correction were evaluated by comparing survival data between patients stratified by deviation category. Δ was calculated as difference between corrected and uncorrected MELD (Δ = MELD_corrected–MELD_original). Patients were divided into those without any score deviation (Δ = 0) and those with at least one score reduced by one point or more following correction (Δ ≤ –1). Kaplan–Meier curves were generated to estimate 30- and 90-day survival probabilities, and log-rank tests were performed for comparison in the ESLD cohort. We employed a Cox proportional hazards regression model to estimate the hazard ratio (HR) associated with score deviations to evaluate their impact on time to death without specifying the baseline hazard function. The model assumes that the hazard ratios remain constant over time, i.e., proportional hazards assumption. The absence of time dependence and overall validity of the model were assessed using Schoenfeld residuals through both visual inspection of residual plots and formal statistical testing.

Additionally, median survival times, the 90-day restricted mean survival time (RMST) and the restricted mean survival time ratio were calculated to quantify survival differences over a fixed time horizon of two years before death. These measures were chosen for their robustness in contexts where the proportional hazards assumption may not hold and to assess whether corrected MELD scores more accurately reflect true mortality risk^42,43^.

Because Kaplan–Meier estimates and the log-rank test may not fully account for competing risks, cumulative incidence functions (CIFs) were used in the SRTR cohort to estimate event probabilities in the presence of competing risks. Group differences were assessed using Gray’s test. For regression analysis, the Fine–Gray subdistribution hazards model was applied to adjust for competing risks and to estimate subdistribution hazard ratios (sHRs), quantifying the effect of covariates on the incidence of specific events.

All analyses were performed in R version 4.3.0 (R Foundation for Statistical Computing, Vienna, Austria) and SAS 9.4 (SAS Institute, Cary, NC), with two-sided *p*-values < 0.05 considered statistically significant. Based on the observed effect sizes, post hoc power analyses yielded type II error probabilities (β) of <0.001.

### Reporting summary

Further information on research design is available in the Nature Portfolio Reporting Summary linked to this article.

## Supplementary information

Supplementary Information

Reporting summary

Transparent Peer Review file

## Source data

Source Data

## Data Availability

The data reported here have been supplied by the Hennepin Healthcare Research Institute (HHRI) as the contractor for the Scientific Registry of Transplant Recipients (SRTR). The interpretation and reporting of these data are the responsibility of the author(s) and in no way should be seen as an official policy of or interpretation by the SRTR or the U.S. Government. To request this data please perform a data requests on the following home pages: https://www.srtr.org/requesting-data/data-requests/; https://www.hrsa.gov/optn/data/data-reports/data-request-instructions. Source data are provided with this paper in a single ZIP-file. The ZIP-file contains all source data as well as scripts and workflows to derive the described analysis. A browser-based file viewer is provided within the ZIP-file to navigate files and to retrace workflows. Source data for experimental derivation and validation of the correction formula and the ESLD study cohort data set are provided as full data sets. Because of ethical and data protection restrictions in our country, individual-level raw data underlying this study cannot be deposited in a public repository. Aggregate data on MELD score distribution and correction for the SRTR cohort are provided in Supplementary Table 3. Source data are provided with this paper.
