# Supplementary material for: Creatinine assay interferences compromises MELD accuracy and may bias liver allocation
Source: Nat Commun. 2026 Jul 23;17:7111. doi: 10.1038/s41467-026-75011-x (PMC13396164; doi:10.1038/s41467-026-75011-x)
Supplement: Supplementary file 1 — Supplementary Information [file 41467_2026_75011_MOESM1_ESM.pdf]

Creatinine Assay Interferences Compromises MELD Accuracy and may Bias Liver Allocation

Supplementary Information

Supplementary Figure 1. Concordance of score deviations among MELD, MELD-Na, MELD 3.0, and reMELD-Na. Numbers in the coloured areas of the Venn-diagram indicate datasets with non-zero changes following modelled creatinine correction. Overlapping areas represent concordant deviations between the respective MELD variants. Data for ESLD and SRTR patients are shown in red and blue, respectively. Statistically significant differences were observed between the two groups in the distribution of score combinations ($\chi^2 = 27.44$, df = 10, $p = 0.0022$). However, the corresponding effect size was negligible (Cramér's V = 0.019).

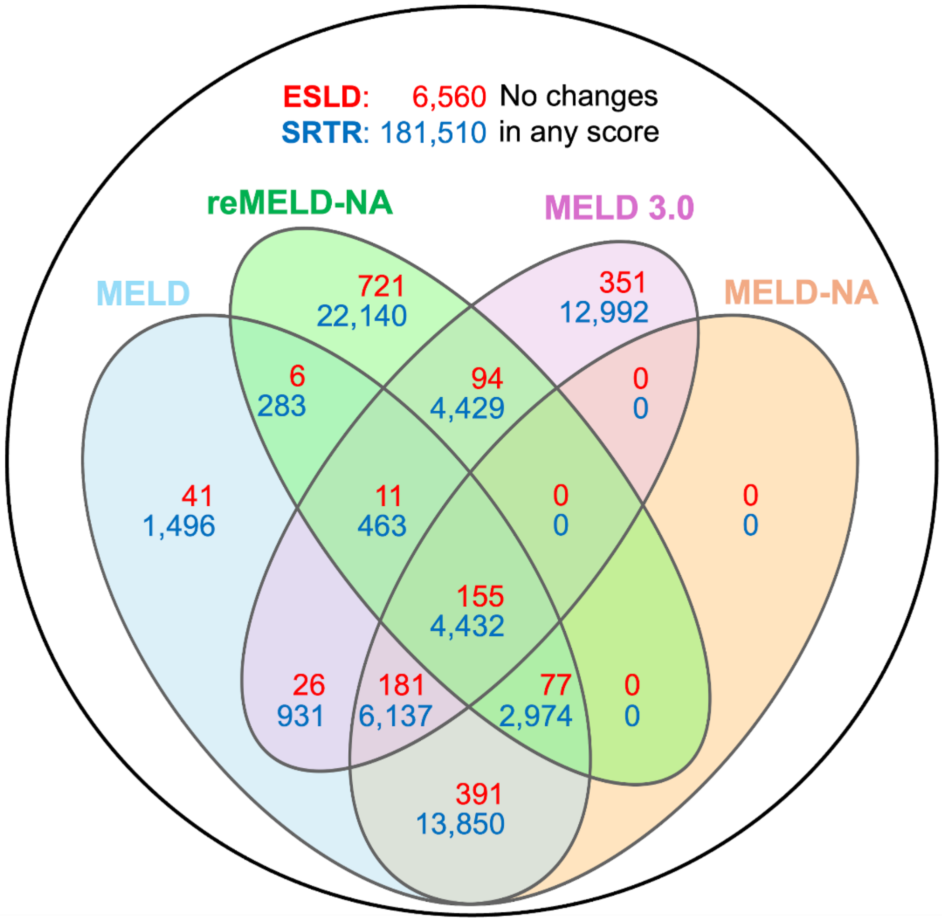

Supplementary Figure 2 (Amendment to Figure 6). Competing risk analysis comparing score variants deviations following creatinine correction in SRTR patients.

Cumulative incidence functions (CIF) are stratified by causes of removal from waitlist: death on waitlist (left column), transplantation (middle column), and other causes for removal (right column) and further grouped by score classes. Green lines: MELD ≤ 15; blue lines: MELD 16 to 25; red lines: MELD > 25. In each panel, patients with no change (Δ =0; full lines) are compared to those with at least one decrease of Δ ≤ – 1 (dashed lines). Detailed statistical analysis for competing risks is provided as amendment to Supplementary Table 3 (Gray's test for competing risks of removal from waitlist) and amendments 1-3 to supplementary Table 4 (Fine-Gray subdistribution hazard for waitlist death, transplantation, and other reasons for waitlist removal). Analyses were performed using SRTR data from **January 2023 through December 2025.**

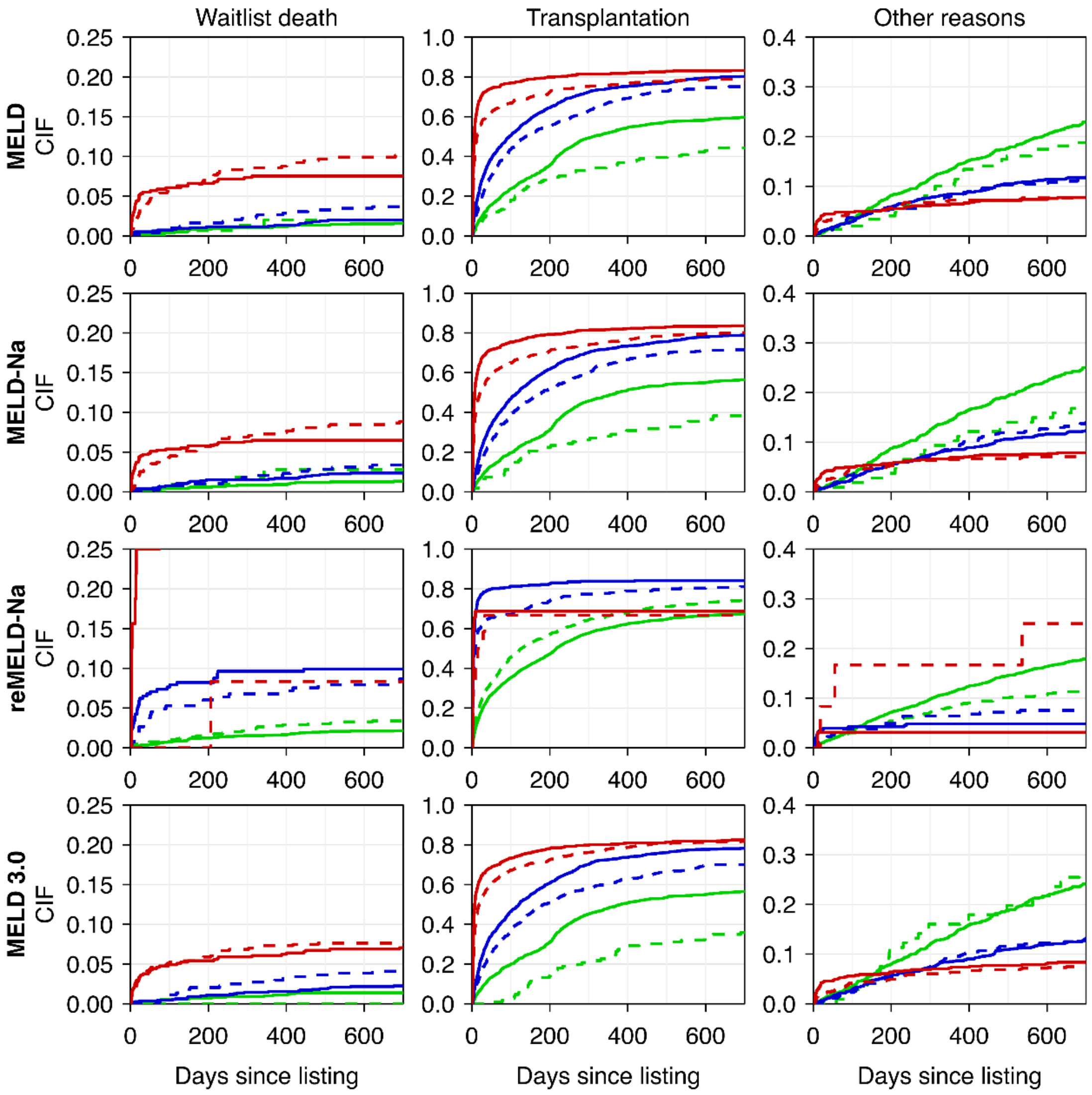

Supplementary tables

Supplementary Table 1. History in ESLD patients regarding evaluation, waitlist registration, transplantation, or complications stratified by survival status.

Clinical courses†	Alive n (%)	Dead n (%)	Total
No evaluation / no codes	1082 (82.4%)	231 (17.6%)	1,313
Evaluation, not eligible	29 (90.6%)	3 (9.4%)	32
Waitlist eligible / registered	4 (80.0%)	1 (20.0%)	5
TX (No complication coded)	12 (92.3%)	1 (7.7%)	13
TX + Complication (Acute ≤28d)	3 (100.0%)	0 (0.0%)	3
TX + Complication (Chronic ≥29d)	4 (100.0%)	0 (0.0%)	4
TX + Complication (Other)	3 (100.0%)	0 (0.0%)	3
Total †	1137 (100.0%)	236 (100.0%)	1,373

†Definitions (coding): Evaluation: OPS 1-920.04/1-920.14 (not eligible), 1-920.24/1-920.34 (eligible/registered); Registration: Z75.67 (no HU; Z75.77 not available in this table); Transplanted: Z94.4; Complications post-LTx: T86.40 (acute ≤28d), T86.41 (chronic ≥29d), T86.49 (other).

Supplementary Table 2: ESLD cohort – score changes by model.

Score	Δ MELD, n (%)					Δ MELD-Na, n (%)					Δ reMELD-Na, n (%)					Δ MELD 3.0, n (%)				
	+2	+1	0	−1	−2	+2	+1	0	−1	−2	+2	+1	0	−1	−2	+2	+1	0	−1	−2
6	0 (0)	0 (0)	963 (100)	0 (0)	0 (0)	0 (0)	0 (0)	731 (100)	0 (0)	0 (0)	0 (0)	101 (10.3)	877 (89.7)	0 (0)	0 (0)	0 (0)	0 (0)	163 (100)	0 (0)	0 (0)
7	0 (0)	9 (0.5)	1801 (99.5)	0 (0)	0 (0)	0 (0)	5 (0.4)	1312 (99.6)	0 (0)	0 (0)	0 (0)	97 (8.9)	987 (91.1)	0 (0)	0 (0)	0 (0)	5 (1.3)	385 (98.7)	0 (0)	0 (0)
8	0 (0)	33 (1.7)	1915 (98.3)	0 (0)	0 (0)	0 (0)	26 (1.8)	1428 (98.2)	0 (0)	0 (0)	0 (0)	101 (9.0)	1015 (90.9)	1 (0.1)	0 (0)	0 (0)	8 (1.6)	500 (98.4)	0 (0)	0 (0)
9	0 (0)	52 (2.9)	1718 (97.1)	0 (0)	0 (0)	0 (0)	44 (3.2)	1351 (96.8)	0 (0)	0 (0)	0 (0)	91 (7.8)	1078 (92.0)	3 (0.3)	0 (0)	0 (0)	9 (1.8)	490 (98.2)	0 (0)	0 (0)
10	0 (0)	32 (2.0)	1530 (97.8)	3 (0.2)	0 (0)	0 (0)	26 (2.0)	1289 (97.9)	2 (0.2)	0 (0)	0 (0)	72 (6.2)	1074 (92.7)	13 (1.1)	0 (0)	0 (0)	12 (2.3)	509 (97.5)	1 (0.2)	0 (0)
11	0 (0)	23 (1.7)	1286 (97.6)	9 (0.7)	0 (0)	0 (0)	26 (2.1)	1201 (97.6)	4 (0.3)	0 (0)	0 (0)	48 (4.3)	1040 (93.8)	21 (1.9)	0 (0)	0 (0)	10 (1.8)	559 (98.1)	1 (0.2)	0 (0)
12	0 (0)	8 (0.7)	1136 (97.6)	20 (1.7)	0 (0)	0 (0)	9 (0.8)	1062 (98.0)	13 (1.2)	0 (0)	0 (0)	33 (2.8)	1098 (93.8)	39 (3.3)	0 (0)	0 (0)	6 (1.1)	520 (98.9)	0 (0)	0 (0)
13	0 (0)	11 (0.9)	1165 (96.2)	35 (2.9)	0 (0)	0 (0)	12 (1.1)	1055 (96.3)	28 (2.6)	0 (0)	0 (0)	17 (1.5)	1054 (93.9)	51 (4.5)	0 (0)	0 (0)	12 (2.4)	491 (96.5)	6 (1.2)	0 (0)
14	0 (0)	7 (0.7)	970 (93.4)	61 (5.9)	0 (0)	0 (0)	10 (1.0)	944 (94.0)	50 (5.0)	0 (0)	0 (0)	12 (1.1)	982 (93.1)	61 (5.8)	0 (0)	0 (0)	8 (1.8)	437 (97.1)	5 (1.1)	0 (0)
15	0 (0)	1 (0.1)	923 (93.3)	65 (6.6)	0 (0)	0 (0)	4 (0.4)	903 (95.0)	44 (4.6)	0 (0)	0 (0)	8 (0.8)	900 (89.8)	94 (9.4)	0 (0)	0 (0)	2 (0.5)	386 (96.3)	13 (3.2)	0 (0)
16	0 (0)	3 (0.3)	782 (88.2)	102 (11.5)	0 (0)	0 (0)	5 (0.6)	769 (92.5)	57 (6.9)	0 (0)	0 (0)	7 (0.8)	741 (85.6)	113 (13.0)	5 (0.6)	0 (0)	1 (0.2)	406 (95.1)	20 (4.7)	0 (0)
17	0 (0)	3 (0.4)	724 (89.4)	83 (10.2)	0 (0)	0 (0)	5 (0.6)	788 (92.3)	61 (7.1)	0 (0)	0 (0)	4 (0.5)	679 (84.6)	119 (14.8)	1 (0.1)	0 (0)	2 (0.5)	370 (92.3)	29 (7.2)	0 (0)
18	0 (0)	3 (0.4)	608 (85.2)	103 (14.4)	0 (0)	0 (0)	4 (0.6)	627 (88.6)	77 (10.9)	0 (0)	0 (0)	3 (0.4)	640 (85.8)	103 (13.8)	0 (0)	0 (0)	2 (0.6)	315 (92.9)	22 (6.5)	0 (0)
19	0 (0)	1 (0.2)	523 (84.8)	93 (15.1)	0 (0)	0 (0)	0 (0)	612 (90.5)	64 (9.5)	0 (0)	0 (0)	0 (0)	489 (84.7)	88 (15.3)	0 (0)	0 (0)	0 (0)	287 (91.4)	27 (8.6)	0 (0)
20	0 (0)	0 (0)	458 (84.8)	82 (15.2)	0 (0)	0 (0)	0 (0)	512 (88.1)	69 (11.9)	0 (0)	0 (0)	0 (0)	440 (83.8)	83 (15.8)	2 (0.4)	0 (0)	0 (0)	285 (89.9)	32 (10.1)	0 (0)
21	0 (0)	0 (0)	373 (79.7)	95 (20.3)	0 (0)	0 (0)	0 (0)	484 (88.0)	66 (12.0)	0 (0)	0 (0)	0 (0)	334 (79.1)	87 (20.6)	1 (0.2)	0 (0)	0 (0)	256 (87.7)	36 (12.3)	0 (0)
22	0 (0)	0 (0)	319 (81.8)	71 (18.2)	0 (0)	0 (0)	1 (0.2)	541 (89.0)	66 (10.9)	0 (0)	0 (0)	0 (0)	284 (77.2)	83 (22.6)	1 (0.3)	0 (0)	0 (0)	196 (84.8)	35 (15.2)	0 (0)
23	0 (0)	0 (0)	279 (75.0)	93 (25.0)	0 (0)	0 (0)	0 (0)	330 (81.7)	74 (18.3)	0 (0)	0 (0)	0 (0)	243 (76.2)	76 (23.8)	0 (0)	0 (0)	1 (0.4)	226 (85.0)	39 (14.7)	0 (0)
24	0 (0)	0 (0)	240 (74.8)	79 (24.6)	2 (0.6)	0 (0)	1 (0.2)	336 (81.8)	73 (17.8)	1 (0.2)	0 (0)	0 (0)	181 (64.0)	101 (35.7)	1 (0.4)	0 (0)	0 (0)	172 (79.3)	45 (20.7)	0 (0)
25	0 (0)	0 (0)	195 (72.0)	76 (28.0)	0 (0)	0 (0)	0 (0)	306 (83.6)	60 (16.4)	0 (0)	0 (0)	0 (0)	146 (73.4)	53 (26.6)	0 (0)	0 (0)	0 (0)	139 (76.8)	42 (23.2)	0 (0)
26	0 (0)	0 (0)	139 (64.4)	77 (35.6)	0 (0)	0 (0)	0 (0)	249 (79.6)	64 (20.4)	0 (0)	0 (0)	0 (0)	115 (68.9)	52 (31.1)	0 (0)	0 (0)	0 (0)	121 (72.9)	45 (27.1)	0 (0)
27	0 (0)	0 (0)	114 (60.6)	74 (39.4)	0 (0)	0 (0)	0 (0)	206 (71.5)	82 (28.5)	0 (0)	0 (0)	0 (0)	95 (64.6)	52 (35.4)	0 (0)	0 (0)	0 (0)	103 (72.0)	40 (28.0)	0 (0)
28	0 (0)	0 (0)	88 (61.1)	56 (38.9)	0 (0)	0 (0)	0 (0)	131 (68.2)	60 (31.2)	1 (0.5)	0 (0)	0 (0)	86 (71.7)	34 (28.3)	0 (0)	0 (0)	0 (0)	102 (68.9)	46 (31.1)	0 (0)
29	0 (0)	0 (0)	72 (52.9)	63 (46.3)	1 (0.7)	0 (0)	0 (0)	112 (66.3)	57 (33.7)	0 (0)	0 (0)	0 (0)	58 (68.2)	27 (31.8)	0 (0)	0 (0)	0 (0)	85 (66.9)	42 (33.1)	0 (0)
30	0 (0)	0 (0)	56 (56.0)	44 (44.0)	0 (0)	0 (0)	0 (0)	111 (71.6)	44 (28.4)	0 (0)	0 (0)	0 (0)	42 (61.8)	26 (38.2)	0 (0)	0 (0)	0 (0)	55 (59.8)	37 (40.2)	0 (0)
31	0 (0)	0 (0)	39 (44.3)	49 (55.7)	0 (0)	0 (0)	0 (0)	60 (57.7)	44 (42.3)	0 (0)	0 (0)	0 (0)	42 (67.7)	20 (32.3)	0 (0)	0 (0)	0 (0)	62 (63.3)	36 (36.7)	0 (0)
32	0 (0)	0 (0)	26 (42.6)	35 (57.4)	0 (0)	0 (0)	0 (0)	55 (56.7)	42 (43.3)	0 (0)	0 (0)	0 (0)	38 (76.0)	12 (24.0)	0 (0)	0 (0)	0 (0)	53 (58.9)	37 (41.1)	0 (0)
33	0 (0)	0 (0)	22 (40.7)	32 (59.3)	0 (0)	0 (0)	0 (0)	54 (64.3)	30 (35.7)	0 (0)	0 (0)	0 (0)	14 (93.3)	1 (6.7)	0 (0)	0 (0)	0 (0)	16 (44.4)	20 (55.6)	0 (0)
34	0 (0)	0 (0)	26 (40.0)	39 (60.0)	0 (0)	0 (0)	0 (0)	41 (54.7)	34 (45.3)	0 (0)	0 (0)	0 (0)	13 (92.9)	1 (7.1)	0 (0)	0 (0)	0 (0)	14 (42.4)	19 (57.6)	0 (0)
35	0 (0)	0 (0)	15 (50.0)	15 (50.0)	0 (0)	0 (0)	0 (0)	33 (61.1)	21 (38.9)	0 (0)	0 (0)	0 (0)	1 (100)	0 (0)	0 (0)	0 (0)	0 (0)	17 (40.5)	25 (59.5)	0 (0)
36	0 (0)	0 (0)	12 (44.4)	15 (55.6)	0 (0)	0 (0)	0 (0)	17 (56.7)	13 (43.3)	0 (0)	0 (0)	0 (0)	1 (100)	0 (0)	0 (0)	0 (0)	0 (0)	22 (61.1)	14 (38.9)	0 (0)
37	0 (0)	0 (0)	9 (42.9)	12 (57.1)	0 (0)	0 (0)	0 (0)	11 (42.3)	15 (57.7)	0 (0)	0 (0)	0 (0)	0 (0)	0 (0)	0 (0)	0 (0)	0 (0)	10 (62.5)	6 (37.5)	0 (0)
38	0 (0)	0 (0)	13 (68.4)	6 (31.6)	0 (0)	0 (0)	0 (0)	14 (73.7)	5 (26.3)	0 (0)	0 (0)	0 (0)	0 (0)	0 (0)	0 (0)	0 (0)	0 (0)	18 (60.0)	12 (40.0)	0 (0)
39	0 (0)	0 (0)	8 (44.4)	10 (55.6)	0 (0)	0 (0)	0 (0)	9 (47.4)	10 (52.6)	0 (0)	0 (0)	0 (0)	0 (0)	0 (0)	0 (0)	0 (0)	0 (0)	3 (27.3)	8 (72.7)	0 (0)
40	0 (0)	0 (0)	26 (100)	0 (0)	0 (0)	0 (0)	0 (0)	20 (100)	0 (0)	0 (0)	0 (0)	0 (0)	0 (0)	0 (0)	0 (0)	0 (0)	0 (0)	23 (100)	0 (0)	0 (0)

Supplementary Table 3: SRTR cohort – score changes by model.

Score	Δ MELD, n (%)					Δ MELD-Na, n (%)					Δ reMELD-Na, n (%)					Δ MELD 3.0, n (%)				
	+2	+1	0	−1	−2	+2	+1	0	−1	−2	+2	+1	0	−1	−2	+2	+1	0	−1	−2
6	0 (0)	0 (0)	1365 (100)	0 (0)	0 (0)	0 (0)	0 (0)	1166 (100)	0 (0)	0 (0)	0 (0)	179 (8.8)	1863 (91.2)	0 (0)	0 (0)	0 (0)	0 (0)	577 (100)	0 (0)	0 (0)
7	0 (0)	279 (7.2)	3576 (92.8)	0 (0)	0 (0)	0 (0)	234 (7.3)	2976 (92.7)	0 (0)	0 (0)	0 (0)	309 (8.6)	3264 (91.4)	0 (0)	0 (0)	0 (0)	6 (0.2)	2482 (99.8)	0 (0)	0 (0)
8	0 (0)	363 (6.8)	5000 (93.2)	0 (0)	0 (0)	0 (0)	285 (6.4)	4135 (93.6)	0 (0)	0 (0)	0 (0)	452 (8.3)	4989 (91.6)	5 (0.1)	0 (0)	0 (0)	45 (1.0)	4317 (99.0)	0 (0)	0 (0)
9	0 (0)	543 (7.4)	6790 (92.6)	0 (0)	0 (0)	0 (0)	440 (7.2)	5644 (92.8)	0 (0)	0 (0)	0 (0)	428 (5.4)	7514 (94.3)	28 (0.4)	0 (0)	0 (0)	174 (3.2)	5215 (96.8)	0 (0)	0 (0)
10	0 (0)	182 (1.8)	9891 (98.1)	14 (0.1)	0 (0)	0 (0)	215 (2.6)	7954 (97.3)	6 (0.1)	0 (0)	0 (0)	448 (4.1)	10480 (95.0)	104 (0.9)	0 (0)	0 (0)	95 (1.3)	7079 (98.5)	16 (0.2)	0 (0)
11	0 (0)	84 (0.6)	12899 (98.0)	180 (1.4)	0 (0)	0 (0)	121 (1.1)	10462 (97.8)	119 (1.1)	0 (0)	0 (0)	267 (2.1)	12213 (96.0)	236 (1.9)	0 (0)	0 (0)	128 (1.4)	8914 (98.1)	45 (0.5)	0 (0)
12	0 (0)	98 (0.6)	15259 (96.8)	413 (2.6)	0 (0)	0 (0)	101 (0.8)	12149 (97.1)	266 (2.1)	0 (0)	0 (0)	198 (1.4)	13578 (95.0)	510 (3.6)	0 (0)	0 (0)	93 (0.8)	10729 (97.9)	136 (1.2)	0 (0)
13	0 (0)	10 (0.1)	16243 (96.8)	530 (3.2)	0 (0)	0 (0)	48 (0.4)	13000 (97.0)	355 (2.6)	0 (0)	0 (0)	239 (1.5)	14748 (93.3)	818 (5.2)	0 (0)	0 (0)	70 (0.6)	11895 (97.5)	234 (1.9)	0 (0)
14	0 (0)	35 (0.2)	15651 (95.3)	740 (4.5)	0 (0)	0 (0)	54 (0.4)	12512 (96.1)	455 (3.5)	0 (0)	0 (0)	126 (0.8)	14583 (92.5)	1054 (6.7)	1 (0.0)	0 (0)	78 (0.6)	12211 (96.8)	321 (2.5)	0 (0)
15	0 (0)	30 (0.2)	14013 (93.6)	929 (6.2)	0 (0)	0 (0)	34 (0.3)	11827 (94.9)	608 (4.9)	0 (0)	0 (0)	61 (0.4)	14587 (90.6)	1452 (9.0)	4 (0.0)	0 (0)	46 (0.4)	11878 (96.0)	455 (3.7)	0 (0)
16	0 (0)	11 (0.1)	12868 (91.8)	1142 (8.1)	0 (0)	0 (0)	22 (0.2)	11139 (93.6)	744 (6.2)	0 (0)	0 (0)	61 (0.4)	14520 (89.3)	1684 (10.4)	2 (0.0)	0 (0)	31 (0.2)	11788 (94.8)	621 (5.0)	0 (0)
17	0 (0)	14 (0.1)	11296 (88.7)	1425 (11.2)	0 (0)	0 (0)	18 (0.1)	12378 (92.5)	991 (7.4)	0 (0)	0 (0)	34 (0.2)	13646 (87.3)	1941 (12.4)	7 (0.0)	0 (0)	33 (0.3)	11298 (92.5)	887 (7.3)	0 (0)
18	0 (0)	3 (0.0)	10580 (88.2)	1415 (11.8)	0 (0)	0 (0)	17 (0.1)	10438 (90.6)	1060 (9.2)	0 (0)	0 (0)	38 (0.3)	12688 (86.6)	1925 (13.1)	6 (0.0)	0 (0)	11 (0.1)	11324 (92.1)	961 (7.8)	0 (0)
19	0 (0)	3 (0.0)	9057 (84.5)	1656 (15.5)	0 (0)	0 (0)	9 (0.1)	9982 (89.4)	1174 (10.5)	0 (0)	0 (0)	4 (0.0)	11868 (84.6)	2144 (15.3)	12 (0.1)	0 (0)	14 (0.1)	11755 (91.2)	1117 (8.7)	2 (0.0)
20	0 (0)	2 (0.0)	10338 (85.5)	1748 (14.5)	2 (0.0)	0 (0)	8 (0.1)	9537 (87.9)	1300 (12.0)	2 (0.0)	0 (0)	3 (0.0)	10683 (82.8)	2208 (17.1)	10 (0.1)	0 (0)	16 (0.1)	11471 (90.4)	1198 (9.4)	0 (0)
21	0 (0)	7 (0.1)	9865 (85.2)	1701 (14.7)	4 (0.0)	0 (0)	15 (0.1)	9399 (87.5)	1321 (12.3)	1 (0.0)	0 (0)	9 (0.1)	9248 (80.6)	2192 (19.1)	18 (0.2)	0 (0)	3 (0.0)	10938 (89.2)	1315 (10.7)	0 (0)
22	0 (0)	2 (0.0)	8386 (83.1)	1700 (16.9)	0 (0)	0 (0)	3 (0.0)	10430 (88.0)	1420 (12.0)	3 (0.0)	0 (0)	2 (0.0)	7986 (78.5)	2169 (21.3)	17 (0.2)	0 (0)	2 (0.0)	9952 (87.3)	1432 (12.6)	8 (0.1)
23	0 (0)	1 (0.0)	7391 (80.6)	1776 (19.4)	0 (0)	0 (0)	6 (0.1)	8124 (84.6)	1476 (15.4)	0 (0)	6 (0.1)	2 (0.0)	6819 (75.2)	2218 (24.4)	28 (0.3)	0 (0)	2 (0.0)	9094 (86.5)	1418 (13.5)	2 (0.0)
24	0 (0)	0 (0)	6021 (79.0)	1595 (20.9)	2 (0.0)	0 (0)	0 (0)	8202 (84.8)	1467 (15.2)	0 (0)	1 (0.0)	8 (0.1)	5674 (74.0)	1961 (25.6)	28 (0.4)	0 (0)	0 (0)	8521 (84.4)	1565 (15.5)	4 (0.0)
25	1 (0.0)	3 (0.0)	4936 (76.6)	1503 (23.3)	0 (0)	0 (0)	2 (0.0)	8279 (85.7)	1375 (14.2)	2 (0.0)	2 (0.0)	11 (0.2)	4468 (73.2)	1602 (26.3)	17 (0.3)	0 (0)	0 (0)	7445 (81.9)	1625 (17.9)	15 (0.2)
26	0 (0)	0 (0)	4138 (75.5)	1339 (24.4)	1 (0.0)	0 (0)	2 (0.0)	6638 (82.7)	1387 (17.3)	1 (0.0)	1 (0.0)	17 (0.3)	3603 (70.2)	1508 (29.4)	2 (0.0)	0 (0)	0 (0)	6607 (80.5)	1589 (19.4)	9 (0.1)
27	0 (0)	0 (0)	3340 (73.1)	1228 (26.9)	4 (0.1)	0 (0)	0 (0)	6140 (82.1)	1338 (17.9)	0 (0)	12 (0.3)	8 (0.2)	2991 (67.0)	1452 (32.5)	0 (0)	1 (0.0)	0 (0)	5724 (77.8)	1625 (22.1)	7 (0.1)
28	0 (0)	4 (0.1)	2817 (71.0)	1147 (28.9)	2 (0.1)	0 (0)	5 (0.1)	3876 (76.7)	1171 (23.2)	1 (0.0)	5 (0.1)	12 (0.3)	2475 (68.0)	1149 (31.6)	0 (0)	0 (0)	3 (0.0)	4851 (76.9)	1438 (22.8)	15 (0.2)
29	0 (0)	3 (0.1)	2348 (69.0)	1047 (30.8)	3 (0.1)	0 (0)	2 (0.0)	4213 (78.8)	1130 (21.1)	1 (0.0)	6 (0.2)	9 (0.3)	2161 (70.6)	885 (28.9)	0 (0)	0 (0)	0 (0)	4219 (74.0)	1471 (25.8)	12 (0.2)
30	0 (0)	3 (0.1)	1989 (67.8)	939 (32.0)	3 (0.1)	0 (0)	3 (0.1)	3958 (77.3)	1154 (22.5)	7 (0.1)	4 (0.2)	13 (0.5)	1861 (72.6)	686 (26.8)	0 (0)	0 (0)	6 (0.1)	3638 (72.5)	1357 (27.1)	14 (0.3)
31	2 (0.1)	8 (0.3)	1548 (65.0)	824 (34.6)	1 (0.0)	0 (0)	8 (0.2)	2172 (67.7)	1030 (32.1)	0 (0)	5 (0.2)	14 (0.6)	1600 (73.7)	552 (25.4)	0 (0)	0 (0)	1 (0.0)	3034 (68.8)	1361 (30.9)	11 (0.2)
32	2 (0.1)	2 (0.1)	1276 (64.6)	693 (35.1)	1 (0.1)	2 (0.1)	1 (0.0)	2613 (75.3)	851 (24.5)	3 (0.1)	11 (0.7)	16 (1.0)	1307 (77.7)	348 (20.7)	0 (0)	0 (0)	3 (0.1)	2371 (67.0)	1158 (32.7)	6 (0.2)
33	2 (0.1)	6 (0.3)	1099 (62.2)	661 (37.4)	0 (0)	3 (0.1)	5 (0.2)	1996 (71.9)	769 (27.7)	2 (0.1)	3 (0.2)	8 (0.6)	1074 (80.4)	251 (18.8)	0 (0)	1 (0.0)	8 (0.3)	2008 (64.9)	1072 (34.6)	5 (0.2)
34	1 (0.1)	1 (0.1)	935 (62.1)	569 (37.8)	0 (0)	1 (0.1)	1 (0.1)	1334 (67.6)	637 (32.3)	0 (0)	3 (0.3)	11 (1.1)	850 (83.8)	150 (14.8)	0 (0)	2 (0.1)	11 (0.4)	1547 (61.8)	942 (37.6)	3 (0.1)
35	0 (0)	0 (0)	950 (64.8)	515 (35.1)	2 (0.1)	1 (0.0)	2 (0.1)	1487 (72.1)	570 (27.6)	2 (0.1)	0 (0)	3 (0.2)	1253 (98.0)	23 (1.8)	0 (0)	1 (0.0)	3 (0.1)	1304 (62.4)	780 (37.3)	2 (0.1)
36	1 (0.1)	2 (0.2)	774 (63.3)	446 (36.5)	0 (0)	1 (0.1)	1 (0.1)	1146 (69.8)	493 (30.0)	0 (0)	0 (0)	0 (0)	251 (100)	0 (0)	0 (0)	6 (0.3)	7 (0.4)	1055 (59.5)	700 (39.5)	4 (0.2)
37	0 (0)	5 (0.4)	782 (68.8)	350 (30.8)	0 (0)	0 (0)	2 (0.1)	976 (70.6)	405 (29.3)	0 (0)	0 (0)	0 (0)	0 (0)	0 (0)	0 (0)	1 (0.1)	15 (1.0)	908 (58.4)	630 (40.5)	1 (0.1)
38	0 (0)	4 (0.4)	723 (71.3)	287 (28.3)	0 (0)	0 (0)	5 (0.4)	889 (74.1)	306 (25.5)	0 (0)	0 (0)	0 (0)	0 (0)	0 (0)	0 (0)	1 (0.1)	9 (0.7)	765 (61.8)	460 (37.2)	2 (0.2)
39	3 (0.3)	3 (0.3)	686 (69.2)	299 (30.2)	0 (0)	3 (0.3)	4 (0.4)	832 (73.1)	299 (26.3)	0 (0)	0 (0)	0 (0)	0 (0)	0 (0)	0 (0)	4 (0.4)	14 (1.4)	644 (62.8)	364 (35.5)	0 (0)
40	0 (0)	7 (0.1)	6241 (99.9)	0 (0)	0 (0)	0 (0)	7 (0.1)	6241 (99.9)	0 (0)	0 (0)	0 (0)	0 (0)	0 (0)	0 (0)	0 (0)	3 (0.1)	22 (0.5)	4695 (99.5)	0 (0)	0 (0)

Supplementary Table 4. Log-rank tests for ESLD patients by score variant and score classes for all-cause 2-years mortality as endpoint.

Score variant	Score class	n (Δ = ±0)	n (Δ ≤ −1)	χ^2 (1 df)	p-value	Hazard Ratio Δ = ±0 vs Δ ≤ −1
MELD	≤15	25	12	0.008	0.930	0.96 (0.43–2.16)
	16–25	31	64	2.876	0.090	0.64 (0.39–1.07)
	>25	11	82	12.183	**<0.001**	0.31 (0.16–0.62)
MELD-Na	≤15	27	7	0.002	0.967	0.98 (0.33–2.93)
	16–25	39	47	0.734	0.392	0.80 (0.49–1.32)
	>25	18	83	7.752	**0.005**	0.46 (0.26–0.80)
reMELD-Na	≤15	18	16	0.964	0.326	0.66 (0.29–1.51)
	16–25	47	54	8.044	**0.005**	0.52 (0.33–0.82)
	>25	15	49	13.427	**<0.001**	0.32 (0.17–0.61)
MELD 3.0	≤15	32	3	0.413	0.521	0.52 (0.07–3.92)
	16–25	44	35	3.970	**0.046**	0.61 (0.37–1.00)
	>25	29	53	3.766	**0.052**	0.61 (0.37–1.00)

Comparison of score deviations, i.e. Δ = ±0 vs Δ ≤ −1 shown in **Figure 5**.

Supplementary Table 5a. Gray's tests (two-sided) for competing risks of removal from waitlist in SRTR patients (1990 – 2022) stratified by MELD variant and score class.

MELD variant	Score class	Event	χ^2 (1 df)	p-value
MELD	≤15	Waitlist death	1.35	0.245
		Transplant	0.75	0.386
		Other removals	13.78	**<0.001**
	16–25	Waitlist death	5.01	**0.025**
		Transplant	2.21	0.137
		Other removals	0.68	0.410
	>25	Waitlist death	1.15	0.283
		Transplant	93.48	**<0.001**
		Other removals	3.16	0.075
MELD-Na	≤15	Waitlist death	2.80	0.094
		Transplant	0.52	0.470
		Other removals	4.69	**0.030**
	16–25	Waitlist death	5.35	**0.021**
		Transplant	3.74	**0.053**
		Other removals	0.29	0.589
	>25	Waitlist death	1.11	0.293
		Transplant	91.61	**<0.001**
		Other removals	2.12	0.146
reMELD-Na	≤15	Waitlist death	3.21	0.073
		Transplant	0.51	0.474
		Other removals	5.25	**0.022**
	16–25	Waitlist death	2.13	0.145
		Transplant	30.76	**<0.001**
		Other removals	0.63	0.428
	>25	Waitlist death	1.22	0.270
		Transplant	51.81	**<0.001**
		Other removals	1.78	0.183
MELD 3.0	≤15	Waitlist death	3.35	0.067
		Transplant	0.61	0.436
		Other removals	4.67	**0.031**
	16–25	Waitlist death	8.77	**0.003**
		Transplant	3.24	**0.072**
		Other removals	0.33	0.567
	>25	Waitlist death	0.22	0.639
		Transplant	90.17	**<0.001**
		Other removals	1.82	0.177

Comparison of score deviations, i.e. $\Delta = \pm 0$ vs $\Delta \leq -1$ shown in **Figure 6**; event types shown in rows indicate competing risks considered.

Supplementary Table 5b (Amendment to Supplementary Table 5a). Gray's tests (two-sided) for competing risks of removal from the liver-transplant waiting list in SRTR patients, stratified by MELD variant and score class. Data shown refer to **Supplementary Figure 2** as amendment to **Figure 6** of the main text.
Analyses were performed using SRTR data from **January 2023 through December 2025**.

**MELD variant**	**Score class**	**Event**	**χ^2 (1 df)**	**p-value**
MELD	≤15	Waitlist death	0.12	0.7335
		Transplant	12.39	**0.0004**
		Other removals	3.69	**0.0549**
	16–25	Waitlist death	1.21	0.2722
		Transplant	4.97	**0.0258**
		Other removals	0.51	0.4767
	>25	Waitlist death	3.71	**0.0542**
		Transplant	19.04	**<0.0001**
		Other removals	0.00	0.9794
MELD-Na	≤15	Waitlist death	0.27	0.6048
		Transplant	11.61	**0.0007**
		Other removals	3.10	0.0784
	16–25	Waitlist death	1.01	0.3154
		Transplant	11.12	**0.0009**
		Other removals	0.09	0.7659
	>25	Waitlist death	3.35	0.0672
		Transplant	23.22	**<0.0001**
		Other removals	0.47	0.4936
reMELD-Na	≤15	Waitlist death	0.09	0.7637
		Transplant	65.84	**<0.0001**
		Other removals	10.47	**0.0012**
	16–25	Waitlist death	0.00	0.9950
		Transplant	7.81	**0.0052**
		Other removals	0.04	0.8460
	>25	Waitlist death	2.63	0.1051
		Transplant	0.01	0.9359
		Other removals	1.72	0.1894
MELD 3.0	≤15	Waitlist death	0.00	0.9764
		Transplant	13.38	**0.0003**
		Other removals	0.01	0.9079
	16–25	Waitlist death	4.70	**0.0301**
		Transplant	17.11	**<0.0001**
		Other removals	0.17	0.6798
	>25	Waitlist death	0.47	0.4940
		Transplant	13.16	**0.0003**
		Other removals	0.08	0.7810

Supplementary Table 6a. Fine–Gray subdistribution hazard (two-sided) for waitlist death by MELD variant and score class in SRTR patients (1990 – 2022).

MELD variant	Score class	N $\Delta = \pm 0$ / $\Delta \leq -1$	n Deaths $\Delta = \pm 0$ / $\Delta \leq -1$	Hazard Ratio $\Delta \leq -1$ / $\Delta = \pm 0$	p-value
MELD	≤15	4977 / 532	528 / 66	1.16 (0.90–1.50)	0.240
	16–25	4229 / 2550	524 / 272	**0.85 (0.73–0.98)**	**0.025**
	>25	3036 / 3888	548 / 766	1.06 (0.95–1.19)	0.279
MELD-Na	≤15	3996 / 348	388 / 44	1.30 (0.96–1.78)	0.091
	16–25	4607 / 2106	596 / 232	**0.84 (0.72–0.97)**	**0.020**
	>25	3639 / 4516	616 / 828	1.06 (0.95–1.17)	0.290
reMELD-Na	≤15	5169 / 466	505 / 58	1.28 (0.98–1.68)	0.071
	16–25	5095 / 3256	698 / 415	0.91 (0.81–1.03)	0.144
	>25	1978 / 3248	397 / 631	0.93 (0.82–1.06)	0.263
MELD 3.0	≤15	3659 / 284	328 / 35	1.38 (0.98–1.95)	0.065
	16–25	5071 / 2040	659 / 215	**0.79 (0.68–0.93)**	**0.003**
	>25	3512 / 4646	613 / 854	1.03 (0.92–1.14)	0.635

Comparison of $\Delta \leq -1$ vs $\Delta = \pm 0$ shown in **Figure 6**. Competing risks for removal from waitlist are considered as indicated in **Supplementary Table 5a.**

Supplementary Table 6b (Amendment to Supplementary Table 6a). Fine–Gray subdistribution hazard models (two-sided) for waitlist death, transplantation, and other removals by MELD variant and score class. Covariate: m1 (Δ≤−1) versus pm (Δ=±0). Data shown refer to **Supplemental Figure 2** as amendment to **Figure 6**.
Analyses were performed using SRTR data from **January 2023 through December 2025**.

MELD variant	Score class	N Δ = ±0 / Δ ≤ −1	Event type within 2023–25	Events (overall)	Subdistribution HR Δ ≤ −1 / Δ = ±0	p-value
MELD	≤15	1383 / 149	Waitlist death	27	1.62 (0.56–4.66)	0.3735
			Transplant	951	0.71 (0.56–0.89)	**0.0031**
			Other removals	554	1.29 (1.02–1.64)	**0.0356**
	16–25	956 / 490	Waitlist death	51	1.89 (1.09–3.28)	**0.0224**
			Transplant	1,176	0.84 (0.75–0.95)	**0.0043**
			Other removals	219	1.01 (0.77–1.34)	0.9272
	>25	544 / 444	Waitlist death	95	1.48 (0.99–2.21)	**0.0588**
			Transplant	815	0.78 (0.69–0.89)	**0.0003**
			Other removals	78	0.99 (0.64–1.55)	0.9730
MELD-Na	≤15	1187 / 107	Waitlist death	21	2.63 (0.89–7.79)	**0.0807**
			Transplant	763	0.67 (0.51–0.88)	**0.0038**
			Other removals	510	1.21 (0.94–1.58)	0.1448
	16–25	1056 / 386	Waitlist death	50	1.54 (0.87–2.75)	0.1394
			Transplant	1,152	0.81 (0.72–0.93)	**0.0018**
			Other removals	240	1.21 (0.92–1.59)	0.1759
	>25	724 / 508	Waitlist death	102	1.42 (0.96–2.09)	**0.0780**
			Transplant	1,028	0.79 (0.70–0.89)	**0.0001**
			Other removals	102	0.91 (0.61–1.35)	0.6446
reMELD-Na	≤15	2601 / 796	Waitlist death	103	1.77 (1.18–2.65)	**0.0058**
			Transplant	2,458	1.26 (1.15–1.38)	**<0.0001**
			Other removals	836	0.61 (0.51–0.74)	**<0.0001**
	16–25	353 / 265	Waitlist death	64	0.95 (0.58–1.55)	0.8367
			Transplant	515	0.78 (0.66–0.92)	**0.0029**
			Other removals	39	1.55 (0.83–2.91)	0.1723
	>25	32 / 12	Waitlist death	10	0.25 (0.04–1.79)	0.1686
			Transplant	30	0.73 (0.38–1.42)	0.3608
			Other removals	4	8.37 (0.87–80.62)	**0.0661**
MELD 3.0	≤15	1091 / 106	Waitlist death	17	0.64 (0.09–4.77)	0.6619
			Transplant	702	0.55 (0.42–0.73)	**<0.0001**
			Other removals	478	1.57 (1.22–2.03)	**0.0004**
	16–25	1108 / 440	Waitlist death	58	2.07 (1.24–3.48)	**0.0057**
			Transplant	1,224	0.80 (0.71–0.90)	**0.0003**
			Other removals	266	1.13 (0.87–1.46)	0.3723
	>25	610 / 629	Waitlist death	101	1.12 (0.76–1.65)	0.5808
			Transplant	1,032	0.84 (0.75–0.95)	**0.0043**
			Other removals	106	0.96 (0.65–1.40)	0.8201

Supplementary Table 7a. Horizon-specific cumulative incidence functions (CIF) at **90 days** since listing, stratified by MELD variant and score class, comparing Δ=±0 versus Δ≤−1. Analyses were performed using SRTR data from **January 2023 through December 2025**.

MELD variant	Score class	N Δ = ±0 / Δ ≤ −1	Event type within 90 days	CIF at horizon Δ = ±0	CIF at horizon Δ ≤ −1	Gray's p-value
MELD	≤15	1383 / 149	Waitlist death	0.0043	0.0000	0.4207
			Transplant	0.2213	0.1611	0.0875
			Other removals	0.0311	0.0201	0.4571
	16–25	956 / 490	Waitlist death	0.0073	0.0102	0.5712
			Transplant	0.4916	0.4061	**0.0008**
			Other removals	0.0262	0.0367	0.2706
	>25	544 / 444	Waitlist death	0.0588	0.0541	0.7112
			Transplant	0.7665	0.6644	**<0.0001**
			Other removals	0.0478	0.0450	0.8127
MELD-Na	≤15	1187 / 107	Waitlist death	0.0042	0.0000	0.5013
			Transplant	0.1845	0.1121	0.0579
			Other removals	0.0337	0.0187	0.4016
	16–25	1056 / 386	Waitlist death	0.0076	0.0078	0.9720
			Transplant	0.4498	0.3627	**0.0020**
			Other removals	0.0218	0.0337	0.2034
	>25	724 / 508	Waitlist death	0.0525	0.0394	0.2687
			Transplant	0.7472	0.6398	**<0.0001**
			Other removals	0.0497	0.0433	0.5764
reMELD-Na	≤15	2601 / 796	Waitlist death	0.0069	0.0101	0.3752
			Transplant	0.3399	0.4309	**<0.0001**
			Other removals	0.0327	0.0289	0.6030
	16–25	353 / 265	Waitlist death	0.0737	0.0491	0.1995
			Transplant	0.8074	0.6717	**<0.0001**
			Other removals	0.0425	0.0377	0.7466
	>25	32 / 12	Waitlist death	0.2812	0.0000	**0.0427**
			Transplant	0.6875	0.6667	0.4335
			Other removals	0.0312	0.1667	0.1912
MELD 3.0	≤15	1091 / 106	Waitlist death	0.0055	0.0000	0.4442
			Transplant	0.1897	0.0283	**<0.0001**
			Other removals	0.0330	0.0283	0.7815
	16–25	1108 / 440	Waitlist death	0.0045	0.0091	0.2876
			Transplant	0.4440	0.3432	**0.0002**
			Other removals	0.0253	0.0205	0.5738
	>25	610 / 629	Waitlist death	0.0475	0.0477	0.9957
			Transplant	0.7262	0.6582	**<0.0001**
			Other removals	0.0557	0.0397	0.1764

Statistical test: two-sided Gray’s test for competing risks.

Supplementary Table 7b. Horizon-specific cumulative incidence functions (CIF) at **365 days** since listing, stratified by MELD variant and score class, comparing Δ=±0 versus Δ≤−1. Analyses were performed using SRTR data from **January 2023 through December 2025**.

MELD variant	Score class	N Δ = ±0 / Δ ≤ −1	Event type within 365 days	CIF at horizon Δ = ±0	CIF at horizon Δ ≤ −1	Gray's p-value
MELD	≤15	1383 / 149	Waitlist death	0.0108	0.0201	0.3209
			Transplant	0.5257	0.3557	**0.0003**
			Other removals	0.1381	0.1141	0.3904
	16–25	956 / 490	Waitlist death	0.0126	0.0265	**0.0550**
			Transplant	0.7448	0.6776	**0.0006**
			Other removals	0.0847	0.0816	0.8310
	>25	544 / 444	Waitlist death	0.0754	0.0856	0.6005
			Transplant	0.8180	0.7613	**0.0001**
			Other removals	0.0643	0.0721	0.6604
MELD-Na	≤15	1187 / 107	Waitlist death	0.0084	0.0280	**0.0523**
			Transplant	0.4920	0.2897	**0.0002**
			Other removals	0.1491	0.1028	0.1855
	16–25	1056 / 386	Waitlist death	0.0161	0.0207	0.5567
			Transplant	0.7235	0.6503	**0.0010**
			Other removals	0.0852	0.0933	0.6415
	>25	724 / 508	Waitlist death	0.0649	0.0728	0.6366
			Transplant	0.8191	0.7579	**<0.0001**
			Other removals	0.0691	0.0650	0.7509
reMELD-Na	≤15	2601 / 796	Waitlist death	0.0158	0.0264	**0.0510**
			Transplant	0.6078	0.6709	**<0.0001**
			Other removals	0.1142	0.0854	**0.0227**
	16–25	353 / 265	Waitlist death	0.0963	0.0679	0.1949
			Transplant	0.8385	0.7811	**0.0008**
			Other removals	0.0482	0.0642	0.4146
	>25	32 / 12	Waitlist death	0.2812	0.0833	**0.0427**
			Transplant	0.6875	0.6667	0.4335
			Other removals	0.0312	0.1667	0.1912
MELD 3.0	≤15	1091 / 106	Waitlist death	0.0101	0.0000	0.2992
			Transplant	0.4885	0.2453	**<0.0001**
			Other removals	0.1421	0.1604	0.6044
	16–25	1108 / 440	Waitlist death	0.0153	0.0273	0.1184
			Transplant	0.7292	0.6091	**<0.0001**
			Other removals	0.0857	0.0955	0.5713
	>25	610 / 629	Waitlist death	0.0607	0.0731	0.3948
			Transplant	0.8033	0.7822	**0.0013**
			Other removals	0.0738	0.0636	0.4514

Statistical test: two-sided Gray’s test for competing risks.

Supplementary Table 8a. Horizon-specific two-sided Fine–Gray subdistribution hazard models at **90 days** since listing for waitlist death, transplantation, and other removals, stratified by MELD variant and score class. Covariate: m1 (Δ≤−1) versus pm (Δ=±0).
Analyses were performed using SRTR data from **January 2023 through December 2025**.

MELD variant	Score class	N Δ = ±0 / Δ ≤ −1	Event type within 90 days	Events (overall)	Subdistribution HR Δ ≤ −1 / Δ = ±0	p-value
MELD	≤15	1383 / 149	Waitlist death	6	0.00 (0.00–0.00)	**<0.0001**
			Transplant	330	0.70 (0.46–1.06)	**0.0926**
			Other removals	46	0.64 (0.20–2.08)	0.4616
	16–25	956 / 490	Waitlist death	12	1.39 (0.44–4.38)	0.5725
			Transplant	669	0.76 (0.64–0.89)	**0.0008**
			Other removals	43	1.40 (0.77–2.57)	0.2720
	>25	544 / 444	Waitlist death	56	0.91 (0.53–1.53)	0.7113
			Transplant	712	0.72 (0.63–0.83)	**<0.0001**
			Other removals	46	0.93 (0.52–1.67)	0.8128
MELD-Na	≤15	1187 / 107	Waitlist death	5	0.00 (0.00–0.00)	**<0.0001**
			Transplant	231	0.58 (0.33–1.03)	**0.0639**
			Other removals	42	0.55 (0.13–2.28)	0.4097
	16–25	1056 / 386	Waitlist death	11	1.02 (0.27–3.85)	0.9720
			Transplant	615	0.75 (0.62–0.90)	**0.0021**
			Other removals	36	1.55 (0.79–3.05)	0.2060
	>25	724 / 508	Waitlist death	58	0.74 (0.43–1.27)	0.2701
			Transplant	866	0.71 (0.62–0.81)	**<0.0001**
			Other removals	58	0.86 (0.51–1.46)	0.5765
reMELD-Na	≤15	2601 / 796	Waitlist death	26	1.45 (0.63–3.34)	0.3777
			Transplant	1,227	1.35 (1.19–1.53)	**<0.0001**
			Other removals	108	0.89 (0.56–1.40)	0.6037
	16–25	353 / 265	Waitlist death	39	0.65 (0.34–1.26)	0.2024
			Transplant	463	0.68 (0.57–0.81)	**<0.0001**
			Other removals	25	0.88 (0.40–1.95)	0.7462
	>25	32 / 12	Waitlist death	9	0.00 (0.00–0.00)	**<0.0001**
			Transplant	30	0.73 (0.38–1.42)	0.3608
			Other removals	3	5.38 (0.50–57.51)	0.1642
MELD 3.0	≤15	1091 / 106	Waitlist death	6	0.00 (0.00–0.00)	**<0.0001**
			Transplant	210	0.14 (0.04–0.42)	**0.0005**
			Other removals	39	0.85 (0.26–2.72)	0.7809
	16–25	1108 / 440	Waitlist death	9	2.01 (0.54–7.48)	0.2969
			Transplant	643	0.71 (0.60–0.86)	**0.0003**
			Other removals	37	0.81 (0.38–1.71)	0.5745
	>25	610 / 629	Waitlist death	59	1.00 (0.60–1.67)	0.9957
			Transplant	857	0.77 (0.67–0.87)	**<0.0001**
			Other removals	59	0.70 (0.42–1.18)	0.1785

Supplementary Table 8b. Horizon-specific two-sided Fine–Gray subdistribution hazard models at **365 days** since listing for waitlist death, transplantation, and other removals, stratified by MELD variant and score class. Covariate: m1 (Δ≤−1) versus pm (Δ=±0).
Analyses were performed using SRTR data from **January 2023 through December 2025**.

MELD variant	Score class	N<br>Δ = ±0 / Δ ≤ −1	Event type within 365 days	Events (overall)	Subdistribution HR<br>Δ ≤ −1 / Δ = ±0	p-value
MELD	≤15	1383 / 149	Waitlist death	18	1.85 (0.54–6.37)	0.3264
			Transplant	780	0.61 (0.46–0.81)	**0.0007**
			Other removals	208	0.81 (0.49–1.32)	0.3906
	16–25	956 / 490	Waitlist death	25	2.12 (0.97–4.64)	**0.0605**
			Transplant	1,044	0.80 (0.70–0.91)	**0.0005**
			Other removals	121	0.96 (0.66–1.40)	0.8308
	>25	544 / 444	Waitlist death	79	1.12 (0.72–1.75)	0.6007
			Transplant	783	0.76 (0.67–0.88)	**0.0001**
			Other removals	67	1.11 (0.69–1.80)	0.6604
MELD-Na	≤15	1187 / 107	Waitlist death	13	3.33 (0.92–12.03)	**0.0658**
			Transplant	615	0.52 (0.36–0.75)	**0.0005**
			Other removals	188	0.67 (0.36–1.22)	0.1907
	16–25	1056 / 386	Waitlist death	25	1.29 (0.56–2.97)	0.5570
			Transplant	1,015	0.79 (0.68–0.91)	**0.0008**
			Other removals	126	1.10 (0.75–1.61)	0.6408
	>25	724 / 508	Waitlist death	84	1.11 (0.72–1.70)	0.6365
			Transplant	978	0.75 (0.67–0.85)	**<0.0001**
			Other removals	83	0.93 (0.60–1.44)	0.7507
reMELD-Na	≤15	2601 / 796	Waitlist death	62	1.68 (0.99–2.84)	**0.0532**
			Transplant	2,115	1.24 (1.12–1.37)	**<0.0001**
			Other removals	365	0.74 (0.57–0.96)	**0.0237**
	16–25	353 / 265	Waitlist death	52	0.69 (0.39–1.21)	0.1965
			Transplant	503	0.75 (0.63–0.88)	**0.0007**
			Other removals	34	1.32 (0.68–2.59)	0.4136
	>25	32 / 12	Waitlist death	10	0.25 (0.04–1.79)	0.1686
			Transplant	30	0.73 (0.38–1.42)	0.3608
			Other removals	3	5.38 (0.50–57.51)	0.1642
MELD 3.0	≤15	1091 / 106	Waitlist death	11	0.00 (0.00–0.00)	**<0.0001**
			Transplant	559	0.41 (0.28–0.60)	**<0.0001**
			Other removals	172	1.14 (0.69–1.88)	0.6028
	16–25	1108 / 440	Waitlist death	29	1.79 (0.85–3.74)	0.1227
			Transplant	1,076	0.73 (0.63–0.83)	**<0.0001**
			Other removals	137	1.11 (0.77–1.59)	0.5702
	>25	610 / 629	Waitlist death	83	1.21 (0.78–1.86)	0.3961
			Transplant	982	0.82 (0.73–0.93)	**0.0014**
			Other removals	85	0.85 (0.56–1.30)	0.4517

Supplementary Table 9. Proportion of female patients within each subgroup: with either no score changes after creatinine correction (Δ = ±0) or a decrease/increase of at least 1 point (Δ ≤ –1, Δ ≥ +1). P-values from two-sided χ^2 tests of sex differences are shown (2×2 contingency tables: Male vs Female by Δ as indicated).

**Cohort**	**Score Variant**	**Females, %**			**p-value, χ^2-test**	
		**(Δ = ±0)**	**(Δ ≤ –1)**	**(Δ ≥ +1)**	**(Δ = ±0) vs (Δ ≤ –1)**	**(Δ = ±0) vs (Δ ≥ +1)**
ESLD, deceased	MELD	41.8	38.5	33.3	0.6546	0.5953
	MELD-Na	40.5	40.3	29.4	1	0.4296
	reMELD-Na	43.8	39.2	34.0	0.5441	0.2826
	MELD 3.0	39.0	40.0	41.7	1	1
ESLD, alive	MELD	51.2	32.1	34.6	**<0.0000**	**0.0018**
	MELD-Na	50.8	31.5	34.9	**<0.0000**	**0.0028**
	reMELD-Na	50.5	32.0	44.2	**<0.0000**	0.0842
	MELD 3.0	48.6	27.7	40.0	**<0.0000**	0.2984
SRTR, deceased	MELD	39.7	42.3	46.9	0.1933	0.3815
	MELD-Na	40.0	42.0	46.9	0.3234	0.4036
	reMELD-Na	36.7	44.8	49.6	**<0.0000**	**0.0088**
	MELD 3.0	38.5	44.1	41.5	**0.0044**	0.8243
SRTR, Liver-Tx survived	MELD	38.4	33.3	29.2	**<0.0000**	**0.0158**
	MELD-Na	38.1	33.4	30.1	**<0.0000**	**0.0431**
	reMELD-Na	36.3	36.2	38.5	0.8904	0.4299
	MELD 3.0	37.5	34.7	30.8	**0.0032**	0.1644

Sex distribution was compared between patients with no relevant MELD change after creatinine correction (Δ = ±0) and those with a ≤ -1-point decrease (Δ ≤ –1) as well as those with a ≥1 point increase (Δ ≥ +1), stratified by survival status. In deceased patients with ESLD, sex distribution did neither differ between patients with Δ = ±0 and those with Δ ≤ –1 or Δ ≥ +1, respectively. Among surviving patients of the ESLD cohort, the proportion of females was consistently and significantly lower in the Δ ≤ –1 group compared to Δ = ±0

across all MELD variants. Noteworthy, the portion of alive female ESLD patients with Δ ≤ –1 was lowest for MELD 3.0, the only score that incorporates sex into its calculation

In the SRTR cohort a significantly lower proportion of female patients surviving after LTx exhibited a with Δ ≤ –1 correction of all MELD variants except reMELD-Na. Among deceased patients in SRTR the proportion of women with Δ ≤ –1 was higher.

than those with Δ = ±0.
