# Supplementary material for: Creatinine assay interferences compromises MELD accuracy and may bias liver allocation
Source: Nat Commun. 2026 Jul 23;17:7111. doi: 10.1038/s41467-026-75011-x (PMC13396164; doi:10.1038/s41467-026-75011-x)
Supplement: Supplementary file 2 — Reporting summary [file 41467_2026_75011_MOESM2_ESM.pdf]

nature portfolio

Corresponding author(s): Prof. Dr. Ali Canbay

Last updated by author(s): May 7, 2026

Reporting Summary

Nature Portfolio wishes to improve the reproducibility of the work that we publish. This form provides structure for consistency and transparency in reporting. For further information on Nature Portfolio policies, see our Editorial Policies and the Editorial Policy Checklist.

Statistics

For all statistical analyses, confirm that the following items are present in the figure legend, table legend, main text, or Methods section.

n/a	Confirmed	
☐	☒	The exact sample size (*n*) for each experimental group/condition, given as a discrete number and unit of measurement
☐	☒	A statement on whether measurements were taken from distinct samples or whether the same sample was measured repeatedly
☐	☒	The statistical test(s) used AND whether they are one- or two-sided<br>*Only common tests should be described solely by name; describe more complex techniques in the Methods section.*
☒	☐	A description of all covariates tested
☒	☐	A description of any assumptions or corrections, such as tests of normality and adjustment for multiple comparisons
☐	☒	A full description of the statistical parameters including central tendency (e.g. means) or other basic estimates (e.g. regression coefficient) AND variation (e.g. standard deviation) or associated estimates of uncertainty (e.g. confidence intervals)
☐	☒	For null hypothesis testing, the test statistic (e.g. *F*, *t*, *r*) with confidence intervals, effect sizes, degrees of freedom and *P* value noted<br>*Give P values as exact values whenever suitable.*
☒	☐	For Bayesian analysis, information on the choice of priors and Markov chain Monte Carlo settings
☐	☒	For hierarchical and complex designs, identification of the appropriate level for tests and full reporting of outcomes
☒	☐	Estimates of effect sizes (e.g. Cohen's *d*, Pearson's *r*), indicating how they were calculated

Our web collection on statistics for biologists contains articles on many of the points above.

Software and code

Policy information about availability of computer code

Data collection	No software was used for data collection.
Data analysis	All analyses were performed in R version 4.3.0 (R Foundation for Statistical Computing, Vienna, Austria), and SAS 9.4 (SAS Institute, Cary, NC). No proprietary codes or skripts were employed, codes and skripts were applied as implemented in R.

For manuscripts utilizing custom algorithms or software that are central to the research but not yet described in published literature, software must be made available to editors and reviewers. We strongly encourage code deposition in a community repository (e.g. GitHub). See the Nature Portfolio guidelines for submitting code & software for further information.

Data

Policy information about availability of data

All manuscripts must include a data availability statement. This statement should provide the following information, where applicable:

- Accession codes, unique identifiers, or web links for publicly available datasets
- A description of any restrictions on data availability
- For clinical datasets or third party data, please ensure that the statement adheres to our policy

The data reported here have been supplied by the Hennepin Healthcare Research Institute (HHRI) as the contractor for the Scientific Registry of Transplant Recipients (SRTR). The interpretation and reporting of these data are the responsibility of the author(s) and in no way should be seen as an official policy of or interpretation by the SRTR or the U.S. Government. To request this data please perform a data requests on the following home pages:

https://www.srtr.org/requesting-data/data-requests/
https://www.hrsa.gov/optn/data/data-reports/data-request-instructions
Source data are provided with this paper in a single ZIP-file. The ZIP-file contains all source data as well as scripts and workflows to derive the described analysis. A browser based file viewer is provided within the ZIP-file to navigate files and to retrace workflows. Source data for experimental derivation and validation of the correction formula and the ESLD study cohort data set are provided as full data sets. Because of ethical and data protection restrictions in our country, individual-level raw data underlying this study cannot be deposited in a public repository. Aggregate data on MELD score distribution and correction for the SRTR cohort are provided in Supplementary Table 3.

Research involving human participants, their data, or biological material

Policy information about studies with human participants or human data. See also policy information about sex, gender (identity/presentation), and sexual orientation and race, ethnicity and racism.

Reporting on sex and gender	No sex or gender based analyses are shown. Information on sex (as biological construct) is given as documented in clinical records as aggregate data.
Reporting on race, ethnicity, or other socially relevant groupings	No race or ethnicity specific analyses or groupings were performed.
Population characteristics	Detailed population characteristics of the 2 large cohorts employed for evaluation of the developed correction formula are given in table 1 of the manuscript.
Recruitment	Data were analysed retrospectively form large patient cohorts.
Ethics oversight	This study was conducted in accordance with the Declaration of Helsinki (2024). Ethical approval was obtained from the institutional review board of Ruhr University Bochum (Register Number: 23-7840). The Inova Health Systems Institutional Review Board classified the study as non-human subject research. Both boards waived the need for informed consent.

Note that full information on the approval of the study protocol must also be provided in the manuscript.

Field-specific reporting

Please select the one below that is the best fit for your research. If you are not sure, read the appropriate sections before making your selection.

☒ Life sciences ☐ Behavioural & social sciences ☐ Ecological, evolutionary & environmental sciences

For a reference copy of the document with all sections, see nature.com/documents/nr-reporting-summary-flat.pdf

Life sciences study design

All studies must disclose on these points even when the disclosure is negative.

Sample size	Sample sizes for different aspects of the study depended on the step of the overall study. In vitro arrays for development of the correction formula contained 396 concentration-combinations of bilirubin and creatinine (n=3, duplicate measurements). Validation of the generated correction model was performed in n = 32 selected patient sera covering the full range of the concentration matrix. Evaluation of correction formular impact on MELD score variants was performed two large patient cohorts: Cohort 1 consisted of end stage liver disease patients (ESLD) comprising 20,359 MELD scores of 1,375 patients; cohort 2 consisted of patients of the Scientific Registry of Transplant Recipients (SRTR) from centers with confirmed Jaffe method for creatinine measurement, comprising 251,637 MELD scores of 20,444 patients. For subanalysis, i.e., survival duration of deceased patients, smaller sample sizes were achieved, which are all detailed for the respective analyses in the manuscript and tables.
Data exclusions	Patients of both cohorts suffering from primary sclerosing cholangitis (ICD-10: K83.0), biliary atresia (ICD-10: Q44.2), benign and malignant liver tumors (ICD-10: D18.03, C22.-), cystic fibrosis (ICD-10: E84.-), and primary hyperoxaluria (ICD-10: E74.8) were excluded from the study due to exceptional MELD-related allocation criteria under the German guideline for waiting list management and organ allocation for liver transplantation
Replication	For generation of the correction model, bilirubin-creatinine concentration arrays were replicated on different days under identical conditions and analyszed on the same day. For details please refer to the method section of the manuscript.
Randomization	No randomization was performed (retrospective data analysis):
Blinding	No blinding was performed (retrospective data analysis):

Reporting for specific materials, systems and methods

We require information from authors about some types of materials, experimental systems and methods used in many studies. Here, indicate whether each material, system or method listed is relevant to your study. If you are not sure if a list item applies to your research, read the appropriate section before selecting a response.

Materials & experimental systems

n/a	Involved in the study
☒	☐ Antibodies
☒	☐ Eukaryotic cell lines
☒	☐ Palaeontology and archaeology
☒	☐ Animals and other organisms
☐	☒ Clinical data
☒	☐ Dual use research of concern
☒	☐ Plants

Methods

n/a	Involved in the study
☒	☐ ChIP-seq
☒	☐ Flow cytometry
☒	☐ MRI-based neuroimaging

Clinical data

Policy information about clinical studies

All manuscripts should comply with the ICMJE guidelines for publication of clinical research and a completed CONSORT checklist must be included with all submissions.

Clinical trial registration

N/A

Study protocol

N/A

Data collection

Cohort 1 consisted of adult inpatients and outpatients with ESLD recruited at a single tertiary care university hospital in Germany. This center functions as a major referral center for liver disease and transplantation. Data were retrospectively extracted from structured electronic health records between May 2006 and April 2025. The pseudonymized dataset included laboratory findings, diagnoses encoded by the international classification of diseases (ICD), and the German Operation and Procedure Code (OPS), the official classification system for surgical procedures, medical interventions and measures.
Cohort 2 comprised data from the Scientific Registry of Transplant Recipients (SRTR). The SRTR data system includes data on all donors, wait-listed candidates, and transplant recipients in the U.S., submitted by the members of the Organ Procurement and Transplantation Network (OPTN). The Health Resources and Services Administration (HRSA), U.S. Department of Health and Human Services provides oversight to the activities of the OPTN and SRTR contractors. Cohort 2 includes patient records from 1990 to 2022, comprising laboratory and clinical data for retrospective calculation of MELD scores.

Outcomes

For individuals of cohort 1 possible outcomes were alive or deceased at end of follow up.
For individuals in cohort 2 who were no longer on the waitlist at the study cutoff (December 2022), the possible outcomes were: transplantation, death, removal due to clinical deterioration (too sick for transplant) or improvement (no longer eligible), refusal, or transfer to another center.

Plants

Seed stocks

N/A

Novel plant genotypes

N/A

Authentication

N/A
