# Supplementary material for: Creatinine assay interferences compromises MELD accuracy and may bias liver allocation
Source: Nat Commun. 2026 Jul 23;17:7111. doi: 10.1038/s41467-026-75011-x (PMC13396164; doi:10.1038/s41467-026-75011-x)
Supplement: Supplementary file 4 — Source Data [file 41467_2026_75011_MOESM4_ESM.zip › figshare_package_FINAL_PUBLIC_DEPOSIT_V1_20260503_002637/00_START_HERE_HTML_NAVIGATOR/index.html]

 
 
 
 
 Public Data Navigator 
 

 

 

 
 
  Public Data Navigator   Creatinine Assay Interference, MELD Accuracy, and Liver Allocation   Browser-readable guide to the public ESLD and simulation data package accompanying the manuscript.  
 
 
  Search  Search files, variables, and indexed file-content values. 
 
 
 All file types  CSV  PDF  TXT  HTML 
  Reset  
  End-user sections  All sections  Start here  1  
 Public primary data  7  
 Manuscript output data  13  
 Public figures  6  
 Workflow readmes  7  
 Technical appendix / request manifests  4   
  Package counts  38 visible files 661 public variables 40.363 MB visible content 97 restricted files listed 93 scripts listed  
 
 
  Study workflow guide  Recommended reading order: experimental data → model derivation → in-silico simulation → clinical ESLD application → SRTR external validation context.    Open the study workflow guide    
  Recommended use 
 
  Public data  Primary datasets and manuscript-ready public data tables.  Primary data   Output data  
  Codebook  Search variable names, definitions, and indexed metadata values.  Variable codebook  
  Technical appendix  Restricted data and scripts are listed here but not included.  Request manifests  
  
  
 Recommended files 
 Variable codebook 
 Section summary 
 Hidden technical/audit files 
 

  Recommended files  Curated end-user file list. Audit logs, patch histories, old dictionaries, intermediate duplicates, and outdated READMEs are hidden by default.   
   File  Section  Output  Type  Size KB  Variables  HTML  Original   
    README PUBLIC DEPOSIT PACKAGE   README_PUBLIC_DEPOSIT_PACKAGE_v01.txt   Start here    txt  1.952  0   Readable HTML view    Original   
   Esld master long   01_primary_data/public/esld_master_long_public.csv   Public primary data    csv  9620.613  43   Readable HTML view    Original   
   F1 simulated surface metadata   01_primary_data/public/f1_simulated_surface_metadata.csv   Public primary data  F1  csv  0.648  2   Readable HTML view    Original   
   F1 simulated surface repository   01_primary_data/public/f1_simulated_surface_repository.csv   Public primary data  F1  csv  4936.887  11   Readable HTML view    Original   
   F1 tb cre experimental array raw   01_primary_data/public/f1_tb_cre_experimental_array_raw.csv   Public primary data  F1  csv  331.003  9   Readable HTML view    Original   
   F1 tb cre experimental validation raw   01_primary_data/public/f1_tb_cre_experimental_validation_raw.csv   Public primary data  F1  csv  2.28  8   Readable HTML view    Original   
   F2 simulated heatmap metadata   01_primary_data/public/f2_simulated_heatmap_metadata.csv   Public primary data  F2  csv  0.545  2   Readable HTML view    Original   
   F2 simulated heatmap repository   01_primary_data/public/f2_simulated_heatmap_repository.csv   Public primary data  F2  csv  7674.803  6   Readable HTML view    Original   
   Expm F1 array input   02_workflows/F1_workflow_v02/submission_ready/public/data/expm_F1_array_input_public.csv   Manuscript output data  F1  csv  598.548  17   Readable HTML view    Original   
   Expm F1 validation input   02_workflows/F1_workflow_v02/submission_ready/public/data/expm_F1_validation_input_public.csv   Manuscript output data  F1  csv  4.949  16   Readable HTML view    Original   
   Slco F1 surface grid   02_workflows/F1_workflow_v02/submission_ready/public/data/slco_F1_surface_grid_public.csv   Manuscript output data  F1  csv  4918.95  11   Readable HTML view    Original   
   Slco F1 surface meta   02_workflows/F1_workflow_v02/submission_ready/public/data/slco_F1_surface_meta_public.csv   Manuscript output data  F1  csv  0.482  2   Readable HTML view    Original   
   Slco F2 heatmap bin   02_workflows/F2_workflow_v01/submission_ready/public/data/slco_F2_heatmap_bin_public.csv   Manuscript output data  F2  csv  7674.803  6   Readable HTML view    Original   
   Slco F2 heatmap meta   02_workflows/F2_workflow_v01/submission_ready/public/data/slco_F2_heatmap_meta_public.csv   Manuscript output data  F2  csv  0.539  2   Readable HTML view    Original   
   Esld F3 score shift aggregate   02_workflows/F3_workflow_v01/submission_ready/public/data/esld_F3_score_shift_aggregate_public.csv   Manuscript output data  F3  csv  6.515  4   Readable HTML view    Original   
   Esld F4 survival stats   02_workflows/F4_workflow_v01/submission_ready/public/data/esld_F4_survival_stats_public.csv   Manuscript output data  F4  csv  2.255  14   Readable HTML view    Original   
   Esld F5 stratified survival meta   02_workflows/F5_workflow_v01/submission_ready/public/data/esld_F5_stratified_survival_meta_public.csv   Manuscript output data  F5  csv  0.424  2   Readable HTML view    Original   
   Esld F5 stratified survival stats   02_workflows/F5_workflow_v01/submission_ready/public/data/esld_F5_stratified_survival_stats_public.csv   Manuscript output data  F5  csv  1.093  8   Readable HTML view    Original   
   Esld F5 stratified survival subject   02_workflows/F5_workflow_v01/submission_ready/public/data/esld_F5_stratified_survival_subject_public.csv   Manuscript output data  F5  csv  119.284  19   Readable HTML view    Original   
   Esld T4 score deviation outcome meta   02_workflows/T4_workflow_v01/submission_ready/public/data/esld_T4_score_deviation_outcome_meta_public.csv   Manuscript output data  T4  csv  0.678  2   Readable HTML view    Original   
   Esld T4 score deviation outcome table   02_workflows/T4_workflow_v01/submission_ready/public/data/esld_T4_score_deviation_outcome_table_public.csv   Manuscript output data  T4  csv  2.566  12   Readable HTML view    Original   
   Slco F1 surface   02_workflows/F1_workflow_v02/submission_ready/public/figures/slco_F1_surface_public.pdf   Public figures  F1  pdf  1506.008  0   Readable HTML view    Original   
   Slco F2 heatmap   02_workflows/F2_workflow_v01/submission_ready/public/figures/slco_F2_heatmap_public.pdf   Public figures  F2  pdf  856.709  0   Readable HTML view    Original   
   F3 ESLD SRTR   02_workflows/F3_workflow_v01/submission_ready/public/figures/F3_ESLD_SRTR_public.pdf   Public figures  F3  pdf  556.029  0   Readable HTML view    Original   
   F4 ESLD SRTR   02_workflows/F4_workflow_v01/submission_ready/public/figures/F4_ESLD_SRTR_public.pdf   Public figures  F4  pdf  817.854  0   Readable HTML view    Original   
   F5 ESLD   02_workflows/F5_workflow_v01/submission_ready/public/figures/F5_ESLD_public.pdf   Public figures  F5  pdf  602.098  0   Readable HTML view    Original   
   F6 SRTR   02_workflows/F6_workflow_v01/submission_ready/public/figures/F6_SRTR_public.pdf   Public figures  F6  pdf  555.652  0   Readable HTML view    Original   
   README F1 submission ready   02_workflows/F1_workflow_v02/submission_ready/README_F1_submission_ready_v02.txt   Workflow readmes  F1  txt  0.677  0   Readable HTML view    Original   
   README F2 submission ready   02_workflows/F2_workflow_v01/submission_ready/README_F2_submission_ready_v01.txt   Workflow readmes  F2  txt  0.289  0   Readable HTML view    Original   
   README F3 submission ready   02_workflows/F3_workflow_v01/submission_ready/README_F3_submission_ready_v01.txt   Workflow readmes  F3  txt  0.328  0   Readable HTML view    Original   
   README F6 submission ready   02_workflows/F6_workflow_v01/submission_ready/README_F6_submission_ready_v01.txt   Workflow readmes  F6  txt  0.466  0   Readable HTML view    Original   
   README T1 submission ready   02_workflows/T1_workflow_v01/submission_ready/README_T1_submission_ready_v01.txt   Workflow readmes  T1  txt  0.385  0   Readable HTML view    Original   
   README T3 submission ready   02_workflows/T3_workflow_v01/submission_ready/README_T3_submission_ready_v01.txt   Workflow readmes  T3  txt  0.471  0   Readable HTML view    Original   
   README T4 submission ready   02_workflows/T4_workflow_v01/submission_ready/README_T4_submission_ready_v01.txt   Workflow readmes  T4  txt  0.719  0   Readable HTML view    Original   
   Public deposit file manifest   00_release_manifests/public_deposit_file_manifest_v01.csv   Technical appendix / request manifests    csv  59.062  0   Readable HTML view    Original   
   Public variable dictionary FINAL CODEBOOK   00_release_manifests/public_variable_dictionary_FINAL_CODEBOOK.csv   Technical appendix / request manifests    csv  339.95  0   Readable HTML view    Original   


   Restricted on request file manifest   00_release_manifests/restricted_on_request_file_manifest_v01.csv   Technical appendix / request manifests    csv  74.085  0   Readable HTML view    Original   
   Script on request file manifest   00_release_manifests/script_on_request_file_manifest_v01.csv   Technical appendix / request manifests    csv  60.976  0   Readable HTML view    Original       

  Public variable codebook   
   Variable  Label  Description  Unit  File  View   
   age_years_first_available  Age at first available public record  Age in years at the first available public ESLD record for the patient or encounter represented in the released data.  years  01_primary_data/public/esld_master_long_public.csv   File view   
  age_years_sample  Age at sample  Age in years at the sample-level public ESLD record.  years  01_primary_data/public/esld_master_long_public.csv   File view   
  albumin_g_dl  Serum albumin concentration  Serum albumin concentration in the public ESLD master data.  g/dL  01_primary_data/public/esld_master_long_public.csv   File view   
  ald  Alcohol-related liver disease etiology flag  Binary etiology indicator for alcohol-related liver disease in the public ESLD cohort.    01_primary_data/public/esld_master_long_public.csv   File view   
  ald_hcv  Combined alcohol-related liver disease and hepatitis C etiology flag  Binary etiology indicator for combined alcohol-related liver disease and hepatitis C in the public ESLD cohort.    01_primary_data/public/esld_master_long_public.csv   File view   
  autoimmune  Autoimmune liver disease etiology flag  Binary etiology indicator for autoimmune liver disease in the public ESLD cohort.    01_primary_data/public/esld_master_long_public.csv   File view   
  crea  Creatinine concentration  Creatinine concentration used in MELD-related score calculation before applying the study-specific correction.  mg/dL  01_primary_data/public/esld_master_long_public.csv   File view   
  crea_corrected  Corrected creatinine concentration  Creatinine concentration after applying the study-specific correction used for recalculated MELD-related scores.  mg/dL  01_primary_data/public/esld_master_long_public.csv   File view   
  date_count  Relative day count  Relative day count used for time alignment; negative/positive values are relative to the analysis anchor and are not calendar dates.    01_primary_data/public/esld_master_long_public.csv   File view   
  date_count_month  Relative month count  Relative month count derived from the relative day count; this is not a calendar month.    01_primary_data/public/esld_master_long_public.csv   File view   
  dead_sample_flag  Dead-sample flag  Binary indicator identifying public ESLD sample records assigned to the deceased-sample subset used in sample-level analyses.    01_primary_data/public/esld_master_long_public.csv   File view   
  death_within_90d  Death within 90 days  Binary indicator for death within 90 days of the analysis anchor.    01_primary_data/public/esld_master_long_public.csv   File view   
  deceased_patient_flag  Deceased patient flag  Binary indicator identifying deceased patients within the released analysis context.    01_primary_data/public/esld_master_long_public.csv   File view   
  delta_eq_0_flag  Zero score-delta flag  Binary indicator equal to 1 when the score delta is exactly zero.    01_primary_data/public/esld_master_long_public.csv   File view   
  delta_gt_0_flag  Positive score-delta flag  Binary indicator equal to 1 when the score delta is positive.    01_primary_data/public/esld_master_long_public.csv   File view   
  delta_le_minus1_flag  Score-delta ≤ −1 flag  Binary indicator equal to 1 when the score delta is less than or equal to −1.    01_primary_data/public/esld_master_long_public.csv   File view   
  dialysis_raw  Dialysis indicator as recorded before score derivation  Binary indicator showing whether dialysis was recorded in the source field before score derivation.    01_primary_data/public/esld_master_long_public.csv   File view   
  encounter_id_public  Public encounter pseudonym  Non-linkable public-release encounter pseudonym used for encounter-level grouping; not an original hospital encounter identifier.    01_primary_data/public/esld_master_long_public.csv   File view   
  etiology_source_current_flag  Current etiology source flag  Binary indicator identifying whether the etiology source is the current source used for the public row.    01_primary_data/public/esld_master_long_public.csv   File view   
  etiology_unclassified  Unclassified liver disease etiology flag  Binary etiology indicator identifying records without a classified liver disease etiology in the public ESLD cohort.    01_primary_data/public/esld_master_long_public.csv   File view   
  hbv  Hepatitis B virus etiology flag  Binary etiology indicator for hepatitis B virus-related liver disease in the public ESLD cohort.    01_primary_data/public/esld_master_long_public.csv   File view   
  hcv  Hepatitis C virus etiology flag  Binary etiology indicator for hepatitis C virus-related liver disease in the public ESLD cohort.    01_primary_data/public/esld_master_long_public.csv   File view   
  icd_source_present  ICD source present flag  Binary indicator showing whether an ICD-derived etiology source was present for the public row.    01_primary_data/public/esld_master_long_public.csv   File view   
  in_t1_cohort  Inclusion flag for Table 1 cohort  Binary indicator identifying records included in the public ESLD Table 1 baseline-characteristics cohort.    01_primary_data/public/esld_master_long_public.csv   File view   
  in_t3_anchor_model  Inclusion flag for Table 3 anchor-model cohort  Binary indicator identifying records included in the Table 3 anchor-model cohort used for creatinine-comparison analyses.    01_primary_data/public/esld_master_long_public.csv   File view   
  in_t4_prevalence_path  Inclusion flag for Table 4 prevalence pathway  Binary indicator identifying records included in the Table 4 prevalence-analysis pathway.    01_primary_data/public/esld_master_long_public.csv   File view   
  in_t4_survival_path  Inclusion flag for Table 4 survival pathway  Binary indicator identifying records included in the Table 4 survival-analysis pathway.    01_primary_data/public/esld_master_long_public.csv   File view   
  inr  International normalized ratio  International normalized ratio of prothrombin time used for MELD-related score calculation.  ratio  01_primary_data/public/esld_master_long_public.csv   File view   
  mash  Metabolic dysfunction-associated steatohepatitis etiology flag  Binary etiology indicator for metabolic dysfunction-associated steatohepatitis in the public ESLD cohort.    01_primary_data/public/esld_master_long_public.csv   File view   
  model  MELD model or score variant  Name of the MELD-related model or score variant represented by the row; expected values include MELD, MELD-Na, reMELD-Na, and MELD 3.0.    01_primary_data/public/esld_master_long_public.csv   File view   
  other  Other liver disease etiology flag  Binary etiology indicator for liver disease etiologies grouped as other in the public ESLD cohort.    01_primary_data/public/esld_master_long_public.csv   File view   
  patient_id  Public patient pseudonym  Non-linkable public-release patient pseudonym used to preserve within-patient grouping in public ESLD data; not an original hospital patient identifier.    01_primary_data/public/esld_master_long_public.csv   File view   
  pbc  Primary biliary cholangitis etiology flag  Binary etiology indicator for primary biliary cholangitis in the public ESLD cohort.    01_primary_data/public/esld_master_long_public.csv   File view   
  sample_day_from_first_sample  Relative sample day  Relative day of the sample measured from the first sample for the public patient/sample sequence; not a calendar date.  relative days  01_primary_data/public/esld_master_long_public.csv   File view   
  sample_group_id_public  Public sample-group pseudonym  Non-linkable public-release sample-group pseudonym used to group samples within the released public data.    01_primary_data/public/esld_master_long_public.csv   File view   
  sample_id  Sample identifier  Identifier of a sample or experimental record within the released public data; not a personal identifier.    01_primary_data/public/esld_master_long_public.csv   File view   
  sample_month_index  Relative sample month index  Relative month index of the sample measured from the first sample for the public patient/sample sequence; not a calendar month.  relative months  01_primary_data/public/esld_master_long_public.csv   File view   
  score_corrected  Corrected score  MELD-related score after creatinine correction or recalculation.  score points  01_primary_data/public/esld_master_long_public.csv   File view   
  score_delta  Score difference  Difference between score variants or scoring approaches, expressed in score points.    01_primary_data/public/esld_master_long_public.csv   File view   
  score_original  Original score  Original MELD-related score before creatinine correction or recalculation.    01_primary_data/public/esld_master_long_public.csv   File view   
  sex  Sex  Sex category represented in the public ESLD data.    01_primary_data/public/esld_master_long_public.csv   File view   
  sodium  Serum sodium concentration  Serum sodium concentration used for MELD-Na, reMELD-Na, or MELD 3.0 score calculation.  mmol/L  01_primary_data/public/esld_master_long_public.csv   File view   
  tbil  Total bilirubin concentration  Total bilirubin concentration used in MELD-related score calculation in the public ESLD data.    01_primary_data/public/esld_master_long_public.csv   File view   
  parameter  Metadata parameter name  Name of a metadata parameter describing the F2 simulated heatmap object, such as figure identity, data origin, grid type, axis variable, or unit/role.    01_primary_data/public/f1_simulated_surface_metadata.csv   File view   
  value  Value  Numerical or character value corresponding to the row-specific variable/metric.    01_primary_data/public/f1_simulated_surface_metadata.csv   File view   
  cre_true_mg_dL  True creatinine concentration  True creatinine concentration used as the gravimetric/reference concentration in F1.    01_primary_data/public/f1_simulated_surface_repository.csv   File view   
  cree_measured_pred_mg_dL  Predicted measured enzymatic creatinine concentration  Predicted measured enzymatic creatinine concentration derived from the interference model.    01_primary_data/public/f1_simulated_surface_repository.csv   File view   
  cree_true_recalc_error  Recalculation error for enzymatic creatinine  Numerical difference between recalculated and expected true enzymatic creatinine values.    01_primary_data/public/f1_simulated_surface_repository.csv   File view   
  cree_true_recalc_mg_dL  Recalculated true enzymatic creatinine concentration  True enzymatic creatinine concentration recalculated from the model.    01_primary_data/public/f1_simulated_surface_repository.csv   File view   
  crej_measured_pred_mg_dL  Predicted measured Jaffe creatinine concentration  Predicted measured Jaffe creatinine concentration derived from the interference model.    01_primary_data/public/f1_simulated_surface_repository.csv   File view   
  crej_true_recalc_error  Recalculation error for Jaffe creatinine  Numerical difference between recalculated and expected true Jaffe creatinine values.    01_primary_data/public/f1_simulated_surface_repository.csv   File view   
  crej_true_recalc_mg_dL  Recalculated true Jaffe creatinine concentration  True Jaffe creatinine concentration recalculated from the model.    01_primary_data/public/f1_simulated_surface_repository.csv   File view   
  delta_cree_to_gravimetry_mg_dL  Enzymatic creatinine deviation from gravimetry  Difference between enzymatic creatinine and the gravimetric/reference value.    01_primary_data/public/f1_simulated_surface_repository.csv   File view   
  delta_crej_to_gravimetry_mg_dL  Jaffe creatinine deviation from gravimetry  Difference between Jaffe creatinine and the gravimetric/reference value.    01_primary_data/public/f1_simulated_surface_repository.csv   File view   
  grid_id  Surface-grid identifier  Identifier of a grid point in the F1 simulated or reconstructed surface data.    01_primary_data/public/f1_simulated_surface_repository.csv   File view   
  tb_mg_dL  Total bilirubin concentration  Total bilirubin concentration used in the creatinine/bilirubin interference model or figure data.    01_primary_data/public/f1_simulated_surface_repository.csv   File view   
  Cre_nominal_grav_mg_dL  Nominal gravimetric creatinine target concentration  Nominal gravimetric target concentration of creatinine used when defining the F1 experimental array.  mg/dL  01_primary_data/public/f1_tb_cre_experimental_array_raw.csv   File view   
  TB_nominal_grav_mg_dL  Nominal gravimetric total bilirubin target concentration  Nominal gravimetric target concentration of total bilirubin used when defining the F1 experimental array.  mg/dL  01_primary_data/public/f1_tb_cre_experimental_array_raw.csv   File view   
  TB_trial_M_mg_dL  Measured total bilirubin concentration in the experimental dataset  Measured total bilirubin concentration in the F1 experimental dataset. In rounded input tables this is the rounded/display value used for model calculation and plotting.  mg/dL  01_primary_data/public/f1_tb_cre_experimental_array_raw.csv   File view   
  array_id  Experimental array identifier  Identifier of the experimental array used in the F1 creatinine/bilirubin interference data.    01_primary_data/public/f1_tb_cre_experimental_array_raw.csv   File view   
  assay  Creatinine assay  Creatinine assay represented by the row, for example enzymatic creatinine (CreE) or Jaffe creatinine (CreJ).    01_primary_data/public/f1_tb_cre_experimental_array_raw.csv   File view   
  plot_correction_mg_dL  Plotted creatinine correction  Creatinine correction value plotted in F1, expressed in mg/dL as the difference between corrected/reference and measured creatinine.  mg/dL  01_primary_data/public/f1_tb_cre_experimental_array_raw.csv   File view   
  preparer_id  Experimental preparer identifier  Identifier of the experimental preparer in F1 raw/validation data, represented as a public technical code.    01_primary_data/public/f1_tb_cre_experimental_array_raw.csv   File view   
  replicate  Experimental replicate number  Replicate number within the F1 experimental dataset.    01_primary_data/public/f1_tb_cre_experimental_array_raw.csv   File view   
  trial_display_Cre_M_mg_dL  Measured creatinine concentration in the experimental dataset  Measured creatinine concentration in the F1 experimental dataset. In rounded input tables this is the rounded/display value used for model calculation and plotting.  mg/dL  01_primary_data/public/f1_tb_cre_experimental_array_raw.csv   File view   
  cre_true_gcidms_mg_dL  GC-IDMS reference creatinine concentration  Creatinine concentration measured by GC-IDMS reference method in the validation data.    01_primary_data/public/f1_tb_cre_experimental_validation_raw.csv   File view   
  cree_corrected_mg_dL  Corrected enzymatic creatinine concentration  Creatinine concentration after applying the enzymatic correction model.    01_primary_data/public/f1_tb_cre_experimental_validation_raw.csv   File view   
  cree_measured_mg_dL  Measured enzymatic creatinine concentration  Measured creatinine concentration using the enzymatic assay.    01_primary_data/public/f1_tb_cre_experimental_validation_raw.csv   File view   
  crej_corrected_mg_dL  Corrected Jaffe creatinine concentration  Creatinine concentration after applying the Jaffe correction model.    01_primary_data/public/f1_tb_cre_experimental_validation_raw.csv   File view   
  crej_measured_mg_dL  Measured Jaffe creatinine concentration  Measured creatinine concentration using the Jaffe assay.    01_primary_data/public/f1_tb_cre_experimental_validation_raw.csv   File view   
  db_measured_mg_dL  Direct bilirubin concentration  Direct bilirubin concentration measured in the experimental or validation data.    01_primary_data/public/f1_tb_cre_experimental_validation_raw.csv   File view   
  sample_id  Sample identifier  Identifier of a sample or experimental record within the released public data; not a personal identifier.    01_primary_data/public/f1_tb_cre_experimental_validation_raw.csv   File view   
  tb_measured_mg_dL  Measured total bilirubin concentration  Measured total bilirubin concentration in the experimental F1 data.    01_primary_data/public/f1_tb_cre_experimental_validation_raw.csv   File view   
  parameter  Metadata parameter name  Name of a metadata parameter describing the F2 simulated heatmap object, such as figure identity, data origin, grid type, axis variable, or unit/role.    01_primary_data/public/f2_simulated_heatmap_metadata.csv   File view   
  value  Value  Numerical or character value corresponding to the row-specific variable/metric.    01_primary_data/public/f2_simulated_heatmap_metadata.csv   File view   
  count_negative  Number of simulated subpoints with negative score shift  Number of simulated subpoints within the F2 heatmap bin where the score shift is negative, defined as score delta ≤ −1.  count  01_primary_data/public/f2_simulated_heatmap_repository.csv   File view   
  model  MELD model or score variant  Name of the MELD-related model or score variant represented by the row; expected values include MELD, MELD-Na, reMELD-Na, and MELD 3.0.    01_primary_data/public/f2_simulated_heatmap_repository.csv   File view   
  n_sub  Number of simulated subpoints per heatmap bin  Number of simulated subpoints aggregated within one F2 heatmap bin.  count  01_primary_data/public/f2_simulated_heatmap_repository.csv   File view   
  pct_negative  Percentage of simulated subpoints with negative score shift  Percentage of simulated subpoints within the F2 heatmap bin where the score shift is negative, defined as score delta ≤ −1.  %  01_primary_data/public/f2_simulated_heatmap_repository.csv   File view   
  x_value  Heatmap x-axis creatinine value  Creatinine value defining the x-axis coordinate of an F2 heatmap bin.  mg/dL  01_primary_data/public/f2_simulated_heatmap_repository.csv   File view   
  y_value  Heatmap y-axis total bilirubin value  Total bilirubin value defining the y-axis coordinate of an F2 heatmap bin.  mg/dL  01_primary_data/public/f2_simulated_heatmap_repository.csv   File view   
  Cre_nominal_grav_mg_dL  Nominal gravimetric creatinine target concentration  Nominal gravimetric target concentration of creatinine used when defining the F1 experimental array.  mg/dL  02_workflows/F1_workflow_v02/data/01_source_loaded_harmonized/expm_F1_array_raw_public.csv    
  TB_nominal_grav_mg_dL  Nominal gravimetric total bilirubin target concentration  Nominal gravimetric target concentration of total bilirubin used when defining the F1 experimental array.  mg/dL  02_workflows/F1_workflow_v02/data/01_source_loaded_harmonized/expm_F1_array_raw_public.csv    
  TB_trial_M_mg_dL  Measured total bilirubin concentration in the experimental dataset  Measured total bilirubin concentration in the F1 experimental dataset. In rounded input tables this is the rounded/display value used for model calculation and plotting.  mg/dL  02_workflows/F1_workflow_v02/data/01_source_loaded_harmonized/expm_F1_array_raw_public.csv    
  array_id  Experimental array identifier  Identifier of the experimental array used in the F1 creatinine/bilirubin interference data.    02_workflows/F1_workflow_v02/data/01_source_loaded_harmonized/expm_F1_array_raw_public.csv    
  assay  Creatinine assay  Creatinine assay represented by the row, for example enzymatic creatinine (CreE) or Jaffe creatinine (CreJ).    02_workflows/F1_workflow_v02/data/01_source_loaded_harmonized/expm_F1_array_raw_public.csv    
  plot_correction_mg_dL  Plotted creatinine correction  Creatinine correction value plotted in F1, expressed in mg/dL as the difference between corrected/reference and measured creatinine.  mg/dL  02_workflows/F1_workflow_v02/data/01_source_loaded_harmonized/expm_F1_array_raw_public.csv    
  preparer_id  Experimental preparer identifier  Identifier of the experimental preparer in F1 raw/validation data, represented as a public technical code.    02_workflows/F1_workflow_v02/data/01_source_loaded_harmonized/expm_F1_array_raw_public.csv    
  replicate  Experimental replicate number  Replicate number within the F1 experimental dataset.    02_workflows/F1_workflow_v02/data/01_source_loaded_harmonized/expm_F1_array_raw_public.csv    
  trial_display_Cre_M_mg_dL  Measured creatinine concentration in the experimental dataset  Measured creatinine concentration in the F1 experimental dataset. In rounded input tables this is the rounded/display value used for model calculation and plotting.  mg/dL  02_workflows/F1_workflow_v02/data/01_source_loaded_harmonized/expm_F1_array_raw_public.csv    
  cre_true_gcidms_mg_dL  GC-IDMS reference creatinine concentration  Creatinine concentration measured by GC-IDMS reference method in the validation data.    02_workflows/F1_workflow_v02/data/01_source_loaded_harmonized/expm_F1_validation_raw_public.csv    
  cree_corrected_mg_dL  Corrected enzymatic creatinine concentration  Creatinine concentration after applying the enzymatic correction model.    02_workflows/F1_workflow_v02/data/01_source_loaded_harmonized/expm_F1_validation_raw_public.csv    
  cree_measured_mg_dL  Measured enzymatic creatinine concentration  Measured creatinine concentration using the enzymatic assay.    02_workflows/F1_workflow_v02/data/01_source_loaded_harmonized/expm_F1_validation_raw_public.csv    
  crej_corrected_mg_dL  Corrected Jaffe creatinine concentration  Creatinine concentration after applying the Jaffe correction model.    02_workflows/F1_workflow_v02/data/01_source_loaded_harmonized/expm_F1_validation_raw_public.csv    
  crej_measured_mg_dL  Measured Jaffe creatinine concentration  Measured creatinine concentration using the Jaffe assay.    02_workflows/F1_workflow_v02/data/01_source_loaded_harmonized/expm_F1_validation_raw_public.csv    
  db_measured_mg_dL  Direct bilirubin concentration  Direct bilirubin concentration measured in the experimental or validation data.    02_workflows/F1_workflow_v02/data/01_source_loaded_harmonized/expm_F1_validation_raw_public.csv    
  sample_id  Sample identifier  Identifier of a sample or experimental record within the released public data; not a personal identifier.    02_workflows/F1_workflow_v02/data/01_source_loaded_harmonized/expm_F1_validation_raw_public.csv    
  tb_measured_mg_dL  Measured total bilirubin concentration  Measured total bilirubin concentration in the experimental F1 data.    02_workflows/F1_workflow_v02/data/01_source_loaded_harmonized/expm_F1_validation_raw_public.csv    
  file_key  Repository file key  Stable repository key identifying a file in a source/load manifest.    02_workflows/F1_workflow_v02/data/01_source_loaded_harmonized/expm_slco_F1_source_load_manifest_public.csv    
  harmonized_loaded_file_name  Loaded harmonized file name  File name of the harmonized public file loaded by the workflow.    02_workflows/F1_workflow_v02/data/01_source_loaded_harmonized/expm_slco_F1_source_load_manifest_public.csv    
  md5  MD5 checksum  MD5 checksum of the referenced file, used for file-integrity checks.  checksum  02_workflows/F1_workflow_v02/data/01_source_loaded_harmonized/expm_slco_F1_source_load_manifest_public.csv    
  n_columns  Number of columns  Number of columns in the referenced public data object.  count  02_workflows/F1_workflow_v02/data/01_source_loaded_harmonized/expm_slco_F1_source_load_manifest_public.csv    
  n_rows  Number of rows  Number of rows in the referenced public data object.  count  02_workflows/F1_workflow_v02/data/01_source_loaded_harmonized/expm_slco_F1_source_load_manifest_public.csv    
  output_exists  Output file existence flag  Logical indicator showing whether the rendered output file exists.    02_workflows/F1_workflow_v02/data/01_source_loaded_harmonized/expm_slco_F1_source_load_manifest_public.csv    
  release_status  Release status  Release-status label indicating the publication status of the row or file object.    02_workflows/F1_workflow_v02/data/01_source_loaded_harmonized/expm_slco_F1_source_load_manifest_public.csv    
  source_path  Source path  Source path recorded by the workflow. For public release this should be package-relative or sanitized if the original value was an absolute local path.    02_workflows/F1_workflow_v02/data/01_source_loaded_harmonized/expm_slco_F1_source_load_manifest_public.csv    
  source_role  Source role  Role of the source file or object in the public workflow.    02_workflows/F1_workflow_v02/data/01_source_loaded_harmonized/expm_slco_F1_source_load_manifest_public.csv    
  parameter  Metadata parameter name  Name of a metadata parameter describing the F2 simulated heatmap object, such as figure identity, data origin, grid type, axis variable, or unit/role.    02_workflows/F1_workflow_v02/data/01_source_loaded_harmonized/expm_slco_F1_step1_run_inputs_public.csv    
  value  Value  Numerical or character value corresponding to the row-specific variable/metric.    02_workflows/F1_workflow_v02/data/01_source_loaded_harmonized/expm_slco_F1_step1_run_inputs_public.csv    
  cre_true_mg_dL  True creatinine concentration  True creatinine concentration used as the gravimetric/reference concentration in F1.    02_workflows/F1_workflow_v02/data/01_source_loaded_harmonized/slco_F1_surface_grid_reference_public.csv    
  cree_measured_pred_mg_dL  Predicted measured enzymatic creatinine concentration  Predicted measured enzymatic creatinine concentration derived from the interference model.    02_workflows/F1_workflow_v02/data/01_source_loaded_harmonized/slco_F1_surface_grid_reference_public.csv    
  cree_true_recalc_error  Recalculation error for enzymatic creatinine  Numerical difference between recalculated and expected true enzymatic creatinine values.    02_workflows/F1_workflow_v02/data/01_source_loaded_harmonized/slco_F1_surface_grid_reference_public.csv    
  cree_true_recalc_mg_dL  Recalculated true enzymatic creatinine concentration  True enzymatic creatinine concentration recalculated from the model.    02_workflows/F1_workflow_v02/data/01_source_loaded_harmonized/slco_F1_surface_grid_reference_public.csv    
  crej_measured_pred_mg_dL  Predicted measured Jaffe creatinine concentration  Predicted measured Jaffe creatinine concentration derived from the interference model.    02_workflows/F1_workflow_v02/data/01_source_loaded_harmonized/slco_F1_surface_grid_reference_public.csv    
  crej_true_recalc_error  Recalculation error for Jaffe creatinine  Numerical difference between recalculated and expected true Jaffe creatinine values.    02_workflows/F1_workflow_v02/data/01_source_loaded_harmonized/slco_F1_surface_grid_reference_public.csv    
  crej_true_recalc_mg_dL  Recalculated true Jaffe creatinine concentration  True Jaffe creatinine concentration recalculated from the model.    02_workflows/F1_workflow_v02/data/01_source_loaded_harmonized/slco_F1_surface_grid_reference_public.csv    
  delta_cree_to_gravimetry_mg_dL  Enzymatic creatinine deviation from gravimetry  Difference between enzymatic creatinine and the gravimetric/reference value.    02_workflows/F1_workflow_v02/data/01_source_loaded_harmonized/slco_F1_surface_grid_reference_public.csv    
  delta_crej_to_gravimetry_mg_dL  Jaffe creatinine deviation from gravimetry  Difference between Jaffe creatinine and the gravimetric/reference value.    02_workflows/F1_workflow_v02/data/01_source_loaded_harmonized/slco_F1_surface_grid_reference_public.csv    
  grid_id  Surface-grid identifier  Identifier of a grid point in the F1 simulated or reconstructed surface data.    02_workflows/F1_workflow_v02/data/01_source_loaded_harmonized/slco_F1_surface_grid_reference_public.csv    
  tb_mg_dL  Total bilirubin concentration  Total bilirubin concentration used in the creatinine/bilirubin interference model or figure data.    02_workflows/F1_workflow_v02/data/01_source_loaded_harmonized/slco_F1_surface_grid_reference_public.csv    
  parameter  Metadata parameter name  Name of a metadata parameter describing the F2 simulated heatmap object, such as figure identity, data origin, grid type, axis variable, or unit/role.    02_workflows/F1_workflow_v02/data/01_source_loaded_harmonized/slco_F1_surface_meta_reference_public.csv    
  value  Value  Numerical or character value corresponding to the row-specific variable/metric.    02_workflows/F1_workflow_v02/data/01_source_loaded_harmonized/slco_F1_surface_meta_reference_public.csv    
  Cre_nominal_grav_mg_dL  Nominal gravimetric creatinine target concentration  Nominal gravimetric target concentration of creatinine used when defining the F1 experimental array.  mg/dL  02_workflows/F1_workflow_v02/data/02a_refined_analysis/expm_F1_array_input_public.csv    
  TB_nominal_grav_mg_dL  Nominal gravimetric total bilirubin target concentration  Nominal gravimetric target concentration of total bilirubin used when defining the F1 experimental array.  mg/dL  02_workflows/F1_workflow_v02/data/02a_refined_analysis/expm_F1_array_input_public.csv    
  TB_trial_M_mg_dL  Measured total bilirubin concentration in the experimental dataset  Measured total bilirubin concentration in the F1 experimental dataset. In rounded input tables this is the rounded/display value used for model calculation and plotting.  mg/dL  02_workflows/F1_workflow_v02/data/02a_refined_analysis/expm_F1_array_input_public.csv    
  TB_trial_M_mg_dL_unrounded  Unrounded measured total bilirubin concentration in the experimental dataset  Measured total bilirubin concentration in the F1 experimental dataset before rounding to the display/model-input precision.  mg/dL  02_workflows/F1_workflow_v02/data/02a_refined_analysis/expm_F1_array_input_public.csv    
  anchor  Anchor/output group  Anchor or output grouping label used by the workflow to identify a specific public output component.    02_workflows/F1_workflow_v02/data/02a_refined_analysis/expm_F1_array_input_public.csv    
  array_id  Experimental array identifier  Identifier of the experimental array used in the F1 creatinine/bilirubin interference data.    02_workflows/F1_workflow_v02/data/02a_refined_analysis/expm_F1_array_input_public.csv    
  assay  Creatinine assay  Creatinine assay represented by the row, for example enzymatic creatinine (CreE) or Jaffe creatinine (CreJ).    02_workflows/F1_workflow_v02/data/02a_refined_analysis/expm_F1_array_input_public.csv    
  data_object  Data object  Name of the data object represented by the row.    02_workflows/F1_workflow_v02/data/02a_refined_analysis/expm_F1_array_input_public.csv    
  domain  Data domain  Workflow or data domain represented by the row.    02_workflows/F1_workflow_v02/data/02a_refined_analysis/expm_F1_array_input_public.csv    
  expm_F1_array_row_id  Experimental array row identifier  Row identifier within the released F1 experimental array table.    02_workflows/F1_workflow_v02/data/02a_refined_analysis/expm_F1_array_input_public.csv    
  plot_correction_mg_dL  Plotted creatinine correction  Creatinine correction value plotted in F1, expressed in mg/dL as the difference between corrected/reference and measured creatinine.  mg/dL  02_workflows/F1_workflow_v02/data/02a_refined_analysis/expm_F1_array_input_public.csv    
  preparer_id  Experimental preparer identifier  Identifier of the experimental preparer in F1 raw/validation data, represented as a public technical code.    02_workflows/F1_workflow_v02/data/02a_refined_analysis/expm_F1_array_input_public.csv    
  release_status  Release status  Release-status label indicating the publication status of the row or file object.    02_workflows/F1_workflow_v02/data/02a_refined_analysis/expm_F1_array_input_public.csv    
  replicate  Experimental replicate number  Replicate number within the F1 experimental dataset.    02_workflows/F1_workflow_v02/data/02a_refined_analysis/expm_F1_array_input_public.csv    
  source_harmonized_file_name  Source harmonized file name  Name of the harmonized source file used to build the released object.    02_workflows/F1_workflow_v02/data/02a_refined_analysis/expm_F1_array_input_public.csv    
  trial_display_Cre_M_mg_dL  Measured creatinine concentration in the experimental dataset  Measured creatinine concentration in the F1 experimental dataset. In rounded input tables this is the rounded/display value used for model calculation and plotting.  mg/dL  02_workflows/F1_workflow_v02/data/02a_refined_analysis/expm_F1_array_input_public.csv    
  trial_display_Cre_M_mg_dL_unrounded  Unrounded measured creatinine concentration in the experimental dataset  Measured creatinine concentration in the F1 experimental dataset before rounding to the display/model-input precision.  mg/dL  02_workflows/F1_workflow_v02/data/02a_refined_analysis/expm_F1_array_input_public.csv    
  unit_or_role  Unit or semantic role  Unit, role, or semantic type corresponding to the row-specific variable/metric.    02_workflows/F1_workflow_v02/data/02a_refined_analysis/expm_F1_array_input_public.csv    
  workflow_step  Workflow step  Workflow step that produced or used the row/object.    02_workflows/F1_workflow_v02/data/02a_refined_analysis/expm_F1_array_input_public.csv    
  assay  Creatinine assay  Creatinine assay represented by the row, for example enzymatic creatinine (CreE) or Jaffe creatinine (CreJ).    02_workflows/F1_workflow_v02/data/02a_refined_analysis/expm_F1_model_coefficients_public.csv    
  coefficient  Model coefficient  Fixed coefficient used in the F1 creatinine/bilirubin correction polynomial.  model coefficient  02_workflows/F1_workflow_v02/data/02a_refined_analysis/expm_F1_model_coefficients_public.csv    
  equation_role  Equation role  Role of the equation represented by the F1 model-coefficient row.    02_workflows/F1_workflow_v02/data/02a_refined_analysis/expm_F1_model_coefficients_public.csv    
  release_status  Release status  Release-status label indicating the publication status of the row or file object.    02_workflows/F1_workflow_v02/data/02a_refined_analysis/expm_F1_model_coefficients_public.csv    
  term  Polynomial model term  Term name in the fixed F1 correction polynomial, for example intercept, bilirubin term, squared bilirubin term, creatinine term, or squared creatinine term.    02_workflows/F1_workflow_v02/data/02a_refined_analysis/expm_F1_model_coefficients_public.csv    
  workflow_step  Workflow step  Workflow step that produced or used the row/object.    02_workflows/F1_workflow_v02/data/02a_refined_analysis/expm_F1_model_coefficients_public.csv    
  max_abs_rounding_difference  Maximum absolute rounding difference  Maximum absolute difference caused by rounding.    02_workflows/F1_workflow_v02/data/02a_refined_analysis/expm_F1_pre_correction_rounding_qc_public.csv    
  max_value_after_rounding  Maximum value after rounding  Maximum value observed after rounding.    02_workflows/F1_workflow_v02/data/02a_refined_analysis/expm_F1_pre_correction_rounding_qc_public.csv    
  mean_abs_rounding_difference  Mean absolute rounding difference  Mean absolute difference caused by rounding.    02_workflows/F1_workflow_v02/data/02a_refined_analysis/expm_F1_pre_correction_rounding_qc_public.csv    
  min_value_after_rounding  Minimum value after rounding  Minimum value observed after rounding.    02_workflows/F1_workflow_v02/data/02a_refined_analysis/expm_F1_pre_correction_rounding_qc_public.csv    
  n_total_values  Total number of values  Total number of values evaluated.    02_workflows/F1_workflow_v02/data/02a_refined_analysis/expm_F1_pre_correction_rounding_qc_public.csv    
  n_values_changed_by_rounding  Number of values changed by rounding  Number of values whose value changed after rounding.    02_workflows/F1_workflow_v02/data/02a_refined_analysis/expm_F1_pre_correction_rounding_qc_public.csv    
  rounding_rule  Rounding rule  Rounding rule applied before correction/model calculation.    02_workflows/F1_workflow_v02/data/02a_refined_analysis/expm_F1_pre_correction_rounding_qc_public.csv    
  variable  Variable represented by row  Name of the variable represented by the row in a long-format table.    02_workflows/F1_workflow_v02/data/02a_refined_analysis/expm_F1_pre_correction_rounding_qc_public.csv    
  metric  Metric  Name of the metric represented by the row.    02_workflows/F1_workflow_v02/data/02a_refined_analysis/expm_F1_refined_dataset_qc_public.csv    
  value  Value  Numerical or character value corresponding to the row-specific variable/metric.    02_workflows/F1_workflow_v02/data/02a_refined_analysis/expm_F1_refined_dataset_qc_public.csv    
  issue  QC or audit issue  Free-text QC or audit note describing a checked issue.    02_workflows/F1_workflow_v02/data/02a_refined_analysis/expm_F1_refined_manual_clarifications_public.csv    
  item  Audit item  Name of the QC or audit item being reported.    02_workflows/F1_workflow_v02/data/02a_refined_analysis/expm_F1_refined_manual_clarifications_public.csv    
  suggested_manual_check  Suggested manual check  Free-text recommendation for a manual QC check made during the public workflow.  mg/dL  02_workflows/F1_workflow_v02/data/02a_refined_analysis/expm_F1_refined_manual_clarifications_public.csv    
  anchor  Anchor/output group  Anchor or output grouping label used by the workflow to identify a specific public output component.    02_workflows/F1_workflow_v02/data/02a_refined_analysis/expm_F1_validation_input_public.csv    
  cre_true_gcidms_mg_dL  GC-IDMS reference creatinine concentration  Creatinine concentration measured by GC-IDMS reference method in the validation data.    02_workflows/F1_workflow_v02/data/02a_refined_analysis/expm_F1_validation_input_public.csv    
  cree_corrected_mg_dL  Corrected enzymatic creatinine concentration  Creatinine concentration after applying the enzymatic correction model.    02_workflows/F1_workflow_v02/data/02a_refined_analysis/expm_F1_validation_input_public.csv    
  cree_measured_mg_dL  Measured enzymatic creatinine concentration  Measured creatinine concentration using the enzymatic assay.    02_workflows/F1_workflow_v02/data/02a_refined_analysis/expm_F1_validation_input_public.csv    
  crej_corrected_mg_dL  Corrected Jaffe creatinine concentration  Creatinine concentration after applying the Jaffe correction model.    02_workflows/F1_workflow_v02/data/02a_refined_analysis/expm_F1_validation_input_public.csv    
  crej_measured_mg_dL  Measured Jaffe creatinine concentration  Measured creatinine concentration using the Jaffe assay.    02_workflows/F1_workflow_v02/data/02a_refined_analysis/expm_F1_validation_input_public.csv    
  data_object  Data object  Name of the data object represented by the row.    02_workflows/F1_workflow_v02/data/02a_refined_analysis/expm_F1_validation_input_public.csv    
  db_measured_mg_dL  Direct bilirubin concentration  Direct bilirubin concentration measured in the experimental or validation data.    02_workflows/F1_workflow_v02/data/02a_refined_analysis/expm_F1_validation_input_public.csv    
  domain  Data domain  Workflow or data domain represented by the row.    02_workflows/F1_workflow_v02/data/02a_refined_analysis/expm_F1_validation_input_public.csv    
  expm_F1_validation_row_id  Experimental validation row identifier  Row identifier within the released F1 experimental validation table.    02_workflows/F1_workflow_v02/data/02a_refined_analysis/expm_F1_validation_input_public.csv    
  release_status  Release status  Release-status label indicating the publication status of the row or file object.    02_workflows/F1_workflow_v02/data/02a_refined_analysis/expm_F1_validation_input_public.csv    
  sample_id  Sample identifier  Identifier of a sample or experimental record within the released public data; not a personal identifier.    02_workflows/F1_workflow_v02/data/02a_refined_analysis/expm_F1_validation_input_public.csv    
  source_harmonized_file_name  Source harmonized file name  Name of the harmonized source file used to build the released object.    02_workflows/F1_workflow_v02/data/02a_refined_analysis/expm_F1_validation_input_public.csv    
  tb_measured_mg_dL  Measured total bilirubin concentration  Measured total bilirubin concentration in the experimental F1 data.    02_workflows/F1_workflow_v02/data/02a_refined_analysis/expm_F1_validation_input_public.csv    
  unit_or_role  Unit or semantic role  Unit, role, or semantic type corresponding to the row-specific variable/metric.    02_workflows/F1_workflow_v02/data/02a_refined_analysis/expm_F1_validation_input_public.csv    
  workflow_step  Workflow step  Workflow step that produced or used the row/object.    02_workflows/F1_workflow_v02/data/02a_refined_analysis/expm_F1_validation_input_public.csv    
  cre_true_mg_dL  True creatinine concentration  True creatinine concentration used as the gravimetric/reference concentration in F1.    02_workflows/F1_workflow_v02/data/02b_figure_content/slco_F1_surface_grid_public.csv    
  cree_measured_pred_mg_dL  Predicted measured enzymatic creatinine concentration  Predicted measured enzymatic creatinine concentration derived from the interference model.    02_workflows/F1_workflow_v02/data/02b_figure_content/slco_F1_surface_grid_public.csv    
  cree_true_recalc_error  Recalculation error for enzymatic creatinine  Numerical difference between recalculated and expected true enzymatic creatinine values.    02_workflows/F1_workflow_v02/data/02b_figure_content/slco_F1_surface_grid_public.csv    
  cree_true_recalc_mg_dL  Recalculated true enzymatic creatinine concentration  True enzymatic creatinine concentration recalculated from the model.    02_workflows/F1_workflow_v02/data/02b_figure_content/slco_F1_surface_grid_public.csv    
  crej_measured_pred_mg_dL  Predicted measured Jaffe creatinine concentration  Predicted measured Jaffe creatinine concentration derived from the interference model.    02_workflows/F1_workflow_v02/data/02b_figure_content/slco_F1_surface_grid_public.csv    
  crej_true_recalc_error  Recalculation error for Jaffe creatinine  Numerical difference between recalculated and expected true Jaffe creatinine values.    02_workflows/F1_workflow_v02/data/02b_figure_content/slco_F1_surface_grid_public.csv    
  crej_true_recalc_mg_dL  Recalculated true Jaffe creatinine concentration  True Jaffe creatinine concentration recalculated from the model.    02_workflows/F1_workflow_v02/data/02b_figure_content/slco_F1_surface_grid_public.csv    
  delta_cree_to_gravimetry_mg_dL  Enzymatic creatinine deviation from gravimetry  Difference between enzymatic creatinine and the gravimetric/reference value.    02_workflows/F1_workflow_v02/data/02b_figure_content/slco_F1_surface_grid_public.csv    
  delta_crej_to_gravimetry_mg_dL  Jaffe creatinine deviation from gravimetry  Difference between Jaffe creatinine and the gravimetric/reference value.    02_workflows/F1_workflow_v02/data/02b_figure_content/slco_F1_surface_grid_public.csv    
  grid_id  Surface-grid identifier  Identifier of a grid point in the F1 simulated or reconstructed surface data.    02_workflows/F1_workflow_v02/data/02b_figure_content/slco_F1_surface_grid_public.csv    
  tb_mg_dL  Total bilirubin concentration  Total bilirubin concentration used in the creatinine/bilirubin interference model or figure data.    02_workflows/F1_workflow_v02/data/02b_figure_content/slco_F1_surface_grid_public.csv    
  parameter  Metadata parameter name  Name of a metadata parameter describing the F2 simulated heatmap object, such as figure identity, data origin, grid type, axis variable, or unit/role.    02_workflows/F1_workflow_v02/data/02b_figure_content/slco_F1_surface_meta_public.csv    
  value  Value  Numerical or character value corresponding to the row-specific variable/metric.    02_workflows/F1_workflow_v02/data/02b_figure_content/slco_F1_surface_meta_public.csv    
  max_abs_difference  Maximum absolute difference  Maximum absolute difference between compared values.    02_workflows/F1_workflow_v02/data/02b_figure_content/slco_F1_surface_rebuild_qc_public.csv    
  mean_abs_difference  Mean absolute difference  Mean absolute difference between compared values.    02_workflows/F1_workflow_v02/data/02b_figure_content/slco_F1_surface_rebuild_qc_public.csv    
  median_abs_difference  Median absolute difference  Median absolute difference between compared values.    02_workflows/F1_workflow_v02/data/02b_figure_content/slco_F1_surface_rebuild_qc_public.csv    
  n_compared  Number of compared values  Number of values included in the comparison.    02_workflows/F1_workflow_v02/data/02b_figure_content/slco_F1_surface_rebuild_qc_public.csv    
  variable  Variable represented by row  Name of the variable represented by the row in a long-format table.    02_workflows/F1_workflow_v02/data/02b_figure_content/slco_F1_surface_rebuild_qc_public.csv    
  metadata_source  Metadata source file  Name of the metadata file used as the source for the row or object.    02_workflows/F1_workflow_v02/data/02b_figure_content/slco_F1_surface_reference_meta_original_public.csv    
  parameter  Metadata parameter name  Name of a metadata parameter describing the F2 simulated heatmap object, such as figure identity, data origin, grid type, axis variable, or unit/role.    02_workflows/F1_workflow_v02/data/02b_figure_content/slco_F1_surface_reference_meta_original_public.csv    
  value  Value  Numerical or character value corresponding to the row-specific variable/metric.    02_workflows/F1_workflow_v02/data/02b_figure_content/slco_F1_surface_reference_meta_original_public.csv    
  exists  File/object existence flag  Logical indicator showing whether the referenced file or workflow object exists.    02_workflows/F1_workflow_v02/data/02b_figure_content/slco_F1_surface_run_inputs_outputs_public.csv    
  item  Audit item  Name of the QC or audit item being reported.    02_workflows/F1_workflow_v02/data/02b_figure_content/slco_F1_surface_run_inputs_outputs_public.csv    
  path  Workflow path  Workflow path recorded in a manifest or audit table. For public release this should refer to a package-relative or otherwise non-sensitive path.  %  02_workflows/F1_workflow_v02/data/02b_figure_content/slco_F1_surface_run_inputs_outputs_public.csv    
  Cre_nominal_grav_mg_dL  Nominal gravimetric creatinine target concentration  Nominal gravimetric target concentration of creatinine used when defining the F1 experimental array.  mg/dL  02_workflows/F1_workflow_v02/submission_ready/public/data/expm_F1_array_input_public.csv   File view   
  TB_nominal_grav_mg_dL  Nominal gravimetric total bilirubin target concentration  Nominal gravimetric target concentration of total bilirubin used when defining the F1 experimental array.  mg/dL  02_workflows/F1_workflow_v02/submission_ready/public/data/expm_F1_array_input_public.csv   File view   
  TB_trial_M_mg_dL  Measured total bilirubin concentration in the experimental dataset  Measured total bilirubin concentration in the F1 experimental dataset. In rounded input tables this is the rounded/display value used for model calculation and plotting.  mg/dL  02_workflows/F1_workflow_v02/submission_ready/public/data/expm_F1_array_input_public.csv   File view   
  anchor  Anchor/output group  Anchor or output grouping label used by the workflow to identify a specific public output component.    02_workflows/F1_workflow_v02/submission_ready/public/data/expm_F1_array_input_public.csv   File view   
  array_id  Experimental array identifier  Identifier of the experimental array used in the F1 creatinine/bilirubin interference data.    02_workflows/F1_workflow_v02/submission_ready/public/data/expm_F1_array_input_public.csv   File view   
  assay  Creatinine assay  Creatinine assay represented by the row, for example enzymatic creatinine (CreE) or Jaffe creatinine (CreJ).    02_workflows/F1_workflow_v02/submission_ready/public/data/expm_F1_array_input_public.csv   File view   
  data_object  Data object  Name of the data object represented by the row.    02_workflows/F1_workflow_v02/submission_ready/public/data/expm_F1_array_input_public.csv   File view   
  domain  Data domain  Workflow or data domain represented by the row.    02_workflows/F1_workflow_v02/submission_ready/public/data/expm_F1_array_input_public.csv   File view   
  expm_F1_array_row_id  Experimental array row identifier  Row identifier within the released F1 experimental array table.    02_workflows/F1_workflow_v02/submission_ready/public/data/expm_F1_array_input_public.csv   File view   
  plot_correction_mg_dL  Plotted creatinine correction  Creatinine correction value plotted in F1, expressed in mg/dL as the difference between corrected/reference and measured creatinine.  mg/dL  02_workflows/F1_workflow_v02/submission_ready/public/data/expm_F1_array_input_public.csv   File view   
  preparer_id  Experimental preparer identifier  Identifier of the experimental preparer in F1 raw/validation data, represented as a public technical code.    02_workflows/F1_workflow_v02/submission_ready/public/data/expm_F1_array_input_public.csv   File view   
  release_status  Release status  Release-status label indicating the publication status of the row or file object.    02_workflows/F1_workflow_v02/submission_ready/public/data/expm_F1_array_input_public.csv   File view   
  replicate  Experimental replicate number  Replicate number within the F1 experimental dataset.    02_workflows/F1_workflow_v02/submission_ready/public/data/expm_F1_array_input_public.csv   File view   
  source_harmonized_file_name  Source harmonized file name  Name of the harmonized source file used to build the released object.    02_workflows/F1_workflow_v02/submission_ready/public/data/expm_F1_array_input_public.csv   File view   
  trial_display_Cre_M_mg_dL  Measured creatinine concentration in the experimental dataset  Measured creatinine concentration in the F1 experimental dataset. In rounded input tables this is the rounded/display value used for model calculation and plotting.  mg/dL  02_workflows/F1_workflow_v02/submission_ready/public/data/expm_F1_array_input_public.csv   File view   
  unit_or_role  Unit or semantic role  Unit, role, or semantic type corresponding to the row-specific variable/metric.    02_workflows/F1_workflow_v02/submission_ready/public/data/expm_F1_array_input_public.csv   File view   
  workflow_step  Workflow step  Workflow step that produced or used the row/object.    02_workflows/F1_workflow_v02/submission_ready/public/data/expm_F1_array_input_public.csv   File view   
  anchor  Anchor/output group  Anchor or output grouping label used by the workflow to identify a specific public output component.    02_workflows/F1_workflow_v02/submission_ready/public/data/expm_F1_validation_input_public.csv   File view   
  cre_true_gcidms_mg_dL  GC-IDMS reference creatinine concentration  Creatinine concentration measured by GC-IDMS reference method in the validation data.    02_workflows/F1_workflow_v02/submission_ready/public/data/expm_F1_validation_input_public.csv   File view   
  cree_corrected_mg_dL  Corrected enzymatic creatinine concentration  Creatinine concentration after applying the enzymatic correction model.    02_workflows/F1_workflow_v02/submission_ready/public/data/expm_F1_validation_input_public.csv   File view   
  cree_measured_mg_dL  Measured enzymatic creatinine concentration  Measured creatinine concentration using the enzymatic assay.    02_workflows/F1_workflow_v02/submission_ready/public/data/expm_F1_validation_input_public.csv   File view   
  crej_corrected_mg_dL  Corrected Jaffe creatinine concentration  Creatinine concentration after applying the Jaffe correction model.    02_workflows/F1_workflow_v02/submission_ready/public/data/expm_F1_validation_input_public.csv   File view   
  crej_measured_mg_dL  Measured Jaffe creatinine concentration  Measured creatinine concentration using the Jaffe assay.    02_workflows/F1_workflow_v02/submission_ready/public/data/expm_F1_validation_input_public.csv   File view   
  data_object  Data object  Name of the data object represented by the row.    02_workflows/F1_workflow_v02/submission_ready/public/data/expm_F1_validation_input_public.csv   File view   
  db_measured_mg_dL  Direct bilirubin concentration  Direct bilirubin concentration measured in the experimental or validation data.    02_workflows/F1_workflow_v02/submission_ready/public/data/expm_F1_validation_input_public.csv   File view   
  domain  Data domain  Workflow or data domain represented by the row.    02_workflows/F1_workflow_v02/submission_ready/public/data/expm_F1_validation_input_public.csv   File view   
  expm_F1_validation_row_id  Experimental validation row identifier  Row identifier within the released F1 experimental validation table.    02_workflows/F1_workflow_v02/submission_ready/public/data/expm_F1_validation_input_public.csv   File view   
  release_status  Release status  Release-status label indicating the publication status of the row or file object.    02_workflows/F1_workflow_v02/submission_ready/public/data/expm_F1_validation_input_public.csv   File view   
  sample_id  Sample identifier  Identifier of a sample or experimental record within the released public data; not a personal identifier.    02_workflows/F1_workflow_v02/submission_ready/public/data/expm_F1_validation_input_public.csv   File view   
  source_harmonized_file_name  Source harmonized file name  Name of the harmonized source file used to build the released object.    02_workflows/F1_workflow_v02/submission_ready/public/data/expm_F1_validation_input_public.csv   File view   
  tb_measured_mg_dL  Measured total bilirubin concentration  Measured total bilirubin concentration in the experimental F1 data.    02_workflows/F1_workflow_v02/submission_ready/public/data/expm_F1_validation_input_public.csv   File view   
  unit_or_role  Unit or semantic role  Unit, role, or semantic type corresponding to the row-specific variable/metric.    02_workflows/F1_workflow_v02/submission_ready/public/data/expm_F1_validation_input_public.csv   File view   
  workflow_step  Workflow step  Workflow step that produced or used the row/object.    02_workflows/F1_workflow_v02/submission_ready/public/data/expm_F1_validation_input_public.csv   File view   
  cre_true_mg_dL  True creatinine concentration  True creatinine concentration used as the gravimetric/reference concentration in F1.    02_workflows/F1_workflow_v02/submission_ready/public/data/slco_F1_surface_grid_public.csv   File view   
  cree_measured_pred_mg_dL  Predicted measured enzymatic creatinine concentration  Predicted measured enzymatic creatinine concentration derived from the interference model.    02_workflows/F1_workflow_v02/submission_ready/public/data/slco_F1_surface_grid_public.csv   File view   
  cree_true_recalc_error  Recalculation error for enzymatic creatinine  Numerical difference between recalculated and expected true enzymatic creatinine values.    02_workflows/F1_workflow_v02/submission_ready/public/data/slco_F1_surface_grid_public.csv   File view   
  cree_true_recalc_mg_dL  Recalculated true enzymatic creatinine concentration  True enzymatic creatinine concentration recalculated from the model.    02_workflows/F1_workflow_v02/submission_ready/public/data/slco_F1_surface_grid_public.csv   File view   
  crej_measured_pred_mg_dL  Predicted measured Jaffe creatinine concentration  Predicted measured Jaffe creatinine concentration derived from the interference model.    02_workflows/F1_workflow_v02/submission_ready/public/data/slco_F1_surface_grid_public.csv   File view   
  crej_true_recalc_error  Recalculation error for Jaffe creatinine  Numerical difference between recalculated and expected true Jaffe creatinine values.    02_workflows/F1_workflow_v02/submission_ready/public/data/slco_F1_surface_grid_public.csv   File view   
  crej_true_recalc_mg_dL  Recalculated true Jaffe creatinine concentration  True Jaffe creatinine concentration recalculated from the model.    02_workflows/F1_workflow_v02/submission_ready/public/data/slco_F1_surface_grid_public.csv   File view   
  delta_cree_to_gravimetry_mg_dL  Enzymatic creatinine deviation from gravimetry  Difference between enzymatic creatinine and the gravimetric/reference value.    02_workflows/F1_workflow_v02/submission_ready/public/data/slco_F1_surface_grid_public.csv   File view   
  delta_crej_to_gravimetry_mg_dL  Jaffe creatinine deviation from gravimetry  Difference between Jaffe creatinine and the gravimetric/reference value.    02_workflows/F1_workflow_v02/submission_ready/public/data/slco_F1_surface_grid_public.csv   File view   
  grid_id  Surface-grid identifier  Identifier of a grid point in the F1 simulated or reconstructed surface data.    02_workflows/F1_workflow_v02/submission_ready/public/data/slco_F1_surface_grid_public.csv   File view   
  tb_mg_dL  Total bilirubin concentration  Total bilirubin concentration used in the creatinine/bilirubin interference model or figure data.    02_workflows/F1_workflow_v02/submission_ready/public/data/slco_F1_surface_grid_public.csv   File view   
  parameter  Metadata parameter name  Name of a metadata parameter describing the F2 simulated heatmap object, such as figure identity, data origin, grid type, axis variable, or unit/role.    02_workflows/F1_workflow_v02/submission_ready/public/data/slco_F1_surface_meta_public.csv   File view   
  value  Value  Numerical or character value corresponding to the row-specific variable/metric.    02_workflows/F1_workflow_v02/submission_ready/public/data/slco_F1_surface_meta_public.csv   File view   
  count_negative  Number of simulated subpoints with negative score shift  Number of simulated subpoints within the F2 heatmap bin where the score shift is negative, defined as score delta ≤ −1.  count  02_workflows/F2_workflow_v01/data/02b_figure_content/slco_F2_heatmap_bin_public.csv    
  model  MELD model or score variant  Name of the MELD-related model or score variant represented by the row; expected values include MELD, MELD-Na, reMELD-Na, and MELD 3.0.    02_workflows/F2_workflow_v01/data/02b_figure_content/slco_F2_heatmap_bin_public.csv    
  n_sub  Number of simulated subpoints per heatmap bin  Number of simulated subpoints aggregated within one F2 heatmap bin.  count  02_workflows/F2_workflow_v01/data/02b_figure_content/slco_F2_heatmap_bin_public.csv    
  pct_negative  Percentage of simulated subpoints with negative score shift  Percentage of simulated subpoints within the F2 heatmap bin where the score shift is negative, defined as score delta ≤ −1.  %  02_workflows/F2_workflow_v01/data/02b_figure_content/slco_F2_heatmap_bin_public.csv    
  x_value  Heatmap x-axis creatinine value  Creatinine value defining the x-axis coordinate of an F2 heatmap bin.  mg/dL  02_workflows/F2_workflow_v01/data/02b_figure_content/slco_F2_heatmap_bin_public.csv    
  y_value  Heatmap y-axis total bilirubin value  Total bilirubin value defining the y-axis coordinate of an F2 heatmap bin.  mg/dL  02_workflows/F2_workflow_v01/data/02b_figure_content/slco_F2_heatmap_bin_public.csv    
  parameter  Metadata parameter name  Name of a metadata parameter describing the F2 simulated heatmap object, such as figure identity, data origin, grid type, axis variable, or unit/role.    02_workflows/F2_workflow_v01/data/02b_figure_content/slco_F2_heatmap_meta_public.csv    
  value  Value  Numerical or character value corresponding to the row-specific variable/metric.    02_workflows/F2_workflow_v01/data/02b_figure_content/slco_F2_heatmap_meta_public.csv    
  count_negative  Number of simulated subpoints with negative score shift  Number of simulated subpoints within the F2 heatmap bin where the score shift is negative, defined as score delta ≤ −1.  count  02_workflows/F2_workflow_v01/submission_ready/public/data/slco_F2_heatmap_bin_public.csv   File view   
  model  MELD model or score variant  Name of the MELD-related model or score variant represented by the row; expected values include MELD, MELD-Na, reMELD-Na, and MELD 3.0.    02_workflows/F2_workflow_v01/submission_ready/public/data/slco_F2_heatmap_bin_public.csv   File view   
  n_sub  Number of simulated subpoints per heatmap bin  Number of simulated subpoints aggregated within one F2 heatmap bin.  count  02_workflows/F2_workflow_v01/submission_ready/public/data/slco_F2_heatmap_bin_public.csv   File view   
  pct_negative  Percentage of simulated subpoints with negative score shift  Percentage of simulated subpoints within the F2 heatmap bin where the score shift is negative, defined as score delta ≤ −1.  %  02_workflows/F2_workflow_v01/submission_ready/public/data/slco_F2_heatmap_bin_public.csv   File view   
  x_value  Heatmap x-axis creatinine value  Creatinine value defining the x-axis coordinate of an F2 heatmap bin.  mg/dL  02_workflows/F2_workflow_v01/submission_ready/public/data/slco_F2_heatmap_bin_public.csv   File view   
  y_value  Heatmap y-axis total bilirubin value  Total bilirubin value defining the y-axis coordinate of an F2 heatmap bin.  mg/dL  02_workflows/F2_workflow_v01/submission_ready/public/data/slco_F2_heatmap_bin_public.csv   File view   
  parameter  Metadata parameter name  Name of a metadata parameter describing the F2 simulated heatmap object, such as figure identity, data origin, grid type, axis variable, or unit/role.    02_workflows/F2_workflow_v01/submission_ready/public/data/slco_F2_heatmap_meta_public.csv   File view   
  value  Value  Numerical or character value corresponding to the row-specific variable/metric.    02_workflows/F2_workflow_v01/submission_ready/public/data/slco_F2_heatmap_meta_public.csv   File view   
  age_years_first_available  Age at first available public record  Age in years at the first available public ESLD record for the patient or encounter represented in the released data.  years  02_workflows/F3_workflow_v01/data/01_source_loaded_harmonized/esld_master_long_public.csv    
  age_years_sample  Age at sample  Age in years at the sample-level public ESLD record.  years  02_workflows/F3_workflow_v01/data/01_source_loaded_harmonized/esld_master_long_public.csv    
  albumin_g_dl  Serum albumin concentration  Serum albumin concentration in the public ESLD master data.  g/dL  02_workflows/F3_workflow_v01/data/01_source_loaded_harmonized/esld_master_long_public.csv    
  ald  Alcohol-related liver disease etiology flag  Binary etiology indicator for alcohol-related liver disease in the public ESLD cohort.    02_workflows/F3_workflow_v01/data/01_source_loaded_harmonized/esld_master_long_public.csv    
  ald_hcv  Combined alcohol-related liver disease and hepatitis C etiology flag  Binary etiology indicator for combined alcohol-related liver disease and hepatitis C in the public ESLD cohort.    02_workflows/F3_workflow_v01/data/01_source_loaded_harmonized/esld_master_long_public.csv    
  autoimmune  Autoimmune liver disease etiology flag  Binary etiology indicator for autoimmune liver disease in the public ESLD cohort.    02_workflows/F3_workflow_v01/data/01_source_loaded_harmonized/esld_master_long_public.csv    
  crea  Creatinine concentration  Creatinine concentration used in MELD-related score calculation before applying the study-specific correction.  mg/dL  02_workflows/F3_workflow_v01/data/01_source_loaded_harmonized/esld_master_long_public.csv    
  crea_corrected  Corrected creatinine concentration  Creatinine concentration after applying the study-specific correction used for recalculated MELD-related scores.  mg/dL  02_workflows/F3_workflow_v01/data/01_source_loaded_harmonized/esld_master_long_public.csv    
  date_count  Relative day count  Relative day count used for time alignment; negative/positive values are relative to the analysis anchor and are not calendar dates.    02_workflows/F3_workflow_v01/data/01_source_loaded_harmonized/esld_master_long_public.csv    
  date_count_month  Relative month count  Relative month count derived from the relative day count; this is not a calendar month.    02_workflows/F3_workflow_v01/data/01_source_loaded_harmonized/esld_master_long_public.csv    
  dead_sample_flag  Dead-sample flag  Binary indicator identifying public ESLD sample records assigned to the deceased-sample subset used in sample-level analyses.    02_workflows/F3_workflow_v01/data/01_source_loaded_harmonized/esld_master_long_public.csv    
  death_within_90d  Death within 90 days  Binary indicator for death within 90 days of the analysis anchor.    02_workflows/F3_workflow_v01/data/01_source_loaded_harmonized/esld_master_long_public.csv    
  deceased_patient_flag  Deceased patient flag  Binary indicator identifying deceased patients within the released analysis context.    02_workflows/F3_workflow_v01/data/01_source_loaded_harmonized/esld_master_long_public.csv    
  delta_eq_0_flag  Zero score-delta flag  Binary indicator equal to 1 when the score delta is exactly zero.    02_workflows/F3_workflow_v01/data/01_source_loaded_harmonized/esld_master_long_public.csv    
  delta_gt_0_flag  Positive score-delta flag  Binary indicator equal to 1 when the score delta is positive.    02_workflows/F3_workflow_v01/data/01_source_loaded_harmonized/esld_master_long_public.csv    
  delta_le_minus1_flag  Score-delta ≤ −1 flag  Binary indicator equal to 1 when the score delta is less than or equal to −1.    02_workflows/F3_workflow_v01/data/01_source_loaded_harmonized/esld_master_long_public.csv    
  dialysis_raw  Dialysis indicator as recorded before score derivation  Binary indicator showing whether dialysis was recorded in the source field before score derivation.    02_workflows/F3_workflow_v01/data/01_source_loaded_harmonized/esld_master_long_public.csv    
  encounter_id_public  Public encounter pseudonym  Non-linkable public-release encounter pseudonym used for encounter-level grouping; not an original hospital encounter identifier.    02_workflows/F3_workflow_v01/data/01_source_loaded_harmonized/esld_master_long_public.csv    
  etiology_source_current_flag  Current etiology source flag  Binary indicator identifying whether the etiology source is the current source used for the public row.    02_workflows/F3_workflow_v01/data/01_source_loaded_harmonized/esld_master_long_public.csv    
  etiology_unclassified  Unclassified liver disease etiology flag  Binary etiology indicator identifying records without a classified liver disease etiology in the public ESLD cohort.    02_workflows/F3_workflow_v01/data/01_source_loaded_harmonized/esld_master_long_public.csv    
  hbv  Hepatitis B virus etiology flag  Binary etiology indicator for hepatitis B virus-related liver disease in the public ESLD cohort.    02_workflows/F3_workflow_v01/data/01_source_loaded_harmonized/esld_master_long_public.csv    
  hcv  Hepatitis C virus etiology flag  Binary etiology indicator for hepatitis C virus-related liver disease in the public ESLD cohort.    02_workflows/F3_workflow_v01/data/01_source_loaded_harmonized/esld_master_long_public.csv    
  icd_source_present  ICD source present flag  Binary indicator showing whether an ICD-derived etiology source was present for the public row.    02_workflows/F3_workflow_v01/data/01_source_loaded_harmonized/esld_master_long_public.csv    
  in_t1_cohort  Inclusion flag for Table 1 cohort  Binary indicator identifying records included in the public ESLD Table 1 baseline-characteristics cohort.    02_workflows/F3_workflow_v01/data/01_source_loaded_harmonized/esld_master_long_public.csv    
  in_t3_anchor_model  Inclusion flag for Table 3 anchor-model cohort  Binary indicator identifying records included in the Table 3 anchor-model cohort used for creatinine-comparison analyses.    02_workflows/F3_workflow_v01/data/01_source_loaded_harmonized/esld_master_long_public.csv    
  in_t4_prevalence_path  Inclusion flag for Table 4 prevalence pathway  Binary indicator identifying records included in the Table 4 prevalence-analysis pathway.    02_workflows/F3_workflow_v01/data/01_source_loaded_harmonized/esld_master_long_public.csv    
  in_t4_survival_path  Inclusion flag for Table 4 survival pathway  Binary indicator identifying records included in the Table 4 survival-analysis pathway.    02_workflows/F3_workflow_v01/data/01_source_loaded_harmonized/esld_master_long_public.csv    
  inr  International normalized ratio  International normalized ratio of prothrombin time used for MELD-related score calculation.  ratio  02_workflows/F3_workflow_v01/data/01_source_loaded_harmonized/esld_master_long_public.csv    
  mash  Metabolic dysfunction-associated steatohepatitis etiology flag  Binary etiology indicator for metabolic dysfunction-associated steatohepatitis in the public ESLD cohort.    02_workflows/F3_workflow_v01/data/01_source_loaded_harmonized/esld_master_long_public.csv    
  model  MELD model or score variant  Name of the MELD-related model or score variant represented by the row; expected values include MELD, MELD-Na, reMELD-Na, and MELD 3.0.    02_workflows/F3_workflow_v01/data/01_source_loaded_harmonized/esld_master_long_public.csv    
  other  Other liver disease etiology flag  Binary etiology indicator for liver disease etiologies grouped as other in the public ESLD cohort.    02_workflows/F3_workflow_v01/data/01_source_loaded_harmonized/esld_master_long_public.csv    
  patient_id  Public patient pseudonym  Non-linkable public-release patient pseudonym used to preserve within-patient grouping in public ESLD data; not an original hospital patient identifier.    02_workflows/F3_workflow_v01/data/01_source_loaded_harmonized/esld_master_long_public.csv    
  pbc  Primary biliary cholangitis etiology flag  Binary etiology indicator for primary biliary cholangitis in the public ESLD cohort.    02_workflows/F3_workflow_v01/data/01_source_loaded_harmonized/esld_master_long_public.csv    
  sample_day_from_first_sample  Relative sample day  Relative day of the sample measured from the first sample for the public patient/sample sequence; not a calendar date.  relative days  02_workflows/F3_workflow_v01/data/01_source_loaded_harmonized/esld_master_long_public.csv    
  sample_group_id_public  Public sample-group pseudonym  Non-linkable public-release sample-group pseudonym used to group samples within the released public data.    02_workflows/F3_workflow_v01/data/01_source_loaded_harmonized/esld_master_long_public.csv    
  sample_id  Sample identifier  Identifier of a sample or experimental record within the released public data; not a personal identifier.    02_workflows/F3_workflow_v01/data/01_source_loaded_harmonized/esld_master_long_public.csv    
  sample_month_index  Relative sample month index  Relative month index of the sample measured from the first sample for the public patient/sample sequence; not a calendar month.  relative months  02_workflows/F3_workflow_v01/data/01_source_loaded_harmonized/esld_master_long_public.csv    
  score_corrected  Corrected score  MELD-related score after creatinine correction or recalculation.  score points  02_workflows/F3_workflow_v01/data/01_source_loaded_harmonized/esld_master_long_public.csv    
  score_delta  Score difference  Difference between score variants or scoring approaches, expressed in score points.    02_workflows/F3_workflow_v01/data/01_source_loaded_harmonized/esld_master_long_public.csv    
  score_original  Original score  Original MELD-related score before creatinine correction or recalculation.    02_workflows/F3_workflow_v01/data/01_source_loaded_harmonized/esld_master_long_public.csv    
  sex  Sex  Sex category represented in the public ESLD data.    02_workflows/F3_workflow_v01/data/01_source_loaded_harmonized/esld_master_long_public.csv    
  sodium  Serum sodium concentration  Serum sodium concentration used for MELD-Na, reMELD-Na, or MELD 3.0 score calculation.  mmol/L  02_workflows/F3_workflow_v01/data/01_source_loaded_harmonized/esld_master_long_public.csv    
  tbil  Total bilirubin concentration  Total bilirubin concentration used in MELD-related score calculation in the public ESLD data.    02_workflows/F3_workflow_v01/data/01_source_loaded_harmonized/esld_master_long_public.csv    
  delta_class  Score-delta class  Encoded score-shift class used in F3 score-shift summaries. p1 denotes a one-point positive score shift; pm denotes the reference/no-decrease category used by the workflow; m1 denotes the minus-one score-shift class; m2 denotes the minus-two score-shift class.    02_workflows/F3_workflow_v01/data/02b_figure_content/esld_F3_score_shift_aggregate_public.csv    
  model  MELD model or score variant  Name of the MELD-related model or score variant represented by the row; expected values include MELD, MELD-Na, reMELD-Na, and MELD 3.0.    02_workflows/F3_workflow_v01/data/02b_figure_content/esld_F3_score_shift_aggregate_public.csv    
  n  Number of observations  Count of observations, patients, samples, events, or rows, depending on the data file.    02_workflows/F3_workflow_v01/data/02b_figure_content/esld_F3_score_shift_aggregate_public.csv    
  x_class  X-axis score class  Score class shown on the x-axis or used as the x-position in the F3 score-shift figure.  score class  02_workflows/F3_workflow_v01/data/02b_figure_content/esld_F3_score_shift_aggregate_public.csv    
  delta_class  Score-delta class  Encoded score-shift class used in F3 score-shift summaries. p1 denotes a one-point positive score shift; pm denotes the reference/no-decrease category used by the workflow; m1 denotes the minus-one score-shift class; m2 denotes the minus-two score-shift class.    02_workflows/F3_workflow_v01/submission_ready/public/data/esld_F3_score_shift_aggregate_public.csv   File view   
  model  MELD model or score variant  Name of the MELD-related model or score variant represented by the row; expected values include MELD, MELD-Na, reMELD-Na, and MELD 3.0.    02_workflows/F3_workflow_v01/submission_ready/public/data/esld_F3_score_shift_aggregate_public.csv   File view   
  n  Number of observations  Count of observations, patients, samples, events, or rows, depending on the data file.    02_workflows/F3_workflow_v01/submission_ready/public/data/esld_F3_score_shift_aggregate_public.csv   File view   
  x_class  X-axis score class  Score class shown on the x-axis or used as the x-position in the F3 score-shift figure.  score class  02_workflows/F3_workflow_v01/submission_ready/public/data/esld_F3_score_shift_aggregate_public.csv   File view   
  age_years_first_available  Age at first available public record  Age in years at the first available public ESLD record for the patient or encounter represented in the released data.  years  02_workflows/F4_workflow_v01/data/01_source_loaded_harmonized/esld_master_long_public.csv    
  age_years_sample  Age at sample  Age in years at the sample-level public ESLD record.  years  02_workflows/F4_workflow_v01/data/01_source_loaded_harmonized/esld_master_long_public.csv    
  albumin_g_dl  Serum albumin concentration  Serum albumin concentration in the public ESLD master data.  g/dL  02_workflows/F4_workflow_v01/data/01_source_loaded_harmonized/esld_master_long_public.csv    
  ald  Alcohol-related liver disease etiology flag  Binary etiology indicator for alcohol-related liver disease in the public ESLD cohort.    02_workflows/F4_workflow_v01/data/01_source_loaded_harmonized/esld_master_long_public.csv    
  ald_hcv  Combined alcohol-related liver disease and hepatitis C etiology flag  Binary etiology indicator for combined alcohol-related liver disease and hepatitis C in the public ESLD cohort.    02_workflows/F4_workflow_v01/data/01_source_loaded_harmonized/esld_master_long_public.csv    
  autoimmune  Autoimmune liver disease etiology flag  Binary etiology indicator for autoimmune liver disease in the public ESLD cohort.    02_workflows/F4_workflow_v01/data/01_source_loaded_harmonized/esld_master_long_public.csv    
  crea  Creatinine concentration  Creatinine concentration used in MELD-related score calculation before applying the study-specific correction.  mg/dL  02_workflows/F4_workflow_v01/data/01_source_loaded_harmonized/esld_master_long_public.csv    
  crea_corrected  Corrected creatinine concentration  Creatinine concentration after applying the study-specific correction used for recalculated MELD-related scores.  mg/dL  02_workflows/F4_workflow_v01/data/01_source_loaded_harmonized/esld_master_long_public.csv    
  date_count  Relative day count  Relative day count used for time alignment; negative/positive values are relative to the analysis anchor and are not calendar dates.    02_workflows/F4_workflow_v01/data/01_source_loaded_harmonized/esld_master_long_public.csv    
  date_count_month  Relative month count  Relative month count derived from the relative day count; this is not a calendar month.    02_workflows/F4_workflow_v01/data/01_source_loaded_harmonized/esld_master_long_public.csv    
  dead_sample_flag  Dead-sample flag  Binary indicator identifying public ESLD sample records assigned to the deceased-sample subset used in sample-level analyses.    02_workflows/F4_workflow_v01/data/01_source_loaded_harmonized/esld_master_long_public.csv    
  death_within_90d  Death within 90 days  Binary indicator for death within 90 days of the analysis anchor.    02_workflows/F4_workflow_v01/data/01_source_loaded_harmonized/esld_master_long_public.csv    
  deceased_patient_flag  Deceased patient flag  Binary indicator identifying deceased patients within the released analysis context.    02_workflows/F4_workflow_v01/data/01_source_loaded_harmonized/esld_master_long_public.csv    
  delta_eq_0_flag  Zero score-delta flag  Binary indicator equal to 1 when the score delta is exactly zero.    02_workflows/F4_workflow_v01/data/01_source_loaded_harmonized/esld_master_long_public.csv    
  delta_gt_0_flag  Positive score-delta flag  Binary indicator equal to 1 when the score delta is positive.    02_workflows/F4_workflow_v01/data/01_source_loaded_harmonized/esld_master_long_public.csv    
  delta_le_minus1_flag  Score-delta ≤ −1 flag  Binary indicator equal to 1 when the score delta is less than or equal to −1.    02_workflows/F4_workflow_v01/data/01_source_loaded_harmonized/esld_master_long_public.csv    
  dialysis_raw  Dialysis indicator as recorded before score derivation  Binary indicator showing whether dialysis was recorded in the source field before score derivation.    02_workflows/F4_workflow_v01/data/01_source_loaded_harmonized/esld_master_long_public.csv    
  encounter_id_public  Public encounter pseudonym  Non-linkable public-release encounter pseudonym used for encounter-level grouping; not an original hospital encounter identifier.    02_workflows/F4_workflow_v01/data/01_source_loaded_harmonized/esld_master_long_public.csv    
  etiology_source_current_flag  Current etiology source flag  Binary indicator identifying whether the etiology source is the current source used for the public row.    02_workflows/F4_workflow_v01/data/01_source_loaded_harmonized/esld_master_long_public.csv    
  etiology_unclassified  Unclassified liver disease etiology flag  Binary etiology indicator identifying records without a classified liver disease etiology in the public ESLD cohort.    02_workflows/F4_workflow_v01/data/01_source_loaded_harmonized/esld_master_long_public.csv    
  hbv  Hepatitis B virus etiology flag  Binary etiology indicator for hepatitis B virus-related liver disease in the public ESLD cohort.    02_workflows/F4_workflow_v01/data/01_source_loaded_harmonized/esld_master_long_public.csv    
  hcv  Hepatitis C virus etiology flag  Binary etiology indicator for hepatitis C virus-related liver disease in the public ESLD cohort.    02_workflows/F4_workflow_v01/data/01_source_loaded_harmonized/esld_master_long_public.csv    
  icd_source_present  ICD source present flag  Binary indicator showing whether an ICD-derived etiology source was present for the public row.    02_workflows/F4_workflow_v01/data/01_source_loaded_harmonized/esld_master_long_public.csv    
  in_t1_cohort  Inclusion flag for Table 1 cohort  Binary indicator identifying records included in the public ESLD Table 1 baseline-characteristics cohort.    02_workflows/F4_workflow_v01/data/01_source_loaded_harmonized/esld_master_long_public.csv    
  in_t3_anchor_model  Inclusion flag for Table 3 anchor-model cohort  Binary indicator identifying records included in the Table 3 anchor-model cohort used for creatinine-comparison analyses.    02_workflows/F4_workflow_v01/data/01_source_loaded_harmonized/esld_master_long_public.csv    
  in_t4_prevalence_path  Inclusion flag for Table 4 prevalence pathway  Binary indicator identifying records included in the Table 4 prevalence-analysis pathway.    02_workflows/F4_workflow_v01/data/01_source_loaded_harmonized/esld_master_long_public.csv    
  in_t4_survival_path  Inclusion flag for Table 4 survival pathway  Binary indicator identifying records included in the Table 4 survival-analysis pathway.    02_workflows/F4_workflow_v01/data/01_source_loaded_harmonized/esld_master_long_public.csv    
  inr  International normalized ratio  International normalized ratio of prothrombin time used for MELD-related score calculation.  ratio  02_workflows/F4_workflow_v01/data/01_source_loaded_harmonized/esld_master_long_public.csv    
  mash  Metabolic dysfunction-associated steatohepatitis etiology flag  Binary etiology indicator for metabolic dysfunction-associated steatohepatitis in the public ESLD cohort.    02_workflows/F4_workflow_v01/data/01_source_loaded_harmonized/esld_master_long_public.csv    
  model  MELD model or score variant  Name of the MELD-related model or score variant represented by the row; expected values include MELD, MELD-Na, reMELD-Na, and MELD 3.0.    02_workflows/F4_workflow_v01/data/01_source_loaded_harmonized/esld_master_long_public.csv    
  other  Other liver disease etiology flag  Binary etiology indicator for liver disease etiologies grouped as other in the public ESLD cohort.    02_workflows/F4_workflow_v01/data/01_source_loaded_harmonized/esld_master_long_public.csv    
  patient_id  Public patient pseudonym  Non-linkable public-release patient pseudonym used to preserve within-patient grouping in public ESLD data; not an original hospital patient identifier.    02_workflows/F4_workflow_v01/data/01_source_loaded_harmonized/esld_master_long_public.csv    
  pbc  Primary biliary cholangitis etiology flag  Binary etiology indicator for primary biliary cholangitis in the public ESLD cohort.    02_workflows/F4_workflow_v01/data/01_source_loaded_harmonized/esld_master_long_public.csv    
  sample_day_from_first_sample  Relative sample day  Relative day of the sample measured from the first sample for the public patient/sample sequence; not a calendar date.  relative days  02_workflows/F4_workflow_v01/data/01_source_loaded_harmonized/esld_master_long_public.csv    
  sample_group_id_public  Public sample-group pseudonym  Non-linkable public-release sample-group pseudonym used to group samples within the released public data.    02_workflows/F4_workflow_v01/data/01_source_loaded_harmonized/esld_master_long_public.csv    
  sample_id  Sample identifier  Identifier of a sample or experimental record within the released public data; not a personal identifier.    02_workflows/F4_workflow_v01/data/01_source_loaded_harmonized/esld_master_long_public.csv    
  sample_month_index  Relative sample month index  Relative month index of the sample measured from the first sample for the public patient/sample sequence; not a calendar month.  relative months  02_workflows/F4_workflow_v01/data/01_source_loaded_harmonized/esld_master_long_public.csv    
  score_corrected  Corrected score  MELD-related score after creatinine correction or recalculation.  score points  02_workflows/F4_workflow_v01/data/01_source_loaded_harmonized/esld_master_long_public.csv    
  score_delta  Score difference  Difference between score variants or scoring approaches, expressed in score points.    02_workflows/F4_workflow_v01/data/01_source_loaded_harmonized/esld_master_long_public.csv    
  score_original  Original score  Original MELD-related score before creatinine correction or recalculation.    02_workflows/F4_workflow_v01/data/01_source_loaded_harmonized/esld_master_long_public.csv    
  sex  Sex  Sex category represented in the public ESLD data.    02_workflows/F4_workflow_v01/data/01_source_loaded_harmonized/esld_master_long_public.csv    
  sodium  Serum sodium concentration  Serum sodium concentration used for MELD-Na, reMELD-Na, or MELD 3.0 score calculation.  mmol/L  02_workflows/F4_workflow_v01/data/01_source_loaded_harmonized/esld_master_long_public.csv    
  tbil  Total bilirubin concentration  Total bilirubin concentration used in MELD-related score calculation in the public ESLD data.    02_workflows/F4_workflow_v01/data/01_source_loaded_harmonized/esld_master_long_public.csv    
  asterisk  Statistical-significance annotation  Text label used to annotate statistical significance in the rendered figure, for example n.s., *, **, ***, or ****.    02_workflows/F4_workflow_v01/data/02b_figure_content/esld_F4_survival_stats_public.csv    
  median_m1  Median value for m1 group  Median row-specific value for the m1 group. m1 denotes the negative score-shift group. In class-level score-shift summaries it denotes the minus-one score-shift class; in grouped survival outputs it is the negative-shift comparison group used by the workflow.  analysis-specific scale  02_workflows/F4_workflow_v01/data/02b_figure_content/esld_F4_survival_stats_public.csv    
  median_pm2  Median value for pm2 group  Median row-specific value for the pm2 group in F4 survival-statistics data. pm2 denotes the F4 comparator group used against m1 in the survival-statistics table; it should be interpreted as the F4 workflow's non-m1/reference comparator group.  analysis-specific scale  02_workflows/F4_workflow_v01/data/02b_figure_content/esld_F4_survival_stats_public.csv    
  model  MELD model or score variant  Name of the MELD-related model or score variant represented by the row; expected values include MELD, MELD-Na, reMELD-Na, and MELD 3.0.    02_workflows/F4_workflow_v01/data/02b_figure_content/esld_F4_survival_stats_public.csv    
  n_m1  Number of observations in m1 group  Count of observations in the m1 group for the row-specific stratum. m1 denotes the negative score-shift group. In class-level score-shift summaries it denotes the minus-one score-shift class; in grouped survival outputs it is the negative-shift comparison group used by the workflow.  count  02_workflows/F4_workflow_v01/data/02b_figure_content/esld_F4_survival_stats_public.csv    
  n_pm2  Number of observations in pm2 group  Count of observations in the pm2 group for the row-specific F4 survival stratum. pm2 denotes the F4 comparator group used against m1 in the survival-statistics table; it should be interpreted as the F4 workflow's non-m1/reference comparator group.  count  02_workflows/F4_workflow_v01/data/02b_figure_content/esld_F4_survival_stats_public.csv    
  p25_m1  25th-percentile value for m1 group  25th percentile of the row-specific value for the m1 group. m1 denotes the negative score-shift group. In class-level score-shift summaries it denotes the minus-one score-shift class; in grouped survival outputs it is the negative-shift comparison group used by the workflow.  analysis-specific scale  02_workflows/F4_workflow_v01/data/02b_figure_content/esld_F4_survival_stats_public.csv    
  p25_pm2  25th-percentile value for pm2 group  25th percentile of the row-specific value for the pm2 group in F4 survival-statistics data. pm2 denotes the F4 comparator group used against m1 in the survival-statistics table; it should be interpreted as the F4 workflow's non-m1/reference comparator group.  analysis-specific scale  02_workflows/F4_workflow_v01/data/02b_figure_content/esld_F4_survival_stats_public.csv    
  p75_m1  75th-percentile value for m1 group  75th percentile of the row-specific value for the m1 group. m1 denotes the negative score-shift group. In class-level score-shift summaries it denotes the minus-one score-shift class; in grouped survival outputs it is the negative-shift comparison group used by the workflow.  analysis-specific scale  02_workflows/F4_workflow_v01/data/02b_figure_content/esld_F4_survival_stats_public.csv    
  p75_pm2  75th-percentile value for pm2 group  75th percentile of the row-specific value for the pm2 group in F4 survival-statistics data. pm2 denotes the F4 comparator group used against m1 in the survival-statistics table; it should be interpreted as the F4 workflow's non-m1/reference comparator group.  analysis-specific scale  02_workflows/F4_workflow_v01/data/02b_figure_content/esld_F4_survival_stats_public.csv    
  p_value  P value  P value from the statistical comparison represented by the row.  probability  02_workflows/F4_workflow_v01/data/02b_figure_content/esld_F4_survival_stats_public.csv    
  score_class_label  Displayed score-class label  Displayed score-class label for the figure/table stratum.  score points  02_workflows/F4_workflow_v01/data/02b_figure_content/esld_F4_survival_stats_public.csv    
  score_class_value  Score-class upper value  Upper score value defining the displayed score class.  score points  02_workflows/F4_workflow_v01/data/02b_figure_content/esld_F4_survival_stats_public.csv    
  y_ast  Y coordinate for significance annotation  Y-axis coordinate used to position the statistical-significance annotation in the F4 survival figure.  analysis-specific y-axis coordinate  02_workflows/F4_workflow_v01/data/02b_figure_content/esld_F4_survival_stats_public.csv    
  asterisk  Statistical-significance annotation  Text label used to annotate statistical significance in the rendered figure, for example n.s., *, **, ***, or ****.    02_workflows/F4_workflow_v01/submission_ready/public/data/esld_F4_survival_stats_public.csv   File view   
  median_m1  Median value for m1 group  Median row-specific value for the m1 group. m1 denotes the negative score-shift group. In class-level score-shift summaries it denotes the minus-one score-shift class; in grouped survival outputs it is the negative-shift comparison group used by the workflow.  analysis-specific scale  02_workflows/F4_workflow_v01/submission_ready/public/data/esld_F4_survival_stats_public.csv   File view   
  median_pm2  Median value for pm2 group  Median row-specific value for the pm2 group in F4 survival-statistics data. pm2 denotes the F4 comparator group used against m1 in the survival-statistics table; it should be interpreted as the F4 workflow's non-m1/reference comparator group.  analysis-specific scale  02_workflows/F4_workflow_v01/submission_ready/public/data/esld_F4_survival_stats_public.csv   File view   
  model  MELD model or score variant  Name of the MELD-related model or score variant represented by the row; expected values include MELD, MELD-Na, reMELD-Na, and MELD 3.0.    02_workflows/F4_workflow_v01/submission_ready/public/data/esld_F4_survival_stats_public.csv   File view   
  n_m1  Number of observations in m1 group  Count of observations in the m1 group for the row-specific stratum. m1 denotes the negative score-shift group. In class-level score-shift summaries it denotes the minus-one score-shift class; in grouped survival outputs it is the negative-shift comparison group used by the workflow.  count  02_workflows/F4_workflow_v01/submission_ready/public/data/esld_F4_survival_stats_public.csv   File view   
  n_pm2  Number of observations in pm2 group  Count of observations in the pm2 group for the row-specific F4 survival stratum. pm2 denotes the F4 comparator group used against m1 in the survival-statistics table; it should be interpreted as the F4 workflow's non-m1/reference comparator group.  count  02_workflows/F4_workflow_v01/submission_ready/public/data/esld_F4_survival_stats_public.csv   File view   
  p25_m1  25th-percentile value for m1 group  25th percentile of the row-specific value for the m1 group. m1 denotes the negative score-shift group. In class-level score-shift summaries it denotes the minus-one score-shift class; in grouped survival outputs it is the negative-shift comparison group used by the workflow.  analysis-specific scale  02_workflows/F4_workflow_v01/submission_ready/public/data/esld_F4_survival_stats_public.csv   File view   
  p25_pm2  25th-percentile value for pm2 group  25th percentile of the row-specific value for the pm2 group in F4 survival-statistics data. pm2 denotes the F4 comparator group used against m1 in the survival-statistics table; it should be interpreted as the F4 workflow's non-m1/reference comparator group.  analysis-specific scale  02_workflows/F4_workflow_v01/submission_ready/public/data/esld_F4_survival_stats_public.csv   File view   
  p75_m1  75th-percentile value for m1 group  75th percentile of the row-specific value for the m1 group. m1 denotes the negative score-shift group. In class-level score-shift summaries it denotes the minus-one score-shift class; in grouped survival outputs it is the negative-shift comparison group used by the workflow.  analysis-specific scale  02_workflows/F4_workflow_v01/submission_ready/public/data/esld_F4_survival_stats_public.csv   File view   
  p75_pm2  75th-percentile value for pm2 group  75th percentile of the row-specific value for the pm2 group in F4 survival-statistics data. pm2 denotes the F4 comparator group used against m1 in the survival-statistics table; it should be interpreted as the F4 workflow's non-m1/reference comparator group.  analysis-specific scale  02_workflows/F4_workflow_v01/submission_ready/public/data/esld_F4_survival_stats_public.csv   File view   
  p_value  P value  P value from the statistical comparison represented by the row.  probability  02_workflows/F4_workflow_v01/submission_ready/public/data/esld_F4_survival_stats_public.csv   File view   
  score_class_label  Displayed score-class label  Displayed score-class label for the figure/table stratum.  score points  02_workflows/F4_workflow_v01/submission_ready/public/data/esld_F4_survival_stats_public.csv   File view   
  score_class_value  Score-class upper value  Upper score value defining the displayed score class.  score points  02_workflows/F4_workflow_v01/submission_ready/public/data/esld_F4_survival_stats_public.csv   File view   
  y_ast  Y coordinate for significance annotation  Y-axis coordinate used to position the statistical-significance annotation in the F4 survival figure.  analysis-specific y-axis coordinate  02_workflows/F4_workflow_v01/submission_ready/public/data/esld_F4_survival_stats_public.csv   File view   
  age_years_first_available  Age at first available public record  Age in years at the first available public ESLD record for the patient or encounter represented in the released data.  years  02_workflows/F5_workflow_v01/data/01_source_loaded_harmonized/esld_master_long_public.csv    
  age_years_sample  Age at sample  Age in years at the sample-level public ESLD record.  years  02_workflows/F5_workflow_v01/data/01_source_loaded_harmonized/esld_master_long_public.csv    
  albumin_g_dl  Serum albumin concentration  Serum albumin concentration in the public ESLD master data.  g/dL  02_workflows/F5_workflow_v01/data/01_source_loaded_harmonized/esld_master_long_public.csv    
  ald  Alcohol-related liver disease etiology flag  Binary etiology indicator for alcohol-related liver disease in the public ESLD cohort.    02_workflows/F5_workflow_v01/data/01_source_loaded_harmonized/esld_master_long_public.csv    
  ald_hcv  Combined alcohol-related liver disease and hepatitis C etiology flag  Binary etiology indicator for combined alcohol-related liver disease and hepatitis C in the public ESLD cohort.    02_workflows/F5_workflow_v01/data/01_source_loaded_harmonized/esld_master_long_public.csv    
  autoimmune  Autoimmune liver disease etiology flag  Binary etiology indicator for autoimmune liver disease in the public ESLD cohort.    02_workflows/F5_workflow_v01/data/01_source_loaded_harmonized/esld_master_long_public.csv    
  crea  Creatinine concentration  Creatinine concentration used in MELD-related score calculation before applying the study-specific correction.  mg/dL  02_workflows/F5_workflow_v01/data/01_source_loaded_harmonized/esld_master_long_public.csv    
  crea_corrected  Corrected creatinine concentration  Creatinine concentration after applying the study-specific correction used for recalculated MELD-related scores.  mg/dL  02_workflows/F5_workflow_v01/data/01_source_loaded_harmonized/esld_master_long_public.csv    
  date_count  Relative day count  Relative day count used for time alignment; negative/positive values are relative to the analysis anchor and are not calendar dates.    02_workflows/F5_workflow_v01/data/01_source_loaded_harmonized/esld_master_long_public.csv    
  date_count_month  Relative month count  Relative month count derived from the relative day count; this is not a calendar month.    02_workflows/F5_workflow_v01/data/01_source_loaded_harmonized/esld_master_long_public.csv    
  dead_sample_flag  Dead-sample flag  Binary indicator identifying public ESLD sample records assigned to the deceased-sample subset used in sample-level analyses.    02_workflows/F5_workflow_v01/data/01_source_loaded_harmonized/esld_master_long_public.csv    
  death_within_90d  Death within 90 days  Binary indicator for death within 90 days of the analysis anchor.    02_workflows/F5_workflow_v01/data/01_source_loaded_harmonized/esld_master_long_public.csv    
  deceased_patient_flag  Deceased patient flag  Binary indicator identifying deceased patients within the released analysis context.    02_workflows/F5_workflow_v01/data/01_source_loaded_harmonized/esld_master_long_public.csv    
  delta_eq_0_flag  Zero score-delta flag  Binary indicator equal to 1 when the score delta is exactly zero.    02_workflows/F5_workflow_v01/data/01_source_loaded_harmonized/esld_master_long_public.csv    
  delta_gt_0_flag  Positive score-delta flag  Binary indicator equal to 1 when the score delta is positive.    02_workflows/F5_workflow_v01/data/01_source_loaded_harmonized/esld_master_long_public.csv    
  delta_le_minus1_flag  Score-delta ≤ −1 flag  Binary indicator equal to 1 when the score delta is less than or equal to −1.    02_workflows/F5_workflow_v01/data/01_source_loaded_harmonized/esld_master_long_public.csv    
  dialysis_raw  Dialysis indicator as recorded before score derivation  Binary indicator showing whether dialysis was recorded in the source field before score derivation.    02_workflows/F5_workflow_v01/data/01_source_loaded_harmonized/esld_master_long_public.csv    
  encounter_id_public  Public encounter pseudonym  Non-linkable public-release encounter pseudonym used for encounter-level grouping; not an original hospital encounter identifier.    02_workflows/F5_workflow_v01/data/01_source_loaded_harmonized/esld_master_long_public.csv    
  etiology_source_current_flag  Current etiology source flag  Binary indicator identifying whether the etiology source is the current source used for the public row.    02_workflows/F5_workflow_v01/data/01_source_loaded_harmonized/esld_master_long_public.csv    
  etiology_unclassified  Unclassified liver disease etiology flag  Binary etiology indicator identifying records without a classified liver disease etiology in the public ESLD cohort.    02_workflows/F5_workflow_v01/data/01_source_loaded_harmonized/esld_master_long_public.csv    
  hbv  Hepatitis B virus etiology flag  Binary etiology indicator for hepatitis B virus-related liver disease in the public ESLD cohort.    02_workflows/F5_workflow_v01/data/01_source_loaded_harmonized/esld_master_long_public.csv    
  hcv  Hepatitis C virus etiology flag  Binary etiology indicator for hepatitis C virus-related liver disease in the public ESLD cohort.    02_workflows/F5_workflow_v01/data/01_source_loaded_harmonized/esld_master_long_public.csv    
  icd_source_present  ICD source present flag  Binary indicator showing whether an ICD-derived etiology source was present for the public row.    02_workflows/F5_workflow_v01/data/01_source_loaded_harmonized/esld_master_long_public.csv    
  in_t1_cohort  Inclusion flag for Table 1 cohort  Binary indicator identifying records included in the public ESLD Table 1 baseline-characteristics cohort.    02_workflows/F5_workflow_v01/data/01_source_loaded_harmonized/esld_master_long_public.csv    
  in_t3_anchor_model  Inclusion flag for Table 3 anchor-model cohort  Binary indicator identifying records included in the Table 3 anchor-model cohort used for creatinine-comparison analyses.    02_workflows/F5_workflow_v01/data/01_source_loaded_harmonized/esld_master_long_public.csv    
  in_t4_prevalence_path  Inclusion flag for Table 4 prevalence pathway  Binary indicator identifying records included in the Table 4 prevalence-analysis pathway.    02_workflows/F5_workflow_v01/data/01_source_loaded_harmonized/esld_master_long_public.csv    
  in_t4_survival_path  Inclusion flag for Table 4 survival pathway  Binary indicator identifying records included in the Table 4 survival-analysis pathway.    02_workflows/F5_workflow_v01/data/01_source_loaded_harmonized/esld_master_long_public.csv    
  inr  International normalized ratio  International normalized ratio of prothrombin time used for MELD-related score calculation.  ratio  02_workflows/F5_workflow_v01/data/01_source_loaded_harmonized/esld_master_long_public.csv    
  mash  Metabolic dysfunction-associated steatohepatitis etiology flag  Binary etiology indicator for metabolic dysfunction-associated steatohepatitis in the public ESLD cohort.    02_workflows/F5_workflow_v01/data/01_source_loaded_harmonized/esld_master_long_public.csv    
  model  MELD model or score variant  Name of the MELD-related model or score variant represented by the row; expected values include MELD, MELD-Na, reMELD-Na, and MELD 3.0.    02_workflows/F5_workflow_v01/data/01_source_loaded_harmonized/esld_master_long_public.csv    
  other  Other liver disease etiology flag  Binary etiology indicator for liver disease etiologies grouped as other in the public ESLD cohort.    02_workflows/F5_workflow_v01/data/01_source_loaded_harmonized/esld_master_long_public.csv    
  patient_id  Public patient pseudonym  Non-linkable public-release patient pseudonym used to preserve within-patient grouping in public ESLD data; not an original hospital patient identifier.    02_workflows/F5_workflow_v01/data/01_source_loaded_harmonized/esld_master_long_public.csv    
  pbc  Primary biliary cholangitis etiology flag  Binary etiology indicator for primary biliary cholangitis in the public ESLD cohort.    02_workflows/F5_workflow_v01/data/01_source_loaded_harmonized/esld_master_long_public.csv    
  sample_day_from_first_sample  Relative sample day  Relative day of the sample measured from the first sample for the public patient/sample sequence; not a calendar date.  relative days  02_workflows/F5_workflow_v01/data/01_source_loaded_harmonized/esld_master_long_public.csv    
  sample_group_id_public  Public sample-group pseudonym  Non-linkable public-release sample-group pseudonym used to group samples within the released public data.    02_workflows/F5_workflow_v01/data/01_source_loaded_harmonized/esld_master_long_public.csv    
  sample_id  Sample identifier  Identifier of a sample or experimental record within the released public data; not a personal identifier.    02_workflows/F5_workflow_v01/data/01_source_loaded_harmonized/esld_master_long_public.csv    
  sample_month_index  Relative sample month index  Relative month index of the sample measured from the first sample for the public patient/sample sequence; not a calendar month.  relative months  02_workflows/F5_workflow_v01/data/01_source_loaded_harmonized/esld_master_long_public.csv    
  score_corrected  Corrected score  MELD-related score after creatinine correction or recalculation.  score points  02_workflows/F5_workflow_v01/data/01_source_loaded_harmonized/esld_master_long_public.csv    
  score_delta  Score difference  Difference between score variants or scoring approaches, expressed in score points.    02_workflows/F5_workflow_v01/data/01_source_loaded_harmonized/esld_master_long_public.csv    
  score_original  Original score  Original MELD-related score before creatinine correction or recalculation.    02_workflows/F5_workflow_v01/data/01_source_loaded_harmonized/esld_master_long_public.csv    
  sex  Sex  Sex category represented in the public ESLD data.    02_workflows/F5_workflow_v01/data/01_source_loaded_harmonized/esld_master_long_public.csv    
  sodium  Serum sodium concentration  Serum sodium concentration used for MELD-Na, reMELD-Na, or MELD 3.0 score calculation.  mmol/L  02_workflows/F5_workflow_v01/data/01_source_loaded_harmonized/esld_master_long_public.csv    
  tbil  Total bilirubin concentration  Total bilirubin concentration used in MELD-related score calculation in the public ESLD data.    02_workflows/F5_workflow_v01/data/01_source_loaded_harmonized/esld_master_long_public.csv    
  parameter  Metadata parameter name  Name of a metadata parameter describing the F2 simulated heatmap object, such as figure identity, data origin, grid type, axis variable, or unit/role.    02_workflows/F5_workflow_v01/data/02b_figure_content/esld_F5_stratified_survival_meta_public.csv    
  value  Value  Numerical or character value corresponding to the row-specific variable/metric.    02_workflows/F5_workflow_v01/data/02b_figure_content/esld_F5_stratified_survival_meta_public.csv    
  chisq  Chi-square statistic  Chi-square statistic for the row-specific survival comparison or stratified test.  chi-square statistic  02_workflows/F5_workflow_v01/data/02b_figure_content/esld_F5_stratified_survival_stats_public.csv    
  chisq_lab  Formatted chi-square label  Formatted display label for the chi-square statistic shown in a table or figure annotation.    02_workflows/F5_workflow_v01/data/02b_figure_content/esld_F5_stratified_survival_stats_public.csv    
  model  MELD model or score variant  Name of the MELD-related model or score variant represented by the row; expected values include MELD, MELD-Na, reMELD-Na, and MELD 3.0.    02_workflows/F5_workflow_v01/data/02b_figure_content/esld_F5_stratified_survival_stats_public.csv    
  n_m1  Number of observations in m1 group  Count of observations in the m1 group for the row-specific stratum. m1 denotes the negative score-shift group. In class-level score-shift summaries it denotes the minus-one score-shift class; in grouped survival outputs it is the negative-shift comparison group used by the workflow.  count  02_workflows/F5_workflow_v01/data/02b_figure_content/esld_F5_stratified_survival_stats_public.csv    
  n_pm  Number of observations in pm group  Count of observations in the pm group for the row-specific stratum. pm denotes the reference group without a negative score shift in grouped score-delta survival outputs; in class-level score-shift summaries it represents the no-decrease/reference category used by the workflow.  count  02_workflows/F5_workflow_v01/data/02b_figure_content/esld_F5_stratified_survival_stats_public.csv    
  p_lab  Formatted p-value label  Formatted p-value label shown in a table or figure annotation.    02_workflows/F5_workflow_v01/data/02b_figure_content/esld_F5_stratified_survival_stats_public.csv    
  pval  P value  P value for the row-specific statistical comparison.  probability  02_workflows/F5_workflow_v01/data/02b_figure_content/esld_F5_stratified_survival_stats_public.csv    
  scr.grp  Score-class stratum  Encoded score-class stratum used in F5; examples include le15, 16to25, and gt25.    02_workflows/F5_workflow_v01/data/02b_figure_content/esld_F5_stratified_survival_stats_public.csv    
  albumin  Serum albumin concentration  Serum albumin concentration used in the public ESLD stratified survival dataset.  g/dL  02_workflows/F5_workflow_v01/data/02b_figure_content/esld_F5_stratified_survival_subject_public.csv    
  bilirubin  Total bilirubin concentration  Total bilirubin concentration used in the public ESLD analysis data.  mg/dL  02_workflows/F5_workflow_v01/data/02b_figure_content/esld_F5_stratified_survival_subject_public.csv    
  case_id  Public case pseudonym  Non-linkable public-release case pseudonym used for case-level grouping; not an original hospital case identifier.    02_workflows/F5_workflow_v01/data/02b_figure_content/esld_F5_stratified_survival_subject_public.csv    
  creatinine_corrected  Corrected creatinine concentration  Corrected creatinine concentration used for recalculated MELD-related scores.  mg/dL  02_workflows/F5_workflow_v01/data/02b_figure_content/esld_F5_stratified_survival_subject_public.csv    
  creatinine_raw  Raw creatinine concentration  Raw measured creatinine concentration before applying the study-specific correction.  mg/dL  02_workflows/F5_workflow_v01/data/02b_figure_content/esld_F5_stratified_survival_subject_public.csv    
  delta.grp  Grouped score-delta comparison group  Grouped score-delta category used in F5 stratified survival analyses. pm denotes the reference group without a negative score shift in grouped score-delta survival outputs; in class-level score-shift summaries it represents the no-decrease/reference category used by the workflow. m1 denotes the negative score-shift group. In class-level score-shift summaries it denotes the minus-one score-shift class; in grouped survival outputs it is the negative-shift comparison group used by the workflow.    02_workflows/F5_workflow_v01/data/02b_figure_content/esld_F5_stratified_survival_subject_public.csv    
  delta_group_label  Displayed score-delta group label  Human-readable version of the grouped score-delta category displayed in the stratified survival figure. It should be read together with delta.grp: pm is the reference/no-negative-score-shift group and m1 is the negative-score-shift comparison group.    02_workflows/F5_workflow_v01/data/02b_figure_content/esld_F5_stratified_survival_subject_public.csv    
  dialysis  Dialysis indicator  Binary dialysis indicator used for MELD-related score calculation or stratified public analyses.    02_workflows/F5_workflow_v01/data/02b_figure_content/esld_F5_stratified_survival_subject_public.csv    
  inr  International normalized ratio  International normalized ratio of prothrombin time used for MELD-related score calculation.  ratio  02_workflows/F5_workflow_v01/data/02b_figure_content/esld_F5_stratified_survival_subject_public.csv    
  model  MELD model or score variant  Name of the MELD-related model or score variant represented by the row; expected values include MELD, MELD-Na, reMELD-Na, and MELD 3.0.    02_workflows/F5_workflow_v01/data/02b_figure_content/esld_F5_stratified_survival_subject_public.csv    
  patient_id  Public patient pseudonym  Non-linkable public-release patient pseudonym used to preserve within-patient grouping in public ESLD data; not an original hospital patient identifier.    02_workflows/F5_workflow_v01/data/02b_figure_content/esld_F5_stratified_survival_subject_public.csv    
  score_class  Score class  Categorical score class used for stratified analyses.    02_workflows/F5_workflow_v01/data/02b_figure_content/esld_F5_stratified_survival_subject_public.csv    
  score_for_class  Score used for class assignment  Score value used to assign the row to a score class.  score points  02_workflows/F5_workflow_v01/data/02b_figure_content/esld_F5_stratified_survival_subject_public.csv    
  scr.grp  Score-class stratum  Encoded score-class stratum used in F5; examples include le15, 16to25, and gt25.    02_workflows/F5_workflow_v01/data/02b_figure_content/esld_F5_stratified_survival_subject_public.csv    
  sex  Sex  Sex category represented in the public ESLD data.    02_workflows/F5_workflow_v01/data/02b_figure_content/esld_F5_stratified_survival_subject_public.csv    
  sodium  Serum sodium concentration  Serum sodium concentration used for MELD-Na, reMELD-Na, or MELD 3.0 score calculation.  mmol/L  02_workflows/F5_workflow_v01/data/02b_figure_content/esld_F5_stratified_survival_subject_public.csv    
  source_file  Source file  Name of the source file used to create the public row or object.    02_workflows/F5_workflow_v01/data/02b_figure_content/esld_F5_stratified_survival_subject_public.csv    
  status  Status flag  Binary or categorical status flag used in a public QC, manifest, or analysis table.    02_workflows/F5_workflow_v01/data/02b_figure_content/esld_F5_stratified_survival_subject_public.csv    
  time  Analysis time  Analysis-specific time scale; interpretation depends on the respective figure/table workflow.    02_workflows/F5_workflow_v01/data/02b_figure_content/esld_F5_stratified_survival_subject_public.csv    
  parameter  Metadata parameter name  Name of a metadata parameter describing the F2 simulated heatmap object, such as figure identity, data origin, grid type, axis variable, or unit/role.    02_workflows/F5_workflow_v01/submission_ready/public/data/esld_F5_stratified_survival_meta_public.csv   File view   
  value  Value  Numerical or character value corresponding to the row-specific variable/metric.    02_workflows/F5_workflow_v01/submission_ready/public/data/esld_F5_stratified_survival_meta_public.csv   File view   
  chisq  Chi-square statistic  Chi-square statistic for the row-specific survival comparison or stratified test.  chi-square statistic  02_workflows/F5_workflow_v01/submission_ready/public/data/esld_F5_stratified_survival_stats_public.csv   File view   
  chisq_lab  Formatted chi-square label  Formatted display label for the chi-square statistic shown in a table or figure annotation.    02_workflows/F5_workflow_v01/submission_ready/public/data/esld_F5_stratified_survival_stats_public.csv   File view   
  model  MELD model or score variant  Name of the MELD-related model or score variant represented by the row; expected values include MELD, MELD-Na, reMELD-Na, and MELD 3.0.    02_workflows/F5_workflow_v01/submission_ready/public/data/esld_F5_stratified_survival_stats_public.csv   File view   
  n_m1  Number of observations in m1 group  Count of observations in the m1 group for the row-specific stratum. m1 denotes the negative score-shift group. In class-level score-shift summaries it denotes the minus-one score-shift class; in grouped survival outputs it is the negative-shift comparison group used by the workflow.  count  02_workflows/F5_workflow_v01/submission_ready/public/data/esld_F5_stratified_survival_stats_public.csv   File view   
  n_pm  Number of observations in pm group  Count of observations in the pm group for the row-specific stratum. pm denotes the reference group without a negative score shift in grouped score-delta survival outputs; in class-level score-shift summaries it represents the no-decrease/reference category used by the workflow.  count  02_workflows/F5_workflow_v01/submission_ready/public/data/esld_F5_stratified_survival_stats_public.csv   File view   
  p_lab  Formatted p-value label  Formatted p-value label shown in a table or figure annotation.    02_workflows/F5_workflow_v01/submission_ready/public/data/esld_F5_stratified_survival_stats_public.csv   File view   
  pval  P value  P value for the row-specific statistical comparison.  probability  02_workflows/F5_workflow_v01/submission_ready/public/data/esld_F5_stratified_survival_stats_public.csv   File view   
  scr.grp  Score-class stratum  Encoded score-class stratum used in F5; examples include le15, 16to25, and gt25.    02_workflows/F5_workflow_v01/submission_ready/public/data/esld_F5_stratified_survival_stats_public.csv   File view   
  albumin  Serum albumin concentration  Serum albumin concentration used in the public ESLD stratified survival dataset.  g/dL  02_workflows/F5_workflow_v01/submission_ready/public/data/esld_F5_stratified_survival_subject_public.csv   File view   
  bilirubin  Total bilirubin concentration  Total bilirubin concentration used in the public ESLD analysis data.  mg/dL  02_workflows/F5_workflow_v01/submission_ready/public/data/esld_F5_stratified_survival_subject_public.csv   File view   
  case_id  Public case pseudonym  Non-linkable public-release case pseudonym used for case-level grouping; not an original hospital case identifier.    02_workflows/F5_workflow_v01/submission_ready/public/data/esld_F5_stratified_survival_subject_public.csv   File view   
  creatinine_corrected  Corrected creatinine concentration  Corrected creatinine concentration used for recalculated MELD-related scores.  mg/dL  02_workflows/F5_workflow_v01/submission_ready/public/data/esld_F5_stratified_survival_subject_public.csv   File view   
  creatinine_raw  Raw creatinine concentration  Raw measured creatinine concentration before applying the study-specific correction.  mg/dL  02_workflows/F5_workflow_v01/submission_ready/public/data/esld_F5_stratified_survival_subject_public.csv   File view   
  delta.grp  Grouped score-delta comparison group  Grouped score-delta category used in F5 stratified survival analyses. pm denotes the reference group without a negative score shift in grouped score-delta survival outputs; in class-level score-shift summaries it represents the no-decrease/reference category used by the workflow. m1 denotes the negative score-shift group. In class-level score-shift summaries it denotes the minus-one score-shift class; in grouped survival outputs it is the negative-shift comparison group used by the workflow.    02_workflows/F5_workflow_v01/submission_ready/public/data/esld_F5_stratified_survival_subject_public.csv   File view   
  delta_group_label  Displayed score-delta group label  Human-readable version of the grouped score-delta category displayed in the stratified survival figure. It should be read together with delta.grp: pm is the reference/no-negative-score-shift group and m1 is the negative-score-shift comparison group.    02_workflows/F5_workflow_v01/submission_ready/public/data/esld_F5_stratified_survival_subject_public.csv   File view   
  dialysis  Dialysis indicator  Binary dialysis indicator used for MELD-related score calculation or stratified public analyses.    02_workflows/F5_workflow_v01/submission_ready/public/data/esld_F5_stratified_survival_subject_public.csv   File view   
  inr  International normalized ratio  International normalized ratio of prothrombin time used for MELD-related score calculation.  ratio  02_workflows/F5_workflow_v01/submission_ready/public/data/esld_F5_stratified_survival_subject_public.csv   File view   
  model  MELD model or score variant  Name of the MELD-related model or score variant represented by the row; expected values include MELD, MELD-Na, reMELD-Na, and MELD 3.0.    02_workflows/F5_workflow_v01/submission_ready/public/data/esld_F5_stratified_survival_subject_public.csv   File view   
  patient_id  Public patient pseudonym  Non-linkable public-release patient pseudonym used to preserve within-patient grouping in public ESLD data; not an original hospital patient identifier.    02_workflows/F5_workflow_v01/submission_ready/public/data/esld_F5_stratified_survival_subject_public.csv   File view   
  score_class  Score class  Categorical score class used for stratified analyses.    02_workflows/F5_workflow_v01/submission_ready/public/data/esld_F5_stratified_survival_subject_public.csv   File view   
  score_for_class  Score used for class assignment  Score value used to assign the row to a score class.  score points  02_workflows/F5_workflow_v01/submission_ready/public/data/esld_F5_stratified_survival_subject_public.csv   File view   
  scr.grp  Score-class stratum  Encoded score-class stratum used in F5; examples include le15, 16to25, and gt25.    02_workflows/F5_workflow_v01/submission_ready/public/data/esld_F5_stratified_survival_subject_public.csv   File view   
  sex  Sex  Sex category represented in the public ESLD data.    02_workflows/F5_workflow_v01/submission_ready/public/data/esld_F5_stratified_survival_subject_public.csv   File view   
  sodium  Serum sodium concentration  Serum sodium concentration used for MELD-Na, reMELD-Na, or MELD 3.0 score calculation.  mmol/L  02_workflows/F5_workflow_v01/submission_ready/public/data/esld_F5_stratified_survival_subject_public.csv   File view   
  source_file  Source file  Name of the source file used to create the public row or object.    02_workflows/F5_workflow_v01/submission_ready/public/data/esld_F5_stratified_survival_subject_public.csv   File view   
  status  Status flag  Binary or categorical status flag used in a public QC, manifest, or analysis table.    02_workflows/F5_workflow_v01/submission_ready/public/data/esld_F5_stratified_survival_subject_public.csv   File view   
  time  Analysis time  Analysis-specific time scale; interpretation depends on the respective figure/table workflow.    02_workflows/F5_workflow_v01/submission_ready/public/data/esld_F5_stratified_survival_subject_public.csv   File view   
  age_years_first_available  Age at first available public record  Age in years at the first available public ESLD record for the patient or encounter represented in the released data.  years  02_workflows/T1_workflow_v01/data/01_source_loaded_harmonized/esld_T1_baseline_patient_public.csv    
  ald  Alcohol-related liver disease etiology flag  Binary etiology indicator for alcohol-related liver disease in the public ESLD cohort.    02_workflows/T1_workflow_v01/data/01_source_loaded_harmonized/esld_T1_baseline_patient_public.csv    
  ald_hcv  Combined alcohol-related liver disease and hepatitis C etiology flag  Binary etiology indicator for combined alcohol-related liver disease and hepatitis C in the public ESLD cohort.    02_workflows/T1_workflow_v01/data/01_source_loaded_harmonized/esld_T1_baseline_patient_public.csv    
  any_dialysis  Any dialysis indicator  Binary indicator showing whether dialysis was present in the public baseline/table cohort.    02_workflows/T1_workflow_v01/data/01_source_loaded_harmonized/esld_T1_baseline_patient_public.csv    
  autoimmune  Autoimmune liver disease etiology flag  Binary etiology indicator for autoimmune liver disease in the public ESLD cohort.    02_workflows/T1_workflow_v01/data/01_source_loaded_harmonized/esld_T1_baseline_patient_public.csv    
  deceased_patient_flag  Deceased patient flag  Binary indicator identifying deceased patients within the released analysis context.    02_workflows/T1_workflow_v01/data/01_source_loaded_harmonized/esld_T1_baseline_patient_public.csv    
  etiology_any  Any etiology assigned  Binary indicator showing whether at least one etiology category is assigned.    02_workflows/T1_workflow_v01/data/01_source_loaded_harmonized/esld_T1_baseline_patient_public.csv    
  etiology_unclassified  Unclassified liver disease etiology flag  Binary etiology indicator identifying records without a classified liver disease etiology in the public ESLD cohort.    02_workflows/T1_workflow_v01/data/01_source_loaded_harmonized/esld_T1_baseline_patient_public.csv    
  hbv  Hepatitis B virus etiology flag  Binary etiology indicator for hepatitis B virus-related liver disease in the public ESLD cohort.    02_workflows/T1_workflow_v01/data/01_source_loaded_harmonized/esld_T1_baseline_patient_public.csv    
  hcv  Hepatitis C virus etiology flag  Binary etiology indicator for hepatitis C virus-related liver disease in the public ESLD cohort.    02_workflows/T1_workflow_v01/data/01_source_loaded_harmonized/esld_T1_baseline_patient_public.csv    
  mash  Metabolic dysfunction-associated steatohepatitis etiology flag  Binary etiology indicator for metabolic dysfunction-associated steatohepatitis in the public ESLD cohort.    02_workflows/T1_workflow_v01/data/01_source_loaded_harmonized/esld_T1_baseline_patient_public.csv    
  n_rows_meld  Number of rows for MELD  Number of rows available for the MELD model or score variant.  count  02_workflows/T1_workflow_v01/data/01_source_loaded_harmonized/esld_T1_baseline_patient_public.csv    
  n_rows_meld3  Number of rows for MELD 3.0  Number of rows available for the MELD 3.0 model or score variant.  count  02_workflows/T1_workflow_v01/data/01_source_loaded_harmonized/esld_T1_baseline_patient_public.csv    
  n_rows_meld_na  Number of rows for MELD-Na  Number of rows available for the MELD-Na model or score variant.  count  02_workflows/T1_workflow_v01/data/01_source_loaded_harmonized/esld_T1_baseline_patient_public.csv    
  n_rows_remeld_na  Number of rows for reMELD-Na  Number of rows available for the reMELD-Na model or score variant.  count  02_workflows/T1_workflow_v01/data/01_source_loaded_harmonized/esld_T1_baseline_patient_public.csv    
  other  Other liver disease etiology flag  Binary etiology indicator for liver disease etiologies grouped as other in the public ESLD cohort.    02_workflows/T1_workflow_v01/data/01_source_loaded_harmonized/esld_T1_baseline_patient_public.csv    
  patient_id  Public patient pseudonym  Non-linkable public-release patient pseudonym used to preserve within-patient grouping in public ESLD data; not an original hospital patient identifier.    02_workflows/T1_workflow_v01/data/01_source_loaded_harmonized/esld_T1_baseline_patient_public.csv    
  pbc  Primary biliary cholangitis etiology flag  Binary etiology indicator for primary biliary cholangitis in the public ESLD cohort.    02_workflows/T1_workflow_v01/data/01_source_loaded_harmonized/esld_T1_baseline_patient_public.csv    
  sex  Sex  Sex category represented in the public ESLD data.    02_workflows/T1_workflow_v01/data/01_source_loaded_harmonized/esld_T1_baseline_patient_public.csv    
  age_years_first_available  Age at first available public record  Age in years at the first available public ESLD record for the patient or encounter represented in the released data.  years  02_workflows/T1_workflow_v01/data/01_source_loaded_harmonized/esld_master_long_public.csv    
  age_years_sample  Age at sample  Age in years at the sample-level public ESLD record.  years  02_workflows/T1_workflow_v01/data/01_source_loaded_harmonized/esld_master_long_public.csv    
  albumin_g_dl  Serum albumin concentration  Serum albumin concentration in the public ESLD master data.  g/dL  02_workflows/T1_workflow_v01/data/01_source_loaded_harmonized/esld_master_long_public.csv    
  ald  Alcohol-related liver disease etiology flag  Binary etiology indicator for alcohol-related liver disease in the public ESLD cohort.    02_workflows/T1_workflow_v01/data/01_source_loaded_harmonized/esld_master_long_public.csv    
  ald_hcv  Combined alcohol-related liver disease and hepatitis C etiology flag  Binary etiology indicator for combined alcohol-related liver disease and hepatitis C in the public ESLD cohort.    02_workflows/T1_workflow_v01/data/01_source_loaded_harmonized/esld_master_long_public.csv    
  autoimmune  Autoimmune liver disease etiology flag  Binary etiology indicator for autoimmune liver disease in the public ESLD cohort.    02_workflows/T1_workflow_v01/data/01_source_loaded_harmonized/esld_master_long_public.csv    
  crea  Creatinine concentration  Creatinine concentration used in MELD-related score calculation before applying the study-specific correction.  mg/dL  02_workflows/T1_workflow_v01/data/01_source_loaded_harmonized/esld_master_long_public.csv    
  crea_corrected  Corrected creatinine concentration  Creatinine concentration after applying the study-specific correction used for recalculated MELD-related scores.  mg/dL  02_workflows/T1_workflow_v01/data/01_source_loaded_harmonized/esld_master_long_public.csv    
  date_count  Relative day count  Relative day count used for time alignment; negative/positive values are relative to the analysis anchor and are not calendar dates.    02_workflows/T1_workflow_v01/data/01_source_loaded_harmonized/esld_master_long_public.csv    
  date_count_month  Relative month count  Relative month count derived from the relative day count; this is not a calendar month.    02_workflows/T1_workflow_v01/data/01_source_loaded_harmonized/esld_master_long_public.csv    
  dead_sample_flag  Dead-sample flag  Binary indicator identifying public ESLD sample records assigned to the deceased-sample subset used in sample-level analyses.    02_workflows/T1_workflow_v01/data/01_source_loaded_harmonized/esld_master_long_public.csv    
  death_within_90d  Death within 90 days  Binary indicator for death within 90 days of the analysis anchor.    02_workflows/T1_workflow_v01/data/01_source_loaded_harmonized/esld_master_long_public.csv    
  deceased_patient_flag  Deceased patient flag  Binary indicator identifying deceased patients within the released analysis context.    02_workflows/T1_workflow_v01/data/01_source_loaded_harmonized/esld_master_long_public.csv    
  delta_eq_0_flag  Zero score-delta flag  Binary indicator equal to 1 when the score delta is exactly zero.    02_workflows/T1_workflow_v01/data/01_source_loaded_harmonized/esld_master_long_public.csv    
  delta_gt_0_flag  Positive score-delta flag  Binary indicator equal to 1 when the score delta is positive.    02_workflows/T1_workflow_v01/data/01_source_loaded_harmonized/esld_master_long_public.csv    
  delta_le_minus1_flag  Score-delta ≤ −1 flag  Binary indicator equal to 1 when the score delta is less than or equal to −1.    02_workflows/T1_workflow_v01/data/01_source_loaded_harmonized/esld_master_long_public.csv    
  dialysis_raw  Dialysis indicator as recorded before score derivation  Binary indicator showing whether dialysis was recorded in the source field before score derivation.    02_workflows/T1_workflow_v01/data/01_source_loaded_harmonized/esld_master_long_public.csv    
  encounter_id_public  Public encounter pseudonym  Non-linkable public-release encounter pseudonym used for encounter-level grouping; not an original hospital encounter identifier.    02_workflows/T1_workflow_v01/data/01_source_loaded_harmonized/esld_master_long_public.csv    
  etiology_source_current_flag  Current etiology source flag  Binary indicator identifying whether the etiology source is the current source used for the public row.    02_workflows/T1_workflow_v01/data/01_source_loaded_harmonized/esld_master_long_public.csv    
  etiology_unclassified  Unclassified liver disease etiology flag  Binary etiology indicator identifying records without a classified liver disease etiology in the public ESLD cohort.    02_workflows/T1_workflow_v01/data/01_source_loaded_harmonized/esld_master_long_public.csv    
  hbv  Hepatitis B virus etiology flag  Binary etiology indicator for hepatitis B virus-related liver disease in the public ESLD cohort.    02_workflows/T1_workflow_v01/data/01_source_loaded_harmonized/esld_master_long_public.csv    
  hcv  Hepatitis C virus etiology flag  Binary etiology indicator for hepatitis C virus-related liver disease in the public ESLD cohort.    02_workflows/T1_workflow_v01/data/01_source_loaded_harmonized/esld_master_long_public.csv    
  icd_source_present  ICD source present flag  Binary indicator showing whether an ICD-derived etiology source was present for the public row.    02_workflows/T1_workflow_v01/data/01_source_loaded_harmonized/esld_master_long_public.csv    
  in_t1_cohort  Inclusion flag for Table 1 cohort  Binary indicator identifying records included in the public ESLD Table 1 baseline-characteristics cohort.    02_workflows/T1_workflow_v01/data/01_source_loaded_harmonized/esld_master_long_public.csv    
  in_t3_anchor_model  Inclusion flag for Table 3 anchor-model cohort  Binary indicator identifying records included in the Table 3 anchor-model cohort used for creatinine-comparison analyses.    02_workflows/T1_workflow_v01/data/01_source_loaded_harmonized/esld_master_long_public.csv    
  in_t4_prevalence_path  Inclusion flag for Table 4 prevalence pathway  Binary indicator identifying records included in the Table 4 prevalence-analysis pathway.    02_workflows/T1_workflow_v01/data/01_source_loaded_harmonized/esld_master_long_public.csv    
  in_t4_survival_path  Inclusion flag for Table 4 survival pathway  Binary indicator identifying records included in the Table 4 survival-analysis pathway.    02_workflows/T1_workflow_v01/data/01_source_loaded_harmonized/esld_master_long_public.csv    
  inr  International normalized ratio  International normalized ratio of prothrombin time used for MELD-related score calculation.  ratio  02_workflows/T1_workflow_v01/data/01_source_loaded_harmonized/esld_master_long_public.csv    
  mash  Metabolic dysfunction-associated steatohepatitis etiology flag  Binary etiology indicator for metabolic dysfunction-associated steatohepatitis in the public ESLD cohort.    02_workflows/T1_workflow_v01/data/01_source_loaded_harmonized/esld_master_long_public.csv    
  model  MELD model or score variant  Name of the MELD-related model or score variant represented by the row; expected values include MELD, MELD-Na, reMELD-Na, and MELD 3.0.    02_workflows/T1_workflow_v01/data/01_source_loaded_harmonized/esld_master_long_public.csv    
  other  Other liver disease etiology flag  Binary etiology indicator for liver disease etiologies grouped as other in the public ESLD cohort.    02_workflows/T1_workflow_v01/data/01_source_loaded_harmonized/esld_master_long_public.csv    
  patient_id  Public patient pseudonym  Non-linkable public-release patient pseudonym used to preserve within-patient grouping in public ESLD data; not an original hospital patient identifier.    02_workflows/T1_workflow_v01/data/01_source_loaded_harmonized/esld_master_long_public.csv    
  pbc  Primary biliary cholangitis etiology flag  Binary etiology indicator for primary biliary cholangitis in the public ESLD cohort.    02_workflows/T1_workflow_v01/data/01_source_loaded_harmonized/esld_master_long_public.csv    
  sample_day_from_first_sample  Relative sample day  Relative day of the sample measured from the first sample for the public patient/sample sequence; not a calendar date.  relative days  02_workflows/T1_workflow_v01/data/01_source_loaded_harmonized/esld_master_long_public.csv    
  sample_group_id_public  Public sample-group pseudonym  Non-linkable public-release sample-group pseudonym used to group samples within the released public data.    02_workflows/T1_workflow_v01/data/01_source_loaded_harmonized/esld_master_long_public.csv    
  sample_id  Sample identifier  Identifier of a sample or experimental record within the released public data; not a personal identifier.    02_workflows/T1_workflow_v01/data/01_source_loaded_harmonized/esld_master_long_public.csv    
  sample_month_index  Relative sample month index  Relative month index of the sample measured from the first sample for the public patient/sample sequence; not a calendar month.  relative months  02_workflows/T1_workflow_v01/data/01_source_loaded_harmonized/esld_master_long_public.csv    
  score_corrected  Corrected score  MELD-related score after creatinine correction or recalculation.  score points  02_workflows/T1_workflow_v01/data/01_source_loaded_harmonized/esld_master_long_public.csv    
  score_delta  Score difference  Difference between score variants or scoring approaches, expressed in score points.    02_workflows/T1_workflow_v01/data/01_source_loaded_harmonized/esld_master_long_public.csv    
  score_original  Original score  Original MELD-related score before creatinine correction or recalculation.    02_workflows/T1_workflow_v01/data/01_source_loaded_harmonized/esld_master_long_public.csv    
  sex  Sex  Sex category represented in the public ESLD data.    02_workflows/T1_workflow_v01/data/01_source_loaded_harmonized/esld_master_long_public.csv    
  sodium  Serum sodium concentration  Serum sodium concentration used for MELD-Na, reMELD-Na, or MELD 3.0 score calculation.  mmol/L  02_workflows/T1_workflow_v01/data/01_source_loaded_harmonized/esld_master_long_public.csv    
  tbil  Total bilirubin concentration  Total bilirubin concentration used in MELD-related score calculation in the public ESLD data.    02_workflows/T1_workflow_v01/data/01_source_loaded_harmonized/esld_master_long_public.csv    
  age_years_first_available  Age at first available public record  Age in years at the first available public ESLD record for the patient or encounter represented in the released data.  years  02_workflows/T3_workflow_v01/data/01_source_loaded_harmonized/esld_master_long_public.csv    
  age_years_sample  Age at sample  Age in years at the sample-level public ESLD record.  years  02_workflows/T3_workflow_v01/data/01_source_loaded_harmonized/esld_master_long_public.csv    
  albumin_g_dl  Serum albumin concentration  Serum albumin concentration in the public ESLD master data.  g/dL  02_workflows/T3_workflow_v01/data/01_source_loaded_harmonized/esld_master_long_public.csv    
  ald  Alcohol-related liver disease etiology flag  Binary etiology indicator for alcohol-related liver disease in the public ESLD cohort.    02_workflows/T3_workflow_v01/data/01_source_loaded_harmonized/esld_master_long_public.csv    
  ald_hcv  Combined alcohol-related liver disease and hepatitis C etiology flag  Binary etiology indicator for combined alcohol-related liver disease and hepatitis C in the public ESLD cohort.    02_workflows/T3_workflow_v01/data/01_source_loaded_harmonized/esld_master_long_public.csv    
  autoimmune  Autoimmune liver disease etiology flag  Binary etiology indicator for autoimmune liver disease in the public ESLD cohort.    02_workflows/T3_workflow_v01/data/01_source_loaded_harmonized/esld_master_long_public.csv    
  crea  Creatinine concentration  Creatinine concentration used in MELD-related score calculation before applying the study-specific correction.  mg/dL  02_workflows/T3_workflow_v01/data/01_source_loaded_harmonized/esld_master_long_public.csv    
  crea_corrected  Corrected creatinine concentration  Creatinine concentration after applying the study-specific correction used for recalculated MELD-related scores.  mg/dL  02_workflows/T3_workflow_v01/data/01_source_loaded_harmonized/esld_master_long_public.csv    
  date_count  Relative day count  Relative day count used for time alignment; negative/positive values are relative to the analysis anchor and are not calendar dates.    02_workflows/T3_workflow_v01/data/01_source_loaded_harmonized/esld_master_long_public.csv    
  date_count_month  Relative month count  Relative month count derived from the relative day count; this is not a calendar month.    02_workflows/T3_workflow_v01/data/01_source_loaded_harmonized/esld_master_long_public.csv    
  dead_sample_flag  Dead-sample flag  Binary indicator identifying public ESLD sample records assigned to the deceased-sample subset used in sample-level analyses.    02_workflows/T3_workflow_v01/data/01_source_loaded_harmonized/esld_master_long_public.csv    
  death_within_90d  Death within 90 days  Binary indicator for death within 90 days of the analysis anchor.    02_workflows/T3_workflow_v01/data/01_source_loaded_harmonized/esld_master_long_public.csv    
  deceased_patient_flag  Deceased patient flag  Binary indicator identifying deceased patients within the released analysis context.    02_workflows/T3_workflow_v01/data/01_source_loaded_harmonized/esld_master_long_public.csv    
  delta_eq_0_flag  Zero score-delta flag  Binary indicator equal to 1 when the score delta is exactly zero.    02_workflows/T3_workflow_v01/data/01_source_loaded_harmonized/esld_master_long_public.csv    
  delta_gt_0_flag  Positive score-delta flag  Binary indicator equal to 1 when the score delta is positive.    02_workflows/T3_workflow_v01/data/01_source_loaded_harmonized/esld_master_long_public.csv    
  delta_le_minus1_flag  Score-delta ≤ −1 flag  Binary indicator equal to 1 when the score delta is less than or equal to −1.    02_workflows/T3_workflow_v01/data/01_source_loaded_harmonized/esld_master_long_public.csv    
  dialysis_raw  Dialysis indicator as recorded before score derivation  Binary indicator showing whether dialysis was recorded in the source field before score derivation.    02_workflows/T3_workflow_v01/data/01_source_loaded_harmonized/esld_master_long_public.csv    
  encounter_id_public  Public encounter pseudonym  Non-linkable public-release encounter pseudonym used for encounter-level grouping; not an original hospital encounter identifier.    02_workflows/T3_workflow_v01/data/01_source_loaded_harmonized/esld_master_long_public.csv    
  etiology_source_current_flag  Current etiology source flag  Binary indicator identifying whether the etiology source is the current source used for the public row.    02_workflows/T3_workflow_v01/data/01_source_loaded_harmonized/esld_master_long_public.csv    
  etiology_unclassified  Unclassified liver disease etiology flag  Binary etiology indicator identifying records without a classified liver disease etiology in the public ESLD cohort.    02_workflows/T3_workflow_v01/data/01_source_loaded_harmonized/esld_master_long_public.csv    
  hbv  Hepatitis B virus etiology flag  Binary etiology indicator for hepatitis B virus-related liver disease in the public ESLD cohort.    02_workflows/T3_workflow_v01/data/01_source_loaded_harmonized/esld_master_long_public.csv    
  hcv  Hepatitis C virus etiology flag  Binary etiology indicator for hepatitis C virus-related liver disease in the public ESLD cohort.    02_workflows/T3_workflow_v01/data/01_source_loaded_harmonized/esld_master_long_public.csv    
  icd_source_present  ICD source present flag  Binary indicator showing whether an ICD-derived etiology source was present for the public row.    02_workflows/T3_workflow_v01/data/01_source_loaded_harmonized/esld_master_long_public.csv    
  in_t1_cohort  Inclusion flag for Table 1 cohort  Binary indicator identifying records included in the public ESLD Table 1 baseline-characteristics cohort.    02_workflows/T3_workflow_v01/data/01_source_loaded_harmonized/esld_master_long_public.csv    
  in_t3_anchor_model  Inclusion flag for Table 3 anchor-model cohort  Binary indicator identifying records included in the Table 3 anchor-model cohort used for creatinine-comparison analyses.    02_workflows/T3_workflow_v01/data/01_source_loaded_harmonized/esld_master_long_public.csv    
  in_t4_prevalence_path  Inclusion flag for Table 4 prevalence pathway  Binary indicator identifying records included in the Table 4 prevalence-analysis pathway.    02_workflows/T3_workflow_v01/data/01_source_loaded_harmonized/esld_master_long_public.csv    
  in_t4_survival_path  Inclusion flag for Table 4 survival pathway  Binary indicator identifying records included in the Table 4 survival-analysis pathway.    02_workflows/T3_workflow_v01/data/01_source_loaded_harmonized/esld_master_long_public.csv    
  inr  International normalized ratio  International normalized ratio of prothrombin time used for MELD-related score calculation.  ratio  02_workflows/T3_workflow_v01/data/01_source_loaded_harmonized/esld_master_long_public.csv    
  mash  Metabolic dysfunction-associated steatohepatitis etiology flag  Binary etiology indicator for metabolic dysfunction-associated steatohepatitis in the public ESLD cohort.    02_workflows/T3_workflow_v01/data/01_source_loaded_harmonized/esld_master_long_public.csv    
  model  MELD model or score variant  Name of the MELD-related model or score variant represented by the row; expected values include MELD, MELD-Na, reMELD-Na, and MELD 3.0.    02_workflows/T3_workflow_v01/data/01_source_loaded_harmonized/esld_master_long_public.csv    
  other  Other liver disease etiology flag  Binary etiology indicator for liver disease etiologies grouped as other in the public ESLD cohort.    02_workflows/T3_workflow_v01/data/01_source_loaded_harmonized/esld_master_long_public.csv    
  patient_id  Public patient pseudonym  Non-linkable public-release patient pseudonym used to preserve within-patient grouping in public ESLD data; not an original hospital patient identifier.    02_workflows/T3_workflow_v01/data/01_source_loaded_harmonized/esld_master_long_public.csv    
  pbc  Primary biliary cholangitis etiology flag  Binary etiology indicator for primary biliary cholangitis in the public ESLD cohort.    02_workflows/T3_workflow_v01/data/01_source_loaded_harmonized/esld_master_long_public.csv    
  sample_day_from_first_sample  Relative sample day  Relative day of the sample measured from the first sample for the public patient/sample sequence; not a calendar date.  relative days  02_workflows/T3_workflow_v01/data/01_source_loaded_harmonized/esld_master_long_public.csv    
  sample_group_id_public  Public sample-group pseudonym  Non-linkable public-release sample-group pseudonym used to group samples within the released public data.    02_workflows/T3_workflow_v01/data/01_source_loaded_harmonized/esld_master_long_public.csv    
  sample_id  Sample identifier  Identifier of a sample or experimental record within the released public data; not a personal identifier.    02_workflows/T3_workflow_v01/data/01_source_loaded_harmonized/esld_master_long_public.csv    
  sample_month_index  Relative sample month index  Relative month index of the sample measured from the first sample for the public patient/sample sequence; not a calendar month.  relative months  02_workflows/T3_workflow_v01/data/01_source_loaded_harmonized/esld_master_long_public.csv    
  score_corrected  Corrected score  MELD-related score after creatinine correction or recalculation.  score points  02_workflows/T3_workflow_v01/data/01_source_loaded_harmonized/esld_master_long_public.csv    
  score_delta  Score difference  Difference between score variants or scoring approaches, expressed in score points.    02_workflows/T3_workflow_v01/data/01_source_loaded_harmonized/esld_master_long_public.csv    
  score_original  Original score  Original MELD-related score before creatinine correction or recalculation.    02_workflows/T3_workflow_v01/data/01_source_loaded_harmonized/esld_master_long_public.csv    
  sex  Sex  Sex category represented in the public ESLD data.    02_workflows/T3_workflow_v01/data/01_source_loaded_harmonized/esld_master_long_public.csv    
  sodium  Serum sodium concentration  Serum sodium concentration used for MELD-Na, reMELD-Na, or MELD 3.0 score calculation.  mmol/L  02_workflows/T3_workflow_v01/data/01_source_loaded_harmonized/esld_master_long_public.csv    
  tbil  Total bilirubin concentration  Total bilirubin concentration used in MELD-related score calculation in the public ESLD data.    02_workflows/T3_workflow_v01/data/01_source_loaded_harmonized/esld_master_long_public.csv    
  age_years_first_available  Age at first available public record  Age in years at the first available public ESLD record for the patient or encounter represented in the released data.  years  02_workflows/T4_workflow_v01/data/01_source_loaded_harmonized/esld_master_long_public.csv    
  age_years_sample  Age at sample  Age in years at the sample-level public ESLD record.  years  02_workflows/T4_workflow_v01/data/01_source_loaded_harmonized/esld_master_long_public.csv    
  albumin_g_dl  Serum albumin concentration  Serum albumin concentration in the public ESLD master data.  g/dL  02_workflows/T4_workflow_v01/data/01_source_loaded_harmonized/esld_master_long_public.csv    
  ald  Alcohol-related liver disease etiology flag  Binary etiology indicator for alcohol-related liver disease in the public ESLD cohort.    02_workflows/T4_workflow_v01/data/01_source_loaded_harmonized/esld_master_long_public.csv    
  ald_hcv  Combined alcohol-related liver disease and hepatitis C etiology flag  Binary etiology indicator for combined alcohol-related liver disease and hepatitis C in the public ESLD cohort.    02_workflows/T4_workflow_v01/data/01_source_loaded_harmonized/esld_master_long_public.csv    
  autoimmune  Autoimmune liver disease etiology flag  Binary etiology indicator for autoimmune liver disease in the public ESLD cohort.    02_workflows/T4_workflow_v01/data/01_source_loaded_harmonized/esld_master_long_public.csv    
  crea  Creatinine concentration  Creatinine concentration used in MELD-related score calculation before applying the study-specific correction.  mg/dL  02_workflows/T4_workflow_v01/data/01_source_loaded_harmonized/esld_master_long_public.csv    
  crea_corrected  Corrected creatinine concentration  Creatinine concentration after applying the study-specific correction used for recalculated MELD-related scores.  mg/dL  02_workflows/T4_workflow_v01/data/01_source_loaded_harmonized/esld_master_long_public.csv    
  date_count  Relative day count  Relative day count used for time alignment; negative/positive values are relative to the analysis anchor and are not calendar dates.    02_workflows/T4_workflow_v01/data/01_source_loaded_harmonized/esld_master_long_public.csv    
  date_count_month  Relative month count  Relative month count derived from the relative day count; this is not a calendar month.    02_workflows/T4_workflow_v01/data/01_source_loaded_harmonized/esld_master_long_public.csv    
  dead_sample_flag  Dead-sample flag  Binary indicator identifying public ESLD sample records assigned to the deceased-sample subset used in sample-level analyses.    02_workflows/T4_workflow_v01/data/01_source_loaded_harmonized/esld_master_long_public.csv    
  death_within_90d  Death within 90 days  Binary indicator for death within 90 days of the analysis anchor.    02_workflows/T4_workflow_v01/data/01_source_loaded_harmonized/esld_master_long_public.csv    
  deceased_patient_flag  Deceased patient flag  Binary indicator identifying deceased patients within the released analysis context.    02_workflows/T4_workflow_v01/data/01_source_loaded_harmonized/esld_master_long_public.csv    
  delta_eq_0_flag  Zero score-delta flag  Binary indicator equal to 1 when the score delta is exactly zero.    02_workflows/T4_workflow_v01/data/01_source_loaded_harmonized/esld_master_long_public.csv    
  delta_gt_0_flag  Positive score-delta flag  Binary indicator equal to 1 when the score delta is positive.    02_workflows/T4_workflow_v01/data/01_source_loaded_harmonized/esld_master_long_public.csv    
  delta_le_minus1_flag  Score-delta ≤ −1 flag  Binary indicator equal to 1 when the score delta is less than or equal to −1.    02_workflows/T4_workflow_v01/data/01_source_loaded_harmonized/esld_master_long_public.csv    
  dialysis_raw  Dialysis indicator as recorded before score derivation  Binary indicator showing whether dialysis was recorded in the source field before score derivation.    02_workflows/T4_workflow_v01/data/01_source_loaded_harmonized/esld_master_long_public.csv    
  encounter_id_public  Public encounter pseudonym  Non-linkable public-release encounter pseudonym used for encounter-level grouping; not an original hospital encounter identifier.    02_workflows/T4_workflow_v01/data/01_source_loaded_harmonized/esld_master_long_public.csv    
  etiology_source_current_flag  Current etiology source flag  Binary indicator identifying whether the etiology source is the current source used for the public row.    02_workflows/T4_workflow_v01/data/01_source_loaded_harmonized/esld_master_long_public.csv    
  etiology_unclassified  Unclassified liver disease etiology flag  Binary etiology indicator identifying records without a classified liver disease etiology in the public ESLD cohort.    02_workflows/T4_workflow_v01/data/01_source_loaded_harmonized/esld_master_long_public.csv    
  hbv  Hepatitis B virus etiology flag  Binary etiology indicator for hepatitis B virus-related liver disease in the public ESLD cohort.    02_workflows/T4_workflow_v01/data/01_source_loaded_harmonized/esld_master_long_public.csv    
  hcv  Hepatitis C virus etiology flag  Binary etiology indicator for hepatitis C virus-related liver disease in the public ESLD cohort.    02_workflows/T4_workflow_v01/data/01_source_loaded_harmonized/esld_master_long_public.csv    
  icd_source_present  ICD source present flag  Binary indicator showing whether an ICD-derived etiology source was present for the public row.    02_workflows/T4_workflow_v01/data/01_source_loaded_harmonized/esld_master_long_public.csv    
  in_t1_cohort  Inclusion flag for Table 1 cohort  Binary indicator identifying records included in the public ESLD Table 1 baseline-characteristics cohort.    02_workflows/T4_workflow_v01/data/01_source_loaded_harmonized/esld_master_long_public.csv    
  in_t3_anchor_model  Inclusion flag for Table 3 anchor-model cohort  Binary indicator identifying records included in the Table 3 anchor-model cohort used for creatinine-comparison analyses.    02_workflows/T4_workflow_v01/data/01_source_loaded_harmonized/esld_master_long_public.csv    
  in_t4_prevalence_path  Inclusion flag for Table 4 prevalence pathway  Binary indicator identifying records included in the Table 4 prevalence-analysis pathway.    02_workflows/T4_workflow_v01/data/01_source_loaded_harmonized/esld_master_long_public.csv    
  in_t4_survival_path  Inclusion flag for Table 4 survival pathway  Binary indicator identifying records included in the Table 4 survival-analysis pathway.    02_workflows/T4_workflow_v01/data/01_source_loaded_harmonized/esld_master_long_public.csv    
  inr  International normalized ratio  International normalized ratio of prothrombin time used for MELD-related score calculation.  ratio  02_workflows/T4_workflow_v01/data/01_source_loaded_harmonized/esld_master_long_public.csv    
  mash  Metabolic dysfunction-associated steatohepatitis etiology flag  Binary etiology indicator for metabolic dysfunction-associated steatohepatitis in the public ESLD cohort.    02_workflows/T4_workflow_v01/data/01_source_loaded_harmonized/esld_master_long_public.csv    
  model  MELD model or score variant  Name of the MELD-related model or score variant represented by the row; expected values include MELD, MELD-Na, reMELD-Na, and MELD 3.0.    02_workflows/T4_workflow_v01/data/01_source_loaded_harmonized/esld_master_long_public.csv    
  other  Other liver disease etiology flag  Binary etiology indicator for liver disease etiologies grouped as other in the public ESLD cohort.    02_workflows/T4_workflow_v01/data/01_source_loaded_harmonized/esld_master_long_public.csv    
  patient_id  Public patient pseudonym  Non-linkable public-release patient pseudonym used to preserve within-patient grouping in public ESLD data; not an original hospital patient identifier.    02_workflows/T4_workflow_v01/data/01_source_loaded_harmonized/esld_master_long_public.csv    
  pbc  Primary biliary cholangitis etiology flag  Binary etiology indicator for primary biliary cholangitis in the public ESLD cohort.    02_workflows/T4_workflow_v01/data/01_source_loaded_harmonized/esld_master_long_public.csv    
  sample_day_from_first_sample  Relative sample day  Relative day of the sample measured from the first sample for the public patient/sample sequence; not a calendar date.  relative days  02_workflows/T4_workflow_v01/data/01_source_loaded_harmonized/esld_master_long_public.csv    
  sample_group_id_public  Public sample-group pseudonym  Non-linkable public-release sample-group pseudonym used to group samples within the released public data.    02_workflows/T4_workflow_v01/data/01_source_loaded_harmonized/esld_master_long_public.csv    
  sample_id  Sample identifier  Identifier of a sample or experimental record within the released public data; not a personal identifier.    02_workflows/T4_workflow_v01/data/01_source_loaded_harmonized/esld_master_long_public.csv    
  sample_month_index  Relative sample month index  Relative month index of the sample measured from the first sample for the public patient/sample sequence; not a calendar month.  relative months  02_workflows/T4_workflow_v01/data/01_source_loaded_harmonized/esld_master_long_public.csv    
  score_corrected  Corrected score  MELD-related score after creatinine correction or recalculation.  score points  02_workflows/T4_workflow_v01/data/01_source_loaded_harmonized/esld_master_long_public.csv    
  score_delta  Score difference  Difference between score variants or scoring approaches, expressed in score points.    02_workflows/T4_workflow_v01/data/01_source_loaded_harmonized/esld_master_long_public.csv    
  score_original  Original score  Original MELD-related score before creatinine correction or recalculation.    02_workflows/T4_workflow_v01/data/01_source_loaded_harmonized/esld_master_long_public.csv    
  sex  Sex  Sex category represented in the public ESLD data.    02_workflows/T4_workflow_v01/data/01_source_loaded_harmonized/esld_master_long_public.csv    
  sodium  Serum sodium concentration  Serum sodium concentration used for MELD-Na, reMELD-Na, or MELD 3.0 score calculation.  mmol/L  02_workflows/T4_workflow_v01/data/01_source_loaded_harmonized/esld_master_long_public.csv    
  tbil  Total bilirubin concentration  Total bilirubin concentration used in MELD-related score calculation in the public ESLD data.    02_workflows/T4_workflow_v01/data/01_source_loaded_harmonized/esld_master_long_public.csv    
  parameter  Metadata parameter name  Name of a metadata parameter describing the F2 simulated heatmap object, such as figure identity, data origin, grid type, axis variable, or unit/role.    02_workflows/T4_workflow_v01/data/02b_table_content/esld_T4_score_deviation_outcome_meta_public.csv    
  value  Value  Numerical or character value corresponding to the row-specific variable/metric.    02_workflows/T4_workflow_v01/data/02b_table_content/esld_T4_score_deviation_outcome_meta_public.csv    
  MELD  MELD result column  Formatted result value for the MELD score variant in the public table/summary row.  formatted table value  02_workflows/T4_workflow_v01/data/02b_table_content/esld_T4_score_deviation_outcome_table_public.csv    
  MELD 3.0  MELD 3.0 result column  Formatted result value for the MELD 3.0 score variant in the public table/summary row.  formatted table value  02_workflows/T4_workflow_v01/data/02b_table_content/esld_T4_score_deviation_outcome_table_public.csv    
  MELD-Na  MELD-Na result column  Formatted result value for the MELD-Na score variant in the public table/summary row.  formatted table value  02_workflows/T4_workflow_v01/data/02b_table_content/esld_T4_score_deviation_outcome_table_public.csv    
  anchor  Anchor/output group  Anchor or output grouping label used by the workflow to identify a specific public output component.    02_workflows/T4_workflow_v01/data/02b_table_content/esld_T4_score_deviation_outcome_table_public.csv    
  data_object  Data object  Name of the data object represented by the row.    02_workflows/T4_workflow_v01/data/02b_table_content/esld_T4_score_deviation_outcome_table_public.csv    
  domain  Data domain  Workflow or data domain represented by the row.    02_workflows/T4_workflow_v01/data/02b_table_content/esld_T4_score_deviation_outcome_table_public.csv    
  reMELD-Na  reMELD-Na result column  Formatted result value for the reMELD-Na score variant in the public table/summary row.  formatted table value  02_workflows/T4_workflow_v01/data/02b_table_content/esld_T4_score_deviation_outcome_table_public.csv    
  release_status  Release status  Release-status label indicating the publication status of the row or file object.    02_workflows/T4_workflow_v01/data/02b_table_content/esld_T4_score_deviation_outcome_table_public.csv    
  row_order  Display row order  Integer row order used to render the public table in the intended display sequence.  relative days  02_workflows/T4_workflow_v01/data/02b_table_content/esld_T4_score_deviation_outcome_table_public.csv    
  section  Table section  Section heading or row group used in the rendered public table.    02_workflows/T4_workflow_v01/data/02b_table_content/esld_T4_score_deviation_outcome_table_public.csv    
  unit_or_role  Unit or semantic role  Unit, role, or semantic type corresponding to the row-specific variable/metric.    02_workflows/T4_workflow_v01/data/02b_table_content/esld_T4_score_deviation_outcome_table_public.csv    
  variable  Variable represented by row  Name of the variable represented by the row in a long-format table.    02_workflows/T4_workflow_v01/data/02b_table_content/esld_T4_score_deviation_outcome_table_public.csv    
  parameter  Metadata parameter name  Name of a metadata parameter describing the F2 simulated heatmap object, such as figure identity, data origin, grid type, axis variable, or unit/role.    02_workflows/T4_workflow_v01/submission_ready/public/data/esld_T4_score_deviation_outcome_meta_public.csv   File view   
  value  Value  Numerical or character value corresponding to the row-specific variable/metric.    02_workflows/T4_workflow_v01/submission_ready/public/data/esld_T4_score_deviation_outcome_meta_public.csv   File view   
  MELD  MELD result column  Formatted result value for the MELD score variant in the public table/summary row.  formatted table value  02_workflows/T4_workflow_v01/submission_ready/public/data/esld_T4_score_deviation_outcome_table_public.csv   File view   
  MELD 3.0  MELD 3.0 result column  Formatted result value for the MELD 3.0 score variant in the public table/summary row.  formatted table value  02_workflows/T4_workflow_v01/submission_ready/public/data/esld_T4_score_deviation_outcome_table_public.csv   File view   
  MELD-Na  MELD-Na result column  Formatted result value for the MELD-Na score variant in the public table/summary row.  formatted table value  02_workflows/T4_workflow_v01/submission_ready/public/data/esld_T4_score_deviation_outcome_table_public.csv   File view   
  anchor  Anchor/output group  Anchor or output grouping label used by the workflow to identify a specific public output component.    02_workflows/T4_workflow_v01/submission_ready/public/data/esld_T4_score_deviation_outcome_table_public.csv   File view   
  data_object  Data object  Name of the data object represented by the row.    02_workflows/T4_workflow_v01/submission_ready/public/data/esld_T4_score_deviation_outcome_table_public.csv   File view   
  domain  Data domain  Workflow or data domain represented by the row.    02_workflows/T4_workflow_v01/submission_ready/public/data/esld_T4_score_deviation_outcome_table_public.csv   File view   
  reMELD-Na  reMELD-Na result column  Formatted result value for the reMELD-Na score variant in the public table/summary row.  formatted table value  02_workflows/T4_workflow_v01/submission_ready/public/data/esld_T4_score_deviation_outcome_table_public.csv   File view   
  release_status  Release status  Release-status label indicating the publication status of the row or file object.    02_workflows/T4_workflow_v01/submission_ready/public/data/esld_T4_score_deviation_outcome_table_public.csv   File view   
  row_order  Display row order  Integer row order used to render the public table in the intended display sequence.  relative days  02_workflows/T4_workflow_v01/submission_ready/public/data/esld_T4_score_deviation_outcome_table_public.csv   File view   
  section  Table section  Section heading or row group used in the rendered public table.    02_workflows/T4_workflow_v01/submission_ready/public/data/esld_T4_score_deviation_outcome_table_public.csv   File view   
  unit_or_role  Unit or semantic role  Unit, role, or semantic type corresponding to the row-specific variable/metric.    02_workflows/T4_workflow_v01/submission_ready/public/data/esld_T4_score_deviation_outcome_table_public.csv   File view   
  variable  Variable represented by row  Name of the variable represented by the row in a long-format table.    02_workflows/T4_workflow_v01/submission_ready/public/data/esld_T4_score_deviation_outcome_table_public.csv   File view       

  Section summary   
   Section  Files  Variables  Size KB   
   Start here  1  0  1.952  
  Public primary data  7  81  22566.779  
  Manuscript output data  13  115  13331.086  
  Public figures  6  0  4894.351  
  Workflow readmes  7  0  3.334  
  Technical appendix / request manifests  5  0  534.67      

  Hidden technical/audit files  Hidden from the recommended list because they are generated logs, older dictionaries, patch histories, intermediate workflow duplicates, system files, or internal audit artifacts.   
   Path  Reason  Type  Size KB   
   02_workflows/F1_workflow_v02/figures/slco_F1_surface_public.pdf  hide_duplicate_workflow_figure  pdf  1506.008  
  02_workflows/F2_workflow_v01/figures/slco_F2_heatmap_public.pdf  hide_duplicate_workflow_figure  pdf  856.709  
  02_workflows/F3_workflow_v01/figures/F3_ESLD_SRTR_public.pdf  hide_duplicate_workflow_figure  pdf  556.029  
  02_workflows/F4_workflow_v01/figures/F4_ESLD_SRTR_public.pdf  hide_duplicate_workflow_figure  pdf  817.854  
  02_workflows/F5_workflow_v01/figures/F5_ESLD_public.pdf  hide_duplicate_workflow_figure  pdf  602.098  
  02_workflows/F6_workflow_v01/figures/F6_SRTR_public.pdf  hide_duplicate_workflow_figure  pdf  555.652  
  02_workflows/F3_workflow_v01/README_F3_workflow_v01.txt  hide_duplicate_workflow_readme  txt  0.436  
  02_workflows/F4_workflow_v01/README_F4_workflow_v01.txt  hide_duplicate_workflow_readme  txt  0.437  
  02_workflows/F5_workflow_v01/README_F5_workflow_v01.txt  hide_duplicate_workflow_readme  txt  0.685  
  02_workflows/F6_workflow_v01/README_F6_workflow_v01.txt  hide_duplicate_workflow_readme  txt  0.618  
  02_workflows/T1_workflow_v01/README_T1_workflow_v01.txt  hide_duplicate_workflow_readme  txt  0.912  
  32_list_final_public_package_contents_v01.R  hide_helper_script  r  5.492  
  02_workflows/F1_workflow_v02/data/01_source_loaded_harmonized/expm_F1_array_raw_public.csv  hide_intermediate_workflow_data  csv  331.003  
  02_workflows/F1_workflow_v02/data/01_source_loaded_harmonized/expm_F1_validation_raw_public.csv  hide_intermediate_workflow_data  csv  2.28  
  02_workflows/F1_workflow_v02/data/01_source_loaded_harmonized/expm_slco_F1_source_load_manifest_public.csv  hide_intermediate_workflow_data  csv  0.981  
  02_workflows/F1_workflow_v02/data/01_source_loaded_harmonized/expm_slco_F1_step1_run_inputs_public.csv  hide_intermediate_workflow_data  csv  0.388  
  02_workflows/F1_workflow_v02/data/01_source_loaded_harmonized/slco_F1_surface_grid_reference_public.csv  hide_intermediate_workflow_data  csv  4936.887  
  02_workflows/F1_workflow_v02/data/01_source_loaded_harmonized/slco_F1_surface_meta_reference_public.csv  hide_intermediate_workflow_data  csv  0.648  
  02_workflows/F1_workflow_v02/data/02a_refined_analysis/expm_F1_array_input_public.csv  hide_intermediate_workflow_data  csv  737.671  
  02_workflows/F1_workflow_v02/data/02a_refined_analysis/expm_F1_model_coefficients_public.csv  hide_intermediate_workflow_data  csv  1.299  
  02_workflows/F1_workflow_v02/data/02a_refined_analysis/expm_F1_pre_correction_rounding_qc_public.csv  hide_intermediate_workflow_data  csv  0.451  
  02_workflows/F1_workflow_v02/data/02a_refined_analysis/expm_F1_refined_dataset_qc_public.csv  hide_intermediate_workflow_data  csv  0.637  
  02_workflows/F1_workflow_v02/data/02a_refined_analysis/expm_F1_refined_manual_clarifications_public.csv  hide_intermediate_workflow_data  csv  0.861  
  02_workflows/F1_workflow_v02/data/02a_refined_analysis/expm_F1_validation_input_public.csv  hide_intermediate_workflow_data  csv  4.949  
  02_workflows/F1_workflow_v02/data/02b_figure_content/slco_F1_surface_grid_public.csv  hide_intermediate_workflow_data  csv  4918.95  
  02_workflows/F1_workflow_v02/data/02b_figure_content/slco_F1_surface_meta_public.csv  hide_intermediate_workflow_data  csv  0.482  
  02_workflows/F1_workflow_v02/data/02b_figure_content/slco_F1_surface_rebuild_qc_public.csv  hide_intermediate_workflow_data  csv  0.934  
  02_workflows/F1_workflow_v02/data/02b_figure_content/slco_F1_surface_reference_meta_original_public.csv  hide_intermediate_workflow_data  csv  0.74  
  02_workflows/F1_workflow_v02/data/02b_figure_content/slco_F1_surface_run_inputs_outputs_public.csv  hide_intermediate_workflow_data  csv  0.563  
  02_workflows/F2_workflow_v01/data/02b_figure_content/slco_F2_heatmap_bin_public.csv  hide_intermediate_workflow_data  csv  7674.803  
  02_workflows/F2_workflow_v01/data/02b_figure_content/slco_F2_heatmap_meta_public.csv  hide_intermediate_workflow_data  csv  0.539  
  02_workflows/F3_workflow_v01/data/01_source_loaded_harmonized/esld_master_long_public.csv  hide_intermediate_workflow_data  csv  9620.613  
  02_workflows/F3_workflow_v01/data/02b_figure_content/esld_F3_score_shift_aggregate_public.csv  hide_intermediate_workflow_data  csv  6.515  
  02_workflows/F4_workflow_v01/data/01_source_loaded_harmonized/esld_master_long_public.csv  hide_intermediate_workflow_data  csv  9620.613  
  02_workflows/F4_workflow_v01/data/02b_figure_content/esld_F4_survival_stats_public.csv  hide_intermediate_workflow_data  csv  2.255  
  02_workflows/F5_workflow_v01/data/01_source_loaded_harmonized/esld_master_long_public.csv  hide_intermediate_workflow_data  csv  9620.613  
  02_workflows/F5_workflow_v01/data/02b_figure_content/esld_F5_stratified_survival_meta_public.csv  hide_intermediate_workflow_data  csv  0.424  
  02_workflows/F5_workflow_v01/data/02b_figure_content/esld_F5_stratified_survival_stats_public.csv  hide_intermediate_workflow_data  csv  1.093  
  02_workflows/F5_workflow_v01/data/02b_figure_content/esld_F5_stratified_survival_subject_public.csv  hide_intermediate_workflow_data  csv  119.284  
  02_workflows/T1_workflow_v01/data/01_source_loaded_harmonized/esld_T1_baseline_patient_public.csv  hide_intermediate_workflow_data  csv  67.969  
  02_workflows/T1_workflow_v01/data/01_source_loaded_harmonized/esld_master_long_public.csv  hide_intermediate_workflow_data  csv  9620.613  
  02_workflows/T3_workflow_v01/data/01_source_loaded_harmonized/esld_master_long_public.csv  hide_intermediate_workflow_data  csv  9620.613  
  02_workflows/T4_workflow_v01/data/01_source_loaded_harmonized/esld_master_long_public.csv  hide_intermediate_workflow_data  csv  9620.613  
  02_workflows/T4_workflow_v01/data/02b_table_content/esld_T4_score_deviation_outcome_meta_public.csv  hide_intermediate_workflow_data  csv  0.678  
  02_workflows/T4_workflow_v01/data/02b_table_content/esld_T4_score_deviation_outcome_table_public.csv  hide_intermediate_workflow_data  csv  2.566  
  00_release_manifests/failed_public_file_copies_v01.csv  hide_internal_audit_manifest  csv  0.899  
  00_release_manifests/final_dictionary_remaining_review_after_global_sanitize_v01.csv  hide_internal_audit_manifest  csv  0.972  
  00_release_manifests/final_global_absolute_path_scan_before_v01.csv  hide_internal_audit_manifest  csv  466.718  
  00_release_manifests/final_global_absolute_path_scan_v01.csv  hide_internal_audit_manifest  csv  0.031  
  00_release_manifests/final_global_path_sanitization_log_v01.csv  hide_internal_audit_manifest  csv  796.206  
  00_release_manifests/final_public_package_upload_readiness_audit_v01.txt  hide_internal_audit_manifest  txt  5.709  
  00_release_manifests/final_public_package_upload_readiness_summary_v01.csv  hide_internal_audit_manifest  csv  0.632  
  00_release_manifests/final_release_file_manifest_closed_v01.csv  hide_internal_audit_manifest  csv  274.4  
  00_release_manifests/final_remaining_root_R_scripts_v01.csv  hide_internal_audit_manifest  csv  0.023  
  00_release_manifests/final_removed_root_helper_R_scripts_v01.csv  hide_internal_audit_manifest  csv  1.399  
  00_release_manifests/final_variable_dictionary_approved_v01.csv  hide_internal_audit_manifest  csv  1697.567  
  00_release_manifests/internal_excluded_file_manifest_v01.csv  hide_internal_audit_manifest  csv  82.845  
  00_release_manifests/missing_public_source_files_v01.csv  hide_internal_audit_manifest  csv  0.899  
  00_release_manifests/not_public_variable_dictionary_v01.csv  hide_internal_audit_manifest  csv  760.736  
  00_release_manifests/public_absolute_path_scan_v01.csv  hide_internal_audit_manifest  csv  105.64  
  00_release_manifests/public_deposit_file_copy_log_v01.csv  hide_internal_audit_manifest  csv  75.441  
  00_release_manifests/public_deposit_package_build_audit_v01.txt  hide_internal_audit_manifest  txt  3.896  
  00_release_manifests/public_deposit_package_build_summary_v01.csv  hide_internal_audit_manifest  csv  0.487  
  00_release_manifests/public_generic_column_names_requiring_mapping_v01.csv  hide_internal_audit_manifest  csv  0.776  
  00_release_manifests/public_path_sanitization_log_v01.csv  hide_internal_audit_manifest  csv  51.985  
  00_release_manifests/public_variable_dictionary_FINAL_CODEBOOK_audit.txt  hide_internal_audit_manifest  txt  3.841  
  00_release_manifests/public_variable_dictionary_FINAL_CODEBOOK_review_rows.csv  hide_internal_audit_manifest  csv  1.044  
  00_release_manifests/public_variable_dictionary_RECOMMENDED.csv  hide_internal_audit_manifest  csv  572.559  
  00_release_manifests/public_variable_dictionary_explanatory_audit_v01.txt  hide_internal_audit_manifest  txt  3.734  
  00_release_manifests/public_variable_dictionary_explanatory_by_status_v01.csv  hide_internal_audit_manifest  csv  0.173  
  00_release_manifests/public_variable_dictionary_explanatory_by_status_v02.csv  hide_internal_audit_manifest  csv  0.148  
  00_release_manifests/public_variable_dictionary_explanatory_patch_audit_v01.txt  hide_internal_audit_manifest  txt  8.206  
  00_release_manifests/public_variable_dictionary_explanatory_patch_audit_v02.txt  hide_internal_audit_manifest  txt  8.665  
  00_release_manifests/public_variable_dictionary_explanatory_v01.csv  hide_internal_audit_manifest  csv  530.68  
  00_release_manifests/public_variable_dictionary_explanatory_v02.csv  hide_internal_audit_manifest  csv  546.594  
  00_release_manifests/public_variable_dictionary_explanatory_v03.csv  hide_internal_audit_manifest  csv  559.98  
  00_release_manifests/public_variable_dictionary_explanatory_v04.csv  hide_internal_audit_manifest  csv  572.559  
  00_release_manifests/public_variable_dictionary_final_precision_audit_v01.txt  hide_internal_audit_manifest  txt  12.948  
  00_release_manifests/public_variable_dictionary_final_precision_by_status_v01.csv  hide_internal_audit_manifest  csv  0.192  
  00_release_manifests/public_variable_dictionary_final_precision_changes_v01.csv  hide_internal_audit_manifest  csv  7.716  
  00_release_manifests/public_variable_dictionary_final_precision_review_v01.csv  hide_internal_audit_manifest  csv  0.15  
  00_release_manifests/public_variable_dictionary_final_precision_summary_v01.csv  hide_internal_audit_manifest  csv  0.419  
  00_release_manifests/public_variable_dictionary_patch_changes_v01.csv  hide_internal_audit_manifest  csv  89.006  
  00_release_manifests/public_variable_dictionary_patch_changes_v02.csv  hide_internal_audit_manifest  csv  89.866  
  00_release_manifests/public_variable_dictionary_precision_by_status_v01.csv  hide_internal_audit_manifest  csv  0.306  
  00_release_manifests/public_variable_dictionary_precision_patch_audit_v01.txt  hide_internal_audit_manifest  txt  12.999  
  00_release_manifests/public_variable_dictionary_precision_patch_changes_v01.csv  hide_internal_audit_manifest  csv  114.999  
  00_release_manifests/public_variable_dictionary_precision_review_v01.csv  hide_internal_audit_manifest  csv  4.576  
  00_release_manifests/public_variable_dictionary_precision_summary_v01.csv  hide_internal_audit_manifest  csv  0.435  
  00_release_manifests/public_variable_dictionary_summary_explanatory_v01.csv  hide_internal_audit_manifest  csv  0.295  
  00_release_manifests/public_variable_dictionary_summary_explanatory_v02.csv  hide_internal_audit_manifest  csv  0.364  
  00_release_manifests/public_variable_dictionary_unresolved_by_file_v01.csv  hide_internal_audit_manifest  csv  0.77  
  00_release_manifests/public_variable_dictionary_unresolved_v01.csv  hide_internal_audit_manifest  csv  21.999  
  00_release_manifests/public_variable_dictionary_unresolved_v02.csv  hide_internal_audit_manifest  csv  1.081  
  00_release_manifests/public_variable_dictionary_v01.csv  hide_internal_audit_manifest  csv  376.47  
  00_release_manifests/restricted_on_request_variable_dictionary_v01.csv  hide_internal_audit_manifest  csv  571.309  
  00_config/01_primary_file_registry_v03.csv  hide_other  csv  4.052  
  02_workflows/F1_workflow_v02/submission_ready/F1_submission_ready_manifest_v02.csv  hide_other  csv  8.39  
  02_workflows/F2_workflow_v01/submission_ready/F2_submission_ready_manifest_v01.csv  hide_other  csv  5.623  
  02_workflows/F2_workflow_v01/submission_ready/public/F2_public_submission_manifest_v01.csv  hide_other  csv  1.051  
  02_workflows/F3_workflow_v01/submission_ready/F3_submission_ready_manifest_v01.csv  hide_other  csv  1.711  
  02_workflows/F3_workflow_v01/submission_ready/public/F3_public_submission_manifest_v01.csv  hide_other  csv  0.438  
  02_workflows/F4_workflow_v01/submission_ready/F4_submission_ready_manifest_v01.csv  hide_other  csv  5.902  
  02_workflows/F4_workflow_v01/submission_ready/public/F4_public_submission_manifest_v01.csv  hide_other  csv  1.454  
  02_workflows/F5_workflow_v01/submission_ready/F5_submission_ready_manifest_v01.csv  hide_other  csv  5.713  
  02_workflows/F5_workflow_v01/submission_ready/public/F5_public_submission_manifest_v01.csv  hide_other  csv  1.458  
  02_workflows/F6_workflow_v01/submission_ready/F6_submission_ready_manifest_v01.csv  hide_other  csv  6.019  
  02_workflows/F6_workflow_v01/submission_ready/public/F6_public_submission_manifest_v01.csv  hide_other  csv  1.049  
  02_workflows/T1_workflow_v01/submission_ready/public/T1_public_submission_manifest_v01.csv  hide_other  csv  0.689  
  02_workflows/T3_workflow_v01/submission_ready/public/T3_public_submission_manifest_v01.csv  hide_other  csv  0.655  
  02_workflows/T4_workflow_v01/submission_ready/public/T4_public_submission_manifest_v01.csv  hide_other  csv  0.691  

  .DS_Store  hide_system_file  [none]  8.004      

 
 
 
 
  
