# Supplementary material for: Creatinine assay interferences compromises MELD accuracy and may bias liver allocation
Source: Nat Commun. 2026 Jul 23;17:7111. doi: 10.1038/s41467-026-75011-x (PMC13396164; doi:10.1038/s41467-026-75011-x)
Supplement: Supplementary file 4 — Source Data [file 41467_2026_75011_MOESM4_ESM.zip › figshare_package_FINAL_PUBLIC_DEPOSIT_V1_20260503_002637/00_START_HERE_HTML_NAVIGATOR/study_workflow_guide.html]

Study Workflow Guide


# Study Workflow Guide

**Creatinine Assay Interference, MELD Accuracy, and Liver Allocation**

Interpretive guide to the analytical layers of the public data package.

← Back to package navigator

## 1. Overview

This public-deposit package provides the public ESLD and simulated data objects required to inspect public-use components of the manuscript analyses. The manuscript contains 6 main figures and 4 main tables.

SRTR cohort data and SRTR-derived data objects are not redistributed in this public package. R scripts are also not included as public-deposit files; they are listed separately as available on request.

Experimental data → model derivation → in-silico simulation (Figures 1–2) → clinical ESLD application (Figures 3–5 and Tables 1–4) → SRTR external validation context (Figure/Table components requiring restricted data)

Table 2 is part of the manuscript text and contains score formulas and parameter boundaries only; it does not contain original data and is therefore not treated as a public data object in this package.

## 2. Experimental data — foundation layer

### 2.1 Experimental array dataset for model derivation

- f1\_tb\_cre\_experimental\_array\_raw.csv  
  01\_primary\_data/public/f1\_tb\_cre\_experimental\_array\_raw.csv

This dataset contains controlled laboratory matrix experiments designed to quantify the effect of bilirubin on creatinine measurements. It is the empirical basis for the correction equations used by the public simulation datasets.

### 2.2 Validation dataset with external reference method

- f1\_tb\_cre\_experimental\_validation\_raw.csv  
  01\_primary\_data/public/f1\_tb\_cre\_experimental\_validation\_raw.csv

This dataset contains patient-derived serum samples used to compare corrected creatinine results with GC-IDMS reference measurements.

## 3. In-silico simulation data — derived layer

### 3.1 Figure 1: continuous bias surface

- f1\_simulated\_surface\_repository.csv  
  01\_primary\_data/public/f1\_simulated\_surface\_repository.csv
- f1\_simulated\_surface\_metadata.csv  
  01\_primary\_data/public/f1\_simulated\_surface\_metadata.csv

These datasets represent a deterministic analytical surface describing creatinine bias as a function of bilirubin and creatinine concentration.

### 3.2 Figure 2: heatmap simulation

- f2\_simulated\_heatmap\_repository.csv  
  01\_primary\_data/public/f2\_simulated\_heatmap\_repository.csv
- f2\_simulated\_heatmap\_metadata.csv  
  01\_primary\_data/public/f2\_simulated\_heatmap\_metadata.csv

These datasets describe how analytical bias propagates into score-level effects. They are fully in-silico and exported as bin-level aggregates.

## 4. Clinical ESLD data — application layer

### 4.1 Core public ESLD dataset

- esld\_master\_long\_public.csv  
  01\_primary\_data/public/esld\_master\_long\_public.csv

This is the canonical public ESLD dataset for clinical analyses. It is structured in long format at sample level and contains pseudonymized identifiers, laboratory values, MELD-related scores, and outcome/grouping indicators.

### 4.2 Public output datasets for main figures and tables

| Manuscript component | Current public package files | Role |
| --- | --- | --- |
| Figure 1 | - f1\_simulated\_surface\_repository.csv 01\_primary\_data/public/f1\_simulated\_surface\_repository.csv - f1\_simulated\_surface\_metadata.csv 01\_primary\_data/public/f1\_simulated\_surface\_metadata.csv | Continuous creatinine-bilirubin bias surface. |
| Figure 2 | - f2\_simulated\_heatmap\_repository.csv 01\_primary\_data/public/f2\_simulated\_heatmap\_repository.csv - f2\_simulated\_heatmap\_metadata.csv 01\_primary\_data/public/f2\_simulated\_heatmap\_metadata.csv | Score-shift propagation across bilirubin-creatinine space. |
| Figure 3 | esld\_F3\_score\_shift\_aggregate\_public.csv 02\_workflows/F3\_workflow\_v01/data/02b\_figure\_content/esld\_F3\_score\_shift\_aggregate\_public.csv | Public ESLD score-shift summaries. |
| Figure 4 | esld\_F4\_survival\_stats\_public.csv 02\_workflows/F4\_workflow\_v01/data/02b\_figure\_content/esld\_F4\_survival\_stats\_public.csv | Public ESLD survival-statistics data. |
| Figure 5 | - esld\_F5\_stratified\_survival\_subject\_public.csv 02\_workflows/F5\_workflow\_v01/data/02b\_figure\_content/esld\_F5\_stratified\_survival\_subject\_public.csv - esld\_F5\_stratified\_survival\_stats\_public.csv 02\_workflows/F5\_workflow\_v01/data/02b\_figure\_content/esld\_F5\_stratified\_survival\_stats\_public.csv - esld\_F5\_stratified\_survival\_meta\_public.csv 02\_workflows/F5\_workflow\_v01/data/02b\_figure\_content/esld\_F5\_stratified\_survival\_meta\_public.csv | Subject-level grouping by score changes and survival/statistical panel data. |
| Figure 6 | F6\_SRTR\_public.pdf 02\_workflows/F6\_workflow\_v01/figures/F6\_SRTR\_public.pdf | Rendered public figure. Underlying SRTR data are restricted/on request. |
| Table 1 | esld\_master\_long\_public.csv 01\_primary\_data/public/esld\_master\_long\_public.csv | The public ESLD component of Table 1 can be derived from the public ESLD master dataset. |
| Table 2 | No public data file required. | Manuscript table listing MELD score variants, formulas, and parameter boundaries. It contains no original data and is available in the main text. |
| Table 3 | esld\_master\_long\_public.csv 01\_primary\_data/public/esld\_master\_long\_public.csv | The public ESLD component of Table 3 can be derived from the public ESLD master dataset. |
| Table 4 | - esld\_T4\_score\_deviation\_outcome\_table\_public.csv 02\_workflows/T4\_workflow\_v01/data/02b\_table\_content/esld\_T4\_score\_deviation\_outcome\_table\_public.csv - esld\_T4\_score\_deviation\_outcome\_meta\_public.csv 02\_workflows/T4\_workflow\_v01/data/02b\_table\_content/esld\_T4\_score\_deviation\_outcome\_meta\_public.csv | Public score-deviation/outcome table and metadata. |

### 4.3 Etiology handling

Etiologies are represented in public variables such as etiology\_unclassified and other. Consult the public codebook for variable-level definitions and coding.

### 4.4 Processing principle

The package separates core public source data from derived public output datasets. Core datasets define reusable public observations; output datasets contain figure/table-specific structure and metadata.

## 5. SRTR data — external validation context

SRTR cohort data and SRTR-derived data objects are not publicly redistributed in this package because of licensing and data-use obligations.

- restricted\_on\_request\_file\_manifest\_v01.csv  
  00\_release\_manifests/restricted\_on\_request\_file\_manifest\_v01.csv

**Role:** External validation and assessment of generalizability. Access must be requested under the applicable SRTR data-use policies.

## 6. Manifests and reproducibility

- public\_variable\_dictionary\_FINAL\_CODEBOOK.csv  
  00\_release\_manifests/public\_variable\_dictionary\_FINAL\_CODEBOOK.csv
- public\_deposit\_file\_manifest\_v01.csv  
  00\_release\_manifests/public\_deposit\_file\_manifest\_v01.csv
- script\_on\_request\_file\_manifest\_v01.csv  
  00\_release\_manifests/script\_on\_request\_file\_manifest\_v01.csv

The final public codebook describes the public variables. The request manifests list restricted data and scripts that are available only upon request. Rebuilding restricted SRTR-dependent results requires restricted-on-request data.
