# Supplementary material for: Creatinine assay interferences compromises MELD accuracy and may bias liver allocation
Source: Nat Commun. 2026 Jul 23;17:7111. doi: 10.1038/s41467-026-75011-x (PMC13396164; doi:10.1038/s41467-026-75011-x)
Supplement: Supplementary file 4 — Source Data [file 41467_2026_75011_MOESM4_ESM.zip › figshare_package_FINAL_PUBLIC_DEPOSIT_V1_20260503_002637/00_START_HERE_HTML_NAVIGATOR/file_views/view_0011_slco_F1_surface_grid_public.html]

02\_workflows/F1\_workflow\_v02/submission\_ready/public/data/slco\_F1\_surface\_grid\_public.csv

# Readable file view

02\_workflows/F1\_workflow\_v02/submission\_ready/public/data/slco\_F1\_surface\_grid\_public.csv

← Back to navigator   |   Open original package file

Section

Manuscript output data

Output

F1

Extension

csv

Size KB

4918.95

Variables

11

## Variables in this file

| Variable | Label | Description | Unit | Type |
| --- | --- | --- | --- | --- |
| cre\_true\_mg\_dL | True creatinine concentration | True creatinine concentration used as the gravimetric/reference concentration in F1. |  | numeric |
| cree\_measured\_pred\_mg\_dL | Predicted measured enzymatic creatinine concentration | Predicted measured enzymatic creatinine concentration derived from the interference model. |  | numeric |
| cree\_true\_recalc\_error | Recalculation error for enzymatic creatinine | Numerical difference between recalculated and expected true enzymatic creatinine values. |  | numeric |
| cree\_true\_recalc\_mg\_dL | Recalculated true enzymatic creatinine concentration | True enzymatic creatinine concentration recalculated from the model. |  | numeric |
| crej\_measured\_pred\_mg\_dL | Predicted measured Jaffe creatinine concentration | Predicted measured Jaffe creatinine concentration derived from the interference model. |  | numeric |
| crej\_true\_recalc\_error | Recalculation error for Jaffe creatinine | Numerical difference between recalculated and expected true Jaffe creatinine values. |  | numeric |
| crej\_true\_recalc\_mg\_dL | Recalculated true Jaffe creatinine concentration | True Jaffe creatinine concentration recalculated from the model. |  | numeric |
| delta\_cree\_to\_gravimetry\_mg\_dL | Enzymatic creatinine deviation from gravimetry | Difference between enzymatic creatinine and the gravimetric/reference value. |  | numeric |
| delta\_crej\_to\_gravimetry\_mg\_dL | Jaffe creatinine deviation from gravimetry | Difference between Jaffe creatinine and the gravimetric/reference value. |  | numeric |
| grid\_id | Surface-grid identifier | Identifier of a grid point in the F1 simulated or reconstructed surface data. |  | integer |
| tb\_mg\_dL | Total bilirubin concentration | Total bilirubin concentration used in the creatinine/bilirubin interference model or figure data. |  | numeric |

## Readable HTML view

Preview shows first 1000 of 34441 rows. Open the original file for full content.

| grid\_id | tb\_mg\_dL | cre\_true\_mg\_dL | crej\_measured\_pred\_mg\_dL | cree\_measured\_pred\_mg\_dL | delta\_crej\_to\_gravimetry\_mg\_dL | delta\_cree\_to\_gravimetry\_mg\_dL | crej\_true\_recalc\_mg\_dL | cree\_true\_recalc\_mg\_dL | crej\_true\_recalc\_error | cree\_true\_recalc\_error |
| --- | --- | --- | --- | --- | --- | --- | --- | --- | --- | --- |
| 1 | 1 | 1 | 0.990463857965053 | 0.902064914425904 | 0.0095361420349469 | 0.097935085574096 | 1 | 1 | 6.66133814775094e-16 | -9.99200722162641e-16 |
| 2 | 1 | 1.05 | 1.04152284217999 | 0.957189139835594 | 0.00847715782000713 | 0.0928108601644061 | 1.05 | 1.04999999999996 | 1.33226762955019e-15 | -4.06341627012807e-14 |
| 3 | 1 | 1.1 | 1.09273908981855 | 1.01231537541398 | 0.00726091018144937 | 0.0876846245860181 | 1.1 | 1.09999999999994 | 2.22044604925031e-16 | -5.97299987248334e-14 |
| 4 | 1 | 1.15 | 1.14411406302484 | 1.06744362138108 | 0.00588593697515893 | 0.0825563786189225 | 1.15 | 1.15000000000002 | 0 | 1.79856129989275e-14 |
| 5 | 1 | 1.2 | 1.19564924674134 | 1.1225738779567 | 0.00435075325865686 | 0.0774261220432957 | 1.2 | 1.20000000000006 | 1.11022302462516e-15 | 6.3504757008559e-14 |
| 6 | 1 | 1.25 | 1.2473461492097 | 1.17770614536087 | 0.00265385079029734 | 0.0722938546391281 | 1.25 | 1.25000000000008 | 1.55431223447522e-15 | 8.10462807976364e-14 |
| 7 | 1 | 1.3 | 1.29920630248577 | 1.23284042381359 | 0.000793697514234193 | 0.0671595761864106 | 1.3 | 1.30000000000004 | -4.44089209850063e-16 | 3.70814490224802e-14 |
| 8 | 1 | 1.35 | 1.35123126296935 | 1.28797671353505 | -0.00123126296935316 | 0.0620232864649488 | 1.35 | 1.35000000000003 | 6.66133814775094e-16 | 2.97539770599542e-14 |
| 9 | 1 | 1.4 | 1.40342261194926 | 1.34311501474527 | -0.00342261194925508 | 0.0568849852547337 | 1.4 | 1.39999999999995 | 0 | -4.64073224293315e-14 |
| 10 | 1 | 1.45 | 1.45578195616405 | 1.39825532766461 | -0.00578195616405286 | 0.051744672335386 | 1.45 | 1.45 | 1.33226762955019e-15 | 1.11022302462516e-15 |
| 11 | 1 | 1.5 | 1.50831092837926 | 1.4533976525131 | -0.00831092837925707 | 0.0466023474868966 | 1.5 | 1.49999999999999 | 0 | -6.43929354282591e-15 |
| 12 | 1 | 1.55 | 1.56101118798142 | 1.50854198951111 | -0.011011187981419 | 0.0414580104888862 | 1.55 | 1.55000000000005 | 1.11022302462516e-15 | 5.15143483426073e-14 |
| 13 | 1 | 1.6 | 1.61388442158975 | 1.56368833887884 | -0.0138844215897536 | 0.0363116611211602 | 1.6 | 1.60000000000009 | 8.88178419700125e-16 | 9.14823772291129e-14 |
| 14 | 1 | 1.65 | 1.66693234368599 | 1.61883670083648 | -0.0169323436859867 | 0.0311632991635247 | 1.65 | 1.64999999999999 | 2.22044604925031e-15 | -7.32747196252603e-15 |
| 15 | 1 | 1.7 | 1.72015669726303 | 1.67398707560477 | -0.0201566972630258 | 0.0260129243952307 | 1.7 | 1.7000000000001 | 1.77635683940025e-15 | 1.01252339845814e-13 |
| 16 | 1 | 1.75 | 1.7735592544932 | 1.72913946340373 | -0.0235592544932013 | 0.0208605365962682 | 1.75 | 1.75000000000006 | 4.44089209850063e-16 | 5.70654634657331e-14 |
| 17 | 1 | 1.8 | 1.82714181741677 | 1.78429386445411 | -0.0271418174167679 | 0.0157061355458885 | 1.8 | 1.80000000000013 | 1.77635683940025e-15 | 1.33226762955019e-13 |
| 18 | 1 | 1.85 | 1.88090621865143 | 1.8394502789761 | -0.0309062186514266 | 0.010549721023897 | 1.85 | 1.85000000000006 | 6.66133814775094e-16 | 6.3726801613484e-14 |
| 19 | 1 | 1.9 | 1.93485432212369 | 1.89460870719046 | -0.0348543221236948 | 0.00539129280954431 | 1.9 | 1.90000000000005 | 8.88178419700125e-16 | 4.9293902293357e-14 |
| 20 | 1 | 1.95 | 1.98898802382289 | 1.94976914931773 | -0.0389880238228877 | 0.000230850682266803 | 1.95 | 1.95000000000009 | 4.44089209850063e-16 | 8.54871728961371e-14 |
| 21 | 1 | 2 | 2.04330925257863 | 2.0049316055785 | -0.0433092525786343 | -0.0049316055785007 | 2 | 2.00000000000013 | 1.33226762955019e-15 | 1.33226762955019e-13 |
| 22 | 1 | 2.05 | 2.09781997086278 | 2.06009607619314 | -0.047819970862784 | -0.010096076193137 | 2.05 | 2.04999999999995 | 8.88178419700125e-16 | -5.24025267623074e-14 |
| 23 | 1 | 2.1 | 2.15252217561668 | 2.11526256138295 | -0.0525221756166845 | -0.0152625613829467 | 2.1 | 2.10000000000009 | 4.44089209850063e-16 | 8.61533067109122e-14 |
| 24 | 1 | 2.15 | 2.20741789910479 | 2.17043106136812 | -0.0574178991047871 | -0.0204310613681233 | 2.15 | 2.15000000000006 | 8.88178419700125e-16 | 6.3504757008559e-14 |
| 25 | 1 | 2.2 | 2.26250920979563 | 2.2256015763696 | -0.0625092097956261 | -0.0256015763696027 | 2.2 | 2.20000000000003 | 8.88178419700125e-16 | 2.97539770599542e-14 |
| 26 | 1 | 2.25 | 2.31779821327126 | 2.28077410660832 | -0.0677982132712587 | -0.0307741066083187 | 2.25 | 2.2500000000001 | 4.44089209850063e-16 | 9.76996261670138e-14 |
| 27 | 1 | 2.3 | 2.37328705316628 | 2.33594865230483 | -0.0732870531662804 | -0.0359486523048349 | 2.3 | 2.30000000000001 | 8.88178419700125e-16 | 6.66133814775094e-15 |
| 28 | 1 | 2.35 | 2.42897791213761 | 2.39112521368027 | -0.078977912137614 | -0.0411252136802709 | 2.35 | 2.34999999999996 | 4.44089209850063e-16 | -3.5527136788005e-14 |
| 29 | 1 | 2.4 | 2.48487301286631 | 2.44630379095556 | -0.0848730128663124 | -0.046303790955561 | 2.4 | 2.39999999999997 | 2.22044604925031e-15 | -2.57571741713036e-14 |
| 30 | 1 | 2.45 | 2.54097461909266 | 2.50148438435164 | -0.090974619092655 | -0.051484384351641 | 2.45 | 2.45 | 4.44089209850063e-16 | 3.10862446895044e-15 |
| 31 | 1 | 2.5 | 2.59728503668596 | 2.55666699408944 | -0.0972850366859555 | -0.056666994089444 | 2.5 | 2.49999999999998 | 0 | -2.04281036531029e-14 |
| 32 | 1 | 2.55 | 2.65380661475043 | 2.61185162039027 | -0.103806614750433 | -0.0618516203902746 | 2.55 | 2.55000000000013 | 1.33226762955019e-15 | 1.32782673745169e-13 |
| 33 | 1 | 2.6 | 2.71054174676872 | 2.6670382634747 | -0.110541746768715 | -0.0670382634746964 | 2.6 | 2.59999999999998 | 8.88178419700125e-16 | -1.68753899743024e-14 |
| 34 | 1 | 2.65 | 2.76749287178452 | 2.72222692356439 | -0.117492871784521 | -0.0722269235643851 | 2.65 | 2.65000000000002 | 0 | 2.22044604925031e-14 |
| 35 | 1 | 2.7 | 2.82466247562617 | 2.77741760088028 | -0.124662475626171 | -0.0774176008802754 | 2.7 | 2.70000000000003 | 8.88178419700125e-16 | 3.41948691584548e-14 |
| 36 | 1 | 2.75 | 2.88205309217267 | 2.83261029564367 | -0.132053092172666 | -0.0826102956436707 | 2.75 | 2.7500000000001 | 4.44089209850063e-16 | 9.9475983006414e-14 |
| 37 | 1 | 2.8 | 2.93966730466418 | 2.88780500807569 | -0.139667304664177 | -0.0878050080756907 | 2.8 | 2.8000000000001 | 0 | 9.72555369571637e-14 |
| 38 | 1 | 2.85 | 2.99750774705883 | 2.94300173839764 | -0.147507747058831 | -0.0930017383976396 | 2.85 | 2.85000000000003 | 8.88178419700125e-16 | 3.46389583683049e-14 |
| 39 | 1 | 2.9 | 3.05557710543784 | 2.99820048683101 | -0.155577105437835 | -0.0982004868310073 | 2.9 | 2.90000000000005 | 8.88178419700125e-16 | 5.24025267623074e-14 |
| 40 | 1 | 2.95 | 3.11387811946105 | 3.05340125359691 | -0.163878119461054 | -0.103401253596914 | 2.95 | 2.94999999999992 | 8.88178419700125e-16 | -8.08242361927114e-14 |
| 41 | 1 | 3 | 3.17241358387526 | 3.10860403891722 | -0.17241358387526 | -0.108604038917217 | 3 | 3.00000000000004 | 8.88178419700125e-16 | 3.50830475781549e-14 |
| 42 | 1 | 3.05 | 3.23118635007743 | 3.16380884301285 | -0.181186350077426 | -0.113808843012853 | 3.05 | 3.04999999999993 | 1.33226762955019e-15 | -7.23865412055602e-14 |
| 43 | 1 | 3.1 | 3.29019932773551 | 3.21901566610587 | -0.190199327735514 | -0.119015666105866 | 3.1 | 3.10000000000009 | 8.88178419700125e-16 | 9.32587340685131e-14 |
| 44 | 1 | 3.15 | 3.34945548646939 | 3.27422450841719 | -0.199455486469387 | -0.124224508417189 | 3.15 | 3.14999999999999 | 0 | -1.46549439250521e-14 |
| 45 | 1 | 3.2 | 3.40895785759457 | 3.32943537016887 | -0.208957857594573 | -0.129435370168868 | 3.2 | 3.20000000000003 | 8.88178419700125e-16 | 2.57571741713036e-14 |
| 46 | 1 | 3.25 | 3.46870953593179 | 3.38464825158239 | -0.218709535931786 | -0.134648251582393 | 3.25 | 3.2500000000001 | 4.44089209850063e-16 | 9.85878045867139e-14 |
| 47 | 1 | 3.3 | 3.52871368168527 | 3.43986315287925 | -0.228713681685272 | -0.139863152879252 | 3.3 | 3.30000000000005 | 4.44089209850063e-16 | 4.79616346638068e-14 |
| 48 | 1 | 3.35 | 3.58897352239318 | 3.49508007428131 | -0.238973522393183 | -0.145080074281305 | 3.35 | 3.35000000000002 | 4.44089209850063e-16 | 2.1316282072803e-14 |
| 49 | 1 | 3.4 | 3.6494923549534 | 3.55029901601023 | -0.2494923549534 | -0.150299016010227 | 3.4 | 3.39999999999996 | 8.88178419700125e-16 | -4.30766533554561e-14 |
| 50 | 1 | 3.45 | 3.7102735477284 | 3.60551997828806 | -0.260273547728403 | -0.155519978288063 | 3.45 | 3.4500000000001 | 8.88178419700125e-16 | 9.54791801177635e-14 |
| 51 | 1 | 3.5 | 3.77132054273299 | 3.66074296133612 | -0.271320542732986 | -0.160742961336116 | 3.5 | 3.49999999999997 | 1.33226762955019e-15 | -3.24185123190546e-14 |
| 52 | 1 | 3.55 | 3.83263685790884 | 3.7159679653768 | -0.282636857908835 | -0.165967965376802 | 3.55 | 3.55000000000007 | 4.44089209850063e-16 | 7.37188088351104e-14 |
| 53 | 1 | 3.6 | 3.89422608949023 | 3.77119499063161 | -0.294226089490234 | -0.171194990631609 | 3.6 | 3.60000000000004 | 4.44089209850063e-16 | 3.95239396766556e-14 |
| 54 | 1 | 3.65 | 3.95609191446539 | 3.82642403732277 | -0.306091914465386 | -0.176424037322769 | 3.65 | 3.65000000000013 | 2.22044604925031e-15 | 1.24789067967868e-13 |
| 55 | 1 | 3.7 | 4.01823809313811 | 3.88165510567195 | -0.318238093138114 | -0.181655105671954 | 3.7 | 3.70000000000005 | 4.44089209850063e-16 | 4.9293902293357e-14 |
| 56 | 1 | 3.75 | 4.08066847179503 | 3.9368881959014 | -0.330668471795034 | -0.186888195901396 | 3.75 | 3.75 | 0 | -8.88178419700125e-16 |
| 57 | 1 | 3.8 | 4.14338698548348 | 3.99212330823314 | -0.343386985483477 | -0.192123308233138 | 3.8 | 3.79999999999995 | 4.44089209850063e-16 | -4.57411886145565e-14 |
| 58 | 1 | 3.85 | 4.20639766090591 | 4.04736044288941 | -0.356397660905907 | -0.19736044288941 | 3.85 | 3.85000000000003 | 4.44089209850063e-16 | 2.88657986402541e-14 |
| 59 | 1 | 3.9 | 4.26970461943683 | 4.10259960009207 | -0.369704619436825 | -0.202599600092071 | 3.9 | 3.89999999999996 | -4.44089209850063e-16 | -3.81916720471054e-14 |
| 60 | 1 | 3.95 | 4.33331208026857 | 4.15784078006372 | -0.383312080268573 | -0.207840780063722 | 3.95 | 3.95000000000013 | 8.88178419700125e-16 | 1.26121335597418e-13 |
| 61 | 1 | 4 | 4.3972243636928 | 4.21308398302604 | -0.397224363692795 | -0.213083983026037 | 4 | 4.00000000000002 | 0 | 2.22044604925031e-14 |
| 62 | 1 | 4.05 | 4.46144589452485 | 4.26832920920162 | -0.411445894524849 | -0.218329209201615 | 4.05 | 4.04999999999995 | 1.77635683940025e-15 | -5.06261699229071e-14 |
| 63 | 1 | 4.1 | 4.52598120567875 | 4.32357645881287 | -0.425981205678754 | -0.223576458812873 | 4.1 | 4.1 | 0 | 3.5527136788005e-15 |
| 64 | 1 | 4.15 | 4.59083494190096 | 4.37882573208204 | -0.440834941900961 | -0.228825732082036 | 4.15 | 4.15000000000007 | 1.77635683940025e-15 | 7.37188088351104e-14 |
| 65 | 1 | 4.2 | 4.65601186367152 | 4.43407702923134 | -0.456011863671521 | -0.234077029231338 | 4.2 | 4.20000000000002 | 8.88178419700125e-16 | 1.59872115546023e-14 |
| 66 | 1 | 4.25 | 4.72151685128205 | 4.48933035048338 | -0.471516851282047 | -0.239330350483378 | 4.25 | 4.24999999999998 | 0 | -1.68753899743024e-14 |
| 67 | 1 | 4.3 | 4.78735490910028 | 4.54458569606075 | -0.487354909100275 | -0.244585696060754 | 4.3 | 4.30000000000009 | 1.77635683940025e-15 | 8.88178419700125e-14 |
| 68 | 1 | 4.35 | 4.85353117003178 | 4.5998430661857 | -0.503531170031783 | -0.249843066185697 | 4.35 | 4.35000000000008 | 8.88178419700125e-16 | 7.8159700933611e-14 |
| 69 | 1 | 4.4 | 4.92005090019017 | 4.65510246108081 | -0.520050900190171 | -0.255102461080806 | 4.4 | 4.39999999999999 | 8.88178419700125e-16 | -7.105427357601e-15 |
| 70 | 1 | 4.45 | 4.98691950378766 | 4.71036388096887 | -0.536919503787661 | -0.260363880968867 | 4.45 | 4.45 | 0 | 3.5527136788005e-15 |
| 71 | 1 | 4.5 | 5.05414252825902 | 4.76562732607248 | -0.55414252825902 | -0.265627326072478 | 4.5 | 4.50000000000008 | 0 | 7.8159700933611e-14 |
| 72 | 1 | 4.55 | 5.12172566963254 | 4.82089279661405 | -0.571725669632535 | -0.270892796614054 | 4.55 | 4.54999999999998 | 1.77635683940025e-15 | -1.77635683940025e-14 |
| 73 | 1 | 4.6 | 5.18967477816275 | 4.87616029281675 | -0.589674778162754 | -0.276160292816752 | 4.6 | 4.60000000000011 | 8.88178419700125e-16 | 1.11910480882216e-13 |
| 74 | 1 | 4.65 | 5.25799586424078 | 4.9314298149028 | -0.607995864240782 | -0.281429814902798 | 4.65 | 4.64999999999999 | 0 | -1.4210854715202e-14 |
| 75 | 1 | 4.7 | 5.32669510459899 | 4.98670136309535 | -0.626695104598992 | -0.28670136309535 | 4.7 | 4.69999999999993 | 8.88178419700125e-16 | -6.83897383169096e-14 |
| 76 | 1 | 4.75 | 5.39577884882831 | 5.04197493761738 | -0.645778848828309 | -0.291974937617376 | 4.75 | 4.75000000000006 | 1.77635683940025e-15 | 6.30606677987089e-14 |
| 77 | 1 | 4.8 | 5.46525362622753 | 5.09725053869129 | -0.665253626227527 | -0.297250538691292 | 4.8 | 4.79999999999996 | 8.88178419700125e-16 | -3.99680288865056e-14 |
| 78 | 1 | 4.85 | 5.53512615300556 | 5.15252816654044 | -0.685126153005555 | -0.302528166540437 | 4.85 | 4.85 | 0 | 3.5527136788005e-15 |
| 79 | 1 | 4.9 | 5.6054033398591 | 5.2078078213876 | -0.705403339859102 | -0.307807821387596 | 4.9 | 4.90000000000003 | 1.77635683940025e-15 | 3.10862446895044e-14 |
| 80 | 1 | 4.95 | 5.67609229994996 | 5.26308950345574 | -0.72609229994996 | -0.313089503455738 | 4.95 | 4.95000000000001 | 2.66453525910038e-15 | 1.24344978758018e-14 |
| 81 | 1 | 5 | 5.747200357308 | 5.31837321296802 | -0.747200357308 | -0.318373212968019 | 5 | 5.00000000000005 | 0 | 4.9737991503207e-14 |
| 82 | 1 | 5.05 | 5.81873505568795 | 5.37365895014741 | -0.768735055687952 | -0.323658950147408 | 5.05 | 5.05000000000004 | 0 | 3.90798504668055e-14 |
| 83 | 1 | 5.1 | 5.89070416791026 | 5.42894671521706 | -0.790704167910259 | -0.32894671521706 | 5.1 | 5.10000000000001 | 8.88178419700125e-16 | 4.44089209850063e-15 |
| 84 | 1 | 5.15 | 5.9631157057188 | 5.48423650840032 | -0.813115705718802 | -0.334236508400315 | 5.15 | 5.15000000000011 | 8.88178419700125e-16 | 1.05693231944315e-13 |
| 85 | 1 | 5.2 | 6.03597793019083 | 5.53952832991996 | -0.835977930190833 | -0.339528329919959 | 5.2 | 5.19999999999996 | 8.88178419700125e-16 | -3.99680288865056e-14 |
| 86 | 1 | 5.25 | 6.10929936273744 | 5.5948221799997 | -0.859299362737438 | -0.344822179999699 | 5.25 | 5.24999999999998 | 1.77635683940025e-15 | -1.68753899743024e-14 |
| 87 | 1 | 5.3 | 6.18308879673596 | 5.65011805886269 | -0.883088796735958 | -0.350118058862693 | 5.3 | 5.30000000000005 | 8.88178419700125e-16 | 5.41788836017076e-14 |
| 88 | 1 | 5.35 | 6.25735530983937 | 5.70541596673209 | -0.907355309839371 | -0.355415966732092 | 5.35 | 5.35000000000002 | 8.88178419700125e-16 | 1.50990331349021e-14 |
| 89 | 1 | 5.4 | 6.33210827701135 | 5.76071590383143 | -0.932108277011351 | -0.360715903831425 | 5.4 | 5.40000000000001 | 8.88178419700125e-16 | 5.32907051820075e-15 |
| 90 | 1 | 5.45 | 6.40735738434006 | 5.81601787038403 | -0.957357384340057 | -0.36601787038403 | 5.45 | 5.44999999999996 | 8.88178419700125e-16 | -4.17443857259059e-14 |
| 91 | 1 | 5.5 | 6.48311264368821 | 5.87132186661362 | -0.98311264368821 | -0.371321866613617 | 5.5 | 5.50000000000011 | 1.77635683940025e-15 | 1.07469588783715e-13 |
| 92 | 1 | 5.55 | 6.55938440824224 | 5.92662789274316 | -1.00938440824224 | -0.376627892743156 | 5.55 | 5.54999999999998 | 1.77635683940025e-15 | -2.30926389122033e-14 |
| 93 | 1 | 5.6 | 6.63618338902886 | 5.98193594899673 | -1.03618338902886 | -0.381935948996726 | 5.6 | 5.60000000000006 | 8.88178419700125e-16 | 5.95079541199084e-14 |
| 94 | 1 | 5.65 | 6.71352067247361 | 6.03724603559748 | -1.06352067247361 | -0.387246035597483 | 5.65 | 5.64999999999998 | 0 | -2.48689957516035e-14 |
| 95 | 1 | 5.7 | 6.79140773908296 | 6.09255815276951 | -1.09140773908296 | -0.392558152769507 | 5.7 | 5.70000000000014 | 8.88178419700125e-16 | 1.4210854715202e-13 |
| 96 | 1 | 5.75 | 6.86985648333904 | 6.14787230073595 | -1.11985648333904 | -0.397872300735953 | 5.75 | 5.75000000000011 | 1.77635683940025e-15 | 1.04805053524615e-13 |
| 97 | 1 | 5.8 | 6.9488792349046 | 6.20318847972072 | -1.1488792349046 | -0.403188479720714 | 5.8 | 5.80000000000004 | 8.88178419700125e-16 | 3.90798504668055e-14 |
| 98 | 1 | 5.85 | 7.02848878124532 | 6.25850668994769 | -1.17848878124532 | -0.408506689947687 | 5.85 | 5.85000000000009 | 1.77635683940025e-15 | 8.5265128291212e-14 |
| 99 | 1 | 5.9 | 7.10869839178688 | 6.3138269316404 | -1.20869839178688 | -0.413826931640397 | 5.9 | 5.90000000000001 | 1.77635683940025e-15 | 1.24344978758018e-14 |
| 100 | 1 | 5.95 | 7.18952184373612 | 6.36914920502292 | -1.23952184373612 | -0.419149205022924 | 5.95 | 5.95000000000005 | 0 | 5.32907051820075e-14 |
| 101 | 1 | 6 | 7.27097344970855 | 6.42447351031898 | -1.27097344970855 | -0.424473510318976 | 6 | 6.00000000000007 | 0 | 6.66133814775094e-14 |
| 102 | 1.1 | 1 | 0.991633909150579 | 0.903491173303202 | 0.00836609084942131 | 0.096508826696798 | 1 | 0.999999999999915 | -2.22044604925031e-16 | -8.4821039081362e-14 |
| 103 | 1.1 | 1.05 | 1.0426964863338 | 0.958615450721475 | 0.0073035136662003 | 0.0913845492785255 | 1.05 | 1.0500000000001 | 0 | 1.01696429055664e-13 |
| 104 | 1.1 | 1.1 | 1.09391636024487 | 1.01374173831381 | 0.00608363975513471 | 0.0862582616861887 | 1.1 | 1.10000000000002 | 6.66133814775094e-16 | 1.53210777398272e-14 |
| 105 | 1.1 | 1.15 | 1.1452949935456 | 1.06887003630059 | 0.00470500645440386 | 0.0811299636994083 | 1.15 | 1.15000000000007 | 4.44089209850063e-16 | 6.66133814775094e-14 |
| 106 | 1.1 | 1.2 | 1.19683387170752 | 1.12400034490145 | 0.00316612829247953 | 0.0759996550985453 | 1.2 | 1.19999999999996 | 1.11022302462516e-15 | -4.13002965160558e-14 |
| 107 | 1.1 | 1.25 | 1.24853450351299 | 1.17913266433678 | 0.00146549648700889 | 0.0708673356632206 | 1.25 | 1.25000000000003 | 2.22044604925031e-15 | 2.93098878501041e-14 |
| 108 | 1.1 | 1.3 | 1.30039842157055 | 1.23426699482639 | -0.000398421570551255 | 0.0657330051736098 | 1.3 | 1.30000000000008 | 1.11022302462516e-15 | 7.63833440942108e-14 |
| 109 | 1.1 | 1.35 | 1.35242718284505 | 1.2894033365903 | -0.00242718284505061 | 0.0605966634097037 | 1.35 | 1.35000000000003 | 4.44089209850063e-16 | 2.97539770599542e-14 |
| 110 | 1.1 | 1.4 | 1.40462236920301 | 1.34454168984869 | -0.00462236920300763 | 0.055458310151308 | 1.4 | 1.39999999999995 | 0 | -4.9293902293357e-14 |
| 111 | 1.1 | 1.45 | 1.45698558797379 | 1.39968205482196 | -0.0069855879737859 | 0.0503179451780436 | 1.45 | 1.45000000000003 | 1.99840144432528e-15 | 3.04201108747293e-14 |
| 112 | 1.1 | 1.5 | 1.50951847252712 | 1.4548244317301 | -0.00951847252712112 | 0.0451755682699013 | 1.5 | 1.50000000000009 | 6.66133814775094e-16 | 9.01501095995627e-14 |
| 113 | 1.1 | 1.55 | 1.56222268286763 | 1.50996882079331 | -0.0122226828676286 | 0.0400311792066868 | 1.55 | 1.55000000000008 | 1.99840144432528e-15 | 8.10462807976364e-14 |
| 114 | 1.1 | 1.6 | 1.61509990624684 | 1.56511522223198 | -0.0150999062468351 | 0.0348847777680208 | 1.6 | 1.60000000000009 | 6.66133814775094e-16 | 8.59312621059871e-14 |
| 115 | 1.1 | 1.65 | 1.66815185779345 | 1.62026363626629 | -0.0181518577934527 | 0.0297363637337089 | 1.65 | 1.64999999999998 | 6.66133814775094e-16 | -1.59872115546023e-14 |
| 116 | 1.1 | 1.7 | 1.72138028116249 | 1.675414063117 | -0.0213802811624897 | 0.0245859368830026 | 1.7 | 1.70000000000012 | 0 | 1.19904086659517e-13 |
| 117 | 1.1 | 1.75 | 1.77478694920395 | 1.73056650300392 | -0.0247869492039483 | 0.0194334969960763 | 1.75 | 1.74999999999997 | 1.11022302462516e-15 | -3.44169137633799e-14 |
| 118 | 1.1 | 1.8 | 1.82837366465179 | 1.78572095614819 | -0.0283736646517918 | 0.0142790438518119 | 1.8 | 1.80000000000013 | 2.22044604925031e-16 | 1.27675647831893e-13 |
| 119 | 1.1 | 1.85 | 1.88214226083398 | 1.84087742276962 | -0.0321422608339823 | 0.00912257723038468 | 1.85 | 1.85 | 2.22044604925031e-16 | 4.2188474935756e-15 |
| 120 | 1.1 | 1.9 | 1.93609460240433 | 1.89603590308933 | -0.0360946024043316 | 0.0039640969106749 | 1.9 | 1.90000000000013 | 2.22044604925031e-16 | 1.30562227695918e-13 |
| 121 | 1.1 | 1.95 | 1.99023258609703 | 1.95119639732733 | -0.0402325860970334 | -0.00119639732732568 | 1.95 | 1.95 | 4.44089209850063e-16 | -1.99840144432528e-15 |
| 122 | 1.1 | 2 | 2.04455814150472 | 2.00635890570474 | -0.0445581415047172 | -0.00635890570473752 | 2 | 2.00000000000007 | 4.44089209850063e-16 | 6.97220059464598e-14 |
| 123 | 1.1 | 2.05 | 2.09907323188094 | 2.06152342844194 | -0.0490732318809375 | -0.0115234284419397 | 2.05 | 2.0500000000001 | 8.88178419700125e-16 | 1.00364161426114e-13 |
| 124 | 1.1 | 2.1 | 2.15377985496803 | 2.11668996575968 | -0.0537798549680315 | -0.0166899657596806 | 2.1 | 2.10000000000014 | 8.88178419700125e-16 | 1.4166445794217e-13 |
| 125 | 1.1 | 2.15 | 2.20868004385135 | 2.17185851787853 | -0.0586800438513495 | -0.0218585178785253 | 2.15 | 2.15000000000005 | 4.44089209850063e-16 | 4.52970994047064e-14 |
| 126 | 1.1 | 2.2 | 2.26377586784088 | 2.22702908501941 | -0.0637758678408784 | -0.0270290850194086 | 2.2 | 2.19999999999996 | 1.77635683940025e-15 | -4.17443857259059e-14 |
| 127 | 1.1 | 2.25 | 2.31906943338133 | 2.28220166740326 | -0.069069433381332 | -0.0322016674032644 | 2.25 | 2.24999999999999 | 1.77635683940025e-15 | -7.105427357601e-15 |
| 128 | 1.1 | 2.3 | 2.37456288499188 | 2.33737626525084 | -0.0745628849918769 | -0.0373762652508418 | 2.3 | 2.30000000000006 | 0 | 5.6399329650958e-14 |
| 129 | 1.1 | 2.35 | 2.43025840623664 | 2.39255287878289 | -0.0802584062366369 | -0.0425528787828902 | 2.35 | 2.35000000000002 | 8.88178419700125e-16 | 1.95399252334028e-14 |
| 130 | 1.1 | 2.4 | 2.48615822072723 | 2.44773150822053 | -0.0861582207272336 | -0.047731508220529 | 2.4 | 2.40000000000005 | 8.88178419700125e-16 | 5.10702591327572e-14 |
| 131 | 1.1 | 2.45 | 2.54226459315868 | 2.50291215378469 | -0.0922645931586845 | -0.0529121537846935 | 2.45 | 2.45000000000012 | 8.88178419700125e-16 | 1.19015908239817e-13 |
| 132 | 1.1 | 2.5 | 2.59857983038 | 2.55809481569613 | -0.0985798303799994 | -0.0580948156961321 | 2.5 | 2.49999999999998 | 1.77635683940025e-15 | -1.82076576038526e-14 |
| 133 | 1.1 | 2.55 | 2.65510628250091 | 2.61327949417633 | -0.105106282500914 | -0.0632794941763346 | 2.55 | 2.55000000000004 | 0 | 3.64153152077051e-14 |
| 134 | 1.1 | 2.6 | 2.71184634403628 | 2.66846618944605 | -0.111846344036276 | -0.0684661894460499 | 2.6 | 2.59999999999997 | 8.88178419700125e-16 | -2.97539770599542e-14 |
| 135 | 1.1 | 2.65 | 2.76880245508963 | 2.72365490172677 | -0.11880245508963 | -0.0736549017267678 | 2.65 | 2.6500000000001 | 8.88178419700125e-16 | 1.04360964314765e-13 |
| 136 | 1.1 | 2.7 | 2.8259771025777 | 2.77884563123924 | -0.125977102577699 | -0.0788456312392385 | 2.7 | 2.70000000000006 | 1.33226762955019e-15 | 5.55111512312578e-14 |
| 137 | 1.1 | 2.75 | 2.88337282149746 | 2.83403837820495 | -0.133372821497455 | -0.0840383782049501 | 2.75 | 2.75000000000007 | 4.44089209850063e-16 | 7.105427357601e-14 |
| 138 | 1.1 | 2.8 | 2.94099219623766 | 2.88923314284502 | -0.140992196237659 | -0.089233142845023 | 2.8 | 2.80000000000003 | 1.77635683940025e-15 | 2.88657986402541e-14 |
| 139 | 1.1 | 2.85 | 2.99883786193671 | 2.94442992538076 | -0.148837861936714 | -0.0944299253807608 | 2.85 | 2.84999999999994 | 2.22044604925031e-15 | -6.48370246381091e-14 |
| 140 | 1.1 | 2.9 | 3.05691250588894 | 2.99962872603384 | -0.156912505888943 | -0.0996287260338384 | 2.9 | 2.9000000000001 | 1.33226762955019e-15 | 9.76996261670138e-14 |
| 141 | 1.1 | 2.95 | 3.11521886900134 | 3.05482954502501 | -0.16521886900134 | -0.104829545025006 | 2.95 | 2.94999999999995 | 1.33226762955019e-15 | -5.19584375524573e-14 |
| 142 | 1.1 | 3 | 3.17375974730306 | 3.11003238257631 | -0.173759747303056 | -0.110032382576307 | 3 | 3.00000000000005 | 8.88178419700125e-16 | 5.41788836017076e-14 |
| 143 | 1.1 | 3.05 | 3.23253799350999 | 3.16523723890868 | -0.182537993509987 | -0.115237238908676 | 3.05 | 3.04999999999994 | 4.44089209850063e-16 | -5.77315972805081e-14 |
| 144 | 1.1 | 3.1 | 3.29155651864691 | 3.22044411424416 | -0.191556518646914 | -0.120444114244158 | 3.1 | 3.10000000000011 | 1.33226762955019e-15 | 1.07025499573865e-13 |
| 145 | 1.1 | 3.15 | 3.35081829372981 | 3.27565300880369 | -0.200818293729814 | -0.125653008803688 | 3.15 | 3.15 | 1.33226762955019e-15 | 1.77635683940025e-15 |
| 146 | 1.1 | 3.2 | 3.41032635151111 | 3.33086392280931 | -0.210326351511114 | -0.130863922809309 | 3.2 | 3.20000000000005 | 8.88178419700125e-16 | 4.66293670342566e-14 |
| 147 | 1.1 | 3.25 | 3.47008378829076 | 3.38607685648233 | -0.220083788290757 | -0.136076856482326 | 3.25 | 3.24999999999996 | 1.77635683940025e-15 | -4.17443857259059e-14 |
| 148 | 1.1 | 3.3 | 3.53009376579615 | 3.44129181004478 | -0.230093765796149 | -0.141291810044784 | 3.3 | 3.30000000000008 | 8.88178419700125e-16 | 8.17124146124115e-14 |
| 149 | 1.1 | 3.35 | 3.59035951313424 | 3.49650878371799 | -0.240359513134239 | -0.146508783717988 | 3.35 | 3.35000000000006 | 0 | 6.12843109593086e-14 |
| 150 | 1.1 | 3.4 | 3.65088432881912 | 3.5517277777238 | -0.250884328819115 | -0.151727777723797 | 3.4 | 3.4 | 0 | 1.77635683940025e-15 |
| 151 | 1.1 | 3.45 | 3.71167158287873 | 3.60694879228407 | -0.261671582878726 | -0.15694879228407 | 3.45 | 3.44999999999997 | 1.77635683940025e-15 | -2.57571741713036e-14 |
| 152 | 1.1 | 3.5 | 3.77272471904454 | 3.66217182762067 | -0.272724719044542 | -0.162171827620667 | 3.5 | 3.50000000000001 | 8.88178419700125e-16 | 1.19904086659517e-14 |
| 153 | 1.1 | 3.55 | 3.83404725702819 | 3.71739688395545 | -0.284047257028189 | -0.167396883955448 | 3.55 | 3.55000000000011 | 1.77635683940025e-15 | 1.11910480882216e-13 |
| 154 | 1.1 | 3.6 | 3.89564279488928 | 3.77262396151009 | -0.295642794889278 | -0.172623961510087 | 3.6 | 3.60000000000007 | 8.88178419700125e-16 | 6.66133814775094e-14 |
| 155 | 1.1 | 3.65 | 3.95751501149899 | 3.82785306050663 | -0.307515011498994 | -0.177853060506628 | 3.65 | 3.64999999999997 | 1.77635683940025e-15 | -3.28626015289046e-14 |
| 156 | 1.1 | 3.7 | 4.01966766910415 | 3.8830841811673 | -0.31966766910415 | -0.183084181167303 | 3.7 | 3.70000000000004 | 0 | 3.46389583683049e-14 |
| 157 | 1.1 | 3.75 | 4.08210461599684 | 3.93831732371397 | -0.332104615996841 | -0.188317323713969 | 3.75 | 3.75000000000012 | 1.77635683940025e-15 | 1.18571819029967e-13 |
| 158 | 1.1 | 3.8 | 4.14482978929497 | 3.99355248836849 | -0.344829789294969 | -0.193552488368486 | 3.8 | 3.80000000000003 | -1.33226762955019e-15 | 3.15303338993544e-14 |
| 159 | 1.1 | 3.85 | 4.20784721783943 | 4.04878967535327 | -0.357847217839427 | -0.19878967535327 | 3.85 | 3.85000000000005 | 4.44089209850063e-16 | 5.41788836017076e-14 |
| 160 | 1.1 | 3.9 | 4.27116102521385 | 4.10402888489037 | -0.371161025213854 | -0.204028884890365 | 3.9 | 3.90000000000009 | 1.77635683940025e-15 | 9.05941988094128e-14 |
| 161 | 1.1 | 3.95 | 4.3347754328935 | 4.15927011720181 | -0.384775432893496 | -0.209270117201815 | 3.95 | 3.95000000000001 | 8.88178419700125e-16 | 1.28785870856518e-14 |
| 162 | 1.1 | 4 | 4.39869476352987 | 4.21451337251003 | -0.398694763529867 | -0.214513372510035 | 4 | 3.99999999999999 | 1.77635683940025e-15 | -1.24344978758018e-14 |
| 163 | 1.1 | 4.05 | 4.46292344437851 | 4.26975865103744 | -0.41292344437851 | -0.219758651037439 | 4.05 | 4.05000000000015 | 8.88178419700125e-16 | 1.46549439250521e-13 |
| 164 | 1.1 | 4.1 | 4.52746601087753 | 4.32500595300589 | -0.427466010877526 | -0.22500595300589 | 4.1 | 4.10000000000009 | 2.66453525910038e-15 | 8.5265128291212e-14 |
| 165 | 1.1 | 4.15 | 4.59232711038504 | 4.38025527863798 | -0.442327110385041 | -0.230255278637982 | 4.15 | 4.15000000000002 | 8.88178419700125e-16 | 2.1316282072803e-14 |
| 166 | 1.1 | 4.2 | 4.65751150608438 | 4.43550662815613 | -0.457511506084376 | -0.235506628156134 | 4.2 | 4.19999999999998 | 8.88178419700125e-16 | -1.77635683940025e-14 |
| 167 | 1.1 | 4.25 | 4.72302408106615 | 4.49076000178276 | -0.473024081066145 | -0.24076000178276 | 4.25 | 4.24999999999995 | 0 | -5.06261699229071e-14 |
| 168 | 1.1 | 4.3 | 4.78886984259722 | 4.54601539974046 | -0.488869842597222 | -0.246015399740458 | 4.3 | 4.30000000000004 | 0 | 3.64153152077051e-14 |
| 169 | 1.1 | 4.35 | 4.85505392658714 | 4.60127282225146 | -0.505053926587138 | -0.25127282225146 | 4.35 | 4.34999999999999 | 1.77635683940025e-15 | -1.15463194561016e-14 |
| 170 | 1.1 | 4.4 | 4.92158160226315 | 4.65653226953855 | -0.521581602263153 | -0.256532269538549 | 4.4 | 4.40000000000001 | 8.88178419700125e-16 | 1.15463194561016e-14 |
| 171 | 1.1 | 4.45 | 4.98845827706608 | 4.71179374182414 | -0.538458277066081 | -0.26179374182414 | 4.45 | 4.44999999999994 | 1.77635683940025e-15 | -5.86197757002083e-14 |
| 172 | 1.1 | 4.5 | 5.05568950177972 | 4.7670572393312 | -0.555689501779718 | -0.267057239331203 | 4.5 | 4.50000000000008 | 0 | 8.26005930321117e-14 |
| 173 | 1.1 | 4.55 | 5.12328097590767 | 4.82232276228197 | -0.573280975907667 | -0.272322762281967 | 4.55 | 4.55000000000003 | 8.88178419700125e-16 | 2.66453525910038e-14 |
| 174 | 1.1 | 4.6 | 5.19123855331225 | 4.8775903108994 | -0.59123855331225 | -0.277590310899404 | 4.6 | 4.60000000000001 | 0 | 5.32907051820075e-15 |
| 175 | 1.1 | 4.65 | 5.25956824813138 | 4.9328598854063 | -0.609568248131381 | -0.282859885406296 | 4.65 | 4.65000000000004 | 8.88178419700125e-16 | 3.90798504668055e-14 |
| 176 | 1.1 | 4.7 | 5.32827624099023 | 4.98813148602525 | -0.628276240990233 | -0.288131486025245 | 4.7 | 4.69999999999995 | 1.77635683940025e-15 | -5.06261699229071e-14 |
| 177 | 1.1 | 4.75 | 5.39736888552593 | 5.0434051129794 | -0.647368885525926 | -0.293405112979404 | 4.75 | 4.75000000000002 | 0 | 1.86517468137026e-14 |
| 178 | 1.1 | 4.8 | 5.4668527152447 | 5.09868076649137 | -0.666852715244703 | -0.298680766491373 | 4.8 | 4.79999999999999 | 0 | -7.105427357601e-15 |
| 179 | 1.1 | 4.85 | 5.53673445073253 | 5.15395844678431 | -0.686734450732528 | -0.303958446784309 | 4.85 | 4.85000000000009 | 0 | 8.5265128291212e-14 |
| 180 | 1.1 | 4.9 | 5.60702100724167 | 5.20923815408081 | -0.707021007241669 | -0.309238154080807 | 4.9 | 4.89999999999996 | 1.77635683940025e-15 | -3.81916720471054e-14 |
| 181 | 1.1 | 4.95 | 5.67771950267745 | 5.2645198886044 | -0.727719502677446 | -0.314519888604398 | 4.95 | 4.9500000000001 | 8.88178419700125e-16 | 9.76996261670138e-14 |
| 182 | 1.1 | 5 | 5.74883726601133 | 5.31980365057768 | -0.748837266011331 | -0.319803650577677 | 5 | 5.00000000000009 | 8.88178419700125e-16 | 9.05941988094128e-14 |
| 183 | 1.1 | 5.05 | 5.82038184614847 | 5.3750894402238 | -0.770381846148466 | -0.325089440223801 | 5.05 | 5.05 | 8.88178419700125e-16 | 0 |
| 184 | 1.1 | 5.1 | 5.89236102128003 | 5.43037725776611 | -0.792361021280026 | -0.330377257766108 | 5.1 | 5.10000000000002 | 8.88178419700125e-16 | 2.1316282072803e-14 |
| 185 | 1.1 | 5.15 | 5.9647828087532 | 5.48566710342776 | -0.8147828087532 | -0.335667103427756 | 5.15 | 5.15000000000014 | 0 | 1.4299672557172e-13 |
| 186 | 1.1 | 5.2 | 6.03765547549423 | 5.54095897743153 | -0.837655475494232 | -0.340958977431527 | 5.2 | 5.19999999999998 | 8.88178419700125e-16 | -1.86517468137026e-14 |
| 187 | 1.1 | 5.25 | 6.11098754902291 | 5.59625288000132 | -0.860987549022912 | -0.346252880001317 | 5.25 | 5.25000000000012 | 0 | 1.18127729820117e-13 |
| 188 | 1.1 | 5.3 | 6.18478782910002 | 5.65154881135991 | -0.884787829100024 | -0.351548811359911 | 5.3 | 5.3000000000001 | 8.88178419700125e-16 | 1.00364161426114e-13 |
| 189 | 1.1 | 5.35 | 6.25906540005281 | 5.70684677173065 | -0.909065400052805 | -0.356846771730647 | 5.35 | 5.34999999999993 | 8.88178419700125e-16 | -6.75015598972095e-14 |
| 190 | 1.1 | 5.4 | 6.33382964382728 | 5.76214676133742 | -0.933829643827278 | -0.362146761337423 | 5.4 | 5.40000000000009 | 8.88178419700125e-16 | 8.88178419700125e-14 |
| 191 | 1.1 | 5.45 | 6.40909025382056 | 5.81744878040302 | -0.95909025382056 | -0.367448780403022 | 5.45 | 5.45 | 0 | -8.88178419700125e-16 |
| 192 | 1.1 | 5.5 | 6.48485724955086 | 5.87275282915134 | -0.984857249550855 | -0.372752829151339 | 5.5 | 5.50000000000006 | 0 | 6.3948846218409e-14 |
| 193 | 1.1 | 5.55 | 6.56114099222802 | 5.92805890780553 | -1.01114099222802 | -0.37805890780553 | 5.55 | 5.54999999999998 | 1.77635683940025e-15 | -2.39808173319034e-14 |
| 194 | 1.1 | 5.6 | 6.63795220129316 | 5.98336701658949 | -1.03795220129316 | -0.383367016589487 | 5.6 | 5.60000000000006 | 8.88178419700125e-16 | 5.59552404411079e-14 |
| 195 | 1.1 | 5.65 | 6.71530197200206 | 6.03867715572655 | -1.06530197200206 | -0.388677155726553 | 5.65 | 5.65000000000009 | 1.77635683940025e-15 | 9.14823772291129e-14 |
| 196 | 1.1 | 5.7 | 6.79320179413407 | 6.09398932544025 | -1.09320179413407 | -0.393989325440251 | 5.7 | 5.7 | 8.88178419700125e-16 | -1.77635683940025e-15 |
| 197 | 1.1 | 5.75 | 6.87166357191575 | 6.14930352595448 | -1.12166357191575 | -0.399303525954478 | 5.75 | 5.74999999999999 | 0 | -9.76996261670138e-15 |
| 198 | 1.1 | 5.8 | 6.95069964525717 | 6.20461975749294 | -1.15069964525717 | -0.404619757492942 | 5.8 | 5.80000000000007 | 1.77635683940025e-15 | 7.19424519957101e-14 |
| 199 | 1.1 | 5.85 | 7.03032281240804 | 6.25993802027917 | -1.18032281240803 | -0.409938020279168 | 5.85 | 5.85000000000005 | 8.88178419700125e-16 | 5.24025267623074e-14 |
| 200 | 1.1 | 5.9 | 7.11054635415148 | 6.31525831453705 | -1.21054635415148 | -0.415258314537052 | 5.9 | 5.90000000000003 | 1.77635683940025e-15 | 2.93098878501041e-14 |
| 201 | 1.1 | 5.95 | 7.19138405966503 | 6.37058064049049 | -1.24138405966503 | -0.420580640490489 | 5.95 | 5.95000000000007 | 8.88178419700125e-16 | 7.105427357601e-14 |
| 202 | 1.1 | 6 | 7.27285025419134 | 6.42590499836319 | -1.27285025419134 | -0.425904998363189 | 6 | 6.00000000000003 | 1.77635683940025e-15 | 3.37507799486048e-14 |
| 203 | 1.2 | 1 | 0.992798943627076 | 0.904916772046331 | 0.00720105637292423 | 0.0950832279536694 | 1 | 0.99999999999995 | 6.66133814775094e-16 | -4.9515946898282e-14 |
| 204 | 1.2 | 1.05 | 1.0438650988766 | 0.960041101448761 | 0.00613490112339798 | 0.0899588985512393 | 1.05 | 1.04999999999995 | 1.33226762955019e-15 | -4.59632332194815e-14 |
| 205 | 1.2 | 1.1 | 1.09508858402476 | 1.01516744103136 | 0.00491141597524081 | 0.0848325589686385 | 1.1 | 1.10000000000006 | 1.11022302462516e-15 | 6.48370246381091e-14 |
| 206 | 1.2 | 1.15 | 1.14647086224908 | 1.07029579101377 | 0.0035291377509199 | 0.0797042089862281 | 1.15 | 1.15000000000002 | 1.33226762955019e-15 | 2.1094237467878e-14 |
| 207 | 1.2 | 1.2 | 1.19801341954809 | 1.12542615161619 | 0.00198658045190614 | 0.0745738483838141 | 1.2 | 1.20000000000003 | 4.44089209850063e-16 | 2.77555756156289e-14 |
| 208 | 1.2 | 1.25 | 1.24971776524278 | 1.18055852305843 | 0.00028223475722422 | 0.0694414769415719 | 1.25 | 1.24999999999992 | 0 | -8.03801469828613e-14 |
| 209 | 1.2 | 1.3 | 1.30158543249224 | 1.23569290556088 | -0.00158543249223775 | 0.0643070944391226 | 1.3 | 1.3 | 4.44089209850063e-16 | -3.77475828372553e-15 |
| 210 | 1.2 | 1.35 | 1.35361797882418 | 1.29082929934336 | -0.00361797882418213 | 0.059170700656642 | 1.35 | 1.35000000000002 | 8.88178419700125e-16 | 1.86517468137026e-14 |
| 211 | 1.2 | 1.4 | 1.40581698668063 | 1.34596770462606 | -0.00581698668062725 | 0.0540322953739354 | 1.4 | 1.40000000000005 | 1.55431223447522e-15 | 4.75175454539567e-14 |
| 212 | 1.2 | 1.45 | 1.45818406397945 | 1.40110812162901 | -0.00818406397944527 | 0.0488918783709942 | 1.45 | 1.44999999999994 | 1.33226762955019e-15 | -6.26165785888588e-14 |
| 213 | 1.2 | 1.5 | 1.51072084469228 | 1.45625055057275 | -0.0107208446922828 | 0.0437494494272539 | 1.5 | 1.50000000000001 | 1.55431223447522e-15 | 1.15463194561016e-14 |
| 214 | 1.2 | 1.55 | 1.56342898943944 | 1.51139499167729 | -0.0134289894394399 | 0.0386050083227052 | 1.55 | 1.55000000000005 | 8.88178419700125e-16 | 5.28466159721575e-14 |
| 215 | 1.2 | 1.6 | 1.61631018610234 | 1.56654144516303 | -0.0163101861023365 | 0.0334585548369688 | 1.6 | 1.60000000000014 | 1.33226762955019e-15 | 1.43218770176645e-13 |
| 216 | 1.2 | 1.65 | 1.66936615045419 | 1.62168991124996 | -0.019366150454186 | 0.0283100887500354 | 1.65 | 1.64999999999999 | 1.77635683940025e-15 | -6.88338275267597e-15 |
| 217 | 1.2 | 1.7 | 1.72259862680956 | 1.67684039015884 | -0.022598626809563 | 0.0231596098411564 | 1.7 | 1.69999999999995 | 1.77635683940025e-15 | -5.37347943918576e-14 |
| 218 | 1.2 | 1.75 | 1.77600938869354 | 1.73199288211005 | -0.0260093886935449 | 0.0180071178899515 | 1.75 | 1.74999999999998 | 1.11022302462516e-15 | -2.37587727269784e-14 |
| 219 | 1.2 | 1.8 | 1.82960023953117 | 1.78714738732396 | -0.0296002395311665 | 0.012852612676042 | 1.8 | 1.80000000000002 | 2.22044604925031e-15 | 1.88737914186277e-14 |
| 220 | 1.2 | 1.85 | 1.88337301335792 | 1.84230390602095 | -0.0333730133579215 | 0.00769609397904869 | 1.85 | 1.84999999999997 | 2.22044604925031e-16 | -2.64233079860787e-14 |
| 221 | 1.2 | 1.9 | 1.93732957555216 | 1.89746243842178 | -0.037329575552155 | 0.00253756157822171 | 1.9 | 1.90000000000004 | 1.33226762955019e-15 | 3.97459842815806e-14 |
| 222 | 1.2 | 1.95 | 1.99147182359009 | 1.95262298474682 | -0.0414718235900924 | -0.00262298474681733 | 1.95 | 1.95000000000004 | 0 | 4.30766533554561e-14 |
| 223 | 1.2 | 2 | 2.04580168782447 | 2.00778554521663 | -0.0458016878244676 | -0.00778554521663377 | 2 | 1.99999999999994 | 1.77635683940025e-15 | -5.72875080706581e-14 |
| 224 | 1.2 | 2.05 | 2.10032113228756 | 2.06295012005216 | -0.0503211322875576 | -0.0129501200521611 | 2.05 | 2.04999999999999 | 1.77635683940025e-15 | -5.32907051820075e-15 |
| 225 | 1.2 | 2.1 | 2.15503215551965 | 2.11811670947396 | -0.0550321555196507 | -0.018116709473964 | 2.1 | 2.10000000000008 | 4.44089209850063e-16 | 8.39328606616618e-14 |
| 226 | 1.2 | 2.15 | 2.20993679142388 | 2.17328531370261 | -0.0599367914238838 | -0.0232853137026066 | 2.15 | 2.15000000000006 | 1.33226762955019e-15 | 5.90638649100583e-14 |
| 227 | 1.2 | 2.2 | 2.2650371101485 | 2.22845593295902 | -0.0650371101484955 | -0.0284559329590239 | 2.2 | 2.20000000000007 | 8.88178419700125e-16 | 6.83897383169096e-14 |
| 228 | 1.2 | 2.25 | 2.3203352189976 | 2.28362856746397 | -0.0703352189976014 | -0.0336285674639649 | 2.25 | 2.25000000000005 | 4.44089209850063e-16 | 5.32907051820075e-14 |
| 229 | 1.2 | 2.3 | 2.37583326337159 | 2.33880321743836 | -0.075833263371591 | -0.0388032174383639 | 2.3 | 2.30000000000009 | 1.33226762955019e-15 | 8.88178419700125e-14 |
| 230 | 1.2 | 2.35 | 2.43153342773835 | 2.39397988310297 | -0.0815334277383508 | -0.0439798831029696 | 2.35 | 2.35000000000005 | 1.33226762955019e-15 | 4.48530101948563e-14 |
| 231 | 1.2 | 2.4 | 2.48743793663657 | 2.4491585646789 | -0.0874379366365674 | -0.0491585646789026 | 2.4 | 2.40000000000009 | 8.88178419700125e-16 | 9.01501095995627e-14 |
| 232 | 1.2 | 2.45 | 2.54354905571239 | 2.50433926238691 | -0.0935490557123919 | -0.0543392623869115 | 2.45 | 2.45000000000002 | 1.77635683940025e-15 | 2.1316282072803e-14 |
| 233 | 1.2 | 2.5 | 2.59986909279084 | 2.5595219764483 | -0.0998690927908399 | -0.0595219764483015 | 2.5 | 2.5000000000001 | -4.44089209850063e-16 | 1.02140518265514e-13 |
| 234 | 1.2 | 2.55 | 2.65640039898339 | 2.61470670708382 | -0.106400398983391 | -0.064706707083821 | 2.55 | 2.55000000000006 | 4.44089209850063e-16 | 5.55111512312578e-14 |
| 235 | 1.2 | 2.6 | 2.71314536983322 | 2.66989345451478 | -0.113145369833219 | -0.0698934545147747 | 2.6 | 2.60000000000007 | 0 | 7.105427357601e-14 |
| 236 | 1.2 | 2.65 | 2.77010644649973 | 2.7250822189621 | -0.120106446499729 | -0.075082218962097 | 2.65 | 2.64999999999997 | 4.44089209850063e-16 | -3.19744231092045e-14 |
| 237 | 1.2 | 2.7 | 2.82728611698396 | 2.78027300064728 | -0.12728611698396 | -0.0802730006472778 | 2.7 | 2.70000000000003 | 8.88178419700125e-16 | 3.10862446895044e-14 |
| 238 | 1.2 | 2.75 | 2.88468691739668 | 2.83546579979125 | -0.134686917396678 | -0.0854657997912516 | 2.75 | 2.75 | 8.88178419700125e-16 | 3.99680288865056e-15 |
| 239 | 1.2 | 2.8 | 2.94231143327092 | 2.89066061661551 | -0.142311433270925 | -0.0906606166155073 | 2.8 | 2.8000000000001 | 4.44089209850063e-16 | 9.9031893796564e-14 |
| 240 | 1.2 | 2.85 | 3.00016230092099 | 2.94585745134098 | -0.150162300920988 | -0.0958574513409789 | 2.85 | 2.84999999999999 | 1.77635683940025e-15 | -1.37667655053519e-14 |
| 241 | 1.2 | 2.9 | 3.05824220884978 | 3.00105630418934 | -0.158242208849777 | -0.101056304189342 | 2.9 | 2.89999999999997 | 1.77635683940025e-15 | -2.75335310107039e-14 |
| 242 | 1.2 | 2.95 | 3.11655389920677 | 3.0562571753819 | -0.166553899206768 | -0.1062571753819 | 2.95 | 2.94999999999999 | 8.88178419700125e-16 | -8.88178419700125e-15 |
| 243 | 1.2 | 3 | 3.17510016929873 | 3.11146006513996 | -0.175100169298725 | -0.111460065139959 | 3 | 2.99999999999994 | -8.88178419700125e-16 | -6.12843109593086e-14 |
| 244 | 1.2 | 3.05 | 3.23388387315557 | 3.16666497368519 | -0.183883873155572 | -0.116664973685192 | 3.05 | 3.05000000000001 | 1.33226762955019e-15 | 1.06581410364015e-14 |
| 245 | 1.2 | 3.1 | 3.29290792315386 | 3.2218719012389 | -0.192907923153864 | -0.121871901238904 | 3.1 | 3.10000000000003 | 1.33226762955019e-15 | 3.15303338993544e-14 |
| 246 | 1.2 | 3.15 | 3.35217529170053 | 3.27708084802277 | -0.202175291700534 | -0.127080848022769 | 3.15 | 3.15000000000012 | 4.44089209850063e-16 | 1.22568621918617e-13 |
| 247 | 1.2 | 3.2 | 3.4116890129796 | 3.33229181425809 | -0.211689012979597 | -0.132291814258092 | 3.2 | 3.20000000000003 | 1.33226762955019e-15 | 3.28626015289046e-14 |
| 248 | 1.2 | 3.25 | 3.47145218476475 | 3.38750480016673 | -0.221452184764751 | -0.137504800166733 | 3.25 | 3.24999999999998 | 1.77635683940025e-15 | -1.82076576038526e-14 |
| 249 | 1.2 | 3.3 | 3.53146797030095 | 3.44271980597037 | -0.231467970300952 | -0.142719805970366 | 3.3 | 3.29999999999998 | 4.44089209850063e-16 | -2.22044604925031e-14 |
| 250 | 1.2 | 3.35 | 3.59173960025818 | 3.49793683189067 | -0.241739600258177 | -0.147936831890665 | 3.35 | 3.35 | 4.44089209850063e-16 | -1.33226762955019e-15 |
| 251 | 1.2 | 3.4 | 3.65227037476078 | 3.55315587814931 | -0.252270374760775 | -0.153155878149305 | 3.4 | 3.39999999999998 | 8.88178419700125e-16 | -1.90958360235527e-14 |
| 252 | 1.2 | 3.45 | 3.71306366549605 | 3.60837694496815 | -0.263063665496047 | -0.158376944968146 | 3.45 | 3.45 | 0 | -3.99680288865056e-15 |
| 253 | 1.2 | 3.5 | 3.77412291790586 | 3.66360003256905 | -0.274122917905864 | -0.163600032569047 | 3.5 | 3.50000000000008 | 1.77635683940025e-15 | 7.54951656745106e-14 |
| 254 | 1.2 | 3.55 | 3.83545165346531 | 3.71882514117368 | -0.285451653465305 | -0.168825141173683 | 3.55 | 3.55000000000005 | 8.88178419700125e-16 | 4.70734562441066e-14 |
| 255 | 1.2 | 3.6 | 3.89705347205266 | 3.7740522710041 | -0.297053472052655 | -0.174052271004098 | 3.6 | 3.60000000000004 | 1.33226762955019e-15 | 3.86357612569555e-14 |
| 256 | 1.2 | 3.65 | 3.95893205441521 | 3.82928142228215 | -0.308932054415208 | -0.179281422282152 | 3.65 | 3.64999999999997 | 0 | -2.88657986402541e-14 |
| 257 | 1.2 | 3.7 | 4.02109116473571 | 3.88451259523008 | -0.321091164735708 | -0.184512595230075 | 3.7 | 3.70000000000007 | 2.22044604925031e-15 | 6.61692922676593e-14 |
| 258 | 1.2 | 3.75 | 4.08353465330443 | 3.93974579006954 | -0.333534653304429 | -0.189745790069541 | 3.75 | 3.75000000000001 | 4.44089209850063e-16 | 4.88498130835069e-15 |
| 259 | 1.2 | 3.8 | 4.14626645930236 | 3.99498100702296 | -0.346266459302361 | -0.194981007022964 | 3.8 | 3.8000000000001 | 1.77635683940025e-15 | 1.00364161426114e-13 |
| 260 | 1.2 | 3.85 | 4.20929061370106 | 4.05021824631202 | -0.35929061370106 | -0.200218246312021 | 3.85 | 3.84999999999996 | 4.44089209850063e-16 | -3.73034936274053e-14 |
| 261 | 1.2 | 3.9 | 4.27261124228535 | 4.10545750815931 | -0.372611242285345 | -0.205457508159308 | 3.9 | 3.9 | 1.77635683940025e-15 | -2.66453525910038e-15 |
| 262 | 1.2 | 3.95 | 4.33623256880513 | 4.16069879278687 | -0.386232568805132 | -0.210698792786872 | 3.95 | 3.95000000000008 | 8.88178419700125e-16 | 7.59392548843607e-14 |
| 263 | 1.2 | 4 | 4.40015891826331 | 4.21594210041676 | -0.400158918263314 | -0.215942100416759 | 4 | 4.00000000000003 | 0 | 3.10862446895044e-14 |
| 264 | 1.2 | 4.05 | 4.46439472034687 | 4.27118743127138 | -0.414394720346865 | -0.221187431271379 | 4.05 | 4.04999999999999 | 0 | -1.06581410364015e-14 |
| 265 | 1.2 | 4.1 | 4.52894451300891 | 4.32643478557315 | -0.42894451300891 | -0.226434785573153 | 4.1 | 4.10000000000005 | 0 | 4.88498130835069e-14 |
| 266 | 1.2 | 4.15 | 4.59381294620993 | 4.3816841635443 | -0.443812946209933 | -0.231684163544304 | 4.15 | 4.15000000000009 | 8.88178419700125e-16 | 9.41469124882133e-14 |
| 267 | 1.2 | 4.2 | 4.65900478582687 | 4.43693556540707 | -0.459004785826869 | -0.236935565407066 | 4.2 | 4.19999999999998 | 8.88178419700125e-16 | -1.86517468137026e-14 |
| 268 | 1.2 | 4.25 | 4.72452491773937 | 4.49218899138422 | -0.474524917739367 | -0.242188991384223 | 4.25 | 4.25000000000003 | 1.77635683940025e-15 | 2.75335310107039e-14 |
| 269 | 1.2 | 4.3 | 4.79037835210316 | 4.547444441698 | -0.490378352103155 | -0.247444441698003 | 4.3 | 4.30000000000001 | 8.88178419700125e-16 | 8.88178419700125e-15 |
| 270 | 1.2 | 4.35 | 4.85657022782106 | 4.60270191657101 | -0.506570227821064 | -0.252701916571009 | 4.35 | 4.35000000000001 | 1.77635683940025e-15 | 6.21724893790088e-15 |
| 271 | 1.2 | 4.4 | 4.92310581722301 | 4.65796141622584 | -0.523105817223009 | -0.257961416225838 | 4.4 | 4.40000000000006 | 0 | 5.59552404411079e-14 |
| 272 | 1.2 | 4.45 | 4.98999053096702 | 4.71322294088491 | -0.539990530967017 | -0.263222940884905 | 4.45 | 4.45 | 0 | -5.32907051820075e-15 |
| 273 | 1.2 | 4.5 | 5.05722992317414 | 4.768486490771 | -0.557229923174138 | -0.268486490770996 | 4.5 | 4.49999999999996 | 8.88178419700125e-16 | -4.35207425653061e-14 |
| 274 | 1.2 | 4.55 | 5.12482969681108 | 4.82375206610689 | -0.574829696811082 | -0.273752066106892 | 4.55 | 4.55000000000004 | 8.88178419700125e-16 | 3.46389583683049e-14 |
| 275 | 1.2 | 4.6 | 5.19279570933533 | 4.87901966711501 | -0.592795709335331 | -0.279019667115014 | 4.6 | 4.59999999999996 | 8.88178419700125e-16 | -4.17443857259059e-14 |
| 276 | 1.2 | 4.65 | 5.26113397861853 | 4.93428929401851 | -0.611133978618526 | -0.284289294018511 | 4.65 | 4.65000000000008 | 8.88178419700125e-16 | 8.08242361927114e-14 |
| 277 | 1.2 | 4.7 | 5.3298506891651 | 4.9895609470398 | -0.629850689165096 | -0.289560947039802 | 4.7 | 4.70000000000006 | 1.77635683940025e-15 | 5.6843418860808e-14 |
| 278 | 1.2 | 4.75 | 5.39895219864431 | 5.04483462640185 | -0.648952198644312 | -0.294834626401854 | 4.75 | 4.75 | 8.88178419700125e-16 | 0 |
| 279 | 1.2 | 4.8 | 5.46844504475531 | 5.10011033232764 | -0.668445044755305 | -0.300110332327637 | 4.8 | 4.79999999999999 | 8.88178419700125e-16 | -1.06581410364015e-14 |
| 280 | 1.2 | 4.85 | 5.53833595244599 | 5.15538806504012 | -0.688335952445988 | -0.305388065040122 | 4.85 | 4.85000000000007 | 8.88178419700125e-16 | 6.83897383169096e-14 |
| 281 | 1.2 | 4.9 | 5.60863184150852 | 5.21066782476209 | -0.708631841508516 | -0.310667824762093 | 4.9 | 4.90000000000007 | 1.77635683940025e-15 | 7.28306304154103e-14 |
| 282 | 1.2 | 4.95 | 5.67933983457547 | 5.26594961171652 | -0.729339834575474 | -0.315949611716521 | 4.95 | 4.94999999999997 | 1.77635683940025e-15 | -2.66453525910038e-14 |
| 283 | 1.2 | 5 | 5.75046726554303 | 5.32123342612674 | -0.750467265543033 | -0.321233426126744 | 5 | 5.00000000000004 | 8.88178419700125e-16 | 3.46389583683049e-14 |
| 284 | 1.2 | 5.05 | 5.82202168844921 | 5.37651926821555 | -0.772021688449208 | -0.326519268215548 | 5.05 | 5.04999999999998 | 1.77635683940025e-15 | -1.59872115546023e-14 |
| 285 | 1.2 | 5.1 | 5.89401088683766 | 5.43180713820627 | -0.794010886837662 | -0.331807138206271 | 5.1 | 5.10000000000001 | 8.88178419700125e-16 | 1.06581410364015e-14 |
| 286 | 1.2 | 5.15 | 5.96644288363993 | 5.48709703632189 | -0.816442883639928 | -0.337097036321885 | 5.15 | 5.14999999999994 | 8.88178419700125e-16 | -6.03961325396085e-14 |
| 287 | 1.2 | 5.2 | 6.03932595161152 | 5.54238896278592 | -0.839325951611517 | -0.342388962785915 | 5.2 | 5.20000000000005 | 8.88178419700125e-16 | 5.32907051820075e-14 |
| 288 | 1.2 | 5.25 | 6.1126686243604 | 5.59768291782133 | -0.862668624360405 | -0.34768291782133 | 5.25 | 5.2500000000001 | 8.88178419700125e-16 | 9.85878045867139e-14 |
| 289 | 1.2 | 5.3 | 6.18647970800944 | 5.65297890165147 | -0.886479708009444 | -0.352978901651469 | 5.3 | 5.30000000000012 | 1.77635683940025e-15 | 1.19015908239817e-13 |
| 290 | 1.2 | 5.35 | 6.26076829353788 | 5.70827691449949 | -0.910768293537879 | -0.358276914499487 | 5.35 | 5.34999999999995 | 8.88178419700125e-16 | -4.79616346638068e-14 |
| 291 | 1.2 | 5.4 | 6.33554376985089 | 5.7635769565891 | -0.935543769850892 | -0.363576956589096 | 5.4 | 5.39999999999991 | 0 | -9.32587340685131e-14 |
| 292 | 1.2 | 5.45 | 6.41081583763039 | 5.81887902814382 | -0.960815837630387 | -0.368879028143819 | 5.45 | 5.45000000000008 | 8.88178419700125e-16 | 7.90478793533112e-14 |
| 293 | 1.2 | 5.5 | 6.48659452402484 | 5.87418312938663 | -0.986594524024837 | -0.374183129386626 | 5.5 | 5.50000000000003 | 8.88178419700125e-16 | 3.10862446895044e-14 |
| 294 | 1.2 | 5.55 | 6.56289019824121 | 5.92948926054141 | -1.01289019824121 | -0.379489260541413 | 5.55 | 5.55000000000012 | 8.88178419700125e-16 | 1.23456800338317e-13 |
| 295 | 1.2 | 5.6 | 6.63971358810755 | 5.98479742183133 | -1.03971358810755 | -0.384797421831332 | 5.6 | 5.60000000000001 | 8.88178419700125e-16 | 9.76996261670138e-15 |
| 296 | 1.2 | 5.65 | 6.71707579768119 | 6.04010761348028 | -1.06707579768119 | -0.390107613480282 | 5.65 | 5.64999999999998 | 1.77635683940025e-15 | -2.22044604925031e-14 |
| 297 | 1.2 | 5.7 | 6.79498832598437 | 6.09541983571179 | -1.09498832598437 | -0.395419835711786 | 5.7 | 5.69999999999994 | 0 | -5.86197757002083e-14 |
| 298 | 1.2 | 5.75 | 6.87346308695674 | 6.15073408874974 | -1.12346308695674 | -0.400734088749739 | 5.75 | 5.75000000000011 | 8.88178419700125e-16 | 1.11022302462516e-13 |
| 299 | 1.2 | 5.8 | 6.95251243072288 | 6.2060503728173 | -1.15251243072288 | -0.406050372817294 | 5.8 | 5.79999999999999 | 8.88178419700125e-16 | -7.105427357601e-15 |
| 300 | 1.2 | 5.85 | 7.03214916628219 | 6.26136868813872 | -1.18214916628219 | -0.41136868813872 | 5.85 | 5.85000000000006 | 8.88178419700125e-16 | 6.12843109593086e-14 |
| 301 | 1.2 | 5.9 | 7.1123865857393 | 6.31668903493754 | -1.21238658573929 | -0.416689034937538 | 5.9 | 5.90000000000008 | 1.77635683940025e-15 | 7.99360577730113e-14 |
| 302 | 1.2 | 5.95 | 7.19323849020472 | 6.37201141343765 | -1.24323849020472 | -0.422011413437646 | 5.95 | 5.95000000000012 | 8.88178419700125e-16 | 1.14575016141316e-13 |
| 303 | 1.2 | 6 | 7.27471921750873 | 6.42733582386275 | -1.27471921750873 | -0.427335823862753 | 6 | 6.00000000000002 | 8.88178419700125e-16 | 2.22044604925031e-14 |
| 304 | 1.3 | 1 | 0.993958960343103 | 0.906341710653254 | 0.00604103965689729 | 0.0936582893467459 | 1 | 0.999999999999952 | 1.11022302462516e-15 | -4.75175454539567e-14 |
| 305 | 1.3 | 1.05 | 1.04502867874735 | 0.961466092016157 | 0.00497132125265454 | 0.0885339079838428 | 1.05 | 1.05000000000004 | 1.99840144432528e-15 | 3.530509218308e-14 |
| 306 | 1.3 | 1.1 | 1.09625576008742 | 1.01659248356478 | 0.003744239912578 | 0.0834075164352179 | 1.1 | 1.1000000000001 | 6.66133814775094e-16 | 1.0413891970984e-13 |
| 307 | 1.3 | 1.15 | 1.14764166805457 | 1.07172088551895 | 0.00235833194542789 | 0.0782791144810473 | 1.15 | 1.15000000000006 | 8.88178419700125e-16 | 6.41708908233341e-14 |
| 308 | 1.3 | 1.2 | 1.19918788917228 | 1.12685129809868 | 0.000812110827715706 | 0.0731487019013219 | 1.2 | 1.19999999999995 | 1.55431223447522e-15 | -4.77395900588817e-14 |
| 309 | 1.3 | 1.25 | 1.25089593329806 | 1.18198372152415 | -0.000895933298060925 | 0.0680162784758474 | 1.25 | 1.24999999999994 | 1.33226762955019e-15 | -6.3504757008559e-14 |
| 310 | 1.3 | 1.3 | 1.30276733413945 | 1.23711815601539 | -0.00276733413944674 | 0.0628818439846144 | 1.3 | 1.29999999999998 | 1.33226762955019e-15 | -1.88737914186277e-14 |
| 311 | 1.3 | 1.35 | 1.35480364978482 | 1.29225460179239 | -0.00480364978481829 | 0.0577453982076139 | 1.35 | 1.35000000000001 | 2.22044604925031e-16 | 1.37667655053519e-14 |
| 312 | 1.3 | 1.4 | 1.40700646324947 | 1.34739305907535 | -0.00700646324946908 | 0.0526069409246512 | 1.4 | 1.40000000000009 | 4.44089209850063e-16 | 9.32587340685131e-14 |
| 313 | 1.3 | 1.45 | 1.45937738303751 | 1.40253352808428 | -0.00937738303750701 | 0.0474664719157181 | 1.45 | 1.45000000000007 | 4.44089209850063e-16 | 7.43849426498855e-14 |
| 314 | 1.3 | 1.5 | 1.51191804372016 | 1.45767600903957 | -0.0119180437201558 | 0.0423239909604345 | 1.5 | 1.50000000000011 | 4.44089209850063e-16 | 1.11022302462516e-13 |
| 315 | 1.3 | 1.55 | 1.56463010653103 | 1.51282050216121 | -0.0146301065310321 | 0.0371794978387914 | 1.55 | 1.54999999999999 | 8.88178419700125e-16 | -1.46549439250521e-14 |
| 316 | 1.3 | 1.6 | 1.61751525997901 | 1.56796700766996 | -0.0175152599790096 | 0.0320329923300395 | 1.6 | 1.60000000000011 | 8.88178419700125e-16 | 1.13908882326541e-13 |
| 317 | 1.3 | 1.65 | 1.67057522047933 | 1.62311552578565 | -0.0205752204793259 | 0.0268844742143546 | 1.65 | 1.65000000000004 | 6.66133814775094e-16 | 3.81916720471054e-14 |
| 318 | 1.3 | 1.7 | 1.72381173300358 | 1.67826605672901 | -0.0238117330035814 | 0.0217339432709875 | 1.7 | 1.7000000000001 | 1.11022302462516e-15 | 1.01252339845814e-13 |
| 319 | 1.3 | 1.75 | 1.77722657174933 | 1.73341860072026 | -0.0272265717493347 | 0.0165813992797437 | 1.75 | 1.75000000000011 | 8.88178419700125e-16 | 1.07913677993565e-13 |
| 320 | 1.3 | 1.8 | 1.83082154083003 | 1.78857315797976 | -0.030821540830027 | 0.0114268420202444 | 1.8 | 1.79999999999999 | 1.11022302462516e-15 | -7.32747196252603e-15 |
| 321 | 1.3 | 1.85 | 1.88459847498598 | 1.84372972872826 | -0.0345984749859796 | 0.00627027127173974 | 1.85 | 1.84999999999999 | 0 | -9.54791801177635e-15 |
| 322 | 1.3 | 1.9 | 1.93855924031728 | 1.89888831318615 | -0.0385592403172808 | 0.00111168681385032 | 1.9 | 1.89999999999996 | 8.88178419700125e-16 | -3.68594044175552e-14 |
| 323 | 1.3 | 1.95 | 1.99270573503935 | 1.95404891157417 | -0.0427057350393543 | -0.0040489115741722 | 1.95 | 1.95000000000007 | 1.99840144432528e-15 | 7.17204073907851e-14 |
| 324 | 1.3 | 2 | 2.04703989026211 | 2.00921152411252 | -0.0470398902621141 | -0.00921152411252324 | 2 | 1.99999999999994 | 8.88178419700125e-16 | -6.19504447740837e-14 |
| 325 | 1.3 | 2.05 | 2.1015636707936 | 2.06437615102232 | -0.051563670793596 | -0.0143761510223217 | 2.05 | 2.04999999999999 | 1.77635683940025e-15 | -1.37667655053519e-14 |
| 326 | 1.3 | 2.1 | 2.15627907596898 | 2.11954279252395 | -0.056279075968984 | -0.0195427925239464 | 2.1 | 2.09999999999993 | 1.33226762955019e-15 | -6.83897383169096e-14 |
| 327 | 1.3 | 2.15 | 2.21118814050608 | 2.17471144883852 | -0.0611881405060779 | -0.0247114488385174 | 2.15 | 2.15000000000013 | 1.77635683940025e-15 | 1.25677246387568e-13 |
| 328 | 1.3 | 2.2 | 2.26629293538818 | 2.22988212018623 | -0.0662929353881787 | -0.0298821201862287 | 2.2 | 2.20000000000004 | 4.44089209850063e-16 | 4.35207425653061e-14 |
| 329 | 1.3 | 2.25 | 2.32159556877553 | 2.28505480678839 | -0.0715955687755265 | -0.0350548067883851 | 2.25 | 2.25000000000013 | 1.33226762955019e-15 | 1.30562227695918e-13 |
| 330 | 1.3 | 2.3 | 2.37709818694637 | 2.34022950886537 | -0.0770981869463738 | -0.0402295088653659 | 2.3 | 2.29999999999996 | 4.44089209850063e-16 | -4.30766533554561e-14 |
| 331 | 1.3 | 2.35 | 2.43280297526895 | 2.39540622663866 | -0.0828029752689492 | -0.0454062266386592 | 2.35 | 2.35000000000006 | 8.88178419700125e-16 | 6.17284001691587e-14 |
| 332 | 1.3 | 2.4 | 2.48871215920548 | 2.45058496032883 | -0.0887121592054774 | -0.0505849603288309 | 2.4 | 2.40000000000011 | 1.33226762955019e-15 | 1.10134124042816e-13 |
| 333 | 1.3 | 2.45 | 2.54482800534963 | 2.50576571015682 | -0.0948280053496324 | -0.0557657101568148 | 2.45 | 2.45000000000007 | 8.88178419700125e-16 | 6.61692922676593e-14 |
| 334 | 1.3 | 2.5 | 2.60115282249875 | 2.56094847634373 | -0.101152822498751 | -0.0609484763437309 | 2.5 | 2.50000000000002 | 1.33226762955019e-15 | 2.39808173319034e-14 |
| 335 | 1.3 | 2.55 | 2.65768896276225 | 2.6161332591107 | -0.107688962762249 | -0.0661332591106976 | 2.55 | 2.55000000000004 | 1.33226762955019e-15 | 4.17443857259059e-14 |
| 336 | 1.3 | 2.6 | 2.71443882270776 | 2.67132005867865 | -0.114438822707758 | -0.0713200586786495 | 2.6 | 2.59999999999997 | 1.33226762955019e-15 | -2.70894418008538e-14 |
| 337 | 1.3 | 2.65 | 2.77140484454654 | 2.72650887526889 | -0.121404844546541 | -0.0765088752688916 | 2.65 | 2.64999999999997 | 4.44089209850063e-16 | -2.97539770599542e-14 |
| 338 | 1.3 | 2.7 | 2.82858951735988 | 2.78169970910254 | -0.128589517359881 | -0.0816997091025433 | 2.7 | 2.69999999999998 | 1.33226762955019e-15 | -1.77635683940025e-14 |
| 339 | 1.3 | 2.75 | 2.88599537836813 | 2.83689256040091 | -0.135995378368134 | -0.086892560400909 | 2.75 | 2.75000000000009 | 8.88178419700125e-16 | 8.74855743404623e-14 |
| 340 | 1.3 | 2.8 | 2.94362501424433 | 2.89208742938492 | -0.14362501424433 | -0.0920874293849225 | 2.8 | 2.79999999999999 | 1.33226762955019e-15 | -5.77315972805081e-15 |
| 341 | 1.3 | 2.85 | 3.00148106247421 | 2.94728431627626 | -0.151481062474212 | -0.0972843162762587 | 2.85 | 2.85000000000004 | 4.44089209850063e-16 | 4.2632564145606e-14 |
| 342 | 1.3 | 2.9 | 3.05956621276475 | 3.00248322129604 | -0.159566212764751 | -0.102483221296036 | 2.9 | 2.90000000000003 | 1.77635683940025e-15 | 3.37507799486048e-14 |
| 343 | 1.3 | 2.95 | 3.11788320850325 | 3.05768414466575 | -0.16788320850325 | -0.107684144665747 | 2.95 | 2.95000000000007 | 4.44089209850063e-16 | 7.01660951563099e-14 |
| 344 | 1.3 | 3 | 3.17643484826931 | 3.11288708660669 | -0.176434848269309 | -0.112887086606693 | 3 | 3.00000000000005 | 1.77635683940025e-15 | 4.61852778244065e-14 |
| 345 | 1.3 | 3.05 | 3.23522398740196 | 3.16809204734036 | -0.185223987401959 | -0.118092047340364 | 3.05 | 3.04999999999999 | 4.44089209850063e-16 | -1.15463194561016e-14 |
| 346 | 1.3 | 3.1 | 3.29425353962451 | 3.22329902708844 | -0.194253539624508 | -0.123299027088437 | 3.1 | 3.10000000000006 | 1.77635683940025e-15 | 5.6399329650958e-14 |
| 347 | 1.3 | 3.15 | 3.35352647872965 | 3.27850802607221 | -0.203526478729647 | -0.128508026072213 | 3.15 | 3.15000000000004 | 8.88178419700125e-16 | 3.50830475781549e-14 |
| 348 | 1.3 | 3.2 | 3.41304584032765 | 3.33371904451337 | -0.213045840327653 | -0.133719044513368 | 3.2 | 3.20000000000001 | 8.88178419700125e-16 | 7.99360577730113e-15 |
| 349 | 1.3 | 3.25 | 3.47281472366052 | 3.38893208263358 | -0.222814723660515 | -0.138932082633577 | 3.25 | 3.25000000000002 | 8.88178419700125e-16 | 2.39808173319034e-14 |
| 350 | 1.3 | 3.3 | 3.53283629348511 | 3.44414714065433 | -0.232836293485113 | -0.144147140654329 | 3.3 | 3.29999999999993 | 1.77635683940025e-15 | -7.37188088351104e-14 |
| 351 | 1.3 | 3.35 | 3.59311378202865 | 3.49936421879767 | -0.243113782028648 | -0.14936421879767 | 3.35 | 3.35000000000003 | 8.88178419700125e-16 | 2.48689957516035e-14 |
| 352 | 1.3 | 3.4 | 3.65365049101979 | 3.55458331728509 | -0.253650491019786 | -0.154583317285086 | 3.4 | 3.40000000000009 | 8.88178419700125e-16 | 8.79296635503124e-14 |
| 353 | 1.3 | 3.45 | 3.71444979379906 | 3.60980443633826 | -0.264449793799064 | -0.159804436338256 | 3.45 | 3.45000000000002 | 1.77635683940025e-15 | 1.90958360235527e-14 |
| 354 | 1.3 | 3.5 | 3.77551513751243 | 3.66502757617922 | -0.275515137512431 | -0.165027576179221 | 3.5 | 3.50000000000002 | 1.33226762955019e-15 | 1.50990331349021e-14 |
| 355 | 1.3 | 3.55 | 3.83685004539195 | 3.72025273702984 | -0.286850045391947 | -0.170252737029842 | 3.55 | 3.55000000000007 | 1.33226762955019e-15 | 7.19424519957101e-14 |
| 356 | 1.3 | 3.6 | 3.89845811912788 | 3.77547991911179 | -0.298458119127881 | -0.175479919111794 | 3.6 | 3.59999999999998 | 1.33226762955019e-15 | -2.04281036531029e-14 |
| 357 | 1.3 | 3.65 | 3.96034304133677 | 3.83070912264731 | -0.310343041336766 | -0.180709122647305 | 3.65 | 3.65 | 1.77635683940025e-15 | -5.32907051820075e-15 |
| 358 | 1.3 | 3.7 | 4.02250857813017 | 3.88594034785824 | -0.32250857813017 | -0.185940347858237 | 3.7 | 3.7 | 4.44089209850063e-16 | 2.66453525910038e-15 |
| 359 | 1.3 | 3.75 | 4.08495858178928 | 3.94117359496663 | -0.334958581789276 | -0.191173594966633 | 3.75 | 3.75000000000002 | 1.77635683940025e-15 | 1.77635683940025e-14 |
| 360 | 1.3 | 3.8 | 4.1476969935506 | 3.99640886419454 | -0.347696993550604 | -0.196408864194537 | 3.8 | 3.80000000000002 | 4.44089209850063e-16 | 1.82076576038526e-14 |
| 361 | 1.3 | 3.85 | 4.21072784650865 | 4.051646155764 | -0.360727846508647 | -0.201646155763995 | 3.85 | 3.84999999999995 | 8.88178419700125e-16 | -5.28466159721575e-14 |
| 362 | 1.3 | 3.9 | 4.27405526864138 | 4.10688546989742 | -0.374055268641381 | -0.206885469897421 | 3.9 | 3.90000000000004 | 4.44089209850063e-16 | 4.35207425653061e-14 |
| 363 | 1.3 | 3.95 | 4.33768348596516 | 4.16212680681668 | -0.387683485965156 | -0.212126806816675 | 3.95 | 3.95000000000001 | 8.88178419700125e-16 | 8.43769498715119e-15 |
| 364 | 1.3 | 4 | 4.40161682582573 | 4.21737016674417 | -0.401616825825728 | -0.217370166744172 | 4 | 4.00000000000001 | 8.88178419700125e-16 | 7.105427357601e-15 |
| 365 | 1.3 | 4.05 | 4.46585972033272 | 4.27261554990214 | -0.415859720332719 | -0.222615549902139 | 4.05 | 4.05 | 1.77635683940025e-15 | 1.77635683940025e-15 |
| 366 | 1.3 | 4.1 | 4.53041670994521 | 4.327862956513 | -0.430416709945211 | -0.227862956512996 | 4.1 | 4.10000000000009 | 0 | 8.79296635503124e-14 |
| 367 | 1.3 | 4.15 | 4.59529244721669 | 4.38311238679878 | -0.445292447216685 | -0.233112386798782 | 4.15 | 4.14999999999998 | 1.77635683940025e-15 | -1.86517468137026e-14 |
| 368 | 1.3 | 4.2 | 4.66049170070802 | 4.43836384098228 | -0.460491700708016 | -0.238363840982284 | 4.2 | 4.20000000000004 | 8.88178419700125e-16 | 3.99680288865056e-14 |
| 369 | 1.3 | 4.25 | 4.72601935907791 | 4.49361731928573 | -0.476019359077911 | -0.243617319285733 | 4.25 | 4.25000000000008 | 8.88178419700125e-16 | 7.54951656745106e-14 |
| 370 | 1.3 | 4.3 | 4.79188043536062 | 4.54887282193154 | -0.491880435360619 | -0.24887282193154 | 4.3 | 4.30000000000003 | 1.77635683940025e-15 | 3.37507799486048e-14 |
| 371 | 1.3 | 4.35 | 4.8580800714416 | 4.60413034914231 | -0.508080071441597 | -0.254130349142311 | 4.35 | 4.34999999999999 | 2.66453525910038e-15 | -6.21724893790088e-15 |
| 372 | 1.3 | 4.4 | 4.92462354274239 | 4.65938990114064 | -0.524623542742385 | -0.259389901140638 | 4.4 | 4.39999999999999 | 8.88178419700125e-16 | -1.15463194561016e-14 |
| 373 | 1.3 | 4.45 | 4.9915162631268 | 4.71465147814913 | -0.541516263126803 | -0.264651478149126 | 4.45 | 4.45000000000002 | 1.77635683940025e-15 | 2.30926389122033e-14 |
| 374 | 1.3 | 4.5 | 5.05876379004133 | 4.76991508039037 | -0.558763790041328 | -0.269915080390375 | 4.5 | 4.50000000000006 | 0 | 6.3948846218409e-14 |
| 375 | 1.3 | 4.55 | 5.12637182990357 | 4.82518070808698 | -0.576371829903571 | -0.27518070808698 | 4.55 | 4.55000000000004 | 0 | 3.5527136788005e-14 |
| 376 | 1.3 | 4.6 | 5.1943462437535 | 4.88044836146173 | -0.5943462437535 | -0.280448361461731 | 4.6 | 4.6 | 8.88178419700125e-16 | -1.77635683940025e-15 |
| 377 | 1.3 | 4.65 | 5.26269305318337 | 4.93571804073759 | -0.612693053183371 | -0.285718040737594 | 4.65 | 4.65000000000014 | 8.88178419700125e-16 | 1.4210854715202e-13 |
| 378 | 1.3 | 4.7 | 5.33141844656328 | 4.9909897461368 | -0.631418446563276 | -0.290989746136801 | 4.7 | 4.69999999999995 | 8.88178419700125e-16 | -5.15143483426073e-14 |
| 379 | 1.3 | 4.75 | 5.40052878558056 | 5.04626347788288 | -0.650528785580555 | -0.296263477882876 | 4.75 | 4.75000000000003 | 8.88178419700125e-16 | 3.37507799486048e-14 |
| 380 | 1.3 | 4.8 | 5.47003061211261 | 5.10153923619823 | -0.670030612112612 | -0.301539236198233 | 4.8 | 4.79999999999998 | 0 | -2.39808173319034e-14 |
| 381 | 1.3 | 4.85 | 5.53993065545417 | 5.15681702130621 | -0.68993065545417 | -0.306817021306214 | 4.85 | 4.85000000000015 | 0 | 1.50990331349021e-13 |
| 382 | 1.3 | 4.9 | 5.61023583992154 | 5.21209683342923 | -0.710235839921543 | -0.312096833429232 | 4.9 | 4.90000000000006 | 8.88178419700125e-16 | 5.77315972805081e-14 |
| 383 | 1.3 | 4.95 | 5.68095329285826 | 5.26737867279063 | -0.73095329285826 | -0.317378672790627 | 4.95 | 4.95 | 8.88178419700125e-16 | 2.66453525910038e-15 |
| 384 | 1.3 | 5 | 5.75209035306825 | 5.32266253961355 | -0.752090353068247 | -0.322662539613553 | 5 | 5.00000000000008 | 0 | 7.99360577730113e-14 |
| 385 | 1.3 | 5.05 | 5.82365457970478 | 5.3779484341208 | -0.773654579704779 | -0.327948434120797 | 5.05 | 5.05000000000002 | 1.77635683940025e-15 | 1.68753899743024e-14 |
| 386 | 1.3 | 5.1 | 5.89565376164571 | 5.4332363565357 | -0.795653761645712 | -0.333236356535696 | 5.1 | 5.10000000000001 | 8.88178419700125e-16 | 4.44089209850063e-15 |
| 387 | 1.3 | 5.15 | 5.96809592738789 | 5.48852630708141 | -0.818095927387889 | -0.338526307081408 | 5.15 | 5.15000000000003 | 0 | 2.93098878501041e-14 |
| 388 | 1.3 | 5.2 | 6.0409893554963 | 5.54381828598109 | -0.840989355496303 | -0.343818285981087 | 5.2 | 5.20000000000004 | 8.88178419700125e-16 | 4.08562073062058e-14 |
| 389 | 1.3 | 5.25 | 6.11434258564651 | 5.59911229345789 | -0.864342585646512 | -0.349112293457885 | 5.25 | 5.24999999999995 | 1.77635683940025e-15 | -4.79616346638068e-14 |
| 390 | 1.3 | 5.3 | 6.188164430302 | 5.65440832973552 | -0.888164430301996 | -0.354408329735516 | 5.3 | 5.30000000000014 | 8.88178419700125e-16 | 1.3944401189292e-13 |
| 391 | 1.3 | 5.35 | 6.26246398707168 | 5.70970639503658 | -0.912463987071675 | -0.359706395036576 | 5.35 | 5.34999999999994 | 1.77635683940025e-15 | -5.95079541199084e-14 |
| 392 | 1.3 | 5.4 | 6.33725065179661 | 5.76500648958515 | -0.937250651796614 | -0.365006489585148 | 5.4 | 5.39999999999999 | 8.88178419700125e-16 | -7.105427357601e-15 |
| 393 | 1.3 | 5.45 | 6.41253413241924 | 5.82030861360439 | -0.96253413241924 | -0.370308613604385 | 5.45 | 5.45000000000006 | 8.88178419700125e-16 | 6.12843109593086e-14 |
| 394 | 1.3 | 5.5 | 6.48832446369299 | 5.87561276731763 | -0.988324463692988 | -0.375612767317628 | 5.5 | 5.50000000000004 | 8.88178419700125e-16 | 3.90798504668055e-14 |
| 395 | 1.3 | 5.55 | 6.56463202279552 | 5.9309189509484 | -1.01463202279552 | -0.380918950948401 | 5.55 | 5.54999999999995 | 0 | -4.61852778244065e-14 |
| 396 | 1.3 | 5.6 | 6.64146754591426 | 5.98622716472041 | -1.04146754591426 | -0.386227164720413 | 5.6 | 5.59999999999996 | 8.88178419700125e-16 | -4.35207425653061e-14 |
| 397 | 1.3 | 5.65 | 6.71884214587927 | 6.04153740885719 | -1.06884214587927 | -0.391537408857192 | 5.65 | 5.65 | 1.77635683940025e-15 | 1.77635683940025e-15 |
| 398 | 1.3 | 5.7 | 6.79676733092558 | 6.09684968358226 | -1.09676733092558 | -0.396849683582261 | 5.7 | 5.7 | 8.88178419700125e-16 | 8.88178419700125e-16 |
| 399 | 1.3 | 5.75 | 6.87525502467445 | 6.15216398911933 | -1.12525502467445 | -0.402163989119329 | 5.75 | 5.75 | 0 | -8.88178419700125e-16 |
| 400 | 1.3 | 5.8 | 6.95431758743203 | 6.20748032569211 | -1.15431758743202 | -0.407480325692108 | 5.8 | 5.8 | 8.88178419700125e-16 | 8.88178419700125e-16 |
| 401 | 1.3 | 5.85 | 7.03396783891292 | 6.26279869352431 | -1.18396783891292 | -0.412798693524306 | 5.85 | 5.84999999999998 | 8.88178419700125e-16 | -2.22044604925031e-14 |
| 402 | 1.3 | 5.9 | 7.11421908250714 | 6.31811909283982 | -1.21421908250714 | -0.41811909283982 | 5.9 | 5.90000000000003 | 1.77635683940025e-15 | 2.93098878501041e-14 |
| 403 | 1.3 | 5.95 | 7.19508513122035 | 6.37344152386236 | -1.24508513122035 | -0.423441523862358 | 5.95 | 5.95000000000005 | 8.88178419700125e-16 | 4.88498130835069e-14 |
| 404 | 1.3 | 6 | 7.27658033543074 | 6.42876598681582 | -1.27658033543073 | -0.428765986815817 | 6 | 6.00000000000006 | 0 | 6.3948846218409e-14 |
| 405 | 1.4 | 1 | 0.995113958251457 | 0.907765989122307 | 0.00488604174854324 | 0.0922340108776927 | 1 | 1.0000000000001 | 1.33226762955019e-15 | 1.03916875104915e-13 |
| 406 | 1.4 | 1.05 | 1.04618722488925 | 0.962890422421444 | 0.00381277511074729 | 0.0871095775785564 | 1.05 | 1.05000000000002 | 1.99840144432528e-15 | 2.48689957516035e-14 |
| 407 | 1.4 | 1.1 | 1.09741788736636 | 1.01801686591204 | 0.00258211263364361 | 0.0819831340879624 | 1.1 | 1.09999999999998 | 1.99840144432528e-15 | -1.90958360235527e-14 |
| 408 | 1.4 | 1.15 | 1.1488074098857 | 1.0731453198141 | 0.00119259011429684 | 0.0768546801859014 | 1.15 | 1.15000000000004 | 1.55431223447522e-15 | 4.41868763800812e-14 |
| 409 | 1.4 | 1.2 | 1.20035727949369 | 1.12827578434745 | -0.000357279493691465 | 0.0717242156525493 | 1.2 | 1.20000000000008 | 4.44089209850063e-16 | 8.28226376370367e-14 |
| 410 | 1.4 | 1.25 | 1.25206900658226 | 1.1834082597321 | -0.00206900658226328 | 0.0665917402678971 | 1.25 | 1.2500000000001 | 1.11022302462516e-15 | 9.59232693276135e-14 |
| 411 | 1.4 | 1.3 | 1.30394412540526 | 1.23854274618806 | -0.00394412540525568 | 0.0614572538119353 | 1.3 | 1.30000000000005 | 1.55431223447522e-15 | 4.72955008490317e-14 |
| 412 | 1.4 | 1.35 | 1.35598419460954 | 1.29367924393553 | -0.0059841946095367 | 0.0563207560644699 | 1.35 | 1.35000000000003 | 6.66133814775094e-16 | 3.04201108747293e-14 |
| 413 | 1.4 | 1.4 | 1.40819079778144 | 1.34881775319451 | -0.00819079778144105 | 0.0511822468054912 | 1.4 | 1.39999999999994 | 1.11022302462516e-15 | -6.32827124036339e-14 |
| 414 | 1.4 | 1.45 | 1.46056554400903 | 1.40395827418556 | -0.0105655440090333 | 0.0460417258144354 | 1.45 | 1.45000000000012 | 1.33226762955019e-15 | 1.22124532708767e-13 |
| 415 | 1.4 | 1.5 | 1.51311006846079 | 1.45910080712834 | -0.0131100684607866 | 0.0408991928716636 | 1.5 | 1.50000000000007 | 1.55431223447522e-15 | 6.97220059464598e-14 |
| 416 | 1.4 | 1.55 | 1.56582603298125 | 1.51424535224339 | -0.0158260329812494 | 0.0357546477566109 | 1.55 | 1.55000000000006 | 1.11022302462516e-15 | 6.30606677987089e-14 |
| 417 | 1.4 | 1.6 | 1.61871512670432 | 1.56939190975092 | -0.0187151267043211 | 0.0306080902490835 | 1.6 | 1.60000000000001 | 8.88178419700125e-16 | 1.46549439250521e-14 |
| 418 | 1.4 | 1.65 | 1.67177906668477 | 1.62454047987148 | -0.0217790666847746 | 0.0254595201285166 | 1.65 | 1.65000000000014 | 1.55431223447522e-15 | 1.36335387423969e-13 |
| 419 | 1.4 | 1.7 | 1.72501959854869 | 1.67969106282491 | -0.0250195985486872 | 0.0203089371750866 | 1.7 | 1.69999999999993 | 0 | -6.86117829218347e-14 |
| 420 | 1.4 | 1.75 | 1.7784384971635 | 1.73484365883233 | -0.0284384971635046 | 0.0151563411676736 | 1.75 | 1.75000000000004 | 1.55431223447522e-15 | 4.2410519540681e-14 |
| 421 | 1.4 | 1.8 | 1.83203756732841 | 1.78999826811373 | -0.0320375673284083 | 0.0100017318862689 | 1.8 | 1.80000000000007 | -2.22044604925031e-16 | 6.75015598972095e-14 |
| 422 | 1.4 | 1.85 | 1.88581864448583 | 1.84515489088969 | -0.0358186444858302 | 0.00484510911030789 | 1.85 | 1.85000000000007 | 2.22044604925031e-16 | 7.26085858104852e-14 |
| 423 | 1.4 | 1.9 | 1.93978359545481 | 1.90031352738077 | -0.0397835954548142 | -0.000313527380773948 | 1.9 | 1.90000000000009 | 1.11022302462516e-15 | 8.68194405256872e-14 |
| 424 | 1.4 | 1.95 | 1.99393431918713 | 1.95547417780754 | -0.0439343191871306 | -0.00547417780754023 | 1.95 | 1.9500000000001 | 6.66133814775094e-16 | 1.02140518265514e-13 |
| 425 | 1.4 | 2 | 2.04827274754697 | 2.01063684239056 | -0.0482727475469709 | -0.0106368423905563 | 2 | 2.00000000000008 | 0 | 7.54951656745106e-14 |
| 426 | 1.4 | 2.05 | 2.10280084611513 | 2.06580152135039 | -0.0528008461151335 | -0.0158015213503857 | 2.05 | 2.04999999999993 | 1.33226762955019e-15 | -7.41628980449605e-14 |
| 427 | 1.4 | 2.1 | 2.15752061501865 | 2.12096821490815 | -0.0575206150186487 | -0.0209682149081476 | 2.1 | 2.10000000000004 | 8.88178419700125e-16 | 3.95239396766556e-14 |
| 428 | 1.4 | 2.15 | 2.21243408978685 | 2.17613692328404 | -0.0624340897868518 | -0.0261369232840365 | 2.15 | 2.14999999999993 | 4.44089209850063e-16 | -7.23865412055602e-14 |
| 429 | 1.4 | 2.2 | 2.26754334223491 | 2.23130764669936 | -0.0675433422349072 | -0.0313076466993576 | 2.2 | 2.20000000000007 | 8.88178419700125e-16 | 7.14983627858601e-14 |
| 430 | 1.4 | 2.25 | 2.32285048137589 | 2.28648038537449 | -0.0728504813758901 | -0.0364803853744897 | 2.25 | 2.25000000000008 | 4.44089209850063e-16 | 7.63833440942108e-14 |
| 431 | 1.4 | 2.3 | 2.37835765436257 | 2.34165513953037 | -0.0783576543625739 | -0.0416551395303668 | 2.3 | 2.30000000000001 | 1.77635683940025e-15 | 1.46549439250521e-14 |
| 432 | 1.4 | 2.35 | 2.43406704746007 | 2.39683190938811 | -0.0840670474600684 | -0.046831909388108 | 2.35 | 2.35000000000009 | 4.44089209850063e-16 | 8.83737527601625e-14 |
| 433 | 1.4 | 2.4 | 2.48998088705062 | 2.45201069516846 | -0.089980887050622 | -0.0520106951684638 | 2.4 | 2.40000000000013 | 4.44089209850063e-16 | 1.31894495325469e-13 |
| 434 | 1.4 | 2.45 | 2.54610144067182 | 2.50719149709218 | -0.0961014406718195 | -0.057191497092183 | 2.45 | 2.44999999999994 | 1.77635683940025e-15 | -6.17284001691587e-14 |
| 435 | 1.4 | 2.5 | 2.6024310180896 | 2.56237431538094 | -0.102431018089605 | -0.0623743153809402 | 2.5 | 2.5000000000001 | 8.88178419700125e-16 | 1.03916875104915e-13 |
| 436 | 1.4 | 2.55 | 2.65897197240754 | 2.61755915025511 | -0.108971972407543 | -0.0675591502551147 | 2.55 | 2.55000000000002 | 8.88178419700125e-16 | 1.73194791841524e-14 |
| 437 | 1.4 | 2.6 | 2.71572670121382 | 2.6727460019362 | -0.115726701213822 | -0.0727460019361952 | 2.6 | 2.60000000000003 | 8.88178419700125e-16 | 3.10862446895044e-14 |
| 438 | 1.4 | 2.65 | 2.77269764776758 | 2.72793487064512 | -0.12269764776758 | -0.0779348706451168 | 2.65 | 2.64999999999996 | 0 | -3.59712259978551e-14 |
| 439 | 1.4 | 2.7 | 2.82988730222624 | 2.78312575660318 | -0.129887302226235 | -0.0831257566031844 | 2.7 | 2.69999999999993 | 8.88178419700125e-16 | -6.97220059464598e-14 |
| 440 | 1.4 | 2.75 | 2.88729820291554 | 2.8383186600317 | -0.137298202915539 | -0.0883186600317019 | 2.75 | 2.75000000000001 | 1.33226762955019e-15 | 7.54951656745106e-15 |
| 441 | 1.4 | 2.8 | 2.94493293764419 | 2.89351358115179 | -0.144932937644194 | -0.0935135811517891 | 2.8 | 2.80000000000007 | 8.88178419700125e-16 | 7.14983627858601e-14 |
| 442 | 1.4 | 2.85 | 3.00279414506498 | 2.94871052018475 | -0.152794145064981 | -0.0987105201847491 | 2.85 | 2.85000000000012 | 8.88178419700125e-16 | 1.23900889548167e-13 |
| 443 | 1.4 | 2.9 | 3.06088451608438 | 3.0039094773517 | -0.160884516084381 | -0.103909477351703 | 2.9 | 2.89999999999997 | 1.33226762955019e-15 | -3.10862446895044e-14 |
| 444 | 1.4 | 2.95 | 3.11920679532287 | 3.05911045287451 | -0.169206795322867 | -0.10911045287451 | 2.95 | 2.95000000000004 | 8.88178419700125e-16 | 3.95239396766556e-14 |
| 445 | 1.4 | 3 | 3.17776378262808 | 3.11431344697429 | -0.177763782628078 | -0.114313446974289 | 3 | 3.00000000000006 | 8.88178419700125e-16 | 6.3504757008559e-14 |
| 446 | 1.4 | 3.05 | 3.23655833464325 | 3.16951845987253 | -0.186558334643245 | -0.119518459872529 | 3.05 | 3.05000000000007 | 4.44089209850063e-16 | 6.57252030578093e-14 |
| 447 | 1.4 | 3.1 | 3.29559336643336 | 3.22472549179072 | -0.195593366433358 | -0.124725491790721 | 3.1 | 3.10000000000004 | 0 | 3.73034936274053e-14 |
| 448 | 1.4 | 3.15 | 3.35487185317169 | 3.27993454295054 | -0.204871853171694 | -0.129934542950539 | 3.15 | 3.1500000000001 | 1.33226762955019e-15 | 9.76996261670138e-14 |
| 449 | 1.4 | 3.2 | 3.41439683188944 | 3.33514561357329 | -0.214396831889442 | -0.135145613573286 | 3.2 | 3.19999999999999 | 0 | -5.77315972805081e-15 |
| 450 | 1.4 | 3.25 | 3.4741714032914 | 3.39035870388082 | -0.224171403291399 | -0.140358703880824 | 3.25 | 3.24999999999994 | 0 | -5.77315972805081e-14 |
| 451 | 1.4 | 3.3 | 3.53419873364072 | 3.44557381409501 | -0.234198733640722 | -0.145573814095011 | 3.3 | 3.30000000000012 | -4.44089209850063e-16 | 1.19015908239817e-13 |
| 452 | 1.4 | 3.35 | 3.59448205671605 | 3.50079094443697 | -0.244482056716053 | -0.150790944436967 | 3.35 | 3.35 | 8.88178419700125e-16 | -3.99680288865056e-15 |
| 453 | 1.4 | 3.4 | 3.65502467584439 | 3.55601009512892 | -0.255024675844394 | -0.156010095128921 | 3.4 | 3.40000000000001 | 8.88178419700125e-16 | 1.15463194561016e-14 |
| 454 | 1.4 | 3.45 | 3.71582996601337 | 3.61123126639255 | -0.265829966013374 | -0.161231266392548 | 3.45 | 3.45000000000007 | 8.88178419700125e-16 | 6.57252030578093e-14 |
| 455 | 1.4 | 3.5 | 3.77690137606672 | 3.66645445844952 | -0.276901376066717 | -0.166454458449523 | 3.5 | 3.50000000000002 | 0 | 2.22044604925031e-14 |
| 456 | 1.4 | 3.55 | 3.83824243098695 | 3.72167967152189 | -0.288242430986952 | -0.171679671521889 | 3.55 | 3.55000000000004 | 1.33226762955019e-15 | 4.13002965160558e-14 |
| 457 | 1.4 | 3.6 | 3.89985673426964 | 3.77690690583151 | -0.299856734269635 | -0.176906905831508 | 3.6 | 3.60000000000008 | 1.33226762955019e-15 | 8.12683254025615e-14 |
| 458 | 1.4 | 3.65 | 3.96174797039364 | 3.83213616160024 | -0.311747970393642 | -0.182136161600237 | 3.65 | 3.65000000000006 | 8.88178419700125e-16 | 6.21724893790088e-14 |
| 459 | 1.4 | 3.7 | 4.02391990739227 | 3.88736743905012 | -0.323919907392273 | -0.187367439050123 | 3.7 | 3.70000000000004 | 4.44089209850063e-16 | 3.5527136788005e-14 |
| 460 | 1.4 | 3.75 | 4.08637639953028 | 3.94260073840321 | -0.336376399530279 | -0.192600738403208 | 3.75 | 3.75000000000002 | 4.44089209850063e-16 | 1.50990331349021e-14 |
| 461 | 1.4 | 3.8 | 4.14912139009219 | 3.99783605988172 | -0.349121390092189 | -0.197836059881724 | 3.8 | 3.80000000000015 | 8.88178419700125e-16 | 1.45661260830821e-13 |
| 462 | 1.4 | 3.85 | 4.21215891428764 | 4.05307340370734 | -0.36215891428764 | -0.203073403707344 | 3.85 | 3.85000000000003 | 0 | 3.41948691584548e-14 |
| 463 | 1.4 | 3.9 | 4.27549310227976 | 4.10831277010248 | -0.375493102279759 | -0.208312770102484 | 3.9 | 3.89999999999992 | 4.44089209850063e-16 | -8.08242361927114e-14 |
| 464 | 1.4 | 3.95 | 4.33912818234305 | 4.16355415928956 | -0.389128182343051 | -0.213554159289558 | 3.95 | 3.95 | 4.44089209850063e-16 | 1.77635683940025e-15 |
| 465 | 1.4 | 4 | 4.4030684841576 | 4.21879757149043 | -0.403068484157604 | -0.218797571490425 | 4 | 3.99999999999995 | 8.88178419700125e-16 | -5.46229728115577e-14 |
| 466 | 1.4 | 4.05 | 4.46731844224688 | 4.27404300692769 | -0.417318442246883 | -0.224043006927685 | 4.05 | 4.05000000000005 | 8.88178419700125e-16 | 4.70734562441066e-14 |
| 467 | 1.4 | 4.1 | 4.53188259956684 | 4.32929046582338 | -0.431882599566841 | -0.229290465823384 | 4.1 | 4.10000000000006 | 0 | 6.30606677987089e-14 |
| 468 | 1.4 | 4.15 | 4.59676561125456 | 4.38453994839994 | -0.446765611254561 | -0.234539948399934 | 4.15 | 4.15000000000005 | 0 | 4.61852778244065e-14 |
| 469 | 1.4 | 4.2 | 4.66197224854518 | 4.43979145487975 | -0.461972248545179 | -0.239791454879752 | 4.2 | 4.20000000000002 | 8.88178419700125e-16 | 1.68753899743024e-14 |
| 470 | 1.4 | 4.25 | 4.72750740286642 | 4.49504498548544 | -0.477507402866421 | -0.245044985485437 | 4.25 | 4.25000000000012 | 8.88178419700125e-16 | 1.22568621918617e-13 |
| 471 | 1.4 | 4.3 | 4.79337609012072 | 4.55030054043903 | -0.493376090120719 | -0.250300540439033 | 4.3 | 4.29999999999997 | 8.88178419700125e-16 | -2.93098878501041e-14 |
| 472 | 1.4 | 4.35 | 4.85958345516545 | 4.6055581199637 | -0.509583455165451 | -0.255558119963697 | 4.35 | 4.35000000000014 | 0 | 1.4210854715202e-13 |
| 473 | 1.4 | 4.4 | 4.92613477650272 | 4.66081772428129 | -0.526134776502717 | -0.260817724281285 | 4.4 | 4.4 | 0 | 2.66453525910038e-15 |
| 474 | 1.4 | 4.45 | 4.99303547119067 | 4.71607935361496 | -0.543035471190672 | -0.266079353614955 | 4.45 | 4.45000000000006 | 8.88178419700125e-16 | 5.50670620214078e-14 |
| 475 | 1.4 | 4.5 | 5.06029109998939 | 4.77134300818712 | -0.560291099989391 | -0.271343008187119 | 4.5 | 4.5000000000001 | 1.77635683940025e-15 | 9.68114477473137e-14 |
| 476 | 1.4 | 4.55 | 5.1279073727551 | 4.82660868822038 | -0.577907372755103 | -0.276608688220378 | 4.55 | 4.55000000000006 | 8.88178419700125e-16 | 5.41788836017076e-14 |
| 477 | 1.4 | 4.6 | 5.19589015409757 | 4.88187639393752 | -0.59589015409757 | -0.28187639393752 | 4.6 | 4.59999999999999 | 1.77635683940025e-15 | -1.15463194561016e-14 |
| 478 | 1.4 | 4.65 | 5.26424546931651 | 4.93714612556151 | -0.614245469316507 | -0.287146125561508 | 4.65 | 4.65000000000008 | 8.88178419700125e-16 | 8.08242361927114e-14 |
| 479 | 1.4 | 4.7 | 5.33297951063404 | 4.99241788331476 | -0.632979510634043 | -0.292417883314762 | 4.7 | 4.69999999999999 | 8.88178419700125e-16 | -1.4210854715202e-14 |
| 480 | 1.4 | 4.75 | 5.40209864374145 | 5.04769166742044 | -0.652098643741454 | -0.297691667420436 | 4.75 | 4.74999999999998 | 8.88178419700125e-16 | -1.59872115546023e-14 |
| 481 | 1.4 | 4.8 | 5.47160941467977 | 5.10296747810131 | -0.671609414679772 | -0.302967478101312 | 4.8 | 4.79999999999998 | 8.88178419700125e-16 | -1.68753899743024e-14 |
| 482 | 1.4 | 4.85 | 5.54151855707532 | 5.15824531558036 | -0.691518557075323 | -0.308245315580364 | 4.85 | 4.85000000000003 | 1.77635683940025e-15 | 2.66453525910038e-14 |
| 483 | 1.4 | 4.9 | 5.61183299975281 | 5.21352518008056 | -0.711832999752813 | -0.313525180080557 | 4.9 | 4.90000000000011 | 1.77635683940025e-15 | 1.13686837721616e-13 |
| 484 | 1.4 | 4.95 | 5.68255987475034 | 5.26880707182468 | -0.732559874750344 | -0.31880707182468 | 4.95 | 4.95000000000005 | 8.88178419700125e-16 | 4.70734562441066e-14 |
| 485 | 1.4 | 5 | 5.75370652576259 | 5.32409099103607 | -0.753706525762589 | -0.324090991036071 | 5 | 5.00000000000009 | 8.88178419700125e-16 | 8.97060203897127e-14 |
| 486 | 1.4 | 5.05 | 5.82528051704042 | 5.37937693793752 | -0.77528051704042 | -0.329376937937515 | 5.05 | 5.04999999999996 | 8.88178419700125e-16 | -3.5527136788005e-14 |
| 487 | 1.4 | 5.1 | 5.89728964277752 | 5.43466491275254 | -0.797289642777523 | -0.334664912752536 | 5.1 | 5.10000000000003 | 8.88178419700125e-16 | 3.10862446895044e-14 |
| 488 | 1.4 | 5.15 | 5.96974193701696 | 5.48995491570411 | -0.819741937016964 | -0.339954915704105 | 5.15 | 5.15000000000011 | 8.88178419700125e-16 | 1.07469588783715e-13 |
| 489 | 1.4 | 5.2 | 6.04264568411335 | 5.54524694701519 | -0.842645684113352 | -0.345246947015191 | 5.2 | 5.19999999999997 | 8.88178419700125e-16 | -2.66453525910038e-14 |
| 490 | 1.4 | 5.25 | 6.11600942978916 | 5.60054100690951 | -0.866009429789161 | -0.350541006909506 | 5.25 | 5.25000000000005 | 8.88178419700125e-16 | 4.52970994047064e-14 |
| 491 | 1.4 | 5.3 | 6.18984199282698 | 5.65583709561002 | -0.889841992826979 | -0.355837095610016 | 5.3 | 5.30000000000003 | 8.88178419700125e-16 | 2.75335310107039e-14 |
| 492 | 1.4 | 5.35 | 6.26415247744299 | 5.71113521334025 | -0.914152477442985 | -0.361135213340249 | 5.35 | 5.35000000000009 | 8.88178419700125e-16 | 9.32587340685131e-14 |
| 493 | 1.4 | 5.4 | 6.33895028639077 | 5.76643536032336 | -0.93895028639077 | -0.366435360323359 | 5.4 | 5.40000000000005 | 8.88178419700125e-16 | 4.44089209850063e-14 |
| 494 | 1.4 | 5.45 | 6.41424513484893 | 5.82173753678287 | -0.964245134848927 | -0.37173753678287 | 5.45 | 5.44999999999998 | 8.88178419700125e-16 | -2.30926389122033e-14 |
| 495 | 1.4 | 5.5 | 6.49004706515045 | 5.87704174294231 | -0.990047065150453 | -0.377041742942309 | 5.5 | 5.49999999999995 | 8.88178419700125e-16 | -4.61852778244065e-14 |
| 496 | 1.4 | 5.55 | 6.5663664624172 | 5.9323479790252 | -1.0163664624172 | -0.382347979025198 | 5.55 | 5.55 | 2.66453525910038e-15 | -2.66453525910038e-15 |
| 497 | 1.4 | 5.6 | 6.64321407116826 | 5.98765624525507 | -1.04321407116826 | -0.387656245255064 | 5.6 | 5.6000000000001 | 2.66453525910038e-15 | 9.41469124882133e-14 |
| 498 | 1.4 | 5.65 | 6.72060101297754 | 6.04296654185525 | -1.07060101297754 | -0.392966541855246 | 5.65 | 5.65000000000003 | 0 | 2.75335310107039e-14 |
| 499 | 1.4 | 5.7 | 6.79853880526261 | 6.09827886904964 | -1.09853880526261 | -0.398278869049641 | 5.7 | 5.70000000000005 | 1.77635683940025e-15 | 4.52970994047064e-14 |
| 500 | 1.4 | 5.75 | 6.87703938129474 | 6.15359322706177 | -1.12703938129474 | -0.40359322706177 | 5.75 | 5.75000000000002 | 1.77635683940025e-15 | 2.04281036531029e-14 |
| 501 | 1.4 | 5.8 | 6.95611511152857 | 6.20890961611535 | -1.15611511152857 | -0.408909616115346 | 5.8 | 5.79999999999996 | 8.88178419700125e-16 | -3.90798504668055e-14 |
| 502 | 1.4 | 5.85 | 7.0357788263593 | 6.26422803643426 | -1.1857788263593 | -0.414228036434263 | 5.85 | 5.85 | 1.77635683940025e-15 | 3.5527136788005e-15 |
| 503 | 1.4 | 5.9 | 7.11604384042602 | 6.31954848824223 | -1.21604384042602 | -0.419548488242231 | 5.9 | 5.90000000000008 | 1.77635683940025e-15 | 7.54951656745106e-14 |
| 504 | 1.4 | 5.95 | 7.19692397859153 | 6.37487097176296 | -1.24692397859153 | -0.424870971762961 | 5.95 | 5.95000000000008 | 1.77635683940025e-15 | 7.63833440942108e-14 |
| 505 | 1.4 | 6 | 7.27843360374206 | 6.43019548722035 | -1.27843360374206 | -0.430195487220346 | 6 | 6.00000000000003 | 0 | 2.57571741713036e-14 |
| 506 | 1.5 | 1 | 0.996263936309178 | 0.90918960745127 | 0.00373606369082224 | 0.0908103925487301 | 1 | 1.00000000000008 | 2.22044604925031e-15 | 8.32667268468867e-14 |
| 507 | 1.5 | 1.05 | 1.04734073624982 | 0.964314092662769 | 0.00265926375017744 | 0.0856859073372306 | 1.05 | 1.04999999999994 | 1.11022302462516e-15 | -6.28386231937839e-14 |
| 508 | 1.5 | 1.1 | 1.09857496479937 | 1.01944058807165 | 0.00142503520062687 | 0.0805594119283521 | 1.1 | 1.10000000000005 | 6.66133814775094e-16 | 4.52970994047064e-14 |
| 509 | 1.5 | 1.15 | 1.14996808667045 | 1.07456909389736 | 3.19133295487717e-05 | 0.0754309061026404 | 1.15 | 1.14999999999998 | 2.22044604925031e-16 | -2.39808173319034e-14 |
| 510 | 1.5 | 1.2 | 1.20152158943031 | 1.12969961036028 | -0.00152158943031222 | 0.0703003896397167 | 1.2 | 1.2000000000001 | 4.44089209850063e-16 | 9.85878045867139e-14 |
| 511 | 1.5 | 1.25 | 1.25323698400324 | 1.18483213768006 | -0.00323698400323713 | 0.0651678623199414 | 1.25 | 1.25000000000008 | 1.55431223447522e-15 | 7.72715225139109e-14 |
| 512 | 1.5 | 1.3 | 1.30511580518721 | 1.23996667607688 | -0.00511580518721333 | 0.0600333239231208 | 1.3 | 1.30000000000004 | 2.22044604925031e-16 | 4.10782519111308e-14 |
| 513 | 1.5 | 1.35 | 1.35715961218542 | 1.29510322577094 | -0.00715961218541983 | 0.05489677422906 | 1.35 | 1.35000000000008 | 1.11022302462516e-15 | 8.43769498715119e-14 |
| 514 | 1.5 | 1.4 | 1.40936998915299 | 1.35024178698225 | -0.00936998915299347 | 0.0497582130177501 | 1.4 | 1.4000000000001 | 1.99840144432528e-15 | 9.61453139325386e-14 |
| 515 | 1.5 | 1.45 | 1.46174854575967 | 1.405382359931 | -0.0117485457596709 | 0.044617640068997 | 1.45 | 1.4500000000001 | 1.11022302462516e-15 | 9.65894031423886e-14 |
| 516 | 1.5 | 1.5 | 1.51429691776884 | 1.46052494483739 | -0.0142969177688441 | 0.0394750551626066 | 1.5 | 1.50000000000007 | 8.88178419700125e-16 | 7.061018436616e-14 |
| 517 | 1.5 | 1.55 | 1.56701676763361 | 1.51566954192162 | -0.0170167676336097 | 0.0343304580783843 | 1.55 | 1.54999999999997 | 6.66133814775094e-16 | -3.41948691584548e-14 |
| 518 | 1.5 | 1.6 | 1.61990978511045 | 1.57081615140423 | -0.0199097851104539 | 0.0291838485957658 | 1.6 | 1.60000000000003 | 1.77635683940025e-15 | 2.93098878501041e-14 |
| 519 | 1.5 | 1.65 | 1.67297768789119 | 1.62596477350526 | -0.0229776878911883 | 0.0240352264947419 | 1.65 | 1.64999999999997 | 1.33226762955019e-15 | -3.15303338993544e-14 |
| 520 | 1.5 | 1.7 | 1.72622222225383 | 1.68111540844525 | -0.0262222222538282 | 0.0188845915547489 | 1.7 | 1.69999999999996 | 8.88178419700125e-16 | -4.35207425653061e-14 |
| 521 | 1.5 | 1.75 | 1.77964516373309 | 1.73626805644459 | -0.0296451637330883 | 0.0137319435554066 | 1.75 | 1.74999999999996 | 6.66133814775094e-16 | -3.59712259978551e-14 |
| 522 | 1.5 | 1.8 | 1.83324831781124 | 1.79142271772366 | -0.0332483178112395 | 0.00857728227633636 | 1.8 | 1.79999999999992 | 2.22044604925031e-16 | -7.57172102794357e-14 |
| 523 | 1.5 | 1.85 | 1.88703352063009 | 1.84657939250321 | -0.0370335206300854 | 0.00342060749678885 | 1.85 | 1.85000000000007 | 4.44089209850063e-16 | 6.92779167366098e-14 |
| 524 | 1.5 | 1.9 | 1.94100263972485 | 1.90173808100343 | -0.041002639724846 | -0.00173808100343087 | 1.9 | 1.90000000000009 | 1.33226762955019e-15 | 9.2370555648813e-14 |
| 525 | 1.5 | 1.95 | 1.99515757478078 | 1.95689878344489 | -0.0451575747807822 | -0.00689878344488593 | 1.95 | 1.94999999999998 | 1.77635683940025e-15 | -1.57651669496772e-14 |
| 526 | 1.5 | 2 | 2.04950025841344 | 2.01206150004851 | -0.0495002584134365 | -0.012061500048512 | 2 | 2.00000000000004 | 0 | 3.68594044175552e-14 |
| 527 | 1.5 | 2.05 | 2.10403265697339 | 2.06722623103469 | -0.0540326569733862 | -0.0172262310346873 | 2.05 | 2.05 | 0 | -8.88178419700125e-16 |
| 528 | 1.5 | 2.1 | 2.15875677137645 | 2.12239297662435 | -0.0587567713764514 | -0.0223929766243467 | 2.1 | 2.10000000000009 | 8.88178419700125e-16 | 8.97060203897127e-14 |
| 529 | 1.5 | 2.15 | 2.21367463796037 | 2.17756173703805 | -0.0636746379603657 | -0.0275617370380541 | 2.15 | 2.15000000000016 | 4.44089209850063e-16 | 1.54543045027822e-13 |
| 530 | 1.5 | 2.2 | 2.26878832936895 | 2.23273251249638 | -0.0687883293689451 | -0.0327325124963749 | 2.2 | 2.2 | 4.44089209850063e-16 | 2.22044604925031e-15 |
| 531 | 1.5 | 2.25 | 2.32409995546483 | 2.28790530322043 | -0.0740999554648267 | -0.0379053032204277 | 2.25 | 2.24999999999991 | 1.77635683940025e-15 | -9.10382880192628e-14 |
| 532 | 1.5 | 2.3 | 2.37961166427192 | 2.34308010943133 | -0.0796116642719218 | -0.0430801094313318 | 2.3 | 2.30000000000011 | 4.44089209850063e-16 | 1.13242748511766e-13 |
| 533 | 1.5 | 2.35 | 2.4353256429488 | 2.39825693134928 | -0.0853256429487952 | -0.0482569313492811 | 2.35 | 2.34999999999998 | 1.77635683940025e-15 | -2.22044604925031e-14 |
| 534 | 1.5 | 2.4 | 2.49124411879417 | 2.45343576919577 | -0.0912441187941679 | -0.0534357691957657 | 2.4 | 2.40000000000001 | 4.44089209850063e-16 | 6.21724893790088e-15 |
| 535 | 1.5 | 2.45 | 2.54736936028592 | 2.50861662319154 | -0.097369360285922 | -0.0586166231915355 | 2.45 | 2.44999999999999 | 1.77635683940025e-15 | -7.99360577730113e-15 |
| 536 | 1.5 | 2.5 | 2.6037036781549 | 2.56379949355771 | -0.103703678154898 | -0.0637994935577093 | 2.5 | 2.50000000000003 | 1.33226762955019e-15 | 2.70894418008538e-14 |
| 537 | 1.5 | 2.55 | 2.66024942649501 | 2.61898438051522 | -0.110249426495006 | -0.068984380515221 | 2.55 | 2.55 | 8.88178419700125e-16 | 0 |
| 538 | 1.5 | 2.6 | 2.71700900391109 | 2.67417128428538 | -0.117009003911086 | -0.0741712842853755 | 2.6 | 2.6000000000001 | 4.44089209850063e-16 | 9.76996261670138e-14 |
| 539 | 1.5 | 2.65 | 2.77398485470615 | 2.72936020508892 | -0.12398485470615 | -0.0793602050889222 | 2.65 | 2.64999999999997 | 4.44089209850063e-16 | -3.06421554796543e-14 |
| 540 | 1.5 | 2.7 | 2.83117947010965 | 2.78455114314754 | -0.131179470109654 | -0.0845511431475359 | 2.7 | 2.70000000000006 | 1.33226762955019e-15 | 6.26165785888588e-14 |
| 541 | 1.5 | 2.75 | 2.88859538954853 | 2.83974409868197 | -0.138595389548525 | -0.0897440986819658 | 2.75 | 2.74999999999995 | 8.88178419700125e-16 | -4.75175454539567e-14 |
| 542 | 1.5 | 2.8 | 2.94623520196283 | 2.89493907191389 | -0.146235201962828 | -0.0949390719138861 | 2.8 | 2.80000000000002 | 4.44089209850063e-16 | 1.50990331349021e-14 |
| 543 | 1.5 | 2.85 | 3.00410154716794 | 2.95013606306442 | -0.154101547167938 | -0.100136063064416 | 2.85 | 2.85000000000009 | 1.33226762955019e-15 | 8.61533067109122e-14 |
| 544 | 1.5 | 2.9 | 3.0621971172653 | 3.00533507235468 | -0.162197117265299 | -0.105335072354675 | 2.9 | 2.89999999999997 | 8.88178419700125e-16 | -3.41948691584548e-14 |
| 545 | 1.5 | 2.95 | 3.12052465810388 | 3.06053610000652 | -0.170524658103879 | -0.110536100006524 | 2.95 | 2.95000000000009 | 4.44089209850063e-16 | 8.83737527601625e-14 |
| 546 | 1.5 | 3 | 3.17908697079456 | 3.1157391462409 | -0.179086970794559 | -0.115739146240896 | 3 | 3.00000000000001 | 4.44089209850063e-16 | 1.19904086659517e-14 |
| 547 | 1.5 | 3.05 | 3.23788691327984 | 3.17094421127965 | -0.187886913279839 | -0.12094421127965 | 3.05 | 3.0500000000001 | 4.44089209850063e-16 | 9.68114477473137e-14 |
| 548 | 1.5 | 3.1 | 3.29692740196133 | 3.22615129534391 | -0.196927401961331 | -0.126151295343908 | 3.1 | 3.1 | 0 | -3.99680288865056e-15 |
| 549 | 1.5 | 3.15 | 3.35621141338768 | 3.28136039865553 | -0.206211413387682 | -0.131360398655527 | 3.15 | 3.15 | 1.33226762955019e-15 | -3.10862446895044e-15 |
| 550 | 1.5 | 3.2 | 3.41574198600566 | 3.336571521436 | -0.21574198600566 | -0.136571521435997 | 3.2 | 3.20000000000001 | 8.88178419700125e-16 | 1.37667655053519e-14 |
| 551 | 1.5 | 3.25 | 3.47552222197736 | 3.39178466390699 | -0.225522221977362 | -0.141784663906993 | 3.25 | 3.25000000000009 | 0 | 9.28146448586631e-14 |
| 552 | 1.5 | 3.3 | 3.53555528906658 | 3.44699982629001 | -0.235555289066576 | -0.146999826290005 | 3.3 | 3.30000000000008 | 8.88178419700125e-16 | 7.54951656745106e-14 |
| 553 | 1.5 | 3.35 | 3.59584442259756 | 3.50221700880689 | -0.245844422597559 | -0.152217008806892 | 3.35 | 3.3500000000001 | 0 | 1.02584607475364e-13 |
| 554 | 1.5 | 3.4 | 3.65639292748968 | 3.55743621167933 | -0.256392927489682 | -0.157436211679328 | 3.4 | 3.40000000000011 | 4.44089209850063e-16 | 1.08357767203415e-13 |
| 555 | 1.5 | 3.45 | 3.71720418037151 | 3.61265743512899 | -0.267204180371512 | -0.162657435128989 | 3.45 | 3.44999999999999 | 0 | -7.54951656745106e-15 |
| 556 | 1.5 | 3.5 | 3.77828163177821 | 3.6678806793781 | -0.278281631778211 | -0.167880679378103 | 3.5 | 3.50000000000012 | 2.22044604925031e-15 | 1.19904086659517e-13 |
| 557 | 1.5 | 3.55 | 3.83962880843625 | 3.72310594464798 | -0.289628808436251 | -0.173105944647975 | 3.55 | 3.54999999999998 | 1.33226762955019e-15 | -1.99840144432528e-14 |
| 558 | 1.5 | 3.6 | 3.90124931563978 | 3.77833323116102 | -0.301249315639776 | -0.17833323116102 | 3.6 | 3.60000000000003 | 4.44089209850063e-16 | 3.33066907387547e-14 |
| 559 | 1.5 | 3.65 | 3.96314683972309 | 3.83356253913891 | -0.31314683972309 | -0.183562539138912 | 3.65 | 3.65000000000003 | 1.33226762955019e-15 | 2.97539770599542e-14 |
| 560 | 1.5 | 3.7 | 4.0253251506341 | 3.8887938688037 | -0.325325150634098 | -0.188793868803697 | 3.7 | 3.70000000000002 | 1.33226762955019e-15 | 2.17603712826531e-14 |
| 561 | 1.5 | 3.75 | 4.08778810461379 | 3.94402722037742 | -0.33778810461379 | -0.194027220377418 | 3.75 | 3.75000000000002 | 1.33226762955019e-15 | 2.1316282072803e-14 |
| 562 | 1.5 | 3.8 | 4.15053964698714 | 3.99926259408212 | -0.350539646987139 | -0.19926259408212 | 3.8 | 3.80000000000001 | 1.33226762955019e-15 | 4.88498130835069e-15 |
| 563 | 1.5 | 3.85 | 4.21358381507113 | 4.05449999014003 | -0.36358381507113 | -0.204499990140033 | 3.85 | 3.85000000000008 | 0 | 8.03801469828613e-14 |
| 564 | 1.5 | 3.9 | 4.27692474120601 | 4.1097394087732 | -0.376924741206006 | -0.209739408773201 | 3.9 | 3.90000000000015 | 1.77635683940025e-15 | 1.50102152929321e-13 |
| 565 | 1.5 | 3.95 | 4.34056665591613 | 4.16498085020367 | -0.390566655916129 | -0.21498085020367 | 3.95 | 3.95000000000008 | 4.44089209850063e-16 | 8.17124146124115e-14 |
| 566 | 1.5 | 4 | 4.40451389120737 | 4.22022431465385 | -0.404513891207372 | -0.220224314653853 | 4 | 4.00000000000004 | 8.88178419700125e-16 | 3.90798504668055e-14 |
| 567 | 1.5 | 4.05 | 4.46877088400821 | 4.27546980234598 | -0.418770884008213 | -0.225469802345979 | 4.05 | 4.04999999999998 | 1.77635683940025e-15 | -2.04281036531029e-14 |
| 568 | 1.5 | 4.1 | 4.53334217976236 | 4.33071731350247 | -0.433342179762358 | -0.230717313502467 | 4.1 | 4.1 | 8.88178419700125e-16 | -8.88178419700125e-16 |
| 569 | 1.5 | 4.15 | 4.59823243618109 | 4.38596684834554 | -0.448232436181086 | -0.235966848345542 | 4.15 | 4.14999999999998 | 8.88178419700125e-16 | -2.22044604925031e-14 |
| 570 | 1.5 | 4.2 | 4.66344642716406 | 4.44121840709762 | -0.463446427164064 | -0.24121840709762 | 4.2 | 4.19999999999994 | 8.88178419700125e-16 | -6.21724893790088e-14 |
| 571 | 1.5 | 4.25 | 4.72898904689801 | 4.4964719899813 | -0.47898904689801 | -0.246471989981302 | 4.25 | 4.25000000000002 | 8.88178419700125e-16 | 2.39808173319034e-14 |
| 572 | 1.5 | 4.3 | 4.79486531414315 | 4.55172759721882 | -0.494865314143146 | -0.251727597218816 | 4.3 | 4.30000000000001 | 8.88178419700125e-16 | 1.15463194561016e-14 |
| 573 | 1.5 | 4.35 | 4.86108037671805 | 4.60698522903276 | -0.511080376718049 | -0.256985229032764 | 4.35 | 4.34999999999998 | 1.77635683940025e-15 | -2.1316282072803e-14 |
| 574 | 1.5 | 4.4 | 4.92763951619427 | 4.66224488564574 | -0.527639516194268 | -0.262244885645742 | 4.4 | 4.39999999999996 | 8.88178419700125e-16 | -4.35207425653061e-14 |
| 575 | 1.5 | 4.45 | 4.99454815281282 | 4.71750656728054 | -0.54454815281282 | -0.267506567280538 | 4.45 | 4.45000000000012 | 8.88178419700125e-16 | 1.16351372980716e-13 |
| 576 | 1.5 | 4.5 | 5.06181185063549 | 4.77277027415938 | -0.561811850635494 | -0.27277027415938 | 4.5 | 4.50000000000008 | 8.88178419700125e-16 | 8.43769498715119e-14 |
| 577 | 1.5 | 4.55 | 5.12943632294484 | 4.82803600650524 | -0.579436322944843 | -0.278036006505238 | 4.55 | 4.55000000000012 | 1.77635683940025e-15 | 1.21680443498917e-13 |
| 578 | 1.5 | 4.6 | 5.19742743790768 | 4.88330376454053 | -0.597427437907681 | -0.283303764540529 | 4.6 | 4.59999999999995 | 1.77635683940025e-15 | -4.88498130835069e-14 |
| 579 | 1.5 | 4.65 | 5.265791224518 | 4.9385735484884 | -0.615791224517996 | -0.288573548488404 | 4.65 | 4.64999999999993 | 1.77635683940025e-15 | -7.37188088351104e-14 |
| 580 | 1.5 | 4.7 | 5.33453387883628 | 4.99384535857165 | -0.634533878836275 | -0.29384535857165 | 4.7 | 4.70000000000003 | 8.88178419700125e-16 | 3.19744231092045e-14 |
| 581 | 1.5 | 4.75 | 5.40366177054356 | 5.04911919501287 | -0.65366177054356 | -0.299119195012866 | 4.75 | 4.75000000000004 | 0 | 4.35207425653061e-14 |
| 582 | 1.5 | 4.8 | 5.47318144982982 | 5.10439505803502 | -0.673181449829818 | -0.304395058035023 | 4.8 | 4.80000000000004 | 0 | 3.90798504668055e-14 |
| 583 | 1.5 | 4.85 | 5.54309965463773 | 5.15967294786109 | -0.69309965463773 | -0.30967294786109 | 4.85 | 4.85000000000006 | 8.88178419700125e-16 | 5.86197757002083e-14 |
| 584 | 1.5 | 4.9 | 5.61342331828458 | 5.21495286471404 | -0.713423318284577 | -0.314952864714035 | 4.9 | 4.9000000000001 | 0 | 1.02140518265514e-13 |
| 585 | 1.5 | 4.95 | 5.68415957748661 | 5.27023480881665 | -0.734159577486605 | -0.320234808816646 | 4.95 | 4.94999999999997 | 8.88178419700125e-16 | -3.10862446895044e-14 |
| 586 | 1.5 | 5 | 5.75531578081218 | 5.32551878039245 | -0.755315780812178 | -0.325518780392446 | 5 | 5.00000000000009 | 1.77635683940025e-15 | 9.05941988094128e-14 |
| 587 | 1.5 | 5.05 | 5.82689949759204 | 5.38080477966404 | -0.77689949759204 | -0.330804779664035 | 5.05 | 5.05000000000002 | 8.88178419700125e-16 | 2.22044604925031e-14 |
| 588 | 1.5 | 5.1 | 5.89891852731729 | 5.43609280685494 | -0.798918527317285 | -0.336092806854937 | 5.1 | 5.10000000000012 | 8.88178419700125e-16 | 1.19015908239817e-13 |
| 589 | 1.5 | 5.15 | 5.97138090955805 | 5.49138286218794 | -0.821380909558044 | -0.341382862187939 | 5.15 | 5.15000000000004 | 0 | 3.46389583683049e-14 |
| 590 | 1.5 | 5.2 | 6.04429493443862 | 5.54667494588656 | -0.844294934438617 | -0.346674945886564 | 5.2 | 5.20000000000005 | 8.88178419700125e-16 | 4.79616346638068e-14 |
| 591 | 1.5 | 5.25 | 6.11766915370765 | 5.60196905817397 | -0.86766915370765 | -0.351969058173968 | 5.25 | 5.25000000000007 | 1.77635683940025e-15 | 7.19424519957101e-14 |
| 592 | 1.5 | 5.3 | 6.19151239244525 | 5.65726519927331 | -0.891512392445248 | -0.357265199273305 | 5.3 | 5.29999999999998 | 1.77635683940025e-15 | -2.04281036531029e-14 |
| 593 | 1.5 | 5.35 | 6.26583376145235 | 5.71256336940829 | -0.915833761452349 | -0.362563369408284 | 5.35 | 5.35000000000011 | 8.88178419700125e-16 | 1.07469588783715e-13 |
| 594 | 1.5 | 5.4 | 6.34064267037163 | 5.76786356880169 | -0.940642670371631 | -0.367863568801693 | 5.4 | 5.39999999999992 | 1.77635683940025e-15 | -7.63833440942108e-14 |
| 595 | 1.5 | 5.45 | 6.41594884159341 | 5.82316579767761 | -0.965948841593406 | -0.373165797677609 | 5.45 | 5.45000000000003 | 1.77635683940025e-15 | 2.48689957516035e-14 |
| 596 | 1.5 | 5.5 | 6.49176232500473 | 5.878470056259 | -0.991762325004731 | -0.378470056259003 | 5.5 | 5.49999999999997 | 0 | -2.8421709430404e-14 |
| 597 | 1.5 | 5.55 | 6.56809351364503 | 5.93377634476977 | -1.01809351364503 | -0.383776344769769 | 5.55 | 5.55000000000012 | 8.88178419700125e-16 | 1.18127729820117e-13 |
| 598 | 1.5 | 5.6 | 6.6449531603373 | 5.98908466343288 | -1.0449531603373 | -0.389084663432878 | 5.6 | 5.59999999999995 | 1.77635683940025e-15 | -4.9737991503207e-14 |
| 599 | 1.5 | 5.65 | 6.72235239537024 | 6.0443950124726 | -1.07235239537024 | -0.394395012472595 | 5.65 | 5.65000000000009 | 8.88178419700125e-16 | 8.61533067109122e-14 |
| 600 | 1.5 | 5.7 | 6.80030274531362 | 6.09970739211189 | -1.10030274531362 | -0.399707392111889 | 5.7 | 5.69999999999994 | 8.88178419700125e-16 | -6.48370246381091e-14 |
| 601 | 1.5 | 5.75 | 6.87881615305697 | 6.15502180257503 | -1.12881615305697 | -0.405021802575026 | 5.75 | 5.75000000000004 | 0 | 4.08562073062058e-14 |
| 602 | 1.5 | 5.8 | 6.95790499917021 | 6.21033824408534 | -1.15790499917021 | -0.410338244085343 | 5.8 | 5.80000000000007 | 8.88178419700125e-16 | 7.28306304154103e-14 |
| 603 | 1.5 | 5.85 | 7.03758212469436 | 6.26565671686656 | -1.18758212469436 | -0.415656716866555 | 5.85 | 5.85 | 8.88178419700125e-16 | 8.88178419700125e-16 |
| 604 | 1.5 | 5.9 | 7.11786085548118 | 6.32097722114274 | -1.21786085548118 | -0.420977221142739 | 5.9 | 5.90000000000009 | 1.77635683940025e-15 | 8.88178419700125e-14 |
| 605 | 1.5 | 5.95 | 7.19875502821238 | 6.37629975713742 | -1.24875502821238 | -0.426299757137419 | 5.95 | 5.95000000000006 | 8.88178419700125e-16 | 6.03961325396085e-14 |
| 606 | 1.5 | 6 | 7.28027901824222 | 6.43162432507468 | -1.28027901824222 | -0.431624325074677 | 6 | 6.00000000000011 | 8.88178419700125e-16 | 1.10134124042816e-13 |
| 607 | 1.6 | 1 | 0.997408893477546 | 0.910612565638476 | 0.00259110652245376 | 0.0893874343615238 | 1 | 1.00000000000007 | 2.22044604925031e-16 | 7.17204073907851e-14 |
| 608 | 1.6 | 1.05 | 1.04848921178084 | 0.965737102738469 | 0.00151078821915918 | 0.0842628972615307 | 1.05 | 1.04999999999995 | 1.77635683940025e-15 | -4.68514116391816e-14 |
| 609 | 1.6 | 1.1 | 1.09972699132861 | 1.02086365004158 | 0.000273008671388686 | 0.0791363499584226 | 1.1 | 1.10000000000014 | 6.66133814775094e-16 | 1.4299672557172e-13 |
| 610 | 1.6 | 1.15 | 1.15112369734116 | 1.07599220776707 | -0.00112369734115947 | 0.0740077922329301 | 1.15 | 1.15000000000004 | 1.33226762955019e-15 | 4.01900734914307e-14 |
| 611 | 1.6 | 1.2 | 1.20268081790454 | 1.13112277613533 | -0.00268081790453567 | 0.0688772238646744 | 1.2 | 1.20000000000001 | 0 | 1.37667655053519e-14 |
| 612 | 1.6 | 1.25 | 1.25439986447327 | 1.18625535536635 | -0.00439986447326546 | 0.0637446446336463 | 1.25 | 1.25000000000006 | 4.44089209850063e-16 | 6.19504447740837e-14 |
| 613 | 1.6 | 1.3 | 1.30628237238735 | 1.24138994567998 | -0.00628237238734486 | 0.0586100543200212 | 1.3 | 1.29999999999998 | -2.22044604925031e-16 | -2.19824158875781e-14 |
| 614 | 1.6 | 1.35 | 1.35832990140406 | 1.29652654729658 | -0.00832990140406476 | 0.0534734527034202 | 1.35 | 1.35000000000002 | 8.88178419700125e-16 | 2.24265050974282e-14 |
| 615 | 1.6 | 1.4 | 1.41054403624513 | 1.35166516043598 | -0.0105440362451292 | 0.0483348395640189 | 1.4 | 1.39999999999992 | 2.22044604925031e-16 | -8.43769498715119e-14 |
| 616 | 1.6 | 1.45 | 1.46292638715967 | 1.40680578531875 | -0.0129263871596661 | 0.0431942146812532 | 1.45 | 1.45000000000001 | 6.66133814775094e-16 | 1.28785870856518e-14 |
| 617 | 1.6 | 1.5 | 1.51547859050364 | 1.4619484221647 | -0.0154785905036425 | 0.0380515778352988 | 1.5 | 1.49999999999996 | 2.22044604925031e-15 | -3.79696274421804e-14 |
| 618 | 1.6 | 1.55 | 1.56820230933631 | 1.51709307119441 | -0.0182023093363131 | 0.0329069288055917 | 1.55 | 1.55000000000005 | 1.33226762955019e-15 | 4.52970994047064e-14 |
| 619 | 1.6 | 1.6 | 1.62109923403431 | 1.57223973262788 | -0.0210992340343101 | 0.0277602673721222 | 1.6 | 1.60000000000001 | 1.33226762955019e-15 | 5.77315972805081e-15 |
| 620 | 1.6 | 1.65 | 1.67417108292399 | 1.62738840668567 | -0.0241710829239925 | 0.0226115933143261 | 1.65 | 1.65000000000005 | 6.66133814775094e-16 | 5.32907051820075e-14 |
| 621 | 1.6 | 1.7 | 1.72741960293276 | 1.68253909358799 | -0.0274196029327578 | 0.0174609064120097 | 1.7 | 1.70000000000002 | 1.99840144432528e-15 | 2.46469511466785e-14 |
| 622 | 1.6 | 1.75 | 1.78084657025998 | 1.73769179355539 | -0.0308465702599796 | 0.0123082064446078 | 1.75 | 1.75000000000006 | 6.66133814775094e-16 | 5.70654634657331e-14 |
| 623 | 1.6 | 1.8 | 1.83445379106835 | 1.79284650680826 | -0.0344537910683487 | 0.00715349319174208 | 1.8 | 1.80000000000008 | 1.99840144432528e-15 | 8.26005930321117e-14 |
| 624 | 1.6 | 1.85 | 1.88824310219631 | 1.84800323356697 | -0.038243102196309 | 0.0019967664330327 | 1.85 | 1.85 | 1.77635683940025e-15 | -2.44249065417534e-15 |
| 625 | 1.6 | 1.9 | 1.94221637189246 | 1.90316197405227 | -0.0422163718924609 | -0.0031619740522697 | 1.9 | 1.9 | 1.77635683940025e-15 | -3.33066907387547e-15 |
| 626 | 1.6 | 1.95 | 1.9963755005727 | 1.95832272848473 | -0.0463755005727016 | -0.00832272848472893 | 1.95 | 1.95000000000007 | 1.77635683940025e-15 | 7.01660951563099e-14 |
| 627 | 1.6 | 2 | 2.050722421601 | 2.01348549708473 | -0.0507224216009998 | -0.0134854970847251 | 2 | 2.00000000000001 | 8.88178419700125e-16 | 5.32907051820075e-15 |
| 628 | 1.6 | 2.05 | 2.10525910209472 | 2.06865028007319 | -0.0552591020947162 | -0.0186502800731923 | 2.05 | 2.05000000000006 | 2.22044604925031e-15 | 5.55111512312578e-14 |
| 629 | 1.6 | 2.1 | 2.15998754375539 | 2.12381707767069 | -0.059987543755387 | -0.0238170776706936 | 2.1 | 2.1000000000001 | 8.88178419700125e-16 | 1.00364161426114e-13 |
| 630 | 1.6 | 2.15 | 2.21490978372602 | 2.1789858900978 | -0.0649097837260162 | -0.0289858900977951 | 2.15 | 2.14999999999999 | 8.88178419700125e-16 | -1.46549439250521e-14 |
| 631 | 1.6 | 2.2 | 2.27002789547586 | 2.23415671757562 | -0.0700278954758558 | -0.0341567175756152 | 2.2 | 2.20000000000002 | 8.88178419700125e-16 | 2.22044604925031e-14 |
| 632 | 1.6 | 2.25 | 2.32534398971381 | 2.28932956032472 | -0.0753439897138071 | -0.039329560324719 | 2.25 | 2.24999999999998 | 0 | -1.77635683940025e-14 |
| 633 | 1.6 | 2.3 | 2.38086021533156 | 2.34450441856623 | -0.0808602153315601 | -0.0445044185662251 | 2.3 | 2.3000000000001 | 4.44089209850063e-16 | 1.03028696685215e-13 |
| 634 | 1.6 | 2.35 | 2.43657876037766 | 2.3996812925207 | -0.0865787603776642 | -0.049681292520698 | 2.35 | 2.35000000000008 | 4.44089209850063e-16 | 8.39328606616618e-14 |
| 635 | 1.6 | 2.4 | 2.49250185306379 | 2.45486018240926 | -0.0925018530637902 | -0.0548601824092567 | 2.4 | 2.40000000000009 | 8.88178419700125e-16 | 8.79296635503124e-14 |
| 636 | 1.6 | 2.45 | 2.54863176280448 | 2.51004108845284 | -0.0986317628044779 | -0.0600410884528371 | 2.45 | 2.45000000000008 | 1.77635683940025e-15 | 7.86037901434611e-14 |
| 637 | 1.6 | 2.5 | 2.60497080129175 | 2.56522401087237 | -0.104970801291751 | -0.0652240108723725 | 2.5 | 2.49999999999998 | 1.33226762955019e-15 | -2.1316282072803e-14 |
| 638 | 1.6 | 2.55 | 2.66152132360605 | 2.62040894988917 | -0.111521323606051 | -0.0704089498891674 | 2.55 | 2.55000000000001 | 1.33226762955019e-15 | 1.11022302462516e-14 |
| 639 | 1.6 | 2.6 | 2.71828572936496 | 2.67559590572416 | -0.118285729364962 | -0.0755959057241555 | 2.6 | 2.60000000000003 | 8.88178419700125e-16 | 2.57571741713036e-14 |
| 640 | 1.6 | 2.65 | 2.77526646391137 | 2.73078487859846 | -0.125266463911365 | -0.0807848785984571 | 2.65 | 2.65000000000001 | 1.33226762955019e-15 | 5.77315972805081e-15 |
| 641 | 1.6 | 2.7 | 2.83246601954263 | 2.78597586873338 | -0.132466019542631 | -0.0859758687333772 | 2.7 | 2.70000000000006 | 8.88178419700125e-16 | 6.3948846218409e-14 |
| 642 | 1.6 | 2.75 | 2.88988693678266 | 2.84116887635003 | -0.139886936782658 | -0.0911688763500345 | 2.75 | 2.75000000000011 | 2.22044604925031e-15 | 1.09690034832965e-13 |
| 643 | 1.6 | 2.8 | 2.94753180569853 | 2.89636390166955 | -0.147531805698529 | -0.0963639016695481 | 2.8 | 2.80000000000001 | 1.33226762955019e-15 | 1.4210854715202e-14 |
| 644 | 1.6 | 2.85 | 3.00540326726378 | 2.95156094491359 | -0.155403267263781 | -0.101560944913593 | 2.85 | 2.85000000000012 | 1.77635683940025e-15 | 1.15463194561016e-13 |
| 645 | 1.6 | 2.9 | 3.06350401477026 | 3.0067600063031 | -0.163504014770258 | -0.106760006303103 | 2.9 | 2.90000000000005 | 1.33226762955019e-15 | 4.52970994047064e-14 |
| 646 | 1.6 | 2.95 | 3.12183679529074 | 3.06196108605975 | -0.171836795290738 | -0.111961086059754 | 2.95 | 2.95000000000007 | 4.44089209850063e-16 | 6.97220059464598e-14 |
| 647 | 1.6 | 3 | 3.18040441119454 | 3.11716418440485 | -0.180404411194536 | -0.117164184404849 | 3 | 3.00000000000008 | 4.44089209850063e-16 | 8.03801469828613e-14 |
| 648 | 1.6 | 3.05 | 3.23920972171848 | 3.17236930155988 | -0.189209721718481 | -0.122369301559877 | 3.05 | 3.0500000000001 | 8.88178419700125e-16 | 1.01696429055664e-13 |
| 649 | 1.6 | 3.1 | 3.29825564459573 | 3.22757643774615 | -0.198255644595731 | -0.127576437746145 | 3.1 | 3.09999999999995 | 8.88178419700125e-16 | -4.66293670342566e-14 |
| 650 | 1.6 | 3.15 | 3.35754515774509 | 3.28278559318551 | -0.20754515774509 | -0.132785593185511 | 3.15 | 3.14999999999992 | 1.33226762955019e-15 | -7.8159700933611e-14 |
| 651 | 1.6 | 3.2 | 3.41708130102354 | 3.33799676809965 | -0.217081301023543 | -0.137996768099649 | 3.2 | 3.20000000000009 | 1.33226762955019e-15 | 8.83737527601625e-14 |
| 652 | 1.6 | 3.25 | 3.47686717804498 | 3.39320996270968 | -0.226867178044981 | -0.143209962709679 | 3.25 | 3.25 | 4.44089209850063e-16 | -4.44089209850063e-15 |
| 653 | 1.6 | 3.3 | 3.53690595806815 | 3.44842517723765 | -0.236905958068153 | -0.148425177237645 | 3.3 | 3.29999999999999 | 1.33226762955019e-15 | -1.46549439250521e-14 |
| 654 | 1.6 | 3.35 | 3.59720087795711 | 3.50364241190522 | -0.247200877957112 | -0.153642411905224 | 3.35 | 3.35000000000003 | 2.22044604925031e-15 | 3.10862446895044e-14 |
| 655 | 1.6 | 3.4 | 3.65775524421759 | 3.55886166693409 | -0.257755244217593 | -0.158861666934087 | 3.4 | 3.40000000000007 | 8.88178419700125e-16 | 6.57252030578093e-14 |
| 656 | 1.6 | 3.45 | 3.71857243511294 | 3.61408294254591 | -0.26857243511294 | -0.164082942545911 | 3.45 | 3.44999999999999 | 0 | -1.11022302462516e-14 |
| 657 | 1.6 | 3.5 | 3.7796559028634 | 3.66930623896274 | -0.279655902863403 | -0.16930623896274 | 3.5 | 3.5 | 8.88178419700125e-16 | -3.99680288865056e-15 |
| 658 | 1.6 | 3.55 | 3.84100917593287 | 3.72453155640643 | -0.291009175932874 | -0.174531556406433 | 3.55 | 3.55000000000008 | 0 | 7.90478793533112e-14 |
| 659 | 1.6 | 3.6 | 3.90263586140735 | 3.77975889509867 | -0.30263586140735 | -0.179758895098665 | 3.6 | 3.60000000000003 | 1.77635683940025e-15 | 2.57571741713036e-14 |
| 660 | 1.6 | 3.65 | 3.96453964746963 | 3.83498825526167 | -0.314539647469629 | -0.184988255261665 | 3.65 | 3.65000000000009 | 4.44089209850063e-16 | 8.97060203897127e-14 |
| 661 | 1.6 | 3.7 | 4.02672430597509 | 3.89021963711711 | -0.326724305975092 | -0.190219637117109 | 3.7 | 3.69999999999999 | 8.88178419700125e-16 | -1.50990331349021e-14 |
| 662 | 1.6 | 3.75 | 4.08919369513361 | 3.94545304088741 | -0.339193695133614 | -0.19545304088741 | 3.75 | 3.75000000000006 | 1.77635683940025e-15 | 5.99520433297585e-14 |
| 663 | 1.6 | 3.8 | 4.15195176230303 | 4.00068846679424 | -0.351951762303025 | -0.200688466794244 | 3.8 | 3.79999999999995 | 1.33226762955019e-15 | -4.57411886145565e-14 |
| 664 | 1.6 | 3.85 | 4.21500254689985 | 4.05592591506021 | -0.365002546899851 | -0.20592591506021 | 3.85 | 3.85000000000011 | 4.44089209850063e-16 | 1.09690034832965e-13 |
| 665 | 1.6 | 3.9 | 4.27835018343338 | 4.1111653859068 | -0.378350183433383 | -0.211165385906797 | 3.9 | 3.89999999999993 | 1.77635683940025e-15 | -7.41628980449605e-14 |
| 666 | 1.6 | 3.95 | 4.34199890466954 | 4.16640687955698 | -0.391998904669543 | -0.216406879556975 | 3.95 | 3.95000000000011 | 0 | 1.05249142734465e-13 |
| 667 | 1.6 | 4 | 4.4059530449314 | 4.22165039623224 | -0.405953044931402 | -0.221650396232235 | 4 | 3.99999999999997 | 1.77635683940025e-15 | -2.53130849614536e-14 |
| 668 | 1.6 | 4.05 | 4.4702170435436 | 4.27689593615536 | -0.420217043543599 | -0.226895936155358 | 4.05 | 4.04999999999999 | 1.77635683940025e-15 | -7.105427357601e-15 |
| 669 | 1.6 | 4.1 | 4.53479544842846 | 4.33214349954858 | -0.434795448428464 | -0.23214349954858 | 4.1 | 4.10000000000009 | 8.88178419700125e-16 | 8.70414851306123e-14 |
| 670 | 1.6 | 4.15 | 4.59969291986203 | 4.38739308663412 | -0.449692919862033 | -0.237393086634124 | 4.15 | 4.15000000000014 | 1.77635683940025e-15 | 1.37667655053519e-13 |
| 671 | 1.6 | 4.2 | 4.66491423439876 | 4.44264469763422 | -0.464914234398756 | -0.242644697634224 | 4.2 | 4.2 | 8.88178419700125e-16 | -3.5527136788005e-15 |
| 672 | 1.6 | 4.25 | 4.73046428897427 | 4.49789833277185 | -0.480464288974273 | -0.247898332771847 | 4.25 | 4.25000000000014 | 0 | 1.4299672557172e-13 |
| 673 | 1.6 | 4.3 | 4.79634810519619 | 4.55315399226886 | -0.496348105196192 | -0.253153992268854 | 4.3 | 4.30000000000002 | 8.88178419700125e-16 | 1.77635683940025e-14 |
| 674 | 1.6 | 4.35 | 4.86257083383352 | 4.60841167634822 | -0.512570833833515 | -0.258411676348215 | 4.35 | 4.35000000000003 | 0 | 2.93098878501041e-14 |
| 675 | 1.6 | 4.4 | 4.92913775951613 | 4.66367138523235 | -0.529137759516131 | -0.263671385232344 | 4.4 | 4.40000000000005 | 1.77635683940025e-15 | 4.52970994047064e-14 |
| 676 | 1.6 | 4.45 | 4.9960543056564 | 4.71893311914384 | -0.546054305656397 | -0.268933119143842 | 4.45 | 4.45000000000007 | 1.77635683940025e-15 | 6.57252030578093e-14 |
| 677 | 1.6 | 4.5 | 5.0633260396059 | 4.77419687830531 | -0.563326039605895 | -0.274196878305307 | 4.5 | 4.50000000000005 | 1.77635683940025e-15 | 5.06261699229071e-14 |
| 678 | 1.6 | 4.55 | 5.13095867806117 | 4.82946266293934 | -0.58095867806117 | -0.279462662939339 | 4.55 | 4.54999999999993 | 0 | -7.37188088351104e-14 |
| 679 | 1.6 | 4.6 | 5.19895809273332 | 4.88473047326909 | -0.598958092733322 | -0.284730473269094 | 4.6 | 4.60000000000008 | 8.88178419700125e-16 | 8.43769498715119e-14 |
| 680 | 1.6 | 4.65 | 5.26733031629737 | 4.9400003095168 | -0.617330316297374 | -0.2900003095168 | 4.65 | 4.65000000000004 | 8.88178419700125e-16 | 3.99680288865056e-14 |
| 681 | 1.6 | 4.7 | 5.33608154863847 | 4.99527217190561 | -0.636081548638466 | -0.295272171905614 | 4.7 | 4.70000000000011 | 8.88178419700125e-16 | 1.11022302462516e-13 |
| 682 | 1.6 | 4.75 | 5.40521816341319 | 5.05054606065813 | -0.655218163413188 | -0.300546060658133 | 4.75 | 4.75000000000007 | 8.88178419700125e-16 | 7.28306304154103e-14 |
| 683 | 1.6 | 4.8 | 5.4747467149457 | 5.10582197599733 | -0.674746714945695 | -0.305821975997329 | 4.8 | 4.8 | 1.77635683940025e-15 | 2.66453525910038e-15 |
| 684 | 1.6 | 4.85 | 5.54467394547974 | 5.16109991814636 | -0.694673945479737 | -0.311099918146358 | 4.85 | 4.85000000000011 | 8.88178419700125e-16 | 1.07469588783715e-13 |
| 685 | 1.6 | 4.9 | 5.6150067928093 | 5.21637988732782 | -0.715006792809299 | -0.316379887327814 | 4.9 | 4.90000000000005 | 0 | 4.88498130835069e-14 |
| 686 | 1.6 | 4.95 | 5.68575239831229 | 5.27166188376486 | -0.735752398312294 | -0.321661883764858 | 4.95 | 4.94999999999996 | 8.88178419700125e-16 | -3.64153152077051e-14 |
| 687 | 1.6 | 5 | 5.75691811541366 | 5.32694590768064 | -0.756918115413661 | -0.326945907680643 | 5 | 4.99999999999995 | 8.88178419700125e-16 | -5.50670620214078e-14 |
| 688 | 1.6 | 5.05 | 5.82851151850624 | 5.38223195929832 | -0.77851151850624 | -0.332231959298322 | 5.05 | 5.05000000000005 | 8.88178419700125e-16 | 4.88498130835069e-14 |
| 689 | 1.6 | 5.1 | 5.90054041236005 | 5.43752003884068 | -0.800540412360046 | -0.337520038840681 | 5.1 | 5.09999999999996 | 1.77635683940025e-15 | -3.64153152077051e-14 |
| 690 | 1.6 | 5.15 | 5.97301284205306 | 5.49281014653125 | -0.823012842053057 | -0.342810146531245 | 5.15 | 5.15000000000001 | 1.77635683940025e-15 | 7.105427357601e-15 |
| 691 | 1.6 | 5.2 | 6.04593710345926 | 5.54810228259299 | -0.845937103459258 | -0.348102282592985 | 5.2 | 5.19999999999996 | 0 | -4.08562073062058e-14 |
| 692 | 1.6 | 5.25 | 6.11932175433267 | 5.60339644724942 | -0.869321754332669 | -0.353396447249423 | 5.25 | 5.25000000000006 | 0 | 6.21724893790088e-14 |
| 693 | 1.6 | 5.3 | 6.19317562602923 | 5.65869264072353 | -0.893175626029234 | -0.358692640723532 | 5.3 | 5.30000000000002 | 8.88178419700125e-16 | 2.39808173319034e-14 |
| 694 | 1.6 | 5.35 | 6.26750783591208 | 5.71399086323883 | -0.917507835912079 | -0.363990863238834 | 5.35 | 5.35000000000001 | 1.77635683940025e-15 | 1.24344978758018e-14 |
| 695 | 1.6 | 5.4 | 6.34232780048943 | 5.76929111501867 | -0.94232780048943 | -0.36929111501867 | 5.4 | 5.4 | 8.88178419700125e-16 | -4.44089209850063e-15 |
| 696 | 1.6 | 5.45 | 6.4176452493388 | 5.82459339628657 | -0.967645249338798 | -0.374593396286566 | 5.45 | 5.45000000000007 | 8.88178419700125e-16 | 6.75015598972095e-14 |
| 697 | 1.6 | 5.5 | 6.4934702398757 | 5.87989770726568 | -0.993470239875698 | -0.379897707265676 | 5.5 | 5.49999999999996 | 0 | -4.44089209850063e-14 |
| 698 | 1.6 | 5.55 | 6.56981317303042 | 5.93520404817989 | -1.01981317303042 | -0.385204048179895 | 5.55 | 5.55000000000001 | 8.88178419700125e-16 | 1.33226762955019e-14 |
| 699 | 1.6 | 5.6 | 6.64668480990196 | 5.99051241925256 | -1.04668480990196 | -0.390512419252561 | 5.6 | 5.60000000000006 | 8.88178419700125e-16 | 5.86197757002083e-14 |
| 700 | 1.6 | 5.65 | 6.72409628946466 | 6.0458228207072 | -1.07409628946465 | -0.395822820707202 | 5.65 | 5.65000000000004 | 8.88178419700125e-16 | 4.08562073062058e-14 |
| 701 | 1.6 | 5.7 | 6.80205914741002 | 6.10113525276753 | -1.10205914741002 | -0.401135252767527 | 5.7 | 5.70000000000004 | 8.88178419700125e-16 | 3.81916720471054e-14 |
| 702 | 1.6 | 5.75 | 6.88058533621399 | 6.15644971565706 | -1.13058533621399 | -0.40644971565706 | 5.75 | 5.74999999999992 | 1.77635683940025e-15 | -7.63833440942108e-14 |
| 703 | 1.6 | 5.8 | 6.9596872465284 | 6.21176620959988 | -1.1596872465284 | -0.411766209599881 | 5.8 | 5.80000000000003 | 0 | 3.37507799486048e-14 |
| 704 | 1.6 | 5.85 | 7.03937773000516 | 6.26708473481933 | -1.18937773000516 | -0.417084734819331 | 5.85 | 5.85 | 0 | 2.66453525910038e-15 |
| 705 | 1.6 | 5.9 | 7.11967012367213 | 6.32240529153949 | -1.21967012367213 | -0.422405291539489 | 5.9 | 5.9000000000001 | 8.88178419700125e-16 | 9.41469124882133e-14 |
| 706 | 1.6 | 5.95 | 7.20057827599158 | 6.37772787998388 | -1.25057827599158 | -0.427727879983881 | 5.95 | 5.95000000000003 | 1.77635683940025e-15 | 3.37507799486048e-14 |
| 707 | 1.6 | 6 | 7.28211657474552 | 6.43305250037659 | -1.28211657474552 | -0.433052500376586 | 6 | 6.00000000000001 | 8.88178419700125e-16 | 8.88178419700125e-15 |
| 708 | 1.7 | 1 | 0.998548828722101 | 0.912034863682076 | 0.00145117127789918 | 0.0879651363179238 | 1 | 1.00000000000008 | -3.33066907387547e-16 | 8.10462807976364e-14 |
| 709 | 1.7 | 1.05 | 1.04963265043837 | 0.967159452646508 | 0.00036734956162543 | 0.082840547353492 | 1.05 | 1.04999999999992 | 2.22044604925031e-15 | -8.21565038222616e-14 |
| 710 | 1.7 | 1.1 | 1.10087396590053 | 1.02228605181979 | -0.000873965900529461 | 0.0777139481802092 | 1.1 | 1.10000000000012 | 1.77635683940025e-15 | 1.18571819029967e-13 |
| 711 | 1.7 | 1.15 | 1.15227424083452 | 1.07741466142119 | -0.00227424083452465 | 0.0725853385788056 | 1.15 | 1.15000000000008 | 2.22044604925031e-15 | 8.26005930321117e-14 |
| 712 | 1.7 | 1.2 | 1.20383496384315 | 1.13254528167091 | -0.00383496384315141 | 0.0674547183290879 | 1.2 | 1.20000000000001 | -2.22044604925031e-16 | 9.32587340685131e-15 |
| 713 | 1.7 | 1.25 | 1.25555764690908 | 1.18767791278914 | -0.00555764690907501 | 0.0623220872108614 | 1.25 | 1.25000000000006 | 4.44089209850063e-16 | 6.41708908233341e-14 |
| 714 | 1.7 | 1.3 | 1.30744382591215 | 1.2428125549957 | -0.00744382591215209 | 0.0571874450043022 | 1.3 | 1.30000000000004 | 1.33226762955019e-15 | 3.88578058618805e-14 |
| 715 | 1.7 | 1.35 | 1.35949506116159 | 1.29794920851078 | -0.00949506116158894 | 0.0520507914892157 | 1.35 | 1.35000000000003 | 1.99840144432528e-15 | 2.57571741713036e-14 |
| 716 | 1.7 | 1.4 | 1.41171293794342 | 1.35308787355459 | -0.0117129379434187 | 0.0469121264454075 | 1.4 | 1.40000000000008 | 8.88178419700125e-16 | 8.01581023779363e-14 |
| 717 | 1.7 | 1.45 | 1.46409906708386 | 1.40822855034713 | -0.0140990670838639 | 0.0417714496528689 | 1.45 | 1.45000000000005 | 6.66133814775094e-16 | 5.21804821573824e-14 |
| 718 | 1.7 | 1.5 | 1.51665508552913 | 1.46337123910878 | -0.0166550855291292 | 0.0366287608912208 | 1.5 | 1.50000000000009 | 8.88178419700125e-16 | 9.28146448586631e-14 |
| 719 | 1.7 | 1.55 | 1.56938265694224 | 1.51851594005955 | -0.0193826569422386 | 0.0314840599404533 | 1.55 | 1.54999999999998 | 0 | -1.97619698383278e-14 |
| 720 | 1.7 | 1.6 | 1.62228347231752 | 1.57366265342 | -0.0222834723175229 | 0.0263373465800028 | 1.6 | 1.59999999999996 | 8.88178419700125e-16 | -4.10782519111308e-14 |
| 721 | 1.7 | 1.65 | 1.67535925061338 | 1.62881137941051 | -0.025359250613384 | 0.0211886205894893 | 1.65 | 1.65000000000007 | 0 | 7.03881397612349e-14 |
| 722 | 1.7 | 1.7 | 1.72861173940404 | 1.6839621182511 | -0.0286117394040435 | 0.0160378817489044 | 1.7 | 1.69999999999998 | 2.22044604925031e-16 | -1.68753899743024e-14 |
| 723 | 1.7 | 1.75 | 1.78204271555093 | 1.7391148701625 | -0.0320427155509304 | 0.0108851298374979 | 1.75 | 1.75 | 1.55431223447522e-15 | 8.88178419700125e-16 |
| 724 | 1.7 | 1.8 | 1.83565398589447 | 1.79426963536511 | -0.0356539858944662 | 0.00573036463489141 | 1.8 | 1.80000000000006 | 1.11022302462516e-15 | 5.48450174164827e-14 |
| 725 | 1.7 | 1.85 | 1.88944738796701 | 1.8494264140793 | -0.0394473879670108 | 0.000573585920705 | 1.85 | 1.85000000000004 | 4.44089209850063e-16 | 4.01900734914307e-14 |
| 726 | 1.7 | 1.9 | 1.94342479072774 | 1.90458520652563 | -0.0434247907277447 | -0.00458520652562555 | 1.9 | 1.89999999999998 | 4.44089209850063e-16 | -1.70974345792274e-14 |
| 727 | 1.7 | 1.95 | 1.99758809532033 | 1.95974601292485 | -0.0475880953203338 | -0.009746012924849 | 1.95 | 1.95000000000004 | 4.44089209850063e-16 | 4.06341627012807e-14 |
| 728 | 1.7 | 2 | 2.05193923585425 | 2.01490883349735 | -0.0519392358542463 | -0.0149088334973455 | 2 | 2 | 1.33226762955019e-15 | -8.88178419700125e-16 |
| 729 | 1.7 | 2.05 | 2.10648018021062 | 2.07007366846405 | -0.0564801802106194 | -0.020073668464049 | 2.05 | 2.05000000000011 | 4.44089209850063e-16 | 1.10578213252666e-13 |
| 730 | 1.7 | 2.1 | 2.16121293087365 | 2.12524051804534 | -0.0612129308736478 | -0.0252405180453383 | 2.1 | 2.10000000000009 | 1.33226762955019e-15 | 8.70414851306123e-14 |
| 731 | 1.7 | 2.15 | 2.21613952578845 | 2.18040938246196 | -0.0661395257884485 | -0.0304093824619631 | 2.15 | 2.14999999999994 | -8.88178419700125e-16 | -6.08402217494586e-14 |
| 732 | 1.7 | 2.2 | 2.2712620392465 | 2.23558026193504 | -0.0712620392465002 | -0.0355802619350438 | 2.2 | 2.19999999999998 | 1.77635683940025e-15 | -2.04281036531029e-14 |
| 733 | 1.7 | 2.25 | 2.32658258279966 | 2.29075315668514 | -0.0765825827996647 | -0.0407531566851436 | 2.25 | 2.24999999999998 | 8.88178419700125e-16 | -2.26485497023532e-14 |
| 734 | 1.7 | 2.3 | 2.38210330620403 | 2.3459280669332 | -0.0821033062040333 | -0.0459280669331967 | 2.3 | 2.3 | 1.77635683940025e-15 | 1.77635683940025e-15 |
| 735 | 1.7 | 2.35 | 2.43782639839468 | 2.40110499289995 | -0.0878263983946805 | -0.051104992899953 | 2.35 | 2.34999999999992 | -4.44089209850063e-16 | -8.17124146124115e-14 |
| 736 | 1.7 | 2.4 | 2.49375408849268 | 2.45628393480672 | -0.0937540884926817 | -0.0562839348067166 | 2.4 | 2.40000000000006 | 1.77635683940025e-15 | 5.90638649100583e-14 |
| 737 | 1.7 | 2.45 | 2.5498886468456 | 2.51146489287405 | -0.0998886468455975 | -0.0614648928740524 | 2.45 | 2.45000000000005 | 4.44089209850063e-16 | 4.79616346638068e-14 |
| 738 | 1.7 | 2.5 | 2.60623238610292 | 2.56664786732308 | -0.106232386102916 | -0.0666478673230793 | 2.5 | 2.49999999999998 | 1.77635683940025e-15 | -2.35367281220533e-14 |
| 739 | 1.7 | 2.55 | 2.66278766232778 | 2.6218328583751 | -0.112787662327781 | -0.0718328583751022 | 2.55 | 2.55000000000007 | -4.44089209850063e-16 | 6.61692922676593e-14 |
| 740 | 1.7 | 2.6 | 2.71955687614662 | 2.67701986625087 | -0.119556876146615 | -0.0770198662508697 | 2.6 | 2.6 | 1.33226762955019e-15 | -1.33226762955019e-15 |
| 741 | 1.7 | 2.65 | 2.77654247393814 | 2.73220889117187 | -0.12654247393814 | -0.0822088911718715 | 2.65 | 2.65000000000009 | 8.88178419700125e-16 | 9.10382880192628e-14 |
| 742 | 1.7 | 2.7 | 2.83374694906353 | 2.78739993335904 | -0.133746949063534 | -0.087399933359043 | 2.7 | 2.70000000000012 | 8.88178419700125e-16 | 1.20792265079217e-13 |
| 743 | 1.7 | 2.75 | 2.89117284313943 | 2.8425929930335 | -0.141172843139426 | -0.0925929930335028 | 2.75 | 2.74999999999999 | 4.44089209850063e-16 | -5.32907051820075e-15 |
| 744 | 1.7 | 2.8 | 2.94882274735559 | 2.89778807041674 | -0.148822747355594 | -0.09778807041674 | 2.8 | 2.79999999999992 | 8.88178419700125e-16 | -8.17124146124115e-14 |
| 745 | 1.7 | 2.85 | 3.00669930383927 | 2.95298516573024 | -0.156699303839273 | -0.102985165730245 | 2.85 | 2.85000000000006 | 1.77635683940025e-15 | 6.3504757008559e-14 |
| 746 | 1.7 | 2.9 | 3.06480520706814 | 3.00818427919495 | -0.164805207068139 | -0.108184279194951 | 2.9 | 2.90000000000006 | 0 | 5.86197757002083e-14 |
| 747 | 1.7 | 2.95 | 3.12314320533409 | 3.06338541103235 | -0.173143205334092 | -0.113385411032348 | 2.95 | 2.95 | 4.44089209850063e-16 | 1.33226762955019e-15 |
| 748 | 1.7 | 3 | 3.18171610226006 | 3.11858856146393 | -0.181716102260059 | -0.118588561463927 | 3 | 2.99999999999995 | 1.33226762955019e-15 | -4.79616346638068e-14 |
| 749 | 1.7 | 3.05 | 3.24052675837224 | 3.17379373071136 | -0.190526758372244 | -0.12379373071136 | 3.05 | 3.0500000000001 | 1.33226762955019e-15 | 9.9475983006414e-14 |
| 750 | 1.7 | 3.1 | 3.29957809273027 | 3.22900091899558 | -0.199578092730272 | -0.129000918995583 | 3.1 | 3.09999999999993 | 1.77635683940025e-15 | -7.105427357601e-14 |
| 751 | 1.7 | 3.15 | 3.35887308461787 | 3.28421012653883 | -0.208873084617874 | -0.134210126538826 | 3.15 | 3.15000000000006 | 1.33226762955019e-15 | 6.08402217494586e-14 |
| 752 | 1.7 | 3.2 | 3.41841477529688 | 3.33942135356221 | -0.218414775296882 | -0.139421353562207 | 3.2 | 3.20000000000007 | 4.44089209850063e-16 | 7.061018436616e-14 |
| 753 | 1.7 | 3.25 | 3.47820626982746 | 3.3946346002874 | -0.228206269827461 | -0.144634600287401 | 3.25 | 3.25000000000001 | 8.88178419700125e-16 | 4.88498130835069e-15 |
| 754 | 1.7 | 3.3 | 3.53825073895764 | 3.44984986693627 | -0.238250738957642 | -0.149849866936268 | 3.3 | 3.30000000000004 | 8.88178419700125e-16 | 3.68594044175552e-14 |
| 755 | 1.7 | 3.35 | 3.59855142108543 | 3.50506715373048 | -0.248551421085431 | -0.155067153730482 | 3.35 | 3.35000000000014 | 1.33226762955019e-15 | 1.37667655053519e-13 |
| 756 | 1.7 | 3.4 | 3.65911162429693 | 3.56028646089153 | -0.259111624296928 | -0.160286460891533 | 3.4 | 3.40000000000007 | -4.44089209850063e-16 | 7.32747196252603e-14 |
| 757 | 1.7 | 3.45 | 3.71993472848407 | 3.61550778864128 | -0.26993472848407 | -0.165507788641281 | 3.45 | 3.44999999999991 | 1.77635683940025e-15 | -9.10382880192628e-14 |
| 758 | 1.7 | 3.5 | 3.78102418754583 | 3.67073113720195 | -0.281024187545827 | -0.170731137201954 | 3.5 | 3.50000000000001 | 8.88178419700125e-16 | 6.66133814775094e-15 |
| 759 | 1.7 | 3.55 | 3.84238353167697 | 3.72595650679504 | -0.292383531676969 | -0.175956506795043 | 3.55 | 3.55000000000002 | 4.44089209850063e-16 | 2.26485497023532e-14 |
| 760 | 1.7 | 3.6 | 3.9040163697486 | 3.78118389764259 | -0.304016369748596 | -0.181183897642592 | 3.6 | 3.60000000000008 | -8.88178419700125e-16 | 7.90478793533112e-14 |
| 761 | 1.7 | 3.65 | 3.96592639178507 | 3.83641330996628 | -0.315926391785069 | -0.186413309966276 | 3.65 | 3.64999999999993 | 8.88178419700125e-16 | -7.37188088351104e-14 |
| 762 | 1.7 | 3.7 | 4.02811737154208 | 3.89164474398869 | -0.32811737154208 | -0.191644743988694 | 3.7 | 3.70000000000011 | 0 | 1.14130926931466e-13 |
| 763 | 1.7 | 3.75 | 4.09059316919102 | 3.94687819993115 | -0.34059316919102 | -0.196878199931151 | 3.75 | 3.74999999999999 | 1.77635683940025e-15 | -1.46549439250521e-14 |
| 764 | 1.7 | 3.8 | 4.15335773411498 | 4.00211367801625 | -0.353357734114979 | -0.202113678016246 | 3.8 | 3.80000000000002 | 4.44089209850063e-16 | 1.55431223447522e-14 |
| 765 | 1.7 | 3.85 | 4.21641510782219 | 4.05735117846584 | -0.36641510782219 | -0.20735117846584 | 3.85 | 3.84999999999998 | 4.44089209850063e-16 | -2.26485497023532e-14 |
| 766 | 1.7 | 3.9 | 4.27976942698292 | 4.11259070150235 | -0.379769426982916 | -0.212590701502346 | 3.9 | 3.9000000000001 | 1.77635683940025e-15 | 1.03916875104915e-13 |
| 767 | 1.7 | 3.95 | 4.3434249265963 | 4.16783224734763 | -0.393424926596302 | -0.217832247347625 | 3.95 | 3.9500000000001 | 1.33226762955019e-15 | 9.50350909079134e-14 |
| 768 | 1.7 | 4 | 4.40738594329402 | 4.22307581622391 | -0.407385943294019 | -0.223075816223905 | 4 | 3.99999999999994 | 0 | -5.59552404411079e-14 |
| 769 | 1.7 | 4.05 | 4.471656918788 | 4.27832140835397 | -0.421656918788001 | -0.228321408353971 | 4.05 | 4.05000000000011 | 0 | 1.11022302462516e-13 |
| 770 | 1.7 | 4.1 | 4.53624240347003 | 4.3335690239595 | -0.436242403470034 | -0.233569023959501 | 4.1 | 4.10000000000002 | 8.88178419700125e-16 | 1.77635683940025e-14 |
| 771 | 1.7 | 4.15 | 4.60114706017145 | 4.38881866326328 | -0.451147060171453 | -0.238818663263275 | 4.15 | 4.15000000000005 | 8.88178419700125e-16 | 4.52970994047064e-14 |
| 772 | 1.7 | 4.2 | 4.66637566809172 | 4.44407032648753 | -0.466375668091724 | -0.244070326487526 | 4.2 | 4.20000000000005 | 8.88178419700125e-16 | 4.88498130835069e-14 |
| 773 | 1.7 | 4.25 | 4.73193312690531 | 4.49932401385467 | -0.481933126905307 | -0.249324013854666 | 4.25 | 4.25 | 8.88178419700125e-16 | 8.88178419700125e-16 |
| 774 | 1.7 | 4.3 | 4.79782446105676 | 4.5545797255873 | -0.497824461056756 | -0.254579725587296 | 4.3 | 4.30000000000001 | 8.88178419700125e-16 | 1.06581410364015e-14 |
| 775 | 1.7 | 4.35 | 4.86405482425472 | 4.60983746190783 | -0.514054824254724 | -0.259837461907832 | 4.35 | 4.34999999999999 | 8.88178419700125e-16 | -1.4210854715202e-14 |
| 776 | 1.7 | 4.4 | 4.93062950417626 | 4.66509722303887 | -0.530629504176257 | -0.265097223038872 | 4.4 | 4.39999999999996 | 8.88178419700125e-16 | -4.08562073062058e-14 |
| 777 | 1.7 | 4.45 | 4.99755392739352 | 4.7203590092032 | -0.547553927393524 | -0.270359009203201 | 4.45 | 4.4500000000001 | 8.88178419700125e-16 | 9.59232693276135e-14 |
| 778 | 1.7 | 4.5 | 5.06483366453595 | 4.77562282062305 | -0.564833664535951 | -0.275622820623048 | 4.5 | 4.50000000000002 | 2.66453525910038e-15 | 2.22044604925031e-14 |
| 779 | 1.7 | 4.55 | 5.1324744357017 | 4.83088865752139 | -0.582474435701702 | -0.280888657521384 | 4.55 | 4.55 | 8.88178419700125e-16 | -1.77635683940025e-15 |
| 780 | 1.7 | 4.6 | 5.20048211613335 | 4.886156520121 | -0.600482116133352 | -0.286156520120995 | 4.6 | 4.60000000000008 | 8.88178419700125e-16 | 7.99360577730113e-14 |
| 781 | 1.7 | 4.65 | 5.26886274217369 | 4.94142640864429 | -0.618862742173689 | -0.291426408644292 | 4.65 | 4.64999999999995 | 0 | -5.32907051820075e-14 |
| 782 | 1.7 | 4.7 | 5.33762251751875 | 4.99669832331462 | -0.637622517518754 | -0.296698323314618 | 4.7 | 4.70000000000008 | 8.88178419700125e-16 | 8.34887714518118e-14 |
| 783 | 1.7 | 4.75 | 5.40676781978643 | 5.0519722643542 | -0.656767819786428 | -0.301972264354202 | 4.75 | 4.74999999999993 | 1.77635683940025e-15 | -6.66133814775094e-14 |
| 784 | 1.7 | 4.8 | 5.47630520742027 | 5.10724823198657 | -0.676305207420272 | -0.307248231986567 | 4.8 | 4.80000000000007 | 1.77635683940025e-15 | 6.92779167366098e-14 |
| 785 | 1.7 | 4.85 | 5.54624142694977 | 5.16252622643413 | -0.696241426949765 | -0.312526226434131 | 4.85 | 4.85000000000003 | 8.88178419700125e-16 | 3.19744231092045e-14 |
| 786 | 1.7 | 4.9 | 5.61658342062967 | 5.21780624792005 | -0.716583420629672 | -0.317806247920045 | 4.9 | 4.89999999999998 | 8.88178419700125e-16 | -1.59872115546023e-14 |
| 787 | 1.7 | 4.95 | 5.68733833448305 | 5.27308829666747 | -0.737338334483047 | -0.323088296667468 | 4.95 | 4.95000000000006 | 8.88178419700125e-16 | 5.86197757002083e-14 |
| 788 | 1.7 | 5 | 5.75851352677424 | 5.32837237289918 | -0.758513526774237 | -0.328372372899181 | 5 | 5.00000000000002 | 8.88178419700125e-16 | 1.50990331349021e-14 |
| 789 | 1.7 | 5.05 | 5.83011657694033 | 5.38365847683853 | -0.780116576940334 | -0.333658476838526 | 5.05 | 5.05000000000008 | 8.88178419700125e-16 | 7.63833440942108e-14 |
| 790 | 1.7 | 5.1 | 5.90215529501174 | 5.43894660870847 | -0.802155295011737 | -0.338946608708471 | 5.1 | 5.1000000000001 | 2.66453525910038e-15 | 9.41469124882133e-14 |
| 791 | 1.7 | 5.15 | 5.97463773155498 | 5.49423676873217 | -0.824637731554977 | -0.344236768732173 | 5.15 | 5.15000000000006 | 0 | 5.41788836017076e-14 |
| 792 | 1.7 | 5.2 | 6.04757218817368 | 5.54952895713297 | -0.847572188173678 | -0.349528957132972 | 5.2 | 5.20000000000007 | 0 | 6.92779167366098e-14 |
| 793 | 1.7 | 5.25 | 6.12096722860633 | 5.60482317413402 | -0.870967228606332 | -0.354823174134021 | 5.25 | 5.25000000000004 | 8.88178419700125e-16 | 4.44089209850063e-14 |
| 794 | 1.7 | 5.3 | 6.19483169046298 | 5.66011941995866 | -0.894831690462979 | -0.360119419958661 | 5.3 | 5.30000000000002 | 8.88178419700125e-16 | 2.30926389122033e-14 |
| 795 | 1.7 | 5.35 | 6.26917469764628 | 5.71541769483023 | -0.919174697646284 | -0.36541769483023 | 5.35 | 5.35 | 1.77635683940025e-15 | 3.5527136788005e-15 |
| 796 | 1.7 | 5.4 | 6.34400567350641 | 5.77071799897207 | -0.944005673506409 | -0.37071799897207 | 5.4 | 5.39999999999995 | 8.88178419700125e-16 | -4.61852778244065e-14 |
| 797 | 1.7 | 5.45 | 6.41933435478343 | 5.82602033260771 | -0.969334354783431 | -0.376020332607705 | 5.45 | 5.44999999999997 | 8.88178419700125e-16 | -3.37507799486048e-14 |
| 798 | 1.7 | 5.5 | 6.49517080639565 | 5.88132469596048 | -0.995170806395648 | -0.381324695960476 | 5.5 | 5.49999999999993 | 8.88178419700125e-16 | -6.57252030578093e-14 |
| 799 | 1.7 | 5.55 | 6.57152543713739 | 5.93663108925409 | -1.02152543713739 | -0.386631089254093 | 5.55 | 5.55000000000005 | 0 | 4.52970994047064e-14 |
| 800 | 1.7 | 5.6 | 6.64840901635565 | 5.99193951271171 | -1.04840901635565 | -0.391939512711707 | 5.6 | 5.59999999999995 | 8.88178419700125e-16 | -5.32907051820075e-14 |
| 801 | 1.7 | 5.65 | 6.72583269168115 | 6.0472499665574 | -1.07583269168115 | -0.397249966557403 | 5.65 | 5.65000000000009 | 8.88178419700125e-16 | 9.05941988094128e-14 |
| 802 | 1.7 | 5.7 | 6.80380800789657 | 6.10256245101433 | -1.10380800789657 | -0.402562451014333 | 5.7 | 5.70000000000005 | 8.88178419700125e-16 | 5.06261699229071e-14 |
| 803 | 1.7 | 5.75 | 6.88234692703223 | 6.15787696630639 | -1.13234692703223 | -0.407876966306393 | 5.75 | 5.75000000000003 | 1.77635683940025e-15 | 3.28626015289046e-14 |
| 804 | 1.7 | 5.8 | 6.9614618497884 | 6.21319351265729 | -1.1614618497884 | -0.413193512657291 | 5.8 | 5.80000000000004 | 1.77635683940025e-15 | 3.99680288865056e-14 |
| 805 | 1.7 | 5.85 | 7.04116563839283 | 6.26851209029074 | -1.19116563839283 | -0.418512090290741 | 5.85 | 5.85000000000004 | 0 | 4.08562073062058e-14 |
| 806 | 1.7 | 5.9 | 7.1214716410127 | 6.32383269943045 | -1.2214716410127 | -0.42383269943045 | 5.9 | 5.89999999999996 | 0 | -3.90798504668055e-14 |
| 807 | 1.7 | 5.95 | 7.20239371785236 | 6.3791553403005 | -1.25239371785236 | -0.429155340300498 | 5.95 | 5.95000000000003 | 8.88178419700125e-16 | 2.75335310107039e-14 |
| 808 | 1.7 | 6 | 7.28394626908116 | 6.4344800131246 | -1.28394626908116 | -0.434480013124595 | 6 | 6.00000000000009 | 1.77635683940025e-15 | 9.32587340685131e-14 |
| 809 | 1.8 | 1 | 0.999683741012632 | 0.913456501580219 | 0.000316258987368423 | 0.0865434984197806 | 1 | 1.00000000000012 | 8.88178419700125e-16 | 1.23456800338317e-13 |
| 810 | 1.8 | 1.05 | 1.05077105118278 | 0.96858114238522 | -0.000771051182782978 | 0.08141885761478 | 1.05 | 1.05000000000001 | 6.66133814775094e-16 | 1.04360964314765e-14 |
| 811 | 1.8 | 1.1 | 1.10201588746591 | 1.02370779340444 | -0.00201588746591197 | 0.076292206595562 | 1.1 | 1.09999999999999 | 4.44089209850063e-16 | -1.50990331349021e-14 |
| 812 | 1.8 | 1.15 | 1.15341971609161 | 1.0788364548577 | -0.0034197160916114 | 0.0711635451423025 | 1.15 | 1.14999999999995 | -2.22044604925031e-16 | -5.26245713672324e-14 |
| 813 | 1.8 | 1.2 | 1.20498402617736 | 1.13396712696519 | -0.00498402617735505 | 0.0660328730348074 | 1.2 | 1.2000000000001 | -4.44089209850063e-16 | 9.74775815620887e-14 |
| 814 | 1.8 | 1.25 | 1.25671033023183 | 1.18909980994656 | -0.00671033023183476 | 0.0609001900534376 | 1.25 | 1.2500000000001 | 8.88178419700125e-16 | 9.54791801177635e-14 |
| 815 | 1.8 | 1.3 | 1.30860016467262 | 1.244234504022 | -0.00860016467261615 | 0.0557654959779987 | 1.3 | 1.30000000000007 | 8.88178419700125e-16 | 6.88338275267597e-14 |
| 816 | 1.8 | 1.35 | 1.36065509035863 | 1.2993712094117 | -0.0106550903586298 | 0.0506287905882965 | 1.35 | 1.35000000000011 | 1.99840144432528e-15 | 1.07913677993565e-13 |
| 817 | 1.8 | 1.4 | 1.41287669313799 | 1.35450992633549 | -0.0128766931379878 | 0.0454900736645065 | 1.4 | 1.39999999999993 | 6.66133814775094e-16 | -6.83897383169096e-14 |
| 818 | 1.8 | 1.45 | 1.46526658441172 | 1.40965065501412 | -0.0152665844117157 | 0.0403493449858801 | 1.45 | 1.45000000000006 | 1.33226762955019e-15 | 6.08402217494586e-14 |
| 819 | 1.8 | 1.5 | 1.51782640171391 | 1.46479339566722 | -0.0178264017139091 | 0.0352066043327777 | 1.5 | 1.49999999999997 | 1.55431223447522e-15 | -2.59792187762287e-14 |
| 820 | 1.8 | 1.55 | 1.57055780930896 | 1.51993814851555 | -0.0205578093089631 | 0.03006185148445 | 1.55 | 1.55000000000012 | 4.44089209850063e-16 | 1.19459997449667e-13 |
| 821 | 1.8 | 1.6 | 1.62346249880646 | 1.57508491377893 | -0.0234624988064573 | 0.024915086221073 | 1.6 | 1.60000000000007 | 4.44089209850063e-16 | 7.01660951563099e-14 |
| 822 | 1.8 | 1.65 | 1.67654218979434 | 1.63023369167792 | -0.0265421897943385 | 0.019766308322082 | 1.65 | 1.65000000000003 | 1.99840144432528e-15 | 3.39728245535298e-14 |
| 823 | 1.8 | 1.7 | 1.72979863049109 | 1.6853844824329 | -0.0297986304910851 | 0.0146155175670983 | 1.7 | 1.70000000000001 | 1.55431223447522e-15 | 1.3988810110277e-14 |
| 824 | 1.8 | 1.75 | 1.78323359841756 | 1.74053728626444 | -0.0332335984175585 | 0.00946271373555718 | 1.75 | 1.75000000000015 | 1.11022302462516e-15 | 1.45439216225896e-13 |
| 825 | 1.8 | 1.8 | 1.83684890108924 | 1.79569210339255 | -0.0368489010892448 | 0.00430789660744946 | 1.8 | 1.80000000000002 | 1.77635683940025e-15 | 2.30926389122033e-14 |
| 826 | 1.8 | 1.85 | 1.89064637672967 | 1.85084893403816 | -0.0406463767296705 | -0.000848934038158999 | 1.85 | 1.85000000000004 | 1.11022302462516e-15 | 4.41868763800812e-14 |
| 827 | 1.8 | 1.9 | 1.94462789500579 | 1.90600777842165 | -0.0446278950057935 | -0.00600777842164768 | 1.9 | 1.90000000000007 | 0 | 6.63913368725844e-14 |
| 828 | 1.8 | 1.95 | 1.99879535778618 | 1.9611686367634 | -0.0487953577861828 | -0.0111686367633956 | 1.95 | 1.94999999999991 | 4.44089209850063e-16 | -8.92619311798626e-14 |
| 829 | 1.8 | 2 | 2.05315069992287 | 2.01633150928452 | -0.0531506999228664 | -0.0163315092845227 | 2 | 2.00000000000003 | 1.33226762955019e-15 | 3.28626015289046e-14 |
| 830 | 1.8 | 2.05 | 2.10769589005776 | 2.07149639620522 | -0.0576958900577602 | -0.0214963962052228 | 2.05 | 2.05000000000001 | 8.88178419700125e-16 | 1.28785870856518e-14 |
| 831 | 1.8 | 2.1 | 2.16243293145463 | 2.12666329774643 | -0.0624329314546301 | -0.0266632977464298 | 2.1 | 2.10000000000007 | 0 | 6.57252030578093e-14 |
| 832 | 1.8 | 2.15 | 2.21736386285757 | 2.18183221412871 | -0.0673638628575706 | -0.0318322141287091 | 2.15 | 2.15000000000003 | 4.44089209850063e-16 | 3.37507799486048e-14 |
| 833 | 1.8 | 2.2 | 2.27249075937705 | 2.23700314557299 | -0.0724907593770472 | -0.0370031455729944 | 2.2 | 2.20000000000006 | 2.22044604925031e-15 | 5.90638649100583e-14 |
| 834 | 1.8 | 2.25 | 2.32781573340459 | 2.29217609230004 | -0.0778157334045893 | -0.0421760923000356 | 2.25 | 2.25000000000008 | 8.88178419700125e-16 | 7.86037901434611e-14 |
| 835 | 1.8 | 2.3 | 2.3833409355573 | 2.34735105453058 | -0.0833409355572998 | -0.0473510545305813 | 2.3 | 2.29999999999999 | 1.77635683940025e-15 | -6.21724893790088e-15 |
| 836 | 1.8 | 2.35 | 2.43906855565332 | 2.40252803248575 | -0.0890685556533177 | -0.0525280324857507 | 2.35 | 2.35 | 0 | 3.10862446895044e-15 |
| 837 | 1.8 | 2.4 | 2.49500082371956 | 2.45770702638648 | -0.0950008237195594 | -0.057707026386479 | 2.4 | 2.4000000000001 | 4.44089209850063e-16 | 1.03472785895065e-13 |
| 838 | 1.8 | 2.45 | 2.55114001103298 | 2.51288803645352 | -0.101140011032979 | -0.0628880364535158 | 2.45 | 2.45000000000009 | 0 | 8.57092175010621e-14 |
| 839 | 1.8 | 2.5 | 2.60748843119678 | 2.56807106290798 | -0.107488431196784 | -0.0680710629079799 | 2.5 | 2.50000000000004 | 8.88178419700125e-16 | 3.90798504668055e-14 |
| 840 | 1.8 | 2.55 | 2.66404844125301 | 2.62325610597099 | -0.114048441253006 | -0.0732561059709909 | 2.55 | 2.55000000000002 | 1.33226762955019e-15 | 1.68753899743024e-14 |
| 841 | 1.8 | 2.6 | 2.72082244283297 | 2.67844316586367 | -0.120822442832969 | -0.0784431658636677 | 2.6 | 2.60000000000004 | 1.33226762955019e-15 | 3.64153152077051e-14 |
| 842 | 1.8 | 2.65 | 2.77781288334722 | 2.73363224280713 | -0.127812883347221 | -0.0836322428071301 | 2.65 | 2.65000000000008 | 1.77635683940025e-15 | 7.68274333040608e-14 |
| 843 | 1.8 | 2.7 | 2.83502225721661 | 2.7888233370225 | -0.135022257216612 | -0.088823337022498 | 2.7 | 2.70000000000008 | 4.44089209850063e-16 | 8.26005930321117e-14 |
| 844 | 1.8 | 2.75 | 2.89245310714628 | 2.84401644873108 | -0.142453107146276 | -0.0940164487310753 | 2.75 | 2.75000000000013 | 0 | 1.28341781646668e-13 |
| 845 | 1.8 | 2.8 | 2.95010802544433 | 2.89921157815398 | -0.15010802544433 | -0.0992115781539815 | 2.8 | 2.80000000000008 | 1.33226762955019e-15 | 8.30446822419617e-14 |
| 846 | 1.8 | 2.85 | 3.00798965538725 | 2.95440872551271 | -0.15798965538725 | -0.104408725512706 | 2.85 | 2.85000000000012 | 1.33226762955019e-15 | 1.16795462190566e-13 |
| 847 | 1.8 | 2.9 | 3.06610069263397 | 3.00960789102837 | -0.16610069263397 | -0.109607891028368 | 2.9 | 2.90000000000003 | 1.33226762955019e-15 | 2.53130849614536e-14 |
| 848 | 1.8 | 2.95 | 3.1244438866908 | 3.06480907492264 | -0.174443886690796 | -0.114809074922643 | 2.95 | 2.95000000000007 | 0 | 7.28306304154103e-14 |
| 849 | 1.8 | 3 | 3.18302204242945 | 3.12001227741665 | -0.18302204242945 | -0.12001227741665 | 3 | 2.99999999999998 | 8.88178419700125e-16 | -1.73194791841524e-14 |
| 850 | 1.8 | 3.05 | 3.24183802166055 | 3.17521749873225 | -0.191838021660548 | -0.125217498732248 | 3.05 | 3.05000000000011 | 4.44089209850063e-16 | 1.11910480882216e-13 |
| 851 | 1.8 | 3.1 | 3.30089474476508 | 3.23042473909056 | -0.200894744765085 | -0.130424739090557 | 3.1 | 3.10000000000011 | 4.44089209850063e-16 | 1.11466391672366e-13 |
| 852 | 1.8 | 3.15 | 3.36019519238648 | 3.28563399871307 | -0.210195192386484 | -0.135633998713066 | 3.15 | 3.14999999999993 | 2.22044604925031e-15 | -7.01660951563099e-14 |
| 853 | 1.8 | 3.2 | 3.41974240718604 | 3.34084527782182 | -0.219742407186039 | -0.14084527782182 | 3.2 | 3.19999999999998 | 1.33226762955019e-15 | -1.90958360235527e-14 |
| 854 | 1.8 | 3.25 | 3.47953949566465 | 3.39605857663812 | -0.229539495664652 | -0.146058576638124 | 3.25 | 3.24999999999997 | 0 | -2.57571741713036e-14 |
| 855 | 1.8 | 3.3 | 3.53958963005395 | 3.45127389538384 | -0.23958963005395 | -0.151273895383836 | 3.3 | 3.30000000000008 | 4.44089209850063e-16 | 8.26005930321117e-14 |
| 856 | 1.8 | 3.35 | 3.59989605028004 | 3.50649123428026 | -0.249896050280041 | -0.156491234280262 | 3.35 | 3.34999999999994 | 4.44089209850063e-16 | -5.95079541199084e-14 |
| 857 | 1.8 | 3.4 | 3.66046206600337 | 3.56171059354963 | -0.26046206600337 | -0.161710593549631 | 3.4 | 3.39999999999998 | 8.88178419700125e-16 | -1.59872115546023e-14 |
| 858 | 1.8 | 3.45 | 3.72129105873826 | 3.61693197341343 | -0.271291058738262 | -0.166931973413432 | 3.45 | 3.44999999999994 | 1.33226762955019e-15 | -5.90638649100583e-14 |
| 859 | 1.8 | 3.5 | 3.78238648405605 | 3.67215537409371 | -0.28238648405605 | -0.17215537409371 | 3.5 | 3.50000000000001 | 8.88178419700125e-16 | 6.21724893790088e-15 |
| 860 | 1.8 | 3.55 | 3.8437518738758 | 3.72738079581214 | -0.293751873875804 | -0.17738079581214 | 3.55 | 3.55 | 1.33226762955019e-15 | 1.77635683940025e-15 |
| 861 | 1.8 | 3.6 | 3.90539083884698 | 3.78260823879077 | -0.305390838846976 | -0.182608238790767 | 3.6 | 3.60000000000005 | 8.88178419700125e-16 | 5.01820807130571e-14 |
| 862 | 1.8 | 3.65 | 3.96730707082852 | 3.83783770325145 | -0.31730707082852 | -0.187837703251449 | 3.65 | 3.65000000000007 | 4.44089209850063e-16 | 6.57252030578093e-14 |
| 863 | 1.8 | 3.7 | 4.02950434546928 | 3.89306918941623 | -0.329504345469281 | -0.193069189416231 | 3.7 | 3.7000000000001 | 8.88178419700125e-16 | 9.68114477473137e-14 |
| 864 | 1.8 | 3.75 | 4.09198652489476 | 3.94830269750697 | -0.341986524894764 | -0.198302697506974 | 3.75 | 3.74999999999999 | 1.77635683940025e-15 | -1.24344978758018e-14 |
| 865 | 1.8 | 3.8 | 4.15475756050571 | 4.00353822774609 | -0.354757560505711 | -0.203538227746091 | 3.8 | 3.80000000000005 | -4.44089209850063e-16 | 4.48530101948563e-14 |
| 866 | 1.8 | 3.85 | 4.21782149589421 | 4.05877578035544 | -0.367821495894209 | -0.208775780355442 | 3.85 | 3.85000000000004 | 1.33226762955019e-15 | 3.95239396766556e-14 |
| 867 | 1.8 | 3.9 | 4.28118246988339 | 4.11401535555726 | -0.381182469883394 | -0.214015355557257 | 3.9 | 3.90000000000004 | 1.77635683940025e-15 | 3.86357612569555e-14 |
| 868 | 1.8 | 3.95 | 4.34484471969728 | 4.16925695357377 | -0.394844719697279 | -0.219256953573766 | 3.95 | 3.95000000000007 | 8.88178419700125e-16 | 7.37188088351104e-14 |
| 869 | 1.8 | 4 | 4.40881258426752 | 4.22450057462701 | -0.408812584267519 | -0.224500574627013 | 4 | 3.99999999999997 | 0 | -2.88657986402541e-14 |
| 870 | 1.8 | 4.05 | 4.47309050768445 | 4.2797462189396 | -0.423090507684448 | -0.229746218939598 | 4.05 | 4.05000000000002 | 1.77635683940025e-15 | 2.1316282072803e-14 |
| 871 | 1.8 | 4.1 | 4.53768304280012 | 4.33499388673357 | -0.437683042800119 | -0.234993886733566 | 4.1 | 4.09999999999998 | 0 | -2.04281036531029e-14 |
| 872 | 1.8 | 4.15 | 4.60259485499169 | 4.39024357823152 | -0.452594854991687 | -0.240243578231515 | 4.15 | 4.15000000000006 | 8.88178419700125e-16 | 6.21724893790088e-14 |
| 873 | 1.8 | 4.2 | 4.66783072609383 | 4.44549529365549 | -0.467830726093834 | -0.245495293655491 | 4.2 | 4.19999999999995 | 1.77635683940025e-15 | -4.9737991503207e-14 |
| 874 | 1.8 | 4.25 | 4.73339555850972 | 4.50074903322846 | -0.483395558509717 | -0.250749033228464 | 4.25 | 4.25000000000012 | 8.88178419700125e-16 | 1.22568621918617e-13 |
| 875 | 1.8 | 4.3 | 4.79929437951037 | 4.55600479717229 | -0.499294379510372 | -0.256004797172293 | 4.3 | 4.30000000000002 | 8.88178419700125e-16 | 1.50990331349021e-14 |
| 876 | 1.8 | 4.35 | 4.86553234573328 | 4.61126258570995 | -0.515532345733281 | -0.261262585709948 | 4.35 | 4.35000000000004 | 8.88178419700125e-16 | 3.90798504668055e-14 |
| 877 | 1.8 | 4.4 | 4.93211474789147 | 4.66652239906384 | -0.532114747891469 | -0.266522399063843 | 4.4 | 4.40000000000006 | 8.88178419700125e-16 | 5.50670620214078e-14 |
| 878 | 1.8 | 4.45 | 4.99904701570532 | 4.72178423745658 | -0.549047015705323 | -0.271784237456579 | 4.45 | 4.45000000000006 | 8.88178419700125e-16 | 6.30606677987089e-14 |
| 879 | 1.8 | 4.5 | 5.06633472307014 | 4.77704810111076 | -0.56633472307014 | -0.277048101110755 | 4.5 | 4.50000000000002 | 8.88178419700125e-16 | 2.30926389122033e-14 |
| 880 | 1.8 | 4.55 | 5.1339835934733 | 4.83231399024916 | -0.583983593473302 | -0.282313990249155 | 4.55 | 4.55000000000003 | 8.88178419700125e-16 | 2.57571741713036e-14 |
| 881 | 1.8 | 4.6 | 5.20199950567601 | 4.88758190509457 | -0.601999505676009 | -0.287581905094566 | 4.6 | 4.60000000000013 | 1.77635683940025e-15 | 1.25233157177718e-13 |
| 882 | 1.8 | 4.65 | 5.27038849967551 | 4.9428518458694 | -0.620388499675505 | -0.292851845869399 | 4.65 | 4.65 | 1.77635683940025e-15 | 2.66453525910038e-15 |
| 883 | 1.8 | 4.7 | 5.33915678296494 | 4.99812381279681 | -0.639156782964935 | -0.298123812796813 | 4.7 | 4.69999999999997 | 8.88178419700125e-16 | -2.66453525910038e-14 |
| 884 | 1.8 | 4.75 | 5.40831073710918 | 5.05339780609959 | -0.658310737109178 | -0.30339780609959 | 4.75 | 4.74999999999998 | 1.77635683940025e-15 | -1.95399252334028e-14 |
| 885 | 1.8 | 4.8 | 5.47785692465637 | 5.1086738260007 | -0.677856924656372 | -0.308673826000701 | 4.8 | 4.8000000000001 | 0 | 9.68114477473137e-14 |
| 886 | 1.8 | 4.85 | 5.54780209640634 | 5.16395187272275 | -0.697802096406337 | -0.313951872722746 | 4.85 | 4.85000000000003 | 1.77635683940025e-15 | 2.48689957516035e-14 |
| 887 | 1.8 | 4.9 | 5.61815319905865 | 5.21923194648906 | -0.718153199058647 | -0.319231946489063 | 4.9 | 4.9000000000001 | 2.66453525910038e-15 | 9.76996261670138e-14 |
| 888 | 1.8 | 4.95 | 5.68891738326491 | 5.27451404752244 | -0.738917383264913 | -0.324514047522437 | 4.95 | 4.95000000000011 | 8.88178419700125e-16 | 1.11022302462516e-13 |
| 889 | 1.8 | 5 | 5.76010201211168 | 5.32979817604584 | -0.760102012111675 | -0.32979817604584 | 5 | 4.99999999999999 | 8.88178419700125e-16 | -8.88178419700125e-15 |
| 890 | 1.8 | 5.05 | 5.83171467006238 | 5.38508433228261 | -0.781714670062379 | -0.335084332282611 | 5.05 | 5.04999999999996 | 8.88178419700125e-16 | -3.99680288865056e-14 |
| 891 | 1.8 | 5.1 | 5.9037631723892 | 5.4403725164559 | -0.803763172389196 | -0.340372516455903 | 5.1 | 5.10000000000004 | 8.88178419700125e-16 | 3.5527136788005e-14 |
| 892 | 1.8 | 5.15 | 5.97625557512788 | 5.49566272878869 | -0.826255575127877 | -0.345662728788689 | 5.15 | 5.15000000000004 | 8.88178419700125e-16 | 3.5527136788005e-14 |
| 893 | 1.8 | 5.2 | 6.04920018559154 | 5.55095496950431 | -0.849200185591535 | -0.350954969504306 | 5.2 | 5.20000000000007 | 8.88178419700125e-16 | 6.92779167366098e-14 |
| 894 | 1.8 | 5.25 | 6.12260557348219 | 5.60624923882591 | -0.872605573482191 | -0.35624923882591 | 5.25 | 5.25000000000005 | 1.77635683940025e-15 | 4.61852778244065e-14 |
| 895 | 1.8 | 5.3 | 6.19648058264216 | 5.66154553697684 | -0.89648058264216 | -0.361545536976841 | 5.3 | 5.3 | 8.88178419700125e-16 | 3.5527136788005e-15 |
| 896 | 1.8 | 5.35 | 6.2708343434909 | 5.71684386418062 | -0.920834343490903 | -0.366843864180623 | 5.35 | 5.35000000000011 | 8.88178419700125e-16 | 1.08357767203415e-13 |
| 897 | 1.8 | 5.4 | 6.34567628619683 | 5.77214422066023 | -0.945676286196828 | -0.372144220660227 | 5.4 | 5.39999999999999 | 8.88178419700125e-16 | -7.99360577730113e-15 |
| 898 | 1.8 | 5.45 | 6.42101615463787 | 5.82744660663955 | -0.971016154637866 | -0.377446606639548 | 5.45 | 5.45000000000009 | 8.88178419700125e-16 | 8.43769498715119e-14 |
| 899 | 1.8 | 5.5 | 6.49686402120931 | 5.88275102234174 | -0.996864021209309 | -0.38275102234174 | 5.5 | 5.50000000000011 | 1.77635683940025e-15 | 1.04805053524615e-13 |
| 900 | 1.8 | 5.55 | 6.57323030254262 | 5.93805746799033 | -1.02323030254262 | -0.388057467990328 | 5.55 | 5.55000000000008 | 8.88178419700125e-16 | 7.54951656745106e-14 |
| 901 | 1.8 | 5.6 | 6.65012577620467 | 5.99336594380884 | -1.05012577620467 | -0.393365943808836 | 5.6 | 5.59999999999998 | 1.77635683940025e-15 | -2.22044604925031e-14 |
| 902 | 1.8 | 5.65 | 6.7275615984532 | 6.04867645002098 | -1.0775615984532 | -0.398676450020976 | 5.65 | 5.64999999999993 | 0 | -7.28306304154103e-14 |
| 903 | 1.8 | 5.7 | 6.80554932313134 | 6.10398898685046 | -1.10554932313134 | -0.403988986850457 | 5.7 | 5.7 | 8.88178419700125e-16 | -1.77635683940025e-15 |
| 904 | 1.8 | 5.75 | 6.88410092179166 | 6.1593035545208 | -1.13410092179166 | -0.409303554520804 | 5.75 | 5.75000000000006 | 0 | 6.21724893790088e-14 |
| 905 | 1.8 | 5.8 | 6.96322880514929 | 6.21462015325573 | -1.16322880514929 | -0.414620153255726 | 5.8 | 5.80000000000012 | 8.88178419700125e-16 | 1.20792265079217e-13 |
| 906 | 1.8 | 5.85 | 7.04294584597255 | 6.26993878327875 | -1.19294584597255 | -0.419938783278749 | 5.85 | 5.84999999999998 | 8.88178419700125e-16 | -2.57571741713036e-14 |
| 907 | 1.8 | 5.9 | 7.12326540353109 | 6.32525944481414 | -1.22326540353109 | -0.425259444814138 | 5.9 | 5.90000000000005 | 0 | 4.88498130835069e-14 |
| 908 | 1.8 | 5.95 | 7.20420134973261 | 6.38058213808542 | -1.25420134973261 | -0.430582138085418 | 5.95 | 5.95000000000007 | 0 | 6.92779167366098e-14 |
| 909 | 1.8 | 6 | 7.28576809709324 | 6.43590686331648 | -1.28576809709324 | -0.435906863316483 | 6 | 6.00000000000006 | 1.77635683940025e-15 | 5.59552404411079e-14 |
| 910 | 1.9 | 1 | 1.00081362932319 | 0.91487747933087 | -0.000813629323185072 | 0.0851225206691296 | 1 | 1.00000000000004 | 1.33226762955019e-15 | 4.19664303308309e-14 |
| 911 | 1.9 | 1.05 | 1.05190441297873 | 0.970002171952385 | -0.00190441297872668 | 0.079997828047615 | 1.05 | 1.04999999999991 | -2.22044604925031e-16 | -9.30366894635881e-14 |
| 912 | 1.9 | 1.1 | 1.10315275497988 | 1.02512887479404 | -0.00315275497988443 | 0.074871125205962 | 1.1 | 1.10000000000009 | 2.22044604925031e-16 | 8.81517081552374e-14 |
| 913 | 1.9 | 1.15 | 1.15456012205786 | 1.0802575880751 | -0.00456012205786438 | 0.0697424119249011 | 1.15 | 1.14999999999998 | 4.44089209850063e-16 | -1.82076576038526e-14 |
| 914 | 1.9 | 1.2 | 1.20612800384275 | 1.13538831201613 | -0.00612800384275203 | 0.0646116879838685 | 1.2 | 1.20000000000012 | 4.44089209850063e-16 | 1.21902488103842e-13 |
| 915 | 1.9 | 1.25 | 1.25785791336716 | 1.19052104683659 | -0.00785791336716235 | 0.0594789531634099 | 1.25 | 1.25 | 1.99840144432528e-15 | 8.88178419700125e-16 |
| 916 | 1.9 | 1.3 | 1.30975138758421 | 1.24565579275704 | -0.00975138758421124 | 0.0543442072429614 | 1.3 | 1.30000000000008 | -4.44089209850063e-16 | 7.97140131680862e-14 |
| 917 | 1.9 | 1.35 | 1.36180998790035 | 1.30079254999712 | -0.0118099879003546 | 0.0492074500028832 | 1.35 | 1.34999999999995 | 4.44089209850063e-16 | -5.50670620214078e-14 |
| 918 | 1.9 | 1.4 | 1.41403530072354 | 1.35593131877739 | -0.0140353007235361 | 0.0440686812226112 | 1.4 | 1.39999999999999 | 2.22044604925031e-16 | -1.4432899320127e-14 |
| 919 | 1.9 | 1.45 | 1.46642893802728 | 1.41107209931786 | -0.0164289380272826 | 0.0389279006821366 | 1.45 | 1.45000000000005 | 1.99840144432528e-15 | 5.08482145278322e-14 |
| 920 | 1.9 | 1.5 | 1.51899253793123 | 1.46621489183874 | -0.0189925379312326 | 0.033785108161265 | 1.5 | 1.50000000000012 | 1.77635683940025e-15 | 1.21014309684142e-13 |
| 921 | 1.9 | 1.55 | 1.57172776529875 | 1.52135969656001 | -0.0217277652987535 | 0.0286403034399869 | 1.55 | 1.54999999999997 | 8.88178419700125e-16 | -2.70894418008538e-14 |
| 922 | 1.9 | 1.6 | 1.62463631235222 | 1.57650651370245 | -0.0246363123522193 | 0.0234934862975533 | 1.6 | 1.60000000000002 | 1.33226762955019e-15 | 1.70974345792274e-14 |
| 923 | 1.9 | 1.65 | 1.67771989930661 | 1.63165534348605 | -0.0277198993066072 | 0.0183446565139545 | 1.65 | 1.64999999999996 | 1.11022302462516e-15 | -4.2632564145606e-14 |
| 924 | 1.9 | 1.7 | 1.7309802750221 | 1.68680618613137 | -0.0309802750220969 | 0.0131938138686272 | 1.7 | 1.69999999999996 | 6.66133814775094e-16 | -3.70814490224802e-14 |
| 925 | 1.9 | 1.75 | 1.78441921767636 | 1.74195904185881 | -0.0344192176763565 | 0.00804095814119088 | 1.75 | 1.75 | 8.88178419700125e-16 | 1.11022302462516e-15 |
| 926 | 1.9 | 1.8 | 1.83803853545725 | 1.79711391088873 | -0.0380385354572477 | 0.00288608911126698 | 1.8 | 1.8 | 8.88178419700125e-16 | 1.55431223447522e-15 |
| 927 | 1.9 | 1.85 | 1.89184006727672 | 1.85227079344171 | -0.0418400672767247 | -0.00227079344170877 | 1.85 | 1.85000000000002 | 8.88178419700125e-16 | 2.37587727269784e-14 |
| 928 | 1.9 | 1.9 | 1.94582568350671 | 1.90742968973812 | -0.0458256835067135 | -0.00742968973811586 | 1.9 | 1.89999999999993 | 6.66133814775094e-16 | -7.41628980449605e-14 |
| 929 | 1.9 | 1.95 | 1.99999728673781 | 1.96259059999889 | -0.0499972867378098 | -0.0125905999988885 | 1.95 | 1.95000000000003 | 1.77635683940025e-15 | 3.01980662698043e-14 |
| 930 | 1.9 | 2 | 2.05435681256166 | 2.01775352444422 | -0.05435681256166 | -0.0177535244442213 | 2 | 1.99999999999995 | 8.88178419700125e-16 | -4.68514116391816e-14 |
| 931 | 1.9 | 2.05 | 2.10890623037795 | 2.07291846329523 | -0.0589062303779526 | -0.0229184632952331 | 2.05 | 2.05000000000011 | 4.44089209850063e-16 | 1.12354570092066e-13 |
| 932 | 1.9 | 2.1 | 2.16364754422695 | 2.12808541677212 | -0.0636475442269462 | -0.0280854167721185 | 2.1 | 2.10000000000005 | 1.77635683940025e-15 | 5.15143483426073e-14 |
| 933 | 1.9 | 2.15 | 2.21858279364855 | 2.183254385096 | -0.0685827936485448 | -0.0332543850959963 | 2.15 | 2.15000000000011 | 4.44089209850063e-16 | 1.14130926931466e-13 |
| 934 | 1.9 | 2.2 | 2.27371405456898 | 2.23842536848743 | -0.0737140545689781 | -0.0384253684874323 | 2.2 | 2.20000000000011 | 0 | 1.07025499573865e-13 |
| 935 | 1.9 | 2.25 | 2.32904344021614 | 2.29359836716736 | -0.0790434402161386 | -0.0435983671673599 | 2.25 | 2.25000000000013 | 1.33226762955019e-15 | 1.34114941374719e-13 |
| 936 | 1.9 | 2.3 | 2.38457310206474 | 2.34877338135653 | -0.0845731020647418 | -0.0487733813565283 | 2.3 | 2.30000000000009 | 1.33226762955019e-15 | 9.41469124882133e-14 |
| 937 | 1.9 | 2.35 | 2.44030523081252 | 2.40395041127587 | -0.0903052308125227 | -0.0539504112758715 | 2.35 | 2.35000000000002 | 8.88178419700125e-16 | 1.90958360235527e-14 |
| 938 | 1.9 | 2.4 | 2.49624205738868 | 2.45912945714651 | -0.0962420573886793 | -0.0591294571465095 | 2.4 | 2.40000000000007 | 1.33226762955019e-15 | 7.061018436616e-14 |
| 939 | 1.9 | 2.45 | 2.55238585399591 | 2.51431051918919 | -0.102385853995905 | -0.0643105191891924 | 2.45 | 2.45000000000004 | -4.44089209850063e-16 | 3.95239396766556e-14 |
| 940 | 1.9 | 2.5 | 2.60873893518739 | 2.56949359762504 | -0.108738935187394 | -0.0694935976250384 | 2.5 | 2.50000000000001 | 0 | 1.4210854715202e-14 |
| 941 | 1.9 | 2.55 | 2.66530365898023 | 2.62467869267517 | -0.11530365898023 | -0.0746786926751679 | 2.55 | 2.55000000000005 | 1.33226762955019e-15 | 4.75175454539567e-14 |
| 942 | 1.9 | 2.6 | 2.72208242800671 | 2.67986580456051 | -0.122082428006709 | -0.0798658045605141 | 2.6 | 2.59999999999999 | 8.88178419700125e-16 | -1.33226762955019e-14 |
| 943 | 1.9 | 2.65 | 2.77907769070517 | 2.73505493350238 | -0.129077690705169 | -0.085054933502382 | 2.65 | 2.64999999999998 | 4.44089209850063e-16 | -2.08721928629529e-14 |
| 944 | 1.9 | 2.7 | 2.836291942552 | 2.79024607972208 | -0.136291942552004 | -0.0902460797220765 | 2.7 | 2.70000000000014 | 8.88178419700125e-16 | 1.35447209004269e-13 |
| 945 | 1.9 | 2.75 | 2.8937277273366 | 2.84543924344035 | -0.143727727336599 | -0.0954392434403468 | 2.75 | 2.75000000000003 | 8.88178419700125e-16 | 2.62012633811537e-14 |
| 946 | 1.9 | 2.8 | 2.95138763848106 | 2.90063442487887 | -0.151387638481057 | -0.100634424878867 | 2.8 | 2.80000000000002 | 1.33226762955019e-15 | 2.22044604925031e-14 |
| 947 | 1.9 | 2.85 | 3.00927432040663 | 2.95583162425876 | -0.15927432040663 | -0.105831624258757 | 2.85 | 2.84999999999996 | 0 | -4.2188474935756e-14 |
| 948 | 1.9 | 2.9 | 3.06739046994892 | 3.01103084180151 | -0.167390469948916 | -0.111030841801505 | 2.9 | 2.89999999999996 | 8.88178419700125e-16 | -3.73034936274053e-14 |
| 949 | 1.9 | 2.95 | 3.12573883782393 | 3.06623207772842 | -0.175738837823925 | -0.116232077728418 | 2.95 | 2.94999999999997 | 4.44089209850063e-16 | -3.50830475781549e-14 |
| 950 | 1.9 | 3 | 3.18432223014732 | 3.12143533226098 | -0.184322230147322 | -0.121435332260984 | 3 | 3.00000000000002 | 8.88178419700125e-16 | 2.26485497023532e-14 |
| 951 | 1.9 | 3.05 | 3.24314351000917 | 3.17664060562051 | -0.193143510009174 | -0.126640605620506 | 3.05 | 3.04999999999999 | 1.33226762955019e-15 | -1.19904086659517e-14 |
| 952 | 1.9 | 3.1 | 3.30220559910673 | 3.23184789802866 | -0.202205599106731 | -0.131847898028661 | 3.1 | 3.10000000000002 | 8.88178419700125e-16 | 1.50990331349021e-14 |
| 953 | 1.9 | 3.15 | 3.36151147943786 | 3.28705720970694 | -0.211511479437864 | -0.137057209706938 | 3.15 | 3.15000000000005 | 0 | 5.10702591327572e-14 |
| 954 | 1.9 | 3.2 | 3.42106419505794 | 3.34226854087682 | -0.22106419505794 | -0.142268540876824 | 3.2 | 3.20000000000001 | 8.88178419700125e-16 | 5.32907051820075e-15 |
| 955 | 1.9 | 3.25 | 3.48086685390305 | 3.39748189176018 | -0.230866853903049 | -0.147481891760183 | 3.25 | 3.25000000000009 | 4.44089209850063e-16 | 9.01501095995627e-14 |
| 956 | 1.9 | 3.3 | 3.54092262968271 | 3.45269726257832 | -0.24092262968271 | -0.152697262578315 | 3.3 | 3.29999999999997 | 1.33226762955019e-15 | -2.62012633811537e-14 |
| 957 | 1.9 | 3.35 | 3.60123476384527 | 3.50791465355345 | -0.25123476384527 | -0.157914653553453 | 3.35 | 3.35000000000013 | 1.33226762955019e-15 | 1.29229960066368e-13 |
| 958 | 1.9 | 3.4 | 3.66180656761948 | 3.56313406490671 | -0.261806567619478 | -0.163134064906714 | 3.4 | 3.39999999999998 | 0 | -1.59872115546023e-14 |
| 959 | 1.9 | 3.45 | 3.72264142413585 | 3.61835549686051 | -0.272641424135853 | -0.168355496860514 | 3.45 | 3.45000000000011 | 0 | 1.04805053524615e-13 |
| 960 | 1.9 | 3.5 | 3.78374279063169 | 3.67357894963616 | -0.283742790631689 | -0.173578949636158 | 3.5 | 3.50000000000001 | 8.88178419700125e-16 | 1.37667655053519e-14 |
| 961 | 1.9 | 3.55 | 3.84511420074378 | 3.72880442345587 | -0.295114200743782 | -0.178804423455874 | 3.55 | 3.55000000000004 | 1.33226762955019e-15 | 3.64153152077051e-14 |
| 962 | 1.9 | 3.6 | 3.90675926689316 | 3.78403191854134 | -0.306759266893161 | -0.184031918541338 | 3.6 | 3.59999999999996 | -4.44089209850063e-16 | -4.35207425653061e-14 |
| 963 | 1.9 | 3.65 | 3.96868168276641 | 3.83926143511478 | -0.318681682766405 | -0.189261435114778 | 3.65 | 3.65000000000003 | 1.77635683940025e-15 | 2.48689957516035e-14 |
| 964 | 1.9 | 3.7 | 4.0308852258983 | 3.89449297339806 | -0.330885225898304 | -0.194492973398055 | 3.7 | 3.70000000000012 | 0 | 1.21236354289067e-13 |
| 965 | 1.9 | 3.75 | 4.09337376036109 | 3.94972653361284 | -0.343373760361092 | -0.199726533612844 | 3.75 | 3.74999999999992 | 8.88178419700125e-16 | -7.99360577730113e-14 |
| 966 | 1.9 | 3.8 | 4.15615123956552 | 4.00496211598193 | -0.35615123956552 | -0.204962115981928 | 3.8 | 3.80000000000006 | 4.44089209850063e-16 | 6.26165785888588e-14 |
| 967 | 1.9 | 3.85 | 4.21922170917965 | 4.0601997207268 | -0.369221709179655 | -0.210199720726798 | 3.85 | 3.84999999999999 | 2.22044604925031e-15 | -1.46549439250521e-14 |
| 968 | 1.9 | 3.9 | 4.28258931017139 | 4.11543934807005 | -0.382589310171388 | -0.215439348070052 | 3.9 | 3.90000000000009 | 8.88178419700125e-16 | 8.61533067109122e-14 |
| 969 | 1.9 | 3.95 | 4.34625828198123 | 4.17068099823355 | -0.396258281981233 | -0.220680998233552 | 3.95 | 3.95000000000006 | 8.88178419700125e-16 | 6.3504757008559e-14 |
| 970 | 1.9 | 4 | 4.41023296583219 | 4.22592467143971 | -0.410232965832186 | -0.22592467143971 | 4 | 4.00000000000008 | -4.44089209850063e-16 | 7.63833440942108e-14 |
| 971 | 1.9 | 4.05 | 4.47451780818406 | 4.28117036791076 | -0.424517808184057 | -0.231170367910758 | 4.05 | 4.05000000000008 | 8.88178419700125e-16 | 8.08242361927114e-14 |
| 972 | 1.9 | 4.1 | 4.53911736433997 | 4.33641808786892 | -0.439117364339969 | -0.236418087868924 | 4.1 | 4.1 | 8.88178419700125e-16 | -3.5527136788005e-15 |
| 973 | 1.9 | 4.15 | 4.60403630221338 | 4.39166783153681 | -0.45403630221338 | -0.241667831536808 | 4.15 | 4.15000000000004 | 1.77635683940025e-15 | 4.08562073062058e-14 |
| 974 | 1.9 | 4.2 | 4.66927940626437 | 4.44691959913664 | -0.469279406264366 | -0.246919599136641 | 4.2 | 4.20000000000006 | 0 | 6.03961325396085e-14 |
| 975 | 1.9 | 4.25 | 4.73485158161464 | 4.50217339089084 | -0.484851581614639 | -0.252173390890836 | 4.25 | 4.25000000000003 | 1.77635683940025e-15 | 2.93098878501041e-14 |
| 976 | 1.9 | 4.3 | 4.80075785835122 | 4.55742920702199 | -0.500757858351221 | -0.257429207021993 | 4.3 | 4.30000000000006 | 8.88178419700125e-16 | 5.59552404411079e-14 |
| 977 | 1.9 | 4.35 | 4.86700339602957 | 4.61268704775253 | -0.51700339602957 | -0.262687047752529 | 4.35 | 4.35000000000004 | 0 | 4.44089209850063e-14 |
| 978 | 1.9 | 4.4 | 4.93359348838747 | 4.66794691330504 | -0.533593488387472 | -0.267946913305039 | 4.4 | 4.40000000000002 | 8.88178419700125e-16 | 2.39808173319034e-14 |
| 979 | 1.9 | 4.45 | 5.00053356828193 | 4.72320880390213 | -0.550533568281929 | -0.273208803902127 | 4.45 | 4.44999999999999 | 0 | -7.105427357601e-15 |
| 980 | 1.9 | 4.5 | 5.06782921286209 | 4.77847271976658 | -0.567829212862089 | -0.278472719766576 | 4.5 | 4.50000000000008 | 1.77635683940025e-15 | 7.90478793533112e-14 |
| 981 | 1.9 | 4.55 | 5.13548614899211 | 4.8337386611208 | -0.585486148992111 | -0.2837386611208 | 4.55 | 4.55000000000003 | 1.77635683940025e-15 | 3.37507799486048e-14 |
| 982 | 1.9 | 4.6 | 5.20351025893894 | 4.88900662818777 | -0.603510258938935 | -0.289006628187772 | 4.6 | 4.60000000000008 | 8.88178419700125e-16 | 8.34887714518118e-14 |
| 983 | 1.9 | 4.65 | 5.27190758634092 | 4.94427662119009 | -0.621907586340923 | -0.294276621190087 | 4.65 | 4.65000000000007 | 0 | 6.92779167366098e-14 |
| 984 | 1.9 | 4.7 | 5.34068434247449 | 4.99954864035053 | -0.64068434247449 | -0.299548640350533 | 4.7 | 4.69999999999998 | 8.88178419700125e-16 | -2.48689957516035e-14 |
| 985 | 1.9 | 4.75 | 5.40984691283717 | 5.05482268589208 | -0.659846912837166 | -0.304822685892079 | 4.75 | 4.74999999999991 | 8.88178419700125e-16 | -9.14823772291129e-14 |
| 986 | 1.9 | 4.8 | 5.47940186406679 | 5.11009875803788 | -0.679401864066793 | -0.31009875803788 | 4.8 | 4.80000000000011 | 1.77635683940025e-15 | 1.10134124042816e-13 |
| 987 | 1.9 | 4.85 | 5.5493559512181 | 5.16537685701017 | -0.699355951218095 | -0.315376857010166 | 4.85 | 4.84999999999995 | 1.77635683940025e-15 | -5.32907051820075e-14 |
| 988 | 1.9 | 4.9 | 5.61971612541944 | 5.22065698303265 | -0.719716125419443 | -0.320656983032644 | 4.9 | 4.90000000000008 | 0 | 8.26005930321117e-14 |
| 989 | 1.9 | 4.95 | 5.69048954193438 | 5.27593913632773 | -0.740489541934378 | -0.325939136327733 | 4.95 | 4.94999999999998 | 8.88178419700125e-16 | -1.95399252334028e-14 |
| 990 | 1.9 | 5 | 5.76168356865435 | 5.33122331711896 | -0.761683568654345 | -0.331223317118956 | 5 | 5.00000000000007 | 8.88178419700125e-16 | 6.83897383169096e-14 |
| 991 | 1.9 | 5.05 | 5.83330579505119 | 5.3865095256291 | -0.783305795051192 | -0.336509525629098 | 5.05 | 5.05000000000006 | 8.88178419700125e-16 | 6.21724893790088e-14 |
| 992 | 1.9 | 5.1 | 5.90536404162021 | 5.44179776208131 | -0.805364041620204 | -0.341797762081312 | 5.1 | 5.09999999999998 | 8.88178419700125e-16 | -1.86517468137026e-14 |
| 993 | 1.9 | 5.15 | 5.97786636984693 | 5.49708802669894 | -0.827866369846932 | -0.34708802669894 | 5.15 | 5.14999999999998 | 1.77635683940025e-15 | -2.39808173319034e-14 |
| 994 | 1.9 | 5.2 | 6.05082109273378 | 5.55238031970514 | -0.850821092733781 | -0.352380319705137 | 5.2 | 5.19999999999999 | 0 | -1.15463194561016e-14 |
| 995 | 1.9 | 5.25 | 6.12423678592528 | 5.60767464132324 | -0.874236785925278 | -0.357674641323242 | 5.25 | 5.25000000000009 | 8.88178419700125e-16 | 9.32587340685131e-14 |
| 996 | 1.9 | 5.3 | 6.19812229947412 | 5.66297099177622 | -0.898122299474118 | -0.362970991776225 | 5.3 | 5.29999999999999 | 8.88178419700125e-16 | -7.99360577730113e-15 |
| 997 | 1.9 | 5.35 | 6.27248677029374 | 5.7182693712878 | -0.92248677029374 | -0.368269371287794 | 5.35 | 5.35000000000002 | 1.77635683940025e-15 | 2.04281036531029e-14 |
| 998 | 1.9 | 5.4 | 6.34733963534702 | 5.77356978008111 | -0.947339635347014 | -0.373569780081107 | 5.4 | 5.39999999999997 | 8.88178419700125e-16 | -2.66453525910038e-14 |
| 999 | 1.9 | 5.45 | 6.42269064562493 | 5.82887221837987 | -0.97269064562493 | -0.378872218379873 | 5.45 | 5.45000000000011 | 8.88178419700125e-16 | 1.12798659301916e-13 |
| 1000 | 1.9 | 5.5 | 6.49854988097389 | 5.88417668640706 | -0.998549880973888 | -0.38417668640706 | 5.5 | 5.49999999999999 | 8.88178419700125e-16 | -7.99360577730113e-15 |
