# Supplementary material for: Creatinine assay interferences compromises MELD accuracy and may bias liver allocation
Source: Nat Commun. 2026 Jul 23;17:7111. doi: 10.1038/s41467-026-75011-x (PMC13396164; doi:10.1038/s41467-026-75011-x)
Supplement: Supplementary file 4 — Source Data [file 41467_2026_75011_MOESM4_ESM.zip › figshare_package_FINAL_PUBLIC_DEPOSIT_V1_20260503_002637/00_START_HERE_HTML_NAVIGATOR/file_views/view_0028_README_F1_submission_ready_v02.html]

02\_workflows/F1\_workflow\_v02/submission\_ready/README\_F1\_submission\_ready\_v02.txt

# Readable file view

02\_workflows/F1\_workflow\_v02/submission\_ready/README\_F1\_submission\_ready\_v02.txt

← Back to navigator   |   Open original package file

Section

Workflow readmes

Output

F1

Extension

txt

Size KB

0.677

Variables

0

## Readable HTML view

```
F1 submission-ready output structure

Use the files under:
  public/data/
  public/figures/

for public submission.

The files under:
  internal/data/
  internal/validation/
  internal/figures/

are retained for local traceability, QC, audit, and workflow documentation.

Important F1 release rule:
  expm_F1_array_input_public.csv is the public curated experimental array dataset.
  In this file, TB_trial_M_mg_dL and trial_display_Cre_M_mg_dL are rounded to two decimal places before correction/model processing.
  Unrounded audit columns are not included in the public file.

Do not use legacy flat folders directly if present from older workflow runs.
Use public/ for the release package.
```
