# Supplementary material for: Creatinine assay interferences compromises MELD accuracy and may bias liver allocation
Source: Nat Commun. 2026 Jul 23;17:7111. doi: 10.1038/s41467-026-75011-x (PMC13396164; doi:10.1038/s41467-026-75011-x)
Supplement: Supplementary file 4 — Source Data [file 41467_2026_75011_MOESM4_ESM.zip › figshare_package_FINAL_PUBLIC_DEPOSIT_V1_20260503_002637/00_START_HERE_HTML_NAVIGATOR/file_views/view_0012_slco_F1_surface_meta_public.html]

02\_workflows/F1\_workflow\_v02/submission\_ready/public/data/slco\_F1\_surface\_meta\_public.csv

# Readable file view

02\_workflows/F1\_workflow\_v02/submission\_ready/public/data/slco\_F1\_surface\_meta\_public.csv

← Back to navigator   |   Open original package file

Section

Manuscript output data

Output

F1

Extension

csv

Size KB

0.482

Variables

2

## Variables in this file

| Variable | Label | Description | Unit | Type |
| --- | --- | --- | --- | --- |
| parameter | Metadata parameter name | Name of a metadata parameter describing the F2 simulated heatmap object, such as figure identity, data origin, grid type, axis variable, or unit/role. |  | character |
| value | Value | Numerical or character value corresponding to the row-specific variable/metric. |  | character |

## Readable HTML view

Showing all 16 rows.

| parameter | value |
| --- | --- |
| dataset\_name | slco\_F1\_surface\_grid\_public |
| domain | slco |
| anchor | F1 |
| data\_object | surface |
| unit\_or\_role | grid |
| release\_status | public |
| source\_model\_coefficients | expm\_F1\_model\_coefficients\_public.csv |
| grid\_tb\_min\_mg\_dL | 1 |
| grid\_tb\_max\_mg\_dL | 35 |
| grid\_tb\_step\_mg\_dL | 0.1 |
| grid\_cre\_true\_min\_mg\_dL | 1 |
| grid\_cre\_true\_max\_mg\_dL | 6 |
| grid\_cre\_true\_step\_mg\_dL | 0.05 |
| n\_grid\_rows | 34441 |
| surface\_reference\_file | slco\_F1\_surface\_grid\_reference\_public.csv |
| surface\_rebuild\_qc\_file | slco\_F1\_surface\_rebuild\_qc\_public.csv |
