# Supplementary material for: Creatinine assay interferences compromises MELD accuracy and may bias liver allocation
Source: Nat Commun. 2026 Jul 23;17:7111. doi: 10.1038/s41467-026-75011-x (PMC13396164; doi:10.1038/s41467-026-75011-x)
Supplement: Supplementary file 4 — Source Data [file 41467_2026_75011_MOESM4_ESM.zip › figshare_package_FINAL_PUBLIC_DEPOSIT_V1_20260503_002637/00_START_HERE_HTML_NAVIGATOR/file_views/view_0004_f1_simulated_surface_repository.html]

01\_primary\_data/public/f1\_simulated\_surface\_repository.csv

# Readable file view

01\_primary\_data/public/f1\_simulated\_surface\_repository.csv

← Back to navigator   |   Open original package file

Section

Public primary data

Output

F1

Extension

csv

Size KB

4936.887

Variables

11

## Variables in this file

| Variable | Label | Description | Unit | Type |
| --- | --- | --- | --- | --- |
| cre\_true\_mg\_dL | True creatinine concentration | True creatinine concentration used as the gravimetric/reference concentration in F1. |  | numeric |
| cree\_measured\_pred\_mg\_dL | Predicted measured enzymatic creatinine concentration | Predicted measured enzymatic creatinine concentration derived from the interference model. |  | numeric |
| cree\_true\_recalc\_error | Recalculation error for enzymatic creatinine | Numerical difference between recalculated and expected true enzymatic creatinine values. |  | numeric |
| cree\_true\_recalc\_mg\_dL | Recalculated true enzymatic creatinine concentration | True enzymatic creatinine concentration recalculated from the model. |  | numeric |
| crej\_measured\_pred\_mg\_dL | Predicted measured Jaffe creatinine concentration | Predicted measured Jaffe creatinine concentration derived from the interference model. |  | numeric |
| crej\_true\_recalc\_error | Recalculation error for Jaffe creatinine | Numerical difference between recalculated and expected true Jaffe creatinine values. |  | numeric |
| crej\_true\_recalc\_mg\_dL | Recalculated true Jaffe creatinine concentration | True Jaffe creatinine concentration recalculated from the model. |  | numeric |
| delta\_cree\_to\_gravimetry\_mg\_dL | Enzymatic creatinine deviation from gravimetry | Difference between enzymatic creatinine and the gravimetric/reference value. |  | numeric |
| delta\_crej\_to\_gravimetry\_mg\_dL | Jaffe creatinine deviation from gravimetry | Difference between Jaffe creatinine and the gravimetric/reference value. |  | numeric |
| grid\_id | Surface-grid identifier | Identifier of a grid point in the F1 simulated or reconstructed surface data. |  | integer |
| tb\_mg\_dL | Total bilirubin concentration | Total bilirubin concentration used in the creatinine/bilirubin interference model or figure data. |  | numeric |

## Readable HTML view

Preview shows first 1000 of 34441 rows. Open the original file for full content.

| grid\_id | tb\_mg\_dL | cre\_true\_mg\_dL | crej\_measured\_pred\_mg\_dL | cree\_measured\_pred\_mg\_dL | delta\_crej\_to\_gravimetry\_mg\_dL | delta\_cree\_to\_gravimetry\_mg\_dL | crej\_true\_recalc\_mg\_dL | cree\_true\_recalc\_mg\_dL | crej\_true\_recalc\_error | cree\_true\_recalc\_error |
| --- | --- | --- | --- | --- | --- | --- | --- | --- | --- | --- |
| 1 | 1 | 1 | 0.990463857965053 | 0.902064914425904 | 0.00953614203494679 | 0.0979350855740958 | 1 | 1 | 6.66133814775094e-16 | -7.7715611723761e-16 |
| 2 | 1 | 1.05 | 1.04152284217999 | 0.957189139835594 | 0.00847715782000691 | 0.092810860164406 | 1.05 | 1.04999999999996 | 1.55431223447522e-15 | -4.06341627012807e-14 |
| 3 | 1 | 1.1 | 1.09273908981855 | 1.01231537541398 | 0.00726091018144937 | 0.0876846245860179 | 1.1 | 1.09999999999994 | 2.22044604925031e-16 | -5.97299987248334e-14 |
| 4 | 1 | 1.15 | 1.14411406302484 | 1.06744362138108 | 0.00588593697515871 | 0.0825563786189223 | 1.15 | 1.15000000000002 | 2.22044604925031e-16 | 1.79856129989275e-14 |
| 5 | 1 | 1.2 | 1.19564924674134 | 1.12257387795652 | 0.00435075325865664 | 0.0774261220434804 | 1.2 | 1.1999999999999 | 1.33226762955019e-15 | -1.03916875104915e-13 |
| 6 | 1 | 1.25 | 1.2473461492097 | 1.17770614536069 | 0.00265385079029912 | 0.0722938546393128 | 1.25 | 1.24999999999991 | 0 | -8.63753513158372e-14 |
| 7 | 1 | 1.3 | 1.29920630248577 | 1.2328404238134 | 0.000793697514233971 | 0.0671595761865953 | 1.3 | 1.29999999999987 | -2.22044604925031e-16 | -1.30340183090993e-13 |
| 8 | 1 | 1.35 | 1.35123126296935 | 1.28797671353505 | -0.00123126296935339 | 0.0620232864649486 | 1.35 | 1.35000000000003 | 6.66133814775094e-16 | 2.99760216648792e-14 |
| 9 | 1 | 1.4 | 1.40342261194926 | 1.34311501474527 | -0.00342261194925508 | 0.0568849852547333 | 1.4 | 1.39999999999995 | 0 | -4.61852778244065e-14 |
| 10 | 1 | 1.45 | 1.45578195616405 | 1.39825532766461 | -0.00578195616405108 | 0.0517446723353858 | 1.45 | 1.45 | -2.22044604925031e-16 | 1.11022302462516e-15 |
| 11 | 1 | 1.5 | 1.50831092837926 | 1.4533976525131 | -0.00831092837925729 | 0.0466023474868964 | 1.5 | 1.49999999999999 | 2.22044604925031e-16 | -6.43929354282591e-15 |
| 12 | 1 | 1.55 | 1.56101118798142 | 1.50854198951093 | -0.011011187981419 | 0.0414580104890709 | 1.55 | 1.54999999999988 | 1.11022302462516e-15 | -1.16129328375791e-13 |
| 13 | 1 | 1.6 | 1.61388442158975 | 1.56368833887866 | -0.0138844215897536 | 0.036311661121345 | 1.6 | 1.59999999999992 | 8.88178419700125e-16 | -7.61612994892857e-14 |
| 14 | 1 | 1.65 | 1.66693234368599 | 1.61883670083648 | -0.016932343685987 | 0.0311632991635245 | 1.65 | 1.64999999999999 | 2.44249065417534e-15 | -7.32747196252603e-15 |
| 15 | 1 | 1.7 | 1.72015669726303 | 1.67398707560458 | -0.020156697263026 | 0.0260129243954155 | 1.7 | 1.69999999999993 | 1.99840144432528e-15 | -6.63913368725844e-14 |
| 16 | 1 | 1.75 | 1.7735592544932 | 1.72913946340355 | -0.0235592544932015 | 0.0208605365964529 | 1.75 | 1.74999999999989 | 6.66133814775094e-16 | -1.10578213252666e-13 |
| 17 | 1 | 1.8 | 1.82714181741677 | 1.78429386445393 | -0.0271418174167681 | 0.0157061355460733 | 1.8 | 1.79999999999997 | 1.99840144432528e-15 | -3.44169137633799e-14 |
| 18 | 1 | 1.85 | 1.88090621865143 | 1.83945027897592 | -0.0309062186514268 | 0.0105497210240817 | 1.85 | 1.8499999999999 | 1.11022302462516e-15 | -1.03916875104915e-13 |
| 19 | 1 | 1.9 | 1.9348543221237 | 1.89460870719046 | -0.034854322123695 | 0.00539129280954409 | 1.9 | 1.90000000000005 | 1.33226762955019e-15 | 4.9293902293357e-14 |
| 20 | 1 | 1.95 | 1.98898802382289 | 1.94976914931755 | -0.0389880238228879 | 0.000230850682451544 | 1.95 | 1.94999999999992 | 8.88178419700125e-16 | -8.21565038222616e-14 |
| 21 | 1 | 2 | 2.04330925257863 | 2.00493160557832 | -0.0433092525786325 | -0.00493160557831596 | 2 | 1.99999999999997 | -2.22044604925031e-16 | -3.41948691584548e-14 |
| 22 | 1 | 2.05 | 2.09781997086278 | 2.06009607619314 | -0.0478199708627844 | -0.0100960761931375 | 2.05 | 2.04999999999995 | 1.33226762955019e-15 | -5.19584375524573e-14 |
| 23 | 1 | 2.1 | 2.15252217561668 | 2.11526256138276 | -0.0525221756166845 | -0.0152625613827619 | 2.1 | 2.09999999999992 | 4.44089209850063e-16 | -8.12683254025615e-14 |
| 24 | 1 | 2.15 | 2.20741789910479 | 2.17043106136794 | -0.0574178991047876 | -0.0204310613679386 | 2.15 | 2.1499999999999 | 1.33226762955019e-15 | -1.04360964314765e-13 |
| 25 | 1 | 2.2 | 2.26250920979563 | 2.22560157636942 | -0.0625092097956266 | -0.025601576369418 | 2.2 | 2.19999999999986 | 1.33226762955019e-15 | -1.38111744263369e-13 |
| 26 | 1 | 2.25 | 2.31779821327126 | 2.28077410660813 | -0.0677982132712591 | -0.030774106608134 | 2.25 | 2.24999999999993 | 8.88178419700125e-16 | -6.97220059464598e-14 |
| 27 | 1 | 2.3 | 2.37328705316628 | 2.33594865230465 | -0.0732870531662804 | -0.0359486523046502 | 2.3 | 2.29999999999984 | 8.88178419700125e-16 | -1.60760293965723e-13 |
| 28 | 1 | 2.35 | 2.42897791213761 | 2.39112521368027 | -0.078977912137614 | -0.0411252136802713 | 2.35 | 2.34999999999997 | 4.44089209850063e-16 | -3.50830475781549e-14 |
| 29 | 1 | 2.4 | 2.48487301286631 | 2.44630379095556 | -0.0848730128663111 | -0.0463037909555615 | 2.4 | 2.39999999999997 | 8.88178419700125e-16 | -2.57571741713036e-14 |
| 30 | 1 | 2.45 | 2.54097461909266 | 2.50148438435164 | -0.0909746190926555 | -0.0514843843516415 | 2.45 | 2.45 | 8.88178419700125e-16 | 3.10862446895044e-15 |
| 31 | 1 | 2.5 | 2.59728503668596 | 2.55666699408944 | -0.0972850366859559 | -0.0566669940894444 | 2.5 | 2.49999999999998 | 4.44089209850063e-16 | -1.99840144432528e-14 |
| 32 | 1 | 2.55 | 2.65380661475043 | 2.61185162039009 | -0.103806614750434 | -0.0618516203900899 | 2.55 | 2.54999999999996 | 1.77635683940025e-15 | -3.50830475781549e-14 |
| 33 | 1 | 2.6 | 2.71054174676872 | 2.6670382634747 | -0.110541746768716 | -0.0670382634746973 | 2.6 | 2.59999999999998 | 1.33226762955019e-15 | -1.64313007644523e-14 |
| 34 | 1 | 2.65 | 2.76749287178452 | 2.72222692356439 | -0.117492871784522 | -0.072226923564386 | 2.65 | 2.65000000000002 | 4.44089209850063e-16 | 2.26485497023532e-14 |
| 35 | 1 | 2.7 | 2.82466247562617 | 2.77741760088009 | -0.124662475626172 | -0.0774176008800906 | 2.7 | 2.69999999999987 | 1.33226762955019e-15 | -1.33670852164869e-13 |
| 36 | 1 | 2.75 | 2.88205309217267 | 2.83261029564349 | -0.132053092172667 | -0.082610295643486 | 2.75 | 2.74999999999993 | 8.88178419700125e-16 | -6.79456491070596e-14 |
| 37 | 1 | 2.8 | 2.93966730466418 | 2.88780500807551 | -0.139667304664177 | -0.0878050080755064 | 2.8 | 2.79999999999993 | 4.44089209850063e-16 | -7.01660951563099e-14 |
| 38 | 1 | 2.85 | 2.99750774705883 | 2.94300173839746 | -0.147507747058831 | -0.0930017383974553 | 2.85 | 2.84999999999987 | 8.88178419700125e-16 | -1.32782673745169e-13 |
| 39 | 1 | 2.9 | 3.05557710543784 | 2.99820048683082 | -0.155577105437836 | -0.0982004868308226 | 2.9 | 2.89999999999989 | 8.88178419700125e-16 | -1.15019105351166e-13 |
| 40 | 1 | 2.95 | 3.11387811946106 | 3.05340125359691 | -0.163878119461055 | -0.103401253596914 | 2.95 | 2.94999999999992 | 1.33226762955019e-15 | -8.08242361927114e-14 |
| 41 | 1 | 3 | 3.17241358387526 | 3.10860403891722 | -0.172413583875261 | -0.108604038917218 | 3 | 3.00000000000004 | 1.33226762955019e-15 | 3.5527136788005e-14 |
| 42 | 1 | 3.05 | 3.23118635007743 | 3.16380884301285 | -0.181186350077425 | -0.113808843012853 | 3.05 | 3.04999999999993 | 4.44089209850063e-16 | -7.23865412055602e-14 |
| 43 | 1 | 3.1 | 3.29019932773551 | 3.21901566610568 | -0.190199327735515 | -0.119015666105681 | 3.1 | 3.09999999999993 | 1.33226762955019e-15 | -7.41628980449605e-14 |
| 44 | 1 | 3.15 | 3.34945548646939 | 3.27422450841719 | -0.199455486469387 | -0.12422450841719 | 3.15 | 3.14999999999999 | 8.88178419700125e-16 | -1.4210854715202e-14 |
| 45 | 1 | 3.2 | 3.40895785759457 | 3.32943537016887 | -0.208957857594573 | -0.129435370168869 | 3.2 | 3.20000000000003 | 1.33226762955019e-15 | 2.57571741713036e-14 |
| 46 | 1 | 3.25 | 3.46870953593179 | 3.38464825158221 | -0.218709535931786 | -0.134648251582208 | 3.25 | 3.24999999999993 | 1.33226762955019e-15 | -6.97220059464598e-14 |
| 47 | 1 | 3.3 | 3.52871368168527 | 3.43986315287925 | -0.228713681685273 | -0.139863152879252 | 3.3 | 3.30000000000005 | 8.88178419700125e-16 | 4.84057238736568e-14 |
| 48 | 1 | 3.35 | 3.58897352239318 | 3.49508007428131 | -0.238973522393184 | -0.145080074281306 | 3.35 | 3.35000000000002 | 8.88178419700125e-16 | 2.1316282072803e-14 |
| 49 | 1 | 3.4 | 3.6494923549534 | 3.55029901601023 | -0.249492354953401 | -0.150299016010228 | 3.4 | 3.39999999999996 | 1.33226762955019e-15 | -4.2632564145606e-14 |
| 50 | 1 | 3.45 | 3.7102735477284 | 3.60551997828788 | -0.260273547728401 | -0.155519978287878 | 3.45 | 3.44999999999993 | -4.44089209850063e-16 | -7.19424519957101e-14 |
| 51 | 1 | 3.5 | 3.77132054273299 | 3.66074296133612 | -0.271320542732986 | -0.160742961336117 | 3.5 | 3.49999999999997 | 1.77635683940025e-15 | -3.19744231092045e-14 |
| 52 | 1 | 3.55 | 3.83263685790883 | 3.71596796537662 | -0.282636857908833 | -0.165967965376617 | 3.55 | 3.54999999999991 | -4.44089209850063e-16 | -9.41469124882133e-14 |
| 53 | 1 | 3.6 | 3.89422608949024 | 3.77119499063143 | -0.294226089490235 | -0.171194990631425 | 3.6 | 3.59999999999987 | 1.33226762955019e-15 | -1.27897692436818e-13 |
| 54 | 1 | 3.65 | 3.95609191446538 | 3.82642403732259 | -0.306091914465384 | -0.176424037322585 | 3.65 | 3.64999999999996 | 8.88178419700125e-16 | -4.2632564145606e-14 |
| 55 | 1 | 3.7 | 4.01823809313812 | 3.88165510567177 | -0.318238093138115 | -0.18165510567177 | 3.7 | 3.69999999999988 | 1.33226762955019e-15 | -1.18127729820117e-13 |
| 56 | 1 | 3.75 | 4.08066847179503 | 3.9368881959014 | -0.330668471795033 | -0.186888195901397 | 3.75 | 3.75 | -8.88178419700125e-16 | -8.88178419700125e-16 |
| 57 | 1 | 3.8 | 4.14338698548348 | 3.99212330823314 | -0.343386985483477 | -0.192123308233138 | 3.8 | 3.79999999999995 | 4.44089209850063e-16 | -4.57411886145565e-14 |
| 58 | 1 | 3.85 | 4.20639766090591 | 4.04736044288941 | -0.356397660905905 | -0.197360442889411 | 3.85 | 3.85000000000003 | -8.88178419700125e-16 | 2.93098878501041e-14 |
| 59 | 1 | 3.9 | 4.26970461943683 | 4.10259960009207 | -0.369704619436825 | -0.202599600092071 | 3.9 | 3.89999999999996 | 0 | -3.86357612569555e-14 |
| 60 | 1 | 3.95 | 4.33331208026857 | 4.15784078006354 | -0.383312080268572 | -0.207840780063537 | 3.95 | 3.94999999999996 | 4.44089209850063e-16 | -4.13002965160558e-14 |
| 61 | 1 | 4 | 4.3972243636928 | 4.21308398302604 | -0.397224363692796 | -0.213083983026038 | 4 | 4.00000000000002 | 8.88178419700125e-16 | 2.30926389122033e-14 |
| 62 | 1 | 4.05 | 4.46144589452485 | 4.26832920920162 | -0.411445894524849 | -0.218329209201615 | 4.05 | 4.04999999999995 | 1.77635683940025e-15 | -5.15143483426073e-14 |
| 63 | 1 | 4.1 | 4.52598120567876 | 4.32357645881287 | -0.425981205678755 | -0.223576458812873 | 4.1 | 4.1 | 1.77635683940025e-15 | 2.66453525910038e-15 |
| 64 | 1 | 4.15 | 4.59083494190096 | 4.37882573208185 | -0.440834941900962 | -0.228825732081852 | 4.15 | 4.14999999999991 | 1.77635683940025e-15 | -9.41469124882133e-14 |
| 65 | 1 | 4.2 | 4.65601186367152 | 4.43407702923134 | -0.456011863671519 | -0.234077029231339 | 4.2 | 4.20000000000002 | -8.88178419700125e-16 | 1.59872115546023e-14 |
| 66 | 1 | 4.25 | 4.72151685128205 | 4.48933035048338 | -0.471516851282048 | -0.239330350483378 | 4.25 | 4.24999999999998 | 8.88178419700125e-16 | -1.68753899743024e-14 |
| 67 | 1 | 4.3 | 4.78735490910028 | 4.54458569606057 | -0.487354909100274 | -0.244585696060569 | 4.3 | 4.29999999999992 | 8.88178419700125e-16 | -7.90478793533112e-14 |
| 68 | 1 | 4.35 | 4.85353117003178 | 4.59984306618551 | -0.503531170031781 | -0.249843066185513 | 4.35 | 4.34999999999991 | 0 | -8.79296635503124e-14 |
| 69 | 1 | 4.4 | 4.92005090019017 | 4.65510246108081 | -0.520050900190172 | -0.255102461080806 | 4.4 | 4.39999999999999 | 1.77635683940025e-15 | -7.99360577730113e-15 |
| 70 | 1 | 4.45 | 4.98691950378766 | 4.71036388096887 | -0.536919503787662 | -0.260363880968868 | 4.45 | 4.45 | 8.88178419700125e-16 | 3.5527136788005e-15 |
| 71 | 1 | 4.5 | 5.05414252825902 | 4.76562732607229 | -0.554142528259021 | -0.265627326072294 | 4.5 | 4.49999999999991 | 8.88178419700125e-16 | -8.79296635503124e-14 |
| 72 | 1 | 4.55 | 5.12172566963254 | 4.82089279661406 | -0.571725669632534 | -0.270892796614055 | 4.55 | 4.54999999999998 | 8.88178419700125e-16 | -1.68753899743024e-14 |
| 73 | 1 | 4.6 | 5.18967477816275 | 4.87616029281657 | -0.589674778162753 | -0.276160292816567 | 4.6 | 4.59999999999995 | 0 | -5.50670620214078e-14 |
| 74 | 1 | 4.65 | 5.25799586424078 | 4.9314298149028 | -0.607995864240783 | -0.281429814902799 | 4.65 | 4.64999999999999 | 8.88178419700125e-16 | -1.33226762955019e-14 |
| 75 | 1 | 4.7 | 5.32669510459899 | 4.98670136309535 | -0.626695104598992 | -0.286701363095351 | 4.7 | 4.69999999999993 | 1.77635683940025e-15 | -6.75015598972095e-14 |
| 76 | 1 | 4.75 | 5.39577884882831 | 5.04197493761719 | -0.645778848828309 | -0.291974937617193 | 4.75 | 4.7499999999999 | 1.77635683940025e-15 | -1.03028696685215e-13 |
| 77 | 1 | 4.8 | 5.46525362622753 | 5.09725053869129 | -0.665253626227526 | -0.297250538691293 | 4.8 | 4.79999999999996 | 8.88178419700125e-16 | -3.90798504668055e-14 |
| 78 | 1 | 4.85 | 5.53512615300556 | 5.15252816654044 | -0.685126153005556 | -0.302528166540439 | 4.85 | 4.85 | 8.88178419700125e-16 | 4.44089209850063e-15 |
| 79 | 1 | 4.9 | 5.6054033398591 | 5.20780782138741 | -0.705403339859101 | -0.307807821387412 | 4.9 | 4.89999999999986 | 8.88178419700125e-16 | -1.36779476633819e-13 |
| 80 | 1 | 4.95 | 5.67609229994996 | 5.26308950345574 | -0.72609229994996 | -0.313089503455739 | 4.95 | 4.95000000000001 | 1.77635683940025e-15 | 1.24344978758018e-14 |
| 81 | 1 | 5 | 5.747200357308 | 5.31837321296784 | -0.747200357308 | -0.318373212967835 | 5 | 4.99999999999988 | 0 | -1.17239551400417e-13 |
| 82 | 1 | 5.05 | 5.81873505568795 | 5.37365895014741 | -0.768735055687952 | -0.32365895014741 | 5.05 | 5.05000000000004 | 8.88178419700125e-16 | 3.99680288865056e-14 |
| 83 | 1 | 5.1 | 5.89070416791026 | 5.42894671521706 | -0.790704167910258 | -0.328946715217061 | 5.1 | 5.10000000000001 | 0 | 4.44089209850063e-15 |
| 84 | 1 | 5.15 | 5.9631157057188 | 5.48423650840013 | -0.813115705718801 | -0.334236508400132 | 5.15 | 5.14999999999994 | 8.88178419700125e-16 | -6.12843109593086e-14 |
| 85 | 1 | 5.2 | 6.03597793019083 | 5.53952832991996 | -0.835977930190833 | -0.339528329919959 | 5.2 | 5.19999999999996 | 8.88178419700125e-16 | -3.99680288865056e-14 |
| 86 | 1 | 5.25 | 6.10929936273744 | 5.5948221799997 | -0.859299362737436 | -0.3448221799997 | 5.25 | 5.24999999999998 | 0 | -1.68753899743024e-14 |
| 87 | 1 | 5.3 | 6.18308879673596 | 5.65011805886251 | -0.883088796735959 | -0.350118058862509 | 5.3 | 5.29999999999989 | 1.77635683940025e-15 | -1.11910480882216e-13 |
| 88 | 1 | 5.35 | 6.25735530983937 | 5.70541596673191 | -0.907355309839372 | -0.355415966731909 | 5.35 | 5.34999999999985 | 1.77635683940025e-15 | -1.51878509768721e-13 |
| 89 | 1 | 5.4 | 6.33210827701135 | 5.76071590383143 | -0.932108277011352 | -0.360715903831427 | 5.4 | 5.40000000000001 | 1.77635683940025e-15 | 6.21724893790088e-15 |
| 90 | 1 | 5.45 | 6.40735738434006 | 5.81601787038403 | -0.957357384340058 | -0.366017870384031 | 5.45 | 5.44999999999996 | 8.88178419700125e-16 | -4.17443857259059e-14 |
| 91 | 1 | 5.5 | 6.48311264368821 | 5.87132186661343 | -0.98311264368821 | -0.371321866613433 | 5.5 | 5.49999999999994 | 8.88178419700125e-16 | -5.95079541199084e-14 |
| 92 | 1 | 5.55 | 6.55938440824224 | 5.92662789274316 | -1.00938440824224 | -0.376627892743157 | 5.55 | 5.54999999999998 | 8.88178419700125e-16 | -2.30926389122033e-14 |
| 93 | 1 | 5.6 | 6.63618338902886 | 5.98193594899673 | -1.03618338902886 | -0.381935948996727 | 5.6 | 5.60000000000006 | 1.77635683940025e-15 | 5.95079541199084e-14 |
| 94 | 1 | 5.65 | 6.71352067247361 | 6.03724603559749 | -1.06352067247361 | -0.387246035597484 | 5.65 | 5.64999999999998 | 8.88178419700125e-16 | -2.48689957516035e-14 |
| 95 | 1 | 5.7 | 6.79140773908296 | 6.09255815276932 | -1.09140773908296 | -0.392558152769324 | 5.7 | 5.69999999999998 | 8.88178419700125e-16 | -2.48689957516035e-14 |
| 96 | 1 | 5.75 | 6.86985648333904 | 6.14787230073577 | -1.11985648333904 | -0.397872300735769 | 5.75 | 5.74999999999994 | 0 | -6.21724893790088e-14 |
| 97 | 1 | 5.8 | 6.9488792349046 | 6.20318847972072 | -1.1488792349046 | -0.403188479720715 | 5.8 | 5.80000000000004 | 1.77635683940025e-15 | 3.90798504668055e-14 |
| 98 | 1 | 5.85 | 7.02848878124532 | 6.2585066899475 | -1.17848878124532 | -0.408506689947504 | 5.85 | 5.84999999999992 | 8.88178419700125e-16 | -8.08242361927114e-14 |
| 99 | 1 | 5.9 | 7.10869839178688 | 6.31382693164021 | -1.20869839178688 | -0.413826931640213 | 5.9 | 5.89999999999985 | 1.77635683940025e-15 | -1.53654866608122e-13 |
| 100 | 1 | 5.95 | 7.18952184373613 | 6.36914920502274 | -1.23952184373612 | -0.41914920502274 | 5.95 | 5.94999999999989 | 8.88178419700125e-16 | -1.13686837721616e-13 |
| 101 | 1 | 6 | 7.27097344970855 | 6.42447351031879 | -1.27097344970854 | -0.424473510318792 | 6 | 5.9999999999999 | 0 | -9.9475983006414e-14 |
| 102 | 1.1 | 1 | 0.991633909150579 | 0.903491173303202 | 0.00836609084942119 | 0.0965088266967978 | 1 | 0.999999999999915 | 0 | -8.4821039081362e-14 |
| 103 | 1.1 | 1.05 | 1.0426964863338 | 0.95861545072129 | 0.00730351366620008 | 0.0913845492787103 | 1.05 | 1.04999999999993 | 2.22044604925031e-16 | -6.59472476627343e-14 |
| 104 | 1.1 | 1.1 | 1.09391636024487 | 1.01374173831363 | 0.00608363975513471 | 0.0862582616863736 | 1.1 | 1.09999999999985 | 6.66133814775094e-16 | -1.52322598978571e-13 |
| 105 | 1.1 | 1.15 | 1.1452949935456 | 1.06887003630041 | 0.00470500645440364 | 0.0811299636995932 | 1.15 | 1.1499999999999 | 6.66133814775094e-16 | -1.01030295240889e-13 |
| 106 | 1.1 | 1.2 | 1.19683387170752 | 1.12400034490145 | 0.00316612829247931 | 0.0759996550985453 | 1.2 | 1.19999999999996 | 1.33226762955019e-15 | -4.13002965160558e-14 |
| 107 | 1.1 | 1.25 | 1.24853450351299 | 1.17913266433678 | 0.00146549648700867 | 0.0708673356632206 | 1.25 | 1.25000000000003 | 2.44249065417534e-15 | 2.93098878501041e-14 |
| 108 | 1.1 | 1.3 | 1.30039842157055 | 1.23426699482621 | -0.000398421570551477 | 0.0657330051737948 | 1.3 | 1.29999999999991 | 1.33226762955019e-15 | -9.12603326241879e-14 |
| 109 | 1.1 | 1.35 | 1.35242718284505 | 1.2894033365903 | -0.00242718284505084 | 0.0605966634097035 | 1.35 | 1.35000000000003 | 6.66133814775094e-16 | 2.99760216648792e-14 |
| 110 | 1.1 | 1.4 | 1.40462236920301 | 1.34454168984869 | -0.00462236920300785 | 0.0554583101513078 | 1.4 | 1.39999999999995 | 2.22044604925031e-16 | -4.9293902293357e-14 |
| 111 | 1.1 | 1.45 | 1.45698558797379 | 1.39968205482196 | -0.0069855879737859 | 0.0503179451780433 | 1.45 | 1.45000000000003 | 1.99840144432528e-15 | 3.06421554796543e-14 |
| 112 | 1.1 | 1.5 | 1.50951847252712 | 1.45482443172991 | -0.00951847252712135 | 0.0451755682700861 | 1.5 | 1.49999999999992 | 8.88178419700125e-16 | -7.74935671188359e-14 |
| 113 | 1.1 | 1.55 | 1.56222268286763 | 1.50996882079331 | -0.0122226828676288 | 0.0400311792066865 | 1.55 | 1.55000000000008 | 1.99840144432528e-15 | 8.10462807976364e-14 |
| 114 | 1.1 | 1.6 | 1.61509990624684 | 1.56511522223179 | -0.0150999062468353 | 0.0348847777682055 | 1.6 | 1.59999999999992 | 8.88178419700125e-16 | -8.17124146124115e-14 |
| 115 | 1.1 | 1.65 | 1.66815185779345 | 1.62026363626629 | -0.018151857793453 | 0.0297363637337087 | 1.65 | 1.64999999999998 | 8.88178419700125e-16 | -1.59872115546023e-14 |
| 116 | 1.1 | 1.7 | 1.72138028116249 | 1.67541406311681 | -0.0213802811624899 | 0.0245859368831873 | 1.7 | 1.69999999999995 | 2.22044604925031e-16 | -4.77395900588817e-14 |
| 117 | 1.1 | 1.75 | 1.77478694920395 | 1.73056650300392 | -0.0247869492039485 | 0.0194334969960761 | 1.75 | 1.74999999999997 | 1.33226762955019e-15 | -3.44169137633799e-14 |
| 118 | 1.1 | 1.8 | 1.82837366465179 | 1.785720956148 | -0.028373664651792 | 0.0142790438519966 | 1.8 | 1.79999999999996 | 4.44089209850063e-16 | -3.99680288865056e-14 |
| 119 | 1.1 | 1.85 | 1.88214226083398 | 1.84087742276962 | -0.0321422608339825 | 0.00912257723038423 | 1.85 | 1.85 | 4.44089209850063e-16 | 4.44089209850063e-15 |
| 120 | 1.1 | 1.9 | 1.93609460240433 | 1.89603590308914 | -0.0360946024043318 | 0.00396409691085964 | 1.9 | 1.89999999999996 | 6.66133814775094e-16 | -3.70814490224802e-14 |
| 121 | 1.1 | 1.95 | 1.99023258609703 | 1.95119639732733 | -0.0402325860970336 | -0.00119639732732613 | 1.95 | 1.95 | 4.44089209850063e-16 | -1.77635683940025e-15 |
| 122 | 1.1 | 2 | 2.04455814150472 | 2.00635890570474 | -0.0445581415047176 | -0.00635890570473796 | 2 | 2.00000000000007 | 8.88178419700125e-16 | 6.97220059464598e-14 |
| 123 | 1.1 | 2.05 | 2.09907323188094 | 2.06152342844175 | -0.0490732318809375 | -0.0115234284417549 | 2.05 | 2.04999999999993 | 8.88178419700125e-16 | -6.75015598972095e-14 |
| 124 | 1.1 | 2.1 | 2.15377985496803 | 2.1166899657595 | -0.053779854968032 | -0.0166899657594959 | 2.1 | 2.09999999999997 | 1.33226762955019e-15 | -2.57571741713036e-14 |
| 125 | 1.1 | 2.15 | 2.20868004385135 | 2.17185851787834 | -0.05868004385135 | -0.0218585178783406 | 2.15 | 2.14999999999988 | 8.88178419700125e-16 | -1.22568621918617e-13 |
| 126 | 1.1 | 2.2 | 2.26377586784088 | 2.22702908501941 | -0.0637758678408771 | -0.027029085019409 | 2.2 | 2.19999999999996 | 8.88178419700125e-16 | -4.13002965160558e-14 |
| 127 | 1.1 | 2.25 | 2.31906943338133 | 2.28220166740327 | -0.069069433381332 | -0.0322016674032648 | 2.25 | 2.24999999999999 | 1.77635683940025e-15 | -6.66133814775094e-15 |
| 128 | 1.1 | 2.3 | 2.37456288499188 | 2.33737626525066 | -0.0745628849918774 | -0.0373762652506571 | 2.3 | 2.29999999999989 | 8.88178419700125e-16 | -1.11022302462516e-13 |
| 129 | 1.1 | 2.35 | 2.43025840623664 | 2.39255287878289 | -0.0802584062366374 | -0.0425528787828906 | 2.35 | 2.35000000000002 | 1.33226762955019e-15 | 1.95399252334028e-14 |
| 130 | 1.1 | 2.4 | 2.48615822072723 | 2.44773150822035 | -0.086158220727234 | -0.0477315082203447 | 2.4 | 2.39999999999988 | 1.33226762955019e-15 | -1.15907283770866e-13 |
| 131 | 1.1 | 2.45 | 2.54226459315868 | 2.50291215378451 | -0.0922645931586845 | -0.0529121537845088 | 2.45 | 2.44999999999995 | 8.88178419700125e-16 | -4.88498130835069e-14 |
| 132 | 1.1 | 2.5 | 2.59857983038 | 2.55809481569613 | -0.0985798303799981 | -0.058094815696133 | 2.5 | 2.49999999999998 | 4.44089209850063e-16 | -1.77635683940025e-14 |
| 133 | 1.1 | 2.55 | 2.65510628250091 | 2.61327949417634 | -0.105106282500914 | -0.0632794941763355 | 2.55 | 2.55000000000004 | 4.44089209850063e-16 | 3.68594044175552e-14 |
| 134 | 1.1 | 2.6 | 2.71184634403628 | 2.66846618944605 | -0.111846344036276 | -0.0684661894460508 | 2.6 | 2.59999999999997 | 1.33226762955019e-15 | -2.93098878501041e-14 |
| 135 | 1.1 | 2.65 | 2.76880245508963 | 2.72365490172658 | -0.118802455089631 | -0.0736549017265835 | 2.65 | 2.64999999999994 | 1.33226762955019e-15 | -6.26165785888588e-14 |
| 136 | 1.1 | 2.7 | 2.8259771025777 | 2.77884563123905 | -0.125977102577699 | -0.0788456312390538 | 2.7 | 2.69999999999989 | 1.77635683940025e-15 | -1.11910480882216e-13 |
| 137 | 1.1 | 2.75 | 2.88337282149746 | 2.83403837820495 | -0.133372821497455 | -0.084038378204951 | 2.75 | 2.75000000000007 | 4.44089209850063e-16 | 7.14983627858601e-14 |
| 138 | 1.1 | 2.8 | 2.94099219623766 | 2.88923314284502 | -0.140992196237658 | -0.0892331428450235 | 2.8 | 2.80000000000003 | 8.88178419700125e-16 | 2.88657986402541e-14 |
| 139 | 1.1 | 2.85 | 2.99883786193671 | 2.94442992538076 | -0.148837861936713 | -0.0944299253807612 | 2.85 | 2.84999999999994 | 4.44089209850063e-16 | -6.48370246381091e-14 |
| 140 | 1.1 | 2.9 | 3.05691250588894 | 2.99962872603365 | -0.156912505888943 | -0.0996287260336541 | 2.9 | 2.89999999999993 | 1.77635683940025e-15 | -6.97220059464598e-14 |
| 141 | 1.1 | 2.95 | 3.11521886900134 | 3.05482954502501 | -0.165218869001338 | -0.104829545025007 | 2.95 | 2.94999999999995 | 0 | -5.15143483426073e-14 |
| 142 | 1.1 | 3 | 3.17375974730306 | 3.11003238257612 | -0.173759747303057 | -0.110032382576123 | 3 | 2.99999999999989 | 1.33226762955019e-15 | -1.12798659301916e-13 |
| 143 | 1.1 | 3.05 | 3.23253799350999 | 3.16523723890868 | -0.182537993509988 | -0.115237238908676 | 3.05 | 3.04999999999994 | 8.88178419700125e-16 | -5.77315972805081e-14 |
| 144 | 1.1 | 3.1 | 3.29155651864691 | 3.22044411424397 | -0.191556518646914 | -0.120444114243974 | 3.1 | 3.09999999999994 | 1.33226762955019e-15 | -5.99520433297585e-14 |
| 145 | 1.1 | 3.15 | 3.35081829372981 | 3.27565300880369 | -0.200818293729812 | -0.125653008803688 | 3.15 | 3.15 | 4.44089209850063e-16 | 1.77635683940025e-15 |
| 146 | 1.1 | 3.2 | 3.41032635151111 | 3.33086392280931 | -0.210326351511114 | -0.130863922809309 | 3.2 | 3.20000000000005 | 8.88178419700125e-16 | 4.66293670342566e-14 |
| 147 | 1.1 | 3.25 | 3.47008378829076 | 3.38607685648233 | -0.220083788290756 | -0.136076856482327 | 3.25 | 3.24999999999996 | 0 | -4.17443857259059e-14 |
| 148 | 1.1 | 3.3 | 3.53009376579615 | 3.4412918100446 | -0.230093765796149 | -0.1412918100446 | 3.3 | 3.29999999999992 | 1.33226762955019e-15 | -8.5265128291212e-14 |
| 149 | 1.1 | 3.35 | 3.59035951313424 | 3.4965087837178 | -0.240359513134239 | -0.146508783717804 | 3.35 | 3.34999999999989 | 4.44089209850063e-16 | -1.06581410364015e-13 |
| 150 | 1.1 | 3.4 | 3.65088432881912 | 3.5517277777238 | -0.250884328819115 | -0.151727777723797 | 3.4 | 3.4 | 4.44089209850063e-16 | 1.77635683940025e-15 |
| 151 | 1.1 | 3.45 | 3.71167158287873 | 3.60694879228407 | -0.261671582878725 | -0.156948792284071 | 3.45 | 3.44999999999998 | 8.88178419700125e-16 | -2.53130849614536e-14 |
| 152 | 1.1 | 3.5 | 3.77272471904454 | 3.66217182762048 | -0.272724719044542 | -0.162171827620483 | 3.5 | 3.49999999999984 | 1.33226762955019e-15 | -1.55431223447522e-13 |
| 153 | 1.1 | 3.55 | 3.83404725702819 | 3.71739688395526 | -0.284047257028188 | -0.167396883955263 | 3.55 | 3.54999999999995 | 8.88178419700125e-16 | -5.55111512312578e-14 |
| 154 | 1.1 | 3.6 | 3.89564279488928 | 3.7726239615099 | -0.295642794889279 | -0.172623961509903 | 3.6 | 3.5999999999999 | 8.88178419700125e-16 | -1.00364161426114e-13 |
| 155 | 1.1 | 3.65 | 3.957515011499 | 3.82785306050663 | -0.307515011498995 | -0.177853060506629 | 3.65 | 3.64999999999997 | 1.77635683940025e-15 | -3.28626015289046e-14 |
| 156 | 1.1 | 3.7 | 4.01966766910415 | 3.88308418116712 | -0.31966766910415 | -0.183084181167118 | 3.7 | 3.69999999999987 | 0 | -1.32782673745169e-13 |
| 157 | 1.1 | 3.75 | 4.08210461599684 | 3.93831732371378 | -0.33210461599684 | -0.188317323713784 | 3.75 | 3.74999999999995 | -4.44089209850063e-16 | -4.88498130835069e-14 |
| 158 | 1.1 | 3.8 | 4.14482978929497 | 3.9935524883683 | -0.34482978929497 | -0.193552488368302 | 3.8 | 3.79999999999987 | 4.44089209850063e-16 | -1.35447209004269e-13 |
| 159 | 1.1 | 3.85 | 4.20784721783943 | 4.04878967535327 | -0.357847217839426 | -0.19878967535327 | 3.85 | 3.85000000000005 | -4.44089209850063e-16 | 5.37347943918576e-14 |
| 160 | 1.1 | 3.9 | 4.27116102521385 | 4.10402888489018 | -0.371161025213852 | -0.20402888489018 | 3.9 | 3.89999999999992 | -4.44089209850063e-16 | -7.68274333040608e-14 |
| 161 | 1.1 | 3.95 | 4.3347754328935 | 4.15927011720182 | -0.384775432893496 | -0.209270117201815 | 3.95 | 3.95000000000001 | 1.33226762955019e-15 | 1.33226762955019e-14 |
| 162 | 1.1 | 4 | 4.39869476352987 | 4.21451337251004 | -0.398694763529865 | -0.214513372510035 | 4 | 3.99999999999999 | 8.88178419700125e-16 | -1.15463194561016e-14 |
| 163 | 1.1 | 4.05 | 4.46292344437851 | 4.26975865103726 | -0.412923444378511 | -0.219758651037255 | 4.05 | 4.04999999999998 | 8.88178419700125e-16 | -1.95399252334028e-14 |
| 164 | 1.1 | 4.1 | 4.52746601087752 | 4.3250059530057 | -0.427466010877525 | -0.225005953005705 | 4.1 | 4.09999999999992 | 0 | -8.26005930321117e-14 |
| 165 | 1.1 | 4.15 | 4.59232711038504 | 4.3802552786378 | -0.442327110385042 | -0.230255278637798 | 4.15 | 4.14999999999986 | 8.88178419700125e-16 | -1.4477308241112e-13 |
| 166 | 1.1 | 4.2 | 4.65751150608438 | 4.43550662815614 | -0.457511506084377 | -0.235506628156135 | 4.2 | 4.19999999999998 | 1.77635683940025e-15 | -1.68753899743024e-14 |
| 167 | 1.1 | 4.25 | 4.72302408106615 | 4.49076000178276 | -0.473024081066145 | -0.24076000178276 | 4.25 | 4.24999999999995 | 8.88178419700125e-16 | -5.06261699229071e-14 |
| 168 | 1.1 | 4.3 | 4.78886984259722 | 4.54601539974046 | -0.488869842597222 | -0.246015399740458 | 4.3 | 4.30000000000004 | 1.77635683940025e-15 | 3.64153152077051e-14 |
| 169 | 1.1 | 4.35 | 4.85505392658714 | 4.60127282225146 | -0.505053926587136 | -0.251272822251461 | 4.35 | 4.34999999999999 | 0 | -1.15463194561016e-14 |
| 170 | 1.1 | 4.4 | 4.92158160226315 | 4.65653226953855 | -0.521581602263151 | -0.256532269538549 | 4.4 | 4.40000000000001 | 0 | 1.06581410364015e-14 |
| 171 | 1.1 | 4.45 | 4.98845827706608 | 4.71179374182414 | -0.538458277066079 | -0.261793741824141 | 4.45 | 4.44999999999994 | 0 | -5.86197757002083e-14 |
| 172 | 1.1 | 4.5 | 5.05568950177972 | 4.76705723933102 | -0.555689501779719 | -0.267057239331018 | 4.5 | 4.49999999999991 | 8.88178419700125e-16 | -8.61533067109122e-14 |
| 173 | 1.1 | 4.55 | 5.12328097590767 | 4.82232276228197 | -0.573280975907668 | -0.272322762281968 | 4.55 | 4.55000000000003 | 1.77635683940025e-15 | 2.75335310107039e-14 |
| 174 | 1.1 | 4.6 | 5.19123855331225 | 4.87759031089941 | -0.591238553312251 | -0.277590310899405 | 4.6 | 4.60000000000001 | 8.88178419700125e-16 | 5.32907051820075e-15 |
| 175 | 1.1 | 4.65 | 5.25956824813138 | 4.93285988540611 | -0.60956824813138 | -0.282859885406112 | 4.65 | 4.64999999999987 | 0 | -1.27897692436818e-13 |
| 176 | 1.1 | 4.7 | 5.32827624099023 | 4.98813148602525 | -0.628276240990231 | -0.288131486025246 | 4.7 | 4.69999999999995 | 8.88178419700125e-16 | -5.06261699229071e-14 |
| 177 | 1.1 | 4.75 | 5.39736888552593 | 5.0434051129794 | -0.647368885525927 | -0.293405112979404 | 4.75 | 4.75000000000002 | 8.88178419700125e-16 | 1.86517468137026e-14 |
| 178 | 1.1 | 4.8 | 5.46685271524471 | 5.09868076649137 | -0.666852715244704 | -0.298680766491374 | 4.8 | 4.79999999999999 | 1.77635683940025e-15 | -7.105427357601e-15 |
| 179 | 1.1 | 4.85 | 5.53673445073253 | 5.15395844678413 | -0.686734450732529 | -0.303958446784125 | 4.85 | 4.84999999999992 | 0 | -8.17124146124115e-14 |
| 180 | 1.1 | 4.9 | 5.60702100724167 | 5.20923815408081 | -0.70702100724167 | -0.309238154080809 | 4.9 | 4.89999999999996 | 1.77635683940025e-15 | -3.73034936274053e-14 |
| 181 | 1.1 | 4.95 | 5.67771950267745 | 5.26451988860421 | -0.727719502677445 | -0.314519888604214 | 4.95 | 4.94999999999993 | 8.88178419700125e-16 | -6.83897383169096e-14 |
| 182 | 1.1 | 5 | 5.74883726601133 | 5.31980365057749 | -0.748837266011329 | -0.319803650577494 | 5 | 4.99999999999992 | 0 | -7.63833440942108e-14 |
| 183 | 1.1 | 5.05 | 5.82038184614846 | 5.3750894402238 | -0.770381846148465 | -0.325089440223803 | 5.05 | 5.05 | 8.88178419700125e-16 | 8.88178419700125e-16 |
| 184 | 1.1 | 5.1 | 5.89236102128003 | 5.43037725776611 | -0.792361021280027 | -0.330377257766109 | 5.1 | 5.10000000000002 | 8.88178419700125e-16 | 2.1316282072803e-14 |
| 185 | 1.1 | 5.15 | 5.9647828087532 | 5.48566710342757 | -0.814782808753201 | -0.335667103427572 | 5.15 | 5.14999999999998 | 8.88178419700125e-16 | -2.39808173319034e-14 |
| 186 | 1.1 | 5.2 | 6.03765547549423 | 5.54095897743153 | -0.837655475494233 | -0.340958977431528 | 5.2 | 5.19999999999998 | 1.77635683940025e-15 | -1.86517468137026e-14 |
| 187 | 1.1 | 5.25 | 6.11098754902291 | 5.59625288000113 | -0.860987549022911 | -0.346252880001133 | 5.25 | 5.24999999999995 | 0 | -4.88498130835069e-14 |
| 188 | 1.1 | 5.3 | 6.18478782910002 | 5.65154881135973 | -0.884787829100024 | -0.351548811359727 | 5.3 | 5.29999999999993 | 8.88178419700125e-16 | -6.66133814775094e-14 |
| 189 | 1.1 | 5.35 | 6.25906540005281 | 5.70684677173065 | -0.909065400052806 | -0.356846771730648 | 5.35 | 5.34999999999993 | 1.77635683940025e-15 | -6.75015598972095e-14 |
| 190 | 1.1 | 5.4 | 6.33382964382728 | 5.76214676133724 | -0.933829643827278 | -0.36214676133724 | 5.4 | 5.39999999999992 | 8.88178419700125e-16 | -7.8159700933611e-14 |
| 191 | 1.1 | 5.45 | 6.40909025382056 | 5.81744878040302 | -0.95909025382056 | -0.367448780403024 | 5.45 | 5.45 | 8.88178419700125e-16 | 0 |
| 192 | 1.1 | 5.5 | 6.48485724955086 | 5.87275282915116 | -0.984857249550856 | -0.372752829151155 | 5.5 | 5.4999999999999 | 8.88178419700125e-16 | -1.03028696685215e-13 |
| 193 | 1.1 | 5.55 | 6.56114099222802 | 5.92805890780553 | -1.01114099222802 | -0.378058907805531 | 5.55 | 5.54999999999998 | 0 | -2.39808173319034e-14 |
| 194 | 1.1 | 5.6 | 6.63795220129316 | 5.98336701658949 | -1.03795220129316 | -0.383367016589488 | 5.6 | 5.60000000000006 | 8.88178419700125e-16 | 5.59552404411079e-14 |
| 195 | 1.1 | 5.65 | 6.71530197200206 | 6.03867715572637 | -1.06530197200206 | -0.388677155726369 | 5.65 | 5.64999999999993 | 1.77635683940025e-15 | -7.46069872548105e-14 |
| 196 | 1.1 | 5.7 | 6.79320179413407 | 6.09398932544025 | -1.09320179413407 | -0.393989325440253 | 5.7 | 5.7 | 8.88178419700125e-16 | -8.88178419700125e-16 |
| 197 | 1.1 | 5.75 | 6.87166357191575 | 6.14930352595448 | -1.12166357191575 | -0.399303525954479 | 5.75 | 5.74999999999999 | 1.77635683940025e-15 | -9.76996261670138e-15 |
| 198 | 1.1 | 5.8 | 6.95069964525717 | 6.20461975749294 | -1.15069964525717 | -0.404619757492942 | 5.8 | 5.80000000000007 | 8.88178419700125e-16 | 7.19424519957101e-14 |
| 199 | 1.1 | 5.85 | 7.03032281240803 | 6.25993802027899 | -1.18032281240803 | -0.409938020278984 | 5.85 | 5.84999999999989 | 8.88178419700125e-16 | -1.15463194561016e-13 |
| 200 | 1.1 | 5.9 | 7.11054635415148 | 6.31525831453687 | -1.21054635415148 | -0.415258314536868 | 5.9 | 5.89999999999986 | 0 | -1.36779476633819e-13 |
| 201 | 1.1 | 5.95 | 7.19138405966503 | 6.37058064049031 | -1.24138405966503 | -0.420580640490305 | 5.95 | 5.9499999999999 | 1.77635683940025e-15 | -9.59232693276135e-14 |
| 202 | 1.1 | 6 | 7.27285025419133 | 6.42590499836301 | -1.27285025419133 | -0.425904998363005 | 6 | 5.99999999999987 | 8.88178419700125e-16 | -1.33226762955019e-13 |
| 203 | 1.2 | 1 | 0.992798943627076 | 0.904916772046331 | 0.00720105637292412 | 0.0950832279536693 | 1 | 0.99999999999995 | 8.88178419700125e-16 | -4.9515946898282e-14 |
| 204 | 1.2 | 1.05 | 1.0438650988766 | 0.960041101448761 | 0.00613490112339798 | 0.0899588985512391 | 1.05 | 1.04999999999995 | 1.33226762955019e-15 | -4.57411886145565e-14 |
| 205 | 1.2 | 1.1 | 1.09508858402476 | 1.01516744103118 | 0.00491141597524081 | 0.0848325589688235 | 1.1 | 1.0999999999999 | 1.11022302462516e-15 | -1.0325074129014e-13 |
| 206 | 1.2 | 1.15 | 1.14647086224908 | 1.07029579101359 | 0.00352913775091968 | 0.0797042089864131 | 1.15 | 1.14999999999985 | 1.55431223447522e-15 | -1.46993528460371e-13 |
| 207 | 1.2 | 1.2 | 1.19801341954809 | 1.12542615161619 | 0.00198658045190592 | 0.0745738483838139 | 1.2 | 1.20000000000003 | 4.44089209850063e-16 | 2.79776202205539e-14 |
| 208 | 1.2 | 1.25 | 1.24971776524278 | 1.18055852305843 | 0.000282234757223998 | 0.0694414769415719 | 1.25 | 1.24999999999992 | 2.22044604925031e-16 | -8.03801469828613e-14 |
| 209 | 1.2 | 1.3 | 1.30158543249224 | 1.23569290556088 | -0.00158543249223775 | 0.0643070944391224 | 1.3 | 1.3 | 4.44089209850063e-16 | -3.5527136788005e-15 |
| 210 | 1.2 | 1.35 | 1.35361797882418 | 1.29082929934317 | -0.00361797882418236 | 0.0591707006568267 | 1.35 | 1.34999999999985 | 1.11022302462516e-15 | -1.48769885299771e-13 |
| 211 | 1.2 | 1.4 | 1.40581698668063 | 1.34596770462606 | -0.00581698668062747 | 0.0540322953739352 | 1.4 | 1.40000000000005 | 1.77635683940025e-15 | 4.75175454539567e-14 |
| 212 | 1.2 | 1.45 | 1.45818406397945 | 1.40110812162901 | -0.00818406397944527 | 0.048891878370994 | 1.45 | 1.44999999999994 | 1.33226762955019e-15 | -6.26165785888588e-14 |
| 213 | 1.2 | 1.5 | 1.51072084469228 | 1.45625055057275 | -0.0107208446922831 | 0.0437494494272535 | 1.5 | 1.50000000000001 | 1.77635683940025e-15 | 1.17683640610267e-14 |
| 214 | 1.2 | 1.55 | 1.56342898943944 | 1.51139499167711 | -0.0134289894394402 | 0.0386050083228899 | 1.55 | 1.54999999999989 | 1.33226762955019e-15 | -1.15019105351166e-13 |
| 215 | 1.2 | 1.6 | 1.61631018610234 | 1.56654144516285 | -0.0163101861023367 | 0.0334585548371535 | 1.6 | 1.59999999999998 | 1.55431223447522e-15 | -2.44249065417534e-14 |
| 216 | 1.2 | 1.65 | 1.66936615045419 | 1.62168991124996 | -0.019366150454186 | 0.0283100887500352 | 1.65 | 1.64999999999999 | 1.77635683940025e-15 | -6.88338275267597e-15 |
| 217 | 1.2 | 1.7 | 1.72259862680956 | 1.67684039015884 | -0.0225986268095633 | 0.0231596098411562 | 1.7 | 1.69999999999995 | 1.99840144432528e-15 | -5.37347943918576e-14 |
| 218 | 1.2 | 1.75 | 1.77600938869355 | 1.73199288211005 | -0.0260093886935451 | 0.0180071178899512 | 1.75 | 1.74999999999998 | 1.33226762955019e-15 | -2.37587727269784e-14 |
| 219 | 1.2 | 1.8 | 1.82960023953116 | 1.78714738732377 | -0.0296002395311648 | 0.0128526126762267 | 1.8 | 1.79999999999985 | 6.66133814775094e-16 | -1.48769885299771e-13 |
| 220 | 1.2 | 1.85 | 1.88337301335792 | 1.84230390602095 | -0.0333730133579218 | 0.00769609397904847 | 1.85 | 1.84999999999997 | 4.44089209850063e-16 | -2.64233079860787e-14 |
| 221 | 1.2 | 1.9 | 1.93732957555215 | 1.89746243842159 | -0.0373295755521534 | 0.00253756157840646 | 1.9 | 1.89999999999987 | -2.22044604925031e-16 | -1.27897692436818e-13 |
| 222 | 1.2 | 1.95 | 1.99147182359009 | 1.95262298474682 | -0.0414718235900926 | -0.00262298474681755 | 1.95 | 1.95000000000004 | 4.44089209850063e-16 | 4.30766533554561e-14 |
| 223 | 1.2 | 2 | 2.04580168782447 | 2.00778554521663 | -0.0458016878244676 | -0.00778554521663377 | 2 | 1.99999999999994 | 1.77635683940025e-15 | -5.72875080706581e-14 |
| 224 | 1.2 | 2.05 | 2.10032113228756 | 2.06295012005216 | -0.0503211322875559 | -0.0129501200521616 | 2.05 | 2.05 | 0 | -4.88498130835069e-15 |
| 225 | 1.2 | 2.1 | 2.15503215551965 | 2.11811670947378 | -0.0550321555196507 | -0.0181167094737793 | 2.1 | 2.09999999999992 | 4.44089209850063e-16 | -8.34887714518118e-14 |
| 226 | 1.2 | 2.15 | 2.20993679142388 | 2.17328531370261 | -0.0599367914238838 | -0.023285313702607 | 2.15 | 2.15000000000006 | 1.33226762955019e-15 | 5.95079541199084e-14 |
| 227 | 1.2 | 2.2 | 2.2650371101485 | 2.22845593295884 | -0.0650371101484955 | -0.0284559329588392 | 2.2 | 2.1999999999999 | 8.88178419700125e-16 | -9.9475983006414e-14 |
| 228 | 1.2 | 2.25 | 2.3203352189976 | 2.28362856746397 | -0.0703352189976014 | -0.0336285674639654 | 2.25 | 2.25000000000005 | 4.44089209850063e-16 | 5.37347943918576e-14 |
| 229 | 1.2 | 2.3 | 2.37583326337159 | 2.33880321743818 | -0.0758332633715915 | -0.0388032174381792 | 2.3 | 2.29999999999992 | 1.77635683940025e-15 | -7.86037901434611e-14 |
| 230 | 1.2 | 2.35 | 2.43153342773835 | 2.39397988310279 | -0.0815334277383513 | -0.0439798831027853 | 2.35 | 2.34999999999988 | 1.77635683940025e-15 | -1.22568621918617e-13 |
| 231 | 1.2 | 2.4 | 2.48743793663657 | 2.44915856467872 | -0.0874379366365674 | -0.0491585646787178 | 2.4 | 2.39999999999992 | 8.88178419700125e-16 | -7.72715225139109e-14 |
| 232 | 1.2 | 2.45 | 2.54354905571239 | 2.50433926238673 | -0.0935490557123901 | -0.0543392623867272 | 2.45 | 2.44999999999985 | 4.44089209850063e-16 | -1.46105350040671e-13 |
| 233 | 1.2 | 2.5 | 2.59986909279084 | 2.55952197644812 | -0.0998690927908403 | -0.0595219764481167 | 2.5 | 2.49999999999993 | 0 | -6.57252030578093e-14 |
| 234 | 1.2 | 2.55 | 2.65640039898339 | 2.61470670708364 | -0.106400398983391 | -0.0647067070836367 | 2.55 | 2.54999999999989 | 8.88178419700125e-16 | -1.11910480882216e-13 |
| 235 | 1.2 | 2.6 | 2.71314536983322 | 2.66989345451459 | -0.113145369833219 | -0.06989345451459 | 2.6 | 2.5999999999999 | 0 | -9.63673585374636e-14 |
| 236 | 1.2 | 2.65 | 2.77010644649973 | 2.7250822189621 | -0.120106446499729 | -0.0750822189620974 | 2.65 | 2.64999999999997 | 8.88178419700125e-16 | -3.15303338993544e-14 |
| 237 | 1.2 | 2.7 | 2.82728611698396 | 2.78027300064728 | -0.127286116983961 | -0.0802730006472783 | 2.7 | 2.70000000000003 | 1.33226762955019e-15 | 3.10862446895044e-14 |
| 238 | 1.2 | 2.75 | 2.88468691739668 | 2.83546579979125 | -0.134686917396678 | -0.085465799791252 | 2.75 | 2.75 | 1.33226762955019e-15 | 4.44089209850063e-15 |
| 239 | 1.2 | 2.8 | 2.94231143327093 | 2.89066061661532 | -0.142311433270925 | -0.0906606166153225 | 2.8 | 2.79999999999993 | 8.88178419700125e-16 | -6.83897383169096e-14 |
| 240 | 1.2 | 2.85 | 3.00016230092099 | 2.94585745134098 | -0.150162300920987 | -0.0958574513409793 | 2.85 | 2.84999999999999 | 0 | -1.37667655053519e-14 |
| 241 | 1.2 | 2.9 | 3.05824220884978 | 3.00105630418934 | -0.158242208849776 | -0.101056304189342 | 2.9 | 2.89999999999997 | 4.44089209850063e-16 | -2.70894418008538e-14 |
| 242 | 1.2 | 2.95 | 3.11655389920677 | 3.0562571753819 | -0.166553899206769 | -0.106257175381901 | 2.95 | 2.94999999999999 | 1.33226762955019e-15 | -8.88178419700125e-15 |
| 243 | 1.2 | 3 | 3.17510016929873 | 3.11146006513996 | -0.175100169298725 | -0.11146006513996 | 3 | 2.99999999999994 | -4.44089209850063e-16 | -6.08402217494586e-14 |
| 244 | 1.2 | 3.05 | 3.23388387315557 | 3.16666497368519 | -0.183883873155572 | -0.116664973685192 | 3.05 | 3.05000000000001 | 1.77635683940025e-15 | 1.06581410364015e-14 |
| 245 | 1.2 | 3.1 | 3.29290792315386 | 3.22187190123872 | -0.192907923153864 | -0.121871901238719 | 3.1 | 3.09999999999986 | 1.33226762955019e-15 | -1.35891298214119e-13 |
| 246 | 1.2 | 3.15 | 3.35217529170053 | 3.27708084802259 | -0.202175291700535 | -0.127080848022585 | 3.15 | 3.14999999999996 | 8.88178419700125e-16 | -4.48530101948563e-14 |
| 247 | 1.2 | 3.2 | 3.4116890129796 | 3.33229181425791 | -0.211689012979595 | -0.132291814257908 | 3.2 | 3.19999999999987 | -4.44089209850063e-16 | -1.34114941374719e-13 |
| 248 | 1.2 | 3.25 | 3.47145218476475 | 3.38750480016673 | -0.221452184764752 | -0.137504800166734 | 3.25 | 3.24999999999998 | 2.22044604925031e-15 | -1.82076576038526e-14 |
| 249 | 1.2 | 3.3 | 3.53146797030095 | 3.44271980597037 | -0.231467970300953 | -0.142719805970366 | 3.3 | 3.29999999999998 | 4.44089209850063e-16 | -2.22044604925031e-14 |
| 250 | 1.2 | 3.35 | 3.59173960025818 | 3.49793683189067 | -0.241739600258178 | -0.147936831890665 | 3.35 | 3.35 | 8.88178419700125e-16 | -1.33226762955019e-15 |
| 251 | 1.2 | 3.4 | 3.65227037476078 | 3.55315587814931 | -0.252270374760775 | -0.153155878149306 | 3.4 | 3.39999999999998 | 1.77635683940025e-15 | -1.86517468137026e-14 |
| 252 | 1.2 | 3.45 | 3.71306366549605 | 3.60837694496815 | -0.263063665496047 | -0.158376944968146 | 3.45 | 3.45 | 4.44089209850063e-16 | -3.99680288865056e-15 |
| 253 | 1.2 | 3.5 | 3.77412291790586 | 3.66360003256886 | -0.274122917905863 | -0.163600032568863 | 3.5 | 3.49999999999991 | 8.88178419700125e-16 | -9.14823772291129e-14 |
| 254 | 1.2 | 3.55 | 3.83545165346531 | 3.7188251411735 | -0.285451653465306 | -0.168825141173499 | 3.55 | 3.54999999999988 | 1.33226762955019e-15 | -1.19904086659517e-13 |
| 255 | 1.2 | 3.6 | 3.89705347205265 | 3.7740522710041 | -0.297053472052653 | -0.174052271004099 | 3.6 | 3.60000000000004 | 0 | 3.86357612569555e-14 |
| 256 | 1.2 | 3.65 | 3.95893205441521 | 3.82928142228215 | -0.308932054415209 | -0.179281422282153 | 3.65 | 3.64999999999997 | 8.88178419700125e-16 | -2.88657986402541e-14 |
| 257 | 1.2 | 3.7 | 4.02109116473571 | 3.88451259522989 | -0.321091164735707 | -0.184512595229891 | 3.7 | 3.6999999999999 | 1.33226762955019e-15 | -1.01252339845814e-13 |
| 258 | 1.2 | 3.75 | 4.08353465330443 | 3.93974579006954 | -0.333534653304429 | -0.189745790069542 | 3.75 | 3.75000000000001 | 1.33226762955019e-15 | 4.88498130835069e-15 |
| 259 | 1.2 | 3.8 | 4.14626645930236 | 3.99498100702278 | -0.346266459302359 | -0.19498100702278 | 3.8 | 3.79999999999993 | 4.44089209850063e-16 | -6.66133814775094e-14 |
| 260 | 1.2 | 3.85 | 4.20929061370106 | 4.05021824631202 | -0.359290613701061 | -0.200218246312021 | 3.85 | 3.84999999999996 | 1.33226762955019e-15 | -3.77475828372553e-14 |
| 261 | 1.2 | 3.9 | 4.27261124228535 | 4.10545750815931 | -0.372611242285345 | -0.205457508159308 | 3.9 | 3.9 | 1.77635683940025e-15 | -2.22044604925031e-15 |
| 262 | 1.2 | 3.95 | 4.33623256880513 | 4.16069879278669 | -0.386232568805132 | -0.210698792786688 | 3.95 | 3.94999999999991 | 1.77635683940025e-15 | -9.14823772291129e-14 |
| 263 | 1.2 | 4 | 4.40015891826331 | 4.21594210041676 | -0.400158918263314 | -0.215942100416759 | 4 | 4.00000000000003 | 0 | 3.01980662698043e-14 |
| 264 | 1.2 | 4.05 | 4.46439472034687 | 4.27118743127138 | -0.414394720346865 | -0.22118743127138 | 4.05 | 4.04999999999999 | 8.88178419700125e-16 | -1.06581410364015e-14 |
| 265 | 1.2 | 4.1 | 4.52894451300891 | 4.32643478557315 | -0.42894451300891 | -0.226434785573153 | 4.1 | 4.10000000000005 | 0 | 4.79616346638068e-14 |
| 266 | 1.2 | 4.15 | 4.59381294620993 | 4.38168416354412 | -0.443812946209933 | -0.231684163544119 | 4.15 | 4.14999999999993 | 8.88178419700125e-16 | -7.37188088351104e-14 |
| 267 | 1.2 | 4.2 | 4.65900478582687 | 4.43693556540707 | -0.459004785826867 | -0.236935565407067 | 4.2 | 4.19999999999998 | -8.88178419700125e-16 | -1.77635683940025e-14 |
| 268 | 1.2 | 4.25 | 4.72452491773937 | 4.49218899138422 | -0.474524917739368 | -0.242188991384224 | 4.25 | 4.25000000000003 | 1.77635683940025e-15 | 2.75335310107039e-14 |
| 269 | 1.2 | 4.3 | 4.79037835210316 | 4.54744444169782 | -0.490378352103154 | -0.247444441697819 | 4.3 | 4.29999999999984 | 0 | -1.58095758706622e-13 |
| 270 | 1.2 | 4.35 | 4.85657022782106 | 4.60270191657101 | -0.506570227821062 | -0.25270191657101 | 4.35 | 4.35000000000001 | 8.88178419700125e-16 | 7.105427357601e-15 |
| 271 | 1.2 | 4.4 | 4.92310581722301 | 4.65796141622565 | -0.52310581722301 | -0.257961416225653 | 4.4 | 4.39999999999989 | 1.77635683940025e-15 | -1.11910480882216e-13 |
| 272 | 1.2 | 4.45 | 4.98999053096702 | 4.71322294088491 | -0.539990530967018 | -0.263222940884906 | 4.45 | 4.45 | 8.88178419700125e-16 | -4.44089209850063e-15 |
| 273 | 1.2 | 4.5 | 5.05722992317414 | 4.768486490771 | -0.557229923174138 | -0.268486490770996 | 4.5 | 4.49999999999996 | 1.77635683940025e-15 | -4.35207425653061e-14 |
| 274 | 1.2 | 4.55 | 5.12482969681108 | 4.82375206610689 | -0.574829696811083 | -0.273752066106893 | 4.55 | 4.55000000000004 | 1.77635683940025e-15 | 3.5527136788005e-14 |
| 275 | 1.2 | 4.6 | 5.19279570933533 | 4.87901966711501 | -0.59279570933533 | -0.279019667115015 | 4.6 | 4.59999999999996 | 0 | -4.17443857259059e-14 |
| 276 | 1.2 | 4.65 | 5.26113397861853 | 4.93428929401833 | -0.611133978618525 | -0.284289294018327 | 4.65 | 4.64999999999992 | 0 | -8.5265128291212e-14 |
| 277 | 1.2 | 4.7 | 5.3298506891651 | 4.98956094703962 | -0.629850689165095 | -0.289560947039617 | 4.7 | 4.69999999999989 | 8.88178419700125e-16 | -1.10134124042816e-13 |
| 278 | 1.2 | 4.75 | 5.39895219864431 | 5.04483462640167 | -0.648952198644313 | -0.29483462640167 | 4.75 | 4.74999999999983 | 1.77635683940025e-15 | -1.66977542903624e-13 |
| 279 | 1.2 | 4.8 | 5.46844504475531 | 5.10011033232764 | -0.668445044755305 | -0.300110332327638 | 4.8 | 4.79999999999999 | 8.88178419700125e-16 | -1.06581410364015e-14 |
| 280 | 1.2 | 4.85 | 5.53833595244599 | 5.15538806503994 | -0.688335952445989 | -0.305388065039939 | 4.85 | 4.8499999999999 | 8.88178419700125e-16 | -9.85878045867139e-14 |
| 281 | 1.2 | 4.9 | 5.60863184150852 | 5.21066782476191 | -0.708631841508515 | -0.310667824761909 | 4.9 | 4.89999999999991 | 8.88178419700125e-16 | -9.41469124882133e-14 |
| 282 | 1.2 | 4.95 | 5.67933983457547 | 5.26594961171652 | -0.729339834575473 | -0.315949611716522 | 4.95 | 4.94999999999997 | 1.77635683940025e-15 | -2.66453525910038e-14 |
| 283 | 1.2 | 5 | 5.75046726554303 | 5.32123342612675 | -0.750467265543032 | -0.321233426126745 | 5 | 5.00000000000004 | 8.88178419700125e-16 | 3.46389583683049e-14 |
| 284 | 1.2 | 5.05 | 5.82202168844921 | 5.37651926821555 | -0.772021688449206 | -0.326519268215549 | 5.05 | 5.04999999999998 | 8.88178419700125e-16 | -1.59872115546023e-14 |
| 285 | 1.2 | 5.1 | 5.89401088683766 | 5.43180713820627 | -0.794010886837662 | -0.331807138206272 | 5.1 | 5.10000000000001 | 8.88178419700125e-16 | 1.06581410364015e-14 |
| 286 | 1.2 | 5.15 | 5.96644288363993 | 5.48709703632189 | -0.816442883639926 | -0.337097036321887 | 5.15 | 5.14999999999994 | 0 | -5.95079541199084e-14 |
| 287 | 1.2 | 5.2 | 6.03932595161152 | 5.54238896278592 | -0.839325951611518 | -0.342388962785916 | 5.2 | 5.20000000000005 | 8.88178419700125e-16 | 5.41788836017076e-14 |
| 288 | 1.2 | 5.25 | 6.11266862436041 | 5.59768291782115 | -0.862668624360405 | -0.347682917821146 | 5.25 | 5.24999999999993 | 1.77635683940025e-15 | -6.83897383169096e-14 |
| 289 | 1.2 | 5.3 | 6.18647970800944 | 5.65297890165129 | -0.886479708009444 | -0.352978901651285 | 5.3 | 5.29999999999995 | 8.88178419700125e-16 | -4.79616346638068e-14 |
| 290 | 1.2 | 5.35 | 6.26076829353788 | 5.70827691449949 | -0.910768293537879 | -0.358276914499489 | 5.35 | 5.34999999999995 | 1.77635683940025e-15 | -4.70734562441066e-14 |
| 291 | 1.2 | 5.4 | 6.33554376985089 | 5.7635769565891 | -0.935543769850892 | -0.363576956589097 | 5.4 | 5.39999999999991 | 8.88178419700125e-16 | -9.32587340685131e-14 |
| 292 | 1.2 | 5.45 | 6.41081583763039 | 5.81887902814364 | -0.960815837630386 | -0.368879028143635 | 5.45 | 5.44999999999991 | 8.88178419700125e-16 | -8.88178419700125e-14 |
| 293 | 1.2 | 5.5 | 6.48659452402484 | 5.87418312938644 | -0.986594524024836 | -0.374183129386442 | 5.5 | 5.49999999999986 | 8.88178419700125e-16 | -1.35891298214119e-13 |
| 294 | 1.2 | 5.55 | 6.56289019824121 | 5.92948926054123 | -1.01289019824121 | -0.379489260541229 | 5.55 | 5.54999999999996 | 8.88178419700125e-16 | -4.35207425653061e-14 |
| 295 | 1.2 | 5.6 | 6.63971358810755 | 5.98479742183115 | -1.03971358810755 | -0.384797421831149 | 5.6 | 5.59999999999984 | 8.88178419700125e-16 | -1.56319401867222e-13 |
| 296 | 1.2 | 5.65 | 6.71707579768119 | 6.04010761348028 | -1.06707579768119 | -0.390107613480284 | 5.65 | 5.64999999999998 | 8.88178419700125e-16 | -2.1316282072803e-14 |
| 297 | 1.2 | 5.7 | 6.79498832598437 | 6.09541983571179 | -1.09498832598437 | -0.395419835711787 | 5.7 | 5.69999999999994 | 8.88178419700125e-16 | -5.86197757002083e-14 |
| 298 | 1.2 | 5.75 | 6.87346308695674 | 6.15073408874956 | -1.12346308695674 | -0.400734088749555 | 5.75 | 5.74999999999994 | 0 | -5.59552404411079e-14 |
| 299 | 1.2 | 5.8 | 6.95251243072288 | 6.2060503728173 | -1.15251243072288 | -0.406050372817296 | 5.8 | 5.79999999999999 | 0 | -6.21724893790088e-15 |
| 300 | 1.2 | 5.85 | 7.03214916628219 | 6.26136868813872 | -1.18214916628219 | -0.411368688138721 | 5.85 | 5.85000000000006 | 8.88178419700125e-16 | 6.12843109593086e-14 |
| 301 | 1.2 | 5.9 | 7.11238658573929 | 6.31668903493736 | -1.21238658573929 | -0.416689034937354 | 5.9 | 5.89999999999991 | 8.88178419700125e-16 | -8.70414851306123e-14 |
| 302 | 1.2 | 5.95 | 7.19323849020472 | 6.37201141343746 | -1.24323849020472 | -0.422011413437462 | 5.95 | 5.94999999999995 | 8.88178419700125e-16 | -5.32907051820075e-14 |
| 303 | 1.2 | 6 | 7.27471921750873 | 6.42733582386275 | -1.27471921750873 | -0.427335823862753 | 6 | 6.00000000000002 | 8.88178419700125e-16 | 2.22044604925031e-14 |
| 304 | 1.3 | 1 | 0.993958960343103 | 0.906341710653254 | 0.00604103965689717 | 0.0936582893467458 | 1 | 0.999999999999952 | 1.33226762955019e-15 | -4.75175454539567e-14 |
| 305 | 1.3 | 1.05 | 1.04502867874734 | 0.961466092016157 | 0.00497132125265631 | 0.0885339079838427 | 1.05 | 1.05000000000004 | 0 | 3.530509218308e-14 |
| 306 | 1.3 | 1.1 | 1.09625576008742 | 1.0165924835646 | 0.00374423991257777 | 0.0834075164354029 | 1.1 | 1.09999999999994 | 8.88178419700125e-16 | -6.3726801613484e-14 |
| 307 | 1.3 | 1.15 | 1.14764166805457 | 1.07172088551895 | 0.00235833194542789 | 0.0782791144810471 | 1.15 | 1.15000000000006 | 8.88178419700125e-16 | 6.43929354282591e-14 |
| 308 | 1.3 | 1.2 | 1.19918788917228 | 1.12685129809868 | 0.000812110827715484 | 0.0731487019013217 | 1.2 | 1.19999999999995 | 1.77635683940025e-15 | -4.75175454539567e-14 |
| 309 | 1.3 | 1.25 | 1.25089593329806 | 1.18198372152415 | -0.000895933298061147 | 0.0680162784758471 | 1.25 | 1.24999999999994 | 1.55431223447522e-15 | -6.32827124036339e-14 |
| 310 | 1.3 | 1.3 | 1.30276733413945 | 1.23711815601539 | -0.00276733413944696 | 0.0628818439846142 | 1.3 | 1.29999999999998 | 1.55431223447522e-15 | -1.86517468137026e-14 |
| 311 | 1.3 | 1.35 | 1.35480364978482 | 1.2922546017922 | -0.00480364978481851 | 0.0577453982077987 | 1.35 | 1.34999999999985 | 4.44089209850063e-16 | -1.53876911213047e-13 |
| 312 | 1.3 | 1.4 | 1.40700646324947 | 1.34739305907516 | -0.0070064632494693 | 0.052606940924836 | 1.4 | 1.39999999999993 | 6.66133814775094e-16 | -7.41628980449605e-14 |
| 313 | 1.3 | 1.45 | 1.45937738303751 | 1.4025335280841 | -0.00937738303750701 | 0.0474664719159028 | 1.45 | 1.44999999999991 | 4.44089209850063e-16 | -9.32587340685131e-14 |
| 314 | 1.3 | 1.5 | 1.51191804372016 | 1.45767600903938 | -0.0119180437201558 | 0.0423239909606192 | 1.5 | 1.49999999999994 | 4.44089209850063e-16 | -5.6621374255883e-14 |
| 315 | 1.3 | 1.55 | 1.56463010653103 | 1.51282050216121 | -0.0146301065310324 | 0.0371794978387912 | 1.55 | 1.54999999999999 | 1.11022302462516e-15 | -1.46549439250521e-14 |
| 316 | 1.3 | 1.6 | 1.61751525997901 | 1.56796700766978 | -0.0175152599790098 | 0.0320329923302243 | 1.6 | 1.59999999999995 | 1.11022302462516e-15 | -5.37347943918576e-14 |
| 317 | 1.3 | 1.65 | 1.67057522047933 | 1.62311552578546 | -0.0205752204793261 | 0.0268844742145393 | 1.65 | 1.64999999999987 | 8.88178419700125e-16 | -1.29674049276218e-13 |
| 318 | 1.3 | 1.7 | 1.72381173300358 | 1.67826605672883 | -0.0238117330035816 | 0.0217339432711723 | 1.7 | 1.69999999999993 | 1.33226762955019e-15 | -6.63913368725844e-14 |
| 319 | 1.3 | 1.75 | 1.77722657174934 | 1.73341860072007 | -0.027226571749335 | 0.0165813992799284 | 1.75 | 1.74999999999994 | 1.11022302462516e-15 | -5.97299987248334e-14 |
| 320 | 1.3 | 1.8 | 1.83082154083003 | 1.78857315797976 | -0.0308215408300272 | 0.0114268420202439 | 1.8 | 1.79999999999999 | 1.33226762955019e-15 | -7.105427357601e-15 |
| 321 | 1.3 | 1.85 | 1.88459847498598 | 1.84372972872826 | -0.0345984749859798 | 0.00627027127173929 | 1.85 | 1.84999999999999 | 0 | -9.32587340685131e-15 |
| 322 | 1.3 | 1.9 | 1.93855924031728 | 1.89888831318615 | -0.038559240317281 | 0.0011116868138501 | 1.9 | 1.89999999999996 | 8.88178419700125e-16 | -3.68594044175552e-14 |
| 323 | 1.3 | 1.95 | 1.99270573503935 | 1.95404891157399 | -0.0427057350393525 | -0.00404891157398746 | 1.95 | 1.9499999999999 | 4.44089209850063e-16 | -9.59232693276135e-14 |
| 324 | 1.3 | 2 | 2.04703989026211 | 2.00921152411252 | -0.0470398902621141 | -0.00921152411252368 | 2 | 1.99999999999994 | 8.88178419700125e-16 | -6.15063555642337e-14 |
| 325 | 1.3 | 2.05 | 2.10156367079359 | 2.06437615102232 | -0.0515636707935947 | -0.0143761510223221 | 2.05 | 2.04999999999999 | 8.88178419700125e-16 | -1.33226762955019e-14 |
| 326 | 1.3 | 2.1 | 2.15627907596898 | 2.11954279252395 | -0.0562790759689822 | -0.0195427925239469 | 2.1 | 2.09999999999993 | -4.44089209850063e-16 | -6.79456491070596e-14 |
| 327 | 1.3 | 2.15 | 2.21118814050608 | 2.17471144883833 | -0.0611881405060784 | -0.0247114488383327 | 2.15 | 2.14999999999996 | 2.22044604925031e-15 | -4.17443857259059e-14 |
| 328 | 1.3 | 2.2 | 2.26629293538818 | 2.22988212018604 | -0.0662929353881787 | -0.029882120186044 | 2.2 | 2.19999999999988 | 4.44089209850063e-16 | -1.23900889548167e-13 |
| 329 | 1.3 | 2.25 | 2.32159556877553 | 2.2850548067882 | -0.071595568775527 | -0.0350548067882004 | 2.25 | 2.24999999999996 | 2.22044604925031e-15 | -3.73034936274053e-14 |
| 330 | 1.3 | 2.3 | 2.37709818694637 | 2.34022950886537 | -0.0770981869463738 | -0.0402295088653659 | 2.3 | 2.29999999999996 | 4.44089209850063e-16 | -4.35207425653061e-14 |
| 331 | 1.3 | 2.35 | 2.43280297526895 | 2.39540622663866 | -0.0828029752689496 | -0.0454062266386597 | 2.35 | 2.35000000000006 | 1.33226762955019e-15 | 6.17284001691587e-14 |
| 332 | 1.3 | 2.4 | 2.48871215920548 | 2.45058496032865 | -0.0887121592054774 | -0.0505849603286461 | 2.4 | 2.39999999999994 | 1.33226762955019e-15 | -5.77315972805081e-14 |
| 333 | 1.3 | 2.45 | 2.54482800534963 | 2.50576571015663 | -0.0948280053496329 | -0.0557657101566305 | 2.45 | 2.4499999999999 | 1.33226762955019e-15 | -1.01252339845814e-13 |
| 334 | 1.3 | 2.5 | 2.60115282249875 | 2.56094847634373 | -0.101152822498749 | -0.0609484763437314 | 2.5 | 2.50000000000002 | 0 | 2.44249065417534e-14 |
| 335 | 1.3 | 2.55 | 2.65768896276225 | 2.61613325911051 | -0.107688962762249 | -0.0661332591105133 | 2.55 | 2.54999999999987 | 1.33226762955019e-15 | -1.25233157177718e-13 |
| 336 | 1.3 | 2.6 | 2.71443882270776 | 2.67132005867865 | -0.114438822707758 | -0.0713200586786504 | 2.6 | 2.59999999999997 | 1.77635683940025e-15 | -2.66453525910038e-14 |
| 337 | 1.3 | 2.65 | 2.77140484454654 | 2.72650887526889 | -0.121404844546542 | -0.0765088752688921 | 2.65 | 2.64999999999997 | 8.88178419700125e-16 | -2.97539770599542e-14 |
| 338 | 1.3 | 2.7 | 2.82858951735988 | 2.78169970910254 | -0.128589517359881 | -0.0816997091025438 | 2.7 | 2.69999999999998 | 1.33226762955019e-15 | -1.77635683940025e-14 |
| 339 | 1.3 | 2.75 | 2.88599537836813 | 2.83689256040072 | -0.135995378368134 | -0.0868925604007242 | 2.75 | 2.74999999999992 | 1.33226762955019e-15 | -7.99360577730113e-14 |
| 340 | 1.3 | 2.8 | 2.94362501424433 | 2.89208742938492 | -0.143625014244328 | -0.092087429384923 | 2.8 | 2.79999999999999 | -4.44089209850063e-16 | -5.77315972805081e-15 |
| 341 | 1.3 | 2.85 | 3.00148106247421 | 2.94728431627607 | -0.151481062474212 | -0.0972843162760739 | 2.85 | 2.84999999999987 | 4.44089209850063e-16 | -1.25677246387568e-13 |
| 342 | 1.3 | 2.9 | 3.05956621276475 | 3.00248322129604 | -0.159566212764751 | -0.102483221296037 | 2.9 | 2.90000000000003 | 1.77635683940025e-15 | 3.41948691584548e-14 |
| 343 | 1.3 | 2.95 | 3.11788320850325 | 3.05768414466556 | -0.16788320850325 | -0.107684144665563 | 2.95 | 2.9499999999999 | 8.88178419700125e-16 | -9.72555369571637e-14 |
| 344 | 1.3 | 3 | 3.17643484826931 | 3.11288708660669 | -0.176434848269308 | -0.112887086606694 | 3 | 3.00000000000005 | 8.88178419700125e-16 | 4.61852778244065e-14 |
| 345 | 1.3 | 3.05 | 3.23522398740196 | 3.16809204734037 | -0.185223987401959 | -0.118092047340365 | 3.05 | 3.04999999999999 | 8.88178419700125e-16 | -1.11022302462516e-14 |
| 346 | 1.3 | 3.1 | 3.29425353962451 | 3.22329902708825 | -0.194253539624506 | -0.123299027088252 | 3.1 | 3.09999999999989 | 4.44089209850063e-16 | -1.11022302462516e-13 |
| 347 | 1.3 | 3.15 | 3.35352647872965 | 3.27850802607221 | -0.203526478729647 | -0.128508026072214 | 3.15 | 3.15000000000004 | 1.33226762955019e-15 | 3.5527136788005e-14 |
| 348 | 1.3 | 3.2 | 3.41304584032765 | 3.33371904451318 | -0.213045840327654 | -0.133719044513184 | 3.2 | 3.19999999999984 | 1.33226762955019e-15 | -1.59428026336172e-13 |
| 349 | 1.3 | 3.25 | 3.47281472366052 | 3.38893208263358 | -0.222814723660516 | -0.138932082633578 | 3.25 | 3.25000000000002 | 1.33226762955019e-15 | 2.44249065417534e-14 |
| 350 | 1.3 | 3.3 | 3.53283629348511 | 3.44414714065433 | -0.232836293485113 | -0.14414714065433 | 3.3 | 3.29999999999993 | 2.22044604925031e-15 | -7.32747196252603e-14 |
| 351 | 1.3 | 3.35 | 3.59311378202865 | 3.49936421879767 | -0.243113782028648 | -0.14936421879767 | 3.35 | 3.35000000000003 | 1.33226762955019e-15 | 2.48689957516035e-14 |
| 352 | 1.3 | 3.4 | 3.65365049101979 | 3.5545833172849 | -0.253650491019787 | -0.154583317284902 | 3.4 | 3.39999999999992 | 1.33226762955019e-15 | -7.90478793533112e-14 |
| 353 | 1.3 | 3.45 | 3.71444979379906 | 3.60980443633826 | -0.264449793799062 | -0.159804436338256 | 3.45 | 3.45000000000002 | 4.44089209850063e-16 | 1.90958360235527e-14 |
| 354 | 1.3 | 3.5 | 3.77551513751243 | 3.66502757617922 | -0.275515137512432 | -0.165027576179221 | 3.5 | 3.50000000000002 | 1.77635683940025e-15 | 1.50990331349021e-14 |
| 355 | 1.3 | 3.55 | 3.83685004539195 | 3.72025273702984 | -0.286850045391946 | -0.170252737029843 | 3.55 | 3.55000000000007 | 4.44089209850063e-16 | 7.19424519957101e-14 |
| 356 | 1.3 | 3.6 | 3.89845811912788 | 3.77547991911179 | -0.298458119127881 | -0.175479919111794 | 3.6 | 3.59999999999998 | 1.33226762955019e-15 | -2.04281036531029e-14 |
| 357 | 1.3 | 3.65 | 3.96034304133676 | 3.83070912264712 | -0.310343041336764 | -0.180709122647121 | 3.65 | 3.64999999999983 | 0 | -1.72750702631674e-13 |
| 358 | 1.3 | 3.7 | 4.02250857813017 | 3.88594034785824 | -0.32250857813017 | -0.185940347858237 | 3.7 | 3.7 | 8.88178419700125e-16 | 2.66453525910038e-15 |
| 359 | 1.3 | 3.75 | 4.08495858178927 | 3.94117359496645 | -0.334958581789274 | -0.191173594966448 | 3.75 | 3.74999999999985 | 4.44089209850063e-16 | -1.49658063719471e-13 |
| 360 | 1.3 | 3.8 | 4.14769699355061 | 3.99640886419454 | -0.347696993550605 | -0.196408864194538 | 3.8 | 3.80000000000002 | 1.33226762955019e-15 | 1.86517468137026e-14 |
| 361 | 1.3 | 3.85 | 4.21072784650865 | 4.051646155764 | -0.360727846508646 | -0.201646155763996 | 3.85 | 3.84999999999995 | 4.44089209850063e-16 | -5.24025267623074e-14 |
| 362 | 1.3 | 3.9 | 4.27405526864138 | 4.10688546989742 | -0.374055268641382 | -0.206885469897422 | 3.9 | 3.90000000000004 | 8.88178419700125e-16 | 4.39648317751562e-14 |
| 363 | 1.3 | 3.95 | 4.33768348596516 | 4.16212680681668 | -0.387683485965155 | -0.212126806816676 | 3.95 | 3.95000000000001 | 0 | 8.88178419700125e-15 |
| 364 | 1.3 | 4 | 4.40161682582573 | 4.21737016674417 | -0.401616825825728 | -0.217370166744172 | 4 | 4.00000000000001 | 1.77635683940025e-15 | 7.105427357601e-15 |
| 365 | 1.3 | 4.05 | 4.46585972033272 | 4.27261554990214 | -0.415859720332719 | -0.22261554990214 | 4.05 | 4.05 | 1.77635683940025e-15 | 2.66453525910038e-15 |
| 366 | 1.3 | 4.1 | 4.53041670994521 | 4.32786295651281 | -0.430416709945212 | -0.227862956512811 | 4.1 | 4.09999999999992 | 8.88178419700125e-16 | -7.99360577730113e-14 |
| 367 | 1.3 | 4.15 | 4.59529244721668 | 4.38311238679878 | -0.445292447216684 | -0.233112386798782 | 4.15 | 4.14999999999998 | 8.88178419700125e-16 | -1.86517468137026e-14 |
| 368 | 1.3 | 4.2 | 4.66049170070802 | 4.43836384098228 | -0.460491700708016 | -0.238363840982284 | 4.2 | 4.20000000000004 | 8.88178419700125e-16 | 3.90798504668055e-14 |
| 369 | 1.3 | 4.25 | 4.72601935907791 | 4.49361731928555 | -0.476019359077911 | -0.243617319285548 | 4.25 | 4.24999999999991 | 1.77635683940025e-15 | -9.14823772291129e-14 |
| 370 | 1.3 | 4.3 | 4.79188043536062 | 4.54887282193136 | -0.491880435360619 | -0.248872821931356 | 4.3 | 4.29999999999987 | 1.77635683940025e-15 | -1.33226762955019e-13 |
| 371 | 1.3 | 4.35 | 4.8580800714416 | 4.60413034914231 | -0.508080071441595 | -0.254130349142311 | 4.35 | 4.34999999999999 | 0 | -7.105427357601e-15 |
| 372 | 1.3 | 4.4 | 4.92462354274239 | 4.65938990114064 | -0.524623542742386 | -0.259389901140639 | 4.4 | 4.39999999999999 | 8.88178419700125e-16 | -1.15463194561016e-14 |
| 373 | 1.3 | 4.45 | 4.9915162631268 | 4.71465147814913 | -0.541516263126801 | -0.264651478149127 | 4.45 | 4.45000000000002 | 8.88178419700125e-16 | 2.39808173319034e-14 |
| 374 | 1.3 | 4.5 | 5.05876379004133 | 4.76991508039019 | -0.558763790041328 | -0.26991508039019 | 4.5 | 4.4999999999999 | 0 | -1.03916875104915e-13 |
| 375 | 1.3 | 4.55 | 5.12637182990357 | 4.82518070808698 | -0.576371829903572 | -0.275180708086981 | 4.55 | 4.55000000000004 | 8.88178419700125e-16 | 3.5527136788005e-14 |
| 376 | 1.3 | 4.6 | 5.1943462437535 | 4.88044836146173 | -0.594346243753501 | -0.280448361461731 | 4.6 | 4.6 | 1.77635683940025e-15 | -8.88178419700125e-16 |
| 377 | 1.3 | 4.65 | 5.26269305318337 | 4.93571804073741 | -0.61269305318337 | -0.28571804073741 | 4.65 | 4.64999999999998 | 0 | -2.57571741713036e-14 |
| 378 | 1.3 | 4.7 | 5.33141844656328 | 4.9909897461368 | -0.631418446563275 | -0.290989746136802 | 4.7 | 4.69999999999995 | 0 | -5.15143483426073e-14 |
| 379 | 1.3 | 4.75 | 5.40052878558056 | 5.04626347788288 | -0.650528785580556 | -0.296263477882877 | 4.75 | 4.75000000000004 | 1.77635683940025e-15 | 3.46389583683049e-14 |
| 380 | 1.3 | 4.8 | 5.47003061211261 | 5.10153923619824 | -0.670030612112613 | -0.301539236198234 | 4.8 | 4.79999999999998 | 0 | -2.39808173319034e-14 |
| 381 | 1.3 | 4.85 | 5.53993065545417 | 5.15681702130603 | -0.68993065545417 | -0.30681702130603 | 4.85 | 4.84999999999998 | 8.88178419700125e-16 | -1.68753899743024e-14 |
| 382 | 1.3 | 4.9 | 5.61023583992154 | 5.21209683342905 | -0.710235839921544 | -0.312096833429048 | 4.9 | 4.89999999999989 | 1.77635683940025e-15 | -1.08357767203415e-13 |
| 383 | 1.3 | 4.95 | 5.68095329285826 | 5.26737867279044 | -0.730953292858261 | -0.317378672790443 | 4.95 | 4.94999999999984 | 1.77635683940025e-15 | -1.65201186064223e-13 |
| 384 | 1.3 | 5 | 5.75209035306825 | 5.32266253961337 | -0.752090353068248 | -0.322662539613369 | 5 | 4.99999999999991 | 8.88178419700125e-16 | -8.70414851306123e-14 |
| 385 | 1.3 | 5.05 | 5.82365457970478 | 5.37794843412061 | -0.77365457970478 | -0.327948434120613 | 5.05 | 5.04999999999985 | 2.66453525910038e-15 | -1.49213974509621e-13 |
| 386 | 1.3 | 5.1 | 5.89565376164571 | 5.4332363565357 | -0.795653761645712 | -0.333236356535698 | 5.1 | 5.10000000000001 | 8.88178419700125e-16 | 5.32907051820075e-15 |
| 387 | 1.3 | 5.15 | 5.96809592738789 | 5.48852630708123 | -0.81809592738789 | -0.338526307081224 | 5.15 | 5.14999999999986 | 8.88178419700125e-16 | -1.3855583347322e-13 |
| 388 | 1.3 | 5.2 | 6.0409893554963 | 5.54381828598109 | -0.840989355496304 | -0.343818285981087 | 5.2 | 5.20000000000004 | 1.77635683940025e-15 | 4.08562073062058e-14 |
| 389 | 1.3 | 5.25 | 6.11434258564651 | 5.59911229345789 | -0.864342585646511 | -0.349112293457887 | 5.25 | 5.24999999999995 | 0 | -4.70734562441066e-14 |
| 390 | 1.3 | 5.3 | 6.188164430302 | 5.65440832973533 | -0.888164430301996 | -0.354408329735332 | 5.3 | 5.29999999999997 | 1.77635683940025e-15 | -2.66453525910038e-14 |
| 391 | 1.3 | 5.35 | 6.26246398707168 | 5.70970639503658 | -0.912463987071674 | -0.359706395036577 | 5.35 | 5.34999999999994 | 8.88178419700125e-16 | -5.95079541199084e-14 |
| 392 | 1.3 | 5.4 | 6.33725065179662 | 5.76500648958515 | -0.937250651796615 | -0.365006489585149 | 5.4 | 5.39999999999999 | 1.77635683940025e-15 | -7.105427357601e-15 |
| 393 | 1.3 | 5.45 | 6.41253413241924 | 5.8203086136042 | -0.962534132419241 | -0.370308613604201 | 5.45 | 5.44999999999989 | 1.77635683940025e-15 | -1.05693231944315e-13 |
| 394 | 1.3 | 5.5 | 6.48832446369299 | 5.87561276731744 | -0.988324463692988 | -0.375612767317444 | 5.5 | 5.49999999999987 | 1.77635683940025e-15 | -1.27897692436818e-13 |
| 395 | 1.3 | 5.55 | 6.56463202279552 | 5.9309189509484 | -1.01463202279552 | -0.380918950948402 | 5.55 | 5.54999999999995 | 8.88178419700125e-16 | -4.61852778244065e-14 |
| 396 | 1.3 | 5.6 | 6.64146754591426 | 5.98622716472042 | -1.04146754591426 | -0.386227164720415 | 5.6 | 5.59999999999996 | 8.88178419700125e-16 | -4.2632564145606e-14 |
| 397 | 1.3 | 5.65 | 6.71884214587927 | 6.04153740885719 | -1.06884214587927 | -0.391537408857193 | 5.65 | 5.65 | 8.88178419700125e-16 | 1.77635683940025e-15 |
| 398 | 1.3 | 5.7 | 6.79676733092558 | 6.09684968358226 | -1.09676733092558 | -0.396849683582262 | 5.7 | 5.7 | 0 | 1.77635683940025e-15 |
| 399 | 1.3 | 5.75 | 6.87525502467445 | 6.15216398911933 | -1.12525502467445 | -0.402163989119331 | 5.75 | 5.75 | 8.88178419700125e-16 | 0 |
| 400 | 1.3 | 5.8 | 6.95431758743202 | 6.20748032569211 | -1.15431758743202 | -0.407480325692108 | 5.8 | 5.8 | 0 | 8.88178419700125e-16 |
| 401 | 1.3 | 5.85 | 7.03396783891292 | 6.26279869352431 | -1.18396783891292 | -0.412798693524308 | 5.85 | 5.84999999999998 | 8.88178419700125e-16 | -2.1316282072803e-14 |
| 402 | 1.3 | 5.9 | 7.11421908250714 | 6.31811909283964 | -1.21421908250714 | -0.418119092839636 | 5.9 | 5.89999999999986 | 0 | -1.37667655053519e-13 |
| 403 | 1.3 | 5.95 | 7.19508513122035 | 6.37344152386236 | -1.24508513122035 | -0.423441523862359 | 5.95 | 5.95000000000005 | 8.88178419700125e-16 | 4.88498130835069e-14 |
| 404 | 1.3 | 6 | 7.27658033543074 | 6.42876598681563 | -1.27658033543074 | -0.428765986815633 | 6 | 5.9999999999999 | 1.77635683940025e-15 | -1.03028696685215e-13 |
| 405 | 1.4 | 1 | 0.995113958251457 | 0.907765989122123 | 0.00488604174854312 | 0.0922340108778775 | 1 | 0.999999999999936 | 1.33226762955019e-15 | -6.3726801613484e-14 |
| 406 | 1.4 | 1.05 | 1.04618722488925 | 0.962890422421259 | 0.00381277511074729 | 0.0871095775787413 | 1.05 | 1.04999999999986 | 1.99840144432528e-15 | -1.42774680966795e-13 |
| 407 | 1.4 | 1.1 | 1.09741788736635 | 1.01801686591204 | 0.00258211263364538 | 0.0819831340879622 | 1.1 | 1.09999999999998 | 4.44089209850063e-16 | -1.88737914186277e-14 |
| 408 | 1.4 | 1.15 | 1.1488074098857 | 1.07314531981391 | 0.00119259011429862 | 0.0768546801860861 | 1.15 | 1.14999999999988 | -2.22044604925031e-16 | -1.23234755733392e-13 |
| 409 | 1.4 | 1.2 | 1.20035727949369 | 1.12827578434727 | -0.000357279493691687 | 0.0717242156527342 | 1.2 | 1.19999999999992 | 6.66133814775094e-16 | -8.4821039081362e-14 |
| 410 | 1.4 | 1.25 | 1.25206900658226 | 1.18340825973192 | -0.0020690065822635 | 0.0665917402680818 | 1.25 | 1.24999999999993 | 1.11022302462516e-15 | -7.14983627858601e-14 |
| 411 | 1.4 | 1.3 | 1.30394412540525 | 1.23854274618807 | -0.00394412540525413 | 0.0614572538119351 | 1.3 | 1.30000000000005 | 2.22044604925031e-16 | 4.72955008490317e-14 |
| 412 | 1.4 | 1.35 | 1.35598419460954 | 1.29367924393553 | -0.00598419460953692 | 0.0563207560644694 | 1.35 | 1.35000000000003 | 8.88178419700125e-16 | 3.06421554796543e-14 |
| 413 | 1.4 | 1.4 | 1.40819079778144 | 1.34881775319451 | -0.00819079778144127 | 0.0511822468054908 | 1.4 | 1.39999999999994 | 1.33226762955019e-15 | -6.30606677987089e-14 |
| 414 | 1.4 | 1.45 | 1.46056554400903 | 1.40395827418538 | -0.0105655440090335 | 0.0460417258146202 | 1.45 | 1.44999999999995 | 1.55431223447522e-15 | -4.52970994047064e-14 |
| 415 | 1.4 | 1.5 | 1.51311006846079 | 1.45910080712815 | -0.0131100684607866 | 0.0408991928718483 | 1.5 | 1.4999999999999 | 1.55431223447522e-15 | -9.81437153768638e-14 |
| 416 | 1.4 | 1.55 | 1.56582603298125 | 1.5142453522432 | -0.0158260329812496 | 0.0357546477567956 | 1.55 | 1.5499999999999 | 1.33226762955019e-15 | -1.0458300891969e-13 |
| 417 | 1.4 | 1.6 | 1.61871512670432 | 1.56939190975073 | -0.0187151267043213 | 0.0306080902492683 | 1.6 | 1.59999999999985 | 1.11022302462516e-15 | -1.53210777398272e-13 |
| 418 | 1.4 | 1.65 | 1.67177906668477 | 1.6245404798713 | -0.0217790666847748 | 0.0254595201287013 | 1.65 | 1.64999999999997 | 1.77635683940025e-15 | -3.15303338993544e-14 |
| 419 | 1.4 | 1.7 | 1.72501959854869 | 1.67969106282491 | -0.0250195985486874 | 0.0203089371750864 | 1.7 | 1.69999999999993 | 2.22044604925031e-16 | -6.86117829218347e-14 |
| 420 | 1.4 | 1.75 | 1.7784384971635 | 1.73484365883233 | -0.0284384971635048 | 0.0151563411676734 | 1.75 | 1.75000000000004 | 1.77635683940025e-15 | 4.2410519540681e-14 |
| 421 | 1.4 | 1.8 | 1.83203756732841 | 1.78999826811355 | -0.0320375673284086 | 0.0100017318864536 | 1.8 | 1.7999999999999 | 0 | -1.00142116821189e-13 |
| 422 | 1.4 | 1.85 | 1.88581864448583 | 1.84515489088969 | -0.0358186444858304 | 0.00484510911030767 | 1.85 | 1.85000000000007 | 4.44089209850063e-16 | 7.26085858104852e-14 |
| 423 | 1.4 | 1.9 | 1.93978359545481 | 1.90031352738059 | -0.0397835954548145 | -0.000313527380589207 | 1.9 | 1.89999999999992 | 1.11022302462516e-15 | -8.08242361927114e-14 |
| 424 | 1.4 | 1.95 | 1.99393431918713 | 1.95547417780736 | -0.0439343191871309 | -0.00547417780735549 | 1.95 | 1.94999999999993 | 6.66133814775094e-16 | -6.55031584528842e-14 |
| 425 | 1.4 | 2 | 2.04827274754697 | 2.01063684239037 | -0.0482727475469709 | -0.0106368423903715 | 2 | 1.99999999999991 | 0 | -9.1926466438963e-14 |
| 426 | 1.4 | 2.05 | 2.10280084611513 | 2.06580152135039 | -0.0528008461151335 | -0.0158015213503857 | 2.05 | 2.04999999999993 | 1.33226762955019e-15 | -7.41628980449605e-14 |
| 427 | 1.4 | 2.1 | 2.15752061501865 | 2.12096821490815 | -0.0575206150186491 | -0.0209682149081476 | 2.1 | 2.10000000000004 | 1.33226762955019e-15 | 3.95239396766556e-14 |
| 428 | 1.4 | 2.15 | 2.21243408978685 | 2.17613692328404 | -0.0624340897868518 | -0.0261369232840369 | 2.15 | 2.14999999999993 | 4.44089209850063e-16 | -7.23865412055602e-14 |
| 429 | 1.4 | 2.2 | 2.26754334223491 | 2.23130764669917 | -0.0675433422349072 | -0.0313076466991729 | 2.2 | 2.1999999999999 | 8.88178419700125e-16 | -9.59232693276135e-14 |
| 430 | 1.4 | 2.25 | 2.32285048137589 | 2.28648038537431 | -0.0728504813758906 | -0.036480385374305 | 2.25 | 2.24999999999991 | 8.88178419700125e-16 | -9.10382880192628e-14 |
| 431 | 1.4 | 2.3 | 2.37835765436257 | 2.34165513953037 | -0.0783576543625721 | -0.0416551395303673 | 2.3 | 2.30000000000001 | 4.44089209850063e-16 | 1.46549439250521e-14 |
| 432 | 1.4 | 2.35 | 2.43406704746007 | 2.39683190938792 | -0.0840670474600689 | -0.0468319093879237 | 2.35 | 2.34999999999992 | 4.44089209850063e-16 | -7.86037901434611e-14 |
| 433 | 1.4 | 2.4 | 2.48998088705062 | 2.45201069516828 | -0.0899808870506225 | -0.0520106951682791 | 2.4 | 2.39999999999996 | 8.88178419700125e-16 | -3.59712259978551e-14 |
| 434 | 1.4 | 2.45 | 2.54610144067182 | 2.50719149709218 | -0.0961014406718181 | -0.0571914970921834 | 2.45 | 2.44999999999994 | 4.44089209850063e-16 | -6.17284001691587e-14 |
| 435 | 1.4 | 2.5 | 2.60243101808961 | 2.56237431538076 | -0.102431018089605 | -0.0623743153807559 | 2.5 | 2.49999999999994 | 1.33226762955019e-15 | -6.30606677987089e-14 |
| 436 | 1.4 | 2.55 | 2.65897197240754 | 2.61755915025512 | -0.108971972407544 | -0.0675591502551152 | 2.55 | 2.55000000000002 | 1.33226762955019e-15 | 1.73194791841524e-14 |
| 437 | 1.4 | 2.6 | 2.71572670121382 | 2.67274600193601 | -0.115726701213822 | -0.0727460019360109 | 2.6 | 2.59999999999986 | 1.33226762955019e-15 | -1.35891298214119e-13 |
| 438 | 1.4 | 2.65 | 2.77269764776758 | 2.72793487064512 | -0.12269764776758 | -0.0779348706451173 | 2.65 | 2.64999999999996 | 4.44089209850063e-16 | -3.59712259978551e-14 |
| 439 | 1.4 | 2.7 | 2.82988730222624 | 2.78312575660319 | -0.129887302226236 | -0.0831257566031849 | 2.7 | 2.69999999999993 | 8.88178419700125e-16 | -6.97220059464598e-14 |
| 440 | 1.4 | 2.75 | 2.88729820291554 | 2.8383186600317 | -0.137298202915538 | -0.0883186600317027 | 2.75 | 2.75000000000001 | 0 | 7.99360577730113e-15 |
| 441 | 1.4 | 2.8 | 2.94493293764419 | 2.8935135811516 | -0.144932937644195 | -0.0935135811516044 | 2.8 | 2.7999999999999 | 1.33226762955019e-15 | -9.68114477473137e-14 |
| 442 | 1.4 | 2.85 | 3.00279414506498 | 2.94871052018457 | -0.15279414506498 | -0.0987105201845648 | 2.85 | 2.84999999999996 | 0 | -4.30766533554561e-14 |
| 443 | 1.4 | 2.9 | 3.06088451608438 | 3.0039094773517 | -0.160884516084382 | -0.103909477351703 | 2.9 | 2.89999999999997 | 1.77635683940025e-15 | -3.10862446895044e-14 |
| 444 | 1.4 | 2.95 | 3.11920679532287 | 3.05911045287451 | -0.169206795322868 | -0.10911045287451 | 2.95 | 2.95000000000004 | 1.33226762955019e-15 | 3.95239396766556e-14 |
| 445 | 1.4 | 3 | 3.17776378262808 | 3.1143134469741 | -0.177763782628078 | -0.114313446974105 | 3 | 2.9999999999999 | 1.33226762955019e-15 | -1.03916875104915e-13 |
| 446 | 1.4 | 3.05 | 3.23655833464325 | 3.16951845987253 | -0.186558334643245 | -0.11951845987253 | 3.05 | 3.05000000000007 | 8.88178419700125e-16 | 6.57252030578093e-14 |
| 447 | 1.4 | 3.1 | 3.29559336643336 | 3.22472549179054 | -0.195593366433359 | -0.124725491790537 | 3.1 | 3.09999999999987 | 8.88178419700125e-16 | -1.29674049276218e-13 |
| 448 | 1.4 | 3.15 | 3.35487185317169 | 3.27993454295035 | -0.204871853171692 | -0.129934542950354 | 3.15 | 3.14999999999993 | -4.44089209850063e-16 | -7.01660951563099e-14 |
| 449 | 1.4 | 3.2 | 3.41439683188944 | 3.33514561357329 | -0.214396831889442 | -0.135145613573287 | 3.2 | 3.2 | 4.44089209850063e-16 | -5.32907051820075e-15 |
| 450 | 1.4 | 3.25 | 3.4741714032914 | 3.39035870388083 | -0.2241714032914 | -0.140358703880825 | 3.25 | 3.24999999999994 | 4.44089209850063e-16 | -5.72875080706581e-14 |
| 451 | 1.4 | 3.3 | 3.53419873364072 | 3.44557381409483 | -0.234198733640722 | -0.145573814094827 | 3.3 | 3.29999999999995 | 0 | -4.79616346638068e-14 |
| 452 | 1.4 | 3.35 | 3.59448205671605 | 3.50079094443697 | -0.244482056716053 | -0.150790944436968 | 3.35 | 3.35 | 1.33226762955019e-15 | -3.99680288865056e-15 |
| 453 | 1.4 | 3.4 | 3.65502467584439 | 3.55601009512892 | -0.255024675844394 | -0.156010095128921 | 3.4 | 3.40000000000001 | 1.33226762955019e-15 | 1.15463194561016e-14 |
| 454 | 1.4 | 3.45 | 3.71582996601338 | 3.61123126639236 | -0.265829966013375 | -0.161231266392364 | 3.45 | 3.4499999999999 | 1.33226762955019e-15 | -1.01696429055664e-13 |
| 455 | 1.4 | 3.5 | 3.77690137606672 | 3.66645445844952 | -0.276901376066718 | -0.166454458449524 | 3.5 | 3.50000000000002 | 0 | 2.22044604925031e-14 |
| 456 | 1.4 | 3.55 | 3.83824243098695 | 3.72167967152171 | -0.288242430986953 | -0.171679671521705 | 3.55 | 3.54999999999987 | 1.33226762955019e-15 | -1.26121335597418e-13 |
| 457 | 1.4 | 3.6 | 3.89985673426964 | 3.77690690583132 | -0.299856734269636 | -0.176906905831324 | 3.6 | 3.59999999999991 | 1.33226762955019e-15 | -8.57092175010621e-14 |
| 458 | 1.4 | 3.65 | 3.96174797039364 | 3.83213616160005 | -0.311747970393643 | -0.182136161600053 | 3.65 | 3.6499999999999 | 8.88178419700125e-16 | -1.04805053524615e-13 |
| 459 | 1.4 | 3.7 | 4.02391990739227 | 3.88736743905012 | -0.323919907392273 | -0.187367439050123 | 3.7 | 3.70000000000004 | 4.44089209850063e-16 | 3.5527136788005e-14 |
| 460 | 1.4 | 3.75 | 4.08637639953028 | 3.94260073840321 | -0.336376399530279 | -0.192600738403209 | 3.75 | 3.75000000000002 | 4.44089209850063e-16 | 1.50990331349021e-14 |
| 461 | 1.4 | 3.8 | 4.14912139009219 | 3.99783605988154 | -0.349121390092189 | -0.197836059881539 | 3.8 | 3.79999999999998 | 8.88178419700125e-16 | -2.17603712826531e-14 |
| 462 | 1.4 | 3.85 | 4.21215891428764 | 4.05307340370735 | -0.362158914287641 | -0.203073403707345 | 3.85 | 3.85000000000003 | 8.88178419700125e-16 | 3.46389583683049e-14 |
| 463 | 1.4 | 3.9 | 4.27549310227976 | 4.10831277010248 | -0.37549310227976 | -0.208312770102484 | 3.9 | 3.89999999999992 | 1.33226762955019e-15 | -8.12683254025615e-14 |
| 464 | 1.4 | 3.95 | 4.33912818234305 | 4.16355415928956 | -0.389128182343051 | -0.213554159289559 | 3.95 | 3.95 | 4.44089209850063e-16 | 2.22044604925031e-15 |
| 465 | 1.4 | 4 | 4.4030684841576 | 4.21879757149043 | -0.403068484157603 | -0.218797571490426 | 4 | 3.99999999999995 | 0 | -5.37347943918576e-14 |
| 466 | 1.4 | 4.05 | 4.46731844224688 | 4.27404300692769 | -0.417318442246883 | -0.224043006927685 | 4.05 | 4.05000000000005 | 1.77635683940025e-15 | 4.61852778244065e-14 |
| 467 | 1.4 | 4.1 | 4.53188259956684 | 4.3292904658232 | -0.431882599566842 | -0.229290465823199 | 4.1 | 4.0999999999999 | 8.88178419700125e-16 | -1.04805053524615e-13 |
| 468 | 1.4 | 4.15 | 4.59676561125456 | 4.38453994839994 | -0.446765611254561 | -0.234539948399934 | 4.15 | 4.15000000000005 | 0 | 4.52970994047064e-14 |
| 469 | 1.4 | 4.2 | 4.66197224854518 | 4.43979145487975 | -0.461972248545177 | -0.239791454879753 | 4.2 | 4.20000000000002 | 0 | 1.68753899743024e-14 |
| 470 | 1.4 | 4.25 | 4.72750740286642 | 4.49504498548525 | -0.477507402866421 | -0.245044985485253 | 4.25 | 4.24999999999995 | 8.88178419700125e-16 | -4.61852778244065e-14 |
| 471 | 1.4 | 4.3 | 4.79337609012072 | 4.55030054043903 | -0.493376090120718 | -0.250300540439033 | 4.3 | 4.29999999999997 | 0 | -3.01980662698043e-14 |
| 472 | 1.4 | 4.35 | 4.85958345516545 | 4.60555811996351 | -0.509583455165452 | -0.255558119963513 | 4.35 | 4.34999999999998 | 8.88178419700125e-16 | -2.48689957516035e-14 |
| 473 | 1.4 | 4.4 | 4.92613477650272 | 4.66081772428129 | -0.526134776502718 | -0.260817724281285 | 4.4 | 4.4 | 8.88178419700125e-16 | 1.77635683940025e-15 |
| 474 | 1.4 | 4.45 | 4.99303547119067 | 4.71607935361477 | -0.543035471190673 | -0.26607935361477 | 4.45 | 4.44999999999989 | 1.77635683940025e-15 | -1.12798659301916e-13 |
| 475 | 1.4 | 4.5 | 5.06029109998939 | 4.77134300818694 | -0.560291099989391 | -0.271343008186935 | 4.5 | 4.49999999999993 | 1.77635683940025e-15 | -7.01660951563099e-14 |
| 476 | 1.4 | 4.55 | 5.1279073727551 | 4.8266086882202 | -0.577907372755102 | -0.276608688220194 | 4.55 | 4.54999999999989 | 0 | -1.12798659301916e-13 |
| 477 | 1.4 | 4.6 | 5.19589015409757 | 4.88187639393752 | -0.595890154097568 | -0.28187639393752 | 4.6 | 4.59999999999999 | 0 | -1.15463194561016e-14 |
| 478 | 1.4 | 4.65 | 5.26424546931651 | 4.93714612556132 | -0.614245469316508 | -0.287146125561324 | 4.65 | 4.64999999999991 | 1.77635683940025e-15 | -8.61533067109122e-14 |
| 479 | 1.4 | 4.7 | 5.33297951063404 | 4.99241788331476 | -0.632979510634044 | -0.292417883314763 | 4.7 | 4.69999999999999 | 8.88178419700125e-16 | -1.33226762955019e-14 |
| 480 | 1.4 | 4.75 | 5.40209864374146 | 5.04769166742044 | -0.652098643741455 | -0.297691667420437 | 4.75 | 4.74999999999998 | 1.77635683940025e-15 | -1.59872115546023e-14 |
| 481 | 1.4 | 4.8 | 5.47160941467977 | 5.10296747810131 | -0.671609414679772 | -0.302967478101313 | 4.8 | 4.79999999999998 | 8.88178419700125e-16 | -1.68753899743024e-14 |
| 482 | 1.4 | 4.85 | 5.54151855707532 | 5.15824531558018 | -0.691518557075322 | -0.30824531558018 | 4.85 | 4.84999999999986 | 8.88178419700125e-16 | -1.4033219031262e-13 |
| 483 | 1.4 | 4.9 | 5.61183299975281 | 5.21352518008037 | -0.711832999752812 | -0.313525180080373 | 4.9 | 4.89999999999995 | 8.88178419700125e-16 | -5.32907051820075e-14 |
| 484 | 1.4 | 4.95 | 5.68255987475035 | 5.2688070718245 | -0.732559874750344 | -0.318807071824496 | 4.95 | 4.94999999999988 | 8.88178419700125e-16 | -1.19904086659517e-13 |
| 485 | 1.4 | 5 | 5.75370652576259 | 5.32409099103589 | -0.753706525762589 | -0.324090991035887 | 5 | 4.99999999999992 | 8.88178419700125e-16 | -7.8159700933611e-14 |
| 486 | 1.4 | 5.05 | 5.82528051704042 | 5.37937693793752 | -0.775280517040421 | -0.329376937937516 | 5.05 | 5.04999999999996 | 1.77635683940025e-15 | -3.5527136788005e-14 |
| 487 | 1.4 | 5.1 | 5.89728964277752 | 5.43466491275235 | -0.797289642777524 | -0.334664912752352 | 5.1 | 5.09999999999987 | 1.77635683940025e-15 | -1.35891298214119e-13 |
| 488 | 1.4 | 5.15 | 5.96974193701696 | 5.48995491570392 | -0.819741937016963 | -0.339954915703921 | 5.15 | 5.14999999999994 | 8.88178419700125e-16 | -5.95079541199084e-14 |
| 489 | 1.4 | 5.2 | 6.04264568411335 | 5.54524694701519 | -0.842645684113353 | -0.345246947015193 | 5.2 | 5.19999999999997 | 1.77635683940025e-15 | -2.57571741713036e-14 |
| 490 | 1.4 | 5.25 | 6.11600942978916 | 5.60054100690951 | -0.86600942978916 | -0.350541006909507 | 5.25 | 5.25000000000005 | 8.88178419700125e-16 | 4.52970994047064e-14 |
| 491 | 1.4 | 5.3 | 6.18984199282698 | 5.65583709560983 | -0.889841992826978 | -0.355837095609832 | 5.3 | 5.29999999999986 | 0 | -1.3944401189292e-13 |
| 492 | 1.4 | 5.35 | 6.26415247744299 | 5.71113521334007 | -0.914152477442985 | -0.361135213340065 | 5.35 | 5.34999999999993 | 8.88178419700125e-16 | -7.37188088351104e-14 |
| 493 | 1.4 | 5.4 | 6.33895028639077 | 5.76643536032318 | -0.938950286390771 | -0.366435360323175 | 5.4 | 5.39999999999988 | 1.77635683940025e-15 | -1.22568621918617e-13 |
| 494 | 1.4 | 5.45 | 6.41424513484893 | 5.82173753678287 | -0.964245134848928 | -0.371737536782871 | 5.45 | 5.44999999999998 | 8.88178419700125e-16 | -2.30926389122033e-14 |
| 495 | 1.4 | 5.5 | 6.49004706515045 | 5.87704174294231 | -0.990047065150454 | -0.37704174294231 | 5.5 | 5.49999999999995 | 1.77635683940025e-15 | -4.61852778244065e-14 |
| 496 | 1.4 | 5.55 | 6.5663664624172 | 5.9323479790252 | -1.0163664624172 | -0.3823479790252 | 5.55 | 5.55 | 8.88178419700125e-16 | -1.77635683940025e-15 |
| 497 | 1.4 | 5.6 | 6.64321407116826 | 5.98765624525488 | -1.04321407116826 | -0.38765624525488 | 5.6 | 5.59999999999993 | 8.88178419700125e-16 | -7.28306304154103e-14 |
| 498 | 1.4 | 5.65 | 6.72060101297754 | 6.04296654185525 | -1.07060101297754 | -0.392966541855247 | 5.65 | 5.65000000000003 | 1.77635683940025e-15 | 2.75335310107039e-14 |
| 499 | 1.4 | 5.7 | 6.79853880526261 | 6.09827886904946 | -1.09853880526261 | -0.398278869049457 | 5.7 | 5.69999999999988 | 2.66453525910038e-15 | -1.21680443498917e-13 |
| 500 | 1.4 | 5.75 | 6.87703938129474 | 6.15359322706159 | -1.12703938129474 | -0.403593227061586 | 5.75 | 5.74999999999985 | 0 | -1.46549439250521e-13 |
| 501 | 1.4 | 5.8 | 6.95611511152857 | 6.20890961611535 | -1.15611511152857 | -0.408909616115347 | 5.8 | 5.79999999999996 | 8.88178419700125e-16 | -3.90798504668055e-14 |
| 502 | 1.4 | 5.85 | 7.0357788263593 | 6.26422803643427 | -1.1857788263593 | -0.414228036434264 | 5.85 | 5.85 | 1.77635683940025e-15 | 3.5527136788005e-15 |
| 503 | 1.4 | 5.9 | 7.11604384042602 | 6.31954848824205 | -1.21604384042602 | -0.419548488242048 | 5.9 | 5.89999999999991 | 8.88178419700125e-16 | -9.05941988094128e-14 |
| 504 | 1.4 | 5.95 | 7.19692397859153 | 6.37487097176278 | -1.24692397859153 | -0.424870971762777 | 5.95 | 5.94999999999991 | 1.77635683940025e-15 | -9.05941988094128e-14 |
| 505 | 1.4 | 6 | 7.27843360374206 | 6.43019548722035 | -1.27843360374206 | -0.430195487220348 | 6 | 6.00000000000003 | 8.88178419700125e-16 | 2.66453525910038e-14 |
| 506 | 1.5 | 1 | 0.996263936309178 | 0.909189607451085 | 0.00373606369082213 | 0.0908103925489151 | 1 | 0.999999999999916 | 2.22044604925031e-15 | -8.43769498715119e-14 |
| 507 | 1.5 | 1.05 | 1.04734073624982 | 0.96431409266277 | 0.00265926375017722 | 0.0856859073372305 | 1.05 | 1.04999999999994 | 1.33226762955019e-15 | -6.28386231937839e-14 |
| 508 | 1.5 | 1.1 | 1.09857496479937 | 1.01944058807165 | 0.00142503520062665 | 0.0805594119283519 | 1.1 | 1.10000000000005 | 8.88178419700125e-16 | 4.52970994047064e-14 |
| 509 | 1.5 | 1.15 | 1.14996808667045 | 1.07456909389736 | 3.19133295485496e-05 | 0.0754309061026404 | 1.15 | 1.14999999999998 | 4.44089209850063e-16 | -2.42028619368284e-14 |
| 510 | 1.5 | 1.2 | 1.20152158943031 | 1.1296996103601 | -0.00152158943031244 | 0.0703003896399015 | 1.2 | 1.19999999999993 | 6.66133814775094e-16 | -6.90558721316847e-14 |
| 511 | 1.5 | 1.25 | 1.25323698400324 | 1.18483213767987 | -0.00323698400323535 | 0.0651678623201264 | 1.25 | 1.24999999999991 | -2.22044604925031e-16 | -9.05941988094128e-14 |
| 512 | 1.5 | 1.3 | 1.30511580518721 | 1.23996667607688 | -0.00511580518721355 | 0.0600333239231206 | 1.3 | 1.30000000000004 | 4.44089209850063e-16 | 4.13002965160558e-14 |
| 513 | 1.5 | 1.35 | 1.35715961218542 | 1.29510322577076 | -0.00715961218541983 | 0.0548967742292448 | 1.35 | 1.34999999999992 | 1.11022302462516e-15 | -8.30446822419617e-14 |
| 514 | 1.5 | 1.4 | 1.40936998915299 | 1.35024178698207 | -0.0093699891529937 | 0.0497582130179348 | 1.4 | 1.39999999999993 | 2.22044604925031e-15 | -7.14983627858601e-14 |
| 515 | 1.5 | 1.45 | 1.46174854575967 | 1.40538235993082 | -0.0117485457596711 | 0.0446176400691818 | 1.45 | 1.44999999999993 | 1.33226762955019e-15 | -7.0832228971085e-14 |
| 516 | 1.5 | 1.5 | 1.51429691776884 | 1.46052494483739 | -0.0142969177688443 | 0.0394750551626062 | 1.5 | 1.50000000000007 | 1.11022302462516e-15 | 7.0832228971085e-14 |
| 517 | 1.5 | 1.55 | 1.56701676763361 | 1.51566954192162 | -0.0170167676336097 | 0.0343304580783839 | 1.55 | 1.54999999999997 | 6.66133814775094e-16 | -3.39728245535298e-14 |
| 518 | 1.5 | 1.6 | 1.61990978511045 | 1.57081615140405 | -0.0199097851104542 | 0.0291838485959506 | 1.6 | 1.59999999999986 | 1.99840144432528e-15 | -1.38333788868295e-13 |
| 519 | 1.5 | 1.65 | 1.67297768789119 | 1.62596477350526 | -0.0229776878911885 | 0.0240352264947417 | 1.65 | 1.64999999999997 | 1.33226762955019e-15 | -3.15303338993544e-14 |
| 520 | 1.5 | 1.7 | 1.72622222225383 | 1.68111540844525 | -0.0262222222538284 | 0.0188845915547486 | 1.7 | 1.69999999999996 | 1.11022302462516e-15 | -4.35207425653061e-14 |
| 521 | 1.5 | 1.75 | 1.77964516373309 | 1.73626805644459 | -0.0296451637330886 | 0.0137319435554064 | 1.75 | 1.74999999999996 | 8.88178419700125e-16 | -3.59712259978551e-14 |
| 522 | 1.5 | 1.8 | 1.83324831781124 | 1.79142271772366 | -0.0332483178112397 | 0.00857728227633614 | 1.8 | 1.79999999999992 | 4.44089209850063e-16 | -7.57172102794357e-14 |
| 523 | 1.5 | 1.85 | 1.88703352063009 | 1.84657939250303 | -0.0370335206300856 | 0.00342060749697359 | 1.85 | 1.8499999999999 | 6.66133814775094e-16 | -9.83657599817889e-14 |
| 524 | 1.5 | 1.9 | 1.94100263972485 | 1.90173808100325 | -0.0410026397248462 | -0.00173808100324613 | 1.9 | 1.89999999999992 | 1.77635683940025e-15 | -7.52731210695856e-14 |
| 525 | 1.5 | 1.95 | 1.99515757478078 | 1.95689878344489 | -0.0451575747807806 | -0.00689878344488615 | 1.95 | 1.94999999999998 | 2.22044604925031e-16 | -1.57651669496772e-14 |
| 526 | 1.5 | 2 | 2.04950025841344 | 2.01206150004833 | -0.0495002584134365 | -0.0120615000483273 | 2 | 1.99999999999987 | 0 | -1.31006316905768e-13 |
| 527 | 1.5 | 2.05 | 2.10403265697339 | 2.06722623103469 | -0.0540326569733867 | -0.0172262310346878 | 2.05 | 2.05 | 0 | -8.88178419700125e-16 |
| 528 | 1.5 | 2.1 | 2.15875677137645 | 2.12239297662416 | -0.0587567713764519 | -0.022392976624162 | 2.1 | 2.09999999999992 | 1.33226762955019e-15 | -7.7715611723761e-14 |
| 529 | 1.5 | 2.15 | 2.21367463796037 | 2.17756173703787 | -0.0636746379603661 | -0.0275617370378694 | 2.15 | 2.14999999999999 | 8.88178419700125e-16 | -1.33226762955019e-14 |
| 530 | 1.5 | 2.2 | 2.26878832936895 | 2.23273251249638 | -0.0687883293689451 | -0.0327325124963753 | 2.2 | 2.2 | 4.44089209850063e-16 | 2.66453525910038e-15 |
| 531 | 1.5 | 2.25 | 2.32409995546483 | 2.28790530322043 | -0.074099955464825 | -0.0379053032204277 | 2.25 | 2.24999999999991 | -4.44089209850063e-16 | -9.14823772291129e-14 |
| 532 | 1.5 | 2.3 | 2.37961166427192 | 2.34308010943115 | -0.0796116642719222 | -0.043080109431147 | 2.3 | 2.29999999999995 | 8.88178419700125e-16 | -5.46229728115577e-14 |
| 533 | 1.5 | 2.35 | 2.4353256429488 | 2.39825693134928 | -0.0853256429487952 | -0.0482569313492816 | 2.35 | 2.34999999999998 | 1.77635683940025e-15 | -2.17603712826531e-14 |
| 534 | 1.5 | 2.4 | 2.49124411879417 | 2.45343576919577 | -0.0912441187941684 | -0.0534357691957661 | 2.4 | 2.40000000000001 | 8.88178419700125e-16 | 6.21724893790088e-15 |
| 535 | 1.5 | 2.45 | 2.54736936028592 | 2.50861662319154 | -0.0973693602859225 | -0.0586166231915359 | 2.45 | 2.44999999999999 | 2.22044604925031e-15 | -7.99360577730113e-15 |
| 536 | 1.5 | 2.5 | 2.6037036781549 | 2.56379949355771 | -0.103703678154898 | -0.0637994935577098 | 2.5 | 2.50000000000003 | 1.33226762955019e-15 | 2.70894418008538e-14 |
| 537 | 1.5 | 2.55 | 2.66024942649501 | 2.61898438051522 | -0.110249426495006 | -0.0689843805152219 | 2.55 | 2.55 | 1.77635683940025e-15 | 4.44089209850063e-16 |
| 538 | 1.5 | 2.6 | 2.71700900391108 | 2.67417128428519 | -0.117009003911084 | -0.0741712842851912 | 2.6 | 2.59999999999993 | -4.44089209850063e-16 | -6.92779167366098e-14 |
| 539 | 1.5 | 2.65 | 2.77398485470615 | 2.72936020508892 | -0.12398485470615 | -0.0793602050889226 | 2.65 | 2.64999999999997 | 4.44089209850063e-16 | -3.06421554796543e-14 |
| 540 | 1.5 | 2.7 | 2.83117947010965 | 2.78455114314754 | -0.131179470109652 | -0.0845511431475363 | 2.7 | 2.70000000000006 | 0 | 6.26165785888588e-14 |
| 541 | 1.5 | 2.75 | 2.88859538954853 | 2.83974409868197 | -0.138595389548525 | -0.0897440986819662 | 2.75 | 2.74999999999995 | 8.88178419700125e-16 | -4.70734562441066e-14 |
| 542 | 1.5 | 2.8 | 2.94623520196283 | 2.8949390719137 | -0.146235201962828 | -0.0949390719137013 | 2.8 | 2.79999999999985 | 8.88178419700125e-16 | -1.52766688188422e-13 |
| 543 | 1.5 | 2.85 | 3.00410154716794 | 2.95013606306423 | -0.154101547167938 | -0.100136063064231 | 2.85 | 2.84999999999992 | 1.33226762955019e-15 | -8.12683254025615e-14 |
| 544 | 1.5 | 2.9 | 3.0621971172653 | 3.00533507235468 | -0.1621971172653 | -0.105335072354676 | 2.9 | 2.89999999999997 | 1.33226762955019e-15 | -3.37507799486048e-14 |
| 545 | 1.5 | 2.95 | 3.12052465810388 | 3.06053610000634 | -0.170524658103879 | -0.110536100006339 | 2.95 | 2.94999999999992 | 8.88178419700125e-16 | -7.90478793533112e-14 |
| 546 | 1.5 | 3 | 3.17908697079456 | 3.11573914624071 | -0.179086970794559 | -0.115739146240711 | 3 | 2.99999999999984 | 8.88178419700125e-16 | -1.55875312657372e-13 |
| 547 | 1.5 | 3.05 | 3.23788691327984 | 3.17094421127947 | -0.187886913279839 | -0.120944211279466 | 3.05 | 3.04999999999993 | 8.88178419700125e-16 | -7.14983627858601e-14 |
| 548 | 1.5 | 3.1 | 3.29692740196133 | 3.22615129534391 | -0.196927401961331 | -0.126151295343908 | 3.1 | 3.1 | 4.44089209850063e-16 | -3.99680288865056e-15 |
| 549 | 1.5 | 3.15 | 3.35621141338768 | 3.28136039865553 | -0.206211413387682 | -0.131360398655527 | 3.15 | 3.15 | 1.77635683940025e-15 | -3.10862446895044e-15 |
| 550 | 1.5 | 3.2 | 3.41574198600566 | 3.336571521436 | -0.215741986005661 | -0.136571521435998 | 3.2 | 3.20000000000001 | 1.33226762955019e-15 | 1.37667655053519e-14 |
| 551 | 1.5 | 3.25 | 3.47552222197736 | 3.39178466390681 | -0.225522221977363 | -0.141784663906809 | 3.25 | 3.24999999999993 | 4.44089209850063e-16 | -7.41628980449605e-14 |
| 552 | 1.5 | 3.3 | 3.53555528906658 | 3.44699982628982 | -0.235555289066576 | -0.146999826289821 | 3.3 | 3.29999999999991 | 1.33226762955019e-15 | -9.14823772291129e-14 |
| 553 | 1.5 | 3.35 | 3.59584442259756 | 3.50221700880671 | -0.24584442259756 | -0.152217008806708 | 3.35 | 3.34999999999994 | 4.44089209850063e-16 | -6.48370246381091e-14 |
| 554 | 1.5 | 3.4 | 3.65639292748968 | 3.55743621167914 | -0.256392927489683 | -0.157436211679144 | 3.4 | 3.39999999999994 | 8.88178419700125e-16 | -5.95079541199084e-14 |
| 555 | 1.5 | 3.45 | 3.71720418037151 | 3.61265743512899 | -0.267204180371512 | -0.162657435128989 | 3.45 | 3.44999999999999 | 4.44089209850063e-16 | -7.54951656745106e-15 |
| 556 | 1.5 | 3.5 | 3.77828163177821 | 3.66788067937792 | -0.27828163177821 | -0.167880679377918 | 3.5 | 3.49999999999995 | 4.44089209850063e-16 | -4.79616346638068e-14 |
| 557 | 1.5 | 3.55 | 3.83962880843625 | 3.72310594464798 | -0.28962880843625 | -0.173105944647975 | 3.55 | 3.54999999999998 | -4.44089209850063e-16 | -1.95399252334028e-14 |
| 558 | 1.5 | 3.6 | 3.90124931563978 | 3.77833323116102 | -0.301249315639777 | -0.17833323116102 | 3.6 | 3.60000000000003 | 1.33226762955019e-15 | 3.33066907387547e-14 |
| 559 | 1.5 | 3.65 | 3.96314683972309 | 3.83356253913873 | -0.313146839723089 | -0.183562539138728 | 3.65 | 3.64999999999986 | 0 | -1.37223565843669e-13 |
| 560 | 1.5 | 3.7 | 4.0253251506341 | 3.8887938688037 | -0.325325150634098 | -0.188793868803697 | 3.7 | 3.70000000000002 | 1.33226762955019e-15 | 2.22044604925031e-14 |
| 561 | 1.5 | 3.75 | 4.08778810461379 | 3.94402722037723 | -0.337788104613791 | -0.194027220377234 | 3.75 | 3.74999999999985 | 1.77635683940025e-15 | -1.45661260830821e-13 |
| 562 | 1.5 | 3.8 | 4.15053964698714 | 3.99926259408212 | -0.35053964698714 | -0.19926259408212 | 3.8 | 3.80000000000001 | 1.77635683940025e-15 | 5.32907051820075e-15 |
| 563 | 1.5 | 3.85 | 4.21358381507113 | 4.05449999013985 | -0.363583815071131 | -0.204499990139848 | 3.85 | 3.84999999999991 | 4.44089209850063e-16 | -8.74855743404623e-14 |
| 564 | 1.5 | 3.9 | 4.27692474120601 | 4.10973940877302 | -0.376924741206006 | -0.209739408773016 | 3.9 | 3.89999999999998 | 1.77635683940025e-15 | -1.73194791841524e-14 |
| 565 | 1.5 | 3.95 | 4.34056665591613 | 4.16498085020349 | -0.390566655916129 | -0.214980850203485 | 3.95 | 3.94999999999992 | 4.44089209850063e-16 | -8.5265128291212e-14 |
| 566 | 1.5 | 4 | 4.40451389120737 | 4.22022431465385 | -0.404513891207372 | -0.220224314653853 | 4 | 4.00000000000004 | 1.77635683940025e-15 | 3.90798504668055e-14 |
| 567 | 1.5 | 4.05 | 4.46877088400821 | 4.27546980234598 | -0.418770884008211 | -0.22546980234598 | 4.05 | 4.04999999999998 | 8.88178419700125e-16 | -2.04281036531029e-14 |
| 568 | 1.5 | 4.1 | 4.53334217976236 | 4.33071731350247 | -0.433342179762358 | -0.230717313502468 | 4.1 | 4.1 | 2.66453525910038e-15 | -8.88178419700125e-16 |
| 569 | 1.5 | 4.15 | 4.59823243618109 | 4.38596684834554 | -0.448232436181087 | -0.235966848345543 | 4.15 | 4.14999999999998 | 1.77635683940025e-15 | -2.1316282072803e-14 |
| 570 | 1.5 | 4.2 | 4.66344642716407 | 4.44121840709762 | -0.463446427164064 | -0.241218407097621 | 4.2 | 4.19999999999994 | 2.66453525910038e-15 | -6.21724893790088e-14 |
| 571 | 1.5 | 4.25 | 4.72898904689801 | 4.4964719899813 | -0.478989046898009 | -0.246471989981303 | 4.25 | 4.25000000000002 | 0 | 2.39808173319034e-14 |
| 572 | 1.5 | 4.3 | 4.79486531414315 | 4.55172759721882 | -0.494865314143147 | -0.251727597218816 | 4.3 | 4.30000000000001 | 1.77635683940025e-15 | 1.24344978758018e-14 |
| 573 | 1.5 | 4.35 | 4.86108037671805 | 4.60698522903276 | -0.511080376718047 | -0.256985229032765 | 4.35 | 4.34999999999998 | 0 | -2.04281036531029e-14 |
| 574 | 1.5 | 4.4 | 4.92763951619427 | 4.66224488564574 | -0.527639516194268 | -0.262244885645742 | 4.4 | 4.39999999999996 | 1.77635683940025e-15 | -4.2632564145606e-14 |
| 575 | 1.5 | 4.45 | 4.99454815281282 | 4.71750656728035 | -0.544548152812821 | -0.267506567280353 | 4.45 | 4.44999999999995 | 1.77635683940025e-15 | -5.15143483426073e-14 |
| 576 | 1.5 | 4.5 | 5.06181185063549 | 4.7727702741592 | -0.561811850635494 | -0.272770274159196 | 4.5 | 4.49999999999992 | 8.88178419700125e-16 | -8.34887714518118e-14 |
| 577 | 1.5 | 4.55 | 5.12943632294484 | 4.82803600650505 | -0.579436322944841 | -0.278036006505054 | 4.55 | 4.54999999999996 | 0 | -4.61852778244065e-14 |
| 578 | 1.5 | 4.6 | 5.19742743790768 | 4.88330376454053 | -0.59742743790768 | -0.28330376454053 | 4.6 | 4.59999999999995 | 8.88178419700125e-16 | -4.88498130835069e-14 |
| 579 | 1.5 | 4.65 | 5.265791224518 | 4.93857354848841 | -0.615791224517994 | -0.288573548488404 | 4.65 | 4.64999999999993 | 8.88178419700125e-16 | -7.28306304154103e-14 |
| 580 | 1.5 | 4.7 | 5.33453387883628 | 4.99384535857165 | -0.634533878836276 | -0.293845358571651 | 4.7 | 4.70000000000003 | 8.88178419700125e-16 | 3.19744231092045e-14 |
| 581 | 1.5 | 4.75 | 5.40366177054356 | 5.04911919501268 | -0.653661770543561 | -0.299119195012683 | 4.75 | 4.74999999999988 | 8.88178419700125e-16 | -1.23456800338317e-13 |
| 582 | 1.5 | 4.8 | 5.47318144982982 | 5.10439505803502 | -0.673181449829819 | -0.304395058035023 | 4.8 | 4.80000000000004 | 8.88178419700125e-16 | 3.90798504668055e-14 |
| 583 | 1.5 | 4.85 | 5.54309965463773 | 5.15967294786091 | -0.69309965463773 | -0.309672947860906 | 4.85 | 4.84999999999989 | 8.88178419700125e-16 | -1.07469588783715e-13 |
| 584 | 1.5 | 4.9 | 5.61342331828458 | 5.21495286471385 | -0.713423318284578 | -0.314952864713852 | 4.9 | 4.89999999999994 | 8.88178419700125e-16 | -6.48370246381091e-14 |
| 585 | 1.5 | 4.95 | 5.68415957748661 | 5.27023480881665 | -0.734159577486605 | -0.320234808816647 | 4.95 | 4.94999999999997 | 8.88178419700125e-16 | -3.10862446895044e-14 |
| 586 | 1.5 | 5 | 5.75531578081218 | 5.32551878039226 | -0.755315780812177 | -0.325518780392262 | 5 | 4.99999999999992 | 8.88178419700125e-16 | -7.63833440942108e-14 |
| 587 | 1.5 | 5.05 | 5.82689949759204 | 5.38080477966404 | -0.776899497592041 | -0.330804779664036 | 5.05 | 5.05000000000002 | 1.77635683940025e-15 | 2.22044604925031e-14 |
| 588 | 1.5 | 5.1 | 5.89891852731729 | 5.43609280685475 | -0.798918527317285 | -0.336092806854754 | 5.1 | 5.09999999999995 | 8.88178419700125e-16 | -4.70734562441066e-14 |
| 589 | 1.5 | 5.15 | 5.97138090955804 | 5.49138286218776 | -0.821380909558044 | -0.341382862187755 | 5.15 | 5.14999999999987 | 0 | -1.33226762955019e-13 |
| 590 | 1.5 | 5.2 | 6.04429493443862 | 5.54667494588657 | -0.844294934438616 | -0.346674945886566 | 5.2 | 5.20000000000005 | 8.88178419700125e-16 | 4.88498130835069e-14 |
| 591 | 1.5 | 5.25 | 6.11766915370765 | 5.60196905817378 | -0.867669153707649 | -0.351969058173784 | 5.25 | 5.24999999999991 | 0 | -9.41469124882133e-14 |
| 592 | 1.5 | 5.3 | 6.19151239244525 | 5.65726519927331 | -0.891512392445246 | -0.357265199273306 | 5.3 | 5.29999999999998 | 8.88178419700125e-16 | -1.95399252334028e-14 |
| 593 | 1.5 | 5.35 | 6.26583376145235 | 5.7125633694081 | -0.91583376145235 | -0.3625633694081 | 5.35 | 5.34999999999994 | 1.77635683940025e-15 | -5.95079541199084e-14 |
| 594 | 1.5 | 5.4 | 6.34064267037163 | 5.76786356880169 | -0.940642670371631 | -0.367863568801694 | 5.4 | 5.39999999999992 | 1.77635683940025e-15 | -7.63833440942108e-14 |
| 595 | 1.5 | 5.45 | 6.41594884159341 | 5.82316579767761 | -0.965948841593405 | -0.37316579767761 | 5.45 | 5.45000000000003 | 8.88178419700125e-16 | 2.57571741713036e-14 |
| 596 | 1.5 | 5.5 | 6.49176232500473 | 5.878470056259 | -0.991762325004731 | -0.378470056259004 | 5.5 | 5.49999999999997 | 0 | -2.8421709430404e-14 |
| 597 | 1.5 | 5.55 | 6.56809351364503 | 5.93377634476959 | -1.01809351364503 | -0.383776344769585 | 5.55 | 5.54999999999995 | 8.88178419700125e-16 | -4.88498130835069e-14 |
| 598 | 1.5 | 5.6 | 6.6449531603373 | 5.98908466343288 | -1.0449531603373 | -0.389084663432879 | 5.6 | 5.59999999999995 | 8.88178419700125e-16 | -4.9737991503207e-14 |
| 599 | 1.5 | 5.65 | 6.72235239537024 | 6.04439501247241 | -1.07235239537024 | -0.394395012472411 | 5.65 | 5.64999999999992 | 1.77635683940025e-15 | -8.08242361927114e-14 |
| 600 | 1.5 | 5.7 | 6.80030274531362 | 6.09970739211189 | -1.10030274531362 | -0.39970739211189 | 5.7 | 5.69999999999994 | 8.88178419700125e-16 | -6.48370246381091e-14 |
| 601 | 1.5 | 5.75 | 6.87881615305697 | 6.15502180257503 | -1.12881615305697 | -0.405021802575027 | 5.75 | 5.75000000000004 | 8.88178419700125e-16 | 4.08562073062058e-14 |
| 602 | 1.5 | 5.8 | 6.95790499917021 | 6.21033824408516 | -1.15790499917021 | -0.41033824408516 | 5.8 | 5.79999999999991 | 1.77635683940025e-15 | -9.32587340685131e-14 |
| 603 | 1.5 | 5.85 | 7.03758212469436 | 6.26565671686656 | -1.18758212469436 | -0.415656716866556 | 5.85 | 5.85 | 0 | 8.88178419700125e-16 |
| 604 | 1.5 | 5.9 | 7.11786085548118 | 6.32097722114256 | -1.21786085548118 | -0.420977221142555 | 5.9 | 5.89999999999992 | 8.88178419700125e-16 | -7.90478793533112e-14 |
| 605 | 1.5 | 5.95 | 7.19875502821238 | 6.37629975713724 | -1.24875502821238 | -0.426299757137235 | 5.95 | 5.94999999999989 | 1.77635683940025e-15 | -1.06581410364015e-13 |
| 606 | 1.5 | 6 | 7.28027901824222 | 6.43162432507449 | -1.28027901824222 | -0.431624325074493 | 6 | 5.99999999999994 | 1.77635683940025e-15 | -5.77315972805081e-14 |
| 607 | 1.6 | 1 | 0.997408893477546 | 0.910612565638291 | 0.00259110652245365 | 0.0893874343617086 | 1 | 0.999999999999904 | 4.44089209850063e-16 | -9.61453139325386e-14 |
| 608 | 1.6 | 1.05 | 1.04848921178084 | 0.965737102738469 | 0.00151078821915918 | 0.0842628972615306 | 1.05 | 1.04999999999995 | 1.77635683940025e-15 | -4.68514116391816e-14 |
| 609 | 1.6 | 1.1 | 1.09972699132861 | 1.02086365004139 | 0.000273008671388686 | 0.0791363499586075 | 1.1 | 1.09999999999998 | 6.66133814775094e-16 | -2.50910403565285e-14 |
| 610 | 1.6 | 1.15 | 1.15112369734116 | 1.07599220776688 | -0.00112369734115947 | 0.074007792233115 | 1.15 | 1.14999999999987 | 1.33226762955019e-15 | -1.27675647831893e-13 |
| 611 | 1.6 | 1.2 | 1.20268081790454 | 1.13112277613514 | -0.00268081790453567 | 0.0688772238648594 | 1.2 | 1.19999999999985 | 0 | -1.53876911213047e-13 |
| 612 | 1.6 | 1.25 | 1.25439986447327 | 1.18625535536617 | -0.00439986447326568 | 0.063744644633831 | 1.25 | 1.24999999999989 | 6.66133814775094e-16 | -1.0547118733939e-13 |
| 613 | 1.6 | 1.3 | 1.30628237238735 | 1.24138994567998 | -0.00628237238734486 | 0.058610054320021 | 1.3 | 1.29999999999998 | -2.22044604925031e-16 | -2.17603712826531e-14 |
| 614 | 1.6 | 1.35 | 1.35832990140407 | 1.29652654729658 | -0.00832990140406498 | 0.05347345270342 | 1.35 | 1.35000000000002 | 1.11022302462516e-15 | 2.26485497023532e-14 |
| 615 | 1.6 | 1.4 | 1.41054403624513 | 1.35166516043598 | -0.0105440362451295 | 0.0483348395640186 | 1.4 | 1.39999999999992 | 4.44089209850063e-16 | -8.43769498715119e-14 |
| 616 | 1.6 | 1.45 | 1.46292638715967 | 1.40680578531875 | -0.0129263871596661 | 0.043194214681253 | 1.45 | 1.45000000000001 | 6.66133814775094e-16 | 1.28785870856518e-14 |
| 617 | 1.6 | 1.5 | 1.51547859050364 | 1.4619484221647 | -0.015478590503641 | 0.0380515778352986 | 1.5 | 1.49999999999996 | 6.66133814775094e-16 | -3.79696274421804e-14 |
| 618 | 1.6 | 1.55 | 1.56820230933631 | 1.51709307119441 | -0.0182023093363133 | 0.0329069288055912 | 1.55 | 1.55000000000005 | 1.55431223447522e-15 | 4.55191440096314e-14 |
| 619 | 1.6 | 1.6 | 1.62109923403431 | 1.57223973262788 | -0.0210992340343104 | 0.0277602673721218 | 1.6 | 1.60000000000001 | 1.55431223447522e-15 | 5.99520433297585e-15 |
| 620 | 1.6 | 1.65 | 1.67417108292399 | 1.62738840668567 | -0.0241710829239927 | 0.0226115933143258 | 1.65 | 1.65000000000005 | 8.88178419700125e-16 | 5.32907051820075e-14 |
| 621 | 1.6 | 1.7 | 1.72741960293276 | 1.68253909358799 | -0.0274196029327578 | 0.0174609064120095 | 1.7 | 1.70000000000002 | 1.99840144432528e-15 | 2.46469511466785e-14 |
| 622 | 1.6 | 1.75 | 1.78084657025998 | 1.73769179355539 | -0.0308465702599798 | 0.0123082064446076 | 1.75 | 1.75000000000006 | 8.88178419700125e-16 | 5.70654634657331e-14 |
| 623 | 1.6 | 1.8 | 1.83445379106835 | 1.79284650680807 | -0.0344537910683489 | 0.00715349319192682 | 1.8 | 1.79999999999992 | 2.22044604925031e-15 | -8.5043083686287e-14 |
| 624 | 1.6 | 1.85 | 1.88824310219631 | 1.84800323356678 | -0.0382431021963072 | 0.00199676643321745 | 1.85 | 1.84999999999983 | 0 | -1.70086167372574e-13 |
| 625 | 1.6 | 1.9 | 1.94221637189246 | 1.90316197405227 | -0.0422163718924611 | -0.00316197405227014 | 1.9 | 1.9 | 1.77635683940025e-15 | -3.10862446895044e-15 |
| 626 | 1.6 | 1.95 | 1.9963755005727 | 1.95832272848454 | -0.0463755005727018 | -0.00832272848454418 | 1.95 | 1.9499999999999 | 1.77635683940025e-15 | -9.74775815620887e-14 |
| 627 | 1.6 | 2 | 2.050722421601 | 2.01348549708473 | -0.0507224216010003 | -0.0134854970847256 | 2 | 2.00000000000001 | 1.33226762955019e-15 | 5.77315972805081e-15 |
| 628 | 1.6 | 2.05 | 2.10525910209471 | 2.06865028007301 | -0.0552591020947144 | -0.0186502800730075 | 2.05 | 2.04999999999989 | 4.44089209850063e-16 | -1.11910480882216e-13 |
| 629 | 1.6 | 2.1 | 2.15998754375539 | 2.12381707767051 | -0.0599875437553874 | -0.0238170776705089 | 2.1 | 2.09999999999993 | 1.33226762955019e-15 | -6.75015598972095e-14 |
| 630 | 1.6 | 2.15 | 2.21490978372602 | 2.1789858900978 | -0.0649097837260166 | -0.0289858900977951 | 2.15 | 2.14999999999999 | 1.33226762955019e-15 | -1.46549439250521e-14 |
| 631 | 1.6 | 2.2 | 2.27002789547586 | 2.23415671757562 | -0.0700278954758562 | -0.0341567175756157 | 2.2 | 2.20000000000002 | 1.33226762955019e-15 | 2.22044604925031e-14 |
| 632 | 1.6 | 2.25 | 2.32534398971381 | 2.28932956032472 | -0.0753439897138075 | -0.0393295603247195 | 2.25 | 2.24999999999998 | 4.44089209850063e-16 | -1.73194791841524e-14 |
| 633 | 1.6 | 2.3 | 2.38086021533156 | 2.34450441856604 | -0.0808602153315601 | -0.0445044185660404 | 2.3 | 2.29999999999994 | 4.44089209850063e-16 | -6.43929354282591e-14 |
| 634 | 1.6 | 2.35 | 2.43657876037766 | 2.39968129252051 | -0.0865787603776642 | -0.0496812925205132 | 2.35 | 2.34999999999992 | 4.44089209850063e-16 | -8.39328606616618e-14 |
| 635 | 1.6 | 2.4 | 2.49250185306379 | 2.45486018240907 | -0.0925018530637907 | -0.0548601824090724 | 2.4 | 2.39999999999992 | 1.33226762955019e-15 | -7.90478793533112e-14 |
| 636 | 1.6 | 2.45 | 2.54863176280448 | 2.51004108845265 | -0.0986317628044762 | -0.0600410884526523 | 2.45 | 2.44999999999991 | 4.44089209850063e-16 | -8.88178419700125e-14 |
| 637 | 1.6 | 2.5 | 2.60497080129175 | 2.56522401087237 | -0.104970801291751 | -0.065224010872373 | 2.5 | 2.49999999999998 | 1.33226762955019e-15 | -2.08721928629529e-14 |
| 638 | 1.6 | 2.55 | 2.66152132360605 | 2.62040894988917 | -0.11152132360605 | -0.0704089498891678 | 2.55 | 2.55000000000001 | 4.44089209850063e-16 | 1.11022302462516e-14 |
| 639 | 1.6 | 2.6 | 2.71828572936496 | 2.67559590572397 | -0.118285729364963 | -0.0755959057239712 | 2.6 | 2.59999999999986 | 1.33226762955019e-15 | -1.4166445794217e-13 |
| 640 | 1.6 | 2.65 | 2.77526646391137 | 2.73078487859846 | -0.125266463911365 | -0.080784878598458 | 2.65 | 2.65000000000001 | 1.77635683940025e-15 | 6.21724893790088e-15 |
| 641 | 1.6 | 2.7 | 2.83246601954263 | 2.78597586873319 | -0.132466019542631 | -0.0859758687331929 | 2.7 | 2.6999999999999 | 8.88178419700125e-16 | -1.03028696685215e-13 |
| 642 | 1.6 | 2.75 | 2.88988693678266 | 2.84116887634985 | -0.139886936782657 | -0.0911688763498497 | 2.75 | 2.74999999999994 | 8.88178419700125e-16 | -5.81756864903582e-14 |
| 643 | 1.6 | 2.8 | 2.94753180569853 | 2.89636390166955 | -0.14753180569853 | -0.0963639016695486 | 2.8 | 2.80000000000001 | 1.77635683940025e-15 | 1.4210854715202e-14 |
| 644 | 1.6 | 2.85 | 3.00540326726378 | 2.95156094491341 | -0.155403267263779 | -0.101560944913408 | 2.85 | 2.84999999999995 | 0 | -5.19584375524573e-14 |
| 645 | 1.6 | 2.9 | 3.06350401477026 | 3.00676000630292 | -0.163504014770257 | -0.106760006302918 | 2.9 | 2.89999999999988 | 0 | -1.22124532708767e-13 |
| 646 | 1.6 | 2.95 | 3.12183679529074 | 3.06196108605975 | -0.171836795290738 | -0.111961086059754 | 2.95 | 2.95000000000007 | 1.33226762955019e-15 | 6.97220059464598e-14 |
| 647 | 1.6 | 3 | 3.18040441119454 | 3.11716418440466 | -0.180404411194537 | -0.117164184404664 | 3 | 2.99999999999991 | 8.88178419700125e-16 | -8.74855743404623e-14 |
| 648 | 1.6 | 3.05 | 3.23920972171848 | 3.17236930155969 | -0.189209721718481 | -0.122369301559693 | 3.05 | 3.04999999999993 | 1.33226762955019e-15 | -6.66133814775094e-14 |
| 649 | 1.6 | 3.1 | 3.29825564459573 | 3.22757643774615 | -0.198255644595732 | -0.127576437746145 | 3.1 | 3.09999999999995 | 1.33226762955019e-15 | -4.66293670342566e-14 |
| 650 | 1.6 | 3.15 | 3.35754515774509 | 3.28278559318551 | -0.207545157745088 | -0.132785593185512 | 3.15 | 3.14999999999992 | 0 | -7.7715611723761e-14 |
| 651 | 1.6 | 3.2 | 3.41708130102354 | 3.33799676809946 | -0.217081301023542 | -0.137996768099464 | 3.2 | 3.19999999999992 | -4.44089209850063e-16 | -7.86037901434611e-14 |
| 652 | 1.6 | 3.25 | 3.47686717804498 | 3.39320996270968 | -0.226867178044981 | -0.14320996270968 | 3.25 | 3.25 | 8.88178419700125e-16 | -4.44089209850063e-15 |
| 653 | 1.6 | 3.3 | 3.53690595806815 | 3.44842517723765 | -0.236905958068151 | -0.148425177237646 | 3.3 | 3.29999999999999 | 0 | -1.4210854715202e-14 |
| 654 | 1.6 | 3.35 | 3.59720087795711 | 3.50364241190504 | -0.247200877957113 | -0.153642411905039 | 3.35 | 3.34999999999986 | 2.22044604925031e-15 | -1.35891298214119e-13 |
| 655 | 1.6 | 3.4 | 3.65775524421759 | 3.5588616669339 | -0.257755244217594 | -0.158861666933903 | 3.4 | 3.3999999999999 | 8.88178419700125e-16 | -1.01252339845814e-13 |
| 656 | 1.6 | 3.45 | 3.71857243511294 | 3.61408294254591 | -0.268572435112941 | -0.164082942545912 | 3.45 | 3.44999999999999 | 4.44089209850063e-16 | -1.11022302462516e-14 |
| 657 | 1.6 | 3.5 | 3.7796559028634 | 3.66930623896274 | -0.279655902863404 | -0.16930623896274 | 3.5 | 3.5 | 1.33226762955019e-15 | -3.99680288865056e-15 |
| 658 | 1.6 | 3.55 | 3.84100917593288 | 3.72453155640625 | -0.291009175932875 | -0.174531556406248 | 3.55 | 3.54999999999991 | 4.44089209850063e-16 | -8.88178419700125e-14 |
| 659 | 1.6 | 3.6 | 3.90263586140735 | 3.77975889509867 | -0.302635861407349 | -0.179758895098666 | 3.6 | 3.60000000000003 | 4.44089209850063e-16 | 2.57571741713036e-14 |
| 660 | 1.6 | 3.65 | 3.96453964746963 | 3.83498825526148 | -0.314539647469629 | -0.184988255261481 | 3.65 | 3.64999999999992 | 1.33226762955019e-15 | -7.8159700933611e-14 |
| 661 | 1.6 | 3.7 | 4.02672430597509 | 3.89021963711711 | -0.326724305975093 | -0.19021963711711 | 3.7 | 3.69999999999999 | 1.77635683940025e-15 | -1.46549439250521e-14 |
| 662 | 1.6 | 3.75 | 4.08919369513361 | 3.94545304088723 | -0.339193695133614 | -0.195453040887226 | 3.75 | 3.74999999999989 | 2.22044604925031e-15 | -1.07469588783715e-13 |
| 663 | 1.6 | 3.8 | 4.15195176230302 | 4.00068846679424 | -0.351951762303024 | -0.200688466794244 | 3.8 | 3.79999999999995 | 0 | -4.61852778244065e-14 |
| 664 | 1.6 | 3.85 | 4.21500254689985 | 4.05592591506003 | -0.365002546899851 | -0.205925915060025 | 3.85 | 3.84999999999994 | 8.88178419700125e-16 | -5.77315972805081e-14 |
| 665 | 1.6 | 3.9 | 4.27835018343338 | 4.1111653859068 | -0.378350183433382 | -0.211165385906797 | 3.9 | 3.89999999999993 | 4.44089209850063e-16 | -7.37188088351104e-14 |
| 666 | 1.6 | 3.95 | 4.34199890466954 | 4.16640687955679 | -0.391998904669543 | -0.216406879556791 | 3.95 | 3.94999999999994 | 4.44089209850063e-16 | -6.17284001691587e-14 |
| 667 | 1.6 | 4 | 4.4059530449314 | 4.22165039623224 | -0.405953044931401 | -0.221650396232235 | 4 | 3.99999999999997 | 8.88178419700125e-16 | -2.62012633811537e-14 |
| 668 | 1.6 | 4.05 | 4.4702170435436 | 4.27689593615536 | -0.420217043543597 | -0.226895936155359 | 4.05 | 4.04999999999999 | -8.88178419700125e-16 | -6.21724893790088e-15 |
| 669 | 1.6 | 4.1 | 4.53479544842846 | 4.3321434995484 | -0.434795448428463 | -0.232143499548395 | 4.1 | 4.09999999999992 | 0 | -7.99360577730113e-14 |
| 670 | 1.6 | 4.15 | 4.59969291986203 | 4.38739308663394 | -0.449692919862032 | -0.237393086633939 | 4.15 | 4.14999999999997 | 8.88178419700125e-16 | -3.01980662698043e-14 |
| 671 | 1.6 | 4.2 | 4.66491423439876 | 4.44264469763422 | -0.464914234398757 | -0.242644697634224 | 4.2 | 4.2 | 8.88178419700125e-16 | -4.44089209850063e-15 |
| 672 | 1.6 | 4.25 | 4.73046428897427 | 4.49789833277166 | -0.480464288974274 | -0.247898332771663 | 4.25 | 4.24999999999998 | 8.88178419700125e-16 | -2.48689957516035e-14 |
| 673 | 1.6 | 4.3 | 4.79634810519619 | 4.55315399226886 | -0.496348105196192 | -0.253153992268854 | 4.3 | 4.30000000000002 | 8.88178419700125e-16 | 1.68753899743024e-14 |
| 674 | 1.6 | 4.35 | 4.86257083383352 | 4.60841167634803 | -0.512570833833516 | -0.258411676348032 | 4.35 | 4.34999999999986 | 0 | -1.37667655053519e-13 |
| 675 | 1.6 | 4.4 | 4.92913775951613 | 4.66367138523235 | -0.52913775951613 | -0.263671385232345 | 4.4 | 4.40000000000005 | 8.88178419700125e-16 | 4.52970994047064e-14 |
| 676 | 1.6 | 4.45 | 4.9960543056564 | 4.71893311914366 | -0.546054305656395 | -0.268933119143657 | 4.45 | 4.4499999999999 | 0 | -1.03028696685215e-13 |
| 677 | 1.6 | 4.5 | 5.06332603960589 | 4.77419687830531 | -0.563326039605894 | -0.274196878305308 | 4.5 | 4.50000000000005 | 8.88178419700125e-16 | 5.06261699229071e-14 |
| 678 | 1.6 | 4.55 | 5.13095867806117 | 4.82946266293934 | -0.580958678061171 | -0.27946266293934 | 4.55 | 4.54999999999993 | 8.88178419700125e-16 | -7.28306304154103e-14 |
| 679 | 1.6 | 4.6 | 5.19895809273332 | 4.88473047326891 | -0.598958092733323 | -0.28473047326891 | 4.6 | 4.59999999999992 | 1.77635683940025e-15 | -8.26005930321117e-14 |
| 680 | 1.6 | 4.65 | 5.26733031629738 | 4.94000030951662 | -0.617330316297375 | -0.290000309516616 | 4.65 | 4.64999999999987 | 1.77635683940025e-15 | -1.27009514017118e-13 |
| 681 | 1.6 | 4.7 | 5.33608154863847 | 4.99527217190543 | -0.636081548638464 | -0.295272171905429 | 4.7 | 4.69999999999994 | 0 | -5.59552404411079e-14 |
| 682 | 1.6 | 4.75 | 5.40521816341319 | 5.05054606065795 | -0.655218163413186 | -0.30054606065795 | 4.75 | 4.74999999999991 | 0 | -9.32587340685131e-14 |
| 683 | 1.6 | 4.8 | 5.4747467149457 | 5.10582197599733 | -0.674746714945695 | -0.30582197599733 | 4.8 | 4.8 | 8.88178419700125e-16 | 2.66453525910038e-15 |
| 684 | 1.6 | 4.85 | 5.54467394547974 | 5.16109991814617 | -0.694673945479738 | -0.311099918146174 | 4.85 | 4.84999999999994 | 1.77635683940025e-15 | -5.95079541199084e-14 |
| 685 | 1.6 | 4.9 | 5.6150067928093 | 5.21637988732782 | -0.7150067928093 | -0.316379887327816 | 4.9 | 4.90000000000005 | 1.77635683940025e-15 | 4.9737991503207e-14 |
| 686 | 1.6 | 4.95 | 5.68575239831229 | 5.27166188376486 | -0.735752398312294 | -0.321661883764859 | 4.95 | 4.94999999999997 | 8.88178419700125e-16 | -3.5527136788005e-14 |
| 687 | 1.6 | 5 | 5.75691811541366 | 5.32694590768064 | -0.756918115413662 | -0.326945907680644 | 5 | 4.99999999999995 | 8.88178419700125e-16 | -5.41788836017076e-14 |
| 688 | 1.6 | 5.05 | 5.82851151850624 | 5.38223195929814 | -0.778511518506238 | -0.332231959298138 | 5.05 | 5.04999999999988 | 0 | -1.17239551400417e-13 |
| 689 | 1.6 | 5.1 | 5.90054041236005 | 5.43752003884068 | -0.800540412360045 | -0.337520038840682 | 5.1 | 5.09999999999997 | 8.88178419700125e-16 | -3.5527136788005e-14 |
| 690 | 1.6 | 5.15 | 5.97301284205306 | 5.49281014653125 | -0.823012842053055 | -0.342810146531247 | 5.15 | 5.15000000000001 | 0 | 7.99360577730113e-15 |
| 691 | 1.6 | 5.2 | 6.04593710345926 | 5.54810228259299 | -0.845937103459259 | -0.348102282592985 | 5.2 | 5.19999999999996 | 8.88178419700125e-16 | -4.08562073062058e-14 |
| 692 | 1.6 | 5.25 | 6.11932175433267 | 5.60339644724942 | -0.869321754332669 | -0.353396447249424 | 5.25 | 5.25000000000006 | 0 | 6.21724893790088e-14 |
| 693 | 1.6 | 5.3 | 6.19317562602923 | 5.65869264072335 | -0.893175626029233 | -0.358692640723348 | 5.3 | 5.29999999999986 | 8.88178419700125e-16 | -1.4299672557172e-13 |
| 694 | 1.6 | 5.35 | 6.26750783591208 | 5.71399086323884 | -0.917507835912078 | -0.363990863238834 | 5.35 | 5.35000000000001 | 8.88178419700125e-16 | 1.24344978758018e-14 |
| 695 | 1.6 | 5.4 | 6.34232780048943 | 5.76929111501867 | -0.942327800489429 | -0.369291115018672 | 5.4 | 5.4 | 0 | -3.5527136788005e-15 |
| 696 | 1.6 | 5.45 | 6.4176452493388 | 5.82459339628657 | -0.967645249338797 | -0.374593396286567 | 5.45 | 5.45000000000007 | 8.88178419700125e-16 | 6.75015598972095e-14 |
| 697 | 1.6 | 5.5 | 6.4934702398757 | 5.87989770726568 | -0.993470239875699 | -0.379897707265677 | 5.5 | 5.49999999999996 | 0 | -4.44089209850063e-14 |
| 698 | 1.6 | 5.55 | 6.56981317303042 | 5.93520404817971 | -1.01981317303042 | -0.385204048179711 | 5.55 | 5.54999999999985 | 8.88178419700125e-16 | -1.53654866608122e-13 |
| 699 | 1.6 | 5.6 | 6.64668480990196 | 5.99051241925238 | -1.04668480990196 | -0.390512419252377 | 5.6 | 5.59999999999989 | 1.77635683940025e-15 | -1.08357767203415e-13 |
| 700 | 1.6 | 5.65 | 6.72409628946465 | 6.04582282070702 | -1.07409628946465 | -0.395822820707018 | 5.65 | 5.64999999999987 | 0 | -1.26121335597418e-13 |
| 701 | 1.6 | 5.7 | 6.80205914741002 | 6.10113525276753 | -1.10205914741002 | -0.401135252767528 | 5.7 | 5.70000000000004 | 1.77635683940025e-15 | 3.81916720471054e-14 |
| 702 | 1.6 | 5.75 | 6.88058533621399 | 6.15644971565706 | -1.13058533621399 | -0.406449715657061 | 5.75 | 5.74999999999992 | 8.88178419700125e-16 | -7.63833440942108e-14 |
| 703 | 1.6 | 5.8 | 6.9596872465284 | 6.21176620959988 | -1.1596872465284 | -0.411766209599882 | 5.8 | 5.80000000000003 | 0 | 3.37507799486048e-14 |
| 704 | 1.6 | 5.85 | 7.03937773000517 | 6.26708473481933 | -1.18937773000516 | -0.417084734819332 | 5.85 | 5.85 | 8.88178419700125e-16 | 2.66453525910038e-15 |
| 705 | 1.6 | 5.9 | 7.11967012367213 | 6.32240529153931 | -1.21967012367213 | -0.422405291539305 | 5.9 | 5.89999999999993 | 8.88178419700125e-16 | -7.28306304154103e-14 |
| 706 | 1.6 | 5.95 | 7.20057827599158 | 6.3777278799837 | -1.25057827599158 | -0.427727879983697 | 5.95 | 5.94999999999987 | 8.88178419700125e-16 | -1.33226762955019e-13 |
| 707 | 1.6 | 6 | 7.28211657474552 | 6.43305250037659 | -1.28211657474552 | -0.433052500376587 | 6 | 6.00000000000001 | 8.88178419700125e-16 | 8.88178419700125e-15 |
| 708 | 1.7 | 1 | 0.998548828722101 | 0.912034863681891 | 0.00145117127789907 | 0.0879651363181086 | 1 | 0.999999999999913 | -1.11022302462516e-16 | -8.67084182232247e-14 |
| 709 | 1.7 | 1.05 | 1.04963265043837 | 0.967159452646508 | 0.000367349561627206 | 0.0828405473534919 | 1.05 | 1.04999999999992 | 6.66133814775094e-16 | -8.21565038222616e-14 |
| 710 | 1.7 | 1.1 | 1.10087396590053 | 1.02228605181961 | -0.000873965900527685 | 0.0777139481803939 | 1.1 | 1.09999999999995 | 0 | -4.88498130835069e-14 |
| 711 | 1.7 | 1.15 | 1.15227424083452 | 1.07741466142101 | -0.00227424083452288 | 0.0725853385789905 | 1.15 | 1.14999999999991 | 4.44089209850063e-16 | -8.54871728961371e-14 |
| 712 | 1.7 | 1.2 | 1.20383496384315 | 1.13254528167091 | -0.00383496384315163 | 0.0674547183290877 | 1.2 | 1.20000000000001 | 0 | 9.32587340685131e-15 |
| 713 | 1.7 | 1.25 | 1.25555764690908 | 1.18767791278895 | -0.00555764690907523 | 0.0623220872110464 | 1.25 | 1.2499999999999 | 6.66133814775094e-16 | -1.0369483049999e-13 |
| 714 | 1.7 | 1.3 | 1.30744382591215 | 1.2428125549957 | -0.00744382591215054 | 0.057187445004302 | 1.3 | 1.30000000000004 | -2.22044604925031e-16 | 3.88578058618805e-14 |
| 715 | 1.7 | 1.35 | 1.35949506116159 | 1.29794920851079 | -0.00949506116158894 | 0.0520507914892152 | 1.35 | 1.35000000000003 | 1.99840144432528e-15 | 2.62012633811537e-14 |
| 716 | 1.7 | 1.4 | 1.41171293794342 | 1.35308787355441 | -0.0117129379434189 | 0.0469121264455923 | 1.4 | 1.39999999999991 | 1.11022302462516e-15 | -8.77076189453874e-14 |
| 717 | 1.7 | 1.45 | 1.46409906708386 | 1.40822855034695 | -0.0140990670838641 | 0.0417714496530537 | 1.45 | 1.44999999999989 | 8.88178419700125e-16 | -1.15019105351166e-13 |
| 718 | 1.7 | 1.5 | 1.51665508552913 | 1.46337123910859 | -0.0166550855291294 | 0.0366287608914055 | 1.5 | 1.49999999999993 | 1.11022302462516e-15 | -7.48290318597356e-14 |
| 719 | 1.7 | 1.55 | 1.56938265694224 | 1.51851594005955 | -0.0193826569422388 | 0.0314840599404531 | 1.55 | 1.54999999999998 | 2.22044604925031e-16 | -1.97619698383278e-14 |
| 720 | 1.7 | 1.6 | 1.62228347231752 | 1.57366265342 | -0.0222834723175231 | 0.0263373465800025 | 1.6 | 1.59999999999996 | 1.11022302462516e-15 | -4.10782519111308e-14 |
| 721 | 1.7 | 1.65 | 1.67535925061338 | 1.62881137941033 | -0.0253592506133842 | 0.0211886205896741 | 1.65 | 1.6499999999999 | 4.44089209850063e-16 | -9.72555369571637e-14 |
| 722 | 1.7 | 1.7 | 1.72861173940404 | 1.6839621182511 | -0.0286117394040437 | 0.0160378817489042 | 1.7 | 1.69999999999998 | 4.44089209850063e-16 | -1.68753899743024e-14 |
| 723 | 1.7 | 1.75 | 1.78204271555093 | 1.7391148701625 | -0.0320427155509306 | 0.0108851298374977 | 1.75 | 1.75 | 1.77635683940025e-15 | 8.88178419700125e-16 |
| 724 | 1.7 | 1.8 | 1.83565398589447 | 1.79426963536492 | -0.0356539858944664 | 0.00573036463507615 | 1.8 | 1.79999999999989 | 1.33226762955019e-15 | -1.12798659301916e-13 |
| 725 | 1.7 | 1.85 | 1.88944738796701 | 1.8494264140793 | -0.039447387967011 | 0.000573585920704778 | 1.85 | 1.85000000000004 | 6.66133814775094e-16 | 4.01900734914307e-14 |
| 726 | 1.7 | 1.9 | 1.94342479072775 | 1.90458520652563 | -0.043424790727745 | -0.00458520652562577 | 1.9 | 1.89999999999998 | 6.66133814775094e-16 | -1.70974345792274e-14 |
| 727 | 1.7 | 1.95 | 1.99758809532033 | 1.95974601292485 | -0.0475880953203343 | -0.00974601292484922 | 1.95 | 1.95000000000004 | 8.88178419700125e-16 | 4.06341627012807e-14 |
| 728 | 1.7 | 2 | 2.05193923585425 | 2.01490883349735 | -0.0519392358542468 | -0.014908833497346 | 2 | 2 | 1.77635683940025e-15 | -4.44089209850063e-16 |
| 729 | 1.7 | 2.05 | 2.10648018021062 | 2.07007366846386 | -0.0564801802106199 | -0.0200736684638643 | 2.05 | 2.04999999999994 | 8.88178419700125e-16 | -5.72875080706581e-14 |
| 730 | 1.7 | 2.1 | 2.16121293087365 | 2.12524051804515 | -0.0612129308736482 | -0.0252405180451536 | 2.1 | 2.09999999999992 | 1.33226762955019e-15 | -8.08242361927114e-14 |
| 731 | 1.7 | 2.15 | 2.21613952578845 | 2.18040938246196 | -0.066139525788449 | -0.0304093824619636 | 2.15 | 2.14999999999994 | -4.44089209850063e-16 | -6.03961325396085e-14 |
| 732 | 1.7 | 2.2 | 2.2712620392465 | 2.23558026193504 | -0.0712620392465002 | -0.0355802619350438 | 2.2 | 2.19999999999998 | 1.77635683940025e-15 | -2.08721928629529e-14 |
| 733 | 1.7 | 2.25 | 2.32658258279967 | 2.29075315668514 | -0.0765825827996651 | -0.0407531566851436 | 2.25 | 2.24999999999998 | 8.88178419700125e-16 | -2.26485497023532e-14 |
| 734 | 1.7 | 2.3 | 2.38210330620403 | 2.3459280669332 | -0.082103306204032 | -0.0459280669331972 | 2.3 | 2.3 | 4.44089209850063e-16 | 2.22044604925031e-15 |
| 735 | 1.7 | 2.35 | 2.43782639839468 | 2.40110499289995 | -0.087826398394681 | -0.0511049928999534 | 2.35 | 2.34999999999992 | -4.44089209850063e-16 | -8.12683254025615e-14 |
| 736 | 1.7 | 2.4 | 2.49375408849268 | 2.45628393480672 | -0.0937540884926817 | -0.056283934806717 | 2.4 | 2.40000000000006 | 1.77635683940025e-15 | 5.95079541199084e-14 |
| 737 | 1.7 | 2.45 | 2.5498886468456 | 2.51146489287387 | -0.099888646845598 | -0.0614648928738677 | 2.45 | 2.44999999999988 | 8.88178419700125e-16 | -1.19459997449667e-13 |
| 738 | 1.7 | 2.5 | 2.60623238610292 | 2.56664786732308 | -0.106232386102917 | -0.0666478673230802 | 2.5 | 2.49999999999998 | 2.22044604925031e-15 | -2.30926389122033e-14 |
| 739 | 1.7 | 2.55 | 2.66278766232778 | 2.62183285837492 | -0.112787662327781 | -0.0718328583749179 | 2.55 | 2.5499999999999 | -4.44089209850063e-16 | -1.00808250635964e-13 |
| 740 | 1.7 | 2.6 | 2.71955687614662 | 2.67701986625087 | -0.119556876146616 | -0.0770198662508701 | 2.6 | 2.6 | 1.77635683940025e-15 | -1.33226762955019e-15 |
| 741 | 1.7 | 2.65 | 2.77654247393814 | 2.73220889117169 | -0.12654247393814 | -0.0822088911716872 | 2.65 | 2.64999999999992 | 1.33226762955019e-15 | -7.63833440942108e-14 |
| 742 | 1.7 | 2.7 | 2.83374694906353 | 2.78739993335886 | -0.133746949063534 | -0.0873999333588587 | 2.7 | 2.69999999999995 | 1.33226762955019e-15 | -4.70734562441066e-14 |
| 743 | 1.7 | 2.75 | 2.89117284313943 | 2.8425929930335 | -0.141172843139427 | -0.0925929930335032 | 2.75 | 2.74999999999999 | 8.88178419700125e-16 | -5.32907051820075e-15 |
| 744 | 1.7 | 2.8 | 2.94882274735559 | 2.89778807041674 | -0.148822747355594 | -0.0977880704167409 | 2.8 | 2.79999999999992 | 1.33226762955019e-15 | -8.12683254025615e-14 |
| 745 | 1.7 | 2.85 | 3.00669930383927 | 2.95298516573006 | -0.156699303839271 | -0.10298516573006 | 2.85 | 2.8499999999999 | 4.44089209850063e-16 | -1.04360964314765e-13 |
| 746 | 1.7 | 2.9 | 3.06480520706814 | 3.00818427919477 | -0.164805207068139 | -0.108184279194766 | 2.9 | 2.89999999999989 | 0 | -1.08801856413265e-13 |
| 747 | 1.7 | 2.95 | 3.12314320533409 | 3.06338541103235 | -0.173143205334092 | -0.113385411032349 | 2.95 | 2.95 | 8.88178419700125e-16 | 1.77635683940025e-15 |
| 748 | 1.7 | 3 | 3.18171610226006 | 3.11858856146393 | -0.181716102260059 | -0.118588561463927 | 3 | 2.99999999999995 | 1.77635683940025e-15 | -4.79616346638068e-14 |
| 749 | 1.7 | 3.05 | 3.24052675837224 | 3.17379373071118 | -0.190526758372243 | -0.123793730711176 | 3.05 | 3.04999999999993 | 4.44089209850063e-16 | -6.75015598972095e-14 |
| 750 | 1.7 | 3.1 | 3.29957809273027 | 3.22900091899558 | -0.199578092730272 | -0.129000918995584 | 3.1 | 3.09999999999993 | 1.77635683940025e-15 | -7.061018436616e-14 |
| 751 | 1.7 | 3.15 | 3.35887308461787 | 3.28421012653883 | -0.208873084617872 | -0.134210126538826 | 3.15 | 3.15000000000006 | 0 | 6.08402217494586e-14 |
| 752 | 1.7 | 3.2 | 3.41841477529688 | 3.33942135356202 | -0.218414775296882 | -0.139421353562022 | 3.2 | 3.1999999999999 | 4.44089209850063e-16 | -9.63673585374636e-14 |
| 753 | 1.7 | 3.25 | 3.47820626982746 | 3.3946346002874 | -0.228206269827462 | -0.144634600287402 | 3.25 | 3.25000000000001 | 8.88178419700125e-16 | 4.88498130835069e-15 |
| 754 | 1.7 | 3.3 | 3.53825073895764 | 3.44984986693608 | -0.238250738957642 | -0.149849866936083 | 3.3 | 3.29999999999987 | 1.33226762955019e-15 | -1.30562227695918e-13 |
| 755 | 1.7 | 3.35 | 3.59855142108543 | 3.5050671537303 | -0.248551421085432 | -0.155067153730298 | 3.35 | 3.34999999999997 | 1.77635683940025e-15 | -2.97539770599542e-14 |
| 756 | 1.7 | 3.4 | 3.65911162429693 | 3.56028646089135 | -0.259111624296928 | -0.160286460891349 | 3.4 | 3.39999999999991 | 0 | -9.41469124882133e-14 |
| 757 | 1.7 | 3.45 | 3.71993472848407 | 3.61550778864128 | -0.269934728484068 | -0.165507788641281 | 3.45 | 3.44999999999991 | 8.88178419700125e-16 | -9.05941988094128e-14 |
| 758 | 1.7 | 3.5 | 3.78102418754583 | 3.67073113720196 | -0.281024187545828 | -0.170731137201955 | 3.5 | 3.50000000000001 | 1.33226762955019e-15 | 7.105427357601e-15 |
| 759 | 1.7 | 3.55 | 3.84238353167697 | 3.72595650679486 | -0.29238353167697 | -0.175956506794859 | 3.55 | 3.54999999999986 | 1.33226762955019e-15 | -1.4432899320127e-13 |
| 760 | 1.7 | 3.6 | 3.9040163697486 | 3.78118389764241 | -0.304016369748596 | -0.181183897642408 | 3.6 | 3.59999999999991 | 0 | -8.79296635503124e-14 |
| 761 | 1.7 | 3.65 | 3.96592639178507 | 3.83641330996628 | -0.31592639178507 | -0.186413309966276 | 3.65 | 3.64999999999993 | 8.88178419700125e-16 | -7.37188088351104e-14 |
| 762 | 1.7 | 3.7 | 4.02811737154208 | 3.89164474398851 | -0.328117371542081 | -0.19164474398851 | 3.7 | 3.69999999999995 | 8.88178419700125e-16 | -5.28466159721575e-14 |
| 763 | 1.7 | 3.75 | 4.09059316919102 | 3.94687819993115 | -0.340593169191019 | -0.196878199931152 | 3.75 | 3.74999999999999 | 4.44089209850063e-16 | -1.46549439250521e-14 |
| 764 | 1.7 | 3.8 | 4.15335773411498 | 4.00211367801625 | -0.353357734114978 | -0.202113678016247 | 3.8 | 3.80000000000002 | -4.44089209850063e-16 | 1.59872115546023e-14 |
| 765 | 1.7 | 3.85 | 4.21641510782219 | 4.05735117846584 | -0.366415107822189 | -0.20735117846584 | 3.85 | 3.84999999999998 | 0 | -2.30926389122033e-14 |
| 766 | 1.7 | 3.9 | 4.27976942698291 | 4.11259070150216 | -0.379769426982914 | -0.212590701502161 | 3.9 | 3.89999999999994 | 0 | -6.3948846218409e-14 |
| 767 | 1.7 | 3.95 | 4.3434249265963 | 4.16783224734744 | -0.393424926596302 | -0.21783224734744 | 3.95 | 3.94999999999993 | 1.33226762955019e-15 | -7.23865412055602e-14 |
| 768 | 1.7 | 4 | 4.40738594329402 | 4.22307581622391 | -0.407385943294017 | -0.223075816223906 | 4 | 3.99999999999995 | -8.88178419700125e-16 | -5.50670620214078e-14 |
| 769 | 1.7 | 4.05 | 4.471656918788 | 4.27832140835379 | -0.421656918788 | -0.228321408353787 | 4.05 | 4.04999999999995 | 0 | -5.59552404411079e-14 |
| 770 | 1.7 | 4.1 | 4.53624240347003 | 4.33356902395932 | -0.436242403470033 | -0.233569023959316 | 4.1 | 4.09999999999985 | 0 | -1.50990331349021e-13 |
| 771 | 1.7 | 4.15 | 4.60114706017145 | 4.38881866326328 | -0.451147060171453 | -0.238818663263276 | 4.15 | 4.15000000000005 | 8.88178419700125e-16 | 4.61852778244065e-14 |
| 772 | 1.7 | 4.2 | 4.66637566809173 | 4.44407032648734 | -0.466375668091724 | -0.244070326487341 | 4.2 | 4.19999999999988 | 1.77635683940025e-15 | -1.19015908239817e-13 |
| 773 | 1.7 | 4.25 | 4.73193312690531 | 4.49932401385467 | -0.481933126905307 | -0.249324013854666 | 4.25 | 4.25 | 8.88178419700125e-16 | 0 |
| 774 | 1.7 | 4.3 | 4.79782446105676 | 4.5545797255873 | -0.497824461056756 | -0.254579725587297 | 4.3 | 4.30000000000001 | 8.88178419700125e-16 | 1.15463194561016e-14 |
| 775 | 1.7 | 4.35 | 4.86405482425472 | 4.60983746190783 | -0.514054824254725 | -0.259837461907833 | 4.35 | 4.34999999999999 | 1.77635683940025e-15 | -1.33226762955019e-14 |
| 776 | 1.7 | 4.4 | 4.93062950417626 | 4.66509722303887 | -0.530629504176258 | -0.265097223038872 | 4.4 | 4.39999999999996 | 1.77635683940025e-15 | -4.17443857259059e-14 |
| 777 | 1.7 | 4.45 | 4.99755392739352 | 4.72035900920302 | -0.547553927393523 | -0.270359009203016 | 4.45 | 4.44999999999993 | 0 | -7.19424519957101e-14 |
| 778 | 1.7 | 4.5 | 5.06483366453595 | 4.77562282062305 | -0.564833664535949 | -0.275622820623049 | 4.5 | 4.50000000000002 | 8.88178419700125e-16 | 2.30926389122033e-14 |
| 779 | 1.7 | 4.55 | 5.1324744357017 | 4.83088865752139 | -0.582474435701703 | -0.280888657521385 | 4.55 | 4.55 | 1.77635683940025e-15 | -1.77635683940025e-15 |
| 780 | 1.7 | 4.6 | 5.20048211613335 | 4.88615652012081 | -0.600482116133353 | -0.286156520120811 | 4.6 | 4.59999999999991 | 1.77635683940025e-15 | -8.79296635503124e-14 |
| 781 | 1.7 | 4.65 | 5.26886274217369 | 4.94142640864429 | -0.61886274217369 | -0.291426408644293 | 4.65 | 4.64999999999995 | 8.88178419700125e-16 | -5.32907051820075e-14 |
| 782 | 1.7 | 4.7 | 5.33762251751875 | 4.99669832331443 | -0.637622517518754 | -0.296698323314434 | 4.7 | 4.69999999999992 | 8.88178419700125e-16 | -8.34887714518118e-14 |
| 783 | 1.7 | 4.75 | 5.40676781978643 | 5.0519722643542 | -0.656767819786426 | -0.301972264354203 | 4.75 | 4.74999999999993 | 8.88178419700125e-16 | -6.66133814775094e-14 |
| 784 | 1.7 | 4.8 | 5.47630520742027 | 5.10724823198638 | -0.676305207420271 | -0.307248231986383 | 4.8 | 4.7999999999999 | 8.88178419700125e-16 | -9.68114477473137e-14 |
| 785 | 1.7 | 4.85 | 5.54624142694977 | 5.16252622643395 | -0.696241426949766 | -0.312526226433947 | 4.85 | 4.84999999999987 | 8.88178419700125e-16 | -1.35003119794419e-13 |
| 786 | 1.7 | 4.9 | 5.61658342062967 | 5.21780624792005 | -0.716583420629673 | -0.317806247920046 | 4.9 | 4.89999999999998 | 1.77635683940025e-15 | -1.59872115546023e-14 |
| 787 | 1.7 | 4.95 | 5.68733833448305 | 5.27308829666728 | -0.737338334483045 | -0.323088296667284 | 4.95 | 4.94999999999989 | -8.88178419700125e-16 | -1.07469588783715e-13 |
| 788 | 1.7 | 5 | 5.75851352677424 | 5.32837237289918 | -0.758513526774236 | -0.328372372899183 | 5 | 5.00000000000002 | 8.88178419700125e-16 | 1.59872115546023e-14 |
| 789 | 1.7 | 5.05 | 5.83011657694034 | 5.38365847683834 | -0.780116576940335 | -0.333658476838342 | 5.05 | 5.04999999999991 | 8.88178419700125e-16 | -9.05941988094128e-14 |
| 790 | 1.7 | 5.1 | 5.90215529501174 | 5.43894660870829 | -0.802155295011735 | -0.338946608708287 | 5.1 | 5.09999999999993 | 8.88178419700125e-16 | -7.19424519957101e-14 |
| 791 | 1.7 | 5.15 | 5.97463773155498 | 5.49423676873199 | -0.824637731554978 | -0.34423676873199 | 5.15 | 5.14999999999989 | 0 | -1.11910480882216e-13 |
| 792 | 1.7 | 5.2 | 6.04757218817368 | 5.54952895713297 | -0.847572188173678 | -0.349528957132973 | 5.2 | 5.20000000000007 | 8.88178419700125e-16 | 6.92779167366098e-14 |
| 793 | 1.7 | 5.25 | 6.12096722860633 | 5.60482317413384 | -0.870967228606331 | -0.354823174133837 | 5.25 | 5.24999999999988 | 0 | -1.21680443498917e-13 |
| 794 | 1.7 | 5.3 | 6.19483169046298 | 5.66011941995848 | -0.894831690462978 | -0.360119419958477 | 5.3 | 5.29999999999986 | 8.88178419700125e-16 | -1.4388490399142e-13 |
| 795 | 1.7 | 5.35 | 6.26917469764628 | 5.71541769483023 | -0.919174697646282 | -0.365417694830231 | 5.35 | 5.35 | 8.88178419700125e-16 | 3.5527136788005e-15 |
| 796 | 1.7 | 5.4 | 6.34400567350641 | 5.77071799897207 | -0.944005673506408 | -0.370717998972071 | 5.4 | 5.39999999999995 | 8.88178419700125e-16 | -4.61852778244065e-14 |
| 797 | 1.7 | 5.45 | 6.41933435478343 | 5.82602033260771 | -0.969334354783432 | -0.376020332607707 | 5.45 | 5.44999999999997 | 1.77635683940025e-15 | -3.28626015289046e-14 |
| 798 | 1.7 | 5.5 | 6.49517080639565 | 5.88132469596048 | -0.995170806395648 | -0.381324695960478 | 5.5 | 5.49999999999994 | 8.88178419700125e-16 | -6.48370246381091e-14 |
| 799 | 1.7 | 5.55 | 6.57152543713739 | 5.93663108925409 | -1.02152543713739 | -0.386631089254093 | 5.55 | 5.55000000000005 | 0 | 4.52970994047064e-14 |
| 800 | 1.7 | 5.6 | 6.64840901635565 | 5.99193951271171 | -1.04840901635565 | -0.391939512711708 | 5.6 | 5.59999999999995 | 0 | -5.24025267623074e-14 |
| 801 | 1.7 | 5.65 | 6.72583269168115 | 6.04724996655722 | -1.07583269168115 | -0.397249966557219 | 5.65 | 5.64999999999993 | 0 | -7.54951656745106e-14 |
| 802 | 1.7 | 5.7 | 6.80380800789657 | 6.10256245101415 | -1.10380800789657 | -0.402562451014149 | 5.7 | 5.69999999999988 | 8.88178419700125e-16 | -1.16351372980716e-13 |
| 803 | 1.7 | 5.75 | 6.88234692703222 | 6.15787696630639 | -1.13234692703222 | -0.407876966306394 | 5.75 | 5.75000000000003 | 8.88178419700125e-16 | 3.28626015289046e-14 |
| 804 | 1.7 | 5.8 | 6.9614618497884 | 6.21319351265711 | -1.1614618497884 | -0.413193512657108 | 5.8 | 5.79999999999988 | 1.77635683940025e-15 | -1.26121335597418e-13 |
| 805 | 1.7 | 5.85 | 7.04116563839283 | 6.26851209029056 | -1.19116563839283 | -0.418512090290557 | 5.85 | 5.84999999999987 | 8.88178419700125e-16 | -1.26121335597418e-13 |
| 806 | 1.7 | 5.9 | 7.1214716410127 | 6.32383269943045 | -1.2214716410127 | -0.42383269943045 | 5.9 | 5.89999999999996 | 8.88178419700125e-16 | -3.90798504668055e-14 |
| 807 | 1.7 | 5.95 | 7.20239371785236 | 6.37915534030031 | -1.25239371785236 | -0.429155340300314 | 5.95 | 5.94999999999986 | 0 | -1.3944401189292e-13 |
| 808 | 1.7 | 6 | 7.28394626908116 | 6.43448001312441 | -1.28394626908116 | -0.434480013124412 | 6 | 5.99999999999993 | 1.77635683940025e-15 | -7.28306304154103e-14 |
| 809 | 1.8 | 1 | 0.999683741012632 | 0.913456501580035 | 0.000316258987368201 | 0.0865434984199655 | 1 | 0.999999999999956 | 1.11022302462516e-15 | -4.41868763800812e-14 |
| 810 | 1.8 | 1.05 | 1.05077105118278 | 0.96858114238522 | -0.0007710511827832 | 0.0814188576147799 | 1.05 | 1.05000000000001 | 8.88178419700125e-16 | 1.04360964314765e-14 |
| 811 | 1.8 | 1.1 | 1.10201588746591 | 1.02370779340444 | -0.00201588746591219 | 0.076292206595562 | 1.1 | 1.09999999999998 | 6.66133814775094e-16 | -1.53210777398272e-14 |
| 812 | 1.8 | 1.15 | 1.15341971609161 | 1.0788364548577 | -0.0034197160916114 | 0.0711635451423023 | 1.15 | 1.14999999999995 | -2.22044604925031e-16 | -5.26245713672324e-14 |
| 813 | 1.8 | 1.2 | 1.20498402617736 | 1.13396712696501 | -0.00498402617735527 | 0.0660328730349924 | 1.2 | 1.19999999999993 | -2.22044604925031e-16 | -7.03881397612349e-14 |
| 814 | 1.8 | 1.25 | 1.25671033023184 | 1.18909980994638 | -0.00671033023183498 | 0.0609001900536224 | 1.25 | 1.24999999999993 | 1.11022302462516e-15 | -7.19424519957101e-14 |
| 815 | 1.8 | 1.3 | 1.30860016467262 | 1.24423450402182 | -0.00860016467261637 | 0.0557654959781835 | 1.3 | 1.2999999999999 | 1.11022302462516e-15 | -9.88098491916389e-14 |
| 816 | 1.8 | 1.35 | 1.36065509035863 | 1.29937120941152 | -0.01065509035863 | 0.0506287905884812 | 1.35 | 1.34999999999994 | 1.99840144432528e-15 | -5.99520433297585e-14 |
| 817 | 1.8 | 1.4 | 1.41287669313799 | 1.35450992633549 | -0.012876693137988 | 0.0454900736645063 | 1.4 | 1.39999999999993 | 8.88178419700125e-16 | -6.83897383169096e-14 |
| 818 | 1.8 | 1.45 | 1.46526658441172 | 1.40965065501394 | -0.0152665844117159 | 0.0403493449860648 | 1.45 | 1.44999999999989 | 1.55431223447522e-15 | -1.0680345496894e-13 |
| 819 | 1.8 | 1.5 | 1.51782640171391 | 1.46479339566722 | -0.0178264017139074 | 0.0352066043327774 | 1.5 | 1.49999999999997 | 0 | -2.59792187762287e-14 |
| 820 | 1.8 | 1.55 | 1.57055780930896 | 1.51993814851537 | -0.0205578093089633 | 0.0300618514846347 | 1.55 | 1.54999999999995 | 6.66133814775094e-16 | -4.81836792687318e-14 |
| 821 | 1.8 | 1.6 | 1.62346249880646 | 1.57508491377874 | -0.0234624988064576 | 0.0249150862212577 | 1.6 | 1.5999999999999 | 4.44089209850063e-16 | -9.74775815620887e-14 |
| 822 | 1.8 | 1.65 | 1.67654218979434 | 1.63023369167792 | -0.0265421897943388 | 0.0197663083220816 | 1.65 | 1.65000000000003 | 2.22044604925031e-15 | 3.41948691584548e-14 |
| 823 | 1.8 | 1.7 | 1.72979863049109 | 1.6853844824329 | -0.0297986304910853 | 0.0146155175670981 | 1.7 | 1.70000000000001 | 1.77635683940025e-15 | 1.3988810110277e-14 |
| 824 | 1.8 | 1.75 | 1.78323359841756 | 1.74053728626426 | -0.0332335984175587 | 0.00946271373574192 | 1.75 | 1.74999999999998 | 1.33226762955019e-15 | -2.22044604925031e-14 |
| 825 | 1.8 | 1.8 | 1.83684890108925 | 1.79569210339255 | -0.036848901089245 | 0.00430789660744924 | 1.8 | 1.80000000000002 | 1.99840144432528e-15 | 2.30926389122033e-14 |
| 826 | 1.8 | 1.85 | 1.89064637672967 | 1.85084893403816 | -0.0406463767296708 | -0.000848934038159221 | 1.85 | 1.85000000000004 | 1.33226762955019e-15 | 4.41868763800812e-14 |
| 827 | 1.8 | 1.9 | 1.94462789500579 | 1.90600777842146 | -0.0446278950057937 | -0.00600777842146294 | 1.9 | 1.8999999999999 | 4.44089209850063e-16 | -1.01252339845814e-13 |
| 828 | 1.8 | 1.95 | 1.99879535778618 | 1.9611686367634 | -0.0487953577861831 | -0.0111686367633959 | 1.95 | 1.94999999999991 | 4.44089209850063e-16 | -8.92619311798626e-14 |
| 829 | 1.8 | 2 | 2.05315069992287 | 2.01633150928452 | -0.0531506999228668 | -0.0163315092845231 | 2 | 2.00000000000003 | 2.22044604925031e-15 | 3.33066907387547e-14 |
| 830 | 1.8 | 2.05 | 2.10769589005776 | 2.07149639620504 | -0.0576958900577607 | -0.0214963962050381 | 2.05 | 2.04999999999985 | 1.77635683940025e-15 | -1.54543045027822e-13 |
| 831 | 1.8 | 2.1 | 2.16243293145463 | 2.12666329774625 | -0.0624329314546306 | -0.0266632977462451 | 2.1 | 2.0999999999999 | 8.88178419700125e-16 | -1.02140518265514e-13 |
| 832 | 1.8 | 2.15 | 2.21736386285757 | 2.18183221412852 | -0.067363862857571 | -0.0318322141285243 | 2.15 | 2.14999999999987 | 8.88178419700125e-16 | -1.33670852164869e-13 |
| 833 | 1.8 | 2.2 | 2.27249075937705 | 2.237003145573 | -0.0724907593770459 | -0.0370031455729949 | 2.2 | 2.20000000000006 | 8.88178419700125e-16 | 5.90638649100583e-14 |
| 834 | 1.8 | 2.25 | 2.32781573340459 | 2.29217609229985 | -0.0778157334045897 | -0.0421760922998509 | 2.25 | 2.24999999999991 | 1.33226762955019e-15 | -8.88178419700125e-14 |
| 835 | 1.8 | 2.3 | 2.3833409355573 | 2.34735105453058 | -0.0833409355572998 | -0.0473510545305818 | 2.3 | 2.29999999999999 | 1.77635683940025e-15 | -5.77315972805081e-15 |
| 836 | 1.8 | 2.35 | 2.43906855565332 | 2.40252803248575 | -0.0890685556533182 | -0.0525280324857511 | 2.35 | 2.35 | 4.44089209850063e-16 | 3.5527136788005e-15 |
| 837 | 1.8 | 2.4 | 2.49500082371956 | 2.4577070263863 | -0.0950008237195599 | -0.0577070263862947 | 2.4 | 2.39999999999994 | 8.88178419700125e-16 | -6.3948846218409e-14 |
| 838 | 1.8 | 2.45 | 2.55114001103298 | 2.51288803645333 | -0.101140011032979 | -0.0628880364533311 | 2.45 | 2.44999999999992 | 4.44089209850063e-16 | -8.17124146124115e-14 |
| 839 | 1.8 | 2.5 | 2.60748843119678 | 2.5680710629078 | -0.107488431196784 | -0.0680710629077956 | 2.5 | 2.49999999999987 | 8.88178419700125e-16 | -1.28341781646668e-13 |
| 840 | 1.8 | 2.55 | 2.66404844125301 | 2.62325610597099 | -0.114048441253006 | -0.0732561059709913 | 2.55 | 2.55000000000002 | 1.33226762955019e-15 | 1.73194791841524e-14 |
| 841 | 1.8 | 2.6 | 2.72082244283297 | 2.67844316586348 | -0.120822442832969 | -0.078443165863483 | 2.6 | 2.59999999999987 | 1.77635683940025e-15 | -1.31450406115619e-13 |
| 842 | 1.8 | 2.65 | 2.77781288334722 | 2.73363224280695 | -0.127812883347219 | -0.0836322428069454 | 2.65 | 2.64999999999991 | 4.44089209850063e-16 | -9.05941988094128e-14 |
| 843 | 1.8 | 2.7 | 2.83502225721661 | 2.78882333702231 | -0.135022257216612 | -0.0888233370223133 | 2.7 | 2.69999999999992 | 8.88178419700125e-16 | -8.4821039081362e-14 |
| 844 | 1.8 | 2.75 | 2.89245310714628 | 2.84401644873089 | -0.142453107146276 | -0.094016448730891 | 2.75 | 2.74999999999996 | 4.44089209850063e-16 | -3.90798504668055e-14 |
| 845 | 1.8 | 2.8 | 2.95010802544433 | 2.8992115781538 | -0.150108025444328 | -0.0992115781537972 | 2.8 | 2.79999999999992 | 0 | -8.39328606616618e-14 |
| 846 | 1.8 | 2.85 | 3.00798965538725 | 2.95440872551252 | -0.157989655387248 | -0.104408725512521 | 2.85 | 2.84999999999995 | 0 | -5.10702591327572e-14 |
| 847 | 1.8 | 2.9 | 3.06610069263397 | 3.00960789102818 | -0.16610069263397 | -0.109607891028184 | 2.9 | 2.89999999999986 | 1.77635683940025e-15 | -1.4255263636187e-13 |
| 848 | 1.8 | 2.95 | 3.1244438866908 | 3.06480907492264 | -0.174443886690797 | -0.114809074922644 | 2.95 | 2.95000000000007 | 4.44089209850063e-16 | 7.28306304154103e-14 |
| 849 | 1.8 | 3 | 3.18302204242945 | 3.12001227741665 | -0.18302204242945 | -0.120012277416651 | 3 | 2.99999999999998 | 1.33226762955019e-15 | -1.68753899743024e-14 |
| 850 | 1.8 | 3.05 | 3.24183802166055 | 3.17521749873206 | -0.191838021660548 | -0.125217498732064 | 3.05 | 3.04999999999995 | 4.44089209850063e-16 | -5.50670620214078e-14 |
| 851 | 1.8 | 3.1 | 3.30089474476509 | 3.23042473909037 | -0.200894744765085 | -0.130424739090373 | 3.1 | 3.09999999999994 | 8.88178419700125e-16 | -5.6399329650958e-14 |
| 852 | 1.8 | 3.15 | 3.36019519238648 | 3.28563399871307 | -0.210195192386483 | -0.135633998713067 | 3.15 | 3.14999999999993 | 4.44089209850063e-16 | -6.97220059464598e-14 |
| 853 | 1.8 | 3.2 | 3.41974240718604 | 3.34084527782182 | -0.21974240718604 | -0.140845277821821 | 3.2 | 3.19999999999998 | 1.77635683940025e-15 | -1.90958360235527e-14 |
| 854 | 1.8 | 3.25 | 3.47953949566465 | 3.39605857663812 | -0.229539495664652 | -0.146058576638124 | 3.25 | 3.24999999999997 | 4.44089209850063e-16 | -2.57571741713036e-14 |
| 855 | 1.8 | 3.3 | 3.53958963005395 | 3.45127389538365 | -0.23958963005395 | -0.151273895383651 | 3.3 | 3.29999999999992 | 1.33226762955019e-15 | -8.4821039081362e-14 |
| 856 | 1.8 | 3.35 | 3.59989605028004 | 3.50649123428026 | -0.249896050280042 | -0.156491234280263 | 3.35 | 3.34999999999994 | 8.88178419700125e-16 | -5.90638649100583e-14 |
| 857 | 1.8 | 3.4 | 3.66046206600337 | 3.56171059354963 | -0.26046206600337 | -0.161710593549631 | 3.4 | 3.39999999999998 | 8.88178419700125e-16 | -1.59872115546023e-14 |
| 858 | 1.8 | 3.45 | 3.72129105873826 | 3.61693197341343 | -0.271291058738263 | -0.166931973413432 | 3.45 | 3.44999999999994 | 1.77635683940025e-15 | -5.90638649100583e-14 |
| 859 | 1.8 | 3.5 | 3.78238648405605 | 3.67215537409371 | -0.282386484056051 | -0.172155374093711 | 3.5 | 3.50000000000001 | 1.33226762955019e-15 | 6.66133814775094e-15 |
| 860 | 1.8 | 3.55 | 3.8437518738758 | 3.72738079581214 | -0.293751873875802 | -0.177380795812141 | 3.55 | 3.55 | 4.44089209850063e-16 | 2.22044604925031e-15 |
| 861 | 1.8 | 3.6 | 3.90539083884698 | 3.78260823879077 | -0.305390838846976 | -0.182608238790767 | 3.6 | 3.60000000000005 | 8.88178419700125e-16 | 5.01820807130571e-14 |
| 862 | 1.8 | 3.65 | 3.96730707082852 | 3.83783770325126 | -0.317307070828521 | -0.187837703251264 | 3.65 | 3.6499999999999 | 1.33226762955019e-15 | -1.02140518265514e-13 |
| 863 | 1.8 | 3.7 | 4.02950434546928 | 3.89306918941605 | -0.329504345469281 | -0.193069189416047 | 3.7 | 3.69999999999993 | 8.88178419700125e-16 | -7.01660951563099e-14 |
| 864 | 1.8 | 3.75 | 4.09198652489476 | 3.94830269750697 | -0.341986524894764 | -0.198302697506974 | 3.75 | 3.74999999999999 | 1.77635683940025e-15 | -1.24344978758018e-14 |
| 865 | 1.8 | 3.8 | 4.15475756050571 | 4.00353822774591 | -0.354757560505712 | -0.203538227745906 | 3.8 | 3.79999999999988 | 4.44089209850063e-16 | -1.22568621918617e-13 |
| 866 | 1.8 | 3.85 | 4.21782149589421 | 4.05877578035544 | -0.367821495894208 | -0.208775780355443 | 3.85 | 3.85000000000004 | 8.88178419700125e-16 | 3.99680288865056e-14 |
| 867 | 1.8 | 3.9 | 4.28118246988339 | 4.11401535555707 | -0.381182469883393 | -0.214015355557073 | 3.9 | 3.89999999999987 | 4.44089209850063e-16 | -1.27897692436818e-13 |
| 868 | 1.8 | 3.95 | 4.34484471969728 | 4.16925695357358 | -0.394844719697278 | -0.219256953573582 | 3.95 | 3.94999999999991 | 4.44089209850063e-16 | -9.32587340685131e-14 |
| 869 | 1.8 | 4 | 4.40881258426752 | 4.22450057462701 | -0.40881258426752 | -0.224500574627014 | 4 | 3.99999999999997 | 8.88178419700125e-16 | -2.88657986402541e-14 |
| 870 | 1.8 | 4.05 | 4.47309050768445 | 4.2797462189396 | -0.423090507684448 | -0.229746218939598 | 4.05 | 4.05000000000002 | 1.77635683940025e-15 | 2.1316282072803e-14 |
| 871 | 1.8 | 4.1 | 4.53768304280012 | 4.33499388673357 | -0.43768304280012 | -0.234993886733567 | 4.1 | 4.09999999999998 | 8.88178419700125e-16 | -2.04281036531029e-14 |
| 872 | 1.8 | 4.15 | 4.60259485499169 | 4.39024357823152 | -0.452594854991687 | -0.240243578231516 | 4.15 | 4.15000000000006 | 8.88178419700125e-16 | 6.21724893790088e-14 |
| 873 | 1.8 | 4.2 | 4.66783072609383 | 4.44549529365549 | -0.467830726093832 | -0.245495293655492 | 4.2 | 4.19999999999995 | 8.88178419700125e-16 | -4.88498130835069e-14 |
| 874 | 1.8 | 4.25 | 4.73339555850972 | 4.50074903322828 | -0.483395558509716 | -0.25074903322828 | 4.25 | 4.24999999999995 | 0 | -4.52970994047064e-14 |
| 875 | 1.8 | 4.3 | 4.79929437951037 | 4.55600479717229 | -0.499294379510373 | -0.256004797172293 | 4.3 | 4.30000000000002 | 1.77635683940025e-15 | 1.59872115546023e-14 |
| 876 | 1.8 | 4.35 | 4.86553234573328 | 4.61126258570976 | -0.515532345733282 | -0.261262585709764 | 4.35 | 4.34999999999987 | 1.77635683940025e-15 | -1.28785870856518e-13 |
| 877 | 1.8 | 4.4 | 4.93211474789147 | 4.66652239906366 | -0.53211474789147 | -0.266522399063659 | 4.4 | 4.39999999999989 | 1.77635683940025e-15 | -1.12798659301916e-13 |
| 878 | 1.8 | 4.45 | 4.99904701570532 | 4.7217842374564 | -0.549047015705324 | -0.271784237456394 | 4.45 | 4.4499999999999 | 1.77635683940025e-15 | -1.03916875104915e-13 |
| 879 | 1.8 | 4.5 | 5.06633472307014 | 4.77704810111076 | -0.566334723070139 | -0.277048101110756 | 4.5 | 4.50000000000002 | 0 | 2.39808173319034e-14 |
| 880 | 1.8 | 4.55 | 5.1339835934733 | 4.83231399024897 | -0.583983593473302 | -0.28231399024897 | 4.55 | 4.54999999999986 | 0 | -1.4210854715202e-13 |
| 881 | 1.8 | 4.6 | 5.20199950567601 | 4.88758190509438 | -0.601999505676008 | -0.287581905094382 | 4.6 | 4.59999999999996 | 8.88178419700125e-16 | -4.08562073062058e-14 |
| 882 | 1.8 | 4.65 | 5.27038849967551 | 4.9428518458694 | -0.620388499675505 | -0.2928518458694 | 4.65 | 4.65 | 1.77635683940025e-15 | 2.66453525910038e-15 |
| 883 | 1.8 | 4.7 | 5.33915678296494 | 4.99812381279681 | -0.639156782964935 | -0.298123812796814 | 4.7 | 4.69999999999997 | 1.77635683940025e-15 | -2.57571741713036e-14 |
| 884 | 1.8 | 4.75 | 5.40831073710918 | 5.05339780609959 | -0.658310737109179 | -0.303397806099591 | 4.75 | 4.74999999999998 | 1.77635683940025e-15 | -1.95399252334028e-14 |
| 885 | 1.8 | 4.8 | 5.47785692465637 | 5.10867382600052 | -0.677856924656373 | -0.308673826000517 | 4.8 | 4.79999999999993 | 8.88178419700125e-16 | -7.01660951563099e-14 |
| 886 | 1.8 | 4.85 | 5.54780209640633 | 5.16395187272275 | -0.697802096406335 | -0.313951872722747 | 4.85 | 4.85000000000003 | 8.88178419700125e-16 | 2.57571741713036e-14 |
| 887 | 1.8 | 4.9 | 5.61815319905865 | 5.21923194648888 | -0.718153199058645 | -0.319231946488879 | 4.9 | 4.89999999999993 | 1.77635683940025e-15 | -6.92779167366098e-14 |
| 888 | 1.8 | 4.95 | 5.68891738326491 | 5.27451404752225 | -0.738917383264914 | -0.324514047522253 | 4.95 | 4.94999999999994 | 1.77635683940025e-15 | -5.59552404411079e-14 |
| 889 | 1.8 | 5 | 5.76010201211168 | 5.32979817604584 | -0.760102012111676 | -0.329798176045842 | 5 | 4.99999999999999 | 1.77635683940025e-15 | -7.99360577730113e-15 |
| 890 | 1.8 | 5.05 | 5.83171467006238 | 5.38508433228261 | -0.78171467006238 | -0.335084332282612 | 5.05 | 5.04999999999996 | 8.88178419700125e-16 | -3.99680288865056e-14 |
| 891 | 1.8 | 5.1 | 5.9037631723892 | 5.44037251645572 | -0.803763172389195 | -0.340372516455719 | 5.1 | 5.09999999999987 | 8.88178419700125e-16 | -1.31450406115619e-13 |
| 892 | 1.8 | 5.15 | 5.97625557512788 | 5.49566272878869 | -0.826255575127878 | -0.34566272878869 | 5.15 | 5.15000000000004 | 1.77635683940025e-15 | 3.5527136788005e-14 |
| 893 | 1.8 | 5.2 | 6.04920018559154 | 5.55095496950412 | -0.849200185591536 | -0.350954969504122 | 5.2 | 5.1999999999999 | 8.88178419700125e-16 | -9.76996261670138e-14 |
| 894 | 1.8 | 5.25 | 6.12260557348219 | 5.60624923882591 | -0.87260557348219 | -0.356249238825912 | 5.25 | 5.25000000000005 | 8.88178419700125e-16 | 4.70734562441066e-14 |
| 895 | 1.8 | 5.3 | 6.19648058264216 | 5.66154553697684 | -0.896480582642161 | -0.361545536976843 | 5.3 | 5.3 | 1.77635683940025e-15 | 4.44089209850063e-15 |
| 896 | 1.8 | 5.35 | 6.2708343434909 | 5.71684386418044 | -0.920834343490903 | -0.366843864180439 | 5.35 | 5.34999999999994 | 8.88178419700125e-16 | -5.86197757002083e-14 |
| 897 | 1.8 | 5.4 | 6.34567628619683 | 5.77214422066023 | -0.945676286196827 | -0.372144220660228 | 5.4 | 5.39999999999999 | 0 | -7.99360577730113e-15 |
| 898 | 1.8 | 5.45 | 6.42101615463787 | 5.82744660663937 | -0.971016154637865 | -0.377446606639364 | 5.45 | 5.44999999999992 | 0 | -8.26005930321117e-14 |
| 899 | 1.8 | 5.5 | 6.49686402120931 | 5.88275102234156 | -0.996864021209307 | -0.382751022341556 | 5.5 | 5.49999999999994 | 8.88178419700125e-16 | -6.30606677987089e-14 |
| 900 | 1.8 | 5.55 | 6.57323030254262 | 5.93805746799033 | -1.02323030254261 | -0.388057467990329 | 5.55 | 5.55000000000008 | 8.88178419700125e-16 | 7.54951656745106e-14 |
| 901 | 1.8 | 5.6 | 6.65012577620467 | 5.99336594380884 | -1.05012577620467 | -0.393365943808838 | 5.6 | 5.59999999999998 | 2.66453525910038e-15 | -2.1316282072803e-14 |
| 902 | 1.8 | 5.65 | 6.72756159845321 | 6.04867645002098 | -1.0775615984532 | -0.398676450020977 | 5.65 | 5.64999999999993 | 8.88178419700125e-16 | -7.28306304154103e-14 |
| 903 | 1.8 | 5.7 | 6.80554932313134 | 6.10398898685046 | -1.10554932313134 | -0.403988986850458 | 5.7 | 5.7 | 0 | -1.77635683940025e-15 |
| 904 | 1.8 | 5.75 | 6.88410092179166 | 6.15930355452062 | -1.13410092179166 | -0.40930355452062 | 5.75 | 5.7499999999999 | 0 | -1.04805053524615e-13 |
| 905 | 1.8 | 5.8 | 6.96322880514929 | 6.21462015325554 | -1.16322880514928 | -0.414620153255542 | 5.8 | 5.79999999999996 | 0 | -4.61852778244065e-14 |
| 906 | 1.8 | 5.85 | 7.04294584597255 | 6.26993878327875 | -1.19294584597255 | -0.41993878327875 | 5.85 | 5.84999999999998 | 8.88178419700125e-16 | -2.48689957516035e-14 |
| 907 | 1.8 | 5.9 | 7.12326540353109 | 6.32525944481414 | -1.22326540353109 | -0.425259444814139 | 5.9 | 5.90000000000005 | 8.88178419700125e-16 | 4.88498130835069e-14 |
| 908 | 1.8 | 5.95 | 7.20420134973261 | 6.38058213808523 | -1.25420134973261 | -0.430582138085234 | 5.95 | 5.9499999999999 | 8.88178419700125e-16 | -9.76996261670138e-14 |
| 909 | 1.8 | 6 | 7.28576809709324 | 6.43590686331648 | -1.28576809709324 | -0.435906863316484 | 6 | 6.00000000000006 | 8.88178419700125e-16 | 5.59552404411079e-14 |
| 910 | 1.9 | 1 | 1.00081362932319 | 0.914877479330685 | -0.000813629323185294 | 0.0851225206693146 | 1 | 0.999999999999874 | 1.55431223447522e-15 | -1.2578826869003e-13 |
| 911 | 1.9 | 1.05 | 1.05190441297873 | 0.970002171952385 | -0.00190441297872668 | 0.0799978280476149 | 1.05 | 1.04999999999991 | -2.22044604925031e-16 | -9.30366894635881e-14 |
| 912 | 1.9 | 1.1 | 1.10315275497988 | 1.02512887479385 | -0.00315275497988465 | 0.0748711252061467 | 1.1 | 1.09999999999992 | 4.44089209850063e-16 | -7.97140131680862e-14 |
| 913 | 1.9 | 1.15 | 1.15456012205786 | 1.0802575880751 | -0.0045601220578646 | 0.0697424119249008 | 1.15 | 1.14999999999998 | 6.66133814775094e-16 | -1.82076576038526e-14 |
| 914 | 1.9 | 1.2 | 1.20612800384275 | 1.13538831201595 | -0.00612800384275225 | 0.0646116879840533 | 1.2 | 1.19999999999995 | 6.66133814775094e-16 | -4.52970994047064e-14 |
| 915 | 1.9 | 1.25 | 1.25785791336716 | 1.19052104683659 | -0.00785791336716057 | 0.0594789531634099 | 1.25 | 1.25 | 2.22044604925031e-16 | 8.88178419700125e-16 |
| 916 | 1.9 | 1.3 | 1.30975138758421 | 1.24565579275685 | -0.00975138758421146 | 0.0543442072431462 | 1.3 | 1.29999999999991 | -2.22044604925031e-16 | -8.79296635503124e-14 |
| 917 | 1.9 | 1.35 | 1.36180998790036 | 1.30079254999712 | -0.0118099879003548 | 0.049207450002883 | 1.35 | 1.34999999999995 | 6.66133814775094e-16 | -5.50670620214078e-14 |
| 918 | 1.9 | 1.4 | 1.41403530072354 | 1.35593131877739 | -0.0140353007235363 | 0.044068681222611 | 1.4 | 1.39999999999999 | 4.44089209850063e-16 | -1.3988810110277e-14 |
| 919 | 1.9 | 1.45 | 1.46642893802728 | 1.41107209931768 | -0.0164289380272828 | 0.0389279006823213 | 1.45 | 1.44999999999988 | 2.22044604925031e-15 | -1.16573417585641e-13 |
| 920 | 1.9 | 1.5 | 1.51899253793123 | 1.46621489183855 | -0.0189925379312308 | 0.0337851081614498 | 1.5 | 1.49999999999995 | 2.22044604925031e-16 | -4.66293670342566e-14 |
| 921 | 1.9 | 1.55 | 1.57172776529875 | 1.52135969656001 | -0.0217277652987538 | 0.0286403034399867 | 1.55 | 1.54999999999997 | 1.11022302462516e-15 | -2.70894418008538e-14 |
| 922 | 1.9 | 1.6 | 1.62463631235222 | 1.57650651370245 | -0.0246363123522195 | 0.0234934862975529 | 1.6 | 1.60000000000002 | 1.55431223447522e-15 | 1.70974345792274e-14 |
| 923 | 1.9 | 1.65 | 1.67771989930661 | 1.63165534348605 | -0.0277198993066075 | 0.0183446565139542 | 1.65 | 1.64999999999996 | 1.33226762955019e-15 | -4.2632564145606e-14 |
| 924 | 1.9 | 1.7 | 1.7309802750221 | 1.68680618613137 | -0.0309802750220971 | 0.0131938138686267 | 1.7 | 1.69999999999996 | 8.88178419700125e-16 | -3.70814490224802e-14 |
| 925 | 1.9 | 1.75 | 1.78441921767636 | 1.74195904185881 | -0.0344192176763567 | 0.00804095814119044 | 1.75 | 1.75 | 8.88178419700125e-16 | 1.55431223447522e-15 |
| 926 | 1.9 | 1.8 | 1.83803853545725 | 1.79711391088873 | -0.0380385354572479 | 0.00288608911126675 | 1.8 | 1.8 | 1.11022302462516e-15 | 1.55431223447522e-15 |
| 927 | 1.9 | 1.85 | 1.89184006727673 | 1.85227079344171 | -0.041840067276725 | -0.002270793441709 | 1.85 | 1.85000000000002 | 1.11022302462516e-15 | 2.37587727269784e-14 |
| 928 | 1.9 | 1.9 | 1.94582568350671 | 1.90742968973812 | -0.0458256835067137 | -0.00742968973811631 | 1.9 | 1.89999999999993 | 1.11022302462516e-15 | -7.37188088351104e-14 |
| 929 | 1.9 | 1.95 | 1.99999728673781 | 1.96259059999889 | -0.04999728673781 | -0.0125905999988887 | 1.95 | 1.95000000000003 | 1.77635683940025e-15 | 3.01980662698043e-14 |
| 930 | 1.9 | 2 | 2.05435681256166 | 2.01775352444422 | -0.0543568125616605 | -0.0177535244442213 | 2 | 1.99999999999995 | 1.33226762955019e-15 | -4.68514116391816e-14 |
| 931 | 1.9 | 2.05 | 2.10890623037795 | 2.07291846329505 | -0.0589062303779526 | -0.0229184632950483 | 2.05 | 2.04999999999994 | 4.44089209850063e-16 | -5.50670620214078e-14 |
| 932 | 1.9 | 2.1 | 2.16364754422695 | 2.12808541677193 | -0.0636475442269462 | -0.0280854167719338 | 2.1 | 2.09999999999988 | 1.77635683940025e-15 | -1.16351372980716e-13 |
| 933 | 1.9 | 2.15 | 2.21858279364855 | 2.18325438509581 | -0.0685827936485448 | -0.0332543850958116 | 2.15 | 2.14999999999995 | 4.44089209850063e-16 | -5.32907051820075e-14 |
| 934 | 1.9 | 2.2 | 2.27371405456898 | 2.23842536848725 | -0.0737140545689785 | -0.0384253684872475 | 2.2 | 2.19999999999994 | 4.44089209850063e-16 | -6.08402217494586e-14 |
| 935 | 1.9 | 2.25 | 2.32904344021614 | 2.29359836716718 | -0.0790434402161386 | -0.0435983671671751 | 2.25 | 2.24999999999997 | 1.33226762955019e-15 | -3.33066907387547e-14 |
| 936 | 1.9 | 2.3 | 2.38457310206474 | 2.34877338135634 | -0.0845731020647405 | -0.0487733813563436 | 2.3 | 2.29999999999993 | 0 | -7.32747196252603e-14 |
| 937 | 1.9 | 2.35 | 2.44030523081252 | 2.40395041127569 | -0.0903052308125232 | -0.0539504112756868 | 2.35 | 2.34999999999985 | 1.33226762955019e-15 | -1.48325796089921e-13 |
| 938 | 1.9 | 2.4 | 2.49624205738868 | 2.45912945714633 | -0.096242057388678 | -0.0591294571463248 | 2.4 | 2.3999999999999 | 4.44089209850063e-16 | -9.72555369571637e-14 |
| 939 | 1.9 | 2.45 | 2.55238585399591 | 2.51431051918901 | -0.102385853995905 | -0.0643105191890077 | 2.45 | 2.44999999999987 | 0 | -1.27897692436818e-13 |
| 940 | 1.9 | 2.5 | 2.60873893518739 | 2.56949359762504 | -0.108738935187394 | -0.0694935976250393 | 2.5 | 2.50000000000001 | 4.44089209850063e-16 | 1.46549439250521e-14 |
| 941 | 1.9 | 2.55 | 2.66530365898023 | 2.62467869267498 | -0.115303658980231 | -0.0746786926749832 | 2.55 | 2.54999999999988 | 1.77635683940025e-15 | -1.19904086659517e-13 |
| 942 | 1.9 | 2.6 | 2.72208242800671 | 2.67986580456051 | -0.122082428006709 | -0.0798658045605145 | 2.6 | 2.59999999999999 | 1.33226762955019e-15 | -1.28785870856518e-14 |
| 943 | 1.9 | 2.65 | 2.77907769070517 | 2.73505493350238 | -0.12907769070517 | -0.0850549335023825 | 2.65 | 2.64999999999998 | 8.88178419700125e-16 | -2.08721928629529e-14 |
| 944 | 1.9 | 2.7 | 2.836291942552 | 2.79024607972189 | -0.136291942552004 | -0.0902460797218922 | 2.7 | 2.69999999999997 | 8.88178419700125e-16 | -3.19744231092045e-14 |
| 945 | 1.9 | 2.75 | 2.8937277273366 | 2.84543924344035 | -0.143727727336599 | -0.0954392434403473 | 2.75 | 2.75000000000003 | 1.33226762955019e-15 | 2.62012633811537e-14 |
| 946 | 1.9 | 2.8 | 2.95138763848106 | 2.90063442487887 | -0.151387638481057 | -0.100634424878868 | 2.8 | 2.80000000000002 | 1.77635683940025e-15 | 2.26485497023532e-14 |
| 947 | 1.9 | 2.85 | 3.00927432040663 | 2.95583162425876 | -0.15927432040663 | -0.105831624258757 | 2.85 | 2.84999999999996 | 4.44089209850063e-16 | -4.2188474935756e-14 |
| 948 | 1.9 | 2.9 | 3.06739046994892 | 3.01103084180151 | -0.167390469948916 | -0.111030841801506 | 2.9 | 2.89999999999996 | 1.33226762955019e-15 | -3.73034936274053e-14 |
| 949 | 1.9 | 2.95 | 3.12573883782393 | 3.06623207772842 | -0.175738837823925 | -0.116232077728418 | 2.95 | 2.94999999999997 | 8.88178419700125e-16 | -3.50830475781549e-14 |
| 950 | 1.9 | 3 | 3.18432223014732 | 3.1214353322608 | -0.184322230147322 | -0.121435332260799 | 3 | 2.99999999999986 | 1.33226762955019e-15 | -1.4521717162097e-13 |
| 951 | 1.9 | 3.05 | 3.24314351000917 | 3.17664060562051 | -0.193143510009172 | -0.126640605620507 | 3.05 | 3.04999999999999 | 0 | -1.15463194561016e-14 |
| 952 | 1.9 | 3.1 | 3.30220559910673 | 3.23184789802848 | -0.202205599106731 | -0.131847898028477 | 3.1 | 3.09999999999985 | 1.33226762955019e-15 | -1.52322598978571e-13 |
| 953 | 1.9 | 3.15 | 3.36151147943786 | 3.28705720970694 | -0.211511479437864 | -0.137057209706938 | 3.15 | 3.15000000000005 | 4.44089209850063e-16 | 5.10702591327572e-14 |
| 954 | 1.9 | 3.2 | 3.42106419505794 | 3.34226854087683 | -0.22106419505794 | -0.142268540876825 | 3.2 | 3.20000000000001 | 8.88178419700125e-16 | 5.77315972805081e-15 |
| 955 | 1.9 | 3.25 | 3.48086685390305 | 3.39748189176 | -0.230866853903049 | -0.147481891759998 | 3.25 | 3.24999999999992 | 8.88178419700125e-16 | -7.72715225139109e-14 |
| 956 | 1.9 | 3.3 | 3.54092262968271 | 3.45269726257832 | -0.24092262968271 | -0.152697262578315 | 3.3 | 3.29999999999997 | 1.77635683940025e-15 | -2.62012633811537e-14 |
| 957 | 1.9 | 3.35 | 3.60123476384527 | 3.50791465355327 | -0.251234763845269 | -0.157914653553268 | 3.35 | 3.34999999999996 | -4.44089209850063e-16 | -3.77475828372553e-14 |
| 958 | 1.9 | 3.4 | 3.66180656761948 | 3.56313406490672 | -0.261806567619479 | -0.163134064906715 | 3.4 | 3.39999999999999 | 4.44089209850063e-16 | -1.55431223447522e-14 |
| 959 | 1.9 | 3.45 | 3.72264142413585 | 3.61835549686033 | -0.272641424135853 | -0.16835549686033 | 3.45 | 3.44999999999994 | 0 | -6.26165785888588e-14 |
| 960 | 1.9 | 3.5 | 3.78374279063169 | 3.67357894963616 | -0.28374279063169 | -0.173578949636159 | 3.5 | 3.50000000000001 | 1.33226762955019e-15 | 1.4210854715202e-14 |
| 961 | 1.9 | 3.55 | 3.84511420074378 | 3.72880442345569 | -0.295114200743782 | -0.17880442345569 | 3.55 | 3.54999999999987 | 1.77635683940025e-15 | -1.31450406115619e-13 |
| 962 | 1.9 | 3.6 | 3.90675926689316 | 3.78403191854134 | -0.306759266893162 | -0.184031918541338 | 3.6 | 3.59999999999996 | 4.44089209850063e-16 | -4.35207425653061e-14 |
| 963 | 1.9 | 3.65 | 3.9686816827664 | 3.83926143511459 | -0.318681682766403 | -0.189261435114594 | 3.65 | 3.64999999999986 | 4.44089209850063e-16 | -1.4210854715202e-13 |
| 964 | 1.9 | 3.7 | 4.03088522589831 | 3.89449297339787 | -0.330885225898305 | -0.194492973397871 | 3.7 | 3.69999999999995 | 8.88178419700125e-16 | -4.57411886145565e-14 |
| 965 | 1.9 | 3.75 | 4.09337376036109 | 3.94972653361284 | -0.343373760361091 | -0.199726533612845 | 3.75 | 3.74999999999992 | 4.44089209850063e-16 | -7.99360577730113e-14 |
| 966 | 1.9 | 3.8 | 4.15615123956552 | 4.00496211598174 | -0.35615123956552 | -0.204962115981744 | 3.8 | 3.7999999999999 | 4.44089209850063e-16 | -1.03916875104915e-13 |
| 967 | 1.9 | 3.85 | 4.21922170917965 | 4.0601997207268 | -0.369221709179653 | -0.210199720726798 | 3.85 | 3.84999999999999 | 8.88178419700125e-16 | -1.50990331349021e-14 |
| 968 | 1.9 | 3.9 | 4.28258931017139 | 4.11543934806987 | -0.382589310171388 | -0.215439348069868 | 3.9 | 3.89999999999992 | 8.88178419700125e-16 | -8.03801469828613e-14 |
| 969 | 1.9 | 3.95 | 4.34625828198123 | 4.17068099823337 | -0.396258281981232 | -0.220680998233367 | 3.95 | 3.9499999999999 | 4.44089209850063e-16 | -1.03916875104915e-13 |
| 970 | 1.9 | 4 | 4.41023296583219 | 4.22592467143971 | -0.410232965832186 | -0.225924671439711 | 4 | 4.00000000000008 | 0 | 7.63833440942108e-14 |
| 971 | 1.9 | 4.05 | 4.47451780818406 | 4.28117036791057 | -0.424517808184058 | -0.231170367910573 | 4.05 | 4.04999999999991 | 1.77635683940025e-15 | -8.70414851306123e-14 |
| 972 | 1.9 | 4.1 | 4.53911736433997 | 4.33641808786874 | -0.439117364339969 | -0.23641808786874 | 4.1 | 4.09999999999983 | 8.88178419700125e-16 | -1.69642078162724e-13 |
| 973 | 1.9 | 4.15 | 4.60403630221338 | 4.39166783153681 | -0.454036302213381 | -0.241667831536809 | 4.15 | 4.15000000000004 | 1.77635683940025e-15 | 4.08562073062058e-14 |
| 974 | 1.9 | 4.2 | 4.66927940626437 | 4.44691959913646 | -0.469279406264367 | -0.246919599136456 | 4.2 | 4.19999999999989 | 8.88178419700125e-16 | -1.07469588783715e-13 |
| 975 | 1.9 | 4.25 | 4.73485158161464 | 4.50217339089084 | -0.484851581614638 | -0.252173390890837 | 4.25 | 4.25000000000003 | 8.88178419700125e-16 | 3.01980662698043e-14 |
| 976 | 1.9 | 4.3 | 4.80075785835122 | 4.55742920702181 | -0.500757858351222 | -0.257429207021809 | 4.3 | 4.29999999999989 | 1.77635683940025e-15 | -1.11022302462516e-13 |
| 977 | 1.9 | 4.35 | 4.86700339602957 | 4.61268704775253 | -0.517003396029571 | -0.262687047752529 | 4.35 | 4.35000000000004 | 8.88178419700125e-16 | 4.35207425653061e-14 |
| 978 | 1.9 | 4.4 | 4.93359348838747 | 4.66794691330486 | -0.533593488387471 | -0.267946913304854 | 4.4 | 4.39999999999986 | 0 | -1.4388490399142e-13 |
| 979 | 1.9 | 4.45 | 5.00053356828193 | 4.72320880390213 | -0.55053356828193 | -0.273208803902127 | 4.45 | 4.44999999999999 | 8.88178419700125e-16 | -6.21724893790088e-15 |
| 980 | 1.9 | 4.5 | 5.06782921286209 | 4.77847271976639 | -0.567829212862088 | -0.278472719766391 | 4.5 | 4.49999999999991 | 8.88178419700125e-16 | -8.88178419700125e-14 |
| 981 | 1.9 | 4.55 | 5.13548614899211 | 4.8337386611208 | -0.58548614899211 | -0.2837386611208 | 4.55 | 4.55000000000004 | 1.77635683940025e-15 | 3.46389583683049e-14 |
| 982 | 1.9 | 4.6 | 5.20351025893894 | 4.88900662818759 | -0.603510258938936 | -0.289006628187587 | 4.6 | 4.59999999999992 | 1.77635683940025e-15 | -8.43769498715119e-14 |
| 983 | 1.9 | 4.65 | 5.27190758634092 | 4.94427662119009 | -0.621907586340924 | -0.294276621190088 | 4.65 | 4.65000000000007 | 8.88178419700125e-16 | 6.92779167366098e-14 |
| 984 | 1.9 | 4.7 | 5.34068434247449 | 4.99954864035053 | -0.64068434247449 | -0.299548640350534 | 4.7 | 4.69999999999998 | 8.88178419700125e-16 | -2.39808173319034e-14 |
| 985 | 1.9 | 4.75 | 5.40984691283717 | 5.05482268589208 | -0.659846912837167 | -0.30482268589208 | 4.75 | 4.74999999999991 | 1.77635683940025e-15 | -9.05941988094128e-14 |
| 986 | 1.9 | 4.8 | 5.47940186406679 | 5.1100987580377 | -0.679401864066794 | -0.310098758037696 | 4.8 | 4.79999999999994 | 1.77635683940025e-15 | -5.6843418860808e-14 |
| 987 | 1.9 | 4.85 | 5.54935595121809 | 5.16537685701017 | -0.699355951218094 | -0.315376857010167 | 4.85 | 4.84999999999995 | 8.88178419700125e-16 | -5.32907051820075e-14 |
| 988 | 1.9 | 4.9 | 5.61971612541944 | 5.22065698303246 | -0.719716125419443 | -0.32065698303246 | 4.9 | 4.89999999999992 | 0 | -8.5265128291212e-14 |
| 989 | 1.9 | 4.95 | 5.69048954193438 | 5.27593913632774 | -0.740489541934378 | -0.325939136327735 | 4.95 | 4.94999999999998 | 8.88178419700125e-16 | -1.86517468137026e-14 |
| 990 | 1.9 | 5 | 5.76168356865434 | 5.33122331711877 | -0.761683568654344 | -0.331223317118773 | 5 | 4.9999999999999 | 0 | -9.9475983006414e-14 |
| 991 | 1.9 | 5.05 | 5.83330579505119 | 5.38650952562891 | -0.783305795051193 | -0.336509525628914 | 5.05 | 5.0499999999999 | 8.88178419700125e-16 | -1.04805053524615e-13 |
| 992 | 1.9 | 5.1 | 5.90536404162021 | 5.44179776208131 | -0.805364041620205 | -0.341797762081313 | 5.1 | 5.09999999999998 | 8.88178419700125e-16 | -1.77635683940025e-14 |
| 993 | 1.9 | 5.15 | 5.97786636984693 | 5.49708802669894 | -0.82786636984693 | -0.347088026698941 | 5.15 | 5.14999999999998 | 8.88178419700125e-16 | -2.39808173319034e-14 |
| 994 | 1.9 | 5.2 | 6.05082109273378 | 5.55238031970514 | -0.850821092733781 | -0.352380319705138 | 5.2 | 5.19999999999999 | 8.88178419700125e-16 | -1.06581410364015e-14 |
| 995 | 1.9 | 5.25 | 6.12423678592528 | 5.60767464132306 | -0.874236785925278 | -0.357674641323058 | 5.25 | 5.24999999999993 | 8.88178419700125e-16 | -7.37188088351104e-14 |
| 996 | 1.9 | 5.3 | 6.19812229947412 | 5.66297099177623 | -0.898122299474117 | -0.362970991776225 | 5.3 | 5.29999999999999 | 0 | -7.105427357601e-15 |
| 997 | 1.9 | 5.35 | 6.27248677029374 | 5.7182693712878 | -0.922486770293739 | -0.368269371287795 | 5.35 | 5.35000000000002 | 8.88178419700125e-16 | 2.04281036531029e-14 |
| 998 | 1.9 | 5.4 | 6.34733963534702 | 5.77356978008111 | -0.947339635347015 | -0.373569780081108 | 5.4 | 5.39999999999997 | 1.77635683940025e-15 | -2.66453525910038e-14 |
| 999 | 1.9 | 5.45 | 6.42269064562493 | 5.82887221837969 | -0.97269064562493 | -0.378872218379689 | 5.45 | 5.44999999999995 | 8.88178419700125e-16 | -5.41788836017076e-14 |
| 1000 | 1.9 | 5.5 | 6.49854988097389 | 5.88417668640706 | -0.998549880973888 | -0.384176686407062 | 5.5 | 5.49999999999999 | 1.77635683940025e-15 | -7.105427357601e-15 |
