# Supplementary material for: Creatinine assay interferences compromises MELD accuracy and may bias liver allocation
Source: Nat Commun. 2026 Jul 23;17:7111. doi: 10.1038/s41467-026-75011-x (PMC13396164; doi:10.1038/s41467-026-75011-x)
Supplement: Supplementary file 4 — Source Data [file 41467_2026_75011_MOESM4_ESM.zip › figshare_package_FINAL_PUBLIC_DEPOSIT_V1_20260503_002637/00_START_HERE_HTML_NAVIGATOR/file_views/view_0007_f2_simulated_heatmap_metadata.html]

01\_primary\_data/public/f2\_simulated\_heatmap\_metadata.csv

# Readable file view

01\_primary\_data/public/f2\_simulated\_heatmap\_metadata.csv

← Back to navigator   |   Open original package file

Section

Public primary data

Output

F2

Extension

csv

Size KB

0.545

Variables

2

## Variables in this file

| Variable | Label | Description | Unit | Type |
| --- | --- | --- | --- | --- |
| parameter | Metadata parameter name | Name of a metadata parameter describing the F2 simulated heatmap object, such as figure identity, data origin, grid type, axis variable, or unit/role. |  | character |
| value | Value | Numerical or character value corresponding to the row-specific variable/metric. |  | character |

## Readable HTML view

Showing all 21 rows.

| parameter | value |
| --- | --- |
| figure | Figure 2 |
| data\_origin | in silico simulation |
| grid\_type | uniform subpoint grid within bins |
| x\_axis\_variable | Creatinine (mg/dL) |
| x\_start | 0.1 |
| x\_end | 5.0 |
| x\_step | 0.02 |
| y\_axis\_variable | Bilirubin (mg/dL) |
| y\_start | 0.1 |
| y\_end | 30.0 |
| y\_step | 0.1 |
| n\_sub\_per\_bin | 1000 |
| sex | M |
| sex\_M | 0 |
| dialysis | 0 |
| inr\_fixed | 1.5 |
| sodium\_fixed | 130 |
| albumin\_fixed | 40 |
| correction\_model;pred\_crea.jaf(TB, Cr) |  |
| repository\_scope | bin-level heatmap data sufficient for figure rebuild |
| note | Point-level simulation not exported because it is unnecessarily large for public figure reconstruction. |
