# Supplementary material for: Creatinine assay interferences compromises MELD accuracy and may bias liver allocation
Source: Nat Commun. 2026 Jul 23;17:7111. doi: 10.1038/s41467-026-75011-x (PMC13396164; doi:10.1038/s41467-026-75011-x)
Supplement: Supplementary file 4 — Source Data [file 41467_2026_75011_MOESM4_ESM.zip › figshare_package_FINAL_PUBLIC_DEPOSIT_V1_20260503_002637/00_START_HERE_HTML_NAVIGATOR/file_views/view_0016_esld_F4_survival_stats_public.html]

02\_workflows/F4\_workflow\_v01/submission\_ready/public/data/esld\_F4\_survival\_stats\_public.csv

# Readable file view

02\_workflows/F4\_workflow\_v01/submission\_ready/public/data/esld\_F4\_survival\_stats\_public.csv

← Back to navigator   |   Open original package file

Section

Manuscript output data

Output

F4

Extension

csv

Size KB

2.255

Variables

14

## Variables in this file

| Variable | Label | Description | Unit | Type |
| --- | --- | --- | --- | --- |
| asterisk | Statistical-significance annotation | Text label used to annotate statistical significance in the rendered figure, for example n.s., \*, \*\*, \*\*\*, or \*\*\*\*. |  | character |
| median\_m1 | Median value for m1 group | Median row-specific value for the m1 group. m1 denotes the negative score-shift group. In class-level score-shift summaries it denotes the minus-one score-shift class; in grouped survival outputs it is the negative-shift comparison group used by the workflow. | analysis-specific scale | numeric |
| median\_pm2 | Median value for pm2 group | Median row-specific value for the pm2 group in F4 survival-statistics data. pm2 denotes the F4 comparator group used against m1 in the survival-statistics table; it should be interpreted as the F4 workflow's non-m1/reference comparator group. | analysis-specific scale | numeric |
| model | MELD model or score variant | Name of the MELD-related model or score variant represented by the row; expected values include MELD, MELD-Na, reMELD-Na, and MELD 3.0. |  | character |
| n\_m1 | Number of observations in m1 group | Count of observations in the m1 group for the row-specific stratum. m1 denotes the negative score-shift group. In class-level score-shift summaries it denotes the minus-one score-shift class; in grouped survival outputs it is the negative-shift comparison group used by the workflow. | count | integer |
| n\_pm2 | Number of observations in pm2 group | Count of observations in the pm2 group for the row-specific F4 survival stratum. pm2 denotes the F4 comparator group used against m1 in the survival-statistics table; it should be interpreted as the F4 workflow's non-m1/reference comparator group. | count | integer |
| p25\_m1 | 25th-percentile value for m1 group | 25th percentile of the row-specific value for the m1 group. m1 denotes the negative score-shift group. In class-level score-shift summaries it denotes the minus-one score-shift class; in grouped survival outputs it is the negative-shift comparison group used by the workflow. | analysis-specific scale | numeric |
| p25\_pm2 | 25th-percentile value for pm2 group | 25th percentile of the row-specific value for the pm2 group in F4 survival-statistics data. pm2 denotes the F4 comparator group used against m1 in the survival-statistics table; it should be interpreted as the F4 workflow's non-m1/reference comparator group. | analysis-specific scale | numeric |
| p75\_m1 | 75th-percentile value for m1 group | 75th percentile of the row-specific value for the m1 group. m1 denotes the negative score-shift group. In class-level score-shift summaries it denotes the minus-one score-shift class; in grouped survival outputs it is the negative-shift comparison group used by the workflow. | analysis-specific scale | numeric |
| p75\_pm2 | 75th-percentile value for pm2 group | 75th percentile of the row-specific value for the pm2 group in F4 survival-statistics data. pm2 denotes the F4 comparator group used against m1 in the survival-statistics table; it should be interpreted as the F4 workflow's non-m1/reference comparator group. | analysis-specific scale | numeric |
| p\_value | P value | P value from the statistical comparison represented by the row. | probability | numeric |
| score\_class\_label | Displayed score-class label | Displayed score-class label for the figure/table stratum. | score points | character |
| score\_class\_value | Score-class upper value | Upper score value defining the displayed score class. | score points | integer |
| y\_ast | Y coordinate for significance annotation | Y-axis coordinate used to position the statistical-significance annotation in the F4 survival figure. | analysis-specific y-axis coordinate | numeric |

## Readable HTML view

Showing all 27 rows.

| model | score\_class\_value | score\_class\_label | n\_pm2 | median\_pm2 | p25\_pm2 | p75\_pm2 | n\_m1 | median\_m1 | p25\_m1 | p75\_m1 | p\_value | asterisk | y\_ast |
| --- | --- | --- | --- | --- | --- | --- | --- | --- | --- | --- | --- | --- | --- |
| MELD | 10 | ≤10 | 405 | 126 | 317 | 31 | 369 | 125 | 273 | 50 | 0.787854614489014 | n.s. | 131 |
| MELD | 15 | 11-15 | 190 | 26 | 97.75 | 12 | 913 | 74 | 247 | 26 | 9.15971812501929e-11 | \*\*\*\* | 79 |
| MELD | 20 | 16-20 | 84 | 15 | 29.75 | 3 | 916 | 33 | 143.25 | 12 | 2.77788222236644e-07 | \*\*\*\* | 38 |
| MELD | 25 | 21-25 | 56 | 9 | 28.25 | 2 | 555 | 17 | 39 | 7 | 0.00360653118106935 | \*\* | 22 |
| MELD | 30 | 26-30 | 31 | 7 | 14.5 | 2 | 305 | 10 | 25 | 4 | 0.114275327333006 | n.s. | 15 |
| MELD | 35 | 31-35 | 9 | 3 | 6 | 2 | 171 | 7 | 17.5 | 2 | 0.109284545248316 | n.s. | 12 |
| MELD | 40 | 36-40 | 8 | 0 | 2.5 | -0.25 | 78 | 3 | 7 | 1 | 0.0488583614103703 | \* | 8 |
| MELD-Na | 10 | ≤10 | 318 | 154 | 319.75 | 42 | 222 | 108.5 | 231 | 62.25 | 0.239141891319107 | n.s. | 159 |
| MELD-Na | 15 | 11-15 | 280 | 38.5 | 226.75 | 14 | 584 | 75.5 | 223.25 | 28.75 | 0.00012669427090306 | \*\*\* | 80.5 |
| MELD-Na | 20 | 16-20 | 147 | 24 | 97.5 | 8.5 | 716 | 58.5 | 199.5 | 17 | 1.6530643473678e-05 | \*\*\*\* | 63.5 |
| MELD-Na | 25 | 21-25 | 106 | 22.5 | 47.75 | 4 | 606 | 26 | 94.75 | 9 | 0.00728411495155192 | \*\* | 31 |
| MELD-Na | 30 | 26-30 | 46 | 13.5 | 28.75 | 4.75 | 363 | 17 | 36 | 6.5 | 0.291781490653651 | n.s. | 22 |
| MELD-Na | 35 | 31-35 | 38 | 7.5 | 14.75 | 4 | 156 | 7 | 22 | 2 | 0.921604783316565 | n.s. | 12.5 |
| MELD-Na | 40 | 36-40 | 7 | 0 | 3 | 0 | 78 | 3 | 9 | 1 | 0.0949724462091512 | n.s. | 8 |
| reMELD-Na | 10 | ≤10 | 503 | 149 | 329.5 | 31 | 276 | 101.5 | 203.5 | 32 | 0.0554512514743742 | n.s. | 154 |
| reMELD-Na | 15 | 11-15 | 208 | 31 | 128.75 | 11.75 | 724 | 81 | 220.25 | 29.75 | 2.0737497351205e-08 | \*\*\*\* | 86 |
| reMELD-Na | 20 | 16-20 | 183 | 12 | 36.5 | 4 | 854 | 42 | 162.75 | 14.25 | 8.65729058747851e-21 | \*\*\*\* | 47 |
| reMELD-Na | 25 | 21-25 | 103 | 9 | 21.5 | 3 | 469 | 20 | 45 | 8 | 1.58139236917944e-08 | \*\*\*\* | 25 |
| reMELD-Na | 30 | 26-30 | 37 | 2 | 9 | 1 | 203 | 8 | 22 | 3 | 0.000348316443633254 | \*\*\* | 13 |
| reMELD-Na | 35 | 31-35 | 9 | 1 | 2 | 0 | 97 | 6 | 16 | 2 | 0.00186433576960212 | \*\* | 11 |
| MELD 3.0 | 10 | ≤10 | 93 | 118 | 275 | 50 | 44 | 153 | 399.5 | 81.75 | 0.376096840909384 | n.s. | 158 |
| MELD 3.0 | 15 | 11-15 | 170 | 61 | 190.75 | 16.25 | 178 | 96.5 | 278.75 | 35.25 | 0.00529262763769945 | \*\* | 101.5 |
| MELD 3.0 | 20 | 16-20 | 124 | 31.5 | 98.5 | 12 | 318 | 46.5 | 198 | 14.25 | 0.035438806906445 | \* | 51.5 |
| MELD 3.0 | 25 | 21-25 | 83 | 15 | 41.5 | 4 | 299 | 20 | 81 | 8 | 0.0170870563414992 | \* | 25 |
| MELD 3.0 | 30 | 26-30 | 54 | 11 | 18.5 | 5.25 | 184 | 15.5 | 38.25 | 6 | 0.033472415830357 | \* | 20.5 |
| MELD 3.0 | 35 | 31-35 | 14 | 5.5 | 15.25 | 3.25 | 114 | 11 | 19.75 | 4.25 | 0.243906274585862 | n.s. | 16 |
| MELD 3.0 | 40 | 36-40 | 17 | 1 | 3 | 0 | 76 | 4 | 18.25 | 1 | 0.00812111867645283 | \*\* | 9 |
