# Supplementary material for: Creatinine assay interferences compromises MELD accuracy and may bias liver allocation
Source: Nat Commun. 2026 Jul 23;17:7111. doi: 10.1038/s41467-026-75011-x (PMC13396164; doi:10.1038/s41467-026-75011-x)
Supplement: Supplementary file 4 — Source Data [file 41467_2026_75011_MOESM4_ESM.zip › figshare_package_FINAL_PUBLIC_DEPOSIT_V1_20260503_002637/00_START_HERE_HTML_NAVIGATOR/file_views/view_0018_esld_F5_stratified_survival_stats_public.html]

02\_workflows/F5\_workflow\_v01/submission\_ready/public/data/esld\_F5\_stratified\_survival\_stats\_public.csv

# Readable file view

02\_workflows/F5\_workflow\_v01/submission\_ready/public/data/esld\_F5\_stratified\_survival\_stats\_public.csv

← Back to navigator   |   Open original package file

Section

Manuscript output data

Output

F5

Extension

csv

Size KB

1.093

Variables

8

## Variables in this file

| Variable | Label | Description | Unit | Type |
| --- | --- | --- | --- | --- |
| chisq | Chi-square statistic | Chi-square statistic for the row-specific survival comparison or stratified test. | chi-square statistic | numeric |
| chisq\_lab | Formatted chi-square label | Formatted display label for the chi-square statistic shown in a table or figure annotation. |  | character |
| model | MELD model or score variant | Name of the MELD-related model or score variant represented by the row; expected values include MELD, MELD-Na, reMELD-Na, and MELD 3.0. |  | character |
| n\_m1 | Number of observations in m1 group | Count of observations in the m1 group for the row-specific stratum. m1 denotes the negative score-shift group. In class-level score-shift summaries it denotes the minus-one score-shift class; in grouped survival outputs it is the negative-shift comparison group used by the workflow. | count | integer |
| n\_pm | Number of observations in pm group | Count of observations in the pm group for the row-specific stratum. pm denotes the reference group without a negative score shift in grouped score-delta survival outputs; in class-level score-shift summaries it represents the no-decrease/reference category used by the workflow. | count | integer |
| p\_lab | Formatted p-value label | Formatted p-value label shown in a table or figure annotation. |  | character |
| pval | P value | P value for the row-specific statistical comparison. | probability | numeric |
| scr.grp | Score-class stratum | Encoded score-class stratum used in F5; examples include le15, 16to25, and gt25. |  | character |

## Readable HTML view

Showing all 12 rows.

| model | scr.grp | n\_pm | n\_m1 | chisq | pval | chisq\_lab | p\_lab |
| --- | --- | --- | --- | --- | --- | --- | --- |
| MELD | le15 | 25 | 12 | 0.00770313568089429 | 0.930061461937626 | χ² = 0.008 | p = 0.930 |
| MELD | 16to25 | 31 | 64 | 2.87624703252109 | 0.0898953142885014 | χ² = 2.876 | p = 0.090 |
| MELD | gt25 | 11 | 82 | 12.1826833766653 | 0.000482352058876657 | χ² = 12.183 | p < 0.001 |
| MELD-Na | le15 | 27 | 7 | 0.00169868292629352 | 0.967124430752549 | χ² = 0.002 | p = 0.967 |
| MELD-Na | 16to25 | 39 | 47 | 0.733720432338734 | 0.391679903320669 | χ² = 0.734 | p = 0.392 |
| MELD-Na | gt25 | 18 | 83 | 7.75171247643523 | 0.00536616335951513 | χ² = 7.752 | p = 0.005 |
| reMELD-Na | le15 | 18 | 16 | 0.963626118400095 | 0.326274967669755 | χ² = 0.964 | p = 0.326 |
| reMELD-Na | 16to25 | 47 | 54 | 8.04403506392806 | 0.00456537302581511 | χ² = 8.044 | p = 0.005 |
| reMELD-Na | gt25 | 15 | 49 | 13.426858720957 | 0.000248047123857103 | χ² = 13.427 | p < 0.001 |
| MELD 3.0 | le15 | 32 | 3 | 0.412726162577639 | 0.520589046088341 | χ² = 0.413 | p = 0.521 |
| MELD 3.0 | 16to25 | 44 | 35 | 3.96959590640388 | 0.0463288891471866 | χ² = 3.970 | p = 0.046 |
| MELD 3.0 | gt25 | 29 | 53 | 3.76637492945154 | 0.0522928491109528 | χ² = 3.766 | p = 0.052 |
