# Supplementary material for: Creatinine assay interferences compromises MELD accuracy and may bias liver allocation
Source: Nat Commun. 2026 Jul 23;17:7111. doi: 10.1038/s41467-026-75011-x (PMC13396164; doi:10.1038/s41467-026-75011-x)
Supplement: Supplementary file 4 — Source Data [file 41467_2026_75011_MOESM4_ESM.zip › figshare_package_FINAL_PUBLIC_DEPOSIT_V1_20260503_002637/00_START_HERE_HTML_NAVIGATOR/file_views/view_0039_script_on_request_file_manifest_v01.html]

00\_release\_manifests/script\_on\_request\_file\_manifest\_v01.csv

# Readable file view

00\_release\_manifests/script\_on\_request\_file\_manifest\_v01.csv

← Back to navigator   |   Open original package file

Section

Technical appendix / request manifests

Output

Extension

csv

Size KB

60.976

Variables

0

## Readable HTML view

Showing all 93 rows.

| relative\_path | file\_name | directory | extension | file\_type | size\_bytes | size\_kb | last\_modified | md5 | access\_tier | data\_origin | used\_for | n\_rows | n\_cols | keep\_in\_repository | needs\_manual\_review | comment | release\_scope | release\_access\_tier | release\_source\_role | release\_decision | output\_tag | registry\_file\_key | registry\_release\_status | registry\_is\_primary | registry\_used\_by\_workflows | registry\_source\_role | final\_release\_tier | final\_release\_decision | final\_policy\_reason | is\_r\_script | is\_srtr\_named | is\_data\_like | is\_rendered\_or\_doc | is\_internal\_table\_template | is\_legacy\_zip | is\_internal\_workflow\_data\_or\_table | is\_internal\_documentation | is\_internal\_primary\_source | final\_release\_tier\_before\_doc\_close | final\_release\_decision\_before\_doc\_close | final\_policy\_reason\_before\_doc\_close | is\_internal\_submission\_manifest | is\_noninternal\_submission\_manifest | is\_workflow\_readme | is\_submission\_ready\_readme | is\_remaining\_manual\_doc |
| --- | --- | --- | --- | --- | --- | --- | --- | --- | --- | --- | --- | --- | --- | --- | --- | --- | --- | --- | --- | --- | --- | --- | --- | --- | --- | --- | --- | --- | --- | --- | --- | --- | --- | --- | --- | --- | --- | --- | --- | --- | --- | --- | --- | --- | --- | --- |
| 00\_config/00\_repository\_paths\_v03.R | 00\_repository\_paths\_v03.R | 00\_config | r | r\_script | 3658 | 3.572 | 2026-05-02T02:10:22Z | 08ef97a9121cbd37f69b7738856cbced | script\_on\_request | script |  |  |  | yes\_default | FALSE |  | release\_candidate | public\_deposit | reproducibility\_script | keep\_public\_deposit |  |  |  |  |  |  | script\_on\_request | keep\_script\_on\_request | R scripts are provided on request, not as public-deposit data. | TRUE | FALSE | FALSE | FALSE | FALSE | FALSE | FALSE | FALSE | FALSE | script\_on\_request | keep\_script\_on\_request | R scripts are provided on request, not as public-deposit data. | FALSE | FALSE | FALSE | FALSE | FALSE |
| 00\_config/02\_helpers\_io\_v03.R | 02\_helpers\_io\_v03.R | 00\_config | r | r\_script | 2031 | 1.983 | 2026-05-01T23:41:30Z | 68a8d4dbb117411a9c9201784961a173 | script\_on\_request | script |  |  |  | yes\_default | FALSE |  | release\_candidate | public\_deposit | reproducibility\_script | keep\_public\_deposit |  |  |  |  |  |  | script\_on\_request | keep\_script\_on\_request | R scripts are provided on request, not as public-deposit data. | TRUE | FALSE | FALSE | FALSE | FALSE | FALSE | FALSE | FALSE | FALSE | script\_on\_request | keep\_script\_on\_request | R scripts are provided on request, not as public-deposit data. | FALSE | FALSE | FALSE | FALSE | FALSE |
| 02\_workflows/F1\_workflow\_v02/code/00\_F1\_config\_helpers\_v02.R | 00\_F1\_config\_helpers\_v02.R | 02\_workflows/F1\_workflow\_v02/code | r | r\_script | 7242 | 7.072 | 2026-05-02T03:47:37Z | 4c6118c4c36956c2961e5d3718cc490d | script\_on\_request | script | F1 |  |  | yes\_default | FALSE |  | release\_candidate | script\_on\_request | reproducibility\_script | keep\_script\_on\_request | F1 |  |  |  |  |  | script\_on\_request | keep\_script\_on\_request | R scripts are provided on request, not as public-deposit data. | TRUE | FALSE | FALSE | FALSE | FALSE | FALSE | FALSE | FALSE | FALSE | script\_on\_request | keep\_script\_on\_request | R scripts are provided on request, not as public-deposit data. | FALSE | FALSE | FALSE | FALSE | FALSE |
| 02\_workflows/F1\_workflow\_v02/code/00\_run\_F1\_workflow\_v02.R | 00\_run\_F1\_workflow\_v02.R | 02\_workflows/F1\_workflow\_v02/code | r | r\_script | 1493 | 1.458 | 2026-05-01T23:41:30Z | 09891e6a0d094622f35c8e7f5b111d37 | script\_on\_request | script | F1 |  |  | yes\_default | FALSE |  | release\_candidate | script\_on\_request | reproducibility\_script | keep\_script\_on\_request | F1 |  |  |  |  |  | script\_on\_request | keep\_script\_on\_request | R scripts are provided on request, not as public-deposit data. | TRUE | FALSE | FALSE | FALSE | FALSE | FALSE | FALSE | FALSE | FALSE | script\_on\_request | keep\_script\_on\_request | R scripts are provided on request, not as public-deposit data. | FALSE | FALSE | FALSE | FALSE | FALSE |
| 02\_workflows/F1\_workflow\_v02/code/01\_F1\_load\_required\_data\_from\_datazip\_v02.R | 01\_F1\_load\_required\_data\_from\_datazip\_v02.R | 02\_workflows/F1\_workflow\_v02/code | r | r\_script | 399 | 0.39 | 2026-05-02T01:16:08Z | a6026ed024703da8e6cbf5b17fb8ca6e | script\_on\_request | script | F1 |  |  | yes\_default | FALSE |  | release\_candidate | script\_on\_request | reproducibility\_script | keep\_script\_on\_request | F1 |  |  |  |  |  | script\_on\_request | keep\_script\_on\_request | R scripts are provided on request, not as public-deposit data. | TRUE | FALSE | FALSE | FALSE | FALSE | FALSE | FALSE | FALSE | FALSE | script\_on\_request | keep\_script\_on\_request | R scripts are provided on request, not as public-deposit data. | FALSE | FALSE | FALSE | FALSE | FALSE |
| 02\_workflows/F1\_workflow\_v02/code/01\_F1\_load\_required\_data\_from\_primary\_v03.R | 01\_F1\_load\_required\_data\_from\_primary\_v03.R | 02\_workflows/F1\_workflow\_v02/code | r | r\_script | 3774 | 3.686 | 2026-05-02T01:16:08Z | fd4a1cbe332db49ea5d651e57772942f | script\_on\_request | script | F1 |  |  | yes\_default | FALSE |  | release\_candidate | script\_on\_request | reproducibility\_script | keep\_script\_on\_request | F1 |  |  |  |  |  | script\_on\_request | keep\_script\_on\_request | R scripts are provided on request, not as public-deposit data. | TRUE | FALSE | FALSE | FALSE | FALSE | FALSE | FALSE | FALSE | FALSE | script\_on\_request | keep\_script\_on\_request | R scripts are provided on request, not as public-deposit data. | FALSE | FALSE | FALSE | FALSE | FALSE |
| 02\_workflows/F1\_workflow\_v02/code/02a\_F1\_build\_refined\_analysis\_datasets\_v02.R | 02a\_F1\_build\_refined\_analysis\_datasets\_v02.R | 02\_workflows/F1\_workflow\_v02/code | r | r\_script | 12941 | 12.638 | 2026-05-02T01:29:36Z | aa4b2a6a2797f12995de053d75a22afd | script\_on\_request | script | F1 |  |  | yes\_default | FALSE |  | release\_candidate | script\_on\_request | reproducibility\_script | keep\_script\_on\_request | F1 |  |  |  |  |  | script\_on\_request | keep\_script\_on\_request | R scripts are provided on request, not as public-deposit data. | TRUE | FALSE | FALSE | FALSE | FALSE | FALSE | FALSE | FALSE | FALSE | script\_on\_request | keep\_script\_on\_request | R scripts are provided on request, not as public-deposit data. | FALSE | FALSE | FALSE | FALSE | FALSE |
| 02\_workflows/F1\_workflow\_v02/code/02b\_F1\_build\_figure\_content\_dataset\_v02.R | 02b\_F1\_build\_figure\_content\_dataset\_v02.R | 02\_workflows/F1\_workflow\_v02/code | r | r\_script | 8857 | 8.649 | 2026-05-02T01:29:36Z | f8028dbad41087403eb56b9c78bd01b9 | script\_on\_request | script | F1 |  |  | yes\_default | FALSE |  | release\_candidate | script\_on\_request | reproducibility\_script | keep\_script\_on\_request | F1 |  |  |  |  |  | script\_on\_request | keep\_script\_on\_request | R scripts are provided on request, not as public-deposit data. | TRUE | FALSE | FALSE | FALSE | FALSE | FALSE | FALSE | FALSE | FALSE | script\_on\_request | keep\_script\_on\_request | R scripts are provided on request, not as public-deposit data. | FALSE | FALSE | FALSE | FALSE | FALSE |
| 02\_workflows/F1\_workflow\_v02/code/03\_F1\_render\_surface\_figure\_v02.R | 03\_F1\_render\_surface\_figure\_v02.R | 02\_workflows/F1\_workflow\_v02/code | r | r\_script | 31720 | 30.977 | 2026-05-01T23:49:52Z | ca22b5306b426d833cf73dffd07d472f | script\_on\_request | script | F1 |  |  | yes\_default | FALSE |  | release\_candidate | script\_on\_request | reproducibility\_script | keep\_script\_on\_request | F1 |  |  |  |  |  | script\_on\_request | keep\_script\_on\_request | R scripts are provided on request, not as public-deposit data. | TRUE | FALSE | FALSE | FALSE | FALSE | FALSE | FALSE | FALSE | FALSE | script\_on\_request | keep\_script\_on\_request | R scripts are provided on request, not as public-deposit data. | FALSE | FALSE | FALSE | FALSE | FALSE |
| 02\_workflows/F1\_workflow\_v02/code/04\_F1\_validate\_and\_document\_outputs\_v02.R | 04\_F1\_validate\_and\_document\_outputs\_v02.R | 02\_workflows/F1\_workflow\_v02/code | r | r\_script | 8143 | 7.952 | 2026-05-02T03:47:37Z | 0e86c775fea000e7f6706d52f8c8a122 | script\_on\_request | script | F1 |  |  | yes\_default | FALSE |  | release\_candidate | script\_on\_request | reproducibility\_script | keep\_script\_on\_request | F1 |  |  |  |  |  | script\_on\_request | keep\_script\_on\_request | R scripts are provided on request, not as public-deposit data. | TRUE | FALSE | FALSE | FALSE | FALSE | FALSE | FALSE | FALSE | FALSE | script\_on\_request | keep\_script\_on\_request | R scripts are provided on request, not as public-deposit data. | FALSE | FALSE | FALSE | FALSE | FALSE |
| 02\_workflows/F1\_workflow\_v02/code/05\_F1\_build\_submission\_ready\_outputs\_v02.R | 05\_F1\_build\_submission\_ready\_outputs\_v02.R | 02\_workflows/F1\_workflow\_v02/code | r | r\_script | 18400 | 17.969 | 2026-05-02T01:29:36Z | 8c06f9a2b8dd7e658f9dd6c4fc48f596 | script\_on\_request | script | F1 |  |  | yes\_default | FALSE |  | release\_candidate | script\_on\_request | reproducibility\_script | keep\_script\_on\_request | F1 |  |  |  |  |  | script\_on\_request | keep\_script\_on\_request | R scripts are provided on request, not as public-deposit data. | TRUE | FALSE | FALSE | FALSE | FALSE | FALSE | FALSE | FALSE | FALSE | script\_on\_request | keep\_script\_on\_request | R scripts are provided on request, not as public-deposit data. | FALSE | FALSE | FALSE | FALSE | FALSE |
| 02\_workflows/F1\_workflow\_v02/code/F1.zip | F1.zip | 02\_workflows/F1\_workflow\_v02/code | zip | other | 29562 | 28.869 | 2026-05-02T03:39:01Z | 98e61f13e34f528d044ecf251ee805fc | script\_on\_request | derived | F1 |  |  | yes\_default | FALSE |  | release\_candidate | script\_on\_request | reproducibility\_script | keep\_script\_on\_request | F1 |  |  |  |  |  | script\_on\_request | keep\_script\_on\_request | Script-on-request file after policy overrides. | FALSE | FALSE | TRUE | FALSE | FALSE | FALSE | FALSE | FALSE | FALSE | script\_on\_request | keep\_script\_on\_request | Script-on-request file after policy overrides. | FALSE | FALSE | FALSE | FALSE | FALSE |
| 02\_workflows/F2\_workflow\_v01/code/00\_F2\_config\_helpers\_v01.R | 00\_F2\_config\_helpers\_v01.R | 02\_workflows/F2\_workflow\_v01/code | r | r\_script | 3057 | 2.985 | 2026-05-02T03:47:37Z | 792a830705c95d6bc5f3751f2007f3e2 | script\_on\_request | script | F2 |  |  | yes\_default | FALSE |  | release\_candidate | script\_on\_request | reproducibility\_script | keep\_script\_on\_request | F2 |  |  |  |  |  | script\_on\_request | keep\_script\_on\_request | R scripts are provided on request, not as public-deposit data. | TRUE | FALSE | FALSE | FALSE | FALSE | FALSE | FALSE | FALSE | FALSE | script\_on\_request | keep\_script\_on\_request | R scripts are provided on request, not as public-deposit data. | FALSE | FALSE | FALSE | FALSE | FALSE |
| 02\_workflows/F2\_workflow\_v01/code/00\_run\_F2\_workflow\_v01.R | 00\_run\_F2\_workflow\_v01.R | 02\_workflows/F2\_workflow\_v01/code | r | r\_script | 1491 | 1.456 | 2026-05-01T23:41:30Z | b2108b324ae8674d24cefc1cce0222aa | script\_on\_request | script | F2 |  |  | yes\_default | FALSE |  | release\_candidate | script\_on\_request | reproducibility\_script | keep\_script\_on\_request | F2 |  |  |  |  |  | script\_on\_request | keep\_script\_on\_request | R scripts are provided on request, not as public-deposit data. | TRUE | FALSE | FALSE | FALSE | FALSE | FALSE | FALSE | FALSE | FALSE | script\_on\_request | keep\_script\_on\_request | R scripts are provided on request, not as public-deposit data. | FALSE | FALSE | FALSE | FALSE | FALSE |
| 02\_workflows/F2\_workflow\_v01/code/01\_F2\_load\_required\_data\_from\_datazip\_v01.R | 01\_F2\_load\_required\_data\_from\_datazip\_v01.R | 02\_workflows/F2\_workflow\_v01/code | r | r\_script | 399 | 0.39 | 2026-05-02T01:16:08Z | a61abb64e248fefeee474490cac1ef1a | script\_on\_request | script | F2 |  |  | yes\_default | FALSE |  | release\_candidate | script\_on\_request | reproducibility\_script | keep\_script\_on\_request | F2 |  |  |  |  |  | script\_on\_request | keep\_script\_on\_request | R scripts are provided on request, not as public-deposit data. | TRUE | FALSE | FALSE | FALSE | FALSE | FALSE | FALSE | FALSE | FALSE | script\_on\_request | keep\_script\_on\_request | R scripts are provided on request, not as public-deposit data. | FALSE | FALSE | FALSE | FALSE | FALSE |
| 02\_workflows/F2\_workflow\_v01/code/01\_F2\_load\_required\_data\_from\_primary\_v03.R | 01\_F2\_load\_required\_data\_from\_primary\_v03.R | 02\_workflows/F2\_workflow\_v01/code | r | r\_script | 3114 | 3.041 | 2026-05-02T01:16:08Z | 53ea2b5ac279f0eeaa4281386291c90d | script\_on\_request | script | F2 |  |  | yes\_default | FALSE |  | release\_candidate | script\_on\_request | reproducibility\_script | keep\_script\_on\_request | F2 |  |  |  |  |  | script\_on\_request | keep\_script\_on\_request | R scripts are provided on request, not as public-deposit data. | TRUE | FALSE | FALSE | FALSE | FALSE | FALSE | FALSE | FALSE | FALSE | script\_on\_request | keep\_script\_on\_request | R scripts are provided on request, not as public-deposit data. | FALSE | FALSE | FALSE | FALSE | FALSE |
| 02\_workflows/F2\_workflow\_v01/code/02a\_F2\_build\_refined\_analysis\_dataset\_v01.R | 02a\_F2\_build\_refined\_analysis\_dataset\_v01.R | 02\_workflows/F2\_workflow\_v01/code | r | r\_script | 3560 | 3.477 | 2026-05-01T23:41:30Z | 47b7cf8751651775429a7a40fb1cf191 | script\_on\_request | script | F2 |  |  | yes\_default | FALSE |  | release\_candidate | script\_on\_request | reproducibility\_script | keep\_script\_on\_request | F2 |  |  |  |  |  | script\_on\_request | keep\_script\_on\_request | R scripts are provided on request, not as public-deposit data. | TRUE | FALSE | FALSE | FALSE | FALSE | FALSE | FALSE | FALSE | FALSE | script\_on\_request | keep\_script\_on\_request | R scripts are provided on request, not as public-deposit data. | FALSE | FALSE | FALSE | FALSE | FALSE |
| 02\_workflows/F2\_workflow\_v01/code/02b\_F2\_build\_figure\_content\_dataset\_v01.R | 02b\_F2\_build\_figure\_content\_dataset\_v01.R | 02\_workflows/F2\_workflow\_v01/code | r | r\_script | 4078 | 3.982 | 2026-05-01T23:41:30Z | 2b0abde770324c7c307b3b6bf3e2850b | script\_on\_request | script | F2 |  |  | yes\_default | FALSE |  | release\_candidate | script\_on\_request | reproducibility\_script | keep\_script\_on\_request | F2 |  |  |  |  |  | script\_on\_request | keep\_script\_on\_request | R scripts are provided on request, not as public-deposit data. | TRUE | FALSE | FALSE | FALSE | FALSE | FALSE | FALSE | FALSE | FALSE | script\_on\_request | keep\_script\_on\_request | R scripts are provided on request, not as public-deposit data. | FALSE | FALSE | FALSE | FALSE | FALSE |
| 02\_workflows/F2\_workflow\_v01/code/03\_F2\_render\_heatmap\_figure\_v01.R | 03\_F2\_render\_heatmap\_figure\_v01.R | 02\_workflows/F2\_workflow\_v01/code | r | r\_script | 21573 | 21.067 | 2026-05-01T23:41:30Z | 0872b74da8cb43576a9539d586395268 | script\_on\_request | script | F2 |  |  | yes\_default | FALSE |  | release\_candidate | script\_on\_request | reproducibility\_script | keep\_script\_on\_request | F2 |  |  |  |  |  | script\_on\_request | keep\_script\_on\_request | R scripts are provided on request, not as public-deposit data. | TRUE | FALSE | FALSE | FALSE | FALSE | FALSE | FALSE | FALSE | FALSE | script\_on\_request | keep\_script\_on\_request | R scripts are provided on request, not as public-deposit data. | FALSE | FALSE | FALSE | FALSE | FALSE |
| 02\_workflows/F2\_workflow\_v01/code/04\_F2\_validate\_and\_document\_outputs\_v01.R | 04\_F2\_validate\_and\_document\_outputs\_v01.R | 02\_workflows/F2\_workflow\_v01/code | r | r\_script | 4983 | 4.866 | 2026-05-01T23:41:30Z | 4a686cb31f4a138ece2de28d39f2bc92 | script\_on\_request | script | F2 |  |  | yes\_default | FALSE |  | release\_candidate | script\_on\_request | reproducibility\_script | keep\_script\_on\_request | F2 |  |  |  |  |  | script\_on\_request | keep\_script\_on\_request | R scripts are provided on request, not as public-deposit data. | TRUE | FALSE | FALSE | FALSE | FALSE | FALSE | FALSE | FALSE | FALSE | script\_on\_request | keep\_script\_on\_request | R scripts are provided on request, not as public-deposit data. | FALSE | FALSE | FALSE | FALSE | FALSE |
| 02\_workflows/F2\_workflow\_v01/code/05\_F2\_build\_submission\_ready\_outputs\_v01.R | 05\_F2\_build\_submission\_ready\_outputs\_v01.R | 02\_workflows/F2\_workflow\_v01/code | r | r\_script | 6205 | 6.06 | 2026-05-01T23:41:30Z | 4b38b6bdddec31db6fd2aeda9cc2364b | script\_on\_request | script | F2 |  |  | yes\_default | FALSE |  | release\_candidate | script\_on\_request | reproducibility\_script | keep\_script\_on\_request | F2 |  |  |  |  |  | script\_on\_request | keep\_script\_on\_request | R scripts are provided on request, not as public-deposit data. | TRUE | FALSE | FALSE | FALSE | FALSE | FALSE | FALSE | FALSE | FALSE | script\_on\_request | keep\_script\_on\_request | R scripts are provided on request, not as public-deposit data. | FALSE | FALSE | FALSE | FALSE | FALSE |
| 02\_workflows/F2\_workflow\_v01/code/F2.zip | F2.zip | 02\_workflows/F2\_workflow\_v01/code | zip | other | 20610 | 20.127 | 2026-05-02T03:39:24Z | cc87c33a853cebc0984db6d8574e9f04 | script\_on\_request | derived | F2 |  |  | yes\_default | FALSE |  | release\_candidate | script\_on\_request | reproducibility\_script | keep\_script\_on\_request | F2 |  |  |  |  |  | script\_on\_request | keep\_script\_on\_request | Script-on-request file after policy overrides. | FALSE | FALSE | TRUE | FALSE | FALSE | FALSE | FALSE | FALSE | FALSE | script\_on\_request | keep\_script\_on\_request | Script-on-request file after policy overrides. | FALSE | FALSE | FALSE | FALSE | FALSE |
| 02\_workflows/F3\_workflow\_v01/code/00\_F3\_config\_helpers\_v01.R | 00\_F3\_config\_helpers\_v01.R | 02\_workflows/F3\_workflow\_v01/code | r | r\_script | 4950 | 4.834 | 2026-05-02T03:47:37Z | 754a34a85524f5829ed399a2f7277cb4 | script\_on\_request | script | F3 |  |  | yes\_default | FALSE |  | release\_candidate | script\_on\_request | reproducibility\_script | keep\_script\_on\_request | F3 |  |  |  |  |  | script\_on\_request | keep\_script\_on\_request | R scripts are provided on request, not as public-deposit data. | TRUE | FALSE | FALSE | FALSE | FALSE | FALSE | FALSE | FALSE | FALSE | script\_on\_request | keep\_script\_on\_request | R scripts are provided on request, not as public-deposit data. | FALSE | FALSE | FALSE | FALSE | FALSE |
| 02\_workflows/F3\_workflow\_v01/code/00\_run\_F3\_workflow\_v01.R | 00\_run\_F3\_workflow\_v01.R | 02\_workflows/F3\_workflow\_v01/code | r | r\_script | 1361 | 1.329 | 2026-05-01T23:41:30Z | 0070e325db2c3ba54816dac69778c592 | script\_on\_request | script | F3 |  |  | yes\_default | FALSE |  | release\_candidate | script\_on\_request | reproducibility\_script | keep\_script\_on\_request | F3 |  |  |  |  |  | script\_on\_request | keep\_script\_on\_request | R scripts are provided on request, not as public-deposit data. | TRUE | FALSE | FALSE | FALSE | FALSE | FALSE | FALSE | FALSE | FALSE | script\_on\_request | keep\_script\_on\_request | R scripts are provided on request, not as public-deposit data. | FALSE | FALSE | FALSE | FALSE | FALSE |
| 02\_workflows/F3\_workflow\_v01/code/01\_F3\_load\_required\_data\_from\_datazip\_v01.R | 01\_F3\_load\_required\_data\_from\_datazip\_v01.R | 02\_workflows/F3\_workflow\_v01/code | r | r\_script | 399 | 0.39 | 2026-05-02T00:47:57Z | 787e305ccba1e9426faa5e24678b13af | script\_on\_request | script | F3 |  |  | yes\_default | FALSE |  | release\_candidate | script\_on\_request | reproducibility\_script | keep\_script\_on\_request | F3 |  |  |  |  |  | script\_on\_request | keep\_script\_on\_request | R scripts are provided on request, not as public-deposit data. | TRUE | FALSE | FALSE | FALSE | FALSE | FALSE | FALSE | FALSE | FALSE | script\_on\_request | keep\_script\_on\_request | R scripts are provided on request, not as public-deposit data. | FALSE | FALSE | FALSE | FALSE | FALSE |
| 02\_workflows/F3\_workflow\_v01/code/01\_F3\_load\_required\_data\_from\_primary\_v03.R | 01\_F3\_load\_required\_data\_from\_primary\_v03.R | 02\_workflows/F3\_workflow\_v01/code | r | r\_script | 2748 | 2.684 | 2026-05-02T09:46:31Z | 4c26a3c85319f89bba09fe13eb59a5e2 | script\_on\_request | script | F3 |  |  | yes\_default | FALSE |  | release\_candidate | script\_on\_request | reproducibility\_script | keep\_script\_on\_request | F3 |  |  |  |  |  | script\_on\_request | keep\_script\_on\_request | R scripts are provided on request, not as public-deposit data. | TRUE | FALSE | FALSE | FALSE | FALSE | FALSE | FALSE | FALSE | FALSE | script\_on\_request | keep\_script\_on\_request | R scripts are provided on request, not as public-deposit data. | FALSE | FALSE | FALSE | FALSE | FALSE |
| 02\_workflows/F3\_workflow\_v01/code/02a\_F3\_build\_refined\_analysis\_datasets\_v01.R | 02a\_F3\_build\_refined\_analysis\_datasets\_v01.R | 02\_workflows/F3\_workflow\_v01/code | r | r\_script | 4491 | 4.386 | 2026-05-02T23:18:13Z | dccbd497cbb04a8be034772b6a1b71b7 | script\_on\_request | script | F3 |  |  | yes\_default | FALSE |  | release\_candidate | script\_on\_request | reproducibility\_script | keep\_script\_on\_request | F3 |  |  |  |  |  | script\_on\_request | keep\_script\_on\_request | R scripts are provided on request, not as public-deposit data. | TRUE | FALSE | FALSE | FALSE | FALSE | FALSE | FALSE | FALSE | FALSE | script\_on\_request | keep\_script\_on\_request | R scripts are provided on request, not as public-deposit data. | FALSE | FALSE | FALSE | FALSE | FALSE |
| 02\_workflows/F3\_workflow\_v01/code/02b\_F3\_build\_figure\_content\_datasets\_v01.R | 02b\_F3\_build\_figure\_content\_datasets\_v01.R | 02\_workflows/F3\_workflow\_v01/code | r | r\_script | 3730 | 3.643 | 2026-05-02T00:47:57Z | 2e7ce6093f7a1df16b5b5885d2b898c0 | script\_on\_request | script | F3 |  |  | yes\_default | FALSE |  | release\_candidate | script\_on\_request | reproducibility\_script | keep\_script\_on\_request | F3 |  |  |  |  |  | script\_on\_request | keep\_script\_on\_request | R scripts are provided on request, not as public-deposit data. | TRUE | FALSE | FALSE | FALSE | FALSE | FALSE | FALSE | FALSE | FALSE | script\_on\_request | keep\_script\_on\_request | R scripts are provided on request, not as public-deposit data. | FALSE | FALSE | FALSE | FALSE | FALSE |
| 02\_workflows/F3\_workflow\_v01/code/03\_F3\_render\_score\_shift\_figure\_v01.R | 03\_F3\_render\_score\_shift\_figure\_v01.R | 02\_workflows/F3\_workflow\_v01/code | r | r\_script | 11464 | 11.195 | 2026-05-01T23:41:30Z | dab2f3a66f41aaa54a5536793aff3d31 | script\_on\_request | script | F3 |  |  | yes\_default | FALSE |  | release\_candidate | script\_on\_request | reproducibility\_script | keep\_script\_on\_request | F3 |  |  |  |  |  | script\_on\_request | keep\_script\_on\_request | R scripts are provided on request, not as public-deposit data. | TRUE | FALSE | FALSE | FALSE | FALSE | FALSE | FALSE | FALSE | FALSE | script\_on\_request | keep\_script\_on\_request | R scripts are provided on request, not as public-deposit data. | FALSE | FALSE | FALSE | FALSE | FALSE |
| 02\_workflows/F3\_workflow\_v01/code/04\_F3\_validate\_and\_document\_outputs\_v01.R | 04\_F3\_validate\_and\_document\_outputs\_v01.R | 02\_workflows/F3\_workflow\_v01/code | r | r\_script | 8763 | 8.558 | 2026-05-02T01:00:07Z | 8aad742b799389fdfc56c4be8fc77a79 | script\_on\_request | script | F3 |  |  | yes\_default | FALSE |  | release\_candidate | script\_on\_request | reproducibility\_script | keep\_script\_on\_request | F3 |  |  |  |  |  | script\_on\_request | keep\_script\_on\_request | R scripts are provided on request, not as public-deposit data. | TRUE | FALSE | FALSE | FALSE | FALSE | FALSE | FALSE | FALSE | FALSE | script\_on\_request | keep\_script\_on\_request | R scripts are provided on request, not as public-deposit data. | FALSE | FALSE | FALSE | FALSE | FALSE |
| 02\_workflows/F3\_workflow\_v01/code/05\_F3\_build\_submission\_ready\_outputs\_v01.R | 05\_F3\_build\_submission\_ready\_outputs\_v01.R | 02\_workflows/F3\_workflow\_v01/code | r | r\_script | 5210 | 5.088 | 2026-05-02T01:00:07Z | 8078006d7b85464d4eddc328c90f0439 | script\_on\_request | script | F3 |  |  | yes\_default | FALSE |  | release\_candidate | script\_on\_request | reproducibility\_script | keep\_script\_on\_request | F3 |  |  |  |  |  | script\_on\_request | keep\_script\_on\_request | R scripts are provided on request, not as public-deposit data. | TRUE | FALSE | FALSE | FALSE | FALSE | FALSE | FALSE | FALSE | FALSE | script\_on\_request | keep\_script\_on\_request | R scripts are provided on request, not as public-deposit data. | FALSE | FALSE | FALSE | FALSE | FALSE |
| 02\_workflows/F3\_workflow\_v01/code/F3.zip | F3.zip | 02\_workflows/F3\_workflow\_v01/code | zip | other | 18859 | 18.417 | 2026-05-02T03:39:51Z | 690e7e847dbc81eb2d0a367e44958e20 | script\_on\_request | derived | F3 |  |  | yes\_default | FALSE |  | release\_candidate | script\_on\_request | reproducibility\_script | keep\_script\_on\_request | F3 |  |  |  |  |  | script\_on\_request | keep\_script\_on\_request | Script-on-request file after policy overrides. | FALSE | FALSE | TRUE | FALSE | FALSE | FALSE | FALSE | FALSE | FALSE | script\_on\_request | keep\_script\_on\_request | Script-on-request file after policy overrides. | FALSE | FALSE | FALSE | FALSE | FALSE |
| 02\_workflows/F4\_workflow\_v01/code/00\_F4\_config\_helpers\_v01.R | 00\_F4\_config\_helpers\_v01.R | 02\_workflows/F4\_workflow\_v01/code | r | r\_script | 2909 | 2.841 | 2026-05-02T03:47:37Z | 6690d530be6c6c7b8c10a66db809481b | script\_on\_request | script | F4 |  |  | yes\_default | FALSE |  | release\_candidate | script\_on\_request | reproducibility\_script | keep\_script\_on\_request | F4 |  |  |  |  |  | script\_on\_request | keep\_script\_on\_request | R scripts are provided on request, not as public-deposit data. | TRUE | FALSE | FALSE | FALSE | FALSE | FALSE | FALSE | FALSE | FALSE | script\_on\_request | keep\_script\_on\_request | R scripts are provided on request, not as public-deposit data. | FALSE | FALSE | FALSE | FALSE | FALSE |
| 02\_workflows/F4\_workflow\_v01/code/00\_run\_F4\_workflow\_v01.R | 00\_run\_F4\_workflow\_v01.R | 02\_workflows/F4\_workflow\_v01/code | r | r\_script | 1351 | 1.319 | 2026-05-01T23:41:30Z | 6f2bd645ae463a999c015a6cd8884497 | script\_on\_request | script | F4 |  |  | yes\_default | FALSE |  | release\_candidate | script\_on\_request | reproducibility\_script | keep\_script\_on\_request | F4 |  |  |  |  |  | script\_on\_request | keep\_script\_on\_request | R scripts are provided on request, not as public-deposit data. | TRUE | FALSE | FALSE | FALSE | FALSE | FALSE | FALSE | FALSE | FALSE | script\_on\_request | keep\_script\_on\_request | R scripts are provided on request, not as public-deposit data. | FALSE | FALSE | FALSE | FALSE | FALSE |
| 02\_workflows/F4\_workflow\_v01/code/01\_F4\_load\_required\_data\_from\_datazip\_v01.R | 01\_F4\_load\_required\_data\_from\_datazip\_v01.R | 02\_workflows/F4\_workflow\_v01/code | r | r\_script | 399 | 0.39 | 2026-05-02T01:36:15Z | e1e7ae4109c46377f0cf35f41bfe0edb | script\_on\_request | script | F4 |  |  | yes\_default | FALSE |  | release\_candidate | script\_on\_request | reproducibility\_script | keep\_script\_on\_request | F4 |  |  |  |  |  | script\_on\_request | keep\_script\_on\_request | R scripts are provided on request, not as public-deposit data. | TRUE | FALSE | FALSE | FALSE | FALSE | FALSE | FALSE | FALSE | FALSE | script\_on\_request | keep\_script\_on\_request | R scripts are provided on request, not as public-deposit data. | FALSE | FALSE | FALSE | FALSE | FALSE |
| 02\_workflows/F4\_workflow\_v01/code/01\_F4\_load\_required\_data\_from\_primary\_v03.R | 01\_F4\_load\_required\_data\_from\_primary\_v03.R | 02\_workflows/F4\_workflow\_v01/code | r | r\_script | 3884 | 3.793 | 2026-05-02T02:02:26Z | ab918a2dc67e53575d665d010418d140 | script\_on\_request | script | F4 |  |  | yes\_default | FALSE |  | release\_candidate | script\_on\_request | reproducibility\_script | keep\_script\_on\_request | F4 |  |  |  |  |  | script\_on\_request | keep\_script\_on\_request | R scripts are provided on request, not as public-deposit data. | TRUE | FALSE | FALSE | FALSE | FALSE | FALSE | FALSE | FALSE | FALSE | script\_on\_request | keep\_script\_on\_request | R scripts are provided on request, not as public-deposit data. | FALSE | FALSE | FALSE | FALSE | FALSE |
| 02\_workflows/F4\_workflow\_v01/code/02a\_F4\_build\_refined\_analysis\_datasets\_v01.R | 02a\_F4\_build\_refined\_analysis\_datasets\_v01.R | 02\_workflows/F4\_workflow\_v01/code | r | r\_script | 15460 | 15.098 | 2026-05-01T23:41:30Z | b0f53cda4fa6af299ae81effc7b37162 | script\_on\_request | script | F4 |  |  | yes\_default | FALSE |  | release\_candidate | script\_on\_request | reproducibility\_script | keep\_script\_on\_request | F4 |  |  |  |  |  | script\_on\_request | keep\_script\_on\_request | R scripts are provided on request, not as public-deposit data. | TRUE | FALSE | FALSE | FALSE | FALSE | FALSE | FALSE | FALSE | FALSE | script\_on\_request | keep\_script\_on\_request | R scripts are provided on request, not as public-deposit data. | FALSE | FALSE | FALSE | FALSE | FALSE |
| 02\_workflows/F4\_workflow\_v01/code/02b\_F4\_build\_figure\_content\_datasets\_v01.R | 02b\_F4\_build\_figure\_content\_datasets\_v01.R | 02\_workflows/F4\_workflow\_v01/code | r | r\_script | 4529 | 4.423 | 2026-05-02T02:02:26Z | b71c77f27415e4ef1b1a1adf3896c182 | script\_on\_request | script | F4 |  |  | yes\_default | FALSE |  | release\_candidate | script\_on\_request | reproducibility\_script | keep\_script\_on\_request | F4 |  |  |  |  |  | script\_on\_request | keep\_script\_on\_request | R scripts are provided on request, not as public-deposit data. | TRUE | FALSE | FALSE | FALSE | FALSE | FALSE | FALSE | FALSE | FALSE | script\_on\_request | keep\_script\_on\_request | R scripts are provided on request, not as public-deposit data. | FALSE | FALSE | FALSE | FALSE | FALSE |
| 02\_workflows/F4\_workflow\_v01/code/03\_F4\_render\_survival\_figure\_v01.R | 03\_F4\_render\_survival\_figure\_v01.R | 02\_workflows/F4\_workflow\_v01/code | r | r\_script | 20886 | 20.396 | 2026-05-02T02:02:26Z | 96c0feb2fd7dcfef01c208003004651f | script\_on\_request | script | F4 |  |  | yes\_default | FALSE |  | release\_candidate | script\_on\_request | reproducibility\_script | keep\_script\_on\_request | F4 |  |  |  |  |  | script\_on\_request | keep\_script\_on\_request | R scripts are provided on request, not as public-deposit data. | TRUE | FALSE | FALSE | FALSE | FALSE | FALSE | FALSE | FALSE | FALSE | script\_on\_request | keep\_script\_on\_request | R scripts are provided on request, not as public-deposit data. | FALSE | FALSE | FALSE | FALSE | FALSE |
| 02\_workflows/F4\_workflow\_v01/code/04\_F4\_validate\_and\_document\_outputs\_v01.R | 04\_F4\_validate\_and\_document\_outputs\_v01.R | 02\_workflows/F4\_workflow\_v01/code | r | r\_script | 5020 | 4.902 | 2026-05-02T02:02:26Z | d46231d411ab6e2f3f9208e651c4386b | script\_on\_request | script | F4 |  |  | yes\_default | FALSE |  | release\_candidate | script\_on\_request | reproducibility\_script | keep\_script\_on\_request | F4 |  |  |  |  |  | script\_on\_request | keep\_script\_on\_request | R scripts are provided on request, not as public-deposit data. | TRUE | FALSE | FALSE | FALSE | FALSE | FALSE | FALSE | FALSE | FALSE | script\_on\_request | keep\_script\_on\_request | R scripts are provided on request, not as public-deposit data. | FALSE | FALSE | FALSE | FALSE | FALSE |
| 02\_workflows/F4\_workflow\_v01/code/05\_F4\_build\_submission\_ready\_outputs\_v01.R | 05\_F4\_build\_submission\_ready\_outputs\_v01.R | 02\_workflows/F4\_workflow\_v01/code | r | r\_script | 7248 | 7.078 | 2026-05-02T02:02:26Z | db4b475ceecb5dd39e8e7b235e386849 | script\_on\_request | script | F4 |  |  | yes\_default | FALSE |  | release\_candidate | script\_on\_request | reproducibility\_script | keep\_script\_on\_request | F4 |  |  |  |  |  | script\_on\_request | keep\_script\_on\_request | R scripts are provided on request, not as public-deposit data. | TRUE | FALSE | FALSE | FALSE | FALSE | FALSE | FALSE | FALSE | FALSE | script\_on\_request | keep\_script\_on\_request | R scripts are provided on request, not as public-deposit data. | FALSE | FALSE | FALSE | FALSE | FALSE |
| 02\_workflows/F4\_workflow\_v01/code/F4.zip | F4.zip | 02\_workflows/F4\_workflow\_v01/code | zip | other | 22749 | 22.216 | 2026-05-02T03:40:10Z | ea5323e6bf626fe41dd328c74916b8e3 | script\_on\_request | derived | F4 |  |  | yes\_default | FALSE |  | release\_candidate | script\_on\_request | reproducibility\_script | keep\_script\_on\_request | F4 |  |  |  |  |  | script\_on\_request | keep\_script\_on\_request | Script-on-request file after policy overrides. | FALSE | FALSE | TRUE | FALSE | FALSE | FALSE | FALSE | FALSE | FALSE | script\_on\_request | keep\_script\_on\_request | Script-on-request file after policy overrides. | FALSE | FALSE | FALSE | FALSE | FALSE |
| 02\_workflows/F5\_workflow\_v01/code/00\_F5\_config\_helpers\_v01.R | 00\_F5\_config\_helpers\_v01.R | 02\_workflows/F5\_workflow\_v01/code | r | r\_script | 2893 | 2.825 | 2026-05-02T03:47:37Z | 64de040356d310cb0d24d178530bcd12 | script\_on\_request | script | F5 |  |  | yes\_default | FALSE |  | release\_candidate | script\_on\_request | reproducibility\_script | keep\_script\_on\_request | F5 |  |  |  |  |  | script\_on\_request | keep\_script\_on\_request | R scripts are provided on request, not as public-deposit data. | TRUE | FALSE | FALSE | FALSE | FALSE | FALSE | FALSE | FALSE | FALSE | script\_on\_request | keep\_script\_on\_request | R scripts are provided on request, not as public-deposit data. | FALSE | FALSE | FALSE | FALSE | FALSE |
| 02\_workflows/F5\_workflow\_v01/code/00\_run\_F5\_workflow\_v01.R | 00\_run\_F5\_workflow\_v01.R | 02\_workflows/F5\_workflow\_v01/code | r | r\_script | 1357 | 1.325 | 2026-05-01T23:41:30Z | 6ed956478204d9e6d60f82b8492675f3 | script\_on\_request | script | F5 |  |  | yes\_default | FALSE |  | release\_candidate | script\_on\_request | reproducibility\_script | keep\_script\_on\_request | F5 |  |  |  |  |  | script\_on\_request | keep\_script\_on\_request | R scripts are provided on request, not as public-deposit data. | TRUE | FALSE | FALSE | FALSE | FALSE | FALSE | FALSE | FALSE | FALSE | script\_on\_request | keep\_script\_on\_request | R scripts are provided on request, not as public-deposit data. | FALSE | FALSE | FALSE | FALSE | FALSE |
| 02\_workflows/F5\_workflow\_v01/code/01\_F5\_load\_required\_data\_from\_datazip\_v01.R | 01\_F5\_load\_required\_data\_from\_datazip\_v01.R | 02\_workflows/F5\_workflow\_v01/code | r | r\_script | 399 | 0.39 | 2026-05-02T01:36:15Z | f0ab691eb2e65ca1307eb20c477bd876 | script\_on\_request | script | F5 |  |  | yes\_default | FALSE |  | release\_candidate | script\_on\_request | reproducibility\_script | keep\_script\_on\_request | F5 |  |  |  |  |  | script\_on\_request | keep\_script\_on\_request | R scripts are provided on request, not as public-deposit data. | TRUE | FALSE | FALSE | FALSE | FALSE | FALSE | FALSE | FALSE | FALSE | script\_on\_request | keep\_script\_on\_request | R scripts are provided on request, not as public-deposit data. | FALSE | FALSE | FALSE | FALSE | FALSE |
| 02\_workflows/F5\_workflow\_v01/code/01\_F5\_load\_required\_data\_from\_primary\_v03.R | 01\_F5\_load\_required\_data\_from\_primary\_v03.R | 02\_workflows/F5\_workflow\_v01/code | r | r\_script | 2725 | 2.661 | 2026-05-02T01:40:58Z | e4eb8e3ba2e71967ed3489691f4d3559 | script\_on\_request | script | F5 |  |  | yes\_default | FALSE |  | release\_candidate | script\_on\_request | reproducibility\_script | keep\_script\_on\_request | F5 |  |  |  |  |  | script\_on\_request | keep\_script\_on\_request | R scripts are provided on request, not as public-deposit data. | TRUE | FALSE | FALSE | FALSE | FALSE | FALSE | FALSE | FALSE | FALSE | script\_on\_request | keep\_script\_on\_request | R scripts are provided on request, not as public-deposit data. | FALSE | FALSE | FALSE | FALSE | FALSE |
| 02\_workflows/F5\_workflow\_v01/code/02a\_F5\_build\_refined\_analysis\_datasets\_v01.R | 02a\_F5\_build\_refined\_analysis\_datasets\_v01.R | 02\_workflows/F5\_workflow\_v01/code | r | r\_script | 8311 | 8.116 | 2026-05-02T23:18:13Z | d91b2c9c9d5450f4bc1ab3ae9786a235 | script\_on\_request | script | F5 |  |  | yes\_default | FALSE |  | release\_candidate | script\_on\_request | reproducibility\_script | keep\_script\_on\_request | F5 |  |  |  |  |  | script\_on\_request | keep\_script\_on\_request | R scripts are provided on request, not as public-deposit data. | TRUE | FALSE | FALSE | FALSE | FALSE | FALSE | FALSE | FALSE | FALSE | script\_on\_request | keep\_script\_on\_request | R scripts are provided on request, not as public-deposit data. | FALSE | FALSE | FALSE | FALSE | FALSE |
| 02\_workflows/F5\_workflow\_v01/code/02b\_F5\_build\_figure\_content\_datasets\_v01.R | 02b\_F5\_build\_figure\_content\_datasets\_v01.R | 02\_workflows/F5\_workflow\_v01/code | r | r\_script | 4367 | 4.265 | 2026-05-02T01:40:58Z | 6a6ddcb2868eef0856bdded99c12b241 | script\_on\_request | script | F5 |  |  | yes\_default | FALSE |  | release\_candidate | script\_on\_request | reproducibility\_script | keep\_script\_on\_request | F5 |  |  |  |  |  | script\_on\_request | keep\_script\_on\_request | R scripts are provided on request, not as public-deposit data. | TRUE | FALSE | FALSE | FALSE | FALSE | FALSE | FALSE | FALSE | FALSE | script\_on\_request | keep\_script\_on\_request | R scripts are provided on request, not as public-deposit data. | FALSE | FALSE | FALSE | FALSE | FALSE |
| 02\_workflows/F5\_workflow\_v01/code/03\_F5\_render\_stratified\_survival\_figure\_v01.R | 03\_F5\_render\_stratified\_survival\_figure\_v01.R | 02\_workflows/F5\_workflow\_v01/code | r | r\_script | 8671 | 8.468 | 2026-05-01T23:41:30Z | d55bbc45cb800c1bddae5512bec328cb | script\_on\_request | script | F5 |  |  | yes\_default | FALSE |  | release\_candidate | script\_on\_request | reproducibility\_script | keep\_script\_on\_request | F5 |  |  |  |  |  | script\_on\_request | keep\_script\_on\_request | R scripts are provided on request, not as public-deposit data. | TRUE | FALSE | FALSE | FALSE | FALSE | FALSE | FALSE | FALSE | FALSE | script\_on\_request | keep\_script\_on\_request | R scripts are provided on request, not as public-deposit data. | FALSE | FALSE | FALSE | FALSE | FALSE |
| 02\_workflows/F5\_workflow\_v01/code/04\_F5\_validate\_and\_document\_outputs\_v01.R | 04\_F5\_validate\_and\_document\_outputs\_v01.R | 02\_workflows/F5\_workflow\_v01/code | r | r\_script | 4255 | 4.155 | 2026-05-01T23:41:30Z | 9900e9f096e4e40e9ee766e4bdf2613c | script\_on\_request | script | F5 |  |  | yes\_default | FALSE |  | release\_candidate | script\_on\_request | reproducibility\_script | keep\_script\_on\_request | F5 |  |  |  |  |  | script\_on\_request | keep\_script\_on\_request | R scripts are provided on request, not as public-deposit data. | TRUE | FALSE | FALSE | FALSE | FALSE | FALSE | FALSE | FALSE | FALSE | script\_on\_request | keep\_script\_on\_request | R scripts are provided on request, not as public-deposit data. | FALSE | FALSE | FALSE | FALSE | FALSE |
| 02\_workflows/F5\_workflow\_v01/code/05\_F5\_build\_submission\_ready\_outputs\_v01.R | 05\_F5\_build\_submission\_ready\_outputs\_v01.R | 02\_workflows/F5\_workflow\_v01/code | r | r\_script | 7208 | 7.039 | 2026-05-02T01:40:58Z | bc05ac1ca778a6b0fe113a1b66e797e6 | script\_on\_request | script | F5 |  |  | yes\_default | FALSE |  | release\_candidate | script\_on\_request | reproducibility\_script | keep\_script\_on\_request | F5 |  |  |  |  |  | script\_on\_request | keep\_script\_on\_request | R scripts are provided on request, not as public-deposit data. | TRUE | FALSE | FALSE | FALSE | FALSE | FALSE | FALSE | FALSE | FALSE | script\_on\_request | keep\_script\_on\_request | R scripts are provided on request, not as public-deposit data. | FALSE | FALSE | FALSE | FALSE | FALSE |
| 02\_workflows/F5\_workflow\_v01/code/F5.zip | F5.zip | 02\_workflows/F5\_workflow\_v01/code | zip | other | 18834 | 18.393 | 2026-05-02T03:40:27Z | ffe846d79fd339f781fea9f23fbdeab8 | script\_on\_request | derived | F5 |  |  | yes\_default | FALSE |  | release\_candidate | script\_on\_request | reproducibility\_script | keep\_script\_on\_request | F5 |  |  |  |  |  | script\_on\_request | keep\_script\_on\_request | Script-on-request file after policy overrides. | FALSE | FALSE | TRUE | FALSE | FALSE | FALSE | FALSE | FALSE | FALSE | script\_on\_request | keep\_script\_on\_request | Script-on-request file after policy overrides. | FALSE | FALSE | FALSE | FALSE | FALSE |
| 02\_workflows/F6\_workflow\_v01/code/00\_F6\_config\_helpers\_v01.R | 00\_F6\_config\_helpers\_v01.R | 02\_workflows/F6\_workflow\_v01/code | r | r\_script | 3641 | 3.556 | 2026-05-02T03:47:37Z | be3ea285572a0c8e8d70e1fa82eecf32 | script\_on\_request | script | F6 |  |  | yes\_default | FALSE |  | release\_candidate | script\_on\_request | reproducibility\_script | keep\_script\_on\_request | F6 |  |  |  |  |  | script\_on\_request | keep\_script\_on\_request | R scripts are provided on request, not as public-deposit data. | TRUE | FALSE | FALSE | FALSE | FALSE | FALSE | FALSE | FALSE | FALSE | script\_on\_request | keep\_script\_on\_request | R scripts are provided on request, not as public-deposit data. | FALSE | FALSE | FALSE | FALSE | FALSE |
| 02\_workflows/F6\_workflow\_v01/code/00\_run\_F6\_workflow\_v01.R | 00\_run\_F6\_workflow\_v01.R | 02\_workflows/F6\_workflow\_v01/code | r | r\_script | 1352 | 1.32 | 2026-05-01T23:41:30Z | e4c7e397fb1ded227b53a69397024ab1 | script\_on\_request | script | F6 |  |  | yes\_default | FALSE |  | release\_candidate | script\_on\_request | reproducibility\_script | keep\_script\_on\_request | F6 |  |  |  |  |  | script\_on\_request | keep\_script\_on\_request | R scripts are provided on request, not as public-deposit data. | TRUE | FALSE | FALSE | FALSE | FALSE | FALSE | FALSE | FALSE | FALSE | script\_on\_request | keep\_script\_on\_request | R scripts are provided on request, not as public-deposit data. | FALSE | FALSE | FALSE | FALSE | FALSE |
| 02\_workflows/F6\_workflow\_v01/code/01\_F6\_load\_required\_data\_from\_datazip\_v01.R | 01\_F6\_load\_required\_data\_from\_datazip\_v01.R | 02\_workflows/F6\_workflow\_v01/code | r | r\_script | 399 | 0.39 | 2026-05-02T01:36:15Z | 7c727a0b9209831cefa337e6ab11a211 | script\_on\_request | script | F6 |  |  | yes\_default | FALSE |  | release\_candidate | script\_on\_request | reproducibility\_script | keep\_script\_on\_request | F6 |  |  |  |  |  | script\_on\_request | keep\_script\_on\_request | R scripts are provided on request, not as public-deposit data. | TRUE | FALSE | FALSE | FALSE | FALSE | FALSE | FALSE | FALSE | FALSE | script\_on\_request | keep\_script\_on\_request | R scripts are provided on request, not as public-deposit data. | FALSE | FALSE | FALSE | FALSE | FALSE |
| 02\_workflows/F6\_workflow\_v01/code/01\_F6\_load\_required\_data\_from\_primary\_v03.R | 01\_F6\_load\_required\_data\_from\_primary\_v03.R | 02\_workflows/F6\_workflow\_v01/code | r | r\_script | 2671 | 2.608 | 2026-05-02T01:36:15Z | 90626778cf0244fc96af91dc284ef355 | script\_on\_request | script | F6 |  |  | yes\_default | FALSE |  | release\_candidate | script\_on\_request | reproducibility\_script | keep\_script\_on\_request | F6 |  |  |  |  |  | script\_on\_request | keep\_script\_on\_request | R scripts are provided on request, not as public-deposit data. | TRUE | FALSE | FALSE | FALSE | FALSE | FALSE | FALSE | FALSE | FALSE | script\_on\_request | keep\_script\_on\_request | R scripts are provided on request, not as public-deposit data. | FALSE | FALSE | FALSE | FALSE | FALSE |
| 02\_workflows/F6\_workflow\_v01/code/02a\_F6\_build\_refined\_analysis\_datasets\_v01.R | 02a\_F6\_build\_refined\_analysis\_datasets\_v01.R | 02\_workflows/F6\_workflow\_v01/code | r | r\_script | 11139 | 10.878 | 2026-05-02T03:08:12Z | 866fc90e58fcd60c0e2cc028baf2c894 | script\_on\_request | script | F6 |  |  | yes\_default | FALSE |  | release\_candidate | script\_on\_request | reproducibility\_script | keep\_script\_on\_request | F6 |  |  |  |  |  | script\_on\_request | keep\_script\_on\_request | R scripts are provided on request, not as public-deposit data. | TRUE | FALSE | FALSE | FALSE | FALSE | FALSE | FALSE | FALSE | FALSE | script\_on\_request | keep\_script\_on\_request | R scripts are provided on request, not as public-deposit data. | FALSE | FALSE | FALSE | FALSE | FALSE |
| 02\_workflows/F6\_workflow\_v01/code/02b\_F6\_build\_figure\_content\_datasets\_v01.R | 02b\_F6\_build\_figure\_content\_datasets\_v01.R | 02\_workflows/F6\_workflow\_v01/code | r | r\_script | 3382 | 3.303 | 2026-05-01T23:41:30Z | ecf2b2eead8e2f4fc9bfcbca95d982e5 | script\_on\_request | script | F6 |  |  | yes\_default | FALSE |  | release\_candidate | script\_on\_request | reproducibility\_script | keep\_script\_on\_request | F6 |  |  |  |  |  | script\_on\_request | keep\_script\_on\_request | R scripts are provided on request, not as public-deposit data. | TRUE | FALSE | FALSE | FALSE | FALSE | FALSE | FALSE | FALSE | FALSE | script\_on\_request | keep\_script\_on\_request | R scripts are provided on request, not as public-deposit data. | FALSE | FALSE | FALSE | FALSE | FALSE |
| 02\_workflows/F6\_workflow\_v01/code/03\_F6\_render\_cif\_figure\_v01.R | 03\_F6\_render\_cif\_figure\_v01.R | 02\_workflows/F6\_workflow\_v01/code | r | r\_script | 10354 | 10.111 | 2026-05-02T03:10:37Z | 86fc7231931e443e20db0884f47117d6 | script\_on\_request | script | F6 |  |  | yes\_default | FALSE |  | release\_candidate | script\_on\_request | reproducibility\_script | keep\_script\_on\_request | F6 |  |  |  |  |  | script\_on\_request | keep\_script\_on\_request | R scripts are provided on request, not as public-deposit data. | TRUE | FALSE | FALSE | FALSE | FALSE | FALSE | FALSE | FALSE | FALSE | script\_on\_request | keep\_script\_on\_request | R scripts are provided on request, not as public-deposit data. | FALSE | FALSE | FALSE | FALSE | FALSE |
| 02\_workflows/F6\_workflow\_v01/code/04\_F6\_validate\_and\_document\_outputs\_v01.R | 04\_F6\_validate\_and\_document\_outputs\_v01.R | 02\_workflows/F6\_workflow\_v01/code | r | r\_script | 5776 | 5.641 | 2026-05-01T23:41:30Z | 96f1f0cd3f20b1ff08c63bacfa0cde77 | script\_on\_request | script | F6 |  |  | yes\_default | FALSE |  | release\_candidate | script\_on\_request | reproducibility\_script | keep\_script\_on\_request | F6 |  |  |  |  |  | script\_on\_request | keep\_script\_on\_request | R scripts are provided on request, not as public-deposit data. | TRUE | FALSE | FALSE | FALSE | FALSE | FALSE | FALSE | FALSE | FALSE | script\_on\_request | keep\_script\_on\_request | R scripts are provided on request, not as public-deposit data. | FALSE | FALSE | FALSE | FALSE | FALSE |
| 02\_workflows/F6\_workflow\_v01/code/05\_F6\_build\_submission\_ready\_outputs\_v01.R | 05\_F6\_build\_submission\_ready\_outputs\_v01.R | 02\_workflows/F6\_workflow\_v01/code | r | r\_script | 8945 | 8.735 | 2026-05-01T23:41:30Z | d4a7c19cb5ab3e410091495c43e0cbc5 | script\_on\_request | script | F6 |  |  | yes\_default | FALSE |  | release\_candidate | script\_on\_request | reproducibility\_script | keep\_script\_on\_request | F6 |  |  |  |  |  | script\_on\_request | keep\_script\_on\_request | R scripts are provided on request, not as public-deposit data. | TRUE | FALSE | FALSE | FALSE | FALSE | FALSE | FALSE | FALSE | FALSE | script\_on\_request | keep\_script\_on\_request | R scripts are provided on request, not as public-deposit data. | FALSE | FALSE | FALSE | FALSE | FALSE |
| 02\_workflows/F6\_workflow\_v01/code/F6.zip | F6.zip | 02\_workflows/F6\_workflow\_v01/code | zip | other | 20441 | 19.962 | 2026-05-02T03:40:48Z | 6156b0eb4332288a49b37ef8aef444e9 | script\_on\_request | derived | F6 |  |  | yes\_default | FALSE |  | release\_candidate | script\_on\_request | reproducibility\_script | keep\_script\_on\_request | F6 |  |  |  |  |  | script\_on\_request | keep\_script\_on\_request | Script-on-request file after policy overrides. | FALSE | FALSE | TRUE | FALSE | FALSE | FALSE | FALSE | FALSE | FALSE | script\_on\_request | keep\_script\_on\_request | Script-on-request file after policy overrides. | FALSE | FALSE | FALSE | FALSE | FALSE |
| 02\_workflows/T1\_workflow\_v01/code/00\_T1\_config\_helpers\_v01.R | 00\_T1\_config\_helpers\_v01.R | 02\_workflows/T1\_workflow\_v01/code | r | r\_script | 5871 | 5.733 | 2026-05-02T03:47:37Z | 378b43a722c4624dc6362cc497de5787 | script\_on\_request | script | T1 |  |  | yes\_default | FALSE |  | release\_candidate | script\_on\_request | reproducibility\_script | keep\_script\_on\_request | T1 |  |  |  |  |  | script\_on\_request | keep\_script\_on\_request | R scripts are provided on request, not as public-deposit data. | TRUE | FALSE | FALSE | FALSE | FALSE | FALSE | FALSE | FALSE | FALSE | script\_on\_request | keep\_script\_on\_request | R scripts are provided on request, not as public-deposit data. | FALSE | FALSE | FALSE | FALSE | FALSE |
| 02\_workflows/T1\_workflow\_v01/code/00\_run\_T1\_workflow\_v01.R | 00\_run\_T1\_workflow\_v01.R | 02\_workflows/T1\_workflow\_v01/code | r | r\_script | 1360 | 1.328 | 2026-05-01T23:41:30Z | 22c6620a122931af811b6a228c84561f | script\_on\_request | script | T1 |  |  | yes\_default | FALSE |  | release\_candidate | script\_on\_request | reproducibility\_script | keep\_script\_on\_request | T1 |  |  |  |  |  | script\_on\_request | keep\_script\_on\_request | R scripts are provided on request, not as public-deposit data. | TRUE | FALSE | FALSE | FALSE | FALSE | FALSE | FALSE | FALSE | FALSE | script\_on\_request | keep\_script\_on\_request | R scripts are provided on request, not as public-deposit data. | FALSE | FALSE | FALSE | FALSE | FALSE |
| 02\_workflows/T1\_workflow\_v01/code/01\_T1\_load\_required\_data\_from\_datazip\_v01.R | 01\_T1\_load\_required\_data\_from\_datazip\_v01.R | 02\_workflows/T1\_workflow\_v01/code | r | r\_script | 399 | 0.39 | 2026-05-02T03:32:22Z | d8c6234066aac572b7fd8e424cf02161 | script\_on\_request | script | T1 |  |  | yes\_default | FALSE |  | release\_candidate | script\_on\_request | reproducibility\_script | keep\_script\_on\_request | T1 |  |  |  |  |  | script\_on\_request | keep\_script\_on\_request | R scripts are provided on request, not as public-deposit data. | TRUE | FALSE | FALSE | FALSE | FALSE | FALSE | FALSE | FALSE | FALSE | script\_on\_request | keep\_script\_on\_request | R scripts are provided on request, not as public-deposit data. | FALSE | FALSE | FALSE | FALSE | FALSE |
| 02\_workflows/T1\_workflow\_v01/code/01\_T1\_load\_required\_data\_from\_primary\_v03.R | 01\_T1\_load\_required\_data\_from\_primary\_v03.R | 02\_workflows/T1\_workflow\_v01/code | r | r\_script | 9550 | 9.326 | 2026-05-02T03:32:22Z | 1be95164b7567e473a9448f3f04f5a52 | script\_on\_request | script | T1 |  |  | yes\_default | FALSE |  | release\_candidate | script\_on\_request | reproducibility\_script | keep\_script\_on\_request | T1 |  |  |  |  |  | script\_on\_request | keep\_script\_on\_request | R scripts are provided on request, not as public-deposit data. | TRUE | FALSE | FALSE | FALSE | FALSE | FALSE | FALSE | FALSE | FALSE | script\_on\_request | keep\_script\_on\_request | R scripts are provided on request, not as public-deposit data. | FALSE | FALSE | FALSE | FALSE | FALSE |
| 02\_workflows/T1\_workflow\_v01/code/02a\_T1\_build\_refined\_analysis\_datasets\_v01.R | 02a\_T1\_build\_refined\_analysis\_datasets\_v01.R | 02\_workflows/T1\_workflow\_v01/code | r | r\_script | 3936 | 3.844 | 2026-05-02T03:47:37Z | 65ed312b3b7a1e1b1ef1af2ed633894a | script\_on\_request | script | T1 |  |  | yes\_default | FALSE |  | release\_candidate | script\_on\_request | reproducibility\_script | keep\_script\_on\_request | T1 |  |  |  |  |  | script\_on\_request | keep\_script\_on\_request | R scripts are provided on request, not as public-deposit data. | TRUE | FALSE | FALSE | FALSE | FALSE | FALSE | FALSE | FALSE | FALSE | script\_on\_request | keep\_script\_on\_request | R scripts are provided on request, not as public-deposit data. | FALSE | FALSE | FALSE | FALSE | FALSE |
| 02\_workflows/T1\_workflow\_v01/code/02b\_T1\_build\_table\_content\_dataset\_v01.R | 02b\_T1\_build\_table\_content\_dataset\_v01.R | 02\_workflows/T1\_workflow\_v01/code | r | r\_script | 37605 | 36.724 | 2026-05-02T20:27:34Z | e252f553ae9d8b189fd09e008b860fe8 | script\_on\_request | script | T1;table\_or\_supplement |  |  | yes\_default | FALSE |  | release\_candidate | script\_on\_request | reproducibility\_script | keep\_script\_on\_request | T1 |  |  |  |  |  | script\_on\_request | keep\_script\_on\_request | R scripts are provided on request, not as public-deposit data. | TRUE | FALSE | FALSE | FALSE | FALSE | FALSE | FALSE | FALSE | FALSE | script\_on\_request | keep\_script\_on\_request | R scripts are provided on request, not as public-deposit data. | FALSE | FALSE | FALSE | FALSE | FALSE |
| 02\_workflows/T1\_workflow\_v01/code/03\_T1\_render\_submitted\_table\_docx\_v01.R | 03\_T1\_render\_submitted\_table\_docx\_v01.R | 02\_workflows/T1\_workflow\_v01/code | r | r\_script | 5471 | 5.343 | 2026-05-01T23:41:30Z | d696b921f32674a4927497ec58da2e41 | script\_on\_request | script | T1;table\_or\_supplement |  |  | yes\_default | FALSE |  | release\_candidate | script\_on\_request | reproducibility\_script | keep\_script\_on\_request | T1 |  |  |  |  |  | script\_on\_request | keep\_script\_on\_request | R scripts are provided on request, not as public-deposit data. | TRUE | FALSE | FALSE | FALSE | FALSE | FALSE | FALSE | FALSE | FALSE | script\_on\_request | keep\_script\_on\_request | R scripts are provided on request, not as public-deposit data. | FALSE | FALSE | FALSE | FALSE | FALSE |
| 02\_workflows/T1\_workflow\_v01/code/04\_T1\_validate\_and\_document\_outputs\_v01.R | 04\_T1\_validate\_and\_document\_outputs\_v01.R | 02\_workflows/T1\_workflow\_v01/code | r | r\_script | 6512 | 6.359 | 2026-05-01T23:41:30Z | d7d78618729a8357dc7faa583e30d6df | script\_on\_request | script | T1 |  |  | yes\_default | FALSE |  | release\_candidate | script\_on\_request | reproducibility\_script | keep\_script\_on\_request | T1 |  |  |  |  |  | script\_on\_request | keep\_script\_on\_request | R scripts are provided on request, not as public-deposit data. | TRUE | FALSE | FALSE | FALSE | FALSE | FALSE | FALSE | FALSE | FALSE | script\_on\_request | keep\_script\_on\_request | R scripts are provided on request, not as public-deposit data. | FALSE | FALSE | FALSE | FALSE | FALSE |
| 02\_workflows/T1\_workflow\_v01/code/05\_T1\_build\_submission\_ready\_outputs\_v01.R | 05\_T1\_build\_submission\_ready\_outputs\_v01.R | 02\_workflows/T1\_workflow\_v01/code | r | r\_script | 8722 | 8.518 | 2026-05-01T23:41:30Z | ce076a3912d6aaf57ea4c0afd72cb1cd | script\_on\_request | script | T1 |  |  | yes\_default | FALSE |  | release\_candidate | script\_on\_request | reproducibility\_script | keep\_script\_on\_request | T1 |  |  |  |  |  | script\_on\_request | keep\_script\_on\_request | R scripts are provided on request, not as public-deposit data. | TRUE | FALSE | FALSE | FALSE | FALSE | FALSE | FALSE | FALSE | FALSE | script\_on\_request | keep\_script\_on\_request | R scripts are provided on request, not as public-deposit data. | FALSE | FALSE | FALSE | FALSE | FALSE |
| 02\_workflows/T1\_workflow\_v01/code/T1.zip | T1.zip | 02\_workflows/T1\_workflow\_v01/code | zip | other | 21655 | 21.147 | 2026-05-02T03:41:09Z | c947b35348e6e0ee9b910620f7e8297a | script\_on\_request | derived | T1 |  |  | yes\_default | FALSE |  | release\_candidate | script\_on\_request | reproducibility\_script | keep\_script\_on\_request | T1 |  |  |  |  |  | script\_on\_request | keep\_script\_on\_request | Script-on-request file after policy overrides. | FALSE | FALSE | TRUE | FALSE | FALSE | FALSE | FALSE | FALSE | FALSE | script\_on\_request | keep\_script\_on\_request | Script-on-request file after policy overrides. | FALSE | FALSE | FALSE | FALSE | FALSE |
| 02\_workflows/T3\_workflow\_v01/code/00\_T3\_config\_helpers\_v01.R | 00\_T3\_config\_helpers\_v01.R | 02\_workflows/T3\_workflow\_v01/code | r | r\_script | 6918 | 6.756 | 2026-05-02T03:47:37Z | c97bfc970a6c7b913897a5410315d5b1 | script\_on\_request | script | T3 |  |  | yes\_default | FALSE |  | release\_candidate | script\_on\_request | reproducibility\_script | keep\_script\_on\_request | T3 |  |  |  |  |  | script\_on\_request | keep\_script\_on\_request | R scripts are provided on request, not as public-deposit data. | TRUE | FALSE | FALSE | FALSE | FALSE | FALSE | FALSE | FALSE | FALSE | script\_on\_request | keep\_script\_on\_request | R scripts are provided on request, not as public-deposit data. | FALSE | FALSE | FALSE | FALSE | FALSE |
| 02\_workflows/T3\_workflow\_v01/code/00\_run\_T3\_workflow\_v01.R | 00\_run\_T3\_workflow\_v01.R | 02\_workflows/T3\_workflow\_v01/code | r | r\_script | 1483 | 1.448 | 2026-05-01T23:41:30Z | 33fcac1a04dc4a1d857d8867aae96762 | script\_on\_request | script | T3 |  |  | yes\_default | FALSE |  | release\_candidate | script\_on\_request | reproducibility\_script | keep\_script\_on\_request | T3 |  |  |  |  |  | script\_on\_request | keep\_script\_on\_request | R scripts are provided on request, not as public-deposit data. | TRUE | FALSE | FALSE | FALSE | FALSE | FALSE | FALSE | FALSE | FALSE | script\_on\_request | keep\_script\_on\_request | R scripts are provided on request, not as public-deposit data. | FALSE | FALSE | FALSE | FALSE | FALSE |
| 02\_workflows/T3\_workflow\_v01/code/01\_T3\_load\_required\_data\_from\_datazip\_v01.R | 01\_T3\_load\_required\_data\_from\_datazip\_v01.R | 02\_workflows/T3\_workflow\_v01/code | r | r\_script | 399 | 0.39 | 2026-05-02T03:23:58Z | a5426fa56a574173c85d3237eb42fa7b | script\_on\_request | script | T3 |  |  | yes\_default | FALSE |  | release\_candidate | script\_on\_request | reproducibility\_script | keep\_script\_on\_request | T3 |  |  |  |  |  | script\_on\_request | keep\_script\_on\_request | R scripts are provided on request, not as public-deposit data. | TRUE | FALSE | FALSE | FALSE | FALSE | FALSE | FALSE | FALSE | FALSE | script\_on\_request | keep\_script\_on\_request | R scripts are provided on request, not as public-deposit data. | FALSE | FALSE | FALSE | FALSE | FALSE |
| 02\_workflows/T3\_workflow\_v01/code/01\_T3\_load\_required\_data\_from\_primary\_v03.R | 01\_T3\_load\_required\_data\_from\_primary\_v03.R | 02\_workflows/T3\_workflow\_v01/code | r | r\_script | 8547 | 8.347 | 2026-05-02T17:16:54Z | 07ba42e1a4dadfdfab8f82b5b11d8f6d | script\_on\_request | script | T3 |  |  | yes\_default | FALSE |  | release\_candidate | script\_on\_request | reproducibility\_script | keep\_script\_on\_request | T3 |  |  |  |  |  | script\_on\_request | keep\_script\_on\_request | R scripts are provided on request, not as public-deposit data. | TRUE | FALSE | FALSE | FALSE | FALSE | FALSE | FALSE | FALSE | FALSE | script\_on\_request | keep\_script\_on\_request | R scripts are provided on request, not as public-deposit data. | FALSE | FALSE | FALSE | FALSE | FALSE |
| 02\_workflows/T3\_workflow\_v01/code/02a\_T3\_build\_refined\_analysis\_datasets\_v01.R | 02a\_T3\_build\_refined\_analysis\_datasets\_v01.R | 02\_workflows/T3\_workflow\_v01/code | r | r\_script | 5553 | 5.423 | 2026-05-02T17:16:54Z | 4882055adbedbf708c98408efd6e741a | script\_on\_request | script | T3 |  |  | yes\_default | FALSE |  | release\_candidate | script\_on\_request | reproducibility\_script | keep\_script\_on\_request | T3 |  |  |  |  |  | script\_on\_request | keep\_script\_on\_request | R scripts are provided on request, not as public-deposit data. | TRUE | FALSE | FALSE | FALSE | FALSE | FALSE | FALSE | FALSE | FALSE | script\_on\_request | keep\_script\_on\_request | R scripts are provided on request, not as public-deposit data. | FALSE | FALSE | FALSE | FALSE | FALSE |
| 02\_workflows/T3\_workflow\_v01/code/02b\_T3\_build\_table\_content\_dataset\_v01.R | 02b\_T3\_build\_table\_content\_dataset\_v01.R | 02\_workflows/T3\_workflow\_v01/code | r | r\_script | 12890 | 12.588 | 2026-05-02T21:13:21Z | 63c5c2dfbce5966dda5820f97adcd5d4 | script\_on\_request | script | T3;table\_or\_supplement |  |  | yes\_default | FALSE |  | release\_candidate | script\_on\_request | reproducibility\_script | keep\_script\_on\_request | T3 |  |  |  |  |  | script\_on\_request | keep\_script\_on\_request | R scripts are provided on request, not as public-deposit data. | TRUE | FALSE | FALSE | FALSE | FALSE | FALSE | FALSE | FALSE | FALSE | script\_on\_request | keep\_script\_on\_request | R scripts are provided on request, not as public-deposit data. | FALSE | FALSE | FALSE | FALSE | FALSE |
| 02\_workflows/T3\_workflow\_v01/code/03\_T3\_render\_submitted\_table\_docx\_v01.R | 03\_T3\_render\_submitted\_table\_docx\_v01.R | 02\_workflows/T3\_workflow\_v01/code | r | r\_script | 4531 | 4.425 | 2026-05-01T23:41:30Z | 9f6895fe13a3393217d92c62f7b3030f | script\_on\_request | script | T3;table\_or\_supplement |  |  | yes\_default | FALSE |  | release\_candidate | script\_on\_request | reproducibility\_script | keep\_script\_on\_request | T3 |  |  |  |  |  | script\_on\_request | keep\_script\_on\_request | R scripts are provided on request, not as public-deposit data. | TRUE | FALSE | FALSE | FALSE | FALSE | FALSE | FALSE | FALSE | FALSE | script\_on\_request | keep\_script\_on\_request | R scripts are provided on request, not as public-deposit data. | FALSE | FALSE | FALSE | FALSE | FALSE |
| 02\_workflows/T3\_workflow\_v01/code/04\_T3\_validate\_and\_document\_outputs\_v01.R | 04\_T3\_validate\_and\_document\_outputs\_v01.R | 02\_workflows/T3\_workflow\_v01/code | r | r\_script | 5708 | 5.574 | 2026-05-01T23:41:30Z | 38d540a1ed8811aab26a20d0512a6211 | script\_on\_request | script | T3 |  |  | yes\_default | FALSE |  | release\_candidate | script\_on\_request | reproducibility\_script | keep\_script\_on\_request | T3 |  |  |  |  |  | script\_on\_request | keep\_script\_on\_request | R scripts are provided on request, not as public-deposit data. | TRUE | FALSE | FALSE | FALSE | FALSE | FALSE | FALSE | FALSE | FALSE | script\_on\_request | keep\_script\_on\_request | R scripts are provided on request, not as public-deposit data. | FALSE | FALSE | FALSE | FALSE | FALSE |
| 02\_workflows/T3\_workflow\_v01/code/05\_T3\_build\_submission\_ready\_outputs\_v01.R | 05\_T3\_build\_submission\_ready\_outputs\_v01.R | 02\_workflows/T3\_workflow\_v01/code | r | r\_script | 7750 | 7.568 | 2026-05-01T23:41:30Z | 9977cd445a71ea491bfbd8afb0248892 | script\_on\_request | script | T3 |  |  | yes\_default | FALSE |  | release\_candidate | script\_on\_request | reproducibility\_script | keep\_script\_on\_request | T3 |  |  |  |  |  | script\_on\_request | keep\_script\_on\_request | R scripts are provided on request, not as public-deposit data. | TRUE | FALSE | FALSE | FALSE | FALSE | FALSE | FALSE | FALSE | FALSE | script\_on\_request | keep\_script\_on\_request | R scripts are provided on request, not as public-deposit data. | FALSE | FALSE | FALSE | FALSE | FALSE |
| 02\_workflows/T3\_workflow\_v01/code/T3.zip | T3.zip | 02\_workflows/T3\_workflow\_v01/code | zip | other | 21639 | 21.132 | 2026-05-02T03:41:38Z | 8fadb725088a191f39cfc7d27f5f6cf5 | script\_on\_request | derived | T3 |  |  | yes\_default | FALSE |  | release\_candidate | script\_on\_request | reproducibility\_script | keep\_script\_on\_request | T3 |  |  |  |  |  | script\_on\_request | keep\_script\_on\_request | Script-on-request file after policy overrides. | FALSE | FALSE | TRUE | FALSE | FALSE | FALSE | FALSE | FALSE | FALSE | script\_on\_request | keep\_script\_on\_request | Script-on-request file after policy overrides. | FALSE | FALSE | FALSE | FALSE | FALSE |
| 02\_workflows/T4\_workflow\_v01/code/00\_run\_T4\_workflow\_v01.R | 00\_run\_T4\_workflow\_v01.R | 02\_workflows/T4\_workflow\_v01/code | r | r\_script | 1543 | 1.507 | 2026-05-01T23:41:30Z | 100eff90fb34bbf0abf02a6cfb945c20 | script\_on\_request | script | T4 |  |  | yes\_default | FALSE |  | release\_candidate | script\_on\_request | reproducibility\_script | keep\_script\_on\_request | T4 |  |  |  |  |  | script\_on\_request | keep\_script\_on\_request | R scripts are provided on request, not as public-deposit data. | TRUE | FALSE | FALSE | FALSE | FALSE | FALSE | FALSE | FALSE | FALSE | script\_on\_request | keep\_script\_on\_request | R scripts are provided on request, not as public-deposit data. | FALSE | FALSE | FALSE | FALSE | FALSE |
| 02\_workflows/T4\_workflow\_v01/code/01\_T4\_load\_required\_data\_from\_datazip\_v01.R | 01\_T4\_load\_required\_data\_from\_datazip\_v01.R | 02\_workflows/T4\_workflow\_v01/code | r | r\_script | 399 | 0.39 | 2026-05-02T00:04:10Z | 7d9dcc6082956372862aac4196e00cc7 | script\_on\_request | script | T4 |  |  | yes\_default | FALSE |  | release\_candidate | script\_on\_request | reproducibility\_script | keep\_script\_on\_request | T4 |  |  |  |  |  | script\_on\_request | keep\_script\_on\_request | R scripts are provided on request, not as public-deposit data. | TRUE | FALSE | FALSE | FALSE | FALSE | FALSE | FALSE | FALSE | FALSE | script\_on\_request | keep\_script\_on\_request | R scripts are provided on request, not as public-deposit data. | FALSE | FALSE | FALSE | FALSE | FALSE |
| 02\_workflows/T4\_workflow\_v01/code/01\_T4\_load\_required\_data\_from\_primary\_v03.R | 01\_T4\_load\_required\_data\_from\_primary\_v03.R | 02\_workflows/T4\_workflow\_v01/code | r | r\_script | 4140 | 4.043 | 2026-05-02T00:04:10Z | 9d7bf130fe008c313e45674a83a1748c | script\_on\_request | script | T4 |  |  | yes\_default | FALSE |  | release\_candidate | script\_on\_request | reproducibility\_script | keep\_script\_on\_request | T4 |  |  |  |  |  | script\_on\_request | keep\_script\_on\_request | R scripts are provided on request, not as public-deposit data. | TRUE | FALSE | FALSE | FALSE | FALSE | FALSE | FALSE | FALSE | FALSE | script\_on\_request | keep\_script\_on\_request | R scripts are provided on request, not as public-deposit data. | FALSE | FALSE | FALSE | FALSE | FALSE |
| 02\_workflows/T4\_workflow\_v01/code/02a\_T4\_build\_refined\_analysis\_datasets\_v01.R | 02a\_T4\_build\_refined\_analysis\_datasets\_v01.R | 02\_workflows/T4\_workflow\_v01/code | r | r\_script | 4833 | 4.72 | 2026-05-01T23:41:30Z | dd920fef0bc1efc2f48c97be255bb9ed | script\_on\_request | script | T4 |  |  | yes\_default | FALSE |  | release\_candidate | script\_on\_request | reproducibility\_script | keep\_script\_on\_request | T4 |  |  |  |  |  | script\_on\_request | keep\_script\_on\_request | R scripts are provided on request, not as public-deposit data. | TRUE | FALSE | FALSE | FALSE | FALSE | FALSE | FALSE | FALSE | FALSE | script\_on\_request | keep\_script\_on\_request | R scripts are provided on request, not as public-deposit data. | FALSE | FALSE | FALSE | FALSE | FALSE |
| 02\_workflows/T4\_workflow\_v01/code/02b\_T4\_build\_table\_content\_dataset\_v01.R | 02b\_T4\_build\_table\_content\_dataset\_v01.R | 02\_workflows/T4\_workflow\_v01/code | r | r\_script | 10626 | 10.377 | 2026-05-01T23:41:30Z | a0f19cc35b244da83bb3dfa6bf365831 | script\_on\_request | script | T4;table\_or\_supplement |  |  | yes\_default | FALSE |  | release\_candidate | script\_on\_request | reproducibility\_script | keep\_script\_on\_request | T4 |  |  |  |  |  | script\_on\_request | keep\_script\_on\_request | R scripts are provided on request, not as public-deposit data. | TRUE | FALSE | FALSE | FALSE | FALSE | FALSE | FALSE | FALSE | FALSE | script\_on\_request | keep\_script\_on\_request | R scripts are provided on request, not as public-deposit data. | FALSE | FALSE | FALSE | FALSE | FALSE |
| 02\_workflows/T4\_workflow\_v01/code/02c\_T4\_build\_srtr\_restricted\_table\_content\_v01.R | 02c\_T4\_build\_srtr\_restricted\_table\_content\_v01.R | 02\_workflows/T4\_workflow\_v01/code | r | r\_script | 45032 | 43.977 | 2026-05-02T00:30:55Z | 5cd8bdd69a5da62b1d7e014b742dd0d9 | restricted\_on\_request | script | T4;table\_or\_supplement |  |  | yes\_default | TRUE |  | release\_candidate | restricted\_on\_request | reproducibility\_script | keep\_restricted\_on\_request | T4 |  |  |  |  |  | script\_on\_request | keep\_script\_on\_request | R scripts are provided on request, not as public-deposit data. | TRUE | TRUE | FALSE | FALSE | FALSE | FALSE | FALSE | FALSE | FALSE | script\_on\_request | keep\_script\_on\_request | R scripts are provided on request, not as public-deposit data. | FALSE | FALSE | FALSE | FALSE | FALSE |
| 02\_workflows/T4\_workflow\_v01/code/03\_T4\_render\_submitted\_table\_docx\_v01.R | 03\_T4\_render\_submitted\_table\_docx\_v01.R | 02\_workflows/T4\_workflow\_v01/code | r | r\_script | 4365 | 4.263 | 2026-05-01T23:41:30Z | 88291251f4e38a85036e258a5d20e37d | script\_on\_request | script | T4;table\_or\_supplement |  |  | yes\_default | FALSE |  | release\_candidate | script\_on\_request | reproducibility\_script | keep\_script\_on\_request | T4 |  |  |  |  |  | script\_on\_request | keep\_script\_on\_request | R scripts are provided on request, not as public-deposit data. | TRUE | FALSE | FALSE | FALSE | FALSE | FALSE | FALSE | FALSE | FALSE | script\_on\_request | keep\_script\_on\_request | R scripts are provided on request, not as public-deposit data. | FALSE | FALSE | FALSE | FALSE | FALSE |
| 02\_workflows/T4\_workflow\_v01/code/04\_T4\_validate\_and\_document\_outputs\_v01.R | 04\_T4\_validate\_and\_document\_outputs\_v01.R | 02\_workflows/T4\_workflow\_v01/code | r | r\_script | 8506 | 8.307 | 2026-05-01T23:49:52Z | 8ea0440a6882d3dfcca1c2913fbd160e | script\_on\_request | script | T4 |  |  | yes\_default | FALSE |  | release\_candidate | script\_on\_request | reproducibility\_script | keep\_script\_on\_request | T4 |  |  |  |  |  | script\_on\_request | keep\_script\_on\_request | R scripts are provided on request, not as public-deposit data. | TRUE | FALSE | FALSE | FALSE | FALSE | FALSE | FALSE | FALSE | FALSE | script\_on\_request | keep\_script\_on\_request | R scripts are provided on request, not as public-deposit data. | FALSE | FALSE | FALSE | FALSE | FALSE |
| 02\_workflows/T4\_workflow\_v01/code/05\_T4\_build\_submission\_ready\_outputs\_v01.R | 05\_T4\_build\_submission\_ready\_outputs\_v01.R | 02\_workflows/T4\_workflow\_v01/code | r | r\_script | 11845 | 11.567 | 2026-05-01T23:49:52Z | 8c0d3685524528e5fa14a3a58e0535ef | script\_on\_request | script | T4 |  |  | yes\_default | FALSE |  | release\_candidate | script\_on\_request | reproducibility\_script | keep\_script\_on\_request | T4 |  |  |  |  |  | script\_on\_request | keep\_script\_on\_request | R scripts are provided on request, not as public-deposit data. | TRUE | FALSE | FALSE | FALSE | FALSE | FALSE | FALSE | FALSE | FALSE | script\_on\_request | keep\_script\_on\_request | R scripts are provided on request, not as public-deposit data. | FALSE | FALSE | FALSE | FALSE | FALSE |
| 02\_workflows/T4\_workflow\_v01/code/99\_verify\_srtr\_T4\_survival\_matrix\_contains\_8\_sources\_v02.R | 99\_verify\_srtr\_T4\_survival\_matrix\_contains\_8\_sources\_v02.R | 02\_workflows/T4\_workflow\_v01/code | r | r\_script | 12934 | 12.631 | 2026-05-01T23:41:30Z | 20e8836b5bb63ef3abf5e184cac85019 | restricted\_on\_request | script | T4 |  |  | yes\_default | TRUE |  | release\_candidate | restricted\_on\_request | reproducibility\_script | keep\_restricted\_on\_request | T4 |  |  |  |  |  | script\_on\_request | keep\_script\_on\_request | R scripts are provided on request, not as public-deposit data. | TRUE | TRUE | FALSE | FALSE | FALSE | FALSE | FALSE | FALSE | FALSE | script\_on\_request | keep\_script\_on\_request | R scripts are provided on request, not as public-deposit data. | FALSE | FALSE | FALSE | FALSE | FALSE |
| 02\_workflows/T4\_workflow\_v01/code/T4.zip | T4.zip | 02\_workflows/T4\_workflow\_v01/code | zip | other | 34818 | 34.002 | 2026-05-02T03:41:58Z | 59e0fd2a2d016731bb3c8d852e1f931c | script\_on\_request | derived | T4 |  |  | yes\_default | FALSE |  | release\_candidate | script\_on\_request | reproducibility\_script | keep\_script\_on\_request | T4 |  |  |  |  |  | script\_on\_request | keep\_script\_on\_request | Script-on-request file after policy overrides. | FALSE | FALSE | TRUE | FALSE | FALSE | FALSE | FALSE | FALSE | FALSE | script\_on\_request | keep\_script\_on\_request | Script-on-request file after policy overrides. | FALSE | FALSE | FALSE | FALSE | FALSE |
