# Supplementary material for: Creatinine assay interferences compromises MELD accuracy and may bias liver allocation
Source: Nat Commun. 2026 Jul 23;17:7111. doi: 10.1038/s41467-026-75011-x (PMC13396164; doi:10.1038/s41467-026-75011-x)
Supplement: Supplementary file 4 — Source Data [file 41467_2026_75011_MOESM4_ESM.zip › figshare_package_FINAL_PUBLIC_DEPOSIT_V1_20260503_002637/00_START_HERE_HTML_NAVIGATOR/file_views/view_0001_README_PUBLIC_DEPOSIT_PACKAGE_v01.html]

README\_PUBLIC\_DEPOSIT\_PACKAGE\_v01.txt

# Readable file view

README\_PUBLIC\_DEPOSIT\_PACKAGE\_v01.txt

← Back to navigator   |   Open original package file

Section

Start here

Output

Extension

txt

Size KB

1.952

Variables

0

## Readable HTML view

```
MELD repository public-deposit package
======================================

Generated: 2026-05-03

Start here
----------
Open the end-user HTML navigator:
00_START_HERE_HTML_NAVIGATOR/index.html

The navigator provides browser-readable views of the public package files, links to the original files, and access to the public variable codebook.

Scope
-----
This public-deposit package contains public ESLD and simulated data objects required to inspect and rebuild public-use figures and tables where the required data are not restricted.

SRTR cohort data and SRTR-derived data objects are not included in this public package. They are listed separately as restricted-on-request files and are available only upon request and in compliance with applicable licence obligations.

R scripts are not included as public-deposit files. They are listed separately as script-on-request files and are available on request.

Recommended public files
------------------------
End-user navigator:
00_START_HERE_HTML_NAVIGATOR/index.html

Public variable codebook:
00_release_manifests/public_variable_dictionary_FINAL_CODEBOOK.csv

Public file manifest:
00_release_manifests/public_deposit_file_manifest_v01.csv

Restricted-on-request file manifest:
00_release_manifests/restricted_on_request_file_manifest_v01.csv

Script-on-request file manifest:
00_release_manifests/script_on_request_file_manifest_v01.csv

Package contents
----------------
Public-deposit files: 94
Public variables documented in the final codebook: 661
Restricted-on-request files listed: 97
Script-on-request files listed: 94

Access policy
-------------
The public package is intended for public inspection and reuse of non-restricted release data. Restricted SRTR data are not redistributed in this public package. Requests for restricted data or scripts should reference the corresponding request manifests in 00_release_manifests/.
```
