# Supplementary material for: Creatinine assay interferences compromises MELD accuracy and may bias liver allocation
Source: Nat Commun. 2026 Jul 23;17:7111. doi: 10.1038/s41467-026-75011-x (PMC13396164; doi:10.1038/s41467-026-75011-x)
Supplement: Supplementary file 4 — Source Data [file 41467_2026_75011_MOESM4_ESM.zip › figshare_package_FINAL_PUBLIC_DEPOSIT_V1_20260503_002637/00_START_HERE_HTML_NAVIGATOR/file_views/view_0017_esld_F5_stratified_survival_meta_public.html]

02\_workflows/F5\_workflow\_v01/submission\_ready/public/data/esld\_F5\_stratified\_survival\_meta\_public.csv

# Readable file view

02\_workflows/F5\_workflow\_v01/submission\_ready/public/data/esld\_F5\_stratified\_survival\_meta\_public.csv

← Back to navigator   |   Open original package file

Section

Manuscript output data

Output

F5

Extension

csv

Size KB

0.424

Variables

2

## Variables in this file

| Variable | Label | Description | Unit | Type |
| --- | --- | --- | --- | --- |
| parameter | Metadata parameter name | Name of a metadata parameter describing the F2 simulated heatmap object, such as figure identity, data origin, grid type, axis variable, or unit/role. |  | character |
| value | Value | Numerical or character value corresponding to the row-specific variable/metric. |  | character |

## Readable HTML view

Showing all 12 rows.

| parameter | value |
| --- | --- |
| dataset\_subject | esld\_F5\_stratified\_survival\_subject\_public |
| dataset\_stats | esld\_F5\_stratified\_survival\_stats\_public |
| domain | esld |
| anchor | F5 |
| data\_object | stratified\_survival |
| unit\_or\_role\_subject | subject |
| unit\_or\_role\_stats | stats |
| release\_status | public |
| source\_master | esld\_master\_long\_public.csv |
| n\_subject\_rows | 841 |
| n\_panel\_stats\_rows | 12 |
| rendering\_figure | F5\_ESLD\_public.pdf |
