# Supplementary material for: Creatinine assay interferences compromises MELD accuracy and may bias liver allocation
Source: Nat Commun. 2026 Jul 23;17:7111. doi: 10.1038/s41467-026-75011-x (PMC13396164; doi:10.1038/s41467-026-75011-x)
Supplement: Supplementary file 4 — Source Data [file 41467_2026_75011_MOESM4_ESM.zip › figshare_package_FINAL_PUBLIC_DEPOSIT_V1_20260503_002637/00_START_HERE_HTML_NAVIGATOR/file_views/view_0009_expm_F1_array_input_public.html]

02\_workflows/F1\_workflow\_v02/submission\_ready/public/data/expm\_F1\_array\_input\_public.csv

# Readable file view

02\_workflows/F1\_workflow\_v02/submission\_ready/public/data/expm\_F1\_array\_input\_public.csv

← Back to navigator   |   Open original package file

Section

Manuscript output data

Output

F1

Extension

csv

Size KB

598.548

Variables

17

## Variables in this file

| Variable | Label | Description | Unit | Type |
| --- | --- | --- | --- | --- |
| Cre\_nominal\_grav\_mg\_dL | Nominal gravimetric creatinine target concentration | Nominal gravimetric target concentration of creatinine used when defining the F1 experimental array. | mg/dL | numeric |
| TB\_nominal\_grav\_mg\_dL | Nominal gravimetric total bilirubin target concentration | Nominal gravimetric target concentration of total bilirubin used when defining the F1 experimental array. | mg/dL | integer |
| TB\_trial\_M\_mg\_dL | Measured total bilirubin concentration in the experimental dataset | Measured total bilirubin concentration in the F1 experimental dataset. In rounded input tables this is the rounded/display value used for model calculation and plotting. | mg/dL | numeric |
| anchor | Anchor/output group | Anchor or output grouping label used by the workflow to identify a specific public output component. |  | character |
| array\_id | Experimental array identifier | Identifier of the experimental array used in the F1 creatinine/bilirubin interference data. |  | character |
| assay | Creatinine assay | Creatinine assay represented by the row, for example enzymatic creatinine (CreE) or Jaffe creatinine (CreJ). |  | character |
| data\_object | Data object | Name of the data object represented by the row. |  | character |
| domain | Data domain | Workflow or data domain represented by the row. |  | character |
| expm\_F1\_array\_row\_id | Experimental array row identifier | Row identifier within the released F1 experimental array table. |  | integer |
| plot\_correction\_mg\_dL | Plotted creatinine correction | Creatinine correction value plotted in F1, expressed in mg/dL as the difference between corrected/reference and measured creatinine. | mg/dL | numeric |
| preparer\_id | Experimental preparer identifier | Identifier of the experimental preparer in F1 raw/validation data, represented as a public technical code. |  | character |
| release\_status | Release status | Release-status label indicating the publication status of the row or file object. |  | character |
| replicate | Experimental replicate number | Replicate number within the F1 experimental dataset. |  | integer |
| source\_harmonized\_file\_name | Source harmonized file name | Name of the harmonized source file used to build the released object. |  | character |
| trial\_display\_Cre\_M\_mg\_dL | Measured creatinine concentration in the experimental dataset | Measured creatinine concentration in the F1 experimental dataset. In rounded input tables this is the rounded/display value used for model calculation and plotting. | mg/dL | numeric |
| unit\_or\_role | Unit or semantic role | Unit, role, or semantic type corresponding to the row-specific variable/metric. |  | character |
| workflow\_step | Workflow step | Workflow step that produced or used the row/object. |  | character |

## Readable HTML view

Preview shows first 1000 of 4200 rows. Open the original file for full content.

| expm\_F1\_array\_row\_id | domain | anchor | data\_object | unit\_or\_role | release\_status | source\_harmonized\_file\_name | workflow\_step | array\_id | preparer\_id | replicate | assay | TB\_nominal\_grav\_mg\_dL | Cre\_nominal\_grav\_mg\_dL | TB\_trial\_M\_mg\_dL | trial\_display\_Cre\_M\_mg\_dL | plot\_correction\_mg\_dL |
| --- | --- | --- | --- | --- | --- | --- | --- | --- | --- | --- | --- | --- | --- | --- | --- | --- |
| 1 | expm | F1 | array | input | public | expm\_F1\_array\_raw\_public.csv | 2a\_refined\_analysis\_dataset | Array\_1 | Prep\_A | 2 | CreE | 27 | 3 | 27.22 | 3.54 | -0.541120525715191 |
| 2 | expm | F1 | array | input | public | expm\_F1\_array\_raw\_public.csv | 2a\_refined\_analysis\_dataset | Array\_1 | Prep\_A | 1 | CreE | 27 | 3 | 27.13 | 3.48 | -0.480865451714561 |
| 3 | expm | F1 | array | input | public | expm\_F1\_array\_raw\_public.csv | 2a\_refined\_analysis\_dataset | Array\_1 | Prep\_A | 2 | CreJ | 27 | 3 | 27.22 | 3.39 | -0.386578220459593 |
| 4 | expm | F1 | array | input | public | expm\_F1\_array\_raw\_public.csv | 2a\_refined\_analysis\_dataset | Array\_1 | Prep\_A | 1 | CreJ | 27 | 3 | 27.13 | 3.32 | -0.317061468810564 |
| 5 | expm | F1 | array | input | public | expm\_F1\_array\_raw\_public.csv | 2a\_refined\_analysis\_dataset | Array\_2 | Prep\_A | 2 | CreE | 27 | 3 | 26.94 | 3.46 | -0.458450885953881 |
| 6 | expm | F1 | array | input | public | expm\_F1\_array\_raw\_public.csv | 2a\_refined\_analysis\_dataset | Array\_2 | Prep\_A | 1 | CreE | 27 | 3 | 27.09 | 3.44 | -0.444941023997581 |
| 7 | expm | F1 | array | input | public | expm\_F1\_array\_raw\_public.csv | 2a\_refined\_analysis\_dataset | Array\_2 | Prep\_A | 2 | CreJ | 27 | 3 | 26.94 | 3.25 | -0.250850883309386 |
| 8 | expm | F1 | array | input | public | expm\_F1\_array\_raw\_public.csv | 2a\_refined\_analysis\_dataset | Array\_2 | Prep\_A | 1 | CreJ | 27 | 3 | 27.09 | 3.36 | -0.356181154200586 |
| 9 | expm | F1 | array | input | public | expm\_F1\_array\_raw\_public.csv | 2a\_refined\_analysis\_dataset | Array\_1 | Prep\_A | 2 | CreJ | 14 | 1.5 | 14.04 | 1.62 | -0.118988270391514 |
| 10 | expm | F1 | array | input | public | expm\_F1\_array\_raw\_public.csv | 2a\_refined\_analysis\_dataset | Array\_1 | Prep\_A | 1 | CreJ | 14 | 1.5 | 13.97 | 1.67 | -0.169119633993204 |
| 11 | expm | F1 | array | input | public | expm\_F1\_array\_raw\_public.csv | 2a\_refined\_analysis\_dataset | Array\_1 | Prep\_A | 2 | CreE | 14 | 1.5 | 14.04 | 1.65 | -0.151671639904077 |
| 12 | expm | F1 | array | input | public | expm\_F1\_array\_raw\_public.csv | 2a\_refined\_analysis\_dataset | Array\_1 | Prep\_A | 1 | CreE | 14 | 1.5 | 13.97 | 1.62 | -0.117354678783357 |
| 13 | expm | F1 | array | input | public | expm\_F1\_array\_raw\_public.csv | 2a\_refined\_analysis\_dataset | Array\_2 | Prep\_A | 2 | CreJ | 14 | 1.5 | 14.05 | 1.62 | -0.118172262759379 |
| 14 | expm | F1 | array | input | public | expm\_F1\_array\_raw\_public.csv | 2a\_refined\_analysis\_dataset | Array\_2 | Prep\_A | 1 | CreJ | 14 | 1.5 | 13.95 | 1.66 | -0.164333157636229 |
| 15 | expm | F1 | array | input | public | expm\_F1\_array\_raw\_public.csv | 2a\_refined\_analysis\_dataset | Array\_2 | Prep\_A | 2 | CreE | 14 | 1.5 | 14.06 | 1.63 | -0.128077453268937 |
| 16 | expm | F1 | array | input | public | expm\_F1\_array\_raw\_public.csv | 2a\_refined\_analysis\_dataset | Array\_2 | Prep\_A | 1 | CreE | 14 | 1.5 | 13.96 | 1.63 | -0.125134268170357 |
| 17 | expm | F1 | array | input | public | expm\_F1\_array\_raw\_public.csv | 2a\_refined\_analysis\_dataset | Array\_1 | Prep\_A | 2 | CreE | 28 | 3 | 28.07 | 3.51 | -0.513187779826595 |
| 18 | expm | F1 | array | input | public | expm\_F1\_array\_raw\_public.csv | 2a\_refined\_analysis\_dataset | Array\_1 | Prep\_A | 1 | CreE | 28 | 3 | 27.97 | 3.49 | -0.493858519229065 |
| 19 | expm | F1 | array | input | public | expm\_F1\_array\_raw\_public.csv | 2a\_refined\_analysis\_dataset | Array\_2 | Prep\_A | 2 | CreE | 28 | 3 | 28.26 | 3.51 | -0.512526062038075 |
| 20 | expm | F1 | array | input | public | expm\_F1\_array\_raw\_public.csv | 2a\_refined\_analysis\_dataset | Array\_2 | Prep\_A | 1 | CreE | 28 | 3 | 28.24 | 3.4 | -0.398363626116995 |
| 21 | expm | F1 | array | input | public | expm\_F1\_array\_raw\_public.csv | 2a\_refined\_analysis\_dataset | Array\_1 | Prep\_A | 1 | CreJ | 28 | 3 | 27.97 | 3.31 | -0.305414308835168 |
| 22 | expm | F1 | array | input | public | expm\_F1\_array\_raw\_public.csv | 2a\_refined\_analysis\_dataset | Array\_1 | Prep\_A | 2 | CreJ | 28 | 3 | 28.07 | 3.22 | -0.224073121688479 |
| 23 | expm | F1 | array | input | public | expm\_F1\_array\_raw\_public.csv | 2a\_refined\_analysis\_dataset | Array\_2 | Prep\_A | 2 | CreJ | 28 | 3 | 28.25 | 3.3 | -0.296779437692968 |
| 24 | expm | F1 | array | input | public | expm\_F1\_array\_raw\_public.csv | 2a\_refined\_analysis\_dataset | Array\_2 | Prep\_A | 1 | CreJ | 28 | 3 | 28.23 | 3.28 | -0.282070106411188 |
| 25 | expm | F1 | array | input | public | expm\_F1\_array\_raw\_public.csv | 2a\_refined\_analysis\_dataset | Array\_2 | Prep\_A | 2 | CreJ | 15 | 1.5 | 15.05 | 1.65 | -0.152195921013691 |
| 26 | expm | F1 | array | input | public | expm\_F1\_array\_raw\_public.csv | 2a\_refined\_analysis\_dataset | Array\_2 | Prep\_A | 1 | CreJ | 15 | 1.5 | 15 | 1.64 | -0.136446367395971 |
| 27 | expm | F1 | array | input | public | expm\_F1\_array\_raw\_public.csv | 2a\_refined\_analysis\_dataset | Array\_2 | Prep\_A | 1 | CreE | 15 | 1.5 | 15 | 1.64 | -0.136199284483749 |
| 28 | expm | F1 | array | input | public | expm\_F1\_array\_raw\_public.csv | 2a\_refined\_analysis\_dataset | Array\_2 | Prep\_A | 2 | CreE | 15 | 1.5 | 15.05 | 1.65 | -0.152348033781639 |
| 29 | expm | F1 | array | input | public | expm\_F1\_array\_raw\_public.csv | 2a\_refined\_analysis\_dataset | Array\_1 | Prep\_A | 2 | CreE | 15 | 1.5 | 14.91 | 1.63 | -0.129434518674189 |
| 30 | expm | F1 | array | input | public | expm\_F1\_array\_raw\_public.csv | 2a\_refined\_analysis\_dataset | Array\_1 | Prep\_A | 1 | CreE | 15 | 1.5 | 15.03 | 1.66 | -0.160141643592769 |
| 31 | expm | F1 | array | input | public | expm\_F1\_array\_raw\_public.csv | 2a\_refined\_analysis\_dataset | Array\_1 | Prep\_A | 2 | CreJ | 15 | 1.5 | 14.91 | 1.54 | -0.0433552206707439 |
| 32 | expm | F1 | array | input | public | expm\_F1\_array\_raw\_public.csv | 2a\_refined\_analysis\_dataset | Array\_1 | Prep\_A | 1 | CreJ | 15 | 1.5 | 15.03 | 1.66 | -0.161509152999964 |
| 33 | expm | F1 | array | input | public | expm\_F1\_array\_raw\_public.csv | 2a\_refined\_analysis\_dataset | Array\_1 | Prep\_A | 2 | CreE | 29 | 3 | 29.04 | 3.41 | -0.406651860659701 |
| 34 | expm | F1 | array | input | public | expm\_F1\_array\_raw\_public.csv | 2a\_refined\_analysis\_dataset | Array\_1 | Prep\_A | 1 | CreE | 29 | 3 | 29.23 | 3.5 | -0.498229344124181 |
| 35 | expm | F1 | array | input | public | expm\_F1\_array\_raw\_public.csv | 2a\_refined\_analysis\_dataset | Array\_2 | Prep\_A | 1 | CreE | 29 | 3 | 29.12 | 3.45 | -0.451398517067751 |
| 36 | expm | F1 | array | input | public | expm\_F1\_array\_raw\_public.csv | 2a\_refined\_analysis\_dataset | Array\_2 | Prep\_A | 2 | CreE | 29 | 3 | 29.18 | 3.5 | -0.50434843279931 |
| 37 | expm | F1 | array | input | public | expm\_F1\_array\_raw\_public.csv | 2a\_refined\_analysis\_dataset | Array\_1 | Prep\_A | 2 | CreJ | 29 | 3 | 29.04 | 3.32 | -0.319649732073259 |
| 38 | expm | F1 | array | input | public | expm\_F1\_array\_raw\_public.csv | 2a\_refined\_analysis\_dataset | Array\_1 | Prep\_A | 1 | CreJ | 29 | 3 | 29.23 | 3.28 | -0.280570676096059 |
| 39 | expm | F1 | array | input | public | expm\_F1\_array\_raw\_public.csv | 2a\_refined\_analysis\_dataset | Array\_2 | Prep\_A | 2 | CreJ | 29 | 3 | 29.18 | 3.29 | -0.291548921330203 |
| 40 | expm | F1 | array | input | public | expm\_F1\_array\_raw\_public.csv | 2a\_refined\_analysis\_dataset | Array\_2 | Prep\_A | 1 | CreJ | 29 | 3 | 29.11 | 3.31 | -0.309885119050053 |
| 41 | expm | F1 | array | input | public | expm\_F1\_array\_raw\_public.csv | 2a\_refined\_analysis\_dataset | Array\_2 | Prep\_A | 2 | CreJ | 16 | 1.5 | 16.02 | 1.68 | -0.175219974626528 |
| 42 | expm | F1 | array | input | public | expm\_F1\_array\_raw\_public.csv | 2a\_refined\_analysis\_dataset | Array\_2 | Prep\_A | 1 | CreJ | 16 | 1.5 | 15.99 | 1.77 | -0.270347604265108 |
| 43 | expm | F1 | array | input | public | expm\_F1\_array\_raw\_public.csv | 2a\_refined\_analysis\_dataset | Array\_1 | Prep\_A | 2 | CreE | 16 | 1.5 | 15.95 | 1.71 | -0.20511069747507 |
| 44 | expm | F1 | array | input | public | expm\_F1\_array\_raw\_public.csv | 2a\_refined\_analysis\_dataset | Array\_1 | Prep\_A | 1 | CreE | 16 | 1.5 | 15.99 | 1.68 | -0.18080267747486 |
| 45 | expm | F1 | array | input | public | expm\_F1\_array\_raw\_public.csv | 2a\_refined\_analysis\_dataset | Array\_2 | Prep\_A | 2 | CreE | 16 | 1.5 | 16.03 | 1.62 | -0.12191408666017 |
| 46 | expm | F1 | array | input | public | expm\_F1\_array\_raw\_public.csv | 2a\_refined\_analysis\_dataset | Array\_2 | Prep\_A | 1 | CreE | 16 | 1.5 | 15.99 | 1.67 | -0.16982539758908 |
| 47 | expm | F1 | array | input | public | expm\_F1\_array\_raw\_public.csv | 2a\_refined\_analysis\_dataset | Array\_1 | Prep\_A | 2 | CreJ | 16 | 1.5 | 15.95 | 1.67 | -0.174455629273199 |
| 48 | expm | F1 | array | input | public | expm\_F1\_array\_raw\_public.csv | 2a\_refined\_analysis\_dataset | Array\_1 | Prep\_A | 1 | CreJ | 16 | 1.5 | 15.99 | 1.66 | -0.16285442016558 |
| 49 | expm | F1 | array | input | public | expm\_F1\_array\_raw\_public.csv | 2a\_refined\_analysis\_dataset | Array\_2 | Prep\_A | 1 | CreJ | 30 | 3 | 30.35 | 3.33 | -0.332180256774908 |
| 50 | expm | F1 | array | input | public | expm\_F1\_array\_raw\_public.csv | 2a\_refined\_analysis\_dataset | Array\_2 | Prep\_A | 2 | CreJ | 30 | 3 | 30.05 | 3.32 | -0.323188515264448 |
| 51 | expm | F1 | array | input | public | expm\_F1\_array\_raw\_public.csv | 2a\_refined\_analysis\_dataset | Array\_2 | Prep\_A | 2 | CreE | 30 | 3 | 30.06 | 3.49 | -0.490709936172323 |
| 52 | expm | F1 | array | input | public | expm\_F1\_array\_raw\_public.csv | 2a\_refined\_analysis\_dataset | Array\_2 | Prep\_A | 1 | CreE | 30 | 3 | 30.35 | 3.51 | -0.509114777665503 |
| 53 | expm | F1 | array | input | public | expm\_F1\_array\_raw\_public.csv | 2a\_refined\_analysis\_dataset | Array\_1 | Prep\_A | 2 | CreJ | 30 | 3 | 30.15 | 3.39 | -0.393611835151979 |
| 54 | expm | F1 | array | input | public | expm\_F1\_array\_raw\_public.csv | 2a\_refined\_analysis\_dataset | Array\_1 | Prep\_A | 1 | CreJ | 30 | 3 | 30 | 3.37 | -0.365745871716079 |
| 55 | expm | F1 | array | input | public | expm\_F1\_array\_raw\_public.csv | 2a\_refined\_analysis\_dataset | Array\_1 | Prep\_A | 1 | CreE | 30 | 3 | 30 | 3.53 | -0.526453819597233 |
| 56 | expm | F1 | array | input | public | expm\_F1\_array\_raw\_public.csv | 2a\_refined\_analysis\_dataset | Array\_1 | Prep\_A | 2 | CreE | 30 | 3 | 30.15 | 3.55 | -0.545101505159623 |
| 57 | expm | F1 | array | input | public | expm\_F1\_array\_raw\_public.csv | 2a\_refined\_analysis\_dataset | Array\_2 | Prep\_A | 2 | CreJ | 17 | 1.5 | 17.07 | 1.65 | -0.150112132520513 |
| 58 | expm | F1 | array | input | public | expm\_F1\_array\_raw\_public.csv | 2a\_refined\_analysis\_dataset | Array\_2 | Prep\_A | 1 | CreJ | 17 | 1.5 | 17.03 | 1.63 | -0.128621702781893 |
| 59 | expm | F1 | array | input | public | expm\_F1\_array\_raw\_public.csv | 2a\_refined\_analysis\_dataset | Array\_2 | Prep\_A | 2 | CreE | 17 | 1.5 | 17.07 | 1.68 | -0.1829927026292 |
| 60 | expm | F1 | array | input | public | expm\_F1\_array\_raw\_public.csv | 2a\_refined\_analysis\_dataset | Array\_2 | Prep\_A | 1 | CreE | 17 | 1.5 | 17.03 | 1.68 | -0.18431572108427 |
| 61 | expm | F1 | array | input | public | expm\_F1\_array\_raw\_public.csv | 2a\_refined\_analysis\_dataset | Array\_1 | Prep\_A | 2 | CreE | 17 | 1.5 | 17.07 | 1.7 | -0.19577309652103 |
| 62 | expm | F1 | array | input | public | expm\_F1\_array\_raw\_public.csv | 2a\_refined\_analysis\_dataset | Array\_1 | Prep\_A | 1 | CreE | 17 | 1.5 | 16.94 | 1.67 | -0.16912147527201 |
| 63 | expm | F1 | array | input | public | expm\_F1\_array\_raw\_public.csv | 2a\_refined\_analysis\_dataset | Array\_1 | Prep\_A | 2 | CreJ | 17 | 1.5 | 17.07 | 1.68 | -0.183373241374002 |
| 64 | expm | F1 | array | input | public | expm\_F1\_array\_raw\_public.csv | 2a\_refined\_analysis\_dataset | Array\_1 | Prep\_A | 1 | CreJ | 17 | 1.5 | 16.94 | 1.65 | -0.150806167589492 |
| 65 | expm | F1 | array | input | public | expm\_F1\_array\_raw\_public.csv | 2a\_refined\_analysis\_dataset | Array\_2 | Prep\_A | 2 | CreE | 31 | 3 | 31.06 | 3.52 | -0.518394699581316 |
| 66 | expm | F1 | array | input | public | expm\_F1\_array\_raw\_public.csv | 2a\_refined\_analysis\_dataset | Array\_2 | Prep\_A | 1 | CreE | 31 | 3 | 31.05 | 3.54 | -0.542913534785716 |
| 67 | expm | F1 | array | input | public | expm\_F1\_array\_raw\_public.csv | 2a\_refined\_analysis\_dataset | Array\_2 | Prep\_A | 1 | CreJ | 31 | 3 | 31.05 | 3.3 | -0.296428525157817 |
| 68 | expm | F1 | array | input | public | expm\_F1\_array\_raw\_public.csv | 2a\_refined\_analysis\_dataset | Array\_2 | Prep\_A | 2 | CreJ | 31 | 3 | 31.06 | 3.25 | -0.245903998713227 |
| 69 | expm | F1 | array | input | public | expm\_F1\_array\_raw\_public.csv | 2a\_refined\_analysis\_dataset | Array\_1 | Prep\_A | 1 | CreJ | 31 | 3 | 31.07 | 3.37 | -0.368666462308452 |
| 70 | expm | F1 | array | input | public | expm\_F1\_array\_raw\_public.csv | 2a\_refined\_analysis\_dataset | Array\_1 | Prep\_A | 2 | CreJ | 31 | 3 | 31.09 | 3.29 | -0.291481187451042 |
| 71 | expm | F1 | array | input | public | expm\_F1\_array\_raw\_public.csv | 2a\_refined\_analysis\_dataset | Array\_1 | Prep\_A | 1 | CreE | 31 | 3 | 31.07 | 3.53 | -0.529652540465026 |
| 72 | expm | F1 | array | input | public | expm\_F1\_array\_raw\_public.csv | 2a\_refined\_analysis\_dataset | Array\_1 | Prep\_A | 2 | CreE | 31 | 3 | 31.09 | 3.54 | -0.539246314716846 |
| 73 | expm | F1 | array | input | public | expm\_F1\_array\_raw\_public.csv | 2a\_refined\_analysis\_dataset | Array\_2 | Prep\_A | 1 | CreJ | 18 | 1.5 | 18.05 | 1.63 | -0.133664925310916 |
| 74 | expm | F1 | array | input | public | expm\_F1\_array\_raw\_public.csv | 2a\_refined\_analysis\_dataset | Array\_2 | Prep\_A | 2 | CreJ | 18 | 1.5 | 18.08 | 1.63 | -0.133735215050376 |
| 75 | expm | F1 | array | input | public | expm\_F1\_array\_raw\_public.csv | 2a\_refined\_analysis\_dataset | Array\_2 | Prep\_A | 1 | CreE | 18 | 1.5 | 18.04 | 1.7 | -0.19655545479304 |
| 76 | expm | F1 | array | input | public | expm\_F1\_array\_raw\_public.csv | 2a\_refined\_analysis\_dataset | Array\_2 | Prep\_A | 2 | CreE | 18 | 1.5 | 18.08 | 1.7 | -0.20109243100682 |
| 77 | expm | F1 | array | input | public | expm\_F1\_array\_raw\_public.csv | 2a\_refined\_analysis\_dataset | Array\_1 | Prep\_A | 2 | CreJ | 18 | 1.5 | 17.94 | 1.55 | -0.0529802466927576 |
| 78 | expm | F1 | array | input | public | expm\_F1\_array\_raw\_public.csv | 2a\_refined\_analysis\_dataset | Array\_1 | Prep\_A | 1 | CreJ | 18 | 1.5 | 17.97 | 1.65 | -0.150536591739148 |
| 79 | expm | F1 | array | input | public | expm\_F1\_array\_raw\_public.csv | 2a\_refined\_analysis\_dataset | Array\_1 | Prep\_A | 2 | CreE | 18 | 1.5 | 17.93 | 1.71 | -0.20734618104594 |
| 80 | expm | F1 | array | input | public | expm\_F1\_array\_raw\_public.csv | 2a\_refined\_analysis\_dataset | Array\_1 | Prep\_A | 1 | CreE | 18 | 1.5 | 17.97 | 1.69 | -0.18997216469488 |
| 81 | expm | F1 | array | input | public | expm\_F1\_array\_raw\_public.csv | 2a\_refined\_analysis\_dataset | Array\_1 | Prep\_A | 1 | CreJ | 32 | 3 | 31.78 | 3.3 | -0.303001399670813 |
| 82 | expm | F1 | array | input | public | expm\_F1\_array\_raw\_public.csv | 2a\_refined\_analysis\_dataset | Array\_1 | Prep\_A | 2 | CreJ | 32 | 3 | 31.93 | 3.31 | -0.312610840495823 |
| 83 | expm | F1 | array | input | public | expm\_F1\_array\_raw\_public.csv | 2a\_refined\_analysis\_dataset | Array\_2 | Prep\_A | 2 | CreJ | 32 | 3 | 32.02 | 3.28 | -0.27649564521116 |
| 84 | expm | F1 | array | input | public | expm\_F1\_array\_raw\_public.csv | 2a\_refined\_analysis\_dataset | Array\_2 | Prep\_A | 1 | CreJ | 32 | 3 | 31.99 | 3.31 | -0.30755293333084 |
| 85 | expm | F1 | array | input | public | expm\_F1\_array\_raw\_public.csv | 2a\_refined\_analysis\_dataset | Array\_2 | Prep\_A | 1 | CreE | 32 | 3 | 31.99 | 3.51 | -0.514149392171567 |
| 86 | expm | F1 | array | input | public | expm\_F1\_array\_raw\_public.csv | 2a\_refined\_analysis\_dataset | Array\_2 | Prep\_A | 2 | CreE | 32 | 3 | 32.02 | 3.51 | -0.505616747193277 |
| 87 | expm | F1 | array | input | public | expm\_F1\_array\_raw\_public.csv | 2a\_refined\_analysis\_dataset | Array\_1 | Prep\_A | 1 | CreE | 32 | 3 | 31.79 | 3.57 | -0.566239931917917 |
| 88 | expm | F1 | array | input | public | expm\_F1\_array\_raw\_public.csv | 2a\_refined\_analysis\_dataset | Array\_1 | Prep\_A | 2 | CreE | 32 | 3 | 31.93 | 3.52 | -0.515646598584297 |
| 89 | expm | F1 | array | input | public | expm\_F1\_array\_raw\_public.csv | 2a\_refined\_analysis\_dataset | Array\_2 | Prep\_A | 1 | CreE | 19 | 1.5 | 19 | 1.73 | -0.226794657439644 |
| 90 | expm | F1 | array | input | public | expm\_F1\_array\_raw\_public.csv | 2a\_refined\_analysis\_dataset | Array\_2 | Prep\_A | 2 | CreE | 19 | 1.5 | 18.94 | 1.71 | -0.207825942023644 |
| 91 | expm | F1 | array | input | public | expm\_F1\_array\_raw\_public.csv | 2a\_refined\_analysis\_dataset | Array\_2 | Prep\_A | 1 | CreJ | 19 | 1.5 | 19 | 1.64 | -0.142114133362172 |
| 92 | expm | F1 | array | input | public | expm\_F1\_array\_raw\_public.csv | 2a\_refined\_analysis\_dataset | Array\_2 | Prep\_A | 2 | CreJ | 19 | 1.5 | 18.94 | 1.35 | 0.154300302677818 |
| 93 | expm | F1 | array | input | public | expm\_F1\_array\_raw\_public.csv | 2a\_refined\_analysis\_dataset | Array\_1 | Prep\_A | 2 | CreE | 19 | 1.5 | 18.96 | 1.68 | -0.179404170971984 |
| 94 | expm | F1 | array | input | public | expm\_F1\_array\_raw\_public.csv | 2a\_refined\_analysis\_dataset | Array\_1 | Prep\_A | 1 | CreE | 19 | 1.5 | 19.07 | 1.7 | -0.198693761381094 |
| 95 | expm | F1 | array | input | public | expm\_F1\_array\_raw\_public.csv | 2a\_refined\_analysis\_dataset | Array\_1 | Prep\_A | 2 | CreJ | 19 | 1.5 | 18.97 | 1.61 | -0.111544840286095 |
| 96 | expm | F1 | array | input | public | expm\_F1\_array\_raw\_public.csv | 2a\_refined\_analysis\_dataset | Array\_1 | Prep\_A | 1 | CreJ | 19 | 1.5 | 19.07 | 1.6 | -0.102205840367885 |
| 97 | expm | F1 | array | input | public | expm\_F1\_array\_raw\_public.csv | 2a\_refined\_analysis\_dataset | Array\_1 | Prep\_A | 1 | CreJ | 33 | 3 | 32.99 | 3.28 | -0.281331541258181 |
| 98 | expm | F1 | array | input | public | expm\_F1\_array\_raw\_public.csv | 2a\_refined\_analysis\_dataset | Array\_1 | Prep\_A | 2 | CreJ | 33 | 3 | 33.28 | 3.35 | -0.351927777946481 |
| 99 | expm | F1 | array | input | public | expm\_F1\_array\_raw\_public.csv | 2a\_refined\_analysis\_dataset | Array\_1 | Prep\_A | 1 | CreE | 33 | 3 | 32.99 | 3.55 | -0.545716715270315 |
| 100 | expm | F1 | array | input | public | expm\_F1\_array\_raw\_public.csv | 2a\_refined\_analysis\_dataset | Array\_1 | Prep\_A | 2 | CreE | 33 | 3 | 33.28 | 3.56 | -0.557666735318025 |
| 101 | expm | F1 | array | input | public | expm\_F1\_array\_raw\_public.csv | 2a\_refined\_analysis\_dataset | Array\_2 | Prep\_A | 1 | CreE | 33 | 3 | 32.79 | 3.57 | -0.570466794290925 |
| 102 | expm | F1 | array | input | public | expm\_F1\_array\_raw\_public.csv | 2a\_refined\_analysis\_dataset | Array\_2 | Prep\_A | 2 | CreE | 33 | 3 | 33.2 | 3.54 | -0.537214539723095 |
| 103 | expm | F1 | array | input | public | expm\_F1\_array\_raw\_public.csv | 2a\_refined\_analysis\_dataset | Array\_2 | Prep\_A | 1 | CreJ | 33 | 3 | 32.79 | 3.21 | -0.208076043735986 |
| 104 | expm | F1 | array | input | public | expm\_F1\_array\_raw\_public.csv | 2a\_refined\_analysis\_dataset | Array\_2 | Prep\_A | 2 | CreJ | 33 | 3 | 33.2 | 3.3 | -0.300543480330736 |
| 105 | expm | F1 | array | input | public | expm\_F1\_array\_raw\_public.csv | 2a\_refined\_analysis\_dataset | Array\_2 | Prep\_A | 1 | CreE | 20 | 1.5 | 20 | 1.71 | -0.213243236509719 |
| 106 | expm | F1 | array | input | public | expm\_F1\_array\_raw\_public.csv | 2a\_refined\_analysis\_dataset | Array\_2 | Prep\_A | 2 | CreE | 20 | 1.5 | 20.05 | 1.71 | -0.211162204362809 |
| 107 | expm | F1 | array | input | public | expm\_F1\_array\_raw\_public.csv | 2a\_refined\_analysis\_dataset | Array\_1 | Prep\_A | 2 | CreE | 20 | 1.5 | 19.95 | 1.67 | -0.165619418950709 |
| 108 | expm | F1 | array | input | public | expm\_F1\_array\_raw\_public.csv | 2a\_refined\_analysis\_dataset | Array\_1 | Prep\_A | 1 | CreE | 20 | 1.5 | 20.2 | 1.73 | -0.227638004243919 |
| 109 | expm | F1 | array | input | public | expm\_F1\_array\_raw\_public.csv | 2a\_refined\_analysis\_dataset | Array\_2 | Prep\_A | 1 | CreJ | 20 | 1.5 | 20 | 1.68 | -0.17560618976989 |
| 110 | expm | F1 | array | input | public | expm\_F1\_array\_raw\_public.csv | 2a\_refined\_analysis\_dataset | Array\_2 | Prep\_A | 2 | CreJ | 20 | 1.5 | 20.05 | 1.62 | -0.11821438862523 |
| 111 | expm | F1 | array | input | public | expm\_F1\_array\_raw\_public.csv | 2a\_refined\_analysis\_dataset | Array\_1 | Prep\_A | 2 | CreJ | 20 | 1.5 | 19.95 | 1.65 | -0.152986723102305 |
| 112 | expm | F1 | array | input | public | expm\_F1\_array\_raw\_public.csv | 2a\_refined\_analysis\_dataset | Array\_1 | Prep\_A | 1 | CreJ | 20 | 1.5 | 20.2 | 1.55 | -0.0499184538088346 |
| 113 | expm | F1 | array | input | public | expm\_F1\_array\_raw\_public.csv | 2a\_refined\_analysis\_dataset | Array\_1 | Prep\_A | 1 | CreE | 34 | 3 | 34.48 | 3.6 | -0.600883509234869 |
| 114 | expm | F1 | array | input | public | expm\_F1\_array\_raw\_public.csv | 2a\_refined\_analysis\_dataset | Array\_1 | Prep\_A | 2 | CreE | 34 | 3 | 34.15 | 3.52 | -0.522252837722519 |
| 115 | expm | F1 | array | input | public | expm\_F1\_array\_raw\_public.csv | 2a\_refined\_analysis\_dataset | Array\_1 | Prep\_A | 2 | CreJ | 34 | 3 | 34.15 | 3.32 | -0.318258869068195 |
| 116 | expm | F1 | array | input | public | expm\_F1\_array\_raw\_public.csv | 2a\_refined\_analysis\_dataset | Array\_1 | Prep\_A | 1 | CreJ | 34 | 3 | 34.48 | 3.27 | -0.268305708653445 |
| 117 | expm | F1 | array | input | public | expm\_F1\_array\_raw\_public.csv | 2a\_refined\_analysis\_dataset | Array\_2 | Prep\_A | 1 | CreE | 34 | 3 | 34.14 | 3.54 | -0.539125942049859 |
| 118 | expm | F1 | array | input | public | expm\_F1\_array\_raw\_public.csv | 2a\_refined\_analysis\_dataset | Array\_2 | Prep\_A | 2 | CreE | 34 | 3 | 34.03 | 3.54 | -0.543526324688749 |
| 119 | expm | F1 | array | input | public | expm\_F1\_array\_raw\_public.csv | 2a\_refined\_analysis\_dataset | Array\_2 | Prep\_A | 2 | CreJ | 34 | 3 | 34.02 | 3.33 | -0.329943878553623 |
| 120 | expm | F1 | array | input | public | expm\_F1\_array\_raw\_public.csv | 2a\_refined\_analysis\_dataset | Array\_2 | Prep\_A | 1 | CreJ | 34 | 3 | 34.14 | 3.25 | -0.246914184224513 |
| 121 | expm | F1 | array | input | public | expm\_F1\_array\_raw\_public.csv | 2a\_refined\_analysis\_dataset | Array\_2 | Prep\_A | 1 | CreE | 21 | 1.5 | 21.14 | 1.75 | -0.249671577461379 |
| 122 | expm | F1 | array | input | public | expm\_F1\_array\_raw\_public.csv | 2a\_refined\_analysis\_dataset | Array\_2 | Prep\_A | 2 | CreE | 21 | 1.5 | 21.09 | 1.72 | -0.216109113033989 |
| 123 | expm | F1 | array | input | public | expm\_F1\_array\_raw\_public.csv | 2a\_refined\_analysis\_dataset | Array\_1 | Prep\_A | 2 | CreE | 21 | 1.5 | 21.03 | 1.71 | -0.205075271061269 |
| 124 | expm | F1 | array | input | public | expm\_F1\_array\_raw\_public.csv | 2a\_refined\_analysis\_dataset | Array\_1 | Prep\_A | 1 | CreE | 21 | 1.5 | 21.06 | 1.73 | -0.232650002951439 |
| 125 | expm | F1 | array | input | public | expm\_F1\_array\_raw\_public.csv | 2a\_refined\_analysis\_dataset | Array\_2 | Prep\_A | 1 | CreJ | 21 | 1.5 | 21.14 | 1.68 | -0.179327329064815 |
| 126 | expm | F1 | array | input | public | expm\_F1\_array\_raw\_public.csv | 2a\_refined\_analysis\_dataset | Array\_2 | Prep\_A | 2 | CreJ | 21 | 1.5 | 21.09 | 1.68 | -0.184561223549515 |
| 127 | expm | F1 | array | input | public | expm\_F1\_array\_raw\_public.csv | 2a\_refined\_analysis\_dataset | Array\_1 | Prep\_A | 1 | CreJ | 21 | 1.5 | 21.06 | 1.65 | -0.151552995070468 |
| 128 | expm | F1 | array | input | public | expm\_F1\_array\_raw\_public.csv | 2a\_refined\_analysis\_dataset | Array\_1 | Prep\_A | 2 | CreJ | 21 | 1.5 | 21.03 | 1.66 | -0.155030480336878 |
| 129 | expm | F1 | array | input | public | expm\_F1\_array\_raw\_public.csv | 2a\_refined\_analysis\_dataset | Array\_2 | Prep\_A | 1 | CreJ | 35 | 3 | 35.28 | 3.3 | -0.296115838539308 |
| 130 | expm | F1 | array | input | public | expm\_F1\_array\_raw\_public.csv | 2a\_refined\_analysis\_dataset | Array\_2 | Prep\_A | 2 | CreJ | 35 | 3 | 35.47 | 3.24 | -0.238971711841908 |
| 131 | expm | F1 | array | input | public | expm\_F1\_array\_raw\_public.csv | 2a\_refined\_analysis\_dataset | Array\_1 | Prep\_A | 2 | CreE | 35 | 3 | 34.98 | 3.58 | -0.581014940279667 |
| 132 | expm | F1 | array | input | public | expm\_F1\_array\_raw\_public.csv | 2a\_refined\_analysis\_dataset | Array\_1 | Prep\_A | 1 | CreE | 35 | 3 | 35.15 | 3.55 | -0.550229158419537 |
| 133 | expm | F1 | array | input | public | expm\_F1\_array\_raw\_public.csv | 2a\_refined\_analysis\_dataset | Array\_1 | Prep\_A | 2 | CreJ | 35 | 3 | 34.97 | 3.25 | -0.25355038340403 |
| 134 | expm | F1 | array | input | public | expm\_F1\_array\_raw\_public.csv | 2a\_refined\_analysis\_dataset | Array\_1 | Prep\_A | 1 | CreJ | 35 | 3 | 35.15 | 3.31 | -0.313910269419861 |
| 135 | expm | F1 | array | input | public | expm\_F1\_array\_raw\_public.csv | 2a\_refined\_analysis\_dataset | Array\_2 | Prep\_A | 1 | CreE | 35 | 3 | 35.28 | 3.57 | -0.571733735538317 |
| 136 | expm | F1 | array | input | public | expm\_F1\_array\_raw\_public.csv | 2a\_refined\_analysis\_dataset | Array\_2 | Prep\_A | 2 | CreE | 35 | 3 | 35.47 | 3.58 | -0.577034304493947 |
| 137 | expm | F1 | array | input | public | expm\_F1\_array\_raw\_public.csv | 2a\_refined\_analysis\_dataset | Array\_2 | Prep\_A | 1 | CreJ | 22 | 1.5 | 22.06 | 1.61 | -0.1097763759216 |
| 138 | expm | F1 | array | input | public | expm\_F1\_array\_raw\_public.csv | 2a\_refined\_analysis\_dataset | Array\_2 | Prep\_A | 2 | CreJ | 22 | 1.5 | 22.05 | 1.7 | -0.20293492406703 |
| 139 | expm | F1 | array | input | public | expm\_F1\_array\_raw\_public.csv | 2a\_refined\_analysis\_dataset | Array\_1 | Prep\_A | 1 | CreJ | 22 | 1.5 | 22.04 | 1.57 | -0.0652446100904756 |
| 140 | expm | F1 | array | input | public | expm\_F1\_array\_raw\_public.csv | 2a\_refined\_analysis\_dataset | Array\_1 | Prep\_A | 2 | CreJ | 22 | 1.5 | 22.03 | 1.66 | -0.155225676148715 |
| 141 | expm | F1 | array | input | public | expm\_F1\_array\_raw\_public.csv | 2a\_refined\_analysis\_dataset | Array\_2 | Prep\_A | 2 | CreE | 22 | 1.5 | 22.05 | 1.73 | -0.232337894810763 |
| 142 | expm | F1 | array | input | public | expm\_F1\_array\_raw\_public.csv | 2a\_refined\_analysis\_dataset | Array\_2 | Prep\_A | 1 | CreE | 22 | 1.5 | 22.06 | 1.74 | -0.243500799717483 |
| 143 | expm | F1 | array | input | public | expm\_F1\_array\_raw\_public.csv | 2a\_refined\_analysis\_dataset | Array\_1 | Prep\_A | 2 | CreE | 22 | 1.5 | 22.03 | 1.77 | -0.268714884695623 |
| 144 | expm | F1 | array | input | public | expm\_F1\_array\_raw\_public.csv | 2a\_refined\_analysis\_dataset | Array\_1 | Prep\_A | 1 | CreE | 22 | 1.5 | 22.04 | 1.72 | -0.215727562144773 |
| 145 | expm | F1 | array | input | public | expm\_F1\_array\_raw\_public.csv | 2a\_refined\_analysis\_dataset | Array\_1 | Prep\_A | 1 | CreE | 23 | 1.5 | 22.99 | 1.78 | -0.280534040922967 |
| 146 | expm | F1 | array | input | public | expm\_F1\_array\_raw\_public.csv | 2a\_refined\_analysis\_dataset | Array\_1 | Prep\_A | 2 | CreE | 23 | 1.5 | 22.89 | 1.76 | -0.258208832754607 |
| 147 | expm | F1 | array | input | public | expm\_F1\_array\_raw\_public.csv | 2a\_refined\_analysis\_dataset | Array\_2 | Prep\_A | 2 | CreE | 23 | 1.5 | 22.98 | 1.77 | -0.270921863775577 |
| 148 | expm | F1 | array | input | public | expm\_F1\_array\_raw\_public.csv | 2a\_refined\_analysis\_dataset | Array\_2 | Prep\_A | 1 | CreE | 23 | 1.5 | 23.19 | 1.75 | -0.250340618998117 |
| 149 | expm | F1 | array | input | public | expm\_F1\_array\_raw\_public.csv | 2a\_refined\_analysis\_dataset | Array\_2 | Prep\_A | 2 | CreJ | 23 | 1.5 | 22.98 | 1.68 | -0.177744370749576 |
| 150 | expm | F1 | array | input | public | expm\_F1\_array\_raw\_public.csv | 2a\_refined\_analysis\_dataset | Array\_2 | Prep\_A | 1 | CreJ | 23 | 1.5 | 23.19 | 1.62 | -0.120232808672676 |
| 151 | expm | F1 | array | input | public | expm\_F1\_array\_raw\_public.csv | 2a\_refined\_analysis\_dataset | Array\_1 | Prep\_A | 1 | CreJ | 23 | 1.5 | 22.99 | 1.66 | -0.161747924655823 |
| 152 | expm | F1 | array | input | public | expm\_F1\_array\_raw\_public.csv | 2a\_refined\_analysis\_dataset | Array\_1 | Prep\_A | 2 | CreJ | 23 | 1.5 | 22.89 | 1.58 | -0.0762189702072231 |
| 153 | expm | F1 | array | input | public | expm\_F1\_array\_raw\_public.csv | 2a\_refined\_analysis\_dataset | Array\_2 | Prep\_A | 2 | CreE | 1 | 3.5 | 1.03 | 3.65 | -0.151645129144694 |
| 154 | expm | F1 | array | input | public | expm\_F1\_array\_raw\_public.csv | 2a\_refined\_analysis\_dataset | Array\_2 | Prep\_A | 1 | CreE | 1 | 3.5 | 1.03 | 3.64 | -0.144579864005745 |
| 155 | expm | F1 | array | input | public | expm\_F1\_array\_raw\_public.csv | 2a\_refined\_analysis\_dataset | Array\_1 | Prep\_A | 1 | CreE | 1 | 3.5 | 0.98 | 3.65 | -0.145716477190275 |
| 156 | expm | F1 | array | input | public | expm\_F1\_array\_raw\_public.csv | 2a\_refined\_analysis\_dataset | Array\_1 | Prep\_A | 2 | CreE | 1 | 3.5 | 1 | 3.66 | -0.159746771168134 |
| 157 | expm | F1 | array | input | public | expm\_F1\_array\_raw\_public.csv | 2a\_refined\_analysis\_dataset | Array\_1 | Prep\_A | 2 | CreJ | 1 | 3.5 | 1 | 3.73 | -0.2322496900894 |
| 158 | expm | F1 | array | input | public | expm\_F1\_array\_raw\_public.csv | 2a\_refined\_analysis\_dataset | Array\_1 | Prep\_A | 1 | CreJ | 1 | 3.5 | 0.98 | 3.76 | -0.26482522850829 |
| 159 | expm | F1 | array | input | public | expm\_F1\_array\_raw\_public.csv | 2a\_refined\_analysis\_dataset | Array\_2 | Prep\_A | 1 | CreJ | 1 | 3.5 | 1.03 | 3.82 | -0.319858426825566 |
| 160 | expm | F1 | array | input | public | expm\_F1\_array\_raw\_public.csv | 2a\_refined\_analysis\_dataset | Array\_2 | Prep\_A | 2 | CreJ | 1 | 3.5 | 1.03 | 3.78 | -0.284585255331216 |
| 161 | expm | F1 | array | input | public | expm\_F1\_array\_raw\_public.csv | 2a\_refined\_analysis\_dataset | Array\_1 | Prep\_A | 2 | CreE | 24 | 1.5 | 24.1 | 1.77 | -0.266381479713985 |
| 162 | expm | F1 | array | input | public | expm\_F1\_array\_raw\_public.csv | 2a\_refined\_analysis\_dataset | Array\_1 | Prep\_A | 1 | CreE | 24 | 1.5 | 24 | 1.8 | -0.297410711502515 |
| 163 | expm | F1 | array | input | public | expm\_F1\_array\_raw\_public.csv | 2a\_refined\_analysis\_dataset | Array\_2 | Prep\_A | 1 | CreJ | 24 | 1.5 | 24.24 | 1.68 | -0.18000657305023 |
| 164 | expm | F1 | array | input | public | expm\_F1\_array\_raw\_public.csv | 2a\_refined\_analysis\_dataset | Array\_2 | Prep\_A | 2 | CreJ | 24 | 1.5 | 24.12 | 1.71 | -0.21004179255945 |
| 165 | expm | F1 | array | input | public | expm\_F1\_array\_raw\_public.csv | 2a\_refined\_analysis\_dataset | Array\_2 | Prep\_A | 1 | CreE | 24 | 1.5 | 24.25 | 1.75 | -0.245066829881405 |
| 166 | expm | F1 | array | input | public | expm\_F1\_array\_raw\_public.csv | 2a\_refined\_analysis\_dataset | Array\_2 | Prep\_A | 2 | CreE | 24 | 1.5 | 24.12 | 1.76 | -0.261368940366515 |
| 167 | expm | F1 | array | input | public | expm\_F1\_array\_raw\_public.csv | 2a\_refined\_analysis\_dataset | Array\_1 | Prep\_A | 1 | CreJ | 24 | 1.5 | 24 | 1.61 | -0.109979781954097 |
| 168 | expm | F1 | array | input | public | expm\_F1\_array\_raw\_public.csv | 2a\_refined\_analysis\_dataset | Array\_1 | Prep\_A | 2 | CreJ | 24 | 1.5 | 24.1 | 1.68 | -0.178748280748287 |
| 169 | expm | F1 | array | input | public | expm\_F1\_array\_raw\_public.csv | 2a\_refined\_analysis\_dataset | Array\_2 | Prep\_A | 2 | CreE | 2 | 3.5 | 2.02 | 3.67 | -0.16514370049096 |
| 170 | expm | F1 | array | input | public | expm\_F1\_array\_raw\_public.csv | 2a\_refined\_analysis\_dataset | Array\_2 | Prep\_A | 1 | CreE | 2 | 3.5 | 2.02 | 3.63 | -0.13449456343362 |
| 171 | expm | F1 | array | input | public | expm\_F1\_array\_raw\_public.csv | 2a\_refined\_analysis\_dataset | Array\_1 | Prep\_A | 2 | CreJ | 2 | 3.5 | 2 | 3.76 | -0.256224344250242 |
| 172 | expm | F1 | array | input | public | expm\_F1\_array\_raw\_public.csv | 2a\_refined\_analysis\_dataset | Array\_1 | Prep\_A | 1 | CreJ | 2 | 3.5 | 2.01 | 3.79 | -0.285338599652942 |
| 173 | expm | F1 | array | input | public | expm\_F1\_array\_raw\_public.csv | 2a\_refined\_analysis\_dataset | Array\_1 | Prep\_A | 1 | CreE | 2 | 3.5 | 2.01 | 3.7 | -0.200583172072041 |
| 174 | expm | F1 | array | input | public | expm\_F1\_array\_raw\_public.csv | 2a\_refined\_analysis\_dataset | Array\_1 | Prep\_A | 2 | CreE | 2 | 3.5 | 2 | 3.72 | -0.21971061188539 |
| 175 | expm | F1 | array | input | public | expm\_F1\_array\_raw\_public.csv | 2a\_refined\_analysis\_dataset | Array\_2 | Prep\_A | 2 | CreJ | 2 | 3.5 | 2.02 | 3.83 | -0.327584893640773 |
| 176 | expm | F1 | array | input | public | expm\_F1\_array\_raw\_public.csv | 2a\_refined\_analysis\_dataset | Array\_2 | Prep\_A | 1 | CreJ | 2 | 3.5 | 2.02 | 3.81 | -0.306683920704483 |
| 177 | expm | F1 | array | input | public | expm\_F1\_array\_raw\_public.csv | 2a\_refined\_analysis\_dataset | Array\_2 | Prep\_A | 2 | CreE | 25 | 1.5 | 25.04 | 1.81 | -0.310861197326068 |
| 178 | expm | F1 | array | input | public | expm\_F1\_array\_raw\_public.csv | 2a\_refined\_analysis\_dataset | Array\_2 | Prep\_A | 1 | CreE | 25 | 1.5 | 25.31 | 1.8 | -0.301139899303348 |
| 179 | expm | F1 | array | input | public | expm\_F1\_array\_raw\_public.csv | 2a\_refined\_analysis\_dataset | Array\_1 | Prep\_A | 2 | CreE | 25 | 1.5 | 24.97 | 1.81 | -0.305931987104278 |
| 180 | expm | F1 | array | input | public | expm\_F1\_array\_raw\_public.csv | 2a\_refined\_analysis\_dataset | Array\_1 | Prep\_A | 1 | CreE | 25 | 1.5 | 25.11 | 1.78 | -0.276950307700148 |
| 181 | expm | F1 | array | input | public | expm\_F1\_array\_raw\_public.csv | 2a\_refined\_analysis\_dataset | Array\_1 | Prep\_A | 2 | CreJ | 25 | 1.5 | 24.97 | 1.63 | -0.125133571571356 |
| 182 | expm | F1 | array | input | public | expm\_F1\_array\_raw\_public.csv | 2a\_refined\_analysis\_dataset | Array\_1 | Prep\_A | 1 | CreJ | 25 | 1.5 | 25.11 | 1.69 | -0.193420012007016 |
| 183 | expm | F1 | array | input | public | expm\_F1\_array\_raw\_public.csv | 2a\_refined\_analysis\_dataset | Array\_2 | Prep\_A | 2 | CreJ | 25 | 1.5 | 25.04 | 1.63 | -0.129630254985357 |
| 184 | expm | F1 | array | input | public | expm\_F1\_array\_raw\_public.csv | 2a\_refined\_analysis\_dataset | Array\_2 | Prep\_A | 1 | CreJ | 25 | 1.5 | 25.31 | 1.7 | -0.204546188710186 |
| 185 | expm | F1 | array | input | public | expm\_F1\_array\_raw\_public.csv | 2a\_refined\_analysis\_dataset | Array\_2 | Prep\_A | 2 | CreJ | 3 | 3.5 | 2.99 | 3.81 | -0.309849610729483 |
| 186 | expm | F1 | array | input | public | expm\_F1\_array\_raw\_public.csv | 2a\_refined\_analysis\_dataset | Array\_2 | Prep\_A | 1 | CreJ | 3 | 3.5 | 2.98 | 3.83 | -0.330948902944713 |
| 187 | expm | F1 | array | input | public | expm\_F1\_array\_raw\_public.csv | 2a\_refined\_analysis\_dataset | Array\_1 | Prep\_A | 2 | CreJ | 3 | 3.5 | 2.98 | 3.81 | -0.308290230920926 |
| 188 | expm | F1 | array | input | public | expm\_F1\_array\_raw\_public.csv | 2a\_refined\_analysis\_dataset | Array\_1 | Prep\_A | 1 | CreJ | 3 | 3.5 | 3.01 | 3.81 | -0.310863513396896 |
| 189 | expm | F1 | array | input | public | expm\_F1\_array\_raw\_public.csv | 2a\_refined\_analysis\_dataset | Array\_2 | Prep\_A | 2 | CreE | 3 | 3.5 | 3 | 3.66 | -0.163775899014177 |
| 190 | expm | F1 | array | input | public | expm\_F1\_array\_raw\_public.csv | 2a\_refined\_analysis\_dataset | Array\_2 | Prep\_A | 1 | CreE | 3 | 3.5 | 2.98 | 3.7 | -0.201811470955346 |
| 191 | expm | F1 | array | input | public | expm\_F1\_array\_raw\_public.csv | 2a\_refined\_analysis\_dataset | Array\_1 | Prep\_A | 1 | CreE | 3 | 3.5 | 3.01 | 3.7 | -0.200590182796927 |
| 192 | expm | F1 | array | input | public | expm\_F1\_array\_raw\_public.csv | 2a\_refined\_analysis\_dataset | Array\_1 | Prep\_A | 2 | CreE | 3 | 3.5 | 2.98 | 3.7 | -0.197038039459176 |
| 193 | expm | F1 | array | input | public | expm\_F1\_array\_raw\_public.csv | 2a\_refined\_analysis\_dataset | Array\_1 | Prep\_A | 1 | CreJ | 26 | 1.5 | 26.01 | 1.63 | -0.129040305348483 |
| 194 | expm | F1 | array | input | public | expm\_F1\_array\_raw\_public.csv | 2a\_refined\_analysis\_dataset | Array\_1 | Prep\_A | 2 | CreJ | 26 | 1.5 | 26.05 | 1.73 | -0.231265900155883 |
| 195 | expm | F1 | array | input | public | expm\_F1\_array\_raw\_public.csv | 2a\_refined\_analysis\_dataset | Array\_2 | Prep\_A | 2 | CreE | 26 | 1.5 | 26.04 | 1.79 | -0.291370619433379 |
| 196 | expm | F1 | array | input | public | expm\_F1\_array\_raw\_public.csv | 2a\_refined\_analysis\_dataset | Array\_2 | Prep\_A | 1 | CreE | 26 | 1.5 | 26.16 | 1.8 | -0.304032070251549 |
| 197 | expm | F1 | array | input | public | expm\_F1\_array\_raw\_public.csv | 2a\_refined\_analysis\_dataset | Array\_1 | Prep\_A | 2 | CreE | 26 | 1.5 | 26.05 | 1.77 | -0.265938188704749 |
| 198 | expm | F1 | array | input | public | expm\_F1\_array\_raw\_public.csv | 2a\_refined\_analysis\_dataset | Array\_1 | Prep\_A | 1 | CreE | 26 | 1.5 | 26.01 | 1.79 | -0.286994518646509 |
| 199 | expm | F1 | array | input | public | expm\_F1\_array\_raw\_public.csv | 2a\_refined\_analysis\_dataset | Array\_2 | Prep\_A | 2 | CreJ | 26 | 1.5 | 26.04 | 1.6 | -0.104201082180848 |
| 200 | expm | F1 | array | input | public | expm\_F1\_array\_raw\_public.csv | 2a\_refined\_analysis\_dataset | Array\_2 | Prep\_A | 1 | CreJ | 26 | 1.5 | 26.16 | 1.62 | -0.119850216899568 |
| 201 | expm | F1 | array | input | public | expm\_F1\_array\_raw\_public.csv | 2a\_refined\_analysis\_dataset | Array\_2 | Prep\_A | 1 | CreE | 4 | 3.5 | 4.03 | 3.69 | -0.190840503248301 |
| 202 | expm | F1 | array | input | public | expm\_F1\_array\_raw\_public.csv | 2a\_refined\_analysis\_dataset | Array\_2 | Prep\_A | 2 | CreE | 4 | 3.5 | 4 | 3.69 | -0.188270715508371 |
| 203 | expm | F1 | array | input | public | expm\_F1\_array\_raw\_public.csv | 2a\_refined\_analysis\_dataset | Array\_1 | Prep\_A | 2 | CreJ | 4 | 3.5 | 4.03 | 3.79 | -0.293058392430988 |
| 204 | expm | F1 | array | input | public | expm\_F1\_array\_raw\_public.csv | 2a\_refined\_analysis\_dataset | Array\_1 | Prep\_A | 1 | CreJ | 4 | 3.5 | 4.03 | 3.94 | -0.435534610351948 |
| 205 | expm | F1 | array | input | public | expm\_F1\_array\_raw\_public.csv | 2a\_refined\_analysis\_dataset | Array\_2 | Prep\_A | 2 | CreJ | 4 | 3.5 | 4 | 3.78 | -0.27770403173586 |
| 206 | expm | F1 | array | input | public | expm\_F1\_array\_raw\_public.csv | 2a\_refined\_analysis\_dataset | Array\_2 | Prep\_A | 1 | CreJ | 4 | 3.5 | 4.03 | 3.83 | -0.330177452355871 |
| 207 | expm | F1 | array | input | public | expm\_F1\_array\_raw\_public.csv | 2a\_refined\_analysis\_dataset | Array\_1 | Prep\_A | 1 | CreE | 4 | 3.5 | 4.03 | 3.7 | -0.199186256307901 |
| 208 | expm | F1 | array | input | public | expm\_F1\_array\_raw\_public.csv | 2a\_refined\_analysis\_dataset | Array\_1 | Prep\_A | 2 | CreE | 4 | 3.5 | 4.03 | 3.72 | -0.218996472857041 |
| 209 | expm | F1 | array | input | public | expm\_F1\_array\_raw\_public.csv | 2a\_refined\_analysis\_dataset | Array\_2 | Prep\_A | 1 | CreE | 27 | 1.5 | 27.12 | 1.82 | -0.319236587282237 |
| 210 | expm | F1 | array | input | public | expm\_F1\_array\_raw\_public.csv | 2a\_refined\_analysis\_dataset | Array\_2 | Prep\_A | 2 | CreE | 27 | 1.5 | 26.93 | 1.82 | -0.322159103947067 |
| 211 | expm | F1 | array | input | public | expm\_F1\_array\_raw\_public.csv | 2a\_refined\_analysis\_dataset | Array\_1 | Prep\_A | 2 | CreE | 27 | 1.5 | 27.02 | 1.82 | -0.317552485387837 |
| 212 | expm | F1 | array | input | public | expm\_F1\_array\_raw\_public.csv | 2a\_refined\_analysis\_dataset | Array\_1 | Prep\_A | 1 | CreE | 27 | 1.5 | 26.67 | 1.81 | -0.314731261827257 |
| 213 | expm | F1 | array | input | public | expm\_F1\_array\_raw\_public.csv | 2a\_refined\_analysis\_dataset | Array\_2 | Prep\_A | 1 | CreJ | 27 | 1.5 | 27.12 | 1.67 | -0.168567533790037 |
| 214 | expm | F1 | array | input | public | expm\_F1\_array\_raw\_public.csv | 2a\_refined\_analysis\_dataset | Array\_2 | Prep\_A | 2 | CreJ | 27 | 1.5 | 26.93 | 1.66 | -0.161923096112117 |
| 215 | expm | F1 | array | input | public | expm\_F1\_array\_raw\_public.csv | 2a\_refined\_analysis\_dataset | Array\_1 | Prep\_A | 1 | CreJ | 27 | 1.5 | 26.68 | 1.61 | -0.114977648010731 |
| 216 | expm | F1 | array | input | public | expm\_F1\_array\_raw\_public.csv | 2a\_refined\_analysis\_dataset | Array\_1 | Prep\_A | 2 | CreJ | 27 | 1.5 | 27.02 | 1.61 | -0.109518072591331 |
| 217 | expm | F1 | array | input | public | expm\_F1\_array\_raw\_public.csv | 2a\_refined\_analysis\_dataset | Array\_1 | Prep\_A | 2 | CreJ | 5 | 3.5 | 5.01 | 3.87 | -0.365181628219607 |
| 218 | expm | F1 | array | input | public | expm\_F1\_array\_raw\_public.csv | 2a\_refined\_analysis\_dataset | Array\_1 | Prep\_A | 1 | CreJ | 5 | 3.5 | 5.03 | 3.83 | -0.326148175882377 |
| 219 | expm | F1 | array | input | public | expm\_F1\_array\_raw\_public.csv | 2a\_refined\_analysis\_dataset | Array\_1 | Prep\_A | 1 | CreE | 5 | 3.5 | 5.02 | 3.72 | -0.221380076695626 |
| 220 | expm | F1 | array | input | public | expm\_F1\_array\_raw\_public.csv | 2a\_refined\_analysis\_dataset | Array\_1 | Prep\_A | 2 | CreE | 5 | 3.5 | 5.01 | 3.7 | -0.197191008593066 |
| 221 | expm | F1 | array | input | public | expm\_F1\_array\_raw\_public.csv | 2a\_refined\_analysis\_dataset | Array\_2 | Prep\_A | 1 | CreJ | 5 | 3.5 | 5.03 | 3.8 | -0.302960424738183 |
| 222 | expm | F1 | array | input | public | expm\_F1\_array\_raw\_public.csv | 2a\_refined\_analysis\_dataset | Array\_2 | Prep\_A | 2 | CreJ | 5 | 3.5 | 5.02 | 3.83 | -0.333903292811503 |
| 223 | expm | F1 | array | input | public | expm\_F1\_array\_raw\_public.csv | 2a\_refined\_analysis\_dataset | Array\_2 | Prep\_A | 1 | CreE | 5 | 3.5 | 5.03 | 3.74 | -0.237636266923146 |
| 224 | expm | F1 | array | input | public | expm\_F1\_array\_raw\_public.csv | 2a\_refined\_analysis\_dataset | Array\_2 | Prep\_A | 2 | CreE | 5 | 3.5 | 5.02 | 3.71 | -0.209770197661356 |
| 225 | expm | F1 | array | input | public | expm\_F1\_array\_raw\_public.csv | 2a\_refined\_analysis\_dataset | Array\_2 | Prep\_A | 2 | CreE | 28 | 1.5 | 27.99 | 1.8 | -0.297372572078582 |
| 226 | expm | F1 | array | input | public | expm\_F1\_array\_raw\_public.csv | 2a\_refined\_analysis\_dataset | Array\_2 | Prep\_A | 1 | CreE | 28 | 1.5 | 28.33 | 1.8 | -0.304817443451512 |
| 227 | expm | F1 | array | input | public | expm\_F1\_array\_raw\_public.csv | 2a\_refined\_analysis\_dataset | Array\_2 | Prep\_A | 2 | CreJ | 28 | 1.5 | 27.99 | 1.65 | -0.151064574386898 |
| 228 | expm | F1 | array | input | public | expm\_F1\_array\_raw\_public.csv | 2a\_refined\_analysis\_dataset | Array\_2 | Prep\_A | 1 | CreJ | 28 | 1.5 | 28.33 | 1.67 | -0.168141683988908 |
| 229 | expm | F1 | array | input | public | expm\_F1\_array\_raw\_public.csv | 2a\_refined\_analysis\_dataset | Array\_1 | Prep\_A | 1 | CreE | 28 | 1.5 | 27.96 | 1.79 | -0.285758888434262 |
| 230 | expm | F1 | array | input | public | expm\_F1\_array\_raw\_public.csv | 2a\_refined\_analysis\_dataset | Array\_1 | Prep\_A | 2 | CreE | 28 | 1.5 | 27.96 | 1.78 | -0.279002618424712 |
| 231 | expm | F1 | array | input | public | expm\_F1\_array\_raw\_public.csv | 2a\_refined\_analysis\_dataset | Array\_1 | Prep\_A | 1 | CreJ | 28 | 1.5 | 27.96 | 1.63 | -0.132949944756938 |
| 232 | expm | F1 | array | input | public | expm\_F1\_array\_raw\_public.csv | 2a\_refined\_analysis\_dataset | Array\_1 | Prep\_A | 2 | CreJ | 28 | 1.5 | 27.96 | 1.71 | -0.207292243885058 |
| 233 | expm | F1 | array | input | public | expm\_F1\_array\_raw\_public.csv | 2a\_refined\_analysis\_dataset | Array\_2 | Prep\_A | 2 | CreE | 6 | 3.5 | 6.02 | 3.73 | -0.23127574645387 |
| 234 | expm | F1 | array | input | public | expm\_F1\_array\_raw\_public.csv | 2a\_refined\_analysis\_dataset | Array\_2 | Prep\_A | 1 | CreE | 6 | 3.5 | 6.04 | 3.75 | -0.253567516459 |
| 235 | expm | F1 | array | input | public | expm\_F1\_array\_raw\_public.csv | 2a\_refined\_analysis\_dataset | Array\_1 | Prep\_A | 1 | CreJ | 6 | 3.5 | 5.99 | 3.8 | -0.304117365638239 |
| 236 | expm | F1 | array | input | public | expm\_F1\_array\_raw\_public.csv | 2a\_refined\_analysis\_dataset | Array\_1 | Prep\_A | 2 | CreJ | 6 | 3.5 | 5.99 | 3.89 | -0.38820693990436 |
| 237 | expm | F1 | array | input | public | expm\_F1\_array\_raw\_public.csv | 2a\_refined\_analysis\_dataset | Array\_2 | Prep\_A | 1 | CreJ | 6 | 3.5 | 6.04 | 3.78 | -0.279160109862246 |
| 238 | expm | F1 | array | input | public | expm\_F1\_array\_raw\_public.csv | 2a\_refined\_analysis\_dataset | Array\_2 | Prep\_A | 2 | CreJ | 6 | 3.5 | 6.02 | 3.82 | -0.321281399629356 |
| 239 | expm | F1 | array | input | public | expm\_F1\_array\_raw\_public.csv | 2a\_refined\_analysis\_dataset | Array\_1 | Prep\_A | 2 | CreE | 6 | 3.5 | 5.99 | 3.73 | -0.22607802825351 |
| 240 | expm | F1 | array | input | public | expm\_F1\_array\_raw\_public.csv | 2a\_refined\_analysis\_dataset | Array\_1 | Prep\_A | 1 | CreE | 6 | 3.5 | 5.99 | 3.73 | -0.232512441881291 |
| 241 | expm | F1 | array | input | public | expm\_F1\_array\_raw\_public.csv | 2a\_refined\_analysis\_dataset | Array\_2 | Prep\_A | 2 | CreE | 29 | 1.5 | 28.98 | 1.82 | -0.317805872537449 |
| 242 | expm | F1 | array | input | public | expm\_F1\_array\_raw\_public.csv | 2a\_refined\_analysis\_dataset | Array\_2 | Prep\_A | 1 | CreE | 29 | 1.5 | 28.96 | 1.87 | -0.367508896936359 |
| 243 | expm | F1 | array | input | public | expm\_F1\_array\_raw\_public.csv | 2a\_refined\_analysis\_dataset | Array\_1 | Prep\_A | 2 | CreJ | 29 | 1.5 | 29.07 | 1.62 | -0.119310131137293 |
| 244 | expm | F1 | array | input | public | expm\_F1\_array\_raw\_public.csv | 2a\_refined\_analysis\_dataset | Array\_1 | Prep\_A | 1 | CreJ | 29 | 1.5 | 28.94 | 1.69 | -0.188033198474533 |
| 245 | expm | F1 | array | input | public | expm\_F1\_array\_raw\_public.csv | 2a\_refined\_analysis\_dataset | Array\_2 | Prep\_A | 1 | CreJ | 29 | 1.5 | 28.96 | 1.65 | -0.149031322982387 |
| 246 | expm | F1 | array | input | public | expm\_F1\_array\_raw\_public.csv | 2a\_refined\_analysis\_dataset | Array\_2 | Prep\_A | 2 | CreJ | 29 | 1.5 | 28.98 | 1.71 | -0.208221337956887 |
| 247 | expm | F1 | array | input | public | expm\_F1\_array\_raw\_public.csv | 2a\_refined\_analysis\_dataset | Array\_1 | Prep\_A | 2 | CreE | 29 | 1.5 | 29.07 | 1.84 | -0.336631003694729 |
| 248 | expm | F1 | array | input | public | expm\_F1\_array\_raw\_public.csv | 2a\_refined\_analysis\_dataset | Array\_1 | Prep\_A | 1 | CreE | 29 | 1.5 | 28.94 | 1.82 | -0.315099143289609 |
| 249 | expm | F1 | array | input | public | expm\_F1\_array\_raw\_public.csv | 2a\_refined\_analysis\_dataset | Array\_1 | Prep\_A | 2 | CreJ | 7 | 3.5 | 7.03 | 3.85 | -0.351141680282973 |
| 250 | expm | F1 | array | input | public | expm\_F1\_array\_raw\_public.csv | 2a\_refined\_analysis\_dataset | Array\_1 | Prep\_A | 1 | CreJ | 7 | 3.5 | 6.98 | 3.81 | -0.310807836285133 |
| 251 | expm | F1 | array | input | public | expm\_F1\_array\_raw\_public.csv | 2a\_refined\_analysis\_dataset | Array\_1 | Prep\_A | 1 | CreE | 7 | 3.5 | 6.98 | 3.69 | -0.192080117160441 |
| 252 | expm | F1 | array | input | public | expm\_F1\_array\_raw\_public.csv | 2a\_refined\_analysis\_dataset | Array\_1 | Prep\_A | 2 | CreE | 7 | 3.5 | 7.03 | 3.75 | -0.246348322897751 |
| 253 | expm | F1 | array | input | public | expm\_F1\_array\_raw\_public.csv | 2a\_refined\_analysis\_dataset | Array\_2 | Prep\_A | 2 | CreJ | 7 | 3.5 | 7 | 3.87 | -0.367954729138508 |
| 254 | expm | F1 | array | input | public | expm\_F1\_array\_raw\_public.csv | 2a\_refined\_analysis\_dataset | Array\_2 | Prep\_A | 1 | CreJ | 7 | 3.5 | 6.96 | 3.85 | -0.354705234537378 |
| 255 | expm | F1 | array | input | public | expm\_F1\_array\_raw\_public.csv | 2a\_refined\_analysis\_dataset | Array\_2 | Prep\_A | 2 | CreE | 7 | 3.5 | 7 | 3.78 | -0.28478567392954 |
| 256 | expm | F1 | array | input | public | expm\_F1\_array\_raw\_public.csv | 2a\_refined\_analysis\_dataset | Array\_2 | Prep\_A | 1 | CreE | 7 | 3.5 | 6.96 | 3.75 | -0.250518423061191 |
| 257 | expm | F1 | array | input | public | expm\_F1\_array\_raw\_public.csv | 2a\_refined\_analysis\_dataset | Array\_2 | Prep\_A | 1 | CreE | 30 | 1.5 | 30.15 | 1.83 | -0.327862277128598 |
| 258 | expm | F1 | array | input | public | expm\_F1\_array\_raw\_public.csv | 2a\_refined\_analysis\_dataset | Array\_2 | Prep\_A | 2 | CreE | 30 | 1.5 | 30.41 | 1.84 | -0.340558717457158 |
| 259 | expm | F1 | array | input | public | expm\_F1\_array\_raw\_public.csv | 2a\_refined\_analysis\_dataset | Array\_1 | Prep\_A | 2 | CreE | 30 | 1.5 | 29.93 | 1.84 | -0.341751424547188 |
| 260 | expm | F1 | array | input | public | expm\_F1\_array\_raw\_public.csv | 2a\_refined\_analysis\_dataset | Array\_1 | Prep\_A | 1 | CreE | 30 | 1.5 | 30.15 | 1.82 | -0.320865058271678 |
| 261 | expm | F1 | array | input | public | expm\_F1\_array\_raw\_public.csv | 2a\_refined\_analysis\_dataset | Array\_1 | Prep\_A | 1 | CreJ | 30 | 1.5 | 30.14 | 1.71 | -0.213346462974143 |
| 262 | expm | F1 | array | input | public | expm\_F1\_array\_raw\_public.csv | 2a\_refined\_analysis\_dataset | Array\_1 | Prep\_A | 2 | CreJ | 30 | 1.5 | 29.93 | 1.64 | -0.143537887454183 |
| 263 | expm | F1 | array | input | public | expm\_F1\_array\_raw\_public.csv | 2a\_refined\_analysis\_dataset | Array\_2 | Prep\_A | 2 | CreJ | 30 | 1.5 | 30.41 | 1.63 | -0.130357720793437 |
| 264 | expm | F1 | array | input | public | expm\_F1\_array\_raw\_public.csv | 2a\_refined\_analysis\_dataset | Array\_2 | Prep\_A | 1 | CreJ | 30 | 1.5 | 30.15 | 1.74 | -0.239050627253498 |
| 265 | expm | F1 | array | input | public | expm\_F1\_array\_raw\_public.csv | 2a\_refined\_analysis\_dataset | Array\_2 | Prep\_A | 1 | CreJ | 8 | 3.5 | 8.01 | 3.87 | -0.37104618231131 |
| 266 | expm | F1 | array | input | public | expm\_F1\_array\_raw\_public.csv | 2a\_refined\_analysis\_dataset | Array\_2 | Prep\_A | 2 | CreJ | 8 | 3.5 | 8.02 | 3.85 | -0.34608836541923 |
| 267 | expm | F1 | array | input | public | expm\_F1\_array\_raw\_public.csv | 2a\_refined\_analysis\_dataset | Array\_1 | Prep\_A | 1 | CreE | 8 | 3.5 | 7.94 | 3.8 | -0.297637207551816 |
| 268 | expm | F1 | array | input | public | expm\_F1\_array\_raw\_public.csv | 2a\_refined\_analysis\_dataset | Array\_1 | Prep\_A | 2 | CreE | 8 | 3.5 | 8 | 3.77 | -0.269091247470235 |
| 269 | expm | F1 | array | input | public | expm\_F1\_array\_raw\_public.csv | 2a\_refined\_analysis\_dataset | Array\_2 | Prep\_A | 1 | CreE | 8 | 3.5 | 8.01 | 3.77 | -0.267344115135455 |
| 270 | expm | F1 | array | input | public | expm\_F1\_array\_raw\_public.csv | 2a\_refined\_analysis\_dataset | Array\_2 | Prep\_A | 2 | CreE | 8 | 3.5 | 8.02 | 3.75 | -0.253100606307465 |
| 271 | expm | F1 | array | input | public | expm\_F1\_array\_raw\_public.csv | 2a\_refined\_analysis\_dataset | Array\_1 | Prep\_A | 1 | CreJ | 8 | 3.5 | 7.94 | 3.83 | -0.331083743437815 |
| 272 | expm | F1 | array | input | public | expm\_F1\_array\_raw\_public.csv | 2a\_refined\_analysis\_dataset | Array\_1 | Prep\_A | 2 | CreJ | 8 | 3.5 | 8 | 3.86 | -0.360846131305326 |
| 273 | expm | F1 | array | input | public | expm\_F1\_array\_raw\_public.csv | 2a\_refined\_analysis\_dataset | Array\_1 | Prep\_A | 1 | CreJ | 31 | 1.5 | 31.18 | 1.59 | -0.0897338759593542 |
| 274 | expm | F1 | array | input | public | expm\_F1\_array\_raw\_public.csv | 2a\_refined\_analysis\_dataset | Array\_1 | Prep\_A | 2 | CreJ | 31 | 1.5 | 30.79 | 1.61 | -0.110882794226924 |
| 275 | expm | F1 | array | input | public | expm\_F1\_array\_raw\_public.csv | 2a\_refined\_analysis\_dataset | Array\_2 | Prep\_A | 2 | CreJ | 31 | 1.5 | 31.17 | 1.58 | -0.075422220741397 |
| 276 | expm | F1 | array | input | public | expm\_F1\_array\_raw\_public.csv | 2a\_refined\_analysis\_dataset | Array\_2 | Prep\_A | 1 | CreJ | 31 | 1.5 | 31.28 | 1.65 | -0.145585843697237 |
| 277 | expm | F1 | array | input | public | expm\_F1\_array\_raw\_public.csv | 2a\_refined\_analysis\_dataset | Array\_2 | Prep\_A | 2 | CreE | 31 | 1.5 | 31.18 | 1.85 | -0.353290328814962 |
| 278 | expm | F1 | array | input | public | expm\_F1\_array\_raw\_public.csv | 2a\_refined\_analysis\_dataset | Array\_2 | Prep\_A | 1 | CreE | 31 | 1.5 | 31.28 | 1.87 | -0.369889316682722 |
| 279 | expm | F1 | array | input | public | expm\_F1\_array\_raw\_public.csv | 2a\_refined\_analysis\_dataset | Array\_1 | Prep\_A | 2 | CreE | 31 | 1.5 | 30.79 | 1.89 | -0.390548218260892 |
| 280 | expm | F1 | array | input | public | expm\_F1\_array\_raw\_public.csv | 2a\_refined\_analysis\_dataset | Array\_1 | Prep\_A | 1 | CreE | 31 | 1.5 | 31.18 | 1.84 | -0.338663804146302 |
| 281 | expm | F1 | array | input | public | expm\_F1\_array\_raw\_public.csv | 2a\_refined\_analysis\_dataset | Array\_1 | Prep\_A | 2 | CreJ | 9 | 3.5 | 9 | 3.84 | -0.338464688644983 |
| 282 | expm | F1 | array | input | public | expm\_F1\_array\_raw\_public.csv | 2a\_refined\_analysis\_dataset | Array\_1 | Prep\_A | 1 | CreJ | 9 | 3.5 | 9.05 | 3.81 | -0.313820665021623 |
| 283 | expm | F1 | array | input | public | expm\_F1\_array\_raw\_public.csv | 2a\_refined\_analysis\_dataset | Array\_2 | Prep\_A | 2 | CreE | 9 | 3.5 | 9.01 | 3.78 | -0.278596258006584 |
| 284 | expm | F1 | array | input | public | expm\_F1\_array\_raw\_public.csv | 2a\_refined\_analysis\_dataset | Array\_2 | Prep\_A | 1 | CreE | 9 | 3.5 | 9.02 | 3.76 | -0.255242825668704 |
| 285 | expm | F1 | array | input | public | expm\_F1\_array\_raw\_public.csv | 2a\_refined\_analysis\_dataset | Array\_2 | Prep\_A | 1 | CreJ | 9 | 3.5 | 9.02 | 3.82 | -0.324490416537737 |
| 286 | expm | F1 | array | input | public | expm\_F1\_array\_raw\_public.csv | 2a\_refined\_analysis\_dataset | Array\_2 | Prep\_A | 2 | CreJ | 9 | 3.5 | 9.01 | 3.86 | -0.355695863346607 |
| 287 | expm | F1 | array | input | public | expm\_F1\_array\_raw\_public.csv | 2a\_refined\_analysis\_dataset | Array\_1 | Prep\_A | 1 | CreE | 9 | 3.5 | 9.05 | 3.81 | -0.305173162613394 |
| 288 | expm | F1 | array | input | public | expm\_F1\_array\_raw\_public.csv | 2a\_refined\_analysis\_dataset | Array\_1 | Prep\_A | 2 | CreE | 9 | 3.5 | 9 | 3.77 | -0.272082750980474 |
| 289 | expm | F1 | array | input | public | expm\_F1\_array\_raw\_public.csv | 2a\_refined\_analysis\_dataset | Array\_1 | Prep\_A | 2 | CreJ | 32 | 1.5 | 32.15 | 1.69 | -0.18605602018935 |
| 290 | expm | F1 | array | input | public | expm\_F1\_array\_raw\_public.csv | 2a\_refined\_analysis\_dataset | Array\_1 | Prep\_A | 1 | CreJ | 32 | 1.5 | 31.74 | 1.6 | -0.0984251942173 |
| 291 | expm | F1 | array | input | public | expm\_F1\_array\_raw\_public.csv | 2a\_refined\_analysis\_dataset | Array\_2 | Prep\_A | 1 | CreE | 32 | 1.5 | 32.1 | 1.89 | -0.387467322396803 |
| 292 | expm | F1 | array | input | public | expm\_F1\_array\_raw\_public.csv | 2a\_refined\_analysis\_dataset | Array\_2 | Prep\_A | 2 | CreE | 32 | 1.5 | 32.26 | 1.86 | -0.364327765952203 |
| 293 | expm | F1 | array | input | public | expm\_F1\_array\_raw\_public.csv | 2a\_refined\_analysis\_dataset | Array\_2 | Prep\_A | 1 | CreJ | 32 | 1.5 | 32.1 | 1.57 | -0.0701137165466057 |
| 294 | expm | F1 | array | input | public | expm\_F1\_array\_raw\_public.csv | 2a\_refined\_analysis\_dataset | Array\_2 | Prep\_A | 2 | CreJ | 32 | 1.5 | 32.26 | 1.62 | -0.115151852369176 |
| 295 | expm | F1 | array | input | public | expm\_F1\_array\_raw\_public.csv | 2a\_refined\_analysis\_dataset | Array\_1 | Prep\_A | 1 | CreE | 32 | 1.5 | 31.74 | 1.9 | -0.401762329241533 |
| 296 | expm | F1 | array | input | public | expm\_F1\_array\_raw\_public.csv | 2a\_refined\_analysis\_dataset | Array\_1 | Prep\_A | 2 | CreE | 32 | 1.5 | 32.15 | 1.85 | -0.350336168565983 |
| 297 | expm | F1 | array | input | public | expm\_F1\_array\_raw\_public.csv | 2a\_refined\_analysis\_dataset | Array\_2 | Prep\_A | 2 | CreJ | 10 | 3.5 | 10.02 | 3.87 | -0.374782986009131 |
| 298 | expm | F1 | array | input | public | expm\_F1\_array\_raw\_public.csv | 2a\_refined\_analysis\_dataset | Array\_2 | Prep\_A | 1 | CreJ | 10 | 3.5 | 10.1 | 3.87 | -0.365719176494671 |
| 299 | expm | F1 | array | input | public | expm\_F1\_array\_raw\_public.csv | 2a\_refined\_analysis\_dataset | Array\_1 | Prep\_A | 2 | CreJ | 10 | 3.5 | 10.02 | 3.79 | -0.28725834735052 |
| 300 | expm | F1 | array | input | public | expm\_F1\_array\_raw\_public.csv | 2a\_refined\_analysis\_dataset | Array\_1 | Prep\_A | 1 | CreJ | 10 | 3.5 | 10.03 | 3.84 | -0.33614818749663 |
| 301 | expm | F1 | array | input | public | expm\_F1\_array\_raw\_public.csv | 2a\_refined\_analysis\_dataset | Array\_2 | Prep\_A | 1 | CreE | 10 | 3.5 | 10.1 | 3.78 | -0.280025837195856 |
| 302 | expm | F1 | array | input | public | expm\_F1\_array\_raw\_public.csv | 2a\_refined\_analysis\_dataset | Array\_2 | Prep\_A | 2 | CreE | 10 | 3.5 | 10.01 | 3.78 | -0.281313830611556 |
| 303 | expm | F1 | array | input | public | expm\_F1\_array\_raw\_public.csv | 2a\_refined\_analysis\_dataset | Array\_1 | Prep\_A | 1 | CreE | 10 | 3.5 | 10.02 | 3.78 | -0.276507114983616 |
| 304 | expm | F1 | array | input | public | expm\_F1\_array\_raw\_public.csv | 2a\_refined\_analysis\_dataset | Array\_1 | Prep\_A | 2 | CreE | 10 | 3.5 | 10.01 | 3.79 | -0.290576674950756 |
| 305 | expm | F1 | array | input | public | expm\_F1\_array\_raw\_public.csv | 2a\_refined\_analysis\_dataset | Array\_2 | Prep\_A | 1 | CreE | 33 | 1.5 | 33.12 | 1.85 | -0.35053799464763 |
| 306 | expm | F1 | array | input | public | expm\_F1\_array\_raw\_public.csv | 2a\_refined\_analysis\_dataset | Array\_2 | Prep\_A | 2 | CreE | 33 | 1.5 | 33 | 1.86 | -0.36200831417099 |
| 307 | expm | F1 | array | input | public | expm\_F1\_array\_raw\_public.csv | 2a\_refined\_analysis\_dataset | Array\_2 | Prep\_A | 2 | CreJ | 33 | 1.5 | 33 | 1.6 | -0.100324934065994 |
| 308 | expm | F1 | array | input | public | expm\_F1\_array\_raw\_public.csv | 2a\_refined\_analysis\_dataset | Array\_2 | Prep\_A | 1 | CreJ | 33 | 1.5 | 33.12 | 1.62 | -0.121481132511474 |
| 309 | expm | F1 | array | input | public | expm\_F1\_array\_raw\_public.csv | 2a\_refined\_analysis\_dataset | Array\_1 | Prep\_A | 2 | CreJ | 33 | 1.5 | 33.15 | 1.68 | -0.17525956769346 |
| 310 | expm | F1 | array | input | public | expm\_F1\_array\_raw\_public.csv | 2a\_refined\_analysis\_dataset | Array\_1 | Prep\_A | 1 | CreJ | 33 | 1.5 | 32.94 | 1.6 | -0.0964998180338301 |
| 311 | expm | F1 | array | input | public | expm\_F1\_array\_raw\_public.csv | 2a\_refined\_analysis\_dataset | Array\_1 | Prep\_A | 2 | CreE | 33 | 1.5 | 33.15 | 1.88 | -0.38205625919029 |
| 312 | expm | F1 | array | input | public | expm\_F1\_array\_raw\_public.csv | 2a\_refined\_analysis\_dataset | Array\_1 | Prep\_A | 1 | CreE | 33 | 1.5 | 32.94 | 1.89 | -0.38518198127293 |
| 313 | expm | F1 | array | input | public | expm\_F1\_array\_raw\_public.csv | 2a\_refined\_analysis\_dataset | Array\_2 | Prep\_A | 2 | CreJ | 11 | 3.5 | 11.11 | 3.88 | -0.384961168680094 |
| 314 | expm | F1 | array | input | public | expm\_F1\_array\_raw\_public.csv | 2a\_refined\_analysis\_dataset | Array\_2 | Prep\_A | 1 | CreJ | 11 | 3.5 | 11.08 | 3.83 | -0.331297082651004 |
| 315 | expm | F1 | array | input | public | expm\_F1\_array\_raw\_public.csv | 2a\_refined\_analysis\_dataset | Array\_1 | Prep\_A | 1 | CreE | 11 | 3.5 | 10.93 | 3.8 | -0.304432590586695 |
| 316 | expm | F1 | array | input | public | expm\_F1\_array\_raw\_public.csv | 2a\_refined\_analysis\_dataset | Array\_1 | Prep\_A | 2 | CreE | 11 | 3.5 | 11.07 | 3.79 | -0.291151231371555 |
| 317 | expm | F1 | array | input | public | expm\_F1\_array\_raw\_public.csv | 2a\_refined\_analysis\_dataset | Array\_2 | Prep\_A | 2 | CreE | 11 | 3.5 | 11.11 | 3.79 | -0.288928817322155 |
| 318 | expm | F1 | array | input | public | expm\_F1\_array\_raw\_public.csv | 2a\_refined\_analysis\_dataset | Array\_2 | Prep\_A | 1 | CreE | 11 | 3.5 | 11.07 | 3.76 | -0.261523045383025 |
| 319 | expm | F1 | array | input | public | expm\_F1\_array\_raw\_public.csv | 2a\_refined\_analysis\_dataset | Array\_1 | Prep\_A | 1 | CreJ | 11 | 3.5 | 10.93 | 3.88 | -0.381087622741449 |
| 320 | expm | F1 | array | input | public | expm\_F1\_array\_raw\_public.csv | 2a\_refined\_analysis\_dataset | Array\_1 | Prep\_A | 2 | CreJ | 11 | 3.5 | 11.07 | 3.89 | -0.392846781172088 |
| 321 | expm | F1 | array | input | public | expm\_F1\_array\_raw\_public.csv | 2a\_refined\_analysis\_dataset | Array\_2 | Prep\_A | 1 | CreE | 34 | 1.5 | 34.39 | 1.86 | -0.362194822089769 |
| 322 | expm | F1 | array | input | public | expm\_F1\_array\_raw\_public.csv | 2a\_refined\_analysis\_dataset | Array\_2 | Prep\_A | 2 | CreE | 34 | 1.5 | 34.17 | 1.93 | -0.430928948043019 |
| 323 | expm | F1 | array | input | public | expm\_F1\_array\_raw\_public.csv | 2a\_refined\_analysis\_dataset | Array\_1 | Prep\_A | 1 | CreJ | 34 | 1.5 | 33.96 | 1.68 | -0.183514164774063 |
| 324 | expm | F1 | array | input | public | expm\_F1\_array\_raw\_public.csv | 2a\_refined\_analysis\_dataset | Array\_1 | Prep\_A | 2 | CreJ | 34 | 1.5 | 34.11 | 1.68 | -0.175549627961833 |
| 325 | expm | F1 | array | input | public | expm\_F1\_array\_raw\_public.csv | 2a\_refined\_analysis\_dataset | Array\_1 | Prep\_A | 2 | CreE | 34 | 1.5 | 34.11 | 1.87 | -0.365042197152659 |
| 326 | expm | F1 | array | input | public | expm\_F1\_array\_raw\_public.csv | 2a\_refined\_analysis\_dataset | Array\_1 | Prep\_A | 1 | CreE | 34 | 1.5 | 33.96 | 1.87 | -0.372039523459699 |
| 327 | expm | F1 | array | input | public | expm\_F1\_array\_raw\_public.csv | 2a\_refined\_analysis\_dataset | Array\_2 | Prep\_A | 2 | CreJ | 34 | 1.5 | 34.17 | 1.6 | -0.103026551273574 |
| 328 | expm | F1 | array | input | public | expm\_F1\_array\_raw\_public.csv | 2a\_refined\_analysis\_dataset | Array\_2 | Prep\_A | 1 | CreJ | 34 | 1.5 | 34.39 | 1.59 | -0.0893662868122343 |
| 329 | expm | F1 | array | input | public | expm\_F1\_array\_raw\_public.csv | 2a\_refined\_analysis\_dataset | Array\_1 | Prep\_A | 1 | CreE | 12 | 3.5 | 12.05 | 3.83 | -0.333641799194336 |
| 330 | expm | F1 | array | input | public | expm\_F1\_array\_raw\_public.csv | 2a\_refined\_analysis\_dataset | Array\_1 | Prep\_A | 2 | CreE | 12 | 3.5 | 12.06 | 3.84 | -0.336717268600696 |
| 331 | expm | F1 | array | input | public | expm\_F1\_array\_raw\_public.csv | 2a\_refined\_analysis\_dataset | Array\_2 | Prep\_A | 2 | CreE | 12 | 3.5 | 12.08 | 3.79 | -0.287322668478406 |
| 332 | expm | F1 | array | input | public | expm\_F1\_array\_raw\_public.csv | 2a\_refined\_analysis\_dataset | Array\_2 | Prep\_A | 1 | CreE | 12 | 3.5 | 11.91 | 3.78 | -0.278471641590596 |
| 333 | expm | F1 | array | input | public | expm\_F1\_array\_raw\_public.csv | 2a\_refined\_analysis\_dataset | Array\_2 | Prep\_A | 1 | CreJ | 12 | 3.5 | 11.91 | 3.86 | -0.362328323817215 |
| 334 | expm | F1 | array | input | public | expm\_F1\_array\_raw\_public.csv | 2a\_refined\_analysis\_dataset | Array\_2 | Prep\_A | 2 | CreJ | 12 | 3.5 | 12.08 | 3.94 | -0.436975984195975 |
| 335 | expm | F1 | array | input | public | expm\_F1\_array\_raw\_public.csv | 2a\_refined\_analysis\_dataset | Array\_1 | Prep\_A | 1 | CreJ | 12 | 3.5 | 12.05 | 3.88 | -0.382583401765394 |
| 336 | expm | F1 | array | input | public | expm\_F1\_array\_raw\_public.csv | 2a\_refined\_analysis\_dataset | Array\_1 | Prep\_A | 2 | CreJ | 12 | 3.5 | 12.07 | 3.88 | -0.382080003145294 |
| 337 | expm | F1 | array | input | public | expm\_F1\_array\_raw\_public.csv | 2a\_refined\_analysis\_dataset | Array\_2 | Prep\_A | 1 | CreE | 35 | 1.5 | 35.29 | 1.92 | -0.418909765119626 |
| 338 | expm | F1 | array | input | public | expm\_F1\_array\_raw\_public.csv | 2a\_refined\_analysis\_dataset | Array\_2 | Prep\_A | 2 | CreE | 35 | 1.5 | 34.97 | 1.87 | -0.374903456885686 |
| 339 | expm | F1 | array | input | public | expm\_F1\_array\_raw\_public.csv | 2a\_refined\_analysis\_dataset | Array\_1 | Prep\_A | 1 | CreE | 35 | 1.5 | 35.18 | 1.88 | -0.375214501635316 |
| 340 | expm | F1 | array | input | public | expm\_F1\_array\_raw\_public.csv | 2a\_refined\_analysis\_dataset | Array\_1 | Prep\_A | 2 | CreE | 35 | 1.5 | 34.96 | 1.93 | -0.426789326021526 |
| 341 | expm | F1 | array | input | public | expm\_F1\_array\_raw\_public.csv | 2a\_refined\_analysis\_dataset | Array\_2 | Prep\_A | 1 | CreJ | 35 | 1.5 | 35.28 | 1.59 | -0.0903683818257326 |
| 342 | expm | F1 | array | input | public | expm\_F1\_array\_raw\_public.csv | 2a\_refined\_analysis\_dataset | Array\_2 | Prep\_A | 2 | CreJ | 35 | 1.5 | 34.97 | 1.6 | -0.0994286578769827 |
| 343 | expm | F1 | array | input | public | expm\_F1\_array\_raw\_public.csv | 2a\_refined\_analysis\_dataset | Array\_1 | Prep\_A | 1 | CreJ | 35 | 1.5 | 35.18 | 1.65 | -0.154452413512362 |
| 344 | expm | F1 | array | input | public | expm\_F1\_array\_raw\_public.csv | 2a\_refined\_analysis\_dataset | Array\_1 | Prep\_A | 2 | CreJ | 35 | 1.5 | 34.96 | 1.6 | -0.101632791279542 |
| 345 | expm | F1 | array | input | public | expm\_F1\_array\_raw\_public.csv | 2a\_refined\_analysis\_dataset | Array\_2 | Prep\_A | 2 | CreJ | 13 | 3.5 | 13 | 3.86 | -0.356625422984413 |
| 346 | expm | F1 | array | input | public | expm\_F1\_array\_raw\_public.csv | 2a\_refined\_analysis\_dataset | Array\_2 | Prep\_A | 1 | CreJ | 13 | 3.5 | 12.95 | 3.96 | -0.464451216604493 |
| 347 | expm | F1 | array | input | public | expm\_F1\_array\_raw\_public.csv | 2a\_refined\_analysis\_dataset | Array\_1 | Prep\_A | 2 | CreJ | 13 | 3.5 | 13 | 3.97 | -0.469257366003048 |
| 348 | expm | F1 | array | input | public | expm\_F1\_array\_raw\_public.csv | 2a\_refined\_analysis\_dataset | Array\_1 | Prep\_A | 1 | CreJ | 13 | 3.5 | 12.87 | 3.84 | -0.343374364249738 |
| 349 | expm | F1 | array | input | public | expm\_F1\_array\_raw\_public.csv | 2a\_refined\_analysis\_dataset | Array\_1 | Prep\_A | 1 | CreE | 13 | 3.5 | 12.87 | 3.84 | -0.340577909426602 |
| 350 | expm | F1 | array | input | public | expm\_F1\_array\_raw\_public.csv | 2a\_refined\_analysis\_dataset | Array\_1 | Prep\_A | 2 | CreE | 13 | 3.5 | 13 | 3.81 | -0.313323658213702 |
| 351 | expm | F1 | array | input | public | expm\_F1\_array\_raw\_public.csv | 2a\_refined\_analysis\_dataset | Array\_2 | Prep\_A | 2 | CreE | 13 | 3.5 | 13 | 3.8 | -0.298312180694382 |
| 352 | expm | F1 | array | input | public | expm\_F1\_array\_raw\_public.csv | 2a\_refined\_analysis\_dataset | Array\_2 | Prep\_A | 1 | CreE | 13 | 3.5 | 12.95 | 3.82 | -0.317596949868652 |
| 353 | expm | F1 | array | input | public | expm\_F1\_array\_raw\_public.csv | 2a\_refined\_analysis\_dataset | Array\_2 | Prep\_A | 2 | CreJ | 14 | 3.5 | 14.14 | 3.93 | -0.43440529728725 |
| 354 | expm | F1 | array | input | public | expm\_F1\_array\_raw\_public.csv | 2a\_refined\_analysis\_dataset | Array\_2 | Prep\_A | 1 | CreJ | 14 | 3.5 | 13.98 | 3.89 | -0.38681072303021 |
| 355 | expm | F1 | array | input | public | expm\_F1\_array\_raw\_public.csv | 2a\_refined\_analysis\_dataset | Array\_2 | Prep\_A | 2 | CreE | 14 | 3.5 | 14.14 | 3.82 | -0.317359191127514 |
| 356 | expm | F1 | array | input | public | expm\_F1\_array\_raw\_public.csv | 2a\_refined\_analysis\_dataset | Array\_2 | Prep\_A | 1 | CreE | 14 | 3.5 | 13.98 | 3.78 | -0.281540120303664 |
| 357 | expm | F1 | array | input | public | expm\_F1\_array\_raw\_public.csv | 2a\_refined\_analysis\_dataset | Array\_1 | Prep\_A | 1 | CreJ | 14 | 3.5 | 14.06 | 3.89 | -0.39075769813368 |
| 358 | expm | F1 | array | input | public | expm\_F1\_array\_raw\_public.csv | 2a\_refined\_analysis\_dataset | Array\_1 | Prep\_A | 2 | CreJ | 14 | 3.5 | 13.97 | 3.91 | -0.414579712485311 |
| 359 | expm | F1 | array | input | public | expm\_F1\_array\_raw\_public.csv | 2a\_refined\_analysis\_dataset | Array\_1 | Prep\_A | 2 | CreE | 14 | 3.5 | 13.97 | 3.85 | -0.350181555325894 |
| 360 | expm | F1 | array | input | public | expm\_F1\_array\_raw\_public.csv | 2a\_refined\_analysis\_dataset | Array\_1 | Prep\_A | 1 | CreE | 14 | 3.5 | 14.06 | 3.84 | -0.337364653853914 |
| 361 | expm | F1 | array | input | public | expm\_F1\_array\_raw\_public.csv | 2a\_refined\_analysis\_dataset | Array\_2 | Prep\_A | 1 | CreE | 1 | 2 | 1.02 | 2.02 | -0.0175812271112337 |
| 362 | expm | F1 | array | input | public | expm\_F1\_array\_raw\_public.csv | 2a\_refined\_analysis\_dataset | Array\_2 | Prep\_A | 2 | CreE | 1 | 2 | 1.02 | 2.04 | -0.0395007978129538 |
| 363 | expm | F1 | array | input | public | expm\_F1\_array\_raw\_public.csv | 2a\_refined\_analysis\_dataset | Array\_2 | Prep\_A | 2 | CreJ | 1 | 2 | 1.02 | 2.08 | -0.0806805810860918 |
| 364 | expm | F1 | array | input | public | expm\_F1\_array\_raw\_public.csv | 2a\_refined\_analysis\_dataset | Array\_2 | Prep\_A | 1 | CreJ | 1 | 2 | 1.02 | 2.09 | -0.0931269150601715 |
| 365 | expm | F1 | array | input | public | expm\_F1\_array\_raw\_public.csv | 2a\_refined\_analysis\_dataset | Array\_1 | Prep\_A | 1 | CreJ | 1 | 2 | 1.01 | 2.12 | -0.121923275735495 |
| 366 | expm | F1 | array | input | public | expm\_F1\_array\_raw\_public.csv | 2a\_refined\_analysis\_dataset | Array\_1 | Prep\_A | 2 | CreJ | 1 | 2 | 1 | 2.06 | -0.056393008810165 |
| 367 | expm | F1 | array | input | public | expm\_F1\_array\_raw\_public.csv | 2a\_refined\_analysis\_dataset | Array\_1 | Prep\_A | 2 | CreE | 1 | 2 | 1 | 2.01 | -0.0058178056935736 |
| 368 | expm | F1 | array | input | public | expm\_F1\_array\_raw\_public.csv | 2a\_refined\_analysis\_dataset | Array\_1 | Prep\_A | 1 | CreE | 1 | 2 | 1.01 | 2.01 | -0.0104154406517738 |
| 369 | expm | F1 | array | input | public | expm\_F1\_array\_raw\_public.csv | 2a\_refined\_analysis\_dataset | Array\_2 | Prep\_A | 2 | CreJ | 15 | 3.5 | 15.08 | 3.85 | -0.350957135698257 |
| 370 | expm | F1 | array | input | public | expm\_F1\_array\_raw\_public.csv | 2a\_refined\_analysis\_dataset | Array\_2 | Prep\_A | 1 | CreJ | 15 | 3.5 | 15.07 | 3.9 | -0.400587672962337 |
| 371 | expm | F1 | array | input | public | expm\_F1\_array\_raw\_public.csv | 2a\_refined\_analysis\_dataset | Array\_2 | Prep\_A | 1 | CreE | 15 | 3.5 | 15.07 | 3.86 | -0.35578650000234 |
| 372 | expm | F1 | array | input | public | expm\_F1\_array\_raw\_public.csv | 2a\_refined\_analysis\_dataset | Array\_2 | Prep\_A | 2 | CreE | 15 | 3.5 | 15.07 | 3.83 | -0.32902835917444 |
| 373 | expm | F1 | array | input | public | expm\_F1\_array\_raw\_public.csv | 2a\_refined\_analysis\_dataset | Array\_1 | Prep\_A | 1 | CreE | 15 | 3.5 | 14.99 | 3.85 | -0.35296139840385 |
| 374 | expm | F1 | array | input | public | expm\_F1\_array\_raw\_public.csv | 2a\_refined\_analysis\_dataset | Array\_1 | Prep\_A | 2 | CreE | 15 | 3.5 | 15.09 | 3.87 | -0.3663287236974 |
| 375 | expm | F1 | array | input | public | expm\_F1\_array\_raw\_public.csv | 2a\_refined\_analysis\_dataset | Array\_1 | Prep\_A | 2 | CreJ | 15 | 3.5 | 15.09 | 3.81 | -0.311803519659037 |
| 376 | expm | F1 | array | input | public | expm\_F1\_array\_raw\_public.csv | 2a\_refined\_analysis\_dataset | Array\_1 | Prep\_A | 1 | CreJ | 15 | 3.5 | 14.99 | 3.89 | -0.394071210412967 |
| 377 | expm | F1 | array | input | public | expm\_F1\_array\_raw\_public.csv | 2a\_refined\_analysis\_dataset | Array\_2 | Prep\_A | 2 | CreE | 2 | 2 | 2.02 | 2.03 | -0.0288748894722173 |
| 378 | expm | F1 | array | input | public | expm\_F1\_array\_raw\_public.csv | 2a\_refined\_analysis\_dataset | Array\_2 | Prep\_A | 1 | CreE | 2 | 2 | 2 | 2 | 0.00244835781509289 |
| 379 | expm | F1 | array | input | public | expm\_F1\_array\_raw\_public.csv | 2a\_refined\_analysis\_dataset | Array\_2 | Prep\_A | 2 | CreJ | 2 | 2 | 2.02 | 2.06 | -0.0624907079747565 |
| 380 | expm | F1 | array | input | public | expm\_F1\_array\_raw\_public.csv | 2a\_refined\_analysis\_dataset | Array\_2 | Prep\_A | 1 | CreJ | 2 | 2 | 2 | 2.01 | -0.00904112215912667 |
| 381 | expm | F1 | array | input | public | expm\_F1\_array\_raw\_public.csv | 2a\_refined\_analysis\_dataset | Array\_1 | Prep\_A | 2 | CreE | 2 | 2 | 1.98 | 2.03 | -0.0300875437730372 |
| 382 | expm | F1 | array | input | public | expm\_F1\_array\_raw\_public.csv | 2a\_refined\_analysis\_dataset | Array\_1 | Prep\_A | 1 | CreE | 2 | 2 | 2.02 | 2.05 | -0.0458152364191373 |
| 383 | expm | F1 | array | input | public | expm\_F1\_array\_raw\_public.csv | 2a\_refined\_analysis\_dataset | Array\_1 | Prep\_A | 2 | CreJ | 2 | 2 | 1.98 | 2.15 | -0.147951933866758 |
| 384 | expm | F1 | array | input | public | expm\_F1\_array\_raw\_public.csv | 2a\_refined\_analysis\_dataset | Array\_1 | Prep\_A | 1 | CreJ | 2 | 2 | 2.02 | 2.08 | -0.0845283930877274 |
| 385 | expm | F1 | array | input | public | expm\_F1\_array\_raw\_public.csv | 2a\_refined\_analysis\_dataset | Array\_2 | Prep\_A | 2 | CreE | 16 | 3.5 | 16.11 | 3.89 | -0.392426983100849 |
| 386 | expm | F1 | array | input | public | expm\_F1\_array\_raw\_public.csv | 2a\_refined\_analysis\_dataset | Array\_2 | Prep\_A | 1 | CreE | 16 | 3.5 | 16.02 | 3.83 | -0.333193497551688 |
| 387 | expm | F1 | array | input | public | expm\_F1\_array\_raw\_public.csv | 2a\_refined\_analysis\_dataset | Array\_1 | Prep\_A | 2 | CreE | 16 | 3.5 | 16.14 | 3.88 | -0.381446136315998 |
| 388 | expm | F1 | array | input | public | expm\_F1\_array\_raw\_public.csv | 2a\_refined\_analysis\_dataset | Array\_1 | Prep\_A | 1 | CreE | 16 | 3.5 | 16.03 | 3.89 | -0.392390891445698 |
| 389 | expm | F1 | array | input | public | expm\_F1\_array\_raw\_public.csv | 2a\_refined\_analysis\_dataset | Array\_1 | Prep\_A | 1 | CreJ | 16 | 3.5 | 16.03 | 3.88 | -0.382613277569507 |
| 390 | expm | F1 | array | input | public | expm\_F1\_array\_raw\_public.csv | 2a\_refined\_analysis\_dataset | Array\_1 | Prep\_A | 2 | CreJ | 16 | 3.5 | 16.14 | 3.89 | -0.393193759926957 |
| 391 | expm | F1 | array | input | public | expm\_F1\_array\_raw\_public.csv | 2a\_refined\_analysis\_dataset | Array\_2 | Prep\_A | 2 | CreJ | 16 | 3.5 | 16.11 | 3.93 | -0.433815619240431 |
| 392 | expm | F1 | array | input | public | expm\_F1\_array\_raw\_public.csv | 2a\_refined\_analysis\_dataset | Array\_2 | Prep\_A | 1 | CreJ | 16 | 3.5 | 16.02 | 3.92 | -0.42242476578053 |
| 393 | expm | F1 | array | input | public | expm\_F1\_array\_raw\_public.csv | 2a\_refined\_analysis\_dataset | Array\_2 | Prep\_A | 2 | CreJ | 3 | 2 | 3.03 | 2.12 | -0.118112119726132 |
| 394 | expm | F1 | array | input | public | expm\_F1\_array\_raw\_public.csv | 2a\_refined\_analysis\_dataset | Array\_2 | Prep\_A | 1 | CreJ | 3 | 2 | 3.01 | 2.05 | -0.0526184394125218 |
| 395 | expm | F1 | array | input | public | expm\_F1\_array\_raw\_public.csv | 2a\_refined\_analysis\_dataset | Array\_1 | Prep\_A | 2 | CreJ | 3 | 2 | 2.94 | 2.12 | -0.11608102260374 |
| 396 | expm | F1 | array | input | public | expm\_F1\_array\_raw\_public.csv | 2a\_refined\_analysis\_dataset | Array\_1 | Prep\_A | 1 | CreJ | 3 | 2 | 3.03 | 2.04 | -0.0360492064127 |
| 397 | expm | F1 | array | input | public | expm\_F1\_array\_raw\_public.csv | 2a\_refined\_analysis\_dataset | Array\_1 | Prep\_A | 2 | CreE | 3 | 2 | 2.94 | 2.04 | -0.0437274513350023 |
| 398 | expm | F1 | array | input | public | expm\_F1\_array\_raw\_public.csv | 2a\_refined\_analysis\_dataset | Array\_1 | Prep\_A | 1 | CreE | 3 | 2 | 3.03 | 2.05 | -0.047111611819342 |
| 399 | expm | F1 | array | input | public | expm\_F1\_array\_raw\_public.csv | 2a\_refined\_analysis\_dataset | Array\_2 | Prep\_A | 1 | CreE | 3 | 2 | 3.01 | 2.04 | -0.040387261750332 |
| 400 | expm | F1 | array | input | public | expm\_F1\_array\_raw\_public.csv | 2a\_refined\_analysis\_dataset | Array\_2 | Prep\_A | 2 | CreE | 3 | 2 | 3.03 | 2.04 | -0.0418544157481424 |
| 401 | expm | F1 | array | input | public | expm\_F1\_array\_raw\_public.csv | 2a\_refined\_analysis\_dataset | Array\_2 | Prep\_A | 2 | CreJ | 17 | 3.5 | 17.03 | 3.92 | -0.416343915538782 |
| 402 | expm | F1 | array | input | public | expm\_F1\_array\_raw\_public.csv | 2a\_refined\_analysis\_dataset | Array\_2 | Prep\_A | 1 | CreJ | 17 | 3.5 | 17.07 | 3.9 | -0.404956625561313 |
| 403 | expm | F1 | array | input | public | expm\_F1\_array\_raw\_public.csv | 2a\_refined\_analysis\_dataset | Array\_1 | Prep\_A | 1 | CreJ | 17 | 3.5 | 17.08 | 3.98 | -0.481888974070882 |
| 404 | expm | F1 | array | input | public | expm\_F1\_array\_raw\_public.csv | 2a\_refined\_analysis\_dataset | Array\_1 | Prep\_A | 2 | CreJ | 17 | 3.5 | 16.99 | 3.92 | -0.415776109802323 |
| 405 | expm | F1 | array | input | public | expm\_F1\_array\_raw\_public.csv | 2a\_refined\_analysis\_dataset | Array\_1 | Prep\_A | 2 | CreE | 17 | 3.5 | 16.99 | 3.88 | -0.37861491530216 |
| 406 | expm | F1 | array | input | public | expm\_F1\_array\_raw\_public.csv | 2a\_refined\_analysis\_dataset | Array\_1 | Prep\_A | 1 | CreE | 17 | 3.5 | 17.07 | 3.89 | -0.38858063588145 |
| 407 | expm | F1 | array | input | public | expm\_F1\_array\_raw\_public.csv | 2a\_refined\_analysis\_dataset | Array\_2 | Prep\_A | 2 | CreE | 17 | 3.5 | 17.03 | 3.88 | -0.38279041250746 |
| 408 | expm | F1 | array | input | public | expm\_F1\_array\_raw\_public.csv | 2a\_refined\_analysis\_dataset | Array\_2 | Prep\_A | 1 | CreE | 17 | 3.5 | 17.07 | 3.84 | -0.3444604097902 |
| 409 | expm | F1 | array | input | public | expm\_F1\_array\_raw\_public.csv | 2a\_refined\_analysis\_dataset | Array\_2 | Prep\_A | 2 | CreE | 4 | 2 | 3.98 | 2.1 | -0.0992928364262822 |
| 410 | expm | F1 | array | input | public | expm\_F1\_array\_raw\_public.csv | 2a\_refined\_analysis\_dataset | Array\_2 | Prep\_A | 1 | CreE | 4 | 2 | 4 | 2.06 | -0.062517492374182 |
| 411 | expm | F1 | array | input | public | expm\_F1\_array\_raw\_public.csv | 2a\_refined\_analysis\_dataset | Array\_2 | Prep\_A | 1 | CreJ | 4 | 2 | 4 | 2.05 | -0.0539628285375695 |
| 412 | expm | F1 | array | input | public | expm\_F1\_array\_raw\_public.csv | 2a\_refined\_analysis\_dataset | Array\_2 | Prep\_A | 2 | CreJ | 4 | 2 | 3.98 | 2.11 | -0.113739328792549 |
| 413 | expm | F1 | array | input | public | expm\_F1\_array\_raw\_public.csv | 2a\_refined\_analysis\_dataset | Array\_1 | Prep\_A | 2 | CreE | 4 | 2 | 4.03 | 2.04 | -0.0350561916519521 |
| 414 | expm | F1 | array | input | public | expm\_F1\_array\_raw\_public.csv | 2a\_refined\_analysis\_dataset | Array\_1 | Prep\_A | 1 | CreE | 4 | 2 | 4 | 2.08 | -0.0797096856917223 |
| 415 | expm | F1 | array | input | public | expm\_F1\_array\_raw\_public.csv | 2a\_refined\_analysis\_dataset | Array\_1 | Prep\_A | 2 | CreJ | 4 | 2 | 4.03 | 2.12 | -0.117409924772151 |
| 416 | expm | F1 | array | input | public | expm\_F1\_array\_raw\_public.csv | 2a\_refined\_analysis\_dataset | Array\_1 | Prep\_A | 1 | CreJ | 4 | 2 | 4 | 2.06 | -0.0569473671661309 |
| 417 | expm | F1 | array | input | public | expm\_F1\_array\_raw\_public.csv | 2a\_refined\_analysis\_dataset | Array\_1 | Prep\_A | 1 | CreE | 18 | 3.5 | 18.14 | 3.91 | -0.406958218145655 |
| 418 | expm | F1 | array | input | public | expm\_F1\_array\_raw\_public.csv | 2a\_refined\_analysis\_dataset | Array\_1 | Prep\_A | 2 | CreE | 18 | 3.5 | 18.09 | 3.89 | -0.386160599396865 |
| 419 | expm | F1 | array | input | public | expm\_F1\_array\_raw\_public.csv | 2a\_refined\_analysis\_dataset | Array\_2 | Prep\_A | 1 | CreJ | 18 | 3.5 | 17.91 | 3.87 | -0.371791662714557 |
| 420 | expm | F1 | array | input | public | expm\_F1\_array\_raw\_public.csv | 2a\_refined\_analysis\_dataset | Array\_2 | Prep\_A | 2 | CreJ | 18 | 3.5 | 18.04 | 3.9 | -0.396425777936017 |
| 421 | expm | F1 | array | input | public | expm\_F1\_array\_raw\_public.csv | 2a\_refined\_analysis\_dataset | Array\_1 | Prep\_A | 1 | CreJ | 18 | 3.5 | 18.14 | 3.93 | -0.43397596345972 |
| 422 | expm | F1 | array | input | public | expm\_F1\_array\_raw\_public.csv | 2a\_refined\_analysis\_dataset | Array\_1 | Prep\_A | 2 | CreJ | 18 | 3.5 | 18.1 | 3.89 | -0.391083285031721 |
| 423 | expm | F1 | array | input | public | expm\_F1\_array\_raw\_public.csv | 2a\_refined\_analysis\_dataset | Array\_2 | Prep\_A | 1 | CreE | 18 | 3.5 | 17.91 | 3.9 | -0.396596897839825 |
| 424 | expm | F1 | array | input | public | expm\_F1\_array\_raw\_public.csv | 2a\_refined\_analysis\_dataset | Array\_2 | Prep\_A | 2 | CreE | 18 | 3.5 | 18.04 | 3.88 | -0.381203893917115 |
| 425 | expm | F1 | array | input | public | expm\_F1\_array\_raw\_public.csv | 2a\_refined\_analysis\_dataset | Array\_2 | Prep\_A | 2 | CreE | 5 | 2 | 5 | 2.1 | -0.0981858844461105 |
| 426 | expm | F1 | array | input | public | expm\_F1\_array\_raw\_public.csv | 2a\_refined\_analysis\_dataset | Array\_2 | Prep\_A | 1 | CreE | 5 | 2 | 5.01 | 2.05 | -0.0487799761132606 |
| 427 | expm | F1 | array | input | public | expm\_F1\_array\_raw\_public.csv | 2a\_refined\_analysis\_dataset | Array\_1 | Prep\_A | 1 | CreJ | 5 | 2 | 4.99 | 2.09 | -0.0869468578260895 |
| 428 | expm | F1 | array | input | public | expm\_F1\_array\_raw\_public.csv | 2a\_refined\_analysis\_dataset | Array\_1 | Prep\_A | 2 | CreJ | 5 | 2 | 5.04 | 2.09 | -0.0891810770073596 |
| 429 | expm | F1 | array | input | public | expm\_F1\_array\_raw\_public.csv | 2a\_refined\_analysis\_dataset | Array\_2 | Prep\_A | 1 | CreJ | 5 | 2 | 5.01 | 2.15 | -0.153039575598483 |
| 430 | expm | F1 | array | input | public | expm\_F1\_array\_raw\_public.csv | 2a\_refined\_analysis\_dataset | Array\_2 | Prep\_A | 2 | CreJ | 5 | 2 | 5 | 2.13 | -0.130642749700573 |
| 431 | expm | F1 | array | input | public | expm\_F1\_array\_raw\_public.csv | 2a\_refined\_analysis\_dataset | Array\_1 | Prep\_A | 1 | CreE | 5 | 2 | 4.99 | 2.08 | -0.0774794220661708 |
| 432 | expm | F1 | array | input | public | expm\_F1\_array\_raw\_public.csv | 2a\_refined\_analysis\_dataset | Array\_1 | Prep\_A | 2 | CreE | 5 | 2 | 5.04 | 2.07 | -0.0678052938060105 |
| 433 | expm | F1 | array | input | public | expm\_F1\_array\_raw\_public.csv | 2a\_refined\_analysis\_dataset | Array\_1 | Prep\_A | 2 | CreE | 19 | 3.5 | 18.98 | 3.94 | -0.437885123756375 |
| 434 | expm | F1 | array | input | public | expm\_F1\_array\_raw\_public.csv | 2a\_refined\_analysis\_dataset | Array\_1 | Prep\_A | 1 | CreE | 19 | 3.5 | 19.24 | 3.9 | -0.396613635850615 |
| 435 | expm | F1 | array | input | public | expm\_F1\_array\_raw\_public.csv | 2a\_refined\_analysis\_dataset | Array\_2 | Prep\_A | 1 | CreE | 19 | 3.5 | 19.18 | 3.92 | -0.423725089419785 |
| 436 | expm | F1 | array | input | public | expm\_F1\_array\_raw\_public.csv | 2a\_refined\_analysis\_dataset | Array\_2 | Prep\_A | 2 | CreE | 19 | 3.5 | 18.96 | 3.93 | -0.428602241950514 |
| 437 | expm | F1 | array | input | public | expm\_F1\_array\_raw\_public.csv | 2a\_refined\_analysis\_dataset | Array\_1 | Prep\_A | 2 | CreJ | 19 | 3.5 | 18.98 | 3.93 | -0.431481983820762 |
| 438 | expm | F1 | array | input | public | expm\_F1\_array\_raw\_public.csv | 2a\_refined\_analysis\_dataset | Array\_1 | Prep\_A | 1 | CreJ | 19 | 3.5 | 19.24 | 4 | -0.495822084773121 |
| 439 | expm | F1 | array | input | public | expm\_F1\_array\_raw\_public.csv | 2a\_refined\_analysis\_dataset | Array\_2 | Prep\_A | 2 | CreJ | 19 | 3.5 | 18.97 | 3.99 | -0.491564272015651 |
| 440 | expm | F1 | array | input | public | expm\_F1\_array\_raw\_public.csv | 2a\_refined\_analysis\_dataset | Array\_2 | Prep\_A | 1 | CreJ | 19 | 3.5 | 19.18 | 3.89 | -0.394302998133051 |
| 441 | expm | F1 | array | input | public | expm\_F1\_array\_raw\_public.csv | 2a\_refined\_analysis\_dataset | Array\_1 | Prep\_A | 2 | CreJ | 6 | 2 | 6.05 | 2.13 | -0.134424399134052 |
| 442 | expm | F1 | array | input | public | expm\_F1\_array\_raw\_public.csv | 2a\_refined\_analysis\_dataset | Array\_1 | Prep\_A | 1 | CreJ | 6 | 2 | 6.02 | 2.11 | -0.108713926718992 |
| 443 | expm | F1 | array | input | public | expm\_F1\_array\_raw\_public.csv | 2a\_refined\_analysis\_dataset | Array\_2 | Prep\_A | 2 | CreE | 6 | 2 | 6.03 | 2.1 | -0.0954949198893909 |
| 444 | expm | F1 | array | input | public | expm\_F1\_array\_raw\_public.csv | 2a\_refined\_analysis\_dataset | Array\_2 | Prep\_A | 1 | CreE | 6 | 2 | 6.01 | 2.06 | -0.0586301267975911 |
| 445 | expm | F1 | array | input | public | expm\_F1\_array\_raw\_public.csv | 2a\_refined\_analysis\_dataset | Array\_2 | Prep\_A | 1 | CreJ | 6 | 2 | 6.01 | 2.06 | -0.0557059924282712 |
| 446 | expm | F1 | array | input | public | expm\_F1\_array\_raw\_public.csv | 2a\_refined\_analysis\_dataset | Array\_2 | Prep\_A | 2 | CreJ | 6 | 2 | 6.03 | 2.12 | -0.119357159309112 |
| 447 | expm | F1 | array | input | public | expm\_F1\_array\_raw\_public.csv | 2a\_refined\_analysis\_dataset | Array\_1 | Prep\_A | 2 | CreE | 6 | 2 | 6.05 | 2.08 | -0.082967943405571 |
| 448 | expm | F1 | array | input | public | expm\_F1\_array\_raw\_public.csv | 2a\_refined\_analysis\_dataset | Array\_1 | Prep\_A | 1 | CreE | 6 | 2 | 6.02 | 2.07 | -0.0678625259437911 |
| 449 | expm | F1 | array | input | public | expm\_F1\_array\_raw\_public.csv | 2a\_refined\_analysis\_dataset | Array\_1 | Prep\_A | 2 | CreJ | 20 | 3.5 | 20.18 | 3.85 | -0.352192906760037 |
| 450 | expm | F1 | array | input | public | expm\_F1\_array\_raw\_public.csv | 2a\_refined\_analysis\_dataset | Array\_1 | Prep\_A | 1 | CreJ | 20 | 3.5 | 19.85 | 3.93 | -0.428747034808957 |
| 451 | expm | F1 | array | input | public | expm\_F1\_array\_raw\_public.csv | 2a\_refined\_analysis\_dataset | Array\_2 | Prep\_A | 2 | CreJ | 20 | 3.5 | 20.07 | 3.97 | -0.466532165228818 |
| 452 | expm | F1 | array | input | public | expm\_F1\_array\_raw\_public.csv | 2a\_refined\_analysis\_dataset | Array\_2 | Prep\_A | 1 | CreJ | 20 | 3.5 | 19.95 | 3.96 | -0.456253169869058 |
| 453 | expm | F1 | array | input | public | expm\_F1\_array\_raw\_public.csv | 2a\_refined\_analysis\_dataset | Array\_2 | Prep\_A | 2 | CreE | 20 | 3.5 | 20.07 | 3.94 | -0.443269482892188 |
| 454 | expm | F1 | array | input | public | expm\_F1\_array\_raw\_public.csv | 2a\_refined\_analysis\_dataset | Array\_2 | Prep\_A | 1 | CreE | 20 | 3.5 | 19.95 | 3.93 | -0.429473292281098 |
| 455 | expm | F1 | array | input | public | expm\_F1\_array\_raw\_public.csv | 2a\_refined\_analysis\_dataset | Array\_1 | Prep\_A | 2 | CreE | 20 | 3.5 | 20.18 | 3.87 | -0.365336977526548 |
| 456 | expm | F1 | array | input | public | expm\_F1\_array\_raw\_public.csv | 2a\_refined\_analysis\_dataset | Array\_1 | Prep\_A | 1 | CreE | 20 | 3.5 | 19.85 | 3.89 | -0.385862957536298 |
| 457 | expm | F1 | array | input | public | expm\_F1\_array\_raw\_public.csv | 2a\_refined\_analysis\_dataset | Array\_1 | Prep\_A | 2 | CreE | 7 | 2 | 7.05 | 2.07 | -0.072880755205067 |
| 458 | expm | F1 | array | input | public | expm\_F1\_array\_raw\_public.csv | 2a\_refined\_analysis\_dataset | Array\_1 | Prep\_A | 1 | CreE | 7 | 2 | 7 | 2.11 | -0.111080518802817 |
| 459 | expm | F1 | array | input | public | expm\_F1\_array\_raw\_public.csv | 2a\_refined\_analysis\_dataset | Array\_2 | Prep\_A | 1 | CreJ | 7 | 2 | 6.97 | 2.14 | -0.136808827462257 |
| 460 | expm | F1 | array | input | public | expm\_F1\_array\_raw\_public.csv | 2a\_refined\_analysis\_dataset | Array\_2 | Prep\_A | 2 | CreJ | 7 | 2 | 7.04 | 2.13 | -0.128440656991537 |
| 461 | expm | F1 | array | input | public | expm\_F1\_array\_raw\_public.csv | 2a\_refined\_analysis\_dataset | Array\_2 | Prep\_A | 2 | CreE | 7 | 2 | 7.04 | 2.09 | -0.0872987321917669 |
| 462 | expm | F1 | array | input | public | expm\_F1\_array\_raw\_public.csv | 2a\_refined\_analysis\_dataset | Array\_2 | Prep\_A | 1 | CreE | 7 | 2 | 6.97 | 2.1 | -0.101412410179277 |
| 463 | expm | F1 | array | input | public | expm\_F1\_array\_raw\_public.csv | 2a\_refined\_analysis\_dataset | Array\_1 | Prep\_A | 1 | CreJ | 7 | 2 | 7 | 2.15 | -0.154426374087848 |
| 464 | expm | F1 | array | input | public | expm\_F1\_array\_raw\_public.csv | 2a\_refined\_analysis\_dataset | Array\_1 | Prep\_A | 2 | CreJ | 7 | 2 | 7.05 | 2.11 | -0.108620737122888 |
| 465 | expm | F1 | array | input | public | expm\_F1\_array\_raw\_public.csv | 2a\_refined\_analysis\_dataset | Array\_2 | Prep\_A | 1 | CreE | 21 | 3.5 | 21.02 | 3.92 | -0.417735653934295 |
| 466 | expm | F1 | array | input | public | expm\_F1\_array\_raw\_public.csv | 2a\_refined\_analysis\_dataset | Array\_2 | Prep\_A | 2 | CreE | 21 | 3.5 | 21.01 | 3.95 | -0.449954118932935 |
| 467 | expm | F1 | array | input | public | expm\_F1\_array\_raw\_public.csv | 2a\_refined\_analysis\_dataset | Array\_1 | Prep\_A | 2 | CreE | 21 | 3.5 | 21.05 | 3.92 | -0.423230946777015 |
| 468 | expm | F1 | array | input | public | expm\_F1\_array\_raw\_public.csv | 2a\_refined\_analysis\_dataset | Array\_1 | Prep\_A | 1 | CreE | 21 | 3.5 | 21.04 | 3.94 | -0.435142809631525 |
| 469 | expm | F1 | array | input | public | expm\_F1\_array\_raw\_public.csv | 2a\_refined\_analysis\_dataset | Array\_1 | Prep\_A | 1 | CreJ | 21 | 3.5 | 21.04 | 3.97 | -0.471499870956099 |
| 470 | expm | F1 | array | input | public | expm\_F1\_array\_raw\_public.csv | 2a\_refined\_analysis\_dataset | Array\_1 | Prep\_A | 2 | CreJ | 21 | 3.5 | 21.05 | 3.89 | -0.390762676168519 |
| 471 | expm | F1 | array | input | public | expm\_F1\_array\_raw\_public.csv | 2a\_refined\_analysis\_dataset | Array\_2 | Prep\_A | 2 | CreJ | 21 | 3.5 | 21.01 | 3.8 | -0.304904250526535 |
| 472 | expm | F1 | array | input | public | expm\_F1\_array\_raw\_public.csv | 2a\_refined\_analysis\_dataset | Array\_2 | Prep\_A | 1 | CreJ | 21 | 3.5 | 21.02 | 3.94 | -0.443892537702765 |
| 473 | expm | F1 | array | input | public | expm\_F1\_array\_raw\_public.csv | 2a\_refined\_analysis\_dataset | Array\_1 | Prep\_A | 1 | CreE | 8 | 2 | 7.96 | 2.1 | -0.103905584834429 |
| 474 | expm | F1 | array | input | public | expm\_F1\_array\_raw\_public.csv | 2a\_refined\_analysis\_dataset | Array\_1 | Prep\_A | 2 | CreE | 8 | 2 | 8.03 | 2.08 | -0.0750176767860995 |
| 475 | expm | F1 | array | input | public | expm\_F1\_array\_raw\_public.csv | 2a\_refined\_analysis\_dataset | Array\_2 | Prep\_A | 2 | CreJ | 8 | 2 | 8.04 | 2.1 | -0.0957537555777193 |
| 476 | expm | F1 | array | input | public | expm\_F1\_array\_raw\_public.csv | 2a\_refined\_analysis\_dataset | Array\_2 | Prep\_A | 1 | CreJ | 8 | 2 | 8.09 | 2.07 | -0.0691056729277593 |
| 477 | expm | F1 | array | input | public | expm\_F1\_array\_raw\_public.csv | 2a\_refined\_analysis\_dataset | Array\_1 | Prep\_A | 2 | CreJ | 8 | 2 | 8.03 | 2.16 | -0.159863204379484 |
| 478 | expm | F1 | array | input | public | expm\_F1\_array\_raw\_public.csv | 2a\_refined\_analysis\_dataset | Array\_1 | Prep\_A | 1 | CreJ | 8 | 2 | 7.95 | 2.06 | -0.0613502605458138 |
| 479 | expm | F1 | array | input | public | expm\_F1\_array\_raw\_public.csv | 2a\_refined\_analysis\_dataset | Array\_2 | Prep\_A | 2 | CreE | 8 | 2 | 8.04 | 2.08 | -0.0803381907211294 |
| 480 | expm | F1 | array | input | public | expm\_F1\_array\_raw\_public.csv | 2a\_refined\_analysis\_dataset | Array\_2 | Prep\_A | 1 | CreE | 8 | 2 | 8.09 | 2.1 | -0.10390092179366 |
| 481 | expm | F1 | array | input | public | expm\_F1\_array\_raw\_public.csv | 2a\_refined\_analysis\_dataset | Array\_1 | Prep\_A | 1 | CreE | 22 | 3.5 | 21.9 | 4.02 | -0.52187192524533 |
| 482 | expm | F1 | array | input | public | expm\_F1\_array\_raw\_public.csv | 2a\_refined\_analysis\_dataset | Array\_1 | Prep\_A | 2 | CreE | 22 | 3.5 | 22.09 | 3.94 | -0.44409985764905 |
| 483 | expm | F1 | array | input | public | expm\_F1\_array\_raw\_public.csv | 2a\_refined\_analysis\_dataset | Array\_1 | Prep\_A | 2 | CreJ | 22 | 3.5 | 22.09 | 3.87 | -0.372019557398927 |
| 484 | expm | F1 | array | input | public | expm\_F1\_array\_raw\_public.csv | 2a\_refined\_analysis\_dataset | Array\_1 | Prep\_A | 1 | CreJ | 22 | 3.5 | 21.89 | 3.89 | -0.389736983562477 |
| 485 | expm | F1 | array | input | public | expm\_F1\_array\_raw\_public.csv | 2a\_refined\_analysis\_dataset | Array\_2 | Prep\_A | 2 | CreJ | 22 | 3.5 | 22.33 | 3.95 | -0.449315029390571 |
| 486 | expm | F1 | array | input | public | expm\_F1\_array\_raw\_public.csv | 2a\_refined\_analysis\_dataset | Array\_2 | Prep\_A | 1 | CreJ | 22 | 3.5 | 22.11 | 3.89 | -0.387687714326931 |
| 487 | expm | F1 | array | input | public | expm\_F1\_array\_raw\_public.csv | 2a\_refined\_analysis\_dataset | Array\_2 | Prep\_A | 1 | CreE | 22 | 3.5 | 22.11 | 3.95 | -0.45149124779194 |
| 488 | expm | F1 | array | input | public | expm\_F1\_array\_raw\_public.csv | 2a\_refined\_analysis\_dataset | Array\_2 | Prep\_A | 2 | CreE | 22 | 3.5 | 22.33 | 3.98 | -0.48256948853875 |
| 489 | expm | F1 | array | input | public | expm\_F1\_array\_raw\_public.csv | 2a\_refined\_analysis\_dataset | Array\_1 | Prep\_A | 1 | CreJ | 9 | 2 | 9.02 | 2.09 | -0.0926981331903032 |
| 490 | expm | F1 | array | input | public | expm\_F1\_array\_raw\_public.csv | 2a\_refined\_analysis\_dataset | Array\_1 | Prep\_A | 2 | CreJ | 9 | 2 | 9.08 | 2.18 | -0.176705414256953 |
| 491 | expm | F1 | array | input | public | expm\_F1\_array\_raw\_public.csv | 2a\_refined\_analysis\_dataset | Array\_2 | Prep\_A | 1 | CreJ | 9 | 2 | 9.04 | 2.1 | -0.104318391110179 |
| 492 | expm | F1 | array | input | public | expm\_F1\_array\_raw\_public.csv | 2a\_refined\_analysis\_dataset | Array\_2 | Prep\_A | 2 | CreJ | 9 | 2 | 9.02 | 2.12 | -0.124793463349549 |
| 493 | expm | F1 | array | input | public | expm\_F1\_array\_raw\_public.csv | 2a\_refined\_analysis\_dataset | Array\_1 | Prep\_A | 1 | CreE | 9 | 2 | 9.02 | 2.12 | -0.123809540151368 |
| 494 | expm | F1 | array | input | public | expm\_F1\_array\_raw\_public.csv | 2a\_refined\_analysis\_dataset | Array\_1 | Prep\_A | 2 | CreE | 9 | 2 | 9.08 | 2.14 | -0.139425747492418 |
| 495 | expm | F1 | array | input | public | expm\_F1\_array\_raw\_public.csv | 2a\_refined\_analysis\_dataset | Array\_2 | Prep\_A | 2 | CreE | 9 | 2 | 9.02 | 2.15 | -0.154665213966348 |
| 496 | expm | F1 | array | input | public | expm\_F1\_array\_raw\_public.csv | 2a\_refined\_analysis\_dataset | Array\_2 | Prep\_A | 1 | CreE | 9 | 2 | 9.05 | 2.11 | -0.105785028824938 |
| 497 | expm | F1 | array | input | public | expm\_F1\_array\_raw\_public.csv | 2a\_refined\_analysis\_dataset | Array\_2 | Prep\_A | 2 | CreE | 23 | 3.5 | 22.89 | 3.96 | -0.457107815063069 |
| 498 | expm | F1 | array | input | public | expm\_F1\_array\_raw\_public.csv | 2a\_refined\_analysis\_dataset | Array\_2 | Prep\_A | 1 | CreE | 23 | 3.5 | 23.03 | 3.98 | -0.482651540997729 |
| 499 | expm | F1 | array | input | public | expm\_F1\_array\_raw\_public.csv | 2a\_refined\_analysis\_dataset | Array\_1 | Prep\_A | 1 | CreE | 23 | 3.5 | 23.14 | 3.96 | -0.463723535347419 |
| 500 | expm | F1 | array | input | public | expm\_F1\_array\_raw\_public.csv | 2a\_refined\_analysis\_dataset | Array\_1 | Prep\_A | 2 | CreE | 23 | 3.5 | 23.12 | 3.96 | -0.459581793879109 |
| 501 | expm | F1 | array | input | public | expm\_F1\_array\_raw\_public.csv | 2a\_refined\_analysis\_dataset | Array\_2 | Prep\_A | 2 | CreJ | 23 | 3.5 | 22.89 | 3.88 | -0.381345211414057 |
| 502 | expm | F1 | array | input | public | expm\_F1\_array\_raw\_public.csv | 2a\_refined\_analysis\_dataset | Array\_2 | Prep\_A | 1 | CreJ | 23 | 3.5 | 23.03 | 3.87 | -0.370500617565337 |
| 503 | expm | F1 | array | input | public | expm\_F1\_array\_raw\_public.csv | 2a\_refined\_analysis\_dataset | Array\_1 | Prep\_A | 2 | CreJ | 23 | 3.5 | 23.13 | 3.95 | -0.45272029352033 |
| 504 | expm | F1 | array | input | public | expm\_F1\_array\_raw\_public.csv | 2a\_refined\_analysis\_dataset | Array\_1 | Prep\_A | 1 | CreJ | 23 | 3.5 | 23.14 | 3.96 | -0.46278764383857 |
| 505 | expm | F1 | array | input | public | expm\_F1\_array\_raw\_public.csv | 2a\_refined\_analysis\_dataset | Array\_2 | Prep\_A | 2 | CreJ | 10 | 2 | 10.08 | 2.13 | -0.125383405227652 |
| 506 | expm | F1 | array | input | public | expm\_F1\_array\_raw\_public.csv | 2a\_refined\_analysis\_dataset | Array\_2 | Prep\_A | 1 | CreJ | 10 | 2 | 10.03 | 2.11 | -0.114433676981882 |
| 507 | expm | F1 | array | input | public | expm\_F1\_array\_raw\_public.csv | 2a\_refined\_analysis\_dataset | Array\_1 | Prep\_A | 2 | CreE | 10 | 2 | 9.96 | 2.14 | -0.143400375929674 |
| 508 | expm | F1 | array | input | public | expm\_F1\_array\_raw\_public.csv | 2a\_refined\_analysis\_dataset | Array\_1 | Prep\_A | 1 | CreE | 10 | 2 | 9.94 | 2.12 | -0.121564762466874 |
| 509 | expm | F1 | array | input | public | expm\_F1\_array\_raw\_public.csv | 2a\_refined\_analysis\_dataset | Array\_1 | Prep\_A | 2 | CreJ | 10 | 2 | 9.96 | 2.1 | -0.10231087785524 |
| 510 | expm | F1 | array | input | public | expm\_F1\_array\_raw\_public.csv | 2a\_refined\_analysis\_dataset | Array\_1 | Prep\_A | 1 | CreJ | 10 | 2 | 9.94 | 2.19 | -0.19055530481607 |
| 511 | expm | F1 | array | input | public | expm\_F1\_array\_raw\_public.csv | 2a\_refined\_analysis\_dataset | Array\_2 | Prep\_A | 1 | CreE | 10 | 2 | 10.03 | 2.12 | -0.123729069917574 |
| 512 | expm | F1 | array | input | public | expm\_F1\_array\_raw\_public.csv | 2a\_refined\_analysis\_dataset | Array\_2 | Prep\_A | 2 | CreE | 10 | 2 | 10.08 | 2.12 | -0.120664305486134 |
| 513 | expm | F1 | array | input | public | expm\_F1\_array\_raw\_public.csv | 2a\_refined\_analysis\_dataset | Array\_1 | Prep\_A | 2 | CreJ | 24 | 3.5 | 23.68 | 3.94 | -0.441445831920587 |
| 514 | expm | F1 | array | input | public | expm\_F1\_array\_raw\_public.csv | 2a\_refined\_analysis\_dataset | Array\_1 | Prep\_A | 1 | CreJ | 24 | 3.5 | 24.13 | 3.97 | -0.467601554080787 |
| 515 | expm | F1 | array | input | public | expm\_F1\_array\_raw\_public.csv | 2a\_refined\_analysis\_dataset | Array\_2 | Prep\_A | 2 | CreE | 24 | 3.5 | 24.23 | 3.96 | -0.455385201165063 |
| 516 | expm | F1 | array | input | public | expm\_F1\_array\_raw\_public.csv | 2a\_refined\_analysis\_dataset | Array\_2 | Prep\_A | 1 | CreE | 24 | 3.5 | 24.16 | 3.98 | -0.484579998712103 |
| 517 | expm | F1 | array | input | public | expm\_F1\_array\_raw\_public.csv | 2a\_refined\_analysis\_dataset | Array\_2 | Prep\_A | 2 | CreJ | 24 | 3.5 | 24.23 | 4 | -0.502036598443521 |
| 518 | expm | F1 | array | input | public | expm\_F1\_array\_raw\_public.csv | 2a\_refined\_analysis\_dataset | Array\_2 | Prep\_A | 1 | CreJ | 24 | 3.5 | 24.16 | 3.93 | -0.426280096179731 |
| 519 | expm | F1 | array | input | public | expm\_F1\_array\_raw\_public.csv | 2a\_refined\_analysis\_dataset | Array\_1 | Prep\_A | 1 | CreE | 24 | 3.5 | 24.13 | 3.98 | -0.479758989087563 |
| 520 | expm | F1 | array | input | public | expm\_F1\_array\_raw\_public.csv | 2a\_refined\_analysis\_dataset | Array\_1 | Prep\_A | 2 | CreE | 24 | 3.5 | 23.68 | 3.99 | -0.494431831331704 |
| 521 | expm | F1 | array | input | public | expm\_F1\_array\_raw\_public.csv | 2a\_refined\_analysis\_dataset | Array\_2 | Prep\_A | 1 | CreJ | 11 | 2 | 10.99 | 2.13 | -0.130254259753649 |
| 522 | expm | F1 | array | input | public | expm\_F1\_array\_raw\_public.csv | 2a\_refined\_analysis\_dataset | Array\_2 | Prep\_A | 2 | CreJ | 11 | 2 | 11.13 | 2.2 | -0.198355497819269 |
| 523 | expm | F1 | array | input | public | expm\_F1\_array\_raw\_public.csv | 2a\_refined\_analysis\_dataset | Array\_2 | Prep\_A | 2 | CreE | 11 | 2 | 11.13 | 2.17 | -0.165064501215993 |
| 524 | expm | F1 | array | input | public | expm\_F1\_array\_raw\_public.csv | 2a\_refined\_analysis\_dataset | Array\_2 | Prep\_A | 1 | CreE | 11 | 2 | 10.99 | 2.17 | -0.168124976084073 |
| 525 | expm | F1 | array | input | public | expm\_F1\_array\_raw\_public.csv | 2a\_refined\_analysis\_dataset | Array\_1 | Prep\_A | 1 | CreE | 11 | 2 | 10.95 | 2.14 | -0.138805617453823 |
| 526 | expm | F1 | array | input | public | expm\_F1\_array\_raw\_public.csv | 2a\_refined\_analysis\_dataset | Array\_1 | Prep\_A | 2 | CreE | 11 | 2 | 11.01 | 2.15 | -0.154279768091413 |
| 527 | expm | F1 | array | input | public | expm\_F1\_array\_raw\_public.csv | 2a\_refined\_analysis\_dataset | Array\_1 | Prep\_A | 1 | CreJ | 11 | 2 | 10.95 | 2.12 | -0.11671499942145 |
| 528 | expm | F1 | array | input | public | expm\_F1\_array\_raw\_public.csv | 2a\_refined\_analysis\_dataset | Array\_1 | Prep\_A | 2 | CreJ | 11 | 2 | 11.01 | 2.15 | -0.15078970209892 |
| 529 | expm | F1 | array | input | public | expm\_F1\_array\_raw\_public.csv | 2a\_refined\_analysis\_dataset | Array\_2 | Prep\_A | 1 | CreE | 25 | 3.5 | 24.86 | 3.98 | -0.480348713973738 |
| 530 | expm | F1 | array | input | public | expm\_F1\_array\_raw\_public.csv | 2a\_refined\_analysis\_dataset | Array\_2 | Prep\_A | 2 | CreE | 25 | 3.5 | 25.01 | 3.99 | -0.494420612250728 |
| 531 | expm | F1 | array | input | public | expm\_F1\_array\_raw\_public.csv | 2a\_refined\_analysis\_dataset | Array\_2 | Prep\_A | 2 | CreJ | 25 | 3.5 | 25.01 | 3.95 | -0.453949738785717 |
| 532 | expm | F1 | array | input | public | expm\_F1\_array\_raw\_public.csv | 2a\_refined\_analysis\_dataset | Array\_2 | Prep\_A | 1 | CreJ | 25 | 3.5 | 24.85 | 3.9 | -0.395665410692807 |
| 533 | expm | F1 | array | input | public | expm\_F1\_array\_raw\_public.csv | 2a\_refined\_analysis\_dataset | Array\_1 | Prep\_A | 1 | CreE | 25 | 3.5 | 24.98 | 3.94 | -0.444775647520168 |
| 534 | expm | F1 | array | input | public | expm\_F1\_array\_raw\_public.csv | 2a\_refined\_analysis\_dataset | Array\_1 | Prep\_A | 2 | CreE | 25 | 3.5 | 25.1 | 3.98 | -0.484627305174128 |
| 535 | expm | F1 | array | input | public | expm\_F1\_array\_raw\_public.csv | 2a\_refined\_analysis\_dataset | Array\_1 | Prep\_A | 1 | CreJ | 25 | 3.5 | 24.98 | 3.94 | -0.442209710596487 |
| 536 | expm | F1 | array | input | public | expm\_F1\_array\_raw\_public.csv | 2a\_refined\_analysis\_dataset | Array\_1 | Prep\_A | 2 | CreJ | 25 | 3.5 | 25.1 | 3.83 | -0.331355424826687 |
| 537 | expm | F1 | array | input | public | expm\_F1\_array\_raw\_public.csv | 2a\_refined\_analysis\_dataset | Array\_2 | Prep\_A | 2 | CreE | 12 | 2 | 11.95 | 2.16 | -0.164700066382387 |
| 538 | expm | F1 | array | input | public | expm\_F1\_array\_raw\_public.csv | 2a\_refined\_analysis\_dataset | Array\_2 | Prep\_A | 1 | CreE | 12 | 2 | 11.96 | 2.13 | -0.125732487362777 |
| 539 | expm | F1 | array | input | public | expm\_F1\_array\_raw\_public.csv | 2a\_refined\_analysis\_dataset | Array\_2 | Prep\_A | 2 | CreJ | 12 | 2 | 11.94 | 2.19 | -0.188528872074352 |
| 540 | expm | F1 | array | input | public | expm\_F1\_array\_raw\_public.csv | 2a\_refined\_analysis\_dataset | Array\_2 | Prep\_A | 1 | CreJ | 12 | 2 | 11.95 | 2.13 | -0.131464768014852 |
| 541 | expm | F1 | array | input | public | expm\_F1\_array\_raw\_public.csv | 2a\_refined\_analysis\_dataset | Array\_1 | Prep\_A | 2 | CreE | 12 | 2 | 11.96 | 2.17 | -0.170950981028387 |
| 542 | expm | F1 | array | input | public | expm\_F1\_array\_raw\_public.csv | 2a\_refined\_analysis\_dataset | Array\_1 | Prep\_A | 1 | CreE | 12 | 2 | 12.04 | 2.21 | -0.213756728522737 |
| 543 | expm | F1 | array | input | public | expm\_F1\_array\_raw\_public.csv | 2a\_refined\_analysis\_dataset | Array\_1 | Prep\_A | 2 | CreJ | 12 | 2 | 11.96 | 2.13 | -0.12799528761405 |
| 544 | expm | F1 | array | input | public | expm\_F1\_array\_raw\_public.csv | 2a\_refined\_analysis\_dataset | Array\_1 | Prep\_A | 1 | CreJ | 12 | 2 | 12.04 | 2.11 | -0.11283248026888 |
| 545 | expm | F1 | array | input | public | expm\_F1\_array\_raw\_public.csv | 2a\_refined\_analysis\_dataset | Array\_2 | Prep\_A | 2 | CreE | 26 | 3.5 | 26.07 | 3.99 | -0.490721379857526 |
| 546 | expm | F1 | array | input | public | expm\_F1\_array\_raw\_public.csv | 2a\_refined\_analysis\_dataset | Array\_2 | Prep\_A | 1 | CreE | 26 | 3.5 | 26.05 | 4.02 | -0.524396879136465 |
| 547 | expm | F1 | array | input | public | expm\_F1\_array\_raw\_public.csv | 2a\_refined\_analysis\_dataset | Array\_2 | Prep\_A | 2 | CreJ | 26 | 3.5 | 26.07 | 3.98 | -0.475244126365145 |
| 548 | expm | F1 | array | input | public | expm\_F1\_array\_raw\_public.csv | 2a\_refined\_analysis\_dataset | Array\_2 | Prep\_A | 1 | CreJ | 26 | 3.5 | 26.05 | 3.9 | -0.404193437386005 |
| 549 | expm | F1 | array | input | public | expm\_F1\_array\_raw\_public.csv | 2a\_refined\_analysis\_dataset | Array\_1 | Prep\_A | 1 | CreJ | 26 | 3.5 | 25.95 | 3.87 | -0.371989054953756 |
| 550 | expm | F1 | array | input | public | expm\_F1\_array\_raw\_public.csv | 2a\_refined\_analysis\_dataset | Array\_1 | Prep\_A | 2 | CreJ | 26 | 3.5 | 26.23 | 3.93 | -0.434457585449676 |
| 551 | expm | F1 | array | input | public | expm\_F1\_array\_raw\_public.csv | 2a\_refined\_analysis\_dataset | Array\_1 | Prep\_A | 2 | CreE | 26 | 3.5 | 26.23 | 4 | -0.502901736427686 |
| 552 | expm | F1 | array | input | public | expm\_F1\_array\_raw\_public.csv | 2a\_refined\_analysis\_dataset | Array\_1 | Prep\_A | 1 | CreE | 26 | 3.5 | 25.96 | 4.01 | -0.508453163070226 |
| 553 | expm | F1 | array | input | public | expm\_F1\_array\_raw\_public.csv | 2a\_refined\_analysis\_dataset | Array\_2 | Prep\_A | 2 | CreJ | 13 | 2 | 13.04 | 2.22 | -0.217910206066644 |
| 554 | expm | F1 | array | input | public | expm\_F1\_array\_raw\_public.csv | 2a\_refined\_analysis\_dataset | Array\_2 | Prep\_A | 1 | CreJ | 13 | 2 | 12.92 | 2.1 | -0.104830976564005 |
| 555 | expm | F1 | array | input | public | expm\_F1\_array\_raw\_public.csv | 2a\_refined\_analysis\_dataset | Array\_1 | Prep\_A | 2 | CreE | 13 | 2 | 12.99 | 2.18 | -0.175382999100035 |
| 556 | expm | F1 | array | input | public | expm\_F1\_array\_raw\_public.csv | 2a\_refined\_analysis\_dataset | Array\_1 | Prep\_A | 1 | CreE | 13 | 2 | 12.99 | 2.19 | -0.187704926028595 |
| 557 | expm | F1 | array | input | public | expm\_F1\_array\_raw\_public.csv | 2a\_refined\_analysis\_dataset | Array\_2 | Prep\_A | 1 | CreE | 13 | 2 | 12.92 | 2.19 | -0.192535492256815 |
| 558 | expm | F1 | array | input | public | expm\_F1\_array\_raw\_public.csv | 2a\_refined\_analysis\_dataset | Array\_2 | Prep\_A | 2 | CreE | 13 | 2 | 13.05 | 2.17 | -0.171255830616225 |
| 559 | expm | F1 | array | input | public | expm\_F1\_array\_raw\_public.csv | 2a\_refined\_analysis\_dataset | Array\_1 | Prep\_A | 2 | CreJ | 13 | 2 | 12.99 | 2.09 | -0.0876951921183427 |
| 560 | expm | F1 | array | input | public | expm\_F1\_array\_raw\_public.csv | 2a\_refined\_analysis\_dataset | Array\_1 | Prep\_A | 1 | CreJ | 13 | 2 | 12.99 | 2.19 | -0.192544182672733 |
| 561 | expm | F1 | array | input | public | expm\_F1\_array\_raw\_public.csv | 2a\_refined\_analysis\_dataset | Array\_2 | Prep\_A | 1 | CreJ | 27 | 3.5 | 27.34 | 3.88 | -0.38053033607286 |
| 562 | expm | F1 | array | input | public | expm\_F1\_array\_raw\_public.csv | 2a\_refined\_analysis\_dataset | Array\_2 | Prep\_A | 2 | CreJ | 27 | 3.5 | 26.93 | 4.03 | -0.53142286805303 |
| 563 | expm | F1 | array | input | public | expm\_F1\_array\_raw\_public.csv | 2a\_refined\_analysis\_dataset | Array\_1 | Prep\_A | 2 | CreJ | 27 | 3.5 | 27.05 | 3.96 | -0.464467024464974 |
| 564 | expm | F1 | array | input | public | expm\_F1\_array\_raw\_public.csv | 2a\_refined\_analysis\_dataset | Array\_1 | Prep\_A | 1 | CreJ | 27 | 3.5 | 26.98 | 3.91 | -0.410979247231884 |
| 565 | expm | F1 | array | input | public | expm\_F1\_array\_raw\_public.csv | 2a\_refined\_analysis\_dataset | Array\_2 | Prep\_A | 2 | CreE | 27 | 3.5 | 26.93 | 4.06 | -0.557507865860973 |
| 566 | expm | F1 | array | input | public | expm\_F1\_array\_raw\_public.csv | 2a\_refined\_analysis\_dataset | Array\_2 | Prep\_A | 1 | CreE | 27 | 3.5 | 27.34 | 4.01 | -0.506329731559593 |
| 567 | expm | F1 | array | input | public | expm\_F1\_array\_raw\_public.csv | 2a\_refined\_analysis\_dataset | Array\_1 | Prep\_A | 1 | CreE | 27 | 3.5 | 26.98 | 3.99 | -0.490813143615283 |
| 568 | expm | F1 | array | input | public | expm\_F1\_array\_raw\_public.csv | 2a\_refined\_analysis\_dataset | Array\_1 | Prep\_A | 2 | CreE | 27 | 3.5 | 27.05 | 4.02 | -0.519218574496994 |
| 569 | expm | F1 | array | input | public | expm\_F1\_array\_raw\_public.csv | 2a\_refined\_analysis\_dataset | Array\_1 | Prep\_A | 2 | CreJ | 14 | 2 | 14.09 | 2.21 | -0.207406436795298 |
| 570 | expm | F1 | array | input | public | expm\_F1\_array\_raw\_public.csv | 2a\_refined\_analysis\_dataset | Array\_1 | Prep\_A | 1 | CreJ | 14 | 2 | 14.04 | 2.12 | -0.121912228697278 |
| 571 | expm | F1 | array | input | public | expm\_F1\_array\_raw\_public.csv | 2a\_refined\_analysis\_dataset | Array\_2 | Prep\_A | 1 | CreE | 14 | 2 | 14.07 | 2.19 | -0.187746035628243 |
| 572 | expm | F1 | array | input | public | expm\_F1\_array\_raw\_public.csv | 2a\_refined\_analysis\_dataset | Array\_2 | Prep\_A | 2 | CreE | 14 | 2 | 14.07 | 2.19 | -0.187027600913813 |
| 573 | expm | F1 | array | input | public | expm\_F1\_array\_raw\_public.csv | 2a\_refined\_analysis\_dataset | Array\_2 | Prep\_A | 1 | CreJ | 14 | 2 | 14.07 | 2.2 | -0.195308334349617 |
| 574 | expm | F1 | array | input | public | expm\_F1\_array\_raw\_public.csv | 2a\_refined\_analysis\_dataset | Array\_2 | Prep\_A | 2 | CreJ | 14 | 2 | 14.07 | 2.19 | -0.189893467139527 |
| 575 | expm | F1 | array | input | public | expm\_F1\_array\_raw\_public.csv | 2a\_refined\_analysis\_dataset | Array\_1 | Prep\_A | 2 | CreE | 14 | 2 | 14.09 | 2.18 | -0.180606941242703 |
| 576 | expm | F1 | array | input | public | expm\_F1\_array\_raw\_public.csv | 2a\_refined\_analysis\_dataset | Array\_1 | Prep\_A | 1 | CreE | 14 | 2 | 14.04 | 2.18 | -0.181510816832623 |
| 577 | expm | F1 | array | input | public | expm\_F1\_array\_raw\_public.csv | 2a\_refined\_analysis\_dataset | Array\_2 | Prep\_A | 2 | CreE | 1 | 0.5 | 1.01 | 0.35 | 0.153188613481158 |
| 578 | expm | F1 | array | input | public | expm\_F1\_array\_raw\_public.csv | 2a\_refined\_analysis\_dataset | Array\_2 | Prep\_A | 1 | CreE | 1 | 0.5 | 1.01 | 0.36 | 0.139472687551918 |
| 579 | expm | F1 | array | input | public | expm\_F1\_array\_raw\_public.csv | 2a\_refined\_analysis\_dataset | Array\_1 | Prep\_A | 1 | CreJ | 1 | 0.5 | 1.01 | 0.51 | -0.00998790296412022 |
| 580 | expm | F1 | array | input | public | expm\_F1\_array\_raw\_public.csv | 2a\_refined\_analysis\_dataset | Array\_1 | Prep\_A | 2 | CreJ | 1 | 0.5 | 1 | 0.49 | 0.00961640829971083 |
| 581 | expm | F1 | array | input | public | expm\_F1\_array\_raw\_public.csv | 2a\_refined\_analysis\_dataset | Array\_1 | Prep\_A | 1 | CreE | 1 | 0.5 | 1.01 | 0.38 | 0.122046702770426 |
| 582 | expm | F1 | array | input | public | expm\_F1\_array\_raw\_public.csv | 2a\_refined\_analysis\_dataset | Array\_1 | Prep\_A | 2 | CreE | 1 | 0.5 | 1.01 | 0.35 | 0.153475381458602 |
| 583 | expm | F1 | array | input | public | expm\_F1\_array\_raw\_public.csv | 2a\_refined\_analysis\_dataset | Array\_2 | Prep\_A | 2 | CreJ | 1 | 0.5 | 1.01 | 0.53 | -0.0312542953093423 |
| 584 | expm | F1 | array | input | public | expm\_F1\_array\_raw\_public.csv | 2a\_refined\_analysis\_dataset | Array\_2 | Prep\_A | 1 | CreJ | 1 | 0.5 | 1.01 | 0.47 | 0.0324321214237016 |
| 585 | expm | F1 | array | input | public | expm\_F1\_array\_raw\_public.csv | 2a\_refined\_analysis\_dataset | Array\_2 | Prep\_A | 2 | CreE | 28 | 3.5 | 27.89 | 4.01 | -0.508782020010599 |
| 586 | expm | F1 | array | input | public | expm\_F1\_array\_raw\_public.csv | 2a\_refined\_analysis\_dataset | Array\_2 | Prep\_A | 1 | CreE | 28 | 3.5 | 28.12 | 4.04 | -0.539456160754719 |
| 587 | expm | F1 | array | input | public | expm\_F1\_array\_raw\_public.csv | 2a\_refined\_analysis\_dataset | Array\_2 | Prep\_A | 2 | CreJ | 28 | 3.5 | 27.89 | 3.91 | -0.410053719327619 |
| 588 | expm | F1 | array | input | public | expm\_F1\_array\_raw\_public.csv | 2a\_refined\_analysis\_dataset | Array\_2 | Prep\_A | 1 | CreJ | 28 | 3.5 | 28.12 | 3.87 | -0.365819740271779 |
| 589 | expm | F1 | array | input | public | expm\_F1\_array\_raw\_public.csv | 2a\_refined\_analysis\_dataset | Array\_1 | Prep\_A | 1 | CreJ | 28 | 3.5 | 27.83 | 3.98 | -0.477786067188732 |
| 590 | expm | F1 | array | input | public | expm\_F1\_array\_raw\_public.csv | 2a\_refined\_analysis\_dataset | Array\_1 | Prep\_A | 2 | CreJ | 28 | 3.5 | 28.16 | 3.94 | -0.438173998753332 |
| 591 | expm | F1 | array | input | public | expm\_F1\_array\_raw\_public.csv | 2a\_refined\_analysis\_dataset | Array\_1 | Prep\_A | 2 | CreE | 28 | 3.5 | 28.16 | 4.01 | -0.510266094123129 |
| 592 | expm | F1 | array | input | public | expm\_F1\_array\_raw\_public.csv | 2a\_refined\_analysis\_dataset | Array\_1 | Prep\_A | 1 | CreE | 28 | 3.5 | 27.83 | 3.98 | -0.478846569217649 |
| 593 | expm | F1 | array | input | public | expm\_F1\_array\_raw\_public.csv | 2a\_refined\_analysis\_dataset | Array\_2 | Prep\_A | 2 | CreJ | 15 | 2 | 15.01 | 2.22 | -0.222909013259233 |
| 594 | expm | F1 | array | input | public | expm\_F1\_array\_raw\_public.csv | 2a\_refined\_analysis\_dataset | Array\_2 | Prep\_A | 1 | CreJ | 15 | 2 | 15.1 | 2.18 | -0.183998022268993 |
| 595 | expm | F1 | array | input | public | expm\_F1\_array\_raw\_public.csv | 2a\_refined\_analysis\_dataset | Array\_2 | Prep\_A | 2 | CreE | 15 | 2 | 15.01 | 2.21 | -0.213762392742466 |
| 596 | expm | F1 | array | input | public | expm\_F1\_array\_raw\_public.csv | 2a\_refined\_analysis\_dataset | Array\_2 | Prep\_A | 1 | CreE | 15 | 2 | 15.1 | 2.2 | -0.200153581829416 |
| 597 | expm | F1 | array | input | public | expm\_F1\_array\_raw\_public.csv | 2a\_refined\_analysis\_dataset | Array\_1 | Prep\_A | 2 | CreJ | 15 | 2 | 14.95 | 2.19 | -0.185063374194024 |
| 598 | expm | F1 | array | input | public | expm\_F1\_array\_raw\_public.csv | 2a\_refined\_analysis\_dataset | Array\_1 | Prep\_A | 1 | CreJ | 15 | 2 | 15.02 | 2.19 | -0.191451389139333 |
| 599 | expm | F1 | array | input | public | expm\_F1\_array\_raw\_public.csv | 2a\_refined\_analysis\_dataset | Array\_1 | Prep\_A | 2 | CreE | 15 | 2 | 14.95 | 2.2 | -0.203347957816516 |
| 600 | expm | F1 | array | input | public | expm\_F1\_array\_raw\_public.csv | 2a\_refined\_analysis\_dataset | Array\_1 | Prep\_A | 1 | CreE | 15 | 2 | 15.02 | 2.21 | -0.208349157375116 |
| 601 | expm | F1 | array | input | public | expm\_F1\_array\_raw\_public.csv | 2a\_refined\_analysis\_dataset | Array\_2 | Prep\_A | 1 | CreJ | 2 | 0.5 | 1.99 | 0.54 | -0.0373245009688943 |
| 602 | expm | F1 | array | input | public | expm\_F1\_array\_raw\_public.csv | 2a\_refined\_analysis\_dataset | Array\_2 | Prep\_A | 2 | CreJ | 2 | 0.5 | 2 | 0.58 | -0.0849655570155613 |
| 603 | expm | F1 | array | input | public | expm\_F1\_array\_raw\_public.csv | 2a\_refined\_analysis\_dataset | Array\_1 | Prep\_A | 2 | CreJ | 2 | 0.5 | 2 | 0.47 | 0.0318680081005279 |
| 604 | expm | F1 | array | input | public | expm\_F1\_array\_raw\_public.csv | 2a\_refined\_analysis\_dataset | Array\_1 | Prep\_A | 1 | CreJ | 2 | 0.5 | 1.98 | 0.53 | -0.0291903193795702 |
| 605 | expm | F1 | array | input | public | expm\_F1\_array\_raw\_public.csv | 2a\_refined\_analysis\_dataset | Array\_2 | Prep\_A | 2 | CreE | 2 | 0.5 | 2 | 0.38 | 0.119032339685936 |
| 606 | expm | F1 | array | input | public | expm\_F1\_array\_raw\_public.csv | 2a\_refined\_analysis\_dataset | Array\_2 | Prep\_A | 1 | CreE | 2 | 0.5 | 1.99 | 0.43 | 0.0713962220375041 |
| 607 | expm | F1 | array | input | public | expm\_F1\_array\_raw\_public.csv | 2a\_refined\_analysis\_dataset | Array\_1 | Prep\_A | 2 | CreE | 2 | 0.5 | 2 | 0.4 | 0.10095689359161 |
| 608 | expm | F1 | array | input | public | expm\_F1\_array\_raw\_public.csv | 2a\_refined\_analysis\_dataset | Array\_1 | Prep\_A | 1 | CreE | 2 | 0.5 | 1.98 | 0.38 | 0.122206358863675 |
| 609 | expm | F1 | array | input | public | expm\_F1\_array\_raw\_public.csv | 2a\_refined\_analysis\_dataset | Array\_2 | Prep\_A | 1 | CreJ | 29 | 3.5 | 29.22 | 3.92 | -0.424704054022082 |
| 610 | expm | F1 | array | input | public | expm\_F1\_array\_raw\_public.csv | 2a\_refined\_analysis\_dataset | Array\_2 | Prep\_A | 2 | CreJ | 29 | 3.5 | 29.05 | 3.87 | -0.366606490074882 |
| 611 | expm | F1 | array | input | public | expm\_F1\_array\_raw\_public.csv | 2a\_refined\_analysis\_dataset | Array\_2 | Prep\_A | 1 | CreE | 29 | 3.5 | 29.22 | 4.06 | -0.556112900658974 |
| 612 | expm | F1 | array | input | public | expm\_F1\_array\_raw\_public.csv | 2a\_refined\_analysis\_dataset | Array\_2 | Prep\_A | 2 | CreE | 29 | 3.5 | 29.05 | 4.04 | -0.535807984860474 |
| 613 | expm | F1 | array | input | public | expm\_F1\_array\_raw\_public.csv | 2a\_refined\_analysis\_dataset | Array\_1 | Prep\_A | 2 | CreE | 29 | 3.5 | 29.07 | 4.03 | -0.529634528167664 |
| 614 | expm | F1 | array | input | public | expm\_F1\_array\_raw\_public.csv | 2a\_refined\_analysis\_dataset | Array\_1 | Prep\_A | 1 | CreE | 29 | 3.5 | 28.59 | 4.06 | -0.559721422931134 |
| 615 | expm | F1 | array | input | public | expm\_F1\_array\_raw\_public.csv | 2a\_refined\_analysis\_dataset | Array\_1 | Prep\_A | 2 | CreJ | 29 | 3.5 | 29.07 | 3.89 | -0.387821582495228 |
| 616 | expm | F1 | array | input | public | expm\_F1\_array\_raw\_public.csv | 2a\_refined\_analysis\_dataset | Array\_1 | Prep\_A | 1 | CreJ | 29 | 3.5 | 28.59 | 4.01 | -0.511890327918067 |
| 617 | expm | F1 | array | input | public | expm\_F1\_array\_raw\_public.csv | 2a\_refined\_analysis\_dataset | Array\_2 | Prep\_A | 2 | CreJ | 16 | 2 | 15.96 | 2.12 | -0.115035380075248 |
| 618 | expm | F1 | array | input | public | expm\_F1\_array\_raw\_public.csv | 2a\_refined\_analysis\_dataset | Array\_2 | Prep\_A | 1 | CreJ | 16 | 2 | 16.08 | 2.27 | -0.268826198818047 |
| 619 | expm | F1 | array | input | public | expm\_F1\_array\_raw\_public.csv | 2a\_refined\_analysis\_dataset | Array\_1 | Prep\_A | 2 | CreJ | 16 | 2 | 15.9 | 2.22 | -0.22053232868309 |
| 620 | expm | F1 | array | input | public | expm\_F1\_array\_raw\_public.csv | 2a\_refined\_analysis\_dataset | Array\_1 | Prep\_A | 1 | CreJ | 16 | 2 | 15.88 | 2.17 | -0.17108536994122 |
| 621 | expm | F1 | array | input | public | expm\_F1\_array\_raw\_public.csv | 2a\_refined\_analysis\_dataset | Array\_1 | Prep\_A | 2 | CreE | 16 | 2 | 15.9 | 2.25 | -0.24537254258036 |
| 622 | expm | F1 | array | input | public | expm\_F1\_array\_raw\_public.csv | 2a\_refined\_analysis\_dataset | Array\_1 | Prep\_A | 1 | CreE | 16 | 2 | 15.89 | 2.2 | -0.198942707168141 |
| 623 | expm | F1 | array | input | public | expm\_F1\_array\_raw\_public.csv | 2a\_refined\_analysis\_dataset | Array\_2 | Prep\_A | 1 | CreE | 16 | 2 | 16.08 | 2.22 | -0.21563986953004 |
| 624 | expm | F1 | array | input | public | expm\_F1\_array\_raw\_public.csv | 2a\_refined\_analysis\_dataset | Array\_2 | Prep\_A | 2 | CreE | 16 | 2 | 15.97 | 2.23 | -0.226992998342521 |
| 625 | expm | F1 | array | input | public | expm\_F1\_array\_raw\_public.csv | 2a\_refined\_analysis\_dataset | Array\_2 | Prep\_A | 1 | CreE | 3 | 0.5 | 3.01 | 0.36 | 0.136696575254697 |
| 626 | expm | F1 | array | input | public | expm\_F1\_array\_raw\_public.csv | 2a\_refined\_analysis\_dataset | Array\_2 | Prep\_A | 2 | CreE | 3 | 0.5 | 2.98 | 0.36 | 0.138847665584461 |
| 627 | expm | F1 | array | input | public | expm\_F1\_array\_raw\_public.csv | 2a\_refined\_analysis\_dataset | Array\_2 | Prep\_A | 2 | CreJ | 3 | 0.5 | 2.98 | 0.6 | -0.0974954880896633 |
| 628 | expm | F1 | array | input | public | expm\_F1\_array\_raw\_public.csv | 2a\_refined\_analysis\_dataset | Array\_2 | Prep\_A | 1 | CreJ | 3 | 0.5 | 3.01 | 0.5 | -0.0018048141968543 |
| 629 | expm | F1 | array | input | public | expm\_F1\_array\_raw\_public.csv | 2a\_refined\_analysis\_dataset | Array\_1 | Prep\_A | 2 | CreE | 3 | 0.5 | 3.01 | 0.39 | 0.10984344619205 |
| 630 | expm | F1 | array | input | public | expm\_F1\_array\_raw\_public.csv | 2a\_refined\_analysis\_dataset | Array\_1 | Prep\_A | 1 | CreE | 3 | 0.5 | 3.02 | 0.43 | 0.0736233666390028 |
| 631 | expm | F1 | array | input | public | expm\_F1\_array\_raw\_public.csv | 2a\_refined\_analysis\_dataset | Array\_1 | Prep\_A | 2 | CreJ | 3 | 0.5 | 3.01 | 0.54 | -0.0354797134223519 |
| 632 | expm | F1 | array | input | public | expm\_F1\_array\_raw\_public.csv | 2a\_refined\_analysis\_dataset | Array\_1 | Prep\_A | 1 | CreJ | 3 | 0.5 | 3.02 | 0.49 | 0.00621968817075208 |
| 633 | expm | F1 | array | input | public | expm\_F1\_array\_raw\_public.csv | 2a\_refined\_analysis\_dataset | Array\_1 | Prep\_A | 2 | CreE | 30 | 3.5 | 29.8 | 4.06 | -0.562771146790162 |
| 634 | expm | F1 | array | input | public | expm\_F1\_array\_raw\_public.csv | 2a\_refined\_analysis\_dataset | Array\_1 | Prep\_A | 1 | CreE | 30 | 3.5 | 30.22 | 3.99 | -0.485831449287202 |
| 635 | expm | F1 | array | input | public | expm\_F1\_array\_raw\_public.csv | 2a\_refined\_analysis\_dataset | Array\_1 | Prep\_A | 2 | CreJ | 30 | 3.5 | 29.8 | 3.99 | -0.485656221414741 |
| 636 | expm | F1 | array | input | public | expm\_F1\_array\_raw\_public.csv | 2a\_refined\_analysis\_dataset | Array\_1 | Prep\_A | 1 | CreJ | 30 | 3.5 | 30.22 | 3.88 | -0.379586183932671 |
| 637 | expm | F1 | array | input | public | expm\_F1\_array\_raw\_public.csv | 2a\_refined\_analysis\_dataset | Array\_2 | Prep\_A | 2 | CreJ | 30 | 3.5 | 30.16 | 3.96 | -0.457863455226649 |
| 638 | expm | F1 | array | input | public | expm\_F1\_array\_raw\_public.csv | 2a\_refined\_analysis\_dataset | Array\_2 | Prep\_A | 1 | CreJ | 30 | 3.5 | 30.18 | 3.91 | -0.405726302910288 |
| 639 | expm | F1 | array | input | public | expm\_F1\_array\_raw\_public.csv | 2a\_refined\_analysis\_dataset | Array\_2 | Prep\_A | 1 | CreE | 30 | 3.5 | 30.18 | 4.03 | -0.531527530508642 |
| 640 | expm | F1 | array | input | public | expm\_F1\_array\_raw\_public.csv | 2a\_refined\_analysis\_dataset | Array\_2 | Prep\_A | 2 | CreE | 30 | 3.5 | 30.16 | 4.04 | -0.543553159623622 |
| 641 | expm | F1 | array | input | public | expm\_F1\_array\_raw\_public.csv | 2a\_refined\_analysis\_dataset | Array\_1 | Prep\_A | 2 | CreJ | 17 | 2 | 17.14 | 2.29 | -0.289609899143169 |
| 642 | expm | F1 | array | input | public | expm\_F1\_array\_raw\_public.csv | 2a\_refined\_analysis\_dataset | Array\_1 | Prep\_A | 1 | CreJ | 17 | 2 | 16.95 | 2.22 | -0.217426197160549 |
| 643 | expm | F1 | array | input | public | expm\_F1\_array\_raw\_public.csv | 2a\_refined\_analysis\_dataset | Array\_2 | Prep\_A | 1 | CreE | 17 | 2 | 17.02 | 2.21 | -0.21440893091272 |
| 644 | expm | F1 | array | input | public | expm\_F1\_array\_raw\_public.csv | 2a\_refined\_analysis\_dataset | Array\_2 | Prep\_A | 2 | CreE | 17 | 2 | 16.97 | 2.26 | -0.257967430645619 |
| 645 | expm | F1 | array | input | public | expm\_F1\_array\_raw\_public.csv | 2a\_refined\_analysis\_dataset | Array\_2 | Prep\_A | 1 | CreJ | 17 | 2 | 17.02 | 2.28 | -0.276008965350695 |
| 646 | expm | F1 | array | input | public | expm\_F1\_array\_raw\_public.csv | 2a\_refined\_analysis\_dataset | Array\_2 | Prep\_A | 2 | CreJ | 17 | 2 | 16.96 | 2.18 | -0.179782719780995 |
| 647 | expm | F1 | array | input | public | expm\_F1\_array\_raw\_public.csv | 2a\_refined\_analysis\_dataset | Array\_1 | Prep\_A | 1 | CreE | 17 | 2 | 16.95 | 2.24 | -0.24224884450566 |
| 648 | expm | F1 | array | input | public | expm\_F1\_array\_raw\_public.csv | 2a\_refined\_analysis\_dataset | Array\_1 | Prep\_A | 2 | CreE | 17 | 2 | 17.14 | 2.22 | -0.2211209205583 |
| 649 | expm | F1 | array | input | public | expm\_F1\_array\_raw\_public.csv | 2a\_refined\_analysis\_dataset | Array\_2 | Prep\_A | 1 | CreE | 4 | 0.5 | 4.04 | 0.44 | 0.0595097060494855 |
| 650 | expm | F1 | array | input | public | expm\_F1\_array\_raw\_public.csv | 2a\_refined\_analysis\_dataset | Array\_2 | Prep\_A | 2 | CreE | 4 | 0.5 | 4.05 | 0.42 | 0.0811848296202585 |
| 651 | expm | F1 | array | input | public | expm\_F1\_array\_raw\_public.csv | 2a\_refined\_analysis\_dataset | Array\_1 | Prep\_A | 2 | CreJ | 4 | 0.5 | 4.03 | 0.56 | -0.0552418022289802 |
| 652 | expm | F1 | array | input | public | expm\_F1\_array\_raw\_public.csv | 2a\_refined\_analysis\_dataset | Array\_1 | Prep\_A | 1 | CreJ | 4 | 0.5 | 4.03 | 0.54 | -0.0409632318606322 |
| 653 | expm | F1 | array | input | public | expm\_F1\_array\_raw\_public.csv | 2a\_refined\_analysis\_dataset | Array\_2 | Prep\_A | 1 | CreJ | 4 | 0.5 | 4.04 | 0.52 | -0.0177421629077716 |
| 654 | expm | F1 | array | input | public | expm\_F1\_array\_raw\_public.csv | 2a\_refined\_analysis\_dataset | Array\_2 | Prep\_A | 2 | CreJ | 4 | 0.5 | 4.05 | 0.55 | -0.0451641376129727 |
| 655 | expm | F1 | array | input | public | expm\_F1\_array\_raw\_public.csv | 2a\_refined\_analysis\_dataset | Array\_1 | Prep\_A | 2 | CreE | 4 | 0.5 | 4.03 | 0.43 | 0.0723624587231365 |
| 656 | expm | F1 | array | input | public | expm\_F1\_array\_raw\_public.csv | 2a\_refined\_analysis\_dataset | Array\_1 | Prep\_A | 1 | CreE | 4 | 0.5 | 4.03 | 0.37 | 0.131087635177926 |
| 657 | expm | F1 | array | input | public | expm\_F1\_array\_raw\_public.csv | 2a\_refined\_analysis\_dataset | Array\_1 | Prep\_A | 1 | CreE | 31 | 3.5 | 31.37 | 4.06 | -0.563042086686483 |
| 658 | expm | F1 | array | input | public | expm\_F1\_array\_raw\_public.csv | 2a\_refined\_analysis\_dataset | Array\_1 | Prep\_A | 2 | CreE | 31 | 3.5 | 30.84 | 4.07 | -0.567791247396094 |
| 659 | expm | F1 | array | input | public | expm\_F1\_array\_raw\_public.csv | 2a\_refined\_analysis\_dataset | Array\_1 | Prep\_A | 2 | CreJ | 31 | 3.5 | 30.84 | 3.92 | -0.417083740649254 |
| 660 | expm | F1 | array | input | public | expm\_F1\_array\_raw\_public.csv | 2a\_refined\_analysis\_dataset | Array\_1 | Prep\_A | 1 | CreJ | 31 | 3.5 | 31.37 | 3.97 | -0.470357512924414 |
| 661 | expm | F1 | array | input | public | expm\_F1\_array\_raw\_public.csv | 2a\_refined\_analysis\_dataset | Array\_2 | Prep\_A | 2 | CreJ | 31 | 3.5 | 31.19 | 3.93 | -0.428097841399609 |
| 662 | expm | F1 | array | input | public | expm\_F1\_array\_raw\_public.csv | 2a\_refined\_analysis\_dataset | Array\_2 | Prep\_A | 1 | CreJ | 31 | 3.5 | 30.9 | 3.95 | -0.449497495784679 |
| 663 | expm | F1 | array | input | public | expm\_F1\_array\_raw\_public.csv | 2a\_refined\_analysis\_dataset | Array\_2 | Prep\_A | 2 | CreE | 31 | 3.5 | 31.19 | 4.07 | -0.565412772766063 |
| 664 | expm | F1 | array | input | public | expm\_F1\_array\_raw\_public.csv | 2a\_refined\_analysis\_dataset | Array\_2 | Prep\_A | 1 | CreE | 31 | 3.5 | 30.89 | 4.04 | -0.544384828053453 |
| 665 | expm | F1 | array | input | public | expm\_F1\_array\_raw\_public.csv | 2a\_refined\_analysis\_dataset | Array\_2 | Prep\_A | 1 | CreE | 18 | 2 | 18.1 | 2.26 | -0.256462261770245 |
| 666 | expm | F1 | array | input | public | expm\_F1\_array\_raw\_public.csv | 2a\_refined\_analysis\_dataset | Array\_2 | Prep\_A | 2 | CreE | 18 | 2 | 18.33 | 2.22 | -0.221732160400526 |
| 667 | expm | F1 | array | input | public | expm\_F1\_array\_raw\_public.csv | 2a\_refined\_analysis\_dataset | Array\_1 | Prep\_A | 1 | CreE | 18 | 2 | 18.04 | 2.26 | -0.255327300252855 |
| 668 | expm | F1 | array | input | public | expm\_F1\_array\_raw\_public.csv | 2a\_refined\_analysis\_dataset | Array\_1 | Prep\_A | 2 | CreE | 18 | 2 | 18.1 | 2.23 | -0.225930564722446 |
| 669 | expm | F1 | array | input | public | expm\_F1\_array\_raw\_public.csv | 2a\_refined\_analysis\_dataset | Array\_2 | Prep\_A | 1 | CreJ | 18 | 2 | 18.1 | 2.19 | -0.190083547177928 |
| 670 | expm | F1 | array | input | public | expm\_F1\_array\_raw\_public.csv | 2a\_refined\_analysis\_dataset | Array\_2 | Prep\_A | 2 | CreJ | 18 | 2 | 18.32 | 2.21 | -0.206265207731268 |
| 671 | expm | F1 | array | input | public | expm\_F1\_array\_raw\_public.csv | 2a\_refined\_analysis\_dataset | Array\_1 | Prep\_A | 2 | CreJ | 18 | 2 | 18.1 | 2.23 | -0.225302756886284 |
| 672 | expm | F1 | array | input | public | expm\_F1\_array\_raw\_public.csv | 2a\_refined\_analysis\_dataset | Array\_1 | Prep\_A | 1 | CreJ | 18 | 2 | 18.04 | 2.17 | -0.167685308663844 |
| 673 | expm | F1 | array | input | public | expm\_F1\_array\_raw\_public.csv | 2a\_refined\_analysis\_dataset | Array\_2 | Prep\_A | 2 | CreJ | 5 | 0.5 | 5.06 | 0.53 | -0.0275414537810853 |
| 674 | expm | F1 | array | input | public | expm\_F1\_array\_raw\_public.csv | 2a\_refined\_analysis\_dataset | Array\_2 | Prep\_A | 1 | CreJ | 5 | 0.5 | 5 | 0.56 | -0.0612572862253094 |
| 675 | expm | F1 | array | input | public | expm\_F1\_array\_raw\_public.csv | 2a\_refined\_analysis\_dataset | Array\_1 | Prep\_A | 2 | CreE | 5 | 0.5 | 4.98 | 0.38 | 0.122447125072096 |
| 676 | expm | F1 | array | input | public | expm\_F1\_array\_raw\_public.csv | 2a\_refined\_analysis\_dataset | Array\_1 | Prep\_A | 1 | CreE | 5 | 0.5 | 5.01 | 0.44 | 0.0582115843792568 |
| 677 | expm | F1 | array | input | public | expm\_F1\_array\_raw\_public.csv | 2a\_refined\_analysis\_dataset | Array\_1 | Prep\_A | 1 | CreJ | 5 | 0.5 | 5.01 | 0.54 | -0.0444950710641624 |
| 678 | expm | F1 | array | input | public | expm\_F1\_array\_raw\_public.csv | 2a\_refined\_analysis\_dataset | Array\_1 | Prep\_A | 2 | CreJ | 5 | 0.5 | 4.98 | 0.54 | -0.0438428486723134 |
| 679 | expm | F1 | array | input | public | expm\_F1\_array\_raw\_public.csv | 2a\_refined\_analysis\_dataset | Array\_2 | Prep\_A | 1 | CreE | 5 | 0.5 | 5 | 0.41 | 0.0888886561800798 |
| 680 | expm | F1 | array | input | public | expm\_F1\_array\_raw\_public.csv | 2a\_refined\_analysis\_dataset | Array\_2 | Prep\_A | 2 | CreE | 5 | 0.5 | 5.06 | 0.44 | 0.0594918719395108 |
| 681 | expm | F1 | array | input | public | expm\_F1\_array\_raw\_public.csv | 2a\_refined\_analysis\_dataset | Array\_1 | Prep\_A | 1 | CreE | 32 | 3.5 | 31.92 | 4.07 | -0.571709904640726 |
| 682 | expm | F1 | array | input | public | expm\_F1\_array\_raw\_public.csv | 2a\_refined\_analysis\_dataset | Array\_1 | Prep\_A | 2 | CreE | 32 | 3.5 | 32.02 | 4.09 | -0.585070287570816 |
| 683 | expm | F1 | array | input | public | expm\_F1\_array\_raw\_public.csv | 2a\_refined\_analysis\_dataset | Array\_2 | Prep\_A | 1 | CreE | 32 | 3.5 | 32.08 | 4.07 | -0.574697320751216 |
| 684 | expm | F1 | array | input | public | expm\_F1\_array\_raw\_public.csv | 2a\_refined\_analysis\_dataset | Array\_2 | Prep\_A | 2 | CreE | 32 | 3.5 | 31.79 | 4.08 | -0.581832974954346 |
| 685 | expm | F1 | array | input | public | expm\_F1\_array\_raw\_public.csv | 2a\_refined\_analysis\_dataset | Array\_2 | Prep\_A | 2 | CreJ | 32 | 3.5 | 31.79 | 3.88 | -0.384412186564778 |
| 686 | expm | F1 | array | input | public | expm\_F1\_array\_raw\_public.csv | 2a\_refined\_analysis\_dataset | Array\_2 | Prep\_A | 1 | CreJ | 32 | 3.5 | 32.08 | 3.87 | -0.370212518928398 |
| 687 | expm | F1 | array | input | public | expm\_F1\_array\_raw\_public.csv | 2a\_refined\_analysis\_dataset | Array\_1 | Prep\_A | 2 | CreJ | 32 | 3.5 | 32.02 | 3.96 | -0.456502645461475 |
| 688 | expm | F1 | array | input | public | expm\_F1\_array\_raw\_public.csv | 2a\_refined\_analysis\_dataset | Array\_1 | Prep\_A | 1 | CreJ | 32 | 3.5 | 31.93 | 3.99 | -0.485846281069195 |
| 689 | expm | F1 | array | input | public | expm\_F1\_array\_raw\_public.csv | 2a\_refined\_analysis\_dataset | Array\_1 | Prep\_A | 1 | CreJ | 19 | 2 | 19.11 | 2.12 | -0.119227882739136 |
| 690 | expm | F1 | array | input | public | expm\_F1\_array\_raw\_public.csv | 2a\_refined\_analysis\_dataset | Array\_1 | Prep\_A | 2 | CreJ | 19 | 2 | 19.04 | 2.16 | -0.162066986522396 |
| 691 | expm | F1 | array | input | public | expm\_F1\_array\_raw\_public.csv | 2a\_refined\_analysis\_dataset | Array\_1 | Prep\_A | 1 | CreE | 19 | 2 | 19.11 | 2.25 | -0.254864152216572 |
| 692 | expm | F1 | array | input | public | expm\_F1\_array\_raw\_public.csv | 2a\_refined\_analysis\_dataset | Array\_1 | Prep\_A | 2 | CreE | 19 | 2 | 19.04 | 2.26 | -0.260255995337622 |
| 693 | expm | F1 | array | input | public | expm\_F1\_array\_raw\_public.csv | 2a\_refined\_analysis\_dataset | Array\_2 | Prep\_A | 1 | CreJ | 19 | 2 | 19.18 | 2.16 | -0.161175270201437 |
| 694 | expm | F1 | array | input | public | expm\_F1\_array\_raw\_public.csv | 2a\_refined\_analysis\_dataset | Array\_2 | Prep\_A | 2 | CreJ | 19 | 2 | 19.07 | 2.14 | -0.142544432566397 |
| 695 | expm | F1 | array | input | public | expm\_F1\_array\_raw\_public.csv | 2a\_refined\_analysis\_dataset | Array\_2 | Prep\_A | 1 | CreE | 19 | 2 | 19.18 | 2.25 | -0.251038116042202 |
| 696 | expm | F1 | array | input | public | expm\_F1\_array\_raw\_public.csv | 2a\_refined\_analysis\_dataset | Array\_2 | Prep\_A | 2 | CreE | 19 | 2 | 19.07 | 2.27 | -0.270515093234232 |
| 697 | expm | F1 | array | input | public | expm\_F1\_array\_raw\_public.csv | 2a\_refined\_analysis\_dataset | Array\_2 | Prep\_A | 2 | CreE | 6 | 0.5 | 6.04 | 0.46 | 0.0391297459266032 |
| 698 | expm | F1 | array | input | public | expm\_F1\_array\_raw\_public.csv | 2a\_refined\_analysis\_dataset | Array\_2 | Prep\_A | 1 | CreE | 6 | 0.5 | 5.98 | 0.41 | 0.0858336272716113 |
| 699 | expm | F1 | array | input | public | expm\_F1\_array\_raw\_public.csv | 2a\_refined\_analysis\_dataset | Array\_1 | Prep\_A | 1 | CreJ | 6 | 0.5 | 6 | 0.58 | -0.0795698036477661 |
| 700 | expm | F1 | array | input | public | expm\_F1\_array\_raw\_public.csv | 2a\_refined\_analysis\_dataset | Array\_1 | Prep\_A | 2 | CreJ | 6 | 0.5 | 6.02 | 0.52 | -0.023571247026493 |
| 701 | expm | F1 | array | input | public | expm\_F1\_array\_raw\_public.csv | 2a\_refined\_analysis\_dataset | Array\_1 | Prep\_A | 2 | CreE | 6 | 0.5 | 6.03 | 0.43 | 0.0654489806131433 |
| 702 | expm | F1 | array | input | public | expm\_F1\_array\_raw\_public.csv | 2a\_refined\_analysis\_dataset | Array\_1 | Prep\_A | 1 | CreE | 6 | 0.5 | 6 | 0.4 | 0.100381485458187 |
| 703 | expm | F1 | array | input | public | expm\_F1\_array\_raw\_public.csv | 2a\_refined\_analysis\_dataset | Array\_2 | Prep\_A | 2 | CreJ | 6 | 0.5 | 6.04 | 0.58 | -0.0791925366702186 |
| 704 | expm | F1 | array | input | public | expm\_F1\_array\_raw\_public.csv | 2a\_refined\_analysis\_dataset | Array\_2 | Prep\_A | 1 | CreJ | 6 | 0.5 | 5.98 | 0.58 | -0.0836858956584016 |
| 705 | expm | F1 | array | input | public | expm\_F1\_array\_raw\_public.csv | 2a\_refined\_analysis\_dataset | Array\_2 | Prep\_A | 2 | CreE | 33 | 3.5 | 33.44 | 4.09 | -0.586008905480046 |
| 706 | expm | F1 | array | input | public | expm\_F1\_array\_raw\_public.csv | 2a\_refined\_analysis\_dataset | Array\_2 | Prep\_A | 1 | CreE | 33 | 3.5 | 33.02 | 4.07 | -0.565890159587116 |
| 707 | expm | F1 | array | input | public | expm\_F1\_array\_raw\_public.csv | 2a\_refined\_analysis\_dataset | Array\_1 | Prep\_A | 2 | CreJ | 33 | 3.5 | 32.92 | 3.9 | -0.402564459887619 |
| 708 | expm | F1 | array | input | public | expm\_F1\_array\_raw\_public.csv | 2a\_refined\_analysis\_dataset | Array\_1 | Prep\_A | 1 | CreJ | 33 | 3.5 | 33.01 | 3.95 | -0.450186792128519 |
| 709 | expm | F1 | array | input | public | expm\_F1\_array\_raw\_public.csv | 2a\_refined\_analysis\_dataset | Array\_1 | Prep\_A | 2 | CreE | 33 | 3.5 | 32.91 | 4.08 | -0.579901811380837 |
| 710 | expm | F1 | array | input | public | expm\_F1\_array\_raw\_public.csv | 2a\_refined\_analysis\_dataset | Array\_1 | Prep\_A | 1 | CreE | 33 | 3.5 | 33.01 | 4.1 | -0.601178528816637 |
| 711 | expm | F1 | array | input | public | expm\_F1\_array\_raw\_public.csv | 2a\_refined\_analysis\_dataset | Array\_2 | Prep\_A | 1 | CreJ | 33 | 3.5 | 33.02 | 3.85 | -0.345237487534129 |
| 712 | expm | F1 | array | input | public | expm\_F1\_array\_raw\_public.csv | 2a\_refined\_analysis\_dataset | Array\_2 | Prep\_A | 2 | CreJ | 33 | 3.5 | 33.44 | 3.85 | -0.349193005300589 |
| 713 | expm | F1 | array | input | public | expm\_F1\_array\_raw\_public.csv | 2a\_refined\_analysis\_dataset | Array\_1 | Prep\_A | 2 | CreE | 20 | 2 | 19.97 | 2.27 | -0.271418099640639 |
| 714 | expm | F1 | array | input | public | expm\_F1\_array\_raw\_public.csv | 2a\_refined\_analysis\_dataset | Array\_1 | Prep\_A | 1 | CreE | 20 | 2 | 19.89 | 2.29 | -0.29273257453282 |
| 715 | expm | F1 | array | input | public | expm\_F1\_array\_raw\_public.csv | 2a\_refined\_analysis\_dataset | Array\_2 | Prep\_A | 2 | CreE | 20 | 2 | 20.21 | 2.28 | -0.2760802388541 |
| 716 | expm | F1 | array | input | public | expm\_F1\_array\_raw\_public.csv | 2a\_refined\_analysis\_dataset | Array\_2 | Prep\_A | 1 | CreE | 20 | 2 | 20.1 | 2.24 | -0.235923974711049 |
| 717 | expm | F1 | array | input | public | expm\_F1\_array\_raw\_public.csv | 2a\_refined\_analysis\_dataset | Array\_2 | Prep\_A | 2 | CreJ | 20 | 2 | 20.21 | 2.14 | -0.143429440908714 |
| 718 | expm | F1 | array | input | public | expm\_F1\_array\_raw\_public.csv | 2a\_refined\_analysis\_dataset | Array\_2 | Prep\_A | 1 | CreJ | 20 | 2 | 20.1 | 2.19 | -0.191749028685765 |
| 719 | expm | F1 | array | input | public | expm\_F1\_array\_raw\_public.csv | 2a\_refined\_analysis\_dataset | Array\_1 | Prep\_A | 2 | CreJ | 20 | 2 | 19.97 | 2.19 | -0.190139922271974 |
| 720 | expm | F1 | array | input | public | expm\_F1\_array\_raw\_public.csv | 2a\_refined\_analysis\_dataset | Array\_1 | Prep\_A | 1 | CreJ | 20 | 2 | 19.89 | 2.17 | -0.169857857963564 |
| 721 | expm | F1 | array | input | public | expm\_F1\_array\_raw\_public.csv | 2a\_refined\_analysis\_dataset | Array\_1 | Prep\_A | 2 | CreJ | 7 | 0.5 | 7.05 | 0.58 | -0.0842962030690657 |
| 722 | expm | F1 | array | input | public | expm\_F1\_array\_raw\_public.csv | 2a\_refined\_analysis\_dataset | Array\_1 | Prep\_A | 1 | CreJ | 7 | 0.5 | 7.02 | 0.57 | -0.0666695577838817 |
| 723 | expm | F1 | array | input | public | expm\_F1\_array\_raw\_public.csv | 2a\_refined\_analysis\_dataset | Array\_2 | Prep\_A | 2 | CreE | 7 | 0.5 | 7 | 0.41 | 0.088738869736622 |
| 724 | expm | F1 | array | input | public | expm\_F1\_array\_raw\_public.csv | 2a\_refined\_analysis\_dataset | Array\_2 | Prep\_A | 1 | CreE | 7 | 0.5 | 7.09 | 0.44 | 0.063320145708831 |
| 725 | expm | F1 | array | input | public | expm\_F1\_array\_raw\_public.csv | 2a\_refined\_analysis\_dataset | Array\_1 | Prep\_A | 2 | CreE | 7 | 0.5 | 7.05 | 0.48 | 0.024751025396538 |
| 726 | expm | F1 | array | input | public | expm\_F1\_array\_raw\_public.csv | 2a\_refined\_analysis\_dataset | Array\_1 | Prep\_A | 1 | CreE | 7 | 0.5 | 7.02 | 0.46 | 0.043615115916233 |
| 727 | expm | F1 | array | input | public | expm\_F1\_array\_raw\_public.csv | 2a\_refined\_analysis\_dataset | Array\_2 | Prep\_A | 1 | CreJ | 7 | 0.5 | 7.08 | 0.46 | 0.043602421172076 |
| 728 | expm | F1 | array | input | public | expm\_F1\_array\_raw\_public.csv | 2a\_refined\_analysis\_dataset | Array\_2 | Prep\_A | 2 | CreJ | 7 | 0.5 | 7 | 0.52 | -0.017347358147776 |
| 729 | expm | F1 | array | input | public | expm\_F1\_array\_raw\_public.csv | 2a\_refined\_analysis\_dataset | Array\_2 | Prep\_A | 2 | CreJ | 34 | 3.5 | 33.92 | 3.78 | -0.281699074969339 |
| 730 | expm | F1 | array | input | public | expm\_F1\_array\_raw\_public.csv | 2a\_refined\_analysis\_dataset | Array\_2 | Prep\_A | 1 | CreJ | 34 | 3.5 | 33.92 | 3.81 | -0.308610171256849 |
| 731 | expm | F1 | array | input | public | expm\_F1\_array\_raw\_public.csv | 2a\_refined\_analysis\_dataset | Array\_1 | Prep\_A | 1 | CreE | 34 | 3.5 | 33.92 | 4.13 | -0.62692119812779 |
| 732 | expm | F1 | array | input | public | expm\_F1\_array\_raw\_public.csv | 2a\_refined\_analysis\_dataset | Array\_1 | Prep\_A | 2 | CreE | 34 | 3.5 | 34 | 4.12 | -0.61716742920497 |
| 733 | expm | F1 | array | input | public | expm\_F1\_array\_raw\_public.csv | 2a\_refined\_analysis\_dataset | Array\_2 | Prep\_A | 2 | CreE | 34 | 3.5 | 33.92 | 4.08 | -0.57522772776813 |
| 734 | expm | F1 | array | input | public | expm\_F1\_array\_raw\_public.csv | 2a\_refined\_analysis\_dataset | Array\_2 | Prep\_A | 1 | CreE | 34 | 3.5 | 33.93 | 4.13 | -0.63009510320328 |
| 735 | expm | F1 | array | input | public | expm\_F1\_array\_raw\_public.csv | 2a\_refined\_analysis\_dataset | Array\_1 | Prep\_A | 2 | CreJ | 34 | 3.5 | 34 | 3.9 | -0.403837137644445 |
| 736 | expm | F1 | array | input | public | expm\_F1\_array\_raw\_public.csv | 2a\_refined\_analysis\_dataset | Array\_1 | Prep\_A | 1 | CreJ | 34 | 3.5 | 33.92 | 3.96 | -0.462593380192674 |
| 737 | expm | F1 | array | input | public | expm\_F1\_array\_raw\_public.csv | 2a\_refined\_analysis\_dataset | Array\_2 | Prep\_A | 1 | CreJ | 21 | 2 | 21.03 | 2.18 | -0.178967115660036 |
| 738 | expm | F1 | array | input | public | expm\_F1\_array\_raw\_public.csv | 2a\_refined\_analysis\_dataset | Array\_2 | Prep\_A | 2 | CreJ | 21 | 2 | 21.03 | 2.14 | -0.136514671808026 |
| 739 | expm | F1 | array | input | public | expm\_F1\_array\_raw\_public.csv | 2a\_refined\_analysis\_dataset | Array\_2 | Prep\_A | 2 | CreE | 21 | 2 | 21.03 | 2.26 | -0.259939771364015 |
| 740 | expm | F1 | array | input | public | expm\_F1\_array\_raw\_public.csv | 2a\_refined\_analysis\_dataset | Array\_2 | Prep\_A | 1 | CreE | 21 | 2 | 21.03 | 2.23 | -0.234038312113664 |
| 741 | expm | F1 | array | input | public | expm\_F1\_array\_raw\_public.csv | 2a\_refined\_analysis\_dataset | Array\_1 | Prep\_A | 2 | CreE | 21 | 2 | 21.08 | 2.28 | -0.278699815102304 |
| 742 | expm | F1 | array | input | public | expm\_F1\_array\_raw\_public.csv | 2a\_refined\_analysis\_dataset | Array\_1 | Prep\_A | 1 | CreE | 21 | 2 | 21.11 | 2.26 | -0.262906322657814 |
| 743 | expm | F1 | array | input | public | expm\_F1\_array\_raw\_public.csv | 2a\_refined\_analysis\_dataset | Array\_1 | Prep\_A | 2 | CreJ | 21 | 2 | 21.08 | 2.19 | -0.191354494855835 |
| 744 | expm | F1 | array | input | public | expm\_F1\_array\_raw\_public.csv | 2a\_refined\_analysis\_dataset | Array\_1 | Prep\_A | 1 | CreJ | 21 | 2 | 21.11 | 2.19 | -0.185111287046945 |
| 745 | expm | F1 | array | input | public | expm\_F1\_array\_raw\_public.csv | 2a\_refined\_analysis\_dataset | Array\_2 | Prep\_A | 2 | CreE | 8 | 0.5 | 7.98 | 0.43 | 0.0730030982139208 |
| 746 | expm | F1 | array | input | public | expm\_F1\_array\_raw\_public.csv | 2a\_refined\_analysis\_dataset | Array\_2 | Prep\_A | 1 | CreE | 8 | 0.5 | 8.02 | 0.48 | 0.0186464782595728 |
| 747 | expm | F1 | array | input | public | expm\_F1\_array\_raw\_public.csv | 2a\_refined\_analysis\_dataset | Array\_2 | Prep\_A | 2 | CreJ | 8 | 0.5 | 7.98 | 0.56 | -0.0588259144918298 |
| 748 | expm | F1 | array | input | public | expm\_F1\_array\_raw\_public.csv | 2a\_refined\_analysis\_dataset | Array\_2 | Prep\_A | 1 | CreJ | 8 | 0.5 | 8.02 | 0.57 | -0.0707014789943229 |
| 749 | expm | F1 | array | input | public | expm\_F1\_array\_raw\_public.csv | 2a\_refined\_analysis\_dataset | Array\_1 | Prep\_A | 1 | CreJ | 8 | 0.5 | 8.04 | 0.54 | -0.0388167114240883 |
| 750 | expm | F1 | array | input | public | expm\_F1\_array\_raw\_public.csv | 2a\_refined\_analysis\_dataset | Array\_1 | Prep\_A | 2 | CreJ | 8 | 0.5 | 8.04 | 0.61 | -0.113247196066779 |
| 751 | expm | F1 | array | input | public | expm\_F1\_array\_raw\_public.csv | 2a\_refined\_analysis\_dataset | Array\_1 | Prep\_A | 1 | CreE | 8 | 0.5 | 8.04 | 0.46 | 0.0427195624407108 |
| 752 | expm | F1 | array | input | public | expm\_F1\_array\_raw\_public.csv | 2a\_refined\_analysis\_dataset | Array\_1 | Prep\_A | 2 | CreE | 8 | 0.5 | 8.04 | 0.41 | 0.0933451598298718 |
| 753 | expm | F1 | array | input | public | expm\_F1\_array\_raw\_public.csv | 2a\_refined\_analysis\_dataset | Array\_1 | Prep\_A | 2 | CreJ | 35 | 3.5 | 34.99 | 3.91 | -0.406541059921764 |
| 754 | expm | F1 | array | input | public | expm\_F1\_array\_raw\_public.csv | 2a\_refined\_analysis\_dataset | Array\_1 | Prep\_A | 1 | CreJ | 35 | 3.5 | 35.19 | 3.96 | -0.455825545025294 |
| 755 | expm | F1 | array | input | public | expm\_F1\_array\_raw\_public.csv | 2a\_refined\_analysis\_dataset | Array\_2 | Prep\_A | 2 | CreJ | 35 | 3.5 | 34.78 | 3.89 | -0.393146497235008 |
| 756 | expm | F1 | array | input | public | expm\_F1\_array\_raw\_public.csv | 2a\_refined\_analysis\_dataset | Array\_2 | Prep\_A | 1 | CreJ | 35 | 3.5 | 34.89 | 3.81 | -0.308907192905768 |
| 757 | expm | F1 | array | input | public | expm\_F1\_array\_raw\_public.csv | 2a\_refined\_analysis\_dataset | Array\_2 | Prep\_A | 2 | CreE | 35 | 3.5 | 34.78 | 4.1 | -0.599901908260596 |
| 758 | expm | F1 | array | input | public | expm\_F1\_array\_raw\_public.csv | 2a\_refined\_analysis\_dataset | Array\_2 | Prep\_A | 1 | CreE | 35 | 3.5 | 34.89 | 4.11 | -0.611305867837765 |
| 759 | expm | F1 | array | input | public | expm\_F1\_array\_raw\_public.csv | 2a\_refined\_analysis\_dataset | Array\_1 | Prep\_A | 1 | CreE | 35 | 3.5 | 35.19 | 4.09 | -0.590266984118405 |
| 760 | expm | F1 | array | input | public | expm\_F1\_array\_raw\_public.csv | 2a\_refined\_analysis\_dataset | Array\_1 | Prep\_A | 2 | CreE | 35 | 3.5 | 34.99 | 4.09 | -0.594468883689915 |
| 761 | expm | F1 | array | input | public | expm\_F1\_array\_raw\_public.csv | 2a\_refined\_analysis\_dataset | Array\_2 | Prep\_A | 1 | CreJ | 22 | 2 | 21.99 | 2.23 | -0.22772523000554 |
| 762 | expm | F1 | array | input | public | expm\_F1\_array\_raw\_public.csv | 2a\_refined\_analysis\_dataset | Array\_2 | Prep\_A | 2 | CreJ | 22 | 2 | 22.05 | 2.26 | -0.25885221824734 |
| 763 | expm | F1 | array | input | public | expm\_F1\_array\_raw\_public.csv | 2a\_refined\_analysis\_dataset | Array\_2 | Prep\_A | 2 | CreE | 22 | 2 | 22.05 | 2.31 | -0.312506226695899 |
| 764 | expm | F1 | array | input | public | expm\_F1\_array\_raw\_public.csv | 2a\_refined\_analysis\_dataset | Array\_2 | Prep\_A | 1 | CreE | 22 | 2 | 22 | 2.32 | -0.315580110713349 |
| 765 | expm | F1 | array | input | public | expm\_F1\_array\_raw\_public.csv | 2a\_refined\_analysis\_dataset | Array\_1 | Prep\_A | 2 | CreJ | 22 | 2 | 22.09 | 2.23 | -0.233501947399291 |
| 766 | expm | F1 | array | input | public | expm\_F1\_array\_raw\_public.csv | 2a\_refined\_analysis\_dataset | Array\_1 | Prep\_A | 1 | CreJ | 22 | 2 | 21.81 | 2.16 | -0.155513992230801 |
| 767 | expm | F1 | array | input | public | expm\_F1\_array\_raw\_public.csv | 2a\_refined\_analysis\_dataset | Array\_1 | Prep\_A | 2 | CreE | 22 | 2 | 22.09 | 2.3 | -0.295457798902869 |
| 768 | expm | F1 | array | input | public | expm\_F1\_array\_raw\_public.csv | 2a\_refined\_analysis\_dataset | Array\_1 | Prep\_A | 1 | CreE | 22 | 2 | 21.81 | 2.3 | -0.302988032753919 |
| 769 | expm | F1 | array | input | public | expm\_F1\_array\_raw\_public.csv | 2a\_refined\_analysis\_dataset | Array\_2 | Prep\_A | 2 | CreJ | 9 | 0.5 | 9.01 | 0.56 | -0.06179558957784 |
| 770 | expm | F1 | array | input | public | expm\_F1\_array\_raw\_public.csv | 2a\_refined\_analysis\_dataset | Array\_2 | Prep\_A | 1 | CreJ | 9 | 0.5 | 9.04 | 0.56 | -0.0580343067189491 |
| 771 | expm | F1 | array | input | public | expm\_F1\_array\_raw\_public.csv | 2a\_refined\_analysis\_dataset | Array\_2 | Prep\_A | 1 | CreE | 9 | 0.5 | 9.04 | 0.45 | 0.0539410942413144 |
| 772 | expm | F1 | array | input | public | expm\_F1\_array\_raw\_public.csv | 2a\_refined\_analysis\_dataset | Array\_2 | Prep\_A | 2 | CreE | 9 | 0.5 | 9.01 | 0.46 | 0.0354517989935074 |
| 773 | expm | F1 | array | input | public | expm\_F1\_array\_raw\_public.csv | 2a\_refined\_analysis\_dataset | Array\_1 | Prep\_A | 1 | CreJ | 9 | 0.5 | 9.02 | 0.61 | -0.10605009756827 |
| 774 | expm | F1 | array | input | public | expm\_F1\_array\_raw\_public.csv | 2a\_refined\_analysis\_dataset | Array\_1 | Prep\_A | 2 | CreJ | 9 | 0.5 | 8.94 | 0.61 | -0.106491243440442 |
| 775 | expm | F1 | array | input | public | expm\_F1\_array\_raw\_public.csv | 2a\_refined\_analysis\_dataset | Array\_1 | Prep\_A | 2 | CreE | 9 | 0.5 | 8.94 | 0.53 | -0.0261969829324317 |
| 776 | expm | F1 | array | input | public | expm\_F1\_array\_raw\_public.csv | 2a\_refined\_analysis\_dataset | Array\_1 | Prep\_A | 1 | CreE | 9 | 0.5 | 9.02 | 0.52 | -0.0232088394639036 |
| 777 | expm | F1 | array | input | public | expm\_F1\_array\_raw\_public.csv | 2a\_refined\_analysis\_dataset | Array\_2 | Prep\_A | 1 | CreJ | 23 | 2 | 22.96 | 2.18 | -0.176849719711585 |
| 778 | expm | F1 | array | input | public | expm\_F1\_array\_raw\_public.csv | 2a\_refined\_analysis\_dataset | Array\_2 | Prep\_A | 2 | CreJ | 23 | 2 | 23.04 | 2.15 | -0.152580077910885 |
| 779 | expm | F1 | array | input | public | expm\_F1\_array\_raw\_public.csv | 2a\_refined\_analysis\_dataset | Array\_1 | Prep\_A | 1 | CreE | 23 | 2 | 22.97 | 2.29 | -0.287769135893794 |
| 780 | expm | F1 | array | input | public | expm\_F1\_array\_raw\_public.csv | 2a\_refined\_analysis\_dataset | Array\_1 | Prep\_A | 2 | CreE | 23 | 2 | 23 | 2.31 | -0.314233601273194 |
| 781 | expm | F1 | array | input | public | expm\_F1\_array\_raw\_public.csv | 2a\_refined\_analysis\_dataset | Array\_2 | Prep\_A | 2 | CreE | 23 | 2 | 23.04 | 2.31 | -0.312424391361444 |
| 782 | expm | F1 | array | input | public | expm\_F1\_array\_raw\_public.csv | 2a\_refined\_analysis\_dataset | Array\_2 | Prep\_A | 1 | CreE | 23 | 2 | 22.96 | 2.29 | -0.286936391869884 |
| 783 | expm | F1 | array | input | public | expm\_F1\_array\_raw\_public.csv | 2a\_refined\_analysis\_dataset | Array\_1 | Prep\_A | 1 | CreJ | 23 | 2 | 22.97 | 2.22 | -0.219017251535559 |
| 784 | expm | F1 | array | input | public | expm\_F1\_array\_raw\_public.csv | 2a\_refined\_analysis\_dataset | Array\_1 | Prep\_A | 2 | CreJ | 23 | 2 | 23 | 2.26 | -0.259472565234689 |
| 785 | expm | F1 | array | input | public | expm\_F1\_array\_raw\_public.csv | 2a\_refined\_analysis\_dataset | Array\_2 | Prep\_A | 2 | CreE | 10 | 0.5 | 10.09 | 0.48 | 0.0169670301653707 |
| 786 | expm | F1 | array | input | public | expm\_F1\_array\_raw\_public.csv | 2a\_refined\_analysis\_dataset | Array\_2 | Prep\_A | 1 | CreE | 10 | 0.5 | 9.98 | 0.5 | -0.00096430691173932 |
| 787 | expm | F1 | array | input | public | expm\_F1\_array\_raw\_public.csv | 2a\_refined\_analysis\_dataset | Array\_1 | Prep\_A | 1 | CreJ | 10 | 0.5 | 10.02 | 0.63 | -0.132486912333568 |
| 788 | expm | F1 | array | input | public | expm\_F1\_array\_raw\_public.csv | 2a\_refined\_analysis\_dataset | Array\_1 | Prep\_A | 2 | CreJ | 10 | 0.5 | 10.01 | 0.54 | -0.0385692239375913 |
| 789 | expm | F1 | array | input | public | expm\_F1\_array\_raw\_public.csv | 2a\_refined\_analysis\_dataset | Array\_2 | Prep\_A | 2 | CreJ | 10 | 0.5 | 10.09 | 0.54 | -0.036318136272214 |
| 790 | expm | F1 | array | input | public | expm\_F1\_array\_raw\_public.csv | 2a\_refined\_analysis\_dataset | Array\_2 | Prep\_A | 1 | CreJ | 10 | 0.5 | 9.98 | 0.57 | -0.069628746852345 |
| 791 | expm | F1 | array | input | public | expm\_F1\_array\_raw\_public.csv | 2a\_refined\_analysis\_dataset | Array\_1 | Prep\_A | 1 | CreE | 10 | 0.5 | 10.02 | 0.5 | 0.00494723008847364 |
| 792 | expm | F1 | array | input | public | expm\_F1\_array\_raw\_public.csv | 2a\_refined\_analysis\_dataset | Array\_1 | Prep\_A | 2 | CreE | 10 | 0.5 | 10.01 | 0.46 | 0.0427726444265086 |
| 793 | expm | F1 | array | input | public | expm\_F1\_array\_raw\_public.csv | 2a\_refined\_analysis\_dataset | Array\_1 | Prep\_A | 1 | CreJ | 1 | 4 | 1.01 | 4.4 | -0.400408188536351 |
| 794 | expm | F1 | array | input | public | expm\_F1\_array\_raw\_public.csv | 2a\_refined\_analysis\_dataset | Array\_1 | Prep\_A | 2 | CreJ | 1 | 4 | 1 | 4.35 | -0.34954659223598 |
| 795 | expm | F1 | array | input | public | expm\_F1\_array\_raw\_public.csv | 2a\_refined\_analysis\_dataset | Array\_2 | Prep\_A | 1 | CreJ | 1 | 4 | 1 | 4.4 | -0.396021452876322 |
| 796 | expm | F1 | array | input | public | expm\_F1\_array\_raw\_public.csv | 2a\_refined\_analysis\_dataset | Array\_2 | Prep\_A | 2 | CreJ | 1 | 4 | 1.02 | 4.45 | -0.450259167250212 |
| 797 | expm | F1 | array | input | public | expm\_F1\_array\_raw\_public.csv | 2a\_refined\_analysis\_dataset | Array\_2 | Prep\_A | 1 | CreE | 1 | 4 | 1 | 4.19 | -0.19016434758136 |
| 798 | expm | F1 | array | input | public | expm\_F1\_array\_raw\_public.csv | 2a\_refined\_analysis\_dataset | Array\_2 | Prep\_A | 2 | CreE | 1 | 4 | 1.01 | 4.22 | -0.21996210714405 |
| 799 | expm | F1 | array | input | public | expm\_F1\_array\_raw\_public.csv | 2a\_refined\_analysis\_dataset | Array\_1 | Prep\_A | 2 | CreE | 1 | 4 | 1 | 4.23 | -0.22550009295639 |
| 800 | expm | F1 | array | input | public | expm\_F1\_array\_raw\_public.csv | 2a\_refined\_analysis\_dataset | Array\_1 | Prep\_A | 1 | CreE | 1 | 4 | 1.01 | 4.19 | -0.19347195254485 |
| 801 | expm | F1 | array | input | public | expm\_F1\_array\_raw\_public.csv | 2a\_refined\_analysis\_dataset | Array\_1 | Prep\_A | 2 | CreJ | 24 | 2 | 23.86 | 2.21 | -0.214630402160895 |
| 802 | expm | F1 | array | input | public | expm\_F1\_array\_raw\_public.csv | 2a\_refined\_analysis\_dataset | Array\_1 | Prep\_A | 1 | CreJ | 24 | 2 | 23.94 | 2.27 | -0.268029478622815 |
| 803 | expm | F1 | array | input | public | expm\_F1\_array\_raw\_public.csv | 2a\_refined\_analysis\_dataset | Array\_1 | Prep\_A | 2 | CreE | 24 | 2 | 23.86 | 2.38 | -0.379800230268306 |
| 804 | expm | F1 | array | input | public | expm\_F1\_array\_raw\_public.csv | 2a\_refined\_analysis\_dataset | Array\_1 | Prep\_A | 1 | CreE | 24 | 2 | 23.94 | 2.34 | -0.344820117225646 |
| 805 | expm | F1 | array | input | public | expm\_F1\_array\_raw\_public.csv | 2a\_refined\_analysis\_dataset | Array\_2 | Prep\_A | 1 | CreE | 24 | 2 | 23.95 | 2.3 | -0.303925108885105 |
| 806 | expm | F1 | array | input | public | expm\_F1\_array\_raw\_public.csv | 2a\_refined\_analysis\_dataset | Array\_2 | Prep\_A | 2 | CreE | 24 | 2 | 24.16 | 2.34 | -0.340619302208236 |
| 807 | expm | F1 | array | input | public | expm\_F1\_array\_raw\_public.csv | 2a\_refined\_analysis\_dataset | Array\_2 | Prep\_A | 1 | CreJ | 24 | 2 | 23.95 | 2.16 | -0.158153345889619 |
| 808 | expm | F1 | array | input | public | expm\_F1\_array\_raw\_public.csv | 2a\_refined\_analysis\_dataset | Array\_2 | Prep\_A | 2 | CreJ | 24 | 2 | 24.16 | 2.15 | -0.147094973996109 |
| 809 | expm | F1 | array | input | public | expm\_F1\_array\_raw\_public.csv | 2a\_refined\_analysis\_dataset | Array\_2 | Prep\_A | 2 | CreJ | 11 | 0.5 | 11.07 | 0.57 | -0.0700659675115348 |
| 810 | expm | F1 | array | input | public | expm\_F1\_array\_raw\_public.csv | 2a\_refined\_analysis\_dataset | Array\_2 | Prep\_A | 1 | CreJ | 11 | 0.5 | 10.98 | 0.6 | -0.102339344859893 |
| 811 | expm | F1 | array | input | public | expm\_F1\_array\_raw\_public.csv | 2a\_refined\_analysis\_dataset | Array\_1 | Prep\_A | 1 | CreE | 11 | 0.5 | 11.01 | 0.48 | 0.0248941449149652 |
| 812 | expm | F1 | array | input | public | expm\_F1\_array\_raw\_public.csv | 2a\_refined\_analysis\_dataset | Array\_1 | Prep\_A | 2 | CreE | 11 | 0.5 | 11 | 0.5 | -0.00365671863623984 |
| 813 | expm | F1 | array | input | public | expm\_F1\_array\_raw\_public.csv | 2a\_refined\_analysis\_dataset | Array\_2 | Prep\_A | 2 | CreE | 11 | 0.5 | 11.07 | 0.46 | 0.0357490752546352 |
| 814 | expm | F1 | array | input | public | expm\_F1\_array\_raw\_public.csv | 2a\_refined\_analysis\_dataset | Array\_2 | Prep\_A | 1 | CreE | 11 | 0.5 | 10.98 | 0.51 | -0.0143355746977758 |
| 815 | expm | F1 | array | input | public | expm\_F1\_array\_raw\_public.csv | 2a\_refined\_analysis\_dataset | Array\_1 | Prep\_A | 2 | CreJ | 11 | 0.5 | 10.99 | 0.67 | -0.166585811828927 |
| 816 | expm | F1 | array | input | public | expm\_F1\_array\_raw\_public.csv | 2a\_refined\_analysis\_dataset | Array\_1 | Prep\_A | 1 | CreJ | 11 | 0.5 | 11.01 | 0.56 | -0.0647863396022076 |
| 817 | expm | F1 | array | input | public | expm\_F1\_array\_raw\_public.csv | 2a\_refined\_analysis\_dataset | Array\_2 | Prep\_A | 1 | CreE | 2 | 4 | 2.02 | 4.23 | -0.228589334775646 |
| 818 | expm | F1 | array | input | public | expm\_F1\_array\_raw\_public.csv | 2a\_refined\_analysis\_dataset | Array\_2 | Prep\_A | 2 | CreE | 2 | 4 | 2.01 | 4.25 | -0.252605231285235 |
| 819 | expm | F1 | array | input | public | expm\_F1\_array\_raw\_public.csv | 2a\_refined\_analysis\_dataset | Array\_2 | Prep\_A | 1 | CreJ | 2 | 4 | 2.02 | 4.43 | -0.433366807935538 |
| 820 | expm | F1 | array | input | public | expm\_F1\_array\_raw\_public.csv | 2a\_refined\_analysis\_dataset | Array\_2 | Prep\_A | 2 | CreJ | 2 | 4 | 2.02 | 4.38 | -0.376437943895549 |
| 821 | expm | F1 | array | input | public | expm\_F1\_array\_raw\_public.csv | 2a\_refined\_analysis\_dataset | Array\_1 | Prep\_A | 1 | CreJ | 2 | 4 | 2.02 | 4.41 | -0.408018277562054 |
| 822 | expm | F1 | array | input | public | expm\_F1\_array\_raw\_public.csv | 2a\_refined\_analysis\_dataset | Array\_1 | Prep\_A | 2 | CreJ | 2 | 4 | 1.99 | 4.44 | -0.438728981722973 |
| 823 | expm | F1 | array | input | public | expm\_F1\_array\_raw\_public.csv | 2a\_refined\_analysis\_dataset | Array\_1 | Prep\_A | 2 | CreE | 2 | 4 | 1.99 | 4.27 | -0.269500600844285 |
| 824 | expm | F1 | array | input | public | expm\_F1\_array\_raw\_public.csv | 2a\_refined\_analysis\_dataset | Array\_1 | Prep\_A | 1 | CreE | 2 | 4 | 2.02 | 4.24 | -0.241469116506265 |
| 825 | expm | F1 | array | input | public | expm\_F1\_array\_raw\_public.csv | 2a\_refined\_analysis\_dataset | Array\_2 | Prep\_A | 1 | CreJ | 25 | 2 | 25.07 | 2.18 | -0.182663697059398 |
| 826 | expm | F1 | array | input | public | expm\_F1\_array\_raw\_public.csv | 2a\_refined\_analysis\_dataset | Array\_2 | Prep\_A | 2 | CreJ | 25 | 2 | 25.2 | 2.22 | -0.223905016068219 |
| 827 | expm | F1 | array | input | public | expm\_F1\_array\_raw\_public.csv | 2a\_refined\_analysis\_dataset | Array\_2 | Prep\_A | 2 | CreE | 25 | 2 | 25.2 | 2.3 | -0.296525329859537 |
| 828 | expm | F1 | array | input | public | expm\_F1\_array\_raw\_public.csv | 2a\_refined\_analysis\_dataset | Array\_2 | Prep\_A | 1 | CreE | 25 | 2 | 25.07 | 2.34 | -0.337199486839567 |
| 829 | expm | F1 | array | input | public | expm\_F1\_array\_raw\_public.csv | 2a\_refined\_analysis\_dataset | Array\_1 | Prep\_A | 2 | CreE | 25 | 2 | 25 | 2.33 | -0.329151283079137 |
| 830 | expm | F1 | array | input | public | expm\_F1\_array\_raw\_public.csv | 2a\_refined\_analysis\_dataset | Array\_1 | Prep\_A | 1 | CreE | 25 | 2 | 25.05 | 2.36 | -0.362898236051387 |
| 831 | expm | F1 | array | input | public | expm\_F1\_array\_raw\_public.csv | 2a\_refined\_analysis\_dataset | Array\_1 | Prep\_A | 1 | CreJ | 25 | 2 | 25.04 | 2.24 | -0.235573694609659 |
| 832 | expm | F1 | array | input | public | expm\_F1\_array\_raw\_public.csv | 2a\_refined\_analysis\_dataset | Array\_1 | Prep\_A | 2 | CreJ | 25 | 2 | 25 | 2.2 | -0.196621377819648 |
| 833 | expm | F1 | array | input | public | expm\_F1\_array\_raw\_public.csv | 2a\_refined\_analysis\_dataset | Array\_1 | Prep\_A | 2 | CreJ | 12 | 0.5 | 12.05 | 0.62 | -0.115464653709074 |
| 834 | expm | F1 | array | input | public | expm\_F1\_array\_raw\_public.csv | 2a\_refined\_analysis\_dataset | Array\_1 | Prep\_A | 1 | CreJ | 12 | 0.5 | 11.96 | 0.69 | -0.189206668422496 |
| 835 | expm | F1 | array | input | public | expm\_F1\_array\_raw\_public.csv | 2a\_refined\_analysis\_dataset | Array\_1 | Prep\_A | 2 | CreE | 12 | 0.5 | 12.05 | 0.5 | 0.00018547500116827 |
| 836 | expm | F1 | array | input | public | expm\_F1\_array\_raw\_public.csv | 2a\_refined\_analysis\_dataset | Array\_1 | Prep\_A | 1 | CreE | 12 | 0.5 | 11.96 | 0.47 | 0.0258453169039423 |
| 837 | expm | F1 | array | input | public | expm\_F1\_array\_raw\_public.csv | 2a\_refined\_analysis\_dataset | Array\_2 | Prep\_A | 2 | CreJ | 12 | 0.5 | 11.99 | 0.62 | -0.117532380095351 |
| 838 | expm | F1 | array | input | public | expm\_F1\_array\_raw\_public.csv | 2a\_refined\_analysis\_dataset | Array\_2 | Prep\_A | 1 | CreJ | 12 | 0.5 | 12.05 | 0.6 | -0.102081224438085 |
| 839 | expm | F1 | array | input | public | expm\_F1\_array\_raw\_public.csv | 2a\_refined\_analysis\_dataset | Array\_2 | Prep\_A | 2 | CreE | 12 | 0.5 | 11.99 | 0.52 | -0.0184958955998857 |
| 840 | expm | F1 | array | input | public | expm\_F1\_array\_raw\_public.csv | 2a\_refined\_analysis\_dataset | Array\_2 | Prep\_A | 1 | CreE | 12 | 0.5 | 12.05 | 0.51 | -0.0109287389947508 |
| 841 | expm | F1 | array | input | public | expm\_F1\_array\_raw\_public.csv | 2a\_refined\_analysis\_dataset | Array\_2 | Prep\_A | 2 | CreE | 3 | 4 | 3 | 4.27 | -0.26714837141069 |
| 842 | expm | F1 | array | input | public | expm\_F1\_array\_raw\_public.csv | 2a\_refined\_analysis\_dataset | Array\_2 | Prep\_A | 1 | CreE | 3 | 4 | 3.03 | 4.24 | -0.24179593663463 |
| 843 | expm | F1 | array | input | public | expm\_F1\_array\_raw\_public.csv | 2a\_refined\_analysis\_dataset | Array\_1 | Prep\_A | 2 | CreE | 3 | 4 | 3 | 4.24 | -0.2360897290396 |
| 844 | expm | F1 | array | input | public | expm\_F1\_array\_raw\_public.csv | 2a\_refined\_analysis\_dataset | Array\_1 | Prep\_A | 1 | CreE | 3 | 4 | 3.05 | 4.24 | -0.2441553005024 |
| 845 | expm | F1 | array | input | public | expm\_F1\_array\_raw\_public.csv | 2a\_refined\_analysis\_dataset | Array\_2 | Prep\_A | 2 | CreJ | 3 | 4 | 3 | 4.41 | -0.410214019155966 |
| 846 | expm | F1 | array | input | public | expm\_F1\_array\_raw\_public.csv | 2a\_refined\_analysis\_dataset | Array\_2 | Prep\_A | 1 | CreJ | 3 | 4 | 3.03 | 4.49 | -0.485637070692335 |
| 847 | expm | F1 | array | input | public | expm\_F1\_array\_raw\_public.csv | 2a\_refined\_analysis\_dataset | Array\_1 | Prep\_A | 1 | CreJ | 3 | 4 | 3.05 | 4.42 | -0.420651876916418 |
| 848 | expm | F1 | array | input | public | expm\_F1\_array\_raw\_public.csv | 2a\_refined\_analysis\_dataset | Array\_1 | Prep\_A | 2 | CreJ | 3 | 4 | 3 | 4.41 | -0.406307734087267 |
| 849 | expm | F1 | array | input | public | expm\_F1\_array\_raw\_public.csv | 2a\_refined\_analysis\_dataset | Array\_2 | Prep\_A | 2 | CreJ | 26 | 2 | 26.02 | 2.22 | -0.222504891861504 |
| 850 | expm | F1 | array | input | public | expm\_F1\_array\_raw\_public.csv | 2a\_refined\_analysis\_dataset | Array\_2 | Prep\_A | 1 | CreJ | 26 | 2 | 26.14 | 2.22 | -0.224520994869074 |
| 851 | expm | F1 | array | input | public | expm\_F1\_array\_raw\_public.csv | 2a\_refined\_analysis\_dataset | Array\_1 | Prep\_A | 1 | CreJ | 26 | 2 | 26.17 | 2.21 | -0.214762225571663 |
| 852 | expm | F1 | array | input | public | expm\_F1\_array\_raw\_public.csv | 2a\_refined\_analysis\_dataset | Array\_1 | Prep\_A | 2 | CreJ | 26 | 2 | 25.99 | 2.25 | -0.246160076854373 |
| 853 | expm | F1 | array | input | public | expm\_F1\_array\_raw\_public.csv | 2a\_refined\_analysis\_dataset | Array\_2 | Prep\_A | 1 | CreE | 26 | 2 | 26.14 | 2.4 | -0.395705454569886 |
| 854 | expm | F1 | array | input | public | expm\_F1\_array\_raw\_public.csv | 2a\_refined\_analysis\_dataset | Array\_2 | Prep\_A | 2 | CreE | 26 | 2 | 26.02 | 2.36 | -0.356428917917796 |
| 855 | expm | F1 | array | input | public | expm\_F1\_array\_raw\_public.csv | 2a\_refined\_analysis\_dataset | Array\_1 | Prep\_A | 2 | CreE | 26 | 2 | 25.99 | 2.33 | -0.333885057588966 |
| 856 | expm | F1 | array | input | public | expm\_F1\_array\_raw\_public.csv | 2a\_refined\_analysis\_dataset | Array\_1 | Prep\_A | 1 | CreE | 26 | 2 | 26.17 | 2.34 | -0.340399621122236 |
| 857 | expm | F1 | array | input | public | expm\_F1\_array\_raw\_public.csv | 2a\_refined\_analysis\_dataset | Array\_1 | Prep\_A | 2 | CreJ | 13 | 0.5 | 13.04 | 0.59 | -0.0852457817692729 |
| 858 | expm | F1 | array | input | public | expm\_F1\_array\_raw\_public.csv | 2a\_refined\_analysis\_dataset | Array\_1 | Prep\_A | 1 | CreJ | 13 | 0.5 | 13.01 | 0.58 | -0.0820299028842899 |
| 859 | expm | F1 | array | input | public | expm\_F1\_array\_raw\_public.csv | 2a\_refined\_analysis\_dataset | Array\_2 | Prep\_A | 1 | CreJ | 13 | 0.5 | 13.07 | 0.62 | -0.117390191257375 |
| 860 | expm | F1 | array | input | public | expm\_F1\_array\_raw\_public.csv | 2a\_refined\_analysis\_dataset | Array\_2 | Prep\_A | 2 | CreJ | 13 | 0.5 | 13.1 | 0.62 | -0.123108959327532 |
| 861 | expm | F1 | array | input | public | expm\_F1\_array\_raw\_public.csv | 2a\_refined\_analysis\_dataset | Array\_2 | Prep\_A | 1 | CreE | 13 | 0.5 | 13.07 | 0.5 | -0.0039940282876304 |
| 862 | expm | F1 | array | input | public | expm\_F1\_array\_raw\_public.csv | 2a\_refined\_analysis\_dataset | Array\_2 | Prep\_A | 2 | CreE | 13 | 0.5 | 13.1 | 0.47 | 0.0275115040809096 |
| 863 | expm | F1 | array | input | public | expm\_F1\_array\_raw\_public.csv | 2a\_refined\_analysis\_dataset | Array\_1 | Prep\_A | 2 | CreE | 13 | 0.5 | 13.04 | 0.55 | -0.0522318715529114 |
| 864 | expm | F1 | array | input | public | expm\_F1\_array\_raw\_public.csv | 2a\_refined\_analysis\_dataset | Array\_1 | Prep\_A | 1 | CreE | 13 | 0.5 | 13.01 | 0.47 | 0.0260108483129596 |
| 865 | expm | F1 | array | input | public | expm\_F1\_array\_raw\_public.csv | 2a\_refined\_analysis\_dataset | Array\_2 | Prep\_A | 2 | CreJ | 4 | 4 | 3.98 | 4.47 | -0.473116801935061 |
| 866 | expm | F1 | array | input | public | expm\_F1\_array\_raw\_public.csv | 2a\_refined\_analysis\_dataset | Array\_2 | Prep\_A | 1 | CreJ | 4 | 4 | 4.05 | 4.46 | -0.464931798320331 |
| 867 | expm | F1 | array | input | public | expm\_F1\_array\_raw\_public.csv | 2a\_refined\_analysis\_dataset | Array\_1 | Prep\_A | 2 | CreJ | 4 | 4 | 3.97 | 4.46 | -0.459871876296077 |
| 868 | expm | F1 | array | input | public | expm\_F1\_array\_raw\_public.csv | 2a\_refined\_analysis\_dataset | Array\_1 | Prep\_A | 1 | CreJ | 4 | 4 | 4.04 | 4.5 | -0.502286501157587 |
| 869 | expm | F1 | array | input | public | expm\_F1\_array\_raw\_public.csv | 2a\_refined\_analysis\_dataset | Array\_2 | Prep\_A | 1 | CreE | 4 | 4 | 4.05 | 4.25 | -0.248978440875867 |
| 870 | expm | F1 | array | input | public | expm\_F1\_array\_raw\_public.csv | 2a\_refined\_analysis\_dataset | Array\_2 | Prep\_A | 2 | CreE | 4 | 4 | 3.98 | 4.23 | -0.231622671918116 |
| 871 | expm | F1 | array | input | public | expm\_F1\_array\_raw\_public.csv | 2a\_refined\_analysis\_dataset | Array\_1 | Prep\_A | 2 | CreE | 4 | 4 | 3.97 | 4.25 | -0.253908211468596 |
| 872 | expm | F1 | array | input | public | expm\_F1\_array\_raw\_public.csv | 2a\_refined\_analysis\_dataset | Array\_1 | Prep\_A | 1 | CreE | 4 | 4 | 4.04 | 4.24 | -0.242931042330657 |
| 873 | expm | F1 | array | input | public | expm\_F1\_array\_raw\_public.csv | 2a\_refined\_analysis\_dataset | Array\_2 | Prep\_A | 1 | CreJ | 27 | 2 | 27.01 | 2.21 | -0.208771372546575 |
| 874 | expm | F1 | array | input | public | expm\_F1\_array\_raw\_public.csv | 2a\_refined\_analysis\_dataset | Array\_2 | Prep\_A | 2 | CreJ | 27 | 2 | 27.12 | 2.2 | -0.196807693684885 |
| 875 | expm | F1 | array | input | public | expm\_F1\_array\_raw\_public.csv | 2a\_refined\_analysis\_dataset | Array\_2 | Prep\_A | 1 | CreE | 27 | 2 | 27.01 | 2.37 | -0.368706631596184 |
| 876 | expm | F1 | array | input | public | expm\_F1\_array\_raw\_public.csv | 2a\_refined\_analysis\_dataset | Array\_2 | Prep\_A | 2 | CreE | 27 | 2 | 27.12 | 2.4 | -0.399242820750364 |
| 877 | expm | F1 | array | input | public | expm\_F1\_array\_raw\_public.csv | 2a\_refined\_analysis\_dataset | Array\_1 | Prep\_A | 2 | CreJ | 27 | 2 | 26.98 | 2.23 | -0.234939737946121 |
| 878 | expm | F1 | array | input | public | expm\_F1\_array\_raw\_public.csv | 2a\_refined\_analysis\_dataset | Array\_1 | Prep\_A | 1 | CreJ | 27 | 2 | 26.84 | 2.21 | -0.213469039257741 |
| 879 | expm | F1 | array | input | public | expm\_F1\_array\_raw\_public.csv | 2a\_refined\_analysis\_dataset | Array\_1 | Prep\_A | 1 | CreE | 27 | 2 | 26.84 | 2.34 | -0.342256931880144 |
| 880 | expm | F1 | array | input | public | expm\_F1\_array\_raw\_public.csv | 2a\_refined\_analysis\_dataset | Array\_1 | Prep\_A | 2 | CreE | 27 | 2 | 26.98 | 2.37 | -0.366722346728504 |
| 881 | expm | F1 | array | input | public | expm\_F1\_array\_raw\_public.csv | 2a\_refined\_analysis\_dataset | Array\_1 | Prep\_A | 2 | CreJ | 14 | 0.5 | 14.01 | 0.56 | -0.0562261402774867 |
| 882 | expm | F1 | array | input | public | expm\_F1\_array\_raw\_public.csv | 2a\_refined\_analysis\_dataset | Array\_1 | Prep\_A | 1 | CreJ | 14 | 0.5 | 14 | 0.64 | -0.141044368831978 |
| 883 | expm | F1 | array | input | public | expm\_F1\_array\_raw\_public.csv | 2a\_refined\_analysis\_dataset | Array\_2 | Prep\_A | 1 | CreJ | 14 | 0.5 | 14.05 | 0.62 | -0.119702025330786 |
| 884 | expm | F1 | array | input | public | expm\_F1\_array\_raw\_public.csv | 2a\_refined\_analysis\_dataset | Array\_2 | Prep\_A | 2 | CreJ | 14 | 0.5 | 13.89 | 0.53 | -0.0325374881147925 |
| 885 | expm | F1 | array | input | public | expm\_F1\_array\_raw\_public.csv | 2a\_refined\_analysis\_dataset | Array\_1 | Prep\_A | 2 | CreE | 14 | 0.5 | 14.01 | 0.52 | -0.0203525100875922 |
| 886 | expm | F1 | array | input | public | expm\_F1\_array\_raw\_public.csv | 2a\_refined\_analysis\_dataset | Array\_1 | Prep\_A | 1 | CreE | 14 | 0.5 | 14 | 0.51 | -0.0133969699139542 |
| 887 | expm | F1 | array | input | public | expm\_F1\_array\_raw\_public.csv | 2a\_refined\_analysis\_dataset | Array\_2 | Prep\_A | 2 | CreE | 14 | 0.5 | 13.9 | 0.52 | -0.0211790081430622 |
| 888 | expm | F1 | array | input | public | expm\_F1\_array\_raw\_public.csv | 2a\_refined\_analysis\_dataset | Array\_2 | Prep\_A | 1 | CreE | 14 | 0.5 | 14.05 | 0.54 | -0.0376466856576592 |
| 889 | expm | F1 | array | input | public | expm\_F1\_array\_raw\_public.csv | 2a\_refined\_analysis\_dataset | Array\_1 | Prep\_A | 2 | CreE | 5 | 4 | 5.03 | 4.23 | -0.232024385603732 |
| 890 | expm | F1 | array | input | public | expm\_F1\_array\_raw\_public.csv | 2a\_refined\_analysis\_dataset | Array\_1 | Prep\_A | 1 | CreE | 5 | 4 | 5 | 4.27 | -0.266699121035262 |
| 891 | expm | F1 | array | input | public | expm\_F1\_array\_raw\_public.csv | 2a\_refined\_analysis\_dataset | Array\_2 | Prep\_A | 2 | CreJ | 5 | 4 | 5.04 | 4.4 | -0.404554281054971 |
| 892 | expm | F1 | array | input | public | expm\_F1\_array\_raw\_public.csv | 2a\_refined\_analysis\_dataset | Array\_2 | Prep\_A | 1 | CreJ | 5 | 4 | 5.05 | 4.46 | -0.461859859418031 |
| 893 | expm | F1 | array | input | public | expm\_F1\_array\_raw\_public.csv | 2a\_refined\_analysis\_dataset | Array\_1 | Prep\_A | 1 | CreJ | 5 | 4 | 5.01 | 4.42 | -0.421758500024192 |
| 894 | expm | F1 | array | input | public | expm\_F1\_array\_raw\_public.csv | 2a\_refined\_analysis\_dataset | Array\_1 | Prep\_A | 2 | CreJ | 5 | 4 | 5.03 | 4.48 | -0.483507371944812 |
| 895 | expm | F1 | array | input | public | expm\_F1\_array\_raw\_public.csv | 2a\_refined\_analysis\_dataset | Array\_2 | Prep\_A | 1 | CreE | 5 | 4 | 5.04 | 4.27 | -0.267604580584252 |
| 896 | expm | F1 | array | input | public | expm\_F1\_array\_raw\_public.csv | 2a\_refined\_analysis\_dataset | Array\_2 | Prep\_A | 2 | CreE | 5 | 4 | 5.04 | 4.29 | -0.285884255412562 |
| 897 | expm | F1 | array | input | public | expm\_F1\_array\_raw\_public.csv | 2a\_refined\_analysis\_dataset | Array\_2 | Prep\_A | 1 | CreJ | 28 | 2 | 28.15 | 2.14 | -0.141677958325185 |
| 898 | expm | F1 | array | input | public | expm\_F1\_array\_raw\_public.csv | 2a\_refined\_analysis\_dataset | Array\_2 | Prep\_A | 2 | CreJ | 28 | 2 | 28 | 2.15 | -0.154536449226665 |
| 899 | expm | F1 | array | input | public | expm\_F1\_array\_raw\_public.csv | 2a\_refined\_analysis\_dataset | Array\_1 | Prep\_A | 1 | CreE | 28 | 2 | 27.66 | 2.41 | -0.414849744669326 |
| 900 | expm | F1 | array | input | public | expm\_F1\_array\_raw\_public.csv | 2a\_refined\_analysis\_dataset | Array\_1 | Prep\_A | 2 | CreE | 28 | 2 | 27.91 | 2.4 | -0.395004820729016 |
| 901 | expm | F1 | array | input | public | expm\_F1\_array\_raw\_public.csv | 2a\_refined\_analysis\_dataset | Array\_1 | Prep\_A | 2 | CreJ | 28 | 2 | 27.91 | 2.22 | -0.223797640295757 |
| 902 | expm | F1 | array | input | public | expm\_F1\_array\_raw\_public.csv | 2a\_refined\_analysis\_dataset | Array\_1 | Prep\_A | 1 | CreJ | 28 | 2 | 27.66 | 2.22 | -0.220474864087937 |
| 903 | expm | F1 | array | input | public | expm\_F1\_array\_raw\_public.csv | 2a\_refined\_analysis\_dataset | Array\_2 | Prep\_A | 2 | CreE | 28 | 2 | 28 | 2.38 | -0.383743000858146 |
| 904 | expm | F1 | array | input | public | expm\_F1\_array\_raw\_public.csv | 2a\_refined\_analysis\_dataset | Array\_2 | Prep\_A | 1 | CreE | 28 | 2 | 28.15 | 2.41 | -0.411455593577226 |
| 905 | expm | F1 | array | input | public | expm\_F1\_array\_raw\_public.csv | 2a\_refined\_analysis\_dataset | Array\_1 | Prep\_A | 2 | CreJ | 15 | 0.5 | 15.06 | 0.59 | -0.0857470334570241 |
| 906 | expm | F1 | array | input | public | expm\_F1\_array\_raw\_public.csv | 2a\_refined\_analysis\_dataset | Array\_1 | Prep\_A | 1 | CreJ | 15 | 0.5 | 14.97 | 0.58 | -0.075115852953846 |
| 907 | expm | F1 | array | input | public | expm\_F1\_array\_raw\_public.csv | 2a\_refined\_analysis\_dataset | Array\_1 | Prep\_A | 2 | CreE | 15 | 0.5 | 15.06 | 0.56 | -0.0633449079507598 |
| 908 | expm | F1 | array | input | public | expm\_F1\_array\_raw\_public.csv | 2a\_refined\_analysis\_dataset | Array\_1 | Prep\_A | 1 | CreE | 15 | 0.5 | 14.97 | 0.54 | -0.0416547806966097 |
| 909 | expm | F1 | array | input | public | expm\_F1\_array\_raw\_public.csv | 2a\_refined\_analysis\_dataset | Array\_2 | Prep\_A | 1 | CreE | 15 | 0.5 | 14.96 | 0.53 | -0.0255981768917597 |
| 910 | expm | F1 | array | input | public | expm\_F1\_array\_raw\_public.csv | 2a\_refined\_analysis\_dataset | Array\_2 | Prep\_A | 2 | CreE | 15 | 0.5 | 15.02 | 0.47 | 0.0345046363711693 |
| 911 | expm | F1 | array | input | public | expm\_F1\_array\_raw\_public.csv | 2a\_refined\_analysis\_dataset | Array\_2 | Prep\_A | 1 | CreJ | 15 | 0.5 | 14.96 | 0.56 | -0.060654333603878 |
| 912 | expm | F1 | array | input | public | expm\_F1\_array\_raw\_public.csv | 2a\_refined\_analysis\_dataset | Array\_2 | Prep\_A | 2 | CreJ | 15 | 0.5 | 15.02 | 0.58 | -0.07826434712599 |
| 913 | expm | F1 | array | input | public | expm\_F1\_array\_raw\_public.csv | 2a\_refined\_analysis\_dataset | Array\_2 | Prep\_A | 2 | CreE | 6 | 4 | 6 | 4.3 | -0.296038342541306 |
| 914 | expm | F1 | array | input | public | expm\_F1\_array\_raw\_public.csv | 2a\_refined\_analysis\_dataset | Array\_2 | Prep\_A | 1 | CreE | 6 | 4 | 6.09 | 4.3 | -0.297070079837426 |
| 915 | expm | F1 | array | input | public | expm\_F1\_array\_raw\_public.csv | 2a\_refined\_analysis\_dataset | Array\_1 | Prep\_A | 2 | CreJ | 6 | 4 | 6 | 4.51 | -0.506857492856271 |
| 916 | expm | F1 | array | input | public | expm\_F1\_array\_raw\_public.csv | 2a\_refined\_analysis\_dataset | Array\_1 | Prep\_A | 1 | CreJ | 6 | 4 | 5.99 | 4.43 | -0.433165858723301 |
| 917 | expm | F1 | array | input | public | expm\_F1\_array\_raw\_public.csv | 2a\_refined\_analysis\_dataset | Array\_2 | Prep\_A | 2 | CreJ | 6 | 4 | 6 | 4.41 | -0.411828755984274 |
| 918 | expm | F1 | array | input | public | expm\_F1\_array\_raw\_public.csv | 2a\_refined\_analysis\_dataset | Array\_2 | Prep\_A | 1 | CreJ | 6 | 4 | 6.09 | 4.48 | -0.475509608701285 |
| 919 | expm | F1 | array | input | public | expm\_F1\_array\_raw\_public.csv | 2a\_refined\_analysis\_dataset | Array\_1 | Prep\_A | 2 | CreE | 6 | 4 | 6 | 4.28 | -0.283357872883256 |
| 920 | expm | F1 | array | input | public | expm\_F1\_array\_raw\_public.csv | 2a\_refined\_analysis\_dataset | Array\_1 | Prep\_A | 1 | CreE | 6 | 4 | 5.99 | 4.3 | -0.295317329140476 |
| 921 | expm | F1 | array | input | public | expm\_F1\_array\_raw\_public.csv | 2a\_refined\_analysis\_dataset | Array\_2 | Prep\_A | 2 | CreE | 29 | 2 | 29.05 | 2.38 | -0.383292084789358 |
| 922 | expm | F1 | array | input | public | expm\_F1\_array\_raw\_public.csv | 2a\_refined\_analysis\_dataset | Array\_2 | Prep\_A | 1 | CreE | 29 | 2 | 29.06 | 2.4 | -0.400552760018079 |
| 923 | expm | F1 | array | input | public | expm\_F1\_array\_raw\_public.csv | 2a\_refined\_analysis\_dataset | Array\_1 | Prep\_A | 2 | CreJ | 29 | 2 | 29.07 | 2.2 | -0.20301214517192 |
| 924 | expm | F1 | array | input | public | expm\_F1\_array\_raw\_public.csv | 2a\_refined\_analysis\_dataset | Array\_1 | Prep\_A | 1 | CreJ | 29 | 2 | 29.2 | 2.23 | -0.2306716278825 |
| 925 | expm | F1 | array | input | public | expm\_F1\_array\_raw\_public.csv | 2a\_refined\_analysis\_dataset | Array\_1 | Prep\_A | 1 | CreE | 29 | 2 | 29.2 | 2.39 | -0.391870749404958 |
| 926 | expm | F1 | array | input | public | expm\_F1\_array\_raw\_public.csv | 2a\_refined\_analysis\_dataset | Array\_1 | Prep\_A | 2 | CreE | 29 | 2 | 29.07 | 2.41 | -0.407478819377999 |
| 927 | expm | F1 | array | input | public | expm\_F1\_array\_raw\_public.csv | 2a\_refined\_analysis\_dataset | Array\_2 | Prep\_A | 2 | CreJ | 29 | 2 | 29.05 | 2.25 | -0.245513795106283 |
| 928 | expm | F1 | array | input | public | expm\_F1\_array\_raw\_public.csv | 2a\_refined\_analysis\_dataset | Array\_2 | Prep\_A | 1 | CreJ | 29 | 2 | 29.06 | 2.2 | -0.199571437574883 |
| 929 | expm | F1 | array | input | public | expm\_F1\_array\_raw\_public.csv | 2a\_refined\_analysis\_dataset | Array\_2 | Prep\_A | 1 | CreE | 16 | 0.5 | 16 | 0.51 | -0.0110850460014629 |
| 930 | expm | F1 | array | input | public | expm\_F1\_array\_raw\_public.csv | 2a\_refined\_analysis\_dataset | Array\_2 | Prep\_A | 2 | CreE | 16 | 0.5 | 15.98 | 0.54 | -0.0372830989335289 |
| 931 | expm | F1 | array | input | public | expm\_F1\_array\_raw\_public.csv | 2a\_refined\_analysis\_dataset | Array\_2 | Prep\_A | 2 | CreJ | 16 | 0.5 | 15.98 | 0.63 | -0.132943346860887 |
| 932 | expm | F1 | array | input | public | expm\_F1\_array\_raw\_public.csv | 2a\_refined\_analysis\_dataset | Array\_2 | Prep\_A | 1 | CreJ | 16 | 0.5 | 15.99 | 0.55 | -0.0480121330816059 |
| 933 | expm | F1 | array | input | public | expm\_F1\_array\_raw\_public.csv | 2a\_refined\_analysis\_dataset | Array\_1 | Prep\_A | 2 | CreE | 16 | 0.5 | 16.01 | 0.57 | -0.071077062560911 |
| 934 | expm | F1 | array | input | public | expm\_F1\_array\_raw\_public.csv | 2a\_refined\_analysis\_dataset | Array\_1 | Prep\_A | 1 | CreE | 16 | 0.5 | 16.24 | 0.53 | -0.0333850775660579 |
| 935 | expm | F1 | array | input | public | expm\_F1\_array\_raw\_public.csv | 2a\_refined\_analysis\_dataset | Array\_1 | Prep\_A | 1 | CreJ | 16 | 0.5 | 16.24 | 0.57 | -0.0652020033074413 |
| 936 | expm | F1 | array | input | public | expm\_F1\_array\_raw\_public.csv | 2a\_refined\_analysis\_dataset | Array\_1 | Prep\_A | 2 | CreJ | 16 | 0.5 | 16.01 | 0.64 | -0.138289923345002 |
| 937 | expm | F1 | array | input | public | expm\_F1\_array\_raw\_public.csv | 2a\_refined\_analysis\_dataset | Array\_2 | Prep\_A | 1 | CreJ | 7 | 4 | 7.01 | 4.46 | -0.458859581716795 |
| 938 | expm | F1 | array | input | public | expm\_F1\_array\_raw\_public.csv | 2a\_refined\_analysis\_dataset | Array\_2 | Prep\_A | 2 | CreJ | 7 | 4 | 7.01 | 4.45 | -0.447570022444725 |
| 939 | expm | F1 | array | input | public | expm\_F1\_array\_raw\_public.csv | 2a\_refined\_analysis\_dataset | Array\_2 | Prep\_A | 2 | CreE | 7 | 4 | 7.01 | 4.32 | -0.317200197047156 |
| 940 | expm | F1 | array | input | public | expm\_F1\_array\_raw\_public.csv | 2a\_refined\_analysis\_dataset | Array\_2 | Prep\_A | 1 | CreE | 7 | 4 | 7.01 | 4.31 | -0.314730976961125 |
| 941 | expm | F1 | array | input | public | expm\_F1\_array\_raw\_public.csv | 2a\_refined\_analysis\_dataset | Array\_1 | Prep\_A | 1 | CreE | 7 | 4 | 7.02 | 4.31 | -0.308694599760336 |
| 942 | expm | F1 | array | input | public | expm\_F1\_array\_raw\_public.csv | 2a\_refined\_analysis\_dataset | Array\_1 | Prep\_A | 2 | CreE | 7 | 4 | 6.98 | 4.28 | -0.280326159448986 |
| 943 | expm | F1 | array | input | public | expm\_F1\_array\_raw\_public.csv | 2a\_refined\_analysis\_dataset | Array\_1 | Prep\_A | 2 | CreJ | 7 | 4 | 6.99 | 4.41 | -0.40878836706448 |
| 944 | expm | F1 | array | input | public | expm\_F1\_array\_raw\_public.csv | 2a\_refined\_analysis\_dataset | Array\_1 | Prep\_A | 1 | CreJ | 7 | 4 | 7.02 | 4.43 | -0.42605233278785 |
| 945 | expm | F1 | array | input | public | expm\_F1\_array\_raw\_public.csv | 2a\_refined\_analysis\_dataset | Array\_2 | Prep\_A | 2 | CreE | 30 | 2 | 30.06 | 2.42 | -0.422250914131612 |
| 946 | expm | F1 | array | input | public | expm\_F1\_array\_raw\_public.csv | 2a\_refined\_analysis\_dataset | Array\_2 | Prep\_A | 1 | CreE | 30 | 2 | 30.17 | 2.39 | -0.386064789509952 |
| 947 | expm | F1 | array | input | public | expm\_F1\_array\_raw\_public.csv | 2a\_refined\_analysis\_dataset | Array\_1 | Prep\_A | 2 | CreE | 30 | 2 | 30.01 | 2.4 | -0.398252397024282 |
| 948 | expm | F1 | array | input | public | expm\_F1\_array\_raw\_public.csv | 2a\_refined\_analysis\_dataset | Array\_1 | Prep\_A | 1 | CreE | 30 | 2 | 30.06 | 2.43 | -0.427264140387792 |
| 949 | expm | F1 | array | input | public | expm\_F1\_array\_raw\_public.csv | 2a\_refined\_analysis\_dataset | Array\_1 | Prep\_A | 1 | CreJ | 30 | 2 | 30.06 | 2.23 | -0.233836810846312 |
| 950 | expm | F1 | array | input | public | expm\_F1\_array\_raw\_public.csv | 2a\_refined\_analysis\_dataset | Array\_1 | Prep\_A | 2 | CreJ | 30 | 2 | 30.01 | 2.17 | -0.174158399756162 |
| 951 | expm | F1 | array | input | public | expm\_F1\_array\_raw\_public.csv | 2a\_refined\_analysis\_dataset | Array\_2 | Prep\_A | 2 | CreJ | 30 | 2 | 30.06 | 2.1 | -0.0962004524514031 |
| 952 | expm | F1 | array | input | public | expm\_F1\_array\_raw\_public.csv | 2a\_refined\_analysis\_dataset | Array\_2 | Prep\_A | 1 | CreJ | 30 | 2 | 30.17 | 2.17 | -0.174473117528633 |
| 953 | expm | F1 | array | input | public | expm\_F1\_array\_raw\_public.csv | 2a\_refined\_analysis\_dataset | Array\_2 | Prep\_A | 1 | CreE | 17 | 0.5 | 17.03 | 0.53 | -0.0267984710484609 |
| 954 | expm | F1 | array | input | public | expm\_F1\_array\_raw\_public.csv | 2a\_refined\_analysis\_dataset | Array\_2 | Prep\_A | 2 | CreE | 17 | 0.5 | 16.97 | 0.54 | -0.0364639175138128 |
| 955 | expm | F1 | array | input | public | expm\_F1\_array\_raw\_public.csv | 2a\_refined\_analysis\_dataset | Array\_2 | Prep\_A | 1 | CreJ | 17 | 0.5 | 17.03 | 0.62 | -0.120149271662586 |
| 956 | expm | F1 | array | input | public | expm\_F1\_array\_raw\_public.csv | 2a\_refined\_analysis\_dataset | Array\_2 | Prep\_A | 2 | CreJ | 17 | 0.5 | 16.97 | 0.62 | -0.120263695890493 |
| 957 | expm | F1 | array | input | public | expm\_F1\_array\_raw\_public.csv | 2a\_refined\_analysis\_dataset | Array\_1 | Prep\_A | 2 | CreJ | 17 | 0.5 | 17.02 | 0.55 | -0.0453709171199458 |
| 958 | expm | F1 | array | input | public | expm\_F1\_array\_raw\_public.csv | 2a\_refined\_analysis\_dataset | Array\_1 | Prep\_A | 1 | CreJ | 17 | 0.5 | 16.98 | 0.64 | -0.13529451213403 |
| 959 | expm | F1 | array | input | public | expm\_F1\_array\_raw\_public.csv | 2a\_refined\_analysis\_dataset | Array\_1 | Prep\_A | 1 | CreE | 17 | 0.5 | 16.98 | 0.54 | -0.0410456295445169 |
| 960 | expm | F1 | array | input | public | expm\_F1\_array\_raw\_public.csv | 2a\_refined\_analysis\_dataset | Array\_1 | Prep\_A | 2 | CreE | 17 | 0.5 | 17.02 | 0.56 | -0.0551314721149928 |
| 961 | expm | F1 | array | input | public | expm\_F1\_array\_raw\_public.csv | 2a\_refined\_analysis\_dataset | Array\_2 | Prep\_A | 2 | CreJ | 8 | 4 | 8.08 | 4.49 | -0.486257097485844 |
| 962 | expm | F1 | array | input | public | expm\_F1\_array\_raw\_public.csv | 2a\_refined\_analysis\_dataset | Array\_2 | Prep\_A | 1 | CreJ | 8 | 4 | 8 | 4.5 | -0.499312876129504 |
| 963 | expm | F1 | array | input | public | expm\_F1\_array\_raw\_public.csv | 2a\_refined\_analysis\_dataset | Array\_2 | Prep\_A | 2 | CreE | 8 | 4 | 8.07 | 4.33 | -0.328319276680163 |
| 964 | expm | F1 | array | input | public | expm\_F1\_array\_raw\_public.csv | 2a\_refined\_analysis\_dataset | Array\_2 | Prep\_A | 1 | CreE | 8 | 4 | 8 | 4.33 | -0.329335230991153 |
| 965 | expm | F1 | array | input | public | expm\_F1\_array\_raw\_public.csv | 2a\_refined\_analysis\_dataset | Array\_1 | Prep\_A | 2 | CreJ | 8 | 4 | 7.96 | 4.5 | -0.498048989698578 |
| 966 | expm | F1 | array | input | public | expm\_F1\_array\_raw\_public.csv | 2a\_refined\_analysis\_dataset | Array\_1 | Prep\_A | 1 | CreJ | 8 | 4 | 8.01 | 4.43 | -0.432867880350388 |
| 967 | expm | F1 | array | input | public | expm\_F1\_array\_raw\_public.csv | 2a\_refined\_analysis\_dataset | Array\_1 | Prep\_A | 1 | CreE | 8 | 4 | 8.01 | 4.33 | -0.325747700205263 |
| 968 | expm | F1 | array | input | public | expm\_F1\_array\_raw\_public.csv | 2a\_refined\_analysis\_dataset | Array\_1 | Prep\_A | 2 | CreE | 8 | 4 | 7.96 | 4.33 | -0.334801245817633 |
| 969 | expm | F1 | array | input | public | expm\_F1\_array\_raw\_public.csv | 2a\_refined\_analysis\_dataset | Array\_2 | Prep\_A | 2 | CreE | 31 | 2 | 30.91 | 2.42 | -0.424085581689929 |
| 970 | expm | F1 | array | input | public | expm\_F1\_array\_raw\_public.csv | 2a\_refined\_analysis\_dataset | Array\_2 | Prep\_A | 1 | CreE | 31 | 2 | 31.39 | 2.41 | -0.410788907254828 |
| 971 | expm | F1 | array | input | public | expm\_F1\_array\_raw\_public.csv | 2a\_refined\_analysis\_dataset | Array\_1 | Prep\_A | 2 | CreE | 31 | 2 | 30.97 | 2.44 | -0.443537395190568 |
| 972 | expm | F1 | array | input | public | expm\_F1\_array\_raw\_public.csv | 2a\_refined\_analysis\_dataset | Array\_1 | Prep\_A | 1 | CreE | 31 | 2 | 30.84 | 2.44 | -0.438080276514108 |
| 973 | expm | F1 | array | input | public | expm\_F1\_array\_raw\_public.csv | 2a\_refined\_analysis\_dataset | Array\_2 | Prep\_A | 2 | CreJ | 31 | 2 | 30.91 | 2.15 | -0.153426029700815 |
| 974 | expm | F1 | array | input | public | expm\_F1\_array\_raw\_public.csv | 2a\_refined\_analysis\_dataset | Array\_2 | Prep\_A | 1 | CreJ | 31 | 2 | 31.39 | 2.17 | -0.170932032889195 |
| 975 | expm | F1 | array | input | public | expm\_F1\_array\_raw\_public.csv | 2a\_refined\_analysis\_dataset | Array\_1 | Prep\_A | 2 | CreJ | 31 | 2 | 30.97 | 2.15 | -0.150465132593669 |
| 976 | expm | F1 | array | input | public | expm\_F1\_array\_raw\_public.csv | 2a\_refined\_analysis\_dataset | Array\_1 | Prep\_A | 1 | CreJ | 31 | 2 | 30.83 | 2.15 | -0.151254530575998 |
| 977 | expm | F1 | array | input | public | expm\_F1\_array\_raw\_public.csv | 2a\_refined\_analysis\_dataset | Array\_2 | Prep\_A | 1 | CreJ | 18 | 0.5 | 18.11 | 0.56 | -0.0609635754061747 |
| 978 | expm | F1 | array | input | public | expm\_F1\_array\_raw\_public.csv | 2a\_refined\_analysis\_dataset | Array\_2 | Prep\_A | 2 | CreJ | 18 | 0.5 | 18.17 | 0.64 | -0.1435304818983 |
| 979 | expm | F1 | array | input | public | expm\_F1\_array\_raw\_public.csv | 2a\_refined\_analysis\_dataset | Array\_2 | Prep\_A | 1 | CreE | 18 | 0.5 | 18.11 | 0.51 | -0.0126850319010789 |
| 980 | expm | F1 | array | input | public | expm\_F1\_array\_raw\_public.csv | 2a\_refined\_analysis\_dataset | Array\_2 | Prep\_A | 2 | CreE | 18 | 0.5 | 18.17 | 0.61 | -0.109144821584332 |
| 981 | expm | F1 | array | input | public | expm\_F1\_array\_raw\_public.csv | 2a\_refined\_analysis\_dataset | Array\_1 | Prep\_A | 2 | CreE | 18 | 0.5 | 17.97 | 0.6 | -0.102730406153779 |
| 982 | expm | F1 | array | input | public | expm\_F1\_array\_raw\_public.csv | 2a\_refined\_analysis\_dataset | Array\_1 | Prep\_A | 1 | CreE | 18 | 0.5 | 17.92 | 0.58 | -0.0848388488212799 |
| 983 | expm | F1 | array | input | public | expm\_F1\_array\_raw\_public.csv | 2a\_refined\_analysis\_dataset | Array\_1 | Prep\_A | 1 | CreJ | 18 | 0.5 | 17.92 | 0.59 | -0.0896476344918656 |
| 984 | expm | F1 | array | input | public | expm\_F1\_array\_raw\_public.csv | 2a\_refined\_analysis\_dataset | Array\_1 | Prep\_A | 2 | CreJ | 18 | 0.5 | 17.97 | 0.55 | -0.0534561293040846 |
| 985 | expm | F1 | array | input | public | expm\_F1\_array\_raw\_public.csv | 2a\_refined\_analysis\_dataset | Array\_2 | Prep\_A | 2 | CreE | 9 | 4 | 9.02 | 4.29 | -0.291017880150233 |
| 986 | expm | F1 | array | input | public | expm\_F1\_array\_raw\_public.csv | 2a\_refined\_analysis\_dataset | Array\_2 | Prep\_A | 1 | CreE | 9 | 4 | 8.96 | 4.3 | -0.301084320417333 |
| 987 | expm | F1 | array | input | public | expm\_F1\_array\_raw\_public.csv | 2a\_refined\_analysis\_dataset | Array\_1 | Prep\_A | 1 | CreJ | 9 | 4 | 8.95 | 4.39 | -0.387553251737493 |
| 988 | expm | F1 | array | input | public | expm\_F1\_array\_raw\_public.csv | 2a\_refined\_analysis\_dataset | Array\_1 | Prep\_A | 2 | CreJ | 9 | 4 | 9.02 | 4.54 | -0.536654129409633 |
| 989 | expm | F1 | array | input | public | expm\_F1\_array\_raw\_public.csv | 2a\_refined\_analysis\_dataset | Array\_1 | Prep\_A | 1 | CreE | 9 | 4 | 8.95 | 4.34 | -0.343401963010162 |
| 990 | expm | F1 | array | input | public | expm\_F1\_array\_raw\_public.csv | 2a\_refined\_analysis\_dataset | Array\_1 | Prep\_A | 2 | CreE | 9 | 4 | 9.02 | 4.35 | -0.351081949455553 |
| 991 | expm | F1 | array | input | public | expm\_F1\_array\_raw\_public.csv | 2a\_refined\_analysis\_dataset | Array\_2 | Prep\_A | 1 | CreJ | 9 | 4 | 8.96 | 4.53 | -0.526680837757853 |
| 992 | expm | F1 | array | input | public | expm\_F1\_array\_raw\_public.csv | 2a\_refined\_analysis\_dataset | Array\_2 | Prep\_A | 2 | CreJ | 9 | 4 | 9.02 | 4.5 | -0.503274772529553 |
| 993 | expm | F1 | array | input | public | expm\_F1\_array\_raw\_public.csv | 2a\_refined\_analysis\_dataset | Array\_2 | Prep\_A | 2 | CreJ | 32 | 2 | 31.97 | 2.17 | -0.169334901465872 |
| 994 | expm | F1 | array | input | public | expm\_F1\_array\_raw\_public.csv | 2a\_refined\_analysis\_dataset | Array\_2 | Prep\_A | 1 | CreJ | 32 | 2 | 32.14 | 2.21 | -0.207689755893862 |
| 995 | expm | F1 | array | input | public | expm\_F1\_array\_raw\_public.csv | 2a\_refined\_analysis\_dataset | Array\_1 | Prep\_A | 2 | CreJ | 32 | 2 | 31.76 | 2.17 | -0.165084583710767 |
| 996 | expm | F1 | array | input | public | expm\_F1\_array\_raw\_public.csv | 2a\_refined\_analysis\_dataset | Array\_1 | Prep\_A | 1 | CreJ | 32 | 2 | 32.15 | 2.21 | -0.205338017913177 |
| 997 | expm | F1 | array | input | public | expm\_F1\_array\_raw\_public.csv | 2a\_refined\_analysis\_dataset | Array\_2 | Prep\_A | 1 | CreE | 32 | 2 | 32.14 | 2.41 | -0.410677040785519 |
| 998 | expm | F1 | array | input | public | expm\_F1\_array\_raw\_public.csv | 2a\_refined\_analysis\_dataset | Array\_2 | Prep\_A | 2 | CreE | 32 | 2 | 31.97 | 2.43 | -0.428160014246849 |
| 999 | expm | F1 | array | input | public | expm\_F1\_array\_raw\_public.csv | 2a\_refined\_analysis\_dataset | Array\_1 | Prep\_A | 2 | CreE | 32 | 2 | 31.76 | 2.41 | -0.405533262055459 |
| 1000 | expm | F1 | array | input | public | expm\_F1\_array\_raw\_public.csv | 2a\_refined\_analysis\_dataset | Array\_1 | Prep\_A | 1 | CreE | 32 | 2 | 32.15 | 2.37 | -0.372457276184619 |
