# Supplementary material for: Creatinine assay interferences compromises MELD accuracy and may bias liver allocation
Source: Nat Commun. 2026 Jul 23;17:7111. doi: 10.1038/s41467-026-75011-x (PMC13396164; doi:10.1038/s41467-026-75011-x)
Supplement: Supplementary file 4 — Source Data [file 41467_2026_75011_MOESM4_ESM.zip › figshare_package_FINAL_PUBLIC_DEPOSIT_V1_20260503_002637/00_START_HERE_HTML_NAVIGATOR/file_views/view_0030_README_F3_submission_ready_v01.html]

02\_workflows/F3\_workflow\_v01/submission\_ready/README\_F3\_submission\_ready\_v01.txt

# Readable file view

02\_workflows/F3\_workflow\_v01/submission\_ready/README\_F3\_submission\_ready\_v01.txt

← Back to navigator   |   Open original package file

Section

Workflow readmes

Output

F3

Extension

txt

Size KB

0.328

Variables

0

## Readable HTML view

```
F3 submission-ready outputs

Public files are located in:
public/data
public/figures

Internal validation files are located in:
internal/data
internal/validation

V3 source logic:
- ESLD aggregate is derived from esld_master_long_public.csv.
- SRTR aggregate uses srtr_F3_score_shift_aggregate_reference_internal.csv as primary source.
```
