# Supplementary material for: Creatinine assay interferences compromises MELD accuracy and may bias liver allocation
Source: Nat Commun. 2026 Jul 23;17:7111. doi: 10.1038/s41467-026-75011-x (PMC13396164; doi:10.1038/s41467-026-75011-x)
Supplement: Supplementary file 4 — Source Data [file 41467_2026_75011_MOESM4_ESM.zip › figshare_package_FINAL_PUBLIC_DEPOSIT_V1_20260503_002637/00_START_HERE_HTML_NAVIGATOR/file_views/view_0038_restricted_on_request_file_manifest_v01.html]

00\_release\_manifests/restricted\_on\_request\_file\_manifest\_v01.csv

# Readable file view

00\_release\_manifests/restricted\_on\_request\_file\_manifest\_v01.csv

← Back to navigator   |   Open original package file

Section

Technical appendix / request manifests

Output

Extension

csv

Size KB

74.085

Variables

0

## Readable HTML view

Showing all 97 rows.

| relative\_path | file\_name | directory | extension | file\_type | size\_bytes | size\_kb | last\_modified | md5 | access\_tier | data\_origin | used\_for | n\_rows | n\_cols | keep\_in\_repository | needs\_manual\_review | comment | release\_scope | release\_access\_tier | release\_source\_role | release\_decision | output\_tag | registry\_file\_key | registry\_release\_status | registry\_is\_primary | registry\_used\_by\_workflows | registry\_source\_role | final\_release\_tier | final\_release\_decision | final\_policy\_reason | is\_r\_script | is\_srtr\_named | is\_data\_like | is\_rendered\_or\_doc | is\_internal\_table\_template | is\_legacy\_zip | is\_internal\_workflow\_data\_or\_table | is\_internal\_documentation | is\_internal\_primary\_source | final\_release\_tier\_before\_doc\_close | final\_release\_decision\_before\_doc\_close | final\_policy\_reason\_before\_doc\_close | is\_internal\_submission\_manifest | is\_noninternal\_submission\_manifest | is\_workflow\_readme | is\_submission\_ready\_readme | is\_remaining\_manual\_doc |
| --- | --- | --- | --- | --- | --- | --- | --- | --- | --- | --- | --- | --- | --- | --- | --- | --- | --- | --- | --- | --- | --- | --- | --- | --- | --- | --- | --- | --- | --- | --- | --- | --- | --- | --- | --- | --- | --- | --- | --- | --- | --- | --- | --- | --- | --- | --- |
| 01\_primary\_data/restricted/provenance/T4\_survival\_matrix\_components/m1\_matrix\_1.csv | m1\_matrix\_1.csv | 01\_primary\_data/restricted/provenance/T4\_survival\_matrix\_components | csv | csv | 9944 | 9.711 | 2025-05-14T07:22:32Z | 4add871bbcd7b24f018d6092c789f6a8 | restricted\_on\_request | primary | T4 | 1 | 9 | yes\_default | TRUE |  | release\_candidate | restricted\_on\_request | primary\_source | keep\_restricted\_on\_request | T4 |  |  |  |  |  | restricted\_on\_request | keep\_restricted\_on\_request | Restricted-on-request file after policy overrides. | FALSE | FALSE | TRUE | FALSE | FALSE | FALSE | FALSE | FALSE | FALSE | restricted\_on\_request | keep\_restricted\_on\_request | Restricted-on-request file after policy overrides. | FALSE | FALSE | FALSE | FALSE | FALSE |
| 01\_primary\_data/restricted/provenance/T4\_survival\_matrix\_components/m1\_matrix\_2.csv | m1\_matrix\_2.csv | 01\_primary\_data/restricted/provenance/T4\_survival\_matrix\_components | csv | csv | 9926 | 9.693 | 2025-05-14T07:23:16Z | b298479905e55451d7dbadbdff4a0f80 | restricted\_on\_request | primary | T4 | 1 | 9 | yes\_default | TRUE |  | release\_candidate | restricted\_on\_request | primary\_source | keep\_restricted\_on\_request | T4 |  |  |  |  |  | restricted\_on\_request | keep\_restricted\_on\_request | Restricted-on-request file after policy overrides. | FALSE | FALSE | TRUE | FALSE | FALSE | FALSE | FALSE | FALSE | FALSE | restricted\_on\_request | keep\_restricted\_on\_request | Restricted-on-request file after policy overrides. | FALSE | FALSE | FALSE | FALSE | FALSE |
| 01\_primary\_data/restricted/provenance/T4\_survival\_matrix\_components/m1\_matrix\_3.csv | m1\_matrix\_3.csv | 01\_primary\_data/restricted/provenance/T4\_survival\_matrix\_components | csv | csv | 9981 | 9.747 | 2025-05-14T07:23:16Z | 94370825c5e38e058ca1291e6078510b | restricted\_on\_request | primary | T4 | 1 | 9 | yes\_default | TRUE |  | release\_candidate | restricted\_on\_request | primary\_source | keep\_restricted\_on\_request | T4 |  |  |  |  |  | restricted\_on\_request | keep\_restricted\_on\_request | Restricted-on-request file after policy overrides. | FALSE | FALSE | TRUE | FALSE | FALSE | FALSE | FALSE | FALSE | FALSE | restricted\_on\_request | keep\_restricted\_on\_request | Restricted-on-request file after policy overrides. | FALSE | FALSE | FALSE | FALSE | FALSE |
| 01\_primary\_data/restricted/provenance/T4\_survival\_matrix\_components/m1\_matrix\_4.csv | m1\_matrix\_4.csv | 01\_primary\_data/restricted/provenance/T4\_survival\_matrix\_components | csv | csv | 9866 | 9.635 | 2025-05-14T07:23:16Z | 0c9c1f26a8d4b83f1d2253e0cf074f09 | restricted\_on\_request | primary | T4 | 1 | 9 | yes\_default | TRUE |  | release\_candidate | restricted\_on\_request | primary\_source | keep\_restricted\_on\_request | T4 |  |  |  |  |  | restricted\_on\_request | keep\_restricted\_on\_request | Restricted-on-request file after policy overrides. | FALSE | FALSE | TRUE | FALSE | FALSE | FALSE | FALSE | FALSE | FALSE | restricted\_on\_request | keep\_restricted\_on\_request | Restricted-on-request file after policy overrides. | FALSE | FALSE | FALSE | FALSE | FALSE |
| 01\_primary\_data/restricted/provenance/T4\_survival\_matrix\_components/pm\_matrix\_1.csv | pm\_matrix\_1.csv | 01\_primary\_data/restricted/provenance/T4\_survival\_matrix\_components | csv | csv | 10098 | 9.861 | 2025-05-14T07:23:16Z | cd30e1ccb4e7ab481206778202fca1d1 | restricted\_on\_request | primary | T4 | 1 | 9 | yes\_default | TRUE |  | release\_candidate | restricted\_on\_request | primary\_source | keep\_restricted\_on\_request | T4 |  |  |  |  |  | restricted\_on\_request | keep\_restricted\_on\_request | Restricted-on-request file after policy overrides. | FALSE | FALSE | TRUE | FALSE | FALSE | FALSE | FALSE | FALSE | FALSE | restricted\_on\_request | keep\_restricted\_on\_request | Restricted-on-request file after policy overrides. | FALSE | FALSE | FALSE | FALSE | FALSE |
| 01\_primary\_data/restricted/provenance/T4\_survival\_matrix\_components/pm\_matrix\_2.csv | pm\_matrix\_2.csv | 01\_primary\_data/restricted/provenance/T4\_survival\_matrix\_components | csv | csv | 10047 | 9.812 | 2025-05-14T07:23:16Z | c0787b060eef6ab89832bad2264d639f | restricted\_on\_request | primary | T4 | 1 | 9 | yes\_default | TRUE |  | release\_candidate | restricted\_on\_request | primary\_source | keep\_restricted\_on\_request | T4 |  |  |  |  |  | restricted\_on\_request | keep\_restricted\_on\_request | Restricted-on-request file after policy overrides. | FALSE | FALSE | TRUE | FALSE | FALSE | FALSE | FALSE | FALSE | FALSE | restricted\_on\_request | keep\_restricted\_on\_request | Restricted-on-request file after policy overrides. | FALSE | FALSE | FALSE | FALSE | FALSE |
| 01\_primary\_data/restricted/provenance/T4\_survival\_matrix\_components/pm\_matrix\_3.csv | pm\_matrix\_3.csv | 01\_primary\_data/restricted/provenance/T4\_survival\_matrix\_components | csv | csv | 9940 | 9.707 | 2025-05-14T07:23:16Z | 957249b7b26268e78f418b947f30ce8e | restricted\_on\_request | primary | T4 | 1 | 9 | yes\_default | TRUE |  | release\_candidate | restricted\_on\_request | primary\_source | keep\_restricted\_on\_request | T4 |  |  |  |  |  | restricted\_on\_request | keep\_restricted\_on\_request | Restricted-on-request file after policy overrides. | FALSE | FALSE | TRUE | FALSE | FALSE | FALSE | FALSE | FALSE | FALSE | restricted\_on\_request | keep\_restricted\_on\_request | Restricted-on-request file after policy overrides. | FALSE | FALSE | FALSE | FALSE | FALSE |
| 01\_primary\_data/restricted/provenance/T4\_survival\_matrix\_components/pm\_matrix\_4.csv | pm\_matrix\_4.csv | 01\_primary\_data/restricted/provenance/T4\_survival\_matrix\_components | csv | csv | 10076 | 9.84 | 2025-05-14T07:23:16Z | c8dfdee2a4fe3ea0433a5b69aec01301 | restricted\_on\_request | primary | T4 | 1 | 9 | yes\_default | TRUE |  | release\_candidate | restricted\_on\_request | primary\_source | keep\_restricted\_on\_request | T4 |  |  |  |  |  | restricted\_on\_request | keep\_restricted\_on\_request | Restricted-on-request file after policy overrides. | FALSE | FALSE | TRUE | FALSE | FALSE | FALSE | FALSE | FALSE | FALSE | restricted\_on\_request | keep\_restricted\_on\_request | Restricted-on-request file after policy overrides. | FALSE | FALSE | FALSE | FALSE | FALSE |
| 01\_primary\_data/restricted/srtr\_F3\_score\_shift\_aggregate\_reference\_internal.csv | srtr\_F3\_score\_shift\_aggregate\_reference\_internal.csv | 01\_primary\_data/restricted | csv | csv | 14332 | 13.996 | 2026-05-01T21:46:43Z | a738a4cddce2c4ca7209ddacb242998b | restricted\_on\_request | primary | F3 | 680 | 4 | yes\_default | TRUE |  | release\_candidate | restricted\_on\_request | primary\_source | keep\_restricted\_on\_request | F3 | srtr\_F3\_score\_shift\_aggregate\_reference\_internal | restricted | TRUE | F3;T4 | SRTR F3/T4 primary aggregate source; restricted. | restricted\_on\_request | keep\_restricted\_on\_request | SRTR-derived data file; provide only on request under licence obligations. | FALSE | TRUE | TRUE | FALSE | FALSE | FALSE | FALSE | FALSE | FALSE | restricted\_on\_request | keep\_restricted\_on\_request | SRTR-derived data file; provide only on request under licence obligations. | FALSE | FALSE | FALSE | FALSE | FALSE |
| 01\_primary\_data/restricted/srtr\_T4\_score\_shift\_counts\_all\_scores\_restricted.csv | srtr\_T4\_score\_shift\_counts\_all\_scores\_restricted.csv | 01\_primary\_data/restricted | csv | csv | 57406 | 56.061 | 2026-05-01T23:39:23Z | f5b54c78ba515a0813aa766447a62d6f | restricted\_on\_request | primary | T4 | 705 | 8 | yes\_default | TRUE |  | release\_candidate | restricted\_on\_request | primary\_source | keep\_restricted\_on\_request | T4 | srtr\_T4\_score\_shift\_counts\_all\_scores\_restricted | restricted | TRUE | T4 | canonical SRTR all-score shift-count primary source; contains broader patient coverage than srtr\_master\_long\_restricted | restricted\_on\_request | keep\_restricted\_on\_request | SRTR-derived data file; provide only on request under licence obligations. | FALSE | TRUE | TRUE | FALSE | FALSE | FALSE | FALSE | FALSE | FALSE | restricted\_on\_request | keep\_restricted\_on\_request | SRTR-derived data file; provide only on request under licence obligations. | FALSE | FALSE | FALSE | FALSE | FALSE |
| 01\_primary\_data/restricted/srtr\_T4\_survival\_matrix\_restricted.csv | srtr\_T4\_survival\_matrix\_restricted.csv | 01\_primary\_data/restricted | csv | csv | 176617 | 172.478 | 2026-05-01T20:54:07Z | 3363b210bc6bf2fd4491ae78cf27e248 | restricted\_on\_request | primary | T4 | 728 | 26 | yes\_default | TRUE |  | release\_candidate | restricted\_on\_request | primary\_source | keep\_restricted\_on\_request | T4 | srtr\_T4\_survival\_matrix\_restricted | restricted | TRUE | T4 | canonical harmonized SRTR T4 survival-matrix primary source; contains all eight pm/m1 matrix files with corrected semantic CI bounds | restricted\_on\_request | keep\_restricted\_on\_request | SRTR-derived data file; provide only on request under licence obligations. | FALSE | TRUE | TRUE | FALSE | FALSE | FALSE | FALSE | FALSE | FALSE | restricted\_on\_request | keep\_restricted\_on\_request | SRTR-derived data file; provide only on request under licence obligations. | FALSE | FALSE | FALSE | FALSE | FALSE |
| 01\_primary\_data/restricted/srtr\_master\_long\_restricted.csv | srtr\_master\_long\_restricted.csv | 01\_primary\_data/restricted | csv | csv | 44648516 | 43602.066 | 2026-04-30T15:41:56Z | 88c4909a08aed3fc79459eb78793a23d | restricted\_on\_request | primary |  | 193191 | 52 | yes\_default | TRUE |  | release\_candidate | restricted\_on\_request | primary\_source | keep\_restricted\_on\_request |  | srtr\_master\_long\_restricted | restricted | TRUE | F4;F6;T1 | canonical SRTR master for workflows that are derivable from SRTR master | restricted\_on\_request | keep\_restricted\_on\_request | SRTR-derived data file; provide only on request under licence obligations. | FALSE | TRUE | TRUE | FALSE | FALSE | FALSE | FALSE | FALSE | FALSE | restricted\_on\_request | keep\_restricted\_on\_request | SRTR-derived data file; provide only on request under licence obligations. | FALSE | FALSE | FALSE | FALSE | FALSE |
| 02\_workflows/F3\_workflow\_v01/data/01\_source\_loaded\_harmonized/srtr\_F3\_score\_shift\_aggregate\_reference\_internal.csv | srtr\_F3\_score\_shift\_aggregate\_reference\_internal.csv | 02\_workflows/F3\_workflow\_v01/data/01\_source\_loaded\_harmonized | csv | csv | 14332 | 13.996 | 2026-05-01T21:46:43Z | a738a4cddce2c4ca7209ddacb242998b | restricted\_on\_request | derived | F3 | 680 | 4 | yes\_default | TRUE |  | release\_candidate | restricted\_on\_request | derived\_or\_workflow\_data | keep\_restricted\_on\_request | F3 |  |  |  |  |  | restricted\_on\_request | keep\_restricted\_on\_request | SRTR-derived data file; provide only on request under licence obligations. | FALSE | TRUE | TRUE | FALSE | FALSE | FALSE | FALSE | FALSE | FALSE | restricted\_on\_request | keep\_restricted\_on\_request | SRTR-derived data file; provide only on request under licence obligations. | FALSE | FALSE | FALSE | FALSE | FALSE |
| 02\_workflows/F3\_workflow\_v01/data/02a\_refined\_analysis/esld\_srtr\_F3\_refined\_dataset\_qc\_internal.csv | esld\_srtr\_F3\_refined\_dataset\_qc\_internal.csv | 02\_workflows/F3\_workflow\_v01/data/02a\_refined\_analysis | csv | csv | 363 | 0.354 | 2026-05-02T09:50:43Z | 6e26808c17aede42ee2d644c8bd2a6e5 | internal\_only | derived | F3 | 12 | 2 | no\_internal\_only\_default | TRUE |  | release\_candidate | restricted\_on\_request | derived\_or\_workflow\_data | keep\_restricted\_on\_request | F3 |  |  |  |  |  | restricted\_on\_request | keep\_restricted\_on\_request | SRTR-derived data file; provide only on request under licence obligations. | FALSE | TRUE | TRUE | FALSE | FALSE | FALSE | FALSE | FALSE | FALSE | restricted\_on\_request | keep\_restricted\_on\_request | SRTR-derived data file; provide only on request under licence obligations. | FALSE | FALSE | FALSE | FALSE | FALSE |
| 02\_workflows/F3\_workflow\_v01/data/02a\_refined\_analysis/esld\_srtr\_F3\_refined\_manual\_clarifications\_internal.csv | esld\_srtr\_F3\_refined\_manual\_clarifications\_internal.csv | 02\_workflows/F3\_workflow\_v01/data/02a\_refined\_analysis | csv | csv | 285 | 0.278 | 2026-05-02T23:18:13Z | ad3d3c9b9bf99795f5fad68f070e624a | restricted\_on\_request | derived | F3 | 2 | 2 | yes\_default | TRUE |  | release\_candidate | restricted\_on\_request | derived\_or\_workflow\_data | keep\_restricted\_on\_request | F3 |  |  |  |  |  | restricted\_on\_request | keep\_restricted\_on\_request | SRTR-derived data file; provide only on request under licence obligations. | FALSE | TRUE | TRUE | FALSE | FALSE | FALSE | FALSE | FALSE | FALSE | restricted\_on\_request | keep\_restricted\_on\_request | SRTR-derived data file; provide only on request under licence obligations. | FALSE | FALSE | FALSE | FALSE | FALSE |
| 02\_workflows/F3\_workflow\_v01/data/02a\_refined\_analysis/srtr\_F3\_score\_shift\_counts\_internal.csv | srtr\_F3\_score\_shift\_counts\_internal.csv | 02\_workflows/F3\_workflow\_v01/data/02a\_refined\_analysis | csv | csv | 14332 | 13.996 | 2026-05-02T09:50:43Z | 28fef57605647f588c80fafacaff09b4 | restricted\_on\_request | derived | F3 | 680 | 4 | yes\_default | TRUE |  | release\_candidate | restricted\_on\_request | derived\_or\_workflow\_data | keep\_restricted\_on\_request | F3 |  |  |  |  |  | restricted\_on\_request | keep\_restricted\_on\_request | SRTR-derived data file; provide only on request under licence obligations. | FALSE | TRUE | TRUE | FALSE | FALSE | FALSE | FALSE | FALSE | FALSE | restricted\_on\_request | keep\_restricted\_on\_request | SRTR-derived data file; provide only on request under licence obligations. | FALSE | FALSE | FALSE | FALSE | FALSE |
| 02\_workflows/F3\_workflow\_v01/data/02b\_figure\_content/esld\_srtr\_F3\_score\_shift\_aggregate\_qc\_internal.csv | esld\_srtr\_F3\_score\_shift\_aggregate\_qc\_internal.csv | 02\_workflows/F3\_workflow\_v01/data/02b\_figure\_content | csv | csv | 350 | 0.342 | 2026-05-02T09:50:43Z | 27efd75697c3d4554da7529e0935e46d | internal\_only | derived | F3 | 12 | 2 | no\_internal\_only\_default | TRUE |  | release\_candidate | restricted\_on\_request | derived\_or\_workflow\_data | keep\_restricted\_on\_request | F3 |  |  |  |  |  | restricted\_on\_request | keep\_restricted\_on\_request | SRTR-derived data file; provide only on request under licence obligations. | FALSE | TRUE | TRUE | FALSE | FALSE | FALSE | FALSE | FALSE | FALSE | restricted\_on\_request | keep\_restricted\_on\_request | SRTR-derived data file; provide only on request under licence obligations. | FALSE | FALSE | FALSE | FALSE | FALSE |
| 02\_workflows/F3\_workflow\_v01/data/02b\_figure\_content/esld\_srtr\_F3\_score\_shift\_meta\_public.csv | esld\_srtr\_F3\_score\_shift\_meta\_public.csv | 02\_workflows/F3\_workflow\_v01/data/02b\_figure\_content | csv | csv | 428 | 0.418 | 2026-05-02T09:50:43Z | 6059a1283e3e5280b196ea6ae6e0d63d | restricted\_on\_request | derived | F3 | 2 | 6 | yes\_default | TRUE |  | release\_candidate | restricted\_on\_request | derived\_or\_workflow\_data | keep\_restricted\_on\_request | F3 |  |  |  |  |  | restricted\_on\_request | keep\_restricted\_on\_request | SRTR-derived data file; provide only on request under licence obligations. | FALSE | TRUE | TRUE | FALSE | FALSE | FALSE | FALSE | FALSE | FALSE | restricted\_on\_request | keep\_restricted\_on\_request | SRTR-derived data file; provide only on request under licence obligations. | FALSE | FALSE | FALSE | FALSE | FALSE |
| 02\_workflows/F3\_workflow\_v01/data/02b\_figure\_content/srtr\_F3\_score\_shift\_aggregate\_public.csv | srtr\_F3\_score\_shift\_aggregate\_public.csv | 02\_workflows/F3\_workflow\_v01/data/02b\_figure\_content | csv | csv | 14332 | 13.996 | 2026-05-02T09:50:43Z | 28fef57605647f588c80fafacaff09b4 | restricted\_on\_request | derived | F3 | 680 | 4 | yes\_default | TRUE |  | release\_candidate | restricted\_on\_request | derived\_or\_workflow\_data | keep\_restricted\_on\_request | F3 |  |  |  |  |  | restricted\_on\_request | keep\_restricted\_on\_request | SRTR-derived data file; provide only on request under licence obligations. | FALSE | TRUE | TRUE | FALSE | FALSE | FALSE | FALSE | FALSE | FALSE | restricted\_on\_request | keep\_restricted\_on\_request | SRTR-derived data file; provide only on request under licence obligations. | FALSE | FALSE | FALSE | FALSE | FALSE |
| 02\_workflows/F3\_workflow\_v01/submission\_ready/internal/data/esld\_srtr\_F3\_refined\_dataset\_qc\_internal.csv | esld\_srtr\_F3\_refined\_dataset\_qc\_internal.csv | 02\_workflows/F3\_workflow\_v01/submission\_ready/internal/data | csv | csv | 363 | 0.354 | 2026-05-02T09:50:43Z | 6e26808c17aede42ee2d644c8bd2a6e5 | internal\_only | derived | F3 | 12 | 2 | no\_internal\_only\_default | TRUE |  | release\_candidate | restricted\_on\_request | derived\_or\_workflow\_data | keep\_restricted\_on\_request | F3 |  |  |  |  |  | restricted\_on\_request | keep\_restricted\_on\_request | SRTR-derived data file; provide only on request under licence obligations. | FALSE | TRUE | TRUE | FALSE | FALSE | FALSE | FALSE | FALSE | FALSE | restricted\_on\_request | keep\_restricted\_on\_request | SRTR-derived data file; provide only on request under licence obligations. | FALSE | FALSE | FALSE | FALSE | FALSE |
| 02\_workflows/F3\_workflow\_v01/submission\_ready/internal/data/esld\_srtr\_F3\_refined\_manual\_clarifications\_internal.csv | esld\_srtr\_F3\_refined\_manual\_clarifications\_internal.csv | 02\_workflows/F3\_workflow\_v01/submission\_ready/internal/data | csv | csv | 285 | 0.278 | 2026-05-02T23:18:13Z | ad3d3c9b9bf99795f5fad68f070e624a | restricted\_on\_request | derived | F3 | 2 | 2 | yes\_default | TRUE |  | release\_candidate | restricted\_on\_request | derived\_or\_workflow\_data | keep\_restricted\_on\_request | F3 |  |  |  |  |  | restricted\_on\_request | keep\_restricted\_on\_request | SRTR-derived data file; provide only on request under licence obligations. | FALSE | TRUE | TRUE | FALSE | FALSE | FALSE | FALSE | FALSE | FALSE | restricted\_on\_request | keep\_restricted\_on\_request | SRTR-derived data file; provide only on request under licence obligations. | FALSE | FALSE | FALSE | FALSE | FALSE |
| 02\_workflows/F3\_workflow\_v01/submission\_ready/internal/data/srtr\_F3\_score\_shift\_counts\_internal.csv | srtr\_F3\_score\_shift\_counts\_internal.csv | 02\_workflows/F3\_workflow\_v01/submission\_ready/internal/data | csv | csv | 14332 | 13.996 | 2026-05-02T09:50:43Z | 28fef57605647f588c80fafacaff09b4 | restricted\_on\_request | derived | F3 | 680 | 4 | yes\_default | TRUE |  | release\_candidate | restricted\_on\_request | derived\_or\_workflow\_data | keep\_restricted\_on\_request | F3 |  |  |  |  |  | restricted\_on\_request | keep\_restricted\_on\_request | SRTR-derived data file; provide only on request under licence obligations. | FALSE | TRUE | TRUE | FALSE | FALSE | FALSE | FALSE | FALSE | FALSE | restricted\_on\_request | keep\_restricted\_on\_request | SRTR-derived data file; provide only on request under licence obligations. | FALSE | FALSE | FALSE | FALSE | FALSE |
| 02\_workflows/F3\_workflow\_v01/submission\_ready/public/data/esld\_srtr\_F3\_score\_shift\_meta\_public.csv | esld\_srtr\_F3\_score\_shift\_meta\_public.csv | 02\_workflows/F3\_workflow\_v01/submission\_ready/public/data | csv | csv | 428 | 0.418 | 2026-05-02T09:50:43Z | 6059a1283e3e5280b196ea6ae6e0d63d | restricted\_on\_request | derived | F3 | 2 | 6 | yes\_default | TRUE |  | release\_candidate | public\_deposit | derived\_or\_workflow\_data | keep\_public\_deposit | F3 |  |  |  |  |  | restricted\_on\_request | keep\_restricted\_on\_request | SRTR-derived data file; provide only on request under licence obligations. | FALSE | TRUE | TRUE | FALSE | FALSE | FALSE | FALSE | FALSE | FALSE | restricted\_on\_request | keep\_restricted\_on\_request | SRTR-derived data file; provide only on request under licence obligations. | FALSE | FALSE | FALSE | FALSE | FALSE |
| 02\_workflows/F3\_workflow\_v01/submission\_ready/public/data/srtr\_F3\_score\_shift\_aggregate\_public.csv | srtr\_F3\_score\_shift\_aggregate\_public.csv | 02\_workflows/F3\_workflow\_v01/submission\_ready/public/data | csv | csv | 14332 | 13.996 | 2026-05-02T09:50:43Z | 28fef57605647f588c80fafacaff09b4 | restricted\_on\_request | derived | F3 | 680 | 4 | yes\_default | TRUE |  | release\_candidate | public\_deposit | derived\_or\_workflow\_data | keep\_public\_deposit | F3 |  |  |  |  |  | restricted\_on\_request | keep\_restricted\_on\_request | SRTR-derived data file; provide only on request under licence obligations. | FALSE | TRUE | TRUE | FALSE | FALSE | FALSE | FALSE | FALSE | FALSE | restricted\_on\_request | keep\_restricted\_on\_request | SRTR-derived data file; provide only on request under licence obligations. | FALSE | FALSE | FALSE | FALSE | FALSE |
| 02\_workflows/F4\_workflow\_v01/data/01\_source\_loaded\_harmonized/esld\_srtr\_F4\_source\_load\_manifest\_internal.csv | esld\_srtr\_F4\_source\_load\_manifest\_internal.csv | 02\_workflows/F4\_workflow\_v01/data/01\_source\_loaded\_harmonized | csv | csv | 1841 | 1.798 | 2026-05-02T02:11:21Z | 0e67c54118abb91992b8cbdc9d6f2e6c | restricted\_on\_request | derived | F4 | 4 | 9 | yes\_default | TRUE |  | release\_candidate | restricted\_on\_request | derived\_or\_workflow\_data | keep\_restricted\_on\_request | F4 |  |  |  |  |  | restricted\_on\_request | keep\_restricted\_on\_request | SRTR-derived data file; provide only on request under licence obligations. | FALSE | TRUE | TRUE | FALSE | FALSE | FALSE | FALSE | FALSE | FALSE | restricted\_on\_request | keep\_restricted\_on\_request | SRTR-derived data file; provide only on request under licence obligations. | FALSE | FALSE | FALSE | FALSE | FALSE |
| 02\_workflows/F4\_workflow\_v01/data/01\_source\_loaded\_harmonized/esld\_srtr\_F4\_step1\_run\_inputs\_internal.csv | esld\_srtr\_F4\_step1\_run\_inputs\_internal.csv | 02\_workflows/F4\_workflow\_v01/data/01\_source\_loaded\_harmonized | csv | csv | 534 | 0.521 | 2026-05-02T02:11:21Z | 2dcdfd79c5e94c72bbc177a528512266 | restricted\_on\_request | derived | F4 | 3 | 2 | yes\_default | TRUE |  | release\_candidate | restricted\_on\_request | derived\_or\_workflow\_data | keep\_restricted\_on\_request | F4 |  |  |  |  |  | restricted\_on\_request | keep\_restricted\_on\_request | SRTR-derived data file; provide only on request under licence obligations. | FALSE | TRUE | TRUE | FALSE | FALSE | FALSE | FALSE | FALSE | FALSE | restricted\_on\_request | keep\_restricted\_on\_request | SRTR-derived data file; provide only on request under licence obligations. | FALSE | FALSE | FALSE | FALSE | FALSE |
| 02\_workflows/F4\_workflow\_v01/data/01\_source\_loaded\_harmonized/srtr\_F4\_survival\_median\_reference\_public.csv | srtr\_F4\_survival\_median\_reference\_public.csv | 02\_workflows/F4\_workflow\_v01/data/01\_source\_loaded\_harmonized | csv | csv | 1014 | 0.99 | 2026-04-18T23:02:32Z | 4f7479a5482294e06d950af23505003a | restricted\_on\_request | derived | F4 | 28 | 7 | yes\_default | TRUE |  | release\_candidate | restricted\_on\_request | derived\_or\_workflow\_data | keep\_restricted\_on\_request | F4 |  |  |  |  |  | restricted\_on\_request | keep\_restricted\_on\_request | SRTR-derived data file; provide only on request under licence obligations. | FALSE | TRUE | TRUE | FALSE | FALSE | FALSE | FALSE | FALSE | FALSE | restricted\_on\_request | keep\_restricted\_on\_request | SRTR-derived data file; provide only on request under licence obligations. | FALSE | FALSE | FALSE | FALSE | FALSE |
| 02\_workflows/F4\_workflow\_v01/data/01\_source\_loaded\_harmonized/srtr\_F4\_survival\_wilcoxon\_reference\_public.csv | srtr\_F4\_survival\_wilcoxon\_reference\_public.csv | 02\_workflows/F4\_workflow\_v01/data/01\_source\_loaded\_harmonized | csv | csv | 1189 | 1.161 | 2026-04-18T23:02:32Z | cb7f4c2bdf6d1953b807999547c73007 | restricted\_on\_request | derived | F4 | 28 | 8 | yes\_default | TRUE |  | release\_candidate | restricted\_on\_request | derived\_or\_workflow\_data | keep\_restricted\_on\_request | F4 |  |  |  |  |  | restricted\_on\_request | keep\_restricted\_on\_request | SRTR-derived data file; provide only on request under licence obligations. | FALSE | TRUE | TRUE | FALSE | FALSE | FALSE | FALSE | FALSE | FALSE | restricted\_on\_request | keep\_restricted\_on\_request | SRTR-derived data file; provide only on request under licence obligations. | FALSE | FALSE | FALSE | FALSE | FALSE |
| 02\_workflows/F4\_workflow\_v01/data/01\_source\_loaded\_harmonized/srtr\_master\_long\_restricted.csv | srtr\_master\_long\_restricted.csv | 02\_workflows/F4\_workflow\_v01/data/01\_source\_loaded\_harmonized | csv | csv | 44648516 | 43602.066 | 2026-04-30T15:41:56Z | 88c4909a08aed3fc79459eb78793a23d | restricted\_on\_request | derived | F4 | 193191 | 52 | yes\_default | TRUE |  | release\_candidate | restricted\_on\_request | derived\_or\_workflow\_data | keep\_restricted\_on\_request | F4 |  |  |  |  |  | restricted\_on\_request | keep\_restricted\_on\_request | SRTR-derived data file; provide only on request under licence obligations. | FALSE | TRUE | TRUE | FALSE | FALSE | FALSE | FALSE | FALSE | FALSE | restricted\_on\_request | keep\_restricted\_on\_request | SRTR-derived data file; provide only on request under licence obligations. | FALSE | FALSE | FALSE | FALSE | FALSE |
| 02\_workflows/F4\_workflow\_v01/data/02b\_figure\_content/esld\_srtr\_F4\_survival\_meta\_public.csv | esld\_srtr\_F4\_survival\_meta\_public.csv | 02\_workflows/F4\_workflow\_v01/data/02b\_figure\_content | csv | csv | 533 | 0.521 | 2026-05-02T02:11:22Z | 53d4700a360c6ade486ca44822c2ccd5 | restricted\_on\_request | derived | F4 | 12 | 2 | yes\_default | TRUE |  | release\_candidate | restricted\_on\_request | derived\_or\_workflow\_data | keep\_restricted\_on\_request | F4 |  |  |  |  |  | restricted\_on\_request | keep\_restricted\_on\_request | SRTR-derived data file; provide only on request under licence obligations. | FALSE | TRUE | TRUE | FALSE | FALSE | FALSE | FALSE | FALSE | FALSE | restricted\_on\_request | keep\_restricted\_on\_request | SRTR-derived data file; provide only on request under licence obligations. | FALSE | FALSE | FALSE | FALSE | FALSE |
| 02\_workflows/F4\_workflow\_v01/data/02b\_figure\_content/srtr\_F4\_survival\_median\_reference\_public.csv | srtr\_F4\_survival\_median\_reference\_public.csv | 02\_workflows/F4\_workflow\_v01/data/02b\_figure\_content | csv | csv | 1143 | 1.116 | 2026-05-02T02:11:22Z | c3cdfffaf37f5c94f3b376b8a23678f8 | restricted\_on\_request | derived | F4 | 28 | 7 | yes\_default | TRUE |  | release\_candidate | restricted\_on\_request | derived\_or\_workflow\_data | keep\_restricted\_on\_request | F4 |  |  |  |  |  | restricted\_on\_request | keep\_restricted\_on\_request | SRTR-derived data file; provide only on request under licence obligations. | FALSE | TRUE | TRUE | FALSE | FALSE | FALSE | FALSE | FALSE | FALSE | restricted\_on\_request | keep\_restricted\_on\_request | SRTR-derived data file; provide only on request under licence obligations. | FALSE | FALSE | FALSE | FALSE | FALSE |
| 02\_workflows/F4\_workflow\_v01/data/02b\_figure\_content/srtr\_F4\_survival\_wilcoxon\_reference\_public.csv | srtr\_F4\_survival\_wilcoxon\_reference\_public.csv | 02\_workflows/F4\_workflow\_v01/data/02b\_figure\_content | csv | csv | 1376 | 1.344 | 2026-05-02T02:11:22Z | ad8ae02fb35334f87b4f63b27387a837 | restricted\_on\_request | derived | F4 | 28 | 8 | yes\_default | TRUE |  | release\_candidate | restricted\_on\_request | derived\_or\_workflow\_data | keep\_restricted\_on\_request | F4 |  |  |  |  |  | restricted\_on\_request | keep\_restricted\_on\_request | SRTR-derived data file; provide only on request under licence obligations. | FALSE | TRUE | TRUE | FALSE | FALSE | FALSE | FALSE | FALSE | FALSE | restricted\_on\_request | keep\_restricted\_on\_request | SRTR-derived data file; provide only on request under licence obligations. | FALSE | FALSE | FALSE | FALSE | FALSE |
| 02\_workflows/F4\_workflow\_v01/submission\_ready/public/data/srtr\_F4\_survival\_median\_reference\_public.csv | srtr\_F4\_survival\_median\_reference\_public.csv | 02\_workflows/F4\_workflow\_v01/submission\_ready/public/data | csv | csv | 1143 | 1.116 | 2026-05-02T02:11:22Z | c3cdfffaf37f5c94f3b376b8a23678f8 | restricted\_on\_request | derived | F4 | 28 | 7 | yes\_default | TRUE |  | release\_candidate | public\_deposit | derived\_or\_workflow\_data | keep\_public\_deposit | F4 |  |  |  |  |  | restricted\_on\_request | keep\_restricted\_on\_request | SRTR-derived data file; provide only on request under licence obligations. | FALSE | TRUE | TRUE | FALSE | FALSE | FALSE | FALSE | FALSE | FALSE | restricted\_on\_request | keep\_restricted\_on\_request | SRTR-derived data file; provide only on request under licence obligations. | FALSE | FALSE | FALSE | FALSE | FALSE |
| 02\_workflows/F4\_workflow\_v01/submission\_ready/public/data/srtr\_F4\_survival\_wilcoxon\_reference\_public.csv | srtr\_F4\_survival\_wilcoxon\_reference\_public.csv | 02\_workflows/F4\_workflow\_v01/submission\_ready/public/data | csv | csv | 1376 | 1.344 | 2026-05-02T02:11:22Z | ad8ae02fb35334f87b4f63b27387a837 | restricted\_on\_request | derived | F4 | 28 | 8 | yes\_default | TRUE |  | release\_candidate | public\_deposit | derived\_or\_workflow\_data | keep\_public\_deposit | F4 |  |  |  |  |  | restricted\_on\_request | keep\_restricted\_on\_request | SRTR-derived data file; provide only on request under licence obligations. | FALSE | TRUE | TRUE | FALSE | FALSE | FALSE | FALSE | FALSE | FALSE | restricted\_on\_request | keep\_restricted\_on\_request | SRTR-derived data file; provide only on request under licence obligations. | FALSE | FALSE | FALSE | FALSE | FALSE |
| 02\_workflows/F6\_workflow\_v01/data/01\_source\_loaded\_harmonized/.~lock.srtr\_F6\_gray\_tests\_stats\_public.csv# | .~lock.srtr\_F6\_gray\_tests\_stats\_public.csv# | 02\_workflows/F6\_workflow\_v01/data/01\_source\_loaded\_harmonized | [none] | other | 115 | 0.112 | 2026-05-02T22:55:18Z | 05af8799e628ef10355f62eb79822d8d | restricted\_on\_request | derived | F6 |  |  | yes\_default | TRUE |  | release\_candidate | restricted\_on\_request | derived\_or\_workflow\_data | keep\_restricted\_on\_request | F6 |  |  |  |  |  | restricted\_on\_request | keep\_restricted\_on\_request | SRTR-derived data file; provide only on request under licence obligations. | FALSE | TRUE | TRUE | FALSE | FALSE | FALSE | FALSE | FALSE | FALSE | restricted\_on\_request | keep\_restricted\_on\_request | SRTR-derived data file; provide only on request under licence obligations. | FALSE | FALSE | FALSE | FALSE | FALSE |
| 02\_workflows/F6\_workflow\_v01/data/01\_source\_loaded\_harmonized/srtr\_F6\_cif\_long\_restricted.csv | srtr\_F6\_cif\_long\_restricted.csv | 02\_workflows/F6\_workflow\_v01/data/01\_source\_loaded\_harmonized | csv | csv | 3131663 | 3058.265 | 2026-05-02T03:11:53Z | f2c3409be09d5d4e6b800f1aadabe51f | restricted\_on\_request | derived | F6 | 48750 | 7 | yes\_default | TRUE |  | release\_candidate | restricted\_on\_request | derived\_or\_workflow\_data | keep\_restricted\_on\_request | F6 |  |  |  |  |  | restricted\_on\_request | keep\_restricted\_on\_request | SRTR-derived data file; provide only on request under licence obligations. | FALSE | TRUE | TRUE | FALSE | FALSE | FALSE | FALSE | FALSE | FALSE | restricted\_on\_request | keep\_restricted\_on\_request | SRTR-derived data file; provide only on request under licence obligations. | FALSE | FALSE | FALSE | FALSE | FALSE |
| 02\_workflows/F6\_workflow\_v01/data/01\_source\_loaded\_harmonized/srtr\_F6\_cif\_panel\_meta\_restricted.csv | srtr\_F6\_cif\_panel\_meta\_restricted.csv | 02\_workflows/F6\_workflow\_v01/data/01\_source\_loaded\_harmonized | csv | csv | 1281 | 1.251 | 2026-05-02T03:11:53Z | e3e58210cd5e8a813c8fa12e5c61e617 | restricted\_on\_request | derived | F6 | 12 | 11 | yes\_default | TRUE |  | release\_candidate | restricted\_on\_request | derived\_or\_workflow\_data | keep\_restricted\_on\_request | F6 |  |  |  |  |  | restricted\_on\_request | keep\_restricted\_on\_request | SRTR-derived data file; provide only on request under licence obligations. | FALSE | TRUE | TRUE | FALSE | FALSE | FALSE | FALSE | FALSE | FALSE | restricted\_on\_request | keep\_restricted\_on\_request | SRTR-derived data file; provide only on request under licence obligations. | FALSE | FALSE | FALSE | FALSE | FALSE |
| 02\_workflows/F6\_workflow\_v01/data/01\_source\_loaded\_harmonized/srtr\_F6\_gray\_tests\_stats\_public.csv | srtr\_F6\_gray\_tests\_stats\_public.csv | 02\_workflows/F6\_workflow\_v01/data/01\_source\_loaded\_harmonized | csv | csv | 3029 | 2.958 | 2026-05-02T03:11:53Z | b57ca4fa99465f55d3ad6cbda8198f8d | restricted\_on\_request | derived | F6 | 36 | 8 | yes\_default | TRUE |  | release\_candidate | restricted\_on\_request | derived\_or\_workflow\_data | keep\_restricted\_on\_request | F6 |  |  |  |  |  | restricted\_on\_request | keep\_restricted\_on\_request | SRTR-derived data file; provide only on request under licence obligations. | FALSE | TRUE | TRUE | FALSE | FALSE | FALSE | FALSE | FALSE | FALSE | restricted\_on\_request | keep\_restricted\_on\_request | SRTR-derived data file; provide only on request under licence obligations. | FALSE | FALSE | FALSE | FALSE | FALSE |
| 02\_workflows/F6\_workflow\_v01/data/01\_source\_loaded\_harmonized/srtr\_F6\_source\_load\_manifest\_internal.csv | srtr\_F6\_source\_load\_manifest\_internal.csv | 02\_workflows/F6\_workflow\_v01/data/01\_source\_loaded\_harmonized | csv | csv | 541 | 0.528 | 2026-05-02T03:11:52Z | 1ea3f2c452f51fc46414cd8f7d45182b | restricted\_on\_request | derived | F6 | 1 | 9 | yes\_default | TRUE |  | release\_candidate | restricted\_on\_request | derived\_or\_workflow\_data | keep\_restricted\_on\_request | F6 |  |  |  |  |  | restricted\_on\_request | keep\_restricted\_on\_request | SRTR-derived data file; provide only on request under licence obligations. | FALSE | TRUE | TRUE | FALSE | FALSE | FALSE | FALSE | FALSE | FALSE | restricted\_on\_request | keep\_restricted\_on\_request | SRTR-derived data file; provide only on request under licence obligations. | FALSE | FALSE | FALSE | FALSE | FALSE |
| 02\_workflows/F6\_workflow\_v01/data/01\_source\_loaded\_harmonized/srtr\_F6\_step1\_run\_inputs\_internal.csv | srtr\_F6\_step1\_run\_inputs\_internal.csv | 02\_workflows/F6\_workflow\_v01/data/01\_source\_loaded\_harmonized | csv | csv | 306 | 0.299 | 2026-05-02T03:11:52Z | ba12e14d516e5d103fcc4da48b3744af | restricted\_on\_request | derived | F6 | 2 | 2 | yes\_default | TRUE |  | release\_candidate | restricted\_on\_request | derived\_or\_workflow\_data | keep\_restricted\_on\_request | F6 |  |  |  |  |  | restricted\_on\_request | keep\_restricted\_on\_request | SRTR-derived data file; provide only on request under licence obligations. | FALSE | TRUE | TRUE | FALSE | FALSE | FALSE | FALSE | FALSE | FALSE | restricted\_on\_request | keep\_restricted\_on\_request | SRTR-derived data file; provide only on request under licence obligations. | FALSE | FALSE | FALSE | FALSE | FALSE |
| 02\_workflows/F6\_workflow\_v01/data/01\_source\_loaded\_harmonized/srtr\_master\_long\_restricted.csv | srtr\_master\_long\_restricted.csv | 02\_workflows/F6\_workflow\_v01/data/01\_source\_loaded\_harmonized | csv | csv | 44648516 | 43602.066 | 2026-04-30T15:41:56Z | 88c4909a08aed3fc79459eb78793a23d | restricted\_on\_request | derived | F6 | 193191 | 52 | yes\_default | TRUE |  | release\_candidate | restricted\_on\_request | derived\_or\_workflow\_data | keep\_restricted\_on\_request | F6 |  |  |  |  |  | restricted\_on\_request | keep\_restricted\_on\_request | SRTR-derived data file; provide only on request under licence obligations. | FALSE | TRUE | TRUE | FALSE | FALSE | FALSE | FALSE | FALSE | FALSE | restricted\_on\_request | keep\_restricted\_on\_request | SRTR-derived data file; provide only on request under licence obligations. | FALSE | FALSE | FALSE | FALSE | FALSE |
| 02\_workflows/F6\_workflow\_v01/data/02a\_refined\_analysis/srtr\_F6\_cif\_long\_restricted.csv | srtr\_F6\_cif\_long\_restricted.csv | 02\_workflows/F6\_workflow\_v01/data/02a\_refined\_analysis | csv | csv | 3131663 | 3058.265 | 2026-05-02T03:11:53Z | f2c3409be09d5d4e6b800f1aadabe51f | restricted\_on\_request | derived | F6 | 48750 | 7 | yes\_default | TRUE |  | release\_candidate | restricted\_on\_request | derived\_or\_workflow\_data | keep\_restricted\_on\_request | F6 |  |  |  |  |  | restricted\_on\_request | keep\_restricted\_on\_request | SRTR-derived data file; provide only on request under licence obligations. | FALSE | TRUE | TRUE | FALSE | FALSE | FALSE | FALSE | FALSE | FALSE | restricted\_on\_request | keep\_restricted\_on\_request | SRTR-derived data file; provide only on request under licence obligations. | FALSE | FALSE | FALSE | FALSE | FALSE |
| 02\_workflows/F6\_workflow\_v01/data/02a\_refined\_analysis/srtr\_F6\_cif\_panel\_meta\_restricted.csv | srtr\_F6\_cif\_panel\_meta\_restricted.csv | 02\_workflows/F6\_workflow\_v01/data/02a\_refined\_analysis | csv | csv | 1281 | 1.251 | 2026-05-02T03:11:53Z | e3e58210cd5e8a813c8fa12e5c61e617 | restricted\_on\_request | derived | F6 | 12 | 11 | yes\_default | TRUE |  | release\_candidate | restricted\_on\_request | derived\_or\_workflow\_data | keep\_restricted\_on\_request | F6 |  |  |  |  |  | restricted\_on\_request | keep\_restricted\_on\_request | SRTR-derived data file; provide only on request under licence obligations. | FALSE | TRUE | TRUE | FALSE | FALSE | FALSE | FALSE | FALSE | FALSE | restricted\_on\_request | keep\_restricted\_on\_request | SRTR-derived data file; provide only on request under licence obligations. | FALSE | FALSE | FALSE | FALSE | FALSE |
| 02\_workflows/F6\_workflow\_v01/data/02a\_refined\_analysis/srtr\_F6\_gray\_tests\_stats\_public.csv | srtr\_F6\_gray\_tests\_stats\_public.csv | 02\_workflows/F6\_workflow\_v01/data/02a\_refined\_analysis | csv | csv | 3029 | 2.958 | 2026-05-02T03:11:53Z | b57ca4fa99465f55d3ad6cbda8198f8d | restricted\_on\_request | derived | F6 | 36 | 8 | yes\_default | TRUE |  | release\_candidate | restricted\_on\_request | derived\_or\_workflow\_data | keep\_restricted\_on\_request | F6 |  |  |  |  |  | restricted\_on\_request | keep\_restricted\_on\_request | SRTR-derived data file; provide only on request under licence obligations. | FALSE | TRUE | TRUE | FALSE | FALSE | FALSE | FALSE | FALSE | FALSE | restricted\_on\_request | keep\_restricted\_on\_request | SRTR-derived data file; provide only on request under licence obligations. | FALSE | FALSE | FALSE | FALSE | FALSE |
| 02\_workflows/F6\_workflow\_v01/data/02a\_refined\_analysis/srtr\_F6\_rebuild\_from\_master\_audit\_internal.csv | srtr\_F6\_rebuild\_from\_master\_audit\_internal.csv | 02\_workflows/F6\_workflow\_v01/data/02a\_refined\_analysis | csv | csv | 882 | 0.861 | 2026-05-02T03:11:53Z | 1dd5b71f98264102cdd4b721bfbaefbc | internal\_only | derived | F6 | 12 | 12 | no\_internal\_only\_default | TRUE |  | release\_candidate | restricted\_on\_request | derived\_or\_workflow\_data | keep\_restricted\_on\_request | F6 |  |  |  |  |  | restricted\_on\_request | keep\_restricted\_on\_request | SRTR-derived data file; provide only on request under licence obligations. | FALSE | TRUE | TRUE | FALSE | FALSE | FALSE | FALSE | FALSE | FALSE | restricted\_on\_request | keep\_restricted\_on\_request | SRTR-derived data file; provide only on request under licence obligations. | FALSE | FALSE | FALSE | FALSE | FALSE |
| 02\_workflows/F6\_workflow\_v01/data/02a\_refined\_analysis/srtr\_F6\_rebuild\_from\_master\_provenance\_internal.csv | srtr\_F6\_rebuild\_from\_master\_provenance\_internal.csv | 02\_workflows/F6\_workflow\_v01/data/02a\_refined\_analysis | csv | csv | 646 | 0.631 | 2026-05-02T03:11:53Z | 00df70e443b32372afcbbb486a73cebd | restricted\_on\_request | derived | F6 | 1 | 8 | yes\_default | TRUE |  | release\_candidate | restricted\_on\_request | derived\_or\_workflow\_data | keep\_restricted\_on\_request | F6 |  |  |  |  |  | restricted\_on\_request | keep\_restricted\_on\_request | SRTR-derived data file; provide only on request under licence obligations. | FALSE | TRUE | TRUE | FALSE | FALSE | FALSE | FALSE | FALSE | FALSE | restricted\_on\_request | keep\_restricted\_on\_request | SRTR-derived data file; provide only on request under licence obligations. | FALSE | FALSE | FALSE | FALSE | FALSE |
| 02\_workflows/F6\_workflow\_v01/data/02a\_refined\_analysis/srtr\_F6\_rebuild\_from\_master\_qc\_internal.csv | srtr\_F6\_rebuild\_from\_master\_qc\_internal.csv | 02\_workflows/F6\_workflow\_v01/data/02a\_refined\_analysis | csv | csv | 404 | 0.395 | 2026-05-02T03:11:53Z | dc58aebf0e295dfc898e1abcf963ae03 | internal\_only | derived | F6 | 6 | 4 | no\_internal\_only\_default | TRUE |  | release\_candidate | restricted\_on\_request | derived\_or\_workflow\_data | keep\_restricted\_on\_request | F6 |  |  |  |  |  | restricted\_on\_request | keep\_restricted\_on\_request | SRTR-derived data file; provide only on request under licence obligations. | FALSE | TRUE | TRUE | FALSE | FALSE | FALSE | FALSE | FALSE | FALSE | restricted\_on\_request | keep\_restricted\_on\_request | SRTR-derived data file; provide only on request under licence obligations. | FALSE | FALSE | FALSE | FALSE | FALSE |
| 02\_workflows/F6\_workflow\_v01/data/02b\_figure\_content/srtr\_F6\_cif\_figure\_meta\_public.csv | srtr\_F6\_cif\_figure\_meta\_public.csv | 02\_workflows/F6\_workflow\_v01/data/02b\_figure\_content | csv | csv | 270 | 0.264 | 2026-05-02T03:11:53Z | 8e3036d40068c2e516526906e925d5ca | restricted\_on\_request | derived | F6 | 8 | 2 | yes\_default | TRUE |  | release\_candidate | restricted\_on\_request | derived\_or\_workflow\_data | keep\_restricted\_on\_request | F6 |  |  |  |  |  | restricted\_on\_request | keep\_restricted\_on\_request | SRTR-derived data file; provide only on request under licence obligations. | FALSE | TRUE | TRUE | FALSE | FALSE | FALSE | FALSE | FALSE | FALSE | restricted\_on\_request | keep\_restricted\_on\_request | SRTR-derived data file; provide only on request under licence obligations. | FALSE | FALSE | FALSE | FALSE | FALSE |
| 02\_workflows/F6\_workflow\_v01/data/02b\_figure\_content/srtr\_F6\_cif\_long\_restricted.csv | srtr\_F6\_cif\_long\_restricted.csv | 02\_workflows/F6\_workflow\_v01/data/02b\_figure\_content | csv | csv | 2741646 | 2677.389 | 2026-05-02T03:11:53Z | a7815a26c11cafc016ed20b394d658ea | restricted\_on\_request | derived | F6 | 48750 | 7 | yes\_default | TRUE |  | release\_candidate | restricted\_on\_request | derived\_or\_workflow\_data | keep\_restricted\_on\_request | F6 |  |  |  |  |  | restricted\_on\_request | keep\_restricted\_on\_request | SRTR-derived data file; provide only on request under licence obligations. | FALSE | TRUE | TRUE | FALSE | FALSE | FALSE | FALSE | FALSE | FALSE | restricted\_on\_request | keep\_restricted\_on\_request | SRTR-derived data file; provide only on request under licence obligations. | FALSE | FALSE | FALSE | FALSE | FALSE |
| 02\_workflows/F6\_workflow\_v01/data/02b\_figure\_content/srtr\_F6\_cif\_panel\_meta\_restricted.csv | srtr\_F6\_cif\_panel\_meta\_restricted.csv | 02\_workflows/F6\_workflow\_v01/data/02b\_figure\_content | csv | csv | 1208 | 1.18 | 2026-05-02T03:11:53Z | a4c4720d4182433217d88092b9eacc60 | restricted\_on\_request | derived | F6 | 12 | 11 | yes\_default | TRUE |  | release\_candidate | restricted\_on\_request | derived\_or\_workflow\_data | keep\_restricted\_on\_request | F6 |  |  |  |  |  | restricted\_on\_request | keep\_restricted\_on\_request | SRTR-derived data file; provide only on request under licence obligations. | FALSE | TRUE | TRUE | FALSE | FALSE | FALSE | FALSE | FALSE | FALSE | restricted\_on\_request | keep\_restricted\_on\_request | SRTR-derived data file; provide only on request under licence obligations. | FALSE | FALSE | FALSE | FALSE | FALSE |
| 02\_workflows/F6\_workflow\_v01/data/02b\_figure\_content/srtr\_F6\_figure\_content\_qc\_internal.csv | srtr\_F6\_figure\_content\_qc\_internal.csv | 02\_workflows/F6\_workflow\_v01/data/02b\_figure\_content | csv | csv | 224 | 0.219 | 2026-05-02T03:11:53Z | ebcfa1ece6baeab0a17d8c0560df6803 | internal\_only | derived | F6 | 3 | 8 | no\_internal\_only\_default | TRUE |  | release\_candidate | restricted\_on\_request | derived\_or\_workflow\_data | keep\_restricted\_on\_request | F6 |  |  |  |  |  | restricted\_on\_request | keep\_restricted\_on\_request | SRTR-derived data file; provide only on request under licence obligations. | FALSE | TRUE | TRUE | FALSE | FALSE | FALSE | FALSE | FALSE | FALSE | restricted\_on\_request | keep\_restricted\_on\_request | SRTR-derived data file; provide only on request under licence obligations. | FALSE | FALSE | FALSE | FALSE | FALSE |
| 02\_workflows/F6\_workflow\_v01/data/02b\_figure\_content/srtr\_F6\_gray\_tests\_stats\_public.csv | srtr\_F6\_gray\_tests\_stats\_public.csv | 02\_workflows/F6\_workflow\_v01/data/02b\_figure\_content | csv | csv | 2722 | 2.658 | 2026-05-02T03:11:53Z | 412448222d35840df92cf73baa52c72d | restricted\_on\_request | derived | F6 | 36 | 8 | yes\_default | TRUE |  | release\_candidate | restricted\_on\_request | derived\_or\_workflow\_data | keep\_restricted\_on\_request | F6 |  |  |  |  |  | restricted\_on\_request | keep\_restricted\_on\_request | SRTR-derived data file; provide only on request under licence obligations. | FALSE | TRUE | TRUE | FALSE | FALSE | FALSE | FALSE | FALSE | FALSE | restricted\_on\_request | keep\_restricted\_on\_request | SRTR-derived data file; provide only on request under licence obligations. | FALSE | FALSE | FALSE | FALSE | FALSE |
| 02\_workflows/F6\_workflow\_v01/submission\_ready/internal/data/srtr\_F6\_cif\_long\_restricted.csv | srtr\_F6\_cif\_long\_restricted.csv | 02\_workflows/F6\_workflow\_v01/submission\_ready/internal/data | csv | csv | 3131663 | 3058.265 | 2026-05-02T03:11:53Z | f2c3409be09d5d4e6b800f1aadabe51f | restricted\_on\_request | derived | F6 | 48750 | 7 | yes\_default | TRUE |  | release\_candidate | restricted\_on\_request | derived\_or\_workflow\_data | keep\_restricted\_on\_request | F6 |  |  |  |  |  | restricted\_on\_request | keep\_restricted\_on\_request | SRTR-derived data file; provide only on request under licence obligations. | FALSE | TRUE | TRUE | FALSE | FALSE | FALSE | FALSE | FALSE | FALSE | restricted\_on\_request | keep\_restricted\_on\_request | SRTR-derived data file; provide only on request under licence obligations. | FALSE | FALSE | FALSE | FALSE | FALSE |
| 02\_workflows/F6\_workflow\_v01/submission\_ready/internal/data/srtr\_F6\_cif\_panel\_meta\_restricted.csv | srtr\_F6\_cif\_panel\_meta\_restricted.csv | 02\_workflows/F6\_workflow\_v01/submission\_ready/internal/data | csv | csv | 1281 | 1.251 | 2026-05-02T03:11:53Z | e3e58210cd5e8a813c8fa12e5c61e617 | restricted\_on\_request | derived | F6 | 12 | 11 | yes\_default | TRUE |  | release\_candidate | restricted\_on\_request | derived\_or\_workflow\_data | keep\_restricted\_on\_request | F6 |  |  |  |  |  | restricted\_on\_request | keep\_restricted\_on\_request | SRTR-derived data file; provide only on request under licence obligations. | FALSE | TRUE | TRUE | FALSE | FALSE | FALSE | FALSE | FALSE | FALSE | restricted\_on\_request | keep\_restricted\_on\_request | SRTR-derived data file; provide only on request under licence obligations. | FALSE | FALSE | FALSE | FALSE | FALSE |
| 02\_workflows/F6\_workflow\_v01/submission\_ready/internal/data/srtr\_F6\_gray\_tests\_stats\_source\_internal.csv | srtr\_F6\_gray\_tests\_stats\_source\_internal.csv | 02\_workflows/F6\_workflow\_v01/submission\_ready/internal/data | csv | csv | 3029 | 2.958 | 2026-05-02T03:11:53Z | b57ca4fa99465f55d3ad6cbda8198f8d | restricted\_on\_request | derived | F6 | 36 | 8 | yes\_default | TRUE |  | release\_candidate | restricted\_on\_request | derived\_or\_workflow\_data | keep\_restricted\_on\_request | F6 |  |  |  |  |  | restricted\_on\_request | keep\_restricted\_on\_request | SRTR-derived data file; provide only on request under licence obligations. | FALSE | TRUE | TRUE | FALSE | FALSE | FALSE | FALSE | FALSE | FALSE | restricted\_on\_request | keep\_restricted\_on\_request | SRTR-derived data file; provide only on request under licence obligations. | FALSE | FALSE | FALSE | FALSE | FALSE |
| 02\_workflows/F6\_workflow\_v01/submission\_ready/public/data/srtr\_F6\_cif\_figure\_meta\_public.csv | srtr\_F6\_cif\_figure\_meta\_public.csv | 02\_workflows/F6\_workflow\_v01/submission\_ready/public/data | csv | csv | 270 | 0.264 | 2026-05-02T03:11:53Z | 8e3036d40068c2e516526906e925d5ca | restricted\_on\_request | derived | F6 | 8 | 2 | yes\_default | TRUE |  | release\_candidate | public\_deposit | derived\_or\_workflow\_data | keep\_public\_deposit | F6 |  |  |  |  |  | restricted\_on\_request | keep\_restricted\_on\_request | SRTR-derived data file; provide only on request under licence obligations. | FALSE | TRUE | TRUE | FALSE | FALSE | FALSE | FALSE | FALSE | FALSE | restricted\_on\_request | keep\_restricted\_on\_request | SRTR-derived data file; provide only on request under licence obligations. | FALSE | FALSE | FALSE | FALSE | FALSE |
| 02\_workflows/F6\_workflow\_v01/submission\_ready/public/data/srtr\_F6\_gray\_tests\_stats\_public.csv | srtr\_F6\_gray\_tests\_stats\_public.csv | 02\_workflows/F6\_workflow\_v01/submission\_ready/public/data | csv | csv | 2722 | 2.658 | 2026-05-02T03:11:53Z | 412448222d35840df92cf73baa52c72d | restricted\_on\_request | derived | F6 | 36 | 8 | yes\_default | TRUE |  | release\_candidate | public\_deposit | derived\_or\_workflow\_data | keep\_public\_deposit | F6 |  |  |  |  |  | restricted\_on\_request | keep\_restricted\_on\_request | SRTR-derived data file; provide only on request under licence obligations. | FALSE | TRUE | TRUE | FALSE | FALSE | FALSE | FALSE | FALSE | FALSE | restricted\_on\_request | keep\_restricted\_on\_request | SRTR-derived data file; provide only on request under licence obligations. | FALSE | FALSE | FALSE | FALSE | FALSE |
| 02\_workflows/F6\_workflow\_v01/submission\_ready/restricted/F6\_restricted\_submission\_manifest\_v01.csv | F6\_restricted\_submission\_manifest\_v01.csv | 02\_workflows/F6\_workflow\_v01/submission\_ready/restricted | csv | csv | 1458 | 1.424 | 2026-05-02T03:11:53Z | 13883576f14aff9da7bd10c8594745d6 | restricted\_on\_request | derived | F6 | 2 | 10 | yes\_default | TRUE |  | release\_candidate | restricted\_on\_request | documentation\_or\_metadata | keep\_restricted\_on\_request | F6 |  |  |  |  |  | restricted\_on\_request | keep\_restricted\_on\_request | Restricted-on-request file after policy overrides. | FALSE | FALSE | TRUE | FALSE | FALSE | FALSE | FALSE | FALSE | FALSE | restricted\_on\_request | keep\_restricted\_on\_request | Restricted-on-request file after policy overrides. | FALSE | FALSE | FALSE | FALSE | FALSE |
| 02\_workflows/F6\_workflow\_v01/submission\_ready/restricted/data/srtr\_F6\_cif\_long\_restricted.csv | srtr\_F6\_cif\_long\_restricted.csv | 02\_workflows/F6\_workflow\_v01/submission\_ready/restricted/data | csv | csv | 2741646 | 2677.389 | 2026-05-02T03:11:53Z | a7815a26c11cafc016ed20b394d658ea | restricted\_on\_request | derived | F6 | 48750 | 7 | yes\_default | TRUE |  | release\_candidate | restricted\_on\_request | derived\_or\_workflow\_data | keep\_restricted\_on\_request | F6 |  |  |  |  |  | restricted\_on\_request | keep\_restricted\_on\_request | SRTR-derived data file; provide only on request under licence obligations. | FALSE | TRUE | TRUE | FALSE | FALSE | FALSE | FALSE | FALSE | FALSE | restricted\_on\_request | keep\_restricted\_on\_request | SRTR-derived data file; provide only on request under licence obligations. | FALSE | FALSE | FALSE | FALSE | FALSE |
| 02\_workflows/F6\_workflow\_v01/submission\_ready/restricted/data/srtr\_F6\_cif\_panel\_meta\_restricted.csv | srtr\_F6\_cif\_panel\_meta\_restricted.csv | 02\_workflows/F6\_workflow\_v01/submission\_ready/restricted/data | csv | csv | 1208 | 1.18 | 2026-05-02T03:11:53Z | a4c4720d4182433217d88092b9eacc60 | restricted\_on\_request | derived | F6 | 12 | 11 | yes\_default | TRUE |  | release\_candidate | restricted\_on\_request | derived\_or\_workflow\_data | keep\_restricted\_on\_request | F6 |  |  |  |  |  | restricted\_on\_request | keep\_restricted\_on\_request | SRTR-derived data file; provide only on request under licence obligations. | FALSE | TRUE | TRUE | FALSE | FALSE | FALSE | FALSE | FALSE | FALSE | restricted\_on\_request | keep\_restricted\_on\_request | SRTR-derived data file; provide only on request under licence obligations. | FALSE | FALSE | FALSE | FALSE | FALSE |
| 02\_workflows/T1\_workflow\_v01/data/01\_source\_loaded\_harmonized/srtr\_master\_long\_restricted.csv | srtr\_master\_long\_restricted.csv | 02\_workflows/T1\_workflow\_v01/data/01\_source\_loaded\_harmonized | csv | csv | 44648516 | 43602.066 | 2026-04-30T15:41:56Z | 88c4909a08aed3fc79459eb78793a23d | restricted\_on\_request | derived | T1 | 193191 | 52 | yes\_default | TRUE |  | release\_candidate | restricted\_on\_request | derived\_or\_workflow\_data | keep\_restricted\_on\_request | T1 |  |  |  |  |  | restricted\_on\_request | keep\_restricted\_on\_request | SRTR-derived data file; provide only on request under licence obligations. | FALSE | TRUE | TRUE | FALSE | FALSE | FALSE | FALSE | FALSE | FALSE | restricted\_on\_request | keep\_restricted\_on\_request | SRTR-derived data file; provide only on request under licence obligations. | FALSE | FALSE | FALSE | FALSE | FALSE |
| 02\_workflows/T1\_workflow\_v01/data/02a\_refined\_analysis/esld\_srtr\_T1\_refined\_dataset\_qc\_internal.csv | esld\_srtr\_T1\_refined\_dataset\_qc\_internal.csv | 02\_workflows/T1\_workflow\_v01/data/02a\_refined\_analysis | csv | csv | 237 | 0.231 | 2026-05-02T20:27:47Z | 75c156283cc38547f2a7f07e1efae97e | internal\_only | derived | T1 | 6 | 2 | no\_internal\_only\_default | TRUE |  | release\_candidate | restricted\_on\_request | derived\_or\_workflow\_data | keep\_restricted\_on\_request | T1 |  |  |  |  |  | restricted\_on\_request | keep\_restricted\_on\_request | SRTR-derived data file; provide only on request under licence obligations. | FALSE | TRUE | TRUE | FALSE | FALSE | FALSE | FALSE | FALSE | FALSE | restricted\_on\_request | keep\_restricted\_on\_request | SRTR-derived data file; provide only on request under licence obligations. | FALSE | FALSE | FALSE | FALSE | FALSE |
| 02\_workflows/T1\_workflow\_v01/data/02a\_refined\_analysis/srtr\_T1\_source\_context\_internal.csv | srtr\_T1\_source\_context\_internal.csv | 02\_workflows/T1\_workflow\_v01/data/02a\_refined\_analysis | csv | csv | 946 | 0.924 | 2026-05-02T20:27:47Z | 4a4875401deb5a259edd09e9b9e767b6 | restricted\_on\_request | derived | T1 | 4 | 2 | yes\_default | TRUE |  | release\_candidate | restricted\_on\_request | derived\_or\_workflow\_data | keep\_restricted\_on\_request | T1 |  |  |  |  |  | restricted\_on\_request | keep\_restricted\_on\_request | SRTR-derived data file; provide only on request under licence obligations. | FALSE | TRUE | TRUE | FALSE | FALSE | FALSE | FALSE | FALSE | FALSE | restricted\_on\_request | keep\_restricted\_on\_request | SRTR-derived data file; provide only on request under licence obligations. | FALSE | FALSE | FALSE | FALSE | FALSE |
| 02\_workflows/T1\_workflow\_v01/data/02b\_table\_content/esld\_srtr\_T1\_etiology\_denominator\_patch\_audit\_internal.csv | esld\_srtr\_T1\_etiology\_denominator\_patch\_audit\_internal.csv | 02\_workflows/T1\_workflow\_v01/data/02b\_table\_content | csv | csv | 2312 | 2.258 | 2026-05-02T20:27:47Z | 4e0164895f653ecdf97474b8dbe46b64 | internal\_only | derived | T1;table\_or\_supplement | 8 | 12 | no\_internal\_only\_default | TRUE |  | release\_candidate | restricted\_on\_request | derived\_or\_workflow\_data | keep\_restricted\_on\_request | T1 |  |  |  |  |  | restricted\_on\_request | keep\_restricted\_on\_request | SRTR-derived data file; provide only on request under licence obligations. | FALSE | TRUE | TRUE | FALSE | FALSE | FALSE | FALSE | FALSE | FALSE | restricted\_on\_request | keep\_restricted\_on\_request | SRTR-derived data file; provide only on request under licence obligations. | FALSE | FALSE | FALSE | FALSE | FALSE |
| 02\_workflows/T1\_workflow\_v01/data/02b\_table\_content/esld\_srtr\_T1\_full\_reference\_table\_override\_internal.csv | esld\_srtr\_T1\_full\_reference\_table\_override\_internal.csv | 02\_workflows/T1\_workflow\_v01/data/02b\_table\_content | csv | csv | 4406 | 4.303 | 2026-05-02T20:27:47Z | 6d71df8a593b4ebb696ea3e34ea4cb79 | restricted\_on\_request | derived | T1;table\_or\_supplement | 23 | 8 | yes\_default | TRUE |  | release\_candidate | restricted\_on\_request | derived\_or\_workflow\_data | keep\_restricted\_on\_request | T1 |  |  |  |  |  | restricted\_on\_request | keep\_restricted\_on\_request | SRTR-derived data file; provide only on request under licence obligations. | FALSE | TRUE | TRUE | FALSE | FALSE | FALSE | FALSE | FALSE | FALSE | restricted\_on\_request | keep\_restricted\_on\_request | SRTR-derived data file; provide only on request under licence obligations. | FALSE | FALSE | FALSE | FALSE | FALSE |
| 02\_workflows/T1\_workflow\_v01/data/02b\_table\_content/esld\_srtr\_T1\_general\_characteristics\_build\_qc\_internal.csv | esld\_srtr\_T1\_general\_characteristics\_build\_qc\_internal.csv | 02\_workflows/T1\_workflow\_v01/data/02b\_table\_content | csv | csv | 1020 | 0.996 | 2026-05-02T20:27:47Z | ae390f026fb9bb64f88a4846eb1e22b7 | internal\_only | derived | T1;table\_or\_supplement | 6 | 4 | no\_internal\_only\_default | TRUE |  | release\_candidate | restricted\_on\_request | derived\_or\_workflow\_data | keep\_restricted\_on\_request | T1 |  |  |  |  |  | restricted\_on\_request | keep\_restricted\_on\_request | SRTR-derived data file; provide only on request under licence obligations. | FALSE | TRUE | TRUE | FALSE | FALSE | FALSE | FALSE | FALSE | FALSE | restricted\_on\_request | keep\_restricted\_on\_request | SRTR-derived data file; provide only on request under licence obligations. | FALSE | FALSE | FALSE | FALSE | FALSE |
| 02\_workflows/T1\_workflow\_v01/data/02b\_table\_content/esld\_srtr\_T1\_general\_characteristics\_compare\_to\_submitted\_internal.csv | esld\_srtr\_T1\_general\_characteristics\_compare\_to\_submitted\_internal.csv | 02\_workflows/T1\_workflow\_v01/data/02b\_table\_content | csv | csv | 5927 | 5.788 | 2026-05-02T20:27:47Z | 4660692bfba7ca207fdcf35e4b7e188b | restricted\_on\_request | derived | T1;table\_or\_supplement | 23 | 11 | yes\_default | TRUE |  | release\_candidate | restricted\_on\_request | derived\_or\_workflow\_data | keep\_restricted\_on\_request | T1 |  |  |  |  |  | restricted\_on\_request | keep\_restricted\_on\_request | SRTR-derived data file; provide only on request under licence obligations. | FALSE | TRUE | TRUE | FALSE | FALSE | FALSE | FALSE | FALSE | FALSE | restricted\_on\_request | keep\_restricted\_on\_request | SRTR-derived data file; provide only on request under licence obligations. | FALSE | FALSE | FALSE | FALSE | FALSE |
| 02\_workflows/T1\_workflow\_v01/data/02b\_table\_content/esld\_srtr\_T1\_general\_characteristics\_meta\_public.csv | esld\_srtr\_T1\_general\_characteristics\_meta\_public.csv | 02\_workflows/T1\_workflow\_v01/data/02b\_table\_content | csv | csv | 647 | 0.632 | 2026-05-02T20:27:47Z | 3f78b474a5a81e3fd94b7309e4eef48f | restricted\_on\_request | derived | T1;table\_or\_supplement | 10 | 2 | yes\_default | TRUE |  | release\_candidate | restricted\_on\_request | derived\_or\_workflow\_data | keep\_restricted\_on\_request | T1 |  |  |  |  |  | restricted\_on\_request | keep\_restricted\_on\_request | SRTR-derived data file; provide only on request under licence obligations. | FALSE | TRUE | TRUE | FALSE | FALSE | FALSE | FALSE | FALSE | FALSE | restricted\_on\_request | keep\_restricted\_on\_request | SRTR-derived data file; provide only on request under licence obligations. | FALSE | FALSE | FALSE | FALSE | FALSE |
| 02\_workflows/T1\_workflow\_v01/data/02b\_table\_content/esld\_srtr\_T1\_general\_characteristics\_public.csv | esld\_srtr\_T1\_general\_characteristics\_public.csv | 02\_workflows/T1\_workflow\_v01/data/02b\_table\_content | csv | csv | 2847 | 2.78 | 2026-05-02T20:27:47Z | 4a9d4eef6746ef68594fd54333d9dd0c | restricted\_on\_request | derived | T1;table\_or\_supplement | 23 | 9 | yes\_default | TRUE |  | release\_candidate | restricted\_on\_request | derived\_or\_workflow\_data | keep\_restricted\_on\_request | T1 |  |  |  |  |  | restricted\_on\_request | keep\_restricted\_on\_request | SRTR-derived data file; provide only on request under licence obligations. | FALSE | TRUE | TRUE | FALSE | FALSE | FALSE | FALSE | FALSE | FALSE | restricted\_on\_request | keep\_restricted\_on\_request | SRTR-derived data file; provide only on request under licence obligations. | FALSE | FALSE | FALSE | FALSE | FALSE |
| 02\_workflows/T1\_workflow\_v01/data/02b\_table\_content/esld\_srtr\_T1\_general\_characteristics\_recalculated\_internal.csv | esld\_srtr\_T1\_general\_characteristics\_recalculated\_internal.csv | 02\_workflows/T1\_workflow\_v01/data/02b\_table\_content | csv | csv | 3378 | 3.299 | 2026-05-02T20:27:47Z | 0c3593bff7d29b78d9685cb5598b7837 | restricted\_on\_request | derived | T1;table\_or\_supplement | 23 | 10 | yes\_default | TRUE |  | release\_candidate | restricted\_on\_request | derived\_or\_workflow\_data | keep\_restricted\_on\_request | T1 |  |  |  |  |  | restricted\_on\_request | keep\_restricted\_on\_request | SRTR-derived data file; provide only on request under licence obligations. | FALSE | TRUE | TRUE | FALSE | FALSE | FALSE | FALSE | FALSE | FALSE | restricted\_on\_request | keep\_restricted\_on\_request | SRTR-derived data file; provide only on request under licence obligations. | FALSE | FALSE | FALSE | FALSE | FALSE |
| 02\_workflows/T1\_workflow\_v01/data/02b\_table\_content/esld\_srtr\_T1\_general\_characteristics\_recalculation\_meta\_internal.csv | esld\_srtr\_T1\_general\_characteristics\_recalculation\_meta\_internal.csv | 02\_workflows/T1\_workflow\_v01/data/02b\_table\_content | csv | csv | 662 | 0.646 | 2026-05-02T20:27:47Z | fe4cc8dfcce6ab4629e8a4befc1e7a2d | restricted\_on\_request | derived | T1;table\_or\_supplement | 15 | 2 | yes\_default | TRUE |  | release\_candidate | restricted\_on\_request | derived\_or\_workflow\_data | keep\_restricted\_on\_request | T1 |  |  |  |  |  | restricted\_on\_request | keep\_restricted\_on\_request | SRTR-derived data file; provide only on request under licence obligations. | FALSE | TRUE | TRUE | FALSE | FALSE | FALSE | FALSE | FALSE | FALSE | restricted\_on\_request | keep\_restricted\_on\_request | SRTR-derived data file; provide only on request under licence obligations. | FALSE | FALSE | FALSE | FALSE | FALSE |
| 02\_workflows/T1\_workflow\_v01/data/02b\_table\_content/esld\_srtr\_T1\_reference\_value\_corrections\_internal.csv | esld\_srtr\_T1\_reference\_value\_corrections\_internal.csv | 02\_workflows/T1\_workflow\_v01/data/02b\_table\_content | csv | csv | 1065 | 1.04 | 2026-05-02T19:05:19Z | 3056cab6e6c0fcd7ad526cb1ceded376 | restricted\_on\_request | derived | T1;table\_or\_supplement | 2 | 11 | yes\_default | TRUE |  | release\_candidate | restricted\_on\_request | derived\_or\_workflow\_data | keep\_restricted\_on\_request | T1 |  |  |  |  |  | restricted\_on\_request | keep\_restricted\_on\_request | SRTR-derived data file; provide only on request under licence obligations. | FALSE | TRUE | TRUE | FALSE | FALSE | FALSE | FALSE | FALSE | FALSE | restricted\_on\_request | keep\_restricted\_on\_request | SRTR-derived data file; provide only on request under licence obligations. | FALSE | FALSE | FALSE | FALSE | FALSE |
| 02\_workflows/T1\_workflow\_v01/data/02b\_table\_content/esld\_srtr\_T1\_sodium\_albumin\_recalc\_patch\_audit\_internal.csv | esld\_srtr\_T1\_sodium\_albumin\_recalc\_patch\_audit\_internal.csv | 02\_workflows/T1\_workflow\_v01/data/02b\_table\_content | csv | csv | 1093 | 1.067 | 2026-05-02T20:27:47Z | bbf0ebc7653faaa97885d99fab84561c | internal\_only | derived | T1;table\_or\_supplement | 2 | 10 | no\_internal\_only\_default | TRUE |  | release\_candidate | restricted\_on\_request | derived\_or\_workflow\_data | keep\_restricted\_on\_request | T1 |  |  |  |  |  | restricted\_on\_request | keep\_restricted\_on\_request | SRTR-derived data file; provide only on request under licence obligations. | FALSE | TRUE | TRUE | FALSE | FALSE | FALSE | FALSE | FALSE | FALSE | restricted\_on\_request | keep\_restricted\_on\_request | SRTR-derived data file; provide only on request under licence obligations. | FALSE | FALSE | FALSE | FALSE | FALSE |
| 02\_workflows/T1\_workflow\_v01/submission\_ready/internal/data/esld\_srtr\_T1\_general\_characteristics\_compare\_to\_submitted\_internal.csv | esld\_srtr\_T1\_general\_characteristics\_compare\_to\_submitted\_internal.csv | 02\_workflows/T1\_workflow\_v01/submission\_ready/internal/data | csv | csv | 5927 | 5.788 | 2026-05-02T20:27:48Z | 4660692bfba7ca207fdcf35e4b7e188b | restricted\_on\_request | derived | T1 | 23 | 11 | yes\_default | TRUE |  | release\_candidate | restricted\_on\_request | derived\_or\_workflow\_data | keep\_restricted\_on\_request | T1 |  |  |  |  |  | restricted\_on\_request | keep\_restricted\_on\_request | SRTR-derived data file; provide only on request under licence obligations. | FALSE | TRUE | TRUE | FALSE | FALSE | FALSE | FALSE | FALSE | FALSE | restricted\_on\_request | keep\_restricted\_on\_request | SRTR-derived data file; provide only on request under licence obligations. | FALSE | FALSE | FALSE | FALSE | FALSE |
| 02\_workflows/T1\_workflow\_v01/submission\_ready/internal/data/esld\_srtr\_T1\_general\_characteristics\_recalculated\_internal.csv | esld\_srtr\_T1\_general\_characteristics\_recalculated\_internal.csv | 02\_workflows/T1\_workflow\_v01/submission\_ready/internal/data | csv | csv | 3378 | 3.299 | 2026-05-02T20:27:48Z | 0c3593bff7d29b78d9685cb5598b7837 | restricted\_on\_request | derived | T1 | 23 | 10 | yes\_default | TRUE |  | release\_candidate | restricted\_on\_request | derived\_or\_workflow\_data | keep\_restricted\_on\_request | T1 |  |  |  |  |  | restricted\_on\_request | keep\_restricted\_on\_request | SRTR-derived data file; provide only on request under licence obligations. | FALSE | TRUE | TRUE | FALSE | FALSE | FALSE | FALSE | FALSE | FALSE | restricted\_on\_request | keep\_restricted\_on\_request | SRTR-derived data file; provide only on request under licence obligations. | FALSE | FALSE | FALSE | FALSE | FALSE |
| 02\_workflows/T1\_workflow\_v01/submission\_ready/internal/data/esld\_srtr\_T1\_general\_characteristics\_recalculation\_meta\_internal.csv | esld\_srtr\_T1\_general\_characteristics\_recalculation\_meta\_internal.csv | 02\_workflows/T1\_workflow\_v01/submission\_ready/internal/data | csv | csv | 662 | 0.646 | 2026-05-02T20:27:48Z | fe4cc8dfcce6ab4629e8a4befc1e7a2d | restricted\_on\_request | derived | T1 | 15 | 2 | yes\_default | TRUE |  | release\_candidate | restricted\_on\_request | derived\_or\_workflow\_data | keep\_restricted\_on\_request | T1 |  |  |  |  |  | restricted\_on\_request | keep\_restricted\_on\_request | SRTR-derived data file; provide only on request under licence obligations. | FALSE | TRUE | TRUE | FALSE | FALSE | FALSE | FALSE | FALSE | FALSE | restricted\_on\_request | keep\_restricted\_on\_request | SRTR-derived data file; provide only on request under licence obligations. | FALSE | FALSE | FALSE | FALSE | FALSE |
| 02\_workflows/T1\_workflow\_v01/submission\_ready/public/data/esld\_srtr\_T1\_general\_characteristics\_meta\_public.csv | esld\_srtr\_T1\_general\_characteristics\_meta\_public.csv | 02\_workflows/T1\_workflow\_v01/submission\_ready/public/data | csv | csv | 647 | 0.632 | 2026-05-02T20:27:48Z | 3f78b474a5a81e3fd94b7309e4eef48f | restricted\_on\_request | derived | T1 | 10 | 2 | yes\_default | TRUE |  | release\_candidate | public\_deposit | derived\_or\_workflow\_data | keep\_public\_deposit | T1 |  |  |  |  |  | restricted\_on\_request | keep\_restricted\_on\_request | SRTR-derived data file; provide only on request under licence obligations. | FALSE | TRUE | TRUE | FALSE | FALSE | FALSE | FALSE | FALSE | FALSE | restricted\_on\_request | keep\_restricted\_on\_request | SRTR-derived data file; provide only on request under licence obligations. | FALSE | FALSE | FALSE | FALSE | FALSE |
| 02\_workflows/T1\_workflow\_v01/submission\_ready/public/data/esld\_srtr\_T1\_general\_characteristics\_public.csv | esld\_srtr\_T1\_general\_characteristics\_public.csv | 02\_workflows/T1\_workflow\_v01/submission\_ready/public/data | csv | csv | 2847 | 2.78 | 2026-05-02T20:27:48Z | 4a9d4eef6746ef68594fd54333d9dd0c | restricted\_on\_request | derived | T1 | 23 | 9 | yes\_default | TRUE |  | release\_candidate | public\_deposit | derived\_or\_workflow\_data | keep\_public\_deposit | T1 |  |  |  |  |  | restricted\_on\_request | keep\_restricted\_on\_request | SRTR-derived data file; provide only on request under licence obligations. | FALSE | TRUE | TRUE | FALSE | FALSE | FALSE | FALSE | FALSE | FALSE | restricted\_on\_request | keep\_restricted\_on\_request | SRTR-derived data file; provide only on request under licence obligations. | FALSE | FALSE | FALSE | FALSE | FALSE |
| 02\_workflows/T3\_workflow\_v01/data/02b\_table\_content/esld\_srtr\_T3\_creatinine\_comparison\_build\_qc\_internal.csv | esld\_srtr\_T3\_creatinine\_comparison\_build\_qc\_internal.csv | 02\_workflows/T3\_workflow\_v01/data/02b\_table\_content | csv | csv | 1476 | 1.441 | 2026-05-02T21:14:25Z | 5612dd3dafcbe725e9bca154c0a00960 | internal\_only | derived | T3;table\_or\_supplement | 6 | 4 | no\_internal\_only\_default | TRUE |  | release\_candidate | restricted\_on\_request | derived\_or\_workflow\_data | keep\_restricted\_on\_request | T3 |  |  |  |  |  | restricted\_on\_request | keep\_restricted\_on\_request | SRTR-derived data file; provide only on request under licence obligations. | FALSE | TRUE | TRUE | FALSE | FALSE | FALSE | FALSE | FALSE | FALSE | restricted\_on\_request | keep\_restricted\_on\_request | SRTR-derived data file; provide only on request under licence obligations. | FALSE | FALSE | FALSE | FALSE | FALSE |
| 02\_workflows/T3\_workflow\_v01/data/02b\_table\_content/esld\_srtr\_T3\_creatinine\_comparison\_meta\_public.csv | esld\_srtr\_T3\_creatinine\_comparison\_meta\_public.csv | 02\_workflows/T3\_workflow\_v01/data/02b\_table\_content | csv | csv | 1041 | 1.017 | 2026-05-02T21:14:25Z | 980587f5b202b799ab283c9920f9a1ad | restricted\_on\_request | derived | T3;table\_or\_supplement | 15 | 2 | yes\_default | TRUE |  | release\_candidate | restricted\_on\_request | derived\_or\_workflow\_data | keep\_restricted\_on\_request | T3 |  |  |  |  |  | restricted\_on\_request | keep\_restricted\_on\_request | SRTR-derived data file; provide only on request under licence obligations. | FALSE | TRUE | TRUE | FALSE | FALSE | FALSE | FALSE | FALSE | FALSE | restricted\_on\_request | keep\_restricted\_on\_request | SRTR-derived data file; provide only on request under licence obligations. | FALSE | FALSE | FALSE | FALSE | FALSE |
| 02\_workflows/T3\_workflow\_v01/data/02b\_table\_content/esld\_srtr\_T3\_creatinine\_comparison\_table\_public.csv | esld\_srtr\_T3\_creatinine\_comparison\_table\_public.csv | 02\_workflows/T3\_workflow\_v01/data/02b\_table\_content | csv | csv | 5337 | 5.212 | 2026-05-02T21:14:25Z | 5fe481717fafc7d2115b64a01112efbe | restricted\_on\_request | derived | T3;table\_or\_supplement | 24 | 15 | yes\_default | TRUE |  | release\_candidate | restricted\_on\_request | derived\_or\_workflow\_data | keep\_restricted\_on\_request | T3 |  |  |  |  |  | restricted\_on\_request | keep\_restricted\_on\_request | SRTR-derived data file; provide only on request under licence obligations. | FALSE | TRUE | TRUE | FALSE | FALSE | FALSE | FALSE | FALSE | FALSE | restricted\_on\_request | keep\_restricted\_on\_request | SRTR-derived data file; provide only on request under licence obligations. | FALSE | FALSE | FALSE | FALSE | FALSE |
| 02\_workflows/T3\_workflow\_v01/submission\_ready/public/data/esld\_srtr\_T3\_creatinine\_comparison\_meta\_public.csv | esld\_srtr\_T3\_creatinine\_comparison\_meta\_public.csv | 02\_workflows/T3\_workflow\_v01/submission\_ready/public/data | csv | csv | 1041 | 1.017 | 2026-05-02T21:14:25Z | 980587f5b202b799ab283c9920f9a1ad | restricted\_on\_request | derived | T3 | 15 | 2 | yes\_default | TRUE |  | release\_candidate | public\_deposit | derived\_or\_workflow\_data | keep\_public\_deposit | T3 |  |  |  |  |  | restricted\_on\_request | keep\_restricted\_on\_request | SRTR-derived data file; provide only on request under licence obligations. | FALSE | TRUE | TRUE | FALSE | FALSE | FALSE | FALSE | FALSE | FALSE | restricted\_on\_request | keep\_restricted\_on\_request | SRTR-derived data file; provide only on request under licence obligations. | FALSE | FALSE | FALSE | FALSE | FALSE |
| 02\_workflows/T3\_workflow\_v01/submission\_ready/public/data/esld\_srtr\_T3\_creatinine\_comparison\_table\_public.csv | esld\_srtr\_T3\_creatinine\_comparison\_table\_public.csv | 02\_workflows/T3\_workflow\_v01/submission\_ready/public/data | csv | csv | 5337 | 5.212 | 2026-05-02T21:14:25Z | 5fe481717fafc7d2115b64a01112efbe | restricted\_on\_request | derived | T3;table\_or\_supplement | 24 | 15 | yes\_default | TRUE |  | release\_candidate | public\_deposit | derived\_or\_workflow\_data | keep\_public\_deposit | T3 |  |  |  |  |  | restricted\_on\_request | keep\_restricted\_on\_request | SRTR-derived data file; provide only on request under licence obligations. | FALSE | TRUE | TRUE | FALSE | FALSE | FALSE | FALSE | FALSE | FALSE | restricted\_on\_request | keep\_restricted\_on\_request | SRTR-derived data file; provide only on request under licence obligations. | FALSE | FALSE | FALSE | FALSE | FALSE |
| 02\_workflows/T4\_workflow\_v01/data/01\_source\_loaded\_harmonized/srtr\_T4\_score\_shift\_counts\_all\_scores\_restricted.csv | srtr\_T4\_score\_shift\_counts\_all\_scores\_restricted.csv | 02\_workflows/T4\_workflow\_v01/data/01\_source\_loaded\_harmonized | csv | csv | 57406 | 56.061 | 2026-05-01T23:39:23Z | f5b54c78ba515a0813aa766447a62d6f | restricted\_on\_request | derived | T4 | 705 | 8 | yes\_default | TRUE |  | release\_candidate | restricted\_on\_request | derived\_or\_workflow\_data | keep\_restricted\_on\_request | T4 |  |  |  |  |  | restricted\_on\_request | keep\_restricted\_on\_request | SRTR-derived data file; provide only on request under licence obligations. | FALSE | TRUE | TRUE | FALSE | FALSE | FALSE | FALSE | FALSE | FALSE | restricted\_on\_request | keep\_restricted\_on\_request | SRTR-derived data file; provide only on request under licence obligations. | FALSE | FALSE | FALSE | FALSE | FALSE |
| 02\_workflows/T4\_workflow\_v01/data/01\_source\_loaded\_harmonized/srtr\_T4\_survival\_matrix\_restricted.csv | srtr\_T4\_survival\_matrix\_restricted.csv | 02\_workflows/T4\_workflow\_v01/data/01\_source\_loaded\_harmonized | csv | csv | 176617 | 172.478 | 2026-05-02T00:32:16Z | 3363b210bc6bf2fd4491ae78cf27e248 | restricted\_on\_request | derived | T4 | 728 | 26 | yes\_default | TRUE |  | release\_candidate | restricted\_on\_request | derived\_or\_workflow\_data | keep\_restricted\_on\_request | T4 |  |  |  |  |  | restricted\_on\_request | keep\_restricted\_on\_request | SRTR-derived data file; provide only on request under licence obligations. | FALSE | TRUE | TRUE | FALSE | FALSE | FALSE | FALSE | FALSE | FALSE | restricted\_on\_request | keep\_restricted\_on\_request | SRTR-derived data file; provide only on request under licence obligations. | FALSE | FALSE | FALSE | FALSE | FALSE |
| 02\_workflows/T4\_workflow\_v01/data/02b\_table\_content/srtr\_T4\_score\_deviation\_outcome\_build\_qc\_internal.csv | srtr\_T4\_score\_deviation\_outcome\_build\_qc\_internal.csv | 02\_workflows/T4\_workflow\_v01/data/02b\_table\_content | csv | csv | 2136 | 2.086 | 2026-05-02T00:32:17Z | 5fcdfb53cefa84bc1b7a76bc8cbdc2cb | internal\_only | derived | T4;table\_or\_supplement | 16 | 4 | no\_internal\_only\_default | TRUE |  | release\_candidate | restricted\_on\_request | derived\_or\_workflow\_data | keep\_restricted\_on\_request | T4 |  |  |  |  |  | restricted\_on\_request | keep\_restricted\_on\_request | SRTR-derived data file; provide only on request under licence obligations. | FALSE | TRUE | TRUE | FALSE | FALSE | FALSE | FALSE | FALSE | FALSE | restricted\_on\_request | keep\_restricted\_on\_request | SRTR-derived data file; provide only on request under licence obligations. | FALSE | FALSE | FALSE | FALSE | FALSE |
| 02\_workflows/T4\_workflow\_v01/data/02b\_table\_content/srtr\_T4\_score\_deviation\_outcome\_long\_internal.csv | srtr\_T4\_score\_deviation\_outcome\_long\_internal.csv | 02\_workflows/T4\_workflow\_v01/data/02b\_table\_content | csv | csv | 10792 | 10.539 | 2026-05-02T00:32:17Z | e4efee9fb4741147f0deb5d4ecfc6f24 | restricted\_on\_request | derived | T4;table\_or\_supplement | 44 | 21 | yes\_default | TRUE |  | release\_candidate | restricted\_on\_request | derived\_or\_workflow\_data | keep\_restricted\_on\_request | T4 |  |  |  |  |  | restricted\_on\_request | keep\_restricted\_on\_request | SRTR-derived data file; provide only on request under licence obligations. | FALSE | TRUE | TRUE | FALSE | FALSE | FALSE | FALSE | FALSE | FALSE | restricted\_on\_request | keep\_restricted\_on\_request | SRTR-derived data file; provide only on request under licence obligations. | FALSE | FALSE | FALSE | FALSE | FALSE |
| 02\_workflows/T4\_workflow\_v01/data/02b\_table\_content/srtr\_T4\_score\_deviation\_outcome\_source\_manifest\_internal.csv | srtr\_T4\_score\_deviation\_outcome\_source\_manifest\_internal.csv | 02\_workflows/T4\_workflow\_v01/data/02b\_table\_content | csv | csv | 929 | 0.907 | 2026-05-02T00:32:17Z | 7a808e50ca363e615de604ab11308541 | restricted\_on\_request | derived | T4;table\_or\_supplement | 2 | 7 | yes\_default | TRUE |  | release\_candidate | restricted\_on\_request | derived\_or\_workflow\_data | keep\_restricted\_on\_request | T4 |  |  |  |  |  | restricted\_on\_request | keep\_restricted\_on\_request | SRTR-derived data file; provide only on request under licence obligations. | FALSE | TRUE | TRUE | FALSE | FALSE | FALSE | FALSE | FALSE | FALSE | restricted\_on\_request | keep\_restricted\_on\_request | SRTR-derived data file; provide only on request under licence obligations. | FALSE | FALSE | FALSE | FALSE | FALSE |
| 02\_workflows/T4\_workflow\_v01/data/02b\_table\_content/srtr\_T4\_score\_deviation\_outcome\_table\_internal.csv | srtr\_T4\_score\_deviation\_outcome\_table\_internal.csv | 02\_workflows/T4\_workflow\_v01/data/02b\_table\_content | csv | csv | 1812 | 1.77 | 2026-05-02T00:32:17Z | 98172d9cfd5ec51116541ef912236c81 | restricted\_on\_request | derived | T4;table\_or\_supplement | 11 | 8 | yes\_default | TRUE |  | release\_candidate | restricted\_on\_request | derived\_or\_workflow\_data | keep\_restricted\_on\_request | T4 |  |  |  |  |  | restricted\_on\_request | keep\_restricted\_on\_request | SRTR-derived data file; provide only on request under licence obligations. | FALSE | TRUE | TRUE | FALSE | FALSE | FALSE | FALSE | FALSE | FALSE | restricted\_on\_request | keep\_restricted\_on\_request | SRTR-derived data file; provide only on request under licence obligations. | FALSE | FALSE | FALSE | FALSE | FALSE |
| 02\_workflows/T4\_workflow\_v01/submission\_ready/internal/data/srtr\_T4\_score\_deviation\_outcome\_long\_internal.csv | srtr\_T4\_score\_deviation\_outcome\_long\_internal.csv | 02\_workflows/T4\_workflow\_v01/submission\_ready/internal/data | csv | csv | 10792 | 10.539 | 2026-05-02T00:32:17Z | e4efee9fb4741147f0deb5d4ecfc6f24 | restricted\_on\_request | derived | T4 | 44 | 21 | yes\_default | TRUE |  | release\_candidate | restricted\_on\_request | derived\_or\_workflow\_data | keep\_restricted\_on\_request | T4 |  |  |  |  |  | restricted\_on\_request | keep\_restricted\_on\_request | SRTR-derived data file; provide only on request under licence obligations. | FALSE | TRUE | TRUE | FALSE | FALSE | FALSE | FALSE | FALSE | FALSE | restricted\_on\_request | keep\_restricted\_on\_request | SRTR-derived data file; provide only on request under licence obligations. | FALSE | FALSE | FALSE | FALSE | FALSE |
| 02\_workflows/T4\_workflow\_v01/submission\_ready/internal/data/srtr\_T4\_score\_deviation\_outcome\_table\_internal.csv | srtr\_T4\_score\_deviation\_outcome\_table\_internal.csv | 02\_workflows/T4\_workflow\_v01/submission\_ready/internal/data | csv | csv | 1812 | 1.77 | 2026-05-02T00:32:17Z | 98172d9cfd5ec51116541ef912236c81 | restricted\_on\_request | derived | T4;table\_or\_supplement | 11 | 8 | yes\_default | TRUE |  | release\_candidate | restricted\_on\_request | derived\_or\_workflow\_data | keep\_restricted\_on\_request | T4 |  |  |  |  |  | restricted\_on\_request | keep\_restricted\_on\_request | SRTR-derived data file; provide only on request under licence obligations. | FALSE | TRUE | TRUE | FALSE | FALSE | FALSE | FALSE | FALSE | FALSE | restricted\_on\_request | keep\_restricted\_on\_request | SRTR-derived data file; provide only on request under licence obligations. | FALSE | FALSE | FALSE | FALSE | FALSE |
| 02\_workflows/T4\_workflow\_v01/submission\_ready/restricted/T4\_restricted\_submission\_manifest\_v01.csv | T4\_restricted\_submission\_manifest\_v01.csv | 02\_workflows/T4\_workflow\_v01/submission\_ready/restricted | csv | csv | 1060 | 1.035 | 2026-05-02T00:32:17Z | ef87495fe5256559fec7f04c67fd5292 | restricted\_on\_request | derived | T4 | 2 | 8 | yes\_default | TRUE |  | release\_candidate | restricted\_on\_request | documentation\_or\_metadata | keep\_restricted\_on\_request | T4 |  |  |  |  |  | restricted\_on\_request | keep\_restricted\_on\_request | Restricted-on-request file after policy overrides. | FALSE | FALSE | TRUE | FALSE | FALSE | FALSE | FALSE | FALSE | FALSE | restricted\_on\_request | keep\_restricted\_on\_request | Restricted-on-request file after policy overrides. | FALSE | FALSE | FALSE | FALSE | FALSE |
| 02\_workflows/T4\_workflow\_v01/submission\_ready/restricted/data/srtr\_T4\_restricted\_source\_manifest.csv | srtr\_T4\_restricted\_source\_manifest.csv | 02\_workflows/T4\_workflow\_v01/submission\_ready/restricted/data | csv | csv | 929 | 0.907 | 2026-05-02T00:32:17Z | 7a808e50ca363e615de604ab11308541 | restricted\_on\_request | derived | T4 | 2 | 7 | yes\_default | TRUE |  | release\_candidate | restricted\_on\_request | derived\_or\_workflow\_data | keep\_restricted\_on\_request | T4 |  |  |  |  |  | restricted\_on\_request | keep\_restricted\_on\_request | SRTR-derived data file; provide only on request under licence obligations. | FALSE | TRUE | TRUE | FALSE | FALSE | FALSE | FALSE | FALSE | FALSE | restricted\_on\_request | keep\_restricted\_on\_request | SRTR-derived data file; provide only on request under licence obligations. | FALSE | FALSE | FALSE | FALSE | FALSE |
| 02\_workflows/T4\_workflow\_v01/submission\_ready/restricted/data/srtr\_T4\_score\_shift\_counts\_all\_scores\_restricted.csv | srtr\_T4\_score\_shift\_counts\_all\_scores\_restricted.csv | 02\_workflows/T4\_workflow\_v01/submission\_ready/restricted/data | csv | csv | 57406 | 56.061 | 2026-05-02T00:32:17Z | f5b54c78ba515a0813aa766447a62d6f | restricted\_on\_request | derived | T4 | 705 | 8 | yes\_default | TRUE |  | release\_candidate | restricted\_on\_request | derived\_or\_workflow\_data | keep\_restricted\_on\_request | T4 |  |  |  |  |  | restricted\_on\_request | keep\_restricted\_on\_request | SRTR-derived data file; provide only on request under licence obligations. | FALSE | TRUE | TRUE | FALSE | FALSE | FALSE | FALSE | FALSE | FALSE | restricted\_on\_request | keep\_restricted\_on\_request | SRTR-derived data file; provide only on request under licence obligations. | FALSE | FALSE | FALSE | FALSE | FALSE |
| 02\_workflows/T4\_workflow\_v01/submission\_ready/restricted/data/srtr\_T4\_survival\_matrix\_restricted.csv | srtr\_T4\_survival\_matrix\_restricted.csv | 02\_workflows/T4\_workflow\_v01/submission\_ready/restricted/data | csv | csv | 176617 | 172.478 | 2026-05-02T00:32:17Z | 3363b210bc6bf2fd4491ae78cf27e248 | restricted\_on\_request | derived | T4 | 728 | 26 | yes\_default | TRUE |  | release\_candidate | restricted\_on\_request | derived\_or\_workflow\_data | keep\_restricted\_on\_request | T4 |  |  |  |  |  | restricted\_on\_request | keep\_restricted\_on\_request | SRTR-derived data file; provide only on request under licence obligations. | FALSE | TRUE | TRUE | FALSE | FALSE | FALSE | FALSE | FALSE | FALSE | restricted\_on\_request | keep\_restricted\_on\_request | SRTR-derived data file; provide only on request under licence obligations. | FALSE | FALSE | FALSE | FALSE | FALSE |
