# Supplementary material for: Creatinine assay interferences compromises MELD accuracy and may bias liver allocation
Source: Nat Commun. 2026 Jul 23;17:7111. doi: 10.1038/s41467-026-75011-x (PMC13396164; doi:10.1038/s41467-026-75011-x)
Supplement: Supplementary file 4 — Source Data [file 41467_2026_75011_MOESM4_ESM.zip › figshare_package_FINAL_PUBLIC_DEPOSIT_V1_20260503_002637/00_START_HERE_HTML_NAVIGATOR/file_views/view_0034_README_T4_submission_ready_v01.html]

02\_workflows/T4\_workflow\_v01/submission\_ready/README\_T4\_submission\_ready\_v01.txt

# Readable file view

02\_workflows/T4\_workflow\_v01/submission\_ready/README\_T4\_submission\_ready\_v01.txt

← Back to navigator   |   Open original package file

Section

Workflow readmes

Output

T4

Extension

txt

Size KB

0.719

Variables

0

## Readable HTML view

```
Table 4 submission-ready outputs

Public release:
The public Table 4 release contains ESLD CSV table content and metadata only.
Rendered DOCX table files are assigned to internal outputs only.

Restricted release:
The restricted Table 4 release contains SRTR-derived source files required to rebuild the SRTR part of Table 4:
- srtr_T4_score_shift_counts_all_scores_restricted.csv
- srtr_T4_survival_matrix_restricted.csv

Internal audit:
The submitted-values DOCX template/copy, recalculated/audit Table 4 files, and SRTR build outputs are internal only.

Use for final public release:
02_workflows/T4_workflow_v01/submission_ready/public/data

Use for restricted release:
02_workflows/T4_workflow_v01/submission_ready/restricted/data
```
