# Supplementary material for: Creatinine assay interferences compromises MELD accuracy and may bias liver allocation
Source: Nat Commun. 2026 Jul 23;17:7111. doi: 10.1038/s41467-026-75011-x (PMC13396164; doi:10.1038/s41467-026-75011-x)
Supplement: Supplementary file 4 — Source Data [file 41467_2026_75011_MOESM4_ESM.zip › figshare_package_FINAL_PUBLIC_DEPOSIT_V1_20260503_002637/00_START_HERE_HTML_NAVIGATOR/file_views/view_0008_f2_simulated_heatmap_repository.html]

01\_primary\_data/public/f2\_simulated\_heatmap\_repository.csv

# Readable file view

01\_primary\_data/public/f2\_simulated\_heatmap\_repository.csv

← Back to navigator   |   Open original package file

Section

Public primary data

Output

F2

Extension

csv

Size KB

7674.803

Variables

6

## Variables in this file

| Variable | Label | Description | Unit | Type |
| --- | --- | --- | --- | --- |
| count\_negative | Number of simulated subpoints with negative score shift | Number of simulated subpoints within the F2 heatmap bin where the score shift is negative, defined as score delta ≤ −1. | count | integer |
| model | MELD model or score variant | Name of the MELD-related model or score variant represented by the row; expected values include MELD, MELD-Na, reMELD-Na, and MELD 3.0. |  | character |
| n\_sub | Number of simulated subpoints per heatmap bin | Number of simulated subpoints aggregated within one F2 heatmap bin. | count | integer |
| pct\_negative | Percentage of simulated subpoints with negative score shift | Percentage of simulated subpoints within the F2 heatmap bin where the score shift is negative, defined as score delta ≤ −1. | % | integer |
| x\_value | Heatmap x-axis creatinine value | Creatinine value defining the x-axis coordinate of an F2 heatmap bin. | mg/dL | numeric |
| y\_value | Heatmap y-axis total bilirubin value | Total bilirubin value defining the y-axis coordinate of an F2 heatmap bin. | mg/dL | numeric |

## Readable HTML view

Preview shows first 1000 of 295200 rows. Open the original file for full content.

| model | x\_value | y\_value | n\_sub | count\_negative | pct\_negative |
| --- | --- | --- | --- | --- | --- |
| MELD | 0.1 | 0.1 | 100 | 0 | 0 |
| MELD | 0.1 | 0.2 | 100 | 0 | 0 |
| MELD | 0.1 | 0.3 | 100 | 0 | 0 |
| MELD | 0.1 | 0.4 | 100 | 0 | 0 |
| MELD | 0.1 | 0.5 | 100 | 0 | 0 |
| MELD | 0.1 | 0.6 | 100 | 0 | 0 |
| MELD | 0.1 | 0.7 | 100 | 0 | 0 |
| MELD | 0.1 | 0.8 | 100 | 0 | 0 |
| MELD | 0.1 | 0.9 | 100 | 0 | 0 |
| MELD | 0.1 | 1 | 100 | 0 | 0 |
| MELD | 0.1 | 1.1 | 100 | 0 | 0 |
| MELD | 0.1 | 1.2 | 100 | 0 | 0 |
| MELD | 0.1 | 1.3 | 100 | 0 | 0 |
| MELD | 0.1 | 1.4 | 100 | 0 | 0 |
| MELD | 0.1 | 1.5 | 100 | 0 | 0 |
| MELD | 0.1 | 1.6 | 100 | 0 | 0 |
| MELD | 0.1 | 1.7 | 100 | 0 | 0 |
| MELD | 0.1 | 1.8 | 100 | 0 | 0 |
| MELD | 0.1 | 1.9 | 100 | 0 | 0 |
| MELD | 0.1 | 2 | 100 | 0 | 0 |
| MELD | 0.1 | 2.1 | 100 | 0 | 0 |
| MELD | 0.1 | 2.2 | 100 | 0 | 0 |
| MELD | 0.1 | 2.3 | 100 | 0 | 0 |
| MELD | 0.1 | 2.4 | 100 | 0 | 0 |
| MELD | 0.1 | 2.5 | 100 | 0 | 0 |
| MELD | 0.1 | 2.6 | 100 | 0 | 0 |
| MELD | 0.1 | 2.7 | 100 | 0 | 0 |
| MELD | 0.1 | 2.8 | 100 | 0 | 0 |
| MELD | 0.1 | 2.9 | 100 | 0 | 0 |
| MELD | 0.1 | 3 | 100 | 0 | 0 |
| MELD | 0.1 | 3.1 | 100 | 0 | 0 |
| MELD | 0.1 | 3.2 | 100 | 0 | 0 |
| MELD | 0.1 | 3.3 | 100 | 0 | 0 |
| MELD | 0.1 | 3.4 | 100 | 0 | 0 |
| MELD | 0.1 | 3.5 | 100 | 0 | 0 |
| MELD | 0.1 | 3.6 | 100 | 0 | 0 |
| MELD | 0.1 | 3.7 | 100 | 0 | 0 |
| MELD | 0.1 | 3.8 | 100 | 0 | 0 |
| MELD | 0.1 | 3.9 | 100 | 0 | 0 |
| MELD | 0.1 | 4 | 100 | 0 | 0 |
| MELD | 0.1 | 4.1 | 100 | 0 | 0 |
| MELD | 0.1 | 4.2 | 100 | 0 | 0 |
| MELD | 0.1 | 4.3 | 100 | 0 | 0 |
| MELD | 0.1 | 4.4 | 100 | 0 | 0 |
| MELD | 0.1 | 4.5 | 100 | 0 | 0 |
| MELD | 0.1 | 4.6 | 100 | 0 | 0 |
| MELD | 0.1 | 4.7 | 100 | 0 | 0 |
| MELD | 0.1 | 4.8 | 100 | 0 | 0 |
| MELD | 0.1 | 4.9 | 100 | 0 | 0 |
| MELD | 0.1 | 5 | 100 | 0 | 0 |
| MELD | 0.1 | 5.1 | 100 | 0 | 0 |
| MELD | 0.1 | 5.2 | 100 | 0 | 0 |
| MELD | 0.1 | 5.3 | 100 | 0 | 0 |
| MELD | 0.1 | 5.4 | 100 | 0 | 0 |
| MELD | 0.1 | 5.5 | 100 | 0 | 0 |
| MELD | 0.1 | 5.6 | 100 | 0 | 0 |
| MELD | 0.1 | 5.7 | 100 | 0 | 0 |
| MELD | 0.1 | 5.8 | 100 | 0 | 0 |
| MELD | 0.1 | 5.9 | 100 | 0 | 0 |
| MELD | 0.1 | 6 | 100 | 0 | 0 |
| MELD | 0.1 | 6.1 | 100 | 0 | 0 |
| MELD | 0.1 | 6.2 | 100 | 0 | 0 |
| MELD | 0.1 | 6.3 | 100 | 0 | 0 |
| MELD | 0.1 | 6.4 | 100 | 0 | 0 |
| MELD | 0.1 | 6.5 | 100 | 0 | 0 |
| MELD | 0.1 | 6.6 | 100 | 0 | 0 |
| MELD | 0.1 | 6.7 | 100 | 0 | 0 |
| MELD | 0.1 | 6.8 | 100 | 0 | 0 |
| MELD | 0.1 | 6.9 | 100 | 0 | 0 |
| MELD | 0.1 | 7 | 100 | 0 | 0 |
| MELD | 0.1 | 7.1 | 100 | 0 | 0 |
| MELD | 0.1 | 7.2 | 100 | 0 | 0 |
| MELD | 0.1 | 7.3 | 100 | 0 | 0 |
| MELD | 0.1 | 7.4 | 100 | 0 | 0 |
| MELD | 0.1 | 7.5 | 100 | 0 | 0 |
| MELD | 0.1 | 7.6 | 100 | 0 | 0 |
| MELD | 0.1 | 7.7 | 100 | 0 | 0 |
| MELD | 0.1 | 7.8 | 100 | 0 | 0 |
| MELD | 0.1 | 7.9 | 100 | 0 | 0 |
| MELD | 0.1 | 8 | 100 | 0 | 0 |
| MELD | 0.1 | 8.1 | 100 | 0 | 0 |
| MELD | 0.1 | 8.2 | 100 | 0 | 0 |
| MELD | 0.1 | 8.3 | 100 | 0 | 0 |
| MELD | 0.1 | 8.4 | 100 | 0 | 0 |
| MELD | 0.1 | 8.5 | 100 | 0 | 0 |
| MELD | 0.1 | 8.6 | 100 | 0 | 0 |
| MELD | 0.1 | 8.7 | 100 | 0 | 0 |
| MELD | 0.1 | 8.8 | 100 | 0 | 0 |
| MELD | 0.1 | 8.9 | 100 | 0 | 0 |
| MELD | 0.1 | 9 | 100 | 0 | 0 |
| MELD | 0.1 | 9.1 | 100 | 0 | 0 |
| MELD | 0.1 | 9.2 | 100 | 0 | 0 |
| MELD | 0.1 | 9.3 | 100 | 0 | 0 |
| MELD | 0.1 | 9.4 | 100 | 0 | 0 |
| MELD | 0.1 | 9.5 | 100 | 0 | 0 |
| MELD | 0.1 | 9.6 | 100 | 0 | 0 |
| MELD | 0.1 | 9.7 | 100 | 0 | 0 |
| MELD | 0.1 | 9.8 | 100 | 0 | 0 |
| MELD | 0.1 | 9.9 | 100 | 0 | 0 |
| MELD | 0.1 | 10 | 100 | 0 | 0 |
| MELD | 0.1 | 10.1 | 100 | 0 | 0 |
| MELD | 0.1 | 10.2 | 100 | 0 | 0 |
| MELD | 0.1 | 10.3 | 100 | 0 | 0 |
| MELD | 0.1 | 10.4 | 100 | 0 | 0 |
| MELD | 0.1 | 10.5 | 100 | 0 | 0 |
| MELD | 0.1 | 10.6 | 100 | 0 | 0 |
| MELD | 0.1 | 10.7 | 100 | 0 | 0 |
| MELD | 0.1 | 10.8 | 100 | 0 | 0 |
| MELD | 0.1 | 10.9 | 100 | 0 | 0 |
| MELD | 0.1 | 11 | 100 | 0 | 0 |
| MELD | 0.1 | 11.1 | 100 | 0 | 0 |
| MELD | 0.1 | 11.2 | 100 | 0 | 0 |
| MELD | 0.1 | 11.3 | 100 | 0 | 0 |
| MELD | 0.1 | 11.4 | 100 | 0 | 0 |
| MELD | 0.1 | 11.5 | 100 | 0 | 0 |
| MELD | 0.1 | 11.6 | 100 | 0 | 0 |
| MELD | 0.1 | 11.7 | 100 | 0 | 0 |
| MELD | 0.1 | 11.8 | 100 | 0 | 0 |
| MELD | 0.1 | 11.9 | 100 | 0 | 0 |
| MELD | 0.1 | 12 | 100 | 0 | 0 |
| MELD | 0.1 | 12.1 | 100 | 0 | 0 |
| MELD | 0.1 | 12.2 | 100 | 0 | 0 |
| MELD | 0.1 | 12.3 | 100 | 0 | 0 |
| MELD | 0.1 | 12.4 | 100 | 0 | 0 |
| MELD | 0.1 | 12.5 | 100 | 0 | 0 |
| MELD | 0.1 | 12.6 | 100 | 0 | 0 |
| MELD | 0.1 | 12.7 | 100 | 0 | 0 |
| MELD | 0.1 | 12.8 | 100 | 0 | 0 |
| MELD | 0.1 | 12.9 | 100 | 0 | 0 |
| MELD | 0.1 | 13 | 100 | 0 | 0 |
| MELD | 0.1 | 13.1 | 100 | 0 | 0 |
| MELD | 0.1 | 13.2 | 100 | 0 | 0 |
| MELD | 0.1 | 13.3 | 100 | 0 | 0 |
| MELD | 0.1 | 13.4 | 100 | 0 | 0 |
| MELD | 0.1 | 13.5 | 100 | 0 | 0 |
| MELD | 0.1 | 13.6 | 100 | 0 | 0 |
| MELD | 0.1 | 13.7 | 100 | 0 | 0 |
| MELD | 0.1 | 13.8 | 100 | 0 | 0 |
| MELD | 0.1 | 13.9 | 100 | 0 | 0 |
| MELD | 0.1 | 14 | 100 | 0 | 0 |
| MELD | 0.1 | 14.1 | 100 | 0 | 0 |
| MELD | 0.1 | 14.2 | 100 | 0 | 0 |
| MELD | 0.1 | 14.3 | 100 | 0 | 0 |
| MELD | 0.1 | 14.4 | 100 | 0 | 0 |
| MELD | 0.1 | 14.5 | 100 | 0 | 0 |
| MELD | 0.1 | 14.6 | 100 | 0 | 0 |
| MELD | 0.1 | 14.7 | 100 | 0 | 0 |
| MELD | 0.1 | 14.8 | 100 | 0 | 0 |
| MELD | 0.1 | 14.9 | 100 | 0 | 0 |
| MELD | 0.1 | 15 | 100 | 0 | 0 |
| MELD | 0.1 | 15.1 | 100 | 0 | 0 |
| MELD | 0.1 | 15.2 | 100 | 0 | 0 |
| MELD | 0.1 | 15.3 | 100 | 0 | 0 |
| MELD | 0.1 | 15.4 | 100 | 0 | 0 |
| MELD | 0.1 | 15.5 | 100 | 0 | 0 |
| MELD | 0.1 | 15.6 | 100 | 0 | 0 |
| MELD | 0.1 | 15.7 | 100 | 0 | 0 |
| MELD | 0.1 | 15.8 | 100 | 0 | 0 |
| MELD | 0.1 | 15.9 | 100 | 0 | 0 |
| MELD | 0.1 | 16 | 100 | 0 | 0 |
| MELD | 0.1 | 16.1 | 100 | 0 | 0 |
| MELD | 0.1 | 16.2 | 100 | 0 | 0 |
| MELD | 0.1 | 16.3 | 100 | 0 | 0 |
| MELD | 0.1 | 16.4 | 100 | 0 | 0 |
| MELD | 0.1 | 16.5 | 100 | 0 | 0 |
| MELD | 0.1 | 16.6 | 100 | 0 | 0 |
| MELD | 0.1 | 16.7 | 100 | 0 | 0 |
| MELD | 0.1 | 16.8 | 100 | 0 | 0 |
| MELD | 0.1 | 16.9 | 100 | 0 | 0 |
| MELD | 0.1 | 17 | 100 | 0 | 0 |
| MELD | 0.1 | 17.1 | 100 | 0 | 0 |
| MELD | 0.1 | 17.2 | 100 | 0 | 0 |
| MELD | 0.1 | 17.3 | 100 | 0 | 0 |
| MELD | 0.1 | 17.4 | 100 | 0 | 0 |
| MELD | 0.1 | 17.5 | 100 | 0 | 0 |
| MELD | 0.1 | 17.6 | 100 | 0 | 0 |
| MELD | 0.1 | 17.7 | 100 | 0 | 0 |
| MELD | 0.1 | 17.8 | 100 | 0 | 0 |
| MELD | 0.1 | 17.9 | 100 | 0 | 0 |
| MELD | 0.1 | 18 | 100 | 0 | 0 |
| MELD | 0.1 | 18.1 | 100 | 0 | 0 |
| MELD | 0.1 | 18.2 | 100 | 0 | 0 |
| MELD | 0.1 | 18.3 | 100 | 0 | 0 |
| MELD | 0.1 | 18.4 | 100 | 0 | 0 |
| MELD | 0.1 | 18.5 | 100 | 0 | 0 |
| MELD | 0.1 | 18.6 | 100 | 0 | 0 |
| MELD | 0.1 | 18.7 | 100 | 0 | 0 |
| MELD | 0.1 | 18.8 | 100 | 0 | 0 |
| MELD | 0.1 | 18.9 | 100 | 0 | 0 |
| MELD | 0.1 | 19 | 100 | 0 | 0 |
| MELD | 0.1 | 19.1 | 100 | 0 | 0 |
| MELD | 0.1 | 19.2 | 100 | 0 | 0 |
| MELD | 0.1 | 19.3 | 100 | 0 | 0 |
| MELD | 0.1 | 19.4 | 100 | 0 | 0 |
| MELD | 0.1 | 19.5 | 100 | 0 | 0 |
| MELD | 0.1 | 19.6 | 100 | 0 | 0 |
| MELD | 0.1 | 19.7 | 100 | 0 | 0 |
| MELD | 0.1 | 19.8 | 100 | 0 | 0 |
| MELD | 0.1 | 19.9 | 100 | 0 | 0 |
| MELD | 0.1 | 20 | 100 | 0 | 0 |
| MELD | 0.1 | 20.1 | 100 | 0 | 0 |
| MELD | 0.1 | 20.2 | 100 | 0 | 0 |
| MELD | 0.1 | 20.3 | 100 | 0 | 0 |
| MELD | 0.1 | 20.4 | 100 | 0 | 0 |
| MELD | 0.1 | 20.5 | 100 | 0 | 0 |
| MELD | 0.1 | 20.6 | 100 | 0 | 0 |
| MELD | 0.1 | 20.7 | 100 | 0 | 0 |
| MELD | 0.1 | 20.8 | 100 | 0 | 0 |
| MELD | 0.1 | 20.9 | 100 | 0 | 0 |
| MELD | 0.1 | 21 | 100 | 0 | 0 |
| MELD | 0.1 | 21.1 | 100 | 0 | 0 |
| MELD | 0.1 | 21.2 | 100 | 0 | 0 |
| MELD | 0.1 | 21.3 | 100 | 0 | 0 |
| MELD | 0.1 | 21.4 | 100 | 0 | 0 |
| MELD | 0.1 | 21.5 | 100 | 0 | 0 |
| MELD | 0.1 | 21.6 | 100 | 0 | 0 |
| MELD | 0.1 | 21.7 | 100 | 0 | 0 |
| MELD | 0.1 | 21.8 | 100 | 0 | 0 |
| MELD | 0.1 | 21.9 | 100 | 0 | 0 |
| MELD | 0.1 | 22 | 100 | 0 | 0 |
| MELD | 0.1 | 22.1 | 100 | 0 | 0 |
| MELD | 0.1 | 22.2 | 100 | 0 | 0 |
| MELD | 0.1 | 22.3 | 100 | 0 | 0 |
| MELD | 0.1 | 22.4 | 100 | 0 | 0 |
| MELD | 0.1 | 22.5 | 100 | 0 | 0 |
| MELD | 0.1 | 22.6 | 100 | 0 | 0 |
| MELD | 0.1 | 22.7 | 100 | 0 | 0 |
| MELD | 0.1 | 22.8 | 100 | 0 | 0 |
| MELD | 0.1 | 22.9 | 100 | 0 | 0 |
| MELD | 0.1 | 23 | 100 | 0 | 0 |
| MELD | 0.1 | 23.1 | 100 | 0 | 0 |
| MELD | 0.1 | 23.2 | 100 | 0 | 0 |
| MELD | 0.1 | 23.3 | 100 | 0 | 0 |
| MELD | 0.1 | 23.4 | 100 | 0 | 0 |
| MELD | 0.1 | 23.5 | 100 | 0 | 0 |
| MELD | 0.1 | 23.6 | 100 | 0 | 0 |
| MELD | 0.1 | 23.7 | 100 | 0 | 0 |
| MELD | 0.1 | 23.8 | 100 | 0 | 0 |
| MELD | 0.1 | 23.9 | 100 | 0 | 0 |
| MELD | 0.1 | 24 | 100 | 0 | 0 |
| MELD | 0.1 | 24.1 | 100 | 0 | 0 |
| MELD | 0.1 | 24.2 | 100 | 0 | 0 |
| MELD | 0.1 | 24.3 | 100 | 0 | 0 |
| MELD | 0.1 | 24.4 | 100 | 0 | 0 |
| MELD | 0.1 | 24.5 | 100 | 0 | 0 |
| MELD | 0.1 | 24.6 | 100 | 0 | 0 |
| MELD | 0.1 | 24.7 | 100 | 0 | 0 |
| MELD | 0.1 | 24.8 | 100 | 0 | 0 |
| MELD | 0.1 | 24.9 | 100 | 0 | 0 |
| MELD | 0.1 | 25 | 100 | 0 | 0 |
| MELD | 0.1 | 25.1 | 100 | 0 | 0 |
| MELD | 0.1 | 25.2 | 100 | 0 | 0 |
| MELD | 0.1 | 25.3 | 100 | 0 | 0 |
| MELD | 0.1 | 25.4 | 100 | 0 | 0 |
| MELD | 0.1 | 25.5 | 100 | 0 | 0 |
| MELD | 0.1 | 25.6 | 100 | 0 | 0 |
| MELD | 0.1 | 25.7 | 100 | 0 | 0 |
| MELD | 0.1 | 25.8 | 100 | 0 | 0 |
| MELD | 0.1 | 25.9 | 100 | 0 | 0 |
| MELD | 0.1 | 26 | 100 | 0 | 0 |
| MELD | 0.1 | 26.1 | 100 | 0 | 0 |
| MELD | 0.1 | 26.2 | 100 | 0 | 0 |
| MELD | 0.1 | 26.3 | 100 | 0 | 0 |
| MELD | 0.1 | 26.4 | 100 | 0 | 0 |
| MELD | 0.1 | 26.5 | 100 | 0 | 0 |
| MELD | 0.1 | 26.6 | 100 | 0 | 0 |
| MELD | 0.1 | 26.7 | 100 | 0 | 0 |
| MELD | 0.1 | 26.8 | 100 | 0 | 0 |
| MELD | 0.1 | 26.9 | 100 | 0 | 0 |
| MELD | 0.1 | 27 | 100 | 0 | 0 |
| MELD | 0.1 | 27.1 | 100 | 0 | 0 |
| MELD | 0.1 | 27.2 | 100 | 0 | 0 |
| MELD | 0.1 | 27.3 | 100 | 0 | 0 |
| MELD | 0.1 | 27.4 | 100 | 0 | 0 |
| MELD | 0.1 | 27.5 | 100 | 0 | 0 |
| MELD | 0.1 | 27.6 | 100 | 0 | 0 |
| MELD | 0.1 | 27.7 | 100 | 0 | 0 |
| MELD | 0.1 | 27.8 | 100 | 0 | 0 |
| MELD | 0.1 | 27.9 | 100 | 0 | 0 |
| MELD | 0.1 | 28 | 100 | 0 | 0 |
| MELD | 0.1 | 28.1 | 100 | 0 | 0 |
| MELD | 0.1 | 28.2 | 100 | 0 | 0 |
| MELD | 0.1 | 28.3 | 100 | 0 | 0 |
| MELD | 0.1 | 28.4 | 100 | 0 | 0 |
| MELD | 0.1 | 28.5 | 100 | 0 | 0 |
| MELD | 0.1 | 28.6 | 100 | 0 | 0 |
| MELD | 0.1 | 28.7 | 100 | 0 | 0 |
| MELD | 0.1 | 28.8 | 100 | 0 | 0 |
| MELD | 0.1 | 28.9 | 100 | 0 | 0 |
| MELD | 0.1 | 29 | 100 | 0 | 0 |
| MELD | 0.1 | 29.1 | 100 | 0 | 0 |
| MELD | 0.1 | 29.2 | 100 | 0 | 0 |
| MELD | 0.1 | 29.3 | 100 | 0 | 0 |
| MELD | 0.1 | 29.4 | 100 | 0 | 0 |
| MELD | 0.1 | 29.5 | 100 | 0 | 0 |
| MELD | 0.1 | 29.6 | 100 | 0 | 0 |
| MELD | 0.1 | 29.7 | 100 | 0 | 0 |
| MELD | 0.1 | 29.8 | 100 | 0 | 0 |
| MELD | 0.1 | 29.9 | 100 | 0 | 0 |
| MELD | 0.1 | 30 | 100 | 0 | 0 |
| MELD | 0.12 | 0.1 | 100 | 0 | 0 |
| MELD | 0.12 | 0.2 | 100 | 0 | 0 |
| MELD | 0.12 | 0.3 | 100 | 0 | 0 |
| MELD | 0.12 | 0.4 | 100 | 0 | 0 |
| MELD | 0.12 | 0.5 | 100 | 0 | 0 |
| MELD | 0.12 | 0.6 | 100 | 0 | 0 |
| MELD | 0.12 | 0.7 | 100 | 0 | 0 |
| MELD | 0.12 | 0.8 | 100 | 0 | 0 |
| MELD | 0.12 | 0.9 | 100 | 0 | 0 |
| MELD | 0.12 | 1 | 100 | 0 | 0 |
| MELD | 0.12 | 1.1 | 100 | 0 | 0 |
| MELD | 0.12 | 1.2 | 100 | 0 | 0 |
| MELD | 0.12 | 1.3 | 100 | 0 | 0 |
| MELD | 0.12 | 1.4 | 100 | 0 | 0 |
| MELD | 0.12 | 1.5 | 100 | 0 | 0 |
| MELD | 0.12 | 1.6 | 100 | 0 | 0 |
| MELD | 0.12 | 1.7 | 100 | 0 | 0 |
| MELD | 0.12 | 1.8 | 100 | 0 | 0 |
| MELD | 0.12 | 1.9 | 100 | 0 | 0 |
| MELD | 0.12 | 2 | 100 | 0 | 0 |
| MELD | 0.12 | 2.1 | 100 | 0 | 0 |
| MELD | 0.12 | 2.2 | 100 | 0 | 0 |
| MELD | 0.12 | 2.3 | 100 | 0 | 0 |
| MELD | 0.12 | 2.4 | 100 | 0 | 0 |
| MELD | 0.12 | 2.5 | 100 | 0 | 0 |
| MELD | 0.12 | 2.6 | 100 | 0 | 0 |
| MELD | 0.12 | 2.7 | 100 | 0 | 0 |
| MELD | 0.12 | 2.8 | 100 | 0 | 0 |
| MELD | 0.12 | 2.9 | 100 | 0 | 0 |
| MELD | 0.12 | 3 | 100 | 0 | 0 |
| MELD | 0.12 | 3.1 | 100 | 0 | 0 |
| MELD | 0.12 | 3.2 | 100 | 0 | 0 |
| MELD | 0.12 | 3.3 | 100 | 0 | 0 |
| MELD | 0.12 | 3.4 | 100 | 0 | 0 |
| MELD | 0.12 | 3.5 | 100 | 0 | 0 |
| MELD | 0.12 | 3.6 | 100 | 0 | 0 |
| MELD | 0.12 | 3.7 | 100 | 0 | 0 |
| MELD | 0.12 | 3.8 | 100 | 0 | 0 |
| MELD | 0.12 | 3.9 | 100 | 0 | 0 |
| MELD | 0.12 | 4 | 100 | 0 | 0 |
| MELD | 0.12 | 4.1 | 100 | 0 | 0 |
| MELD | 0.12 | 4.2 | 100 | 0 | 0 |
| MELD | 0.12 | 4.3 | 100 | 0 | 0 |
| MELD | 0.12 | 4.4 | 100 | 0 | 0 |
| MELD | 0.12 | 4.5 | 100 | 0 | 0 |
| MELD | 0.12 | 4.6 | 100 | 0 | 0 |
| MELD | 0.12 | 4.7 | 100 | 0 | 0 |
| MELD | 0.12 | 4.8 | 100 | 0 | 0 |
| MELD | 0.12 | 4.9 | 100 | 0 | 0 |
| MELD | 0.12 | 5 | 100 | 0 | 0 |
| MELD | 0.12 | 5.1 | 100 | 0 | 0 |
| MELD | 0.12 | 5.2 | 100 | 0 | 0 |
| MELD | 0.12 | 5.3 | 100 | 0 | 0 |
| MELD | 0.12 | 5.4 | 100 | 0 | 0 |
| MELD | 0.12 | 5.5 | 100 | 0 | 0 |
| MELD | 0.12 | 5.6 | 100 | 0 | 0 |
| MELD | 0.12 | 5.7 | 100 | 0 | 0 |
| MELD | 0.12 | 5.8 | 100 | 0 | 0 |
| MELD | 0.12 | 5.9 | 100 | 0 | 0 |
| MELD | 0.12 | 6 | 100 | 0 | 0 |
| MELD | 0.12 | 6.1 | 100 | 0 | 0 |
| MELD | 0.12 | 6.2 | 100 | 0 | 0 |
| MELD | 0.12 | 6.3 | 100 | 0 | 0 |
| MELD | 0.12 | 6.4 | 100 | 0 | 0 |
| MELD | 0.12 | 6.5 | 100 | 0 | 0 |
| MELD | 0.12 | 6.6 | 100 | 0 | 0 |
| MELD | 0.12 | 6.7 | 100 | 0 | 0 |
| MELD | 0.12 | 6.8 | 100 | 0 | 0 |
| MELD | 0.12 | 6.9 | 100 | 0 | 0 |
| MELD | 0.12 | 7 | 100 | 0 | 0 |
| MELD | 0.12 | 7.1 | 100 | 0 | 0 |
| MELD | 0.12 | 7.2 | 100 | 0 | 0 |
| MELD | 0.12 | 7.3 | 100 | 0 | 0 |
| MELD | 0.12 | 7.4 | 100 | 0 | 0 |
| MELD | 0.12 | 7.5 | 100 | 0 | 0 |
| MELD | 0.12 | 7.6 | 100 | 0 | 0 |
| MELD | 0.12 | 7.7 | 100 | 0 | 0 |
| MELD | 0.12 | 7.8 | 100 | 0 | 0 |
| MELD | 0.12 | 7.9 | 100 | 0 | 0 |
| MELD | 0.12 | 8 | 100 | 0 | 0 |
| MELD | 0.12 | 8.1 | 100 | 0 | 0 |
| MELD | 0.12 | 8.2 | 100 | 0 | 0 |
| MELD | 0.12 | 8.3 | 100 | 0 | 0 |
| MELD | 0.12 | 8.4 | 100 | 0 | 0 |
| MELD | 0.12 | 8.5 | 100 | 0 | 0 |
| MELD | 0.12 | 8.6 | 100 | 0 | 0 |
| MELD | 0.12 | 8.7 | 100 | 0 | 0 |
| MELD | 0.12 | 8.8 | 100 | 0 | 0 |
| MELD | 0.12 | 8.9 | 100 | 0 | 0 |
| MELD | 0.12 | 9 | 100 | 0 | 0 |
| MELD | 0.12 | 9.1 | 100 | 0 | 0 |
| MELD | 0.12 | 9.2 | 100 | 0 | 0 |
| MELD | 0.12 | 9.3 | 100 | 0 | 0 |
| MELD | 0.12 | 9.4 | 100 | 0 | 0 |
| MELD | 0.12 | 9.5 | 100 | 0 | 0 |
| MELD | 0.12 | 9.6 | 100 | 0 | 0 |
| MELD | 0.12 | 9.7 | 100 | 0 | 0 |
| MELD | 0.12 | 9.8 | 100 | 0 | 0 |
| MELD | 0.12 | 9.9 | 100 | 0 | 0 |
| MELD | 0.12 | 10 | 100 | 0 | 0 |
| MELD | 0.12 | 10.1 | 100 | 0 | 0 |
| MELD | 0.12 | 10.2 | 100 | 0 | 0 |
| MELD | 0.12 | 10.3 | 100 | 0 | 0 |
| MELD | 0.12 | 10.4 | 100 | 0 | 0 |
| MELD | 0.12 | 10.5 | 100 | 0 | 0 |
| MELD | 0.12 | 10.6 | 100 | 0 | 0 |
| MELD | 0.12 | 10.7 | 100 | 0 | 0 |
| MELD | 0.12 | 10.8 | 100 | 0 | 0 |
| MELD | 0.12 | 10.9 | 100 | 0 | 0 |
| MELD | 0.12 | 11 | 100 | 0 | 0 |
| MELD | 0.12 | 11.1 | 100 | 0 | 0 |
| MELD | 0.12 | 11.2 | 100 | 0 | 0 |
| MELD | 0.12 | 11.3 | 100 | 0 | 0 |
| MELD | 0.12 | 11.4 | 100 | 0 | 0 |
| MELD | 0.12 | 11.5 | 100 | 0 | 0 |
| MELD | 0.12 | 11.6 | 100 | 0 | 0 |
| MELD | 0.12 | 11.7 | 100 | 0 | 0 |
| MELD | 0.12 | 11.8 | 100 | 0 | 0 |
| MELD | 0.12 | 11.9 | 100 | 0 | 0 |
| MELD | 0.12 | 12 | 100 | 0 | 0 |
| MELD | 0.12 | 12.1 | 100 | 0 | 0 |
| MELD | 0.12 | 12.2 | 100 | 0 | 0 |
| MELD | 0.12 | 12.3 | 100 | 0 | 0 |
| MELD | 0.12 | 12.4 | 100 | 0 | 0 |
| MELD | 0.12 | 12.5 | 100 | 0 | 0 |
| MELD | 0.12 | 12.6 | 100 | 0 | 0 |
| MELD | 0.12 | 12.7 | 100 | 0 | 0 |
| MELD | 0.12 | 12.8 | 100 | 0 | 0 |
| MELD | 0.12 | 12.9 | 100 | 0 | 0 |
| MELD | 0.12 | 13 | 100 | 0 | 0 |
| MELD | 0.12 | 13.1 | 100 | 0 | 0 |
| MELD | 0.12 | 13.2 | 100 | 0 | 0 |
| MELD | 0.12 | 13.3 | 100 | 0 | 0 |
| MELD | 0.12 | 13.4 | 100 | 0 | 0 |
| MELD | 0.12 | 13.5 | 100 | 0 | 0 |
| MELD | 0.12 | 13.6 | 100 | 0 | 0 |
| MELD | 0.12 | 13.7 | 100 | 0 | 0 |
| MELD | 0.12 | 13.8 | 100 | 0 | 0 |
| MELD | 0.12 | 13.9 | 100 | 0 | 0 |
| MELD | 0.12 | 14 | 100 | 0 | 0 |
| MELD | 0.12 | 14.1 | 100 | 0 | 0 |
| MELD | 0.12 | 14.2 | 100 | 0 | 0 |
| MELD | 0.12 | 14.3 | 100 | 0 | 0 |
| MELD | 0.12 | 14.4 | 100 | 0 | 0 |
| MELD | 0.12 | 14.5 | 100 | 0 | 0 |
| MELD | 0.12 | 14.6 | 100 | 0 | 0 |
| MELD | 0.12 | 14.7 | 100 | 0 | 0 |
| MELD | 0.12 | 14.8 | 100 | 0 | 0 |
| MELD | 0.12 | 14.9 | 100 | 0 | 0 |
| MELD | 0.12 | 15 | 100 | 0 | 0 |
| MELD | 0.12 | 15.1 | 100 | 0 | 0 |
| MELD | 0.12 | 15.2 | 100 | 0 | 0 |
| MELD | 0.12 | 15.3 | 100 | 0 | 0 |
| MELD | 0.12 | 15.4 | 100 | 0 | 0 |
| MELD | 0.12 | 15.5 | 100 | 0 | 0 |
| MELD | 0.12 | 15.6 | 100 | 0 | 0 |
| MELD | 0.12 | 15.7 | 100 | 0 | 0 |
| MELD | 0.12 | 15.8 | 100 | 0 | 0 |
| MELD | 0.12 | 15.9 | 100 | 0 | 0 |
| MELD | 0.12 | 16 | 100 | 0 | 0 |
| MELD | 0.12 | 16.1 | 100 | 0 | 0 |
| MELD | 0.12 | 16.2 | 100 | 0 | 0 |
| MELD | 0.12 | 16.3 | 100 | 0 | 0 |
| MELD | 0.12 | 16.4 | 100 | 0 | 0 |
| MELD | 0.12 | 16.5 | 100 | 0 | 0 |
| MELD | 0.12 | 16.6 | 100 | 0 | 0 |
| MELD | 0.12 | 16.7 | 100 | 0 | 0 |
| MELD | 0.12 | 16.8 | 100 | 0 | 0 |
| MELD | 0.12 | 16.9 | 100 | 0 | 0 |
| MELD | 0.12 | 17 | 100 | 0 | 0 |
| MELD | 0.12 | 17.1 | 100 | 0 | 0 |
| MELD | 0.12 | 17.2 | 100 | 0 | 0 |
| MELD | 0.12 | 17.3 | 100 | 0 | 0 |
| MELD | 0.12 | 17.4 | 100 | 0 | 0 |
| MELD | 0.12 | 17.5 | 100 | 0 | 0 |
| MELD | 0.12 | 17.6 | 100 | 0 | 0 |
| MELD | 0.12 | 17.7 | 100 | 0 | 0 |
| MELD | 0.12 | 17.8 | 100 | 0 | 0 |
| MELD | 0.12 | 17.9 | 100 | 0 | 0 |
| MELD | 0.12 | 18 | 100 | 0 | 0 |
| MELD | 0.12 | 18.1 | 100 | 0 | 0 |
| MELD | 0.12 | 18.2 | 100 | 0 | 0 |
| MELD | 0.12 | 18.3 | 100 | 0 | 0 |
| MELD | 0.12 | 18.4 | 100 | 0 | 0 |
| MELD | 0.12 | 18.5 | 100 | 0 | 0 |
| MELD | 0.12 | 18.6 | 100 | 0 | 0 |
| MELD | 0.12 | 18.7 | 100 | 0 | 0 |
| MELD | 0.12 | 18.8 | 100 | 0 | 0 |
| MELD | 0.12 | 18.9 | 100 | 0 | 0 |
| MELD | 0.12 | 19 | 100 | 0 | 0 |
| MELD | 0.12 | 19.1 | 100 | 0 | 0 |
| MELD | 0.12 | 19.2 | 100 | 0 | 0 |
| MELD | 0.12 | 19.3 | 100 | 0 | 0 |
| MELD | 0.12 | 19.4 | 100 | 0 | 0 |
| MELD | 0.12 | 19.5 | 100 | 0 | 0 |
| MELD | 0.12 | 19.6 | 100 | 0 | 0 |
| MELD | 0.12 | 19.7 | 100 | 0 | 0 |
| MELD | 0.12 | 19.8 | 100 | 0 | 0 |
| MELD | 0.12 | 19.9 | 100 | 0 | 0 |
| MELD | 0.12 | 20 | 100 | 0 | 0 |
| MELD | 0.12 | 20.1 | 100 | 0 | 0 |
| MELD | 0.12 | 20.2 | 100 | 0 | 0 |
| MELD | 0.12 | 20.3 | 100 | 0 | 0 |
| MELD | 0.12 | 20.4 | 100 | 0 | 0 |
| MELD | 0.12 | 20.5 | 100 | 0 | 0 |
| MELD | 0.12 | 20.6 | 100 | 0 | 0 |
| MELD | 0.12 | 20.7 | 100 | 0 | 0 |
| MELD | 0.12 | 20.8 | 100 | 0 | 0 |
| MELD | 0.12 | 20.9 | 100 | 0 | 0 |
| MELD | 0.12 | 21 | 100 | 0 | 0 |
| MELD | 0.12 | 21.1 | 100 | 0 | 0 |
| MELD | 0.12 | 21.2 | 100 | 0 | 0 |
| MELD | 0.12 | 21.3 | 100 | 0 | 0 |
| MELD | 0.12 | 21.4 | 100 | 0 | 0 |
| MELD | 0.12 | 21.5 | 100 | 0 | 0 |
| MELD | 0.12 | 21.6 | 100 | 0 | 0 |
| MELD | 0.12 | 21.7 | 100 | 0 | 0 |
| MELD | 0.12 | 21.8 | 100 | 0 | 0 |
| MELD | 0.12 | 21.9 | 100 | 0 | 0 |
| MELD | 0.12 | 22 | 100 | 0 | 0 |
| MELD | 0.12 | 22.1 | 100 | 0 | 0 |
| MELD | 0.12 | 22.2 | 100 | 0 | 0 |
| MELD | 0.12 | 22.3 | 100 | 0 | 0 |
| MELD | 0.12 | 22.4 | 100 | 0 | 0 |
| MELD | 0.12 | 22.5 | 100 | 0 | 0 |
| MELD | 0.12 | 22.6 | 100 | 0 | 0 |
| MELD | 0.12 | 22.7 | 100 | 0 | 0 |
| MELD | 0.12 | 22.8 | 100 | 0 | 0 |
| MELD | 0.12 | 22.9 | 100 | 0 | 0 |
| MELD | 0.12 | 23 | 100 | 0 | 0 |
| MELD | 0.12 | 23.1 | 100 | 0 | 0 |
| MELD | 0.12 | 23.2 | 100 | 0 | 0 |
| MELD | 0.12 | 23.3 | 100 | 0 | 0 |
| MELD | 0.12 | 23.4 | 100 | 0 | 0 |
| MELD | 0.12 | 23.5 | 100 | 0 | 0 |
| MELD | 0.12 | 23.6 | 100 | 0 | 0 |
| MELD | 0.12 | 23.7 | 100 | 0 | 0 |
| MELD | 0.12 | 23.8 | 100 | 0 | 0 |
| MELD | 0.12 | 23.9 | 100 | 0 | 0 |
| MELD | 0.12 | 24 | 100 | 0 | 0 |
| MELD | 0.12 | 24.1 | 100 | 0 | 0 |
| MELD | 0.12 | 24.2 | 100 | 0 | 0 |
| MELD | 0.12 | 24.3 | 100 | 0 | 0 |
| MELD | 0.12 | 24.4 | 100 | 0 | 0 |
| MELD | 0.12 | 24.5 | 100 | 0 | 0 |
| MELD | 0.12 | 24.6 | 100 | 0 | 0 |
| MELD | 0.12 | 24.7 | 100 | 0 | 0 |
| MELD | 0.12 | 24.8 | 100 | 0 | 0 |
| MELD | 0.12 | 24.9 | 100 | 0 | 0 |
| MELD | 0.12 | 25 | 100 | 0 | 0 |
| MELD | 0.12 | 25.1 | 100 | 0 | 0 |
| MELD | 0.12 | 25.2 | 100 | 0 | 0 |
| MELD | 0.12 | 25.3 | 100 | 0 | 0 |
| MELD | 0.12 | 25.4 | 100 | 0 | 0 |
| MELD | 0.12 | 25.5 | 100 | 0 | 0 |
| MELD | 0.12 | 25.6 | 100 | 0 | 0 |
| MELD | 0.12 | 25.7 | 100 | 0 | 0 |
| MELD | 0.12 | 25.8 | 100 | 0 | 0 |
| MELD | 0.12 | 25.9 | 100 | 0 | 0 |
| MELD | 0.12 | 26 | 100 | 0 | 0 |
| MELD | 0.12 | 26.1 | 100 | 0 | 0 |
| MELD | 0.12 | 26.2 | 100 | 0 | 0 |
| MELD | 0.12 | 26.3 | 100 | 0 | 0 |
| MELD | 0.12 | 26.4 | 100 | 0 | 0 |
| MELD | 0.12 | 26.5 | 100 | 0 | 0 |
| MELD | 0.12 | 26.6 | 100 | 0 | 0 |
| MELD | 0.12 | 26.7 | 100 | 0 | 0 |
| MELD | 0.12 | 26.8 | 100 | 0 | 0 |
| MELD | 0.12 | 26.9 | 100 | 0 | 0 |
| MELD | 0.12 | 27 | 100 | 0 | 0 |
| MELD | 0.12 | 27.1 | 100 | 0 | 0 |
| MELD | 0.12 | 27.2 | 100 | 0 | 0 |
| MELD | 0.12 | 27.3 | 100 | 0 | 0 |
| MELD | 0.12 | 27.4 | 100 | 0 | 0 |
| MELD | 0.12 | 27.5 | 100 | 0 | 0 |
| MELD | 0.12 | 27.6 | 100 | 0 | 0 |
| MELD | 0.12 | 27.7 | 100 | 0 | 0 |
| MELD | 0.12 | 27.8 | 100 | 0 | 0 |
| MELD | 0.12 | 27.9 | 100 | 0 | 0 |
| MELD | 0.12 | 28 | 100 | 0 | 0 |
| MELD | 0.12 | 28.1 | 100 | 0 | 0 |
| MELD | 0.12 | 28.2 | 100 | 0 | 0 |
| MELD | 0.12 | 28.3 | 100 | 0 | 0 |
| MELD | 0.12 | 28.4 | 100 | 0 | 0 |
| MELD | 0.12 | 28.5 | 100 | 0 | 0 |
| MELD | 0.12 | 28.6 | 100 | 0 | 0 |
| MELD | 0.12 | 28.7 | 100 | 0 | 0 |
| MELD | 0.12 | 28.8 | 100 | 0 | 0 |
| MELD | 0.12 | 28.9 | 100 | 0 | 0 |
| MELD | 0.12 | 29 | 100 | 0 | 0 |
| MELD | 0.12 | 29.1 | 100 | 0 | 0 |
| MELD | 0.12 | 29.2 | 100 | 0 | 0 |
| MELD | 0.12 | 29.3 | 100 | 0 | 0 |
| MELD | 0.12 | 29.4 | 100 | 0 | 0 |
| MELD | 0.12 | 29.5 | 100 | 0 | 0 |
| MELD | 0.12 | 29.6 | 100 | 0 | 0 |
| MELD | 0.12 | 29.7 | 100 | 0 | 0 |
| MELD | 0.12 | 29.8 | 100 | 0 | 0 |
| MELD | 0.12 | 29.9 | 100 | 0 | 0 |
| MELD | 0.12 | 30 | 100 | 0 | 0 |
| MELD | 0.14 | 0.1 | 100 | 0 | 0 |
| MELD | 0.14 | 0.2 | 100 | 0 | 0 |
| MELD | 0.14 | 0.3 | 100 | 0 | 0 |
| MELD | 0.14 | 0.4 | 100 | 0 | 0 |
| MELD | 0.14 | 0.5 | 100 | 0 | 0 |
| MELD | 0.14 | 0.6 | 100 | 0 | 0 |
| MELD | 0.14 | 0.7 | 100 | 0 | 0 |
| MELD | 0.14 | 0.8 | 100 | 0 | 0 |
| MELD | 0.14 | 0.9 | 100 | 0 | 0 |
| MELD | 0.14 | 1 | 100 | 0 | 0 |
| MELD | 0.14 | 1.1 | 100 | 0 | 0 |
| MELD | 0.14 | 1.2 | 100 | 0 | 0 |
| MELD | 0.14 | 1.3 | 100 | 0 | 0 |
| MELD | 0.14 | 1.4 | 100 | 0 | 0 |
| MELD | 0.14 | 1.5 | 100 | 0 | 0 |
| MELD | 0.14 | 1.6 | 100 | 0 | 0 |
| MELD | 0.14 | 1.7 | 100 | 0 | 0 |
| MELD | 0.14 | 1.8 | 100 | 0 | 0 |
| MELD | 0.14 | 1.9 | 100 | 0 | 0 |
| MELD | 0.14 | 2 | 100 | 0 | 0 |
| MELD | 0.14 | 2.1 | 100 | 0 | 0 |
| MELD | 0.14 | 2.2 | 100 | 0 | 0 |
| MELD | 0.14 | 2.3 | 100 | 0 | 0 |
| MELD | 0.14 | 2.4 | 100 | 0 | 0 |
| MELD | 0.14 | 2.5 | 100 | 0 | 0 |
| MELD | 0.14 | 2.6 | 100 | 0 | 0 |
| MELD | 0.14 | 2.7 | 100 | 0 | 0 |
| MELD | 0.14 | 2.8 | 100 | 0 | 0 |
| MELD | 0.14 | 2.9 | 100 | 0 | 0 |
| MELD | 0.14 | 3 | 100 | 0 | 0 |
| MELD | 0.14 | 3.1 | 100 | 0 | 0 |
| MELD | 0.14 | 3.2 | 100 | 0 | 0 |
| MELD | 0.14 | 3.3 | 100 | 0 | 0 |
| MELD | 0.14 | 3.4 | 100 | 0 | 0 |
| MELD | 0.14 | 3.5 | 100 | 0 | 0 |
| MELD | 0.14 | 3.6 | 100 | 0 | 0 |
| MELD | 0.14 | 3.7 | 100 | 0 | 0 |
| MELD | 0.14 | 3.8 | 100 | 0 | 0 |
| MELD | 0.14 | 3.9 | 100 | 0 | 0 |
| MELD | 0.14 | 4 | 100 | 0 | 0 |
| MELD | 0.14 | 4.1 | 100 | 0 | 0 |
| MELD | 0.14 | 4.2 | 100 | 0 | 0 |
| MELD | 0.14 | 4.3 | 100 | 0 | 0 |
| MELD | 0.14 | 4.4 | 100 | 0 | 0 |
| MELD | 0.14 | 4.5 | 100 | 0 | 0 |
| MELD | 0.14 | 4.6 | 100 | 0 | 0 |
| MELD | 0.14 | 4.7 | 100 | 0 | 0 |
| MELD | 0.14 | 4.8 | 100 | 0 | 0 |
| MELD | 0.14 | 4.9 | 100 | 0 | 0 |
| MELD | 0.14 | 5 | 100 | 0 | 0 |
| MELD | 0.14 | 5.1 | 100 | 0 | 0 |
| MELD | 0.14 | 5.2 | 100 | 0 | 0 |
| MELD | 0.14 | 5.3 | 100 | 0 | 0 |
| MELD | 0.14 | 5.4 | 100 | 0 | 0 |
| MELD | 0.14 | 5.5 | 100 | 0 | 0 |
| MELD | 0.14 | 5.6 | 100 | 0 | 0 |
| MELD | 0.14 | 5.7 | 100 | 0 | 0 |
| MELD | 0.14 | 5.8 | 100 | 0 | 0 |
| MELD | 0.14 | 5.9 | 100 | 0 | 0 |
| MELD | 0.14 | 6 | 100 | 0 | 0 |
| MELD | 0.14 | 6.1 | 100 | 0 | 0 |
| MELD | 0.14 | 6.2 | 100 | 0 | 0 |
| MELD | 0.14 | 6.3 | 100 | 0 | 0 |
| MELD | 0.14 | 6.4 | 100 | 0 | 0 |
| MELD | 0.14 | 6.5 | 100 | 0 | 0 |
| MELD | 0.14 | 6.6 | 100 | 0 | 0 |
| MELD | 0.14 | 6.7 | 100 | 0 | 0 |
| MELD | 0.14 | 6.8 | 100 | 0 | 0 |
| MELD | 0.14 | 6.9 | 100 | 0 | 0 |
| MELD | 0.14 | 7 | 100 | 0 | 0 |
| MELD | 0.14 | 7.1 | 100 | 0 | 0 |
| MELD | 0.14 | 7.2 | 100 | 0 | 0 |
| MELD | 0.14 | 7.3 | 100 | 0 | 0 |
| MELD | 0.14 | 7.4 | 100 | 0 | 0 |
| MELD | 0.14 | 7.5 | 100 | 0 | 0 |
| MELD | 0.14 | 7.6 | 100 | 0 | 0 |
| MELD | 0.14 | 7.7 | 100 | 0 | 0 |
| MELD | 0.14 | 7.8 | 100 | 0 | 0 |
| MELD | 0.14 | 7.9 | 100 | 0 | 0 |
| MELD | 0.14 | 8 | 100 | 0 | 0 |
| MELD | 0.14 | 8.1 | 100 | 0 | 0 |
| MELD | 0.14 | 8.2 | 100 | 0 | 0 |
| MELD | 0.14 | 8.3 | 100 | 0 | 0 |
| MELD | 0.14 | 8.4 | 100 | 0 | 0 |
| MELD | 0.14 | 8.5 | 100 | 0 | 0 |
| MELD | 0.14 | 8.6 | 100 | 0 | 0 |
| MELD | 0.14 | 8.7 | 100 | 0 | 0 |
| MELD | 0.14 | 8.8 | 100 | 0 | 0 |
| MELD | 0.14 | 8.9 | 100 | 0 | 0 |
| MELD | 0.14 | 9 | 100 | 0 | 0 |
| MELD | 0.14 | 9.1 | 100 | 0 | 0 |
| MELD | 0.14 | 9.2 | 100 | 0 | 0 |
| MELD | 0.14 | 9.3 | 100 | 0 | 0 |
| MELD | 0.14 | 9.4 | 100 | 0 | 0 |
| MELD | 0.14 | 9.5 | 100 | 0 | 0 |
| MELD | 0.14 | 9.6 | 100 | 0 | 0 |
| MELD | 0.14 | 9.7 | 100 | 0 | 0 |
| MELD | 0.14 | 9.8 | 100 | 0 | 0 |
| MELD | 0.14 | 9.9 | 100 | 0 | 0 |
| MELD | 0.14 | 10 | 100 | 0 | 0 |
| MELD | 0.14 | 10.1 | 100 | 0 | 0 |
| MELD | 0.14 | 10.2 | 100 | 0 | 0 |
| MELD | 0.14 | 10.3 | 100 | 0 | 0 |
| MELD | 0.14 | 10.4 | 100 | 0 | 0 |
| MELD | 0.14 | 10.5 | 100 | 0 | 0 |
| MELD | 0.14 | 10.6 | 100 | 0 | 0 |
| MELD | 0.14 | 10.7 | 100 | 0 | 0 |
| MELD | 0.14 | 10.8 | 100 | 0 | 0 |
| MELD | 0.14 | 10.9 | 100 | 0 | 0 |
| MELD | 0.14 | 11 | 100 | 0 | 0 |
| MELD | 0.14 | 11.1 | 100 | 0 | 0 |
| MELD | 0.14 | 11.2 | 100 | 0 | 0 |
| MELD | 0.14 | 11.3 | 100 | 0 | 0 |
| MELD | 0.14 | 11.4 | 100 | 0 | 0 |
| MELD | 0.14 | 11.5 | 100 | 0 | 0 |
| MELD | 0.14 | 11.6 | 100 | 0 | 0 |
| MELD | 0.14 | 11.7 | 100 | 0 | 0 |
| MELD | 0.14 | 11.8 | 100 | 0 | 0 |
| MELD | 0.14 | 11.9 | 100 | 0 | 0 |
| MELD | 0.14 | 12 | 100 | 0 | 0 |
| MELD | 0.14 | 12.1 | 100 | 0 | 0 |
| MELD | 0.14 | 12.2 | 100 | 0 | 0 |
| MELD | 0.14 | 12.3 | 100 | 0 | 0 |
| MELD | 0.14 | 12.4 | 100 | 0 | 0 |
| MELD | 0.14 | 12.5 | 100 | 0 | 0 |
| MELD | 0.14 | 12.6 | 100 | 0 | 0 |
| MELD | 0.14 | 12.7 | 100 | 0 | 0 |
| MELD | 0.14 | 12.8 | 100 | 0 | 0 |
| MELD | 0.14 | 12.9 | 100 | 0 | 0 |
| MELD | 0.14 | 13 | 100 | 0 | 0 |
| MELD | 0.14 | 13.1 | 100 | 0 | 0 |
| MELD | 0.14 | 13.2 | 100 | 0 | 0 |
| MELD | 0.14 | 13.3 | 100 | 0 | 0 |
| MELD | 0.14 | 13.4 | 100 | 0 | 0 |
| MELD | 0.14 | 13.5 | 100 | 0 | 0 |
| MELD | 0.14 | 13.6 | 100 | 0 | 0 |
| MELD | 0.14 | 13.7 | 100 | 0 | 0 |
| MELD | 0.14 | 13.8 | 100 | 0 | 0 |
| MELD | 0.14 | 13.9 | 100 | 0 | 0 |
| MELD | 0.14 | 14 | 100 | 0 | 0 |
| MELD | 0.14 | 14.1 | 100 | 0 | 0 |
| MELD | 0.14 | 14.2 | 100 | 0 | 0 |
| MELD | 0.14 | 14.3 | 100 | 0 | 0 |
| MELD | 0.14 | 14.4 | 100 | 0 | 0 |
| MELD | 0.14 | 14.5 | 100 | 0 | 0 |
| MELD | 0.14 | 14.6 | 100 | 0 | 0 |
| MELD | 0.14 | 14.7 | 100 | 0 | 0 |
| MELD | 0.14 | 14.8 | 100 | 0 | 0 |
| MELD | 0.14 | 14.9 | 100 | 0 | 0 |
| MELD | 0.14 | 15 | 100 | 0 | 0 |
| MELD | 0.14 | 15.1 | 100 | 0 | 0 |
| MELD | 0.14 | 15.2 | 100 | 0 | 0 |
| MELD | 0.14 | 15.3 | 100 | 0 | 0 |
| MELD | 0.14 | 15.4 | 100 | 0 | 0 |
| MELD | 0.14 | 15.5 | 100 | 0 | 0 |
| MELD | 0.14 | 15.6 | 100 | 0 | 0 |
| MELD | 0.14 | 15.7 | 100 | 0 | 0 |
| MELD | 0.14 | 15.8 | 100 | 0 | 0 |
| MELD | 0.14 | 15.9 | 100 | 0 | 0 |
| MELD | 0.14 | 16 | 100 | 0 | 0 |
| MELD | 0.14 | 16.1 | 100 | 0 | 0 |
| MELD | 0.14 | 16.2 | 100 | 0 | 0 |
| MELD | 0.14 | 16.3 | 100 | 0 | 0 |
| MELD | 0.14 | 16.4 | 100 | 0 | 0 |
| MELD | 0.14 | 16.5 | 100 | 0 | 0 |
| MELD | 0.14 | 16.6 | 100 | 0 | 0 |
| MELD | 0.14 | 16.7 | 100 | 0 | 0 |
| MELD | 0.14 | 16.8 | 100 | 0 | 0 |
| MELD | 0.14 | 16.9 | 100 | 0 | 0 |
| MELD | 0.14 | 17 | 100 | 0 | 0 |
| MELD | 0.14 | 17.1 | 100 | 0 | 0 |
| MELD | 0.14 | 17.2 | 100 | 0 | 0 |
| MELD | 0.14 | 17.3 | 100 | 0 | 0 |
| MELD | 0.14 | 17.4 | 100 | 0 | 0 |
| MELD | 0.14 | 17.5 | 100 | 0 | 0 |
| MELD | 0.14 | 17.6 | 100 | 0 | 0 |
| MELD | 0.14 | 17.7 | 100 | 0 | 0 |
| MELD | 0.14 | 17.8 | 100 | 0 | 0 |
| MELD | 0.14 | 17.9 | 100 | 0 | 0 |
| MELD | 0.14 | 18 | 100 | 0 | 0 |
| MELD | 0.14 | 18.1 | 100 | 0 | 0 |
| MELD | 0.14 | 18.2 | 100 | 0 | 0 |
| MELD | 0.14 | 18.3 | 100 | 0 | 0 |
| MELD | 0.14 | 18.4 | 100 | 0 | 0 |
| MELD | 0.14 | 18.5 | 100 | 0 | 0 |
| MELD | 0.14 | 18.6 | 100 | 0 | 0 |
| MELD | 0.14 | 18.7 | 100 | 0 | 0 |
| MELD | 0.14 | 18.8 | 100 | 0 | 0 |
| MELD | 0.14 | 18.9 | 100 | 0 | 0 |
| MELD | 0.14 | 19 | 100 | 0 | 0 |
| MELD | 0.14 | 19.1 | 100 | 0 | 0 |
| MELD | 0.14 | 19.2 | 100 | 0 | 0 |
| MELD | 0.14 | 19.3 | 100 | 0 | 0 |
| MELD | 0.14 | 19.4 | 100 | 0 | 0 |
| MELD | 0.14 | 19.5 | 100 | 0 | 0 |
| MELD | 0.14 | 19.6 | 100 | 0 | 0 |
| MELD | 0.14 | 19.7 | 100 | 0 | 0 |
| MELD | 0.14 | 19.8 | 100 | 0 | 0 |
| MELD | 0.14 | 19.9 | 100 | 0 | 0 |
| MELD | 0.14 | 20 | 100 | 0 | 0 |
| MELD | 0.14 | 20.1 | 100 | 0 | 0 |
| MELD | 0.14 | 20.2 | 100 | 0 | 0 |
| MELD | 0.14 | 20.3 | 100 | 0 | 0 |
| MELD | 0.14 | 20.4 | 100 | 0 | 0 |
| MELD | 0.14 | 20.5 | 100 | 0 | 0 |
| MELD | 0.14 | 20.6 | 100 | 0 | 0 |
| MELD | 0.14 | 20.7 | 100 | 0 | 0 |
| MELD | 0.14 | 20.8 | 100 | 0 | 0 |
| MELD | 0.14 | 20.9 | 100 | 0 | 0 |
| MELD | 0.14 | 21 | 100 | 0 | 0 |
| MELD | 0.14 | 21.1 | 100 | 0 | 0 |
| MELD | 0.14 | 21.2 | 100 | 0 | 0 |
| MELD | 0.14 | 21.3 | 100 | 0 | 0 |
| MELD | 0.14 | 21.4 | 100 | 0 | 0 |
| MELD | 0.14 | 21.5 | 100 | 0 | 0 |
| MELD | 0.14 | 21.6 | 100 | 0 | 0 |
| MELD | 0.14 | 21.7 | 100 | 0 | 0 |
| MELD | 0.14 | 21.8 | 100 | 0 | 0 |
| MELD | 0.14 | 21.9 | 100 | 0 | 0 |
| MELD | 0.14 | 22 | 100 | 0 | 0 |
| MELD | 0.14 | 22.1 | 100 | 0 | 0 |
| MELD | 0.14 | 22.2 | 100 | 0 | 0 |
| MELD | 0.14 | 22.3 | 100 | 0 | 0 |
| MELD | 0.14 | 22.4 | 100 | 0 | 0 |
| MELD | 0.14 | 22.5 | 100 | 0 | 0 |
| MELD | 0.14 | 22.6 | 100 | 0 | 0 |
| MELD | 0.14 | 22.7 | 100 | 0 | 0 |
| MELD | 0.14 | 22.8 | 100 | 0 | 0 |
| MELD | 0.14 | 22.9 | 100 | 0 | 0 |
| MELD | 0.14 | 23 | 100 | 0 | 0 |
| MELD | 0.14 | 23.1 | 100 | 0 | 0 |
| MELD | 0.14 | 23.2 | 100 | 0 | 0 |
| MELD | 0.14 | 23.3 | 100 | 0 | 0 |
| MELD | 0.14 | 23.4 | 100 | 0 | 0 |
| MELD | 0.14 | 23.5 | 100 | 0 | 0 |
| MELD | 0.14 | 23.6 | 100 | 0 | 0 |
| MELD | 0.14 | 23.7 | 100 | 0 | 0 |
| MELD | 0.14 | 23.8 | 100 | 0 | 0 |
| MELD | 0.14 | 23.9 | 100 | 0 | 0 |
| MELD | 0.14 | 24 | 100 | 0 | 0 |
| MELD | 0.14 | 24.1 | 100 | 0 | 0 |
| MELD | 0.14 | 24.2 | 100 | 0 | 0 |
| MELD | 0.14 | 24.3 | 100 | 0 | 0 |
| MELD | 0.14 | 24.4 | 100 | 0 | 0 |
| MELD | 0.14 | 24.5 | 100 | 0 | 0 |
| MELD | 0.14 | 24.6 | 100 | 0 | 0 |
| MELD | 0.14 | 24.7 | 100 | 0 | 0 |
| MELD | 0.14 | 24.8 | 100 | 0 | 0 |
| MELD | 0.14 | 24.9 | 100 | 0 | 0 |
| MELD | 0.14 | 25 | 100 | 0 | 0 |
| MELD | 0.14 | 25.1 | 100 | 0 | 0 |
| MELD | 0.14 | 25.2 | 100 | 0 | 0 |
| MELD | 0.14 | 25.3 | 100 | 0 | 0 |
| MELD | 0.14 | 25.4 | 100 | 0 | 0 |
| MELD | 0.14 | 25.5 | 100 | 0 | 0 |
| MELD | 0.14 | 25.6 | 100 | 0 | 0 |
| MELD | 0.14 | 25.7 | 100 | 0 | 0 |
| MELD | 0.14 | 25.8 | 100 | 0 | 0 |
| MELD | 0.14 | 25.9 | 100 | 0 | 0 |
| MELD | 0.14 | 26 | 100 | 0 | 0 |
| MELD | 0.14 | 26.1 | 100 | 0 | 0 |
| MELD | 0.14 | 26.2 | 100 | 0 | 0 |
| MELD | 0.14 | 26.3 | 100 | 0 | 0 |
| MELD | 0.14 | 26.4 | 100 | 0 | 0 |
| MELD | 0.14 | 26.5 | 100 | 0 | 0 |
| MELD | 0.14 | 26.6 | 100 | 0 | 0 |
| MELD | 0.14 | 26.7 | 100 | 0 | 0 |
| MELD | 0.14 | 26.8 | 100 | 0 | 0 |
| MELD | 0.14 | 26.9 | 100 | 0 | 0 |
| MELD | 0.14 | 27 | 100 | 0 | 0 |
| MELD | 0.14 | 27.1 | 100 | 0 | 0 |
| MELD | 0.14 | 27.2 | 100 | 0 | 0 |
| MELD | 0.14 | 27.3 | 100 | 0 | 0 |
| MELD | 0.14 | 27.4 | 100 | 0 | 0 |
| MELD | 0.14 | 27.5 | 100 | 0 | 0 |
| MELD | 0.14 | 27.6 | 100 | 0 | 0 |
| MELD | 0.14 | 27.7 | 100 | 0 | 0 |
| MELD | 0.14 | 27.8 | 100 | 0 | 0 |
| MELD | 0.14 | 27.9 | 100 | 0 | 0 |
| MELD | 0.14 | 28 | 100 | 0 | 0 |
| MELD | 0.14 | 28.1 | 100 | 0 | 0 |
| MELD | 0.14 | 28.2 | 100 | 0 | 0 |
| MELD | 0.14 | 28.3 | 100 | 0 | 0 |
| MELD | 0.14 | 28.4 | 100 | 0 | 0 |
| MELD | 0.14 | 28.5 | 100 | 0 | 0 |
| MELD | 0.14 | 28.6 | 100 | 0 | 0 |
| MELD | 0.14 | 28.7 | 100 | 0 | 0 |
| MELD | 0.14 | 28.8 | 100 | 0 | 0 |
| MELD | 0.14 | 28.9 | 100 | 0 | 0 |
| MELD | 0.14 | 29 | 100 | 0 | 0 |
| MELD | 0.14 | 29.1 | 100 | 0 | 0 |
| MELD | 0.14 | 29.2 | 100 | 0 | 0 |
| MELD | 0.14 | 29.3 | 100 | 0 | 0 |
| MELD | 0.14 | 29.4 | 100 | 0 | 0 |
| MELD | 0.14 | 29.5 | 100 | 0 | 0 |
| MELD | 0.14 | 29.6 | 100 | 0 | 0 |
| MELD | 0.14 | 29.7 | 100 | 0 | 0 |
| MELD | 0.14 | 29.8 | 100 | 0 | 0 |
| MELD | 0.14 | 29.9 | 100 | 0 | 0 |
| MELD | 0.14 | 30 | 100 | 0 | 0 |
| MELD | 0.16 | 0.1 | 100 | 0 | 0 |
| MELD | 0.16 | 0.2 | 100 | 0 | 0 |
| MELD | 0.16 | 0.3 | 100 | 0 | 0 |
| MELD | 0.16 | 0.4 | 100 | 0 | 0 |
| MELD | 0.16 | 0.5 | 100 | 0 | 0 |
| MELD | 0.16 | 0.6 | 100 | 0 | 0 |
| MELD | 0.16 | 0.7 | 100 | 0 | 0 |
| MELD | 0.16 | 0.8 | 100 | 0 | 0 |
| MELD | 0.16 | 0.9 | 100 | 0 | 0 |
| MELD | 0.16 | 1 | 100 | 0 | 0 |
| MELD | 0.16 | 1.1 | 100 | 0 | 0 |
| MELD | 0.16 | 1.2 | 100 | 0 | 0 |
| MELD | 0.16 | 1.3 | 100 | 0 | 0 |
| MELD | 0.16 | 1.4 | 100 | 0 | 0 |
| MELD | 0.16 | 1.5 | 100 | 0 | 0 |
| MELD | 0.16 | 1.6 | 100 | 0 | 0 |
| MELD | 0.16 | 1.7 | 100 | 0 | 0 |
| MELD | 0.16 | 1.8 | 100 | 0 | 0 |
| MELD | 0.16 | 1.9 | 100 | 0 | 0 |
| MELD | 0.16 | 2 | 100 | 0 | 0 |
| MELD | 0.16 | 2.1 | 100 | 0 | 0 |
| MELD | 0.16 | 2.2 | 100 | 0 | 0 |
| MELD | 0.16 | 2.3 | 100 | 0 | 0 |
| MELD | 0.16 | 2.4 | 100 | 0 | 0 |
| MELD | 0.16 | 2.5 | 100 | 0 | 0 |
| MELD | 0.16 | 2.6 | 100 | 0 | 0 |
| MELD | 0.16 | 2.7 | 100 | 0 | 0 |
| MELD | 0.16 | 2.8 | 100 | 0 | 0 |
| MELD | 0.16 | 2.9 | 100 | 0 | 0 |
| MELD | 0.16 | 3 | 100 | 0 | 0 |
| MELD | 0.16 | 3.1 | 100 | 0 | 0 |
| MELD | 0.16 | 3.2 | 100 | 0 | 0 |
| MELD | 0.16 | 3.3 | 100 | 0 | 0 |
| MELD | 0.16 | 3.4 | 100 | 0 | 0 |
| MELD | 0.16 | 3.5 | 100 | 0 | 0 |
| MELD | 0.16 | 3.6 | 100 | 0 | 0 |
| MELD | 0.16 | 3.7 | 100 | 0 | 0 |
| MELD | 0.16 | 3.8 | 100 | 0 | 0 |
| MELD | 0.16 | 3.9 | 100 | 0 | 0 |
| MELD | 0.16 | 4 | 100 | 0 | 0 |
| MELD | 0.16 | 4.1 | 100 | 0 | 0 |
| MELD | 0.16 | 4.2 | 100 | 0 | 0 |
| MELD | 0.16 | 4.3 | 100 | 0 | 0 |
| MELD | 0.16 | 4.4 | 100 | 0 | 0 |
| MELD | 0.16 | 4.5 | 100 | 0 | 0 |
| MELD | 0.16 | 4.6 | 100 | 0 | 0 |
| MELD | 0.16 | 4.7 | 100 | 0 | 0 |
| MELD | 0.16 | 4.8 | 100 | 0 | 0 |
| MELD | 0.16 | 4.9 | 100 | 0 | 0 |
| MELD | 0.16 | 5 | 100 | 0 | 0 |
| MELD | 0.16 | 5.1 | 100 | 0 | 0 |
| MELD | 0.16 | 5.2 | 100 | 0 | 0 |
| MELD | 0.16 | 5.3 | 100 | 0 | 0 |
| MELD | 0.16 | 5.4 | 100 | 0 | 0 |
| MELD | 0.16 | 5.5 | 100 | 0 | 0 |
| MELD | 0.16 | 5.6 | 100 | 0 | 0 |
| MELD | 0.16 | 5.7 | 100 | 0 | 0 |
| MELD | 0.16 | 5.8 | 100 | 0 | 0 |
| MELD | 0.16 | 5.9 | 100 | 0 | 0 |
| MELD | 0.16 | 6 | 100 | 0 | 0 |
| MELD | 0.16 | 6.1 | 100 | 0 | 0 |
| MELD | 0.16 | 6.2 | 100 | 0 | 0 |
| MELD | 0.16 | 6.3 | 100 | 0 | 0 |
| MELD | 0.16 | 6.4 | 100 | 0 | 0 |
| MELD | 0.16 | 6.5 | 100 | 0 | 0 |
| MELD | 0.16 | 6.6 | 100 | 0 | 0 |
| MELD | 0.16 | 6.7 | 100 | 0 | 0 |
| MELD | 0.16 | 6.8 | 100 | 0 | 0 |
| MELD | 0.16 | 6.9 | 100 | 0 | 0 |
| MELD | 0.16 | 7 | 100 | 0 | 0 |
| MELD | 0.16 | 7.1 | 100 | 0 | 0 |
| MELD | 0.16 | 7.2 | 100 | 0 | 0 |
| MELD | 0.16 | 7.3 | 100 | 0 | 0 |
| MELD | 0.16 | 7.4 | 100 | 0 | 0 |
| MELD | 0.16 | 7.5 | 100 | 0 | 0 |
| MELD | 0.16 | 7.6 | 100 | 0 | 0 |
| MELD | 0.16 | 7.7 | 100 | 0 | 0 |
| MELD | 0.16 | 7.8 | 100 | 0 | 0 |
| MELD | 0.16 | 7.9 | 100 | 0 | 0 |
| MELD | 0.16 | 8 | 100 | 0 | 0 |
| MELD | 0.16 | 8.1 | 100 | 0 | 0 |
| MELD | 0.16 | 8.2 | 100 | 0 | 0 |
| MELD | 0.16 | 8.3 | 100 | 0 | 0 |
| MELD | 0.16 | 8.4 | 100 | 0 | 0 |
| MELD | 0.16 | 8.5 | 100 | 0 | 0 |
| MELD | 0.16 | 8.6 | 100 | 0 | 0 |
| MELD | 0.16 | 8.7 | 100 | 0 | 0 |
| MELD | 0.16 | 8.8 | 100 | 0 | 0 |
| MELD | 0.16 | 8.9 | 100 | 0 | 0 |
| MELD | 0.16 | 9 | 100 | 0 | 0 |
| MELD | 0.16 | 9.1 | 100 | 0 | 0 |
| MELD | 0.16 | 9.2 | 100 | 0 | 0 |
| MELD | 0.16 | 9.3 | 100 | 0 | 0 |
| MELD | 0.16 | 9.4 | 100 | 0 | 0 |
| MELD | 0.16 | 9.5 | 100 | 0 | 0 |
| MELD | 0.16 | 9.6 | 100 | 0 | 0 |
| MELD | 0.16 | 9.7 | 100 | 0 | 0 |
| MELD | 0.16 | 9.8 | 100 | 0 | 0 |
| MELD | 0.16 | 9.9 | 100 | 0 | 0 |
| MELD | 0.16 | 10 | 100 | 0 | 0 |
