# Supplementary material for: Creatinine assay interferences compromises MELD accuracy and may bias liver allocation
Source: Nat Commun. 2026 Jul 23;17:7111. doi: 10.1038/s41467-026-75011-x (PMC13396164; doi:10.1038/s41467-026-75011-x)
Supplement: Supplementary file 4 — Source Data [file 41467_2026_75011_MOESM4_ESM.zip › figshare_package_FINAL_PUBLIC_DEPOSIT_V1_20260503_002637/00_START_HERE_HTML_NAVIGATOR/file_views/view_0014_slco_F2_heatmap_meta_public.html]

02\_workflows/F2\_workflow\_v01/submission\_ready/public/data/slco\_F2\_heatmap\_meta\_public.csv

# Readable file view

02\_workflows/F2\_workflow\_v01/submission\_ready/public/data/slco\_F2\_heatmap\_meta\_public.csv

← Back to navigator   |   Open original package file

Section

Manuscript output data

Output

F2

Extension

csv

Size KB

0.539

Variables

2

## Variables in this file

| Variable | Label | Description | Unit | Type |
| --- | --- | --- | --- | --- |
| parameter | Metadata parameter name | Name of a metadata parameter describing the F2 simulated heatmap object, such as figure identity, data origin, grid type, axis variable, or unit/role. |  | character |
| value | Value | Numerical or character value corresponding to the row-specific variable/metric. |  | character |

## Readable HTML view

Showing all 22 rows.

| parameter | value |
| --- | --- |
| dataset\_name | slco\_F2\_heatmap\_bin\_public |
| domain | slco |
| anchor | F2 |
| data\_object | heatmap |
| unit\_or\_role | bin |
| release\_status | public |
| source\_input\_file | slco\_F2\_heatmap\_input\_internal.csv |
| x\_axis | x\_value |
| x\_axis\_label | Creatinine (mg/dL) |
| x\_min | 0.1 |
| x\_max | 5 |
| x\_unique\_values | 246 |
| y\_axis | y\_value |
| y\_axis\_label | Bilirubin (mg/dL) |
| y\_min | 0.1 |
| y\_max | 30 |
| y\_unique\_values | 300 |
| fill\_variable | pct\_negative |
| fill\_label | Percentage of subpoints with score delta <= -1 |
| models | MELD | MELD-Na | reMELD-Na | MELD 3.0 |
| n\_rows | 295200 |
| render\_script | 03\_F2\_render\_heatmap\_figure\_v01.R |
