# Supplementary material for: Creatinine assay interferences compromises MELD accuracy and may bias liver allocation
Source: Nat Commun. 2026 Jul 23;17:7111. doi: 10.1038/s41467-026-75011-x (PMC13396164; doi:10.1038/s41467-026-75011-x)
Supplement: Supplementary file 4 — Source Data [file 41467_2026_75011_MOESM4_ESM.zip › figshare_package_FINAL_PUBLIC_DEPOSIT_V1_20260503_002637/00_START_HERE_HTML_NAVIGATOR/file_views/view_0005_f1_tb_cre_experimental_array_raw.html]

01\_primary\_data/public/f1\_tb\_cre\_experimental\_array\_raw.csv

# Readable file view

01\_primary\_data/public/f1\_tb\_cre\_experimental\_array\_raw.csv

← Back to navigator   |   Open original package file

Section

Public primary data

Output

F1

Extension

csv

Size KB

331.003

Variables

9

## Variables in this file

| Variable | Label | Description | Unit | Type |
| --- | --- | --- | --- | --- |
| Cre\_nominal\_grav\_mg\_dL | Nominal gravimetric creatinine target concentration | Nominal gravimetric target concentration of creatinine used when defining the F1 experimental array. | mg/dL | numeric |
| TB\_nominal\_grav\_mg\_dL | Nominal gravimetric total bilirubin target concentration | Nominal gravimetric target concentration of total bilirubin used when defining the F1 experimental array. | mg/dL | integer |
| TB\_trial\_M\_mg\_dL | Measured total bilirubin concentration in the experimental dataset | Measured total bilirubin concentration in the F1 experimental dataset. In rounded input tables this is the rounded/display value used for model calculation and plotting. | mg/dL | numeric |
| array\_id | Experimental array identifier | Identifier of the experimental array used in the F1 creatinine/bilirubin interference data. |  | character |
| assay | Creatinine assay | Creatinine assay represented by the row, for example enzymatic creatinine (CreE) or Jaffe creatinine (CreJ). |  | character |
| plot\_correction\_mg\_dL | Plotted creatinine correction | Creatinine correction value plotted in F1, expressed in mg/dL as the difference between corrected/reference and measured creatinine. | mg/dL | numeric |
| preparer\_id | Experimental preparer identifier | Identifier of the experimental preparer in F1 raw/validation data, represented as a public technical code. |  | character |
| replicate | Experimental replicate number | Replicate number within the F1 experimental dataset. |  | integer |
| trial\_display\_Cre\_M\_mg\_dL | Measured creatinine concentration in the experimental dataset | Measured creatinine concentration in the F1 experimental dataset. In rounded input tables this is the rounded/display value used for model calculation and plotting. | mg/dL | numeric |

## Readable HTML view

Preview shows first 1000 of 4200 rows. Open the original file for full content.

| array\_id | preparer\_id | replicate | assay | TB\_nominal\_grav\_mg\_dL | Cre\_nominal\_grav\_mg\_dL | TB\_trial\_M\_mg\_dL | trial\_display\_Cre\_M\_mg\_dL | plot\_correction\_mg\_dL |
| --- | --- | --- | --- | --- | --- | --- | --- | --- |
| Array\_1 | Prep\_A | 2 | CreE | 27 | 3 | 27.2227640681025 | 3.54112052571519 | -0.541120525715191 |
| Array\_1 | Prep\_A | 1 | CreE | 27 | 3 | 27.1297714113581 | 3.48086545171456 | -0.480865451714561 |
| Array\_1 | Prep\_A | 2 | CreJ | 27 | 3 | 27.2238964489877 | 3.38657822045959 | -0.386578220459593 |
| Array\_1 | Prep\_A | 1 | CreJ | 27 | 3 | 27.1309037922433 | 3.31706146881056 | -0.317061468810564 |
| Array\_2 | Prep\_A | 2 | CreE | 27 | 3 | 26.9427373034614 | 3.45845088595388 | -0.458450885953881 |
| Array\_2 | Prep\_A | 1 | CreE | 27 | 3 | 27.0883874463113 | 3.44494102399758 | -0.444941023997581 |
| Array\_2 | Prep\_A | 2 | CreJ | 27 | 3 | 26.9408989462757 | 3.25085088330939 | -0.250850883309386 |
| Array\_2 | Prep\_A | 1 | CreJ | 27 | 3 | 27.0865490891256 | 3.35618115420059 | -0.356181154200586 |
| Array\_1 | Prep\_A | 2 | CreJ | 14 | 1.5 | 14.0405223698577 | 1.61898827039151 | -0.118988270391514 |
| Array\_1 | Prep\_A | 1 | CreJ | 14 | 1.5 | 13.9731345470821 | 1.6691196339932 | -0.169119633993204 |
| Array\_1 | Prep\_A | 2 | CreE | 14 | 1.5 | 14.0423697323553 | 1.65167163990408 | -0.151671639904077 |
| Array\_1 | Prep\_A | 1 | CreE | 14 | 1.5 | 13.9749819095797 | 1.61735467878336 | -0.117354678783357 |
| Array\_2 | Prep\_A | 2 | CreJ | 14 | 1.5 | 14.0544349504726 | 1.61817226275938 | -0.118172262759379 |
| Array\_2 | Prep\_A | 1 | CreJ | 14 | 1.5 | 13.9536370559732 | 1.66433315763623 | -0.164333157636229 |
| Array\_2 | Prep\_A | 2 | CreE | 14 | 1.5 | 14.056282364532 | 1.62807745326894 | -0.128077453268937 |
| Array\_2 | Prep\_A | 1 | CreE | 14 | 1.5 | 13.9554844700326 | 1.62513426817036 | -0.125134268170357 |
| Array\_1 | Prep\_A | 2 | CreE | 28 | 3 | 28.0708837474689 | 3.5131877798266 | -0.513187779826595 |
| Array\_1 | Prep\_A | 1 | CreE | 28 | 3 | 27.9677062606411 | 3.49385851922907 | -0.493858519229065 |
| Array\_2 | Prep\_A | 2 | CreE | 28 | 3 | 28.2558230259916 | 3.51252606203808 | -0.512526062038075 |
| Array\_2 | Prep\_A | 1 | CreE | 28 | 3 | 28.2356597302308 | 3.398363626117 | -0.398363626116995 |
| Array\_1 | Prep\_A | 1 | CreJ | 28 | 3 | 27.9658659510949 | 3.30541430883517 | -0.305414308835168 |
| Array\_1 | Prep\_A | 2 | CreJ | 28 | 3 | 28.0690434379227 | 3.22407312168848 | -0.224073121688479 |
| Array\_2 | Prep\_A | 2 | CreJ | 28 | 3 | 28.2539844744108 | 3.29677943769297 | -0.296779437692968 |
| Array\_2 | Prep\_A | 1 | CreJ | 28 | 3 | 28.23382117865 | 3.28207010641119 | -0.282070106411188 |
| Array\_2 | Prep\_A | 2 | CreJ | 15 | 1.5 | 15.0485725629229 | 1.65219592101369 | -0.152195921013691 |
| Array\_2 | Prep\_A | 1 | CreJ | 15 | 1.5 | 15.0014441869439 | 1.63644636739597 | -0.136446367395971 |
| Array\_2 | Prep\_A | 1 | CreE | 15 | 1.5 | 15.0032919090534 | 1.63619928448375 | -0.136199284483749 |
| Array\_2 | Prep\_A | 2 | CreE | 15 | 1.5 | 15.0504202850324 | 1.65234803378164 | -0.152348033781639 |
| Array\_1 | Prep\_A | 2 | CreE | 15 | 1.5 | 14.9071548679041 | 1.62943451867419 | -0.129434518674189 |
| Array\_1 | Prep\_A | 1 | CreE | 15 | 1.5 | 15.0274976228685 | 1.66014164359277 | -0.160141643592769 |
| Array\_1 | Prep\_A | 2 | CreJ | 15 | 1.5 | 14.9082740015751 | 1.54335522067074 | -0.0433552206707439 |
| Array\_1 | Prep\_A | 1 | CreJ | 15 | 1.5 | 15.0286167565395 | 1.66150915299996 | -0.161509152999964 |
| Array\_1 | Prep\_A | 2 | CreE | 29 | 3 | 29.0445886455963 | 3.4066518606597 | -0.406651860659701 |
| Array\_1 | Prep\_A | 1 | CreE | 29 | 3 | 29.2300887361234 | 3.49822934412418 | -0.498229344124181 |
| Array\_2 | Prep\_A | 1 | CreE | 29 | 3 | 29.1155285371394 | 3.45139851706775 | -0.451398517067751 |
| Array\_2 | Prep\_A | 2 | CreE | 29 | 3 | 29.1839025983353 | 3.50434843279931 | -0.50434843279931 |
| Array\_1 | Prep\_A | 2 | CreJ | 29 | 3 | 29.0427510818117 | 3.31964973207326 | -0.319649732073259 |
| Array\_1 | Prep\_A | 1 | CreJ | 29 | 3 | 29.2282511723388 | 3.28057067609606 | -0.280570676096059 |
| Array\_2 | Prep\_A | 2 | CreJ | 29 | 3 | 29.1820650702812 | 3.2915489213302 | -0.291548921330203 |
| Array\_2 | Prep\_A | 1 | CreJ | 29 | 3 | 29.1136910090852 | 3.30988511905005 | -0.309885119050053 |
| Array\_2 | Prep\_A | 2 | CreJ | 16 | 1.5 | 16.0245789869479 | 1.67521997462653 | -0.175219974626528 |
| Array\_2 | Prep\_A | 1 | CreJ | 16 | 1.5 | 15.9861862110729 | 1.77034760426511 | -0.270347604265108 |
| Array\_1 | Prep\_A | 2 | CreE | 16 | 1.5 | 15.9508600580198 | 1.70511069747507 | -0.20511069747507 |
| Array\_1 | Prep\_A | 1 | CreE | 16 | 1.5 | 15.9916443763298 | 1.68080267747486 | -0.18080267747486 |
| Array\_2 | Prep\_A | 2 | CreE | 16 | 1.5 | 16.0264245841629 | 1.62191408666017 | -0.12191408666017 |
| Array\_2 | Prep\_A | 1 | CreE | 16 | 1.5 | 15.9880318082879 | 1.66982539758908 | -0.16982539758908 |
| Array\_1 | Prep\_A | 2 | CreJ | 16 | 1.5 | 15.9490133989677 | 1.6744556292732 | -0.174455629273199 |
| Array\_1 | Prep\_A | 1 | CreJ | 16 | 1.5 | 15.9897977172777 | 1.66285442016558 | -0.16285442016558 |
| Array\_2 | Prep\_A | 1 | CreJ | 30 | 3 | 30.3453474082191 | 3.33218025677491 | -0.332180256774908 |
| Array\_2 | Prep\_A | 2 | CreJ | 30 | 3 | 30.0542957885671 | 3.32318851526445 | -0.323188515264448 |
| Array\_2 | Prep\_A | 2 | CreE | 30 | 3 | 30.0558334799325 | 3.49070993617232 | -0.490709936172323 |
| Array\_2 | Prep\_A | 1 | CreE | 30 | 3 | 30.3468850995845 | 3.5091147776655 | -0.509114777665503 |
| Array\_1 | Prep\_A | 2 | CreJ | 30 | 3 | 30.1493953361159 | 3.39361183515198 | -0.393611835151979 |
| Array\_1 | Prep\_A | 1 | CreJ | 30 | 3 | 30.0004986954669 | 3.36574587171608 | -0.365745871716079 |
| Array\_1 | Prep\_A | 1 | CreE | 30 | 3 | 29.9993619170577 | 3.52645381959723 | -0.526453819597233 |
| Array\_1 | Prep\_A | 2 | CreE | 30 | 3 | 30.1482585577067 | 3.54510150515962 | -0.545101505159623 |
| Array\_2 | Prep\_A | 2 | CreJ | 17 | 1.5 | 17.0720068879795 | 1.65011213252051 | -0.150112132520513 |
| Array\_2 | Prep\_A | 1 | CreJ | 17 | 1.5 | 17.0309911505681 | 1.62862170278189 | -0.128621702781893 |
| Array\_2 | Prep\_A | 2 | CreE | 17 | 1.5 | 17.0724194225428 | 1.6829927026292 | -0.1829927026292 |
| Array\_2 | Prep\_A | 1 | CreE | 17 | 1.5 | 17.0314036851314 | 1.68431572108427 | -0.18431572108427 |
| Array\_1 | Prep\_A | 2 | CreE | 17 | 1.5 | 17.07012787024 | 1.69577309652103 | -0.19577309652103 |
| Array\_1 | Prep\_A | 1 | CreE | 17 | 1.5 | 16.9369580247236 | 1.66912147527201 | -0.16912147527201 |
| Array\_1 | Prep\_A | 2 | CreJ | 17 | 1.5 | 17.0682818435985 | 1.683373241374 | -0.183373241374002 |
| Array\_1 | Prep\_A | 1 | CreJ | 17 | 1.5 | 16.9351119980821 | 1.65080616758949 | -0.150806167589492 |
| Array\_2 | Prep\_A | 2 | CreE | 31 | 3 | 31.062156948219 | 3.51839469958132 | -0.518394699581316 |
| Array\_2 | Prep\_A | 1 | CreE | 31 | 3 | 31.050206000277502 | 3.54291353478572 | -0.542913534785716 |
| Array\_2 | Prep\_A | 1 | CreJ | 31 | 3 | 31.0483693691696 | 3.29642852515782 | -0.296428525157817 |
| Array\_2 | Prep\_A | 2 | CreJ | 31 | 3 | 31.0603203171111 | 3.24590399871323 | -0.245903998713227 |
| Array\_1 | Prep\_A | 1 | CreJ | 31 | 3 | 31.0736523191123 | 3.36866646230845 | -0.368666462308452 |
| Array\_1 | Prep\_A | 2 | CreJ | 31 | 3 | 31.0870004024983 | 3.29148118745104 | -0.291481187451042 |
| Array\_1 | Prep\_A | 1 | CreE | 31 | 3 | 31.0726271258079 | 3.52965254046503 | -0.529652540465026 |
| Array\_1 | Prep\_A | 2 | CreE | 31 | 3 | 31.0859752091939 | 3.53924631471685 | -0.539246314716846 |
| Array\_2 | Prep\_A | 1 | CreJ | 18 | 1.5 | 18.0458754216208 | 1.63366492531092 | -0.133664925310916 |
| Array\_2 | Prep\_A | 2 | CreJ | 18 | 1.5 | 18.0847233940363 | 1.63373521505038 | -0.133735215050376 |
| Array\_2 | Prep\_A | 1 | CreE | 18 | 1.5 | 18.0447546374035 | 1.69655545479304 | -0.19655545479304 |
| Array\_2 | Prep\_A | 2 | CreE | 18 | 1.5 | 18.083602609819 | 1.70109243100682 | -0.20109243100682 |
| Array\_1 | Prep\_A | 2 | CreJ | 18 | 1.5 | 17.9356696932976 | 1.55298024669276 | -0.0529802466927576 |
| Array\_1 | Prep\_A | 1 | CreJ | 18 | 1.5 | 17.9707759256787 | 1.65053659173915 | -0.150536591739148 |
| Array\_1 | Prep\_A | 2 | CreE | 18 | 1.5 | 17.9345488726656 | 1.70734618104594 | -0.20734618104594 |
| Array\_1 | Prep\_A | 1 | CreE | 18 | 1.5 | 17.9696551050467 | 1.68997216469488 | -0.18997216469488 |
| Array\_1 | Prep\_A | 1 | CreJ | 32 | 3 | 31.7832824303764 | 3.30300139967081 | -0.303001399670813 |
| Array\_1 | Prep\_A | 2 | CreJ | 32 | 3 | 31.9274366698266 | 3.31261084049582 | -0.312610840495823 |
| Array\_2 | Prep\_A | 2 | CreJ | 32 | 3 | 32.0201906654968 | 3.27649564521116 | -0.27649564521116 |
| Array\_2 | Prep\_A | 1 | CreJ | 32 | 3 | 31.9929674057969 | 3.30755293333084 | -0.30755293333084 |
| Array\_2 | Prep\_A | 1 | CreE | 32 | 3 | 31.9948030011885 | 3.51414939217157 | -0.514149392171567 |
| Array\_2 | Prep\_A | 2 | CreE | 32 | 3 | 32.0220262608884 | 3.50561674719328 | -0.505616747193277 |
| Array\_1 | Prep\_A | 1 | CreE | 32 | 3 | 31.7851171984213 | 3.56623993191792 | -0.566239931917917 |
| Array\_1 | Prep\_A | 2 | CreE | 32 | 3 | 31.9292714378715 | 3.5156465985843 | -0.515646598584297 |
| Array\_2 | Prep\_A | 1 | CreE | 19 | 1.5 | 19.0021187943973 | 1.72679465743964 | -0.226794657439644 |
| Array\_2 | Prep\_A | 2 | CreE | 19 | 1.5 | 18.9431542471888 | 1.70782594202364 | -0.207825942023644 |
| Array\_2 | Prep\_A | 1 | CreJ | 19 | 1.5 | 19.0032421357207 | 1.64211413336217 | -0.142114133362172 |
| Array\_2 | Prep\_A | 2 | CreJ | 19 | 1.5 | 18.9442775885122 | 1.34569969732218 | 0.154300302677818 |
| Array\_1 | Prep\_A | 2 | CreE | 19 | 1.5 | 18.9643045249774 | 1.67940417097198 | -0.179404170971984 |
| Array\_1 | Prep\_A | 1 | CreE | 19 | 1.5 | 19.0717631060454 | 1.69869376138109 | -0.198693761381094 |
| Array\_1 | Prep\_A | 2 | CreJ | 19 | 1.5 | 18.9654258773716 | 1.61154484028609 | -0.111544840286095 |
| Array\_1 | Prep\_A | 1 | CreJ | 19 | 1.5 | 19.0728844584396 | 1.60220584036788 | -0.102205840367885 |
| Array\_1 | Prep\_A | 1 | CreJ | 33 | 3 | 32.9944969178462 | 3.28133154125818 | -0.281331541258181 |
| Array\_1 | Prep\_A | 2 | CreJ | 33 | 3 | 33.2842112274736 | 3.35192777794648 | -0.351927777946481 |
| Array\_1 | Prep\_A | 1 | CreE | 33 | 3 | 32.9933632474377 | 3.54571671527032 | -0.545716715270315 |
| Array\_1 | Prep\_A | 2 | CreE | 33 | 3 | 33.2830775570651 | 3.55766673531803 | -0.557666735318025 |
| Array\_2 | Prep\_A | 1 | CreE | 33 | 3 | 32.7936444891605 | 3.57046679429093 | -0.570466794290925 |
| Array\_2 | Prep\_A | 2 | CreE | 33 | 3 | 33.1980202447905 | 3.5372145397231 | -0.537214539723095 |
| Array\_2 | Prep\_A | 1 | CreJ | 33 | 3 | 32.7918069948593 | 3.20807604373599 | -0.208076043735986 |
| Array\_2 | Prep\_A | 2 | CreJ | 33 | 3 | 33.1961827504893 | 3.30054348033074 | -0.300543480330736 |
| Array\_2 | Prep\_A | 1 | CreE | 20 | 1.5 | 19.997833605011 | 1.71324323650972 | -0.213243236509719 |
| Array\_2 | Prep\_A | 2 | CreE | 20 | 1.5 | 20.0511953062174 | 1.71116220436281 | -0.211162204362809 |
| Array\_1 | Prep\_A | 2 | CreE | 20 | 1.5 | 19.9509782041155 | 1.66561941895071 | -0.165619418950709 |
| Array\_1 | Prep\_A | 1 | CreE | 20 | 1.5 | 20.1998346823266 | 1.72763800424392 | -0.227638004243919 |
| Array\_2 | Prep\_A | 1 | CreJ | 20 | 1.5 | 19.9988102166055 | 1.67560618976989 | -0.17560618976989 |
| Array\_2 | Prep\_A | 2 | CreJ | 20 | 1.5 | 20.0521719178119 | 1.61821438862523 | -0.11821438862523 |
| Array\_1 | Prep\_A | 2 | CreJ | 20 | 1.5 | 19.9520999340623 | 1.6529867231023 | -0.152986723102305 |
| Array\_1 | Prep\_A | 1 | CreJ | 20 | 1.5 | 20.2009564122734 | 1.54991845380883 | -0.0499184538088346 |
| Array\_1 | Prep\_A | 1 | CreE | 34 | 3 | 34.4789532385066 | 3.60088350923487 | -0.600883509234869 |
| Array\_1 | Prep\_A | 2 | CreE | 34 | 3 | 34.150285830789 | 3.52225283772252 | -0.522252837722519 |
| Array\_1 | Prep\_A | 2 | CreJ | 34 | 3 | 34.1484515140082 | 3.3182588690682 | -0.318258869068195 |
| Array\_1 | Prep\_A | 1 | CreJ | 34 | 3 | 34.4771189217257 | 3.26830570865345 | -0.268305708653445 |
| Array\_2 | Prep\_A | 1 | CreE | 34 | 3 | 34.1381266038546 | 3.53912594204986 | -0.539125942049859 |
| Array\_2 | Prep\_A | 2 | CreE | 34 | 3 | 34.0252637227787 | 3.54352632468875 | -0.543526324688749 |
| Array\_2 | Prep\_A | 2 | CreJ | 34 | 3 | 34.023428848426 | 3.32994387855362 | -0.329943878553623 |
| Array\_2 | Prep\_A | 1 | CreJ | 34 | 3 | 34.1362917295019 | 3.24691418422451 | -0.246914184224513 |
| Array\_2 | Prep\_A | 1 | CreE | 21 | 1.5 | 21.137233802049 | 1.74967157746138 | -0.249671577461379 |
| Array\_2 | Prep\_A | 2 | CreE | 21 | 1.5 | 21.0913524145679 | 1.71610911303399 | -0.216109113033989 |
| Array\_1 | Prep\_A | 2 | CreE | 21 | 1.5 | 21.0307039926531 | 1.70507527106127 | -0.205075271061269 |
| Array\_1 | Prep\_A | 1 | CreE | 21 | 1.5 | 21.0648212479997 | 1.73265000295144 | -0.232650002951439 |
| Array\_2 | Prep\_A | 1 | CreJ | 21 | 1.5 | 21.1353880874114 | 1.67932732906481 | -0.179327329064815 |
| Array\_2 | Prep\_A | 2 | CreJ | 21 | 1.5 | 21.0895066999304 | 1.68456122354951 | -0.184561223549515 |
| Array\_1 | Prep\_A | 1 | CreJ | 21 | 1.5 | 21.0645300784666 | 1.65155299507047 | -0.151552995070468 |
| Array\_1 | Prep\_A | 2 | CreJ | 21 | 1.5 | 21.03041282312 | 1.65503048033688 | -0.155030480336878 |
| Array\_2 | Prep\_A | 1 | CreJ | 35 | 3 | 35.2808942494946 | 3.29611583853931 | -0.296115838539308 |
| Array\_2 | Prep\_A | 2 | CreJ | 35 | 3 | 35.4653689545104 | 3.23897171184191 | -0.238971711841908 |
| Array\_1 | Prep\_A | 2 | CreE | 35 | 3 | 34.9756885306074 | 3.58101494027967 | -0.581014940279667 |
| Array\_1 | Prep\_A | 1 | CreE | 35 | 3 | 35.1517646933065 | 3.55022915841954 | -0.550229158419537 |
| Array\_1 | Prep\_A | 2 | CreJ | 35 | 3 | 34.9738547098436 | 3.25355038340403 | -0.25355038340403 |
| Array\_1 | Prep\_A | 1 | CreJ | 35 | 3 | 35.1499308725427 | 3.31391026941986 | -0.313910269419861 |
| Array\_2 | Prep\_A | 1 | CreE | 35 | 3 | 35.2827285884784 | 3.57173373553832 | -0.571733735538317 |
| Array\_2 | Prep\_A | 2 | CreE | 35 | 3 | 35.4672032934943 | 3.57703430449395 | -0.577034304493947 |
| Array\_2 | Prep\_A | 1 | CreJ | 22 | 1.5 | 22.0569001869919 | 1.6097763759216 | -0.1097763759216 |
| Array\_2 | Prep\_A | 2 | CreJ | 22 | 1.5 | 22.0514473623761 | 1.70293492406703 | -0.20293492406703 |
| Array\_1 | Prep\_A | 1 | CreJ | 22 | 1.5 | 22.0394113287824 | 1.56524461009048 | -0.0652446100904756 |
| Array\_1 | Prep\_A | 2 | CreJ | 22 | 1.5 | 22.0346691885046 | 1.65522567614872 | -0.155225676148715 |
| Array\_2 | Prep\_A | 2 | CreE | 22 | 1.5 | 22.0518739341742 | 1.73233789481076 | -0.232337894810763 |
| Array\_2 | Prep\_A | 1 | CreE | 22 | 1.5 | 22.05732675879 | 1.74350079971748 | -0.243500799717483 |
| Array\_1 | Prep\_A | 2 | CreE | 22 | 1.5 | 22.0335475411583 | 1.76871488469562 | -0.268714884695623 |
| Array\_1 | Prep\_A | 1 | CreE | 22 | 1.5 | 22.0382896814361 | 1.71572756214477 | -0.215727562144773 |
| Array\_1 | Prep\_A | 1 | CreE | 23 | 1.5 | 22.9867229544026 | 1.78053404092297 | -0.280534040922967 |
| Array\_1 | Prep\_A | 2 | CreE | 23 | 1.5 | 22.889366546332 | 1.75820883275461 | -0.258208832754607 |
| Array\_2 | Prep\_A | 2 | CreE | 23 | 1.5 | 22.9756084515827 | 1.77092186377558 | -0.270921863775577 |
| Array\_2 | Prep\_A | 1 | CreE | 23 | 1.5 | 23.1905270566643 | 1.75034061899812 | -0.250340618998117 |
| Array\_2 | Prep\_A | 2 | CreJ | 23 | 1.5 | 22.9762774892584 | 1.67774437074958 | -0.177744370749576 |
| Array\_2 | Prep\_A | 1 | CreJ | 23 | 1.5 | 23.19119609434 | 1.62023280867268 | -0.120232808672676 |
| Array\_1 | Prep\_A | 1 | CreJ | 23 | 1.5 | 22.9878445537317 | 1.66174792465582 | -0.161747924655823 |
| Array\_1 | Prep\_A | 2 | CreJ | 23 | 1.5 | 22.8904881456611 | 1.57621897020722 | -0.0762189702072231 |
| Array\_2 | Prep\_A | 2 | CreE | 1 | 3.5 | 1.03388872345755 | 3.65164512914469 | -0.151645129144694 |
| Array\_2 | Prep\_A | 1 | CreE | 1 | 3.5 | 1.02956745909463 | 3.64457986400574 | -0.144579864005745 |
| Array\_1 | Prep\_A | 1 | CreE | 1 | 3.5 | 0.979904083413194 | 3.64571647719027 | -0.145716477190275 |
| Array\_1 | Prep\_A | 2 | CreE | 1 | 3.5 | 1.0020246615841 | 3.65974677116813 | -0.159746771168134 |
| Array\_1 | Prep\_A | 2 | CreJ | 1 | 3.5 | 1.00311806772468 | 3.7322496900894 | -0.2322496900894 |
| Array\_1 | Prep\_A | 1 | CreJ | 1 | 3.5 | 0.980997489553775 | 3.76482522850829 | -0.26482522850829 |
| Array\_2 | Prep\_A | 1 | CreJ | 1 | 3.5 | 1.02770169306617 | 3.81985842682557 | -0.319858426825566 |
| Array\_2 | Prep\_A | 2 | CreJ | 1 | 3.5 | 1.03202295742909 | 3.78458525533122 | -0.284585255331216 |
| Array\_1 | Prep\_A | 2 | CreE | 24 | 1.5 | 24.1019973737768 | 1.76638147971398 | -0.266381479713985 |
| Array\_1 | Prep\_A | 1 | CreE | 24 | 1.5 | 23.9950700342478 | 1.79741071150251 | -0.297410711502515 |
| Array\_2 | Prep\_A | 1 | CreJ | 24 | 1.5 | 24.2441700579128 | 1.68000657305023 | -0.18000657305023 |
| Array\_2 | Prep\_A | 2 | CreJ | 24 | 1.5 | 24.1208280963982 | 1.71004179255945 | -0.21004179255945 |
| Array\_2 | Prep\_A | 1 | CreE | 24 | 1.5 | 24.2460169204099 | 1.7450668298814 | -0.245066829881405 |
| Array\_2 | Prep\_A | 2 | CreE | 24 | 1.5 | 24.1226749588953 | 1.76136894036652 | -0.261368940366515 |
| Array\_1 | Prep\_A | 1 | CreJ | 24 | 1.5 | 23.9960695452165 | 1.6099797819541 | -0.109979781954097 |
| Array\_1 | Prep\_A | 2 | CreJ | 24 | 1.5 | 24.1029968847455 | 1.67874828074829 | -0.178748280748287 |
| Array\_2 | Prep\_A | 2 | CreE | 2 | 3.5 | 2.01995654036665 | 3.66514370049096 | -0.16514370049096 |
| Array\_2 | Prep\_A | 1 | CreE | 2 | 3.5 | 2.02412069398652 | 3.63449456343362 | -0.13449456343362 |
| Array\_1 | Prep\_A | 2 | CreJ | 2 | 3.5 | 2.00339098455704 | 3.75622434425024 | -0.256224344250242 |
| Array\_1 | Prep\_A | 1 | CreJ | 2 | 3.5 | 2.01360588958182 | 3.78533859965294 | -0.285338599652942 |
| Array\_1 | Prep\_A | 1 | CreE | 2 | 3.5 | 2.01251696390969 | 3.70058317207204 | -0.200583172072041 |
| Array\_1 | Prep\_A | 2 | CreE | 2 | 3.5 | 2.00230205888491 | 3.71971061188539 | -0.21971061188539 |
| Array\_2 | Prep\_A | 2 | CreJ | 2 | 3.5 | 2.01808498580782 | 3.82758489364077 | -0.327584893640773 |
| Array\_2 | Prep\_A | 1 | CreJ | 2 | 3.5 | 2.02224913942769 | 3.80668392070448 | -0.306683920704483 |
| Array\_2 | Prep\_A | 2 | CreE | 25 | 1.5 | 25.0413320842648 | 1.81086119732607 | -0.310861197326068 |
| Array\_2 | Prep\_A | 1 | CreE | 25 | 1.5 | 25.3118854398346 | 1.80113989930335 | -0.301139899303348 |
| Array\_1 | Prep\_A | 2 | CreE | 25 | 1.5 | 24.9732091456492 | 1.80593198710428 | -0.305931987104278 |
| Array\_1 | Prep\_A | 1 | CreE | 25 | 1.5 | 25.112059154222 | 1.77695030770015 | -0.276950307700148 |
| Array\_1 | Prep\_A | 2 | CreJ | 25 | 1.5 | 24.971362404679 | 1.62513357157136 | -0.125133571571356 |
| Array\_1 | Prep\_A | 1 | CreJ | 25 | 1.5 | 25.1102124132518 | 1.69342001200702 | -0.193420012007016 |
| Array\_2 | Prep\_A | 2 | CreJ | 25 | 1.5 | 25.0394852690954 | 1.62963025498536 | -0.129630254985357 |
| Array\_2 | Prep\_A | 1 | CreJ | 25 | 1.5 | 25.3100386246652 | 1.70454618871019 | -0.204546188710186 |
| Array\_2 | Prep\_A | 2 | CreJ | 3 | 3.5 | 2.99469206307471 | 3.80984961072948 | -0.309849610729483 |
| Array\_2 | Prep\_A | 1 | CreJ | 3 | 3.5 | 2.98215996713363 | 3.83094890294471 | -0.330948902944713 |
| Array\_1 | Prep\_A | 2 | CreJ | 3 | 3.5 | 2.97960763875924 | 3.80829023092093 | -0.308290230920926 |
| Array\_1 | Prep\_A | 1 | CreJ | 3 | 3.5 | 3.00614922460798 | 3.8108635133969 | -0.310863513396896 |
| Array\_2 | Prep\_A | 2 | CreE | 3 | 3.5 | 2.99587875882383 | 3.66377589901418 | -0.163775899014177 |
| Array\_2 | Prep\_A | 1 | CreE | 3 | 3.5 | 2.98334666288275 | 3.70181147095535 | -0.201811470955346 |
| Array\_1 | Prep\_A | 1 | CreE | 3 | 3.5 | 3.00505204173629 | 3.70059018279693 | -0.200590182796927 |
| Array\_1 | Prep\_A | 2 | CreE | 3 | 3.5 | 2.97851045588755 | 3.69703803945918 | -0.197038039459176 |
| Array\_1 | Prep\_A | 1 | CreJ | 26 | 1.5 | 26.006851766246 | 1.62904030534848 | -0.129040305348483 |
| Array\_1 | Prep\_A | 2 | CreJ | 26 | 1.5 | 26.0491420581244 | 1.7312659001558801 | -0.231265900155883 |
| Array\_2 | Prep\_A | 2 | CreE | 26 | 1.5 | 26.0432888900395 | 1.79137061943338 | -0.291370619433379 |
| Array\_2 | Prep\_A | 1 | CreE | 26 | 1.5 | 26.1574058251557 | 1.80403207025155 | -0.304032070251549 |
| Array\_1 | Prep\_A | 2 | CreE | 26 | 1.5 | 26.0509901295851 | 1.76593818870475 | -0.265938188704749 |
| Array\_1 | Prep\_A | 1 | CreE | 26 | 1.5 | 26.0086998377067 | 1.78699451864651 | -0.286994518646509 |
| Array\_2 | Prep\_A | 2 | CreJ | 26 | 1.5 | 26.0444085779686 | 1.60420108218085 | -0.104201082180848 |
| Array\_2 | Prep\_A | 1 | CreJ | 26 | 1.5 | 26.1585255130848 | 1.61985021689957 | -0.119850216899568 |
| Array\_2 | Prep\_A | 1 | CreE | 4 | 3.5 | 4.03311614137008 | 3.6908405032483 | -0.190840503248301 |
| Array\_2 | Prep\_A | 2 | CreE | 4 | 3.5 | 3.99515756090611 | 3.68827071550837 | -0.188270715508371 |
| Array\_1 | Prep\_A | 2 | CreJ | 4 | 3.5 | 4.02916635591591 | 3.79305839243099 | -0.293058392430988 |
| Array\_1 | Prep\_A | 1 | CreJ | 4 | 3.5 | 4.02935494700498 | 3.93553461035195 | -0.435534610351948 |
| Array\_2 | Prep\_A | 2 | CreJ | 4 | 3.5 | 3.99625575868634 | 3.77770403173586 | -0.27770403173586 |
| Array\_2 | Prep\_A | 1 | CreJ | 4 | 3.5 | 4.03421433915031 | 3.83017745235587 | -0.330177452355871 |
| Array\_1 | Prep\_A | 1 | CreE | 4 | 3.5 | 4.03121493387315 | 3.6991862563079 | -0.199186256307901 |
| Array\_1 | Prep\_A | 2 | CreE | 4 | 3.5 | 4.03102634278408 | 3.71899647285704 | -0.218996472857041 |
| Array\_2 | Prep\_A | 1 | CreE | 27 | 1.5 | 27.1209790311119 | 1.81923658728224 | -0.31923658728223703 |
| Array\_2 | Prep\_A | 2 | CreE | 27 | 1.5 | 26.927232996281 | 1.82215910394707 | -0.322159103947067 |
| Array\_1 | Prep\_A | 2 | CreE | 27 | 1.5 | 27.0197389129872 | 1.81755248538784 | -0.317552485387837 |
| Array\_1 | Prep\_A | 1 | CreE | 27 | 1.5 | 26.6740836973345 | 1.81473126182726 | -0.314731261827257 |
| Array\_2 | Prep\_A | 1 | CreJ | 27 | 1.5 | 27.1191313509328 | 1.66856753379004 | -0.168567533790037 |
| Array\_2 | Prep\_A | 2 | CreJ | 27 | 1.5 | 26.9253853161019 | 1.66192309611212 | -0.161923096112117 |
| Array\_1 | Prep\_A | 1 | CreJ | 27 | 1.5 | 26.6750326051297 | 1.61497764801073 | -0.114977648010731 |
| Array\_1 | Prep\_A | 2 | CreJ | 27 | 1.5 | 27.0206878207824 | 1.60951807259133 | -0.109518072591331 |
| Array\_1 | Prep\_A | 2 | CreJ | 5 | 3.5 | 5.00970599411778 | 3.86518162821961 | -0.365181628219607 |
| Array\_1 | Prep\_A | 1 | CreJ | 5 | 3.5 | 5.02529773897391 | 3.82614817588238 | -0.326148175882377 |
| Array\_1 | Prep\_A | 1 | CreE | 5 | 3.5 | 5.02419372893272 | 3.72138007669563 | -0.221380076695626 |
| Array\_1 | Prep\_A | 2 | CreE | 5 | 3.5 | 5.00860198407659 | 3.69719100859307 | -0.197191008593066 |
| Array\_2 | Prep\_A | 1 | CreJ | 5 | 3.5 | 5.0348292192903 | 3.80296042473818 | -0.302960424738183 |
| Array\_2 | Prep\_A | 2 | CreJ | 5 | 3.5 | 5.01628087598922 | 3.8339032928115 | -0.333903292811503 |
| Array\_2 | Prep\_A | 1 | CreE | 5 | 3.5 | 5.03372914708176 | 3.73763626692315 | -0.237636266923146 |
| Array\_2 | Prep\_A | 2 | CreE | 5 | 3.5 | 5.01518080378068 | 3.70977019766136 | -0.209770197661356 |
| Array\_2 | Prep\_A | 2 | CreE | 28 | 1.5 | 27.9897268119224 | 1.79737257207858 | -0.297372572078582 |
| Array\_2 | Prep\_A | 1 | CreE | 28 | 1.5 | 28.3306357061179 | 1.80481744345151 | -0.304817443451512 |
| Array\_2 | Prep\_A | 2 | CreJ | 28 | 1.5 | 27.9878742835675 | 1.6510645743869 | -0.151064574386898 |
| Array\_2 | Prep\_A | 1 | CreJ | 28 | 1.5 | 28.328783177763 | 1.66814168398891 | -0.168141683988908 |
| Array\_1 | Prep\_A | 1 | CreE | 28 | 1.5 | 27.9610173830246 | 1.78575888843426 | -0.285758888434262 |
| Array\_1 | Prep\_A | 2 | CreE | 28 | 1.5 | 27.9641590885407 | 1.77900261842471 | -0.279002618424712 |
| Array\_1 | Prep\_A | 1 | CreJ | 28 | 1.5 | 27.9591648581538 | 1.63294994475694 | -0.132949944756938 |
| Array\_1 | Prep\_A | 2 | CreJ | 28 | 1.5 | 27.9623065636699 | 1.70729224388506 | -0.207292243885058 |
| Array\_2 | Prep\_A | 2 | CreE | 6 | 3.5 | 6.01935782266994 | 3.73127574645387 | -0.23127574645387 |
| Array\_2 | Prep\_A | 1 | CreE | 6 | 3.5 | 6.04244718756767 | 3.753567516459 | -0.253567516459 |
| Array\_1 | Prep\_A | 1 | CreJ | 6 | 3.5 | 5.9906534432693 | 3.80411736563824 | -0.304117365638239 |
| Array\_1 | Prep\_A | 2 | CreJ | 6 | 3.5 | 5.99172220774234 | 3.88820693990436 | -0.38820693990436 |
| Array\_2 | Prep\_A | 1 | CreJ | 6 | 3.5 | 6.04354071650777 | 3.77916010986225 | -0.279160109862246 |
| Array\_2 | Prep\_A | 2 | CreJ | 6 | 3.5 | 6.02045135161004 | 3.82128139962936 | -0.321281399629356 |
| Array\_1 | Prep\_A | 2 | CreE | 6 | 3.5 | 5.9906220771934 | 3.72607802825351 | -0.22607802825351 |
| Array\_1 | Prep\_A | 1 | CreE | 6 | 3.5 | 5.98955331272036 | 3.73251244188129 | -0.232512441881291 |
| Array\_2 | Prep\_A | 2 | CreE | 29 | 1.5 | 28.977365431914 | 1.81780587253745 | -0.317805872537449 |
| Array\_2 | Prep\_A | 1 | CreE | 29 | 1.5 | 28.9601612600774 | 1.86750889693636 | -0.367508896936359 |
| Array\_1 | Prep\_A | 2 | CreJ | 29 | 1.5 | 29.065595448882 | 1.61931013113729 | -0.119310131137293 |
| Array\_1 | Prep\_A | 1 | CreJ | 29 | 1.5 | 28.9393145513526 | 1.68803319847453 | -0.188033198474533 |
| Array\_2 | Prep\_A | 1 | CreJ | 29 | 1.5 | 28.9583099986285 | 1.64903132298239 | -0.149031322982387 |
| Array\_2 | Prep\_A | 2 | CreJ | 29 | 1.5 | 28.9755141704651 | 1.70822133795689 | -0.208221337956887 |
| Array\_1 | Prep\_A | 2 | CreE | 29 | 1.5 | 29.067446362516 | 1.83663100369473 | -0.336631003694729 |
| Array\_1 | Prep\_A | 1 | CreE | 29 | 1.5 | 28.9411654649865 | 1.81509914328961 | -0.315099143289609 |
| Array\_1 | Prep\_A | 2 | CreJ | 7 | 3.5 | 7.02629724511668 | 3.85114168028297 | -0.351141680282973 |
| Array\_1 | Prep\_A | 1 | CreJ | 7 | 3.5 | 6.97958792776083 | 3.81080783628513 | -0.310807836285133 |
| Array\_1 | Prep\_A | 1 | CreE | 7 | 3.5 | 6.97848422461156 | 3.69208011716044 | -0.192080117160441 |
| Array\_1 | Prep\_A | 2 | CreE | 7 | 3.5 | 7.02519354196741 | 3.74634832289775 | -0.246348322897751 |
| Array\_2 | Prep\_A | 2 | CreJ | 7 | 3.5 | 7.00357296274211 | 3.86795472913851 | -0.367954729138508 |
| Array\_2 | Prep\_A | 1 | CreJ | 7 | 3.5 | 6.95978021910096 | 3.85470523453738 | -0.354705234537378 |
| Array\_2 | Prep\_A | 2 | CreE | 7 | 3.5 | 7.00246501299474 | 3.78478567392954 | -0.28478567392954 |
| Array\_2 | Prep\_A | 1 | CreE | 7 | 3.5 | 6.95867226935359 | 3.75051842306119 | -0.250518423061191 |
| Array\_2 | Prep\_A | 1 | CreE | 30 | 1.5 | 30.1522301651882 | 1.8278622771286 | -0.327862277128598 |
| Array\_2 | Prep\_A | 2 | CreE | 30 | 1.5 | 30.4112492374347 | 1.84055871745716 | -0.340558717457158 |
| Array\_1 | Prep\_A | 2 | CreE | 30 | 1.5 | 29.9331892873093 | 1.84175142454719 | -0.341751424547188 |
| Array\_1 | Prep\_A | 1 | CreE | 30 | 1.5 | 30.1454135178473 | 1.82086505827168 | -0.320865058271678 |
| Array\_1 | Prep\_A | 1 | CreJ | 30 | 1.5 | 30.1435589146328 | 1.71334646297414 | -0.213346462974143 |
| Array\_1 | Prep\_A | 2 | CreJ | 30 | 1.5 | 29.9313346840949 | 1.64353788745418 | -0.143537887454183 |
| Array\_2 | Prep\_A | 2 | CreJ | 30 | 1.5 | 30.4093945654822 | 1.63035772079344 | -0.130357720793437 |
| Array\_2 | Prep\_A | 1 | CreJ | 30 | 1.5 | 30.1503754932358 | 1.7390506272535 | -0.239050627253498 |
| Array\_2 | Prep\_A | 1 | CreJ | 8 | 3.5 | 8.00892948960882 | 3.87104618231131 | -0.37104618231131 |
| Array\_2 | Prep\_A | 2 | CreJ | 8 | 3.5 | 8.02460751573183 | 3.84608836541923 | -0.34608836541923 |
| Array\_1 | Prep\_A | 1 | CreE | 8 | 3.5 | 7.94366882503391 | 3.79763720755182 | -0.297637207551816 |
| Array\_1 | Prep\_A | 2 | CreE | 8 | 3.5 | 8.00277595218769 | 3.76909124747024 | -0.269091247470235 |
| Array\_2 | Prep\_A | 1 | CreE | 8 | 3.5 | 8.00782898299165 | 3.76734411513546 | -0.267344115135455 |
| Array\_2 | Prep\_A | 2 | CreE | 8 | 3.5 | 8.02350700911465 | 3.75310060630747 | -0.253100606307465 |
| Array\_1 | Prep\_A | 1 | CreJ | 8 | 3.5 | 7.94476777152592 | 3.83108374343782 | -0.331083743437815 |
| Array\_1 | Prep\_A | 2 | CreJ | 8 | 3.5 | 8.0038748986797 | 3.86084613130533 | -0.360846131305326 |
| Array\_1 | Prep\_A | 1 | CreJ | 31 | 1.5 | 31.1759544563513 | 1.58973387595935 | -0.0897338759593542 |
| Array\_1 | Prep\_A | 2 | CreJ | 31 | 1.5 | 30.7879424573273 | 1.61088279422692 | -0.110882794226924 |
| Array\_2 | Prep\_A | 2 | CreJ | 31 | 1.5 | 31.1743064342095 | 1.5754222207414 | -0.075422220741397 |
| Array\_2 | Prep\_A | 1 | CreJ | 31 | 1.5 | 31.2820939040684 | 1.64558584369724 | -0.145585843697237 |
| Array\_2 | Prep\_A | 2 | CreE | 31 | 1.5 | 31.1761586058509 | 1.85329032881496 | -0.353290328814962 |
| Array\_2 | Prep\_A | 1 | CreE | 31 | 1.5 | 31.2839460757098 | 1.86988931668272 | -0.369889316682722 |
| Array\_1 | Prep\_A | 2 | CreE | 31 | 1.5 | 30.7897943626197 | 1.89054821826089 | -0.390548218260892 |
| Array\_1 | Prep\_A | 1 | CreE | 31 | 1.5 | 31.1778063616437 | 1.8386638041463 | -0.338663804146302 |
| Array\_1 | Prep\_A | 2 | CreJ | 9 | 3.5 | 9.00108763241217 | 3.83846468864498 | -0.338464688644983 |
| Array\_1 | Prep\_A | 1 | CreJ | 9 | 3.5 | 9.04647446916186 | 3.81382066502162 | -0.313820665021623 |
| Array\_2 | Prep\_A | 2 | CreE | 9 | 3.5 | 9.00900851427506 | 3.77859625800658 | -0.278596258006584 |
| Array\_2 | Prep\_A | 1 | CreE | 9 | 3.5 | 9.0235736394875 | 3.7552428256687 | -0.255242825668704 |
| Array\_2 | Prep\_A | 1 | CreJ | 9 | 3.5 | 9.02467615527238 | 3.82449041653774 | -0.324490416537737 |
| Array\_2 | Prep\_A | 2 | CreJ | 9 | 3.5 | 9.01011103005994 | 3.85569586334661 | -0.355695863346607 |
| Array\_1 | Prep\_A | 1 | CreE | 9 | 3.5 | 9.0453737943974 | 3.80517316261339 | -0.305173162613394 |
| Array\_1 | Prep\_A | 2 | CreE | 9 | 3.5 | 8.99998695764771 | 3.77208275098047 | -0.272082750980474 |
| Array\_1 | Prep\_A | 2 | CreJ | 32 | 1.5 | 32.1450164197438 | 1.68605602018935 | -0.18605602018935 |
| Array\_1 | Prep\_A | 1 | CreJ | 32 | 1.5 | 31.7355536901344 | 1.5984251942173 | -0.0984251942173 |
| Array\_2 | Prep\_A | 1 | CreE | 32 | 1.5 | 32.098446325136 | 1.8874673223968 | -0.387467322396803 |
| Array\_2 | Prep\_A | 2 | CreE | 32 | 1.5 | 32.2620034367095 | 1.8643277659522 | -0.364327765952203 |
| Array\_2 | Prep\_A | 1 | CreJ | 32 | 1.5 | 32.0965914732906 | 1.57011371654661 | -0.0701137165466057 |
| Array\_2 | Prep\_A | 2 | CreJ | 32 | 1.5 | 32.2601485848641 | 1.61515185236918 | -0.115151852369176 |
| Array\_1 | Prep\_A | 1 | CreE | 32 | 1.5 | 31.737409532512 | 1.90176232924153 | -0.401762329241533 |
| Array\_1 | Prep\_A | 2 | CreE | 32 | 1.5 | 32.1468722621214 | 1.85033616856598 | -0.350336168565983 |
| Array\_2 | Prep\_A | 2 | CreJ | 10 | 3.5 | 10.0156576926851 | 3.87478298600913 | -0.374782986009131 |
| Array\_2 | Prep\_A | 1 | CreJ | 10 | 3.5 | 10.1046458615282 | 3.86571917649467 | -0.365719176494671 |
| Array\_1 | Prep\_A | 2 | CreJ | 10 | 3.5 | 10.0152732612636 | 3.78725834735052 | -0.28725834735052 |
| Array\_1 | Prep\_A | 1 | CreJ | 10 | 3.5 | 10.0251760711788 | 3.83614818749663 | -0.33614818749663 |
| Array\_2 | Prep\_A | 1 | CreE | 10 | 3.5 | 10.1035380008291 | 3.78002583719586 | -0.280025837195856 |
| Array\_2 | Prep\_A | 2 | CreE | 10 | 3.5 | 10.014549831986 | 3.78131383061156 | -0.281313830611556 |
| Array\_1 | Prep\_A | 1 | CreE | 10 | 3.5 | 10.0240755715252 | 3.77650711498362 | -0.276507114983616 |
| Array\_1 | Prep\_A | 2 | CreE | 10 | 3.5 | 10.01417276161 | 3.79057667495076 | -0.290576674950756 |
| Array\_2 | Prep\_A | 1 | CreE | 33 | 1.5 | 33.1238900232092 | 1.85053799464763 | -0.35053799464763 |
| Array\_2 | Prep\_A | 2 | CreE | 33 | 1.5 | 32.9991120066278 | 1.86200831417099 | -0.36200831417099 |
| Array\_2 | Prep\_A | 2 | CreJ | 33 | 1.5 | 32.9972517257631 | 1.60032493406599 | -0.100324934065994 |
| Array\_2 | Prep\_A | 1 | CreJ | 33 | 1.5 | 33.1220297423445 | 1.62148113251147 | -0.121481132511474 |
| Array\_1 | Prep\_A | 2 | CreJ | 33 | 1.5 | 33.1493021411266 | 1.67525956769346 | -0.17525956769346 |
| Array\_1 | Prep\_A | 1 | CreJ | 33 | 1.5 | 32.9386508206712 | 1.59649981803383 | -0.0964998180338301 |
| Array\_1 | Prep\_A | 2 | CreE | 33 | 1.5 | 33.1511629355926 | 1.88205625919029 | -0.38205625919029 |
| Array\_1 | Prep\_A | 1 | CreE | 33 | 1.5 | 32.9405116151372 | 1.88518198127293 | -0.38518198127293 |
| Array\_2 | Prep\_A | 2 | CreJ | 11 | 3.5 | 11.106182391164 | 3.88496116868009 | -0.384961168680094 |
| Array\_2 | Prep\_A | 1 | CreJ | 11 | 3.5 | 11.0759881708666 | 3.831297082651 | -0.331297082651004 |
| Array\_1 | Prep\_A | 1 | CreE | 11 | 3.5 | 10.9281227674732 | 3.8044325905867 | -0.304432590586695 |
| Array\_1 | Prep\_A | 2 | CreE | 11 | 3.5 | 11.0707531584685 | 3.79115123137156 | -0.291151231371555 |
| Array\_2 | Prep\_A | 2 | CreE | 11 | 3.5 | 11.1050720904744 | 3.78892881732215 | -0.288928817322155 |
| Array\_2 | Prep\_A | 1 | CreE | 11 | 3.5 | 11.074877870177 | 3.76152304538303 | -0.261523045383025 |
| Array\_1 | Prep\_A | 1 | CreJ | 11 | 3.5 | 10.9292369543382 | 3.88108762274145 | -0.381087622741449 |
| Array\_1 | Prep\_A | 2 | CreJ | 11 | 3.5 | 11.0718673453335 | 3.89284678117209 | -0.392846781172088 |
| Array\_2 | Prep\_A | 1 | CreE | 34 | 1.5 | 34.3904445733969 | 1.86219482208977 | -0.362194822089769 |
| Array\_2 | Prep\_A | 2 | CreE | 34 | 1.5 | 34.1722446497158 | 1.93092894804302 | -0.430928948043019 |
| Array\_1 | Prep\_A | 1 | CreJ | 34 | 1.5 | 33.9601288895866 | 1.68351416477406 | -0.183514164774063 |
| Array\_1 | Prep\_A | 2 | CreJ | 34 | 1.5 | 34.1056799473184 | 1.67554962796183 | -0.175549627961833 |
| Array\_1 | Prep\_A | 2 | CreE | 34 | 1.5 | 34.107545327631 | 1.86504219715266 | -0.365042197152659 |
| Array\_1 | Prep\_A | 1 | CreE | 34 | 1.5 | 33.9619942698992 | 1.8720395234597 | -0.372039523459699 |
| Array\_2 | Prep\_A | 2 | CreJ | 34 | 1.5 | 34.1703810866935 | 1.60302655127357 | -0.103026551273574 |
| Array\_2 | Prep\_A | 1 | CreJ | 34 | 1.5 | 34.3885810103746 | 1.58936628681223 | -0.0893662868122343 |
| Array\_1 | Prep\_A | 1 | CreE | 12 | 3.5 | 12.052002836834 | 3.83364179919434 | -0.333641799194336 |
| Array\_1 | Prep\_A | 2 | CreE | 12 | 3.5 | 12.0642812842392 | 3.8367172686007 | -0.336717268600696 |
| Array\_2 | Prep\_A | 2 | CreE | 12 | 3.5 | 12.0768800116113 | 3.78732266847841 | -0.287322668478406 |
| Array\_2 | Prep\_A | 1 | CreE | 12 | 3.5 | 11.9063689641918 | 3.7784716415906 | -0.278471641590596 |
| Array\_2 | Prep\_A | 1 | CreJ | 12 | 3.5 | 11.9074856118283 | 3.86232832381721 | -0.362328323817215 |
| Array\_2 | Prep\_A | 2 | CreJ | 12 | 3.5 | 12.0779966592478 | 3.93697598419598 | -0.436975984195975 |
| Array\_1 | Prep\_A | 1 | CreJ | 12 | 3.5 | 12.0531170835572 | 3.88258340176539 | -0.382583401765394 |
| Array\_1 | Prep\_A | 2 | CreJ | 12 | 3.5 | 12.0653955309624 | 3.88208000314529 | -0.382080003145294 |
| Array\_2 | Prep\_A | 1 | CreE | 35 | 1.5 | 35.2850830890654 | 1.91890976511963 | -0.418909765119626 |
| Array\_2 | Prep\_A | 2 | CreE | 35 | 1.5 | 34.9676868071448 | 1.87490345688569 | -0.374903456885686 |
| Array\_1 | Prep\_A | 1 | CreE | 35 | 1.5 | 35.1783398858572 | 1.87521450163532 | -0.375214501635316 |
| Array\_1 | Prep\_A | 2 | CreE | 35 | 1.5 | 34.9599231980899 | 1.92678932602153 | -0.426789326021526 |
| Array\_2 | Prep\_A | 1 | CreJ | 35 | 1.5 | 35.2832178359869 | 1.59036838182573 | -0.0903683818257326 |
| Array\_2 | Prep\_A | 2 | CreJ | 35 | 1.5 | 34.9658215540663 | 1.59942865787698 | -0.0994286578769827 |
| Array\_1 | Prep\_A | 1 | CreJ | 35 | 1.5 | 35.1764737918777 | 1.65445241351236 | -0.154452413512362 |
| Array\_1 | Prep\_A | 2 | CreJ | 35 | 1.5 | 34.9580571041104 | 1.60163279127954 | -0.101632791279542 |
| Array\_2 | Prep\_A | 2 | CreJ | 13 | 3.5 | 13.0049783239775 | 3.85662542298441 | -0.356625422984413 |
| Array\_2 | Prep\_A | 1 | CreJ | 13 | 3.5 | 12.9491350406392 | 3.96445121660449 | -0.464451216604493 |
| Array\_1 | Prep\_A | 2 | CreJ | 13 | 3.5 | 12.9985455702557 | 3.96925736600305 | -0.469257366003048 |
| Array\_1 | Prep\_A | 1 | CreJ | 13 | 3.5 | 12.8720506313047 | 3.84337436424974 | -0.343374364249738 |
| Array\_1 | Prep\_A | 1 | CreE | 13 | 3.5 | 12.8709357362009 | 3.8405779094266 | -0.340577909426602 |
| Array\_1 | Prep\_A | 2 | CreE | 13 | 3.5 | 12.9974306751519 | 3.8133236582137 | -0.313323658213702 |
| Array\_2 | Prep\_A | 2 | CreE | 13 | 3.5 | 13.0038630176534 | 3.79831218069438 | -0.298312180694382 |
| Array\_2 | Prep\_A | 1 | CreE | 13 | 3.5 | 12.9480197343151 | 3.81759694986865 | -0.317596949868652 |
| Array\_2 | Prep\_A | 2 | CreJ | 14 | 3.5 | 14.1377384698352 | 3.93440529728725 | -0.43440529728725 |
| Array\_2 | Prep\_A | 1 | CreJ | 14 | 3.5 | 13.9812517534921 | 3.88681072303021 | -0.38681072303021 |
| Array\_2 | Prep\_A | 2 | CreE | 14 | 3.5 | 14.1366182192652 | 3.81735919112751 | -0.317359191127514 |
| Array\_2 | Prep\_A | 1 | CreE | 14 | 3.5 | 13.9801315029221 | 3.78154012030366 | -0.281540120303664 |
| Array\_1 | Prep\_A | 1 | CreJ | 14 | 3.5 | 14.0585398723784 | 3.89075769813368 | -0.39075769813368 |
| Array\_1 | Prep\_A | 2 | CreJ | 14 | 3.5 | 13.9716922331456 | 3.91457971248531 | -0.414579712485311 |
| Array\_1 | Prep\_A | 2 | CreE | 14 | 3.5 | 13.9705728895108 | 3.85018155532589 | -0.350181555325894 |
| Array\_1 | Prep\_A | 1 | CreE | 14 | 3.5 | 14.0574205287436 | 3.83736465385391 | -0.337364653853914 |
| Array\_2 | Prep\_A | 1 | CreE | 1 | 2 | 1.01694241638999 | 2.01758122711123 | -0.0175812271112337 |
| Array\_2 | Prep\_A | 2 | CreE | 1 | 2 | 1.02307989677109 | 2.03950079781295 | -0.0395007978129538 |
| Array\_2 | Prep\_A | 2 | CreJ | 1 | 2 | 1.02121296290163 | 2.08068058108609 | -0.0806805810860918 |
| Array\_2 | Prep\_A | 1 | CreJ | 1 | 2 | 1.01507548252053 | 2.09312691506017 | -0.0931269150601715 |
| Array\_1 | Prep\_A | 1 | CreJ | 1 | 2 | 1.00884546156274 | 2.1219232757354902 | -0.121923275735495 |
| Array\_1 | Prep\_A | 2 | CreJ | 1 | 2 | 0.995220011094833 | 2.05639300881017 | -0.056393008810165 |
| Array\_1 | Prep\_A | 2 | CreE | 1 | 2 | 0.997086672680762 | 2.00581780569357 | -0.0058178056935736 |
| Array\_1 | Prep\_A | 1 | CreE | 1 | 2 | 1.01071212314866 | 2.01041544065177 | -0.0104154406517738 |
| Array\_2 | Prep\_A | 2 | CreJ | 15 | 3.5 | 15.0756444625619 | 3.85095713569826 | -0.350957135698257 |
| Array\_2 | Prep\_A | 1 | CreJ | 15 | 3.5 | 15.0742050794592 | 3.90058767296234 | -0.400587672962337 |
| Array\_2 | Prep\_A | 1 | CreE | 15 | 3.5 | 15.0730917771986 | 3.85578650000234 | -0.35578650000234 |
| Array\_2 | Prep\_A | 2 | CreE | 15 | 3.5 | 15.0745311603013 | 3.82902835917444 | -0.32902835917444 |
| Array\_1 | Prep\_A | 1 | CreE | 15 | 3.5 | 14.9868584673725 | 3.85296139840385 | -0.35296139840385 |
| Array\_1 | Prep\_A | 2 | CreE | 15 | 3.5 | 15.0925938731971 | 3.8663287236974 | -0.3663287236974 |
| Array\_1 | Prep\_A | 2 | CreJ | 15 | 3.5 | 15.0937046192655 | 3.81180351965904 | -0.311803519659037 |
| Array\_1 | Prep\_A | 1 | CreJ | 15 | 3.5 | 14.9879692134409 | 3.89407121041297 | -0.394071210412967 |
| Array\_2 | Prep\_A | 2 | CreE | 2 | 2 | 2.01986556540154 | 2.02887488947222 | -0.0288748894722173 |
| Array\_2 | Prep\_A | 1 | CreE | 2 | 2 | 2.00294907044386 | 1.99755164218491 | 0.00244835781509289 |
| Array\_2 | Prep\_A | 2 | CreJ | 2 | 2 | 2.01799330628166 | 2.06249070797476 | -0.0624907079747565 |
| Array\_2 | Prep\_A | 1 | CreJ | 2 | 2 | 2.00107681132398 | 2.0090411221591302 | -0.00904112215912667 |
| Array\_1 | Prep\_A | 2 | CreE | 2 | 2 | 1.97881488814176 | 2.03008754377304 | -0.0300875437730372 |
| Array\_1 | Prep\_A | 1 | CreE | 2 | 2 | 2.0175138519393 | 2.04581523641914 | -0.0458152364191373 |
| Array\_1 | Prep\_A | 2 | CreJ | 2 | 2 | 1.97694872530811 | 2.14795193386676 | -0.147951933866758 |
| Array\_1 | Prep\_A | 1 | CreJ | 2 | 2 | 2.01564768910565 | 2.08452839308773 | -0.0845283930877274 |
| Array\_2 | Prep\_A | 2 | CreE | 16 | 3.5 | 16.1069405422984 | 3.89242698310085 | -0.392426983100849 |
| Array\_2 | Prep\_A | 1 | CreE | 16 | 3.5 | 16.0235444839913 | 3.83319349755169 | -0.333193497551688 |
| Array\_1 | Prep\_A | 2 | CreE | 16 | 3.5 | 16.1363356460223 | 3.881446136316 | -0.381446136315998 |
| Array\_1 | Prep\_A | 1 | CreE | 16 | 3.5 | 16.0295903485606 | 3.8923908914457 | -0.392390891445698 |
| Array\_1 | Prep\_A | 1 | CreJ | 16 | 3.5 | 16.0307050881517 | 3.88261327756951 | -0.382613277569507 |
| Array\_1 | Prep\_A | 2 | CreJ | 16 | 3.5 | 16.1374503856134 | 3.89319375992696 | -0.393193759926957 |
| Array\_2 | Prep\_A | 2 | CreJ | 16 | 3.5 | 16.1080600413011 | 3.93381561924043 | -0.433815619240431 |
| Array\_2 | Prep\_A | 1 | CreJ | 16 | 3.5 | 16.024663982994 | 3.92242476578053 | -0.42242476578053 |
| Array\_2 | Prep\_A | 2 | CreJ | 3 | 2 | 3.02754658880075 | 2.11811211972613 | -0.118112119726132 |
| Array\_2 | Prep\_A | 1 | CreJ | 3 | 2 | 3.00671358439426 | 2.05261843941252 | -0.0526184394125218 |
| Array\_1 | Prep\_A | 2 | CreJ | 3 | 2 | 2.94231099144499 | 2.11608102260374 | -0.11608102260374 |
| Array\_1 | Prep\_A | 1 | CreJ | 3 | 2 | 3.02679049760692 | 2.0360492064127 | -0.0360492064127 |
| Array\_1 | Prep\_A | 2 | CreE | 3 | 2 | 2.94417970788066 | 2.043727451335 | -0.0437274513350023 |
| Array\_1 | Prep\_A | 1 | CreE | 3 | 2 | 3.02865921404259 | 2.04711161181934 | -0.047111611819342 |
| Array\_2 | Prep\_A | 1 | CreE | 3 | 2 | 3.00858173262858 | 2.04038726175033 | -0.040387261750332 |
| Array\_2 | Prep\_A | 2 | CreE | 3 | 2 | 3.02941473703507 | 2.04185441574814 | -0.0418544157481424 |
| Array\_2 | Prep\_A | 2 | CreJ | 17 | 3.5 | 17.0328706312026 | 3.91634391553878 | -0.416343915538782 |
| Array\_2 | Prep\_A | 1 | CreJ | 17 | 3.5 | 17.0723223371818 | 3.90495662556131 | -0.404956625561313 |
| Array\_1 | Prep\_A | 1 | CreJ | 17 | 3.5 | 17.075235705976 | 3.98188897407088 | -0.481888974070882 |
| Array\_1 | Prep\_A | 2 | CreJ | 17 | 3.5 | 16.992419399544 | 3.91577610980232 | -0.415776109802323 |
| Array\_1 | Prep\_A | 2 | CreE | 17 | 3.5 | 16.9912939303266 | 3.87861491530216 | -0.37861491530216 |
| Array\_1 | Prep\_A | 1 | CreE | 17 | 3.5 | 17.0741102367586 | 3.88858063588145 | -0.38858063588145 |
| Array\_2 | Prep\_A | 2 | CreE | 17 | 3.5 | 17.0317497085359 | 3.88279041250746 | -0.38279041250746 |
| Array\_2 | Prep\_A | 1 | CreE | 17 | 3.5 | 17.0712014145151 | 3.8444604097902 | -0.3444604097902 |
| Array\_2 | Prep\_A | 2 | CreE | 4 | 2 | 3.97759271333441 | 2.09929283642628 | -0.0992928364262822 |
| Array\_2 | Prep\_A | 1 | CreE | 4 | 2 | 4.00076163327134 | 2.06251749237418 | -0.062517492374182 |
| Array\_2 | Prep\_A | 1 | CreJ | 4 | 2 | 3.99889518347576 | 2.05396282853757 | -0.0539628285375695 |
| Array\_2 | Prep\_A | 2 | CreJ | 4 | 2 | 3.97572626353883 | 2.11373932879255 | -0.113739328792549 |
| Array\_1 | Prep\_A | 2 | CreE | 4 | 2 | 4.03052284897275 | 2.03505619165195 | -0.0350561916519521 |
| Array\_1 | Prep\_A | 1 | CreE | 4 | 2 | 3.9973533996257 | 2.07970968569172 | -0.0797096856917223 |
| Array\_1 | Prep\_A | 2 | CreJ | 4 | 2 | 4.02865658038999 | 2.11740992477215 | -0.117409924772151 |
| Array\_1 | Prep\_A | 1 | CreJ | 4 | 2 | 3.99548713104294 | 2.05694736716613 | -0.0569473671661309 |
| Array\_1 | Prep\_A | 1 | CreE | 18 | 3.5 | 18.1421464363415 | 3.90695821814566 | -0.406958218145655 |
| Array\_1 | Prep\_A | 2 | CreE | 18 | 3.5 | 18.0941831971508 | 3.88616059939687 | -0.386160599396865 |
| Array\_2 | Prep\_A | 1 | CreJ | 18 | 3.5 | 17.9130455943525 | 3.87179166271456 | -0.371791662714557 |
| Array\_2 | Prep\_A | 2 | CreJ | 18 | 3.5 | 18.0369077271024 | 3.89642577793602 | -0.396425777936017 |
| Array\_1 | Prep\_A | 1 | CreJ | 18 | 3.5 | 18.1432683209454 | 3.93397596345972 | -0.43397596345972 |
| Array\_1 | Prep\_A | 2 | CreJ | 18 | 3.5 | 18.0953050817547 | 3.89108328503172 | -0.391083285031721 |
| Array\_2 | Prep\_A | 1 | CreE | 18 | 3.5 | 17.9119268022989 | 3.89659689783983 | -0.396596897839825 |
| Array\_2 | Prep\_A | 2 | CreE | 18 | 3.5 | 18.0357889350488 | 3.88120389391712 | -0.381203893917115 |
| Array\_2 | Prep\_A | 2 | CreE | 5 | 2 | 4.99803645862351 | 2.09818588444611 | -0.0981858844461105 |
| Array\_2 | Prep\_A | 1 | CreE | 5 | 2 | 5.01108182214834 | 2.04877997611326 | -0.0487799761132606 |
| Array\_1 | Prep\_A | 1 | CreJ | 5 | 2 | 4.98982879622861 | 2.08694685782609 | -0.0869468578260895 |
| Array\_1 | Prep\_A | 2 | CreJ | 5 | 2 | 5.03820890338223 | 2.08918107700736 | -0.0891810770073596 |
| Array\_2 | Prep\_A | 1 | CreJ | 5 | 2 | 5.00922201164521 | 2.15303957559848 | -0.153039575598483 |
| Array\_2 | Prep\_A | 2 | CreJ | 5 | 2 | 4.99617664812038 | 2.13064274970057 | -0.130642749700573 |
| Array\_1 | Prep\_A | 1 | CreE | 5 | 2 | 4.99169228497517 | 2.07747942206617 | -0.0774794220661708 |
| Array\_1 | Prep\_A | 2 | CreE | 5 | 2 | 5.04007239212879 | 2.06780529380601 | -0.0678052938060105 |
| Array\_1 | Prep\_A | 2 | CreE | 19 | 3.5 | 18.9810638086671 | 3.93788512375637 | -0.437885123756375 |
| Array\_1 | Prep\_A | 1 | CreE | 19 | 3.5 | 19.2405919928373 | 3.89661363585061 | -0.396613635850615 |
| Array\_2 | Prep\_A | 1 | CreE | 19 | 3.5 | 19.1758123957662 | 3.92372508941978 | -0.423725089419785 |
| Array\_2 | Prep\_A | 2 | CreE | 19 | 3.5 | 18.9647156884327 | 3.92860224195051 | -0.428602241950514 |
| Array\_1 | Prep\_A | 2 | CreJ | 19 | 3.5 | 18.9821910287858 | 3.93148198382076 | -0.431481983820762 |
| Array\_1 | Prep\_A | 1 | CreJ | 19 | 3.5 | 19.241719212956 | 3.99582208477312 | -0.495822084773121 |
| Array\_2 | Prep\_A | 2 | CreJ | 19 | 3.5 | 18.9658406318694 | 3.99156427201565 | -0.491564272015651 |
| Array\_2 | Prep\_A | 1 | CreJ | 19 | 3.5 | 19.1769373392029 | 3.89430299813305 | -0.394302998133051 |
| Array\_1 | Prep\_A | 2 | CreJ | 6 | 2 | 6.04598370448462 | 2.13442439913405 | -0.134424399134052 |
| Array\_1 | Prep\_A | 1 | CreJ | 6 | 2 | 6.01576495640159 | 2.10871392671899 | -0.108713926718992 |
| Array\_2 | Prep\_A | 2 | CreE | 6 | 2 | 6.03471011645602 | 2.09549491988939 | -0.0954949198893909 |
| Array\_2 | Prep\_A | 1 | CreE | 6 | 2 | 6.01010340756224 | 2.05863012679759 | -0.0586301267975911 |
| Array\_2 | Prep\_A | 1 | CreJ | 6 | 2 | 6.00824242569425 | 2.05570599242827 | -0.0557059924282712 |
| Array\_2 | Prep\_A | 2 | CreJ | 6 | 2 | 6.03284913458803 | 2.11935715930911 | -0.119357159309112 |
| Array\_1 | Prep\_A | 2 | CreE | 6 | 2 | 6.04784259645724 | 2.08296794340557 | -0.082967943405571 |
| Array\_1 | Prep\_A | 1 | CreE | 6 | 2 | 6.01762384837421 | 2.06786252594379 | -0.0678625259437911 |
| Array\_1 | Prep\_A | 2 | CreJ | 20 | 3.5 | 20.1812940395429 | 3.85219290676004 | -0.352192906760037 |
| Array\_1 | Prep\_A | 1 | CreJ | 20 | 3.5 | 19.847563422819 | 3.92874703480896 | -0.428747034808957 |
| Array\_2 | Prep\_A | 2 | CreJ | 20 | 3.5 | 20.0707007746171 | 3.96653216522882 | -0.466532165228818 |
| Array\_2 | Prep\_A | 1 | CreJ | 20 | 3.5 | 19.9514058907068 | 3.95625316986906 | -0.456253169869058 |
| Array\_2 | Prep\_A | 2 | CreE | 20 | 3.5 | 20.0695711578346 | 3.94326948289219 | -0.443269482892188 |
| Array\_2 | Prep\_A | 1 | CreE | 20 | 3.5 | 19.9502762739243 | 3.9294732922811 | -0.429473292281098 |
| Array\_1 | Prep\_A | 2 | CreE | 20 | 3.5 | 20.180875345154 | 3.86533697752655 | -0.365336977526548 |
| Array\_1 | Prep\_A | 1 | CreE | 20 | 3.5 | 19.8471447284301 | 3.8858629575363 | -0.385862957536298 |
| Array\_1 | Prep\_A | 2 | CreE | 7 | 2 | 7.05476461489203 | 2.07288075520507 | -0.072880755205067 |
| Array\_1 | Prep\_A | 1 | CreE | 7 | 2 | 6.99952570219378 | 2.11108051880282 | -0.111080518802817 |
| Array\_2 | Prep\_A | 1 | CreJ | 7 | 2 | 6.96547066506629 | 2.13680882746226 | -0.136808827462257 |
| Array\_2 | Prep\_A | 2 | CreJ | 7 | 2 | 7.03613524718836 | 2.12844065699154 | -0.128440656991537 |
| Array\_2 | Prep\_A | 2 | CreE | 7 | 2 | 7.03799197958379 | 2.08729873219177 | -0.0872987321917669 |
| Array\_2 | Prep\_A | 1 | CreE | 7 | 2 | 6.96732739746172 | 2.10141241017928 | -0.101412410179277 |
| Array\_1 | Prep\_A | 1 | CreJ | 7 | 2 | 6.99766889263689 | 2.15442637408785 | -0.154426374087848 |
| Array\_1 | Prep\_A | 2 | CreJ | 7 | 2 | 7.05290780533514 | 2.10862073712289 | -0.108620737122888 |
| Array\_2 | Prep\_A | 1 | CreE | 21 | 3.5 | 21.0185221953032 | 3.91773565393429 | -0.417735653934295 |
| Array\_2 | Prep\_A | 2 | CreE | 21 | 3.5 | 21.0104617736501 | 3.94995411893293 | -0.449954118932935 |
| Array\_1 | Prep\_A | 2 | CreE | 21 | 3.5 | 21.0523829940127 | 3.92323094677702 | -0.423230946777015 |
| Array\_1 | Prep\_A | 1 | CreE | 21 | 3.5 | 21.0400339161028 | 3.93514280963153 | -0.435142809631525 |
| Array\_1 | Prep\_A | 1 | CreJ | 21 | 3.5 | 21.0411600082246 | 3.9714998709561 | -0.471499870956099 |
| Array\_1 | Prep\_A | 2 | CreJ | 21 | 3.5 | 21.0535090861345 | 3.89076267616852 | -0.390762676168519 |
| Array\_2 | Prep\_A | 2 | CreJ | 21 | 3.5 | 21.0086147942281 | 3.80490425052654 | -0.304904250526535 |
| Array\_2 | Prep\_A | 1 | CreJ | 21 | 3.5 | 21.0166752158812 | 3.94389253770277 | -0.443892537702765 |
| Array\_1 | Prep\_A | 1 | CreE | 8 | 2 | 7.95621005876221 | 2.10390558483443 | -0.103905584834429 |
| Array\_1 | Prep\_A | 2 | CreE | 8 | 2 | 8.02847055658119 | 2.0750176767861 | -0.0750176767860995 |
| Array\_2 | Prep\_A | 2 | CreJ | 8 | 2 | 8.03893290626914 | 2.09575375557772 | -0.0957537555777193 |
| Array\_2 | Prep\_A | 1 | CreJ | 8 | 2 | 8.08733992055299 | 2.06910567292776 | -0.0691056729277593 |
| Array\_1 | Prep\_A | 2 | CreJ | 8 | 2 | 8.02661559375596 | 2.15986320437948 | -0.159863204379484 |
| Array\_1 | Prep\_A | 1 | CreJ | 8 | 2 | 7.95435509593698 | 2.06135026054581 | -0.0613502605458138 |
| Array\_2 | Prep\_A | 2 | CreE | 8 | 2 | 8.04078962939418 | 2.08033819072113 | -0.0803381907211294 |
| Array\_2 | Prep\_A | 1 | CreE | 8 | 2 | 8.08919664367803 | 2.10390092179366 | -0.10390092179366 |
| Array\_1 | Prep\_A | 1 | CreE | 22 | 3.5 | 21.8964439497281 | 4.02187192524533 | -0.52187192524533 |
| Array\_1 | Prep\_A | 2 | CreE | 22 | 3.5 | 22.0925228835233 | 3.94409985764905 | -0.44409985764905 |
| Array\_1 | Prep\_A | 2 | CreJ | 22 | 3.5 | 22.0906757883022 | 3.87201955739893 | -0.372019557398927 |
| Array\_1 | Prep\_A | 1 | CreJ | 22 | 3.5 | 21.894596854507 | 3.88973698356248 | -0.389736983562477 |
| Array\_2 | Prep\_A | 2 | CreJ | 22 | 3.5 | 22.3329157865886 | 3.94931502939057 | -0.449315029390571 |
| Array\_2 | Prep\_A | 1 | CreJ | 22 | 3.5 | 22.1090495799647 | 3.88768771432693 | -0.387687714326931 |
| Array\_2 | Prep\_A | 1 | CreE | 22 | 3.5 | 22.1100009738146 | 3.95149124779194 | -0.45149124779194 |
| Array\_2 | Prep\_A | 2 | CreE | 22 | 3.5 | 22.3338671804385 | 3.98256948853875 | -0.48256948853875 |
| Array\_1 | Prep\_A | 1 | CreJ | 9 | 2 | 9.01863156155453 | 2.0926981331903 | -0.0926981331903032 |
| Array\_1 | Prep\_A | 2 | CreJ | 9 | 2 | 9.07991219201499 | 2.17670541425695 | -0.176705414256953 |
| Array\_2 | Prep\_A | 1 | CreJ | 9 | 2 | 9.04477949846897 | 2.10431839111018 | -0.104318391110179 |
| Array\_2 | Prep\_A | 2 | CreJ | 9 | 2 | 9.01807301251543 | 2.12479346334955 | -0.124793463349549 |
| Array\_1 | Prep\_A | 1 | CreE | 9 | 2 | 9.02048667390639 | 2.12380954015137 | -0.123809540151368 |
| Array\_1 | Prep\_A | 2 | CreE | 9 | 2 | 9.08176730436685 | 2.13942574749242 | -0.139425747492418 |
| Array\_2 | Prep\_A | 2 | CreE | 9 | 2 | 9.01992929437951 | 2.15466521396635 | -0.154665213966348 |
| Array\_2 | Prep\_A | 1 | CreE | 9 | 2 | 9.04663578033305 | 2.10578502882494 | -0.105785028824938 |
| Array\_2 | Prep\_A | 2 | CreE | 23 | 3.5 | 22.894504827728 | 3.95710781506307 | -0.457107815063069 |
| Array\_2 | Prep\_A | 1 | CreE | 23 | 3.5 | 23.0340164040054 | 3.98265154099773 | -0.482651540997729 |
| Array\_1 | Prep\_A | 1 | CreE | 23 | 3.5 | 23.1406593848626 | 3.96372353534742 | -0.463723535347419 |
| Array\_1 | Prep\_A | 2 | CreE | 23 | 3.5 | 23.1243796853558 | 3.95958179387911 | -0.459581793879109 |
| Array\_2 | Prep\_A | 2 | CreJ | 23 | 3.5 | 22.8926597735668 | 3.88134521141406 | -0.381345211414057 |
| Array\_2 | Prep\_A | 1 | CreJ | 23 | 3.5 | 23.0321713498442 | 3.87050061756534 | -0.370500617565337 |
| Array\_1 | Prep\_A | 2 | CreJ | 23 | 3.5 | 23.1255103596289 | 3.95272029352033 | -0.45272029352033 |
| Array\_1 | Prep\_A | 1 | CreJ | 23 | 3.5 | 23.1417900591357 | 3.96278764383857 | -0.46278764383857 |
| Array\_2 | Prep\_A | 2 | CreJ | 10 | 2 | 10.0827467262353 | 2.12538340522765 | -0.125383405227652 |
| Array\_2 | Prep\_A | 1 | CreJ | 10 | 2 | 10.0317274039254 | 2.11443367698188 | -0.114433676981882 |
| Array\_1 | Prep\_A | 2 | CreE | 10 | 2 | 9.95725396603933 | 2.14340037592967 | -0.143400375929674 |
| Array\_1 | Prep\_A | 1 | CreE | 10 | 2 | 9.94428256926329 | 2.12156476246687 | -0.121564762466874 |
| Array\_1 | Prep\_A | 2 | CreJ | 10 | 2 | 9.95540272384077 | 2.10231087785524 | -0.10231087785524 |
| Array\_1 | Prep\_A | 1 | CreJ | 10 | 2 | 9.94243132706473 | 2.19055530481607 | -0.19055530481607 |
| Array\_2 | Prep\_A | 1 | CreE | 10 | 2 | 10.0335802636808 | 2.12372906991757 | -0.123729069917574 |
| Array\_2 | Prep\_A | 2 | CreE | 10 | 2 | 10.0845995859907 | 2.12066430548613 | -0.120664305486134 |
| Array\_1 | Prep\_A | 2 | CreJ | 24 | 3.5 | 23.6798520378079 | 3.94144583192059 | -0.441445831920587 |
| Array\_1 | Prep\_A | 1 | CreJ | 24 | 3.5 | 24.1298084647565 | 3.96760155408079 | -0.467601554080787 |
| Array\_2 | Prep\_A | 2 | CreE | 24 | 3.5 | 24.2331114395457 | 3.95538520116506 | -0.455385201165063 |
| Array\_2 | Prep\_A | 1 | CreE | 24 | 3.5 | 24.1582064703623 | 3.9845799987121 | -0.484579998712103 |
| Array\_2 | Prep\_A | 2 | CreJ | 24 | 3.5 | 24.2342439821227 | 4.00203659844352 | -0.502036598443521 |
| Array\_2 | Prep\_A | 1 | CreJ | 24 | 3.5 | 24.1593390129393 | 3.92628009617973 | -0.426280096179731 |
| Array\_1 | Prep\_A | 1 | CreE | 24 | 3.5 | 24.128677105661 | 3.97975898908756 | -0.479758989087563 |
| Array\_1 | Prep\_A | 2 | CreE | 24 | 3.5 | 23.6787206787124 | 3.9944318313317 | -0.494431831331704 |
| Array\_2 | Prep\_A | 1 | CreJ | 11 | 2 | 10.9904385385208 | 2.13025425975365 | -0.130254259753649 |
| Array\_2 | Prep\_A | 2 | CreJ | 11 | 2 | 11.1273438508352 | 2.19835549781927 | -0.198355497819269 |
| Array\_2 | Prep\_A | 2 | CreE | 11 | 2 | 11.1291954251637 | 2.16506450121599 | -0.165064501215993 |
| Array\_2 | Prep\_A | 1 | CreE | 11 | 2 | 10.9922901128493 | 2.16812497608407 | -0.168124976084073 |
| Array\_1 | Prep\_A | 1 | CreE | 11 | 2 | 10.9485323699337 | 2.13880561745382 | -0.138805617453823 |
| Array\_1 | Prep\_A | 2 | CreE | 11 | 2 | 11.014737203529 | 2.15427976809141 | -0.154279768091413 |
| Array\_1 | Prep\_A | 1 | CreJ | 11 | 2 | 10.946679267101 | 2.11671499942145 | -0.11671499942145 |
| Array\_1 | Prep\_A | 2 | CreJ | 11 | 2 | 11.0128841006963 | 2.15078970209892 | -0.15078970209892 |
| Array\_2 | Prep\_A | 1 | CreE | 25 | 3.5 | 24.8564389965588 | 3.98034871397374 | -0.480348713973738 |
| Array\_2 | Prep\_A | 2 | CreE | 25 | 3.5 | 25.0072871570554 | 3.99442061225073 | -0.494420612250728 |
| Array\_2 | Prep\_A | 2 | CreJ | 25 | 3.5 | 25.0054508308134 | 3.95394973878572 | -0.453949738785717 |
| Array\_2 | Prep\_A | 1 | CreJ | 25 | 3.5 | 24.8546026703168 | 3.89566541069281 | -0.395665410692807 |
| Array\_1 | Prep\_A | 1 | CreE | 25 | 3.5 | 24.9796471682386 | 3.94477564752017 | -0.444775647520168 |
| Array\_1 | Prep\_A | 2 | CreE | 25 | 3.5 | 25.0993453550104 | 3.98462730517413 | -0.484627305174128 |
| Array\_1 | Prep\_A | 1 | CreJ | 25 | 3.5 | 24.9778072217586 | 3.94220971059649 | -0.442209710596487 |
| Array\_1 | Prep\_A | 2 | CreJ | 25 | 3.5 | 25.0975054085304 | 3.83135542482669 | -0.331355424826687 |
| Array\_2 | Prep\_A | 2 | CreE | 12 | 2 | 11.946187366202 | 2.16470006638239 | -0.164700066382387 |
| Array\_2 | Prep\_A | 1 | CreE | 12 | 2 | 11.9562016934418 | 2.12573248736278 | -0.125732487362777 |
| Array\_2 | Prep\_A | 2 | CreJ | 12 | 2 | 11.9443374466784 | 2.18852887207435 | -0.188528872074352 |
| Array\_2 | Prep\_A | 1 | CreJ | 12 | 2 | 11.9543517739182 | 2.13146476801485 | -0.131464768014852 |
| Array\_1 | Prep\_A | 2 | CreE | 12 | 2 | 11.9615846470985 | 2.17095098102839 | -0.170950981028387 |
| Array\_1 | Prep\_A | 1 | CreE | 12 | 2 | 12.0408922380554 | 2.21375672852274 | -0.213756728522737 |
| Array\_1 | Prep\_A | 2 | CreJ | 12 | 2 | 11.962699938779 | 2.12799528761405 | -0.12799528761405 |
| Array\_1 | Prep\_A | 1 | CreJ | 12 | 2 | 12.0420075297359 | 2.11283248026888 | -0.11283248026888 |
| Array\_2 | Prep\_A | 2 | CreE | 26 | 3.5 | 26.0717378675648 | 3.99072137985753 | -0.490721379857526 |
| Array\_2 | Prep\_A | 1 | CreE | 26 | 3.5 | 26.0480799737574 | 4.02439687913647 | -0.524396879136465 |
| Array\_2 | Prep\_A | 2 | CreJ | 26 | 3.5 | 26.0717434161186 | 3.97524412636514 | -0.475244126365145 |
| Array\_2 | Prep\_A | 1 | CreJ | 26 | 3.5 | 26.0480855223112 | 3.904193437386 | -0.404193437386005 |
| Array\_1 | Prep\_A | 1 | CreJ | 26 | 3.5 | 25.9537428636794 | 3.87198905495376 | -0.371989054953756 |
| Array\_1 | Prep\_A | 2 | CreJ | 26 | 3.5 | 26.2273152997489 | 3.93445758544968 | -0.434457585449676 |
| Array\_1 | Prep\_A | 2 | CreE | 26 | 3.5 | 26.2291531978661 | 4.00290173642769 | -0.502901736427686 |
| Array\_1 | Prep\_A | 1 | CreE | 26 | 3.5 | 25.9555807617966 | 4.00845316307023 | -0.508453163070226 |
| Array\_2 | Prep\_A | 2 | CreJ | 13 | 2 | 13.0437716286479 | 2.21791020606664 | -0.217910206066644 |
| Array\_2 | Prep\_A | 1 | CreJ | 13 | 2 | 12.9186053809883 | 2.104830976564 | -0.104830976564005 |
| Array\_1 | Prep\_A | 2 | CreE | 13 | 2 | 12.9910363230792 | 2.17538299910003 | -0.175382999100035 |
| Array\_1 | Prep\_A | 1 | CreE | 13 | 2 | 12.9934044530318 | 2.18770492602859 | -0.187704926028595 |
| Array\_2 | Prep\_A | 1 | CreE | 13 | 2 | 12.9204535456046 | 2.19253549225681 | -0.192535492256815 |
| Array\_2 | Prep\_A | 2 | CreE | 13 | 2 | 13.0456197932642 | 2.17125583061622 | -0.171255830616225 |
| Array\_1 | Prep\_A | 2 | CreJ | 13 | 2 | 12.9921543400391 | 2.08769519211834 | -0.0876951921183427 |
| Array\_1 | Prep\_A | 1 | CreJ | 13 | 2 | 12.9945224699917 | 2.19254418267273 | -0.192544182672733 |
| Array\_2 | Prep\_A | 1 | CreJ | 27 | 3.5 | 27.3448011591692 | 3.88053033607286 | -0.38053033607286 |
| Array\_2 | Prep\_A | 2 | CreJ | 27 | 3.5 | 26.9348030230401 | 4.03142286805303 | -0.53142286805303 |
| Array\_1 | Prep\_A | 2 | CreJ | 27 | 3.5 | 27.0483248540721 | 3.96446702446497 | -0.464467024464974 |
| Array\_1 | Prep\_A | 1 | CreJ | 27 | 3.5 | 26.9797695681642 | 3.91097924723188 | -0.410979247231884 |
| Array\_2 | Prep\_A | 2 | CreE | 27 | 3.5 | 26.9336666739162 | 4.05750786586097 | -0.557507865860973 |
| Array\_2 | Prep\_A | 1 | CreE | 27 | 3.5 | 27.3436648100453 | 4.00632973155959 | -0.506329731559593 |
| Array\_1 | Prep\_A | 1 | CreE | 27 | 3.5 | 26.9798799372327 | 3.99081314361528 | -0.490813143615283 |
| Array\_1 | Prep\_A | 2 | CreE | 27 | 3.5 | 27.0484352231406 | 4.01921857449699 | -0.519218574496994 |
| Array\_1 | Prep\_A | 2 | CreJ | 14 | 2 | 14.0921831399934 | 2.2074064367953 | -0.207406436795298 |
| Array\_1 | Prep\_A | 1 | CreJ | 14 | 2 | 14.0352872171779 | 2.12191222869728 | -0.121912228697278 |
| Array\_2 | Prep\_A | 1 | CreE | 14 | 2 | 14.0736564046881 | 2.18774603562824 | -0.187746035628243 |
| Array\_2 | Prep\_A | 2 | CreE | 14 | 2 | 14.0706031031434 | 2.18702760091381 | -0.187027600913813 |
| Array\_2 | Prep\_A | 1 | CreJ | 14 | 2 | 14.0718104756217 | 2.19530833434962 | -0.195308334349617 |
| Array\_2 | Prep\_A | 2 | CreJ | 14 | 2 | 14.068757174077 | 2.18989346713953 | -0.189893467139527 |
| Array\_1 | Prep\_A | 2 | CreE | 14 | 2 | 14.0934402502643 | 2.1806069412427 | -0.180606941242703 |
| Array\_1 | Prep\_A | 1 | CreE | 14 | 2 | 14.0365443274488 | 2.18151081683262 | -0.181510816832623 |
| Array\_2 | Prep\_A | 2 | CreE | 1 | 0.5 | 1.00767589593454 | 0.346811386518842 | 0.153188613481158 |
| Array\_2 | Prep\_A | 1 | CreE | 1 | 0.5 | 1.01272093992418 | 0.360527312448082 | 0.139472687551918 |
| Array\_1 | Prep\_A | 1 | CreJ | 1 | 0.5 | 1.00676838074222 | 0.50998790296412 | -0.00998790296412022 |
| Array\_1 | Prep\_A | 2 | CreJ | 1 | 0.5 | 1.00489706103694 | 0.490383591700289 | 0.00961640829971083 |
| Array\_1 | Prep\_A | 1 | CreE | 1 | 0.5 | 1.0079036700895 | 0.377953297229574 | 0.122046702770426 |
| Array\_1 | Prep\_A | 2 | CreE | 1 | 0.5 | 1.00603235038422 | 0.346524618541398 | 0.153475381458602 |
| Array\_2 | Prep\_A | 2 | CreJ | 1 | 0.5 | 1.00654067225755 | 0.531254295309342 | -0.0312542953093423 |
| Array\_2 | Prep\_A | 1 | CreJ | 1 | 0.5 | 1.01158571624719 | 0.467567878576298 | 0.0324321214237016 |
| Array\_2 | Prep\_A | 2 | CreE | 28 | 3.5 | 27.8878776186521 | 4.0087820200106 | -0.508782020010599 |
| Array\_2 | Prep\_A | 1 | CreE | 28 | 3.5 | 28.1174596821025 | 4.03945616075472 | -0.539456160754719 |
| Array\_2 | Prep\_A | 2 | CreJ | 28 | 3.5 | 27.8860413555131 | 3.91005371932762 | -0.410053719327619 |
| Array\_2 | Prep\_A | 1 | CreJ | 28 | 3.5 | 28.1156234189635 | 3.86581974027178 | -0.365819740271779 |
| Array\_1 | Prep\_A | 1 | CreJ | 28 | 3.5 | 27.8318153726536 | 3.97778606718873 | -0.477786067188732 |
| Array\_1 | Prep\_A | 2 | CreJ | 28 | 3.5 | 28.1599804999184 | 3.93817399875333 | -0.438173998753332 |
| Array\_1 | Prep\_A | 2 | CreE | 28 | 3.5 | 28.1588428060271 | 4.01026609412313 | -0.510266094123129 |
| Array\_1 | Prep\_A | 1 | CreE | 28 | 3.5 | 27.8306776787623 | 3.97884656921765 | -0.478846569217649 |
| Array\_2 | Prep\_A | 2 | CreJ | 15 | 2 | 15.0112099512194 | 2.22290901325923 | -0.222909013259233 |
| Array\_2 | Prep\_A | 1 | CreJ | 15 | 2 | 15.0961949949891 | 2.18399802226899 | -0.183998022268993 |
| Array\_2 | Prep\_A | 2 | CreE | 15 | 2 | 15.0130555156752 | 2.21376239274247 | -0.213762392742466 |
| Array\_2 | Prep\_A | 1 | CreE | 15 | 2 | 15.0980405594449 | 2.20015358182942 | -0.200153581829416 |
| Array\_1 | Prep\_A | 2 | CreJ | 15 | 2 | 14.9459545163153 | 2.18506337419402 | -0.185063374194024 |
| Array\_1 | Prep\_A | 1 | CreJ | 15 | 2 | 15.0228897237245 | 2.19145138913933 | -0.191451389139333 |
| Array\_1 | Prep\_A | 2 | CreE | 15 | 2 | 14.9478007320195 | 2.20334795781652 | -0.203347957816516 |
| Array\_1 | Prep\_A | 1 | CreE | 15 | 2 | 15.0247359394287 | 2.20834915737512 | -0.208349157375116 |
| Array\_2 | Prep\_A | 1 | CreJ | 2 | 0.5 | 1.98588771353854 | 0.537324500968894 | -0.0373245009688943 |
| Array\_2 | Prep\_A | 2 | CreJ | 2 | 0.5 | 2.00183241041607 | 0.584965557015561 | -0.0849655570155613 |
| Array\_1 | Prep\_A | 2 | CreJ | 2 | 0.5 | 2.00257641914736 | 0.468131991899472 | 0.0318680081005279 |
| Array\_1 | Prep\_A | 1 | CreJ | 2 | 0.5 | 1.98093055215752 | 0.52919031937957 | -0.0291903193795702 |
| Array\_2 | Prep\_A | 2 | CreE | 2 | 0.5 | 2.00296713939821 | 0.380967660314064 | 0.119032339685936 |
| Array\_2 | Prep\_A | 1 | CreE | 2 | 0.5 | 1.98702244252068 | 0.428603777962496 | 0.0713962220375041 |
| Array\_1 | Prep\_A | 2 | CreE | 2 | 0.5 | 2.00371047183236 | 0.39904310640839 | 0.10095689359161 |
| Array\_1 | Prep\_A | 1 | CreE | 2 | 0.5 | 1.98206460484252 | 0.377793641136325 | 0.122206358863675 |
| Array\_2 | Prep\_A | 1 | CreJ | 29 | 3.5 | 29.2217852433148 | 3.92470405402208 | -0.424704054022082 |
| Array\_2 | Prep\_A | 2 | CreJ | 29 | 3.5 | 29.0474704546787 | 3.86660649007488 | -0.366606490074882 |
| Array\_2 | Prep\_A | 1 | CreE | 29 | 3.5 | 29.2236193953799 | 4.05611290065897 | -0.556112900658974 |
| Array\_2 | Prep\_A | 2 | CreE | 29 | 3.5 | 29.0493046067438 | 4.03580798486047 | -0.535807984860474 |
| Array\_1 | Prep\_A | 2 | CreE | 29 | 3.5 | 29.0655519968492 | 4.02963452816766 | -0.529634528167664 |
| Array\_1 | Prep\_A | 1 | CreE | 29 | 3.5 | 28.587934173562 | 4.05972142293113 | -0.559721422931134 |
| Array\_1 | Prep\_A | 2 | CreJ | 29 | 3.5 | 29.0666899676155 | 3.88782158249523 | -0.387821582495228 |
| Array\_1 | Prep\_A | 1 | CreJ | 29 | 3.5 | 28.5890721443283 | 4.01189032791807 | -0.511890327918067 |
| Array\_2 | Prep\_A | 2 | CreJ | 16 | 2 | 15.963634335783 | 2.11503538007525 | -0.115035380075248 |
| Array\_2 | Prep\_A | 1 | CreJ | 16 | 2 | 16.0764019237143 | 2.26882619881805 | -0.268826198818047 |
| Array\_1 | Prep\_A | 2 | CreJ | 16 | 2 | 15.8986807910646 | 2.22053232868309 | -0.22053232868309 |
| Array\_1 | Prep\_A | 1 | CreJ | 16 | 2 | 15.8844676916062 | 2.17108536994122 | -0.17108536994122 |
| Array\_1 | Prep\_A | 2 | CreE | 16 | 2 | 15.9005256366637 | 2.24537254258036 | -0.24537254258036 |
| Array\_1 | Prep\_A | 1 | CreE | 16 | 2 | 15.8863125372053 | 2.19894270716814 | -0.198942707168141 |
| Array\_2 | Prep\_A | 1 | CreE | 16 | 2 | 16.0782467345036 | 2.21563986953004 | -0.21563986953004 |
| Array\_2 | Prep\_A | 2 | CreE | 16 | 2 | 15.9654791465723 | 2.22699299834252 | -0.226992998342521 |
| Array\_2 | Prep\_A | 1 | CreE | 3 | 0.5 | 3.00849527749114 | 0.363303424745303 | 0.136696575254697 |
| Array\_2 | Prep\_A | 2 | CreE | 3 | 0.5 | 2.98096704118301 | 0.361152334415539 | 0.138847665584461 |
| Array\_2 | Prep\_A | 2 | CreJ | 3 | 0.5 | 2.97983788374028 | 0.597495488089663 | -0.0974954880896633 |
| Array\_2 | Prep\_A | 1 | CreJ | 3 | 0.5 | 3.00736612004841 | 0.501804814196854 | -0.0018048141968543 |
| Array\_1 | Prep\_A | 2 | CreE | 3 | 0.5 | 3.00822131671603 | 0.39015655380795 | 0.10984344619205 |
| Array\_1 | Prep\_A | 1 | CreE | 3 | 0.5 | 3.01796580431023 | 0.426376633360997 | 0.0736233666390028 |
| Array\_1 | Prep\_A | 2 | CreJ | 3 | 0.5 | 3.00709255597663 | 0.535479713422352 | -0.0354797134223519 |
| Array\_1 | Prep\_A | 1 | CreJ | 3 | 0.5 | 3.01683704357083 | 0.493780311829248 | 0.00621968817075208 |
| Array\_1 | Prep\_A | 2 | CreE | 30 | 3.5 | 29.7956081371564 | 4.06277114679016 | -0.562771146790162 |
| Array\_1 | Prep\_A | 1 | CreE | 30 | 3.5 | 30.2204809166956 | 3.9858314492872 | -0.485831449287202 |
| Array\_1 | Prep\_A | 2 | CreJ | 30 | 3.5 | 29.7967458394485 | 3.98565622141474 | -0.485656221414741 |
| Array\_1 | Prep\_A | 1 | CreJ | 30 | 3.5 | 30.2216186189877 | 3.87958618393267 | -0.379586183932671 |
| Array\_2 | Prep\_A | 2 | CreJ | 30 | 3.5 | 30.1643105990472 | 3.95786345522665 | -0.457863455226649 |
| Array\_2 | Prep\_A | 1 | CreJ | 30 | 3.5 | 30.1806458799486 | 3.90572630291029 | -0.405726302910288 |
| Array\_2 | Prep\_A | 1 | CreE | 30 | 3.5 | 30.1795081312352 | 4.03152753050864 | -0.531527530508642 |
| Array\_2 | Prep\_A | 2 | CreE | 30 | 3.5 | 30.1631728503338 | 4.04355315962362 | -0.543553159623622 |
| Array\_1 | Prep\_A | 2 | CreJ | 17 | 2 | 17.1406523169589 | 2.28960989914317 | -0.289609899143169 |
| Array\_1 | Prep\_A | 1 | CreJ | 17 | 2 | 16.945716801287 | 2.21742619716055 | -0.217426197160549 |
| Array\_2 | Prep\_A | 1 | CreE | 17 | 2 | 17.0222515042225 | 2.21440893091272 | -0.21440893091272 |
| Array\_2 | Prep\_A | 2 | CreE | 17 | 2 | 16.9666080730208 | 2.25796743064562 | -0.257967430645619 |
| Array\_2 | Prep\_A | 1 | CreJ | 17 | 2 | 17.0204081717601 | 2.27600896535069 | -0.276008965350695 |
| Array\_2 | Prep\_A | 2 | CreJ | 17 | 2 | 16.9647647405584 | 2.17978271978099 | -0.179782719780995 |
| Array\_1 | Prep\_A | 1 | CreE | 17 | 2 | 16.9475590974576 | 2.24224884450566 | -0.24224884450566 |
| Array\_1 | Prep\_A | 2 | CreE | 17 | 2 | 17.1424946131295 | 2.2211209205583 | -0.2211209205583 |
| Array\_2 | Prep\_A | 1 | CreE | 4 | 0.5 | 4.03852309901995 | 0.440490293950515 | 0.0595097060494855 |
| Array\_2 | Prep\_A | 2 | CreE | 4 | 0.5 | 4.05140410258245 | 0.418815170379742 | 0.0811848296202585 |
| Array\_1 | Prep\_A | 2 | CreJ | 4 | 0.5 | 4.02674194014227 | 0.55524180222898 | -0.0552418022289802 |
| Array\_1 | Prep\_A | 1 | CreJ | 4 | 0.5 | 4.02957456622017 | 0.540963231860632 | -0.0409632318606322 |
| Array\_2 | Prep\_A | 1 | CreJ | 4 | 0.5 | 4.03739511951021 | 0.517742162907772 | -0.0177421629077716 |
| Array\_2 | Prep\_A | 2 | CreJ | 4 | 0.5 | 4.05027612307271 | 0.545164137612973 | -0.0451641376129727 |
| Array\_1 | Prep\_A | 2 | CreE | 4 | 0.5 | 4.02787022759116 | 0.427637541276864 | 0.0723624587231365 |
| Array\_1 | Prep\_A | 1 | CreE | 4 | 0.5 | 4.03070285366906 | 0.368912364822074 | 0.131087635177926 |
| Array\_1 | Prep\_A | 1 | CreE | 31 | 3.5 | 31.3709617482234 | 4.06304208668648 | -0.563042086686483 |
| Array\_1 | Prep\_A | 2 | CreE | 31 | 3.5 | 30.8411033879338 | 4.06779124739609 | -0.567791247396094 |
| Array\_1 | Prep\_A | 2 | CreJ | 31 | 3.5 | 30.8422436084624 | 3.91708374064925 | -0.417083740649254 |
| Array\_1 | Prep\_A | 1 | CreJ | 31 | 3.5 | 31.372101968752 | 3.97035751292441 | -0.470357512924414 |
| Array\_2 | Prep\_A | 2 | CreJ | 31 | 3.5 | 31.191270891413 | 3.92809784139961 | -0.428097841399609 |
| Array\_2 | Prep\_A | 1 | CreJ | 31 | 3.5 | 30.8960258100717 | 3.94949749578468 | -0.449497495784679 |
| Array\_2 | Prep\_A | 2 | CreE | 31 | 3.5 | 31.190131260586 | 4.06541277276606 | -0.565412772766063 |
| Array\_2 | Prep\_A | 1 | CreE | 31 | 3.5 | 30.8948861792447 | 4.04438482805345 | -0.544384828053453 |
| Array\_2 | Prep\_A | 1 | CreE | 18 | 2 | 18.0976884070967 | 2.25646226177025 | -0.256462261770245 |
| Array\_2 | Prep\_A | 2 | CreE | 18 | 2 | 18.3253126951447 | 2.22173216040053 | -0.221732160400526 |
| Array\_1 | Prep\_A | 1 | CreE | 18 | 2 | 18.0429977806516 | 2.25532730025286 | -0.255327300252855 |
| Array\_1 | Prep\_A | 2 | CreE | 18 | 2 | 18.097427237669 | 2.22593056472245 | -0.225930564722446 |
| Array\_2 | Prep\_A | 1 | CreJ | 18 | 2 | 18.0972137119856 | 2.19008354717793 | -0.190083547177928 |
| Array\_2 | Prep\_A | 2 | CreJ | 18 | 2 | 18.3248380000336 | 2.20626520773127 | -0.206265207731268 |
| Array\_1 | Prep\_A | 2 | CreJ | 18 | 2 | 18.0973060189273 | 2.22530275688628 | -0.225302756886284 |
| Array\_1 | Prep\_A | 1 | CreJ | 18 | 2 | 18.0428765619099 | 2.16768530866384 | -0.167685308663844 |
| Array\_2 | Prep\_A | 2 | CreJ | 5 | 0.5 | 5.06190450184126 | 0.527541453781085 | -0.0275414537810853 |
| Array\_2 | Prep\_A | 1 | CreJ | 5 | 0.5 | 4.99660610683809 | 0.561257286225309 | -0.0612572862253094 |
| Array\_1 | Prep\_A | 2 | CreE | 5 | 0.5 | 4.98366253461456 | 0.377552874927904 | 0.122447125072096 |
| Array\_1 | Prep\_A | 1 | CreE | 5 | 0.5 | 5.01054136178092 | 0.441788415620743 | 0.0582115843792568 |
| Array\_1 | Prep\_A | 1 | CreJ | 5 | 0.5 | 5.00941696095578 | 0.544495071064162 | -0.0444950710641624 |
| Array\_1 | Prep\_A | 2 | CreJ | 5 | 0.5 | 4.98253813378942 | 0.543842848672313 | -0.0438428486723134 |
| Array\_2 | Prep\_A | 1 | CreE | 5 | 0.5 | 4.99773047417601 | 0.41111134381992 | 0.0888886561800798 |
| Array\_2 | Prep\_A | 2 | CreE | 5 | 0.5 | 5.06302886917918 | 0.440508128060489 | 0.0594918719395108 |
| Array\_1 | Prep\_A | 1 | CreE | 32 | 3.5 | 31.9246271478017 | 4.07170990464073 | -0.571709904640726 |
| Array\_1 | Prep\_A | 2 | CreE | 32 | 3.5 | 32.0228558536346 | 4.08507028757082 | -0.585070287570816 |
| Array\_2 | Prep\_A | 1 | CreE | 32 | 3.5 | 32.0777272190295 | 4.07469732075122 | -0.574697320751216 |
| Array\_2 | Prep\_A | 2 | CreE | 32 | 3.5 | 31.7933952593367 | 4.08183297495435 | -0.581832974954346 |
| Array\_2 | Prep\_A | 2 | CreJ | 32 | 3.5 | 31.7915635591534 | 3.88441218656478 | -0.384412186564778 |
| Array\_2 | Prep\_A | 1 | CreJ | 32 | 3.5 | 32.0758955188462 | 3.8702125189284 | -0.370212518928398 |
| Array\_1 | Prep\_A | 2 | CreJ | 32 | 3.5 | 32.0240001008516 | 3.95650264546148 | -0.456502645461475 |
| Array\_1 | Prep\_A | 1 | CreJ | 32 | 3.5 | 31.9257713950187 | 3.9858462810692 | -0.485846281069195 |
| Array\_1 | Prep\_A | 1 | CreJ | 19 | 2 | 19.1088790071445 | 2.11922788273914 | -0.119227882739136 |
| Array\_1 | Prep\_A | 2 | CreJ | 19 | 2 | 19.0392560370507 | 2.1620669865224 | -0.162066986522396 |
| Array\_1 | Prep\_A | 1 | CreE | 19 | 2 | 19.1077576803377 | 2.25486415221657 | -0.254864152216572 |
| Array\_1 | Prep\_A | 2 | CreE | 19 | 2 | 19.0381347102439 | 2.26025599533762 | -0.260255995337622 |
| Array\_2 | Prep\_A | 1 | CreJ | 19 | 2 | 19.1818026932973 | 2.16117527020144 | -0.161175270201437 |
| Array\_2 | Prep\_A | 2 | CreJ | 19 | 2 | 19.0737216405593 | 2.1425444325664 | -0.142544432566397 |
| Array\_2 | Prep\_A | 1 | CreE | 19 | 2 | 19.1806810768711 | 2.2510381160422 | -0.251038116042202 |
| Array\_2 | Prep\_A | 2 | CreE | 19 | 2 | 19.0726000241331 | 2.27051509323423 | -0.270515093234232 |
| Array\_2 | Prep\_A | 2 | CreE | 6 | 0.5 | 6.03672177572849 | 0.460870254073397 | 0.0391297459266032 |
| Array\_2 | Prep\_A | 1 | CreE | 6 | 0.5 | 5.97825611952018 | 0.414166372728389 | 0.0858336272716113 |
| Array\_1 | Prep\_A | 1 | CreJ | 6 | 0.5 | 5.99622137504997 | 0.579569803647766 | -0.0795698036477661 |
| Array\_1 | Prep\_A | 2 | CreJ | 6 | 0.5 | 6.02487489060807 | 0.523571247026493 | -0.023571247026493 |
| Array\_1 | Prep\_A | 2 | CreE | 6 | 0.5 | 6.02599541189247 | 0.434551019386857 | 0.0654489806131433 |
| Array\_1 | Prep\_A | 1 | CreE | 6 | 0.5 | 5.99734189633437 | 0.399618514541813 | 0.100381485458187 |
| Array\_2 | Prep\_A | 2 | CreJ | 6 | 0.5 | 6.03560055160651 | 0.579192536670219 | -0.0791925366702186 |
| Array\_2 | Prep\_A | 1 | CreJ | 6 | 0.5 | 5.9771348953982 | 0.583685895658402 | -0.0836858956584016 |
| Array\_2 | Prep\_A | 2 | CreE | 33 | 3.5 | 33.440916494446 | 4.08600890548005 | -0.586008905480046 |
| Array\_2 | Prep\_A | 1 | CreE | 33 | 3.5 | 33.0191184447103 | 4.06589015958712 | -0.565890159587116 |
| Array\_1 | Prep\_A | 2 | CreJ | 33 | 3.5 | 32.9153030799187 | 3.90256445988762 | -0.402564459887619 |
| Array\_1 | Prep\_A | 1 | CreJ | 33 | 3.5 | 33.009658614829 | 3.95018679212852 | -0.450186792128519 |
| Array\_1 | Prep\_A | 2 | CreE | 33 | 3.5 | 32.9141620077198 | 4.07990181138084 | -0.579901811380837 |
| Array\_1 | Prep\_A | 1 | CreE | 33 | 3.5 | 33.0085175426301 | 4.10117852881664 | -0.601178528816637 |
| Array\_2 | Prep\_A | 1 | CreJ | 33 | 3.5 | 33.0172858420847 | 3.84523748753413 | -0.345237487534129 |
| Array\_2 | Prep\_A | 2 | CreJ | 33 | 3.5 | 33.4390838918204 | 3.84919300530059 | -0.349193005300589 |
| Array\_1 | Prep\_A | 2 | CreE | 20 | 2 | 19.9668208749372 | 2.27141809964064 | -0.271418099640639 |
| Array\_1 | Prep\_A | 1 | CreE | 20 | 2 | 19.8860451348674 | 2.29273257453282 | -0.29273257453282 |
| Array\_2 | Prep\_A | 2 | CreE | 20 | 2 | 20.208883274082 | 2.2760802388541 | -0.2760802388541 |
| Array\_2 | Prep\_A | 1 | CreE | 20 | 2 | 20.1029517421925 | 2.23592397471105 | -0.235923974711049 |
| Array\_2 | Prep\_A | 2 | CreJ | 20 | 2 | 20.2100059907188 | 2.14342944090871 | -0.143429440908714 |
| Array\_2 | Prep\_A | 1 | CreJ | 20 | 2 | 20.1040744588293 | 2.19174902868576 | -0.191749028685765 |
| Array\_1 | Prep\_A | 2 | CreJ | 20 | 2 | 19.9679439722485 | 2.19013992227197 | -0.190139922271974 |
| Array\_1 | Prep\_A | 1 | CreJ | 20 | 2 | 19.8871682321787 | 2.16985785796356 | -0.169857857963564 |
| Array\_1 | Prep\_A | 2 | CreJ | 7 | 0.5 | 7.04656001872733 | 0.584296203069066 | -0.0842962030690657 |
| Array\_1 | Prep\_A | 1 | CreJ | 7 | 0.5 | 7.01874738929166 | 0.566669557783882 | -0.0666695577838817 |
| Array\_2 | Prep\_A | 2 | CreE | 7 | 0.5 | 7.00102918020356 | 0.411261130263378 | 0.088738869736622 |
| Array\_2 | Prep\_A | 1 | CreE | 7 | 0.5 | 7.08575157576771 | 0.436679854291169 | 0.063320145708831 |
| Array\_1 | Prep\_A | 2 | CreE | 7 | 0.5 | 7.04767905739942 | 0.475248974603462 | 0.024751025396538 |
| Array\_1 | Prep\_A | 1 | CreE | 7 | 0.5 | 7.01986642796375 | 0.456384884083767 | 0.043615115916233 |
| Array\_2 | Prep\_A | 1 | CreJ | 7 | 0.5 | 7.08463525751025 | 0.456397578827924 | 0.043602421172076 |
| Array\_2 | Prep\_A | 2 | CreJ | 7 | 0.5 | 6.9999128619461 | 0.517347358147776 | -0.017347358147776 |
| Array\_2 | Prep\_A | 2 | CreJ | 34 | 3.5 | 33.9171744136365 | 3.78169907496934 | -0.281699074969339 |
| Array\_2 | Prep\_A | 1 | CreJ | 34 | 3.5 | 33.9238211583182 | 3.80861017125685 | -0.308610171256849 |
| Array\_1 | Prep\_A | 1 | CreE | 34 | 3.5 | 33.9172460946782 | 4.12692119812779 | -0.62692119812779 |
| Array\_1 | Prep\_A | 2 | CreE | 34 | 3.5 | 34.0015897544842 | 4.11716742920497 | -0.61716742920497 |
| Array\_2 | Prep\_A | 2 | CreE | 34 | 3.5 | 33.9190112704696 | 4.07522772776813 | -0.57522772776813 |
| Array\_2 | Prep\_A | 1 | CreE | 34 | 3.5 | 33.9256580151513 | 4.13009510320328 | -0.63009510320328 |
| Array\_1 | Prep\_A | 2 | CreJ | 34 | 3.5 | 34.0027319870939 | 3.90383713764444 | -0.403837137644445 |
| Array\_1 | Prep\_A | 1 | CreJ | 34 | 3.5 | 33.9183883272879 | 3.96259338019267 | -0.462593380192674 |
| Array\_2 | Prep\_A | 1 | CreJ | 21 | 2 | 21.0269792513226 | 2.17896711566004 | -0.178967115660036 |
| Array\_2 | Prep\_A | 2 | CreJ | 21 | 2 | 21.0281137951889 | 2.13651467180803 | -0.136514671808026 |
| Array\_2 | Prep\_A | 2 | CreE | 21 | 2 | 21.0269910628743 | 2.25993977136401 | -0.259939771364015 |
| Array\_2 | Prep\_A | 1 | CreE | 21 | 2 | 21.025856519008 | 2.23403831211366 | -0.234038312113664 |
| Array\_1 | Prep\_A | 2 | CreE | 21 | 2 | 21.0828678569367 | 2.2786998151023 | -0.278699815102304 |
| Array\_1 | Prep\_A | 1 | CreE | 21 | 2 | 21.1068357325109 | 2.26290632265781 | -0.262906322657814 |
| Array\_1 | Prep\_A | 2 | CreJ | 21 | 2 | 21.083991384871702 | 2.19135449485583 | -0.191354494855835 |
| Array\_1 | Prep\_A | 1 | CreJ | 21 | 2 | 21.1079592604459 | 2.18511128704695 | -0.185111287046945 |
| Array\_2 | Prep\_A | 2 | CreE | 8 | 0.5 | 7.98073324464826 | 0.426996901786079 | 0.0730030982139208 |
| Array\_2 | Prep\_A | 1 | CreE | 8 | 0.5 | 8.01765688424735 | 0.481353521740427 | 0.0186464782595728 |
| Array\_2 | Prep\_A | 2 | CreJ | 8 | 0.5 | 7.97961747716105 | 0.55882591449183 | -0.0588259144918298 |
| Array\_2 | Prep\_A | 1 | CreJ | 8 | 0.5 | 8.01654111676014 | 0.570701478994323 | -0.0707014789943229 |
| Array\_1 | Prep\_A | 1 | CreJ | 8 | 0.5 | 8.03721126038475 | 0.538816711424088 | -0.0388167114240883 |
| Array\_1 | Prep\_A | 2 | CreJ | 8 | 0.5 | 8.04211692381157 | 0.613247196066779 | -0.113247196066779 |
| Array\_1 | Prep\_A | 1 | CreE | 8 | 0.5 | 8.03832724826461 | 0.457280437559289 | 0.0427195624407108 |
| Array\_1 | Prep\_A | 2 | CreE | 8 | 0.5 | 8.04323291169143 | 0.406654840170128 | 0.0933451598298718 |
| Array\_1 | Prep\_A | 2 | CreJ | 35 | 3.5 | 34.9895083515375 | 3.90654105992176 | -0.406541059921764 |
| Array\_1 | Prep\_A | 1 | CreJ | 35 | 3.5 | 35.1898833263887 | 3.95582554502529 | -0.455825545025294 |
| Array\_2 | Prep\_A | 2 | CreJ | 35 | 3.5 | 34.7813885376086 | 3.89314649723501 | -0.393146497235008 |
| Array\_2 | Prep\_A | 1 | CreJ | 35 | 3.5 | 34.8890595978455 | 3.80890719290577 | -0.308907192905768 |
| Array\_2 | Prep\_A | 2 | CreE | 35 | 3.5 | 34.7832191810608 | 4.0999019082606 | -0.599901908260596 |
| Array\_2 | Prep\_A | 1 | CreE | 35 | 3.5 | 34.8908902412977 | 4.11130586783777 | -0.611305867837765 |
| Array\_1 | Prep\_A | 1 | CreE | 35 | 3.5 | 35.1887393255244 | 4.09026698411841 | -0.590266984118405 |
| Array\_1 | Prep\_A | 2 | CreE | 35 | 3.5 | 34.9883643506732 | 4.09446888368992 | -0.594468883689915 |
| Array\_2 | Prep\_A | 1 | CreJ | 22 | 2 | 21.9946663801613 | 2.22772523000554 | -0.22772523000554 |
| Array\_2 | Prep\_A | 2 | CreJ | 22 | 2 | 22.0514726020391 | 2.25885221824734 | -0.25885221824734 |
| Array\_2 | Prep\_A | 2 | CreE | 22 | 2 | 22.053314623609 | 2.3125062266959 | -0.312506226695899 |
| Array\_2 | Prep\_A | 1 | CreE | 22 | 2 | 21.9965084017312 | 2.31558011071335 | -0.315580110713349 |
| Array\_1 | Prep\_A | 2 | CreJ | 22 | 2 | 22.0903647288022 | 2.23350194739929 | -0.233501947399291 |
| Array\_1 | Prep\_A | 1 | CreJ | 22 | 2 | 21.8098724917485 | 2.1555139922308 | -0.155513992230801 |
| Array\_1 | Prep\_A | 2 | CreE | 22 | 2 | 22.0892409733603 | 2.29545779890287 | -0.295457798902869 |
| Array\_1 | Prep\_A | 1 | CreE | 22 | 2 | 21.8087487363066 | 2.30298803275392 | -0.302988032753919 |
| Array\_2 | Prep\_A | 2 | CreJ | 9 | 0.5 | 9.01170567810725 | 0.56179558957784 | -0.06179558957784 |
| Array\_2 | Prep\_A | 1 | CreJ | 9 | 0.5 | 9.03609120515526 | 0.558034306718949 | -0.0580343067189491 |
| Array\_2 | Prep\_A | 1 | CreE | 9 | 0.5 | 9.03720784369771 | 0.446058905758686 | 0.0539410942413144 |
| Array\_2 | Prep\_A | 2 | CreE | 9 | 0.5 | 9.0128223166497 | 0.464548201006493 | 0.0354517989935074 |
| Array\_1 | Prep\_A | 1 | CreJ | 9 | 0.5 | 9.02148819080579 | 0.60605009756827 | -0.10605009756827 |
| Array\_1 | Prep\_A | 2 | CreJ | 9 | 0.5 | 8.93912339084057 | 0.606491243440442 | -0.106491243440442 |
| Array\_1 | Prep\_A | 2 | CreE | 9 | 0.5 | 8.94024147206413 | 0.526196982932432 | -0.0261969829324317 |
| Array\_1 | Prep\_A | 1 | CreE | 9 | 0.5 | 9.02260627202935 | 0.523208839463904 | -0.0232088394639036 |
| Array\_2 | Prep\_A | 1 | CreJ | 23 | 2 | 22.9630991228744 | 2.17684971971159 | -0.176849719711585 |
| Array\_2 | Prep\_A | 2 | CreJ | 23 | 2 | 23.0417999578153 | 2.15258007791089 | -0.152580077910885 |
| Array\_1 | Prep\_A | 1 | CreE | 23 | 2 | 22.9748059051133 | 2.28776913589379 | -0.287769135893794 |
| Array\_1 | Prep\_A | 2 | CreE | 23 | 2 | 23.0036479556956 | 2.31423360127319 | -0.314233601273194 |
| Array\_2 | Prep\_A | 2 | CreE | 23 | 2 | 23.040676953709202 | 2.31242439136144 | -0.312424391361444 |
| Array\_2 | Prep\_A | 1 | CreE | 23 | 2 | 22.9619761187683 | 2.28693639186988 | -0.286936391869884 |
| Array\_1 | Prep\_A | 1 | CreJ | 23 | 2 | 22.9729637016541 | 2.21901725153556 | -0.219017251535559 |
| Array\_1 | Prep\_A | 2 | CreJ | 23 | 2 | 23.0018057522364 | 2.25947256523469 | -0.259472565234689 |
| Array\_2 | Prep\_A | 2 | CreE | 10 | 0.5 | 10.0940457943169 | 0.483032969834629 | 0.0169670301653707 |
| Array\_2 | Prep\_A | 1 | CreE | 10 | 0.5 | 9.97635693711473 | 0.500964306911739 | -9.6430691173932e-4 |
| Array\_1 | Prep\_A | 1 | CreJ | 10 | 0.5 | 10.0187518885354 | 0.632486912333568 | -0.132486912333568 |
| Array\_1 | Prep\_A | 2 | CreJ | 10 | 0.5 | 10.0107690077451 | 0.538569223937591 | -0.0385692239375913 |
| Array\_2 | Prep\_A | 2 | CreJ | 10 | 0.5 | 10.0929328903988 | 0.536318136272214 | -0.036318136272214 |
| Array\_2 | Prep\_A | 1 | CreJ | 10 | 0.5 | 9.97524403319662 | 0.569628746852345 | -0.069628746852345 |
| Array\_1 | Prep\_A | 1 | CreE | 10 | 0.5 | 10.0198658574688 | 0.495052769911526 | 0.00494723008847364 |
| Array\_1 | Prep\_A | 2 | CreE | 10 | 0.5 | 10.0118829766785 | 0.457227355573491 | 0.0427726444265086 |
| Array\_1 | Prep\_A | 1 | CreJ | 1 | 4 | 1.00926930372321 | 4.40040818853635 | -0.400408188536351 |
| Array\_1 | Prep\_A | 2 | CreJ | 1 | 4 | 0.99646069712931 | 4.34954659223598 | -0.34954659223598 |
| Array\_2 | Prep\_A | 1 | CreJ | 1 | 4 | 1.00316563754409 | 4.39602145287632 | -0.396021452876322 |
| Array\_2 | Prep\_A | 2 | CreJ | 1 | 4 | 1.0159663267283 | 4.45025916725021 | -0.450259167250212 |
| Array\_2 | Prep\_A | 1 | CreE | 1 | 4 | 1.0020686447816 | 4.19016434758136 | -0.19016434758136 |
| Array\_2 | Prep\_A | 2 | CreE | 1 | 4 | 1.01486933396581 | 4.21996210714405 | -0.21996210714405 |
| Array\_1 | Prep\_A | 2 | CreE | 1 | 4 | 0.995372091732521 | 4.22550009295639 | -0.22550009295639 |
| Array\_1 | Prep\_A | 1 | CreE | 1 | 4 | 1.00818069832642 | 4.19347195254485 | -0.19347195254485 |
| Array\_1 | Prep\_A | 2 | CreJ | 24 | 2 | 23.855073286063 | 2.2146304021609 | -0.214630402160895 |
| Array\_1 | Prep\_A | 1 | CreJ | 24 | 2 | 23.9418970805671 | 2.26802947862282 | -0.268029478622815 |
| Array\_1 | Prep\_A | 2 | CreE | 24 | 2 | 23.8569152213817 | 2.37980023026831 | -0.379800230268306 |
| Array\_1 | Prep\_A | 1 | CreE | 24 | 2 | 23.9437390158858 | 2.34482011722565 | -0.344820117225646 |
| Array\_2 | Prep\_A | 1 | CreE | 24 | 2 | 23.9528026356806 | 2.30392510888511 | -0.303925108885105 |
| Array\_2 | Prep\_A | 2 | CreE | 24 | 2 | 24.1551158417812 | 2.34061930220824 | -0.340619302208236 |
| Array\_2 | Prep\_A | 1 | CreJ | 24 | 2 | 23.9539257303967 | 2.15815334588962 | -0.158153345889619 |
| Array\_2 | Prep\_A | 2 | CreJ | 24 | 2 | 24.1562389364973 | 2.14709497399611 | -0.147094973996109 |
| Array\_2 | Prep\_A | 2 | CreJ | 11 | 0.5 | 11.0709739073731 | 0.570065967511535 | -0.0700659675115348 |
| Array\_2 | Prep\_A | 1 | CreJ | 11 | 0.5 | 10.9821463446005 | 0.602339344859893 | -0.102339344859893 |
| Array\_1 | Prep\_A | 1 | CreE | 11 | 0.5 | 11.0081192496705 | 0.475105855085035 | 0.0248941449149652 |
| Array\_1 | Prep\_A | 2 | CreE | 11 | 0.5 | 10.9959451599412 | 0.50365671863624 | -0.00365671863623984 |
| Array\_2 | Prep\_A | 2 | CreE | 11 | 0.5 | 11.072084836787 | 0.464250924745365 | 0.0357490752546352 |
| Array\_2 | Prep\_A | 1 | CreE | 11 | 0.5 | 10.9832572740144 | 0.514335574697776 | -0.0143355746977758 |
| Array\_1 | Prep\_A | 2 | CreJ | 11 | 0.5 | 10.9948333188197 | 0.666585811828927 | -0.166585811828927 |
| Array\_1 | Prep\_A | 1 | CreJ | 11 | 0.5 | 11.007007408549 | 0.564786339602208 | -0.0647863396022076 |
| Array\_2 | Prep\_A | 1 | CreE | 2 | 4 | 2.01935614349209 | 4.22858933477565 | -0.228589334775646 |
| Array\_2 | Prep\_A | 2 | CreE | 2 | 4 | 2.01479110750504 | 4.25260523128524 | -0.252605231285235 |
| Array\_2 | Prep\_A | 1 | CreJ | 2 | 4 | 2.0204443384632498 | 4.43336680793554 | -0.433366807935538 |
| Array\_2 | Prep\_A | 2 | CreJ | 2 | 4 | 2.0158793024762 | 4.37643794389555 | -0.376437943895549 |
| Array\_1 | Prep\_A | 1 | CreJ | 2 | 4 | 2.01655856801766 | 4.40801827756205 | -0.408018277562054 |
| Array\_1 | Prep\_A | 2 | CreJ | 2 | 4 | 1.99045026971308 | 4.43872898172297 | -0.438728981722973 |
| Array\_1 | Prep\_A | 2 | CreE | 2 | 4 | 1.98935882020432 | 4.26950060084429 | -0.269500600844285 |
| Array\_1 | Prep\_A | 1 | CreE | 2 | 4 | 2.0154671185089 | 4.24146911650627 | -0.241469116506265 |
| Array\_2 | Prep\_A | 1 | CreJ | 25 | 2 | 25.0723835943197 | 2.1826636970594 | -0.182663697059398 |
| Array\_2 | Prep\_A | 2 | CreJ | 25 | 2 | 25.2015314618893 | 2.22390501606822 | -0.223905016068219 |
| Array\_2 | Prep\_A | 2 | CreE | 25 | 2 | 25.203375006719 | 2.29652532985954 | -0.296525329859537 |
| Array\_2 | Prep\_A | 1 | CreE | 25 | 2 | 25.0742271391494 | 2.33719948683957 | -0.337199486839567 |
| Array\_1 | Prep\_A | 2 | CreE | 25 | 2 | 25.0045401025494 | 2.32915128307914 | -0.329151283079137 |
| Array\_1 | Prep\_A | 1 | CreE | 25 | 2 | 25.0458935249157 | 2.36289823605139 | -0.362898236051387 |
| Array\_1 | Prep\_A | 1 | CreJ | 25 | 2 | 25.0440502767652 | 2.23557369460966 | -0.235573694609659 |
| Array\_1 | Prep\_A | 2 | CreJ | 25 | 2 | 25.0026968543989 | 2.19662137781965 | -0.196621377819648 |
| Array\_1 | Prep\_A | 2 | CreJ | 12 | 0.5 | 12.0455706091362 | 0.615464653709074 | -0.115464653709074 |
| Array\_1 | Prep\_A | 1 | CreJ | 12 | 0.5 | 11.9617197182765 | 0.689206668422496 | -0.189206668422496 |
| Array\_1 | Prep\_A | 2 | CreE | 12 | 0.5 | 12.0466831459728 | 0.499814524998832 | 1.8547500116827e-4 |
| Array\_1 | Prep\_A | 1 | CreE | 12 | 0.5 | 11.9628322551131 | 0.474154683096058 | 0.0258453169039423 |
| Array\_2 | Prep\_A | 2 | CreJ | 12 | 0.5 | 11.9870456527447 | 0.617532380095351 | -0.117532380095351 |
| Array\_2 | Prep\_A | 1 | CreJ | 12 | 0.5 | 12.047175590169 | 0.602081224438085 | -0.102081224438085 |
| Array\_2 | Prep\_A | 2 | CreE | 12 | 0.5 | 11.9881567845129 | 0.518495895599886 | -0.0184958955998857 |
| Array\_2 | Prep\_A | 1 | CreE | 12 | 0.5 | 12.0482867219372 | 0.510928738994751 | -0.0109287389947508 |
| Array\_2 | Prep\_A | 2 | CreE | 3 | 4 | 3.00303943496544 | 4.26714837141069 | -0.26714837141069 |
| Array\_2 | Prep\_A | 1 | CreE | 3 | 4 | 3.02607182567109 | 4.24179593663463 | -0.24179593663463 |
| Array\_1 | Prep\_A | 2 | CreE | 3 | 4 | 2.99546517017109 | 4.2360897290396 | -0.2360897290396 |
| Array\_1 | Prep\_A | 1 | CreE | 3 | 4 | 3.04512201073932 | 4.2441553005024 | -0.2441553005024 |
| Array\_2 | Prep\_A | 2 | CreJ | 3 | 4 | 3.00413750865732 | 4.41021401915597 | -0.410214019155966 |
| Array\_2 | Prep\_A | 1 | CreJ | 3 | 4 | 3.02716989936297 | 4.48563707069234 | -0.485637070692335 |
| Array\_1 | Prep\_A | 1 | CreJ | 3 | 4 | 3.0462141312661 | 4.42065187691642 | -0.420651876916418 |
| Array\_1 | Prep\_A | 2 | CreJ | 3 | 4 | 2.99655729069787 | 4.40630773408727 | -0.406307734087267 |
| Array\_2 | Prep\_A | 2 | CreJ | 26 | 2 | 26.0226029097432 | 2.2225048918615 | -0.222504891861504 |
| Array\_2 | Prep\_A | 1 | CreJ | 26 | 2 | 26.1353205189347 | 2.22452099486907 | -0.224520994869074 |
| Array\_1 | Prep\_A | 1 | CreJ | 26 | 2 | 26.1661059064677 | 2.21476222557166 | -0.214762225571663 |
| Array\_1 | Prep\_A | 2 | CreJ | 26 | 2 | 25.9890393593011 | 2.24616007685437 | -0.246160076854373 |
| Array\_2 | Prep\_A | 1 | CreE | 26 | 2 | 26.1371636743423 | 2.39570545456989 | -0.395705454569886 |
| Array\_2 | Prep\_A | 2 | CreE | 26 | 2 | 26.0244460651508 | 2.3564289179178 | -0.356428917917796 |
| Array\_1 | Prep\_A | 2 | CreE | 26 | 2 | 25.9908823386806 | 2.33388505758897 | -0.33388505758896603 |
| Array\_1 | Prep\_A | 1 | CreE | 26 | 2 | 26.1679488858472 | 2.34039962112224 | -0.340399621122236 |
| Array\_1 | Prep\_A | 2 | CreJ | 13 | 0.5 | 13.0367156465639 | 0.585245781769273 | -0.0852457817692729 |
| Array\_1 | Prep\_A | 1 | CreJ | 13 | 0.5 | 13.0064143303294 | 0.58202990288429 | -0.0820299028842899 |
| Array\_2 | Prep\_A | 1 | CreJ | 13 | 0.5 | 13.0665849575943 | 0.617390191257375 | -0.117390191257375 |
| Array\_2 | Prep\_A | 2 | CreJ | 13 | 0.5 | 13.0966999273588 | 0.623108959327532 | -0.123108959327532 |
| Array\_2 | Prep\_A | 1 | CreE | 13 | 0.5 | 13.0676956049281 | 0.50399402828763 | -0.0039940282876304 |
| Array\_2 | Prep\_A | 2 | CreE | 13 | 0.5 | 13.0978105746926 | 0.47248849591909 | 0.0275115040809096 |
| Array\_1 | Prep\_A | 2 | CreE | 13 | 0.5 | 13.0378247870019 | 0.552231871552911 | -0.0522318715529114 |
| Array\_1 | Prep\_A | 1 | CreE | 13 | 0.5 | 13.0075234707674 | 0.47398915168704 | 0.0260108483129596 |
| Array\_2 | Prep\_A | 2 | CreJ | 4 | 4 | 3.98066153545955 | 4.47311680193506 | -0.473116801935061 |
| Array\_2 | Prep\_A | 1 | CreJ | 4 | 4 | 4.04969557188391 | 4.46493179832033 | -0.464931798320331 |
| Array\_1 | Prep\_A | 2 | CreJ | 4 | 4 | 3.9663435780307 | 4.45987187629608 | -0.459871876296077 |
| Array\_1 | Prep\_A | 1 | CreJ | 4 | 4 | 4.04359007394619 | 4.50228650115759 | -0.502286501157587 |
| Array\_2 | Prep\_A | 1 | CreE | 4 | 4 | 4.04858893968247 | 4.24897844087587 | -0.248978440875867 |
| Array\_2 | Prep\_A | 2 | CreE | 4 | 4 | 3.9795549032581 | 4.23162267191812 | -0.231622671918116 |
| Array\_1 | Prep\_A | 2 | CreE | 4 | 4 | 3.96523482036359 | 4.2539082114686 | -0.253908211468596 |
| Array\_1 | Prep\_A | 1 | CreE | 4 | 4 | 4.04248131627909 | 4.24293104233066 | -0.242931042330657 |
| Array\_2 | Prep\_A | 1 | CreJ | 27 | 2 | 27.0090860734972 | 2.20877137254658 | -0.208771372546575 |
| Array\_2 | Prep\_A | 2 | CreJ | 27 | 2 | 27.1210479709712 | 2.19680769368488 | -0.196807693684885 |
| Array\_2 | Prep\_A | 1 | CreE | 27 | 2 | 27.010929731974 | 2.36870663159618 | -0.368706631596184 |
| Array\_2 | Prep\_A | 2 | CreE | 27 | 2 | 27.122891629448 | 2.39924282075036 | -0.399242820750364 |
| Array\_1 | Prep\_A | 2 | CreJ | 27 | 2 | 26.9830127076762 | 2.23493973794612 | -0.234939737946121 |
| Array\_1 | Prep\_A | 1 | CreJ | 27 | 2 | 26.8401675645598 | 2.21346903925774 | -0.213469039257741 |
| Array\_1 | Prep\_A | 1 | CreE | 27 | 2 | 26.8420107928233 | 2.34225693188014 | -0.342256931880144 |
| Array\_1 | Prep\_A | 2 | CreE | 27 | 2 | 26.9848559359397 | 2.3667223467285 | -0.366722346728504 |
| Array\_1 | Prep\_A | 2 | CreJ | 14 | 0.5 | 14.0091715894993 | 0.556226140277487 | -0.0562261402774867 |
| Array\_1 | Prep\_A | 1 | CreJ | 14 | 0.5 | 13.9965557201149 | 0.641044368831978 | -0.141044368831978 |
| Array\_2 | Prep\_A | 1 | CreJ | 14 | 0.5 | 14.0451702516336 | 0.619702025330787 | -0.119702025330786 |
| Array\_2 | Prep\_A | 2 | CreJ | 14 | 0.5 | 13.8939244394048 | 0.532537488114793 | -0.0325374881147925 |
| Array\_1 | Prep\_A | 2 | CreE | 14 | 0.5 | 14.0102804865798 | 0.520352510087592 | -0.0203525100875922 |
| Array\_1 | Prep\_A | 1 | CreE | 14 | 0.5 | 13.9976646171954 | 0.513396969913954 | -0.0133969699139542 |
| Array\_2 | Prep\_A | 2 | CreE | 14 | 0.5 | 13.8950323083639 | 0.521179008143062 | -0.0211790081430622 |
| Array\_2 | Prep\_A | 1 | CreE | 14 | 0.5 | 14.0462781205927 | 0.537646685657659 | -0.0376466856576592 |
| Array\_1 | Prep\_A | 2 | CreE | 5 | 4 | 5.02936919539691 | 4.23202438560373 | -0.232024385603732 |
| Array\_1 | Prep\_A | 1 | CreE | 5 | 4 | 5.00399071842502 | 4.26669912103526 | -0.266699121035262 |
| Array\_2 | Prep\_A | 2 | CreJ | 5 | 4 | 5.04108813293696 | 4.40455428105497 | -0.404554281054971 |
| Array\_2 | Prep\_A | 1 | CreJ | 5 | 4 | 5.04502831740167 | 4.46185985941803 | -0.461859859418031 |
| Array\_1 | Prep\_A | 1 | CreJ | 5 | 4 | 5.00509224449587 | 4.42175850002419 | -0.421758500024192 |
| Array\_1 | Prep\_A | 2 | CreJ | 5 | 4 | 5.03047072146776 | 4.48350737194481 | -0.483507371944812 |
| Array\_2 | Prep\_A | 1 | CreE | 5 | 4 | 5.04393011773091 | 4.26760458058425 | -0.267604580584252 |
| Array\_2 | Prep\_A | 2 | CreE | 5 | 4 | 5.0399899332662 | 4.28588425541256 | -0.285884255412562 |
| Array\_2 | Prep\_A | 1 | CreJ | 28 | 2 | 28.1456299824641 | 2.14167795832518 | -0.141677958325185 |
| Array\_2 | Prep\_A | 2 | CreJ | 28 | 2 | 28.0025503860298 | 2.15453644922666 | -0.154536449226665 |
| Array\_1 | Prep\_A | 1 | CreE | 28 | 2 | 27.6592431770126 | 2.41484974466933 | -0.414849744669326 |
| Array\_1 | Prep\_A | 2 | CreE | 28 | 2 | 27.9149547639907 | 2.39500482072902 | -0.395004820729016 |
| Array\_1 | Prep\_A | 2 | CreJ | 28 | 2 | 27.9131117255545 | 2.22379764029576 | -0.223797640295757 |
| Array\_1 | Prep\_A | 1 | CreJ | 28 | 2 | 27.6574001385764 | 2.22047486408794 | -0.220474864087937 |
| Array\_2 | Prep\_A | 2 | CreE | 28 | 2 | 28.0043945496649 | 2.38374300085815 | -0.383743000858146 |
| Array\_2 | Prep\_A | 1 | CreE | 28 | 2 | 28.1474741460992 | 2.41145559357723 | -0.411455593577226 |
| Array\_1 | Prep\_A | 2 | CreJ | 15 | 0.5 | 15.0617951861229 | 0.585747033457024 | -0.0857470334570241 |
| Array\_1 | Prep\_A | 1 | CreJ | 15 | 0.5 | 14.966086044251 | 0.575115852953846 | -0.075115852953846 |
| Array\_1 | Prep\_A | 2 | CreE | 15 | 0.5 | 15.0629025369824 | 0.56334490795076 | -0.0633449079507598 |
| Array\_1 | Prep\_A | 1 | CreE | 15 | 0.5 | 14.9671933951105 | 0.54165478069661 | -0.0416547806966097 |
| Array\_2 | Prep\_A | 1 | CreE | 15 | 0.5 | 14.963099058236 | 0.52559817689176 | -0.0255981768917597 |
| Array\_2 | Prep\_A | 2 | CreE | 15 | 0.5 | 15.0178648081239 | 0.465495363628831 | 0.0345046363711693 |
| Array\_2 | Prep\_A | 1 | CreJ | 15 | 0.5 | 14.9619922607732 | 0.560654333603878 | -0.060654333603878 |
| Array\_2 | Prep\_A | 2 | CreJ | 15 | 0.5 | 15.0167580106611 | 0.57826434712599 | -0.07826434712599 |
| Array\_2 | Prep\_A | 2 | CreE | 6 | 4 | 5.9980764920208 | 4.29603834254131 | -0.296038342541306 |
| Array\_2 | Prep\_A | 1 | CreE | 6 | 4 | 6.08586290164389 | 4.29707007983743 | -0.297070079837426 |
| Array\_1 | Prep\_A | 2 | CreJ | 6 | 4 | 5.99829037216625 | 4.50685749285627 | -0.506857492856271 |
| Array\_1 | Prep\_A | 1 | CreJ | 6 | 4 | 5.99244096878259 | 4.4331658587233 | -0.433165858723301 |
| Array\_2 | Prep\_A | 2 | CreJ | 6 | 4 | 5.99916975997826 | 4.41182875598427 | -0.411828755984274 |
| Array\_2 | Prep\_A | 1 | CreJ | 6 | 4 | 6.08695616960135 | 4.47550960870128 | -0.475509608701285 |
| Array\_1 | Prep\_A | 2 | CreE | 6 | 4 | 5.99719258456647 | 4.28335787288326 | -0.283357872883256 |
| Array\_1 | Prep\_A | 1 | CreE | 6 | 4 | 5.99134318118281 | 4.29531732914048 | -0.295317329140476 |
| Array\_2 | Prep\_A | 2 | CreE | 29 | 2 | 29.048000082802 | 2.38329208478936 | -0.383292084789358 |
| Array\_2 | Prep\_A | 1 | CreE | 29 | 2 | 29.0628806639134 | 2.40055276001808 | -0.400552760018079 |
| Array\_1 | Prep\_A | 2 | CreJ | 29 | 2 | 29.0709058814288 | 2.20301214517192 | -0.20301214517192 |
| Array\_1 | Prep\_A | 1 | CreJ | 29 | 2 | 29.1957615195311 | 2.2306716278825 | -0.2306716278825 |
| Array\_1 | Prep\_A | 1 | CreE | 29 | 2 | 29.1976055896205 | 2.39187074940496 | -0.391870749404958 |
| Array\_1 | Prep\_A | 2 | CreE | 29 | 2 | 29.0727499515182 | 2.407478819378 | -0.407478819377999 |
| Array\_2 | Prep\_A | 2 | CreJ | 29 | 2 | 29.0461561294422 | 2.24551379510628 | -0.245513795106283 |
| Array\_2 | Prep\_A | 1 | CreJ | 29 | 2 | 29.0610367105536 | 2.19957143757488 | -0.199571437574883 |
| Array\_2 | Prep\_A | 1 | CreE | 16 | 0.5 | 15.9958384920153 | 0.511085046001463 | -0.0110850460014629 |
| Array\_2 | Prep\_A | 2 | CreE | 16 | 0.5 | 15.9838159949649 | 0.537283098933529 | -0.0372830989335289 |
| Array\_2 | Prep\_A | 2 | CreJ | 16 | 0.5 | 15.9827084874515 | 0.632943346860887 | -0.132943346860887 |
| Array\_2 | Prep\_A | 1 | CreJ | 16 | 0.5 | 15.9947309845019 | 0.548012133081606 | -0.0480121330816059 |
| Array\_1 | Prep\_A | 2 | CreE | 16 | 0.5 | 16.0070275199123 | 0.571077062560911 | -0.071077062560911 |
| Array\_1 | Prep\_A | 1 | CreE | 16 | 0.5 | 16.2439525993622 | 0.533385077566058 | -0.0333850775660579 |
| Array\_1 | Prep\_A | 1 | CreJ | 16 | 0.5 | 16.2428445302663 | 0.565202003307441 | -0.0652020033074413 |
| Array\_1 | Prep\_A | 2 | CreJ | 16 | 0.5 | 16.0059194508164 | 0.638289923345002 | -0.138289923345002 |
| Array\_2 | Prep\_A | 1 | CreJ | 7 | 4 | 7.00969952646319 | 4.4588595817168 | -0.458859581716795 |
| Array\_2 | Prep\_A | 2 | CreJ | 7 | 4 | 7.0127241157021 | 4.44757002244473 | -0.447570022444725 |
| Array\_2 | Prep\_A | 2 | CreE | 7 | 4 | 7.01162959865422 | 4.31720019704716 | -0.317200197047156 |
| Array\_2 | Prep\_A | 1 | CreE | 7 | 4 | 7.00860500941531 | 4.31473097696113 | -0.314730976961125 |
| Array\_1 | Prep\_A | 1 | CreE | 7 | 4 | 7.01751781464709 | 4.30869459976034 | -0.308694599760336 |
| Array\_1 | Prep\_A | 2 | CreE | 7 | 4 | 6.98478073063093 | 4.28032615944899 | -0.280326159448986 |
| Array\_1 | Prep\_A | 2 | CreJ | 7 | 4 | 6.98586965143064 | 4.40878836706448 | -0.40878836706448 |
| Array\_1 | Prep\_A | 1 | CreJ | 7 | 4 | 7.0186067354468 | 4.42605233278785 | -0.42605233278785 |
| Array\_2 | Prep\_A | 2 | CreE | 30 | 2 | 30.0598527606048 | 2.42225091413161 | -0.422250914131612 |
| Array\_2 | Prep\_A | 1 | CreE | 30 | 2 | 30.1722737942669 | 2.38606478950995 | -0.386064789509952 |
| Array\_1 | Prep\_A | 2 | CreE | 30 | 2 | 30.0093623353696 | 2.39825239702428 | -0.398252397024282 |
| Array\_1 | Prep\_A | 1 | CreE | 30 | 2 | 30.061860901061 | 2.42726414038779 | -0.427264140387792 |
| Array\_1 | Prep\_A | 1 | CreJ | 30 | 2 | 30.0600151278428 | 2.23383681084631 | -0.233836810846312 |
| Array\_1 | Prep\_A | 2 | CreJ | 30 | 2 | 30.0075165621514 | 2.17415839975616 | -0.174158399756162 |
| Array\_2 | Prep\_A | 2 | CreJ | 30 | 2 | 30.058006293732 | 2.0962004524514 | -0.0962004524514031 |
| Array\_2 | Prep\_A | 1 | CreJ | 30 | 2 | 30.1704273273941 | 2.17447311752863 | -0.174473117528633 |
| Array\_2 | Prep\_A | 1 | CreE | 17 | 0.5 | 17.0263702792858 | 0.526798471048461 | -0.0267984710484609 |
| Array\_2 | Prep\_A | 2 | CreE | 17 | 0.5 | 16.9715682112488 | 0.536463917513813 | -0.0364639175138128 |
| Array\_2 | Prep\_A | 1 | CreJ | 17 | 0.5 | 17.0252610366611 | 0.620149271662586 | -0.120149271662586 |
| Array\_2 | Prep\_A | 2 | CreJ | 17 | 0.5 | 16.9704589686241 | 0.620263695890493 | -0.120263695890493 |
| Array\_1 | Prep\_A | 2 | CreJ | 17 | 0.5 | 17.0191417602828 | 0.545370917119946 | -0.0453709171199458 |
| Array\_1 | Prep\_A | 1 | CreJ | 17 | 0.5 | 16.9836886043661 | 0.63529451213403 | -0.13529451213403 |
| Array\_1 | Prep\_A | 1 | CreE | 17 | 0.5 | 16.983648458931 | 0.541045629544517 | -0.0410456295445169 |
| Array\_1 | Prep\_A | 2 | CreE | 17 | 0.5 | 17.0191016148477 | 0.555131472114993 | -0.0551314721149928 |
| Array\_2 | Prep\_A | 2 | CreJ | 8 | 4 | 8.07515760042338 | 4.48625709748584 | -0.486257097485844 |
| Array\_2 | Prep\_A | 1 | CreJ | 8 | 4 | 8.00380145419493 | 4.4993128761295 | -0.499312876129504 |
| Array\_2 | Prep\_A | 2 | CreE | 8 | 4 | 8.07405566848656 | 4.32831927668016 | -0.328319276680163 |
| Array\_2 | Prep\_A | 1 | CreE | 8 | 4 | 8.00269952225811 | 4.32933523099115 | -0.329335230991153 |
| Array\_1 | Prep\_A | 2 | CreJ | 8 | 4 | 7.95618532321449 | 4.49804898969858 | -0.498048989698578 |
| Array\_1 | Prep\_A | 1 | CreJ | 8 | 4 | 8.00777086850374 | 4.43286788035039 | -0.432867880350388 |
| Array\_1 | Prep\_A | 1 | CreE | 8 | 4 | 8.00667297375667 | 4.32574770020526 | -0.325747700205263 |
| Array\_1 | Prep\_A | 2 | CreE | 8 | 4 | 7.95508742846742 | 4.33480124581763 | -0.334801245817633 |
| Array\_2 | Prep\_A | 2 | CreE | 31 | 2 | 30.9143072102308 | 2.42408558168993 | -0.424085581689929 |
| Array\_2 | Prep\_A | 1 | CreE | 31 | 2 | 31.3892846152146 | 2.41078890725483 | -0.410788907254828 |
| Array\_1 | Prep\_A | 2 | CreE | 31 | 2 | 30.9711892441469 | 2.44353739519057 | -0.443537395190568 |
| Array\_1 | Prep\_A | 1 | CreE | 31 | 2 | 30.8366337417029 | 2.43808027651411 | -0.438080276514108 |
| Array\_2 | Prep\_A | 2 | CreJ | 31 | 2 | 30.9124605629254 | 2.15342602970082 | -0.153426029700815 |
| Array\_2 | Prep\_A | 1 | CreJ | 31 | 2 | 31.3874379679092 | 2.1709320328892 | -0.170932032889195 |
| Array\_1 | Prep\_A | 2 | CreJ | 31 | 2 | 30.9693424787836 | 2.15046513259367 | -0.150465132593669 |
| Array\_1 | Prep\_A | 1 | CreJ | 31 | 2 | 30.8347869763396 | 2.151254530576 | -0.151254530575998 |
| Array\_2 | Prep\_A | 1 | CreJ | 18 | 0.5 | 18.1108887986678 | 0.560963575406175 | -0.0609635754061747 |
| Array\_2 | Prep\_A | 2 | CreJ | 18 | 0.5 | 18.1657731759969 | 0.6435304818983 | -0.1435304818983 |
| Array\_2 | Prep\_A | 1 | CreE | 18 | 0.5 | 18.1118764324459 | 0.512685031901079 | -0.0126850319010789 |
| Array\_2 | Prep\_A | 2 | CreE | 18 | 0.5 | 18.166760809775 | 0.609144821584332 | -0.109144821584332 |
| Array\_1 | Prep\_A | 2 | CreE | 18 | 0.5 | 17.9713137816786 | 0.602730406153779 | -0.102730406153779 |
| Array\_1 | Prep\_A | 1 | CreE | 18 | 0.5 | 17.9206877917801 | 0.58483884882128 | -0.0848388488212799 |
| Array\_1 | Prep\_A | 1 | CreJ | 18 | 0.5 | 17.9225485998672 | 0.589647634491866 | -0.0896476344918656 |
| Array\_1 | Prep\_A | 2 | CreJ | 18 | 0.5 | 17.9731745897657 | 0.553456129304085 | -0.0534561293040846 |
| Array\_2 | Prep\_A | 2 | CreE | 9 | 4 | 9.01984894308119 | 4.29101788015023 | -0.291017880150233 |
| Array\_2 | Prep\_A | 1 | CreE | 9 | 4 | 8.95609792321245 | 4.30108432041733 | -0.301084320417333 |
| Array\_1 | Prep\_A | 1 | CreJ | 9 | 4 | 8.94839837765285 | 4.38755325173749 | -0.387553251737493 |
| Array\_1 | Prep\_A | 2 | CreJ | 9 | 4 | 9.02464799328541 | 4.53665412940963 | -0.536654129409633 |
| Array\_1 | Prep\_A | 1 | CreE | 9 | 4 | 8.9472948777471 | 4.34340196301016 | -0.343401963010162 |
| Array\_1 | Prep\_A | 2 | CreE | 9 | 4 | 9.02354449337966 | 4.35108194945555 | -0.351081949455553 |
| Array\_2 | Prep\_A | 1 | CreJ | 9 | 4 | 8.95720943838395 | 4.52668083775785 | -0.526680837757853 |
| Array\_2 | Prep\_A | 2 | CreJ | 9 | 4 | 9.02096045825269 | 4.50327477252955 | -0.503274772529553 |
| Array\_2 | Prep\_A | 2 | CreJ | 32 | 2 | 31.9702387693546 | 2.16933490146587 | -0.169334901465872 |
| Array\_2 | Prep\_A | 1 | CreJ | 32 | 2 | 32.139862875977 | 2.20768975589386 | -0.207689755893862 |
| Array\_1 | Prep\_A | 2 | CreJ | 32 | 2 | 31.7625024154108 | 2.16508458371077 | -0.165084583710767 |
| Array\_1 | Prep\_A | 1 | CreJ | 32 | 2 | 32.1517850521398 | 2.20533801791318 | -0.205338017913177 |
| Array\_2 | Prep\_A | 1 | CreE | 32 | 2 | 32.1417136385851 | 2.41067704078552 | -0.410677040785519 |
| Array\_2 | Prep\_A | 2 | CreE | 32 | 2 | 31.9720895319627 | 2.42816001424685 | -0.428160014246849 |
| Array\_1 | Prep\_A | 2 | CreE | 32 | 2 | 31.7643532172718 | 2.40553326205546 | -0.405533262055459 |
| Array\_1 | Prep\_A | 1 | CreE | 32 | 2 | 32.1536358540008 | 2.37245727618462 | -0.372457276184619 |
