# Supplementary material for: Creatinine assay interferences compromises MELD accuracy and may bias liver allocation
Source: Nat Commun. 2026 Jul 23;17:7111. doi: 10.1038/s41467-026-75011-x (PMC13396164; doi:10.1038/s41467-026-75011-x)
Supplement: Supplementary file 4 — Source Data [file 41467_2026_75011_MOESM4_ESM.zip › figshare_package_FINAL_PUBLIC_DEPOSIT_V1_20260503_002637/00_START_HERE_HTML_NAVIGATOR/file_views/view_0019_esld_F5_stratified_survival_subject_public.html]

02\_workflows/F5\_workflow\_v01/submission\_ready/public/data/esld\_F5\_stratified\_survival\_subject\_public.csv

# Readable file view

02\_workflows/F5\_workflow\_v01/submission\_ready/public/data/esld\_F5\_stratified\_survival\_subject\_public.csv

← Back to navigator   |   Open original package file

Section

Manuscript output data

Output

F5

Extension

csv

Size KB

119.284

Variables

19

## Variables in this file

| Variable | Label | Description | Unit | Type |
| --- | --- | --- | --- | --- |
| albumin | Serum albumin concentration | Serum albumin concentration used in the public ESLD stratified survival dataset. | g/dL | numeric |
| bilirubin | Total bilirubin concentration | Total bilirubin concentration used in the public ESLD analysis data. | mg/dL | numeric |
| case\_id | Public case pseudonym | Non-linkable public-release case pseudonym used for case-level grouping; not an original hospital case identifier. |  | integer |
| creatinine\_corrected | Corrected creatinine concentration | Corrected creatinine concentration used for recalculated MELD-related scores. | mg/dL | numeric |
| creatinine\_raw | Raw creatinine concentration | Raw measured creatinine concentration before applying the study-specific correction. | mg/dL | numeric |
| delta.grp | Grouped score-delta comparison group | Grouped score-delta category used in F5 stratified survival analyses. pm denotes the reference group without a negative score shift in grouped score-delta survival outputs; in class-level score-shift summaries it represents the no-decrease/reference category used by the workflow. m1 denotes the negative score-shift group. In class-level score-shift summaries it denotes the minus-one score-shift class; in grouped survival outputs it is the negative-shift comparison group used by the workflow. |  | character |
| delta\_group\_label | Displayed score-delta group label | Human-readable version of the grouped score-delta category displayed in the stratified survival figure. It should be read together with delta.grp: pm is the reference/no-negative-score-shift group and m1 is the negative-score-shift comparison group. |  | character |
| dialysis | Dialysis indicator | Binary dialysis indicator used for MELD-related score calculation or stratified public analyses. |  | integer |
| inr | International normalized ratio | International normalized ratio of prothrombin time used for MELD-related score calculation. | ratio | numeric |
| model | MELD model or score variant | Name of the MELD-related model or score variant represented by the row; expected values include MELD, MELD-Na, reMELD-Na, and MELD 3.0. |  | character |
| patient\_id | Public patient pseudonym | Non-linkable public-release patient pseudonym used to preserve within-patient grouping in public ESLD data; not an original hospital patient identifier. |  | character |
| score\_class | Score class | Categorical score class used for stratified analyses. |  | character |
| score\_for\_class | Score used for class assignment | Score value used to assign the row to a score class. | score points | integer |
| scr.grp | Score-class stratum | Encoded score-class stratum used in F5; examples include le15, 16to25, and gt25. |  | character |
| sex | Sex | Sex category represented in the public ESLD data. |  | character |
| sodium | Serum sodium concentration | Serum sodium concentration used for MELD-Na, reMELD-Na, or MELD 3.0 score calculation. | mmol/L | numeric |
| source\_file | Source file | Name of the source file used to create the public row or object. |  | character |
| status | Status flag | Binary or categorical status flag used in a public QC, manifest, or analysis table. |  | integer |
| time | Analysis time | Analysis-specific time scale; interpretation depends on the respective figure/table workflow. |  | integer |

## Readable HTML view

Showing all 841 rows.

| case\_id | patient\_id | model | creatinine\_raw | creatinine\_corrected | inr | bilirubin | sodium | albumin | sex | dialysis | delta.grp | scr.grp | time | status | score\_class | delta\_group\_label | source\_file | score\_for\_class |
| --- | --- | --- | --- | --- | --- | --- | --- | --- | --- | --- | --- | --- | --- | --- | --- | --- | --- | --- |
| 1 | P001277 | MELD | 0.68 | 0.698460325475166 | 1.15 | 0.5 |  |  | M | 0 | pm | le15 | 1 | 1 | ≤ 15 | Δ score = ± 0 | esld\_master\_long\_public.csv | 8 |
| 2 | P000525 | MELD | 1.02 | 1.02098453959733 | 1.22 | 1.7 |  |  | M | 0 | pm | le15 | 6 | 1 | ≤ 15 | Δ score = ± 0 | esld\_master\_long\_public.csv | 11 |
| 3 | P000302 | MELD | 0.64 | 0.657275352328835 | 1.79 | 0.6 |  |  | F | 0 | pm | le15 | 7 | 1 | ≤ 15 | Δ score = ± 0 | esld\_master\_long\_public.csv | 13 |
| 4 | P001040 | MELD | 1.55 | 1.54121277346097 | 1.23 | 0.86 |  |  | F | 0 | pm | le15 | 7 | 1 | ≤ 15 | Δ score = ± 0 | esld\_master\_long\_public.csv | 13 |
| 5 | P000988 | MELD | 1.05 | 1.05977483679343 | 1.69 | 0.87 |  |  | M | 0 | pm | le15 | 13 | 1 | ≤ 15 | Δ score = ± 0 | esld\_master\_long\_public.csv | 13 |
| 6 | P000384 | MELD | 0.58 | 0.587866490762143 | 1.82 | 1.4 |  |  | F | 0 | pm | le15 | 15 | 1 | ≤ 15 | Δ score = ± 0 | esld\_master\_long\_public.csv | 14 |
| 7 | P000766 | MELD | 0.75 | 0.765790396402186 | 1.04 | 0.71 |  |  | M | 0 | pm | le15 | 19 | 1 | ≤ 15 | Δ score = ± 0 | esld\_master\_long\_public.csv | 7 |
| 8 | P000386 | MELD | 0.72 | 0.731402868916093 | 1.35 | 1.1 |  |  | M | 0 | pm | le15 | 24 | 1 | ≤ 15 | Δ score = ± 0 | esld\_master\_long\_public.csv | 10 |
| 9 | P000496 | MELD | 1.68 | 1.66581135829394 | 1.08 | 0.7 |  |  | F | 0 | pm | le15 | 24 | 1 | ≤ 15 | Δ score = ± 0 | esld\_master\_long\_public.csv | 12 |
| 10 | P000131 | MELD | 1.05 | 1.05597848096179 | 1.8 | 1.2 |  |  | F | 0 | pm | le15 | 25 | 1 | ≤ 15 | Δ score = ± 0 | esld\_master\_long\_public.csv | 14 |
| 11 | P000116 | MELD | 0.98 | 0.992029762213455 | 1.09 | 0.8 |  |  | M | 0 | pm | le15 | 26 | 1 | ≤ 15 | Δ score = ± 0 | esld\_master\_long\_public.csv | 7 |
| 12 | P000961 | MELD | 0.31 | 0.325840952994798 | 1.14 | 0.41 |  |  | F | 0 | pm | le15 | 28 | 1 | ≤ 15 | Δ score = ± 0 | esld\_master\_long\_public.csv | 8 |
| 13 | P000506 | MELD | 0.67 | 0.669179586872747 | 1.44 | 2.2 |  |  | M | 0 | pm | le15 | 46 | 1 | ≤ 15 | Δ score = ± 0 | esld\_master\_long\_public.csv | 13 |
| 14 | P000503 | MELD | 1.29 | 1.29508728750764 | 1.17 | 0.66 |  |  | M | 0 | pm | le15 | 120 | 1 | ≤ 15 | Δ score = ± 0 | esld\_master\_long\_public.csv | 11 |
| 15 | P000966 | MELD | 1.4 | 1.39662552916786 | 1.37 | 1.01 |  |  | M | 0 | pm | le15 | 192 | 1 | ≤ 15 | Δ score = ± 0 | esld\_master\_long\_public.csv | 13 |
| 16 | P000070 | MELD | 0.94 | 0.955059411832967 | 1.77 | 0.6 |  |  | F | 0 | pm | le15 | 294 | 1 | ≤ 15 | Δ score = ± 0 | esld\_master\_long\_public.csv | 13 |
| 17 | P000362 | MELD | 1.22 | 1.230574356631 | 1.07 | 0.4 |  |  | F | 0 | pm | le15 | 413 | 1 | ≤ 15 | Δ score = ± 0 | esld\_master\_long\_public.csv | 9 |
| 18 | P000155 | MELD | 0.405 | 0.417982545133566 | 1.8 | 0.8 |  |  | M | 0 | pm | le15 | 730 | 0 | ≤ 15 | Δ score = ± 0 | esld\_master\_long\_public.csv | 13 |
| 19 | P000218 | MELD | 1.325 | 1.33282854531669 | 1.48 | 0.315 |  |  | M | 0 | pm | le15 | 730 | 0 | ≤ 15 | Δ score = ± 0 | esld\_master\_long\_public.csv | 14 |
| 20 | P000429 | MELD | 1.22 | 1.23187807173977 | 1.16 | 0.29 |  |  | M | 0 | pm | le15 | 730 | 0 | ≤ 15 | Δ score = ± 0 | esld\_master\_long\_public.csv | 10 |
| 21 | P000497 | MELD | 0.94 | 0.954707936587699 | 1.16 | 0.63 |  |  | M | 0 | pm | le15 | 730 | 0 | ≤ 15 | Δ score = ± 0 | esld\_master\_long\_public.csv | 8 |
| 22 | P000507 | MELD | 0.64 | 0.661885818438325 | 1.33 | 0.21 |  |  | F | 0 | pm | le15 | 730 | 0 | ≤ 15 | Δ score = ± 0 | esld\_master\_long\_public.csv | 10 |
| 23 | P000576 | MELD | 1.535 | 1.5327276273706 | 1.33 | 0.37 |  |  | M | 0 | pm | le15 | 730 | 0 | ≤ 15 | Δ score = ± 0 | esld\_master\_long\_public.csv | 14 |
| 24 | P000593 | MELD | 1.07 | 1.08105354861449 | 1.44 | 0.72 |  |  | M | 0 | pm | le15 | 730 | 0 | ≤ 15 | Δ score = ± 0 | esld\_master\_long\_public.csv | 11 |
| 25 | P000718 | MELD | 0.75 | 0.769431684013923 | 1.73 | 0.4 |  |  | M | 0 | pm | le15 | 730 | 0 | ≤ 15 | Δ score = ± 0 | esld\_master\_long\_public.csv | 13 |
| 26 | P001285 | MELD | 0.9 | 0.916247241802327 | 1.23 | 0.55 |  |  | M | 0 | m1 | le15 | 5 | 1 | ≤ 15 | Δ score ≤ -1 | esld\_master\_long\_public.csv | 9 |
| 27 | P001317 | MELD | 0.82 | 0.825558269310575 | 1.57 | 1.56 |  |  | F | 0 | m1 | le15 | 13 | 1 | ≤ 15 | Δ score ≤ -1 | esld\_master\_long\_public.csv | 13 |
| 28 | P000038 | MELD | 0.47 | 0.473369420702739 | 1.64 | 1.72 |  |  | F | 0 | m1 | le15 | 40 | 1 | ≤ 15 | Δ score ≤ -1 | esld\_master\_long\_public.csv | 14 |
| 29 | P000828 | MELD | 1.38 | 1.38072286319533 | 1.39 | 0.73 |  |  | M | 0 | m1 | le15 | 64 | 1 | ≤ 15 | Δ score ≤ -1 | esld\_master\_long\_public.csv | 13 |
| 30 | P000402 | MELD | 0.23 | 0.228045643722108 | 1.24 | 1.8 |  |  | M | 0 | m1 | le15 | 66 | 1 | ≤ 15 | Δ score ≤ -1 | esld\_master\_long\_public.csv | 11 |
| 31 | P001329 | MELD | 1.23 | 1.22274385466638 | 1.27 | 1.93 |  |  | F | 0 | m1 | le15 | 73 | 1 | ≤ 15 | Δ score ≤ -1 | esld\_master\_long\_public.csv | 14 |
| 32 | P000243 | MELD | 0.795 | 0.800881059739979 | 1.4 | 1.55 |  |  | M | 0 | m1 | le15 | 119 | 1 | ≤ 15 | Δ score ≤ -1 | esld\_master\_long\_public.csv | 12 |
| 33 | P000594 | MELD | 1.06 | 1.05154308510941 | 1.24 | 2.48 |  |  | M | 0 | m1 | le15 | 298 | 1 | ≤ 15 | Δ score ≤ -1 | esld\_master\_long\_public.csv | 13 |
| 34 | P000253 | MELD | 1.44 | 1.44422391650995 | 1.28 | 0.21 |  |  | F | 0 | m1 | le15 | 601 | 1 | ≤ 15 | Δ score ≤ -1 | esld\_master\_long\_public.csv | 13 |
| 35 | P000104 | MELD | 0.98 | 0.995656984709247 | 1.6 | 0.49 |  |  | F | 0 | m1 | le15 | 730 | 0 | ≤ 15 | Δ score ≤ -1 | esld\_master\_long\_public.csv | 12 |
| 36 | P000312 | MELD | 0.53 | 0.538338983740448 | 1.36 | 1.33 |  |  | M | 0 | m1 | le15 | 730 | 0 | ≤ 15 | Δ score ≤ -1 | esld\_master\_long\_public.csv | 11 |
| 37 | P000626 | MELD | 0.68 | 0.682028708290505 | 1.48 | 1.94 |  |  | M | 0 | m1 | le15 | 730 | 0 | ≤ 15 | Δ score ≤ -1 | esld\_master\_long\_public.csv | 13 |
| 38 | P000164 | MELD | 2.3 | 2.23786201906189 | 1.495 | 0.67 |  |  | F | 0 | pm | 16to25 | 0 | 1 | 16 - 25 | Δ score = ± 0 | esld\_master\_long\_public.csv | 19 |
| 39 | P000661 | MELD | 2.02 | 1.98623796901758 | 2.14 | 0.35 |  |  | M | 0 | pm | 16to25 | 1 | 1 | 16 - 25 | Δ score = ± 0 | esld\_master\_long\_public.csv | 22 |
| 40 | P000319 | MELD | 1.38333333333333 | 1.37792174700726 | 1.59333333333333 | 1.25 |  |  | F | 0 | pm | 16to25 | 2 | 1 | 16 - 25 | Δ score = ± 0 | esld\_master\_long\_public.csv | 16 |
| 41 | P000500 | MELD | 1.46 | 1.41975252540341 | 1.935 | 4.2 |  |  | M | 0 | pm | 16to25 | 2 | 1 | 16 - 25 | Δ score = ± 0 | esld\_master\_long\_public.csv | 23 |
| 42 | P001348 | MELD | 2.92 | 2.7915702249002 | 1.13 | 0.28 |  |  | F | 0 | pm | 16to25 | 2 | 1 | 16 - 25 | Δ score = ± 0 | esld\_master\_long\_public.csv | 18 |
| 43 | P000051 | MELD | 1.83 | 1.78794862075361 | 1.2 | 2.32 |  |  | M | 0 | pm | 16to25 | 3 | 1 | 16 - 25 | Δ score = ± 0 | esld\_master\_long\_public.csv | 17 |
| 44 | P001254 | MELD | 2.02 | 1.90525228005634 | 0 | 8.65 |  |  | M | 0 | pm | 16to25 | 3 | 1 | 16 - 25 | Δ score = ± 0 | esld\_master\_long\_public.csv | 21 |
| 45 | P001301 | MELD | 2.62 | 2.49437469190787 | 1.32 | 3.37 |  |  | M | 0 | pm | 16to25 | 3 | 1 | 16 - 25 | Δ score = ± 0 | esld\_master\_long\_public.csv | 23 |
| 46 | P000665 | MELD | 2.38 | 2.30765687029206 | 1.64 | 0.87 |  |  | F | 0 | pm | 16to25 | 4 | 1 | 16 - 25 | Δ score = ± 0 | esld\_master\_long\_public.csv | 20 |
| 47 | P000943 | MELD | 3.03 | 2.8774022541663 | 1.31 | 1.07 |  |  | M | 0 | pm | 16to25 | 5 | 1 | 16 - 25 | Δ score = ± 0 | esld\_master\_long\_public.csv | 20 |
| 48 | P000396 | MELD | 0.93 | 0.917485369101848 | 1.72 | 3.1 |  |  | M | 0 | pm | 16to25 | 8 | 1 | 16 - 25 | Δ score = ± 0 | esld\_master\_long\_public.csv | 17 |
| 49 | P000903 | MELD | 0.75 | 0.733971158022278 | 2.2 | 3.62 |  |  | M | 0 | pm | 16to25 | 8 | 1 | 16 - 25 | Δ score = ± 0 | esld\_master\_long\_public.csv | 20 |
| 50 | P000210 | MELD | 3.03 | 2.88639627973574 | 1.9 | 0.3 |  |  | M | 0 | pm | 16to25 | 10 | 1 | 16 - 25 | Δ score = ± 0 | esld\_master\_long\_public.csv | 24 |
| 51 | P000598 | MELD | 0.38 | 0.318034206950338 | 1.63 | 8.5 |  |  | F | 0 | pm | 16to25 | 22 | 1 | 16 - 25 | Δ score = ± 0 | esld\_master\_long\_public.csv | 20 |
| 52 | P000157 | MELD | 1.73 | 1.68000319321039 | 1.9 | 3.7 |  |  | F | 0 | pm | 16to25 | 31 | 1 | 16 - 25 | Δ score = ± 0 | esld\_master\_long\_public.csv | 24 |
| 53 | P000065 | MELD | 1.51 | 1.50534242218578 | 1.02 | 0.68 |  |  | M | 1 | pm | 16to25 | 48 | 1 | 16 - 25 | Δ score = ± 0 | esld\_master\_long\_public.csv | 20 |
| 54 | P000274 | MELD | 1.59 | 1.56840982135296 | 1.44 | 1.8 |  |  | M | 0 | pm | 16to25 | 75 | 1 | 16 - 25 | Δ score = ± 0 | esld\_master\_long\_public.csv | 17 |
| 55 | P000523 | MELD | 1.04 | 1.0405547447764 | 2 | 1.7 |  |  | F | 0 | pm | 16to25 | 82 | 1 | 16 - 25 | Δ score = ± 0 | esld\_master\_long\_public.csv | 17 |
| 56 | P000140 | MELD | 1.11 | 1.04850589020649 | 2.26 | 8 |  |  | M | 0 | pm | 16to25 | 146 | 1 | 16 - 25 | Δ score = ± 0 | esld\_master\_long\_public.csv | 24 |
| 57 | P001060 | MELD | 2.27 | 2.20345569382691 | 1.38 | 1.3 |  |  | M | 0 | pm | 16to25 | 152 | 1 | 16 - 25 | Δ score = ± 0 | esld\_master\_long\_public.csv | 19 |
| 58 | P000169 | MELD | 1.79 | 1.77158848879538 | 1.63 | 0.47 |  |  | F | 0 | pm | 16to25 | 158 | 1 | 16 - 25 | Δ score = ± 0 | esld\_master\_long\_public.csv | 17 |
| 59 | P001208 | MELD | 1.585 | 1.55596784652806 | 2.65333333333333 | 2.505 |  |  | M | 0 | pm | 16to25 | 316 | 1 | 16 - 25 | Δ score = ± 0 | esld\_master\_long\_public.csv | 25 |
| 60 | P001162 | MELD | 1.49 | 1.46471517319051 | 1.19 | 2.61 |  |  | F | 0 | pm | 16to25 | 358 | 1 | 16 - 25 | Δ score = ± 0 | esld\_master\_long\_public.csv | 16 |
| 61 | P000015 | MELD | 1.96 | 1.87899575814967 | 1.45 | 5.24 |  |  | M | 0 | pm | 16to25 | 730 | 0 | 16 - 25 | Δ score = ± 0 | esld\_master\_long\_public.csv | 23 |
| 62 | P000035 | MELD | 1.61 | 1.59213766196358 | 1.92 | 1.37 |  |  | F | 0 | pm | 16to25 | 730 | 0 | 16 - 25 | Δ score = ± 0 | esld\_master\_long\_public.csv | 19 |
| 63 | P000073 | MELD | 1.24 | 1.22234728941079 | 1.56 | 2.86 |  |  | M | 0 | pm | 16to25 | 730 | 0 | 16 - 25 | Δ score = ± 0 | esld\_master\_long\_public.csv | 17 |
| 64 | P000091 | MELD | 1.19 | 1.19695444758187 | 2.41 | 0.79 |  |  | F | 0 | pm | 16to25 | 730 | 0 | 16 - 25 | Δ score = ± 0 | esld\_master\_long\_public.csv | 18 |
| 65 | P000108 | MELD | 2.52 | 2.4160440175503 | 1.51 | 2.38 |  |  | F | 0 | pm | 16to25 | 730 | 0 | 16 - 25 | Δ score = ± 0 | esld\_master\_long\_public.csv | 23 |
| 66 | P000220 | MELD | 0.99 | 0.991808637222224 | 1.9325 | 1.68 |  |  | F | 0 | pm | 16to25 | 730 | 0 | 16 - 25 | Δ score = ± 0 | esld\_master\_long\_public.csv | 16 |
| 67 | P000265 | MELD | 1.22 | 1.17976671415079 | 1.64 | 5.185 |  |  | F | 0 | pm | 16to25 | 730 | 0 | 16 - 25 | Δ score = ± 0 | esld\_master\_long\_public.csv | 20 |
| 68 | P000532 | MELD | 1.91 | 1.87739592391313 | 1.8 | 0.97 |  |  | F | 0 | pm | 16to25 | 730 | 0 | 16 - 25 | Δ score = ± 0 | esld\_master\_long\_public.csv | 19 |
| 69 | P000487 | MELD | 2 | 1.89272451929947 | 1.42 | 7.9 |  |  | M | 0 | m1 | 16to25 | 1 | 1 | 16 - 25 | Δ score ≤ -1 | esld\_master\_long\_public.csv | 25 |
| 70 | P000336 | MELD | 2 | 1.96487292632333 | 1.49 | 0.6 |  |  | M | 0 | m1 | 16to25 | 4 | 1 | 16 - 25 | Δ score ≤ -1 | esld\_master\_long\_public.csv | 18 |
| 71 | P000376 | MELD | 3.97 | 3.66504898584215 | 1.04 | 0.7 |  |  | F | 0 | m1 | 16to25 | 5 | 1 | 16 - 25 | Δ score ≤ -1 | esld\_master\_long\_public.csv | 20 |
| 72 | P000649 | MELD | 3.16 | 2.99253496943502 | 1.36 | 0.75 |  |  | F | 0 | m1 | 16to25 | 6 | 1 | 16 - 25 | Δ score ≤ -1 | esld\_master\_long\_public.csv | 21 |
| 73 | P000682 | MELD | 1.6 | 1.58189821954639 | 2.11 | 1.44 |  |  | M | 0 | m1 | 16to25 | 6 | 1 | 16 - 25 | Δ score ≤ -1 | esld\_master\_long\_public.csv | 21 |
| 74 | P000024 | MELD | 2.34 | 2.24487038165096 | 1.15 | 3.31 |  |  | M | 0 | m1 | 16to25 | 9 | 1 | 16 - 25 | Δ score ≤ -1 | esld\_master\_long\_public.csv | 21 |
| 75 | P000514 | MELD | 1.07 | 1.01665109671129 | 1.51 | 7.13 |  |  | M | 0 | m1 | 16to25 | 9 | 1 | 16 - 25 | Δ score ≤ -1 | esld\_master\_long\_public.csv | 19 |
| 76 | P000380 | MELD | 3.42 | 3.21183636496828 | 1.87 | 0.8 |  |  | M | 0 | m1 | 16to25 | 10 | 1 | 16 - 25 | Δ score ≤ -1 | esld\_master\_long\_public.csv | 25 |
| 77 | P000191 | MELD | 3.61 | 3.37398887833063 | 1.45 | 0.46 |  |  | M | 0 | m1 | 16to25 | 11 | 1 | 16 - 25 | Δ score ≤ -1 | esld\_master\_long\_public.csv | 23 |
| 78 | P000361 | MELD | 2.73 | 2.61842297819206 | 2.26 | 0.9 |  |  | F | 0 | m1 | 16to25 | 13 | 1 | 16 - 25 | Δ score ≤ -1 | esld\_master\_long\_public.csv | 25 |
| 79 | P001273 | MELD | 2.47 | 2.3818221593811 | 1.33 | 1.44 |  |  | M | 0 | m1 | 16to25 | 20 | 1 | 16 - 25 | Δ score ≤ -1 | esld\_master\_long\_public.csv | 20 |
| 80 | P001189 | MELD | 0.53 | 0.457931298853161 | 2.31 | 10.13 |  |  | F | 0 | m1 | 16to25 | 22 | 1 | 16 - 25 | Δ score ≤ -1 | esld\_master\_long\_public.csv | 25 |
| 81 | P001176 | MELD | 1.34 | 1.32675030514996 | 1.65 | 2.11 |  |  | M | 0 | m1 | 16to25 | 23 | 1 | 16 - 25 | Δ score ≤ -1 | esld\_master\_long\_public.csv | 18 |
| 82 | P000257 | MELD | 2.29 | 2.15275604518725 | 1.3 | 8.5 |  |  | M | 0 | m1 | 16to25 | 25 | 1 | 16 - 25 | Δ score ≤ -1 | esld\_master\_long\_public.csv | 25 |
| 83 | P000522 | MELD | 3.11666666666667 | 2.94466366091965 | 1.63 | 1.7 |  |  | M | 0 | m1 | 16to25 | 28 | 1 | 16 - 25 | Δ score ≤ -1 | esld\_master\_long\_public.csv | 25 |
| 84 | P001261 | MELD | 0.82 | 0.767854671246883 | 2.54 | 7.48 |  |  | F | 0 | m1 | 16to25 | 32 | 1 | 16 - 25 | Δ score ≤ -1 | esld\_master\_long\_public.csv | 24 |
| 85 | P000602 | MELD | 1.37 | 1.351295758735 | 1.9 | 2.5 |  |  | F | 0 | m1 | 16to25 | 35 | 1 | 16 - 25 | Δ score ≤ -1 | esld\_master\_long\_public.csv | 20 |
| 86 | P000441 | MELD | 3.11 | 2.94460386895077 | 1.83 | 1.2 |  |  | M | 0 | m1 | 16to25 | 40 | 1 | 16 - 25 | Δ score ≤ -1 | esld\_master\_long\_public.csv | 25 |
| 87 | P000750 | MELD | 2.53 | 2.43682307393645 | 1.57 | 1.31 |  |  | F | 0 | m1 | 16to25 | 40 | 1 | 16 - 25 | Δ score ≤ -1 | esld\_master\_long\_public.csv | 21 |
| 88 | P000050 | MELD | 1.82 | 1.72382661844864 | 1.07 | 8.16 |  |  | M | 0 | m1 | 16to25 | 41 | 1 | 16 - 25 | Δ score ≤ -1 | esld\_master\_long\_public.csv | 21 |
| 89 | P000179 | MELD | 0.54 | 0.499984913783214 | 1.65 | 6.1 |  |  | F | 0 | m1 | 16to25 | 46 | 1 | 16 - 25 | Δ score ≤ -1 | esld\_master\_long\_public.csv | 19 |
| 90 | P000678 | MELD | 0.81 | 0.807576356313816 | 2.22 | 2.29 |  |  | F | 0 | m1 | 16to25 | 47 | 1 | 16 - 25 | Δ score ≤ -1 | esld\_master\_long\_public.csv | 18 |
| 91 | P001159 | MELD | 1.54 | 1.47799706868196 | 1.75 | 6.09 |  |  | F | 0 | m1 | 16to25 | 47 | 1 | 16 - 25 | Δ score ≤ -1 | esld\_master\_long\_public.csv | 24 |
| 92 | P001117 | MELD | 1.9 | 1.82131333361556 | 1.65 | 5.47 |  |  | M | 0 | m1 | 16to25 | 71 | 1 | 16 - 25 | Δ score ≤ -1 | esld\_master\_long\_public.csv | 25 |
| 93 | P000292 | MELD | 1.45 | 1.44221138454306 | 1.57 | 1.2 |  |  | F | 0 | m1 | 16to25 | 81 | 1 | 16 - 25 | Δ score ≤ -1 | esld\_master\_long\_public.csv | 16 |
| 94 | P000433 | MELD | 1.92 | 1.8674565708872 | 1.15 | 2.7 |  |  | M | 0 | m1 | 16to25 | 89 | 1 | 16 - 25 | Δ score ≤ -1 | esld\_master\_long\_public.csv | 18 |
| 95 | P000297 | MELD | 0.71 | 0.623188920331771 | 1.56 | 12.54 |  |  | F | 0 | m1 | 16to25 | 110 | 1 | 16 - 25 | Δ score ≤ -1 | esld\_master\_long\_public.csv | 21 |
| 96 | P000629 | MELD | 5.96 | 5.15482505408464 | 1.43 | 0.47 |  |  | M | 0 | m1 | 16to25 | 111 | 1 | 16 - 25 | Δ score ≤ -1 | esld\_master\_long\_public.csv | 24 |
| 97 | P000092 | MELD | 1.49 | 1.45952773957017 | 1.515 | 3.1 |  |  | M | 0 | m1 | 16to25 | 113 | 1 | 16 - 25 | Δ score ≤ -1 | esld\_master\_long\_public.csv | 19 |
| 98 | P000859 | MELD | 1.2 | 1.13252039458619 | 1.9 | 8.43 |  |  | M | 0 | m1 | 16to25 | 158 | 1 | 16 - 25 | Δ score ≤ -1 | esld\_master\_long\_public.csv | 23 |
| 99 | P000189 | MELD | 2.27 | 2.19894781783425 | 2.03 | 1.7 |  |  | M | 0 | m1 | 16to25 | 163 | 1 | 16 - 25 | Δ score ≤ -1 | esld\_master\_long\_public.csv | 24 |
| 100 | P000424 | MELD | 1.94 | 1.90367789392987 | 1.49 | 1.1 |  |  | M | 0 | m1 | 16to25 | 163 | 1 | 16 - 25 | Δ score ≤ -1 | esld\_master\_long\_public.csv | 18 |
| 101 | P000159 | MELD | 2.04 | 1.97500201145955 | 1.46 | 3 |  |  | M | 0 | m1 | 16to25 | 169 | 1 | 16 - 25 | Δ score ≤ -1 | esld\_master\_long\_public.csv | 22 |
| 102 | P001116 | MELD | 0.88 | 0.81857852265579 | 2.29 | 8.56 |  |  | M | 0 | m1 | 16to25 | 189 | 1 | 16 - 25 | Δ score ≤ -1 | esld\_master\_long\_public.csv | 24 |
| 103 | P000627 | MELD | 4.08 | 3.75414377879156 | 1.19 | 0.63 |  |  | M | 0 | m1 | 16to25 | 202 | 1 | 16 - 25 | Δ score ≤ -1 | esld\_master\_long\_public.csv | 22 |
| 104 | P001266 | MELD | 12.51 | 8.40855359864775 | 1.38 | 0.25 |  |  | M | 0 | m1 | 16to25 | 249 | 1 | 16 - 25 | Δ score ≤ -1 | esld\_master\_long\_public.csv | 23 |
| 105 | P000300 | MELD | 1.77 | 1.73990009546554 | 1.465 | 1.6 |  |  | F | 0 | m1 | 16to25 | 296 | 1 | 16 - 25 | Δ score ≤ -1 | esld\_master\_long\_public.csv | 18 |
| 106 | P000858 | MELD | 0.88 | 0.835182372333962 | 2.61 | 6.54 |  |  | M | 0 | m1 | 16to25 | 551 | 1 | 16 - 25 | Δ score ≤ -1 | esld\_master\_long\_public.csv | 24 |
| 107 | P000320 | MELD | 2.88 | 2.75304803420211 | 1.21 | 0.6 |  |  | M | 0 | m1 | 16to25 | 619 | 1 | 16 - 25 | Δ score ≤ -1 | esld\_master\_long\_public.csv | 19 |
| 108 | P000440 | MELD | 3.27 | 3.06751465504505 | 1.21 | 2.4 |  |  | M | 0 | m1 | 16to25 | 658 | 1 | 16 - 25 | Δ score ≤ -1 | esld\_master\_long\_public.csv | 23 |
| 109 | P001129 | MELD | 2.03 | 1.9753641583731 | 1.31 | 2.11 |  |  | F | 0 | m1 | 16to25 | 713 | 1 | 16 - 25 | Δ score ≤ -1 | esld\_master\_long\_public.csv | 19 |
| 110 | P000011 | MELD | 3.25 | 3.04144999400473 | 1.13 | 3.26 |  |  | F | 0 | m1 | 16to25 | 730 | 0 | 16 - 25 | Δ score ≤ -1 | esld\_master\_long\_public.csv | 24 |
| 111 | P000019 | MELD | 4.16 | 3.82060446704423 | 1.55 | 0.4 |  |  | M | 0 | m1 | 16to25 | 730 | 0 | 16 - 25 | Δ score ≤ -1 | esld\_master\_long\_public.csv | 25 |
| 112 | P000115 | MELD | 3.97 | 3.65927550619595 | 1.39 | 1.2 |  |  | F | 0 | m1 | 16to25 | 730 | 0 | 16 - 25 | Δ score ≤ -1 | esld\_master\_long\_public.csv | 24 |
| 113 | P000126 | MELD | 2.21 | 2.134655635035 | 1.64 | 2.6 |  |  | M | 0 | m1 | 16to25 | 730 | 0 | 16 - 25 | Δ score ≤ -1 | esld\_master\_long\_public.csv | 23 |
| 114 | P000130 | MELD | 0.82 | 0.705314619080917 | 1.71 | 19.38 |  |  | M | 0 | m1 | 16to25 | 730 | 0 | 16 - 25 | Δ score ≤ -1 | esld\_master\_long\_public.csv | 24 |
| 115 | P000143 | MELD | 1.37 | 1.37137375331679 | 1.77 | 0.71 |  |  | M | 0 | m1 | 16to25 | 730 | 0 | 16 - 25 | Δ score ≤ -1 | esld\_master\_long\_public.csv | 16 |
| 116 | P000177 | MELD | 4.61 | 4.17197929982629 | 1.26 | 0.42 |  |  | F | 0 | m1 | 16to25 | 730 | 0 | 16 - 25 | Δ score ≤ -1 | esld\_master\_long\_public.csv | 22 |
| 117 | P000195 | MELD | 1.89 | 1.86000441431409 | 1.33 | 0.87 |  |  | F | 0 | m1 | 16to25 | 730 | 0 | 16 - 25 | Δ score ≤ -1 | esld\_master\_long\_public.csv | 16 |
| 118 | P000202 | MELD | 3.65 | 3.40654651456607 | 1.44 | 0.5 |  |  | M | 0 | m1 | 16to25 | 730 | 0 | 16 - 25 | Δ score ≤ -1 | esld\_master\_long\_public.csv | 23 |
| 119 | P000213 | MELD | 0.93 | 0.907274343761223 | 3.35 | 4.1 |  |  | F | 0 | m1 | 16to25 | 730 | 0 | 16 - 25 | Δ score ≤ -1 | esld\_master\_long\_public.csv | 25 |
| 120 | P000260 | MELD | 1.45 | 1.4022616446585 | 1.305 | 5.02 |  |  | M | 0 | m1 | 16to25 | 730 | 0 | 16 - 25 | Δ score ≤ -1 | esld\_master\_long\_public.csv | 19 |
| 121 | P000285 | MELD | 0.66 | 0.552008354530637 | 1.55 | 17.24 |  |  | M | 0 | m1 | 16to25 | 730 | 0 | 16 - 25 | Δ score ≤ -1 | esld\_master\_long\_public.csv | 22 |
| 122 | P000290 | MELD | 1.29 | 1.248138744936 | 1.82 | 5.1 |  |  | F | 0 | m1 | 16to25 | 730 | 0 | 16 - 25 | Δ score ≤ -1 | esld\_master\_long\_public.csv | 22 |
| 123 | P000321 | MELD | 2.09 | 2.03869321898837 | 1.64 | 1.37 |  |  | M | 0 | m1 | 16to25 | 730 | 0 | 16 - 25 | Δ score ≤ -1 | esld\_master\_long\_public.csv | 20 |
| 124 | P000365 | MELD | 2.225 | 2.16082941528465 | 1.67 | 1.46 |  |  | F | 0 | m1 | 16to25 | 730 | 0 | 16 - 25 | Δ score ≤ -1 | esld\_master\_long\_public.csv | 21 |
| 125 | P000454 | MELD | 2.29 | 2.21704893210422 | 1.73 | 1.7 |  |  | M | 0 | m1 | 16to25 | 730 | 0 | 16 - 25 | Δ score ≤ -1 | esld\_master\_long\_public.csv | 23 |
| 126 | P000494 | MELD | 1.26 | 1.22694053926999 | 2.405 | 4.3 |  |  | F | 0 | m1 | 16to25 | 730 | 1 | 16 - 25 | Δ score ≤ -1 | esld\_master\_long\_public.csv | 24 |
| 127 | P000561 | MELD | 2.26 | 2.18899655017485 | 1.56 | 1.78 |  |  | F | 0 | m1 | 16to25 | 730 | 0 | 16 - 25 | Δ score ≤ -1 | esld\_master\_long\_public.csv | 21 |
| 128 | P000623 | MELD | 1.41 | 1.32687198304717 | 1.69 | 9.46 |  |  | F | 0 | m1 | 16to25 | 730 | 0 | 16 - 25 | Δ score ≤ -1 | esld\_master\_long\_public.csv | 24 |
| 129 | P000692 | MELD | 1.68 | 1.6652283389116 | 2.34 | 0.75 |  |  | M | 0 | m1 | 16to25 | 730 | 0 | 16 - 25 | Δ score ≤ -1 | esld\_master\_long\_public.csv | 21 |
| 130 | P000727 | MELD | 1.89 | 1.8481156599397 | 1.42 | 1.92 |  |  | M | 0 | m1 | 16to25 | 730 | 0 | 16 - 25 | Δ score ≤ -1 | esld\_master\_long\_public.csv | 19 |
| 131 | P000794 | MELD | 2.46 | 2.38079727907689 | 1.12 | 0.75 |  |  | M | 0 | m1 | 16to25 | 730 | 0 | 16 - 25 | Δ score ≤ -1 | esld\_master\_long\_public.csv | 16 |
| 132 | P000958 | MELD | 2.8 | 2.67978185268242 | 1.6 | 0.9 |  |  | M | 0 | m1 | 16to25 | 730 | 0 | 16 - 25 | Δ score ≤ -1 | esld\_master\_long\_public.csv | 22 |
| 133 | P001067 | MELD | 0.766666666666667 | 0.748280092804634 | 3.90285714285714 | 3.84333333333333 |  |  | F | 0 | pm | gt25 | 0 | 1 | > 25 | Δ score = ± 0 | esld\_master\_long\_public.csv | 27 |
| 134 | P001324 | MELD | 4.71 | 4.11007701578553 | 4.91 | 24.05 |  |  | M | 0 | pm | gt25 | 0 | 1 | > 25 | Δ score = ± 0 | esld\_master\_long\_public.csv | 40 |
| 135 | P000844 | MELD | 1.865 | 1.81335453090402 | 2.47 | 2.99 |  |  | M | 1 | pm | gt25 | 1 | 1 | > 25 | Δ score = ± 0 | esld\_master\_long\_public.csv | 34 |
| 136 | P000264 | MELD | 6.2 | 5.31574786694408 | 2.07 | 0.7 |  |  | M | 0 | pm | gt25 | 2 | 1 | > 25 | Δ score = ± 0 | esld\_master\_long\_public.csv | 28 |
| 137 | P000480 | MELD | 2.33 | 2.23803827709083 | 2.255 | 3.1 |  |  | M | 0 | pm | gt25 | 2 | 1 | > 25 | Δ score = ± 0 | esld\_master\_long\_public.csv | 28 |
| 138 | P001158 | MELD | 2.04 | 1.98237633431562 | 2.64333333333333 | 2.31 |  |  | F | 0 | pm | gt25 | 8 | 1 | > 25 | Δ score = ± 0 | esld\_master\_long\_public.csv | 27 |
| 139 | P000389 | MELD | 3.03 | 2.87477370154041 | 5.99 | 1.3 |  |  | M | 0 | pm | gt25 | 9 | 1 | > 25 | Δ score = ± 0 | esld\_master\_long\_public.csv | 38 |
| 140 | P000485 | MELD | 6.07 | 5.177317196471 | 2.03 | 5.5 |  |  | M | 0 | pm | gt25 | 9 | 1 | > 25 | Δ score = ± 0 | esld\_master\_long\_public.csv | 34 |
| 141 | P001369 | MELD | 7.01 | 5.81433345566015 | 3.45 | 3.3 |  |  | M | 0 | pm | gt25 | 15 | 1 | > 25 | Δ score = ± 0 | esld\_master\_long\_public.csv | 38 |
| 142 | P001196 | MELD | 0.97 | 0.887587546683377 | 2.49 | 36.35 |  |  | F | 0 | pm | gt25 | 30 | 1 | > 25 | Δ score = ± 0 | esld\_master\_long\_public.csv | 30 |
| 143 | P000647 | MELD | 2.615 | 2.49987865571779 | 2.68 | 2.43 |  |  | F | 0 | pm | gt25 | 730 | 0 | > 25 | Δ score = ± 0 | esld\_master\_long\_public.csv | 30 |
| 144 | P000620 | MELD | 1.915 | 1.78274785030804 | 3.68 | 12.46 |  |  | F | 0 | m1 | gt25 | 0 | 1 | > 25 | Δ score ≤ -1 | esld\_master\_long\_public.csv | 37 |
| 145 | P000456 | MELD | 3.13 | 2.94729653449257 | 4.82 | 2.5 |  |  | F | 0 | m1 | gt25 | 1 | 1 | > 25 | Δ score ≤ -1 | esld\_master\_long\_public.csv | 38 |
| 146 | P000168 | MELD | 3.14 | 2.89139954843583 | 2.015 | 9.7 |  |  | F | 0 | m1 | gt25 | 2 | 1 | > 25 | Δ score ≤ -1 | esld\_master\_long\_public.csv | 34 |
| 147 | P000680 | MELD | 4.31 | 3.91061764448838 | 2.2 | 2.95 |  |  | F | 0 | m1 | gt25 | 3 | 1 | > 25 | Δ score ≤ -1 | esld\_master\_long\_public.csv | 33 |
| 148 | P000800 | MELD | 1.89 | 1.78178838992938 | 5.285 | 9.09 |  |  | F | 0 | m1 | gt25 | 3 | 1 | > 25 | Δ score ≤ -1 | esld\_master\_long\_public.csv | 40 |
| 149 | P000432 | MELD | 3.65 | 3.3577206142473 | 2.25 | 5.1 |  |  | M | 0 | m1 | gt25 | 4 | 1 | > 25 | Δ score ≤ -1 | esld\_master\_long\_public.csv | 34 |
| 150 | P001229 | MELD | 3.66 | 3.4166793350743 | 2.69 | 0.34 |  |  | M | 1 | m1 | gt25 | 4 | 1 | > 25 | Δ score ≤ -1 | esld\_master\_long\_public.csv | 31 |
| 151 | P001310 | MELD | 1.92 | 1.83901719263343 | 2.2 | 5.56 |  |  | M | 0 | m1 | gt25 | 4 | 1 | > 25 | Δ score ≤ -1 | esld\_master\_long\_public.csv | 28 |
| 152 | P000162 | MELD | 2.16 | 1.97602523002878 | 1.79 | 22.1 |  |  | M | 0 | m1 | gt25 | 5 | 1 | > 25 | Δ score ≤ -1 | esld\_master\_long\_public.csv | 32 |
| 153 | P001212 | MELD | 1.39 | 1.29879475437444 | 4.6 | 10.75 |  |  | M | 0 | m1 | gt25 | 6 | 1 | > 25 | Δ score ≤ -1 | esld\_master\_long\_public.csv | 36 |
| 154 | P001370 | MELD | 2.35 | 2.26243747069092 | 2.03 | 2.5 |  |  | M | 0 | m1 | gt25 | 6 | 1 | > 25 | Δ score ≤ -1 | esld\_master\_long\_public.csv | 26 |
| 155 | P001177 | MELD | 2.49 | 2.30390531507049 | 4.93 | 12.79 |  |  | M | 0 | m1 | gt25 | 7 | 1 | > 25 | Δ score ≤ -1 | esld\_master\_long\_public.csv | 40 |
| 156 | P001203 | MELD | 1.83 | 1.73740882629503 | 1.68 | 7.63 |  |  | M | 0 | m1 | gt25 | 7 | 1 | > 25 | Δ score ≤ -1 | esld\_master\_long\_public.csv | 26 |
| 157 | P001319 | MELD | 1.89 | 1.74145436066875 | 2.5 | 16.24 |  |  | F | 1 | m1 | gt25 | 7 | 1 | > 25 | Δ score ≤ -1 | esld\_master\_long\_public.csv | 40 |
| 158 | P001366 | MELD | 3.50333333333333 | 3.26899124879486 | 1.52666666666667 | 1.9 |  |  | M | 0 | m1 | gt25 | 7 | 1 | > 25 | Δ score ≤ -1 | esld\_master\_long\_public.csv | 26 |
| 159 | P000201 | MELD | 5.255 | 4.52397347601369 | 2.77 | 29.2 |  |  | F | 0 | m1 | gt25 | 9 | 1 | > 25 | Δ score ≤ -1 | esld\_master\_long\_public.csv | 40 |
| 160 | P000315 | MELD | 2.1 | 1.97754687021265 | 3.19 | 8.8 |  |  | M | 0 | m1 | gt25 | 12 | 1 | > 25 | Δ score ≤ -1 | esld\_master\_long\_public.csv | 35 |
| 161 | P001051 | MELD | 4.68 | 4.16057670476513 | 1.84 | 6.79 |  |  | M | 0 | m1 | gt25 | 12 | 1 | > 25 | Δ score ≤ -1 | esld\_master\_long\_public.csv | 34 |
| 162 | P000689 | MELD | 4.21 | 3.76921229063832 | 1.66 | 10.11 |  |  | F | 0 | m1 | gt25 | 13 | 1 | > 25 | Δ score ≤ -1 | esld\_master\_long\_public.csv | 34 |
| 163 | P001142 | MELD | 1.545 | 1.40357750769417 | 3.11 | 22.615 |  |  | F | 0 | m1 | gt25 | 13 | 1 | > 25 | Δ score ≤ -1 | esld\_master\_long\_public.csv | 35 |
| 164 | P001334 | MELD | 1.7 | 1.65867128122365 | 2.86 | 3.04 |  |  | F | 0 | m1 | gt25 | 13 | 1 | > 25 | Δ score ≤ -1 | esld\_master\_long\_public.csv | 27 |
| 165 | P000873 | MELD | 4.84 | 4.22433662146392 | 1.71 | 31.74 |  |  | M | 0 | m1 | gt25 | 14 | 1 | > 25 | Δ score ≤ -1 | esld\_master\_long\_public.csv | 39 |
| 166 | P001181 | MELD | 1.49 | 1.35194080851258 | 1.73 | 25.84 |  |  | M | 0 | m1 | gt25 | 14 | 1 | > 25 | Δ score ≤ -1 | esld\_master\_long\_public.csv | 29 |
| 167 | P000112 | MELD | 1.295 | 1.18837620081537 | 2.05 | 14.08 |  |  | M | 0 | m1 | gt25 | 15 | 1 | > 25 | Δ score ≤ -1 | esld\_master\_long\_public.csv | 27 |
| 168 | P000442 | MELD | 5.35 | 4.66895920445532 | 1.36 | 5.7 |  |  | M | 0 | m1 | gt25 | 15 | 1 | > 25 | Δ score ≤ -1 | esld\_master\_long\_public.csv | 30 |
| 169 | P001024 | MELD | 2.6 | 2.39164624491039 | 5.43 | 14.78 |  |  | F | 0 | m1 | gt25 | 17 | 1 | > 25 | Δ score ≤ -1 | esld\_master\_long\_public.csv | 40 |
| 170 | P000397 | MELD | 2.81 | 2.60764585682596 | 2.18 | 9.5 |  |  | M | 0 | m1 | gt25 | 18 | 1 | > 25 | Δ score ≤ -1 | esld\_master\_long\_public.csv | 34 |
| 171 | P000865 | MELD | 1.19 | 1.06515063758319 | 3.28 | 20.84 |  |  | F | 0 | m1 | gt25 | 18 | 1 | > 25 | Δ score ≤ -1 | esld\_master\_long\_public.csv | 33 |
| 172 | P000176 | MELD | 2.55 | 2.3671885060304 | 2.58 | 11.15 |  |  | M | 0 | m1 | gt25 | 19 | 1 | > 25 | Δ score ≤ -1 | esld\_master\_long\_public.csv | 35 |
| 173 | P001217 | MELD | 1.495 | 1.41226793731349 | 1.555 | 38.835 |  |  | M | 0 | m1 | gt25 | 19 | 1 | > 25 | Δ score ≤ -1 | esld\_master\_long\_public.csv | 29 |
| 174 | P000084 | MELD | 1.89 | 1.74918190373269 | 2.26 | 33.27 |  |  | M | 0 | m1 | gt25 | 20 | 1 | > 25 | Δ score ≤ -1 | esld\_master\_long\_public.csv | 35 |
| 175 | P000120 | MELD | 1.95 | 1.91407496391572 | 5.76 | 1 |  |  | M | 0 | m1 | gt25 | 22 | 1 | > 25 | Δ score ≤ -1 | esld\_master\_long\_public.csv | 32 |
| 176 | P000641 | MELD | 1.15 | 1.04130791805534 | 2.52 | 15.58 |  |  | M | 0 | m1 | gt25 | 22 | 1 | > 25 | Δ score ≤ -1 | esld\_master\_long\_public.csv | 28 |
| 177 | P001171 | MELD | 2.34 | 2.16385888489141 | 2.58 | 13.82 |  |  | M | 0 | m1 | gt25 | 22 | 1 | > 25 | Δ score ≤ -1 | esld\_master\_long\_public.csv | 35 |
| 178 | P000438 | MELD | 1.34 | 1.29528391306445 | 2.62 | 5.2 |  |  | F | 0 | m1 | gt25 | 33 | 1 | > 25 | Δ score ≤ -1 | esld\_master\_long\_public.csv | 26 |
| 179 | P000696 | MELD | 3.03 | 2.78446056521364 | 2.355 | 11.59 |  |  | M | 1 | m1 | gt25 | 33 | 1 | > 25 | Δ score ≤ -1 | esld\_master\_long\_public.csv | 39 |
| 180 | P000739 | MELD | 2.92 | 2.75336529906707 | 1.895 | 3.75 |  |  | M | 0 | m1 | gt25 | 37 | 1 | > 25 | Δ score ≤ -1 | esld\_master\_long\_public.csv | 29 |
| 181 | P001200 | MELD | 2.74 | 2.49438823364993 | 5.14 | 23.79 |  |  | M | 0 | m1 | gt25 | 38 | 1 | > 25 | Δ score ≤ -1 | esld\_master\_long\_public.csv | 40 |
| 182 | P000318 | MELD | 1.13 | 1.00462235284229 | 1.7 | 24.1 |  |  | F | 0 | m1 | gt25 | 42 | 1 | > 25 | Δ score ≤ -1 | esld\_master\_long\_public.csv | 26 |
| 183 | P000740 | MELD | 2.16 | 2.08309798108392 | 4.05 | 3.17 |  |  | F | 0 | m1 | gt25 | 52 | 1 | > 25 | Δ score ≤ -1 | esld\_master\_long\_public.csv | 34 |
| 184 | P000781 | MELD | 2.5 | 2.38294446725591 | 1.67 | 3.84 |  |  | M | 0 | m1 | gt25 | 52 | 1 | > 25 | Δ score ≤ -1 | esld\_master\_long\_public.csv | 26 |
| 185 | P001146 | MELD | 2.985 | 2.76373688529807 | 2.58 | 8.95 |  |  | F | 0 | m1 | gt25 | 55 | 1 | > 25 | Δ score ≤ -1 | esld\_master\_long\_public.csv | 36 |
| 186 | P000194 | MELD | 2.75 | 2.5467843756506 | 5.14 | 10.7 |  |  | M | 0 | m1 | gt25 | 62 | 1 | > 25 | Δ score ≤ -1 | esld\_master\_long\_public.csv | 40 |
| 187 | P000141 | MELD | 3.03 | 2.82072307690323 | 3.18 | 6.7 |  |  | F | 0 | m1 | gt25 | 63 | 1 | > 25 | Δ score ≤ -1 | esld\_master\_long\_public.csv | 37 |
| 188 | P000228 | MELD | 4.075 | 3.72434013363439 | 1.98 | 2.95 |  |  | M | 0 | m1 | gt25 | 68 | 1 | > 25 | Δ score ≤ -1 | esld\_master\_long\_public.csv | 31 |
| 189 | P000847 | MELD | 1.82 | 1.67246734762944 | 3.26 | 17.33 |  |  | M | 0 | m1 | gt25 | 73 | 1 | > 25 | Δ score ≤ -1 | esld\_master\_long\_public.csv | 36 |
| 190 | P000840 | MELD | 1.86 | 1.75512013291459 | 2.18 | 8.93 |  |  | F | 0 | m1 | gt25 | 96 | 1 | > 25 | Δ score ≤ -1 | esld\_master\_long\_public.csv | 29 |
| 191 | P000571 | MELD | 4.09 | 3.63211295757681 | 3.9 | 19 |  |  | F | 0 | m1 | gt25 | 107 | 1 | > 25 | Δ score ≤ -1 | esld\_master\_long\_public.csv | 40 |
| 192 | P000577 | MELD | 4.71 | 4.2400091234591 | 1.62 | 1.15 |  |  | M | 0 | m1 | gt25 | 109 | 1 | > 25 | Δ score ≤ -1 | esld\_master\_long\_public.csv | 26 |
| 193 | P000425 | MELD | 2.73 | 2.51263121514642 | 1.7 | 13.5 |  |  | M | 0 | m1 | gt25 | 116 | 1 | > 25 | Δ score ≤ -1 | esld\_master\_long\_public.csv | 32 |
| 194 | P000534 | MELD | 7.77 | 6.29275482358055 | 3.37 | 1.56 |  |  | M | 0 | m1 | gt25 | 120 | 1 | > 25 | Δ score ≤ -1 | esld\_master\_long\_public.csv | 35 |
| 195 | P000955 | MELD | 3.47 | 3.1881030251959 | 1.91 | 7.38 |  |  | F | 0 | m1 | gt25 | 150 | 1 | > 25 | Δ score ≤ -1 | esld\_master\_long\_public.csv | 33 |
| 196 | P001042 | MELD | 2.84 | 2.67966835168512 | 3.14 | 4.16 |  |  | F | 0 | m1 | gt25 | 155 | 1 | > 25 | Δ score ≤ -1 | esld\_master\_long\_public.csv | 35 |
| 197 | P001145 | MELD | 2.83 | 2.63291110427294 | 5.93 | 8.46 |  |  | M | 0 | m1 | gt25 | 205 | 1 | > 25 | Δ score ≤ -1 | esld\_master\_long\_public.csv | 40 |
| 198 | P000233 | MELD | 3.08 | 2.88229341452655 | 2.1 | 4.67 |  |  | F | 0 | m1 | gt25 | 225 | 1 | > 25 | Δ score ≤ -1 | esld\_master\_long\_public.csv | 31 |
| 199 | P000565 | MELD | 2.95 | 2.71589130555848 | 1.49 | 36.2 |  |  | F | 0 | m1 | gt25 | 273 | 1 | > 25 | Δ score ≤ -1 | esld\_master\_long\_public.csv | 35 |
| 200 | P000138 | MELD | 1.33 | 1.2068676673072 | 1.69 | 29.8 |  |  | M | 0 | m1 | gt25 | 320 | 1 | > 25 | Δ score ≤ -1 | esld\_master\_long\_public.csv | 28 |
| 201 | P000694 | MELD | 2.39 | 2.30597639647277 | 2.24 | 1.81 |  |  | M | 0 | m1 | gt25 | 322 | 1 | > 25 | Δ score ≤ -1 | esld\_master\_long\_public.csv | 26 |
| 202 | P001070 | MELD | 1.85 | 1.74410263671729 | 2.62666666666667 | 9.16 |  |  | F | 0 | m1 | gt25 | 471 | 1 | > 25 | Δ score ≤ -1 | esld\_master\_long\_public.csv | 32 |
| 203 | P000912 | MELD | 4.59 | 4.08893397889789 | 2.34 | 7.1 |  |  | M | 0 | m1 | gt25 | 616 | 1 | > 25 | Δ score ≤ -1 | esld\_master\_long\_public.csv | 37 |
| 204 | P001014 | MELD | 2.59 | 2.38556772526454 | 2.62 | 14.19 |  |  | M | 0 | m1 | gt25 | 666 | 1 | > 25 | Δ score ≤ -1 | esld\_master\_long\_public.csv | 36 |
| 205 | P000006 | MELD | 1.86 | 1.76337882658825 | 1.62 | 7.87 |  |  | M | 0 | m1 | gt25 | 730 | 0 | > 25 | Δ score ≤ -1 | esld\_master\_long\_public.csv | 26 |
| 206 | P000027 | MELD | 3.28 | 3.07105850131161 | 1.63 | 2.86 |  |  | M | 0 | m1 | gt25 | 730 | 0 | > 25 | Δ score ≤ -1 | esld\_master\_long\_public.csv | 27 |
| 207 | P000041 | MELD | 3.45 | 3.23660342588408 | 2.13 | 0.83 |  |  | F | 0 | m1 | gt25 | 730 | 0 | > 25 | Δ score ≤ -1 | esld\_master\_long\_public.csv | 27 |
| 208 | P000069 | MELD | 1.4 | 1.33086008495746 | 2.55 | 7.7 |  |  | F | 0 | m1 | gt25 | 730 | 0 | > 25 | Δ score ≤ -1 | esld\_master\_long\_public.csv | 28 |
| 209 | P000071 | MELD | 2.75 | 2.52038256890317 | 2.07 | 15.59 |  |  | M | 0 | m1 | gt25 | 730 | 0 | > 25 | Δ score ≤ -1 | esld\_master\_long\_public.csv | 35 |
| 210 | P000093 | MELD | 2.165 | 2.0180358542989 | 4.585 | 11.54 |  |  | F | 0 | m1 | gt25 | 730 | 0 | > 25 | Δ score ≤ -1 | esld\_master\_long\_public.csv | 40 |
| 211 | P000102 | MELD | 2.56 | 2.38689845315044 | 3.6 | 9.56 |  |  | M | 0 | m1 | gt25 | 730 | 0 | > 25 | Δ score ≤ -1 | esld\_master\_long\_public.csv | 38 |
| 212 | P000129 | MELD | 2.29 | 2.1059431928805 | 2.21 | 16.78 |  |  | M | 0 | m1 | gt25 | 730 | 0 | > 25 | Δ score ≤ -1 | esld\_master\_long\_public.csv | 34 |
| 213 | P000148 | MELD | 3.4 | 3.11596053060185 | 3.34 | 9.1 |  |  | M | 0 | m1 | gt25 | 730 | 0 | > 25 | Δ score ≤ -1 | esld\_master\_long\_public.csv | 40 |
| 214 | P000197 | MELD | 2.89 | 2.74167404972661 | 2.25 | 2.38 |  |  | M | 0 | m1 | gt25 | 730 | 0 | > 25 | Δ score ≤ -1 | esld\_master\_long\_public.csv | 29 |
| 215 | P000284 | MELD | 1.18 | 1.0856087709612 | 2.08 | 35.2 |  |  | F | 0 | m1 | gt25 | 730 | 0 | > 25 | Δ score ≤ -1 | esld\_master\_long\_public.csv | 30 |
| 216 | P000413 | MELD | 2.51 | 2.34487422997289 | 3.47 | 9.22 |  |  | F | 0 | m1 | gt25 | 730 | 0 | > 25 | Δ score ≤ -1 | esld\_master\_long\_public.csv | 38 |
| 217 | P000513 | MELD | 3.82 | 3.42904959645688 | 3.32 | 14.8 |  |  | F | 0 | m1 | gt25 | 730 | 0 | > 25 | Δ score ≤ -1 | esld\_master\_long\_public.csv | 40 |
| 218 | P000559 | MELD | 2.28 | 2.20533530443375 | 2.53 | 1.94 |  |  | M | 0 | m1 | gt25 | 730 | 0 | > 25 | Δ score ≤ -1 | esld\_master\_long\_public.csv | 27 |
| 219 | P000615 | MELD | 2.39333333333333 | 2.3128355042854 | 1.63333333333333 | 1.465 |  |  | F | 1 | m1 | gt25 | 730 | 0 | > 25 | Δ score ≤ -1 | esld\_master\_long\_public.csv | 27 |
| 220 | P000633 | MELD | 1.38 | 1.28189387049087 | 2.96 | 11.91 |  |  | M | 0 | m1 | gt25 | 730 | 0 | > 25 | Δ score ≤ -1 | esld\_master\_long\_public.csv | 31 |
| 221 | P000705 | MELD | 3.27 | 3.06536154225263 | 2.45 | 2.6 |  |  | M | 0 | m1 | gt25 | 730 | 0 | > 25 | Δ score ≤ -1 | esld\_master\_long\_public.csv | 31 |
| 222 | P000755 | MELD | 2.8 | 2.60523710385799 | 2.89 | 8.65 |  |  | F | 0 | m1 | gt25 | 730 | 0 | > 25 | Δ score ≤ -1 | esld\_master\_long\_public.csv | 36 |
| 223 | P000760 | MELD | 4.615 | 4.06176843504607 | 2.38 | 14.02 |  |  | M | 0 | m1 | gt25 | 730 | 0 | > 25 | Δ score ≤ -1 | esld\_master\_long\_public.csv | 39 |
| 224 | P000849 | MELD | 2.16 | 2.04013980704436 | 2.1275 | 7.81 |  |  | M | 0 | m1 | gt25 | 730 | 0 | > 25 | Δ score ≤ -1 | esld\_master\_long\_public.csv | 30 |
| 225 | P000867 | MELD | 3.59 | 3.23818684544348 | 2.02 | 15.24 |  |  | M | 0 | m1 | gt25 | 730 | 0 | > 25 | Δ score ≤ -1 | esld\_master\_long\_public.csv | 37 |
| 226 | P001277 | MELD-Na | 0.32 | 0.337230654948723 | 1.55 | 0.31 | 143 |  | M | 0 | pm | le15 | 1 | 1 | ≤ 15 | Δ score = ± 0 | esld\_master\_long\_public.csv | 11 |
| 227 | P000525 | MELD-Na | 1.02 | 1.02098453959733 | 1.22 | 1.7 | 142 |  | M | 0 | pm | le15 | 6 | 1 | ≤ 15 | Δ score = ± 0 | esld\_master\_long\_public.csv | 11 |
| 228 | P000302 | MELD-Na | 0.64 | 0.657275352328835 | 1.79 | 0.6 | 138 |  | F | 0 | pm | le15 | 7 | 1 | ≤ 15 | Δ score = ± 0 | esld\_master\_long\_public.csv | 13 |
| 229 | P001040 | MELD-Na | 1.55 | 1.54121277346097 | 1.23 | 0.86 | 143 |  | F | 0 | pm | le15 | 7 | 1 | ≤ 15 | Δ score = ± 0 | esld\_master\_long\_public.csv | 13 |
| 230 | P000384 | MELD-Na | 0.58 | 0.587866490762143 | 1.82 | 1.4 | 151 |  | F | 0 | pm | le15 | 15 | 1 | ≤ 15 | Δ score = ± 0 | esld\_master\_long\_public.csv | 14 |
| 231 | P000038 | MELD-Na | 0.47 | 0.473369420702739 | 1.64 | 1.72 | 145 |  | F | 0 | pm | le15 | 22 | 1 | ≤ 15 | Δ score = ± 0 | esld\_master\_long\_public.csv | 14 |
| 232 | P000496 | MELD-Na | 1.68 | 1.66581135829394 | 1.08 | 0.7 | 140 |  | F | 0 | pm | le15 | 24 | 1 | ≤ 15 | Δ score = ± 0 | esld\_master\_long\_public.csv | 12 |
| 233 | P000131 | MELD-Na | 1.05 | 1.05597848096179 | 1.8 | 1.2 | 153 |  | F | 0 | pm | le15 | 25 | 1 | ≤ 15 | Δ score = ± 0 | esld\_master\_long\_public.csv | 14 |
| 234 | P000116 | MELD-Na | 0.98 | 0.992029762213455 | 1.09 | 0.8 | 156 |  | M | 0 | pm | le15 | 26 | 1 | ≤ 15 | Δ score = ± 0 | esld\_master\_long\_public.csv | 7 |
| 235 | P000961 | MELD-Na | 0.31 | 0.325840952994798 | 1.14 | 0.41 | 141 |  | F | 0 | pm | le15 | 28 | 1 | ≤ 15 | Δ score = ± 0 | esld\_master\_long\_public.csv | 8 |
| 236 | P000503 | MELD-Na | 1.29 | 1.29508728750764 | 1.17 | 0.66 | 142 |  | M | 0 | pm | le15 | 120 | 1 | ≤ 15 | Δ score = ± 0 | esld\_master\_long\_public.csv | 11 |
| 237 | P000966 | MELD-Na | 1.4 | 1.39662552916786 | 1.37 | 1.01 | 136 |  | M | 0 | pm | le15 | 192 | 1 | ≤ 15 | Δ score = ± 0 | esld\_master\_long\_public.csv | 14 |
| 238 | P000070 | MELD-Na | 0.94 | 0.955059411832967 | 1.77 | 0.6 | 139 |  | F | 0 | pm | le15 | 294 | 1 | ≤ 15 | Δ score = ± 0 | esld\_master\_long\_public.csv | 13 |
| 239 | P000594 | MELD-Na | 1.06 | 1.05154308510941 | 1.24 | 2.48 | 140 |  | M | 0 | pm | le15 | 298 | 1 | ≤ 15 | Δ score = ± 0 | esld\_master\_long\_public.csv | 13 |
| 240 | P000362 | MELD-Na | 1.22 | 1.230574356631 | 1.07 | 0.4 | 133 |  | F | 0 | pm | le15 | 413 | 1 | ≤ 15 | Δ score = ± 0 | esld\_master\_long\_public.csv | 13 |
| 241 | P000253 | MELD-Na | 1.445 | 1.44146970478353 | 1.26 | 0.85 | 139 |  | F | 0 | pm | le15 | 601 | 1 | ≤ 15 | Δ score = ± 0 | esld\_master\_long\_public.csv | 13 |
| 242 | P000104 | MELD-Na | 0.98 | 0.995656984709247 | 1.6 | 0.49 | 148 |  | F | 0 | pm | le15 | 730 | 0 | ≤ 15 | Δ score = ± 0 | esld\_master\_long\_public.csv | 12 |
| 243 | P000155 | MELD-Na | 0.405 | 0.417982545133566 | 1.8 | 0.8 | 143 |  | M | 0 | pm | le15 | 730 | 0 | ≤ 15 | Δ score = ± 0 | esld\_master\_long\_public.csv | 13 |
| 244 | P000220 | MELD-Na | 0.5 | 0.512427224422537 | 1.36 | 0.95 | 137 |  | F | 0 | pm | le15 | 730 | 0 | ≤ 15 | Δ score = ± 0 | esld\_master\_long\_public.csv | 10 |
| 245 | P000429 | MELD-Na | 1.22 | 1.23187807173977 | 1.16 | 0.29 | 152 |  | M | 0 | pm | le15 | 730 | 0 | ≤ 15 | Δ score = ± 0 | esld\_master\_long\_public.csv | 10 |
| 246 | P000497 | MELD-Na | 0.8 | 0.81546514571348 | 1.08 | 0.71 | 139 |  | M | 0 | pm | le15 | 730 | 0 | ≤ 15 | Δ score = ± 0 | esld\_master\_long\_public.csv | 7 |
| 247 | P000507 | MELD-Na | 0.64 | 0.661885818438325 | 1.33 | 0.21 | 147 |  | F | 0 | pm | le15 | 730 | 0 | ≤ 15 | Δ score = ± 0 | esld\_master\_long\_public.csv | 10 |
| 248 | P000576 | MELD-Na | 1.535 | 1.5327276273706 | 1.33 | 0.37 | 140.5 |  | M | 0 | pm | le15 | 730 | 0 | ≤ 15 | Δ score = ± 0 | esld\_master\_long\_public.csv | 14 |
| 249 | P000593 | MELD-Na | 1.07 | 1.08105354861449 | 1.44 | 0.72 | 135 |  | M | 0 | pm | le15 | 730 | 0 | ≤ 15 | Δ score = ± 0 | esld\_master\_long\_public.csv | 13 |
| 250 | P000623 | MELD-Na | 0.646666666666667 | 0.657178304926113 | 1.20666666666667 | 1.185 | 140.5 |  | F | 0 | pm | le15 | 730 | 0 | ≤ 15 | Δ score = ± 0 | esld\_master\_long\_public.csv | 9 |
| 251 | P000705 | MELD-Na | 1.515 | 1.51026850902176 | 1.46 | 0.665 | 141 |  | M | 0 | pm | le15 | 730 | 0 | ≤ 15 | Δ score = ± 0 | esld\_master\_long\_public.csv | 15 |
| 252 | P000718 | MELD-Na | 0.75 | 0.769431684013923 | 1.73 | 0.4 | 144 |  | M | 0 | pm | le15 | 730 | 0 | ≤ 15 | Δ score = ± 0 | esld\_master\_long\_public.csv | 13 |
| 253 | P001285 | MELD-Na | 0.9 | 0.916247241802327 | 1.23 | 0.55 | 143 |  | M | 0 | m1 | le15 | 5 | 1 | ≤ 15 | Δ score ≤ -1 | esld\_master\_long\_public.csv | 9 |
| 254 | P000828 | MELD-Na | 1.38 | 1.38072286319533 | 1.39 | 0.73 | 152 |  | M | 0 | m1 | le15 | 64 | 1 | ≤ 15 | Δ score ≤ -1 | esld\_master\_long\_public.csv | 13 |
| 255 | P000402 | MELD-Na | 0.23 | 0.228045643722108 | 1.24 | 1.8 | 138 |  | M | 0 | m1 | le15 | 66 | 1 | ≤ 15 | Δ score ≤ -1 | esld\_master\_long\_public.csv | 11 |
| 256 | P001329 | MELD-Na | 1.23 | 1.22274385466638 | 1.27 | 1.93 | 136 |  | F | 0 | m1 | le15 | 73 | 1 | ≤ 15 | Δ score ≤ -1 | esld\_master\_long\_public.csv | 15 |
| 257 | P000260 | MELD-Na | 1.46 | 1.46010047914932 | 1.22 | 0.48 | 140 |  | M | 0 | m1 | le15 | 730 | 0 | ≤ 15 | Δ score ≤ -1 | esld\_master\_long\_public.csv | 12 |
| 258 | P000312 | MELD-Na | 0.53 | 0.538338983740448 | 1.36 | 1.33 | 137 |  | M | 0 | m1 | le15 | 730 | 0 | ≤ 15 | Δ score ≤ -1 | esld\_master\_long\_public.csv | 11 |
| 259 | P000626 | MELD-Na | 0.68 | 0.682028708290505 | 1.48 | 1.94 | 136 |  | M | 0 | m1 | le15 | 730 | 0 | ≤ 15 | Δ score ≤ -1 | esld\_master\_long\_public.csv | 14 |
| 260 | P000164 | MELD-Na | 2.3 | 2.23786201906189 | 1.495 | 0.67 | 128.5 |  | F | 0 | pm | 16to25 | 0 | 1 | 16 - 25 | Δ score = ± 0 | esld\_master\_long\_public.csv | 25 |
| 261 | P000661 | MELD-Na | 2.02 | 1.98623796901758 | 2.14 | 0.35 | 143 |  | M | 0 | pm | 16to25 | 1 | 1 | 16 - 25 | Δ score = ± 0 | esld\_master\_long\_public.csv | 22 |
| 262 | P000844 | MELD-Na | 2.08 | 2.02193667277054 | 1.68 | 2.05 | 142 |  | M | 0 | pm | 16to25 | 1 | 1 | 16 - 25 | Δ score = ± 0 | esld\_master\_long\_public.csv | 22 |
| 263 | P000319 | MELD-Na | 1.38333333333333 | 1.37792174700726 | 1.59333333333333 | 1.25 | 140 |  | F | 0 | pm | 16to25 | 2 | 1 | 16 - 25 | Δ score = ± 0 | esld\_master\_long\_public.csv | 16 |
| 264 | P000500 | MELD-Na | 1.46 | 1.41975252540341 | 1.935 | 4.2 | 139.5 |  | M | 0 | pm | 16to25 | 2 | 1 | 16 - 25 | Δ score = ± 0 | esld\_master\_long\_public.csv | 23 |
| 265 | P001348 | MELD-Na | 2.92 | 2.7915702249002 | 1.13 | 0.28 | 133 |  | F | 0 | pm | 16to25 | 2 | 1 | 16 - 25 | Δ score = ± 0 | esld\_master\_long\_public.csv | 21 |
| 266 | P001254 | MELD-Na | 2.02 | 1.90525228005634 | 0 | 8.65 | 143 |  | M | 0 | pm | 16to25 | 3 | 1 | 16 - 25 | Δ score = ± 0 | esld\_master\_long\_public.csv | 21 |
| 267 | P000665 | MELD-Na | 2.38 | 2.30765687029206 | 1.64 | 0.87 | 143 |  | F | 0 | pm | 16to25 | 4 | 1 | 16 - 25 | Δ score = ± 0 | esld\_master\_long\_public.csv | 20 |
| 268 | P000943 | MELD-Na | 3.03 | 2.8774022541663 | 1.31 | 1.07 | 139 |  | M | 0 | pm | 16to25 | 5 | 1 | 16 - 25 | Δ score = ± 0 | esld\_master\_long\_public.csv | 20 |
| 269 | P000396 | MELD-Na | 0.93 | 0.917485369101848 | 1.72 | 3.1 | 139 |  | M | 0 | pm | 16to25 | 8 | 1 | 16 - 25 | Δ score = ± 0 | esld\_master\_long\_public.csv | 17 |
| 270 | P000903 | MELD-Na | 0.75 | 0.733971158022278 | 2.2 | 3.62 | 144 |  | M | 0 | pm | 16to25 | 8 | 1 | 16 - 25 | Δ score = ± 0 | esld\_master\_long\_public.csv | 20 |
| 271 | P001158 | MELD-Na | 0.99 | 0.985300801450023 | 1.85 | 2.27 | 146.5 |  | F | 0 | pm | 16to25 | 8 | 1 | 16 - 25 | Δ score = ± 0 | esld\_master\_long\_public.csv | 16 |
| 272 | P000210 | MELD-Na | 3.03 | 2.88639627973574 | 1.9 | 0.3 | 144 |  | M | 0 | pm | 16to25 | 10 | 1 | 16 - 25 | Δ score = ± 0 | esld\_master\_long\_public.csv | 24 |
| 273 | P001051 | MELD-Na | 2.55 | 2.46094587701674 | 2.15 | 0.76 | 137 |  | M | 0 | pm | 16to25 | 12 | 1 | 16 - 25 | Δ score = ± 0 | esld\_master\_long\_public.csv | 24 |
| 274 | P000988 | MELD-Na | 1.05 | 1.05977483679343 | 1.69 | 0.87 | 129 |  | M | 0 | pm | 16to25 | 13 | 1 | 16 - 25 | Δ score = ± 0 | esld\_master\_long\_public.csv | 20 |
| 275 | P000598 | MELD-Na | 0.38 | 0.318034206950338 | 1.63 | 8.5 | 141 |  | F | 0 | pm | 16to25 | 22 | 1 | 16 - 25 | Δ score = ± 0 | esld\_master\_long\_public.csv | 20 |
| 276 | P000386 | MELD-Na | 0.72 | 0.731402868916093 | 1.35 | 1.1 | 122 |  | M | 0 | pm | 16to25 | 24 | 1 | 16 - 25 | Δ score = ± 0 | esld\_master\_long\_public.csv | 22 |
| 277 | P000179 | MELD-Na | 0.54 | 0.499984913783214 | 1.65 | 6.1 | 138 |  | F | 0 | pm | 16to25 | 46 | 1 | 16 - 25 | Δ score = ± 0 | esld\_master\_long\_public.csv | 19 |
| 278 | P000506 | MELD-Na | 0.67 | 0.669179586872747 | 1.44 | 2.2 | 132 |  | M | 0 | pm | 16to25 | 46 | 1 | 16 - 25 | Δ score = ± 0 | esld\_master\_long\_public.csv | 17 |
| 279 | P000065 | MELD-Na | 1.51 | 1.50534242218578 | 1.02 | 0.68 | 142 |  | M | 1 | pm | 16to25 | 48 | 1 | 16 - 25 | Δ score = ± 0 | esld\_master\_long\_public.csv | 20 |
| 280 | P000274 | MELD-Na | 1.59 | 1.56840982135296 | 1.44 | 1.8 | 144 |  | M | 0 | pm | 16to25 | 75 | 1 | 16 - 25 | Δ score = ± 0 | esld\_master\_long\_public.csv | 17 |
| 281 | P000523 | MELD-Na | 1.04 | 1.0405547447764 | 2 | 1.7 | 131 |  | F | 0 | pm | 16to25 | 82 | 1 | 16 - 25 | Δ score = ± 0 | esld\_master\_long\_public.csv | 22 |
| 282 | P001060 | MELD-Na | 2.27 | 2.20345569382691 | 1.38 | 1.3 | 141 |  | M | 0 | pm | 16to25 | 152 | 1 | 16 - 25 | Δ score = ± 0 | esld\_master\_long\_public.csv | 19 |
| 283 | P000169 | MELD-Na | 1.79 | 1.77158848879538 | 1.63 | 0.47 | 138 |  | F | 0 | pm | 16to25 | 158 | 1 | 16 - 25 | Δ score = ± 0 | esld\_master\_long\_public.csv | 17 |
| 284 | P000859 | MELD-Na | 1.2 | 1.13252039458619 | 1.9 | 8.43 | 135 |  | M | 0 | pm | 16to25 | 158 | 1 | 16 - 25 | Δ score = ± 0 | esld\_master\_long\_public.csv | 24 |
| 285 | P000159 | MELD-Na | 2.04 | 1.97500201145955 | 1.46 | 3 | 133 |  | M | 0 | pm | 16to25 | 169 | 1 | 16 - 25 | Δ score = ± 0 | esld\_master\_long\_public.csv | 24 |
| 286 | P001208 | MELD-Na | 0.8 | 0.802380357076246 | 2.34 | 1.86 | 137.5 |  | M | 0 | pm | 16to25 | 316 | 1 | 16 - 25 | Δ score = ± 0 | esld\_master\_long\_public.csv | 18 |
| 287 | P001162 | MELD-Na | 1.49 | 1.46471517319051 | 1.19 | 2.61 | 134 |  | F | 0 | pm | 16to25 | 358 | 1 | 16 - 25 | Δ score = ± 0 | esld\_master\_long\_public.csv | 18 |
| 288 | P001014 | MELD-Na | 0.64 | 0.633767803063514 | 1.61 | 2.7 | 136 |  | M | 0 | pm | 16to25 | 666 | 1 | 16 - 25 | Δ score = ± 0 | esld\_master\_long\_public.csv | 17 |
| 289 | P000015 | MELD-Na | 1.96 | 1.87899575814967 | 1.45 | 5.24 | 164 |  | M | 0 | pm | 16to25 | 730 | 0 | 16 - 25 | Δ score = ± 0 | esld\_master\_long\_public.csv | 23 |
| 290 | P000035 | MELD-Na | 1.61 | 1.59213766196358 | 1.92 | 1.37 | 143 |  | F | 0 | pm | 16to25 | 730 | 0 | 16 - 25 | Δ score = ± 0 | esld\_master\_long\_public.csv | 19 |
| 291 | P000073 | MELD-Na | 1.24 | 1.22234728941079 | 1.56 | 2.86 | 145 |  | M | 0 | pm | 16to25 | 730 | 0 | 16 - 25 | Δ score = ± 0 | esld\_master\_long\_public.csv | 17 |
| 292 | P000091 | MELD-Na | 1.19 | 1.19695444758187 | 2.41 | 0.79 | 137.5 |  | F | 0 | pm | 16to25 | 730 | 0 | 16 - 25 | Δ score = ± 0 | esld\_master\_long\_public.csv | 18 |
| 293 | P000108 | MELD-Na | 2.52 | 2.4160440175503 | 1.51 | 2.38 | 149 |  | F | 0 | pm | 16to25 | 730 | 0 | 16 - 25 | Δ score = ± 0 | esld\_master\_long\_public.csv | 23 |
| 294 | P000218 | MELD-Na | 2.29 | 2.23317938629221 | 1.62 | 0.3 | 135 |  | M | 0 | pm | 16to25 | 730 | 0 | 16 - 25 | Δ score = ± 0 | esld\_master\_long\_public.csv | 21 |
| 295 | P000265 | MELD-Na | 1.22 | 1.17976671415079 | 1.64 | 5.185 | 134 |  | F | 0 | pm | 16to25 | 730 | 0 | 16 - 25 | Δ score = ± 0 | esld\_master\_long\_public.csv | 22 |
| 296 | P000532 | MELD-Na | 1.91 | 1.87739592391313 | 1.8 | 0.97 | 139 |  | F | 0 | pm | 16to25 | 730 | 0 | 16 - 25 | Δ score = ± 0 | esld\_master\_long\_public.csv | 19 |
| 297 | P000692 | MELD-Na | 1.68 | 1.6652283389116 | 2.34 | 0.75 | 132 |  | M | 0 | pm | 16to25 | 730 | 0 | 16 - 25 | Δ score = ± 0 | esld\_master\_long\_public.csv | 24 |
| 298 | P000727 | MELD-Na | 1.89 | 1.8481156599397 | 1.42 | 1.92 | 130 |  | M | 0 | pm | 16to25 | 730 | 0 | 16 - 25 | Δ score = ± 0 | esld\_master\_long\_public.csv | 24 |
| 299 | P000487 | MELD-Na | 2 | 1.89272451929947 | 1.42 | 7.9 | 154 |  | M | 0 | m1 | 16to25 | 1 | 1 | 16 - 25 | Δ score ≤ -1 | esld\_master\_long\_public.csv | 25 |
| 300 | P000336 | MELD-Na | 2 | 1.96487292632333 | 1.49 | 0.6 | 134 |  | M | 0 | m1 | 16to25 | 4 | 1 | 16 - 25 | Δ score ≤ -1 | esld\_master\_long\_public.csv | 20 |
| 301 | P001229 | MELD-Na | 3.01 | 2.86774354004668 | 1.24 | 0.42 | 137 |  | M | 0 | m1 | 16to25 | 4 | 1 | 16 - 25 | Δ score ≤ -1 | esld\_master\_long\_public.csv | 19 |
| 302 | P000376 | MELD-Na | 3.97 | 3.66504898584215 | 1.04 | 0.7 | 149 |  | F | 0 | m1 | 16to25 | 5 | 1 | 16 - 25 | Δ score ≤ -1 | esld\_master\_long\_public.csv | 20 |
| 303 | P000649 | MELD-Na | 3.16 | 2.99253496943502 | 1.36 | 0.75 | 153 |  | F | 0 | m1 | 16to25 | 6 | 1 | 16 - 25 | Δ score ≤ -1 | esld\_master\_long\_public.csv | 21 |
| 304 | P000682 | MELD-Na | 1.6 | 1.58189821954639 | 2.11 | 1.44 | 140 |  | M | 0 | m1 | 16to25 | 6 | 1 | 16 - 25 | Δ score ≤ -1 | esld\_master\_long\_public.csv | 21 |
| 305 | P000024 | MELD-Na | 2.34 | 2.24487038165096 | 1.15 | 3.31 | 148 |  | M | 0 | m1 | 16to25 | 9 | 1 | 16 - 25 | Δ score ≤ -1 | esld\_master\_long\_public.csv | 21 |
| 306 | P000514 | MELD-Na | 1.07 | 1.01665109671129 | 1.51 | 7.13 | 154 |  | M | 0 | m1 | 16to25 | 9 | 1 | 16 - 25 | Δ score ≤ -1 | esld\_master\_long\_public.csv | 19 |
| 307 | P000380 | MELD-Na | 3.42 | 3.21183636496828 | 1.87 | 0.8 | 139 |  | M | 0 | m1 | 16to25 | 10 | 1 | 16 - 25 | Δ score ≤ -1 | esld\_master\_long\_public.csv | 25 |
| 308 | P000191 | MELD-Na | 3.61 | 3.37398887833063 | 1.45 | 0.46 | 146 |  | M | 0 | m1 | 16to25 | 11 | 1 | 16 - 25 | Δ score ≤ -1 | esld\_master\_long\_public.csv | 23 |
| 309 | P000361 | MELD-Na | 2.73 | 2.61842297819206 | 2.26 | 0.9 | 145 |  | F | 0 | m1 | 16to25 | 13 | 1 | 16 - 25 | Δ score ≤ -1 | esld\_master\_long\_public.csv | 25 |
| 310 | P001317 | MELD-Na | 3.09 | 2.93382423766075 | 1.66 | 0.65 | 138 |  | F | 0 | m1 | 16to25 | 13 | 1 | 16 - 25 | Δ score ≤ -1 | esld\_master\_long\_public.csv | 23 |
| 311 | P001273 | MELD-Na | 2.47 | 2.3818221593811 | 1.33 | 1.44 | 138 |  | M | 0 | m1 | 16to25 | 20 | 1 | 16 - 25 | Δ score ≤ -1 | esld\_master\_long\_public.csv | 20 |
| 312 | P000522 | MELD-Na | 3.11666666666667 | 2.94466366091965 | 1.63 | 1.7 | 148.333333333333 |  | M | 0 | m1 | 16to25 | 28 | 1 | 16 - 25 | Δ score ≤ -1 | esld\_master\_long\_public.csv | 25 |
| 313 | P001261 | MELD-Na | 2.05 | 1.98704768379053 | 1.86 | 2.73 | 138 |  | F | 0 | m1 | 16to25 | 32 | 1 | 16 - 25 | Δ score ≤ -1 | esld\_master\_long\_public.csv | 24 |
| 314 | P000602 | MELD-Na | 1.37 | 1.351295758735 | 1.9 | 2.5 | 140 |  | F | 0 | m1 | 16to25 | 35 | 1 | 16 - 25 | Δ score ≤ -1 | esld\_master\_long\_public.csv | 20 |
| 315 | P000441 | MELD-Na | 3.11 | 2.94460386895077 | 1.83 | 1.2 | 158 |  | M | 0 | m1 | 16to25 | 40 | 1 | 16 - 25 | Δ score ≤ -1 | esld\_master\_long\_public.csv | 25 |
| 316 | P000750 | MELD-Na | 2.53 | 2.43682307393645 | 1.57 | 1.31 | 147 |  | F | 0 | m1 | 16to25 | 40 | 1 | 16 - 25 | Δ score ≤ -1 | esld\_master\_long\_public.csv | 21 |
| 317 | P000678 | MELD-Na | 0.81 | 0.807576356313816 | 2.22 | 2.29 | 133.5 |  | F | 0 | m1 | 16to25 | 47 | 1 | 16 - 25 | Δ score ≤ -1 | esld\_master\_long\_public.csv | 21 |
| 318 | P001159 | MELD-Na | 1.215 | 1.16841908553479 | 1.49875 | 5.89 | 141 |  | F | 0 | m1 | 16to25 | 47 | 1 | 16 - 25 | Δ score ≤ -1 | esld\_master\_long\_public.csv | 20 |
| 319 | P001117 | MELD-Na | 1.9 | 1.82131333361556 | 1.65 | 5.47 | 137 |  | M | 0 | m1 | 16to25 | 71 | 1 | 16 - 25 | Δ score ≤ -1 | esld\_master\_long\_public.csv | 25 |
| 320 | P000292 | MELD-Na | 1.45 | 1.44221138454306 | 1.57 | 1.2 | 140 |  | F | 0 | m1 | 16to25 | 81 | 1 | 16 - 25 | Δ score ≤ -1 | esld\_master\_long\_public.csv | 16 |
| 321 | P000433 | MELD-Na | 1.92 | 1.8674565708872 | 1.15 | 2.7 | 137 |  | M | 0 | m1 | 16to25 | 89 | 1 | 16 - 25 | Δ score ≤ -1 | esld\_master\_long\_public.csv | 18 |
| 322 | P000297 | MELD-Na | 0.72 | 0.671902719315829 | 1.46 | 7.07 | 139 |  | F | 0 | m1 | 16to25 | 110 | 1 | 16 - 25 | Δ score ≤ -1 | esld\_master\_long\_public.csv | 18 |
| 323 | P000092 | MELD-Na | 1.49 | 1.45952773957017 | 1.515 | 3.1 | 136 |  | M | 0 | m1 | 16to25 | 113 | 1 | 16 - 25 | Δ score ≤ -1 | esld\_master\_long\_public.csv | 20 |
| 324 | P000243 | MELD-Na | 0.795 | 0.800881059739979 | 1.4 | 1.55 | 129.5 |  | M | 0 | m1 | 16to25 | 119 | 1 | 16 - 25 | Δ score ≤ -1 | esld\_master\_long\_public.csv | 19 |
| 325 | P000627 | MELD-Na | 4.08 | 3.75414377879156 | 1.19 | 0.63 | 139 |  | M | 0 | m1 | 16to25 | 202 | 1 | 16 - 25 | Δ score ≤ -1 | esld\_master\_long\_public.csv | 22 |
| 326 | P001266 | MELD-Na | 12.51 | 8.40855359864775 | 1.38 | 0.25 | 133 |  | M | 0 | m1 | 16to25 | 249 | 1 | 16 - 25 | Δ score ≤ -1 | esld\_master\_long\_public.csv | 25 |
| 327 | P000300 | MELD-Na | 1.77 | 1.73990009546554 | 1.465 | 1.6 | 136 |  | F | 0 | m1 | 16to25 | 296 | 1 | 16 - 25 | Δ score ≤ -1 | esld\_master\_long\_public.csv | 19 |
| 328 | P000320 | MELD-Na | 2.88 | 2.75304803420211 | 1.21 | 0.6 | 143 |  | M | 0 | m1 | 16to25 | 619 | 1 | 16 - 25 | Δ score ≤ -1 | esld\_master\_long\_public.csv | 19 |
| 329 | P000440 | MELD-Na | 3.27 | 3.06751465504505 | 1.21 | 2.4 | 137 |  | M | 0 | m1 | 16to25 | 658 | 1 | 16 - 25 | Δ score ≤ -1 | esld\_master\_long\_public.csv | 23 |
| 330 | P001129 | MELD-Na | 2.03 | 1.9753641583731 | 1.31 | 2.11 | 166 |  | F | 0 | m1 | 16to25 | 713 | 1 | 16 - 25 | Δ score ≤ -1 | esld\_master\_long\_public.csv | 19 |
| 331 | P000019 | MELD-Na | 4.16 | 3.82060446704423 | 1.55 | 0.4 | 141 |  | M | 0 | m1 | 16to25 | 730 | 0 | 16 - 25 | Δ score ≤ -1 | esld\_master\_long\_public.csv | 25 |
| 332 | P000027 | MELD-Na | 2.94 | 2.78877108851964 | 1.43 | 2.04 | 139 |  | M | 0 | m1 | 16to25 | 730 | 0 | 16 - 25 | Δ score ≤ -1 | esld\_master\_long\_public.csv | 23 |
| 333 | P000115 | MELD-Na | 3.97 | 3.65927550619595 | 1.39 | 1.2 | 146 |  | F | 0 | m1 | 16to25 | 730 | 0 | 16 - 25 | Δ score ≤ -1 | esld\_master\_long\_public.csv | 24 |
| 334 | P000126 | MELD-Na | 2.21 | 2.134655635035 | 1.64 | 2.6 | 146 |  | M | 0 | m1 | 16to25 | 730 | 0 | 16 - 25 | Δ score ≤ -1 | esld\_master\_long\_public.csv | 23 |
| 335 | P000143 | MELD-Na | 1.37 | 1.37137375331679 | 1.77 | 0.71 | 150 |  | M | 0 | m1 | 16to25 | 730 | 0 | 16 - 25 | Δ score ≤ -1 | esld\_master\_long\_public.csv | 16 |
| 336 | P000195 | MELD-Na | 1.89 | 1.86000441431409 | 1.33 | 0.87 | 150.5 |  | F | 0 | m1 | 16to25 | 730 | 0 | 16 - 25 | Δ score ≤ -1 | esld\_master\_long\_public.csv | 16 |
| 337 | P000202 | MELD-Na | 3.65 | 3.40654651456607 | 1.44 | 0.5 | 140 |  | M | 0 | m1 | 16to25 | 730 | 0 | 16 - 25 | Δ score ≤ -1 | esld\_master\_long\_public.csv | 23 |
| 338 | P000213 | MELD-Na | 0.93 | 0.907274343761223 | 3.35 | 4.1 | 142 |  | F | 0 | m1 | 16to25 | 730 | 0 | 16 - 25 | Δ score ≤ -1 | esld\_master\_long\_public.csv | 25 |
| 339 | P000285 | MELD-Na | 0.66 | 0.552008354530637 | 1.55 | 17.24 | 158.5 |  | M | 0 | m1 | 16to25 | 730 | 0 | 16 - 25 | Δ score ≤ -1 | esld\_master\_long\_public.csv | 22 |
| 340 | P000290 | MELD-Na | 1.29 | 1.248138744936 | 1.82 | 5.1 | 145 |  | F | 0 | m1 | 16to25 | 730 | 0 | 16 - 25 | Δ score ≤ -1 | esld\_master\_long\_public.csv | 22 |
| 341 | P000321 | MELD-Na | 2.09 | 2.03869321898837 | 1.64 | 1.37 | 134 |  | M | 0 | m1 | 16to25 | 730 | 0 | 16 - 25 | Δ score ≤ -1 | esld\_master\_long\_public.csv | 22 |
| 342 | P000365 | MELD-Na | 2.225 | 2.16082941528465 | 1.67 | 1.46 | 145.5 |  | F | 0 | m1 | 16to25 | 730 | 0 | 16 - 25 | Δ score ≤ -1 | esld\_master\_long\_public.csv | 21 |
| 343 | P000494 | MELD-Na | 1.26 | 1.22694053926999 | 2.405 | 4.3 | 142 |  | F | 0 | m1 | 16to25 | 730 | 1 | 16 - 25 | Δ score ≤ -1 | esld\_master\_long\_public.csv | 24 |
| 344 | P000794 | MELD-Na | 2.46 | 2.38079727907689 | 1.12 | 0.75 | 135 |  | M | 0 | m1 | 16to25 | 730 | 0 | 16 - 25 | Δ score ≤ -1 | esld\_master\_long\_public.csv | 18 |
| 345 | P000958 | MELD-Na | 2.8 | 2.67978185268242 | 1.6 | 0.9 | 132 |  | M | 0 | m1 | 16to25 | 730 | 0 | 16 - 25 | Δ score ≤ -1 | esld\_master\_long\_public.csv | 25 |
| 346 | P001067 | MELD-Na | 0.766666666666667 | 0.748280092804634 | 3.90285714285714 | 3.84333333333333 | 138 |  | F | 0 | pm | gt25 | 0 | 1 | > 25 | Δ score = ± 0 | esld\_master\_long\_public.csv | 27 |
| 347 | P001324 | MELD-Na | 3.94 | 3.534002269821 | 4.86 | 34.45 | 132 |  | M | 1 | pm | gt25 | 0 | 1 | > 25 | Δ score = ± 0 | esld\_master\_long\_public.csv | 40 |
| 348 | P000264 | MELD-Na | 6.2 | 5.31574786694408 | 2.07 | 0.7 | 147 |  | M | 0 | pm | gt25 | 2 | 1 | > 25 | Δ score = ± 0 | esld\_master\_long\_public.csv | 28 |
| 349 | P000480 | MELD-Na | 2.33 | 2.23803827709083 | 2.255 | 3.1 | 141 |  | M | 0 | pm | gt25 | 2 | 1 | > 25 | Δ score = ± 0 | esld\_master\_long\_public.csv | 28 |
| 350 | P001301 | MELD-Na | 2.62 | 2.49437469190787 | 1.32 | 3.37 | 129 |  | M | 0 | pm | gt25 | 3 | 1 | > 25 | Δ score = ± 0 | esld\_master\_long\_public.csv | 27 |
| 351 | P000389 | MELD-Na | 3.03 | 2.87477370154041 | 5.99 | 1.3 | 129 |  | M | 0 | pm | gt25 | 9 | 1 | > 25 | Δ score = ± 0 | esld\_master\_long\_public.csv | 39 |
| 352 | P000485 | MELD-Na | 6.07 | 5.177317196471 | 2.03 | 5.5 | 136 |  | M | 0 | pm | gt25 | 9 | 1 | > 25 | Δ score = ± 0 | esld\_master\_long\_public.csv | 34 |
| 353 | P000442 | MELD-Na | 5.35 | 4.66895920445532 | 1.36 | 5.7 | 141 |  | M | 0 | pm | gt25 | 15 | 1 | > 25 | Δ score = ± 0 | esld\_master\_long\_public.csv | 30 |
| 354 | P001369 | MELD-Na | 7.01 | 5.81433345566015 | 3.45 | 3.3 | 138 |  | M | 0 | pm | gt25 | 15 | 1 | > 25 | Δ score = ± 0 | esld\_master\_long\_public.csv | 38 |
| 355 | P000865 | MELD-Na | 3.13 | 2.94451452732188 | 1.365 | 2.76 | 134 |  | F | 1 | pm | gt25 | 18 | 1 | > 25 | Δ score = ± 0 | esld\_master\_long\_public.csv | 28 |
| 356 | P000176 | MELD-Na | 1.71 | 1.64605366806371 | 2.49 | 5.255 | 138 |  | M | 0 | pm | gt25 | 19 | 1 | > 25 | Δ score = ± 0 | esld\_master\_long\_public.csv | 28 |
| 357 | P000641 | MELD-Na | 1.15 | 1.04130791805534 | 2.52 | 15.58 | 120 |  | M | 0 | pm | gt25 | 22 | 1 | > 25 | Δ score = ± 0 | esld\_master\_long\_public.csv | 33 |
| 358 | P001196 | MELD-Na | 0.97 | 0.887587546683377 | 2.49 | 36.35 | 122 |  | F | 0 | pm | gt25 | 30 | 1 | > 25 | Δ score = ± 0 | esld\_master\_long\_public.csv | 34 |
| 359 | P000157 | MELD-Na | 1.73 | 1.68000319321039 | 1.9 | 3.7 | 130 |  | F | 0 | pm | gt25 | 31 | 1 | > 25 | Δ score = ± 0 | esld\_master\_long\_public.csv | 28 |
| 360 | P001200 | MELD-Na | 3.12 | 2.93799017571626 | 2.12 | 2.57 | 124 |  | M | 0 | pm | gt25 | 38 | 1 | > 25 | Δ score = ± 0 | esld\_master\_long\_public.csv | 33 |
| 361 | P000140 | MELD-Na | 1.11 | 1.04850589020649 | 2.26 | 8 | 126 |  | M | 0 | pm | gt25 | 146 | 1 | > 25 | Δ score = ± 0 | esld\_master\_long\_public.csv | 30 |
| 362 | P000647 | MELD-Na | 2.615 | 2.49987865571779 | 2.68 | 2.43 | 136 |  | F | 0 | pm | gt25 | 730 | 0 | > 25 | Δ score = ± 0 | esld\_master\_long\_public.csv | 30 |
| 363 | P000867 | MELD-Na | 2.35 | 2.17605094133548 | 1.77 | 13.21 | 126 |  | M | 0 | pm | gt25 | 730 | 0 | > 25 | Δ score = ± 0 | esld\_master\_long\_public.csv | 34 |
| 364 | P000620 | MELD-Na | 1.915 | 1.78274785030804 | 3.68 | 12.46 | 127 |  | F | 0 | m1 | gt25 | 0 | 1 | > 25 | Δ score ≤ -1 | esld\_master\_long\_public.csv | 38 |
| 365 | P000456 | MELD-Na | 3.13 | 2.94729653449257 | 4.82 | 2.5 | 143 |  | F | 0 | m1 | gt25 | 1 | 1 | > 25 | Δ score ≤ -1 | esld\_master\_long\_public.csv | 38 |
| 366 | P001181 | MELD-Na | 1.49 | 1.35194080851258 | 1.73 | 25.84 | 142 |  | M | 0 | m1 | gt25 | 1 | 1 | > 25 | Δ score ≤ -1 | esld\_master\_long\_public.csv | 29 |
| 367 | P000168 | MELD-Na | 3.14 | 2.89139954843583 | 2.015 | 9.7 | 139.5 |  | F | 0 | m1 | gt25 | 2 | 1 | > 25 | Δ score ≤ -1 | esld\_master\_long\_public.csv | 34 |
| 368 | P000680 | MELD-Na | 4.31 | 3.91061764448838 | 2.2 | 2.95 | 123 |  | F | 0 | m1 | gt25 | 3 | 1 | > 25 | Δ score ≤ -1 | esld\_master\_long\_public.csv | 36 |
| 369 | P000800 | MELD-Na | 1.89 | 1.78178838992938 | 5.285 | 9.09 | 140 |  | F | 0 | m1 | gt25 | 3 | 1 | > 25 | Δ score ≤ -1 | esld\_master\_long\_public.csv | 40 |
| 370 | P000432 | MELD-Na | 3.65 | 3.3577206142473 | 2.25 | 5.1 | 141 |  | M | 0 | m1 | gt25 | 4 | 1 | > 25 | Δ score ≤ -1 | esld\_master\_long\_public.csv | 34 |
| 371 | P001310 | MELD-Na | 2.41 | 2.3096015157557 | 2.56 | 3.14 | 134 |  | M | 0 | m1 | gt25 | 4 | 1 | > 25 | Δ score ≤ -1 | esld\_master\_long\_public.csv | 31 |
| 372 | P000162 | MELD-Na | 2.16 | 1.97602523002878 | 1.79 | 22.1 | 136 |  | M | 0 | m1 | gt25 | 5 | 1 | > 25 | Δ score ≤ -1 | esld\_master\_long\_public.csv | 32 |
| 373 | P001177 | MELD-Na | 2.49 | 2.30390531507049 | 4.93 | 12.79 | 144 |  | M | 0 | m1 | gt25 | 5 | 1 | > 25 | Δ score ≤ -1 | esld\_master\_long\_public.csv | 40 |
| 374 | P001212 | MELD-Na | 1.39 | 1.29879475437444 | 4.6 | 10.75 | 135 |  | M | 0 | m1 | gt25 | 6 | 1 | > 25 | Δ score ≤ -1 | esld\_master\_long\_public.csv | 36 |
| 375 | P001370 | MELD-Na | 2.35 | 2.26243747069092 | 2.03 | 2.5 | 141 |  | M | 0 | m1 | gt25 | 6 | 1 | > 25 | Δ score ≤ -1 | esld\_master\_long\_public.csv | 26 |
| 376 | P001203 | MELD-Na | 1.83 | 1.73740882629503 | 1.68 | 7.63 | 143 |  | M | 0 | m1 | gt25 | 7 | 1 | > 25 | Δ score ≤ -1 | esld\_master\_long\_public.csv | 26 |
| 377 | P001319 | MELD-Na | 2.63 | 2.43069793320467 | 2.05 | 35.34 | 125 |  | F | 1 | m1 | gt25 | 7 | 1 | > 25 | Δ score ≤ -1 | esld\_master\_long\_public.csv | 40 |
| 378 | P001366 | MELD-Na | 3.50333333333333 | 3.26899124879486 | 1.52666666666667 | 1.9 | 148 |  | M | 0 | m1 | gt25 | 7 | 1 | > 25 | Δ score ≤ -1 | esld\_master\_long\_public.csv | 26 |
| 379 | P000201 | MELD-Na | 5.255 | 4.52397347601369 | 2.77 | 29.2 | 127 |  | F | 0 | m1 | gt25 | 9 | 1 | > 25 | Δ score ≤ -1 | esld\_master\_long\_public.csv | 40 |
| 380 | P000315 | MELD-Na | 2.1 | 1.97754687021265 | 3.19 | 8.8 | 153 |  | M | 0 | m1 | gt25 | 12 | 1 | > 25 | Δ score ≤ -1 | esld\_master\_long\_public.csv | 35 |
| 381 | P000689 | MELD-Na | 4.21 | 3.76921229063832 | 1.66 | 10.11 | 126 |  | F | 0 | m1 | gt25 | 13 | 1 | > 25 | Δ score ≤ -1 | esld\_master\_long\_public.csv | 36 |
| 382 | P001142 | MELD-Na | 1.545 | 1.40357750769417 | 3.11 | 22.615 | 127.5 |  | F | 0 | m1 | gt25 | 13 | 1 | > 25 | Δ score ≤ -1 | esld\_master\_long\_public.csv | 37 |
| 383 | P001334 | MELD-Na | 3.6 | 3.33644226386934 | 2.36 | 3.09 | 122 |  | F | 1 | m1 | gt25 | 13 | 1 | > 25 | Δ score ≤ -1 | esld\_master\_long\_public.csv | 36 |
| 384 | P000873 | MELD-Na | 4.84 | 4.22433662146392 | 1.71 | 31.74 | 129 |  | M | 0 | m1 | gt25 | 14 | 1 | > 25 | Δ score ≤ -1 | esld\_master\_long\_public.csv | 39 |
| 385 | P000112 | MELD-Na | 1.295 | 1.18837620081537 | 2.05 | 14.08 | 143.5 |  | M | 0 | m1 | gt25 | 15 | 1 | > 25 | Δ score ≤ -1 | esld\_master\_long\_public.csv | 27 |
| 386 | P001024 | MELD-Na | 2.6 | 2.39164624491039 | 5.43 | 14.78 | 139 |  | F | 0 | m1 | gt25 | 17 | 1 | > 25 | Δ score ≤ -1 | esld\_master\_long\_public.csv | 40 |
| 387 | P001217 | MELD-Na | 1.495 | 1.41226793731349 | 1.555 | 38.835 | 141 |  | M | 0 | m1 | gt25 | 17 | 1 | > 25 | Δ score ≤ -1 | esld\_master\_long\_public.csv | 29 |
| 388 | P000397 | MELD-Na | 2.81 | 2.60764585682596 | 2.18 | 9.5 | 147 |  | M | 0 | m1 | gt25 | 18 | 1 | > 25 | Δ score ≤ -1 | esld\_master\_long\_public.csv | 34 |
| 389 | P000120 | MELD-Na | 1.95 | 1.91407496391572 | 5.76 | 1 | 141 |  | M | 0 | m1 | gt25 | 22 | 1 | > 25 | Δ score ≤ -1 | esld\_master\_long\_public.csv | 32 |
| 390 | P001171 | MELD-Na | 2.34 | 2.16385888489141 | 2.58 | 13.82 | 138 |  | M | 0 | m1 | gt25 | 22 | 1 | > 25 | Δ score ≤ -1 | esld\_master\_long\_public.csv | 35 |
| 391 | P001189 | MELD-Na | 0.53 | 0.457931298853161 | 2.31 | 10.13 | 135 |  | F | 0 | m1 | gt25 | 22 | 1 | > 25 | Δ score ≤ -1 | esld\_master\_long\_public.csv | 26 |
| 392 | P001176 | MELD-Na | 1.34 | 1.32675030514996 | 1.65 | 2.11 | 120 |  | M | 0 | m1 | gt25 | 23 | 1 | > 25 | Δ score ≤ -1 | esld\_master\_long\_public.csv | 27 |
| 393 | P000257 | MELD-Na | 2.29 | 2.15275604518725 | 1.3 | 8.5 | 134 |  | M | 0 | m1 | gt25 | 25 | 1 | > 25 | Δ score ≤ -1 | esld\_master\_long\_public.csv | 26 |
| 394 | P000438 | MELD-Na | 1.34 | 1.29528391306445 | 2.62 | 5.2 | 142 |  | F | 0 | m1 | gt25 | 33 | 1 | > 25 | Δ score ≤ -1 | esld\_master\_long\_public.csv | 26 |
| 395 | P000696 | MELD-Na | 3.03 | 2.78446056521364 | 2.355 | 11.59 | 130 |  | M | 1 | m1 | gt25 | 33 | 1 | > 25 | Δ score ≤ -1 | esld\_master\_long\_public.csv | 39 |
| 396 | P000739 | MELD-Na | 2.92 | 2.75336529906707 | 1.895 | 3.75 | 147 |  | M | 0 | m1 | gt25 | 37 | 1 | > 25 | Δ score ≤ -1 | esld\_master\_long\_public.csv | 29 |
| 397 | P000050 | MELD-Na | 1.99 | 1.92275324971491 | 2.19 | 3.6 | 145 |  | M | 0 | m1 | gt25 | 41 | 1 | > 25 | Δ score ≤ -1 | esld\_master\_long\_public.csv | 27 |
| 398 | P000318 | MELD-Na | 1.13 | 1.00462235284229 | 1.7 | 24.1 | 129 |  | F | 0 | m1 | gt25 | 42 | 1 | > 25 | Δ score ≤ -1 | esld\_master\_long\_public.csv | 30 |
| 399 | P000740 | MELD-Na | 2.16 | 2.08309798108392 | 4.05 | 3.17 | 134 |  | F | 0 | m1 | gt25 | 52 | 1 | > 25 | Δ score ≤ -1 | esld\_master\_long\_public.csv | 35 |
| 400 | P000781 | MELD-Na | 2.5 | 2.38294446725591 | 1.67 | 3.84 | 126.5 |  | M | 0 | m1 | gt25 | 52 | 1 | > 25 | Δ score ≤ -1 | esld\_master\_long\_public.csv | 31 |
| 401 | P001146 | MELD-Na | 2.985 | 2.76373688529807 | 2.58 | 8.95 | 139 |  | F | 0 | m1 | gt25 | 55 | 1 | > 25 | Δ score ≤ -1 | esld\_master\_long\_public.csv | 36 |
| 402 | P000194 | MELD-Na | 2.75 | 2.5467843756506 | 5.14 | 10.7 | 136 |  | M | 0 | m1 | gt25 | 62 | 1 | > 25 | Δ score ≤ -1 | esld\_master\_long\_public.csv | 40 |
| 403 | P000141 | MELD-Na | 3.03 | 2.82072307690323 | 3.18 | 6.7 | 145 |  | F | 0 | m1 | gt25 | 63 | 1 | > 25 | Δ score ≤ -1 | esld\_master\_long\_public.csv | 37 |
| 404 | P000228 | MELD-Na | 4.075 | 3.72434013363439 | 1.98 | 2.95 | 122 |  | M | 0 | m1 | gt25 | 68 | 1 | > 25 | Δ score ≤ -1 | esld\_master\_long\_public.csv | 35 |
| 405 | P000847 | MELD-Na | 1.82 | 1.67246734762944 | 3.26 | 17.33 | 129 |  | M | 0 | m1 | gt25 | 73 | 1 | > 25 | Δ score ≤ -1 | esld\_master\_long\_public.csv | 37 |
| 406 | P000840 | MELD-Na | 1.86 | 1.75512013291459 | 2.18 | 8.93 | 130 |  | F | 0 | m1 | gt25 | 96 | 1 | > 25 | Δ score ≤ -1 | esld\_master\_long\_public.csv | 32 |
| 407 | P000571 | MELD-Na | 4.09 | 3.63211295757681 | 3.9 | 19 | 157 |  | F | 0 | m1 | gt25 | 107 | 1 | > 25 | Δ score ≤ -1 | esld\_master\_long\_public.csv | 40 |
| 408 | P000577 | MELD-Na | 4.71 | 4.2400091234591 | 1.62 | 1.15 | 145 |  | M | 0 | m1 | gt25 | 109 | 1 | > 25 | Δ score ≤ -1 | esld\_master\_long\_public.csv | 26 |
| 409 | P000629 | MELD-Na | 5.96 | 5.15482505408464 | 1.43 | 0.47 | 134 |  | M | 0 | m1 | gt25 | 111 | 1 | > 25 | Δ score ≤ -1 | esld\_master\_long\_public.csv | 26 |
| 410 | P000425 | MELD-Na | 2.73 | 2.51263121514642 | 1.7 | 13.5 | 132 |  | M | 0 | m1 | gt25 | 116 | 1 | > 25 | Δ score ≤ -1 | esld\_master\_long\_public.csv | 33 |
| 411 | P000534 | MELD-Na | 7.77 | 6.29275482358055 | 3.37 | 1.56 | 169 |  | M | 0 | m1 | gt25 | 120 | 1 | > 25 | Δ score ≤ -1 | esld\_master\_long\_public.csv | 35 |
| 412 | P000955 | MELD-Na | 3.47 | 3.1881030251959 | 1.91 | 7.38 | 146.5 |  | F | 0 | m1 | gt25 | 150 | 1 | > 25 | Δ score ≤ -1 | esld\_master\_long\_public.csv | 33 |
| 413 | P001042 | MELD-Na | 2.84 | 2.67966835168512 | 3.14 | 4.16 | 136 |  | F | 0 | m1 | gt25 | 155 | 1 | > 25 | Δ score ≤ -1 | esld\_master\_long\_public.csv | 35 |
| 414 | P000189 | MELD-Na | 2.27 | 2.19894781783425 | 2.03 | 1.7 | 131 |  | M | 0 | m1 | gt25 | 163 | 1 | > 25 | Δ score ≤ -1 | esld\_master\_long\_public.csv | 27 |
| 415 | P000424 | MELD-Na | 1.94 | 1.90367789392987 | 1.49 | 1.1 | 119 |  | M | 0 | m1 | gt25 | 163 | 1 | > 25 | Δ score ≤ -1 | esld\_master\_long\_public.csv | 27 |
| 416 | P001116 | MELD-Na | 0.88 | 0.81857852265579 | 2.29 | 8.56 | 134 |  | M | 0 | m1 | gt25 | 189 | 1 | > 25 | Δ score ≤ -1 | esld\_master\_long\_public.csv | 26 |
| 417 | P001145 | MELD-Na | 2.83 | 2.63291110427294 | 5.93 | 8.46 | 148 |  | M | 0 | m1 | gt25 | 205 | 1 | > 25 | Δ score ≤ -1 | esld\_master\_long\_public.csv | 40 |
| 418 | P000233 | MELD-Na | 3.08 | 2.88229341452655 | 2.1 | 4.67 | 119 |  | F | 0 | m1 | gt25 | 225 | 1 | > 25 | Δ score ≤ -1 | esld\_master\_long\_public.csv | 35 |
| 419 | P000565 | MELD-Na | 2.95 | 2.71589130555848 | 1.49 | 36.2 | 140 |  | F | 0 | m1 | gt25 | 273 | 1 | > 25 | Δ score ≤ -1 | esld\_master\_long\_public.csv | 35 |
| 420 | P000138 | MELD-Na | 1.33 | 1.2068676673072 | 1.69 | 29.8 | 143 |  | M | 0 | m1 | gt25 | 320 | 1 | > 25 | Δ score ≤ -1 | esld\_master\_long\_public.csv | 28 |
| 421 | P000694 | MELD-Na | 2.39 | 2.30597639647277 | 2.24 | 1.81 | 139 |  | M | 0 | m1 | gt25 | 322 | 1 | > 25 | Δ score ≤ -1 | esld\_master\_long\_public.csv | 26 |
| 422 | P001070 | MELD-Na | 1.04 | 1.00796974982539 | 2.132 | 4.84 | 143 |  | F | 1 | m1 | gt25 | 471 | 1 | > 25 | Δ score ≤ -1 | esld\_master\_long\_public.csv | 34 |
| 423 | P000858 | MELD-Na | 0.663333333333333 | 0.632377176098575 | 4.28666666666667 | 5.16333333333333 | 131 |  | M | 0 | m1 | gt25 | 551 | 1 | > 25 | Δ score ≤ -1 | esld\_master\_long\_public.csv | 31 |
| 424 | P000912 | MELD-Na | 4.59 | 4.08893397889789 | 2.34 | 7.1 | 135 |  | M | 0 | m1 | gt25 | 616 | 1 | > 25 | Δ score ≤ -1 | esld\_master\_long\_public.csv | 37 |
| 425 | P000006 | MELD-Na | 1.86 | 1.76337882658825 | 1.62 | 7.87 | 142 |  | M | 0 | m1 | gt25 | 730 | 0 | > 25 | Δ score ≤ -1 | esld\_master\_long\_public.csv | 26 |
| 426 | P000011 | MELD-Na | 3.25 | 3.04144999400473 | 1.13 | 3.26 | 126 |  | F | 0 | m1 | gt25 | 730 | 0 | > 25 | Δ score ≤ -1 | esld\_master\_long\_public.csv | 30 |
| 427 | P000041 | MELD-Na | 3.45 | 3.23660342588408 | 2.13 | 0.83 | 143 |  | F | 0 | m1 | gt25 | 730 | 0 | > 25 | Δ score ≤ -1 | esld\_master\_long\_public.csv | 27 |
| 428 | P000069 | MELD-Na | 1.4 | 1.33086008495746 | 2.55 | 7.7 | 138 |  | F | 0 | m1 | gt25 | 730 | 0 | > 25 | Δ score ≤ -1 | esld\_master\_long\_public.csv | 28 |
| 429 | P000071 | MELD-Na | 2.75 | 2.52038256890317 | 2.07 | 15.59 | 134 |  | M | 0 | m1 | gt25 | 730 | 0 | > 25 | Δ score ≤ -1 | esld\_master\_long\_public.csv | 35 |
| 430 | P000093 | MELD-Na | 2.165 | 2.0180358542989 | 4.585 | 11.54 | 139 |  | F | 0 | m1 | gt25 | 730 | 0 | > 25 | Δ score ≤ -1 | esld\_master\_long\_public.csv | 40 |
| 431 | P000102 | MELD-Na | 2.56 | 2.38689845315044 | 3.6 | 9.56 | 137 |  | M | 0 | m1 | gt25 | 730 | 0 | > 25 | Δ score ≤ -1 | esld\_master\_long\_public.csv | 38 |
| 432 | P000129 | MELD-Na | 2.29 | 2.1059431928805 | 2.21 | 16.78 | 147 |  | M | 0 | m1 | gt25 | 730 | 0 | > 25 | Δ score ≤ -1 | esld\_master\_long\_public.csv | 34 |
| 433 | P000130 | MELD-Na | 0.82 | 0.705314619080917 | 1.71 | 19.38 | 127.5 |  | M | 0 | m1 | gt25 | 730 | 0 | > 25 | Δ score ≤ -1 | esld\_master\_long\_public.csv | 29 |
| 434 | P000148 | MELD-Na | 3.4 | 3.11596053060185 | 3.34 | 9.1 | 156 |  | M | 0 | m1 | gt25 | 730 | 0 | > 25 | Δ score ≤ -1 | esld\_master\_long\_public.csv | 40 |
| 435 | P000197 | MELD-Na | 2.89 | 2.74167404972661 | 2.25 | 2.38 | 134 |  | M | 0 | m1 | gt25 | 730 | 0 | > 25 | Δ score ≤ -1 | esld\_master\_long\_public.csv | 30 |
| 436 | P000284 | MELD-Na | 1.18 | 1.0856087709612 | 2.08 | 35.2 | 153 |  | F | 0 | m1 | gt25 | 730 | 0 | > 25 | Δ score ≤ -1 | esld\_master\_long\_public.csv | 30 |
| 437 | P000413 | MELD-Na | 3.03333333333333 | 2.79369845685236 | 2.23666666666667 | 10.6 | 137 |  | F | 0 | m1 | gt25 | 730 | 0 | > 25 | Δ score ≤ -1 | esld\_master\_long\_public.csv | 35 |
| 438 | P000454 | MELD-Na | 2.29 | 2.21704893210422 | 1.73 | 1.7 | 132 |  | M | 0 | m1 | gt25 | 730 | 0 | > 25 | Δ score ≤ -1 | esld\_master\_long\_public.csv | 26 |
| 439 | P000513 | MELD-Na | 3.82 | 3.42904959645688 | 3.32 | 14.8 | 132 |  | F | 0 | m1 | gt25 | 730 | 0 | > 25 | Δ score ≤ -1 | esld\_master\_long\_public.csv | 40 |
| 440 | P000559 | MELD-Na | 2.28 | 2.20533530443375 | 2.53 | 1.94 | 148 |  | M | 0 | m1 | gt25 | 730 | 0 | > 25 | Δ score ≤ -1 | esld\_master\_long\_public.csv | 27 |
| 441 | P000561 | MELD-Na | 2.26 | 2.18899655017485 | 1.56 | 1.78 | 125 |  | F | 0 | m1 | gt25 | 730 | 0 | > 25 | Δ score ≤ -1 | esld\_master\_long\_public.csv | 29 |
| 442 | P000615 | MELD-Na | 2.39333333333333 | 2.3128355042854 | 1.63333333333333 | 1.465 | 146.5 |  | F | 1 | m1 | gt25 | 730 | 0 | > 25 | Δ score ≤ -1 | esld\_master\_long\_public.csv | 27 |
| 443 | P000633 | MELD-Na | 1.64 | 1.5370936408271 | 1.78 | 10.63 | 152 |  | M | 1 | m1 | gt25 | 730 | 0 | > 25 | Δ score ≤ -1 | esld\_master\_long\_public.csv | 35 |
| 444 | P000755 | MELD-Na | 2.8 | 2.60523710385799 | 2.89 | 8.65 | 136 |  | F | 0 | m1 | gt25 | 730 | 0 | > 25 | Δ score ≤ -1 | esld\_master\_long\_public.csv | 36 |
| 445 | P000760 | MELD-Na | 4.615 | 4.06176843504607 | 2.38 | 14.02 | 106.5 |  | M | 0 | m1 | gt25 | 730 | 0 | > 25 | Δ score ≤ -1 | esld\_master\_long\_public.csv | 39 |
| 446 | P000849 | MELD-Na | 2.16 | 2.04013980704436 | 2.1275 | 7.81 | 139 |  | M | 0 | m1 | gt25 | 730 | 0 | > 25 | Δ score ≤ -1 | esld\_master\_long\_public.csv | 30 |
| 447 | P001277 | reMELD-Na | 0.32 | 0.337230654948723 | 1.55 | 0.31 | 143 |  | M |  | pm | le15 | 1 | 1 | ≤ 15 | Δ score = ± 0 | esld\_master\_long\_public.csv | 6 |
| 448 | P001348 | reMELD-Na | 2.92 | 2.7915702249002 | 1.13 | 0.28 | 133 |  | F |  | pm | le15 | 2 | 1 | ≤ 15 | Δ score = ± 0 | esld\_master\_long\_public.csv | 14 |
| 449 | P000525 | reMELD-Na | 1.02 | 1.02098453959733 | 1.22 | 1.7 | 142 |  | M |  | pm | le15 | 6 | 1 | ≤ 15 | Δ score = ± 0 | esld\_master\_long\_public.csv | 12 |
| 450 | P000302 | reMELD-Na | 0.64 | 0.657275352328835 | 1.79 | 0.6 | 138 |  | F |  | pm | le15 | 7 | 1 | ≤ 15 | Δ score = ± 0 | esld\_master\_long\_public.csv | 9 |
| 451 | P001040 | reMELD-Na | 1.55 | 1.54121277346097 | 1.23 | 0.86 | 143 |  | F |  | pm | le15 | 7 | 1 | ≤ 15 | Δ score = ± 0 | esld\_master\_long\_public.csv | 13 |
| 452 | P000384 | reMELD-Na | 0.58 | 0.587866490762143 | 1.82 | 1.4 | 151 |  | F |  | pm | le15 | 15 | 1 | ≤ 15 | Δ score = ± 0 | esld\_master\_long\_public.csv | 12 |
| 453 | P000131 | reMELD-Na | 1.05 | 1.05597848096179 | 1.8 | 1.2 | 153 |  | F |  | pm | le15 | 25 | 1 | ≤ 15 | Δ score = ± 0 | esld\_master\_long\_public.csv | 15 |
| 454 | P000961 | reMELD-Na | 0.31 | 0.325840952994798 | 1.14 | 0.41 | 141 |  | F |  | pm | le15 | 28 | 1 | ≤ 15 | Δ score = ± 0 | esld\_master\_long\_public.csv | 4 |
| 455 | P000179 | reMELD-Na | 0.54 | 0.499984913783214 | 1.65 | 6.1 | 138 |  | F |  | pm | le15 | 46 | 1 | ≤ 15 | Δ score = ± 0 | esld\_master\_long\_public.csv | 15 |
| 456 | P000506 | reMELD-Na | 0.67 | 0.669179586872747 | 1.44 | 2.2 | 132 |  | M |  | pm | le15 | 46 | 1 | ≤ 15 | Δ score = ± 0 | esld\_master\_long\_public.csv | 14 |
| 457 | P000065 | reMELD-Na | 1.51 | 1.50534242218578 | 1.02 | 0.68 | 142 |  | M |  | pm | le15 | 48 | 1 | ≤ 15 | Δ score = ± 0 | esld\_master\_long\_public.csv | 11 |
| 458 | P001329 | reMELD-Na | 1.23 | 1.22274385466638 | 1.27 | 1.93 | 136 |  | F |  | pm | le15 | 73 | 1 | ≤ 15 | Δ score = ± 0 | esld\_master\_long\_public.csv | 15 |
| 459 | P000253 | reMELD-Na | 1.445 | 1.44146970478353 | 1.26 | 0.85 | 139 |  | F |  | pm | le15 | 601 | 1 | ≤ 15 | Δ score = ± 0 | esld\_master\_long\_public.csv | 13 |
| 460 | P000220 | reMELD-Na | 0.5 | 0.512427224422537 | 1.36 | 0.95 | 137 |  | F |  | pm | le15 | 730 | 0 | ≤ 15 | Δ score = ± 0 | esld\_master\_long\_public.csv | 8 |
| 461 | P000576 | reMELD-Na | 1.535 | 1.5327276273706 | 1.33 | 0.37 | 140.5 |  | M |  | pm | le15 | 730 | 0 | ≤ 15 | Δ score = ± 0 | esld\_master\_long\_public.csv | 12 |
| 462 | P000623 | reMELD-Na | 0.646666666666667 | 0.657178304926113 | 1.20666666666667 | 1.185 | 140.5 |  | F |  | pm | le15 | 730 | 0 | ≤ 15 | Δ score = ± 0 | esld\_master\_long\_public.csv | 7 |
| 463 | P000705 | reMELD-Na | 1.515 | 1.51026850902176 | 1.46 | 0.665 | 141 |  | M |  | pm | le15 | 730 | 0 | ≤ 15 | Δ score = ± 0 | esld\_master\_long\_public.csv | 14 |
| 464 | P000718 | reMELD-Na | 0.75 | 0.769431684013923 | 1.73 | 0.4 | 144 |  | M |  | pm | le15 | 730 | 0 | ≤ 15 | Δ score = ± 0 | esld\_master\_long\_public.csv | 8 |
| 465 | P001254 | reMELD-Na | 2.02 | 1.90525228005634 | 0 | 8.65 | 143 |  | M |  | m1 | le15 | 3 | 1 | ≤ 15 | Δ score ≤ -1 | esld\_master\_long\_public.csv | 1 |
| 466 | P000376 | reMELD-Na | 3.97 | 3.66504898584215 | 1.04 | 0.7 | 149 |  | F |  | m1 | le15 | 5 | 1 | ≤ 15 | Δ score ≤ -1 | esld\_master\_long\_public.csv | 15 |
| 467 | P000038 | reMELD-Na | 0.47 | 0.473369420702739 | 1.64 | 1.72 | 145 |  | F |  | m1 | le15 | 22 | 1 | ≤ 15 | Δ score ≤ -1 | esld\_master\_long\_public.csv | 11 |
| 468 | P000496 | reMELD-Na | 1.68 | 1.66581135829394 | 1.08 | 0.7 | 140 |  | F |  | m1 | le15 | 24 | 1 | ≤ 15 | Δ score ≤ -1 | esld\_master\_long\_public.csv | 12 |
| 469 | P000828 | reMELD-Na | 1.38 | 1.38072286319533 | 1.39 | 0.73 | 152 |  | M |  | m1 | le15 | 64 | 1 | ≤ 15 | Δ score ≤ -1 | esld\_master\_long\_public.csv | 13 |
| 470 | P000402 | reMELD-Na | 0.23 | 0.228045643722108 | 1.24 | 1.8 | 138 |  | M |  | m1 | le15 | 66 | 1 | ≤ 15 | Δ score ≤ -1 | esld\_master\_long\_public.csv | 9 |
| 471 | P000297 | reMELD-Na | 0.72 | 0.671902719315829 | 1.46 | 7.07 | 139 |  | F |  | m1 | le15 | 110 | 1 | ≤ 15 | Δ score ≤ -1 | esld\_master\_long\_public.csv | 15 |
| 472 | P000243 | reMELD-Na | 0.795 | 0.800881059739979 | 1.4 | 1.55 | 129.5 |  | M |  | m1 | le15 | 119 | 1 | ≤ 15 | Δ score ≤ -1 | esld\_master\_long\_public.csv | 15 |
| 473 | P000966 | reMELD-Na | 1.4 | 1.39662552916786 | 1.37 | 1.01 | 136 |  | M |  | m1 | le15 | 192 | 1 | ≤ 15 | Δ score ≤ -1 | esld\_master\_long\_public.csv | 15 |
| 474 | P000594 | reMELD-Na | 1.06 | 1.05154308510941 | 1.24 | 2.48 | 140 |  | M |  | m1 | le15 | 298 | 1 | ≤ 15 | Δ score ≤ -1 | esld\_master\_long\_public.csv | 13 |
| 475 | P000104 | reMELD-Na | 0.98 | 0.995656984709247 | 1.6 | 0.49 | 148 |  | F |  | m1 | le15 | 730 | 0 | ≤ 15 | Δ score ≤ -1 | esld\_master\_long\_public.csv | 10 |
| 476 | P000143 | reMELD-Na | 1.37 | 1.37137375331679 | 1.77 | 0.71 | 150 |  | M |  | m1 | le15 | 730 | 0 | ≤ 15 | Δ score ≤ -1 | esld\_master\_long\_public.csv | 15 |
| 477 | P000177 | reMELD-Na | 1.6 | 1.58958232878517 | 1.24 | 0.77 | 138 |  | F |  | m1 | le15 | 730 | 0 | ≤ 15 | Δ score ≤ -1 | esld\_master\_long\_public.csv | 14 |
| 478 | P000260 | reMELD-Na | 1.46 | 1.46010047914932 | 1.22 | 0.48 | 140 |  | M |  | m1 | le15 | 730 | 0 | ≤ 15 | Δ score ≤ -1 | esld\_master\_long\_public.csv | 11 |
| 479 | P000399 | reMELD-Na | 1.68 | 1.66768548963456 | 1.14 | 0.54 | 142 |  | M |  | m1 | le15 | 730 | 0 | ≤ 15 | Δ score ≤ -1 | esld\_master\_long\_public.csv | 12 |
| 480 | P000626 | reMELD-Na | 0.68 | 0.682028708290505 | 1.48 | 1.94 | 136 |  | M |  | m1 | le15 | 730 | 0 | ≤ 15 | Δ score ≤ -1 | esld\_master\_long\_public.csv | 12 |
| 481 | P000164 | reMELD-Na | 2.3 | 2.23786201906189 | 1.495 | 0.67 | 128.5 |  | F |  | pm | 16to25 | 0 | 1 | 16 - 25 | Δ score = ± 0 | esld\_master\_long\_public.csv | 19 |
| 482 | P001067 | reMELD-Na | 0.766666666666667 | 0.748280092804634 | 3.90285714285714 | 3.84333333333333 | 138 |  | F |  | pm | 16to25 | 0 | 1 | 16 - 25 | Δ score = ± 0 | esld\_master\_long\_public.csv | 19 |
| 483 | P000661 | reMELD-Na | 2.02 | 1.98623796901758 | 2.14 | 0.35 | 143 |  | M |  | pm | 16to25 | 1 | 1 | 16 - 25 | Δ score = ± 0 | esld\_master\_long\_public.csv | 18 |
| 484 | P000264 | reMELD-Na | 6.2 | 5.31574786694408 | 2.07 | 0.7 | 147 |  | M |  | pm | 16to25 | 2 | 1 | 16 - 25 | Δ score = ± 0 | esld\_master\_long\_public.csv | 22 |
| 485 | P000319 | reMELD-Na | 1.38333333333333 | 1.37792174700726 | 1.59333333333333 | 1.25 | 140 |  | F |  | pm | 16to25 | 2 | 1 | 16 - 25 | Δ score = ± 0 | esld\_master\_long\_public.csv | 16 |
| 486 | P000500 | reMELD-Na | 1.46 | 1.41975252540341 | 1.935 | 4.2 | 139.5 |  | M |  | pm | 16to25 | 2 | 1 | 16 - 25 | Δ score = ± 0 | esld\_master\_long\_public.csv | 22 |
| 487 | P001301 | reMELD-Na | 2.62 | 2.49437469190787 | 1.32 | 3.37 | 129 |  | M |  | pm | 16to25 | 3 | 1 | 16 - 25 | Δ score = ± 0 | esld\_master\_long\_public.csv | 23 |
| 488 | P000336 | reMELD-Na | 2 | 1.96487292632333 | 1.49 | 0.6 | 134 |  | M |  | pm | 16to25 | 4 | 1 | 16 - 25 | Δ score = ± 0 | esld\_master\_long\_public.csv | 17 |
| 489 | P000665 | reMELD-Na | 2.38 | 2.30765687029206 | 1.64 | 0.87 | 143 |  | F |  | pm | 16to25 | 4 | 1 | 16 - 25 | Δ score = ± 0 | esld\_master\_long\_public.csv | 20 |
| 490 | P001229 | reMELD-Na | 3.01 | 2.86774354004668 | 1.24 | 0.42 | 137 |  | M |  | pm | 16to25 | 4 | 1 | 16 - 25 | Δ score = ± 0 | esld\_master\_long\_public.csv | 16 |
| 491 | P000943 | reMELD-Na | 3.03 | 2.8774022541663 | 1.31 | 1.07 | 139 |  | M |  | pm | 16to25 | 5 | 1 | 16 - 25 | Δ score = ± 0 | esld\_master\_long\_public.csv | 19 |
| 492 | P000682 | reMELD-Na | 1.6 | 1.58189821954639 | 2.11 | 1.44 | 140 |  | M |  | pm | 16to25 | 6 | 1 | 16 - 25 | Δ score = ± 0 | esld\_master\_long\_public.csv | 20 |
| 493 | P001370 | reMELD-Na | 2.35 | 2.26243747069092 | 2.03 | 2.5 | 141 |  | M |  | pm | 16to25 | 6 | 1 | 16 - 25 | Δ score = ± 0 | esld\_master\_long\_public.csv | 25 |
| 494 | P001203 | reMELD-Na | 1.83 | 1.73740882629503 | 1.68 | 7.63 | 143 |  | M |  | pm | 16to25 | 7 | 1 | 16 - 25 | Δ score = ± 0 | esld\_master\_long\_public.csv | 24 |
| 495 | P001366 | reMELD-Na | 3.50333333333333 | 3.26899124879486 | 1.52666666666667 | 1.9 | 148 |  | M |  | pm | 16to25 | 7 | 1 | 16 - 25 | Δ score = ± 0 | esld\_master\_long\_public.csv | 22 |
| 496 | P000903 | reMELD-Na | 0.75 | 0.733971158022278 | 2.2 | 3.62 | 144 |  | M |  | pm | 16to25 | 8 | 1 | 16 - 25 | Δ score = ± 0 | esld\_master\_long\_public.csv | 17 |
| 497 | P001158 | reMELD-Na | 0.99 | 0.985300801450023 | 1.85 | 2.27 | 146.5 |  | F |  | pm | 16to25 | 8 | 1 | 16 - 25 | Δ score = ± 0 | esld\_master\_long\_public.csv | 16 |
| 498 | P000210 | reMELD-Na | 3.03 | 2.88639627973574 | 1.9 | 0.3 | 144 |  | M |  | pm | 16to25 | 10 | 1 | 16 - 25 | Δ score = ± 0 | esld\_master\_long\_public.csv | 19 |
| 499 | P000380 | reMELD-Na | 3.42 | 3.21183636496828 | 1.87 | 0.8 | 139 |  | M |  | pm | 16to25 | 10 | 1 | 16 - 25 | Δ score = ± 0 | esld\_master\_long\_public.csv | 21 |
| 500 | P001051 | reMELD-Na | 2.55 | 2.46094587701674 | 2.15 | 0.76 | 137 |  | M |  | pm | 16to25 | 12 | 1 | 16 - 25 | Δ score = ± 0 | esld\_master\_long\_public.csv | 23 |
| 501 | P000361 | reMELD-Na | 2.73 | 2.61842297819206 | 2.26 | 0.9 | 145 |  | F |  | pm | 16to25 | 13 | 1 | 16 - 25 | Δ score = ± 0 | esld\_master\_long\_public.csv | 24 |
| 502 | P001317 | reMELD-Na | 3.09 | 2.93382423766075 | 1.66 | 0.65 | 138 |  | F |  | pm | 16to25 | 13 | 1 | 16 - 25 | Δ score = ± 0 | esld\_master\_long\_public.csv | 20 |
| 503 | P000442 | reMELD-Na | 5.35 | 4.66895920445532 | 1.36 | 5.7 | 141 |  | M |  | pm | 16to25 | 15 | 1 | 16 - 25 | Δ score = ± 0 | esld\_master\_long\_public.csv | 24 |
| 504 | P000865 | reMELD-Na | 3.13 | 2.94451452732188 | 1.365 | 2.76 | 134 |  | F |  | pm | 16to25 | 18 | 1 | 16 - 25 | Δ score = ± 0 | esld\_master\_long\_public.csv | 22 |
| 505 | P001273 | reMELD-Na | 2.47 | 2.3818221593811 | 1.33 | 1.44 | 138 |  | M |  | pm | 16to25 | 20 | 1 | 16 - 25 | Δ score = ± 0 | esld\_master\_long\_public.csv | 20 |
| 506 | P000120 | reMELD-Na | 1.95 | 1.91407496391572 | 5.76 | 1 | 141 |  | M |  | pm | 16to25 | 22 | 1 | 16 - 25 | Δ score = ± 0 | esld\_master\_long\_public.csv | 23 |
| 507 | P000386 | reMELD-Na | 0.72 | 0.731402868916093 | 1.35 | 1.1 | 122 |  | M |  | pm | 16to25 | 24 | 1 | 16 - 25 | Δ score = ± 0 | esld\_master\_long\_public.csv | 16 |
| 508 | P000522 | reMELD-Na | 3.11666666666667 | 2.94466366091965 | 1.63 | 1.7 | 148.333333333333 |  | M |  | pm | 16to25 | 28 | 1 | 16 - 25 | Δ score = ± 0 | esld\_master\_long\_public.csv | 22 |
| 509 | P001261 | reMELD-Na | 2.05 | 1.98704768379053 | 1.86 | 2.73 | 138 |  | F |  | pm | 16to25 | 32 | 1 | 16 - 25 | Δ score = ± 0 | esld\_master\_long\_public.csv | 23 |
| 510 | P001159 | reMELD-Na | 1.215 | 1.16841908553479 | 1.49875 | 5.89 | 141 |  | F |  | pm | 16to25 | 47 | 1 | 16 - 25 | Δ score = ± 0 | esld\_master\_long\_public.csv | 19 |
| 511 | P000274 | reMELD-Na | 1.59 | 1.56840982135296 | 1.44 | 1.8 | 144 |  | M |  | pm | 16to25 | 75 | 1 | 16 - 25 | Δ score = ± 0 | esld\_master\_long\_public.csv | 17 |
| 512 | P000629 | reMELD-Na | 5.96 | 5.15482505408464 | 1.43 | 0.47 | 134 |  | M |  | pm | 16to25 | 111 | 1 | 16 - 25 | Δ score = ± 0 | esld\_master\_long\_public.csv | 18 |
| 513 | P000169 | reMELD-Na | 1.79 | 1.77158848879538 | 1.63 | 0.47 | 138 |  | F |  | pm | 16to25 | 158 | 1 | 16 - 25 | Δ score = ± 0 | esld\_master\_long\_public.csv | 16 |
| 514 | P000159 | reMELD-Na | 2.04 | 1.97500201145955 | 1.46 | 3 | 133 |  | M |  | pm | 16to25 | 169 | 1 | 16 - 25 | Δ score = ± 0 | esld\_master\_long\_public.csv | 22 |
| 515 | P000627 | reMELD-Na | 4.08 | 3.75414377879156 | 1.19 | 0.63 | 139 |  | M |  | pm | 16to25 | 202 | 1 | 16 - 25 | Δ score = ± 0 | esld\_master\_long\_public.csv | 16 |
| 516 | P001208 | reMELD-Na | 0.8 | 0.802380357076246 | 2.34 | 1.86 | 137.5 |  | M |  | pm | 16to25 | 316 | 1 | 16 - 25 | Δ score = ± 0 | esld\_master\_long\_public.csv | 16 |
| 517 | P001162 | reMELD-Na | 1.49 | 1.46471517319051 | 1.19 | 2.61 | 134 |  | F |  | pm | 16to25 | 358 | 1 | 16 - 25 | Δ score = ± 0 | esld\_master\_long\_public.csv | 17 |
| 518 | P000440 | reMELD-Na | 3.27 | 3.06751465504505 | 1.21 | 2.4 | 137 |  | M |  | pm | 16to25 | 658 | 1 | 16 - 25 | Δ score = ± 0 | esld\_master\_long\_public.csv | 21 |
| 519 | P000011 | reMELD-Na | 3.25 | 3.04144999400473 | 1.13 | 3.26 | 126 |  | F |  | pm | 16to25 | 730 | 0 | 16 - 25 | Δ score = ± 0 | esld\_master\_long\_public.csv | 22 |
| 520 | P000015 | reMELD-Na | 1.96 | 1.87899575814967 | 1.45 | 5.24 | 164 |  | M |  | pm | 16to25 | 730 | 0 | 16 - 25 | Δ score = ± 0 | esld\_master\_long\_public.csv | 22 |
| 521 | P000035 | reMELD-Na | 1.61 | 1.59213766196358 | 1.92 | 1.37 | 143 |  | F |  | pm | 16to25 | 730 | 0 | 16 - 25 | Δ score = ± 0 | esld\_master\_long\_public.csv | 19 |
| 522 | P000115 | reMELD-Na | 3.97 | 3.65927550619595 | 1.39 | 1.2 | 146 |  | F |  | pm | 16to25 | 730 | 0 | 16 - 25 | Δ score = ± 0 | esld\_master\_long\_public.csv | 20 |
| 523 | P000218 | reMELD-Na | 2.29 | 2.23317938629221 | 1.62 | 0.3 | 135 |  | M |  | pm | 16to25 | 730 | 0 | 16 - 25 | Δ score = ± 0 | esld\_master\_long\_public.csv | 17 |
| 524 | P000365 | reMELD-Na | 2.225 | 2.16082941528465 | 1.67 | 1.46 | 145.5 |  | F |  | pm | 16to25 | 730 | 0 | 16 - 25 | Δ score = ± 0 | esld\_master\_long\_public.csv | 21 |
| 525 | P000454 | reMELD-Na | 2.29 | 2.21704893210422 | 1.73 | 1.7 | 132 |  | M |  | pm | 16to25 | 730 | 0 | 16 - 25 | Δ score = ± 0 | esld\_master\_long\_public.csv | 23 |
| 526 | P000532 | reMELD-Na | 1.91 | 1.87739592391313 | 1.8 | 0.97 | 139 |  | F |  | pm | 16to25 | 730 | 0 | 16 - 25 | Δ score = ± 0 | esld\_master\_long\_public.csv | 19 |
| 527 | P000727 | reMELD-Na | 1.89 | 1.8481156599397 | 1.42 | 1.92 | 130 |  | M |  | pm | 16to25 | 730 | 0 | 16 - 25 | Δ score = ± 0 | esld\_master\_long\_public.csv | 20 |
| 528 | P000487 | reMELD-Na | 2 | 1.89272451929947 | 1.42 | 7.9 | 154 |  | M |  | m1 | 16to25 | 1 | 1 | 16 - 25 | Δ score ≤ -1 | esld\_master\_long\_public.csv | 24 |
| 529 | P000844 | reMELD-Na | 2.08 | 2.02193667277054 | 1.68 | 2.05 | 142 |  | M |  | m1 | 16to25 | 1 | 1 | 16 - 25 | Δ score ≤ -1 | esld\_master\_long\_public.csv | 22 |
| 530 | P000649 | reMELD-Na | 3.16 | 2.99253496943502 | 1.36 | 0.75 | 153 |  | F |  | m1 | 16to25 | 6 | 1 | 16 - 25 | Δ score ≤ -1 | esld\_master\_long\_public.csv | 18 |
| 531 | P000024 | reMELD-Na | 2.34 | 2.24487038165096 | 1.15 | 3.31 | 148 |  | M |  | m1 | 16to25 | 9 | 1 | 16 - 25 | Δ score ≤ -1 | esld\_master\_long\_public.csv | 20 |
| 532 | P000514 | reMELD-Na | 1.07 | 1.01665109671129 | 1.51 | 7.13 | 154 |  | M |  | m1 | 16to25 | 9 | 1 | 16 - 25 | Δ score ≤ -1 | esld\_master\_long\_public.csv | 18 |
| 533 | P000191 | reMELD-Na | 3.61 | 3.37398887833063 | 1.45 | 0.46 | 146 |  | M |  | m1 | 16to25 | 11 | 1 | 16 - 25 | Δ score ≤ -1 | esld\_master\_long\_public.csv | 17 |
| 534 | P000112 | reMELD-Na | 1.295 | 1.18837620081537 | 2.05 | 14.08 | 143.5 |  | M |  | m1 | 16to25 | 15 | 1 | 16 - 25 | Δ score ≤ -1 | esld\_master\_long\_public.csv | 25 |
| 535 | P000598 | reMELD-Na | 0.38 | 0.318034206950338 | 1.63 | 8.5 | 141 |  | F |  | m1 | 16to25 | 22 | 1 | 16 - 25 | Δ score ≤ -1 | esld\_master\_long\_public.csv | 16 |
| 536 | P001189 | reMELD-Na | 0.53 | 0.457931298853161 | 2.31 | 10.13 | 135 |  | F |  | m1 | 16to25 | 22 | 1 | 16 - 25 | Δ score ≤ -1 | esld\_master\_long\_public.csv | 21 |
| 537 | P001176 | reMELD-Na | 1.34 | 1.32675030514996 | 1.65 | 2.11 | 120 |  | M |  | m1 | 16to25 | 23 | 1 | 16 - 25 | Δ score ≤ -1 | esld\_master\_long\_public.csv | 23 |
| 538 | P000257 | reMELD-Na | 2.29 | 2.15275604518725 | 1.3 | 8.5 | 134 |  | M |  | m1 | 16to25 | 25 | 1 | 16 - 25 | Δ score ≤ -1 | esld\_master\_long\_public.csv | 25 |
| 539 | P000157 | reMELD-Na | 1.73 | 1.68000319321039 | 1.9 | 3.7 | 130 |  | F |  | m1 | 16to25 | 31 | 1 | 16 - 25 | Δ score ≤ -1 | esld\_master\_long\_public.csv | 25 |
| 540 | P000438 | reMELD-Na | 1.34 | 1.29528391306445 | 2.62 | 5.2 | 142 |  | F |  | m1 | 16to25 | 33 | 1 | 16 - 25 | Δ score ≤ -1 | esld\_master\_long\_public.csv | 25 |
| 541 | P000602 | reMELD-Na | 1.37 | 1.351295758735 | 1.9 | 2.5 | 140 |  | F |  | m1 | 16to25 | 35 | 1 | 16 - 25 | Δ score ≤ -1 | esld\_master\_long\_public.csv | 20 |
| 542 | P000441 | reMELD-Na | 3.11 | 2.94460386895077 | 1.83 | 1.2 | 158 |  | M |  | m1 | 16to25 | 40 | 1 | 16 - 25 | Δ score ≤ -1 | esld\_master\_long\_public.csv | 22 |
| 543 | P000750 | reMELD-Na | 2.53 | 2.43682307393645 | 1.57 | 1.31 | 147 |  | F |  | m1 | 16to25 | 40 | 1 | 16 - 25 | Δ score ≤ -1 | esld\_master\_long\_public.csv | 21 |
| 544 | P000050 | reMELD-Na | 1.99 | 1.92275324971491 | 2.19 | 3.6 | 145 |  | M |  | m1 | 16to25 | 41 | 1 | 16 - 25 | Δ score ≤ -1 | esld\_master\_long\_public.csv | 25 |
| 545 | P000678 | reMELD-Na | 0.81 | 0.807576356313816 | 2.22 | 2.29 | 133.5 |  | F |  | m1 | 16to25 | 47 | 1 | 16 - 25 | Δ score ≤ -1 | esld\_master\_long\_public.csv | 18 |
| 546 | P001117 | reMELD-Na | 1.9 | 1.82131333361556 | 1.65 | 5.47 | 137 |  | M |  | m1 | 16to25 | 71 | 1 | 16 - 25 | Δ score ≤ -1 | esld\_master\_long\_public.csv | 24 |
| 547 | P000523 | reMELD-Na | 1.04 | 1.0405547447764 | 2 | 1.7 | 131 |  | F |  | m1 | 16to25 | 82 | 1 | 16 - 25 | Δ score ≤ -1 | esld\_master\_long\_public.csv | 19 |
| 548 | P000433 | reMELD-Na | 1.92 | 1.8674565708872 | 1.15 | 2.7 | 137 |  | M |  | m1 | 16to25 | 89 | 1 | 16 - 25 | Δ score ≤ -1 | esld\_master\_long\_public.csv | 18 |
| 549 | P000577 | reMELD-Na | 4.71 | 4.2400091234591 | 1.62 | 1.15 | 145 |  | M |  | m1 | 16to25 | 109 | 1 | 16 - 25 | Δ score ≤ -1 | esld\_master\_long\_public.csv | 21 |
| 550 | P000092 | reMELD-Na | 1.49 | 1.45952773957017 | 1.515 | 3.1 | 136 |  | M |  | m1 | 16to25 | 113 | 1 | 16 - 25 | Δ score ≤ -1 | esld\_master\_long\_public.csv | 19 |
| 551 | P001060 | reMELD-Na | 2.27 | 2.20345569382691 | 1.38 | 1.3 | 141 |  | M |  | m1 | 16to25 | 152 | 1 | 16 - 25 | Δ score ≤ -1 | esld\_master\_long\_public.csv | 19 |
| 552 | P000859 | reMELD-Na | 1.2 | 1.13252039458619 | 1.9 | 8.43 | 135 |  | M |  | m1 | 16to25 | 158 | 1 | 16 - 25 | Δ score ≤ -1 | esld\_master\_long\_public.csv | 23 |
| 553 | P000189 | reMELD-Na | 2.27 | 2.19894781783425 | 2.03 | 1.7 | 131 |  | M |  | m1 | 16to25 | 163 | 1 | 16 - 25 | Δ score ≤ -1 | esld\_master\_long\_public.csv | 24 |
| 554 | P000424 | reMELD-Na | 1.94 | 1.90367789392987 | 1.49 | 1.1 | 119 |  | M |  | m1 | 16to25 | 163 | 1 | 16 - 25 | Δ score ≤ -1 | esld\_master\_long\_public.csv | 21 |
| 555 | P001116 | reMELD-Na | 0.88 | 0.81857852265579 | 2.29 | 8.56 | 134 |  | M |  | m1 | 16to25 | 189 | 1 | 16 - 25 | Δ score ≤ -1 | esld\_master\_long\_public.csv | 23 |
| 556 | P001266 | reMELD-Na | 12.51 | 8.40855359864775 | 1.38 | 0.25 | 133 |  | M |  | m1 | 16to25 | 249 | 1 | 16 - 25 | Δ score ≤ -1 | esld\_master\_long\_public.csv | 16 |
| 557 | P000300 | reMELD-Na | 1.77 | 1.73990009546554 | 1.465 | 1.6 | 136 |  | F |  | m1 | 16to25 | 296 | 1 | 16 - 25 | Δ score ≤ -1 | esld\_master\_long\_public.csv | 19 |
| 558 | P000138 | reMELD-Na | 1.33 | 1.2068676673072 | 1.69 | 29.8 | 143 |  | M |  | m1 | 16to25 | 320 | 1 | 16 - 25 | Δ score ≤ -1 | esld\_master\_long\_public.csv | 25 |
| 559 | P000694 | reMELD-Na | 2.39 | 2.30597639647277 | 2.24 | 1.81 | 139 |  | M |  | m1 | 16to25 | 322 | 1 | 16 - 25 | Δ score ≤ -1 | esld\_master\_long\_public.csv | 25 |
| 560 | P001070 | reMELD-Na | 1.04 | 1.00796974982539 | 2.132 | 4.84 | 143 |  | F |  | m1 | 16to25 | 471 | 1 | 16 - 25 | Δ score ≤ -1 | esld\_master\_long\_public.csv | 20 |
| 561 | P000858 | reMELD-Na | 0.663333333333333 | 0.632377176098575 | 4.28666666666667 | 5.16333333333333 | 131 |  | M |  | m1 | 16to25 | 551 | 1 | 16 - 25 | Δ score ≤ -1 | esld\_master\_long\_public.csv | 23 |
| 562 | P001129 | reMELD-Na | 2.03 | 1.9753641583731 | 1.31 | 2.11 | 166 |  | F |  | m1 | 16to25 | 713 | 1 | 16 - 25 | Δ score ≤ -1 | esld\_master\_long\_public.csv | 19 |
| 563 | P000006 | reMELD-Na | 1.86 | 1.76337882658825 | 1.62 | 7.87 | 142 |  | M |  | m1 | 16to25 | 730 | 0 | 16 - 25 | Δ score ≤ -1 | esld\_master\_long\_public.csv | 24 |
| 564 | P000027 | reMELD-Na | 2.94 | 2.78877108851964 | 1.43 | 2.04 | 139 |  | M |  | m1 | 16to25 | 730 | 0 | 16 - 25 | Δ score ≤ -1 | esld\_master\_long\_public.csv | 22 |
| 565 | P000073 | reMELD-Na | 1.24 | 1.22234728941079 | 1.56 | 2.86 | 145 |  | M |  | m1 | 16to25 | 730 | 0 | 16 - 25 | Δ score ≤ -1 | esld\_master\_long\_public.csv | 17 |
| 566 | P000108 | reMELD-Na | 2.52 | 2.4160440175503 | 1.51 | 2.38 | 149 |  | F |  | m1 | 16to25 | 730 | 0 | 16 - 25 | Δ score ≤ -1 | esld\_master\_long\_public.csv | 23 |
| 567 | P000126 | reMELD-Na | 2.21 | 2.134655635035 | 1.64 | 2.6 | 146 |  | M |  | m1 | 16to25 | 730 | 0 | 16 - 25 | Δ score ≤ -1 | esld\_master\_long\_public.csv | 23 |
| 568 | P000130 | reMELD-Na | 0.82 | 0.705314619080917 | 1.71 | 19.38 | 127.5 |  | M |  | m1 | 16to25 | 730 | 0 | 16 - 25 | Δ score ≤ -1 | esld\_master\_long\_public.csv | 25 |
| 569 | P000195 | reMELD-Na | 1.89 | 1.86000441431409 | 1.33 | 0.87 | 150.5 |  | F |  | m1 | 16to25 | 730 | 0 | 16 - 25 | Δ score ≤ -1 | esld\_master\_long\_public.csv | 16 |
| 570 | P000202 | reMELD-Na | 3.65 | 3.40654651456607 | 1.44 | 0.5 | 140 |  | M |  | m1 | 16to25 | 730 | 0 | 16 - 25 | Δ score ≤ -1 | esld\_master\_long\_public.csv | 18 |
| 571 | P000213 | reMELD-Na | 0.93 | 0.907274343761223 | 3.35 | 4.1 | 142 |  | F |  | m1 | 16to25 | 730 | 0 | 16 - 25 | Δ score ≤ -1 | esld\_master\_long\_public.csv | 21 |
| 572 | P000265 | reMELD-Na | 1.22 | 1.17976671415079 | 1.64 | 5.185 | 134 |  | F |  | m1 | 16to25 | 730 | 0 | 16 - 25 | Δ score ≤ -1 | esld\_master\_long\_public.csv | 21 |
| 573 | P000285 | reMELD-Na | 0.66 | 0.552008354530637 | 1.55 | 17.24 | 158.5 |  | M |  | m1 | 16to25 | 730 | 0 | 16 - 25 | Δ score ≤ -1 | esld\_master\_long\_public.csv | 18 |
| 574 | P000290 | reMELD-Na | 1.29 | 1.248138744936 | 1.82 | 5.1 | 145 |  | F |  | m1 | 16to25 | 730 | 0 | 16 - 25 | Δ score ≤ -1 | esld\_master\_long\_public.csv | 21 |
| 575 | P000434 | reMELD-Na | 1.73 | 1.70904405271139 | 1.76 | 1.02 | 137 |  | M |  | m1 | 16to25 | 730 | 0 | 16 - 25 | Δ score ≤ -1 | esld\_master\_long\_public.csv | 19 |
| 576 | P000494 | reMELD-Na | 1.26 | 1.22694053926999 | 2.405 | 4.3 | 142 |  | F |  | m1 | 16to25 | 730 | 1 | 16 - 25 | Δ score ≤ -1 | esld\_master\_long\_public.csv | 23 |
| 577 | P000561 | reMELD-Na | 2.26 | 2.18899655017485 | 1.56 | 1.78 | 125 |  | F |  | m1 | 16to25 | 730 | 0 | 16 - 25 | Δ score ≤ -1 | esld\_master\_long\_public.csv | 23 |
| 578 | P000615 | reMELD-Na | 2.39333333333333 | 2.3128355042854 | 1.63333333333333 | 1.465 | 146.5 |  | F |  | m1 | 16to25 | 730 | 0 | 16 - 25 | Δ score ≤ -1 | esld\_master\_long\_public.csv | 22 |
| 579 | P000633 | reMELD-Na | 1.64 | 1.5370936408271 | 1.78 | 10.63 | 152 |  | M |  | m1 | 16to25 | 730 | 0 | 16 - 25 | Δ score ≤ -1 | esld\_master\_long\_public.csv | 25 |
| 580 | P000692 | reMELD-Na | 1.68 | 1.6652283389116 | 2.34 | 0.75 | 132 |  | M |  | m1 | 16to25 | 730 | 0 | 16 - 25 | Δ score ≤ -1 | esld\_master\_long\_public.csv | 21 |
| 581 | P000958 | reMELD-Na | 2.8 | 2.67978185268242 | 1.6 | 0.9 | 132 |  | M |  | m1 | 16to25 | 730 | 0 | 16 - 25 | Δ score ≤ -1 | esld\_master\_long\_public.csv | 21 |
| 582 | P000620 | reMELD-Na | 1.915 | 1.78274785030804 | 3.68 | 12.46 | 127 |  | F |  | pm | gt25 | 0 | 1 | > 25 | Δ score = ± 0 | esld\_master\_long\_public.csv | 32 |
| 583 | P001324 | reMELD-Na | 3.94 | 3.534002269821 | 4.86 | 34.45 | 132 |  | M |  | pm | gt25 | 0 | 1 | > 25 | Δ score = ± 0 | esld\_master\_long\_public.csv | 35 |
| 584 | P000456 | reMELD-Na | 3.13 | 2.94729653449257 | 4.82 | 2.5 | 143 |  | F |  | pm | gt25 | 1 | 1 | > 25 | Δ score = ± 0 | esld\_master\_long\_public.csv | 28 |
| 585 | P000680 | reMELD-Na | 4.31 | 3.91061764448838 | 2.2 | 2.95 | 123 |  | F |  | pm | gt25 | 3 | 1 | > 25 | Δ score = ± 0 | esld\_master\_long\_public.csv | 28 |
| 586 | P001319 | reMELD-Na | 2.63 | 2.43069793320467 | 2.05 | 35.34 | 125 |  | F |  | pm | gt25 | 7 | 1 | > 25 | Δ score = ± 0 | esld\_master\_long\_public.csv | 34 |
| 587 | P000389 | reMELD-Na | 3.03 | 2.87477370154041 | 5.99 | 1.3 | 129 |  | M |  | pm | gt25 | 9 | 1 | > 25 | Δ score = ± 0 | esld\_master\_long\_public.csv | 27 |
| 588 | P000485 | reMELD-Na | 6.07 | 5.177317196471 | 2.03 | 5.5 | 136 |  | M |  | pm | gt25 | 9 | 1 | > 25 | Δ score = ± 0 | esld\_master\_long\_public.csv | 28 |
| 589 | P001334 | reMELD-Na | 3.6 | 3.33644226386934 | 2.36 | 3.09 | 122 |  | F |  | pm | gt25 | 13 | 1 | > 25 | Δ score = ± 0 | esld\_master\_long\_public.csv | 29 |
| 590 | P001369 | reMELD-Na | 7.01 | 5.81433345566015 | 3.45 | 3.3 | 138 |  | M |  | pm | gt25 | 15 | 1 | > 25 | Δ score = ± 0 | esld\_master\_long\_public.csv | 29 |
| 591 | P000397 | reMELD-Na | 2.81 | 2.60764585682596 | 2.18 | 9.5 | 147 |  | M |  | pm | gt25 | 18 | 1 | > 25 | Δ score = ± 0 | esld\_master\_long\_public.csv | 30 |
| 592 | P000176 | reMELD-Na | 1.71 | 1.64605366806371 | 2.49 | 5.255 | 138 |  | M |  | pm | gt25 | 19 | 1 | > 25 | Δ score = ± 0 | esld\_master\_long\_public.csv | 26 |
| 593 | P001200 | reMELD-Na | 3.12 | 2.93799017571626 | 2.12 | 2.57 | 124 |  | M |  | pm | gt25 | 38 | 1 | > 25 | Δ score = ± 0 | esld\_master\_long\_public.csv | 27 |
| 594 | P001146 | reMELD-Na | 2.985 | 2.76373688529807 | 2.58 | 8.95 | 139 |  | F |  | pm | gt25 | 55 | 1 | > 25 | Δ score = ± 0 | esld\_master\_long\_public.csv | 32 |
| 595 | P000840 | reMELD-Na | 1.86 | 1.75512013291459 | 2.18 | 8.93 | 130 |  | F |  | pm | gt25 | 96 | 1 | > 25 | Δ score = ± 0 | esld\_master\_long\_public.csv | 29 |
| 596 | P000140 | reMELD-Na | 1.11 | 1.04850589020649 | 2.26 | 8 | 126 |  | M |  | pm | gt25 | 146 | 1 | > 25 | Δ score = ± 0 | esld\_master\_long\_public.csv | 27 |
| 597 | P001181 | reMELD-Na | 1.49 | 1.35194080851258 | 1.73 | 25.84 | 142 |  | M |  | m1 | gt25 | 1 | 1 | > 25 | Δ score ≤ -1 | esld\_master\_long\_public.csv | 26 |
| 598 | P000168 | reMELD-Na | 3.14 | 2.89139954843583 | 2.015 | 9.7 | 139.5 |  | F |  | m1 | gt25 | 2 | 1 | > 25 | Δ score ≤ -1 | esld\_master\_long\_public.csv | 30 |
| 599 | P000480 | reMELD-Na | 2.33 | 2.23803827709083 | 2.255 | 3.1 | 141 |  | M |  | m1 | gt25 | 2 | 1 | > 25 | Δ score ≤ -1 | esld\_master\_long\_public.csv | 27 |
| 600 | P000800 | reMELD-Na | 1.89 | 1.78178838992938 | 5.285 | 9.09 | 140 |  | F |  | m1 | gt25 | 3 | 1 | > 25 | Δ score ≤ -1 | esld\_master\_long\_public.csv | 29 |
| 601 | P000432 | reMELD-Na | 3.65 | 3.3577206142473 | 2.25 | 5.1 | 141 |  | M |  | m1 | gt25 | 4 | 1 | > 25 | Δ score ≤ -1 | esld\_master\_long\_public.csv | 29 |
| 602 | P001310 | reMELD-Na | 2.41 | 2.3096015157557 | 2.56 | 3.14 | 134 |  | M |  | m1 | gt25 | 4 | 1 | > 25 | Δ score ≤ -1 | esld\_master\_long\_public.csv | 29 |
| 603 | P000162 | reMELD-Na | 2.16 | 1.97602523002878 | 1.79 | 22.1 | 136 |  | M |  | m1 | gt25 | 5 | 1 | > 25 | Δ score ≤ -1 | esld\_master\_long\_public.csv | 30 |
| 604 | P001177 | reMELD-Na | 2.49 | 2.30390531507049 | 4.93 | 12.79 | 144 |  | M |  | m1 | gt25 | 5 | 1 | > 25 | Δ score ≤ -1 | esld\_master\_long\_public.csv | 33 |
| 605 | P001212 | reMELD-Na | 1.39 | 1.29879475437444 | 4.6 | 10.75 | 135 |  | M |  | m1 | gt25 | 6 | 1 | > 25 | Δ score ≤ -1 | esld\_master\_long\_public.csv | 28 |
| 606 | P000201 | reMELD-Na | 5.255 | 4.52397347601369 | 2.77 | 29.2 | 127 |  | F |  | m1 | gt25 | 9 | 1 | > 25 | Δ score ≤ -1 | esld\_master\_long\_public.csv | 36 |
| 607 | P000315 | reMELD-Na | 2.1 | 1.97754687021265 | 3.19 | 8.8 | 153 |  | M |  | m1 | gt25 | 12 | 1 | > 25 | Δ score ≤ -1 | esld\_master\_long\_public.csv | 30 |
| 608 | P000689 | reMELD-Na | 4.21 | 3.76921229063832 | 1.66 | 10.11 | 126 |  | F |  | m1 | gt25 | 13 | 1 | > 25 | Δ score ≤ -1 | esld\_master\_long\_public.csv | 29 |
| 609 | P001142 | reMELD-Na | 1.545 | 1.40357750769417 | 3.11 | 22.615 | 127.5 |  | F |  | m1 | gt25 | 13 | 1 | > 25 | Δ score ≤ -1 | esld\_master\_long\_public.csv | 33 |
| 610 | P000873 | reMELD-Na | 4.84 | 4.22433662146392 | 1.71 | 31.74 | 129 |  | M |  | m1 | gt25 | 14 | 1 | > 25 | Δ score ≤ -1 | esld\_master\_long\_public.csv | 32 |
| 611 | P001024 | reMELD-Na | 2.6 | 2.39164624491039 | 5.43 | 14.78 | 139 |  | F |  | m1 | gt25 | 17 | 1 | > 25 | Δ score ≤ -1 | esld\_master\_long\_public.csv | 33 |
| 612 | P001217 | reMELD-Na | 1.495 | 1.41226793731349 | 1.555 | 38.835 | 141 |  | M |  | m1 | gt25 | 17 | 1 | > 25 | Δ score ≤ -1 | esld\_master\_long\_public.csv | 26 |
| 613 | P000641 | reMELD-Na | 1.15 | 1.04130791805534 | 2.52 | 15.58 | 120 |  | M |  | m1 | gt25 | 22 | 1 | > 25 | Δ score ≤ -1 | esld\_master\_long\_public.csv | 32 |
| 614 | P001171 | reMELD-Na | 2.34 | 2.16385888489141 | 2.58 | 13.82 | 138 |  | M |  | m1 | gt25 | 22 | 1 | > 25 | Δ score ≤ -1 | esld\_master\_long\_public.csv | 32 |
| 615 | P001196 | reMELD-Na | 0.97 | 0.887587546683377 | 2.49 | 36.35 | 122 |  | F |  | m1 | gt25 | 30 | 1 | > 25 | Δ score ≤ -1 | esld\_master\_long\_public.csv | 33 |
| 616 | P000696 | reMELD-Na | 3.03 | 2.78446056521364 | 2.355 | 11.59 | 130 |  | M |  | m1 | gt25 | 33 | 1 | > 25 | Δ score ≤ -1 | esld\_master\_long\_public.csv | 32 |
| 617 | P000318 | reMELD-Na | 1.13 | 1.00462235284229 | 1.7 | 24.1 | 129 |  | F |  | m1 | gt25 | 42 | 1 | > 25 | Δ score ≤ -1 | esld\_master\_long\_public.csv | 27 |
| 618 | P000740 | reMELD-Na | 2.16 | 2.08309798108392 | 4.05 | 3.17 | 134 |  | F |  | m1 | gt25 | 52 | 1 | > 25 | Δ score ≤ -1 | esld\_master\_long\_public.csv | 28 |
| 619 | P000194 | reMELD-Na | 2.75 | 2.5467843756506 | 5.14 | 10.7 | 136 |  | M |  | m1 | gt25 | 62 | 1 | > 25 | Δ score ≤ -1 | esld\_master\_long\_public.csv | 32 |
| 620 | P000141 | reMELD-Na | 3.03 | 2.82072307690323 | 3.18 | 6.7 | 145 |  | F |  | m1 | gt25 | 63 | 1 | > 25 | Δ score ≤ -1 | esld\_master\_long\_public.csv | 31 |
| 621 | P000228 | reMELD-Na | 4.075 | 3.72434013363439 | 1.98 | 2.95 | 122 |  | M |  | m1 | gt25 | 68 | 1 | > 25 | Δ score ≤ -1 | esld\_master\_long\_public.csv | 27 |
| 622 | P000847 | reMELD-Na | 1.82 | 1.67246734762944 | 3.26 | 17.33 | 129 |  | M |  | m1 | gt25 | 73 | 1 | > 25 | Δ score ≤ -1 | esld\_master\_long\_public.csv | 33 |
| 623 | P000571 | reMELD-Na | 4.09 | 3.63211295757681 | 3.9 | 19 | 157 |  | F |  | m1 | gt25 | 107 | 1 | > 25 | Δ score ≤ -1 | esld\_master\_long\_public.csv | 34 |
| 624 | P000425 | reMELD-Na | 2.73 | 2.51263121514642 | 1.7 | 13.5 | 132 |  | M |  | m1 | gt25 | 116 | 1 | > 25 | Δ score ≤ -1 | esld\_master\_long\_public.csv | 29 |
| 625 | P000534 | reMELD-Na | 7.77 | 6.29275482358055 | 3.37 | 1.56 | 169 |  | M |  | m1 | gt25 | 120 | 1 | > 25 | Δ score ≤ -1 | esld\_master\_long\_public.csv | 27 |
| 626 | P000955 | reMELD-Na | 3.47 | 3.1881030251959 | 1.91 | 7.38 | 146.5 |  | F |  | m1 | gt25 | 150 | 1 | > 25 | Δ score ≤ -1 | esld\_master\_long\_public.csv | 28 |
| 627 | P001042 | reMELD-Na | 2.84 | 2.67966835168512 | 3.14 | 4.16 | 136 |  | F |  | m1 | gt25 | 155 | 1 | > 25 | Δ score ≤ -1 | esld\_master\_long\_public.csv | 30 |
| 628 | P001145 | reMELD-Na | 2.83 | 2.63291110427294 | 5.93 | 8.46 | 148 |  | M |  | m1 | gt25 | 205 | 1 | > 25 | Δ score ≤ -1 | esld\_master\_long\_public.csv | 32 |
| 629 | P000233 | reMELD-Na | 3.08 | 2.88229341452655 | 2.1 | 4.67 | 119 |  | F |  | m1 | gt25 | 225 | 1 | > 25 | Δ score ≤ -1 | esld\_master\_long\_public.csv | 29 |
| 630 | P000565 | reMELD-Na | 2.95 | 2.71589130555848 | 1.49 | 36.2 | 140 |  | F |  | m1 | gt25 | 273 | 1 | > 25 | Δ score ≤ -1 | esld\_master\_long\_public.csv | 30 |
| 631 | P000912 | reMELD-Na | 4.59 | 4.08893397889789 | 2.34 | 7.1 | 135 |  | M |  | m1 | gt25 | 616 | 1 | > 25 | Δ score ≤ -1 | esld\_master\_long\_public.csv | 30 |
| 632 | P000069 | reMELD-Na | 1.4 | 1.33086008495746 | 2.55 | 7.7 | 138 |  | F |  | m1 | gt25 | 730 | 0 | > 25 | Δ score ≤ -1 | esld\_master\_long\_public.csv | 26 |
| 633 | P000071 | reMELD-Na | 2.75 | 2.52038256890317 | 2.07 | 15.59 | 134 |  | M |  | m1 | gt25 | 730 | 0 | > 25 | Δ score ≤ -1 | esld\_master\_long\_public.csv | 32 |
| 634 | P000093 | reMELD-Na | 2.165 | 2.0180358542989 | 4.585 | 11.54 | 139 |  | F |  | m1 | gt25 | 730 | 0 | > 25 | Δ score ≤ -1 | esld\_master\_long\_public.csv | 31 |
| 635 | P000102 | reMELD-Na | 2.56 | 2.38689845315044 | 3.6 | 9.56 | 137 |  | M |  | m1 | gt25 | 730 | 0 | > 25 | Δ score ≤ -1 | esld\_master\_long\_public.csv | 32 |
| 636 | P000129 | reMELD-Na | 2.29 | 2.1059431928805 | 2.21 | 16.78 | 147 |  | M |  | m1 | gt25 | 730 | 0 | > 25 | Δ score ≤ -1 | esld\_master\_long\_public.csv | 31 |
| 637 | P000148 | reMELD-Na | 3.4 | 3.11596053060185 | 3.34 | 9.1 | 156 |  | M |  | m1 | gt25 | 730 | 0 | > 25 | Δ score ≤ -1 | esld\_master\_long\_public.csv | 32 |
| 638 | P000197 | reMELD-Na | 2.89 | 2.74167404972661 | 2.25 | 2.38 | 134 |  | M |  | m1 | gt25 | 730 | 0 | > 25 | Δ score ≤ -1 | esld\_master\_long\_public.csv | 27 |
| 639 | P000284 | reMELD-Na | 1.18 | 1.0856087709612 | 2.08 | 35.2 | 153 |  | F |  | m1 | gt25 | 730 | 0 | > 25 | Δ score ≤ -1 | esld\_master\_long\_public.csv | 26 |
| 640 | P000413 | reMELD-Na | 3.03333333333333 | 2.79369845685236 | 2.23666666666667 | 10.6 | 137 |  | F |  | m1 | gt25 | 730 | 0 | > 25 | Δ score ≤ -1 | esld\_master\_long\_public.csv | 31 |
| 641 | P000513 | reMELD-Na | 3.82 | 3.42904959645688 | 3.32 | 14.8 | 132 |  | F |  | m1 | gt25 | 730 | 0 | > 25 | Δ score ≤ -1 | esld\_master\_long\_public.csv | 34 |
| 642 | P000559 | reMELD-Na | 2.28 | 2.20533530443375 | 2.53 | 1.94 | 148 |  | M |  | m1 | gt25 | 730 | 0 | > 25 | Δ score ≤ -1 | esld\_master\_long\_public.csv | 26 |
| 643 | P000755 | reMELD-Na | 2.8 | 2.60523710385799 | 2.89 | 8.65 | 136 |  | F |  | m1 | gt25 | 730 | 0 | > 25 | Δ score ≤ -1 | esld\_master\_long\_public.csv | 32 |
| 644 | P000760 | reMELD-Na | 4.615 | 4.06176843504607 | 2.38 | 14.02 | 106.5 |  | M |  | m1 | gt25 | 730 | 0 | > 25 | Δ score ≤ -1 | esld\_master\_long\_public.csv | 33 |
| 645 | P000867 | reMELD-Na | 2.35 | 2.17605094133548 | 1.77 | 13.21 | 126 |  | M |  | m1 | gt25 | 730 | 0 | > 25 | Δ score ≤ -1 | esld\_master\_long\_public.csv | 30 |
| 646 | P000116 | MELD 3.0 | 0.98 | 0.992029762213455 | 1.09 | 0.8 | 156 | 2.86 | M |  | pm | le15 | 7 | 1 | ≤ 15 | Δ score = ± 0 | esld\_master\_long\_public.csv | 8 |
| 647 | P000384 | MELD 3.0 | 0.49 | 0.491585611916996 | 1.44 | 1.9 | 157 | 3.4 | F |  | pm | le15 | 14 | 1 | ≤ 15 | Δ score = ± 0 | esld\_master\_long\_public.csv | 14 |
| 648 | P000496 | MELD 3.0 | 1.68 | 1.66581135829394 | 1.08 | 0.7 | 140 | 2.21 | F |  | pm | le15 | 17 | 1 | ≤ 15 | Δ score = ± 0 | esld\_master\_long\_public.csv | 15 |
| 649 | P000386 | MELD 3.0 | 0.64 | 0.654940754169378 | 1.31 | 0.8 | 134 | 2.89 | M |  | pm | le15 | 21 | 1 | ≤ 15 | Δ score = ± 0 | esld\_master\_long\_public.csv | 12 |
| 650 | P000961 | MELD 3.0 | 0.41 | 0.428220732051151 | 1.08 | 0.36 | 133 | 2.53 | F |  | pm | le15 | 25 | 1 | ≤ 15 | Δ score = ± 0 | esld\_master\_long\_public.csv | 13 |
| 651 | P000402 | MELD 3.0 | 0.24 | 0.241656138544348 | 1.4 | 1.5 | 141 | 0.66 | M |  | pm | le15 | 36 | 1 | ≤ 15 | Δ score = ± 0 | esld\_master\_long\_public.csv | 15 |
| 652 | P000750 | MELD 3.0 | 1.65 | 1.63375086049673 | 1.24 | 1.03 | 148 | 3.5 | F |  | pm | le15 | 40 | 1 | ≤ 15 | Δ score = ± 0 | esld\_master\_long\_public.csv | 15 |
| 653 | P000503 | MELD 3.0 | 0.74 | 0.749838641527572 | 1.1 | 1.23 | 137 | 3.31 | M |  | pm | le15 | 52 | 1 | ≤ 15 | Δ score = ± 0 | esld\_master\_long\_public.csv | 8 |
| 654 | P000312 | MELD 3.0 | 0.48 | 0.494891506741215 | 1.31 | 0.72 | 137 | 1.92 | M |  | pm | le15 | 94 | 1 | ≤ 15 | Δ score = ± 0 | esld\_master\_long\_public.csv | 11 |
| 655 | P000243 | MELD 3.0 | 0.63 | 0.635786792986197 | 1.32 | 1.6 | 138 | 2.79 | M |  | pm | le15 | 95 | 1 | ≤ 15 | Δ score = ± 0 | esld\_master\_long\_public.csv | 12 |
| 656 | P000497 | MELD 3.0 | 0.8 | 0.81546514571348 | 1.08 | 0.71 | 139 | 3.3 | M |  | pm | le15 | 142 | 1 | ≤ 15 | Δ score = ± 0 | esld\_master\_long\_public.csv | 7 |
| 657 | P000424 | MELD 3.0 | 0.88 | 0.881111260181458 | 1.73 | 1.9 | 145 | 4.12 | M |  | pm | le15 | 150 | 1 | ≤ 15 | Δ score = ± 0 | esld\_master\_long\_public.csv | 14 |
| 658 | P000169 | MELD 3.0 | 0.69 | 0.70703893439735 | 1.04 | 0.62 | 138 | 3.97 | F |  | pm | le15 | 158 | 1 | ≤ 15 | Δ score = ± 0 | esld\_master\_long\_public.csv | 8 |
| 659 | P001162 | MELD 3.0 | 0.94 | 0.941576768356667 | 1.22 | 1.78 | 138 | 2.08 | F |  | pm | le15 | 169 | 1 | ≤ 15 | Δ score = ± 0 | esld\_master\_long\_public.csv | 14 |
| 660 | P001250 | MELD 3.0 | 0.68 | 0.69763739346383 | 0.89 | 0.57 | 139 | 3.4 | M |  | pm | le15 | 351 | 1 | ≤ 15 | Δ score = ± 0 | esld\_master\_long\_public.csv | 6 |
| 661 | P000623 | MELD 3.0 | 0.67 | 0.689292014205374 | 1.11 | 0.43 | 139 | 3.57 | F |  | pm | le15 | 452 | 1 | ≤ 15 | Δ score = ± 0 | esld\_master\_long\_public.csv | 8 |
| 662 | P000465 | MELD 3.0 | 0.73 | 0.748340747513733 | 1.15 | 0.5 | 139 | 3.31 | M |  | pm | le15 | 481 | 1 | ≤ 15 | Δ score = ± 0 | esld\_master\_long\_public.csv | 8 |
| 663 | P000440 | MELD 3.0 | 0.78 | 0.784308651994027 | 1.15 | 1.7 | 139 | 2.76 | M |  | pm | le15 | 645 | 1 | ≤ 15 | Δ score = ± 0 | esld\_master\_long\_public.csv | 11 |
| 664 | P000019 | MELD 3.0 | 0.59 | 0.608305354896653 | 1.21 | 0.5 | 144 | 2.56 | M |  | pm | le15 | 730 | 0 | ≤ 15 | Δ score = ± 0 | esld\_master\_long\_public.csv | 9 |
| 665 | P000035 | MELD 3.0 | 0.79 | 0.801374154111208 | 0.99 | 1.07 | 151 | 3.11 | F |  | pm | le15 | 730 | 0 | ≤ 15 | Δ score = ± 0 | esld\_master\_long\_public.csv | 8 |
| 666 | P000041 | MELD 3.0 | 0.83 | 0.844267656176916 | 1.75 | 0.79 | 147 | 4.08 | F |  | pm | le15 | 730 | 0 | ≤ 15 | Δ score = ± 0 | esld\_master\_long\_public.csv | 12 |
| 667 | P000044 | MELD 3.0 | 0.98 | 0.99814166071287 | 1.145 | 0.28 | 138.5 | 2.55 | M |  | pm | le15 | 730 | 0 | ≤ 15 | Δ score = ± 0 | esld\_master\_long\_public.csv | 9 |
| 668 | P000069 | MELD 3.0 | 0.47 | 0.481534413392301 | 1.37 | 1 | 140 | 2.84 | F |  | pm | le15 | 730 | 0 | ≤ 15 | Δ score = ± 0 | esld\_master\_long\_public.csv | 11 |
| 669 | P000104 | MELD 3.0 | 0.98 | 0.995656984709247 | 1.6 | 0.49 | 148 | 0.48 | F |  | pm | le15 | 730 | 0 | ≤ 15 | Δ score = ± 0 | esld\_master\_long\_public.csv | 15 |
| 670 | P000108 | MELD 3.0 | 0.71 | 0.713637443202232 | 1.08 | 1.79 | 137 | 3.33 | F |  | pm | le15 | 730 | 0 | ≤ 15 | Δ score = ± 0 | esld\_master\_long\_public.csv | 11 |
| 671 | P000155 | MELD 3.0 | 0.26 | 0.273664809442108 | 1.73 | 0.5 | 145 | 1.86 | M |  | pm | le15 | 730 | 0 | ≤ 15 | Δ score = ± 0 | esld\_master\_long\_public.csv | 14 |
| 672 | P000220 | MELD 3.0 | 0.59 | 0.602136036915465 | 1.36 | 1.03 | 138 | 3.19 | F |  | pm | le15 | 730 | 0 | ≤ 15 | Δ score = ± 0 | esld\_master\_long\_public.csv | 11 |
| 673 | P000399 | MELD 3.0 | 1.36 | 1.36330660221376 | 1.03 | 0.58 | 140 | 3.57 | M |  | pm | le15 | 730 | 0 | ≤ 15 | Δ score = ± 0 | esld\_master\_long\_public.csv | 10 |
| 674 | P000429 | MELD 3.0 | 0.76 | 0.779378386603455 | 1.25 | 0.4 | 140 | 2.23 | M |  | pm | le15 | 730 | 0 | ≤ 15 | Δ score = ± 0 | esld\_master\_long\_public.csv | 10 |
| 675 | P000434 | MELD 3.0 | 1.25 | 1.25581032613515 | 1.47 | 0.72 | 138 | 3.29 | M |  | pm | le15 | 730 | 0 | ≤ 15 | Δ score = ± 0 | esld\_master\_long\_public.csv | 12 |
| 676 | P000507 | MELD 3.0 | 0.64 | 0.661885818438325 | 1.33 | 0.21 | 147 | 2.35 | F |  | pm | le15 | 730 | 0 | ≤ 15 | Δ score = ± 0 | esld\_master\_long\_public.csv | 12 |
| 677 | P000576 | MELD 3.0 | 1.535 | 1.5327276273706 | 1.33 | 0.37 | 140.5 | 2.91 | M |  | pm | le15 | 730 | 0 | ≤ 15 | Δ score = ± 0 | esld\_master\_long\_public.csv | 14 |
| 678 | P000828 | MELD 3.0 | 1.38 | 1.38072286319533 | 1.39 | 0.73 | 152 | 4.27 | M |  | m1 | le15 | 63 | 1 | ≤ 15 | Δ score ≤ -1 | esld\_master\_long\_public.csv | 13 |
| 679 | P000143 | MELD 3.0 | 1.17 | 1.17825909790607 | 1.505 | 0.73 | 146 | 2.29 | M |  | m1 | le15 | 730 | 0 | ≤ 15 | Δ score ≤ -1 | esld\_master\_long\_public.csv | 13 |
| 680 | P000626 | MELD 3.0 | 0.95 | 0.94213468106991 | 1.34 | 2.63 | 138 | 4.29 | M |  | m1 | le15 | 730 | 0 | ≤ 15 | Δ score ≤ -1 | esld\_master\_long\_public.csv | 13 |
| 681 | P000027 | MELD 3.0 | 2.94 | 2.78877108851964 | 1.43 | 2.04 | 139 | 2.05 | M |  | pm | 16to25 | 1 | 1 | 16 - 25 | Δ score = ± 0 | esld\_master\_long\_public.csv | 24 |
| 682 | P000202 | MELD 3.0 | 3.43 | 3.22490343982659 | 1.46 | 0.4 | 140 | 1.57 | M |  | pm | 16to25 | 1 | 1 | 16 - 25 | Δ score = ± 0 | esld\_master\_long\_public.csv | 21 |
| 683 | P000661 | MELD 3.0 | 2.02 | 1.98623796901758 | 2.14 | 0.35 | 143 | 2.54 | M |  | pm | 16to25 | 1 | 1 | 16 - 25 | Δ score = ± 0 | esld\_master\_long\_public.csv | 21 |
| 684 | P000500 | MELD 3.0 | 1.46 | 1.41975252540341 | 1.935 | 4.2 | 139.5 | 2.1 | M |  | pm | 16to25 | 2 | 1 | 16 - 25 | Δ score = ± 0 | esld\_master\_long\_public.csv | 24 |
| 685 | P001348 | MELD 3.0 | 2.92 | 2.7915702249002 | 1.13 | 0.28 | 133 | 2.04 | F |  | pm | 16to25 | 2 | 1 | 16 - 25 | Δ score = ± 0 | esld\_master\_long\_public.csv | 23 |
| 686 | P001370 | MELD 3.0 | 2.35 | 2.26460066600373 | 1.68 | 2.3 | 138.333333333333 | 2.33 | M |  | pm | 16to25 | 2 | 1 | 16 - 25 | Δ score = ± 0 | esld\_master\_long\_public.csv | 24 |
| 687 | P001229 | MELD 3.0 | 3.41 | 3.20873397805339 | 1.24 | 0.35 | 136 | 1.23 | M |  | pm | 16to25 | 4 | 1 | 16 - 25 | Δ score = ± 0 | esld\_master\_long\_public.csv | 21 |
| 688 | P000302 | MELD 3.0 | 0.64 | 0.657275352328835 | 1.79 | 0.6 | 138 | 1.71 | F |  | pm | 16to25 | 7 | 1 | 16 - 25 | Δ score = ± 0 | esld\_master\_long\_public.csv | 16 |
| 689 | P000321 | MELD 3.0 | 2.09 | 2.03869321898837 | 1.64 | 1.37 | 134 | 1.42 | M |  | pm | 16to25 | 7 | 1 | 16 - 25 | Δ score = ± 0 | esld\_master\_long\_public.csv | 23 |
| 690 | P001366 | MELD 3.0 | 2.1425 | 2.06996098554576 | 1.6 | 2.9 | 146 | 2.38 | M |  | pm | 16to25 | 7 | 1 | 16 - 25 | Δ score = ± 0 | esld\_master\_long\_public.csv | 24 |
| 691 | P000396 | MELD 3.0 | 0.93 | 0.917485369101848 | 1.72 | 3.1 | 139 | 1.81 | M |  | pm | 16to25 | 8 | 1 | 16 - 25 | Δ score = ± 0 | esld\_master\_long\_public.csv | 19 |
| 692 | P000380 | MELD 3.0 | 3.09 | 2.92861109172601 | 1.87 | 1.1 | 153 | 2.67 | M |  | pm | 16to25 | 10 | 1 | 16 - 25 | Δ score = ± 0 | esld\_master\_long\_public.csv | 24 |
| 693 | P000361 | MELD 3.0 | 1.44 | 1.43153057889578 | 1.46 | 1.3 | 139 | 1.49 | F |  | pm | 16to25 | 13 | 1 | 16 - 25 | Δ score = ± 0 | esld\_master\_long\_public.csv | 18 |
| 694 | P001317 | MELD 3.0 | 3.09 | 2.93382423766075 | 1.66 | 0.65 | 138 | 3.4 | F |  | pm | 16to25 | 13 | 1 | 16 - 25 | Δ score = ± 0 | esld\_master\_long\_public.csv | 24 |
| 695 | P000265 | MELD 3.0 | 0.66 | 0.628698158794473 | 1.73 | 5.2 | 137 | 2.94 | F |  | pm | 16to25 | 15 | 1 | 16 - 25 | Δ score = ± 0 | esld\_master\_long\_public.csv | 21 |
| 696 | P000598 | MELD 3.0 | 0.38 | 0.318034206950338 | 1.63 | 8.5 | 141 | 3.09 | F |  | pm | 16to25 | 20 | 1 | 16 - 25 | Δ score = ± 0 | esld\_master\_long\_public.csv | 22 |
| 697 | P000038 | MELD 3.0 | 0.92 | 0.895544435985299 | 1.51 | 4.29 | 165 | 3.82 | F |  | pm | 16to25 | 22 | 1 | 16 - 25 | Δ score = ± 0 | esld\_master\_long\_public.csv | 18 |
| 698 | P000065 | MELD 3.0 | 5.6 | 4.90269373770657 | 1.37 | 0.5 | 144 | 2.24 | M |  | pm | 16to25 | 22 | 1 | 16 - 25 | Δ score = ± 0 | esld\_master\_long\_public.csv | 21 |
| 699 | P000257 | MELD 3.0 | 0.87 | 0.80610988441736 | 1.27 | 8.9 | 132 | 1.79 | M |  | pm | 16to25 | 24 | 1 | 16 - 25 | Δ score = ± 0 | esld\_master\_long\_public.csv | 23 |
| 700 | P000522 | MELD 3.0 | 3.11666666666667 | 2.94466366091965 | 1.63 | 1.7 | 148.333333333333 | 2.11 | M |  | pm | 16to25 | 28 | 1 | 16 - 25 | Δ score = ± 0 | esld\_master\_long\_public.csv | 25 |
| 701 | P000441 | MELD 3.0 | 1.5 | 1.49212367465952 | 1.55 | 1 | 151 | 1.95 | M |  | pm | 16to25 | 34 | 1 | 16 - 25 | Δ score = ± 0 | esld\_master\_long\_public.csv | 16 |
| 702 | P000234 | MELD 3.0 | 0.72 | 0.737318814103564 | 1.24 | 0.59 | 131 | 1.85 | M |  | pm | 16to25 | 38 | 1 | 16 - 25 | Δ score = ± 0 | esld\_master\_long\_public.csv | 16 |
| 703 | P000433 | MELD 3.0 | 1.92 | 1.8674565708872 | 1.15 | 2.7 | 137 | 2.22 | M |  | pm | 16to25 | 42 | 1 | 16 - 25 | Δ score = ± 0 | esld\_master\_long\_public.csv | 20 |
| 704 | P000506 | MELD 3.0 | 0.67 | 0.669179586872747 | 1.44 | 2.2 | 132 | 2.57 | M |  | pm | 16to25 | 45 | 1 | 16 - 25 | Δ score = ± 0 | esld\_master\_long\_public.csv | 18 |
| 705 | P001159 | MELD 3.0 | 1.215 | 1.16841908553479 | 1.49875 | 5.89 | 141 | 2.78 | F |  | pm | 16to25 | 47 | 1 | 16 - 25 | Δ score = ± 0 | esld\_master\_long\_public.csv | 22 |
| 706 | P000292 | MELD 3.0 | 1.23 | 1.23439577158049 | 1.56 | 0.9 | 135 | 1.78 | F |  | pm | 16to25 | 71 | 1 | 16 - 25 | Δ score = ± 0 | esld\_master\_long\_public.csv | 18 |
| 707 | P000847 | MELD 3.0 | 1.21 | 1.21667391183768 | 1.82 | 0.76 | 128 | 3.2 | M |  | pm | 16to25 | 73 | 1 | 16 - 25 | Δ score = ± 0 | esld\_master\_long\_public.csv | 21 |
| 708 | P000523 | MELD 3.0 | 1.04 | 1.0405547447764 | 2 | 1.7 | 131 | 2.48 | F |  | pm | 16to25 | 81 | 1 | 16 - 25 | Δ score = ± 0 | esld\_master\_long\_public.csv | 22 |
| 709 | P000840 | MELD 3.0 | 0.85 | 0.832359285331602 | 1.16 | 3.7 | 136 | 3.17 | F |  | pm | 16to25 | 96 | 1 | 16 - 25 | Δ score = ± 0 | esld\_master\_long\_public.csv | 16 |
| 710 | P000138 | MELD 3.0 | 2.31 | 2.23512654091965 | 1.56 | 1.7 | 134 | 3 | M |  | pm | 16to25 | 144 | 1 | 16 - 25 | Δ score = ± 0 | esld\_master\_long\_public.csv | 24 |
| 711 | P001060 | MELD 3.0 | 2.23 | 2.17468028041557 | 1.32 | 0.65 | 140 | 2.11 | M |  | pm | 16to25 | 152 | 1 | 16 - 25 | Δ score = ± 0 | esld\_master\_long\_public.csv | 18 |
| 712 | P000189 | MELD 3.0 | 1.58 | 1.55673724903048 | 1.35 | 2 | 130 | 3.01 | M |  | pm | 16to25 | 158 | 1 | 16 - 25 | Δ score = ± 0 | esld\_master\_long\_public.csv | 22 |
| 713 | P000966 | MELD 3.0 | 1.59 | 1.57792717684032 | 1.4 | 0.96 | 140 | 2.01 | M |  | pm | 16to25 | 180 | 1 | 16 - 25 | Δ score = ± 0 | esld\_master\_long\_public.csv | 16 |
| 714 | P000627 | MELD 3.0 | 4.28 | 3.9128247396731 | 1.36 | 0.63 | 146 | 3.23 | M |  | pm | 16to25 | 200 | 1 | 16 - 25 | Δ score = ± 0 | esld\_master\_long\_public.csv | 21 |
| 715 | P000594 | MELD 3.0 | 0.665 | 0.628511257953676 | 1 | 5.76 | 131 | 2.24 | M |  | pm | 16to25 | 292 | 1 | 16 - 25 | Δ score = ± 0 | esld\_master\_long\_public.csv | 19 |
| 716 | P001208 | MELD 3.0 | 0.77 | 0.74882123118003 | 2.08 | 4.12 | 142 | 3.78 | M |  | pm | 16to25 | 316 | 1 | 16 - 25 | Δ score = ± 0 | esld\_master\_long\_public.csv | 19 |
| 717 | P000152 | MELD 3.0 | 1.21 | 1.21815270352521 | 1.40666666666667 | 0.633333333333333 | 134 | 3.125 | F |  | pm | 16to25 | 506 | 1 | 16 - 25 | Δ score = ± 0 | esld\_master\_long\_public.csv | 16 |
| 718 | P001014 | MELD 3.0 | 0.64 | 0.633767803063514 | 1.61 | 2.7 | 136 | 2.14 | M |  | pm | 16to25 | 666 | 1 | 16 - 25 | Δ score = ± 0 | esld\_master\_long\_public.csv | 18 |
| 719 | P001129 | MELD 3.0 | 2.03 | 1.9753641583731 | 1.31 | 2.11 | 166 | 2.92 | F |  | pm | 16to25 | 713 | 1 | 16 - 25 | Δ score = ± 0 | esld\_master\_long\_public.csv | 21 |
| 720 | P000073 | MELD 3.0 | 1.21 | 1.19049642308766 | 1.48 | 3.13 | 141 | 2.73 | M |  | pm | 16to25 | 730 | 0 | 16 - 25 | Δ score = ± 0 | esld\_master\_long\_public.csv | 18 |
| 721 | P000091 | MELD 3.0 | 1.19 | 1.19695444758187 | 2.41 | 0.79 | 137.5 | 2.11 | F |  | pm | 16to25 | 730 | 0 | 16 - 25 | Δ score = ± 0 | esld\_master\_long\_public.csv | 19 |
| 722 | P000177 | MELD 3.0 | 1.6 | 1.58958232878517 | 1.24 | 0.77 | 138 | 1.84 | F |  | pm | 16to25 | 730 | 0 | 16 - 25 | Δ score = ± 0 | esld\_master\_long\_public.csv | 16 |
| 723 | P000218 | MELD 3.0 | 0.93 | 0.941724148439998 | 1.55 | 0.9 | 133 | 1.72 | M |  | pm | 16to25 | 730 | 0 | 16 - 25 | Δ score = ± 0 | esld\_master\_long\_public.csv | 17 |
| 724 | P000692 | MELD 3.0 | 1.28 | 1.28850929216819 | 1.4 | 0.4 | 130 | 1.95 | M |  | pm | 16to25 | 730 | 0 | 16 - 25 | Δ score = ± 0 | esld\_master\_long\_public.csv | 20 |
| 725 | P001254 | MELD 3.0 | 2.02 | 1.90525228005634 | 0 | 8.65 | 143 | 1.64 | M |  | m1 | 16to25 | 3 | 1 | 16 - 25 | Δ score ≤ -1 | esld\_master\_long\_public.csv | 25 |
| 726 | P000682 | MELD 3.0 | 1.6 | 1.58189821954639 | 2.11 | 1.44 | 140 | 1.9 | M |  | m1 | 16to25 | 6 | 1 | 16 - 25 | Δ score ≤ -1 | esld\_master\_long\_public.csv | 21 |
| 727 | P000024 | MELD 3.0 | 1.62 | 1.58429858972254 | 1.25 | 2.95 | 150 | 3.37 | M |  | m1 | 16to25 | 8 | 1 | 16 - 25 | Δ score ≤ -1 | esld\_master\_long\_public.csv | 18 |
| 728 | P000191 | MELD 3.0 | 3.61 | 3.37398887833063 | 1.45 | 0.46 | 146 | 2.02 | M |  | m1 | 16to25 | 8 | 1 | 16 - 25 | Δ score ≤ -1 | esld\_master\_long\_public.csv | 21 |
| 729 | P000210 | MELD 3.0 | 2.82 | 2.70310917095607 | 1.56 | 0.4 | 142 | 1.78 | M |  | m1 | 16to25 | 9 | 1 | 16 - 25 | Δ score ≤ -1 | esld\_master\_long\_public.csv | 22 |
| 730 | P000514 | MELD 3.0 | 1.07 | 1.01665109671129 | 1.51 | 7.13 | 154 | 2.85 | M |  | m1 | 16to25 | 9 | 1 | 16 - 25 | Δ score ≤ -1 | esld\_master\_long\_public.csv | 21 |
| 731 | P000115 | MELD 3.0 | 3.97 | 3.65927550619595 | 1.39 | 1.2 | 146 | 4.06 | F |  | m1 | 16to25 | 10 | 1 | 16 - 25 | Δ score ≤ -1 | esld\_master\_long\_public.csv | 23 |
| 732 | P001273 | MELD 3.0 | 2.47 | 2.3818221593811 | 1.33 | 1.44 | 138 | 3.26 | M |  | m1 | 16to25 | 20 | 1 | 16 - 25 | Δ score ≤ -1 | esld\_master\_long\_public.csv | 20 |
| 733 | P000274 | MELD 3.0 | 1.59 | 1.56840982135296 | 1.44 | 1.8 | 144 | 2.08 | M |  | m1 | 16to25 | 30 | 1 | 16 - 25 | Δ score ≤ -1 | esld\_master\_long\_public.csv | 19 |
| 734 | P000602 | MELD 3.0 | 1.68 | 1.64347362873844 | 2.28 | 2.7 | 139 | 3.46 | F |  | m1 | 16to25 | 35 | 1 | 16 - 25 | Δ score ≤ -1 | esld\_master\_long\_public.csv | 25 |
| 735 | P000678 | MELD 3.0 | 0.81 | 0.807576356313816 | 2.22 | 2.29 | 133.5 | 4.95 | F |  | m1 | 16to25 | 47 | 1 | 16 - 25 | Δ score ≤ -1 | esld\_master\_long\_public.csv | 21 |
| 736 | P000727 | MELD 3.0 | 1.79 | 1.7512939216756 | 1.31 | 2.26 | 129 | 2.3 | M |  | m1 | 16to25 | 59 | 1 | 16 - 25 | Δ score ≤ -1 | esld\_master\_long\_public.csv | 25 |
| 737 | P000228 | MELD 3.0 | 2.01 | 1.95272056446502 | 1.65 | 2.5 | 138 | 5.52 | M |  | m1 | 16to25 | 64 | 1 | 16 - 25 | Δ score ≤ -1 | esld\_master\_long\_public.csv | 23 |
| 738 | P001329 | MELD 3.0 | 1.37 | 1.35685175794452 | 1.25 | 1.99 | 136 | 2.7 | F |  | m1 | 16to25 | 73 | 1 | 16 - 25 | Δ score ≤ -1 | esld\_master\_long\_public.csv | 18 |
| 739 | P000577 | MELD 3.0 | 4.71 | 4.2400091234591 | 1.62 | 1.15 | 145 | 3.1 | M |  | m1 | 16to25 | 108 | 1 | 16 - 25 | Δ score ≤ -1 | esld\_master\_long\_public.csv | 23 |
| 740 | P000297 | MELD 3.0 | 0.72 | 0.671902719315829 | 1.46 | 7.07 | 139 | 2.39 | F |  | m1 | 16to25 | 110 | 1 | 16 - 25 | Δ score ≤ -1 | esld\_master\_long\_public.csv | 22 |
| 741 | P000092 | MELD 3.0 | 1.49 | 1.45952773957017 | 1.515 | 3.1 | 136 | 2.57 | M |  | m1 | 16to25 | 113 | 1 | 16 - 25 | Δ score ≤ -1 | esld\_master\_long\_public.csv | 21 |
| 742 | P000859 | MELD 3.0 | 1.2 | 1.13252039458619 | 1.9 | 8.43 | 135 | 3.03 | M |  | m1 | 16to25 | 154 | 1 | 16 - 25 | Δ score ≤ -1 | esld\_master\_long\_public.csv | 25 |
| 743 | P001116 | MELD 3.0 | 0.88 | 0.81857852265579 | 2.29 | 8.56 | 134 | 3.92 | M |  | m1 | 16to25 | 189 | 1 | 16 - 25 | Δ score ≤ -1 | esld\_master\_long\_public.csv | 24 |
| 744 | P001266 | MELD 3.0 | 12.51 | 8.40855359864775 | 1.38 | 0.25 | 133 | 2.56 | M |  | m1 | 16to25 | 249 | 1 | 16 - 25 | Δ score ≤ -1 | esld\_master\_long\_public.csv | 24 |
| 745 | P000195 | MELD 3.0 | 1.89 | 1.86000441431409 | 1.33 | 0.87 | 150.5 | 4.48 | F |  | m1 | 16to25 | 259 | 1 | 16 - 25 | Δ score ≤ -1 | esld\_master\_long\_public.csv | 17 |
| 746 | P000300 | MELD 3.0 | 1.605 | 1.58203195733145 | 2.1075 | 1.85 | 135 | 2.45 | F |  | m1 | 16to25 | 296 | 1 | 16 - 25 | Δ score ≤ -1 | esld\_master\_long\_public.csv | 25 |
| 747 | P000694 | MELD 3.0 | 3.97 | 3.6492008422251 | 1.49 | 2.1 | 139 | 3.55 | M |  | m1 | 16to25 | 322 | 1 | 16 - 25 | Δ score ≤ -1 | esld\_master\_long\_public.csv | 25 |
| 748 | P000260 | MELD 3.0 | 1.86 | 1.77471706119377 | 0.9 | 6.52 | 138 | 2.63 | M |  | m1 | 16to25 | 371 | 1 | 16 - 25 | Δ score ≤ -1 | esld\_master\_long\_public.csv | 22 |
| 749 | P001070 | MELD 3.0 | 1.04 | 1.00796974982539 | 2.132 | 4.84 | 143 | 2.82 | F |  | m1 | 16to25 | 471 | 1 | 16 - 25 | Δ score ≤ -1 | esld\_master\_long\_public.csv | 23 |
| 750 | P000320 | MELD 3.0 | 2.58 | 2.48712981290216 | 1.3 | 0.8 | 152 | 3.43 | M |  | m1 | 16to25 | 619 | 1 | 16 - 25 | Δ score ≤ -1 | esld\_master\_long\_public.csv | 19 |
| 751 | P000365 | MELD 3.0 | 2.225 | 2.16082941528465 | 1.67 | 1.46 | 145.5 | 2.68 | F |  | m1 | 16to25 | 715 | 1 | 16 - 25 | Δ score ≤ -1 | esld\_master\_long\_public.csv | 23 |
| 752 | P000130 | MELD 3.0 | 0.47 | 0.40430067572555 | 1.51 | 9.16 | 134 | 3.02 | M |  | m1 | 16to25 | 730 | 0 | 16 - 25 | Δ score ≤ -1 | esld\_master\_long\_public.csv | 22 |
| 753 | P000285 | MELD 3.0 | 0.66 | 0.552008354530637 | 1.55 | 17.24 | 158.5 | 4.38 | M |  | m1 | 16to25 | 730 | 0 | 16 - 25 | Δ score ≤ -1 | esld\_master\_long\_public.csv | 23 |
| 754 | P000290 | MELD 3.0 | 1.07 | 1.03083291141513 | 1.885 | 5.525 | 148 | 4.93 | F |  | m1 | 16to25 | 730 | 0 | 16 - 25 | Δ score ≤ -1 | esld\_master\_long\_public.csv | 22 |
| 755 | P000532 | MELD 3.0 | 1.91 | 1.87739592391313 | 1.8 | 0.97 | 139 | 3.42 | F |  | m1 | 16to25 | 730 | 0 | 16 - 25 | Δ score ≤ -1 | esld\_master\_long\_public.csv | 20 |
| 756 | P000561 | MELD 3.0 | 1.9 | 1.85541019164658 | 1.29 | 2.1 | 132 | 2.4 | F |  | m1 | 16to25 | 730 | 0 | 16 - 25 | Δ score ≤ -1 | esld\_master\_long\_public.csv | 24 |
| 757 | P000615 | MELD 3.0 | 2.39333333333333 | 2.3128355042854 | 1.63333333333333 | 1.465 | 146.5 | 2.21 | F |  | m1 | 16to25 | 730 | 0 | 16 - 25 | Δ score ≤ -1 | esld\_master\_long\_public.csv | 24 |
| 758 | P000794 | MELD 3.0 | 2.46 | 2.38079727907689 | 1.12 | 0.75 | 135 | 3.07 | M |  | m1 | 16to25 | 730 | 0 | 16 - 25 | Δ score ≤ -1 | esld\_master\_long\_public.csv | 19 |
| 759 | P000958 | MELD 3.0 | 2.53 | 2.44152529003779 | 1.53 | 0.9 | 138 | 2.96 | M |  | m1 | 16to25 | 730 | 0 | 16 - 25 | Δ score ≤ -1 | esld\_master\_long\_public.csv | 20 |
| 760 | P000164 | MELD 3.0 | 2.3 | 2.23786201906189 | 1.495 | 0.67 | 128.5 | 0.97 | F |  | pm | gt25 | 0 | 1 | > 25 | Δ score = ± 0 | esld\_master\_long\_public.csv | 28 |
| 761 | P000456 | MELD 3.0 | 3.13 | 2.94729653449257 | 4.82 | 2.5 | 143 | 1.91 | F |  | pm | gt25 | 0 | 1 | > 25 | Δ score = ± 0 | esld\_master\_long\_public.csv | 38 |
| 762 | P000487 | MELD 3.0 | 2 | 1.89272451929947 | 1.42 | 7.9 | 154 | 2.22 | M |  | pm | gt25 | 0 | 1 | > 25 | Δ score = ± 0 | esld\_master\_long\_public.csv | 27 |
| 763 | P000620 | MELD 3.0 | 1.915 | 1.78274785030804 | 3.68 | 12.46 | 127 | 2.18 | F |  | pm | gt25 | 0 | 1 | > 25 | Δ score = ± 0 | esld\_master\_long\_public.csv | 40 |
| 764 | P000740 | MELD 3.0 | 2.16 | 2.08309798108392 | 4.05 | 3.17 | 134 | 2.24 | F |  | pm | gt25 | 0 | 1 | > 25 | Δ score = ± 0 | esld\_master\_long\_public.csv | 36 |
| 765 | P001324 | MELD 3.0 | 3.94 | 3.534002269821 | 4.86 | 34.45 | 132 | 2.1 | M |  | pm | gt25 | 0 | 1 | > 25 | Δ score = ± 0 | esld\_master\_long\_public.csv | 40 |
| 766 | P000480 | MELD 3.0 | 2.33 | 2.23803827709083 | 2.255 | 3.1 | 141 | 2.3 | M |  | pm | gt25 | 1 | 1 | > 25 | Δ score = ± 0 | esld\_master\_long\_public.csv | 28 |
| 767 | P000800 | MELD 3.0 | 1.89 | 1.78178838992938 | 5.285 | 9.09 | 140 | 2.3 | F |  | pm | gt25 | 3 | 1 | > 25 | Δ score = ± 0 | esld\_master\_long\_public.csv | 40 |
| 768 | P001301 | MELD 3.0 | 2.62 | 2.49437469190787 | 1.32 | 3.37 | 129 | 2.4 | M |  | pm | gt25 | 3 | 1 | > 25 | Δ score = ± 0 | esld\_master\_long\_public.csv | 29 |
| 769 | P000432 | MELD 3.0 | 4.11 | 3.73222010295745 | 1.88666666666667 | 4.95 | 137.333333333333 | 1.74 | M |  | pm | gt25 | 4 | 1 | > 25 | Δ score = ± 0 | esld\_master\_long\_public.csv | 31 |
| 770 | P001310 | MELD 3.0 | 2.08 | 2.01436548917569 | 2.28 | 2.75 | 128 | 2.1 | M |  | pm | gt25 | 4 | 1 | > 25 | Δ score = ± 0 | esld\_master\_long\_public.csv | 32 |
| 771 | P000201 | MELD 3.0 | 2.31 | 2.11330339464056 | 2.62 | 26.6 | 131 | 2.1 | F |  | pm | gt25 | 5 | 1 | > 25 | Δ score = ± 0 | esld\_master\_long\_public.csv | 40 |
| 772 | P000389 | MELD 3.0 | 3.03 | 2.87477370154041 | 5.99 | 1.3 | 129 | 2.41 | M |  | pm | gt25 | 9 | 1 | > 25 | Δ score = ± 0 | esld\_master\_long\_public.csv | 40 |
| 773 | P001369 | MELD 3.0 | 5.2 | 4.59581564619204 | 2.27 | 2.1 | 138 | 2.43 | M |  | pm | gt25 | 11 | 1 | > 25 | Δ score = ± 0 | esld\_master\_long\_public.csv | 29 |
| 774 | P000689 | MELD 3.0 | 4.21 | 3.76921229063832 | 1.66 | 10.11 | 126 | 3.37 | F |  | pm | gt25 | 13 | 1 | > 25 | Δ score = ± 0 | esld\_master\_long\_public.csv | 38 |
| 775 | P000179 | MELD 3.0 | 1.415 | 1.37287405212234 | 2.05 | 4.6 | 122.5 | 2.09 | F |  | pm | gt25 | 18 | 1 | > 25 | Δ score = ± 0 | esld\_master\_long\_public.csv | 32 |
| 776 | P000176 | MELD 3.0 | 1.71 | 1.64605366806371 | 2.49 | 5.255 | 138 | 2.13 | M |  | pm | gt25 | 19 | 1 | > 25 | Δ score = ± 0 | esld\_master\_long\_public.csv | 29 |
| 777 | P001196 | MELD 3.0 | 0.54 | 0.496621681453642 | 2.48 | 41.23 | 127 | 4.11 | F |  | pm | gt25 | 30 | 1 | > 25 | Δ score = ± 0 | esld\_master\_long\_public.csv | 32 |
| 778 | P000438 | MELD 3.0 | 1.34 | 1.29528391306445 | 2.62 | 5.2 | 142 | 0.86 | F |  | pm | gt25 | 32 | 1 | > 25 | Δ score = ± 0 | esld\_master\_long\_public.csv | 29 |
| 779 | P001117 | MELD 3.0 | 1.9 | 1.82131333361556 | 1.65 | 5.47 | 137 | 2.05 | M |  | pm | gt25 | 71 | 1 | > 25 | Δ score = ± 0 | esld\_master\_long\_public.csv | 26 |
| 780 | P000140 | MELD 3.0 | 1.11 | 1.04850589020649 | 2.26 | 8 | 126 | 1.85 | M |  | pm | gt25 | 146 | 1 | > 25 | Δ score = ± 0 | esld\_master\_long\_public.csv | 30 |
| 781 | P001042 | MELD 3.0 | 2.26 | 2.17982643668975 | 2.05 | 2.62 | 137 | 1.8 | F |  | pm | gt25 | 155 | 1 | > 25 | Δ score = ± 0 | esld\_master\_long\_public.csv | 28 |
| 782 | P000159 | MELD 3.0 | 1.71 | 1.66226250811711 | 1.53 | 3.6 | 131 | 1.85 | M |  | pm | gt25 | 163 | 1 | > 25 | Δ score = ± 0 | esld\_master\_long\_public.csv | 26 |
| 783 | P000454 | MELD 3.0 | 2.29 | 2.21704893210422 | 1.73 | 1.7 | 132 | 1.5 | M |  | pm | gt25 | 315 | 1 | > 25 | Δ score = ± 0 | esld\_master\_long\_public.csv | 27 |
| 784 | P000912 | MELD 3.0 | 4.59 | 4.08893397889789 | 2.34 | 7.1 | 135 | 1.9 | M |  | pm | gt25 | 616 | 1 | > 25 | Δ score = ± 0 | esld\_master\_long\_public.csv | 35 |
| 785 | P000494 | MELD 3.0 | 1.26 | 1.22694053926999 | 2.405 | 4.3 | 142 | 2.41 | F |  | pm | gt25 | 710 | 1 | > 25 | Δ score = ± 0 | esld\_master\_long\_public.csv | 26 |
| 786 | P000148 | MELD 3.0 | 3.4 | 3.11596053060185 | 3.34 | 9.1 | 156 | 2.12 | M |  | pm | gt25 | 730 | 0 | > 25 | Δ score = ± 0 | esld\_master\_long\_public.csv | 39 |
| 787 | P000197 | MELD 3.0 | 2.255 | 2.1859716067306 | 1.95 | 1.645 | 132.5 | 2.2 | M |  | pm | gt25 | 730 | 0 | > 25 | Δ score = ± 0 | esld\_master\_long\_public.csv | 27 |
| 788 | P000760 | MELD 3.0 | 4.615 | 4.06176843504607 | 2.38 | 14.02 | 106.5 | 2.98 | M |  | pm | gt25 | 730 | 0 | > 25 | Δ score = ± 0 | esld\_master\_long\_public.csv | 40 |
| 789 | P000168 | MELD 3.0 | 3.14 | 2.89139954843583 | 2.015 | 9.7 | 139.5 | 3.125 | F |  | m1 | gt25 | 1 | 1 | > 25 | Δ score ≤ -1 | esld\_master\_long\_public.csv | 36 |
| 790 | P001181 | MELD 3.0 | 1.49 | 1.35194080851258 | 1.73 | 25.84 | 142 | 3.38 | M |  | m1 | gt25 | 1 | 1 | > 25 | Δ score ≤ -1 | esld\_master\_long\_public.csv | 30 |
| 791 | P000680 | MELD 3.0 | 4.31 | 3.91061764448838 | 2.2 | 2.95 | 123 | 2.47 | F |  | m1 | gt25 | 2 | 1 | > 25 | Δ score ≤ -1 | esld\_master\_long\_public.csv | 38 |
| 792 | P000413 | MELD 3.0 | 3.03333333333333 | 2.79369845685236 | 2.23666666666667 | 10.6 | 137 | 2.35 | F |  | m1 | gt25 | 5 | 1 | > 25 | Δ score ≤ -1 | esld\_master\_long\_public.csv | 36 |
| 793 | P001177 | MELD 3.0 | 2.49 | 2.30390531507049 | 4.93 | 12.79 | 144 | 2.87 | M |  | m1 | gt25 | 5 | 1 | > 25 | Δ score ≤ -1 | esld\_master\_long\_public.csv | 40 |
| 794 | P001203 | MELD 3.0 | 1.85 | 1.75775585001493 | 1.3 | 7.42 | 137 | 1.92 | M |  | m1 | gt25 | 6 | 1 | > 25 | Δ score ≤ -1 | esld\_master\_long\_public.csv | 26 |
| 795 | P001212 | MELD 3.0 | 1.39 | 1.29879475437444 | 4.6 | 10.75 | 135 | 2.43 | M |  | m1 | gt25 | 6 | 1 | > 25 | Δ score ≤ -1 | esld\_master\_long\_public.csv | 36 |
| 796 | P001319 | MELD 3.0 | 2.02 | 1.88391366221174 | 1.84 | 35.96 | 128 | 2.49 | F |  | m1 | gt25 | 7 | 1 | > 25 | Δ score ≤ -1 | esld\_master\_long\_public.csv | 37 |
| 797 | P000315 | MELD 3.0 | 2.1 | 1.97754687021265 | 3.19 | 8.8 | 153 | 1.32 | M |  | m1 | gt25 | 9 | 1 | > 25 | Δ score ≤ -1 | esld\_master\_long\_public.csv | 36 |
| 798 | P000112 | MELD 3.0 | 1.295 | 1.18837620081537 | 2.05 | 14.08 | 143.5 | 3.72 | M |  | m1 | gt25 | 11 | 1 | > 25 | Δ score ≤ -1 | esld\_master\_long\_public.csv | 27 |
| 799 | P000213 | MELD 3.0 | 0.83 | 0.812565778334357 | 2.22 | 3.7 | 130 | 2.68 | F |  | m1 | gt25 | 12 | 1 | > 25 | Δ score ≤ -1 | esld\_master\_long\_public.csv | 26 |
| 800 | P000873 | MELD 3.0 | 1.92 | 1.75694196298264 | 1.9 | 20.84 | 134 | 3.12 | M |  | m1 | gt25 | 12 | 1 | > 25 | Δ score ≤ -1 | esld\_master\_long\_public.csv | 33 |
| 801 | P000865 | MELD 3.0 | 3.13 | 2.94451452732188 | 1.365 | 2.76 | 134 | 2.23 | F |  | m1 | gt25 | 13 | 1 | > 25 | Δ score ≤ -1 | esld\_master\_long\_public.csv | 29 |
| 802 | P001142 | MELD 3.0 | 1.545 | 1.40357750769417 | 3.11 | 22.615 | 127.5 | 4.105 | F |  | m1 | gt25 | 13 | 1 | > 25 | Δ score ≤ -1 | esld\_master\_long\_public.csv | 37 |
| 803 | P001334 | MELD 3.0 | 3.6 | 3.33644226386934 | 2.36 | 3.09 | 122 | 3.53 | F |  | m1 | gt25 | 13 | 1 | > 25 | Δ score ≤ -1 | esld\_master\_long\_public.csv | 39 |
| 804 | P001217 | MELD 3.0 | 1.5 | 1.38400560418474 | 1.435 | 33.52 | 136.5 | 3.235 | M |  | m1 | gt25 | 16 | 1 | > 25 | Δ score ≤ -1 | esld\_master\_long\_public.csv | 30 |
| 805 | P001024 | MELD 3.0 | 2.6 | 2.39164624491039 | 5.43 | 14.78 | 139 | 3.56 | F |  | m1 | gt25 | 17 | 1 | > 25 | Δ score ≤ -1 | esld\_master\_long\_public.csv | 40 |
| 806 | P001171 | MELD 3.0 | 2.34 | 2.16385888489141 | 2.58 | 13.82 | 138 | 3.64 | M |  | m1 | gt25 | 21 | 1 | > 25 | Δ score ≤ -1 | esld\_master\_long\_public.csv | 36 |
| 807 | P000641 | MELD 3.0 | 1.21 | 1.10988715621097 | 2.6 | 13.38 | 118 | 3.1 | M |  | m1 | gt25 | 22 | 1 | > 25 | Δ score ≤ -1 | esld\_master\_long\_public.csv | 32 |
| 808 | P001189 | MELD 3.0 | 0.53 | 0.457931298853161 | 2.31 | 10.13 | 135 | 3.07 | F |  | m1 | gt25 | 22 | 1 | > 25 | Δ score ≤ -1 | esld\_master\_long\_public.csv | 27 |
| 809 | P001176 | MELD 3.0 | 1.53 | 1.4884273985883 | 1.66 | 3.99 | 117 | 2.45 | M |  | m1 | gt25 | 23 | 1 | > 25 | Δ score ≤ -1 | esld\_master\_long\_public.csv | 29 |
| 810 | P001200 | MELD 3.0 | 3.12 | 2.93799017571626 | 2.12 | 2.57 | 124 | 3.705 | M |  | m1 | gt25 | 24 | 1 | > 25 | Δ score ≤ -1 | esld\_master\_long\_public.csv | 36 |
| 811 | P000696 | MELD 3.0 | 3.03 | 2.78446056521364 | 2.355 | 11.59 | 130 | 3.02 | M |  | m1 | gt25 | 32 | 1 | > 25 | Δ score ≤ -1 | esld\_master\_long\_public.csv | 39 |
| 812 | P001261 | MELD 3.0 | 2.05 | 1.98704768379053 | 1.86 | 2.73 | 138 | 3.75 | F |  | m1 | gt25 | 32 | 1 | > 25 | Δ score ≤ -1 | esld\_master\_long\_public.csv | 26 |
| 813 | P000318 | MELD 3.0 | 1.13 | 1.00462235284229 | 1.7 | 24.1 | 129 | 2.61 | F |  | m1 | gt25 | 34 | 1 | > 25 | Δ score ≤ -1 | esld\_master\_long\_public.csv | 30 |
| 814 | P000739 | MELD 3.0 | 2.92 | 2.75336529906707 | 1.895 | 3.75 | 147 | 2.8 | M |  | m1 | gt25 | 37 | 1 | > 25 | Δ score ≤ -1 | esld\_master\_long\_public.csv | 30 |
| 815 | P000129 | MELD 3.0 | 2.29 | 2.1059431928805 | 2.21 | 16.78 | 147 | 2.61 | M |  | m1 | gt25 | 44 | 1 | > 25 | Δ score ≤ -1 | esld\_master\_long\_public.csv | 36 |
| 816 | P000141 | MELD 3.0 | 3.03 | 2.82072307690323 | 3.18 | 6.7 | 145 | 2.44 | F |  | m1 | gt25 | 50 | 1 | > 25 | Δ score ≤ -1 | esld\_master\_long\_public.csv | 39 |
| 817 | P000781 | MELD 3.0 | 2.5 | 2.38294446725591 | 1.67 | 3.84 | 126.5 | 1.78 | M |  | m1 | gt25 | 52 | 1 | > 25 | Δ score ≤ -1 | esld\_master\_long\_public.csv | 33 |
| 818 | P001146 | MELD 3.0 | 2.985 | 2.76373688529807 | 2.58 | 8.95 | 139 | 2.53 | F |  | m1 | gt25 | 55 | 1 | > 25 | Δ score ≤ -1 | esld\_master\_long\_public.csv | 38 |
| 819 | P000194 | MELD 3.0 | 2.75 | 2.5467843756506 | 5.14 | 10.7 | 136 | 2.34 | M |  | m1 | gt25 | 62 | 1 | > 25 | Δ score ≤ -1 | esld\_master\_long\_public.csv | 40 |
| 820 | P000571 | MELD 3.0 | 1.86 | 1.73499717555769 | 1.76 | 11.9 | 167 | 2.96 | F |  | m1 | gt25 | 107 | 1 | > 25 | Δ score ≤ -1 | esld\_master\_long\_public.csv | 31 |
| 821 | P000425 | MELD 3.0 | 2.73 | 2.51263121514642 | 1.7 | 13.5 | 132 | 2.29 | M |  | m1 | gt25 | 110 | 1 | > 25 | Δ score ≤ -1 | esld\_master\_long\_public.csv | 35 |
| 822 | P000633 | MELD 3.0 | 1.64 | 1.5370936408271 | 1.78 | 10.63 | 152 | 3.32 | M |  | m1 | gt25 | 132 | 1 | > 25 | Δ score ≤ -1 | esld\_master\_long\_public.csv | 28 |
| 823 | P000955 | MELD 3.0 | 3.47 | 3.1881030251959 | 1.91 | 7.38 | 146.5 | 3.65 | F |  | m1 | gt25 | 149 | 1 | > 25 | Δ score ≤ -1 | esld\_master\_long\_public.csv | 35 |
| 824 | P001145 | MELD 3.0 | 2.83 | 2.63291110427294 | 5.93 | 8.46 | 148 | 3.09 | M |  | m1 | gt25 | 198 | 1 | > 25 | Δ score ≤ -1 | esld\_master\_long\_public.csv | 40 |
| 825 | P000233 | MELD 3.0 | 3.08 | 2.88229341452655 | 2.1 | 4.67 | 119 | 2.27 | F |  | m1 | gt25 | 225 | 1 | > 25 | Δ score ≤ -1 | esld\_master\_long\_public.csv | 39 |
| 826 | P000565 | MELD 3.0 | 2.95 | 2.71589130555848 | 1.49 | 36.2 | 140 | 4.84 | F |  | m1 | gt25 | 273 | 1 | > 25 | Δ score ≤ -1 | esld\_master\_long\_public.csv | 39 |
| 827 | P000015 | MELD 3.0 | 1.85 | 1.76247464478455 | 1.66 | 6.86 | 154 | 2.595 | M |  | m1 | gt25 | 294 | 1 | > 25 | Δ score ≤ -1 | esld\_master\_long\_public.csv | 27 |
| 828 | P000858 | MELD 3.0 | 0.663333333333333 | 0.632377176098575 | 4.28666666666667 | 5.16333333333333 | 131 | 3.88 | M |  | m1 | gt25 | 509 | 1 | > 25 | Δ score ≤ -1 | esld\_master\_long\_public.csv | 29 |
| 829 | P000559 | MELD 3.0 | 2.23666666666667 | 2.15934404898939 | 2.21666666666667 | 2.55666666666667 | 147 | 2.225 | M |  | m1 | gt25 | 729 | 1 | > 25 | Δ score ≤ -1 | esld\_master\_long\_public.csv | 27 |
| 830 | P000006 | MELD 3.0 | 1.86 | 1.76337882658825 | 1.62 | 7.87 | 142 | 1.76 | M |  | m1 | gt25 | 730 | 0 | > 25 | Δ score ≤ -1 | esld\_master\_long\_public.csv | 28 |
| 831 | P000011 | MELD 3.0 | 3.25 | 3.04144999400473 | 1.13 | 3.26 | 126 | 2.62 | F |  | m1 | gt25 | 730 | 0 | > 25 | Δ score ≤ -1 | esld\_master\_long\_public.csv | 32 |
| 832 | P000071 | MELD 3.0 | 2.75 | 2.52038256890317 | 2.07 | 15.59 | 134 | 2.78 | M |  | m1 | gt25 | 730 | 0 | > 25 | Δ score ≤ -1 | esld\_master\_long\_public.csv | 37 |
| 833 | P000093 | MELD 3.0 | 2.165 | 2.0180358542989 | 4.585 | 11.54 | 139 | 3.04 | F |  | m1 | gt25 | 730 | 0 | > 25 | Δ score ≤ -1 | esld\_master\_long\_public.csv | 40 |
| 834 | P000102 | MELD 3.0 | 2.56 | 2.38689845315044 | 3.6 | 9.56 | 137 | 1.8 | M |  | m1 | gt25 | 730 | 0 | > 25 | Δ score ≤ -1 | esld\_master\_long\_public.csv | 39 |
| 835 | P000126 | MELD 3.0 | 2.4 | 2.28591504264576 | 2.41 | 4.6 | 133 | 1.75 | M |  | m1 | gt25 | 730 | 0 | > 25 | Δ score ≤ -1 | esld\_master\_long\_public.csv | 33 |
| 836 | P000284 | MELD 3.0 | 1.61 | 1.53992478639526 | 2.01 | 41.13 | 144 | 3.94 | F |  | m1 | gt25 | 730 | 0 | > 25 | Δ score ≤ -1 | esld\_master\_long\_public.csv | 36 |
| 837 | P000513 | MELD 3.0 | 3.82 | 3.42904959645688 | 3.32 | 14.8 | 132 | 5.01 | F |  | m1 | gt25 | 730 | 0 | > 25 | Δ score ≤ -1 | esld\_master\_long\_public.csv | 40 |
| 838 | P000647 | MELD 3.0 | 2.615 | 2.49987865571779 | 2.68 | 2.43 | 136 | 3.11 | F |  | m1 | gt25 | 730 | 0 | > 25 | Δ score ≤ -1 | esld\_master\_long\_public.csv | 32 |
| 839 | P000755 | MELD 3.0 | 2.8 | 2.60523710385799 | 2.89 | 8.65 | 136 | 3.11 | F |  | m1 | gt25 | 730 | 0 | > 25 | Δ score ≤ -1 | esld\_master\_long\_public.csv | 39 |
| 840 | P000849 | MELD 3.0 | 2.16 | 2.04013980704436 | 2.1275 | 7.81 | 139 | 2.75 | M |  | m1 | gt25 | 730 | 0 | > 25 | Δ score ≤ -1 | esld\_master\_long\_public.csv | 31 |
| 841 | P000867 | MELD 3.0 | 2.35 | 2.17605094133548 | 1.77 | 13.21 | 126 | 3.35 | M |  | m1 | gt25 | 730 | 0 | > 25 | Δ score ≤ -1 | esld\_master\_long\_public.csv | 35 |
