# Supplementary material for: Creatinine assay interferences compromises MELD accuracy and may bias liver allocation
Source: Nat Commun. 2026 Jul 23;17:7111. doi: 10.1038/s41467-026-75011-x (PMC13396164; doi:10.1038/s41467-026-75011-x)
Supplement: Supplementary file 4 — Source Data [file 41467_2026_75011_MOESM4_ESM.zip › figshare_package_FINAL_PUBLIC_DEPOSIT_V1_20260503_002637/00_START_HERE_HTML_NAVIGATOR/file_views/view_0021_esld_T4_score_deviation_outcome_table_public.html]

02\_workflows/T4\_workflow\_v01/submission\_ready/public/data/esld\_T4\_score\_deviation\_outcome\_table\_public.csv

# Readable file view

02\_workflows/T4\_workflow\_v01/submission\_ready/public/data/esld\_T4\_score\_deviation\_outcome\_table\_public.csv

← Back to navigator   |   Open original package file

Section

Manuscript output data

Output

T4

Extension

csv

Size KB

2.566

Variables

12

## Variables in this file

| Variable | Label | Description | Unit | Type |
| --- | --- | --- | --- | --- |
| MELD | MELD result column | Formatted result value for the MELD score variant in the public table/summary row. | formatted table value | character |
| MELD 3.0 | MELD 3.0 result column | Formatted result value for the MELD 3.0 score variant in the public table/summary row. | formatted table value | character |
| MELD-Na | MELD-Na result column | Formatted result value for the MELD-Na score variant in the public table/summary row. | formatted table value | character |
| anchor | Anchor/output group | Anchor or output grouping label used by the workflow to identify a specific public output component. |  | character |
| data\_object | Data object | Name of the data object represented by the row. |  | character |
| domain | Data domain | Workflow or data domain represented by the row. |  | character |
| reMELD-Na | reMELD-Na result column | Formatted result value for the reMELD-Na score variant in the public table/summary row. | formatted table value | character |
| release\_status | Release status | Release-status label indicating the publication status of the row or file object. |  | character |
| row\_order | Display row order | Integer row order used to render the public table in the intended display sequence. | relative days | integer |
| section | Table section | Section heading or row group used in the rendered public table. |  | character |
| unit\_or\_role | Unit or semantic role | Unit, role, or semantic type corresponding to the row-specific variable/metric. |  | character |
| variable | Variable represented by row | Name of the variable represented by the row in a long-format table. |  | character |

## Readable HTML view

Showing all 13 rows.

| row\_order | section | variable | MELD | MELD-Na | reMELD-Na | MELD 3.0 | domain | anchor | data\_object | unit\_or\_role | release\_status |
| --- | --- | --- | --- | --- | --- | --- | --- | --- | --- | --- | --- |
| 1 | Overall prevalence of score Δ ≤ −1, % | alive | 6.2 (5.8–6.6) | 5.7 (5.4–6.1) | 6.9 (6.5–7.3) | 6.7 (6.2–7.4) | esld | T4 | score\_deviation\_outcome | table | public |
| 2 | Overall prevalence of score Δ ≤ −1, % | deceased | 12.8 (11.9–13.8) | 10.7 (9.9–11.6) | 9.0 (8.2–9.8) | 14.5 (13.0–16.1) | esld | T4 | score\_deviation\_outcome | table | public |
| 3 | Odds ratio | deceased vs alive | 2.222 (1.997–2.472) | 1.978 (1.757–2.225) | 1.330 (1.178–1.501) | 2.346 (1.999–2.750) | esld | T4 | score\_deviation\_outcome | table | public |
| 4 | 30 days survival probability | Score Δ ≤ −1 | 0.677 (0.608–0.754) | 0.672 (0.597–0.755) | 0.714 (0.638–0.800) | 0.659 (0.569–0.764) | esld | T4 | score\_deviation\_outcome | table | public |
| 5 | 30 days survival probability | Score Δ = 0 | 0.463 (0.357–0.599) | 0.524 (0.427–0.642) | 0.412 (0.318–0.536) | 0.590 (0.504–0.692) | esld | T4 | score\_deviation\_outcome | table | public |
| 6 | 90 days survival probability | Score Δ ≤ −1 | 0.506 (0.434–0.591) | 0.496 (0.419–0.588) | 0.555 (0.472–0.652) | 0.495 (0.402–0.609) | esld | T4 | score\_deviation\_outcome | table | public |
| 7 | 90 days survival probability | Score Δ = 0 | 0.388 (0.287–0.524) | 0.440 (0.346–0.561) | 0.300 (0.215–0.419) | 0.467 (0.380–0.573) | esld | T4 | score\_deviation\_outcome | table | public |
| 8 | 90 days restricted mean survival time (RMST90) | Score Δ ≤ −1 | 58.2 (52.7–63.8) | 57.5 (51.4–63.6) | 61.9 (55.6–68.3) | 56.6 (49.2–64.0) | esld | T4 | score\_deviation\_outcome | table | public |
| 9 | 90 days restricted mean survival time (RMST90) | Score Δ = 0 | 44.3 (34.9–53.7) | 49.1 (40.8–57.4) | 38.2 (30.1–46.3) | 52.2 (44.9–59.6) | esld | T4 | score\_deviation\_outcome | table | public |
| 10 | RMST90-ratio | Δ ≤ −1 / Δ = 0 | 1.32 (1.04–1.66) | 1.17 (0.96–1.43) | 1.62 (1.28–2.05) | 1.08 (0.89–1.31) | esld | T4 | score\_deviation\_outcome | table | public |
| 11 | 90 days hazard ratio | Δ ≤ −1 vs. Δ = 0 | 0.754 (0.540–1.052) | 0.884 (0.642–1.218) | 0.548 (0.395–0.760) | 0.880 (0.639–1.211) | esld | T4 | score\_deviation\_outcome | table | public |
| 12 | Model sample size | ESLD samples, N | 20,359 | 19,213 | 19,213 | 8,614 | esld | T4 | score\_deviation\_outcome | table | public |
| 13 | Model sample size | note | submitted Table 4 values | submitted Table 4 values | submitted Table 4 values | submitted Table 4 values | esld | T4 | score\_deviation\_outcome | table | public |
