# Supplementary material for: Creatinine assay interferences compromises MELD accuracy and may bias liver allocation
Source: Nat Commun. 2026 Jul 23;17:7111. doi: 10.1038/s41467-026-75011-x (PMC13396164; doi:10.1038/s41467-026-75011-x)
Supplement: Supplementary file 4 — Source Data [file 41467_2026_75011_MOESM4_ESM.zip › figshare_package_FINAL_PUBLIC_DEPOSIT_V1_20260503_002637/00_START_HERE_HTML_NAVIGATOR/file_views/view_0023_slco_F2_heatmap_public.html]

02\_workflows/F2\_workflow\_v01/submission\_ready/public/figures/slco\_F2\_heatmap\_public.pdf

# Readable file view

02\_workflows/F2\_workflow\_v01/submission\_ready/public/figures/slco\_F2\_heatmap\_public.pdf

← Back to navigator   |   Open original package file

Section

Public figures

Output

F2

Extension

pdf

Size KB

856.709

Variables

0

## Readable HTML view

If the PDF does not display, use the direct file link above.
