# Supplementary material for: Creatinine assay interferences compromises MELD accuracy and may bias liver allocation
Source: Nat Commun. 2026 Jul 23;17:7111. doi: 10.1038/s41467-026-75011-x (PMC13396164; doi:10.1038/s41467-026-75011-x)
Supplement: Supplementary file 4 — Source Data [file 41467_2026_75011_MOESM4_ESM.zip › figshare_package_FINAL_PUBLIC_DEPOSIT_V1_20260503_002637/00_START_HERE_HTML_NAVIGATOR/file_views/view_0003_f1_simulated_surface_metadata.html]

01\_primary\_data/public/f1\_simulated\_surface\_metadata.csv

# Readable file view

01\_primary\_data/public/f1\_simulated\_surface\_metadata.csv

← Back to navigator   |   Open original package file

Section

Public primary data

Output

F1

Extension

csv

Size KB

0.648

Variables

2

## Variables in this file

| Variable | Label | Description | Unit | Type |
| --- | --- | --- | --- | --- |
| parameter | Metadata parameter name | Name of a metadata parameter describing the F2 simulated heatmap object, such as figure identity, data origin, grid type, axis variable, or unit/role. |  | character |
| value | Value | Numerical or character value corresponding to the row-specific variable/metric. |  | character |

## Readable HTML view

Showing all 20 rows.

| parameter | value |
| --- | --- |
| dataset\_name | f1\_simulated\_surface\_repository |
| cohort | SIMULATED |
| data\_type | in\_silico surface grid |
| row\_definition;One row = one equation-defined grid point (tb\_mg\_dL, cre\_true\_mg\_dL) |  |
| tb\_range\_mg\_dL | 1 to 35 |
| cre\_true\_range\_mg\_dL | 1 to 6 |
| tb\_step\_mg\_dL | 0.1 |
| cre\_step\_mg\_dL | 0.05 |
| delta\_definition | delta = cre\_true\_mg\_dL - predicted measured creatinine |
| equation\_form;""""cre\_true = a + b\*TB + c\*TB^2 + d\*cre\_measured + e\*cre\_measured^2; inverted to measured creatinine by quadratic solution"""" |  |
| CreJ\_a | 0.0115 |
| CreJ\_b | -0.012 |
| CreJ\_c | 0.00025 |
| CreJ\_d | 1.039 |
| CreJ\_e | -0.0294 |
| CreE\_a | 0.1945 |
| CreE\_b | -0.013 |
| CreE\_c | 3e-05 |
| CreE\_d | 0.9076 |
| CreE\_e | -3e-04grouping\_rule\_registry |
