# Supplementary material for: Creatinine assay interferences compromises MELD accuracy and may bias liver allocation
Source: Nat Commun. 2026 Jul 23;17:7111. doi: 10.1038/s41467-026-75011-x (PMC13396164; doi:10.1038/s41467-026-75011-x)
Supplement: Supplementary file 4 — Source Data [file 41467_2026_75011_MOESM4_ESM.zip › figshare_package_FINAL_PUBLIC_DEPOSIT_V1_20260503_002637/00_START_HERE_HTML_NAVIGATOR/file_views/view_0033_README_T3_submission_ready_v01.html]

02\_workflows/T3\_workflow\_v01/submission\_ready/README\_T3\_submission\_ready\_v01.txt

# Readable file view

02\_workflows/T3\_workflow\_v01/submission\_ready/README\_T3\_submission\_ready\_v01.txt

← Back to navigator   |   Open original package file

Section

Workflow readmes

Output

T3

Extension

txt

Size KB

0.471

Variables

0

## Readable HTML view

```
Table 3 submission-ready outputs

Public release:
The public Table 3 release contains CSV table content and metadata only.
Rendered DOCX table files and raw/recalculation audit files are assigned to internal outputs only.

Internal audit:
Internal outputs contain the submitted DOCX template copy, ESLD raw source layer, recalculated summaries, statistical checks, DOCX renders, and QC files.

Use for final public release:
02_workflows/T3_workflow_v01/submission_ready/public/data
```
