# Supplementary material for: Creatinine assay interferences compromises MELD accuracy and may bias liver allocation
Source: Nat Commun. 2026 Jul 23;17:7111. doi: 10.1038/s41467-026-75011-x (PMC13396164; doi:10.1038/s41467-026-75011-x)
Supplement: Supplementary file 4 — Source Data [file 41467_2026_75011_MOESM4_ESM.zip › figshare_package_FINAL_PUBLIC_DEPOSIT_V1_20260503_002637/00_START_HERE_HTML_NAVIGATOR/file_views/view_0015_esld_F3_score_shift_aggregate_public.html]

02\_workflows/F3\_workflow\_v01/submission\_ready/public/data/esld\_F3\_score\_shift\_aggregate\_public.csv

# Readable file view

02\_workflows/F3\_workflow\_v01/submission\_ready/public/data/esld\_F3\_score\_shift\_aggregate\_public.csv

← Back to navigator   |   Open original package file

Section

Manuscript output data

Output

F3

Extension

csv

Size KB

6.515

Variables

4

## Variables in this file

| Variable | Label | Description | Unit | Type |
| --- | --- | --- | --- | --- |
| delta\_class | Score-delta class | Encoded score-shift class used in F3 score-shift summaries. p1 denotes a one-point positive score shift; pm denotes the reference/no-decrease category used by the workflow; m1 denotes the minus-one score-shift class; m2 denotes the minus-two score-shift class. |  | character |
| model | MELD model or score variant | Name of the MELD-related model or score variant represented by the row; expected values include MELD, MELD-Na, reMELD-Na, and MELD 3.0. |  | character |
| n | Number of observations | Count of observations, patients, samples, events, or rows, depending on the data file. |  | integer |
| x\_class | X-axis score class | Score class shown on the x-axis or used as the x-position in the F3 score-shift figure. | score class | integer |

## Readable HTML view

Showing all 315 rows.

| model | x\_class | delta\_class | n |
| --- | --- | --- | --- |
| MELD | 6 | pm | 963 |
| MELD | 7 | p1 | 9 |
| MELD | 7 | pm | 1801 |
| MELD | 8 | p1 | 33 |
| MELD | 8 | pm | 1915 |
| MELD | 9 | p1 | 52 |
| MELD | 9 | pm | 1718 |
| MELD | 10 | m1 | 3 |
| MELD | 10 | p1 | 32 |
| MELD | 10 | pm | 1530 |
| MELD | 11 | m1 | 9 |
| MELD | 11 | p1 | 23 |
| MELD | 11 | pm | 1286 |
| MELD | 12 | m1 | 20 |
| MELD | 12 | p1 | 8 |
| MELD | 12 | pm | 1136 |
| MELD | 13 | m1 | 35 |
| MELD | 13 | p1 | 11 |
| MELD | 13 | pm | 1165 |
| MELD | 14 | m1 | 61 |
| MELD | 14 | p1 | 7 |
| MELD | 14 | pm | 970 |
| MELD | 15 | m1 | 65 |
| MELD | 15 | p1 | 1 |
| MELD | 15 | pm | 923 |
| MELD | 16 | m1 | 102 |
| MELD | 16 | p1 | 3 |
| MELD | 16 | pm | 782 |
| MELD | 17 | m1 | 83 |
| MELD | 17 | p1 | 3 |
| MELD | 17 | pm | 724 |
| MELD | 18 | m1 | 103 |
| MELD | 18 | p1 | 3 |
| MELD | 18 | pm | 608 |
| MELD | 19 | m1 | 93 |
| MELD | 19 | p1 | 1 |
| MELD | 19 | pm | 523 |
| MELD | 20 | m1 | 82 |
| MELD | 20 | pm | 458 |
| MELD | 21 | m1 | 95 |
| MELD | 21 | pm | 373 |
| MELD | 22 | m1 | 71 |
| MELD | 22 | pm | 319 |
| MELD | 23 | m1 | 93 |
| MELD | 23 | pm | 279 |
| MELD | 24 | m1 | 79 |
| MELD | 24 | m2 | 2 |
| MELD | 24 | pm | 240 |
| MELD | 25 | m1 | 76 |
| MELD | 25 | pm | 195 |
| MELD | 26 | m1 | 77 |
| MELD | 26 | pm | 139 |
| MELD | 27 | m1 | 74 |
| MELD | 27 | pm | 114 |
| MELD | 28 | m1 | 56 |
| MELD | 28 | pm | 88 |
| MELD | 29 | m1 | 63 |
| MELD | 29 | m2 | 1 |
| MELD | 29 | pm | 72 |
| MELD | 30 | m1 | 44 |
| MELD | 30 | pm | 56 |
| MELD | 31 | m1 | 49 |
| MELD | 31 | pm | 39 |
| MELD | 32 | m1 | 35 |
| MELD | 32 | pm | 26 |
| MELD | 33 | m1 | 32 |
| MELD | 33 | pm | 22 |
| MELD | 34 | m1 | 39 |
| MELD | 34 | pm | 26 |
| MELD | 35 | m1 | 15 |
| MELD | 35 | pm | 15 |
| MELD | 36 | m1 | 15 |
| MELD | 36 | pm | 12 |
| MELD | 37 | m1 | 12 |
| MELD | 37 | pm | 9 |
| MELD | 38 | m1 | 6 |
| MELD | 38 | pm | 13 |
| MELD | 39 | m1 | 10 |
| MELD | 39 | pm | 8 |
| MELD | 40 | pm | 26 |
| MELD 3.0 | 6 | pm | 163 |
| MELD 3.0 | 7 | p1 | 5 |
| MELD 3.0 | 7 | pm | 385 |
| MELD 3.0 | 8 | p1 | 8 |
| MELD 3.0 | 8 | pm | 500 |
| MELD 3.0 | 9 | p1 | 9 |
| MELD 3.0 | 9 | pm | 490 |
| MELD 3.0 | 10 | m1 | 1 |
| MELD 3.0 | 10 | p1 | 12 |
| MELD 3.0 | 10 | pm | 509 |
| MELD 3.0 | 11 | m1 | 1 |
| MELD 3.0 | 11 | p1 | 10 |
| MELD 3.0 | 11 | pm | 559 |
| MELD 3.0 | 12 | p1 | 6 |
| MELD 3.0 | 12 | pm | 520 |
| MELD 3.0 | 13 | m1 | 6 |
| MELD 3.0 | 13 | p1 | 12 |
| MELD 3.0 | 13 | pm | 491 |
| MELD 3.0 | 14 | m1 | 5 |
| MELD 3.0 | 14 | p1 | 8 |
| MELD 3.0 | 14 | pm | 437 |
| MELD 3.0 | 15 | m1 | 13 |
| MELD 3.0 | 15 | p1 | 2 |
| MELD 3.0 | 15 | pm | 386 |
| MELD 3.0 | 16 | m1 | 20 |
| MELD 3.0 | 16 | p1 | 1 |
| MELD 3.0 | 16 | pm | 406 |
| MELD 3.0 | 17 | m1 | 29 |
| MELD 3.0 | 17 | p1 | 2 |
| MELD 3.0 | 17 | pm | 370 |
| MELD 3.0 | 18 | m1 | 22 |
| MELD 3.0 | 18 | p1 | 2 |
| MELD 3.0 | 18 | pm | 315 |
| MELD 3.0 | 19 | m1 | 27 |
| MELD 3.0 | 19 | pm | 287 |
| MELD 3.0 | 20 | m1 | 32 |
| MELD 3.0 | 20 | pm | 285 |
| MELD 3.0 | 21 | m1 | 36 |
| MELD 3.0 | 21 | pm | 256 |
| MELD 3.0 | 22 | m1 | 35 |
| MELD 3.0 | 22 | pm | 196 |
| MELD 3.0 | 23 | m1 | 39 |
| MELD 3.0 | 23 | p1 | 1 |
| MELD 3.0 | 23 | pm | 226 |
| MELD 3.0 | 24 | m1 | 45 |
| MELD 3.0 | 24 | pm | 172 |
| MELD 3.0 | 25 | m1 | 42 |
| MELD 3.0 | 25 | pm | 139 |
| MELD 3.0 | 26 | m1 | 45 |
| MELD 3.0 | 26 | pm | 121 |
| MELD 3.0 | 27 | m1 | 40 |
| MELD 3.0 | 27 | pm | 103 |
| MELD 3.0 | 28 | m1 | 46 |
| MELD 3.0 | 28 | pm | 102 |
| MELD 3.0 | 29 | m1 | 42 |
| MELD 3.0 | 29 | pm | 85 |
| MELD 3.0 | 30 | m1 | 37 |
| MELD 3.0 | 30 | pm | 55 |
| MELD 3.0 | 31 | m1 | 36 |
| MELD 3.0 | 31 | pm | 62 |
| MELD 3.0 | 32 | m1 | 37 |
| MELD 3.0 | 32 | pm | 53 |
| MELD 3.0 | 33 | m1 | 20 |
| MELD 3.0 | 33 | pm | 16 |
| MELD 3.0 | 34 | m1 | 19 |
| MELD 3.0 | 34 | pm | 14 |
| MELD 3.0 | 35 | m1 | 25 |
| MELD 3.0 | 35 | pm | 17 |
| MELD 3.0 | 36 | m1 | 14 |
| MELD 3.0 | 36 | pm | 22 |
| MELD 3.0 | 37 | m1 | 6 |
| MELD 3.0 | 37 | pm | 10 |
| MELD 3.0 | 38 | m1 | 12 |
| MELD 3.0 | 38 | pm | 18 |
| MELD 3.0 | 39 | m1 | 8 |
| MELD 3.0 | 39 | pm | 3 |
| MELD 3.0 | 40 | pm | 23 |
| MELD-Na | 6 | pm | 731 |
| MELD-Na | 7 | p1 | 5 |
| MELD-Na | 7 | pm | 1312 |
| MELD-Na | 8 | p1 | 26 |
| MELD-Na | 8 | pm | 1428 |
| MELD-Na | 9 | p1 | 44 |
| MELD-Na | 9 | pm | 1351 |
| MELD-Na | 10 | m1 | 2 |
| MELD-Na | 10 | p1 | 26 |
| MELD-Na | 10 | pm | 1289 |
| MELD-Na | 11 | m1 | 4 |
| MELD-Na | 11 | p1 | 26 |
| MELD-Na | 11 | pm | 1201 |
| MELD-Na | 12 | m1 | 13 |
| MELD-Na | 12 | p1 | 9 |
| MELD-Na | 12 | pm | 1062 |
| MELD-Na | 13 | m1 | 28 |
| MELD-Na | 13 | p1 | 12 |
| MELD-Na | 13 | pm | 1055 |
| MELD-Na | 14 | m1 | 50 |
| MELD-Na | 14 | p1 | 10 |
| MELD-Na | 14 | pm | 944 |
| MELD-Na | 15 | m1 | 44 |
| MELD-Na | 15 | p1 | 4 |
| MELD-Na | 15 | pm | 903 |
| MELD-Na | 16 | m1 | 57 |
| MELD-Na | 16 | p1 | 5 |
| MELD-Na | 16 | pm | 769 |
| MELD-Na | 17 | m1 | 61 |
| MELD-Na | 17 | p1 | 5 |
| MELD-Na | 17 | pm | 788 |
| MELD-Na | 18 | m1 | 77 |
| MELD-Na | 18 | p1 | 4 |
| MELD-Na | 18 | pm | 627 |
| MELD-Na | 19 | m1 | 64 |
| MELD-Na | 19 | pm | 612 |
| MELD-Na | 20 | m1 | 69 |
| MELD-Na | 20 | pm | 512 |
| MELD-Na | 21 | m1 | 66 |
| MELD-Na | 21 | pm | 484 |
| MELD-Na | 22 | m1 | 66 |
| MELD-Na | 22 | p1 | 1 |
| MELD-Na | 22 | pm | 541 |
| MELD-Na | 23 | m1 | 74 |
| MELD-Na | 23 | pm | 330 |
| MELD-Na | 24 | m1 | 73 |
| MELD-Na | 24 | m2 | 1 |
| MELD-Na | 24 | p1 | 1 |
| MELD-Na | 24 | pm | 336 |
| MELD-Na | 25 | m1 | 60 |
| MELD-Na | 25 | pm | 306 |
| MELD-Na | 26 | m1 | 64 |
| MELD-Na | 26 | pm | 249 |
| MELD-Na | 27 | m1 | 82 |
| MELD-Na | 27 | pm | 206 |
| MELD-Na | 28 | m1 | 60 |
| MELD-Na | 28 | m2 | 1 |
| MELD-Na | 28 | pm | 131 |
| MELD-Na | 29 | m1 | 57 |
| MELD-Na | 29 | pm | 112 |
| MELD-Na | 30 | m1 | 44 |
| MELD-Na | 30 | pm | 111 |
| MELD-Na | 31 | m1 | 44 |
| MELD-Na | 31 | pm | 60 |
| MELD-Na | 32 | m1 | 42 |
| MELD-Na | 32 | pm | 55 |
| MELD-Na | 33 | m1 | 30 |
| MELD-Na | 33 | pm | 54 |
| MELD-Na | 34 | m1 | 34 |
| MELD-Na | 34 | pm | 41 |
| MELD-Na | 35 | m1 | 21 |
| MELD-Na | 35 | pm | 33 |
| MELD-Na | 36 | m1 | 13 |
| MELD-Na | 36 | pm | 17 |
| MELD-Na | 37 | m1 | 15 |
| MELD-Na | 37 | pm | 11 |
| MELD-Na | 38 | m1 | 5 |
| MELD-Na | 38 | pm | 14 |
| MELD-Na | 39 | m1 | 10 |
| MELD-Na | 39 | pm | 9 |
| MELD-Na | 40 | pm | 20 |
| reMELD-Na | 6 | p1 | 101 |
| reMELD-Na | 6 | pm | 877 |
| reMELD-Na | 7 | p1 | 97 |
| reMELD-Na | 7 | pm | 987 |
| reMELD-Na | 8 | m1 | 1 |
| reMELD-Na | 8 | p1 | 101 |
| reMELD-Na | 8 | pm | 1015 |
| reMELD-Na | 9 | m1 | 3 |
| reMELD-Na | 9 | p1 | 91 |
| reMELD-Na | 9 | pm | 1078 |
| reMELD-Na | 10 | m1 | 13 |
| reMELD-Na | 10 | p1 | 72 |
| reMELD-Na | 10 | pm | 1074 |
| reMELD-Na | 11 | m1 | 21 |
| reMELD-Na | 11 | p1 | 48 |
| reMELD-Na | 11 | pm | 1040 |
| reMELD-Na | 12 | m1 | 39 |
| reMELD-Na | 12 | p1 | 33 |
| reMELD-Na | 12 | pm | 1098 |
| reMELD-Na | 13 | m1 | 51 |
| reMELD-Na | 13 | p1 | 17 |
| reMELD-Na | 13 | pm | 1054 |
| reMELD-Na | 14 | m1 | 61 |
| reMELD-Na | 14 | p1 | 12 |
| reMELD-Na | 14 | pm | 982 |
| reMELD-Na | 15 | m1 | 94 |
| reMELD-Na | 15 | p1 | 8 |
| reMELD-Na | 15 | pm | 900 |
| reMELD-Na | 16 | m1 | 113 |
| reMELD-Na | 16 | m2 | 5 |
| reMELD-Na | 16 | p1 | 7 |
| reMELD-Na | 16 | pm | 741 |
| reMELD-Na | 17 | m1 | 119 |
| reMELD-Na | 17 | m2 | 1 |
| reMELD-Na | 17 | p1 | 4 |
| reMELD-Na | 17 | pm | 679 |
| reMELD-Na | 18 | m1 | 103 |
| reMELD-Na | 18 | p1 | 3 |
| reMELD-Na | 18 | pm | 640 |
| reMELD-Na | 19 | m1 | 88 |
| reMELD-Na | 19 | pm | 489 |
| reMELD-Na | 20 | m1 | 83 |
| reMELD-Na | 20 | m2 | 2 |
| reMELD-Na | 20 | pm | 440 |
| reMELD-Na | 21 | m1 | 87 |
| reMELD-Na | 21 | m2 | 1 |
| reMELD-Na | 21 | pm | 334 |
| reMELD-Na | 22 | m1 | 83 |
| reMELD-Na | 22 | m2 | 1 |
| reMELD-Na | 22 | pm | 284 |
| reMELD-Na | 23 | m1 | 76 |
| reMELD-Na | 23 | pm | 243 |
| reMELD-Na | 24 | m1 | 101 |
| reMELD-Na | 24 | m2 | 1 |
| reMELD-Na | 24 | pm | 181 |
| reMELD-Na | 25 | m1 | 53 |
| reMELD-Na | 25 | pm | 146 |
| reMELD-Na | 26 | m1 | 52 |
| reMELD-Na | 26 | pm | 115 |
| reMELD-Na | 27 | m1 | 52 |
| reMELD-Na | 27 | pm | 95 |
| reMELD-Na | 28 | m1 | 34 |
| reMELD-Na | 28 | pm | 86 |
| reMELD-Na | 29 | m1 | 27 |
| reMELD-Na | 29 | pm | 58 |
| reMELD-Na | 30 | m1 | 26 |
| reMELD-Na | 30 | pm | 42 |
| reMELD-Na | 31 | m1 | 20 |
| reMELD-Na | 31 | pm | 42 |
| reMELD-Na | 32 | m1 | 12 |
| reMELD-Na | 32 | pm | 38 |
| reMELD-Na | 33 | m1 | 1 |
| reMELD-Na | 33 | pm | 14 |
| reMELD-Na | 34 | m1 | 1 |
| reMELD-Na | 34 | pm | 13 |
| reMELD-Na | 35 | pm | 1 |
| reMELD-Na | 36 | pm | 1 |
