# Supplementary material for: Creatinine assay interferences compromises MELD accuracy and may bias liver allocation
Source: Nat Commun. 2026 Jul 23;17:7111. doi: 10.1038/s41467-026-75011-x (PMC13396164; doi:10.1038/s41467-026-75011-x)
Supplement: Supplementary file 4 — Source Data [file 41467_2026_75011_MOESM4_ESM.zip › figshare_package_FINAL_PUBLIC_DEPOSIT_V1_20260503_002637/00_START_HERE_HTML_NAVIGATOR/file_views/view_0035_public_deposit_file_manifest_v01.html]

00\_release\_manifests/public\_deposit\_file\_manifest\_v01.csv

# Readable file view

00\_release\_manifests/public\_deposit\_file\_manifest\_v01.csv

← Back to navigator   |   Open original package file

Section

Technical appendix / request manifests

Output

Extension

csv

Size KB

59.062

Variables

0

## Readable HTML view

Showing all 94 rows.

| relative\_path | file\_name | directory | extension | file\_type | size\_bytes | size\_kb | last\_modified | md5 | access\_tier | data\_origin | used\_for | n\_rows | n\_cols | keep\_in\_repository | needs\_manual\_review | comment | release\_scope | release\_access\_tier | release\_source\_role | release\_decision | output\_tag | registry\_file\_key | registry\_release\_status | registry\_is\_primary | registry\_used\_by\_workflows | registry\_source\_role | final\_release\_tier | final\_release\_decision | final\_policy\_reason | is\_r\_script | is\_srtr\_named | is\_data\_like | is\_rendered\_or\_doc | is\_internal\_table\_template | is\_legacy\_zip | is\_internal\_workflow\_data\_or\_table | is\_internal\_documentation | is\_internal\_primary\_source | final\_release\_tier\_before\_doc\_close | final\_release\_decision\_before\_doc\_close | final\_policy\_reason\_before\_doc\_close | is\_internal\_submission\_manifest | is\_noninternal\_submission\_manifest | is\_workflow\_readme | is\_submission\_ready\_readme | is\_remaining\_manual\_doc |
| --- | --- | --- | --- | --- | --- | --- | --- | --- | --- | --- | --- | --- | --- | --- | --- | --- | --- | --- | --- | --- | --- | --- | --- | --- | --- | --- | --- | --- | --- | --- | --- | --- | --- | --- | --- | --- | --- | --- | --- | --- | --- | --- | --- | --- | --- | --- |
| 00\_config/01\_primary\_file\_registry\_v03.csv | 01\_primary\_file\_registry\_v03.csv | 00\_config | csv | csv | 4149 | 4.052 | 2026-05-02T09:46:31Z | 628025805c2e9de1df7a0991e61d2054 | manual\_review | manual\_review |  | 13 | 9 | manual\_review | TRUE |  | release\_candidate | public\_deposit | documentation\_or\_metadata | keep\_public\_deposit |  |  |  |  |  |  | public\_deposit | keep\_public\_deposit | Public-deposit file after policy overrides. | FALSE | FALSE | TRUE | FALSE | FALSE | FALSE | FALSE | FALSE | FALSE | public\_deposit | keep\_public\_deposit | Public-deposit file after policy overrides. | FALSE | FALSE | FALSE | FALSE | FALSE |
| 01\_primary\_data/public/esld\_master\_long\_public.csv | esld\_master\_long\_public.csv | 01\_primary\_data/public | csv | csv | 9851508 | 9620.613 | 2026-05-02T23:18:12Z | 8228407e927ff10ad6e4740d0d5cd48b | public | primary |  | 67399 | 43 | yes\_default | FALSE |  | release\_candidate | public\_deposit | primary\_source | keep\_public\_deposit |  | esld\_master\_long\_public | public | TRUE | F3;F4;F5;T1;T2;T3;T4 | canonical ESLD master source; ESLD Fig 3 aggregate and ESLD table files are derived from this | public\_deposit | keep\_public\_deposit | Public-deposit file after policy overrides. | FALSE | FALSE | TRUE | FALSE | FALSE | FALSE | FALSE | FALSE | FALSE | public\_deposit | keep\_public\_deposit | Public-deposit file after policy overrides. | FALSE | FALSE | FALSE | FALSE | FALSE |
| 01\_primary\_data/public/f1\_simulated\_surface\_metadata.csv | f1\_simulated\_surface\_metadata.csv | 01\_primary\_data/public | csv | csv | 664 | 0.648 | 2026-04-19T20:30:45Z | ee2dfece0c0751ab1643f0e896edea9b | public | primary | F1 | 9 | 2 | yes\_default | FALSE |  | release\_candidate | public\_deposit | primary\_source | keep\_public\_deposit | F1 | f1\_simulated\_surface\_metadata | public | TRUE | F1 | F1 simulated surface metadata primary source | public\_deposit | keep\_public\_deposit | Public-deposit file after policy overrides. | FALSE | FALSE | TRUE | FALSE | FALSE | FALSE | FALSE | FALSE | FALSE | public\_deposit | keep\_public\_deposit | Public-deposit file after policy overrides. | FALSE | FALSE | FALSE | FALSE | FALSE |
| 01\_primary\_data/public/f1\_simulated\_surface\_repository.csv | f1\_simulated\_surface\_repository.csv | 01\_primary\_data/public | csv | csv | 5055372 | 4936.887 | 2026-04-16T16:32:54Z | 87713a0d6868c6ac52ff20ea850e3d58 | public | primary | F1 | 34441 | 11 | yes\_default | FALSE |  | release\_candidate | public\_deposit | primary\_source | keep\_public\_deposit | F1 | f1\_simulated\_surface\_repository | public | TRUE | F1 | F1 simulated surface primary source | public\_deposit | keep\_public\_deposit | Public-deposit file after policy overrides. | FALSE | FALSE | TRUE | FALSE | FALSE | FALSE | FALSE | FALSE | FALSE | public\_deposit | keep\_public\_deposit | Public-deposit file after policy overrides. | FALSE | FALSE | FALSE | FALSE | FALSE |
| 01\_primary\_data/public/f1\_tb\_cre\_experimental\_array\_raw.csv | f1\_tb\_cre\_experimental\_array\_raw.csv | 01\_primary\_data/public | csv | csv | 338947 | 331.003 | 2026-04-22T16:53:59Z | b8ffb94bc127381e0963db2211ba44b8 | public | primary | F1 | 4200 | 9 | yes\_default | FALSE |  | release\_candidate | public\_deposit | primary\_source | keep\_public\_deposit | F1 | f1\_tb\_cre\_experimental\_array\_raw | public | TRUE | F1 | F1 experimental array raw source | public\_deposit | keep\_public\_deposit | Public-deposit file after policy overrides. | FALSE | FALSE | TRUE | FALSE | FALSE | FALSE | FALSE | FALSE | FALSE | public\_deposit | keep\_public\_deposit | Public-deposit file after policy overrides. | FALSE | FALSE | FALSE | FALSE | FALSE |
| 01\_primary\_data/public/f1\_tb\_cre\_experimental\_validation\_raw.csv | f1\_tb\_cre\_experimental\_validation\_raw.csv | 01\_primary\_data/public | csv | csv | 2335 | 2.28 | 2026-04-23T00:35:04Z | 30e9eb0a37dab1d84dbff1649b1b9e07 | internal\_only | primary | F1 | 32 | 8 | no\_internal\_only\_default | FALSE |  | release\_candidate | public\_deposit | primary\_source | keep\_public\_deposit | F1 | f1\_tb\_cre\_experimental\_validation\_raw | public | TRUE | F1 | F1 experimental validation raw source | public\_deposit | keep\_public\_deposit | Public-deposit file after policy overrides. | FALSE | FALSE | TRUE | FALSE | FALSE | FALSE | FALSE | FALSE | FALSE | public\_deposit | keep\_public\_deposit | Public-deposit file after policy overrides. | FALSE | FALSE | FALSE | FALSE | FALSE |
| 01\_primary\_data/public/f2\_simulated\_heatmap\_metadata.csv | f2\_simulated\_heatmap\_metadata.csv | 01\_primary\_data/public | csv | csv | 558 | 0.545 | 2026-04-19T20:30:45Z | df833ec1562a6c70ca8dc8338c7e625d | public | primary | F2 | 21 | 2 | yes\_default | FALSE |  | release\_candidate | public\_deposit | primary\_source | keep\_public\_deposit | F2 | f2\_simulated\_heatmap\_metadata | public | TRUE | F2 | F2 simulated heatmap metadata primary source | public\_deposit | keep\_public\_deposit | Public-deposit file after policy overrides. | FALSE | FALSE | TRUE | FALSE | FALSE | FALSE | FALSE | FALSE | FALSE | public\_deposit | keep\_public\_deposit | Public-deposit file after policy overrides. | FALSE | FALSE | FALSE | FALSE | FALSE |
| 01\_primary\_data/public/f2\_simulated\_heatmap\_repository.csv | f2\_simulated\_heatmap\_repository.csv | 01\_primary\_data/public | csv | csv | 7858998 | 7674.803 | 2026-04-17T19:20:15Z | 69355977324161fce080931369bd51f9 | public | primary | F2 | 295200 | 6 | yes\_default | FALSE |  | release\_candidate | public\_deposit | primary\_source | keep\_public\_deposit | F2 | f2\_simulated\_heatmap\_repository | public | TRUE | F2 | F2 simulated heatmap primary source | public\_deposit | keep\_public\_deposit | Public-deposit file after policy overrides. | FALSE | FALSE | TRUE | FALSE | FALSE | FALSE | FALSE | FALSE | FALSE | public\_deposit | keep\_public\_deposit | Public-deposit file after policy overrides. | FALSE | FALSE | FALSE | FALSE | FALSE |
| 02\_workflows/F1\_workflow\_v02/data/01\_source\_loaded\_harmonized/expm\_F1\_array\_raw\_public.csv | expm\_F1\_array\_raw\_public.csv | 02\_workflows/F1\_workflow\_v02/data/01\_source\_loaded\_harmonized | csv | csv | 338947 | 331.003 | 2026-04-22T16:53:59Z | b8ffb94bc127381e0963db2211ba44b8 | public | derived | F1 | 4200 | 9 | yes\_default | FALSE |  | release\_candidate | public\_deposit | derived\_or\_workflow\_data | keep\_public\_deposit | F1 |  |  |  |  |  | public\_deposit | keep\_public\_deposit | Public-deposit file after policy overrides. | FALSE | FALSE | TRUE | FALSE | FALSE | FALSE | FALSE | FALSE | FALSE | public\_deposit | keep\_public\_deposit | Public-deposit file after policy overrides. | FALSE | FALSE | FALSE | FALSE | FALSE |
| 02\_workflows/F1\_workflow\_v02/data/01\_source\_loaded\_harmonized/expm\_F1\_validation\_raw\_public.csv | expm\_F1\_validation\_raw\_public.csv | 02\_workflows/F1\_workflow\_v02/data/01\_source\_loaded\_harmonized | csv | csv | 2335 | 2.28 | 2026-04-23T00:35:04Z | 30e9eb0a37dab1d84dbff1649b1b9e07 | internal\_only | derived | F1 | 32 | 8 | no\_internal\_only\_default | FALSE |  | release\_candidate | public\_deposit | derived\_or\_workflow\_data | keep\_public\_deposit | F1 |  |  |  |  |  | public\_deposit | keep\_public\_deposit | Public-deposit file after policy overrides. | FALSE | FALSE | TRUE | FALSE | FALSE | FALSE | FALSE | FALSE | FALSE | public\_deposit | keep\_public\_deposit | Public-deposit file after policy overrides. | FALSE | FALSE | FALSE | FALSE | FALSE |
| 02\_workflows/F1\_workflow\_v02/data/01\_source\_loaded\_harmonized/expm\_slco\_F1\_source\_load\_manifest\_public.csv | expm\_slco\_F1\_source\_load\_manifest\_public.csv | 02\_workflows/F1\_workflow\_v02/data/01\_source\_loaded\_harmonized | csv | csv | 1698 | 1.658 | 2026-05-02T01:30:05Z | 468a7c790812e70d5f1b33a02258494a | public | derived | F1 | 4 | 9 | yes\_default | FALSE |  | release\_candidate | public\_deposit | derived\_or\_workflow\_data | keep\_public\_deposit | F1 |  |  |  |  |  | public\_deposit | keep\_public\_deposit | Public-deposit file after policy overrides. | FALSE | FALSE | TRUE | FALSE | FALSE | FALSE | FALSE | FALSE | FALSE | public\_deposit | keep\_public\_deposit | Public-deposit file after policy overrides. | FALSE | FALSE | FALSE | FALSE | FALSE |
| 02\_workflows/F1\_workflow\_v02/data/01\_source\_loaded\_harmonized/expm\_slco\_F1\_step1\_run\_inputs\_public.csv | expm\_slco\_F1\_step1\_run\_inputs\_public.csv | 02\_workflows/F1\_workflow\_v02/data/01\_source\_loaded\_harmonized | csv | csv | 1024 | 1 | 2026-05-02T01:30:05Z | 3459100ae284c27b3496897366c4c5d0 | public | derived | F1 | 5 | 2 | yes\_default | FALSE |  | release\_candidate | public\_deposit | derived\_or\_workflow\_data | keep\_public\_deposit | F1 |  |  |  |  |  | public\_deposit | keep\_public\_deposit | Public-deposit file after policy overrides. | FALSE | FALSE | TRUE | FALSE | FALSE | FALSE | FALSE | FALSE | FALSE | public\_deposit | keep\_public\_deposit | Public-deposit file after policy overrides. | FALSE | FALSE | FALSE | FALSE | FALSE |
| 02\_workflows/F1\_workflow\_v02/data/01\_source\_loaded\_harmonized/slco\_F1\_surface\_grid\_reference\_public.csv | slco\_F1\_surface\_grid\_reference\_public.csv | 02\_workflows/F1\_workflow\_v02/data/01\_source\_loaded\_harmonized | csv | csv | 5055372 | 4936.887 | 2026-04-16T16:32:54Z | 87713a0d6868c6ac52ff20ea850e3d58 | public | derived | F1 | 34441 | 11 | yes\_default | FALSE |  | release\_candidate | public\_deposit | derived\_or\_workflow\_data | keep\_public\_deposit | F1 |  |  |  |  |  | public\_deposit | keep\_public\_deposit | Public-deposit file after policy overrides. | FALSE | FALSE | TRUE | FALSE | FALSE | FALSE | FALSE | FALSE | FALSE | public\_deposit | keep\_public\_deposit | Public-deposit file after policy overrides. | FALSE | FALSE | FALSE | FALSE | FALSE |
| 02\_workflows/F1\_workflow\_v02/data/01\_source\_loaded\_harmonized/slco\_F1\_surface\_meta\_reference\_public.csv | slco\_F1\_surface\_meta\_reference\_public.csv | 02\_workflows/F1\_workflow\_v02/data/01\_source\_loaded\_harmonized | csv | csv | 664 | 0.648 | 2026-04-19T20:30:45Z | ee2dfece0c0751ab1643f0e896edea9b | public | derived | F1 | 9 | 2 | yes\_default | FALSE |  | release\_candidate | public\_deposit | derived\_or\_workflow\_data | keep\_public\_deposit | F1 |  |  |  |  |  | public\_deposit | keep\_public\_deposit | Public-deposit file after policy overrides. | FALSE | FALSE | TRUE | FALSE | FALSE | FALSE | FALSE | FALSE | FALSE | public\_deposit | keep\_public\_deposit | Public-deposit file after policy overrides. | FALSE | FALSE | FALSE | FALSE | FALSE |
| 02\_workflows/F1\_workflow\_v02/data/02a\_refined\_analysis/expm\_F1\_array\_input\_public.csv | expm\_F1\_array\_input\_public.csv | 02\_workflows/F1\_workflow\_v02/data/02a\_refined\_analysis | csv | csv | 755375 | 737.671 | 2026-05-02T01:30:05Z | 48545523b96fdb9fce1e19bdf1689efc | public | derived | F1 | 4200 | 19 | yes\_default | FALSE |  | release\_candidate | public\_deposit | derived\_or\_workflow\_data | keep\_public\_deposit | F1 |  |  |  |  |  | public\_deposit | keep\_public\_deposit | Public-deposit file after policy overrides. | FALSE | FALSE | TRUE | FALSE | FALSE | FALSE | FALSE | FALSE | FALSE | public\_deposit | keep\_public\_deposit | Public-deposit file after policy overrides. | FALSE | FALSE | FALSE | FALSE | FALSE |
| 02\_workflows/F1\_workflow\_v02/data/02a\_refined\_analysis/expm\_F1\_model\_coefficients\_public.csv | expm\_F1\_model\_coefficients\_public.csv | 02\_workflows/F1\_workflow\_v02/data/02a\_refined\_analysis | csv | csv | 1330 | 1.299 | 2026-05-02T01:30:05Z | c05403544122bdf248154bfbf8559b9f | public | derived | F1 | 10 | 6 | yes\_default | FALSE |  | release\_candidate | public\_deposit | derived\_or\_workflow\_data | keep\_public\_deposit | F1 |  |  |  |  |  | public\_deposit | keep\_public\_deposit | Public-deposit file after policy overrides. | FALSE | FALSE | TRUE | FALSE | FALSE | FALSE | FALSE | FALSE | FALSE | public\_deposit | keep\_public\_deposit | Public-deposit file after policy overrides. | FALSE | FALSE | FALSE | FALSE | FALSE |
| 02\_workflows/F1\_workflow\_v02/data/02a\_refined\_analysis/expm\_F1\_pre\_correction\_rounding\_qc\_public.csv | expm\_F1\_pre\_correction\_rounding\_qc\_public.csv | 02\_workflows/F1\_workflow\_v02/data/02a\_refined\_analysis | csv | csv | 462 | 0.451 | 2026-05-02T01:30:05Z | ca3266c1f5df19844f2079981510ff28 | internal\_only | derived | F1 | 2 | 8 | no\_internal\_only\_default | FALSE |  | release\_candidate | public\_deposit | derived\_or\_workflow\_data | keep\_public\_deposit | F1 |  |  |  |  |  | public\_deposit | keep\_public\_deposit | Public-deposit file after policy overrides. | FALSE | FALSE | TRUE | FALSE | FALSE | FALSE | FALSE | FALSE | FALSE | public\_deposit | keep\_public\_deposit | Public-deposit file after policy overrides. | FALSE | FALSE | FALSE | FALSE | FALSE |
| 02\_workflows/F1\_workflow\_v02/data/02a\_refined\_analysis/expm\_F1\_refined\_dataset\_qc\_public.csv | expm\_F1\_refined\_dataset\_qc\_public.csv | 02\_workflows/F1\_workflow\_v02/data/02a\_refined\_analysis | csv | csv | 652 | 0.637 | 2026-05-02T01:30:05Z | 812231b8c2303741846747de6999d3c7 | internal\_only | derived | F1 | 18 | 2 | no\_internal\_only\_default | FALSE |  | release\_candidate | public\_deposit | derived\_or\_workflow\_data | keep\_public\_deposit | F1 |  |  |  |  |  | public\_deposit | keep\_public\_deposit | Public-deposit file after policy overrides. | FALSE | FALSE | TRUE | FALSE | FALSE | FALSE | FALSE | FALSE | FALSE | public\_deposit | keep\_public\_deposit | Public-deposit file after policy overrides. | FALSE | FALSE | FALSE | FALSE | FALSE |
| 02\_workflows/F1\_workflow\_v02/data/02a\_refined\_analysis/expm\_F1\_refined\_manual\_clarifications\_public.csv | expm\_F1\_refined\_manual\_clarifications\_public.csv | 02\_workflows/F1\_workflow\_v02/data/02a\_refined\_analysis | csv | csv | 882 | 0.861 | 2026-05-02T01:30:05Z | 8c4c1d35703560f1e9341b3df38ffad4 | public | derived | F1 | 3 | 3 | yes\_default | FALSE |  | release\_candidate | public\_deposit | derived\_or\_workflow\_data | keep\_public\_deposit | F1 |  |  |  |  |  | public\_deposit | keep\_public\_deposit | Public-deposit file after policy overrides. | FALSE | FALSE | TRUE | FALSE | FALSE | FALSE | FALSE | FALSE | FALSE | public\_deposit | keep\_public\_deposit | Public-deposit file after policy overrides. | FALSE | FALSE | FALSE | FALSE | FALSE |
| 02\_workflows/F1\_workflow\_v02/data/02a\_refined\_analysis/expm\_F1\_validation\_input\_public.csv | expm\_F1\_validation\_input\_public.csv | 02\_workflows/F1\_workflow\_v02/data/02a\_refined\_analysis | csv | csv | 5068 | 4.949 | 2026-05-02T01:30:05Z | 2a1c7ff9a9b2d7b913576fc64c1af459 | internal\_only | derived | F1 | 32 | 16 | no\_internal\_only\_default | FALSE |  | release\_candidate | public\_deposit | derived\_or\_workflow\_data | keep\_public\_deposit | F1 |  |  |  |  |  | public\_deposit | keep\_public\_deposit | Public-deposit file after policy overrides. | FALSE | FALSE | TRUE | FALSE | FALSE | FALSE | FALSE | FALSE | FALSE | public\_deposit | keep\_public\_deposit | Public-deposit file after policy overrides. | FALSE | FALSE | FALSE | FALSE | FALSE |
| 02\_workflows/F1\_workflow\_v02/data/02b\_figure\_content/slco\_F1\_surface\_grid\_public.csv | slco\_F1\_surface\_grid\_public.csv | 02\_workflows/F1\_workflow\_v02/data/02b\_figure\_content | csv | csv | 5037005 | 4918.95 | 2026-05-02T01:30:05Z | 7043e4517bc15ae9142ae09b0631e890 | public | derived | F1 | 34441 | 11 | yes\_default | FALSE |  | release\_candidate | public\_deposit | derived\_or\_workflow\_data | keep\_public\_deposit | F1 |  |  |  |  |  | public\_deposit | keep\_public\_deposit | Public-deposit file after policy overrides. | FALSE | FALSE | TRUE | FALSE | FALSE | FALSE | FALSE | FALSE | FALSE | public\_deposit | keep\_public\_deposit | Public-deposit file after policy overrides. | FALSE | FALSE | FALSE | FALSE | FALSE |
| 02\_workflows/F1\_workflow\_v02/data/02b\_figure\_content/slco\_F1\_surface\_meta\_public.csv | slco\_F1\_surface\_meta\_public.csv | 02\_workflows/F1\_workflow\_v02/data/02b\_figure\_content | csv | csv | 494 | 0.482 | 2026-05-02T01:30:05Z | 63cd6a8340d66c32e5915d2dadcc2378 | public | derived | F1 | 16 | 2 | yes\_default | FALSE |  | release\_candidate | public\_deposit | derived\_or\_workflow\_data | keep\_public\_deposit | F1 |  |  |  |  |  | public\_deposit | keep\_public\_deposit | Public-deposit file after policy overrides. | FALSE | FALSE | TRUE | FALSE | FALSE | FALSE | FALSE | FALSE | FALSE | public\_deposit | keep\_public\_deposit | Public-deposit file after policy overrides. | FALSE | FALSE | FALSE | FALSE | FALSE |
| 02\_workflows/F1\_workflow\_v02/data/02b\_figure\_content/slco\_F1\_surface\_rebuild\_qc\_public.csv | slco\_F1\_surface\_rebuild\_qc\_public.csv | 02\_workflows/F1\_workflow\_v02/data/02b\_figure\_content | csv | csv | 956 | 0.934 | 2026-05-02T01:30:05Z | c1c5a45987d8b24e318b2857d5b3529a | internal\_only | derived | F1 | 10 | 5 | no\_internal\_only\_default | FALSE |  | release\_candidate | public\_deposit | derived\_or\_workflow\_data | keep\_public\_deposit | F1 |  |  |  |  |  | public\_deposit | keep\_public\_deposit | Public-deposit file after policy overrides. | FALSE | FALSE | TRUE | FALSE | FALSE | FALSE | FALSE | FALSE | FALSE | public\_deposit | keep\_public\_deposit | Public-deposit file after policy overrides. | FALSE | FALSE | FALSE | FALSE | FALSE |
| 02\_workflows/F1\_workflow\_v02/data/02b\_figure\_content/slco\_F1\_surface\_reference\_meta\_original\_public.csv | slco\_F1\_surface\_reference\_meta\_original\_public.csv | 02\_workflows/F1\_workflow\_v02/data/02b\_figure\_content | csv | csv | 758 | 0.74 | 2026-05-02T01:30:05Z | 28bbb6eeab2bcd8799b28d088fee5ec2 | public | derived | F1 | 9 | 3 | yes\_default | FALSE |  | release\_candidate | public\_deposit | derived\_or\_workflow\_data | keep\_public\_deposit | F1 |  |  |  |  |  | public\_deposit | keep\_public\_deposit | Public-deposit file after policy overrides. | FALSE | FALSE | TRUE | FALSE | FALSE | FALSE | FALSE | FALSE | FALSE | public\_deposit | keep\_public\_deposit | Public-deposit file after policy overrides. | FALSE | FALSE | FALSE | FALSE | FALSE |
| 02\_workflows/F1\_workflow\_v02/data/02b\_figure\_content/slco\_F1\_surface\_run\_inputs\_outputs\_public.csv | slco\_F1\_surface\_run\_inputs\_outputs\_public.csv | 02\_workflows/F1\_workflow\_v02/data/02b\_figure\_content | csv | csv | 1357 | 1.325 | 2026-05-02T01:30:05Z | 8f9e627475762fd28c3c1276f469fc88 | public | derived | F1 | 5 | 3 | yes\_default | FALSE |  | release\_candidate | public\_deposit | derived\_or\_workflow\_data | keep\_public\_deposit | F1 |  |  |  |  |  | public\_deposit | keep\_public\_deposit | Public-deposit file after policy overrides. | FALSE | FALSE | TRUE | FALSE | FALSE | FALSE | FALSE | FALSE | FALSE | public\_deposit | keep\_public\_deposit | Public-deposit file after policy overrides. | FALSE | FALSE | FALSE | FALSE | FALSE |
| 02\_workflows/F1\_workflow\_v02/figures/slco\_F1\_surface\_public.pdf | slco\_F1\_surface\_public.pdf | 02\_workflows/F1\_workflow\_v02/figures | pdf | pdf | 1542152 | 1506.008 | 2026-05-02T01:30:14Z | 6de20aaebcce1d88c93b705cf4bd8152 | public | rendered\_output | F1 |  |  | yes\_default | FALSE |  | release\_candidate | public\_deposit | rendered\_figure | keep\_public\_deposit | F1 |  |  |  |  |  | public\_deposit | keep\_public\_deposit | Public-deposit file after policy overrides. | FALSE | FALSE | FALSE | TRUE | FALSE | FALSE | FALSE | FALSE | FALSE | public\_deposit | keep\_public\_deposit | Public-deposit file after policy overrides. | FALSE | FALSE | FALSE | FALSE | FALSE |
| 02\_workflows/F1\_workflow\_v02/submission\_ready/F1\_submission\_ready\_manifest\_v02.csv | F1\_submission\_ready\_manifest\_v02.csv | 02\_workflows/F1\_workflow\_v02/submission\_ready | csv | csv | 15124 | 14.77 | 2026-05-02T01:30:14Z | 3ed9b43000dcf410355e62e80576d9c0 | public | derived | F1 | 21 | 12 | yes\_default | FALSE |  | release\_candidate | manual\_review | documentation\_or\_metadata | manual\_review | F1 |  |  |  |  |  | public\_deposit | keep\_public\_submission\_ready\_manifest | Non-internal submission-ready manifest kept as public documentation/metadata. | FALSE | FALSE | TRUE | FALSE | FALSE | FALSE | FALSE | FALSE | FALSE | manual\_review | manual\_review\_required | No automatic final policy rule applied. | FALSE | TRUE | FALSE | FALSE | TRUE |
| 02\_workflows/F1\_workflow\_v02/submission\_ready/README\_F1\_submission\_ready\_v02.txt | README\_F1\_submission\_ready\_v02.txt | 02\_workflows/F1\_workflow\_v02/submission\_ready | txt | text\_table\_or\_text | 693 | 0.677 | 2026-05-02T01:30:14Z | f5d66cca875ad3e26170da9e649747f2 | public | derived | F1 | 1 | 13 | yes\_default | FALSE |  | release\_candidate | manual\_review | documentation\_or\_metadata | manual\_review | F1 |  |  |  |  |  | public\_deposit | keep\_public\_workflow\_readme | Workflow README kept as public documentation/metadata. | FALSE | FALSE | TRUE | FALSE | FALSE | FALSE | FALSE | FALSE | FALSE | manual\_review | manual\_review\_required | No automatic final policy rule applied. | FALSE | FALSE | TRUE | TRUE | TRUE |
| 02\_workflows/F1\_workflow\_v02/submission\_ready/public/F1\_public\_submission\_manifest\_v02.csv | F1\_public\_submission\_manifest\_v02.csv | 02\_workflows/F1\_workflow\_v02/submission\_ready/public | csv | csv | 3309 | 3.231 | 2026-05-02T01:30:14Z | a18df481d8a36383805720b63eb3feb0 | public | derived | F1 | 5 | 10 | yes\_default | FALSE |  | release\_candidate | public\_deposit | documentation\_or\_metadata | keep\_public\_deposit | F1 |  |  |  |  |  | public\_deposit | keep\_public\_deposit | Public-deposit file after policy overrides. | FALSE | FALSE | TRUE | FALSE | FALSE | FALSE | FALSE | FALSE | FALSE | public\_deposit | keep\_public\_deposit | Public-deposit file after policy overrides. | FALSE | FALSE | FALSE | FALSE | FALSE |
| 02\_workflows/F1\_workflow\_v02/submission\_ready/public/data/expm\_F1\_array\_input\_public.csv | expm\_F1\_array\_input\_public.csv | 02\_workflows/F1\_workflow\_v02/submission\_ready/public/data | csv | csv | 612913 | 598.548 | 2026-05-02T01:30:14Z | 28054a92825a550b9e2a0149689b4b69 | public | derived | F1 | 4200 | 17 | yes\_default | FALSE |  | release\_candidate | public\_deposit | derived\_or\_workflow\_data | keep\_public\_deposit | F1 |  |  |  |  |  | public\_deposit | keep\_public\_deposit | Public-deposit file after policy overrides. | FALSE | FALSE | TRUE | FALSE | FALSE | FALSE | FALSE | FALSE | FALSE | public\_deposit | keep\_public\_deposit | Public-deposit file after policy overrides. | FALSE | FALSE | FALSE | FALSE | FALSE |
| 02\_workflows/F1\_workflow\_v02/submission\_ready/public/data/expm\_F1\_validation\_input\_public.csv | expm\_F1\_validation\_input\_public.csv | 02\_workflows/F1\_workflow\_v02/submission\_ready/public/data | csv | csv | 5068 | 4.949 | 2026-05-02T01:30:14Z | 2a1c7ff9a9b2d7b913576fc64c1af459 | internal\_only | derived | F1 | 32 | 16 | no\_internal\_only\_default | FALSE |  | release\_candidate | public\_deposit | derived\_or\_workflow\_data | keep\_public\_deposit | F1 |  |  |  |  |  | public\_deposit | keep\_public\_deposit | Public-deposit file after policy overrides. | FALSE | FALSE | TRUE | FALSE | FALSE | FALSE | FALSE | FALSE | FALSE | public\_deposit | keep\_public\_deposit | Public-deposit file after policy overrides. | FALSE | FALSE | FALSE | FALSE | FALSE |
| 02\_workflows/F1\_workflow\_v02/submission\_ready/public/data/slco\_F1\_surface\_grid\_public.csv | slco\_F1\_surface\_grid\_public.csv | 02\_workflows/F1\_workflow\_v02/submission\_ready/public/data | csv | csv | 5037005 | 4918.95 | 2026-05-02T01:30:14Z | 7043e4517bc15ae9142ae09b0631e890 | public | derived | F1 | 34441 | 11 | yes\_default | FALSE |  | release\_candidate | public\_deposit | derived\_or\_workflow\_data | keep\_public\_deposit | F1 |  |  |  |  |  | public\_deposit | keep\_public\_deposit | Public-deposit file after policy overrides. | FALSE | FALSE | TRUE | FALSE | FALSE | FALSE | FALSE | FALSE | FALSE | public\_deposit | keep\_public\_deposit | Public-deposit file after policy overrides. | FALSE | FALSE | FALSE | FALSE | FALSE |
| 02\_workflows/F1\_workflow\_v02/submission\_ready/public/data/slco\_F1\_surface\_meta\_public.csv | slco\_F1\_surface\_meta\_public.csv | 02\_workflows/F1\_workflow\_v02/submission\_ready/public/data | csv | csv | 494 | 0.482 | 2026-05-02T01:30:14Z | 63cd6a8340d66c32e5915d2dadcc2378 | public | derived | F1 | 16 | 2 | yes\_default | FALSE |  | release\_candidate | public\_deposit | derived\_or\_workflow\_data | keep\_public\_deposit | F1 |  |  |  |  |  | public\_deposit | keep\_public\_deposit | Public-deposit file after policy overrides. | FALSE | FALSE | TRUE | FALSE | FALSE | FALSE | FALSE | FALSE | FALSE | public\_deposit | keep\_public\_deposit | Public-deposit file after policy overrides. | FALSE | FALSE | FALSE | FALSE | FALSE |
| 02\_workflows/F1\_workflow\_v02/submission\_ready/public/figures/slco\_F1\_surface\_public.pdf | slco\_F1\_surface\_public.pdf | 02\_workflows/F1\_workflow\_v02/submission\_ready/public/figures | pdf | pdf | 1542152 | 1506.008 | 2026-05-02T01:30:14Z | 6de20aaebcce1d88c93b705cf4bd8152 | public | rendered\_output | F1 |  |  | yes\_default | FALSE |  | release\_candidate | public\_deposit | rendered\_figure | keep\_public\_deposit | F1 |  |  |  |  |  | public\_deposit | keep\_public\_deposit | Public-deposit file after policy overrides. | FALSE | FALSE | FALSE | TRUE | FALSE | FALSE | FALSE | FALSE | FALSE | public\_deposit | keep\_public\_deposit | Public-deposit file after policy overrides. | FALSE | FALSE | FALSE | FALSE | FALSE |
| 02\_workflows/F2\_workflow\_v01/data/02b\_figure\_content/slco\_F2\_heatmap\_bin\_public.csv | slco\_F2\_heatmap\_bin\_public.csv | 02\_workflows/F2\_workflow\_v01/data/02b\_figure\_content | csv | csv | 7858998 | 7674.803 | 2026-05-02T01:33:01Z | 93e06eeefac7f60a0cd987630232561c | public | derived | F2 | 295200 | 6 | yes\_default | FALSE |  | release\_candidate | public\_deposit | derived\_or\_workflow\_data | keep\_public\_deposit | F2 |  |  |  |  |  | public\_deposit | keep\_public\_deposit | Public-deposit file after policy overrides. | FALSE | FALSE | TRUE | FALSE | FALSE | FALSE | FALSE | FALSE | FALSE | public\_deposit | keep\_public\_deposit | Public-deposit file after policy overrides. | FALSE | FALSE | FALSE | FALSE | FALSE |
| 02\_workflows/F2\_workflow\_v01/data/02b\_figure\_content/slco\_F2\_heatmap\_meta\_public.csv | slco\_F2\_heatmap\_meta\_public.csv | 02\_workflows/F2\_workflow\_v01/data/02b\_figure\_content | csv | csv | 552 | 0.539 | 2026-05-02T01:33:01Z | d6bec83444bffd55eb65533a93ad5c5f | public | derived | F2 | 22 | 2 | yes\_default | FALSE |  | release\_candidate | public\_deposit | derived\_or\_workflow\_data | keep\_public\_deposit | F2 |  |  |  |  |  | public\_deposit | keep\_public\_deposit | Public-deposit file after policy overrides. | FALSE | FALSE | TRUE | FALSE | FALSE | FALSE | FALSE | FALSE | FALSE | public\_deposit | keep\_public\_deposit | Public-deposit file after policy overrides. | FALSE | FALSE | FALSE | FALSE | FALSE |
| 02\_workflows/F2\_workflow\_v01/figures/slco\_F2\_heatmap\_public.pdf | slco\_F2\_heatmap\_public.pdf | 02\_workflows/F2\_workflow\_v01/figures | pdf | pdf | 877270 | 856.709 | 2026-05-02T01:33:03Z | a300627a40ff5d92e416ad2fdd7da6f9 | public | rendered\_output | F2 |  |  | yes\_default | FALSE |  | release\_candidate | public\_deposit | rendered\_figure | keep\_public\_deposit | F2 |  |  |  |  |  | public\_deposit | keep\_public\_deposit | Public-deposit file after policy overrides. | FALSE | FALSE | FALSE | TRUE | FALSE | FALSE | FALSE | FALSE | FALSE | public\_deposit | keep\_public\_deposit | Public-deposit file after policy overrides. | FALSE | FALSE | FALSE | FALSE | FALSE |
| 02\_workflows/F2\_workflow\_v01/submission\_ready/F2\_submission\_ready\_manifest\_v01.csv | F2\_submission\_ready\_manifest\_v01.csv | 02\_workflows/F2\_workflow\_v01/submission\_ready | csv | csv | 10090 | 9.854 | 2026-05-02T01:33:03Z | 9a714c11f127689b919994d3f34a21d5 | public | derived | F2 | 14 | 12 | yes\_default | FALSE |  | release\_candidate | manual\_review | documentation\_or\_metadata | manual\_review | F2 |  |  |  |  |  | public\_deposit | keep\_public\_submission\_ready\_manifest | Non-internal submission-ready manifest kept as public documentation/metadata. | FALSE | FALSE | TRUE | FALSE | FALSE | FALSE | FALSE | FALSE | FALSE | manual\_review | manual\_review\_required | No automatic final policy rule applied. | FALSE | TRUE | FALSE | FALSE | TRUE |
| 02\_workflows/F2\_workflow\_v01/submission\_ready/README\_F2\_submission\_ready\_v01.txt | README\_F2\_submission\_ready\_v01.txt | 02\_workflows/F2\_workflow\_v01/submission\_ready | txt | text\_table\_or\_text | 296 | 0.289 | 2026-05-02T01:33:03Z | bd8ca0253fd5c12541633d4990502d74 | public | derived | F2 | 0 | 3 | yes\_default | FALSE |  | release\_candidate | manual\_review | documentation\_or\_metadata | manual\_review | F2 |  |  |  |  |  | public\_deposit | keep\_public\_workflow\_readme | Workflow README kept as public documentation/metadata. | FALSE | FALSE | TRUE | FALSE | FALSE | FALSE | FALSE | FALSE | FALSE | manual\_review | manual\_review\_required | No automatic final policy rule applied. | FALSE | FALSE | TRUE | TRUE | TRUE |
| 02\_workflows/F2\_workflow\_v01/submission\_ready/public/F2\_public\_submission\_manifest\_v01.csv | F2\_public\_submission\_manifest\_v01.csv | 02\_workflows/F2\_workflow\_v01/submission\_ready/public | csv | csv | 2012 | 1.965 | 2026-05-02T01:33:03Z | e40767cc9d3957d1686cc714d7c24287 | public | derived | F2 | 3 | 10 | yes\_default | FALSE |  | release\_candidate | public\_deposit | documentation\_or\_metadata | keep\_public\_deposit | F2 |  |  |  |  |  | public\_deposit | keep\_public\_deposit | Public-deposit file after policy overrides. | FALSE | FALSE | TRUE | FALSE | FALSE | FALSE | FALSE | FALSE | FALSE | public\_deposit | keep\_public\_deposit | Public-deposit file after policy overrides. | FALSE | FALSE | FALSE | FALSE | FALSE |
| 02\_workflows/F2\_workflow\_v01/submission\_ready/public/data/slco\_F2\_heatmap\_bin\_public.csv | slco\_F2\_heatmap\_bin\_public.csv | 02\_workflows/F2\_workflow\_v01/submission\_ready/public/data | csv | csv | 7858998 | 7674.803 | 2026-05-02T01:33:03Z | 93e06eeefac7f60a0cd987630232561c | public | derived | F2 | 295200 | 6 | yes\_default | FALSE |  | release\_candidate | public\_deposit | derived\_or\_workflow\_data | keep\_public\_deposit | F2 |  |  |  |  |  | public\_deposit | keep\_public\_deposit | Public-deposit file after policy overrides. | FALSE | FALSE | TRUE | FALSE | FALSE | FALSE | FALSE | FALSE | FALSE | public\_deposit | keep\_public\_deposit | Public-deposit file after policy overrides. | FALSE | FALSE | FALSE | FALSE | FALSE |
| 02\_workflows/F2\_workflow\_v01/submission\_ready/public/data/slco\_F2\_heatmap\_meta\_public.csv | slco\_F2\_heatmap\_meta\_public.csv | 02\_workflows/F2\_workflow\_v01/submission\_ready/public/data | csv | csv | 552 | 0.539 | 2026-05-02T01:33:03Z | d6bec83444bffd55eb65533a93ad5c5f | public | derived | F2 | 22 | 2 | yes\_default | FALSE |  | release\_candidate | public\_deposit | derived\_or\_workflow\_data | keep\_public\_deposit | F2 |  |  |  |  |  | public\_deposit | keep\_public\_deposit | Public-deposit file after policy overrides. | FALSE | FALSE | TRUE | FALSE | FALSE | FALSE | FALSE | FALSE | FALSE | public\_deposit | keep\_public\_deposit | Public-deposit file after policy overrides. | FALSE | FALSE | FALSE | FALSE | FALSE |
| 02\_workflows/F2\_workflow\_v01/submission\_ready/public/figures/slco\_F2\_heatmap\_public.pdf | slco\_F2\_heatmap\_public.pdf | 02\_workflows/F2\_workflow\_v01/submission\_ready/public/figures | pdf | pdf | 877270 | 856.709 | 2026-05-02T01:33:03Z | a300627a40ff5d92e416ad2fdd7da6f9 | public | rendered\_output | F2 |  |  | yes\_default | FALSE |  | release\_candidate | public\_deposit | rendered\_figure | keep\_public\_deposit | F2 |  |  |  |  |  | public\_deposit | keep\_public\_deposit | Public-deposit file after policy overrides. | FALSE | FALSE | FALSE | TRUE | FALSE | FALSE | FALSE | FALSE | FALSE | public\_deposit | keep\_public\_deposit | Public-deposit file after policy overrides. | FALSE | FALSE | FALSE | FALSE | FALSE |
| 02\_workflows/F3\_workflow\_v01/README\_F3\_workflow\_v01.txt | README\_F3\_workflow\_v01.txt | 02\_workflows/F3\_workflow\_v01 | txt | text\_table\_or\_text | 720 | 0.703 | 2026-04-30T14:01:54Z | 8ebddb4a72afe11cfba90f6257e315ea | public | derived | F3 |  |  | yes\_default | FALSE |  | release\_candidate | manual\_review | documentation\_or\_metadata | manual\_review | F3 |  |  |  |  |  | public\_deposit | keep\_public\_workflow\_readme | Workflow README kept as public documentation/metadata. | FALSE | FALSE | TRUE | FALSE | FALSE | FALSE | FALSE | FALSE | FALSE | manual\_review | manual\_review\_required | No automatic final policy rule applied. | FALSE | FALSE | TRUE | FALSE | TRUE |
| 02\_workflows/F3\_workflow\_v01/data/01\_source\_loaded\_harmonized/esld\_master\_long\_public.csv | esld\_master\_long\_public.csv | 02\_workflows/F3\_workflow\_v01/data/01\_source\_loaded\_harmonized | csv | csv | 9851508 | 9620.613 | 2026-05-02T23:18:12Z | 8228407e927ff10ad6e4740d0d5cd48b | public | derived | F3 | 67399 | 43 | yes\_default | FALSE |  | release\_candidate | public\_deposit | derived\_or\_workflow\_data | keep\_public\_deposit | F3 |  |  |  |  |  | public\_deposit | keep\_public\_deposit | Public-deposit file after policy overrides. | FALSE | FALSE | TRUE | FALSE | FALSE | FALSE | FALSE | FALSE | FALSE | public\_deposit | keep\_public\_deposit | Public-deposit file after policy overrides. | FALSE | FALSE | FALSE | FALSE | FALSE |
| 02\_workflows/F3\_workflow\_v01/data/02b\_figure\_content/esld\_F3\_score\_shift\_aggregate\_public.csv | esld\_F3\_score\_shift\_aggregate\_public.csv | 02\_workflows/F3\_workflow\_v01/data/02b\_figure\_content | csv | csv | 6671 | 6.515 | 2026-05-02T09:50:43Z | 9e9ace861cb329c001b4fcf732aef69c | public | derived | F3 | 315 | 4 | yes\_default | FALSE |  | release\_candidate | public\_deposit | derived\_or\_workflow\_data | keep\_public\_deposit | F3 |  |  |  |  |  | public\_deposit | keep\_public\_deposit | Public-deposit file after policy overrides. | FALSE | FALSE | TRUE | FALSE | FALSE | FALSE | FALSE | FALSE | FALSE | public\_deposit | keep\_public\_deposit | Public-deposit file after policy overrides. | FALSE | FALSE | FALSE | FALSE | FALSE |
| 02\_workflows/F3\_workflow\_v01/figures/F3\_ESLD\_SRTR\_public.pdf | F3\_ESLD\_SRTR\_public.pdf | 02\_workflows/F3\_workflow\_v01/figures | pdf | pdf | 569374 | 556.029 | 2026-05-02T09:50:44Z | f8b24dda2b8388ecfc6b61ab02e80c27 | restricted\_on\_request | rendered\_output | F3 |  |  | yes\_default | TRUE |  | release\_candidate | public\_deposit | rendered\_figure | keep\_public\_deposit | F3 |  |  |  |  |  | public\_deposit | keep\_public\_deposit | Public-deposit file after policy overrides. | FALSE | TRUE | FALSE | TRUE | FALSE | FALSE | FALSE | FALSE | FALSE | public\_deposit | keep\_public\_deposit | Public-deposit file after policy overrides. | FALSE | FALSE | FALSE | FALSE | FALSE |
| 02\_workflows/F3\_workflow\_v01/submission\_ready/F3\_submission\_ready\_manifest\_v01.csv | F3\_submission\_ready\_manifest\_v01.csv | 02\_workflows/F3\_workflow\_v01/submission\_ready | csv | csv | 1752 | 1.711 | 2026-05-02T09:50:44Z | c524b4a4a7c9ae655d310516ff16a166 | public | derived | F3 | 15 | 6 | yes\_default | FALSE |  | release\_candidate | manual\_review | documentation\_or\_metadata | manual\_review | F3 |  |  |  |  |  | public\_deposit | keep\_public\_submission\_ready\_manifest | Non-internal submission-ready manifest kept as public documentation/metadata. | FALSE | FALSE | TRUE | FALSE | FALSE | FALSE | FALSE | FALSE | FALSE | manual\_review | manual\_review\_required | No automatic final policy rule applied. | FALSE | TRUE | FALSE | FALSE | TRUE |
| 02\_workflows/F3\_workflow\_v01/submission\_ready/README\_F3\_submission\_ready\_v01.txt | README\_F3\_submission\_ready\_v01.txt | 02\_workflows/F3\_workflow\_v01/submission\_ready | txt | text\_table\_or\_text | 336 | 0.328 | 2026-05-02T09:50:44Z | 1f67873b1c86188d38ce46eb6cd2dd66 | public | derived | F3 | 1 | 8 | yes\_default | FALSE |  | release\_candidate | manual\_review | documentation\_or\_metadata | manual\_review | F3 |  |  |  |  |  | public\_deposit | keep\_public\_workflow\_readme | Workflow README kept as public documentation/metadata. | FALSE | FALSE | TRUE | FALSE | FALSE | FALSE | FALSE | FALSE | FALSE | manual\_review | manual\_review\_required | No automatic final policy rule applied. | FALSE | FALSE | TRUE | TRUE | TRUE |
| 02\_workflows/F3\_workflow\_v01/submission\_ready/public/F3\_public\_submission\_manifest\_v01.csv | F3\_public\_submission\_manifest\_v01.csv | 02\_workflows/F3\_workflow\_v01/submission\_ready/public | csv | csv | 449 | 0.438 | 2026-05-02T09:50:44Z | 17a134e04f2847572a292674f992bc29 | public | derived | F3 | 4 | 5 | yes\_default | FALSE |  | release\_candidate | public\_deposit | documentation\_or\_metadata | keep\_public\_deposit | F3 |  |  |  |  |  | public\_deposit | keep\_public\_deposit | Public-deposit file after policy overrides. | FALSE | FALSE | TRUE | FALSE | FALSE | FALSE | FALSE | FALSE | FALSE | public\_deposit | keep\_public\_deposit | Public-deposit file after policy overrides. | FALSE | FALSE | FALSE | FALSE | FALSE |
| 02\_workflows/F3\_workflow\_v01/submission\_ready/public/data/esld\_F3\_score\_shift\_aggregate\_public.csv | esld\_F3\_score\_shift\_aggregate\_public.csv | 02\_workflows/F3\_workflow\_v01/submission\_ready/public/data | csv | csv | 6671 | 6.515 | 2026-05-02T09:50:43Z | 9e9ace861cb329c001b4fcf732aef69c | public | derived | F3 | 315 | 4 | yes\_default | FALSE |  | release\_candidate | public\_deposit | derived\_or\_workflow\_data | keep\_public\_deposit | F3 |  |  |  |  |  | public\_deposit | keep\_public\_deposit | Public-deposit file after policy overrides. | FALSE | FALSE | TRUE | FALSE | FALSE | FALSE | FALSE | FALSE | FALSE | public\_deposit | keep\_public\_deposit | Public-deposit file after policy overrides. | FALSE | FALSE | FALSE | FALSE | FALSE |
| 02\_workflows/F3\_workflow\_v01/submission\_ready/public/figures/F3\_ESLD\_SRTR\_public.pdf | F3\_ESLD\_SRTR\_public.pdf | 02\_workflows/F3\_workflow\_v01/submission\_ready/public/figures | pdf | pdf | 569374 | 556.029 | 2026-05-02T09:50:44Z | f8b24dda2b8388ecfc6b61ab02e80c27 | restricted\_on\_request | rendered\_output | F3 |  |  | yes\_default | TRUE |  | release\_candidate | public\_deposit | rendered\_figure | keep\_public\_deposit | F3 |  |  |  |  |  | public\_deposit | keep\_public\_deposit | Public-deposit file after policy overrides. | FALSE | TRUE | FALSE | TRUE | FALSE | FALSE | FALSE | FALSE | FALSE | public\_deposit | keep\_public\_deposit | Public-deposit file after policy overrides. | FALSE | FALSE | FALSE | FALSE | FALSE |
| 02\_workflows/F4\_workflow\_v01/README\_F4\_workflow\_v01.txt | README\_F4\_workflow\_v01.txt | 02\_workflows/F4\_workflow\_v01 | txt | text\_table\_or\_text | 447 | 0.437 | 2026-04-30T12:11:40Z | b976db4271a8f5d859f540e16d517a74 | public | derived | F4 | 13 | 1 | yes\_default | FALSE |  | release\_candidate | manual\_review | documentation\_or\_metadata | manual\_review | F4 |  |  |  |  |  | public\_deposit | keep\_public\_workflow\_readme | Workflow README kept as public documentation/metadata. | FALSE | FALSE | TRUE | FALSE | FALSE | FALSE | FALSE | FALSE | FALSE | manual\_review | manual\_review\_required | No automatic final policy rule applied. | FALSE | FALSE | TRUE | FALSE | TRUE |
| 02\_workflows/F4\_workflow\_v01/data/01\_source\_loaded\_harmonized/esld\_master\_long\_public.csv | esld\_master\_long\_public.csv | 02\_workflows/F4\_workflow\_v01/data/01\_source\_loaded\_harmonized | csv | csv | 9851508 | 9620.613 | 2026-05-02T23:18:12Z | 8228407e927ff10ad6e4740d0d5cd48b | public | derived | F4 | 67399 | 43 | yes\_default | FALSE |  | release\_candidate | public\_deposit | derived\_or\_workflow\_data | keep\_public\_deposit | F4 |  |  |  |  |  | public\_deposit | keep\_public\_deposit | Public-deposit file after policy overrides. | FALSE | FALSE | TRUE | FALSE | FALSE | FALSE | FALSE | FALSE | FALSE | public\_deposit | keep\_public\_deposit | Public-deposit file after policy overrides. | FALSE | FALSE | FALSE | FALSE | FALSE |
| 02\_workflows/F4\_workflow\_v01/data/02b\_figure\_content/esld\_F4\_survival\_stats\_public.csv | esld\_F4\_survival\_stats\_public.csv | 02\_workflows/F4\_workflow\_v01/data/02b\_figure\_content | csv | csv | 2309 | 2.255 | 2026-05-02T02:11:22Z | 33d16cd47150ae66d00d8a83b4b0c3ab | public | derived | F4 | 27 | 14 | yes\_default | FALSE |  | release\_candidate | public\_deposit | derived\_or\_workflow\_data | keep\_public\_deposit | F4 |  |  |  |  |  | public\_deposit | keep\_public\_deposit | Public-deposit file after policy overrides. | FALSE | FALSE | TRUE | FALSE | FALSE | FALSE | FALSE | FALSE | FALSE | public\_deposit | keep\_public\_deposit | Public-deposit file after policy overrides. | FALSE | FALSE | FALSE | FALSE | FALSE |
| 02\_workflows/F4\_workflow\_v01/figures/F4\_ESLD\_SRTR\_public.pdf | F4\_ESLD\_SRTR\_public.pdf | 02\_workflows/F4\_workflow\_v01/figures | pdf | pdf | 837483 | 817.854 | 2026-05-02T02:11:22Z | 66ff2e5f3a6621ee2b581cc02aa6a6a9 | restricted\_on\_request | rendered\_output | F4 |  |  | yes\_default | TRUE |  | release\_candidate | public\_deposit | rendered\_figure | keep\_public\_deposit | F4 |  |  |  |  |  | public\_deposit | keep\_public\_deposit | Public-deposit file after policy overrides. | FALSE | TRUE | FALSE | TRUE | FALSE | FALSE | FALSE | FALSE | FALSE | public\_deposit | keep\_public\_deposit | Public-deposit file after policy overrides. | FALSE | FALSE | FALSE | FALSE | FALSE |
| 02\_workflows/F4\_workflow\_v01/submission\_ready/F4\_submission\_ready\_manifest\_v01.csv | F4\_submission\_ready\_manifest\_v01.csv | 02\_workflows/F4\_workflow\_v01/submission\_ready | csv | csv | 11006 | 10.748 | 2026-05-02T02:11:22Z | 084184caa0b53fd9f4fd2b0f275e2435 | public | derived | F4 | 15 | 12 | yes\_default | FALSE |  | release\_candidate | manual\_review | documentation\_or\_metadata | manual\_review | F4 |  |  |  |  |  | public\_deposit | keep\_public\_submission\_ready\_manifest | Non-internal submission-ready manifest kept as public documentation/metadata. | FALSE | FALSE | TRUE | FALSE | FALSE | FALSE | FALSE | FALSE | FALSE | manual\_review | manual\_review\_required | No automatic final policy rule applied. | FALSE | TRUE | FALSE | FALSE | TRUE |
| 02\_workflows/F4\_workflow\_v01/submission\_ready/public/F4\_public\_submission\_manifest\_v01.csv | F4\_public\_submission\_manifest\_v01.csv | 02\_workflows/F4\_workflow\_v01/submission\_ready/public | csv | csv | 2816 | 2.75 | 2026-05-02T02:11:22Z | ba26a26357f0be2cd909d30095f6ae0f | public | derived | F4 | 4 | 10 | yes\_default | FALSE |  | release\_candidate | public\_deposit | documentation\_or\_metadata | keep\_public\_deposit | F4 |  |  |  |  |  | public\_deposit | keep\_public\_deposit | Public-deposit file after policy overrides. | FALSE | FALSE | TRUE | FALSE | FALSE | FALSE | FALSE | FALSE | FALSE | public\_deposit | keep\_public\_deposit | Public-deposit file after policy overrides. | FALSE | FALSE | FALSE | FALSE | FALSE |
| 02\_workflows/F4\_workflow\_v01/submission\_ready/public/data/esld\_F4\_survival\_stats\_public.csv | esld\_F4\_survival\_stats\_public.csv | 02\_workflows/F4\_workflow\_v01/submission\_ready/public/data | csv | csv | 2309 | 2.255 | 2026-05-02T02:11:22Z | 33d16cd47150ae66d00d8a83b4b0c3ab | public | derived | F4 | 27 | 14 | yes\_default | FALSE |  | release\_candidate | public\_deposit | derived\_or\_workflow\_data | keep\_public\_deposit | F4 |  |  |  |  |  | public\_deposit | keep\_public\_deposit | Public-deposit file after policy overrides. | FALSE | FALSE | TRUE | FALSE | FALSE | FALSE | FALSE | FALSE | FALSE | public\_deposit | keep\_public\_deposit | Public-deposit file after policy overrides. | FALSE | FALSE | FALSE | FALSE | FALSE |
| 02\_workflows/F4\_workflow\_v01/submission\_ready/public/figures/F4\_ESLD\_SRTR\_public.pdf | F4\_ESLD\_SRTR\_public.pdf | 02\_workflows/F4\_workflow\_v01/submission\_ready/public/figures | pdf | pdf | 837483 | 817.854 | 2026-05-02T02:11:22Z | 66ff2e5f3a6621ee2b581cc02aa6a6a9 | restricted\_on\_request | rendered\_output | F4 |  |  | yes\_default | TRUE |  | release\_candidate | public\_deposit | rendered\_figure | keep\_public\_deposit | F4 |  |  |  |  |  | public\_deposit | keep\_public\_deposit | Public-deposit file after policy overrides. | FALSE | TRUE | FALSE | TRUE | FALSE | FALSE | FALSE | FALSE | FALSE | public\_deposit | keep\_public\_deposit | Public-deposit file after policy overrides. | FALSE | FALSE | FALSE | FALSE | FALSE |
| 02\_workflows/F5\_workflow\_v01/README\_F5\_workflow\_v01.txt | README\_F5\_workflow\_v01.txt | 02\_workflows/F5\_workflow\_v01 | txt | text\_table\_or\_text | 975 | 0.952 | 2026-04-30T14:37:07Z | f9733312a2ebff5ac2a6b89de943363f | public | derived | F5 |  |  | yes\_default | FALSE |  | release\_candidate | manual\_review | documentation\_or\_metadata | manual\_review | F5 |  |  |  |  |  | public\_deposit | keep\_public\_workflow\_readme | Workflow README kept as public documentation/metadata. | FALSE | FALSE | TRUE | FALSE | FALSE | FALSE | FALSE | FALSE | FALSE | manual\_review | manual\_review\_required | No automatic final policy rule applied. | FALSE | FALSE | TRUE | FALSE | TRUE |
| 02\_workflows/F5\_workflow\_v01/data/01\_source\_loaded\_harmonized/esld\_master\_long\_public.csv | esld\_master\_long\_public.csv | 02\_workflows/F5\_workflow\_v01/data/01\_source\_loaded\_harmonized | csv | csv | 9851508 | 9620.613 | 2026-05-02T23:18:13Z | 8228407e927ff10ad6e4740d0d5cd48b | public | derived | F5 | 67399 | 43 | yes\_default | FALSE |  | release\_candidate | public\_deposit | derived\_or\_workflow\_data | keep\_public\_deposit | F5 |  |  |  |  |  | public\_deposit | keep\_public\_deposit | Public-deposit file after policy overrides. | FALSE | FALSE | TRUE | FALSE | FALSE | FALSE | FALSE | FALSE | FALSE | public\_deposit | keep\_public\_deposit | Public-deposit file after policy overrides. | FALSE | FALSE | FALSE | FALSE | FALSE |
| 02\_workflows/F5\_workflow\_v01/data/02b\_figure\_content/esld\_F5\_stratified\_survival\_meta\_public.csv | esld\_F5\_stratified\_survival\_meta\_public.csv | 02\_workflows/F5\_workflow\_v01/data/02b\_figure\_content | csv | csv | 434 | 0.424 | 2026-05-02T01:41:36Z | 91f3a3d116ae7b1e3930facc0dafe562 | public | derived | F5 | 12 | 2 | yes\_default | FALSE |  | release\_candidate | public\_deposit | derived\_or\_workflow\_data | keep\_public\_deposit | F5 |  |  |  |  |  | public\_deposit | keep\_public\_deposit | Public-deposit file after policy overrides. | FALSE | FALSE | TRUE | FALSE | FALSE | FALSE | FALSE | FALSE | FALSE | public\_deposit | keep\_public\_deposit | Public-deposit file after policy overrides. | FALSE | FALSE | FALSE | FALSE | FALSE |
| 02\_workflows/F5\_workflow\_v01/data/02b\_figure\_content/esld\_F5\_stratified\_survival\_stats\_public.csv | esld\_F5\_stratified\_survival\_stats\_public.csv | 02\_workflows/F5\_workflow\_v01/data/02b\_figure\_content | csv | csv | 1119 | 1.093 | 2026-05-02T01:41:36Z | a2935cfa9107cbfc3bd687c572f65c38 | public | derived | F5 | 12 | 8 | yes\_default | FALSE |  | release\_candidate | public\_deposit | derived\_or\_workflow\_data | keep\_public\_deposit | F5 |  |  |  |  |  | public\_deposit | keep\_public\_deposit | Public-deposit file after policy overrides. | FALSE | FALSE | TRUE | FALSE | FALSE | FALSE | FALSE | FALSE | FALSE | public\_deposit | keep\_public\_deposit | Public-deposit file after policy overrides. | FALSE | FALSE | FALSE | FALSE | FALSE |
| 02\_workflows/F5\_workflow\_v01/data/02b\_figure\_content/esld\_F5\_stratified\_survival\_subject\_public.csv | esld\_F5\_stratified\_survival\_subject\_public.csv | 02\_workflows/F5\_workflow\_v01/data/02b\_figure\_content | csv | csv | 122147 | 119.284 | 2026-05-02T01:41:36Z | d4865d62db7ce845d162bdd848dc4ce2 | public | derived | F5 | 841 | 19 | yes\_default | FALSE |  | release\_candidate | public\_deposit | derived\_or\_workflow\_data | keep\_public\_deposit | F5 |  |  |  |  |  | public\_deposit | keep\_public\_deposit | Public-deposit file after policy overrides. | FALSE | FALSE | TRUE | FALSE | FALSE | FALSE | FALSE | FALSE | FALSE | public\_deposit | keep\_public\_deposit | Public-deposit file after policy overrides. | FALSE | FALSE | FALSE | FALSE | FALSE |
| 02\_workflows/F5\_workflow\_v01/figures/F5\_ESLD\_public.pdf | F5\_ESLD\_public.pdf | 02\_workflows/F5\_workflow\_v01/figures | pdf | pdf | 616548 | 602.098 | 2026-05-02T01:41:36Z | 136a8ddede064a07771670ea2ccddf45 | public | rendered\_output | F5 |  |  | yes\_default | FALSE |  | release\_candidate | public\_deposit | rendered\_figure | keep\_public\_deposit | F5 |  |  |  |  |  | public\_deposit | keep\_public\_deposit | Public-deposit file after policy overrides. | FALSE | FALSE | FALSE | TRUE | FALSE | FALSE | FALSE | FALSE | FALSE | public\_deposit | keep\_public\_deposit | Public-deposit file after policy overrides. | FALSE | FALSE | FALSE | FALSE | FALSE |
| 02\_workflows/F5\_workflow\_v01/submission\_ready/F5\_submission\_ready\_manifest\_v01.csv | F5\_submission\_ready\_manifest\_v01.csv | 02\_workflows/F5\_workflow\_v01/submission\_ready | csv | csv | 10483 | 10.237 | 2026-05-02T01:41:36Z | d52c6273c3d9c4a425dae83ce8a6fd4a | public | derived | F5 | 14 | 12 | yes\_default | FALSE |  | release\_candidate | manual\_review | documentation\_or\_metadata | manual\_review | F5 |  |  |  |  |  | public\_deposit | keep\_public\_submission\_ready\_manifest | Non-internal submission-ready manifest kept as public documentation/metadata. | FALSE | FALSE | TRUE | FALSE | FALSE | FALSE | FALSE | FALSE | FALSE | manual\_review | manual\_review\_required | No automatic final policy rule applied. | FALSE | TRUE | FALSE | FALSE | TRUE |
| 02\_workflows/F5\_workflow\_v01/submission\_ready/public/F5\_public\_submission\_manifest\_v01.csv | F5\_public\_submission\_manifest\_v01.csv | 02\_workflows/F5\_workflow\_v01/submission\_ready/public | csv | csv | 2820 | 2.754 | 2026-05-02T01:41:36Z | 8e75867808215c69fd8292a7f4768c6c | public | derived | F5 | 4 | 10 | yes\_default | FALSE |  | release\_candidate | public\_deposit | documentation\_or\_metadata | keep\_public\_deposit | F5 |  |  |  |  |  | public\_deposit | keep\_public\_deposit | Public-deposit file after policy overrides. | FALSE | FALSE | TRUE | FALSE | FALSE | FALSE | FALSE | FALSE | FALSE | public\_deposit | keep\_public\_deposit | Public-deposit file after policy overrides. | FALSE | FALSE | FALSE | FALSE | FALSE |
| 02\_workflows/F5\_workflow\_v01/submission\_ready/public/data/esld\_F5\_stratified\_survival\_meta\_public.csv | esld\_F5\_stratified\_survival\_meta\_public.csv | 02\_workflows/F5\_workflow\_v01/submission\_ready/public/data | csv | csv | 434 | 0.424 | 2026-05-02T01:41:36Z | 91f3a3d116ae7b1e3930facc0dafe562 | public | derived | F5 | 12 | 2 | yes\_default | FALSE |  | release\_candidate | public\_deposit | derived\_or\_workflow\_data | keep\_public\_deposit | F5 |  |  |  |  |  | public\_deposit | keep\_public\_deposit | Public-deposit file after policy overrides. | FALSE | FALSE | TRUE | FALSE | FALSE | FALSE | FALSE | FALSE | FALSE | public\_deposit | keep\_public\_deposit | Public-deposit file after policy overrides. | FALSE | FALSE | FALSE | FALSE | FALSE |
| 02\_workflows/F5\_workflow\_v01/submission\_ready/public/data/esld\_F5\_stratified\_survival\_stats\_public.csv | esld\_F5\_stratified\_survival\_stats\_public.csv | 02\_workflows/F5\_workflow\_v01/submission\_ready/public/data | csv | csv | 1119 | 1.093 | 2026-05-02T01:41:36Z | a2935cfa9107cbfc3bd687c572f65c38 | public | derived | F5 | 12 | 8 | yes\_default | FALSE |  | release\_candidate | public\_deposit | derived\_or\_workflow\_data | keep\_public\_deposit | F5 |  |  |  |  |  | public\_deposit | keep\_public\_deposit | Public-deposit file after policy overrides. | FALSE | FALSE | TRUE | FALSE | FALSE | FALSE | FALSE | FALSE | FALSE | public\_deposit | keep\_public\_deposit | Public-deposit file after policy overrides. | FALSE | FALSE | FALSE | FALSE | FALSE |
| 02\_workflows/F5\_workflow\_v01/submission\_ready/public/data/esld\_F5\_stratified\_survival\_subject\_public.csv | esld\_F5\_stratified\_survival\_subject\_public.csv | 02\_workflows/F5\_workflow\_v01/submission\_ready/public/data | csv | csv | 122147 | 119.284 | 2026-05-02T01:41:36Z | d4865d62db7ce845d162bdd848dc4ce2 | public | derived | F5 | 841 | 19 | yes\_default | FALSE |  | release\_candidate | public\_deposit | derived\_or\_workflow\_data | keep\_public\_deposit | F5 |  |  |  |  |  | public\_deposit | keep\_public\_deposit | Public-deposit file after policy overrides. | FALSE | FALSE | TRUE | FALSE | FALSE | FALSE | FALSE | FALSE | FALSE | public\_deposit | keep\_public\_deposit | Public-deposit file after policy overrides. | FALSE | FALSE | FALSE | FALSE | FALSE |
| 02\_workflows/F5\_workflow\_v01/submission\_ready/public/figures/F5\_ESLD\_public.pdf | F5\_ESLD\_public.pdf | 02\_workflows/F5\_workflow\_v01/submission\_ready/public/figures | pdf | pdf | 616548 | 602.098 | 2026-05-02T01:41:36Z | 136a8ddede064a07771670ea2ccddf45 | public | rendered\_output | F5 |  |  | yes\_default | FALSE |  | release\_candidate | public\_deposit | rendered\_figure | keep\_public\_deposit | F5 |  |  |  |  |  | public\_deposit | keep\_public\_deposit | Public-deposit file after policy overrides. | FALSE | FALSE | FALSE | TRUE | FALSE | FALSE | FALSE | FALSE | FALSE | public\_deposit | keep\_public\_deposit | Public-deposit file after policy overrides. | FALSE | FALSE | FALSE | FALSE | FALSE |
| 02\_workflows/F6\_workflow\_v01/README\_F6\_workflow\_v01.txt | README\_F6\_workflow\_v01.txt | 02\_workflows/F6\_workflow\_v01 | txt | text\_table\_or\_text | 907 | 0.886 | 2026-04-30T12:58:14Z | 74a79476f5426f8eec7c0af3be7410c2 | public | derived | F6 | 0 | 3 | yes\_default | FALSE |  | release\_candidate | manual\_review | documentation\_or\_metadata | manual\_review | F6 |  |  |  |  |  | public\_deposit | keep\_public\_workflow\_readme | Workflow README kept as public documentation/metadata. | FALSE | FALSE | TRUE | FALSE | FALSE | FALSE | FALSE | FALSE | FALSE | manual\_review | manual\_review\_required | No automatic final policy rule applied. | FALSE | FALSE | TRUE | FALSE | TRUE |
| 02\_workflows/F6\_workflow\_v01/figures/F6\_SRTR\_public.pdf | F6\_SRTR\_public.pdf | 02\_workflows/F6\_workflow\_v01/figures | pdf | pdf | 568988 | 555.652 | 2026-05-02T03:11:53Z | 363a49e6865292f51c335342b4e50772 | restricted\_on\_request | rendered\_output | F6 |  |  | yes\_default | TRUE |  | release\_candidate | public\_deposit | rendered\_figure | keep\_public\_deposit | F6 |  |  |  |  |  | public\_deposit | keep\_public\_deposit | Public-deposit file after policy overrides. | FALSE | TRUE | FALSE | TRUE | FALSE | FALSE | FALSE | FALSE | FALSE | public\_deposit | keep\_public\_deposit | Public-deposit file after policy overrides. | FALSE | FALSE | FALSE | FALSE | FALSE |
| 02\_workflows/F6\_workflow\_v01/submission\_ready/F6\_submission\_ready\_manifest\_v01.csv | F6\_submission\_ready\_manifest\_v01.csv | 02\_workflows/F6\_workflow\_v01/submission\_ready | csv | csv | 11135 | 10.874 | 2026-05-02T03:11:53Z | 31f0fe575e38946b6f3dadfa5de2a47e | public | derived | F6 | 16 | 12 | yes\_default | FALSE |  | release\_candidate | manual\_review | documentation\_or\_metadata | manual\_review | F6 |  |  |  |  |  | public\_deposit | keep\_public\_submission\_ready\_manifest | Non-internal submission-ready manifest kept as public documentation/metadata. | FALSE | FALSE | TRUE | FALSE | FALSE | FALSE | FALSE | FALSE | FALSE | manual\_review | manual\_review\_required | No automatic final policy rule applied. | FALSE | TRUE | FALSE | FALSE | TRUE |
| 02\_workflows/F6\_workflow\_v01/submission\_ready/README\_F6\_submission\_ready\_v01.txt | README\_F6\_submission\_ready\_v01.txt | 02\_workflows/F6\_workflow\_v01/submission\_ready | txt | text\_table\_or\_text | 477 | 0.466 | 2026-05-02T03:11:53Z | a193c8f8a36df7b9d2e14fb8372013d7 | public | derived | F6 | 0 | 24 | yes\_default | FALSE |  | release\_candidate | manual\_review | documentation\_or\_metadata | manual\_review | F6 |  |  |  |  |  | public\_deposit | keep\_public\_workflow\_readme | Workflow README kept as public documentation/metadata. | FALSE | FALSE | TRUE | FALSE | FALSE | FALSE | FALSE | FALSE | FALSE | manual\_review | manual\_review\_required | No automatic final policy rule applied. | FALSE | FALSE | TRUE | TRUE | TRUE |
| 02\_workflows/F6\_workflow\_v01/submission\_ready/public/F6\_public\_submission\_manifest\_v01.csv | F6\_public\_submission\_manifest\_v01.csv | 02\_workflows/F6\_workflow\_v01/submission\_ready/public | csv | csv | 2010 | 1.963 | 2026-05-02T03:11:53Z | 64711b5a57aded6fe83d18b7a7dd288a | public | derived | F6 | 3 | 10 | yes\_default | FALSE |  | release\_candidate | public\_deposit | documentation\_or\_metadata | keep\_public\_deposit | F6 |  |  |  |  |  | public\_deposit | keep\_public\_deposit | Public-deposit file after policy overrides. | FALSE | FALSE | TRUE | FALSE | FALSE | FALSE | FALSE | FALSE | FALSE | public\_deposit | keep\_public\_deposit | Public-deposit file after policy overrides. | FALSE | FALSE | FALSE | FALSE | FALSE |
| 02\_workflows/F6\_workflow\_v01/submission\_ready/public/figures/F6\_SRTR\_public.pdf | F6\_SRTR\_public.pdf | 02\_workflows/F6\_workflow\_v01/submission\_ready/public/figures | pdf | pdf | 568988 | 555.652 | 2026-05-02T03:11:53Z | 363a49e6865292f51c335342b4e50772 | restricted\_on\_request | rendered\_output | F6 |  |  | yes\_default | TRUE |  | release\_candidate | public\_deposit | rendered\_figure | keep\_public\_deposit | F6 |  |  |  |  |  | public\_deposit | keep\_public\_deposit | Public-deposit file after policy overrides. | FALSE | TRUE | FALSE | TRUE | FALSE | FALSE | FALSE | FALSE | FALSE | public\_deposit | keep\_public\_deposit | Public-deposit file after policy overrides. | FALSE | FALSE | FALSE | FALSE | FALSE |
| 02\_workflows/T1\_workflow\_v01/README\_T1\_workflow\_v01.txt | README\_T1\_workflow\_v01.txt | 02\_workflows/T1\_workflow\_v01 | txt | text\_table\_or\_text | 1071 | 1.046 | 2026-04-30T15:33:29Z | 20040f67e0c4ee67a7e491986c7f6024 | public | derived | T1 | 1 | 3 | yes\_default | FALSE |  | release\_candidate | manual\_review | documentation\_or\_metadata | manual\_review | T1 |  |  |  |  |  | public\_deposit | keep\_public\_workflow\_readme | Workflow README kept as public documentation/metadata. | FALSE | FALSE | TRUE | FALSE | FALSE | FALSE | FALSE | FALSE | FALSE | manual\_review | manual\_review\_required | No automatic final policy rule applied. | FALSE | FALSE | TRUE | FALSE | TRUE |
| 02\_workflows/T1\_workflow\_v01/data/01\_source\_loaded\_harmonized/esld\_T1\_baseline\_patient\_public.csv | esld\_T1\_baseline\_patient\_public.csv | 02\_workflows/T1\_workflow\_v01/data/01\_source\_loaded\_harmonized | csv | csv | 69600 | 67.969 | 2026-05-02T20:27:46Z | 5d5b1fa7a2a3845550ca78b467c41b7c | public | derived | T1 | 1375 | 19 | yes\_default | FALSE |  | release\_candidate | public\_deposit | derived\_or\_workflow\_data | keep\_public\_deposit | T1 |  |  |  |  |  | public\_deposit | keep\_public\_deposit | Public-deposit file after policy overrides. | FALSE | FALSE | TRUE | FALSE | FALSE | FALSE | FALSE | FALSE | FALSE | public\_deposit | keep\_public\_deposit | Public-deposit file after policy overrides. | FALSE | FALSE | FALSE | FALSE | FALSE |
| 02\_workflows/T1\_workflow\_v01/data/01\_source\_loaded\_harmonized/esld\_master\_long\_public.csv | esld\_master\_long\_public.csv | 02\_workflows/T1\_workflow\_v01/data/01\_source\_loaded\_harmonized | csv | csv | 9851508 | 9620.613 | 2026-05-02T23:18:13Z | 8228407e927ff10ad6e4740d0d5cd48b | public | derived | T1 | 67399 | 43 | yes\_default | FALSE |  | release\_candidate | public\_deposit | derived\_or\_workflow\_data | keep\_public\_deposit | T1 |  |  |  |  |  | public\_deposit | keep\_public\_deposit | Public-deposit file after policy overrides. | FALSE | FALSE | TRUE | FALSE | FALSE | FALSE | FALSE | FALSE | FALSE | public\_deposit | keep\_public\_deposit | Public-deposit file after policy overrides. | FALSE | FALSE | FALSE | FALSE | FALSE |
| 02\_workflows/T1\_workflow\_v01/submission\_ready/README\_T1\_submission\_ready\_v01.txt | README\_T1\_submission\_ready\_v01.txt | 02\_workflows/T1\_workflow\_v01/submission\_ready | txt | text\_table\_or\_text | 550 | 0.537 | 2026-05-02T20:27:48Z | 5c48ce4c0a9f0c619123f99c97705e76 | public | derived | T1 | 10 | 1 | yes\_default | FALSE |  | release\_candidate | manual\_review | documentation\_or\_metadata | manual\_review | T1 |  |  |  |  |  | public\_deposit | keep\_public\_workflow\_readme | Workflow README kept as public documentation/metadata. | FALSE | FALSE | TRUE | FALSE | FALSE | FALSE | FALSE | FALSE | FALSE | manual\_review | manual\_review\_required | No automatic final policy rule applied. | FALSE | FALSE | TRUE | TRUE | TRUE |
| 02\_workflows/T1\_workflow\_v01/submission\_ready/public/T1\_public\_submission\_manifest\_v01.csv | T1\_public\_submission\_manifest\_v01.csv | 02\_workflows/T1\_workflow\_v01/submission\_ready/public | csv | csv | 1021 | 0.997 | 2026-05-02T20:27:48Z | 50ceb725e1005aaef4026b224fd3e84d | public | derived | T1 | 2 | 8 | yes\_default | FALSE |  | release\_candidate | public\_deposit | documentation\_or\_metadata | keep\_public\_deposit | T1 |  |  |  |  |  | public\_deposit | keep\_public\_deposit | Public-deposit file after policy overrides. | FALSE | FALSE | TRUE | FALSE | FALSE | FALSE | FALSE | FALSE | FALSE | public\_deposit | keep\_public\_deposit | Public-deposit file after policy overrides. | FALSE | FALSE | FALSE | FALSE | FALSE |
| 02\_workflows/T3\_workflow\_v01/data/01\_source\_loaded\_harmonized/esld\_master\_long\_public.csv | esld\_master\_long\_public.csv | 02\_workflows/T3\_workflow\_v01/data/01\_source\_loaded\_harmonized | csv | csv | 9851508 | 9620.613 | 2026-05-02T23:18:13Z | 8228407e927ff10ad6e4740d0d5cd48b | public | derived | T3 | 67399 | 43 | yes\_default | FALSE |  | release\_candidate | public\_deposit | derived\_or\_workflow\_data | keep\_public\_deposit | T3 |  |  |  |  |  | public\_deposit | keep\_public\_deposit | Public-deposit file after policy overrides. | FALSE | FALSE | TRUE | FALSE | FALSE | FALSE | FALSE | FALSE | FALSE | public\_deposit | keep\_public\_deposit | Public-deposit file after policy overrides. | FALSE | FALSE | FALSE | FALSE | FALSE |
| 02\_workflows/T3\_workflow\_v01/submission\_ready/README\_T3\_submission\_ready\_v01.txt | README\_T3\_submission\_ready\_v01.txt | 02\_workflows/T3\_workflow\_v01/submission\_ready | txt | text\_table\_or\_text | 638 | 0.623 | 2026-05-02T21:14:25Z | 896dfb22fbd13cdbe4049651bf404bfe | public | derived | T3 | 10 | 1 | yes\_default | FALSE |  | release\_candidate | manual\_review | documentation\_or\_metadata | manual\_review | T3 |  |  |  |  |  | public\_deposit | keep\_public\_workflow\_readme | Workflow README kept as public documentation/metadata. | FALSE | FALSE | TRUE | FALSE | FALSE | FALSE | FALSE | FALSE | FALSE | manual\_review | manual\_review\_required | No automatic final policy rule applied. | FALSE | FALSE | TRUE | TRUE | TRUE |
| 02\_workflows/T3\_workflow\_v01/submission\_ready/public/T3\_public\_submission\_manifest\_v01.csv | T3\_public\_submission\_manifest\_v01.csv | 02\_workflows/T3\_workflow\_v01/submission\_ready/public | csv | csv | 986 | 0.963 | 2026-05-02T21:14:25Z | 5f44456d11292898dbf5ba9d4db9324e | public | derived | T3 | 2 | 8 | yes\_default | FALSE |  | release\_candidate | public\_deposit | documentation\_or\_metadata | keep\_public\_deposit | T3 |  |  |  |  |  | public\_deposit | keep\_public\_deposit | Public-deposit file after policy overrides. | FALSE | FALSE | TRUE | FALSE | FALSE | FALSE | FALSE | FALSE | FALSE | public\_deposit | keep\_public\_deposit | Public-deposit file after policy overrides. | FALSE | FALSE | FALSE | FALSE | FALSE |
| 02\_workflows/T4\_workflow\_v01/data/01\_source\_loaded\_harmonized/esld\_master\_long\_public.csv | esld\_master\_long\_public.csv | 02\_workflows/T4\_workflow\_v01/data/01\_source\_loaded\_harmonized | csv | csv | 9851508 | 9620.613 | 2026-05-02T23:18:13Z | 8228407e927ff10ad6e4740d0d5cd48b | public | derived | T4 | 67399 | 43 | yes\_default | FALSE |  | release\_candidate | public\_deposit | derived\_or\_workflow\_data | keep\_public\_deposit | T4 |  |  |  |  |  | public\_deposit | keep\_public\_deposit | Public-deposit file after policy overrides. | FALSE | FALSE | TRUE | FALSE | FALSE | FALSE | FALSE | FALSE | FALSE | public\_deposit | keep\_public\_deposit | Public-deposit file after policy overrides. | FALSE | FALSE | FALSE | FALSE | FALSE |
| 02\_workflows/T4\_workflow\_v01/data/02b\_table\_content/esld\_T4\_score\_deviation\_outcome\_meta\_public.csv | esld\_T4\_score\_deviation\_outcome\_meta\_public.csv | 02\_workflows/T4\_workflow\_v01/data/02b\_table\_content | csv | csv | 694 | 0.678 | 2026-05-02T00:32:16Z | da8cb16231868249ed6a58148548d6d8 | public | derived | T4;table\_or\_supplement | 10 | 2 | yes\_default | FALSE |  | release\_candidate | public\_deposit | derived\_or\_workflow\_data | keep\_public\_deposit | T4 |  |  |  |  |  | public\_deposit | keep\_public\_deposit | Public-deposit file after policy overrides. | FALSE | FALSE | TRUE | FALSE | FALSE | FALSE | FALSE | FALSE | FALSE | public\_deposit | keep\_public\_deposit | Public-deposit file after policy overrides. | FALSE | FALSE | FALSE | FALSE | FALSE |
| 02\_workflows/T4\_workflow\_v01/data/02b\_table\_content/esld\_T4\_score\_deviation\_outcome\_table\_public.csv | esld\_T4\_score\_deviation\_outcome\_table\_public.csv | 02\_workflows/T4\_workflow\_v01/data/02b\_table\_content | csv | csv | 2628 | 2.566 | 2026-05-02T00:32:16Z | c7c6b8bd97bb8df75be2ffa87ec0c281 | public | derived | T4;table\_or\_supplement | 13 | 12 | yes\_default | FALSE |  | release\_candidate | public\_deposit | derived\_or\_workflow\_data | keep\_public\_deposit | T4 |  |  |  |  |  | public\_deposit | keep\_public\_deposit | Public-deposit file after policy overrides. | FALSE | FALSE | TRUE | FALSE | FALSE | FALSE | FALSE | FALSE | FALSE | public\_deposit | keep\_public\_deposit | Public-deposit file after policy overrides. | FALSE | FALSE | FALSE | FALSE | FALSE |
| 02\_workflows/T4\_workflow\_v01/submission\_ready/README\_T4\_submission\_ready\_v01.txt | README\_T4\_submission\_ready\_v01.txt | 02\_workflows/T4\_workflow\_v01/submission\_ready | txt | text\_table\_or\_text | 1048 | 1.023 | 2026-05-02T00:32:17Z | af763a666a55c1a1cc38c3dba503c572 | public | derived | T4 | 11 | 18 | yes\_default | FALSE |  | release\_candidate | manual\_review | documentation\_or\_metadata | manual\_review | T4 |  |  |  |  |  | public\_deposit | keep\_public\_workflow\_readme | Workflow README kept as public documentation/metadata. | FALSE | FALSE | TRUE | FALSE | FALSE | FALSE | FALSE | FALSE | FALSE | manual\_review | manual\_review\_required | No automatic final policy rule applied. | FALSE | FALSE | TRUE | TRUE | TRUE |
| 02\_workflows/T4\_workflow\_v01/submission\_ready/public/T4\_public\_submission\_manifest\_v01.csv | T4\_public\_submission\_manifest\_v01.csv | 02\_workflows/T4\_workflow\_v01/submission\_ready/public | csv | csv | 1023 | 0.999 | 2026-05-02T00:32:17Z | 6250c9dc4dfe6e0325806479aff6639b | public | derived | T4 | 2 | 8 | yes\_default | FALSE |  | release\_candidate | public\_deposit | documentation\_or\_metadata | keep\_public\_deposit | T4 |  |  |  |  |  | public\_deposit | keep\_public\_deposit | Public-deposit file after policy overrides. | FALSE | FALSE | TRUE | FALSE | FALSE | FALSE | FALSE | FALSE | FALSE | public\_deposit | keep\_public\_deposit | Public-deposit file after policy overrides. | FALSE | FALSE | FALSE | FALSE | FALSE |
| 02\_workflows/T4\_workflow\_v01/submission\_ready/public/data/esld\_T4\_score\_deviation\_outcome\_meta\_public.csv | esld\_T4\_score\_deviation\_outcome\_meta\_public.csv | 02\_workflows/T4\_workflow\_v01/submission\_ready/public/data | csv | csv | 694 | 0.678 | 2026-05-02T00:32:17Z | da8cb16231868249ed6a58148548d6d8 | public | derived | T4 | 10 | 2 | yes\_default | FALSE |  | release\_candidate | public\_deposit | derived\_or\_workflow\_data | keep\_public\_deposit | T4 |  |  |  |  |  | public\_deposit | keep\_public\_deposit | Public-deposit file after policy overrides. | FALSE | FALSE | TRUE | FALSE | FALSE | FALSE | FALSE | FALSE | FALSE | public\_deposit | keep\_public\_deposit | Public-deposit file after policy overrides. | FALSE | FALSE | FALSE | FALSE | FALSE |
| 02\_workflows/T4\_workflow\_v01/submission\_ready/public/data/esld\_T4\_score\_deviation\_outcome\_table\_public.csv | esld\_T4\_score\_deviation\_outcome\_table\_public.csv | 02\_workflows/T4\_workflow\_v01/submission\_ready/public/data | csv | csv | 2628 | 2.566 | 2026-05-02T00:32:17Z | c7c6b8bd97bb8df75be2ffa87ec0c281 | public | derived | T4;table\_or\_supplement | 13 | 12 | yes\_default | FALSE |  | release\_candidate | public\_deposit | derived\_or\_workflow\_data | keep\_public\_deposit | T4 |  |  |  |  |  | public\_deposit | keep\_public\_deposit | Public-deposit file after policy overrides. | FALSE | FALSE | TRUE | FALSE | FALSE | FALSE | FALSE | FALSE | FALSE | public\_deposit | keep\_public\_deposit | Public-deposit file after policy overrides. | FALSE | FALSE | FALSE | FALSE | FALSE |
