# Supplementary material for: Creatinine assay interferences compromises MELD accuracy and may bias liver allocation
Source: Nat Commun. 2026 Jul 23;17:7111. doi: 10.1038/s41467-026-75011-x (PMC13396164; doi:10.1038/s41467-026-75011-x)
Supplement: Supplementary file 4 — Source Data [file 41467_2026_75011_MOESM4_ESM.zip › figshare_package_FINAL_PUBLIC_DEPOSIT_V1_20260503_002637/00_START_HERE_HTML_NAVIGATOR/file_views/view_0031_README_F6_submission_ready_v01.html]

02\_workflows/F6\_workflow\_v01/submission\_ready/README\_F6\_submission\_ready\_v01.txt

# Readable file view

02\_workflows/F6\_workflow\_v01/submission\_ready/README\_F6\_submission\_ready\_v01.txt

← Back to navigator   |   Open original package file

Section

Workflow readmes

Output

F6

Extension

txt

Size KB

0.466

Variables

0

## Readable HTML view

```
F6 submission-ready output structure

Public files are under public/data and public/figures.
Restricted SRTR-derived rendering inputs are under restricted/data and must not be publicly released unless explicitly permitted by the applicable data-use agreement.
Internal validation/QC files are under internal/validation.

F6 differs from ESLD-only figures because the CIF long dataset required to render the figure is SRTR-derived and classified as restricted in this workflow.
```
