# Supplementary material for: Creatinine assay interferences compromises MELD accuracy and may bias liver allocation
Source: Nat Commun. 2026 Jul 23;17:7111. doi: 10.1038/s41467-026-75011-x (PMC13396164; doi:10.1038/s41467-026-75011-x)
Supplement: Supplementary file 4 — Source Data [file 41467_2026_75011_MOESM4_ESM.zip › figshare_package_FINAL_PUBLIC_DEPOSIT_V1_20260503_002637/00_START_HERE_HTML_NAVIGATOR/file_views/view_0002_esld_master_long_public.html]

01\_primary\_data/public/esld\_master\_long\_public.csv

# Readable file view

01\_primary\_data/public/esld\_master\_long\_public.csv

← Back to navigator   |   Open original package file

Section

Public primary data

Output

Extension

csv

Size KB

9620.613

Variables

43

## Variables in this file

| Variable | Label | Description | Unit | Type |
| --- | --- | --- | --- | --- |
| age\_years\_first\_available | Age at first available public record | Age in years at the first available public ESLD record for the patient or encounter represented in the released data. | years | integer |
| age\_years\_sample | Age at sample | Age in years at the sample-level public ESLD record. | years | integer |
| albumin\_g\_dl | Serum albumin concentration | Serum albumin concentration in the public ESLD master data. | g/dL | numeric |
| ald | Alcohol-related liver disease etiology flag | Binary etiology indicator for alcohol-related liver disease in the public ESLD cohort. |  | integer |
| ald\_hcv | Combined alcohol-related liver disease and hepatitis C etiology flag | Binary etiology indicator for combined alcohol-related liver disease and hepatitis C in the public ESLD cohort. |  | integer |
| autoimmune | Autoimmune liver disease etiology flag | Binary etiology indicator for autoimmune liver disease in the public ESLD cohort. |  | integer |
| crea | Creatinine concentration | Creatinine concentration used in MELD-related score calculation before applying the study-specific correction. | mg/dL | numeric |
| crea\_corrected | Corrected creatinine concentration | Creatinine concentration after applying the study-specific correction used for recalculated MELD-related scores. | mg/dL | numeric |
| date\_count | Relative day count | Relative day count used for time alignment; negative/positive values are relative to the analysis anchor and are not calendar dates. |  | integer |
| date\_count\_month | Relative month count | Relative month count derived from the relative day count; this is not a calendar month. |  | integer |
| dead\_sample\_flag | Dead-sample flag | Binary indicator identifying public ESLD sample records assigned to the deceased-sample subset used in sample-level analyses. |  | integer |
| death\_within\_90d | Death within 90 days | Binary indicator for death within 90 days of the analysis anchor. |  | logical |
| deceased\_patient\_flag | Deceased patient flag | Binary indicator identifying deceased patients within the released analysis context. |  | integer |
| delta\_eq\_0\_flag | Zero score-delta flag | Binary indicator equal to 1 when the score delta is exactly zero. |  | integer |
| delta\_gt\_0\_flag | Positive score-delta flag | Binary indicator equal to 1 when the score delta is positive. |  | integer |
| delta\_le\_minus1\_flag | Score-delta ≤ −1 flag | Binary indicator equal to 1 when the score delta is less than or equal to −1. |  | integer |
| dialysis\_raw | Dialysis indicator as recorded before score derivation | Binary indicator showing whether dialysis was recorded in the source field before score derivation. |  | integer |
| encounter\_id\_public | Public encounter pseudonym | Non-linkable public-release encounter pseudonym used for encounter-level grouping; not an original hospital encounter identifier. |  | character |
| etiology\_source\_current\_flag | Current etiology source flag | Binary indicator identifying whether the etiology source is the current source used for the public row. |  | integer |
| etiology\_unclassified | Unclassified liver disease etiology flag | Binary etiology indicator identifying records without a classified liver disease etiology in the public ESLD cohort. |  | integer |
| hbv | Hepatitis B virus etiology flag | Binary etiology indicator for hepatitis B virus-related liver disease in the public ESLD cohort. |  | integer |
| hcv | Hepatitis C virus etiology flag | Binary etiology indicator for hepatitis C virus-related liver disease in the public ESLD cohort. |  | integer |
| icd\_source\_present | ICD source present flag | Binary indicator showing whether an ICD-derived etiology source was present for the public row. |  | integer |
| in\_t1\_cohort | Inclusion flag for Table 1 cohort | Binary indicator identifying records included in the public ESLD Table 1 baseline-characteristics cohort. |  | integer |
| in\_t3\_anchor\_model | Inclusion flag for Table 3 anchor-model cohort | Binary indicator identifying records included in the Table 3 anchor-model cohort used for creatinine-comparison analyses. |  | integer |
| in\_t4\_prevalence\_path | Inclusion flag for Table 4 prevalence pathway | Binary indicator identifying records included in the Table 4 prevalence-analysis pathway. |  | integer |
| in\_t4\_survival\_path | Inclusion flag for Table 4 survival pathway | Binary indicator identifying records included in the Table 4 survival-analysis pathway. |  | integer |
| inr | International normalized ratio | International normalized ratio of prothrombin time used for MELD-related score calculation. | ratio | numeric |
| mash | Metabolic dysfunction-associated steatohepatitis etiology flag | Binary etiology indicator for metabolic dysfunction-associated steatohepatitis in the public ESLD cohort. |  | integer |
| model | MELD model or score variant | Name of the MELD-related model or score variant represented by the row; expected values include MELD, MELD-Na, reMELD-Na, and MELD 3.0. |  | character |
| other | Other liver disease etiology flag | Binary etiology indicator for liver disease etiologies grouped as other in the public ESLD cohort. |  | integer |
| patient\_id | Public patient pseudonym | Non-linkable public-release patient pseudonym used to preserve within-patient grouping in public ESLD data; not an original hospital patient identifier. |  | character |
| pbc | Primary biliary cholangitis etiology flag | Binary etiology indicator for primary biliary cholangitis in the public ESLD cohort. |  | integer |
| sample\_day\_from\_first\_sample | Relative sample day | Relative day of the sample measured from the first sample for the public patient/sample sequence; not a calendar date. | relative days | integer |
| sample\_group\_id\_public | Public sample-group pseudonym | Non-linkable public-release sample-group pseudonym used to group samples within the released public data. |  | character |
| sample\_id | Sample identifier | Identifier of a sample or experimental record within the released public data; not a personal identifier. |  | character |
| sample\_month\_index | Relative sample month index | Relative month index of the sample measured from the first sample for the public patient/sample sequence; not a calendar month. | relative months | integer |
| score\_corrected | Corrected score | MELD-related score after creatinine correction or recalculation. | score points | integer |
| score\_delta | Score difference | Difference between score variants or scoring approaches, expressed in score points. |  | integer |
| score\_original | Original score | Original MELD-related score before creatinine correction or recalculation. |  | integer |
| sex | Sex | Sex category represented in the public ESLD data. |  | character |
| sodium | Serum sodium concentration | Serum sodium concentration used for MELD-Na, reMELD-Na, or MELD 3.0 score calculation. | mmol/L | numeric |
| tbil | Total bilirubin concentration | Total bilirubin concentration used in MELD-related score calculation in the public ESLD data. |  | numeric |

## Readable HTML view

Preview shows first 1000 of 67399 rows. Open the original file for full content.

| patient\_id | sample\_group\_id\_public | sample\_id | encounter\_id\_public | model | sample\_day\_from\_first\_sample | sample\_month\_index | sex | age\_years\_sample | age\_years\_first\_available | crea | crea\_corrected | tbil | inr | sodium | albumin\_g\_dl | dialysis\_raw | score\_original | score\_corrected | score\_delta | delta\_le\_minus1\_flag | delta\_eq\_0\_flag | delta\_gt\_0\_flag | dead\_sample\_flag | deceased\_patient\_flag | death\_within\_90d | date\_count | date\_count\_month | icd\_source\_present | ald | mash | hbv | hcv | ald\_hcv | autoimmune | pbc | other | etiology\_unclassified | etiology\_source\_current\_flag | in\_t1\_cohort | in\_t3\_anchor\_model | in\_t4\_prevalence\_path | in\_t4\_survival\_path |
| --- | --- | --- | --- | --- | --- | --- | --- | --- | --- | --- | --- | --- | --- | --- | --- | --- | --- | --- | --- | --- | --- | --- | --- | --- | --- | --- | --- | --- | --- | --- | --- | --- | --- | --- | --- | --- | --- | --- | --- | --- | --- | --- |
| P000001 | G0000001 | S0000001 | E001181 | MELD | 0 | 0 | M | 65 | 65 | 1.91 | 1.87705004446668 | 1 | 1.14 |  |  | 0 | 14 | 14 | 0 | 0 | 1 | 0 | 0 | 0 |  |  |  | 1 | 0 | 0 | 0 | 0 | 0 | 0 | 0 | 1 | 0 | 1 | 1 | 1 | 1 | 0 |
| P000001 | G0000001 | S0000002 | E001181 | MELD-Na | 0 | 0 | M | 65 | 65 | 1.91 | 1.87705004446668 | 1 | 1.14 | 131 |  | 0 | 19 | 19 | 0 | 0 | 1 | 0 | 0 | 0 |  |  |  | 1 | 0 | 0 | 0 | 0 | 0 | 0 | 0 | 1 | 0 | 1 | 1 | 0 | 1 | 0 |
| P000001 | G0000001 | S0000003 | E001181 | reMELD-Na | 0 | 0 | M | 65 | 65 | 1.91 | 1.87705004446668 | 1 | 1.14 | 131 |  |  | 16 | 16 | 0 | 0 | 1 | 0 | 0 | 0 |  |  |  | 1 | 0 | 0 | 0 | 0 | 0 | 0 | 0 | 1 | 0 | 1 | 1 | 0 | 1 | 0 |
| P000002 | G0000002 | S0000004 | E000008 | MELD | 0 | 0 | F | 65 | 65 | 1.42 | 1.41586432303417 | 1 | 0.91 |  |  | 0 | 10 | 10 | 0 | 0 | 1 | 0 | 0 | 0 |  |  |  | 1 | 1 | 1 | 0 | 0 | 0 | 0 | 0 | 0 | 0 | 1 | 1 | 1 | 1 | 0 |
| P000002 | G0000002 | S0000005 | E000008 | MELD 3.0 | 0 | 0 | F | 65 | 65 | 1.42 | 1.41586432303417 | 1 | 0.91 | 134 | 3.3 |  | 14 | 14 | 0 | 0 | 1 | 0 | 0 | 0 |  |  |  | 1 | 1 | 1 | 0 | 0 | 0 | 0 | 0 | 0 | 0 | 1 | 1 | 0 | 1 | 0 |
| P000002 | G0000002 | S0000006 | E000008 | MELD-Na | 0 | 0 | F | 65 | 65 | 1.42 | 1.41586432303417 | 1 | 0.91 | 134 |  | 0 | 13 | 13 | 0 | 0 | 1 | 0 | 0 | 0 |  |  |  | 1 | 1 | 1 | 0 | 0 | 0 | 0 | 0 | 0 | 0 | 1 | 1 | 0 | 1 | 0 |
| P000002 | G0000002 | S0000007 | E000008 | reMELD-Na | 0 | 0 | F | 65 | 65 | 1.42 | 1.41586432303417 | 1 | 0.91 | 134 |  |  | 11 | 11 | 0 | 0 | 1 | 0 | 0 | 0 |  |  |  | 1 | 1 | 1 | 0 | 0 | 0 | 0 | 0 | 0 | 0 | 1 | 1 | 0 | 1 | 0 |
| P000002 | G0000003 | S0000008 | E000008 | MELD | 42 | 1 | F | 66 | 65 | 1.31 | 1.31039363454933 | 1 | 1 |  |  | 0 | 9 | 9 | 0 | 0 | 1 | 0 | 0 | 0 |  |  |  | 1 | 1 | 1 | 0 | 0 | 0 | 0 | 0 | 0 | 0 | 1 | 1 | 1 | 1 | 0 |
| P000002 | G0000003 | S0000009 | E000008 | MELD 3.0 | 42 | 1 | F | 66 | 65 | 1.31 | 1.31039363454933 | 1 | 1 | 132 | 6.15 |  | 14 | 14 | 0 | 0 | 1 | 0 | 0 | 0 |  |  |  | 1 | 1 | 1 | 0 | 0 | 0 | 0 | 0 | 0 | 0 | 1 | 1 | 0 | 1 | 0 |
| P000002 | G0000003 | S0000010 | E000008 | MELD-Na | 42 | 1 | F | 66 | 65 | 1.31 | 1.31039363454933 | 1 | 1 | 132 |  | 0 | 14 | 14 | 0 | 0 | 1 | 0 | 0 | 0 |  |  |  | 1 | 1 | 1 | 0 | 0 | 0 | 0 | 0 | 0 | 0 | 1 | 1 | 0 | 1 | 0 |
| P000002 | G0000003 | S0000011 | E000008 | reMELD-Na | 42 | 1 | F | 66 | 65 | 1.31 | 1.31039363454933 | 1 | 1 | 132 |  |  | 12 | 12 | 0 | 0 | 1 | 0 | 0 | 0 |  |  |  | 1 | 1 | 1 | 0 | 0 | 0 | 0 | 0 | 0 | 0 | 1 | 1 | 0 | 1 | 0 |
| P000002 | G0000004 | S0000012 | E000041 | MELD | 91 | 2 | F | 66 | 65 | 1.3 | 1.30192482788903 | 0.9 | 1.04 |  |  | 0 | 9 | 9 | 0 | 0 | 1 | 0 | 0 | 0 |  |  |  | 1 | 1 | 1 | 0 | 0 | 0 | 0 | 0 | 0 | 0 | 1 | 1 | 1 | 1 | 0 |
| P000002 | G0000004 | S0000013 | E000041 | MELD 3.0 | 91 | 2 | F | 66 | 65 | 1.3 | 1.30192482788903 | 0.9 | 1.04 | 133 | 3.14 |  | 14 | 14 | 0 | 0 | 1 | 0 | 0 | 0 |  |  |  | 1 | 1 | 1 | 0 | 0 | 0 | 0 | 0 | 0 | 0 | 1 | 1 | 0 | 1 | 0 |
| P000002 | G0000004 | S0000014 | E000041 | MELD-Na | 91 | 2 | F | 66 | 65 | 1.3 | 1.30192482788903 | 0.9 | 1.04 | 133 |  | 0 | 13 | 13 | 0 | 0 | 1 | 0 | 0 | 0 |  |  |  | 1 | 1 | 1 | 0 | 0 | 0 | 0 | 0 | 0 | 0 | 1 | 1 | 0 | 1 | 0 |
| P000002 | G0000004 | S0000015 | E000041 | reMELD-Na | 91 | 2 | F | 66 | 65 | 1.3 | 1.30192482788903 | 0.9 | 1.04 | 133 |  |  | 12 | 12 | 0 | 0 | 1 | 0 | 0 | 0 |  |  |  | 1 | 1 | 1 | 0 | 0 | 0 | 0 | 0 | 0 | 0 | 1 | 1 | 0 | 1 | 0 |
| P000002 | G0000005 | S0000016 | E000041 | MELD | 118 | 3 | F | 66 | 65 | 1.28 | 1.28150549768982 | 1 | 0.98 |  |  | 0 | 9 | 9 | 0 | 0 | 1 | 0 | 0 | 0 |  |  |  | 1 | 1 | 1 | 0 | 0 | 0 | 0 | 0 | 0 | 0 | 1 | 1 | 1 | 1 | 0 |
| P000002 | G0000005 | S0000017 | E000041 | MELD 3.0 | 118 | 3 | F | 66 | 65 | 1.28 | 1.28150549768982 | 1 | 0.98 | 140 | 2.8 |  | 11 | 11 | 0 | 0 | 1 | 0 | 0 | 0 |  |  |  | 1 | 1 | 1 | 0 | 0 | 0 | 0 | 0 | 0 | 0 | 1 | 1 | 0 | 1 | 0 |
| P000002 | G0000005 | S0000018 | E000041 | MELD-Na | 118 | 3 | F | 66 | 65 | 1.28 | 1.28150549768982 | 1 | 0.98 | 140 |  | 0 | 9 | 9 | 0 | 0 | 1 | 0 | 0 | 0 |  |  |  | 1 | 1 | 1 | 0 | 0 | 0 | 0 | 0 | 0 | 0 | 1 | 1 | 0 | 1 | 0 |
| P000002 | G0000005 | S0000019 | E000041 | reMELD-Na | 118 | 3 | F | 66 | 65 | 1.28 | 1.28150549768982 | 1 | 0.98 | 140 |  |  | 10 | 10 | 0 | 0 | 1 | 0 | 0 | 0 |  |  |  | 1 | 1 | 1 | 0 | 0 | 0 | 0 | 0 | 0 | 0 | 1 | 1 | 0 | 1 | 0 |
| P000002 | G0000006 | S0000020 | E000100 | MELD | 133 | 4 | F | 66 | 65 | 1.33 | 1.32847335596843 | 1.1 | 1.05 |  |  | 0 | 10 | 10 | 0 | 0 | 1 | 0 | 0 | 0 |  |  |  | 1 | 1 | 1 | 0 | 0 | 0 | 0 | 0 | 0 | 0 | 1 | 1 | 1 | 1 | 0 |
| P000002 | G0000006 | S0000021 | E000100 | MELD-Na | 133 | 4 | F | 66 | 65 | 1.33 | 1.32847335596843 | 1.1 | 1.05 | 140 |  | 0 | 10 | 10 | 0 | 0 | 1 | 0 | 0 | 0 |  |  |  | 1 | 1 | 1 | 0 | 0 | 0 | 0 | 0 | 0 | 0 | 1 | 1 | 0 | 1 | 0 |
| P000002 | G0000006 | S0000022 | E000100 | reMELD-Na | 133 | 4 | F | 66 | 65 | 1.33 | 1.32847335596843 | 1.1 | 1.05 | 140 |  |  | 11 | 11 | 0 | 0 | 1 | 0 | 0 | 0 |  |  |  | 1 | 1 | 1 | 0 | 0 | 0 | 0 | 0 | 0 | 0 | 1 | 1 | 0 | 1 | 0 |
| P000002 | G0000007 | S0000023 | E000126 | MELD | 161 | 5 | F | 66 | 65 | 0.96 | 0.972389040670756 | 0.8 | 0.96 |  |  | 0 | 6 | 6 | 0 | 0 | 1 | 0 | 0 | 0 |  |  |  | 1 | 1 | 1 | 0 | 0 | 0 | 0 | 0 | 0 | 0 | 1 | 1 | 1 | 1 | 0 |
| P000002 | G0000007 | S0000024 | E000126 | MELD-Na | 161 | 5 | F | 66 | 65 | 0.96 | 0.972389040670756 | 0.8 | 0.96 | 143 |  | 0 | 6 | 6 | 0 | 0 | 1 | 0 | 0 | 0 |  |  |  | 1 | 1 | 1 | 0 | 0 | 0 | 0 | 0 | 0 | 0 | 1 | 1 | 0 | 1 | 0 |
| P000002 | G0000007 | S0000025 | E000126 | reMELD-Na | 161 | 5 | F | 66 | 65 | 0.96 | 0.972389040670756 | 0.8 | 0.96 | 143 |  |  | 7 | 7 | 0 | 0 | 1 | 0 | 0 | 0 |  |  |  | 1 | 1 | 1 | 0 | 0 | 0 | 0 | 0 | 0 | 0 | 1 | 1 | 0 | 1 | 0 |
| P000002 | G0000008 | S0000026 | E000126 | MELD | 175 | 5 | F | 66 | 65 | 1.07 | 1.08245672731506 | 0.6 | 0.96 |  |  | 0 | 7 | 7 | 0 | 0 | 1 | 0 | 0 | 0 |  |  |  | 1 | 1 | 1 | 0 | 0 | 0 | 0 | 0 | 0 | 0 | 1 | 1 | 1 | 1 | 0 |
| P000002 | G0000008 | S0000027 | E000126 | MELD-Na | 175 | 5 | F | 66 | 65 | 1.07 | 1.08245672731506 | 0.6 | 0.96 | 140 |  | 0 | 7 | 7 | 0 | 0 | 1 | 0 | 0 | 0 |  |  |  | 1 | 1 | 1 | 0 | 0 | 0 | 0 | 0 | 0 | 0 | 1 | 1 | 0 | 1 | 0 |
| P000002 | G0000008 | S0000028 | E000126 | reMELD-Na | 175 | 5 | F | 66 | 65 | 1.07 | 1.08245672731506 | 0.6 | 0.96 | 140 |  |  | 7 | 7 | 0 | 0 | 1 | 0 | 0 | 0 |  |  |  | 1 | 1 | 1 | 0 | 0 | 0 | 0 | 0 | 0 | 0 | 1 | 1 | 0 | 1 | 0 |
| P000002 | G0000009 | S0000029 | E000126 | MELD | 189 | 6 | F | 66 | 65 | 1.12 | 1.13002143873471 | 0.7 | 0.91 |  |  | 0 | 8 | 8 | 0 | 0 | 1 | 0 | 0 | 0 |  |  |  | 1 | 1 | 1 | 0 | 0 | 0 | 0 | 0 | 0 | 0 | 1 | 1 | 1 | 1 | 0 |
| P000002 | G0000009 | S0000030 | E000126 | MELD-Na | 189 | 6 | F | 66 | 65 | 1.12 | 1.13002143873471 | 0.7 | 0.91 | 143 |  | 0 | 8 | 8 | 0 | 0 | 1 | 0 | 0 | 0 |  |  |  | 1 | 1 | 1 | 0 | 0 | 0 | 0 | 0 | 0 | 0 | 1 | 1 | 0 | 1 | 0 |
| P000002 | G0000009 | S0000031 | E000126 | reMELD-Na | 189 | 6 | F | 66 | 65 | 1.12 | 1.13002143873471 | 0.7 | 0.91 | 143 |  |  | 7 | 7 | 0 | 0 | 1 | 0 | 0 | 0 |  |  |  | 1 | 1 | 1 | 0 | 0 | 0 | 0 | 0 | 0 | 0 | 1 | 1 | 0 | 1 | 0 |
| P000002 | G0000010 | S0000032 | E000126 | MELD | 203 | 6 | F | 66 | 65 | 1.18 | 1.18714417764045 | 0.8 | 0.98 |  |  | 0 | 8 | 8 | 0 | 0 | 1 | 0 | 0 | 0 |  |  |  | 1 | 1 | 1 | 0 | 0 | 0 | 0 | 0 | 0 | 0 | 1 | 1 | 1 | 1 | 0 |
| P000002 | G0000010 | S0000033 | E000126 | MELD-Na | 203 | 6 | F | 66 | 65 | 1.18 | 1.18714417764045 | 0.8 | 0.98 | 141 |  | 0 | 8 | 8 | 0 | 0 | 1 | 0 | 0 | 0 |  |  |  | 1 | 1 | 1 | 0 | 0 | 0 | 0 | 0 | 0 | 0 | 1 | 1 | 0 | 1 | 0 |
| P000002 | G0000010 | S0000034 | E000126 | reMELD-Na | 203 | 6 | F | 66 | 65 | 1.18 | 1.18714417764045 | 0.8 | 0.98 | 141 |  |  | 9 | 9 | 0 | 0 | 1 | 0 | 0 | 0 |  |  |  | 1 | 1 | 1 | 0 | 0 | 0 | 0 | 0 | 0 | 0 | 1 | 1 | 0 | 1 | 0 |
| P000002 | G0000011 | S0000035 | E000126 | MELD | 206 | 6 | F | 66 | 65 | 1.08 | 1.09221538626823 | 0.6 | 0.96 |  |  | 0 | 7 | 7 | 0 | 0 | 1 | 0 | 0 | 0 |  |  |  | 1 | 1 | 1 | 0 | 0 | 0 | 0 | 0 | 0 | 0 | 1 | 1 | 1 | 1 | 0 |
| P000002 | G0000011 | S0000036 | E000126 | MELD-Na | 206 | 6 | F | 66 | 65 | 1.08 | 1.09221538626823 | 0.6 | 0.96 | 142 |  | 0 | 7 | 7 | 0 | 0 | 1 | 0 | 0 | 0 |  |  |  | 1 | 1 | 1 | 0 | 0 | 0 | 0 | 0 | 0 | 0 | 1 | 1 | 0 | 1 | 0 |
| P000002 | G0000011 | S0000037 | E000126 | reMELD-Na | 206 | 6 | F | 66 | 65 | 1.08 | 1.09221538626823 | 0.6 | 0.96 | 142 |  |  | 7 | 7 | 0 | 0 | 1 | 0 | 0 | 0 |  |  |  | 1 | 1 | 1 | 0 | 0 | 0 | 0 | 0 | 0 | 0 | 1 | 1 | 0 | 1 | 0 |
| P000002 | G0000012 | S0000038 | E000126 | MELD | 210 | 6 | F | 66 | 65 | 1.62 | 1.61163143070933 | 0.5 | 0.91 |  |  | 0 | 11 | 11 | 0 | 0 | 1 | 0 | 0 | 0 |  |  |  | 1 | 1 | 1 | 0 | 0 | 0 | 0 | 0 | 0 | 0 | 1 | 1 | 1 | 1 | 0 |
| P000002 | G0000012 | S0000039 | E000126 | MELD-Na | 210 | 6 | F | 66 | 65 | 1.62 | 1.61163143070933 | 0.5 | 0.91 | 140 |  | 0 | 11 | 11 | 0 | 0 | 1 | 0 | 0 | 0 |  |  |  | 1 | 1 | 1 | 0 | 0 | 0 | 0 | 0 | 0 | 0 | 1 | 1 | 0 | 1 | 0 |
| P000002 | G0000012 | S0000040 | E000126 | reMELD-Na | 210 | 6 | F | 66 | 65 | 1.62 | 1.61163143070933 | 0.5 | 0.91 | 140 |  |  | 9 | 9 | 0 | 0 | 1 | 0 | 0 | 0 |  |  |  | 1 | 1 | 1 | 0 | 0 | 0 | 0 | 0 | 0 | 0 | 1 | 1 | 0 | 1 | 0 |
| P000002 | G0000013 | S0000041 | E000151 | MELD | 212 | 6 | F | 66 | 65 | 1.18 | 1.19183353900015 | 0.4 | 1 |  |  | 0 | 8 | 8 | 0 | 0 | 1 | 0 | 0 | 0 |  |  |  | 1 | 1 | 1 | 0 | 0 | 0 | 0 | 0 | 0 | 0 | 1 | 1 | 1 | 1 | 0 |
| P000002 | G0000013 | S0000042 | E000151 | MELD-Na | 212 | 6 | F | 66 | 65 | 1.18 | 1.19183353900015 | 0.4 | 1 | 143 |  | 0 | 8 | 8 | 0 | 0 | 1 | 0 | 0 | 0 |  |  |  | 1 | 1 | 1 | 0 | 0 | 0 | 0 | 0 | 0 | 0 | 1 | 1 | 0 | 1 | 0 |
| P000002 | G0000013 | S0000043 | E000151 | reMELD-Na | 212 | 6 | F | 66 | 65 | 1.18 | 1.19183353900015 | 0.4 | 1 | 143 |  |  | 7 | 7 | 0 | 0 | 1 | 0 | 0 | 0 |  |  |  | 1 | 1 | 1 | 0 | 0 | 0 | 0 | 0 | 0 | 0 | 1 | 1 | 0 | 1 | 0 |
| P000002 | G0000014 | S0000044 | E000157 | MELD | 224 | 7 | F | 66 | 65 | 1.19 | 1.2003476557231 | 0.5 | 0.92 |  |  | 0 | 8 | 8 | 0 | 0 | 1 | 0 | 0 | 0 |  |  |  | 1 | 1 | 1 | 0 | 0 | 0 | 0 | 0 | 0 | 0 | 1 | 1 | 1 | 1 | 0 |
| P000002 | G0000014 | S0000045 | E000157 | MELD-Na | 224 | 7 | F | 66 | 65 | 1.19 | 1.2003476557231 | 0.5 | 0.92 | 145 |  | 0 | 8 | 8 | 0 | 0 | 1 | 0 | 0 | 0 |  |  |  | 1 | 1 | 1 | 0 | 0 | 0 | 0 | 0 | 0 | 0 | 1 | 1 | 0 | 1 | 0 |
| P000002 | G0000014 | S0000046 | E000157 | reMELD-Na | 224 | 7 | F | 66 | 65 | 1.19 | 1.2003476557231 | 0.5 | 0.92 | 145 |  |  | 7 | 7 | 0 | 0 | 1 | 0 | 0 | 0 |  |  |  | 1 | 1 | 1 | 0 | 0 | 0 | 0 | 0 | 0 | 0 | 1 | 1 | 0 | 1 | 0 |
| P000002 | G0000015 | S0000047 | E000157 | MELD | 252 | 8 | F | 66 | 65 | 1.18 | 1.18947877579991 | 0.6 | 1.07 |  |  | 0 | 9 | 9 | 0 | 0 | 1 | 0 | 0 | 0 |  |  |  | 1 | 1 | 1 | 0 | 0 | 0 | 0 | 0 | 0 | 0 | 1 | 1 | 1 | 1 | 0 |
| P000002 | G0000015 | S0000048 | E000157 | MELD-Na | 252 | 8 | F | 66 | 65 | 1.18 | 1.18947877579991 | 0.6 | 1.07 | 135 |  | 0 | 11 | 11 | 0 | 0 | 1 | 0 | 0 | 0 |  |  |  | 1 | 1 | 1 | 0 | 0 | 0 | 0 | 0 | 0 | 0 | 1 | 1 | 0 | 1 | 0 |
| P000002 | G0000015 | S0000049 | E000157 | reMELD-Na | 252 | 8 | F | 66 | 65 | 1.18 | 1.18947877579991 | 0.6 | 1.07 | 135 |  |  | 10 | 10 | 0 | 0 | 1 | 0 | 0 | 0 |  |  |  | 1 | 1 | 1 | 0 | 0 | 0 | 0 | 0 | 0 | 0 | 1 | 1 | 0 | 1 | 0 |
| P000002 | G0000016 | S0000050 | E000157 | MELD | 259 | 8 | F | 66 | 65 | 1.06 | 1.0750469551985 | 0.4 | 1 |  |  | 0 | 7 | 7 | 0 | 0 | 1 | 0 | 0 | 0 |  |  |  | 1 | 1 | 1 | 0 | 0 | 0 | 0 | 0 | 0 | 0 | 1 | 1 | 1 | 1 | 0 |
| P000002 | G0000016 | S0000051 | E000157 | MELD-Na | 259 | 8 | F | 66 | 65 | 1.06 | 1.0750469551985 | 0.4 | 1 | 142 |  | 0 | 7 | 7 | 0 | 0 | 1 | 0 | 0 | 0 |  |  |  | 1 | 1 | 1 | 0 | 0 | 0 | 0 | 0 | 0 | 0 | 1 | 1 | 0 | 1 | 0 |
| P000002 | G0000016 | S0000052 | E000157 | reMELD-Na | 259 | 8 | F | 66 | 65 | 1.06 | 1.0750469551985 | 0.4 | 1 | 142 |  |  | 6 | 6 | 0 | 0 | 1 | 0 | 0 | 0 |  |  |  | 1 | 1 | 1 | 0 | 0 | 0 | 0 | 0 | 0 | 0 | 1 | 1 | 0 | 1 | 0 |
| P000002 | G0000017 | S0000053 | E000157 | MELD | 286 | 9 | F | 66 | 65 | 1.23 | 1.24024487012963 | 0.4 | 0.9 |  |  | 0 | 8 | 8 | 0 | 0 | 1 | 0 | 0 | 0 |  |  |  | 1 | 1 | 1 | 0 | 0 | 0 | 0 | 0 | 0 | 0 | 1 | 1 | 1 | 1 | 0 |
| P000002 | G0000017 | S0000054 | E000157 | MELD 3.0 | 286 | 9 | F | 66 | 65 | 1.23 | 1.24024487012963 | 0.4 | 0.9 | 142 | 3.26 |  | 10 | 10 | 0 | 0 | 1 | 0 | 0 | 0 |  |  |  | 1 | 1 | 1 | 0 | 0 | 0 | 0 | 0 | 0 | 0 | 1 | 1 | 0 | 1 | 0 |
| P000002 | G0000017 | S0000055 | E000157 | MELD-Na | 286 | 9 | F | 66 | 65 | 1.23 | 1.24024487012963 | 0.4 | 0.9 | 142 |  | 0 | 8 | 8 | 0 | 0 | 1 | 0 | 0 | 0 |  |  |  | 1 | 1 | 1 | 0 | 0 | 0 | 0 | 0 | 0 | 0 | 1 | 1 | 0 | 1 | 0 |
| P000002 | G0000017 | S0000056 | E000157 | reMELD-Na | 286 | 9 | F | 66 | 65 | 1.23 | 1.24024487012963 | 0.4 | 0.9 | 142 |  |  | 6 | 6 | 0 | 0 | 1 | 0 | 0 | 0 |  |  |  | 1 | 1 | 1 | 0 | 0 | 0 | 0 | 0 | 0 | 0 | 1 | 1 | 0 | 1 | 0 |
| P000002 | G0000018 | S0000057 | E000219 | MELD | 314 | 10 | F | 66 | 65 | 1.57 | 1.56436599048894 | 0.5 | 1.01 |  |  | 0 | 11 | 11 | 0 | 0 | 1 | 0 | 0 | 0 |  |  |  | 1 | 1 | 1 | 0 | 0 | 0 | 0 | 0 | 0 | 0 | 1 | 1 | 1 | 1 | 0 |
| P000002 | G0000018 | S0000058 | E000219 | MELD 3.0 | 314 | 10 | F | 66 | 65 | 1.57 | 1.56436599048894 | 0.5 | 1.01 | 137 | 3.19 |  | 13 | 13 | 0 | 0 | 1 | 0 | 0 | 0 |  |  |  | 1 | 1 | 1 | 0 | 0 | 0 | 0 | 0 | 0 | 0 | 1 | 1 | 0 | 1 | 0 |
| P000002 | G0000018 | S0000059 | E000219 | MELD-Na | 314 | 10 | F | 66 | 65 | 1.57 | 1.56436599048894 | 0.5 | 1.01 | 137 |  | 0 | 11 | 11 | 0 | 0 | 1 | 0 | 0 | 0 |  |  |  | 1 | 1 | 1 | 0 | 0 | 0 | 0 | 0 | 0 | 0 | 1 | 1 | 0 | 1 | 0 |
| P000002 | G0000018 | S0000060 | E000219 | reMELD-Na | 314 | 10 | F | 66 | 65 | 1.57 | 1.56436599048894 | 0.5 | 1.01 | 137 |  |  | 10 | 10 | 0 | 0 | 1 | 0 | 0 | 0 |  |  |  | 1 | 1 | 1 | 0 | 0 | 0 | 0 | 0 | 0 | 0 | 1 | 1 | 0 | 1 | 0 |
| P000002 | G0000019 | S0000061 | E000219 | MELD | 329 | 10 | F | 66 | 65 | 1.32 | 1.32349047234353 | 0.7 | 0.95 |  |  | 0 | 9 | 9 | 0 | 0 | 1 | 0 | 0 | 0 |  |  |  | 1 | 1 | 1 | 0 | 0 | 0 | 0 | 0 | 0 | 0 | 1 | 1 | 1 | 1 | 0 |
| P000002 | G0000019 | S0000062 | E000219 | MELD 3.0 | 329 | 10 | F | 66 | 65 | 1.32 | 1.32349047234353 | 0.7 | 0.95 | 137 | 3.36 |  | 11 | 11 | 0 | 0 | 1 | 0 | 0 | 0 |  |  |  | 1 | 1 | 1 | 0 | 0 | 0 | 0 | 0 | 0 | 0 | 1 | 1 | 0 | 1 | 0 |
| P000002 | G0000019 | S0000063 | E000219 | MELD-Na | 329 | 10 | F | 66 | 65 | 1.32 | 1.32349047234353 | 0.7 | 0.95 | 137 |  | 0 | 9 | 9 | 0 | 0 | 1 | 0 | 0 | 0 |  |  |  | 1 | 1 | 1 | 0 | 0 | 0 | 0 | 0 | 0 | 0 | 1 | 1 | 0 | 1 | 0 |
| P000002 | G0000019 | S0000064 | E000219 | reMELD-Na | 329 | 10 | F | 66 | 65 | 1.32 | 1.32349047234353 | 0.7 | 0.95 | 137 |  |  | 9 | 9 | 0 | 0 | 1 | 0 | 0 | 0 |  |  |  | 1 | 1 | 1 | 0 | 0 | 0 | 0 | 0 | 0 | 0 | 1 | 1 | 0 | 1 | 0 |
| P000002 | G0000020 | S0000065 | E000219 | MELD | 342 | 11 | F | 66 | 65 | 1.17 | 1.18095374145313 | 0.5 | 0.91 |  |  | 0 | 8 | 8 | 0 | 0 | 1 | 0 | 0 | 0 |  |  |  | 1 | 1 | 1 | 0 | 0 | 0 | 0 | 0 | 0 | 0 | 1 | 1 | 1 | 1 | 0 |
| P000002 | G0000020 | S0000066 | E000219 | MELD 3.0 | 342 | 11 | F | 66 | 65 | 1.17 | 1.18095374145313 | 0.5 | 0.91 | 140 | 3.34 |  | 9 | 9 | 0 | 0 | 1 | 0 | 0 | 0 |  |  |  | 1 | 1 | 1 | 0 | 0 | 0 | 0 | 0 | 0 | 0 | 1 | 1 | 0 | 1 | 0 |
| P000002 | G0000020 | S0000067 | E000219 | MELD-Na | 342 | 11 | F | 66 | 65 | 1.17 | 1.18095374145313 | 0.5 | 0.91 | 140 |  | 0 | 8 | 8 | 0 | 0 | 1 | 0 | 0 | 0 |  |  |  | 1 | 1 | 1 | 0 | 0 | 0 | 0 | 0 | 0 | 0 | 1 | 1 | 0 | 1 | 0 |
| P000002 | G0000020 | S0000068 | E000219 | reMELD-Na | 342 | 11 | F | 66 | 65 | 1.17 | 1.18095374145313 | 0.5 | 0.91 | 140 |  |  | 7 | 7 | 0 | 0 | 1 | 0 | 0 | 0 |  |  |  | 1 | 1 | 1 | 0 | 0 | 0 | 0 | 0 | 0 | 0 | 1 | 1 | 0 | 1 | 0 |
| P000002 | G0000021 | S0000069 | E000219 | MELD | 384 | 12 | F | 66 | 65 | 1.42 | 1.41934353460248 | 0.7 | 0.93 |  |  | 0 | 10 | 10 | 0 | 0 | 1 | 0 | 0 | 0 |  |  |  | 1 | 1 | 1 | 0 | 0 | 0 | 0 | 0 | 0 | 0 | 1 | 1 | 1 | 1 | 0 |
| P000002 | G0000021 | S0000070 | E000219 | MELD-Na | 384 | 12 | F | 66 | 65 | 1.42 | 1.41934353460248 | 0.7 | 0.93 | 146 |  | 0 | 10 | 10 | 0 | 0 | 1 | 0 | 0 | 0 |  |  |  | 1 | 1 | 1 | 0 | 0 | 0 | 0 | 0 | 0 | 0 | 1 | 1 | 0 | 1 | 0 |
| P000002 | G0000021 | S0000071 | E000219 | reMELD-Na | 384 | 12 | F | 66 | 65 | 1.42 | 1.41934353460248 | 0.7 | 0.93 | 146 |  |  | 9 | 9 | 0 | 0 | 1 | 0 | 0 | 0 |  |  |  | 1 | 1 | 1 | 0 | 0 | 0 | 0 | 0 | 0 | 0 | 1 | 1 | 0 | 1 | 0 |
| P000002 | G0000022 | S0000072 | E000289 | MELD | 420 | 13 | F | 67 | 65 | 1.23 | 1.23789010692939 | 0.6 | 0.92 |  |  | 0 | 8 | 8 | 0 | 0 | 1 | 0 | 0 | 0 |  |  |  | 1 | 1 | 1 | 0 | 0 | 0 | 0 | 0 | 0 | 0 | 1 | 1 | 1 | 1 | 0 |
| P000002 | G0000022 | S0000073 | E000289 | MELD-Na | 420 | 13 | F | 67 | 65 | 1.23 | 1.23789010692939 | 0.6 | 0.92 | 144 |  | 0 | 8 | 8 | 0 | 0 | 1 | 0 | 0 | 0 |  |  |  | 1 | 1 | 1 | 0 | 0 | 0 | 0 | 0 | 0 | 0 | 1 | 1 | 0 | 1 | 0 |
| P000002 | G0000022 | S0000074 | E000289 | reMELD-Na | 420 | 13 | F | 67 | 65 | 1.23 | 1.23789010692939 | 0.6 | 0.92 | 144 |  |  | 8 | 8 | 0 | 0 | 1 | 0 | 0 | 0 |  |  |  | 1 | 1 | 1 | 0 | 0 | 0 | 0 | 0 | 0 | 0 | 1 | 1 | 0 | 1 | 0 |
| P000002 | G0000023 | S0000075 | E000289 | MELD | 455 | 14 | F | 67 | 65 | 1.16 | 1.1724278720029 | 0.4 | 0.89 |  |  | 0 | 8 | 8 | 0 | 0 | 1 | 0 | 0 | 0 |  |  |  | 1 | 1 | 1 | 0 | 0 | 0 | 0 | 0 | 0 | 0 | 1 | 1 | 1 | 1 | 0 |
| P000002 | G0000023 | S0000076 | E000289 | MELD-Na | 455 | 14 | F | 67 | 65 | 1.16 | 1.1724278720029 | 0.4 | 0.89 | 136 |  | 0 | 9 | 9 | 0 | 0 | 1 | 0 | 0 | 0 |  |  |  | 1 | 1 | 1 | 0 | 0 | 0 | 0 | 0 | 0 | 0 | 1 | 1 | 0 | 1 | 0 |
| P000002 | G0000023 | S0000077 | E000289 | reMELD-Na | 455 | 14 | F | 67 | 65 | 1.16 | 1.1724278720029 | 0.4 | 0.89 | 136 |  |  | 6 | 6 | 0 | 0 | 1 | 0 | 0 | 0 |  |  |  | 1 | 1 | 1 | 0 | 0 | 0 | 0 | 0 | 0 | 0 | 1 | 1 | 0 | 1 | 0 |
| P000002 | G0000024 | S0000078 | E000344 | MELD | 506 | 16 | F | 67 | 65 | 1.22 | 1.22821959343076 | 0.6 | 1.1 |  |  | 0 | 9 | 9 | 0 | 0 | 1 | 0 | 0 | 0 |  |  |  | 1 | 1 | 1 | 0 | 0 | 0 | 0 | 0 | 0 | 0 | 1 | 1 | 1 | 1 | 0 |
| P000002 | G0000024 | S0000079 | E000344 | MELD-Na | 506 | 16 | F | 67 | 65 | 1.22 | 1.22821959343076 | 0.6 | 1.1 | 145 |  | 0 | 9 | 9 | 0 | 0 | 1 | 0 | 0 | 0 |  |  |  | 1 | 1 | 1 | 0 | 0 | 0 | 0 | 0 | 0 | 0 | 1 | 1 | 0 | 1 | 0 |
| P000002 | G0000024 | S0000080 | E000344 | reMELD-Na | 506 | 16 | F | 67 | 65 | 1.22 | 1.22821959343076 | 0.6 | 1.1 | 145 |  |  | 9 | 9 | 0 | 0 | 1 | 0 | 0 | 0 |  |  |  | 1 | 1 | 1 | 0 | 0 | 0 | 0 | 0 | 0 | 0 | 1 | 1 | 0 | 1 | 0 |
| P000002 | G0000025 | S0000081 | E000344 | MELD | 538 | 17 | F | 67 | 65 | 1.29 | 1.29696464525478 | 0.5 | 0.91 |  |  | 0 | 9 | 9 | 0 | 0 | 1 | 0 | 0 | 0 |  |  |  | 1 | 1 | 1 | 0 | 0 | 0 | 0 | 0 | 0 | 0 | 1 | 1 | 1 | 1 | 0 |
| P000002 | G0000025 | S0000082 | E000344 | MELD-Na | 538 | 17 | F | 67 | 65 | 1.29 | 1.29696464525478 | 0.5 | 0.91 | 142 |  | 0 | 9 | 9 | 0 | 0 | 1 | 0 | 0 | 0 |  |  |  | 1 | 1 | 1 | 0 | 0 | 0 | 0 | 0 | 0 | 0 | 1 | 1 | 0 | 1 | 0 |
| P000002 | G0000025 | S0000083 | E000344 | reMELD-Na | 538 | 17 | F | 67 | 65 | 1.29 | 1.29696464525478 | 0.5 | 0.91 | 142 |  |  | 7 | 7 | 0 | 0 | 1 | 0 | 0 | 0 |  |  |  | 1 | 1 | 1 | 0 | 0 | 0 | 0 | 0 | 0 | 0 | 1 | 1 | 0 | 1 | 0 |
| P000002 | G0000026 | S0000084 | E000344 | MELD | 567 | 18 | F | 67 | 65 | 1.24 | 1.24990950726461 | 0.4 | 0.98 |  |  | 0 | 8 | 9 | 1 | 0 | 0 | 1 | 0 | 0 |  |  |  | 1 | 1 | 1 | 0 | 0 | 0 | 0 | 0 | 0 | 0 | 1 | 1 | 1 | 1 | 0 |
| P000002 | G0000026 | S0000085 | E000344 | MELD-Na | 567 | 18 | F | 67 | 65 | 1.24 | 1.24990950726461 | 0.4 | 0.98 | 141 |  | 0 | 8 | 9 | 1 | 0 | 0 | 1 | 0 | 0 |  |  |  | 1 | 1 | 1 | 0 | 0 | 0 | 0 | 0 | 0 | 0 | 1 | 1 | 0 | 1 | 0 |
| P000002 | G0000026 | S0000086 | E000344 | reMELD-Na | 567 | 18 | F | 67 | 65 | 1.24 | 1.24990950726461 | 0.4 | 0.98 | 141 |  |  | 7 | 7 | 0 | 0 | 1 | 0 | 0 | 0 |  |  |  | 1 | 1 | 1 | 0 | 0 | 0 | 0 | 0 | 0 | 0 | 1 | 1 | 0 | 1 | 0 |
| P000002 | G0000027 | S0000087 | E000399 | MELD | 595 | 19 | F | 67 | 65 | 1.26 | 1.27040609593409 | 0.3 | 0.97 |  |  | 0 | 9 | 9 | 0 | 0 | 1 | 0 | 0 | 0 |  |  |  | 1 | 1 | 1 | 0 | 0 | 0 | 0 | 0 | 0 | 0 | 1 | 1 | 1 | 1 | 0 |
| P000002 | G0000027 | S0000088 | E000399 | MELD-Na | 595 | 19 | F | 67 | 65 | 1.26 | 1.27040609593409 | 0.3 | 0.97 | 144 |  | 0 | 9 | 9 | 0 | 0 | 1 | 0 | 0 | 0 |  |  |  | 1 | 1 | 1 | 0 | 0 | 0 | 0 | 0 | 0 | 0 | 1 | 1 | 0 | 1 | 0 |
| P000002 | G0000027 | S0000089 | E000399 | reMELD-Na | 595 | 19 | F | 67 | 65 | 1.26 | 1.27040609593409 | 0.3 | 0.97 | 144 |  |  | 6 | 6 | 0 | 0 | 1 | 0 | 0 | 0 |  |  |  | 1 | 1 | 1 | 0 | 0 | 0 | 0 | 0 | 0 | 0 | 1 | 1 | 0 | 1 | 0 |
| P000002 | G0000028 | S0000090 | E000449 | MELD | 681 | 22 | F | 67 | 65 | 1.18 | 1.19183353900015 | 0.4 | 0.91 |  |  | 0 | 8 | 8 | 0 | 0 | 1 | 0 | 0 | 0 |  |  |  | 1 | 1 | 1 | 0 | 0 | 0 | 0 | 0 | 0 | 0 | 1 | 1 | 1 | 1 | 0 |
| P000002 | G0000028 | S0000091 | E000449 | MELD-Na | 681 | 22 | F | 67 | 65 | 1.18 | 1.19183353900015 | 0.4 | 0.91 | 142 |  | 0 | 8 | 8 | 0 | 0 | 1 | 0 | 0 | 0 |  |  |  | 1 | 1 | 1 | 0 | 0 | 0 | 0 | 0 | 0 | 0 | 1 | 1 | 0 | 1 | 0 |
| P000002 | G0000028 | S0000092 | E000449 | reMELD-Na | 681 | 22 | F | 67 | 65 | 1.18 | 1.19183353900015 | 0.4 | 0.91 | 142 |  |  | 6 | 6 | 0 | 0 | 1 | 0 | 0 | 0 |  |  |  | 1 | 1 | 1 | 0 | 0 | 0 | 0 | 0 | 0 | 0 | 1 | 1 | 0 | 1 | 0 |
| P000002 | G0000029 | S0000093 | E000449 | MELD | 709 | 23 | F | 67 | 65 | 1.06 | 1.07623189868891 | 0.3 | 0.97 |  |  | 0 | 7 | 7 | 0 | 0 | 1 | 0 | 0 | 0 |  |  |  | 1 | 1 | 1 | 0 | 0 | 0 | 0 | 0 | 0 | 0 | 1 | 1 | 1 | 1 | 0 |
| P000002 | G0000029 | S0000094 | E000449 | MELD-Na | 709 | 23 | F | 67 | 65 | 1.06 | 1.07623189868891 | 0.3 | 0.97 | 143 |  | 0 | 7 | 7 | 0 | 0 | 1 | 0 | 0 | 0 |  |  |  | 1 | 1 | 1 | 0 | 0 | 0 | 0 | 0 | 0 | 0 | 1 | 1 | 0 | 1 | 0 |
| P000002 | G0000029 | S0000095 | E000449 | reMELD-Na | 709 | 23 | F | 67 | 65 | 1.06 | 1.07623189868891 | 0.3 | 0.97 | 143 |  |  | 5 | 5 | 0 | 0 | 1 | 0 | 0 | 0 |  |  |  | 1 | 1 | 1 | 0 | 0 | 0 | 0 | 0 | 0 | 0 | 1 | 1 | 0 | 1 | 0 |
| P000002 | G0000030 | S0000096 | E000500 | MELD | 821 | 26 | F | 68 | 65 | 1.29 | 1.298144547485 | 0.4 | 0.92 |  |  | 0 | 9 | 9 | 0 | 0 | 1 | 0 | 0 | 0 |  |  |  | 1 | 1 | 1 | 0 | 0 | 0 | 0 | 0 | 0 | 0 | 1 | 1 | 1 | 1 | 0 |
| P000002 | G0000030 | S0000097 | E000500 | MELD-Na | 821 | 26 | F | 68 | 65 | 1.29 | 1.298144547485 | 0.4 | 0.92 | 142 |  | 0 | 9 | 9 | 0 | 0 | 1 | 0 | 0 | 0 |  |  |  | 1 | 1 | 1 | 0 | 0 | 0 | 0 | 0 | 0 | 0 | 1 | 1 | 0 | 1 | 0 |
| P000002 | G0000030 | S0000098 | E000500 | reMELD-Na | 821 | 26 | F | 68 | 65 | 1.29 | 1.298144547485 | 0.4 | 0.92 | 142 |  |  | 7 | 7 | 0 | 0 | 1 | 0 | 0 | 0 |  |  |  | 1 | 1 | 1 | 0 | 0 | 0 | 0 | 0 | 0 | 0 | 1 | 1 | 0 | 1 | 0 |
| P000002 | G0000031 | S0000099 | E000564 | MELD | 877 | 28 | F | 68 | 65 | 1.25 | 1.26075321152637 | 0.3 | 0.98 |  |  | 0 | 9 | 9 | 0 | 0 | 1 | 0 | 0 | 0 |  |  |  | 1 | 1 | 1 | 0 | 0 | 0 | 0 | 0 | 0 | 0 | 1 | 1 | 1 | 1 | 0 |
| P000002 | G0000031 | S0000100 | E000564 | MELD-Na | 877 | 28 | F | 68 | 65 | 1.25 | 1.26075321152637 | 0.3 | 0.98 | 140 |  | 0 | 9 | 9 | 0 | 0 | 1 | 0 | 0 | 0 |  |  |  | 1 | 1 | 1 | 0 | 0 | 0 | 0 | 0 | 0 | 0 | 1 | 1 | 0 | 1 | 0 |
| P000002 | G0000031 | S0000101 | E000564 | reMELD-Na | 877 | 28 | F | 68 | 65 | 1.25 | 1.26075321152637 | 0.3 | 0.98 | 140 |  |  | 6 | 6 | 0 | 0 | 1 | 0 | 0 | 0 |  |  |  | 1 | 1 | 1 | 0 | 0 | 0 | 0 | 0 | 0 | 0 | 1 | 1 | 0 | 1 | 0 |
| P000002 | G0000032 | S0000102 | E000564 | MELD | 933 | 30 | F | 68 | 65 | 1.27 | 1.27768825825753 | 0.5 | 0.98 |  |  | 0 | 9 | 9 | 0 | 0 | 1 | 0 | 0 | 0 |  |  |  | 1 | 1 | 1 | 0 | 0 | 0 | 0 | 0 | 0 | 0 | 1 | 1 | 1 | 1 | 0 |
| P000002 | G0000032 | S0000103 | E000564 | MELD-Na | 933 | 30 | F | 68 | 65 | 1.27 | 1.27768825825753 | 0.5 | 0.98 | 142 |  | 0 | 9 | 9 | 0 | 0 | 1 | 0 | 0 | 0 |  |  |  | 1 | 1 | 1 | 0 | 0 | 0 | 0 | 0 | 0 | 0 | 1 | 1 | 0 | 1 | 0 |
| P000002 | G0000032 | S0000104 | E000564 | reMELD-Na | 933 | 30 | F | 68 | 65 | 1.27 | 1.27768825825753 | 0.5 | 0.98 | 142 |  |  | 8 | 8 | 0 | 0 | 1 | 0 | 0 | 0 |  |  |  | 1 | 1 | 1 | 0 | 0 | 0 | 0 | 0 | 0 | 0 | 1 | 1 | 0 | 1 | 0 |
| P000002 | G0000033 | S0000105 | E000620 | MELD | 989 | 32 | F | 68 | 65 | 1.48 | 1.48128283290378 | 0.3 | 0.89 |  |  | 0 | 10 | 10 | 0 | 0 | 1 | 0 | 0 | 0 |  |  |  | 1 | 1 | 1 | 0 | 0 | 0 | 0 | 0 | 0 | 0 | 1 | 1 | 1 | 1 | 0 |
| P000002 | G0000033 | S0000106 | E000620 | MELD 3.0 | 989 | 32 | F | 68 | 65 | 1.48 | 1.48128283290378 | 0.3 | 0.89 | 141 | 3.67 |  | 12 | 12 | 0 | 0 | 1 | 0 | 0 | 0 |  |  |  | 1 | 1 | 1 | 0 | 0 | 0 | 0 | 0 | 0 | 0 | 1 | 1 | 0 | 1 | 0 |
| P000002 | G0000033 | S0000107 | E000620 | MELD-Na | 989 | 32 | F | 68 | 65 | 1.48 | 1.48128283290378 | 0.3 | 0.89 | 141 |  | 0 | 10 | 10 | 0 | 0 | 1 | 0 | 0 | 0 |  |  |  | 1 | 1 | 1 | 0 | 0 | 0 | 0 | 0 | 0 | 0 | 1 | 1 | 0 | 1 | 0 |
| P000002 | G0000033 | S0000108 | E000620 | reMELD-Na | 989 | 32 | F | 68 | 65 | 1.48 | 1.48128283290378 | 0.3 | 0.89 | 141 |  |  | 7 | 7 | 0 | 0 | 1 | 0 | 0 | 0 |  |  |  | 1 | 1 | 1 | 0 | 0 | 0 | 0 | 0 | 0 | 0 | 1 | 1 | 0 | 1 | 0 |
| P000002 | G0000034 | S0000109 | E000645 | MELD | 1045 | 34 | F | 68 | 65 | 1.23 | 1.24142981362004 | 0.3 | 0.92 |  |  | 0 | 8 | 8 | 0 | 0 | 1 | 0 | 0 | 0 |  |  |  | 1 | 1 | 1 | 0 | 0 | 0 | 0 | 0 | 0 | 0 | 1 | 1 | 1 | 1 | 0 |
| P000002 | G0000034 | S0000110 | E000645 | MELD 3.0 | 1045 | 34 | F | 68 | 65 | 1.23 | 1.24142981362004 | 0.3 | 0.92 | 144 | 5.19 |  | 10 | 10 | 0 | 0 | 1 | 0 | 0 | 0 |  |  |  | 1 | 1 | 1 | 0 | 0 | 0 | 0 | 0 | 0 | 0 | 1 | 1 | 0 | 1 | 0 |
| P000002 | G0000034 | S0000111 | E000645 | MELD-Na | 1045 | 34 | F | 68 | 65 | 1.23 | 1.24142981362004 | 0.3 | 0.92 | 144 |  | 0 | 8 | 8 | 0 | 0 | 1 | 0 | 0 | 0 |  |  |  | 1 | 1 | 1 | 0 | 0 | 0 | 0 | 0 | 0 | 0 | 1 | 1 | 0 | 1 | 0 |
| P000002 | G0000034 | S0000112 | E000645 | reMELD-Na | 1045 | 34 | F | 68 | 65 | 1.23 | 1.24142981362004 | 0.3 | 0.92 | 144 |  |  | 6 | 6 | 0 | 0 | 1 | 0 | 0 | 0 |  |  |  | 1 | 1 | 1 | 0 | 0 | 0 | 0 | 0 | 0 | 0 | 1 | 1 | 0 | 1 | 0 |
| P000002 | G0000035 | S0000113 | E000645 | MELD | 1094 | 35 | F | 68 | 65 | 0.99 | 1.00653066979905 | 0.4 | 0.93 |  |  | 0 | 6 | 6 | 0 | 0 | 1 | 0 | 0 | 0 |  |  |  | 1 | 1 | 1 | 0 | 0 | 0 | 0 | 0 | 0 | 0 | 1 | 1 | 1 | 1 | 0 |
| P000002 | G0000035 | S0000114 | E000645 | MELD 3.0 | 1094 | 35 | F | 68 | 65 | 0.99 | 1.00653066979905 | 0.4 | 0.93 | 141 | 3.69 |  | 7 | 7 | 0 | 0 | 1 | 0 | 0 | 0 |  |  |  | 1 | 1 | 1 | 0 | 0 | 0 | 0 | 0 | 0 | 0 | 1 | 1 | 0 | 1 | 0 |
| P000002 | G0000035 | S0000115 | E000645 | MELD-Na | 1094 | 35 | F | 68 | 65 | 0.99 | 1.00653066979905 | 0.4 | 0.93 | 141 |  | 0 | 6 | 6 | 0 | 0 | 1 | 0 | 0 | 0 |  |  |  | 1 | 1 | 1 | 0 | 0 | 0 | 0 | 0 | 0 | 0 | 1 | 1 | 0 | 1 | 0 |
| P000002 | G0000035 | S0000116 | E000645 | reMELD-Na | 1094 | 35 | F | 68 | 65 | 0.99 | 1.00653066979905 | 0.4 | 0.93 | 141 |  |  | 5 | 5 | 0 | 0 | 1 | 0 | 0 | 0 |  |  |  | 1 | 1 | 1 | 0 | 0 | 0 | 0 | 0 | 0 | 0 | 1 | 1 | 0 | 1 | 0 |
| P000002 | G0000036 | S0000117 | E000703 | MELD | 1213 | 39 | F | 69 | 65 | 1.1 | 1.11288993605368 | 0.5 | 0.94 |  |  | 0 | 7 | 7 | 0 | 0 | 1 | 0 | 0 | 0 |  |  |  | 1 | 1 | 1 | 0 | 0 | 0 | 0 | 0 | 0 | 0 | 1 | 1 | 1 | 1 | 0 |
| P000002 | G0000036 | S0000118 | E000703 | MELD-Na | 1213 | 39 | F | 69 | 65 | 1.1 | 1.11288993605368 | 0.5 | 0.94 | 141 |  | 0 | 7 | 7 | 0 | 0 | 1 | 0 | 0 | 0 |  |  |  | 1 | 1 | 1 | 0 | 0 | 0 | 0 | 0 | 0 | 0 | 1 | 1 | 0 | 1 | 0 |
| P000002 | G0000036 | S0000119 | E000703 | reMELD-Na | 1213 | 39 | F | 69 | 65 | 1.1 | 1.11288993605368 | 0.5 | 0.94 | 141 |  |  | 6 | 6 | 0 | 0 | 1 | 0 | 0 | 0 |  |  |  | 1 | 1 | 1 | 0 | 0 | 0 | 0 | 0 | 0 | 0 | 1 | 1 | 0 | 1 | 0 |
| P000002 | G0000037 | S0000120 | E000766 | MELD | 1287 | 42 | F | 69 | 65 | 1.3 | 1.30777392643817 | 0.4 | 0.89 |  |  | 0 | 9 | 9 | 0 | 0 | 1 | 0 | 0 | 0 |  |  |  | 1 | 1 | 1 | 0 | 0 | 0 | 0 | 0 | 0 | 0 | 1 | 1 | 1 | 1 | 0 |
| P000002 | G0000037 | S0000121 | E000766 | MELD-Na | 1287 | 42 | F | 69 | 65 | 1.3 | 1.30777392643817 | 0.4 | 0.89 | 141 |  | 0 | 9 | 9 | 0 | 0 | 1 | 0 | 0 | 0 |  |  |  | 1 | 1 | 1 | 0 | 0 | 0 | 0 | 0 | 0 | 0 | 1 | 1 | 0 | 1 | 0 |
| P000002 | G0000037 | S0000122 | E000766 | reMELD-Na | 1287 | 42 | F | 69 | 65 | 1.3 | 1.30777392643817 | 0.4 | 0.89 | 141 |  |  | 7 | 7 | 0 | 0 | 1 | 0 | 0 | 0 |  |  |  | 1 | 1 | 1 | 0 | 0 | 0 | 0 | 0 | 0 | 0 | 1 | 1 | 0 | 1 | 0 |
| P000002 | G0000038 | S0000123 | E000766 | MELD | 1296 | 42 | F | 69 | 65 | 1.39 | 1.39535884414345 | 0.3 | 0.95 |  |  | 0 | 10 | 10 | 0 | 0 | 1 | 0 | 0 | 0 |  |  |  | 1 | 1 | 1 | 0 | 0 | 0 | 0 | 0 | 0 | 0 | 1 | 1 | 1 | 1 | 0 |
| P000002 | G0000038 | S0000124 | E000766 | MELD-Na | 1296 | 42 | F | 69 | 65 | 1.39 | 1.39535884414345 | 0.3 | 0.95 | 140 |  | 0 | 10 | 10 | 0 | 0 | 1 | 0 | 0 | 0 |  |  |  | 1 | 1 | 1 | 0 | 0 | 0 | 0 | 0 | 0 | 0 | 1 | 1 | 0 | 1 | 0 |
| P000002 | G0000038 | S0000125 | E000766 | reMELD-Na | 1296 | 42 | F | 69 | 65 | 1.39 | 1.39535884414345 | 0.3 | 0.95 | 140 |  |  | 7 | 7 | 0 | 0 | 1 | 0 | 0 | 0 |  |  |  | 1 | 1 | 1 | 0 | 0 | 0 | 0 | 0 | 0 | 0 | 1 | 1 | 0 | 1 | 0 |
| P000002 | G0000039 | S0000126 | E000766 | MELD | 1298 | 42 | F | 69 | 65 | 1.02 | 1.03593005029491 | 0.4 | 0.9 |  |  | 0 | 7 | 7 | 0 | 0 | 1 | 0 | 0 | 0 |  |  |  | 1 | 1 | 1 | 0 | 0 | 0 | 0 | 0 | 0 | 0 | 1 | 1 | 1 | 1 | 0 |
| P000002 | G0000039 | S0000127 | E000766 | MELD-Na | 1298 | 42 | F | 69 | 65 | 1.02 | 1.03593005029491 | 0.4 | 0.9 | 142 |  | 0 | 7 | 7 | 0 | 0 | 1 | 0 | 0 | 0 |  |  |  | 1 | 1 | 1 | 0 | 0 | 0 | 0 | 0 | 0 | 0 | 1 | 1 | 0 | 1 | 0 |
| P000002 | G0000039 | S0000128 | E000766 | reMELD-Na | 1298 | 42 | F | 69 | 65 | 1.02 | 1.03593005029491 | 0.4 | 0.9 | 142 |  |  | 5 | 5 | 0 | 0 | 1 | 0 | 0 | 0 |  |  |  | 1 | 1 | 1 | 0 | 0 | 0 | 0 | 0 | 0 | 0 | 1 | 1 | 0 | 1 | 0 |
| P000002 | G0000040 | S0000129 | E000766 | MELD | 1301 | 42 | F | 69 | 65 | 1.13 | 1.14327529877976 | 0.4 | 0.85 |  |  | 0 | 8 | 8 | 0 | 0 | 1 | 0 | 0 | 0 |  |  |  | 1 | 1 | 1 | 0 | 0 | 0 | 0 | 0 | 0 | 0 | 1 | 1 | 1 | 1 | 0 |
| P000002 | G0000040 | S0000130 | E000766 | MELD-Na | 1301 | 42 | F | 69 | 65 | 1.13 | 1.14327529877976 | 0.4 | 0.85 | 144 |  | 0 | 8 | 8 | 0 | 0 | 1 | 0 | 0 | 0 |  |  |  | 1 | 1 | 1 | 0 | 0 | 0 | 0 | 0 | 0 | 0 | 1 | 1 | 0 | 1 | 0 |
| P000002 | G0000040 | S0000131 | E000766 | reMELD-Na | 1301 | 42 | F | 69 | 65 | 1.13 | 1.14327529877976 | 0.4 | 0.85 | 144 |  |  | 5 | 5 | 0 | 0 | 1 | 0 | 0 | 0 |  |  |  | 1 | 1 | 1 | 0 | 0 | 0 | 0 | 0 | 0 | 0 | 1 | 1 | 0 | 1 | 0 |
| P000002 | G0000041 | S0000132 | E000766 | MELD | 1303 | 42 | F | 69 | 65 | 1.13 | 1.14327529877976 | 0.4 | 0.9 |  |  | 0 | 8 | 8 | 0 | 0 | 1 | 0 | 0 | 0 |  |  |  | 1 | 1 | 1 | 0 | 0 | 0 | 0 | 0 | 0 | 0 | 1 | 1 | 1 | 1 | 0 |
| P000002 | G0000041 | S0000133 | E000766 | MELD-Na | 1303 | 42 | F | 69 | 65 | 1.13 | 1.14327529877976 | 0.4 | 0.9 | 142 |  | 0 | 8 | 8 | 0 | 0 | 1 | 0 | 0 | 0 |  |  |  | 1 | 1 | 1 | 0 | 0 | 0 | 0 | 0 | 0 | 0 | 1 | 1 | 0 | 1 | 0 |
| P000002 | G0000041 | S0000134 | E000766 | reMELD-Na | 1303 | 42 | F | 69 | 65 | 1.13 | 1.14327529877976 | 0.4 | 0.9 | 142 |  |  | 5 | 6 | 1 | 0 | 0 | 1 | 0 | 0 |  |  |  | 1 | 1 | 1 | 0 | 0 | 0 | 0 | 0 | 0 | 0 | 1 | 1 | 0 | 1 | 0 |
| P000002 | G0000042 | S0000135 | E000766 | MELD | 1305 | 42 | F | 69 | 65 | 1.17 | 1.18331858717376 | 0.3 | 0.88 |  |  | 0 | 8 | 8 | 0 | 0 | 1 | 0 | 0 | 0 |  |  |  | 1 | 1 | 1 | 0 | 0 | 0 | 0 | 0 | 0 | 0 | 1 | 1 | 1 | 1 | 0 |
| P000002 | G0000042 | S0000136 | E000766 | MELD-Na | 1305 | 42 | F | 69 | 65 | 1.17 | 1.18331858717376 | 0.3 | 0.88 | 139 |  | 0 | 8 | 8 | 0 | 0 | 1 | 0 | 0 | 0 |  |  |  | 1 | 1 | 1 | 0 | 0 | 0 | 0 | 0 | 0 | 0 | 1 | 1 | 0 | 1 | 0 |
| P000002 | G0000042 | S0000137 | E000766 | reMELD-Na | 1305 | 42 | F | 69 | 65 | 1.17 | 1.18331858717376 | 0.3 | 0.88 | 139 |  |  | 5 | 5 | 0 | 0 | 1 | 0 | 0 | 0 |  |  |  | 1 | 1 | 1 | 0 | 0 | 0 | 0 | 0 | 0 | 0 | 1 | 1 | 0 | 1 | 0 |
| P000002 | G0000043 | S0000138 | E000795 | MELD | 1402 | 46 | F | 69 | 65 | 1.24 | 1.25228443550563 | 0.2 | 0.88 |  |  | 0 | 8 | 9 | 1 | 0 | 0 | 1 | 0 | 0 |  |  |  | 1 | 1 | 1 | 0 | 0 | 0 | 0 | 0 | 0 | 0 | 1 | 1 | 1 | 1 | 0 |
| P000002 | G0000043 | S0000139 | E000795 | MELD 3.0 | 1402 | 46 | F | 69 | 65 | 1.24 | 1.25228443550563 | 0.2 | 0.88 | 141 | 3.61 |  | 10 | 10 | 0 | 0 | 1 | 0 | 0 | 0 |  |  |  | 1 | 1 | 1 | 0 | 0 | 0 | 0 | 0 | 0 | 0 | 1 | 1 | 0 | 1 | 0 |
| P000002 | G0000043 | S0000140 | E000795 | MELD-Na | 1402 | 46 | F | 69 | 65 | 1.24 | 1.25228443550563 | 0.2 | 0.88 | 141 |  | 0 | 8 | 9 | 1 | 0 | 0 | 1 | 0 | 0 |  |  |  | 1 | 1 | 1 | 0 | 0 | 0 | 0 | 0 | 0 | 0 | 1 | 1 | 0 | 1 | 0 |
| P000002 | G0000043 | S0000141 | E000795 | reMELD-Na | 1402 | 46 | F | 69 | 65 | 1.24 | 1.25228443550563 | 0.2 | 0.88 | 141 |  |  | 5 | 5 | 0 | 0 | 1 | 0 | 0 | 0 |  |  |  | 1 | 1 | 1 | 0 | 0 | 0 | 0 | 0 | 0 | 0 | 1 | 1 | 0 | 1 | 0 |
| P000002 | G0000044 | S0000142 | E000853 | MELD | 1465 | 48 | F | 69 | 65 | 1.38 | 1.38578235246301 | 0.3 | 0.93 |  |  | 0 | 10 | 10 | 0 | 0 | 1 | 0 | 0 | 0 |  |  |  | 1 | 1 | 1 | 0 | 0 | 0 | 0 | 0 | 0 | 0 | 1 | 1 | 1 | 1 | 0 |
| P000002 | G0000044 | S0000143 | E000853 | MELD 3.0 | 1465 | 48 | F | 69 | 65 | 1.38 | 1.38578235246301 | 0.3 | 0.93 | 141 | 3.7 |  | 11 | 11 | 0 | 0 | 1 | 0 | 0 | 0 |  |  |  | 1 | 1 | 1 | 0 | 0 | 0 | 0 | 0 | 0 | 0 | 1 | 1 | 0 | 1 | 0 |
| P000002 | G0000044 | S0000144 | E000853 | MELD-Na | 1465 | 48 | F | 69 | 65 | 1.38 | 1.38578235246301 | 0.3 | 0.93 | 141 |  | 0 | 10 | 10 | 0 | 0 | 1 | 0 | 0 | 0 |  |  |  | 1 | 1 | 1 | 0 | 0 | 0 | 0 | 0 | 0 | 0 | 1 | 1 | 0 | 1 | 0 |
| P000002 | G0000044 | S0000145 | E000853 | reMELD-Na | 1465 | 48 | F | 69 | 65 | 1.38 | 1.38578235246301 | 0.3 | 0.93 | 141 |  |  | 7 | 7 | 0 | 0 | 1 | 0 | 0 | 0 |  |  |  | 1 | 1 | 1 | 0 | 0 | 0 | 0 | 0 | 0 | 0 | 1 | 1 | 0 | 1 | 0 |
| P000002 | G0000045 | S0000146 | E000906 | MELD | 1675 | 55 | F | 70 | 65 | 0.95 | 0.968434170204059 | 0.3 | 0.92 |  |  | 0 | 6 | 6 | 0 | 0 | 1 | 0 | 0 | 0 |  |  |  | 1 | 1 | 1 | 0 | 0 | 0 | 0 | 0 | 0 | 0 | 1 | 1 | 1 | 1 | 0 |
| P000002 | G0000045 | S0000147 | E000906 | MELD 3.0 | 1675 | 55 | F | 70 | 65 | 0.95 | 0.968434170204059 | 0.3 | 0.92 | 139 | 3.21 |  | 8 | 8 | 0 | 0 | 1 | 0 | 0 | 0 |  |  |  | 1 | 1 | 1 | 0 | 0 | 0 | 0 | 0 | 0 | 0 | 1 | 1 | 0 | 1 | 0 |
| P000002 | G0000045 | S0000148 | E000906 | MELD-Na | 1675 | 55 | F | 70 | 65 | 0.95 | 0.968434170204059 | 0.3 | 0.92 | 139 |  | 0 | 6 | 6 | 0 | 0 | 1 | 0 | 0 | 0 |  |  |  | 1 | 1 | 1 | 0 | 0 | 0 | 0 | 0 | 0 | 0 | 1 | 1 | 0 | 1 | 0 |
| P000002 | G0000045 | S0000149 | E000906 | reMELD-Na | 1675 | 55 | F | 70 | 65 | 0.95 | 0.968434170204059 | 0.3 | 0.92 | 139 |  |  | 3 | 3 | 0 | 0 | 1 | 0 | 0 | 0 |  |  |  | 1 | 1 | 1 | 0 | 0 | 0 | 0 | 0 | 0 | 0 | 1 | 1 | 0 | 1 | 0 |
| P000002 | G0000046 | S0000150 | E000984 | MELD | 1766 | 58 | F | 70 | 65 | 1.62 | 1.61518626118056 | 0.2 | 0.96 |  |  | 0 | 11 | 11 | 0 | 0 | 1 | 0 | 0 | 0 |  |  |  | 1 | 1 | 1 | 0 | 0 | 0 | 0 | 0 | 0 | 0 | 1 | 1 | 1 | 1 | 0 |
| P000002 | G0000046 | S0000151 | E000984 | MELD 3.0 | 1766 | 58 | F | 70 | 65 | 1.62 | 1.61518626118056 | 0.2 | 0.96 | 139 | 3.31 |  | 13 | 13 | 0 | 0 | 1 | 0 | 0 | 0 |  |  |  | 1 | 1 | 1 | 0 | 0 | 0 | 0 | 0 | 0 | 0 | 1 | 1 | 0 | 1 | 0 |
| P000002 | G0000046 | S0000152 | E000984 | MELD-Na | 1766 | 58 | F | 70 | 65 | 1.62 | 1.61518626118056 | 0.2 | 0.96 | 139 |  | 0 | 11 | 11 | 0 | 0 | 1 | 0 | 0 | 0 |  |  |  | 1 | 1 | 1 | 0 | 0 | 0 | 0 | 0 | 0 | 0 | 1 | 1 | 0 | 1 | 0 |
| P000002 | G0000046 | S0000153 | E000984 | reMELD-Na | 1766 | 58 | F | 70 | 65 | 1.62 | 1.61518626118056 | 0.2 | 0.96 | 139 |  |  | 8 | 8 | 0 | 0 | 1 | 0 | 0 | 0 |  |  |  | 1 | 1 | 1 | 0 | 0 | 0 | 0 | 0 | 0 | 0 | 1 | 1 | 0 | 1 | 0 |
| P000002 | G0000047 | S0000154 | E001023 | MELD | 1840 | 60 | F | 70 | 65 | 1.06 | 1.07742188343952 | 0.2 | 1.05 |  |  | 0 | 8 | 8 | 0 | 0 | 1 | 0 | 0 | 0 |  |  |  | 1 | 1 | 1 | 0 | 0 | 0 | 0 | 0 | 0 | 0 | 1 | 1 | 1 | 1 | 0 |
| P000002 | G0000047 | S0000155 | E001023 | MELD-Na | 1840 | 60 | F | 70 | 65 | 1.06 | 1.07742188343952 | 0.2 | 1.05 | 135 |  | 0 | 10 | 10 | 0 | 0 | 1 | 0 | 0 | 0 |  |  |  | 1 | 1 | 1 | 0 | 0 | 0 | 0 | 0 | 0 | 0 | 1 | 1 | 0 | 1 | 0 |
| P000002 | G0000047 | S0000156 | E001023 | reMELD-Na | 1840 | 60 | F | 70 | 65 | 1.06 | 1.07742188343952 | 0.2 | 1.05 | 135 |  |  | 7 | 7 | 0 | 0 | 1 | 0 | 0 | 0 |  |  |  | 1 | 1 | 1 | 0 | 0 | 0 | 0 | 0 | 0 | 0 | 1 | 1 | 0 | 1 | 0 |
| P000002 | G0000048 | S0000157 | E001023 | MELD | 1841 | 60 | F | 70 | 65 | 0.99 | 1.00771561328946 | 0.3 | 1.04 |  |  | 0 | 7 | 7 | 0 | 0 | 1 | 0 | 0 | 0 |  |  |  | 1 | 1 | 1 | 0 | 0 | 0 | 0 | 0 | 0 | 0 | 1 | 1 | 1 | 1 | 0 |
| P000002 | G0000048 | S0000158 | E001023 | MELD 3.0 | 1841 | 60 | F | 70 | 65 | 0.99 | 1.00771561328946 | 0.3 | 1.04 | 138 | 2.74 |  | 9 | 9 | 0 | 0 | 1 | 0 | 0 | 0 |  |  |  | 1 | 1 | 1 | 0 | 0 | 0 | 0 | 0 | 0 | 0 | 1 | 1 | 0 | 1 | 0 |
| P000002 | G0000048 | S0000159 | E001023 | MELD-Na | 1841 | 60 | F | 70 | 65 | 0.99 | 1.00771561328946 | 0.3 | 1.04 | 138 |  | 0 | 7 | 7 | 0 | 0 | 1 | 0 | 0 | 0 |  |  |  | 1 | 1 | 1 | 0 | 0 | 0 | 0 | 0 | 0 | 0 | 1 | 1 | 0 | 1 | 0 |
| P000002 | G0000048 | S0000160 | E001023 | reMELD-Na | 1841 | 60 | F | 70 | 65 | 0.99 | 1.00771561328946 | 0.3 | 1.04 | 138 |  |  | 5 | 5 | 0 | 0 | 1 | 0 | 0 | 0 |  |  |  | 1 | 1 | 1 | 0 | 0 | 0 | 0 | 0 | 0 | 0 | 1 | 1 | 0 | 1 | 0 |
| P000002 | G0000049 | S0000161 | E001023 | MELD | 1849 | 60 | F | 70 | 65 | 0.97 | 0.988086644474032 | 0.3 | 0.92 |  |  | 0 | 6 | 6 | 0 | 0 | 1 | 0 | 0 | 0 |  |  |  | 1 | 1 | 1 | 0 | 0 | 0 | 0 | 0 | 0 | 0 | 1 | 1 | 1 | 1 | 0 |
| P000002 | G0000049 | S0000162 | E001023 | MELD-Na | 1849 | 60 | F | 70 | 65 | 0.97 | 0.988086644474032 | 0.3 | 0.92 | 141 |  | 0 | 6 | 6 | 0 | 0 | 1 | 0 | 0 | 0 |  |  |  | 1 | 1 | 1 | 0 | 0 | 0 | 0 | 0 | 0 | 0 | 1 | 1 | 0 | 1 | 0 |
| P000002 | G0000049 | S0000163 | E001023 | reMELD-Na | 1849 | 60 | F | 70 | 65 | 0.97 | 0.988086644474032 | 0.3 | 0.92 | 141 |  |  | 3 | 4 | 1 | 0 | 0 | 1 | 0 | 0 |  |  |  | 1 | 1 | 1 | 0 | 0 | 0 | 0 | 0 | 0 | 0 | 1 | 1 | 0 | 1 | 0 |
| P000002 | G0000050 | S0000164 | E001191 | MELD | 2155 | 70 | F | 71 | 65 | 1.17 | 1.18213364368334 | 0.4 | 2.13 |  |  | 0 | 16 | 16 | 0 | 0 | 1 | 0 | 0 | 0 |  |  |  | 1 | 1 | 1 | 0 | 0 | 0 | 0 | 0 | 0 | 0 | 1 | 1 | 1 | 1 | 0 |
| P000002 | G0000050 | S0000165 | E001191 | MELD 3.0 | 2155 | 70 | F | 71 | 65 | 1.17 | 1.18213364368334 | 0.4 | 2.13 | 143 | 2.69 |  | 17 | 17 | 0 | 0 | 1 | 0 | 0 | 0 |  |  |  | 1 | 1 | 1 | 0 | 0 | 0 | 0 | 0 | 0 | 0 | 1 | 1 | 0 | 1 | 0 |
| P000002 | G0000050 | S0000166 | E001191 | MELD-Na | 2155 | 70 | F | 71 | 65 | 1.17 | 1.18213364368334 | 0.4 | 2.13 | 143 |  | 0 | 16 | 16 | 0 | 0 | 1 | 0 | 0 | 0 |  |  |  | 1 | 1 | 1 | 0 | 0 | 0 | 0 | 0 | 0 | 0 | 1 | 1 | 0 | 1 | 0 |
| P000002 | G0000050 | S0000167 | E001191 | reMELD-Na | 2155 | 70 | F | 71 | 65 | 1.17 | 1.18213364368334 | 0.4 | 2.13 | 143 |  |  | 14 | 14 | 0 | 0 | 1 | 0 | 0 | 0 |  |  |  | 1 | 1 | 1 | 0 | 0 | 0 | 0 | 0 | 0 | 0 | 1 | 1 | 0 | 1 | 0 |
| P000002 | G0000051 | S0000168 | E001191 | MELD | 2158 | 70 | F | 71 | 65 | 1.14 | 1.1518187973209 | 0.5 | 2.18 |  |  | 0 | 16 | 17 | 1 | 0 | 0 | 1 | 0 | 0 |  |  |  | 1 | 1 | 1 | 0 | 0 | 0 | 0 | 0 | 0 | 0 | 1 | 1 | 1 | 1 | 0 |
| P000002 | G0000051 | S0000169 | E001191 | MELD-Na | 2158 | 70 | F | 71 | 65 | 1.14 | 1.1518187973209 | 0.5 | 2.18 | 147 |  | 0 | 16 | 17 | 1 | 0 | 0 | 1 | 0 | 0 |  |  |  | 1 | 1 | 1 | 0 | 0 | 0 | 0 | 0 | 0 | 0 | 1 | 1 | 0 | 1 | 0 |
| P000002 | G0000051 | S0000170 | E001191 | reMELD-Na | 2158 | 70 | F | 71 | 65 | 1.14 | 1.1518187973209 | 0.5 | 2.18 | 147 |  |  | 15 | 15 | 0 | 0 | 1 | 0 | 0 | 0 |  |  |  | 1 | 1 | 1 | 0 | 0 | 0 | 0 | 0 | 0 | 0 | 1 | 1 | 0 | 1 | 0 |
| P000002 | G0000052 | S0000171 | E001191 | MELD | 2162 | 71 | F | 71 | 65 | 1.4 | 1.40492945946026 | 0.3 | 3.06 |  |  | 0 | 22 | 22 | 0 | 0 | 1 | 0 | 0 | 0 |  |  |  | 1 | 1 | 1 | 0 | 0 | 0 | 0 | 0 | 0 | 0 | 1 | 1 | 1 | 1 | 0 |
| P000002 | G0000052 | S0000172 | E001191 | MELD-Na | 2162 | 71 | F | 71 | 65 | 1.4 | 1.40492945946026 | 0.3 | 3.06 | 146 |  | 0 | 22 | 22 | 0 | 0 | 1 | 0 | 0 | 0 |  |  |  | 1 | 1 | 1 | 0 | 0 | 0 | 0 | 0 | 0 | 0 | 1 | 1 | 0 | 1 | 0 |
| P000002 | G0000052 | S0000173 | E001191 | reMELD-Na | 2162 | 71 | F | 71 | 65 | 1.4 | 1.40492945946026 | 0.3 | 3.06 | 146 |  |  | 17 | 17 | 0 | 0 | 1 | 0 | 0 | 0 |  |  |  | 1 | 1 | 1 | 0 | 0 | 0 | 0 | 0 | 0 | 0 | 1 | 1 | 0 | 1 | 0 |
| P000002 | G0000053 | S0000174 | E001349 | MELD | 2424 | 79 | F | 72 | 65 | 1.91 | 1.87590038979764 | 1.1 | 0.99 |  |  | 0 | 13 | 13 | 0 | 0 | 1 | 0 | 0 | 0 |  |  |  | 1 | 1 | 1 | 0 | 0 | 0 | 0 | 0 | 0 | 0 | 1 | 1 | 1 | 1 | 0 |
| P000002 | G0000053 | S0000175 | E001349 | MELD-Na | 2424 | 79 | F | 72 | 65 | 1.91 | 1.87590038979764 | 1.1 | 0.99 | 142 |  | 0 | 13 | 13 | 0 | 0 | 1 | 0 | 0 | 0 |  |  |  | 1 | 1 | 1 | 0 | 0 | 0 | 0 | 0 | 0 | 0 | 1 | 1 | 0 | 1 | 0 |
| P000002 | G0000053 | S0000176 | E001349 | reMELD-Na | 2424 | 79 | F | 72 | 65 | 1.91 | 1.87590038979764 | 1.1 | 0.99 | 142 |  |  | 14 | 14 | 0 | 0 | 1 | 0 | 0 | 0 |  |  |  | 1 | 1 | 1 | 0 | 0 | 0 | 0 | 0 | 0 | 0 | 1 | 1 | 0 | 1 | 0 |
| P000002 | G0000054 | S0000177 | E001349 | MELD | 2432 | 79 | F | 72 | 65 | 1.13 | 1.14092053557952 | 0.6 | 0.91 |  |  | 0 | 8 | 8 | 0 | 0 | 1 | 0 | 0 | 0 |  |  |  | 1 | 1 | 1 | 0 | 0 | 0 | 0 | 0 | 0 | 0 | 1 | 1 | 1 | 1 | 0 |
| P000002 | G0000054 | S0000178 | E001349 | MELD-Na | 2432 | 79 | F | 72 | 65 | 1.13 | 1.14092053557952 | 0.6 | 0.91 | 148 |  | 0 | 8 | 8 | 0 | 0 | 1 | 0 | 0 | 0 |  |  |  | 1 | 1 | 1 | 0 | 0 | 0 | 0 | 0 | 0 | 0 | 1 | 1 | 0 | 1 | 0 |
| P000002 | G0000054 | S0000179 | E001349 | reMELD-Na | 2432 | 79 | F | 72 | 65 | 1.13 | 1.14092053557952 | 0.6 | 0.91 | 148 |  |  | 7 | 7 | 0 | 0 | 1 | 0 | 0 | 0 |  |  |  | 1 | 1 | 1 | 0 | 0 | 0 | 0 | 0 | 0 | 0 | 1 | 1 | 0 | 1 | 0 |
| P000002 | G0000055 | S0000180 | E001410 | MELD | 2533 | 83 | F | 72 | 65 | 2.81 | 2.69437299927563 | 0.4 | 1.4 |  |  | 0 | 20 | 20 | 0 | 0 | 1 | 0 | 0 | 0 |  |  |  | 1 | 1 | 1 | 0 | 0 | 0 | 0 | 0 | 0 | 0 | 1 | 1 | 1 | 1 | 0 |
| P000002 | G0000055 | S0000181 | E001410 | MELD-Na | 2533 | 83 | F | 72 | 65 | 2.81 | 2.69437299927563 | 0.4 | 1.4 | 133 |  | 0 | 23 | 23 | 0 | 0 | 1 | 0 | 0 | 0 |  |  |  | 1 | 1 | 1 | 0 | 0 | 0 | 0 | 0 | 0 | 0 | 1 | 1 | 0 | 1 | 0 |
| P000002 | G0000055 | S0000182 | E001410 | reMELD-Na | 2533 | 83 | F | 72 | 65 | 2.81 | 2.69437299927563 | 0.4 | 1.4 | 133 |  |  | 17 | 17 | 0 | 0 | 1 | 0 | 0 | 0 |  |  |  | 1 | 1 | 1 | 0 | 0 | 0 | 0 | 0 | 0 | 0 | 1 | 1 | 0 | 1 | 0 |
| P000002 | G0000056 | S0000183 | E001410 | MELD | 2534 | 83 | F | 72 | 65 | 2.16 | 2.11273713781676 | 0.5 | 1.38 |  |  | 0 | 17 | 17 | 0 | 0 | 1 | 0 | 0 | 0 |  |  |  | 1 | 1 | 1 | 0 | 0 | 0 | 0 | 0 | 0 | 0 | 1 | 1 | 1 | 1 | 0 |
| P000002 | G0000056 | S0000184 | E001410 | MELD-Na | 2534 | 83 | F | 72 | 65 | 2.16 | 2.11273713781676 | 0.5 | 1.38 | 139.5 |  | 0 | 17 | 17 | 0 | 0 | 1 | 0 | 0 | 0 |  |  |  | 1 | 1 | 1 | 0 | 0 | 0 | 0 | 0 | 0 | 0 | 1 | 1 | 0 | 1 | 0 |
| P000002 | G0000056 | S0000185 | E001410 | reMELD-Na | 2534 | 83 | F | 72 | 65 | 2.16 | 2.11273713781676 | 0.5 | 1.38 | 139.5 |  |  | 16 | 16 | 0 | 0 | 1 | 0 | 0 | 0 |  |  |  | 1 | 1 | 1 | 0 | 0 | 0 | 0 | 0 | 0 | 0 | 1 | 1 | 0 | 1 | 0 |
| P000002 | G0000057 | S0000186 | E001410 | MELD | 2535 | 83 | F | 72 | 65 | 1.58 | 1.57619567698092 | 0.3 | 1.36 |  |  | 0 | 14 | 14 | 0 | 0 | 1 | 0 | 0 | 0 |  |  |  | 1 | 1 | 1 | 0 | 0 | 0 | 0 | 0 | 0 | 0 | 1 | 1 | 1 | 1 | 0 |
| P000002 | G0000057 | S0000187 | E001410 | MELD 3.0 | 2535 | 83 | F | 72 | 65 | 1.58 | 1.57619567698092 | 0.3 | 1.36 | 146 | 2.51 |  | 16 | 16 | 0 | 0 | 1 | 0 | 0 | 0 |  |  |  | 1 | 1 | 1 | 0 | 0 | 0 | 0 | 0 | 0 | 0 | 1 | 1 | 0 | 1 | 0 |
| P000002 | G0000057 | S0000188 | E001410 | MELD-Na | 2535 | 83 | F | 72 | 65 | 1.58 | 1.57619567698092 | 0.3 | 1.36 | 146 |  | 0 | 14 | 14 | 0 | 0 | 1 | 0 | 0 | 0 |  |  |  | 1 | 1 | 1 | 0 | 0 | 0 | 0 | 0 | 0 | 0 | 1 | 1 | 0 | 1 | 0 |
| P000002 | G0000057 | S0000189 | E001410 | reMELD-Na | 2535 | 83 | F | 72 | 65 | 1.58 | 1.57619567698092 | 0.3 | 1.36 | 146 |  |  | 11 | 11 | 0 | 0 | 1 | 0 | 0 | 0 |  |  |  | 1 | 1 | 1 | 0 | 0 | 0 | 0 | 0 | 0 | 0 | 1 | 1 | 0 | 1 | 0 |
| P000002 | G0000058 | S0000190 | E001410 | MELD | 2536 | 83 | F | 72 | 65 | 1.41 | 1.41449419841343 | 0.3 | 1.27 |  |  | 0 | 12 | 12 | 0 | 0 | 1 | 0 | 0 | 0 |  |  |  | 1 | 1 | 1 | 0 | 0 | 0 | 0 | 0 | 0 | 0 | 1 | 1 | 1 | 1 | 0 |
| P000002 | G0000058 | S0000191 | E001410 | MELD 3.0 | 2536 | 83 | F | 72 | 65 | 1.41 | 1.41449419841343 | 0.3 | 1.27 | 148 | 3.39 |  | 13 | 13 | 0 | 0 | 1 | 0 | 0 | 0 |  |  |  | 1 | 1 | 1 | 0 | 0 | 0 | 0 | 0 | 0 | 0 | 1 | 1 | 0 | 1 | 0 |
| P000002 | G0000058 | S0000192 | E001410 | MELD-Na | 2536 | 83 | F | 72 | 65 | 1.41 | 1.41449419841343 | 0.3 | 1.27 | 148 |  | 0 | 12 | 12 | 0 | 0 | 1 | 0 | 0 | 0 |  |  |  | 1 | 1 | 1 | 0 | 0 | 0 | 0 | 0 | 0 | 0 | 1 | 1 | 0 | 1 | 0 |
| P000002 | G0000058 | S0000193 | E001410 | reMELD-Na | 2536 | 83 | F | 72 | 65 | 1.41 | 1.41449419841343 | 0.3 | 1.27 | 148 |  |  | 10 | 10 | 0 | 0 | 1 | 0 | 0 | 0 |  |  |  | 1 | 1 | 1 | 0 | 0 | 0 | 0 | 0 | 0 | 0 | 1 | 1 | 0 | 1 | 0 |
| P000002 | G0000059 | S0000194 | E001410 | MELD | 2537 | 83 | F | 72 | 65 | 1.39 | 1.39417390065304 | 0.4 | 1.35 |  |  | 0 | 13 | 13 | 0 | 0 | 1 | 0 | 0 | 0 |  |  |  | 1 | 1 | 1 | 0 | 0 | 0 | 0 | 0 | 0 | 0 | 1 | 1 | 1 | 1 | 0 |
| P000002 | G0000059 | S0000195 | E001410 | MELD 3.0 | 2537 | 83 | F | 72 | 65 | 1.39 | 1.39417390065304 | 0.4 | 1.35 | 151 | 2.78 |  | 15 | 15 | 0 | 0 | 1 | 0 | 0 | 0 |  |  |  | 1 | 1 | 1 | 0 | 0 | 0 | 0 | 0 | 0 | 0 | 1 | 1 | 0 | 1 | 0 |
| P000002 | G0000059 | S0000196 | E001410 | MELD-Na | 2537 | 83 | F | 72 | 65 | 1.39 | 1.39417390065304 | 0.4 | 1.35 | 151 |  | 0 | 13 | 13 | 0 | 0 | 1 | 0 | 0 | 0 |  |  |  | 1 | 1 | 1 | 0 | 0 | 0 | 0 | 0 | 0 | 0 | 1 | 1 | 0 | 1 | 0 |
| P000002 | G0000059 | S0000197 | E001410 | reMELD-Na | 2537 | 83 | F | 72 | 65 | 1.39 | 1.39417390065304 | 0.4 | 1.35 | 151 |  |  | 11 | 11 | 0 | 0 | 1 | 0 | 0 | 0 |  |  |  | 1 | 1 | 1 | 0 | 0 | 0 | 0 | 0 | 0 | 0 | 1 | 1 | 0 | 1 | 0 |
| P000002 | G0000060 | S0000198 | E001410 | MELD | 2541 | 83 | F | 72 | 65 | 1.23 | 1.24142981362004 | 0.3 | 1.44 |  |  | 0 | 12 | 13 | 1 | 0 | 0 | 1 | 0 | 0 |  |  |  | 1 | 1 | 1 | 0 | 0 | 0 | 0 | 0 | 0 | 0 | 1 | 1 | 1 | 1 | 0 |
| P000002 | G0000060 | S0000199 | E001410 | MELD 3.0 | 2541 | 83 | F | 72 | 65 | 1.23 | 1.24142981362004 | 0.3 | 1.44 | 146 | 3.21 |  | 13 | 13 | 0 | 0 | 1 | 0 | 0 | 0 |  |  |  | 1 | 1 | 1 | 0 | 0 | 0 | 0 | 0 | 0 | 0 | 1 | 1 | 0 | 1 | 0 |
| P000002 | G0000060 | S0000200 | E001410 | MELD-Na | 2541 | 83 | F | 72 | 65 | 1.23 | 1.24142981362004 | 0.3 | 1.44 | 146 |  | 0 | 12 | 13 | 1 | 0 | 0 | 1 | 0 | 0 |  |  |  | 1 | 1 | 1 | 0 | 0 | 0 | 0 | 0 | 0 | 0 | 1 | 1 | 0 | 1 | 0 |
| P000002 | G0000060 | S0000201 | E001410 | reMELD-Na | 2541 | 83 | F | 72 | 65 | 1.23 | 1.24142981362004 | 0.3 | 1.44 | 146 |  |  | 10 | 10 | 0 | 0 | 1 | 0 | 0 | 0 |  |  |  | 1 | 1 | 1 | 0 | 0 | 0 | 0 | 0 | 0 | 0 | 1 | 1 | 0 | 1 | 0 |
| P000002 | G0000061 | S0000202 | E001410 | MELD | 2543 | 83 | F | 72 | 65 | 1.305 | 1.30673731372925 | 0.9 | 1.76 |  |  | 0 | 15 | 15 | 0 | 0 | 1 | 0 | 0 | 0 |  |  |  | 1 | 1 | 1 | 0 | 0 | 0 | 0 | 0 | 0 | 0 | 1 | 1 | 1 | 1 | 0 |
| P000002 | G0000061 | S0000203 | E001410 | MELD-Na | 2543 | 83 | F | 72 | 65 | 1.305 | 1.30673731372925 | 0.9 | 1.76 | 143 |  | 0 | 15 | 15 | 0 | 0 | 1 | 0 | 0 | 0 |  |  |  | 1 | 1 | 1 | 0 | 0 | 0 | 0 | 0 | 0 | 0 | 1 | 1 | 0 | 1 | 0 |
| P000002 | G0000061 | S0000204 | E001410 | reMELD-Na | 2543 | 83 | F | 72 | 65 | 1.305 | 1.30673731372925 | 0.9 | 1.76 | 143 |  |  | 16 | 16 | 0 | 0 | 1 | 0 | 0 | 0 |  |  |  | 1 | 1 | 1 | 0 | 0 | 0 | 0 | 0 | 0 | 0 | 1 | 1 | 0 | 1 | 0 |
| P000002 | G0000062 | S0000205 | E001410 | MELD | 2544 | 83 | F | 72 | 65 | 1.84 | 1.81903398990924 | 0.4 | 1.41 |  |  | 1 | 24 | 24 | 0 | 0 | 1 | 0 | 0 | 0 |  |  |  | 1 | 1 | 1 | 0 | 0 | 0 | 0 | 0 | 0 | 0 | 1 | 1 | 1 | 1 | 0 |
| P000002 | G0000062 | S0000206 | E001410 | MELD-Na | 2544 | 83 | F | 72 | 65 | 1.84 | 1.81903398990924 | 0.4 | 1.41 | 141 |  | 1 | 24 | 24 | 0 | 0 | 1 | 0 | 0 | 0 |  |  |  | 1 | 1 | 1 | 0 | 0 | 0 | 0 | 0 | 0 | 0 | 1 | 1 | 0 | 1 | 0 |
| P000002 | G0000062 | S0000207 | E001410 | reMELD-Na | 2544 | 83 | F | 72 | 65 | 1.84 | 1.81903398990924 | 0.4 | 1.41 | 141 |  |  | 14 | 14 | 0 | 0 | 1 | 0 | 0 | 0 |  |  |  | 1 | 1 | 1 | 0 | 0 | 0 | 0 | 0 | 0 | 0 | 1 | 1 | 0 | 1 | 0 |
| P000002 | G0000063 | S0000208 | E001410 | MELD | 2547 | 83 | F | 72 | 65 | 0.79 | 0.805658653280166 | 0.7 | 1.13 |  |  | 0 | 8 | 8 | 0 | 0 | 1 | 0 | 0 | 0 |  |  |  | 1 | 1 | 1 | 0 | 0 | 0 | 0 | 0 | 0 | 0 | 1 | 1 | 1 | 1 | 0 |
| P000002 | G0000063 | S0000209 | E001410 | MELD 3.0 | 2547 | 83 | F | 72 | 65 | 0.79 | 0.805658653280166 | 0.7 | 1.13 | 144 | 3.32 |  | 9 | 9 | 0 | 0 | 1 | 0 | 0 | 0 |  |  |  | 1 | 1 | 1 | 0 | 0 | 0 | 0 | 0 | 0 | 0 | 1 | 1 | 0 | 1 | 0 |
| P000002 | G0000063 | S0000210 | E001410 | MELD-Na | 2547 | 83 | F | 72 | 65 | 0.79 | 0.805658653280166 | 0.7 | 1.13 | 144 |  | 0 | 8 | 8 | 0 | 0 | 1 | 0 | 0 | 0 |  |  |  | 1 | 1 | 1 | 0 | 0 | 0 | 0 | 0 | 0 | 0 | 1 | 1 | 0 | 1 | 0 |
| P000002 | G0000063 | S0000211 | E001410 | reMELD-Na | 2547 | 83 | F | 72 | 65 | 0.79 | 0.805658653280166 | 0.7 | 1.13 | 144 |  |  | 6 | 6 | 0 | 0 | 1 | 0 | 0 | 0 |  |  |  | 1 | 1 | 1 | 0 | 0 | 0 | 0 | 0 | 0 | 0 | 1 | 1 | 0 | 1 | 0 |
| P000002 | G0000064 | S0000212 | E001410 | MELD | 2549 | 83 | F | 72 | 65 | 0.7 | 0.714920664252023 | 0.8 | 1.36 |  |  | 0 | 10 | 10 | 0 | 0 | 1 | 0 | 0 | 0 |  |  |  | 1 | 1 | 1 | 0 | 0 | 0 | 0 | 0 | 0 | 0 | 1 | 1 | 1 | 1 | 0 |
| P000002 | G0000064 | S0000213 | E001410 | MELD-Na | 2549 | 83 | F | 72 | 65 | 0.7 | 0.714920664252023 | 0.8 | 1.36 | 146 |  | 0 | 10 | 10 | 0 | 0 | 1 | 0 | 0 | 0 |  |  |  | 1 | 1 | 1 | 0 | 0 | 0 | 0 | 0 | 0 | 0 | 1 | 1 | 0 | 1 | 0 |
| P000002 | G0000064 | S0000214 | E001410 | reMELD-Na | 2549 | 83 | F | 72 | 65 | 0.7 | 0.714920664252023 | 0.8 | 1.36 | 146 |  |  | 7 | 7 | 0 | 0 | 1 | 0 | 0 | 0 |  |  |  | 1 | 1 | 1 | 0 | 0 | 0 | 0 | 0 | 0 | 0 | 1 | 1 | 0 | 1 | 0 |
| P000002 | G0000065 | S0000215 | E001410 | MELD | 2550 | 83 | F | 72 | 65 | 0.72 | 0.732552523585138 | 1 | 1.36 |  |  | 0 | 10 | 10 | 0 | 0 | 1 | 0 | 0 | 0 |  |  |  | 1 | 1 | 1 | 0 | 0 | 0 | 0 | 0 | 0 | 0 | 1 | 1 | 1 | 1 | 0 |
| P000002 | G0000065 | S0000216 | E001410 | MELD 3.0 | 2550 | 83 | F | 72 | 65 | 0.72 | 0.732552523585138 | 1 | 1.36 | 148 | 3.02 |  | 11 | 11 | 0 | 0 | 1 | 0 | 0 | 0 |  |  |  | 1 | 1 | 1 | 0 | 0 | 0 | 0 | 0 | 0 | 0 | 1 | 1 | 0 | 1 | 0 |
| P000002 | G0000065 | S0000217 | E001410 | MELD-Na | 2550 | 83 | F | 72 | 65 | 0.72 | 0.732552523585138 | 1 | 1.36 | 148 |  | 0 | 10 | 10 | 0 | 0 | 1 | 0 | 0 | 0 |  |  |  | 1 | 1 | 1 | 0 | 0 | 0 | 0 | 0 | 0 | 0 | 1 | 1 | 0 | 1 | 0 |
| P000002 | G0000065 | S0000218 | E001410 | reMELD-Na | 2550 | 83 | F | 72 | 65 | 0.72 | 0.732552523585138 | 1 | 1.36 | 148 |  |  | 8 | 8 | 0 | 0 | 1 | 0 | 0 | 0 |  |  |  | 1 | 1 | 1 | 0 | 0 | 0 | 0 | 0 | 0 | 0 | 1 | 1 | 0 | 1 | 0 |
| P000002 | G0000066 | S0000219 | E001410 | MELD | 2554 | 83 | F | 72 | 65 | 1.38 | 1.37874831042347 | 0.9 | 1.43 |  |  | 0 | 14 | 14 | 0 | 0 | 1 | 0 | 0 | 0 |  |  |  | 1 | 1 | 1 | 0 | 0 | 0 | 0 | 0 | 0 | 0 | 1 | 1 | 1 | 1 | 0 |
| P000002 | G0000066 | S0000220 | E001410 | MELD 3.0 | 2554 | 83 | F | 72 | 65 | 1.38 | 1.37874831042347 | 0.9 | 1.43 | 146 | 2.75 |  | 15 | 15 | 0 | 0 | 1 | 0 | 0 | 0 |  |  |  | 1 | 1 | 1 | 0 | 0 | 0 | 0 | 0 | 0 | 0 | 1 | 1 | 0 | 1 | 0 |
| P000002 | G0000066 | S0000221 | E001410 | MELD-Na | 2554 | 83 | F | 72 | 65 | 1.38 | 1.37874831042347 | 0.9 | 1.43 | 146 |  | 0 | 14 | 14 | 0 | 0 | 1 | 0 | 0 | 0 |  |  |  | 1 | 1 | 1 | 0 | 0 | 0 | 0 | 0 | 0 | 0 | 1 | 1 | 0 | 1 | 0 |
| P000002 | G0000066 | S0000222 | E001410 | reMELD-Na | 2554 | 83 | F | 72 | 65 | 1.38 | 1.37874831042347 | 0.9 | 1.43 | 146 |  |  | 14 | 14 | 0 | 0 | 1 | 0 | 0 | 0 |  |  |  | 1 | 1 | 1 | 0 | 0 | 0 | 0 | 0 | 0 | 0 | 1 | 1 | 0 | 1 | 0 |
| P000002 | G0000067 | S0000223 | E001410 | MELD | 2555 | 83 | F | 72 | 65 | 1.135 | 1.14113393923252 | 1 | 1.895 |  |  | 0 | 15 | 15 | 0 | 0 | 1 | 0 | 0 | 0 |  |  |  | 1 | 1 | 1 | 0 | 0 | 0 | 0 | 0 | 0 | 0 | 1 | 1 | 1 | 1 | 0 |
| P000002 | G0000067 | S0000224 | E001410 | MELD-Na | 2555 | 83 | F | 72 | 65 | 1.135 | 1.14113393923252 | 1 | 1.895 | 145 |  | 0 | 15 | 15 | 0 | 0 | 1 | 0 | 0 | 0 |  |  |  | 1 | 1 | 1 | 0 | 0 | 0 | 0 | 0 | 0 | 0 | 1 | 1 | 0 | 1 | 0 |
| P000002 | G0000067 | S0000225 | E001410 | reMELD-Na | 2555 | 83 | F | 72 | 65 | 1.135 | 1.14113393923252 | 1 | 1.895 | 145 |  |  | 15 | 15 | 0 | 0 | 1 | 0 | 0 | 0 |  |  |  | 1 | 1 | 1 | 0 | 0 | 0 | 0 | 0 | 0 | 0 | 1 | 1 | 0 | 1 | 0 |
| P000002 | G0000068 | S0000226 | E001410 | MELD | 2556 | 83 | F | 72 | 65 | 1.32 | 1.32116595670446 | 0.9 | 2.28 |  |  | 0 | 18 | 18 | 0 | 0 | 1 | 0 | 0 | 0 |  |  |  | 1 | 1 | 1 | 0 | 0 | 0 | 0 | 0 | 0 | 0 | 1 | 1 | 1 | 1 | 0 |
| P000002 | G0000068 | S0000227 | E001410 | MELD-Na | 2556 | 83 | F | 72 | 65 | 1.32 | 1.32116595670446 | 0.9 | 2.28 | 148 |  | 0 | 18 | 18 | 0 | 0 | 1 | 0 | 0 | 0 |  |  |  | 1 | 1 | 1 | 0 | 0 | 0 | 0 | 0 | 0 | 0 | 1 | 1 | 0 | 1 | 0 |
| P000002 | G0000068 | S0000228 | E001410 | reMELD-Na | 2556 | 83 | F | 72 | 65 | 1.32 | 1.32116595670446 | 0.9 | 2.28 | 148 |  |  | 18 | 18 | 0 | 0 | 1 | 0 | 0 | 0 |  |  |  | 1 | 1 | 1 | 0 | 0 | 0 | 0 | 0 | 0 | 0 | 1 | 1 | 0 | 1 | 0 |
| P000002 | G0000069 | S0000229 | E001410 | MELD | 2557 | 84 | F | 72 | 65 | 1.11 | 1.11912150678646 | 0.8 | 1.68 |  |  | 0 | 13 | 13 | 0 | 0 | 1 | 0 | 0 | 0 |  |  |  | 1 | 1 | 1 | 0 | 0 | 0 | 0 | 0 | 0 | 0 | 1 | 1 | 1 | 1 | 0 |
| P000002 | G0000069 | S0000230 | E001410 | MELD 3.0 | 2557 | 84 | F | 72 | 65 | 1.11 | 1.11912150678646 | 0.8 | 1.68 | 147 | 2.15 |  | 15 | 16 | 1 | 0 | 0 | 1 | 0 | 0 |  |  |  | 1 | 1 | 1 | 0 | 0 | 0 | 0 | 0 | 0 | 0 | 1 | 1 | 0 | 1 | 0 |
| P000002 | G0000069 | S0000231 | E001410 | MELD-Na | 2557 | 84 | F | 72 | 65 | 1.11 | 1.11912150678646 | 0.8 | 1.68 | 147 |  | 0 | 13 | 13 | 0 | 0 | 1 | 0 | 0 | 0 |  |  |  | 1 | 1 | 1 | 0 | 0 | 0 | 0 | 0 | 0 | 0 | 1 | 1 | 0 | 1 | 0 |
| P000002 | G0000069 | S0000232 | E001410 | reMELD-Na | 2557 | 84 | F | 72 | 65 | 1.11 | 1.11912150678646 | 0.8 | 1.68 | 147 |  |  | 13 | 13 | 0 | 0 | 1 | 0 | 0 | 0 |  |  |  | 1 | 1 | 1 | 0 | 0 | 0 | 0 | 0 | 0 | 0 | 1 | 1 | 0 | 1 | 0 |
| P000002 | G0000070 | S0000233 | E001410 | MELD | 2558 | 84 | F | 72 | 65 | 0.83 | 0.841836972069986 | 1 | 1.37 |  |  | 0 | 10 | 10 | 0 | 0 | 1 | 0 | 0 | 0 |  |  |  | 1 | 1 | 1 | 0 | 0 | 0 | 0 | 0 | 0 | 0 | 1 | 1 | 1 | 1 | 0 |
| P000002 | G0000070 | S0000234 | E001410 | MELD-Na | 2558 | 84 | F | 72 | 65 | 0.83 | 0.841836972069986 | 1 | 1.37 | 145 |  | 0 | 10 | 10 | 0 | 0 | 1 | 0 | 0 | 0 |  |  |  | 1 | 1 | 1 | 0 | 0 | 0 | 0 | 0 | 0 | 0 | 1 | 1 | 0 | 1 | 0 |
| P000002 | G0000070 | S0000235 | E001410 | reMELD-Na | 2558 | 84 | F | 72 | 65 | 0.83 | 0.841836972069986 | 1 | 1.37 | 145 |  |  | 9 | 10 | 1 | 0 | 0 | 1 | 0 | 0 |  |  |  | 1 | 1 | 1 | 0 | 0 | 0 | 0 | 0 | 0 | 0 | 1 | 1 | 0 | 1 | 0 |
| P000002 | G0000071 | S0000236 | E001410 | MELD | 2559 | 84 | F | 72 | 65 | 0.37 | 0.376664965452254 | 1.3 | 1.25 |  |  | 0 | 10 | 10 | 0 | 0 | 1 | 0 | 0 | 0 |  |  |  | 1 | 1 | 1 | 0 | 0 | 0 | 0 | 0 | 0 | 0 | 1 | 1 | 1 | 1 | 0 |
| P000002 | G0000071 | S0000237 | E001410 | MELD-Na | 2559 | 84 | F | 72 | 65 | 0.37 | 0.376664965452254 | 1.3 | 1.25 | 147 |  | 0 | 10 | 10 | 0 | 0 | 1 | 0 | 0 | 0 |  |  |  | 1 | 1 | 1 | 0 | 0 | 0 | 0 | 0 | 0 | 0 | 1 | 1 | 0 | 1 | 0 |
| P000002 | G0000071 | S0000238 | E001410 | reMELD-Na | 2559 | 84 | F | 72 | 65 | 0.37 | 0.376664965452254 | 1.3 | 1.25 | 147 |  |  | 8 | 8 | 0 | 0 | 1 | 0 | 0 | 0 |  |  |  | 1 | 1 | 1 | 0 | 0 | 0 | 0 | 0 | 0 | 0 | 1 | 1 | 0 | 1 | 0 |
| P000002 | G0000072 | S0000239 | E001410 | MELD | 2560 | 84 | F | 72 | 65 | 0.71 | 0.714640599321851 | 1.7 | 1.28 |  |  | 0 | 11 | 11 | 0 | 0 | 1 | 0 | 0 | 0 |  |  |  | 1 | 1 | 1 | 0 | 0 | 0 | 0 | 0 | 0 | 0 | 1 | 1 | 1 | 1 | 0 |
| P000002 | G0000072 | S0000240 | E001410 | MELD-Na | 2560 | 84 | F | 72 | 65 | 0.71 | 0.714640599321851 | 1.7 | 1.28 | 146 |  | 0 | 11 | 11 | 0 | 0 | 1 | 0 | 0 | 0 |  |  |  | 1 | 1 | 1 | 0 | 0 | 0 | 0 | 0 | 0 | 0 | 1 | 1 | 0 | 1 | 0 |
| P000002 | G0000072 | S0000241 | E001410 | reMELD-Na | 2560 | 84 | F | 72 | 65 | 0.71 | 0.714640599321851 | 1.7 | 1.28 | 146 |  |  | 9 | 9 | 0 | 0 | 1 | 0 | 0 | 0 |  |  |  | 1 | 1 | 1 | 0 | 0 | 0 | 0 | 0 | 0 | 0 | 1 | 1 | 0 | 1 | 0 |
| P000002 | G0000073 | S0000242 | E001410 | MELD | 2561 | 84 | F | 72 | 65 | 0.85 | 0.851465072412863 | 1.9 | 1.55 |  |  | 0 | 14 | 14 | 0 | 0 | 1 | 0 | 0 | 0 |  |  |  | 1 | 1 | 1 | 0 | 0 | 0 | 0 | 0 | 0 | 0 | 1 | 1 | 1 | 1 | 0 |
| P000002 | G0000073 | S0000243 | E001410 | MELD 3.0 | 2561 | 84 | F | 72 | 65 | 0.85 | 0.851465072412863 | 1.9 | 1.55 | 148 | 4.38 |  | 14 | 14 | 0 | 0 | 1 | 0 | 0 | 0 |  |  |  | 1 | 1 | 1 | 0 | 0 | 0 | 0 | 0 | 0 | 0 | 1 | 1 | 0 | 1 | 0 |
| P000002 | G0000073 | S0000244 | E001410 | MELD-Na | 2561 | 84 | F | 72 | 65 | 0.85 | 0.851465072412863 | 1.9 | 1.55 | 148 |  | 0 | 14 | 14 | 0 | 0 | 1 | 0 | 0 | 0 |  |  |  | 1 | 1 | 1 | 0 | 0 | 0 | 0 | 0 | 0 | 0 | 1 | 1 | 0 | 1 | 0 |
| P000002 | G0000073 | S0000245 | E001410 | reMELD-Na | 2561 | 84 | F | 72 | 65 | 0.85 | 0.851465072412863 | 1.9 | 1.55 | 148 |  |  | 13 | 13 | 0 | 0 | 1 | 0 | 0 | 0 |  |  |  | 1 | 1 | 1 | 0 | 0 | 0 | 0 | 0 | 0 | 0 | 1 | 1 | 0 | 1 | 0 |
| P000002 | G0000074 | S0000246 | E001410 | MELD | 2562 | 84 | F | 72 | 65 | 0.8 | 0.798639505991204 | 2.2 | 1.4 |  |  | 0 | 13 | 13 | 0 | 0 | 1 | 0 | 0 | 0 |  |  |  | 1 | 1 | 1 | 0 | 0 | 0 | 0 | 0 | 0 | 0 | 1 | 1 | 1 | 1 | 0 |
| P000002 | G0000074 | S0000247 | E001410 | MELD-Na | 2562 | 84 | F | 72 | 65 | 0.8 | 0.798639505991204 | 2.2 | 1.4 | 146 |  | 0 | 13 | 13 | 0 | 0 | 1 | 0 | 0 | 0 |  |  |  | 1 | 1 | 1 | 0 | 0 | 0 | 0 | 0 | 0 | 0 | 1 | 1 | 0 | 1 | 0 |
| P000002 | G0000074 | S0000248 | E001410 | reMELD-Na | 2562 | 84 | F | 72 | 65 | 0.8 | 0.798639505991204 | 2.2 | 1.4 | 146 |  |  | 12 | 12 | 0 | 0 | 1 | 0 | 0 | 0 |  |  |  | 1 | 1 | 1 | 0 | 0 | 0 | 0 | 0 | 0 | 0 | 1 | 1 | 0 | 1 | 0 |
| P000002 | G0000075 | S0000249 | E001410 | MELD | 2563 | 84 | F | 72 | 65 | 2.3 | 2.22609067469375 | 1.7 | 1.33 |  |  | 1 | 25 | 25 | 0 | 0 | 1 | 0 | 0 | 0 |  |  |  | 1 | 1 | 1 | 0 | 0 | 0 | 0 | 0 | 0 | 0 | 1 | 1 | 1 | 1 | 0 |
| P000002 | G0000075 | S0000250 | E001410 | MELD 3.0 | 2563 | 84 | F | 72 | 65 | 2.3 | 2.22609067469375 | 1.7 | 1.33 | 145 | 4.13 |  | 22 | 21 | -1 | 1 | 0 | 0 | 0 | 0 |  |  |  | 1 | 1 | 1 | 0 | 0 | 0 | 0 | 0 | 0 | 0 | 1 | 1 | 0 | 1 | 0 |
| P000002 | G0000075 | S0000251 | E001410 | MELD-Na | 2563 | 84 | F | 72 | 65 | 2.3 | 2.22609067469375 | 1.7 | 1.33 | 145 |  | 1 | 25 | 25 | 0 | 0 | 1 | 0 | 0 | 0 |  |  |  | 1 | 1 | 1 | 0 | 0 | 0 | 0 | 0 | 0 | 0 | 1 | 1 | 0 | 1 | 0 |
| P000002 | G0000075 | S0000252 | E001410 | reMELD-Na | 2563 | 84 | F | 72 | 65 | 2.3 | 2.22609067469375 | 1.7 | 1.33 | 145 |  |  | 20 | 19 | -1 | 1 | 0 | 0 | 0 | 0 |  |  |  | 1 | 1 | 1 | 0 | 0 | 0 | 0 | 0 | 0 | 0 | 1 | 1 | 0 | 1 | 0 |
| P000002 | G0000076 | S0000253 | E001410 | MELD | 2565 | 84 | F | 72 | 65 | 3.39 | 3.18205926509402 | 1.2 | 1.265 |  |  | 1 | 23 | 23 | 0 | 0 | 1 | 0 | 0 | 0 |  |  |  | 1 | 1 | 1 | 0 | 0 | 0 | 0 | 0 | 0 | 0 | 1 | 1 | 1 | 1 | 0 |
| P000002 | G0000076 | S0000254 | E001410 | MELD-Na | 2565 | 84 | F | 72 | 65 | 3.39 | 3.18205926509402 | 1.2 | 1.265 | 141 |  | 1 | 23 | 23 | 0 | 0 | 1 | 0 | 0 | 0 |  |  |  | 1 | 1 | 1 | 0 | 0 | 0 | 0 | 0 | 0 | 0 | 1 | 1 | 0 | 1 | 0 |
| P000002 | G0000076 | S0000255 | E001410 | reMELD-Na | 2565 | 84 | F | 72 | 65 | 3.39 | 3.18205926509402 | 1.2 | 1.265 | 141 |  |  | 19 | 19 | 0 | 0 | 1 | 0 | 0 | 0 |  |  |  | 1 | 1 | 1 | 0 | 0 | 0 | 0 | 0 | 0 | 0 | 1 | 1 | 0 | 1 | 0 |
| P000002 | G0000077 | S0000256 | E001410 | MELD | 2568 | 84 | F | 72 | 65 | 4.56 | 4.12673161251076 | 1 | 1.18 |  |  | 1 | 22 | 22 | 0 | 0 | 1 | 0 | 0 | 0 |  |  |  | 1 | 1 | 1 | 0 | 0 | 0 | 0 | 0 | 0 | 0 | 1 | 1 | 1 | 1 | 0 |
| P000002 | G0000077 | S0000257 | E001410 | MELD-Na | 2568 | 84 | F | 72 | 65 | 4.56 | 4.12673161251076 | 1 | 1.18 | 138 |  | 1 | 22 | 22 | 0 | 0 | 1 | 0 | 0 | 0 |  |  |  | 1 | 1 | 1 | 0 | 0 | 0 | 0 | 0 | 0 | 0 | 1 | 1 | 0 | 1 | 0 |
| P000002 | G0000077 | S0000258 | E001410 | reMELD-Na | 2568 | 84 | F | 72 | 65 | 4.56 | 4.12673161251076 | 1 | 1.18 | 138 |  |  | 18 | 18 | 0 | 0 | 1 | 0 | 0 | 0 |  |  |  | 1 | 1 | 1 | 0 | 0 | 0 | 0 | 0 | 0 | 0 | 1 | 1 | 0 | 1 | 0 |
| P000002 | G0000078 | S0000259 | E001410 | MELD | 2575 | 84 | F | 72 | 65 | 3.22 | 3.04304805863219 | 0.8 | 1.045 |  |  | 1 | 20 | 20 | 0 | 0 | 1 | 0 | 0 | 0 |  |  |  | 1 | 1 | 1 | 0 | 0 | 0 | 0 | 0 | 0 | 0 | 1 | 1 | 1 | 1 | 0 |
| P000002 | G0000078 | S0000260 | E001410 | MELD-Na | 2575 | 84 | F | 72 | 65 | 3.22 | 3.04304805863219 | 0.8 | 1.045 | 134 |  | 1 | 22 | 22 | 0 | 0 | 1 | 0 | 0 | 0 |  |  |  | 1 | 1 | 1 | 0 | 0 | 0 | 0 | 0 | 0 | 0 | 1 | 1 | 0 | 1 | 0 |
| P000002 | G0000078 | S0000261 | E001410 | reMELD-Na | 2575 | 84 | F | 72 | 65 | 3.22 | 3.04304805863219 | 0.8 | 1.045 | 134 |  |  | 16 | 16 | 0 | 0 | 1 | 0 | 0 | 0 |  |  |  | 1 | 1 | 1 | 0 | 0 | 0 | 0 | 0 | 0 | 0 | 1 | 1 | 0 | 1 | 0 |
| P000002 | G0000079 | S0000262 | E001410 | MELD | 2581 | 84 | F | 72 | 65 | 3.61 | 3.36884832607085 | 0.9 | 1.06 |  |  | 0 | 19 | 19 | 0 | 0 | 1 | 0 | 0 | 0 |  |  |  | 1 | 1 | 1 | 0 | 0 | 0 | 0 | 0 | 0 | 0 | 1 | 1 | 1 | 1 | 0 |
| P000002 | G0000079 | S0000263 | E001410 | MELD-Na | 2581 | 84 | F | 72 | 65 | 3.61 | 3.36884832607085 | 0.9 | 1.06 | 141 |  | 0 | 19 | 19 | 0 | 0 | 1 | 0 | 0 | 0 |  |  |  | 1 | 1 | 1 | 0 | 0 | 0 | 0 | 0 | 0 | 0 | 1 | 1 | 0 | 1 | 0 |
| P000002 | G0000079 | S0000264 | E001410 | reMELD-Na | 2581 | 84 | F | 72 | 65 | 3.61 | 3.36884832607085 | 0.9 | 1.06 | 141 |  |  | 16 | 16 | 0 | 0 | 1 | 0 | 0 | 0 |  |  |  | 1 | 1 | 1 | 0 | 0 | 0 | 0 | 0 | 0 | 0 | 1 | 1 | 0 | 1 | 0 |
| P000002 | G0000080 | S0000265 | E001410 | MELD | 2582 | 84 | F | 72 | 65 | 3.83 | 3.55050400022998 | 0.8 | 1.08 |  |  | 1 | 21 | 21 | 0 | 0 | 1 | 0 | 0 | 0 |  |  |  | 1 | 1 | 1 | 0 | 0 | 0 | 0 | 0 | 0 | 0 | 1 | 1 | 1 | 1 | 0 |
| P000002 | G0000080 | S0000266 | E001410 | MELD-Na | 2582 | 84 | F | 72 | 65 | 3.83 | 3.55050400022998 | 0.8 | 1.08 | 140 |  | 1 | 21 | 21 | 0 | 0 | 1 | 0 | 0 | 0 |  |  |  | 1 | 1 | 1 | 0 | 0 | 0 | 0 | 0 | 0 | 0 | 1 | 1 | 0 | 1 | 0 |
| P000002 | G0000080 | S0000267 | E001410 | reMELD-Na | 2582 | 84 | F | 72 | 65 | 3.83 | 3.55050400022998 | 0.8 | 1.08 | 140 |  |  | 16 | 16 | 0 | 0 | 1 | 0 | 0 | 0 |  |  |  | 1 | 1 | 1 | 0 | 0 | 0 | 0 | 0 | 0 | 0 | 1 | 1 | 0 | 1 | 0 |
| P000002 | G0000081 | S0000268 | E001463 | MELD | 2599 | 85 | F | 73 | 65 | 1.61 | 1.6010152344225 | 0.6 | 1.99 |  |  | 1 | 27 | 27 | 0 | 0 | 1 | 0 | 0 | 0 |  |  |  | 1 | 1 | 1 | 0 | 0 | 0 | 0 | 0 | 0 | 0 | 1 | 1 | 1 | 1 | 0 |
| P000002 | G0000081 | S0000269 | E001463 | MELD 3.0 | 2599 | 85 | F | 73 | 65 | 1.61 | 1.6010152344225 | 0.6 | 1.99 | 140 | 2.2 |  | 20 | 20 | 0 | 0 | 1 | 0 | 0 | 0 |  |  |  | 1 | 1 | 1 | 0 | 0 | 0 | 0 | 0 | 0 | 0 | 1 | 1 | 0 | 1 | 0 |
| P000002 | G0000081 | S0000270 | E001463 | MELD-Na | 2599 | 85 | F | 73 | 65 | 1.61 | 1.6010152344225 | 0.6 | 1.99 | 140 |  | 1 | 27 | 27 | 0 | 0 | 1 | 0 | 0 | 0 |  |  |  | 1 | 1 | 1 | 0 | 0 | 0 | 0 | 0 | 0 | 0 | 1 | 1 | 0 | 1 | 0 |
| P000002 | G0000081 | S0000271 | E001463 | reMELD-Na | 2599 | 85 | F | 73 | 65 | 1.61 | 1.6010152344225 | 0.6 | 1.99 | 140 |  |  | 17 | 17 | 0 | 0 | 1 | 0 | 0 | 0 |  |  |  | 1 | 1 | 1 | 0 | 0 | 0 | 0 | 0 | 0 | 0 | 1 | 1 | 0 | 1 | 0 |
| P000002 | G0000082 | S0000272 | E001463 | MELD | 2603 | 85 | F | 73 | 65 | 2.13 | 2.08183801455505 | 0.8 | 1.67 |  |  | 1 | 25 | 25 | 0 | 0 | 1 | 0 | 0 | 0 |  |  |  | 1 | 1 | 1 | 0 | 0 | 0 | 0 | 0 | 0 | 0 | 1 | 1 | 1 | 1 | 0 |
| P000002 | G0000082 | S0000273 | E001463 | MELD-Na | 2603 | 85 | F | 73 | 65 | 2.13 | 2.08183801455505 | 0.8 | 1.67 | 138 |  | 1 | 25 | 25 | 0 | 0 | 1 | 0 | 0 | 0 |  |  |  | 1 | 1 | 1 | 0 | 0 | 0 | 0 | 0 | 0 | 0 | 1 | 1 | 0 | 1 | 0 |
| P000002 | G0000082 | S0000274 | E001463 | reMELD-Na | 2603 | 85 | F | 73 | 65 | 2.13 | 2.08183801455505 | 0.8 | 1.67 | 138 |  |  | 19 | 19 | 0 | 0 | 1 | 0 | 0 | 0 |  |  |  | 1 | 1 | 1 | 0 | 0 | 0 | 0 | 0 | 0 | 0 | 1 | 1 | 0 | 1 | 0 |
| P000002 | G0000083 | S0000275 | E001463 | MELD | 2604 | 85 | F | 73 | 65 | 1.76 | 1.74084842628292 | 0.7 | 1.96 |  |  | 0 | 19 | 19 | 0 | 0 | 1 | 0 | 0 | 0 |  |  |  | 1 | 1 | 1 | 0 | 0 | 0 | 0 | 0 | 0 | 0 | 1 | 1 | 1 | 1 | 0 |
| P000002 | G0000083 | S0000276 | E001463 | MELD-Na | 2604 | 85 | F | 73 | 65 | 1.76 | 1.74084842628292 | 0.7 | 1.96 | 137 |  | 0 | 19 | 19 | 0 | 0 | 1 | 0 | 0 | 0 |  |  |  | 1 | 1 | 1 | 0 | 0 | 0 | 0 | 0 | 0 | 0 | 1 | 1 | 0 | 1 | 0 |
| P000002 | G0000083 | S0000277 | E001463 | reMELD-Na | 2604 | 85 | F | 73 | 65 | 1.76 | 1.74084842628292 | 0.7 | 1.96 | 137 |  |  | 19 | 19 | 0 | 0 | 1 | 0 | 0 | 0 |  |  |  | 1 | 1 | 1 | 0 | 0 | 0 | 0 | 0 | 0 | 0 | 1 | 1 | 0 | 1 | 0 |
| P000002 | G0000084 | S0000278 | E001463 | MELD | 2605 | 85 | F | 73 | 65 | 1.79 | 1.77006018648861 | 0.6 | 2.01 |  |  | 1 | 28 | 28 | 0 | 0 | 1 | 0 | 0 | 0 |  |  |  | 1 | 1 | 1 | 0 | 0 | 0 | 0 | 0 | 0 | 0 | 1 | 1 | 1 | 1 | 0 |
| P000002 | G0000084 | S0000279 | E001463 | MELD-Na | 2605 | 85 | F | 73 | 65 | 1.79 | 1.77006018648861 | 0.6 | 2.01 | 142 |  | 1 | 28 | 28 | 0 | 0 | 1 | 0 | 0 | 0 |  |  |  | 1 | 1 | 1 | 0 | 0 | 0 | 0 | 0 | 0 | 0 | 1 | 1 | 0 | 1 | 0 |
| P000002 | G0000084 | S0000280 | E001463 | reMELD-Na | 2605 | 85 | F | 73 | 65 | 1.79 | 1.77006018648861 | 0.6 | 2.01 | 142 |  |  | 18 | 18 | 0 | 0 | 1 | 0 | 0 | 0 |  |  |  | 1 | 1 | 1 | 0 | 0 | 0 | 0 | 0 | 0 | 0 | 1 | 1 | 0 | 1 | 0 |
| P000002 | G0000085 | S0000281 | E001463 | MELD | 2608 | 85 | F | 73 | 65 | 0.63 | 0.648432994345689 | 0.5 | 2.48 |  |  | 0 | 17 | 17 | 0 | 0 | 1 | 0 | 0 | 0 |  |  |  | 1 | 1 | 1 | 0 | 0 | 0 | 0 | 0 | 0 | 0 | 1 | 1 | 1 | 1 | 0 |
| P000002 | G0000085 | S0000282 | E001463 | MELD-Na | 2608 | 85 | F | 73 | 65 | 0.63 | 0.648432994345689 | 0.5 | 2.48 | 140 |  | 0 | 17 | 17 | 0 | 0 | 1 | 0 | 0 | 0 |  |  |  | 1 | 1 | 1 | 0 | 0 | 0 | 0 | 0 | 0 | 0 | 1 | 1 | 0 | 1 | 0 |
| P000002 | G0000085 | S0000283 | E001463 | reMELD-Na | 2608 | 85 | F | 73 | 65 | 0.63 | 0.648432994345689 | 0.5 | 2.48 | 140 |  |  | 12 | 12 | 0 | 0 | 1 | 0 | 0 | 0 |  |  |  | 1 | 1 | 1 | 0 | 0 | 0 | 0 | 0 | 0 | 0 | 1 | 1 | 0 | 1 | 0 |
| P000002 | G0000086 | S0000284 | E001463 | MELD | 2609 | 85 | F | 73 | 65 | 0.43 | 0.446854578918692 | 0.5 | 2.52 |  |  | 0 | 17 | 17 | 0 | 0 | 1 | 0 | 0 | 0 |  |  |  | 1 | 1 | 1 | 0 | 0 | 0 | 0 | 0 | 0 | 0 | 1 | 1 | 1 | 1 | 0 |
| P000002 | G0000086 | S0000285 | E001463 | MELD-Na | 2609 | 85 | F | 73 | 65 | 0.43 | 0.446854578918692 | 0.5 | 2.52 | 143 |  | 0 | 17 | 17 | 0 | 0 | 1 | 0 | 0 | 0 |  |  |  | 1 | 1 | 1 | 0 | 0 | 0 | 0 | 0 | 0 | 0 | 1 | 1 | 0 | 1 | 0 |
| P000002 | G0000086 | S0000286 | E001463 | reMELD-Na | 2609 | 85 | F | 73 | 65 | 0.43 | 0.446854578918692 | 0.5 | 2.52 | 143 |  |  | 12 | 12 | 0 | 0 | 1 | 0 | 0 | 0 |  |  |  | 1 | 1 | 1 | 0 | 0 | 0 | 0 | 0 | 0 | 0 | 1 | 1 | 0 | 1 | 0 |
| P000002 | G0000087 | S0000287 | E001463 | MELD | 2610 | 85 | F | 73 | 65 | 0.33 | 0.345183916659739 | 0.5 | 2.36 |  |  | 0 | 16 | 16 | 0 | 0 | 1 | 0 | 0 | 0 |  |  |  | 1 | 1 | 1 | 0 | 0 | 0 | 0 | 0 | 0 | 0 | 1 | 1 | 1 | 1 | 0 |
| P000002 | G0000087 | S0000288 | E001463 | MELD 3.0 | 2610 | 85 | F | 73 | 65 | 0.33 | 0.345183916659739 | 0.5 | 2.36 | 145 | 2.26 |  | 17 | 17 | 0 | 0 | 1 | 0 | 0 | 0 |  |  |  | 1 | 1 | 1 | 0 | 0 | 0 | 0 | 0 | 0 | 0 | 1 | 1 | 0 | 1 | 0 |
| P000002 | G0000087 | S0000289 | E001463 | MELD-Na | 2610 | 85 | F | 73 | 65 | 0.33 | 0.345183916659739 | 0.5 | 2.36 | 145 |  | 0 | 16 | 16 | 0 | 0 | 1 | 0 | 0 | 0 |  |  |  | 1 | 1 | 1 | 0 | 0 | 0 | 0 | 0 | 0 | 0 | 1 | 1 | 0 | 1 | 0 |
| P000002 | G0000087 | S0000290 | E001463 | reMELD-Na | 2610 | 85 | F | 73 | 65 | 0.33 | 0.345183916659739 | 0.5 | 2.36 | 145 |  |  | 11 | 11 | 0 | 0 | 1 | 0 | 0 | 0 |  |  |  | 1 | 1 | 1 | 0 | 0 | 0 | 0 | 0 | 0 | 0 | 1 | 1 | 0 | 1 | 0 |
| P000002 | G0000088 | S0000291 | E001463 | MELD | 2611 | 85 | F | 73 | 65 | 0.34 | 0.354202565551976 | 0.6 | 2.07 |  |  | 0 | 15 | 15 | 0 | 0 | 1 | 0 | 0 | 0 |  |  |  | 1 | 1 | 1 | 0 | 0 | 0 | 0 | 0 | 0 | 0 | 1 | 1 | 1 | 1 | 0 |
| P000002 | G0000088 | S0000292 | E001463 | MELD-Na | 2611 | 85 | F | 73 | 65 | 0.34 | 0.354202565551976 | 0.6 | 2.07 | 139 |  | 0 | 15 | 15 | 0 | 0 | 1 | 0 | 0 | 0 |  |  |  | 1 | 1 | 1 | 0 | 0 | 0 | 0 | 0 | 0 | 0 | 1 | 1 | 0 | 1 | 0 |
| P000002 | G0000088 | S0000293 | E001463 | reMELD-Na | 2611 | 85 | F | 73 | 65 | 0.34 | 0.354202565551976 | 0.6 | 2.07 | 139 |  |  | 10 | 10 | 0 | 0 | 1 | 0 | 0 | 0 |  |  |  | 1 | 1 | 1 | 0 | 0 | 0 | 0 | 0 | 0 | 0 | 1 | 1 | 0 | 1 | 0 |
| P000002 | G0000089 | S0000294 | E001463 | MELD | 2612 | 85 | F | 73 | 65 | 0.41 | 0.426567457375992 | 0.5 | 2.26 |  |  | 0 | 16 | 16 | 0 | 0 | 1 | 0 | 0 | 0 |  |  |  | 1 | 1 | 1 | 0 | 0 | 0 | 0 | 0 | 0 | 0 | 1 | 1 | 1 | 1 | 0 |
| P000002 | G0000089 | S0000295 | E001463 | MELD-Na | 2612 | 85 | F | 73 | 65 | 0.41 | 0.426567457375992 | 0.5 | 2.26 | 147 |  | 0 | 16 | 16 | 0 | 0 | 1 | 0 | 0 | 0 |  |  |  | 1 | 1 | 1 | 0 | 0 | 0 | 0 | 0 | 0 | 0 | 1 | 1 | 0 | 1 | 0 |
| P000002 | G0000089 | S0000296 | E001463 | reMELD-Na | 2612 | 85 | F | 73 | 65 | 0.41 | 0.426567457375992 | 0.5 | 2.26 | 147 |  |  | 11 | 11 | 0 | 0 | 1 | 0 | 0 | 0 |  |  |  | 1 | 1 | 1 | 0 | 0 | 0 | 0 | 0 | 0 | 0 | 1 | 1 | 0 | 1 | 0 |
| P000002 | G0000090 | S0000297 | E001463 | MELD | 2613 | 85 | F | 73 | 65 | 0.4 | 0.417594984289405 | 0.4 | 1.72 |  |  | 0 | 13 | 13 | 0 | 0 | 1 | 0 | 0 | 0 |  |  |  | 1 | 1 | 1 | 0 | 0 | 0 | 0 | 0 | 0 | 0 | 1 | 1 | 1 | 1 | 0 |
| P000002 | G0000090 | S0000298 | E001463 | MELD 3.0 | 2613 | 85 | F | 73 | 65 | 0.4 | 0.417594984289405 | 0.4 | 1.72 | 142 | 1.97 |  | 15 | 15 | 0 | 0 | 1 | 0 | 0 | 0 |  |  |  | 1 | 1 | 1 | 0 | 0 | 0 | 0 | 0 | 0 | 0 | 1 | 1 | 0 | 1 | 0 |
| P000002 | G0000090 | S0000299 | E001463 | MELD-Na | 2613 | 85 | F | 73 | 65 | 0.4 | 0.417594984289405 | 0.4 | 1.72 | 142 |  | 0 | 13 | 13 | 0 | 0 | 1 | 0 | 0 | 0 |  |  |  | 1 | 1 | 1 | 0 | 0 | 0 | 0 | 0 | 0 | 0 | 1 | 1 | 0 | 1 | 0 |
| P000002 | G0000090 | S0000300 | E001463 | reMELD-Na | 2613 | 85 | F | 73 | 65 | 0.4 | 0.417594984289405 | 0.4 | 1.72 | 142 |  |  | 7 | 7 | 0 | 0 | 1 | 0 | 0 | 0 |  |  |  | 1 | 1 | 1 | 0 | 0 | 0 | 0 | 0 | 0 | 0 | 1 | 1 | 0 | 1 | 0 |
| P000002 | G0000091 | S0000301 | E001463 | MELD | 2614 | 85 | F | 73 | 65 | 0.38 | 0.397272604564887 | 0.4 | 1.28 |  |  | 0 | 9 | 9 | 0 | 0 | 1 | 0 | 0 | 0 |  |  |  | 1 | 1 | 1 | 0 | 0 | 0 | 0 | 0 | 0 | 0 | 1 | 1 | 1 | 1 | 0 |
| P000002 | G0000091 | S0000302 | E001463 | MELD-Na | 2614 | 85 | F | 73 | 65 | 0.38 | 0.397272604564887 | 0.4 | 1.28 | 150 |  | 0 | 9 | 9 | 0 | 0 | 1 | 0 | 0 | 0 |  |  |  | 1 | 1 | 1 | 0 | 0 | 0 | 0 | 0 | 0 | 0 | 1 | 1 | 0 | 1 | 0 |
| P000002 | G0000091 | S0000303 | E001463 | reMELD-Na | 2614 | 85 | F | 73 | 65 | 0.38 | 0.397272604564887 | 0.4 | 1.28 | 150 |  |  | 5 | 5 | 0 | 0 | 1 | 0 | 0 | 0 |  |  |  | 1 | 1 | 1 | 0 | 0 | 0 | 0 | 0 | 0 | 0 | 1 | 1 | 0 | 1 | 0 |
| P000002 | G0000092 | S0000304 | E001463 | MELD | 2615 | 85 | F | 73 | 65 | 0.38 | 0.397272604564887 | 0.4 | 1.21 |  |  | 0 | 9 | 9 | 0 | 0 | 1 | 0 | 0 | 0 |  |  |  | 1 | 1 | 1 | 0 | 0 | 0 | 0 | 0 | 0 | 0 | 1 | 1 | 1 | 1 | 0 |
| P000002 | G0000092 | S0000305 | E001463 | MELD-Na | 2615 | 85 | F | 73 | 65 | 0.38 | 0.397272604564887 | 0.4 | 1.21 | 148 |  | 0 | 9 | 9 | 0 | 0 | 1 | 0 | 0 | 0 |  |  |  | 1 | 1 | 1 | 0 | 0 | 0 | 0 | 0 | 0 | 0 | 1 | 1 | 0 | 1 | 0 |
| P000002 | G0000092 | S0000306 | E001463 | reMELD-Na | 2615 | 85 | F | 73 | 65 | 0.38 | 0.397272604564887 | 0.4 | 1.21 | 148 |  |  | 4 | 4 | 0 | 0 | 1 | 0 | 0 | 0 |  |  |  | 1 | 1 | 1 | 0 | 0 | 0 | 0 | 0 | 0 | 0 | 1 | 1 | 0 | 1 | 0 |
| P000002 | G0000093 | S0000307 | E001463 | MELD | 2616 | 85 | F | 73 | 65 | 0.34 | 0.356557328752215 | 0.4 | 1.22 |  |  | 0 | 9 | 9 | 0 | 0 | 1 | 0 | 0 | 0 |  |  |  | 1 | 1 | 1 | 0 | 0 | 0 | 0 | 0 | 0 | 0 | 1 | 1 | 1 | 1 | 0 |
| P000002 | G0000093 | S0000308 | E001463 | MELD-Na | 2616 | 85 | F | 73 | 65 | 0.34 | 0.356557328752215 | 0.4 | 1.22 | 156 |  | 0 | 9 | 9 | 0 | 0 | 1 | 0 | 0 | 0 |  |  |  | 1 | 1 | 1 | 0 | 0 | 0 | 0 | 0 | 0 | 0 | 1 | 1 | 0 | 1 | 0 |
| P000002 | G0000093 | S0000309 | E001463 | reMELD-Na | 2616 | 85 | F | 73 | 65 | 0.34 | 0.356557328752215 | 0.4 | 1.22 | 156 |  |  | 4 | 4 | 0 | 0 | 1 | 0 | 0 | 0 |  |  |  | 1 | 1 | 1 | 0 | 0 | 0 | 0 | 0 | 0 | 0 | 1 | 1 | 0 | 1 | 0 |
| P000002 | G0000094 | S0000310 | E001463 | MELD | 2617 | 85 | F | 73 | 65 | 0.81 | 0.83020869758698 | 0.3 | 1.33 |  |  | 0 | 10 | 10 | 0 | 0 | 1 | 0 | 0 | 0 |  |  |  | 1 | 1 | 1 | 0 | 0 | 0 | 0 | 0 | 0 | 0 | 1 | 1 | 1 | 1 | 0 |
| P000002 | G0000094 | S0000311 | E001463 | MELD 3.0 | 2617 | 85 | F | 73 | 65 | 0.81 | 0.83020869758698 | 0.3 | 1.33 | 146 | 1.71 |  | 13 | 13 | 0 | 0 | 1 | 0 | 0 | 0 |  |  |  | 1 | 1 | 1 | 0 | 0 | 0 | 0 | 0 | 0 | 0 | 1 | 1 | 0 | 1 | 0 |
| P000002 | G0000094 | S0000312 | E001463 | MELD-Na | 2617 | 85 | F | 73 | 65 | 0.81 | 0.83020869758698 | 0.3 | 1.33 | 146 |  | 0 | 10 | 10 | 0 | 0 | 1 | 0 | 0 | 0 |  |  |  | 1 | 1 | 1 | 0 | 0 | 0 | 0 | 0 | 0 | 0 | 1 | 1 | 0 | 1 | 0 |
| P000002 | G0000094 | S0000313 | E001463 | reMELD-Na | 2617 | 85 | F | 73 | 65 | 0.81 | 0.83020869758698 | 0.3 | 1.33 | 146 |  |  | 5 | 6 | 1 | 0 | 0 | 1 | 0 | 0 |  |  |  | 1 | 1 | 1 | 0 | 0 | 0 | 0 | 0 | 0 | 0 | 1 | 1 | 0 | 1 | 0 |
| P000002 | G0000095 | S0000314 | E001463 | MELD | 2618 | 86 | F | 73 | 65 | 1.39 | 1.39535884414345 | 0.3 | 1.49 |  |  | 0 | 14 | 14 | 0 | 0 | 1 | 0 | 0 | 0 |  |  |  | 1 | 1 | 1 | 0 | 0 | 0 | 0 | 0 | 0 | 0 | 1 | 1 | 1 | 1 | 0 |
| P000002 | G0000095 | S0000315 | E001463 | MELD-Na | 2618 | 86 | F | 73 | 65 | 1.39 | 1.39535884414345 | 0.3 | 1.49 | 145 |  | 0 | 14 | 14 | 0 | 0 | 1 | 0 | 0 | 0 |  |  |  | 1 | 1 | 1 | 0 | 0 | 0 | 0 | 0 | 0 | 0 | 1 | 1 | 0 | 1 | 0 |
| P000002 | G0000095 | S0000316 | E001463 | reMELD-Na | 2618 | 86 | F | 73 | 65 | 1.39 | 1.39535884414345 | 0.3 | 1.49 | 145 |  |  | 11 | 11 | 0 | 0 | 1 | 0 | 0 | 0 |  |  |  | 1 | 1 | 1 | 0 | 0 | 0 | 0 | 0 | 0 | 0 | 1 | 1 | 0 | 1 | 0 |
| P000002 | G0000096 | S0000317 | E001463 | MELD | 2619 | 86 | F | 73 | 65 | 1.64 | 1.63167637448224 | 0.4 | 1.72 |  |  | 1 | 26 | 26 | 0 | 0 | 1 | 0 | 0 | 0 |  |  |  | 1 | 1 | 1 | 0 | 0 | 0 | 0 | 0 | 0 | 0 | 1 | 1 | 1 | 1 | 0 |
| P000002 | G0000096 | S0000318 | E001463 | MELD-Na | 2619 | 86 | F | 73 | 65 | 1.64 | 1.63167637448224 | 0.4 | 1.72 | 148 |  | 1 | 26 | 26 | 0 | 0 | 1 | 0 | 0 | 0 |  |  |  | 1 | 1 | 1 | 0 | 0 | 0 | 0 | 0 | 0 | 0 | 1 | 1 | 0 | 1 | 0 |
| P000002 | G0000096 | S0000319 | E001463 | reMELD-Na | 2619 | 86 | F | 73 | 65 | 1.64 | 1.63167637448224 | 0.4 | 1.72 | 148 |  |  | 15 | 15 | 0 | 0 | 1 | 0 | 0 | 0 |  |  |  | 1 | 1 | 1 | 0 | 0 | 0 | 0 | 0 | 0 | 0 | 1 | 1 | 0 | 1 | 0 |
| P000002 | G0000097 | S0000320 | E001463 | MELD | 2620 | 86 | F | 73 | 65 | 1.04 | 1.05550025547398 | 0.4 | 1.47 |  |  | 0 | 11 | 11 | 0 | 0 | 1 | 0 | 0 | 0 |  |  |  | 1 | 1 | 1 | 0 | 0 | 0 | 0 | 0 | 0 | 0 | 1 | 1 | 1 | 1 | 0 |
| P000002 | G0000097 | S0000321 | E001463 | MELD 3.0 | 2620 | 86 | F | 73 | 65 | 1.04 | 1.05550025547398 | 0.4 | 1.47 | 146 | 1.78 |  | 14 | 14 | 0 | 0 | 1 | 0 | 0 | 0 |  |  |  | 1 | 1 | 1 | 0 | 0 | 0 | 0 | 0 | 0 | 0 | 1 | 1 | 0 | 1 | 0 |
| P000002 | G0000097 | S0000322 | E001463 | MELD-Na | 2620 | 86 | F | 73 | 65 | 1.04 | 1.05550025547398 | 0.4 | 1.47 | 146 |  | 0 | 11 | 11 | 0 | 0 | 1 | 0 | 0 | 0 |  |  |  | 1 | 1 | 1 | 0 | 0 | 0 | 0 | 0 | 0 | 0 | 1 | 1 | 0 | 1 | 0 |
| P000002 | G0000097 | S0000323 | E001463 | reMELD-Na | 2620 | 86 | F | 73 | 65 | 1.04 | 1.05550025547398 | 0.4 | 1.47 | 146 |  |  | 9 | 10 | 1 | 0 | 0 | 1 | 0 | 0 |  |  |  | 1 | 1 | 1 | 0 | 0 | 0 | 0 | 0 | 0 | 0 | 1 | 1 | 0 | 1 | 0 |
| P000002 | G0000098 | S0000324 | E001463 | MELD | 2621 | 86 | F | 73 | 65 | 0.98 | 0.997904067063564 | 0.3 | 1.22 |  |  | 0 | 9 | 9 | 0 | 0 | 1 | 0 | 0 | 0 |  |  |  | 1 | 1 | 1 | 0 | 0 | 0 | 0 | 0 | 0 | 0 | 1 | 1 | 1 | 1 | 0 |
| P000002 | G0000098 | S0000325 | E001463 | MELD-Na | 2621 | 86 | F | 73 | 65 | 0.98 | 0.997904067063564 | 0.3 | 1.22 | 147 |  | 0 | 9 | 9 | 0 | 0 | 1 | 0 | 0 | 0 |  |  |  | 1 | 1 | 1 | 0 | 0 | 0 | 0 | 0 | 0 | 0 | 1 | 1 | 0 | 1 | 0 |
| P000002 | G0000098 | S0000326 | E001463 | reMELD-Na | 2621 | 86 | F | 73 | 65 | 0.98 | 0.997904067063564 | 0.3 | 1.22 | 147 |  |  | 6 | 6 | 0 | 0 | 1 | 0 | 0 | 0 |  |  |  | 1 | 1 | 1 | 0 | 0 | 0 | 0 | 0 | 0 | 0 | 1 | 1 | 0 | 1 | 0 |
| P000002 | G0000099 | S0000327 | E001463 | MELD | 2622 | 86 | F | 73 | 65 | 0.485 | 0.503702865391333 | 0.4 | 1.105 |  |  | 0 | 8 | 8 | 0 | 0 | 1 | 0 | 0 | 0 |  |  |  | 1 | 1 | 1 | 0 | 0 | 0 | 0 | 0 | 0 | 0 | 1 | 1 | 1 | 1 | 0 |
| P000002 | G0000099 | S0000328 | E001463 | MELD-Na | 2622 | 86 | F | 73 | 65 | 0.485 | 0.503702865391333 | 0.4 | 1.105 | 145 |  | 0 | 8 | 8 | 0 | 0 | 1 | 0 | 0 | 0 |  |  |  | 1 | 1 | 1 | 0 | 0 | 0 | 0 | 0 | 0 | 0 | 1 | 1 | 0 | 1 | 0 |
| P000002 | G0000099 | S0000329 | E001463 | reMELD-Na | 2622 | 86 | F | 73 | 65 | 0.485 | 0.503702865391333 | 0.4 | 1.105 | 145 |  |  | 3 | 3 | 0 | 0 | 1 | 0 | 0 | 0 |  |  |  | 1 | 1 | 1 | 0 | 0 | 0 | 0 | 0 | 0 | 0 | 1 | 1 | 0 | 1 | 0 |
| P000002 | G0000100 | S0000330 | E001463 | MELD | 2623 | 86 | F | 73 | 65 | 0.4 | 0.416415082059188 | 0.5 | 1.62 |  |  | 0 | 12 | 12 | 0 | 0 | 1 | 0 | 0 | 0 |  |  |  | 1 | 1 | 1 | 0 | 0 | 0 | 0 | 0 | 0 | 0 | 1 | 1 | 1 | 1 | 0 |
| P000002 | G0000100 | S0000331 | E001463 | MELD-Na | 2623 | 86 | F | 73 | 65 | 0.4 | 0.416415082059188 | 0.5 | 1.62 | 145 |  | 0 | 12 | 12 | 0 | 0 | 1 | 0 | 0 | 0 |  |  |  | 1 | 1 | 1 | 0 | 0 | 0 | 0 | 0 | 0 | 0 | 1 | 1 | 0 | 1 | 0 |
| P000002 | G0000100 | S0000332 | E001463 | reMELD-Na | 2623 | 86 | F | 73 | 65 | 0.4 | 0.416415082059188 | 0.5 | 1.62 | 145 |  |  | 7 | 7 | 0 | 0 | 1 | 0 | 0 | 0 |  |  |  | 1 | 1 | 1 | 0 | 0 | 0 | 0 | 0 | 0 | 0 | 1 | 1 | 0 | 1 | 0 |
| P000002 | G0000101 | S0000333 | E001463 | MELD | 2624 | 86 | F | 73 | 65 | 0.41 | 0.42774735960621 | 0.4 | 1.51 |  |  | 0 | 11 | 11 | 0 | 0 | 1 | 0 | 0 | 0 |  |  |  | 1 | 1 | 1 | 0 | 0 | 0 | 0 | 0 | 0 | 0 | 1 | 1 | 1 | 1 | 0 |
| P000002 | G0000101 | S0000334 | E001463 | MELD 3.0 | 2624 | 86 | F | 73 | 65 | 0.41 | 0.42774735960621 | 0.4 | 1.51 | 144 | 1.83 |  | 14 | 14 | 0 | 0 | 1 | 0 | 0 | 0 |  |  |  | 1 | 1 | 1 | 0 | 0 | 0 | 0 | 0 | 0 | 0 | 1 | 1 | 0 | 1 | 0 |
| P000002 | G0000101 | S0000335 | E001463 | MELD-Na | 2624 | 86 | F | 73 | 65 | 0.41 | 0.42774735960621 | 0.4 | 1.51 | 144 |  | 0 | 11 | 11 | 0 | 0 | 1 | 0 | 0 | 0 |  |  |  | 1 | 1 | 1 | 0 | 0 | 0 | 0 | 0 | 0 | 0 | 1 | 1 | 0 | 1 | 0 |
| P000002 | G0000101 | S0000336 | E001463 | reMELD-Na | 2624 | 86 | F | 73 | 65 | 0.41 | 0.42774735960621 | 0.4 | 1.51 | 144 |  |  | 6 | 6 | 0 | 0 | 1 | 0 | 0 | 0 |  |  |  | 1 | 1 | 1 | 0 | 0 | 0 | 0 | 0 | 0 | 0 | 1 | 1 | 0 | 1 | 0 |
| P000002 | G0000102 | S0000337 | E001463 | MELD | 2625 | 86 | F | 73 | 65 | 0.33 | 0.347548762380368 | 0.3 | 1.55 |  |  | 0 | 11 | 11 | 0 | 0 | 1 | 0 | 0 | 0 |  |  |  | 1 | 1 | 1 | 0 | 0 | 0 | 0 | 0 | 0 | 0 | 1 | 1 | 1 | 1 | 0 |
| P000002 | G0000102 | S0000338 | E001463 | MELD-Na | 2625 | 86 | F | 73 | 65 | 0.33 | 0.347548762380368 | 0.3 | 1.55 | 150 |  | 0 | 11 | 11 | 0 | 0 | 1 | 0 | 0 | 0 |  |  |  | 1 | 1 | 1 | 0 | 0 | 0 | 0 | 0 | 0 | 0 | 1 | 1 | 0 | 1 | 0 |
| P000002 | G0000102 | S0000339 | E001463 | reMELD-Na | 2625 | 86 | F | 73 | 65 | 0.33 | 0.347548762380368 | 0.3 | 1.55 | 150 |  |  | 6 | 6 | 0 | 0 | 1 | 0 | 0 | 0 |  |  |  | 1 | 1 | 1 | 0 | 0 | 0 | 0 | 0 | 0 | 0 | 1 | 1 | 0 | 1 | 0 |
| P000002 | G0000103 | S0000340 | E001463 | MELD | 2626 | 86 | F | 73 | 65 | 0.31 | 0.327144113564941 | 0.3 | 1.5 |  |  | 0 | 11 | 11 | 0 | 0 | 1 | 0 | 0 | 0 |  |  |  | 1 | 1 | 1 | 0 | 0 | 0 | 0 | 0 | 0 | 0 | 1 | 1 | 1 | 1 | 0 |
| P000002 | G0000103 | S0000341 | E001463 | MELD-Na | 2626 | 86 | F | 73 | 65 | 0.31 | 0.327144113564941 | 0.3 | 1.5 | 146 |  | 0 | 11 | 11 | 0 | 0 | 1 | 0 | 0 | 0 |  |  |  | 1 | 1 | 1 | 0 | 0 | 0 | 0 | 0 | 0 | 0 | 1 | 1 | 0 | 1 | 0 |
| P000002 | G0000103 | S0000342 | E001463 | reMELD-Na | 2626 | 86 | F | 73 | 65 | 0.31 | 0.327144113564941 | 0.3 | 1.5 | 146 |  |  | 5 | 5 | 0 | 0 | 1 | 0 | 0 | 0 |  |  |  | 1 | 1 | 1 | 0 | 0 | 0 | 0 | 0 | 0 | 0 | 1 | 1 | 0 | 1 | 0 |
| P000002 | G0000104 | S0000343 | E001463 | MELD | 2627 | 86 | F | 73 | 65 | 0.38 | 0.399647532805907 | 0.2 | 1.9 |  |  | 0 | 14 | 14 | 0 | 0 | 1 | 0 | 0 | 0 |  |  |  | 1 | 1 | 1 | 0 | 0 | 0 | 0 | 0 | 0 | 0 | 1 | 1 | 1 | 1 | 0 |
| P000002 | G0000104 | S0000344 | E001463 | MELD 3.0 | 2627 | 86 | F | 73 | 65 | 0.38 | 0.399647532805907 | 0.2 | 1.9 | 146 | 1.56 |  | 17 | 17 | 0 | 0 | 1 | 0 | 0 | 0 |  |  |  | 1 | 1 | 1 | 0 | 0 | 0 | 0 | 0 | 0 | 0 | 1 | 1 | 0 | 1 | 0 |
| P000002 | G0000104 | S0000345 | E001463 | MELD-Na | 2627 | 86 | F | 73 | 65 | 0.38 | 0.399647532805907 | 0.2 | 1.9 | 146 |  | 0 | 14 | 14 | 0 | 0 | 1 | 0 | 0 | 0 |  |  |  | 1 | 1 | 1 | 0 | 0 | 0 | 0 | 0 | 0 | 0 | 1 | 1 | 0 | 1 | 0 |
| P000002 | G0000104 | S0000346 | E001463 | reMELD-Na | 2627 | 86 | F | 73 | 65 | 0.38 | 0.399647532805907 | 0.2 | 1.9 | 146 |  |  | 7 | 7 | 0 | 0 | 1 | 0 | 0 | 0 |  |  |  | 1 | 1 | 1 | 0 | 0 | 0 | 0 | 0 | 0 | 0 | 1 | 1 | 0 | 1 | 0 |
| P000002 | G0000105 | S0000347 | E001463 | MELD | 2628 | 86 | F | 73 | 65 | 0.35 | 0.366744962250838 | 0.4 | 1.5 |  |  | 0 | 11 | 11 | 0 | 0 | 1 | 0 | 0 | 0 |  |  |  | 1 | 1 | 1 | 0 | 0 | 0 | 0 | 0 | 0 | 0 | 1 | 1 | 1 | 1 | 0 |
| P000002 | G0000105 | S0000348 | E001463 | MELD-Na | 2628 | 86 | F | 73 | 65 | 0.35 | 0.366744962250838 | 0.4 | 1.5 | 143 |  | 0 | 11 | 11 | 0 | 0 | 1 | 0 | 0 | 0 |  |  |  | 1 | 1 | 1 | 0 | 0 | 0 | 0 | 0 | 0 | 0 | 1 | 1 | 0 | 1 | 0 |
| P000002 | G0000105 | S0000349 | E001463 | reMELD-Na | 2628 | 86 | F | 73 | 65 | 0.35 | 0.366744962250838 | 0.4 | 1.5 | 143 |  |  | 6 | 6 | 0 | 0 | 1 | 0 | 0 | 0 |  |  |  | 1 | 1 | 1 | 0 | 0 | 0 | 0 | 0 | 0 | 0 | 1 | 1 | 0 | 1 | 0 |
| P000002 | G0000106 | S0000350 | E001463 | MELD | 2629 | 86 | F | 73 | 65 | 0.4 | 0.417594984289405 | 0.4 | 1.53 |  |  | 0 | 11 | 11 | 0 | 0 | 1 | 0 | 0 | 0 |  |  |  | 1 | 1 | 1 | 0 | 0 | 0 | 0 | 0 | 0 | 0 | 1 | 1 | 1 | 1 | 0 |
| P000002 | G0000106 | S0000351 | E001463 | MELD-Na | 2629 | 86 | F | 73 | 65 | 0.4 | 0.417594984289405 | 0.4 | 1.53 | 143 |  | 0 | 11 | 11 | 0 | 0 | 1 | 0 | 0 | 0 |  |  |  | 1 | 1 | 1 | 0 | 0 | 0 | 0 | 0 | 0 | 0 | 1 | 1 | 0 | 1 | 0 |
| P000002 | G0000106 | S0000352 | E001463 | reMELD-Na | 2629 | 86 | F | 73 | 65 | 0.4 | 0.417594984289405 | 0.4 | 1.53 | 143 |  |  | 6 | 6 | 0 | 0 | 1 | 0 | 0 | 0 |  |  |  | 1 | 1 | 1 | 0 | 0 | 0 | 0 | 0 | 0 | 0 | 1 | 1 | 0 | 1 | 0 |
| P000002 | G0000107 | S0000353 | E001463 | MELD | 2630 | 86 | F | 73 | 65 | 0.42 | 0.43907880204979 | 0.3 | 1.59 |  |  | 0 | 12 | 12 | 0 | 0 | 1 | 0 | 0 | 0 |  |  |  | 1 | 1 | 1 | 0 | 0 | 0 | 0 | 0 | 0 | 0 | 1 | 1 | 1 | 1 | 0 |
| P000002 | G0000107 | S0000354 | E001463 | MELD-Na | 2630 | 86 | F | 73 | 65 | 0.42 | 0.43907880204979 | 0.3 | 1.59 | 141 |  | 0 | 12 | 12 | 0 | 0 | 1 | 0 | 0 | 0 |  |  |  | 1 | 1 | 1 | 0 | 0 | 0 | 0 | 0 | 0 | 0 | 1 | 1 | 0 | 1 | 0 |
| P000002 | G0000107 | S0000355 | E001463 | reMELD-Na | 2630 | 86 | F | 73 | 65 | 0.42 | 0.43907880204979 | 0.3 | 1.59 | 141 |  |  | 6 | 6 | 0 | 0 | 1 | 0 | 0 | 0 |  |  |  | 1 | 1 | 1 | 0 | 0 | 0 | 0 | 0 | 0 | 0 | 1 | 1 | 0 | 1 | 0 |
| P000002 | G0000108 | S0000356 | E001463 | MELD | 2631 | 86 | F | 73 | 65 | 0.43 | 0.449219424639322 | 0.3 | 1.77 |  |  | 0 | 13 | 13 | 0 | 0 | 1 | 0 | 0 | 0 |  |  |  | 1 | 1 | 1 | 0 | 0 | 0 | 0 | 0 | 0 | 0 | 1 | 1 | 1 | 1 | 0 |
| P000002 | G0000108 | S0000357 | E001463 | MELD 3.0 | 2631 | 86 | F | 73 | 65 | 0.43 | 0.449219424639322 | 0.3 | 1.77 | 136 | 2.14 |  | 16 | 16 | 0 | 0 | 1 | 0 | 0 | 0 |  |  |  | 1 | 1 | 1 | 0 | 0 | 0 | 0 | 0 | 0 | 0 | 1 | 1 | 0 | 1 | 0 |
| P000002 | G0000108 | S0000358 | E001463 | MELD-Na | 2631 | 86 | F | 73 | 65 | 0.43 | 0.449219424639322 | 0.3 | 1.77 | 136 |  | 0 | 14 | 14 | 0 | 0 | 1 | 0 | 0 | 0 |  |  |  | 1 | 1 | 1 | 0 | 0 | 0 | 0 | 0 | 0 | 0 | 1 | 1 | 0 | 1 | 0 |
| P000002 | G0000108 | S0000359 | E001463 | reMELD-Na | 2631 | 86 | F | 73 | 65 | 0.43 | 0.449219424639322 | 0.3 | 1.77 | 136 |  |  | 8 | 8 | 0 | 0 | 1 | 0 | 0 | 0 |  |  |  | 1 | 1 | 1 | 0 | 0 | 0 | 0 | 0 | 0 | 0 | 1 | 1 | 0 | 1 | 0 |
| P000002 | G0000109 | S0000360 | E001463 | MELD | 2632 | 86 | F | 73 | 65 | 0.46 | 0.478421090735686 | 0.4 | 2.14 |  |  | 0 | 15 | 15 | 0 | 0 | 1 | 0 | 0 | 0 |  |  |  | 1 | 1 | 1 | 0 | 0 | 0 | 0 | 0 | 0 | 0 | 1 | 1 | 1 | 1 | 0 |
| P000002 | G0000109 | S0000361 | E001463 | MELD-Na | 2632 | 86 | F | 73 | 65 | 0.46 | 0.478421090735686 | 0.4 | 2.14 | 139 |  | 0 | 15 | 15 | 0 | 0 | 1 | 0 | 0 | 0 |  |  |  | 1 | 1 | 1 | 0 | 0 | 0 | 0 | 0 | 0 | 0 | 1 | 1 | 0 | 1 | 0 |
| P000002 | G0000109 | S0000362 | E001463 | reMELD-Na | 2632 | 86 | F | 73 | 65 | 0.46 | 0.478421090735686 | 0.4 | 2.14 | 139 |  |  | 9 | 9 | 0 | 0 | 1 | 0 | 0 | 0 |  |  |  | 1 | 1 | 1 | 0 | 0 | 0 | 0 | 0 | 0 | 0 | 1 | 1 | 0 | 1 | 0 |
| P000002 | G0000110 | S0000363 | E001463 | MELD | 2633 | 86 | F | 73 | 65 | 0.76 | 0.774689025243759 | 0.8 | 2.26 |  |  | 0 | 16 | 16 | 0 | 0 | 1 | 0 | 0 | 0 |  |  |  | 1 | 1 | 1 | 0 | 0 | 0 | 0 | 0 | 0 | 0 | 1 | 1 | 1 | 1 | 0 |
| P000002 | G0000110 | S0000364 | E001463 | MELD-Na | 2633 | 86 | F | 73 | 65 | 0.76 | 0.774689025243759 | 0.8 | 2.26 | 143 |  | 0 | 16 | 16 | 0 | 0 | 1 | 0 | 0 | 0 |  |  |  | 1 | 1 | 1 | 0 | 0 | 0 | 0 | 0 | 0 | 0 | 1 | 1 | 0 | 1 | 0 |
| P000002 | G0000110 | S0000365 | E001463 | reMELD-Na | 2633 | 86 | F | 73 | 65 | 0.76 | 0.774689025243759 | 0.8 | 2.26 | 143 |  |  | 13 | 13 | 0 | 0 | 1 | 0 | 0 | 0 |  |  |  | 1 | 1 | 1 | 0 | 0 | 0 | 0 | 0 | 0 | 0 | 1 | 1 | 0 | 1 | 0 |
| P000002 | G0000111 | S0000366 | E001463 | MELD | 2634 | 86 | F | 73 | 65 | 0.59 | 0.605960674216805 | 0.7 | 1.665 |  |  | 0 | 12 | 12 | 0 | 0 | 1 | 0 | 0 | 0 |  |  |  | 1 | 1 | 1 | 0 | 0 | 0 | 0 | 0 | 0 | 0 | 1 | 1 | 1 | 1 | 0 |
| P000002 | G0000111 | S0000367 | E001463 | MELD 3.0 | 2634 | 86 | F | 73 | 65 | 0.59 | 0.605960674216805 | 0.7 | 1.665 | 140 | 3 |  | 13 | 13 | 0 | 0 | 1 | 0 | 0 | 0 |  |  |  | 1 | 1 | 1 | 0 | 0 | 0 | 0 | 0 | 0 | 0 | 1 | 1 | 0 | 1 | 0 |
| P000002 | G0000111 | S0000368 | E001463 | MELD-Na | 2634 | 86 | F | 73 | 65 | 0.59 | 0.605960674216805 | 0.7 | 1.665 | 140 |  | 0 | 12 | 12 | 0 | 0 | 1 | 0 | 0 | 0 |  |  |  | 1 | 1 | 1 | 0 | 0 | 0 | 0 | 0 | 0 | 0 | 1 | 1 | 0 | 1 | 0 |
| P000002 | G0000111 | S0000369 | E001463 | reMELD-Na | 2634 | 86 | F | 73 | 65 | 0.59 | 0.605960674216805 | 0.7 | 1.665 | 140 |  |  | 9 | 9 | 0 | 0 | 1 | 0 | 0 | 0 |  |  |  | 1 | 1 | 1 | 0 | 0 | 0 | 0 | 0 | 0 | 0 | 1 | 1 | 0 | 1 | 0 |
| P000002 | G0000112 | S0000370 | E001463 | MELD | 2635 | 86 | F | 73 | 65 | 0.64 | 0.658450213298857 | 0.5 | 2.29 |  |  | 0 | 16 | 16 | 0 | 0 | 1 | 0 | 0 | 0 |  |  |  | 1 | 1 | 1 | 0 | 0 | 0 | 0 | 0 | 0 | 0 | 1 | 1 | 1 | 1 | 0 |
| P000002 | G0000112 | S0000371 | E001463 | MELD-Na | 2635 | 86 | F | 73 | 65 | 0.64 | 0.658450213298857 | 0.5 | 2.29 | 146 |  | 0 | 16 | 16 | 0 | 0 | 1 | 0 | 0 | 0 |  |  |  | 1 | 1 | 1 | 0 | 0 | 0 | 0 | 0 | 0 | 0 | 1 | 1 | 0 | 1 | 0 |
| P000002 | G0000112 | S0000372 | E001463 | reMELD-Na | 2635 | 86 | F | 73 | 65 | 0.64 | 0.658450213298857 | 0.5 | 2.29 | 146 |  |  | 11 | 11 | 0 | 0 | 1 | 0 | 0 | 0 |  |  |  | 1 | 1 | 1 | 0 | 0 | 0 | 0 | 0 | 0 | 0 | 1 | 1 | 0 | 1 | 0 |
| P000002 | G0000113 | S0000373 | E001463 | MELD | 2636 | 86 | F | 73 | 65 | 0.51 | 0.530132856264666 | 0.3 | 1.98 |  |  | 0 | 14 | 14 | 0 | 0 | 1 | 0 | 0 | 0 |  |  |  | 1 | 1 | 1 | 0 | 0 | 0 | 0 | 0 | 0 | 0 | 1 | 1 | 1 | 1 | 0 |
| P000002 | G0000113 | S0000374 | E001463 | MELD-Na | 2636 | 86 | F | 73 | 65 | 0.51 | 0.530132856264666 | 0.3 | 1.98 | 145 |  | 0 | 14 | 14 | 0 | 0 | 1 | 0 | 0 | 0 |  |  |  | 1 | 1 | 1 | 0 | 0 | 0 | 0 | 0 | 0 | 0 | 1 | 1 | 0 | 1 | 0 |
| P000002 | G0000113 | S0000375 | E001463 | reMELD-Na | 2636 | 86 | F | 73 | 65 | 0.51 | 0.530132856264666 | 0.3 | 1.98 | 145 |  |  | 8 | 8 | 0 | 0 | 1 | 0 | 0 | 0 |  |  |  | 1 | 1 | 1 | 0 | 0 | 0 | 0 | 0 | 0 | 0 | 1 | 1 | 0 | 1 | 0 |
| P000002 | G0000114 | S0000376 | E001463 | MELD | 2637 | 86 | F | 73 | 65 | 0.52 | 0.539035648091058 | 0.4 | 1.62 |  |  | 0 | 12 | 12 | 0 | 0 | 1 | 0 | 0 | 0 |  |  |  | 1 | 1 | 1 | 0 | 0 | 0 | 0 | 0 | 0 | 0 | 1 | 1 | 1 | 1 | 0 |
| P000002 | G0000114 | S0000377 | E001463 | MELD-Na | 2637 | 86 | F | 73 | 65 | 0.52 | 0.539035648091058 | 0.4 | 1.62 | 143 |  | 0 | 12 | 12 | 0 | 0 | 1 | 0 | 0 | 0 |  |  |  | 1 | 1 | 1 | 0 | 0 | 0 | 0 | 0 | 0 | 0 | 1 | 1 | 0 | 1 | 0 |
| P000002 | G0000114 | S0000378 | E001463 | reMELD-Na | 2637 | 86 | F | 73 | 65 | 0.52 | 0.539035648091058 | 0.4 | 1.62 | 143 |  |  | 7 | 7 | 0 | 0 | 1 | 0 | 0 | 0 |  |  |  | 1 | 1 | 1 | 0 | 0 | 0 | 0 | 0 | 0 | 0 | 1 | 1 | 0 | 1 | 0 |
| P000002 | G0000115 | S0000379 | E001463 | MELD | 2638 | 86 | F | 73 | 65 | 0.39 | 0.408621676099377 | 0.3 | 1.69 |  |  | 0 | 12 | 12 | 0 | 0 | 1 | 0 | 0 | 0 |  |  |  | 1 | 1 | 1 | 0 | 0 | 0 | 0 | 0 | 0 | 0 | 1 | 1 | 1 | 1 | 0 |
| P000002 | G0000115 | S0000380 | E001463 | MELD 3.0 | 2638 | 86 | F | 73 | 65 | 0.39 | 0.408621676099377 | 0.3 | 1.69 | 143 | 2.39 |  | 14 | 14 | 0 | 0 | 1 | 0 | 0 | 0 |  |  |  | 1 | 1 | 1 | 0 | 0 | 0 | 0 | 0 | 0 | 0 | 1 | 1 | 0 | 1 | 0 |
| P000002 | G0000115 | S0000381 | E001463 | MELD-Na | 2638 | 86 | F | 73 | 65 | 0.39 | 0.408621676099377 | 0.3 | 1.69 | 143 |  | 0 | 12 | 12 | 0 | 0 | 1 | 0 | 0 | 0 |  |  |  | 1 | 1 | 1 | 0 | 0 | 0 | 0 | 0 | 0 | 0 | 1 | 1 | 0 | 1 | 0 |
| P000002 | G0000115 | S0000382 | E001463 | reMELD-Na | 2638 | 86 | F | 73 | 65 | 0.39 | 0.408621676099377 | 0.3 | 1.69 | 143 |  |  | 6 | 6 | 0 | 0 | 1 | 0 | 0 | 0 |  |  |  | 1 | 1 | 1 | 0 | 0 | 0 | 0 | 0 | 0 | 0 | 1 | 1 | 0 | 1 | 0 |
| P000002 | G0000116 | S0000383 | E001463 | MELD | 2639 | 86 | F | 73 | 65 | 0.4 | 0.418779927779817 | 0.3 | 1.94 |  |  | 0 | 14 | 14 | 0 | 0 | 1 | 0 | 0 | 0 |  |  |  | 1 | 1 | 1 | 0 | 0 | 0 | 0 | 0 | 0 | 0 | 1 | 1 | 1 | 1 | 0 |
| P000002 | G0000116 | S0000384 | E001463 | MELD-Na | 2639 | 86 | F | 73 | 65 | 0.4 | 0.418779927779817 | 0.3 | 1.94 | 145 |  | 0 | 14 | 14 | 0 | 0 | 1 | 0 | 0 | 0 |  |  |  | 1 | 1 | 1 | 0 | 0 | 0 | 0 | 0 | 0 | 0 | 1 | 1 | 0 | 1 | 0 |
| P000002 | G0000116 | S0000385 | E001463 | reMELD-Na | 2639 | 86 | F | 73 | 65 | 0.4 | 0.418779927779817 | 0.3 | 1.94 | 145 |  |  | 8 | 8 | 0 | 0 | 1 | 0 | 0 | 0 |  |  |  | 1 | 1 | 1 | 0 | 0 | 0 | 0 | 0 | 0 | 0 | 1 | 1 | 0 | 1 | 0 |
| P000002 | G0000117 | S0000386 | E001463 | MELD | 2640 | 86 | F | 73 | 65 | 0.39 | 0.409811660849985 | 0.2 | 1.77 |  |  | 0 | 13 | 13 | 0 | 0 | 1 | 0 | 0 | 0 |  |  |  | 1 | 1 | 1 | 0 | 0 | 0 | 0 | 0 | 0 | 0 | 1 | 1 | 1 | 1 | 0 |
| P000002 | G0000117 | S0000387 | E001463 | MELD-Na | 2640 | 86 | F | 73 | 65 | 0.39 | 0.409811660849985 | 0.2 | 1.77 | 148 |  | 0 | 13 | 13 | 0 | 0 | 1 | 0 | 0 | 0 |  |  |  | 1 | 1 | 1 | 0 | 0 | 0 | 0 | 0 | 0 | 0 | 1 | 1 | 0 | 1 | 0 |
| P000002 | G0000117 | S0000388 | E001463 | reMELD-Na | 2640 | 86 | F | 73 | 65 | 0.39 | 0.409811660849985 | 0.2 | 1.77 | 148 |  |  | 7 | 7 | 0 | 0 | 1 | 0 | 0 | 0 |  |  |  | 1 | 1 | 1 | 0 | 0 | 0 | 0 | 0 | 0 | 0 | 1 | 1 | 0 | 1 | 0 |
| P000002 | G0000118 | S0000389 | E001463 | MELD | 2641 | 86 | F | 73 | 65 | 0.37 | 0.388287543647586 | 0.3 | 1.24 |  |  | 0 | 9 | 9 | 0 | 0 | 1 | 0 | 0 | 0 |  |  |  | 1 | 1 | 1 | 0 | 0 | 0 | 0 | 0 | 0 | 0 | 1 | 1 | 1 | 1 | 0 |
| P000002 | G0000118 | S0000390 | E001463 | MELD 3.0 | 2641 | 86 | F | 73 | 65 | 0.37 | 0.388287543647586 | 0.3 | 1.24 | 147 | 2.43 |  | 11 | 11 | 0 | 0 | 1 | 0 | 0 | 0 |  |  |  | 1 | 1 | 1 | 0 | 0 | 0 | 0 | 0 | 0 | 0 | 1 | 1 | 0 | 1 | 0 |
| P000002 | G0000118 | S0000391 | E001463 | MELD-Na | 2641 | 86 | F | 73 | 65 | 0.37 | 0.388287543647586 | 0.3 | 1.24 | 147 |  | 0 | 9 | 9 | 0 | 0 | 1 | 0 | 0 | 0 |  |  |  | 1 | 1 | 1 | 0 | 0 | 0 | 0 | 0 | 0 | 0 | 1 | 1 | 0 | 1 | 0 |
| P000002 | G0000118 | S0000392 | E001463 | reMELD-Na | 2641 | 86 | F | 73 | 65 | 0.37 | 0.388287543647586 | 0.3 | 1.24 | 147 |  |  | 3 | 3 | 0 | 0 | 1 | 0 | 0 | 0 |  |  |  | 1 | 1 | 1 | 0 | 0 | 0 | 0 | 0 | 0 | 0 | 1 | 1 | 0 | 1 | 0 |
| P000002 | G0000119 | S0000393 | E001463 | MELD | 2642 | 86 | F | 73 | 65 | 0.37 | 0.387102600157174 | 0.4 | 1.76 |  |  | 0 | 13 | 13 | 0 | 0 | 1 | 0 | 0 | 0 |  |  |  | 1 | 1 | 1 | 0 | 0 | 0 | 0 | 0 | 0 | 0 | 1 | 1 | 1 | 1 | 0 |
| P000002 | G0000119 | S0000394 | E001463 | MELD-Na | 2642 | 86 | F | 73 | 65 | 0.37 | 0.387102600157174 | 0.4 | 1.76 | 123 |  | 0 | 24 | 24 | 0 | 0 | 1 | 0 | 0 | 0 |  |  |  | 1 | 1 | 1 | 0 | 0 | 0 | 0 | 0 | 0 | 0 | 1 | 1 | 0 | 1 | 0 |
| P000002 | G0000119 | S0000395 | E001463 | reMELD-Na | 2642 | 86 | F | 73 | 65 | 0.37 | 0.387102600157174 | 0.4 | 1.76 | 123 |  |  | 15 | 15 | 0 | 0 | 1 | 0 | 0 | 0 |  |  |  | 1 | 1 | 1 | 0 | 0 | 0 | 0 | 0 | 0 | 0 | 1 | 1 | 0 | 1 | 0 |
| P000002 | G0000120 | S0000396 | E001463 | MELD | 2643 | 86 | F | 73 | 65 | 0.39 | 0.406256830378747 | 0.5 | 1.86 |  |  | 0 | 13 | 13 | 0 | 0 | 1 | 0 | 0 | 0 |  |  |  | 1 | 1 | 1 | 0 | 0 | 0 | 0 | 0 | 0 | 0 | 1 | 1 | 1 | 1 | 0 |
| P000002 | G0000120 | S0000397 | E001463 | MELD-Na | 2643 | 86 | F | 73 | 65 | 0.39 | 0.406256830378747 | 0.5 | 1.86 | 149 |  | 0 | 13 | 13 | 0 | 0 | 1 | 0 | 0 | 0 |  |  |  | 1 | 1 | 1 | 0 | 0 | 0 | 0 | 0 | 0 | 0 | 1 | 1 | 0 | 1 | 0 |
| P000002 | G0000120 | S0000398 | E001463 | reMELD-Na | 2643 | 86 | F | 73 | 65 | 0.39 | 0.406256830378747 | 0.5 | 1.86 | 149 |  |  | 9 | 9 | 0 | 0 | 1 | 0 | 0 | 0 |  |  |  | 1 | 1 | 1 | 0 | 0 | 0 | 0 | 0 | 0 | 0 | 1 | 1 | 0 | 1 | 0 |
| P000002 | G0000121 | S0000399 | E001463 | MELD | 2644 | 86 | F | 73 | 65 | 0.39 | 0.409811660849985 | 0.2 | 1.42 |  |  | 0 | 10 | 10 | 0 | 0 | 1 | 0 | 0 | 0 |  |  |  | 1 | 1 | 1 | 0 | 0 | 0 | 0 | 0 | 0 | 0 | 1 | 1 | 1 | 1 | 0 |
| P000002 | G0000121 | S0000400 | E001463 | MELD-Na | 2644 | 86 | F | 73 | 65 | 0.39 | 0.409811660849985 | 0.2 | 1.42 | 149 |  | 0 | 10 | 10 | 0 | 0 | 1 | 0 | 0 | 0 |  |  |  | 1 | 1 | 1 | 0 | 0 | 0 | 0 | 0 | 0 | 0 | 1 | 1 | 0 | 1 | 0 |
| P000002 | G0000121 | S0000401 | E001463 | reMELD-Na | 2644 | 86 | F | 73 | 65 | 0.39 | 0.409811660849985 | 0.2 | 1.42 | 149 |  |  | 5 | 5 | 0 | 0 | 1 | 0 | 0 | 0 |  |  |  | 1 | 1 | 1 | 0 | 0 | 0 | 0 | 0 | 0 | 0 | 1 | 1 | 0 | 1 | 0 |
| P000002 | G0000122 | S0000402 | E001463 | MELD | 2645 | 86 | F | 73 | 65 | 0.41 | 0.42774735960621 | 0.4 | 1.59 |  |  | 0 | 12 | 12 | 0 | 0 | 1 | 0 | 0 | 0 |  |  |  | 1 | 1 | 1 | 0 | 0 | 0 | 0 | 0 | 0 | 0 | 1 | 1 | 1 | 1 | 0 |
| P000002 | G0000122 | S0000403 | E001463 | MELD 3.0 | 2645 | 86 | F | 73 | 65 | 0.41 | 0.42774735960621 | 0.4 | 1.59 | 147 | 1.97 |  | 14 | 14 | 0 | 0 | 1 | 0 | 0 | 0 |  |  |  | 1 | 1 | 1 | 0 | 0 | 0 | 0 | 0 | 0 | 0 | 1 | 1 | 0 | 1 | 0 |
| P000002 | G0000122 | S0000404 | E001463 | MELD-Na | 2645 | 86 | F | 73 | 65 | 0.41 | 0.42774735960621 | 0.4 | 1.59 | 147 |  | 0 | 12 | 12 | 0 | 0 | 1 | 0 | 0 | 0 |  |  |  | 1 | 1 | 1 | 0 | 0 | 0 | 0 | 0 | 0 | 0 | 1 | 1 | 0 | 1 | 0 |
| P000002 | G0000122 | S0000405 | E001463 | reMELD-Na | 2645 | 86 | F | 73 | 65 | 0.41 | 0.42774735960621 | 0.4 | 1.59 | 147 |  |  | 7 | 7 | 0 | 0 | 1 | 0 | 0 | 0 |  |  |  | 1 | 1 | 1 | 0 | 0 | 0 | 0 | 0 | 0 | 0 | 1 | 1 | 0 | 1 | 0 |
| P000002 | G0000123 | S0000406 | E001463 | MELD | 2646 | 86 | F | 73 | 65 | 0.35 | 0.366744962250838 | 0.4 | 1.35 |  |  | 0 | 10 | 10 | 0 | 0 | 1 | 0 | 0 | 0 |  |  |  | 1 | 1 | 1 | 0 | 0 | 0 | 0 | 0 | 0 | 0 | 1 | 1 | 1 | 1 | 0 |
| P000002 | G0000123 | S0000407 | E001463 | MELD-Na | 2646 | 86 | F | 73 | 65 | 0.35 | 0.366744962250838 | 0.4 | 1.35 | 147 |  | 0 | 10 | 10 | 0 | 0 | 1 | 0 | 0 | 0 |  |  |  | 1 | 1 | 1 | 0 | 0 | 0 | 0 | 0 | 0 | 0 | 1 | 1 | 0 | 1 | 0 |
| P000002 | G0000123 | S0000408 | E001463 | reMELD-Na | 2646 | 86 | F | 73 | 65 | 0.35 | 0.366744962250838 | 0.4 | 1.35 | 147 |  |  | 5 | 5 | 0 | 0 | 1 | 0 | 0 | 0 |  |  |  | 1 | 1 | 1 | 0 | 0 | 0 | 0 | 0 | 0 | 0 | 1 | 1 | 0 | 1 | 0 |
| P000003 | G0000124 | S0000409 | E000071 | MELD | 0 | 0 | F | 67 | 67 | 0.93 | 0.939419797841713 | 1.1 | 1 |  |  | 0 | 7 | 7 | 0 | 0 | 1 | 0 | 0 | 0 |  |  |  | 1 | 0 | 0 | 0 | 1 | 0 | 0 | 0 | 0 | 0 | 1 | 1 | 1 | 1 | 0 |
| P000003 | G0000124 | S0000410 | E000071 | MELD 3.0 | 0 | 0 | F | 67 | 67 | 0.93 | 0.939419797841713 | 1.1 | 1 | 144 | 4.02 |  | 8 | 8 | 0 | 0 | 1 | 0 | 0 | 0 |  |  |  | 1 | 0 | 0 | 0 | 1 | 0 | 0 | 0 | 0 | 0 | 1 | 1 | 0 | 1 | 0 |
| P000003 | G0000124 | S0000411 | E000071 | MELD-Na | 0 | 0 | F | 67 | 67 | 0.93 | 0.939419797841713 | 1.1 | 1 | 144 |  | 0 | 7 | 7 | 0 | 0 | 1 | 0 | 0 | 0 |  |  |  | 1 | 0 | 0 | 0 | 1 | 0 | 0 | 0 | 0 | 0 | 1 | 1 | 0 | 1 | 0 |
| P000003 | G0000124 | S0000412 | E000071 | reMELD-Na | 0 | 0 | F | 67 | 67 | 0.93 | 0.939419797841713 | 1.1 | 1 | 144 |  |  | 8 | 8 | 0 | 0 | 1 | 0 | 0 | 0 |  |  |  | 1 | 0 | 0 | 0 | 1 | 0 | 0 | 0 | 0 | 0 | 1 | 1 | 0 | 1 | 0 |
| P000003 | G0000125 | S0000413 | E000071 | MELD | 28 | 0 | F | 67 | 67 | 0.86 | 0.868084577793852 | 1.3 | 1.17 |  |  | 0 | 9 | 9 | 0 | 0 | 1 | 0 | 0 | 0 |  |  |  | 1 | 0 | 0 | 0 | 1 | 0 | 0 | 0 | 0 | 0 | 1 | 1 | 1 | 1 | 0 |
| P000003 | G0000125 | S0000414 | E000071 | MELD 3.0 | 28 | 0 | F | 67 | 67 | 0.86 | 0.868084577793852 | 1.3 | 1.17 | 146 | 4.81 |  | 10 | 10 | 0 | 0 | 1 | 0 | 0 | 0 |  |  |  | 1 | 0 | 0 | 0 | 1 | 0 | 0 | 0 | 0 | 0 | 1 | 1 | 0 | 1 | 0 |
| P000003 | G0000125 | S0000415 | E000071 | MELD-Na | 28 | 0 | F | 67 | 67 | 0.86 | 0.868084577793852 | 1.3 | 1.17 | 146 |  | 0 | 9 | 9 | 0 | 0 | 1 | 0 | 0 | 0 |  |  |  | 1 | 0 | 0 | 0 | 1 | 0 | 0 | 0 | 0 | 0 | 1 | 1 | 0 | 1 | 0 |
| P000003 | G0000125 | S0000416 | E000071 | reMELD-Na | 28 | 0 | F | 67 | 67 | 0.86 | 0.868084577793852 | 1.3 | 1.17 | 146 |  |  | 9 | 9 | 0 | 0 | 1 | 0 | 0 | 0 |  |  |  | 1 | 0 | 0 | 0 | 1 | 0 | 0 | 0 | 0 | 0 | 1 | 1 | 0 | 1 | 0 |
| P000003 | G0000126 | S0000417 | E000106 | MELD | 59 | 1 | F | 67 | 67 | 0.52 | 0.533186549541926 | 0.9 | 1.09 |  |  | 0 | 7 | 7 | 0 | 0 | 1 | 0 | 0 | 0 |  |  |  | 1 | 0 | 0 | 0 | 1 | 0 | 0 | 0 | 0 | 0 | 1 | 1 | 1 | 1 | 0 |
| P000003 | G0000126 | S0000418 | E000106 | MELD 3.0 | 59 | 1 | F | 67 | 67 | 0.52 | 0.533186549541926 | 0.9 | 1.09 | 141 | 3.48 |  | 8 | 8 | 0 | 0 | 1 | 0 | 0 | 0 |  |  |  | 1 | 0 | 0 | 0 | 1 | 0 | 0 | 0 | 0 | 0 | 1 | 1 | 0 | 1 | 0 |
| P000003 | G0000126 | S0000419 | E000106 | MELD-Na | 59 | 1 | F | 67 | 67 | 0.52 | 0.533186549541926 | 0.9 | 1.09 | 141 |  | 0 | 7 | 7 | 0 | 0 | 1 | 0 | 0 | 0 |  |  |  | 1 | 0 | 0 | 0 | 1 | 0 | 0 | 0 | 0 | 0 | 1 | 1 | 0 | 1 | 0 |
| P000003 | G0000126 | S0000420 | E000106 | reMELD-Na | 59 | 1 | F | 67 | 67 | 0.52 | 0.533186549541926 | 0.9 | 1.09 | 141 |  |  | 5 | 5 | 0 | 0 | 1 | 0 | 0 | 0 |  |  |  | 1 | 0 | 0 | 0 | 1 | 0 | 0 | 0 | 0 | 0 | 1 | 1 | 0 | 1 | 0 |
| P000003 | G0000127 | S0000421 | E000142 | MELD | 107 | 3 | F | 67 | 67 | 0.84 | 0.852891359679667 | 0.9 | 1.08 |  |  | 0 | 7 | 7 | 0 | 0 | 1 | 0 | 0 | 0 |  |  |  | 1 | 0 | 0 | 0 | 1 | 0 | 0 | 0 | 0 | 0 | 1 | 1 | 1 | 1 | 0 |
| P000003 | G0000127 | S0000422 | E000142 | MELD-Na | 107 | 3 | F | 67 | 67 | 0.84 | 0.852891359679667 | 0.9 | 1.08 | 140 |  | 0 | 7 | 7 | 0 | 0 | 1 | 0 | 0 | 0 |  |  |  | 1 | 0 | 0 | 0 | 1 | 0 | 0 | 0 | 0 | 0 | 1 | 1 | 0 | 1 | 0 |
| P000003 | G0000127 | S0000423 | E000142 | reMELD-Na | 107 | 3 | F | 67 | 67 | 0.84 | 0.852891359679667 | 0.9 | 1.08 | 140 |  |  | 7 | 7 | 0 | 0 | 1 | 0 | 0 | 0 |  |  |  | 1 | 0 | 0 | 0 | 1 | 0 | 0 | 0 | 0 | 0 | 1 | 1 | 0 | 1 | 0 |
| P000003 | G0000128 | S0000424 | E000142 | MELD | 119 | 3 | F | 67 | 67 | 0.82 | 0.834245837144585 | 0.8 | 1.06 |  |  | 0 | 7 | 7 | 0 | 0 | 1 | 0 | 0 | 0 |  |  |  | 1 | 0 | 0 | 0 | 1 | 0 | 0 | 0 | 0 | 0 | 1 | 1 | 1 | 1 | 0 |
| P000003 | G0000128 | S0000425 | E000142 | MELD 3.0 | 119 | 3 | F | 67 | 67 | 0.82 | 0.834245837144585 | 0.8 | 1.06 | 148 | 3.51 |  | 8 | 8 | 0 | 0 | 1 | 0 | 0 | 0 |  |  |  | 1 | 0 | 0 | 0 | 1 | 0 | 0 | 0 | 0 | 0 | 1 | 1 | 0 | 1 | 0 |
| P000003 | G0000128 | S0000426 | E000142 | MELD-Na | 119 | 3 | F | 67 | 67 | 0.82 | 0.834245837144585 | 0.8 | 1.06 | 148 |  | 0 | 7 | 7 | 0 | 0 | 1 | 0 | 0 | 0 |  |  |  | 1 | 0 | 0 | 0 | 1 | 0 | 0 | 0 | 0 | 0 | 1 | 1 | 0 | 1 | 0 |
| P000003 | G0000128 | S0000427 | E000142 | reMELD-Na | 119 | 3 | F | 67 | 67 | 0.82 | 0.834245837144585 | 0.8 | 1.06 | 148 |  |  | 6 | 6 | 0 | 0 | 1 | 0 | 0 | 0 |  |  |  | 1 | 0 | 0 | 0 | 1 | 0 | 0 | 0 | 0 | 0 | 1 | 1 | 0 | 1 | 0 |
| P000004 | G0000129 | S0000428 | E000113 | MELD | 0 | 0 | F | 21 | 21 | 0.95 | 0.96606932448343 | 0.5 | 0.97 |  |  | 0 | 6 | 6 | 0 | 0 | 1 | 0 | 0 | 0 |  |  |  | 1 | 0 | 1 | 1 | 1 | 0 | 0 | 0 | 0 | 0 | 1 | 1 | 1 | 1 | 0 |
| P000004 | G0000129 | S0000429 | E000113 | MELD-Na | 0 | 0 | F | 21 | 21 | 0.95 | 0.96606932448343 | 0.5 | 0.97 | 141 |  | 0 | 6 | 6 | 0 | 0 | 1 | 0 | 0 | 0 |  |  |  | 1 | 0 | 1 | 1 | 1 | 0 | 0 | 0 | 0 | 0 | 1 | 1 | 0 | 1 | 0 |
| P000004 | G0000129 | S0000430 | E000113 | reMELD-Na | 0 | 0 | F | 21 | 21 | 0.95 | 0.96606932448343 | 0.5 | 0.97 | 141 |  |  | 5 | 5 | 0 | 0 | 1 | 0 | 0 | 0 |  |  |  | 1 | 0 | 1 | 1 | 1 | 0 | 0 | 0 | 0 | 0 | 1 | 1 | 0 | 1 | 0 |
| P000004 | G0000130 | S0000431 | E000220 | MELD | 165 | 5 | F | 22 | 21 | 0.97 | 0.983377118073554 | 0.7 | 1.05 |  |  | 0 | 7 | 7 | 0 | 0 | 1 | 0 | 0 | 0 |  |  |  | 1 | 0 | 1 | 1 | 1 | 0 | 0 | 0 | 0 | 0 | 1 | 1 | 1 | 1 | 0 |
| P000004 | G0000130 | S0000432 | E000220 | MELD-Na | 165 | 5 | F | 22 | 21 | 0.97 | 0.983377118073554 | 0.7 | 1.05 | 142 |  | 0 | 7 | 7 | 0 | 0 | 1 | 0 | 0 | 0 |  |  |  | 1 | 0 | 1 | 1 | 1 | 0 | 0 | 0 | 0 | 0 | 1 | 1 | 0 | 1 | 0 |
| P000004 | G0000130 | S0000433 | E000220 | reMELD-Na | 165 | 5 | F | 22 | 21 | 0.97 | 0.983377118073554 | 0.7 | 1.05 | 142 |  |  | 7 | 7 | 0 | 0 | 1 | 0 | 0 | 0 |  |  |  | 1 | 0 | 1 | 1 | 1 | 0 | 0 | 0 | 0 | 0 | 1 | 1 | 0 | 1 | 0 |
| P000004 | G0000131 | S0000434 | E000402 | MELD | 446 | 14 | F | 22 | 21 | 1.01 | 1.02378136995969 | 0.6 | 0.95 |  |  | 0 | 7 | 7 | 0 | 0 | 1 | 0 | 0 | 0 |  |  |  | 1 | 0 | 1 | 1 | 1 | 0 | 0 | 0 | 0 | 0 | 1 | 1 | 1 | 1 | 0 |
| P000004 | G0000131 | S0000435 | E000402 | MELD 3.0 | 446 | 14 | F | 22 | 21 | 1.01 | 1.02378136995969 | 0.6 | 0.95 | 141 | 3.78 |  | 7 | 8 | 1 | 0 | 0 | 1 | 0 | 0 |  |  |  | 1 | 0 | 1 | 1 | 1 | 0 | 0 | 0 | 0 | 0 | 1 | 1 | 0 | 1 | 0 |
| P000004 | G0000131 | S0000436 | E000402 | MELD-Na | 446 | 14 | F | 22 | 21 | 1.01 | 1.02378136995969 | 0.6 | 0.95 | 141 |  | 0 | 7 | 7 | 0 | 0 | 1 | 0 | 0 | 0 |  |  |  | 1 | 0 | 1 | 1 | 1 | 0 | 0 | 0 | 0 | 0 | 1 | 1 | 0 | 1 | 0 |
| P000004 | G0000131 | S0000437 | E000402 | reMELD-Na | 446 | 14 | F | 22 | 21 | 1.01 | 1.02378136995969 | 0.6 | 0.95 | 141 |  |  | 6 | 6 | 0 | 0 | 1 | 0 | 0 | 0 |  |  |  | 1 | 0 | 1 | 1 | 1 | 0 | 0 | 0 | 0 | 0 | 1 | 1 | 0 | 1 | 0 |
| P000004 | G0000132 | S0000438 | E000479 | MELD | 588 | 19 | F | 23 | 21 | 0.97 | 0.98454693778338 | 0.6 | 1.09 |  |  | 0 | 7 | 7 | 0 | 0 | 1 | 0 | 0 | 0 |  |  |  | 1 | 0 | 1 | 1 | 1 | 0 | 0 | 0 | 0 | 0 | 1 | 1 | 1 | 1 | 0 |
| P000004 | G0000132 | S0000439 | E000479 | MELD 3.0 | 588 | 19 | F | 23 | 21 | 0.97 | 0.98454693778338 | 0.6 | 1.09 | 141 | 4.48 |  | 8 | 8 | 0 | 0 | 1 | 0 | 0 | 0 |  |  |  | 1 | 0 | 1 | 1 | 1 | 0 | 0 | 0 | 0 | 0 | 1 | 1 | 0 | 1 | 0 |
| P000004 | G0000132 | S0000440 | E000479 | MELD-Na | 588 | 19 | F | 23 | 21 | 0.97 | 0.98454693778338 | 0.6 | 1.09 | 141 |  | 0 | 7 | 7 | 0 | 0 | 1 | 0 | 0 | 0 |  |  |  | 1 | 0 | 1 | 1 | 1 | 0 | 0 | 0 | 0 | 0 | 1 | 1 | 0 | 1 | 0 |
| P000004 | G0000132 | S0000441 | E000479 | reMELD-Na | 588 | 19 | F | 23 | 21 | 0.97 | 0.98454693778338 | 0.6 | 1.09 | 141 |  |  | 7 | 7 | 0 | 0 | 1 | 0 | 0 | 0 |  |  |  | 1 | 0 | 1 | 1 | 1 | 0 | 0 | 0 | 0 | 0 | 1 | 1 | 0 | 1 | 0 |
| P000004 | G0000133 | S0000442 | E000625 | MELD | 859 | 28 | F | 23 | 21 | 0.98 | 0.994364360372913 | 0.6 | 0.96 |  |  | 0 | 6 | 6 | 0 | 0 | 1 | 0 | 0 | 0 |  |  |  | 1 | 0 | 1 | 1 | 1 | 0 | 0 | 0 | 0 | 0 | 1 | 1 | 1 | 1 | 0 |
| P000004 | G0000133 | S0000443 | E000625 | MELD-Na | 859 | 28 | F | 23 | 21 | 0.98 | 0.994364360372913 | 0.6 | 0.96 | 134 |  | 0 | 9 | 9 | 0 | 0 | 1 | 0 | 0 | 0 |  |  |  | 1 | 0 | 1 | 1 | 1 | 0 | 0 | 0 | 0 | 0 | 1 | 1 | 0 | 1 | 0 |
| P000004 | G0000133 | S0000444 | E000625 | reMELD-Na | 859 | 28 | F | 23 | 21 | 0.98 | 0.994364360372913 | 0.6 | 0.96 | 134 |  |  | 8 | 8 | 0 | 0 | 1 | 0 | 0 | 0 |  |  |  | 1 | 0 | 1 | 1 | 1 | 0 | 0 | 0 | 0 | 0 | 1 | 1 | 0 | 1 | 0 |
| P000005 | G0000134 | S0000445 | E000015 | MELD | 0 | 0 | F | 56 | 56 | 0.52 | 0.535511065180993 | 0.7 | 1.29 |  |  | 0 | 9 | 9 | 0 | 0 | 1 | 0 | 0 | 0 |  |  |  | 1 | 1 | 0 | 0 | 0 | 0 | 0 | 0 | 0 | 0 | 1 | 1 | 1 | 1 | 0 |
| P000005 | G0000134 | S0000446 | E000015 | MELD-Na | 0 | 0 | F | 56 | 56 | 0.52 | 0.535511065180993 | 0.7 | 1.29 | 137 |  | 0 | 9 | 9 | 0 | 0 | 1 | 0 | 0 | 0 |  |  |  | 1 | 1 | 0 | 0 | 0 | 0 | 0 | 0 | 0 | 0 | 1 | 1 | 0 | 1 | 0 |
| P000005 | G0000134 | S0000447 | E000015 | reMELD-Na | 0 | 0 | F | 56 | 56 | 0.52 | 0.535511065180993 | 0.7 | 1.29 | 137 |  |  | 7 | 7 | 0 | 0 | 1 | 0 | 0 | 0 |  |  |  | 1 | 1 | 0 | 0 | 0 | 0 | 0 | 0 | 0 | 0 | 1 | 1 | 0 | 1 | 0 |
| P000005 | G0000135 | S0000448 | E000015 | MELD | 1 | 0 | F | 56 | 56 | 0.54 | 0.555668906723693 | 0.7 | 1.18 |  |  | 0 | 8 | 8 | 0 | 0 | 1 | 0 | 0 | 0 |  |  |  | 1 | 1 | 0 | 0 | 0 | 0 | 0 | 0 | 0 | 0 | 1 | 1 | 1 | 1 | 0 |
| P000005 | G0000135 | S0000449 | E000015 | MELD-Na | 1 | 0 | F | 56 | 56 | 0.54 | 0.555668906723693 | 0.7 | 1.18 | 136 |  | 0 | 9 | 9 | 0 | 0 | 1 | 0 | 0 | 0 |  |  |  | 1 | 1 | 0 | 0 | 0 | 0 | 0 | 0 | 0 | 0 | 1 | 1 | 0 | 1 | 0 |
| P000005 | G0000135 | S0000450 | E000015 | reMELD-Na | 1 | 0 | F | 56 | 56 | 0.54 | 0.555668906723693 | 0.7 | 1.18 | 136 |  |  | 6 | 6 | 0 | 0 | 1 | 0 | 0 | 0 |  |  |  | 1 | 1 | 0 | 0 | 0 | 0 | 0 | 0 | 0 | 0 | 1 | 1 | 0 | 1 | 0 |
| P000005 | G0000136 | S0000451 | E000015 | MELD | 4 | 0 | F | 56 | 56 | 0.51 | 0.524258551414557 | 0.8 | 1.23 |  |  | 0 | 9 | 9 | 0 | 0 | 1 | 0 | 0 | 0 |  |  |  | 1 | 1 | 0 | 0 | 0 | 0 | 0 | 0 | 0 | 0 | 1 | 1 | 1 | 1 | 0 |
| P000005 | G0000136 | S0000452 | E000015 | MELD-Na | 4 | 0 | F | 56 | 56 | 0.51 | 0.524258551414557 | 0.8 | 1.23 | 139 |  | 0 | 9 | 9 | 0 | 0 | 1 | 0 | 0 | 0 |  |  |  | 1 | 1 | 0 | 0 | 0 | 0 | 0 | 0 | 0 | 0 | 1 | 1 | 0 | 1 | 0 |
| P000005 | G0000136 | S0000453 | E000015 | reMELD-Na | 4 | 0 | F | 56 | 56 | 0.51 | 0.524258551414557 | 0.8 | 1.23 | 139 |  |  | 6 | 6 | 0 | 0 | 1 | 0 | 0 | 0 |  |  |  | 1 | 1 | 0 | 0 | 0 | 0 | 0 | 0 | 0 | 0 | 1 | 1 | 0 | 1 | 0 |
| P000005 | G0000137 | S0000454 | E000015 | MELD | 6 | 0 | F | 56 | 56 | 0.51 | 0.525423329864188 | 0.7 | 1.28 |  |  | 0 | 9 | 9 | 0 | 0 | 1 | 0 | 0 | 0 |  |  |  | 1 | 1 | 0 | 0 | 0 | 0 | 0 | 0 | 0 | 0 | 1 | 1 | 1 | 1 | 0 |
| P000005 | G0000137 | S0000455 | E000015 | MELD-Na | 6 | 0 | F | 56 | 56 | 0.51 | 0.525423329864188 | 0.7 | 1.28 | 138 |  | 0 | 9 | 9 | 0 | 0 | 1 | 0 | 0 | 0 |  |  |  | 1 | 1 | 0 | 0 | 0 | 0 | 0 | 0 | 0 | 0 | 1 | 1 | 0 | 1 | 0 |
| P000005 | G0000137 | S0000456 | E000015 | reMELD-Na | 6 | 0 | F | 56 | 56 | 0.51 | 0.525423329864188 | 0.7 | 1.28 | 138 |  |  | 6 | 6 | 0 | 0 | 1 | 0 | 0 | 0 |  |  |  | 1 | 1 | 0 | 0 | 0 | 0 | 0 | 0 | 0 | 0 | 1 | 1 | 0 | 1 | 0 |
| P000005 | G0000138 | S0000457 | E000001 | MELD | 50 | 1 | F | 56 | 56 | 0.64 | 0.643570238983816 | 1.8 | 1.46 |  |  | 0 | 13 | 13 | 0 | 0 | 1 | 0 | 0 | 0 |  |  |  | 1 | 1 | 0 | 0 | 0 | 0 | 0 | 0 | 0 | 0 | 1 | 1 | 1 | 1 | 0 |
| P000005 | G0000138 | S0000458 | E000001 | MELD 3.0 | 50 | 1 | F | 56 | 56 | 0.64 | 0.643570238983816 | 1.8 | 1.46 | 136 | 7.13 |  | 14 | 14 | 0 | 0 | 1 | 0 | 0 | 0 |  |  |  | 1 | 1 | 0 | 0 | 0 | 0 | 0 | 0 | 0 | 0 | 1 | 1 | 0 | 1 | 0 |
| P000005 | G0000138 | S0000459 | E000001 | MELD-Na | 50 | 1 | F | 56 | 56 | 0.64 | 0.643570238983816 | 1.8 | 1.46 | 136 |  | 0 | 14 | 14 | 0 | 0 | 1 | 0 | 0 | 0 |  |  |  | 1 | 1 | 0 | 0 | 0 | 0 | 0 | 0 | 0 | 0 | 1 | 1 | 0 | 1 | 0 |
| P000005 | G0000138 | S0000460 | E000001 | reMELD-Na | 50 | 1 | F | 56 | 56 | 0.64 | 0.643570238983816 | 1.8 | 1.46 | 136 |  |  | 11 | 11 | 0 | 0 | 1 | 0 | 0 | 0 |  |  |  | 1 | 1 | 0 | 0 | 0 | 0 | 0 | 0 | 0 | 0 | 1 | 1 | 0 | 1 | 0 |
| P000005 | G0000139 | S0000461 | E000046 | MELD | 67 | 2 | F | 56 | 56 | 0.58 | 0.584493145657939 | 1.7 | 1.48 |  |  | 0 | 13 | 13 | 0 | 0 | 1 | 0 | 0 | 0 |  |  |  | 1 | 1 | 0 | 0 | 0 | 0 | 0 | 0 | 0 | 0 | 1 | 1 | 1 | 1 | 0 |
| P000005 | G0000139 | S0000462 | E000046 | MELD-Na | 67 | 2 | F | 56 | 56 | 0.58 | 0.584493145657939 | 1.7 | 1.48 | 139 |  | 0 | 13 | 13 | 0 | 0 | 1 | 0 | 0 | 0 |  |  |  | 1 | 1 | 0 | 0 | 0 | 0 | 0 | 0 | 0 | 0 | 1 | 1 | 0 | 1 | 0 |
| P000005 | G0000139 | S0000463 | E000046 | reMELD-Na | 67 | 2 | F | 56 | 56 | 0.58 | 0.584493145657939 | 1.7 | 1.48 | 139 |  |  | 10 | 10 | 0 | 0 | 1 | 0 | 0 | 0 |  |  |  | 1 | 1 | 0 | 0 | 0 | 0 | 0 | 0 | 0 | 0 | 1 | 1 | 0 | 1 | 0 |
| P000005 | G0000140 | S0000464 | E000486 | MELD | 757 | 24 | F | 58 | 56 | 0.68 | 0.683580351160125 | 1.8 | 1.42 |  |  | 0 | 13 | 13 | 0 | 0 | 1 | 0 | 0 | 0 |  |  |  | 1 | 1 | 0 | 0 | 0 | 0 | 0 | 0 | 0 | 0 | 1 | 1 | 1 | 1 | 0 |
| P000005 | G0000140 | S0000465 | E000486 | MELD-Na | 757 | 24 | F | 58 | 56 | 0.68 | 0.683580351160125 | 1.8 | 1.42 | 138 |  | 0 | 13 | 13 | 0 | 0 | 1 | 0 | 0 | 0 |  |  |  | 1 | 1 | 0 | 0 | 0 | 0 | 0 | 0 | 0 | 0 | 1 | 1 | 0 | 1 | 0 |
| P000005 | G0000140 | S0000466 | E000486 | reMELD-Na | 757 | 24 | F | 58 | 56 | 0.68 | 0.683580351160125 | 1.8 | 1.42 | 138 |  |  | 10 | 10 | 0 | 0 | 1 | 0 | 0 | 0 |  |  |  | 1 | 1 | 0 | 0 | 0 | 0 | 0 | 0 | 0 | 0 | 1 | 1 | 0 | 1 | 0 |
| P000005 | G0000141 | S0000467 | E001115 | MELD | 2048 | 67 | F | 61 | 56 | 0.63 | 0.633553020030648 | 1.8 | 1.41 |  |  | 0 | 13 | 13 | 0 | 0 | 1 | 0 | 0 | 0 |  |  |  | 1 | 1 | 0 | 0 | 0 | 0 | 0 | 0 | 0 | 0 | 1 | 1 | 1 | 1 | 0 |
| P000005 | G0000141 | S0000468 | E001115 | MELD 3.0 | 2048 | 67 | F | 61 | 56 | 0.63 | 0.633553020030648 | 1.8 | 1.41 | 138 | 4 |  | 13 | 13 | 0 | 0 | 1 | 0 | 0 | 0 |  |  |  | 1 | 1 | 0 | 0 | 0 | 0 | 0 | 0 | 0 | 0 | 1 | 1 | 0 | 1 | 0 |
| P000005 | G0000141 | S0000469 | E001115 | MELD-Na | 2048 | 67 | F | 61 | 56 | 0.63 | 0.633553020030648 | 1.8 | 1.41 | 138 |  | 0 | 13 | 13 | 0 | 0 | 1 | 0 | 0 | 0 |  |  |  | 1 | 1 | 0 | 0 | 0 | 0 | 0 | 0 | 0 | 0 | 1 | 1 | 0 | 1 | 0 |
| P000005 | G0000141 | S0000470 | E001115 | reMELD-Na | 2048 | 67 | F | 61 | 56 | 0.63 | 0.633553020030648 | 1.8 | 1.41 | 138 |  |  | 10 | 10 | 0 | 0 | 1 | 0 | 0 | 0 |  |  |  | 1 | 1 | 0 | 0 | 0 | 0 | 0 | 0 | 0 | 0 | 1 | 1 | 0 | 1 | 0 |
| P000005 | G0000142 | S0000471 | E002489 | MELD | 4183 | 137 | F | 67 | 56 | 0.76 | 0.762430647087395 | 1.88 | 1.17 |  |  | 0 | 11 | 11 | 0 | 0 | 1 | 0 | 0 | 0 |  |  |  | 1 | 1 | 0 | 0 | 0 | 0 | 0 | 0 | 0 | 0 | 1 | 1 | 1 | 1 | 0 |
| P000005 | G0000142 | S0000472 | E002489 | MELD-Na | 4183 | 137 | F | 67 | 56 | 0.76 | 0.762430647087395 | 1.88 | 1.17 | 134 |  | 0 | 14 | 14 | 0 | 0 | 1 | 0 | 0 | 0 |  |  |  | 1 | 1 | 0 | 0 | 0 | 0 | 0 | 0 | 0 | 0 | 1 | 1 | 0 | 1 | 0 |
| P000005 | G0000142 | S0000473 | E002489 | reMELD-Na | 4183 | 137 | F | 67 | 56 | 0.76 | 0.762430647087395 | 1.88 | 1.17 | 134 |  |  | 11 | 11 | 0 | 0 | 1 | 0 | 0 | 0 |  |  |  | 1 | 1 | 0 | 0 | 0 | 0 | 0 | 0 | 0 | 0 | 1 | 1 | 0 | 1 | 0 |
| P000005 | G0000143 | S0000474 | E002489 | MELD | 4187 | 137 | F | 67 | 56 | 0.65 | 0.65424959613073 | 1.74 | 1.14 |  |  | 0 | 10 | 10 | 0 | 0 | 1 | 0 | 0 | 0 |  |  |  | 1 | 1 | 0 | 0 | 0 | 0 | 0 | 0 | 0 | 0 | 1 | 1 | 1 | 1 | 0 |
| P000005 | G0000143 | S0000475 | E002489 | MELD-Na | 4187 | 137 | F | 67 | 56 | 0.65 | 0.65424959613073 | 1.74 | 1.14 | 135 |  | 0 | 12 | 12 | 0 | 0 | 1 | 0 | 0 | 0 |  |  |  | 1 | 1 | 0 | 0 | 0 | 0 | 0 | 0 | 0 | 0 | 1 | 1 | 0 | 1 | 0 |
| P000005 | G0000143 | S0000476 | E002489 | reMELD-Na | 4187 | 137 | F | 67 | 56 | 0.65 | 0.65424959613073 | 1.74 | 1.14 | 135 |  |  | 9 | 9 | 0 | 0 | 1 | 0 | 0 | 0 |  |  |  | 1 | 1 | 0 | 0 | 0 | 0 | 0 | 0 | 0 | 0 | 1 | 1 | 0 | 1 | 0 |
| P000005 | G0000144 | S0000477 | E003603 | MELD | 5013 | 164 | F | 70 | 56 | 0.49 | 0.498658520657092 | 1.27 | 1.365 |  |  | 0 | 11 | 11 | 0 | 0 | 1 | 0 | 0 | 0 |  |  |  | 1 | 1 | 0 | 0 | 0 | 0 | 0 | 0 | 0 | 0 | 1 | 1 | 1 | 1 | 0 |
| P000005 | G0000145 | S0000478 | E003603 | MELD | 5014 | 164 | F | 70 | 56 | 0.61 | 0.615174392277491 | 1.65 | 1.285 |  |  | 0 | 11 | 11 | 0 | 0 | 1 | 0 | 0 | 0 |  |  |  | 1 | 1 | 0 | 0 | 0 | 0 | 0 | 0 | 0 | 0 | 1 | 1 | 1 | 1 | 0 |
| P000005 | G0000146 | S0000479 | E003603 | MELD | 5015 | 164 | F | 70 | 56 | 0.53 | 0.53228057395541 | 1.87 | 1.25 |  |  | 0 | 11 | 11 | 0 | 0 | 1 | 0 | 0 | 0 |  |  |  | 1 | 1 | 0 | 0 | 0 | 0 | 0 | 0 | 0 | 0 | 1 | 1 | 1 | 1 | 0 |
| P000005 | G0000147 | S0000480 | E003603 | MELD | 5019 | 164 | F | 70 | 56 | 0.52 | 0.523866860923125 | 1.72 | 1.2 |  |  | 0 | 11 | 11 | 0 | 0 | 1 | 0 | 0 | 0 |  |  |  | 1 | 1 | 0 | 0 | 0 | 0 | 0 | 0 | 0 | 0 | 1 | 1 | 1 | 1 | 0 |
| P000005 | G0000147 | S0000481 | E003603 | MELD 3.0 | 5019 | 164 | F | 70 | 56 | 0.52 | 0.523866860923125 | 1.72 | 1.2 | 137 | 2.04 |  | 14 | 14 | 0 | 0 | 1 | 0 | 0 | 0 |  |  |  | 1 | 1 | 0 | 0 | 0 | 0 | 0 | 0 | 0 | 0 | 1 | 1 | 0 | 1 | 0 |
| P000005 | G0000147 | S0000482 | E003603 | MELD-Na | 5019 | 164 | F | 70 | 56 | 0.52 | 0.523866860923125 | 1.72 | 1.2 | 137 |  | 0 | 11 | 11 | 0 | 0 | 1 | 0 | 0 | 0 |  |  |  | 1 | 1 | 0 | 0 | 0 | 0 | 0 | 0 | 0 | 0 | 1 | 1 | 0 | 1 | 0 |
| P000005 | G0000147 | S0000483 | E003603 | reMELD-Na | 5019 | 164 | F | 70 | 56 | 0.52 | 0.523866860923125 | 1.72 | 1.2 | 137 |  |  | 9 | 9 | 0 | 0 | 1 | 0 | 0 | 0 |  |  |  | 1 | 1 | 0 | 0 | 0 | 0 | 0 | 0 | 0 | 0 | 1 | 1 | 0 | 1 | 0 |
| P000005 | G0000148 | S0000484 | E003603 | MELD | 5022 | 164 | F | 70 | 56 | 0.37 | 0.370598388825699 | 1.84 | 1.29 |  |  | 0 | 12 | 12 | 0 | 0 | 1 | 0 | 0 | 0 |  |  |  | 1 | 1 | 0 | 0 | 0 | 0 | 0 | 0 | 0 | 0 | 1 | 1 | 1 | 1 | 0 |
| P000005 | G0000148 | S0000485 | E003603 | MELD-Na | 5022 | 164 | F | 70 | 56 | 0.37 | 0.370598388825699 | 1.84 | 1.29 | 134 |  | 0 | 15 | 15 | 0 | 0 | 1 | 0 | 0 | 0 |  |  |  | 1 | 1 | 0 | 0 | 0 | 0 | 0 | 0 | 0 | 0 | 1 | 1 | 0 | 1 | 0 |
| P000005 | G0000148 | S0000486 | E003603 | reMELD-Na | 5022 | 164 | F | 70 | 56 | 0.37 | 0.370598388825699 | 1.84 | 1.29 | 134 |  |  | 11 | 11 | 0 | 0 | 1 | 0 | 0 | 0 |  |  |  | 1 | 1 | 0 | 0 | 0 | 0 | 0 | 0 | 0 | 0 | 1 | 1 | 0 | 1 | 0 |
| P000005 | G0000149 | S0000487 | E003603 | MELD | 5023 | 165 | F | 70 | 56 | 0.5 | 0.500580816633786 | 2 | 1.3 |  |  | 0 | 12 | 12 | 0 | 0 | 1 | 0 | 0 | 0 |  |  |  | 1 | 1 | 0 | 0 | 0 | 0 | 0 | 0 | 0 | 0 | 1 | 1 | 1 | 1 | 0 |
| P000005 | G0000149 | S0000488 | E003603 | MELD-Na | 5023 | 165 | F | 70 | 56 | 0.5 | 0.500580816633786 | 2 | 1.3 | 136 |  | 0 | 13 | 13 | 0 | 0 | 1 | 0 | 0 | 0 |  |  |  | 1 | 1 | 0 | 0 | 0 | 0 | 0 | 0 | 0 | 0 | 1 | 1 | 0 | 1 | 0 |
| P000005 | G0000149 | S0000489 | E003603 | reMELD-Na | 5023 | 165 | F | 70 | 56 | 0.5 | 0.500580816633786 | 2 | 1.3 | 136 |  |  | 11 | 11 | 0 | 0 | 1 | 0 | 0 | 0 |  |  |  | 1 | 1 | 0 | 0 | 0 | 0 | 0 | 0 | 0 | 0 | 1 | 1 | 0 | 1 | 0 |
| P000005 | G0000150 | S0000490 | E003603 | MELD | 5028 | 165 | F | 70 | 56 | 0.65 | 0.651257822595162 | 2.01 | 1.37 |  |  | 0 | 13 | 13 | 0 | 0 | 1 | 0 | 0 | 0 |  |  |  | 1 | 1 | 0 | 0 | 0 | 0 | 0 | 0 | 0 | 0 | 1 | 1 | 1 | 1 | 0 |
| P000005 | G0000150 | S0000491 | E003603 | MELD-Na | 5028 | 165 | F | 70 | 56 | 0.65 | 0.651257822595162 | 2.01 | 1.37 | 133 |  | 0 | 17 | 17 | 0 | 0 | 1 | 0 | 0 | 0 |  |  |  | 1 | 1 | 0 | 0 | 0 | 0 | 0 | 0 | 0 | 0 | 1 | 1 | 0 | 1 | 0 |
| P000005 | G0000150 | S0000492 | E003603 | reMELD-Na | 5028 | 165 | F | 70 | 56 | 0.65 | 0.651257822595162 | 2.01 | 1.37 | 133 |  |  | 13 | 13 | 0 | 0 | 1 | 0 | 0 | 0 |  |  |  | 1 | 1 | 0 | 0 | 0 | 0 | 0 | 0 | 0 | 0 | 1 | 1 | 0 | 1 | 0 |
| P000005 | G0000151 | S0000493 | E003603 | MELD | 5029 | 165 | F | 70 | 56 | 0.66 | 0.668527966155284 | 1.36 | 1.41 |  |  | 0 | 11 | 11 | 0 | 0 | 1 | 0 | 0 | 0 |  |  |  | 1 | 1 | 0 | 0 | 0 | 0 | 0 | 0 | 0 | 0 | 1 | 1 | 1 | 1 | 0 |
| P000005 | G0000151 | S0000494 | E003603 | MELD-Na | 5029 | 165 | F | 70 | 56 | 0.66 | 0.668527966155284 | 1.36 | 1.41 | 134 |  | 0 | 14 | 14 | 0 | 0 | 1 | 0 | 0 | 0 |  |  |  | 1 | 1 | 0 | 0 | 0 | 0 | 0 | 0 | 0 | 0 | 1 | 1 | 0 | 1 | 0 |
| P000005 | G0000151 | S0000495 | E003603 | reMELD-Na | 5029 | 165 | F | 70 | 56 | 0.66 | 0.668527966155284 | 1.36 | 1.41 | 134 |  |  | 11 | 11 | 0 | 0 | 1 | 0 | 0 | 0 |  |  |  | 1 | 1 | 0 | 0 | 0 | 0 | 0 | 0 | 0 | 0 | 1 | 1 | 0 | 1 | 0 |
| P000005 | G0000152 | S0000496 | E003603 | MELD | 5030 | 165 | F | 70 | 56 | 0.68 | 0.683025058708859 | 1.85 | 1.23 |  |  | 0 | 11 | 11 | 0 | 0 | 1 | 0 | 0 | 0 |  |  |  | 1 | 1 | 0 | 0 | 0 | 0 | 0 | 0 | 0 | 0 | 1 | 1 | 1 | 1 | 0 |
| P000005 | G0000152 | S0000497 | E003603 | MELD 3.0 | 5030 | 165 | F | 70 | 56 | 0.68 | 0.683025058708859 | 1.85 | 1.23 | 135 | 2.31 |  | 16 | 16 | 0 | 0 | 1 | 0 | 0 | 0 |  |  |  | 1 | 1 | 0 | 0 | 0 | 0 | 0 | 0 | 0 | 0 | 1 | 1 | 0 | 1 | 0 |
| P000005 | G0000152 | S0000498 | E003603 | MELD-Na | 5030 | 165 | F | 70 | 56 | 0.68 | 0.683025058708859 | 1.85 | 1.23 | 135 |  | 0 | 13 | 13 | 0 | 0 | 1 | 0 | 0 | 0 |  |  |  | 1 | 1 | 0 | 0 | 0 | 0 | 0 | 0 | 0 | 0 | 1 | 1 | 0 | 1 | 0 |
| P000005 | G0000152 | S0000499 | E003603 | reMELD-Na | 5030 | 165 | F | 70 | 56 | 0.68 | 0.683025058708859 | 1.85 | 1.23 | 135 |  |  | 10 | 10 | 0 | 0 | 1 | 0 | 0 | 0 |  |  |  | 1 | 1 | 0 | 0 | 0 | 0 | 0 | 0 | 0 | 0 | 1 | 1 | 0 | 1 | 0 |
| P000005 | G0000153 | S0000500 | E003603 | MELD | 5033 | 165 | F | 70 | 56 | 0.58 | 0.590941293853129 | 1.13 | 1.22 |  |  | 0 | 9 | 9 | 0 | 0 | 1 | 0 | 0 | 0 |  |  |  | 1 | 1 | 0 | 0 | 0 | 0 | 0 | 0 | 0 | 0 | 1 | 1 | 1 | 1 | 0 |
| P000005 | G0000153 | S0000501 | E003603 | MELD 3.0 | 5033 | 165 | F | 70 | 56 | 0.58 | 0.590941293853129 | 1.13 | 1.22 | 135.5 | 2.05 |  | 14 | 14 | 0 | 0 | 1 | 0 | 0 | 0 |  |  |  | 1 | 1 | 0 | 0 | 0 | 0 | 0 | 0 | 0 | 0 | 1 | 1 | 0 | 1 | 0 |
| P000005 | G0000153 | S0000502 | E003603 | MELD-Na | 5033 | 165 | F | 70 | 56 | 0.58 | 0.590941293853129 | 1.13 | 1.22 | 135.5 |  | 0 | 11 | 11 | 0 | 0 | 1 | 0 | 0 | 0 |  |  |  | 1 | 1 | 0 | 0 | 0 | 0 | 0 | 0 | 0 | 0 | 1 | 1 | 0 | 1 | 0 |
| P000005 | G0000153 | S0000503 | E003603 | reMELD-Na | 5033 | 165 | F | 70 | 56 | 0.58 | 0.590941293853129 | 1.13 | 1.22 | 135.5 |  |  | 8 | 8 | 0 | 0 | 1 | 0 | 0 | 0 |  |  |  | 1 | 1 | 0 | 0 | 0 | 0 | 0 | 0 | 0 | 0 | 1 | 1 | 0 | 1 | 0 |
| P000005 | G0000154 | S0000504 | E003603 | MELD | 5034 | 165 | F | 70 | 56 | 0.58 | 0.591744464124499 | 1.06 | 1.16 |  |  | 0 | 8 | 8 | 0 | 0 | 1 | 0 | 0 | 0 |  |  |  | 1 | 1 | 0 | 0 | 0 | 0 | 0 | 0 | 0 | 0 | 1 | 1 | 1 | 1 | 0 |
| P000005 | G0000154 | S0000505 | E003603 | MELD 3.0 | 5034 | 165 | F | 70 | 56 | 0.58 | 0.591744464124499 | 1.06 | 1.16 | 134 | 2.05 |  | 14 | 14 | 0 | 0 | 1 | 0 | 0 | 0 |  |  |  | 1 | 1 | 0 | 0 | 0 | 0 | 0 | 0 | 0 | 0 | 1 | 1 | 0 | 1 | 0 |
| P000005 | G0000154 | S0000506 | E003603 | MELD-Na | 5034 | 165 | F | 70 | 56 | 0.58 | 0.591744464124499 | 1.06 | 1.16 | 134 |  | 0 | 11 | 11 | 0 | 0 | 1 | 0 | 0 | 0 |  |  |  | 1 | 1 | 0 | 0 | 0 | 0 | 0 | 0 | 0 | 0 | 1 | 1 | 0 | 1 | 0 |
| P000005 | G0000154 | S0000507 | E003603 | reMELD-Na | 5034 | 165 | F | 70 | 56 | 0.58 | 0.591744464124499 | 1.06 | 1.16 | 134 |  |  | 9 | 9 | 0 | 0 | 1 | 0 | 0 | 0 |  |  |  | 1 | 1 | 0 | 0 | 0 | 0 | 0 | 0 | 0 | 0 | 1 | 1 | 0 | 1 | 0 |
| P000005 | G0000155 | S0000508 | E003603 | MELD | 5035 | 165 | F | 70 | 56 | 0.61 | 0.621407380431245 | 1.1 | 1.16 |  |  | 0 | 8 | 8 | 0 | 0 | 1 | 0 | 0 | 0 |  |  |  | 1 | 1 | 0 | 0 | 0 | 0 | 0 | 0 | 0 | 0 | 1 | 1 | 1 | 1 | 0 |
| P000005 | G0000155 | S0000509 | E003603 | MELD-Na | 5035 | 165 | F | 70 | 56 | 0.61 | 0.621407380431245 | 1.1 | 1.16 | 131 |  | 0 | 14 | 14 | 0 | 0 | 1 | 0 | 0 | 0 |  |  |  | 1 | 1 | 0 | 0 | 0 | 0 | 0 | 0 | 0 | 0 | 1 | 1 | 0 | 1 | 0 |
| P000005 | G0000155 | S0000510 | E003603 | reMELD-Na | 5035 | 165 | F | 70 | 56 | 0.61 | 0.621407380431245 | 1.1 | 1.16 | 131 |  |  | 10 | 10 | 0 | 0 | 1 | 0 | 0 | 0 |  |  |  | 1 | 1 | 0 | 0 | 0 | 0 | 0 | 0 | 0 | 0 | 1 | 1 | 0 | 1 | 0 |
| P000005 | G0000156 | S0000511 | E003665 | MELD | 5045 | 165 | F | 70 | 56 | 0.39 | 0.402050923984384 | 0.86 | 1.41 |  |  | 0 | 10 | 10 | 0 | 0 | 1 | 0 | 0 | 0 |  |  |  | 1 | 1 | 0 | 0 | 0 | 0 | 0 | 0 | 0 | 0 | 1 | 1 | 1 | 1 | 0 |
| P000005 | G0000157 | S0000512 | E003665 | MELD | 5046 | 165 | F | 70 | 56 | 0.46 | 0.476535666972233 | 0.56 | 1.27 |  |  | 0 | 9 | 9 | 0 | 0 | 1 | 0 | 0 | 0 |  |  |  | 1 | 1 | 0 | 0 | 0 | 0 | 0 | 0 | 0 | 0 | 1 | 1 | 1 | 1 | 0 |
| P000005 | G0000158 | S0000513 | E003665 | MELD | 5047 | 165 | F | 70 | 56 | 0.44 | 0.454061625082399 | 0.75 | 1.31 |  |  | 0 | 9 | 9 | 0 | 0 | 1 | 0 | 0 | 0 |  |  |  | 1 | 1 | 0 | 0 | 0 | 0 | 0 | 0 | 0 | 0 | 1 | 1 | 1 | 1 | 0 |
| P000006 | G0000159 | S0000514 | E000061 | MELD | 0 | 0 | M | 63 | 63 | 1.08 | 1.09457014946847 | 0.4 | 1.13 |  |  | 0 | 9 | 9 | 0 | 0 | 1 | 0 | 1 | 1 |  | -3021 | -99 | 1 | 0 | 1 | 0 | 0 | 0 | 0 | 1 | 0 | 0 | 1 | 1 | 1 | 1 | 1 |
| P000006 | G0000159 | S0000515 | E000061 | MELD-Na | 0 | 0 | M | 63 | 63 | 1.08 | 1.09457014946847 | 0.4 | 1.13 | 134 |  | 0 | 12 | 12 | 0 | 0 | 1 | 0 | 1 | 1 |  | -3021 | -99 | 1 | 0 | 1 | 0 | 0 | 0 | 0 | 1 | 0 | 0 | 1 | 1 | 0 | 1 | 1 |
| P000006 | G0000159 | S0000516 | E000061 | reMELD-Na | 0 | 0 | M | 63 | 63 | 1.08 | 1.09457014946847 | 0.4 | 1.13 | 134 |  |  | 9 | 9 | 0 | 0 | 1 | 0 | 1 | 1 |  | -3021 | -99 | 1 | 0 | 1 | 0 | 0 | 0 | 0 | 1 | 0 | 0 | 1 | 1 | 0 | 1 | 1 |
| P000006 | G0000160 | S0000517 | E000061 | MELD | 188 | 6 | M | 64 | 63 | 0.92 | 0.936546540351198 | 0.5 | 1.16 |  |  | 0 | 8 | 8 | 0 | 0 | 1 | 0 | 1 | 1 |  | -2833 | -93 | 1 | 0 | 1 | 0 | 0 | 0 | 0 | 1 | 0 | 0 | 1 | 1 | 1 | 1 | 1 |
| P000006 | G0000160 | S0000518 | E000061 | MELD-Na | 188 | 6 | M | 64 | 63 | 0.92 | 0.936546540351198 | 0.5 | 1.16 | 142 |  | 0 | 8 | 8 | 0 | 0 | 1 | 0 | 1 | 1 |  | -2833 | -93 | 1 | 0 | 1 | 0 | 0 | 0 | 0 | 1 | 0 | 0 | 1 | 1 | 0 | 1 | 1 |
| P000006 | G0000160 | S0000519 | E000061 | reMELD-Na | 188 | 6 | M | 64 | 63 | 0.92 | 0.936546540351198 | 0.5 | 1.16 | 142 |  |  | 7 | 7 | 0 | 0 | 1 | 0 | 1 | 1 |  | -2833 | -93 | 1 | 0 | 1 | 0 | 0 | 0 | 0 | 1 | 0 | 0 | 1 | 1 | 0 | 1 | 1 |
| P000006 | G0000161 | S0000520 | E000061 | MELD | 223 | 7 | M | 64 | 63 | 1.02 | 1.03593005029491 | 0.4 | 1.19 |  |  | 0 | 9 | 9 | 0 | 0 | 1 | 0 | 1 | 1 |  | -2798 | -92 | 1 | 0 | 1 | 0 | 0 | 0 | 0 | 1 | 0 | 0 | 1 | 1 | 1 | 1 | 1 |
| P000006 | G0000161 | S0000521 | E000061 | MELD-Na | 223 | 7 | M | 64 | 63 | 1.02 | 1.03593005029491 | 0.4 | 1.19 | 139 |  | 0 | 9 | 9 | 0 | 0 | 1 | 0 | 1 | 1 |  | -2798 | -92 | 1 | 0 | 1 | 0 | 0 | 0 | 0 | 1 | 0 | 0 | 1 | 1 | 0 | 1 | 1 |
| P000006 | G0000161 | S0000522 | E000061 | reMELD-Na | 223 | 7 | M | 64 | 63 | 1.02 | 1.03593005029491 | 0.4 | 1.19 | 139 |  |  | 7 | 7 | 0 | 0 | 1 | 0 | 1 | 1 |  | -2798 | -92 | 1 | 0 | 1 | 0 | 0 | 0 | 0 | 1 | 0 | 0 | 1 | 1 | 0 | 1 | 1 |
| P000006 | G0000162 | S0000523 | E000061 | MELD | 228 | 7 | M | 64 | 63 | 1.38 | 1.38224264577236 | 0.6 | 1.13 |  |  | 0 | 11 | 11 | 0 | 0 | 1 | 0 | 1 | 1 |  | -2793 | -92 | 1 | 0 | 1 | 0 | 0 | 0 | 0 | 1 | 0 | 0 | 1 | 1 | 1 | 1 | 1 |
| P000006 | G0000162 | S0000524 | E000061 | MELD-Na | 228 | 7 | M | 64 | 63 | 1.38 | 1.38224264577236 | 0.6 | 1.13 | 138 |  | 0 | 11 | 11 | 0 | 0 | 1 | 0 | 1 | 1 |  | -2793 | -92 | 1 | 0 | 1 | 0 | 0 | 0 | 0 | 1 | 0 | 0 | 1 | 1 | 0 | 1 | 1 |
| P000006 | G0000162 | S0000525 | E000061 | reMELD-Na | 228 | 7 | M | 64 | 63 | 1.38 | 1.38224264577236 | 0.6 | 1.13 | 138 |  |  | 11 | 11 | 0 | 0 | 1 | 0 | 1 | 1 |  | -2793 | -92 | 1 | 0 | 1 | 0 | 0 | 0 | 0 | 1 | 0 | 0 | 1 | 1 | 0 | 1 | 1 |
| P000006 | G0000163 | S0000526 | E000061 | MELD | 230 | 7 | M | 64 | 63 | 1.08 | 1.09221538626823 | 0.6 | 1.13 |  |  | 0 | 9 | 9 | 0 | 0 | 1 | 0 | 1 | 1 |  | -2791 | -92 | 1 | 0 | 1 | 0 | 0 | 0 | 0 | 1 | 0 | 0 | 1 | 1 | 1 | 1 | 1 |
| P000006 | G0000163 | S0000527 | E000061 | MELD-Na | 230 | 7 | M | 64 | 63 | 1.08 | 1.09221538626823 | 0.6 | 1.13 | 138 |  | 0 | 9 | 9 | 0 | 0 | 1 | 0 | 1 | 1 |  | -2791 | -92 | 1 | 0 | 1 | 0 | 0 | 0 | 0 | 1 | 0 | 0 | 1 | 1 | 0 | 1 | 1 |
| P000006 | G0000163 | S0000528 | E000061 | reMELD-Na | 230 | 7 | M | 64 | 63 | 1.08 | 1.09221538626823 | 0.6 | 1.13 | 138 |  |  | 8 | 9 | 1 | 0 | 0 | 1 | 1 | 1 |  | -2791 | -92 | 1 | 0 | 1 | 0 | 0 | 0 | 0 | 1 | 0 | 0 | 1 | 1 | 0 | 1 | 1 |
| P000006 | G0000164 | S0000529 | E000061 | MELD | 236 | 7 | M | 64 | 63 | 1.05 | 1.06527654351806 | 0.4 | 1.1 |  |  | 0 | 8 | 8 | 0 | 0 | 1 | 0 | 1 | 1 |  | -2785 | -91 | 1 | 0 | 1 | 0 | 0 | 0 | 0 | 1 | 0 | 0 | 1 | 1 | 1 | 1 | 1 |
| P000006 | G0000164 | S0000530 | E000061 | MELD-Na | 236 | 7 | M | 64 | 63 | 1.05 | 1.06527654351806 | 0.4 | 1.1 | 139 |  | 0 | 8 | 8 | 0 | 0 | 1 | 0 | 1 | 1 |  | -2785 | -91 | 1 | 0 | 1 | 0 | 0 | 0 | 0 | 1 | 0 | 0 | 1 | 1 | 0 | 1 | 1 |
| P000006 | G0000164 | S0000531 | E000061 | reMELD-Na | 236 | 7 | M | 64 | 63 | 1.05 | 1.06527654351806 | 0.4 | 1.1 | 139 |  |  | 7 | 7 | 0 | 0 | 1 | 0 | 1 | 1 |  | -2785 | -91 | 1 | 0 | 1 | 0 | 0 | 0 | 0 | 1 | 0 | 0 | 1 | 1 | 0 | 1 | 1 |
| P000006 | G0000165 | S0000532 | E000314 | MELD | 363 | 11 | M | 64 | 63 | 1.64 | 1.63286131797265 | 0.3 | 1.05 |  |  | 0 | 12 | 12 | 0 | 0 | 1 | 0 | 1 | 1 |  | -2658 | -87 | 1 | 0 | 1 | 0 | 0 | 0 | 0 | 1 | 0 | 0 | 1 | 1 | 1 | 1 | 1 |
| P000006 | G0000165 | S0000533 | E000314 | MELD-Na | 363 | 11 | M | 64 | 63 | 1.64 | 1.63286131797265 | 0.3 | 1.05 | 132 |  | 0 | 17 | 17 | 0 | 0 | 1 | 0 | 1 | 1 |  | -2658 | -87 | 1 | 0 | 1 | 0 | 0 | 0 | 0 | 1 | 0 | 0 | 1 | 1 | 0 | 1 | 1 |
| P000006 | G0000165 | S0000534 | E000314 | reMELD-Na | 363 | 11 | M | 64 | 63 | 1.64 | 1.63286131797265 | 0.3 | 1.05 | 132 |  |  | 11 | 11 | 0 | 0 | 1 | 0 | 1 | 1 |  | -2658 | -87 | 1 | 0 | 1 | 0 | 0 | 0 | 0 | 1 | 0 | 0 | 1 | 1 | 0 | 1 | 1 |
| P000006 | G0000166 | S0000535 | E000314 | MELD | 379 | 12 | M | 64 | 63 | 1.39 | 1.39299399842282 | 0.5 | 1.14 |  |  | 0 | 11 | 11 | 0 | 0 | 1 | 0 | 1 | 1 |  | -2642 | -87 | 1 | 0 | 1 | 0 | 0 | 0 | 0 | 1 | 0 | 0 | 1 | 1 | 1 | 1 | 1 |
| P000006 | G0000166 | S0000536 | E000314 | MELD 3.0 | 379 | 12 | M | 64 | 63 | 1.39 | 1.39299399842282 | 0.5 | 1.14 | 133 | 4.1 |  | 14 | 14 | 0 | 0 | 1 | 0 | 1 | 1 |  | -2642 | -87 | 1 | 0 | 1 | 0 | 0 | 0 | 0 | 1 | 0 | 0 | 1 | 1 | 0 | 1 | 1 |
| P000006 | G0000166 | S0000537 | E000314 | MELD-Na | 379 | 12 | M | 64 | 63 | 1.39 | 1.39299399842282 | 0.5 | 1.14 | 133 |  | 0 | 15 | 15 | 0 | 0 | 1 | 0 | 1 | 1 |  | -2642 | -87 | 1 | 0 | 1 | 0 | 0 | 0 | 0 | 1 | 0 | 0 | 1 | 1 | 0 | 1 | 1 |
| P000006 | G0000166 | S0000538 | E000314 | reMELD-Na | 379 | 12 | M | 64 | 63 | 1.39 | 1.39299399842282 | 0.5 | 1.14 | 133 |  |  | 12 | 12 | 0 | 0 | 1 | 0 | 1 | 1 |  | -2642 | -87 | 1 | 0 | 1 | 0 | 0 | 0 | 0 | 1 | 0 | 0 | 1 | 1 | 0 | 1 | 1 |
| P000006 | G0000167 | S0000539 | E000314 | MELD | 392 | 12 | M | 64 | 63 | 1.49 | 1.49080056094786 | 0.3 | 1.08 |  |  | 0 | 11 | 11 | 0 | 0 | 1 | 0 | 1 | 1 |  | -2629 | -86 | 1 | 0 | 1 | 0 | 0 | 0 | 0 | 1 | 0 | 0 | 1 | 1 | 1 | 1 | 1 |
| P000006 | G0000167 | S0000540 | E000314 | MELD-Na | 392 | 12 | M | 64 | 63 | 1.49 | 1.49080056094786 | 0.3 | 1.08 | 141 |  | 0 | 11 | 11 | 0 | 0 | 1 | 0 | 1 | 1 |  | -2629 | -86 | 1 | 0 | 1 | 0 | 0 | 0 | 0 | 1 | 0 | 0 | 1 | 1 | 0 | 1 | 1 |
| P000006 | G0000167 | S0000541 | E000314 | reMELD-Na | 392 | 12 | M | 64 | 63 | 1.49 | 1.49080056094786 | 0.3 | 1.08 | 141 |  |  | 9 | 9 | 0 | 0 | 1 | 0 | 1 | 1 |  | -2629 | -86 | 1 | 0 | 1 | 0 | 0 | 0 | 0 | 1 | 0 | 0 | 1 | 1 | 0 | 1 | 1 |
| P000006 | G0000168 | S0000542 | E000314 | MELD | 400 | 13 | M | 64 | 63 | 1.17 | 1.18331858717376 | 0.3 | 1.12 |  |  | 0 | 9 | 9 | 0 | 0 | 1 | 0 | 1 | 1 |  | -2621 | -86 | 1 | 0 | 1 | 0 | 0 | 0 | 0 | 1 | 0 | 0 | 1 | 1 | 1 | 1 | 1 |
| P000006 | G0000168 | S0000543 | E000314 | MELD-Na | 400 | 13 | M | 64 | 63 | 1.17 | 1.18331858717376 | 0.3 | 1.12 | 140 |  | 0 | 9 | 9 | 0 | 0 | 1 | 0 | 1 | 1 |  | -2621 | -86 | 1 | 0 | 1 | 0 | 0 | 0 | 0 | 1 | 0 | 0 | 1 | 1 | 0 | 1 | 1 |
| P000006 | G0000168 | S0000544 | E000314 | reMELD-Na | 400 | 13 | M | 64 | 63 | 1.17 | 1.18331858717376 | 0.3 | 1.12 | 140 |  |  | 7 | 7 | 0 | 0 | 1 | 0 | 1 | 1 |  | -2621 | -86 | 1 | 0 | 1 | 0 | 0 | 0 | 0 | 1 | 0 | 0 | 1 | 1 | 0 | 1 | 1 |
| P000006 | G0000169 | S0000545 | E000314 | MELD | 417 | 13 | M | 64 | 63 | 1.17 | 1.18213364368334 | 0.4 | 1.12 |  |  | 0 | 9 | 9 | 0 | 0 | 1 | 0 | 1 | 1 |  | -2604 | -85 | 1 | 0 | 1 | 0 | 0 | 0 | 0 | 1 | 0 | 0 | 1 | 1 | 1 | 1 | 1 |
| P000006 | G0000169 | S0000546 | E000314 | MELD-Na | 417 | 13 | M | 64 | 63 | 1.17 | 1.18213364368334 | 0.4 | 1.12 | 140 |  | 0 | 9 | 9 | 0 | 0 | 1 | 0 | 1 | 1 |  | -2604 | -85 | 1 | 0 | 1 | 0 | 0 | 0 | 0 | 1 | 0 | 0 | 1 | 1 | 0 | 1 | 1 |
| P000006 | G0000169 | S0000547 | E000314 | reMELD-Na | 417 | 13 | M | 64 | 63 | 1.17 | 1.18213364368334 | 0.4 | 1.12 | 140 |  |  | 8 | 8 | 0 | 0 | 1 | 0 | 1 | 1 |  | -2604 | -85 | 1 | 0 | 1 | 0 | 0 | 0 | 0 | 1 | 0 | 0 | 1 | 1 | 0 | 1 | 1 |
| P000006 | G0000170 | S0000548 | E000314 | MELD | 463 | 15 | M | 64 | 63 | 1.12 | 1.13354602164478 | 0.4 | 1.15 |  |  | 0 | 9 | 9 | 0 | 0 | 1 | 0 | 1 | 1 |  | -2558 | -84 | 1 | 0 | 1 | 0 | 0 | 0 | 0 | 1 | 0 | 0 | 1 | 1 | 1 | 1 | 1 |
| P000006 | G0000170 | S0000549 | E000314 | MELD 3.0 | 463 | 15 | M | 64 | 63 | 1.12 | 1.13354602164478 | 0.4 | 1.15 | 141 | 4.33 |  | 9 | 9 | 0 | 0 | 1 | 0 | 1 | 1 |  | -2558 | -84 | 1 | 0 | 1 | 0 | 0 | 0 | 0 | 1 | 0 | 0 | 1 | 1 | 0 | 1 | 1 |
| P000006 | G0000170 | S0000550 | E000314 | MELD-Na | 463 | 15 | M | 64 | 63 | 1.12 | 1.13354602164478 | 0.4 | 1.15 | 141 |  | 0 | 9 | 9 | 0 | 0 | 1 | 0 | 1 | 1 |  | -2558 | -84 | 1 | 0 | 1 | 0 | 0 | 0 | 0 | 1 | 0 | 0 | 1 | 1 | 0 | 1 | 1 |
| P000006 | G0000170 | S0000551 | E000314 | reMELD-Na | 463 | 15 | M | 64 | 63 | 1.12 | 1.13354602164478 | 0.4 | 1.15 | 141 |  |  | 8 | 8 | 0 | 0 | 1 | 0 | 1 | 1 |  | -2558 | -84 | 1 | 0 | 1 | 0 | 0 | 0 | 0 | 1 | 0 | 0 | 1 | 1 | 0 | 1 | 1 |
| P000006 | G0000171 | S0000552 | E000314 | MELD | 466 | 15 | M | 64 | 63 | 1.22 | 1.230574356631 | 0.4 | 1.19 |  |  | 0 | 10 | 10 | 0 | 0 | 1 | 0 | 1 | 1 |  | -2555 | -84 | 1 | 0 | 1 | 0 | 0 | 0 | 0 | 1 | 0 | 0 | 1 | 1 | 1 | 1 | 1 |
| P000006 | G0000171 | S0000553 | E000314 | MELD 3.0 | 466 | 15 | M | 64 | 63 | 1.22 | 1.230574356631 | 0.4 | 1.19 | 137 | 4.26 |  | 10 | 10 | 0 | 0 | 1 | 0 | 1 | 1 |  | -2555 | -84 | 1 | 0 | 1 | 0 | 0 | 0 | 0 | 1 | 0 | 0 | 1 | 1 | 0 | 1 | 1 |
| P000006 | G0000171 | S0000554 | E000314 | MELD-Na | 466 | 15 | M | 64 | 63 | 1.22 | 1.230574356631 | 0.4 | 1.19 | 137 |  | 0 | 10 | 10 | 0 | 0 | 1 | 0 | 1 | 1 |  | -2555 | -84 | 1 | 0 | 1 | 0 | 0 | 0 | 0 | 1 | 0 | 0 | 1 | 1 | 0 | 1 | 1 |
| P000006 | G0000171 | S0000555 | E000314 | reMELD-Na | 466 | 15 | M | 64 | 63 | 1.22 | 1.230574356631 | 0.4 | 1.19 | 137 |  |  | 9 | 9 | 0 | 0 | 1 | 0 | 1 | 1 |  | -2555 | -84 | 1 | 0 | 1 | 0 | 0 | 0 | 0 | 1 | 0 | 0 | 1 | 1 | 0 | 1 | 1 |
| P000006 | G0000172 | S0000556 | E000314 | MELD | 531 | 17 | M | 64 | 63 | 1.08 | 1.09457014946847 | 0.4 | 1.25 |  |  | 0 | 10 | 10 | 0 | 0 | 1 | 0 | 1 | 1 |  | -2490 | -82 | 1 | 0 | 1 | 0 | 0 | 0 | 0 | 1 | 0 | 0 | 1 | 1 | 1 | 1 | 1 |
| P000006 | G0000172 | S0000557 | E000314 | MELD 3.0 | 531 | 17 | M | 64 | 63 | 1.08 | 1.09457014946847 | 0.4 | 1.25 | 133 | 2.88 |  | 13 | 13 | 0 | 0 | 1 | 0 | 1 | 1 |  | -2490 | -82 | 1 | 0 | 1 | 0 | 0 | 0 | 0 | 1 | 0 | 0 | 1 | 1 | 0 | 1 | 1 |
| P000006 | G0000172 | S0000558 | E000314 | MELD-Na | 531 | 17 | M | 64 | 63 | 1.08 | 1.09457014946847 | 0.4 | 1.25 | 133 |  | 0 | 14 | 14 | 0 | 0 | 1 | 0 | 1 | 1 |  | -2490 | -82 | 1 | 0 | 1 | 0 | 0 | 0 | 0 | 1 | 0 | 0 | 1 | 1 | 0 | 1 | 1 |
| P000006 | G0000172 | S0000559 | E000314 | reMELD-Na | 531 | 17 | M | 64 | 63 | 1.08 | 1.09457014946847 | 0.4 | 1.25 | 133 |  |  | 10 | 10 | 0 | 0 | 1 | 0 | 1 | 1 |  | -2490 | -82 | 1 | 0 | 1 | 0 | 0 | 0 | 0 | 1 | 0 | 0 | 1 | 1 | 0 | 1 | 1 |
| P000006 | G0000173 | S0000560 | E000314 | MELD | 547 | 17 | M | 64 | 63 | 0.88 | 0.899465404804611 | 0.3 | 1.23 |  |  | 0 | 9 | 9 | 0 | 0 | 1 | 0 | 1 | 1 |  | -2474 | -81 | 1 | 0 | 1 | 0 | 0 | 0 | 0 | 1 | 0 | 0 | 1 | 1 | 1 | 1 | 1 |
| P000006 | G0000173 | S0000561 | E000314 | MELD-Na | 547 | 17 | M | 64 | 63 | 0.88 | 0.899465404804611 | 0.3 | 1.23 | 137 |  | 0 | 9 | 9 | 0 | 0 | 1 | 0 | 1 | 1 |  | -2474 | -81 | 1 | 0 | 1 | 0 | 0 | 0 | 0 | 1 | 0 | 0 | 1 | 1 | 0 | 1 | 1 |
| P000006 | G0000173 | S0000562 | E000314 | reMELD-Na | 547 | 17 | M | 64 | 63 | 0.88 | 0.899465404804611 | 0.3 | 1.23 | 137 |  |  | 6 | 6 | 0 | 0 | 1 | 0 | 1 | 1 |  | -2474 | -81 | 1 | 0 | 1 | 0 | 0 | 0 | 0 | 1 | 0 | 0 | 1 | 1 | 0 | 1 | 1 |
| P000006 | G0000174 | S0000563 | E000314 | MELD | 554 | 18 | M | 65 | 63 | 1.22 | 1.23175930012141 | 0.3 | 1.19 |  |  | 0 | 10 | 10 | 0 | 0 | 1 | 0 | 1 | 1 |  | -2467 | -81 | 1 | 0 | 1 | 0 | 0 | 0 | 0 | 1 | 0 | 0 | 1 | 1 | 1 | 1 | 1 |
| P000006 | G0000174 | S0000564 | E000314 | MELD-Na | 554 | 18 | M | 65 | 63 | 1.22 | 1.23175930012141 | 0.3 | 1.19 | 135 |  | 0 | 12 | 12 | 0 | 0 | 1 | 0 | 1 | 1 |  | -2467 | -81 | 1 | 0 | 1 | 0 | 0 | 0 | 0 | 1 | 0 | 0 | 1 | 1 | 0 | 1 | 1 |
| P000006 | G0000174 | S0000565 | E000314 | reMELD-Na | 554 | 18 | M | 65 | 63 | 1.22 | 1.23175930012141 | 0.3 | 1.19 | 135 |  |  | 9 | 9 | 0 | 0 | 1 | 0 | 1 | 1 |  | -2467 | -81 | 1 | 0 | 1 | 0 | 0 | 0 | 0 | 1 | 0 | 0 | 1 | 1 | 0 | 1 | 1 |
| P000006 | G0000175 | S0000566 | E000522 | MELD | 699 | 22 | M | 65 | 63 | 1.06 | 1.07742188343952 | 0.2 | 1.13 |  |  | 0 | 8 | 9 | 1 | 0 | 0 | 1 | 1 | 1 |  | -2322 | -76 | 1 | 0 | 1 | 0 | 0 | 0 | 0 | 1 | 0 | 0 | 1 | 1 | 1 | 1 | 1 |
| P000006 | G0000175 | S0000567 | E000522 | MELD-Na | 699 | 22 | M | 65 | 63 | 1.06 | 1.07742188343952 | 0.2 | 1.13 | 137 |  | 0 | 8 | 9 | 1 | 0 | 0 | 1 | 1 | 1 |  | -2322 | -76 | 1 | 0 | 1 | 0 | 0 | 0 | 0 | 1 | 0 | 0 | 1 | 1 | 0 | 1 | 1 |
| P000006 | G0000175 | S0000568 | E000522 | reMELD-Na | 699 | 22 | M | 65 | 63 | 1.06 | 1.07742188343952 | 0.2 | 1.13 | 137 |  |  | 7 | 7 | 0 | 0 | 1 | 0 | 1 | 1 |  | -2322 | -76 | 1 | 0 | 1 | 0 | 0 | 0 | 0 | 1 | 0 | 0 | 1 | 1 | 0 | 1 | 1 |
| P000006 | G0000176 | S0000569 | E000522 | MELD | 789 | 25 | M | 65 | 63 | 1.14 | 1.15537362779213 | 0.2 | 1.19 |  |  | 0 | 10 | 10 | 0 | 0 | 1 | 0 | 1 | 1 |  | -2232 | -73 | 1 | 0 | 1 | 0 | 0 | 0 | 0 | 1 | 0 | 0 | 1 | 1 | 1 | 1 | 1 |
| P000006 | G0000176 | S0000570 | E000522 | MELD-Na | 789 | 25 | M | 65 | 63 | 1.14 | 1.15537362779213 | 0.2 | 1.19 | 138 |  | 0 | 10 | 10 | 0 | 0 | 1 | 0 | 1 | 1 |  | -2232 | -73 | 1 | 0 | 1 | 0 | 0 | 0 | 0 | 1 | 0 | 0 | 1 | 1 | 0 | 1 | 1 |
| P000006 | G0000176 | S0000571 | E000522 | reMELD-Na | 789 | 25 | M | 65 | 63 | 1.14 | 1.15537362779213 | 0.2 | 1.19 | 138 |  |  | 7 | 7 | 0 | 0 | 1 | 0 | 1 | 1 |  | -2232 | -73 | 1 | 0 | 1 | 0 | 0 | 0 | 0 | 1 | 0 | 0 | 1 | 1 | 0 | 1 | 1 |
| P000006 | G0000177 | S0000572 | E000522 | MELD | 858 | 28 | M | 65 | 63 | 1.56 | 1.55607517558417 | 0.4 | 1.28 |  |  | 0 | 13 | 13 | 0 | 0 | 1 | 0 | 1 | 1 |  | -2163 | -71 | 1 | 0 | 1 | 0 | 0 | 0 | 0 | 1 | 0 | 0 | 1 | 1 | 1 | 1 | 1 |
| P000006 | G0000177 | S0000573 | E000522 | MELD-Na | 858 | 28 | M | 65 | 63 | 1.56 | 1.55607517558417 | 0.4 | 1.28 | 138 |  | 0 | 13 | 13 | 0 | 0 | 1 | 0 | 1 | 1 |  | -2163 | -71 | 1 | 0 | 1 | 0 | 0 | 0 | 0 | 1 | 0 | 0 | 1 | 1 | 0 | 1 | 1 |
| P000006 | G0000177 | S0000574 | E000522 | reMELD-Na | 858 | 28 | M | 65 | 63 | 1.56 | 1.55607517558417 | 0.4 | 1.28 | 138 |  |  | 12 | 12 | 0 | 0 | 1 | 0 | 1 | 1 |  | -2163 | -71 | 1 | 0 | 1 | 0 | 0 | 0 | 0 | 1 | 0 | 0 | 1 | 1 | 0 | 1 | 1 |
| P000006 | G0000178 | S0000575 | E000710 | MELD | 1058 | 34 | M | 66 | 63 | 1.36 | 1.36661174001122 | 0.3 | 1.13 |  |  | 0 | 11 | 11 | 0 | 0 | 1 | 0 | 1 | 1 |  | -1963 | -64 | 1 | 0 | 1 | 0 | 0 | 0 | 0 | 1 | 0 | 0 | 1 | 1 | 1 | 1 | 1 |
| P000006 | G0000178 | S0000576 | E000710 | MELD-Na | 1058 | 34 | M | 66 | 63 | 1.36 | 1.36661174001122 | 0.3 | 1.13 | 141 |  | 0 | 11 | 11 | 0 | 0 | 1 | 0 | 1 | 1 |  | -1963 | -64 | 1 | 0 | 1 | 0 | 0 | 0 | 0 | 1 | 0 | 0 | 1 | 1 | 0 | 1 | 1 |
| P000006 | G0000178 | S0000577 | E000710 | reMELD-Na | 1058 | 34 | M | 66 | 63 | 1.36 | 1.36661174001122 | 0.3 | 1.13 | 141 |  |  | 8 | 8 | 0 | 0 | 1 | 0 | 1 | 1 |  | -1963 | -64 | 1 | 0 | 1 | 0 | 0 | 0 | 0 | 1 | 0 | 0 | 1 | 1 | 0 | 1 | 1 |
| P000006 | G0000179 | S0000578 | E000710 | MELD | 1278 | 41 | M | 66 | 63 | 1.26 | 1.26922115244367 | 0.4 | 1.07 |  |  | 0 | 9 | 9 | 0 | 0 | 1 | 0 | 1 | 1 |  | -1743 | -57 | 1 | 0 | 1 | 0 | 0 | 0 | 0 | 1 | 0 | 0 | 1 | 1 | 1 | 1 | 1 |
| P000006 | G0000179 | S0000579 | E000710 | MELD-Na | 1278 | 41 | M | 66 | 63 | 1.26 | 1.26922115244367 | 0.4 | 1.07 | 138 |  | 0 | 9 | 9 | 0 | 0 | 1 | 0 | 1 | 1 |  | -1743 | -57 | 1 | 0 | 1 | 0 | 0 | 0 | 0 | 1 | 0 | 0 | 1 | 1 | 0 | 1 | 1 |
| P000006 | G0000179 | S0000580 | E000710 | reMELD-Na | 1278 | 41 | M | 66 | 63 | 1.26 | 1.26922115244367 | 0.4 | 1.07 | 138 |  |  | 8 | 8 | 0 | 0 | 1 | 0 | 1 | 1 |  | -1743 | -57 | 1 | 0 | 1 | 0 | 0 | 0 | 0 | 1 | 0 | 0 | 1 | 1 | 0 | 1 | 1 |
| P000006 | G0000180 | S0000581 | E000710 | MELD | 1294 | 42 | M | 67 | 63 | 1.11 | 1.12381086814615 | 0.4 | 1.08 |  |  | 0 | 8 | 8 | 0 | 0 | 1 | 0 | 1 | 1 |  | -1727 | -57 | 1 | 0 | 1 | 0 | 0 | 0 | 0 | 1 | 0 | 0 | 1 | 1 | 1 | 1 | 1 |
| P000006 | G0000180 | S0000582 | E000710 | MELD-Na | 1294 | 42 | M | 67 | 63 | 1.11 | 1.12381086814615 | 0.4 | 1.08 | 138 |  | 0 | 8 | 8 | 0 | 0 | 1 | 0 | 1 | 1 |  | -1727 | -57 | 1 | 0 | 1 | 0 | 0 | 0 | 0 | 1 | 0 | 0 | 1 | 1 | 0 | 1 | 1 |
| P000006 | G0000180 | S0000583 | E000710 | reMELD-Na | 1294 | 42 | M | 67 | 63 | 1.11 | 1.12381086814615 | 0.4 | 1.08 | 138 |  |  | 7 | 7 | 0 | 0 | 1 | 0 | 1 | 1 |  | -1727 | -57 | 1 | 0 | 1 | 0 | 0 | 0 | 0 | 1 | 0 | 0 | 1 | 1 | 0 | 1 | 1 |
| P000006 | G0000181 | S0000584 | E000710 | MELD | 1323 | 43 | M | 67 | 63 | 1.01 | 1.01913233868156 | 1 | 1.14 |  |  | 0 | 8 | 8 | 0 | 0 | 1 | 0 | 1 | 1 |  | -1698 | -56 | 1 | 0 | 1 | 0 | 0 | 0 | 0 | 1 | 0 | 0 | 1 | 1 | 1 | 1 | 1 |
| P000006 | G0000181 | S0000585 | E000710 | MELD-Na | 1323 | 43 | M | 67 | 63 | 1.01 | 1.01913233868156 | 1 | 1.14 | 134 |  | 0 | 11 | 11 | 0 | 0 | 1 | 0 | 1 | 1 |  | -1698 | -56 | 1 | 0 | 1 | 0 | 0 | 0 | 0 | 1 | 0 | 0 | 1 | 1 | 0 | 1 | 1 |
| P000006 | G0000181 | S0000586 | E000710 | reMELD-Na | 1323 | 43 | M | 67 | 63 | 1.01 | 1.01913233868156 | 1 | 1.14 | 134 |  |  | 11 | 11 | 0 | 0 | 1 | 0 | 1 | 1 |  | -1698 | -56 | 1 | 0 | 1 | 0 | 0 | 0 | 0 | 1 | 0 | 0 | 1 | 1 | 0 | 1 | 1 |
| P000006 | G0000182 | S0000587 | E001048 | MELD | 1782 | 58 | M | 68 | 63 | 1.61 | 1.6010152344225 | 0.6 | 1.17 |  |  | 0 | 13 | 13 | 0 | 0 | 1 | 0 | 1 | 1 |  | -1239 | -41 | 1 | 0 | 1 | 0 | 0 | 0 | 0 | 1 | 0 | 0 | 1 | 1 | 1 | 1 | 1 |
| P000006 | G0000182 | S0000588 | E001048 | MELD-Na | 1782 | 58 | M | 68 | 63 | 1.61 | 1.6010152344225 | 0.6 | 1.17 | 131 |  | 0 | 18 | 18 | 0 | 0 | 1 | 0 | 1 | 1 |  | -1239 | -41 | 1 | 0 | 1 | 0 | 0 | 0 | 0 | 1 | 0 | 0 | 1 | 1 | 0 | 1 | 1 |
| P000006 | G0000182 | S0000589 | E001048 | reMELD-Na | 1782 | 58 | M | 68 | 63 | 1.61 | 1.6010152344225 | 0.6 | 1.17 | 131 |  |  | 14 | 14 | 0 | 0 | 1 | 0 | 1 | 1 |  | -1239 | -41 | 1 | 0 | 1 | 0 | 0 | 0 | 0 | 1 | 0 | 0 | 1 | 1 | 0 | 1 | 1 |
| P000006 | G0000183 | S0000590 | E001165 | MELD | 2010 | 66 | M | 68 | 63 | 1.12 | 1.12310838693986 | 1.3 | 1.11 |  |  | 0 | 10 | 10 | 0 | 0 | 1 | 0 | 1 | 1 |  | -1011 | -33 | 1 | 0 | 1 | 0 | 0 | 0 | 0 | 1 | 0 | 0 | 1 | 1 | 1 | 1 | 1 |
| P000006 | G0000183 | S0000591 | E001165 | MELD-Na | 2010 | 66 | M | 68 | 63 | 1.12 | 1.12310838693986 | 1.3 | 1.11 | 137 |  | 0 | 10 | 10 | 0 | 0 | 1 | 0 | 1 | 1 |  | -1011 | -33 | 1 | 0 | 1 | 0 | 0 | 0 | 0 | 1 | 0 | 0 | 1 | 1 | 0 | 1 | 1 |
| P000006 | G0000183 | S0000592 | E001165 | reMELD-Na | 2010 | 66 | M | 68 | 63 | 1.12 | 1.12310838693986 | 1.3 | 1.11 | 137 |  |  | 11 | 11 | 0 | 0 | 1 | 0 | 1 | 1 |  | -1011 | -33 | 1 | 0 | 1 | 0 | 0 | 0 | 0 | 1 | 0 | 0 | 1 | 1 | 0 | 1 | 1 |
| P000006 | G0000184 | S0000593 | E001048 | MELD | 2055 | 67 | M | 69 | 63 | 2.84 | 2.71587452386635 | 0.8 | 1.2 |  |  | 0 | 18 | 18 | 0 | 0 | 1 | 0 | 1 | 1 |  | -966 | -32 | 1 | 0 | 1 | 0 | 0 | 0 | 0 | 1 | 0 | 0 | 1 | 1 | 1 | 1 | 1 |
| P000006 | G0000184 | S0000594 | E001048 | MELD-Na | 2055 | 67 | M | 69 | 63 | 2.84 | 2.71587452386635 | 0.8 | 1.2 | 132 |  | 0 | 22 | 22 | 0 | 0 | 1 | 0 | 1 | 1 |  | -966 | -32 | 1 | 0 | 1 | 0 | 0 | 0 | 0 | 1 | 0 | 0 | 1 | 1 | 0 | 1 | 1 |
| P000006 | G0000184 | S0000595 | E001048 | reMELD-Na | 2055 | 67 | M | 69 | 63 | 2.84 | 2.71587452386635 | 0.8 | 1.2 | 132 |  |  | 18 | 18 | 0 | 0 | 1 | 0 | 1 | 1 |  | -966 | -32 | 1 | 0 | 1 | 0 | 0 | 0 | 0 | 1 | 0 | 0 | 1 | 1 | 0 | 1 | 1 |
| P000006 | G0000185 | S0000596 | E001048 | MELD | 2101 | 69 | M | 69 | 63 | 1.15 | 1.1315845578666 | 3.2 | 1.16 |  |  | 0 | 14 | 14 | 0 | 0 | 1 | 0 | 1 | 1 |  | -920 | -30 | 1 | 0 | 1 | 0 | 0 | 0 | 0 | 1 | 0 | 0 | 1 | 1 | 1 | 1 | 1 |
| P000006 | G0000185 | S0000597 | E001048 | MELD 3.0 | 2101 | 69 | M | 69 | 63 | 1.15 | 1.1315845578666 | 3.2 | 1.16 | 130 | 3.63 |  | 18 | 18 | 0 | 0 | 1 | 0 | 1 | 1 |  | -920 | -30 | 1 | 0 | 1 | 0 | 0 | 0 | 0 | 1 | 0 | 0 | 1 | 1 | 0 | 1 | 1 |
| P000006 | G0000185 | S0000598 | E001048 | MELD-Na | 2101 | 69 | M | 69 | 63 | 1.15 | 1.1315845578666 | 3.2 | 1.16 | 130 |  | 0 | 20 | 20 | 0 | 0 | 1 | 0 | 1 | 1 |  | -920 | -30 | 1 | 0 | 1 | 0 | 0 | 0 | 0 | 1 | 0 | 0 | 1 | 1 | 0 | 1 | 1 |
| P000006 | G0000185 | S0000599 | E001048 | reMELD-Na | 2101 | 69 | M | 69 | 63 | 1.15 | 1.1315845578666 | 3.2 | 1.16 | 130 |  |  | 17 | 17 | 0 | 0 | 1 | 0 | 1 | 1 |  | -920 | -30 | 1 | 0 | 1 | 0 | 0 | 0 | 0 | 1 | 0 | 0 | 1 | 1 | 0 | 1 | 1 |
| P000006 | G0000186 | S0000600 | E001216 | MELD | 2103 | 69 | M | 69 | 63 | 0.81 | 0.7771370668057 | 5.3 | 1.2 |  |  | 0 | 15 | 15 | 0 | 0 | 1 | 0 | 1 | 1 |  | -918 | -30 | 1 | 0 | 1 | 0 | 0 | 0 | 0 | 1 | 0 | 0 | 1 | 1 | 1 | 1 | 1 |
| P000006 | G0000186 | S0000601 | E001216 | MELD-Na | 2103 | 69 | M | 69 | 63 | 0.81 | 0.7771370668057 | 5.3 | 1.2 | 133 |  | 0 | 18 | 18 | 0 | 0 | 1 | 0 | 1 | 1 |  | -918 | -30 | 1 | 0 | 1 | 0 | 0 | 0 | 0 | 1 | 0 | 0 | 1 | 1 | 0 | 1 | 1 |
| P000006 | G0000186 | S0000602 | E001216 | reMELD-Na | 2103 | 69 | M | 69 | 63 | 0.81 | 0.7771370668057 | 5.3 | 1.2 | 133 |  |  | 15 | 15 | 0 | 0 | 1 | 0 | 1 | 1 |  | -918 | -30 | 1 | 0 | 1 | 0 | 0 | 0 | 0 | 1 | 0 | 0 | 1 | 1 | 0 | 1 | 1 |
| P000006 | G0000187 | S0000603 | E001216 | MELD | 2104 | 69 | M | 69 | 63 | 0.85 | 0.780469877251253 | 9.7 | 1.21 |  |  | 0 | 17 | 17 | 0 | 0 | 1 | 0 | 1 | 1 |  | -917 | -30 | 1 | 0 | 1 | 0 | 0 | 0 | 0 | 1 | 0 | 0 | 1 | 1 | 1 | 1 | 1 |
| P000006 | G0000187 | S0000604 | E001216 | MELD 3.0 | 2104 | 69 | M | 69 | 63 | 0.85 | 0.780469877251253 | 9.7 | 1.21 | 136 | 3.07 |  | 19 | 19 | 0 | 0 | 1 | 0 | 1 | 1 |  | -917 | -30 | 1 | 0 | 1 | 0 | 0 | 0 | 0 | 1 | 0 | 0 | 1 | 1 | 0 | 1 | 1 |
| P000006 | G0000187 | S0000605 | E001216 | MELD-Na | 2104 | 69 | M | 69 | 63 | 0.85 | 0.780469877251253 | 9.7 | 1.21 | 136 |  | 0 | 18 | 18 | 0 | 0 | 1 | 0 | 1 | 1 |  | -917 | -30 | 1 | 0 | 1 | 0 | 0 | 0 | 0 | 1 | 0 | 0 | 1 | 1 | 0 | 1 | 1 |
| P000006 | G0000187 | S0000606 | E001216 | reMELD-Na | 2104 | 69 | M | 69 | 63 | 0.85 | 0.780469877251253 | 9.7 | 1.21 | 136 |  |  | 16 | 15 | -1 | 1 | 0 | 0 | 1 | 1 |  | -917 | -30 | 1 | 0 | 1 | 0 | 0 | 0 | 0 | 1 | 0 | 0 | 1 | 1 | 0 | 1 | 1 |
| P000006 | G0000188 | S0000607 | E001216 | MELD | 2105 | 69 | M | 69 | 63 | 1.03 | 0.948703067650545 | 11 | 1.33 |  |  | 0 | 19 | 19 | 0 | 0 | 1 | 0 | 1 | 1 |  | -916 | -30 | 1 | 0 | 1 | 0 | 0 | 0 | 0 | 1 | 0 | 0 | 1 | 1 | 1 | 1 | 1 |
| P000006 | G0000188 | S0000608 | E001216 | MELD-Na | 2105 | 69 | M | 69 | 63 | 1.03 | 0.948703067650545 | 11 | 1.33 | 133 |  | 0 | 22 | 22 | 0 | 0 | 1 | 0 | 1 | 1 |  | -916 | -30 | 1 | 0 | 1 | 0 | 0 | 0 | 0 | 1 | 0 | 0 | 1 | 1 | 0 | 1 | 1 |
| P000006 | G0000188 | S0000609 | E001216 | reMELD-Na | 2105 | 69 | M | 69 | 63 | 1.03 | 0.948703067650545 | 11 | 1.33 | 133 |  |  | 20 | 20 | 0 | 0 | 1 | 0 | 1 | 1 |  | -916 | -30 | 1 | 0 | 1 | 0 | 0 | 0 | 0 | 1 | 0 | 0 | 1 | 1 | 0 | 1 | 1 |
| P000006 | G0000189 | S0000610 | E001216 | MELD | 2106 | 69 | M | 69 | 63 | 0.82 | 0.732196667919897 | 12.6 | 1.3 |  |  | 0 | 19 | 19 | 0 | 0 | 1 | 0 | 1 | 1 |  | -915 | -30 | 1 | 0 | 1 | 0 | 0 | 0 | 0 | 1 | 0 | 0 | 1 | 1 | 1 | 1 | 1 |
| P000006 | G0000189 | S0000611 | E001216 | MELD-Na | 2106 | 69 | M | 69 | 63 | 0.82 | 0.732196667919897 | 12.6 | 1.3 | 139 |  | 0 | 19 | 19 | 0 | 0 | 1 | 0 | 1 | 1 |  | -915 | -30 | 1 | 0 | 1 | 0 | 0 | 0 | 0 | 1 | 0 | 0 | 1 | 1 | 0 | 1 | 1 |
| P000006 | G0000189 | S0000612 | E001216 | reMELD-Na | 2106 | 69 | M | 69 | 63 | 0.82 | 0.732196667919897 | 12.6 | 1.3 | 139 |  |  | 16 | 15 | -1 | 1 | 0 | 0 | 1 | 1 |  | -915 | -30 | 1 | 0 | 1 | 0 | 0 | 0 | 0 | 1 | 0 | 0 | 1 | 1 | 0 | 1 | 1 |
| P000006 | G0000190 | S0000613 | E001216 | MELD | 2107 | 69 | M | 69 | 63 | 0.87 | 0.766912638097485 | 15.6 | 1.2 |  |  | 0 | 19 | 19 | 0 | 0 | 1 | 0 | 1 | 1 |  | -914 | -30 | 1 | 0 | 1 | 0 | 0 | 0 | 0 | 1 | 0 | 0 | 1 | 1 | 1 | 1 | 1 |
| P000006 | G0000190 | S0000614 | E001216 | MELD-Na | 2107 | 69 | M | 69 | 63 | 0.87 | 0.766912638097485 | 15.6 | 1.2 | 139 |  | 0 | 19 | 19 | 0 | 0 | 1 | 0 | 1 | 1 |  | -914 | -30 | 1 | 0 | 1 | 0 | 0 | 0 | 0 | 1 | 0 | 0 | 1 | 1 | 0 | 1 | 1 |
| P000006 | G0000190 | S0000615 | E001216 | reMELD-Na | 2107 | 69 | M | 69 | 63 | 0.87 | 0.766912638097485 | 15.6 | 1.2 | 139 |  |  | 17 | 16 | -1 | 1 | 0 | 0 | 1 | 1 |  | -914 | -30 | 1 | 0 | 1 | 0 | 0 | 0 | 0 | 1 | 0 | 0 | 1 | 1 | 0 | 1 | 1 |
| P000006 | G0000191 | S0000616 | E001216 | MELD | 2108 | 69 | M | 69 | 63 | 0.91 | 0.809872655630438 | 14.8 | 1.25 |  |  | 0 | 19 | 19 | 0 | 0 | 1 | 0 | 1 | 1 |  | -913 | -30 | 1 | 0 | 1 | 0 | 0 | 0 | 0 | 1 | 0 | 0 | 1 | 1 | 1 | 1 | 1 |
| P000006 | G0000191 | S0000617 | E001216 | MELD-Na | 2108 | 69 | M | 69 | 63 | 0.91 | 0.809872655630438 | 14.8 | 1.25 | 137 |  | 0 | 19 | 19 | 0 | 0 | 1 | 0 | 1 | 1 |  | -913 | -30 | 1 | 0 | 1 | 0 | 0 | 0 | 0 | 1 | 0 | 0 | 1 | 1 | 0 | 1 | 1 |
| P000006 | G0000191 | S0000618 | E001216 | reMELD-Na | 2108 | 69 | M | 69 | 63 | 0.91 | 0.809872655630438 | 14.8 | 1.25 | 137 |  |  | 18 | 17 | -1 | 1 | 0 | 0 | 1 | 1 |  | -913 | -30 | 1 | 0 | 1 | 0 | 0 | 0 | 0 | 1 | 0 | 0 | 1 | 1 | 0 | 1 | 1 |
| P000006 | G0000192 | S0000619 | E001216 | MELD | 2109 | 69 | M | 69 | 63 | 0.82 | 0.725750969256223 | 13.8 | 1.31 |  |  | 0 | 19 | 19 | 0 | 0 | 1 | 0 | 1 | 1 |  | -912 | -30 | 1 | 0 | 1 | 0 | 0 | 0 | 0 | 1 | 0 | 0 | 1 | 1 | 1 | 1 | 1 |
| P000006 | G0000192 | S0000620 | E001216 | MELD-Na | 2109 | 69 | M | 69 | 63 | 0.82 | 0.725750969256223 | 13.8 | 1.31 | 138 |  | 0 | 19 | 19 | 0 | 0 | 1 | 0 | 1 | 1 |  | -912 | -30 | 1 | 0 | 1 | 0 | 0 | 0 | 0 | 1 | 0 | 0 | 1 | 1 | 0 | 1 | 1 |
| P000006 | G0000192 | S0000621 | E001216 | reMELD-Na | 2109 | 69 | M | 69 | 63 | 0.82 | 0.725750969256223 | 13.8 | 1.31 | 138 |  |  | 17 | 16 | -1 | 1 | 0 | 0 | 1 | 1 |  | -912 | -30 | 1 | 0 | 1 | 0 | 0 | 0 | 0 | 1 | 0 | 0 | 1 | 1 | 0 | 1 | 1 |
| P000006 | G0000193 | S0000622 | E001216 | MELD | 2110 | 69 | M | 69 | 63 | 0.72 | 0.624384842920478 | 14.2 | 1.46 |  |  | 0 | 21 | 21 | 0 | 0 | 1 | 0 | 1 | 1 |  | -911 | -30 | 1 | 0 | 1 | 0 | 0 | 0 | 0 | 1 | 0 | 0 | 1 | 1 | 1 | 1 | 1 |
| P000006 | G0000193 | S0000623 | E001216 | MELD-Na | 2110 | 69 | M | 69 | 63 | 0.72 | 0.624384842920478 | 14.2 | 1.46 | 138 |  | 0 | 21 | 21 | 0 | 0 | 1 | 0 | 1 | 1 |  | -911 | -30 | 1 | 0 | 1 | 0 | 0 | 0 | 0 | 1 | 0 | 0 | 1 | 1 | 0 | 1 | 1 |
| P000006 | G0000193 | S0000624 | E001216 | reMELD-Na | 2110 | 69 | M | 69 | 63 | 0.72 | 0.624384842920478 | 14.2 | 1.46 | 138 |  |  | 17 | 16 | -1 | 1 | 0 | 0 | 1 | 1 |  | -911 | -30 | 1 | 0 | 1 | 0 | 0 | 0 | 0 | 1 | 0 | 0 | 1 | 1 | 0 | 1 | 1 |
| P000006 | G0000194 | S0000625 | E001216 | MELD | 2111 | 69 | M | 69 | 63 | 0.87 | 0.776763413493526 | 13.5 | 1.54 |  |  | 0 | 21 | 21 | 0 | 0 | 1 | 0 | 1 | 1 |  | -910 | -30 | 1 | 0 | 1 | 0 | 0 | 0 | 0 | 1 | 0 | 0 | 1 | 1 | 1 | 1 | 1 |
| P000006 | G0000194 | S0000626 | E001216 | MELD-Na | 2111 | 69 | M | 69 | 63 | 0.87 | 0.776763413493526 | 13.5 | 1.54 | 139 |  | 0 | 21 | 21 | 0 | 0 | 1 | 0 | 1 | 1 |  | -910 | -30 | 1 | 0 | 1 | 0 | 0 | 0 | 0 | 1 | 0 | 0 | 1 | 1 | 0 | 1 | 1 |
| P000006 | G0000194 | S0000627 | E001216 | reMELD-Na | 2111 | 69 | M | 69 | 63 | 0.87 | 0.776763413493526 | 13.5 | 1.54 | 139 |  |  | 19 | 18 | -1 | 1 | 0 | 0 | 1 | 1 |  | -910 | -30 | 1 | 0 | 1 | 0 | 0 | 0 | 0 | 1 | 0 | 0 | 1 | 1 | 0 | 1 | 1 |
| P000006 | G0000195 | S0000628 | E001216 | MELD | 2112 | 69 | M | 69 | 63 | 0.91 | 0.820570362739301 | 12.7 | 1.66 |  |  | 0 | 22 | 22 | 0 | 0 | 1 | 0 | 1 | 1 |  | -909 | -30 | 1 | 0 | 1 | 0 | 0 | 0 | 0 | 1 | 0 | 0 | 1 | 1 | 1 | 1 | 1 |
| P000006 | G0000195 | S0000629 | E001216 | MELD-Na | 2112 | 69 | M | 69 | 63 | 0.91 | 0.820570362739301 | 12.7 | 1.66 | 138 |  | 0 | 22 | 22 | 0 | 0 | 1 | 0 | 1 | 1 |  | -909 | -30 | 1 | 0 | 1 | 0 | 0 | 0 | 0 | 1 | 0 | 0 | 1 | 1 | 0 | 1 | 1 |
| P000006 | G0000195 | S0000630 | E001216 | reMELD-Na | 2112 | 69 | M | 69 | 63 | 0.91 | 0.820570362739301 | 12.7 | 1.66 | 138 |  |  | 20 | 19 | -1 | 1 | 0 | 0 | 1 | 1 |  | -909 | -30 | 1 | 0 | 1 | 0 | 0 | 0 | 0 | 1 | 0 | 0 | 1 | 1 | 0 | 1 | 1 |
| P000006 | G0000196 | S0000631 | E001216 | MELD | 2114 | 69 | M | 69 | 63 | 0.97 | 0.879038474686669 | 12.8 | 1.35 |  |  | 0 | 19 | 19 | 0 | 0 | 1 | 0 | 1 | 1 |  | -907 | -30 | 1 | 0 | 1 | 0 | 0 | 0 | 0 | 1 | 0 | 0 | 1 | 1 | 1 | 1 | 1 |
| P000006 | G0000196 | S0000632 | E001216 | MELD-Na | 2114 | 69 | M | 69 | 63 | 0.97 | 0.879038474686669 | 12.8 | 1.35 | 141 |  | 0 | 19 | 19 | 0 | 0 | 1 | 0 | 1 | 1 |  | -907 | -30 | 1 | 0 | 1 | 0 | 0 | 0 | 0 | 1 | 0 | 0 | 1 | 1 | 0 | 1 | 1 |
| P000006 | G0000196 | S0000633 | E001216 | reMELD-Na | 2114 | 69 | M | 69 | 63 | 0.97 | 0.879038474686669 | 12.8 | 1.35 | 141 |  |  | 18 | 17 | -1 | 1 | 0 | 0 | 1 | 1 |  | -907 | -30 | 1 | 0 | 1 | 0 | 0 | 0 | 0 | 1 | 0 | 0 | 1 | 1 | 0 | 1 | 1 |
| P000006 | G0000197 | S0000634 | E001216 | MELD | 2118 | 69 | M | 69 | 63 | 0.92 | 0.817077190732223 | 15.4 | 1.24 |  |  | 0 | 19 | 19 | 0 | 0 | 1 | 0 | 1 | 1 |  | -903 | -30 | 1 | 0 | 1 | 0 | 0 | 0 | 0 | 1 | 0 | 0 | 1 | 1 | 1 | 1 | 1 |
| P000006 | G0000197 | S0000635 | E001216 | MELD-Na | 2118 | 69 | M | 69 | 63 | 0.92 | 0.817077190732223 | 15.4 | 1.24 | 139 |  | 0 | 19 | 19 | 0 | 0 | 1 | 0 | 1 | 1 |  | -903 | -30 | 1 | 0 | 1 | 0 | 0 | 0 | 0 | 1 | 0 | 0 | 1 | 1 | 0 | 1 | 1 |
| P000006 | G0000197 | S0000636 | E001216 | reMELD-Na | 2118 | 69 | M | 69 | 63 | 0.92 | 0.817077190732223 | 15.4 | 1.24 | 139 |  |  | 18 | 16 | -2 | 1 | 0 | 0 | 1 | 1 |  | -903 | -30 | 1 | 0 | 1 | 0 | 0 | 0 | 0 | 1 | 0 | 0 | 1 | 1 | 0 | 1 | 1 |
| P000006 | G0000198 | S0000637 | E001216 | MELD | 2119 | 69 | M | 69 | 63 | 1 | 0.903661525037479 | 13.7 | 1.27 |  |  | 0 | 19 | 19 | 0 | 0 | 1 | 0 | 1 | 1 |  | -902 | -30 | 1 | 0 | 1 | 0 | 0 | 0 | 0 | 1 | 0 | 0 | 1 | 1 | 1 | 1 | 1 |
| P000006 | G0000198 | S0000638 | E001216 | MELD-Na | 2119 | 69 | M | 69 | 63 | 1 | 0.903661525037479 | 13.7 | 1.27 | 140 |  | 0 | 19 | 19 | 0 | 0 | 1 | 0 | 1 | 1 |  | -902 | -30 | 1 | 0 | 1 | 0 | 0 | 0 | 0 | 1 | 0 | 0 | 1 | 1 | 0 | 1 | 1 |
| P000006 | G0000198 | S0000639 | E001216 | reMELD-Na | 2119 | 69 | M | 69 | 63 | 1 | 0.903661525037479 | 13.7 | 1.27 | 140 |  |  | 18 | 17 | -1 | 1 | 0 | 0 | 1 | 1 |  | -902 | -30 | 1 | 0 | 1 | 0 | 0 | 0 | 0 | 1 | 0 | 0 | 1 | 1 | 0 | 1 | 1 |
| P000006 | G0000199 | S0000640 | E001216 | MELD | 2122 | 69 | M | 69 | 63 | 0.94 | 0.839872070875855 | 14.7 | 1.29 |  |  | 0 | 19 | 19 | 0 | 0 | 1 | 0 | 1 | 1 |  | -899 | -29 | 1 | 0 | 1 | 0 | 0 | 0 | 0 | 1 | 0 | 0 | 1 | 1 | 1 | 1 | 1 |
| P000006 | G0000199 | S0000641 | E001216 | MELD 3.0 | 2122 | 69 | M | 69 | 63 | 0.94 | 0.839872070875855 | 14.7 | 1.29 | 139 | 2.76 |  | 22 | 22 | 0 | 0 | 1 | 0 | 1 | 1 |  | -899 | -29 | 1 | 0 | 1 | 0 | 0 | 0 | 0 | 1 | 0 | 0 | 1 | 1 | 0 | 1 | 1 |
| P000006 | G0000199 | S0000642 | E001216 | MELD-Na | 2122 | 69 | M | 69 | 63 | 0.94 | 0.839872070875855 | 14.7 | 1.29 | 139 |  | 0 | 19 | 19 | 0 | 0 | 1 | 0 | 1 | 1 |  | -899 | -29 | 1 | 0 | 1 | 0 | 0 | 0 | 0 | 1 | 0 | 0 | 1 | 1 | 0 | 1 | 1 |
| P000006 | G0000199 | S0000643 | E001216 | reMELD-Na | 2122 | 69 | M | 69 | 63 | 0.94 | 0.839872070875855 | 14.7 | 1.29 | 139 |  |  | 18 | 17 | -1 | 1 | 0 | 0 | 1 | 1 |  | -899 | -29 | 1 | 0 | 1 | 0 | 0 | 0 | 0 | 1 | 0 | 0 | 1 | 1 | 0 | 1 | 1 |
| P000006 | G0000200 | S0000644 | E001216 | MELD | 2125 | 69 | M | 69 | 63 | 0.99 | 0.908229630344509 | 11.2 | 1.41 |  |  | 0 | 19 | 19 | 0 | 0 | 1 | 0 | 1 | 1 |  | -896 | -29 | 1 | 0 | 1 | 0 | 0 | 0 | 0 | 1 | 0 | 0 | 1 | 1 | 1 | 1 | 1 |
| P000006 | G0000200 | S0000645 | E001216 | MELD-Na | 2125 | 69 | M | 69 | 63 | 0.99 | 0.908229630344509 | 11.2 | 1.41 | 137 |  | 0 | 19 | 19 | 0 | 0 | 1 | 0 | 1 | 1 |  | -896 | -29 | 1 | 0 | 1 | 0 | 0 | 0 | 0 | 1 | 0 | 0 | 1 | 1 | 0 | 1 | 1 |
| P000006 | G0000200 | S0000646 | E001216 | reMELD-Na | 2125 | 69 | M | 69 | 63 | 0.99 | 0.908229630344509 | 11.2 | 1.41 | 137 |  |  | 19 | 18 | -1 | 1 | 0 | 0 | 1 | 1 |  | -896 | -29 | 1 | 0 | 1 | 0 | 0 | 0 | 0 | 1 | 0 | 0 | 1 | 1 | 0 | 1 | 1 |
| P000006 | G0000201 | S0000647 | E001216 | MELD | 2127 | 69 | M | 69 | 63 | 1.04 | 0.971708115836011 | 9.1 | 1.38 |  |  | 0 | 19 | 18 | -1 | 1 | 0 | 0 | 1 | 1 |  | -894 | -29 | 1 | 0 | 1 | 0 | 0 | 0 | 0 | 1 | 0 | 0 | 1 | 1 | 1 | 1 | 1 |
| P000006 | G0000201 | S0000648 | E001216 | MELD 3.0 | 2127 | 69 | M | 69 | 63 | 1.04 | 0.971708115836011 | 9.1 | 1.38 | 138 | 2.59 |  | 21 | 21 | 0 | 0 | 1 | 0 | 1 | 1 |  | -894 | -29 | 1 | 0 | 1 | 0 | 0 | 0 | 0 | 1 | 0 | 0 | 1 | 1 | 0 | 1 | 1 |
| P000006 | G0000201 | S0000649 | E001216 | MELD-Na | 2127 | 69 | M | 69 | 63 | 1.04 | 0.971708115836011 | 9.1 | 1.38 | 138 |  | 0 | 19 | 18 | -1 | 1 | 0 | 0 | 1 | 1 |  | -894 | -29 | 1 | 0 | 1 | 0 | 0 | 0 | 0 | 1 | 0 | 0 | 1 | 1 | 0 | 1 | 1 |
| P000006 | G0000201 | S0000650 | E001216 | reMELD-Na | 2127 | 69 | M | 69 | 63 | 1.04 | 0.971708115836011 | 9.1 | 1.38 | 138 |  |  | 18 | 17 | -1 | 1 | 0 | 0 | 1 | 1 |  | -894 | -29 | 1 | 0 | 1 | 0 | 0 | 0 | 0 | 1 | 0 | 0 | 1 | 1 | 0 | 1 | 1 |
| P000006 | G0000202 | S0000651 | E001216 | MELD | 2129 | 69 | M | 69 | 63 | 1.06 | 0.994270478535359 | 8.7 | 1.29 |  |  | 0 | 18 | 17 | -1 | 1 | 0 | 0 | 1 | 1 |  | -892 | -29 | 1 | 0 | 1 | 0 | 0 | 0 | 0 | 1 | 0 | 0 | 1 | 1 | 1 | 1 | 1 |
| P000006 | G0000202 | S0000652 | E001216 | MELD 3.0 | 2129 | 69 | M | 69 | 63 | 1.06 | 0.994270478535359 | 8.7 | 1.29 | 136 | 2.78 |  | 20 | 20 | 0 | 0 | 1 | 0 | 1 | 1 |  | -892 | -29 | 1 | 0 | 1 | 0 | 0 | 0 | 0 | 1 | 0 | 0 | 1 | 1 | 0 | 1 | 1 |
| P000006 | G0000202 | S0000653 | E001216 | MELD-Na | 2129 | 69 | M | 69 | 63 | 1.06 | 0.994270478535359 | 8.7 | 1.29 | 136 |  | 0 | 19 | 18 | -1 | 1 | 0 | 0 | 1 | 1 |  | -892 | -29 | 1 | 0 | 1 | 0 | 0 | 0 | 0 | 1 | 0 | 0 | 1 | 1 | 0 | 1 | 1 |
| P000006 | G0000202 | S0000654 | E001216 | reMELD-Na | 2129 | 69 | M | 69 | 63 | 1.06 | 0.994270478535359 | 8.7 | 1.29 | 136 |  |  | 18 | 18 | 0 | 0 | 1 | 0 | 1 | 1 |  | -892 | -29 | 1 | 0 | 1 | 0 | 0 | 0 | 0 | 1 | 0 | 0 | 1 | 1 | 0 | 1 | 1 |
| P000006 | G0000203 | S0000655 | E001216 | MELD | 2136 | 70 | M | 69 | 63 | 1.57 | 1.5117984857272 | 5.5 | 1.24 |  |  | 0 | 20 | 19 | -1 | 1 | 0 | 0 | 1 | 1 |  | -885 | -29 | 1 | 0 | 1 | 0 | 0 | 0 | 0 | 1 | 0 | 0 | 1 | 1 | 1 | 1 | 1 |
| P000006 | G0000203 | S0000656 | E001216 | MELD 3.0 | 2136 | 70 | M | 69 | 63 | 1.57 | 1.5117984857272 | 5.5 | 1.24 | 134 | 2.8 |  | 23 | 22 | -1 | 1 | 0 | 0 | 1 | 1 |  | -885 | -29 | 1 | 0 | 1 | 0 | 0 | 0 | 0 | 1 | 0 | 0 | 1 | 1 | 0 | 1 | 1 |
| P000006 | G0000203 | S0000657 | E001216 | MELD-Na | 2136 | 70 | M | 69 | 63 | 1.57 | 1.5117984857272 | 5.5 | 1.24 | 134 |  | 0 | 22 | 21 | -1 | 1 | 0 | 0 | 1 | 1 |  | -885 | -29 | 1 | 0 | 1 | 0 | 0 | 0 | 0 | 1 | 0 | 0 | 1 | 1 | 0 | 1 | 1 |
| P000006 | G0000203 | S0000658 | E001216 | reMELD-Na | 2136 | 70 | M | 69 | 63 | 1.57 | 1.5117984857272 | 5.5 | 1.24 | 134 |  |  | 20 | 20 | 0 | 0 | 1 | 0 | 1 | 1 |  | -885 | -29 | 1 | 0 | 1 | 0 | 0 | 0 | 0 | 1 | 0 | 0 | 1 | 1 | 0 | 1 | 1 |
| P000006 | G0000204 | S0000659 | E001216 | MELD | 2143 | 70 | M | 69 | 63 | 0.77 | 0.743135674886446 | 4.7 | 1.22 |  |  | 0 | 15 | 15 | 0 | 0 | 1 | 0 | 1 | 1 |  | -878 | -29 | 1 | 0 | 1 | 0 | 0 | 0 | 0 | 1 | 0 | 0 | 1 | 1 | 1 | 1 | 1 |
| P000006 | G0000204 | S0000660 | E001216 | MELD-Na | 2143 | 70 | M | 69 | 63 | 0.77 | 0.743135674886446 | 4.7 | 1.22 | 134 |  | 0 | 17 | 17 | 0 | 0 | 1 | 0 | 1 | 1 |  | -878 | -29 | 1 | 0 | 1 | 0 | 0 | 0 | 0 | 1 | 0 | 0 | 1 | 1 | 0 | 1 | 1 |
| P000006 | G0000204 | S0000661 | E001216 | reMELD-Na | 2143 | 70 | M | 69 | 63 | 0.77 | 0.743135674886446 | 4.7 | 1.22 | 134 |  |  | 14 | 14 | 0 | 0 | 1 | 0 | 1 | 1 |  | -878 | -29 | 1 | 0 | 1 | 0 | 0 | 0 | 0 | 1 | 0 | 0 | 1 | 1 | 0 | 1 | 1 |
| P000006 | G0000205 | S0000662 | E001216 | MELD | 2146 | 70 | M | 69 | 63 | 0.77 | 0.750018726464626 | 4 | 1.21 |  |  | 0 | 14 | 14 | 0 | 0 | 1 | 0 | 1 | 1 |  | -875 | -29 | 1 | 0 | 1 | 0 | 0 | 0 | 0 | 1 | 0 | 0 | 1 | 1 | 1 | 1 | 1 |
| P000006 | G0000205 | S0000663 | E001216 | MELD-Na | 2146 | 70 | M | 69 | 63 | 0.77 | 0.750018726464626 | 4 | 1.21 | 135 |  | 0 | 16 | 16 | 0 | 0 | 1 | 0 | 1 | 1 |  | -875 | -29 | 1 | 0 | 1 | 0 | 0 | 0 | 0 | 1 | 0 | 0 | 1 | 1 | 0 | 1 | 1 |
| P000006 | G0000205 | S0000664 | E001216 | reMELD-Na | 2146 | 70 | M | 69 | 63 | 0.77 | 0.750018726464626 | 4 | 1.21 | 135 |  |  | 13 | 13 | 0 | 0 | 1 | 0 | 1 | 1 |  | -875 | -29 | 1 | 0 | 1 | 0 | 0 | 0 | 0 | 1 | 0 | 0 | 1 | 1 | 0 | 1 | 1 |
| P000006 | G0000206 | S0000665 | E001243 | MELD | 2183 | 71 | M | 69 | 63 | 1.21 | 1.20262448230872 | 2 | 1.42 |  |  | 0 | 15 | 15 | 0 | 0 | 1 | 0 | 1 | 1 |  | -838 | -27 | 1 | 0 | 1 | 0 | 0 | 0 | 0 | 1 | 0 | 0 | 1 | 1 | 1 | 1 | 1 |
| P000006 | G0000206 | S0000666 | E001243 | MELD-Na | 2183 | 71 | M | 69 | 63 | 1.21 | 1.20262448230872 | 2 | 1.42 | 135 |  | 0 | 17 | 17 | 0 | 0 | 1 | 0 | 1 | 1 |  | -838 | -27 | 1 | 0 | 1 | 0 | 0 | 0 | 0 | 1 | 0 | 0 | 1 | 1 | 0 | 1 | 1 |
| P000006 | G0000206 | S0000667 | E001243 | reMELD-Na | 2183 | 71 | M | 69 | 63 | 1.21 | 1.20262448230872 | 2 | 1.42 | 135 |  |  | 16 | 16 | 0 | 0 | 1 | 0 | 1 | 1 |  | -838 | -27 | 1 | 0 | 1 | 0 | 0 | 0 | 0 | 1 | 0 | 0 | 1 | 1 | 0 | 1 | 1 |
| P000006 | G0000207 | S0000668 | E001243 | MELD | 2195 | 72 | M | 69 | 63 | 1.57 | 1.54617316619204 | 2.1 | 1.3 |  |  | 0 | 16 | 16 | 0 | 0 | 1 | 0 | 1 | 1 |  | -826 | -27 | 1 | 0 | 1 | 0 | 0 | 0 | 0 | 1 | 0 | 0 | 1 | 1 | 1 | 1 | 1 |
| P000006 | G0000207 | S0000669 | E001243 | MELD-Na | 2195 | 72 | M | 69 | 63 | 1.57 | 1.54617316619204 | 2.1 | 1.3 | 136 |  | 0 | 17 | 17 | 0 | 0 | 1 | 0 | 1 | 1 |  | -826 | -27 | 1 | 0 | 1 | 0 | 0 | 0 | 0 | 1 | 0 | 0 | 1 | 1 | 0 | 1 | 1 |
| P000006 | G0000207 | S0000670 | E001243 | reMELD-Na | 2195 | 72 | M | 69 | 63 | 1.57 | 1.54617316619204 | 2.1 | 1.3 | 136 |  |  | 17 | 17 | 0 | 0 | 1 | 0 | 1 | 1 |  | -826 | -27 | 1 | 0 | 1 | 0 | 0 | 0 | 0 | 1 | 0 | 0 | 1 | 1 | 0 | 1 | 1 |
| P000006 | G0000208 | S0000671 | E001266 | MELD | 2202 | 72 | M | 69 | 63 | 1.4 | 1.38768463942459 | 1.8 | 1.13 |  |  | 0 | 13 | 13 | 0 | 0 | 1 | 0 | 1 | 1 |  | -819 | -27 | 1 | 0 | 1 | 0 | 0 | 0 | 0 | 1 | 0 | 0 | 1 | 1 | 1 | 1 | 1 |
| P000006 | G0000208 | S0000672 | E001266 | MELD 3.0 | 2202 | 72 | M | 69 | 63 | 1.4 | 1.38768463942459 | 1.8 | 1.13 | 136 | 3.17 |  | 15 | 15 | 0 | 0 | 1 | 0 | 1 | 1 |  | -819 | -27 | 1 | 0 | 1 | 0 | 0 | 0 | 0 | 1 | 0 | 0 | 1 | 1 | 0 | 1 | 1 |
| P000006 | G0000208 | S0000673 | E001266 | MELD-Na | 2202 | 72 | M | 69 | 63 | 1.4 | 1.38768463942459 | 1.8 | 1.13 | 136 |  | 0 | 14 | 14 | 0 | 0 | 1 | 0 | 1 | 1 |  | -819 | -27 | 1 | 0 | 1 | 0 | 0 | 0 | 0 | 1 | 0 | 0 | 1 | 1 | 0 | 1 | 1 |
| P000006 | G0000208 | S0000674 | E001266 | reMELD-Na | 2202 | 72 | M | 69 | 63 | 1.4 | 1.38768463942459 | 1.8 | 1.13 | 136 |  |  | 15 | 14 | -1 | 1 | 0 | 0 | 1 | 1 |  | -819 | -27 | 1 | 0 | 1 | 0 | 0 | 0 | 0 | 1 | 0 | 0 | 1 | 1 | 0 | 1 | 1 |
| P000006 | G0000209 | S0000675 | E001266 | MELD | 2205 | 72 | M | 69 | 63 | 1.62 | 1.5956421318068 | 1.9 | 1.14 |  |  | 0 | 15 | 15 | 0 | 0 | 1 | 0 | 1 | 1 |  | -816 | -27 | 1 | 0 | 1 | 0 | 0 | 0 | 0 | 1 | 0 | 0 | 1 | 1 | 1 | 1 | 1 |
| P000006 | G0000209 | S0000676 | E001266 | MELD-Na | 2205 | 72 | M | 69 | 63 | 1.62 | 1.5956421318068 | 1.9 | 1.14 | 140 |  | 0 | 15 | 15 | 0 | 0 | 1 | 0 | 1 | 1 |  | -816 | -27 | 1 | 0 | 1 | 0 | 0 | 0 | 0 | 1 | 0 | 0 | 1 | 1 | 0 | 1 | 1 |
| P000006 | G0000209 | S0000677 | E001266 | reMELD-Na | 2205 | 72 | M | 69 | 63 | 1.62 | 1.5956421318068 | 1.9 | 1.14 | 140 |  |  | 15 | 15 | 0 | 0 | 1 | 0 | 1 | 1 |  | -816 | -27 | 1 | 0 | 1 | 0 | 0 | 0 | 0 | 1 | 0 | 0 | 1 | 1 | 0 | 1 | 1 |
| P000006 | G0000210 | S0000678 | E001266 | MELD | 2206 | 72 | M | 69 | 63 | 1.62 | 1.5956421318068 | 1.9 | 1.06 |  |  | 0 | 14 | 14 | 0 | 0 | 1 | 0 | 1 | 1 |  | -815 | -27 | 1 | 0 | 1 | 0 | 0 | 0 | 0 | 1 | 0 | 0 | 1 | 1 | 1 | 1 | 1 |
| P000006 | G0000210 | S0000679 | E001266 | MELD 3.0 | 2206 | 72 | M | 69 | 63 | 1.62 | 1.5956421318068 | 1.9 | 1.06 | 140 | 3.64 |  | 15 | 15 | 0 | 0 | 1 | 0 | 1 | 1 |  | -815 | -27 | 1 | 0 | 1 | 0 | 0 | 0 | 0 | 1 | 0 | 0 | 1 | 1 | 0 | 1 | 1 |
| P000006 | G0000210 | S0000680 | E001266 | MELD-Na | 2206 | 72 | M | 69 | 63 | 1.62 | 1.5956421318068 | 1.9 | 1.06 | 140 |  | 0 | 14 | 14 | 0 | 0 | 1 | 0 | 1 | 1 |  | -815 | -27 | 1 | 0 | 1 | 0 | 0 | 0 | 0 | 1 | 0 | 0 | 1 | 1 | 0 | 1 | 1 |
| P000006 | G0000210 | S0000681 | E001266 | reMELD-Na | 2206 | 72 | M | 69 | 63 | 1.62 | 1.5956421318068 | 1.9 | 1.06 | 140 |  |  | 15 | 15 | 0 | 0 | 1 | 0 | 1 | 1 |  | -815 | -27 | 1 | 0 | 1 | 0 | 0 | 0 | 0 | 1 | 0 | 0 | 1 | 1 | 0 | 1 | 1 |
| P000006 | G0000211 | S0000682 | E001266 | MELD | 2211 | 72 | M | 69 | 63 | 1.15 | 1.14889012036912 | 1.6 | 1.14 |  |  | 0 | 11 | 11 | 0 | 0 | 1 | 0 | 1 | 1 |  | -810 | -27 | 1 | 0 | 1 | 0 | 0 | 0 | 0 | 1 | 0 | 0 | 1 | 1 | 1 | 1 | 1 |
| P000006 | G0000211 | S0000683 | E001266 | MELD 3.0 | 2211 | 72 | M | 69 | 63 | 1.15 | 1.14889012036912 | 1.6 | 1.14 | 136 | 2.67 |  | 13 | 13 | 0 | 0 | 1 | 0 | 1 | 1 |  | -810 | -27 | 1 | 0 | 1 | 0 | 0 | 0 | 0 | 1 | 0 | 0 | 1 | 1 | 0 | 1 | 1 |
| P000006 | G0000211 | S0000684 | E001266 | MELD-Na | 2211 | 72 | M | 69 | 63 | 1.15 | 1.14889012036912 | 1.6 | 1.14 | 136 |  | 0 | 12 | 12 | 0 | 0 | 1 | 0 | 1 | 1 |  | -810 | -27 | 1 | 0 | 1 | 0 | 0 | 0 | 0 | 1 | 0 | 0 | 1 | 1 | 0 | 1 | 1 |
| P000006 | G0000211 | S0000685 | E001266 | reMELD-Na | 2211 | 72 | M | 69 | 63 | 1.15 | 1.14889012036912 | 1.6 | 1.14 | 136 |  |  | 13 | 13 | 0 | 0 | 1 | 0 | 1 | 1 |  | -810 | -27 | 1 | 0 | 1 | 0 | 0 | 0 | 0 | 1 | 0 | 0 | 1 | 1 | 0 | 1 | 1 |
| P000006 | G0000212 | S0000686 | E001243 | MELD | 2218 | 72 | M | 69 | 63 | 1.83 | 1.79928429988752 | 1.3 | 1.33 |  |  | 0 | 16 | 16 | 0 | 0 | 1 | 0 | 1 | 1 |  | -803 | -26 | 1 | 0 | 1 | 0 | 0 | 0 | 0 | 1 | 0 | 0 | 1 | 1 | 1 | 1 | 1 |
| P000006 | G0000212 | S0000687 | E001243 | MELD-Na | 2218 | 72 | M | 69 | 63 | 1.83 | 1.79928429988752 | 1.3 | 1.33 | 141 |  | 0 | 16 | 16 | 0 | 0 | 1 | 0 | 1 | 1 |  | -803 | -26 | 1 | 0 | 1 | 0 | 0 | 0 | 0 | 1 | 0 | 0 | 1 | 1 | 0 | 1 | 1 |
| P000006 | G0000212 | S0000688 | E001243 | reMELD-Na | 2218 | 72 | M | 69 | 63 | 1.83 | 1.79928429988752 | 1.3 | 1.33 | 141 |  |  | 17 | 17 | 0 | 0 | 1 | 0 | 1 | 1 |  | -803 | -26 | 1 | 0 | 1 | 0 | 0 | 0 | 0 | 1 | 0 | 0 | 1 | 1 | 0 | 1 | 1 |
| P000006 | G0000213 | S0000689 | E001280 | MELD | 2219 | 72 | M | 69 | 63 | 1.735 | 1.70826267022479 | 1.5 | 1.23 |  |  | 0 | 16 | 15 | -1 | 1 | 0 | 0 | 1 | 1 |  | -802 | -26 | 1 | 0 | 1 | 0 | 0 | 0 | 0 | 1 | 0 | 0 | 1 | 1 | 1 | 1 | 1 |
| P000006 | G0000213 | S0000690 | E001280 | MELD 3.0 | 2219 | 72 | M | 69 | 63 | 1.735 | 1.70826267022479 | 1.5 | 1.23 | 142 | 2.71 |  | 17 | 16 | -1 | 1 | 0 | 0 | 1 | 1 |  | -802 | -26 | 1 | 0 | 1 | 0 | 0 | 0 | 0 | 1 | 0 | 0 | 1 | 1 | 0 | 1 | 1 |
| P000006 | G0000213 | S0000691 | E001280 | MELD-Na | 2219 | 72 | M | 69 | 63 | 1.735 | 1.70826267022479 | 1.5 | 1.23 | 142 |  | 0 | 16 | 15 | -1 | 1 | 0 | 0 | 1 | 1 |  | -802 | -26 | 1 | 0 | 1 | 0 | 0 | 0 | 0 | 1 | 0 | 0 | 1 | 1 | 0 | 1 | 1 |
| P000006 | G0000213 | S0000692 | E001280 | reMELD-Na | 2219 | 72 | M | 69 | 63 | 1.735 | 1.70826267022479 | 1.5 | 1.23 | 142 |  |  | 16 | 16 | 0 | 0 | 1 | 0 | 1 | 1 |  | -802 | -26 | 1 | 0 | 1 | 0 | 0 | 0 | 0 | 1 | 0 | 0 | 1 | 1 | 0 | 1 | 1 |
| P000006 | G0000214 | S0000693 | E001280 | MELD | 2220 | 72 | M | 69 | 63 | 1.7 | 1.67655830078969 | 1.4 | 1.17 |  |  | 0 | 15 | 14 | -1 | 1 | 0 | 0 | 1 | 1 |  | -801 | -26 | 1 | 0 | 1 | 0 | 0 | 0 | 0 | 1 | 0 | 0 | 1 | 1 | 1 | 1 | 1 |
| P000006 | G0000214 | S0000694 | E001280 | MELD 3.0 | 2220 | 72 | M | 69 | 63 | 1.7 | 1.67655830078969 | 1.4 | 1.17 | 138 | 2.92 |  | 15 | 15 | 0 | 0 | 1 | 0 | 1 | 1 |  | -801 | -26 | 1 | 0 | 1 | 0 | 0 | 0 | 0 | 1 | 0 | 0 | 1 | 1 | 0 | 1 | 1 |
| P000006 | G0000214 | S0000695 | E001280 | MELD-Na | 2220 | 72 | M | 69 | 63 | 1.7 | 1.67655830078969 | 1.4 | 1.17 | 138 |  | 0 | 15 | 14 | -1 | 1 | 0 | 0 | 1 | 1 |  | -801 | -26 | 1 | 0 | 1 | 0 | 0 | 0 | 0 | 1 | 0 | 0 | 1 | 1 | 0 | 1 | 1 |
| P000006 | G0000214 | S0000696 | E001280 | reMELD-Na | 2220 | 72 | M | 69 | 63 | 1.7 | 1.67655830078969 | 1.4 | 1.17 | 138 |  |  | 15 | 15 | 0 | 0 | 1 | 0 | 1 | 1 |  | -801 | -26 | 1 | 0 | 1 | 0 | 0 | 0 | 0 | 1 | 0 | 0 | 1 | 1 | 0 | 1 | 1 |
| P000006 | G0000215 | S0000697 | E001280 | MELD | 2224 | 73 | M | 69 | 63 | 1 | 1.00703827710504 | 1.2 | 1.14 |  |  | 0 | 9 | 9 | 0 | 0 | 1 | 0 | 1 | 1 |  | -797 | -26 | 1 | 0 | 1 | 0 | 0 | 0 | 0 | 1 | 0 | 0 | 1 | 1 | 1 | 1 | 1 |
| P000006 | G0000215 | S0000698 | E001280 | MELD-Na | 2224 | 73 | M | 69 | 63 | 1 | 1.00703827710504 | 1.2 | 1.14 | 137.5 |  | 0 | 9 | 9 | 0 | 0 | 1 | 0 | 1 | 1 |  | -797 | -26 | 1 | 0 | 1 | 0 | 0 | 0 | 0 | 1 | 0 | 0 | 1 | 1 | 0 | 1 | 1 |
| P000006 | G0000215 | S0000699 | E001280 | reMELD-Na | 2224 | 73 | M | 69 | 63 | 1 | 1.00703827710504 | 1.2 | 1.14 | 137.5 |  |  | 10 | 10 | 0 | 0 | 1 | 0 | 1 | 1 |  | -797 | -26 | 1 | 0 | 1 | 0 | 0 | 0 | 0 | 1 | 0 | 0 | 1 | 1 | 0 | 1 | 1 |
| P000006 | G0000216 | S0000700 | E001280 | MELD | 2226 | 73 | M | 69 | 63 | 0.89 | 0.899997322029041 | 1.1 | 1.2 |  |  | 0 | 9 | 9 | 0 | 0 | 1 | 0 | 1 | 1 |  | -795 | -26 | 1 | 0 | 1 | 0 | 0 | 0 | 0 | 1 | 0 | 0 | 1 | 1 | 1 | 1 | 1 |
| P000006 | G0000216 | S0000701 | E001280 | MELD-Na | 2226 | 73 | M | 69 | 63 | 0.89 | 0.899997322029041 | 1.1 | 1.2 | 138 |  | 0 | 9 | 9 | 0 | 0 | 1 | 0 | 1 | 1 |  | -795 | -26 | 1 | 0 | 1 | 0 | 0 | 0 | 0 | 1 | 0 | 0 | 1 | 1 | 0 | 1 | 1 |
| P000006 | G0000216 | S0000702 | E001280 | reMELD-Na | 2226 | 73 | M | 69 | 63 | 0.89 | 0.899997322029041 | 1.1 | 1.2 | 138 |  |  | 9 | 9 | 0 | 0 | 1 | 0 | 1 | 1 |  | -795 | -26 | 1 | 0 | 1 | 0 | 0 | 0 | 0 | 1 | 0 | 0 | 1 | 1 | 0 | 1 | 1 |
| P000006 | G0000217 | S0000703 | E001243 | MELD | 2231 | 73 | M | 69 | 63 | 1.12 | 1.12310838693986 | 1.3 | 1.17 |  |  | 0 | 10 | 10 | 0 | 0 | 1 | 0 | 1 | 1 |  | -790 | -26 | 1 | 0 | 1 | 0 | 0 | 0 | 0 | 1 | 0 | 0 | 1 | 1 | 1 | 1 | 1 |
| P000006 | G0000217 | S0000704 | E001243 | MELD-Na | 2231 | 73 | M | 69 | 63 | 1.12 | 1.12310838693986 | 1.3 | 1.17 | 135 |  | 0 | 12 | 12 | 0 | 0 | 1 | 0 | 1 | 1 |  | -790 | -26 | 1 | 0 | 1 | 0 | 0 | 0 | 0 | 1 | 0 | 0 | 1 | 1 | 0 | 1 | 1 |
| P000006 | G0000217 | S0000705 | E001243 | reMELD-Na | 2231 | 73 | M | 69 | 63 | 1.12 | 1.12310838693986 | 1.3 | 1.17 | 135 |  |  | 12 | 12 | 0 | 0 | 1 | 0 | 1 | 1 |  | -790 | -26 | 1 | 0 | 1 | 0 | 0 | 0 | 0 | 1 | 0 | 0 | 1 | 1 | 0 | 1 | 1 |
| P000006 | G0000218 | S0000706 | E001304 | MELD | 2243 | 73 | M | 69 | 63 | 1.25 | 1.24239906690322 | 1.9 | 1.2 |  |  | 0 | 13 | 13 | 0 | 0 | 1 | 0 | 1 | 1 |  | -778 | -26 | 1 | 0 | 1 | 0 | 0 | 0 | 0 | 1 | 0 | 0 | 1 | 1 | 1 | 1 | 1 |
| P000006 | G0000218 | S0000707 | E001304 | MELD-Na | 2243 | 73 | M | 69 | 63 | 1.25 | 1.24239906690322 | 1.9 | 1.2 | 140 |  | 0 | 13 | 13 | 0 | 0 | 1 | 0 | 1 | 1 |  | -778 | -26 | 1 | 0 | 1 | 0 | 0 | 0 | 0 | 1 | 0 | 0 | 1 | 1 | 0 | 1 | 1 |
| P000006 | G0000218 | S0000708 | E001304 | reMELD-Na | 2243 | 73 | M | 69 | 63 | 1.25 | 1.24239906690322 | 1.9 | 1.2 | 140 |  |  | 14 | 14 | 0 | 0 | 1 | 0 | 1 | 1 |  | -778 | -26 | 1 | 0 | 1 | 0 | 0 | 0 | 0 | 1 | 0 | 0 | 1 | 1 | 0 | 1 | 1 |
| P000006 | G0000219 | S0000709 | E001307 | MELD | 2252 | 73 | M | 69 | 63 | 1.06 | 1.05132767300497 | 2.5 | 1.31 |  |  | 0 | 13 | 13 | 0 | 0 | 1 | 0 | 1 | 1 |  | -769 | -25 | 1 | 0 | 1 | 0 | 0 | 0 | 0 | 1 | 0 | 0 | 1 | 1 | 1 | 1 | 1 |
| P000006 | G0000219 | S0000710 | E001307 | MELD-Na | 2252 | 73 | M | 69 | 63 | 1.06 | 1.05132767300497 | 2.5 | 1.31 | 138 |  | 0 | 13 | 13 | 0 | 0 | 1 | 0 | 1 | 1 |  | -769 | -25 | 1 | 0 | 1 | 0 | 0 | 0 | 0 | 1 | 0 | 0 | 1 | 1 | 0 | 1 | 1 |
| P000006 | G0000219 | S0000711 | E001307 | reMELD-Na | 2252 | 73 | M | 69 | 63 | 1.06 | 1.05132767300497 | 2.5 | 1.31 | 138 |  |  | 14 | 14 | 0 | 0 | 1 | 0 | 1 | 1 |  | -769 | -25 | 1 | 0 | 1 | 0 | 0 | 0 | 0 | 1 | 0 | 0 | 1 | 1 | 0 | 1 | 1 |
| P000006 | G0000220 | S0000712 | E001307 | MELD | 2253 | 74 | M | 69 | 63 | 0.98 | 0.97192580561351 | 2.6 | 1.24 |  |  | 0 | 12 | 12 | 0 | 0 | 1 | 0 | 1 | 1 |  | -768 | -25 | 1 | 0 | 1 | 0 | 0 | 0 | 0 | 1 | 0 | 0 | 1 | 1 | 1 | 1 | 1 |
| P000006 | G0000220 | S0000713 | E001307 | MELD-Na | 2253 | 74 | M | 69 | 63 | 0.98 | 0.97192580561351 | 2.6 | 1.24 | 139 |  | 0 | 12 | 12 | 0 | 0 | 1 | 0 | 1 | 1 |  | -768 | -25 | 1 | 0 | 1 | 0 | 0 | 0 | 0 | 1 | 0 | 0 | 1 | 1 | 0 | 1 | 1 |
| P000006 | G0000220 | S0000714 | E001307 | reMELD-Na | 2253 | 74 | M | 69 | 63 | 0.98 | 0.97192580561351 | 2.6 | 1.24 | 139 |  |  | 13 | 13 | 0 | 0 | 1 | 0 | 1 | 1 |  | -768 | -25 | 1 | 0 | 1 | 0 | 0 | 0 | 0 | 1 | 0 | 0 | 1 | 1 | 0 | 1 | 1 |
| P000006 | G0000221 | S0000715 | E001307 | MELD | 2255 | 74 | M | 69 | 63 | 1 | 0.999167138528565 | 1.9 | 1.31 |  |  | 0 | 12 | 12 | 0 | 0 | 1 | 0 | 1 | 1 |  | -766 | -25 | 1 | 0 | 1 | 0 | 0 | 0 | 0 | 1 | 0 | 0 | 1 | 1 | 1 | 1 | 1 |
| P000006 | G0000221 | S0000716 | E001307 | MELD-Na | 2255 | 74 | M | 69 | 63 | 1 | 0.999167138528565 | 1.9 | 1.31 | 135 |  | 0 | 14 | 14 | 0 | 0 | 1 | 0 | 1 | 1 |  | -766 | -25 | 1 | 0 | 1 | 0 | 0 | 0 | 0 | 1 | 0 | 0 | 1 | 1 | 0 | 1 | 1 |
| P000006 | G0000221 | S0000717 | E001307 | reMELD-Na | 2255 | 74 | M | 69 | 63 | 1 | 0.999167138528565 | 1.9 | 1.31 | 135 |  |  | 14 | 14 | 0 | 0 | 1 | 0 | 1 | 1 |  | -766 | -25 | 1 | 0 | 1 | 0 | 0 | 0 | 0 | 1 | 0 | 0 | 1 | 1 | 0 | 1 | 1 |
| P000006 | G0000222 | S0000718 | E001243 | MELD | 2266 | 74 | M | 69 | 63 | 1.35 | 1.33645994922234 | 2.1 | 1.31 |  |  | 0 | 15 | 15 | 0 | 0 | 1 | 0 | 1 | 1 |  | -755 | -25 | 1 | 0 | 1 | 0 | 0 | 0 | 0 | 1 | 0 | 0 | 1 | 1 | 1 | 1 | 1 |
| P000006 | G0000222 | S0000719 | E001243 | MELD-Na | 2266 | 74 | M | 69 | 63 | 1.35 | 1.33645994922234 | 2.1 | 1.31 | 139 |  | 0 | 15 | 15 | 0 | 0 | 1 | 0 | 1 | 1 |  | -755 | -25 | 1 | 0 | 1 | 0 | 0 | 0 | 0 | 1 | 0 | 0 | 1 | 1 | 0 | 1 | 1 |
| P000006 | G0000222 | S0000720 | E001243 | reMELD-Na | 2266 | 74 | M | 69 | 63 | 1.35 | 1.33645994922234 | 2.1 | 1.31 | 139 |  |  | 16 | 15 | -1 | 1 | 0 | 0 | 1 | 1 |  | -755 | -25 | 1 | 0 | 1 | 0 | 0 | 0 | 0 | 1 | 0 | 0 | 1 | 1 | 0 | 1 | 1 |
| P000006 | G0000223 | S0000721 | E001314 | MELD | 2268 | 74 | M | 69 | 63 | 1 | 0.995869412327292 | 2.2 | 1.21 |  |  | 0 | 12 | 12 | 0 | 0 | 1 | 0 | 1 | 1 |  | -753 | -25 | 1 | 0 | 1 | 0 | 0 | 0 | 0 | 1 | 0 | 0 | 1 | 1 | 1 | 1 | 1 |
| P000006 | G0000223 | S0000722 | E001314 | MELD 3.0 | 2268 | 74 | M | 69 | 63 | 1 | 0.995869412327292 | 2.2 | 1.21 | 136 | 3.14 |  | 13 | 13 | 0 | 0 | 1 | 0 | 1 | 1 |  | -753 | -25 | 1 | 0 | 1 | 0 | 0 | 0 | 0 | 1 | 0 | 0 | 1 | 1 | 0 | 1 | 1 |
| P000006 | G0000223 | S0000723 | E001314 | MELD-Na | 2268 | 74 | M | 69 | 63 | 1 | 0.995869412327292 | 2.2 | 1.21 | 136 |  | 0 | 13 | 13 | 0 | 0 | 1 | 0 | 1 | 1 |  | -753 | -25 | 1 | 0 | 1 | 0 | 0 | 0 | 0 | 1 | 0 | 0 | 1 | 1 | 0 | 1 | 1 |
| P000006 | G0000223 | S0000724 | E001314 | reMELD-Na | 2268 | 74 | M | 69 | 63 | 1 | 0.995869412327292 | 2.2 | 1.21 | 136 |  |  | 13 | 13 | 0 | 0 | 1 | 0 | 1 | 1 |  | -753 | -25 | 1 | 0 | 1 | 0 | 0 | 0 | 0 | 1 | 0 | 0 | 1 | 1 | 0 | 1 | 1 |
| P000006 | G0000224 | S0000725 | E001243 | MELD | 2274 | 74 | M | 69 | 63 | 1.02 | 1.02549241559 | 1.3 | 1.22 |  |  | 0 | 10 | 10 | 0 | 0 | 1 | 0 | 1 | 1 |  | -747 | -24 | 1 | 0 | 1 | 0 | 0 | 0 | 0 | 1 | 0 | 0 | 1 | 1 | 1 | 1 | 1 |
| P000006 | G0000224 | S0000726 | E001243 | MELD-Na | 2274 | 74 | M | 69 | 63 | 1.02 | 1.02549241559 | 1.3 | 1.22 | 131 |  | 0 | 16 | 16 | 0 | 0 | 1 | 0 | 1 | 1 |  | -747 | -24 | 1 | 0 | 1 | 0 | 0 | 0 | 0 | 1 | 0 | 0 | 1 | 1 | 0 | 1 | 1 |
| P000006 | G0000224 | S0000727 | E001243 | reMELD-Na | 2274 | 74 | M | 69 | 63 | 1.02 | 1.02549241559 | 1.3 | 1.22 | 131 |  |  | 14 | 14 | 0 | 0 | 1 | 0 | 1 | 1 |  | -747 | -24 | 1 | 0 | 1 | 0 | 0 | 0 | 0 | 1 | 0 | 0 | 1 | 1 | 0 | 1 | 1 |
| P000006 | G0000225 | S0000728 | E001243 | MELD | 2276 | 74 | M | 69 | 63 | 1.73 | 1.70357652620275 | 1.5 | 1.32 |  |  | 0 | 16 | 16 | 0 | 0 | 1 | 0 | 1 | 1 |  | -745 | -24 | 1 | 0 | 1 | 0 | 0 | 0 | 0 | 1 | 0 | 0 | 1 | 1 | 1 | 1 | 1 |
| P000006 | G0000225 | S0000729 | E001243 | MELD-Na | 2276 | 74 | M | 69 | 63 | 1.73 | 1.70357652620275 | 1.5 | 1.32 | 130 |  | 0 | 22 | 22 | 0 | 0 | 1 | 0 | 1 | 1 |  | -745 | -24 | 1 | 0 | 1 | 0 | 0 | 0 | 0 | 1 | 0 | 0 | 1 | 1 | 0 | 1 | 1 |
| P000006 | G0000225 | S0000730 | E001243 | reMELD-Na | 2276 | 74 | M | 69 | 63 | 1.73 | 1.70357652620275 | 1.5 | 1.32 | 130 |  |  | 18 | 18 | 0 | 0 | 1 | 0 | 1 | 1 |  | -745 | -24 | 1 | 0 | 1 | 0 | 0 | 0 | 0 | 1 | 0 | 0 | 1 | 1 | 0 | 1 | 1 |
| P000006 | G0000226 | S0000731 | E001243 | MELD | 2282 | 74 | M | 69 | 63 | 1.39 | 1.38260173505966 | 1.4 | 1.22 |  |  | 0 | 13 | 13 | 0 | 0 | 1 | 0 | 1 | 1 |  | -739 | -24 | 1 | 0 | 1 | 0 | 0 | 0 | 0 | 1 | 0 | 0 | 1 | 1 | 1 | 1 | 1 |
| P000006 | G0000226 | S0000732 | E001243 | MELD-Na | 2282 | 74 | M | 69 | 63 | 1.39 | 1.38260173505966 | 1.4 | 1.22 | 135 |  | 0 | 15 | 15 | 0 | 0 | 1 | 0 | 1 | 1 |  | -739 | -24 | 1 | 0 | 1 | 0 | 0 | 0 | 0 | 1 | 0 | 0 | 1 | 1 | 0 | 1 | 1 |
| P000006 | G0000226 | S0000733 | E001243 | reMELD-Na | 2282 | 74 | M | 69 | 63 | 1.39 | 1.38260173505966 | 1.4 | 1.22 | 135 |  |  | 15 | 15 | 0 | 0 | 1 | 0 | 1 | 1 |  | -739 | -24 | 1 | 0 | 1 | 0 | 0 | 0 | 0 | 1 | 0 | 0 | 1 | 1 | 0 | 1 | 1 |
| P000006 | G0000227 | S0000734 | E001243 | MELD | 2300 | 75 | M | 69 | 63 | 1.43 | 1.39212220051053 | 4.1 | 1.17 |  |  | 0 | 17 | 17 | 0 | 0 | 1 | 0 | 1 | 1 |  | -721 | -24 | 1 | 0 | 1 | 0 | 0 | 0 | 0 | 1 | 0 | 0 | 1 | 1 | 1 | 1 | 1 |
| P000006 | G0000227 | S0000735 | E001243 | MELD-Na | 2300 | 75 | M | 69 | 63 | 1.43 | 1.39212220051053 | 4.1 | 1.17 | 129 |  | 0 | 23 | 23 | 0 | 0 | 1 | 0 | 1 | 1 |  | -721 | -24 | 1 | 0 | 1 | 0 | 0 | 0 | 0 | 1 | 0 | 0 | 1 | 1 | 0 | 1 | 1 |
| P000006 | G0000227 | S0000736 | E001243 | reMELD-Na | 2300 | 75 | M | 69 | 63 | 1.43 | 1.39212220051053 | 4.1 | 1.17 | 129 |  |  | 19 | 19 | 0 | 0 | 1 | 0 | 1 | 1 |  | -721 | -24 | 1 | 0 | 1 | 0 | 0 | 0 | 0 | 1 | 0 | 0 | 1 | 1 | 0 | 1 | 1 |
| P000006 | G0000228 | S0000737 | E001333 | MELD | 2301 | 75 | M | 69 | 63 | 1.48 | 1.44484148310334 | 3.6 | 1.17 |  |  | 0 | 17 | 17 | 0 | 0 | 1 | 0 | 1 | 1 |  | -720 | -24 | 1 | 0 | 1 | 0 | 0 | 0 | 0 | 1 | 0 | 0 | 1 | 1 | 1 | 1 | 1 |
| P000006 | G0000228 | S0000738 | E001333 | MELD 3.0 | 2301 | 75 | M | 69 | 63 | 1.48 | 1.44484148310334 | 3.6 | 1.17 | 137 | 3.3 |  | 18 | 18 | 0 | 0 | 1 | 0 | 1 | 1 |  | -720 | -24 | 1 | 0 | 1 | 0 | 0 | 0 | 0 | 1 | 0 | 0 | 1 | 1 | 0 | 1 | 1 |
| P000006 | G0000228 | S0000739 | E001333 | MELD-Na | 2301 | 75 | M | 69 | 63 | 1.48 | 1.44484148310334 | 3.6 | 1.17 | 137 |  | 0 | 17 | 17 | 0 | 0 | 1 | 0 | 1 | 1 |  | -720 | -24 | 1 | 0 | 1 | 0 | 0 | 0 | 0 | 1 | 0 | 0 | 1 | 1 | 0 | 1 | 1 |
| P000006 | G0000228 | S0000740 | E001333 | reMELD-Na | 2301 | 75 | M | 69 | 63 | 1.48 | 1.44484148310334 | 3.6 | 1.17 | 137 |  |  | 17 | 17 | 0 | 0 | 1 | 0 | 1 | 1 |  | -720 | -24 | 1 | 0 | 1 | 0 | 0 | 0 | 0 | 1 | 0 | 0 | 1 | 1 | 0 | 1 | 1 |
| P000006 | G0000229 | S0000741 | E001243 | MELD | 2318 | 76 | M | 69 | 63 | 1.45 | 1.43656393640174 | 1.7 | 1.18 |  |  | 0 | 14 | 14 | 0 | 0 | 1 | 0 | 1 | 1 |  | -703 | -23 | 1 | 0 | 1 | 0 | 0 | 0 | 0 | 1 | 0 | 0 | 1 | 1 | 1 | 1 | 1 |
| P000006 | G0000229 | S0000742 | E001243 | MELD-Na | 2318 | 76 | M | 69 | 63 | 1.45 | 1.43656393640174 | 1.7 | 1.18 | 130 |  | 0 | 20 | 20 | 0 | 0 | 1 | 0 | 1 | 1 |  | -703 | -23 | 1 | 0 | 1 | 0 | 0 | 0 | 0 | 1 | 0 | 0 | 1 | 1 | 0 | 1 | 1 |
| P000006 | G0000229 | S0000743 | E001243 | reMELD-Na | 2318 | 76 | M | 69 | 63 | 1.45 | 1.43656393640174 | 1.7 | 1.18 | 130 |  |  | 17 | 17 | 0 | 0 | 1 | 0 | 1 | 1 |  | -703 | -23 | 1 | 0 | 1 | 0 | 0 | 0 | 0 | 1 | 0 | 0 | 1 | 1 | 0 | 1 | 1 |
| P000006 | G0000230 | S0000744 | E001243 | MELD | 2332 | 76 | M | 69 | 63 | 1.37 | 1.35895516438326 | 1.8 | 1.16 |  |  | 0 | 13 | 13 | 0 | 0 | 1 | 0 | 1 | 1 |  | -689 | -23 | 1 | 0 | 1 | 0 | 0 | 0 | 0 | 1 | 0 | 0 | 1 | 1 | 1 | 1 | 1 |
| P000006 | G0000230 | S0000745 | E001243 | MELD-Na | 2332 | 76 | M | 69 | 63 | 1.37 | 1.35895516438326 | 1.8 | 1.16 | 134 |  | 0 | 16 | 16 | 0 | 0 | 1 | 0 | 1 | 1 |  | -689 | -23 | 1 | 0 | 1 | 0 | 0 | 0 | 0 | 1 | 0 | 0 | 1 | 1 | 0 | 1 | 1 |
| P000006 | G0000230 | S0000746 | E001243 | reMELD-Na | 2332 | 76 | M | 69 | 63 | 1.37 | 1.35895516438326 | 1.8 | 1.16 | 134 |  |  | 15 | 15 | 0 | 0 | 1 | 0 | 1 | 1 |  | -689 | -23 | 1 | 0 | 1 | 0 | 0 | 0 | 0 | 1 | 0 | 0 | 1 | 1 | 0 | 1 | 1 |
| P000006 | G0000231 | S0000747 | E001368 | MELD | 2358 | 77 | M | 69 | 63 | 1.25 | 1.23692806286874 | 2.4 | 1.32 |  |  | 0 | 15 | 15 | 0 | 0 | 1 | 0 | 1 | 1 |  | -663 | -22 | 1 | 0 | 1 | 0 | 0 | 0 | 0 | 1 | 0 | 0 | 1 | 1 | 1 | 1 | 1 |
| P000006 | G0000231 | S0000748 | E001368 | MELD-Na | 2358 | 77 | M | 69 | 63 | 1.25 | 1.23692806286874 | 2.4 | 1.32 | 135 |  | 0 | 17 | 17 | 0 | 0 | 1 | 0 | 1 | 1 |  | -663 | -22 | 1 | 0 | 1 | 0 | 0 | 0 | 0 | 1 | 0 | 0 | 1 | 1 | 0 | 1 | 1 |
| P000006 | G0000231 | S0000749 | E001368 | reMELD-Na | 2358 | 77 | M | 69 | 63 | 1.25 | 1.23692806286874 | 2.4 | 1.32 | 135 |  |  | 16 | 16 | 0 | 0 | 1 | 0 | 1 | 1 |  | -663 | -22 | 1 | 0 | 1 | 0 | 0 | 0 | 0 | 1 | 0 | 0 | 1 | 1 | 0 | 1 | 1 |
| P000006 | G0000232 | S0000750 | E001373 | MELD | 2367 | 77 | M | 69 | 63 | 1.5 | 1.47540818694436 | 2.5 | 1.14 |  |  | 0 | 15 | 15 | 0 | 0 | 1 | 0 | 1 | 1 |  | -654 | -21 | 1 | 0 | 1 | 0 | 0 | 0 | 0 | 1 | 0 | 0 | 1 | 1 | 1 | 1 | 1 |
| P000006 | G0000232 | S0000751 | E001373 | MELD-Na | 2367 | 77 | M | 69 | 63 | 1.5 | 1.47540818694436 | 2.5 | 1.14 | 132 |  | 0 | 19 | 19 | 0 | 0 | 1 | 0 | 1 | 1 |  | -654 | -21 | 1 | 0 | 1 | 0 | 0 | 0 | 0 | 1 | 0 | 0 | 1 | 1 | 0 | 1 | 1 |
| P000006 | G0000232 | S0000752 | E001373 | reMELD-Na | 2367 | 77 | M | 69 | 63 | 1.5 | 1.47540818694436 | 2.5 | 1.14 | 132 |  |  | 17 | 17 | 0 | 0 | 1 | 0 | 1 | 1 |  | -654 | -21 | 1 | 0 | 1 | 0 | 0 | 0 | 0 | 1 | 0 | 0 | 1 | 1 | 0 | 1 | 1 |
| P000006 | G0000233 | S0000753 | E001373 | MELD | 2368 | 77 | M | 69 | 63 | 1.35 | 1.3299703632839 | 2.7 | 1.175 |  |  | 0 | 15 | 15 | 0 | 0 | 1 | 0 | 1 | 1 |  | -653 | -21 | 1 | 0 | 1 | 0 | 0 | 0 | 0 | 1 | 0 | 0 | 1 | 1 | 1 | 1 | 1 |
| P000006 | G0000233 | S0000754 | E001373 | MELD 3.0 | 2368 | 77 | M | 69 | 63 | 1.35 | 1.3299703632839 | 2.7 | 1.175 | 137.333333333333 | 3.215 |  | 16 | 16 | 0 | 0 | 1 | 0 | 1 | 1 |  | -653 | -21 | 1 | 0 | 1 | 0 | 0 | 0 | 0 | 1 | 0 | 0 | 1 | 1 | 0 | 1 | 1 |
| P000006 | G0000233 | S0000755 | E001373 | MELD-Na | 2368 | 77 | M | 69 | 63 | 1.35 | 1.3299703632839 | 2.7 | 1.175 | 137.333333333333 |  | 0 | 15 | 15 | 0 | 0 | 1 | 0 | 1 | 1 |  | -653 | -21 | 1 | 0 | 1 | 0 | 0 | 0 | 0 | 1 | 0 | 0 | 1 | 1 | 0 | 1 | 1 |
| P000006 | G0000233 | S0000756 | E001373 | reMELD-Na | 2368 | 77 | M | 69 | 63 | 1.35 | 1.3299703632839 | 2.7 | 1.175 | 137.333333333333 |  |  | 15 | 15 | 0 | 0 | 1 | 0 | 1 | 1 |  | -653 | -21 | 1 | 0 | 1 | 0 | 0 | 0 | 0 | 1 | 0 | 0 | 1 | 1 | 0 | 1 | 1 |
| P000006 | G0000234 | S0000757 | E001373 | MELD | 2369 | 77 | M | 69 | 63 | 1.1 | 1.09912432758631 | 1.7 | 1.25 |  |  | 0 | 12 | 12 | 0 | 0 | 1 | 0 | 1 | 1 |  | -652 | -21 | 1 | 0 | 1 | 0 | 0 | 0 | 0 | 1 | 0 | 0 | 1 | 1 | 1 | 1 | 1 |
| P000006 | G0000234 | S0000758 | E001373 | MELD-Na | 2369 | 77 | M | 69 | 63 | 1.1 | 1.09912432758631 | 1.7 | 1.25 | 138 |  | 0 | 12 | 12 | 0 | 0 | 1 | 0 | 1 | 1 |  | -652 | -21 | 1 | 0 | 1 | 0 | 0 | 0 | 0 | 1 | 0 | 0 | 1 | 1 | 0 | 1 | 1 |
| P000006 | G0000234 | S0000759 | E001373 | reMELD-Na | 2369 | 77 | M | 69 | 63 | 1.1 | 1.09912432758631 | 1.7 | 1.25 | 138 |  |  | 13 | 13 | 0 | 0 | 1 | 0 | 1 | 1 |  | -652 | -21 | 1 | 0 | 1 | 0 | 0 | 0 | 0 | 1 | 0 | 0 | 1 | 1 | 0 | 1 | 1 |
| P000006 | G0000235 | S0000760 | E001373 | MELD | 2370 | 77 | M | 69 | 63 | 1.18 | 1.17688802830257 | 1.7 | 1.13 |  |  | 0 | 11 | 11 | 0 | 0 | 1 | 0 | 1 | 1 |  | -651 | -21 | 1 | 0 | 1 | 0 | 0 | 0 | 0 | 1 | 0 | 0 | 1 | 1 | 1 | 1 | 1 |
| P000006 | G0000235 | S0000761 | E001373 | MELD 3.0 | 2370 | 77 | M | 69 | 63 | 1.18 | 1.17688802830257 | 1.7 | 1.13 | 135 | 3.36 |  | 13 | 13 | 0 | 0 | 1 | 0 | 1 | 1 |  | -651 | -21 | 1 | 0 | 1 | 0 | 0 | 0 | 0 | 1 | 0 | 0 | 1 | 1 | 0 | 1 | 1 |
| P000006 | G0000235 | S0000762 | E001373 | MELD-Na | 2370 | 77 | M | 69 | 63 | 1.18 | 1.17688802830257 | 1.7 | 1.13 | 135 |  | 0 | 13 | 13 | 0 | 0 | 1 | 0 | 1 | 1 |  | -651 | -21 | 1 | 0 | 1 | 0 | 0 | 0 | 0 | 1 | 0 | 0 | 1 | 1 | 0 | 1 | 1 |
| P000006 | G0000235 | S0000763 | E001373 | reMELD-Na | 2370 | 77 | M | 69 | 63 | 1.18 | 1.17688802830257 | 1.7 | 1.13 | 135 |  |  | 13 | 13 | 0 | 0 | 1 | 0 | 1 | 1 |  | -651 | -21 | 1 | 0 | 1 | 0 | 0 | 0 | 0 | 1 | 0 | 0 | 1 | 1 | 0 | 1 | 1 |
| P000006 | G0000236 | S0000764 | E001391 | MELD | 2393 | 78 | M | 70 | 63 | 2.03 | 1.9754738053105 | 2.1 | 1.19 |  |  | 0 | 18 | 18 | 0 | 0 | 1 | 0 | 1 | 1 |  | -628 | -21 | 1 | 0 | 1 | 0 | 0 | 0 | 0 | 1 | 0 | 0 | 1 | 1 | 1 | 1 | 1 |
| P000006 | G0000236 | S0000765 | E001391 | MELD-Na | 2393 | 78 | M | 70 | 63 | 2.03 | 1.9754738053105 | 2.1 | 1.19 | 136 |  | 0 | 19 | 19 | 0 | 0 | 1 | 0 | 1 | 1 |  | -628 | -21 | 1 | 0 | 1 | 0 | 0 | 0 | 0 | 1 | 0 | 0 | 1 | 1 | 0 | 1 | 1 |
| P000006 | G0000236 | S0000766 | E001391 | reMELD-Na | 2393 | 78 | M | 70 | 63 | 2.03 | 1.9754738053105 | 2.1 | 1.19 | 136 |  |  | 18 | 18 | 0 | 0 | 1 | 0 | 1 | 1 |  | -628 | -21 | 1 | 0 | 1 | 0 | 0 | 0 | 0 | 1 | 0 | 0 | 1 | 1 | 0 | 1 | 1 |
| P000006 | G0000237 | S0000767 | E001391 | MELD | 2394 | 78 | M | 70 | 63 | 1.65 | 1.62282659624811 | 2 | 1.22 |  |  | 0 | 16 | 16 | 0 | 0 | 1 | 0 | 1 | 1 |  | -627 | -21 | 1 | 0 | 1 | 0 | 0 | 0 | 0 | 1 | 0 | 0 | 1 | 1 | 1 | 1 | 1 |
| P000006 | G0000237 | S0000768 | E001391 | MELD 3.0 | 2394 | 78 | M | 70 | 63 | 1.65 | 1.62282659624811 | 2 | 1.22 | 137 | 2.98 |  | 17 | 17 | 0 | 0 | 1 | 0 | 1 | 1 |  | -627 | -21 | 1 | 0 | 1 | 0 | 0 | 0 | 0 | 1 | 0 | 0 | 1 | 1 | 0 | 1 | 1 |
| P000006 | G0000237 | S0000769 | E001391 | MELD-Na | 2394 | 78 | M | 70 | 63 | 1.65 | 1.62282659624811 | 2 | 1.22 | 137 |  | 0 | 16 | 16 | 0 | 0 | 1 | 0 | 1 | 1 |  | -627 | -21 | 1 | 0 | 1 | 0 | 0 | 0 | 0 | 1 | 0 | 0 | 1 | 1 | 0 | 1 | 1 |
| P000006 | G0000237 | S0000770 | E001391 | reMELD-Na | 2394 | 78 | M | 70 | 63 | 1.65 | 1.62282659624811 | 2 | 1.22 | 137 |  |  | 17 | 17 | 0 | 0 | 1 | 0 | 1 | 1 |  | -627 | -21 | 1 | 0 | 1 | 0 | 0 | 0 | 0 | 1 | 0 | 0 | 1 | 1 | 0 | 1 | 1 |
| P000006 | G0000238 | S0000771 | E001391 | MELD | 2395 | 78 | M | 70 | 63 | 1.49 | 1.46805953057674 | 2.3 | 1.29 |  |  | 0 | 16 | 16 | 0 | 0 | 1 | 0 | 1 | 1 |  | -626 | -21 | 1 | 0 | 1 | 0 | 0 | 0 | 0 | 1 | 0 | 0 | 1 | 1 | 1 | 1 | 1 |
| P000006 | G0000238 | S0000772 | E001391 | MELD-Na | 2395 | 78 | M | 70 | 63 | 1.49 | 1.46805953057674 | 2.3 | 1.29 | 140 |  | 0 | 16 | 16 | 0 | 0 | 1 | 0 | 1 | 1 |  | -626 | -21 | 1 | 0 | 1 | 0 | 0 | 0 | 0 | 1 | 0 | 0 | 1 | 1 | 0 | 1 | 1 |
| P000006 | G0000238 | S0000773 | E001391 | reMELD-Na | 2395 | 78 | M | 70 | 63 | 1.49 | 1.46805953057674 | 2.3 | 1.29 | 140 |  |  | 17 | 16 | -1 | 1 | 0 | 0 | 1 | 1 |  | -626 | -21 | 1 | 0 | 1 | 0 | 0 | 0 | 0 | 1 | 0 | 0 | 1 | 1 | 0 | 1 | 1 |
| P000006 | G0000239 | S0000774 | E001391 | MELD | 2396 | 78 | M | 70 | 63 | 1.19 | 1.18658204725573 | 1.7 | 1.23 |  |  | 0 | 12 | 12 | 0 | 0 | 1 | 0 | 1 | 1 |  | -625 | -20 | 1 | 0 | 1 | 0 | 0 | 0 | 0 | 1 | 0 | 0 | 1 | 1 | 1 | 1 | 1 |
| P000006 | G0000239 | S0000775 | E001391 | MELD-Na | 2396 | 78 | M | 70 | 63 | 1.19 | 1.18658204725573 | 1.7 | 1.23 | 134 |  | 0 | 15 | 15 | 0 | 0 | 1 | 0 | 1 | 1 |  | -625 | -20 | 1 | 0 | 1 | 0 | 0 | 0 | 0 | 1 | 0 | 0 | 1 | 1 | 0 | 1 | 1 |
| P000006 | G0000239 | S0000776 | E001391 | reMELD-Na | 2396 | 78 | M | 70 | 63 | 1.19 | 1.18658204725573 | 1.7 | 1.23 | 134 |  |  | 14 | 14 | 0 | 0 | 1 | 0 | 1 | 1 |  | -625 | -20 | 1 | 0 | 1 | 0 | 0 | 0 | 0 | 1 | 0 | 0 | 1 | 1 | 0 | 1 | 1 |
| P000006 | G0000240 | S0000777 | E001403 | MELD | 2411 | 79 | M | 70 | 63 | 1.51 | 1.49146424332195 | 1.9 | 1.35 |  |  | 0 | 16 | 16 | 0 | 0 | 1 | 0 | 1 | 1 |  | -610 | -20 | 1 | 0 | 1 | 0 | 0 | 0 | 0 | 1 | 0 | 0 | 1 | 1 | 1 | 1 | 1 |
| P000006 | G0000240 | S0000778 | E001403 | MELD-Na | 2411 | 79 | M | 70 | 63 | 1.51 | 1.49146424332195 | 1.9 | 1.35 | 141 |  | 0 | 16 | 16 | 0 | 0 | 1 | 0 | 1 | 1 |  | -610 | -20 | 1 | 0 | 1 | 0 | 0 | 0 | 0 | 1 | 0 | 0 | 1 | 1 | 0 | 1 | 1 |
| P000006 | G0000240 | S0000779 | E001403 | reMELD-Na | 2411 | 79 | M | 70 | 63 | 1.51 | 1.49146424332195 | 1.9 | 1.35 | 141 |  |  | 16 | 16 | 0 | 0 | 1 | 0 | 1 | 1 |  | -610 | -20 | 1 | 0 | 1 | 0 | 0 | 0 | 0 | 1 | 0 | 0 | 1 | 1 | 0 | 1 | 1 |
| P000006 | G0000241 | S0000780 | E001403 | MELD | 2412 | 79 | M | 70 | 63 | 1.27 | 1.26729599489437 | 1.4 | 1.38 |  |  | 0 | 14 | 14 | 0 | 0 | 1 | 0 | 1 | 1 |  | -609 | -20 | 1 | 0 | 1 | 0 | 0 | 0 | 0 | 1 | 0 | 0 | 1 | 1 | 1 | 1 | 1 |
| P000006 | G0000241 | S0000781 | E001403 | MELD 3.0 | 2412 | 79 | M | 70 | 63 | 1.27 | 1.26729599489437 | 1.4 | 1.38 | 138 | 2.65 |  | 14 | 14 | 0 | 0 | 1 | 0 | 1 | 1 |  | -609 | -20 | 1 | 0 | 1 | 0 | 0 | 0 | 0 | 1 | 0 | 0 | 1 | 1 | 0 | 1 | 1 |
| P000006 | G0000241 | S0000782 | E001403 | MELD-Na | 2412 | 79 | M | 70 | 63 | 1.27 | 1.26729599489437 | 1.4 | 1.38 | 138 |  | 0 | 14 | 14 | 0 | 0 | 1 | 0 | 1 | 1 |  | -609 | -20 | 1 | 0 | 1 | 0 | 0 | 0 | 0 | 1 | 0 | 0 | 1 | 1 | 0 | 1 | 1 |
| P000006 | G0000241 | S0000783 | E001403 | reMELD-Na | 2412 | 79 | M | 70 | 63 | 1.27 | 1.26729599489437 | 1.4 | 1.38 | 138 |  |  | 14 | 14 | 0 | 0 | 1 | 0 | 1 | 1 |  | -609 | -20 | 1 | 0 | 1 | 0 | 0 | 0 | 0 | 1 | 0 | 0 | 1 | 1 | 0 | 1 | 1 |
| P000006 | G0000242 | S0000784 | E001403 | MELD | 2413 | 79 | M | 70 | 63 | 1.33 | 1.32618917041093 | 1.3 | 1.15 |  |  | 0 | 12 | 12 | 0 | 0 | 1 | 0 | 1 | 1 |  | -608 | -20 | 1 | 0 | 1 | 0 | 0 | 0 | 0 | 1 | 0 | 0 | 1 | 1 | 1 | 1 | 1 |
| P000006 | G0000242 | S0000785 | E001403 | MELD 3.0 | 2413 | 79 | M | 70 | 63 | 1.33 | 1.32618917041093 | 1.3 | 1.15 | 133 | 3.55 |  | 15 | 15 | 0 | 0 | 1 | 0 | 1 | 1 |  | -608 | -20 | 1 | 0 | 1 | 0 | 0 | 0 | 0 | 1 | 0 | 0 | 1 | 1 | 0 | 1 | 1 |
| P000006 | G0000242 | S0000786 | E001403 | MELD-Na | 2413 | 79 | M | 70 | 63 | 1.33 | 1.32618917041093 | 1.3 | 1.15 | 133 |  | 0 | 16 | 16 | 0 | 0 | 1 | 0 | 1 | 1 |  | -608 | -20 | 1 | 0 | 1 | 0 | 0 | 0 | 0 | 1 | 0 | 0 | 1 | 1 | 0 | 1 | 1 |
| P000006 | G0000242 | S0000787 | E001403 | reMELD-Na | 2413 | 79 | M | 70 | 63 | 1.33 | 1.32618917041093 | 1.3 | 1.15 | 133 |  |  | 14 | 14 | 0 | 0 | 1 | 0 | 1 | 1 |  | -608 | -20 | 1 | 0 | 1 | 0 | 0 | 0 | 0 | 1 | 0 | 0 | 1 | 1 | 0 | 1 | 1 |
| P000006 | G0000243 | S0000788 | E001403 | MELD | 2415 | 79 | M | 70 | 63 | 1.2 | 1.20421190606448 | 1 | 1.25 |  |  | 0 | 11 | 11 | 0 | 0 | 1 | 0 | 1 | 1 |  | -606 | -20 | 1 | 0 | 1 | 0 | 0 | 0 | 0 | 1 | 0 | 0 | 1 | 1 | 1 | 1 | 1 |
| P000006 | G0000243 | S0000789 | E001403 | MELD-Na | 2415 | 79 | M | 70 | 63 | 1.2 | 1.20421190606448 | 1 | 1.25 | 134 |  | 0 | 14 | 14 | 0 | 0 | 1 | 0 | 1 | 1 |  | -606 | -20 | 1 | 0 | 1 | 0 | 0 | 0 | 0 | 1 | 0 | 0 | 1 | 1 | 0 | 1 | 1 |
| P000006 | G0000243 | S0000790 | E001403 | reMELD-Na | 2415 | 79 | M | 70 | 63 | 1.2 | 1.20421190606448 | 1 | 1.25 | 134 |  |  | 13 | 13 | 0 | 0 | 1 | 0 | 1 | 1 |  | -606 | -20 | 1 | 0 | 1 | 0 | 0 | 0 | 0 | 1 | 0 | 0 | 1 | 1 | 0 | 1 | 1 |
| P000006 | G0000244 | S0000791 | E001417 | MELD | 2433 | 79 | M | 70 | 63 | 1.72 | 1.69307538251788 | 1.6 | 1.31 |  |  | 0 | 16 | 16 | 0 | 0 | 1 | 0 | 1 | 1 |  | -588 | -19 | 1 | 0 | 1 | 0 | 0 | 0 | 0 | 1 | 0 | 0 | 1 | 1 | 1 | 1 | 1 |
| P000006 | G0000244 | S0000792 | E001417 | MELD-Na | 2433 | 79 | M | 70 | 63 | 1.72 | 1.69307538251788 | 1.6 | 1.31 | 134 |  | 0 | 18 | 18 | 0 | 0 | 1 | 0 | 1 | 1 |  | -588 | -19 | 1 | 0 | 1 | 0 | 0 | 0 | 0 | 1 | 0 | 0 | 1 | 1 | 0 | 1 | 1 |
| P000006 | G0000244 | S0000793 | E001417 | reMELD-Na | 2433 | 79 | M | 70 | 63 | 1.72 | 1.69307538251788 | 1.6 | 1.31 | 134 |  |  | 18 | 18 | 0 | 0 | 1 | 0 | 1 | 1 |  | -588 | -19 | 1 | 0 | 1 | 0 | 0 | 0 | 0 | 1 | 0 | 0 | 1 | 1 | 0 | 1 | 1 |
| P000006 | G0000245 | S0000794 | E001417 | MELD | 2436 | 80 | M | 70 | 63 | 1.26 | 1.25539504885397 | 1.6 | 1.3 |  |  | 0 | 13 | 13 | 0 | 0 | 1 | 0 | 1 | 1 |  | -585 | -19 | 1 | 0 | 1 | 0 | 0 | 0 | 0 | 1 | 0 | 0 | 1 | 1 | 1 | 1 | 1 |
| P000006 | G0000245 | S0000795 | E001417 | MELD-Na | 2436 | 80 | M | 70 | 63 | 1.26 | 1.25539504885397 | 1.6 | 1.3 | 139 |  | 0 | 13 | 13 | 0 | 0 | 1 | 0 | 1 | 1 |  | -585 | -19 | 1 | 0 | 1 | 0 | 0 | 0 | 0 | 1 | 0 | 0 | 1 | 1 | 0 | 1 | 1 |
| P000006 | G0000245 | S0000796 | E001417 | reMELD-Na | 2436 | 80 | M | 70 | 63 | 1.26 | 1.25539504885397 | 1.6 | 1.3 | 139 |  |  | 14 | 14 | 0 | 0 | 1 | 0 | 1 | 1 |  | -585 | -19 | 1 | 0 | 1 | 0 | 0 | 0 | 0 | 1 | 0 | 0 | 1 | 1 | 0 | 1 | 1 |
| P000006 | G0000246 | S0000797 | E001417 | MELD | 2437 | 80 | M | 70 | 63 | 1.09 | 1.09275076646462 | 1.4 | 1.4 |  |  | 0 | 12 | 12 | 0 | 0 | 1 | 0 | 1 | 1 |  | -584 | -19 | 1 | 0 | 1 | 0 | 0 | 0 | 0 | 1 | 0 | 0 | 1 | 1 | 1 | 1 | 1 |
| P000006 | G0000246 | S0000798 | E001417 | MELD-Na | 2437 | 80 | M | 70 | 63 | 1.09 | 1.09275076646462 | 1.4 | 1.4 | 136 |  | 0 | 13 | 13 | 0 | 0 | 1 | 0 | 1 | 1 |  | -584 | -19 | 1 | 0 | 1 | 0 | 0 | 0 | 0 | 1 | 0 | 0 | 1 | 1 | 0 | 1 | 1 |
| P000006 | G0000246 | S0000799 | E001417 | reMELD-Na | 2437 | 80 | M | 70 | 63 | 1.09 | 1.09275076646462 | 1.4 | 1.4 | 136 |  |  | 14 | 14 | 0 | 0 | 1 | 0 | 1 | 1 |  | -584 | -19 | 1 | 0 | 1 | 0 | 0 | 0 | 0 | 1 | 0 | 0 | 1 | 1 | 0 | 1 | 1 |
| P000006 | G0000247 | S0000800 | E001243 | MELD | 2448 | 80 | M | 70 | 63 | 1.94 | 1.89577146653202 | 1.8 | 1.37 |  |  | 0 | 19 | 18 | -1 | 1 | 0 | 0 | 1 | 1 |  | -573 | -19 | 1 | 0 | 1 | 0 | 0 | 0 | 0 | 1 | 0 | 0 | 1 | 1 | 1 | 1 | 1 |
| P000006 | G0000247 | S0000801 | E001243 | MELD 3.0 | 2448 | 80 | M | 70 | 63 | 1.94 | 1.89577146653202 | 1.8 | 1.37 | 133 | 3.22 |  | 22 | 22 | 0 | 0 | 1 | 0 | 1 | 1 |  | -573 | -19 | 1 | 0 | 1 | 0 | 0 | 0 | 0 | 1 | 0 | 0 | 1 | 1 | 0 | 1 | 1 |
| P000006 | G0000247 | S0000802 | E001243 | MELD-Na | 2448 | 80 | M | 70 | 63 | 1.94 | 1.89577146653202 | 1.8 | 1.37 | 133 |  | 0 | 22 | 21 | -1 | 1 | 0 | 0 | 1 | 1 |  | -573 | -19 | 1 | 0 | 1 | 0 | 0 | 0 | 0 | 1 | 0 | 0 | 1 | 1 | 0 | 1 | 1 |
| P000006 | G0000247 | S0000803 | E001243 | reMELD-Na | 2448 | 80 | M | 70 | 63 | 1.94 | 1.89577146653202 | 1.8 | 1.37 | 133 |  |  | 19 | 19 | 0 | 0 | 1 | 0 | 1 | 1 |  | -573 | -19 | 1 | 0 | 1 | 0 | 0 | 0 | 0 | 1 | 0 | 0 | 1 | 1 | 0 | 1 | 1 |
| P000006 | G0000248 | S0000804 | E001433 | MELD | 2449 | 80 | M | 70 | 63 | 2.13 | 2.06935817478201 | 1.9 | 1.4 |  |  | 0 | 20 | 20 | 0 | 0 | 1 | 0 | 1 | 1 |  | -572 | -19 | 1 | 0 | 1 | 0 | 0 | 0 | 0 | 1 | 0 | 0 | 1 | 1 | 1 | 1 | 1 |
| P000006 | G0000248 | S0000805 | E001433 | MELD-Na | 2449 | 80 | M | 70 | 63 | 2.13 | 2.06935817478201 | 1.9 | 1.4 | 135 |  | 0 | 21 | 21 | 0 | 0 | 1 | 0 | 1 | 1 |  | -572 | -19 | 1 | 0 | 1 | 0 | 0 | 0 | 0 | 1 | 0 | 0 | 1 | 1 | 0 | 1 | 1 |
| P000006 | G0000248 | S0000806 | E001433 | reMELD-Na | 2449 | 80 | M | 70 | 63 | 2.13 | 2.06935817478201 | 1.9 | 1.4 | 135 |  |  | 20 | 20 | 0 | 0 | 1 | 0 | 1 | 1 |  | -572 | -19 | 1 | 0 | 1 | 0 | 0 | 0 | 0 | 1 | 0 | 0 | 1 | 1 | 0 | 1 | 1 |
| P000006 | G0000249 | S0000807 | E001433 | MELD | 2450 | 80 | M | 70 | 63 | 1.55 | 1.52722585555843 | 2.1 | 1.52 |  |  | 0 | 18 | 18 | 0 | 0 | 1 | 0 | 1 | 1 |  | -571 | -19 | 1 | 0 | 1 | 0 | 0 | 0 | 0 | 1 | 0 | 0 | 1 | 1 | 1 | 1 | 1 |
| P000006 | G0000249 | S0000808 | E001433 | MELD-Na | 2450 | 80 | M | 70 | 63 | 1.55 | 1.52722585555843 | 2.1 | 1.52 | 129 |  | 0 | 24 | 24 | 0 | 0 | 1 | 0 | 1 | 1 |  | -571 | -19 | 1 | 0 | 1 | 0 | 0 | 0 | 0 | 1 | 0 | 0 | 1 | 1 | 0 | 1 | 1 |
| P000006 | G0000249 | S0000809 | E001433 | reMELD-Na | 2450 | 80 | M | 70 | 63 | 1.55 | 1.52722585555843 | 2.1 | 1.52 | 129 |  |  | 20 | 20 | 0 | 0 | 1 | 0 | 1 | 1 |  | -571 | -19 | 1 | 0 | 1 | 0 | 0 | 0 | 0 | 1 | 0 | 0 | 1 | 1 | 0 | 1 | 1 |
| P000006 | G0000250 | S0000810 | E001433 | MELD | 2451 | 80 | M | 70 | 63 | 1.48 | 1.47194444026596 | 1.1 | 1.4 |  |  | 0 | 14 | 14 | 0 | 0 | 1 | 0 | 1 | 1 |  | -570 | -19 | 1 | 0 | 1 | 0 | 0 | 0 | 0 | 1 | 0 | 0 | 1 | 1 | 1 | 1 | 1 |
| P000006 | G0000250 | S0000811 | E001433 | MELD-Na | 2451 | 80 | M | 70 | 63 | 1.48 | 1.47194444026596 | 1.1 | 1.4 | 135 |  | 0 | 16 | 16 | 0 | 0 | 1 | 0 | 1 | 1 |  | -570 | -19 | 1 | 0 | 1 | 0 | 0 | 0 | 0 | 1 | 0 | 0 | 1 | 1 | 0 | 1 | 1 |
| P000006 | G0000250 | S0000812 | E001433 | reMELD-Na | 2451 | 80 | M | 70 | 63 | 1.48 | 1.47194444026596 | 1.1 | 1.4 | 135 |  |  | 16 | 16 | 0 | 0 | 1 | 0 | 1 | 1 |  | -570 | -19 | 1 | 0 | 1 | 0 | 0 | 0 | 0 | 1 | 0 | 0 | 1 | 1 | 0 | 1 | 1 |
| P000006 | G0000251 | S0000813 | E001433 | MELD | 2454 | 80 | M | 70 | 63 | 0.95 | 0.959095777566231 | 1.1 | 1.41 |  |  | 0 | 11 | 11 | 0 | 0 | 1 | 0 | 1 | 1 |  | -567 | -19 | 1 | 0 | 1 | 0 | 0 | 0 | 0 | 1 | 0 | 0 | 1 | 1 | 1 | 1 | 1 |
| P000006 | G0000251 | S0000814 | E001433 | MELD-Na | 2454 | 80 | M | 70 | 63 | 0.95 | 0.959095777566231 | 1.1 | 1.41 | 140 |  | 0 | 11 | 11 | 0 | 0 | 1 | 0 | 1 | 1 |  | -567 | -19 | 1 | 0 | 1 | 0 | 0 | 0 | 0 | 1 | 0 | 0 | 1 | 1 | 0 | 1 | 1 |
| P000006 | G0000251 | S0000815 | E001433 | reMELD-Na | 2454 | 80 | M | 70 | 63 | 0.95 | 0.959095777566231 | 1.1 | 1.41 | 140 |  |  | 11 | 11 | 0 | 0 | 1 | 0 | 1 | 1 |  | -567 | -19 | 1 | 0 | 1 | 0 | 0 | 0 | 0 | 1 | 0 | 0 | 1 | 1 | 0 | 1 | 1 |
| P000006 | G0000252 | S0000816 | E001450 | MELD | 2464 | 80 | M | 70 | 63 | 2.14 | 2.07629041379534 | 2.1 | 1.21 |  |  | 0 | 19 | 18 | -1 | 1 | 0 | 0 | 1 | 1 |  | -557 | -18 | 1 | 0 | 1 | 0 | 0 | 0 | 0 | 1 | 0 | 0 | 1 | 1 | 1 | 1 | 1 |
| P000006 | G0000252 | S0000817 | E001450 | MELD-Na | 2464 | 80 | M | 70 | 63 | 2.14 | 2.07629041379534 | 2.1 | 1.21 | 136 |  | 0 | 20 | 19 | -1 | 1 | 0 | 0 | 1 | 1 |  | -557 | -18 | 1 | 0 | 1 | 0 | 0 | 0 | 0 | 1 | 0 | 0 | 1 | 1 | 0 | 1 | 1 |
| P000006 | G0000252 | S0000818 | E001450 | reMELD-Na | 2464 | 80 | M | 70 | 63 | 2.14 | 2.07629041379534 | 2.1 | 1.21 | 136 |  |  | 19 | 19 | 0 | 0 | 1 | 0 | 1 | 1 |  | -557 | -18 | 1 | 0 | 1 | 0 | 0 | 0 | 0 | 1 | 0 | 0 | 1 | 1 | 0 | 1 | 1 |
| P000006 | G0000253 | S0000819 | E001450 | MELD | 2475 | 81 | M | 70 | 63 | 1.34 | 1.32906347748173 | 1.9 | 1.28 |  |  | 0 | 14 | 14 | 0 | 0 | 1 | 0 | 1 | 1 |  | -546 | -18 | 1 | 0 | 1 | 0 | 0 | 0 | 0 | 1 | 0 | 0 | 1 | 1 | 1 | 1 | 1 |
| P000006 | G0000253 | S0000820 | E001450 | MELD-Na | 2475 | 81 | M | 70 | 63 | 1.34 | 1.32906347748173 | 1.9 | 1.28 | 136 |  | 0 | 15 | 15 | 0 | 0 | 1 | 0 | 1 | 1 |  | -546 | -18 | 1 | 0 | 1 | 0 | 0 | 0 | 0 | 1 | 0 | 0 | 1 | 1 | 0 | 1 | 1 |
| P000006 | G0000253 | S0000821 | E001450 | reMELD-Na | 2475 | 81 | M | 70 | 63 | 1.34 | 1.32906347748173 | 1.9 | 1.28 | 136 |  |  | 16 | 15 | -1 | 1 | 0 | 0 | 1 | 1 |  | -546 | -18 | 1 | 0 | 1 | 0 | 0 | 0 | 0 | 1 | 0 | 0 | 1 | 1 | 0 | 1 | 1 |
| P000006 | G0000254 | S0000822 | E001450 | MELD | 2476 | 81 | M | 70 | 63 | 1.23 | 1.2219713856696 | 2 | 1.21 |  |  | 0 | 13 | 13 | 0 | 0 | 1 | 0 | 1 | 1 |  | -545 | -18 | 1 | 0 | 1 | 0 | 0 | 0 | 0 | 1 | 0 | 0 | 1 | 1 | 1 | 1 | 1 |
| P000006 | G0000254 | S0000823 | E001450 | MELD-Na | 2476 | 81 | M | 70 | 63 | 1.23 | 1.2219713856696 | 2 | 1.21 | 140 |  | 0 | 13 | 13 | 0 | 0 | 1 | 0 | 1 | 1 |  | -545 | -18 | 1 | 0 | 1 | 0 | 0 | 0 | 0 | 1 | 0 | 0 | 1 | 1 | 0 | 1 | 1 |
| P000006 | G0000254 | S0000824 | E001450 | reMELD-Na | 2476 | 81 | M | 70 | 63 | 1.23 | 1.2219713856696 | 2 | 1.21 | 140 |  |  | 14 | 14 | 0 | 0 | 1 | 0 | 1 | 1 |  | -545 | -18 | 1 | 0 | 1 | 0 | 0 | 0 | 0 | 1 | 0 | 0 | 1 | 1 | 0 | 1 | 1 |
| P000006 | G0000255 | S0000825 | E001450 | MELD | 2489 | 81 | M | 70 | 63 | 1.53 | 1.51380166240768 | 1.6 | 1.19 |  |  | 0 | 14 | 14 | 0 | 0 | 1 | 0 | 1 | 1 |  | -532 | -17 | 1 | 0 | 1 | 0 | 0 | 0 | 0 | 1 | 0 | 0 | 1 | 1 | 1 | 1 | 1 |
| P000006 | G0000255 | S0000826 | E001450 | MELD 3.0 | 2489 | 81 | M | 70 | 63 | 1.53 | 1.51380166240768 | 1.6 | 1.19 | 133 | 3.27 |  | 18 | 17 | -1 | 1 | 0 | 0 | 1 | 1 |  | -532 | -17 | 1 | 0 | 1 | 0 | 0 | 0 | 0 | 1 | 0 | 0 | 1 | 1 | 0 | 1 | 1 |
| P000006 | G0000255 | S0000827 | E001450 | MELD-Na | 2489 | 81 | M | 70 | 63 | 1.53 | 1.51380166240768 | 1.6 | 1.19 | 133 |  | 0 | 17 | 17 | 0 | 0 | 1 | 0 | 1 | 1 |  | -532 | -17 | 1 | 0 | 1 | 0 | 0 | 0 | 0 | 1 | 0 | 0 | 1 | 1 | 0 | 1 | 1 |
| P000006 | G0000255 | S0000828 | E001450 | reMELD-Na | 2489 | 81 | M | 70 | 63 | 1.53 | 1.51380166240768 | 1.6 | 1.19 | 133 |  |  | 16 | 16 | 0 | 0 | 1 | 0 | 1 | 1 |  | -532 | -17 | 1 | 0 | 1 | 0 | 0 | 0 | 0 | 1 | 0 | 0 | 1 | 1 | 0 | 1 | 1 |
| P000006 | G0000256 | S0000829 | E001499 | MELD | 2525 | 82 | M | 70 | 63 | 1.81 | 1.780642560163 | 1.3 | 1.34 |  |  | 0 | 16 | 16 | 0 | 0 | 1 | 0 | 1 | 1 |  | -496 | -16 | 1 | 0 | 1 | 0 | 0 | 0 | 0 | 1 | 0 | 0 | 1 | 1 | 1 | 1 | 1 |
| P000006 | G0000256 | S0000830 | E001499 | MELD-Na | 2525 | 82 | M | 70 | 63 | 1.81 | 1.780642560163 | 1.3 | 1.34 | 130 |  | 0 | 22 | 22 | 0 | 0 | 1 | 0 | 1 | 1 |  | -496 | -16 | 1 | 0 | 1 | 0 | 0 | 0 | 0 | 1 | 0 | 0 | 1 | 1 | 0 | 1 | 1 |
| P000006 | G0000256 | S0000831 | E001499 | reMELD-Na | 2525 | 82 | M | 70 | 63 | 1.81 | 1.780642560163 | 1.3 | 1.34 | 130 |  |  | 18 | 18 | 0 | 0 | 1 | 0 | 1 | 1 |  | -496 | -16 | 1 | 0 | 1 | 0 | 0 | 0 | 0 | 1 | 0 | 0 | 1 | 1 | 0 | 1 | 1 |
| P000006 | G0000257 | S0000832 | E001499 | MELD | 2527 | 83 | M | 70 | 63 | 1.3 | 1.29507227121652 | 1.5 | 1.23 |  |  | 0 | 13 | 13 | 0 | 0 | 1 | 0 | 1 | 1 |  | -494 | -16 | 1 | 0 | 1 | 0 | 0 | 0 | 0 | 1 | 0 | 0 | 1 | 1 | 1 | 1 | 1 |
| P000006 | G0000257 | S0000833 | E001499 | MELD-Na | 2527 | 83 | M | 70 | 63 | 1.3 | 1.29507227121652 | 1.5 | 1.23 | 132 |  | 0 | 17 | 17 | 0 | 0 | 1 | 0 | 1 | 1 |  | -494 | -16 | 1 | 0 | 1 | 0 | 0 | 0 | 0 | 1 | 0 | 0 | 1 | 1 | 0 | 1 | 1 |
| P000006 | G0000257 | S0000834 | E001499 | reMELD-Na | 2527 | 83 | M | 70 | 63 | 1.3 | 1.29507227121652 | 1.5 | 1.23 | 132 |  |  | 15 | 15 | 0 | 0 | 1 | 0 | 1 | 1 |  | -494 | -16 | 1 | 0 | 1 | 0 | 0 | 0 | 0 | 1 | 0 | 0 | 1 | 1 | 0 | 1 | 1 |
| P000006 | G0000258 | S0000835 | E001499 | MELD | 2535 | 83 | M | 70 | 63 | 1.24 | 1.2360834036749 | 1.6 | 1.3 |  |  | 0 | 13 | 13 | 0 | 0 | 1 | 0 | 1 | 1 |  | -486 | -16 | 1 | 0 | 1 | 0 | 0 | 0 | 0 | 1 | 0 | 0 | 1 | 1 | 1 | 1 | 1 |
| P000006 | G0000258 | S0000836 | E001499 | MELD-Na | 2535 | 83 | M | 70 | 63 | 1.24 | 1.2360834036749 | 1.6 | 1.3 | 141 |  | 0 | 13 | 13 | 0 | 0 | 1 | 0 | 1 | 1 |  | -486 | -16 | 1 | 0 | 1 | 0 | 0 | 0 | 0 | 1 | 0 | 0 | 1 | 1 | 0 | 1 | 1 |
| P000006 | G0000258 | S0000837 | E001499 | reMELD-Na | 2535 | 83 | M | 70 | 63 | 1.24 | 1.2360834036749 | 1.6 | 1.3 | 141 |  |  | 14 | 14 | 0 | 0 | 1 | 0 | 1 | 1 |  | -486 | -16 | 1 | 0 | 1 | 0 | 0 | 0 | 0 | 1 | 0 | 0 | 1 | 1 | 0 | 1 | 1 |
| P000006 | G0000259 | S0000838 | E001499 | MELD | 2545 | 83 | M | 70 | 63 | 1.27 | 1.26059467602773 | 2 | 1.44 |  |  | 0 | 15 | 15 | 0 | 0 | 1 | 0 | 1 | 1 |  | -476 | -16 | 1 | 0 | 1 | 0 | 0 | 0 | 0 | 1 | 0 | 0 | 1 | 1 | 1 | 1 | 1 |
| P000006 | G0000259 | S0000839 | E001499 | MELD 3.0 | 2545 | 83 | M | 70 | 63 | 1.27 | 1.26059467602773 | 2 | 1.44 | 134 | 3.51 |  | 17 | 17 | 0 | 0 | 1 | 0 | 1 | 1 |  | -476 | -16 | 1 | 0 | 1 | 0 | 0 | 0 | 0 | 1 | 0 | 0 | 1 | 1 | 0 | 1 | 1 |
| P000006 | G0000259 | S0000840 | E001499 | MELD-Na | 2545 | 83 | M | 70 | 63 | 1.27 | 1.26059467602773 | 2 | 1.44 | 134 |  | 0 | 17 | 17 | 0 | 0 | 1 | 0 | 1 | 1 |  | -476 | -16 | 1 | 0 | 1 | 0 | 0 | 0 | 0 | 1 | 0 | 0 | 1 | 1 | 0 | 1 | 1 |
| P000006 | G0000259 | S0000841 | E001499 | reMELD-Na | 2545 | 83 | M | 70 | 63 | 1.27 | 1.26059467602773 | 2 | 1.44 | 134 |  |  | 17 | 17 | 0 | 0 | 1 | 0 | 1 | 1 |  | -476 | -16 | 1 | 0 | 1 | 0 | 0 | 0 | 0 | 1 | 0 | 0 | 1 | 1 | 0 | 1 | 1 |
| P000006 | G0000260 | S0000842 | E001517 | MELD | 2559 | 84 | M | 70 | 63 | 1.36 | 1.3438707096401 | 2.3 | 1.36 |  |  | 0 | 16 | 16 | 0 | 0 | 1 | 0 | 1 | 1 |  | -462 | -15 | 1 | 0 | 1 | 0 | 0 | 0 | 0 | 1 | 0 | 0 | 1 | 1 | 1 | 1 | 1 |
| P000006 | G0000260 | S0000843 | E001517 | MELD-Na | 2559 | 84 | M | 70 | 63 | 1.36 | 1.3438707096401 | 2.3 | 1.36 | 129 |  | 0 | 22 | 22 | 0 | 0 | 1 | 0 | 1 | 1 |  | -462 | -15 | 1 | 0 | 1 | 0 | 0 | 0 | 0 | 1 | 0 | 0 | 1 | 1 | 0 | 1 | 1 |
| P000006 | G0000260 | S0000844 | E001517 | reMELD-Na | 2559 | 84 | M | 70 | 63 | 1.36 | 1.3438707096401 | 2.3 | 1.36 | 129 |  |  | 19 | 19 | 0 | 0 | 1 | 0 | 1 | 1 |  | -462 | -15 | 1 | 0 | 1 | 0 | 0 | 0 | 0 | 1 | 0 | 0 | 1 | 1 | 0 | 1 | 1 |
| P000006 | G0000261 | S0000845 | E001531 | MELD | 2561 | 84 | M | 70 | 63 | 1.57 | 1.53861962700787 | 2.8 | 1.38 |  |  | 0 | 18 | 18 | 0 | 0 | 1 | 0 | 1 | 1 |  | -460 | -15 | 1 | 0 | 1 | 0 | 0 | 0 | 0 | 1 | 0 | 0 | 1 | 1 | 1 | 1 | 1 |
| P000006 | G0000261 | S0000846 | E001531 | MELD-Na | 2561 | 84 | M | 70 | 63 | 1.57 | 1.53861962700787 | 2.8 | 1.38 | 129.5 |  | 0 | 23 | 23 | 0 | 0 | 1 | 0 | 1 | 1 |  | -460 | -15 | 1 | 0 | 1 | 0 | 0 | 0 | 0 | 1 | 0 | 0 | 1 | 1 | 0 | 1 | 1 |
| P000006 | G0000261 | S0000847 | E001531 | reMELD-Na | 2561 | 84 | M | 70 | 63 | 1.57 | 1.53861962700787 | 2.8 | 1.38 | 129.5 |  |  | 20 | 20 | 0 | 0 | 1 | 0 | 1 | 1 |  | -460 | -15 | 1 | 0 | 1 | 0 | 0 | 0 | 0 | 1 | 0 | 0 | 1 | 1 | 0 | 1 | 1 |
| P000006 | G0000262 | S0000848 | E001531 | MELD | 2562 | 84 | M | 70 | 63 | 1.3 | 1.28084766072688 | 2.8 | 1.45 |  |  | 0 | 17 | 17 | 0 | 0 | 1 | 0 | 1 | 1 |  | -459 | -15 | 1 | 0 | 1 | 0 | 0 | 0 | 0 | 1 | 0 | 0 | 1 | 1 | 1 | 1 | 1 |
| P000006 | G0000262 | S0000849 | E001531 | MELD 3.0 | 2562 | 84 | M | 70 | 63 | 1.3 | 1.28084766072688 | 2.8 | 1.45 | 136 | 3.04 |  | 18 | 18 | 0 | 0 | 1 | 0 | 1 | 1 |  | -459 | -15 | 1 | 0 | 1 | 0 | 0 | 0 | 0 | 1 | 0 | 0 | 1 | 1 | 0 | 1 | 1 |
| P000006 | G0000262 | S0000850 | E001531 | MELD-Na | 2562 | 84 | M | 70 | 63 | 1.3 | 1.28084766072688 | 2.8 | 1.45 | 136 |  | 0 | 18 | 18 | 0 | 0 | 1 | 0 | 1 | 1 |  | -459 | -15 | 1 | 0 | 1 | 0 | 0 | 0 | 0 | 1 | 0 | 0 | 1 | 1 | 0 | 1 | 1 |
| P000006 | G0000262 | S0000851 | E001531 | reMELD-Na | 2562 | 84 | M | 70 | 63 | 1.3 | 1.28084766072688 | 2.8 | 1.45 | 136 |  |  | 18 | 17 | -1 | 1 | 0 | 0 | 1 | 1 |  | -459 | -15 | 1 | 0 | 1 | 0 | 0 | 0 | 0 | 1 | 0 | 0 | 1 | 1 | 0 | 1 | 1 |
| P000006 | G0000263 | S0000852 | E001531 | MELD | 2563 | 84 | M | 70 | 63 | 1.12 | 1.1034581437575 | 3.1 | 1.41 |  |  | 0 | 16 | 15 | -1 | 1 | 0 | 0 | 1 | 1 |  | -458 | -15 | 1 | 0 | 1 | 0 | 0 | 0 | 0 | 1 | 0 | 0 | 1 | 1 | 1 | 1 | 1 |
| P000006 | G0000263 | S0000853 | E001531 | MELD 3.0 | 2563 | 84 | M | 70 | 63 | 1.12 | 1.1034581437575 | 3.1 | 1.41 | 140.5 | 3.08 |  | 16 | 16 | 0 | 0 | 1 | 0 | 1 | 1 |  | -458 | -15 | 1 | 0 | 1 | 0 | 0 | 0 | 0 | 1 | 0 | 0 | 1 | 1 | 0 | 1 | 1 |
| P000006 | G0000263 | S0000854 | E001531 | MELD-Na | 2563 | 84 | M | 70 | 63 | 1.12 | 1.1034581437575 | 3.1 | 1.41 | 140.5 |  | 0 | 16 | 15 | -1 | 1 | 0 | 0 | 1 | 1 |  | -458 | -15 | 1 | 0 | 1 | 0 | 0 | 0 | 0 | 1 | 0 | 0 | 1 | 1 | 0 | 1 | 1 |
| P000006 | G0000263 | S0000855 | E001531 | reMELD-Na | 2563 | 84 | M | 70 | 63 | 1.12 | 1.1034581437575 | 3.1 | 1.41 | 140.5 |  |  | 16 | 16 | 0 | 0 | 1 | 0 | 1 | 1 |  | -458 | -15 | 1 | 0 | 1 | 0 | 0 | 0 | 0 | 1 | 0 | 0 | 1 | 1 | 0 | 1 | 1 |
| P000006 | G0000264 | S0000856 | E001531 | MELD | 2564 | 84 | M | 70 | 63 | 1.2 | 1.19074877320884 | 2.2 | 1.38 |  |  | 0 | 15 | 15 | 0 | 0 | 1 | 0 | 1 | 1 |  | -457 | -15 | 1 | 0 | 1 | 0 | 0 | 0 | 0 | 1 | 0 | 0 | 1 | 1 | 1 | 1 | 1 |
| P000006 | G0000264 | S0000857 | E001531 | MELD 3.0 | 2564 | 84 | M | 70 | 63 | 1.2 | 1.19074877320884 | 2.2 | 1.38 | 137 | 2.85 |  | 16 | 15 | -1 | 1 | 0 | 0 | 1 | 1 |  | -457 | -15 | 1 | 0 | 1 | 0 | 0 | 0 | 0 | 1 | 0 | 0 | 1 | 1 | 0 | 1 | 1 |
| P000006 | G0000264 | S0000858 | E001531 | MELD-Na | 2564 | 84 | M | 70 | 63 | 1.2 | 1.19074877320884 | 2.2 | 1.38 | 137 |  | 0 | 15 | 15 | 0 | 0 | 1 | 0 | 1 | 1 |  | -457 | -15 | 1 | 0 | 1 | 0 | 0 | 0 | 0 | 1 | 0 | 0 | 1 | 1 | 0 | 1 | 1 |
| P000006 | G0000264 | S0000859 | E001531 | reMELD-Na | 2564 | 84 | M | 70 | 63 | 1.2 | 1.19074877320884 | 2.2 | 1.38 | 137 |  |  | 15 | 15 | 0 | 0 | 1 | 0 | 1 | 1 |  | -457 | -15 | 1 | 0 | 1 | 0 | 0 | 0 | 0 | 1 | 0 | 0 | 1 | 1 | 0 | 1 | 1 |
| P000006 | G0000265 | S0000860 | E001531 | MELD | 2565 | 84 | M | 70 | 63 | 0.94 | 0.940244973900466 | 1.9 | 1.51 |  |  | 0 | 13 | 13 | 0 | 0 | 1 | 0 | 1 | 1 |  | -456 | -15 | 1 | 0 | 1 | 0 | 0 | 0 | 0 | 1 | 0 | 0 | 1 | 1 | 1 | 1 | 1 |
| P000006 | G0000265 | S0000861 | E001531 | MELD 3.0 | 2565 | 84 | M | 70 | 63 | 0.94 | 0.940244973900466 | 1.9 | 1.51 | 133 | 2.98 |  | 16 | 16 | 0 | 0 | 1 | 0 | 1 | 1 |  | -456 | -15 | 1 | 0 | 1 | 0 | 0 | 0 | 0 | 1 | 0 | 0 | 1 | 1 | 0 | 1 | 1 |
| P000006 | G0000265 | S0000862 | E001531 | MELD-Na | 2565 | 84 | M | 70 | 63 | 0.94 | 0.940244973900466 | 1.9 | 1.51 | 133 |  | 0 | 17 | 17 | 0 | 0 | 1 | 0 | 1 | 1 |  | -456 | -15 | 1 | 0 | 1 | 0 | 0 | 0 | 0 | 1 | 0 | 0 | 1 | 1 | 0 | 1 | 1 |
| P000006 | G0000265 | S0000863 | E001531 | reMELD-Na | 2565 | 84 | M | 70 | 63 | 0.94 | 0.940244973900466 | 1.9 | 1.51 | 133 |  |  | 15 | 15 | 0 | 0 | 1 | 0 | 1 | 1 |  | -456 | -15 | 1 | 0 | 1 | 0 | 0 | 0 | 0 | 1 | 0 | 0 | 1 | 1 | 0 | 1 | 1 |
| P000006 | G0000266 | S0000864 | E001531 | MELD | 2566 | 84 | M | 70 | 63 | 0.91 | 0.917436127105147 | 1.3 | 1.33 |  |  | 0 | 11 | 11 | 0 | 0 | 1 | 0 | 1 | 1 |  | -455 | -15 | 1 | 0 | 1 | 0 | 0 | 0 | 0 | 1 | 0 | 0 | 1 | 1 | 1 | 1 | 1 |
| P000006 | G0000266 | S0000865 | E001531 | MELD 3.0 | 2566 | 84 | M | 70 | 63 | 0.91 | 0.917436127105147 | 1.3 | 1.33 | 130 | 2.61 |  | 17 | 17 | 0 | 0 | 1 | 0 | 1 | 1 |  | -455 | -15 | 1 | 0 | 1 | 0 | 0 | 0 | 0 | 1 | 0 | 0 | 1 | 1 | 0 | 1 | 1 |
| P000006 | G0000266 | S0000866 | E001531 | MELD-Na | 2566 | 84 | M | 70 | 63 | 0.91 | 0.917436127105147 | 1.3 | 1.33 | 130 |  | 0 | 18 | 18 | 0 | 0 | 1 | 0 | 1 | 1 |  | -455 | -15 | 1 | 0 | 1 | 0 | 0 | 0 | 0 | 1 | 0 | 0 | 1 | 1 | 0 | 1 | 1 |
| P000006 | G0000266 | S0000867 | E001531 | reMELD-Na | 2566 | 84 | M | 70 | 63 | 0.91 | 0.917436127105147 | 1.3 | 1.33 | 130 |  |  | 14 | 14 | 0 | 0 | 1 | 0 | 1 | 1 |  | -455 | -15 | 1 | 0 | 1 | 0 | 0 | 0 | 0 | 1 | 0 | 0 | 1 | 1 | 0 | 1 | 1 |
| P000006 | G0000267 | S0000868 | E001531 | MELD | 2574 | 84 | M | 70 | 63 | 1.01 | 1.01343447793829 | 1.5 | 1.25 |  |  | 0 | 11 | 11 | 0 | 0 | 1 | 0 | 1 | 1 |  | -447 | -15 | 1 | 0 | 1 | 0 | 0 | 0 | 0 | 1 | 0 | 0 | 1 | 1 | 1 | 1 | 1 |
| P000006 | G0000267 | S0000869 | E001531 | MELD 3.0 | 2574 | 84 | M | 70 | 63 | 1.01 | 1.01343447793829 | 1.5 | 1.25 | 140 | 3.88 |  | 10 | 10 | 0 | 0 | 1 | 0 | 1 | 1 |  | -447 | -15 | 1 | 0 | 1 | 0 | 0 | 0 | 0 | 1 | 0 | 0 | 1 | 1 | 0 | 1 | 1 |
| P000006 | G0000267 | S0000870 | E001531 | MELD-Na | 2574 | 84 | M | 70 | 63 | 1.01 | 1.01343447793829 | 1.5 | 1.25 | 140 |  | 0 | 11 | 11 | 0 | 0 | 1 | 0 | 1 | 1 |  | -447 | -15 | 1 | 0 | 1 | 0 | 0 | 0 | 0 | 1 | 0 | 0 | 1 | 1 | 0 | 1 | 1 |
| P000006 | G0000267 | S0000871 | E001531 | reMELD-Na | 2574 | 84 | M | 70 | 63 | 1.01 | 1.01343447793829 | 1.5 | 1.25 | 140 |  |  | 12 | 12 | 0 | 0 | 1 | 0 | 1 | 1 |  | -447 | -15 | 1 | 0 | 1 | 0 | 0 | 0 | 0 | 1 | 0 | 0 | 1 | 1 | 0 | 1 | 1 |
| P000006 | G0000268 | S0000872 | E001539 | MELD | 2580 | 84 | M | 70 | 63 | 1.5 | 1.47220120342661 | 2.8 | 1.46 |  |  | 0 | 18 | 18 | 0 | 0 | 1 | 0 | 1 | 1 |  | -441 | -14 | 1 | 0 | 1 | 0 | 0 | 0 | 0 | 1 | 0 | 0 | 1 | 1 | 1 | 1 | 1 |
| P000006 | G0000268 | S0000873 | E001539 | MELD-Na | 2580 | 84 | M | 70 | 63 | 1.5 | 1.47220120342661 | 2.8 | 1.46 | 144 |  | 0 | 18 | 18 | 0 | 0 | 1 | 0 | 1 | 1 |  | -441 | -14 | 1 | 0 | 1 | 0 | 0 | 0 | 0 | 1 | 0 | 0 | 1 | 1 | 0 | 1 | 1 |
| P000006 | G0000268 | S0000874 | E001539 | reMELD-Na | 2580 | 84 | M | 70 | 63 | 1.5 | 1.47220120342661 | 2.8 | 1.46 | 144 |  |  | 18 | 18 | 0 | 0 | 1 | 0 | 1 | 1 |  | -441 | -14 | 1 | 0 | 1 | 0 | 0 | 0 | 0 | 1 | 0 | 0 | 1 | 1 | 0 | 1 | 1 |
| P000006 | G0000269 | S0000875 | E001517 | MELD | 2582 | 84 | M | 70 | 63 | 1.57 | 1.53545801483188 | 3.1 | 1.41 |  |  | 0 | 19 | 19 | 0 | 0 | 1 | 0 | 1 | 1 |  | -439 | -14 | 1 | 0 | 1 | 0 | 0 | 0 | 0 | 1 | 0 | 0 | 1 | 1 | 1 | 1 | 1 |
| P000006 | G0000269 | S0000876 | E001517 | MELD-Na | 2582 | 84 | M | 70 | 63 | 1.57 | 1.53545801483188 | 3.1 | 1.41 | 137 |  | 0 | 19 | 19 | 0 | 0 | 1 | 0 | 1 | 1 |  | -439 | -14 | 1 | 0 | 1 | 0 | 0 | 0 | 0 | 1 | 0 | 0 | 1 | 1 | 0 | 1 | 1 |
| P000006 | G0000269 | S0000877 | E001517 | reMELD-Na | 2582 | 84 | M | 70 | 63 | 1.57 | 1.53545801483188 | 3.1 | 1.41 | 137 |  |  | 19 | 19 | 0 | 0 | 1 | 0 | 1 | 1 |  | -439 | -14 | 1 | 0 | 1 | 0 | 0 | 0 | 0 | 1 | 0 | 0 | 1 | 1 | 0 | 1 | 1 |
| P000006 | G0000270 | S0000878 | E001517 | MELD | 2584 | 84 | M | 70 | 63 | 1.6 | 1.57345585860828 | 2.2 | 1.25 |  |  | 0 | 16 | 16 | 0 | 0 | 1 | 0 | 1 | 1 |  | -437 | -14 | 1 | 0 | 1 | 0 | 0 | 0 | 0 | 1 | 0 | 0 | 1 | 1 | 1 | 1 | 1 |
| P000006 | G0000270 | S0000879 | E001517 | MELD-Na | 2584 | 84 | M | 70 | 63 | 1.6 | 1.57345585860828 | 2.2 | 1.25 | 128 |  | 0 | 23 | 23 | 0 | 0 | 1 | 0 | 1 | 1 |  | -437 | -14 | 1 | 0 | 1 | 0 | 0 | 0 | 0 | 1 | 0 | 0 | 1 | 1 | 0 | 1 | 1 |
| P000006 | G0000270 | S0000880 | E001517 | reMELD-Na | 2584 | 84 | M | 70 | 63 | 1.6 | 1.57345585860828 | 2.2 | 1.25 | 128 |  |  | 19 | 19 | 0 | 0 | 1 | 0 | 1 | 1 |  | -437 | -14 | 1 | 0 | 1 | 0 | 0 | 0 | 0 | 1 | 0 | 0 | 1 | 1 | 0 | 1 | 1 |
| P000006 | G0000271 | S0000881 | E001517 | MELD | 2590 | 85 | M | 70 | 63 | 1.22 | 1.20901826975029 | 2.3 | 1.25 |  |  | 0 | 14 | 14 | 0 | 0 | 1 | 0 | 1 | 1 |  | -431 | -14 | 1 | 0 | 1 | 0 | 0 | 0 | 0 | 1 | 0 | 0 | 1 | 1 | 1 | 1 | 1 |
| P000006 | G0000271 | S0000882 | E001517 | MELD-Na | 2590 | 85 | M | 70 | 63 | 1.22 | 1.20901826975029 | 2.3 | 1.25 | 140 |  | 0 | 14 | 14 | 0 | 0 | 1 | 0 | 1 | 1 |  | -431 | -14 | 1 | 0 | 1 | 0 | 0 | 0 | 0 | 1 | 0 | 0 | 1 | 1 | 0 | 1 | 1 |
| P000006 | G0000271 | S0000883 | E001517 | reMELD-Na | 2590 | 85 | M | 70 | 63 | 1.22 | 1.20901826975029 | 2.3 | 1.25 | 140 |  |  | 14 | 14 | 0 | 0 | 1 | 0 | 1 | 1 |  | -431 | -14 | 1 | 0 | 1 | 0 | 0 | 0 | 0 | 1 | 0 | 0 | 1 | 1 | 0 | 1 | 1 |
| P000006 | G0000272 | S0000884 | E001517 | MELD | 2597 | 85 | M | 70 | 63 | 1.14 | 1.1325317722171 | 2.2 | 1.33 |  |  | 0 | 14 | 14 | 0 | 0 | 1 | 0 | 1 | 1 |  | -424 | -14 | 1 | 0 | 1 | 0 | 0 | 0 | 0 | 1 | 0 | 0 | 1 | 1 | 1 | 1 | 1 |
| P000006 | G0000272 | S0000885 | E001517 | MELD-Na | 2597 | 85 | M | 70 | 63 | 1.14 | 1.1325317722171 | 2.2 | 1.33 | 147 |  | 0 | 14 | 14 | 0 | 0 | 1 | 0 | 1 | 1 |  | -424 | -14 | 1 | 0 | 1 | 0 | 0 | 0 | 0 | 1 | 0 | 0 | 1 | 1 | 0 | 1 | 1 |
| P000006 | G0000272 | S0000886 | E001517 | reMELD-Na | 2597 | 85 | M | 70 | 63 | 1.14 | 1.1325317722171 | 2.2 | 1.33 | 147 |  |  | 14 | 14 | 0 | 0 | 1 | 0 | 1 | 1 |  | -424 | -14 | 1 | 0 | 1 | 0 | 0 | 0 | 0 | 1 | 0 | 0 | 1 | 1 | 0 | 1 | 1 |
| P000006 | G0000273 | S0000887 | E001561 | MELD | 2605 | 85 | M | 70 | 63 | 1.35 | 1.33755919128943 | 2 | 1.28 |  |  | 0 | 15 | 15 | 0 | 0 | 1 | 0 | 1 | 1 |  | -416 | -14 | 1 | 0 | 1 | 0 | 0 | 0 | 0 | 1 | 0 | 0 | 1 | 1 | 1 | 1 | 1 |
| P000006 | G0000273 | S0000888 | E001561 | MELD-Na | 2605 | 85 | M | 70 | 63 | 1.35 | 1.33755919128943 | 2 | 1.28 | 136 |  | 0 | 16 | 16 | 0 | 0 | 1 | 0 | 1 | 1 |  | -416 | -14 | 1 | 0 | 1 | 0 | 0 | 0 | 0 | 1 | 0 | 0 | 1 | 1 | 0 | 1 | 1 |
| P000006 | G0000273 | S0000889 | E001561 | reMELD-Na | 2605 | 85 | M | 70 | 63 | 1.35 | 1.33755919128943 | 2 | 1.28 | 136 |  |  | 16 | 16 | 0 | 0 | 1 | 0 | 1 | 1 |  | -416 | -14 | 1 | 0 | 1 | 0 | 0 | 0 | 0 | 1 | 0 | 0 | 1 | 1 | 0 | 1 | 1 |
| P000006 | G0000274 | S0000890 | E001561 | MELD | 2608 | 85 | M | 70 | 63 | 1.25 | 1.23692806286874 | 2.4 | 1.18 |  |  | 0 | 14 | 14 | 0 | 0 | 1 | 0 | 1 | 1 |  | -413 | -14 | 1 | 0 | 1 | 0 | 0 | 0 | 0 | 1 | 0 | 0 | 1 | 1 | 1 | 1 | 1 |
| P000006 | G0000274 | S0000891 | E001561 | MELD-Na | 2608 | 85 | M | 70 | 63 | 1.25 | 1.23692806286874 | 2.4 | 1.18 | 127 |  | 0 | 23 | 23 | 0 | 0 | 1 | 0 | 1 | 1 |  | -413 | -14 | 1 | 0 | 1 | 0 | 0 | 0 | 0 | 1 | 0 | 0 | 1 | 1 | 0 | 1 | 1 |
| P000006 | G0000274 | S0000892 | E001561 | reMELD-Na | 2608 | 85 | M | 70 | 63 | 1.25 | 1.23692806286874 | 2.4 | 1.18 | 127 |  |  | 18 | 18 | 0 | 0 | 1 | 0 | 1 | 1 |  | -413 | -14 | 1 | 0 | 1 | 0 | 0 | 0 | 0 | 1 | 0 | 0 | 1 | 1 | 0 | 1 | 1 |
| P000006 | G0000275 | S0000893 | E001561 | MELD | 2615 | 85 | M | 70 | 63 | 1 | 0.998062855201279 | 2 | 1.3 |  |  | 0 | 12 | 12 | 0 | 0 | 1 | 0 | 1 | 1 |  | -406 | -13 | 1 | 0 | 1 | 0 | 0 | 0 | 0 | 1 | 0 | 0 | 1 | 1 | 1 | 1 | 1 |
| P000006 | G0000275 | S0000894 | E001561 | MELD-Na | 2615 | 85 | M | 70 | 63 | 1 | 0.998062855201279 | 2 | 1.3 | 136 |  | 0 | 13 | 13 | 0 | 0 | 1 | 0 | 1 | 1 |  | -406 | -13 | 1 | 0 | 1 | 0 | 0 | 0 | 0 | 1 | 0 | 0 | 1 | 1 | 0 | 1 | 1 |
| P000006 | G0000275 | S0000895 | E001561 | reMELD-Na | 2615 | 85 | M | 70 | 63 | 1 | 0.998062855201279 | 2 | 1.3 | 136 |  |  | 13 | 13 | 0 | 0 | 1 | 0 | 1 | 1 |  | -406 | -13 | 1 | 0 | 1 | 0 | 0 | 0 | 0 | 1 | 0 | 0 | 1 | 1 | 0 | 1 | 1 |
| P000006 | G0000276 | S0000896 | E001517 | MELD | 2617 | 85 | M | 70 | 63 | 1.13 | 1.12944919519005 | 1.6 | 1.28 |  |  | 0 | 12 | 12 | 0 | 0 | 1 | 0 | 1 | 1 |  | -404 | -13 | 1 | 0 | 1 | 0 | 0 | 0 | 0 | 1 | 0 | 0 | 1 | 1 | 1 | 1 | 1 |
| P000006 | G0000276 | S0000897 | E001517 | MELD-Na | 2617 | 85 | M | 70 | 63 | 1.13 | 1.12944919519005 | 1.6 | 1.28 | 141 |  | 0 | 12 | 12 | 0 | 0 | 1 | 0 | 1 | 1 |  | -404 | -13 | 1 | 0 | 1 | 0 | 0 | 0 | 0 | 1 | 0 | 0 | 1 | 1 | 0 | 1 | 1 |
| P000006 | G0000276 | S0000898 | E001517 | reMELD-Na | 2617 | 85 | M | 70 | 63 | 1.13 | 1.12944919519005 | 1.6 | 1.28 | 141 |  |  | 13 | 13 | 0 | 0 | 1 | 0 | 1 | 1 |  | -404 | -13 | 1 | 0 | 1 | 0 | 0 | 0 | 0 | 1 | 0 | 0 | 1 | 1 | 0 | 1 | 1 |
| P000006 | G0000277 | S0000899 | E001570 | MELD | 2618 | 86 | M | 70 | 63 | 1.5 | 1.48642581391625 | 1.5 | 1.33 |  |  | 0 | 15 | 15 | 0 | 0 | 1 | 0 | 1 | 1 |  | -403 | -13 | 1 | 0 | 1 | 0 | 0 | 0 | 0 | 1 | 0 | 0 | 1 | 1 | 1 | 1 | 1 |
| P000006 | G0000277 | S0000900 | E001570 | MELD-Na | 2618 | 86 | M | 70 | 63 | 1.5 | 1.48642581391625 | 1.5 | 1.33 | 139 |  | 0 | 15 | 15 | 0 | 0 | 1 | 0 | 1 | 1 |  | -403 | -13 | 1 | 0 | 1 | 0 | 0 | 0 | 0 | 1 | 0 | 0 | 1 | 1 | 0 | 1 | 1 |
| P000006 | G0000277 | S0000901 | E001570 | reMELD-Na | 2618 | 86 | M | 70 | 63 | 1.5 | 1.48642581391625 | 1.5 | 1.33 | 139 |  |  | 16 | 16 | 0 | 0 | 1 | 0 | 1 | 1 |  | -403 | -13 | 1 | 0 | 1 | 0 | 0 | 0 | 0 | 1 | 0 | 0 | 1 | 1 | 0 | 1 | 1 |
| P000006 | G0000278 | S0000902 | E001570 | MELD | 2619 | 86 | M | 70 | 63 | 1.37 | 1.36118893733881 | 1.6 | 1.41 |  |  | 0 | 15 | 15 | 0 | 0 | 1 | 0 | 1 | 1 |  | -402 | -13 | 1 | 0 | 1 | 0 | 0 | 0 | 0 | 1 | 0 | 0 | 1 | 1 | 1 | 1 | 1 |
| P000006 | G0000278 | S0000903 | E001570 | MELD-Na | 2619 | 86 | M | 70 | 63 | 1.37 | 1.36118893733881 | 1.6 | 1.41 | 138 |  | 0 | 15 | 15 | 0 | 0 | 1 | 0 | 1 | 1 |  | -402 | -13 | 1 | 0 | 1 | 0 | 0 | 0 | 0 | 1 | 0 | 0 | 1 | 1 | 0 | 1 | 1 |
| P000006 | G0000278 | S0000904 | E001570 | reMELD-Na | 2619 | 86 | M | 70 | 63 | 1.37 | 1.36118893733881 | 1.6 | 1.41 | 138 |  |  | 16 | 15 | -1 | 1 | 0 | 0 | 1 | 1 |  | -402 | -13 | 1 | 0 | 1 | 0 | 0 | 0 | 0 | 1 | 0 | 0 | 1 | 1 | 0 | 1 | 1 |
| P000006 | G0000279 | S0000905 | E001570 | MELD | 2622 | 86 | M | 70 | 63 | 0.95 | 0.956811592008728 | 1.3 | 1.4 |  |  | 0 | 11 | 11 | 0 | 0 | 1 | 0 | 1 | 1 |  | -399 | -13 | 1 | 0 | 1 | 0 | 0 | 0 | 0 | 1 | 0 | 0 | 1 | 1 | 1 | 1 | 1 |
| P000006 | G0000279 | S0000906 | E001570 | MELD-Na | 2622 | 86 | M | 70 | 63 | 0.95 | 0.956811592008728 | 1.3 | 1.4 | 139 |  | 0 | 11 | 11 | 0 | 0 | 1 | 0 | 1 | 1 |  | -399 | -13 | 1 | 0 | 1 | 0 | 0 | 0 | 0 | 1 | 0 | 0 | 1 | 1 | 0 | 1 | 1 |
| P000006 | G0000279 | S0000907 | E001570 | reMELD-Na | 2622 | 86 | M | 70 | 63 | 0.95 | 0.956811592008728 | 1.3 | 1.4 | 139 |  |  | 12 | 12 | 0 | 0 | 1 | 0 | 1 | 1 |  | -399 | -13 | 1 | 0 | 1 | 0 | 0 | 0 | 0 | 1 | 0 | 0 | 1 | 1 | 0 | 1 | 1 |
| P000006 | G0000280 | S0000908 | E001570 | MELD | 2623 | 86 | M | 70 | 63 | 0.82 | 0.827363032910903 | 1.4 | 1.34 |  |  | 0 | 11 | 11 | 0 | 0 | 1 | 0 | 1 | 1 |  | -398 | -13 | 1 | 0 | 1 | 0 | 0 | 0 | 0 | 1 | 0 | 0 | 1 | 1 | 1 | 1 | 1 |
| P000006 | G0000280 | S0000909 | E001570 | MELD-Na | 2623 | 86 | M | 70 | 63 | 0.82 | 0.827363032910903 | 1.4 | 1.34 | 140 |  | 0 | 11 | 11 | 0 | 0 | 1 | 0 | 1 | 1 |  | -398 | -13 | 1 | 0 | 1 | 0 | 0 | 0 | 0 | 1 | 0 | 0 | 1 | 1 | 0 | 1 | 1 |
| P000006 | G0000280 | S0000910 | E001570 | reMELD-Na | 2623 | 86 | M | 70 | 63 | 0.82 | 0.827363032910903 | 1.4 | 1.34 | 140 |  |  | 10 | 10 | 0 | 0 | 1 | 0 | 1 | 1 |  | -398 | -13 | 1 | 0 | 1 | 0 | 0 | 0 | 0 | 1 | 0 | 0 | 1 | 1 | 0 | 1 | 1 |
| P000006 | G0000281 | S0000911 | E001570 | MELD | 2626 | 86 | M | 70 | 63 | 1.12 | 1.11748614509952 | 1.8 | 1.36 |  |  | 0 | 13 | 13 | 0 | 0 | 1 | 0 | 1 | 1 |  | -395 | -13 | 1 | 0 | 1 | 0 | 0 | 0 | 0 | 1 | 0 | 0 | 1 | 1 | 1 | 1 | 1 |
| P000006 | G0000281 | S0000912 | E001570 | MELD 3.0 | 2626 | 86 | M | 70 | 63 | 1.12 | 1.11748614509952 | 1.8 | 1.36 | 141 | 2.76 |  | 14 | 14 | 0 | 0 | 1 | 0 | 1 | 1 |  | -395 | -13 | 1 | 0 | 1 | 0 | 0 | 0 | 0 | 1 | 0 | 0 | 1 | 1 | 0 | 1 | 1 |
| P000006 | G0000281 | S0000913 | E001570 | MELD-Na | 2626 | 86 | M | 70 | 63 | 1.12 | 1.11748614509952 | 1.8 | 1.36 | 141 |  | 0 | 13 | 13 | 0 | 0 | 1 | 0 | 1 | 1 |  | -395 | -13 | 1 | 0 | 1 | 0 | 0 | 0 | 0 | 1 | 0 | 0 | 1 | 1 | 0 | 1 | 1 |
| P000006 | G0000281 | S0000914 | E001570 | reMELD-Na | 2626 | 86 | M | 70 | 63 | 1.12 | 1.11748614509952 | 1.8 | 1.36 | 141 |  |  | 14 | 14 | 0 | 0 | 1 | 0 | 1 | 1 |  | -395 | -13 | 1 | 0 | 1 | 0 | 0 | 0 | 0 | 1 | 0 | 0 | 1 | 1 | 0 | 1 | 1 |
| P000006 | G0000282 | S0000915 | E001570 | MELD | 2629 | 86 | M | 70 | 63 | 1.01 | 1.01683807060366 | 1.2 | 1.31 |  |  | 0 | 10 | 10 | 0 | 0 | 1 | 0 | 1 | 1 |  | -392 | -13 | 1 | 0 | 1 | 0 | 0 | 0 | 0 | 1 | 0 | 0 | 1 | 1 | 1 | 1 | 1 |
| P000006 | G0000282 | S0000916 | E001570 | MELD 3.0 | 2629 | 86 | M | 70 | 63 | 1.01 | 1.01683807060366 | 1.2 | 1.31 | 143 | 3.57 |  | 9 | 9 | 0 | 0 | 1 | 0 | 1 | 1 |  | -392 | -13 | 1 | 0 | 1 | 0 | 0 | 0 | 0 | 1 | 0 | 0 | 1 | 1 | 0 | 1 | 1 |
| P000006 | G0000282 | S0000917 | E001570 | MELD-Na | 2629 | 86 | M | 70 | 63 | 1.01 | 1.01683807060366 | 1.2 | 1.31 | 143 |  | 0 | 10 | 10 | 0 | 0 | 1 | 0 | 1 | 1 |  | -392 | -13 | 1 | 0 | 1 | 0 | 0 | 0 | 0 | 1 | 0 | 0 | 1 | 1 | 0 | 1 | 1 |
| P000006 | G0000282 | S0000918 | E001570 | reMELD-Na | 2629 | 86 | M | 70 | 63 | 1.01 | 1.01683807060366 | 1.2 | 1.31 | 143 |  |  | 11 | 11 | 0 | 0 | 1 | 0 | 1 | 1 |  | -392 | -13 | 1 | 0 | 1 | 0 | 0 | 0 | 0 | 1 | 0 | 0 | 1 | 1 | 0 | 1 | 1 |
| P000006 | G0000283 | S0000919 | E001574 | MELD | 2635 | 86 | M | 70 | 63 | 2.06 | 2.00635261033368 | 1.8 | 1.36 |  |  | 0 | 19 | 19 | 0 | 0 | 1 | 0 | 1 | 1 |  | -386 | -13 | 1 | 0 | 1 | 0 | 0 | 0 | 0 | 1 | 0 | 0 | 1 | 1 | 1 | 1 | 1 |
| P000006 | G0000283 | S0000920 | E001574 | MELD-Na | 2635 | 86 | M | 70 | 63 | 2.06 | 2.00635261033368 | 1.8 | 1.36 | 141 |  | 0 | 19 | 19 | 0 | 0 | 1 | 0 | 1 | 1 |  | -386 | -13 | 1 | 0 | 1 | 0 | 0 | 0 | 0 | 1 | 0 | 0 | 1 | 1 | 0 | 1 | 1 |
| P000006 | G0000283 | S0000921 | E001574 | reMELD-Na | 2635 | 86 | M | 70 | 63 | 2.06 | 2.00635261033368 | 1.8 | 1.36 | 141 |  |  | 19 | 19 | 0 | 0 | 1 | 0 | 1 | 1 |  | -386 | -13 | 1 | 0 | 1 | 0 | 0 | 0 | 0 | 1 | 0 | 0 | 1 | 1 | 0 | 1 | 1 |
| P000006 | G0000284 | S0000922 | E001574 | MELD | 2636 | 86 | M | 70 | 63 | 1.23 | 1.22641876653992 | 1.6 | 1.28 |  |  | 0 | 13 | 13 | 0 | 0 | 1 | 0 | 1 | 1 |  | -385 | -13 | 1 | 0 | 1 | 0 | 0 | 0 | 0 | 1 | 0 | 0 | 1 | 1 | 1 | 1 | 1 |
| P000006 | G0000284 | S0000923 | E001574 | MELD-Na | 2636 | 86 | M | 70 | 63 | 1.23 | 1.22641876653992 | 1.6 | 1.28 | 141 |  | 0 | 13 | 13 | 0 | 0 | 1 | 0 | 1 | 1 |  | -385 | -13 | 1 | 0 | 1 | 0 | 0 | 0 | 0 | 1 | 0 | 0 | 1 | 1 | 0 | 1 | 1 |
| P000006 | G0000284 | S0000924 | E001574 | reMELD-Na | 2636 | 86 | M | 70 | 63 | 1.23 | 1.22641876653992 | 1.6 | 1.28 | 141 |  |  | 14 | 14 | 0 | 0 | 1 | 0 | 1 | 1 |  | -385 | -13 | 1 | 0 | 1 | 0 | 0 | 0 | 0 | 1 | 0 | 0 | 1 | 1 | 0 | 1 | 1 |
| P000006 | G0000285 | S0000925 | E001517 | MELD | 2645 | 86 | M | 70 | 63 | 1.5 | 1.47975474261077 | 2.1 | 1.41 |  |  | 0 | 17 | 17 | 0 | 0 | 1 | 0 | 1 | 1 |  | -376 | -12 | 1 | 0 | 1 | 0 | 0 | 0 | 0 | 1 | 0 | 0 | 1 | 1 | 1 | 1 | 1 |
| P000006 | G0000285 | S0000926 | E001517 | MELD-Na | 2645 | 86 | M | 70 | 63 | 1.5 | 1.47975474261077 | 2.1 | 1.41 | 133 |  | 0 | 20 | 20 | 0 | 0 | 1 | 0 | 1 | 1 |  | -376 | -12 | 1 | 0 | 1 | 0 | 0 | 0 | 0 | 1 | 0 | 0 | 1 | 1 | 0 | 1 | 1 |
| P000006 | G0000285 | S0000927 | E001517 | reMELD-Na | 2645 | 86 | M | 70 | 63 | 1.5 | 1.47975474261077 | 2.1 | 1.41 | 133 |  |  | 18 | 18 | 0 | 0 | 1 | 0 | 1 | 1 |  | -376 | -12 | 1 | 0 | 1 | 0 | 0 | 0 | 0 | 1 | 0 | 0 | 1 | 1 | 0 | 1 | 1 |
| P000006 | G0000286 | S0000928 | E001586 | MELD | 2652 | 87 | M | 70 | 63 | 1.54 | 1.51884262776326 | 2 | 1.25 |  |  | 0 | 16 | 16 | 0 | 0 | 1 | 0 | 1 | 1 |  | -369 | -12 | 1 | 0 | 1 | 0 | 0 | 0 | 0 | 1 | 0 | 0 | 1 | 1 | 1 | 1 | 1 |
| P000006 | G0000286 | S0000929 | E001586 | MELD 3.0 | 2652 | 87 | M | 70 | 63 | 1.54 | 1.51884262776326 | 2 | 1.25 | 134 | 3.61 |  | 18 | 18 | 0 | 0 | 1 | 0 | 1 | 1 |  | -369 | -12 | 1 | 0 | 1 | 0 | 0 | 0 | 0 | 1 | 0 | 0 | 1 | 1 | 0 | 1 | 1 |
| P000006 | G0000286 | S0000930 | E001586 | MELD-Na | 2652 | 87 | M | 70 | 63 | 1.54 | 1.51884262776326 | 2 | 1.25 | 134 |  | 0 | 18 | 18 | 0 | 0 | 1 | 0 | 1 | 1 |  | -369 | -12 | 1 | 0 | 1 | 0 | 0 | 0 | 0 | 1 | 0 | 0 | 1 | 1 | 0 | 1 | 1 |
| P000006 | G0000286 | S0000931 | E001586 | reMELD-Na | 2652 | 87 | M | 70 | 63 | 1.54 | 1.51884262776326 | 2 | 1.25 | 134 |  |  | 17 | 17 | 0 | 0 | 1 | 0 | 1 | 1 |  | -369 | -12 | 1 | 0 | 1 | 0 | 0 | 0 | 0 | 1 | 0 | 0 | 1 | 1 | 0 | 1 | 1 |
| P000006 | G0000287 | S0000932 | E001586 | MELD | 2653 | 87 | M | 70 | 63 | 1.31 | 1.28419331030618 | 3.4 | 1.3 |  |  | 0 | 17 | 16 | -1 | 1 | 0 | 0 | 1 | 1 |  | -368 | -12 | 1 | 0 | 1 | 0 | 0 | 0 | 0 | 1 | 0 | 0 | 1 | 1 | 1 | 1 | 1 |
| P000006 | G0000287 | S0000933 | E001586 | MELD-Na | 2653 | 87 | M | 70 | 63 | 1.31 | 1.28419331030618 | 3.4 | 1.3 | 141 |  | 0 | 17 | 16 | -1 | 1 | 0 | 0 | 1 | 1 |  | -368 | -12 | 1 | 0 | 1 | 0 | 0 | 0 | 0 | 1 | 0 | 0 | 1 | 1 | 0 | 1 | 1 |
| P000006 | G0000287 | S0000934 | E001586 | reMELD-Na | 2653 | 87 | M | 70 | 63 | 1.31 | 1.28419331030618 | 3.4 | 1.3 | 141 |  |  | 17 | 16 | -1 | 1 | 0 | 0 | 1 | 1 |  | -368 | -12 | 1 | 0 | 1 | 0 | 0 | 0 | 0 | 1 | 0 | 0 | 1 | 1 | 0 | 1 | 1 |
| P000006 | G0000288 | S0000935 | E001517 | MELD | 2666 | 87 | M | 70 | 63 | 1.45 | 1.43768334350961 | 1.6 | 1.29 |  |  | 0 | 15 | 15 | 0 | 0 | 1 | 0 | 1 | 1 |  | -355 | -12 | 1 | 0 | 1 | 0 | 0 | 0 | 0 | 1 | 0 | 0 | 1 | 1 | 1 | 1 | 1 |
| P000006 | G0000288 | S0000936 | E001517 | MELD-Na | 2666 | 87 | M | 70 | 63 | 1.45 | 1.43768334350961 | 1.6 | 1.29 | 134 |  | 0 | 17 | 17 | 0 | 0 | 1 | 0 | 1 | 1 |  | -355 | -12 | 1 | 0 | 1 | 0 | 0 | 0 | 0 | 1 | 0 | 0 | 1 | 1 | 0 | 1 | 1 |
| P000006 | G0000288 | S0000937 | E001517 | reMELD-Na | 2666 | 87 | M | 70 | 63 | 1.45 | 1.43768334350961 | 1.6 | 1.29 | 134 |  |  | 16 | 16 | 0 | 0 | 1 | 0 | 1 | 1 |  | -355 | -12 | 1 | 0 | 1 | 0 | 0 | 0 | 0 | 1 | 0 | 0 | 1 | 1 | 0 | 1 | 1 |
| P000006 | G0000289 | S0000938 | E001596 | MELD | 2673 | 87 | M | 70 | 63 | 1.73 | 1.69472209454368 | 2.3 | 1.3 |  |  | 0 | 18 | 18 | 0 | 0 | 1 | 0 | 1 | 1 |  | -348 | -11 | 1 | 0 | 1 | 0 | 0 | 0 | 0 | 1 | 0 | 0 | 1 | 1 | 1 | 1 | 1 |
| P000006 | G0000289 | S0000939 | E001596 | MELD-Na | 2673 | 87 | M | 70 | 63 | 1.73 | 1.69472209454368 | 2.3 | 1.3 | 135 |  | 0 | 19 | 19 | 0 | 0 | 1 | 0 | 1 | 1 |  | -348 | -11 | 1 | 0 | 1 | 0 | 0 | 0 | 0 | 1 | 0 | 0 | 1 | 1 | 0 | 1 | 1 |
| P000006 | G0000289 | S0000940 | E001596 | reMELD-Na | 2673 | 87 | M | 70 | 63 | 1.73 | 1.69472209454368 | 2.3 | 1.3 | 135 |  |  | 18 | 18 | 0 | 0 | 1 | 0 | 1 | 1 |  | -348 | -11 | 1 | 0 | 1 | 0 | 0 | 0 | 0 | 1 | 0 | 0 | 1 | 1 | 0 | 1 | 1 |
| P000006 | G0000290 | S0000941 | E001596 | MELD | 2674 | 87 | M | 70 | 63 | 1.26 | 1.25205195131093 | 1.9 | 1.36 |  |  | 0 | 15 | 14 | -1 | 1 | 0 | 0 | 1 | 1 |  | -347 | -11 | 1 | 0 | 1 | 0 | 0 | 0 | 0 | 1 | 0 | 0 | 1 | 1 | 1 | 1 | 1 |
| P000006 | G0000290 | S0000942 | E001596 | MELD 3.0 | 2674 | 87 | M | 70 | 63 | 1.26 | 1.25205195131093 | 1.9 | 1.36 | 136 | 2.96 |  | 16 | 16 | 0 | 0 | 1 | 0 | 1 | 1 |  | -347 | -11 | 1 | 0 | 1 | 0 | 0 | 0 | 0 | 1 | 0 | 0 | 1 | 1 | 0 | 1 | 1 |
| P000006 | G0000290 | S0000943 | E001596 | MELD-Na | 2674 | 87 | M | 70 | 63 | 1.26 | 1.25205195131093 | 1.9 | 1.36 | 136 |  | 0 | 16 | 15 | -1 | 1 | 0 | 0 | 1 | 1 |  | -347 | -11 | 1 | 0 | 1 | 0 | 0 | 0 | 0 | 1 | 0 | 0 | 1 | 1 | 0 | 1 | 1 |
| P000006 | G0000290 | S0000944 | E001596 | reMELD-Na | 2674 | 87 | M | 70 | 63 | 1.26 | 1.25205195131093 | 1.9 | 1.36 | 136 |  |  | 16 | 16 | 0 | 0 | 1 | 0 | 1 | 1 |  | -347 | -11 | 1 | 0 | 1 | 0 | 0 | 0 | 0 | 1 | 0 | 0 | 1 | 1 | 0 | 1 | 1 |
| P000006 | G0000291 | S0000945 | E001596 | MELD | 2679 | 88 | M | 70 | 63 | 1.1 | 1.09800996173864 | 1.8 | 1.29 |  |  | 0 | 12 | 12 | 0 | 0 | 1 | 0 | 1 | 1 |  | -342 | -11 | 1 | 0 | 1 | 0 | 0 | 0 | 0 | 1 | 0 | 0 | 1 | 1 | 1 | 1 | 1 |
| P000006 | G0000291 | S0000946 | E001596 | MELD 3.0 | 2679 | 88 | M | 70 | 63 | 1.1 | 1.09800996173864 | 1.8 | 1.29 | 132 | 2.93 |  | 16 | 16 | 0 | 0 | 1 | 0 | 1 | 1 |  | -342 | -11 | 1 | 0 | 1 | 0 | 0 | 0 | 0 | 1 | 0 | 0 | 1 | 1 | 0 | 1 | 1 |
| P000006 | G0000291 | S0000947 | E001596 | MELD-Na | 2679 | 88 | M | 70 | 63 | 1.1 | 1.09800996173864 | 1.8 | 1.29 | 132 |  | 0 | 17 | 17 | 0 | 0 | 1 | 0 | 1 | 1 |  | -342 | -11 | 1 | 0 | 1 | 0 | 0 | 0 | 0 | 1 | 0 | 0 | 1 | 1 | 0 | 1 | 1 |
| P000006 | G0000291 | S0000948 | E001596 | reMELD-Na | 2679 | 88 | M | 70 | 63 | 1.1 | 1.09800996173864 | 1.8 | 1.29 | 132 |  |  | 15 | 15 | 0 | 0 | 1 | 0 | 1 | 1 |  | -342 | -11 | 1 | 0 | 1 | 0 | 0 | 0 | 0 | 1 | 0 | 0 | 1 | 1 | 0 | 1 | 1 |
| P000006 | G0000292 | S0000949 | E001517 | MELD | 2685 | 88 | M | 70 | 63 | 1.45 | 1.43768334350961 | 1.6 | 1.46 |  |  | 0 | 16 | 16 | 0 | 0 | 1 | 0 | 1 | 1 |  | -336 | -11 | 1 | 0 | 1 | 0 | 0 | 0 | 0 | 1 | 0 | 0 | 1 | 1 | 1 | 1 | 1 |
| P000006 | G0000292 | S0000950 | E001517 | MELD-Na | 2685 | 88 | M | 70 | 63 | 1.45 | 1.43768334350961 | 1.6 | 1.46 | 132 |  | 0 | 20 | 20 | 0 | 0 | 1 | 0 | 1 | 1 |  | -336 | -11 | 1 | 0 | 1 | 0 | 0 | 0 | 0 | 1 | 0 | 0 | 1 | 1 | 0 | 1 | 1 |
| P000006 | G0000292 | S0000951 | E001517 | reMELD-Na | 2685 | 88 | M | 70 | 63 | 1.45 | 1.43768334350961 | 1.6 | 1.46 | 132 |  |  | 18 | 18 | 0 | 0 | 1 | 0 | 1 | 1 |  | -336 | -11 | 1 | 0 | 1 | 0 | 0 | 0 | 0 | 1 | 0 | 0 | 1 | 1 | 0 | 1 | 1 |
| P000006 | G0000293 | S0000952 | E001604 | MELD | 2688 | 88 | M | 70 | 63 | 1.71 | 1.67705198709313 | 2.2 | 1.36 |  |  | 0 | 18 | 18 | 0 | 0 | 1 | 0 | 1 | 1 |  | -333 | -11 | 1 | 0 | 1 | 0 | 0 | 0 | 0 | 1 | 0 | 0 | 1 | 1 | 1 | 1 | 1 |
| P000006 | G0000293 | S0000953 | E001604 | MELD-Na | 2688 | 88 | M | 70 | 63 | 1.71 | 1.67705198709313 | 2.2 | 1.36 | 132 |  | 0 | 22 | 22 | 0 | 0 | 1 | 0 | 1 | 1 |  | -333 | -11 | 1 | 0 | 1 | 0 | 0 | 0 | 0 | 1 | 0 | 0 | 1 | 1 | 0 | 1 | 1 |
| P000006 | G0000293 | S0000954 | E001604 | reMELD-Na | 2688 | 88 | M | 70 | 63 | 1.71 | 1.67705198709313 | 2.2 | 1.36 | 132 |  |  | 19 | 19 | 0 | 0 | 1 | 0 | 1 | 1 |  | -333 | -11 | 1 | 0 | 1 | 0 | 0 | 0 | 0 | 1 | 0 | 0 | 1 | 1 | 0 | 1 | 1 |
| P000006 | G0000294 | S0000955 | E001604 | MELD | 2689 | 88 | M | 70 | 63 | 1.38 | 1.36742820783986 | 1.9 | 1.4 |  |  | 0 | 16 | 16 | 0 | 0 | 1 | 0 | 1 | 1 |  | -332 | -11 | 1 | 0 | 1 | 0 | 0 | 0 | 0 | 1 | 0 | 0 | 1 | 1 | 1 | 1 | 1 |
| P000006 | G0000294 | S0000956 | E001604 | MELD 3.0 | 2689 | 88 | M | 70 | 63 | 1.38 | 1.36742820783986 | 1.9 | 1.4 | 134 | 2.86 |  | 18 | 18 | 0 | 0 | 1 | 0 | 1 | 1 |  | -332 | -11 | 1 | 0 | 1 | 0 | 0 | 0 | 0 | 1 | 0 | 0 | 1 | 1 | 0 | 1 | 1 |
| P000006 | G0000294 | S0000957 | E001604 | MELD-Na | 2689 | 88 | M | 70 | 63 | 1.38 | 1.36742820783986 | 1.9 | 1.4 | 134 |  | 0 | 18 | 18 | 0 | 0 | 1 | 0 | 1 | 1 |  | -332 | -11 | 1 | 0 | 1 | 0 | 0 | 0 | 0 | 1 | 0 | 0 | 1 | 1 | 0 | 1 | 1 |
| P000006 | G0000294 | S0000958 | E001604 | reMELD-Na | 2689 | 88 | M | 70 | 63 | 1.38 | 1.36742820783986 | 1.9 | 1.4 | 134 |  |  | 17 | 17 | 0 | 0 | 1 | 0 | 1 | 1 |  | -332 | -11 | 1 | 0 | 1 | 0 | 0 | 0 | 0 | 1 | 0 | 0 | 1 | 1 | 0 | 1 | 1 |
| P000006 | G0000295 | S0000959 | E001604 | MELD | 2692 | 88 | M | 70 | 63 | 1 | 1.00363468443966 | 1.5 | 1.36 |  |  | 0 | 11 | 11 | 0 | 0 | 1 | 0 | 1 | 1 |  | -329 | -11 | 1 | 0 | 1 | 0 | 0 | 0 | 0 | 1 | 0 | 0 | 1 | 1 | 1 | 1 | 1 |
| P000006 | G0000295 | S0000960 | E001604 | MELD-Na | 2692 | 88 | M | 70 | 63 | 1 | 1.00363468443966 | 1.5 | 1.36 | 132 |  | 0 | 16 | 16 | 0 | 0 | 1 | 0 | 1 | 1 |  | -329 | -11 | 1 | 0 | 1 | 0 | 0 | 0 | 0 | 1 | 0 | 0 | 1 | 1 | 0 | 1 | 1 |
| P000006 | G0000295 | S0000961 | E001604 | reMELD-Na | 2692 | 88 | M | 70 | 63 | 1 | 1.00363468443966 | 1.5 | 1.36 | 132 |  |  | 15 | 15 | 0 | 0 | 1 | 0 | 1 | 1 |  | -329 | -11 | 1 | 0 | 1 | 0 | 0 | 0 | 0 | 1 | 0 | 0 | 1 | 1 | 0 | 1 | 1 |
| P000006 | G0000296 | S0000962 | E001517 | MELD | 2695 | 88 | M | 70 | 63 | 1.13 | 1.13283766407484 | 1.3 | 1.29 |  |  | 0 | 11 | 11 | 0 | 0 | 1 | 0 | 1 | 1 |  | -326 | -11 | 1 | 0 | 1 | 0 | 0 | 0 | 0 | 1 | 0 | 0 | 1 | 1 | 1 | 1 | 1 |
| P000006 | G0000296 | S0000963 | E001517 | MELD-Na | 2695 | 88 | M | 70 | 63 | 1.13 | 1.13283766407484 | 1.3 | 1.29 | 132 |  | 0 | 16 | 16 | 0 | 0 | 1 | 0 | 1 | 1 |  | -326 | -11 | 1 | 0 | 1 | 0 | 0 | 0 | 0 | 1 | 0 | 0 | 1 | 1 | 0 | 1 | 1 |
| P000006 | G0000296 | S0000964 | E001517 | reMELD-Na | 2695 | 88 | M | 70 | 63 | 1.13 | 1.13283766407484 | 1.3 | 1.29 | 132 |  |  | 14 | 14 | 0 | 0 | 1 | 0 | 1 | 1 |  | -326 | -11 | 1 | 0 | 1 | 0 | 0 | 0 | 0 | 1 | 0 | 0 | 1 | 1 | 0 | 1 | 1 |
| P000006 | G0000297 | S0000965 | E001517 | MELD | 2701 | 88 | M | 70 | 63 | 1.3 | 1.28950044197814 | 2 | 1.31 |  |  | 0 | 15 | 15 | 0 | 0 | 1 | 0 | 1 | 1 |  | -320 | -10 | 1 | 0 | 1 | 0 | 0 | 0 | 0 | 1 | 0 | 0 | 1 | 1 | 1 | 1 | 1 |
| P000006 | G0000297 | S0000966 | E001517 | MELD-Na | 2701 | 88 | M | 70 | 63 | 1.3 | 1.28950044197814 | 2 | 1.31 | 132 |  | 0 | 19 | 19 | 0 | 0 | 1 | 0 | 1 | 1 |  | -320 | -10 | 1 | 0 | 1 | 0 | 0 | 0 | 0 | 1 | 0 | 0 | 1 | 1 | 0 | 1 | 1 |
| P000006 | G0000297 | S0000967 | E001517 | reMELD-Na | 2701 | 88 | M | 70 | 63 | 1.3 | 1.28950044197814 | 2 | 1.31 | 132 |  |  | 17 | 17 | 0 | 0 | 1 | 0 | 1 | 1 |  | -320 | -10 | 1 | 0 | 1 | 0 | 0 | 0 | 0 | 1 | 0 | 0 | 1 | 1 | 0 | 1 | 1 |
| P000006 | G0000298 | S0000968 | E001517 | MELD | 2706 | 88 | M | 70 | 63 | 1.59 | 1.56619621343819 | 2 | 1.28 |  |  | 0 | 16 | 16 | 0 | 0 | 1 | 0 | 1 | 1 |  | -315 | -10 | 1 | 0 | 1 | 0 | 0 | 0 | 0 | 1 | 0 | 0 | 1 | 1 | 1 | 1 | 1 |
| P000006 | G0000298 | S0000969 | E001517 | MELD-Na | 2706 | 88 | M | 70 | 63 | 1.59 | 1.56619621343819 | 2 | 1.28 | 129 |  | 0 | 22 | 22 | 0 | 0 | 1 | 0 | 1 | 1 |  | -315 | -10 | 1 | 0 | 1 | 0 | 0 | 0 | 0 | 1 | 0 | 0 | 1 | 1 | 0 | 1 | 1 |
| P000006 | G0000298 | S0000970 | E001517 | reMELD-Na | 2706 | 88 | M | 70 | 63 | 1.59 | 1.56619621343819 | 2 | 1.28 | 129 |  |  | 19 | 19 | 0 | 0 | 1 | 0 | 1 | 1 |  | -315 | -10 | 1 | 0 | 1 | 0 | 0 | 0 | 0 | 1 | 0 | 0 | 1 | 1 | 0 | 1 | 1 |
| P000006 | G0000299 | S0000971 | E001621 | MELD | 2708 | 88 | M | 70 | 63 | 1.67 | 1.62984198618174 | 3.1 | 1.54 |  |  | 0 | 20 | 20 | 0 | 0 | 1 | 0 | 1 | 1 |  | -313 | -10 | 1 | 0 | 1 | 0 | 0 | 0 | 0 | 1 | 0 | 0 | 1 | 1 | 1 | 1 | 1 |
| P000006 | G0000299 | S0000972 | E001621 | MELD-Na | 2708 | 88 | M | 70 | 63 | 1.67 | 1.62984198618174 | 3.1 | 1.54 | 133 |  | 0 | 23 | 23 | 0 | 0 | 1 | 0 | 1 | 1 |  | -313 | -10 | 1 | 0 | 1 | 0 | 0 | 0 | 0 | 1 | 0 | 0 | 1 | 1 | 0 | 1 | 1 |
| P000006 | G0000299 | S0000973 | E001621 | reMELD-Na | 2708 | 88 | M | 70 | 63 | 1.67 | 1.62984198618174 | 3.1 | 1.54 | 133 |  |  | 21 | 21 | 0 | 0 | 1 | 0 | 1 | 1 |  | -313 | -10 | 1 | 0 | 1 | 0 | 0 | 0 | 0 | 1 | 0 | 0 | 1 | 1 | 0 | 1 | 1 |
| P000006 | G0000300 | S0000974 | E001621 | MELD | 2709 | 89 | M | 70 | 63 | 1.53 | 1.49858871757525 | 3 | 1.48 |  |  | 0 | 19 | 19 | 0 | 0 | 1 | 0 | 1 | 1 |  | -312 | -10 | 1 | 0 | 1 | 0 | 0 | 0 | 0 | 1 | 0 | 0 | 1 | 1 | 1 | 1 | 1 |
| P000006 | G0000300 | S0000975 | E001621 | MELD-Na | 2709 | 89 | M | 70 | 63 | 1.53 | 1.49858871757525 | 3 | 1.48 | 130.5 |  | 0 | 24 | 24 | 0 | 0 | 1 | 0 | 1 | 1 |  | -312 | -10 | 1 | 0 | 1 | 0 | 0 | 0 | 0 | 1 | 0 | 0 | 1 | 1 | 0 | 1 | 1 |
| P000006 | G0000300 | S0000976 | E001621 | reMELD-Na | 2709 | 89 | M | 70 | 63 | 1.53 | 1.49858871757525 | 3 | 1.48 | 130.5 |  |  | 21 | 21 | 0 | 0 | 1 | 0 | 1 | 1 |  | -312 | -10 | 1 | 0 | 1 | 0 | 0 | 0 | 0 | 1 | 0 | 0 | 1 | 1 | 0 | 1 | 1 |
| P000006 | G0000301 | S0000977 | E001621 | MELD | 2710 | 89 | M | 70 | 63 | 1.44 | 1.41932800843348 | 2.4 | 1.26 |  |  | 0 | 16 | 16 | 0 | 0 | 1 | 0 | 1 | 1 |  | -311 | -10 | 1 | 0 | 1 | 0 | 0 | 0 | 0 | 1 | 0 | 0 | 1 | 1 | 1 | 1 | 1 |
| P000006 | G0000301 | S0000978 | E001621 | MELD-Na | 2710 | 89 | M | 70 | 63 | 1.44 | 1.41932800843348 | 2.4 | 1.26 | 132 |  | 0 | 20 | 20 | 0 | 0 | 1 | 0 | 1 | 1 |  | -311 | -10 | 1 | 0 | 1 | 0 | 0 | 0 | 0 | 1 | 0 | 0 | 1 | 1 | 0 | 1 | 1 |
| P000006 | G0000301 | S0000979 | E001621 | reMELD-Na | 2710 | 89 | M | 70 | 63 | 1.44 | 1.41932800843348 | 2.4 | 1.26 | 132 |  |  | 18 | 18 | 0 | 0 | 1 | 0 | 1 | 1 |  | -311 | -10 | 1 | 0 | 1 | 0 | 0 | 0 | 0 | 1 | 0 | 0 | 1 | 1 | 0 | 1 | 1 |
| P000006 | G0000302 | S0000980 | E001621 | MELD | 2713 | 89 | M | 70 | 63 | 0.93 | 0.924933041821912 | 2.4 | 1.31 |  |  | 0 | 13 | 13 | 0 | 0 | 1 | 0 | 1 | 1 |  | -308 | -10 | 1 | 0 | 1 | 0 | 0 | 0 | 0 | 1 | 0 | 0 | 1 | 1 | 1 | 1 | 1 |
| P000006 | G0000302 | S0000981 | E001621 | MELD 3.0 | 2713 | 89 | M | 70 | 63 | 0.93 | 0.924933041821912 | 2.4 | 1.31 | 133 | 2.65 |  | 16 | 16 | 0 | 0 | 1 | 0 | 1 | 1 |  | -308 | -10 | 1 | 0 | 1 | 0 | 0 | 0 | 0 | 1 | 0 | 0 | 1 | 1 | 0 | 1 | 1 |
| P000006 | G0000302 | S0000982 | E001621 | MELD-Na | 2713 | 89 | M | 70 | 63 | 0.93 | 0.924933041821912 | 2.4 | 1.31 | 133 |  | 0 | 17 | 17 | 0 | 0 | 1 | 0 | 1 | 1 |  | -308 | -10 | 1 | 0 | 1 | 0 | 0 | 0 | 0 | 1 | 0 | 0 | 1 | 1 | 0 | 1 | 1 |
| P000006 | G0000302 | S0000983 | E001621 | reMELD-Na | 2713 | 89 | M | 70 | 63 | 0.93 | 0.924933041821912 | 2.4 | 1.31 | 133 |  |  | 15 | 15 | 0 | 0 | 1 | 0 | 1 | 1 |  | -308 | -10 | 1 | 0 | 1 | 0 | 0 | 0 | 0 | 1 | 0 | 0 | 1 | 1 | 0 | 1 | 1 |
| P000006 | G0000303 | S0000984 | E001630 | MELD | 2726 | 89 | M | 70 | 63 | 1.21 | 1.20152524024163 | 2.1 | 1.31 |  |  | 0 | 14 | 14 | 0 | 0 | 1 | 0 | 1 | 1 |  | -295 | -10 | 1 | 0 | 1 | 0 | 0 | 0 | 0 | 1 | 0 | 0 | 1 | 1 | 1 | 1 | 1 |
| P000006 | G0000303 | S0000985 | E001630 | MELD 3.0 | 2726 | 89 | M | 70 | 63 | 1.21 | 1.20152524024163 | 2.1 | 1.31 | 128 | 3.41 |  | 20 | 20 | 0 | 0 | 1 | 0 | 1 | 1 |  | -295 | -10 | 1 | 0 | 1 | 0 | 0 | 0 | 0 | 1 | 0 | 0 | 1 | 1 | 0 | 1 | 1 |
| P000006 | G0000303 | S0000986 | E001630 | MELD-Na | 2726 | 89 | M | 70 | 63 | 1.21 | 1.20152524024163 | 2.1 | 1.31 | 128 |  | 0 | 22 | 22 | 0 | 0 | 1 | 0 | 1 | 1 |  | -295 | -10 | 1 | 0 | 1 | 0 | 0 | 0 | 0 | 1 | 0 | 0 | 1 | 1 | 0 | 1 | 1 |
| P000006 | G0000303 | S0000987 | E001630 | reMELD-Na | 2726 | 89 | M | 70 | 63 | 1.21 | 1.20152524024163 | 2.1 | 1.31 | 128 |  |  | 18 | 18 | 0 | 0 | 1 | 0 | 1 | 1 |  | -295 | -10 | 1 | 0 | 1 | 0 | 0 | 0 | 0 | 1 | 0 | 0 | 1 | 1 | 0 | 1 | 1 |
| P000006 | G0000304 | S0000988 | E001517 | MELD | 2738 | 89 | M | 70 | 63 | 0.97 | 0.97307559739391 | 1.6 | 1.22 |  |  | 0 | 10 | 10 | 0 | 0 | 1 | 0 | 1 | 1 |  | -283 | -9 | 1 | 0 | 1 | 0 | 0 | 0 | 0 | 1 | 0 | 0 | 1 | 1 | 1 | 1 | 1 |
| P000006 | G0000304 | S0000989 | E001517 | MELD-Na | 2738 | 89 | M | 70 | 63 | 0.97 | 0.97307559739391 | 1.6 | 1.22 | 136 |  | 0 | 11 | 11 | 0 | 0 | 1 | 0 | 1 | 1 |  | -283 | -9 | 1 | 0 | 1 | 0 | 0 | 0 | 0 | 1 | 0 | 0 | 1 | 1 | 0 | 1 | 1 |
| P000006 | G0000304 | S0000990 | E001517 | reMELD-Na | 2738 | 89 | M | 70 | 63 | 0.97 | 0.97307559739391 | 1.6 | 1.22 | 136 |  |  | 12 | 12 | 0 | 0 | 1 | 0 | 1 | 1 |  | -283 | -9 | 1 | 0 | 1 | 0 | 0 | 0 | 0 | 1 | 0 | 0 | 1 | 1 | 0 | 1 | 1 |
| P000006 | G0000305 | S0000991 | E001640 | MELD | 2743 | 90 | M | 71 | 63 | 1.22 | 1.21230087217098 | 2 | 1.33 |  |  | 0 | 14 | 14 | 0 | 0 | 1 | 0 | 1 | 1 |  | -278 | -9 | 1 | 0 | 1 | 0 | 0 | 0 | 0 | 1 | 0 | 0 | 1 | 1 | 1 | 1 | 1 |
| P000006 | G0000305 | S0000992 | E001640 | MELD-Na | 2743 | 90 | M | 71 | 63 | 1.22 | 1.21230087217098 | 2 | 1.33 | 135 |  | 0 | 16 | 16 | 0 | 0 | 1 | 0 | 1 | 1 |  | -278 | -9 | 1 | 0 | 1 | 0 | 0 | 0 | 0 | 1 | 0 | 0 | 1 | 1 | 0 | 1 | 1 |
| P000006 | G0000305 | S0000993 | E001640 | reMELD-Na | 2743 | 90 | M | 71 | 63 | 1.22 | 1.21230087217098 | 2 | 1.33 | 135 |  |  | 16 | 16 | 0 | 0 | 1 | 0 | 1 | 1 |  | -278 | -9 | 1 | 0 | 1 | 0 | 0 | 0 | 0 | 1 | 0 | 0 | 1 | 1 | 0 | 1 | 1 |
| P000006 | G0000306 | S0000994 | E001517 | MELD | 2756 | 90 | M | 71 | 63 | 1.08 | 1.07717968836946 | 1.92 | 1.25 |  |  | 0 | 12 | 12 | 0 | 0 | 1 | 0 | 1 | 1 |  | -265 | -9 | 1 | 0 | 1 | 0 | 0 | 0 | 0 | 1 | 0 | 0 | 1 | 1 | 1 | 1 | 1 |
| P000006 | G0000306 | S0000995 | E001517 | MELD-Na | 2756 | 90 | M | 71 | 63 | 1.08 | 1.07717968836946 | 1.92 | 1.25 | 130 |  | 0 | 18 | 18 | 0 | 0 | 1 | 0 | 1 | 1 |  | -265 | -9 | 1 | 0 | 1 | 0 | 0 | 0 | 0 | 1 | 0 | 0 | 1 | 1 | 0 | 1 | 1 |
| P000006 | G0000306 | S0000996 | E001517 | reMELD-Na | 2756 | 90 | M | 71 | 63 | 1.08 | 1.07717968836946 | 1.92 | 1.25 | 130 |  |  | 16 | 16 | 0 | 0 | 1 | 0 | 1 | 1 |  | -265 | -9 | 1 | 0 | 1 | 0 | 0 | 0 | 0 | 1 | 0 | 0 | 1 | 1 | 0 | 1 | 1 |
| P000006 | G0000307 | S0000997 | E001654 | MELD | 2764 | 90 | M | 71 | 63 | 1.26 | 1.24453501120394 | 2.59 | 1.3 |  |  | 0 | 15 | 15 | 0 | 0 | 1 | 0 | 1 | 1 |  | -257 | -8 | 1 | 0 | 1 | 0 | 0 | 0 | 0 | 1 | 0 | 0 | 1 | 1 | 1 | 1 | 1 |
| P000006 | G0000307 | S0000998 | E001654 | MELD 3.0 | 2764 | 90 | M | 71 | 63 | 1.26 | 1.24453501120394 | 2.59 | 1.3 | 131 | 3.1 |  | 19 | 19 | 0 | 0 | 1 | 0 | 1 | 1 |  | -257 | -8 | 1 | 0 | 1 | 0 | 0 | 0 | 0 | 1 | 0 | 0 | 1 | 1 | 0 | 1 | 1 |
| P000006 | G0000307 | S0000999 | E001654 | MELD-Na | 2764 | 90 | M | 71 | 63 | 1.26 | 1.24453501120394 | 2.59 | 1.3 | 131 |  | 0 | 20 | 20 | 0 | 0 | 1 | 0 | 1 | 1 |  | -257 | -8 | 1 | 0 | 1 | 0 | 0 | 0 | 0 | 1 | 0 | 0 | 1 | 1 | 0 | 1 | 1 |
| P000006 | G0000307 | S0001000 | E001654 | reMELD-Na | 2764 | 90 | M | 71 | 63 | 1.26 | 1.24453501120394 | 2.59 | 1.3 | 131 |  |  | 18 | 18 | 0 | 0 | 1 | 0 | 1 | 1 |  | -257 | -8 | 1 | 0 | 1 | 0 | 0 | 0 | 0 | 1 | 0 | 0 | 1 | 1 | 0 | 1 | 1 |
