# Supplementary material for: Creatinine assay interferences compromises MELD accuracy and may bias liver allocation
Source: Nat Commun. 2026 Jul 23;17:7111. doi: 10.1038/s41467-026-75011-x (PMC13396164; doi:10.1038/s41467-026-75011-x)
Supplement: Supplementary file 4 — Source Data [file 41467_2026_75011_MOESM4_ESM.zip › figshare_package_FINAL_PUBLIC_DEPOSIT_V1_20260503_002637/00_START_HERE_HTML_NAVIGATOR/file_views/view_0020_esld_T4_score_deviation_outcome_meta_public.html]

02\_workflows/T4\_workflow\_v01/submission\_ready/public/data/esld\_T4\_score\_deviation\_outcome\_meta\_public.csv

# Readable file view

02\_workflows/T4\_workflow\_v01/submission\_ready/public/data/esld\_T4\_score\_deviation\_outcome\_meta\_public.csv

← Back to navigator   |   Open original package file

Section

Manuscript output data

Output

T4

Extension

csv

Size KB

0.678

Variables

2

## Variables in this file

| Variable | Label | Description | Unit | Type |
| --- | --- | --- | --- | --- |
| parameter | Metadata parameter name | Name of a metadata parameter describing the F2 simulated heatmap object, such as figure identity, data origin, grid type, axis variable, or unit/role. |  | character |
| value | Value | Numerical or character value corresponding to the row-specific variable/metric. |  | character |

## Readable HTML view

Showing all 10 rows.

| parameter | value |
| --- | --- |
| dataset\_name | esld\_T4\_score\_deviation\_outcome\_table\_public |
| domain | esld |
| anchor | T4 |
| data\_object | score\_deviation\_outcome |
| unit\_or\_role | table |
| release\_status | public |
| source\_public\_values | submitted Table 4 DOCX values preserved exactly |
| title | Table 4. Score deviations (Δ) of ≤ −1 stratified by outcome (alive/dead within 90 days after MELD calculation); subanalysis of 30- and 90-days survival indicators in deceased ESLD patients only. Data are presented as point estimates with 95% confidence intervals in parentheses. |
| n\_table\_rows | 13 |
| public\_model\_columns | MELD (N=20,359); MELD-Na (N=19,213); reMELD-Na (N=19,213); MELD 3.0 (N=8,614) |
