# Supplementary material for: Creatinine assay interferences compromises MELD accuracy and may bias liver allocation
Source: Nat Commun. 2026 Jul 23;17:7111. doi: 10.1038/s41467-026-75011-x (PMC13396164; doi:10.1038/s41467-026-75011-x)
Supplement: Supplementary file 4 — Source Data [file 41467_2026_75011_MOESM4_ESM.zip › figshare_package_FINAL_PUBLIC_DEPOSIT_V1_20260503_002637/00_START_HERE_HTML_NAVIGATOR/file_views/view_0010_expm_F1_validation_input_public.html]

02\_workflows/F1\_workflow\_v02/submission\_ready/public/data/expm\_F1\_validation\_input\_public.csv

# Readable file view

02\_workflows/F1\_workflow\_v02/submission\_ready/public/data/expm\_F1\_validation\_input\_public.csv

← Back to navigator   |   Open original package file

Section

Manuscript output data

Output

F1

Extension

csv

Size KB

4.949

Variables

16

## Variables in this file

| Variable | Label | Description | Unit | Type |
| --- | --- | --- | --- | --- |
| anchor | Anchor/output group | Anchor or output grouping label used by the workflow to identify a specific public output component. |  | character |
| cre\_true\_gcidms\_mg\_dL | GC-IDMS reference creatinine concentration | Creatinine concentration measured by GC-IDMS reference method in the validation data. |  | numeric |
| cree\_corrected\_mg\_dL | Corrected enzymatic creatinine concentration | Creatinine concentration after applying the enzymatic correction model. |  | numeric |
| cree\_measured\_mg\_dL | Measured enzymatic creatinine concentration | Measured creatinine concentration using the enzymatic assay. |  | numeric |
| crej\_corrected\_mg\_dL | Corrected Jaffe creatinine concentration | Creatinine concentration after applying the Jaffe correction model. |  | numeric |
| crej\_measured\_mg\_dL | Measured Jaffe creatinine concentration | Measured creatinine concentration using the Jaffe assay. |  | numeric |
| data\_object | Data object | Name of the data object represented by the row. |  | character |
| db\_measured\_mg\_dL | Direct bilirubin concentration | Direct bilirubin concentration measured in the experimental or validation data. |  | numeric |
| domain | Data domain | Workflow or data domain represented by the row. |  | character |
| expm\_F1\_validation\_row\_id | Experimental validation row identifier | Row identifier within the released F1 experimental validation table. |  | integer |
| release\_status | Release status | Release-status label indicating the publication status of the row or file object. |  | character |
| sample\_id | Sample identifier | Identifier of a sample or experimental record within the released public data; not a personal identifier. |  | integer |
| source\_harmonized\_file\_name | Source harmonized file name | Name of the harmonized source file used to build the released object. |  | character |
| tb\_measured\_mg\_dL | Measured total bilirubin concentration | Measured total bilirubin concentration in the experimental F1 data. |  | numeric |
| unit\_or\_role | Unit or semantic role | Unit, role, or semantic type corresponding to the row-specific variable/metric. |  | character |
| workflow\_step | Workflow step | Workflow step that produced or used the row/object. |  | character |

## Readable HTML view

Showing all 32 rows.

| expm\_F1\_validation\_row\_id | domain | anchor | data\_object | unit\_or\_role | release\_status | source\_harmonized\_file\_name | workflow\_step | sample\_id | tb\_measured\_mg\_dL | db\_measured\_mg\_dL | cre\_true\_gcidms\_mg\_dL | cree\_measured\_mg\_dL | crej\_measured\_mg\_dL | cree\_corrected\_mg\_dL | crej\_corrected\_mg\_dL |
| --- | --- | --- | --- | --- | --- | --- | --- | --- | --- | --- | --- | --- | --- | --- | --- |
| 1 | expm | F1 | validation | input | public | expm\_F1\_validation\_raw\_public.csv | 2a\_refined\_analysis\_dataset | 1 | 9.263 | 7.411 | 0.82 | 0.75 | 0.95 | 0.68240141154 | 0.8822513 |
| 2 | expm | F1 | validation | input | public | expm\_F1\_validation\_raw\_public.csv | 2a\_refined\_analysis\_dataset | 2 | 4.852 | 3.874 | 0.86 | 0.78 | 0.98 | 0.81680053664 | 0.9490736 |
| 3 | expm | F1 | validation | input | public | expm\_F1\_validation\_raw\_public.csv | 2a\_refined\_analysis\_dataset | 3 | 16.121 | 13.015 | 0.63 | 0.52 | 0.78 | 0.35207418306 | 0.6756628 |
| 4 | expm | F1 | validation | input | public | expm\_F1\_validation\_raw\_public.csv | 2a\_refined\_analysis\_dataset | 4 | 4.419 | 3.245 | 0.94 | 0.89 | 0.92 | 0.93410841026 | 0.8942763 |
| 5 | expm | F1 | validation | input | public | expm\_F1\_validation\_raw\_public.csv | 2a\_refined\_analysis\_dataset | 5 | 14.415 | 11.224 | 0.9 | 0.8 | 1.06 | 0.6516394885 | 0.9588407 |
| 6 | expm | F1 | validation | input | public | expm\_F1\_validation\_raw\_public.csv | 2a\_refined\_analysis\_dataset | 6 | 3.82 | 3.222 | 2.31 | 2.16 | 2.51 | 2.176252904 | 2.3920647 |
| 7 | expm | F1 | validation | input | public | expm\_F1\_validation\_raw\_public.csv | 2a\_refined\_analysis\_dataset | 7 | 10.275 | 8.719 | 0.96 | 0.88 | 1.13 | 0.7899974925 | 1.0510911 |
| 8 | expm | F1 | validation | input | public | expm\_F1\_validation\_raw\_public.csv | 2a\_refined\_analysis\_dataset | 8 | 0.243 | 0.118 | 0.66 | 0.66 | 0.97 | 0.82791189234 | 0.9887643 |
| 9 | expm | F1 | validation | input | public | expm\_F1\_validation\_raw\_public.csv | 2a\_refined\_analysis\_dataset | 9 | 0.68 | 0.51 | 0.8 | 0.76 | 0.75 | 0.911525504 | 0.7661407 |
| 10 | expm | F1 | validation | input | public | expm\_F1\_validation\_raw\_public.csv | 2a\_refined\_analysis\_dataset | 10 | 1.957 | 1.244 | 1.27 | 1.2 | 1.5 | 1.30006870034 | 1.4813282 |
| 11 | expm | F1 | validation | input | public | expm\_F1\_validation\_raw\_public.csv | 2a\_refined\_analysis\_dataset | 11 | 5.239 | 3.447 | 1.2 | 1.3 | 1.39 | 1.31061409986 | 1.3428587 |
| 12 | expm | F1 | validation | input | public | expm\_F1\_validation\_raw\_public.csv | 2a\_refined\_analysis\_dataset | 12 | 2.076 | 0.536 | 0.97 | 0.93 | 1 | 1.03542663216 | 0.997227 |
| 13 | expm | F1 | validation | input | public | expm\_F1\_validation\_raw\_public.csv | 2a\_refined\_analysis\_dataset | 13 | 7.378 | 1.989 | 1.31 | 1.32 | 1.28 | 1.28145270344 | 1.2182734 |
| 14 | expm | F1 | validation | input | public | expm\_F1\_validation\_raw\_public.csv | 2a\_refined\_analysis\_dataset | 14 | 14.577 | 7.938 | 0.96 | 0.87 | 1.15 | 0.71751441314 | 1.0457456 |
| 15 | expm | F1 | validation | input | public | expm\_F1\_validation\_raw\_public.csv | 2a\_refined\_analysis\_dataset | 15 | 11.172 | 9.176 | 1.46 | 1.37 | 1.6 | 1.24895568544 | 1.4958006 |
| 16 | expm | F1 | validation | input | public | expm\_F1\_validation\_raw\_public.csv | 2a\_refined\_analysis\_dataset | 16 | 7.014 | 6.554 | 1.48 | 1.45 | 1.59 | 1.41492598936 | 1.5172907 |
| 17 | expm | F1 | validation | input | public | expm\_F1\_validation\_raw\_public.csv | 2a\_refined\_analysis\_dataset | 17 | 10.853 | 8.535 | 2.57 | 2.39 | 2.75 | 2.23827090194 | 2.5457756 |
| 18 | expm | F1 | validation | input | public | expm\_F1\_validation\_raw\_public.csv | 2a\_refined\_analysis\_dataset | 18 | 22.221 | 14.076 | 1.65 | 1.43 | 1.93 | 1.18861107506 | 1.7645829 |
| 19 | expm | F1 | validation | input | public | expm\_F1\_validation\_raw\_public.csv | 2a\_refined\_analysis\_dataset | 19 | 14.149 | 8.354 | 1.43 | 1.18 | 1.41 | 1.02270015266 | 1.2983867 |
| 20 | expm | F1 | validation | input | public | expm\_F1\_validation\_raw\_public.csv | 2a\_refined\_analysis\_dataset | 20 | 28.515 | 22.912 | 0.89 | 0.7 | 1.04 | 0.4783989485 | 0.9223061 |
| 21 | expm | F1 | validation | input | public | expm\_F1\_validation\_raw\_public.csv | 2a\_refined\_analysis\_dataset | 21 | 10.447 | 7.504 | 1.31 | 1.13 | 1.44 | 1.02899735394 | 1.348614 |
| 22 | expm | F1 | validation | input | public | expm\_F1\_validation\_raw\_public.csv | 2a\_refined\_analysis\_dataset | 22 | 17.579 | 13.228 | 1.59 | 1.52 | 1.93 | 1.30720501906 | 1.773838 |
| 23 | expm | F1 | validation | input | public | expm\_F1\_validation\_raw\_public.csv | 2a\_refined\_analysis\_dataset | 23 | 7.788 | 5.857 | 1.13 | 1.14 | 1.31 | 1.09877366304 | 1.2437983 |
| 24 | expm | F1 | validation | input | public | expm\_F1\_validation\_raw\_public.csv | 2a\_refined\_analysis\_dataset | 24 | 16.372 | 6.565 | 2.42 | 2.32 | 2.46 | 2.08930397344 | 2.2603508 |
| 25 | expm | F1 | validation | input | public | expm\_F1\_validation\_raw\_public.csv | 2a\_refined\_analysis\_dataset | 25 | 6.268 | 4.33 | 1.99 | 1.94 | 2.04 | 1.90455748384 | 1.9433359 |
| 26 | expm | F1 | validation | input | public | expm\_F1\_validation\_raw\_public.csv | 2a\_refined\_analysis\_dataset | 26 | 0.83 | 0.372 | 1.63 | 1.69 | 1.81 | 1.806418694 | 1.7860424 |
| 27 | expm | F1 | validation | input | public | expm\_F1\_validation\_raw\_public.csv | 2a\_refined\_analysis\_dataset | 27 | 0.107 | 0.078 | 1.4 | 1.59 | 1.82 | 1.73182497634 | 1.8038901 |
| 28 | expm | F1 | validation | input | public | expm\_F1\_validation\_raw\_public.csv | 2a\_refined\_analysis\_dataset | 28 | 0.376 | 0.133 | 4.26 | 4.35 | 4.87 | 4.37540180816 | 4.3702289 |
| 29 | expm | F1 | validation | input | public | expm\_F1\_validation\_raw\_public.csv | 2a\_refined\_analysis\_dataset | 29 | 0.15 | 0.08 | 2.55 | 2.41 | 3.02 | 2.52122527 | 2.8795704 |
| 30 | expm | F1 | validation | input | public | expm\_F1\_validation\_raw\_public.csv | 2a\_refined\_analysis\_dataset | 30 | 0.901 | 0.364 | 2.68 | 2.65 | 2.93 | 2.72943928866 | 2.7929585 |
| 31 | expm | F1 | validation | input | public | expm\_F1\_validation\_raw\_public.csv | 2a\_refined\_analysis\_dataset | 31 | 0.972 | 0.587 | 3.07 | 3.03 | 3.21 | 3.09261093744 | 3.0325553 |
| 32 | expm | F1 | validation | input | public | expm\_F1\_validation\_raw\_public.csv | 2a\_refined\_analysis\_dataset | 32 | 11.915 | 6.937 | 3.14 | 2.96 | 3.32 | 2.7695938685 | 3.0296918 |
