# Supplementary material for: Creatinine assay interferences compromises MELD accuracy and may bias liver allocation
Source: Nat Commun. 2026 Jul 23;17:7111. doi: 10.1038/s41467-026-75011-x (PMC13396164; doi:10.1038/s41467-026-75011-x)
Supplement: Supplementary file 4 — Source Data [file 41467_2026_75011_MOESM4_ESM.zip › figshare_package_FINAL_PUBLIC_DEPOSIT_V1_20260503_002637/00_START_HERE_HTML_NAVIGATOR/file_views/view_0037_public_variable_dictionary_FINAL_CODEBOOK_summary.html]

00\_release\_manifests/public\_variable\_dictionary\_FINAL\_CODEBOOK\_summary.csv| recommended\_dictionary\_rows | 661 | info |
| final\_codebook\_rows | 661 | info |
| nonpublic\_status\_rows | 0 | pass |
| nonpublic\_tier\_rows | 0 | pass |
| restricted\_internal\_or\_srtr\_path\_rows | 0 | pass |
| absolute\_local\_path\_rows | 0 | pass |
| generic\_variable\_name\_rows | 0 | pass |
| empty\_description\_rows | 0 | pass |
| imprecise\_description\_rows | 0 | pass |
| public\_dictionary\_action\_nonacceptable\_rows | 0 | pass |
| final\_review\_rows | 0 | pass |
