# Supplementary material for: Creatinine assay interferences compromises MELD accuracy and may bias liver allocation
Source: Nat Commun. 2026 Jul 23;17:7111. doi: 10.1038/s41467-026-75011-x (PMC13396164; doi:10.1038/s41467-026-75011-x)
Supplement: Supplementary file 4 — Source Data [file 41467_2026_75011_MOESM4_ESM.zip › figshare_package_FINAL_PUBLIC_DEPOSIT_V1_20260503_002637/00_START_HERE_HTML_NAVIGATOR/file_views/view_0029_README_F2_submission_ready_v01.html]

02\_workflows/F2\_workflow\_v01/submission\_ready/README\_F2\_submission\_ready\_v01.txt

# Readable file view

02\_workflows/F2\_workflow\_v01/submission\_ready/README\_F2\_submission\_ready\_v01.txt

← Back to navigator   |   Open original package file

Section

Workflow readmes

Output

F2

Extension

txt

Size KB

0.289

Variables

0

## Readable HTML view

```
F2 submission-ready output structure

Use public/data/ and public/figures/ for public submission.

Public F2 files:
  slco_F2_heatmap_bin_public.csv
  slco_F2_heatmap_meta_public.csv
  slco_F2_heatmap_public.pdf

Internal files are retained only for traceability, QC, and workflow documentation.
```
