# Supplementary material for: Creatinine assay interferences compromises MELD accuracy and may bias liver allocation
Source: Nat Commun. 2026 Jul 23;17:7111. doi: 10.1038/s41467-026-75011-x (PMC13396164; doi:10.1038/s41467-026-75011-x)
Supplement: Supplementary file 4 — Source Data [file 41467_2026_75011_MOESM4_ESM.zip › figshare_package_FINAL_PUBLIC_DEPOSIT_V1_20260503_002637/00_START_HERE_HTML_NAVIGATOR/file_views/view_0006_f1_tb_cre_experimental_validation_raw.html]

01\_primary\_data/public/f1\_tb\_cre\_experimental\_validation\_raw.csv

# Readable file view

01\_primary\_data/public/f1\_tb\_cre\_experimental\_validation\_raw.csv

← Back to navigator   |   Open original package file

Section

Public primary data

Output

F1

Extension

csv

Size KB

2.28

Variables

8

## Variables in this file

| Variable | Label | Description | Unit | Type |
| --- | --- | --- | --- | --- |
| cre\_true\_gcidms\_mg\_dL | GC-IDMS reference creatinine concentration | Creatinine concentration measured by GC-IDMS reference method in the validation data. |  | numeric |
| cree\_corrected\_mg\_dL | Corrected enzymatic creatinine concentration | Creatinine concentration after applying the enzymatic correction model. |  | numeric |
| cree\_measured\_mg\_dL | Measured enzymatic creatinine concentration | Measured creatinine concentration using the enzymatic assay. |  | numeric |
| crej\_corrected\_mg\_dL | Corrected Jaffe creatinine concentration | Creatinine concentration after applying the Jaffe correction model. |  | numeric |
| crej\_measured\_mg\_dL | Measured Jaffe creatinine concentration | Measured creatinine concentration using the Jaffe assay. |  | numeric |
| db\_measured\_mg\_dL | Direct bilirubin concentration | Direct bilirubin concentration measured in the experimental or validation data. |  | numeric |
| sample\_id | Sample identifier | Identifier of a sample or experimental record within the released public data; not a personal identifier. |  | integer |
| tb\_measured\_mg\_dL | Measured total bilirubin concentration | Measured total bilirubin concentration in the experimental F1 data. |  | numeric |

## Readable HTML view

Showing all 32 rows.

| sample\_id | tb\_measured\_mg\_dL | db\_measured\_mg\_dL | cre\_true\_gcidms\_mg\_dL | cree\_measured\_mg\_dL | crej\_measured\_mg\_dL | cree\_corrected\_mg\_dL | crej\_corrected\_mg\_dL |
| --- | --- | --- | --- | --- | --- | --- | --- |
| 1 | 9.263 | 7.4110000000000005 | 0.8200000000000001 | 0.75 | 0.9500000000000001 | 0.6824014115400001 | 0.8822513 |
| 2 | 4.852 | 3.874 | 0.86 | 0.78 | 0.98 | 0.8168005366400001 | 0.9490736 |
| 3 | 16.121 | 13.015 | 0.63 | 0.52 | 0.78 | 0.35207418306 | 0.6756628 |
| 4 | 4.4190000000000005 | 3.245 | 0.9400000000000001 | 0.89 | 0.92 | 0.93410841026 | 0.8942762999999999 |
| 5 | 14.415000000000001 | 11.224 | 0.9 | 0.8 | 1.06 | 0.6516394885000001 | 0.9588407 |
| 6 | 3.8200000000000003 | 3.222 | 2.31 | 2.16 | 2.5100000000000002 | 2.176252904 | 2.3920646999999997 |
| 7 | 10.275 | 8.719 | 0.96 | 0.88 | 1.1300000000000001 | 0.7899974925 | 1.0510911 |
| 8 | 0.243 | 0.11800000000000001 | 0.66 | 0.66 | 0.97 | 0.8279118923400001 | 0.9887642999999999 |
| 9 | 0.68 | 0.51 | 0.8 | 0.76 | 0.75 | 0.911525504 | 0.7661407 |
| 10 | 1.957 | 1.244 | 1.27 | 1.2 | 1.5 | 1.30006870034 | 1.4813281999999999 |
| 11 | 5.239 | 3.447 | 1.2 | 1.3 | 1.3900000000000001 | 1.31061409986 | 1.3428586999999998 |
| 12 | 2.076 | 0.536 | 0.97 | 0.93 | 1 | 1.03542663216 | 0.997227 |
| 13 | 7.378 | 1.989 | 1.31 | 1.32 | 1.28 | 1.28145270344 | 1.2182734 |
| 14 | 14.577 | 7.938 | 0.96 | 0.87 | 1.1500000000000001 | 0.71751441314 | 1.0457456 |
| 15 | 11.172 | 9.176 | 1.46 | 1.37 | 1.6 | 1.2489556854400001 | 1.4958006 |
| 16 | 7.014 | 6.554 | 1.48 | 1.45 | 1.59 | 1.41492598936 | 1.5172907 |
| 17 | 10.853 | 8.535 | 2.57 | 2.39 | 2.75 | 2.23827090194 | 2.5457756 |
| 18 | 22.221 | 14.076 | 1.6500000000000001 | 1.43 | 1.93 | 1.18861107506 | 1.7645829 |
| 19 | 14.149000000000001 | 8.354000000000001 | 1.43 | 1.18 | 1.41 | 1.0227001526600001 | 1.2983867 |
| 20 | 28.515 | 22.912 | 0.89 | 0.7 | 1.04 | 0.4783989484999999 | 0.9223060999999999 |
| 21 | 10.447000000000001 | 7.5040000000000004 | 1.31 | 1.13 | 1.44 | 1.0289973539400004 | 1.348614 |
| 22 | 17.579 | 13.228 | 1.59 | 1.52 | 1.93 | 1.30720501906 | 1.773838 |
| 23 | 7.788 | 5.857 | 1.1300000000000001 | 1.14 | 1.31 | 1.0987736630400002 | 1.2437983 |
| 24 | 16.372 | 6.565 | 2.42 | 2.32 | 2.46 | 2.0893039734400003 | 2.2603508 |
| 25 | 6.268 | 4.33 | 1.99 | 1.94 | 2.04 | 1.9045574838400001 | 1.9433359 |
| 26 | 0.8300000000000001 | 0.372 | 1.6300000000000001 | 1.69 | 1.81 | 1.806418694 | 1.7860424 |
| 27 | 0.107 | 0.078 | 1.4000000000000001 | 1.59 | 1.82 | 1.73182497634 | 1.8038900999999998 |
| 28 | 0.376 | 0.133 | 4.26 | 4.35 | 4.87 | 4.375401808160001 | 4.3702289 |
| 29 | 0.15 | 0.08 | 2.5500000000000003 | 2.41 | 3.02 | 2.52122527 | 2.8795704 |
| 30 | 0.901 | 0.364 | 2.68 | 2.65 | 2.93 | 2.72943928866 | 2.7929584999999997 |
| 31 | 0.972 | 0.587 | 3.0700000000000003 | 3.03 | 3.21 | 3.092610937440001 | 3.0325553 |
| 32 | 11.915000000000001 | 6.937 | 3.14 | 2.96 | 3.3200000000000003 | 2.7695938685 | 3.0296917999999997 |
