# Supplementary material for: Creatinine assay interferences compromises MELD accuracy and may bias liver allocation
Source: Nat Commun. 2026 Jul 23;17:7111. doi: 10.1038/s41467-026-75011-x (PMC13396164; doi:10.1038/s41467-026-75011-x)
Supplement: Supplementary file 4 — Source Data [file 41467_2026_75011_MOESM4_ESM.zip › figshare_package_FINAL_PUBLIC_DEPOSIT_V1_20260503_002637/00_release_manifests/FINAL_PUBLIC_PACKAGE_NAVIGATOR_v02.html]

Final Public Package Navigator


# Final Public Package Navigator

Browsable index of public package files, release manifests, and public variable definitions.

**Search**

Search across visible file paths, variables, labels, and descriptions.


All file types
CSV
PDF
TXT
HTML
Reset

**Folders**
All folders
00\_config 1
00\_release\_manifests 56
01\_primary\_data 7
02\_workflows 85
[root] 1
[root] 2

**On-request content**

Restricted and script files are listed in manifests but not included as public data.

**97** restricted-on-request files listed

**94** script-on-request files listed

**101** internal/excluded files listed


## Overview

152

Public package files

661

Public variables

128.19

Total size MB

97

Restricted files listed

94

Scripts on request listed

Files
Variables
Folder summary
File types

## Package files

| File path and details | Section | Type | Size KB | Variables |
| --- | --- | --- | --- | --- |
| .DS\_Store **Section:** other\_public\_package\_content **Directory:** . **Extension:** [none] **Size KB:** 8.004 **Last modified:** 2026-05-03 01:48:32 **MD5:** 84042dda2b5af761cfd0ece46d9e2d1f **Variables in codebook:** 0 | other\_public\_package\_content | [none] | 8.004 | 0 |
| 00\_config/01\_primary\_file\_registry\_v03.csv **Section:** other\_public\_package\_content **Directory:** 00\_config **Extension:** csv **Size KB:** 4.052 **Last modified:** 2026-05-02 09:46:31 **MD5:** 628025805c2e9de1df7a0991e61d2054 **Variables in codebook:** 0 | other\_public\_package\_content | csv | 4.052 | 0 |
| README\_PUBLIC\_DEPOSIT\_PACKAGE\_v01.txt **Section:** package\_readme **Directory:** . **Extension:** txt **Size KB:** 1.952 **Last modified:** 2026-05-03 02:03:16 **MD5:** 3a04cf12c7723019a3e0ec303cafc579 **Variables in codebook:** 0 | package\_readme | txt | 1.952 | 0 |
| README\_repository\_package\_v03.txt **Section:** package\_readme **Directory:** . **Extension:** txt **Size KB:** 1.788 **Last modified:** 2026-05-01 23:41:30 **MD5:** 17f3c4a9bf6179a86f3a09dadf62a986 **Variables in codebook:** 0 | package\_readme | txt | 1.788 | 0 |
| 01\_primary\_data/public/esld\_master\_long\_public.csv **Section:** public\_primary\_data **Directory:** 01\_primary\_data/public **Extension:** csv **Size KB:** 9620.613 **Last modified:** 2026-05-02 23:18:12 **MD5:** 8228407e927ff10ad6e4740d0d5cd48b **Variables in codebook:** 43 | public\_primary\_data | csv | 9620.613 | 43 |
| 01\_primary\_data/public/f1\_simulated\_surface\_metadata.csv **Section:** public\_primary\_data **Directory:** 01\_primary\_data/public **Extension:** csv **Size KB:** 0.648 **Last modified:** 2026-04-19 20:30:45 **MD5:** ee2dfece0c0751ab1643f0e896edea9b **Variables in codebook:** 2 | public\_primary\_data | csv | 0.648 | 2 |
| 01\_primary\_data/public/f1\_simulated\_surface\_repository.csv **Section:** public\_primary\_data **Directory:** 01\_primary\_data/public **Extension:** csv **Size KB:** 4936.887 **Last modified:** 2026-04-16 16:32:54 **MD5:** 87713a0d6868c6ac52ff20ea850e3d58 **Variables in codebook:** 11 | public\_primary\_data | csv | 4936.887 | 11 |
| 01\_primary\_data/public/f1\_tb\_cre\_experimental\_array\_raw.csv **Section:** public\_primary\_data **Directory:** 01\_primary\_data/public **Extension:** csv **Size KB:** 331.003 **Last modified:** 2026-04-22 16:53:59 **MD5:** b8ffb94bc127381e0963db2211ba44b8 **Variables in codebook:** 9 | public\_primary\_data | csv | 331.003 | 9 |
| 01\_primary\_data/public/f1\_tb\_cre\_experimental\_validation\_raw.csv **Section:** public\_primary\_data **Directory:** 01\_primary\_data/public **Extension:** csv **Size KB:** 2.28 **Last modified:** 2026-04-23 00:35:04 **MD5:** 30e9eb0a37dab1d84dbff1649b1b9e07 **Variables in codebook:** 8 | public\_primary\_data | csv | 2.28 | 8 |
| 01\_primary\_data/public/f2\_simulated\_heatmap\_metadata.csv **Section:** public\_primary\_data **Directory:** 01\_primary\_data/public **Extension:** csv **Size KB:** 0.545 **Last modified:** 2026-04-19 20:30:45 **MD5:** df833ec1562a6c70ca8dc8338c7e625d **Variables in codebook:** 2 | public\_primary\_data | csv | 0.545 | 2 |
| 01\_primary\_data/public/f2\_simulated\_heatmap\_repository.csv **Section:** public\_primary\_data **Directory:** 01\_primary\_data/public **Extension:** csv **Size KB:** 7674.803 **Last modified:** 2026-04-17 19:20:15 **MD5:** 69355977324161fce080931369bd51f9 **Variables in codebook:** 6 | public\_primary\_data | csv | 7674.803 | 6 |
| 02\_workflows/F1\_workflow\_v02/data/01\_source\_loaded\_harmonized/expm\_F1\_array\_raw\_public.csv **Section:** public\_workflow\_output\_or\_documentation **Directory:** 02\_workflows/F1\_workflow\_v02/data/01\_source\_loaded\_harmonized **Extension:** csv **Size KB:** 331.003 **Last modified:** 2026-04-22 16:53:59 **MD5:** b8ffb94bc127381e0963db2211ba44b8 **Variables in codebook:** 9 | public\_workflow\_output\_or\_documentation | csv | 331.003 | 9 |
| 02\_workflows/F1\_workflow\_v02/data/01\_source\_loaded\_harmonized/expm\_F1\_validation\_raw\_public.csv **Section:** public\_workflow\_output\_or\_documentation **Directory:** 02\_workflows/F1\_workflow\_v02/data/01\_source\_loaded\_harmonized **Extension:** csv **Size KB:** 2.28 **Last modified:** 2026-04-23 00:35:04 **MD5:** 30e9eb0a37dab1d84dbff1649b1b9e07 **Variables in codebook:** 8 | public\_workflow\_output\_or\_documentation | csv | 2.28 | 8 |
| 02\_workflows/F1\_workflow\_v02/data/01\_source\_loaded\_harmonized/expm\_slco\_F1\_source\_load\_manifest\_public.csv **Section:** public\_workflow\_output\_or\_documentation **Directory:** 02\_workflows/F1\_workflow\_v02/data/01\_source\_loaded\_harmonized **Extension:** csv **Size KB:** 0.981 **Last modified:** 2026-05-03 01:59:36 **MD5:** 68a1686231833acdbc4fd95965648281 **Variables in codebook:** 9 | public\_workflow\_output\_or\_documentation | csv | 0.981 | 9 |
| 02\_workflows/F1\_workflow\_v02/data/01\_source\_loaded\_harmonized/expm\_slco\_F1\_step1\_run\_inputs\_public.csv **Section:** public\_workflow\_output\_or\_documentation **Directory:** 02\_workflows/F1\_workflow\_v02/data/01\_source\_loaded\_harmonized **Extension:** csv **Size KB:** 0.388 **Last modified:** 2026-05-03 02:01:58 **MD5:** 4c7e7ed723c1f6ad29616256e07323fc **Variables in codebook:** 2 | public\_workflow\_output\_or\_documentation | csv | 0.388 | 2 |
| 02\_workflows/F1\_workflow\_v02/data/01\_source\_loaded\_harmonized/slco\_F1\_surface\_grid\_reference\_public.csv **Section:** public\_workflow\_output\_or\_documentation **Directory:** 02\_workflows/F1\_workflow\_v02/data/01\_source\_loaded\_harmonized **Extension:** csv **Size KB:** 4936.887 **Last modified:** 2026-04-16 16:32:54 **MD5:** 87713a0d6868c6ac52ff20ea850e3d58 **Variables in codebook:** 11 | public\_workflow\_output\_or\_documentation | csv | 4936.887 | 11 |
| 02\_workflows/F1\_workflow\_v02/data/01\_source\_loaded\_harmonized/slco\_F1\_surface\_meta\_reference\_public.csv **Section:** public\_workflow\_output\_or\_documentation **Directory:** 02\_workflows/F1\_workflow\_v02/data/01\_source\_loaded\_harmonized **Extension:** csv **Size KB:** 0.648 **Last modified:** 2026-04-19 20:30:45 **MD5:** ee2dfece0c0751ab1643f0e896edea9b **Variables in codebook:** 2 | public\_workflow\_output\_or\_documentation | csv | 0.648 | 2 |
| 02\_workflows/F1\_workflow\_v02/data/02a\_refined\_analysis/expm\_F1\_array\_input\_public.csv **Section:** public\_workflow\_output\_or\_documentation **Directory:** 02\_workflows/F1\_workflow\_v02/data/02a\_refined\_analysis **Extension:** csv **Size KB:** 737.671 **Last modified:** 2026-05-02 01:30:05 **MD5:** 48545523b96fdb9fce1e19bdf1689efc **Variables in codebook:** 19 | public\_workflow\_output\_or\_documentation | csv | 737.671 | 19 |
| 02\_workflows/F1\_workflow\_v02/data/02a\_refined\_analysis/expm\_F1\_model\_coefficients\_public.csv **Section:** public\_workflow\_output\_or\_documentation **Directory:** 02\_workflows/F1\_workflow\_v02/data/02a\_refined\_analysis **Extension:** csv **Size KB:** 1.299 **Last modified:** 2026-05-02 01:30:05 **MD5:** c05403544122bdf248154bfbf8559b9f **Variables in codebook:** 6 | public\_workflow\_output\_or\_documentation | csv | 1.299 | 6 |
| 02\_workflows/F1\_workflow\_v02/data/02a\_refined\_analysis/expm\_F1\_pre\_correction\_rounding\_qc\_public.csv **Section:** public\_workflow\_output\_or\_documentation **Directory:** 02\_workflows/F1\_workflow\_v02/data/02a\_refined\_analysis **Extension:** csv **Size KB:** 0.451 **Last modified:** 2026-05-02 01:30:05 **MD5:** ca3266c1f5df19844f2079981510ff28 **Variables in codebook:** 8 | public\_workflow\_output\_or\_documentation | csv | 0.451 | 8 |
| 02\_workflows/F1\_workflow\_v02/data/02a\_refined\_analysis/expm\_F1\_refined\_dataset\_qc\_public.csv **Section:** public\_workflow\_output\_or\_documentation **Directory:** 02\_workflows/F1\_workflow\_v02/data/02a\_refined\_analysis **Extension:** csv **Size KB:** 0.637 **Last modified:** 2026-05-02 01:30:05 **MD5:** 812231b8c2303741846747de6999d3c7 **Variables in codebook:** 2 | public\_workflow\_output\_or\_documentation | csv | 0.637 | 2 |
| 02\_workflows/F1\_workflow\_v02/data/02a\_refined\_analysis/expm\_F1\_refined\_manual\_clarifications\_public.csv **Section:** public\_workflow\_output\_or\_documentation **Directory:** 02\_workflows/F1\_workflow\_v02/data/02a\_refined\_analysis **Extension:** csv **Size KB:** 0.861 **Last modified:** 2026-05-02 01:30:05 **MD5:** 8c4c1d35703560f1e9341b3df38ffad4 **Variables in codebook:** 3 | public\_workflow\_output\_or\_documentation | csv | 0.861 | 3 |
| 02\_workflows/F1\_workflow\_v02/data/02a\_refined\_analysis/expm\_F1\_validation\_input\_public.csv **Section:** public\_workflow\_output\_or\_documentation **Directory:** 02\_workflows/F1\_workflow\_v02/data/02a\_refined\_analysis **Extension:** csv **Size KB:** 4.949 **Last modified:** 2026-05-02 01:30:05 **MD5:** 2a1c7ff9a9b2d7b913576fc64c1af459 **Variables in codebook:** 16 | public\_workflow\_output\_or\_documentation | csv | 4.949 | 16 |
| 02\_workflows/F1\_workflow\_v02/data/02b\_figure\_content/slco\_F1\_surface\_grid\_public.csv **Section:** public\_workflow\_output\_or\_documentation **Directory:** 02\_workflows/F1\_workflow\_v02/data/02b\_figure\_content **Extension:** csv **Size KB:** 4918.95 **Last modified:** 2026-05-02 01:30:05 **MD5:** 7043e4517bc15ae9142ae09b0631e890 **Variables in codebook:** 11 | public\_workflow\_output\_or\_documentation | csv | 4918.95 | 11 |
| 02\_workflows/F1\_workflow\_v02/data/02b\_figure\_content/slco\_F1\_surface\_meta\_public.csv **Section:** public\_workflow\_output\_or\_documentation **Directory:** 02\_workflows/F1\_workflow\_v02/data/02b\_figure\_content **Extension:** csv **Size KB:** 0.482 **Last modified:** 2026-05-02 01:30:05 **MD5:** 63cd6a8340d66c32e5915d2dadcc2378 **Variables in codebook:** 2 | public\_workflow\_output\_or\_documentation | csv | 0.482 | 2 |
| 02\_workflows/F1\_workflow\_v02/data/02b\_figure\_content/slco\_F1\_surface\_rebuild\_qc\_public.csv **Section:** public\_workflow\_output\_or\_documentation **Directory:** 02\_workflows/F1\_workflow\_v02/data/02b\_figure\_content **Extension:** csv **Size KB:** 0.934 **Last modified:** 2026-05-02 01:30:05 **MD5:** c1c5a45987d8b24e318b2857d5b3529a **Variables in codebook:** 5 | public\_workflow\_output\_or\_documentation | csv | 0.934 | 5 |
| 02\_workflows/F1\_workflow\_v02/data/02b\_figure\_content/slco\_F1\_surface\_reference\_meta\_original\_public.csv **Section:** public\_workflow\_output\_or\_documentation **Directory:** 02\_workflows/F1\_workflow\_v02/data/02b\_figure\_content **Extension:** csv **Size KB:** 0.74 **Last modified:** 2026-05-02 01:30:05 **MD5:** 28bbb6eeab2bcd8799b28d088fee5ec2 **Variables in codebook:** 3 | public\_workflow\_output\_or\_documentation | csv | 0.74 | 3 |
| 02\_workflows/F1\_workflow\_v02/data/02b\_figure\_content/slco\_F1\_surface\_run\_inputs\_outputs\_public.csv **Section:** public\_workflow\_output\_or\_documentation **Directory:** 02\_workflows/F1\_workflow\_v02/data/02b\_figure\_content **Extension:** csv **Size KB:** 0.563 **Last modified:** 2026-05-03 01:59:36 **MD5:** 960195fb64df37593cc1af1c29e793a1 **Variables in codebook:** 3 | public\_workflow\_output\_or\_documentation | csv | 0.563 | 3 |
| 02\_workflows/F1\_workflow\_v02/figures/slco\_F1\_surface\_public.pdf **Section:** public\_workflow\_output\_or\_documentation **Directory:** 02\_workflows/F1\_workflow\_v02/figures **Extension:** pdf **Size KB:** 1506.008 **Last modified:** 2026-05-02 01:30:14 **MD5:** 6de20aaebcce1d88c93b705cf4bd8152 **Variables in codebook:** 0 | public\_workflow\_output\_or\_documentation | pdf | 1506.008 | 0 |
| 02\_workflows/F1\_workflow\_v02/submission\_ready/F1\_submission\_ready\_manifest\_v02.csv **Section:** public\_workflow\_output\_or\_documentation **Directory:** 02\_workflows/F1\_workflow\_v02/submission\_ready **Extension:** csv **Size KB:** 8.39 **Last modified:** 2026-05-03 02:02:03 **MD5:** e4086d397409f8893f974735f8652773 **Variables in codebook:** 0 | public\_workflow\_output\_or\_documentation | csv | 8.39 | 0 |
| 02\_workflows/F1\_workflow\_v02/submission\_ready/README\_F1\_submission\_ready\_v02.txt **Section:** public\_workflow\_output\_or\_documentation **Directory:** 02\_workflows/F1\_workflow\_v02/submission\_ready **Extension:** txt **Size KB:** 0.677 **Last modified:** 2026-05-02 01:30:14 **MD5:** f5d66cca875ad3e26170da9e649747f2 **Variables in codebook:** 0 | public\_workflow\_output\_or\_documentation | txt | 0.677 | 0 |
| 02\_workflows/F1\_workflow\_v02/submission\_ready/public/data/expm\_F1\_array\_input\_public.csv **Section:** public\_workflow\_output\_or\_documentation **Directory:** 02\_workflows/F1\_workflow\_v02/submission\_ready/public/data **Extension:** csv **Size KB:** 598.548 **Last modified:** 2026-05-02 01:30:14 **MD5:** 28054a92825a550b9e2a0149689b4b69 **Variables in codebook:** 17 | public\_workflow\_output\_or\_documentation | csv | 598.548 | 17 |
| 02\_workflows/F1\_workflow\_v02/submission\_ready/public/data/expm\_F1\_validation\_input\_public.csv **Section:** public\_workflow\_output\_or\_documentation **Directory:** 02\_workflows/F1\_workflow\_v02/submission\_ready/public/data **Extension:** csv **Size KB:** 4.949 **Last modified:** 2026-05-02 01:30:14 **MD5:** 2a1c7ff9a9b2d7b913576fc64c1af459 **Variables in codebook:** 16 | public\_workflow\_output\_or\_documentation | csv | 4.949 | 16 |
| 02\_workflows/F1\_workflow\_v02/submission\_ready/public/data/slco\_F1\_surface\_grid\_public.csv **Section:** public\_workflow\_output\_or\_documentation **Directory:** 02\_workflows/F1\_workflow\_v02/submission\_ready/public/data **Extension:** csv **Size KB:** 4918.95 **Last modified:** 2026-05-02 01:30:14 **MD5:** 7043e4517bc15ae9142ae09b0631e890 **Variables in codebook:** 11 | public\_workflow\_output\_or\_documentation | csv | 4918.95 | 11 |
| 02\_workflows/F1\_workflow\_v02/submission\_ready/public/data/slco\_F1\_surface\_meta\_public.csv **Section:** public\_workflow\_output\_or\_documentation **Directory:** 02\_workflows/F1\_workflow\_v02/submission\_ready/public/data **Extension:** csv **Size KB:** 0.482 **Last modified:** 2026-05-02 01:30:14 **MD5:** 63cd6a8340d66c32e5915d2dadcc2378 **Variables in codebook:** 2 | public\_workflow\_output\_or\_documentation | csv | 0.482 | 2 |
| 02\_workflows/F1\_workflow\_v02/submission\_ready/public/figures/slco\_F1\_surface\_public.pdf **Section:** public\_workflow\_output\_or\_documentation **Directory:** 02\_workflows/F1\_workflow\_v02/submission\_ready/public/figures **Extension:** pdf **Size KB:** 1506.008 **Last modified:** 2026-05-02 01:30:14 **MD5:** 6de20aaebcce1d88c93b705cf4bd8152 **Variables in codebook:** 0 | public\_workflow\_output\_or\_documentation | pdf | 1506.008 | 0 |
| 02\_workflows/F2\_workflow\_v01/data/02b\_figure\_content/slco\_F2\_heatmap\_bin\_public.csv **Section:** public\_workflow\_output\_or\_documentation **Directory:** 02\_workflows/F2\_workflow\_v01/data/02b\_figure\_content **Extension:** csv **Size KB:** 7674.803 **Last modified:** 2026-05-02 01:33:01 **MD5:** 93e06eeefac7f60a0cd987630232561c **Variables in codebook:** 6 | public\_workflow\_output\_or\_documentation | csv | 7674.803 | 6 |
| 02\_workflows/F2\_workflow\_v01/data/02b\_figure\_content/slco\_F2\_heatmap\_meta\_public.csv **Section:** public\_workflow\_output\_or\_documentation **Directory:** 02\_workflows/F2\_workflow\_v01/data/02b\_figure\_content **Extension:** csv **Size KB:** 0.539 **Last modified:** 2026-05-02 01:33:01 **MD5:** d6bec83444bffd55eb65533a93ad5c5f **Variables in codebook:** 2 | public\_workflow\_output\_or\_documentation | csv | 0.539 | 2 |
| 02\_workflows/F2\_workflow\_v01/figures/slco\_F2\_heatmap\_public.pdf **Section:** public\_workflow\_output\_or\_documentation **Directory:** 02\_workflows/F2\_workflow\_v01/figures **Extension:** pdf **Size KB:** 856.709 **Last modified:** 2026-05-02 01:33:03 **MD5:** a300627a40ff5d92e416ad2fdd7da6f9 **Variables in codebook:** 0 | public\_workflow\_output\_or\_documentation | pdf | 856.709 | 0 |
| 02\_workflows/F2\_workflow\_v01/submission\_ready/F2\_submission\_ready\_manifest\_v01.csv **Section:** public\_workflow\_output\_or\_documentation **Directory:** 02\_workflows/F2\_workflow\_v01/submission\_ready **Extension:** csv **Size KB:** 5.623 **Last modified:** 2026-05-03 02:02:27 **MD5:** 017ed86553baf44bc67fbbdea726f157 **Variables in codebook:** 0 | public\_workflow\_output\_or\_documentation | csv | 5.623 | 0 |
| 02\_workflows/F2\_workflow\_v01/submission\_ready/README\_F2\_submission\_ready\_v01.txt **Section:** public\_workflow\_output\_or\_documentation **Directory:** 02\_workflows/F2\_workflow\_v01/submission\_ready **Extension:** txt **Size KB:** 0.289 **Last modified:** 2026-05-02 01:33:03 **MD5:** bd8ca0253fd5c12541633d4990502d74 **Variables in codebook:** 0 | public\_workflow\_output\_or\_documentation | txt | 0.289 | 0 |
| 02\_workflows/F2\_workflow\_v01/submission\_ready/public/F2\_public\_submission\_manifest\_v01.csv **Section:** public\_workflow\_output\_or\_documentation **Directory:** 02\_workflows/F2\_workflow\_v01/submission\_ready/public **Extension:** csv **Size KB:** 1.051 **Last modified:** 2026-05-03 02:02:47 **MD5:** 371fe08bba06b58bdce3fcfbf727418f **Variables in codebook:** 0 | public\_workflow\_output\_or\_documentation | csv | 1.051 | 0 |
| 02\_workflows/F2\_workflow\_v01/submission\_ready/public/data/slco\_F2\_heatmap\_bin\_public.csv **Section:** public\_workflow\_output\_or\_documentation **Directory:** 02\_workflows/F2\_workflow\_v01/submission\_ready/public/data **Extension:** csv **Size KB:** 7674.803 **Last modified:** 2026-05-02 01:33:03 **MD5:** 93e06eeefac7f60a0cd987630232561c **Variables in codebook:** 6 | public\_workflow\_output\_or\_documentation | csv | 7674.803 | 6 |
| 02\_workflows/F2\_workflow\_v01/submission\_ready/public/data/slco\_F2\_heatmap\_meta\_public.csv **Section:** public\_workflow\_output\_or\_documentation **Directory:** 02\_workflows/F2\_workflow\_v01/submission\_ready/public/data **Extension:** csv **Size KB:** 0.539 **Last modified:** 2026-05-02 01:33:03 **MD5:** d6bec83444bffd55eb65533a93ad5c5f **Variables in codebook:** 2 | public\_workflow\_output\_or\_documentation | csv | 0.539 | 2 |
| 02\_workflows/F2\_workflow\_v01/submission\_ready/public/figures/slco\_F2\_heatmap\_public.pdf **Section:** public\_workflow\_output\_or\_documentation **Directory:** 02\_workflows/F2\_workflow\_v01/submission\_ready/public/figures **Extension:** pdf **Size KB:** 856.709 **Last modified:** 2026-05-02 01:33:03 **MD5:** a300627a40ff5d92e416ad2fdd7da6f9 **Variables in codebook:** 0 | public\_workflow\_output\_or\_documentation | pdf | 856.709 | 0 |
| 02\_workflows/F3\_workflow\_v01/README\_F3\_workflow\_v01.txt **Section:** public\_workflow\_output\_or\_documentation **Directory:** 02\_workflows/F3\_workflow\_v01 **Extension:** txt **Size KB:** 0.436 **Last modified:** 2026-05-03 02:02:52 **MD5:** b3a9fd1cb5f13f91342f645acd133b87 **Variables in codebook:** 0 | public\_workflow\_output\_or\_documentation | txt | 0.436 | 0 |
| 02\_workflows/F3\_workflow\_v01/data/01\_source\_loaded\_harmonized/esld\_master\_long\_public.csv **Section:** public\_workflow\_output\_or\_documentation **Directory:** 02\_workflows/F3\_workflow\_v01/data/01\_source\_loaded\_harmonized **Extension:** csv **Size KB:** 9620.613 **Last modified:** 2026-05-02 23:18:12 **MD5:** 8228407e927ff10ad6e4740d0d5cd48b **Variables in codebook:** 43 | public\_workflow\_output\_or\_documentation | csv | 9620.613 | 43 |
| 02\_workflows/F3\_workflow\_v01/data/02b\_figure\_content/esld\_F3\_score\_shift\_aggregate\_public.csv **Section:** public\_workflow\_output\_or\_documentation **Directory:** 02\_workflows/F3\_workflow\_v01/data/02b\_figure\_content **Extension:** csv **Size KB:** 6.515 **Last modified:** 2026-05-02 09:50:43 **MD5:** 9e9ace861cb329c001b4fcf732aef69c **Variables in codebook:** 4 | public\_workflow\_output\_or\_documentation | csv | 6.515 | 4 |
| 02\_workflows/F3\_workflow\_v01/figures/F3\_ESLD\_SRTR\_public.pdf **Section:** public\_workflow\_output\_or\_documentation **Directory:** 02\_workflows/F3\_workflow\_v01/figures **Extension:** pdf **Size KB:** 556.029 **Last modified:** 2026-05-02 09:50:44 **MD5:** f8b24dda2b8388ecfc6b61ab02e80c27 **Variables in codebook:** 0 | public\_workflow\_output\_or\_documentation | pdf | 556.029 | 0 |
| 02\_workflows/F3\_workflow\_v01/submission\_ready/F3\_submission\_ready\_manifest\_v01.csv **Section:** public\_workflow\_output\_or\_documentation **Directory:** 02\_workflows/F3\_workflow\_v01/submission\_ready **Extension:** csv **Size KB:** 1.711 **Last modified:** 2026-05-02 09:50:44 **MD5:** c524b4a4a7c9ae655d310516ff16a166 **Variables in codebook:** 0 | public\_workflow\_output\_or\_documentation | csv | 1.711 | 0 |
| 02\_workflows/F3\_workflow\_v01/submission\_ready/README\_F3\_submission\_ready\_v01.txt **Section:** public\_workflow\_output\_or\_documentation **Directory:** 02\_workflows/F3\_workflow\_v01/submission\_ready **Extension:** txt **Size KB:** 0.328 **Last modified:** 2026-05-02 09:50:44 **MD5:** 1f67873b1c86188d38ce46eb6cd2dd66 **Variables in codebook:** 0 | public\_workflow\_output\_or\_documentation | txt | 0.328 | 0 |
| 02\_workflows/F3\_workflow\_v01/submission\_ready/public/F3\_public\_submission\_manifest\_v01.csv **Section:** public\_workflow\_output\_or\_documentation **Directory:** 02\_workflows/F3\_workflow\_v01/submission\_ready/public **Extension:** csv **Size KB:** 0.438 **Last modified:** 2026-05-02 09:50:44 **MD5:** 17a134e04f2847572a292674f992bc29 **Variables in codebook:** 0 | public\_workflow\_output\_or\_documentation | csv | 0.438 | 0 |
| 02\_workflows/F3\_workflow\_v01/submission\_ready/public/data/esld\_F3\_score\_shift\_aggregate\_public.csv **Section:** public\_workflow\_output\_or\_documentation **Directory:** 02\_workflows/F3\_workflow\_v01/submission\_ready/public/data **Extension:** csv **Size KB:** 6.515 **Last modified:** 2026-05-02 09:50:43 **MD5:** 9e9ace861cb329c001b4fcf732aef69c **Variables in codebook:** 4 | public\_workflow\_output\_or\_documentation | csv | 6.515 | 4 |
| 02\_workflows/F3\_workflow\_v01/submission\_ready/public/figures/F3\_ESLD\_SRTR\_public.pdf **Section:** public\_workflow\_output\_or\_documentation **Directory:** 02\_workflows/F3\_workflow\_v01/submission\_ready/public/figures **Extension:** pdf **Size KB:** 556.029 **Last modified:** 2026-05-02 09:50:44 **MD5:** f8b24dda2b8388ecfc6b61ab02e80c27 **Variables in codebook:** 0 | public\_workflow\_output\_or\_documentation | pdf | 556.029 | 0 |
| 02\_workflows/F4\_workflow\_v01/README\_F4\_workflow\_v01.txt **Section:** public\_workflow\_output\_or\_documentation **Directory:** 02\_workflows/F4\_workflow\_v01 **Extension:** txt **Size KB:** 0.437 **Last modified:** 2026-04-30 12:11:40 **MD5:** b976db4271a8f5d859f540e16d517a74 **Variables in codebook:** 0 | public\_workflow\_output\_or\_documentation | txt | 0.437 | 0 |
| 02\_workflows/F4\_workflow\_v01/data/01\_source\_loaded\_harmonized/esld\_master\_long\_public.csv **Section:** public\_workflow\_output\_or\_documentation **Directory:** 02\_workflows/F4\_workflow\_v01/data/01\_source\_loaded\_harmonized **Extension:** csv **Size KB:** 9620.613 **Last modified:** 2026-05-02 23:18:12 **MD5:** 8228407e927ff10ad6e4740d0d5cd48b **Variables in codebook:** 43 | public\_workflow\_output\_or\_documentation | csv | 9620.613 | 43 |
| 02\_workflows/F4\_workflow\_v01/data/02b\_figure\_content/esld\_F4\_survival\_stats\_public.csv **Section:** public\_workflow\_output\_or\_documentation **Directory:** 02\_workflows/F4\_workflow\_v01/data/02b\_figure\_content **Extension:** csv **Size KB:** 2.255 **Last modified:** 2026-05-02 02:11:22 **MD5:** 33d16cd47150ae66d00d8a83b4b0c3ab **Variables in codebook:** 14 | public\_workflow\_output\_or\_documentation | csv | 2.255 | 14 |
| 02\_workflows/F4\_workflow\_v01/figures/F4\_ESLD\_SRTR\_public.pdf **Section:** public\_workflow\_output\_or\_documentation **Directory:** 02\_workflows/F4\_workflow\_v01/figures **Extension:** pdf **Size KB:** 817.854 **Last modified:** 2026-05-02 02:11:22 **MD5:** 66ff2e5f3a6621ee2b581cc02aa6a6a9 **Variables in codebook:** 0 | public\_workflow\_output\_or\_documentation | pdf | 817.854 | 0 |
| 02\_workflows/F4\_workflow\_v01/submission\_ready/F4\_submission\_ready\_manifest\_v01.csv **Section:** public\_workflow\_output\_or\_documentation **Directory:** 02\_workflows/F4\_workflow\_v01/submission\_ready **Extension:** csv **Size KB:** 5.902 **Last modified:** 2026-05-03 02:02:57 **MD5:** 4c6a84eda2bf24b137e6953a1e742eb4 **Variables in codebook:** 0 | public\_workflow\_output\_or\_documentation | csv | 5.902 | 0 |
| 02\_workflows/F4\_workflow\_v01/submission\_ready/public/F4\_public\_submission\_manifest\_v01.csv **Section:** public\_workflow\_output\_or\_documentation **Directory:** 02\_workflows/F4\_workflow\_v01/submission\_ready/public **Extension:** csv **Size KB:** 1.454 **Last modified:** 2026-05-03 02:02:57 **MD5:** 9329f622689f84814eb381fbc9db82f0 **Variables in codebook:** 0 | public\_workflow\_output\_or\_documentation | csv | 1.454 | 0 |
| 02\_workflows/F4\_workflow\_v01/submission\_ready/public/data/esld\_F4\_survival\_stats\_public.csv **Section:** public\_workflow\_output\_or\_documentation **Directory:** 02\_workflows/F4\_workflow\_v01/submission\_ready/public/data **Extension:** csv **Size KB:** 2.255 **Last modified:** 2026-05-02 02:11:22 **MD5:** 33d16cd47150ae66d00d8a83b4b0c3ab **Variables in codebook:** 14 | public\_workflow\_output\_or\_documentation | csv | 2.255 | 14 |
| 02\_workflows/F4\_workflow\_v01/submission\_ready/public/figures/F4\_ESLD\_SRTR\_public.pdf **Section:** public\_workflow\_output\_or\_documentation **Directory:** 02\_workflows/F4\_workflow\_v01/submission\_ready/public/figures **Extension:** pdf **Size KB:** 817.854 **Last modified:** 2026-05-02 02:11:22 **MD5:** 66ff2e5f3a6621ee2b581cc02aa6a6a9 **Variables in codebook:** 0 | public\_workflow\_output\_or\_documentation | pdf | 817.854 | 0 |
| 02\_workflows/F5\_workflow\_v01/README\_F5\_workflow\_v01.txt **Section:** public\_workflow\_output\_or\_documentation **Directory:** 02\_workflows/F5\_workflow\_v01 **Extension:** txt **Size KB:** 0.685 **Last modified:** 2026-05-03 02:03:01 **MD5:** e0bd3d490b3abe68c9dc9f2b787efdd0 **Variables in codebook:** 0 | public\_workflow\_output\_or\_documentation | txt | 0.685 | 0 |
| 02\_workflows/F5\_workflow\_v01/data/01\_source\_loaded\_harmonized/esld\_master\_long\_public.csv **Section:** public\_workflow\_output\_or\_documentation **Directory:** 02\_workflows/F5\_workflow\_v01/data/01\_source\_loaded\_harmonized **Extension:** csv **Size KB:** 9620.613 **Last modified:** 2026-05-02 23:18:13 **MD5:** 8228407e927ff10ad6e4740d0d5cd48b **Variables in codebook:** 43 | public\_workflow\_output\_or\_documentation | csv | 9620.613 | 43 |
| 02\_workflows/F5\_workflow\_v01/data/02b\_figure\_content/esld\_F5\_stratified\_survival\_meta\_public.csv **Section:** public\_workflow\_output\_or\_documentation **Directory:** 02\_workflows/F5\_workflow\_v01/data/02b\_figure\_content **Extension:** csv **Size KB:** 0.424 **Last modified:** 2026-05-02 01:41:36 **MD5:** 91f3a3d116ae7b1e3930facc0dafe562 **Variables in codebook:** 2 | public\_workflow\_output\_or\_documentation | csv | 0.424 | 2 |
| 02\_workflows/F5\_workflow\_v01/data/02b\_figure\_content/esld\_F5\_stratified\_survival\_stats\_public.csv **Section:** public\_workflow\_output\_or\_documentation **Directory:** 02\_workflows/F5\_workflow\_v01/data/02b\_figure\_content **Extension:** csv **Size KB:** 1.093 **Last modified:** 2026-05-02 01:41:36 **MD5:** a2935cfa9107cbfc3bd687c572f65c38 **Variables in codebook:** 8 | public\_workflow\_output\_or\_documentation | csv | 1.093 | 8 |
| 02\_workflows/F5\_workflow\_v01/data/02b\_figure\_content/esld\_F5\_stratified\_survival\_subject\_public.csv **Section:** public\_workflow\_output\_or\_documentation **Directory:** 02\_workflows/F5\_workflow\_v01/data/02b\_figure\_content **Extension:** csv **Size KB:** 119.284 **Last modified:** 2026-05-02 01:41:36 **MD5:** d4865d62db7ce845d162bdd848dc4ce2 **Variables in codebook:** 19 | public\_workflow\_output\_or\_documentation | csv | 119.284 | 19 |
| 02\_workflows/F5\_workflow\_v01/figures/F5\_ESLD\_public.pdf **Section:** public\_workflow\_output\_or\_documentation **Directory:** 02\_workflows/F5\_workflow\_v01/figures **Extension:** pdf **Size KB:** 602.098 **Last modified:** 2026-05-02 01:41:36 **MD5:** 136a8ddede064a07771670ea2ccddf45 **Variables in codebook:** 0 | public\_workflow\_output\_or\_documentation | pdf | 602.098 | 0 |
| 02\_workflows/F5\_workflow\_v01/submission\_ready/F5\_submission\_ready\_manifest\_v01.csv **Section:** public\_workflow\_output\_or\_documentation **Directory:** 02\_workflows/F5\_workflow\_v01/submission\_ready **Extension:** csv **Size KB:** 5.713 **Last modified:** 2026-05-03 02:03:01 **MD5:** 4bc75000400ff5794a20aa2839c2911d **Variables in codebook:** 0 | public\_workflow\_output\_or\_documentation | csv | 5.713 | 0 |
| 02\_workflows/F5\_workflow\_v01/submission\_ready/public/F5\_public\_submission\_manifest\_v01.csv **Section:** public\_workflow\_output\_or\_documentation **Directory:** 02\_workflows/F5\_workflow\_v01/submission\_ready/public **Extension:** csv **Size KB:** 1.458 **Last modified:** 2026-05-03 02:03:02 **MD5:** 729f3f0fe8c82c66559e6a4fcac1661b **Variables in codebook:** 0 | public\_workflow\_output\_or\_documentation | csv | 1.458 | 0 |
| 02\_workflows/F5\_workflow\_v01/submission\_ready/public/data/esld\_F5\_stratified\_survival\_meta\_public.csv **Section:** public\_workflow\_output\_or\_documentation **Directory:** 02\_workflows/F5\_workflow\_v01/submission\_ready/public/data **Extension:** csv **Size KB:** 0.424 **Last modified:** 2026-05-02 01:41:36 **MD5:** 91f3a3d116ae7b1e3930facc0dafe562 **Variables in codebook:** 2 | public\_workflow\_output\_or\_documentation | csv | 0.424 | 2 |
| 02\_workflows/F5\_workflow\_v01/submission\_ready/public/data/esld\_F5\_stratified\_survival\_stats\_public.csv **Section:** public\_workflow\_output\_or\_documentation **Directory:** 02\_workflows/F5\_workflow\_v01/submission\_ready/public/data **Extension:** csv **Size KB:** 1.093 **Last modified:** 2026-05-02 01:41:36 **MD5:** a2935cfa9107cbfc3bd687c572f65c38 **Variables in codebook:** 8 | public\_workflow\_output\_or\_documentation | csv | 1.093 | 8 |
| 02\_workflows/F5\_workflow\_v01/submission\_ready/public/data/esld\_F5\_stratified\_survival\_subject\_public.csv **Section:** public\_workflow\_output\_or\_documentation **Directory:** 02\_workflows/F5\_workflow\_v01/submission\_ready/public/data **Extension:** csv **Size KB:** 119.284 **Last modified:** 2026-05-02 01:41:36 **MD5:** d4865d62db7ce845d162bdd848dc4ce2 **Variables in codebook:** 19 | public\_workflow\_output\_or\_documentation | csv | 119.284 | 19 |
| 02\_workflows/F5\_workflow\_v01/submission\_ready/public/figures/F5\_ESLD\_public.pdf **Section:** public\_workflow\_output\_or\_documentation **Directory:** 02\_workflows/F5\_workflow\_v01/submission\_ready/public/figures **Extension:** pdf **Size KB:** 602.098 **Last modified:** 2026-05-02 01:41:36 **MD5:** 136a8ddede064a07771670ea2ccddf45 **Variables in codebook:** 0 | public\_workflow\_output\_or\_documentation | pdf | 602.098 | 0 |
| 02\_workflows/F6\_workflow\_v01/README\_F6\_workflow\_v01.txt **Section:** public\_workflow\_output\_or\_documentation **Directory:** 02\_workflows/F6\_workflow\_v01 **Extension:** txt **Size KB:** 0.618 **Last modified:** 2026-05-03 02:03:02 **MD5:** e8ed745bfee80fe3e4ad741db1f743b3 **Variables in codebook:** 0 | public\_workflow\_output\_or\_documentation | txt | 0.618 | 0 |
| 02\_workflows/F6\_workflow\_v01/figures/F6\_SRTR\_public.pdf **Section:** public\_workflow\_output\_or\_documentation **Directory:** 02\_workflows/F6\_workflow\_v01/figures **Extension:** pdf **Size KB:** 555.652 **Last modified:** 2026-05-02 03:11:53 **MD5:** 363a49e6865292f51c335342b4e50772 **Variables in codebook:** 0 | public\_workflow\_output\_or\_documentation | pdf | 555.652 | 0 |
| 02\_workflows/F6\_workflow\_v01/submission\_ready/F6\_submission\_ready\_manifest\_v01.csv **Section:** public\_workflow\_output\_or\_documentation **Directory:** 02\_workflows/F6\_workflow\_v01/submission\_ready **Extension:** csv **Size KB:** 6.019 **Last modified:** 2026-05-03 02:03:02 **MD5:** 6b44b4eff55f55d223e929fddea85caa **Variables in codebook:** 0 | public\_workflow\_output\_or\_documentation | csv | 6.019 | 0 |
| 02\_workflows/F6\_workflow\_v01/submission\_ready/README\_F6\_submission\_ready\_v01.txt **Section:** public\_workflow\_output\_or\_documentation **Directory:** 02\_workflows/F6\_workflow\_v01/submission\_ready **Extension:** txt **Size KB:** 0.466 **Last modified:** 2026-05-02 03:11:53 **MD5:** a193c8f8a36df7b9d2e14fb8372013d7 **Variables in codebook:** 0 | public\_workflow\_output\_or\_documentation | txt | 0.466 | 0 |
| 02\_workflows/F6\_workflow\_v01/submission\_ready/public/F6\_public\_submission\_manifest\_v01.csv **Section:** public\_workflow\_output\_or\_documentation **Directory:** 02\_workflows/F6\_workflow\_v01/submission\_ready/public **Extension:** csv **Size KB:** 1.049 **Last modified:** 2026-05-03 02:03:02 **MD5:** 7829901750447160109c71b246f41a75 **Variables in codebook:** 0 | public\_workflow\_output\_or\_documentation | csv | 1.049 | 0 |
| 02\_workflows/F6\_workflow\_v01/submission\_ready/public/figures/F6\_SRTR\_public.pdf **Section:** public\_workflow\_output\_or\_documentation **Directory:** 02\_workflows/F6\_workflow\_v01/submission\_ready/public/figures **Extension:** pdf **Size KB:** 555.652 **Last modified:** 2026-05-02 03:11:53 **MD5:** 363a49e6865292f51c335342b4e50772 **Variables in codebook:** 0 | public\_workflow\_output\_or\_documentation | pdf | 555.652 | 0 |
| 02\_workflows/T1\_workflow\_v01/README\_T1\_workflow\_v01.txt **Section:** public\_workflow\_output\_or\_documentation **Directory:** 02\_workflows/T1\_workflow\_v01 **Extension:** txt **Size KB:** 0.912 **Last modified:** 2026-05-03 02:03:06 **MD5:** e9f276de625615b50531fbbff3c76ee3 **Variables in codebook:** 0 | public\_workflow\_output\_or\_documentation | txt | 0.912 | 0 |
| 02\_workflows/T1\_workflow\_v01/data/01\_source\_loaded\_harmonized/esld\_T1\_baseline\_patient\_public.csv **Section:** public\_workflow\_output\_or\_documentation **Directory:** 02\_workflows/T1\_workflow\_v01/data/01\_source\_loaded\_harmonized **Extension:** csv **Size KB:** 67.969 **Last modified:** 2026-05-02 20:27:46 **MD5:** 5d5b1fa7a2a3845550ca78b467c41b7c **Variables in codebook:** 19 | public\_workflow\_output\_or\_documentation | csv | 67.969 | 19 |
| 02\_workflows/T1\_workflow\_v01/data/01\_source\_loaded\_harmonized/esld\_master\_long\_public.csv **Section:** public\_workflow\_output\_or\_documentation **Directory:** 02\_workflows/T1\_workflow\_v01/data/01\_source\_loaded\_harmonized **Extension:** csv **Size KB:** 9620.613 **Last modified:** 2026-05-02 23:18:13 **MD5:** 8228407e927ff10ad6e4740d0d5cd48b **Variables in codebook:** 43 | public\_workflow\_output\_or\_documentation | csv | 9620.613 | 43 |
| 02\_workflows/T1\_workflow\_v01/submission\_ready/README\_T1\_submission\_ready\_v01.txt **Section:** public\_workflow\_output\_or\_documentation **Directory:** 02\_workflows/T1\_workflow\_v01/submission\_ready **Extension:** txt **Size KB:** 0.385 **Last modified:** 2026-05-03 02:03:06 **MD5:** 57936d05a6cfd63bddc49c8b1394ba6c **Variables in codebook:** 0 | public\_workflow\_output\_or\_documentation | txt | 0.385 | 0 |
| 02\_workflows/T1\_workflow\_v01/submission\_ready/public/T1\_public\_submission\_manifest\_v01.csv **Section:** public\_workflow\_output\_or\_documentation **Directory:** 02\_workflows/T1\_workflow\_v01/submission\_ready/public **Extension:** csv **Size KB:** 0.689 **Last modified:** 2026-05-03 02:03:06 **MD5:** ea3042f1b950b0575a01c0f098c7b00d **Variables in codebook:** 0 | public\_workflow\_output\_or\_documentation | csv | 0.689 | 0 |
| 02\_workflows/T3\_workflow\_v01/data/01\_source\_loaded\_harmonized/esld\_master\_long\_public.csv **Section:** public\_workflow\_output\_or\_documentation **Directory:** 02\_workflows/T3\_workflow\_v01/data/01\_source\_loaded\_harmonized **Extension:** csv **Size KB:** 9620.613 **Last modified:** 2026-05-02 23:18:13 **MD5:** 8228407e927ff10ad6e4740d0d5cd48b **Variables in codebook:** 43 | public\_workflow\_output\_or\_documentation | csv | 9620.613 | 43 |
| 02\_workflows/T3\_workflow\_v01/submission\_ready/README\_T3\_submission\_ready\_v01.txt **Section:** public\_workflow\_output\_or\_documentation **Directory:** 02\_workflows/T3\_workflow\_v01/submission\_ready **Extension:** txt **Size KB:** 0.471 **Last modified:** 2026-05-03 02:03:11 **MD5:** 09cb40ad6bf172aeb6faca7a3f38cb59 **Variables in codebook:** 0 | public\_workflow\_output\_or\_documentation | txt | 0.471 | 0 |
| 02\_workflows/T3\_workflow\_v01/submission\_ready/public/T3\_public\_submission\_manifest\_v01.csv **Section:** public\_workflow\_output\_or\_documentation **Directory:** 02\_workflows/T3\_workflow\_v01/submission\_ready/public **Extension:** csv **Size KB:** 0.655 **Last modified:** 2026-05-03 02:03:11 **MD5:** b7f01899cd334092e745ec6a99723853 **Variables in codebook:** 0 | public\_workflow\_output\_or\_documentation | csv | 0.655 | 0 |
| 02\_workflows/T4\_workflow\_v01/data/01\_source\_loaded\_harmonized/esld\_master\_long\_public.csv **Section:** public\_workflow\_output\_or\_documentation **Directory:** 02\_workflows/T4\_workflow\_v01/data/01\_source\_loaded\_harmonized **Extension:** csv **Size KB:** 9620.613 **Last modified:** 2026-05-02 23:18:13 **MD5:** 8228407e927ff10ad6e4740d0d5cd48b **Variables in codebook:** 43 | public\_workflow\_output\_or\_documentation | csv | 9620.613 | 43 |
| 02\_workflows/T4\_workflow\_v01/data/02b\_table\_content/esld\_T4\_score\_deviation\_outcome\_meta\_public.csv **Section:** public\_workflow\_output\_or\_documentation **Directory:** 02\_workflows/T4\_workflow\_v01/data/02b\_table\_content **Extension:** csv **Size KB:** 0.678 **Last modified:** 2026-05-02 00:32:16 **MD5:** da8cb16231868249ed6a58148548d6d8 **Variables in codebook:** 2 | public\_workflow\_output\_or\_documentation | csv | 0.678 | 2 |
| 02\_workflows/T4\_workflow\_v01/data/02b\_table\_content/esld\_T4\_score\_deviation\_outcome\_table\_public.csv **Section:** public\_workflow\_output\_or\_documentation **Directory:** 02\_workflows/T4\_workflow\_v01/data/02b\_table\_content **Extension:** csv **Size KB:** 2.566 **Last modified:** 2026-05-02 00:32:16 **MD5:** c7c6b8bd97bb8df75be2ffa87ec0c281 **Variables in codebook:** 12 | public\_workflow\_output\_or\_documentation | csv | 2.566 | 12 |
| 02\_workflows/T4\_workflow\_v01/submission\_ready/README\_T4\_submission\_ready\_v01.txt **Section:** public\_workflow\_output\_or\_documentation **Directory:** 02\_workflows/T4\_workflow\_v01/submission\_ready **Extension:** txt **Size KB:** 0.719 **Last modified:** 2026-05-03 02:03:16 **MD5:** dabc0dfce5571c27a02b2265df4c5f66 **Variables in codebook:** 0 | public\_workflow\_output\_or\_documentation | txt | 0.719 | 0 |
| 02\_workflows/T4\_workflow\_v01/submission\_ready/public/T4\_public\_submission\_manifest\_v01.csv **Section:** public\_workflow\_output\_or\_documentation **Directory:** 02\_workflows/T4\_workflow\_v01/submission\_ready/public **Extension:** csv **Size KB:** 0.691 **Last modified:** 2026-05-03 02:03:16 **MD5:** 37698cb9743c533b96988553cb8c29a2 **Variables in codebook:** 0 | public\_workflow\_output\_or\_documentation | csv | 0.691 | 0 |
| 02\_workflows/T4\_workflow\_v01/submission\_ready/public/data/esld\_T4\_score\_deviation\_outcome\_meta\_public.csv **Section:** public\_workflow\_output\_or\_documentation **Directory:** 02\_workflows/T4\_workflow\_v01/submission\_ready/public/data **Extension:** csv **Size KB:** 0.678 **Last modified:** 2026-05-02 00:32:17 **MD5:** da8cb16231868249ed6a58148548d6d8 **Variables in codebook:** 2 | public\_workflow\_output\_or\_documentation | csv | 0.678 | 2 |
| 02\_workflows/T4\_workflow\_v01/submission\_ready/public/data/esld\_T4\_score\_deviation\_outcome\_table\_public.csv **Section:** public\_workflow\_output\_or\_documentation **Directory:** 02\_workflows/T4\_workflow\_v01/submission\_ready/public/data **Extension:** csv **Size KB:** 2.566 **Last modified:** 2026-05-02 00:32:17 **MD5:** c7c6b8bd97bb8df75be2ffa87ec0c281 **Variables in codebook:** 12 | public\_workflow\_output\_or\_documentation | csv | 2.566 | 12 |
| 00\_release\_manifests/failed\_public\_file\_copies\_v01.csv **Section:** release\_manifest\_or\_codebook **Directory:** 00\_release\_manifests **Extension:** csv **Size KB:** 0.899 **Last modified:** 2026-05-03 00:26:37 **MD5:** c9b759622702f225885033e3774e46b1 **Variables in codebook:** 0 | release\_manifest\_or\_codebook | csv | 0.899 | 0 |
| 00\_release\_manifests/final\_dictionary\_remaining\_review\_after\_global\_sanitize\_v01.csv **Section:** release\_manifest\_or\_codebook **Directory:** 00\_release\_manifests **Extension:** csv **Size KB:** 0.972 **Last modified:** 2026-05-03 02:03:19 **MD5:** 248695a5a2734a817cf0d6384d51dfe2 **Variables in codebook:** 0 | release\_manifest\_or\_codebook | csv | 0.972 | 0 |
| 00\_release\_manifests/final\_global\_absolute\_path\_scan\_before\_v01.csv **Section:** release\_manifest\_or\_codebook **Directory:** 00\_release\_manifests **Extension:** csv **Size KB:** 466.718 **Last modified:** 2026-05-03 02:03:19 **MD5:** b4ba8ea1589b9367b698049e8b4563e8 **Variables in codebook:** 0 | release\_manifest\_or\_codebook | csv | 466.718 | 0 |
| 00\_release\_manifests/final\_global\_absolute\_path\_scan\_v01.csv **Section:** release\_manifest\_or\_codebook **Directory:** 00\_release\_manifests **Extension:** csv **Size KB:** 0.031 **Last modified:** 2026-05-03 02:03:19 **MD5:** 7263f19145b829d45776a54755d7e281 **Variables in codebook:** 0 | release\_manifest\_or\_codebook | csv | 0.031 | 0 |
| 00\_release\_manifests/final\_global\_path\_sanitization\_log\_v01.csv **Section:** release\_manifest\_or\_codebook **Directory:** 00\_release\_manifests **Extension:** csv **Size KB:** 796.206 **Last modified:** 2026-05-03 02:03:19 **MD5:** b1bd0753f25ef8c8ff8d03fe15720295 **Variables in codebook:** 0 | release\_manifest\_or\_codebook | csv | 796.206 | 0 |
| 00\_release\_manifests/final\_public\_package\_upload\_readiness\_audit\_v01.txt **Section:** release\_manifest\_or\_codebook **Directory:** 00\_release\_manifests **Extension:** txt **Size KB:** 5.709 **Last modified:** 2026-05-03 02:03:19 **MD5:** 2d5669e3dc7f37c956eeac7cc307295c **Variables in codebook:** 0 | release\_manifest\_or\_codebook | txt | 5.709 | 0 |
| 00\_release\_manifests/final\_public\_package\_upload\_readiness\_summary\_v01.csv **Section:** release\_manifest\_or\_codebook **Directory:** 00\_release\_manifests **Extension:** csv **Size KB:** 0.632 **Last modified:** 2026-05-03 02:03:19 **MD5:** 6fdbb9d3ca7880510b2e0b067f4da2df **Variables in codebook:** 0 | release\_manifest\_or\_codebook | csv | 0.632 | 0 |
| 00\_release\_manifests/final\_release\_file\_manifest\_closed\_v01.csv **Section:** release\_manifest\_or\_codebook **Directory:** 00\_release\_manifests **Extension:** csv **Size KB:** 274.4 **Last modified:** 2026-05-03 00:26:37 **MD5:** c4576a7f18289c5098f064bce89076b6 **Variables in codebook:** 0 | release\_manifest\_or\_codebook | csv | 274.4 | 0 |
| 00\_release\_manifests/final\_remaining\_root\_R\_scripts\_v01.csv **Section:** release\_manifest\_or\_codebook **Directory:** 00\_release\_manifests **Extension:** csv **Size KB:** 0.023 **Last modified:** 2026-05-03 02:03:19 **MD5:** 5931700348139d13d6eba3c1b5f2203d **Variables in codebook:** 0 | release\_manifest\_or\_codebook | csv | 0.023 | 0 |
| 00\_release\_manifests/final\_removed\_root\_helper\_R\_scripts\_v01.csv **Section:** release\_manifest\_or\_codebook **Directory:** 00\_release\_manifests **Extension:** csv **Size KB:** 1.399 **Last modified:** 2026-05-03 02:03:19 **MD5:** db509e02d607c6b20d721c17a6cb9ce9 **Variables in codebook:** 0 | release\_manifest\_or\_codebook | csv | 1.399 | 0 |
| 00\_release\_manifests/final\_variable\_dictionary\_approved\_v01.csv **Section:** release\_manifest\_or\_codebook **Directory:** 00\_release\_manifests **Extension:** csv **Size KB:** 1697.567 **Last modified:** 2026-05-03 02:01:28 **MD5:** d01e8f414a6f3745b0085d862ebe5b13 **Variables in codebook:** 0 | release\_manifest\_or\_codebook | csv | 1697.567 | 0 |
| 00\_release\_manifests/internal\_excluded\_file\_manifest\_v01.csv **Section:** release\_manifest\_or\_codebook **Directory:** 00\_release\_manifests **Extension:** csv **Size KB:** 82.845 **Last modified:** 2026-05-03 00:26:37 **MD5:** 3277cb18f6d32885cc4ee8dde001aaea **Variables in codebook:** 0 | release\_manifest\_or\_codebook | csv | 82.845 | 0 |
| 00\_release\_manifests/missing\_public\_source\_files\_v01.csv **Section:** release\_manifest\_or\_codebook **Directory:** 00\_release\_manifests **Extension:** csv **Size KB:** 0.899 **Last modified:** 2026-05-03 00:26:37 **MD5:** c9b759622702f225885033e3774e46b1 **Variables in codebook:** 0 | release\_manifest\_or\_codebook | csv | 0.899 | 0 |
| 00\_release\_manifests/not\_public\_variable\_dictionary\_v01.csv **Section:** release\_manifest\_or\_codebook **Directory:** 00\_release\_manifests **Extension:** csv **Size KB:** 760.736 **Last modified:** 2026-05-03 02:01:29 **MD5:** d3b878eb603e910ea3367c3f97d236ff **Variables in codebook:** 0 | release\_manifest\_or\_codebook | csv | 760.736 | 0 |
| 00\_release\_manifests/public\_absolute\_path\_scan\_v01.csv **Section:** release\_manifest\_or\_codebook **Directory:** 00\_release\_manifests **Extension:** csv **Size KB:** 105.64 **Last modified:** 2026-05-03 02:01:29 **MD5:** c53ebd957ea5894b2c62dd18f144f221 **Variables in codebook:** 0 | release\_manifest\_or\_codebook | csv | 105.64 | 0 |
| 00\_release\_manifests/public\_deposit\_file\_copy\_log\_v01.csv **Section:** release\_manifest\_or\_codebook **Directory:** 00\_release\_manifests **Extension:** csv **Size KB:** 75.441 **Last modified:** 2026-05-03 02:01:29 **MD5:** 4ee604086002c1c7bdc1c912e456fe26 **Variables in codebook:** 0 | release\_manifest\_or\_codebook | csv | 75.441 | 0 |
| 00\_release\_manifests/public\_deposit\_file\_manifest\_v01.csv **Section:** release\_manifest\_or\_codebook **Directory:** 00\_release\_manifests **Extension:** csv **Size KB:** 59.062 **Last modified:** 2026-05-03 00:26:37 **MD5:** d9b9d7b5412a55082d0cd4ff5a7fc8be **Variables in codebook:** 0 | release\_manifest\_or\_codebook | csv | 59.062 | 0 |
| 00\_release\_manifests/public\_deposit\_package\_build\_audit\_v01.txt **Section:** release\_manifest\_or\_codebook **Directory:** 00\_release\_manifests **Extension:** txt **Size KB:** 3.896 **Last modified:** 2026-05-03 02:01:29 **MD5:** 51b7f8ea4b9c5804c7c31c5e77ad39af **Variables in codebook:** 0 | release\_manifest\_or\_codebook | txt | 3.896 | 0 |
| 00\_release\_manifests/public\_deposit\_package\_build\_summary\_v01.csv **Section:** release\_manifest\_or\_codebook **Directory:** 00\_release\_manifests **Extension:** csv **Size KB:** 0.487 **Last modified:** 2026-05-03 02:01:29 **MD5:** 6ec3c5776930552ebef14a899f1b03af **Variables in codebook:** 0 | release\_manifest\_or\_codebook | csv | 0.487 | 0 |
| 00\_release\_manifests/public\_generic\_column\_names\_requiring\_mapping\_v01.csv **Section:** release\_manifest\_or\_codebook **Directory:** 00\_release\_manifests **Extension:** csv **Size KB:** 0.776 **Last modified:** 2026-05-03 01:20:22 **MD5:** fd48670b3cced3540e884462c85157c9 **Variables in codebook:** 0 | release\_manifest\_or\_codebook | csv | 0.776 | 0 |
| 00\_release\_manifests/public\_path\_sanitization\_log\_v01.csv **Section:** release\_manifest\_or\_codebook **Directory:** 00\_release\_manifests **Extension:** csv **Size KB:** 51.985 **Last modified:** 2026-05-03 02:01:29 **MD5:** fcfab0404f88bbd19d100259f58d481f **Variables in codebook:** 0 | release\_manifest\_or\_codebook | csv | 51.985 | 0 |
| 00\_release\_manifests/public\_variable\_dictionary\_FINAL\_CODEBOOK.csv **Section:** release\_manifest\_or\_codebook **Directory:** 00\_release\_manifests **Extension:** csv **Size KB:** 339.95 **Last modified:** 2026-05-03 09:48:19 **MD5:** 387dbd82bd4fd7288c436a890d165326 **Variables in codebook:** 0 | release\_manifest\_or\_codebook | csv | 339.95 | 0 |
| 00\_release\_manifests/public\_variable\_dictionary\_FINAL\_CODEBOOK\_audit.txt **Section:** release\_manifest\_or\_codebook **Directory:** 00\_release\_manifests **Extension:** txt **Size KB:** 3.841 **Last modified:** 2026-05-03 09:48:19 **MD5:** 0ccfcb4b09d856fed50667c193e9aeca **Variables in codebook:** 0 | release\_manifest\_or\_codebook | txt | 3.841 | 0 |
| 00\_release\_manifests/public\_variable\_dictionary\_FINAL\_CODEBOOK\_review\_rows.csv **Section:** release\_manifest\_or\_codebook **Directory:** 00\_release\_manifests **Extension:** csv **Size KB:** 1.044 **Last modified:** 2026-05-03 09:48:19 **MD5:** 992c0b97fc0db2d59ef19b56f8ee9a78 **Variables in codebook:** 0 | release\_manifest\_or\_codebook | csv | 1.044 | 0 |
| 00\_release\_manifests/public\_variable\_dictionary\_FINAL\_CODEBOOK\_summary.csv **Section:** release\_manifest\_or\_codebook **Directory:** 00\_release\_manifests **Extension:** csv **Size KB:** 0.598 **Last modified:** 2026-05-03 09:48:19 **MD5:** c3600f4a261d7f4d7daf42a21e4f7519 **Variables in codebook:** 0 | release\_manifest\_or\_codebook | csv | 0.598 | 0 |
| 00\_release\_manifests/public\_variable\_dictionary\_RECOMMENDED.csv **Section:** release\_manifest\_or\_codebook **Directory:** 00\_release\_manifests **Extension:** csv **Size KB:** 572.559 **Last modified:** 2026-05-03 02:01:29 **MD5:** a7e4cac9b02294cc78c627768254e0cf **Variables in codebook:** 0 | release\_manifest\_or\_codebook | csv | 572.559 | 0 |
| 00\_release\_manifests/public\_variable\_dictionary\_explanatory\_audit\_v01.txt **Section:** release\_manifest\_or\_codebook **Directory:** 00\_release\_manifests **Extension:** txt **Size KB:** 3.734 **Last modified:** 2026-05-03 02:01:29 **MD5:** 479e38593e50bd1970dd5d3ad0b35fef **Variables in codebook:** 0 | release\_manifest\_or\_codebook | txt | 3.734 | 0 |
| 00\_release\_manifests/public\_variable\_dictionary\_explanatory\_by\_status\_v01.csv **Section:** release\_manifest\_or\_codebook **Directory:** 00\_release\_manifests **Extension:** csv **Size KB:** 0.173 **Last modified:** 2026-05-03 01:20:22 **MD5:** b2295997b5e0b04740d14ea0a9368a3e **Variables in codebook:** 0 | release\_manifest\_or\_codebook | csv | 0.173 | 0 |
| 00\_release\_manifests/public\_variable\_dictionary\_explanatory\_by\_status\_v02.csv **Section:** release\_manifest\_or\_codebook **Directory:** 00\_release\_manifests **Extension:** csv **Size KB:** 0.148 **Last modified:** 2026-05-03 01:30:09 **MD5:** 44ce42ac4b43f55e739ede4f1ede77ae **Variables in codebook:** 0 | release\_manifest\_or\_codebook | csv | 0.148 | 0 |
| 00\_release\_manifests/public\_variable\_dictionary\_explanatory\_patch\_audit\_v01.txt **Section:** release\_manifest\_or\_codebook **Directory:** 00\_release\_manifests **Extension:** txt **Size KB:** 8.206 **Last modified:** 2026-05-03 02:01:29 **MD5:** 609874efd1b1c244b69c8555384fad3a **Variables in codebook:** 0 | release\_manifest\_or\_codebook | txt | 8.206 | 0 |
| 00\_release\_manifests/public\_variable\_dictionary\_explanatory\_patch\_audit\_v02.txt **Section:** release\_manifest\_or\_codebook **Directory:** 00\_release\_manifests **Extension:** txt **Size KB:** 8.665 **Last modified:** 2026-05-03 02:01:29 **MD5:** 1837d24aba83f22d5c065a611c105e14 **Variables in codebook:** 0 | release\_manifest\_or\_codebook | txt | 8.665 | 0 |
| 00\_release\_manifests/public\_variable\_dictionary\_explanatory\_v01.csv **Section:** release\_manifest\_or\_codebook **Directory:** 00\_release\_manifests **Extension:** csv **Size KB:** 530.68 **Last modified:** 2026-05-03 02:01:29 **MD5:** be305db6e914186adb3f714ada2234ce **Variables in codebook:** 0 | release\_manifest\_or\_codebook | csv | 530.68 | 0 |
| 00\_release\_manifests/public\_variable\_dictionary\_explanatory\_v02.csv **Section:** release\_manifest\_or\_codebook **Directory:** 00\_release\_manifests **Extension:** csv **Size KB:** 546.594 **Last modified:** 2026-05-03 02:01:29 **MD5:** 3c50f3fb0d71979cb93e4c7c8c4bb140 **Variables in codebook:** 0 | release\_manifest\_or\_codebook | csv | 546.594 | 0 |
| 00\_release\_manifests/public\_variable\_dictionary\_explanatory\_v03.csv **Section:** release\_manifest\_or\_codebook **Directory:** 00\_release\_manifests **Extension:** csv **Size KB:** 559.98 **Last modified:** 2026-05-03 02:01:29 **MD5:** da3f02f93b8a53174c54b6879291ec38 **Variables in codebook:** 0 | release\_manifest\_or\_codebook | csv | 559.98 | 0 |
| 00\_release\_manifests/public\_variable\_dictionary\_explanatory\_v04.csv **Section:** release\_manifest\_or\_codebook **Directory:** 00\_release\_manifests **Extension:** csv **Size KB:** 572.559 **Last modified:** 2026-05-03 02:01:29 **MD5:** a7e4cac9b02294cc78c627768254e0cf **Variables in codebook:** 0 | release\_manifest\_or\_codebook | csv | 572.559 | 0 |
| 00\_release\_manifests/public\_variable\_dictionary\_final\_precision\_audit\_v01.txt **Section:** release\_manifest\_or\_codebook **Directory:** 00\_release\_manifests **Extension:** txt **Size KB:** 12.948 **Last modified:** 2026-05-03 02:01:29 **MD5:** 76d80f99407691739cc1a6c1734b061d **Variables in codebook:** 0 | release\_manifest\_or\_codebook | txt | 12.948 | 0 |
| 00\_release\_manifests/public\_variable\_dictionary\_final\_precision\_by\_status\_v01.csv **Section:** release\_manifest\_or\_codebook **Directory:** 00\_release\_manifests **Extension:** csv **Size KB:** 0.192 **Last modified:** 2026-05-03 01:59:40 **MD5:** 437e4dcf0da51e33a2f8109cff77ac17 **Variables in codebook:** 0 | release\_manifest\_or\_codebook | csv | 0.192 | 0 |
| 00\_release\_manifests/public\_variable\_dictionary\_final\_precision\_changes\_v01.csv **Section:** release\_manifest\_or\_codebook **Directory:** 00\_release\_manifests **Extension:** csv **Size KB:** 7.716 **Last modified:** 2026-05-03 02:01:29 **MD5:** bef7351a8b38261ec140cd7834af8553 **Variables in codebook:** 0 | release\_manifest\_or\_codebook | csv | 7.716 | 0 |
| 00\_release\_manifests/public\_variable\_dictionary\_final\_precision\_review\_v01.csv **Section:** release\_manifest\_or\_codebook **Directory:** 00\_release\_manifests **Extension:** csv **Size KB:** 0.15 **Last modified:** 2026-05-03 01:59:40 **MD5:** 7a59f4898872f36fb499a2433d98663d **Variables in codebook:** 0 | release\_manifest\_or\_codebook | csv | 0.15 | 0 |
| 00\_release\_manifests/public\_variable\_dictionary\_final\_precision\_summary\_v01.csv **Section:** release\_manifest\_or\_codebook **Directory:** 00\_release\_manifests **Extension:** csv **Size KB:** 0.419 **Last modified:** 2026-05-03 02:01:29 **MD5:** 73b5b054bd8e664e5ad0e25e9f03fa69 **Variables in codebook:** 0 | release\_manifest\_or\_codebook | csv | 0.419 | 0 |
| 00\_release\_manifests/public\_variable\_dictionary\_patch\_changes\_v01.csv **Section:** release\_manifest\_or\_codebook **Directory:** 00\_release\_manifests **Extension:** csv **Size KB:** 89.006 **Last modified:** 2026-05-03 01:24:44 **MD5:** 6aa3e11db5505ab3074d0649ebe2c2c6 **Variables in codebook:** 0 | release\_manifest\_or\_codebook | csv | 89.006 | 0 |
| 00\_release\_manifests/public\_variable\_dictionary\_patch\_changes\_v02.csv **Section:** release\_manifest\_or\_codebook **Directory:** 00\_release\_manifests **Extension:** csv **Size KB:** 89.866 **Last modified:** 2026-05-03 01:30:09 **MD5:** 54371296aace3a20a18ca18397481130 **Variables in codebook:** 0 | release\_manifest\_or\_codebook | csv | 89.866 | 0 |
| 00\_release\_manifests/public\_variable\_dictionary\_precision\_by\_status\_v01.csv **Section:** release\_manifest\_or\_codebook **Directory:** 00\_release\_manifests **Extension:** csv **Size KB:** 0.306 **Last modified:** 2026-05-03 01:56:40 **MD5:** 61eac49f2b1345126892902e10b5895e **Variables in codebook:** 0 | release\_manifest\_or\_codebook | csv | 0.306 | 0 |
| 00\_release\_manifests/public\_variable\_dictionary\_precision\_patch\_audit\_v01.txt **Section:** release\_manifest\_or\_codebook **Directory:** 00\_release\_manifests **Extension:** txt **Size KB:** 12.999 **Last modified:** 2026-05-03 02:01:29 **MD5:** 3c5319ac4c1943b60491a9aaebd2ad76 **Variables in codebook:** 0 | release\_manifest\_or\_codebook | txt | 12.999 | 0 |
| 00\_release\_manifests/public\_variable\_dictionary\_precision\_patch\_changes\_v01.csv **Section:** release\_manifest\_or\_codebook **Directory:** 00\_release\_manifests **Extension:** csv **Size KB:** 114.999 **Last modified:** 2026-05-03 01:56:40 **MD5:** aefc39306e1a3a94eff0fd21f42ca24f **Variables in codebook:** 0 | release\_manifest\_or\_codebook | csv | 114.999 | 0 |
| 00\_release\_manifests/public\_variable\_dictionary\_precision\_review\_v01.csv **Section:** release\_manifest\_or\_codebook **Directory:** 00\_release\_manifests **Extension:** csv **Size KB:** 4.576 **Last modified:** 2026-05-03 02:01:29 **MD5:** 5595e2266499258082deade2510f7e42 **Variables in codebook:** 0 | release\_manifest\_or\_codebook | csv | 4.576 | 0 |
| 00\_release\_manifests/public\_variable\_dictionary\_precision\_summary\_v01.csv **Section:** release\_manifest\_or\_codebook **Directory:** 00\_release\_manifests **Extension:** csv **Size KB:** 0.435 **Last modified:** 2026-05-03 02:01:29 **MD5:** 3b769195bbe656c1523f7d0edeb50982 **Variables in codebook:** 0 | release\_manifest\_or\_codebook | csv | 0.435 | 0 |
| 00\_release\_manifests/public\_variable\_dictionary\_summary\_explanatory\_v01.csv **Section:** release\_manifest\_or\_codebook **Directory:** 00\_release\_manifests **Extension:** csv **Size KB:** 0.295 **Last modified:** 2026-05-03 02:01:29 **MD5:** 27b10702f4ea6e193de53bec74aef84e **Variables in codebook:** 0 | release\_manifest\_or\_codebook | csv | 0.295 | 0 |
| 00\_release\_manifests/public\_variable\_dictionary\_summary\_explanatory\_v02.csv **Section:** release\_manifest\_or\_codebook **Directory:** 00\_release\_manifests **Extension:** csv **Size KB:** 0.364 **Last modified:** 2026-05-03 02:01:29 **MD5:** 1bb6ba3f7e43fe103872622d2ac6f5d7 **Variables in codebook:** 0 | release\_manifest\_or\_codebook | csv | 0.364 | 0 |
| 00\_release\_manifests/public\_variable\_dictionary\_unresolved\_by\_file\_v01.csv **Section:** release\_manifest\_or\_codebook **Directory:** 00\_release\_manifests **Extension:** csv **Size KB:** 0.77 **Last modified:** 2026-05-03 01:20:22 **MD5:** a7aa8a7941c0d4cd2a3c60657ff82304 **Variables in codebook:** 0 | release\_manifest\_or\_codebook | csv | 0.77 | 0 |
| 00\_release\_manifests/public\_variable\_dictionary\_unresolved\_v01.csv **Section:** release\_manifest\_or\_codebook **Directory:** 00\_release\_manifests **Extension:** csv **Size KB:** 21.999 **Last modified:** 2026-05-03 01:20:22 **MD5:** cb1b7898a2c26af0af7ee8f028de8cbb **Variables in codebook:** 0 | release\_manifest\_or\_codebook | csv | 21.999 | 0 |
| 00\_release\_manifests/public\_variable\_dictionary\_unresolved\_v02.csv **Section:** release\_manifest\_or\_codebook **Directory:** 00\_release\_manifests **Extension:** csv **Size KB:** 1.081 **Last modified:** 2026-05-03 01:30:09 **MD5:** f69120bf7299c678f926f10160b1f9bd **Variables in codebook:** 0 | release\_manifest\_or\_codebook | csv | 1.081 | 0 |
| 00\_release\_manifests/public\_variable\_dictionary\_v01.csv **Section:** release\_manifest\_or\_codebook **Directory:** 00\_release\_manifests **Extension:** csv **Size KB:** 376.47 **Last modified:** 2026-05-03 02:01:29 **MD5:** 46926c0df8bcd34f26c7c2b840644e38 **Variables in codebook:** 0 | release\_manifest\_or\_codebook | csv | 376.47 | 0 |
| 00\_release\_manifests/restricted\_on\_request\_file\_manifest\_v01.csv **Section:** release\_manifest\_or\_codebook **Directory:** 00\_release\_manifests **Extension:** csv **Size KB:** 74.085 **Last modified:** 2026-05-03 00:26:37 **MD5:** 19d7dfd5b86069a125d540dfa558fd53 **Variables in codebook:** 0 | release\_manifest\_or\_codebook | csv | 74.085 | 0 |
| 00\_release\_manifests/restricted\_on\_request\_variable\_dictionary\_v01.csv **Section:** release\_manifest\_or\_codebook **Directory:** 00\_release\_manifests **Extension:** csv **Size KB:** 571.309 **Last modified:** 2026-05-03 02:01:29 **MD5:** bf4aee2716ec59fd45b95169c7237a67 **Variables in codebook:** 0 | release\_manifest\_or\_codebook | csv | 571.309 | 0 |
| 00\_release\_manifests/script\_on\_request\_file\_manifest\_v01.csv **Section:** release\_manifest\_or\_codebook **Directory:** 00\_release\_manifests **Extension:** csv **Size KB:** 60.976 **Last modified:** 2026-05-03 00:26:37 **MD5:** 9c8a314ffb45aa3dfcab5ace607369b1 **Variables in codebook:** 0 | release\_manifest\_or\_codebook | csv | 60.976 | 0 |

## Public variable codebook

| Variable and definition | File | Unit | Type |
| --- | --- | --- | --- |
| age\_years\_first\_available — Age at first available public record **File:** 01\_primary\_data/public/esld\_master\_long\_public.csv **Recommended public name:** age\_years\_first\_available **Description:** Age in years at the first available public ESLD record for the patient or encounter represented in the released data. **Unit:** years **Value coding:** Integer age in years. **Data type:** integer **Example values:** 65 | 67 | 21 | 56 | 63 **Allowed values:**  **Missing-value coding:** NA/blank as read by fread **n rows / missing / unique:** 67399 / 0 / 79 **Release status:** public **Definition status:** manual\_exact\_definition | 01\_primary\_data/public/esld\_master\_long\_public.csv | years | integer |
| age\_years\_sample — Age at sample **File:** 01\_primary\_data/public/esld\_master\_long\_public.csv **Recommended public name:** age\_years\_sample **Description:** Age in years at the sample-level public ESLD record. **Unit:** years **Value coding:** Integer age in years. **Data type:** integer **Example values:** 65 | 66 | 67 | 68 | 69 **Allowed values:**  **Missing-value coding:** NA/blank as read by fread **n rows / missing / unique:** 67399 / 0 / 84 **Release status:** public **Definition status:** manual\_precision\_definition | 01\_primary\_data/public/esld\_master\_long\_public.csv | years | integer |
| albumin\_g\_dl — Serum albumin concentration **File:** 01\_primary\_data/public/esld\_master\_long\_public.csv **Recommended public name:** albumin\_g\_dl **Description:** Serum albumin concentration in the public ESLD master data. **Unit:** g/dL **Value coding:** Numeric concentration in g/dL. **Data type:** numeric **Example values:** 3.3 | 6.15 | 3.14 | 2.8 | 3.26 **Allowed values:**  **Missing-value coding:** NA/blank as read by fread **n rows / missing / unique:** 67399 / 58785 / 580 **Release status:** public **Definition status:** manual\_precision\_definition | 01\_primary\_data/public/esld\_master\_long\_public.csv | g/dL | numeric |
| ald — Alcohol-related liver disease etiology flag **File:** 01\_primary\_data/public/esld\_master\_long\_public.csv **Recommended public name:** ald **Description:** Binary etiology indicator for alcohol-related liver disease in the public ESLD cohort. **Unit:**  **Value coding:** 0 = no; 1 = yes. **Data type:** integer **Example values:** 0 | 1 **Allowed values:**  **Missing-value coding:** NA/blank as read by fread **n rows / missing / unique:** 67399 / 0 / 2 **Release status:** public **Definition status:** manual\_exact\_definition | 01\_primary\_data/public/esld\_master\_long\_public.csv |  | integer |
| ald\_hcv — Combined alcohol-related liver disease and hepatitis C etiology flag **File:** 01\_primary\_data/public/esld\_master\_long\_public.csv **Recommended public name:** ald\_hcv **Description:** Binary etiology indicator for combined alcohol-related liver disease and hepatitis C in the public ESLD cohort. **Unit:**  **Value coding:** 0 = no; 1 = yes. **Data type:** integer **Example values:** 0 | 1 **Allowed values:**  **Missing-value coding:** NA/blank as read by fread **n rows / missing / unique:** 67399 / 0 / 2 **Release status:** public **Definition status:** manual\_exact\_definition | 01\_primary\_data/public/esld\_master\_long\_public.csv |  | integer |
| autoimmune — Autoimmune liver disease etiology flag **File:** 01\_primary\_data/public/esld\_master\_long\_public.csv **Recommended public name:** autoimmune **Description:** Binary etiology indicator for autoimmune liver disease in the public ESLD cohort. **Unit:**  **Value coding:** 0 = no; 1 = yes. **Data type:** integer **Example values:** 0 | 1 **Allowed values:**  **Missing-value coding:** NA/blank as read by fread **n rows / missing / unique:** 67399 / 0 / 2 **Release status:** public **Definition status:** manual\_exact\_definition | 01\_primary\_data/public/esld\_master\_long\_public.csv |  | integer |
| crea — Creatinine concentration **File:** 01\_primary\_data/public/esld\_master\_long\_public.csv **Recommended public name:** crea **Description:** Creatinine concentration used in MELD-related score calculation before applying the study-specific correction. **Unit:** mg/dL **Value coding:** Numeric concentration in mg/dL. **Data type:** numeric **Example values:** 1.91 | 1.42 | 1.31 | 1.3 | 1.28 **Allowed values:**  **Missing-value coding:** NA/blank as read by fread **n rows / missing / unique:** 67399 / 0 / 975 **Release status:** public **Definition status:** manual\_exact\_definition | 01\_primary\_data/public/esld\_master\_long\_public.csv | mg/dL | numeric |
| crea\_corrected — Corrected creatinine concentration **File:** 01\_primary\_data/public/esld\_master\_long\_public.csv **Recommended public name:** crea\_corrected **Description:** Creatinine concentration after applying the study-specific correction used for recalculated MELD-related scores. **Unit:** mg/dL **Value coding:** Numeric concentration in mg/dL. **Data type:** numeric **Example values:** 1.87705004446668 | 1.41586432303417 | 1.31039363454933 | 1.30192482788903 | 1.28150549768982 **Allowed values:**  **Missing-value coding:** NA/blank as read by fread **n rows / missing / unique:** 67399 / 0 / 15868 **Release status:** public **Definition status:** manual\_exact\_definition | 01\_primary\_data/public/esld\_master\_long\_public.csv | mg/dL | numeric |
| date\_count — Relative day count **File:** 01\_primary\_data/public/esld\_master\_long\_public.csv **Recommended public name:** date\_count **Description:** Relative day count used for time alignment; negative/positive values are relative to the analysis anchor and are not calendar dates. **Unit:**  **Value coding:** See example\_values and allowed\_values. **Data type:** integer **Example values:** -3021 | -2833 | -2798 | -2793 | -2791 **Allowed values:**  **Missing-value coding:** NA/blank as read by fread **n rows / missing / unique:** 67399 / 50970 / 1450 **Release status:** public **Definition status:** exact\_definition | 01\_primary\_data/public/esld\_master\_long\_public.csv |  | integer |
| date\_count\_month — Relative month count **File:** 01\_primary\_data/public/esld\_master\_long\_public.csv **Recommended public name:** date\_count\_month **Description:** Relative month count derived from the relative day count; this is not a calendar month. **Unit:**  **Value coding:** See example\_values and allowed\_values. **Data type:** integer **Example values:** -99 | -93 | -92 | -91 | -87 **Allowed values:**  **Missing-value coding:** NA/blank as read by fread **n rows / missing / unique:** 67399 / 50970 / 147 **Release status:** public **Definition status:** exact\_definition | 01\_primary\_data/public/esld\_master\_long\_public.csv |  | integer |
| dead\_sample\_flag — Dead-sample flag **File:** 01\_primary\_data/public/esld\_master\_long\_public.csv **Recommended public name:** dead\_sample\_flag **Description:** Binary indicator identifying public ESLD sample records assigned to the deceased-sample subset used in sample-level analyses. **Unit:**  **Value coding:** 0 = not assigned to deceased-sample subset; 1 = assigned to deceased-sample subset. **Data type:** integer **Example values:** 0 | 1 **Allowed values:**  **Missing-value coding:** NA/blank as read by fread **n rows / missing / unique:** 67399 / 0 / 2 **Release status:** public **Definition status:** manual\_final\_definition | 01\_primary\_data/public/esld\_master\_long\_public.csv |  | integer |
| death\_within\_90d — Death within 90 days **File:** 01\_primary\_data/public/esld\_master\_long\_public.csv **Recommended public name:** death\_within\_90d **Description:** Binary indicator for death within 90 days of the analysis anchor. **Unit:**  **Value coding:** 0 = no; 1 = yes **Data type:** logical **Example values:**  **Allowed values:**  **Missing-value coding:** NA/blank as read by fread **n rows / missing / unique:** 67399 / 67399 / 0 **Release status:** public **Definition status:** exact\_definition | 01\_primary\_data/public/esld\_master\_long\_public.csv |  | logical |
| deceased\_patient\_flag — Deceased patient flag **File:** 01\_primary\_data/public/esld\_master\_long\_public.csv **Recommended public name:** deceased\_patient\_flag **Description:** Binary indicator identifying deceased patients within the released analysis context. **Unit:**  **Value coding:** 0 = no; 1 = yes **Data type:** integer **Example values:** 0 | 1 **Allowed values:**  **Missing-value coding:** NA/blank as read by fread **n rows / missing / unique:** 67399 / 0 / 2 **Release status:** public **Definition status:** exact\_definition | 01\_primary\_data/public/esld\_master\_long\_public.csv |  | integer |
| delta\_eq\_0\_flag — Zero score-delta flag **File:** 01\_primary\_data/public/esld\_master\_long\_public.csv **Recommended public name:** delta\_eq\_0\_flag **Description:** Binary indicator equal to 1 when the score delta is exactly zero. **Unit:**  **Value coding:** 0 = no; 1 = yes. **Data type:** integer **Example values:** 1 | 0 **Allowed values:**  **Missing-value coding:** NA/blank as read by fread **n rows / missing / unique:** 67399 / 0 / 2 **Release status:** public **Definition status:** manual\_precision\_definition | 01\_primary\_data/public/esld\_master\_long\_public.csv |  | integer |
| delta\_gt\_0\_flag — Positive score-delta flag **File:** 01\_primary\_data/public/esld\_master\_long\_public.csv **Recommended public name:** delta\_gt\_0\_flag **Description:** Binary indicator equal to 1 when the score delta is positive. **Unit:**  **Value coding:** 0 = no; 1 = yes. **Data type:** integer **Example values:** 0 | 1 **Allowed values:**  **Missing-value coding:** NA/blank as read by fread **n rows / missing / unique:** 67399 / 0 / 2 **Release status:** public **Definition status:** manual\_precision\_definition | 01\_primary\_data/public/esld\_master\_long\_public.csv |  | integer |
| delta\_le\_minus1\_flag — Score-delta ≤ −1 flag **File:** 01\_primary\_data/public/esld\_master\_long\_public.csv **Recommended public name:** delta\_le\_minus1\_flag **Description:** Binary indicator equal to 1 when the score delta is less than or equal to −1. **Unit:**  **Value coding:** 0 = no; 1 = yes. **Data type:** integer **Example values:** 0 | 1 **Allowed values:**  **Missing-value coding:** NA/blank as read by fread **n rows / missing / unique:** 67399 / 0 / 2 **Release status:** public **Definition status:** manual\_precision\_definition | 01\_primary\_data/public/esld\_master\_long\_public.csv |  | integer |
| dialysis\_raw — Dialysis indicator as recorded before score derivation **File:** 01\_primary\_data/public/esld\_master\_long\_public.csv **Recommended public name:** dialysis\_raw **Description:** Binary indicator showing whether dialysis was recorded in the source field before score derivation. **Unit:**  **Value coding:** 0 = no dialysis recorded; 1 = dialysis recorded. **Data type:** integer **Example values:** 0 | 1 **Allowed values:**  **Missing-value coding:** NA/blank as read by fread **n rows / missing / unique:** 67399 / 27827 / 2 **Release status:** public **Definition status:** manual\_exact\_definition | 01\_primary\_data/public/esld\_master\_long\_public.csv |  | integer |
| encounter\_id\_public — Public encounter pseudonym **File:** 01\_primary\_data/public/esld\_master\_long\_public.csv **Recommended public name:** encounter\_id\_public **Description:** Non-linkable public-release encounter pseudonym used for encounter-level grouping; not an original hospital encounter identifier. **Unit:**  **Value coding:** See example\_values and allowed\_values. **Data type:** character **Example values:** E001181 | E000008 | E000041 | E000100 | E000126 **Allowed values:** E001181 | E000008 | E000041 | E000100 | E000126 | E000151 | E000157 | E000219 | E000289 | E000344 **Missing-value coding:** NA/blank as read by fread **n rows / missing / unique:** 67399 / 0 / 5045 **Release status:** public **Definition status:** exact\_definition | 01\_primary\_data/public/esld\_master\_long\_public.csv |  | character |
| etiology\_source\_current\_flag — Current etiology source flag **File:** 01\_primary\_data/public/esld\_master\_long\_public.csv **Recommended public name:** etiology\_source\_current\_flag **Description:** Binary indicator identifying whether the etiology source is the current source used for the public row. **Unit:**  **Value coding:** 0 = no; 1 = yes. **Data type:** integer **Example values:** 1 **Allowed values:**  **Missing-value coding:** NA/blank as read by fread **n rows / missing / unique:** 67399 / 0 / 1 **Release status:** public **Definition status:** manual\_precision\_definition | 01\_primary\_data/public/esld\_master\_long\_public.csv |  | integer |
| etiology\_unclassified — Unclassified liver disease etiology flag **File:** 01\_primary\_data/public/esld\_master\_long\_public.csv **Recommended public name:** etiology\_unclassified **Description:** Binary etiology indicator identifying records without a classified liver disease etiology in the public ESLD cohort. **Unit:**  **Value coding:** 0 = no; 1 = yes. **Data type:** integer **Example values:** 0 **Allowed values:**  **Missing-value coding:** NA/blank as read by fread **n rows / missing / unique:** 67399 / 0 / 1 **Release status:** public **Definition status:** manual\_exact\_definition | 01\_primary\_data/public/esld\_master\_long\_public.csv |  | integer |
| hbv — Hepatitis B virus etiology flag **File:** 01\_primary\_data/public/esld\_master\_long\_public.csv **Recommended public name:** hbv **Description:** Binary etiology indicator for hepatitis B virus-related liver disease in the public ESLD cohort. **Unit:**  **Value coding:** 0 = no; 1 = yes. **Data type:** integer **Example values:** 0 | 1 **Allowed values:**  **Missing-value coding:** NA/blank as read by fread **n rows / missing / unique:** 67399 / 0 / 2 **Release status:** public **Definition status:** manual\_exact\_definition | 01\_primary\_data/public/esld\_master\_long\_public.csv |  | integer |
| hcv — Hepatitis C virus etiology flag **File:** 01\_primary\_data/public/esld\_master\_long\_public.csv **Recommended public name:** hcv **Description:** Binary etiology indicator for hepatitis C virus-related liver disease in the public ESLD cohort. **Unit:**  **Value coding:** 0 = no; 1 = yes. **Data type:** integer **Example values:** 0 | 1 **Allowed values:**  **Missing-value coding:** NA/blank as read by fread **n rows / missing / unique:** 67399 / 0 / 2 **Release status:** public **Definition status:** manual\_exact\_definition | 01\_primary\_data/public/esld\_master\_long\_public.csv |  | integer |
| icd\_source\_present — ICD source present flag **File:** 01\_primary\_data/public/esld\_master\_long\_public.csv **Recommended public name:** icd\_source\_present **Description:** Binary indicator showing whether an ICD-derived etiology source was present for the public row. **Unit:**  **Value coding:** 0 = no; 1 = yes. **Data type:** integer **Example values:** 1 | 0 **Allowed values:**  **Missing-value coding:** NA/blank as read by fread **n rows / missing / unique:** 67399 / 0 / 2 **Release status:** public **Definition status:** manual\_precision\_definition | 01\_primary\_data/public/esld\_master\_long\_public.csv |  | integer |
| in\_t1\_cohort — Inclusion flag for Table 1 cohort **File:** 01\_primary\_data/public/esld\_master\_long\_public.csv **Recommended public name:** in\_t1\_cohort **Description:** Binary indicator identifying records included in the public ESLD Table 1 baseline-characteristics cohort. **Unit:**  **Value coding:** 0 = not included; 1 = included. **Data type:** integer **Example values:** 1 **Allowed values:**  **Missing-value coding:** NA/blank as read by fread **n rows / missing / unique:** 67399 / 0 / 1 **Release status:** public **Definition status:** manual\_exact\_definition | 01\_primary\_data/public/esld\_master\_long\_public.csv |  | integer |
| in\_t3\_anchor\_model — Inclusion flag for Table 3 anchor-model cohort **File:** 01\_primary\_data/public/esld\_master\_long\_public.csv **Recommended public name:** in\_t3\_anchor\_model **Description:** Binary indicator identifying records included in the Table 3 anchor-model cohort used for creatinine-comparison analyses. **Unit:**  **Value coding:** 0 = not included; 1 = included. **Data type:** integer **Example values:** 1 | 0 **Allowed values:**  **Missing-value coding:** NA/blank as read by fread **n rows / missing / unique:** 67399 / 0 / 2 **Release status:** public **Definition status:** manual\_exact\_definition | 01\_primary\_data/public/esld\_master\_long\_public.csv |  | integer |
| in\_t4\_prevalence\_path — Inclusion flag for Table 4 prevalence pathway **File:** 01\_primary\_data/public/esld\_master\_long\_public.csv **Recommended public name:** in\_t4\_prevalence\_path **Description:** Binary indicator identifying records included in the Table 4 prevalence-analysis pathway. **Unit:**  **Value coding:** 0 = not included; 1 = included. **Data type:** integer **Example values:** 1 **Allowed values:**  **Missing-value coding:** NA/blank as read by fread **n rows / missing / unique:** 67399 / 0 / 1 **Release status:** public **Definition status:** manual\_exact\_definition | 01\_primary\_data/public/esld\_master\_long\_public.csv |  | integer |
| in\_t4\_survival\_path — Inclusion flag for Table 4 survival pathway **File:** 01\_primary\_data/public/esld\_master\_long\_public.csv **Recommended public name:** in\_t4\_survival\_path **Description:** Binary indicator identifying records included in the Table 4 survival-analysis pathway. **Unit:**  **Value coding:** 0 = not included; 1 = included. **Data type:** integer **Example values:** 0 | 1 **Allowed values:**  **Missing-value coding:** NA/blank as read by fread **n rows / missing / unique:** 67399 / 0 / 2 **Release status:** public **Definition status:** manual\_exact\_definition | 01\_primary\_data/public/esld\_master\_long\_public.csv |  | integer |
| inr — International normalized ratio **File:** 01\_primary\_data/public/esld\_master\_long\_public.csv **Recommended public name:** inr **Description:** International normalized ratio of prothrombin time used for MELD-related score calculation. **Unit:** ratio **Value coding:** Numeric INR value. **Data type:** numeric **Example values:** 1.14 | 0.91 | 1 | 1.04 | 0.98 **Allowed values:**  **Missing-value coding:** NA/blank as read by fread **n rows / missing / unique:** 67399 / 0 / 786 **Release status:** public **Definition status:** manual\_exact\_definition | 01\_primary\_data/public/esld\_master\_long\_public.csv | ratio | numeric |
| mash — Metabolic dysfunction-associated steatohepatitis etiology flag **File:** 01\_primary\_data/public/esld\_master\_long\_public.csv **Recommended public name:** mash **Description:** Binary etiology indicator for metabolic dysfunction-associated steatohepatitis in the public ESLD cohort. **Unit:**  **Value coding:** 0 = no; 1 = yes. **Data type:** integer **Example values:** 0 | 1 **Allowed values:**  **Missing-value coding:** NA/blank as read by fread **n rows / missing / unique:** 67399 / 0 / 2 **Release status:** public **Definition status:** manual\_exact\_definition | 01\_primary\_data/public/esld\_master\_long\_public.csv |  | integer |
| model — MELD model or score variant **File:** 01\_primary\_data/public/esld\_master\_long\_public.csv **Recommended public name:** model **Description:** Name of the MELD-related model or score variant represented by the row; expected values include MELD, MELD-Na, reMELD-Na, and MELD 3.0. **Unit:**  **Value coding:** Categorical score/model label, for example MELD, MELD-Na, reMELD-Na, or MELD 3.0. **Data type:** character **Example values:** MELD | MELD-Na | reMELD-Na | MELD 3.0 **Allowed values:** MELD | MELD-Na | reMELD-Na | MELD 3.0 **Missing-value coding:** NA/blank as read by fread **n rows / missing / unique:** 67399 / 0 / 4 **Release status:** public **Definition status:** manual\_exact\_definition | 01\_primary\_data/public/esld\_master\_long\_public.csv |  | character |
| other — Other liver disease etiology flag **File:** 01\_primary\_data/public/esld\_master\_long\_public.csv **Recommended public name:** other **Description:** Binary etiology indicator for liver disease etiologies grouped as other in the public ESLD cohort. **Unit:**  **Value coding:** 0 = no; 1 = yes. **Data type:** integer **Example values:** 1 | 0 **Allowed values:**  **Missing-value coding:** NA/blank as read by fread **n rows / missing / unique:** 67399 / 0 / 2 **Release status:** public **Definition status:** manual\_exact\_definition | 01\_primary\_data/public/esld\_master\_long\_public.csv |  | integer |
| patient\_id — Public patient pseudonym **File:** 01\_primary\_data/public/esld\_master\_long\_public.csv **Recommended public name:** patient\_id **Description:** Non-linkable public-release patient pseudonym used to preserve within-patient grouping in public ESLD data; not an original hospital patient identifier. **Unit:**  **Value coding:** See example\_values and allowed\_values. **Data type:** character **Example values:** P000001 | P000002 | P000003 | P000004 | P000005 **Allowed values:** P000001 | P000002 | P000003 | P000004 | P000005 | P000006 | P000007 | P000008 | P000009 | P000010 **Missing-value coding:** NA/blank as read by fread **n rows / missing / unique:** 67399 / 0 / 1375 **Release status:** public **Definition status:** exact\_definition | 01\_primary\_data/public/esld\_master\_long\_public.csv |  | character |
| pbc — Primary biliary cholangitis etiology flag **File:** 01\_primary\_data/public/esld\_master\_long\_public.csv **Recommended public name:** pbc **Description:** Binary etiology indicator for primary biliary cholangitis in the public ESLD cohort. **Unit:**  **Value coding:** 0 = no; 1 = yes. **Data type:** integer **Example values:** 0 | 1 **Allowed values:**  **Missing-value coding:** NA/blank as read by fread **n rows / missing / unique:** 67399 / 0 / 2 **Release status:** public **Definition status:** manual\_exact\_definition | 01\_primary\_data/public/esld\_master\_long\_public.csv |  | integer |
| sample\_day\_from\_first\_sample — Relative sample day **File:** 01\_primary\_data/public/esld\_master\_long\_public.csv **Recommended public name:** sample\_day\_from\_first\_sample **Description:** Relative day of the sample measured from the first sample for the public patient/sample sequence; not a calendar date. **Unit:** relative months **Value coding:** Integer or numeric relative day count. **Data type:** integer **Example values:** 0 | 42 | 91 | 118 | 133 **Allowed values:**  **Missing-value coding:** NA/blank as read by fread **n rows / missing / unique:** 67399 / 0 / 3438 **Release status:** public **Definition status:** manual\_precision\_definition | 01\_primary\_data/public/esld\_master\_long\_public.csv | relative months | integer |
| sample\_group\_id\_public — Public sample-group pseudonym **File:** 01\_primary\_data/public/esld\_master\_long\_public.csv **Recommended public name:** sample\_group\_id\_public **Description:** Non-linkable public-release sample-group pseudonym used to group samples within the released public data. **Unit:**  **Value coding:** See example\_values and allowed\_values. **Data type:** character **Example values:** G0000001 | G0000002 | G0000003 | G0000004 | G0000005 **Allowed values:** G0000001 | G0000002 | G0000003 | G0000004 | G0000005 | G0000006 | G0000007 | G0000008 | G0000009 | G0000010 **Missing-value coding:** NA/blank as read by fread **n rows / missing / unique:** 67399 / 0 / 20359 **Release status:** public **Definition status:** exact\_definition | 01\_primary\_data/public/esld\_master\_long\_public.csv |  | character |
| sample\_id — Sample identifier **File:** 01\_primary\_data/public/esld\_master\_long\_public.csv **Recommended public name:** sample\_id **Description:** Identifier of a sample or experimental record within the released public data; not a personal identifier. **Unit:**  **Value coding:** See example\_values and allowed\_values. **Data type:** character **Example values:** S0000001 | S0000002 | S0000003 | S0000004 | S0000005 **Allowed values:** S0000001 | S0000002 | S0000003 | S0000004 | S0000005 | S0000006 | S0000007 | S0000008 | S0000009 | S0000010 **Missing-value coding:** NA/blank as read by fread **n rows / missing / unique:** 67399 / 0 / 67399 **Release status:** public **Definition status:** exact\_definition | 01\_primary\_data/public/esld\_master\_long\_public.csv |  | character |
| sample\_month\_index — Relative sample month index **File:** 01\_primary\_data/public/esld\_master\_long\_public.csv **Recommended public name:** sample\_month\_index **Description:** Relative month index of the sample measured from the first sample for the public patient/sample sequence; not a calendar month. **Unit:** score points **Value coding:** Integer or numeric relative month index. **Data type:** integer **Example values:** 0 | 1 | 2 | 3 | 4 **Allowed values:**  **Missing-value coding:** NA/blank as read by fread **n rows / missing / unique:** 67399 / 0 / 177 **Release status:** public **Definition status:** manual\_precision\_definition | 01\_primary\_data/public/esld\_master\_long\_public.csv | score points | integer |
| score\_corrected — Corrected score **File:** 01\_primary\_data/public/esld\_master\_long\_public.csv **Recommended public name:** score\_corrected **Description:** MELD-related score after creatinine correction or recalculation. **Unit:** score points **Value coding:** Numeric score. **Data type:** integer **Example values:** 14 | 19 | 16 | 10 | 13 **Allowed values:**  **Missing-value coding:** NA/blank as read by fread **n rows / missing / unique:** 67399 / 0 / 40 **Release status:** public **Definition status:** manual\_precision\_definition | 01\_primary\_data/public/esld\_master\_long\_public.csv | score points | integer |
| score\_delta — Score difference **File:** 01\_primary\_data/public/esld\_master\_long\_public.csv **Recommended public name:** score\_delta **Description:** Difference between score variants or scoring approaches, expressed in score points. **Unit:**  **Value coding:** See example\_values and allowed\_values. **Data type:** integer **Example values:** 0 | 1 | -1 | -2 **Allowed values:**  **Missing-value coding:** NA/blank as read by fread **n rows / missing / unique:** 67399 / 0 / 4 **Release status:** public **Definition status:** exact\_definition | 01\_primary\_data/public/esld\_master\_long\_public.csv |  | integer |
| score\_original — Original score **File:** 01\_primary\_data/public/esld\_master\_long\_public.csv **Recommended public name:** score\_original **Description:** Original MELD-related score before creatinine correction or recalculation. **Unit:**  **Value coding:** Numeric score. **Data type:** integer **Example values:** 14 | 19 | 16 | 10 | 13 **Allowed values:**  **Missing-value coding:** NA/blank as read by fread **n rows / missing / unique:** 67399 / 0 / 40 **Release status:** public **Definition status:** manual\_precision\_definition | 01\_primary\_data/public/esld\_master\_long\_public.csv |  | integer |
| sex — Sex **File:** 01\_primary\_data/public/esld\_master\_long\_public.csv **Recommended public name:** sex **Description:** Sex category represented in the public ESLD data. **Unit:**  **Value coding:** M = male; F = female. **Data type:** character **Example values:** M | F **Allowed values:** M | F **Missing-value coding:** NA/blank as read by fread **n rows / missing / unique:** 67399 / 0 / 2 **Release status:** public **Definition status:** manual\_exact\_definition | 01\_primary\_data/public/esld\_master\_long\_public.csv |  | character |
| sodium — Serum sodium concentration **File:** 01\_primary\_data/public/esld\_master\_long\_public.csv **Recommended public name:** sodium **Description:** Serum sodium concentration used for MELD-Na, reMELD-Na, or MELD 3.0 score calculation. **Unit:** mmol/L **Value coding:** Numeric concentration in mmol/L. **Data type:** numeric **Example values:** 131 | 134 | 132 | 133 | 140 **Allowed values:**  **Missing-value coding:** NA/blank as read by fread **n rows / missing / unique:** 67399 / 20359 / 188 **Release status:** public **Definition status:** manual\_exact\_definition | 01\_primary\_data/public/esld\_master\_long\_public.csv | mmol/L | numeric |
| tbil — Total bilirubin concentration **File:** 01\_primary\_data/public/esld\_master\_long\_public.csv **Recommended public name:** tbil **Description:** Total bilirubin concentration used in MELD-related score calculation in the public ESLD data. **Unit:**  **Value coding:** Numeric concentration in mg/dL. **Data type:** numeric **Example values:** 1 | 0.9 | 1.1 | 0.8 | 0.6 **Allowed values:**  **Missing-value coding:** NA/blank as read by fread **n rows / missing / unique:** 67399 / 0 / 2029 **Release status:** public **Definition status:** manual\_precision\_definition | 01\_primary\_data/public/esld\_master\_long\_public.csv |  | numeric |
| parameter — Metadata parameter name **File:** 01\_primary\_data/public/f1\_simulated\_surface\_metadata.csv **Recommended public name:** parameter **Description:** Name of a metadata parameter describing the F2 simulated heatmap object, such as figure identity, data origin, grid type, axis variable, or unit/role. **Unit:**  **Value coding:** Categorical metadata key; value is given in the corresponding value column or file-specific metadata field. **Data type:** character **Example values:** dataset\_name | cohort | data\_type | """"""""""""""""""""""""""""""""""""""""""""""""""""""""""""""""""""""""""""""""""""""""""""""""""""""""""""""""""""""""""""""""""""""""""""""""""""""""""""""""""""""""""""""""""""""""""""""""""""""""""""""""""""""""""""""""""""""""""""""""""""""""""""""""""""""""""""""""""""""""""""""""""""""""""""""""""""""""""""""""""""""""""""""""""""""""""""""""""""""""""""""""""""""""""""""""""""""""""""""""""""""""""""""""""""""""""""""""""""""""""""""""""""""""""""""""""""""""""""""""""""""""""""""""""""""""""""""""""""""""""""""""""""""""""""""""""""""""""""""""""""""""""""""""""""""""""""""""""""""""""""""""""""""""""""""""""""""""""""""""""""""""""""""""""""""""""""""""""""""""""""""""""""""""""""""""""""""""""""""""""""""""""""""""""""""""""""""""""""""""""""""""""""""""""""""""""""""""""""""""""""""""""""""""""""""""""""""""""""""""""""""""""""""""""""""""""""""""""""""""""""""""""""""""""""""""""""""""""""""""""""""""""""""""""""""""""""""""""""""""""""""""""""""""""""""""""""""""""""""""""""""""""""""""""""""""""""""""""""""""""""""""""""""""""""""""""""""""""""""""""""""""""""""""""""""""""""""""""""""""""""""""""""""""""""""""""""""""""""""""""""""""""""""""""""""""""""""""""""""""""""""""""""""""""""""""""""""""""""""""""""""""""""""""""""""""""""""""""""""""""""""""""""""""""""""""""""""""""""""""""""""""""""""""""""""""""""""""""""""""""""""""""""""""""""""""""""""""""""""""""""""""""""""""""""""""""""""""""""""""""""""""""""""""""""""""""""""""""""""""""""""""""""""""""""""""""""""""""""""""""""""""""""""""""""""""""""""""""""""""""""""""""""""""""""""""""""""""""""""""""""""""""""""""""""""""""""""""""""""""""""""""""""""""""""""""""""""""""""""""""""""""""""""""""""""""""""""""""""""""""""""""""""""""""""""""""""""""""""""""""""""""""""""""""""""""""""""""""""""""""""""""""""""""""""""""""""""""""""""""""""""""""""""""""""""""""""""""""""""""""""""""""""""""""""""""""""""""""""""""""""""""""""""""""""""""""""""""""""""""""""""""""""""""""""""""""""""""""""""""""""""""""""""""""""""""""""""""""""""""""""""""""""""""""""""""""""""""""""""""""""""""""""""""""""""""""""""""""""""""""""""""""""""""""""""""""""""""""""""""""""""""""""""""""""""""""""""""""""""""""""""""""""""""""""""""""""""""""""""""""""""""""""""""""""""""""""""""""""""""""""""""""""""""""""""""""""""""""""""""""""""""""""""""""""""""""""""""""""""""""""""""""""""""""""""""""""""""""""""""""""""""""""""""""""""""""""""""""""""""""""""""""""""""""""""""""""""""""""""""""""""""""""""""""""""""""""""""""""""""""""""""""""""""""""""""""""""""""""""""""""""""""""""""""""""""""""""""""""""""""""""""""""""""""""""""""""""""""""""""""""""""""""""""""""""""""""""""""""""""""""""""""""""""""""""""""""""""""""""""""""""""""""""""""""""""""""""""""""""""""""""""""""""""""""""""""""""""""""""""""""""""""""""""""""""""""""""""""""""""""""""""""""""""""""""""""""""""""""""""""""""""""""""""""""""""""""""""""""""""""""""""""""""""""""""""""""""""""""""""""""""""""""""""""""""""""""""""""""""""""""""""""""""""""""""""""""""""""""""""""""""""""""""""""""""""""""""""""""""""""""""""""""""""""""""""""""""""""""""""""""""""""""""""""""""""""""""""""""""""""""""""""""""""""""""""""""""""""""""""""""""""""""""""""""""""""""""""""""""""""""""""""""""""""""""""""""""""""""""""""""""""""""""""""""""""""""""""""""""""""""""""""""""""""""""""""""""""""""""""""""""""""""""""""""""""""""""""""""""""""""""""""""""""""""""""""""""""""""""""""""""""""""""""""""""""""""""""""""""""""""""""""""""""""""""""""""""""""""""""""""""""""""""""""""""""""""""""""""""""""""""""""""""""""""""""""""""""""""""""""""""""""""""""""""""""""""""""""""""""""""""""""""""""""""""""""""""""""""""""""""""""""""""""""""""""""""""""""""""""""""""""""""""""""""""""""""""""""""""""""""""""""""""""""""""""""""""""""""""""""""""""""""""""""""""""""""""""""""""""""""""""""""""""""""""""""""""""""""""""""""""""""""""""""""""""""""""""""""""""""""""""""""""""""""""""""""""""""""""""""""""""""""""""""""""""""""""""""""""""""""""""""""""""""""""""""""""""""""""""""""""""""""""""""""""""""""""""""""""""""""""""""""""""row\_definition | tb\_range\_mg\_dL **Allowed values:** dataset\_name | cohort | data\_type | """"""""""""""""""""""""""""""""""""""""""""""""""""""""""""""""""""""""""""""""""""""""""""""""""""""""""""""""""""""""""""""""""""""""""""""""""""""""""""""""""""""""""""""""""""""""""""""""""""""""""""""""""""""""""""""""""""""""""""""""""""""""""""""""""""""""""""""""""""""""""""""""""""""""""""""""""""""""""""""""""""""""""""""""""""""""""""""""""""""""""""""""""""""""""""""""""""""""""""""""""""""""""""""""""""""""""""""""""""""""""""""""""""""""""""""""""""""""""""""""""""""""""""""""""""""""""""""""""""""""""""""""""""""""""""""""""""""""""""""""""""""""""""""""""""""""""""""""""""""""""""""""""""""""""""""""""""""""""""""""""""""""""""""""""""""""""""""""""""""""""""""""""""""""""""""""""""""""""""""""""""""""""""""""""""""""""""""""""""""""""""""""""""""""""""""""""""""""""""""""""""""""""""""""""""""""""""""""""""""""""""""""""""""""""""""""""""""""""""""""""""""""""""""""""""""""""""""""""""""""""""""""""""""""""""""""""""""""""""""""""""""""""""""""""""""""""""""""""""""""""""""""""""""""""""""""""""""""""""""""""""""""""""""""""""""""""""""""""""""""""""""""""""""""""""""""""""""""""""""""""""""""""""""""""""""""""""""""""""""""""""""""""""""""""""""""""""""""""""""""""""""""""""""""""""""""""""""""""""""""""""""""""""""""""""""""""""""""""""""""""""""""""""""""""""""""""""""""""""""""""""""""""""""""""""""""""""""""""""""""""""""""""""""""""""""""""""""""""""""""""""""""""""""""""""""""""""""""""""""""""""""""""""""""""""""""""""""""""""""""""""""""""""""""""""""""""""""""""""""""""""""""""""""""""""""""""""""""""""""""""""""""""""""""""""""""""""""""""""""""""""""""""""""""""""""""""""""""""""""""""""""""""""""""""""""""""""""""""""""""""""""""""""""""""""""""""""""""""""""""""""""""""""""""""""""""""""""""""""""""""""""""""""""""""""""""""""""""""""""""""""""""""""""""""""""""""""""""""""""""""""""""""""""""""""""""""""""""""""""""""""""""""""""""""""""""""""""""""""""""""""""""""""""""""""""""""""""""""""""""""""""""""""""""""""""""""""""""""""""""""""""""""""""""""""""""""""""""""""""""""""""""""""""""""""""""""""""""""""""""""""""""""""""""""""""""""""""""""""""""""""""""""""""""""""""""""""""""""""""""""""""""""""""""""""""""""""""""""""""""""""""""""""""""""""""""""""""""""""""""""""""""""""""""""""""""""""""""""""""""""""""""""""""""""""""""""""""""""""""""""""""""""""""""""""""""""""""""""""""""""""""""""""""""""""""""""""""""""""""""""""""""""""""""""""""""""""""""""""""""""""""""""""""""""""""""""""""""""""""""""""""""""""""""""""""""""""""""""""""""""""""""""""""""""""""""""""""""""""""""""""""""""""""""""""""""""""""""""""""""""""""""""""""""""""""""""""""""""""""""""""""""""""""""""""""""""""""""""""""""""""""""""""""""""""""""""""""""""""""""""""""""""""""""""""""""""""""""""""""""""""""""""""""""""""""""""""""""""""""""""""""""""""""""""""""""""""""""""""""""""""""""""""""""""""""""""""""""""""""""""""""""""""""""""""""""""""""""""""""""""""""""""""""""""""""""""""""""""""""""""""""""""""""""""""""""""""""""""""""""""""""""""""""""""""""""""""""""""""""""""""""""""""""""""""""""""""""""""""""""""""""""""""""""""""""""""""""""""""""""""""""""""""""""""""""""""""""""""""""""""""""""""""""""""""""""""""""""""""""""""""""""""""""""""""""""""""""""""""""""""""""""""""""""""""""""""""""""""""""""""""""""""""""""""""""""""""""""""""""""""""""""""""""""""""""""""""""""""""""""""""""""""""""""""""""""""""""""""""""""""""""""""""""""""""""""""""""""""""""""""""""""""""""""""""""""""""""""""""""""""""""""""""""""""""""""""""""""""""""""""""""""""""""""""""""""""""""""""""""""""""""""""""""""""""""""""""""""""""""""""""""""""""""""""""""""""""""""""""""""""""""""""""""""""""""""""""""""""""""""""""""""""""""""""""""""""""""""""""""""""""""""""""""""""""""""""""""""""""""""""""""""""""""""""""""""""""""""""""""""""""""""""""""""""""""""""""""""""""""""""""""""""""""""""""""""""""""""""""""""""""""""""""""""""""""""""""""""""""""""""""""""""""""""""""""""""""""""""""""""""""""""""""""""""""""""""""""""""""""""""""""""""""""""""""""""""""""""""""""""""""""""""""""""""""""""""""""""""""""""""""""""""""""""row\_definition | tb\_range\_mg\_dL | cre\_true\_range\_mg\_dL | tb\_step\_mg\_dL | cre\_step\_mg\_dL | delta\_definition **Missing-value coding:** NA/blank as read by fread **n rows / missing / unique:** 9 / 0 / 9 **Release status:** public **Definition status:** manual\_exact\_definition | 01\_primary\_data/public/f1\_simulated\_surface\_metadata.csv |  | character |
| value — Value **File:** 01\_primary\_data/public/f1\_simulated\_surface\_metadata.csv **Recommended public name:** value **Description:** Numerical or character value corresponding to the row-specific variable/metric. **Unit:**  **Value coding:** See example\_values and allowed\_values. **Data type:** character **Example values:** f1\_simulated\_surface\_repository | SIMULATED | in\_silico surface grid | One row = one equation-defined grid point (tb\_mg\_... **Allowed values:** f1\_simulated\_surface\_repository | SIMULATED | in\_silico surface grid | One row = one equation-defined grid point (tb\_mg\_dL, cre\_true\_mg\_dL)"""""""""""""""""""""""""""""""""""""""""""""""""""""""""""""""""""""""""""""""""""""""""""""""""""""""""""""""""""""""""""""""""""""""""""""""""""""""""""""""""""""""""""""""""""""""""""""""""""""""""""""""""""""""""""""""""""""""""""""""""""""""""""""""""""""""""""""""""""""""""""""""""""""""""""""""""""""""""""""""""""""""""""""""""""""""""""""""""""""""""""""""""""""""""""""""""""""""""""""""""""""""""""""""""""""""""""""""""""""""""""""""""""""""""""""""""""""""""""""""""""""""""""""""""""""""""""""""""""""""""""""""""""""""""""""""""""""""""""""""""""""""""""""""""""""""""""""""""""""""""""""""""""""""""""""""""""""""""""""""""""""""""""""""""""""""""""""""""""""""""""""""""""""""""""""""""""""""""""""""""""""""""""""""""""""""""""""""""""""""""""""""""""""""""""""""""""""""""""""""""""""""""""""""""""""""""""""""""""""""""""""""""""""""""""""""""""""""""""""""""""""""""""""""""""""""""""""""""""""""""""""""""""""""""""""""""""""""""""""""""""""""""""""""""""""""""""""""""""""""""""""""""""""""""""""""""""""""""""""""""""""""""""""""""""""""""""""""""""""""""""""""""""""""""""""""""""""""""""""""""""""""""""""""""""""""""""""""""""""""""""""""""""""""""""""""""""""""""""""""""""""""""""""""""""""""""""""""""""""""""""""""""""""""""""""""""""""""""""""""""""""""""""""""""""""""""""""""""""""""""""""""""""""""""""""""""""""""""""""""""""""""""""""""""""""""""""""""""""""""""""""""""""""""""""""""""""""""""""""""""""""""""""""""""""""""""""""""""""""""""""""""""""""""""""""""""""""""""""""""""""""""""""""""""""""""""""""""""""""""""""""""""""""""""""""""""""""""""""""""""""""""""""""""""""""""""""""""""""""""""""""""""""""""""""""""""""""""""""""""""""""""""""""""""""""""""""""""""""""""""""""""""""""""""""""""""""""""""""""""""""""""""""""""""""""""""""""""""""""""""""""""""""""""""""""""""""""""""""""""""""""""""""""""""""""""""""""""""""""""""""""""""""""""""""""""""""""""""""""""""""""""""""""""""""""""""""""""""""""""""""""""""""""""""""""""""""""""""""""""""""""""""""""""""""""""""""""""""""""""""""""""""""""""""""""""""""""""""""""""""""""""""""""""""""""""""""""""""""""""""""""""""""""""""""""""""""""""""""""""""""""""""""""""""""""""""""""""""""""""""""""""""""""""""""""""""""""""""""""""""""""""""""""""""""""""""""""""""""""""""""""""""""""""""""""""""""""""""""""""""""""""""""""""""""""""""""""""""""""""""""""""""""""""""""""""""""""""""""""""""""""""""""""""""""""""""""""""""""""""""""""""""""""""""""""""""""""""""""""""""""""""""""""""""""""""""""""""""""""""""""""""""""""""""""""""""""""""""""""""""""""""""""""""""""""""""""""""""""""""""""""""""""""""""""""""""""""""""""""""""""""""""""""""""""""""""""""""""""""""""""""""""""""""""""""""""""""""""""""""""""""""""""""""""""""""""""""""""""""""""""""""""""""""""""""""""""""""""""""""""""""""""""""""""""""""""""""""""""""""""""""""""""""""""""""""""""""""""""""""""""""""""""""""""""""""""""""""""""""""""""""""""""""""""""""""""""""""""""""""""""""""""""""""""""""""""""""""""""""""""""""""""""""""""""""""""""""""""""""""""""""""""""""""""""""""""""""""""""""""""""""""""""""""""""""""""""""""""""""""""""""""""""""""""""""""""""""""""""""""""""""""""""""""""""""""""""""""""""""""""""""""""""""""""""""""""""""""""""""""""""""""""""""""""""""""""""""""""""""""""""""""""""""""""""""""""""""""""""""""""""""""""""""""""""""""""""""""""""""""""""""""""""""""""""""""""""""""""""""""""""""""""""""""""""""""""""""""""""""""""""""""""""""""""""""""""""""""""""""""""""""""""""""""""""""""""""""""""""""""""""""""""""""""""""""""""""""""""""""""""""""""""""""""""""""""""""""""""""""""""""""""""""""""""""""""""""""""""""""""""""""""""""""""""""""""""""""""""""""""""""""""""""""""""""""""""""""""""""""""""""""""""""""""""""""""""""""""""""""""""""""""""""""""""""""""""""""""""""""""""""""""""""""""""""""""""""""""""""""""""""""""""""""""""""""""""""""""""""""""""""""""""""""""""""""""""""""""""""""""""""""""""""""""""""""""""""""""""""""""""""""""""""""""""""""""""""""""""""""""""""""""""""""""""""""""""""""""""""""""""""""""""""""""""""""""""""""""""""""""""""""""""""""""" | 1 to 35 | 1 to 6 | 0.1 | 0.05 | delta = cre\_true\_mg\_dL - predicted measured creatinine **Missing-value coding:** NA/blank as read by fread **n rows / missing / unique:** 9 / 0 / 9 **Release status:** public **Definition status:** exact\_definition | 01\_primary\_data/public/f1\_simulated\_surface\_metadata.csv |  | character |
| cre\_true\_mg\_dL — True creatinine concentration **File:** 01\_primary\_data/public/f1\_simulated\_surface\_repository.csv **Recommended public name:** cre\_true\_mg\_dL **Description:** True creatinine concentration used as the gravimetric/reference concentration in F1. **Unit:**  **Value coding:** See example\_values and allowed\_values. **Data type:** numeric **Example values:** 1 | 1.05 | 1.1 | 1.15 | 1.2 **Allowed values:**  **Missing-value coding:** NA/blank as read by fread **n rows / missing / unique:** 34441 / 0 / 101 **Release status:** public **Definition status:** exact\_definition | 01\_primary\_data/public/f1\_simulated\_surface\_repository.csv |  | numeric |
| cree\_measured\_pred\_mg\_dL — Predicted measured enzymatic creatinine concentration **File:** 01\_primary\_data/public/f1\_simulated\_surface\_repository.csv **Recommended public name:** cree\_measured\_pred\_mg\_dL **Description:** Predicted measured enzymatic creatinine concentration derived from the interference model. **Unit:**  **Value coding:** See example\_values and allowed\_values. **Data type:** numeric **Example values:** 0.902064914425904 | 0.957189139835594 | 1.01231537541398 | 1.06744362138108 | 1.12257387795652 **Allowed values:**  **Missing-value coding:** NA/blank as read by fread **n rows / missing / unique:** 34441 / 0 / 34441 **Release status:** public **Definition status:** exact\_definition | 01\_primary\_data/public/f1\_simulated\_surface\_repository.csv |  | numeric |
| cree\_true\_recalc\_error — Recalculation error for enzymatic creatinine **File:** 01\_primary\_data/public/f1\_simulated\_surface\_repository.csv **Recommended public name:** cree\_true\_recalc\_error **Description:** Numerical difference between recalculated and expected true enzymatic creatinine values. **Unit:**  **Value coding:** See example\_values and allowed\_values. **Data type:** numeric **Example values:** -7.7715611723761e-16 | -4.06341627012807e-14 | -5.97299987248334e-14 | 1.79856129989275e-14 | -1.03916875104915e-13 **Allowed values:**  **Missing-value coding:** NA/blank as read by fread **n rows / missing / unique:** 34441 / 0 / 1240 **Release status:** public **Definition status:** exact\_definition | 01\_primary\_data/public/f1\_simulated\_surface\_repository.csv |  | numeric |
| cree\_true\_recalc\_mg\_dL — Recalculated true enzymatic creatinine concentration **File:** 01\_primary\_data/public/f1\_simulated\_surface\_repository.csv **Recommended public name:** cree\_true\_recalc\_mg\_dL **Description:** True enzymatic creatinine concentration recalculated from the model. **Unit:**  **Value coding:** See example\_values and allowed\_values. **Data type:** numeric **Example values:** 1 | 1.04999999999996 | 1.09999999999994 | 1.15000000000002 | 1.1999999999999 **Allowed values:**  **Missing-value coding:** NA/blank as read by fread **n rows / missing / unique:** 34441 / 0 / 2657 **Release status:** public **Definition status:** exact\_definition | 01\_primary\_data/public/f1\_simulated\_surface\_repository.csv |  | numeric |
| crej\_measured\_pred\_mg\_dL — Predicted measured Jaffe creatinine concentration **File:** 01\_primary\_data/public/f1\_simulated\_surface\_repository.csv **Recommended public name:** crej\_measured\_pred\_mg\_dL **Description:** Predicted measured Jaffe creatinine concentration derived from the interference model. **Unit:**  **Value coding:** See example\_values and allowed\_values. **Data type:** numeric **Example values:** 0.990463857965053 | 1.04152284217999 | 1.09273908981855 | 1.14411406302484 | 1.19564924674134 **Allowed values:**  **Missing-value coding:** NA/blank as read by fread **n rows / missing / unique:** 34441 / 0 / 22885 **Release status:** public **Definition status:** exact\_definition | 01\_primary\_data/public/f1\_simulated\_surface\_repository.csv |  | numeric |
| crej\_true\_recalc\_error — Recalculation error for Jaffe creatinine **File:** 01\_primary\_data/public/f1\_simulated\_surface\_repository.csv **Recommended public name:** crej\_true\_recalc\_error **Description:** Numerical difference between recalculated and expected true Jaffe creatinine values. **Unit:**  **Value coding:** See example\_values and allowed\_values. **Data type:** numeric **Example values:** 6.66133814775094e-16 | 1.55431223447522e-15 | 2.22044604925031e-16 | 1.33226762955019e-15 | 0 **Allowed values:**  **Missing-value coding:** NA/blank as read by fread **n rows / missing / unique:** 34441 / 0 / 23 **Release status:** public **Definition status:** exact\_definition | 01\_primary\_data/public/f1\_simulated\_surface\_repository.csv |  | numeric |
| crej\_true\_recalc\_mg\_dL — Recalculated true Jaffe creatinine concentration **File:** 01\_primary\_data/public/f1\_simulated\_surface\_repository.csv **Recommended public name:** crej\_true\_recalc\_mg\_dL **Description:** True Jaffe creatinine concentration recalculated from the model. **Unit:**  **Value coding:** See example\_values and allowed\_values. **Data type:** numeric **Example values:** 1 | 1.05 | 1.1 | 1.15 | 1.2 **Allowed values:**  **Missing-value coding:** NA/blank as read by fread **n rows / missing / unique:** 34441 / 0 / 101 **Release status:** public **Definition status:** exact\_definition | 01\_primary\_data/public/f1\_simulated\_surface\_repository.csv |  | numeric |
| delta\_cree\_to\_gravimetry\_mg\_dL — Enzymatic creatinine deviation from gravimetry **File:** 01\_primary\_data/public/f1\_simulated\_surface\_repository.csv **Recommended public name:** delta\_cree\_to\_gravimetry\_mg\_dL **Description:** Difference between enzymatic creatinine and the gravimetric/reference value. **Unit:**  **Value coding:** See example\_values and allowed\_values. **Data type:** numeric **Example values:** 0.0979350855740958 | 0.092810860164406 | 0.0876846245860179 | 0.0825563786189223 | 0.0774261220434804 **Allowed values:**  **Missing-value coding:** NA/blank as read by fread **n rows / missing / unique:** 34441 / 0 / 34441 **Release status:** public **Definition status:** exact\_definition | 01\_primary\_data/public/f1\_simulated\_surface\_repository.csv |  | numeric |
| delta\_crej\_to\_gravimetry\_mg\_dL — Jaffe creatinine deviation from gravimetry **File:** 01\_primary\_data/public/f1\_simulated\_surface\_repository.csv **Recommended public name:** delta\_crej\_to\_gravimetry\_mg\_dL **Description:** Difference between Jaffe creatinine and the gravimetric/reference value. **Unit:**  **Value coding:** See example\_values and allowed\_values. **Data type:** numeric **Example values:** 0.00953614203494679 | 0.00847715782000691 | 0.00726091018144937 | 0.00588593697515871 | 0.00435075325865664 **Allowed values:**  **Missing-value coding:** NA/blank as read by fread **n rows / missing / unique:** 34441 / 0 / 23475 **Release status:** public **Definition status:** exact\_definition | 01\_primary\_data/public/f1\_simulated\_surface\_repository.csv |  | numeric |
| grid\_id — Surface-grid identifier **File:** 01\_primary\_data/public/f1\_simulated\_surface\_repository.csv **Recommended public name:** grid\_id **Description:** Identifier of a grid point in the F1 simulated or reconstructed surface data. **Unit:**  **Value coding:** See example\_values and allowed\_values. **Data type:** integer **Example values:** 1 | 2 | 3 | 4 | 5 **Allowed values:**  **Missing-value coding:** NA/blank as read by fread **n rows / missing / unique:** 34441 / 0 / 34441 **Release status:** public **Definition status:** exact\_definition | 01\_primary\_data/public/f1\_simulated\_surface\_repository.csv |  | integer |
| tb\_mg\_dL — Total bilirubin concentration **File:** 01\_primary\_data/public/f1\_simulated\_surface\_repository.csv **Recommended public name:** tb\_mg\_dL **Description:** Total bilirubin concentration used in the creatinine/bilirubin interference model or figure data. **Unit:**  **Value coding:** See example\_values and allowed\_values. **Data type:** numeric **Example values:** 1 | 1.1 | 1.2 | 1.3 | 1.4 **Allowed values:**  **Missing-value coding:** NA/blank as read by fread **n rows / missing / unique:** 34441 / 0 / 341 **Release status:** public **Definition status:** exact\_definition | 01\_primary\_data/public/f1\_simulated\_surface\_repository.csv |  | numeric |
| Cre\_nominal\_grav\_mg\_dL — Nominal gravimetric creatinine target concentration **File:** 01\_primary\_data/public/f1\_tb\_cre\_experimental\_array\_raw.csv **Recommended public name:** Cre\_nominal\_grav\_mg\_dL **Description:** Nominal gravimetric target concentration of creatinine used when defining the F1 experimental array. **Unit:** mg/dL **Value coding:** Numeric concentration in mg/dL. **Data type:** numeric **Example values:** 3 | 1.5 | 3.5 | 2 | 0.5 **Allowed values:**  **Missing-value coding:** NA/blank as read by fread **n rows / missing / unique:** 4200 / 0 / 10 **Release status:** public **Definition status:** manual\_precision\_definition | 01\_primary\_data/public/f1\_tb\_cre\_experimental\_array\_raw.csv | mg/dL | numeric |
| TB\_nominal\_grav\_mg\_dL — Nominal gravimetric total bilirubin target concentration **File:** 01\_primary\_data/public/f1\_tb\_cre\_experimental\_array\_raw.csv **Recommended public name:** TB\_nominal\_grav\_mg\_dL **Description:** Nominal gravimetric target concentration of total bilirubin used when defining the F1 experimental array. **Unit:** mg/dL **Value coding:** Numeric concentration in mg/dL. **Data type:** integer **Example values:** 27 | 14 | 28 | 15 | 29 **Allowed values:**  **Missing-value coding:** NA/blank as read by fread **n rows / missing / unique:** 4200 / 0 / 35 **Release status:** public **Definition status:** manual\_precision\_definition | 01\_primary\_data/public/f1\_tb\_cre\_experimental\_array\_raw.csv | mg/dL | integer |
| TB\_trial\_M\_mg\_dL — Measured total bilirubin concentration in the experimental dataset **File:** 01\_primary\_data/public/f1\_tb\_cre\_experimental\_array\_raw.csv **Recommended public name:** TB\_trial\_M\_mg\_dL **Description:** Measured total bilirubin concentration in the F1 experimental dataset. In rounded input tables this is the rounded/display value used for model calculation and plotting. **Unit:** mg/dL **Value coding:** Numeric concentration in mg/dL. **Data type:** numeric **Example values:** 27.2227640681025 | 27.1297714113581 | 27.2238964489877 | 27.1309037922433 | 26.9427373034614 **Allowed values:**  **Missing-value coding:** NA/blank as read by fread **n rows / missing / unique:** 4200 / 0 / 4200 **Release status:** public **Definition status:** manual\_precision\_definition | 01\_primary\_data/public/f1\_tb\_cre\_experimental\_array\_raw.csv | mg/dL | numeric |
| array\_id — Experimental array identifier **File:** 01\_primary\_data/public/f1\_tb\_cre\_experimental\_array\_raw.csv **Recommended public name:** array\_id **Description:** Identifier of the experimental array used in the F1 creatinine/bilirubin interference data. **Unit:**  **Value coding:** See example\_values and allowed\_values. **Data type:** character **Example values:** Array\_1 | Array\_2 | Array\_3 **Allowed values:** Array\_1 | Array\_2 | Array\_3 **Missing-value coding:** NA/blank as read by fread **n rows / missing / unique:** 4200 / 0 / 3 **Release status:** public **Definition status:** exact\_definition | 01\_primary\_data/public/f1\_tb\_cre\_experimental\_array\_raw.csv |  | character |
| assay — Creatinine assay **File:** 01\_primary\_data/public/f1\_tb\_cre\_experimental\_array\_raw.csv **Recommended public name:** assay **Description:** Creatinine assay represented by the row, for example enzymatic creatinine (CreE) or Jaffe creatinine (CreJ). **Unit:**  **Value coding:** Categorical assay label, e.g. CreE or CreJ. **Data type:** character **Example values:** CreE | CreJ **Allowed values:** CreE | CreJ **Missing-value coding:** NA/blank as read by fread **n rows / missing / unique:** 4200 / 0 / 2 **Release status:** public **Definition status:** manual\_precision\_definition | 01\_primary\_data/public/f1\_tb\_cre\_experimental\_array\_raw.csv |  | character |
| plot\_correction\_mg\_dL — Plotted creatinine correction **File:** 01\_primary\_data/public/f1\_tb\_cre\_experimental\_array\_raw.csv **Recommended public name:** plot\_correction\_mg\_dL **Description:** Creatinine correction value plotted in F1, expressed as the difference between corrected/reference and measured creatinine. **Unit:** probability **Value coding:** Numeric correction in mg/dL. **Data type:** numeric **Example values:** -0.541120525715191 | -0.480865451714561 | -0.386578220459593 | -0.317061468810564 | -0.458450885953881 **Allowed values:**  **Missing-value coding:** NA/blank as read by fread **n rows / missing / unique:** 4200 / 0 / 4200 **Release status:** public **Definition status:** manual\_precision\_definition | 01\_primary\_data/public/f1\_tb\_cre\_experimental\_array\_raw.csv | probability | numeric |
| preparer\_id — Experimental preparer identifier **File:** 01\_primary\_data/public/f1\_tb\_cre\_experimental\_array\_raw.csv **Recommended public name:** preparer\_id **Description:** Identifier of the experimental preparer in F1 raw/validation data, represented as a public technical code. **Unit:**  **Value coding:** See example\_values and allowed\_values. **Data type:** character **Example values:** Prep\_A | Prep\_B **Allowed values:** Prep\_A | Prep\_B **Missing-value coding:** NA/blank as read by fread **n rows / missing / unique:** 4200 / 0 / 2 **Release status:** public **Definition status:** exact\_definition | 01\_primary\_data/public/f1\_tb\_cre\_experimental\_array\_raw.csv |  | character |
| replicate — Experimental replicate number **File:** 01\_primary\_data/public/f1\_tb\_cre\_experimental\_array\_raw.csv **Recommended public name:** replicate **Description:** Replicate number within the F1 experimental dataset. **Unit:** display order **Value coding:** Integer replicate number. **Data type:** integer **Example values:** 2 | 1 **Allowed values:**  **Missing-value coding:** NA/blank as read by fread **n rows / missing / unique:** 4200 / 0 / 2 **Release status:** public **Definition status:** manual\_precision\_definition | 01\_primary\_data/public/f1\_tb\_cre\_experimental\_array\_raw.csv | display order | integer |
| trial\_display\_Cre\_M\_mg\_dL — Measured creatinine concentration in the experimental dataset **File:** 01\_primary\_data/public/f1\_tb\_cre\_experimental\_array\_raw.csv **Recommended public name:** trial\_display\_Cre\_M\_mg\_dL **Description:** Measured creatinine concentration in the F1 experimental dataset. In rounded input tables this is the rounded/display value used for model calculation and plotting. **Unit:** mg/dL **Value coding:** Numeric concentration in mg/dL. **Data type:** numeric **Example values:** 3.54112052571519 | 3.48086545171456 | 3.38657822045959 | 3.31706146881056 | 3.45845088595388 **Allowed values:**  **Missing-value coding:** NA/blank as read by fread **n rows / missing / unique:** 4200 / 0 / 4200 **Release status:** public **Definition status:** manual\_precision\_definition | 01\_primary\_data/public/f1\_tb\_cre\_experimental\_array\_raw.csv | mg/dL | numeric |
| cre\_true\_gcidms\_mg\_dL — GC-IDMS reference creatinine concentration **File:** 01\_primary\_data/public/f1\_tb\_cre\_experimental\_validation\_raw.csv **Recommended public name:** cre\_true\_gcidms\_mg\_dL **Description:** Creatinine concentration measured by GC-IDMS reference method in the validation data. **Unit:**  **Value coding:** See example\_values and allowed\_values. **Data type:** numeric **Example values:** 0.82 | 0.86 | 0.63 | 0.94 | 0.9 **Allowed values:**  **Missing-value coding:** NA/blank as read by fread **n rows / missing / unique:** 32 / 0 / 30 **Release status:** public **Definition status:** exact\_definition | 01\_primary\_data/public/f1\_tb\_cre\_experimental\_validation\_raw.csv |  | numeric |
| cree\_corrected\_mg\_dL — Corrected enzymatic creatinine concentration **File:** 01\_primary\_data/public/f1\_tb\_cre\_experimental\_validation\_raw.csv **Recommended public name:** cree\_corrected\_mg\_dL **Description:** Creatinine concentration after applying the enzymatic correction model. **Unit:**  **Value coding:** See example\_values and allowed\_values. **Data type:** numeric **Example values:** 0.68240141154 | 0.81680053664 | 0.35207418306 | 0.93410841026 | 0.6516394885 **Allowed values:**  **Missing-value coding:** NA/blank as read by fread **n rows / missing / unique:** 32 / 0 / 32 **Release status:** public **Definition status:** exact\_definition | 01\_primary\_data/public/f1\_tb\_cre\_experimental\_validation\_raw.csv |  | numeric |
| cree\_measured\_mg\_dL — Measured enzymatic creatinine concentration **File:** 01\_primary\_data/public/f1\_tb\_cre\_experimental\_validation\_raw.csv **Recommended public name:** cree\_measured\_mg\_dL **Description:** Measured creatinine concentration using the enzymatic assay. **Unit:**  **Value coding:** See example\_values and allowed\_values. **Data type:** numeric **Example values:** 0.75 | 0.78 | 0.52 | 0.89 | 0.8 **Allowed values:**  **Missing-value coding:** NA/blank as read by fread **n rows / missing / unique:** 32 / 0 / 32 **Release status:** public **Definition status:** exact\_definition | 01\_primary\_data/public/f1\_tb\_cre\_experimental\_validation\_raw.csv |  | numeric |
| crej\_corrected\_mg\_dL — Corrected Jaffe creatinine concentration **File:** 01\_primary\_data/public/f1\_tb\_cre\_experimental\_validation\_raw.csv **Recommended public name:** crej\_corrected\_mg\_dL **Description:** Creatinine concentration after applying the Jaffe correction model. **Unit:**  **Value coding:** See example\_values and allowed\_values. **Data type:** numeric **Example values:** 0.8822513 | 0.9490736 | 0.6756628 | 0.8942763 | 0.9588407 **Allowed values:**  **Missing-value coding:** NA/blank as read by fread **n rows / missing / unique:** 32 / 0 / 32 **Release status:** public **Definition status:** exact\_definition | 01\_primary\_data/public/f1\_tb\_cre\_experimental\_validation\_raw.csv |  | numeric |
| crej\_measured\_mg\_dL — Measured Jaffe creatinine concentration **File:** 01\_primary\_data/public/f1\_tb\_cre\_experimental\_validation\_raw.csv **Recommended public name:** crej\_measured\_mg\_dL **Description:** Measured creatinine concentration using the Jaffe assay. **Unit:**  **Value coding:** See example\_values and allowed\_values. **Data type:** numeric **Example values:** 0.95 | 0.98 | 0.78 | 0.92 | 1.06 **Allowed values:**  **Missing-value coding:** NA/blank as read by fread **n rows / missing / unique:** 32 / 0 / 31 **Release status:** public **Definition status:** exact\_definition | 01\_primary\_data/public/f1\_tb\_cre\_experimental\_validation\_raw.csv |  | numeric |
| db\_measured\_mg\_dL — Direct bilirubin concentration **File:** 01\_primary\_data/public/f1\_tb\_cre\_experimental\_validation\_raw.csv **Recommended public name:** db\_measured\_mg\_dL **Description:** Direct bilirubin concentration measured in the experimental or validation data. **Unit:**  **Value coding:** See example\_values and allowed\_values. **Data type:** numeric **Example values:** 7.411 | 3.874 | 13.015 | 3.245 | 11.224 **Allowed values:**  **Missing-value coding:** NA/blank as read by fread **n rows / missing / unique:** 32 / 0 / 32 **Release status:** public **Definition status:** exact\_definition | 01\_primary\_data/public/f1\_tb\_cre\_experimental\_validation\_raw.csv |  | numeric |
| sample\_id — Sample identifier **File:** 01\_primary\_data/public/f1\_tb\_cre\_experimental\_validation\_raw.csv **Recommended public name:** sample\_id **Description:** Identifier of a sample or experimental record within the released public data; not a personal identifier. **Unit:**  **Value coding:** See example\_values and allowed\_values. **Data type:** integer **Example values:** 1 | 2 | 3 | 4 | 5 **Allowed values:**  **Missing-value coding:** NA/blank as read by fread **n rows / missing / unique:** 32 / 0 / 32 **Release status:** public **Definition status:** exact\_definition | 01\_primary\_data/public/f1\_tb\_cre\_experimental\_validation\_raw.csv |  | integer |
| tb\_measured\_mg\_dL — Measured total bilirubin concentration **File:** 01\_primary\_data/public/f1\_tb\_cre\_experimental\_validation\_raw.csv **Recommended public name:** tb\_measured\_mg\_dL **Description:** Measured total bilirubin concentration in the experimental F1 data. **Unit:**  **Value coding:** See example\_values and allowed\_values. **Data type:** numeric **Example values:** 9.263 | 4.852 | 16.121 | 4.419 | 14.415 **Allowed values:**  **Missing-value coding:** NA/blank as read by fread **n rows / missing / unique:** 32 / 0 / 32 **Release status:** public **Definition status:** exact\_definition | 01\_primary\_data/public/f1\_tb\_cre\_experimental\_validation\_raw.csv |  | numeric |
| parameter — Metadata parameter name **File:** 01\_primary\_data/public/f2\_simulated\_heatmap\_metadata.csv **Recommended public name:** parameter **Description:** Name of a metadata parameter describing the F2 simulated heatmap object, such as figure identity, data origin, grid type, axis variable, or unit/role. **Unit:**  **Value coding:** Categorical metadata key; value is given in the corresponding value column or file-specific metadata field. **Data type:** character **Example values:** figure | data\_origin | grid\_type | x\_axis\_variable | x\_start **Allowed values:** figure | data\_origin | grid\_type | x\_axis\_variable | x\_start | x\_end | x\_step | y\_axis\_variable | y\_start | y\_end **Missing-value coding:** NA/blank as read by fread **n rows / missing / unique:** 21 / 0 / 21 **Release status:** public **Definition status:** manual\_exact\_definition | 01\_primary\_data/public/f2\_simulated\_heatmap\_metadata.csv |  | character |
| value — Value **File:** 01\_primary\_data/public/f2\_simulated\_heatmap\_metadata.csv **Recommended public name:** value **Description:** Numerical or character value corresponding to the row-specific variable/metric. **Unit:**  **Value coding:** See example\_values and allowed\_values. **Data type:** character **Example values:** Figure 2 | in silico simulation | uniform subpoint grid within bins | Creatinine (mg/dL) | 0.1 **Allowed values:** Figure 2 | in silico simulation | uniform subpoint grid within bins | Creatinine (mg/dL) | 0.1 | 5.0 | 0.02 | Bilirubin (mg/dL) | 30.0 | 1000 **Missing-value coding:** NA/blank as read by fread **n rows / missing / unique:** 21 / 0 / 18 **Release status:** public **Definition status:** exact\_definition | 01\_primary\_data/public/f2\_simulated\_heatmap\_metadata.csv |  | character |
| count\_negative — Number of simulated subpoints with negative score shift **File:** 01\_primary\_data/public/f2\_simulated\_heatmap\_repository.csv **Recommended public name:** count\_negative **Description:** Number of simulated subpoints within the F2 heatmap bin where the score shift is negative, defined as score delta ≤ −1. **Unit:** count **Value coding:** Integer count. **Data type:** integer **Example values:** 0 | 1 | 17 | 20 | 4 **Allowed values:**  **Missing-value coding:** NA/blank as read by fread **n rows / missing / unique:** 295200 / 0 / 101 **Release status:** public **Definition status:** manual\_precision\_definition | 01\_primary\_data/public/f2\_simulated\_heatmap\_repository.csv | count | integer |
| model — MELD model or score variant **File:** 01\_primary\_data/public/f2\_simulated\_heatmap\_repository.csv **Recommended public name:** model **Description:** Name of the MELD-related model or score variant represented by the row; expected values include MELD, MELD-Na, reMELD-Na, and MELD 3.0. **Unit:**  **Value coding:** Categorical score/model label, for example MELD, MELD-Na, reMELD-Na, or MELD 3.0. **Data type:** character **Example values:** MELD | MELD-Na | reMELD-Na | MELD 3.0 **Allowed values:** MELD | MELD-Na | reMELD-Na | MELD 3.0 **Missing-value coding:** NA/blank as read by fread **n rows / missing / unique:** 295200 / 0 / 4 **Release status:** public **Definition status:** manual\_exact\_definition | 01\_primary\_data/public/f2\_simulated\_heatmap\_repository.csv |  | character |
| n\_sub — Number of simulated subpoints per heatmap bin **File:** 01\_primary\_data/public/f2\_simulated\_heatmap\_repository.csv **Recommended public name:** n\_sub **Description:** Number of simulated subpoints aggregated within one F2 heatmap bin. **Unit:** count **Value coding:** Integer count. **Data type:** integer **Example values:** 100 **Allowed values:**  **Missing-value coding:** NA/blank as read by fread **n rows / missing / unique:** 295200 / 0 / 1 **Release status:** public **Definition status:** manual\_precision\_definition | 01\_primary\_data/public/f2\_simulated\_heatmap\_repository.csv | count | integer |
| pct\_negative — Percentage of simulated subpoints with negative score shift **File:** 01\_primary\_data/public/f2\_simulated\_heatmap\_repository.csv **Recommended public name:** pct\_negative **Description:** Percentage of simulated subpoints within the F2 heatmap bin where the score shift is negative, defined as score delta ≤ −1. **Unit:** mg/dL **Value coding:** 0–100 percentage scale. **Data type:** integer **Example values:** 0 | 1 | 17 | 20 | 4 **Allowed values:**  **Missing-value coding:** NA/blank as read by fread **n rows / missing / unique:** 295200 / 0 / 101 **Release status:** public **Definition status:** manual\_precision\_definition | 01\_primary\_data/public/f2\_simulated\_heatmap\_repository.csv | mg/dL | integer |
| x\_value — Heatmap x-axis creatinine value **File:** 01\_primary\_data/public/f2\_simulated\_heatmap\_repository.csv **Recommended public name:** x\_value **Description:** Creatinine value defining the x-axis coordinate of an F2 heatmap bin. **Unit:** mg/dL **Value coding:** Numeric creatinine value in mg/dL. **Data type:** numeric **Example values:** 0.1 | 0.12 | 0.14 | 0.16 | 0.18 **Allowed values:**  **Missing-value coding:** NA/blank as read by fread **n rows / missing / unique:** 295200 / 0 / 246 **Release status:** public **Definition status:** manual\_precision\_definition | 01\_primary\_data/public/f2\_simulated\_heatmap\_repository.csv | mg/dL | numeric |
| y\_value — Heatmap y-axis total bilirubin value **File:** 01\_primary\_data/public/f2\_simulated\_heatmap\_repository.csv **Recommended public name:** y\_value **Description:** Total bilirubin value defining the y-axis coordinate of an F2 heatmap bin. **Unit:** analysis-specific y-axis coordinate **Value coding:** Numeric total bilirubin value in mg/dL. **Data type:** numeric **Example values:** 0.1 | 0.2 | 0.3 | 0.4 | 0.5 **Allowed values:**  **Missing-value coding:** NA/blank as read by fread **n rows / missing / unique:** 295200 / 0 / 300 **Release status:** public **Definition status:** manual\_precision\_definition | 01\_primary\_data/public/f2\_simulated\_heatmap\_repository.csv | analysis-specific y-axis coordinate | numeric |
| Cre\_nominal\_grav\_mg\_dL — Nominal gravimetric creatinine target concentration **File:** 02\_workflows/F1\_workflow\_v02/data/01\_source\_loaded\_harmonized/expm\_F1\_array\_raw\_public.csv **Recommended public name:** Cre\_nominal\_grav\_mg\_dL **Description:** Nominal gravimetric target concentration of creatinine used when defining the F1 experimental array. **Unit:** mg/dL **Value coding:** Numeric concentration in mg/dL. **Data type:** numeric **Example values:** 3 | 1.5 | 3.5 | 2 | 0.5 **Allowed values:**  **Missing-value coding:** NA/blank as read by fread **n rows / missing / unique:** 4200 / 0 / 10 **Release status:** public **Definition status:** manual\_precision\_definition | 02\_workflows/F1\_workflow\_v02/data/01\_source\_loaded\_harmonized/expm\_F1\_array\_raw\_public.csv | mg/dL | numeric |
| TB\_nominal\_grav\_mg\_dL — Nominal gravimetric total bilirubin target concentration **File:** 02\_workflows/F1\_workflow\_v02/data/01\_source\_loaded\_harmonized/expm\_F1\_array\_raw\_public.csv **Recommended public name:** TB\_nominal\_grav\_mg\_dL **Description:** Nominal gravimetric target concentration of total bilirubin used when defining the F1 experimental array. **Unit:** mg/dL **Value coding:** Numeric concentration in mg/dL. **Data type:** integer **Example values:** 27 | 14 | 28 | 15 | 29 **Allowed values:**  **Missing-value coding:** NA/blank as read by fread **n rows / missing / unique:** 4200 / 0 / 35 **Release status:** public **Definition status:** manual\_precision\_definition | 02\_workflows/F1\_workflow\_v02/data/01\_source\_loaded\_harmonized/expm\_F1\_array\_raw\_public.csv | mg/dL | integer |
| TB\_trial\_M\_mg\_dL — Measured total bilirubin concentration in the experimental dataset **File:** 02\_workflows/F1\_workflow\_v02/data/01\_source\_loaded\_harmonized/expm\_F1\_array\_raw\_public.csv **Recommended public name:** TB\_trial\_M\_mg\_dL **Description:** Measured total bilirubin concentration in the F1 experimental dataset. In rounded input tables this is the rounded/display value used for model calculation and plotting. **Unit:** mg/dL **Value coding:** Numeric concentration in mg/dL. **Data type:** numeric **Example values:** 27.2227640681025 | 27.1297714113581 | 27.2238964489877 | 27.1309037922433 | 26.9427373034614 **Allowed values:**  **Missing-value coding:** NA/blank as read by fread **n rows / missing / unique:** 4200 / 0 / 4200 **Release status:** public **Definition status:** manual\_precision\_definition | 02\_workflows/F1\_workflow\_v02/data/01\_source\_loaded\_harmonized/expm\_F1\_array\_raw\_public.csv | mg/dL | numeric |
| array\_id — Experimental array identifier **File:** 02\_workflows/F1\_workflow\_v02/data/01\_source\_loaded\_harmonized/expm\_F1\_array\_raw\_public.csv **Recommended public name:** array\_id **Description:** Identifier of the experimental array used in the F1 creatinine/bilirubin interference data. **Unit:**  **Value coding:** See example\_values and allowed\_values. **Data type:** character **Example values:** Array\_1 | Array\_2 | Array\_3 **Allowed values:** Array\_1 | Array\_2 | Array\_3 **Missing-value coding:** NA/blank as read by fread **n rows / missing / unique:** 4200 / 0 / 3 **Release status:** public **Definition status:** exact\_definition | 02\_workflows/F1\_workflow\_v02/data/01\_source\_loaded\_harmonized/expm\_F1\_array\_raw\_public.csv |  | character |
| assay — Creatinine assay **File:** 02\_workflows/F1\_workflow\_v02/data/01\_source\_loaded\_harmonized/expm\_F1\_array\_raw\_public.csv **Recommended public name:** assay **Description:** Creatinine assay represented by the row, for example enzymatic creatinine (CreE) or Jaffe creatinine (CreJ). **Unit:**  **Value coding:** Categorical assay label, e.g. CreE or CreJ. **Data type:** character **Example values:** CreE | CreJ **Allowed values:** CreE | CreJ **Missing-value coding:** NA/blank as read by fread **n rows / missing / unique:** 4200 / 0 / 2 **Release status:** public **Definition status:** manual\_precision\_definition | 02\_workflows/F1\_workflow\_v02/data/01\_source\_loaded\_harmonized/expm\_F1\_array\_raw\_public.csv |  | character |
| plot\_correction\_mg\_dL — Plotted creatinine correction **File:** 02\_workflows/F1\_workflow\_v02/data/01\_source\_loaded\_harmonized/expm\_F1\_array\_raw\_public.csv **Recommended public name:** plot\_correction\_mg\_dL **Description:** Creatinine correction value plotted in F1, expressed as the difference between corrected/reference and measured creatinine. **Unit:** probability **Value coding:** Numeric correction in mg/dL. **Data type:** numeric **Example values:** -0.541120525715191 | -0.480865451714561 | -0.386578220459593 | -0.317061468810564 | -0.458450885953881 **Allowed values:**  **Missing-value coding:** NA/blank as read by fread **n rows / missing / unique:** 4200 / 0 / 4200 **Release status:** public **Definition status:** manual\_precision\_definition | 02\_workflows/F1\_workflow\_v02/data/01\_source\_loaded\_harmonized/expm\_F1\_array\_raw\_public.csv | probability | numeric |
| preparer\_id — Experimental preparer identifier **File:** 02\_workflows/F1\_workflow\_v02/data/01\_source\_loaded\_harmonized/expm\_F1\_array\_raw\_public.csv **Recommended public name:** preparer\_id **Description:** Identifier of the experimental preparer in F1 raw/validation data, represented as a public technical code. **Unit:**  **Value coding:** See example\_values and allowed\_values. **Data type:** character **Example values:** Prep\_A | Prep\_B **Allowed values:** Prep\_A | Prep\_B **Missing-value coding:** NA/blank as read by fread **n rows / missing / unique:** 4200 / 0 / 2 **Release status:** public **Definition status:** exact\_definition | 02\_workflows/F1\_workflow\_v02/data/01\_source\_loaded\_harmonized/expm\_F1\_array\_raw\_public.csv |  | character |
| replicate — Experimental replicate number **File:** 02\_workflows/F1\_workflow\_v02/data/01\_source\_loaded\_harmonized/expm\_F1\_array\_raw\_public.csv **Recommended public name:** replicate **Description:** Replicate number within the F1 experimental dataset. **Unit:** display order **Value coding:** Integer replicate number. **Data type:** integer **Example values:** 2 | 1 **Allowed values:**  **Missing-value coding:** NA/blank as read by fread **n rows / missing / unique:** 4200 / 0 / 2 **Release status:** public **Definition status:** manual\_precision\_definition | 02\_workflows/F1\_workflow\_v02/data/01\_source\_loaded\_harmonized/expm\_F1\_array\_raw\_public.csv | display order | integer |
| trial\_display\_Cre\_M\_mg\_dL — Measured creatinine concentration in the experimental dataset **File:** 02\_workflows/F1\_workflow\_v02/data/01\_source\_loaded\_harmonized/expm\_F1\_array\_raw\_public.csv **Recommended public name:** trial\_display\_Cre\_M\_mg\_dL **Description:** Measured creatinine concentration in the F1 experimental dataset. In rounded input tables this is the rounded/display value used for model calculation and plotting. **Unit:** mg/dL **Value coding:** Numeric concentration in mg/dL. **Data type:** numeric **Example values:** 3.54112052571519 | 3.48086545171456 | 3.38657822045959 | 3.31706146881056 | 3.45845088595388 **Allowed values:**  **Missing-value coding:** NA/blank as read by fread **n rows / missing / unique:** 4200 / 0 / 4200 **Release status:** public **Definition status:** manual\_precision\_definition | 02\_workflows/F1\_workflow\_v02/data/01\_source\_loaded\_harmonized/expm\_F1\_array\_raw\_public.csv | mg/dL | numeric |
| cre\_true\_gcidms\_mg\_dL — GC-IDMS reference creatinine concentration **File:** 02\_workflows/F1\_workflow\_v02/data/01\_source\_loaded\_harmonized/expm\_F1\_validation\_raw\_public.csv **Recommended public name:** cre\_true\_gcidms\_mg\_dL **Description:** Creatinine concentration measured by GC-IDMS reference method in the validation data. **Unit:**  **Value coding:** See example\_values and allowed\_values. **Data type:** numeric **Example values:** 0.82 | 0.86 | 0.63 | 0.94 | 0.9 **Allowed values:**  **Missing-value coding:** NA/blank as read by fread **n rows / missing / unique:** 32 / 0 / 30 **Release status:** public **Definition status:** exact\_definition | 02\_workflows/F1\_workflow\_v02/data/01\_source\_loaded\_harmonized/expm\_F1\_validation\_raw\_public.csv |  | numeric |
| cree\_corrected\_mg\_dL — Corrected enzymatic creatinine concentration **File:** 02\_workflows/F1\_workflow\_v02/data/01\_source\_loaded\_harmonized/expm\_F1\_validation\_raw\_public.csv **Recommended public name:** cree\_corrected\_mg\_dL **Description:** Creatinine concentration after applying the enzymatic correction model. **Unit:**  **Value coding:** See example\_values and allowed\_values. **Data type:** numeric **Example values:** 0.68240141154 | 0.81680053664 | 0.35207418306 | 0.93410841026 | 0.6516394885 **Allowed values:**  **Missing-value coding:** NA/blank as read by fread **n rows / missing / unique:** 32 / 0 / 32 **Release status:** public **Definition status:** exact\_definition | 02\_workflows/F1\_workflow\_v02/data/01\_source\_loaded\_harmonized/expm\_F1\_validation\_raw\_public.csv |  | numeric |
| cree\_measured\_mg\_dL — Measured enzymatic creatinine concentration **File:** 02\_workflows/F1\_workflow\_v02/data/01\_source\_loaded\_harmonized/expm\_F1\_validation\_raw\_public.csv **Recommended public name:** cree\_measured\_mg\_dL **Description:** Measured creatinine concentration using the enzymatic assay. **Unit:**  **Value coding:** See example\_values and allowed\_values. **Data type:** numeric **Example values:** 0.75 | 0.78 | 0.52 | 0.89 | 0.8 **Allowed values:**  **Missing-value coding:** NA/blank as read by fread **n rows / missing / unique:** 32 / 0 / 32 **Release status:** public **Definition status:** exact\_definition | 02\_workflows/F1\_workflow\_v02/data/01\_source\_loaded\_harmonized/expm\_F1\_validation\_raw\_public.csv |  | numeric |
| crej\_corrected\_mg\_dL — Corrected Jaffe creatinine concentration **File:** 02\_workflows/F1\_workflow\_v02/data/01\_source\_loaded\_harmonized/expm\_F1\_validation\_raw\_public.csv **Recommended public name:** crej\_corrected\_mg\_dL **Description:** Creatinine concentration after applying the Jaffe correction model. **Unit:**  **Value coding:** See example\_values and allowed\_values. **Data type:** numeric **Example values:** 0.8822513 | 0.9490736 | 0.6756628 | 0.8942763 | 0.9588407 **Allowed values:**  **Missing-value coding:** NA/blank as read by fread **n rows / missing / unique:** 32 / 0 / 32 **Release status:** public **Definition status:** exact\_definition | 02\_workflows/F1\_workflow\_v02/data/01\_source\_loaded\_harmonized/expm\_F1\_validation\_raw\_public.csv |  | numeric |
| crej\_measured\_mg\_dL — Measured Jaffe creatinine concentration **File:** 02\_workflows/F1\_workflow\_v02/data/01\_source\_loaded\_harmonized/expm\_F1\_validation\_raw\_public.csv **Recommended public name:** crej\_measured\_mg\_dL **Description:** Measured creatinine concentration using the Jaffe assay. **Unit:**  **Value coding:** See example\_values and allowed\_values. **Data type:** numeric **Example values:** 0.95 | 0.98 | 0.78 | 0.92 | 1.06 **Allowed values:**  **Missing-value coding:** NA/blank as read by fread **n rows / missing / unique:** 32 / 0 / 31 **Release status:** public **Definition status:** exact\_definition | 02\_workflows/F1\_workflow\_v02/data/01\_source\_loaded\_harmonized/expm\_F1\_validation\_raw\_public.csv |  | numeric |
| db\_measured\_mg\_dL — Direct bilirubin concentration **File:** 02\_workflows/F1\_workflow\_v02/data/01\_source\_loaded\_harmonized/expm\_F1\_validation\_raw\_public.csv **Recommended public name:** db\_measured\_mg\_dL **Description:** Direct bilirubin concentration measured in the experimental or validation data. **Unit:**  **Value coding:** See example\_values and allowed\_values. **Data type:** numeric **Example values:** 7.411 | 3.874 | 13.015 | 3.245 | 11.224 **Allowed values:**  **Missing-value coding:** NA/blank as read by fread **n rows / missing / unique:** 32 / 0 / 32 **Release status:** public **Definition status:** exact\_definition | 02\_workflows/F1\_workflow\_v02/data/01\_source\_loaded\_harmonized/expm\_F1\_validation\_raw\_public.csv |  | numeric |
| sample\_id — Sample identifier **File:** 02\_workflows/F1\_workflow\_v02/data/01\_source\_loaded\_harmonized/expm\_F1\_validation\_raw\_public.csv **Recommended public name:** sample\_id **Description:** Identifier of a sample or experimental record within the released public data; not a personal identifier. **Unit:**  **Value coding:** See example\_values and allowed\_values. **Data type:** integer **Example values:** 1 | 2 | 3 | 4 | 5 **Allowed values:**  **Missing-value coding:** NA/blank as read by fread **n rows / missing / unique:** 32 / 0 / 32 **Release status:** public **Definition status:** exact\_definition | 02\_workflows/F1\_workflow\_v02/data/01\_source\_loaded\_harmonized/expm\_F1\_validation\_raw\_public.csv |  | integer |
| tb\_measured\_mg\_dL — Measured total bilirubin concentration **File:** 02\_workflows/F1\_workflow\_v02/data/01\_source\_loaded\_harmonized/expm\_F1\_validation\_raw\_public.csv **Recommended public name:** tb\_measured\_mg\_dL **Description:** Measured total bilirubin concentration in the experimental F1 data. **Unit:**  **Value coding:** See example\_values and allowed\_values. **Data type:** numeric **Example values:** 9.263 | 4.852 | 16.121 | 4.419 | 14.415 **Allowed values:**  **Missing-value coding:** NA/blank as read by fread **n rows / missing / unique:** 32 / 0 / 32 **Release status:** public **Definition status:** exact\_definition | 02\_workflows/F1\_workflow\_v02/data/01\_source\_loaded\_harmonized/expm\_F1\_validation\_raw\_public.csv |  | numeric |
| file\_key — Repository file key **File:** 02\_workflows/F1\_workflow\_v02/data/01\_source\_loaded\_harmonized/expm\_slco\_F1\_source\_load\_manifest\_public.csv **Recommended public name:** file\_key **Description:** Stable repository key identifying a file in a source/load manifest. **Unit:**  **Value coding:** Categorical repository key. **Data type:** character **Example values:** f1\_tb\_cre\_experimental\_array\_raw | f1\_tb\_cre\_experimental\_validation\_raw | f1\_simulated\_surface\_repository | f1\_simulate... **Allowed values:** f1\_tb\_cre\_experimental\_array\_raw | f1\_tb\_cre\_experimental\_validation\_raw | f1\_simulated\_surface\_repository | f1\_simulated\_surface\_metadata **Missing-value coding:** NA/blank as read by fread **n rows / missing / unique:** 4 / 0 / 4 **Release status:** public **Definition status:** manual\_precision\_definition | 02\_workflows/F1\_workflow\_v02/data/01\_source\_loaded\_harmonized/expm\_slco\_F1\_source\_load\_manifest\_public.csv |  | character |
| harmonized\_loaded\_file\_name — Loaded harmonized file name **File:** 02\_workflows/F1\_workflow\_v02/data/01\_source\_loaded\_harmonized/expm\_slco\_F1\_source\_load\_manifest\_public.csv **Recommended public name:** harmonized\_loaded\_file\_name **Description:** File name of the harmonized public file loaded by the workflow. **Unit:**  **Value coding:** File name. **Data type:** character **Example values:** expm\_F1\_array\_raw\_public.csv | expm\_F1\_validation\_raw\_public.csv | slco\_F1\_surface\_grid\_reference\_public.csv | slco\_F1\_s... **Allowed values:** expm\_F1\_array\_raw\_public.csv | expm\_F1\_validation\_raw\_public.csv | slco\_F1\_surface\_grid\_reference\_public.csv | slco\_F1\_surface\_meta\_reference\_public.csv **Missing-value coding:** NA/blank as read by fread **n rows / missing / unique:** 4 / 0 / 4 **Release status:** public **Definition status:** manual\_precision\_definition | 02\_workflows/F1\_workflow\_v02/data/01\_source\_loaded\_harmonized/expm\_slco\_F1\_source\_load\_manifest\_public.csv |  | character |
| md5 — MD5 checksum **File:** 02\_workflows/F1\_workflow\_v02/data/01\_source\_loaded\_harmonized/expm\_slco\_F1\_source\_load\_manifest\_public.csv **Recommended public name:** md5 **Description:** MD5 checksum of the referenced file, used for file-integrity checks. **Unit:** checksum **Value coding:** MD5 checksum string. **Data type:** character **Example values:** b8ffb94bc127381e0963db2211ba44b8 | 30e9eb0a37dab1d84dbff1649b1b9e07 | 87713a0d6868c6ac52ff20ea850e3d58 | ee2dfece0c0751a... **Allowed values:** b8ffb94bc127381e0963db2211ba44b8 | 30e9eb0a37dab1d84dbff1649b1b9e07 | 87713a0d6868c6ac52ff20ea850e3d58 | ee2dfece0c0751ab1643f0e896edea9b **Missing-value coding:** NA/blank as read by fread **n rows / missing / unique:** 4 / 0 / 4 **Release status:** public **Definition status:** manual\_precision\_definition | 02\_workflows/F1\_workflow\_v02/data/01\_source\_loaded\_harmonized/expm\_slco\_F1\_source\_load\_manifest\_public.csv | checksum | character |
| n\_columns — Number of columns **File:** 02\_workflows/F1\_workflow\_v02/data/01\_source\_loaded\_harmonized/expm\_slco\_F1\_source\_load\_manifest\_public.csv **Recommended public name:** n\_columns **Description:** Number of columns in the referenced public data object. **Unit:** count **Value coding:** Integer count. **Data type:** integer **Example values:** 9 | 8 | 11 | 2 **Allowed values:**  **Missing-value coding:** NA/blank as read by fread **n rows / missing / unique:** 4 / 0 / 4 **Release status:** public **Definition status:** manual\_precision\_definition | 02\_workflows/F1\_workflow\_v02/data/01\_source\_loaded\_harmonized/expm\_slco\_F1\_source\_load\_manifest\_public.csv | count | integer |
| n\_rows — Number of rows **File:** 02\_workflows/F1\_workflow\_v02/data/01\_source\_loaded\_harmonized/expm\_slco\_F1\_source\_load\_manifest\_public.csv **Recommended public name:** n\_rows **Description:** Number of rows in the referenced public data object. **Unit:** count **Value coding:** Integer count. **Data type:** integer **Example values:** 4200 | 32 | 34441 | 9 **Allowed values:**  **Missing-value coding:** NA/blank as read by fread **n rows / missing / unique:** 4 / 0 / 4 **Release status:** public **Definition status:** manual\_precision\_definition | 02\_workflows/F1\_workflow\_v02/data/01\_source\_loaded\_harmonized/expm\_slco\_F1\_source\_load\_manifest\_public.csv | count | integer |
| output\_exists — Output file existence flag **File:** 02\_workflows/F1\_workflow\_v02/data/01\_source\_loaded\_harmonized/expm\_slco\_F1\_source\_load\_manifest\_public.csv **Recommended public name:** output\_exists **Description:** Logical indicator showing whether the rendered output file exists. **Unit:**  **Value coding:** TRUE/FALSE. **Data type:** logical **Example values:** TRUE **Allowed values:** TRUE **Missing-value coding:** NA/blank as read by fread **n rows / missing / unique:** 4 / 0 / 1 **Release status:** public **Definition status:** manual\_precision\_definition | 02\_workflows/F1\_workflow\_v02/data/01\_source\_loaded\_harmonized/expm\_slco\_F1\_source\_load\_manifest\_public.csv |  | logical |
| release\_status — Release status **File:** 02\_workflows/F1\_workflow\_v02/data/01\_source\_loaded\_harmonized/expm\_slco\_F1\_source\_load\_manifest\_public.csv **Recommended public name:** release\_status **Description:** Release-status label indicating the publication status of the row or file object. **Unit:**  **Value coding:** Release status of the row/object, for example public. **Data type:** character **Example values:** public **Allowed values:** public **Missing-value coding:** NA/blank as read by fread **n rows / missing / unique:** 4 / 0 / 1 **Release status:** public **Definition status:** exact\_definition | 02\_workflows/F1\_workflow\_v02/data/01\_source\_loaded\_harmonized/expm\_slco\_F1\_source\_load\_manifest\_public.csv |  | character |
| source\_path — Source path **File:** 02\_workflows/F1\_workflow\_v02/data/01\_source\_loaded\_harmonized/expm\_slco\_F1\_source\_load\_manifest\_public.csv **Recommended public name:** source\_path **Description:** Source path recorded by the workflow. For public release this should be package-relative or sanitized if the original value was an absolute local path. **Unit:**  **Value coding:** Path string; prefer package-relative or sanitized values in the public package. **Data type:** character **Example values:** 01\_primary\_data/public/f1\_tb\_cre\_experimental\_array\_raw.csv | 01\_primary\_data/public/f1\_tb\_cre\_experimental\_validation\_raw.csv | 01\_primary\_data/public/f1\_simulated\_surface\_repository.csv | 01\_primary\_data/public/f1\_simulated\_surface\_metadata.csv **Allowed values:** 01\_primary\_data/public/f1\_tb\_cre\_experimental\_array\_raw.csv | LOCAL\_PATH\_REMOVED **Missing-value coding:** NA/blank as read by fread **n rows / missing / unique:** 4 / 0 / 4 **Release status:** public **Definition status:** manual\_precision\_definition | 02\_workflows/F1\_workflow\_v02/data/01\_source\_loaded\_harmonized/expm\_slco\_F1\_source\_load\_manifest\_public.csv |  | character |
| source\_role — Source role **File:** 02\_workflows/F1\_workflow\_v02/data/01\_source\_loaded\_harmonized/expm\_slco\_F1\_source\_load\_manifest\_public.csv **Recommended public name:** source\_role **Description:** Role of the source file or object in the public workflow. **Unit:**  **Value coding:** Character source-role label. **Data type:** character **Example values:** F1 experimental array raw primary source | F1 experimental validation raw primary source | F1 simulated surface primary ... **Allowed values:** F1 experimental array raw primary source | F1 experimental validation raw primary source | F1 simulated surface primary source | F1 simulated surface metadata primary source **Missing-value coding:** NA/blank as read by fread **n rows / missing / unique:** 4 / 0 / 4 **Release status:** public **Definition status:** manual\_precision\_definition | 02\_workflows/F1\_workflow\_v02/data/01\_source\_loaded\_harmonized/expm\_slco\_F1\_source\_load\_manifest\_public.csv |  | character |
| parameter — Metadata parameter name **File:** 02\_workflows/F1\_workflow\_v02/data/01\_source\_loaded\_harmonized/expm\_slco\_F1\_step1\_run\_inputs\_public.csv **Recommended public name:** parameter **Description:** Name of a metadata parameter describing the F2 simulated heatmap object, such as figure identity, data origin, grid type, axis variable, or unit/role. **Unit:**  **Value coding:** Categorical metadata key; value is given in the corresponding value column or file-specific metadata field. **Data type:** character **Example values:** source\_layer | f1\_array\_raw | f1\_validation\_raw | f1\_surface\_repository | f1\_surface\_metadata **Allowed values:** source\_layer | f1\_array\_raw | f1\_validation\_raw | f1\_surface\_repository | f1\_surface\_metadata **Missing-value coding:** NA/blank as read by fread **n rows / missing / unique:** 5 / 0 / 5 **Release status:** public **Definition status:** manual\_exact\_definition | 02\_workflows/F1\_workflow\_v02/data/01\_source\_loaded\_harmonized/expm\_slco\_F1\_step1\_run\_inputs\_public.csv |  | character |
| value — Value **File:** 02\_workflows/F1\_workflow\_v02/data/01\_source\_loaded\_harmonized/expm\_slco\_F1\_step1\_run\_inputs\_public.csv **Recommended public name:** value **Description:** Numerical or character value corresponding to the row-specific variable/metric. **Unit:**  **Value coding:** See example\_values and allowed\_values. **Data type:** character **Example values:** V3 central primary-data layer | LOCAL\_PATH\_REMOVED **Allowed values:** V3 central primary-data layer | 01\_primary\_data/public/f1\_tb\_cre\_experimental\_array\_raw.csv | /... **Missing-value coding:** NA/blank as read by fread **n rows / missing / unique:** 5 / 0 / 5 **Release status:** public **Definition status:** exact\_definition | 02\_workflows/F1\_workflow\_v02/data/01\_source\_loaded\_harmonized/expm\_slco\_F1\_step1\_run\_inputs\_public.csv |  | character |
| cre\_true\_mg\_dL — True creatinine concentration **File:** 02\_workflows/F1\_workflow\_v02/data/01\_source\_loaded\_harmonized/slco\_F1\_surface\_grid\_reference\_public.csv **Recommended public name:** cre\_true\_mg\_dL **Description:** True creatinine concentration used as the gravimetric/reference concentration in F1. **Unit:**  **Value coding:** See example\_values and allowed\_values. **Data type:** numeric **Example values:** 1 | 1.05 | 1.1 | 1.15 | 1.2 **Allowed values:**  **Missing-value coding:** NA/blank as read by fread **n rows / missing / unique:** 34441 / 0 / 101 **Release status:** public **Definition status:** exact\_definition | 02\_workflows/F1\_workflow\_v02/data/01\_source\_loaded\_harmonized/slco\_F1\_surface\_grid\_reference\_public.csv |  | numeric |
| cree\_measured\_pred\_mg\_dL — Predicted measured enzymatic creatinine concentration **File:** 02\_workflows/F1\_workflow\_v02/data/01\_source\_loaded\_harmonized/slco\_F1\_surface\_grid\_reference\_public.csv **Recommended public name:** cree\_measured\_pred\_mg\_dL **Description:** Predicted measured enzymatic creatinine concentration derived from the interference model. **Unit:**  **Value coding:** See example\_values and allowed\_values. **Data type:** numeric **Example values:** 0.902064914425904 | 0.957189139835594 | 1.01231537541398 | 1.06744362138108 | 1.12257387795652 **Allowed values:**  **Missing-value coding:** NA/blank as read by fread **n rows / missing / unique:** 34441 / 0 / 34441 **Release status:** public **Definition status:** exact\_definition | 02\_workflows/F1\_workflow\_v02/data/01\_source\_loaded\_harmonized/slco\_F1\_surface\_grid\_reference\_public.csv |  | numeric |
| cree\_true\_recalc\_error — Recalculation error for enzymatic creatinine **File:** 02\_workflows/F1\_workflow\_v02/data/01\_source\_loaded\_harmonized/slco\_F1\_surface\_grid\_reference\_public.csv **Recommended public name:** cree\_true\_recalc\_error **Description:** Numerical difference between recalculated and expected true enzymatic creatinine values. **Unit:**  **Value coding:** See example\_values and allowed\_values. **Data type:** numeric **Example values:** -7.7715611723761e-16 | -4.06341627012807e-14 | -5.97299987248334e-14 | 1.79856129989275e-14 | -1.03916875104915e-13 **Allowed values:**  **Missing-value coding:** NA/blank as read by fread **n rows / missing / unique:** 34441 / 0 / 1240 **Release status:** public **Definition status:** exact\_definition | 02\_workflows/F1\_workflow\_v02/data/01\_source\_loaded\_harmonized/slco\_F1\_surface\_grid\_reference\_public.csv |  | numeric |
| cree\_true\_recalc\_mg\_dL — Recalculated true enzymatic creatinine concentration **File:** 02\_workflows/F1\_workflow\_v02/data/01\_source\_loaded\_harmonized/slco\_F1\_surface\_grid\_reference\_public.csv **Recommended public name:** cree\_true\_recalc\_mg\_dL **Description:** True enzymatic creatinine concentration recalculated from the model. **Unit:**  **Value coding:** See example\_values and allowed\_values. **Data type:** numeric **Example values:** 1 | 1.04999999999996 | 1.09999999999994 | 1.15000000000002 | 1.1999999999999 **Allowed values:**  **Missing-value coding:** NA/blank as read by fread **n rows / missing / unique:** 34441 / 0 / 2657 **Release status:** public **Definition status:** exact\_definition | 02\_workflows/F1\_workflow\_v02/data/01\_source\_loaded\_harmonized/slco\_F1\_surface\_grid\_reference\_public.csv |  | numeric |
| crej\_measured\_pred\_mg\_dL — Predicted measured Jaffe creatinine concentration **File:** 02\_workflows/F1\_workflow\_v02/data/01\_source\_loaded\_harmonized/slco\_F1\_surface\_grid\_reference\_public.csv **Recommended public name:** crej\_measured\_pred\_mg\_dL **Description:** Predicted measured Jaffe creatinine concentration derived from the interference model. **Unit:**  **Value coding:** See example\_values and allowed\_values. **Data type:** numeric **Example values:** 0.990463857965053 | 1.04152284217999 | 1.09273908981855 | 1.14411406302484 | 1.19564924674134 **Allowed values:**  **Missing-value coding:** NA/blank as read by fread **n rows / missing / unique:** 34441 / 0 / 22885 **Release status:** public **Definition status:** exact\_definition | 02\_workflows/F1\_workflow\_v02/data/01\_source\_loaded\_harmonized/slco\_F1\_surface\_grid\_reference\_public.csv |  | numeric |
| crej\_true\_recalc\_error — Recalculation error for Jaffe creatinine **File:** 02\_workflows/F1\_workflow\_v02/data/01\_source\_loaded\_harmonized/slco\_F1\_surface\_grid\_reference\_public.csv **Recommended public name:** crej\_true\_recalc\_error **Description:** Numerical difference between recalculated and expected true Jaffe creatinine values. **Unit:**  **Value coding:** See example\_values and allowed\_values. **Data type:** numeric **Example values:** 6.66133814775094e-16 | 1.55431223447522e-15 | 2.22044604925031e-16 | 1.33226762955019e-15 | 0 **Allowed values:**  **Missing-value coding:** NA/blank as read by fread **n rows / missing / unique:** 34441 / 0 / 23 **Release status:** public **Definition status:** exact\_definition | 02\_workflows/F1\_workflow\_v02/data/01\_source\_loaded\_harmonized/slco\_F1\_surface\_grid\_reference\_public.csv |  | numeric |
| crej\_true\_recalc\_mg\_dL — Recalculated true Jaffe creatinine concentration **File:** 02\_workflows/F1\_workflow\_v02/data/01\_source\_loaded\_harmonized/slco\_F1\_surface\_grid\_reference\_public.csv **Recommended public name:** crej\_true\_recalc\_mg\_dL **Description:** True Jaffe creatinine concentration recalculated from the model. **Unit:**  **Value coding:** See example\_values and allowed\_values. **Data type:** numeric **Example values:** 1 | 1.05 | 1.1 | 1.15 | 1.2 **Allowed values:**  **Missing-value coding:** NA/blank as read by fread **n rows / missing / unique:** 34441 / 0 / 101 **Release status:** public **Definition status:** exact\_definition | 02\_workflows/F1\_workflow\_v02/data/01\_source\_loaded\_harmonized/slco\_F1\_surface\_grid\_reference\_public.csv |  | numeric |
| delta\_cree\_to\_gravimetry\_mg\_dL — Enzymatic creatinine deviation from gravimetry **File:** 02\_workflows/F1\_workflow\_v02/data/01\_source\_loaded\_harmonized/slco\_F1\_surface\_grid\_reference\_public.csv **Recommended public name:** delta\_cree\_to\_gravimetry\_mg\_dL **Description:** Difference between enzymatic creatinine and the gravimetric/reference value. **Unit:**  **Value coding:** See example\_values and allowed\_values. **Data type:** numeric **Example values:** 0.0979350855740958 | 0.092810860164406 | 0.0876846245860179 | 0.0825563786189223 | 0.0774261220434804 **Allowed values:**  **Missing-value coding:** NA/blank as read by fread **n rows / missing / unique:** 34441 / 0 / 34441 **Release status:** public **Definition status:** exact\_definition | 02\_workflows/F1\_workflow\_v02/data/01\_source\_loaded\_harmonized/slco\_F1\_surface\_grid\_reference\_public.csv |  | numeric |
| delta\_crej\_to\_gravimetry\_mg\_dL — Jaffe creatinine deviation from gravimetry **File:** 02\_workflows/F1\_workflow\_v02/data/01\_source\_loaded\_harmonized/slco\_F1\_surface\_grid\_reference\_public.csv **Recommended public name:** delta\_crej\_to\_gravimetry\_mg\_dL **Description:** Difference between Jaffe creatinine and the gravimetric/reference value. **Unit:**  **Value coding:** See example\_values and allowed\_values. **Data type:** numeric **Example values:** 0.00953614203494679 | 0.00847715782000691 | 0.00726091018144937 | 0.00588593697515871 | 0.00435075325865664 **Allowed values:**  **Missing-value coding:** NA/blank as read by fread **n rows / missing / unique:** 34441 / 0 / 23475 **Release status:** public **Definition status:** exact\_definition | 02\_workflows/F1\_workflow\_v02/data/01\_source\_loaded\_harmonized/slco\_F1\_surface\_grid\_reference\_public.csv |  | numeric |
| grid\_id — Surface-grid identifier **File:** 02\_workflows/F1\_workflow\_v02/data/01\_source\_loaded\_harmonized/slco\_F1\_surface\_grid\_reference\_public.csv **Recommended public name:** grid\_id **Description:** Identifier of a grid point in the F1 simulated or reconstructed surface data. **Unit:**  **Value coding:** See example\_values and allowed\_values. **Data type:** integer **Example values:** 1 | 2 | 3 | 4 | 5 **Allowed values:**  **Missing-value coding:** NA/blank as read by fread **n rows / missing / unique:** 34441 / 0 / 34441 **Release status:** public **Definition status:** exact\_definition | 02\_workflows/F1\_workflow\_v02/data/01\_source\_loaded\_harmonized/slco\_F1\_surface\_grid\_reference\_public.csv |  | integer |
| tb\_mg\_dL — Total bilirubin concentration **File:** 02\_workflows/F1\_workflow\_v02/data/01\_source\_loaded\_harmonized/slco\_F1\_surface\_grid\_reference\_public.csv **Recommended public name:** tb\_mg\_dL **Description:** Total bilirubin concentration used in the creatinine/bilirubin interference model or figure data. **Unit:**  **Value coding:** See example\_values and allowed\_values. **Data type:** numeric **Example values:** 1 | 1.1 | 1.2 | 1.3 | 1.4 **Allowed values:**  **Missing-value coding:** NA/blank as read by fread **n rows / missing / unique:** 34441 / 0 / 341 **Release status:** public **Definition status:** exact\_definition | 02\_workflows/F1\_workflow\_v02/data/01\_source\_loaded\_harmonized/slco\_F1\_surface\_grid\_reference\_public.csv |  | numeric |
| parameter — Metadata parameter name **File:** 02\_workflows/F1\_workflow\_v02/data/01\_source\_loaded\_harmonized/slco\_F1\_surface\_meta\_reference\_public.csv **Recommended public name:** parameter **Description:** Name of a metadata parameter describing the F2 simulated heatmap object, such as figure identity, data origin, grid type, axis variable, or unit/role. **Unit:**  **Value coding:** Categorical metadata key; value is given in the corresponding value column or file-specific metadata field. **Data type:** character **Example values:** dataset\_name | cohort | data\_type | """"""""""""""""""""""""""""""""""""""""""""""""""""""""""""""""""""""""""""""""""""""""""""""""""""""""""""""""""""""""""""""""""""""""""""""""""""""""""""""""""""""""""""""""""""""""""""""""""""""""""""""""""""""""""""""""""""""""""""""""""""""""""""""""""""""""""""""""""""""""""""""""""""""""""""""""""""""""""""""""""""""""""""""""""""""""""""""""""""""""""""""""""""""""""""""""""""""""""""""""""""""""""""""""""""""""""""""""""""""""""""""""""""""""""""""""""""""""""""""""""""""""""""""""""""""""""""""""""""""""""""""""""""""""""""""""""""""""""""""""""""""""""""""""""""""""""""""""""""""""""""""""""""""""""""""""""""""""""""""""""""""""""""""""""""""""""""""""""""""""""""""""""""""""""""""""""""""""""""""""""""""""""""""""""""""""""""""""""""""""""""""""""""""""""""""""""""""""""""""""""""""""""""""""""""""""""""""""""""""""""""""""""""""""""""""""""""""""""""""""""""""""""""""""""""""""""""""""""""""""""""""""""""""""""""""""""""""""""""""""""""""""""""""""""""""""""""""""""""""""""""""""""""""""""""""""""""""""""""""""""""""""""""""""""""""""""""""""""""""""""""""""""""""""""""""""""""""""""""""""""""""""""""""""""""""""""""""""""""""""""""""""""""""""""""""""""""""""""""""""""""""""""""""""""""""""""""""""""""""""""""""""""""""""""""""""""""""""""""""""""""""""""""""""""""""""""""""""""""""""""""""""""""""""""""""""""""""""""""""""""""""""""""""""""""""""""""""""""""""""""""""""""""""""""""""""""""""""""""""""""""""""""""""""""""""""""""""""""""""""""""""""""""""""""""""""""""""""""""""""""""""""""""""""""""""""""""""""""""""""""""""""""""""""""""""""""""""""""""""""""""""""""""""""""""""""""""""""""""""""""""""""""""""""""""""""""""""""""""""""""""""""""""""""""""""""""""""""""""""""""""""""""""""""""""""""""""""""""""""""""""""""""""""""""""""""""""""""""""""""""""""""""""""""""""""""""""""""""""""""""""""""""""""""""""""""""""""""""""""""""""""""""""""""""""""""""""""""""""""""""""""""""""""""""""""""""""""""""""""""""""""""""""""""""""""""""""""""""""""""""""""""""""""""""""""""""""""""""""""""""""""""""""""""""""""""""""""""""""""""""""""""""""""""""""""""""""""""""""""""""""""""""""""""""""""""""""""""""""""""""""""""""""""""""""""""""""""""""""""""""""""""""""""""""""""""""""""""""""""""""""""""""""""""""""""""""""""""""""""""""""""""""""""""""""""""""""""""""""""""""""""""""""""""""""""""""""""""""""""""""""""""""""""""""""""""""""""""""""""""""""""""""""""""""""""""""""""""""""""""""""""""""""""""""""""""""""""""""""""""""""""""""""""""""""""""""""""""""""""""""""""""""""""""""""""""""""""""""""""""""""""""""""""""""""""""""""""""""""""""""""""""""""""""""""""""""""""""""""""""""""""""""""""""""""""""""""""""""""""""""""""""""""""""""""""""""""""""""""""""""""""""""""""""""""""""""""""""""""""""""""""""""""""""""""""""""""""""""""""""""""""""""""""""""""""""""""""""""""""""""""""""""""""""""""""""""""""""""""""""""""""""""""""""""""""""""""""""""""""""""""""""""""""""""""""""""""""""""""""""""""""""""""""""""""""""""""""""""""""""""""""""""""""""""""""""""""""""""""""""""""""""""""""""""""""""""""""""""""""""""""""""""""""""""""""""""""""""""""""""""""""""""""""""""""""""""""""""""""""""""""""""""""""""""""""""""""""""""""""""""""""""""""""""""""""""""""""""""""""""""""""""""""""""""""""""""""""""""""""""""""""""""""""""""""""""""""""""""""""""""""""""""""""""""""""""""""""""""""""""""""""""""""""""""""""""""""""""""""""""""""""""""""""""""""""""""""""""""""""""""""""""""""""""""""""""""""""""""""""""""""""""""""""""""""""""""""""""""""""""""""""""""""""""""""""""""""""""""""""""""""""""""""""""""""""""""""""""""""""""""""""""""""""""""""""""""""""""""""""""""""""""""""""""""""""""""""""""""""""""""""""""""""""""""""""""""""""""""""""""""""""""""""""""""""""""""""""""""""""""""""""""""""""""""""""""""""""""""""""""""""""""""""""""""""""""""""""""""""""""""""""""""""""""""""""""""""""""""""""""""""""""""""""""""""""""""""""""""""""""""""""""""""""""""""""""""""""""""""""""""""""""""""""""""""""""""""""""""""""""""""""""""""""""""""""""""""""""""""""""""""""""""""""""""""""""""""""""""""""""""""""""""""""""row\_definition | tb\_range\_mg\_dL **Allowed values:** dataset\_name | cohort | data\_type | """"""""""""""""""""""""""""""""""""""""""""""""""""""""""""""""""""""""""""""""""""""""""""""""""""""""""""""""""""""""""""""""""""""""""""""""""""""""""""""""""""""""""""""""""""""""""""""""""""""""""""""""""""""""""""""""""""""""""""""""""""""""""""""""""""""""""""""""""""""""""""""""""""""""""""""""""""""""""""""""""""""""""""""""""""""""""""""""""""""""""""""""""""""""""""""""""""""""""""""""""""""""""""""""""""""""""""""""""""""""""""""""""""""""""""""""""""""""""""""""""""""""""""""""""""""""""""""""""""""""""""""""""""""""""""""""""""""""""""""""""""""""""""""""""""""""""""""""""""""""""""""""""""""""""""""""""""""""""""""""""""""""""""""""""""""""""""""""""""""""""""""""""""""""""""""""""""""""""""""""""""""""""""""""""""""""""""""""""""""""""""""""""""""""""""""""""""""""""""""""""""""""""""""""""""""""""""""""""""""""""""""""""""""""""""""""""""""""""""""""""""""""""""""""""""""""""""""""""""""""""""""""""""""""""""""""""""""""""""""""""""""""""""""""""""""""""""""""""""""""""""""""""""""""""""""""""""""""""""""""""""""""""""""""""""""""""""""""""""""""""""""""""""""""""""""""""""""""""""""""""""""""""""""""""""""""""""""""""""""""""""""""""""""""""""""""""""""""""""""""""""""""""""""""""""""""""""""""""""""""""""""""""""""""""""""""""""""""""""""""""""""""""""""""""""""""""""""""""""""""""""""""""""""""""""""""""""""""""""""""""""""""""""""""""""""""""""""""""""""""""""""""""""""""""""""""""""""""""""""""""""""""""""""""""""""""""""""""""""""""""""""""""""""""""""""""""""""""""""""""""""""""""""""""""""""""""""""""""""""""""""""""""""""""""""""""""""""""""""""""""""""""""""""""""""""""""""""""""""""""""""""""""""""""""""""""""""""""""""""""""""""""""""""""""""""""""""""""""""""""""""""""""""""""""""""""""""""""""""""""""""""""""""""""""""""""""""""""""""""""""""""""""""""""""""""""""""""""""""""""""""""""""""""""""""""""""""""""""""""""""""""""""""""""""""""""""""""""""""""""""""""""""""""""""""""""""""""""""""""""""""""""""""""""""""""""""""""""""""""""""""""""""""""""""""""""""""""""""""""""""""""""""""""""""""""""""""""""""""""""""""""""""""""""""""""""""""""""""""""""""""""""""""""""""""""""""""""""""""""""""""""""""""""""""""""""""""""""""""""""""""""""""""""""""""""""""""""""""""""""""""""""""""""""""""""""""""""""""""""""""""""""""""""""""""""""""""""""""""""""""""""""""""""""""""""""""""""""""""""""""""""""""""""""""""""""""""""""""""""""""""""""""""""""""""""""""""""""""""""""""""""""""""""""""""""""""""""""""""""""""""""""""""""""""""""""""""""""""""""""""""""""""""""""""""""""""""""""""""""""""""""""""""""""""""""""""""""""""""""""""""""""""""""""""""""""""""""""""""""""""""""""""""""""""""""""""""""""""""""""""""""""""""""""""""""""""""""""""""""""""""""""""""""""""""""""""""""""""""""""""""""""""""""""""""""""""""""""""""""""""""""""""""""""""""""""""""""""""""""""""""""""""""""""""""""""""""""""""""""""""""""""""""""""""""""""""""""""""""""""""""""""""""""""""""""""""""""""""""""""""""""""""""""""""""""""""""""""""""""""""""""""""""""""""""""""""""""""""""""""""""""""""""""""""""""""""""""""""""""""""""""""""""""""""""""""""""""""""""""""""""""""""""""""""""""""""""""""""""""""""""""""""""""""""""""""""""""""""""""""""""""""""""""""""""""""""""""""""""""""""""""""""""""""""""""""""""""""""""""""""""""""""""""""""""""""""""""""""""""""""""""""""""""""""""""""""""""""""""""""""""""""""""""""""""""""""""""""""""""""""""""""""""""""""""""""""""""""""""""""""""""""""""""""""""""""""""""""""""""""""""""""""""""""""""""""""""""""""""""""""""""""""""""""""""""""""""""""""""""""""""""""""""""""""""""""""""""""""""""""""""""""""""""""""""""""""""""""""""""""""""""""""""""""""""""""""""""""""""""""""""""""""""""""""""""""""""""""""""""""""""""""""""""""""""""""""""""""""""""""""""""""""""""""""""""""""""""""""""""""""""""""""""""""""""""""""""""""""""""""""""""""""""""""""""""""""""""""""""""""""""""""""""""""""""""""""""""""""""""""""""""""""""""""""""""""""""""""""""""""""""""""""""""""""""""""""""""""""""""""""""""""""""""""""""""""""""""""""""""""""""""""""""""""""""""""""""""""""""""""""""""""""""""""row\_definition | tb\_range\_mg\_dL | cre\_true\_range\_mg\_dL | tb\_step\_mg\_dL | cre\_step\_mg\_dL | delta\_definition **Missing-value coding:** NA/blank as read by fread **n rows / missing / unique:** 9 / 0 / 9 **Release status:** public **Definition status:** manual\_exact\_definition | 02\_workflows/F1\_workflow\_v02/data/01\_source\_loaded\_harmonized/slco\_F1\_surface\_meta\_reference\_public.csv |  | character |
| value — Value **File:** 02\_workflows/F1\_workflow\_v02/data/01\_source\_loaded\_harmonized/slco\_F1\_surface\_meta\_reference\_public.csv **Recommended public name:** value **Description:** Numerical or character value corresponding to the row-specific variable/metric. **Unit:**  **Value coding:** See example\_values and allowed\_values. **Data type:** character **Example values:** f1\_simulated\_surface\_repository | SIMULATED | in\_silico surface grid | One row = one equation-defined grid point (tb\_mg\_... **Allowed values:** f1\_simulated\_surface\_repository | SIMULATED | in\_silico surface grid | One row = one equation-defined grid point (tb\_mg\_dL, cre\_true\_mg\_dL)"""""""""""""""""""""""""""""""""""""""""""""""""""""""""""""""""""""""""""""""""""""""""""""""""""""""""""""""""""""""""""""""""""""""""""""""""""""""""""""""""""""""""""""""""""""""""""""""""""""""""""""""""""""""""""""""""""""""""""""""""""""""""""""""""""""""""""""""""""""""""""""""""""""""""""""""""""""""""""""""""""""""""""""""""""""""""""""""""""""""""""""""""""""""""""""""""""""""""""""""""""""""""""""""""""""""""""""""""""""""""""""""""""""""""""""""""""""""""""""""""""""""""""""""""""""""""""""""""""""""""""""""""""""""""""""""""""""""""""""""""""""""""""""""""""""""""""""""""""""""""""""""""""""""""""""""""""""""""""""""""""""""""""""""""""""""""""""""""""""""""""""""""""""""""""""""""""""""""""""""""""""""""""""""""""""""""""""""""""""""""""""""""""""""""""""""""""""""""""""""""""""""""""""""""""""""""""""""""""""""""""""""""""""""""""""""""""""""""""""""""""""""""""""""""""""""""""""""""""""""""""""""""""""""""""""""""""""""""""""""""""""""""""""""""""""""""""""""""""""""""""""""""""""""""""""""""""""""""""""""""""""""""""""""""""""""""""""""""""""""""""""""""""""""""""""""""""""""""""""""""""""""""""""""""""""""""""""""""""""""""""""""""""""""""""""""""""""""""""""""""""""""""""""""""""""""""""""""""""""""""""""""""""""""""""""""""""""""""""""""""""""""""""""""""""""""""""""""""""""""""""""""""""""""""""""""""""""""""""""""""""""""""""""""""""""""""""""""""""""""""""""""""""""""""""""""""""""""""""""""""""""""""""""""""""""""""""""""""""""""""""""""""""""""""""""""""""""""""""""""""""""""""""""""""""""""""""""""""""""""""""""""""""""""""""""""""""""""""""""""""""""""""""""""""""""""""""""""""""""""""""""""""""""""""""""""""""""""""""""""""""""""""""""""""""""""""""""""""""""""""""""""""""""""""""""""""""""""""""""""""""""""""""""""""""""""""""""""""""""""""""""""""""""""""""""""""""""""""""""""""""""""""""""""""""""""""""""""""""""""""""""""""""""""""""""""""""""""""""""""""""""""""""""""""""""""""""""""""""""""""""""""""""""""""""""""""""""""""""""""""""""""""""""""""""""""""""""""""""""""""""""""""""""""""""""""""""""""""""""""""""""""""""""""""""""""""""""""""""""""""""""""""""""""""""""""""""""""""""""""""""""""""""""""""""""""""""""""""""""""""""""""""""""""""""""""""""""""""""""""""""""""""""""""""""""""""""""""""""""""""""""""""""""""""""""""""""""""""""""""""""""""""""""""""""""""""""""""""""""""""""""""""""""""""""""""""""""""""""""""""""""""""""""""""""""""""""""""""""""""""""""""""""""""""""""""""""""""""""""""""""""""""""""""""""""""""""""""""""""""""""""""""""""""""""""""""""""""""""""""""""""""""""""""""""""""""""""""""""""""""""""""""""""""""""""""""""""""""""""""""""""""""""""""""""""""""""""""""""""""""""""""""""""""""""""""""""""""""""""""""""""""""""""""""""""""""""""""""""""""""""""""""""""""""""""""""""""""""""""""""""""""""""""""""""""""""""""""""""""""""""""""""""""""""""""""""""""""""""""""""""""""""""""""""""""""""""""""""""""""""""""""""""""""""""""""""""""""""""""""""""""""""""""""""""""""""""""""""""""""""""""""""""""""""""""""""""""""""""""""""""""""""""""""""""""""""""""""""""""""""""""""""""""""""""""""""""""""""""""""""""""""""""""""""""""""""""""""""""""""""""""""""""""""""""""""""""""""""""""""""""""""""""""""""""""""""""""""""""""""""""""""""""""""""""""""""""""""""""""""""""""""""""""""""""""""""""""""""""""""""""""""""""""""""""""""""""""""""""""""""""""""""""""""""""""""""""""""""""""""""""""""""""""""""""""""""""""""""""""""""""""""""""""""""""""""""""""""""""""""""""""""""""""""""""""""""""""""""""""""""""""""""""""""""""""""""""""""""""""""""""""""""""""""""""""""""""""""""""""""""""""""""""""""""""""""""""""""""""""""""""""""""""""""""""""""""""""""""""""""""""""""""""""""""""""""""""""""""""""""""""""""""""""""""""""""""""""""""""""""""""""""""""""""""""""""""""""""""""""""""""""""""""""""""""""""""""""""""""""""""""""""""""""""""""""""""""""""""""""""""""""""""""""""""""""""""""""""""""""""""""""""""""""""""""""""""""""""""""""""""""""""""""""""""""""""""""""""""""""""""""""""""""""""""""""""""""""""""""""""""""""""""""""""""""""""""""""""""""""""""""""""""""""""" | 1 to 35 | 1 to 6 | 0.1 | 0.05 | delta = cre\_true\_mg\_dL - predicted measured creatinine **Missing-value coding:** NA/blank as read by fread **n rows / missing / unique:** 9 / 0 / 9 **Release status:** public **Definition status:** exact\_definition | 02\_workflows/F1\_workflow\_v02/data/01\_source\_loaded\_harmonized/slco\_F1\_surface\_meta\_reference\_public.csv |  | character |
| Cre\_nominal\_grav\_mg\_dL — Nominal gravimetric creatinine target concentration **File:** 02\_workflows/F1\_workflow\_v02/data/02a\_refined\_analysis/expm\_F1\_array\_input\_public.csv **Recommended public name:** Cre\_nominal\_grav\_mg\_dL **Description:** Nominal gravimetric target concentration of creatinine used when defining the F1 experimental array. **Unit:** mg/dL **Value coding:** Numeric concentration in mg/dL. **Data type:** numeric **Example values:** 3 | 1.5 | 3.5 | 2 | 0.5 **Allowed values:**  **Missing-value coding:** NA/blank as read by fread **n rows / missing / unique:** 4200 / 0 / 10 **Release status:** public **Definition status:** manual\_precision\_definition | 02\_workflows/F1\_workflow\_v02/data/02a\_refined\_analysis/expm\_F1\_array\_input\_public.csv | mg/dL | numeric |
| TB\_nominal\_grav\_mg\_dL — Nominal gravimetric total bilirubin target concentration **File:** 02\_workflows/F1\_workflow\_v02/data/02a\_refined\_analysis/expm\_F1\_array\_input\_public.csv **Recommended public name:** TB\_nominal\_grav\_mg\_dL **Description:** Nominal gravimetric target concentration of total bilirubin used when defining the F1 experimental array. **Unit:** mg/dL **Value coding:** Numeric concentration in mg/dL. **Data type:** integer **Example values:** 27 | 14 | 28 | 15 | 29 **Allowed values:**  **Missing-value coding:** NA/blank as read by fread **n rows / missing / unique:** 4200 / 0 / 35 **Release status:** public **Definition status:** manual\_precision\_definition | 02\_workflows/F1\_workflow\_v02/data/02a\_refined\_analysis/expm\_F1\_array\_input\_public.csv | mg/dL | integer |
| TB\_trial\_M\_mg\_dL — Measured total bilirubin concentration in the experimental dataset **File:** 02\_workflows/F1\_workflow\_v02/data/02a\_refined\_analysis/expm\_F1\_array\_input\_public.csv **Recommended public name:** TB\_trial\_M\_mg\_dL **Description:** Measured total bilirubin concentration in the F1 experimental dataset. In rounded input tables this is the rounded/display value used for model calculation and plotting. **Unit:** mg/dL **Value coding:** Numeric concentration in mg/dL. **Data type:** numeric **Example values:** 27.22 | 27.13 | 26.94 | 27.09 | 14.04 **Allowed values:**  **Missing-value coding:** NA/blank as read by fread **n rows / missing / unique:** 4200 / 0 / 1104 **Release status:** public **Definition status:** manual\_precision\_definition | 02\_workflows/F1\_workflow\_v02/data/02a\_refined\_analysis/expm\_F1\_array\_input\_public.csv | mg/dL | numeric |
| TB\_trial\_M\_mg\_dL\_unrounded — Unrounded measured total bilirubin concentration in the experimental dataset **File:** 02\_workflows/F1\_workflow\_v02/data/02a\_refined\_analysis/expm\_F1\_array\_input\_public.csv **Recommended public name:** TB\_trial\_M\_mg\_dL\_unrounded **Description:** Measured total bilirubin concentration in the F1 experimental dataset before rounding to the display/model-input precision. **Unit:** mg/dL **Value coding:** Numeric concentration in mg/dL before rounding. **Data type:** numeric **Example values:** 27.2227640681025 | 27.1297714113581 | 27.2238964489877 | 27.1309037922433 | 26.9427373034614 **Allowed values:**  **Missing-value coding:** NA/blank as read by fread **n rows / missing / unique:** 4200 / 0 / 4200 **Release status:** public **Definition status:** manual\_precision\_definition | 02\_workflows/F1\_workflow\_v02/data/02a\_refined\_analysis/expm\_F1\_array\_input\_public.csv | mg/dL | numeric |
| anchor — Anchor/output group **File:** 02\_workflows/F1\_workflow\_v02/data/02a\_refined\_analysis/expm\_F1\_array\_input\_public.csv **Recommended public name:** anchor **Description:** Anchor or output grouping label used by the workflow to identify a specific public output component. **Unit:**  **Value coding:** See example\_values and allowed\_values. **Data type:** character **Example values:** F1 **Allowed values:** F1 **Missing-value coding:** NA/blank as read by fread **n rows / missing / unique:** 4200 / 0 / 1 **Release status:** public **Definition status:** exact\_definition | 02\_workflows/F1\_workflow\_v02/data/02a\_refined\_analysis/expm\_F1\_array\_input\_public.csv |  | character |
| array\_id — Experimental array identifier **File:** 02\_workflows/F1\_workflow\_v02/data/02a\_refined\_analysis/expm\_F1\_array\_input\_public.csv **Recommended public name:** array\_id **Description:** Identifier of the experimental array used in the F1 creatinine/bilirubin interference data. **Unit:**  **Value coding:** See example\_values and allowed\_values. **Data type:** character **Example values:** Array\_1 | Array\_2 | Array\_3 **Allowed values:** Array\_1 | Array\_2 | Array\_3 **Missing-value coding:** NA/blank as read by fread **n rows / missing / unique:** 4200 / 0 / 3 **Release status:** public **Definition status:** exact\_definition | 02\_workflows/F1\_workflow\_v02/data/02a\_refined\_analysis/expm\_F1\_array\_input\_public.csv |  | character |
| assay — Creatinine assay **File:** 02\_workflows/F1\_workflow\_v02/data/02a\_refined\_analysis/expm\_F1\_array\_input\_public.csv **Recommended public name:** assay **Description:** Creatinine assay represented by the row, for example enzymatic creatinine (CreE) or Jaffe creatinine (CreJ). **Unit:**  **Value coding:** Categorical assay label, e.g. CreE or CreJ. **Data type:** character **Example values:** CreE | CreJ **Allowed values:** CreE | CreJ **Missing-value coding:** NA/blank as read by fread **n rows / missing / unique:** 4200 / 0 / 2 **Release status:** public **Definition status:** manual\_precision\_definition | 02\_workflows/F1\_workflow\_v02/data/02a\_refined\_analysis/expm\_F1\_array\_input\_public.csv |  | character |
| data\_object — Data object **File:** 02\_workflows/F1\_workflow\_v02/data/02a\_refined\_analysis/expm\_F1\_array\_input\_public.csv **Recommended public name:** data\_object **Description:** Name of the data object represented by the row. **Unit:**  **Value coding:** Categorical descriptor; see allowed/example values. **Data type:** character **Example values:** array **Allowed values:** array **Missing-value coding:** NA/blank as read by fread **n rows / missing / unique:** 4200 / 0 / 1 **Release status:** public **Definition status:** exact\_definition | 02\_workflows/F1\_workflow\_v02/data/02a\_refined\_analysis/expm\_F1\_array\_input\_public.csv |  | character |
| domain — Data domain **File:** 02\_workflows/F1\_workflow\_v02/data/02a\_refined\_analysis/expm\_F1\_array\_input\_public.csv **Recommended public name:** domain **Description:** Workflow or data domain represented by the row. **Unit:**  **Value coding:** Categorical descriptor; see allowed/example values. **Data type:** character **Example values:** expm **Allowed values:** expm **Missing-value coding:** NA/blank as read by fread **n rows / missing / unique:** 4200 / 0 / 1 **Release status:** public **Definition status:** exact\_definition | 02\_workflows/F1\_workflow\_v02/data/02a\_refined\_analysis/expm\_F1\_array\_input\_public.csv |  | character |
| expm\_F1\_array\_row\_id — Experimental array row identifier **File:** 02\_workflows/F1\_workflow\_v02/data/02a\_refined\_analysis/expm\_F1\_array\_input\_public.csv **Recommended public name:** expm\_F1\_array\_row\_id **Description:** Row identifier within the released F1 experimental array table. **Unit:**  **Value coding:** See example\_values and allowed\_values. **Data type:** integer **Example values:** 1 | 2 | 3 | 4 | 5 **Allowed values:**  **Missing-value coding:** NA/blank as read by fread **n rows / missing / unique:** 4200 / 0 / 4200 **Release status:** public **Definition status:** exact\_definition | 02\_workflows/F1\_workflow\_v02/data/02a\_refined\_analysis/expm\_F1\_array\_input\_public.csv |  | integer |
| plot\_correction\_mg\_dL — Plotted creatinine correction **File:** 02\_workflows/F1\_workflow\_v02/data/02a\_refined\_analysis/expm\_F1\_array\_input\_public.csv **Recommended public name:** plot\_correction\_mg\_dL **Description:** Creatinine correction value plotted in F1, expressed as the difference between corrected/reference and measured creatinine. **Unit:** probability **Value coding:** Numeric correction in mg/dL. **Data type:** numeric **Example values:** -0.541120525715191 | -0.480865451714561 | -0.386578220459593 | -0.317061468810564 | -0.458450885953881 **Allowed values:**  **Missing-value coding:** NA/blank as read by fread **n rows / missing / unique:** 4200 / 0 / 4200 **Release status:** public **Definition status:** manual\_precision\_definition | 02\_workflows/F1\_workflow\_v02/data/02a\_refined\_analysis/expm\_F1\_array\_input\_public.csv | probability | numeric |
| preparer\_id — Experimental preparer identifier **File:** 02\_workflows/F1\_workflow\_v02/data/02a\_refined\_analysis/expm\_F1\_array\_input\_public.csv **Recommended public name:** preparer\_id **Description:** Identifier of the experimental preparer in F1 raw/validation data, represented as a public technical code. **Unit:**  **Value coding:** See example\_values and allowed\_values. **Data type:** character **Example values:** Prep\_A | Prep\_B **Allowed values:** Prep\_A | Prep\_B **Missing-value coding:** NA/blank as read by fread **n rows / missing / unique:** 4200 / 0 / 2 **Release status:** public **Definition status:** exact\_definition | 02\_workflows/F1\_workflow\_v02/data/02a\_refined\_analysis/expm\_F1\_array\_input\_public.csv |  | character |
| release\_status — Release status **File:** 02\_workflows/F1\_workflow\_v02/data/02a\_refined\_analysis/expm\_F1\_array\_input\_public.csv **Recommended public name:** release\_status **Description:** Release-status label indicating the publication status of the row or file object. **Unit:**  **Value coding:** Release status of the row/object, for example public. **Data type:** character **Example values:** public **Allowed values:** public **Missing-value coding:** NA/blank as read by fread **n rows / missing / unique:** 4200 / 0 / 1 **Release status:** public **Definition status:** exact\_definition | 02\_workflows/F1\_workflow\_v02/data/02a\_refined\_analysis/expm\_F1\_array\_input\_public.csv |  | character |
| replicate — Experimental replicate number **File:** 02\_workflows/F1\_workflow\_v02/data/02a\_refined\_analysis/expm\_F1\_array\_input\_public.csv **Recommended public name:** replicate **Description:** Replicate number within the F1 experimental dataset. **Unit:** display order **Value coding:** Integer replicate number. **Data type:** integer **Example values:** 2 | 1 **Allowed values:**  **Missing-value coding:** NA/blank as read by fread **n rows / missing / unique:** 4200 / 0 / 2 **Release status:** public **Definition status:** manual\_precision\_definition | 02\_workflows/F1\_workflow\_v02/data/02a\_refined\_analysis/expm\_F1\_array\_input\_public.csv | display order | integer |
| source\_harmonized\_file\_name — Source harmonized file name **File:** 02\_workflows/F1\_workflow\_v02/data/02a\_refined\_analysis/expm\_F1\_array\_input\_public.csv **Recommended public name:** source\_harmonized\_file\_name **Description:** Name of the harmonized source file used to build the released object. **Unit:**  **Value coding:** See example\_values and allowed\_values. **Data type:** character **Example values:** expm\_F1\_array\_raw\_public.csv **Allowed values:** expm\_F1\_array\_raw\_public.csv **Missing-value coding:** NA/blank as read by fread **n rows / missing / unique:** 4200 / 0 / 1 **Release status:** public **Definition status:** exact\_definition | 02\_workflows/F1\_workflow\_v02/data/02a\_refined\_analysis/expm\_F1\_array\_input\_public.csv |  | character |
| trial\_display\_Cre\_M\_mg\_dL — Measured creatinine concentration in the experimental dataset **File:** 02\_workflows/F1\_workflow\_v02/data/02a\_refined\_analysis/expm\_F1\_array\_input\_public.csv **Recommended public name:** trial\_display\_Cre\_M\_mg\_dL **Description:** Measured creatinine concentration in the F1 experimental dataset. In rounded input tables this is the rounded/display value used for model calculation and plotting. **Unit:** mg/dL **Value coding:** Numeric concentration in mg/dL. **Data type:** numeric **Example values:** 3.54 | 3.48 | 3.39 | 3.32 | 3.46 **Allowed values:**  **Missing-value coding:** NA/blank as read by fread **n rows / missing / unique:** 4200 / 0 / 540 **Release status:** public **Definition status:** manual\_precision\_definition | 02\_workflows/F1\_workflow\_v02/data/02a\_refined\_analysis/expm\_F1\_array\_input\_public.csv | mg/dL | numeric |
| trial\_display\_Cre\_M\_mg\_dL\_unrounded — Unrounded measured creatinine concentration in the experimental dataset **File:** 02\_workflows/F1\_workflow\_v02/data/02a\_refined\_analysis/expm\_F1\_array\_input\_public.csv **Recommended public name:** trial\_display\_Cre\_M\_mg\_dL\_unrounded **Description:** Measured creatinine concentration in the F1 experimental dataset before rounding to the display/model-input precision. **Unit:** mg/dL **Value coding:** Numeric concentration in mg/dL before rounding. **Data type:** numeric **Example values:** 3.54112052571519 | 3.48086545171456 | 3.38657822045959 | 3.31706146881056 | 3.45845088595388 **Allowed values:**  **Missing-value coding:** NA/blank as read by fread **n rows / missing / unique:** 4200 / 0 / 4200 **Release status:** public **Definition status:** manual\_precision\_definition | 02\_workflows/F1\_workflow\_v02/data/02a\_refined\_analysis/expm\_F1\_array\_input\_public.csv | mg/dL | numeric |
| unit\_or\_role — Unit or semantic role **File:** 02\_workflows/F1\_workflow\_v02/data/02a\_refined\_analysis/expm\_F1\_array\_input\_public.csv **Recommended public name:** unit\_or\_role **Description:** Unit, role, or semantic type corresponding to the row-specific variable/metric. **Unit:**  **Value coding:** Categorical descriptor; see allowed/example values. **Data type:** character **Example values:** input **Allowed values:** input **Missing-value coding:** NA/blank as read by fread **n rows / missing / unique:** 4200 / 0 / 1 **Release status:** public **Definition status:** exact\_definition | 02\_workflows/F1\_workflow\_v02/data/02a\_refined\_analysis/expm\_F1\_array\_input\_public.csv |  | character |
| workflow\_step — Workflow step **File:** 02\_workflows/F1\_workflow\_v02/data/02a\_refined\_analysis/expm\_F1\_array\_input\_public.csv **Recommended public name:** workflow\_step **Description:** Workflow step that produced or used the row/object. **Unit:**  **Value coding:** Categorical descriptor; see allowed/example values. **Data type:** character **Example values:** 2a\_refined\_analysis\_dataset **Allowed values:** 2a\_refined\_analysis\_dataset **Missing-value coding:** NA/blank as read by fread **n rows / missing / unique:** 4200 / 0 / 1 **Release status:** public **Definition status:** exact\_definition | 02\_workflows/F1\_workflow\_v02/data/02a\_refined\_analysis/expm\_F1\_array\_input\_public.csv |  | character |
| assay — Creatinine assay **File:** 02\_workflows/F1\_workflow\_v02/data/02a\_refined\_analysis/expm\_F1\_model\_coefficients\_public.csv **Recommended public name:** assay **Description:** Creatinine assay represented by the row, for example enzymatic creatinine (CreE) or Jaffe creatinine (CreJ). **Unit:**  **Value coding:** Categorical assay label, e.g. CreE or CreJ. **Data type:** character **Example values:** CreJ | CreE **Allowed values:** CreJ | CreE **Missing-value coding:** NA/blank as read by fread **n rows / missing / unique:** 10 / 0 / 2 **Release status:** public **Definition status:** manual\_precision\_definition | 02\_workflows/F1\_workflow\_v02/data/02a\_refined\_analysis/expm\_F1\_model\_coefficients\_public.csv |  | character |
| coefficient — Model coefficient **File:** 02\_workflows/F1\_workflow\_v02/data/02a\_refined\_analysis/expm\_F1\_model\_coefficients\_public.csv **Recommended public name:** coefficient **Description:** Fixed coefficient used in the F1 creatinine/bilirubin correction polynomial. **Unit:** model coefficient **Value coding:** Numeric model coefficient. **Data type:** numeric **Example values:** 0.0115 | -0.012 | 0.00025 | 1.039 | -0.0294 **Allowed values:**  **Missing-value coding:** NA/blank as read by fread **n rows / missing / unique:** 10 / 0 / 10 **Release status:** public **Definition status:** manual\_precision\_definition | 02\_workflows/F1\_workflow\_v02/data/02a\_refined\_analysis/expm\_F1\_model\_coefficients\_public.csv | model coefficient | numeric |
| equation\_role — Equation role **File:** 02\_workflows/F1\_workflow\_v02/data/02a\_refined\_analysis/expm\_F1\_model\_coefficients\_public.csv **Recommended public name:** equation\_role **Description:** Role of the equation represented by the F1 model-coefficient row. **Unit:**  **Value coding:** Categorical equation-role label. **Data type:** character **Example values:** true\_creatinine\_from\_measured\_creatinine\_and\_total\_bilirubin **Allowed values:** true\_creatinine\_from\_measured\_creatinine\_and\_total\_bilirubin **Missing-value coding:** NA/blank as read by fread **n rows / missing / unique:** 10 / 0 / 1 **Release status:** public **Definition status:** manual\_precision\_definition | 02\_workflows/F1\_workflow\_v02/data/02a\_refined\_analysis/expm\_F1\_model\_coefficients\_public.csv |  | character |
| release\_status — Release status **File:** 02\_workflows/F1\_workflow\_v02/data/02a\_refined\_analysis/expm\_F1\_model\_coefficients\_public.csv **Recommended public name:** release\_status **Description:** Release-status label indicating the publication status of the row or file object. **Unit:**  **Value coding:** Release status of the row/object, for example public. **Data type:** character **Example values:** public **Allowed values:** public **Missing-value coding:** NA/blank as read by fread **n rows / missing / unique:** 10 / 0 / 1 **Release status:** public **Definition status:** exact\_definition | 02\_workflows/F1\_workflow\_v02/data/02a\_refined\_analysis/expm\_F1\_model\_coefficients\_public.csv |  | character |
| term — Polynomial model term **File:** 02\_workflows/F1\_workflow\_v02/data/02a\_refined\_analysis/expm\_F1\_model\_coefficients\_public.csv **Recommended public name:** term **Description:** Term name in the fixed F1 correction polynomial, for example intercept, bilirubin term, squared bilirubin term, creatinine term, or squared creatinine term. **Unit:** score class **Value coding:** Categorical polynomial term label. **Data type:** character **Example values:** intercept | tb\_mg\_dL | tb\_mg\_dL\_squared | cre\_measured\_mg\_dL | cre\_measured\_mg\_dL\_squared **Allowed values:** intercept | tb\_mg\_dL | tb\_mg\_dL\_squared | cre\_measured\_mg\_dL | cre\_measured\_mg\_dL\_squared **Missing-value coding:** NA/blank as read by fread **n rows / missing / unique:** 10 / 0 / 5 **Release status:** public **Definition status:** manual\_precision\_definition | 02\_workflows/F1\_workflow\_v02/data/02a\_refined\_analysis/expm\_F1\_model\_coefficients\_public.csv | score class | character |
| workflow\_step — Workflow step **File:** 02\_workflows/F1\_workflow\_v02/data/02a\_refined\_analysis/expm\_F1\_model\_coefficients\_public.csv **Recommended public name:** workflow\_step **Description:** Workflow step that produced or used the row/object. **Unit:**  **Value coding:** Categorical descriptor; see allowed/example values. **Data type:** character **Example values:** 2a\_refined\_model\_coefficients **Allowed values:** 2a\_refined\_model\_coefficients **Missing-value coding:** NA/blank as read by fread **n rows / missing / unique:** 10 / 0 / 1 **Release status:** public **Definition status:** exact\_definition | 02\_workflows/F1\_workflow\_v02/data/02a\_refined\_analysis/expm\_F1\_model\_coefficients\_public.csv |  | character |
| max\_abs\_rounding\_difference — Maximum absolute rounding difference **File:** 02\_workflows/F1\_workflow\_v02/data/02a\_refined\_analysis/expm\_F1\_pre\_correction\_rounding\_qc\_public.csv **Recommended public name:** max\_abs\_rounding\_difference **Description:** Maximum absolute difference caused by rounding. **Unit:**  **Value coding:** See example\_values and allowed\_values. **Data type:** numeric **Example values:** 0.00499893919266015 | 0.00499854811828993 **Allowed values:**  **Missing-value coding:** NA/blank as read by fread **n rows / missing / unique:** 2 / 0 / 2 **Release status:** public **Definition status:** exact\_definition | 02\_workflows/F1\_workflow\_v02/data/02a\_refined\_analysis/expm\_F1\_pre\_correction\_rounding\_qc\_public.csv |  | numeric |
| max\_value\_after\_rounding — Maximum value after rounding **File:** 02\_workflows/F1\_workflow\_v02/data/02a\_refined\_analysis/expm\_F1\_pre\_correction\_rounding\_qc\_public.csv **Recommended public name:** max\_value\_after\_rounding **Description:** Maximum value observed after rounding. **Unit:**  **Value coding:** See example\_values and allowed\_values. **Data type:** numeric **Example values:** 35.58 | 6.09 **Allowed values:**  **Missing-value coding:** NA/blank as read by fread **n rows / missing / unique:** 2 / 0 / 2 **Release status:** public **Definition status:** exact\_definition | 02\_workflows/F1\_workflow\_v02/data/02a\_refined\_analysis/expm\_F1\_pre\_correction\_rounding\_qc\_public.csv |  | numeric |
| mean\_abs\_rounding\_difference — Mean absolute rounding difference **File:** 02\_workflows/F1\_workflow\_v02/data/02a\_refined\_analysis/expm\_F1\_pre\_correction\_rounding\_qc\_public.csv **Recommended public name:** mean\_abs\_rounding\_difference **Description:** Mean absolute difference caused by rounding. **Unit:**  **Value coding:** See example\_values and allowed\_values. **Data type:** numeric **Example values:** 0.00250339547922569 | 0.00252476009694654 **Allowed values:**  **Missing-value coding:** NA/blank as read by fread **n rows / missing / unique:** 2 / 0 / 2 **Release status:** public **Definition status:** exact\_definition | 02\_workflows/F1\_workflow\_v02/data/02a\_refined\_analysis/expm\_F1\_pre\_correction\_rounding\_qc\_public.csv |  | numeric |
| min\_value\_after\_rounding — Minimum value after rounding **File:** 02\_workflows/F1\_workflow\_v02/data/02a\_refined\_analysis/expm\_F1\_pre\_correction\_rounding\_qc\_public.csv **Recommended public name:** min\_value\_after\_rounding **Description:** Minimum value observed after rounding. **Unit:**  **Value coding:** See example\_values and allowed\_values. **Data type:** numeric **Example values:** 0.98 | 0.35 **Allowed values:**  **Missing-value coding:** NA/blank as read by fread **n rows / missing / unique:** 2 / 0 / 2 **Release status:** public **Definition status:** exact\_definition | 02\_workflows/F1\_workflow\_v02/data/02a\_refined\_analysis/expm\_F1\_pre\_correction\_rounding\_qc\_public.csv |  | numeric |
| n\_total\_values — Total number of values **File:** 02\_workflows/F1\_workflow\_v02/data/02a\_refined\_analysis/expm\_F1\_pre\_correction\_rounding\_qc\_public.csv **Recommended public name:** n\_total\_values **Description:** Total number of values evaluated. **Unit:**  **Value coding:** See example\_values and allowed\_values. **Data type:** integer **Example values:** 4200 **Allowed values:**  **Missing-value coding:** NA/blank as read by fread **n rows / missing / unique:** 2 / 0 / 1 **Release status:** public **Definition status:** exact\_definition | 02\_workflows/F1\_workflow\_v02/data/02a\_refined\_analysis/expm\_F1\_pre\_correction\_rounding\_qc\_public.csv |  | integer |
| n\_values\_changed\_by\_rounding — Number of values changed by rounding **File:** 02\_workflows/F1\_workflow\_v02/data/02a\_refined\_analysis/expm\_F1\_pre\_correction\_rounding\_qc\_public.csv **Recommended public name:** n\_values\_changed\_by\_rounding **Description:** Number of values whose value changed after rounding. **Unit:**  **Value coding:** See example\_values and allowed\_values. **Data type:** integer **Example values:** 4200 **Allowed values:**  **Missing-value coding:** NA/blank as read by fread **n rows / missing / unique:** 2 / 0 / 1 **Release status:** public **Definition status:** exact\_definition | 02\_workflows/F1\_workflow\_v02/data/02a\_refined\_analysis/expm\_F1\_pre\_correction\_rounding\_qc\_public.csv |  | integer |
| rounding\_rule — Rounding rule **File:** 02\_workflows/F1\_workflow\_v02/data/02a\_refined\_analysis/expm\_F1\_pre\_correction\_rounding\_qc\_public.csv **Recommended public name:** rounding\_rule **Description:** Rounding rule applied before correction/model calculation. **Unit:**  **Value coding:** See example\_values and allowed\_values. **Data type:** character **Example values:** round to 2 decimal places before correction/model calculation **Allowed values:** round to 2 decimal places before correction/model calculation **Missing-value coding:** NA/blank as read by fread **n rows / missing / unique:** 2 / 0 / 1 **Release status:** public **Definition status:** exact\_definition | 02\_workflows/F1\_workflow\_v02/data/02a\_refined\_analysis/expm\_F1\_pre\_correction\_rounding\_qc\_public.csv |  | character |
| variable — Variable represented by row **File:** 02\_workflows/F1\_workflow\_v02/data/02a\_refined\_analysis/expm\_F1\_pre\_correction\_rounding\_qc\_public.csv **Recommended public name:** variable **Description:** Name of the variable represented by the row in a long-format table. **Unit:**  **Value coding:** Categorical descriptor; see allowed/example values. **Data type:** character **Example values:** TB\_trial\_M\_mg\_dL | trial\_display\_Cre\_M\_mg\_dL **Allowed values:** TB\_trial\_M\_mg\_dL | trial\_display\_Cre\_M\_mg\_dL **Missing-value coding:** NA/blank as read by fread **n rows / missing / unique:** 2 / 0 / 2 **Release status:** public **Definition status:** exact\_definition | 02\_workflows/F1\_workflow\_v02/data/02a\_refined\_analysis/expm\_F1\_pre\_correction\_rounding\_qc\_public.csv |  | character |
| metric — Metric **File:** 02\_workflows/F1\_workflow\_v02/data/02a\_refined\_analysis/expm\_F1\_refined\_dataset\_qc\_public.csv **Recommended public name:** metric **Description:** Name of the metric represented by the row. **Unit:**  **Value coding:** Categorical descriptor; see allowed/example values. **Data type:** character **Example values:** array\_rows | array\_columns | array\_unique\_arrays | array\_unique\_preparers | array\_unique\_assays **Allowed values:** array\_rows | array\_columns | array\_unique\_arrays | array\_unique\_preparers | array\_unique\_assays | array\_min\_tb\_trial\_mg\_dL\_after\_rounding | array\_max\_tb\_trial\_mg\_dL\_after\_rounding | array\_min\_cre\_trial\_display\_mg\_dL\_after\_rounding | array\_max\_cre\_tri... **Missing-value coding:** NA/blank as read by fread **n rows / missing / unique:** 18 / 0 / 18 **Release status:** public **Definition status:** exact\_definition | 02\_workflows/F1\_workflow\_v02/data/02a\_refined\_analysis/expm\_F1\_refined\_dataset\_qc\_public.csv |  | character |
| value — Value **File:** 02\_workflows/F1\_workflow\_v02/data/02a\_refined\_analysis/expm\_F1\_refined\_dataset\_qc\_public.csv **Recommended public name:** value **Description:** Numerical or character value corresponding to the row-specific variable/metric. **Unit:**  **Value coding:** See example\_values and allowed\_values. **Data type:** numeric **Example values:** 4200 | 19 | 3 | 2 | 0.98 **Allowed values:**  **Missing-value coding:** NA/blank as read by fread **n rows / missing / unique:** 18 / 0 / 15 **Release status:** public **Definition status:** exact\_definition | 02\_workflows/F1\_workflow\_v02/data/02a\_refined\_analysis/expm\_F1\_refined\_dataset\_qc\_public.csv |  | numeric |
| issue — QC or audit issue **File:** 02\_workflows/F1\_workflow\_v02/data/02a\_refined\_analysis/expm\_F1\_refined\_manual\_clarifications\_public.csv **Recommended public name:** issue **Description:** Free-text QC or audit note describing a checked issue. **Unit:**  **Value coding:** Free text. **Data type:** character **Example values:** Coefficients are used as fixed manuscript/model coefficients; this script does not refit regression equations from the r... **Allowed values:** Coefficients are used as fixed manuscript/model coefficients; this script does not refit regression equations from the raw experimental array. | TB\_trial\_M\_mg\_dL and trial\_display\_Cre\_M\_mg\_dL are rounded to 2 decimal places in the refined array datas... **Missing-value coding:** NA/blank as read by fread **n rows / missing / unique:** 3 / 0 / 3 **Release status:** public **Definition status:** manual\_precision\_definition | 02\_workflows/F1\_workflow\_v02/data/02a\_refined\_analysis/expm\_F1\_refined\_manual\_clarifications\_public.csv |  | character |
| item — Audit item **File:** 02\_workflows/F1\_workflow\_v02/data/02a\_refined\_analysis/expm\_F1\_refined\_manual\_clarifications\_public.csv **Recommended public name:** item **Description:** Name of the QC or audit item being reported. **Unit:**  **Value coding:** Categorical audit item. **Data type:** character **Example values:** model\_coefficients | pre\_correction\_rounding | plot\_correction\_mg\_dL **Allowed values:** model\_coefficients | pre\_correction\_rounding | plot\_correction\_mg\_dL **Missing-value coding:** NA/blank as read by fread **n rows / missing / unique:** 3 / 0 / 3 **Release status:** public **Definition status:** manual\_precision\_definition | 02\_workflows/F1\_workflow\_v02/data/02a\_refined\_analysis/expm\_F1\_refined\_manual\_clarifications\_public.csv |  | character |
| suggested\_manual\_check — Suggested manual check **File:** 02\_workflows/F1\_workflow\_v02/data/02a\_refined\_analysis/expm\_F1\_refined\_manual\_clarifications\_public.csv **Recommended public name:** suggested\_manual\_check **Description:** Free-text recommendation for a manual QC check made during the public workflow. **Unit:** mg/dL **Value coding:** Free text. **Data type:** character **Example values:** Confirm that fixed coefficients are the final accepted coefficients for Figure 1 reproduction. | Confirm that two-decima... **Allowed values:** Confirm that fixed coefficients are the final accepted coefficients for Figure 1 reproduction. | Confirm that two-decimal rounding is the intended final analytical precision for both variables. | Confirm whether plot\_correction\_mg\_dL should remain so... **Missing-value coding:** NA/blank as read by fread **n rows / missing / unique:** 3 / 0 / 3 **Release status:** public **Definition status:** manual\_precision\_definition | 02\_workflows/F1\_workflow\_v02/data/02a\_refined\_analysis/expm\_F1\_refined\_manual\_clarifications\_public.csv | mg/dL | character |
| anchor — Anchor/output group **File:** 02\_workflows/F1\_workflow\_v02/data/02a\_refined\_analysis/expm\_F1\_validation\_input\_public.csv **Recommended public name:** anchor **Description:** Anchor or output grouping label used by the workflow to identify a specific public output component. **Unit:**  **Value coding:** See example\_values and allowed\_values. **Data type:** character **Example values:** F1 **Allowed values:** F1 **Missing-value coding:** NA/blank as read by fread **n rows / missing / unique:** 32 / 0 / 1 **Release status:** public **Definition status:** exact\_definition | 02\_workflows/F1\_workflow\_v02/data/02a\_refined\_analysis/expm\_F1\_validation\_input\_public.csv |  | character |
| cre\_true\_gcidms\_mg\_dL — GC-IDMS reference creatinine concentration **File:** 02\_workflows/F1\_workflow\_v02/data/02a\_refined\_analysis/expm\_F1\_validation\_input\_public.csv **Recommended public name:** cre\_true\_gcidms\_mg\_dL **Description:** Creatinine concentration measured by GC-IDMS reference method in the validation data. **Unit:**  **Value coding:** See example\_values and allowed\_values. **Data type:** numeric **Example values:** 0.82 | 0.86 | 0.63 | 0.94 | 0.9 **Allowed values:**  **Missing-value coding:** NA/blank as read by fread **n rows / missing / unique:** 32 / 0 / 30 **Release status:** public **Definition status:** exact\_definition | 02\_workflows/F1\_workflow\_v02/data/02a\_refined\_analysis/expm\_F1\_validation\_input\_public.csv |  | numeric |
| cree\_corrected\_mg\_dL — Corrected enzymatic creatinine concentration **File:** 02\_workflows/F1\_workflow\_v02/data/02a\_refined\_analysis/expm\_F1\_validation\_input\_public.csv **Recommended public name:** cree\_corrected\_mg\_dL **Description:** Creatinine concentration after applying the enzymatic correction model. **Unit:**  **Value coding:** See example\_values and allowed\_values. **Data type:** numeric **Example values:** 0.68240141154 | 0.81680053664 | 0.35207418306 | 0.93410841026 | 0.6516394885 **Allowed values:**  **Missing-value coding:** NA/blank as read by fread **n rows / missing / unique:** 32 / 0 / 32 **Release status:** public **Definition status:** exact\_definition | 02\_workflows/F1\_workflow\_v02/data/02a\_refined\_analysis/expm\_F1\_validation\_input\_public.csv |  | numeric |
| cree\_measured\_mg\_dL — Measured enzymatic creatinine concentration **File:** 02\_workflows/F1\_workflow\_v02/data/02a\_refined\_analysis/expm\_F1\_validation\_input\_public.csv **Recommended public name:** cree\_measured\_mg\_dL **Description:** Measured creatinine concentration using the enzymatic assay. **Unit:**  **Value coding:** See example\_values and allowed\_values. **Data type:** numeric **Example values:** 0.75 | 0.78 | 0.52 | 0.89 | 0.8 **Allowed values:**  **Missing-value coding:** NA/blank as read by fread **n rows / missing / unique:** 32 / 0 / 32 **Release status:** public **Definition status:** exact\_definition | 02\_workflows/F1\_workflow\_v02/data/02a\_refined\_analysis/expm\_F1\_validation\_input\_public.csv |  | numeric |
| crej\_corrected\_mg\_dL — Corrected Jaffe creatinine concentration **File:** 02\_workflows/F1\_workflow\_v02/data/02a\_refined\_analysis/expm\_F1\_validation\_input\_public.csv **Recommended public name:** crej\_corrected\_mg\_dL **Description:** Creatinine concentration after applying the Jaffe correction model. **Unit:**  **Value coding:** See example\_values and allowed\_values. **Data type:** numeric **Example values:** 0.8822513 | 0.9490736 | 0.6756628 | 0.8942763 | 0.9588407 **Allowed values:**  **Missing-value coding:** NA/blank as read by fread **n rows / missing / unique:** 32 / 0 / 32 **Release status:** public **Definition status:** exact\_definition | 02\_workflows/F1\_workflow\_v02/data/02a\_refined\_analysis/expm\_F1\_validation\_input\_public.csv |  | numeric |
| crej\_measured\_mg\_dL — Measured Jaffe creatinine concentration **File:** 02\_workflows/F1\_workflow\_v02/data/02a\_refined\_analysis/expm\_F1\_validation\_input\_public.csv **Recommended public name:** crej\_measured\_mg\_dL **Description:** Measured creatinine concentration using the Jaffe assay. **Unit:**  **Value coding:** See example\_values and allowed\_values. **Data type:** numeric **Example values:** 0.95 | 0.98 | 0.78 | 0.92 | 1.06 **Allowed values:**  **Missing-value coding:** NA/blank as read by fread **n rows / missing / unique:** 32 / 0 / 31 **Release status:** public **Definition status:** exact\_definition | 02\_workflows/F1\_workflow\_v02/data/02a\_refined\_analysis/expm\_F1\_validation\_input\_public.csv |  | numeric |
| data\_object — Data object **File:** 02\_workflows/F1\_workflow\_v02/data/02a\_refined\_analysis/expm\_F1\_validation\_input\_public.csv **Recommended public name:** data\_object **Description:** Name of the data object represented by the row. **Unit:**  **Value coding:** Categorical descriptor; see allowed/example values. **Data type:** character **Example values:** validation **Allowed values:** validation **Missing-value coding:** NA/blank as read by fread **n rows / missing / unique:** 32 / 0 / 1 **Release status:** public **Definition status:** exact\_definition | 02\_workflows/F1\_workflow\_v02/data/02a\_refined\_analysis/expm\_F1\_validation\_input\_public.csv |  | character |
| db\_measured\_mg\_dL — Direct bilirubin concentration **File:** 02\_workflows/F1\_workflow\_v02/data/02a\_refined\_analysis/expm\_F1\_validation\_input\_public.csv **Recommended public name:** db\_measured\_mg\_dL **Description:** Direct bilirubin concentration measured in the experimental or validation data. **Unit:**  **Value coding:** See example\_values and allowed\_values. **Data type:** numeric **Example values:** 7.411 | 3.874 | 13.015 | 3.245 | 11.224 **Allowed values:**  **Missing-value coding:** NA/blank as read by fread **n rows / missing / unique:** 32 / 0 / 32 **Release status:** public **Definition status:** exact\_definition | 02\_workflows/F1\_workflow\_v02/data/02a\_refined\_analysis/expm\_F1\_validation\_input\_public.csv |  | numeric |
| domain — Data domain **File:** 02\_workflows/F1\_workflow\_v02/data/02a\_refined\_analysis/expm\_F1\_validation\_input\_public.csv **Recommended public name:** domain **Description:** Workflow or data domain represented by the row. **Unit:**  **Value coding:** Categorical descriptor; see allowed/example values. **Data type:** character **Example values:** expm **Allowed values:** expm **Missing-value coding:** NA/blank as read by fread **n rows / missing / unique:** 32 / 0 / 1 **Release status:** public **Definition status:** exact\_definition | 02\_workflows/F1\_workflow\_v02/data/02a\_refined\_analysis/expm\_F1\_validation\_input\_public.csv |  | character |
| expm\_F1\_validation\_row\_id — Experimental validation row identifier **File:** 02\_workflows/F1\_workflow\_v02/data/02a\_refined\_analysis/expm\_F1\_validation\_input\_public.csv **Recommended public name:** expm\_F1\_validation\_row\_id **Description:** Row identifier within the released F1 experimental validation table. **Unit:**  **Value coding:** See example\_values and allowed\_values. **Data type:** integer **Example values:** 1 | 2 | 3 | 4 | 5 **Allowed values:**  **Missing-value coding:** NA/blank as read by fread **n rows / missing / unique:** 32 / 0 / 32 **Release status:** public **Definition status:** exact\_definition | 02\_workflows/F1\_workflow\_v02/data/02a\_refined\_analysis/expm\_F1\_validation\_input\_public.csv |  | integer |
| release\_status — Release status **File:** 02\_workflows/F1\_workflow\_v02/data/02a\_refined\_analysis/expm\_F1\_validation\_input\_public.csv **Recommended public name:** release\_status **Description:** Release-status label indicating the publication status of the row or file object. **Unit:**  **Value coding:** Release status of the row/object, for example public. **Data type:** character **Example values:** public **Allowed values:** public **Missing-value coding:** NA/blank as read by fread **n rows / missing / unique:** 32 / 0 / 1 **Release status:** public **Definition status:** exact\_definition | 02\_workflows/F1\_workflow\_v02/data/02a\_refined\_analysis/expm\_F1\_validation\_input\_public.csv |  | character |
| sample\_id — Sample identifier **File:** 02\_workflows/F1\_workflow\_v02/data/02a\_refined\_analysis/expm\_F1\_validation\_input\_public.csv **Recommended public name:** sample\_id **Description:** Identifier of a sample or experimental record within the released public data; not a personal identifier. **Unit:**  **Value coding:** See example\_values and allowed\_values. **Data type:** integer **Example values:** 1 | 2 | 3 | 4 | 5 **Allowed values:**  **Missing-value coding:** NA/blank as read by fread **n rows / missing / unique:** 32 / 0 / 32 **Release status:** public **Definition status:** exact\_definition | 02\_workflows/F1\_workflow\_v02/data/02a\_refined\_analysis/expm\_F1\_validation\_input\_public.csv |  | integer |
| source\_harmonized\_file\_name — Source harmonized file name **File:** 02\_workflows/F1\_workflow\_v02/data/02a\_refined\_analysis/expm\_F1\_validation\_input\_public.csv **Recommended public name:** source\_harmonized\_file\_name **Description:** Name of the harmonized source file used to build the released object. **Unit:**  **Value coding:** See example\_values and allowed\_values. **Data type:** character **Example values:** expm\_F1\_validation\_raw\_public.csv **Allowed values:** expm\_F1\_validation\_raw\_public.csv **Missing-value coding:** NA/blank as read by fread **n rows / missing / unique:** 32 / 0 / 1 **Release status:** public **Definition status:** exact\_definition | 02\_workflows/F1\_workflow\_v02/data/02a\_refined\_analysis/expm\_F1\_validation\_input\_public.csv |  | character |
| tb\_measured\_mg\_dL — Measured total bilirubin concentration **File:** 02\_workflows/F1\_workflow\_v02/data/02a\_refined\_analysis/expm\_F1\_validation\_input\_public.csv **Recommended public name:** tb\_measured\_mg\_dL **Description:** Measured total bilirubin concentration in the experimental F1 data. **Unit:**  **Value coding:** See example\_values and allowed\_values. **Data type:** numeric **Example values:** 9.263 | 4.852 | 16.121 | 4.419 | 14.415 **Allowed values:**  **Missing-value coding:** NA/blank as read by fread **n rows / missing / unique:** 32 / 0 / 32 **Release status:** public **Definition status:** exact\_definition | 02\_workflows/F1\_workflow\_v02/data/02a\_refined\_analysis/expm\_F1\_validation\_input\_public.csv |  | numeric |
| unit\_or\_role — Unit or semantic role **File:** 02\_workflows/F1\_workflow\_v02/data/02a\_refined\_analysis/expm\_F1\_validation\_input\_public.csv **Recommended public name:** unit\_or\_role **Description:** Unit, role, or semantic type corresponding to the row-specific variable/metric. **Unit:**  **Value coding:** Categorical descriptor; see allowed/example values. **Data type:** character **Example values:** input **Allowed values:** input **Missing-value coding:** NA/blank as read by fread **n rows / missing / unique:** 32 / 0 / 1 **Release status:** public **Definition status:** exact\_definition | 02\_workflows/F1\_workflow\_v02/data/02a\_refined\_analysis/expm\_F1\_validation\_input\_public.csv |  | character |
| workflow\_step — Workflow step **File:** 02\_workflows/F1\_workflow\_v02/data/02a\_refined\_analysis/expm\_F1\_validation\_input\_public.csv **Recommended public name:** workflow\_step **Description:** Workflow step that produced or used the row/object. **Unit:**  **Value coding:** Categorical descriptor; see allowed/example values. **Data type:** character **Example values:** 2a\_refined\_analysis\_dataset **Allowed values:** 2a\_refined\_analysis\_dataset **Missing-value coding:** NA/blank as read by fread **n rows / missing / unique:** 32 / 0 / 1 **Release status:** public **Definition status:** exact\_definition | 02\_workflows/F1\_workflow\_v02/data/02a\_refined\_analysis/expm\_F1\_validation\_input\_public.csv |  | character |
| cre\_true\_mg\_dL — True creatinine concentration **File:** 02\_workflows/F1\_workflow\_v02/data/02b\_figure\_content/slco\_F1\_surface\_grid\_public.csv **Recommended public name:** cre\_true\_mg\_dL **Description:** True creatinine concentration used as the gravimetric/reference concentration in F1. **Unit:**  **Value coding:** See example\_values and allowed\_values. **Data type:** numeric **Example values:** 1 | 1.05 | 1.1 | 1.15 | 1.2 **Allowed values:**  **Missing-value coding:** NA/blank as read by fread **n rows / missing / unique:** 34441 / 0 / 101 **Release status:** public **Definition status:** exact\_definition | 02\_workflows/F1\_workflow\_v02/data/02b\_figure\_content/slco\_F1\_surface\_grid\_public.csv |  | numeric |
| cree\_measured\_pred\_mg\_dL — Predicted measured enzymatic creatinine concentration **File:** 02\_workflows/F1\_workflow\_v02/data/02b\_figure\_content/slco\_F1\_surface\_grid\_public.csv **Recommended public name:** cree\_measured\_pred\_mg\_dL **Description:** Predicted measured enzymatic creatinine concentration derived from the interference model. **Unit:**  **Value coding:** See example\_values and allowed\_values. **Data type:** numeric **Example values:** 0.902064914425904 | 0.957189139835594 | 1.01231537541398 | 1.06744362138108 | 1.1225738779567 **Allowed values:**  **Missing-value coding:** NA/blank as read by fread **n rows / missing / unique:** 34441 / 0 / 34441 **Release status:** public **Definition status:** exact\_definition | 02\_workflows/F1\_workflow\_v02/data/02b\_figure\_content/slco\_F1\_surface\_grid\_public.csv |  | numeric |
| cree\_true\_recalc\_error — Recalculation error for enzymatic creatinine **File:** 02\_workflows/F1\_workflow\_v02/data/02b\_figure\_content/slco\_F1\_surface\_grid\_public.csv **Recommended public name:** cree\_true\_recalc\_error **Description:** Numerical difference between recalculated and expected true enzymatic creatinine values. **Unit:**  **Value coding:** See example\_values and allowed\_values. **Data type:** numeric **Example values:** -9.99200722162641e-16 | -4.06341627012807e-14 | -5.97299987248334e-14 | 1.79856129989275e-14 | 6.3504757008559e-14 **Allowed values:**  **Missing-value coding:** NA/blank as read by fread **n rows / missing / unique:** 34441 / 0 / 1163 **Release status:** public **Definition status:** exact\_definition | 02\_workflows/F1\_workflow\_v02/data/02b\_figure\_content/slco\_F1\_surface\_grid\_public.csv |  | numeric |
| cree\_true\_recalc\_mg\_dL — Recalculated true enzymatic creatinine concentration **File:** 02\_workflows/F1\_workflow\_v02/data/02b\_figure\_content/slco\_F1\_surface\_grid\_public.csv **Recommended public name:** cree\_true\_recalc\_mg\_dL **Description:** True enzymatic creatinine concentration recalculated from the model. **Unit:**  **Value coding:** See example\_values and allowed\_values. **Data type:** numeric **Example values:** 1 | 1.04999999999996 | 1.09999999999994 | 1.15000000000002 | 1.20000000000006 **Allowed values:**  **Missing-value coding:** NA/blank as read by fread **n rows / missing / unique:** 34441 / 0 / 2603 **Release status:** public **Definition status:** exact\_definition | 02\_workflows/F1\_workflow\_v02/data/02b\_figure\_content/slco\_F1\_surface\_grid\_public.csv |  | numeric |
| crej\_measured\_pred\_mg\_dL — Predicted measured Jaffe creatinine concentration **File:** 02\_workflows/F1\_workflow\_v02/data/02b\_figure\_content/slco\_F1\_surface\_grid\_public.csv **Recommended public name:** crej\_measured\_pred\_mg\_dL **Description:** Predicted measured Jaffe creatinine concentration derived from the interference model. **Unit:**  **Value coding:** See example\_values and allowed\_values. **Data type:** numeric **Example values:** 0.990463857965053 | 1.04152284217999 | 1.09273908981855 | 1.14411406302484 | 1.19564924674134 **Allowed values:**  **Missing-value coding:** NA/blank as read by fread **n rows / missing / unique:** 34441 / 0 / 22878 **Release status:** public **Definition status:** exact\_definition | 02\_workflows/F1\_workflow\_v02/data/02b\_figure\_content/slco\_F1\_surface\_grid\_public.csv |  | numeric |
| crej\_true\_recalc\_error — Recalculation error for Jaffe creatinine **File:** 02\_workflows/F1\_workflow\_v02/data/02b\_figure\_content/slco\_F1\_surface\_grid\_public.csv **Recommended public name:** crej\_true\_recalc\_error **Description:** Numerical difference between recalculated and expected true Jaffe creatinine values. **Unit:**  **Value coding:** See example\_values and allowed\_values. **Data type:** numeric **Example values:** 6.66133814775094e-16 | 1.33226762955019e-15 | 2.22044604925031e-16 | 0 | 1.11022302462516e-15 **Allowed values:**  **Missing-value coding:** NA/blank as read by fread **n rows / missing / unique:** 34441 / 0 / 22 **Release status:** public **Definition status:** exact\_definition | 02\_workflows/F1\_workflow\_v02/data/02b\_figure\_content/slco\_F1\_surface\_grid\_public.csv |  | numeric |
| crej\_true\_recalc\_mg\_dL — Recalculated true Jaffe creatinine concentration **File:** 02\_workflows/F1\_workflow\_v02/data/02b\_figure\_content/slco\_F1\_surface\_grid\_public.csv **Recommended public name:** crej\_true\_recalc\_mg\_dL **Description:** True Jaffe creatinine concentration recalculated from the model. **Unit:**  **Value coding:** See example\_values and allowed\_values. **Data type:** numeric **Example values:** 1 | 1.05 | 1.1 | 1.15 | 1.2 **Allowed values:**  **Missing-value coding:** NA/blank as read by fread **n rows / missing / unique:** 34441 / 0 / 101 **Release status:** public **Definition status:** exact\_definition | 02\_workflows/F1\_workflow\_v02/data/02b\_figure\_content/slco\_F1\_surface\_grid\_public.csv |  | numeric |
| delta\_cree\_to\_gravimetry\_mg\_dL — Enzymatic creatinine deviation from gravimetry **File:** 02\_workflows/F1\_workflow\_v02/data/02b\_figure\_content/slco\_F1\_surface\_grid\_public.csv **Recommended public name:** delta\_cree\_to\_gravimetry\_mg\_dL **Description:** Difference between enzymatic creatinine and the gravimetric/reference value. **Unit:**  **Value coding:** See example\_values and allowed\_values. **Data type:** numeric **Example values:** 0.097935085574096 | 0.0928108601644061 | 0.0876846245860181 | 0.0825563786189225 | 0.0774261220432957 **Allowed values:**  **Missing-value coding:** NA/blank as read by fread **n rows / missing / unique:** 34441 / 0 / 34441 **Release status:** public **Definition status:** exact\_definition | 02\_workflows/F1\_workflow\_v02/data/02b\_figure\_content/slco\_F1\_surface\_grid\_public.csv |  | numeric |
| delta\_crej\_to\_gravimetry\_mg\_dL — Jaffe creatinine deviation from gravimetry **File:** 02\_workflows/F1\_workflow\_v02/data/02b\_figure\_content/slco\_F1\_surface\_grid\_public.csv **Recommended public name:** delta\_crej\_to\_gravimetry\_mg\_dL **Description:** Difference between Jaffe creatinine and the gravimetric/reference value. **Unit:**  **Value coding:** See example\_values and allowed\_values. **Data type:** numeric **Example values:** 0.0095361420349469 | 0.00847715782000713 | 0.00726091018144937 | 0.00588593697515893 | 0.00435075325865686 **Allowed values:**  **Missing-value coding:** NA/blank as read by fread **n rows / missing / unique:** 34441 / 0 / 23473 **Release status:** public **Definition status:** exact\_definition | 02\_workflows/F1\_workflow\_v02/data/02b\_figure\_content/slco\_F1\_surface\_grid\_public.csv |  | numeric |
| grid\_id — Surface-grid identifier **File:** 02\_workflows/F1\_workflow\_v02/data/02b\_figure\_content/slco\_F1\_surface\_grid\_public.csv **Recommended public name:** grid\_id **Description:** Identifier of a grid point in the F1 simulated or reconstructed surface data. **Unit:**  **Value coding:** See example\_values and allowed\_values. **Data type:** integer **Example values:** 1 | 2 | 3 | 4 | 5 **Allowed values:**  **Missing-value coding:** NA/blank as read by fread **n rows / missing / unique:** 34441 / 0 / 34441 **Release status:** public **Definition status:** exact\_definition | 02\_workflows/F1\_workflow\_v02/data/02b\_figure\_content/slco\_F1\_surface\_grid\_public.csv |  | integer |
| tb\_mg\_dL — Total bilirubin concentration **File:** 02\_workflows/F1\_workflow\_v02/data/02b\_figure\_content/slco\_F1\_surface\_grid\_public.csv **Recommended public name:** tb\_mg\_dL **Description:** Total bilirubin concentration used in the creatinine/bilirubin interference model or figure data. **Unit:**  **Value coding:** See example\_values and allowed\_values. **Data type:** numeric **Example values:** 1 | 1.1 | 1.2 | 1.3 | 1.4 **Allowed values:**  **Missing-value coding:** NA/blank as read by fread **n rows / missing / unique:** 34441 / 0 / 341 **Release status:** public **Definition status:** exact\_definition | 02\_workflows/F1\_workflow\_v02/data/02b\_figure\_content/slco\_F1\_surface\_grid\_public.csv |  | numeric |
| parameter — Metadata parameter name **File:** 02\_workflows/F1\_workflow\_v02/data/02b\_figure\_content/slco\_F1\_surface\_meta\_public.csv **Recommended public name:** parameter **Description:** Name of a metadata parameter describing the F2 simulated heatmap object, such as figure identity, data origin, grid type, axis variable, or unit/role. **Unit:**  **Value coding:** Categorical metadata key; value is given in the corresponding value column or file-specific metadata field. **Data type:** character **Example values:** dataset\_name | domain | anchor | data\_object | unit\_or\_role **Allowed values:** dataset\_name | domain | anchor | data\_object | unit\_or\_role | release\_status | source\_model\_coefficients | grid\_tb\_min\_mg\_dL | grid\_tb\_max\_mg\_dL | grid\_tb\_step\_mg\_dL **Missing-value coding:** NA/blank as read by fread **n rows / missing / unique:** 16 / 0 / 16 **Release status:** public **Definition status:** manual\_exact\_definition | 02\_workflows/F1\_workflow\_v02/data/02b\_figure\_content/slco\_F1\_surface\_meta\_public.csv |  | character |
| value — Value **File:** 02\_workflows/F1\_workflow\_v02/data/02b\_figure\_content/slco\_F1\_surface\_meta\_public.csv **Recommended public name:** value **Description:** Numerical or character value corresponding to the row-specific variable/metric. **Unit:**  **Value coding:** See example\_values and allowed\_values. **Data type:** character **Example values:** slco\_F1\_surface\_grid\_public | slco | F1 | surface | grid **Allowed values:** slco\_F1\_surface\_grid\_public | slco | F1 | surface | grid | public | expm\_F1\_model\_coefficients\_public.csv | 1 | 35 | 0.1 **Missing-value coding:** NA/blank as read by fread **n rows / missing / unique:** 16 / 0 / 15 **Release status:** public **Definition status:** exact\_definition | 02\_workflows/F1\_workflow\_v02/data/02b\_figure\_content/slco\_F1\_surface\_meta\_public.csv |  | character |
| max\_abs\_difference — Maximum absolute difference **File:** 02\_workflows/F1\_workflow\_v02/data/02b\_figure\_content/slco\_F1\_surface\_rebuild\_qc\_public.csv **Recommended public name:** max\_abs\_difference **Description:** Maximum absolute difference between compared values. **Unit:**  **Value coding:** See example\_values and allowed\_values. **Data type:** numeric **Example values:** 7.105427357601e-15 | 8.88178419700125e-16 | 6.66133814775094e-15 | 1.89848137210902e-13 | 1.85435000688017e-13 **Allowed values:**  **Missing-value coding:** NA/blank as read by fread **n rows / missing / unique:** 10 / 0 / 9 **Release status:** public **Definition status:** exact\_definition | 02\_workflows/F1\_workflow\_v02/data/02b\_figure\_content/slco\_F1\_surface\_rebuild\_qc\_public.csv |  | numeric |
| mean\_abs\_difference — Mean absolute difference **File:** 02\_workflows/F1\_workflow\_v02/data/02b\_figure\_content/slco\_F1\_surface\_rebuild\_qc\_public.csv **Recommended public name:** mean\_abs\_difference **Description:** Mean absolute difference between compared values. **Unit:**  **Value coding:** See example\_values and allowed\_values. **Data type:** numeric **Example values:** 1.92091373762115e-16 | 1.09923071745065e-16 | 2.59235674005511e-15 | 8.49343468811224e-14 | 9.91687996527855e-16 **Allowed values:**  **Missing-value coding:** NA/blank as read by fread **n rows / missing / unique:** 10 / 0 / 10 **Release status:** public **Definition status:** exact\_definition | 02\_workflows/F1\_workflow\_v02/data/02b\_figure\_content/slco\_F1\_surface\_rebuild\_qc\_public.csv |  | numeric |
| median\_abs\_difference — Median absolute difference **File:** 02\_workflows/F1\_workflow\_v02/data/02b\_figure\_content/slco\_F1\_surface\_rebuild\_qc\_public.csv **Recommended public name:** median\_abs\_difference **Description:** Median absolute difference between compared values. **Unit:**  **Value coding:** See example\_values and allowed\_values. **Data type:** numeric **Example values:** 0 | 2.66453525910038e-15 | 5.32907051820075e-15 | 6.66133814775094e-16 | 1.49880108324396e-15 **Allowed values:**  **Missing-value coding:** NA/blank as read by fread **n rows / missing / unique:** 10 / 0 / 9 **Release status:** public **Definition status:** exact\_definition | 02\_workflows/F1\_workflow\_v02/data/02b\_figure\_content/slco\_F1\_surface\_rebuild\_qc\_public.csv |  | numeric |
| n\_compared — Number of compared values **File:** 02\_workflows/F1\_workflow\_v02/data/02b\_figure\_content/slco\_F1\_surface\_rebuild\_qc\_public.csv **Recommended public name:** n\_compared **Description:** Number of values included in the comparison. **Unit:**  **Value coding:** See example\_values and allowed\_values. **Data type:** integer **Example values:** 34441 **Allowed values:**  **Missing-value coding:** NA/blank as read by fread **n rows / missing / unique:** 10 / 0 / 1 **Release status:** public **Definition status:** exact\_definition | 02\_workflows/F1\_workflow\_v02/data/02b\_figure\_content/slco\_F1\_surface\_rebuild\_qc\_public.csv |  | integer |
| variable — Variable represented by row **File:** 02\_workflows/F1\_workflow\_v02/data/02b\_figure\_content/slco\_F1\_surface\_rebuild\_qc\_public.csv **Recommended public name:** variable **Description:** Name of the variable represented by the row in a long-format table. **Unit:**  **Value coding:** Categorical descriptor; see allowed/example values. **Data type:** character **Example values:** tb\_mg\_dL | cre\_true\_mg\_dL | crej\_measured\_pred\_mg\_dL | cree\_measured\_pred\_mg\_dL | delta\_crej\_to\_gravimetry\_mg\_dL **Allowed values:** tb\_mg\_dL | cre\_true\_mg\_dL | crej\_measured\_pred\_mg\_dL | cree\_measured\_pred\_mg\_dL | delta\_crej\_to\_gravimetry\_mg\_dL | delta\_cree\_to\_gravimetry\_mg\_dL | crej\_true\_recalc\_mg\_dL | cree\_true\_recalc\_mg\_dL | crej\_true\_recalc\_error | cree\_true\_recalc\_error **Missing-value coding:** NA/blank as read by fread **n rows / missing / unique:** 10 / 0 / 10 **Release status:** public **Definition status:** exact\_definition | 02\_workflows/F1\_workflow\_v02/data/02b\_figure\_content/slco\_F1\_surface\_rebuild\_qc\_public.csv |  | character |
| metadata\_source — Metadata source file **File:** 02\_workflows/F1\_workflow\_v02/data/02b\_figure\_content/slco\_F1\_surface\_reference\_meta\_original\_public.csv **Recommended public name:** metadata\_source **Description:** Name of the metadata file used as the source for the row or object. **Unit:**  **Value coding:** File name. **Data type:** character **Example values:** slco\_F1\_surface\_meta\_reference\_public.csv **Allowed values:** slco\_F1\_surface\_meta\_reference\_public.csv **Missing-value coding:** NA/blank as read by fread **n rows / missing / unique:** 9 / 0 / 1 **Release status:** public **Definition status:** manual\_precision\_definition | 02\_workflows/F1\_workflow\_v02/data/02b\_figure\_content/slco\_F1\_surface\_reference\_meta\_original\_public.csv |  | character |
| parameter — Metadata parameter name **File:** 02\_workflows/F1\_workflow\_v02/data/02b\_figure\_content/slco\_F1\_surface\_reference\_meta\_original\_public.csv **Recommended public name:** parameter **Description:** Name of a metadata parameter describing the F2 simulated heatmap object, such as figure identity, data origin, grid type, axis variable, or unit/role. **Unit:**  **Value coding:** Categorical metadata key; value is given in the corresponding value column or file-specific metadata field. **Data type:** character **Example values:** dataset\_name | cohort | data\_type | """"""""""""""""""""""""""""""""""""""""""""""""""""""""""""""""""""""""""""""""""""""""""""""""""""""""""""""""""""""""""""""""""""""""""""""""""""""""""""""""""""""""""""""""""""""""""""""""""""""""""""""""""""""""""""""""""""""""""""""""""""""""""""""""""""""""""""""""""""""""""""""""""""""""""""""""""""""""""""""""""""""""""""""""""""""""""""""""""""""""""""""""""""""""""""""""""""""""""""""""""""""""""""""""""""""""""""""""""""""""""""""""""""""""""""""""""""""""""""""""""""""""""""""""""""""""""""""""""""""""""""""""""""""""""""""""""""""""""""""""""""""""""""""""""""""""""""""""""""""""""""""""""""""""""""""""""""""""""""""""""""""""""""""""""""""""""""""""""""""""""""""""""""""""""""""""""""""""""""""""""""""""""""""""""""""""""""""""""""""""""""""""""""""""""""""""""""""""""""""""""""""""""""""""""""""""""""""""""""""""""""""""""""""""""""""""""""""""""""""""""""""""""""""""""""""""""""""""""""""""""""""""""""""""""""""""""""""""""""""""""""""""""""""""""""""""""""""""""""""""""""""""""""""""""""""""""""""""""""""""""""""""""""""""""""""""""""""""""""""""""""""""""""""""""""""""""""""""""""""""""""""""""""""""""""""""""""""""""""""""""""""""""""""""""""""""""""""""""""""""""""""""""""""""""""""""""""""""""""""""""""""""""""""""""""""""""""""""""""""""""""""""""""""""""""""""""""""""""""""""""""""""""""""""""""""""""""""""""""""""""""""""""""""""""""""""""""""""""""""""""""""""""""""""""""""""""""""""""""""""""""""""""""""""""""""""""""""""""""""""""""""""""""""""""""""""""""""""""""""""""""""""""""""""""""""""""""""""""""""""""""""""""""""""""""""""""""""""""""""""""""""""""""""""""""""""""""""""""""""""""""""""""""""""""""""""""""""""""""""""""""""""""""""""""""""""""""""""""""""""""""""""""""""""""""""""""""""""""""""""""""""""""""""""""""""""""""""""""""""""""""""""""""""""""""""""""""""""""""""""""""""""""""""""""""""""""""""""""""""""""""""""""""""""""""""""""""""""""""""""""""""""""""""""""""""""""""""""""""""""""""""""""""""""""""""""""""""""""""""""""""""""""""""""""""""""""""""""""""""""""""""""""""""""""""""""""""""""""""""""""""""""""""""""""""""""""""""""""""""""""""""""""""""""""""""""""""""""""""""""""""""""""""""""""""""""""""""""""""""""""""""""""""""""""""""""""""""""""""""""""""""""""""""""""""""""""""""""""""""""""""""""""""""""""""""""""""""""""""""""""""""""""""""""""""""""""""""""""""""""""""""""""""""""""""""""""""""""""""""""""""""""""""""""""""""""""""""""""""""""""""""""""""""""""""""""""""""""""""""""""""""""""""""""""""""""""""""""""""""""""""""""""""""""""""""""""""""""""""""""""""""""""""""""""""""""""""""""""""""""""""""""""""""""""""""""""""""""""""""""""""""""""""""""""""""""""""""""""""""""""""""""""""""""""""""""""""""""""""""""""""""""""""""""""""""""""""""""""""""""""""""""""""""""""""""""""""""""""""""""""""""""""""""""""""""""""""""""""""""""""""""""""""""""""""""""""""""""""""""""""""""""""""""""""""""""""""""""""""""""""""""""""""""""""""""""""""""""""""""""""""""""""""""""""""""""""""""""""""""""""""""""""""""""""""""""""""""""""""""""""""""""""""""""""""""""""""""""""""""""""""""""""""""""""""""""""""""""""""""""""""""""""""""""""""""""""""""""""""""""""""""""""""""""""""""""""""""""""""""""""""""""""""""""""""""""""""""""""""""""""""""""""""""""""""""""""""""""""""""""""""""""""""""""""""""""""""""""""""""""""""""""""""""""""""""""""""""""""""""""""""""""""""""""""""""""""""""""""""""""""""""""""""""""""""""""""""""""""""""""""""""""""""""""""""""""""""""""""""""""""""""""""""""""""""""""""""""""""""""""""""""""""""""""""""""""""""""""""""""""""""""""""""""""""""""""""""""""""""""""""""""""""""""""""""""""""""""""""""""""""""""""""""""""""""""""""""""""""""""""""""""""""""""""""""""""""""""""""""""""""""""""""""""""""""""""""""""""""""""""""""""""""""""""""""""""""""""""""""""""""""""""""""""""""""""""""""""""""""""""""""""""""""""""""""""""""""""""""""""""""""""""""""""""""""""""""""""""""""""""""""""""""""""""""""""""""""""""""""""""""""""""""""""""""""""""""""""""""""""""""""""""""""""""""""""""""""""""""""""""""""""""""""""""""""""""""""""""""""""""""""""""""""""""""""""""""""""""""""""""""""""""""""""""""""""""""""""""""""""""""""""""""""""""""""""""""""""""""""""""""""""""""""""""""""""""""""""""""""""""""""""""""""""""""""""""""""""""""""""""""""""""""""""""""""""""""""""""""""""""""""""""""""""""""""""""""""""""""""""""""""""""""""""""""""""""""""""""""""""""""""""""""""""""""""""""""""""""""""""""""""""""""""""""""""""""""""""""""""""""""""""""""""""""""""""""""""""""""""""""""""""""""""""""""""""""""""""""""""""""""""""""""""""""""""""""""""""""""""""""""""""""""""""""""""""""""""""""""""""""""""""""""""""""""""""""""""""""""""""""""""""""""""""""""""""""""""""""""""""""""""""""""""""""""""""""""""""""""""""""""""""""""""""""""""""""""""""""""""""""""""""""""""""""""""""""""""""""""""""""""""""""""""""""""""""""""""""""""""""""""""""""""""""""""""""""""""""""""""""""""""""""""""""""""""""""""""""""""""""""""""""""""""""""""""""""""""""""""""""""""""""""""""""""""""""""""""""""""""""""""""""""""""""""""""""""""""""""""""""""""""""""""""""""""""""""""""""""""""""""""""""""""""""""""""""""""""""""""""""""""""""""""""""""""""""""""""""""""""""""""""""""""""""""""""""""""""""""""""""""""""""""""""""""""""""""""""""""""""""""""""""""""""""""""""""""""""""""""""""""""""""""""""""""""""""""""""""""""""""""""""""""""""""""""""""""""""""""""""""""""""""""""""""""""""""""""""""""""""""""""""""""""""""""""""""""""""""""""""""""""""""""""""""""""""""""""""""""""""""""""""""""""""""""""""""""""""""""""""""""""""""""""""""""""""""""""""""""""""""""""""""""""""""""""""""""""""""""""""""""""""""""""""""""""""""""""""""""""""""""""""""""""""""""""""""""""""""""""""""""""""""""""""""""""""""""""""""""""""""""""""""""""""""""""""""""""""""""""""""""""""""""""""""""""""""""""""""""""""""""""""""""""""""""""""""""""""""""""""""""""""""""""""""""""""""""""""""""""""""""""""""""""""""""""""""""""""""""""""""""""""""""""""""""""""""""""""""""""""""""""""""""""""""""""""""""""""""""""""""""""""""""""""""""""""""""""""""""""""""""""""""""""""""""""""""""""""""""""""""""""""""""""""""""""""""""""""""""""""""""""""""""""""""""""""""""""""""""""""""""""""""""""""""""""""""""""""""""""""""""""""""""""""""""""""""""""""""""""""""""""""""""""""""""""""""""""""""""""""""""""""""""""""""""""""""""""""""""""""""""""""""""""""""""""""""""""""""""""""""""""""""""""""""""""""""""""""""""""""""""""""""""""""""""""""""""""""""""""""""""""""""""""""""""""""""""""""""""""""""""""""""""""""""""""""""""""""""""""""""""""""""""""""""""""""""""""""""""""""""""""""""""""""""""""""""""""""""""""""""""""""""""""""""""""""""""""""""""""""""""""""""""""""""""""""""""""""""""""""""""""""""""""""""""""""""""""""""""""""""""""""""""""""""""""""""""""""""""""""""""""""""""""""""""""""""""""""""""""""""""""""""""""""""""""""""""""""""""""""""""""""""""""""""""""""""""""""""""""""""""""""""""""""""""""""""""""""""""""""""""""""""""""""""""""""""""""""""""""""""""""""""""""""""""""""""""""""""""""""""""""""""""""""""""""""""""""""""""""""""""""""""""""""""""""""""""""""""""""""""""""""""""""""""""""""""""""""""""""""""""""""""""""""""""""""""""""""""""""""""""""""""""""""""""""""""""""""""""""""""""""""""""""""""""""""""""""""""""""""""""""""""""""""""""""""""""""""""""""""""""""""""""""""""""""""""""""""""""""""""""""""""""""""""""""""""""""""""""""""""""""""""""""""""""""""""""""""""""""""""""""""""""""""""""""""""""""""""""""""""""""""""""""""""""""""""""""""""""""""""""""""""""""""""""""""""""""""""""""""""""""""""""""""""""""""""""""""""""""""""""""""""""""""""""""""""""""""""""""""""""""""""""""""""""""""""""""""""""""""""""""""""""""""""""""""""""""""""""""""""""""""""""""""""""""""""""""""""""""""""""""""""""""""""""""""""""""""""""""""""""""""""""""""""""""""""""""""""""""""""""""""""""""""""""""""""""""""""""""""""""""""""""""""""""""""""""""""""""""""""""""""""""""""""""""""""""""""""""""""""""""""""""""""""""""""""""""""""""""""""""""""""""""""""""""""""""""""""""""""""""""""""""""""""""""""""""""""""""""""""""""""""""""""""""""""""""""""""""""""""""""""""""""""""""""""""""""""""""""""""""""""""""""""""""""""""""""""""""row\_definition | tb\_range\_mg\_dL **Allowed values:** dataset\_name | cohort | data\_type | """"""""""""""""""""""""""""""""""""""""""""""""""""""""""""""""""""""""""""""""""""""""""""""""""""""""""""""""""""""""""""""""""""""""""""""""""""""""""""""""""""""""""""""""""""""""""""""""""""""""""""""""""""""""""""""""""""""""""""""""""""""""""""""""""""""""""""""""""""""""""""""""""""""""""""""""""""""""""""""""""""""""""""""""""""""""""""""""""""""""""""""""""""""""""""""""""""""""""""""""""""""""""""""""""""""""""""""""""""""""""""""""""""""""""""""""""""""""""""""""""""""""""""""""""""""""""""""""""""""""""""""""""""""""""""""""""""""""""""""""""""""""""""""""""""""""""""""""""""""""""""""""""""""""""""""""""""""""""""""""""""""""""""""""""""""""""""""""""""""""""""""""""""""""""""""""""""""""""""""""""""""""""""""""""""""""""""""""""""""""""""""""""""""""""""""""""""""""""""""""""""""""""""""""""""""""""""""""""""""""""""""""""""""""""""""""""""""""""""""""""""""""""""""""""""""""""""""""""""""""""""""""""""""""""""""""""""""""""""""""""""""""""""""""""""""""""""""""""""""""""""""""""""""""""""""""""""""""""""""""""""""""""""""""""""""""""""""""""""""""""""""""""""""""""""""""""""""""""""""""""""""""""""""""""""""""""""""""""""""""""""""""""""""""""""""""""""""""""""""""""""""""""""""""""""""""""""""""""""""""""""""""""""""""""""""""""""""""""""""""""""""""""""""""""""""""""""""""""""""""""""""""""""""""""""""""""""""""""""""""""""""""""""""""""""""""""""""""""""""""""""""""""""""""""""""""""""""""""""""""""""""""""""""""""""""""""""""""""""""""""""""""""""""""""""""""""""""""""""""""""""""""""""""""""""""""""""""""""""""""""""""""""""""""""""""""""""""""""""""""""""""""""""""""""""""""""""""""""""""""""""""""""""""""""""""""""""""""""""""""""""""""""""""""""""""""""""""""""""""""""""""""""""""""""""""""""""""""""""""""""""""""""""""""""""""""""""""""""""""""""""""""""""""""""""""""""""""""""""""""""""""""""""""""""""""""""""""""""""""""""""""""""""""""""""""""""""""""""""""""""""""""""""""""""""""""""""""""""""""""""""""""""""""""""""""""""""""""""""""""""""""""""""""""""""""""""""""""""""""""""""""""""""""""""""""""""""""""""""""""""""""""""""""""""""""""""""""""""""""""""""""""""""""""""""""""""""""""""""""""""""""""""""""""""""""""""""""""""""""""""""""""""""""""""""""""""""""""""""""""""""""""""""""""""""""""""""""""""""""""""""""""""""""""""""""""""""""""""""""""""""""""""""""""""""""""""""""""""""""""""""""""""""""""""""""""""""""""""""""""""""""""""""""""""""""""""""""""""""""""""""""""""""""""""""""""""""""""""""""""""""""""""""""""""""""""""""""""""""""""""""""""""""""""""""""""""""""""""""""""""""""""""""""""""""""""""""""""""""""""""""""""""""""""""""""""""""""""""""""""""""""""""""""""""""""""""""""""""""""""""""""""""""""""""""""""""""""""""""""""""""""""""""""""""""""""""""""""""""""""""""""""""""""""""""""""""""""""""""""""""""""""""""""""""""""""""""""""""""""""""""""""""""""""""""""""""""""""""""""""""""""""""""""""""""""""""""""""""""""""""""""""""""""""""""""""""""""""""""""""""""""""""""""""""""""""""""""""""""""""""""""""""""""""""""""""""""""""""""""""""""""""""""""""""""""""""""""""""""""""""""""""""""""""""""""""""""""""""""""""""""""""""""""""""""""""""""""""""""""""""""""""""""""""""""""""""""""""""""""""""""""""""""""""""""""""""""""""""""""""""""""""""""""""""""""""""""""""""""""""""""""""""""""""""""""""""""""""""""""""""""""""""""""""""""""""""""""""""""""""""""""""""""""""""""""""""""""""""""""""""""""""""""""""""""""""""""""""""""""""""""""""""""""""""""""""""""""""""""""""""""""""""""""""""""""""""""""""""""""""""""""""""""""""""""""""""""""""""""""""""""""""""""""""""""""""""""""""""""""""""""""""""""""""""""""""""""""""""""""""""""""""""""""""""""""""""""""""""""""""""""""""""""""""""""""""""""""""""""""""""""""""""""""""""""""""""""""""""""""""""""""""""""""""""""""""""""""""""""""""""""""""""""""""""""""""""""""""""""""""""""""""""""""""""""""""""""""""""""""""""""""""""""""""""""""""""""""""""""""""""""""""""""""""""""""""""""""""""""""""""""""""""""""""""""""""""""""""""""""""""""""""""""""""""""""""""""""""""""""""""""""""""""""""""""""""""""""""""""""""""""""""""""""""""""""""""""""""""""""""""""""""""""""""""""""""""""""""""""""""""""""""""""""""""""""""""""""""""""""""""""""""""""""""""""""""""""""""""""""""""""""""""""""""""""""""""""""""""""""""""""""""""""""""""""""""""""""""""""""""""""""""""""""""""""""""""""""""""""""""""""""""""""""""""""""""""""""""""""""""""""""""""""""""""""""""""""""""""""""""""""""""""""""""""""""""""""""""""""""""""""""""""""""""""""""""""""""""""""""""""""""""""""""""""""""""""""""""""""""""""""""""""""""""""""""""""""""""""""""""""""""""""""""""""""""""""""""""""""""""""""""""""""""""""""""""""""""""""""""""""""""""""""""""""""""""""""""""""""""""""""""""""""""""""""""""""""""""""""""""""""""""""""""""""""""""""""""""""""""""""""""""""""""""""""""""""""""""""""""""""""""""""""""""""""""""""""""""""""""""""""""""""""""""""""""""""""""""""""""""""""""""""""""""""""""""""""""""""""""""""""""""""""""""""""""""""""""""""""""""""""""""""""""""""""""""""""""""""""""""""""""""""""""""""""""""""""""""""""""""""""""""""""""""""""""""""""""""""""""""""""""""""""""""""""""""""""""""""""""""""""""""""""""""""""""""""""""""""""""""""""""""""""""""""""""""""""""""""""""""""""""""""""""""""""""""""""""""""""""""""""""""""""""""""""""""""""""""""""""""""""""""""""""""""""""""""""""""""""""""""""""""""""""""""""""""""""""""""""""""""""""""""""""""""""""""""""""""""""""""""""""""""""""""""""""""""""""""""""""""""""""""""""""""""""""""""""""""""""""""""""""""""""""""""""""""""""""""""""""""""""""""""""""""""""""""""""""""""""""""""""""""""""""""""""""""""""""""""""""""""""""""""""""""""""""""""""""""""""""""""""""""""""""""""""""""""""""""""""""""""""""""""""""""""""""""""""""""""""""""""""""""""""""""""""""""""""""""""""""""""""""""""""""""""""""""""""""""""""""""""""""""""""""""""""""""""""""""""""""""""""""""""""""""""""""""""""""""""""""""""""""""""""""""""""""""""""""""""""""""""""""""""""""""""""""""""""""""""""""""""""""""""""""""""""""""""""""""""""""""""""""""""""""""""""""""""""""""""""""""""""""""""""""""""""""""""""""""""""""""""""""""""""""""""""""""""""""""""""""""""""""""""""""""""""""""""""""""""""""""""""""""""""""""""""""""""""""""""""""""""""""""""""""""""""""""""""""""""""""""""""""""""""""""""""""""""""""""""""""""""""""""""""""""""""""""""""""""""""""""""""""""""""""""""""""""""""""""""""""""""""""""""""""""""""""""""""""""""""""""""""""""""""""""""""""""""""""""""""""""""""""""""""""""""""""""""""""""""""""""""""""""""""""""""""""""""""""""""""""""""""""""""""""""""""""""""""""""""""""""""""""""""""""""""""""""""""""""""""""""""""""""""""""""""""""""""""""""""""""""""""""""""""""""""""""""""""""""""""""""""""""""""""""""""""""""""""""""""""""""""""""""""""""""""""""""""""""""""""""""""""""""""""""""""""""""""""""""""""""""""""""""""""""""""""""""""""""""""""""""""""""""""""""""""""""""""""""""""""""""""""""""""""""""""""""""""""""""""""""""""""""""""""""""""""""""""""""""""""""""""""""""""""""""""""""""""""""""""""""""""""""""""""""""""""""""""""""""""""""""""""""""""""""""""""""""""""""""""""""""""""""""""""""""""""""""""""""""""""""""""""""""""""""""""""""""""""""""""""""""""""""""""""""""""""""""""""""""""""""""""""""""""""""""""""""""""""""""""""""""""""""""""""""""""""""""""""""""""""""""""""""""""""""""""""""""""""""""""""""""""""""""""""""""""""""""""""""""""""""""""""""""""""""""""""""""""""""""""""""""""""""""""""""""""""""""""""""""""""""""""""""""""""""""""""""""""""""""""""""""""""""""""""""""""""""""""""""""""""""""""""""""""""""""""""""""""""""""""""""""""""""""""""""""""""""""""""""""""""""""""""""""""""""""""""""""""""""""""""""""""""""""""""""""""""""""""""""""""""""""""""""""""""""""""""""""""""""""""""""""""""""""""""""""""""""""""""""""""""""""""""""""""""""""""""""""""""""""""""""""""""""""""""""""""""""""""""""""""""""""""""""""""""""""""""""""""""""""""""""""""""""""""""""""""""""""""""""""""""""""""""""""""""""""""""""""""""""""""""""""""""""""""""""""""""""""""""""""""""""""""""""""""""""""""""""""""""""""""""""""""""""""""""""""""""""""""""""""""""""""""""""""""""""""""""""""""""""""""""""""""""""""""""""""""""""""""""""""row\_definition | tb\_range\_mg\_dL | cre\_true\_range\_mg\_dL | tb\_step\_mg\_dL | cre\_step\_mg\_dL | delta\_definition **Missing-value coding:** NA/blank as read by fread **n rows / missing / unique:** 9 / 0 / 9 **Release status:** public **Definition status:** manual\_exact\_definition | 02\_workflows/F1\_workflow\_v02/data/02b\_figure\_content/slco\_F1\_surface\_reference\_meta\_original\_public.csv |  | character |
| value — Value **File:** 02\_workflows/F1\_workflow\_v02/data/02b\_figure\_content/slco\_F1\_surface\_reference\_meta\_original\_public.csv **Recommended public name:** value **Description:** Numerical or character value corresponding to the row-specific variable/metric. **Unit:**  **Value coding:** See example\_values and allowed\_values. **Data type:** character **Example values:** f1\_simulated\_surface\_repository | SIMULATED | in\_silico surface grid | One row = one equation-defined grid point (tb\_mg\_... **Allowed values:** f1\_simulated\_surface\_repository | SIMULATED | in\_silico surface grid | One row = one equation-defined grid point (tb\_mg\_dL, cre\_true\_mg\_dL)"""""""""""""""""""""""""""""""""""""""""""""""""""""""""""""""""""""""""""""""""""""""""""""""""""""""""""""""""""""""""""""""""""""""""""""""""""""""""""""""""""""""""""""""""""""""""""""""""""""""""""""""""""""""""""""""""""""""""""""""""""""""""""""""""""""""""""""""""""""""""""""""""""""""""""""""""""""""""""""""""""""""""""""""""""""""""""""""""""""""""""""""""""""""""""""""""""""""""""""""""""""""""""""""""""""""""""""""""""""""""""""""""""""""""""""""""""""""""""""""""""""""""""""""""""""""""""""""""""""""""""""""""""""""""""""""""""""""""""""""""""""""""""""""""""""""""""""""""""""""""""""""""""""""""""""""""""""""""""""""""""""""""""""""""""""""""""""""""""""""""""""""""""""""""""""""""""""""""""""""""""""""""""""""""""""""""""""""""""""""""""""""""""""""""""""""""""""""""""""""""""""""""""""""""""""""""""""""""""""""""""""""""""""""""""""""""""""""""""""""""""""""""""""""""""""""""""""""""""""""""""""""""""""""""""""""""""""""""""""""""""""""""""""""""""""""""""""""""""""""""""""""""""""""""""""""""""""""""""""""""""""""""""""""""""""""""""""""""""""""""""""""""""""""""""""""""""""""""""""""""""""""""""""""""""""""""""""""""""""""""""""""""""""""""""""""""""""""""""""""""""""""""""""""""""""""""""""""""""""""""""""""""""""""""""""""""""""""""""""""""""""""""""""""""""""""""""""""""""""""""""""""""""""""""""""""""""""""""""""""""""""""""""""""""""""""""""""""""""""""""""""""""""""""""""""""""""""""""""""""""""""""""""""""""""""""""""""""""""""""""""""""""""""""""""""""""""""""""""""""""""""""""""""""""""""""""""""""""""""""""""""""""""""""""""""""""""""""""""""""""""""""""""""""""""""""""""""""""""""""""""""""""""""""""""""""""""""""""""""""""""""""""""""""""""""""""""""""""""""""""""""""""""""""""""""""""""""""""""""""""""""""""""""""""""""""""""""""""""""""""""""""""""""""""""""""""""""""""""""""""""""""""""""""""""""""""""""""""""""""""""""""""""""""""""""""""""""""""""""""""""""""""""""""""""""""""""""""""""""""""""""""""""""""""""""""""""""""""""""""""""""""""""""""""""""""""""""""""""""""""""""""""""""""""""""""""""""""""""""""""""""""""""""""""""""""""""""""""""""""""""""""""""""""""""""""""""""""""""""""""""""""""""""""""""""""""""""""""""""""""""""""""""""""""""""""""""""""""""""""""""""""""""""""""""""""""""""""""""""""""""""""""""""""""""""""""""""""""""""""""""""""""""""""""""""""""""""""""""""""""""""""""""""""""""""""""""""""""""""""""""""""""""""""""""""""""""""""""""""""""""""""""""""""""""""""""""""""""""""""""""""""""""""""""""""""""""""""""""""""""""""""""""""""""""""""""""""""""""""""""""""""""""""""""""""""""""""""""""""""""""""""""""""""""""""""""""""""""""""""""""""""""""""""""""""""""""""""""""""""""""""""""""""""""""""""""""""""""""""""""""""""""""""""""""""""""""""""""""""""""""""""""""""""""""""""""""""""""""""""""""""""""""""""""""""""""""""""""""""""""""""""""""""""""""""""""""""""""""""""""""""""""""""""""""""""""""""""""""""""""""""""""""""""""""""""""""""""""""""""""""""""""""""""""""""""""""""""""""""""""""""""""""""""""""""""""""""""""""""""""""""""""""""""""""""""""""""""""""""""""""""""""""""""""""""""""""""""""""""""""""""""""""""""""""""""""""""""""""""""""""""""""""""""""""""""""""""""""""""""""""""""""""""""""""""""""""""""""""""""""""""""""""""""""""""""""""""""""""""""""""""""""""""""""""""""""""""""""""""""""""""""""""""""""""""""""""""""""""""""""""""""""""""""""""""""""""""""""""""""""""""""""""""""""""""""""""""""""""""""""""""""""""""""""""""""""""""""""""""""""""""""""""""""""""""""""""""""""""""""""""""""""""""""""""""""""""""""""""""""""""""""""""""""""""""""""""""""""""""""""""""""""""""""""""""""""""""""""""""""""""""""""""""""""""""""""""""""""""""""""""""""""""""""""""""""""""""""""""""""""""""""""""""""""""""""""""""""""""""""""""""""""""""""""""""""""""""""""""""""""""""""""""""""""""""""""""""""""""""""""""""""""""""""""""""""""""""""""""""""""""""""""""""""""""""""""""""""""""""""""""""""""""""""""""""""""""""""""""""""""""""""""""""""""""""""""""""""""""""""""""""""""""""""""""""""""""""""""""""""""""""""""""""""""""""""""""""""""""""""""""""""""""""""""""""""""""""""""""""""""""""""""""""""""""""""""""""""""""""""""""""""""""""""""""""""""""""""""""""""""""""""""""""""""""""""""""""""""""""""""""""""""""""""""""""""""""""""""""""""""""""""""""""""""""""""""""""""""""""""""""""""""""""""""""""""""""""""""""""""""""""""""""""""""""""""""""""""""""""""""""""""""""""""""""""""""""""""""""""""""""""""""""""""""""""""""""""""""""""""""""""""""""""""""""""""""""""""""""""""""""""""""""""""""""""""""""""""""""""""""""""""""""""""""""""""""""""""""""""""""""""""""""""""""""""""""""""""""""""""""""""""""""""""""""""""""""""""""""""""""""""""""""""""""""""""""""""""""""""""""""""""""""""""""""""""""""""""""""""""""""""""""""""""""""""""""""""""""""""""""""""""""""""""""""""""""""""""""""""""""""""""""""""""""""""""""""""""""""""""""""""""""""""""""""""""""""""""""""""""""""""""""""""""""""""""""""""""""""""""""""""""""""""""""""""""""""""""""""""""""""""""""""""""""""""""""""""""""""""""""""""""""""""""""""""""""""""""""""""""""""""""""""""""""""""""""""""""""""""""""""""""""""""""""""""""""""""""""""""""""""""""""""""""""""""""""""""""""""""""""""""""""""""""""""""""""""""""""""""""""""""""""""""""""""""""""""""""""""""""""""""""""""""""""""""""""""""""""""""""""""""""""""""""""""""""""""""""""""""""""""""""""""""""""""""""""""""""""""""""""""""""""""""""""""""""""""""""""""""""""""""""""""""""""""""""""""""""""""""""""""""""""""""""""""""""""""""""""""""""""""""""""""""""""""""""""""""""""""""""""""""""""""""""""""""""""""""""""""""""""""""""""""""""""""""""""""""""""""""""""""""""""""""""""""""""""""""""""""""""""""""""""""""""""""""""""""""""""""""""""""""""""""""""""""""""""""""""""""""""""""""""""""""""""""""""""""""""""""""""""""""""""""""""""""""""""""""""""""""""""""""""""""""""""""""""""""""""""""""""""""""""""""""""""""""""""""""""""""""""""""""""""""""""""""""""""""""""""""""""""""""""""""""""""""""""""""""""""""""""""""""""""""""""""""""""""""""""""""""""""""""""""""""""""""""""""""""""""""""""""""""""""""""""""""""""""""""""""""""""""""""""""""""""""""""""""""""""""""""""""""""""""""""""""""""""""""""""""""""""""""""""""""""""""""""""""""""""""""""""""""""""""""""""""""""""""""""""""""""""""""""""""""""""""""""""""""""""""""""""""""""""""""""""""""""""""""""""""""""""""""""""""""""""""""""""""""""""""""""""""""""""""""""""""""""""""""""""""""""""""""""""""""""""""""""""""""""""""""""""""""""""""""""""""""""""""""""""""""""""""""""""""""""""""""""""""""""""""""""""""""""""""""""""""""""""""""""""""""""""""""""""""""""""""""""""""""""""""""""""""""""""""""""""""""""""""""""""""""""""""""""""""""""""""""""""""""""""""""""""""""""""""""""""""""""""""""""""""""""""""""""""""""""""""""""""""""""""""""""""""""""""""""""""""""""""""""""""""""""""""""""""""""""""""""""""""""""""""""""""""""""""""""""""""""""""""""""""""""""""""""""""""""""""""""""""""""""""""""""""""""""""""""""""""""""""""""""""""""""""""""""""""""""""""""""""""""""""""""""""""""""""""""""""""""""""""""""""""""""""""""""""""""""""""""""""""""""""""""""""""""""""""""""""""""""""""""""""""""""""""""""""""""""""""""""""""""""""""""""""""""""""""""""""""""""""""""""""""""""""""""""""""""""""""""""""""""""""""""""""""""""""""""""""""""""""""""""""""""""""""""""""""""""""""""""""""""""""""""""""""""""""""""""""""""""""""""""""""""""""""""""""""""""""""""""""""""""""""""""""""""""""""""""""""""""""""""""""""""""""""""""""""""""""""""""""""""""""""""""""""""""""""""""""""""""""""""""""""""""""""""""""""""""""""""""""""""""""""""""""""""""""""""""""""""""""""""""""""""""""""""""""""""""""""""""""""""""""""""""""""""""""""""""""""""""""""""""""""""""""""""""""""""""""""""""""""""""""""""""""""""""""""""""""""""""""""""""""""""""""""""""""""""""""""""""""""""""""""""""""""""""""""""""""""""""""""""""""""""""""""""""""""""""""""""""""""""""""""""""""""""""""""""""""""""""""""""""""""""""""""""""""""""""""""""""""""""""""""""""""""""""""""""""""""""""""""""""""""""""""""""""""""""""""""""""""""""""""""""""""""""""""""""""""""""""""""""""""""""""""""""""""""""""""""""""""""""""""""""""""""""""""""""""""""""""""""""""""" | 1 to 35 | 1 to 6 | 0.1 | 0.05 | delta = cre\_true\_mg\_dL - predicted measured creatinine **Missing-value coding:** NA/blank as read by fread **n rows / missing / unique:** 9 / 0 / 9 **Release status:** public **Definition status:** exact\_definition | 02\_workflows/F1\_workflow\_v02/data/02b\_figure\_content/slco\_F1\_surface\_reference\_meta\_original\_public.csv |  | character |
| exists — File/object existence flag **File:** 02\_workflows/F1\_workflow\_v02/data/02b\_figure\_content/slco\_F1\_surface\_run\_inputs\_outputs\_public.csv **Recommended public name:** exists **Description:** Logical indicator showing whether the referenced file or workflow object exists. **Unit:**  **Value coding:** TRUE/FALSE. **Data type:** logical **Example values:** TRUE **Allowed values:** TRUE **Missing-value coding:** NA/blank as read by fread **n rows / missing / unique:** 5 / 0 / 1 **Release status:** public **Definition status:** manual\_precision\_definition | 02\_workflows/F1\_workflow\_v02/data/02b\_figure\_content/slco\_F1\_surface\_run\_inputs\_outputs\_public.csv |  | logical |
| item — Audit item **File:** 02\_workflows/F1\_workflow\_v02/data/02b\_figure\_content/slco\_F1\_surface\_run\_inputs\_outputs\_public.csv **Recommended public name:** item **Description:** Name of the QC or audit item being reported. **Unit:**  **Value coding:** Categorical audit item. **Data type:** character **Example values:** coef\_path | surface\_reference\_path | surface\_out | surface\_meta\_out | surface\_qc\_out **Allowed values:** coef\_path | surface\_reference\_path | surface\_out | surface\_meta\_out | surface\_qc\_out **Missing-value coding:** NA/blank as read by fread **n rows / missing / unique:** 5 / 0 / 5 **Release status:** public **Definition status:** manual\_precision\_definition | 02\_workflows/F1\_workflow\_v02/data/02b\_figure\_content/slco\_F1\_surface\_run\_inputs\_outputs\_public.csv |  | character |
| path — Workflow path **File:** 02\_workflows/F1\_workflow\_v02/data/02b\_figure\_content/slco\_F1\_surface\_run\_inputs\_outputs\_public.csv **Recommended public name:** path **Description:** Workflow path recorded in a manifest or audit table. For public release this should refer to a package-relative or otherwise non-sensitive path. **Unit:** % **Value coding:** Path string; prefer package-relative or sanitized values in the public package. **Data type:** character **Example values:** 02\_workflows/F1\_workflow\_v02/data/02a\_refined\_analysis/expm\_F1\_model\_coefficients\_public.csv | 02\_workflows/F1\_workflow\_v02/data/01\_source\_loaded\_harmonized/slco\_F1\_surface\_grid\_reference\_public.csv | 02\_workflows/F1\_workflow\_v02/data/02b\_figure\_cont... **Allowed values:** 02\_workflows/F1\_workflow\_v02/data/02a\_refined\_analysis/expm\_F1\_model\_coefficients\_public.csv | ... **Missing-value coding:** NA/blank as read by fread **n rows / missing / unique:** 5 / 0 / 5 **Release status:** public **Definition status:** manual\_precision\_definition | 02\_workflows/F1\_workflow\_v02/data/02b\_figure\_content/slco\_F1\_surface\_run\_inputs\_outputs\_public.csv | % | character |
| Cre\_nominal\_grav\_mg\_dL — Nominal gravimetric creatinine target concentration **File:** 02\_workflows/F1\_workflow\_v02/submission\_ready/public/data/expm\_F1\_array\_input\_public.csv **Recommended public name:** Cre\_nominal\_grav\_mg\_dL **Description:** Nominal gravimetric target concentration of creatinine used when defining the F1 experimental array. **Unit:** mg/dL **Value coding:** Numeric concentration in mg/dL. **Data type:** numeric **Example values:** 3 | 1.5 | 3.5 | 2 | 0.5 **Allowed values:**  **Missing-value coding:** NA/blank as read by fread **n rows / missing / unique:** 4200 / 0 / 10 **Release status:** public **Definition status:** manual\_precision\_definition | 02\_workflows/F1\_workflow\_v02/submission\_ready/public/data/expm\_F1\_array\_input\_public.csv | mg/dL | numeric |
| TB\_nominal\_grav\_mg\_dL — Nominal gravimetric total bilirubin target concentration **File:** 02\_workflows/F1\_workflow\_v02/submission\_ready/public/data/expm\_F1\_array\_input\_public.csv **Recommended public name:** TB\_nominal\_grav\_mg\_dL **Description:** Nominal gravimetric target concentration of total bilirubin used when defining the F1 experimental array. **Unit:** mg/dL **Value coding:** Numeric concentration in mg/dL. **Data type:** integer **Example values:** 27 | 14 | 28 | 15 | 29 **Allowed values:**  **Missing-value coding:** NA/blank as read by fread **n rows / missing / unique:** 4200 / 0 / 35 **Release status:** public **Definition status:** manual\_precision\_definition | 02\_workflows/F1\_workflow\_v02/submission\_ready/public/data/expm\_F1\_array\_input\_public.csv | mg/dL | integer |
| TB\_trial\_M\_mg\_dL — Measured total bilirubin concentration in the experimental dataset **File:** 02\_workflows/F1\_workflow\_v02/submission\_ready/public/data/expm\_F1\_array\_input\_public.csv **Recommended public name:** TB\_trial\_M\_mg\_dL **Description:** Measured total bilirubin concentration in the F1 experimental dataset. In rounded input tables this is the rounded/display value used for model calculation and plotting. **Unit:** mg/dL **Value coding:** Numeric concentration in mg/dL. **Data type:** numeric **Example values:** 27.22 | 27.13 | 26.94 | 27.09 | 14.04 **Allowed values:**  **Missing-value coding:** NA/blank as read by fread **n rows / missing / unique:** 4200 / 0 / 1104 **Release status:** public **Definition status:** manual\_precision\_definition | 02\_workflows/F1\_workflow\_v02/submission\_ready/public/data/expm\_F1\_array\_input\_public.csv | mg/dL | numeric |
| anchor — Anchor/output group **File:** 02\_workflows/F1\_workflow\_v02/submission\_ready/public/data/expm\_F1\_array\_input\_public.csv **Recommended public name:** anchor **Description:** Anchor or output grouping label used by the workflow to identify a specific public output component. **Unit:**  **Value coding:** See example\_values and allowed\_values. **Data type:** character **Example values:** F1 **Allowed values:** F1 **Missing-value coding:** NA/blank as read by fread **n rows / missing / unique:** 4200 / 0 / 1 **Release status:** public **Definition status:** exact\_definition | 02\_workflows/F1\_workflow\_v02/submission\_ready/public/data/expm\_F1\_array\_input\_public.csv |  | character |
| array\_id — Experimental array identifier **File:** 02\_workflows/F1\_workflow\_v02/submission\_ready/public/data/expm\_F1\_array\_input\_public.csv **Recommended public name:** array\_id **Description:** Identifier of the experimental array used in the F1 creatinine/bilirubin interference data. **Unit:**  **Value coding:** See example\_values and allowed\_values. **Data type:** character **Example values:** Array\_1 | Array\_2 | Array\_3 **Allowed values:** Array\_1 | Array\_2 | Array\_3 **Missing-value coding:** NA/blank as read by fread **n rows / missing / unique:** 4200 / 0 / 3 **Release status:** public **Definition status:** exact\_definition | 02\_workflows/F1\_workflow\_v02/submission\_ready/public/data/expm\_F1\_array\_input\_public.csv |  | character |
| assay — Creatinine assay **File:** 02\_workflows/F1\_workflow\_v02/submission\_ready/public/data/expm\_F1\_array\_input\_public.csv **Recommended public name:** assay **Description:** Creatinine assay represented by the row, for example enzymatic creatinine (CreE) or Jaffe creatinine (CreJ). **Unit:**  **Value coding:** Categorical assay label, e.g. CreE or CreJ. **Data type:** character **Example values:** CreE | CreJ **Allowed values:** CreE | CreJ **Missing-value coding:** NA/blank as read by fread **n rows / missing / unique:** 4200 / 0 / 2 **Release status:** public **Definition status:** manual\_precision\_definition | 02\_workflows/F1\_workflow\_v02/submission\_ready/public/data/expm\_F1\_array\_input\_public.csv |  | character |
| data\_object — Data object **File:** 02\_workflows/F1\_workflow\_v02/submission\_ready/public/data/expm\_F1\_array\_input\_public.csv **Recommended public name:** data\_object **Description:** Name of the data object represented by the row. **Unit:**  **Value coding:** Categorical descriptor; see allowed/example values. **Data type:** character **Example values:** array **Allowed values:** array **Missing-value coding:** NA/blank as read by fread **n rows / missing / unique:** 4200 / 0 / 1 **Release status:** public **Definition status:** exact\_definition | 02\_workflows/F1\_workflow\_v02/submission\_ready/public/data/expm\_F1\_array\_input\_public.csv |  | character |
| domain — Data domain **File:** 02\_workflows/F1\_workflow\_v02/submission\_ready/public/data/expm\_F1\_array\_input\_public.csv **Recommended public name:** domain **Description:** Workflow or data domain represented by the row. **Unit:**  **Value coding:** Categorical descriptor; see allowed/example values. **Data type:** character **Example values:** expm **Allowed values:** expm **Missing-value coding:** NA/blank as read by fread **n rows / missing / unique:** 4200 / 0 / 1 **Release status:** public **Definition status:** exact\_definition | 02\_workflows/F1\_workflow\_v02/submission\_ready/public/data/expm\_F1\_array\_input\_public.csv |  | character |
| expm\_F1\_array\_row\_id — Experimental array row identifier **File:** 02\_workflows/F1\_workflow\_v02/submission\_ready/public/data/expm\_F1\_array\_input\_public.csv **Recommended public name:** expm\_F1\_array\_row\_id **Description:** Row identifier within the released F1 experimental array table. **Unit:**  **Value coding:** See example\_values and allowed\_values. **Data type:** integer **Example values:** 1 | 2 | 3 | 4 | 5 **Allowed values:**  **Missing-value coding:** NA/blank as read by fread **n rows / missing / unique:** 4200 / 0 / 4200 **Release status:** public **Definition status:** exact\_definition | 02\_workflows/F1\_workflow\_v02/submission\_ready/public/data/expm\_F1\_array\_input\_public.csv |  | integer |
| plot\_correction\_mg\_dL — Plotted creatinine correction **File:** 02\_workflows/F1\_workflow\_v02/submission\_ready/public/data/expm\_F1\_array\_input\_public.csv **Recommended public name:** plot\_correction\_mg\_dL **Description:** Creatinine correction value plotted in F1, expressed as the difference between corrected/reference and measured creatinine. **Unit:** probability **Value coding:** Numeric correction in mg/dL. **Data type:** numeric **Example values:** -0.541120525715191 | -0.480865451714561 | -0.386578220459593 | -0.317061468810564 | -0.458450885953881 **Allowed values:**  **Missing-value coding:** NA/blank as read by fread **n rows / missing / unique:** 4200 / 0 / 4200 **Release status:** public **Definition status:** manual\_precision\_definition | 02\_workflows/F1\_workflow\_v02/submission\_ready/public/data/expm\_F1\_array\_input\_public.csv | probability | numeric |
| preparer\_id — Experimental preparer identifier **File:** 02\_workflows/F1\_workflow\_v02/submission\_ready/public/data/expm\_F1\_array\_input\_public.csv **Recommended public name:** preparer\_id **Description:** Identifier of the experimental preparer in F1 raw/validation data, represented as a public technical code. **Unit:**  **Value coding:** See example\_values and allowed\_values. **Data type:** character **Example values:** Prep\_A | Prep\_B **Allowed values:** Prep\_A | Prep\_B **Missing-value coding:** NA/blank as read by fread **n rows / missing / unique:** 4200 / 0 / 2 **Release status:** public **Definition status:** exact\_definition | 02\_workflows/F1\_workflow\_v02/submission\_ready/public/data/expm\_F1\_array\_input\_public.csv |  | character |
| release\_status — Release status **File:** 02\_workflows/F1\_workflow\_v02/submission\_ready/public/data/expm\_F1\_array\_input\_public.csv **Recommended public name:** release\_status **Description:** Release-status label indicating the publication status of the row or file object. **Unit:**  **Value coding:** Release status of the row/object, for example public. **Data type:** character **Example values:** public **Allowed values:** public **Missing-value coding:** NA/blank as read by fread **n rows / missing / unique:** 4200 / 0 / 1 **Release status:** public **Definition status:** exact\_definition | 02\_workflows/F1\_workflow\_v02/submission\_ready/public/data/expm\_F1\_array\_input\_public.csv |  | character |
| replicate — Experimental replicate number **File:** 02\_workflows/F1\_workflow\_v02/submission\_ready/public/data/expm\_F1\_array\_input\_public.csv **Recommended public name:** replicate **Description:** Replicate number within the F1 experimental dataset. **Unit:** display order **Value coding:** Integer replicate number. **Data type:** integer **Example values:** 2 | 1 **Allowed values:**  **Missing-value coding:** NA/blank as read by fread **n rows / missing / unique:** 4200 / 0 / 2 **Release status:** public **Definition status:** manual\_precision\_definition | 02\_workflows/F1\_workflow\_v02/submission\_ready/public/data/expm\_F1\_array\_input\_public.csv | display order | integer |
| source\_harmonized\_file\_name — Source harmonized file name **File:** 02\_workflows/F1\_workflow\_v02/submission\_ready/public/data/expm\_F1\_array\_input\_public.csv **Recommended public name:** source\_harmonized\_file\_name **Description:** Name of the harmonized source file used to build the released object. **Unit:**  **Value coding:** See example\_values and allowed\_values. **Data type:** character **Example values:** expm\_F1\_array\_raw\_public.csv **Allowed values:** expm\_F1\_array\_raw\_public.csv **Missing-value coding:** NA/blank as read by fread **n rows / missing / unique:** 4200 / 0 / 1 **Release status:** public **Definition status:** exact\_definition | 02\_workflows/F1\_workflow\_v02/submission\_ready/public/data/expm\_F1\_array\_input\_public.csv |  | character |
| trial\_display\_Cre\_M\_mg\_dL — Measured creatinine concentration in the experimental dataset **File:** 02\_workflows/F1\_workflow\_v02/submission\_ready/public/data/expm\_F1\_array\_input\_public.csv **Recommended public name:** trial\_display\_Cre\_M\_mg\_dL **Description:** Measured creatinine concentration in the F1 experimental dataset. In rounded input tables this is the rounded/display value used for model calculation and plotting. **Unit:** mg/dL **Value coding:** Numeric concentration in mg/dL. **Data type:** numeric **Example values:** 3.54 | 3.48 | 3.39 | 3.32 | 3.46 **Allowed values:**  **Missing-value coding:** NA/blank as read by fread **n rows / missing / unique:** 4200 / 0 / 540 **Release status:** public **Definition status:** manual\_precision\_definition | 02\_workflows/F1\_workflow\_v02/submission\_ready/public/data/expm\_F1\_array\_input\_public.csv | mg/dL | numeric |
| unit\_or\_role — Unit or semantic role **File:** 02\_workflows/F1\_workflow\_v02/submission\_ready/public/data/expm\_F1\_array\_input\_public.csv **Recommended public name:** unit\_or\_role **Description:** Unit, role, or semantic type corresponding to the row-specific variable/metric. **Unit:**  **Value coding:** Categorical descriptor; see allowed/example values. **Data type:** character **Example values:** input **Allowed values:** input **Missing-value coding:** NA/blank as read by fread **n rows / missing / unique:** 4200 / 0 / 1 **Release status:** public **Definition status:** exact\_definition | 02\_workflows/F1\_workflow\_v02/submission\_ready/public/data/expm\_F1\_array\_input\_public.csv |  | character |
| workflow\_step — Workflow step **File:** 02\_workflows/F1\_workflow\_v02/submission\_ready/public/data/expm\_F1\_array\_input\_public.csv **Recommended public name:** workflow\_step **Description:** Workflow step that produced or used the row/object. **Unit:**  **Value coding:** Categorical descriptor; see allowed/example values. **Data type:** character **Example values:** 2a\_refined\_analysis\_dataset **Allowed values:** 2a\_refined\_analysis\_dataset **Missing-value coding:** NA/blank as read by fread **n rows / missing / unique:** 4200 / 0 / 1 **Release status:** public **Definition status:** exact\_definition | 02\_workflows/F1\_workflow\_v02/submission\_ready/public/data/expm\_F1\_array\_input\_public.csv |  | character |
| anchor — Anchor/output group **File:** 02\_workflows/F1\_workflow\_v02/submission\_ready/public/data/expm\_F1\_validation\_input\_public.csv **Recommended public name:** anchor **Description:** Anchor or output grouping label used by the workflow to identify a specific public output component. **Unit:**  **Value coding:** See example\_values and allowed\_values. **Data type:** character **Example values:** F1 **Allowed values:** F1 **Missing-value coding:** NA/blank as read by fread **n rows / missing / unique:** 32 / 0 / 1 **Release status:** public **Definition status:** exact\_definition | 02\_workflows/F1\_workflow\_v02/submission\_ready/public/data/expm\_F1\_validation\_input\_public.csv |  | character |
| cre\_true\_gcidms\_mg\_dL — GC-IDMS reference creatinine concentration **File:** 02\_workflows/F1\_workflow\_v02/submission\_ready/public/data/expm\_F1\_validation\_input\_public.csv **Recommended public name:** cre\_true\_gcidms\_mg\_dL **Description:** Creatinine concentration measured by GC-IDMS reference method in the validation data. **Unit:**  **Value coding:** See example\_values and allowed\_values. **Data type:** numeric **Example values:** 0.82 | 0.86 | 0.63 | 0.94 | 0.9 **Allowed values:**  **Missing-value coding:** NA/blank as read by fread **n rows / missing / unique:** 32 / 0 / 30 **Release status:** public **Definition status:** exact\_definition | 02\_workflows/F1\_workflow\_v02/submission\_ready/public/data/expm\_F1\_validation\_input\_public.csv |  | numeric |
| cree\_corrected\_mg\_dL — Corrected enzymatic creatinine concentration **File:** 02\_workflows/F1\_workflow\_v02/submission\_ready/public/data/expm\_F1\_validation\_input\_public.csv **Recommended public name:** cree\_corrected\_mg\_dL **Description:** Creatinine concentration after applying the enzymatic correction model. **Unit:**  **Value coding:** See example\_values and allowed\_values. **Data type:** numeric **Example values:** 0.68240141154 | 0.81680053664 | 0.35207418306 | 0.93410841026 | 0.6516394885 **Allowed values:**  **Missing-value coding:** NA/blank as read by fread **n rows / missing / unique:** 32 / 0 / 32 **Release status:** public **Definition status:** exact\_definition | 02\_workflows/F1\_workflow\_v02/submission\_ready/public/data/expm\_F1\_validation\_input\_public.csv |  | numeric |
| cree\_measured\_mg\_dL — Measured enzymatic creatinine concentration **File:** 02\_workflows/F1\_workflow\_v02/submission\_ready/public/data/expm\_F1\_validation\_input\_public.csv **Recommended public name:** cree\_measured\_mg\_dL **Description:** Measured creatinine concentration using the enzymatic assay. **Unit:**  **Value coding:** See example\_values and allowed\_values. **Data type:** numeric **Example values:** 0.75 | 0.78 | 0.52 | 0.89 | 0.8 **Allowed values:**  **Missing-value coding:** NA/blank as read by fread **n rows / missing / unique:** 32 / 0 / 32 **Release status:** public **Definition status:** exact\_definition | 02\_workflows/F1\_workflow\_v02/submission\_ready/public/data/expm\_F1\_validation\_input\_public.csv |  | numeric |
| crej\_corrected\_mg\_dL — Corrected Jaffe creatinine concentration **File:** 02\_workflows/F1\_workflow\_v02/submission\_ready/public/data/expm\_F1\_validation\_input\_public.csv **Recommended public name:** crej\_corrected\_mg\_dL **Description:** Creatinine concentration after applying the Jaffe correction model. **Unit:**  **Value coding:** See example\_values and allowed\_values. **Data type:** numeric **Example values:** 0.8822513 | 0.9490736 | 0.6756628 | 0.8942763 | 0.9588407 **Allowed values:**  **Missing-value coding:** NA/blank as read by fread **n rows / missing / unique:** 32 / 0 / 32 **Release status:** public **Definition status:** exact\_definition | 02\_workflows/F1\_workflow\_v02/submission\_ready/public/data/expm\_F1\_validation\_input\_public.csv |  | numeric |
| crej\_measured\_mg\_dL — Measured Jaffe creatinine concentration **File:** 02\_workflows/F1\_workflow\_v02/submission\_ready/public/data/expm\_F1\_validation\_input\_public.csv **Recommended public name:** crej\_measured\_mg\_dL **Description:** Measured creatinine concentration using the Jaffe assay. **Unit:**  **Value coding:** See example\_values and allowed\_values. **Data type:** numeric **Example values:** 0.95 | 0.98 | 0.78 | 0.92 | 1.06 **Allowed values:**  **Missing-value coding:** NA/blank as read by fread **n rows / missing / unique:** 32 / 0 / 31 **Release status:** public **Definition status:** exact\_definition | 02\_workflows/F1\_workflow\_v02/submission\_ready/public/data/expm\_F1\_validation\_input\_public.csv |  | numeric |
| data\_object — Data object **File:** 02\_workflows/F1\_workflow\_v02/submission\_ready/public/data/expm\_F1\_validation\_input\_public.csv **Recommended public name:** data\_object **Description:** Name of the data object represented by the row. **Unit:**  **Value coding:** Categorical descriptor; see allowed/example values. **Data type:** character **Example values:** validation **Allowed values:** validation **Missing-value coding:** NA/blank as read by fread **n rows / missing / unique:** 32 / 0 / 1 **Release status:** public **Definition status:** exact\_definition | 02\_workflows/F1\_workflow\_v02/submission\_ready/public/data/expm\_F1\_validation\_input\_public.csv |  | character |
| db\_measured\_mg\_dL — Direct bilirubin concentration **File:** 02\_workflows/F1\_workflow\_v02/submission\_ready/public/data/expm\_F1\_validation\_input\_public.csv **Recommended public name:** db\_measured\_mg\_dL **Description:** Direct bilirubin concentration measured in the experimental or validation data. **Unit:**  **Value coding:** See example\_values and allowed\_values. **Data type:** numeric **Example values:** 7.411 | 3.874 | 13.015 | 3.245 | 11.224 **Allowed values:**  **Missing-value coding:** NA/blank as read by fread **n rows / missing / unique:** 32 / 0 / 32 **Release status:** public **Definition status:** exact\_definition | 02\_workflows/F1\_workflow\_v02/submission\_ready/public/data/expm\_F1\_validation\_input\_public.csv |  | numeric |
| domain — Data domain **File:** 02\_workflows/F1\_workflow\_v02/submission\_ready/public/data/expm\_F1\_validation\_input\_public.csv **Recommended public name:** domain **Description:** Workflow or data domain represented by the row. **Unit:**  **Value coding:** Categorical descriptor; see allowed/example values. **Data type:** character **Example values:** expm **Allowed values:** expm **Missing-value coding:** NA/blank as read by fread **n rows / missing / unique:** 32 / 0 / 1 **Release status:** public **Definition status:** exact\_definition | 02\_workflows/F1\_workflow\_v02/submission\_ready/public/data/expm\_F1\_validation\_input\_public.csv |  | character |
| expm\_F1\_validation\_row\_id — Experimental validation row identifier **File:** 02\_workflows/F1\_workflow\_v02/submission\_ready/public/data/expm\_F1\_validation\_input\_public.csv **Recommended public name:** expm\_F1\_validation\_row\_id **Description:** Row identifier within the released F1 experimental validation table. **Unit:**  **Value coding:** See example\_values and allowed\_values. **Data type:** integer **Example values:** 1 | 2 | 3 | 4 | 5 **Allowed values:**  **Missing-value coding:** NA/blank as read by fread **n rows / missing / unique:** 32 / 0 / 32 **Release status:** public **Definition status:** exact\_definition | 02\_workflows/F1\_workflow\_v02/submission\_ready/public/data/expm\_F1\_validation\_input\_public.csv |  | integer |
| release\_status — Release status **File:** 02\_workflows/F1\_workflow\_v02/submission\_ready/public/data/expm\_F1\_validation\_input\_public.csv **Recommended public name:** release\_status **Description:** Release-status label indicating the publication status of the row or file object. **Unit:**  **Value coding:** Release status of the row/object, for example public. **Data type:** character **Example values:** public **Allowed values:** public **Missing-value coding:** NA/blank as read by fread **n rows / missing / unique:** 32 / 0 / 1 **Release status:** public **Definition status:** exact\_definition | 02\_workflows/F1\_workflow\_v02/submission\_ready/public/data/expm\_F1\_validation\_input\_public.csv |  | character |
| sample\_id — Sample identifier **File:** 02\_workflows/F1\_workflow\_v02/submission\_ready/public/data/expm\_F1\_validation\_input\_public.csv **Recommended public name:** sample\_id **Description:** Identifier of a sample or experimental record within the released public data; not a personal identifier. **Unit:**  **Value coding:** See example\_values and allowed\_values. **Data type:** integer **Example values:** 1 | 2 | 3 | 4 | 5 **Allowed values:**  **Missing-value coding:** NA/blank as read by fread **n rows / missing / unique:** 32 / 0 / 32 **Release status:** public **Definition status:** exact\_definition | 02\_workflows/F1\_workflow\_v02/submission\_ready/public/data/expm\_F1\_validation\_input\_public.csv |  | integer |
| source\_harmonized\_file\_name — Source harmonized file name **File:** 02\_workflows/F1\_workflow\_v02/submission\_ready/public/data/expm\_F1\_validation\_input\_public.csv **Recommended public name:** source\_harmonized\_file\_name **Description:** Name of the harmonized source file used to build the released object. **Unit:**  **Value coding:** See example\_values and allowed\_values. **Data type:** character **Example values:** expm\_F1\_validation\_raw\_public.csv **Allowed values:** expm\_F1\_validation\_raw\_public.csv **Missing-value coding:** NA/blank as read by fread **n rows / missing / unique:** 32 / 0 / 1 **Release status:** public **Definition status:** exact\_definition | 02\_workflows/F1\_workflow\_v02/submission\_ready/public/data/expm\_F1\_validation\_input\_public.csv |  | character |
| tb\_measured\_mg\_dL — Measured total bilirubin concentration **File:** 02\_workflows/F1\_workflow\_v02/submission\_ready/public/data/expm\_F1\_validation\_input\_public.csv **Recommended public name:** tb\_measured\_mg\_dL **Description:** Measured total bilirubin concentration in the experimental F1 data. **Unit:**  **Value coding:** See example\_values and allowed\_values. **Data type:** numeric **Example values:** 9.263 | 4.852 | 16.121 | 4.419 | 14.415 **Allowed values:**  **Missing-value coding:** NA/blank as read by fread **n rows / missing / unique:** 32 / 0 / 32 **Release status:** public **Definition status:** exact\_definition | 02\_workflows/F1\_workflow\_v02/submission\_ready/public/data/expm\_F1\_validation\_input\_public.csv |  | numeric |
| unit\_or\_role — Unit or semantic role **File:** 02\_workflows/F1\_workflow\_v02/submission\_ready/public/data/expm\_F1\_validation\_input\_public.csv **Recommended public name:** unit\_or\_role **Description:** Unit, role, or semantic type corresponding to the row-specific variable/metric. **Unit:**  **Value coding:** Categorical descriptor; see allowed/example values. **Data type:** character **Example values:** input **Allowed values:** input **Missing-value coding:** NA/blank as read by fread **n rows / missing / unique:** 32 / 0 / 1 **Release status:** public **Definition status:** exact\_definition | 02\_workflows/F1\_workflow\_v02/submission\_ready/public/data/expm\_F1\_validation\_input\_public.csv |  | character |
| workflow\_step — Workflow step **File:** 02\_workflows/F1\_workflow\_v02/submission\_ready/public/data/expm\_F1\_validation\_input\_public.csv **Recommended public name:** workflow\_step **Description:** Workflow step that produced or used the row/object. **Unit:**  **Value coding:** Categorical descriptor; see allowed/example values. **Data type:** character **Example values:** 2a\_refined\_analysis\_dataset **Allowed values:** 2a\_refined\_analysis\_dataset **Missing-value coding:** NA/blank as read by fread **n rows / missing / unique:** 32 / 0 / 1 **Release status:** public **Definition status:** exact\_definition | 02\_workflows/F1\_workflow\_v02/submission\_ready/public/data/expm\_F1\_validation\_input\_public.csv |  | character |
| cre\_true\_mg\_dL — True creatinine concentration **File:** 02\_workflows/F1\_workflow\_v02/submission\_ready/public/data/slco\_F1\_surface\_grid\_public.csv **Recommended public name:** cre\_true\_mg\_dL **Description:** True creatinine concentration used as the gravimetric/reference concentration in F1. **Unit:**  **Value coding:** See example\_values and allowed\_values. **Data type:** numeric **Example values:** 1 | 1.05 | 1.1 | 1.15 | 1.2 **Allowed values:**  **Missing-value coding:** NA/blank as read by fread **n rows / missing / unique:** 34441 / 0 / 101 **Release status:** public **Definition status:** exact\_definition | 02\_workflows/F1\_workflow\_v02/submission\_ready/public/data/slco\_F1\_surface\_grid\_public.csv |  | numeric |
| cree\_measured\_pred\_mg\_dL — Predicted measured enzymatic creatinine concentration **File:** 02\_workflows/F1\_workflow\_v02/submission\_ready/public/data/slco\_F1\_surface\_grid\_public.csv **Recommended public name:** cree\_measured\_pred\_mg\_dL **Description:** Predicted measured enzymatic creatinine concentration derived from the interference model. **Unit:**  **Value coding:** See example\_values and allowed\_values. **Data type:** numeric **Example values:** 0.902064914425904 | 0.957189139835594 | 1.01231537541398 | 1.06744362138108 | 1.1225738779567 **Allowed values:**  **Missing-value coding:** NA/blank as read by fread **n rows / missing / unique:** 34441 / 0 / 34441 **Release status:** public **Definition status:** exact\_definition | 02\_workflows/F1\_workflow\_v02/submission\_ready/public/data/slco\_F1\_surface\_grid\_public.csv |  | numeric |
| cree\_true\_recalc\_error — Recalculation error for enzymatic creatinine **File:** 02\_workflows/F1\_workflow\_v02/submission\_ready/public/data/slco\_F1\_surface\_grid\_public.csv **Recommended public name:** cree\_true\_recalc\_error **Description:** Numerical difference between recalculated and expected true enzymatic creatinine values. **Unit:**  **Value coding:** See example\_values and allowed\_values. **Data type:** numeric **Example values:** -9.99200722162641e-16 | -4.06341627012807e-14 | -5.97299987248334e-14 | 1.79856129989275e-14 | 6.3504757008559e-14 **Allowed values:**  **Missing-value coding:** NA/blank as read by fread **n rows / missing / unique:** 34441 / 0 / 1163 **Release status:** public **Definition status:** exact\_definition | 02\_workflows/F1\_workflow\_v02/submission\_ready/public/data/slco\_F1\_surface\_grid\_public.csv |  | numeric |
| cree\_true\_recalc\_mg\_dL — Recalculated true enzymatic creatinine concentration **File:** 02\_workflows/F1\_workflow\_v02/submission\_ready/public/data/slco\_F1\_surface\_grid\_public.csv **Recommended public name:** cree\_true\_recalc\_mg\_dL **Description:** True enzymatic creatinine concentration recalculated from the model. **Unit:**  **Value coding:** See example\_values and allowed\_values. **Data type:** numeric **Example values:** 1 | 1.04999999999996 | 1.09999999999994 | 1.15000000000002 | 1.20000000000006 **Allowed values:**  **Missing-value coding:** NA/blank as read by fread **n rows / missing / unique:** 34441 / 0 / 2603 **Release status:** public **Definition status:** exact\_definition | 02\_workflows/F1\_workflow\_v02/submission\_ready/public/data/slco\_F1\_surface\_grid\_public.csv |  | numeric |
| crej\_measured\_pred\_mg\_dL — Predicted measured Jaffe creatinine concentration **File:** 02\_workflows/F1\_workflow\_v02/submission\_ready/public/data/slco\_F1\_surface\_grid\_public.csv **Recommended public name:** crej\_measured\_pred\_mg\_dL **Description:** Predicted measured Jaffe creatinine concentration derived from the interference model. **Unit:**  **Value coding:** See example\_values and allowed\_values. **Data type:** numeric **Example values:** 0.990463857965053 | 1.04152284217999 | 1.09273908981855 | 1.14411406302484 | 1.19564924674134 **Allowed values:**  **Missing-value coding:** NA/blank as read by fread **n rows / missing / unique:** 34441 / 0 / 22878 **Release status:** public **Definition status:** exact\_definition | 02\_workflows/F1\_workflow\_v02/submission\_ready/public/data/slco\_F1\_surface\_grid\_public.csv |  | numeric |
| crej\_true\_recalc\_error — Recalculation error for Jaffe creatinine **File:** 02\_workflows/F1\_workflow\_v02/submission\_ready/public/data/slco\_F1\_surface\_grid\_public.csv **Recommended public name:** crej\_true\_recalc\_error **Description:** Numerical difference between recalculated and expected true Jaffe creatinine values. **Unit:**  **Value coding:** See example\_values and allowed\_values. **Data type:** numeric **Example values:** 6.66133814775094e-16 | 1.33226762955019e-15 | 2.22044604925031e-16 | 0 | 1.11022302462516e-15 **Allowed values:**  **Missing-value coding:** NA/blank as read by fread **n rows / missing / unique:** 34441 / 0 / 22 **Release status:** public **Definition status:** exact\_definition | 02\_workflows/F1\_workflow\_v02/submission\_ready/public/data/slco\_F1\_surface\_grid\_public.csv |  | numeric |
| crej\_true\_recalc\_mg\_dL — Recalculated true Jaffe creatinine concentration **File:** 02\_workflows/F1\_workflow\_v02/submission\_ready/public/data/slco\_F1\_surface\_grid\_public.csv **Recommended public name:** crej\_true\_recalc\_mg\_dL **Description:** True Jaffe creatinine concentration recalculated from the model. **Unit:**  **Value coding:** See example\_values and allowed\_values. **Data type:** numeric **Example values:** 1 | 1.05 | 1.1 | 1.15 | 1.2 **Allowed values:**  **Missing-value coding:** NA/blank as read by fread **n rows / missing / unique:** 34441 / 0 / 101 **Release status:** public **Definition status:** exact\_definition | 02\_workflows/F1\_workflow\_v02/submission\_ready/public/data/slco\_F1\_surface\_grid\_public.csv |  | numeric |
| delta\_cree\_to\_gravimetry\_mg\_dL — Enzymatic creatinine deviation from gravimetry **File:** 02\_workflows/F1\_workflow\_v02/submission\_ready/public/data/slco\_F1\_surface\_grid\_public.csv **Recommended public name:** delta\_cree\_to\_gravimetry\_mg\_dL **Description:** Difference between enzymatic creatinine and the gravimetric/reference value. **Unit:**  **Value coding:** See example\_values and allowed\_values. **Data type:** numeric **Example values:** 0.097935085574096 | 0.0928108601644061 | 0.0876846245860181 | 0.0825563786189225 | 0.0774261220432957 **Allowed values:**  **Missing-value coding:** NA/blank as read by fread **n rows / missing / unique:** 34441 / 0 / 34441 **Release status:** public **Definition status:** exact\_definition | 02\_workflows/F1\_workflow\_v02/submission\_ready/public/data/slco\_F1\_surface\_grid\_public.csv |  | numeric |
| delta\_crej\_to\_gravimetry\_mg\_dL — Jaffe creatinine deviation from gravimetry **File:** 02\_workflows/F1\_workflow\_v02/submission\_ready/public/data/slco\_F1\_surface\_grid\_public.csv **Recommended public name:** delta\_crej\_to\_gravimetry\_mg\_dL **Description:** Difference between Jaffe creatinine and the gravimetric/reference value. **Unit:**  **Value coding:** See example\_values and allowed\_values. **Data type:** numeric **Example values:** 0.0095361420349469 | 0.00847715782000713 | 0.00726091018144937 | 0.00588593697515893 | 0.00435075325865686 **Allowed values:**  **Missing-value coding:** NA/blank as read by fread **n rows / missing / unique:** 34441 / 0 / 23473 **Release status:** public **Definition status:** exact\_definition | 02\_workflows/F1\_workflow\_v02/submission\_ready/public/data/slco\_F1\_surface\_grid\_public.csv |  | numeric |
| grid\_id — Surface-grid identifier **File:** 02\_workflows/F1\_workflow\_v02/submission\_ready/public/data/slco\_F1\_surface\_grid\_public.csv **Recommended public name:** grid\_id **Description:** Identifier of a grid point in the F1 simulated or reconstructed surface data. **Unit:**  **Value coding:** See example\_values and allowed\_values. **Data type:** integer **Example values:** 1 | 2 | 3 | 4 | 5 **Allowed values:**  **Missing-value coding:** NA/blank as read by fread **n rows / missing / unique:** 34441 / 0 / 34441 **Release status:** public **Definition status:** exact\_definition | 02\_workflows/F1\_workflow\_v02/submission\_ready/public/data/slco\_F1\_surface\_grid\_public.csv |  | integer |
| tb\_mg\_dL — Total bilirubin concentration **File:** 02\_workflows/F1\_workflow\_v02/submission\_ready/public/data/slco\_F1\_surface\_grid\_public.csv **Recommended public name:** tb\_mg\_dL **Description:** Total bilirubin concentration used in the creatinine/bilirubin interference model or figure data. **Unit:**  **Value coding:** See example\_values and allowed\_values. **Data type:** numeric **Example values:** 1 | 1.1 | 1.2 | 1.3 | 1.4 **Allowed values:**  **Missing-value coding:** NA/blank as read by fread **n rows / missing / unique:** 34441 / 0 / 341 **Release status:** public **Definition status:** exact\_definition | 02\_workflows/F1\_workflow\_v02/submission\_ready/public/data/slco\_F1\_surface\_grid\_public.csv |  | numeric |
| parameter — Metadata parameter name **File:** 02\_workflows/F1\_workflow\_v02/submission\_ready/public/data/slco\_F1\_surface\_meta\_public.csv **Recommended public name:** parameter **Description:** Name of a metadata parameter describing the F2 simulated heatmap object, such as figure identity, data origin, grid type, axis variable, or unit/role. **Unit:**  **Value coding:** Categorical metadata key; value is given in the corresponding value column or file-specific metadata field. **Data type:** character **Example values:** dataset\_name | domain | anchor | data\_object | unit\_or\_role **Allowed values:** dataset\_name | domain | anchor | data\_object | unit\_or\_role | release\_status | source\_model\_coefficients | grid\_tb\_min\_mg\_dL | grid\_tb\_max\_mg\_dL | grid\_tb\_step\_mg\_dL **Missing-value coding:** NA/blank as read by fread **n rows / missing / unique:** 16 / 0 / 16 **Release status:** public **Definition status:** manual\_exact\_definition | 02\_workflows/F1\_workflow\_v02/submission\_ready/public/data/slco\_F1\_surface\_meta\_public.csv |  | character |
| value — Value **File:** 02\_workflows/F1\_workflow\_v02/submission\_ready/public/data/slco\_F1\_surface\_meta\_public.csv **Recommended public name:** value **Description:** Numerical or character value corresponding to the row-specific variable/metric. **Unit:**  **Value coding:** See example\_values and allowed\_values. **Data type:** character **Example values:** slco\_F1\_surface\_grid\_public | slco | F1 | surface | grid **Allowed values:** slco\_F1\_surface\_grid\_public | slco | F1 | surface | grid | public | expm\_F1\_model\_coefficients\_public.csv | 1 | 35 | 0.1 **Missing-value coding:** NA/blank as read by fread **n rows / missing / unique:** 16 / 0 / 15 **Release status:** public **Definition status:** exact\_definition | 02\_workflows/F1\_workflow\_v02/submission\_ready/public/data/slco\_F1\_surface\_meta\_public.csv |  | character |
| count\_negative — Number of simulated subpoints with negative score shift **File:** 02\_workflows/F2\_workflow\_v01/data/02b\_figure\_content/slco\_F2\_heatmap\_bin\_public.csv **Recommended public name:** count\_negative **Description:** Number of simulated subpoints within the F2 heatmap bin where the score shift is negative, defined as score delta ≤ −1. **Unit:** count **Value coding:** Integer count. **Data type:** integer **Example values:** 0 | 1 | 17 | 20 | 4 **Allowed values:**  **Missing-value coding:** NA/blank as read by fread **n rows / missing / unique:** 295200 / 0 / 101 **Release status:** public **Definition status:** manual\_precision\_definition | 02\_workflows/F2\_workflow\_v01/data/02b\_figure\_content/slco\_F2\_heatmap\_bin\_public.csv | count | integer |
| model — MELD model or score variant **File:** 02\_workflows/F2\_workflow\_v01/data/02b\_figure\_content/slco\_F2\_heatmap\_bin\_public.csv **Recommended public name:** model **Description:** Name of the MELD-related model or score variant represented by the row; expected values include MELD, MELD-Na, reMELD-Na, and MELD 3.0. **Unit:**  **Value coding:** Categorical score/model label, for example MELD, MELD-Na, reMELD-Na, or MELD 3.0. **Data type:** character **Example values:** MELD | MELD 3.0 | MELD-Na | reMELD-Na **Allowed values:** MELD | MELD 3.0 | MELD-Na | reMELD-Na **Missing-value coding:** NA/blank as read by fread **n rows / missing / unique:** 295200 / 0 / 4 **Release status:** public **Definition status:** manual\_exact\_definition | 02\_workflows/F2\_workflow\_v01/data/02b\_figure\_content/slco\_F2\_heatmap\_bin\_public.csv |  | character |
| n\_sub — Number of simulated subpoints per heatmap bin **File:** 02\_workflows/F2\_workflow\_v01/data/02b\_figure\_content/slco\_F2\_heatmap\_bin\_public.csv **Recommended public name:** n\_sub **Description:** Number of simulated subpoints aggregated within one F2 heatmap bin. **Unit:** count **Value coding:** Integer count. **Data type:** integer **Example values:** 100 **Allowed values:**  **Missing-value coding:** NA/blank as read by fread **n rows / missing / unique:** 295200 / 0 / 1 **Release status:** public **Definition status:** manual\_precision\_definition | 02\_workflows/F2\_workflow\_v01/data/02b\_figure\_content/slco\_F2\_heatmap\_bin\_public.csv | count | integer |
| pct\_negative — Percentage of simulated subpoints with negative score shift **File:** 02\_workflows/F2\_workflow\_v01/data/02b\_figure\_content/slco\_F2\_heatmap\_bin\_public.csv **Recommended public name:** pct\_negative **Description:** Percentage of simulated subpoints within the F2 heatmap bin where the score shift is negative, defined as score delta ≤ −1. **Unit:** mg/dL **Value coding:** 0–100 percentage scale. **Data type:** integer **Example values:** 0 | 1 | 17 | 20 | 4 **Allowed values:**  **Missing-value coding:** NA/blank as read by fread **n rows / missing / unique:** 295200 / 0 / 101 **Release status:** public **Definition status:** manual\_precision\_definition | 02\_workflows/F2\_workflow\_v01/data/02b\_figure\_content/slco\_F2\_heatmap\_bin\_public.csv | mg/dL | integer |
| x\_value — Heatmap x-axis creatinine value **File:** 02\_workflows/F2\_workflow\_v01/data/02b\_figure\_content/slco\_F2\_heatmap\_bin\_public.csv **Recommended public name:** x\_value **Description:** Creatinine value defining the x-axis coordinate of an F2 heatmap bin. **Unit:** mg/dL **Value coding:** Numeric creatinine value in mg/dL. **Data type:** numeric **Example values:** 0.1 | 0.12 | 0.14 | 0.16 | 0.18 **Allowed values:**  **Missing-value coding:** NA/blank as read by fread **n rows / missing / unique:** 295200 / 0 / 246 **Release status:** public **Definition status:** manual\_precision\_definition | 02\_workflows/F2\_workflow\_v01/data/02b\_figure\_content/slco\_F2\_heatmap\_bin\_public.csv | mg/dL | numeric |
| y\_value — Heatmap y-axis total bilirubin value **File:** 02\_workflows/F2\_workflow\_v01/data/02b\_figure\_content/slco\_F2\_heatmap\_bin\_public.csv **Recommended public name:** y\_value **Description:** Total bilirubin value defining the y-axis coordinate of an F2 heatmap bin. **Unit:** analysis-specific y-axis coordinate **Value coding:** Numeric total bilirubin value in mg/dL. **Data type:** numeric **Example values:** 0.1 | 0.2 | 0.3 | 0.4 | 0.5 **Allowed values:**  **Missing-value coding:** NA/blank as read by fread **n rows / missing / unique:** 295200 / 0 / 300 **Release status:** public **Definition status:** manual\_precision\_definition | 02\_workflows/F2\_workflow\_v01/data/02b\_figure\_content/slco\_F2\_heatmap\_bin\_public.csv | analysis-specific y-axis coordinate | numeric |
| parameter — Metadata parameter name **File:** 02\_workflows/F2\_workflow\_v01/data/02b\_figure\_content/slco\_F2\_heatmap\_meta\_public.csv **Recommended public name:** parameter **Description:** Name of a metadata parameter describing the F2 simulated heatmap object, such as figure identity, data origin, grid type, axis variable, or unit/role. **Unit:**  **Value coding:** Categorical metadata key; value is given in the corresponding value column or file-specific metadata field. **Data type:** character **Example values:** dataset\_name | domain | anchor | data\_object | unit\_or\_role **Allowed values:** dataset\_name | domain | anchor | data\_object | unit\_or\_role | release\_status | source\_input\_file | x\_axis | x\_axis\_label | x\_min **Missing-value coding:** NA/blank as read by fread **n rows / missing / unique:** 22 / 0 / 22 **Release status:** public **Definition status:** manual\_exact\_definition | 02\_workflows/F2\_workflow\_v01/data/02b\_figure\_content/slco\_F2\_heatmap\_meta\_public.csv |  | character |
| value — Value **File:** 02\_workflows/F2\_workflow\_v01/data/02b\_figure\_content/slco\_F2\_heatmap\_meta\_public.csv **Recommended public name:** value **Description:** Numerical or character value corresponding to the row-specific variable/metric. **Unit:**  **Value coding:** See example\_values and allowed\_values. **Data type:** character **Example values:** slco\_F2\_heatmap\_bin\_public | slco | F2 | heatmap | bin **Allowed values:** slco\_F2\_heatmap\_bin\_public | slco | F2 | heatmap | bin | public | slco\_F2\_heatmap\_input\_internal.csv | x\_value | Creatinine (mg/dL) | 0.1 **Missing-value coding:** NA/blank as read by fread **n rows / missing / unique:** 22 / 0 / 21 **Release status:** public **Definition status:** exact\_definition | 02\_workflows/F2\_workflow\_v01/data/02b\_figure\_content/slco\_F2\_heatmap\_meta\_public.csv |  | character |
| count\_negative — Number of simulated subpoints with negative score shift **File:** 02\_workflows/F2\_workflow\_v01/submission\_ready/public/data/slco\_F2\_heatmap\_bin\_public.csv **Recommended public name:** count\_negative **Description:** Number of simulated subpoints within the F2 heatmap bin where the score shift is negative, defined as score delta ≤ −1. **Unit:** count **Value coding:** Integer count. **Data type:** integer **Example values:** 0 | 1 | 17 | 20 | 4 **Allowed values:**  **Missing-value coding:** NA/blank as read by fread **n rows / missing / unique:** 295200 / 0 / 101 **Release status:** public **Definition status:** manual\_precision\_definition | 02\_workflows/F2\_workflow\_v01/submission\_ready/public/data/slco\_F2\_heatmap\_bin\_public.csv | count | integer |
| model — MELD model or score variant **File:** 02\_workflows/F2\_workflow\_v01/submission\_ready/public/data/slco\_F2\_heatmap\_bin\_public.csv **Recommended public name:** model **Description:** Name of the MELD-related model or score variant represented by the row; expected values include MELD, MELD-Na, reMELD-Na, and MELD 3.0. **Unit:**  **Value coding:** Categorical score/model label, for example MELD, MELD-Na, reMELD-Na, or MELD 3.0. **Data type:** character **Example values:** MELD | MELD 3.0 | MELD-Na | reMELD-Na **Allowed values:** MELD | MELD 3.0 | MELD-Na | reMELD-Na **Missing-value coding:** NA/blank as read by fread **n rows / missing / unique:** 295200 / 0 / 4 **Release status:** public **Definition status:** manual\_exact\_definition | 02\_workflows/F2\_workflow\_v01/submission\_ready/public/data/slco\_F2\_heatmap\_bin\_public.csv |  | character |
| n\_sub — Number of simulated subpoints per heatmap bin **File:** 02\_workflows/F2\_workflow\_v01/submission\_ready/public/data/slco\_F2\_heatmap\_bin\_public.csv **Recommended public name:** n\_sub **Description:** Number of simulated subpoints aggregated within one F2 heatmap bin. **Unit:** count **Value coding:** Integer count. **Data type:** integer **Example values:** 100 **Allowed values:**  **Missing-value coding:** NA/blank as read by fread **n rows / missing / unique:** 295200 / 0 / 1 **Release status:** public **Definition status:** manual\_precision\_definition | 02\_workflows/F2\_workflow\_v01/submission\_ready/public/data/slco\_F2\_heatmap\_bin\_public.csv | count | integer |
| pct\_negative — Percentage of simulated subpoints with negative score shift **File:** 02\_workflows/F2\_workflow\_v01/submission\_ready/public/data/slco\_F2\_heatmap\_bin\_public.csv **Recommended public name:** pct\_negative **Description:** Percentage of simulated subpoints within the F2 heatmap bin where the score shift is negative, defined as score delta ≤ −1. **Unit:** mg/dL **Value coding:** 0–100 percentage scale. **Data type:** integer **Example values:** 0 | 1 | 17 | 20 | 4 **Allowed values:**  **Missing-value coding:** NA/blank as read by fread **n rows / missing / unique:** 295200 / 0 / 101 **Release status:** public **Definition status:** manual\_precision\_definition | 02\_workflows/F2\_workflow\_v01/submission\_ready/public/data/slco\_F2\_heatmap\_bin\_public.csv | mg/dL | integer |
| x\_value — Heatmap x-axis creatinine value **File:** 02\_workflows/F2\_workflow\_v01/submission\_ready/public/data/slco\_F2\_heatmap\_bin\_public.csv **Recommended public name:** x\_value **Description:** Creatinine value defining the x-axis coordinate of an F2 heatmap bin. **Unit:** mg/dL **Value coding:** Numeric creatinine value in mg/dL. **Data type:** numeric **Example values:** 0.1 | 0.12 | 0.14 | 0.16 | 0.18 **Allowed values:**  **Missing-value coding:** NA/blank as read by fread **n rows / missing / unique:** 295200 / 0 / 246 **Release status:** public **Definition status:** manual\_precision\_definition | 02\_workflows/F2\_workflow\_v01/submission\_ready/public/data/slco\_F2\_heatmap\_bin\_public.csv | mg/dL | numeric |
| y\_value — Heatmap y-axis total bilirubin value **File:** 02\_workflows/F2\_workflow\_v01/submission\_ready/public/data/slco\_F2\_heatmap\_bin\_public.csv **Recommended public name:** y\_value **Description:** Total bilirubin value defining the y-axis coordinate of an F2 heatmap bin. **Unit:** analysis-specific y-axis coordinate **Value coding:** Numeric total bilirubin value in mg/dL. **Data type:** numeric **Example values:** 0.1 | 0.2 | 0.3 | 0.4 | 0.5 **Allowed values:**  **Missing-value coding:** NA/blank as read by fread **n rows / missing / unique:** 295200 / 0 / 300 **Release status:** public **Definition status:** manual\_precision\_definition | 02\_workflows/F2\_workflow\_v01/submission\_ready/public/data/slco\_F2\_heatmap\_bin\_public.csv | analysis-specific y-axis coordinate | numeric |
| parameter — Metadata parameter name **File:** 02\_workflows/F2\_workflow\_v01/submission\_ready/public/data/slco\_F2\_heatmap\_meta\_public.csv **Recommended public name:** parameter **Description:** Name of a metadata parameter describing the F2 simulated heatmap object, such as figure identity, data origin, grid type, axis variable, or unit/role. **Unit:**  **Value coding:** Categorical metadata key; value is given in the corresponding value column or file-specific metadata field. **Data type:** character **Example values:** dataset\_name | domain | anchor | data\_object | unit\_or\_role **Allowed values:** dataset\_name | domain | anchor | data\_object | unit\_or\_role | release\_status | source\_input\_file | x\_axis | x\_axis\_label | x\_min **Missing-value coding:** NA/blank as read by fread **n rows / missing / unique:** 22 / 0 / 22 **Release status:** public **Definition status:** manual\_exact\_definition | 02\_workflows/F2\_workflow\_v01/submission\_ready/public/data/slco\_F2\_heatmap\_meta\_public.csv |  | character |
| value — Value **File:** 02\_workflows/F2\_workflow\_v01/submission\_ready/public/data/slco\_F2\_heatmap\_meta\_public.csv **Recommended public name:** value **Description:** Numerical or character value corresponding to the row-specific variable/metric. **Unit:**  **Value coding:** See example\_values and allowed\_values. **Data type:** character **Example values:** slco\_F2\_heatmap\_bin\_public | slco | F2 | heatmap | bin **Allowed values:** slco\_F2\_heatmap\_bin\_public | slco | F2 | heatmap | bin | public | slco\_F2\_heatmap\_input\_internal.csv | x\_value | Creatinine (mg/dL) | 0.1 **Missing-value coding:** NA/blank as read by fread **n rows / missing / unique:** 22 / 0 / 21 **Release status:** public **Definition status:** exact\_definition | 02\_workflows/F2\_workflow\_v01/submission\_ready/public/data/slco\_F2\_heatmap\_meta\_public.csv |  | character |
| age\_years\_first\_available — Age at first available public record **File:** 02\_workflows/F3\_workflow\_v01/data/01\_source\_loaded\_harmonized/esld\_master\_long\_public.csv **Recommended public name:** age\_years\_first\_available **Description:** Age in years at the first available public ESLD record for the patient or encounter represented in the released data. **Unit:** years **Value coding:** Integer age in years. **Data type:** integer **Example values:** 65 | 67 | 21 | 56 | 63 **Allowed values:**  **Missing-value coding:** NA/blank as read by fread **n rows / missing / unique:** 67399 / 0 / 79 **Release status:** public **Definition status:** manual\_exact\_definition | 02\_workflows/F3\_workflow\_v01/data/01\_source\_loaded\_harmonized/esld\_master\_long\_public.csv | years | integer |
| age\_years\_sample — Age at sample **File:** 02\_workflows/F3\_workflow\_v01/data/01\_source\_loaded\_harmonized/esld\_master\_long\_public.csv **Recommended public name:** age\_years\_sample **Description:** Age in years at the sample-level public ESLD record. **Unit:** years **Value coding:** Integer age in years. **Data type:** integer **Example values:** 65 | 66 | 67 | 68 | 69 **Allowed values:**  **Missing-value coding:** NA/blank as read by fread **n rows / missing / unique:** 67399 / 0 / 84 **Release status:** public **Definition status:** manual\_precision\_definition | 02\_workflows/F3\_workflow\_v01/data/01\_source\_loaded\_harmonized/esld\_master\_long\_public.csv | years | integer |
| albumin\_g\_dl — Serum albumin concentration **File:** 02\_workflows/F3\_workflow\_v01/data/01\_source\_loaded\_harmonized/esld\_master\_long\_public.csv **Recommended public name:** albumin\_g\_dl **Description:** Serum albumin concentration in the public ESLD master data. **Unit:** g/dL **Value coding:** Numeric concentration in g/dL. **Data type:** numeric **Example values:** 3.3 | 6.15 | 3.14 | 2.8 | 3.26 **Allowed values:**  **Missing-value coding:** NA/blank as read by fread **n rows / missing / unique:** 67399 / 58785 / 580 **Release status:** public **Definition status:** manual\_precision\_definition | 02\_workflows/F3\_workflow\_v01/data/01\_source\_loaded\_harmonized/esld\_master\_long\_public.csv | g/dL | numeric |
| ald — Alcohol-related liver disease etiology flag **File:** 02\_workflows/F3\_workflow\_v01/data/01\_source\_loaded\_harmonized/esld\_master\_long\_public.csv **Recommended public name:** ald **Description:** Binary etiology indicator for alcohol-related liver disease in the public ESLD cohort. **Unit:**  **Value coding:** 0 = no; 1 = yes. **Data type:** integer **Example values:** 0 | 1 **Allowed values:**  **Missing-value coding:** NA/blank as read by fread **n rows / missing / unique:** 67399 / 0 / 2 **Release status:** public **Definition status:** manual\_exact\_definition | 02\_workflows/F3\_workflow\_v01/data/01\_source\_loaded\_harmonized/esld\_master\_long\_public.csv |  | integer |
| ald\_hcv — Combined alcohol-related liver disease and hepatitis C etiology flag **File:** 02\_workflows/F3\_workflow\_v01/data/01\_source\_loaded\_harmonized/esld\_master\_long\_public.csv **Recommended public name:** ald\_hcv **Description:** Binary etiology indicator for combined alcohol-related liver disease and hepatitis C in the public ESLD cohort. **Unit:**  **Value coding:** 0 = no; 1 = yes. **Data type:** integer **Example values:** 0 | 1 **Allowed values:**  **Missing-value coding:** NA/blank as read by fread **n rows / missing / unique:** 67399 / 0 / 2 **Release status:** public **Definition status:** manual\_exact\_definition | 02\_workflows/F3\_workflow\_v01/data/01\_source\_loaded\_harmonized/esld\_master\_long\_public.csv |  | integer |
| autoimmune — Autoimmune liver disease etiology flag **File:** 02\_workflows/F3\_workflow\_v01/data/01\_source\_loaded\_harmonized/esld\_master\_long\_public.csv **Recommended public name:** autoimmune **Description:** Binary etiology indicator for autoimmune liver disease in the public ESLD cohort. **Unit:**  **Value coding:** 0 = no; 1 = yes. **Data type:** integer **Example values:** 0 | 1 **Allowed values:**  **Missing-value coding:** NA/blank as read by fread **n rows / missing / unique:** 67399 / 0 / 2 **Release status:** public **Definition status:** manual\_exact\_definition | 02\_workflows/F3\_workflow\_v01/data/01\_source\_loaded\_harmonized/esld\_master\_long\_public.csv |  | integer |
| crea — Creatinine concentration **File:** 02\_workflows/F3\_workflow\_v01/data/01\_source\_loaded\_harmonized/esld\_master\_long\_public.csv **Recommended public name:** crea **Description:** Creatinine concentration used in MELD-related score calculation before applying the study-specific correction. **Unit:** mg/dL **Value coding:** Numeric concentration in mg/dL. **Data type:** numeric **Example values:** 1.91 | 1.42 | 1.31 | 1.3 | 1.28 **Allowed values:**  **Missing-value coding:** NA/blank as read by fread **n rows / missing / unique:** 67399 / 0 / 975 **Release status:** public **Definition status:** manual\_exact\_definition | 02\_workflows/F3\_workflow\_v01/data/01\_source\_loaded\_harmonized/esld\_master\_long\_public.csv | mg/dL | numeric |
| crea\_corrected — Corrected creatinine concentration **File:** 02\_workflows/F3\_workflow\_v01/data/01\_source\_loaded\_harmonized/esld\_master\_long\_public.csv **Recommended public name:** crea\_corrected **Description:** Creatinine concentration after applying the study-specific correction used for recalculated MELD-related scores. **Unit:** mg/dL **Value coding:** Numeric concentration in mg/dL. **Data type:** numeric **Example values:** 1.87705004446668 | 1.41586432303417 | 1.31039363454933 | 1.30192482788903 | 1.28150549768982 **Allowed values:**  **Missing-value coding:** NA/blank as read by fread **n rows / missing / unique:** 67399 / 0 / 15868 **Release status:** public **Definition status:** manual\_exact\_definition | 02\_workflows/F3\_workflow\_v01/data/01\_source\_loaded\_harmonized/esld\_master\_long\_public.csv | mg/dL | numeric |
| date\_count — Relative day count **File:** 02\_workflows/F3\_workflow\_v01/data/01\_source\_loaded\_harmonized/esld\_master\_long\_public.csv **Recommended public name:** date\_count **Description:** Relative day count used for time alignment; negative/positive values are relative to the analysis anchor and are not calendar dates. **Unit:**  **Value coding:** See example\_values and allowed\_values. **Data type:** integer **Example values:** -3021 | -2833 | -2798 | -2793 | -2791 **Allowed values:**  **Missing-value coding:** NA/blank as read by fread **n rows / missing / unique:** 67399 / 50970 / 1450 **Release status:** public **Definition status:** exact\_definition | 02\_workflows/F3\_workflow\_v01/data/01\_source\_loaded\_harmonized/esld\_master\_long\_public.csv |  | integer |
| date\_count\_month — Relative month count **File:** 02\_workflows/F3\_workflow\_v01/data/01\_source\_loaded\_harmonized/esld\_master\_long\_public.csv **Recommended public name:** date\_count\_month **Description:** Relative month count derived from the relative day count; this is not a calendar month. **Unit:**  **Value coding:** See example\_values and allowed\_values. **Data type:** integer **Example values:** -99 | -93 | -92 | -91 | -87 **Allowed values:**  **Missing-value coding:** NA/blank as read by fread **n rows / missing / unique:** 67399 / 50970 / 147 **Release status:** public **Definition status:** exact\_definition | 02\_workflows/F3\_workflow\_v01/data/01\_source\_loaded\_harmonized/esld\_master\_long\_public.csv |  | integer |
| dead\_sample\_flag — Dead-sample flag **File:** 02\_workflows/F3\_workflow\_v01/data/01\_source\_loaded\_harmonized/esld\_master\_long\_public.csv **Recommended public name:** dead\_sample\_flag **Description:** Binary indicator identifying public ESLD sample records assigned to the deceased-sample subset used in sample-level analyses. **Unit:**  **Value coding:** 0 = not assigned to deceased-sample subset; 1 = assigned to deceased-sample subset. **Data type:** integer **Example values:** 0 | 1 **Allowed values:**  **Missing-value coding:** NA/blank as read by fread **n rows / missing / unique:** 67399 / 0 / 2 **Release status:** public **Definition status:** manual\_final\_definition | 02\_workflows/F3\_workflow\_v01/data/01\_source\_loaded\_harmonized/esld\_master\_long\_public.csv |  | integer |
| death\_within\_90d — Death within 90 days **File:** 02\_workflows/F3\_workflow\_v01/data/01\_source\_loaded\_harmonized/esld\_master\_long\_public.csv **Recommended public name:** death\_within\_90d **Description:** Binary indicator for death within 90 days of the analysis anchor. **Unit:**  **Value coding:** 0 = no; 1 = yes **Data type:** logical **Example values:**  **Allowed values:**  **Missing-value coding:** NA/blank as read by fread **n rows / missing / unique:** 67399 / 67399 / 0 **Release status:** public **Definition status:** exact\_definition | 02\_workflows/F3\_workflow\_v01/data/01\_source\_loaded\_harmonized/esld\_master\_long\_public.csv |  | logical |
| deceased\_patient\_flag — Deceased patient flag **File:** 02\_workflows/F3\_workflow\_v01/data/01\_source\_loaded\_harmonized/esld\_master\_long\_public.csv **Recommended public name:** deceased\_patient\_flag **Description:** Binary indicator identifying deceased patients within the released analysis context. **Unit:**  **Value coding:** 0 = no; 1 = yes **Data type:** integer **Example values:** 0 | 1 **Allowed values:**  **Missing-value coding:** NA/blank as read by fread **n rows / missing / unique:** 67399 / 0 / 2 **Release status:** public **Definition status:** exact\_definition | 02\_workflows/F3\_workflow\_v01/data/01\_source\_loaded\_harmonized/esld\_master\_long\_public.csv |  | integer |
| delta\_eq\_0\_flag — Zero score-delta flag **File:** 02\_workflows/F3\_workflow\_v01/data/01\_source\_loaded\_harmonized/esld\_master\_long\_public.csv **Recommended public name:** delta\_eq\_0\_flag **Description:** Binary indicator equal to 1 when the score delta is exactly zero. **Unit:**  **Value coding:** 0 = no; 1 = yes. **Data type:** integer **Example values:** 1 | 0 **Allowed values:**  **Missing-value coding:** NA/blank as read by fread **n rows / missing / unique:** 67399 / 0 / 2 **Release status:** public **Definition status:** manual\_precision\_definition | 02\_workflows/F3\_workflow\_v01/data/01\_source\_loaded\_harmonized/esld\_master\_long\_public.csv |  | integer |
| delta\_gt\_0\_flag — Positive score-delta flag **File:** 02\_workflows/F3\_workflow\_v01/data/01\_source\_loaded\_harmonized/esld\_master\_long\_public.csv **Recommended public name:** delta\_gt\_0\_flag **Description:** Binary indicator equal to 1 when the score delta is positive. **Unit:**  **Value coding:** 0 = no; 1 = yes. **Data type:** integer **Example values:** 0 | 1 **Allowed values:**  **Missing-value coding:** NA/blank as read by fread **n rows / missing / unique:** 67399 / 0 / 2 **Release status:** public **Definition status:** manual\_precision\_definition | 02\_workflows/F3\_workflow\_v01/data/01\_source\_loaded\_harmonized/esld\_master\_long\_public.csv |  | integer |
| delta\_le\_minus1\_flag — Score-delta ≤ −1 flag **File:** 02\_workflows/F3\_workflow\_v01/data/01\_source\_loaded\_harmonized/esld\_master\_long\_public.csv **Recommended public name:** delta\_le\_minus1\_flag **Description:** Binary indicator equal to 1 when the score delta is less than or equal to −1. **Unit:**  **Value coding:** 0 = no; 1 = yes. **Data type:** integer **Example values:** 0 | 1 **Allowed values:**  **Missing-value coding:** NA/blank as read by fread **n rows / missing / unique:** 67399 / 0 / 2 **Release status:** public **Definition status:** manual\_precision\_definition | 02\_workflows/F3\_workflow\_v01/data/01\_source\_loaded\_harmonized/esld\_master\_long\_public.csv |  | integer |
| dialysis\_raw — Dialysis indicator as recorded before score derivation **File:** 02\_workflows/F3\_workflow\_v01/data/01\_source\_loaded\_harmonized/esld\_master\_long\_public.csv **Recommended public name:** dialysis\_raw **Description:** Binary indicator showing whether dialysis was recorded in the source field before score derivation. **Unit:**  **Value coding:** 0 = no dialysis recorded; 1 = dialysis recorded. **Data type:** integer **Example values:** 0 | 1 **Allowed values:**  **Missing-value coding:** NA/blank as read by fread **n rows / missing / unique:** 67399 / 27827 / 2 **Release status:** public **Definition status:** manual\_exact\_definition | 02\_workflows/F3\_workflow\_v01/data/01\_source\_loaded\_harmonized/esld\_master\_long\_public.csv |  | integer |
| encounter\_id\_public — Public encounter pseudonym **File:** 02\_workflows/F3\_workflow\_v01/data/01\_source\_loaded\_harmonized/esld\_master\_long\_public.csv **Recommended public name:** encounter\_id\_public **Description:** Non-linkable public-release encounter pseudonym used for encounter-level grouping; not an original hospital encounter identifier. **Unit:**  **Value coding:** See example\_values and allowed\_values. **Data type:** character **Example values:** E001181 | E000008 | E000041 | E000100 | E000126 **Allowed values:** E001181 | E000008 | E000041 | E000100 | E000126 | E000151 | E000157 | E000219 | E000289 | E000344 **Missing-value coding:** NA/blank as read by fread **n rows / missing / unique:** 67399 / 0 / 5045 **Release status:** public **Definition status:** exact\_definition | 02\_workflows/F3\_workflow\_v01/data/01\_source\_loaded\_harmonized/esld\_master\_long\_public.csv |  | character |
| etiology\_source\_current\_flag — Current etiology source flag **File:** 02\_workflows/F3\_workflow\_v01/data/01\_source\_loaded\_harmonized/esld\_master\_long\_public.csv **Recommended public name:** etiology\_source\_current\_flag **Description:** Binary indicator identifying whether the etiology source is the current source used for the public row. **Unit:**  **Value coding:** 0 = no; 1 = yes. **Data type:** integer **Example values:** 1 **Allowed values:**  **Missing-value coding:** NA/blank as read by fread **n rows / missing / unique:** 67399 / 0 / 1 **Release status:** public **Definition status:** manual\_precision\_definition | 02\_workflows/F3\_workflow\_v01/data/01\_source\_loaded\_harmonized/esld\_master\_long\_public.csv |  | integer |
| etiology\_unclassified — Unclassified liver disease etiology flag **File:** 02\_workflows/F3\_workflow\_v01/data/01\_source\_loaded\_harmonized/esld\_master\_long\_public.csv **Recommended public name:** etiology\_unclassified **Description:** Binary etiology indicator identifying records without a classified liver disease etiology in the public ESLD cohort. **Unit:**  **Value coding:** 0 = no; 1 = yes. **Data type:** integer **Example values:** 0 **Allowed values:**  **Missing-value coding:** NA/blank as read by fread **n rows / missing / unique:** 67399 / 0 / 1 **Release status:** public **Definition status:** manual\_exact\_definition | 02\_workflows/F3\_workflow\_v01/data/01\_source\_loaded\_harmonized/esld\_master\_long\_public.csv |  | integer |
| hbv — Hepatitis B virus etiology flag **File:** 02\_workflows/F3\_workflow\_v01/data/01\_source\_loaded\_harmonized/esld\_master\_long\_public.csv **Recommended public name:** hbv **Description:** Binary etiology indicator for hepatitis B virus-related liver disease in the public ESLD cohort. **Unit:**  **Value coding:** 0 = no; 1 = yes. **Data type:** integer **Example values:** 0 | 1 **Allowed values:**  **Missing-value coding:** NA/blank as read by fread **n rows / missing / unique:** 67399 / 0 / 2 **Release status:** public **Definition status:** manual\_exact\_definition | 02\_workflows/F3\_workflow\_v01/data/01\_source\_loaded\_harmonized/esld\_master\_long\_public.csv |  | integer |
| hcv — Hepatitis C virus etiology flag **File:** 02\_workflows/F3\_workflow\_v01/data/01\_source\_loaded\_harmonized/esld\_master\_long\_public.csv **Recommended public name:** hcv **Description:** Binary etiology indicator for hepatitis C virus-related liver disease in the public ESLD cohort. **Unit:**  **Value coding:** 0 = no; 1 = yes. **Data type:** integer **Example values:** 0 | 1 **Allowed values:**  **Missing-value coding:** NA/blank as read by fread **n rows / missing / unique:** 67399 / 0 / 2 **Release status:** public **Definition status:** manual\_exact\_definition | 02\_workflows/F3\_workflow\_v01/data/01\_source\_loaded\_harmonized/esld\_master\_long\_public.csv |  | integer |
| icd\_source\_present — ICD source present flag **File:** 02\_workflows/F3\_workflow\_v01/data/01\_source\_loaded\_harmonized/esld\_master\_long\_public.csv **Recommended public name:** icd\_source\_present **Description:** Binary indicator showing whether an ICD-derived etiology source was present for the public row. **Unit:**  **Value coding:** 0 = no; 1 = yes. **Data type:** integer **Example values:** 1 | 0 **Allowed values:**  **Missing-value coding:** NA/blank as read by fread **n rows / missing / unique:** 67399 / 0 / 2 **Release status:** public **Definition status:** manual\_precision\_definition | 02\_workflows/F3\_workflow\_v01/data/01\_source\_loaded\_harmonized/esld\_master\_long\_public.csv |  | integer |
| in\_t1\_cohort — Inclusion flag for Table 1 cohort **File:** 02\_workflows/F3\_workflow\_v01/data/01\_source\_loaded\_harmonized/esld\_master\_long\_public.csv **Recommended public name:** in\_t1\_cohort **Description:** Binary indicator identifying records included in the public ESLD Table 1 baseline-characteristics cohort. **Unit:**  **Value coding:** 0 = not included; 1 = included. **Data type:** integer **Example values:** 1 **Allowed values:**  **Missing-value coding:** NA/blank as read by fread **n rows / missing / unique:** 67399 / 0 / 1 **Release status:** public **Definition status:** manual\_exact\_definition | 02\_workflows/F3\_workflow\_v01/data/01\_source\_loaded\_harmonized/esld\_master\_long\_public.csv |  | integer |
| in\_t3\_anchor\_model — Inclusion flag for Table 3 anchor-model cohort **File:** 02\_workflows/F3\_workflow\_v01/data/01\_source\_loaded\_harmonized/esld\_master\_long\_public.csv **Recommended public name:** in\_t3\_anchor\_model **Description:** Binary indicator identifying records included in the Table 3 anchor-model cohort used for creatinine-comparison analyses. **Unit:**  **Value coding:** 0 = not included; 1 = included. **Data type:** integer **Example values:** 1 | 0 **Allowed values:**  **Missing-value coding:** NA/blank as read by fread **n rows / missing / unique:** 67399 / 0 / 2 **Release status:** public **Definition status:** manual\_exact\_definition | 02\_workflows/F3\_workflow\_v01/data/01\_source\_loaded\_harmonized/esld\_master\_long\_public.csv |  | integer |
| in\_t4\_prevalence\_path — Inclusion flag for Table 4 prevalence pathway **File:** 02\_workflows/F3\_workflow\_v01/data/01\_source\_loaded\_harmonized/esld\_master\_long\_public.csv **Recommended public name:** in\_t4\_prevalence\_path **Description:** Binary indicator identifying records included in the Table 4 prevalence-analysis pathway. **Unit:**  **Value coding:** 0 = not included; 1 = included. **Data type:** integer **Example values:** 1 **Allowed values:**  **Missing-value coding:** NA/blank as read by fread **n rows / missing / unique:** 67399 / 0 / 1 **Release status:** public **Definition status:** manual\_exact\_definition | 02\_workflows/F3\_workflow\_v01/data/01\_source\_loaded\_harmonized/esld\_master\_long\_public.csv |  | integer |
| in\_t4\_survival\_path — Inclusion flag for Table 4 survival pathway **File:** 02\_workflows/F3\_workflow\_v01/data/01\_source\_loaded\_harmonized/esld\_master\_long\_public.csv **Recommended public name:** in\_t4\_survival\_path **Description:** Binary indicator identifying records included in the Table 4 survival-analysis pathway. **Unit:**  **Value coding:** 0 = not included; 1 = included. **Data type:** integer **Example values:** 0 | 1 **Allowed values:**  **Missing-value coding:** NA/blank as read by fread **n rows / missing / unique:** 67399 / 0 / 2 **Release status:** public **Definition status:** manual\_exact\_definition | 02\_workflows/F3\_workflow\_v01/data/01\_source\_loaded\_harmonized/esld\_master\_long\_public.csv |  | integer |
| inr — International normalized ratio **File:** 02\_workflows/F3\_workflow\_v01/data/01\_source\_loaded\_harmonized/esld\_master\_long\_public.csv **Recommended public name:** inr **Description:** International normalized ratio of prothrombin time used for MELD-related score calculation. **Unit:** ratio **Value coding:** Numeric INR value. **Data type:** numeric **Example values:** 1.14 | 0.91 | 1 | 1.04 | 0.98 **Allowed values:**  **Missing-value coding:** NA/blank as read by fread **n rows / missing / unique:** 67399 / 0 / 786 **Release status:** public **Definition status:** manual\_exact\_definition | 02\_workflows/F3\_workflow\_v01/data/01\_source\_loaded\_harmonized/esld\_master\_long\_public.csv | ratio | numeric |
| mash — Metabolic dysfunction-associated steatohepatitis etiology flag **File:** 02\_workflows/F3\_workflow\_v01/data/01\_source\_loaded\_harmonized/esld\_master\_long\_public.csv **Recommended public name:** mash **Description:** Binary etiology indicator for metabolic dysfunction-associated steatohepatitis in the public ESLD cohort. **Unit:**  **Value coding:** 0 = no; 1 = yes. **Data type:** integer **Example values:** 0 | 1 **Allowed values:**  **Missing-value coding:** NA/blank as read by fread **n rows / missing / unique:** 67399 / 0 / 2 **Release status:** public **Definition status:** manual\_exact\_definition | 02\_workflows/F3\_workflow\_v01/data/01\_source\_loaded\_harmonized/esld\_master\_long\_public.csv |  | integer |
| model — MELD model or score variant **File:** 02\_workflows/F3\_workflow\_v01/data/01\_source\_loaded\_harmonized/esld\_master\_long\_public.csv **Recommended public name:** model **Description:** Name of the MELD-related model or score variant represented by the row; expected values include MELD, MELD-Na, reMELD-Na, and MELD 3.0. **Unit:**  **Value coding:** Categorical score/model label, for example MELD, MELD-Na, reMELD-Na, or MELD 3.0. **Data type:** character **Example values:** MELD | MELD-Na | reMELD-Na | MELD 3.0 **Allowed values:** MELD | MELD-Na | reMELD-Na | MELD 3.0 **Missing-value coding:** NA/blank as read by fread **n rows / missing / unique:** 67399 / 0 / 4 **Release status:** public **Definition status:** manual\_exact\_definition | 02\_workflows/F3\_workflow\_v01/data/01\_source\_loaded\_harmonized/esld\_master\_long\_public.csv |  | character |
| other — Other liver disease etiology flag **File:** 02\_workflows/F3\_workflow\_v01/data/01\_source\_loaded\_harmonized/esld\_master\_long\_public.csv **Recommended public name:** other **Description:** Binary etiology indicator for liver disease etiologies grouped as other in the public ESLD cohort. **Unit:**  **Value coding:** 0 = no; 1 = yes. **Data type:** integer **Example values:** 1 | 0 **Allowed values:**  **Missing-value coding:** NA/blank as read by fread **n rows / missing / unique:** 67399 / 0 / 2 **Release status:** public **Definition status:** manual\_exact\_definition | 02\_workflows/F3\_workflow\_v01/data/01\_source\_loaded\_harmonized/esld\_master\_long\_public.csv |  | integer |
| patient\_id — Public patient pseudonym **File:** 02\_workflows/F3\_workflow\_v01/data/01\_source\_loaded\_harmonized/esld\_master\_long\_public.csv **Recommended public name:** patient\_id **Description:** Non-linkable public-release patient pseudonym used to preserve within-patient grouping in public ESLD data; not an original hospital patient identifier. **Unit:**  **Value coding:** See example\_values and allowed\_values. **Data type:** character **Example values:** P000001 | P000002 | P000003 | P000004 | P000005 **Allowed values:** P000001 | P000002 | P000003 | P000004 | P000005 | P000006 | P000007 | P000008 | P000009 | P000010 **Missing-value coding:** NA/blank as read by fread **n rows / missing / unique:** 67399 / 0 / 1375 **Release status:** public **Definition status:** exact\_definition | 02\_workflows/F3\_workflow\_v01/data/01\_source\_loaded\_harmonized/esld\_master\_long\_public.csv |  | character |
| pbc — Primary biliary cholangitis etiology flag **File:** 02\_workflows/F3\_workflow\_v01/data/01\_source\_loaded\_harmonized/esld\_master\_long\_public.csv **Recommended public name:** pbc **Description:** Binary etiology indicator for primary biliary cholangitis in the public ESLD cohort. **Unit:**  **Value coding:** 0 = no; 1 = yes. **Data type:** integer **Example values:** 0 | 1 **Allowed values:**  **Missing-value coding:** NA/blank as read by fread **n rows / missing / unique:** 67399 / 0 / 2 **Release status:** public **Definition status:** manual\_exact\_definition | 02\_workflows/F3\_workflow\_v01/data/01\_source\_loaded\_harmonized/esld\_master\_long\_public.csv |  | integer |
| sample\_day\_from\_first\_sample — Relative sample day **File:** 02\_workflows/F3\_workflow\_v01/data/01\_source\_loaded\_harmonized/esld\_master\_long\_public.csv **Recommended public name:** sample\_day\_from\_first\_sample **Description:** Relative day of the sample measured from the first sample for the public patient/sample sequence; not a calendar date. **Unit:** relative months **Value coding:** Integer or numeric relative day count. **Data type:** integer **Example values:** 0 | 42 | 91 | 118 | 133 **Allowed values:**  **Missing-value coding:** NA/blank as read by fread **n rows / missing / unique:** 67399 / 0 / 3438 **Release status:** public **Definition status:** manual\_precision\_definition | 02\_workflows/F3\_workflow\_v01/data/01\_source\_loaded\_harmonized/esld\_master\_long\_public.csv | relative months | integer |
| sample\_group\_id\_public — Public sample-group pseudonym **File:** 02\_workflows/F3\_workflow\_v01/data/01\_source\_loaded\_harmonized/esld\_master\_long\_public.csv **Recommended public name:** sample\_group\_id\_public **Description:** Non-linkable public-release sample-group pseudonym used to group samples within the released public data. **Unit:**  **Value coding:** See example\_values and allowed\_values. **Data type:** character **Example values:** G0000001 | G0000002 | G0000003 | G0000004 | G0000005 **Allowed values:** G0000001 | G0000002 | G0000003 | G0000004 | G0000005 | G0000006 | G0000007 | G0000008 | G0000009 | G0000010 **Missing-value coding:** NA/blank as read by fread **n rows / missing / unique:** 67399 / 0 / 20359 **Release status:** public **Definition status:** exact\_definition | 02\_workflows/F3\_workflow\_v01/data/01\_source\_loaded\_harmonized/esld\_master\_long\_public.csv |  | character |
| sample\_id — Sample identifier **File:** 02\_workflows/F3\_workflow\_v01/data/01\_source\_loaded\_harmonized/esld\_master\_long\_public.csv **Recommended public name:** sample\_id **Description:** Identifier of a sample or experimental record within the released public data; not a personal identifier. **Unit:**  **Value coding:** See example\_values and allowed\_values. **Data type:** character **Example values:** S0000001 | S0000002 | S0000003 | S0000004 | S0000005 **Allowed values:** S0000001 | S0000002 | S0000003 | S0000004 | S0000005 | S0000006 | S0000007 | S0000008 | S0000009 | S0000010 **Missing-value coding:** NA/blank as read by fread **n rows / missing / unique:** 67399 / 0 / 67399 **Release status:** public **Definition status:** exact\_definition | 02\_workflows/F3\_workflow\_v01/data/01\_source\_loaded\_harmonized/esld\_master\_long\_public.csv |  | character |
| sample\_month\_index — Relative sample month index **File:** 02\_workflows/F3\_workflow\_v01/data/01\_source\_loaded\_harmonized/esld\_master\_long\_public.csv **Recommended public name:** sample\_month\_index **Description:** Relative month index of the sample measured from the first sample for the public patient/sample sequence; not a calendar month. **Unit:** score points **Value coding:** Integer or numeric relative month index. **Data type:** integer **Example values:** 0 | 1 | 2 | 3 | 4 **Allowed values:**  **Missing-value coding:** NA/blank as read by fread **n rows / missing / unique:** 67399 / 0 / 177 **Release status:** public **Definition status:** manual\_precision\_definition | 02\_workflows/F3\_workflow\_v01/data/01\_source\_loaded\_harmonized/esld\_master\_long\_public.csv | score points | integer |
| score\_corrected — Corrected score **File:** 02\_workflows/F3\_workflow\_v01/data/01\_source\_loaded\_harmonized/esld\_master\_long\_public.csv **Recommended public name:** score\_corrected **Description:** MELD-related score after creatinine correction or recalculation. **Unit:** score points **Value coding:** Numeric score. **Data type:** integer **Example values:** 14 | 19 | 16 | 10 | 13 **Allowed values:**  **Missing-value coding:** NA/blank as read by fread **n rows / missing / unique:** 67399 / 0 / 40 **Release status:** public **Definition status:** manual\_precision\_definition | 02\_workflows/F3\_workflow\_v01/data/01\_source\_loaded\_harmonized/esld\_master\_long\_public.csv | score points | integer |
| score\_delta — Score difference **File:** 02\_workflows/F3\_workflow\_v01/data/01\_source\_loaded\_harmonized/esld\_master\_long\_public.csv **Recommended public name:** score\_delta **Description:** Difference between score variants or scoring approaches, expressed in score points. **Unit:**  **Value coding:** See example\_values and allowed\_values. **Data type:** integer **Example values:** 0 | 1 | -1 | -2 **Allowed values:**  **Missing-value coding:** NA/blank as read by fread **n rows / missing / unique:** 67399 / 0 / 4 **Release status:** public **Definition status:** exact\_definition | 02\_workflows/F3\_workflow\_v01/data/01\_source\_loaded\_harmonized/esld\_master\_long\_public.csv |  | integer |
| score\_original — Original score **File:** 02\_workflows/F3\_workflow\_v01/data/01\_source\_loaded\_harmonized/esld\_master\_long\_public.csv **Recommended public name:** score\_original **Description:** Original MELD-related score before creatinine correction or recalculation. **Unit:**  **Value coding:** Numeric score. **Data type:** integer **Example values:** 14 | 19 | 16 | 10 | 13 **Allowed values:**  **Missing-value coding:** NA/blank as read by fread **n rows / missing / unique:** 67399 / 0 / 40 **Release status:** public **Definition status:** manual\_precision\_definition | 02\_workflows/F3\_workflow\_v01/data/01\_source\_loaded\_harmonized/esld\_master\_long\_public.csv |  | integer |
| sex — Sex **File:** 02\_workflows/F3\_workflow\_v01/data/01\_source\_loaded\_harmonized/esld\_master\_long\_public.csv **Recommended public name:** sex **Description:** Sex category represented in the public ESLD data. **Unit:**  **Value coding:** M = male; F = female. **Data type:** character **Example values:** M | F **Allowed values:** M | F **Missing-value coding:** NA/blank as read by fread **n rows / missing / unique:** 67399 / 0 / 2 **Release status:** public **Definition status:** manual\_exact\_definition | 02\_workflows/F3\_workflow\_v01/data/01\_source\_loaded\_harmonized/esld\_master\_long\_public.csv |  | character |
| sodium — Serum sodium concentration **File:** 02\_workflows/F3\_workflow\_v01/data/01\_source\_loaded\_harmonized/esld\_master\_long\_public.csv **Recommended public name:** sodium **Description:** Serum sodium concentration used for MELD-Na, reMELD-Na, or MELD 3.0 score calculation. **Unit:** mmol/L **Value coding:** Numeric concentration in mmol/L. **Data type:** numeric **Example values:** 131 | 134 | 132 | 133 | 140 **Allowed values:**  **Missing-value coding:** NA/blank as read by fread **n rows / missing / unique:** 67399 / 20359 / 188 **Release status:** public **Definition status:** manual\_exact\_definition | 02\_workflows/F3\_workflow\_v01/data/01\_source\_loaded\_harmonized/esld\_master\_long\_public.csv | mmol/L | numeric |
| tbil — Total bilirubin concentration **File:** 02\_workflows/F3\_workflow\_v01/data/01\_source\_loaded\_harmonized/esld\_master\_long\_public.csv **Recommended public name:** tbil **Description:** Total bilirubin concentration used in MELD-related score calculation in the public ESLD data. **Unit:**  **Value coding:** Numeric concentration in mg/dL. **Data type:** numeric **Example values:** 1 | 0.9 | 1.1 | 0.8 | 0.6 **Allowed values:**  **Missing-value coding:** NA/blank as read by fread **n rows / missing / unique:** 67399 / 0 / 2029 **Release status:** public **Definition status:** manual\_precision\_definition | 02\_workflows/F3\_workflow\_v01/data/01\_source\_loaded\_harmonized/esld\_master\_long\_public.csv |  | numeric |
| delta\_class — Score-delta class **File:** 02\_workflows/F3\_workflow\_v01/data/02b\_figure\_content/esld\_F3\_score\_shift\_aggregate\_public.csv **Recommended public name:** delta\_class **Description:** Encoded score-delta class used in F3 score-shift summaries, for example pm, p1, m1, or m2. **Unit:**  **Value coding:** Categorical score-delta class as encoded by the F3 workflow. **Data type:** character **Example values:** pm | p1 | m1 | m2 **Allowed values:** pm | p1 | m1 | m2 **Missing-value coding:** NA/blank as read by fread **n rows / missing / unique:** 315 / 0 / 4 **Release status:** public **Definition status:** manual\_precision\_definition | 02\_workflows/F3\_workflow\_v01/data/02b\_figure\_content/esld\_F3\_score\_shift\_aggregate\_public.csv |  | character |
| model — MELD model or score variant **File:** 02\_workflows/F3\_workflow\_v01/data/02b\_figure\_content/esld\_F3\_score\_shift\_aggregate\_public.csv **Recommended public name:** model **Description:** Name of the MELD-related model or score variant represented by the row; expected values include MELD, MELD-Na, reMELD-Na, and MELD 3.0. **Unit:**  **Value coding:** Categorical score/model label, for example MELD, MELD-Na, reMELD-Na, or MELD 3.0. **Data type:** character **Example values:** MELD | MELD 3.0 | MELD-Na | reMELD-Na **Allowed values:** MELD | MELD 3.0 | MELD-Na | reMELD-Na **Missing-value coding:** NA/blank as read by fread **n rows / missing / unique:** 315 / 0 / 4 **Release status:** public **Definition status:** manual\_exact\_definition | 02\_workflows/F3\_workflow\_v01/data/02b\_figure\_content/esld\_F3\_score\_shift\_aggregate\_public.csv |  | character |
| n — Number of observations **File:** 02\_workflows/F3\_workflow\_v01/data/02b\_figure\_content/esld\_F3\_score\_shift\_aggregate\_public.csv **Recommended public name:** n **Description:** Count of observations, patients, samples, events, or rows, depending on the data file. **Unit:**  **Value coding:** See example\_values and allowed\_values. **Data type:** integer **Example values:** 963 | 9 | 1801 | 33 | 1915 **Allowed values:**  **Missing-value coding:** NA/blank as read by fread **n rows / missing / unique:** 315 / 0 / 173 **Release status:** public **Definition status:** exact\_definition | 02\_workflows/F3\_workflow\_v01/data/02b\_figure\_content/esld\_F3\_score\_shift\_aggregate\_public.csv |  | integer |
| x\_class — X-axis score class **File:** 02\_workflows/F3\_workflow\_v01/data/02b\_figure\_content/esld\_F3\_score\_shift\_aggregate\_public.csv **Recommended public name:** x\_class **Description:** Score class shown on the x-axis or used as the x-position in the F3 score-shift figure. **Unit:** mg/dL **Value coding:** Score-class label or value. **Data type:** integer **Example values:** 6 | 7 | 8 | 9 | 10 **Allowed values:**  **Missing-value coding:** NA/blank as read by fread **n rows / missing / unique:** 315 / 0 / 35 **Release status:** public **Definition status:** manual\_precision\_definition | 02\_workflows/F3\_workflow\_v01/data/02b\_figure\_content/esld\_F3\_score\_shift\_aggregate\_public.csv | mg/dL | integer |
| delta\_class — Score-delta class **File:** 02\_workflows/F3\_workflow\_v01/submission\_ready/public/data/esld\_F3\_score\_shift\_aggregate\_public.csv **Recommended public name:** delta\_class **Description:** Encoded score-delta class used in F3 score-shift summaries, for example pm, p1, m1, or m2. **Unit:**  **Value coding:** Categorical score-delta class as encoded by the F3 workflow. **Data type:** character **Example values:** pm | p1 | m1 | m2 **Allowed values:** pm | p1 | m1 | m2 **Missing-value coding:** NA/blank as read by fread **n rows / missing / unique:** 315 / 0 / 4 **Release status:** public **Definition status:** manual\_precision\_definition | 02\_workflows/F3\_workflow\_v01/submission\_ready/public/data/esld\_F3\_score\_shift\_aggregate\_public.csv |  | character |
| model — MELD model or score variant **File:** 02\_workflows/F3\_workflow\_v01/submission\_ready/public/data/esld\_F3\_score\_shift\_aggregate\_public.csv **Recommended public name:** model **Description:** Name of the MELD-related model or score variant represented by the row; expected values include MELD, MELD-Na, reMELD-Na, and MELD 3.0. **Unit:**  **Value coding:** Categorical score/model label, for example MELD, MELD-Na, reMELD-Na, or MELD 3.0. **Data type:** character **Example values:** MELD | MELD 3.0 | MELD-Na | reMELD-Na **Allowed values:** MELD | MELD 3.0 | MELD-Na | reMELD-Na **Missing-value coding:** NA/blank as read by fread **n rows / missing / unique:** 315 / 0 / 4 **Release status:** public **Definition status:** manual\_exact\_definition | 02\_workflows/F3\_workflow\_v01/submission\_ready/public/data/esld\_F3\_score\_shift\_aggregate\_public.csv |  | character |
| n — Number of observations **File:** 02\_workflows/F3\_workflow\_v01/submission\_ready/public/data/esld\_F3\_score\_shift\_aggregate\_public.csv **Recommended public name:** n **Description:** Count of observations, patients, samples, events, or rows, depending on the data file. **Unit:**  **Value coding:** See example\_values and allowed\_values. **Data type:** integer **Example values:** 963 | 9 | 1801 | 33 | 1915 **Allowed values:**  **Missing-value coding:** NA/blank as read by fread **n rows / missing / unique:** 315 / 0 / 173 **Release status:** public **Definition status:** exact\_definition | 02\_workflows/F3\_workflow\_v01/submission\_ready/public/data/esld\_F3\_score\_shift\_aggregate\_public.csv |  | integer |
| x\_class — X-axis score class **File:** 02\_workflows/F3\_workflow\_v01/submission\_ready/public/data/esld\_F3\_score\_shift\_aggregate\_public.csv **Recommended public name:** x\_class **Description:** Score class shown on the x-axis or used as the x-position in the F3 score-shift figure. **Unit:** mg/dL **Value coding:** Score-class label or value. **Data type:** integer **Example values:** 6 | 7 | 8 | 9 | 10 **Allowed values:**  **Missing-value coding:** NA/blank as read by fread **n rows / missing / unique:** 315 / 0 / 35 **Release status:** public **Definition status:** manual\_precision\_definition | 02\_workflows/F3\_workflow\_v01/submission\_ready/public/data/esld\_F3\_score\_shift\_aggregate\_public.csv | mg/dL | integer |
| age\_years\_first\_available — Age at first available public record **File:** 02\_workflows/F4\_workflow\_v01/data/01\_source\_loaded\_harmonized/esld\_master\_long\_public.csv **Recommended public name:** age\_years\_first\_available **Description:** Age in years at the first available public ESLD record for the patient or encounter represented in the released data. **Unit:** years **Value coding:** Integer age in years. **Data type:** integer **Example values:** 65 | 67 | 21 | 56 | 63 **Allowed values:**  **Missing-value coding:** NA/blank as read by fread **n rows / missing / unique:** 67399 / 0 / 79 **Release status:** public **Definition status:** manual\_exact\_definition | 02\_workflows/F4\_workflow\_v01/data/01\_source\_loaded\_harmonized/esld\_master\_long\_public.csv | years | integer |
| age\_years\_sample — Age at sample **File:** 02\_workflows/F4\_workflow\_v01/data/01\_source\_loaded\_harmonized/esld\_master\_long\_public.csv **Recommended public name:** age\_years\_sample **Description:** Age in years at the sample-level public ESLD record. **Unit:** years **Value coding:** Integer age in years. **Data type:** integer **Example values:** 65 | 66 | 67 | 68 | 69 **Allowed values:**  **Missing-value coding:** NA/blank as read by fread **n rows / missing / unique:** 67399 / 0 / 84 **Release status:** public **Definition status:** manual\_precision\_definition | 02\_workflows/F4\_workflow\_v01/data/01\_source\_loaded\_harmonized/esld\_master\_long\_public.csv | years | integer |
| albumin\_g\_dl — Serum albumin concentration **File:** 02\_workflows/F4\_workflow\_v01/data/01\_source\_loaded\_harmonized/esld\_master\_long\_public.csv **Recommended public name:** albumin\_g\_dl **Description:** Serum albumin concentration in the public ESLD master data. **Unit:** g/dL **Value coding:** Numeric concentration in g/dL. **Data type:** numeric **Example values:** 3.3 | 6.15 | 3.14 | 2.8 | 3.26 **Allowed values:**  **Missing-value coding:** NA/blank as read by fread **n rows / missing / unique:** 67399 / 58785 / 580 **Release status:** public **Definition status:** manual\_precision\_definition | 02\_workflows/F4\_workflow\_v01/data/01\_source\_loaded\_harmonized/esld\_master\_long\_public.csv | g/dL | numeric |
| ald — Alcohol-related liver disease etiology flag **File:** 02\_workflows/F4\_workflow\_v01/data/01\_source\_loaded\_harmonized/esld\_master\_long\_public.csv **Recommended public name:** ald **Description:** Binary etiology indicator for alcohol-related liver disease in the public ESLD cohort. **Unit:**  **Value coding:** 0 = no; 1 = yes. **Data type:** integer **Example values:** 0 | 1 **Allowed values:**  **Missing-value coding:** NA/blank as read by fread **n rows / missing / unique:** 67399 / 0 / 2 **Release status:** public **Definition status:** manual\_exact\_definition | 02\_workflows/F4\_workflow\_v01/data/01\_source\_loaded\_harmonized/esld\_master\_long\_public.csv |  | integer |
| ald\_hcv — Combined alcohol-related liver disease and hepatitis C etiology flag **File:** 02\_workflows/F4\_workflow\_v01/data/01\_source\_loaded\_harmonized/esld\_master\_long\_public.csv **Recommended public name:** ald\_hcv **Description:** Binary etiology indicator for combined alcohol-related liver disease and hepatitis C in the public ESLD cohort. **Unit:**  **Value coding:** 0 = no; 1 = yes. **Data type:** integer **Example values:** 0 | 1 **Allowed values:**  **Missing-value coding:** NA/blank as read by fread **n rows / missing / unique:** 67399 / 0 / 2 **Release status:** public **Definition status:** manual\_exact\_definition | 02\_workflows/F4\_workflow\_v01/data/01\_source\_loaded\_harmonized/esld\_master\_long\_public.csv |  | integer |
| autoimmune — Autoimmune liver disease etiology flag **File:** 02\_workflows/F4\_workflow\_v01/data/01\_source\_loaded\_harmonized/esld\_master\_long\_public.csv **Recommended public name:** autoimmune **Description:** Binary etiology indicator for autoimmune liver disease in the public ESLD cohort. **Unit:**  **Value coding:** 0 = no; 1 = yes. **Data type:** integer **Example values:** 0 | 1 **Allowed values:**  **Missing-value coding:** NA/blank as read by fread **n rows / missing / unique:** 67399 / 0 / 2 **Release status:** public **Definition status:** manual\_exact\_definition | 02\_workflows/F4\_workflow\_v01/data/01\_source\_loaded\_harmonized/esld\_master\_long\_public.csv |  | integer |
| crea — Creatinine concentration **File:** 02\_workflows/F4\_workflow\_v01/data/01\_source\_loaded\_harmonized/esld\_master\_long\_public.csv **Recommended public name:** crea **Description:** Creatinine concentration used in MELD-related score calculation before applying the study-specific correction. **Unit:** mg/dL **Value coding:** Numeric concentration in mg/dL. **Data type:** numeric **Example values:** 1.91 | 1.42 | 1.31 | 1.3 | 1.28 **Allowed values:**  **Missing-value coding:** NA/blank as read by fread **n rows / missing / unique:** 67399 / 0 / 975 **Release status:** public **Definition status:** manual\_exact\_definition | 02\_workflows/F4\_workflow\_v01/data/01\_source\_loaded\_harmonized/esld\_master\_long\_public.csv | mg/dL | numeric |
| crea\_corrected — Corrected creatinine concentration **File:** 02\_workflows/F4\_workflow\_v01/data/01\_source\_loaded\_harmonized/esld\_master\_long\_public.csv **Recommended public name:** crea\_corrected **Description:** Creatinine concentration after applying the study-specific correction used for recalculated MELD-related scores. **Unit:** mg/dL **Value coding:** Numeric concentration in mg/dL. **Data type:** numeric **Example values:** 1.87705004446668 | 1.41586432303417 | 1.31039363454933 | 1.30192482788903 | 1.28150549768982 **Allowed values:**  **Missing-value coding:** NA/blank as read by fread **n rows / missing / unique:** 67399 / 0 / 15868 **Release status:** public **Definition status:** manual\_exact\_definition | 02\_workflows/F4\_workflow\_v01/data/01\_source\_loaded\_harmonized/esld\_master\_long\_public.csv | mg/dL | numeric |
| date\_count — Relative day count **File:** 02\_workflows/F4\_workflow\_v01/data/01\_source\_loaded\_harmonized/esld\_master\_long\_public.csv **Recommended public name:** date\_count **Description:** Relative day count used for time alignment; negative/positive values are relative to the analysis anchor and are not calendar dates. **Unit:**  **Value coding:** See example\_values and allowed\_values. **Data type:** integer **Example values:** -3021 | -2833 | -2798 | -2793 | -2791 **Allowed values:**  **Missing-value coding:** NA/blank as read by fread **n rows / missing / unique:** 67399 / 50970 / 1450 **Release status:** public **Definition status:** exact\_definition | 02\_workflows/F4\_workflow\_v01/data/01\_source\_loaded\_harmonized/esld\_master\_long\_public.csv |  | integer |
| date\_count\_month — Relative month count **File:** 02\_workflows/F4\_workflow\_v01/data/01\_source\_loaded\_harmonized/esld\_master\_long\_public.csv **Recommended public name:** date\_count\_month **Description:** Relative month count derived from the relative day count; this is not a calendar month. **Unit:**  **Value coding:** See example\_values and allowed\_values. **Data type:** integer **Example values:** -99 | -93 | -92 | -91 | -87 **Allowed values:**  **Missing-value coding:** NA/blank as read by fread **n rows / missing / unique:** 67399 / 50970 / 147 **Release status:** public **Definition status:** exact\_definition | 02\_workflows/F4\_workflow\_v01/data/01\_source\_loaded\_harmonized/esld\_master\_long\_public.csv |  | integer |
| dead\_sample\_flag — Dead-sample flag **File:** 02\_workflows/F4\_workflow\_v01/data/01\_source\_loaded\_harmonized/esld\_master\_long\_public.csv **Recommended public name:** dead\_sample\_flag **Description:** Binary indicator identifying public ESLD sample records assigned to the deceased-sample subset used in sample-level analyses. **Unit:**  **Value coding:** 0 = not assigned to deceased-sample subset; 1 = assigned to deceased-sample subset. **Data type:** integer **Example values:** 0 | 1 **Allowed values:**  **Missing-value coding:** NA/blank as read by fread **n rows / missing / unique:** 67399 / 0 / 2 **Release status:** public **Definition status:** manual\_final\_definition | 02\_workflows/F4\_workflow\_v01/data/01\_source\_loaded\_harmonized/esld\_master\_long\_public.csv |  | integer |
| death\_within\_90d — Death within 90 days **File:** 02\_workflows/F4\_workflow\_v01/data/01\_source\_loaded\_harmonized/esld\_master\_long\_public.csv **Recommended public name:** death\_within\_90d **Description:** Binary indicator for death within 90 days of the analysis anchor. **Unit:**  **Value coding:** 0 = no; 1 = yes **Data type:** logical **Example values:**  **Allowed values:**  **Missing-value coding:** NA/blank as read by fread **n rows / missing / unique:** 67399 / 67399 / 0 **Release status:** public **Definition status:** exact\_definition | 02\_workflows/F4\_workflow\_v01/data/01\_source\_loaded\_harmonized/esld\_master\_long\_public.csv |  | logical |
| deceased\_patient\_flag — Deceased patient flag **File:** 02\_workflows/F4\_workflow\_v01/data/01\_source\_loaded\_harmonized/esld\_master\_long\_public.csv **Recommended public name:** deceased\_patient\_flag **Description:** Binary indicator identifying deceased patients within the released analysis context. **Unit:**  **Value coding:** 0 = no; 1 = yes **Data type:** integer **Example values:** 0 | 1 **Allowed values:**  **Missing-value coding:** NA/blank as read by fread **n rows / missing / unique:** 67399 / 0 / 2 **Release status:** public **Definition status:** exact\_definition | 02\_workflows/F4\_workflow\_v01/data/01\_source\_loaded\_harmonized/esld\_master\_long\_public.csv |  | integer |
| delta\_eq\_0\_flag — Zero score-delta flag **File:** 02\_workflows/F4\_workflow\_v01/data/01\_source\_loaded\_harmonized/esld\_master\_long\_public.csv **Recommended public name:** delta\_eq\_0\_flag **Description:** Binary indicator equal to 1 when the score delta is exactly zero. **Unit:**  **Value coding:** 0 = no; 1 = yes. **Data type:** integer **Example values:** 1 | 0 **Allowed values:**  **Missing-value coding:** NA/blank as read by fread **n rows / missing / unique:** 67399 / 0 / 2 **Release status:** public **Definition status:** manual\_precision\_definition | 02\_workflows/F4\_workflow\_v01/data/01\_source\_loaded\_harmonized/esld\_master\_long\_public.csv |  | integer |
| delta\_gt\_0\_flag — Positive score-delta flag **File:** 02\_workflows/F4\_workflow\_v01/data/01\_source\_loaded\_harmonized/esld\_master\_long\_public.csv **Recommended public name:** delta\_gt\_0\_flag **Description:** Binary indicator equal to 1 when the score delta is positive. **Unit:**  **Value coding:** 0 = no; 1 = yes. **Data type:** integer **Example values:** 0 | 1 **Allowed values:**  **Missing-value coding:** NA/blank as read by fread **n rows / missing / unique:** 67399 / 0 / 2 **Release status:** public **Definition status:** manual\_precision\_definition | 02\_workflows/F4\_workflow\_v01/data/01\_source\_loaded\_harmonized/esld\_master\_long\_public.csv |  | integer |
| delta\_le\_minus1\_flag — Score-delta ≤ −1 flag **File:** 02\_workflows/F4\_workflow\_v01/data/01\_source\_loaded\_harmonized/esld\_master\_long\_public.csv **Recommended public name:** delta\_le\_minus1\_flag **Description:** Binary indicator equal to 1 when the score delta is less than or equal to −1. **Unit:**  **Value coding:** 0 = no; 1 = yes. **Data type:** integer **Example values:** 0 | 1 **Allowed values:**  **Missing-value coding:** NA/blank as read by fread **n rows / missing / unique:** 67399 / 0 / 2 **Release status:** public **Definition status:** manual\_precision\_definition | 02\_workflows/F4\_workflow\_v01/data/01\_source\_loaded\_harmonized/esld\_master\_long\_public.csv |  | integer |
| dialysis\_raw — Dialysis indicator as recorded before score derivation **File:** 02\_workflows/F4\_workflow\_v01/data/01\_source\_loaded\_harmonized/esld\_master\_long\_public.csv **Recommended public name:** dialysis\_raw **Description:** Binary indicator showing whether dialysis was recorded in the source field before score derivation. **Unit:**  **Value coding:** 0 = no dialysis recorded; 1 = dialysis recorded. **Data type:** integer **Example values:** 0 | 1 **Allowed values:**  **Missing-value coding:** NA/blank as read by fread **n rows / missing / unique:** 67399 / 27827 / 2 **Release status:** public **Definition status:** manual\_exact\_definition | 02\_workflows/F4\_workflow\_v01/data/01\_source\_loaded\_harmonized/esld\_master\_long\_public.csv |  | integer |
| encounter\_id\_public — Public encounter pseudonym **File:** 02\_workflows/F4\_workflow\_v01/data/01\_source\_loaded\_harmonized/esld\_master\_long\_public.csv **Recommended public name:** encounter\_id\_public **Description:** Non-linkable public-release encounter pseudonym used for encounter-level grouping; not an original hospital encounter identifier. **Unit:**  **Value coding:** See example\_values and allowed\_values. **Data type:** character **Example values:** E001181 | E000008 | E000041 | E000100 | E000126 **Allowed values:** E001181 | E000008 | E000041 | E000100 | E000126 | E000151 | E000157 | E000219 | E000289 | E000344 **Missing-value coding:** NA/blank as read by fread **n rows / missing / unique:** 67399 / 0 / 5045 **Release status:** public **Definition status:** exact\_definition | 02\_workflows/F4\_workflow\_v01/data/01\_source\_loaded\_harmonized/esld\_master\_long\_public.csv |  | character |
| etiology\_source\_current\_flag — Current etiology source flag **File:** 02\_workflows/F4\_workflow\_v01/data/01\_source\_loaded\_harmonized/esld\_master\_long\_public.csv **Recommended public name:** etiology\_source\_current\_flag **Description:** Binary indicator identifying whether the etiology source is the current source used for the public row. **Unit:**  **Value coding:** 0 = no; 1 = yes. **Data type:** integer **Example values:** 1 **Allowed values:**  **Missing-value coding:** NA/blank as read by fread **n rows / missing / unique:** 67399 / 0 / 1 **Release status:** public **Definition status:** manual\_precision\_definition | 02\_workflows/F4\_workflow\_v01/data/01\_source\_loaded\_harmonized/esld\_master\_long\_public.csv |  | integer |
| etiology\_unclassified — Unclassified liver disease etiology flag **File:** 02\_workflows/F4\_workflow\_v01/data/01\_source\_loaded\_harmonized/esld\_master\_long\_public.csv **Recommended public name:** etiology\_unclassified **Description:** Binary etiology indicator identifying records without a classified liver disease etiology in the public ESLD cohort. **Unit:**  **Value coding:** 0 = no; 1 = yes. **Data type:** integer **Example values:** 0 **Allowed values:**  **Missing-value coding:** NA/blank as read by fread **n rows / missing / unique:** 67399 / 0 / 1 **Release status:** public **Definition status:** manual\_exact\_definition | 02\_workflows/F4\_workflow\_v01/data/01\_source\_loaded\_harmonized/esld\_master\_long\_public.csv |  | integer |
| hbv — Hepatitis B virus etiology flag **File:** 02\_workflows/F4\_workflow\_v01/data/01\_source\_loaded\_harmonized/esld\_master\_long\_public.csv **Recommended public name:** hbv **Description:** Binary etiology indicator for hepatitis B virus-related liver disease in the public ESLD cohort. **Unit:**  **Value coding:** 0 = no; 1 = yes. **Data type:** integer **Example values:** 0 | 1 **Allowed values:**  **Missing-value coding:** NA/blank as read by fread **n rows / missing / unique:** 67399 / 0 / 2 **Release status:** public **Definition status:** manual\_exact\_definition | 02\_workflows/F4\_workflow\_v01/data/01\_source\_loaded\_harmonized/esld\_master\_long\_public.csv |  | integer |
| hcv — Hepatitis C virus etiology flag **File:** 02\_workflows/F4\_workflow\_v01/data/01\_source\_loaded\_harmonized/esld\_master\_long\_public.csv **Recommended public name:** hcv **Description:** Binary etiology indicator for hepatitis C virus-related liver disease in the public ESLD cohort. **Unit:**  **Value coding:** 0 = no; 1 = yes. **Data type:** integer **Example values:** 0 | 1 **Allowed values:**  **Missing-value coding:** NA/blank as read by fread **n rows / missing / unique:** 67399 / 0 / 2 **Release status:** public **Definition status:** manual\_exact\_definition | 02\_workflows/F4\_workflow\_v01/data/01\_source\_loaded\_harmonized/esld\_master\_long\_public.csv |  | integer |
| icd\_source\_present — ICD source present flag **File:** 02\_workflows/F4\_workflow\_v01/data/01\_source\_loaded\_harmonized/esld\_master\_long\_public.csv **Recommended public name:** icd\_source\_present **Description:** Binary indicator showing whether an ICD-derived etiology source was present for the public row. **Unit:**  **Value coding:** 0 = no; 1 = yes. **Data type:** integer **Example values:** 1 | 0 **Allowed values:**  **Missing-value coding:** NA/blank as read by fread **n rows / missing / unique:** 67399 / 0 / 2 **Release status:** public **Definition status:** manual\_precision\_definition | 02\_workflows/F4\_workflow\_v01/data/01\_source\_loaded\_harmonized/esld\_master\_long\_public.csv |  | integer |
| in\_t1\_cohort — Inclusion flag for Table 1 cohort **File:** 02\_workflows/F4\_workflow\_v01/data/01\_source\_loaded\_harmonized/esld\_master\_long\_public.csv **Recommended public name:** in\_t1\_cohort **Description:** Binary indicator identifying records included in the public ESLD Table 1 baseline-characteristics cohort. **Unit:**  **Value coding:** 0 = not included; 1 = included. **Data type:** integer **Example values:** 1 **Allowed values:**  **Missing-value coding:** NA/blank as read by fread **n rows / missing / unique:** 67399 / 0 / 1 **Release status:** public **Definition status:** manual\_exact\_definition | 02\_workflows/F4\_workflow\_v01/data/01\_source\_loaded\_harmonized/esld\_master\_long\_public.csv |  | integer |
| in\_t3\_anchor\_model — Inclusion flag for Table 3 anchor-model cohort **File:** 02\_workflows/F4\_workflow\_v01/data/01\_source\_loaded\_harmonized/esld\_master\_long\_public.csv **Recommended public name:** in\_t3\_anchor\_model **Description:** Binary indicator identifying records included in the Table 3 anchor-model cohort used for creatinine-comparison analyses. **Unit:**  **Value coding:** 0 = not included; 1 = included. **Data type:** integer **Example values:** 1 | 0 **Allowed values:**  **Missing-value coding:** NA/blank as read by fread **n rows / missing / unique:** 67399 / 0 / 2 **Release status:** public **Definition status:** manual\_exact\_definition | 02\_workflows/F4\_workflow\_v01/data/01\_source\_loaded\_harmonized/esld\_master\_long\_public.csv |  | integer |
| in\_t4\_prevalence\_path — Inclusion flag for Table 4 prevalence pathway **File:** 02\_workflows/F4\_workflow\_v01/data/01\_source\_loaded\_harmonized/esld\_master\_long\_public.csv **Recommended public name:** in\_t4\_prevalence\_path **Description:** Binary indicator identifying records included in the Table 4 prevalence-analysis pathway. **Unit:**  **Value coding:** 0 = not included; 1 = included. **Data type:** integer **Example values:** 1 **Allowed values:**  **Missing-value coding:** NA/blank as read by fread **n rows / missing / unique:** 67399 / 0 / 1 **Release status:** public **Definition status:** manual\_exact\_definition | 02\_workflows/F4\_workflow\_v01/data/01\_source\_loaded\_harmonized/esld\_master\_long\_public.csv |  | integer |
| in\_t4\_survival\_path — Inclusion flag for Table 4 survival pathway **File:** 02\_workflows/F4\_workflow\_v01/data/01\_source\_loaded\_harmonized/esld\_master\_long\_public.csv **Recommended public name:** in\_t4\_survival\_path **Description:** Binary indicator identifying records included in the Table 4 survival-analysis pathway. **Unit:**  **Value coding:** 0 = not included; 1 = included. **Data type:** integer **Example values:** 0 | 1 **Allowed values:**  **Missing-value coding:** NA/blank as read by fread **n rows / missing / unique:** 67399 / 0 / 2 **Release status:** public **Definition status:** manual\_exact\_definition | 02\_workflows/F4\_workflow\_v01/data/01\_source\_loaded\_harmonized/esld\_master\_long\_public.csv |  | integer |
| inr — International normalized ratio **File:** 02\_workflows/F4\_workflow\_v01/data/01\_source\_loaded\_harmonized/esld\_master\_long\_public.csv **Recommended public name:** inr **Description:** International normalized ratio of prothrombin time used for MELD-related score calculation. **Unit:** ratio **Value coding:** Numeric INR value. **Data type:** numeric **Example values:** 1.14 | 0.91 | 1 | 1.04 | 0.98 **Allowed values:**  **Missing-value coding:** NA/blank as read by fread **n rows / missing / unique:** 67399 / 0 / 786 **Release status:** public **Definition status:** manual\_exact\_definition | 02\_workflows/F4\_workflow\_v01/data/01\_source\_loaded\_harmonized/esld\_master\_long\_public.csv | ratio | numeric |
| mash — Metabolic dysfunction-associated steatohepatitis etiology flag **File:** 02\_workflows/F4\_workflow\_v01/data/01\_source\_loaded\_harmonized/esld\_master\_long\_public.csv **Recommended public name:** mash **Description:** Binary etiology indicator for metabolic dysfunction-associated steatohepatitis in the public ESLD cohort. **Unit:**  **Value coding:** 0 = no; 1 = yes. **Data type:** integer **Example values:** 0 | 1 **Allowed values:**  **Missing-value coding:** NA/blank as read by fread **n rows / missing / unique:** 67399 / 0 / 2 **Release status:** public **Definition status:** manual\_exact\_definition | 02\_workflows/F4\_workflow\_v01/data/01\_source\_loaded\_harmonized/esld\_master\_long\_public.csv |  | integer |
| model — MELD model or score variant **File:** 02\_workflows/F4\_workflow\_v01/data/01\_source\_loaded\_harmonized/esld\_master\_long\_public.csv **Recommended public name:** model **Description:** Name of the MELD-related model or score variant represented by the row; expected values include MELD, MELD-Na, reMELD-Na, and MELD 3.0. **Unit:**  **Value coding:** Categorical score/model label, for example MELD, MELD-Na, reMELD-Na, or MELD 3.0. **Data type:** character **Example values:** MELD | MELD-Na | reMELD-Na | MELD 3.0 **Allowed values:** MELD | MELD-Na | reMELD-Na | MELD 3.0 **Missing-value coding:** NA/blank as read by fread **n rows / missing / unique:** 67399 / 0 / 4 **Release status:** public **Definition status:** manual\_exact\_definition | 02\_workflows/F4\_workflow\_v01/data/01\_source\_loaded\_harmonized/esld\_master\_long\_public.csv |  | character |
| other — Other liver disease etiology flag **File:** 02\_workflows/F4\_workflow\_v01/data/01\_source\_loaded\_harmonized/esld\_master\_long\_public.csv **Recommended public name:** other **Description:** Binary etiology indicator for liver disease etiologies grouped as other in the public ESLD cohort. **Unit:**  **Value coding:** 0 = no; 1 = yes. **Data type:** integer **Example values:** 1 | 0 **Allowed values:**  **Missing-value coding:** NA/blank as read by fread **n rows / missing / unique:** 67399 / 0 / 2 **Release status:** public **Definition status:** manual\_exact\_definition | 02\_workflows/F4\_workflow\_v01/data/01\_source\_loaded\_harmonized/esld\_master\_long\_public.csv |  | integer |
| patient\_id — Public patient pseudonym **File:** 02\_workflows/F4\_workflow\_v01/data/01\_source\_loaded\_harmonized/esld\_master\_long\_public.csv **Recommended public name:** patient\_id **Description:** Non-linkable public-release patient pseudonym used to preserve within-patient grouping in public ESLD data; not an original hospital patient identifier. **Unit:**  **Value coding:** See example\_values and allowed\_values. **Data type:** character **Example values:** P000001 | P000002 | P000003 | P000004 | P000005 **Allowed values:** P000001 | P000002 | P000003 | P000004 | P000005 | P000006 | P000007 | P000008 | P000009 | P000010 **Missing-value coding:** NA/blank as read by fread **n rows / missing / unique:** 67399 / 0 / 1375 **Release status:** public **Definition status:** exact\_definition | 02\_workflows/F4\_workflow\_v01/data/01\_source\_loaded\_harmonized/esld\_master\_long\_public.csv |  | character |
| pbc — Primary biliary cholangitis etiology flag **File:** 02\_workflows/F4\_workflow\_v01/data/01\_source\_loaded\_harmonized/esld\_master\_long\_public.csv **Recommended public name:** pbc **Description:** Binary etiology indicator for primary biliary cholangitis in the public ESLD cohort. **Unit:**  **Value coding:** 0 = no; 1 = yes. **Data type:** integer **Example values:** 0 | 1 **Allowed values:**  **Missing-value coding:** NA/blank as read by fread **n rows / missing / unique:** 67399 / 0 / 2 **Release status:** public **Definition status:** manual\_exact\_definition | 02\_workflows/F4\_workflow\_v01/data/01\_source\_loaded\_harmonized/esld\_master\_long\_public.csv |  | integer |
| sample\_day\_from\_first\_sample — Relative sample day **File:** 02\_workflows/F4\_workflow\_v01/data/01\_source\_loaded\_harmonized/esld\_master\_long\_public.csv **Recommended public name:** sample\_day\_from\_first\_sample **Description:** Relative day of the sample measured from the first sample for the public patient/sample sequence; not a calendar date. **Unit:** relative months **Value coding:** Integer or numeric relative day count. **Data type:** integer **Example values:** 0 | 42 | 91 | 118 | 133 **Allowed values:**  **Missing-value coding:** NA/blank as read by fread **n rows / missing / unique:** 67399 / 0 / 3438 **Release status:** public **Definition status:** manual\_precision\_definition | 02\_workflows/F4\_workflow\_v01/data/01\_source\_loaded\_harmonized/esld\_master\_long\_public.csv | relative months | integer |
| sample\_group\_id\_public — Public sample-group pseudonym **File:** 02\_workflows/F4\_workflow\_v01/data/01\_source\_loaded\_harmonized/esld\_master\_long\_public.csv **Recommended public name:** sample\_group\_id\_public **Description:** Non-linkable public-release sample-group pseudonym used to group samples within the released public data. **Unit:**  **Value coding:** See example\_values and allowed\_values. **Data type:** character **Example values:** G0000001 | G0000002 | G0000003 | G0000004 | G0000005 **Allowed values:** G0000001 | G0000002 | G0000003 | G0000004 | G0000005 | G0000006 | G0000007 | G0000008 | G0000009 | G0000010 **Missing-value coding:** NA/blank as read by fread **n rows / missing / unique:** 67399 / 0 / 20359 **Release status:** public **Definition status:** exact\_definition | 02\_workflows/F4\_workflow\_v01/data/01\_source\_loaded\_harmonized/esld\_master\_long\_public.csv |  | character |
| sample\_id — Sample identifier **File:** 02\_workflows/F4\_workflow\_v01/data/01\_source\_loaded\_harmonized/esld\_master\_long\_public.csv **Recommended public name:** sample\_id **Description:** Identifier of a sample or experimental record within the released public data; not a personal identifier. **Unit:**  **Value coding:** See example\_values and allowed\_values. **Data type:** character **Example values:** S0000001 | S0000002 | S0000003 | S0000004 | S0000005 **Allowed values:** S0000001 | S0000002 | S0000003 | S0000004 | S0000005 | S0000006 | S0000007 | S0000008 | S0000009 | S0000010 **Missing-value coding:** NA/blank as read by fread **n rows / missing / unique:** 67399 / 0 / 67399 **Release status:** public **Definition status:** exact\_definition | 02\_workflows/F4\_workflow\_v01/data/01\_source\_loaded\_harmonized/esld\_master\_long\_public.csv |  | character |
| sample\_month\_index — Relative sample month index **File:** 02\_workflows/F4\_workflow\_v01/data/01\_source\_loaded\_harmonized/esld\_master\_long\_public.csv **Recommended public name:** sample\_month\_index **Description:** Relative month index of the sample measured from the first sample for the public patient/sample sequence; not a calendar month. **Unit:** score points **Value coding:** Integer or numeric relative month index. **Data type:** integer **Example values:** 0 | 1 | 2 | 3 | 4 **Allowed values:**  **Missing-value coding:** NA/blank as read by fread **n rows / missing / unique:** 67399 / 0 / 177 **Release status:** public **Definition status:** manual\_precision\_definition | 02\_workflows/F4\_workflow\_v01/data/01\_source\_loaded\_harmonized/esld\_master\_long\_public.csv | score points | integer |
| score\_corrected — Corrected score **File:** 02\_workflows/F4\_workflow\_v01/data/01\_source\_loaded\_harmonized/esld\_master\_long\_public.csv **Recommended public name:** score\_corrected **Description:** MELD-related score after creatinine correction or recalculation. **Unit:** score points **Value coding:** Numeric score. **Data type:** integer **Example values:** 14 | 19 | 16 | 10 | 13 **Allowed values:**  **Missing-value coding:** NA/blank as read by fread **n rows / missing / unique:** 67399 / 0 / 40 **Release status:** public **Definition status:** manual\_precision\_definition | 02\_workflows/F4\_workflow\_v01/data/01\_source\_loaded\_harmonized/esld\_master\_long\_public.csv | score points | integer |
| score\_delta — Score difference **File:** 02\_workflows/F4\_workflow\_v01/data/01\_source\_loaded\_harmonized/esld\_master\_long\_public.csv **Recommended public name:** score\_delta **Description:** Difference between score variants or scoring approaches, expressed in score points. **Unit:**  **Value coding:** See example\_values and allowed\_values. **Data type:** integer **Example values:** 0 | 1 | -1 | -2 **Allowed values:**  **Missing-value coding:** NA/blank as read by fread **n rows / missing / unique:** 67399 / 0 / 4 **Release status:** public **Definition status:** exact\_definition | 02\_workflows/F4\_workflow\_v01/data/01\_source\_loaded\_harmonized/esld\_master\_long\_public.csv |  | integer |
| score\_original — Original score **File:** 02\_workflows/F4\_workflow\_v01/data/01\_source\_loaded\_harmonized/esld\_master\_long\_public.csv **Recommended public name:** score\_original **Description:** Original MELD-related score before creatinine correction or recalculation. **Unit:**  **Value coding:** Numeric score. **Data type:** integer **Example values:** 14 | 19 | 16 | 10 | 13 **Allowed values:**  **Missing-value coding:** NA/blank as read by fread **n rows / missing / unique:** 67399 / 0 / 40 **Release status:** public **Definition status:** manual\_precision\_definition | 02\_workflows/F4\_workflow\_v01/data/01\_source\_loaded\_harmonized/esld\_master\_long\_public.csv |  | integer |
| sex — Sex **File:** 02\_workflows/F4\_workflow\_v01/data/01\_source\_loaded\_harmonized/esld\_master\_long\_public.csv **Recommended public name:** sex **Description:** Sex category represented in the public ESLD data. **Unit:**  **Value coding:** M = male; F = female. **Data type:** character **Example values:** M | F **Allowed values:** M | F **Missing-value coding:** NA/blank as read by fread **n rows / missing / unique:** 67399 / 0 / 2 **Release status:** public **Definition status:** manual\_exact\_definition | 02\_workflows/F4\_workflow\_v01/data/01\_source\_loaded\_harmonized/esld\_master\_long\_public.csv |  | character |
| sodium — Serum sodium concentration **File:** 02\_workflows/F4\_workflow\_v01/data/01\_source\_loaded\_harmonized/esld\_master\_long\_public.csv **Recommended public name:** sodium **Description:** Serum sodium concentration used for MELD-Na, reMELD-Na, or MELD 3.0 score calculation. **Unit:** mmol/L **Value coding:** Numeric concentration in mmol/L. **Data type:** numeric **Example values:** 131 | 134 | 132 | 133 | 140 **Allowed values:**  **Missing-value coding:** NA/blank as read by fread **n rows / missing / unique:** 67399 / 20359 / 188 **Release status:** public **Definition status:** manual\_exact\_definition | 02\_workflows/F4\_workflow\_v01/data/01\_source\_loaded\_harmonized/esld\_master\_long\_public.csv | mmol/L | numeric |
| tbil — Total bilirubin concentration **File:** 02\_workflows/F4\_workflow\_v01/data/01\_source\_loaded\_harmonized/esld\_master\_long\_public.csv **Recommended public name:** tbil **Description:** Total bilirubin concentration used in MELD-related score calculation in the public ESLD data. **Unit:**  **Value coding:** Numeric concentration in mg/dL. **Data type:** numeric **Example values:** 1 | 0.9 | 1.1 | 0.8 | 0.6 **Allowed values:**  **Missing-value coding:** NA/blank as read by fread **n rows / missing / unique:** 67399 / 0 / 2029 **Release status:** public **Definition status:** manual\_precision\_definition | 02\_workflows/F4\_workflow\_v01/data/01\_source\_loaded\_harmonized/esld\_master\_long\_public.csv |  | numeric |
| asterisk — Statistical-significance annotation **File:** 02\_workflows/F4\_workflow\_v01/data/02b\_figure\_content/esld\_F4\_survival\_stats\_public.csv **Recommended public name:** asterisk **Description:** Text label used to annotate statistical significance in the rendered figure, for example n.s., \*, \*\*, \*\*\*, or \*\*\*\*. **Unit:**  **Value coding:** Categorical display label such as n.s., \*, \*\*, \*\*\*, or \*\*\*\*. **Data type:** character **Example values:** n.s. | \*\*\*\* | \*\* | \* | \*\*\* **Allowed values:** n.s. | \*\*\*\* | \*\* | \* | \*\*\* **Missing-value coding:** NA/blank as read by fread **n rows / missing / unique:** 27 / 0 / 5 **Release status:** public **Definition status:** manual\_precision\_definition | 02\_workflows/F4\_workflow\_v01/data/02b\_figure\_content/esld\_F4\_survival\_stats\_public.csv |  | character |
| median\_m1 — Median value for m1 group **File:** 02\_workflows/F4\_workflow\_v01/data/02b\_figure\_content/esld\_F4\_survival\_stats\_public.csv **Recommended public name:** median\_m1 **Description:** Median row-specific value for the m1 group in F4 survival-statistics data. **Unit:** analysis-specific scale **Value coding:** Numeric row-specific value. **Data type:** numeric **Example values:** 125 | 74 | 33 | 17 | 10 **Allowed values:**  **Missing-value coding:** NA/blank as read by fread **n rows / missing / unique:** 27 / 0 / 23 **Release status:** public **Definition status:** manual\_precision\_definition | 02\_workflows/F4\_workflow\_v01/data/02b\_figure\_content/esld\_F4\_survival\_stats\_public.csv | analysis-specific scale | numeric |
| median\_pm2 — Median value for pm2 group **File:** 02\_workflows/F4\_workflow\_v01/data/02b\_figure\_content/esld\_F4\_survival\_stats\_public.csv **Recommended public name:** median\_pm2 **Description:** Median row-specific value for the pm2 group in F4 survival-statistics data. **Unit:** analysis-specific scale **Value coding:** Numeric row-specific value. **Data type:** numeric **Example values:** 126 | 26 | 15 | 9 | 7 **Allowed values:**  **Missing-value coding:** NA/blank as read by fread **n rows / missing / unique:** 27 / 0 / 23 **Release status:** public **Definition status:** manual\_precision\_definition | 02\_workflows/F4\_workflow\_v01/data/02b\_figure\_content/esld\_F4\_survival\_stats\_public.csv | analysis-specific scale | numeric |
| model — MELD model or score variant **File:** 02\_workflows/F4\_workflow\_v01/data/02b\_figure\_content/esld\_F4\_survival\_stats\_public.csv **Recommended public name:** model **Description:** Name of the MELD-related model or score variant represented by the row; expected values include MELD, MELD-Na, reMELD-Na, and MELD 3.0. **Unit:**  **Value coding:** Categorical score/model label, for example MELD, MELD-Na, reMELD-Na, or MELD 3.0. **Data type:** character **Example values:** MELD | MELD-Na | reMELD-Na | MELD 3.0 **Allowed values:** MELD | MELD-Na | reMELD-Na | MELD 3.0 **Missing-value coding:** NA/blank as read by fread **n rows / missing / unique:** 27 / 0 / 4 **Release status:** public **Definition status:** manual\_exact\_definition | 02\_workflows/F4\_workflow\_v01/data/02b\_figure\_content/esld\_F4\_survival\_stats\_public.csv |  | character |
| n\_m1 — Number of observations in m1 group **File:** 02\_workflows/F4\_workflow\_v01/data/02b\_figure\_content/esld\_F4\_survival\_stats\_public.csv **Recommended public name:** n\_m1 **Description:** Number of observations in the m1 comparison group for the row-specific figure/table stratum. **Unit:** count **Value coding:** Integer count. **Data type:** integer **Example values:** 369 | 913 | 916 | 555 | 305 **Allowed values:**  **Missing-value coding:** NA/blank as read by fread **n rows / missing / unique:** 27 / 0 / 26 **Release status:** public **Definition status:** manual\_precision\_definition | 02\_workflows/F4\_workflow\_v01/data/02b\_figure\_content/esld\_F4\_survival\_stats\_public.csv | count | integer |
| n\_pm2 — Number of observations in pm2 group **File:** 02\_workflows/F4\_workflow\_v01/data/02b\_figure\_content/esld\_F4\_survival\_stats\_public.csv **Recommended public name:** n\_pm2 **Description:** Number of observations in the pm2 comparison group for the row-specific F4 stratum. **Unit:** count **Value coding:** Integer count. **Data type:** integer **Example values:** 405 | 190 | 84 | 56 | 31 **Allowed values:**  **Missing-value coding:** NA/blank as read by fread **n rows / missing / unique:** 27 / 0 / 26 **Release status:** public **Definition status:** manual\_precision\_definition | 02\_workflows/F4\_workflow\_v01/data/02b\_figure\_content/esld\_F4\_survival\_stats\_public.csv | count | integer |
| p25\_m1 — 25th-percentile value for m1 group **File:** 02\_workflows/F4\_workflow\_v01/data/02b\_figure\_content/esld\_F4\_survival\_stats\_public.csv **Recommended public name:** p25\_m1 **Description:** 25th-percentile row-specific value for the m1 group in F4 survival-statistics data. **Unit:** analysis-specific scale **Value coding:** Numeric row-specific value. **Data type:** numeric **Example values:** 273 | 247 | 143.25 | 39 | 25 **Allowed values:**  **Missing-value coding:** NA/blank as read by fread **n rows / missing / unique:** 27 / 0 / 26 **Release status:** public **Definition status:** manual\_precision\_definition | 02\_workflows/F4\_workflow\_v01/data/02b\_figure\_content/esld\_F4\_survival\_stats\_public.csv | analysis-specific scale | numeric |
| p25\_pm2 — 25th-percentile value for pm2 group **File:** 02\_workflows/F4\_workflow\_v01/data/02b\_figure\_content/esld\_F4\_survival\_stats\_public.csv **Recommended public name:** p25\_pm2 **Description:** 25th-percentile row-specific value for the pm2 group in F4 survival-statistics data. **Unit:** analysis-specific scale **Value coding:** Numeric row-specific value. **Data type:** numeric **Example values:** 317 | 97.75 | 29.75 | 28.25 | 14.5 **Allowed values:**  **Missing-value coding:** NA/blank as read by fread **n rows / missing / unique:** 27 / 0 / 26 **Release status:** public **Definition status:** manual\_precision\_definition | 02\_workflows/F4\_workflow\_v01/data/02b\_figure\_content/esld\_F4\_survival\_stats\_public.csv | analysis-specific scale | numeric |
| p75\_m1 — 75th-percentile value for m1 group **File:** 02\_workflows/F4\_workflow\_v01/data/02b\_figure\_content/esld\_F4\_survival\_stats\_public.csv **Recommended public name:** p75\_m1 **Description:** 75th-percentile row-specific value for the m1 group in F4 survival-statistics data. **Unit:** analysis-specific scale **Value coding:** Numeric row-specific value. **Data type:** numeric **Example values:** 50 | 26 | 12 | 7 | 4 **Allowed values:**  **Missing-value coding:** NA/blank as read by fread **n rows / missing / unique:** 27 / 0 / 21 **Release status:** public **Definition status:** manual\_precision\_definition | 02\_workflows/F4\_workflow\_v01/data/02b\_figure\_content/esld\_F4\_survival\_stats\_public.csv | analysis-specific scale | numeric |
| p75\_pm2 — 75th-percentile value for pm2 group **File:** 02\_workflows/F4\_workflow\_v01/data/02b\_figure\_content/esld\_F4\_survival\_stats\_public.csv **Recommended public name:** p75\_pm2 **Description:** 75th-percentile row-specific value for the pm2 group in F4 survival-statistics data. **Unit:** analysis-specific scale **Value coding:** Numeric row-specific value. **Data type:** numeric **Example values:** 31 | 12 | 3 | 2 | -0.25 **Allowed values:**  **Missing-value coding:** NA/blank as read by fread **n rows / missing / unique:** 27 / 0 / 17 **Release status:** public **Definition status:** manual\_precision\_definition | 02\_workflows/F4\_workflow\_v01/data/02b\_figure\_content/esld\_F4\_survival\_stats\_public.csv | analysis-specific scale | numeric |
| p\_value — P value **File:** 02\_workflows/F4\_workflow\_v01/data/02b\_figure\_content/esld\_F4\_survival\_stats\_public.csv **Recommended public name:** p\_value **Description:** P value from the statistical comparison represented by the row. **Unit:**  **Value coding:** Probability value from the corresponding statistical test. **Data type:** numeric **Example values:** 0.787854614489014 | 9.15971812501929e-11 | 2.77788222236644e-07 | 0.00360653118106935 | 0.114275327333006 **Allowed values:**  **Missing-value coding:** NA/blank as read by fread **n rows / missing / unique:** 27 / 0 / 27 **Release status:** public **Definition status:** exact\_definition | 02\_workflows/F4\_workflow\_v01/data/02b\_figure\_content/esld\_F4\_survival\_stats\_public.csv |  | numeric |
| score\_class\_label — Displayed score-class label **File:** 02\_workflows/F4\_workflow\_v01/data/02b\_figure\_content/esld\_F4\_survival\_stats\_public.csv **Recommended public name:** score\_class\_label **Description:** Displayed score-class label for the figure/table stratum. **Unit:** score points **Value coding:** Categorical score-class label. **Data type:** character **Example values:** ≤10 | 11-15 | 16-20 | 21-25 | 26-30 **Allowed values:** ≤10 | 11-15 | 16-20 | 21-25 | 26-30 | 31-35 | 36-40 **Missing-value coding:** NA/blank as read by fread **n rows / missing / unique:** 27 / 0 / 7 **Release status:** public **Definition status:** manual\_precision\_definition | 02\_workflows/F4\_workflow\_v01/data/02b\_figure\_content/esld\_F4\_survival\_stats\_public.csv | score points | character |
| score\_class\_value — Score-class upper value **File:** 02\_workflows/F4\_workflow\_v01/data/02b\_figure\_content/esld\_F4\_survival\_stats\_public.csv **Recommended public name:** score\_class\_value **Description:** Upper score value defining the displayed score class. **Unit:** score points **Value coding:** Numeric score-class upper value. **Data type:** integer **Example values:** 10 | 15 | 20 | 25 | 30 **Allowed values:**  **Missing-value coding:** NA/blank as read by fread **n rows / missing / unique:** 27 / 0 / 7 **Release status:** public **Definition status:** manual\_precision\_definition | 02\_workflows/F4\_workflow\_v01/data/02b\_figure\_content/esld\_F4\_survival\_stats\_public.csv | score points | integer |
| y\_ast — Y coordinate for significance annotation **File:** 02\_workflows/F4\_workflow\_v01/data/02b\_figure\_content/esld\_F4\_survival\_stats\_public.csv **Recommended public name:** y\_ast **Description:** Y-axis coordinate used to position the statistical-significance annotation in the F4 survival figure. **Unit:** formatted table value **Value coding:** Numeric y-axis coordinate for annotation placement. **Data type:** numeric **Example values:** 131 \| 79 \| 38 \| 22 \| 15 **Allowed values:**  **Missing-value coding:** NA/blank as read by fread **n rows / missing / unique:** 27 / 0 / 24 **Release status:** public **Definition status:** manual\_precision\_definition | 02\_workflows/F4\_workflow\_v01/data/02b\_figure\_content/esld\_F4\_survival\_stats\_public.csv | formatted table value | numeric |
| asterisk — Statistical-significance annotation **File:** 02\_workflows/F4\_workflow\_v01/submission\_ready/public/data/esld\_F4\_survival\_stats\_public.csv **Recommended public name:** asterisk **Description:** Text label used to annotate statistical significance in the rendered figure, for example n.s., \*, \*\*, \*\*\*, or \*\*\*\*. **Unit:**  **Value coding:** Categorical display label such as n.s., \*, \*\*, \*\*\*, or \*\*\*\*. **Data type:** character **Example values:** n.s. \| \*\*\*\* \| \*\* \| \* \| \*\*\* **Allowed values:** n.s. \| \*\*\*\* \| \*\* \| \* \| \*\*\* **Missing-value coding:** NA/blank as read by fread **n rows / missing / unique:** 27 / 0 / 5 **Release status:** public **Definition status:** manual\_precision\_definition | 02\_workflows/F4\_workflow\_v01/submission\_ready/public/data/esld\_F4\_survival\_stats\_public.csv |  | character |
| median\_m1 — Median value for m1 group **File:** 02\_workflows/F4\_workflow\_v01/submission\_ready/public/data/esld\_F4\_survival\_stats\_public.csv **Recommended public name:** median\_m1 **Description:** Median row-specific value for the m1 group in F4 survival-statistics data. **Unit:** analysis-specific scale **Value coding:** Numeric row-specific value. **Data type:** numeric **Example values:** 125 \| 74 \| 33 \| 17 \| 10 **Allowed values:**  **Missing-value coding:** NA/blank as read by fread **n rows / missing / unique:** 27 / 0 / 23 **Release status:** public **Definition status:** manual\_precision\_definition | 02\_workflows/F4\_workflow\_v01/submission\_ready/public/data/esld\_F4\_survival\_stats\_public.csv | analysis-specific scale | numeric |
| median\_pm2 — Median value for pm2 group **File:** 02\_workflows/F4\_workflow\_v01/submission\_ready/public/data/esld\_F4\_survival\_stats\_public.csv **Recommended public name:** median\_pm2 **Description:** Median row-specific value for the pm2 group in F4 survival-statistics data. **Unit:** analysis-specific scale **Value coding:** Numeric row-specific value. **Data type:** numeric **Example values:** 126 \| 26 \| 15 \| 9 \| 7 **Allowed values:**  **Missing-value coding:** NA/blank as read by fread **n rows / missing / unique:** 27 / 0 / 23 **Release status:** public **Definition status:** manual\_precision\_definition | 02\_workflows/F4\_workflow\_v01/submission\_ready/public/data/esld\_F4\_survival\_stats\_public.csv | analysis-specific scale | numeric |
| model — MELD model or score variant **File:** 02\_workflows/F4\_workflow\_v01/submission\_ready/public/data/esld\_F4\_survival\_stats\_public.csv **Recommended public name:** model **Description:** Name of the MELD-related model or score variant represented by the row; expected values include MELD, MELD-Na, reMELD-Na, and MELD 3.0. **Unit:**  **Value coding:** Categorical score/model label, for example MELD, MELD-Na, reMELD-Na, or MELD 3.0. **Data type:** character **Example values:** MELD \| MELD-Na \| reMELD-Na \| MELD 3.0 **Allowed values:** MELD \| MELD-Na \| reMELD-Na \| MELD 3.0 **Missing-value coding:** NA/blank as read by fread **n rows / missing / unique:** 27 / 0 / 4 **Release status:** public **Definition status:** manual\_exact\_definition | 02\_workflows/F4\_workflow\_v01/submission\_ready/public/data/esld\_F4\_survival\_stats\_public.csv |  | character |
| n\_m1 — Number of observations in m1 group **File:** 02\_workflows/F4\_workflow\_v01/submission\_ready/public/data/esld\_F4\_survival\_stats\_public.csv **Recommended public name:** n\_m1 **Description:** Number of observations in the m1 comparison group for the row-specific figure/table stratum. **Unit:** count **Value coding:** Integer count. **Data type:** integer **Example values:** 369 \| 913 \| 916 \| 555 \| 305 **Allowed values:**  **Missing-value coding:** NA/blank as read by fread **n rows / missing / unique:** 27 / 0 / 26 **Release status:** public **Definition status:** manual\_precision\_definition | 02\_workflows/F4\_workflow\_v01/submission\_ready/public/data/esld\_F4\_survival\_stats\_public.csv | count | integer |
| n\_pm2 — Number of observations in pm2 group **File:** 02\_workflows/F4\_workflow\_v01/submission\_ready/public/data/esld\_F4\_survival\_stats\_public.csv **Recommended public name:** n\_pm2 **Description:** Number of observations in the pm2 comparison group for the row-specific F4 stratum. **Unit:** count **Value coding:** Integer count. **Data type:** integer **Example values:** 405 \| 190 \| 84 \| 56 \| 31 **Allowed values:**  **Missing-value coding:** NA/blank as read by fread **n rows / missing / unique:** 27 / 0 / 26 **Release status:** public **Definition status:** manual\_precision\_definition | 02\_workflows/F4\_workflow\_v01/submission\_ready/public/data/esld\_F4\_survival\_stats\_public.csv | count | integer |
| p25\_m1 — 25th-percentile value for m1 group **File:** 02\_workflows/F4\_workflow\_v01/submission\_ready/public/data/esld\_F4\_survival\_stats\_public.csv **Recommended public name:** p25\_m1 **Description:** 25th-percentile row-specific value for the m1 group in F4 survival-statistics data. **Unit:** analysis-specific scale **Value coding:** Numeric row-specific value. **Data type:** numeric **Example values:** 273 \| 247 \| 143.25 \| 39 \| 25 **Allowed values:**  **Missing-value coding:** NA/blank as read by fread **n rows / missing / unique:** 27 / 0 / 26 **Release status:** public **Definition status:** manual\_precision\_definition | 02\_workflows/F4\_workflow\_v01/submission\_ready/public/data/esld\_F4\_survival\_stats\_public.csv | analysis-specific scale | numeric |
| p25\_pm2 — 25th-percentile value for pm2 group **File:** 02\_workflows/F4\_workflow\_v01/submission\_ready/public/data/esld\_F4\_survival\_stats\_public.csv **Recommended public name:** p25\_pm2 **Description:** 25th-percentile row-specific value for the pm2 group in F4 survival-statistics data. **Unit:** analysis-specific scale **Value coding:** Numeric row-specific value. **Data type:** numeric **Example values:** 317 \| 97.75 \| 29.75 \| 28.25 \| 14.5 **Allowed values:**  **Missing-value coding:** NA/blank as read by fread **n rows / missing / unique:** 27 / 0 / 26 **Release status:** public **Definition status:** manual\_precision\_definition | 02\_workflows/F4\_workflow\_v01/submission\_ready/public/data/esld\_F4\_survival\_stats\_public.csv | analysis-specific scale | numeric |
| p75\_m1 — 75th-percentile value for m1 group **File:** 02\_workflows/F4\_workflow\_v01/submission\_ready/public/data/esld\_F4\_survival\_stats\_public.csv **Recommended public name:** p75\_m1 **Description:** 75th-percentile row-specific value for the m1 group in F4 survival-statistics data. **Unit:** analysis-specific scale **Value coding:** Numeric row-specific value. **Data type:** numeric **Example values:** 50 \| 26 \| 12 \| 7 \| 4 **Allowed values:**  **Missing-value coding:** NA/blank as read by fread **n rows / missing / unique:** 27 / 0 / 21 **Release status:** public **Definition status:** manual\_precision\_definition | 02\_workflows/F4\_workflow\_v01/submission\_ready/public/data/esld\_F4\_survival\_stats\_public.csv | analysis-specific scale | numeric |
| p75\_pm2 — 75th-percentile value for pm2 group **File:** 02\_workflows/F4\_workflow\_v01/submission\_ready/public/data/esld\_F4\_survival\_stats\_public.csv **Recommended public name:** p75\_pm2 **Description:** 75th-percentile row-specific value for the pm2 group in F4 survival-statistics data. **Unit:** analysis-specific scale **Value coding:** Numeric row-specific value. **Data type:** numeric **Example values:** 31 \| 12 \| 3 \| 2 \| -0.25 **Allowed values:**  **Missing-value coding:** NA/blank as read by fread **n rows / missing / unique:** 27 / 0 / 17 **Release status:** public **Definition status:** manual\_precision\_definition | 02\_workflows/F4\_workflow\_v01/submission\_ready/public/data/esld\_F4\_survival\_stats\_public.csv | analysis-specific scale | numeric |
| p\_value — P value **File:** 02\_workflows/F4\_workflow\_v01/submission\_ready/public/data/esld\_F4\_survival\_stats\_public.csv **Recommended public name:** p\_value **Description:** P value from the statistical comparison represented by the row. **Unit:**  **Value coding:** Probability value from the corresponding statistical test. **Data type:** numeric **Example values:** 0.787854614489014 \| 9.15971812501929e-11 \| 2.77788222236644e-07 \| 0.00360653118106935 \| 0.114275327333006 **Allowed values:**  **Missing-value coding:** NA/blank as read by fread **n rows / missing / unique:** 27 / 0 / 27 **Release status:** public **Definition status:** exact\_definition | 02\_workflows/F4\_workflow\_v01/submission\_ready/public/data/esld\_F4\_survival\_stats\_public.csv |  | numeric |
| score\_class\_label — Displayed score-class label **File:** 02\_workflows/F4\_workflow\_v01/submission\_ready/public/data/esld\_F4\_survival\_stats\_public.csv **Recommended public name:** score\_class\_label **Description:** Displayed score-class label for the figure/table stratum. **Unit:** score points **Value coding:** Categorical score-class label. **Data type:** character **Example values:** ≤10 \| 11-15 \| 16-20 \| 21-25 \| 26-30 **Allowed values:** ≤10 \| 11-15 \| 16-20 \| 21-25 \| 26-30 \| 31-35 \| 36-40 **Missing-value coding:** NA/blank as read by fread **n rows / missing / unique:** 27 / 0 / 7 **Release status:** public **Definition status:** manual\_precision\_definition | 02\_workflows/F4\_workflow\_v01/submission\_ready/public/data/esld\_F4\_survival\_stats\_public.csv | score points | character |
| score\_class\_value — Score-class upper value **File:** 02\_workflows/F4\_workflow\_v01/submission\_ready/public/data/esld\_F4\_survival\_stats\_public.csv **Recommended public name:** score\_class\_value **Description:** Upper score value defining the displayed score class. **Unit:** score points **Value coding:** Numeric score-class upper value. **Data type:** integer **Example values:** 10 \| 15 \| 20 \| 25 \| 30 **Allowed values:**  **Missing-value coding:** NA/blank as read by fread **n rows / missing / unique:** 27 / 0 / 7 **Release status:** public **Definition status:** manual\_precision\_definition | 02\_workflows/F4\_workflow\_v01/submission\_ready/public/data/esld\_F4\_survival\_stats\_public.csv | score points | integer |
| y\_ast — Y coordinate for significance annotation **File:** 02\_workflows/F4\_workflow\_v01/submission\_ready/public/data/esld\_F4\_survival\_stats\_public.csv **Recommended public name:** y\_ast **Description:** Y-axis coordinate used to position the statistical-significance annotation in the F4 survival figure. **Unit:** formatted table value **Value coding:** Numeric y-axis coordinate for annotation placement. **Data type:** numeric **Example values:** 131 \| 79 \| 38 \| 22 \| 15 **Allowed values:**  **Missing-value coding:** NA/blank as read by fread **n rows / missing / unique:** 27 / 0 / 24 **Release status:** public **Definition status:** manual\_precision\_definition | 02\_workflows/F4\_workflow\_v01/submission\_ready/public/data/esld\_F4\_survival\_stats\_public.csv | formatted table value | numeric |
| age\_years\_first\_available — Age at first available public record **File:** 02\_workflows/F5\_workflow\_v01/data/01\_source\_loaded\_harmonized/esld\_master\_long\_public.csv **Recommended public name:** age\_years\_first\_available **Description:** Age in years at the first available public ESLD record for the patient or encounter represented in the released data. **Unit:** years **Value coding:** Integer age in years. **Data type:** integer **Example values:** 65 \| 67 \| 21 \| 56 \| 63 **Allowed values:**  **Missing-value coding:** NA/blank as read by fread **n rows / missing / unique:** 67399 / 0 / 79 **Release status:** public **Definition status:** manual\_exact\_definition | 02\_workflows/F5\_workflow\_v01/data/01\_source\_loaded\_harmonized/esld\_master\_long\_public.csv | years | integer |
| age\_years\_sample — Age at sample **File:** 02\_workflows/F5\_workflow\_v01/data/01\_source\_loaded\_harmonized/esld\_master\_long\_public.csv **Recommended public name:** age\_years\_sample **Description:** Age in years at the sample-level public ESLD record. **Unit:** years **Value coding:** Integer age in years. **Data type:** integer **Example values:** 65 \| 66 \| 67 \| 68 \| 69 **Allowed values:**  **Missing-value coding:** NA/blank as read by fread **n rows / missing / unique:** 67399 / 0 / 84 **Release status:** public **Definition status:** manual\_precision\_definition | 02\_workflows/F5\_workflow\_v01/data/01\_source\_loaded\_harmonized/esld\_master\_long\_public.csv | years | integer |
| albumin\_g\_dl — Serum albumin concentration **File:** 02\_workflows/F5\_workflow\_v01/data/01\_source\_loaded\_harmonized/esld\_master\_long\_public.csv **Recommended public name:** albumin\_g\_dl **Description:** Serum albumin concentration in the public ESLD master data. **Unit:** g/dL **Value coding:** Numeric concentration in g/dL. **Data type:** numeric **Example values:** 3.3 \| 6.15 \| 3.14 \| 2.8 \| 3.26 **Allowed values:**  **Missing-value coding:** NA/blank as read by fread **n rows / missing / unique:** 67399 / 58785 / 580 **Release status:** public **Definition status:** manual\_precision\_definition | 02\_workflows/F5\_workflow\_v01/data/01\_source\_loaded\_harmonized/esld\_master\_long\_public.csv | g/dL | numeric |
| ald — Alcohol-related liver disease etiology flag **File:** 02\_workflows/F5\_workflow\_v01/data/01\_source\_loaded\_harmonized/esld\_master\_long\_public.csv **Recommended public name:** ald **Description:** Binary etiology indicator for alcohol-related liver disease in the public ESLD cohort. **Unit:**  **Value coding:** 0 = no; 1 = yes. **Data type:** integer **Example values:** 0 \| 1 **Allowed values:**  **Missing-value coding:** NA/blank as read by fread **n rows / missing / unique:** 67399 / 0 / 2 **Release status:** public **Definition status:** manual\_exact\_definition | 02\_workflows/F5\_workflow\_v01/data/01\_source\_loaded\_harmonized/esld\_master\_long\_public.csv |  | integer |
| ald\_hcv — Combined alcohol-related liver disease and hepatitis C etiology flag **File:** 02\_workflows/F5\_workflow\_v01/data/01\_source\_loaded\_harmonized/esld\_master\_long\_public.csv **Recommended public name:** ald\_hcv **Description:** Binary etiology indicator for combined alcohol-related liver disease and hepatitis C in the public ESLD cohort. **Unit:**  **Value coding:** 0 = no; 1 = yes. **Data type:** integer **Example values:** 0 \| 1 **Allowed values:**  **Missing-value coding:** NA/blank as read by fread **n rows / missing / unique:** 67399 / 0 / 2 **Release status:** public **Definition status:** manual\_exact\_definition | 02\_workflows/F5\_workflow\_v01/data/01\_source\_loaded\_harmonized/esld\_master\_long\_public.csv |  | integer |
| autoimmune — Autoimmune liver disease etiology flag **File:** 02\_workflows/F5\_workflow\_v01/data/01\_source\_loaded\_harmonized/esld\_master\_long\_public.csv **Recommended public name:** autoimmune **Description:** Binary etiology indicator for autoimmune liver disease in the public ESLD cohort. **Unit:**  **Value coding:** 0 = no; 1 = yes. **Data type:** integer **Example values:** 0 \| 1 **Allowed values:**  **Missing-value coding:** NA/blank as read by fread **n rows / missing / unique:** 67399 / 0 / 2 **Release status:** public **Definition status:** manual\_exact\_definition | 02\_workflows/F5\_workflow\_v01/data/01\_source\_loaded\_harmonized/esld\_master\_long\_public.csv |  | integer |
| crea — Creatinine concentration **File:** 02\_workflows/F5\_workflow\_v01/data/01\_source\_loaded\_harmonized/esld\_master\_long\_public.csv **Recommended public name:** crea **Description:** Creatinine concentration used in MELD-related score calculation before applying the study-specific correction. **Unit:** mg/dL **Value coding:** Numeric concentration in mg/dL. **Data type:** numeric **Example values:** 1.91 \| 1.42 \| 1.31 \| 1.3 \| 1.28 **Allowed values:**  **Missing-value coding:** NA/blank as read by fread **n rows / missing / unique:** 67399 / 0 / 975 **Release status:** public **Definition status:** manual\_exact\_definition | 02\_workflows/F5\_workflow\_v01/data/01\_source\_loaded\_harmonized/esld\_master\_long\_public.csv | mg/dL | numeric |
| crea\_corrected — Corrected creatinine concentration **File:** 02\_workflows/F5\_workflow\_v01/data/01\_source\_loaded\_harmonized/esld\_master\_long\_public.csv **Recommended public name:** crea\_corrected **Description:** Creatinine concentration after applying the study-specific correction used for recalculated MELD-related scores. **Unit:** mg/dL **Value coding:** Numeric concentration in mg/dL. **Data type:** numeric **Example values:** 1.87705004446668 \| 1.41586432303417 \| 1.31039363454933 \| 1.30192482788903 \| 1.28150549768982 **Allowed values:**  **Missing-value coding:** NA/blank as read by fread **n rows / missing / unique:** 67399 / 0 / 15868 **Release status:** public **Definition status:** manual\_exact\_definition | 02\_workflows/F5\_workflow\_v01/data/01\_source\_loaded\_harmonized/esld\_master\_long\_public.csv | mg/dL | numeric |
| date\_count — Relative day count **File:** 02\_workflows/F5\_workflow\_v01/data/01\_source\_loaded\_harmonized/esld\_master\_long\_public.csv **Recommended public name:** date\_count **Description:** Relative day count used for time alignment; negative/positive values are relative to the analysis anchor and are not calendar dates. **Unit:**  **Value coding:** See example\_values and allowed\_values. **Data type:** integer **Example values:** -3021 \| -2833 \| -2798 \| -2793 \| -2791 **Allowed values:**  **Missing-value coding:** NA/blank as read by fread **n rows / missing / unique:** 67399 / 50970 / 1450 **Release status:** public **Definition status:** exact\_definition | 02\_workflows/F5\_workflow\_v01/data/01\_source\_loaded\_harmonized/esld\_master\_long\_public.csv |  | integer |
| date\_count\_month — Relative month count **File:** 02\_workflows/F5\_workflow\_v01/data/01\_source\_loaded\_harmonized/esld\_master\_long\_public.csv **Recommended public name:** date\_count\_month **Description:** Relative month count derived from the relative day count; this is not a calendar month. **Unit:**  **Value coding:** See example\_values and allowed\_values. **Data type:** integer **Example values:** -99 \| -93 \| -92 \| -91 \| -87 **Allowed values:**  **Missing-value coding:** NA/blank as read by fread **n rows / missing / unique:** 67399 / 50970 / 147 **Release status:** public **Definition status:** exact\_definition | 02\_workflows/F5\_workflow\_v01/data/01\_source\_loaded\_harmonized/esld\_master\_long\_public.csv |  | integer |
| dead\_sample\_flag — Dead-sample flag **File:** 02\_workflows/F5\_workflow\_v01/data/01\_source\_loaded\_harmonized/esld\_master\_long\_public.csv **Recommended public name:** dead\_sample\_flag **Description:** Binary indicator identifying public ESLD sample records assigned to the deceased-sample subset used in sample-level analyses. **Unit:**  **Value coding:** 0 = not assigned to deceased-sample subset; 1 = assigned to deceased-sample subset. **Data type:** integer **Example values:** 0 \| 1 **Allowed values:**  **Missing-value coding:** NA/blank as read by fread **n rows / missing / unique:** 67399 / 0 / 2 **Release status:** public **Definition status:** manual\_final\_definition | 02\_workflows/F5\_workflow\_v01/data/01\_source\_loaded\_harmonized/esld\_master\_long\_public.csv |  | integer |
| death\_within\_90d — Death within 90 days **File:** 02\_workflows/F5\_workflow\_v01/data/01\_source\_loaded\_harmonized/esld\_master\_long\_public.csv **Recommended public name:** death\_within\_90d **Description:** Binary indicator for death within 90 days of the analysis anchor. **Unit:**  **Value coding:** 0 = no; 1 = yes **Data type:** logical **Example values:**  **Allowed values:**  **Missing-value coding:** NA/blank as read by fread **n rows / missing / unique:** 67399 / 67399 / 0 **Release status:** public **Definition status:** exact\_definition | 02\_workflows/F5\_workflow\_v01/data/01\_source\_loaded\_harmonized/esld\_master\_long\_public.csv |  | logical |
| deceased\_patient\_flag — Deceased patient flag **File:** 02\_workflows/F5\_workflow\_v01/data/01\_source\_loaded\_harmonized/esld\_master\_long\_public.csv **Recommended public name:** deceased\_patient\_flag **Description:** Binary indicator identifying deceased patients within the released analysis context. **Unit:**  **Value coding:** 0 = no; 1 = yes **Data type:** integer **Example values:** 0 \| 1 **Allowed values:**  **Missing-value coding:** NA/blank as read by fread **n rows / missing / unique:** 67399 / 0 / 2 **Release status:** public **Definition status:** exact\_definition | 02\_workflows/F5\_workflow\_v01/data/01\_source\_loaded\_harmonized/esld\_master\_long\_public.csv |  | integer |
| delta\_eq\_0\_flag — Zero score-delta flag **File:** 02\_workflows/F5\_workflow\_v01/data/01\_source\_loaded\_harmonized/esld\_master\_long\_public.csv **Recommended public name:** delta\_eq\_0\_flag **Description:** Binary indicator equal to 1 when the score delta is exactly zero. **Unit:**  **Value coding:** 0 = no; 1 = yes. **Data type:** integer **Example values:** 1 \| 0 **Allowed values:**  **Missing-value coding:** NA/blank as read by fread **n rows / missing / unique:** 67399 / 0 / 2 **Release status:** public **Definition status:** manual\_precision\_definition | 02\_workflows/F5\_workflow\_v01/data/01\_source\_loaded\_harmonized/esld\_master\_long\_public.csv |  | integer |
| delta\_gt\_0\_flag — Positive score-delta flag **File:** 02\_workflows/F5\_workflow\_v01/data/01\_source\_loaded\_harmonized/esld\_master\_long\_public.csv **Recommended public name:** delta\_gt\_0\_flag **Description:** Binary indicator equal to 1 when the score delta is positive. **Unit:**  **Value coding:** 0 = no; 1 = yes. **Data type:** integer **Example values:** 0 \| 1 **Allowed values:**  **Missing-value coding:** NA/blank as read by fread **n rows / missing / unique:** 67399 / 0 / 2 **Release status:** public **Definition status:** manual\_precision\_definition | 02\_workflows/F5\_workflow\_v01/data/01\_source\_loaded\_harmonized/esld\_master\_long\_public.csv |  | integer |
| delta\_le\_minus1\_flag — Score-delta ≤ −1 flag **File:** 02\_workflows/F5\_workflow\_v01/data/01\_source\_loaded\_harmonized/esld\_master\_long\_public.csv **Recommended public name:** delta\_le\_minus1\_flag **Description:** Binary indicator equal to 1 when the score delta is less than or equal to −1. **Unit:**  **Value coding:** 0 = no; 1 = yes. **Data type:** integer **Example values:** 0 \| 1 **Allowed values:**  **Missing-value coding:** NA/blank as read by fread **n rows / missing / unique:** 67399 / 0 / 2 **Release status:** public **Definition status:** manual\_precision\_definition | 02\_workflows/F5\_workflow\_v01/data/01\_source\_loaded\_harmonized/esld\_master\_long\_public.csv |  | integer |
| dialysis\_raw — Dialysis indicator as recorded before score derivation **File:** 02\_workflows/F5\_workflow\_v01/data/01\_source\_loaded\_harmonized/esld\_master\_long\_public.csv **Recommended public name:** dialysis\_raw **Description:** Binary indicator showing whether dialysis was recorded in the source field before score derivation. **Unit:**  **Value coding:** 0 = no dialysis recorded; 1 = dialysis recorded. **Data type:** integer **Example values:** 0 \| 1 **Allowed values:**  **Missing-value coding:** NA/blank as read by fread **n rows / missing / unique:** 67399 / 27827 / 2 **Release status:** public **Definition status:** manual\_exact\_definition | 02\_workflows/F5\_workflow\_v01/data/01\_source\_loaded\_harmonized/esld\_master\_long\_public.csv |  | integer |
| encounter\_id\_public — Public encounter pseudonym **File:** 02\_workflows/F5\_workflow\_v01/data/01\_source\_loaded\_harmonized/esld\_master\_long\_public.csv **Recommended public name:** encounter\_id\_public **Description:** Non-linkable public-release encounter pseudonym used for encounter-level grouping; not an original hospital encounter identifier. **Unit:**  **Value coding:** See example\_values and allowed\_values. **Data type:** character **Example values:** E001181 \| E000008 \| E000041 \| E000100 \| E000126 **Allowed values:** E001181 \| E000008 \| E000041 \| E000100 \| E000126 \| E000151 \| E000157 \| E000219 \| E000289 \| E000344 **Missing-value coding:** NA/blank as read by fread **n rows / missing / unique:** 67399 / 0 / 5045 **Release status:** public **Definition status:** exact\_definition | 02\_workflows/F5\_workflow\_v01/data/01\_source\_loaded\_harmonized/esld\_master\_long\_public.csv |  | character |
| etiology\_source\_current\_flag — Current etiology source flag **File:** 02\_workflows/F5\_workflow\_v01/data/01\_source\_loaded\_harmonized/esld\_master\_long\_public.csv **Recommended public name:** etiology\_source\_current\_flag **Description:** Binary indicator identifying whether the etiology source is the current source used for the public row. **Unit:**  **Value coding:** 0 = no; 1 = yes. **Data type:** integer **Example values:** 1 **Allowed values:**  **Missing-value coding:** NA/blank as read by fread **n rows / missing / unique:** 67399 / 0 / 1 **Release status:** public **Definition status:** manual\_precision\_definition | 02\_workflows/F5\_workflow\_v01/data/01\_source\_loaded\_harmonized/esld\_master\_long\_public.csv |  | integer |
| etiology\_unclassified — Unclassified liver disease etiology flag **File:** 02\_workflows/F5\_workflow\_v01/data/01\_source\_loaded\_harmonized/esld\_master\_long\_public.csv **Recommended public name:** etiology\_unclassified **Description:** Binary etiology indicator identifying records without a classified liver disease etiology in the public ESLD cohort. **Unit:**  **Value coding:** 0 = no; 1 = yes. **Data type:** integer **Example values:** 0 **Allowed values:**  **Missing-value coding:** NA/blank as read by fread **n rows / missing / unique:** 67399 / 0 / 1 **Release status:** public **Definition status:** manual\_exact\_definition | 02\_workflows/F5\_workflow\_v01/data/01\_source\_loaded\_harmonized/esld\_master\_long\_public.csv |  | integer |
| hbv — Hepatitis B virus etiology flag **File:** 02\_workflows/F5\_workflow\_v01/data/01\_source\_loaded\_harmonized/esld\_master\_long\_public.csv **Recommended public name:** hbv **Description:** Binary etiology indicator for hepatitis B virus-related liver disease in the public ESLD cohort. **Unit:**  **Value coding:** 0 = no; 1 = yes. **Data type:** integer **Example values:** 0 \| 1 **Allowed values:**  **Missing-value coding:** NA/blank as read by fread **n rows / missing / unique:** 67399 / 0 / 2 **Release status:** public **Definition status:** manual\_exact\_definition | 02\_workflows/F5\_workflow\_v01/data/01\_source\_loaded\_harmonized/esld\_master\_long\_public.csv |  | integer |
| hcv — Hepatitis C virus etiology flag **File:** 02\_workflows/F5\_workflow\_v01/data/01\_source\_loaded\_harmonized/esld\_master\_long\_public.csv **Recommended public name:** hcv **Description:** Binary etiology indicator for hepatitis C virus-related liver disease in the public ESLD cohort. **Unit:**  **Value coding:** 0 = no; 1 = yes. **Data type:** integer **Example values:** 0 \| 1 **Allowed values:**  **Missing-value coding:** NA/blank as read by fread **n rows / missing / unique:** 67399 / 0 / 2 **Release status:** public **Definition status:** manual\_exact\_definition | 02\_workflows/F5\_workflow\_v01/data/01\_source\_loaded\_harmonized/esld\_master\_long\_public.csv |  | integer |
| icd\_source\_present — ICD source present flag **File:** 02\_workflows/F5\_workflow\_v01/data/01\_source\_loaded\_harmonized/esld\_master\_long\_public.csv **Recommended public name:** icd\_source\_present **Description:** Binary indicator showing whether an ICD-derived etiology source was present for the public row. **Unit:**  **Value coding:** 0 = no; 1 = yes. **Data type:** integer **Example values:** 1 \| 0 **Allowed values:**  **Missing-value coding:** NA/blank as read by fread **n rows / missing / unique:** 67399 / 0 / 2 **Release status:** public **Definition status:** manual\_precision\_definition | 02\_workflows/F5\_workflow\_v01/data/01\_source\_loaded\_harmonized/esld\_master\_long\_public.csv |  | integer |
| in\_t1\_cohort — Inclusion flag for Table 1 cohort **File:** 02\_workflows/F5\_workflow\_v01/data/01\_source\_loaded\_harmonized/esld\_master\_long\_public.csv **Recommended public name:** in\_t1\_cohort **Description:** Binary indicator identifying records included in the public ESLD Table 1 baseline-characteristics cohort. **Unit:**  **Value coding:** 0 = not included; 1 = included. **Data type:** integer **Example values:** 1 **Allowed values:**  **Missing-value coding:** NA/blank as read by fread **n rows / missing / unique:** 67399 / 0 / 1 **Release status:** public **Definition status:** manual\_exact\_definition | 02\_workflows/F5\_workflow\_v01/data/01\_source\_loaded\_harmonized/esld\_master\_long\_public.csv |  | integer |
| in\_t3\_anchor\_model — Inclusion flag for Table 3 anchor-model cohort **File:** 02\_workflows/F5\_workflow\_v01/data/01\_source\_loaded\_harmonized/esld\_master\_long\_public.csv **Recommended public name:** in\_t3\_anchor\_model **Description:** Binary indicator identifying records included in the Table 3 anchor-model cohort used for creatinine-comparison analyses. **Unit:**  **Value coding:** 0 = not included; 1 = included. **Data type:** integer **Example values:** 1 \| 0 **Allowed values:**  **Missing-value coding:** NA/blank as read by fread **n rows / missing / unique:** 67399 / 0 / 2 **Release status:** public **Definition status:** manual\_exact\_definition | 02\_workflows/F5\_workflow\_v01/data/01\_source\_loaded\_harmonized/esld\_master\_long\_public.csv |  | integer |
| in\_t4\_prevalence\_path — Inclusion flag for Table 4 prevalence pathway **File:** 02\_workflows/F5\_workflow\_v01/data/01\_source\_loaded\_harmonized/esld\_master\_long\_public.csv **Recommended public name:** in\_t4\_prevalence\_path **Description:** Binary indicator identifying records included in the Table 4 prevalence-analysis pathway. **Unit:**  **Value coding:** 0 = not included; 1 = included. **Data type:** integer **Example values:** 1 **Allowed values:**  **Missing-value coding:** NA/blank as read by fread **n rows / missing / unique:** 67399 / 0 / 1 **Release status:** public **Definition status:** manual\_exact\_definition | 02\_workflows/F5\_workflow\_v01/data/01\_source\_loaded\_harmonized/esld\_master\_long\_public.csv |  | integer |
| in\_t4\_survival\_path — Inclusion flag for Table 4 survival pathway **File:** 02\_workflows/F5\_workflow\_v01/data/01\_source\_loaded\_harmonized/esld\_master\_long\_public.csv **Recommended public name:** in\_t4\_survival\_path **Description:** Binary indicator identifying records included in the Table 4 survival-analysis pathway. **Unit:**  **Value coding:** 0 = not included; 1 = included. **Data type:** integer **Example values:** 0 \| 1 **Allowed values:**  **Missing-value coding:** NA/blank as read by fread **n rows / missing / unique:** 67399 / 0 / 2 **Release status:** public **Definition status:** manual\_exact\_definition | 02\_workflows/F5\_workflow\_v01/data/01\_source\_loaded\_harmonized/esld\_master\_long\_public.csv |  | integer |
| inr — International normalized ratio **File:** 02\_workflows/F5\_workflow\_v01/data/01\_source\_loaded\_harmonized/esld\_master\_long\_public.csv **Recommended public name:** inr **Description:** International normalized ratio of prothrombin time used for MELD-related score calculation. **Unit:** ratio **Value coding:** Numeric INR value. **Data type:** numeric **Example values:** 1.14 \| 0.91 \| 1 \| 1.04 \| 0.98 **Allowed values:**  **Missing-value coding:** NA/blank as read by fread **n rows / missing / unique:** 67399 / 0 / 786 **Release status:** public **Definition status:** manual\_exact\_definition | 02\_workflows/F5\_workflow\_v01/data/01\_source\_loaded\_harmonized/esld\_master\_long\_public.csv | ratio | numeric |
| mash — Metabolic dysfunction-associated steatohepatitis etiology flag **File:** 02\_workflows/F5\_workflow\_v01/data/01\_source\_loaded\_harmonized/esld\_master\_long\_public.csv **Recommended public name:** mash **Description:** Binary etiology indicator for metabolic dysfunction-associated steatohepatitis in the public ESLD cohort. **Unit:**  **Value coding:** 0 = no; 1 = yes. **Data type:** integer **Example values:** 0 \| 1 **Allowed values:**  **Missing-value coding:** NA/blank as read by fread **n rows / missing / unique:** 67399 / 0 / 2 **Release status:** public **Definition status:** manual\_exact\_definition | 02\_workflows/F5\_workflow\_v01/data/01\_source\_loaded\_harmonized/esld\_master\_long\_public.csv |  | integer |
| model — MELD model or score variant **File:** 02\_workflows/F5\_workflow\_v01/data/01\_source\_loaded\_harmonized/esld\_master\_long\_public.csv **Recommended public name:** model **Description:** Name of the MELD-related model or score variant represented by the row; expected values include MELD, MELD-Na, reMELD-Na, and MELD 3.0. **Unit:**  **Value coding:** Categorical score/model label, for example MELD, MELD-Na, reMELD-Na, or MELD 3.0. **Data type:** character **Example values:** MELD \| MELD-Na \| reMELD-Na \| MELD 3.0 **Allowed values:** MELD \| MELD-Na \| reMELD-Na \| MELD 3.0 **Missing-value coding:** NA/blank as read by fread **n rows / missing / unique:** 67399 / 0 / 4 **Release status:** public **Definition status:** manual\_exact\_definition | 02\_workflows/F5\_workflow\_v01/data/01\_source\_loaded\_harmonized/esld\_master\_long\_public.csv |  | character |
| other — Other liver disease etiology flag **File:** 02\_workflows/F5\_workflow\_v01/data/01\_source\_loaded\_harmonized/esld\_master\_long\_public.csv **Recommended public name:** other **Description:** Binary etiology indicator for liver disease etiologies grouped as other in the public ESLD cohort. **Unit:**  **Value coding:** 0 = no; 1 = yes. **Data type:** integer **Example values:** 1 \| 0 **Allowed values:**  **Missing-value coding:** NA/blank as read by fread **n rows / missing / unique:** 67399 / 0 / 2 **Release status:** public **Definition status:** manual\_exact\_definition | 02\_workflows/F5\_workflow\_v01/data/01\_source\_loaded\_harmonized/esld\_master\_long\_public.csv |  | integer |
| patient\_id — Public patient pseudonym **File:** 02\_workflows/F5\_workflow\_v01/data/01\_source\_loaded\_harmonized/esld\_master\_long\_public.csv **Recommended public name:** patient\_id **Description:** Non-linkable public-release patient pseudonym used to preserve within-patient grouping in public ESLD data; not an original hospital patient identifier. **Unit:**  **Value coding:** See example\_values and allowed\_values. **Data type:** character **Example values:** P000001 \| P000002 \| P000003 \| P000004 \| P000005 **Allowed values:** P000001 \| P000002 \| P000003 \| P000004 \| P000005 \| P000006 \| P000007 \| P000008 \| P000009 \| P000010 **Missing-value coding:** NA/blank as read by fread **n rows / missing / unique:** 67399 / 0 / 1375 **Release status:** public **Definition status:** exact\_definition | 02\_workflows/F5\_workflow\_v01/data/01\_source\_loaded\_harmonized/esld\_master\_long\_public.csv |  | character |
| pbc — Primary biliary cholangitis etiology flag **File:** 02\_workflows/F5\_workflow\_v01/data/01\_source\_loaded\_harmonized/esld\_master\_long\_public.csv **Recommended public name:** pbc **Description:** Binary etiology indicator for primary biliary cholangitis in the public ESLD cohort. **Unit:**  **Value coding:** 0 = no; 1 = yes. **Data type:** integer **Example values:** 0 \| 1 **Allowed values:**  **Missing-value coding:** NA/blank as read by fread **n rows / missing / unique:** 67399 / 0 / 2 **Release status:** public **Definition status:** manual\_exact\_definition | 02\_workflows/F5\_workflow\_v01/data/01\_source\_loaded\_harmonized/esld\_master\_long\_public.csv |  | integer |
| sample\_day\_from\_first\_sample — Relative sample day **File:** 02\_workflows/F5\_workflow\_v01/data/01\_source\_loaded\_harmonized/esld\_master\_long\_public.csv **Recommended public name:** sample\_day\_from\_first\_sample **Description:** Relative day of the sample measured from the first sample for the public patient/sample sequence; not a calendar date. **Unit:** relative months **Value coding:** Integer or numeric relative day count. **Data type:** integer **Example values:** 0 \| 42 \| 91 \| 118 \| 133 **Allowed values:**  **Missing-value coding:** NA/blank as read by fread **n rows / missing / unique:** 67399 / 0 / 3438 **Release status:** public **Definition status:** manual\_precision\_definition | 02\_workflows/F5\_workflow\_v01/data/01\_source\_loaded\_harmonized/esld\_master\_long\_public.csv | relative months | integer |
| sample\_group\_id\_public — Public sample-group pseudonym **File:** 02\_workflows/F5\_workflow\_v01/data/01\_source\_loaded\_harmonized/esld\_master\_long\_public.csv **Recommended public name:** sample\_group\_id\_public **Description:** Non-linkable public-release sample-group pseudonym used to group samples within the released public data. **Unit:**  **Value coding:** See example\_values and allowed\_values. **Data type:** character **Example values:** G0000001 \| G0000002 \| G0000003 \| G0000004 \| G0000005 **Allowed values:** G0000001 \| G0000002 \| G0000003 \| G0000004 \| G0000005 \| G0000006 \| G0000007 \| G0000008 \| G0000009 \| G0000010 **Missing-value coding:** NA/blank as read by fread **n rows / missing / unique:** 67399 / 0 / 20359 **Release status:** public **Definition status:** exact\_definition | 02\_workflows/F5\_workflow\_v01/data/01\_source\_loaded\_harmonized/esld\_master\_long\_public.csv |  | character |
| sample\_id — Sample identifier **File:** 02\_workflows/F5\_workflow\_v01/data/01\_source\_loaded\_harmonized/esld\_master\_long\_public.csv **Recommended public name:** sample\_id **Description:** Identifier of a sample or experimental record within the released public data; not a personal identifier. **Unit:**  **Value coding:** See example\_values and allowed\_values. **Data type:** character **Example values:** S0000001 \| S0000002 \| S0000003 \| S0000004 \| S0000005 **Allowed values:** S0000001 \| S0000002 \| S0000003 \| S0000004 \| S0000005 \| S0000006 \| S0000007 \| S0000008 \| S0000009 \| S0000010 **Missing-value coding:** NA/blank as read by fread **n rows / missing / unique:** 67399 / 0 / 67399 **Release status:** public **Definition status:** exact\_definition | 02\_workflows/F5\_workflow\_v01/data/01\_source\_loaded\_harmonized/esld\_master\_long\_public.csv |  | character |
| sample\_month\_index — Relative sample month index **File:** 02\_workflows/F5\_workflow\_v01/data/01\_source\_loaded\_harmonized/esld\_master\_long\_public.csv **Recommended public name:** sample\_month\_index **Description:** Relative month index of the sample measured from the first sample for the public patient/sample sequence; not a calendar month. **Unit:** score points **Value coding:** Integer or numeric relative month index. **Data type:** integer **Example values:** 0 \| 1 \| 2 \| 3 \| 4 **Allowed values:**  **Missing-value coding:** NA/blank as read by fread **n rows / missing / unique:** 67399 / 0 / 177 **Release status:** public **Definition status:** manual\_precision\_definition | 02\_workflows/F5\_workflow\_v01/data/01\_source\_loaded\_harmonized/esld\_master\_long\_public.csv | score points | integer |
| score\_corrected — Corrected score **File:** 02\_workflows/F5\_workflow\_v01/data/01\_source\_loaded\_harmonized/esld\_master\_long\_public.csv **Recommended public name:** score\_corrected **Description:** MELD-related score after creatinine correction or recalculation. **Unit:** score points **Value coding:** Numeric score. **Data type:** integer **Example values:** 14 \| 19 \| 16 \| 10 \| 13 **Allowed values:**  **Missing-value coding:** NA/blank as read by fread **n rows / missing / unique:** 67399 / 0 / 40 **Release status:** public **Definition status:** manual\_precision\_definition | 02\_workflows/F5\_workflow\_v01/data/01\_source\_loaded\_harmonized/esld\_master\_long\_public.csv | score points | integer |
| score\_delta — Score difference **File:** 02\_workflows/F5\_workflow\_v01/data/01\_source\_loaded\_harmonized/esld\_master\_long\_public.csv **Recommended public name:** score\_delta **Description:** Difference between score variants or scoring approaches, expressed in score points. **Unit:**  **Value coding:** See example\_values and allowed\_values. **Data type:** integer **Example values:** 0 \| 1 \| -1 \| -2 **Allowed values:**  **Missing-value coding:** NA/blank as read by fread **n rows / missing / unique:** 67399 / 0 / 4 **Release status:** public **Definition status:** exact\_definition | 02\_workflows/F5\_workflow\_v01/data/01\_source\_loaded\_harmonized/esld\_master\_long\_public.csv |  | integer |
| score\_original — Original score **File:** 02\_workflows/F5\_workflow\_v01/data/01\_source\_loaded\_harmonized/esld\_master\_long\_public.csv **Recommended public name:** score\_original **Description:** Original MELD-related score before creatinine correction or recalculation. **Unit:**  **Value coding:** Numeric score. **Data type:** integer **Example values:** 14 \| 19 \| 16 \| 10 \| 13 **Allowed values:**  **Missing-value coding:** NA/blank as read by fread **n rows / missing / unique:** 67399 / 0 / 40 **Release status:** public **Definition status:** manual\_precision\_definition | 02\_workflows/F5\_workflow\_v01/data/01\_source\_loaded\_harmonized/esld\_master\_long\_public.csv |  | integer |
| sex — Sex **File:** 02\_workflows/F5\_workflow\_v01/data/01\_source\_loaded\_harmonized/esld\_master\_long\_public.csv **Recommended public name:** sex **Description:** Sex category represented in the public ESLD data. **Unit:**  **Value coding:** M = male; F = female. **Data type:** character **Example values:** M \| F **Allowed values:** M \| F **Missing-value coding:** NA/blank as read by fread **n rows / missing / unique:** 67399 / 0 / 2 **Release status:** public **Definition status:** manual\_exact\_definition | 02\_workflows/F5\_workflow\_v01/data/01\_source\_loaded\_harmonized/esld\_master\_long\_public.csv |  | character |
| sodium — Serum sodium concentration **File:** 02\_workflows/F5\_workflow\_v01/data/01\_source\_loaded\_harmonized/esld\_master\_long\_public.csv **Recommended public name:** sodium **Description:** Serum sodium concentration used for MELD-Na, reMELD-Na, or MELD 3.0 score calculation. **Unit:** mmol/L **Value coding:** Numeric concentration in mmol/L. **Data type:** numeric **Example values:** 131 \| 134 \| 132 \| 133 \| 140 **Allowed values:**  **Missing-value coding:** NA/blank as read by fread **n rows / missing / unique:** 67399 / 20359 / 188 **Release status:** public **Definition status:** manual\_exact\_definition | 02\_workflows/F5\_workflow\_v01/data/01\_source\_loaded\_harmonized/esld\_master\_long\_public.csv | mmol/L | numeric |
| tbil — Total bilirubin concentration **File:** 02\_workflows/F5\_workflow\_v01/data/01\_source\_loaded\_harmonized/esld\_master\_long\_public.csv **Recommended public name:** tbil **Description:** Total bilirubin concentration used in MELD-related score calculation in the public ESLD data. **Unit:**  **Value coding:** Numeric concentration in mg/dL. **Data type:** numeric **Example values:** 1 \| 0.9 \| 1.1 \| 0.8 \| 0.6 **Allowed values:**  **Missing-value coding:** NA/blank as read by fread **n rows / missing / unique:** 67399 / 0 / 2029 **Release status:** public **Definition status:** manual\_precision\_definition | 02\_workflows/F5\_workflow\_v01/data/01\_source\_loaded\_harmonized/esld\_master\_long\_public.csv |  | numeric |
| parameter — Metadata parameter name **File:** 02\_workflows/F5\_workflow\_v01/data/02b\_figure\_content/esld\_F5\_stratified\_survival\_meta\_public.csv **Recommended public name:** parameter **Description:** Name of a metadata parameter describing the F2 simulated heatmap object, such as figure identity, data origin, grid type, axis variable, or unit/role. **Unit:**  **Value coding:** Categorical metadata key; value is given in the corresponding value column or file-specific metadata field. **Data type:** character **Example values:** dataset\_subject \| dataset\_stats \| domain \| anchor \| data\_object **Allowed values:** dataset\_subject \| dataset\_stats \| domain \| anchor \| data\_object \| unit\_or\_role\_subject \| unit\_or\_role\_stats \| release\_status \| source\_master \| n\_subject\_rows **Missing-value coding:** NA/blank as read by fread **n rows / missing / unique:** 12 / 0 / 12 **Release status:** public **Definition status:** manual\_exact\_definition | 02\_workflows/F5\_workflow\_v01/data/02b\_figure\_content/esld\_F5\_stratified\_survival\_meta\_public.csv |  | character |
| value — Value **File:** 02\_workflows/F5\_workflow\_v01/data/02b\_figure\_content/esld\_F5\_stratified\_survival\_meta\_public.csv **Recommended public name:** value **Description:** Numerical or character value corresponding to the row-specific variable/metric. **Unit:**  **Value coding:** See example\_values and allowed\_values. **Data type:** character **Example values:** esld\_F5\_stratified\_survival\_subject\_public \| esld\_F5\_stratified\_survival\_stats\_public \| esld \| F5 \| stratified\_survival **Allowed values:** esld\_F5\_stratified\_survival\_subject\_public \| esld\_F5\_stratified\_survival\_stats\_public \| esld \| F5 \| stratified\_survival \| subject \| stats \| public \| esld\_master\_long\_public.csv \| 841 **Missing-value coding:** NA/blank as read by fread **n rows / missing / unique:** 12 / 0 / 12 **Release status:** public **Definition status:** exact\_definition | 02\_workflows/F5\_workflow\_v01/data/02b\_figure\_content/esld\_F5\_stratified\_survival\_meta\_public.csv |  | character |
| chisq — Chi-square statistic **File:** 02\_workflows/F5\_workflow\_v01/data/02b\_figure\_content/esld\_F5\_stratified\_survival\_stats\_public.csv **Recommended public name:** chisq **Description:** Chi-square statistic for the row-specific survival comparison or stratified test. **Unit:** chi-square statistic **Value coding:** Numeric chi-square statistic. **Data type:** numeric **Example values:** 0.00770313568089429 \| 2.87624703252109 \| 12.1826833766653 \| 0.00169868292629352 \| 0.733720432338734 **Allowed values:**  **Missing-value coding:** NA/blank as read by fread **n rows / missing / unique:** 12 / 0 / 12 **Release status:** public **Definition status:** manual\_precision\_definition | 02\_workflows/F5\_workflow\_v01/data/02b\_figure\_content/esld\_F5\_stratified\_survival\_stats\_public.csv | chi-square statistic | numeric |
| chisq\_lab — Formatted chi-square label **File:** 02\_workflows/F5\_workflow\_v01/data/02b\_figure\_content/esld\_F5\_stratified\_survival\_stats\_public.csv **Recommended public name:** chisq\_lab **Description:** Formatted display label for the chi-square statistic shown in a table or figure annotation. **Unit:**  **Value coding:** Formatted character label. **Data type:** character **Example values:** χ² = 0.008 \| χ² = 2.876 \| χ² = 12.183 \| χ² = 0.002 \| χ² = 0.734 **Allowed values:** χ² = 0.008 \| χ² = 2.876 \| χ² = 12.183 \| χ² = 0.002 \| χ² = 0.734 \| χ² = 7.752 \| χ² = 0.964 \| χ² = 8.044 \| χ² = 13.427 \| χ² = 0.413 **Missing-value coding:** NA/blank as read by fread **n rows / missing / unique:** 12 / 0 / 12 **Release status:** public **Definition status:** manual\_precision\_definition | 02\_workflows/F5\_workflow\_v01/data/02b\_figure\_content/esld\_F5\_stratified\_survival\_stats\_public.csv |  | character |
| model — MELD model or score variant **File:** 02\_workflows/F5\_workflow\_v01/data/02b\_figure\_content/esld\_F5\_stratified\_survival\_stats\_public.csv **Recommended public name:** model **Description:** Name of the MELD-related model or score variant represented by the row; expected values include MELD, MELD-Na, reMELD-Na, and MELD 3.0. **Unit:**  **Value coding:** Categorical score/model label, for example MELD, MELD-Na, reMELD-Na, or MELD 3.0. **Data type:** character **Example values:** MELD \| MELD-Na \| reMELD-Na \| MELD 3.0 **Allowed values:** MELD \| MELD-Na \| reMELD-Na \| MELD 3.0 **Missing-value coding:** NA/blank as read by fread **n rows / missing / unique:** 12 / 0 / 4 **Release status:** public **Definition status:** manual\_exact\_definition | 02\_workflows/F5\_workflow\_v01/data/02b\_figure\_content/esld\_F5\_stratified\_survival\_stats\_public.csv |  | character |
| n\_m1 — Number of observations in m1 group **File:** 02\_workflows/F5\_workflow\_v01/data/02b\_figure\_content/esld\_F5\_stratified\_survival\_stats\_public.csv **Recommended public name:** n\_m1 **Description:** Number of observations in the m1 comparison group for the row-specific figure/table stratum. **Unit:** count **Value coding:** Integer count. **Data type:** integer **Example values:** 12 \| 64 \| 82 \| 7 \| 47 **Allowed values:**  **Missing-value coding:** NA/blank as read by fread **n rows / missing / unique:** 12 / 0 / 12 **Release status:** public **Definition status:** manual\_precision\_definition | 02\_workflows/F5\_workflow\_v01/data/02b\_figure\_content/esld\_F5\_stratified\_survival\_stats\_public.csv | count | integer |
| n\_pm — Number of observations in pm group **File:** 02\_workflows/F5\_workflow\_v01/data/02b\_figure\_content/esld\_F5\_stratified\_survival\_stats\_public.csv **Recommended public name:** n\_pm **Description:** Number of observations in the pm comparison group for the row-specific figure/table stratum. **Unit:** count **Value coding:** Integer count. **Data type:** integer **Example values:** 25 \| 31 \| 11 \| 27 \| 39 **Allowed values:**  **Missing-value coding:** NA/blank as read by fread **n rows / missing / unique:** 12 / 0 / 11 **Release status:** public **Definition status:** manual\_precision\_definition | 02\_workflows/F5\_workflow\_v01/data/02b\_figure\_content/esld\_F5\_stratified\_survival\_stats\_public.csv | count | integer |
| p\_lab — Formatted p-value label **File:** 02\_workflows/F5\_workflow\_v01/data/02b\_figure\_content/esld\_F5\_stratified\_survival\_stats\_public.csv **Recommended public name:** p\_lab **Description:** Formatted p-value label shown in a table or figure annotation. **Unit:**  **Value coding:** Formatted p-value label. **Data type:** character **Example values:** p = 0.930 \| p = 0.090 \| p < 0.001 \| p = 0.967 \| p = 0.392 **Allowed values:** p = 0.930 \| p = 0.090 \| p < 0.001 \| p = 0.967 \| p = 0.392 \| p = 0.005 \| p = 0.326 \| p = 0.521 \| p = 0.046 \| p = 0.052 **Missing-value coding:** NA/blank as read by fread **n rows / missing / unique:** 12 / 0 / 10 **Release status:** public **Definition status:** manual\_precision\_definition | 02\_workflows/F5\_workflow\_v01/data/02b\_figure\_content/esld\_F5\_stratified\_survival\_stats\_public.csv |  | character |
| pval — P value **File:** 02\_workflows/F5\_workflow\_v01/data/02b\_figure\_content/esld\_F5\_stratified\_survival\_stats\_public.csv **Recommended public name:** pval **Description:** P value for the row-specific statistical comparison. **Unit:** replicate number **Value coding:** Numeric probability value. **Data type:** numeric **Example values:** 0.930061461937626 \| 0.0898953142885014 \| 0.000482352058876657 \| 0.967124430752549 \| 0.391679903320669 **Allowed values:**  **Missing-value coding:** NA/blank as read by fread **n rows / missing / unique:** 12 / 0 / 12 **Release status:** public **Definition status:** manual\_precision\_definition | 02\_workflows/F5\_workflow\_v01/data/02b\_figure\_content/esld\_F5\_stratified\_survival\_stats\_public.csv | replicate number | numeric |
| scr.grp — Score-class stratum **File:** 02\_workflows/F5\_workflow\_v01/data/02b\_figure\_content/esld\_F5\_stratified\_survival\_stats\_public.csv **Recommended public name:** scr.grp **Description:** Encoded score-class stratum used in F5; examples include le15, 16to25, and gt25. **Unit:**  **Value coding:** Categorical stratum code. **Data type:** character **Example values:** le15 \| 16to25 \| gt25 **Allowed values:** le15 \| 16to25 \| gt25 **Missing-value coding:** NA/blank as read by fread **n rows / missing / unique:** 12 / 0 / 3 **Release status:** public **Definition status:** manual\_precision\_definition | 02\_workflows/F5\_workflow\_v01/data/02b\_figure\_content/esld\_F5\_stratified\_survival\_stats\_public.csv |  | character |
| albumin — Serum albumin concentration **File:** 02\_workflows/F5\_workflow\_v01/data/02b\_figure\_content/esld\_F5\_stratified\_survival\_subject\_public.csv **Recommended public name:** albumin **Description:** Serum albumin concentration used in the public ESLD stratified survival dataset. **Unit:** g/dL **Value coding:** Numeric concentration in g/dL. **Data type:** numeric **Example values:** 2.86 \| 3.4 \| 2.21 \| 2.89 \| 2.53 **Allowed values:**  **Missing-value coding:** NA/blank as read by fread **n rows / missing / unique:** 841 / 645 / 149 **Release status:** public **Definition status:** manual\_final\_definition | 02\_workflows/F5\_workflow\_v01/data/02b\_figure\_content/esld\_F5\_stratified\_survival\_subject\_public.csv | g/dL | numeric |
| bilirubin — Total bilirubin concentration **File:** 02\_workflows/F5\_workflow\_v01/data/02b\_figure\_content/esld\_F5\_stratified\_survival\_subject\_public.csv **Recommended public name:** bilirubin **Description:** Total bilirubin concentration used in the public ESLD analysis data. **Unit:** mg/dL **Value coding:** Numeric concentration in mg/dL. **Data type:** numeric **Example values:** 0.5 \| 1.7 \| 0.6 \| 0.86 \| 0.87 **Allowed values:**  **Missing-value coding:** NA/blank as read by fread **n rows / missing / unique:** 841 / 0 / 254 **Release status:** public **Definition status:** manual\_precision\_definition | 02\_workflows/F5\_workflow\_v01/data/02b\_figure\_content/esld\_F5\_stratified\_survival\_subject\_public.csv | mg/dL | numeric |
| case\_id — Public case pseudonym **File:** 02\_workflows/F5\_workflow\_v01/data/02b\_figure\_content/esld\_F5\_stratified\_survival\_subject\_public.csv **Recommended public name:** case\_id **Description:** Non-linkable public-release case pseudonym used for case-level grouping; not an original hospital case identifier. **Unit:**  **Value coding:** See example\_values and allowed\_values. **Data type:** integer **Example values:** 1 \| 2 \| 3 \| 4 \| 5 **Allowed values:**  **Missing-value coding:** NA/blank as read by fread **n rows / missing / unique:** 841 / 0 / 841 **Release status:** public **Definition status:** exact\_definition | 02\_workflows/F5\_workflow\_v01/data/02b\_figure\_content/esld\_F5\_stratified\_survival\_subject\_public.csv |  | integer |
| creatinine\_corrected — Corrected creatinine concentration **File:** 02\_workflows/F5\_workflow\_v01/data/02b\_figure\_content/esld\_F5\_stratified\_survival\_subject\_public.csv **Recommended public name:** creatinine\_corrected **Description:** Corrected creatinine concentration used for recalculated MELD-related scores. **Unit:** mg/dL **Value coding:** Numeric concentration in mg/dL. **Data type:** numeric **Example values:** 0.698460325475166 \| 1.02098453959733 \| 0.657275352328835 \| 1.54121277346097 \| 1.05977483679343 **Allowed values:**  **Missing-value coding:** NA/blank as read by fread **n rows / missing / unique:** 841 / 0 / 349 **Release status:** public **Definition status:** manual\_precision\_definition | 02\_workflows/F5\_workflow\_v01/data/02b\_figure\_content/esld\_F5\_stratified\_survival\_subject\_public.csv | mg/dL | numeric |
| creatinine\_raw — Raw creatinine concentration **File:** 02\_workflows/F5\_workflow\_v01/data/02b\_figure\_content/esld\_F5\_stratified\_survival\_subject\_public.csv **Recommended public name:** creatinine\_raw **Description:** Raw measured creatinine concentration before applying the study-specific correction. **Unit:** mg/dL **Value coding:** Numeric concentration in mg/dL. **Data type:** numeric **Example values:** 0.68 \| 1.02 \| 0.64 \| 1.55 \| 1.05 **Allowed values:**  **Missing-value coding:** NA/blank as read by fread **n rows / missing / unique:** 841 / 0 / 240 **Release status:** public **Definition status:** manual\_precision\_definition | 02\_workflows/F5\_workflow\_v01/data/02b\_figure\_content/esld\_F5\_stratified\_survival\_subject\_public.csv | mg/dL | numeric |
| delta.grp — Score-delta comparison group **File:** 02\_workflows/F5\_workflow\_v01/data/02b\_figure\_content/esld\_F5\_stratified\_survival\_subject\_public.csv **Recommended public name:** delta.grp **Description:** Encoded score-delta comparison group used in F5; examples include pm and m1 as defined by the workflow grouping. **Unit:**  **Value coding:** Categorical group code as encoded by the F5 workflow. **Data type:** character **Example values:** pm \| m1 **Allowed values:** pm \| m1 **Missing-value coding:** NA/blank as read by fread **n rows / missing / unique:** 841 / 0 / 2 **Release status:** public **Definition status:** manual\_precision\_definition | 02\_workflows/F5\_workflow\_v01/data/02b\_figure\_content/esld\_F5\_stratified\_survival\_subject\_public.csv |  | character |
| delta\_group\_label — Displayed score-delta group label **File:** 02\_workflows/F5\_workflow\_v01/data/02b\_figure\_content/esld\_F5\_stratified\_survival\_subject\_public.csv **Recommended public name:** delta\_group\_label **Description:** Human-readable score-delta group label displayed in the stratified survival figure. **Unit:**  **Value coding:** Display label. **Data type:** character **Example values:** Δ score = ± 0 \| Δ score ≤ -1 **Allowed values:** Δ score = ± 0 \| Δ score ≤ -1 **Missing-value coding:** NA/blank as read by fread **n rows / missing / unique:** 841 / 0 / 2 **Release status:** public **Definition status:** manual\_precision\_definition | 02\_workflows/F5\_workflow\_v01/data/02b\_figure\_content/esld\_F5\_stratified\_survival\_subject\_public.csv |  | character |
| dialysis — Dialysis indicator **File:** 02\_workflows/F5\_workflow\_v01/data/02b\_figure\_content/esld\_F5\_stratified\_survival\_subject\_public.csv **Recommended public name:** dialysis **Description:** Binary dialysis indicator used for MELD-related score calculation or stratified public analyses. **Unit:**  **Value coding:** 0 = no; 1 = yes. **Data type:** integer **Example values:** 0 \| 1 **Allowed values:**  **Missing-value coding:** NA/blank as read by fread **n rows / missing / unique:** 841 / 395 / 2 **Release status:** public **Definition status:** manual\_precision\_definition | 02\_workflows/F5\_workflow\_v01/data/02b\_figure\_content/esld\_F5\_stratified\_survival\_subject\_public.csv |  | integer |
| inr — International normalized ratio **File:** 02\_workflows/F5\_workflow\_v01/data/02b\_figure\_content/esld\_F5\_stratified\_survival\_subject\_public.csv **Recommended public name:** inr **Description:** International normalized ratio of prothrombin time used for MELD-related score calculation. **Unit:** ratio **Value coding:** Numeric INR value. **Data type:** numeric **Example values:** 1.15 \| 1.22 \| 1.79 \| 1.23 \| 1.69 **Allowed values:**  **Missing-value coding:** NA/blank as read by fread **n rows / missing / unique:** 841 / 0 / 196 **Release status:** public **Definition status:** manual\_exact\_definition | 02\_workflows/F5\_workflow\_v01/data/02b\_figure\_content/esld\_F5\_stratified\_survival\_subject\_public.csv | ratio | numeric |
| model — MELD model or score variant **File:** 02\_workflows/F5\_workflow\_v01/data/02b\_figure\_content/esld\_F5\_stratified\_survival\_subject\_public.csv **Recommended public name:** model **Description:** Name of the MELD-related model or score variant represented by the row; expected values include MELD, MELD-Na, reMELD-Na, and MELD 3.0. **Unit:**  **Value coding:** Categorical score/model label, for example MELD, MELD-Na, reMELD-Na, or MELD 3.0. **Data type:** character **Example values:** MELD \| MELD-Na \| reMELD-Na \| MELD 3.0 **Allowed values:** MELD \| MELD-Na \| reMELD-Na \| MELD 3.0 **Missing-value coding:** NA/blank as read by fread **n rows / missing / unique:** 841 / 0 / 4 **Release status:** public **Definition status:** manual\_exact\_definition | 02\_workflows/F5\_workflow\_v01/data/02b\_figure\_content/esld\_F5\_stratified\_survival\_subject\_public.csv |  | character |
| patient\_id — Public patient pseudonym **File:** 02\_workflows/F5\_workflow\_v01/data/02b\_figure\_content/esld\_F5\_stratified\_survival\_subject\_public.csv **Recommended public name:** patient\_id **Description:** Non-linkable public-release patient pseudonym used to preserve within-patient grouping in public ESLD data; not an original hospital patient identifier. **Unit:**  **Value coding:** See example\_values and allowed\_values. **Data type:** character **Example values:** P001277 \| P000525 \| P000302 \| P001040 \| P000988 **Allowed values:** P001277 \| P000525 \| P000302 \| P001040 \| P000988 \| P000384 \| P000766 \| P000386 \| P000496 \| P000131 **Missing-value coding:** NA/blank as read by fread **n rows / missing / unique:** 841 / 0 / 232 **Release status:** public **Definition status:** exact\_definition | 02\_workflows/F5\_workflow\_v01/data/02b\_figure\_content/esld\_F5\_stratified\_survival\_subject\_public.csv |  | character |
| score\_class — Score class **File:** 02\_workflows/F5\_workflow\_v01/data/02b\_figure\_content/esld\_F5\_stratified\_survival\_subject\_public.csv **Recommended public name:** score\_class **Description:** Categorical score class used for stratified analyses. **Unit:**  **Value coding:** Score or category class as represented in the data file. **Data type:** character **Example values:** ≤ 15 \| 16 - 25 \| > 25 **Allowed values:** ≤ 15 \| 16 - 25 \| > 25 **Missing-value coding:** NA/blank as read by fread **n rows / missing / unique:** 841 / 0 / 3 **Release status:** public **Definition status:** exact\_definition | 02\_workflows/F5\_workflow\_v01/data/02b\_figure\_content/esld\_F5\_stratified\_survival\_subject\_public.csv |  | character |
| score\_for\_class — Score used for class assignment **File:** 02\_workflows/F5\_workflow\_v01/data/02b\_figure\_content/esld\_F5\_stratified\_survival\_subject\_public.csv **Recommended public name:** score\_for\_class **Description:** Score value used to assign the row to a score class. **Unit:** score points **Value coding:** Numeric score. **Data type:** integer **Example values:** 8 \| 11 \| 13 \| 14 \| 7 **Allowed values:**  **Missing-value coding:** NA/blank as read by fread **n rows / missing / unique:** 841 / 0 / 37 **Release status:** public **Definition status:** manual\_precision\_definition | 02\_workflows/F5\_workflow\_v01/data/02b\_figure\_content/esld\_F5\_stratified\_survival\_subject\_public.csv | score points | integer |
| scr.grp — Score-class stratum **File:** 02\_workflows/F5\_workflow\_v01/data/02b\_figure\_content/esld\_F5\_stratified\_survival\_subject\_public.csv **Recommended public name:** scr.grp **Description:** Encoded score-class stratum used in F5; examples include le15, 16to25, and gt25. **Unit:**  **Value coding:** Categorical stratum code. **Data type:** character **Example values:** le15 \| 16to25 \| gt25 **Allowed values:** le15 \| 16to25 \| gt25 **Missing-value coding:** NA/blank as read by fread **n rows / missing / unique:** 841 / 0 / 3 **Release status:** public **Definition status:** manual\_precision\_definition | 02\_workflows/F5\_workflow\_v01/data/02b\_figure\_content/esld\_F5\_stratified\_survival\_subject\_public.csv |  | character |
| sex — Sex **File:** 02\_workflows/F5\_workflow\_v01/data/02b\_figure\_content/esld\_F5\_stratified\_survival\_subject\_public.csv **Recommended public name:** sex **Description:** Sex category represented in the public ESLD data. **Unit:**  **Value coding:** M = male; F = female. **Data type:** character **Example values:** M \| F **Allowed values:** M \| F **Missing-value coding:** NA/blank as read by fread **n rows / missing / unique:** 841 / 0 / 2 **Release status:** public **Definition status:** manual\_exact\_definition | 02\_workflows/F5\_workflow\_v01/data/02b\_figure\_content/esld\_F5\_stratified\_survival\_subject\_public.csv |  | character |
| sodium — Serum sodium concentration **File:** 02\_workflows/F5\_workflow\_v01/data/02b\_figure\_content/esld\_F5\_stratified\_survival\_subject\_public.csv **Recommended public name:** sodium **Description:** Serum sodium concentration used for MELD-Na, reMELD-Na, or MELD 3.0 score calculation. **Unit:** mmol/L **Value coding:** Numeric concentration in mmol/L. **Data type:** numeric **Example values:** 143 \| 142 \| 138 \| 151 \| 145 **Allowed values:**  **Missing-value coding:** NA/blank as read by fread **n rows / missing / unique:** 841 / 225 / 66 **Release status:** public **Definition status:** manual\_exact\_definition | 02\_workflows/F5\_workflow\_v01/data/02b\_figure\_content/esld\_F5\_stratified\_survival\_subject\_public.csv | mmol/L | numeric |
| source\_file — Source file **File:** 02\_workflows/F5\_workflow\_v01/data/02b\_figure\_content/esld\_F5\_stratified\_survival\_subject\_public.csv **Recommended public name:** source\_file **Description:** Name of the source file used to create the public row or object. **Unit:**  **Value coding:** File name. **Data type:** character **Example values:** esld\_master\_long\_public.csv **Allowed values:** esld\_master\_long\_public.csv **Missing-value coding:** NA/blank as read by fread **n rows / missing / unique:** 841 / 0 / 1 **Release status:** public **Definition status:** manual\_precision\_definition | 02\_workflows/F5\_workflow\_v01/data/02b\_figure\_content/esld\_F5\_stratified\_survival\_subject\_public.csv |  | character |
| status — Status flag **File:** 02\_workflows/F5\_workflow\_v01/data/02b\_figure\_content/esld\_F5\_stratified\_survival\_subject\_public.csv **Recommended public name:** status **Description:** Binary or categorical status flag used in a public QC, manifest, or analysis table. **Unit:**  **Value coding:** Coding as represented in the data file. **Data type:** integer **Example values:** 1 \| 0 **Allowed values:**  **Missing-value coding:** NA/blank as read by fread **n rows / missing / unique:** 841 / 0 / 2 **Release status:** public **Definition status:** manual\_precision\_definition | 02\_workflows/F5\_workflow\_v01/data/02b\_figure\_content/esld\_F5\_stratified\_survival\_subject\_public.csv |  | integer |
| time — Analysis time **File:** 02\_workflows/F5\_workflow\_v01/data/02b\_figure\_content/esld\_F5\_stratified\_survival\_subject\_public.csv **Recommended public name:** time **Description:** Analysis-specific time scale; interpretation depends on the respective figure/table workflow. **Unit:**  **Value coding:** See example\_values and allowed\_values. **Data type:** integer **Example values:** 1 \| 6 \| 7 \| 13 \| 15 **Allowed values:**  **Missing-value coding:** NA/blank as read by fread **n rows / missing / unique:** 841 / 0 / 124 **Release status:** public **Definition status:** exact\_definition | 02\_workflows/F5\_workflow\_v01/data/02b\_figure\_content/esld\_F5\_stratified\_survival\_subject\_public.csv |  | integer |
| parameter — Metadata parameter name **File:** 02\_workflows/F5\_workflow\_v01/submission\_ready/public/data/esld\_F5\_stratified\_survival\_meta\_public.csv **Recommended public name:** parameter **Description:** Name of a metadata parameter describing the F2 simulated heatmap object, such as figure identity, data origin, grid type, axis variable, or unit/role. **Unit:**  **Value coding:** Categorical metadata key; value is given in the corresponding value column or file-specific metadata field. **Data type:** character **Example values:** dataset\_subject \| dataset\_stats \| domain \| anchor \| data\_object **Allowed values:** dataset\_subject \| dataset\_stats \| domain \| anchor \| data\_object \| unit\_or\_role\_subject \| unit\_or\_role\_stats \| release\_status \| source\_master \| n\_subject\_rows **Missing-value coding:** NA/blank as read by fread **n rows / missing / unique:** 12 / 0 / 12 **Release status:** public **Definition status:** manual\_exact\_definition | 02\_workflows/F5\_workflow\_v01/submission\_ready/public/data/esld\_F5\_stratified\_survival\_meta\_public.csv |  | character |
| value — Value **File:** 02\_workflows/F5\_workflow\_v01/submission\_ready/public/data/esld\_F5\_stratified\_survival\_meta\_public.csv **Recommended public name:** value **Description:** Numerical or character value corresponding to the row-specific variable/metric. **Unit:**  **Value coding:** See example\_values and allowed\_values. **Data type:** character **Example values:** esld\_F5\_stratified\_survival\_subject\_public \| esld\_F5\_stratified\_survival\_stats\_public \| esld \| F5 \| stratified\_survival **Allowed values:** esld\_F5\_stratified\_survival\_subject\_public \| esld\_F5\_stratified\_survival\_stats\_public \| esld \| F5 \| stratified\_survival \| subject \| stats \| public \| esld\_master\_long\_public.csv \| 841 **Missing-value coding:** NA/blank as read by fread **n rows / missing / unique:** 12 / 0 / 12 **Release status:** public **Definition status:** exact\_definition | 02\_workflows/F5\_workflow\_v01/submission\_ready/public/data/esld\_F5\_stratified\_survival\_meta\_public.csv |  | character |
| chisq — Chi-square statistic **File:** 02\_workflows/F5\_workflow\_v01/submission\_ready/public/data/esld\_F5\_stratified\_survival\_stats\_public.csv **Recommended public name:** chisq **Description:** Chi-square statistic for the row-specific survival comparison or stratified test. **Unit:** chi-square statistic **Value coding:** Numeric chi-square statistic. **Data type:** numeric **Example values:** 0.00770313568089429 \| 2.87624703252109 \| 12.1826833766653 \| 0.00169868292629352 \| 0.733720432338734 **Allowed values:**  **Missing-value coding:** NA/blank as read by fread **n rows / missing / unique:** 12 / 0 / 12 **Release status:** public **Definition status:** manual\_precision\_definition | 02\_workflows/F5\_workflow\_v01/submission\_ready/public/data/esld\_F5\_stratified\_survival\_stats\_public.csv | chi-square statistic | numeric |
| chisq\_lab — Formatted chi-square label **File:** 02\_workflows/F5\_workflow\_v01/submission\_ready/public/data/esld\_F5\_stratified\_survival\_stats\_public.csv **Recommended public name:** chisq\_lab **Description:** Formatted display label for the chi-square statistic shown in a table or figure annotation. **Unit:**  **Value coding:** Formatted character label. **Data type:** character **Example values:** χ² = 0.008 \| χ² = 2.876 \| χ² = 12.183 \| χ² = 0.002 \| χ² = 0.734 **Allowed values:** χ² = 0.008 \| χ² = 2.876 \| χ² = 12.183 \| χ² = 0.002 \| χ² = 0.734 \| χ² = 7.752 \| χ² = 0.964 \| χ² = 8.044 \| χ² = 13.427 \| χ² = 0.413 **Missing-value coding:** NA/blank as read by fread **n rows / missing / unique:** 12 / 0 / 12 **Release status:** public **Definition status:** manual\_precision\_definition | 02\_workflows/F5\_workflow\_v01/submission\_ready/public/data/esld\_F5\_stratified\_survival\_stats\_public.csv |  | character |
| model — MELD model or score variant **File:** 02\_workflows/F5\_workflow\_v01/submission\_ready/public/data/esld\_F5\_stratified\_survival\_stats\_public.csv **Recommended public name:** model **Description:** Name of the MELD-related model or score variant represented by the row; expected values include MELD, MELD-Na, reMELD-Na, and MELD 3.0. **Unit:**  **Value coding:** Categorical score/model label, for example MELD, MELD-Na, reMELD-Na, or MELD 3.0. **Data type:** character **Example values:** MELD \| MELD-Na \| reMELD-Na \| MELD 3.0 **Allowed values:** MELD \| MELD-Na \| reMELD-Na \| MELD 3.0 **Missing-value coding:** NA/blank as read by fread **n rows / missing / unique:** 12 / 0 / 4 **Release status:** public **Definition status:** manual\_exact\_definition | 02\_workflows/F5\_workflow\_v01/submission\_ready/public/data/esld\_F5\_stratified\_survival\_stats\_public.csv |  | character |
| n\_m1 — Number of observations in m1 group **File:** 02\_workflows/F5\_workflow\_v01/submission\_ready/public/data/esld\_F5\_stratified\_survival\_stats\_public.csv **Recommended public name:** n\_m1 **Description:** Number of observations in the m1 comparison group for the row-specific figure/table stratum. **Unit:** count **Value coding:** Integer count. **Data type:** integer **Example values:** 12 \| 64 \| 82 \| 7 \| 47 **Allowed values:**  **Missing-value coding:** NA/blank as read by fread **n rows / missing / unique:** 12 / 0 / 12 **Release status:** public **Definition status:** manual\_precision\_definition | 02\_workflows/F5\_workflow\_v01/submission\_ready/public/data/esld\_F5\_stratified\_survival\_stats\_public.csv | count | integer |
| n\_pm — Number of observations in pm group **File:** 02\_workflows/F5\_workflow\_v01/submission\_ready/public/data/esld\_F5\_stratified\_survival\_stats\_public.csv **Recommended public name:** n\_pm **Description:** Number of observations in the pm comparison group for the row-specific figure/table stratum. **Unit:** count **Value coding:** Integer count. **Data type:** integer **Example values:** 25 \| 31 \| 11 \| 27 \| 39 **Allowed values:**  **Missing-value coding:** NA/blank as read by fread **n rows / missing / unique:** 12 / 0 / 11 **Release status:** public **Definition status:** manual\_precision\_definition | 02\_workflows/F5\_workflow\_v01/submission\_ready/public/data/esld\_F5\_stratified\_survival\_stats\_public.csv | count | integer |
| p\_lab — Formatted p-value label **File:** 02\_workflows/F5\_workflow\_v01/submission\_ready/public/data/esld\_F5\_stratified\_survival\_stats\_public.csv **Recommended public name:** p\_lab **Description:** Formatted p-value label shown in a table or figure annotation. **Unit:**  **Value coding:** Formatted p-value label. **Data type:** character **Example values:** p = 0.930 \| p = 0.090 \| p < 0.001 \| p = 0.967 \| p = 0.392 **Allowed values:** p = 0.930 \| p = 0.090 \| p < 0.001 \| p = 0.967 \| p = 0.392 \| p = 0.005 \| p = 0.326 \| p = 0.521 \| p = 0.046 \| p = 0.052 **Missing-value coding:** NA/blank as read by fread **n rows / missing / unique:** 12 / 0 / 10 **Release status:** public **Definition status:** manual\_precision\_definition | 02\_workflows/F5\_workflow\_v01/submission\_ready/public/data/esld\_F5\_stratified\_survival\_stats\_public.csv |  | character |
| pval — P value **File:** 02\_workflows/F5\_workflow\_v01/submission\_ready/public/data/esld\_F5\_stratified\_survival\_stats\_public.csv **Recommended public name:** pval **Description:** P value for the row-specific statistical comparison. **Unit:** replicate number **Value coding:** Numeric probability value. **Data type:** numeric **Example values:** 0.930061461937626 \| 0.0898953142885014 \| 0.000482352058876657 \| 0.967124430752549 \| 0.391679903320669 **Allowed values:**  **Missing-value coding:** NA/blank as read by fread **n rows / missing / unique:** 12 / 0 / 12 **Release status:** public **Definition status:** manual\_precision\_definition | 02\_workflows/F5\_workflow\_v01/submission\_ready/public/data/esld\_F5\_stratified\_survival\_stats\_public.csv | replicate number | numeric |
| scr.grp — Score-class stratum **File:** 02\_workflows/F5\_workflow\_v01/submission\_ready/public/data/esld\_F5\_stratified\_survival\_stats\_public.csv **Recommended public name:** scr.grp **Description:** Encoded score-class stratum used in F5; examples include le15, 16to25, and gt25. **Unit:**  **Value coding:** Categorical stratum code. **Data type:** character **Example values:** le15 \| 16to25 \| gt25 **Allowed values:** le15 \| 16to25 \| gt25 **Missing-value coding:** NA/blank as read by fread **n rows / missing / unique:** 12 / 0 / 3 **Release status:** public **Definition status:** manual\_precision\_definition | 02\_workflows/F5\_workflow\_v01/submission\_ready/public/data/esld\_F5\_stratified\_survival\_stats\_public.csv |  | character |
| albumin — Serum albumin concentration **File:** 02\_workflows/F5\_workflow\_v01/submission\_ready/public/data/esld\_F5\_stratified\_survival\_subject\_public.csv **Recommended public name:** albumin **Description:** Serum albumin concentration used in the public ESLD stratified survival dataset. **Unit:** g/dL **Value coding:** Numeric concentration in g/dL. **Data type:** numeric **Example values:** 2.86 \| 3.4 \| 2.21 \| 2.89 \| 2.53 **Allowed values:**  **Missing-value coding:** NA/blank as read by fread **n rows / missing / unique:** 841 / 645 / 149 **Release status:** public **Definition status:** manual\_final\_definition | 02\_workflows/F5\_workflow\_v01/submission\_ready/public/data/esld\_F5\_stratified\_survival\_subject\_public.csv | g/dL | numeric |
| bilirubin — Total bilirubin concentration **File:** 02\_workflows/F5\_workflow\_v01/submission\_ready/public/data/esld\_F5\_stratified\_survival\_subject\_public.csv **Recommended public name:** bilirubin **Description:** Total bilirubin concentration used in the public ESLD analysis data. **Unit:** mg/dL **Value coding:** Numeric concentration in mg/dL. **Data type:** numeric **Example values:** 0.5 \| 1.7 \| 0.6 \| 0.86 \| 0.87 **Allowed values:**  **Missing-value coding:** NA/blank as read by fread **n rows / missing / unique:** 841 / 0 / 254 **Release status:** public **Definition status:** manual\_precision\_definition | 02\_workflows/F5\_workflow\_v01/submission\_ready/public/data/esld\_F5\_stratified\_survival\_subject\_public.csv | mg/dL | numeric |
| case\_id — Public case pseudonym **File:** 02\_workflows/F5\_workflow\_v01/submission\_ready/public/data/esld\_F5\_stratified\_survival\_subject\_public.csv **Recommended public name:** case\_id **Description:** Non-linkable public-release case pseudonym used for case-level grouping; not an original hospital case identifier. **Unit:**  **Value coding:** See example\_values and allowed\_values. **Data type:** integer **Example values:** 1 \| 2 \| 3 \| 4 \| 5 **Allowed values:**  **Missing-value coding:** NA/blank as read by fread **n rows / missing / unique:** 841 / 0 / 841 **Release status:** public **Definition status:** exact\_definition | 02\_workflows/F5\_workflow\_v01/submission\_ready/public/data/esld\_F5\_stratified\_survival\_subject\_public.csv |  | integer |
| creatinine\_corrected — Corrected creatinine concentration **File:** 02\_workflows/F5\_workflow\_v01/submission\_ready/public/data/esld\_F5\_stratified\_survival\_subject\_public.csv **Recommended public name:** creatinine\_corrected **Description:** Corrected creatinine concentration used for recalculated MELD-related scores. **Unit:** mg/dL **Value coding:** Numeric concentration in mg/dL. **Data type:** numeric **Example values:** 0.698460325475166 \| 1.02098453959733 \| 0.657275352328835 \| 1.54121277346097 \| 1.05977483679343 **Allowed values:**  **Missing-value coding:** NA/blank as read by fread **n rows / missing / unique:** 841 / 0 / 349 **Release status:** public **Definition status:** manual\_precision\_definition | 02\_workflows/F5\_workflow\_v01/submission\_ready/public/data/esld\_F5\_stratified\_survival\_subject\_public.csv | mg/dL | numeric |
| creatinine\_raw — Raw creatinine concentration **File:** 02\_workflows/F5\_workflow\_v01/submission\_ready/public/data/esld\_F5\_stratified\_survival\_subject\_public.csv **Recommended public name:** creatinine\_raw **Description:** Raw measured creatinine concentration before applying the study-specific correction. **Unit:** mg/dL **Value coding:** Numeric concentration in mg/dL. **Data type:** numeric **Example values:** 0.68 \| 1.02 \| 0.64 \| 1.55 \| 1.05 **Allowed values:**  **Missing-value coding:** NA/blank as read by fread **n rows / missing / unique:** 841 / 0 / 240 **Release status:** public **Definition status:** manual\_precision\_definition | 02\_workflows/F5\_workflow\_v01/submission\_ready/public/data/esld\_F5\_stratified\_survival\_subject\_public.csv | mg/dL | numeric |
| delta.grp — Score-delta comparison group **File:** 02\_workflows/F5\_workflow\_v01/submission\_ready/public/data/esld\_F5\_stratified\_survival\_subject\_public.csv **Recommended public name:** delta.grp **Description:** Encoded score-delta comparison group used in F5; examples include pm and m1 as defined by the workflow grouping. **Unit:**  **Value coding:** Categorical group code as encoded by the F5 workflow. **Data type:** character **Example values:** pm \| m1 **Allowed values:** pm \| m1 **Missing-value coding:** NA/blank as read by fread **n rows / missing / unique:** 841 / 0 / 2 **Release status:** public **Definition status:** manual\_precision\_definition | 02\_workflows/F5\_workflow\_v01/submission\_ready/public/data/esld\_F5\_stratified\_survival\_subject\_public.csv |  | character |
| delta\_group\_label — Displayed score-delta group label **File:** 02\_workflows/F5\_workflow\_v01/submission\_ready/public/data/esld\_F5\_stratified\_survival\_subject\_public.csv **Recommended public name:** delta\_group\_label **Description:** Human-readable score-delta group label displayed in the stratified survival figure. **Unit:**  **Value coding:** Display label. **Data type:** character **Example values:** Δ score = ± 0 \| Δ score ≤ -1 **Allowed values:** Δ score = ± 0 \| Δ score ≤ -1 **Missing-value coding:** NA/blank as read by fread **n rows / missing / unique:** 841 / 0 / 2 **Release status:** public **Definition status:** manual\_precision\_definition | 02\_workflows/F5\_workflow\_v01/submission\_ready/public/data/esld\_F5\_stratified\_survival\_subject\_public.csv |  | character |
| dialysis — Dialysis indicator **File:** 02\_workflows/F5\_workflow\_v01/submission\_ready/public/data/esld\_F5\_stratified\_survival\_subject\_public.csv **Recommended public name:** dialysis **Description:** Binary dialysis indicator used for MELD-related score calculation or stratified public analyses. **Unit:**  **Value coding:** 0 = no; 1 = yes. **Data type:** integer **Example values:** 0 \| 1 **Allowed values:**  **Missing-value coding:** NA/blank as read by fread **n rows / missing / unique:** 841 / 395 / 2 **Release status:** public **Definition status:** manual\_precision\_definition | 02\_workflows/F5\_workflow\_v01/submission\_ready/public/data/esld\_F5\_stratified\_survival\_subject\_public.csv |  | integer |
| inr — International normalized ratio **File:** 02\_workflows/F5\_workflow\_v01/submission\_ready/public/data/esld\_F5\_stratified\_survival\_subject\_public.csv **Recommended public name:** inr **Description:** International normalized ratio of prothrombin time used for MELD-related score calculation. **Unit:** ratio **Value coding:** Numeric INR value. **Data type:** numeric **Example values:** 1.15 \| 1.22 \| 1.79 \| 1.23 \| 1.69 **Allowed values:**  **Missing-value coding:** NA/blank as read by fread **n rows / missing / unique:** 841 / 0 / 196 **Release status:** public **Definition status:** manual\_exact\_definition | 02\_workflows/F5\_workflow\_v01/submission\_ready/public/data/esld\_F5\_stratified\_survival\_subject\_public.csv | ratio | numeric |
| model — MELD model or score variant **File:** 02\_workflows/F5\_workflow\_v01/submission\_ready/public/data/esld\_F5\_stratified\_survival\_subject\_public.csv **Recommended public name:** model **Description:** Name of the MELD-related model or score variant represented by the row; expected values include MELD, MELD-Na, reMELD-Na, and MELD 3.0. **Unit:**  **Value coding:** Categorical score/model label, for example MELD, MELD-Na, reMELD-Na, or MELD 3.0. **Data type:** character **Example values:** MELD \| MELD-Na \| reMELD-Na \| MELD 3.0 **Allowed values:** MELD \| MELD-Na \| reMELD-Na \| MELD 3.0 **Missing-value coding:** NA/blank as read by fread **n rows / missing / unique:** 841 / 0 / 4 **Release status:** public **Definition status:** manual\_exact\_definition | 02\_workflows/F5\_workflow\_v01/submission\_ready/public/data/esld\_F5\_stratified\_survival\_subject\_public.csv |  | character |
| patient\_id — Public patient pseudonym **File:** 02\_workflows/F5\_workflow\_v01/submission\_ready/public/data/esld\_F5\_stratified\_survival\_subject\_public.csv **Recommended public name:** patient\_id **Description:** Non-linkable public-release patient pseudonym used to preserve within-patient grouping in public ESLD data; not an original hospital patient identifier. **Unit:**  **Value coding:** See example\_values and allowed\_values. **Data type:** character **Example values:** P001277 \| P000525 \| P000302 \| P001040 \| P000988 **Allowed values:** P001277 \| P000525 \| P000302 \| P001040 \| P000988 \| P000384 \| P000766 \| P000386 \| P000496 \| P000131 **Missing-value coding:** NA/blank as read by fread **n rows / missing / unique:** 841 / 0 / 232 **Release status:** public **Definition status:** exact\_definition | 02\_workflows/F5\_workflow\_v01/submission\_ready/public/data/esld\_F5\_stratified\_survival\_subject\_public.csv |  | character |
| score\_class — Score class **File:** 02\_workflows/F5\_workflow\_v01/submission\_ready/public/data/esld\_F5\_stratified\_survival\_subject\_public.csv **Recommended public name:** score\_class **Description:** Categorical score class used for stratified analyses. **Unit:**  **Value coding:** Score or category class as represented in the data file. **Data type:** character **Example values:** ≤ 15 \| 16 - 25 \| > 25 **Allowed values:** ≤ 15 \| 16 - 25 \| > 25 **Missing-value coding:** NA/blank as read by fread **n rows / missing / unique:** 841 / 0 / 3 **Release status:** public **Definition status:** exact\_definition | 02\_workflows/F5\_workflow\_v01/submission\_ready/public/data/esld\_F5\_stratified\_survival\_subject\_public.csv |  | character |
| score\_for\_class — Score used for class assignment **File:** 02\_workflows/F5\_workflow\_v01/submission\_ready/public/data/esld\_F5\_stratified\_survival\_subject\_public.csv **Recommended public name:** score\_for\_class **Description:** Score value used to assign the row to a score class. **Unit:** score points **Value coding:** Numeric score. **Data type:** integer **Example values:** 8 \| 11 \| 13 \| 14 \| 7 **Allowed values:**  **Missing-value coding:** NA/blank as read by fread **n rows / missing / unique:** 841 / 0 / 37 **Release status:** public **Definition status:** manual\_precision\_definition | 02\_workflows/F5\_workflow\_v01/submission\_ready/public/data/esld\_F5\_stratified\_survival\_subject\_public.csv | score points | integer |
| scr.grp — Score-class stratum **File:** 02\_workflows/F5\_workflow\_v01/submission\_ready/public/data/esld\_F5\_stratified\_survival\_subject\_public.csv **Recommended public name:** scr.grp **Description:** Encoded score-class stratum used in F5; examples include le15, 16to25, and gt25. **Unit:**  **Value coding:** Categorical stratum code. **Data type:** character **Example values:** le15 \| 16to25 \| gt25 **Allowed values:** le15 \| 16to25 \| gt25 **Missing-value coding:** NA/blank as read by fread **n rows / missing / unique:** 841 / 0 / 3 **Release status:** public **Definition status:** manual\_precision\_definition | 02\_workflows/F5\_workflow\_v01/submission\_ready/public/data/esld\_F5\_stratified\_survival\_subject\_public.csv |  | character |
| sex — Sex **File:** 02\_workflows/F5\_workflow\_v01/submission\_ready/public/data/esld\_F5\_stratified\_survival\_subject\_public.csv **Recommended public name:** sex **Description:** Sex category represented in the public ESLD data. **Unit:**  **Value coding:** M = male; F = female. **Data type:** character **Example values:** M \| F **Allowed values:** M \| F **Missing-value coding:** NA/blank as read by fread **n rows / missing / unique:** 841 / 0 / 2 **Release status:** public **Definition status:** manual\_exact\_definition | 02\_workflows/F5\_workflow\_v01/submission\_ready/public/data/esld\_F5\_stratified\_survival\_subject\_public.csv |  | character |
| sodium — Serum sodium concentration **File:** 02\_workflows/F5\_workflow\_v01/submission\_ready/public/data/esld\_F5\_stratified\_survival\_subject\_public.csv **Recommended public name:** sodium **Description:** Serum sodium concentration used for MELD-Na, reMELD-Na, or MELD 3.0 score calculation. **Unit:** mmol/L **Value coding:** Numeric concentration in mmol/L. **Data type:** numeric **Example values:** 143 \| 142 \| 138 \| 151 \| 145 **Allowed values:**  **Missing-value coding:** NA/blank as read by fread **n rows / missing / unique:** 841 / 225 / 66 **Release status:** public **Definition status:** manual\_exact\_definition | 02\_workflows/F5\_workflow\_v01/submission\_ready/public/data/esld\_F5\_stratified\_survival\_subject\_public.csv | mmol/L | numeric |
| source\_file — Source file **File:** 02\_workflows/F5\_workflow\_v01/submission\_ready/public/data/esld\_F5\_stratified\_survival\_subject\_public.csv **Recommended public name:** source\_file **Description:** Name of the source file used to create the public row or object. **Unit:**  **Value coding:** File name. **Data type:** character **Example values:** esld\_master\_long\_public.csv **Allowed values:** esld\_master\_long\_public.csv **Missing-value coding:** NA/blank as read by fread **n rows / missing / unique:** 841 / 0 / 1 **Release status:** public **Definition status:** manual\_precision\_definition | 02\_workflows/F5\_workflow\_v01/submission\_ready/public/data/esld\_F5\_stratified\_survival\_subject\_public.csv |  | character |
| status — Status flag **File:** 02\_workflows/F5\_workflow\_v01/submission\_ready/public/data/esld\_F5\_stratified\_survival\_subject\_public.csv **Recommended public name:** status **Description:** Binary or categorical status flag used in a public QC, manifest, or analysis table. **Unit:**  **Value coding:** Coding as represented in the data file. **Data type:** integer **Example values:** 1 \| 0 **Allowed values:**  **Missing-value coding:** NA/blank as read by fread **n rows / missing / unique:** 841 / 0 / 2 **Release status:** public **Definition status:** manual\_precision\_definition | 02\_workflows/F5\_workflow\_v01/submission\_ready/public/data/esld\_F5\_stratified\_survival\_subject\_public.csv |  | integer |
| time — Analysis time **File:** 02\_workflows/F5\_workflow\_v01/submission\_ready/public/data/esld\_F5\_stratified\_survival\_subject\_public.csv **Recommended public name:** time **Description:** Analysis-specific time scale; interpretation depends on the respective figure/table workflow. **Unit:**  **Value coding:** See example\_values and allowed\_values. **Data type:** integer **Example values:** 1 \| 6 \| 7 \| 13 \| 15 **Allowed values:**  **Missing-value coding:** NA/blank as read by fread **n rows / missing / unique:** 841 / 0 / 124 **Release status:** public **Definition status:** exact\_definition | 02\_workflows/F5\_workflow\_v01/submission\_ready/public/data/esld\_F5\_stratified\_survival\_subject\_public.csv |  | integer |
| age\_years\_first\_available — Age at first available public record **File:** 02\_workflows/T1\_workflow\_v01/data/01\_source\_loaded\_harmonized/esld\_T1\_baseline\_patient\_public.csv **Recommended public name:** age\_years\_first\_available **Description:** Age in years at the first available public ESLD record for the patient or encounter represented in the released data. **Unit:** years **Value coding:** Integer age in years. **Data type:** integer **Example values:** 65 \| 67 \| 21 \| 56 \| 63 **Allowed values:**  **Missing-value coding:** NA/blank as read by fread **n rows / missing / unique:** 1375 / 0 / 79 **Release status:** public **Definition status:** manual\_exact\_definition | 02\_workflows/T1\_workflow\_v01/data/01\_source\_loaded\_harmonized/esld\_T1\_baseline\_patient\_public.csv | years | integer |
| ald — Alcohol-related liver disease etiology flag **File:** 02\_workflows/T1\_workflow\_v01/data/01\_source\_loaded\_harmonized/esld\_T1\_baseline\_patient\_public.csv **Recommended public name:** ald **Description:** Binary etiology indicator for alcohol-related liver disease in the public ESLD cohort. **Unit:**  **Value coding:** 0 = no; 1 = yes. **Data type:** integer **Example values:** 0 \| 1 **Allowed values:**  **Missing-value coding:** NA/blank as read by fread **n rows / missing / unique:** 1375 / 0 / 2 **Release status:** public **Definition status:** manual\_exact\_definition | 02\_workflows/T1\_workflow\_v01/data/01\_source\_loaded\_harmonized/esld\_T1\_baseline\_patient\_public.csv |  | integer |
| ald\_hcv — Combined alcohol-related liver disease and hepatitis C etiology flag **File:** 02\_workflows/T1\_workflow\_v01/data/01\_source\_loaded\_harmonized/esld\_T1\_baseline\_patient\_public.csv **Recommended public name:** ald\_hcv **Description:** Binary etiology indicator for combined alcohol-related liver disease and hepatitis C in the public ESLD cohort. **Unit:**  **Value coding:** 0 = no; 1 = yes. **Data type:** integer **Example values:** 0 \| 1 **Allowed values:**  **Missing-value coding:** NA/blank as read by fread **n rows / missing / unique:** 1375 / 0 / 2 **Release status:** public **Definition status:** manual\_exact\_definition | 02\_workflows/T1\_workflow\_v01/data/01\_source\_loaded\_harmonized/esld\_T1\_baseline\_patient\_public.csv |  | integer |
| any\_dialysis — Any dialysis indicator **File:** 02\_workflows/T1\_workflow\_v01/data/01\_source\_loaded\_harmonized/esld\_T1\_baseline\_patient\_public.csv **Recommended public name:** any\_dialysis **Description:** Binary indicator showing whether dialysis was present in the public baseline/table cohort. **Unit:**  **Value coding:** 0 = no; 1 = yes. **Data type:** integer **Example values:** 0 \| 1 **Allowed values:**  **Missing-value coding:** NA/blank as read by fread **n rows / missing / unique:** 1375 / 0 / 2 **Release status:** public **Definition status:** manual\_precision\_definition | 02\_workflows/T1\_workflow\_v01/data/01\_source\_loaded\_harmonized/esld\_T1\_baseline\_patient\_public.csv |  | integer |
| autoimmune — Autoimmune liver disease etiology flag **File:** 02\_workflows/T1\_workflow\_v01/data/01\_source\_loaded\_harmonized/esld\_T1\_baseline\_patient\_public.csv **Recommended public name:** autoimmune **Description:** Binary etiology indicator for autoimmune liver disease in the public ESLD cohort. **Unit:**  **Value coding:** 0 = no; 1 = yes. **Data type:** integer **Example values:** 0 \| 1 **Allowed values:**  **Missing-value coding:** NA/blank as read by fread **n rows / missing / unique:** 1375 / 0 / 2 **Release status:** public **Definition status:** manual\_exact\_definition | 02\_workflows/T1\_workflow\_v01/data/01\_source\_loaded\_harmonized/esld\_T1\_baseline\_patient\_public.csv |  | integer |
| deceased\_patient\_flag — Deceased patient flag **File:** 02\_workflows/T1\_workflow\_v01/data/01\_source\_loaded\_harmonized/esld\_T1\_baseline\_patient\_public.csv **Recommended public name:** deceased\_patient\_flag **Description:** Binary indicator identifying deceased patients within the released analysis context. **Unit:**  **Value coding:** 0 = no; 1 = yes **Data type:** integer **Example values:** 0 \| 1 **Allowed values:**  **Missing-value coding:** NA/blank as read by fread **n rows / missing / unique:** 1375 / 0 / 2 **Release status:** public **Definition status:** exact\_definition | 02\_workflows/T1\_workflow\_v01/data/01\_source\_loaded\_harmonized/esld\_T1\_baseline\_patient\_public.csv |  | integer |
| etiology\_any — Any etiology assigned **File:** 02\_workflows/T1\_workflow\_v01/data/01\_source\_loaded\_harmonized/esld\_T1\_baseline\_patient\_public.csv **Recommended public name:** etiology\_any **Description:** Binary indicator showing whether at least one etiology category is assigned. **Unit:**  **Value coding:** 0 = no; 1 = yes. **Data type:** integer **Example values:** 1 \| 0 **Allowed values:**  **Missing-value coding:** NA/blank as read by fread **n rows / missing / unique:** 1375 / 0 / 2 **Release status:** public **Definition status:** manual\_precision\_definition | 02\_workflows/T1\_workflow\_v01/data/01\_source\_loaded\_harmonized/esld\_T1\_baseline\_patient\_public.csv |  | integer |
| etiology\_unclassified — Unclassified liver disease etiology flag **File:** 02\_workflows/T1\_workflow\_v01/data/01\_source\_loaded\_harmonized/esld\_T1\_baseline\_patient\_public.csv **Recommended public name:** etiology\_unclassified **Description:** Binary etiology indicator identifying records without a classified liver disease etiology in the public ESLD cohort. **Unit:**  **Value coding:** 0 = no; 1 = yes. **Data type:** integer **Example values:** 0 **Allowed values:**  **Missing-value coding:** NA/blank as read by fread **n rows / missing / unique:** 1375 / 0 / 1 **Release status:** public **Definition status:** manual\_exact\_definition | 02\_workflows/T1\_workflow\_v01/data/01\_source\_loaded\_harmonized/esld\_T1\_baseline\_patient\_public.csv |  | integer |
| hbv — Hepatitis B virus etiology flag **File:** 02\_workflows/T1\_workflow\_v01/data/01\_source\_loaded\_harmonized/esld\_T1\_baseline\_patient\_public.csv **Recommended public name:** hbv **Description:** Binary etiology indicator for hepatitis B virus-related liver disease in the public ESLD cohort. **Unit:**  **Value coding:** 0 = no; 1 = yes. **Data type:** integer **Example values:** 0 \| 1 **Allowed values:**  **Missing-value coding:** NA/blank as read by fread **n rows / missing / unique:** 1375 / 0 / 2 **Release status:** public **Definition status:** manual\_exact\_definition | 02\_workflows/T1\_workflow\_v01/data/01\_source\_loaded\_harmonized/esld\_T1\_baseline\_patient\_public.csv |  | integer |
| hcv — Hepatitis C virus etiology flag **File:** 02\_workflows/T1\_workflow\_v01/data/01\_source\_loaded\_harmonized/esld\_T1\_baseline\_patient\_public.csv **Recommended public name:** hcv **Description:** Binary etiology indicator for hepatitis C virus-related liver disease in the public ESLD cohort. **Unit:**  **Value coding:** 0 = no; 1 = yes. **Data type:** integer **Example values:** 0 \| 1 **Allowed values:**  **Missing-value coding:** NA/blank as read by fread **n rows / missing / unique:** 1375 / 0 / 2 **Release status:** public **Definition status:** manual\_exact\_definition | 02\_workflows/T1\_workflow\_v01/data/01\_source\_loaded\_harmonized/esld\_T1\_baseline\_patient\_public.csv |  | integer |
| mash — Metabolic dysfunction-associated steatohepatitis etiology flag **File:** 02\_workflows/T1\_workflow\_v01/data/01\_source\_loaded\_harmonized/esld\_T1\_baseline\_patient\_public.csv **Recommended public name:** mash **Description:** Binary etiology indicator for metabolic dysfunction-associated steatohepatitis in the public ESLD cohort. **Unit:**  **Value coding:** 0 = no; 1 = yes. **Data type:** integer **Example values:** 0 \| 1 **Allowed values:**  **Missing-value coding:** NA/blank as read by fread **n rows / missing / unique:** 1375 / 0 / 2 **Release status:** public **Definition status:** manual\_exact\_definition | 02\_workflows/T1\_workflow\_v01/data/01\_source\_loaded\_harmonized/esld\_T1\_baseline\_patient\_public.csv |  | integer |
| n\_rows\_meld — Number of rows for MELD **File:** 02\_workflows/T1\_workflow\_v01/data/01\_source\_loaded\_harmonized/esld\_T1\_baseline\_patient\_public.csv **Recommended public name:** n\_rows\_meld **Description:** Number of rows available for the MELD model or score variant. **Unit:** count **Value coding:** Integer count. **Data type:** integer **Example values:** 1 \| 122 \| 5 \| 25 \| 180 **Allowed values:**  **Missing-value coding:** NA/blank as read by fread **n rows / missing / unique:** 1375 / 0 / 94 **Release status:** public **Definition status:** manual\_precision\_definition | 02\_workflows/T1\_workflow\_v01/data/01\_source\_loaded\_harmonized/esld\_T1\_baseline\_patient\_public.csv | count | integer |
| n\_rows\_meld3 — Number of rows for MELD 3.0 **File:** 02\_workflows/T1\_workflow\_v01/data/01\_source\_loaded\_harmonized/esld\_T1\_baseline\_patient\_public.csv **Recommended public name:** n\_rows\_meld3 **Description:** Number of rows available for the MELD 3.0 model or score variant. **Unit:** count **Value coding:** Integer count. **Data type:** integer **Example values:** 0 \| 39 \| 4 \| 2 \| 6 **Allowed values:**  **Missing-value coding:** NA/blank as read by fread **n rows / missing / unique:** 1375 / 0 / 60 **Release status:** public **Definition status:** manual\_precision\_definition | 02\_workflows/T1\_workflow\_v01/data/01\_source\_loaded\_harmonized/esld\_T1\_baseline\_patient\_public.csv | count | integer |
| n\_rows\_meld\_na — Number of rows for MELD-Na **File:** 02\_workflows/T1\_workflow\_v01/data/01\_source\_loaded\_harmonized/esld\_T1\_baseline\_patient\_public.csv **Recommended public name:** n\_rows\_meld\_na **Description:** Number of rows available for the MELD-Na model or score variant. **Unit:** count **Value coding:** Integer count. **Data type:** integer **Example values:** 1 \| 122 \| 5 \| 19 \| 176 **Allowed values:**  **Missing-value coding:** NA/blank as read by fread **n rows / missing / unique:** 1375 / 0 / 94 **Release status:** public **Definition status:** manual\_precision\_definition | 02\_workflows/T1\_workflow\_v01/data/01\_source\_loaded\_harmonized/esld\_T1\_baseline\_patient\_public.csv | count | integer |
| n\_rows\_remeld\_na — Number of rows for reMELD-Na **File:** 02\_workflows/T1\_workflow\_v01/data/01\_source\_loaded\_harmonized/esld\_T1\_baseline\_patient\_public.csv **Recommended public name:** n\_rows\_remeld\_na **Description:** Number of rows available for the reMELD-Na model or score variant. **Unit:** count **Value coding:** Integer count. **Data type:** integer **Example values:** 1 \| 122 \| 5 \| 19 \| 176 **Allowed values:**  **Missing-value coding:** NA/blank as read by fread **n rows / missing / unique:** 1375 / 0 / 94 **Release status:** public **Definition status:** manual\_precision\_definition | 02\_workflows/T1\_workflow\_v01/data/01\_source\_loaded\_harmonized/esld\_T1\_baseline\_patient\_public.csv | count | integer |
| other — Other liver disease etiology flag **File:** 02\_workflows/T1\_workflow\_v01/data/01\_source\_loaded\_harmonized/esld\_T1\_baseline\_patient\_public.csv **Recommended public name:** other **Description:** Binary etiology indicator for liver disease etiologies grouped as other in the public ESLD cohort. **Unit:**  **Value coding:** 0 = no; 1 = yes. **Data type:** integer **Example values:** 1 \| 0 **Allowed values:**  **Missing-value coding:** NA/blank as read by fread **n rows / missing / unique:** 1375 / 0 / 2 **Release status:** public **Definition status:** manual\_exact\_definition | 02\_workflows/T1\_workflow\_v01/data/01\_source\_loaded\_harmonized/esld\_T1\_baseline\_patient\_public.csv |  | integer |
| patient\_id — Public patient pseudonym **File:** 02\_workflows/T1\_workflow\_v01/data/01\_source\_loaded\_harmonized/esld\_T1\_baseline\_patient\_public.csv **Recommended public name:** patient\_id **Description:** Non-linkable public-release patient pseudonym used to preserve within-patient grouping in public ESLD data; not an original hospital patient identifier. **Unit:**  **Value coding:** See example\_values and allowed\_values. **Data type:** character **Example values:** P000001 \| P000002 \| P000003 \| P000004 \| P000005 **Allowed values:** P000001 \| P000002 \| P000003 \| P000004 \| P000005 \| P000006 \| P000007 \| P000008 \| P000009 \| P000010 **Missing-value coding:** NA/blank as read by fread **n rows / missing / unique:** 1375 / 0 / 1375 **Release status:** public **Definition status:** exact\_definition | 02\_workflows/T1\_workflow\_v01/data/01\_source\_loaded\_harmonized/esld\_T1\_baseline\_patient\_public.csv |  | character |
| pbc — Primary biliary cholangitis etiology flag **File:** 02\_workflows/T1\_workflow\_v01/data/01\_source\_loaded\_harmonized/esld\_T1\_baseline\_patient\_public.csv **Recommended public name:** pbc **Description:** Binary etiology indicator for primary biliary cholangitis in the public ESLD cohort. **Unit:**  **Value coding:** 0 = no; 1 = yes. **Data type:** integer **Example values:** 0 \| 1 **Allowed values:**  **Missing-value coding:** NA/blank as read by fread **n rows / missing / unique:** 1375 / 0 / 2 **Release status:** public **Definition status:** manual\_exact\_definition | 02\_workflows/T1\_workflow\_v01/data/01\_source\_loaded\_harmonized/esld\_T1\_baseline\_patient\_public.csv |  | integer |
| sex — Sex **File:** 02\_workflows/T1\_workflow\_v01/data/01\_source\_loaded\_harmonized/esld\_T1\_baseline\_patient\_public.csv **Recommended public name:** sex **Description:** Sex category represented in the public ESLD data. **Unit:**  **Value coding:** M = male; F = female. **Data type:** character **Example values:** M \| F **Allowed values:** M \| F **Missing-value coding:** NA/blank as read by fread **n rows / missing / unique:** 1375 / 0 / 2 **Release status:** public **Definition status:** manual\_exact\_definition | 02\_workflows/T1\_workflow\_v01/data/01\_source\_loaded\_harmonized/esld\_T1\_baseline\_patient\_public.csv |  | character |
| age\_years\_first\_available — Age at first available public record **File:** 02\_workflows/T1\_workflow\_v01/data/01\_source\_loaded\_harmonized/esld\_master\_long\_public.csv **Recommended public name:** age\_years\_first\_available **Description:** Age in years at the first available public ESLD record for the patient or encounter represented in the released data. **Unit:** years **Value coding:** Integer age in years. **Data type:** integer **Example values:** 65 \| 67 \| 21 \| 56 \| 63 **Allowed values:**  **Missing-value coding:** NA/blank as read by fread **n rows / missing / unique:** 67399 / 0 / 79 **Release status:** public **Definition status:** manual\_exact\_definition | 02\_workflows/T1\_workflow\_v01/data/01\_source\_loaded\_harmonized/esld\_master\_long\_public.csv | years | integer |
| age\_years\_sample — Age at sample **File:** 02\_workflows/T1\_workflow\_v01/data/01\_source\_loaded\_harmonized/esld\_master\_long\_public.csv **Recommended public name:** age\_years\_sample **Description:** Age in years at the sample-level public ESLD record. **Unit:** years **Value coding:** Integer age in years. **Data type:** integer **Example values:** 65 \| 66 \| 67 \| 68 \| 69 **Allowed values:**  **Missing-value coding:** NA/blank as read by fread **n rows / missing / unique:** 67399 / 0 / 84 **Release status:** public **Definition status:** manual\_precision\_definition | 02\_workflows/T1\_workflow\_v01/data/01\_source\_loaded\_harmonized/esld\_master\_long\_public.csv | years | integer |
| albumin\_g\_dl — Serum albumin concentration **File:** 02\_workflows/T1\_workflow\_v01/data/01\_source\_loaded\_harmonized/esld\_master\_long\_public.csv **Recommended public name:** albumin\_g\_dl **Description:** Serum albumin concentration in the public ESLD master data. **Unit:** g/dL **Value coding:** Numeric concentration in g/dL. **Data type:** numeric **Example values:** 3.3 \| 6.15 \| 3.14 \| 2.8 \| 3.26 **Allowed values:**  **Missing-value coding:** NA/blank as read by fread **n rows / missing / unique:** 67399 / 58785 / 580 **Release status:** public **Definition status:** manual\_precision\_definition | 02\_workflows/T1\_workflow\_v01/data/01\_source\_loaded\_harmonized/esld\_master\_long\_public.csv | g/dL | numeric |
| ald — Alcohol-related liver disease etiology flag **File:** 02\_workflows/T1\_workflow\_v01/data/01\_source\_loaded\_harmonized/esld\_master\_long\_public.csv **Recommended public name:** ald **Description:** Binary etiology indicator for alcohol-related liver disease in the public ESLD cohort. **Unit:**  **Value coding:** 0 = no; 1 = yes. **Data type:** integer **Example values:** 0 \| 1 **Allowed values:**  **Missing-value coding:** NA/blank as read by fread **n rows / missing / unique:** 67399 / 0 / 2 **Release status:** public **Definition status:** manual\_exact\_definition | 02\_workflows/T1\_workflow\_v01/data/01\_source\_loaded\_harmonized/esld\_master\_long\_public.csv |  | integer |
| ald\_hcv — Combined alcohol-related liver disease and hepatitis C etiology flag **File:** 02\_workflows/T1\_workflow\_v01/data/01\_source\_loaded\_harmonized/esld\_master\_long\_public.csv **Recommended public name:** ald\_hcv **Description:** Binary etiology indicator for combined alcohol-related liver disease and hepatitis C in the public ESLD cohort. **Unit:**  **Value coding:** 0 = no; 1 = yes. **Data type:** integer **Example values:** 0 \| 1 **Allowed values:**  **Missing-value coding:** NA/blank as read by fread **n rows / missing / unique:** 67399 / 0 / 2 **Release status:** public **Definition status:** manual\_exact\_definition | 02\_workflows/T1\_workflow\_v01/data/01\_source\_loaded\_harmonized/esld\_master\_long\_public.csv |  | integer |
| autoimmune — Autoimmune liver disease etiology flag **File:** 02\_workflows/T1\_workflow\_v01/data/01\_source\_loaded\_harmonized/esld\_master\_long\_public.csv **Recommended public name:** autoimmune **Description:** Binary etiology indicator for autoimmune liver disease in the public ESLD cohort. **Unit:**  **Value coding:** 0 = no; 1 = yes. **Data type:** integer **Example values:** 0 \| 1 **Allowed values:**  **Missing-value coding:** NA/blank as read by fread **n rows / missing / unique:** 67399 / 0 / 2 **Release status:** public **Definition status:** manual\_exact\_definition | 02\_workflows/T1\_workflow\_v01/data/01\_source\_loaded\_harmonized/esld\_master\_long\_public.csv |  | integer |
| crea — Creatinine concentration **File:** 02\_workflows/T1\_workflow\_v01/data/01\_source\_loaded\_harmonized/esld\_master\_long\_public.csv **Recommended public name:** crea **Description:** Creatinine concentration used in MELD-related score calculation before applying the study-specific correction. **Unit:** mg/dL **Value coding:** Numeric concentration in mg/dL. **Data type:** numeric **Example values:** 1.91 \| 1.42 \| 1.31 \| 1.3 \| 1.28 **Allowed values:**  **Missing-value coding:** NA/blank as read by fread **n rows / missing / unique:** 67399 / 0 / 975 **Release status:** public **Definition status:** manual\_exact\_definition | 02\_workflows/T1\_workflow\_v01/data/01\_source\_loaded\_harmonized/esld\_master\_long\_public.csv | mg/dL | numeric |
| crea\_corrected — Corrected creatinine concentration **File:** 02\_workflows/T1\_workflow\_v01/data/01\_source\_loaded\_harmonized/esld\_master\_long\_public.csv **Recommended public name:** crea\_corrected **Description:** Creatinine concentration after applying the study-specific correction used for recalculated MELD-related scores. **Unit:** mg/dL **Value coding:** Numeric concentration in mg/dL. **Data type:** numeric **Example values:** 1.87705004446668 \| 1.41586432303417 \| 1.31039363454933 \| 1.30192482788903 \| 1.28150549768982 **Allowed values:**  **Missing-value coding:** NA/blank as read by fread **n rows / missing / unique:** 67399 / 0 / 15868 **Release status:** public **Definition status:** manual\_exact\_definition | 02\_workflows/T1\_workflow\_v01/data/01\_source\_loaded\_harmonized/esld\_master\_long\_public.csv | mg/dL | numeric |
| date\_count — Relative day count **File:** 02\_workflows/T1\_workflow\_v01/data/01\_source\_loaded\_harmonized/esld\_master\_long\_public.csv **Recommended public name:** date\_count **Description:** Relative day count used for time alignment; negative/positive values are relative to the analysis anchor and are not calendar dates. **Unit:**  **Value coding:** See example\_values and allowed\_values. **Data type:** integer **Example values:** -3021 \| -2833 \| -2798 \| -2793 \| -2791 **Allowed values:**  **Missing-value coding:** NA/blank as read by fread **n rows / missing / unique:** 67399 / 50970 / 1450 **Release status:** public **Definition status:** exact\_definition | 02\_workflows/T1\_workflow\_v01/data/01\_source\_loaded\_harmonized/esld\_master\_long\_public.csv |  | integer |
| date\_count\_month — Relative month count **File:** 02\_workflows/T1\_workflow\_v01/data/01\_source\_loaded\_harmonized/esld\_master\_long\_public.csv **Recommended public name:** date\_count\_month **Description:** Relative month count derived from the relative day count; this is not a calendar month. **Unit:**  **Value coding:** See example\_values and allowed\_values. **Data type:** integer **Example values:** -99 \| -93 \| -92 \| -91 \| -87 **Allowed values:**  **Missing-value coding:** NA/blank as read by fread **n rows / missing / unique:** 67399 / 50970 / 147 **Release status:** public **Definition status:** exact\_definition | 02\_workflows/T1\_workflow\_v01/data/01\_source\_loaded\_harmonized/esld\_master\_long\_public.csv |  | integer |
| dead\_sample\_flag — Dead-sample flag **File:** 02\_workflows/T1\_workflow\_v01/data/01\_source\_loaded\_harmonized/esld\_master\_long\_public.csv **Recommended public name:** dead\_sample\_flag **Description:** Binary indicator identifying public ESLD sample records assigned to the deceased-sample subset used in sample-level analyses. **Unit:**  **Value coding:** 0 = not assigned to deceased-sample subset; 1 = assigned to deceased-sample subset. **Data type:** integer **Example values:** 0 \| 1 **Allowed values:**  **Missing-value coding:** NA/blank as read by fread **n rows / missing / unique:** 67399 / 0 / 2 **Release status:** public **Definition status:** manual\_final\_definition | 02\_workflows/T1\_workflow\_v01/data/01\_source\_loaded\_harmonized/esld\_master\_long\_public.csv |  | integer |
| death\_within\_90d — Death within 90 days **File:** 02\_workflows/T1\_workflow\_v01/data/01\_source\_loaded\_harmonized/esld\_master\_long\_public.csv **Recommended public name:** death\_within\_90d **Description:** Binary indicator for death within 90 days of the analysis anchor. **Unit:**  **Value coding:** 0 = no; 1 = yes **Data type:** logical **Example values:**  **Allowed values:**  **Missing-value coding:** NA/blank as read by fread **n rows / missing / unique:** 67399 / 67399 / 0 **Release status:** public **Definition status:** exact\_definition | 02\_workflows/T1\_workflow\_v01/data/01\_source\_loaded\_harmonized/esld\_master\_long\_public.csv |  | logical |
| deceased\_patient\_flag — Deceased patient flag **File:** 02\_workflows/T1\_workflow\_v01/data/01\_source\_loaded\_harmonized/esld\_master\_long\_public.csv **Recommended public name:** deceased\_patient\_flag **Description:** Binary indicator identifying deceased patients within the released analysis context. **Unit:**  **Value coding:** 0 = no; 1 = yes **Data type:** integer **Example values:** 0 \| 1 **Allowed values:**  **Missing-value coding:** NA/blank as read by fread **n rows / missing / unique:** 67399 / 0 / 2 **Release status:** public **Definition status:** exact\_definition | 02\_workflows/T1\_workflow\_v01/data/01\_source\_loaded\_harmonized/esld\_master\_long\_public.csv |  | integer |
| delta\_eq\_0\_flag — Zero score-delta flag **File:** 02\_workflows/T1\_workflow\_v01/data/01\_source\_loaded\_harmonized/esld\_master\_long\_public.csv **Recommended public name:** delta\_eq\_0\_flag **Description:** Binary indicator equal to 1 when the score delta is exactly zero. **Unit:**  **Value coding:** 0 = no; 1 = yes. **Data type:** integer **Example values:** 1 \| 0 **Allowed values:**  **Missing-value coding:** NA/blank as read by fread **n rows / missing / unique:** 67399 / 0 / 2 **Release status:** public **Definition status:** manual\_precision\_definition | 02\_workflows/T1\_workflow\_v01/data/01\_source\_loaded\_harmonized/esld\_master\_long\_public.csv |  | integer |
| delta\_gt\_0\_flag — Positive score-delta flag **File:** 02\_workflows/T1\_workflow\_v01/data/01\_source\_loaded\_harmonized/esld\_master\_long\_public.csv **Recommended public name:** delta\_gt\_0\_flag **Description:** Binary indicator equal to 1 when the score delta is positive. **Unit:**  **Value coding:** 0 = no; 1 = yes. **Data type:** integer **Example values:** 0 \| 1 **Allowed values:**  **Missing-value coding:** NA/blank as read by fread **n rows / missing / unique:** 67399 / 0 / 2 **Release status:** public **Definition status:** manual\_precision\_definition | 02\_workflows/T1\_workflow\_v01/data/01\_source\_loaded\_harmonized/esld\_master\_long\_public.csv |  | integer |
| delta\_le\_minus1\_flag — Score-delta ≤ −1 flag **File:** 02\_workflows/T1\_workflow\_v01/data/01\_source\_loaded\_harmonized/esld\_master\_long\_public.csv **Recommended public name:** delta\_le\_minus1\_flag **Description:** Binary indicator equal to 1 when the score delta is less than or equal to −1. **Unit:**  **Value coding:** 0 = no; 1 = yes. **Data type:** integer **Example values:** 0 \| 1 **Allowed values:**  **Missing-value coding:** NA/blank as read by fread **n rows / missing / unique:** 67399 / 0 / 2 **Release status:** public **Definition status:** manual\_precision\_definition | 02\_workflows/T1\_workflow\_v01/data/01\_source\_loaded\_harmonized/esld\_master\_long\_public.csv |  | integer |
| dialysis\_raw — Dialysis indicator as recorded before score derivation **File:** 02\_workflows/T1\_workflow\_v01/data/01\_source\_loaded\_harmonized/esld\_master\_long\_public.csv **Recommended public name:** dialysis\_raw **Description:** Binary indicator showing whether dialysis was recorded in the source field before score derivation. **Unit:**  **Value coding:** 0 = no dialysis recorded; 1 = dialysis recorded. **Data type:** integer **Example values:** 0 \| 1 **Allowed values:**  **Missing-value coding:** NA/blank as read by fread **n rows / missing / unique:** 67399 / 27827 / 2 **Release status:** public **Definition status:** manual\_exact\_definition | 02\_workflows/T1\_workflow\_v01/data/01\_source\_loaded\_harmonized/esld\_master\_long\_public.csv |  | integer |
| encounter\_id\_public — Public encounter pseudonym **File:** 02\_workflows/T1\_workflow\_v01/data/01\_source\_loaded\_harmonized/esld\_master\_long\_public.csv **Recommended public name:** encounter\_id\_public **Description:** Non-linkable public-release encounter pseudonym used for encounter-level grouping; not an original hospital encounter identifier. **Unit:**  **Value coding:** See example\_values and allowed\_values. **Data type:** character **Example values:** E001181 \| E000008 \| E000041 \| E000100 \| E000126 **Allowed values:** E001181 \| E000008 \| E000041 \| E000100 \| E000126 \| E000151 \| E000157 \| E000219 \| E000289 \| E000344 **Missing-value coding:** NA/blank as read by fread **n rows / missing / unique:** 67399 / 0 / 5045 **Release status:** public **Definition status:** exact\_definition | 02\_workflows/T1\_workflow\_v01/data/01\_source\_loaded\_harmonized/esld\_master\_long\_public.csv |  | character |
| etiology\_source\_current\_flag — Current etiology source flag **File:** 02\_workflows/T1\_workflow\_v01/data/01\_source\_loaded\_harmonized/esld\_master\_long\_public.csv **Recommended public name:** etiology\_source\_current\_flag **Description:** Binary indicator identifying whether the etiology source is the current source used for the public row. **Unit:**  **Value coding:** 0 = no; 1 = yes. **Data type:** integer **Example values:** 1 **Allowed values:**  **Missing-value coding:** NA/blank as read by fread **n rows / missing / unique:** 67399 / 0 / 1 **Release status:** public **Definition status:** manual\_precision\_definition | 02\_workflows/T1\_workflow\_v01/data/01\_source\_loaded\_harmonized/esld\_master\_long\_public.csv |  | integer |
| etiology\_unclassified — Unclassified liver disease etiology flag **File:** 02\_workflows/T1\_workflow\_v01/data/01\_source\_loaded\_harmonized/esld\_master\_long\_public.csv **Recommended public name:** etiology\_unclassified **Description:** Binary etiology indicator identifying records without a classified liver disease etiology in the public ESLD cohort. **Unit:**  **Value coding:** 0 = no; 1 = yes. **Data type:** integer **Example values:** 0 **Allowed values:**  **Missing-value coding:** NA/blank as read by fread **n rows / missing / unique:** 67399 / 0 / 1 **Release status:** public **Definition status:** manual\_exact\_definition | 02\_workflows/T1\_workflow\_v01/data/01\_source\_loaded\_harmonized/esld\_master\_long\_public.csv |  | integer |
| hbv — Hepatitis B virus etiology flag **File:** 02\_workflows/T1\_workflow\_v01/data/01\_source\_loaded\_harmonized/esld\_master\_long\_public.csv **Recommended public name:** hbv **Description:** Binary etiology indicator for hepatitis B virus-related liver disease in the public ESLD cohort. **Unit:**  **Value coding:** 0 = no; 1 = yes. **Data type:** integer **Example values:** 0 \| 1 **Allowed values:**  **Missing-value coding:** NA/blank as read by fread **n rows / missing / unique:** 67399 / 0 / 2 **Release status:** public **Definition status:** manual\_exact\_definition | 02\_workflows/T1\_workflow\_v01/data/01\_source\_loaded\_harmonized/esld\_master\_long\_public.csv |  | integer |
| hcv — Hepatitis C virus etiology flag **File:** 02\_workflows/T1\_workflow\_v01/data/01\_source\_loaded\_harmonized/esld\_master\_long\_public.csv **Recommended public name:** hcv **Description:** Binary etiology indicator for hepatitis C virus-related liver disease in the public ESLD cohort. **Unit:**  **Value coding:** 0 = no; 1 = yes. **Data type:** integer **Example values:** 0 \| 1 **Allowed values:**  **Missing-value coding:** NA/blank as read by fread **n rows / missing / unique:** 67399 / 0 / 2 **Release status:** public **Definition status:** manual\_exact\_definition | 02\_workflows/T1\_workflow\_v01/data/01\_source\_loaded\_harmonized/esld\_master\_long\_public.csv |  | integer |
| icd\_source\_present — ICD source present flag **File:** 02\_workflows/T1\_workflow\_v01/data/01\_source\_loaded\_harmonized/esld\_master\_long\_public.csv **Recommended public name:** icd\_source\_present **Description:** Binary indicator showing whether an ICD-derived etiology source was present for the public row. **Unit:**  **Value coding:** 0 = no; 1 = yes. **Data type:** integer **Example values:** 1 \| 0 **Allowed values:**  **Missing-value coding:** NA/blank as read by fread **n rows / missing / unique:** 67399 / 0 / 2 **Release status:** public **Definition status:** manual\_precision\_definition | 02\_workflows/T1\_workflow\_v01/data/01\_source\_loaded\_harmonized/esld\_master\_long\_public.csv |  | integer |
| in\_t1\_cohort — Inclusion flag for Table 1 cohort **File:** 02\_workflows/T1\_workflow\_v01/data/01\_source\_loaded\_harmonized/esld\_master\_long\_public.csv **Recommended public name:** in\_t1\_cohort **Description:** Binary indicator identifying records included in the public ESLD Table 1 baseline-characteristics cohort. **Unit:**  **Value coding:** 0 = not included; 1 = included. **Data type:** integer **Example values:** 1 **Allowed values:**  **Missing-value coding:** NA/blank as read by fread **n rows / missing / unique:** 67399 / 0 / 1 **Release status:** public **Definition status:** manual\_exact\_definition | 02\_workflows/T1\_workflow\_v01/data/01\_source\_loaded\_harmonized/esld\_master\_long\_public.csv |  | integer |
| in\_t3\_anchor\_model — Inclusion flag for Table 3 anchor-model cohort **File:** 02\_workflows/T1\_workflow\_v01/data/01\_source\_loaded\_harmonized/esld\_master\_long\_public.csv **Recommended public name:** in\_t3\_anchor\_model **Description:** Binary indicator identifying records included in the Table 3 anchor-model cohort used for creatinine-comparison analyses. **Unit:**  **Value coding:** 0 = not included; 1 = included. **Data type:** integer **Example values:** 1 \| 0 **Allowed values:**  **Missing-value coding:** NA/blank as read by fread **n rows / missing / unique:** 67399 / 0 / 2 **Release status:** public **Definition status:** manual\_exact\_definition | 02\_workflows/T1\_workflow\_v01/data/01\_source\_loaded\_harmonized/esld\_master\_long\_public.csv |  | integer |
| in\_t4\_prevalence\_path — Inclusion flag for Table 4 prevalence pathway **File:** 02\_workflows/T1\_workflow\_v01/data/01\_source\_loaded\_harmonized/esld\_master\_long\_public.csv **Recommended public name:** in\_t4\_prevalence\_path **Description:** Binary indicator identifying records included in the Table 4 prevalence-analysis pathway. **Unit:**  **Value coding:** 0 = not included; 1 = included. **Data type:** integer **Example values:** 1 **Allowed values:**  **Missing-value coding:** NA/blank as read by fread **n rows / missing / unique:** 67399 / 0 / 1 **Release status:** public **Definition status:** manual\_exact\_definition | 02\_workflows/T1\_workflow\_v01/data/01\_source\_loaded\_harmonized/esld\_master\_long\_public.csv |  | integer |
| in\_t4\_survival\_path — Inclusion flag for Table 4 survival pathway **File:** 02\_workflows/T1\_workflow\_v01/data/01\_source\_loaded\_harmonized/esld\_master\_long\_public.csv **Recommended public name:** in\_t4\_survival\_path **Description:** Binary indicator identifying records included in the Table 4 survival-analysis pathway. **Unit:**  **Value coding:** 0 = not included; 1 = included. **Data type:** integer **Example values:** 0 \| 1 **Allowed values:**  **Missing-value coding:** NA/blank as read by fread **n rows / missing / unique:** 67399 / 0 / 2 **Release status:** public **Definition status:** manual\_exact\_definition | 02\_workflows/T1\_workflow\_v01/data/01\_source\_loaded\_harmonized/esld\_master\_long\_public.csv |  | integer |
| inr — International normalized ratio **File:** 02\_workflows/T1\_workflow\_v01/data/01\_source\_loaded\_harmonized/esld\_master\_long\_public.csv **Recommended public name:** inr **Description:** International normalized ratio of prothrombin time used for MELD-related score calculation. **Unit:** ratio **Value coding:** Numeric INR value. **Data type:** numeric **Example values:** 1.14 \| 0.91 \| 1 \| 1.04 \| 0.98 **Allowed values:**  **Missing-value coding:** NA/blank as read by fread **n rows / missing / unique:** 67399 / 0 / 786 **Release status:** public **Definition status:** manual\_exact\_definition | 02\_workflows/T1\_workflow\_v01/data/01\_source\_loaded\_harmonized/esld\_master\_long\_public.csv | ratio | numeric |
| mash — Metabolic dysfunction-associated steatohepatitis etiology flag **File:** 02\_workflows/T1\_workflow\_v01/data/01\_source\_loaded\_harmonized/esld\_master\_long\_public.csv **Recommended public name:** mash **Description:** Binary etiology indicator for metabolic dysfunction-associated steatohepatitis in the public ESLD cohort. **Unit:**  **Value coding:** 0 = no; 1 = yes. **Data type:** integer **Example values:** 0 \| 1 **Allowed values:**  **Missing-value coding:** NA/blank as read by fread **n rows / missing / unique:** 67399 / 0 / 2 **Release status:** public **Definition status:** manual\_exact\_definition | 02\_workflows/T1\_workflow\_v01/data/01\_source\_loaded\_harmonized/esld\_master\_long\_public.csv |  | integer |
| model — MELD model or score variant **File:** 02\_workflows/T1\_workflow\_v01/data/01\_source\_loaded\_harmonized/esld\_master\_long\_public.csv **Recommended public name:** model **Description:** Name of the MELD-related model or score variant represented by the row; expected values include MELD, MELD-Na, reMELD-Na, and MELD 3.0. **Unit:**  **Value coding:** Categorical score/model label, for example MELD, MELD-Na, reMELD-Na, or MELD 3.0. **Data type:** character **Example values:** MELD \| MELD-Na \| reMELD-Na \| MELD 3.0 **Allowed values:** MELD \| MELD-Na \| reMELD-Na \| MELD 3.0 **Missing-value coding:** NA/blank as read by fread **n rows / missing / unique:** 67399 / 0 / 4 **Release status:** public **Definition status:** manual\_exact\_definition | 02\_workflows/T1\_workflow\_v01/data/01\_source\_loaded\_harmonized/esld\_master\_long\_public.csv |  | character |
| other — Other liver disease etiology flag **File:** 02\_workflows/T1\_workflow\_v01/data/01\_source\_loaded\_harmonized/esld\_master\_long\_public.csv **Recommended public name:** other **Description:** Binary etiology indicator for liver disease etiologies grouped as other in the public ESLD cohort. **Unit:**  **Value coding:** 0 = no; 1 = yes. **Data type:** integer **Example values:** 1 \| 0 **Allowed values:**  **Missing-value coding:** NA/blank as read by fread **n rows / missing / unique:** 67399 / 0 / 2 **Release status:** public **Definition status:** manual\_exact\_definition | 02\_workflows/T1\_workflow\_v01/data/01\_source\_loaded\_harmonized/esld\_master\_long\_public.csv |  | integer |
| patient\_id — Public patient pseudonym **File:** 02\_workflows/T1\_workflow\_v01/data/01\_source\_loaded\_harmonized/esld\_master\_long\_public.csv **Recommended public name:** patient\_id **Description:** Non-linkable public-release patient pseudonym used to preserve within-patient grouping in public ESLD data; not an original hospital patient identifier. **Unit:**  **Value coding:** See example\_values and allowed\_values. **Data type:** character **Example values:** P000001 \| P000002 \| P000003 \| P000004 \| P000005 **Allowed values:** P000001 \| P000002 \| P000003 \| P000004 \| P000005 \| P000006 \| P000007 \| P000008 \| P000009 \| P000010 **Missing-value coding:** NA/blank as read by fread **n rows / missing / unique:** 67399 / 0 / 1375 **Release status:** public **Definition status:** exact\_definition | 02\_workflows/T1\_workflow\_v01/data/01\_source\_loaded\_harmonized/esld\_master\_long\_public.csv |  | character |
| pbc — Primary biliary cholangitis etiology flag **File:** 02\_workflows/T1\_workflow\_v01/data/01\_source\_loaded\_harmonized/esld\_master\_long\_public.csv **Recommended public name:** pbc **Description:** Binary etiology indicator for primary biliary cholangitis in the public ESLD cohort. **Unit:**  **Value coding:** 0 = no; 1 = yes. **Data type:** integer **Example values:** 0 \| 1 **Allowed values:**  **Missing-value coding:** NA/blank as read by fread **n rows / missing / unique:** 67399 / 0 / 2 **Release status:** public **Definition status:** manual\_exact\_definition | 02\_workflows/T1\_workflow\_v01/data/01\_source\_loaded\_harmonized/esld\_master\_long\_public.csv |  | integer |
| sample\_day\_from\_first\_sample — Relative sample day **File:** 02\_workflows/T1\_workflow\_v01/data/01\_source\_loaded\_harmonized/esld\_master\_long\_public.csv **Recommended public name:** sample\_day\_from\_first\_sample **Description:** Relative day of the sample measured from the first sample for the public patient/sample sequence; not a calendar date. **Unit:** relative months **Value coding:** Integer or numeric relative day count. **Data type:** integer **Example values:** 0 \| 42 \| 91 \| 118 \| 133 **Allowed values:**  **Missing-value coding:** NA/blank as read by fread **n rows / missing / unique:** 67399 / 0 / 3438 **Release status:** public **Definition status:** manual\_precision\_definition | 02\_workflows/T1\_workflow\_v01/data/01\_source\_loaded\_harmonized/esld\_master\_long\_public.csv | relative months | integer |
| sample\_group\_id\_public — Public sample-group pseudonym **File:** 02\_workflows/T1\_workflow\_v01/data/01\_source\_loaded\_harmonized/esld\_master\_long\_public.csv **Recommended public name:** sample\_group\_id\_public **Description:** Non-linkable public-release sample-group pseudonym used to group samples within the released public data. **Unit:**  **Value coding:** See example\_values and allowed\_values. **Data type:** character **Example values:** G0000001 \| G0000002 \| G0000003 \| G0000004 \| G0000005 **Allowed values:** G0000001 \| G0000002 \| G0000003 \| G0000004 \| G0000005 \| G0000006 \| G0000007 \| G0000008 \| G0000009 \| G0000010 **Missing-value coding:** NA/blank as read by fread **n rows / missing / unique:** 67399 / 0 / 20359 **Release status:** public **Definition status:** exact\_definition | 02\_workflows/T1\_workflow\_v01/data/01\_source\_loaded\_harmonized/esld\_master\_long\_public.csv |  | character |
| sample\_id — Sample identifier **File:** 02\_workflows/T1\_workflow\_v01/data/01\_source\_loaded\_harmonized/esld\_master\_long\_public.csv **Recommended public name:** sample\_id **Description:** Identifier of a sample or experimental record within the released public data; not a personal identifier. **Unit:**  **Value coding:** See example\_values and allowed\_values. **Data type:** character **Example values:** S0000001 \| S0000002 \| S0000003 \| S0000004 \| S0000005 **Allowed values:** S0000001 \| S0000002 \| S0000003 \| S0000004 \| S0000005 \| S0000006 \| S0000007 \| S0000008 \| S0000009 \| S0000010 **Missing-value coding:** NA/blank as read by fread **n rows / missing / unique:** 67399 / 0 / 67399 **Release status:** public **Definition status:** exact\_definition | 02\_workflows/T1\_workflow\_v01/data/01\_source\_loaded\_harmonized/esld\_master\_long\_public.csv |  | character |
| sample\_month\_index — Relative sample month index **File:** 02\_workflows/T1\_workflow\_v01/data/01\_source\_loaded\_harmonized/esld\_master\_long\_public.csv **Recommended public name:** sample\_month\_index **Description:** Relative month index of the sample measured from the first sample for the public patient/sample sequence; not a calendar month. **Unit:** score points **Value coding:** Integer or numeric relative month index. **Data type:** integer **Example values:** 0 \| 1 \| 2 \| 3 \| 4 **Allowed values:**  **Missing-value coding:** NA/blank as read by fread **n rows / missing / unique:** 67399 / 0 / 177 **Release status:** public **Definition status:** manual\_precision\_definition | 02\_workflows/T1\_workflow\_v01/data/01\_source\_loaded\_harmonized/esld\_master\_long\_public.csv | score points | integer |
| score\_corrected — Corrected score **File:** 02\_workflows/T1\_workflow\_v01/data/01\_source\_loaded\_harmonized/esld\_master\_long\_public.csv **Recommended public name:** score\_corrected **Description:** MELD-related score after creatinine correction or recalculation. **Unit:** score points **Value coding:** Numeric score. **Data type:** integer **Example values:** 14 \| 19 \| 16 \| 10 \| 13 **Allowed values:**  **Missing-value coding:** NA/blank as read by fread **n rows / missing / unique:** 67399 / 0 / 40 **Release status:** public **Definition status:** manual\_precision\_definition | 02\_workflows/T1\_workflow\_v01/data/01\_source\_loaded\_harmonized/esld\_master\_long\_public.csv | score points | integer |
| score\_delta — Score difference **File:** 02\_workflows/T1\_workflow\_v01/data/01\_source\_loaded\_harmonized/esld\_master\_long\_public.csv **Recommended public name:** score\_delta **Description:** Difference between score variants or scoring approaches, expressed in score points. **Unit:**  **Value coding:** See example\_values and allowed\_values. **Data type:** integer **Example values:** 0 \| 1 \| -1 \| -2 **Allowed values:**  **Missing-value coding:** NA/blank as read by fread **n rows / missing / unique:** 67399 / 0 / 4 **Release status:** public **Definition status:** exact\_definition | 02\_workflows/T1\_workflow\_v01/data/01\_source\_loaded\_harmonized/esld\_master\_long\_public.csv |  | integer |
| score\_original — Original score **File:** 02\_workflows/T1\_workflow\_v01/data/01\_source\_loaded\_harmonized/esld\_master\_long\_public.csv **Recommended public name:** score\_original **Description:** Original MELD-related score before creatinine correction or recalculation. **Unit:**  **Value coding:** Numeric score. **Data type:** integer **Example values:** 14 \| 19 \| 16 \| 10 \| 13 **Allowed values:**  **Missing-value coding:** NA/blank as read by fread **n rows / missing / unique:** 67399 / 0 / 40 **Release status:** public **Definition status:** manual\_precision\_definition | 02\_workflows/T1\_workflow\_v01/data/01\_source\_loaded\_harmonized/esld\_master\_long\_public.csv |  | integer |
| sex — Sex **File:** 02\_workflows/T1\_workflow\_v01/data/01\_source\_loaded\_harmonized/esld\_master\_long\_public.csv **Recommended public name:** sex **Description:** Sex category represented in the public ESLD data. **Unit:**  **Value coding:** M = male; F = female. **Data type:** character **Example values:** M \| F **Allowed values:** M \| F **Missing-value coding:** NA/blank as read by fread **n rows / missing / unique:** 67399 / 0 / 2 **Release status:** public **Definition status:** manual\_exact\_definition | 02\_workflows/T1\_workflow\_v01/data/01\_source\_loaded\_harmonized/esld\_master\_long\_public.csv |  | character |
| sodium — Serum sodium concentration **File:** 02\_workflows/T1\_workflow\_v01/data/01\_source\_loaded\_harmonized/esld\_master\_long\_public.csv **Recommended public name:** sodium **Description:** Serum sodium concentration used for MELD-Na, reMELD-Na, or MELD 3.0 score calculation. **Unit:** mmol/L **Value coding:** Numeric concentration in mmol/L. **Data type:** numeric **Example values:** 131 \| 134 \| 132 \| 133 \| 140 **Allowed values:**  **Missing-value coding:** NA/blank as read by fread **n rows / missing / unique:** 67399 / 20359 / 188 **Release status:** public **Definition status:** manual\_exact\_definition | 02\_workflows/T1\_workflow\_v01/data/01\_source\_loaded\_harmonized/esld\_master\_long\_public.csv | mmol/L | numeric |
| tbil — Total bilirubin concentration **File:** 02\_workflows/T1\_workflow\_v01/data/01\_source\_loaded\_harmonized/esld\_master\_long\_public.csv **Recommended public name:** tbil **Description:** Total bilirubin concentration used in MELD-related score calculation in the public ESLD data. **Unit:**  **Value coding:** Numeric concentration in mg/dL. **Data type:** numeric **Example values:** 1 \| 0.9 \| 1.1 \| 0.8 \| 0.6 **Allowed values:**  **Missing-value coding:** NA/blank as read by fread **n rows / missing / unique:** 67399 / 0 / 2029 **Release status:** public **Definition status:** manual\_precision\_definition | 02\_workflows/T1\_workflow\_v01/data/01\_source\_loaded\_harmonized/esld\_master\_long\_public.csv |  | numeric |
| age\_years\_first\_available — Age at first available public record **File:** 02\_workflows/T3\_workflow\_v01/data/01\_source\_loaded\_harmonized/esld\_master\_long\_public.csv **Recommended public name:** age\_years\_first\_available **Description:** Age in years at the first available public ESLD record for the patient or encounter represented in the released data. **Unit:** years **Value coding:** Integer age in years. **Data type:** integer **Example values:** 65 \| 67 \| 21 \| 56 \| 63 **Allowed values:**  **Missing-value coding:** NA/blank as read by fread **n rows / missing / unique:** 67399 / 0 / 79 **Release status:** public **Definition status:** manual\_exact\_definition | 02\_workflows/T3\_workflow\_v01/data/01\_source\_loaded\_harmonized/esld\_master\_long\_public.csv | years | integer |
| age\_years\_sample — Age at sample **File:** 02\_workflows/T3\_workflow\_v01/data/01\_source\_loaded\_harmonized/esld\_master\_long\_public.csv **Recommended public name:** age\_years\_sample **Description:** Age in years at the sample-level public ESLD record. **Unit:** years **Value coding:** Integer age in years. **Data type:** integer **Example values:** 65 \| 66 \| 67 \| 68 \| 69 **Allowed values:**  **Missing-value coding:** NA/blank as read by fread **n rows / missing / unique:** 67399 / 0 / 84 **Release status:** public **Definition status:** manual\_precision\_definition | 02\_workflows/T3\_workflow\_v01/data/01\_source\_loaded\_harmonized/esld\_master\_long\_public.csv | years | integer |
| albumin\_g\_dl — Serum albumin concentration **File:** 02\_workflows/T3\_workflow\_v01/data/01\_source\_loaded\_harmonized/esld\_master\_long\_public.csv **Recommended public name:** albumin\_g\_dl **Description:** Serum albumin concentration in the public ESLD master data. **Unit:** g/dL **Value coding:** Numeric concentration in g/dL. **Data type:** numeric **Example values:** 3.3 \| 6.15 \| 3.14 \| 2.8 \| 3.26 **Allowed values:**  **Missing-value coding:** NA/blank as read by fread **n rows / missing / unique:** 67399 / 58785 / 580 **Release status:** public **Definition status:** manual\_precision\_definition | 02\_workflows/T3\_workflow\_v01/data/01\_source\_loaded\_harmonized/esld\_master\_long\_public.csv | g/dL | numeric |
| ald — Alcohol-related liver disease etiology flag **File:** 02\_workflows/T3\_workflow\_v01/data/01\_source\_loaded\_harmonized/esld\_master\_long\_public.csv **Recommended public name:** ald **Description:** Binary etiology indicator for alcohol-related liver disease in the public ESLD cohort. **Unit:**  **Value coding:** 0 = no; 1 = yes. **Data type:** integer **Example values:** 0 \| 1 **Allowed values:**  **Missing-value coding:** NA/blank as read by fread **n rows / missing / unique:** 67399 / 0 / 2 **Release status:** public **Definition status:** manual\_exact\_definition | 02\_workflows/T3\_workflow\_v01/data/01\_source\_loaded\_harmonized/esld\_master\_long\_public.csv |  | integer |
| ald\_hcv — Combined alcohol-related liver disease and hepatitis C etiology flag **File:** 02\_workflows/T3\_workflow\_v01/data/01\_source\_loaded\_harmonized/esld\_master\_long\_public.csv **Recommended public name:** ald\_hcv **Description:** Binary etiology indicator for combined alcohol-related liver disease and hepatitis C in the public ESLD cohort. **Unit:**  **Value coding:** 0 = no; 1 = yes. **Data type:** integer **Example values:** 0 \| 1 **Allowed values:**  **Missing-value coding:** NA/blank as read by fread **n rows / missing / unique:** 67399 / 0 / 2 **Release status:** public **Definition status:** manual\_exact\_definition | 02\_workflows/T3\_workflow\_v01/data/01\_source\_loaded\_harmonized/esld\_master\_long\_public.csv |  | integer |
| autoimmune — Autoimmune liver disease etiology flag **File:** 02\_workflows/T3\_workflow\_v01/data/01\_source\_loaded\_harmonized/esld\_master\_long\_public.csv **Recommended public name:** autoimmune **Description:** Binary etiology indicator for autoimmune liver disease in the public ESLD cohort. **Unit:**  **Value coding:** 0 = no; 1 = yes. **Data type:** integer **Example values:** 0 \| 1 **Allowed values:**  **Missing-value coding:** NA/blank as read by fread **n rows / missing / unique:** 67399 / 0 / 2 **Release status:** public **Definition status:** manual\_exact\_definition | 02\_workflows/T3\_workflow\_v01/data/01\_source\_loaded\_harmonized/esld\_master\_long\_public.csv |  | integer |
| crea — Creatinine concentration **File:** 02\_workflows/T3\_workflow\_v01/data/01\_source\_loaded\_harmonized/esld\_master\_long\_public.csv **Recommended public name:** crea **Description:** Creatinine concentration used in MELD-related score calculation before applying the study-specific correction. **Unit:** mg/dL **Value coding:** Numeric concentration in mg/dL. **Data type:** numeric **Example values:** 1.91 \| 1.42 \| 1.31 \| 1.3 \| 1.28 **Allowed values:**  **Missing-value coding:** NA/blank as read by fread **n rows / missing / unique:** 67399 / 0 / 975 **Release status:** public **Definition status:** manual\_exact\_definition | 02\_workflows/T3\_workflow\_v01/data/01\_source\_loaded\_harmonized/esld\_master\_long\_public.csv | mg/dL | numeric |
| crea\_corrected — Corrected creatinine concentration **File:** 02\_workflows/T3\_workflow\_v01/data/01\_source\_loaded\_harmonized/esld\_master\_long\_public.csv **Recommended public name:** crea\_corrected **Description:** Creatinine concentration after applying the study-specific correction used for recalculated MELD-related scores. **Unit:** mg/dL **Value coding:** Numeric concentration in mg/dL. **Data type:** numeric **Example values:** 1.87705004446668 \| 1.41586432303417 \| 1.31039363454933 \| 1.30192482788903 \| 1.28150549768982 **Allowed values:**  **Missing-value coding:** NA/blank as read by fread **n rows / missing / unique:** 67399 / 0 / 15868 **Release status:** public **Definition status:** manual\_exact\_definition | 02\_workflows/T3\_workflow\_v01/data/01\_source\_loaded\_harmonized/esld\_master\_long\_public.csv | mg/dL | numeric |
| date\_count — Relative day count **File:** 02\_workflows/T3\_workflow\_v01/data/01\_source\_loaded\_harmonized/esld\_master\_long\_public.csv **Recommended public name:** date\_count **Description:** Relative day count used for time alignment; negative/positive values are relative to the analysis anchor and are not calendar dates. **Unit:**  **Value coding:** See example\_values and allowed\_values. **Data type:** integer **Example values:** -3021 \| -2833 \| -2798 \| -2793 \| -2791 **Allowed values:**  **Missing-value coding:** NA/blank as read by fread **n rows / missing / unique:** 67399 / 50970 / 1450 **Release status:** public **Definition status:** exact\_definition | 02\_workflows/T3\_workflow\_v01/data/01\_source\_loaded\_harmonized/esld\_master\_long\_public.csv |  | integer |
| date\_count\_month — Relative month count **File:** 02\_workflows/T3\_workflow\_v01/data/01\_source\_loaded\_harmonized/esld\_master\_long\_public.csv **Recommended public name:** date\_count\_month **Description:** Relative month count derived from the relative day count; this is not a calendar month. **Unit:**  **Value coding:** See example\_values and allowed\_values. **Data type:** integer **Example values:** -99 \| -93 \| -92 \| -91 \| -87 **Allowed values:**  **Missing-value coding:** NA/blank as read by fread **n rows / missing / unique:** 67399 / 50970 / 147 **Release status:** public **Definition status:** exact\_definition | 02\_workflows/T3\_workflow\_v01/data/01\_source\_loaded\_harmonized/esld\_master\_long\_public.csv |  | integer |
| dead\_sample\_flag — Dead-sample flag **File:** 02\_workflows/T3\_workflow\_v01/data/01\_source\_loaded\_harmonized/esld\_master\_long\_public.csv **Recommended public name:** dead\_sample\_flag **Description:** Binary indicator identifying public ESLD sample records assigned to the deceased-sample subset used in sample-level analyses. **Unit:**  **Value coding:** 0 = not assigned to deceased-sample subset; 1 = assigned to deceased-sample subset. **Data type:** integer **Example values:** 0 \| 1 **Allowed values:**  **Missing-value coding:** NA/blank as read by fread **n rows / missing / unique:** 67399 / 0 / 2 **Release status:** public **Definition status:** manual\_final\_definition | 02\_workflows/T3\_workflow\_v01/data/01\_source\_loaded\_harmonized/esld\_master\_long\_public.csv |  | integer |
| death\_within\_90d — Death within 90 days **File:** 02\_workflows/T3\_workflow\_v01/data/01\_source\_loaded\_harmonized/esld\_master\_long\_public.csv **Recommended public name:** death\_within\_90d **Description:** Binary indicator for death within 90 days of the analysis anchor. **Unit:**  **Value coding:** 0 = no; 1 = yes **Data type:** logical **Example values:**  **Allowed values:**  **Missing-value coding:** NA/blank as read by fread **n rows / missing / unique:** 67399 / 67399 / 0 **Release status:** public **Definition status:** exact\_definition | 02\_workflows/T3\_workflow\_v01/data/01\_source\_loaded\_harmonized/esld\_master\_long\_public.csv |  | logical |
| deceased\_patient\_flag — Deceased patient flag **File:** 02\_workflows/T3\_workflow\_v01/data/01\_source\_loaded\_harmonized/esld\_master\_long\_public.csv **Recommended public name:** deceased\_patient\_flag **Description:** Binary indicator identifying deceased patients within the released analysis context. **Unit:**  **Value coding:** 0 = no; 1 = yes **Data type:** integer **Example values:** 0 \| 1 **Allowed values:**  **Missing-value coding:** NA/blank as read by fread **n rows / missing / unique:** 67399 / 0 / 2 **Release status:** public **Definition status:** exact\_definition | 02\_workflows/T3\_workflow\_v01/data/01\_source\_loaded\_harmonized/esld\_master\_long\_public.csv |  | integer |
| delta\_eq\_0\_flag — Zero score-delta flag **File:** 02\_workflows/T3\_workflow\_v01/data/01\_source\_loaded\_harmonized/esld\_master\_long\_public.csv **Recommended public name:** delta\_eq\_0\_flag **Description:** Binary indicator equal to 1 when the score delta is exactly zero. **Unit:**  **Value coding:** 0 = no; 1 = yes. **Data type:** integer **Example values:** 1 \| 0 **Allowed values:**  **Missing-value coding:** NA/blank as read by fread **n rows / missing / unique:** 67399 / 0 / 2 **Release status:** public **Definition status:** manual\_precision\_definition | 02\_workflows/T3\_workflow\_v01/data/01\_source\_loaded\_harmonized/esld\_master\_long\_public.csv |  | integer |
| delta\_gt\_0\_flag — Positive score-delta flag **File:** 02\_workflows/T3\_workflow\_v01/data/01\_source\_loaded\_harmonized/esld\_master\_long\_public.csv **Recommended public name:** delta\_gt\_0\_flag **Description:** Binary indicator equal to 1 when the score delta is positive. **Unit:**  **Value coding:** 0 = no; 1 = yes. **Data type:** integer **Example values:** 0 \| 1 **Allowed values:**  **Missing-value coding:** NA/blank as read by fread **n rows / missing / unique:** 67399 / 0 / 2 **Release status:** public **Definition status:** manual\_precision\_definition | 02\_workflows/T3\_workflow\_v01/data/01\_source\_loaded\_harmonized/esld\_master\_long\_public.csv |  | integer |
| delta\_le\_minus1\_flag — Score-delta ≤ −1 flag **File:** 02\_workflows/T3\_workflow\_v01/data/01\_source\_loaded\_harmonized/esld\_master\_long\_public.csv **Recommended public name:** delta\_le\_minus1\_flag **Description:** Binary indicator equal to 1 when the score delta is less than or equal to −1. **Unit:**  **Value coding:** 0 = no; 1 = yes. **Data type:** integer **Example values:** 0 \| 1 **Allowed values:**  **Missing-value coding:** NA/blank as read by fread **n rows / missing / unique:** 67399 / 0 / 2 **Release status:** public **Definition status:** manual\_precision\_definition | 02\_workflows/T3\_workflow\_v01/data/01\_source\_loaded\_harmonized/esld\_master\_long\_public.csv |  | integer |
| dialysis\_raw — Dialysis indicator as recorded before score derivation **File:** 02\_workflows/T3\_workflow\_v01/data/01\_source\_loaded\_harmonized/esld\_master\_long\_public.csv **Recommended public name:** dialysis\_raw **Description:** Binary indicator showing whether dialysis was recorded in the source field before score derivation. **Unit:**  **Value coding:** 0 = no dialysis recorded; 1 = dialysis recorded. **Data type:** integer **Example values:** 0 \| 1 **Allowed values:**  **Missing-value coding:** NA/blank as read by fread **n rows / missing / unique:** 67399 / 27827 / 2 **Release status:** public **Definition status:** manual\_exact\_definition | 02\_workflows/T3\_workflow\_v01/data/01\_source\_loaded\_harmonized/esld\_master\_long\_public.csv |  | integer |
| encounter\_id\_public — Public encounter pseudonym **File:** 02\_workflows/T3\_workflow\_v01/data/01\_source\_loaded\_harmonized/esld\_master\_long\_public.csv **Recommended public name:** encounter\_id\_public **Description:** Non-linkable public-release encounter pseudonym used for encounter-level grouping; not an original hospital encounter identifier. **Unit:**  **Value coding:** See example\_values and allowed\_values. **Data type:** character **Example values:** E001181 \| E000008 \| E000041 \| E000100 \| E000126 **Allowed values:** E001181 \| E000008 \| E000041 \| E000100 \| E000126 \| E000151 \| E000157 \| E000219 \| E000289 \| E000344 **Missing-value coding:** NA/blank as read by fread **n rows / missing / unique:** 67399 / 0 / 5045 **Release status:** public **Definition status:** exact\_definition | 02\_workflows/T3\_workflow\_v01/data/01\_source\_loaded\_harmonized/esld\_master\_long\_public.csv |  | character |
| etiology\_source\_current\_flag — Current etiology source flag **File:** 02\_workflows/T3\_workflow\_v01/data/01\_source\_loaded\_harmonized/esld\_master\_long\_public.csv **Recommended public name:** etiology\_source\_current\_flag **Description:** Binary indicator identifying whether the etiology source is the current source used for the public row. **Unit:**  **Value coding:** 0 = no; 1 = yes. **Data type:** integer **Example values:** 1 **Allowed values:**  **Missing-value coding:** NA/blank as read by fread **n rows / missing / unique:** 67399 / 0 / 1 **Release status:** public **Definition status:** manual\_precision\_definition | 02\_workflows/T3\_workflow\_v01/data/01\_source\_loaded\_harmonized/esld\_master\_long\_public.csv |  | integer |
| etiology\_unclassified — Unclassified liver disease etiology flag **File:** 02\_workflows/T3\_workflow\_v01/data/01\_source\_loaded\_harmonized/esld\_master\_long\_public.csv **Recommended public name:** etiology\_unclassified **Description:** Binary etiology indicator identifying records without a classified liver disease etiology in the public ESLD cohort. **Unit:**  **Value coding:** 0 = no; 1 = yes. **Data type:** integer **Example values:** 0 **Allowed values:**  **Missing-value coding:** NA/blank as read by fread **n rows / missing / unique:** 67399 / 0 / 1 **Release status:** public **Definition status:** manual\_exact\_definition | 02\_workflows/T3\_workflow\_v01/data/01\_source\_loaded\_harmonized/esld\_master\_long\_public.csv |  | integer |
| hbv — Hepatitis B virus etiology flag **File:** 02\_workflows/T3\_workflow\_v01/data/01\_source\_loaded\_harmonized/esld\_master\_long\_public.csv **Recommended public name:** hbv **Description:** Binary etiology indicator for hepatitis B virus-related liver disease in the public ESLD cohort. **Unit:**  **Value coding:** 0 = no; 1 = yes. **Data type:** integer **Example values:** 0 \| 1 **Allowed values:**  **Missing-value coding:** NA/blank as read by fread **n rows / missing / unique:** 67399 / 0 / 2 **Release status:** public **Definition status:** manual\_exact\_definition | 02\_workflows/T3\_workflow\_v01/data/01\_source\_loaded\_harmonized/esld\_master\_long\_public.csv |  | integer |
| hcv — Hepatitis C virus etiology flag **File:** 02\_workflows/T3\_workflow\_v01/data/01\_source\_loaded\_harmonized/esld\_master\_long\_public.csv **Recommended public name:** hcv **Description:** Binary etiology indicator for hepatitis C virus-related liver disease in the public ESLD cohort. **Unit:**  **Value coding:** 0 = no; 1 = yes. **Data type:** integer **Example values:** 0 \| 1 **Allowed values:**  **Missing-value coding:** NA/blank as read by fread **n rows / missing / unique:** 67399 / 0 / 2 **Release status:** public **Definition status:** manual\_exact\_definition | 02\_workflows/T3\_workflow\_v01/data/01\_source\_loaded\_harmonized/esld\_master\_long\_public.csv |  | integer |
| icd\_source\_present — ICD source present flag **File:** 02\_workflows/T3\_workflow\_v01/data/01\_source\_loaded\_harmonized/esld\_master\_long\_public.csv **Recommended public name:** icd\_source\_present **Description:** Binary indicator showing whether an ICD-derived etiology source was present for the public row. **Unit:**  **Value coding:** 0 = no; 1 = yes. **Data type:** integer **Example values:** 1 \| 0 **Allowed values:**  **Missing-value coding:** NA/blank as read by fread **n rows / missing / unique:** 67399 / 0 / 2 **Release status:** public **Definition status:** manual\_precision\_definition | 02\_workflows/T3\_workflow\_v01/data/01\_source\_loaded\_harmonized/esld\_master\_long\_public.csv |  | integer |
| in\_t1\_cohort — Inclusion flag for Table 1 cohort **File:** 02\_workflows/T3\_workflow\_v01/data/01\_source\_loaded\_harmonized/esld\_master\_long\_public.csv **Recommended public name:** in\_t1\_cohort **Description:** Binary indicator identifying records included in the public ESLD Table 1 baseline-characteristics cohort. **Unit:**  **Value coding:** 0 = not included; 1 = included. **Data type:** integer **Example values:** 1 **Allowed values:**  **Missing-value coding:** NA/blank as read by fread **n rows / missing / unique:** 67399 / 0 / 1 **Release status:** public **Definition status:** manual\_exact\_definition | 02\_workflows/T3\_workflow\_v01/data/01\_source\_loaded\_harmonized/esld\_master\_long\_public.csv |  | integer |
| in\_t3\_anchor\_model — Inclusion flag for Table 3 anchor-model cohort **File:** 02\_workflows/T3\_workflow\_v01/data/01\_source\_loaded\_harmonized/esld\_master\_long\_public.csv **Recommended public name:** in\_t3\_anchor\_model **Description:** Binary indicator identifying records included in the Table 3 anchor-model cohort used for creatinine-comparison analyses. **Unit:**  **Value coding:** 0 = not included; 1 = included. **Data type:** integer **Example values:** 1 \| 0 **Allowed values:**  **Missing-value coding:** NA/blank as read by fread **n rows / missing / unique:** 67399 / 0 / 2 **Release status:** public **Definition status:** manual\_exact\_definition | 02\_workflows/T3\_workflow\_v01/data/01\_source\_loaded\_harmonized/esld\_master\_long\_public.csv |  | integer |
| in\_t4\_prevalence\_path — Inclusion flag for Table 4 prevalence pathway **File:** 02\_workflows/T3\_workflow\_v01/data/01\_source\_loaded\_harmonized/esld\_master\_long\_public.csv **Recommended public name:** in\_t4\_prevalence\_path **Description:** Binary indicator identifying records included in the Table 4 prevalence-analysis pathway. **Unit:**  **Value coding:** 0 = not included; 1 = included. **Data type:** integer **Example values:** 1 **Allowed values:**  **Missing-value coding:** NA/blank as read by fread **n rows / missing / unique:** 67399 / 0 / 1 **Release status:** public **Definition status:** manual\_exact\_definition | 02\_workflows/T3\_workflow\_v01/data/01\_source\_loaded\_harmonized/esld\_master\_long\_public.csv |  | integer |
| in\_t4\_survival\_path — Inclusion flag for Table 4 survival pathway **File:** 02\_workflows/T3\_workflow\_v01/data/01\_source\_loaded\_harmonized/esld\_master\_long\_public.csv **Recommended public name:** in\_t4\_survival\_path **Description:** Binary indicator identifying records included in the Table 4 survival-analysis pathway. **Unit:**  **Value coding:** 0 = not included; 1 = included. **Data type:** integer **Example values:** 0 \| 1 **Allowed values:**  **Missing-value coding:** NA/blank as read by fread **n rows / missing / unique:** 67399 / 0 / 2 **Release status:** public **Definition status:** manual\_exact\_definition | 02\_workflows/T3\_workflow\_v01/data/01\_source\_loaded\_harmonized/esld\_master\_long\_public.csv |  | integer |
| inr — International normalized ratio **File:** 02\_workflows/T3\_workflow\_v01/data/01\_source\_loaded\_harmonized/esld\_master\_long\_public.csv **Recommended public name:** inr **Description:** International normalized ratio of prothrombin time used for MELD-related score calculation. **Unit:** ratio **Value coding:** Numeric INR value. **Data type:** numeric **Example values:** 1.14 \| 0.91 \| 1 \| 1.04 \| 0.98 **Allowed values:**  **Missing-value coding:** NA/blank as read by fread **n rows / missing / unique:** 67399 / 0 / 786 **Release status:** public **Definition status:** manual\_exact\_definition | 02\_workflows/T3\_workflow\_v01/data/01\_source\_loaded\_harmonized/esld\_master\_long\_public.csv | ratio | numeric |
| mash — Metabolic dysfunction-associated steatohepatitis etiology flag **File:** 02\_workflows/T3\_workflow\_v01/data/01\_source\_loaded\_harmonized/esld\_master\_long\_public.csv **Recommended public name:** mash **Description:** Binary etiology indicator for metabolic dysfunction-associated steatohepatitis in the public ESLD cohort. **Unit:**  **Value coding:** 0 = no; 1 = yes. **Data type:** integer **Example values:** 0 \| 1 **Allowed values:**  **Missing-value coding:** NA/blank as read by fread **n rows / missing / unique:** 67399 / 0 / 2 **Release status:** public **Definition status:** manual\_exact\_definition | 02\_workflows/T3\_workflow\_v01/data/01\_source\_loaded\_harmonized/esld\_master\_long\_public.csv |  | integer |
| model — MELD model or score variant **File:** 02\_workflows/T3\_workflow\_v01/data/01\_source\_loaded\_harmonized/esld\_master\_long\_public.csv **Recommended public name:** model **Description:** Name of the MELD-related model or score variant represented by the row; expected values include MELD, MELD-Na, reMELD-Na, and MELD 3.0. **Unit:**  **Value coding:** Categorical score/model label, for example MELD, MELD-Na, reMELD-Na, or MELD 3.0. **Data type:** character **Example values:** MELD \| MELD-Na \| reMELD-Na \| MELD 3.0 **Allowed values:** MELD \| MELD-Na \| reMELD-Na \| MELD 3.0 **Missing-value coding:** NA/blank as read by fread **n rows / missing / unique:** 67399 / 0 / 4 **Release status:** public **Definition status:** manual\_exact\_definition | 02\_workflows/T3\_workflow\_v01/data/01\_source\_loaded\_harmonized/esld\_master\_long\_public.csv |  | character |
| other — Other liver disease etiology flag **File:** 02\_workflows/T3\_workflow\_v01/data/01\_source\_loaded\_harmonized/esld\_master\_long\_public.csv **Recommended public name:** other **Description:** Binary etiology indicator for liver disease etiologies grouped as other in the public ESLD cohort. **Unit:**  **Value coding:** 0 = no; 1 = yes. **Data type:** integer **Example values:** 1 \| 0 **Allowed values:**  **Missing-value coding:** NA/blank as read by fread **n rows / missing / unique:** 67399 / 0 / 2 **Release status:** public **Definition status:** manual\_exact\_definition | 02\_workflows/T3\_workflow\_v01/data/01\_source\_loaded\_harmonized/esld\_master\_long\_public.csv |  | integer |
| patient\_id — Public patient pseudonym **File:** 02\_workflows/T3\_workflow\_v01/data/01\_source\_loaded\_harmonized/esld\_master\_long\_public.csv **Recommended public name:** patient\_id **Description:** Non-linkable public-release patient pseudonym used to preserve within-patient grouping in public ESLD data; not an original hospital patient identifier. **Unit:**  **Value coding:** See example\_values and allowed\_values. **Data type:** character **Example values:** P000001 \| P000002 \| P000003 \| P000004 \| P000005 **Allowed values:** P000001 \| P000002 \| P000003 \| P000004 \| P000005 \| P000006 \| P000007 \| P000008 \| P000009 \| P000010 **Missing-value coding:** NA/blank as read by fread **n rows / missing / unique:** 67399 / 0 / 1375 **Release status:** public **Definition status:** exact\_definition | 02\_workflows/T3\_workflow\_v01/data/01\_source\_loaded\_harmonized/esld\_master\_long\_public.csv |  | character |
| pbc — Primary biliary cholangitis etiology flag **File:** 02\_workflows/T3\_workflow\_v01/data/01\_source\_loaded\_harmonized/esld\_master\_long\_public.csv **Recommended public name:** pbc **Description:** Binary etiology indicator for primary biliary cholangitis in the public ESLD cohort. **Unit:**  **Value coding:** 0 = no; 1 = yes. **Data type:** integer **Example values:** 0 \| 1 **Allowed values:**  **Missing-value coding:** NA/blank as read by fread **n rows / missing / unique:** 67399 / 0 / 2 **Release status:** public **Definition status:** manual\_exact\_definition | 02\_workflows/T3\_workflow\_v01/data/01\_source\_loaded\_harmonized/esld\_master\_long\_public.csv |  | integer |
| sample\_day\_from\_first\_sample — Relative sample day **File:** 02\_workflows/T3\_workflow\_v01/data/01\_source\_loaded\_harmonized/esld\_master\_long\_public.csv **Recommended public name:** sample\_day\_from\_first\_sample **Description:** Relative day of the sample measured from the first sample for the public patient/sample sequence; not a calendar date. **Unit:** relative months **Value coding:** Integer or numeric relative day count. **Data type:** integer **Example values:** 0 \| 42 \| 91 \| 118 \| 133 **Allowed values:**  **Missing-value coding:** NA/blank as read by fread **n rows / missing / unique:** 67399 / 0 / 3438 **Release status:** public **Definition status:** manual\_precision\_definition | 02\_workflows/T3\_workflow\_v01/data/01\_source\_loaded\_harmonized/esld\_master\_long\_public.csv | relative months | integer |
| sample\_group\_id\_public — Public sample-group pseudonym **File:** 02\_workflows/T3\_workflow\_v01/data/01\_source\_loaded\_harmonized/esld\_master\_long\_public.csv **Recommended public name:** sample\_group\_id\_public **Description:** Non-linkable public-release sample-group pseudonym used to group samples within the released public data. **Unit:**  **Value coding:** See example\_values and allowed\_values. **Data type:** character **Example values:** G0000001 \| G0000002 \| G0000003 \| G0000004 \| G0000005 **Allowed values:** G0000001 \| G0000002 \| G0000003 \| G0000004 \| G0000005 \| G0000006 \| G0000007 \| G0000008 \| G0000009 \| G0000010 **Missing-value coding:** NA/blank as read by fread **n rows / missing / unique:** 67399 / 0 / 20359 **Release status:** public **Definition status:** exact\_definition | 02\_workflows/T3\_workflow\_v01/data/01\_source\_loaded\_harmonized/esld\_master\_long\_public.csv |  | character |
| sample\_id — Sample identifier **File:** 02\_workflows/T3\_workflow\_v01/data/01\_source\_loaded\_harmonized/esld\_master\_long\_public.csv **Recommended public name:** sample\_id **Description:** Identifier of a sample or experimental record within the released public data; not a personal identifier. **Unit:**  **Value coding:** See example\_values and allowed\_values. **Data type:** character **Example values:** S0000001 \| S0000002 \| S0000003 \| S0000004 \| S0000005 **Allowed values:** S0000001 \| S0000002 \| S0000003 \| S0000004 \| S0000005 \| S0000006 \| S0000007 \| S0000008 \| S0000009 \| S0000010 **Missing-value coding:** NA/blank as read by fread **n rows / missing / unique:** 67399 / 0 / 67399 **Release status:** public **Definition status:** exact\_definition | 02\_workflows/T3\_workflow\_v01/data/01\_source\_loaded\_harmonized/esld\_master\_long\_public.csv |  | character |
| sample\_month\_index — Relative sample month index **File:** 02\_workflows/T3\_workflow\_v01/data/01\_source\_loaded\_harmonized/esld\_master\_long\_public.csv **Recommended public name:** sample\_month\_index **Description:** Relative month index of the sample measured from the first sample for the public patient/sample sequence; not a calendar month. **Unit:** score points **Value coding:** Integer or numeric relative month index. **Data type:** integer **Example values:** 0 \| 1 \| 2 \| 3 \| 4 **Allowed values:**  **Missing-value coding:** NA/blank as read by fread **n rows / missing / unique:** 67399 / 0 / 177 **Release status:** public **Definition status:** manual\_precision\_definition | 02\_workflows/T3\_workflow\_v01/data/01\_source\_loaded\_harmonized/esld\_master\_long\_public.csv | score points | integer |
| score\_corrected — Corrected score **File:** 02\_workflows/T3\_workflow\_v01/data/01\_source\_loaded\_harmonized/esld\_master\_long\_public.csv **Recommended public name:** score\_corrected **Description:** MELD-related score after creatinine correction or recalculation. **Unit:** score points **Value coding:** Numeric score. **Data type:** integer **Example values:** 14 \| 19 \| 16 \| 10 \| 13 **Allowed values:**  **Missing-value coding:** NA/blank as read by fread **n rows / missing / unique:** 67399 / 0 / 40 **Release status:** public **Definition status:** manual\_precision\_definition | 02\_workflows/T3\_workflow\_v01/data/01\_source\_loaded\_harmonized/esld\_master\_long\_public.csv | score points | integer |
| score\_delta — Score difference **File:** 02\_workflows/T3\_workflow\_v01/data/01\_source\_loaded\_harmonized/esld\_master\_long\_public.csv **Recommended public name:** score\_delta **Description:** Difference between score variants or scoring approaches, expressed in score points. **Unit:**  **Value coding:** See example\_values and allowed\_values. **Data type:** integer **Example values:** 0 \| 1 \| -1 \| -2 **Allowed values:**  **Missing-value coding:** NA/blank as read by fread **n rows / missing / unique:** 67399 / 0 / 4 **Release status:** public **Definition status:** exact\_definition | 02\_workflows/T3\_workflow\_v01/data/01\_source\_loaded\_harmonized/esld\_master\_long\_public.csv |  | integer |
| score\_original — Original score **File:** 02\_workflows/T3\_workflow\_v01/data/01\_source\_loaded\_harmonized/esld\_master\_long\_public.csv **Recommended public name:** score\_original **Description:** Original MELD-related score before creatinine correction or recalculation. **Unit:**  **Value coding:** Numeric score. **Data type:** integer **Example values:** 14 \| 19 \| 16 \| 10 \| 13 **Allowed values:**  **Missing-value coding:** NA/blank as read by fread **n rows / missing / unique:** 67399 / 0 / 40 **Release status:** public **Definition status:** manual\_precision\_definition | 02\_workflows/T3\_workflow\_v01/data/01\_source\_loaded\_harmonized/esld\_master\_long\_public.csv |  | integer |
| sex — Sex **File:** 02\_workflows/T3\_workflow\_v01/data/01\_source\_loaded\_harmonized/esld\_master\_long\_public.csv **Recommended public name:** sex **Description:** Sex category represented in the public ESLD data. **Unit:**  **Value coding:** M = male; F = female. **Data type:** character **Example values:** M \| F **Allowed values:** M \| F **Missing-value coding:** NA/blank as read by fread **n rows / missing / unique:** 67399 / 0 / 2 **Release status:** public **Definition status:** manual\_exact\_definition | 02\_workflows/T3\_workflow\_v01/data/01\_source\_loaded\_harmonized/esld\_master\_long\_public.csv |  | character |
| sodium — Serum sodium concentration **File:** 02\_workflows/T3\_workflow\_v01/data/01\_source\_loaded\_harmonized/esld\_master\_long\_public.csv **Recommended public name:** sodium **Description:** Serum sodium concentration used for MELD-Na, reMELD-Na, or MELD 3.0 score calculation. **Unit:** mmol/L **Value coding:** Numeric concentration in mmol/L. **Data type:** numeric **Example values:** 131 \| 134 \| 132 \| 133 \| 140 **Allowed values:**  **Missing-value coding:** NA/blank as read by fread **n rows / missing / unique:** 67399 / 20359 / 188 **Release status:** public **Definition status:** manual\_exact\_definition | 02\_workflows/T3\_workflow\_v01/data/01\_source\_loaded\_harmonized/esld\_master\_long\_public.csv | mmol/L | numeric |
| tbil — Total bilirubin concentration **File:** 02\_workflows/T3\_workflow\_v01/data/01\_source\_loaded\_harmonized/esld\_master\_long\_public.csv **Recommended public name:** tbil **Description:** Total bilirubin concentration used in MELD-related score calculation in the public ESLD data. **Unit:**  **Value coding:** Numeric concentration in mg/dL. **Data type:** numeric **Example values:** 1 \| 0.9 \| 1.1 \| 0.8 \| 0.6 **Allowed values:**  **Missing-value coding:** NA/blank as read by fread **n rows / missing / unique:** 67399 / 0 / 2029 **Release status:** public **Definition status:** manual\_precision\_definition | 02\_workflows/T3\_workflow\_v01/data/01\_source\_loaded\_harmonized/esld\_master\_long\_public.csv |  | numeric |
| age\_years\_first\_available — Age at first available public record **File:** 02\_workflows/T4\_workflow\_v01/data/01\_source\_loaded\_harmonized/esld\_master\_long\_public.csv **Recommended public name:** age\_years\_first\_available **Description:** Age in years at the first available public ESLD record for the patient or encounter represented in the released data. **Unit:** years **Value coding:** Integer age in years. **Data type:** integer **Example values:** 65 \| 67 \| 21 \| 56 \| 63 **Allowed values:**  **Missing-value coding:** NA/blank as read by fread **n rows / missing / unique:** 67399 / 0 / 79 **Release status:** public **Definition status:** manual\_exact\_definition | 02\_workflows/T4\_workflow\_v01/data/01\_source\_loaded\_harmonized/esld\_master\_long\_public.csv | years | integer |
| age\_years\_sample — Age at sample **File:** 02\_workflows/T4\_workflow\_v01/data/01\_source\_loaded\_harmonized/esld\_master\_long\_public.csv **Recommended public name:** age\_years\_sample **Description:** Age in years at the sample-level public ESLD record. **Unit:** years **Value coding:** Integer age in years. **Data type:** integer **Example values:** 65 \| 66 \| 67 \| 68 \| 69 **Allowed values:**  **Missing-value coding:** NA/blank as read by fread **n rows / missing / unique:** 67399 / 0 / 84 **Release status:** public **Definition status:** manual\_precision\_definition | 02\_workflows/T4\_workflow\_v01/data/01\_source\_loaded\_harmonized/esld\_master\_long\_public.csv | years | integer |
| albumin\_g\_dl — Serum albumin concentration **File:** 02\_workflows/T4\_workflow\_v01/data/01\_source\_loaded\_harmonized/esld\_master\_long\_public.csv **Recommended public name:** albumin\_g\_dl **Description:** Serum albumin concentration in the public ESLD master data. **Unit:** g/dL **Value coding:** Numeric concentration in g/dL. **Data type:** numeric **Example values:** 3.3 \| 6.15 \| 3.14 \| 2.8 \| 3.26 **Allowed values:**  **Missing-value coding:** NA/blank as read by fread **n rows / missing / unique:** 67399 / 58785 / 580 **Release status:** public **Definition status:** manual\_precision\_definition | 02\_workflows/T4\_workflow\_v01/data/01\_source\_loaded\_harmonized/esld\_master\_long\_public.csv | g/dL | numeric |
| ald — Alcohol-related liver disease etiology flag **File:** 02\_workflows/T4\_workflow\_v01/data/01\_source\_loaded\_harmonized/esld\_master\_long\_public.csv **Recommended public name:** ald **Description:** Binary etiology indicator for alcohol-related liver disease in the public ESLD cohort. **Unit:**  **Value coding:** 0 = no; 1 = yes. **Data type:** integer **Example values:** 0 \| 1 **Allowed values:**  **Missing-value coding:** NA/blank as read by fread **n rows / missing / unique:** 67399 / 0 / 2 **Release status:** public **Definition status:** manual\_exact\_definition | 02\_workflows/T4\_workflow\_v01/data/01\_source\_loaded\_harmonized/esld\_master\_long\_public.csv |  | integer |
| ald\_hcv — Combined alcohol-related liver disease and hepatitis C etiology flag **File:** 02\_workflows/T4\_workflow\_v01/data/01\_source\_loaded\_harmonized/esld\_master\_long\_public.csv **Recommended public name:** ald\_hcv **Description:** Binary etiology indicator for combined alcohol-related liver disease and hepatitis C in the public ESLD cohort. **Unit:**  **Value coding:** 0 = no; 1 = yes. **Data type:** integer **Example values:** 0 \| 1 **Allowed values:**  **Missing-value coding:** NA/blank as read by fread **n rows / missing / unique:** 67399 / 0 / 2 **Release status:** public **Definition status:** manual\_exact\_definition | 02\_workflows/T4\_workflow\_v01/data/01\_source\_loaded\_harmonized/esld\_master\_long\_public.csv |  | integer |
| autoimmune — Autoimmune liver disease etiology flag **File:** 02\_workflows/T4\_workflow\_v01/data/01\_source\_loaded\_harmonized/esld\_master\_long\_public.csv **Recommended public name:** autoimmune **Description:** Binary etiology indicator for autoimmune liver disease in the public ESLD cohort. **Unit:**  **Value coding:** 0 = no; 1 = yes. **Data type:** integer **Example values:** 0 \| 1 **Allowed values:**  **Missing-value coding:** NA/blank as read by fread **n rows / missing / unique:** 67399 / 0 / 2 **Release status:** public **Definition status:** manual\_exact\_definition | 02\_workflows/T4\_workflow\_v01/data/01\_source\_loaded\_harmonized/esld\_master\_long\_public.csv |  | integer |
| crea — Creatinine concentration **File:** 02\_workflows/T4\_workflow\_v01/data/01\_source\_loaded\_harmonized/esld\_master\_long\_public.csv **Recommended public name:** crea **Description:** Creatinine concentration used in MELD-related score calculation before applying the study-specific correction. **Unit:** mg/dL **Value coding:** Numeric concentration in mg/dL. **Data type:** numeric **Example values:** 1.91 \| 1.42 \| 1.31 \| 1.3 \| 1.28 **Allowed values:**  **Missing-value coding:** NA/blank as read by fread **n rows / missing / unique:** 67399 / 0 / 975 **Release status:** public **Definition status:** manual\_exact\_definition | 02\_workflows/T4\_workflow\_v01/data/01\_source\_loaded\_harmonized/esld\_master\_long\_public.csv | mg/dL | numeric |
| crea\_corrected — Corrected creatinine concentration **File:** 02\_workflows/T4\_workflow\_v01/data/01\_source\_loaded\_harmonized/esld\_master\_long\_public.csv **Recommended public name:** crea\_corrected **Description:** Creatinine concentration after applying the study-specific correction used for recalculated MELD-related scores. **Unit:** mg/dL **Value coding:** Numeric concentration in mg/dL. **Data type:** numeric **Example values:** 1.87705004446668 \| 1.41586432303417 \| 1.31039363454933 \| 1.30192482788903 \| 1.28150549768982 **Allowed values:**  **Missing-value coding:** NA/blank as read by fread **n rows / missing / unique:** 67399 / 0 / 15868 **Release status:** public **Definition status:** manual\_exact\_definition | 02\_workflows/T4\_workflow\_v01/data/01\_source\_loaded\_harmonized/esld\_master\_long\_public.csv | mg/dL | numeric |
| date\_count — Relative day count **File:** 02\_workflows/T4\_workflow\_v01/data/01\_source\_loaded\_harmonized/esld\_master\_long\_public.csv **Recommended public name:** date\_count **Description:** Relative day count used for time alignment; negative/positive values are relative to the analysis anchor and are not calendar dates. **Unit:**  **Value coding:** See example\_values and allowed\_values. **Data type:** integer **Example values:** -3021 \| -2833 \| -2798 \| -2793 \| -2791 **Allowed values:**  **Missing-value coding:** NA/blank as read by fread **n rows / missing / unique:** 67399 / 50970 / 1450 **Release status:** public **Definition status:** exact\_definition | 02\_workflows/T4\_workflow\_v01/data/01\_source\_loaded\_harmonized/esld\_master\_long\_public.csv |  | integer |
| date\_count\_month — Relative month count **File:** 02\_workflows/T4\_workflow\_v01/data/01\_source\_loaded\_harmonized/esld\_master\_long\_public.csv **Recommended public name:** date\_count\_month **Description:** Relative month count derived from the relative day count; this is not a calendar month. **Unit:**  **Value coding:** See example\_values and allowed\_values. **Data type:** integer **Example values:** -99 \| -93 \| -92 \| -91 \| -87 **Allowed values:**  **Missing-value coding:** NA/blank as read by fread **n rows / missing / unique:** 67399 / 50970 / 147 **Release status:** public **Definition status:** exact\_definition | 02\_workflows/T4\_workflow\_v01/data/01\_source\_loaded\_harmonized/esld\_master\_long\_public.csv |  | integer |
| dead\_sample\_flag — Dead-sample flag **File:** 02\_workflows/T4\_workflow\_v01/data/01\_source\_loaded\_harmonized/esld\_master\_long\_public.csv **Recommended public name:** dead\_sample\_flag **Description:** Binary indicator identifying public ESLD sample records assigned to the deceased-sample subset used in sample-level analyses. **Unit:**  **Value coding:** 0 = not assigned to deceased-sample subset; 1 = assigned to deceased-sample subset. **Data type:** integer **Example values:** 0 \| 1 **Allowed values:**  **Missing-value coding:** NA/blank as read by fread **n rows / missing / unique:** 67399 / 0 / 2 **Release status:** public **Definition status:** manual\_final\_definition | 02\_workflows/T4\_workflow\_v01/data/01\_source\_loaded\_harmonized/esld\_master\_long\_public.csv |  | integer |
| death\_within\_90d — Death within 90 days **File:** 02\_workflows/T4\_workflow\_v01/data/01\_source\_loaded\_harmonized/esld\_master\_long\_public.csv **Recommended public name:** death\_within\_90d **Description:** Binary indicator for death within 90 days of the analysis anchor. **Unit:**  **Value coding:** 0 = no; 1 = yes **Data type:** logical **Example values:**  **Allowed values:**  **Missing-value coding:** NA/blank as read by fread **n rows / missing / unique:** 67399 / 67399 / 0 **Release status:** public **Definition status:** exact\_definition | 02\_workflows/T4\_workflow\_v01/data/01\_source\_loaded\_harmonized/esld\_master\_long\_public.csv |  | logical |
| deceased\_patient\_flag — Deceased patient flag **File:** 02\_workflows/T4\_workflow\_v01/data/01\_source\_loaded\_harmonized/esld\_master\_long\_public.csv **Recommended public name:** deceased\_patient\_flag **Description:** Binary indicator identifying deceased patients within the released analysis context. **Unit:**  **Value coding:** 0 = no; 1 = yes **Data type:** integer **Example values:** 0 \| 1 **Allowed values:**  **Missing-value coding:** NA/blank as read by fread **n rows / missing / unique:** 67399 / 0 / 2 **Release status:** public **Definition status:** exact\_definition | 02\_workflows/T4\_workflow\_v01/data/01\_source\_loaded\_harmonized/esld\_master\_long\_public.csv |  | integer |
| delta\_eq\_0\_flag — Zero score-delta flag **File:** 02\_workflows/T4\_workflow\_v01/data/01\_source\_loaded\_harmonized/esld\_master\_long\_public.csv **Recommended public name:** delta\_eq\_0\_flag **Description:** Binary indicator equal to 1 when the score delta is exactly zero. **Unit:**  **Value coding:** 0 = no; 1 = yes. **Data type:** integer **Example values:** 1 \| 0 **Allowed values:**  **Missing-value coding:** NA/blank as read by fread **n rows / missing / unique:** 67399 / 0 / 2 **Release status:** public **Definition status:** manual\_precision\_definition | 02\_workflows/T4\_workflow\_v01/data/01\_source\_loaded\_harmonized/esld\_master\_long\_public.csv |  | integer |
| delta\_gt\_0\_flag — Positive score-delta flag **File:** 02\_workflows/T4\_workflow\_v01/data/01\_source\_loaded\_harmonized/esld\_master\_long\_public.csv **Recommended public name:** delta\_gt\_0\_flag **Description:** Binary indicator equal to 1 when the score delta is positive. **Unit:**  **Value coding:** 0 = no; 1 = yes. **Data type:** integer **Example values:** 0 \| 1 **Allowed values:**  **Missing-value coding:** NA/blank as read by fread **n rows / missing / unique:** 67399 / 0 / 2 **Release status:** public **Definition status:** manual\_precision\_definition | 02\_workflows/T4\_workflow\_v01/data/01\_source\_loaded\_harmonized/esld\_master\_long\_public.csv |  | integer |
| delta\_le\_minus1\_flag — Score-delta ≤ −1 flag **File:** 02\_workflows/T4\_workflow\_v01/data/01\_source\_loaded\_harmonized/esld\_master\_long\_public.csv **Recommended public name:** delta\_le\_minus1\_flag **Description:** Binary indicator equal to 1 when the score delta is less than or equal to −1. **Unit:**  **Value coding:** 0 = no; 1 = yes. **Data type:** integer **Example values:** 0 \| 1 **Allowed values:**  **Missing-value coding:** NA/blank as read by fread **n rows / missing / unique:** 67399 / 0 / 2 **Release status:** public **Definition status:** manual\_precision\_definition | 02\_workflows/T4\_workflow\_v01/data/01\_source\_loaded\_harmonized/esld\_master\_long\_public.csv |  | integer |
| dialysis\_raw — Dialysis indicator as recorded before score derivation **File:** 02\_workflows/T4\_workflow\_v01/data/01\_source\_loaded\_harmonized/esld\_master\_long\_public.csv **Recommended public name:** dialysis\_raw **Description:** Binary indicator showing whether dialysis was recorded in the source field before score derivation. **Unit:**  **Value coding:** 0 = no dialysis recorded; 1 = dialysis recorded. **Data type:** integer **Example values:** 0 \| 1 **Allowed values:**  **Missing-value coding:** NA/blank as read by fread **n rows / missing / unique:** 67399 / 27827 / 2 **Release status:** public **Definition status:** manual\_exact\_definition | 02\_workflows/T4\_workflow\_v01/data/01\_source\_loaded\_harmonized/esld\_master\_long\_public.csv |  | integer |
| encounter\_id\_public — Public encounter pseudonym **File:** 02\_workflows/T4\_workflow\_v01/data/01\_source\_loaded\_harmonized/esld\_master\_long\_public.csv **Recommended public name:** encounter\_id\_public **Description:** Non-linkable public-release encounter pseudonym used for encounter-level grouping; not an original hospital encounter identifier. **Unit:**  **Value coding:** See example\_values and allowed\_values. **Data type:** character **Example values:** E001181 \| E000008 \| E000041 \| E000100 \| E000126 **Allowed values:** E001181 \| E000008 \| E000041 \| E000100 \| E000126 \| E000151 \| E000157 \| E000219 \| E000289 \| E000344 **Missing-value coding:** NA/blank as read by fread **n rows / missing / unique:** 67399 / 0 / 5045 **Release status:** public **Definition status:** exact\_definition | 02\_workflows/T4\_workflow\_v01/data/01\_source\_loaded\_harmonized/esld\_master\_long\_public.csv |  | character |
| etiology\_source\_current\_flag — Current etiology source flag **File:** 02\_workflows/T4\_workflow\_v01/data/01\_source\_loaded\_harmonized/esld\_master\_long\_public.csv **Recommended public name:** etiology\_source\_current\_flag **Description:** Binary indicator identifying whether the etiology source is the current source used for the public row. **Unit:**  **Value coding:** 0 = no; 1 = yes. **Data type:** integer **Example values:** 1 **Allowed values:**  **Missing-value coding:** NA/blank as read by fread **n rows / missing / unique:** 67399 / 0 / 1 **Release status:** public **Definition status:** manual\_precision\_definition | 02\_workflows/T4\_workflow\_v01/data/01\_source\_loaded\_harmonized/esld\_master\_long\_public.csv |  | integer |
| etiology\_unclassified — Unclassified liver disease etiology flag **File:** 02\_workflows/T4\_workflow\_v01/data/01\_source\_loaded\_harmonized/esld\_master\_long\_public.csv **Recommended public name:** etiology\_unclassified **Description:** Binary etiology indicator identifying records without a classified liver disease etiology in the public ESLD cohort. **Unit:**  **Value coding:** 0 = no; 1 = yes. **Data type:** integer **Example values:** 0 **Allowed values:**  **Missing-value coding:** NA/blank as read by fread **n rows / missing / unique:** 67399 / 0 / 1 **Release status:** public **Definition status:** manual\_exact\_definition | 02\_workflows/T4\_workflow\_v01/data/01\_source\_loaded\_harmonized/esld\_master\_long\_public.csv |  | integer |
| hbv — Hepatitis B virus etiology flag **File:** 02\_workflows/T4\_workflow\_v01/data/01\_source\_loaded\_harmonized/esld\_master\_long\_public.csv **Recommended public name:** hbv **Description:** Binary etiology indicator for hepatitis B virus-related liver disease in the public ESLD cohort. **Unit:**  **Value coding:** 0 = no; 1 = yes. **Data type:** integer **Example values:** 0 \| 1 **Allowed values:**  **Missing-value coding:** NA/blank as read by fread **n rows / missing / unique:** 67399 / 0 / 2 **Release status:** public **Definition status:** manual\_exact\_definition | 02\_workflows/T4\_workflow\_v01/data/01\_source\_loaded\_harmonized/esld\_master\_long\_public.csv |  | integer |
| hcv — Hepatitis C virus etiology flag **File:** 02\_workflows/T4\_workflow\_v01/data/01\_source\_loaded\_harmonized/esld\_master\_long\_public.csv **Recommended public name:** hcv **Description:** Binary etiology indicator for hepatitis C virus-related liver disease in the public ESLD cohort. **Unit:**  **Value coding:** 0 = no; 1 = yes. **Data type:** integer **Example values:** 0 \| 1 **Allowed values:**  **Missing-value coding:** NA/blank as read by fread **n rows / missing / unique:** 67399 / 0 / 2 **Release status:** public **Definition status:** manual\_exact\_definition | 02\_workflows/T4\_workflow\_v01/data/01\_source\_loaded\_harmonized/esld\_master\_long\_public.csv |  | integer |
| icd\_source\_present — ICD source present flag **File:** 02\_workflows/T4\_workflow\_v01/data/01\_source\_loaded\_harmonized/esld\_master\_long\_public.csv **Recommended public name:** icd\_source\_present **Description:** Binary indicator showing whether an ICD-derived etiology source was present for the public row. **Unit:**  **Value coding:** 0 = no; 1 = yes. **Data type:** integer **Example values:** 1 \| 0 **Allowed values:**  **Missing-value coding:** NA/blank as read by fread **n rows / missing / unique:** 67399 / 0 / 2 **Release status:** public **Definition status:** manual\_precision\_definition | 02\_workflows/T4\_workflow\_v01/data/01\_source\_loaded\_harmonized/esld\_master\_long\_public.csv |  | integer |
| in\_t1\_cohort — Inclusion flag for Table 1 cohort **File:** 02\_workflows/T4\_workflow\_v01/data/01\_source\_loaded\_harmonized/esld\_master\_long\_public.csv **Recommended public name:** in\_t1\_cohort **Description:** Binary indicator identifying records included in the public ESLD Table 1 baseline-characteristics cohort. **Unit:**  **Value coding:** 0 = not included; 1 = included. **Data type:** integer **Example values:** 1 **Allowed values:**  **Missing-value coding:** NA/blank as read by fread **n rows / missing / unique:** 67399 / 0 / 1 **Release status:** public **Definition status:** manual\_exact\_definition | 02\_workflows/T4\_workflow\_v01/data/01\_source\_loaded\_harmonized/esld\_master\_long\_public.csv |  | integer |
| in\_t3\_anchor\_model — Inclusion flag for Table 3 anchor-model cohort **File:** 02\_workflows/T4\_workflow\_v01/data/01\_source\_loaded\_harmonized/esld\_master\_long\_public.csv **Recommended public name:** in\_t3\_anchor\_model **Description:** Binary indicator identifying records included in the Table 3 anchor-model cohort used for creatinine-comparison analyses. **Unit:**  **Value coding:** 0 = not included; 1 = included. **Data type:** integer **Example values:** 1 \| 0 **Allowed values:**  **Missing-value coding:** NA/blank as read by fread **n rows / missing / unique:** 67399 / 0 / 2 **Release status:** public **Definition status:** manual\_exact\_definition | 02\_workflows/T4\_workflow\_v01/data/01\_source\_loaded\_harmonized/esld\_master\_long\_public.csv |  | integer |
| in\_t4\_prevalence\_path — Inclusion flag for Table 4 prevalence pathway **File:** 02\_workflows/T4\_workflow\_v01/data/01\_source\_loaded\_harmonized/esld\_master\_long\_public.csv **Recommended public name:** in\_t4\_prevalence\_path **Description:** Binary indicator identifying records included in the Table 4 prevalence-analysis pathway. **Unit:**  **Value coding:** 0 = not included; 1 = included. **Data type:** integer **Example values:** 1 **Allowed values:**  **Missing-value coding:** NA/blank as read by fread **n rows / missing / unique:** 67399 / 0 / 1 **Release status:** public **Definition status:** manual\_exact\_definition | 02\_workflows/T4\_workflow\_v01/data/01\_source\_loaded\_harmonized/esld\_master\_long\_public.csv |  | integer |
| in\_t4\_survival\_path — Inclusion flag for Table 4 survival pathway **File:** 02\_workflows/T4\_workflow\_v01/data/01\_source\_loaded\_harmonized/esld\_master\_long\_public.csv **Recommended public name:** in\_t4\_survival\_path **Description:** Binary indicator identifying records included in the Table 4 survival-analysis pathway. **Unit:**  **Value coding:** 0 = not included; 1 = included. **Data type:** integer **Example values:** 0 \| 1 **Allowed values:**  **Missing-value coding:** NA/blank as read by fread **n rows / missing / unique:** 67399 / 0 / 2 **Release status:** public **Definition status:** manual\_exact\_definition | 02\_workflows/T4\_workflow\_v01/data/01\_source\_loaded\_harmonized/esld\_master\_long\_public.csv |  | integer |
| inr — International normalized ratio **File:** 02\_workflows/T4\_workflow\_v01/data/01\_source\_loaded\_harmonized/esld\_master\_long\_public.csv **Recommended public name:** inr **Description:** International normalized ratio of prothrombin time used for MELD-related score calculation. **Unit:** ratio **Value coding:** Numeric INR value. **Data type:** numeric **Example values:** 1.14 \| 0.91 \| 1 \| 1.04 \| 0.98 **Allowed values:**  **Missing-value coding:** NA/blank as read by fread **n rows / missing / unique:** 67399 / 0 / 786 **Release status:** public **Definition status:** manual\_exact\_definition | 02\_workflows/T4\_workflow\_v01/data/01\_source\_loaded\_harmonized/esld\_master\_long\_public.csv | ratio | numeric |
| mash — Metabolic dysfunction-associated steatohepatitis etiology flag **File:** 02\_workflows/T4\_workflow\_v01/data/01\_source\_loaded\_harmonized/esld\_master\_long\_public.csv **Recommended public name:** mash **Description:** Binary etiology indicator for metabolic dysfunction-associated steatohepatitis in the public ESLD cohort. **Unit:**  **Value coding:** 0 = no; 1 = yes. **Data type:** integer **Example values:** 0 \| 1 **Allowed values:**  **Missing-value coding:** NA/blank as read by fread **n rows / missing / unique:** 67399 / 0 / 2 **Release status:** public **Definition status:** manual\_exact\_definition | 02\_workflows/T4\_workflow\_v01/data/01\_source\_loaded\_harmonized/esld\_master\_long\_public.csv |  | integer |
| model — MELD model or score variant **File:** 02\_workflows/T4\_workflow\_v01/data/01\_source\_loaded\_harmonized/esld\_master\_long\_public.csv **Recommended public name:** model **Description:** Name of the MELD-related model or score variant represented by the row; expected values include MELD, MELD-Na, reMELD-Na, and MELD 3.0. **Unit:**  **Value coding:** Categorical score/model label, for example MELD, MELD-Na, reMELD-Na, or MELD 3.0. **Data type:** character **Example values:** MELD \| MELD-Na \| reMELD-Na \| MELD 3.0 **Allowed values:** MELD \| MELD-Na \| reMELD-Na \| MELD 3.0 **Missing-value coding:** NA/blank as read by fread **n rows / missing / unique:** 67399 / 0 / 4 **Release status:** public **Definition status:** manual\_exact\_definition | 02\_workflows/T4\_workflow\_v01/data/01\_source\_loaded\_harmonized/esld\_master\_long\_public.csv |  | character |
| other — Other liver disease etiology flag **File:** 02\_workflows/T4\_workflow\_v01/data/01\_source\_loaded\_harmonized/esld\_master\_long\_public.csv **Recommended public name:** other **Description:** Binary etiology indicator for liver disease etiologies grouped as other in the public ESLD cohort. **Unit:**  **Value coding:** 0 = no; 1 = yes. **Data type:** integer **Example values:** 1 \| 0 **Allowed values:**  **Missing-value coding:** NA/blank as read by fread **n rows / missing / unique:** 67399 / 0 / 2 **Release status:** public **Definition status:** manual\_exact\_definition | 02\_workflows/T4\_workflow\_v01/data/01\_source\_loaded\_harmonized/esld\_master\_long\_public.csv |  | integer |
| patient\_id — Public patient pseudonym **File:** 02\_workflows/T4\_workflow\_v01/data/01\_source\_loaded\_harmonized/esld\_master\_long\_public.csv **Recommended public name:** patient\_id **Description:** Non-linkable public-release patient pseudonym used to preserve within-patient grouping in public ESLD data; not an original hospital patient identifier. **Unit:**  **Value coding:** See example\_values and allowed\_values. **Data type:** character **Example values:** P000001 \| P000002 \| P000003 \| P000004 \| P000005 **Allowed values:** P000001 \| P000002 \| P000003 \| P000004 \| P000005 \| P000006 \| P000007 \| P000008 \| P000009 \| P000010 **Missing-value coding:** NA/blank as read by fread **n rows / missing / unique:** 67399 / 0 / 1375 **Release status:** public **Definition status:** exact\_definition | 02\_workflows/T4\_workflow\_v01/data/01\_source\_loaded\_harmonized/esld\_master\_long\_public.csv |  | character |
| pbc — Primary biliary cholangitis etiology flag **File:** 02\_workflows/T4\_workflow\_v01/data/01\_source\_loaded\_harmonized/esld\_master\_long\_public.csv **Recommended public name:** pbc **Description:** Binary etiology indicator for primary biliary cholangitis in the public ESLD cohort. **Unit:**  **Value coding:** 0 = no; 1 = yes. **Data type:** integer **Example values:** 0 \| 1 **Allowed values:**  **Missing-value coding:** NA/blank as read by fread **n rows / missing / unique:** 67399 / 0 / 2 **Release status:** public **Definition status:** manual\_exact\_definition | 02\_workflows/T4\_workflow\_v01/data/01\_source\_loaded\_harmonized/esld\_master\_long\_public.csv |  | integer |
| sample\_day\_from\_first\_sample — Relative sample day **File:** 02\_workflows/T4\_workflow\_v01/data/01\_source\_loaded\_harmonized/esld\_master\_long\_public.csv **Recommended public name:** sample\_day\_from\_first\_sample **Description:** Relative day of the sample measured from the first sample for the public patient/sample sequence; not a calendar date. **Unit:** relative months **Value coding:** Integer or numeric relative day count. **Data type:** integer **Example values:** 0 \| 42 \| 91 \| 118 \| 133 **Allowed values:**  **Missing-value coding:** NA/blank as read by fread **n rows / missing / unique:** 67399 / 0 / 3438 **Release status:** public **Definition status:** manual\_precision\_definition | 02\_workflows/T4\_workflow\_v01/data/01\_source\_loaded\_harmonized/esld\_master\_long\_public.csv | relative months | integer |
| sample\_group\_id\_public — Public sample-group pseudonym **File:** 02\_workflows/T4\_workflow\_v01/data/01\_source\_loaded\_harmonized/esld\_master\_long\_public.csv **Recommended public name:** sample\_group\_id\_public **Description:** Non-linkable public-release sample-group pseudonym used to group samples within the released public data. **Unit:**  **Value coding:** See example\_values and allowed\_values. **Data type:** character **Example values:** G0000001 \| G0000002 \| G0000003 \| G0000004 \| G0000005 **Allowed values:** G0000001 \| G0000002 \| G0000003 \| G0000004 \| G0000005 \| G0000006 \| G0000007 \| G0000008 \| G0000009 \| G0000010 **Missing-value coding:** NA/blank as read by fread **n rows / missing / unique:** 67399 / 0 / 20359 **Release status:** public **Definition status:** exact\_definition | 02\_workflows/T4\_workflow\_v01/data/01\_source\_loaded\_harmonized/esld\_master\_long\_public.csv |  | character |
| sample\_id — Sample identifier **File:** 02\_workflows/T4\_workflow\_v01/data/01\_source\_loaded\_harmonized/esld\_master\_long\_public.csv **Recommended public name:** sample\_id **Description:** Identifier of a sample or experimental record within the released public data; not a personal identifier. **Unit:**  **Value coding:** See example\_values and allowed\_values. **Data type:** character **Example values:** S0000001 \| S0000002 \| S0000003 \| S0000004 \| S0000005 **Allowed values:** S0000001 \| S0000002 \| S0000003 \| S0000004 \| S0000005 \| S0000006 \| S0000007 \| S0000008 \| S0000009 \| S0000010 **Missing-value coding:** NA/blank as read by fread **n rows / missing / unique:** 67399 / 0 / 67399 **Release status:** public **Definition status:** exact\_definition | 02\_workflows/T4\_workflow\_v01/data/01\_source\_loaded\_harmonized/esld\_master\_long\_public.csv |  | character |
| sample\_month\_index — Relative sample month index **File:** 02\_workflows/T4\_workflow\_v01/data/01\_source\_loaded\_harmonized/esld\_master\_long\_public.csv **Recommended public name:** sample\_month\_index **Description:** Relative month index of the sample measured from the first sample for the public patient/sample sequence; not a calendar month. **Unit:** score points **Value coding:** Integer or numeric relative month index. **Data type:** integer **Example values:** 0 \| 1 \| 2 \| 3 \| 4 **Allowed values:**  **Missing-value coding:** NA/blank as read by fread **n rows / missing / unique:** 67399 / 0 / 177 **Release status:** public **Definition status:** manual\_precision\_definition | 02\_workflows/T4\_workflow\_v01/data/01\_source\_loaded\_harmonized/esld\_master\_long\_public.csv | score points | integer |
| score\_corrected — Corrected score **File:** 02\_workflows/T4\_workflow\_v01/data/01\_source\_loaded\_harmonized/esld\_master\_long\_public.csv **Recommended public name:** score\_corrected **Description:** MELD-related score after creatinine correction or recalculation. **Unit:** score points **Value coding:** Numeric score. **Data type:** integer **Example values:** 14 \| 19 \| 16 \| 10 \| 13 **Allowed values:**  **Missing-value coding:** NA/blank as read by fread **n rows / missing / unique:** 67399 / 0 / 40 **Release status:** public **Definition status:** manual\_precision\_definition | 02\_workflows/T4\_workflow\_v01/data/01\_source\_loaded\_harmonized/esld\_master\_long\_public.csv | score points | integer |
| score\_delta — Score difference **File:** 02\_workflows/T4\_workflow\_v01/data/01\_source\_loaded\_harmonized/esld\_master\_long\_public.csv **Recommended public name:** score\_delta **Description:** Difference between score variants or scoring approaches, expressed in score points. **Unit:**  **Value coding:** See example\_values and allowed\_values. **Data type:** integer **Example values:** 0 \| 1 \| -1 \| -2 **Allowed values:**  **Missing-value coding:** NA/blank as read by fread **n rows / missing / unique:** 67399 / 0 / 4 **Release status:** public **Definition status:** exact\_definition | 02\_workflows/T4\_workflow\_v01/data/01\_source\_loaded\_harmonized/esld\_master\_long\_public.csv |  | integer |
| score\_original — Original score **File:** 02\_workflows/T4\_workflow\_v01/data/01\_source\_loaded\_harmonized/esld\_master\_long\_public.csv **Recommended public name:** score\_original **Description:** Original MELD-related score before creatinine correction or recalculation. **Unit:**  **Value coding:** Numeric score. **Data type:** integer **Example values:** 14 \| 19 \| 16 \| 10 \| 13 **Allowed values:**  **Missing-value coding:** NA/blank as read by fread **n rows / missing / unique:** 67399 / 0 / 40 **Release status:** public **Definition status:** manual\_precision\_definition | 02\_workflows/T4\_workflow\_v01/data/01\_source\_loaded\_harmonized/esld\_master\_long\_public.csv |  | integer |
| sex — Sex **File:** 02\_workflows/T4\_workflow\_v01/data/01\_source\_loaded\_harmonized/esld\_master\_long\_public.csv **Recommended public name:** sex **Description:** Sex category represented in the public ESLD data. **Unit:**  **Value coding:** M = male; F = female. **Data type:** character **Example values:** M \| F **Allowed values:** M \| F **Missing-value coding:** NA/blank as read by fread **n rows / missing / unique:** 67399 / 0 / 2 **Release status:** public **Definition status:** manual\_exact\_definition | 02\_workflows/T4\_workflow\_v01/data/01\_source\_loaded\_harmonized/esld\_master\_long\_public.csv |  | character |
| sodium — Serum sodium concentration **File:** 02\_workflows/T4\_workflow\_v01/data/01\_source\_loaded\_harmonized/esld\_master\_long\_public.csv **Recommended public name:** sodium **Description:** Serum sodium concentration used for MELD-Na, reMELD-Na, or MELD 3.0 score calculation. **Unit:** mmol/L **Value coding:** Numeric concentration in mmol/L. **Data type:** numeric **Example values:** 131 \| 134 \| 132 \| 133 \| 140 **Allowed values:**  **Missing-value coding:** NA/blank as read by fread **n rows / missing / unique:** 67399 / 20359 / 188 **Release status:** public **Definition status:** manual\_exact\_definition | 02\_workflows/T4\_workflow\_v01/data/01\_source\_loaded\_harmonized/esld\_master\_long\_public.csv | mmol/L | numeric |
| tbil — Total bilirubin concentration **File:** 02\_workflows/T4\_workflow\_v01/data/01\_source\_loaded\_harmonized/esld\_master\_long\_public.csv **Recommended public name:** tbil **Description:** Total bilirubin concentration used in MELD-related score calculation in the public ESLD data. **Unit:**  **Value coding:** Numeric concentration in mg/dL. **Data type:** numeric **Example values:** 1 \| 0.9 \| 1.1 \| 0.8 \| 0.6 **Allowed values:**  **Missing-value coding:** NA/blank as read by fread **n rows / missing / unique:** 67399 / 0 / 2029 **Release status:** public **Definition status:** manual\_precision\_definition | 02\_workflows/T4\_workflow\_v01/data/01\_source\_loaded\_harmonized/esld\_master\_long\_public.csv |  | numeric |
| parameter — Metadata parameter name **File:** 02\_workflows/T4\_workflow\_v01/data/02b\_table\_content/esld\_T4\_score\_deviation\_outcome\_meta\_public.csv **Recommended public name:** parameter **Description:** Name of a metadata parameter describing the F2 simulated heatmap object, such as figure identity, data origin, grid type, axis variable, or unit/role. **Unit:**  **Value coding:** Categorical metadata key; value is given in the corresponding value column or file-specific metadata field. **Data type:** character **Example values:** dataset\_name \| domain \| anchor \| data\_object \| unit\_or\_role **Allowed values:** dataset\_name \| domain \| anchor \| data\_object \| unit\_or\_role \| release\_status \| source\_public\_values \| title \| n\_table\_rows \| public\_model\_columns **Missing-value coding:** NA/blank as read by fread **n rows / missing / unique:** 10 / 0 / 10 **Release status:** public **Definition status:** manual\_exact\_definition | 02\_workflows/T4\_workflow\_v01/data/02b\_table\_content/esld\_T4\_score\_deviation\_outcome\_meta\_public.csv |  | character |
| value — Value **File:** 02\_workflows/T4\_workflow\_v01/data/02b\_table\_content/esld\_T4\_score\_deviation\_outcome\_meta\_public.csv **Recommended public name:** value **Description:** Numerical or character value corresponding to the row-specific variable/metric. **Unit:**  **Value coding:** See example\_values and allowed\_values. **Data type:** character **Example values:** esld\_T4\_score\_deviation\_outcome\_table\_public \| esld \| T4 \| score\_deviation\_outcome \| table **Allowed values:** esld\_T4\_score\_deviation\_outcome\_table\_public \| esld \| T4 \| score\_deviation\_outcome \| table \| public \| submitted Table 4 DOCX values preserved exactly \| Table 4. Score deviations (Δ) of ≤ −1 stratified by outcome (alive/dead within 90 days after MELD ... **Missing-value coding:** NA/blank as read by fread **n rows / missing / unique:** 10 / 0 / 10 **Release status:** public **Definition status:** exact\_definition | 02\_workflows/T4\_workflow\_v01/data/02b\_table\_content/esld\_T4\_score\_deviation\_outcome\_meta\_public.csv |  | character |
| MELD — MELD result column **File:** 02\_workflows/T4\_workflow\_v01/data/02b\_table\_content/esld\_T4\_score\_deviation\_outcome\_table\_public.csv **Recommended public name:** MELD **Description:** Formatted result value for the MELD score variant in the public table/summary row. **Unit:** mg/dL **Value coding:** Formatted character or numeric table value. **Data type:** character **Example values:** 6.2 (5.8–6.6) \| 12.8 (11.9–13.8) \| 2.222 (1.997–2.472) \| 0.677 (0.608–0.754) \| 0.463 (0.357–0.599) **Allowed values:** 6.2 (5.8–6.6) \| 12.8 (11.9–13.8) \| 2.222 (1.997–2.472) \| 0.677 (0.608–0.754) \| 0.463 (0.357–0.599) \| 0.506 (0.434–0.591) \| 0.388 (0.287–0.524) \| 58.2 (52.7–63.8) \| 44.3 (34.9–53.7) \| 1.32 (1.04–1.66) **Missing-value coding:** NA/blank as read by fread **n rows / missing / unique:** 13 / 0 / 13 **Release status:** public **Definition status:** manual\_precision\_definition | 02\_workflows/T4\_workflow\_v01/data/02b\_table\_content/esld\_T4\_score\_deviation\_outcome\_table\_public.csv | mg/dL | character |
| MELD 3.0 — MELD 3.0 result column **File:** 02\_workflows/T4\_workflow\_v01/data/02b\_table\_content/esld\_T4\_score\_deviation\_outcome\_table\_public.csv **Recommended public name:** MELD 3.0 **Description:** Formatted result value for the MELD 3.0 score variant in the public table/summary row. **Unit:** formatted table value **Value coding:** Formatted character or numeric table value. **Data type:** character **Example values:** 6.7 (6.2–7.4) \| 14.5 (13.0–16.1) \| 2.346 (1.999–2.750) \| 0.659 (0.569–0.764) \| 0.590 (0.504–0.692) **Allowed values:** 6.7 (6.2–7.4) \| 14.5 (13.0–16.1) \| 2.346 (1.999–2.750) \| 0.659 (0.569–0.764) \| 0.590 (0.504–0.692) \| 0.495 (0.402–0.609) \| 0.467 (0.380–0.573) \| 56.6 (49.2–64.0) \| 52.2 (44.9–59.6) \| 1.08 (0.89–1.31) **Missing-value coding:** NA/blank as read by fread **n rows / missing / unique:** 13 / 0 / 13 **Release status:** public **Definition status:** manual\_precision\_definition | 02\_workflows/T4\_workflow\_v01/data/02b\_table\_content/esld\_T4\_score\_deviation\_outcome\_table\_public.csv | formatted table value | character |
| MELD-Na — MELD-Na result column **File:** 02\_workflows/T4\_workflow\_v01/data/02b\_table\_content/esld\_T4\_score\_deviation\_outcome\_table\_public.csv **Recommended public name:** MELD-Na **Description:** Formatted result value for the MELD-Na score variant in the public table/summary row. **Unit:** formatted table value **Value coding:** Formatted character or numeric table value. **Data type:** character **Example values:** 5.7 (5.4–6.1) \| 10.7 (9.9–11.6) \| 1.978 (1.757–2.225) \| 0.672 (0.597–0.755) \| 0.524 (0.427–0.642) **Allowed values:** 5.7 (5.4–6.1) \| 10.7 (9.9–11.6) \| 1.978 (1.757–2.225) \| 0.672 (0.597–0.755) \| 0.524 (0.427–0.642) \| 0.496 (0.419–0.588) \| 0.440 (0.346–0.561) \| 57.5 (51.4–63.6) \| 49.1 (40.8–57.4) \| 1.17 (0.96–1.43) **Missing-value coding:** NA/blank as read by fread **n rows / missing / unique:** 13 / 0 / 13 **Release status:** public **Definition status:** manual\_precision\_definition | 02\_workflows/T4\_workflow\_v01/data/02b\_table\_content/esld\_T4\_score\_deviation\_outcome\_table\_public.csv | formatted table value | character |
| anchor — Anchor/output group **File:** 02\_workflows/T4\_workflow\_v01/data/02b\_table\_content/esld\_T4\_score\_deviation\_outcome\_table\_public.csv **Recommended public name:** anchor **Description:** Anchor or output grouping label used by the workflow to identify a specific public output component. **Unit:**  **Value coding:** See example\_values and allowed\_values. **Data type:** character **Example values:** T4 **Allowed values:** T4 **Missing-value coding:** NA/blank as read by fread **n rows / missing / unique:** 13 / 0 / 1 **Release status:** public **Definition status:** exact\_definition | 02\_workflows/T4\_workflow\_v01/data/02b\_table\_content/esld\_T4\_score\_deviation\_outcome\_table\_public.csv |  | character |
| data\_object — Data object **File:** 02\_workflows/T4\_workflow\_v01/data/02b\_table\_content/esld\_T4\_score\_deviation\_outcome\_table\_public.csv **Recommended public name:** data\_object **Description:** Name of the data object represented by the row. **Unit:**  **Value coding:** Categorical descriptor; see allowed/example values. **Data type:** character **Example values:** score\_deviation\_outcome **Allowed values:** score\_deviation\_outcome **Missing-value coding:** NA/blank as read by fread **n rows / missing / unique:** 13 / 0 / 1 **Release status:** public **Definition status:** exact\_definition | 02\_workflows/T4\_workflow\_v01/data/02b\_table\_content/esld\_T4\_score\_deviation\_outcome\_table\_public.csv |  | character |
| domain — Data domain **File:** 02\_workflows/T4\_workflow\_v01/data/02b\_table\_content/esld\_T4\_score\_deviation\_outcome\_table\_public.csv **Recommended public name:** domain **Description:** Workflow or data domain represented by the row. **Unit:**  **Value coding:** Categorical descriptor; see allowed/example values. **Data type:** character **Example values:** esld **Allowed values:** esld **Missing-value coding:** NA/blank as read by fread **n rows / missing / unique:** 13 / 0 / 1 **Release status:** public **Definition status:** exact\_definition | 02\_workflows/T4\_workflow\_v01/data/02b\_table\_content/esld\_T4\_score\_deviation\_outcome\_table\_public.csv |  | character |
| reMELD-Na — reMELD-Na result column **File:** 02\_workflows/T4\_workflow\_v01/data/02b\_table\_content/esld\_T4\_score\_deviation\_outcome\_table\_public.csv **Recommended public name:** reMELD-Na **Description:** Formatted result value for the reMELD-Na score variant in the public table/summary row. **Unit:** formatted table value **Value coding:** Formatted character or numeric table value. **Data type:** character **Example values:** 6.9 (6.5–7.3) \| 9.0 (8.2–9.8) \| 1.330 (1.178–1.501) \| 0.714 (0.638–0.800) \| 0.412 (0.318–0.536) **Allowed values:** 6.9 (6.5–7.3) \| 9.0 (8.2–9.8) \| 1.330 (1.178–1.501) \| 0.714 (0.638–0.800) \| 0.412 (0.318–0.536) \| 0.555 (0.472–0.652) \| 0.300 (0.215–0.419) \| 61.9 (55.6–68.3) \| 38.2 (30.1–46.3) \| 1.62 (1.28–2.05) **Missing-value coding:** NA/blank as read by fread **n rows / missing / unique:** 13 / 0 / 13 **Release status:** public **Definition status:** manual\_precision\_definition | 02\_workflows/T4\_workflow\_v01/data/02b\_table\_content/esld\_T4\_score\_deviation\_outcome\_table\_public.csv | formatted table value | character |
| release\_status — Release status **File:** 02\_workflows/T4\_workflow\_v01/data/02b\_table\_content/esld\_T4\_score\_deviation\_outcome\_table\_public.csv **Recommended public name:** release\_status **Description:** Release-status label indicating the publication status of the row or file object. **Unit:**  **Value coding:** Release status of the row/object, for example public. **Data type:** character **Example values:** public **Allowed values:** public **Missing-value coding:** NA/blank as read by fread **n rows / missing / unique:** 13 / 0 / 1 **Release status:** public **Definition status:** exact\_definition | 02\_workflows/T4\_workflow\_v01/data/02b\_table\_content/esld\_T4\_score\_deviation\_outcome\_table\_public.csv |  | character |
| row\_order — Display row order **File:** 02\_workflows/T4\_workflow\_v01/data/02b\_table\_content/esld\_T4\_score\_deviation\_outcome\_table\_public.csv **Recommended public name:** row\_order **Description:** Integer row order used to render the public table in the intended display sequence. **Unit:** relative days **Value coding:** Integer display order. **Data type:** integer **Example values:** 1 \| 2 \| 3 \| 4 \| 5 **Allowed values:**  **Missing-value coding:** NA/blank as read by fread **n rows / missing / unique:** 13 / 0 / 13 **Release status:** public **Definition status:** manual\_precision\_definition | 02\_workflows/T4\_workflow\_v01/data/02b\_table\_content/esld\_T4\_score\_deviation\_outcome\_table\_public.csv | relative days | integer |
| section — Table section **File:** 02\_workflows/T4\_workflow\_v01/data/02b\_table\_content/esld\_T4\_score\_deviation\_outcome\_table\_public.csv **Recommended public name:** section **Description:** Section heading or row group used in the rendered public table. **Unit:**  **Value coding:** Character table-section label. **Data type:** character **Example values:** Overall prevalence of score Δ ≤ −1, % \| Odds ratio \| 30 days survival probability \| 90 days survival probability \| 90 da... **Allowed values:** Overall prevalence of score Δ ≤ −1, % \| Odds ratio \| 30 days survival probability \| 90 days survival probability \| 90 days restricted mean survival time (RMST90) \| RMST90-ratio \| 90 days hazard ratio \| Model sample size **Missing-value coding:** NA/blank as read by fread **n rows / missing / unique:** 13 / 0 / 8 **Release status:** public **Definition status:** manual\_precision\_definition | 02\_workflows/T4\_workflow\_v01/data/02b\_table\_content/esld\_T4\_score\_deviation\_outcome\_table\_public.csv |  | character |
| unit\_or\_role — Unit or semantic role **File:** 02\_workflows/T4\_workflow\_v01/data/02b\_table\_content/esld\_T4\_score\_deviation\_outcome\_table\_public.csv **Recommended public name:** unit\_or\_role **Description:** Unit, role, or semantic type corresponding to the row-specific variable/metric. **Unit:**  **Value coding:** Categorical descriptor; see allowed/example values. **Data type:** character **Example values:** table **Allowed values:** table **Missing-value coding:** NA/blank as read by fread **n rows / missing / unique:** 13 / 0 / 1 **Release status:** public **Definition status:** exact\_definition | 02\_workflows/T4\_workflow\_v01/data/02b\_table\_content/esld\_T4\_score\_deviation\_outcome\_table\_public.csv |  | character |
| variable — Variable represented by row **File:** 02\_workflows/T4\_workflow\_v01/data/02b\_table\_content/esld\_T4\_score\_deviation\_outcome\_table\_public.csv **Recommended public name:** variable **Description:** Name of the variable represented by the row in a long-format table. **Unit:**  **Value coding:** Categorical descriptor; see allowed/example values. **Data type:** character **Example values:** alive \| deceased \| deceased vs alive \| Score Δ ≤ −1 \| Score Δ = 0 **Allowed values:** alive \| deceased \| deceased vs alive \| Score Δ ≤ −1 \| Score Δ = 0 \| Δ ≤ −1 / Δ = 0 \| Δ ≤ −1 vs. Δ = 0 \| ESLD samples, N \| note **Missing-value coding:** NA/blank as read by fread **n rows / missing / unique:** 13 / 0 / 9 **Release status:** public **Definition status:** exact\_definition | 02\_workflows/T4\_workflow\_v01/data/02b\_table\_content/esld\_T4\_score\_deviation\_outcome\_table\_public.csv |  | character |
| parameter — Metadata parameter name **File:** 02\_workflows/T4\_workflow\_v01/submission\_ready/public/data/esld\_T4\_score\_deviation\_outcome\_meta\_public.csv **Recommended public name:** parameter **Description:** Name of a metadata parameter describing the F2 simulated heatmap object, such as figure identity, data origin, grid type, axis variable, or unit/role. **Unit:**  **Value coding:** Categorical metadata key; value is given in the corresponding value column or file-specific metadata field. **Data type:** character **Example values:** dataset\_name \| domain \| anchor \| data\_object \| unit\_or\_role **Allowed values:** dataset\_name \| domain \| anchor \| data\_object \| unit\_or\_role \| release\_status \| source\_public\_values \| title \| n\_table\_rows \| public\_model\_columns **Missing-value coding:** NA/blank as read by fread **n rows / missing / unique:** 10 / 0 / 10 **Release status:** public **Definition status:** manual\_exact\_definition | 02\_workflows/T4\_workflow\_v01/submission\_ready/public/data/esld\_T4\_score\_deviation\_outcome\_meta\_public.csv |  | character |
| value — Value **File:** 02\_workflows/T4\_workflow\_v01/submission\_ready/public/data/esld\_T4\_score\_deviation\_outcome\_meta\_public.csv **Recommended public name:** value **Description:** Numerical or character value corresponding to the row-specific variable/metric. **Unit:**  **Value coding:** See example\_values and allowed\_values. **Data type:** character **Example values:** esld\_T4\_score\_deviation\_outcome\_table\_public \| esld \| T4 \| score\_deviation\_outcome \| table **Allowed values:** esld\_T4\_score\_deviation\_outcome\_table\_public \| esld \| T4 \| score\_deviation\_outcome \| table \| public \| submitted Table 4 DOCX values preserved exactly \| Table 4. Score deviations (Δ) of ≤ −1 stratified by outcome (alive/dead within 90 days after MELD ... **Missing-value coding:** NA/blank as read by fread **n rows / missing / unique:** 10 / 0 / 10 **Release status:** public **Definition status:** exact\_definition | 02\_workflows/T4\_workflow\_v01/submission\_ready/public/data/esld\_T4\_score\_deviation\_outcome\_meta\_public.csv |  | character |
| MELD — MELD result column **File:** 02\_workflows/T4\_workflow\_v01/submission\_ready/public/data/esld\_T4\_score\_deviation\_outcome\_table\_public.csv **Recommended public name:** MELD **Description:** Formatted result value for the MELD score variant in the public table/summary row. **Unit:** mg/dL **Value coding:** Formatted character or numeric table value. **Data type:** character **Example values:** 6.2 (5.8–6.6) \| 12.8 (11.9–13.8) \| 2.222 (1.997–2.472) \| 0.677 (0.608–0.754) \| 0.463 (0.357–0.599) **Allowed values:** 6.2 (5.8–6.6) \| 12.8 (11.9–13.8) \| 2.222 (1.997–2.472) \| 0.677 (0.608–0.754) \| 0.463 (0.357–0.599) \| 0.506 (0.434–0.591) \| 0.388 (0.287–0.524) \| 58.2 (52.7–63.8) \| 44.3 (34.9–53.7) \| 1.32 (1.04–1.66) **Missing-value coding:** NA/blank as read by fread **n rows / missing / unique:** 13 / 0 / 13 **Release status:** public **Definition status:** manual\_precision\_definition | 02\_workflows/T4\_workflow\_v01/submission\_ready/public/data/esld\_T4\_score\_deviation\_outcome\_table\_public.csv | mg/dL | character |
| MELD 3.0 — MELD 3.0 result column **File:** 02\_workflows/T4\_workflow\_v01/submission\_ready/public/data/esld\_T4\_score\_deviation\_outcome\_table\_public.csv **Recommended public name:** MELD 3.0 **Description:** Formatted result value for the MELD 3.0 score variant in the public table/summary row. **Unit:** formatted table value **Value coding:** Formatted character or numeric table value. **Data type:** character **Example values:** 6.7 (6.2–7.4) \| 14.5 (13.0–16.1) \| 2.346 (1.999–2.750) \| 0.659 (0.569–0.764) \| 0.590 (0.504–0.692) **Allowed values:** 6.7 (6.2–7.4) \| 14.5 (13.0–16.1) \| 2.346 (1.999–2.750) \| 0.659 (0.569–0.764) \| 0.590 (0.504–0.692) \| 0.495 (0.402–0.609) \| 0.467 (0.380–0.573) \| 56.6 (49.2–64.0) \| 52.2 (44.9–59.6) \| 1.08 (0.89–1.31) **Missing-value coding:** NA/blank as read by fread **n rows / missing / unique:** 13 / 0 / 13 **Release status:** public **Definition status:** manual\_precision\_definition | 02\_workflows/T4\_workflow\_v01/submission\_ready/public/data/esld\_T4\_score\_deviation\_outcome\_table\_public.csv | formatted table value | character |
| MELD-Na — MELD-Na result column **File:** 02\_workflows/T4\_workflow\_v01/submission\_ready/public/data/esld\_T4\_score\_deviation\_outcome\_table\_public.csv **Recommended public name:** MELD-Na **Description:** Formatted result value for the MELD-Na score variant in the public table/summary row. **Unit:** formatted table value **Value coding:** Formatted character or numeric table value. **Data type:** character **Example values:** 5.7 (5.4–6.1) \| 10.7 (9.9–11.6) \| 1.978 (1.757–2.225) \| 0.672 (0.597–0.755) \| 0.524 (0.427–0.642) **Allowed values:** 5.7 (5.4–6.1) \| 10.7 (9.9–11.6) \| 1.978 (1.757–2.225) \| 0.672 (0.597–0.755) \| 0.524 (0.427–0.642) \| 0.496 (0.419–0.588) \| 0.440 (0.346–0.561) \| 57.5 (51.4–63.6) \| 49.1 (40.8–57.4) \| 1.17 (0.96–1.43) **Missing-value coding:** NA/blank as read by fread **n rows / missing / unique:** 13 / 0 / 13 **Release status:** public **Definition status:** manual\_precision\_definition | 02\_workflows/T4\_workflow\_v01/submission\_ready/public/data/esld\_T4\_score\_deviation\_outcome\_table\_public.csv | formatted table value | character |
| anchor — Anchor/output group **File:** 02\_workflows/T4\_workflow\_v01/submission\_ready/public/data/esld\_T4\_score\_deviation\_outcome\_table\_public.csv **Recommended public name:** anchor **Description:** Anchor or output grouping label used by the workflow to identify a specific public output component. **Unit:**  **Value coding:** See example\_values and allowed\_values. **Data type:** character **Example values:** T4 **Allowed values:** T4 **Missing-value coding:** NA/blank as read by fread **n rows / missing / unique:** 13 / 0 / 1 **Release status:** public **Definition status:** exact\_definition | 02\_workflows/T4\_workflow\_v01/submission\_ready/public/data/esld\_T4\_score\_deviation\_outcome\_table\_public.csv |  | character |
| data\_object — Data object **File:** 02\_workflows/T4\_workflow\_v01/submission\_ready/public/data/esld\_T4\_score\_deviation\_outcome\_table\_public.csv **Recommended public name:** data\_object **Description:** Name of the data object represented by the row. **Unit:**  **Value coding:** Categorical descriptor; see allowed/example values. **Data type:** character **Example values:** score\_deviation\_outcome **Allowed values:** score\_deviation\_outcome **Missing-value coding:** NA/blank as read by fread **n rows / missing / unique:** 13 / 0 / 1 **Release status:** public **Definition status:** exact\_definition | 02\_workflows/T4\_workflow\_v01/submission\_ready/public/data/esld\_T4\_score\_deviation\_outcome\_table\_public.csv |  | character |
| domain — Data domain **File:** 02\_workflows/T4\_workflow\_v01/submission\_ready/public/data/esld\_T4\_score\_deviation\_outcome\_table\_public.csv **Recommended public name:** domain **Description:** Workflow or data domain represented by the row. **Unit:**  **Value coding:** Categorical descriptor; see allowed/example values. **Data type:** character **Example values:** esld **Allowed values:** esld **Missing-value coding:** NA/blank as read by fread **n rows / missing / unique:** 13 / 0 / 1 **Release status:** public **Definition status:** exact\_definition | 02\_workflows/T4\_workflow\_v01/submission\_ready/public/data/esld\_T4\_score\_deviation\_outcome\_table\_public.csv |  | character |
| reMELD-Na — reMELD-Na result column **File:** 02\_workflows/T4\_workflow\_v01/submission\_ready/public/data/esld\_T4\_score\_deviation\_outcome\_table\_public.csv **Recommended public name:** reMELD-Na **Description:** Formatted result value for the reMELD-Na score variant in the public table/summary row. **Unit:** formatted table value **Value coding:** Formatted character or numeric table value. **Data type:** character **Example values:** 6.9 (6.5–7.3) \| 9.0 (8.2–9.8) \| 1.330 (1.178–1.501) \| 0.714 (0.638–0.800) \| 0.412 (0.318–0.536) **Allowed values:** 6.9 (6.5–7.3) \| 9.0 (8.2–9.8) \| 1.330 (1.178–1.501) \| 0.714 (0.638–0.800) \| 0.412 (0.318–0.536) \| 0.555 (0.472–0.652) \| 0.300 (0.215–0.419) \| 61.9 (55.6–68.3) \| 38.2 (30.1–46.3) \| 1.62 (1.28–2.05) **Missing-value coding:** NA/blank as read by fread **n rows / missing / unique:** 13 / 0 / 13 **Release status:** public **Definition status:** manual\_precision\_definition | 02\_workflows/T4\_workflow\_v01/submission\_ready/public/data/esld\_T4\_score\_deviation\_outcome\_table\_public.csv | formatted table value | character |
| release\_status — Release status **File:** 02\_workflows/T4\_workflow\_v01/submission\_ready/public/data/esld\_T4\_score\_deviation\_outcome\_table\_public.csv **Recommended public name:** release\_status **Description:** Release-status label indicating the publication status of the row or file object. **Unit:**  **Value coding:** Release status of the row/object, for example public. **Data type:** character **Example values:** public **Allowed values:** public **Missing-value coding:** NA/blank as read by fread **n rows / missing / unique:** 13 / 0 / 1 **Release status:** public **Definition status:** exact\_definition | 02\_workflows/T4\_workflow\_v01/submission\_ready/public/data/esld\_T4\_score\_deviation\_outcome\_table\_public.csv |  | character |
| row\_order — Display row order **File:** 02\_workflows/T4\_workflow\_v01/submission\_ready/public/data/esld\_T4\_score\_deviation\_outcome\_table\_public.csv **Recommended public name:** row\_order **Description:** Integer row order used to render the public table in the intended display sequence. **Unit:** relative days **Value coding:** Integer display order. **Data type:** integer **Example values:** 1 \| 2 \| 3 \| 4 \| 5 **Allowed values:**  **Missing-value coding:** NA/blank as read by fread **n rows / missing / unique:** 13 / 0 / 13 **Release status:** public **Definition status:** manual\_precision\_definition | 02\_workflows/T4\_workflow\_v01/submission\_ready/public/data/esld\_T4\_score\_deviation\_outcome\_table\_public.csv | relative days | integer |
| section — Table section **File:** 02\_workflows/T4\_workflow\_v01/submission\_ready/public/data/esld\_T4\_score\_deviation\_outcome\_table\_public.csv **Recommended public name:** section **Description:** Section heading or row group used in the rendered public table. **Unit:**  **Value coding:** Character table-section label. **Data type:** character **Example values:** Overall prevalence of score Δ ≤ −1, % \| Odds ratio \| 30 days survival probability \| 90 days survival probability \| 90 da... **Allowed values:** Overall prevalence of score Δ ≤ −1, % \| Odds ratio \| 30 days survival probability \| 90 days survival probability \| 90 days restricted mean survival time (RMST90) \| RMST90-ratio \| 90 days hazard ratio \| Model sample size **Missing-value coding:** NA/blank as read by fread **n rows / missing / unique:** 13 / 0 / 8 **Release status:** public **Definition status:** manual\_precision\_definition | 02\_workflows/T4\_workflow\_v01/submission\_ready/public/data/esld\_T4\_score\_deviation\_outcome\_table\_public.csv |  | character |
| unit\_or\_role — Unit or semantic role **File:** 02\_workflows/T4\_workflow\_v01/submission\_ready/public/data/esld\_T4\_score\_deviation\_outcome\_table\_public.csv **Recommended public name:** unit\_or\_role **Description:** Unit, role, or semantic type corresponding to the row-specific variable/metric. **Unit:**  **Value coding:** Categorical descriptor; see allowed/example values. **Data type:** character **Example values:** table **Allowed values:** table **Missing-value coding:** NA/blank as read by fread **n rows / missing / unique:** 13 / 0 / 1 **Release status:** public **Definition status:** exact\_definition | 02\_workflows/T4\_workflow\_v01/submission\_ready/public/data/esld\_T4\_score\_deviation\_outcome\_table\_public.csv |  | character |
| variable — Variable represented by row **File:** 02\_workflows/T4\_workflow\_v01/submission\_ready/public/data/esld\_T4\_score\_deviation\_outcome\_table\_public.csv **Recommended public name:** variable **Description:** Name of the variable represented by the row in a long-format table. **Unit:**  **Value coding:** Categorical descriptor; see allowed/example values. **Data type:** character **Example values:** alive \| deceased \| deceased vs alive \| Score Δ ≤ −1 \| Score Δ = 0 **Allowed values:** alive \| deceased \| deceased vs alive \| Score Δ ≤ −1 \| Score Δ = 0 \| Δ ≤ −1 / Δ = 0 \| Δ ≤ −1 vs. Δ = 0 \| ESLD samples, N \| note **Missing-value coding:** NA/blank as read by fread **n rows / missing / unique:** 13 / 0 / 9 **Release status:** public **Definition status:** exact\_definition | 02\_workflows/T4\_workflow\_v01/submission\_ready/public/data/esld\_T4\_score\_deviation\_outcome\_table\_public.csv |  | character |

## Folder summary

| Top folder | Section | Files | Variables | Size KB |
| --- | --- | --- | --- | --- |
| 00\_config | other\_public\_package\_content | 1 | 0 | 4.052 |
| 00\_release\_manifests | release\_manifest\_or\_codebook | 56 | 0 | 8976.016 |
| 01\_primary\_data | public\_primary\_data | 7 | 81 | 22566.779 |
| 02\_workflows | public\_workflow\_output\_or\_documentation | 85 | 580 | 99708.288 |
| [root] | other\_public\_package\_content | 1 | 0 | 8.004 |
| [root] | package\_readme | 2 | 0 | 3.74 |

## File type summary

| Extension | Files | Size KB |
| --- | --- | --- |
| [none] | 1 | 8.004 |
| csv | 117 | 121400.015 |
| pdf | 12 | 9788.701 |
| txt | 22 | 70.159 |
