# Supplementary figures and images for: Creatinine assay interferences compromises MELD accuracy and may bias liver allocation
Source: Nat Commun. 2026 Jul 23;17:7111. doi: 10.1038/s41467-026-75011-x (PMC13396164; doi:10.1038/s41467-026-75011-x)

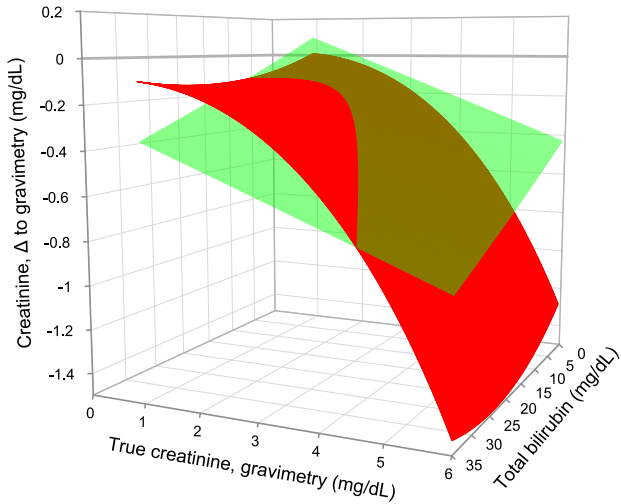

Supplement: Supplementary file 4 — Source Data [file 41467_2026_75011_MOESM4_ESM.zip › figshare_package_FINAL_PUBLIC_DEPOSIT_V1_20260503_002637/02_workflows/F1_workflow_v02/submission_ready/public/figures/slco_F1_surface_public.pdf]

Portion of deviations within score classes after creatinine correction (%)

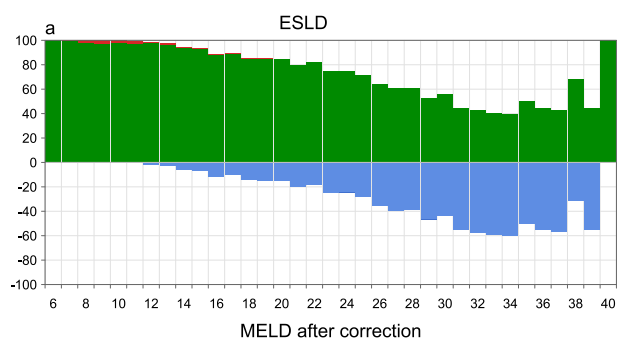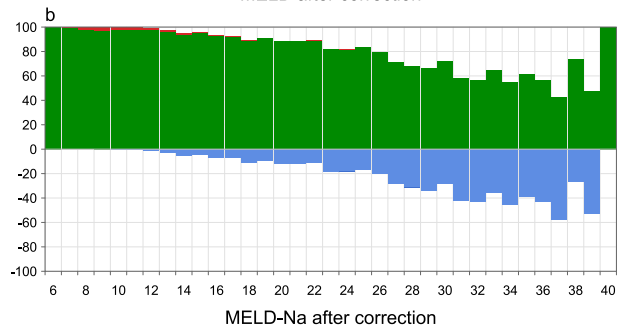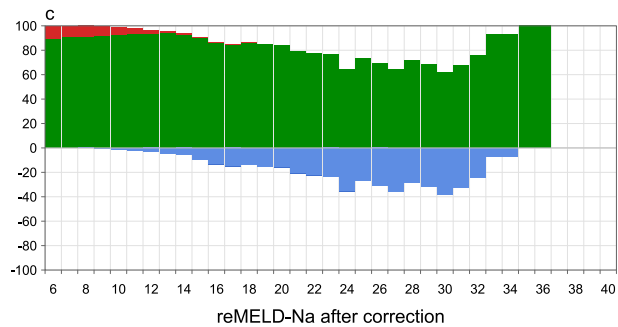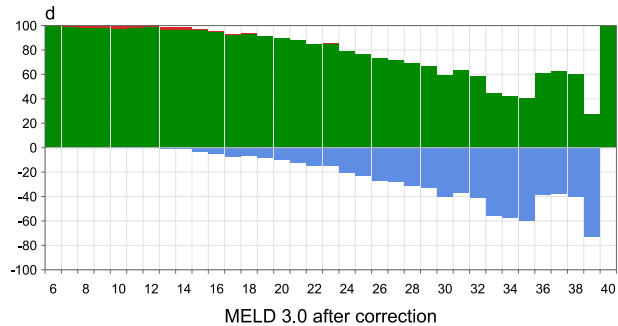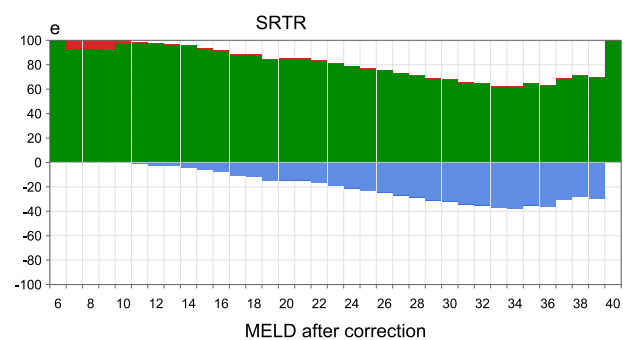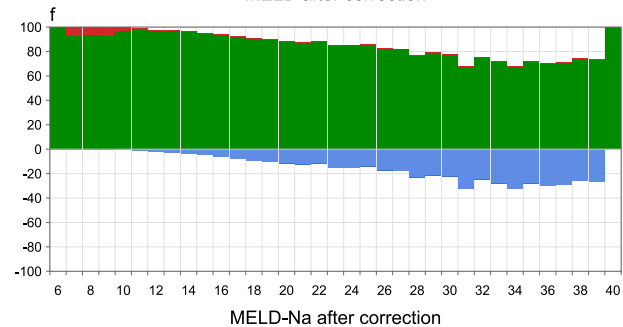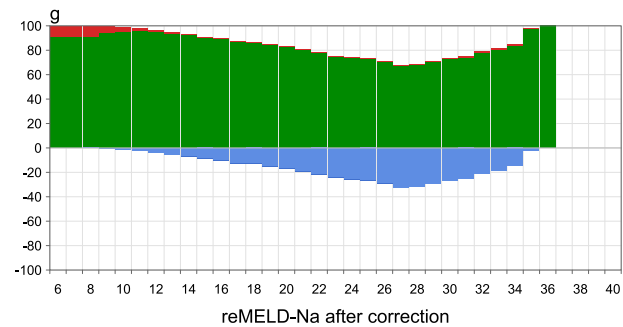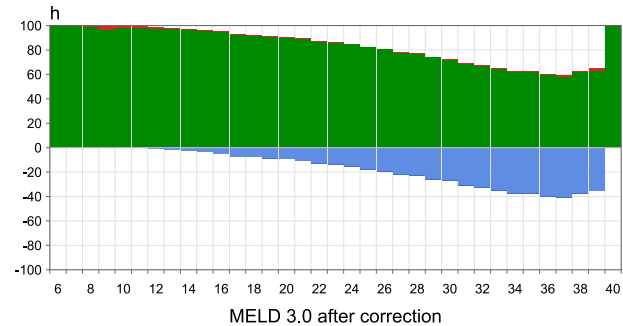

Supplement: Supplementary file 4 — Source Data [file 41467_2026_75011_MOESM4_ESM.zip › figshare_package_FINAL_PUBLIC_DEPOSIT_V1_20260503_002637/02_workflows/F3_workflow_v01/submission_ready/public/figures/F3_ESLD_SRTR_public.pdf]

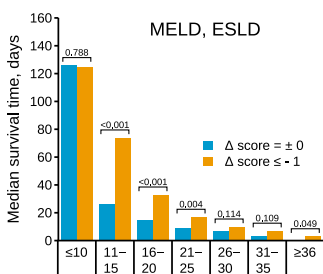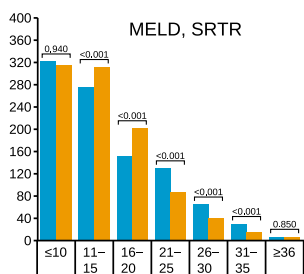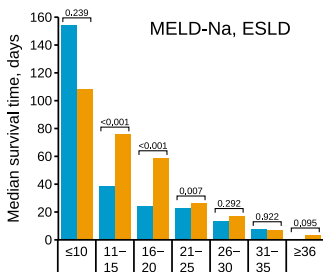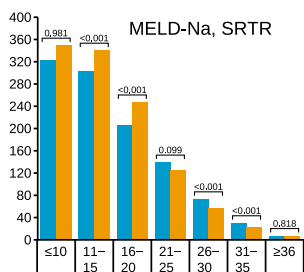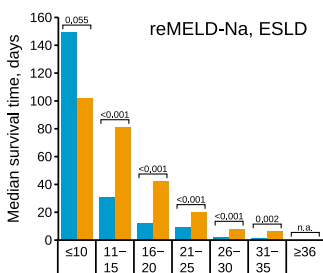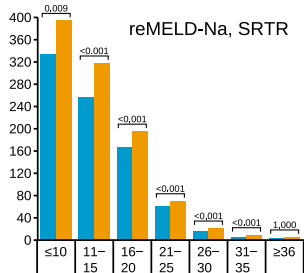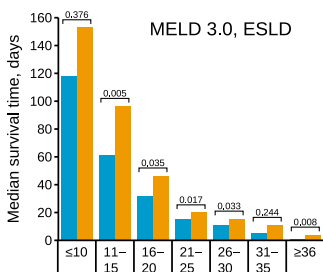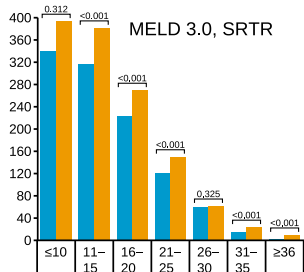

Score class

Score class

Supplement: Supplementary file 4 — Source Data [file 41467_2026_75011_MOESM4_ESM.zip › figshare_package_FINAL_PUBLIC_DEPOSIT_V1_20260503_002637/02_workflows/F4_workflow_v01/submission_ready/public/figures/F4_ESLD_SRTR_public.pdf]

Score class:  $\leq 15$ 

MELD

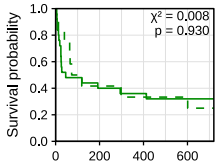

Score class: 16 - 25

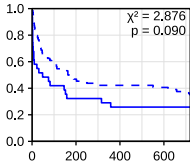Score class:  $> 25$ 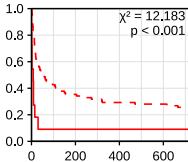

MELD-Na

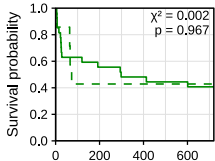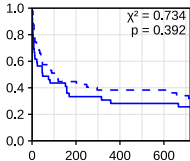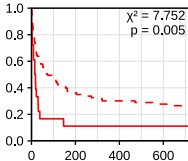

reMELD-Na

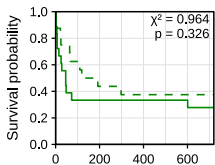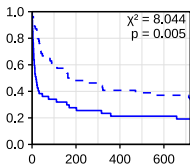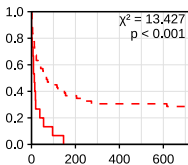

MELD 3.0

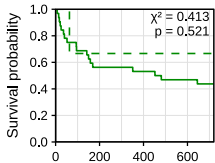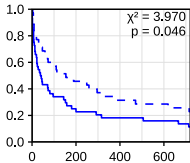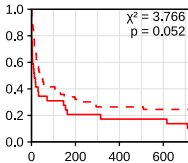

Supplement: Supplementary file 4 — Source Data [file 41467_2026_75011_MOESM4_ESM.zip › figshare_package_FINAL_PUBLIC_DEPOSIT_V1_20260503_002637/02_workflows/F5_workflow_v01/submission_ready/public/figures/F5_ESLD_public.pdf]
