# Supplementary material for: Creatinine assay interferences compromises MELD accuracy and may bias liver allocation
Source: Nat Commun. 2026 Jul 23;17:7111. doi: 10.1038/s41467-026-75011-x (PMC13396164; doi:10.1038/s41467-026-75011-x)
Supplement: Supplementary file 3 — Transparent Peer Review file [file 41467_2026_75011_MOESM3_ESM.pdf]

nature portfolio

Peer Review File

Creatinine Assay Interferences Compromises MELD Accuracy and may Bias Liver Allocation

Corresponding Author: Professor Ali Canbay

This file contains all reviewer reports in order by version, followed by all author rebuttals in order by version.

Version 0:

Reviewer comments:

Reviewer #1

(Remarks to the Author)
The authors aimed to develop an assay-specific correction for creatinine measurements to better estimate true levels and evaluate performance of the correction across the MELD spectrum. This is an important topic with global applicability given the widespread use of MELD calculated with creatinine. The authors nicely characterize the landscape of inaccuracy in creatinine measurement and developed a correction. This is a thorough investigation with robust study methods, and the clinical relevance of the results demonstrated.

Cohort 1 was derived from tertiary care. Can the authors provide more details on the origin of this cohort? What was the country of origin? Were the data obtained from a single center?

For the Kaplan-Meier curves (Figure 6), the text indicates "consistently higher survival in patients with a score reduction of delta ≤-1" yet the figure shows the score change 0 (yellow lines) with higher survival. Are the colors switched in the figure legend?

In the discussion, the authors highlight sex-related disparities in organ allocation and the changing demographics of the liver transplant waiting list (aging population with more cases of alcohol and metabolic associated liver disease). Are any of these groups more impacted by this creatinine bias, particularly by sex?

Reviewer #2

(Remarks to the Author)
The authors investigated possible discrepancies in MELD score due to difference of creatinine measurement and bilirubin interference. The Jaffe assays might cause falsely higher creatinine levels especially in patients with high bilirubin. The authors were concerned that MELD score might falsely increase due to inaccuracy of creatinine values.

The methodologies and results for the reference model to estimate true creatine values independent bilirubin interference are appropriate. They developed the model which was validated with independent samples.

Possible impact of inaccurate creatinine values measured by Jaffe assays was analyzed for each MELD score model, including MELD, MELDNa, MELD3.0, and reMELD-Na.

They compared survival between those with or without deviation of score <-1, which showed that patients deviating <-1 had significantly longer median survival.

1. In the analysis of SRTR data, they identified patients whose creatine was measured by Jaffe assay based on survey. Please show the survey results, including response rate, UNOS regions.
2. Cohort 2 covers 1990 to 2022. How could the authors evaluate waitlist data in 1990's when MELD allocation system was not implemented?
3. There were only 12 centers included in the SRTR data. Please specify their location and transplant volume as long as confidentiality for those transplant centers is secured.

4. Patients alive vs patients deceased. Please clarify the timing of patient death on the waitlist. Deceased within 90 days? I could not find its definition. This should be more clearly stated everywhere. For example, labeling it “patients deceased within 90 days after listing”
5. Waitlist survival analysis should be done by competing risk analyses using Gray test and Fine Gray test. Events for “Removal from the waitlist” include death (too sick), transplant, improvement, and others. Those events should be considered competing risk events. Kaplan Meier and log rank test are not appropriate to compare the survival. 90-day patient “survival” was around 50%. Waitlist mortality was reported at much lower rate. Majority of patients listed for transplant were actually transplanted. Therefore, Figure 6 data became inaccurate due to the flaw of its statistical methodology.
6. Figure 6. Survival analysis should be done according to the MELD score range, too.
7. I noticed that there was huge differences in median survival time between the ESLD and SRTR cohorts pretty much in all meld categories. Could you please comment on why there were so much differencs?
8. Possible changes in MELD score would range -2 to +2 (Figure 4). Let’s say these inaccurate MELD scores are corrected with the appropriate creatinine measurement. The patients’ MELD class might not change too much with only -2 points. The majority of patients might have had -1 point of MELD score. How do the authors explain that “inaccurately measured creatinine” caused “significant difference in waitlist survival” between those alive and deceased?
9. Figure 5. By closely looking at all bars and comparisons, actually score<-1 group had longer median survival time in the ESLD cohort (Cohort 1) in all MELD classes but this was not the case in Cohort 2 (SRTR) (i.e. MELD class 21-25, 26-30, 31-35 in MELD, MELD Na. Please comment on those findings.
10. Why did the authors exclude all PSC patients? Many of PSC patients were not given MELD exception. If needing to exclude patients with MELD exception, this should not be based on disease condition. There are data for MELD exception status in SRTR database. I am not familiar with European allocation system and MELD exception. Please clarify how the exclusion criteria were decided and applied.

Reviewer #3

(Remarks to the Author)
Kaya et al. report on a model they developed to account for interference of bilirubin and creatinine measurement which are both laboratory values that are accounted for in the MELD score. The MELD score has clinical relevance as it is used to prioritize patients on the liver transplant waiting list as well as prognosticate for those with end stage liver disease.
In it’s current form, this report needs to be reworked prior to further review and is not suitable for publication in this journal. Specifically, it is difficult to understand from the abstract and introduction how the study accounts for interference, and what the findings mean. In fact the abstract is a bit misleading, since it does not discuss the exact number of patients on which model-based expectations were experimentally assessed (from what I can can gather, this is n=32). This is, in fact, the premise of the report and is not clearly described. Rather, the abstract highlights the large number of patients from SRTR data in which they adjustments were theoretically applied. For transparency, it is important for the authors to state the very limited number of patients samples that their model is derived from in the abstract and how they came about their interference adjustment model. Additionally, interpretation of their data in current form is challenging to discern. For example, they state that deceased patients with a transient score decrease after correction had longer median survival compared to those with stable scores – this is very confusing.

Version 1:

Reviewer comments:

Reviewer #1

(Remarks to the Author)
The authors addressed all of my concerns.

Reviewer #2

(Remarks to the Author)
All of my questions and concerns were answered and addressed appropriately.
Of note, the findings of this study are “scientifically” appropriate. However, my remaining concern is clinical applicability of this methodology and its impact on our transplant practice. To address this, I would like see if the authors can adopt this correction to the more recent cohort in SRTR. Over the last 3 years, the transplant number in the US has dramatically increased, mainly due to expansion of the donor pool. Based on our recent observation, higher MELD patients, like over 25, got transplanted very quickly. Wait time in patients with MELD 15-25 has become shorter and shorter.
With that, readers will be interested in seeing possible impact of MELD correction on their current practice. Since this MELD correction would occurs mainly in those with high MELD, how does this change their waitlist outcomes (currently shown in Figure 6)?
My suggestion is creating a subgroup using the SRTR only for patients who were listed for transplant between 2022-2024, and looking at 90-day and 365-day waitlist outcomes using the same methodologies (Gray and Fine Gray).
Currently Figure 6 includes over 600 days waitlist outcomes according to MELD categories. As you know, MELD is designed to predict 90-day outcomes and it fluctuates. Therefore, I do not recommend looking at such a long-term outcomes.

Minor point
Figure3. Violet (+2) or Dark Blue (-2) is almost invisible. I cannot tell where they are even with zooming in. These actual

numbers (percentage) should be described in figure legend.

Reviewer #3

(Remarks to the Author)
no further comments

Version 2:

Reviewer comments:

Reviewer #2

(Remarks to the Author)
No further comments

Version 3:

Reviewer comments:

Reviewer #2

(Remarks to the Author)
The authors appropriately addressed the questions and concerns. No more comments. Thank you for the opportunity to review the work.

Open Access This Peer Review File is licensed under a Creative Commons Attribution 4.0 International License, which permits use, sharing, adaptation, distribution and reproduction in any medium or format, as long as you give appropriate credit to the original author(s) and the source, provide a link to the Creative Commons license, and indicate if changes were made.
In cases where reviewers are anonymous, credit should be given to 'Anonymous Referee' and the source.
The images or other third party material in this Peer Review File are included in the article’s Creative Commons license, unless indicated otherwise in a credit line to the material. If material is not included in the article’s Creative Commons license and your intended use is not permitted by statutory regulation or exceeds the permitted use, you will need to obtain permission directly from the copyright holder.
To view a copy of this license, visit https://creativecommons.org/licenses/by/4.0/

<u>POINT-BY-POINT RESPONSE TO REVIEWERS</u>

(NCOMMS-25-45407A)

<u>Reviewer #1 (Remarks to the Author):</u>

We would like to thank the reviewer for their encouraging assessment of our manuscript, for acknowledging the importance of the topic, and for the constructive comments.

Comment 1: Cohort 1 was derived from tertiary care. Can the authors provide more details on the origin of this cohort? What was the country of origin? Were the data obtained from a single center?

Response: We thank the reviewer for pointing out the omitted information. Cohort 1 was derived from a single tertiary care university hospital in Germany. This center functions as a major referral center for patients with advanced liver disease and serves both inpatient and outpatient populations. We have expanded information on this cohort in the Methods section (P22 L8-11):

"Cohort 1 consisted of adult inpatients and outpatients with ESLD recruited at a single tertiary care university hospital in Germany. This center functions as a major referral center for liver disease and transplantation. Data were retrospectively extracted from structured electronic health records between May 2006 and April 2025."

Comment 2: For the Kaplan-Meier curves (Figure 6), the text indicates "consistently higher survival in patients with a score reduction of delta ≤-1" yet the figure shows the score change 0 (yellow lines) with higher survival. Are the colors switched in the figure legend?

Response: We appreciate the reviewer's careful inspection of Figure 6 and confirm that their observation was correct: in the original submission, the color assignments in the figure legend were inadvertently reversed for the two groups (Δ score ≤ –1 and Δ score = ±0).

However, following Reviewer 2's methodological recommendation to use competing-risk analyses, Figure 6 has been completely revised. The updated figure now presents cumulative incidence functions (CIFs) for SRTR cohort instead of Kaplan–Meier survival curves. The respective Kaplan–Meier survival curve of the ESLD cohort are presented in Figure 5. In the new version:

Solid vs. dashed lines distinguish Δ score groups (Δ = ±0 vs. Δ ≤ –1).

Colors represent MELD score categories (≤15, 16–25, >25).

The legend and line styles have been carefully verified to avoid any inconsistency.

This comprehensive update both corrects the original legend error noted by the reviewer and aligns the figure with the revised statistical methodology described in our response to Reviewer 2. Corresponding updates were made to the figure legend and Results section to maintain full consistency between the figure, legend, and text.

Comment 3: In the discussion, the authors highlight sex-related disparities in organ allocation and the changing demographics of the liver transplant waiting list (aging population with more cases of alcohol and metabolic associated liver disease). Are any of these groups more impacted by this creatinine bias, particularly by sex?

Response: This important comment of the reviewer touches on a clinically and scientifically highly relevant aspect of MELD-based allocation. The sex-specific impact of assay-related creatinine bias is indeed a question of broad interest and potential clinical consequence. To address the reviewer's question, we have performed a detailed sex-stratified analysis in both, the ESLD and the SRTR cohort:

Following the reviewer’s suggestion, we compared sex distribution between patients with no relevant MELD change after creatinine correction (Δ = ±0) and those with a ≤ -1-point decrease (Δ ≤ –1) as well as those with a ≥1 point increase (Δ ≥ +1), stratified by survival status. The results are summarized in Table 1 below. In deceased patients with ESLD, sex distribution did neither differ between patients with Δ = ±0 and those with Δ ≤ –1 or Δ ≥ +1, respectively. In contrast, among surviving patients of the ESLD cohort, the proportion of females was consistently and significantly lower in the Δ ≤ –1 group compared to Δ = ±0 across all MELD variants. Noteworthy, the portion of alive female ESLD patients with Δ ≤ –1 was lowest for MELD 3.0, the only score that incorporates sex into its calculation

In the SRTR cohort a significantly lower proportion of female patients surviving after LTx exhibited a with Δ ≤ –1 correction of all MELD variants except reMELD-Na. Among deceased patients in SRTR the proportion of women with Δ ≤ –1 was higher.

than those with Δ = ±0. This rather counterintuitive finding warrants further analyses.

Table 1 (for reviewers only): Proportion of female patients within each subgroup: with either no score changes after creatinine correction (Δ = ±0) or a decrease/increase of at least 1 point (Δ ≤ –1, Δ ≥ +1). P-values from χ^2 tests of sex differences are shown (2×2 contingency tables: Male vs Female by Δ as indicated)

**Cohort**	**Score Variant**	**Females, %**			**p-value, χ^2-test**	
		**(Δ = ±0)**	**(Δ ≤ –1)**	**(Δ ≥ +1)**	**(Δ = ±0) vs (Δ ≤ –1)**	**(Δ = ±0) vs (Δ ≥ +1)**
ESLD, deceased	MELD	41.8	38.5	33.3	0.6546	0.5953
	MELD-Na	40.5	40.3	29.4	1	0.4296
	ReMELD-Na	43.8	39.2	34.0	0.5441	0.2826
	MELD 3.0	39.0	40.0	41.7	1	1
ESLD, alive	MELD	51.2	32.1	34.6	**<0.0000**	**0.0018**
	MELD-Na	50.8	31.5	34.9	**<0.0000**	**0.0028**
	ReMELD-Na	50.5	32.0	44.2	**<0.0000**	0.0842
	MELD 3.0	48.6	27.7	40.0	**<0.0000**	0.2984
SRTR, deceased	MELD	39.7	42.3	46.9	0.1933	0.3815
	MELD-Na	40.0	42.0	46.9	0.3234	0.4036
	ReMELD	36.7	44.8	49.6	**<0.0000**	**0.0088**
	MELD 3.0	38.5	44.1	41.5	**0.0044**	0.8243
SRTR, Liver-Tx survived	MELD	38.4	33.3	29.2	**<0.0000**	**0.0158**
	MELD-Na	38.1	33.4	30.1	**<0.0000**	**0.0431**
	ReMELD-Na	36.3	36.2	38.5	0.8904	0.4299
	MELD 3.0	37.5	34.7	30.8	**0.0032**	0.1644

Overall these findings indicate that surviving female ESLD and SRTR patients are disproportionately underrepresented among those whose MELD scores decrease after

creatinine correction. In other words: men may receive more often an erroneously higher score than women, systematically biasing waitlist prioritization. While previous MELD versions may have disadvantaged women, hypothetically due to lower creatinine from lower muscle mass, recent adjustments (e.g., sex inclusion in MELD 3.0) may partially correct or overcorrect this bias.

However, our analysis focuses solely on analytical bias from creatinine measurement and does not address other known sex-related disparities in transplant access (e.g., referral timing, listing, organ size matching). Nonetheless, these results underscore the importance of sex-stratified validation of MELD-based scores to ensure that bias removal in one domain does not unintentionally introduce inequities in another. We consider these findings substantial enough to merit further detailed analyses, where the full range of MELD-related outcomes can be reanalyzed after stratification by sex, including survival analyses, Δ-score distributions, and allocation implications. This will allow us to address the broader question of how assay-specific creatinine bias interacts with sex, muscle mass, MELD variants, and allocation policies in detail.

Since the present manuscript focuses on the development and validation of the bilirubin–creatinine interference correction model, we believe that a full set of sex-specific analyses lies beyond its scope. We have therefore reserved these analyses for a dedicated follow-up paper, if the findings should warrant it. Nonetheless, we acknowledge their importance in the discussion, adding the following statement related to our findings, and hope that the reviewer can agree on this approach (P16 L12 – P17 L2):

"In our study, score corrections of $\Delta \leq -1$ in the non-deceased population occurred more frequently in men than in women (data not shown), likely reflecting physiologically higher creatinine levels in men and indicating a potential sex related bias. This trend was absent among deceased patients, possibly due to more advanced disease states influenced by

additional pathological factors. Whether the proposed creatinine correction could further enhance fairness in score variants that do not account for sex will require more detailed future analyses.”

Reviewer #2 (Remarks to the Author):

We thank the reviewer for their rigorous and thorough assessment of our manuscript. In particular, we believe that their comments helped improve the clarity of the data presentation and statistical analyses. We also conducted additional analyses to address the reviewer's remarks on statistics.

Comment 1: In the analysis of SRTR data, they identified patients whose creatine was measured by Jaffe assay based on survey. Please show the survey results, including response rate, UNOS regions.

Comment 3: There were only 12 centers included in the SRTR data. Please specify their location and transplant volume as long as confidentiality for those transplant centers is secured.

Response: The reviewer is raising an interesting point and we are grateful for the opportunity to clarify our approach. To identify SRTR patients whose creatinine measurements were performed using the Jaffe method, we reviewed the list of centers derived from the UNOS regions. We used publicly available information from laboratory service websites and departmental contact pages. When this information was not available, we contacted centers directly by e-mail or phone, obtaining responses from 21 centers (response rate 29%). Across all sources, we obtained suitable laboratory methodology information from 42 centers, of which 8 were identified as using the Jaffe method. Direct contacts revealed an additional 4 centers using Jaffe, for a total of 12 transplant centers. These 12 centers were located across 9 OPTN regions (Regions 2, 3, 4, 5, 7, 8, 9, 10, and 11) and had transplant volumes ranging from approximately 140 to over 3,200 candidates per center during the study period (1990–2022).

Due to confidentiality agreements and the sensitive nature of identifying individual transplant programs and their laboratory methodologies, the names of the centers and detailed survey responses cannot be disclosed in the manuscript. Instead, we present the information in aggregated form for the reviewer's reference. This approach protects institutional identities while maintaining transparency regarding geographic coverage and methodology distribution in our analysis. To this end, we included the following text in the Methods section:
"Twelve centres of the OPTN met the predefined inclusion criteria, contributing a total of 20,444 patient records. Creatinine measurement methods were available from 42 centers, of which 8 used the Jaffe method. An additional 21 centers were contacted directly, yielding a 29% response rate and identifying 4 further centers using the Jaffe method. In total, 12 transplant centers were included in the analysis, spanning 9 OPTN regions." (P22 L23 – P 23 L3).

We hope the reviewer finds this approach acceptable.

Comment 2: Cohort 2 covers 1990 to 2022. How could the authors evaluate waitlist data in 1990's when MELD allocation system was not implemented?

Response: The reviewer is of course correct that the MELD score was not implemented for liver allocation until 2002. However, the SRTR dataset includes comprehensive laboratory data back to 1990, including all parameters necessary to retrospectively calculate MELD scores for earlier periods. Only 4% of all MELD data points in our analysis were from the pre-2002 era, limiting the influence of these retrospectively calculated scores on our findings. All Cohort 2 data, irrespective of time period, came from centers that explicitly confirmed the use of the Jaffe method for creatinine measurement, ensuring methodological consistency and exclusion of non-Jaffe results. Notably, during the 1990s the Jaffe method was the standard

approach in U.S. clinical laboratories, as enzymatic assays were not yet widely available, further reducing any risk of assay misclassification.

It is important to note that the retrospectively derived MELD scores were used solely to explore theoretical MELD deviations arising from assay-specific creatinine differences. These were not included in analyses to evaluate allocation decisions. All outcome-related analyses (e.g., waitlist removal, transplant, or death) were interpreted in the context of the allocation practices relevant at the time. These clarifications have been added to the revised Methods section (P22 L20–23) to avoid any confusion:

“Cohort 2 includes patient records from 1990 to 2022, comprising laboratory and clinical data for retrospective calculation of MELD scores. Since MELD was not implemented for liver allocation before 2002, these retrospectively derived scores were not used in any analyses related to allocation decisions.”

Comment 4: Patients alive vs patients deceased. Please clarify the timing of patient death on the waitlist. Deceased within 90 days? I could not find its definition. This should be more clearly stated everywhere. For example, labeling it “patients deceased within 90 days after listing”

Response: We thank the reviewer for highlighting the need to clarify our definition of 'deceased patients.' This definition indeed varies between analyses. In the previous Figure 6, the survival curves illustrated 90-day survival rates after the MELD measurement. In Figure 4 (previously Figure 5) and in the new Figure 5 median survival times can also exceed 90 days because the MELD values in this analysis were obtained up to two years before death. This broader window was chosen deliberately, as MELD scores in clinical practice are calculated repeatedly over months to years before transplantation or death, and fixed cut-offs guide decisions such as waiting-list prioritization or evaluation intervals. Including scores up to two

years before death therefore mirrors real-world use and allowed us to assess the impact of creatinine correction in both short- and medium-term prognostic contexts. Table 4 complements these figures by showing:

- The prevalence of relevant score deviations ($\Delta \leq -1$) in alive vs. deceased patients (without restricting survival time);
- Direct comparisons of 30-day and 90-day survival probabilities, restricted mean survival times, and hazard ratios.

This combined approach was chosen to comprehensively explore the effect of MELD correction across different prognostic horizons, from immediate to longer-term outcomes, while reflecting how MELD is actually used in transplant decision-making.

These more detailed information on survival or “patients deceased”, respectively, have been incorporated into the *Results* section so that readers can readily distinguish between short- and long-term survival analyses and understand the rationale for including each time frame. (P9 L16-17; L23; L24-25; P10 L8; P23 L23 – P24 L2).

Comment 5: Waitlist survival analysis should be done by competing risk analyses using Gray test and Fine Gray test. Events for “Removal from the waitlist” include death (too sick), transplant, improvement, and others. Those events should be considered competing risk events. Kaplan Meier and log rank test are not appropriate to compare the survival. 90-day patient “survival” was around 50%. Waitlist mortality was reported at much lower rate. Majority of patients listed for transplant were actually transplanted. Therefore, Figure 6 data became inaccurate due to the flaw of its statistical methodology.

Comment 6: Figure 6. Survival analysis should be done according to the MELD score range, too.

Response: We thank the reviewer for highlighting these important statistical/methodological issues. In the SRTR cohort**,** we agree that our initial use of Kaplan–Meier and log-rank tests was not fully appropriate for analyzing waitlist outcomes. We therefore reanalyzed waitlist outcomes with competing-risk methods: Gray's test to compare cumulative incidence functions and Fine–Gray regression for subdistribution hazard ratios, stratified by MELD categories (≤15, 16–25, >25) in line with your comment 6. Competing events for removal from the waitlist were defined as (i) transplantation, (ii) waitlist death, and (iii) other removals (e.g., improvement, refusal, deterioration/too sick, transplant at another center, loss to follow-up); candidates removed in error were censored.

The revised Figure 6 displays cumulative incidence functions for visual comparison, and the detailed statistical results are provided in Supplementary Tables 3 and 4.

Gray's tests showed a significantly lower cumulative incidence of waitlist death for $\Delta \le -1$ in the 16–25 class (MELD χ^2=5.61, p=0.018; MELD-Na χ^2=6.22, p=0.013; MELD 3.0 χ^2=9.89, p=0.002), while ≤15 and >25 were not significant. Fine–Gray confirmed these findings in the 16–25 class with subdistribution HRs <1 (MELD 0.84, 95% CI 0.73–0.98, p=0.022; MELD-Na 0.83, 0.71–0.97, p=0.016; MELD 3.0 0.79, 0.67–0.92, p=0.002). Transplant as a competing event differed strongly in the >25 class across MELD variants (p<0.001), consistent with expected listing dynamics.

For the ESLD cohort, the endpoint was all-cause mortality with near-complete follow-up and negligible competing events (5 patients were on a waitlist, 23 underwent transplantation—<2% of 1,375—and only 1 of 233 waitlisted patients died on the list). Under these conditions, competing-risk modeling is not informative, and Kaplan–Meier estimates with log-rank tests are appropriate; however, we re-analyzed the data stratified by MELD categories, according to comment 6. The results show no significant differences in ≤15 and 16–25, but a markedly lower hazard for $\Delta \le -1$ in the >25 class for MELD (χ^2=12.01, p<0.001; HR 0.28, 95% CI

0.13–0.60), MELD-Na (χ^2=10.87, p<0.001; HR 0.37, 0.20–0.68), and reMELD-Na (χ^2=14.39, p<0.001; HR 0.28, 0.14–0.56); MELD 3.0 showed the same direction with a borderline result (χ^2=2.75, p=0.097; HR 0.64, 0.38–1.09).

Figure 6 revision: In ESLD, where competing events were rare, we show Kaplan-Meier curves (Figure 5) with log-rank tests (Supplementary Table 2). In SRTR, cumulative incidence functions are required, because competing events are frequent (Figure 6, Gray/Fine–Gray in Supplementary Tables 3 and 4).

While the most pronounced and statistically significant differences were observed in the MELD 16–25 range**,** the direction of effect was uniformly present across all score categories and MELD variants in the SRTR cohort**,** and to a lesser extent also in the ESLD cohort. Even when not reaching conventional significance thresholds, the effect estimates were consistently below unity (e.g., reMELD-Na in the 16–25 class, subdistribution HR 0.91, 95% CI 0.80–1.02). These results show that correcting creatinine for bilirubin-related interference aligns MELD values more closely with true biological risk, improving agreement between scores and observed outcomes. Our analysis does not suggest routine clinical use of a correction function but demonstrates that different creatinine methods (Jaffé vs. enzymatic) introduce systematic bias into MELD scoring with measurable impact on patient outcomes. This highlights the need for robust, harmonized diagnostics to ensure fairness in liver allocation. We appreciate the reviewer's comment, which prompted us to reapply appropriate statistical methods. Following reanalysis, the effects became even clearer—both visually in the revised plots and statistically in the supplementary tables—thereby strengthening the evidence that creatinine assay interference has direct clinical consequences for MELD-based allocation.

Comment 7: I noticed that there was huge differences in median survival time between the ESLD and SRTR cohorts pretty much in all meld categories. Could you please comment on why there were so much differences?

Response: The reviewer is again correct in this observation. The differences in median survival times between Cohort 1 (ESLD) and Cohort 2 (SRTR) are largely explained by intrinsic differences between the cohorts (see also response to comments 5 and 6) and in some part by different reasons for waitlist removal. As the reviewer also noted in comment 5, the varying causes for removal introduce competing risks and informative censoring in SRTR. In the SRTR subgroup of Jaffe-confirmed centers, more than 50% of candidates were removed due to transplantation (deceased donor, n=10,778; living donor, n=794), with additional removals due to improvement (n=1,128) or transfer (n=328). By contrast, Cohort 1 includes ESLD patients not necessarily listed for transplant (actually only 2 % of the 1.373 patients in this cohort), with fewer removals for transplantation and a higher proportion of removals due to clinical deterioration or death. This may reflect an older patient population, which showed a higher proportion of deaths independent of transplant listing.

Death on the waitlist was the event of interest for SRTR in our initial Kaplan-Meyer analyses, which was limited to those patients, who deceased while on the transplant list. In contrast the Kaplan-Meyer analysis for the ESLD cohort had overall mortality as main outcome, since not all patients were listed for transplantation. To address these issues and the intrinsic differences of the two cohorts, we now

(i) More clearly point out the differences between the clinical background of the cohorts (P22 L8-11; L20 - P23 L7);

(ii) clarified censoring rules and endpoints for transplant-listed patients in the Methods section (P23, L19–23) as follows: “For individuals in cohort 2 who were no longer on the waitlist at the study cutoff (December 2022), the possible outcomes were:

transplantation, death, removal due to clinical deterioration (too sick for transplant) or improvement (no longer eligible), refusal, or transfer to another center.";

(iii) for the SRTR cohort provided a new competing-risks analysis (Fine–Gray subdistribution hazards) for death on the waitlist with transplant as a competing event (new Figure 6 and Supplementary Tables 3 and 4), as also mentioned in our responses to comments 5 and 6. These results are consistent with the Gray analyses but appropriately account for the high transplant rate in SRTR;

(iv) for the ESLD cohort provide a new Figure (Figure 5) as Kaplan-Meier curve alongside a table with log rank test for all-cause mortality as endpoint separated by score class (Supplementary Table 2; see response to comment 6).

Comment 8: Possible changes in MELD score would range -2 to +2 (Figure 4). Let's say these inaccurate MELD scores are corrected with the appropriate creatinine measurement. The patients' MELD class might not change too much with only -2 points. The majority of patients might have had -1 point of MELD score. How do the authors explain that "inaccurately measured creatinine" caused "significant difference in waitlist survival" between those alive and deceased?

Response: We thank the reviewer for this important comment, as it alludes to the potential impact and clinical consequence from our findings. It is correct that the majority of deviations amounted to –1 MELD point. While this may appear small in absolute terms, even a one-point shift can have clinically and systemically relevant consequences. At the individual level, it may move a patient across MELD categories, thereby altering the required frequency of recertification (e.g. 16–20 points: every 30 days vs. 10–15 points: every 90 days). More

importantly, MELD serves as the rank-ordering metric for organ allocation. In the context of a scarce donor organ supply, even a one-point difference can influence prioritization when patients are otherwise similar. In a hypothetical allocation scenario, a patient with an inaccurately elevated MELD score could receive an organ ahead of another candidate. Although such cases may be rare at the individual level, the consequences at the system level are more profound: if entire groups of patients are evaluated with assay methods that systematically bias creatinine and thereby MELD scores, this creates a consistent inequity in organ access (i.e., between transplant centers applying Jaffe vs. enzymatic creatinine measurement). Our findings therefore highlight that differences in laboratory assay principles are not a minor technical issue but a source of measurable allocation bias, underscoring the need for harmonization or correction measures to minimize unfairness and improve equity in transplantation. We have tried to clarify this aspect of our findings and expanded on potential clinical and systemic impact in the revised Discussion (P14 L15 – P15 L7; P15 L8 – P15 L17).

Comment 9: Figure 5. By closely looking at all bars and comparisons, actually score<-1 group had longer median survival time in the ESLD cohort (Cohort 1) in all MELD classes but this was not the case in Cohort 2 (SRTR) (i.e. MELD class 21-25, 26-30, 31-35 in MELD, MELD Na. Please comment on those findings.

Response: The reviewer points out an important and critical observation. In the ESLD cohort, patients with MELD scores requiring correction ($\Delta \leq -1$) generally exhibited longer median survival times across most score classes. However, exceptions were observed in the SRTR cohort. The pattern was less consistent, and in some MELD and MELD-Na categories at higher scores (e.g., 21–25, but most prevalent for 26–30, 31–35), survival differences reversed direction and reached statistical significance. These findings are in line with the

observed crossing CIFs in the >25 class (Figure 6) and likely reflect cohort- and selection-related factors. The ESLD cohort is smaller and not waitlist-based, which may have accentuated survival differences at lower MELD scores.

The SRTR cohort shown in Figure 4 comprises patients with waitlist death subject to allocation and removal criteria. These structural differences may contribute to the apparent discrepancies between cohorts, especially in intermediate and high MELD strata where removal due to transplantation or deterioration occurs at higher frequency. The new competing risk analyses suggested by the reviewer (comment 5) within different MELD classes (comment 6) underscores that in intermediate (16 – 25) and high (> 25) MELD strata patients with a score correction of -1 have a significantly lower risk of death on the waitlist. This would fit well into the overall picture that the correction of creatinine also would reduce overestimation of risk by an incorrect MELD. These findings and the clinical differences between cohort 1 and 2 are now discussed on P15 L15 – P16 L4

Most importantly, the existence of these measurable survival differences - regardless of direction or magnitude - demonstrates that assay-specific creatinine bias can systematically influence MELD scoring. Even modest deviations may shift patients between recertification categories or alter relative prioritization for organ allocation. This underlines the central message of our study: analytical bias in creatinine measurement is not merely a methodological concern, but one with direct implications for fairness and equity in transplant access.

Overall, these findings do not contradict our main conclusion: the potential for assay-related creatinine differences to influence MELD-based risk stratification underscores the need for harmonization or restriction to uniform assay types to ensure fairer organ allocation.

Comment 10: Why did the authors exclude all PSC patients? Many of PSC patients were not given MELD exception. If needing to exclude patients with MELD exception, this should not

be based on disease condition. There are data for MELD exception status in SRTR database. I am not familiar with European allocation system and MELD exception. Please clarify how the exclusion criteria were decided and applied.

Response: We appreciate this comment as it shows the inconsistent handling of this matter between global transplant systems, which again emphasizes the importance for standardization of as many procedures related to organ allocation and transplantation as possible. In the Eurotransplant system the MELD score alone is not considered a sufficient indication for liver transplantation in primary sclerosing cholangitis (PSC), given the disease's complexity and potentially severe prognosis. In Germany and other Eurotransplant countries, PSC patients meeting two of the following three criteria may be assigned a *matchMELD* score, which corresponds to an estimated 15 % mortality risk within the following two months:

1. Documented dominant strictures (significant biliary narrowing with increased cancer risk),
2. At least two spontaneously occurring, clinically confirmed sepsis episodes within six months unrelated to interventions (e.g., ERCP),
3. A reduction in body mass index (BMI) of more than 10 % within one year.

The *matchMELD* score is increased every three months to reflect a 10 % rise in mortality risk. This process differs from the U.S. system, where MELD exceptions are recorded as part of the SRTR database, and exception status can be analyzed directly. Due to the unique considerations in *matchMELD* assignment and the complexity of MELD scoring in PSC patients, we excluded PSC from both cohorts to maintain comparability and avoid introducing allocation-specific biases. In addition, one of the MELD variants we evaluated, reMELD-Na, is used exclusively in Eurotransplant countries, further reinforcing the need to restrict our analysis to patient groups that meet European allocation and exception criteria.

We have added a brief description of the Eurotransplant allocation system and the rationale for excluding PSC patients to the Methods section to acknowledge the reviewer's point (P24 L4-8):

"Patients of both cohorts suffering from primary sclerosing cholangitis (ICD-10: K83.0), biliary atresia (ICD-10: Q44.2), benign and malignant liver tumors (ICD-10: D18.03, C22.-), cystic fibrosis (ICD-10: E84.-), and primary hyperoxaluria (ICD-10: E74.8) were excluded from the study due to their exceptional MELD-related allocation criteria following the local guideline for waiting list management and organ allocation for liver transplantation."

Reviewer #3 (Remarks to the Author):

We thank the Reviewer for their rigorous study of our manuscript and their feedback. In particular we would like to apologize for the confusing representation of the experimental approach to derive our model. Please find below detailed responses to the different aspects of your comment. To facilitate review, we have structured our responses to follow the four key concerns raised in your comments:

1. clarification of how the study accounts for bilirubin–creatinine interference,
2. explicit description of the validation cohort size and scope (n=32),
3. clarification of how the interference adjustment model was developed, and
4. clarification of the Δ-score survival findings.

In addition, we added a fifth point summarizing how we improved transparency and clarity of the overall workflow presentation in the manuscript. We believe that the revisions we implemented in the manuscript due to these comments substantially strengthened clarity, transparency, and reproducibility of the manuscript. These revisions clarify the experimental design, validation approach, data interpretation, and application of the model, ensuring that the study's rationale, workflow, and findings are now clearly presented in a manner suitable for transparent evaluation and reproducible application.

Comment part 1: "Specifically, it is difficult to understand from the abstract and introduction how the study accounts for interference, and what the findings mean."

Response: We appreciate the reviewer's observation that the abstract would benefit from a clearer description of how the interference-correction model was derived. We now explain clearly in the Abstract and Introduction that the model was experimentally derived from a gravimetrically defined two-dimensional creatinine–bilirubin concentration matrix spanning the full clinical range (0–5 mg/dL creatinine, 0–35 mg/dL bilirubin). This means, no patient

samples were employed in this step at all, instead the creatinine–bilirubin concentration matrix was gravimetrically prepared from the pure reagents *in vitro*. Certified reference creatinine was dissolved in a serum-free matrix (0–5 mg/dL in 0.5 mg/dL increments), and bilirubin was gravimetrically quantified and added stepwise (0–35 mg/dL in 1 mg/dL increments), producing an array of 396 concentration combinations in which each creatinine–bilirubin combination had a known true creatinine value. This procedure was independently repeated on different days.

Both Jaffe and enzymatic creatinine methods were systematically tested under these conditions, and the deviations from the gravimetric "true" values were quantified and fitted by method-specific polynomial functions (Equations 1 and 2). This procedure enables any measured creatinine–bilirubin pair to be translated back to its corresponding true creatinine value (Figure 1), thereby removing bilirubin-related bias. To our knowledge, this is the first implementation of such a fully mapped correction framework for MELD score calculation. The corresponding clarifications have been implemented in the **Abstract, Results,** and **Methods** (Results P5 L7-9; P5 L22 -P6 L2; Methods P20 L10-24; P21 L21 – P22 L5).

Revised Abstract:

"The MELD score is central to liver transplant allocation and survival assessment in end-stage liver disease (ESLD). However, standards for creatinine measurement and bilirubin interference are lacking. We developed a correction model for true serum creatinine and bilirubin interference in artificial *in vitro* matrices (396 data points per array) and validated it against ID-GCMS in 32 representative patient samples to exclude serum matrix effects, which confirmed the reported Jaffe bias. The model, designed to quantify and correct methodological bias in four MELD variants, was applied to retrospective data from two large patient cohorts**:** the Scientific Registry of Transplant Recipients (SRTR; n=20,444; 251,637 MELD data points) and 1,375 ESLD patients (20,359 data points). After creatinine correction,

score deviations of Δ+2 to Δ–2 occurred in 10.9–13.8% and 7.9–11.5% of samples, respectively. Deceased patients were more likely to show Δ–1 deviations, with odds ratios of 5.373 (SRTR) and 3.129 (ESLD). A transient score decrease (Δ–1) after correction affected transplantation incidence in SRTR and corresponded to significant lower mortality metrics across MELD classes in patients of both cohorts. These findings underscore the need for harmonized interference-resistant assays to ensure fairness in liver allocation and reduced assay associated bias."

These revisions ensure that the experimental basis of the model and its role in supporting the broader analyses are fully transparent to the reader already from the abstract, despite the word limit of the journal.

Comment part 2: "In fact the abstract is a bit misleading, since it does not discuss the exact number of patients on which model-based expectations were experimentally assessed (from what I can gather, this is n=32). This is, in fact, the premise of the report and is not clearly described. Rather, the abstract highlights the large number of patients from SRTR data in which they adjustments were theoretically applied."

Response: We agree with the reviewer that the multiple steps to derive, validate, and test our model were not sufficiently detailed in the original submission. While in the above response we tried to clarify model derivation, the $n = 32$ refers exclusively to the validation phase of the model. These patient samples were selected to cover the full clinically relevant creatinine–bilirubin range observed in our target population (creatinine: 0.63–4.26 mg/dL; bilirubin: 0.107–28.5 mg/dL), ensuring inclusion of low, intermediate, and high values for both parameters.

In these samples true creatinine values were determined using two independent reference procedures:
(i) gravimetric preparation employing certified reference materials for model derivation, and
(ii) isotope dilution GC-MS (ID-GCMS) at the German Reference Institute for Bioanalytics for validation. These procedures are not comparable to routine laboratory measurements and ensured that both the model and its validation rested on acknowledged reference standards. Analysis of these 32 samples at reference-method level required approximately 12 working days of hands-on analytical work, underscoring the exceptional analytical rigor of this validation step. We would like to mention that other studies, performing ID-GCMS for assessment of creatinine in patient sera employed less than 20 (https://pubmed.ncbi.nlm.nih.gov/24508629/; otherwise nondescript sera with different bilirubin concentrations, including icteric samples) or 40 sera (https://pubmed.ncbi.nlm.nih.gov/26519089/; all icteric residual sera from clinical routine). To our knowledge no other study has analyzed sera of patients on a transplant waitlist with Jaffe assay, enzymatic creatinine measurement and ID-GCMS in parallel, as performed by us. This validation was not intended to statistically reproduce the model in a large patient cohort. Instead, the use of real patient sera confirmed that the correction functions derived from the artificial creatinine–bilirubin matrix remained valid in the presence of additional serum matrix effects. By employing nationally recognized reference methods and carefully targeted sample selection, we minimized this critical risk and verified that the experimentally derived correction behaved as intended under real-world clinical conditions.

Importantly, this analytical confirmation is distinct from the subsequent clinical validation, which evaluated whether the correction had clinical relevance by applying it to patient cohorts (ESLD and SRTR) and demonstrating consistency between corrected MELD scores and actual survival outcomes.

Comparison with routine Jaffe and enzymatic results before and after correction confirmed the expected performance of both methods:

– For the Jaffe assay, proportional bias was reduced from 7.19% to 0.26% after correction, and precision improved by 10.2%.
– For the enzymatic assay, agreement with ID-GCMS was already high, and only negligible additional improvement was achieved after correction (bias change from 0.89% to 0.004%).

Changes in the manuscript: We revised the Abstract to clearly state that validation of the correction formula was performed in 32 patient samples against ID-GCMS to exclude an impact of potential matrix effect (P2 L6-7). The Results and Methods were updated to clarify the rationale for the validation step, the reference procedures applied, and the representativeness of the sample set (Results P5 L22 -P6 L2; Methods P21 L21 – P22 L5).

Comment part 3: For transparency, it is important for the authors to state the very limited number of patients samples that their model is derived from in the abstract and how they came about their interference adjustment model.

Response: Of course, the reviewer is correct that we have to be fully transparent on how the interference adjustment model was derived, validated, and tested. To briefly summarize the two previous responses (comment parts 1 and 2):

The process comprised

- experimental derivation (Step 1): Gravimetric preparation of a two-dimensional creatinine–bilirubin matrix spanning the full clinical range (0–5 mg/dL creatinine, 0–35 mg/dL bilirubin, resulting in 396 combinations per array) from pure reagents, analyzed by both Jaffe and enzymatic assays on a uniform analyzer platform.

Deviations from gravimetric truth were captured and fitted using method-specific polynomial functions (Equations 1 and 2).

- validation in patient matrices (Step 2): Independent testing of 32 patient sera by isotope-dilution GC-MS confirmed that the correction functions remained valid in real clinical matrices, demonstrating that the model was not compromised by serum matrix effects absent from artificial samples.

To clarify, the interference-adjustment was derived from gravimetrically defined creatinine–bilirubin arrays with 396 creatinine-bilirubin concentration combinations per array, repeated on multiple days to ensure reproducibility. The 32 patient sera were used solely for analytical validation with ID-GCMS to confirm that the equations remain accurate in real serum without introducing matrix-related bias.

To our knowledge, this is the first application of a structured two-step strategy, combining a fully mapped artificial matrix with reference-standard validation in patient serum, to correct bilirubin–creatinine interference in MELD scoring.

Changes in the manuscript: We explicitly state in the Abstract that the model derivation is based on gravimetrically defined sample combinations (396 per array, repeated) and that n=32 refers exclusively to ID-GCMS validation in patient sera (P2 L4–7). The Methods provide the full procedural details and step sizes (P20 L10-24; P21 L21 – P22 L5).

Comment part 4: Additionally, interpretation of their data in current form is challenging to discern. For example, they state that deceased patients with a transient score decrease after correction had longer median survival compared to those with stable scores – this is very confusing.

Response: We thank the reviewer for pointing out that this result was difficult to interpret in its previous form. In the revised manuscript, we now define Δ at first mention as the difference between corrected and uncorrected MELD (P8 L9-10) and in the statistics part (P24 L23 – P25 L1). We also rephrased a part of the results on survival duration in patients where score correction reduced the MELD (Δ = –1) compared to patients where score correction did not affect the MELD (Δ = 0). We hope that this part is now more clear (P9 L26 – P10 L3):

"In both cohorts, patients with decreased scores (Δ ≤ –1), indicating creatinine overestimation in the uncorrected score, consistently presented significantly longer median survival compared to patients with unchanged scores (Δ = 0), particularly within intermediate MELD ranges."

We also added an explanatory note in the Discussion that this effect most likely reflects the removal of analytical bias, which had previously inflated MELD-based risk classification (P15 L8-15):

"To address this, we conducted a targeted analysis of the subgroup of deceased patients. Both, median overall survival times and 90-day restricted mean survival times were longer among deceased ESLD and SRTR patients who experienced a MELD score reduction of Δ ≤ –1 following creatinine correction, compared to those whose scores remained unchanged. This result indicates that initial MELD values were inflated by falsely elevated creatinine measurements, leading to artificially higher scores. Adjusting creatinine toward its true value could provide a more accurate reflection of disease severity and prognosis, as the down-corrected MELD scores were associated with longer survival times."

Point-by-point response to comments

Reviewer #1 (Remarks to the Author):

We again thank the reviewer for their rigorous study of our manuscript and their constructive feedback. We are convinced that the respective changes and improvements in our manuscript have deepened the clinical relevance and enhanced representation of contained information.

Reviewer #2 (Remarks to the Author):

Thank you for revisiting our submission and for the generally positive feedback and the critical assessment.

Comment 1a: Of note, the findings of this study are "scientifically" appropriate. However, my remaining concern is clinical applicability of this methodology and its impact on our transplant practice. To address this, I would like see if the authors can adopt this correction to the more recent cohort in SRTR. Over the last 3 years, the transplant number in the US has dramatically increased, mainly due to expansion of the donor pool. Based on our recent observation, higher MELD patients, like over 25, got transplanted very quickly. Wait time in patients with MELD 15-25 has become shorter and shorter.
With that, readers will be interested in seeing possible impact of MELD correction on their current practice.

Response: Thank you for these very insightful considerations regarding recent changes of the transplant situation in the USA. On the one hand this very encouraging development might not be transferable to other regions and population, i.e. the Eurotransplant regions. On the other hand, we would like to highlight, that the correction formula we developed would be a temporary solution to address the differences in creatinine measurement. As we stated in the discussion and the conclusion of the manuscript the ideal way to address this issue and to

avoid any potential unfairness in the allocation procedure would be the harmonized utilization of the enzymatic creatinine assessment throughout all transplant centers. However, we believe that this is beyond the scope of this manuscript and is rather an issue of medical / transplant policy makers and stakeholders to address this appropriately. Our manuscript can only demonstrate that there are significant differences in survival / outcomes when correcting for the different measurement methods, indicating unfairness in the organ allocation between transplant centers.

Comment 1b: Since this MELD correction would occurs mainly in those with high MELD, how does this change their waitlist outcomes (currently shown in Figure 6)?
My suggestion is creating a subgroup using the SRTR only for patients who were listed for transplant between 2022-2024, and looking at 90-day and 365-day waitlist outcomes using the same methodologies (Gray and Fine Gray).
Currently Figure 6 includes over 600 days waitlist outcomes according to MELD categories. As you know, MELD is designed to predict 90-day outcomes and it fluctuates. Therefore, I do not recommend looking at such a long-term outcomes.

Response: We thank the reviewer for raising this point and for highlighting the clinical aspect of our findings. Unfortunately, we cannot provide the requested subgroup analysis for 2022-2024 since we have no access to the data of this period. If we would be able to access this data, we of course would have included an analysis of this most recent time frame.

Regarding the time frame for waitlist outcomes the reviewer is of course correct that the MELD was designed to predict 90-day outcomes. However, especially individuals with a low to moderately elevated MELD, as in the majority of cases in the SRTR dataset, actually exhibit much longer survival durations. As the reviewer also noted, the MELD 15–25 interval

is a critical range. We demonstrated a significant effect within this MELD range and the 600 day interval. We chose to conduct our analysis over a 600-day period because survival within this timeframe is common in clinical practice. As can be gathered from Figure 6 the 90-day survival in the intermediate (16-25) and high (>25) MELD classes would be similarly different between corrected and uncorrected values. Since we have shown that the creatinine method can make a difference from –2 to +2, this could have an impact on the order of allocation. In addition, we demonstrated clinical significance through the prolonged median survival time observed in patients who were down-corrected. This implies potentially unfair access to liver transplantation for those patients solely due to the applied creatinine measurement method. We thus hope the reviewer can agree on presentation of Figure 6 in the present form.

Minor point

Figure3. Violet (+2) or Dark Blue (-2) is almost invisible. I cannot tell where they are even with zooming in. These actual numbers (percentage) should be described in figure legend.

Response: We agree with the reviewer that the representation of these groups is difficult to discern. However, this is mostly due to the very low number of individuals affected by a +2 or -2 deviations. In the respective datasets for each score variant these make up between 0 and 0.01 % of the data. The highest proportions are found in the reMELD-Na of the SRTR group with 0.1 % (Δ +2) and 0.11 % (Δ -2). To provide more detailed information, as probably intended by the reviewer, we now added two supplementary tables (5 for ESLD and 6 for SRTR) including absolute numbers and percentages for each score variant and individual score. In the Figure legend for Figure 6 we added percentages for individuals affected by any positive (+1/2) or negative (-1/2) score deviation.

Reviewer #3 (Remarks to the Author):

Once again we thank the Reviewer for their thorough assessment and constructive comments on our manuscript. We believe that the resulting revisions improved information content and clarity.
