# Supplementary material for: Creatinine assay interferences compromises MELD accuracy and may bias liver allocation
Source: Nat Commun. 2026 Jul 23;17:7111. doi: 10.1038/s41467-026-75011-x (PMC13396164; doi:10.1038/s41467-026-75011-x)
Supplement: Supplementary file 4 — Source Data [file 41467_2026_75011_MOESM4_ESM.zip › figshare_package_FINAL_PUBLIC_DEPOSIT_V1_20260503_002637/00_START_HERE_HTML_NAVIGATOR/file_views/view_0036_public_variable_dictionary_FINAL_CODEBOOK.html]

     00_release_manifests/public_variable_dictionary_FINAL_CODEBOOK.csv  
 
    Readable file view  00_release_manifests/public_variable_dictionary_FINAL_CODEBOOK.csv      ← Back to navigator    |    Open original package file    Section  Technical appendix / request manifests  Output    Extension  csv  Size KB  339.95  Variables  0     Readable HTML view  Showing all 661 rows.      file  variable_name  recommended_public_variable_name  variable_label  variable_description  unit  value_coding  data_type  example_values  allowed_values  missing_value_coding  n_rows  n_missing  n_unique  release_status  file_release_tier  file_role  file_type  definition_status  unit_detected  class      01_primary_data/public/esld_master_long_public.csv  age_years_first_available  age_years_first_available  Age at first available public record  Age in years at the first available public ESLD record for the patient or encounter represented in the released data.  years  Integer age in years.  integer  65 | 67 | 21 | 56 | 63    NA/blank as read by fread  67399  0  79  public  public_deposit  primary_source  csv  manual_exact_definition      
  01_primary_data/public/esld_master_long_public.csv  age_years_sample  age_years_sample  Age at sample  Age in years at the sample-level public ESLD record.  years  Integer age in years.  integer  65 | 66 | 67 | 68 | 69    NA/blank as read by fread  67399  0  84  public  public_deposit  primary_source  csv  manual_precision_definition      
  01_primary_data/public/esld_master_long_public.csv  albumin_g_dl  albumin_g_dl  Serum albumin concentration  Serum albumin concentration in the public ESLD master data.  g/dL  Numeric concentration in g/dL.  numeric  3.3 | 6.15 | 3.14 | 2.8 | 3.26    NA/blank as read by fread  67399  58785  580  public  public_deposit  primary_source  csv  manual_precision_definition      
  01_primary_data/public/esld_master_long_public.csv  ald  ald  Alcohol-related liver disease etiology flag  Binary etiology indicator for alcohol-related liver disease in the public ESLD cohort.    0 = no; 1 = yes.  integer  0 | 1    NA/blank as read by fread  67399  0  2  public  public_deposit  primary_source  csv  manual_exact_definition      
  01_primary_data/public/esld_master_long_public.csv  ald_hcv  ald_hcv  Combined alcohol-related liver disease and hepatitis C etiology flag  Binary etiology indicator for combined alcohol-related liver disease and hepatitis C in the public ESLD cohort.    0 = no; 1 = yes.  integer  0 | 1    NA/blank as read by fread  67399  0  2  public  public_deposit  primary_source  csv  manual_exact_definition      
  01_primary_data/public/esld_master_long_public.csv  autoimmune  autoimmune  Autoimmune liver disease etiology flag  Binary etiology indicator for autoimmune liver disease in the public ESLD cohort.    0 = no; 1 = yes.  integer  0 | 1    NA/blank as read by fread  67399  0  2  public  public_deposit  primary_source  csv  manual_exact_definition      
  01_primary_data/public/esld_master_long_public.csv  crea  crea  Creatinine concentration  Creatinine concentration used in MELD-related score calculation before applying the study-specific correction.  mg/dL  Numeric concentration in mg/dL.  numeric  1.91 | 1.42 | 1.31 | 1.3 | 1.28    NA/blank as read by fread  67399  0  975  public  public_deposit  primary_source  csv  manual_exact_definition      
  01_primary_data/public/esld_master_long_public.csv  crea_corrected  crea_corrected  Corrected creatinine concentration  Creatinine concentration after applying the study-specific correction used for recalculated MELD-related scores.  mg/dL  Numeric concentration in mg/dL.  numeric  1.87705004446668 | 1.41586432303417 | 1.31039363454933 | 1.30192482788903 | 1.28150549768982    NA/blank as read by fread  67399  0  15868  public  public_deposit  primary_source  csv  manual_exact_definition      
  01_primary_data/public/esld_master_long_public.csv  date_count  date_count  Relative day count  Relative day count used for time alignment; negative/positive values are relative to the analysis anchor and are not calendar dates.    See example_values and allowed_values.  integer  -3021 | -2833 | -2798 | -2793 | -2791    NA/blank as read by fread  67399  50970  1450  public  public_deposit  primary_source  csv  exact_definition      
  01_primary_data/public/esld_master_long_public.csv  date_count_month  date_count_month  Relative month count  Relative month count derived from the relative day count; this is not a calendar month.    See example_values and allowed_values.  integer  -99 | -93 | -92 | -91 | -87    NA/blank as read by fread  67399  50970  147  public  public_deposit  primary_source  csv  exact_definition      
  01_primary_data/public/esld_master_long_public.csv  dead_sample_flag  dead_sample_flag  Dead-sample flag  Binary indicator identifying public ESLD sample records assigned to the deceased-sample subset used in sample-level analyses.    0 = not assigned to deceased-sample subset; 1 = assigned to deceased-sample subset.  integer  0 | 1    NA/blank as read by fread  67399  0  2  public  public_deposit  primary_source  csv  manual_final_definition      
  01_primary_data/public/esld_master_long_public.csv  death_within_90d  death_within_90d  Death within 90 days  Binary indicator for death within 90 days of the analysis anchor.    0 = no; 1 = yes  logical      NA/blank as read by fread  67399  67399  0  public  public_deposit  primary_source  csv  exact_definition      
  01_primary_data/public/esld_master_long_public.csv  deceased_patient_flag  deceased_patient_flag  Deceased patient flag  Binary indicator identifying deceased patients within the released analysis context.    0 = no; 1 = yes  integer  0 | 1    NA/blank as read by fread  67399  0  2  public  public_deposit  primary_source  csv  exact_definition      
  01_primary_data/public/esld_master_long_public.csv  delta_eq_0_flag  delta_eq_0_flag  Zero score-delta flag  Binary indicator equal to 1 when the score delta is exactly zero.    0 = no; 1 = yes.  integer  1 | 0    NA/blank as read by fread  67399  0  2  public  public_deposit  primary_source  csv  manual_precision_definition      
  01_primary_data/public/esld_master_long_public.csv  delta_gt_0_flag  delta_gt_0_flag  Positive score-delta flag  Binary indicator equal to 1 when the score delta is positive.    0 = no; 1 = yes.  integer  0 | 1    NA/blank as read by fread  67399  0  2  public  public_deposit  primary_source  csv  manual_precision_definition      
  01_primary_data/public/esld_master_long_public.csv  delta_le_minus1_flag  delta_le_minus1_flag  Score-delta ≤ −1 flag  Binary indicator equal to 1 when the score delta is less than or equal to −1.    0 = no; 1 = yes.  integer  0 | 1    NA/blank as read by fread  67399  0  2  public  public_deposit  primary_source  csv  manual_precision_definition      
  01_primary_data/public/esld_master_long_public.csv  dialysis_raw  dialysis_raw  Dialysis indicator as recorded before score derivation  Binary indicator showing whether dialysis was recorded in the source field before score derivation.    0 = no dialysis recorded; 1 = dialysis recorded.  integer  0 | 1    NA/blank as read by fread  67399  27827  2  public  public_deposit  primary_source  csv  manual_exact_definition      
  01_primary_data/public/esld_master_long_public.csv  encounter_id_public  encounter_id_public  Public encounter pseudonym  Non-linkable public-release encounter pseudonym used for encounter-level grouping; not an original hospital encounter identifier.    See example_values and allowed_values.  character  E001181 | E000008 | E000041 | E000100 | E000126  E001181 | E000008 | E000041 | E000100 | E000126 | E000151 | E000157 | E000219 | E000289 | E000344  NA/blank as read by fread  67399  0  5045  public  public_deposit  primary_source  csv  exact_definition      
  01_primary_data/public/esld_master_long_public.csv  etiology_source_current_flag  etiology_source_current_flag  Current etiology source flag  Binary indicator identifying whether the etiology source is the current source used for the public row.    0 = no; 1 = yes.  integer  1    NA/blank as read by fread  67399  0  1  public  public_deposit  primary_source  csv  manual_precision_definition      
  01_primary_data/public/esld_master_long_public.csv  etiology_unclassified  etiology_unclassified  Unclassified liver disease etiology flag  Binary etiology indicator identifying records without a classified liver disease etiology in the public ESLD cohort.    0 = no; 1 = yes.  integer  0    NA/blank as read by fread  67399  0  1  public  public_deposit  primary_source  csv  manual_exact_definition      
  01_primary_data/public/esld_master_long_public.csv  hbv  hbv  Hepatitis B virus etiology flag  Binary etiology indicator for hepatitis B virus-related liver disease in the public ESLD cohort.    0 = no; 1 = yes.  integer  0 | 1    NA/blank as read by fread  67399  0  2  public  public_deposit  primary_source  csv  manual_exact_definition      
  01_primary_data/public/esld_master_long_public.csv  hcv  hcv  Hepatitis C virus etiology flag  Binary etiology indicator for hepatitis C virus-related liver disease in the public ESLD cohort.    0 = no; 1 = yes.  integer  0 | 1    NA/blank as read by fread  67399  0  2  public  public_deposit  primary_source  csv  manual_exact_definition      
  01_primary_data/public/esld_master_long_public.csv  icd_source_present  icd_source_present  ICD source present flag  Binary indicator showing whether an ICD-derived etiology source was present for the public row.    0 = no; 1 = yes.  integer  1 | 0    NA/blank as read by fread  67399  0  2  public  public_deposit  primary_source  csv  manual_precision_definition      
  01_primary_data/public/esld_master_long_public.csv  in_t1_cohort  in_t1_cohort  Inclusion flag for Table 1 cohort  Binary indicator identifying records included in the public ESLD Table 1 baseline-characteristics cohort.    0 = not included; 1 = included.  integer  1    NA/blank as read by fread  67399  0  1  public  public_deposit  primary_source  csv  manual_exact_definition      
  01_primary_data/public/esld_master_long_public.csv  in_t3_anchor_model  in_t3_anchor_model  Inclusion flag for Table 3 anchor-model cohort  Binary indicator identifying records included in the Table 3 anchor-model cohort used for creatinine-comparison analyses.    0 = not included; 1 = included.  integer  1 | 0    NA/blank as read by fread  67399  0  2  public  public_deposit  primary_source  csv  manual_exact_definition      
  01_primary_data/public/esld_master_long_public.csv  in_t4_prevalence_path  in_t4_prevalence_path  Inclusion flag for Table 4 prevalence pathway  Binary indicator identifying records included in the Table 4 prevalence-analysis pathway.    0 = not included; 1 = included.  integer  1    NA/blank as read by fread  67399  0  1  public  public_deposit  primary_source  csv  manual_exact_definition      
  01_primary_data/public/esld_master_long_public.csv  in_t4_survival_path  in_t4_survival_path  Inclusion flag for Table 4 survival pathway  Binary indicator identifying records included in the Table 4 survival-analysis pathway.    0 = not included; 1 = included.  integer  0 | 1    NA/blank as read by fread  67399  0  2  public  public_deposit  primary_source  csv  manual_exact_definition      
  01_primary_data/public/esld_master_long_public.csv  inr  inr  International normalized ratio  International normalized ratio of prothrombin time used for MELD-related score calculation.  ratio  Numeric INR value.  numeric  1.14 | 0.91 | 1 | 1.04 | 0.98    NA/blank as read by fread  67399  0  786  public  public_deposit  primary_source  csv  manual_exact_definition      
  01_primary_data/public/esld_master_long_public.csv  mash  mash  Metabolic dysfunction-associated steatohepatitis etiology flag  Binary etiology indicator for metabolic dysfunction-associated steatohepatitis in the public ESLD cohort.    0 = no; 1 = yes.  integer  0 | 1    NA/blank as read by fread  67399  0  2  public  public_deposit  primary_source  csv  manual_exact_definition      
  01_primary_data/public/esld_master_long_public.csv  model  model  MELD model or score variant  Name of the MELD-related model or score variant represented by the row; expected values include MELD, MELD-Na, reMELD-Na, and MELD 3.0.    Categorical score/model label, for example MELD, MELD-Na, reMELD-Na, or MELD 3.0.  character  MELD | MELD-Na | reMELD-Na | MELD 3.0  MELD | MELD-Na | reMELD-Na | MELD 3.0  NA/blank as read by fread  67399  0  4  public  public_deposit  primary_source  csv  manual_exact_definition      
  01_primary_data/public/esld_master_long_public.csv  other  other  Other liver disease etiology flag  Binary etiology indicator for liver disease etiologies grouped as other in the public ESLD cohort.    0 = no; 1 = yes.  integer  1 | 0    NA/blank as read by fread  67399  0  2  public  public_deposit  primary_source  csv  manual_exact_definition      
  01_primary_data/public/esld_master_long_public.csv  patient_id  patient_id  Public patient pseudonym  Non-linkable public-release patient pseudonym used to preserve within-patient grouping in public ESLD data; not an original hospital patient identifier.    See example_values and allowed_values.  character  P000001 | P000002 | P000003 | P000004 | P000005  P000001 | P000002 | P000003 | P000004 | P000005 | P000006 | P000007 | P000008 | P000009 | P000010  NA/blank as read by fread  67399  0  1375  public  public_deposit  primary_source  csv  exact_definition      
  01_primary_data/public/esld_master_long_public.csv  pbc  pbc  Primary biliary cholangitis etiology flag  Binary etiology indicator for primary biliary cholangitis in the public ESLD cohort.    0 = no; 1 = yes.  integer  0 | 1    NA/blank as read by fread  67399  0  2  public  public_deposit  primary_source  csv  manual_exact_definition      
  01_primary_data/public/esld_master_long_public.csv  sample_day_from_first_sample  sample_day_from_first_sample  Relative sample day  Relative day of the sample measured from the first sample for the public patient/sample sequence; not a calendar date.  relative days  Integer or numeric relative day count.  integer  0 | 42 | 91 | 118 | 133    NA/blank as read by fread  67399  0  3438  public  public_deposit  primary_source  csv  manual_precision_definition  relative days    
  01_primary_data/public/esld_master_long_public.csv  sample_group_id_public  sample_group_id_public  Public sample-group pseudonym  Non-linkable public-release sample-group pseudonym used to group samples within the released public data.    See example_values and allowed_values.  character  G0000001 | G0000002 | G0000003 | G0000004 | G0000005  G0000001 | G0000002 | G0000003 | G0000004 | G0000005 | G0000006 | G0000007 | G0000008 | G0000009 | G0000010  NA/blank as read by fread  67399  0  20359  public  public_deposit  primary_source  csv  exact_definition      
  01_primary_data/public/esld_master_long_public.csv  sample_id  sample_id  Sample identifier  Identifier of a sample or experimental record within the released public data; not a personal identifier.    See example_values and allowed_values.  character  S0000001 | S0000002 | S0000003 | S0000004 | S0000005  S0000001 | S0000002 | S0000003 | S0000004 | S0000005 | S0000006 | S0000007 | S0000008 | S0000009 | S0000010  NA/blank as read by fread  67399  0  67399  public  public_deposit  primary_source  csv  exact_definition      
  01_primary_data/public/esld_master_long_public.csv  sample_month_index  sample_month_index  Relative sample month index  Relative month index of the sample measured from the first sample for the public patient/sample sequence; not a calendar month.  relative months  Integer or numeric relative month index.  integer  0 | 1 | 2 | 3 | 4    NA/blank as read by fread  67399  0  177  public  public_deposit  primary_source  csv  manual_precision_definition  relative months    
  01_primary_data/public/esld_master_long_public.csv  score_corrected  score_corrected  Corrected score  MELD-related score after creatinine correction or recalculation.  score points  Numeric score.  integer  14 | 19 | 16 | 10 | 13    NA/blank as read by fread  67399  0  40  public  public_deposit  primary_source  csv  manual_precision_definition      
  01_primary_data/public/esld_master_long_public.csv  score_delta  score_delta  Score difference  Difference between score variants or scoring approaches, expressed in score points.    See example_values and allowed_values.  integer  0 | 1 | -1 | -2    NA/blank as read by fread  67399  0  4  public  public_deposit  primary_source  csv  exact_definition      
  01_primary_data/public/esld_master_long_public.csv  score_original  score_original  Original score  Original MELD-related score before creatinine correction or recalculation.    Numeric score.  integer  14 | 19 | 16 | 10 | 13    NA/blank as read by fread  67399  0  40  public  public_deposit  primary_source  csv  manual_precision_definition      
  01_primary_data/public/esld_master_long_public.csv  sex  sex  Sex  Sex category represented in the public ESLD data.    M = male; F = female.  character  M | F  M | F  NA/blank as read by fread  67399  0  2  public  public_deposit  primary_source  csv  manual_exact_definition      
  01_primary_data/public/esld_master_long_public.csv  sodium  sodium  Serum sodium concentration  Serum sodium concentration used for MELD-Na, reMELD-Na, or MELD 3.0 score calculation.  mmol/L  Numeric concentration in mmol/L.  numeric  131 | 134 | 132 | 133 | 140    NA/blank as read by fread  67399  20359  188  public  public_deposit  primary_source  csv  manual_exact_definition      
  01_primary_data/public/esld_master_long_public.csv  tbil  tbil  Total bilirubin concentration  Total bilirubin concentration used in MELD-related score calculation in the public ESLD data.    Numeric concentration in mg/dL.  numeric  1 | 0.9 | 1.1 | 0.8 | 0.6    NA/blank as read by fread  67399  0  2029  public  public_deposit  primary_source  csv  manual_precision_definition      
  01_primary_data/public/f1_simulated_surface_metadata.csv  parameter  parameter  Metadata parameter name  Name of a metadata parameter describing the F2 simulated heatmap object, such as figure identity, data origin, grid type, axis variable, or unit/role.    Categorical metadata key; value is given in the corresponding value column or file-specific metadata field.  character  dataset_name | cohort | data_type | """""""""""""""""""""""""""""""""""""""""""""""""""""""""""""""""""""""""""""""""""""""""""""""""""""""""""""""""""""""""""""""""""""""""""""""""""""""""""""""""""""""""""""""""""""""""""""""""""""""""""""""""""""""""""""""""""""""""""""""""""""""""""""""""""""""""""""""""""""""""""""""""""""""""""""""""""""""""""""""""""""""""""""""""""""""""""""""""""""""""""""""""""""""""""""""""""""""""""""""""""""""""""""""""""""""""""""""""""""""""""""""""""""""""""""""""""""""""""""""""""""""""""""""""""""""""""""""""""""""""""""""""""""""""""""""""""""""""""""""""""""""""""""""""""""""""""""""""""""""""""""""""""""""""""""""""""""""""""""""""""""""""""""""""""""""""""""""""""""""""""""""""""""""""""""""""""""""""""""""""""""""""""""""""""""""""""""""""""""""""""""""""""""""""""""""""""""""""""""""""""""""""""""""""""""""""""""""""""""""""""""""""""""""""""""""""""""""""""""""""""""""""""""""""""""""""""""""""""""""""""""""""""""""""""""""""""""""""""""""""""""""""""""""""""""""""""""""""""""""""""""""""""""""""""""""""""""""""""""""""""""""""""""""""""""""""""""""""""""""""""""""""""""""""""""""""""""""""""""""""""""""""""""""""""""""""""""""""""""""""""""""""""""""""""""""""""""""""""""""""""""""""""""""""""""""""""""""""""""""""""""""""""""""""""""""""""""""""""""""""""""""""""""""""""""""""""""""""""""""""""""""""""""""""""""""""""""""""""""""""""""""""""""""""""""""""""""""""""""""""""""""""""""""""""""""""""""""""""""""""""""""""""""""""""""""""""""""""""""""""""""""""""""""""""""""""""""""""""""""""""""""""""""""""""""""""""""""""""""""""""""""""""""""""""""""""""""""""""""""""""""""""""""""""""""""""""""""""""""""""""""""""""""""""""""""""""""""""""""""""""""""""""""""""""""""""""""""""""""""""""""""""""""""""""""""""""""""""""""""""""""""""""""""""""""""""""""""""""""""""""""""""""""""""""""""""""""""""""""""""""""""""""""""""""""""""""""""""""""""""""""""""""""""""""""""""""""""""""""""""""""""""""""""""""""""""""""""""""""""""""""""""""""""""""""""""""""""""""""""""""""""""""""""""""""""""""""""""""""""""""""""""""""""""""""""""""""""""""""""""""""""""""""""""""""""""""""""""""""""""""""""""""""""""""""""""""""""""""""""""""""""""""""""""""""""""""""""""""""""""""""""""""""""""""""""""""""""""""""""""""""""""""""""""""""""""""""""""""""""""""""""""""""""""""""""""""""""""""""""""""""""""""""""""""""""""""""""""""""""""""""""""""""""""""""""""""""""""""""""""""""""""""""""""""""""""""""""""""""""""""""""""""""""""""""""""""""""""""""""""""""""""""""""""""""""""""""""""""""""""""""""""""""""""""""""""""""""""""""""""""""""""""""""""""""""""""""""""""""""""""""""""""""""""""""""""""""""""""""""""""""""""""""""""""""""""""""""""""""""""""""""""""""""""""""""""""""""""""""""""""""""""""""""""""""""""""""""""""""""""""""""""""""""""""""""""""""""""""""""""""""""""""""""""""""""""""""""""""""""""""""""""""""""""""""""""""""""""""""""""""""""""""""""""""""""""""""""""""""""""""""""""""""""""""""""""""""""""""""""""""""""""""""""""""""""""""""""""""""""""""""""""""""""""""""""""""""""""""""""""""""""""""""""""""""""""""""""""""""""""""""""""""""""""""""""""""""""""""""""""""""""""""""""""""""""""""""""""""""""""""""""""""""""""""""""""""""""""""""""""""""""""""""""""""""""""""""""""""""""""""""""""""""""""""""""""""""""""""""""""""""""""""""""""""""""""""""""""""""""""""""""""""""""""""""""""""""""""""""""""""""""""""""""""""""""""""""""""""""""""""""""""""""""""""""""""""""""""""""""""""""""""""""""""""""""""""""""""""""""""""""""""""""""""""""""""""""""""""""""""""""""""""""""""""""""""""""""""""""""""""""""""""""""""""""""""""""""""""""""""""""""""""""""""""""""""""""""""""""""""""""""""""""""""""""""""""""""""""""""""""""""""""""""""""""""""""""""""""""""""""""""""""""""""""""""""""""""""""""""""""""""""""""""""""""""""""""""""""""""""""""""""""""""""""""""""""""""""""""""""""""""""""""""""""""""""""""""""""""""""""""""""""""""""""""""""""""""""""""""""""""""""""""""""""""""""""""""""""""""""""""""""""""""""""""""""""""""""""""""""""""""""""""""""""""""""""""""""""""""""""""""""""""""""""""""""""""""""""""""""""""""""""""""""""""""""""""""""""""""""""""""""""""""""""""""""""""""""""""""""""""""""""""""""""""""""""""""""""""""""""""""""""""""""""""""""""""""""""""""""""""""""""""""""""""""""""""""""""""""""""""""""""""""""""""""""""""""""""""""""""""""""""""""""""""""""""""""""""""""""""""""""""""""""""""""""""""""""""""""""""""""""""""""""""""""""""""""""""""""""""""""""""""""""""""""""""""""""""""""""""""""""""""""""""""""""""""""""""""""""""""""""""""""""""""""""""""""""""""""""""""""""""""""""""""""""""""""""""""""""""""""""""""""""""""""""""""""""""""""""""""""""""""""""""""""""""""""""""""""""""""""""""""""""""""""""""""""""""""""""""""""""""""""""""""""""""""""""""""""""""""""""""""""""""""""""""""""""""""""""""""""""""""""""""""""""""""""""""""""""""""""""""""""""""""""""""""""""""""""""""""""""""""""""""""""""""""""""""""""""""""""""""""""""""""""""""""""""""""""""""""""""""""""""""""""""""""""""""""""""""""""""""""""""""""""""""""""""""""""""""""""""""""""""""""""""""""""""""""""""""""""""""""""""""""""""""""""""""""""""""""""""""""""""""""""""""""""""""""""""""""""""""""""""""""""""""""""""""""""""""""""""""""""""""""""""""""""""""""""""""""""""""""""""""""""""""""""""""""""""""""""""""""""""""""""""""""""""""""""""""""""""""""""""""""""""""""""""""""""""""""""""""""""""""""""""""""""""""""""""""""""""""""""""""""""""""""""""""""""""""""""""""""""""""""""""""""""""""""""""""""""""""""""""""""""""""""""""""""""""""""""""""""""""""""""""""""""""""""""""""""""""""""""""""""""""""""""""""""""""""""""""""""""""""""""""""""""""""""""""""""""""""""""""""""""""""""""""""""""""""""""""""""""""""""""""""""""""""""""""""""""""""""""""""""""""""""""""""""""""""""""""""""""""""""""""""""""""""""""""""""""""""""""""""""""""""""""""""""""""""""""""""""""""""""""""""""""""""""""""""""""""""""""""""""""""""""""""""""""""""""""""""""""""""""""""""""""""""""""""""""""""""""""""""""""""""""""""""""""""""""""""""""""""""""""""""""""""""""""""""""""""""""""""""""""""""""""""""""""""""""""""""""""""""""""""""""""""""""""""""""""""""""""""""""""""""""""""""""""""""""""""""""""""""""""""""""""""""""""""""""""""""""""""""""""""""""""""""""""""""""""""""""""""""""""""""""""""""""""""""""""""""""""""""""""""""""""""""""""""""""""""""""""""""""""""""""""""""""""""""""""""""""""""""""""""""""""""""""""""""""""""""""""""""""""""""""""""""""""""""""""""""""""""""""""""""""""""""""""""""""""""""""""""""""""""""""""""""""""""""""""""""""""""""""""""""""""""""""""""""""""""""""""""""""""""""""""""""""""""""""""""""""""""""""""""""""""""""""""""""""""""""""""""""""""""""""""""""""""""""""""""""""""""""""""""""""""""""""""""""""""""""""""""""""""""""""""""""""""""""""""""""""""""""""""""""""""""""""""""""""""""""""""""""""""""""""""""""""""""""""""""""""""""""""""""""""""""""""""""""""""""""""""""""""""""""""""""""""""""""""""""""""""""""""""""""""""""""""""""""""""""""""""""""""""""""""""""""""""""""""""""""""""""""""""""""""""""""""""""""""""""""""""""""""""""""""""""""""""""""""""""""""""""""""""""""""""""""""""""""""""""""""""""""""""""""""""""""""""""""""""""""""""""""""""""""""""""""""""""""""""""""""""""""""""""""""""""""""""""""""""""""""""""""""""""""""""""""""""""""""""""""""""""""""""""""""""""""""""""""""""""""""""""""""""""""""""""""""""""""""""""""""""""""""""""""""""""""""""""""""""""""""""""""""""""""""""""""""""""""""""""""""""""""""""""""""""""""""""""""""""""""""""""""""""""""""""""""""""""""""""""""""""""""""""""""""""""""""""""""""""""""""""""""""""""""""""""""""""""""""""""""""""""""""""""""""""""""""""""""""""""""""""""""""""""""""""""""""""""""""""""""""""""""""""""""""""""""""""""""""""""""""""""""""""""""""""""""""""""""""""""""""""""""""""""""""""""""""""""""""""""""""""""""""""""""""""""""""""""""""""""""""""""""""""""""""""""""""""""""""""""""""""""""""""""""""""""""""""""""""""""""""""""""""""""""""""""""""""""""""""""""""""""""""""""""""""""""""""""""""""""""""""""""""""""""""""""""""""""""""""""""""""""""""""""""""""""""""""""""""""""""""""""""""""""""""""""""""""""""""""""""""""""""""""""""""""""""""""""""""""""""""""""""""""""""""""""""""""""""""""""""""""""""""""""""""""""""""""""""""""""""""""""""""""""""""""""""""""""""""""""""""""""""""""""""""""""""""""""""""""""""""""""""""""""""""""""""""""""""""""""""""""""""""""""""""""""""""""""""""""""""""""""""""""""""""""""""""""""""""""""""""""""""""""""""""""""""""""""""""""""""""""""""""""""""""""""""""""""""""""""""""""""""""""""""""""""""""""""""""""""""""""""""""""""""""""""""""""""""""""""""""""""""""""""""""""""""""""""""""""""""""""""""""""""""""""""""""""""""""""""""""""""""""""""""""""""""""""""""""""""""""""""""""""""""""""""""""""""""""""""""""""""""""""""""""""""""""""""""""""""""""""""""""""""""""""""""""""""""""""""""""""""""""""""""""""""""""""""""""""""""""""""""""""""""""""""""""""""""""""""""""""""""""""""""""""""""""""""""""""""""""""""""""""""""""""""""""""""""""""""""""""""""""""""""""""""""""""""""""""""""""""""""""""""""""""""""""""""""""""""""""""""""""""""""""""""""""""""""""""""""""""""""""""""""""""""""""""""""""""""""""""""""""""""""""""""""""""""""""""""""""""""""""""""""""""""""""""""""""""""""""""""""""""""""""""""""""""""""""""""""""""""""""""""""""""""""""""""""""""""""""""""""""""""""""""""""""""""""""""""""""""""""""""""""""""""""""""""""""""""""""""""""""""""""""""""""""""""""""""""""""""""""""""""""""""""""""""""""""""""""""""""""""""""""""""""""""""""""""""""""""""""""""""""""""""""""""""""""""""""""""""""""""""""""""""""""""""""""""""""""""""""""""""""""""""""""""""""""""""""""""""""""""""""""""""""""""""""""""""""""""""""""""""""""""""""""""""""""""""""""""""""""""""""""""""""""""""""""""""""""""""""""""""""""""""""""""""""""""""""""""""""""""""""""""""""""""""""""""""""""""""""""""""""""""""""""""""""""""""""""""""""""""""""""""""""""""""""""""""""""""""""""""""""""""""""""""""""""""""""""""""""""""""""""""""""""""""""""""""""""""""""""""""""""""""""""""""""""""""""""""""""""""""""""""""""""""""""""""""""""""""""""""""""""""""""""""""""""""""""""""""""""""""""""""""""""""""""""""""""""""""""""""""""""""""""""""""""""""""""""""""""""""""""""""""""""""""""""""""""""""""""""""""""""""""""""""""""""""""""""""""""""""""""""""""""""""""""""""""""""""""""""""""""""""""""""""""""""""""""""""""""""""""""""""""""""""""""""""""""""""""""""""""""""""""""""""""""""""""""""""""""""""""""""""""""""""""""""""""""""""""""""""""""""""""""""""""""""""""""""""""""""""""""""""""""""""""""""""""""""""""""""""""""""""""""""""""""""""""""""""""""""""""""""""""""""""""""""""""""""""""""""""""""""""""""""""""""""""""""""""""""""""""""""""""""""""""""""""""""""""""""""""""""""""""""""""""""""""""""""""""""""""""""""""""""""""""""""""""""""""""""""""""""""""""""""""""""""""""""""""""""""""""""""""""""""""""""""""""""""""""""""""""""""""""""""""""""""""""""""""""""""""""""""""""""""""""""""""""""""""""""""""""""""""""""""""""""""""""""""""""""""""""""""""""""""""""""""""""""""""""""""""""""""""""""""""""""""""""""""""""""""""""""""""""""""""""""""""""""""""""""""""""""""""""""""""""""""""""""""""""""""""""""""""""""""""""""""""""""""""""""""""""""""""""""""""""""""""""""""""""""""""""""""""""""""""""""""""""""""""""""""""""""""""""""""""""""""""""""""""""""""""""""""""""""""""""""""""""""""""""""""""""""""""""""""""""""""""""""""""""""""""""""""""""""""""""""""""""""""""""""""""""""""""""""""""""""""""""""""""""""""""""""""""""""""""""""""""""""""""""""""""""""""""""""""""""""""""""""""""""""""""""""""""""""""""""""""""""""""""""""""""""""""""""""""""""""""""""""""""""""""""""""""""""""""""""""""""""""""""""""""""""""""""""""""""""""""""""""""""""""""""""""""""""""""""""""""""""""""""""""""""""""""""""""""""""""""""""""""""""""""""""""""""""""""""""""""""""""""""""""""""""""""""""""""""""""""""""""""""""""""""""""""""""""""""""""""""""""""""""""""""""""""""""""""""""""""""""""""""""""""""""""""""""""""""""""""""""""""""""""""""""""""""""""""""""""""""""""""""""""""""""""""""""""""""""""""""""""""""""""""""""""""""""""""""""""""""""""""""""""""""""""""""""""""""""""""""""""""""""""""""""""""""""""""""""""""""""""""""""""""""""""""""""""""""""""""""""""""""""""""""""""""""""""""""""""""""""""""""""""""""""""""""""""""""""""""""""""""""""""""""""""""""""""""""""""""""""""""""""""""""""""""""""""""""""""""""""""""""""""""""""""""""""""""""""""""""""""""""""""""""""""""""""""""""""""""""""""""""""""""""""""""""""""""""""""""""""""""""""""""""""""""""""""""""""""""""""""""""""""""""""""""""""""""""""""""""""""""""""""""""""""""""""""""""""""""""""""""""""""""""""""""""""""""""""""""""""""""""""""""""""""""""""""""""""""""""""""""""""""""""""""""""""""""""""""""""""""""""""""""""""""""""""""""""""""""""""""""""""""""""""""""""""""""""""""""""""""""""""""""""""""""""""""""""""""""""""""""""""""""""""""""""""""""""""""""""""""""""""""""""""""""""""""""""""""""""""""""""""""""""""""""""""""""""""""""""""""""""""""""""""""""""""""""""""""""""""""""""""""""""""""""""""""""""""""""""""""""""""""""""""""""""""""""""""""""""""""""""""""""""""""""""""""""""""""""""""""""""""""""""""""""""""""""""""""""""""""""""""""""""""""""""""""""""""""""""""""""""""""""""""""""""""""""""""""""""""""""""""""""""""""""""""""""""""""""""""""""""""""""""""""""""""""""""""""""""""""""""""""""""""""""""""""""""""""""""""""""""""""""""""""""""""""""""""""""""""""""""""""""""""""""""""""""""""""""""""""""""""""""""""""""""""""""""""""""""""""""""""""""""""""""""""""""""""""""""""""""""""""""""""""""""""""""""""""""""""""""""""""""""""""""""""""""""""""""""""""""""""""""""""""""""""""""""""""""""""""""""""""""""""""""""""""""""""""""""""""""""""""""""""""""""""""""""""""""""""""""""""""""""""""""""""""""""""""""""""""""""""""""""""""""""""""""""""""""""""""""""""""""""""""""""""""""""""""""""""""""""""""""""""""""""""""""""""""""""""""""""""""""""""""""""""""""""""""""""""""""""""""""""""""""""""""""""""""""""""""""""""""""""""""""""""""""""""""""""""""""""""""""""""""""""""""""""""""""""""""""""""""""""""""""""""""""""""""""""""""""""""""""""""""""""""""""""""""""""""""""""""""""""""""""""""""""""""""""""""""""""""""""""""""""""""""""""""""""""""""""""""""""""""""""""""""""""""""""""""""""""""""""""""""""""""""""""""""""""""""""""""""""""""""""""""""""""""""""""""""""""""""""""""""""""""""""""""""""""""""""""""""""""""""""""""""""""""""""""""""""""""""""""""""""""""""""""""""""""""""""""""""""""""""""""""""""""""""""""""""""""""""""""""""""""""""""""""""""""""""""""""""""""""""""""""""""""""""""""""""""""""""""""""""""""""""""""""""""""""""""""""""""""""""""""""""""""""""""""""""""""""""""""""""""""""""""""""""""""""""""""""""""""""""""""""""""""""""""""""""""""""""""""""""""""""""""""""""""""""""""""""""""""""""""""""""""""""""""""""""""""""""""""""""""""""""""""""""""""""""""""""""""""""""""""""""""""""""""""""""""""""""""""""""""""""""""""""""""""""""""""""""""""""""""""""""""""""""""""""""""""""""""""""""""""""""""""""""""""""""""""""""""""""""""""""""""""""""""""""""""""""""""""""""""""""""""""""""""""""""""""""""""""""""""""""""""""""""""""""""""""""""""""""""""""""""""""""""""""""""""""""""""""""""""""""""""""""""""""""""""""""""""""""""""""""""""""""""""""""""""""""""""""""""""""""""""""""""""""""""""""""""""""""""""""""""""""""""""""""""""""""""""""""""""""""""""""""""""""""""""""""""""""""""""""""""""""""""""""""""""""""""""""""""""""""""""""""""""""""""""""""""""""""""""""""""""""""""""""""""""""""""""""""""""""""""""""""""""""""""""""""""""""""""""""""""""""""""""""""""""""""""""""""""""""""""""""""""""""""""""""""""""""""""""""""""""""""""""""""""""""""""""""""""""""""""""""""""""""""""""""""""""""""""""""""""""""""""""""""""""""""""""""""""""""""""""""""""""""""""""""""""""""""""""""""""""""""""""""""""""""""""""""""""""""""""""""""""""""""""""""""""""""""""""""""""""""""""""""""""""""""""""""""""""""""""""""""""""""""""""""""""""""""""""""""""""""""""""""""""""""""""""""""""""""""""""""""""""""""""""""""""""""""""""""""""""""""""""""""""""""""""""""""""""""""""""""""""""""""""""""""""""""""""""""""""""""""""""""""""""""""""""""""""""""""""""""""""""""""""""""""""""""""""""""""""""""""""""""""""""""""""""""""""""""""""""""""""""""""""""""""""""""""""""""""""""""""""""""""""""""""""""""""""""""""""""""""""""""""""""""""""""""""""""""""""""""""""""""""""""""""""""""""""""""""""""""""""""""""""""""""""""""""""""""""""""""""""""""""""""""""""""""""""""""""""""""""""""""""""""""""""""""""""""""""""""""""""""""""""""""""""""""""""""""""""""""""""""""""""""""""""""""""""""""""""""""""""""""""""""""""""""""""""""""""""""""""""""""""""""""""""""""""""""""""""""""""""""""""""""""""""""""""""""""""""""""""""""""""""""""""""""""""""""""""""""""""""""""""""""""""""""""""""""""""""""""""""""""""""""""""""""""""""""""""""""""""""""""""""""""""""""""""""""""""""""""""""""""""""""""""""""""""""""""""""""""""""""""""""""""""""""""""""""""""""""""""""""""""""""""""""""""""""""""""""""""""""""""""""""""""""""""""""""""""""""""""""""""""""""""""""""""""""""""""""""""""""""""""""""""""""""""""""""""""""""""""""""""""""""""""""""""""""""""""""""""""""""""""""""""""""""""""""""""""""""""""""""""""""""""""""""""""""""""""""""""""""""""""""""""""""""""""""""""""""""""""""""""""""""""""""""""""""""""""""""""""""""""""""""""""""""""""""""""""""""""""""""""""""""""""""""""""""""""""""""""""""""""""""""""""""""""""""""""""""""""""""""""""""""""""""""""""""""""""""""""""""""""""""""""""""""""""""""""""""""""""""""""""""""""""""""""""""""""""""""""""""""""""""""""""""""""""""""""""""""""""""""""""""""""""""""""""""""""""""""""""""""""""""""""""""""""""""""""""""""""""""""""""""""""""""""""""""""""""""""""""""""""""""""""""""""""""""""""""""""""""""""""""""""""""""""""""""""""""""""""""""""""""""""""""""""""""""""""""""""""""""""""""""""""""""""""""""""""""""""""""""""""""""""""""""""""""""""""""""""""""""""""""""""""""""""""""""""""""""""""""""""""""""""""""""""""""""""""""""""""""""""""""""""""""""""""""""""""""""""""""""""""""""""""""""""""""""""""""""""""""""""""""""""""""""""""""""""""""""""""""""""""""""""""""""""""""""""""""""""""""""""""""""""""""""""""""""""""""""""""""""""""""""""""""""""""""""""""""""""""""""""""""""""""""""""""""""""""""""""""""""""""""""""""""""""""""""""""""""""""""""""""""""""""""""""""""""""""""""""""""""""""""""""""""""""""""""""""""""""""""""""""""""""""""""""""""""""""""""""""""""""""""""""""""""""""""""""""""""""""""""""""""""""""""""""""""""""""""""""""""""""""""""""""""""""""""""""""""""""""""""""""""""""""""""""""""""""""""""""""""""""""""""""""""""""""""""""""""""""""""""""""""""""""""""""""""""""""""""""""""""""""""""""""""""""""""""""""""""""""""""""""""""""""""""""""""""""""""""""""""""""""""""""""""""""""""""""""""""""""""""""""""""""""""""""""""""""""""""""""""""""""""""""""""""""""""""""""""""""""""""""""""""""""""""""""""""""""""""""""""""""""""""""""""""""""""""""""""""""""""""""""""""""""""""""""""""""""""""""""""""""""""""""""""""""""""""""""""""""""""""""""""""""""""""""""""""""""""""""""""""""""""""""""""""""""""""""""""""""""""""""""""""""""""""""""""""""""""""""""""""""""""""""""""""""""""""""""""""""""""""""""""""""""""""""""""""""""""""""""""""""""""""""""""""""""""""""""""""""""""""""""""""""""""""""""""""""""""""""""""""""""""""""""""""""""""""""""""""""""""""""""""""""""""""""""""""""""""""""""""""""""""""""""""""""""""""""""""""""""""""""""""""""""""""""""""""""""""""""""""""""""""""""""""""""""""""""""""""""""""""""""""""""""""""""""""""""""""""""""""""""""""""""""""""""""""""""""""""""""""""""""""""""""""""""""""""""""""""""""""""""""""""""""""""""""""""""""""""""""""""""""""""""""""""""""""""""""""""""""""""""""""""""""""""""""""""""""""""""""""""""""""""""""""""""""""""""""""""""""""""""""""""""""""""""""""""""""""""""""""""""""""""""""""""""""""""""""""""""""""""""""""""""""""""""""""""""""""""""""""""""""""""""""""""""""""""""""""""""""""""""""""""""""""""""""""""""""""""""""""""""""""""""""""""""""""""""""""""""""""""""""""""""""""""""""""""""""""""""""""""""""""""""""""""""""""""""""""""""""""""""""""""""""""""""""""""""""""""""""""""""""""""""""""""""""""""""""""""""""""""""""""""""""""""""""""""""""""""""""""""""""""""""""""""""""""""""""""""""""""""""""""""""""""""""""""""""""""""""""""""""""""""""""""""""""""""""""""""""""""""""""""""""""""""""""""""""""""""""""""""""""""""""""""""""""""""""""""""""""""""""""""""""""""""""""""""""""""""""""""""""""""""""""""""""""""""""""""""""""""""""""""""""""""""""""""""""""""""""""""""""""""""""""""""""""""""""""""""""""""""""""""""""""""""""""""""""""""""""""""""""""""""""""""""""""""""""""""""""""""""""""""""""""""""""""""""""""""""""""""""""""""""""""""""""""""""""""""""""""""""""""""""""""""""""""""""""""""""""""""""""""""""""""""""""""""""""""""""""""""""""""""""""""""""""""""""""""""""""""""""""""""""""""""""""""""""""""""""""""""""""""""""""""""""""""""""""""""""""""""""""""""""""""""""""""""""""""""""""""""""""""""""""""""""""""""""""""""""""""""""""""""""""""""""""""""""""""""""""""""""""""""""""""""""""""""""""""""""""""""""""""""""""""""""""""""""""""""""""""""""""""""""""""""""""""""""""""""""""""""""""""""""""""""""""""""""""""""""""""""""""""""""""""""""""""""""""""""""""""""""""""""""""""""""""""""""""""""""""""""""""""""""""""""""""""""""""""""""""""""""""""""""""""""""""""""""""""""""""""""""""""""""""""""""""""""""""""""""""""""""""""""""""""""""""""""""""""""""""""""""""""""""""""""""""""""""""""""""""""""""""""""""""""""""""""""""""""""""""""""""""""""""""""""""""""""""""""""""""""""""""""""""""""""""""""""""""""""""""""""""""""""""""""""""""""""""""""""""""""""""""""""""""""""""""""""""""""""""""""""""""""""""""""""""""""""""""""""""""""""""""""""""""""""""""""""""""""""""""""""""""""""""""""""""""""""""""""""""""""""""""""""""""""""""""""""""""""""""""""""""""""""""""""""""""""""""""""""""""""""""""""""""""""""""""""""""""""""""""""""""""""""""""""""""""""""""""""""""""""""""""""""""""""""""""""""""""""""""""""""""""""""""""""""""""""""""""""""""""""""""""""""""""""""""""""""""""""""""""""""""""""""""""""""""""""""""""""""""""""""""""""""""""""""""""""""""""""""""""""""""""""""""""""""""""""""""""""""""""""""""""""""""""""""""""""""""""""""""""""""""""""""""""""""""""""""""""""""""""""""""""""""""""""""""""""""""""""""""""""""""""""""""""""""""""""""""""""""""""""""""""""""""""""""""""""""""""""""""""""""""""""""""""""""""""""""""""""""""""""""""""""""""""""""""""""""""""""""""""""""""""""""""""""""""""""""""""""""""""""""""""""""""""""""""""""""""""""""""""""""""""""""""""""""""""""""""""""""""""""""""""""""""""""""""""""""""""""""""""""""""""""""""""""""""""""""""""""""""""""""""""""""""""""""""""""""""""""""""""""""""""""""""""""""""""""""""""""""""""""""""""""""""""""""""""""""""""""""""""""""""""""""""""""""""""""""""""""""""""""""""""""""""""""""""""""""""""""""""""""""""""""""""""""""""""""""""""""""""""""""""""""""""""""""""""""""""""""""""""""""""""""""""""""""""""""""""""""""""""""""""""""""""""""""""""""""""""""""""""""""""""""""""""""""""""""""""""""""""""""""""""""""""""""""""""""""""""""""""""""""""""""""""""""""""""""""""""""""""""""""""""""""""""""""""""""""""""""""""""""""""""""""""""""""""""""""""""""""""""""""""""""""""""""""""""""""""""""""""""""""""""""""""""""""""""""""""""""""""""""""""""""""""""""""""""""""""""""""""""""""""""""""""""""""""""""""""""""""""""""""""""""""""""""""""""""""""""""""""""""""""""""""""""""""""""""""""""""""""""""""""""""""""""""""""""""""""""""""""""""""""""""""""""""""""""""""""""""""""""""""""""""""""""""""""""""""""""""""""""""""""""""""""""""""""""""""""""""""""""""""""""""""""""""""""""""""""""""""""""""""""""""""""""""""""""""""""""""""""""""""""""""""""""""""""""""""""""""""""""""""""""""""""""""""""""""""""""""""""""""""""""""""""""""""""""""""""""""""""""""""""""""""""""""""""""""""""""""""""""""""""""""""""""""""""""""""""""""""""""""""""""""""""""""""""""""""""""""""""""""""""""""""""""""""""""""""""""""""""""""""""""""""""""""""""""""""""""""""""""""""""""""""""""""""""""""""""""""""""""""""""""""""""""""""""""""""""""""""""""""""""""""""""""""""""""""""""""""""""""""""""""""""""""""""""""""""""""""""""""""""""""""""""""""""""""""""""""""""""""""""""""""""""""""""""""""""""""""""""""""""""""""""""""""""""""""""""""""""""""""""""""""""""""""""""""""""""""""""""""""""""""""""""""""""""""""""""""""""""""""""""""""""""""""""""""""""""""""""""""""""""""""""""""""""""""""""""""""""""""""""""""""""""""""""""""""""""""""""""""""""""""""""""""""""""""""""""""""""""""""""""""""""""""""""""""""""""""""""""""""""""""""""""""""""""""""""""""""""""""""""""""""""""""""""""""""""""""""""""""""""""""""""""""""""""""""""""""""""""""""""""""""""""""""""""""""""""""""""""""""""""""""""""""""""""""""""""""""""""""""""""""""""""""""""""""""""""""""""""""""""""""""""""""""""""""""""""""""""""""""""""""""""""""""""""""""""""""""""""""""""""""""""""""""""""""""""""""""""""""""""""""""""""""""""""""""""""""""""""""""""""""""""""""""""""""""""""""""""""""""""""""""""""""""""""""""""""""""""""""""""""""""""""""""""""""""""""""""""""""""""""""""""""""""""""""""""""""""""""""""""""""""""""""""""""""""""""""""""""""""""""""""""""""""""""""""""""""""""""""""""""""""""""""""""""""""""""""""""""""""""""""""""""""""""""""""""""""""""""""""""""""""""""""""""""""""""""""""""""""""""""""""""""""""""""""""""""""""""""""""""""""""""""""""""""""""""""""""""""""""""""""""""""""""""""""""""""""""""""""""""""""""""""""""""""""""""""""""""""""""""""""""""""""""""""""""""""""""""""""""""""""""""""""""""""""""""""""""""""""""""""""""""""""""""""""""""""""""""""""""""""""""""""""""""""""""""""""""""""""""""""""""""""""""""""""""""""""""""""""""""""""""""""""""""""""""""""""""""""""""""""""""""""""""""""""""""""""""""""""""""""""""""""""""""""""""""""""""""""""""""""""""""""""""""""""""""""""""""""""""""""""""""""""""""""""""""""""""""""""""""""""""""""""""""""""""""""""""""""""""""""""""""""""""""""""""""""""""""""""""""""""""""""""""""""""""""""""""""""""""""""""""""""""""""""""""""""""""""""""""""""""""""""""""""""""""""""""""""""""""""""""""""""""""""""""""""""""""""""""""""""""""""""""""""""""""""""""""""""""""""""""""""""""""""""""""""""""""""""""""""""""""""""""""""""""""""""""""""""""""""""""""""""""""""""""""""""""""""""""""""""""""""""""""""""""""""""""""""""""""""""""""""""""""""""""""""""""""""""""""""""""""""""""""""""""""""""""""""""""""""""""""""""""""""""""""""""""""""""""""""""""""""""""""""""""""""""""""""""""""""""""""""""""""""""""""""""""""""""""""""""""""""""""""""""""""""""""""""""""""""""""""""""""""""""""""""""""""""""""""""""""""""""""""""""""""""""""""""""""""""""""""""""""""""""""""""""""""""""""""""""""""""""""""""""""""""""""""""""""""""""""""""""""""""""""""""""""""""""""""""""""""""""""""""""""""""""""""""""""""""""""""""""""""""""""""""""""""""""""""""""""""""""""""""""""""""""""""""""""""""""""""""""""""""""""""""""""""""""""""""""""""""""""""""""""""""""""""""""""""""""""""""""""""""""""""""""""""""""""""""""""""""""""""""""""""""""""""""""""""""""""""""""""""""""""""""""""""""""""""""""""""""""""""""""""""""""""""""""""""""""""""""""""""""""""""""""""""""""""""""""""""""""""""""""""""""""""""""""""""""""""""""""""""""""""""""""""""""""""""""""""""""""""""""""""""""""""""""""""""""""""""""""""""""""""""""""""""""""""""""""""""""""""""""""""""""""""""""""""""""""""""""""""""""""""""""""""""""""""""""""""""""""""""""""""""""""""""""""""""""""""""""""""""""""""""""""""""""""""""""""""""""""""""""""""""""""""""""""""""""""""""""""""""""""""""""""""""""""""""""""""""""""""""""""""""""""""""""""""""""""""""""""""""""""""""""""""""""""""""""""""""""""""""""""""""""""""""""""""""""""""""""""""""""""""""""""""""""""""""""""""""""""""""""""""""""""""""""""""""""""""""""""""""""""""""""""""""""""""""""""""""""""""""""""""""""""""""""""""""""""""""""""""""""""""""""""""""""""""""""""""""""""""""""""""""""""""""""""""""""""""""""""""""""""""""""""""""""""""""""""""""""""""""""""""""""""""""""""""""""""""""""""""""""""""""""""""""""""""""""""""""""""""""""""""""""""""""""""""""""""""""""""""""""""""""""""""""""""""""""""""""""""""""""""""""""""""""""""""""""""""""""""""""""""""""""""""""""""""""""""""""""""""""""""""""""""""""""""""""""""""""""""""""""""""""""""""""""""""""""""""""""""""""""""""""""""""""""""""""""""""""""""""""""""""""""""""""""""""""""""""""""""""""""""""""""""""""""""""""""""""""""""""""""""""""""""""""""""""""""""""""""""""""""""""""""""""""""""""""""""""""""""""""""""""""""""""""""""""""""""""""""""""""""""""""""""""""""""""""""""""""""""""""""""""""""""""""""""""""""""""""""""""""""""""""""""""""""""""""""""""""""""""""""""""""""""""""""""""""""""""""""""""""""""""""""""""""""""""""""""""""""""""""""""""""""""""""""""""""""""""""""""""""""""""""""""""""""""""""""""""""""""""""""""""""""""""""""""""""""""""""""""""""""""""""""""""""""""""""""""""""""""""""""""""""""""""""""""""""""""""""""""""""""""""""""""""""""""""""""""""""""""""""""""""""""""""""""""""""""""""""""""""""""""""""""""""""""""""""""""""""""""""""""""""""""""""""""""""""""""""""""""""""""""""""""""""""""""""""""""""""""""""""""""""""""""""""""""""""""""""""""""""""""""""""""""""""""""""""""""""""""""""""""""""""""""""""""""""""""""""""""""""""""""""""""""""""""""""""""""""""""""""""""""""""""""""""""""""""""""""""""""""""""""""""""""""""""""""""""""""""""""""""""""""""""""""""""""""""""""""""""""""""""""""""""""""""""""""""""""""""""""""""""""""""""""""""""""""""""""""""""""""""""""""""""""""""""""""""""""""""""""""""""""""""""""""""""""""""""""""""""""""""""""""""""""""""""""""""""""""""""""""""""""""""""""""""""""""""""""""""""""""""""""""""""""""""""""""""""""""""""""""""""""""""""""""""""""""""""""""""""""""""""""""""""""""""""""""""""""""""""""""""""""""""""""""""""""""""""""""""""""""""""""""""""""""""""""""""""""""""""""""""""""""""""""""""""""""""""""""""""""""""""""""""""""""""""""""""""""""""""""""""""""""""""""""""""""""""""""""""""""""""""""""""""""""""""""""""""""""""""""""""""""""""""""""""""""""""""""""""""""""""""""""""""""""""""""""""""""""""""""""""""""""""""""""""""""""""""""""""""""""""""""""""""""""""""""""""""""""""""""""""""""""""""""""""""""""""""""""""""""""""""""""""""""""""""""""""""""""""""""""""""""""""""""""""""""""""""""""""""""""""""""""""""""""""""""""""""""""""""""""""""""""""""""""""""""""""""""""""""""""""""""""""""""""""""""""""""""""""""""""""""""""""""""""""""""""""""""""""""""""""""""""""""""""""""""""""""""""""""""""""""""""""""""""""""""""""""""""""""""""""""""""""""""""""""""""""""""""""""""""""""""""""""""""""""""""""""""""""""""""""""""""""""""""""""""""""""""""""""""""""""""""""""""""""""""""""""""""""""""""""""""""""""""""""""""""""""""""""""""""""""""""""""""""""""""""""""""""""""""""""""""""""""""""""""""""""""""""""""""""""""""""""""""""""""""""""""""""""""""""""""""""""""""""""""""""""""""""""""""""""""""""""""""""""""""""""""""""""""""""""""""""""""""""""""""""""""""""""""""""""""""""""""""""""""""""""""""""""""""""""""""""""""""""""""""""""""""""""""""""""""""""""""""""""""""""""""""""""""""""""""""""""""""""""""""""""""""""""""""""""""""""""""""""""""""""""""""""""""""""""""""""""""""""""""""""""""""""""""""""""""""""""""""""""""""""""""""""""""""""""""""""""""""""""""""""""""""""""""""""""""""""""""""""""""""""""""""""""""""""""""""""""""""""""""""""""""""""""""""""""""""""""""""""""""""""""""""""""""""""""""""""""""""""""""""""""""""""""""""""""""""""""""""""""""""""""""""""""""""""""""""""""""""""""""""""""""""""""""""""""""""""""""""""""""""""""""""""""""""""""""""""""""""""""""""""""""""""""""""""""""""""""""""""""""""""""""""""""""""""""""""""""""""""""""""""""""""""""""""""""""""""""""""""""""""""""""""""""""""""""""""""""""""""""""""""""""""""""""""""""""""""""""""""""""""""""""""""""""""""""""""""""""""""""""""""""""""""""""""""""""""""""""""""""""""""""""""""""""""""""""""""""""""""""""""""""""""""""""""""""""""""""""""""""""""""""""""""""""""""""""""""""""""""""""""""""""""""""""""""""""""""""""""""""""""""""""""""""""""""""""""""""""""""""""""""""""""""""""""""""""""""""""""""""""""""""""""""""""""""""""""""""""""""""""""""""""""""""""""""""""""""""""""""""""""""""""""""""""""""""""""""""""""""""""""""""""""""""""""""""""""""""""""""""""""""""""""""""""""""""""""""""""""""""""""""""""""""""""""""""""""""""""""""""""""""""""""""""""""""""""""""""""""""""""""""""""""""""""""""""""""""""""""""""""""""""""""""""""""""""""""""""""""""""""""""""""""""""""""""""""""""""""""""""""""""""""""""""""""""""""""""""""""""""""""""""""""""""""""""""""""""""""""""""""""""""""""""""""""""""""""""""""""""""""""""""""""""""""""""""""""""""""""""""""""""""""""""""""""""""""""""""""""""""""""""""""""""""""""""""""""""""""""""""""""""""""""""""""""""""""""""""""""""""""""""""""""""""""""""""""""""""""""""""""""""""""""""""""""""""""""""""""""""""""""""""""""""""""""""""""""""""""""""""""""""""""""""""""""""""""""""""""""""""""""""""""""""""""""""""""""""""""""""""""""""""""""""""""""""""""""""""""""""""""""""""""""""""""""""""""""""""""""""""""""""""""""""""""""""""""""""""""""""""""""""""""""""""""""""""""""""""""""""""""""""""""""""""""""""""""""""""""""""""""""""""""""""""""""""""""""""""""""""""""""""""""""""""""""""""""""""""""""""""""""""""""""""""""""""""""""""""""""""""""""""""""""""""""""""""""""""""""""""""""""""""""""""""""""""""""""""""""""""""""""""""""""""""""""""""""""""""""""""""""""""""""""""""""""""""""""""""""""""""""""""""""""""""""""""""""""""""""""""""""""""""""""""""""""""""""""""""""""""""""""""""""""""""""""""""""""""""""""""""""""""""""""""""""""""""""""""""""""""""""""""""""""""""""""""""""""""""""""""""""""""""""""""""""""""""""""""""""""""""""""""""""""""""""""""""""""""""""""""""""""""""""""""""""""""""""""""""""""""""""""""""""""""""""""""""""""""""""""""""""""""""""""""""""""""""""""""""""""""""""""""""""""""""""""""""""""""""""""""""""""""""""""""""""""""""""""""""""""""""""""""""""""""""""""""""""""""""""""""""""""""""""""""""""""""""""""""""""""""""""""""""""""""""""""""""""""""""""""""""""""""""""""""""""""""""""""""""""""""""""""""""""""""""""""""""""""""""""""""""""""""""""""""""""""""""""""""""""""""""""""""""""""""""""""""""""""""""""""""""""""""""""""""""""""""""""""""""""""""""""""""""""""""""""""""""""""""""""""""""""""""""""""""""""""""""""""""""""""""""""""""""""""""""""""""""""""""""""""""""""""""""""""""""""""""""""""""""""""""""""""""""""""""""""""""""""""""""""""""""""""""""""""""""""""""""""""""""""""""""""""""""""""""""""""""""""""""""""""""""""""""""""""""""""""""""""""""""""""""""""""""""""""""""""""""""""""""""""""""""""""""""""""""""""""""""""""""""""""""""""""""""""""""""""""""""""""""""""""""""""""""""""""""""""""""""""""""""""""""""""""""""""""""""""""""""""""""""""""""""""""""""""""""""""""""""""""""""""""""""""""""""""""""""""""""""""""""""""""""""""""""""""""""""""""""""""""""""""""""""""""""""""""""""""""""""""""""""""""""""""""""""""""""""""""""""""""""""""""""""""""""""""""""""""""""""""""""""""""""""""""""""""""""""""""""""""""""""""""""""""""""""""""""""""""""""""""""""""""""""""""""""""""""""""""""""""""""""""""""""""""""""""""""""""""""""""""""""""""""""""""""""""""""""""""""""""""""""""""""""""""""""""""""""""""""""""""""""""""""""""""""""""""""""""""""""""""""""""""""""""""""""""""""""""""""""""""""""""""""""""""""""""""""""""""""""""""""""""""""""""""""""""""""""""""""""""""""""""""""""""""""""""""""""""""""""""""""""""""""""""""""""""""""""""""""""""""""""""""""""""""""""""""""""""""""""""""""""""""""""""""""""""""""""""""""""""""""""""""""""""""""""""""""""""""""""""""""""""""""""""""""""""""""""""""""""""""""""""""""""""""""""""""""""""""""""""""""""""""""""""""""""""""""""""""""""""""""""""""""""""""""""""""""""""""""""""""""""""""""""""""""""""""""""""""""""""""""""""""""""""""""""""""""""""""""""""""""""""""""""""""""""""""""""""""""""""""""""""""""""""""""""""""""""""""""""""""""""""""""""""""""""""""""""""""""""""""""""""""""""""""""""""""""""""""""""""""""""""""""""""""""""""""""""""""""""""""""""""""""""""""""""""""""""""""""""""""""""""""""""""""""""""""""""""""""""""""""""""""""""""""""""""""""""""""""""""""""""""""""""""""""""""""""""""""""""""""""""""""""""""""""""""""""""""""""""""""""""""""""""""""""""""""""""""""""""""""""""""""""""""""""""""""""""""""""""""""""""""""""""""""""""""""""""""""""""""""""""""""""""""""""""""""""""""""""""""""""""""""""""""""""""""""""""""""""""""""""""""""""""""""""""""""""""""""""""""""""""""""""""""""""""""""""""""""""""""""""""""""""""""""""""""""""""""""""""""""""""""""""""""""""""""""""""""""""""""""""""""""""""""""""""""""""""""""""""""""""""""""""""""""""""""""""""""""""""""""""""""""""""""""""""""""""""""""""""""""""""""""""""""""""""""""""""""""""""""""""""""""""""""""""""""""""""""""""""""""""""""""""""""""""""""""""""""""""""""""""""""""""""""""""""""""""""""""""""""""""""""""""""""""""""""""""""""""""""""""""""""""""""""""""""""""""""""""""""""""""""""""""""""""""""""""""""""""""""""""""""""""""""""""""""""""""""""""""""""""""""""""""""""""""""""""""""""""""""""""""""""""""""""""""""""""""""""""""""""""""""""""""""""""""""""""""""""""""""""""""""""""""""""""""""""""""""""""""""""""""""""""""""""""""""""""""""""""""""""""""""""""""""""""""""""""""""""""""""""""""""""""""""""""""""""""""""""""""""""""""""""""""""""""""""""""""""""""""""""""""""""""""""""""""""""""""""""""""""""""""""""""""""""""""""""""""""""""""""""""""""""""""""""""""""""""""""""""""""""""""""""""""""""""""""""""""""""""""""""""""""""""""""""""""""""""""""""""""""""""""""""""""""""""""""""""""""""""""""""""""""""""""""""""""""""""""""""""""""""""""""""""""""""""""""""""""""""""""""""""""""""""""""""""""""""""""""""""""""""""""""""""""""""""""""""""""""""""""""""""""""""""""""""""""""""""""""""""""""""""""""""""""""""""""""""""""""""""""""""""""""""""""""""""""""""""""""""""""""""""""""""""""""""""""""""""""""""""""""""""""""""""""""""""""""""""""""""""""""""""""""""""""""""""""""""""""""""""""""""""""""""""""""""""""""""""""""""""""""""""""""""""""""""""""""""""""""""""""""""""""""""""""""""""""""""""""""""""""""""""""""""""""""""""""""""""""""""""""""""""""""""""""""""""""""""""""""""""""""""""""""""""""""""""""""""""""""""""""""""""""""""""""""""""""""""""""""""""""""""""""""""""""""""""""""""""""""""""""""""""""""""""""""""""""""""""""""""""""""""""""""""""""""""""""""""""""""""""""""""""""""""""""""""""""""""""""""""""""""""""""""""""""""""""""""""""""""""""""""""""""""""""""""""""""""""""""""""""""""""""""""""""""""""""""""""""""""""""""""""""""""""""""""""""""""""""""""""""""""""""""""""""""""""""""""""""""""""""""""""""""""""""""""""""""""""""""""""""""""""""""""""""""""""""""""""""""""""""""""""""""""""""""""""""""""""""""""""""""""""""""""""""""""""""""""""""""""""""""""""""""""""""""""""""""""""""""""""""""""""""""""""""""""""""""""""""""""""""""""""""""""""""""""""""""""""""""""""""""""""""""""""""""""""""""""""""""""""""""""""""""""""""""""""""""""""""""""""""""""""""""""""""""""""""""""""""""""""""""""""""""""""""""""""""""""""""""""""""""""""""""""""""""""""""""""""""""""""""""""""""""""""""""""""""""""""""""""""""""""""""""""""""""""""""""""""""""""""""""""""""""""""""""""""""""""""""""""""""""""""""""""""""""""""""""""""""""""""""""""""""""""""""""""""""""""""""""""""""""""""""""""""""""""""""""""""""""""""""""""""""""""""""""""""""""""""""""""""""""""""""""""""""""""""""""""""""""""""""""""""""""""""""""""""""""""""""""""""""""""""""""""""""""""""""""""""""""""""""""""""""""""""""""""""""""""""""""""""""""""""""""""""""""""""""""""""""""""""""""""""""""""""""""""""""""""""""""""""""""""""""""""""""""""""""""""""""""""""""""""""""""""""""""""""""""""""""""""""""""""""""""""""""""""""""""""""""""""""""""""""""""""""""""""""""""""""""""""""""""""""""""""""""""""""""""""""""""""""""""""""""""""""""""""""""""""""""""""""""""""""""""""""""""""""""""""""""""""""""""""""""""""""""""""""""""""""""""""""""""""""""""""""""""""""""""""""""""""""""""""""""""""""""""""""""""""""""""""""""""""""""""""""""""""""""""""""""""""""""""""""""""""""""""""""""""""""""""""""""""""""""""""""""""""""""""""""""""""""""""""""""""""""""""""""""""""""""""""""""""""""""""""""""""""""""""""""""""""""""""""""""""""""""""""""""""""""""""""""""""""""""""""""""""""""""""""""""""""""""""""""""""""""""""""""""""""""""""""""""""""""""""""""""""""""""""""""""""""""""""""""""""""""""""""""""""""""""""""""""""""""""""""""""""""""""""""""""""""""""""""""""""""""""""""""""""""""""""""""""""""""""""""""""""""""""""""""""""""""""""""""""""""""""""""""""""""""""""""""""""""""""""""""""""""""""""""""""""""""""""""""""""""""""""""""""""""""""""""""""""""""""""""""""""""""""""""""""""""""""""""""""""""""""""""""""""""""""""""""""""""""""""""""""""""""""""""""""""""""""""""""""""""""""""""""""""""""""""""""""""""""""""""""""""""""""""""""""""""""""""""""""""""""""""""""""""""""""""""""""""""""""""""""""""""""""""""""""""""""""""""""""""""""""""""""""""""""""""""""""""""""""""""""""""""""""""""""""""""""""""""""""""""""""""""""""""""""""""""""""""""""""""""""""""""""""""""""""""""""""""""""""""""""""""""""""""""""""""""""""""""""""""""""""""""""""""""""""""""""""""""""""""""""""""""""""""""""""""""""""""""""""""""""""""""""""""""""""""""""""""""""""""""""""""""""""""""""""""""""""""""""""""""""""""""""""""""""""""""""""""""""""""""""""""""""""""""""""""""""""""""""""""""""""""""""""""""""""""""""""""""""""""""""""""""""""""""""""""""""""""""""""""""""""""""""""""""""""""""""""""""""""""""""""""""""""""""""""""""""""""""""""""""""""""""""""""""""""""""""""""""""""""""""""""""""""""""""""""""""""""""""""""""""""""""""""""""""""""""""""""""""""""""""""""""""""""""""""""""""""""""""""""""""""""""""""""""""""""""""""""""""""""""""""""""""""""""""""""""""""""""""""""""""""""""""""""""""""""""""""""""""""""""""""""""""""""""""""""""""""""""""""""""""""""""""""""""""""""""""""""""""""""""""""""""""""""""""""""""""""""""""""""""""""""""""""""""""""""""""""""""""""""""""""""""""""""""""""""""""""""""""""""""""""""""""""""""""""""""""""""""""""""""""""""""""""""""""""""""""""""""""""""""""""""""""""""""""""""""""""""""""""""""""""""""""""""""""""""""""""""""""""""""""""""""""""""""""""""""""""""""""""""""""""""""""""""""""""""""""""""""""""""""""""""""""""""""""""""""""""""""""""""""""""""""""""""""""""""""""""""""""""""""""""""""""""""""""""""""""""""""""""""""""""""""""""""""""""""""""""""""""""""""""""""""""""""""""""""""""""""""""""""""""""""""""""""""""""""""""""""""""""""""""""""""""""""""""""""""""""""""""""""""""""""""""""""""""""""""""""""""""""""""""""""""""""""""""""""""""""""""""""""""""""""""""""""""""""""""""""""""""""""""""""""""""""""""""""""""""""""""""""""""""""""""""""""""""""""""""""""""""""""""""""""""""""""""""""""""""""""""""""""""""""""""""""""""""""""""""""""""""""""""""""""""""""""""""""""""""""""""""""""""""""""""""""""""""""""""""""""""""""""""""""""""""""""""""""""""""""""""""""""""""""""""""""""""""""""""""""""""""""""""""""""""""""""""""""""""""""""""""""""""""""""""""""""""""""""""""""""""""""""""""""""""""""""""""""""""""""""""""""""""""""""""""""""""""""""""""""""""""""""""""""""""""""""""""""""""""""""""""""""""""""""""""""""""""""""""""""""""""""""""""""""""""""""""""""""""""""""""""""""""""""""""""""""""""""""""""""""""""""""""""""""""""""""""""""""""""""""""""""""""""""""""""""""""""""""""""""""""""""""""""""""""""""""""""""""""""""""""""""""""""""""""""""""""""""""""""""""""""""""""""""""""""""""""""""""""""""""""""""""""""""""""""""""""""""""""""""""""""""""""""""""""""""""""""""""""""""""""""""""""""""""""""""""""""""""""""""""""""""""""""""""""""""""""""""""""""""""""""""""""""""""""""""""""""""""""""""""""""""""""""""""""""""""""""""""""""""""""""""""""""""""""""""""""""""""""""""""""""""""""""""""""""""""""""""""""""""""""""""""""""""""""""""""""""""""""""""""""""""""""""""""""""""""""""""""""""""""""""""""""""""""""""""""""""""""""""""""""""""""""""""""""""""""""""""""""""""""""""""""""""""""""""""""""""""""""""""""""""""""""""""""""""""""""""""""""""""""""""""""""""""""""""""""""""""""""""""""""""""""""""""""""""""""""""""""""""""""""""""""""""""""""""""""""""""""""""""""""""""""""""""""""""""""""""""""""""""""""""""""""""""""""""""""""""""""""""""""""""""""""""""""""""""""""""""""""""""""""""""""""""""""""""""""""""""""""""""""""""""""""""""""""""""""""""""""""""""""""""""""""""""""""""""""""""""""""""""""""""""""""""""""""""""""""""""""""""""""""""""""""""""""""""""""""""""""""""""""""""""""""""""""""""""""""""""""""""""""""""""""""""""""""""""""""""""""""""""""""""""""""""""""""""""""""""""""""""""""""""""""""""""""""""""""""""""""""""""""""""""""""""""""""""""""""""""""""""""""""""""""""""""""""""""""""""""""""""""""""""""""""""""""""""""""""""""""""""""""""""""""""""""""""""""""""""""""""""""""""""""""""""""""""""""""""""""""""""""""""""""""""""""""""""""""""""""""""""""""""""""""""""""""""""""""""""""""""""""""""""""""""""""""""""""""""""""""""""""""""""""""""""""""""""""""""""""""""""""""""""""""""""""""""""""""""""""""""""""""""""""""""""""""""""""""""""""""""""""""""""""""""""""""""""""""""""""""""""""""""""""""""""""""""""""""""""""""""""""""""""""""""""""""""""""""""""""""""""""""""""""""""""""""""""""""""""""""""""""""""""""""""""""""""""""""""""""""""""""""""""""""""""""""""""""""""""""""""""""""""""""""""""""""""""""""""""""""""""""""""""""""""""""""""""""""""""""""""""""""""""""""""""""""""""""""""""""""""""""""""""""""""""""""""""""""""""""""""""""""""""""""""""""""""""""""""""""""""""""""""""""""""""""""""""""""""""""""""""""""""""""""""""""""""""""""""""""""""""""""""""""""""""""""""""""""""""""""""""""""""""""""""""""""""""""""""""""""""""""""""""""""""""""""""""""""""""""""""""""""""""""""""""""""""""""""""""""""""""""""""""""""""""""""""""""""""""""""""""""""""""""""""""""""""""""""""""""""""""""""""""""""""""""""""""""""""""""""""""""""""""""""""""""""""""""""""""""""""""""""""""""""""""""""""""""""""""""""""""""""""""""""""""""""""""""""""""""""""""""""""""""""""""""""""""""""""""""""""""""""""""""""""""""""""""""""""""""""""""""""""""""""""""""""""""""""""""""""""""""""""""""""""""""""""""""""""""""""""""""""""""""""""""""""""""""""""""""""""""""""""""""""""""""""""""""""""""""""""""""""""""""""""""""""""""""""""""""""""""""""""""""""""""""""""""""""""""""""""""""""""""""""""""""""""""""""""""""""""""""""""""""""""""""""""""""""""""""""""""""""""""""""""""""""""""""""""""""""""""""""""""""""""""""""""""""""""""""""""""""""""""""""""""""""""""""""""""""""""""""""""""""""""""""""""""""""""""""""""""""""""""""""""""""""""""""""""""""""""""""""""""""""""""""""""""""""""""""""""""""""""""""""""""""""""""""""""""""""""""""""""""""""""""""""""""""""""""""""""""""""""""""""""""""""""""""""""""""""""""""""""""""""""""""""""""""""""""""""""""""""""""""""""""""""""""""""""""""""""""""""""""""""""""""""""""""""""""""""""""""""""""""""""""""""""""""""""""""""""""""""""""""""""""""""""""""""""""""""""""""""""""""""""""""""""""""""""""""""""""""""""""""""""""""""""""""""""""""""""""""""""""""""""""""""""""""""""""""""""""""""""""""""""""""""""""""""""""""""""""""""""""""""""""""""""""""""""""""""""""""""""""""""""""""""""""""""""""""""""""""""""""""""""""""""""""""""""""""""""""""""""""""""""""""""""""""""""""""""""""""""""""""""""""""""""""""""""""""""""""""""""""""""""""""""""""""""""""""""""""""""""""""""""""""""""""""""""""""""""""""""""""""""""""""""""""""""""""""""""""""""""""""""""""""""""""""""""""""""""""""""""""""""""""""""""""""""""""""""""""""""""""""""""""""""""""""""""""""""""""""""""""""""""""""""""""""""""""""""""""""""""""""""""""""""""""""""""""""""""""""""""""""""""""""""""""""""""""""""""""""""""""""""""""""""""""""""""""""""""""""""""""""""""""""""""""""""""""""""""""""""""""""""""""""""""""""""""""""""""""""""""""""""""""""""""""""""""""""""""""""""""""""""""""""""""""""""""""""""""""""""""""""""""""""""""""""""""""""""""""""""""""""""""""""""""""""""""""""""""""""""""""""""""""""""""""""""""""""""""""""""""""""""""""""""""""""""""""""""""""""""""""""""""""""""""""""""""""""""""""""""""""""""""""""""""""""""""""""""""""""""""""""""""""""""""""""""""""""""""""""""""""""""""""""""""""""""""""""""""""""""""""""""""""""""""""""""""""""""""""""""""""""""""""""""""""""""""""""""""""""""""""""""""""""""""""""""""""""""""""""""""""""""""""""""""""""""""""""""""""""""""""""""""""""""""""""""""""""""""""""""""""""""""""""""""""""""""""""""""""""""""""""""""""""""""""""""""""""""""""""""""""""""""""""""""""""""""""""""""""""""""""""""""""""""""""""""""""""""""""""""""""""""""""""""""""""""""""""""""""""""""""""""""""""""""""""""""""""""""""""""""""""""""""""""""""""""""""""""""""""""""""""""""""""""""""""""""""""""""""""""""""""""""""""""""""""""""""""""""""""""""""""""""""""""""""""""""""""""""""""""""""""""""""""""""""""""""""""""""""""""""""""""""""""""""""""""""""""""""""""""""""""""""""""""""""""""""""""""""""""""""""""""""""""""""""""""""""""""""""""""""""""""""""""""""""""""""""""""""""""""""""""""""""""""""""""""""""""""""""""""""""""""""""""""""""""""""""""""""""""""""""""""""""""""""""""""""""""""""""""""""""""""""""""""""""""""""""""""""""""""""""""""""""""""""""""""""""""""""""""""""""""""""""""""""""""""""""""""""""""""""""""""""""""""""""""""""""""""""""""""""""""""""""""""""""""""""""""""""""""""""""""""""""""""""""""""""""""""""""""""""""""""""""""""""""""""""""""""""""""""""""""""""""""""""""""""""""""""""""""""""""""""""""""""""""""""""""""""""""""""""""""""""""""""""""""""""""""""""""""""""""""""""""""""""""""""""""""""""""""""""""""""""""""""""""""""""""""""""""""""""""""""""""""""""""""""""""""""""""""""""""""""""""""""""""""""""""""""""""""""""""""""""""""""""""""""""""""""""""""""""""""""""""""""""""""""""""""""""""""""""""""""""""""""""""""""""""""""""""""""""""""""""""""""""""""""""""""""""""""""""""""""""""""""""""""""""""""""""""""""""""""""""""""""""""""""""""""""""""""""""""""""""""""""""""""""""""""""""""""""""""""""""""""""""""""""""""""""""""""""""""""""""""""""""""""""""""""""""""""""""""""""""""""""""""""""""""""""""""""""""""""""""""""""""""""""""""""""""""""""""""""""""""""""""""""""""""""""""""""""""""""""""""""""""""""""""""""""""""""""""""""""""""""""""""""""""""""""""""""""""""""""""""""""""""""""""""""""""""""""""""""""""""""""""""""""""""""""""""""""""""""""""""""""""""""""""""""""""""""""""""""""""""""""""""""""""""""""""""""""""""""""""""""""""""""""""""""""""""""""""""""""""""""""""""""""""""""""""""""""""""""""""""""""""""""""""""""""""""""""""""""""""""""""""""""""""""""""""""""""""""""""""""""""""""""""""""""""""""""""""""""""""""""""""""""""""""""""""""""""""""""""""""""""""""""""""""""""""""""""""""""""""""""""""""""""""""""""""""""""""""""""""""""""""""""""""""""""""""""""""""""""""""""""""""""""""""""""""""""""""""""""""""""""""""""""""""""""""""""""""""""""""""""""""""""""""""""""""""""""""""""""""""""""""""""""""""""""""""""""""""""""""""""""""""""""""""""""""""""""""""""""""""""""""""""""""""""""""""""""""""""""""""""""""""""""""""""""""""""""""""""""""""""""""""""""""""""""""""""""""""""""""""""""""""""""""""""""""""""""""""""""""""""""""""""""""""""""""""""""""""""""""""""""""""""""""""""""""""""""""""""""""""""""""""""""""""""""""""""""""""""""""""""""""""""""""""""""""""""""""""""""""""""""""""""""""""""""""""""""""""""""""""""""""""""""""""""""""""""""""""""""""""""""""""""""""""""""""""""""""""""""""""""""""""""""""""""""""""""""""""""""""""""""""""""""""""""""""""""""""""""""""""""""""""""""""""""""""""""""""""""""""""""""""""""""""""""""""""""""""""""""""""""""""""""""""""""""""""""""""""""""""""""""""""""""""""""""""""""""""""""""""""""""""""""""""""""""""""""""""""""""""""""""""""""""""""""""""""""""""""""""""""""""""""""""""""""""""""""""""""""""""""""""""""""""""""""""""""""""""""""""""""""""""""""""""""""""""""""""""""""""""""""""""""""""""""""""""""""""""""""""""""""""""""""""""""""""""""""""""""""""""""""""""""""""""""""""""""""""""""""""""""""""""""""""""""""""""""""""""""""""""""""""""""""""""""""""""""""""""""""""""""""""""""""""""""""""""""""""""""""""""""""""""""""""""""""""""""""""""""""""""""""""""""""""""""""""""""""""""""""""""""""""""""""""""""""""""""""""""""""""""""""""""""""""""""""""""""""""""""""""""""""""""""""""""""""""""""""""""""""""""""""""""""""""""""""""""""""""""""""""""""""""""""""""""""""""""""""""""""""""""""""""""""""""""""""""""""""""""""""""""""""""""""""""""""""""""""""""""""""""""""""""""""""""""""""""""""""""""""""""""""""""""""""""""""""""""""""""""""""""""""""""""""""""""""""""""""""""""""""""""""""""""""""""""""""""""""""""""""""""""""""""""""""""""""""""""""""""""""""""""""""""""""""""""""""""""""""""""""""""""""""""""""""""""""""""""""""""""""""""""""""""""""""""""""""""""""""""""""""""""""""""""""""""""""""""""""""""""""""""""""""""""""""""""""""""""""""""""""""""""""""""""""""""""""""""""""""""""""""""""""""""""""""""""""""""""""""""""""""""""""""""""""""""""""""""""""""""""""""""""""""""""""""""""""""""""""""""""""""""""""""""""""""""""""""""""""""""""""""""""""""""""""""""""""""""""""""""""""""""""""""""""""""""""""""""""""""""""""""""""""""""""""""""""""""""""""""""""""""""""""""""""""""""""""""""""""""""""""""""""""""""""""""""""""""""""""""""""""""""""""""""""""""""""""""""""""""""""""""""""""""""""""""""""""""""""""""""""""""""""""""""""""""""""""""""""""""""""""""""""""""""""""""""""""""""""""""""""""""""""""""""""""""""""""""""""""""""""""""""""""""""""""""""""""""""""""""""""""""""""""""""""""""""""""""""""""""""""""""""""""""""""""""""""""""""""""""""""""""""""""""""""""""""""""""""""""""""""""""""""""""""""""""""""""""""""""""""""""""""""""""""""""""""""""""""""""""""""""""""""""""""""""""""""""""""""""""""""""""""""""""""""""""""""""""""""""""""""""""""""""""""""""""""""""""""""""""""""""""""""""""""""""""""""""""""""""""""""""""""""""""""""""""""""""""""""""""""""""""""""""""""""""""""""""""""""""""""""""""""""""""""""""""""""""""""""""""""""""""""""""""""""""""""""""""""""""""""""""""""""""""""""""""""""""""""""""""""""""""""""""""""""""""""""""""""""""""""""""""""""""""""""""""""""""""""""""""""""""""""""""""""""""""""""""""""""""""""""""""""""""""""""""""""""""""""""""""""""""""""""""""""""""""""""""""""""""""""""""""""""""""""""""""""""""""""""""""""""""""""""""""""""""""""""""""""""""""""""""""""""""""""""""""""""""""""""""""""""""""""""""""""""""""""""""""""""""""""""""""""""""""""""""""""""""""""""""""""""""""""""""""""""""""""""""""""""""""""""""""""""""""""""""""""""""""""""""""""""""""""""""""""""""""""""""""""""""""""""""""""""""""""""""""""""""""""""""""""""""""""""""""""""""""""""""""""""""""""""""""""""""""""""""""""""""""""""""""""""""""""""""""""""""""""""""""""""""""""""""""""""""""""""""""""""""""""""""""""""""""""""""""""""""""""""""""""""""""""""""""""""""""""""""""""""""""""""""""""""""""""""""""""""""""""""""""""""""""""""""""""""""""""""""""""""""""""""""""""""""""""""""""""""""""""""""""""""""""""""""""""""""""""""""""""""""""""""""""""""""""""""""""""""""""""""""""""""""""""""""""""""""""""""""""""""""""""""""""""""""""""""""""""""""""""""""""""""""""""""""""""""""""""""""""""""""""""""""""""""""""""""""""""""""""""""""""""""""""""""""""""""""""""""""""""""""""""""""""""""""""""""""""""""""""""""""""""""""""""""""""""""""""""""""""""""""""""""""""""""""""""""""""""""""""""""""""""""""""""""""""""""""""""""""""""""""""""""""""""""""""""""""""""""""""""""""""""""""""""""""""""""""""""""""""""""""""""""""""""""""""""""""""""""""""""""""""""""""""""""""""""""""""""""""""""""""""""""""""""""""""""""""""""""""""""""""""""""""""""""""""""""""""""""""""""""""""""""""""""""""""""""""""""""""""""""""""""""""""""""""""""""""""""""""""""""""""""""""""""""""""""""""""""""""""""""""""""""""""""""""""""""""""""""""""""""""""""""""""""""""""""""""""""""""""""""""""""""""""""""""""""""""""""""""""""""""""""""""""""""""""""""""""""""""""""""""""""""""""""""""""""""""""""""""""""""""""""""""""""""""""""""""""""""""""""""""""""""""""""""""""""""""""""""""""""""""""""""""""""""""""""""""""""""""""""""""""""""""""""""""""""""""""""""""""""""""""""""""""""""""""""""""""""""""""""""""""""""""""""""""""""""""""""""""""""""""""""""""""""""""""""""""""""""""""""""""""""""""""""""""""""""""""""""""""""""""""""""""""""""""""""""""""""""""""""""""""""""""""""""""""""""""""""""""""""""""""""""""""""""""""""""""""""""""""""""""""""""""""""""""""""""""""""""""""""""""""""""""""""""""""""""""""""""""""""""""""""""""""""""""""""""""""""""""""""""""""""""""""""""""""""""""""""""""""""""""""""""""""""""""""""""""""""""""""""""""""""""""""""""""""""""""""""""""""""""""""""""""""""""""""""""""""""""""""""""""""""""""""""""""""""""""""""""""""""""""""""""""""""""""""""""""""""""""""""""""""""""""""""""""""""""""""""""""""""""""""""""""""""""""""""""""""""""""""""""""""""""""""""""""""""""""""""""""""""""""""""""""""""""""""""""""""""""""""""""""""""""""""""""""""""""""""""""""""""""""""""""""""""""""""""""""""""""""""""""""""""""""""""""""""""""""""""""""""""""""""""""""""""""""""""""""""""""""""""""""""""""""""""""""""""""""""""""""""""""""""""""""""""""""""""""""""""""""""""""""""""""""""""""""""""""""""""""""""""""""""""""""""""""""""""""""""""""""""""""""""""""""""""""""""""""""""""""""""""""""""""""""""""""""""""""""""""""""""""""""""""""""""""""""""""""""""""""""""""""""""""""""""""""""""""""""""""""""""""""""""""""""""""""""""""""""""""""""""""""""""""""""""""""""""""""""""""""""""""""""""""""""""""""""""""""""""""""""""""""""""""""""""""""""""""""""""""""""""""""""""""""""""""""""""""""""""""""""""""""""""""""""""""""""""""""""""""""""""""""""""""""""""""""""""""""""""""""""""""""""""""""""""""""""""""""""""""""""""""""""""""""""""""""""""""""""""""""""""""""""""""""""""""""""""""""""""""""""""""""""""""""""""""""""""""""""""""""""""""""""""""""""""""""""""""""""""""""""""""""""""""""""""""""""""""""""""""""""""""""""""""""""""""""""""""""""""""""""""""""""""""""""""""""""""""""""""""""""""""""""""""""""""""""""""""""""""""""""""""""""""""""""""""""""""""""""""""""""""""""""""""""""""""""""""""""""""""""""""""""""""""""""""""""""""""""""""""""""""""""""""""""""""""""""""""""""""""""""""""""""""""""""""""""""""""""""""""""""""""""""""""""""""""""""""""""""""""""""""""""""""""""""""""""""""""""""""""""""""""""""""""""""""""""""""""""""""""""""""""""""""""""""""""""""""""""""""""""""""""""""""""""""""""""""""""""""""""""""""""""""""""""""""""""""""""""""""""""""""""""""""""""""""""""""""""""""""""""""""""""""""""""""""""""""""""""""""""""""""""""""""""""""""""""""""""""""""""""""""""""""""""""""""""""""""""""""""""""""""""""""""""""""""""""""""""""""""""""""""""""""""""""""""""""""""""""""""""""""""""""""""""""""""""""""""""""""""""""""""""""""""""""""""""""""""""""""""""""""""""""""""""""""""""""""""""""""""""""""""""""""""""""""""""""""""""""""""""""""""""""""""""""""""""""""""""""""""""""""""""""""""""""""""""""""""""""""""""""""""""""""""""""""""""""""""""""""""""""""""""""""""""""""""""""""""""""""""""""""""""""""""""""""""""""""""""""""""""""""""""""""""""""""""""""""""""""""""""""""""""""""""""""""""""""""""""""""""""""""""""""""""""""""""""""""""""""""""""""""""""""""""""""""""""""""""""""""""""""""""""""""""""""""""""""""""""""""""""""""""""""""""""""""""""""""""""""""""""""""""""""""""""""""""""""""""""""""""""""""""""""""""""""""""""""""""""""""""""""""""""""""""""""""""""""""""""""""""""""""""""""""""""""""""""""""""""""""""""""""""""""""""""""""""""""""""""""""""""""""""""""""""""""""""""""""""""""""""""""""""""""""""""""""""""""""""""""""""""""""""""""""""""""""""""""""""""""""""""""""""""""""""""""""""""""""""""""""""""""""""""""""""""""""""""""""""""""""""""""""""""""""""""""""""""""""""""""""""""""""""""""""""""""""""""""""""""""""""""""""""""""""""""""""""""""""""""""""""""""""""""""""""""""""""""""""""""""""""""""""""""""""""""""""""""""""""""""""""""""""""""""""""""""""""""""""""""""""""""""""""""""""""""""""""""""""""""""""""""""""""""""""""""""""""""""""""""""""""""""""""""""""""""""""""""""""""""""""""""""""""""""""""""""""""""""""""""""""""""""""""""""""""""""""""""""""""""""""""""""""""""""""""""""""""""""""""""""""""""""""""""""""""""""""""""""""""""""""""""""""""""""""""""""""""""""""""""""""""""""""""""""""""""""""""""""""""""""""""""""""""""""""""""""""""""""""""""""""""""""""""""""""""""""""""""""""""""""""""""""""""""""""""""""""""""""""""""""""""""""""""""""""""""""""""""""""""""""""""""""""""""""""""""""""""""""""""""""""""""""""""""""""""""""""""""""""""""""""""""""""""""""""""""""""""""""""""""""""""""""""""""""""""""""""""""""""""""""""""""""""""""""""""""""""""""""""""""""""""""""""""""""""""""""""""""""""""""""""""""""""""""""""""""""""""""""""""""""""""""""""""""""""""""""""""""""""""""""""""""""""""""""""""""""""""""""""""""""""""""""""""""""""""""""""""""""""""""""""""""""""""""""""""""""""""""""""""""""""""""""""""""""""""""""""""""""""""""""""""""""""""""""""""""""""""""""""""""""""""""""""""""""""""""""""""""""""""""""""""""""""""""""""""""""""""""""""""""""""""""""""""""""""""""""""""""""""""""""""""""""""""""""""""""""""""""""""""""""""""""""""""""""""""""""""""""""""""""""""""""""""""""""""""""""""""""""""""""""""""""""""""""""""""""""""""""""""""""""""""""""""""""""""""""""""""""""""""""""""""""""""""""""""""""""""""""""""""""""""""""""""""""""""""""""""""""""""""""""""""""""""""""""""""""""""""""""""""""""""""""""""""""""""""""""""""""""""""""""""""""""""""""""""""""""""""""""""""""""""""""""""""""""""""""""""""""""""""""""""""""""""""""""""""""""""""""""""""""""""""""""""""""""""""""""""""""""""""""""""""""""""""""""""""""""""""""""""""""""""""""""""""""""""""""""""""""""""""""""""""""""""""""""""""""""""""""""""""""""""""""""""""""""""""""""""""""""""""""""""""""""""""""""""""""""""""""""""""""""""""""""""""""""""""""""""""""""""""""""""""""""""""""""""""""""""""""""""""""""""""""""""""""""""""""""""""""""""""""""""""""""""""""""""""""""""""""""""""""""""""""""""""""""""""""""""""""""""""""""""""""""""""""""""""""""""""""""""""""""""""""""""""""""""""""""""""""""""""""""""""""""""""""""""""""""""""""""""""""""""""""""""""""""""""""""""""""""""""""""""""""""""""""""""""""""""""""""""""""""""""""""""""""""""""""""""""""""""""""""""""""""""""""""""""""""""""""""""""""""""""""""""""""""""""""""""""""""""""""""""""""""""""""""""""""""""""""""""""""""""""""""""""""""""""""""""""""""""""""""""""""""""""""""""""""""""""""""""""""""""""""""""""""""""""""""""""""""""""""""""""""""""""""""""""""""""""""""""""""""""""""""""""""""""""""""""""""""""""""""""""""""""""""""""""""""""""""""""""""""""""""""""""""""""""""""""""""""""""""""""""""""""""""""""""""""""""""""""""""""""""""""""""""""""""""""""""""""""""""""""""""""""""""""""""""""""""""""""""""""""""""""""""""""""""""""""""""""""""""""""""""""""""""""""""""""""""""""""""""""""""""""""""""""""""""""""""""""""""""""""""""""""""""""""""""""""""""""""""""""""""""""""""""""""""""""""""""""""""""""""""""""""""""""""""""""""""""""""""""""""""""""""""""""""""""""""""""""""""""""""""""""""""""""""""""""""""""""""""""""""""""""""""""""""""""""""""""""""""""""""""""""""""""""""""""""""""""""""""""""""""""""""""""""""""""""""""""""""""""""""""""""""""""""""""""""""""""""""""""""""""""""""""""""""""""""""""""""""""""""""""""""""""""""""""""""""""""""""""""""""""""""""""""""""""""""""""""""""""""""""""""""""""""""""""""""""""""""""""""""""""""""""""""""""""""""""""""""""""""""""""""""""""""""""""""""""""""""""""""""""""""""""""""""""""""""""""""""""""""""""""""""""""""""""""""""""""""""""""""""""""""""""""""""""""""""""""""""""""""""""""""""""""""""""""""""""""""""""""""""""""""""""""""""""""""""""""""""""""""""""""""""""""""""""""""""""""""""""""""""""""""""""""""""""""""""""""""""""""""""""""""""""""""""""""""""""""""""""""""""""""""""""""""""""""""""""""""""""""""""""""""""""""""""""""""""""""""""""""""""""""""""""""""""""""""""""""""""""""""""""""""""""""""""""""""""""""""""""""""""""""""""""""""""""""""""""""""""""""""""""""""""""""""""""""""""""""""""""""""""""""""""""""""""""""""""""""""""""""""""""""""""""""""""""""""""""""""""""""""""""""""""""""""""""""""""""""""""""""""""""""""""""""""""""""""""""""""""""""""""""""""""""""""""""""""""""""""""""""""""""""""""""""""""""""""""""""""""""""""""""""""""""""""""""""""""""""""""""""""""""""""""""""""""""""""""""""""""""""""""""""""""""""""""""""""""""""""""""""""""""""""""""""""""""""""""""""""""""""""""""""""""""""""""""""""""""""""""""""""""""""""""""""""""""""""""""""""""""""""""""""""""""""""""""""""""""""""""""""""""""""""""""""""""""""""""""""""""""""""""""""""""""""""""""""""""""""""""""""""""""""""""""""""""""""""""""""""""""""""""""""""""""""""""""""""""""""""""""""""""""""""""""""""""""""""""""""""""""""""""""""""""""""""""""""""""""""""""""""""""""""""""""""""""""""""""""""""""""""""""""""""""""""""""""""""""""""""""""""""""""""""""""""""""""""""""""""""""""""""""""""""""""""""""""""""""""""""""""""""""""""""""""""""""""""""""""""""""""""""""""""""""""""""""""""""""""""""""""""""""""""""""""""""""""""""""""""""""""""""""""""""""""""""""""""""""""""""""""""""""""""""""""""""""""""""""""""""""""""""""""""""""""""""""""""""""""""""""""""""""""""""""""""""""""""""""""""""""""""""""""""""""""""""""""""""""""""""""""""""""""""""""""""""""""""""""""""""""""""""""""""""""""""""""""""""""""""""""""""""""""""""""""""""""""""""""""""""""""""""""""""""""""""""""""""""""""""""""""""""""""""""""""""""""""""""""""""""""""""""""""""""""""""""""""""""""""""""""""""""""""""""""""""""""""""""""""""""""""""""""""""""""""""""""""""""""""""""""""""""""""""""""""""""""""""""""""""""""""""""""""""""""""""""""""""""""""""""""""""""""""""""""""""""""""""""""""""""""""""""""""""""""""""""""""""""""""""""""""""""""""""""""""""""""""""""""""""""""""""""""""""""""""""""""""""""""""""""""""""""""""""""""""""""""""""""""""""""""""""""""""""""""""""""""""""""""""""""""""""""""""""""""""""""""""""""""""""""""""""""""""""""""""""""""""""""""""""""""""""""""""""""""""""""""""""""""""""""""""""""""""""""""""""""""""""""""""""""""""""""""""""""""""""""""""""""""""""""""""""""""""""""""""""""""""""""""""""""""""""""""""""""""""""""""""""""""""""""""""""""""""""""""""""""""""""""""""""""""""""""""""""""""""""""""""""""""""""""""""""""""""""""""""""""""""""""""""""""""""""""""""""""""""""""""""""""""""""""""""""""""""""""""""""""""""""""""""""""""""""""""""""""""""""""""""""""""""""""""""""""""""""""""""""""""""""""""""""""""""""""""""""""""""""""""""""""""""""""""""""""""""""""""""""""""""""""""""""""""""""""""""""""""""""""""""""""""""""""""""""""""""""""""""""""""""""""""""""""""""""""""""""""""""""""""""""""""""""""""""""""""""""""""""""""""""""""""""""""""""""""""""""""""""""""""""""""""""""""""""""""""""""""""""""""""""""""""""""""""""""""""""""""""""""""""""""""""""""""""""""""""""""""""""""""""""""""""""""""""""""""""""""""""""""""""""""""""""""""""""""""""""""""""""""""""""""""""""""""""""""""""""""""""""""""""""""""""""""""""""""""""""""""""""""""""""""""""""""""""""""""""""""""""""""""""""""""""""""""""""""""""""""""""""""""""""""""""""""""""""""""""""""""""""""""""""""""""""""""""""""""""""""""""""""""""""""""""""""""""""""""""""""""""""""""""""""""""""""""""""""""""""""""""""""""""""""""""""""""""""""""""""""""""""""""""""""""""""""""""""""""""""""""""""""""""""""""""""""""""""""""""""""""""""""""""""""""""""""""""""""""""""""""""""""""""""""""""""""""""""""""""""""""""""""""""""""""""""""""""""""""""""""""""""""""""""""""""""""""""""""""""""""""""""""""""""""""""""""""""""""""""""""""""""""""""""""""""""""""""""""""""""""""""""""""""""""""""""""""""""""""""""""""""""""""""""""""""""""""""""""""""""""""""""""""""""""""""""""""""""""""""""""""""""""""""""""""""""""""""""""""""""""""""""""""""""""""""""""""""""""""""""""""""""""""""""""""""""""""""""""""""""""""""""""""""""""""""""""""""""""""""""""""""""""""""""""""""""""""""""""""""""""""""""""""""""""""""""""""""""""""""""""""""""""""""""""""""""""""""""""""""""""""""""""""""""""""""""""""""""""""""""""""""""""""""""""""""""""""""""""""""""""""""""""""""""""""""""""""""""""""""""""""""""""""""""""""""""""""""""""""""""""""""""""""""""""""""""""""""""""""""""""""""""""""""""""""""""""""""""""""""""""""""""""""""""""""""""""""""""""""""""""""""""""""""""""""""""""""""""""""""""""""""""""""""""""""""""""""""""""""""""""""""""""""""""""""""""""""""""""""""""""""""""""""""""""""""""""""""""""""""""""""""""""""""""""""""""""""""""""""""""""""""""""""""""""""""""""""""""""""""""""""""""""""""""""""""""""""""""""""""""""""""""""""""""""""""""""""""""""""""""""""""""""""""""""""""""""""""""""""""""""""""""""""""""""""""""""""""""""""""""""""""""""""""""""""""""""""""""""""""""""""""""""""""""""""""""""""""""""""""""""""""""""""""""""""""""""""""""""""""""""""""""""""""""""""""""""""""""""""""""""""""""""""""""""""""""""""""""""""""""""""""""""""""""""""""""""""""""""""""""""""""""""""""""""""""""""""""""""""""""""""""""""""""""""""""""""""""""""""""""""""""""""""""""""""""""""""""""""""""""""""""""""""""""""""""""""""""""""""""""""""""""""""""""""""""""""""""""""""""""""""""""""""""""""""""""""""""""""""""""""""""""""""""""""""""""""""""""""""""""""""""""""""""""""""""""""""""""""""""""""""""""""""""""""""""""""""""""""""""""""""""""""""""""""""""""""""""""""""""""""""""""""""""""""""""""""""""""""""""""""""""""""""""""""""""""""""""""""""""""""""""""""""""""""""""""""""""""""""""""""""""""""""""""""""""""""""""""""""""""""""""""""""""""""""""""""""""""""""""""""""""""""""""""""""""""""""""""""""""""""""""""""""""""""""""""""""""""""""""""""""""""""""""""""""""""""""""""""""""""""""""""""""""""""""""""""""""""""""""""""""""""""""""""""""""""""""""""""""""""""""""""""""""""""""""""""""""""""""""""""""""""""""""""""""""""""""""""""""""""""""""""""""""""""""""""""""""""""""""""""""""""""""""""""""""""""""""""""""""""""""""""""""""""""""""""""""""""""""""""""""""""""""""""""""""""""""""""""""""""""""""""""""""""""""""""""""""""""""""""""""""""""""""""""""""""""""""""""""""""""""""""""""""""""""""""""""""""""""""""""""""""""""""""""""""""""""""""""""""""""""""""""""""""""""""""""""""""""""""""""""""""""""""""""""""""""""""""""""""""""""""""""""""""""""""""""""""""""""""""""""""""""""""""""""""""""""""""""""""""""""""""""""""""""""""""""""""""""""""""""""""""""""""""""""""""""""""""""""""""""""""""""""""""""""""""""""""""""""""""""""""""""""""""""""""""""""""""""""""""""""""""""""""""""""""""""""""""""""""""""""""""""""""""""""""""""""""""""""""""""""""""""""""""""""""""""""""""""""""""""""""""""""""""""""""""""""""""""""""""""""""""""""""""""""""""""""""""""""""""""""""""""""""""""""""""""""""""""""""""""""""""""""""""""""""""""""""""""""""""""""""""""""""""""""""""""""""""""""""""""""""""""""""""""""""""""""""""""""""""""""""""""""""""""""""""""""""""""""""""""""""""""""""""""""""""""""""""""""""""""""""""""""""""""""""""""""""""""""""""""""""""""""""""""""""""""""""""""""""""""""""""""""""""""""""""""""""""""""""""""""""""""""""""""""""""""""""""""""""""""""""""""""""""""""""""""""""""""""""""""""""""""""""""""""""""""""""""""""""""""""""""""""""""""""""""""""""""""""""""""""""""""""""""""""""""""""""""""""""""""""""""""""""""""""""""""""""""""""""""""""""""""""""""""""""""""""""""""""""""""""""""""""""""""""""""""""""""""""""""""""""""""""""""""""""""""""""""""""""""""""""""""""""""""""""""""""""""""""""""""""""""""""""""""""""""""""""""""""""""""""""""""""""""""""""""""""""""""""""""""""""""""""""""""""""""""""""""""""""""""""""""""""""""""""""""""""""""""""""""""""""""""""""""""""""""""""""""""""""""""""""""""""""""""""""""""""""""""""""""""""""""""""""""""""""""""""""""""""""""""""""""""""""""""""""""""""""""""""""""""""""""""""""""""""""""""""""""""""""""""""""""""""""""""""""""""""""""""""""""""""""""""""""""""""""""""""""""""""""""""""""""""""""""""""""""""""""""""""""""""""""""""""""""""""""""""""""""""""""""""""""""""""""""""""""""""""""""""""""""""""""""""""""""""""""""""""""""""""""""""""""""""""""""""""""""""""""""""""""""""""""""""""""""""""""""""""""""""""""""""""""""""""""""""""""""""""""""""""""""""""""""""""""""""""""""""""""""""""""""""""""""""""""""""""""""""""""""""""""""""""""""""""""""""""""""""""""""""""""""""""""""""""""""""""""""""""""""""""""""""""""""""""""""""""""""""""""""""""""""""""""""""""""""""""""""""""""""""""""""""""""""""""""""""""""""""""""""""""""""""""""""""""""""""""""""""""""""""""""""""""""""""""""""""""""""""""""""""""""""""""""""""""""""""""""""""""""""""""""""""""""""""""""""""""""""""""""""""""""""""""""""""""""""""""""""""""""""""""""""""""""""""""""""""""""""""""""""""""""""""""""""""""""""""""""""""""""""""""""""""""""""""""""""""""""""""""""""""""""""""""""""""""""""""""""""""""""""""""""""""""""""""""""""""""""""""""""""""""""""""""""""""""""""""""""""""""""""""""""""""""""""""""""""""""""""""""""""""""""""""""""""""""""""""""""""""""""""""""""""""""""""""""""""""""""""""""""""""""""""""""""""""""""""""""""""""""""""""""""""""""""""""""""""""""""""""""""""""""""""""""""""""""""""""""""""""""""""""""""""""""""""""""""""""""""""""""""""""""""""""""""""""""""""""""""""""""""""""""""""""""""""""""""""""""""""""""""""""""""""""""""""""""""""""""""""""""""""""""""""""""""""""""""""""""""""""""""""""""""""""""""""""""""""""""""""""""""""""""""""""""""""""""""""""""""""""""""""""""""""""""""""""""""""""""""""""""""""""""""""""""""""""""""""""""""""""""""""""""""""""""""""""""""""""""""""""""""""""""""""""""""""""""""""""""""""""""""""""""""""""""""""""""""""""""""""""""""""""""""""""""""""""""""""""""""""""""""""""""""""""""""""""""""""""""""""""""""""""""""""""""""""""""""""""""""""""""""""""""""""""""""""""""""""""""""""""""""""""""""""""""""""""""""""""""""""""""""""""""""""""""""""""""""""""""""""""""""""""""""""""""""""""""""""""""""""""""""""""""""""""""""""""""""""""""""""""""""""""""""""""""""""""""""""""""""""""""""""""""""""""""""""""""""""""""""""""""""""""""""""""""""""""""""""""""""""""""""""""""""""""""""""""""""""""""""""""""""""""""""""""""""""""""""""""""""""""""""""""""""""""""""""""""""""""""""""""""""""""""""""""""""""""""""""""""""""""""""""""""""""""""""""""""""""""""""""""""""""""""""""""""""""""""""""""""""""""""""""""""""""""""""""""""""""""""""""""""""""""""""""""""""""""""""""""""""""""""""""""""""""""""""""""""""""""""""""""""""""""""""""""""""""""""""""""""""""""""""""""""""""""""""""""""""""""""""""""""""""""""""""""""""""""""""""""""""""""""""""""""""""""""""""""""""""""""""""""""""""""""""""""""""""""""""""""""""""""""""""""""""""""""""""""""""""""""""""""""""""""""""""""""""""""""""""""""""""""""""""""""""""""""""""""""""""""""""""""""""""""""""""""""""""""""""""""""""""""""""""""""""""""""""""""""""""""""""""""""""""""""""""""""""""""""""""""""""""""""""""""""""""""""""""""""""""""""""""""""""""""""""""""""""""""""""""""""""""""""""""""""""""""""""""""""""""""""""""""""""""""""""""""""""""""""""""""""""""""""""""""""""""""""""""""""""""""""""""""""""""""""""""""""""""""""""""""""""""""""""""""""""""""""""""""""""""""""""""""""""""""""""""""""""""""""""""""""""""""""""""""""""""""""""""""""""""""""""""""""""""""""""""""""""""""""""""""""""""""""""""""""""""""""""""""""""""""""""""""""""""""""""""""""""""""""""""""""""""""""""""""""""""""""""""""""""""""""""""""""""""""""""""""""""""""""""""""""""""""""""""""""""""""""""""""""""""""""""""""""""""""""""""""""""""""""""""""""""""""""""""""""""""""""""""""""""""""""""""""""""""""""""""""""""""""""""""""""""""""""""""""""""""""""""""""""""""""""""""""""""""""""""""""""""""""""""""""""""""""""""""""""""""""""""""""""""""""""""""""""""""""""""""""""""""""""""""""""""""""""""""""""""""""""""""""""""""""""""""""""""""""""""""""""""""""""""""""""""""""""""""""""""""""""""""""""""""""""""""""""""""""""""""""""""""""""""""""""""""""""""""""""""""""""""""""""""""""""""""""""""""""""""""""""""""""""""""""""""""""""""""""""""""""""""""""""""""""""""""""""""""""""""""""""""""""""""""""""""""""""""""""""""""""""""""""""""""""""""""""""""""""""""""""""""""""""""""""""""""""""""""""""""""""""""""""""""""""""""""""""""""""""""""""""""""""""""""""""""""""""""""""""""""""""""""""""""""""""""""""""""""""""""""""""""""""""""""""""""""""""""""""""""""""""""""""""""""""""""""""""""""""""""""""""""""""""""""""""""""""""""""""""""""""""""""""""""""""""""""""""""""""""""""""""""""""""""""""""""""""""""""""""""""""""""""""""""""""""""""""""""""""""""""""""""""""""""""""""""""""""""""""""""""""""""""""""""""""""""""""""""""""""""""""""""""""""""""""""""""""""""""""""""""""""""""""""""""""""""""""""""""""""""""""""""""""""""""""""""""""""""""""""""""""""""""""""""""""""""""""""""""""""""""""""""""""""""""""""""""""""""""""""""""""""""""""""""""""""""""""""""""""""""""""""""""""""""""""""""""""""""""""""""""""""""""""""""""""""""""""""""""""""""""""""""""""""""""""""""""""""""""""""""""""""""""""""""""""""""""""""""""""""""""""""""""""""""""""""""""""""""""""""""""""""""""""""""""""""""""""""""""""""""""""""""""""""""""""""""""""""""""""""""""""""""""""""""""""""""""""""""""""""""""""""""""""""""""""""""""""""""""""""""""""""""""""""""""""""""""""""""""""""""""""""""""""""""""""""""""""""""""""""""""""""""""""""""""""""""""""""""""""""""""""""""""""""""""""""""""""""""""""""""""""""""""""""""""""""""""""""""""""""""""""""""""""""""""""""""""""""""""""""""""""""""""""""""""""""""""""""""""""""""""""""""""""""""""""""""""""""""""""""""""""""""""""""""""""""""""""""""""""""""""""""""""""""""""""""""""""""""""""""""""""""""""""""""""""""""""""""""""""""""""""""""""""""""""""""""""""""""""""""""""""""""""""""""""""""""""""""""""""""""""""""""""""""""""""""""""""""""""""""""""""""""""""""""""""""""""""""""""""""""""""""""""""""""""""""""""""""""""""""""""""""""""""""""""""""""""""""""""""""""""""""""""""""""""""""""""""""""""""""""""""""""""""""""""""""""""""""""""""""""""""""""""""""""""""""""""""""""""""""""""""""""""""""""""""""""""""""""""""""""""""""""""""""""""""""""""""""""""""""""""""""""""""""""""""""""""""""""""""""""""""""""""""""""""""""""""""""""""""""""""""""""""""""""""""""""""""""""""""""""""""""""""""""""""""""""""""""""""""""""""""""""""""""""""""""""""""""""""""""""""""""""""""""""""""""""""""""""""""""""""""""""""""""""""""""""""""""""""""""""""""""""""""""""""""""""""""""""""""""""""""""""""""""""""""""""""""""""""""""""""""""""""""""""""""""""""""""""""""""""""""""""""""""""""""""""""""""""""""""""""""""""""""""""""""""""""""""""""""""""""""""""""""""""""""""""""""""""""""""""""""""""""""""""""""""""""""""""""""""""""""""""""""""""""""""""""""""""""""""""""""""""""""""""""""""""""""""""""""""""""""""""""""""""""""""""""""""""""""""""""""""""""""""""""""""""""""""""""""""""""""""""""""""""""""""""""""""""""""""""""""""""""""""""""""""""""""""""""""""""""""""""""""""""""""""""""""""""""""""""""""""""""""""""""""""""""""""""""""""""""""""""""""""""""""""""""""""""""""""""""""""""""""""""""""""""""""""""""""""""""""""""""""""""""""""""""""""""""""""""""""""""""""""""""""""""""""""""""""""""""""""""""""""""""""""""""""""""""""""""""""""""""""""""""""""""""""""""""""""""""""""""""""""""""""""""""""""""""""""""""""""""""""""""""""""""""""""""""""""""""""""""""""""""""""""""""""""""""""""""""""""""""""""""""""""""""""""""""""""""""""""""""""""""""""""""""""""""""""""""""""""""""""""""""""""""""""""""""""""""""""""""""""""""""""""""""""""""""""""""""""""""""""""""""""""""""""""""""""""""""""""""""""""""""""""""""""""""""""""""""""""""""""""""""""""""""""""""""""""""""""""""""""""""""""""""""""""""""""""""""""""""""""""""""""""""""""""""""""""""""""""""""""""""""""""""""""""""""""""""""""""""""""""""""""""""""""""""""""""""""""""""""""""""""""""""""""""""""""""""""""""""""""""""""""""""""""""""""""""""""""""""""""""""""""""""""""""""""""""""""""""""""""""""""""""""""""""""""""""""""""""""""""""""""""""""""""""""""""""""""""""""""""""""""""""""""""""""""""""""""""""""""""""""""""""""""""""""""""""""""""""""""""""""""""""""""""""""""""""""""""""""""""""""""""""""""""""""""""""""""""""""""""""""""""""""""""""""""""""""""""""""""""""""""""""""""""""""""""""""""""""""""""""""""""""""""""""""""""""""""""""""""""""""""""""""""""""""""""""""""""""""""""""""""""""""""""""""""""""""""""""""""""""""""""""""""""""""""""""""""""""""""""""""""""""""""""""""""""""""""""""""""""""""""""""""""""""""""""""""""""""""""""""""""""""""""""""""""""""""""""""""""""""""""""""""""""""""""""""""""""""""""""""""""""""""""""""""""""""""""""""""""""""""""""""""""""""""""""""""""""""""""""""""""""""""""""""""""""""""""""""""""""""""""""""""""""""""""""""""""""""""""""""""""""""""""""""""""""""""""""""""""""""""""""""""""""""""""""""""""""""""""""""""""""""""""""""""""""""""""""""""""""""""""""""""""""""""""""""""""""""""""""""""""""""""""""""""""""""""""""""""""""""""""""""""""""""""""""""""""""""""""""""""""""""""""""""""""""""""""""""""""""""""""""""""""""""""""""""""""""""""""""""""""""""""""""""""""""""""""""""""""""""""""""""""""""""""""""""""""""""""""""""""""""""""""""""""""""""""""""""""""""""""""""""""""""""""""""""""""""""""""""""""""""""""""""""""""""""""""""""""""""""""""""""""""""""""""""""""""""""""""""""""""""""""""""""""""""""""""""""""""""""""""""""""""""""""""""""""""""""""""""""""""""""""""""""""""""""""""""""""""""""""""""""""""""""""""""""""""""""""""""""""""""""""""""""""""""""""""""""""""""""""""""""""""""""""""""""""""""""""""""""""""""""""""""""""""""""""""""""""""""""""""""""""""""""""""""""""""""""""""""""""""""""""""""""""""""""""""""""""""""""""""""""""""""""""""""""""""""""""""""""""""""""""""""""""""""""""""""""""""""""""""""""""""""""""""""""""""""""""""""""""""""""""""""""""""""""""""""""""""""""""""""""""""""""""""""""""""""""""""""""""""""""""""""""""""""""""""""""""""""""""""""""""""""""""""""""""""""""""""""""""""""""""""""""""""""""""""""""""""""""""""""""""""""""""""""""""""""""""""""""""""""""""""""""""""""""""""""""""""""""""""""""""""""""""""""""""""""""""""""""""""""""""""""""""""""""""""""""""""""""""""""""""""""""""""""""""""""""""""""""""""""""""""""""""""""""""""""""""""""""""""""""""""""""""""""""""""""""""""""""""""""""""""""""""""""""""""""""""""""""""""""""""""""""""""""""""""""""""""""""""""""""""""""""""""""""""""""""""""""""""""""""""""""""""""""""""""""""""""""""""""""""""""""""""""""""""""""""""""""""""""""""""""""""""""""""""""""""""""""""""""""""""""""""""""""""""""""""""""""""""""""""""""""""""""""""""""""""""""""""""""""""""""""""""""""""""""""""""""""""""""""""""""""""""""""""""""""""""""""""""""""""""""""""""""""""""""""""""""""""""""""""""""""""""""""""""""""""""""""""""""""""""""""""""""""""""""""""""""""""""""""""""""""""""""""""""""""""""""""""""""""""""""""""""""""""""""""""""""""""""""""""""""""""""""""""""""""""""""""""""""""""""""""""""""""""""""""""""""""""""""""""""""""""""""""""""""""""""""""""""""""""""""""""""""""""""""""""""""""""""""""""""""""""""""""""""""""""""""""""""""""""""""""""""""""""""""""""""""""""""""""""""""""""""""""""""""""""""""""""""""""""""""""""""""""""""""""""""""""""""""""""""""""""""""""""""""""""""""""""""""""""""""""""""""""""""""""""""""""""""""""""""""""""""""""""""""""""""""""""""""""""""""""""""""""""""""""""""""""""""""""""""""""""""""""""""""""""""""""""""""""""""""""""""""""""""""""""""""""""""""""""""""""""""""""""""""""""""""""""""""""""""""""""""""""""""""""""""""""""""""""""""""""""""""""""""""""""""""""""""""""""""""""""""""""""""""""""""""""""""""""""""""""""""""""""""""""""""""""""""""""""""""""""""""""""""""""""""""""""""""""""""""""""""""""""""""""""""""""""""""""""""""""""""""""""""""""""""""""""""""""""""""""""""""""""""""""""""""""""""""""""""""""""""""""""""""""""""""""""""""""""""""""""""""""""""""""""""""""""""""""""""""""""""""""""""""""""""""""""""""""""""""""""""""""""""""""""""""""""""""""""""""""""""""""""""""""""""""""""""""""""""""""""""""""""""""""""""""""""""""""""""""""""""""""""""""""""""""""""""""""""""""""""""""""""""""""""""""""""""""""""""""""""""""""""""""""""""""""""""""""""""""""""""""""""""""""""""""""""""""""""""""""""""""""""""""""""""""""""""""""""""""""""""""""""""""""""""""""""""""""""""""""""""""""""""""""""""""""""""""""""""""""""""""""""""""""""""""""""""""""""""""""""""""""""""""""""""""""""""""""""""""""""""""""""""""""""""""""""""""""""""""""""""""""""""""""""""""""""""""""""""""""""""""""""""""""""""""""""""""""""""""""""""""""""""""""""""""""""""""""""""""""""""""""""""""""""""""""""""""""""""""""""""""""""""""""""""""""""""""""""""""""""""""""""""""""""""""""""""""""""""""""""""""""""""""""""""""""""""""""""""""""""""""""""""""""""""""""""""""""""""""""""""""""""""""""""""""""""""""""""""""""""""""""""""""""""""""""""""""""""""""""""""""""""""""""""""""""""""""""""""""""""""""""""""""""""""""""""""""""""""""""""""""""""""""""""""""""""""""""""""""""""""""""""""""""""""""""""""""""""""""""""""""""""""""""""""""""""""""""""""""""""""""""""""""""""""""""""""""""""""""""""""""""""""""""""""""""""""""""""""""""""""""""""""""""""""""""""""""""""""""""""""""""""""""""""""""""""""""""""""""""""""""""""""""""""""""""""""""""""""""""""""""""""""""""""""""""""""""""""""""""""""""""""""""""""""""""""""""""""""""""""""""""""""""""""""""""""""""""""""""""""""""""""""""""""""""""""""""""""""""""""""""""""""""""""""""""""""""""""""""""""""""""""""""""""""""""""""""""""""""""""""""""""""""""""""""""""""""""""""""""""""""""""""""""""""""""""""""""""""""""""""""""""""""""""""""""""""""""""""""""""""""""""""""""""""""""""""""""""""""""""""""""""""""""""""""""""""""""""""""""""""""""""""""""""""""""""""""""""""""""""""""""""""""""""""""""""""""""""""""""""""""""""""""""""""""""""""""""""""""""""""""""""""""""""""""""""""""""""""""""""""""""""""""""""""""""""""""""""""""""""""""""""""""""""""""""""""""""""""""""""""""""""""""""""""""""""""""""""""""""""""""""""""""""""""""""""""""""""""""""""""""""""""""""""""""""""""""""""""""""""""""""""""""""""""""""""""""""""""""""""""""""""""""""""""""""""""""""""""""""""""""""""""""""""""""""""""""""""""""""""""""""""""""""""""""""""""""""""""""""""""""""""""""""""""""""""""""""""""""""""""""""""""""""""""""""""""""""""""""""""""""""""""""""""""""""""""""""""""""""""""""""""""""""""""""""""""""""""""""""""""""""""""""""""""""""""""""""""""""""""""""""""""""""""""""""""""""""""""""""""""""""""""""""""""""""""""""""""""""""""""""""""""""""""""""""""""""""""""""""""""""""""""""""""""""""""""""""""""""""""""""""""""""""""""""""""""""""""""""""""""""""""""""""""""""""""""""""""""""""""""""""""""""""""""""""""""""""""""""""""""""""""""""""""""""""""""""""""""""""""""""""""""""""""""""""""""""""""""""""""""""""""""""""""""""""""""""""""""""""""""""""""""""""""""""""""""""""""""""""""""""""""""""""""""""""""""""""""""""""""""""""""""""""""""""""""""""""""""""""""""""""""""""""""""""""""""""""""""""""""""""""""""""""""""""""""""""""""""""""""""""""""""""""""""""""""""""""""""""""""""""""""""""""""""""""""""""""""""""""""""""""""""""""""""""""""""""""""""""""""""""""""""""""""""""""""""""""""""""""""""""""""""""""""""""""""""""""""""""""""""""""""""""""""""""""""""""""""""""""""""""""""""""""""""""""""""""""""""""""""""""""""""""""""""""""""""""""""""""""""""""""""""""""""""""""""""""""""""""""""""""""""""""""""""""""""""""""""""""""""""""""""""""""""""""""""""""""""""""""""""""""""""""""""""""""""""""""""""""""""""""""""""""""""""""""""""""""""""""""""""""""""""""""""""""""""""""""""""""""""""""""""""""""""""""""""""""""""""""""""""""""""""""""""""""""""""""""""""""""""""""""""""""""""""""""""""""""""""""""""""""""""""""""""""""""""""""""""""""""""""""""""""""""""""""""""""""""""""""""""""""""""""""""""""""""""""""""""""""""""""""""""""""""""""""""""""""""""""""""""""""""""""""""""""""""""""""""""""""""""""""""""""""""""""""""""""""""""""""""""""""""""""""""""""""""""""""""""""""""""""""""""""""""""""""""""""""""""""""""""""""""""""""""""""""""""""""""""""""""""""""""""""""""""""""""""""""""""""""""""""""""""""""""""""""""""""""""""""""""""""""""""""""""""""""""""""""""""""""""""""""""""""""""""""""""""""""""""""""""""""""""""""""""""""""""""""""""""""""""""""""""""""""""""""""""""""""""""""""""""""""""""""""""""""""""""""""""""""""""""""""""""""""""""""""""""""""""""""""""""""""""""""""""""""""""""""""""""""""""""""""""""""""""""""""""""""""""""""""""""""""""""""""""""""""""""""""""""""""""""""""""""""""""""""""""""""""""""""""""""""""""""""""""""""""""""""""""""""""""""""""""""""""""""""""""""""""""""""""""""""""""""""""""""""""""""""""""""""""""""""""""""""""""""""""""""""""""""""""""""""""""""""""""""""""""""""""""""""""""""""""""""""""""""""""""""""""""""""""""""""""""""""""""""""""""""""""""""""""""""""""""""""""""""""""""""""""""""""""""""""""""""""""""""""""""""""""""""""""""""""""""""""""""""""""""""""""""""""""""""""""""""""""""""""""""""""""""""""""""""""""""""""""""""""""""""""""""""""""""""""""""""""""""""""""""""""""""""""""""""""""""""""""""""""""""""""""""""""""""""""""""""""""""""""""""""""""""""""""""""""""""""""""""""""""""""""""""""""""""""""""""""""""""""""""""""""""""""""""""""""""""""""""""""""""""""""""""""""""""""""""""""""""""""""""""""""""""""""""""""""""""""""""""""""""""""""""""""""""""""""""""""""""""""""""""""""""""""""""""""""""""""""""""""""""""""""""""""""""""""""""""""""""""""""""""""""""""""""""""""""""""""""""""""""""""""""""""""""""""""""""""""""""""""""""""""""""""""""""""""""""""""""""""""""""""""""""""""""""""""""""""""""""""""""""""""""""""""""""""""""""""""""""""""""""""""""""""""""""""""""""""""""""""""""""""""""""""""""""""""""""""""""""""""""""""""""""""""""""""""""""""""""""""""""""""""""""""""""""""""""""""""""""""""""""""""""""""""""""""""""""""""""""""""""""""""""""""""""""""""""""""""""""""""""""""""""""""""""""""""""""""""""""""""""""""""""""""""""""""""""""""""""""""""""""""""""""""""""""""""""""""""""""""""""""""""""""""""""""""""""""""""""""""""""""""""""""""""""""""""""""""""""""""""""""""""""""""""""""""""""""""""""""""""""""""""""""""""""""""""""""""""""""""""""""""""""""""""""""""""""""""""""""""""""""""""""""""""""""""""""""""""""""""""""""""""""""""""""""""""""""""""""""""""""""""""""""""""""""""""""""""""""""""""""""""""""""""""""""""""""""""""""""""""""""""""""""""""""""""""""""""""""""""""""""""""""""""""""""""""""""""""""""""""""""""""""""""""""""""""""""""""""""""""""""""""""""""""""""""""""""""""""""""""""""""""""""""""""""""""""""""""""""""""""""""""""""""""""""""""""""""""""""""""""""""""""""""""""""""""""""""""""""""""""""""""""""""""""""""""""""""""""""""""""""""""""""""""""""""""""""""""""""""""""""""""""""""""""""""""""""""""""""""""""""""""""""""""""""""""""""""""""""""""""""""""""""""""""""""""""""""""""""""""""""""""""""""""""""""""""""""""""""""""""""""""""""""""""""""""""""""""""""""""""""""""""""""""""""""""""""""""""""""""""""""""""""""""""""""""""""""""""""""""""""""""""""""""""""""""""""""""""""""""""""""""""""""""""""""""""""""""""""""""""""""""""""""""""""""""""""""""""""""""""""""""""""""""""""""""""""""""""""""""""""""""""""""""""""""""""""""""""""""""""""""""""""""""""""""""""""""""""""""""""""""""""""""""""""""""""""""""""""""""""""""""""""""""""""""""""""""""""""""""""""""""""""""""""""""""""""""""""""""""""""""""""""""""""""""""""""""""""""""""""""""""""""""""""""""""""""""""""""""""""""""""""""""""""""""""""""""""""""""""""""""""""""""""""""""""""""""""""""""""""""""""""""""""""""""""""""""""""""""""""""""""""""""""""""""""""""""""""""""""""""""""""""""""""""""""""""""""""""""""""""""""""""""""""""""""""""""""""""""""""""""""""""""""""""""""""""""""""""""""""""""""""""""""""""""""""""""""""""""""""""""""""""""""""""""""""""""""""""""""""""""""""""""""""""""""""""""""""""""""""""""""""""""""""""""""""""""""""""""""""""""""""""""""""""""""""""""""""""""""""""""""""""""""""""""""""""""""""""""""""""""""""""""""""""""""""""""""""""""""""""""""""""""""""""""""""""""""""""""""""""""""""""""""""""""""""""""""""""""""""""""""""""""""""""""""""""""""""""""""""""""""""""""""""""""""""""""""""""""""""""""""""""""""""""""""""""""""""""""""""""""""""""""""""""""""""""""""""""""""""""""""""""""""""""""""""""""""""""""""""""""""""""""""""""""""""""""""""""""""""""""""""""""""""""""""""""""""""""""""""""""""""""""""""""""""""""""""""""""""""""""""""""""""""""""""""""""""""""""""""""""""""""""""""""""""""""""""""""""""""""""""""""""""""""""""""""""""""""""""""""""""""""""""""""""""""""""""""""""""""""""""""""""""""""""""""""""""""""""""""""""""""""""""""""""""""""""""""""""""""""""""""""""""""""""""""""""""""""""""""""""""""""""""""""""""""""""""""""""""""""""""""""""""""""""""""""""""""""""""""""""""""""""""""""""""""""""""""""""""""""""""""""""""""""""""""""""""""""""""""""""""""""""""""""""""""""""""""""""""""""""""""""""""""""""""""""""""""""""""""""""""""""""""""""""""""""""""""""""""""""""""""""""""""""""""""""""""""""""""""""""""""""""""""""""""""""""""""""""""""""""""""""""""""""""""""""""""""""""""""""""""""""""""""""""""""""""""""""""""""""""""""""""""""""""""""""""""""""""""""""""""""""""""""""""""""""""""""""""""""""""""""""""""""""""""""""""""""""""""""""""""""""""""""""""""""""""""""""""""""""""""""""""""""""""""""""""""""""""""""""""""""""""""""""""""""""""""""""""""""""""""""""""""""""""""""""""""""""""""""""""""""""""""""""""""""""""""""""""""""""""""""""""""""""""""""""""""""""""""""""""""""""""""""""""""""""""""""""""""""""""""""""""""""""""""""""""""""""""""""""""""""""""""""""""""""""""""""""""""""""""""""""""""""""""""""""""""""""""""""""""""""""""""""""""""""""""""""""""""""""""""""""""""""""""""""""""""""""""""""""""""""""""""""""""""""""""""""""""""""""""""""""""""""""""""""""""""""""""""""""""""""""""""""""""""""""""""""""""""""""""""""""""""""""""""""""""""""""""""""""""""""""""""""""""""""""""""""""""""""""""""""""""""""""""""""""""""""""""""""""""""""""""""""""""""""""""""""""""""""""""""""""""""""""""""""""""""""""""""""""""""""""""""""""""""""""""""""""""""""""""""""""""""""""""""""""""""""""""""""""""""""""""""""""""""""""""""""""""""""""""""""""""""""""""""""""""""""""""""""""""""""""""""""""""""""""""""""""""""""""""""""""""""""""""""""""""""""""""""""""""""""""""""""""""""""""""""""""""""""""""""""""""""""""""""""""""""""""""""""""""""""""""""""""""""""""""""""""""""""""""""""""""""""""""""""""""""""""""""""""""""""""""""""""""""""""""""""""""""""""""""""""""""""""""""""""""""""""""""""""""""""""""""""""""""""""""""""""""""""""""""""""""""""""""""""""""""""""""""""""""""""""""""""""""""""""""""""""""""""""""""""""""""""""""""""""""""""""""""""""""""""""""""""""""""""""""""""""""""""""""""""""""""""""""""""""""""""""""""""""""""""""""""""""""""""""""""""""""""""""""""""""""""""""""""""""""""""""""""""""""""""""""""""""""""""""""""""""""""""""""""""""""""""""""""""""""""""""""""""""""""""""""""""""""""""""""""""""""""""""""""""""""""""""""""""""""""""""""""""""""""""""""""""""""""""""""""""""""""""""""""""""""""""""""""""""""""""""""""""""""""""""""""""""""""""""""""""""""""""""""""""""""""""""""""""""""""""""""""""""""""""""""""""""""""""""""""""""""""""""""""""""""""""""""""""""""""""""""""""""""""""""""""""""""""""""""""""""""""""""""""""""""""""""""""""""""""""""""""""""""""""""""""""""""""""""""""""""""""""""""""""""""""""""""""""""""""""""""""""""""""""""""""""""""""""""""""""""""""""""""""""""""""""""""""""""""""""""""""""""""""""""""""""""""""""""""""""""""""""""""""""""""""""""""""""""""""""""""""""""""""""""""""""""""""""""""""""""""""""""""""""""""""""""""""""""""""""""""""""""""""""""""""""""""""""""""""""""""""""""""""""""""""""""""""""""""""""""""""""""""""""""""""""""""""""""""""""""""""""""""""""""""""""""""""""""""""""""""""""""""""""""""""""""""""""""""""""""""""""""""""""""""""""""""""""""""""""""""""""""""""""""""""""""""""""""""""""""""""""""""""""""""""""""""""""""""""""""""""""""""""""""""""""""""""""""""""""""""""""""""""""""""""""""""""""""""""""""""""""""""""""""""""""""""""""""""""""""""""""""""""""""""""""""""""""""""""""""""""""""""""""""""""""""""""""""""""""""""""""""""""""""""""""""""""""""""""""""""""""""""""""""""""""""""""""""""""""""""""""""""""""""""""""""""""""""""""""""""""""""""""""""""""""""""""""""""""""""""""""""""""""""""""""""""""""""""""""""""""""""""""""""""""""""""""""""""""""""""""""""""""""""""""""""""""""""""""""""""""""""""""""""""""""""""""""""""""""""""""""""""""""""""""""""""""""""""""""""""""""""""""""""""""""""""""""""""""""""""""""""""""""""""""""""""""""""""""""""""""""""""""""""""""""""""""""""""""""""""""""""""""""""""""""""""""""""""""""""""""""""""""""""""""""""""""""""""""""""""""""""""""""""""""""""""""""""""""""""""""""""""""""""""""""""""""""""""""""""""""""""""""""""""""""""""""""""""""""""""""""""""""""""""""""""""""""""""""""""""""""""""""""""""""""""""""""""""""""""""""""""""""""""""""""""""""""""""""""""""""""""""""""""""""""""""""""""""""""""""""""""""""""""""""""""""""""""""""""""""""""""""""""""""""""""""""""""""""""""""""""""""""""""""""""""""""""""""""""""""""""""""""""""""""""""""""""""""""""""""""""""""""""""""""""""""""""""""""""""""""""""""""""""""""""""""""""""""""""""""""""""""""""""""""""""""""""""""""""""""""""""""""""""""""""""""""""""""""""""""""""""""""""""""""""""""""""""""""""""""""""""""""""""""""""""""""""""""""""""""""""""""""""""""""""""""""""""""""""""""""""""""""""""""""""""""""""""""""""""""""""""""""""""""""""""""""""""""""""""""""""""""""""""""""""""""""""""""""""""""""""""""""""""""""""""""""""""""""""""""""""""""""""""""""""""""""""""""""""""""""""""""""""""""""""""""""""""""""""""""""""""""""""""""""""""""""""""""""""""""""""""""""""""""""""""""""""""""""""""""""""""""""""""""""""""""""""""""""""""""""""""""""""""""""""""""""""""""""""""""""""""""""""""""""""""""""""""""""""""""""""""""""""""""""""""""""""""""""""""""""""""""""""""""""""""""""""""""""""""""""""""""""""""""""""""""""""""""""""""""""""""""""""""""""""""""""""""""""""""""""""""""""""""""""""""""""""""""""""""""""""""""""""""""""""""""""""""""""""""""""""""""""""""""""""""""""""""""""""""""""""""""""""""""""""""""""""""""""""""""""""""""""""""""""""""""""""""""""""""""""""""""""""""""""""""""""""""""""""""""""""""""""""""""""""""""""""""""""""""""""""""""""""""""""""""""""""""""""""""""""""""""""""""""""""""""""""""""""""""""""""""""""""""""""""""""""""""""""""""""""""""""""""""""""""""""""""""""""""""""""""""""""""""""""""""""""""""""""""""""""""""""""""""""""""""""""""""""""""""""""""""""""""""""""""""""""""""""""""""""""""""""""""""""""""""""""""""""""""""""""""""""""""""""""""""""""""""""""""""""""""""""""""""""""""""""""""""""""""""""""""""""""""""""""""""""""""""""""""""""""""""""""""""""""""""""""""""""""""""""""""""""""""""""""""""""""""""""""""""""""""""""""""""""""""""""""""""""""""""""""""""""""""""""""""""""""""""""""""""""""""""""""""""""""""""""""""""""""""""""""""""""""""""""""""""""""""""""""""""""""""""""""""""""""""""""""""""""""""""""""""""""""""""""""""""""""""""""""""""""""""""""""""""""""""""""""""""""""""""""""""""""""""""""""""""""""""""""""""""""""""""""""""""""""""""""""""""""""""""""""""""""""""""""""""""""""""""""""""""""""""""""""""""""""""""""""""""""""""""""""""""""""""""""""""""""""""""""""""""""""""""""""""""""""""""""""""""""""""""""""""""""""""""""""""""""""""""""""""""""""""""""""""""""""""""""""""""""""""""""""""""""""""""""""""""""""""""""""""""""""""""""""""""""""""""""""""""""""""""""""""""""""""""""""""""""""""""""""""""""""""""""""""""""""""""""""""""""""""""""""""""""""""""""""""""""""""""""""""""""""""""""""""""""""""""""""""""""""""""""""""""""""""""""""""""""""""""""""""""""""""""""""""""""""""""""""""""""""""""""""""""""""""""""""""""""""""""""""""""""""""""""""""""""""""""""""""""""""""""""""""""""""""""""""""""""""""""""""""""""""""""""""""""""""""""""""""""""""""""""""""""""""""""""""""""""""""""""""""""""""""""""""""""""""""""""""""""""""""""""""""""""""""""""""""""""""""""""""""""""""""""""""""""""""""""""""""""""""""""""""""""""""""""""""""""""""""""""""""""""""""""""""""""""""""""""""""""""""""""""""""""""""""""""""""""""""""""""""""""""""""""""""""""""""""""""""""""""""""""""""""""""""""""""""""""""""""""""""""""""""""""""""""""""""""""""""""""""""""""""""""""""""""""""""""""""""""""""""""""""""""""""""""""""""""""""""""""""""""""""""""""""""""""""""""""""""""""""""""""""""""""""""""""""""""""""""""""""""""""""""""""""""""""""""""""""""""""""""""""""""""""""""""""""""""""""""""""""""""""""""""""""""""""""""""""""""""""""""""""""""""""""""""""""""""""""""""""""""""""""""""""""""""""""""""""""""""""""""""""""""""""""""""""""""""""""""""""""""""""""""""""""""""""""""""""""""""""""""""""""""""""""""""""""""""""""""""""""""""""""""""""""""""""""""""""""""""""""""""""""""""""""""""""""""""""""""""""""""""""""""""""""""""""""""""""""""""""""""""""""""""""""""""""""""""""""""""""""""""""""""""""""""""""""""""""""""""""""""""""""""""""""""""""""""""""""""""""""""""""""""""""""""""""""""""""""""""""""""""""""""""""""""""""""""""""""""""""""""""""""""""""""""""""""""""""""""""""""""""""""""""""""""""""""""""""""""""""""""""""""""""""""""""""""""""""""""""""""""""""""""""""""""""""""""""""""""""""""""""""""""""""""""""""""""""""""""""""""""""""""""""""""""""""""""""""""""""""""""""""""""""""""""""""""""""""""""""""""""""""""""""""""""""""""""""""""""""""""""""""""""""""""""""""""""""""""""""""""""""""""""""""""""""""""""""""""""""""""""""""""""""""""""""""""""""""""""""""""""""""""""""""""""""""""""""""""""""""""""""""""""""""""""""""""""""""""""""""""""""""""""""""""""""""""""""""""""""""""""""""""""""""""""""""""""""""""""""""""""""""""""""""""""""""""""""""""""""""""""""""""""""""""""""""""""""""""""""""""""""""""""""""""""""""""""""""""""""""""""""""""""""""""""""""""""""""""""""""""""""""""""""""""""""""""""""""""""""""""""""""""""""""""""""""""""""""""""""""""""""""""""""""""""""""""""""""""""""""""""""""""""""""""""""""""""""""""""""""""""""""""""""""""""""""""""""""""""""""""""""""""""""""""""""""""""""""""""""""""""""""""""""""""""""""""""""""""""""""""""""""""""""""""""""""""""""""""""""""""""""""""""""""""""""""""""""""""""""""""""""""""""""""""""""""""""""""""""""""""""""""""""""""""""""""""""""""""""""""""""""""""""""""""""""""""""""""""""""""""""""""""""""""""""""""""""""""""""""""""""""""""""""""""""""""""""""""""""""""""""""""""""""""""""""""""""""""""""""""""""""""""""""""""""""""""""""""""""""""""""""""""""""""""""""""""""""""""""""""""""""""""""""""""""""""""""""""""""""""""""""""""""""""""""""""""""""""""""""""""""""""""""""""""""""""""""""""""""""""""""""""""""""""""""""""""""""""""""""""""""""""""""""""""""""""""""""""""""""""""""""""""""""""""""""""""""""""""""""""""""""""""""""""""""""""""""""""""""""""""""""""""""""""""""""""""""""""""""""""""""""""""""""""""""""""""""""""""""""""""""""""""""""""""""""""""""""""""""""""""""""""""""""""""""""""""""""""""""""""""""""""""""""""""""""""""""""""""""""""""""""""""""""""""""""""""""""""""""""""""""""""""""""""""""""""""""""""""""""""""""""""""""""""""""""""""""""""""""""""""""""""""""""""""""""""""""""""""""""""""""""""""""""""""""""""""""""""""""""""""""""""""""""""""""""""""""""""""""""""""""""""""""""""""""""""""""""""""""""""""""""""""""""""""""""""""""""""""""""""""""""""""""""""""""""""""""""""""""""""""""""""""""""""""""""""""""""""""""""""""""""""""""""""""""""""""""""""""""""""""""""""""""""""""""""""""""""""""""""""""""""""""""""""""""""""""""""""""""""""""""""""""""""""""""""""""""""""""""""""""""""""""""""""""""""""""""""""""""""""""""""""""""""""""""""""""""""""""""""""""""""""""""""""""""""""""""""""""""""""""""""""""""""""""""""""""""""""""""""""""""""""""""""""""""""""""""""""""""""""""""""""""""""""""""""""""""""""""""""""""""""""""""""""""""""""""""""""""""""""""""""""""""""""""""""""""""""""""""""""""""""""""""""""""""""""""""""""""""""""""""""""""""""""""""""""""""""""""""""""""""""""""""""""""""""""""""""""""""""""""""""""""""""""""""""""""""""""""""""""""""""""""""""""""""""""""""""""""""""""""""""""""""""""""""""""""""""""""""""""""""""""""""""""""""""""""""""""""""""""""""""""""""""""""""""""""""""""""""""""""""""""""""""""""""""""""""""""""""""""""""""""""""""""""""""""""""""""""""""""""""""""""""""""""""""""""""""""""""""""""""""""""""""""""""""""""""""""""""""""""""""""""""""""""""""""""""""""""""""""""""""""""""""""""""""""""""""""""""""""""""""""""""""""""""""""""""""""""""""""""""""""""""""""""""""""""""""""""""""""""""""""""""""""""""""""""""""""""""""""""""""""""""""""""""""""""""""""""""""""""""""""""""""""""""""""""""""""""""""""""""""""""""""""""""""""""""""""""""""""""""""""""""""""""""""""""""""""""""""""""""""""""""""""""""""""""""""""""""""""""""""""""""""""""""""""""""""""""""""""""""""""""""""""""""""""""""""""""""""""""""""""""""""""""""""""""""""""""""""""""""""""""""""""""""""""""""""""""""""""""""""""""""""""""""""""""""""""""""""""""""""""""""""""""""""""""""""""""""""""""""""""""""""""""""""""""""""""""""""""""""""""""""""""""""""""""""""""""""""""""""""""""""""""""""""""""""""""""""""""""""""""""""""""""""""""""""""""""""""""""""""""""""""""""""""""""""""""""""""""""""""""""""""""""""""""""""""""""""""""""""""""""""""""""""""""""""""""""""""""""""""""""""""""""""""""""""""""""""""""""""""""""""""""""""""""""""""""""""""""""""""""""""""""""""""""""""""""""""""""""""""""""""""""""""""""""""""""""""""""""""""""""""""""""""""""""""""""""""""""""""""""""""""""""""""""""""""""""""""""""""""""""""""""""""""""""""""""""""""""""""""""""""""""""""""""""""""""""""""""""""""""""""""""""""""""""""""""""""""""""""""""""""""""""""""""""""""""""""""""""""""""""""""""""""""""""""""""""""""""""""""""""""""""""""""""""""""""""""""""""""""""""""""""""""""""""""""""""""""""""""""""""""""""""""""""""""""""""""""""""""""""""""""""""""""""""""""""""""""""""""""""""""""""""""""""""""""""""""""""""""""""""""""""""""""""""""""""""""""""""""""""""""""""""""""""""""""""""""""""""""""""""""""""""""""""""""""""""""""""""""""""""""""""""""""""""""""""""""""""""""""""""""""""""""""""""""""""""""""""""""""""""""""""""""""""""""""""""""""""""""""""""""""""""""""""""""""""""""""""""""""""""""""""""""""""""""""""""""""""""""""""""""""""""""""""""""""""""""""""""""""""""""""""""""""""""""""""""""""""""""""""""""""""""""""""""""""""""""""""""""""""""""""""""""""""""""""""""""""""""""""""""""""""""""""""""""""""""""""""""""""""""""""""""""""""""""""""""""""""""""""""""""""""""""""""""""""""""""""""""""""""""""""""""""""""""""""""""""""""""""""""""""""""""""""""""""""""""""""""""""""""""""""""""""""""""""""""""""""""""""""""""""""""""""""""""""""""""""""""""""""""""""""""""""""""""""""""""""""""""""""""""""""""""""""""""""""""""""""""""""""""""""""""""""""""""""""""""""""""""""""""""""""""""""""""""""""""""""""""""""""""""""""""""""""""""""""""""""""""""""""""""""""""""""""""""""""""""""""""""""""""""""""""""""""""""""""""""""""""""""""""""""""""""""""""""""""""""""""""""""""""""""""""""""""""""""""""""""""""""""""""""""""""""""""""""""""""""""""""""""""""""""""""""""""""""""""""""""""""""""""""""""""""""""""""""""""""""""""""""""""""""""""""""""""""""""""""""""""""""""""""""""""""""""""""""""""""""""""""""""""""""""""""""""""""""""""""""""""""""""""""""""""""""""""""""""""""""""""""""""""""""""""""""""""""""""""""""""""""""""""""""""""""""""""""""""""""""""""""""""""""""""""""""""""""""""""""""""""""""""""""""""""""""""""""""""""""""""""""""""""""""""""""""""""""""""""""""""""""""""""""""""""""""""""""""""""""""""""""""""""""""""""""""""""""""""""""""""""""""""""""""""""""""""""""""""""""""""""""""""""""""""""""""""""""""""""""""""""""""""""""""""""""""""""""""""""""""""""""""""""""""""""""""""""""""""""""""""""""""""""""""""""""""""""""""""""""""""""""""""""""""""""""""""""""""""""""""""""""""""""""""""""""""""""""""""""""""""""""""""""""""""""""""""""""""""""""""""""""""""""""""""""""""""""""""""""""""""""""""""""""""""""""""""""""""""""""""""""""""""""""""""""""""""""""""""""""""""""""""""""""""""""""""""""""""""""""""""""""""""""""""""""""""""""""""""""""""""""""""""""""""""""""""""""""""""""""""""""""""""""""""""""""""""""""""""""""""""""""""""""""""""""""""""""""""""""""""""""""""""""""""""""""""""""""""""""""""""""""""""""""""""""""""""""""""""""""""""""""""""""""""""""""""""""""""""""""""""""""""""""""""""""""""""""""""""""""""""""""""""""""""""""""""""""""""""""""""""""""""""""""""""""""""""""""""""""""""""""""""""""""""""""""""""""""""""""""""""""""""""""""""""""""""""""""""""""""""""""""""""""""""""""""""""""""""""""""""""""""""""""""""""""""""""""""""""""""""""""""""""""""""""""""""""""""""""""""""""""""""""""""""""""""""""""""""""""""""""""""""""""""""""""""""""""""""""""""""""""""""""""""""""""""""""""""""""""""""""""""""""""""""""""""""""""""""""""""""""""""""""""""""""""""""""""""""""""""""""""""""""""""""""""""""""""""""""""""""""""""""""""""""""""""""""""""""""""""""""""""""""""""""""""""""""""""""""""""""""""""""""""""""""""""""""""""""""""""""""""""""""""""""""""""""""""""""""""""""""""""""""""""""""""""""""""""""""""""""""""""""""""""""""""""""""""""""""""""""""""""""""""""""""""""""""""""""""""""""""""""""""""""""""""""""""""""""""""""""""""""""""""""""""""""""""""""""""""""""""""""""""""""""""""""""""""""""""""""""""""""""""""""""""""""""""""""""""""""""""""""""""""""""""""""""""""""""""""""""""""""""""""""""""""""""""""""""""""""""""""""""""""""""""""""""""""""""""""""""""""""""""""""""""""""""""""""""""""""""""""""""""""""""""""""""""""""""""""""""""""""""""""""""""""""""""""""""""""""""""""""""""""""""""""""""""""""""""""""""""""""""""""""""""""""""""""""""""""""""""""""""""""""""""""""""""""""""""""""""""""""""""""""""""""""""""""""""""""""""""""""""""""""""""""""""""""""""""""""""""""""""""""""""""""""""""""""""""""""""""""""""""""""""""""""""""""""""""""""""""""""""""""""""""""""""""""""""""""""""""""""""""""""""""""""""""""""""""""""""""""""""""""""""""""""""""""""""""""""""""""""""""""""""""""""""""""""""""""""""""""""""""""""""""""""""""""""""""""""""""""""""""""""""""""""""""""""""""""""""""""""""""""""""""""""""""""""""""""""""""""""""""""""""""""""""""""""""""""""""""""""""""""""""""""""""""""""""""""""""""""""""""""""""""""""""""""""""""""""""""""""""""""""""""""""""""""""""""""""""""""""""""""""""""""""""""""""""""""""""""""""""""""""""""""""""""""""""""""""""""""""""""""""""""""""""""""""""""""""""""""""""""""""""""""""""""""""""""""""""""""""""""""""""""""""""""""""""""""""""""""""""""""""""""""""""""""""""""""""""""""""""""""""""""""""""""""""""""""""""""""""""""""""""""""""""""""""""""""""""""""""""""""""""""""""""""""""""""""""""""""""""""""""""""""""""""""""""""""""""""""""""""""""""""""""""""""""""""""""""""""""""""""""""""""""""""""""""""""""""""""""""""""""""""""""""""""""""""""""""""""""""""""""""""""""""""""""""""""""""""""""""""""""""""""""""""""""""""""""""""""""""""""""""""""""""""""""""""""""""""""""""""""""""""""""""""""""""""""""""""""""""""""""""""""""""""""""""""""""""""""""""""""""""""""""""""""""""""""""""""""""""""""""""""""""""""""""""""""""""""""""""""""""""""""""""""""""""""""""""""""""""""""""""""""""""""""""""""""""""""""""""""""""""""""""""""""""""""""""""""""""""""""""""""""""""""""""""""""""""""""""""""""""""""""""""""""""""""""""""""""""""""""""""""""""""""""""""""""""""""""""""""""""""""""""""""""""""""""""""""""""""""""""""""""""""""""""""""""""""""""""""""""""""""""""""""""""""""""""""""""""""""""""""""""""""""""""""""""""""""""""""""""""""""""""""""""""""""""""""""""""""""""""""""""""""""""""""""""""""""""""""""""""""""""""""""""""""""""""""""""""""""""""""""""""""""""""""""""""""""""""""""""""""""""""""""""""""""""""""""""""""""""""""""""""""""""""""""""""""""""""""""""""""""""""""""""""""""""""""""""""""""""""""""""""""""""""""""""""""""""""""""""""""""""""""""""""""""""""""""""""""""""""""""""""""""""""""""""""""""""""""""""""""""""""""""""""""""""""""""""""""""""""""""""""""""""""""""""""""""""""""""""""""""""""""""""""""""""""""""""""""""""""""""""""""""""""""""""""""""""""""""""""""""""""""""""""""""""""""""""""""""""""""""""""""""""""""""""""""""""""""""""""""""""""""""""""""""""""""""""""""""""""""""""""""""""""""""""""""""""""""""""""""""""""""""""""""""""""""""""""""""""""""""""""""""""""""""""""""""""""""""""""""""""""""""""""""""""""""""""""""""""""""""""""""""""""""""""""""""""""""""""""""""""""""""""""""""""""""""""""""""""""""""""""""""""""""""""""""""""""""""""""""""""""""""""""""""""""""""""""""""""""""""""""""""""""""""""""""""""""""""""""""""""""""""""""""""""""""""""""""""""""""""""""""""""""""""""""""""""""""""""""""""""""""""""""""""""""""""""""""""""""""""""""""""""""""""""""""""""""""""""""""""""""""""""""""""""""""""""""""""""""""""""""""""""""""""""""""""""""""""""""""""""""""""""""""""""""""""""""""""""""""""""""""""""""""""""""""""""""""""""""""""""""""""""""""""""""""""""""""""""""""""""""""""""""""""""""""""""""""""""""""""""""""""""""""""""""""""""""""""""""""""""""""""""""""""""""""""""""""""""""""""""""""""""""""""""""""""""""""""""""""""""""""""""""""""""""""""""""""""""""""""""""""""""""""""""""""""""""""""""""""""""""""""""""""""""""""""""""""""""""""""""""""""""""""""""""""""""""""""""""""""""""""""""""""""""""""""""""""""""""""""""""""""""""""""""""""""""""""""""""""""""""""""""""""""""""""""""""""""""""""""""""""""""""""""""""""""""""""""""""""""""""""""""""""""""""""""""""""""""""""""""""""""""""""""""""""""""""""""""""""""""""""""""""""""""""""""""""""""""""""""""""""""""""""""""""""""""""""""""""""""""""""""""""""""""""""""""""""""""""""""""""""""""""""""""""""""""""""""""""""""""""""""""""""""""""""""""""""""""""""""""""""""""""""""""""""""""""""""""""""""""""""""""""""""""""""""""""""""""""""""""""""""""""""""""""""""""""""""""""""""""""""""""""""""""""""""""""""""""""""""""""""""""""""""""""""""""""""""""""""""""""""""""""""""""""""""""""""""""""""""""""""""""""""""""""""""""""""""""""""""""""""""""""""""""""""""""""""""""""""""""""""""""""""""""""""""""""""""""""""""""""""""""""""""""""""""""""""""""""""""""""""""""""""""""""""""""""""""""""""""""""""""""""""""""""""""""""""""""""""""""""""""""""""""""""""""""""""""""""""""""""""""""""""""""""""""""""""""""""""""""""""""""""""""""""""""""""""""""""""""""""""""""""""""""""""""""""""""""""""""""""""""""""""""""""""""""""""""""""""""""""""""""""""""""""""""""""""""""""""""""""""""""""""""""""""""""""""""""""""""""""""""""""""""""""""""""""""""""""""""""""""""""""""""""""""""""""""""""""""""""""""""""""""""""""""""""""""""""""""""""""""""""""""""""""""""""""""""""""""""""""""""""""""""""""""""""""""""""""""""""""""""""""""""""""""""""""""""""""""""""""""""""""""""""""""""""""""""""""""""""""""""""""""""""""""""""""""""""""""""""""""""""""""""""""""""""""""""""""""""""""""""""""""""""""""""""""""""""""""""""""""""""""""""""""""""""""""""""""""""""""""""""""""""""""""""""""""""""""""""""""""""""""""""""""""""""""""""""""""""""""""""""""""""""""""""""""""""""""""""""""""""""""""""""""""""""""""""""""""""""""""""""""""""""""""""""""""""""""""""""""""""""""""""""""""""""""""""""""""""""""""""""""""""""""""""""""""""""""""""""""""""""""""""""""""""""""""""""""""""""""""""""""""""""""""""""""""""""""""""""""""""""""""""""""""""""""""""""""""""""""""""""""""""""""""""""""""""""""""""""""""""""""""""""""""""""""""""""""""""""""""""""""""""""""""""""""""""""""""""""""""""""""""""""""""""""""""""""""""""""""""""""""""""""""""""""""""""""""""""""""""""""""""""""""""""""""""""""""""""""""""""""""""""""""""""""""""""""""""""""""""""""""""""""""""""""""""""""""""""""""""""""""""""""""""""""""""""""""""""""""""""""""""""""""""""""""""""""""""""""""""""""""""""""""""""""""""""""""""""""""""""""""""""""""""""""""""""""""""""""""""""""""""""""""""""""""""""""""""""""""""""""""""""""""""""""""""""""""""""""""""""""""""""""""""""""""""""""""""""""""""""""""""""""""""""""""""""""""""""""""""""""""""""""""""""""""""""""""""""""""""""""""""""""""""""""""""""""""""""""""""""""""""""""""""""""""""""""""""""""""""""""""""""""""""""""""""""""""""""""""""""""""""""""""""""""""""""""""""""""""""""""""""""""""""""""""""""""""""""""""""""""""""""""""""""""""""""""""""""""""""""""""""""""""""""""""""""""""""""""""""""""""""""""""""""""""""""""""""""""""""""""""""""""""""""""""""""""""""""""""""""""""""""""""""""""""""""""""""""""""""""""""""""""""""""""""""""""""""""""""""""""""""""""""""""""""""""""""""""""""""""""""""""""""""""""""""""""""""""""""""""""""""""""""""""""""""""""""""""""""""""""""""""""""""""""""""""""""""""""""""""""""""""""""""""""""""""""""""""""""""""""""""""""""""""""""""""""""""""""""""""""""""""""""""""""""""""""""""""""""""""""""""""""""""""""""""""""""""""""""""""""""""""""""""""""""""""""""""""""""""""""""""""""""""""""""""""""""""""""""""""""""""""""""""""""""""""""""""""""""""""""""""""""""""""""""""""""""""""""""""""""""""""""""""""""""""""""""""""""""""""""""""""""""""""""""""""""""""""""""""""""""""""""""""""""""""""""""""""""""""""""""""""""""""""""""""""""""""""""""""""""""""""""""""""""""""""""""""""""""""""""""""""""""""""""""""""""""""""""""""""""""""""""""""""""""""""""""""""""""""""""""""""""""""""""""""""""""""""""""""""""""""""""""""""""""""""""""""""""""""""""""""""""""""""""""""""""""""""""""""""""""""""""""""""""""""""""""""""""""""""""""""""""""""""""""""""""""""""""""""""""""""""""""""""""""""""""""""""""""""""""""""""""""""""""""""""""""""""""""""""""""""""""""""""""""""""""""""""""""""""""""""""""""""""""""""""""""""""""""""""""""""""""""""""""""""""""""""""""""""""""""""""""""""""""""""""""""""""""""""""""""""""""""""""""""""""""""""""""""""""""""""""""""""""""""""""""""""""""""""""""""""""""""""""""""""""""""""""""""""""""""""""""""""""""""""""""""""""""""""""""""""""""""""""""""""""""""""""""""""""""""""""""""""""""""""""""""""""""""""""""""""""""""""""""""""""""""""""""""""""""""""""""""""""""""""""""""""""""""""""""""""""""""""""""""""""""""""""""""""""""""""""""""""""""""""""""""""""""""""""""""""""""""""""""""""""""""""""""""""""""""""""""""""""""""""""""""""""""""""""""""""""""""""""""""""""""""""""""""""""""""""""""""""""""""""""""""""""""""""""""""""""""""""""""""""""""""""""""""""""""""""""""""""""""""""""""""""""""""""""""""""""""""""""""""""""""""""""""""""""""""""""""""""""""""""""""""""""""""""""""""""""""""""""""""""""""""""""""""""""""""""""""""""""""""""""""""""""""""""""""""""""""""""""""""""""""""""""""""""""""""""""""""""""""""""""""""""""""""""""""""""""""""""""""""""""""""""""""""""""""""""""""""""""""""""""""""""""""""""""""""""""""""""""""""""""""""""""""""""""""""""""""""""""""""""""""""""""""""""""""""""""""""""""""""""""""""""""""""""""""""""""""""""""""""""""""""""""""""""""""""""""""""""""""""""""""""""""""""""""""""""""""""""""""""""""""""""""""""""""""""""""""""""""""""""""""""""""""""""""""""""""""""""""""""""""""""""""""""""""""""""""""""""""""""""""""""""""""""""""""""""""""""""""""""""""""""""""""""""""""""""""""""""""""""""""""""""""""""""""""""""""""""""""""""""""""""""""""""""""""""""""""""""""""""""""""""""""""""""""""""""""""""""""""""""""""""""""""""""""""""""""""""""""""""""""""""""""""""""""""""""""""""""""""""""""""""""""""""""""""""""""""""""""""""""""""""""""""""""""""""""""""""""""""""""""""""""""""""""""""""""""""""""""""""""""""""""""""""""""""""""""""""""""""""""""""""""""""""""""""""""""""""""""""""""""""""""""""""""""""""""""""""""""""""""""""""""""""""""""""""""""""""""""""""""""""""""""""""""""""""""""""""""""""""""""""""""""""""""""""""""""""""""""""""""""""""""""""""""""""""""""""""""""""""""""""""""""""""""""""""""""""""""""""""""""""""""""""""""""""""""""""""""""""""""""""""""""""""""""""""""""""""""""""""""""""""""""""""""""""""""""""""""""""""""""""""""""""""""""""""""""""""""""""""""""""""""""""""""""""""""""""""""""""""""""""""""""""""""""""""""""""""""""""""""""""""""""""""""""""""""""""""""""""""""""""""""""""""""""""""""""""""""""""""""""""""""""""""""""""""""""""""""""""""""""""""""""""""""""""""""""""""""""""""""""""""""""""""""""""""""""""""""""""""""""""""""""""""""""""""""""""""""""""""""""""""""""""""""""""""""""""""""""""""""""""""""""""""""""""""""""""""""""""""""""""""""""""""""""""""""""""""""""""""""""""""""""""""""""""""""""""""""""""""""""""""""""""""""""""""""""""""""""""""""""""""""""""""""""""""""""""""""""""""""""""""""""""""""""""""""""""""""""""""""""""""""""""""""""""""""""""""""""""""""""""""""""""""""""""""""""""""""""""""""""""""""""""""""""""""""""""""""""""""""""""""""""""""""""""""""""""""""""""""""""""""""""""""""""""""""""""""""""""""""""""""""""""""""""""""""""""""""""""""""""""""""""""""""""""""""""""""""""""""""""""""""""""""""""""""""""""""""""""""""""""""""""""""""""""""""""""""""""""""""""""""""""""""""""""""""""""""""""""""""""""""""""""""""""""""""""""""""""""""""""""""""""""""""""""""""""""""""""""""""""""""""""""""""""""""""""""""""""""""""""""""""""""""""""""""""""""""""""""""""""""""""""""""""""""""""""""""""""""""""""""""""""""""""""""""""""""""""""""""""""""""""""""""""""""""""""""""""""""""""""""""""""""""""""""""""""""""""""""""""""""""""""""""""""""""""""""""""""""""""""""""""""""""""""""""""""""""""""""""""""""""""""""""""""""""""""""""""""""""""""""""""""""""""""""""""""""""""""""""""""""""""""""""""""""""""""""""""""""""""""""""""""""""""""""""""""""""""""""""""""""""""""""""""""""""""""""""""""""""""""""""""""""""""""""""""""""""""""""""""""""""""""""""""""""""""""""""""""""""""""""""""""""""""""""""""""""""""""""""""""""""""""""""""""""""""""""""""""""""""""""""""""""""""""""""""""""""""""""""""""""""""""""""""""""""""""""""""""""""""""""""""""""""""""""""""""""""""""""""""""""""""""""""""""""""""""""""""""""""""""""""""""""""""""""""""""""""""""""""""""""""""""""""""""""""""""""""""""""""""""""""""""""""""""""""""""""""""""""""""""""""""""""""""""""""""""""""""""""""""""""""""""""""""""""""""""""""""""""""""""""""""""""""""""""""""""""""""""""""""""""""""""""""""""""""""""""""""""""""""""""""""""""""""""""""""""""""""""""""""""""""""""""""""""""""""""""""""""""""""""""""""""""""""""""""""""""""""""""""""""""""""""""""""""""""""""""""""""""""""""""""""""""""""""""""""""""""""""""""""""""""""""""""""""""""""""""""""""""""""""""""""""""""""""""""""""""""""""""""""""""""""""""""""""""""""""""""""""""""""""""""""""""""""""""""""""""""""""""""""""""""""""""""""""""""""""""""""""""""""""""""""""""""""""""""""""""""""""""""""""""""""""""""""""""""""""""""""""""""""""""""""""""""""""""""""""""""""""""""""""""""""""""""""""""""""""""""""""""""""""""""""""""""""""""""""""""""""""""""""""""""""""""""""""""""""""""""""""""""""""""""""""""""""""""""""""""""""""""""""""""""""""""""""""""""""""""""""""""""""""""""""""""""""""""""""""""""""""""""""""""""""""""""""""""""""""""""""""""""""""""""""""""""""""""""""""""""""""""""""""""""""""""""""""""""""""""""""""""""""""""""""""""""""""""""""""""""""""""""""""""""""""""""""""""""""""""""""""""""""""""""""""""""""""""""""""""""""""""""""""""""""""""""""""""""""""""""""""""""""""""""""""""""""""""""""""""""""""""""""""""""""""""""""""""""""""""""""""""""""""""""""""""""""""""""""""""""""""""""""""""""""""""""""""""""""""""""""""""""""""""""""""""""""""""""""""""""""""""""""""""""""""""""""""""""""""""""""""""""""""""""""""""""""""""""""""""""""""""""""""""""""""""""""""""""""""""""""""""""""""""""""""""""""""""""""""""""""""""""""""""""""""""""""""""""""""""""""""""""""""""""""""""""""""""""""""""""""""""""""""""""""""""""""""""""""""""""""""""""""""""""""""""""""""""""""""""""""""""""""""""""""""""""""""""""""""""""""""""""""""""""""""""""""""""""""""""""""""""""""""""""""""""""""""""""""""""""""""""""""""""""""""""""""""""""""""""""""""""""""""""""""""""""""""""""""""""""""""""""""""""""""""""""""""""""""""""""""""""""""""""""""""""""""""""""""""""""""""""""""""""""""""""""""""""""""""""""""""""""""""""""""""""""""""""""""""""""""""""""""""""""""""""""""""""""""""""""""""""""""""""""""""""""""""""""""""""""""""""""""""""""""""""""""""""""""""""""""""""""""""""""""""""""""""""""""""""""""""""""""""""""""""""""""""""""""""""""""""""""""""""""""""""""""""""""""""""""""""""""""""""""""""""""""""""""""""""""""""""""""""""""""""""""""""""""""""""""""""""""""""""""""""""""""""""""""""""""""""""""""""""""""""""""""""""""""""""""""""""""""""""""""""""""""""""""""""""""""""""""""""""""""""""""""""""""""""""""""""""""""""""""""""""""""""""""""""""""""""""""""""""""""""""""""""""""""""""""""""""""""""""""""""""""""""""""""""""""""""""""""""""""""""""""""""""""""""""""""""""""""""""""""""""""""""""""""""""""""""""""""""""""""""""""""""""""""""""""""""""""""""""""""""""""""""""""""""""""""""""""""""""""""""""""""""""""""""""""""""""""""""""""""""""""""""""""""""""""""""""""""""""""""""""""""""""""""""""""""""""""""""""""""""""""""""""""""""""""""""""""""""""""""""""""""""""""""""""""""""""""""""""""""""""""""""""""""""""""""""""""""""""""""""""""""""""""""""""""""""""""""""""""""""""""""""""""""""""""""""""""""""""""""""""""""""""""""""""""""""""""""""""""""""""""""""""""""""""""""""""""""""""""""""""""""""""""""""""""""""""""""""""""""""""""""""""""""""""""""""""""""""""""""""""""""""""""""""""""""""""""""""""""""""""""""""""""""""""""""""""""""""""""""""""""""""""""""""""""""""""""""""""""""""""""""""""""""""""""""""""""""""""""""""""""""""""""""""""""""""""""""""""""""""""""""""""""""""""""""""""""""""""""""""""""""""""""""""""""""""""""""""""""""""""""""""""""""""""""""""""""""""""""""""""""""""""""""""""""""""""""""""""""""""""""""""""""""""""""""""""""""""""""""""""""""""""""""""""""""""""""""""""""""""""""""""""""""""""""""""""""""""""""""""""""""""""""""""""""""""""""""""""""""""""""""""""""""""""""""""""""""""""""""""""""""""""""""""""""""""""""""""""""""""""""""""""""""""""""""""""""""""""""""""""""""""""""""""""""""""""""""""""""""""""""""""""""""""""""""""""""""""""""""""""""""""""""""""""""""""""""""""""""""""""""""""""""""""""""""""""""""""""""""""""""""""""""""""""""""""""""""""""""""""""""""""""""""""""""""""""""""""""""""""""""""""""""""""""""""""""""""""""""""""""""""""""""""""""""""""""""""""""""""""""""""""""""""""""""""""""""""""""""""""""""""""""""""""""""""""""""""""""""""""""""""""""""""""""""""""""""""""""""""""""""""""""""""""""""""""""""""""""""""""""""""""""""""""""""""""""""""""""""""""""""""""""""""""""""""""""""""""""""""""""""""""""""""""""""""""""""""""""""""""""""""""""""""""""""""""""""""""""""""""""""""""""""""""""""""""""""""""""""""""""""""""""""""""""""""""""""""""""""""""""""""""""""""""""""""""""""""""""""""""""""""""""""""""""""""""""""""""""""""""""""""""""""""""""""""""""""""""""""""""""""""""""""""""""""""""""""""""""""""""""""""""""""""""""""""""""""""""""""""""""""""""""""""""""""""""""""""""""""""""""""""""""""""""""""""""""""""""""""""""""""""""""""""""""""""""""""""""""""""""""""""""""""""""""""""""""""""""""""""""""""""""""""""""""""""""""""""""""""""""""""""""""""""""""""""""""""""""""""""""""""""""""""""""""""""""""""""""""""""""""""""""""""""""""""""""""""""""""""""""""""""""""""""""""""""""""""""""""""""""""""""""""""""""""""""""""""""""""""""""""""""""""""""""""""""""""""""""""""""""""""""""""""""""""""""""""""""""""""""""""""""""""""""""""""""""""""""""""""""""""""""""""""""""""""""""""""""""""""""""""""""""""""""""""""""""""""""""""""""""""""""""""""""""""""""""""""""""""""""""""""""""""""""""""""""""""""""""""""""""""""""""""""""""""""""""""""""""""""""""""""""""""""""""""""""""""""""""""""""""""""""""""""""""""""""""""""""""""""""""""""""""""""""""""""""""""""""""""""""""""""""""""""""""""""""""""""""""""""""""""""""""""""""""""""""""""""""""""""""""""""""""""""""""""""""""""""""""""""""""""""""""""""""""""""""""""""""""""""""""""""""""""""""""""""""""""""""""""""""""""""""""""""""""""""""""""""""""""""""""""""""""""""""""""""""""""""""""""""""""""""""""""""""""""""""""""""""""""""""""""""""""""""""""""""""""""""""""""""""""""""""""""""""""""""""""""""""""""""""""""""""""""""""""""""""""""""""""""""""""""""""""""""""""""""""""""""""""""""""""""""""""""""""""""""""""""""""""""""""""""""""""""""""""""""""""""""""""""""""""""""""""""""""""""""""""""""""""""""""""""""""""""""""""""""""""""""""""""""""""""""""""""""""""""""""""""""""""""""""""""""""""""""""""""""""""""""""""""""""""""""""""""""""""""""""""""""""""""""""""""""""""""""""""""""""""""""""""""""""""""""""""""""""""""""""""""""""""""""""""""""""""""""""""""""""""""""""""""""""""""""""""""""""""""""""""""""""""""""""""""""""""""""""""""""""""""""""""""""""""""""""""""""""""""""""""""""""""""""""""""""""""""""""""""""""""""""""""""""""""""""""""""""""""""""""""""""""""""""""""""""""""""""""""""""""""""""""""""""""""""""""""""""""""""""""""""""""""""""""""""""""""""""""""""""""""""""""""""""""""""""""""""""""""""""""""""""""""""""""""""""""""""""""""""""""""""""""""""""""""""""""""""""""""""""""""""""""""""""""""""""""""""""""""""""""""""""""""""""""""""""""""""""""""""""""""""""""""""""""""""""""""""""""""""""""""""""""""""""""""""""""""""""""""""""""""""""""""""""""""""""""""""""""""""""""""""""""""""""""""""""""""""""""""""""""""""""""""""""""""""""""""""""""""""""""""""""""""""""""""""""""""""""""""""""""""""""""""""""""""""""""""""""""""""""""""""""""""""""""""""""""""""""""""""""""""""""""""""""""""""""""""""""""""""""""""""""""""""""""""""""""""""""""""""""""""""""""""""""""""""""""""""""""""""""""""""""""""""""""""""""""""""""""""""""""""""""""""""""""""""""""""""""""""""""""""""""""""""""""""""""""""""""""""""""""""""""""""""""""""""""""""""""""""""""""""""""""""""""""""""""""""""""""""""""""""""""""""""""""""""""""""""""""""""""""""""""""""""""""""""""""""""""""""""""""""""""""""""""""""""""""""""""""""""""""""""""""""""""""""""""""""""""""""""""""""""""""""""""""""""""""""""""""""""""""""""""""""""""""""""""""""""""""""""""""""""""""""""""""""""""""""""""""""""""""""""""""""""""""""""""""""""""""""""""""""""""""""""""""""""""""""""""""""""""""""""""""""""""""""""""""""""""""""""""""""""""""""""""""""""""""""""""""""""""""""""""""""""""""""""""""""""""""""""""""""""""""""""""""""""""""""""""""""""""""""""""""""""""""""""""""""""""""""""""""""""""""""""""""""""""""""""""""""""""""""""""""""""""""""""""""""""""""""""""""""""""""""""""""""""""""""""""""""""""""""""""""""""""""""""""""""""""""""""""""""""""""""""""""""""""""""""""""""""""""""""""""""""""""""""""""""""""""""""""""""""""""""""""""""""""""""""""""""""""""""""""""""""""""""""""""""""""""""""""""""""""""""""""""""""""""""""""""""""""""""""""""""""""""""""""""""""""""""""""""""""""""""""""""""""""""""""""""""""""""""""""""""""""""""""""""""""""""""""""""""""""""""""""""""""""""""""""""""""""""""""""""""""""""""""""""""""""""""""""""""""""""""""""""""""""""""""""""""""""""""""""""""""""""""""""""""""""""""""""""""""""""""""""""""""""""""""""""""""""""""""""""""""""""""""""""""""""""""""""""""""""""""""""""""""""""""""""""""""""""""""""""""""""""""""""""""""""""""""""""""""""""""""""""""""""""""""""""""""""""""""""""""""""""""""""""""""""""""""""""""""""""""""""""""""""""""""""""""""""""""""""""""""""""""""""""""""""""""""""""""""""""""""""""""""""""""""""""""""""""""""""""""""""""""""""""""""""""""""""""""""""""""""""""""""""""""""""""""""""""""""""""""""""""""""""""""""""""""""""""""""""""""""""""""""""""""""""""""""""""""""""""""""""""""""""""""""""""""""""""""""""""""""""""""""""""""""""""""""""""""""""""""""""""""""""""""""""""""""""""""""""""""""""""""""""""""""""""""""""""""""""""""""""""""""""""""""""""""""""""""""""""""""""""""""""""""""""""""""""""""""""""""""""""""""""""""""""""""""""""""""""""""""""""""""""""""""""""""""""""""""""""""""""""""""""""""""""""""""""""""""""""""""""""""""""""""""""""""""""""""""""""""""""""""""""""""""""""""""""""""""""""""""""""""""""""""""""""""""""""""""""""""""""""""""""""""""""""""""""""""""""""""""""""""""""""""""""""""""""""""""""""""""""""""""""""""""""""""""""""""""""""""""""""""""""""""""""""""""""""""""""""""""""""""""""""""""""""""""""""""""""""""""""""""""""""""""""""""""""""""""""""""""""""""""""""""""""""""""""""""""""""""""""""""""""""""""""""""""""""""""""""""""""""""""""""""""""""""""""""""""""""""""""""""""""""""""""""""""""""""""""""""""""""""""""""""""""""""""""""""""""""""""""""""""""""""""""""""""""""""""""""""""""""""""""""""""""""""""""""""""""""""""""""""""""""""""""""""""""""""""""""""""""""""""""""""""""""""""""""""""""""""""""""""""""""""""""""""""""""""""""""""""""""""""""""""""""""""""""""""""""""""""""""""""""""""""""""""""""""""""""""""""""""""""""""""""""""""""""""""""""""""""""""""""""""""""""""""""""""""""""""""""""""""""""""""""""""""""""""""""""""""""""""""""""""""""""""""""""""""""""""""""""""""""""""""""""""""""""""""""""""""""""""""""""""""""""""""""""""""""""""""""""""""""""""""""""""""""""""""""""""""""""""""""""""""""""""""""""""""""""""""""""""""""""""""""""""""""""""""""""""""""""""""""""""""""""""""""""""""""""""""""""""""""""""""""""""""""""""""""""""""""""""""""""""""""""""""""""""""""""""""""""""""""""""""""""""""""""""""""""""""""""""""""""""""""""""""""""""""""""""""""""""""""""""""""""""""""""""""""""""""""""""""""""""""""""""""""""""""""""""""""""""""""""""""""""""""""""""""""""""""""""""""""""""""""""""""""""""""""""""""""""""""""""""""""""""""""""""""""""""""""""""""""""""""""""""""""""""""""""""""""""""""""""""""""""""""""""""""""""""""""""""""""""""""""""""""""""""""""""""""""""""""""""""""""""""""""""""""""""""""""""""""""""""""""""""""""""""""""""""""""""""""""""""""""""""""""""""""""""""""""""""""""""""""""""""""""""""""""""""""""""""""""""""""""""""""""""""""""""""""""""""""""""""""""""""""""""""""""""""""""""""""""""""""""""""""""""""""""""""""""""""""""""""""""""""""""""""""""""""""""""""""""""""""""""""""""""""""""""""""""""""""""""""""""""""""""""""""""""""""""""""""""""""""""""""""""""""""""""""""""""""""""""""""""""""""""""""""""""""""""""""""""""""""""""""""""""""""""""""""""""""""""""""""""""""""""""""""""""""""""""""""""""""""""""""""""""""""""""""""""""""""""""""""""""""""""""""""""""""""""""""""""""""""""""""""""""""""""""""""""""""""""""""""""""""""""""""""""""""""""""""""""""""""""""""""""""""""""""""""""""""""""""""""""""""""""""""""""""""""""""""""""""""""""""""""""""""""""""""""""""""""""""""""""""""""""""""""""""""""""""""""""""""""""""""""""""""""""""""""""""""""""""""""""""""""""""""""""""""""""""""""""""""""""""""""""""""""""""""""""""""""""""""""""""""""""""""""""""""""""""""""""""""""""""""""""""""""""""""""""""""""""""""""""""""""""""""""""""""""""""""""""""""""""""""""""""""""""""""""""""""""""""""""""""""""""""""""""""""""""""""""""""""""""""""""""""""""""""""""""""""""""""""""""""""""""""""""""""""""""""""""""""""""""""""""""""""""""""""""""""""""""""""""""""""""""""""""""""""""""""""""""""""""""""""""""""""""""""""""""""""""""""""""""""""""""""""""""""""""""""""""""""""""""""""""""""""""""""""""""""""""""""""""""""""""""""""""""""""""""""""""""""""""""""""""""""""""""""""""""""""""""""""""""""""""""""""""""""""""""""""""""""""""""""""""""""""""""""""""""""""""""""""""""""""""""""""""""""""""""""""""""""""""""""""""""""""""""""""""""""""""""""""""""""""""""""""""""""""""""""""""""""""""""""""""""""""""""""""""""""""""""""""""""""""""""""""""""""""""""""""""""""""""""""""""""""""""""""""""""""""""""""""""""""""""""""""""""""""""""""""""""""""""""""""""""""""""""""""""""""""""""""""""""""""""""""""""""""""""""""""""""""""""""""""""""""""""""""""""""""""""""""""""""""""""""""""""""""""""""""""""""""""""""""""""""""""""""""""""""""""""""""""""""""""""""""""""""""""""""""""""""""""""""""""""""""""""""""""""""""""""""""""""""""""""""""""""""""""""""""""""""""""""""""""""""""""""""""""""""""""""""""""""""""""""""""""""""""""""""""""""""""""""""""""""""""""""""""""""""""""""""""""""""""""""""""""""""""""""""""""""""""""""""""""""""""""""""""""""""""""""""""""""""""""""""""""""""""""""""""""""""""""""""""""""""""""""""""""""""""""""""""""""""""""""""""""""""""""""""""""""""""""""""""""""""""""""""""""""""""""""""""""""""""""""""""""""""""""""""""""""""""""""""""""""""""""""""""""""""""""""""""""""""""""""""""""""""""""""""""""""""""""""""""""""""""""""""""""""""""""""""""""""""""""""""""""""""""""""""""""""""""""""""""""""""""""""""""""""""""""""""""""""""""""""""""""""""""""""""""""""""""""""""""""""""""""""""""""""""""""""""""""""""""""""""""""""""""""""""""""""""""""""""""""""""""""""""""""""""""""""""""""""""""""""""""""""""""""""""""""""""""""""""""""""""""""""""""""""""""""""""""""""""""""""""""""""""""""""""""""""""""""""""""""""""""""""""""""""""""""""""""""""""""""""""""""""""""""""""""""""""""""""""""""""""""""""""""""""""""""""""""""""""""""""""""""""""""""""""""""""""""""""""""""""""""""""""""""""""""""""""""""""""""""""""""""""""""""""""""""""""""""""""""""""""""""""""""""""""""""""""""""""""""""""""""""""""""""""""""""""""""""""""""""""""""""""""""""""""""""""""""""""""""""""""""""""""""""""""""""""""""""""""""""""""""""""""""""""""""""""""""""""""""""""""""""""""""""""""""""""""""""""""""""""""""""""""""""""""""""""""""""""""""""""""""""""""""""""""""""""""""""""""""""""""""""""""""""""""""""""""""""""""""""""""""""""""""""""""""""""""""""""""""""""""""""""""""""""""""""""""""""""""""""""""""""""""""""""""""""""""""""""""""""""""""""""""""""""""""""""""""""""""""""""""""""""""""""""""""""""""""""""""""""""""""""""""""""""""""""""""""""""""""""""""""""""""""""""""""""""""""""""""""""""""""""""""""""""""""""""""""""""""""""""""""""""""""""""""""""""""""""""""""""""""""""""""""""""""""""""""""""""""""""""""""""""""""""""""""""""""""""""""""""""""""""""""""""""""""""""""""""""""""""""""""""""""""""""""""""""""""""""""""""""""""""""""""""""""""""""""""""""""""""""""""""""""""""""""""""""""""""""""""""""""""""""""""""""""""""""""""""""""""""""""""""""""""""""""""""""""""""""""""""""""""""""""""""""""""""""""""""""""""""""""""""""""""""""""""""""""""""""""""""""""""""""""""""""""""""""""""""""""""""""""""""""""""""""""""""""""""""""""""""""""""""""""""""""""""""""""""""""""""""""""""""""""""""""""""""""""""""""""""""""""""""""""""""""""""""""""""""""""""""""""""""""""""""""""""""""""""""""""""""""""""""""""""""""""""""""""""""""""""""""""""""""""""""""""""""""""""""""""""""""""""""""""""""""""""""""""""""""""""""""""""""""""""""""""""""""""""""""""""""""""""""""""""""""""""""""""""""""""""""""""""""""""""""""""""""""""""""""""""""""""""""""""""""""""""""""""""""""""""""""""""""""""""""""""""""""""""""""""""""""""""""""""""""""""""""""""""""""""""""""""""""""""""""""""""""""""""""""""""""""""""""""""""""""""""""""""""""""""""""""""""""""""""""""""""""""""""""""""""""""""""""""""""""""""""""""""""""""""""""""""""""""""""""""""""""""""""""""""""""""""""""""""""""""""""""""""""""""""""""""""""""""""""""""""""""""""""""""""""""""""""""""""""""""""""""""""""""""""""""""""""""""""""""""""""""""""""""""""""""""""""""""""""""""""""""""""""""""""""""""""""""""""""""""""""""""""""""""""""""""""""""""""""""""""""""""""""""""""""""""""""""""""""""""""""""""""""""""""""""""""""""""""""""""""""""""""""""""""""""""""""""""""""""""""""""""""""""""""""""""""""""""""""""""""""""""""""""""""""""""""""""""""""""""""""""""""""""""""""""""""""""""""""""""""""""""""""""""""""""""""""""""""""""""""""""""""""""""""""""""""""""""""""""""""""""""""""""""""""""""""""""""""""""""""""""""""""""""""""""""""""""""""""""""""""""""""""""""""""""""""""""""""""""""""""""""""""""""""""""""""""""""""""""""""""""""""""""""""""""""""""""""""""""""""""""""""""""""""""""""""""""""""""""""""""""""""""""""""""""""""""""""""""""""""""""""""""""""""""""""""""""""""""""""""""""""""""""""""""""""""""""""""""""""""""""""""""""""""""""""""""""""""""""""""""""""""""""""""""""""""""""""""""""""""""""""""""""""""""""""""""""""""""""""""""""""""""""""""""""""""""""""""""""""""""""""""""""""""""""""""""""""""""""""""""""""""""""""""""""""""""""""""""""""""""""""""""""""""""""""""""""""""""""""""""""""""""""""""""""""""""""""""""""""""""""""""""""""""""""""""""""""""""""""""""""""""""""""""""""""""""""""""""""""""""""""""""""""""""""""""""""""""""""""""""""""""""""""""""""""""""""""""""""""""""""""""""""""""""""""""""""""""""""""""""""""""""""""""""""""""""""""""""""""""""""""""""""""""""""""""""""""""""""""""""""""""""""""""""""""""""""""""""""""""""""""""""""""""""""""""""""""""""""""""""""""""""""""""""""""""""""""""""""""""""""""""""""""""""""""""""""""""""""""""""""""""""""""""""""""""""""""""""""""""""""""""""""""""""""""""""""""""""""""""""""""""""""""""""""""""""""""""""""""""""""""""""""""""""""""""""""""""""""""""""""""""""""""""""""""""""""""""""""""""""""""""""""""""""""""""""""""""""""""""""""""""""""""""""""""""""""""""""""""""""""""""""""""""""""""""""""""""""""""""""""""""""""""""""""""""""""""""""""""""""""""""""""""""""""""""""""""""""""""""""""""""""""""""""""""""""""""""""""""""""""""""""""""""""""""""""""""""""""""""""""""""""""""""""""""""""""""""""""""""""""""""""""""""""""""""""""""""""""""""""""""""""""""""""""""""""""""""""""""""""""""""""""""""""""""""""""""""""""""""""""""""""""""""""""""""""""""""""""""""""""""""""""""""""""""""""""""""""""""""""""""""""""""""""""""""""""""""""""""""""""""""""""""""""""""""""""""""""""""""""""""""""""""""""""""""""""""""""""""""""""""""""""""""""""""""""""""""""""""""""""""""""""""""""""""""""""""""""""""""""""""""""""""""""""""""""""""""""""""""""""""""""""""""""""""""""""""""""""""""""""""""""""""""""""""""""""""""""""""""""""""""""""""""""""""""""""""""""""""""""""""""""""""""""""""""""""""""""""""""""""""""""""""""""""""""""""""""""""""""""""""""""""""""""""""""""""""""""""""""""""""""""""""""""""""""""""""""""""""""""""""""""""""""""""""""""""""""""""""""""""""""""""""""""""""""""""""""""""""""""""""""""""""""""""""""""""""""""""""""""""""""""""""""""""""""""""""""""""""""""""""""""""""""""""""""""""""""""""""""""""""""""""""""""""""""""""""""""""""""""""""""""""""""""""""""""""""""""""""""""""""""""""""""""""""""""""""""""""""""""""""""""""""""""""""""""""""""""""""""""""""""""""""""""""""""""""""""""""""""""""""""""""""""""""""""""""""""""""""""""""""""""""""""""""""""""""""""""""""""""""""""""""""""""""""""""""""""""""""""""""""""""""""""""""""""""""""""""""""""""""""""""""""""""""""""""""""""""""""""""""""""""""""""""""""""""""""""""""""""""""""""""""""""""""""""""""""""""""""""""""""""""""""""""""""""""""""""""""""""""""""""""""""""""""""""""""""""""""""""""""""""""""""""""""""""""""""""""""""""""""""""""""""""""""""""""""""""""""""""""""""""""""""""""""""""""""""""""""""""""""""""""""""""""""""""""""""""""""""""""""""""""""""""""""""""""""""""""""""""""""""""""""""""""""""""""""""""""""""""""""""""""""""""""""""""""""""""""""""""""""""""""""""""""""""""""""""""""""""""""""""""""""""""""""""""""""""""""""""""""""""""""""""""""""""""""""""""""""""""""""""""""""""""""""""""""""""""""""""""""""""""""""""""""""""""""""""""""""""""""""""""""""""""""""""""""""""""""""""""""""""""""""""""""""""""""""""""""""""""""""""""""""""""""""""""""""""""""""""""""""""""""""""""""""""""""""""""""""""""""""""""""""""""""""""""""""""""""""""""""""""""""""""""""""""""""""""""""""""""""""""""""""""""""""""""""""""""""""""""""""""""""""""""""""""""""""""""""""""""""""""""""""""""""""""""""""""""""""""""""""""""""""""""""""""""""""""""""""""""""""""""""""""""""""""""""""""""""""""""""""""""""""""""""""""""""""""""""""""""""""""""""""""""""""""""""""""""""""""""""""""""""""""""""""""""""""""""""""""""""""""""""""""""""""""""""""""""""""""""""""""""""""""""""""""""""""""""""""""""""""""""""""""""""""""""""""""""""""""""""""""""""""""""""""""""""""""""""""""""""""""""""""""""""""""""""""""""""""""""""""""""""""""""""""""""""""""""""""""""""""""""""""""""""""""""""""""""""""""""""""""""""""""""""""""""""""""""""""""""""""""""""""""""""""""""""""""""""""""""""""""""""""""""""""""""""""""""""""""""""""""""""""""""""""""""""""""""""""""""""""""""""""""""""""""""""""""""""""""""""""""""""""""""""""""""""""""""""""""""""""""""""""""""""""""""""""""""""""""""""""""""""""""""""""""""""""""""""""""""""""""""""""""""""""""""""""""""""""""""""""""""""""""""""""""""""""""""""""""""""""""""""""""""""""""""""""""""""""""""""""""""""""""""""""""""""""""""""""""""""""""""""""""""""""""""""""""""""""""""""""""""""""""""""""""""""""""""""""""""""""""""""""""""""""""""""""""""""""""""""""""""""""""""""""""""""""""""""""""""""""""""""""""""""""""""""""""""""""""""""""""""""""""""""""""""""""""""""""""""""""""""""""""""""""""""""""""""""""""""""""""""""""""""""""""""""""""""""""""""""""""""""""""""""""""""""""""""""""""""""""""""""""""""""""""""""""""""""""""""""""""""""""""""""""""""""""""""""""""""""""""""""""""""""""""""""""""""""""""""""""""""""""""""""""""""""""""""""""""""""""""""""""""""""""""""""""""""""""""""""""""""""""""""""""""""""""""""""""""""""""""""""""""""""""""""""""""""""""""""""""""""""""""""""""""""""""""""""""""""""""""""""""""""""""""""""""""""""""""""""""""""""""""""""""""""""""""""""""""""""""""""""""""""""""""""""""""""""""""""""""""""""""""""""""""""""""""""""""""""""""""""""""""""""""""""""""""""""""""""""""""""""""""""""""""""""""""""""""""""""""""""""""""""""""""""""""""""""""""""""""""""""""""""""""""""""""""""""""""""""""""""""""""""""""""""""""""""""""""""""""""""""""""""""""""""""""""""""""""""""""""""""""""""""""""""""""""""""""""""""""""""""""""""""""""""""""""""""""""""""""""""""""""""""""""""""""""""""""""""""""""""""""""""""""""""""""""""""""""""""""""""""""""""""""""""""""""""""""""""""""""""""""""""""""""""""""""""""""""""""""""""""""""""""""""""""""""""""""""""""""""""""""""""""""""""""""""""""""""""""""""""""""""""""""""""""""""""""""""""""""""""""""""""""""""""""""""""""""""""""""""""""""""""""""""""""""""""""""""""""""""""""""""""""""""""""""""""""""""""""""""""""""""""""""""""""""""""""""""""""""""""""""""""""""""""""""""""""""""""""""""""""""""""""""""""""""""""""""""""""""""""""""""""""""""""""""""""""""""""""""""""""""""""""""""""""""""""""""""""""""""""""""""""""""""""""""""""""""""""""""""""""""""""""""""""""""""""""""""""""""""""""""""""""""""""""""""""""""""""""""""""""""""""""""""""""""""""""""""""""""""""""""""""""""""""""""""""""""""""""""""""""""""""""""""""""""""""""""""""""""""""""""""""""""""""""""""""""""""""""""""""""""""""""""""""""""""""""""""""""""""""""""""""""""""""""""""""""""""""""""""""""""""""""""""""""""""""""""""""""""""""""""""""""""""""""""""""""""""""""""""""""""""""""""""""""""""""""""""""""""""""""""""""""""""""""""""""""""""""""""""""""""""""""""""""""""""""""""""""""""""""""""""""""""""""""""""""""""""""""""""""""""""""""""""""""""""""""""""""""""""""""""""""""""""""""""""""""""""""""""""""""""""""""""""""""""""""""""""""""""""""""""""""""""""""""""""""""""""""""""""""""""""""""""""""""""""""""""""""""""""""""""""""""""""""""""""""""""""""""""""""""""""""""""""""""""""""""""""""""""""""""""""""""""""""""""""""""""""""""""""""""""""""""""""""""""""""""""""""""""""""""""""""""""""""""""""""""""""""""""""""""""""""""""""""""""""""""""""""""""""""""""""""""""""""""""""""""""""""""""""""""""""""""""""""""""""""""""""""""""""""""""""""""""""""""""""""""""""""""""""""""""""""""""""""""""""""""""""""""""""""""""""""""""""""""""""""""""""""""""""""""""""""""""""""""""""""""""""""""""""""""""""""""""""""""""""""""""""""""""""""""""""""""""""""""""""""""""""""""""""""""""""""""""""""""""""""""""""""""""""""""""""""""""""""""""""""""""""""""""""""""""""""""""""""""""""""""""""""""""""""""""""""""""""""""""""""""""""""""""""""""""""""""""""""""""""""""""""""""""""""""""""""""""""""""""""""""""""""""""""""""""""""""""""""""""""""""""""""""""""""""""""""""""""""""""""""""""""""""""""""""""""""""""""""""""""""""""""""""""""""""""""""""""""""""""""""""""""""""""""""""""""""""""""""""""""""""""""""""""""""""""""""""""""""""""""""""""""""""""""""""""""""""""""""""""""""""""""""""""""""""""""""""""""""""""""""""""""""""""""""""""""""""""""""""""""""""""""""""""""""""""""""""""""""""""""""""""""""""""""""""""""""""""""""""""""""""""""""""""""""""""""""""""""""""""""""""""""""""""""""""""""""""""""""""""""""""""""""""""""""""""""""""""""""""""""""""""""""""""""""""""""""""""""""""""""""""""""""""""""""""""""""""""""""""""""""""""""""""""""""""""""""""""""""""""""""""""""""""""""""""""""""""""""""""""""""""""""""""""""""""""""""""""""""""""""""""""""""""""""""""""""""""""""""""""""""""""""""""""""""""""""""""""""""""""""""""""""""""""""""""""""""""""""""""""""""""""""""""""""""""""""""""""""""""""""""""""""""""""""""""""""""""""""""""""""""""""""""""""""""""""""""""""""""""""""""""""""""""""""""""""""""""""""""""""""""""""""""""""""""""""""""""""""""""""""""""""""""""""""""""""""""""""""""""""""""""""""""""""""""""""""""""""""""""""""""""""""""""""""""""""""""""""""""""""""""""""""""""""""""""""""""""""""""""""""""""""""""""""""""""""""""""""""""""""""""""""""""""""""""""""""""""""""""""""""""""""""""""""""""""""""""""""""""""""""""""""""""""""""""""""""""""""""""""""""""""""""""""""""""""""""""""""""""""""""""""""""""""""""""""""""""""""""""""""""""""""""""""""""""""""""""""""""""""""""""""""""""""""""""""""""""""""""""""""""""""""""""""""""""""""""""""""""""""""""""""""""""""""""""""""""""""""""""""""""""""""""""""""""""""""""""""""""""""""""""""""""""""""""""""""""""""""""""""""""""""""""""""""""""""""""""""""""""""""""""""""""""""""""""""""""""""""""""""""""""""""""""""""""""""""""""""""""""""""""""""""""""""""""""""""""""""""""""""""""""""""""""""""""""""""""""""""""""""""""""""""""""""""""""""""""""""""""""""""""""""""""""""""""""""""""""""""""""""""""""""""""""""""""""""""""""""""""""""""""""""""""""""""""""""""""""""""""""""""""""""""""""""""""""""""""""""""""""""""""""""""""""""""""""""""""""""""""""""""""""""""""""""""""""""""""""""""""""""""""""""""""""""""""""""""""""""""""""""""""""""""""""""""""""""""""""""""""""""""""""""""""""""""""""""""""""""""""""""""""""""""""""""""""""""""""""""""""""""""""""""""""""""""""""""""""""""""""""""""""""""""""""""""""""""""""""""""""""""""""""""""""""""""""""""""""""""""""""""""""""""""""""""""""""""""""""""""""""""""""""""""""""""""""""""""""""""""""""""""""""""""""""""""""""""""""""""""""""""""""""""""""""""""""""""""""""""""""""""""""""""""""""""""""""""""""""""""""""""""""""""""""""""""""""""""""""""""""""""""""""""""""""""""""""""""""""""""""""""""""""""""""""""""""""""""""""""""""""""""""""""""""""""""""""""""""""""""""""""""""""""""""""""""""""""""""""""""""""""""""""""""""""""""""""""""""""""""""""""""""""""""""""""""""""""""""""""""""""""""""""""""""""""""""""""""""""""""""""""""""""""""""""""""""""""""""""""""""""""""""""""""""""""""""""""""""""""""""""""""""""""""""""""""""""""""""""""""""""""""""""""""""""""""""""""""""""""""""""""""""""""""""""""""""""""""""""""""""""""""""""""""""""""""""""""""""""""""""""""""""""""""""""""""""""""""""""""""""""""""""""""""""""""""""""""""""""""""""""""""""""""""""""""""""""""""""""""""""""""""""""""""""""""""""""""""""""""""""""""""""""""""""""""""""""""""""""""""""""""""""""""""""""""""""""""""""""""""""""""""""""""""""""""""""""""""""""""""""""""""""""""""""""""""""""""""""""""""""""""""""""""""""""""""""""""""""""""""""""""""""""""""""""""""""""""""""""""""""""""""""""""""""""""""""""""""""""""""""""""""""""""""""""""""""""""""""""""""""""""""""""""""""""""""""""""""""""""""""""""""""""""""""""""""""""""""""""""""""""""""""""""""""""""""""""""""""""""""""""""""""""""""""""""""""""""""""""""""""""""""""""""""""""""""""""""""""""""""""""""""""""""""""""""""""""""""""""""""""""""""""""""""""""""""""""""""""""""""""""""""""""""""""""""""""""""""""""""""""""""""""""""""""""""""""""""""""""""""""""""""""""""""""""""""""""""""""""""""""""""""""""""""""""""""""""""""""""""""""""""""""""""""""""""""""""""""""""""""""""""""""""""""""""""""""""""""""""""""""""""""""""""""""""""""""""""""""""""""""""""""""""""""""""""""""""""""""""""""""""""""""""""""""""""""""""""""""""""""""""""""""""""""""""""""""""""""""""""""""""""""""""""""""""""""""""""""""""""""""""""""""""""""""""""""""""""""""""""""""""""""""""""""""""""""""""""""""""""""""""""""""""""""""""""""""""""""""""""""""""""""""""""""""""""""""""""""""""""""""""""""""""""""""""""""""""""""""""""""""""""""""""""""""""""""""""""""""""""""""""""""""""""""""""""""""""""""""""""""""""""""""""""""""""""""""""""""""""""""""""""""""""""""""""""""""""""""""""""""""""""""""""""""""""""""""""""""""""""""""""""""""""""""""""""""""""""""""""""""""""""""""""""""""""""""""""""""""""""""""""""""""""""""""""""""""""""""""""""""""""""""""""""""""""""""""""""""""""""""""""""""""""""""""""""""""""""""""""""""""""""""""""""""""""""""""""""""""""""""""""""""""""""""""""""""""""""""""""""""""""""""""""""""""""""""""""""""""""""""""""""""""""""""""""""""""""""""""""""""""""""""""""""""""""""""""""""""""""""""""""""""""""""""""""""""""""""""""""""""""""""""""""""""""""""""""""""""""""""""""""""""""""""""""""""""""""""""""""""""""""""""""""""""""""""""""""""""""""""""""""""""""""""""""""""""""""""""""""""""""""""""""""""""""""""""""""""""""""""""""""""""""""""""""""""""""""""""""""""""""""""""""""""""""""""""""""""""""""""""""""""""""""""""""""""""""""""""""""""""""""""""""""""""""""""""""""""""""""""""""""""""""""""""""""""""""""""""""""""""""""""""""""""""""""""""""""""""""""""""""""""""""""""""""""""""""""""""""""""""""""""""""""""""""""""""""""""""""""""""""""""""""""""""""""""""""""""""""""""""""""""""""""""""""""""""""""""""""""""""""""""""""""""""""""""""""""""""""""""""""""""""""""""""""""""""""""""""""""""""""""""""""""""""""""""""""""""""""""""""""""""""""""""""""""""""""""""""""""""""""""""""""""""""""""""""""""""""""""""""""""""""""""""""""""""""""""""""""""""""""""""""""""""""""""""""""""""""""""""""""""""""""""""""""""""""""""""""""""""""""""""""""""""""""""""""""""""""""""""""""""""""""""""""""""""""""""""""""""""""""""""""""""""""""""""""""""""""""""""""""""""""""""""""""""""""""""""""""""""""""""""""""""""""""""""""""""""""""""""""""""""""""""""""""""""""""""""""""""""""""""""""""""""""""""""""""""""""""""""""""""""""""""""""""""""""""""""""""""""""""""""""""""""""""""""""""""""""""""""""""""""""""""""""""""""""""""""""""""""""""""""""""""""""""""""""""""""""""""""""""""""""""""""""""""""""""""""""""""""""""""""""""""""""""""""""""""""""""""""""""""""""""""""""""""""""""""""""""""""""""""""""""""""""""""""""""""""""""""""""""""""""""""""""""""""""""""""""""""""""""""""""""""""""""""""""""""""""""""""""""""""""""""""""""""""""""""""""""""""""""""""""""""""""""""""""""""""""""""""""""""""""""""""""""""""""""""""""""""""""""""""""""""""""""""""""""""""""""""""""""""""""""""""""""""""""""""""""""""""""""""""""""""""""""""""""""""""""""""""""""""""""""""""""""""""""""""""""""""""""""""""""""""""""""""""""""""""""""""""""""""""""""""""""""""""""""""""""""""""""""""""""""""""""""""""""""""""""""""""""""""""""""""""""""""""""""""""""""""""""""""""""""""""""""""""""""""""""""""""""""""""""""""""""""""""""""""""""""""""""""""""""""""""""""""""""""""""""""""""""""""""""""""""""""""""""""""""""""""""""""""""""""""""""""""""""""""""""""""""""""""""""""""""""""""""""""""""""""""""""""""""""""""""""""""""""""""""""""""""""""""""""""""""""""""""""""""""""""""""""""""""""""""""""""""""""""""""""""""""""""""""""""""""""""""""""""""""""""""""""""""""""""""""""""""""""""""""""""""""""""""""""""""""""""""""""""""""""""""""""""""""""""""""""""""""""""""""""""""""""""""""""""""""""""""""""""""""""""""""""""""""""""""""""""""""""""""""""""""""""""""""""""""""""""""""""""""""""""""""""""""""""""""""""""""""""""""""""""""""""""""""""""""""""""""""""""""""""""""""""""""""""""""""""""""""""""""""""""""""""""""""""""""""""""""""""""""""""""""""""""""""""""""""""""""""""""""""""""""""""""""""""""""""""""""""""""""""""""""""""""""""""""""""""""""""""""""""""""""""""""""""""""""""""""""""""""""""""""""""""""""""""""""""""""""""""""""""""""""""""""""""""""""""""""""""""""""""""""""""""""""""""""""""""""""""""""""""""""""""""""""""""""""""""""""""""""""""""""""""""""""""""""""""""""""""""""""""""""""""""""""""""""""""""""""""""""""""""""""""""""""""""""""""""""""""""""""""""""""""""""""""""""""""""""""""""""""""""""""""""""""""""""""""""""""""""""""""""""""""""""""""""""""""""""""""""""""""""""""""""""""""""""""""""""""""""""""""""""""""""""""""""""""""""""""""""""""""""""""""""""""""""""""""""""""""""""""""""""""""""""""""""""""""""""""""""""""""""""""""""""""""""""""""""""""""""""""""""""""""""""""""""""""""""""""""""""""""""""""""""""""""""""""""""""""""""""""""""""""""""""""""""""""""""""""""""""""""""""""""""""""""""""""""""""""""""""""""""""""""""""""""""""""""""""""""""""""""""""""""""""""""""""""""""""""""""""""""""""""""""""""""""""""""""""""""""""""""""""""""""""""""""""""""""""""""""""""""""""""""""""""""""""""""""""""""""""""""""""""""""""""""""""""""""""""""""""""""""""""""""""""""""""""""""""""""""""""""""""""""""""""""""""""""""""""""""""""""""""""""""""""""""""""""""""""""""""""""""""""""""""""""""""""""""""""""""""""""""""""""""""""""""""""""""""""""""""""""""""""""""""""""""""""""""""""""""""""""""""""""""""""""""""""""""""""""""""""""""""""""""""""""""""""""""""""""""""""""""""""""""""""""""""""""""""""""""""""""""""""""""""""""""""""""""""""""""""""""""""""""""""""""""""""""""""""""""""""""""""""""""""""""""""""""""""""""""""""""""""""""""""""""""""""""""""""""""""""""""""""""""""""""""""""""""""""""""""""""""""""""""""""""""""""""""""""""""""""""""""""""""""""""""""""""""""""""""""""""""""""""""""""""""""""""""""""""""""""""""""""""""""""""""""""""""""""""""""""""""""""""""""""""""""""""""""""""""""""""""""""""""""""""""""""""""""""""""""""""""""""""""""""""""""""""""""""""""""""""""""""""""""""""""""""""""""""""""""""""""""""""""""""""""""""""""""""""""""""""""""""""""""""""""""""""""""""""""""""""""""""""""""""""""""""""""""""""""""""""""""""""""""""""""""""""""""""""""""""""""""""""""""""""""""""""""""""""""""""""""""""""""""""""""""""""""""""""""""""""""""""""""""""""""""""""""""""""""""""""""""""""""""""""""""""""""""""""""""""""""""""""""""""""""""""""""""""""""""""""""""""""""""""""""""""""""""""""""""""""""""""""""""""""""""""""""""""""""""""""""""""""""""""""""""""""""""""""""""""""""""""""""""""""""""""""""""""""""""""""""""""""""""""""""""""""""""""""""""""""""""""""""""""""""""""""""""""""""""""""""""""""""""""""""""""""""""""""""""""""""""""""""""""""""""""""""""""""""""""""""""""""""""""""""""""""""""""""""""""""""""""""""""""""""""""""""""""""""""""""""""""""""""""""""""""""""""""""""""""""""""""""""""""""""""""""""""""""""""""""""""""""""""""""""""""""""""""""""""""""""""""""""""""""""""""""""""""""""""""""""""""""""""""""""""""""""""""""""""""""""""""""""""""""""""""""""""""""""""""""""""""""""""""""""""""""""""""""""""""""""""""""""""""""""""""""""""""""""""""""""""""""""""""""""""""""""""""""""""""""""""""""""""""""""""""""""""""""""""""""""""""""""""""""""""""""""""""""""""""""""""""""""""""""""""""""""""""""""""""""""""""""""""""""""""""""""""""""""""""""""""""""""""""""""""""""""""""""""""""""""""""""""""""""""""""""""""""""""""""""""""""""""""""""""""""""""""""""""""""""""""""""""""""""""""""""""""""""""""""""""""""""""""""""""""""""""""""""""""""""""""""""""""""""""""""""""""""""""""""""""""""""""""""""""""""""""""""""""""""""""""""""""""""""""""""""""""""""""""""""""""""""""""""""""""""""""""""""""""""""""""""""""""""""""""""""""""""""""""""""""""""""""""""""""""""""""""""""""""""""""""""""""""""""""""""""""""""""""""""""""""""""""""""""""""""""""""""""""""""""""""""""""""""""""""""""""""""""""""""""""""""""""""""""""""""""""""""""""""""""""""""""""""""""""""""""""""""""""""""""""""""""""""""""""""""""""""""""""""""""""""""""""""""""""""""""""""""""""""""""""""""""""""""""""""""""""""""""""""""""""""""""""""""""""""""""""""""""""""""""""""""""""""""""""""""""""""""""""""""""""""""""""""""""""""""""""""""""""""""""""""""""""""""""""""""""""""""""""""""""""""""""""""""""""""""""""""""""""""""""""""""""""""""""""""""""""""""""""""""""""""""""""""""""""""""""""""""""""""""""""""""""""""""""""""""""""""""""""""""""""""""""""""""""""""""""""""""""""""""""""""""""""""""""""""""""""""""""""""""""""""""""""""""""""""""""""""""""""""""""""""""""""""""""""""""""""""""""""""""""""""""""""""""""""""""""""""""""""""""""""""""""""""""""""""""""""""""""""""""""""""""""""""""""""""""""""""""""""""""""""""""""""""""""""""""""""""""""""""""""""""""""""""""""""""""""""""""""""""""""""""""""""""""""""""""""""""""""""""""""""""""""""""""""""""""""""""""""""""""""""""""""""""""""""""""""""""""""""""""""""""""""""""""""""""""""""""""""""""""""""""""""""""""""""""""""""""""""""""""""""""""""""""""""""""""""""""""""""""""""""""""""""""""""""""""""""""""""""""""""""""""""""""""""""""""""""""""""""""""""""""""""""""""""""""""""""""""""""""""""""""""""""""""""""""""""""""""""""""""""""""""""""""""""""""""""""""""""""""""""""""""""""""""""""""""""""""""""""""""""""""""""""""""""""""""""""""""""""""""""""""""""""""""""""""""""""""""""""""""""""""""""""""""""""""""""""""""""""""""""""""""""""""""""""""""""""""""""""""""""""""""""""""""""""""""""""""""""""""""""""""""""""""""""""""""""""""""""""""""""""""""""""""""""""""""""""""""""""""""""""""""""""""""""""""""""""""""""""""""""""""""""""""""""""""""""""""""""""""""""""""""""""""""""""""""""""""""""""""""""""""""""""""""""""""""""""""""""""""""""""""""""""""""""""""""""""""""""""""""""""""""""""""""""""""""""""""""""""""""""""""""""""""""""""""""""""""""""""""""""""""""""""""""""""""""""""""""""""""""""""""""""""""""""""""""""""""""""""""""""""""""""""""""""""""""""""""""""""""""""""""""""""""""""""""""""""""""""""""""""""""""""""""""""""""""""""""""""""""""""""""""""""""""""""""""""""""""""""""""""""""""""""""""""""""""""""""""""""""""""""""""""""""""""""""""""""""""""""""""""""""""""""""""""""""""""""""""""""""""""""""""""""""""""""""""""""""""""""""""""""""""""""""""""""""""""""""""""""""""""""""""""""""""""""""""""""""""""""""""""""""""""""""""""""""""""""""""""""""""""""""""""""""""""""""""""""""""""""""""""""""""""""""""""""""""""""""""""""""""""""""""""""""""""""""""""""""""""""""""""""""""""""""""""""""""""""""""""""""""""""""""""""""""""""""""""""""""""""""""""""""""""""""""""""""""""""""""""""""""""""""""""""""""""""""""""""""""""""""""""""""""""""""""""""""""""""""""""""""""""""""""""""""""""""""""""""""""""""""""""""""""""""""""""""""""""""""""""""""""""""""""""""""""""""""""""""""""""""""""""""""""""""""""""""""""""""""""""""""""""""""""""""""""""""""""""""""""""""""""""""""""""""""""""""""""""""""""""""""""""""""""""""""""""""""""""""""""""""""""""""""""""""""""""""""""""""""""""""""""""""""""""""""""""""""""""""""""""""""""""""""""""""""""""""""""""""""""""""""""""""""""""""""""""""""""""""""""""""""""""""""""""""""""""""""""""""""""""""""""""""""""""""""""""""""""""""""""""""""""""""""""""""""""""""""""""""""""""""""""""""""""""""""""""""""""""""""""""""""""""""""""""""""""""""""""""""""""""""""""""""""""""""""""""""""""""""""""""""""""""""""""""""""""""""""""""""""""""""""""""""""""""""""""""""""""""""""""""""""""""""""""""""""""""""""""""""""""""""""""""""""""""""""""""""""""""""""""""""""""""""""""""""""""""""""""""""""""""""""""""""""""""""""""""""""""""""""""""""""""""""""""""""""""""""""""""""""""""""""""""""""""""""""""""""""""""""""""""""""""""""""""""""""""""""""""""""""""""""""""""""""""""""""""""""""""""""""""""""""""""""""""""""""""""""""""""""""""""""""""""""""""""""""""""""""""""""""""""""""""""""""""""""""""""""""""""""""""""""""""""""""""""""""""""""""""""""""""""""""""""""""""""""""""""""""""""""""""""""""""""""""""""""""""""""""""""""""""""""""""""""""""""""""""""""""""""""""""""""""""""""""""""""""""""""""""""""""""""""""""""""""""""""""""""""""""""""""""""""""""""""""""""""""""""""""""""""""""""""""""""""""""""""""""""""""""""""""""""""""""""""""""""""""""""""""""""""""""""""""""""""""""""""""""""""""""""""""""""""""""""""""""""""""""""""""""""""""""""""""""""""""""""""""""""""""""""""""""""""""""""""""""""""""""""""""""""""""""""""""""""""""""""""""""""""""""""""""""""""""""""""""""""""""""""""""""""""""""""""""""""""""""""""""""""""""""""""""""""""""""""""""""""""""""""""""""""""""""""""""""""""""""""""""""""""""""""""""""""""""""""""""""""""""""""""""""""""""""""""""""""""""""""""""""""""""""""""""""""""""""""""""""""""""""""""""""""""""""""""""""""""""""""""""""""""""""""""""""""""""""""""""""""""""""""""""""""""""""""""""""""""""""""""""""""""""""""""""""""""""""""""""""""""""""""""""""""""""""""""""""""""""""""""""""""""""""""""""""""""""""""""""""""""""""""""""""""""""""""""""""""""""""""""""""""""""""""""""""""""""""""""""""""""""""""""""""""""""""""""""""""""""""""""""""""""""""""""""""""""""""""""""""""""""""""""""""""""""""""""""""""""""""""""""""""""""""""""""""""""""""""""""""""""""""""""""""""""""""""""""""""""""""""""""""""""""""""""""""""""""""""""""""""""""""""""""""""""""""""""""""""""""""""""""""""""""""""""""""""""""""""""""""""""""""""""""""""""""""""""""""""""""""""""""""""""""""""""""""""""""""""""""""""""""""""""""""""""""""""""""""""""""""""""""""""""""""""""""""""""""""""""""""""""""""""""""""""""""""""""""""""""""""""""""""""""""""""""""""""""""""""""""""""""""""""""""""""""""""""""""""""""""""""""""""""""""""""""""""""""""""""""""""""""""""""""""""""""""""""""""""""""""""""""""""""""""""""""""""""""""""""""""""""""""""""""""""""""""""""""""""""""""""""""""""""""""""""""""""""""""""""""""""""""""""""""""""""""""""""""""""""""""""""""""""""""""""""""""""""""""""""""""""""""""""""""""""""""""""""""""""""""""""""""""""""""""""""""""""""""""""""""""""""""""""""""""""""""""""""""""""""""""""""""""""""""""""""""""""""""""""""""""""""""""""""""""""""""""""""""""""""""""""""""""""""""""""""""""""""""""""""""""""""""""""""""""""""""""""""""""""""""""""""""""""""""""""""""""""""""""""""""""""""""""""""""""""""""""""""""""""""""""""""""""""""""""""""""""""""""""""""""""""""""""""""""""""""""""""""""""""""""""""""""""""""""""""""""""""""""""""""""""""""""""""""""""""""""""""""""""""""""""""""""""""""""""""""""""""""""""""""""""""""""""""""""""""""""""""""""""""""""""""""""""""""""""""""""""""""""""""""""""""""""""""""""""""""""""""""""""""""""""""""""""""""""""""""""""""""""""""""""""""""""""""""""""""""""""""""""""""""""""""""""""""""""""""""""""""""""""""""""""""""""""""""""""""""""""""""""""""""""""""""""""""""""""""""""""""""""""""""""""""""""""""""""""""""""""""""""""""""""""""""""""""""""""""""""""""""""""""""""""""""""""""""""""""""""""""""""""""""""""""""""""""""""""""""""""""""""""""""""""""""""""""""""""""""""""""""""""""""""""""""""""""""""""""""""""""""""""""""""""""""""""""""""""""""""""""""""""""""""""""""""""""""""""""""""""""""""""""""""""""""""""""""""""""""""""""""""""""""""""""""""""""""""""""""""""""""""""""""""""""""""""""""""""""""""""""""""""""""""""""""""""""""""""""""""""""""""""""""""""""""""""""""""""""""""""""""""""""""""""""""""""""""""""""""""""""""""""""""""""""""""""""""""""""""""""""""""""""""""""""""""""""""""""""""""""""""""""""""""""""""""""""""""""""""""""""""""""""""""""""""""""""""""""""""""""""""""""""""""""""""""""""""""""""""""""""""""""""""""""""""""""""""""""""""""""""""""""""""""""""""""""""""""""""""""""""""""""""""""""""""""""""""""""""""""""""""""""""""""""""""""""""""""""""""""""""""""""""""""""""""""""""""""""""""""""""""""""""""""""""""""""""""""""""""""""""""""""""""""""""""""""""""""""""""""""""""""""""""""""""""""""""""""""""""""""""""""""""""""""""""""""""""""""""""""""""""""""""""""""""""""""""""""""""""""""""""""""""""""""""""""""""""""""""""""""""""""""""""""""""""""""""""""""""""""""""""""""""""""""""""""""""""""""""""""""""""""""""""""""""""""""""""""""""""""""""""""""""""""""""""""""""""""""""""""""""""""""""""""""""""""""""""""""""""""""""""""""""""""""""""""""""""""""""""""""""""""""""""""""""""""""""""""""""""""""""""""""""""""""""""""""""""""""""""""""""""""""""""""""""""""""""""""""""""""""""""""""""""""""""""""""""""""""""""""""""""""""""""""""""""""""""""""""""""""""""""""""""""""""""""""""""""""""""""""""""""""""""""""""""""""""""""""""""""""""""""""""""""""""""""""""""""""""""""""""""""""""""""""""""""""""""""""""""""""""""""""""""""""""""""""""""""""""""""""""""""""""""""""""""""""""""""""""""""""""""""""""""""""""""""""""""""""""""""""""""""""""""""""""""""""""""""""""""""""""""""""""""""""""""""""""""""""""""""""""""""""""""""""""""""""""""""""""""""""""""""""""""""""""""""""""""""""""""""""""""""""""""""""""""""""""""""""""""""""""""""""""""""""""""""""""""""""""""""""""""""""""""""""""""""""""""""""""""""""""""""""""""""""""""""""""""""""""""""""""""""""""""""""""""""""""""""""""""""""""""""""""""""""""""""""""""""""""""""""""""""""""""""""""""""""""""""""""""""""""""""""""""""""""""""""""""""""""""""""""""""""""""""""""""""""""""""""""""""""""""""""""""""""""""""""""""""""""""""""""""""""""""""""""""""""""""""""""""""""""""""""""""""""""""""""""""""""""""""""""""""""""""""""""""""""""""""""""""""""""""""""""""""""""""""""""""""""""""""""""""""""""""""""""""""""""""""""""""""""""""""""""""""""""""""""""""""""""""""""""""""""""""""""""""""""""""""""""""""""""""""""""""""""""""""""""""""""""""""""""""""""""""""""""""""""""""""""""""""""""""""""""""""""""""""""""""""""""""""""""""""""""""""""""""""""""""""""""""""""""""""""""""""""""""""""""""""""""""""""""""""""""""""""""""""""""""""""""""""""""""""""""""""""""""""""""""""""""""""""""""""""""""""""""""""""""""""""""""""""""""""""""""""""""""""""""""""""""""""""""""""""""""""""""""""""""""""""""""""""""""""""""""""""""""""""""""""""""""""""""""""""""""""""""""""""""""""""""""""""""""""""""""""""""""""""""""""""""""""""""""""""""""""""""""""""""""""""""""""""""""""""""""""""""""""""""""""""""""""""""""""""""""""""""""""""""""""""""""""""""""""""""""""""""""""""""""""""""""""""""""""""""""""""""""""""""""""""""""""""""""""""""""""""""""""""""""""""""""""""""""""""""""""""""""""""""""""""""""""""""""""""""""""""""""""""""""""""""""""""""""""""""""""""""""""""""""""""""""""""""""""""""""""""""""""""""""""""""""""""""""""""""""""""""""""""""""""""""""""""""""""""""""""""""""""""""""""""""""""""""""""""""""""""""""""""""""""""""""""""""""""""""""""""""""""""""""""""""""""""""""""""""""""""""""""""""""""""""""""""""""""""""""""""""""""""""""""""""""""""""""""""""""""""""""""""""""""""""""""""""""""""""""""""""""""""""""""""""""""""""""""""""""""""""""""""""""""""""""""""""""""""""""""""""""""""""""""""""""""""""""""""""""""""""""""""""""""""""""""""""""""""""""""""""""""""""""""""""""""""""""""""""""""""""""""""""""""""""""""""""""""""""""""""""""""""""""""""""""""""""""""""""""""""""""""""""""""""""""""""""""""""""""""""""""""""""""""""""""""""""""""""""""""""""""""""""""""""""""""""""""""""""""""""""""""""""""""""""""""""""""""""""""""""""""""""""""""""""""""""""""""""""""""""""""""""""""""""""""""""""""""""""""""""""""""""""""""""""""""""""""""""""""""""""""""""""""""""""""""""""""""""""""""""""""""""""""""""""""""""""""""""""""""""""""""""""""""""""""""""""""""""""""""""""""""""""""""""""""""""""""""""""""""""""""""""""""""""""""""""""""""""""""""""""""""""""""""""""""""""""""""""""""""""""""""""""""""""""""""""""""""""""""""""""""""""""""""""""""""""""""""""""""""""""""""""""""""""""""""""""""""""""""""""""""""""""""""""""""""""""""""""""""""""""""""""""""""""""""""""""""""""""""""""""""""""""""""""""""""""""""""""""""""""""""""""""""""""""""""""""""""""""""""""""""""""""""""""""""""""""""""""""""""""""""""""""""""""""""""""""""""""""""""""""""""""""""""""""""""""""""""""""""""""""""""""""""""""""""""""""""""""""""""""""""""""""""""""""""""""""""""""""""""""""""""""""""""""""""""""""""""""""""""""""""""""""""""""""""""""""""""""""""""""""""""""""""""""""""""""""""""""""""""""""""""""""""""""""""""""""""""""""""""""""""""""""""""""""""""""""""""""""""""""""""""""""""""""""""""""""""""""""""""""""""""""""""""""""""""""""""""""""""""""""""""""""""""""""""""""""""""""""""""""""""""""""""""""""""""""""""""""""""""""""""""""""""""""""""""""""""""""""""""""""""""""""""""""""""""""""""""""""""""""""""""""""""""""""""""""""""""""""""""""""""""""""""""""""""""""""""""""""""""""""""""""""""""""""""""""""""""""""""""""""""""""""""""""""""""""""""""""""""""""""""""""""""""""""""""""""""""""""""""""""""""""""""""""""""""""""""""""""""""""""""""""""""""""""""""""""""""""""""""""""""""""""""""""""""""""""""""""""""""""""""""""""""""""""""""""""""""""""""""""""""""""""""""""""""""""""""""""""""""""""""""""""""""""""""""""""""""""""""""""""""""""""""""""""""""""""""""""""""""""""""""""""""""""""""""""""""""""""""""""""""""""""""""""""""""""""""""""""""""""""""""""""""""""""""""""""""""""""""""""""""""""""""""""""""""""""""""""""""""""""""""""""""""""""""""""""""""""""""""""""""""""""""""""""""""""""""""""""""""""""""""""""""""""""""""""""""""""""""""""""""""""""""""""""""""""""""""""""""""""""""""""""""""""""""""""""""""""""""""""""""""""""""""""""""""""""""""""""""""""""""""""""""""""""""""""""""""""""""""""""""""""""""""""""""""""""""""""""""""""""""""""""""""""""""""""""""""""""""""""""""""""""""""""""""""""""""""""""""""""""""""""""""""""""""""""""""""""""""""""""""""""""""""""""""""""""""""""""""""""""""""""""""""""""""""""""""""""""""""""""""""""""""""""""""""""""""""""""""""""""""""""""""""""""""""""""""""""""""""""""""""""""""""""""""""""""""""""""""""""""""""""""""""""""""""""""""""""""""""""""""""""""""""""""""""""""""""""""""""""""""""""""""""""""""""""""""""""""""""""""""""""""""""""""""""""""""""""""""""""""""""""""""""""""""""""""""""""""""""""""""""""""""""""""""""""""""""""""""""""""""""""""""""""""""""""""""""""""""""""""""""""""""""""""""""""""""""""""""""""""""""""""""""""""""""""""""""""""""""""""""""""""""""""""""""""""""""""""""""""""""""""""""""""""""""""""""""""""""""""""""""""""""""""""""""""""""""""""""""""""""""""""""""""""""""""""""""""""""""""""""""""""""""""""""""""""""""""""""""""""""""""""""""""""""""""""""""""""""""""""""""""""""""""""""""""""""""""""""""""""""""""""""""""""""""""""""""""""""""""""""""""""""""""""""""""""""""""""""""""""""""""""""""""""""""""""""""""""""""""""""""""""""""""""""""""""""""""""""""""""""""""""""""""""""""""""""""""""""""""""""""""""""""""""""""""""""""""""""""""""""""""""""""""""""""""""""""""""""""""""""""""""""""""""""""""""""""""""""""""""""""""""""""""""""""""""""""""""""""""""""""""""""""""""""""""""""""""""""""""""""""""""""""""""""""""""""""""""""""""""""""""""""""""""""""""""""""""""""""""""""""""""""""""""""""""""""""""""""""""""""""""""""""""""""""""""""""""""""""""""""""""""""""""""""""""""""""""""""""""""""""""""""""""""""""""""""""""""""""""""""""""""""""""""""""""""""""""""""""""""""""""""""""""""""""""""""""""""""""""""""""""""""""""""""""""""""""""""""""""""""""""""""""""""""""""""""""""""""""""""""""""""""""""""""""""""""""""""""""""""""""""""""""""""""""""""""""""""""""""""""""""""""""""""""""""""""""""""""""""""""""""""""""""""""""""""""""""""""""""""""""""""""""""""""""""""""""""""""""""""""""""""""""""""""""""""""""""""""""""""""""""""""""""""""""""""""""""""""""""""""""""""""""""""""""""""""""""""""""""""""""""""""""""""""""""""""""""""""""""""""""""""""""""""""""""""""""""""""""""""""""""""""""""""""""""""""""""""""""""""""""""""""""""""""""""""""""""""""""""""""""""""""""""""""""""""""""""""""""""""""""""""""""""""""""""""""""""""""""""""""""""""""""""""""""""""""""""""""""""""""""""""""""""""""""""""""""""""""""""""""""""""""""""""""""""""""""""""""""""""""""""""""""""""""""""""""""""""""""""""""""""""""""""""""""""""""""""""""""""""""""""""""""""""""""""""""""""""""""""""""""""""""""""""""""""""""""""""""""""""""""""""""""""""""""""""""""""""""""""""""""""""""""""""""""""""""""""""""""""""""""""""""""""""""""""""""""""""""""""""""""""""""""""""""""""""""""""""""""""""""""""""""""""""""""""""""""""""""""""""""""""""""""""""""""""""""""""""""""""""""""""""""""""""""""""""""""""""""""""""""""""""""""""""""""""""""""""""""""""""""""""""""""""""""""""""""""""""""""""""""""""""""""""""""""""""""""""""""""""""""""""""""""""""""""""""""""""""""""""""""""""""""""""""""""""""""""""""""""""""""""""""""""""""""""""""""""""""""""""""""""""""""""""""""""""""""""""""""""""""""""""""""""""""""""""""""""""""""""""""""""""""""""""""""""""""""""""""""""""""""""""""""""""""""""""""""""""""""""""""""""""""""""""""""""""""""""""""""""""""""""""""""""""""""""""""""""""""""""""""""""""""""""""""""""""""""""""""""""""""""""""""""""""""""""""""""""""""""""""""""""""""""""""""""""""""""""""""""""""""""""""""""""""""""""""""""""""""""""""""""""""""""""""""""""""""""""""""""""""""""""""""""""""""""""""""""""""""""""""""""""""""""""""""""""""""""""""""""""""""""""""""""""""""""""""""""""""""""""""""""""""""""""""""""""""""""""""""""""""""""""""""""""""""""""""""""""""""""""""""""""""""""""""""""""""""""""""""""""""""""""""""""""""""""""""""""""""""""""""""""""""""""""""""""""""""""""""""""""""""""""""""""""""""""""""""""""""""""""""""""""""""""""""""""""""""""""""""""""""""""""""""""""""""""""""""""""""""""""""""""""""""""""""""""""""""""""""""""""""""""""""""""""""""""""""""""""""""""""""""""""""""""""""""""""""""""""""""""""""""""""""""""""""""""""""""""""""""""""""""""""""""""""""""""""""""""""""""""""""""""""""""""""""""""""""""""""""""""""""""""""""""""""""""""""""""""""""""""""""""""""""""""""""""""""""""""""""""""""""""""""""""""""""""""""""""""""""""""""""""""""""""""""""""""""""""""""""""""""""""""""""""""""""""""""""""""""""""""""""""""""""""""""""""""""""""""""""""""""""""""""""""""""""""""""""""""""""""""""""""""""""""""""""""""""""""""""""""""""""""""""""""""""""""""""""""""""""""""""""""""""""""""""""""""""""""""""""""""""""""""""""""""""""""""""""""""""""""""""""""""""""""""""""""""""""""""""""""""""""""""""""""""""""""""""""""""""""""""""""""""""""""""""""""""""""""""""""""""""""""""""""""""""""""""""""""""""""""""""""""""""""""""""""""""""""""""""""""""""""""""""""""""""""""""""""""""""""""""""""""""""""""""""""""""""""""""""""""""""""""""""""""""""""""""""""""""""""""""""""""""""""""""""""""""""""""""""""""""""""""""""""""""""""""""""""""""""""""""""""""""""""""""""""""""""""""""""""""""""""""""""""""""""""""""""""""""""""""""""""""""""""""""""""""""""""""""""""""""""""""""""""""""""""""""""""""""""""""""""""""""""""""""""""""""""""""""""""""""""""""""""""""""""""""""""""""""""""""""""""""""""""""""""""""""""""""""""""""""""""""""""""""""""""""""""""""""""""""""""""""""""""""""""""""""""""""""""""""""""""""""""""""""""""""""""""""""""""""""""""""""""""""""""""""""""""""""""""""""""""""""""""""""""""""""""""""""""""""""""""""""""""""""""""""""""""""""""""""""""""""""""""""""""""""""""""""""""""""""""""""""""""""""""""""""""""""""""""""""""""""""""""""""""""""""""""""""""""""""""""""""""""""""""""""""""""""""""""""""""""""""""""""""""""""""""""""""""""""""""""""""""""""""""""""""""""""""""""""""""""""""""""""""""""""""""""""""""""""""""""""""""""""""""""""""""""""""""""""""""""""""""""""""""""""""""""""""""""""""""""""""""""""""""""""""""""""""""""""""""""""""""""""""""""""""""""""""""""""""""""""""""""""""""""""""""""""""""""""""""""""""""""""""""""""""""""""""""""""""""""""""""""""""""""""""""""""""""""""""""""""""""""""""""""""""""""""""""""""""""""""""""""""""""""""""""""""""""""""""""""""""""""""""""""""""""""""""""""""""""""""""""""""""""""""""""""""""""""""""""""""""""""""""""""""""""""""""""""""""""""""""""""""""""""""""""""""""""""""""""""""""""""""""""""""""""""""""""""""""""""""""""""""""""""""""""""""""""""""""""""""""""""""""""""""""""""""""""""""""""""""""""""""""""""""""""""""""""""""""""""""""""""""""""""""""""""""""""""""""""""""""""""""""""""""""""""""""""""""""""""""""""""""""""""""""""""""""""""""""""""""""""""""""""""""""""""""""""""""""""""""""""""""""""""""""""""""""""""""""""""""""""""""""""""""""""""""""""""""""""""""""""""""""""""""""""""""""""""""""""""""""""""""""""""""""""""""""""""""""""""""""""""""""""""""""""""""""""""""""""""""""""""""""""""""""""""""""""""""""""""""""""""""""""""""""""""""""""""""""""""""""""""""""""""""""""""""""""""""""""""""""""""""""""""""""""""""""""""""""""""""""""""""""""""""""""""""""""""""""""""""""""""""""""""""""""""""""""""""""""""""""""""""""""""""""""""""""""""""""""""""""""""""""""""""""""""""""""""""""""""""""""""""""""""""""""""""""""""""""""""""""""""""""""""""""""""""""""""""""""""""""""""""""""""""""""""""""""""""""""""""""""""""""""""""""""""""""""""""""""""""""""""""""""""""""""""""""""""""""""""""""""""""""""""""""""""""""""""""""""""""""""""""""""""""""""""""""""""""""""""""""""""""""""""""""""""""""""""""""""""""""""""""""""""""""""""""""""""""""""""""""""""""""""""""""""""""""""""""""""""""""""""""""""""""""""""""""""""""""""""""""""""""""""""""""""""""""""""""""""""""""""""""""""""""""""""""""""""""""""""""""""""""""""""""""""""""""""""""""""""""""""""""""""""""""""""""""""""""""""""""""""""""""""""""""""""""""""""""""""""""""""""""""""""""""""""""""""""""""""""""""""""""""""""""""""""""""""""""""""""""""""""""""""""""""""""""""""""""""""""""""""""""""""""""""""""""""""""""""""""""""""""""""""""""""""""""""""""""""""""""""""""""""""""""""""""""""""""""""""""""""""""""""""""""""""""""""""""""""""""""""""""""""""""""""""""""""""""""""""""""""""""""""""""""""""""""""""""""""""""""""""""""""""""""""""""""""""""""""""""""""""""""""""""""""""""""""""""""""""""""""""""""""""""""""""""""""""""""""""""""""""""""""""""""""""""""""""""""""""""""""""""""""""""""""""""""""""""""""""""""""""""""""""""""""""""""""""""""""""""""""""""""""""""""""""""""""""""""""""""""""""""""""""""""""""""""""""""""""""""""""""""""""""""""""""""""""""""""""""""""""""""""""""""""""""""""""""""""""""""""""""""""""""""""""""""""""""""""""""""""""""""""""""""""""""""""""""""""""""""""""""""""""""""""""""""""""""""""""""""""""""""""""""""""""""""""""""""""""""""""""""""""""""""""""""""""""""""""""""""""""""""""""""""""""""""""""""""""""""""""""""""""""""""""""""""""""""""""""""""""""""""""""""""""""""""""""""""""""""""""""""""""""""""""""""""""""""""""""""""""""""""""""""""""""""""""""""""""""""""""""""""""""""""""""""""""""""""""""""""""""""""""""""""""""""""""""""""""""""""""""""""""""""""""""""""""""""""""""""""""""""""""""""""""""""""""""""""""""""""""""""""""""""""""""""""""""""""""""""""""""""""""""""""""""""""""""""""""""""""""""""""""""""""""""""""""""""""""""""""""""""""""""""""""""""""""""""""""""""""""""""""""""""""""""""""""""""""""""""""""""""""""""""""""""""""""""""""""""""""""""""""""""""""""""""""""""""""""""""""""""""""""""""""""""""""""""""""""""""""""""""""""""""""""""""""""""""""""""""""""""""""""""""""""""""""""""""""""""""""""""""""""""""""""""""""""""""""""""""""""""""""""""""""""""""""""""""""""""""""""""""""""""""""""""""""""""""""""""""""""""""""""""""""""""""""""""""""""""""""""""""""""""""""""""""""""""""""""""""""""""""""""""""""""""""""""""""""""""""""""""""""""""""""""""""""""""""""""""""""""""""""""""""""""""""""""""""""""""""""""""""""""""""""""""""""""""""""""""""""""""""""""""""""""""""""""""""""""""""""""""""""""""""""""""""""""""""""""""""""""""""""""""""""""""""""""""""""""""""""""""""""""""""""""""""""""""""""""""""""""""""""""""""""""""""""""""""""""""""""""""""""""""""""""""""""""""""""""""""""""""""""""""""""""""""""""""""""""""""""""""""""""""""""""""""""""""""""""""""""""""""""""""""""""""""""""""""""""""""""""""""""""""""""""""""""""""""""""""""""""""""""""""""""""""""""""""""""""""""""""""""""""""""""""""""""""""""""""""""""""""""""""""""""""""""""""""""""""""""""""""""""""""""""""""""""""""""""""""""""""""""""""""""""""""""""""""""""""""""""""""""""""""""""""""""""""""""""""""""""""""""""""""""""""""""""""""""""""""""""""""""""""""""""""""""""""""""""""""""""""""""""""""""""""""""""""""""""""""""""""""""""""""""""""""""""""""""""""""""""""""""""""""""""""""""""""""""""""""""""""""""""""""""""""""""""""""""""""""""""""""""""""""""""""""""""""""""""""""""""""""""""""""""""""""""""""""""""""""""""""""""""""""""""""""""""""""""""""""""""""""""""""""""""""""""""""""""""""""""""""""""""""""""""""""""""""""""""""""""""""""""""""""""""""""""""""""""""""""""""""""""""""""""""""""""""""""""""""""""""""""""""""""""""""""""""""""""""""""""""""""""""""""""""""""""""""""""""""""""""""""""""""""""""""""""""""""""""""""""""""""""""""""""""""""""""""""""""""""""""""""""""""""""""""""""""""""""""""""""""""""""""""""""""""""""""""""""""""""""""""""""""""""""""""""""""""""""""""""""""""""""""""""""""""""""""""""""""""""""""""""""""""""""""""""""""""""""""""""""""""""""""""""""""""""""""""""""""""""""""""""""""""""""""""""""""""""""""""""""""""""""""""""""""""""""""""""""""""""""""""""""""""""""""""""""""""""""""""""""""""""""""""""""""""""""""""""""""""""""""""""""""""""""""""""""""""""""""""""""""""""""""""""""""""""""""""""""""""""""""""""""""""""""""""""""""""""""""""""""""""""""""""""""""""""""""""""""""""""""""""""""""""""""""""""""""""""""""""""""""""""""""""""""""""""""""""""""""""""""""""""""""""""""""""""""""""""""""""""""""""""""""""""""""""""""""""""""""""""""""""""""""""""""""""""""""""""""""""""""""""""""""""""""""""""""""""""""""""""""""""""""""""""""""""""""""""""""""""""""""""""""""""""""""""""""""""""""""""""""""""""""""""""""""""""""""""""""""""""""""""""""""""""""""""""""""""""""""""""""""""""""""""""""""""""""""""""""""""""""""""""""""""""""""""""""""""""""""""""""""""""""""""""""""""""""""""""""""""""""""""""""""""""""""""""""""""""""""""""""""""""""""""""""""""""""""""""""""""""""""""""""""""""""""""""""""""""""""""""""""""""""""""""""""""""""""""""""""""""""""""""""""""""""""""""""""""""""""""""""""""""""""""""""""""""""""""""""""""""""""""""""""""""""""""""""""""""""""""""""""""""""""""""""""""""""""""""""""""""""""""""""""""""""""""""""""""""""""""""""""""""""""""""""""""""""""""""""""""""""""""""""""""""""""""""""""""""""""""""""""""""""""""""""""""""""""""""""""""""""""""""""""""""""""""""""""""""""""""""""""""""""""""""""""""""""""""""""""""""""""""""""""""""""""""""""""""""""""""""""""""""""""""""""""""""""""""""""""""""""""""""""""""""""""""""""""""""""""""""""""""""""""""""""""""""""""""""""""""""""""""""""""""""""""""""""""""""""""""""""""""""""""""""""""""""""""""""""""""""""""""""""""""""""""""""""""""""""""""""""""""""""""""""""""""""""""""""""""""""""""""""""""""""""""""""""""""""""""""""""""""""""""""""""""""""""""""""""""""""""""""""""""""""""""""""""""""""""""""""""""""""""""""""""""""""""""""""""""""""""""""""""""""""""""""""""""""""""""""""""""""""""""""""""""""""""""""""""""""""""""""""""""""""""""""""""""""""""""""""""""""""""""""""""""""""""""""""""""""""""""""""""""""""""""""""""""""""""""""""""""""""""""""""""""""""""""""""""""""""""""""""""""""""""""""""""""""""""""""""""""""""""""""""""""""""""""""""""""""""""""""""""""""""""""""""""""""""""""""""""""""""""""""""""""""""""""""""""""""""""""""""""""""""""""""""""""""""""""""""""""""""""""""""""""""""""""""""""""""""""""""""""""""""""""""""""""""""""""""""""""""""""""""""""""""""""""""""""""""""""""""""""""""""""""""""""""""""""""""""""""""""""""""""""""""""""""""""""""""""""""""""""""""""""""""""""""""""""""""""""""""""""""""""""""""""""""""""""""""""""""""""""""""""""""""""""""""""""""""""""""""""""""""""""""""""""""""""""""""""""""""""""""""""""""""""""""""""""""""""""""""""""""""""""""""""""""""""""""""""""""""""""""""""""""""""""""""""""""""""""""""""""""""""""""""""""""""""""""""""""""""""""""""""""""""""""""""""""""""""""""""""""""""""""""""""""""""""""""""""""""""""""""""""""""""""""""""""""""""""""""""""""""""""""""""""""""""""""""""""""""""""""""""""""""""""""""""""""""""""""""""""""""""""""""""""""""""""""""""""""""""""""""""""""""""""""""""""""""""""""""""""""""""""""""""""""""""""""""""""""""""""""""""""""""""""""""""""""""""""""""""""""""""""""""""""""""""""""""""""""""""""""""""""""""""""""""""""""""""""""""""""""""""""""""""""""""""""""""""""""""""""""""""""""""""""""""""""""""""""""""""""""""""""""""""""""""""""""""""""""""""""""""""""""""""""""""""""""""""""""""""""""""""""""""""""""""""""""""""""""""""""""""""""""""""""""""""""""""""""""""""""""""""""""""""""""""""""""""""""""""""""""""""""""""""""""""""""""""""""""""""""""""""""""""""""""""""""""""""""""""""""""""""""""""""""""""""""""""""""""""""""""""""""""""""""""""""""""""""""""""""""""""""""""""""""""""""""""""""""""""""""""""""""""""""""""""""""""""""""""""""""""""""""""""""""""""""""""""""""""""""""""""""""""""""""""""""""""""""""""""""""""""""""""""""""""""""""""""""""""""""""""""""""""""""""""""""""""""""""""""""""""""""""""""""""""""""""""""""""""""""""""""""""""""""""""""""""""""""""""""""""""""""""""""""""""""""""""""""""""""""""""""""""""""""""""""""""""""""""""""""""""""""""""""""""""""""""""""""""""""""""""""""""""""""""""""""""""""""""""""""""""""""""""""""""""""""""""""""""""""""""""""""""""""""""""""""""""""""""""""""""""""""""""""""""""""""""""""""""""""""""""""""""""""""""""""""""""""""""""""""""""""""""""""""""""""""""""""""""""""""""""""""""""""""""""""""""""""""""""""""""""""""""""""""""""""""""""""""""""""""""""""""""""""""""""""""""""""""""""""""""""""""""""""""""""""""""""""""""""""""""""""""""""""""""""""""""""""""""""""""""""""""""""""""""""""""""""""""""""""""""""""""""""""""""""""""""""""""""""""""""""""""""""""""""""""""""""""""""""""""""""""""""""""""""""""""""""""""""""""""""""""""""""""""""""""""""""""""""""""""""""""""""""""""""""""""""""""""""""""""""""""""""""""""""""""""""""""""""""""""""""""""""""""""""""""""""""""""""""""""""""""""""""""""""""""""""""""""""""""""""""""""""""""""""""""""""""""""""""""""""""""""""""""""""""""""""""""""""""""""""""""""""""""""""""""""""""""""""""""""""""""""""""""""""""""""""""""""""""""""""""""""""""""""""""""""""""""""""""""""""""""""""""""""""""""""""""""""""""""""""""""""""""""""""""""""""""""""""""""""""""""""""""""""""""""""""""""""""""""""""""""""""""""""""""""""""""""""""""""""""""""""""""""""""""""""""""""""""""""""""""""""""""""""""""""""""""""""""""""""""""""""""""""""""""""""""""""""""""""""""""""""""""""""""""""""""""""""""""""""""""""""""""""""""""""""""""""""""""""""""""""""""""""""""""""""""""""""""""""""""""""""""""""""""""""""""""""""""""""""""""""""""""""""""""""""""""""""""""""""""""""""""""""""""""""""""""""""""""""""""""""""""""""""""""""""""""""""""""""""""""""""""""""""""""""""""""""""""""""""""""""""""""""""""""""""""""""""""""""""""""""""""""""""""""""""""""""""""""""""""""""""""""""""""""""""""""""""""""""""""""""""""""""""""""""""""""""""""""""""""""""""""""""""""""""""""""""""""""""""""""""""""""""""""""""""""""""""""""""""""""""""""""""""""""""""""""""""""""""""""""""""""""""""""""""""""""""""""""""""""""""""""""""""""""""""""""""""""""""""""""""""""""""""""""""""""""""""""""""""""""""""""""""""""""""""""""""""""""""""""""""""""""""""""""""""""""""""""""""""""""""""""""""""""""""""""""""""""""""""""""""""""""""""""""""""""""""""""""""""""""""""""""""""""""""""""""""""""""""""""""""""""""""""""""""""""""""""""""""""""""""""""""""""""""""""""""""""""""""""""""""""""""""""""""""""""""""""""""""""""""""""""""""""""""""""""""""""""""""""""""""""""""""""""""""""""""""""""""""""""""""""""""""""""""""""""""""""""""""""""""""""""""""""""""""""""""""""""""""""""""""""""""""""""""""""""""""""""""""""""""""""""""""""""""""""""""""""""""""""""""""""""""""""""""""""""""""""""""""""""""""""""""""""""""""""""""""""""""""""""""""""""""""""""""""""""""""""""""""""""""""""""""""""""""""""""""""""""""""""""""""""""""""""""""""""""""""""""""""""""""""""""""""""""""""""""""""""""""""""""""""""""""""""""""""""""""""""""""""""""""""""""""""""""""""""""""""""""""""""""""""""""""""""""""""""""""""""""""""""""""""""""""""""""""""""""""""""""""""""""""""""""""""""""""""""""""""""""""""""""""""""""""""""""""""""""""""""""""""""""""""""""""""""""""""""""""""""""""""""""""""""""""""""""""""""""""""""""""""""""""""""""""""""""""""""""""""""""""""""""""""""""""""""""""""""""""""""""""""""""""""""""""""""""""""""""""""""""""""""""""""""""""""""""""""""""""""""""""""""""""""""""""""""""""""""""""""""""""""""""""""""""""""""""""""""""""""""""""""""""""""""""""""""""""""""""""""""""""""""""""""""""""""""""""""""""""""""""""""""""""""""""""""""""""""""""""""""""""""""""""""""""""""""""""""""""""""""""""""""""""""""""""""""""""""""""""""""""""""""""""""""""""""""""""""""""""""""""""""""""""""""""""""""""""""""""""""""""""""""""""""""""""""""""""""""""""""""""""""""""""""""""""""""""""""""""""""""""""""""""""""""""""""""""""""""""""""""""""""""""""""""""""""""""""""""""""""""""""""""""""""""""""""""""""""""""""""""""""""""""""""""""""""""""""""""""""""""""""""""""""""""""""""""""""""""""""""""""""""""""""""""""""""""""""""""""""""""""""""""""""""""""""""""""""""""""""""""""""""""""""""""""""""""""""""""""""""""""""""""""""""""""""""""""""""""""""""""""""""""""""""""""""""""""""""""""""""""""""""""""""""""""""""""""""""""""""""""""""""""""""""""""""""""""""""""""""""""""""""""""""""""""""""""""""""""""""""""""""""""""""""""""""""""""""""""""""""""""""""""""""""""""""""""""""""""""""""""""""""""""""""""""""""""""""""""""""""""""""""""""""""""""""""""""""""""""""""""""""""""""""""""""""""""""""""""""""""""""""""""""""""""""""""""""""""""""""""""""""""""""""""""""""""""""""""""""""""""""""""""""""""""""""""""""""""""""""""""""""""""""""""""""""""""""""""""""""""""""""""""""""""""""""""""""""""""""""""""""""""""""""""""""""""""""""""""""""""""""""""""""""""""""""""""""""""""""""""""""""""""""""""""""""""""""""""""""""""""""""""""""""""""""""""""""""""""""""""""""""""""""""""""""""""""""""""""""""""""""""""""""""""""""""""""""""""""""""""""""""""""""""""""""""""""""""""""""""""""""""""""""""""""""""""""""""""""""""""""""""""""""""""""""""""""""""""""""""""""""""""""""""""""""""""""""""""""""""""""""""""""""""""""""""""""""""""""""""""""""""""""""""""""""""""""""""""""""""""""""""""""""""""""""""""""""""""""""""""""""""""""""""""""""""""""""""""""""""""""""""""""""""""""""""""""""""""""""""""""""""""""""""""""""""""""""""""""""""""""""""""""""""""""""""""""""""""""""""""""""""""""""""""""""""""""""""""""""""""""""""""""""""""""""""""""""""""""""""""""""""""""""""""""""""""""""""""""""""""""""""""""""""""""""""""""""""""""""""""""""""""""""""""""""""""""""""""""""""""""""""""""""""""""""""""""""""""""""""""""""""""""""""""""""""""""""""""""""""""""""""""""""""""""""""""""""""""""""""""""""""""""""""""""""""""""""""""""""""""""""""""""""""""""""""""""""""""""""""""""""""""""""""""""""""""""""""""""""""""""""""""""""""""""""""""""""""""""""""""""""""""""""""""""""""""""""""""""""""""""""""""""""""""""""""""""""""""""""""""""""""""""""""""""""""""""""""""""""""""""""""""""""""""""""""""""""""""""""""""""""""""""""""""""""""""""""""""""""""""""""""""""""""""""""""""""""""""""""""""""""""""""""""""""""""""""""""""""""""""""""""""""""""""""""""""""""""""""""""""""""""""""""""""""""""""""""""""""""""""""""""""""""""""""""""""""""""""""""""""""""""""""""""""""""""""""""""""""""""""""""""""""""""""""""""""""""""""""""""""""""""""""""""""""""""""""""""""""""""""""""""""""""""""""""""""""""""""""""""""""""""""""""""""""""""""""""""""""""""""""""""""""""""""""""""""""""""""""""""""""""""""""""""""""""""""""""""""""""""""""""""""""""""""""""""""""""""""""""""""""""""""""""""""""""""""""""""""""""""""""""""""""""""""""""""""""""""""""""""""""""""""""""""""""""""""""""""""""""""""""""""""""""""""""""""""""""""""""""""""""""""""""""""""""""""""""""""""""""""""""""""""""""""""""""""""""""""""""""""""""""""""""""""""""""""""""""""""""""""""""""""""""""""""""""""""""""""""""""""""""""""""""""""""""""""""""""""""""""""""""""""""""""""""""""""""""""""""""""""""""""""""""""""""""""""""""""""""""""""""""""""""""""""""""""""""""""""""""""""""""""""""""""""""""""""""""""""""""""""""""""""""""""""""""""""""""""""""""""""""""""""""""""""""""""""""""""""""""""""""""""""""""""""""""""""""""""""""""""""""""""""""""""""""""""""""""""""""""""""""""""""""""""""""""""""""""""""""""""""""""""""""""""""""""""""""""""""""""""""""""""""""""""""""""""""""""""""""""""""""""""""""""""""""""""""""""""""""""""""""""""""""""""""""""""""""""""""""""""""""""""""""""""""""""""""""""""""""""""""""""""""""""""""""""""""""""""""""""""""""""""""""""""""""""""""""""""""""""""""""""""""""""""""""""""""""""""""""""""""""""""""""""""""""""""""""""""""""""""""""""""""""""""""""""""""""""""""""""""""""""""""""""""""""""""""""""""""""""""""""""""""""""""""""""""""""""""""""""""""""""""""""""""""""""""""""""""""""""""""""""""""""""""""""""""""""""""""""""""""""""""""""""""""""""""""""""""""""""""""""""""""""""""""""""""""""""""""""""""""""""""""""""""""""""""""""""""""""""""""""""""""""""""""""""""""""""""""""""""""""""""""""""""""""""""""""""""""""""""""""""""""""""""""""""""""""""""""""""""""""""""""""""""""""""""""""""""""""""""""""""""""""""""""""""""""""""""""""""""""""""""""""""""""""""""""""""""""""""""""""""""""""""""""""""""""""""""""""""""""""""""""""""""""""""""""""""""""""""""""""""""""""""""""""""""""""""""""""""""""""""""""""""""""""""""""""""""""""""""""""""""""""""""""""""""""""""""""""""""""""""""""""""""""""""""""""""""""""""""""""""""""""""""""""""""""""""""""""""""""""""""""""""""""""""""""""""""""""""""""""""""""""""""""""""""""""""""""""""""""""""""""""""""""""""""""""""""""""""""""""""""""""""""""""""""""""""""""""""""""""""""""""""""""""""""""""""""""""""""""""""""""""""""""""""""""""""""""""""""""""""""""""""""""""""""""""""""""""""""""""""""""""""""""""""""""""""""""""""""""""""""""""""""""""""""""""""""""""""""""""""""""""""""""""""""""""""""""""""""""""""""""""""""""""""""""""""""""""""""""""""""""""""""""""""""""""""""""""""""""""""""""""""""""""""""""""""""""""""""""""""""""""""""""""""""""""""""""""""""""""""""""""""""""""""""""""""""""""""""""""""""""""""""""""""""""""""""""""""""""""""""""""""""""""""""""""""""""""""""""""""""""""""""""""""""""""""""""""""""""""""""""""""""""""""""""""""""""""""""""""""""""""""""""""""""""""""""""""""""""""""""""""""""""""""""""""""""""""""""""""""""""""""""""""""""""""""""""""""""""""""""""""""""""""""""""""""""""""""""""""""""""""""""""""""""""""""""""""""""""""""""""""""""""""""""""""""""""""""""""""""""""""""""""""""""""""""""""""""""""""""""""""""""""""""""""""""""""""""""""""""""""""""""""""""""""""""""""""""""""""""""""""""""""""""""""""""""""""""""""""""""""""""""""""""""""""""""""""""""""""""""""""""""""""""""""""""""""""""""""""""""""""""""""""""""""""""""""""""""""""""""""""""""""""""""""""""""""""""""""""""""""""""""""""""""""""""""""""""""""""""""""""""""""""""""""""""""""""""""""""""""""""""""""""""""""""""""""""""""""""""""""""""""""""""""""""""""""""""""""""""""""""""""""""""""""""""""""""""""""""""""""""""""""""""""""""""""""""""""""""""""""""""""""""""""""""""""""""""""""""""""""""""""""""""""""""""""""""""""""""""""""""""""""""""""""""""""""""""""""""""""""""""""""""""""""""""""""""""""""""""""""""""""""""""""""""""""""""""""""""""""""""""""""""""""""""""""""""""""""""""""""""""""""""""""""""""""""""""""""""""""""""""""""""""""""""""""""""""""""""""""""""""""""""""""""""""""""""""""""""""""""""""""""""""""""""""""""""""""""""""""""""""""""""""""""""""""""""""""""""""""""""""""""""""""""""""""""""""""""""""""""""""""""""""""""""""""""""""""""""""""""""""""""""""""""""""""""""""""""""""""""""""""""""""""""""""""""""""""""""""""""""""""""""""""""""""""""""""""""""""""""""""""""""""""""""""""""""""""""""""""""""""""""""""""""""""""""""""""""""""""""""""""""""""""""""""""""""""""""""""""""""""""""""""""""""""""""""""""""""""""""""""""""""""""""""""""""""""""""""""""""""""""""""""""""""""""""""""""""""""""""""""""""""""""""""""""""""""""""""""""""""""""""""""""""""""""""""""""""""""""""""""""""""""""""""""""""""""""""""""""""""""""""""""""""""""""""""""""""""""""""""""""""""""""""""""""""""""""""""""""""""""""""""""""""""""""""""""""""""""""""""""""""""""""""""""""""""""""""""""""""""""""""""""""""""""""""""""""""""""""""""""""""""""""""""""""""""""""""""""""""""""""""""""""""""""""""""""""""""""""""""""""""""""""""""""""""""""""""""""""""""""""""""""""""""""""""""""""""""""""""""""""""""""""""""""""""""""""""""""""""""""""""""""""""""""""""""""""""""""""""""""""""""""""""""""""""""""""""""""""""""""""""""""""""""""""""""""""""""""""""""""""""""""""""""""""""""""""""""""""""""""""""""""""""""""""""""""""""""""""""""""""""""""""""""""""""""""""""""""""""""""""""""""""""""""""""""""""""""""""""""""""""""""""""""""""""""""""""""""""""""""""""""""""""""""""""""""""""""""""""""""""""""""""""""""""""""""""""""""""""""""""""""""""""""""""""""""""""""""""""""""""""""""""""""""""""""""""""""""""""""""""""""""""""""""""""""""""""""""""""""""""""""""""""""""""""""""""""""""""""""""""""""""""""""""""""""""""""""""""""""""""""""""""""""""""""""""""""""""""""""""""""""""""""""""""""""""""""""""""""""""""""""""""""""""""""""""""""""""""""""""""""""""""""""""""""""""""""""""""""""""""""""""""""""""""""""""""""""""""""""""""""""""""""""""""""""""""""""""""""""""""""""""""""""""""""""""""""""""""""""""""""""""""""""""""""""""""""""""""""""""""""""""""""""""""""""""""""""""""""""""""""""""""""""""""""""""""""""""""""""""""""""""""""""""""""""""""""""""""""""""""""""""""""""""""""""""""""""""""""""""""""""""""""""""""""""""""""""""""""""""""""""""""""""""""""""""""""""""""""""""""""""""""""""""""""""""""""""""""""""""""""""""""""""""""""""""""""""""""""""""""""""""""""""""""""""""""""""""""""""""""""""""""""""""""""""""""""""""""""""""""""""""""""""""""""""""""""""""""""""""""""""""""""""""""""""""""""""""""""""""""""""""""""""""""""""""""""""""""""""""""""""""""""""""""""""""""""""""""""""""""""""""""""""""""""""""""""""""""""""""""""""""""""""""""""""""""""""""""""""""""""""""""""""""""""""""""""""""""""""""""""""""""""""""""""""""""""""""""""""""""""""""""""""""""""""""""""""""""""""""""""""""""""""""""""""""""""""""""""""""""""""""""""""""""""""""""""""""""""""""""""""""""""""""""""""""""""""""""""""""""""""""""""""""""""""""""""""""""""""""""""""""""""""""""""""""""""""""""""""""""""""""""""""""""""""""""""""""""""""""""""""""""""""""""""""""""""""""""""""""""""""""""""""""""""""""""""""""""""""""""""""""""""""""""""""""""""""""""""""""""""""""""""""""""""""""""""""""""""""""""""""""""""""""""""""""""""""""""""""""""""""""""""""""""""""""""""""""""""""""""""""""""""""""""""""""""""""""""""""""""""""""""""""""""""""""""""""""""""""""""""""""""""""""""""""""""""""""""""""""""""""""""""""""""""""""""""""""""""""""""""""""""""""""""""""""""""""""""""""""""""""""""""""""""""""""""""""""""""""""""""""""""""""""""""""""""""""""""""""""""""""""""""""""""""""""""""""""""""""""""""""""""""""""""""""""""""""""""""""""""""""""""""""""""""""""""""""""""""""""""""""""""""""""""""""""""""""""""""""""""""""""""""""""""""""""""""""""""""""""""""""""""""""""""""""""""""""""""""""""""""""""""""""""""""""""""""""""""""""""""""""""""""""""""""""""""""""""""""""""""""""""""""""""""""""""""""""""""""""""""""""""""""""""""""""""""""""""""""""""""""""""""""""""""""""""""""""""""""""""""""""""""""""""""""""""""""""""""""""""""""""""""""""""""""""""""""""""""""""""""""""""""""""""""""""""""""""""""""""""""""""""""""""""""""""""""""""""""""""""""""""""""""""""""""""""""""""""""""""""""""""""""""""""""""""""""""""""""""""""""""""""""""""""""""""""""""""""""""""""""""""""""""""""""""""""""""""""""""""""""""""""""""""""""""""""""""""""""""""""""""""""""""""""""""""""""""""""""""""""""""""""""""""""""""""""""""""""""""""""""""""""""""""""""""""""""""""""""""""""""""""""""""""""""""""""""""""""""""""""""""""""""""""""""""""""""""""""""""""""""""""""""""""""""""""""""""""""""""""""""""""""""""""""""""""""""""""""""""""""""""""""""""""""""""""""""""""""""""""""""""""""""""""""""""""""""""""""""""""""""""""""""""""""""""""""""""""""""""""""""""""""""""""""""""""""""""""""""""""""""""""""""""""""""""""""""""""""""""""""""""""""""""""""""""""""""""""""""""""""""""""""""""""""""""""""""""""""""""""""""""""""""""""""""""""""""""""""""""""""""""""""""""""""""""""""""""""""""""""""""""""""""""""""""""""""""""""""""""""""""""""""""""""""""""""""""""""""""""""""""""""""""""""""""""""""""""""""""""""""""""""""""""""""""""""""""""""""""""""""""""""""""""""""""""""""""""""""""""""""""""""""""""""""""""""""""""""""""""""""""""""""""""""""""""""""""""""""""""""""""""""""""""""""""""""""""""""""""""""""""""""""""""""""""""""""""""""""""""""""""""""""""""""""""""""""""""""""""""""""""""""""""""""""""""""""""""""""""""""""""""""""""""""""""""""""""""""""""""""""""""""""""""""""""""""""""""""""""""""""""""""""""""""""""""""""""""""""""""""""""""""""""""""""""""""""""""""""""""""""""""""""""""""""""""""""""""""""""""""""""""""""""""""""""""""""""""""""""""""""""""""""""""""""""""""""""""""""""""""""""""""""""""""""""""""""""""""""""""""""""""""""""""""""""""""""""""""""""""""""""""""""""""""""""""""""""""""""""""""""""""""""""""""""""""""""""""""""""""""""""""""""""""""""""""""""""""""""""""""""""""""""""""""""""""""""""""""""""""""""""""""""""""""""""""""""""""""""""""""""""""""""""""""""""""""""""""""""""""""""""""""""""""""""""""""""""""""""""""""""""""""""""""""""""""""""""""""""""""""""""""""""""""""""""""""""""""""""""""""""""""""""""""""""""""""""""""""""""""""""""""""""""""""""""""""""""""""""""""""""""""""""""""""""""""""""""""""""""""""""""""""""""""""""""""""""""""""""""""""""""""""""""""""""""""""""""""""""""""""""""""""""""""""""""""""""""""""""""""""""""""""""""""""""""""""""""""""""""""""""""""""""""""""""""""""""""""""""""""""""""""""""""""""""""""""""""""""""""""""""""""""""""""""""""""""""""""""""""""""""""""""""""""""""""""""""""""""""""""""""""""""""""""""""""""""""""""""""""""""""""""""""""""""""""""""""""""""""""""""""""""""""""""""""""""""""""""""""""""""""""""""""""""""""""""""""""""""""""""""""""""""""""""""""""""""""""""""""""""""""""""""""""""""""""""""""""""""""""""""""""""""""""""""""""""""""""""""""""""""""""""""""""""""""""""""""""""""""""""""""""""""""""""""""""""""""""""""""""""""""""""""""""""""""""""""""""""""""""""""""""""""""""""""""""""""""""""""""""""""""""""""""""""""""""""""""""""""""""""""""""""""""""""""""""""""""""""""""""""""""""""""""""""""""""""""""""""""""""""""""""""""""""""""""""""""""""""""""""""""""""""""""""""""""""""""""""""""""""""""""""""""""""""""""""""""""""""""""""""""""""""""""""""""""""""""""""""""""""""""""""""""""""""""""""""""""""""""""""""""""""""""""""""""""""""""""""""""""""""""""""""""""""""""""""""""""""""""""""""""""""""""""""""""""""""""""""""""""""""""""""""""""""""""""""""""""""""""""""""""""""""""""""""""""""""""""""""""""""""""""""""""""""""""""""""""""""""""""""""""""""""""""""""""""""""""""""""""""""""""""""""""""""""""""""""""""""""""""""""""""""""""""""""""""""""""""""""""""""""""""""""""""""""""""""""""""""""""""""""""""""""""""""""""""""""""""""""""""""""""""""""""""""""""""""""""""""""""""""""""""""""""""""""""""""""""""""""""""""""""""""""""""""""""""""""""""""""""""""""""""""""""""""""""""""""""""""""""""""""""""""""""""""""""""""""""""""""""""""""""""""""""""""""""""""""""""""""""""""""""""""""""""""""""""""""""""""""""""""""""""""""""""""""""""""""""""""""""""""""""""""""""""""""""""""""""""""""""""""""""""""""""""""""""""""""""""""""""""""""""""""""""""""""""""""""""""""""""""""""""""""""""""""""""""""""""""""""""""""""""""""""""""""""""""""""""""""""""""""""""""""""""""""""""""""""""""""""""""""""""""""""""""""""""""""""""""""""""""""""""""""""""""""""""""""""""""""""""""""""""""""""""""""""""""""""""""""""""""""""""""""""""""""""""""""""""""""""""""""""""""""""""""""""""""""""""""""""""""""""""""""""""""""""""""""""""""""""""""""""""""""""""""""""""""""""""""""""""""""""""""""""""""""""""""""""""""""""""""""""""""""""""""""""""""""""""""""""""""""""""""""""""""""""""""""""""""""""""""""""""""""""""""""""""""""""""""""""""""""""""""""""""""""""""""""""""""""""""""""""""""""""""""""""""""""""""""""""""""""""""""""""""""""""""""""""""""""""""""""""""""""""""""""""""""""""""""""""""""""""""""""""""""""""""""""""""""""""""""""""""""""""""""""""""""""""""""""""""""""""""""""""""""""""""""""""""""""""""""""""""""""""""""""""""""""""""""""""""""""""""""""""""""""""""""""""""""""""""""""""""""""""""""""""""""""""""""""""""""""""""""""""""""""""""""""""""""""""""""""""""""""""""""""""""""""""""""""""""""""""""""""""""""""""""""""""""""""""""""""""""""""""""""""""""""""""""""""""""""""""""""""""""""""""""""""""""""""""""""""""""""""""""""""""""""""""""""""""""""""""""""""""""""""""""""""""""""""""""""""""""""""""""""""""""""""""""""""""""""""""""""""""""""""""""""""""""""""""""""""""""""""""""""""""""""""""""""""""""""""""""""""""""""""""""""""""""""""""""""""""""""""""""""""""""""""""""""""""""""""""""""""""""""""""""""""""""""""""""""""""""""""""""""""""""""""""""""""""""""""""""""""""""""""""""""""""""""""""""""""""""""""""""""""""""""""""""""""""""""""""""""""""""""""""""""""""""""""""""""""""""""""""""""""""""""""""""""""""""""""""""""""""""""""""""""""""""""""""""""""""""""""""""""""""""""""""""""""""""""""""""""""""""""""""""""""""""""""""""""""""""""""""""""""""""""""""""""""""""""""""""""""""""""""""""""""""""""""""""""""""""""""""""""""""""""""""""""""""""""""""""""""""""""""""""""""""""""""""""""""""""""""""""""""""""""""""""""""""""""""""""""""""""""""""""""""""""""""""""""""""""""""""""""""""""""""""""""""""""""""""""""""""""""""""""""""""""""""""""""""""""""""""""""""""""""""""""""""""""""""""""""""""""""""""""""""""""""""""""""""""""""""""""""""""""""""""""""""""""""""""""""""""""""""""""""""""""""""""""""""""""""""""""""""""""""""""""""""""""""""""""""""""""""""""""""""""""""""""""""""""""""""""""""""""""""""""""""""""""""""""""""""""""""""""""""""""""""""""""""""""""""""""""""""""""""""""""""""""""""""""""""""""""""""""""""""""""""""""""""""""""""""""""""""""""""""""""""""""""""""""""""""""""""""""""""""""""""""""""""""""""""""""""""""""""""""""""""""""""""""""""""""""""""""""""""""""""""""""""""""""""""""""""""""""""""""""""""""""""""""""""""""""""""""""""""""""""""""""""""""""""""""""""""""""""""""""""""""""""""""""""""""""""""""""""""""""""""""""""""""""""""""""""""""""""""""""""""""""""""""""""""""""""""""""""""""""""""""""""""""""""""""""""""""""""""""""""""""""""""""""""""""""""""""""""""""""""""""""""""""""""""""""""""""""""""""""""""""""""""""""""""""""""""""""""""""""""""""""""""""""""""""""""""""""""""""""""""""""""""""""""""""""""""""""""""""""""""""""""""""""""""""""""""""""""""""""""""""""""""""""""""""""""""""""""""""""""""""""""""""""""""""""""""""""""""""""""""""""""""""""""""""""""""""""""""""""""""""""""""""""""""""""""""""""""""""""""""""""""""""""""""""""""""""""""""""""""""""""""""""""""""""""""""""""""""""""""""""""""""""""""""""""""""""""""""""""""""""""""""""""""""""""""""""""""""""""""""""""""""""""""""""""""""""""""""""""""""""""""""""""""""""""""""""""""""""""""""""""""""""""""""""""""""""""""""""""""""""""""""""""""""""""""""""""""""""""""""""""""""""""""""""""""""""""""""""""""""""""""""""""""""""""""""""""""""""""""""""""""""""""""""""""""""""""""""""""""""""""""""""""""""""""""""""""""""""""""""""""""""""""""""""""""""""""""""""""""""""""""""""""""""""""""""""""""""""""""""""""""""""""""""""""""""""""""""""""""""""""""""""""""""""""""""""""""""""""""""""""""""""""""""""""""""""""""""""""""""""""""""""""""""""""""""""""""""""""""""""""""""""""""""""""""""""""""""""""""""""""""""""""""""""""""""""""""""""""""""""""""""""""""""""""""""""""""""""""""""""""""""""""""""""""""""""""""""""""""""""""""""""""""""""""""""""""""""""""""""""""""""""""""""""""""""""""""""""""""""""""""""""""""""""""""""""""""""""""""""""""""""""""""""""""""""""""""""""""""""""""""""""""""""""""""""""""""""""""""""""""""""""""""""""""""""""""""""""""""""""""""""""""""""""""""""""""""""""""""""""""""""""""""""""""""""""""""""""""""""""""""""""""""""""""""""""""""""""""""""""""""""""""""""""""""""""""""""""""""""""""""""""""""""""""""""""""""""""""""""""""""""""""""""""""""""""""""""""""""""""""""""""""""""""""""""""""""""""""""""""""""""""""""""""""""""""""""""""""""""""""""""""""""""""""""""""""""""""""""""""""""""""""""""""""""""""""""""""""""""""""""""""""""""""""""""""""""""""""""""""""""""""""""""""""""""""""""""""""""""""""""""""""""""""""""""""""""""""""""""""""""""""""""""""""""""""""""""""""""""""""""""""""""""""""""""""""""""""""""""""""""""""""""""""""""""""""""""""""""""""""""""""""""""""""""""""""""""""""""""""""""""""""""""""""""""""""""""""""""""""""""""""""""""""""""""""""""""""""""""""""""""""""""""""""""""""""""""""""""""""""""""""""""""""""""""""""""""""""""""""""""""""""""""""""""""""""""""""""""""""""""""""""""""""""""""""""""""""""""""""""""""""""""""""""""""""""""""""""""""""""""""""""""""""""""""""""""""""""""""""""""""""""""""""""""""""""""""""""""""""""""""""""""""""""""""""""""""""""""""""""""""""""""""""""""""""""""""""""""""""""""""""""""""""""""""""""""""""""""""""""""""""""""""""""""""""""""""""""""""""""""""""""""""""""""""""""""""""""""""""""""""""""""""""""""""""""""""""""""""""""""""""""""""""""""""""""""""""""""""""""""""""""""""""""""""""""""""""""""""""""""""""""""""""""""""""""""""""""""""""""""""""""""""""""""""""""""""""""""""""""""""""""""""""""""""""""""""""""""""""""""""""""""""""""""""""""""""""""""""""""""""""""""""""""""""""""""""""""""""""""""""""""""""""""""""""""""""""""""""""""""""""""""""""""""""""""""""""""""""""""""""""""""""""""""""""""""""""""""""""""""""""""""""""""""""""""""""""""""""""""""""""""""""""""""""""""""""""""""""""""""""""""""""""""""""""""""""""""""""""""""""""""""""""""""""""""""""""""""""""""""""""""""""""""""""""""""""""""""""""""""""""""""""""""""""""""""""""""""""""""""""""""""""""""""""""""""""""""""""""""""""""""""""""""""""""""""""""""""""""""""""""""""""""""""""""""""""""""""""""""""""""""""""""""""""""""""""""""""""""""""""""""""""""""""""""""""""""""""""""""""""""""""""""""""""""""""""""""""""""""""""""""""""""""""""""""""""""""""""""""""""""""""""""""""""""""""""""""""""""""""""""""""""""""""""""""""""""""""""""""""""""""""""""""""""""""""""""""""""""""""""""""""""""""""""""""""""""""""""""""""""""""""""""""""""""""""""""""""""""""""""""""""""""""""""""""""""""""""""""""""""""""""""""""""""""""""""""""""""""""""""""""""""""""""""""""""""""""""""""""""""""""""""""""""""""""""""""""""""""""""""""""""""""""""""""""""""""""""""""""""""""""""""""""""""""""""""""""""""""""""""""""""""""""""""""""""""""""""""""""""""""""""""""""""""""""""""""""""""""""""""""""""""""""""""""""""""""""""""""""""""""""""""""""""""""""""""""""""""""""""""""""""""""""""""""""""""""""""""""""""""""""""""""""""""""""""""""""""""""""""""""""""""""""""""""""""""""""""""""""""""""""""""""""""""""""""""""""""""""""""""""""""""""""""""""""""""""""""""""""""""""""""""""""""""""""""""""""""""""""""""""""""""""""""""""""""""""""""""""""""""""""""""""""""""""""""""""""""""""""""""""""""""""""""""""""""""""""""""""""""""""""""""""""""""""""""""""""""""""""""""""""""""""""""""""""""""""""""""""""""""""""""""""""""""""""""""""""""""""""""""""""""""""""""""""""""""""""""""""""""""""""""""""""""""""""""""""""""""""""""""""""""""""""""""""""""""""""""""""""""""""""""""""""""""""""""""""""""""""""""""""""""""""""""""""""""""""""""""""""""""""""""""""""""""""""""""""""""""""""""""""""""""""""""""""""""""""""""""""""""""""""""""""""""""""""""""""""""""""""""""""""""""""""""""""""""""""""""""""""""""""""""""""""""""""""""""""""""""""""""""""""""""""""""""""""""""""""""""""""""""""""""""""""""""""""""""""""""""""""""""""""""""""""""""""""""""""""""""""""""""""""""""""""""""""""""""""""""""""""""""""""""""""""""""""""""""""""""""""""""""""""""""""""""""""""""""""""""""""""""""""""""""""""""""""""""""""""""""""""""""""""""""""""""""""""""""""""""""""""""""""""""""""""""""""""""""""""""""""""""""""""""""""""""""""""""""""""""""""""""""""""""""""""""""""""""""""""""""""""""""""""""""""""""""""""""""""""""""""""""""""""""""""""""""""""""""""""""""""""""""""""""""""""""""""""""""""""""""""""""""""""""""""""""""""""""""""""""""""""""""""""""""""""""""""""""""""""""""""""""""""""""""""""""""""""""""""""""""""""""""""""""""""""""""""""""""""""""""""""""""""""""""""""""""""""""""""""""""""""""""""""""""""""""""""""""""""""""""""""""""""""""""""""""""""""""""""""""""""""""""""""""""""""""""""""""""""""""""""""""""""""""""""""""""""""""""""""""""""""""""""""""""""""""""""""""""""""""""""""""""""""""""""""""""""""""""""""""""""""""""""""""""""""""""""""""""""""""""""""""""""""""""""""""""""""""""""""""""""""""""""""""""""""""""""""""""""""""""""""""""""""""""""""""""""""""""""""""""""""""""""""""""""""""""""""""""""""""""""""""""""""""""""""""""""""""""""""""""""""""""""""""""""""""""""""""""""""""""""""""""""""""""""""""""""""""""""""""""""""""""""""""""""""""""""""""""""""""""""""""""""""""""""""""""""""""""""""""""""""""""""""""""""""""""""""""""""""""""""""""""""""""""""""""""""""""""""""""""""""""""""""""""""""""""""""""""""""""""""""""""""""""""""""""""""""""""""""""""""""""""""""""""""""""""""""""""""""""""""""""""""""""""""""""""""""""""""""""""""""""""""""""""""""""""""""""""""""""""""""""""""""""""""""""""""""""""""""""""""""""""""""""""""""""""""""""""""""""""""""""""""""""""""""""""""""""""""""""""""""""""""""""""""""""""""""""""""""""""""""""""""""""""""""""""""""""""""""""""""""""""""""""""""""""""""""""""""""""""""""""""""""""""""""""""""""""""""""""""""""""""""""""""""""""""""""""""""""""""""""""""""""""""""""""""""""""""""""""""""""""""""""""""""""""""""""""""""""""""""""""""""""""""""""""""""""""""""""""""""""""""""""""""""""""""""""""""""""""""""""""""""""""""""""""""""""""""""""""""""""""""""""""""""""""""""""""""""""""""""""""""""""""""""""""""""""""""""""""""""""""""""""""""""""""""""""""""""""""""""""""""""""""""""""""""""""""""""""""""""""""""""""""""""""""""""""""""""""""""""""""""""""""""""""""""""""""""""""""""""""""""""""""""""""""""""""""""""""""""""""""""""""""""""""""""""""""""""""""""""""""""""""""""""""""""""""""""""""""""""""""""""""""""""""""""""""""""""""""""""""""""""""""""""""""""""""""""""""""""""""""""""""""""""""""""""""""""""""""""""""""""""""""""""""""""""""""""""""""""""""""""""""""""""""""""""""""""""""""""""""""""""""""""""""""""""""""""""""""""""""""""""""""""""""""""""""""""""""""""""""""""""""""""""""""""""""""""""""""""""""""""""""""""""""""""""""""""""""""""""""""""""""""""""""""""""""""""""""""""""""""""""""""""""""""""""""""""""""""""""""""""""""""""""""""""""""""""""""""""""""""""""""""""""""""""""""""""""""""""""""""""""""""""""""""""""""""""""""""""""""""""""""""""""""""""""""""""""""""""""""""""""""""""""""""""""""""""""""""""""""""""""""""""""""""""""""""""""""""""""""""""""""""""""""""""""""""""""""""""""""""""""""""""""""""""""""""""""""""""""""""""""""""""""""""""""""""""""""""""""""""""""""""""""""""""""""""""""""""""""""""""""""""""""""""""""""""""""""""""""""""""""""""""""""""""""""""""""""""""""""""""""""""""""""""""""""""""""""""""""""""""""""""""""""""""""""""""""""""""""""""""""""""""""""""""""""""""""""""""""""""""""""""""""""""""""""""""""""""""""""""""""""""""""""""""""""""""""""""""""""""""""""""""""""""""""""""""""""""""""""""""""""""""""""""""""""""""""""""""""""""""""""""""""""""""""""""""""""""""""""""""""""""""""""""""""""""""""""""""""""""""""""""""""""""""""""""""""""""""""""""""""""""""""""""""""""""""""""""""""""""""""""""""""""""""""""""""""""""""""""""""""""""""""""""""""""""""""""""""""""""""""""""""""""""""""""""""""""""""""""""""""""""""""""""""""""""""""""""""""""""""""""""""""""""""""""""""""""""""""""""""""""""""""""""""""""""""""""""""""""""""""""""""""""""""""""""""""""""""""""""""""""""""""""""""""""""""""""""""""""""""""""""""""""""""""""""""""""""""""""""""""""""""""""""""""""""""""""""""""""""""""""""""""""""""""""""""""""""""""""""""""""""""""""""""""""""""""""""""""""""""""""""""""""""""""""""""""""""""""""""""""""""""""""""""""""""""""""""""""""""""""""""""""""""""""""""""""""""""""""""""""""""""""""""""""""""""""""""""""""""""""""""""""""""""""""""""""""""""""""""""""""""""""""""""""""""""""""""""""""""""""""""""""""""""""""""""""""""""""""""""""""""""""""""""""""""""""""""""""""""""""""""""""""""""""""""""""""""""""""""""""""""""""""""""""""""""""""""""""""""""""""""""""""""""""""""""""""""""""""""""""""""""""""""""""""""""""""""""""""""""""""""""""""""""""""""""""""""""""""""""""""""""""""""""""""""""""""""""""""""""""""""""""""""""""""""""""""""""""""""""""""""""""""""""""""""""""""""""""""""""""""""""""""""""""""""""""""""""""""""""""""""""""""""""""""""""""""""""""""""""""""""""""""""""""""""""""""""""""""""""""""""""""""""""""""""""""""""""""""""""""""""""""""""""""""""""""""""""""""""""""""""""""""""""""""""""""""""""""""""""""""""""""""""""""""""""""""""""""""""""""""""""""""""""""""""""""""""""""""""""""""""""""""""""""""""""""""""""""""""""""""""""""""""""""""""""""""""""""""""""""""""""""""""""""""""""""""""""""""""""""""""""""""""""""""""""""""""""""""""""""""""""""""""""""""""""""""""""""""""""""""""""""""""""""""""""""""""""""""""""""""""""""""""""""""""""""""""""""""""""""""""""""""""""""""""""""""""""""""""""""""""""""""""""""""""""""""""""""""""""""""""""""""""""""""""""""""""""""""""""""""""""""""""""""""""""""""""""""""""""""""""""""""""""""""""""""""""""""""""""""""""""""""""""""""""""""""""""""""""""""""""""""""""""""""""""""""""""""""""""""""""""""""""""""""""""""""""""""""""""""""""""""""""""""""""""""""""""""""""""""""""""""""""""""""""""""""""""""""""""""""""""""""""""""""""""""""""""""""""""""""""""""""""""""""""""""""""""""""""""""""""""""""""""""""""""""""""""""""""""""""""""""""""""""""""""""""""""""""""""""""""""""""""""""""""""""""""""""""""""""""""""""""""""""""""""""""""""""""""""""""""""""""""""""""""""""""""""""""""""""""""""""""""""""""""""""""""""""""""""""""""""""""""""""""""""""""""""""""""""""""""""""""""""""""""""""""""""""""""""""""""""""""""""""""""""""""""""""""""""""""""""""""""""""""""""""""""""""""""""""""""""""""""""""""""""""""""""""""""""""""""""""""""""""""""""""""""""""""""""""""""""""""""""""""""""""""""""""""""""""""""""""""""""""""""""""""""""""""""""""""""""""""""""""""""""""""""""""""""""""""""""""""""""""""""""""""""""""""""""""""""""""""""""""""""""""""""""""""""""""""""""""""""""""""""""""""""""""""""""""""""""""""""""""""""""""""""""""""""""""""""""""""""""""""""""""""""""""""""""""""""""""""""""""""""""""""""""""""""""""""""""""""""""""""""""""""""""""""""""""""""""""""""""""""""""""""""""""""""""""""""""""""""""""""""""""""""""""""""""""""""""""""""""""""""""""""""""""""""""""""""""""""""""""""""""""""""""""""""""""""""""""""""""""""""""""""""""""""""""""""""""""""""""""""""""""""""""""""""""""""""""""""""""""""""""""""""""""""""""""""""""""""""""""""""""""""""""""""""""""""""""""""""""""""""""""""""""""""""""""""""""""""""""""""""""""""""""""""""""""""""""""""""""""""""""""""""""""""""""""""""""""""""""""""""""""""""""""""""""""""""""""""""""""""""""""""""""""""""""""""""""""""""""""""""""""""""""""""""""""""""""""""""""""""""""""""""""""""""""""""""""""""""""""""""""""""""""""""""""""""""""""""""""""""""""""""""""""""""""""""""""""""""""""""""""""""""""""""""""""""""""""""""""""""""""""""""""""""""""""""""""""""""""""""""""""""""""""""""""""""""""""""""""""""""""""""""""""""""""""""""""""""""""""""""""""""""""""""""""""""""""""""""""""""""""""""""""""""""""""""""""""""""""""""""""""""""""""""""""""""""""""""""""""""""""""""""""""""""""""""""""""""""""""""""""""""""""""""""""""""""""""""""""""""""""""""""""""""""""""""""""""""""""""""""""""""""""""""""""""""""""""""""""""""""""""""""""""""""""""""""""""""""""""""""""""""""""""""""""""""""""""""""""""""""""""""""""""""""""""""""""""""""""""""""""""""""""""""""""""""""""""""""""""""""""""""""""""""""""""""""""""""""""""""""""""""""""""""""""""""""""""""""""""""""""""""""""""""""""""""""""""""""""""""""""""""""""""""""""""""""""""""""""""""""""""""""""""""""""""""""""""""""""""""""""""""""""""""""""""""""""""""""""""""""""""""""""""""""""""""""""""""""""""""""""""""""""""""""""""""""""""""""""""""""""""""""""""""""""""""""""""""""""""""""""""""""""""""""""""""""""""""""""""""""""""""""""""""""""""""""""""""""""""""""""""""""""""""""""""""""""""""""""""""""""""""""""""""""""""""""""""""""""""""""""""""""""""""""""""""""""""""""""""""""""""""""""""""""""""""""""""""""""""""""""""""""""""""""""""""""""""""""""""""""""""""""""""""""""""""""""""""""""""""""""""""""""""""""""""""""""""""""""""""""""""""""""""""""""""""""""""""""""""""""""""""""""""""""""""""""""""""""""""""""""""""""""""""""""""""""""""""""""""""""""""""""""""""""""""""""""""""""""""""""""""""""""""""""""""""""""""""""""""""""""""""""""""""""""""""""""""""""""""""""""""""""""""""""""""""""""""""""""""""""""""""""""""""""""""""""""""""""""""""""""""""""""""""""""""""""""""""""""""""""""""""""""""""""""""""""""""""""""""""""""""""""""""""""""""""""""""""""""""""""""""""""""""""""""""""""""""""""""""""""""""""""""""""""""""""""""""""""""""""""""""""""""""""""""""""""""""""""""""""""""""""""""""""""""""""""""""""""""""""""""""""""""""""""""""""""""""""""""""""""""""""""""""""""""""""""""""""""""""""""""""""""""""""""""""""""""""""""""""""""""""""""""""""""""""""""""""""""""""""""""""""""""""""""""""""""""""""""""""""""""""""""""""""""""""""""""""""""""""""""""""""""""""""""""""""""""""""""""""""""""""""""""""""""""""""""""""""""""""""""""""""""""""""""""""""""""""""""""""""""""""""""""""""""""""""""""""""""""""""""""""""""""""""""""""""""""""""""""""""""""""""""""""""""""""""""""""""""""""""""""""""""""""""""""""""""""""""""""""""""""""""""""""""""""""""""""""""""""""""""""""""""""""""""""""""""""""""""""""""""""""""""""""""""""""""""""""""""""""""""""""""""""""""""""""""""""""""""""""""""""""""""""""""""""""""""""""""""""""""""""""""""""""""""""""""""""""""""""""""""""""""""""""""""""""""""""""""""""""""""""""""""""""""""""""""""""""""""""""""""""""""""""""""""""""""""""""""""""""""""""""""""""""""""""""""""""""""""""""""""""""""""""""""""""""""""""""""""""""""""""""""""""""""""""""""""""""""""""""""""""""""""""""""""""""""""""""""""""""""""""""""""""""""""""""""""""""""""""""""""""""""""""""""""""""""""""""""""""""""""""""""""""""""""""""""""""""""""""""""""""""""""""""""""""""""""""""""""""""""""""""""""""""""""""""""""""""""""""""""""""""""""""""""""""""""""""""""""""""""""""""""""""""""""""""""""""""""""""""""""""""""""""""""""""""""""""""""""""""""""""""""""""""""""""""""""""""""""""""""""""""""""""""""""""""""""""""""""""""""""""""""""""""""""""""""""""""""""""""""""""""""""""""""""""""""""""""""""""""""""""""""""""""""""""""""""""""""""""""""""""""""""""""""""""""""""""""""""""""""""""""""""""""""""""""""""""""""""""""""""""""""""""""""""""""""""""""""""""""""""""""""""""""""""""""""""""""""""""""""""""""""""""""""""""""""""""""""""""""""""""""""""""""""""""""""""""""""""""""""""""""""""""""""""""""""""""""""""""""""""""""""""""""""""""""""""""""""""""""""""""""""""""""""""""""""""""""""""""""""""""""""""""""""""""""""""""""""""""""""""""""""""""""""""""""""""""""""""""""""""""""""""""""""""""""""""""""""""""""""""""""""""""""""""""""""""""""""""""""""""""""""""""""""""""""""""""""""""""""""""""""""""""""""""""""""""""""""""""""""""""""""""""""""""""""""""""""""""""""""""""""""""""""""""""""""""""""""""""""""""""""""""""""""""""""""""""""""""""""""""""""""""""""""""""""""""""""""""""""""""""""""""""""""""""""""""""""""""""""""""""""""""""""""""""""""""""""""""""""""""""""""""""""""""""""""""""""""""""""""""""""""""""""""""""""""""""""""""""""""""""""""""""""""""""""""""""""""""""""""""""""""""""""""""""""""""""""""""""""""""""""""""""""""""""""""""""""""""""""""""""""""""""""""""""""""""""""""""""""""""""""""""""""""""""""""""""""""""""""""""""""""""""""""""""""""""""""""""""""""""""""""""""""""""""""""""""""""""""""""""""""""""""""""""""""""""""""""""""""""""""""""""""""""""""""""""""""""""""""""""""""""""""""""""""""""""""""""""""""""""""""""""""""""""""""""""""""""""""""""""""""""""""""""""""""""""""""""""""""""""""""""""""""""""""""""""""""""""""""""""""""""""""""""""""""""""""""""""""""""""""""""""""""""""""""""""""""""""""""""""""""""""""""""""""""""""""""""""""""""""""""""""""""""""""""""""""""""""""""""""""""""""""""""""""""""""""""""""""""""""""""""""""""""""""""""""""""""""""""""""""""""""""""""""""""""""""""""""""""""""""""""""""""""""""""""""""""""""""""""""""""""""""""""""""""""""""""""""""""""""""""""""""""""""""""""""""""""""""""""""""""""""""""""""""""""""""""""""""""""""""""""""""""""""""""""""""""""""""""""""""""""""""""""""""""""""""""""""""""""""""""""""""""""""""""""""""""""""""""""""""""""""""""""""""""""""""""""""""""""""""""""""""""""""""""""""""""""""""""""""""""""""""""""""""""""""""""""""""""""""""""""""""""""""""""""""""""""""""""""""""""""""""""""""""""""""""""""""""""""""""""""""""""""""""""""""""""""""""""""""""""""""""""""""""""""""""""""""""""""""""""""""""""""""""""""""""""""""""""""""""""""""""""""""""""""""""""""""""""""""""""""""""""""""""""""""""""""""""""""""""""""""""""""""""""""""""""""""""""""""""""""""""""""""""""""""""""""""""""""""""""""""""""""""""""""""""""""""""""""""""""""""""""""""""""""""""""""""""""""""""""""""""""""""""""""""""""""""""""""""""""""""""""""""""""""""""""""""""""""""""""""""""""""""""""""""""""""""""""""""""""""""""""""""""""""""""""""""""""""""""""""""""""""""""""""""""""""""""""""""""""""""""""""""""""""""""""""""""""""""""""""""""""""""""""""""""""""""""""""""""""""""""""""""""""""""""""""""""""""""""""""""""""""""""""""""""""""""""""""""""""""""""""""""""""""""""""""""""""""""""""""""""""""""""""""""""""""""""""""""""""""""""""""""""""""""""""""""""""""""""""""""""""""""""""""""""""""""""""""""""""""""""""""""""""""""""""""""""""""""""""""""""""""""""""""""""""""""""""""""""""""""""""""""""""""""""""""""""""""""""""""""""""""""""""""""""""""""""""""""""""""""""""""""""""""""""""""""""""""""""""""""""""""""""""""""""""""""""""""""""""""""""""""""""""""""""""""""""""""""""""""""""""""""""""""""""""""""""""""""""""""""""""""""""""""""""""""""""""""""""""""""""""""""""""""""""""""""""""""""""""""""""""""""""""""""""""""""""""""""""""""""""""""""""""""""""""""""""""""""""""""""""""""""""""""""""""""""""""""""""""""""""""""""""""""""""""""""""""""""""""""""""""""""""""""""""""""""""""""""""""""""""""""""""""""""""""""""""""""""""""""""""""""""""""""""""""""""""""""""""""""""""""""""""""""""""""""""""""""""""""""""""""""""""""""""""""""""""""""""""""""""""""""""""""""""""""""""""""""""""""""""""""""""""""""""""""""""""""""""""""""""""""""""""""""""""""""""""""""""""""""""""""""""""""""""""""""""""""""""""""""""""""""""""""""""""""""""""""""""""""""""""""""""""""""""""""""""""""""""""""""""""""""""""""""""""""""""""""""""""""""""""""""""""""""""""""""""""""""""""""""""""""""""""""""""""""""""""""""""""""""""""""""""""""""""""""""""""""""""""""""""""""""""""""""""""""""""""""""""""""""""""""""""""""""""""""""""""""""""""""""""""""""""""""""""""""""""""""""""""""""""""""""""""""""""""""""""""""""""""""""""""""""""""""""""""""""""""""""""""""""""""""""""""""""""""""""""""""""""""""""""""""""""""""""""""""""""""""""""""""""""""""""""""""""""""""""""""""""""""""""""""""""""""""""""""""""""""""""""""""""""""""""""""""""""""""""""""""""""""""""""""""""""""""""""""""""""""""""""""""""""""""""""""""""""""""""""""""""""""""""""""""""""""""""""""""""""""""""""""""""""""""""""""""""""""""""""""""""""""""""""""""""""""""""""""""""""""""""""""""""""""""""""""""""""""""""""""""""""""""""""""""""""""""""""""""""""""""""""""""""""""""""""""""""""""""""""""""""""""""""""""""""""""""""""""""""""""""""""""""""""""""""""""""""""""""""""""""""""""""""""""""""""""""""""""""""""""""""""""""""""""""""""""""""""""""""""""""""""""""""""""""""""""""""""""""""""""""""""""""""""""""""""""""""""""""""""""""""""""""""""""""""""""""""""""""""""""""""""""""""""""""""""""""""""""""""""""""""""""""""""""""""""""""""""""""""""""""""""""""""""""""""""""""""""""""""""""""""""""""""""""""""""""""""""""""""""""""""""""""""""""""""""""""""""""""""""""""""""""""""""""""""""""""""""""""""""""""""""""""""""""""""""""""""""""""""""""""""""""""""""""""""""""""""""""""""""""""""""""""""""""""""""""""""""""""""""""""""""""""""""""""""""""""""""""""""""""""""""""""""""""""""""""""""""""""""""""""""""""""""""""""""""""""""""""""""""""""""""""""""""""""""""""""""""""""""""""""""""""""""""""""""""""""""""""""""""""""""""""""""""""""""""""""""""""""""""""""""""""""""""""""""""""""""""""""""""""""""""""""""""""""""""""""""""""""""""""""""""""""""""""""""""""""""""""""""""""""""""""""""""""""""""""""""""""""""""""""""""""""""""""""""""""""""""""""""""""""""""""""""""""""""""""""""""""""""""""""""""""""""""""""""""""""""""""""""""""""""""""""""""""""""""""""""""""""""""""""""""""""""""""""""""""""""""""""""""""""""""""""""""""""""""""""""""""""""""""""""""""""""""""""""""""""""""""""""""""""""""""""""""""""""""""""""""""""""""""""""""""""""""""""""""""""""""""""""""""""""""""""""""""""""""""""""""""""""""""""""""""""""""""""""""""""""""""""""""""""""""""""""""""""""""""""""""""""""""""""""""""""""""""""""""""""""""""""""""""""""""""""""""""""""""""""""""""""""""""""""""""""""""""""""""""""""""""""""""""""""""""""""""""""""""""""""""""""""""""""""""""""""""""""""""""""""""""""""""""""""""""""""""""""""""""""""""""""""""""""""""""""""""""""""""""""""""""""""""""""""""""""""""""""""""""""""""""""""""""""""""""""""""""""""""""""""""""""""""""""""""""""""""""""""""""""""""""""""""""""""""""""""""""""""""""""""""""""""""""""""""""""""""""""""""""""""""""""""""""""""""""""""""""""""""""""""""""""""""""""""""""""""""""""""""""""""""""""""""""""""""""""""""""""""""""""""""""""""""""""""""""""""""""""""""""""""""""""""""""""""""""""""""""""""""""""""""""""""""""""""""""""""""""""""""""""""""""""""""""""""""""""""""""""""""""""""""""""""""""""""""""""""""""""""""""""""""""""""""""""""""""""""""""""""""""""""""""""""""""""""""""""""""""""""""""""""""""""""""""""""""""""""""""""""""""""""""""""""""""""""""""""""""""""""""""""""""""""""""""""""""""""""""""""""""""""""""""""""""""""""""""""""""""""""""""""""""""""""""""""""""""""""""""""""""""""""""""""""""""""""""""""""""""""""""""""""""""""""""""""""""""""""""""""""""""""""""""""""""""""""""""""""""""""""""""""""""""""""""""""""""""""""""""""""""""""""""""""""""""""""""""""""""""""""""""""""""""""""""""""""""""""""""""""""""""""""""""""""""""""""""""""""""""""""""""""""""""""""""""""""""""""""""""""""""""""""""""""""""""""""""""""""""""""""""""""""""""""""""""""""""""""""""""""""""""""""""""""""""""""""""""""""""""""""""""""""""""""""""""""""""""""""""""""""""""""""""""""""""""""""""""""""""""""""""""""""""""""""""""""""""""""""""""""""""""""""""""""""""""""""""""""""""""""""""""""""""""""""""""""""""""""""""""""""""""""""""""""""""""""""""""""""""""""""""""""""""""""""""""""""""""""""""""""""""""""""""""""""""""""""""""""""""""""""""""""""""""""""""""""""""""""""""""""""""""""""""""""""""""""""""""""""""""""""""""""""""""""""""""""""""""""""""""""""""""""""""""""""""""""""""""""""""""""""""""""""""""""""""""""""""""""""""""""""""""""""""""""""""""""""""""""""""""""""""""""""""""""""""""""""""""""""""""""""""""""""""""""""""""""""""""""""""""""""""""""""""""""""""""""""""""""""""""""""""""""""""""""""""""""""""""""""""""""""""""""""""""""""""""""""""""""""""""""""""""""""""""""""""""""""""""""""""""""""""""""""""""""""""""""""""""""""""""""""""""""""""""""""""""""""""""""""""""""""""""""""""""""""""""""""""""""""""""""""""""""""""""""""""""""""""""""""""""""""""""""""""""""""""""""""""""""""""""""""""""""""""""""""""""""""""""""""""""""""""""""""""""""""""""""""""""""""""""""""""""""""""""""""""""""""""""""""""""""""""""""""""""""""""""""""""""""""""""""""""""""""""""""""""""""""""""""""""""""""""""""""""""""""""""""""""""""""""""""""""""""""""""""""""""""""""""""""""""""""""""""""""""""""""""""""""""""""""""""""""""""""""""""""""""""""""""""""""""""""""""""""""""""""""""""""""""""""""""""""""""""""""""""""""""""""""""""""""""""""""""""""""""""""""""""""""""""""""""""""""""""""""""""""""""""""""""""""""""""""""""""""""""""""""""""""""""""""""""""""""""""""""""""""""""""""""""""""""""""""""""""""""""""""""""""""""""""""""""""""""""""""""""""""""""""""""""""""""""""""""""""""""""""""""""""""""""""""""""""""""""""""""""""""""""""""""""""""""""""""""""""""""""""""""""""""""""""""""""""""""""""""""""""""""""""""""""""""""""""""""""""""""""""""""""""""""""""""""""""""""""""""""""""""""""""""""""""""""""""""""""""""""""""""""""""""""""""""""""""""""""""""""""""""""""""""""""""""""""""""""""""""""""""""""""""""""""""""""""""""""""""""""""""""""""""""""""""""""""""""""""""""""""""""""""""""""""""""""""""""""""""""""""""""""""""""""""""""""""""""""""""""""""""""""""""""""""""""""""""""""""""""""""""""""""""""""""""""""""""""""""""""""""""""""""""""""""""""""""""""""""""""""""""""""""""""""""""""""""""""""""""""""""""""""""""""""""""""""""""""""""""""""""""""""""""""""""""""""""""""""""""""""""""""""""""""""""""""""""""""""""""""""""""""""""""""""""""""""""""""""""""""""""""""""""""""""""""""""""""""""""""""""""""""""""""""""""""""""""""""""""""""""""""""""""""""""""""""""""""""""""""""""""""""""""""""""""""""""""""""""""""""""""""""""""""""""""""""""""""""""""""""""""""""""""""""""""""""""""""""""""""""""""""""""""""""""""""""""""""""""""""""""""""""""""""""""""""""""""""""""""""""""""""""""""""""""""""""""""""""""""""""""""""""""""""""""""""""""""""""""""""""""""""""""""""""""""""""""""""""""""""""""""""""""""""""""""""""""""""""""""""""""""""""""""""""""""""""""""""""""""""""""""""""""""""""""""""""""""""""""""""""""""""""""""""""""""""""""""""""""""""""""""""""""""""""""""""""""""""""""""""""""""""""""""""""""""""""""""""""""""""""""""""""""""""""""""""""""""""""""""""""""""""""""""""""""""""""""""""""""""""""""""""""""""""""""""""""""""""""""""""""""""""""""""""""""""""""""""""""""""""""""""""""""""""""""""""""""""""""""""""""""""""""""""""""""""""""""""""""""""""""""""""""""""""""""""""""""""""""""""""""""""""""""""""""""""""""""""""""""""""""""""""""""""""""""""""""""""""""""""""""""""""""""""""""""""""""""""""""""""""""""""""""""""""""""""""""""""""""""""""""""""""""""""""""""""""""""""""""""""""""""""""""""""""""""""""""""""""""""""""""""""""""""""""""""""""""""""""""""""""""""""""""""""""""""""""""""""""""""""""""""""""""""""""""""""""""""""""""""""""""""""""""""""""""""""""""""""""""""""""""""""""""""""""""""""""""""""""""""""""""""""""""""""""""""""""""""""""""""""""""""""""""""""""""""""""""""""""""""""""""""""""""""""""""""""""""""""""""""""""""""""""""""""""""""""""""""""""""""""""""""""""""""""""""""""""""""""""""""""""""""""""""""""""""""""""""""""""""""""""""""""""""""""""""""""""""""""""""""""""""""""""""""""""""""""""""""""""""""""""""""""""""""""""""""""""""""""""""""""""""""""""""""""""""""""""""""""""""""""""""""""""""""""""""""""""""""""""""""""""""""""""""""""""""""""""""""""""""""""""""""""""""""""""""""""""""""""""""""""""""""""""""""""""""""""""""""""""""""""""""""""""""""""""""""""""""""""""""""""""""""""""""""""""""""""""""""""""""""""""""""""""""""""""""""""""""""""""""""""""""""""""""""""""""""""""""""""""""""""""""""""""""""""""""""""""""""""""""""""""""""""""""""""""""""""""""""""""""""""""""""""""""""""""""""""""""""""""""""""""""""""""""""""""""""""""""""""""""""""""""""""""""""""""""""""""""""""""""""""""""""""""""""""""""""""""""""""""""""""""""""""""""""""""""""""""""""""""""""""""""""""""""""""""""""""""""""""""""""""""""""""""""""""""""""""""""""""""""""""""""""""""""""""""""""""""""""""""""""""""""""""""""""""""""""""""""""""""""""""""""""""""""""""""""""""""""""""""""""""""""""""""""""""""""""""""""""""""""""""""""""""""""""""""""""""""""""""""""""""""""""""""""""""""""""""""""""""""""""""""""""""""""""""""""""""""""""""""""""""""""""""""""""""""""""""""""""""""""""""""""""""""""""""""""""""""""""""""""""""""""""""""""""""""""""""""""""""""""""""""""""""""""""""""""""""""""""""""""""""""""""""""""""""""""""""""""""""""""""""""""""""""""""""""""""""""""""""""""""""""""""""""""""""""""""""""""""""""""""""""""""""""""""""""""""""""""""""""""""""""""""""""""""""""""""""""""""""""""""""""""""""""""""""""""""""""""""""""""""""""""""""""""""""""""""""""""""""""""""""""""""""""""""""""""""""""""""""""""""""""""""""""""""""""""""""""""""""""""""""""""""""""""""""""""""""""""""""""""""""""""""""""""""""""""""""""""""""""""""""""""""""""""""""""""""""""""""""""""""""""""""""""""""""""""""""""""""""""""""""""""""""""""""""""""""""""""""""""""""""""""""""""""""""""""""""""""""""""""""""""""""""""""""""""""""""""""""""""""""""""""""""""""""""""""""""""""""""""""""""""""""""""""""""""""""""""""""""""""""""""""""""""""""""""""""""""""""""""""""""""""""""""""""""""""""""""""""""""""""""""""""""""""""""""""""""""""""""""""""""""""""""""""""""""""""""""""""""""""""""""""""""""""""""""""""""""""""""""""""""""""""""""""""""""""""""""""""""""""""""""""""""""""""""""""""""""""""""""""""""""""""""""""""""""""""""""""""""""""""""""""""""""""""""""""""""""""""""""""""""""""""""""""""""""""""""""""""""""""""""""""""""""""""""""""""""""""""""""""""""""""""""""""""""""""""""""""""""""""""""""""""""""""""""""""""""""""""""""""""""""""""""""""""""""""""""""""""""""""""""""""""""""""""""""""""""""""""""""""""""""""""""""""""""""""""""""""""""""""""""""""""""""""""""""""""""""""""""""""""""""""""""""""""""""""""""""""""""""""""""""""""""""""""""""""""""""""""""""""""""""""""""""""""""""""""""""""""""""""""""""""""""""""""""""""""""""""""""""""""""""""""""""""""""""""""""""""""""""""""""""""""""""""""""""""""""""""""""""""""""""""""""""""""""""""""""""""""""""""""""""""""""""""""""""""""""""""""""""""""""""""""""""""""""""""""""""""""""""""""""""""""""""""""""""""""""""""""""""""""""""""""""""""""""""""""""""""""""""""""""""""""""""""""""""""""""""""""""""""""""""""""""""""""""""""""""""""""""""""""""""""""""""""""""""""""""""""""""""""""""""""""""""""""""""""""""""""""""""""""""""""""""""""""""""""""""""""""""""""""""""""""""""""""""""""""""""""""""""""""""""""""""""""""""""""""""""""""""""""""""""""""""""""""""""""""""""""""""""""""""""""""""""""""""""""""""""""""""""""""""""""""""""""""""""""""""""""""""""""""""""""""""""""""""""""""""""""""""""""""""""""""""""""""""""""""""""""""""""""""""""""""""""""""""""""""""""""""""""""""""""""""""""""""""""""""""""""""""""""""""""""""""""""""""""""""""""""""""""""""""""""""""""""""""""""""""""""""""""""""""""""""""""""""""""""""""""""""""""""""""""""""""""""""""""""""""""""""""""""""""""""""""""""""""""""""""""""""""""""""""""""""""""""""""""""""""""""""""""""""""""""""""""""""""""""""""""""""""""""""""""""""""""""""""""""""""""""""""""""""""""""""""""""""""""""""""""""""""""""""""""""""""""""""""""""""""""""""""""""""""""""""""""""""""""""""""""""""""""""""""""""""""""""""""""""""""""""""""""""""""""""""""""""""""""""""""""""""""""""""""""""""""""""""""""""""""""""""""""""""""""""""""""""""""""""""""""""""""""""""""""""""""""""""""""""""""""""""""""""""""""""""""""""""""""""""""""""""""""""""""""""""""""""""""""""""""""""""""""""""""""""""""""""""""""""""""""""""""""""""""""""""""""""""""""""""""""""""""""""""""""""""""""""""""""""""""""""""""""""""""""""""""""""""""""""""""""""""""""""""""""""""""""""""""""""""""""""""""""""""""""""""""""""""""""""""""""""""""""""""""""""""""""""""""""""""""""""""""""""""""""""""""""""""""""""""""""""""""""""""""""""""""""""""""""""""""""""""""""""""""""""""""""""""""""""""""""""""""""""""""""""""""""""""""""""""""""""""""""""""""""""""""""""""""""""""""""""""""""""""""""""""""""""""""""""""""""""""""""""""""""""""""""""""""""""""""""""""""""""""""""""""""""""""""""""""""""""""""""""""""""""""""""""""""""""""""""""""""""""""""""""""""""""""""""""""""""""""""""""""""""""""""""""""""""""""""""""""""""""""""""""""""""""""""""""""""""""""""""""""""""""""""""""""""""""""""""""""""""""""""""""""""""""""""""""""""""""""""""""""""""""""""""""""""""""""""""""""""""""""""""""""""""""""""""""""""""""""""""""""""""""""""""""""""""""""""""""""""""""""""""""""""""""""""""""""""""""""""""""""""""""""""""""""""""""""""""""""""""""""""""""""""""""""""""""""""""""""""""""""""""""""""""""""""""""""""""""""""""""""""""""""""""""""""""""""""""""""""""""""""""""""""""""""""""""""""""""""""""""""""""""""""""""""""""""""""""""""""""""""""""""""""""""""""""""""""""""""""""""""""""""""""""""""""""""""""""""""""""""""""""""""""""""""""""""""""""""""""""""""""""""""""""""""""""""""""""""""""""""""""""""""""""""""""""""""""""""""""""""""""""""""""""""""""""""""""""""""""""""""""""""""""""""""""""""""""""""""""""""""""""""""""""""""""""""""""""""""""""""""""""""""""""""""""""""""""""""""""""""""""""""""""""""""""""""""""""""""""""""""""""""""""""""""""""""""""""""""""""""""""""""""""""""""""""""""""""""""""""""""""""""""""""""""""""""""""""""""""""""""""""""""""""""""""""""""""""""""""""""""""""""""""""""""""""""""""""""""""""""""""""""""""""""""""""""""""""""""""""""""""""""""""""""""""""""""""""""""""""""""""""""""""""""""""""""""""""""""""""""""""""""""""""""""""""""""""""""""""""""""""""""""""""""""""""""""""""""""""""""""""""""""""""""""""""""""""""""""""""""""""""""""""""""""""""""""""""""""""""""""""""""""""""""""""""""""""""""""""""""""""""""""""""""""""""""""""""""""""""""""""""""""""""""""""""""""""""""""""""""""""""""""""""""""""""""""""""""""""""""""""""""""""""""""""""""""""""""""""""""""""""""""""""""""""""""""""""""""""""""""""""""""""""""""""""""""""""""""""""""""""""""""""""""""""""""""""""""""""""""""""""""""""""""""""""""""""""""""""""""""""""""""""""""""""""""""""""""""""""""""""""""""""""""""""""""""""""""""""""""""""""""""""""""""""""""""""""""""""""""""""""""""""""""""""""""""""""""""""""""""""""""""""""""""""""""""""""""""""""""""""""""""""""""""""""""""""""""""""""""""""""""""""""""""""""""""""""""""""""""""""""""""""""""""""""""""""""""""""""""""""""""""""""""""""""""""""""""""""""""""""""""""""""""""""""""""""""""""""""""""""""""""""""""""""""""""""""""""""""""""""""""""""""""""""""""""""""""""""""""""""""""""""""""""""""""""""""""""""""""""""""""""""""""""""""""""""""""""""""""""""""""""""""""""""""""""""""""""""""""""""""""""""""""""""""""""""""""""""""""""""""""""""""""""""""""""""""""""""""""""""""""""""""""""""""""""""""""""""""""""""""""""""""""""""""""""""""""""""""""""""""""""""""""""""""""""""""""""""""""""""""""""""""""""""""""""""""""""""""""""""""""""""""""""""""""""""""""""""""""""""""""""""""""""""""""""""""""""""""""""""""""""""""""""""""""""""""""""""""""""""""""""""""""""""""""""""""""""""""""""""""""""""""""""""""""""""""""""""""""""""""""""""""""""""""""""""""""""""""""""""""""""""""""""""""""""""""""""""""""""""""""""""""""""""""""""""""""""""""""""""""""""""""""""""""""""""""""""""""""""""""""""""""""""""""""""""""""""""""""""""""""""""""""""""""""""""""""""""""""""""""""""""""""""""""""""""""""""""""""""""""""""""""""""""""""""""""""""""""""""""""""""""""""""""""""""""""""""""""""""""""""""""""""""""""""""""""""""""""""""""""""""""""""""""""""""""""""""""""""""""""""""""""""""""""""""""""""""""""""""""""""""""""""""""""""""""""""""""""""""""""""""""""""""""""""""""""""""""""""""""""""""""""""""""""""""""""""""""""""""""""""""""""""""""""""""""""""""""""""""""""""""""""""""""""""""""""""""""""""""""""""""""""""""""""""""""""""""""""""""""""""""""""""""""""""""""""""""""""""""""""""""""""""""""""""""""""""""""""""""""""""""""""""""""""""""""""""""""""""""""""""""""""""""""""""""""""""""""""""""""""""""""""""""""""""""""""""""""""""""""""""""""""""""""""""""""""""""""""""""""""""""""""""""""""""""""""""""""""""""""""""""""""""""""""""""""""""""""""""""""""""""""""""""""""""""""""""""""""""""""""""""""""""""""""""""""""""""""""""""""""""""""""""""""""""""""""""""""""""""""""""""""""""""""""""""""""""""""""""""""""""""""""""""""""""""""""""""""""""""""""""""""""""""""""""""""""""""""""""""""""""""""""""""""""""""""""""""""""""""""""""""""""""""""""""""""""""""""""""""""""""""""""""""""""""""""""""""""""""""""""""""""""""""""""""""""""""""""""""""""""""""""""""""""""""""""""""""""""""""""""""""""""""""""""""""""""""""""""""""""""""""""""""""""""""""""""""""""""""""""""""""""""""""""""""""""""""""""""""""""""""""""""""""""""""""""""""""""""""""""""""""""""""""""""""""""""""""""""""""""""""""""""""""""""""""""""""""""""""""""""""""""""""""""""""""""""""""""""""""""""""""""""""""""""""""""""""""""""""""""""""""""""""""""""""""""""""""""""""""""""""""""""""""""""""""""""""""""""""""""""""""""""""""""""""""""""""""""""""""""""""""""""""""""""""""""""""""""""""""""""""""""""""""""""""""""""""""""""""""""""""""""""""""""""""""""""""""""""""""""""""""""""""""""""""""""""""""""""""""""""""""""""""""""""""""""""""""""""""""""""""""""""""""""""""""""""""""""""""""""""""""""""""""""""""""""""""""""""""""""""""""""""""""""""""""""""""""""""""""""""""""""""""""""""""""""""""""""""""""""""""""""""""""""""""""""""""""""""""""""""""""""""""""""""""""""""""""""""""""""""""""""""""""""""""""""""""""""""""""""""""""""""""""""""""""""""""""""""""""""""""""""""""""""""""""""""""""""""""""""""""""""""""""""""""""""""""""""""""""""""""""""""""""""""""""""""""""""""""""""""""""""""""""""""""""""""""""""""""""""""""""""""""""""""""""""""""""""""""""""""""""""""""""""""""""""""""""""""""""""""""""""""""""""""""""""""""""""""""""""""""""""""""""""""""""""""""""""""""""""""""""""""""""""""""""""""""""""""""""""""""""""""""""""""""""""""""""""""""""""""""""""""""""""""""""""""""""""""""""""""""""""""""""""""""""""""""""""""""""""""""""""""""""""""""""""""""""""""""""""""""""""""""""""""""""""""""""""""""""""""""""""""""""""""""""""""""""""""""""""""""""""""""""""""""""""""""""""""""""""""""""""""""""""""""""""""""""""""""""""""""""""""""""""""""""""""""""""""""""""""""""""""""""""""""""""""""""""""""""""""""""""""""""""""""""""""""""""""""""""""""""""""""""""""""""""""""""""""""""""""""""""""""""""""""""""""""""""""""""""""""""""""""""""""""""""""""""""""""""""""""""""""""""""""""""""""""""""""""""""""""""""""""""""""""""""""""""""""""""""""""""""""""""""""""""""""""""""""""""""""""""""""""""""""""""""""""""""""""""""""""""""""""""""""""""""""""""""""""""""""""""""""""""""""""""""""""""""""""""""""""""""""""""""""""""""""""""""""""""""""""""""""""""""""""""""""""""""""""""""""""""""""""""""""""""""""""""""""""""""""""""""""""""""""""""""""""""""""""""""""""""""""""""""""""""""""""""""""""""""""""""""""""""""""""""""""""""""""""""""""""""""""""""""""""""""""""""""""""""""""""""""""""""""""""""""""""""""""""""""""""""""""""""""""""""""""""""""""""""""""""""""""""""""""""""""""""""""""""""""""""""""""""""""""""""""""""""""""""""""""""""""""""""""""""""""""""""""""""""""""""""""""""""""""""""""""""""""""""""""""""""""""""""""""""""""""""""""""""""""""""""""""""""""""""""""""""""""""""""""""""""""""""""""""""""""""""""""""""""""""""""""""""""""""""""""""""""""""""""""""""""""""""""""""""""""""""""""""""""""""""""""""""""""""""""""""""""""""""""""""""""""""""""""""""""""""""""""""""""""""""""""""""""""""""""""""""""""""""""""""""""""""""""""""""""""""""""""""""""""""""""""""""""""""""""""""""""""""""""""""""""""""""""""""""""""""""""""""""""""""""""""""""""""""""""""""""""""""""""""""""""""""""""""""""""""""""""""""""""""""""""""""""""""""""""""""""""""""""""""""""""""""""""""""""""""""""""""""""""""""""""""""""""""""""""""""""""""""""""""""""""""""""""""""""""""""""""""""""""""""""""""""""""""""""""""""""""""""""""""""""""""""""""""""""""""""""""""""""""""""""""""""""""""""""""""""""""""""""""""""""""""""""""""""""""""""""""""""""""""""""""""""""""""""""""""""""""""""""""""""""""""""""""""""""""""""""""""""""""""""""""""""""""""""""""""""""""""""""""""""""""""""""""""""""""""""""""""""""""""""""""""""""""""""""""""""""""""""""""""""""""""""""""""""""""""""""""""""""""""""""""""""""""""""""""""""""""""""""""""""""""""""""""""""""""""""""""""""""""""""""""""""""""""""""""""""""""""""""""""""""""""""""""""""""""""""""""""""""""""""""""""""""""""""""""""""""""""""""""""""""""""""""""""""""""""""""""""""""""""""""""""""""""""""""""""""""""""""""""""""""""""""""""""""""""""""""""""""""""""""""""""""""""""""""""""""""""""""""""""""""""""""""""""""""""""""""""""""""""""""""""""""""""""""""""""""""""""""""""""""""""""""""""""""""""""""""""""""""""""""""""""""""""""""""""""""""""""""""""""""""""""""""""""""""""""""""""""""""""""""""""""""""""""""""""""""""""""""""""""""""""""""""""""""""""""""""""""""""""""""""""""""""""""""""""""""""""""""""""""""""""""""""""""""""""""""""""""""""""""""""""""""""""""""""""""""""""""""""""""""""""""""""""""""""""""""""""""""""""""""""""""""""""""""""""""""""""""""""""""""""""""""""""""""""""""""""""""""""""""""""""""""""""""""""""""""""""""""""""""""""""""""""""""""""""""""""""""""""""""""""""""""""""""""""""""""""""""""""""""""""""""""""""""""""""""""""""""""""""""""""""""""""""""""""""""""""""""""""""""""""""""""""""""""""""""""""""""""""""""""""""""""""""""""""""""""""""""""""""""""""""""""""""""""""""""""""""""""""""""""""""""""""""""""""""""""""""""""""""""""""""""""""""""""""""""""""""""""""""""""""""""""""""""""""""""""""""""""""""""""""""""""""""""""""""""""""""""""""""""""""""""""""""""""""""""""""""""""""""""""""""""""""""""""""""""""""""""""""""""""""""""""""""""""""""""""""""""""""""""""""""""""""""""""""""""""""""""""""""""""""""""""""""""""""""""""""""""""""""""""""""""""""""""""""""""""""""""""""""""""""""""""""""""""""""""""""""""""""""""""""""""""""""""""""""""""""""""""""""""""""""""""""""""""""""""""""""""""""""""""""""""""""""""""""""""""""""""""""""""""""""""""""""""""""""""""""""""""""""""""""""""""""""""""""""""""""""""""""""""""""""""""""""""""""""""""""""""""""""""""""""""""""""""""""""""""""""""""""""""""""""""""""""""""""""""""""""""""""""""""""""""""""""""""""""""""""""""""""""""""""""""""""""""""""""""""""""""""""""""""""""""""""""""""""""""""""""""""""""""""""""""""""""""""""""""""""""""""""""""""""""""""""""""""""""""""""""""""""""""""""""""""""""""""""""""""""""""""""""""""""""""""""""""""""""""""""""""""""""""""""""""""""""""""""""""""""""""""""""""""""""""""""""""""""""""""""""""""""""""""""""""""""""""""""""""""""""""""""""""""""""""""""""""""""""""""""""""""""""""""""""""""""""""""""""""""""""""""""""""""""""""""""""""""""""""""""""""""""""""""""""""""""""""""""""""""""""""""""""""""""""""""""""""""""""""""""""""""""""""""""""""""""""""""""""""""""""""""""""""""""""""""""""""""""""""""""""""""""""""""""""""""""""""""""""""""""""""""""""""""""""""""""""""""""""""""""""""""""""""""""""""""""""""""""""""""""""""""""""""""""""""""""""""""""""""""""""""""""""""""""""""""""""""""""""""""""""""""""""""""""""""""""""""""""""""""""""""""""""""""""""""""""""""""""""""""""""""""""""""""""""""""""""""""""""""""""""""""""""""""""""""""""""""""""""""""""""""""""""""""""""""""""""""""""""""""""""""""""""""""""""""""""""""""""""""""""""""""""""""""""""""""""""""""""""""""""""""""""""""""""""""""""""""""""""""""""""""""""""""""""""""""""""""""""""""""""""""""""""""""""""""""""""""""""""""""""""""""""""""""""""""""""""""""""""""""""""""""""""""""""""""""""""""""""""""""""""""""""""""""""""""""""""""""""""""""""""""""""""""""""""""""""""""""""""""""""""""""""""""""""""""""""""""""""""""""""""""""""""""""""""""""""""""""""""""""""""""""""""""""""""""""""""""""""""""""""""""""""""""""""""""""""""""""""""""""""""""""""""""""""""""""""""""""""""""""""""""""""""""""""""""""""""""""""""""""""""""""""""""""""""""""""""""""""""""""""""""""""""""""""""""""""""""""""""""""""""""""""""""""""""""""""""""""""""""""""""""""""""""""""""""""""""""""""""""""""""""""""""""""""""""""""""""""""""""""""""""""""""""""""""""""""""""""""""""""""""""""""""""""""""""""""""""""""""""""""""""""""""""""""""""""""""""""""""""""""""""""""""""""""""""""""""""""""""""""""""""""""""""""""""""""""""""""""""""""""""""""""""""""""""""""""""""""""""""""""""""""""""""""""""""""""""""""""""""""""""""""""""""""""""""""""""""""""""""""""""""""""""""""""""""""""""""""""""""""""""""""""""""""""""""""""""""""""""""""""""""""""""""""""""""""""""""""""""""""""""""""""""""""""""""""""""""""""""""""""""""""""""""""""""""""""""""""""""""""""""""""""""""""""""""""""""""""""""""""""""""""""""""""""""""""""""""""""""""""""""""""""""""""""""""""""""""""""""""""""""""""""""""""""""""""""""""""""""""""""""""""""""""""""""""""""""""""""""""""""""""""""""""""""""""""""""""""""""""""""""""""""""""""""""""""""""""""""""""""""""""""""""""""""""""""""""""""""""""""""""""""""""""""""""""""""""""""""""""""""""""""""""""""""""""""""""""""""""""""""""""""""""""""""""""""""""""""""""""""""""""""""""""""""""""""""""""""""""""""""""""""""""""""""""""""""""""""""""""""""""""""""""""""""""""""""""""""""""""""""""""""""""""""""""""""""""""""""""""""""""""""""""""""""""""""""""""""""""""""""""""""""""""""""""""""""""""""""""""""""""""""""""""""""""""""""""""""""""""""""""""""""""""""""""""""""""""""""""""""""""""""""""""""""""""""""""""""""""""""""""""""""""""""""""""""""""""""""""""""""""""""""""""""""""""""""""""""""""""""""""""""""""""""""""""""""""""""""""""""""""""""""""""""""""""""""""""""""""""""""""""""""""""""""""""""""""""""""""""""""""""""""""""""""""""""""""""""""""""""""""""""""""""""""""""""""""""""""""""""""""""""""""""""""""""""""""""""""""""""""""""""""""""""""""""""""""""""""""""""""""""""""""""""""""""""""""""""""""""""""""""""""""""""""""""""""""""""""""""""""""""""""""""""""""""""""""""""""""""""""""""""""""""""""""""""""""""""""""""""""""""""""""""""""""""""""""""""""""""""""""""""""""""""""""""""""""""""""""""""""""""""""""""""""""""""""""""""""""""""""""""""""""""""""""""""""""""""""""""""""""""""""""""""""""""""""""""""""""""""""""""""""""""""""""""""""""""""""""""""""""""""""""""""""""""""""""""""""""""""""""""""""""""""""""""""""""""""""""""""""""""""""""""""""""""""""""""""""""""""""""""""""""""""""""""""""""""""""""""""""""""""""""""""""""""""""""""""""""""""""""""""""""""""""""""""""""""""""""""""""""""""""""""""""""""""""""""""""""""""""""""""""""""""""""""""""""""""""""""""""""""""""""""""""""""""""""""""""""""""""""""""""""""""""""""""""""""""""""""""""""""""""""""""""""""""""""""""""""""""""""""""""""""""""""""""""""""""""""""""""""""""""""""""""""""""""""""""""""""""""""""""""""""""""""""""""""""""""""""""""""""""""""""""""""""""""""""""""""""""""""""""""""""""""""""""""""""""""""""""""""""""""""""""""""""""""""""""""""""""""""""""""""""""""""""""""""""""""""""""""""""""""""""""""""""""""""""""""""""""""""""""""""""""""""""""""""""""""""""""""""""""""""""""""""""""""""""""""""""""""""""""""""""""""""""""""""""""""""""""""""""""""""""""""""""""""""""""""""""""""""""""""""""""""""""""""""""""""""""""""""""""""""""""""""""""""""""""""""""""""""""""""""""""""""""""""""""""""""""""""""""""""""""""""""""""""""""""""""""""""""""""""""""""""""""""""""""""""""""""""""""""""""""""""""""""""""""""""""""""""""""""""""""""""""""""""""""""""""""""""""""""""""""""""""""""""""""""""""""""""""""""""""""""""""""""""""""""""""""""""""""""""""""""""""""""""""""""""""""""""""""""""""""""""""""""""""""""""""""""""""""""""""""""""""""""""""""""""""""""""""""""""""""""""""""""""""""""""""""""""""""""""""""""""""""""""""""""""""""""""""""""""""""""""""""""""""""""""""""""""""""""""""""""""""""""""""""""""""""""""""""""""""""""""""""""""""""""""""""""""""""""""""""""""""""""""""""""""""""""""""""""""""""""""""""""""""""""""""""""""""""""""""""""""""""""""""""""""""""""""""""""""""""""""""""""""""""""""""""""""""""""""""""""""""""""""""""""""""""""""""""""""""""""""""""""""""""""""""""""""""""""""""""""""""""""""""""""""""""""""""""""""""""""""""""""""""""""""""""""""""""""""""""""""""""""""""""""""""""""""""""""""""""""""""""""""""""""""""""""""""""""""""""""""""""""""""""""""""""""""""""""""""""""""""""""""""""""""""""""""""""""""""""""""""""""""""""""""""""""""""""""""""""""""""""""""""""""""""""""""""""""""""""""""""""""""""""""""""""""""""""""""""""""""""""""""""""""""""""""""""""""""""""""""""""""""""""""""""""""""""""""""""""""""""""""""""""""""""""""""""""""""""""""""""""""""""""""""""""""""""""""""""""""""""""""""""""""""""""""""""""""""""""""""""""""""""""""""""""""""""""""""""""""""""""""""""""""""""""""""""""""""""""""""""""""""""""""""""""""""""""""""""""""""""""""""""""""""""""""""""""""""""""""""""""""""""""""""""""""""""""""""""""""""""""""""""""""""""""""""""""""""""""""""""""""""""""""""""""""""""""""""""""""""""""""""""""""""""""""""""""""""""""""""""""""""""""""""""""""""""""""""""""""""""""""""""""""""""""""""""""""""""""""""""""""""""""""""""""""""""""""""""""""""""""""""""""""""""""""""""""""""""""""""""""""""""""""""""""""""""""""""""""""""""""""""""""""""""""""""""""""""""""""""""""""""""""""""""""""""""""""""""""""""""""""""""""""""""""""""""""""""""""""""""""""""""""""""""""""""""""""""""""""""""""""""""""""""""""""""""""""""""""""""""""""""""""""""""""""""""""""""""""""""""""""""""""""""""""""""""""""""""""""""""""""""""""""""""""""""""""""""""""""""""""""""""""""""""""""""""""""""""""""""""""""""""""""""""""""""""""""""""""""""""""""""""""""""""""""""""""""""""""""""""""""""""""""""""""""""""""""""""""""""""""""""""""""""""""""""""""""""""""""""""""""""""""""""""""""""""""""""""""""""""""""""""""""""""""""""""""""""""""""""""""""""""""""""""""""""""""""""""""""""""""""""""""""""""""""""""""""""""""""""""""""""""""""""""""""""""""""""""""""""""""""""""""""""""""""""""""""""""""""""""""""""""""""""""""""""""""""""""""""""""""""""""""""""""""""""""""""""""""""""""""""""""""""""""""""""""""""""""""""""""""""""""""""""""""""""""""""""""""""""""""""""""""""""""""""""""""""""""""""""""""""""""""""""""""""""""""""""""""""""""""""""""""""""""""""""""""""""""""""""""""""""""""""""""""""""""""""""""""""""""""""""""""""""""""""""""""""""""""""""""""""""""""""""""""""""""""""""""""""""""""""""""""""""""""""""""""""""""""""""""""""""""""""""""""""""""""""""""""""""""""""""""""""""""""""""""""""""""""""""""""""""""""""""""""""""""""""""""""""""""""""""""""""""""""""""""""""""""""""""""""""""""""""""""""""""""""""""""""""""""""""""""""""""""""""""""""""""""""""""""""""""""""""""""""""""""""""""""""""""""""""""""""""""""""""""""""""""""""""""""""""""""""""""""""""""""""""""""""""""""""""""""""""""""""""""""""""""""""""""""""""""""""""""""""""""""""""""""""""""""""""""""""""""""""""""""""""""""""""""""""""""""""""""""""""""""""""""""""""""""""""""""""""""""""""""""""""""""""""""""""""""""""""""""""""""""""""""""""""""""""""""""""""""""""""""""""""""""""""""""""""""""""""""""""""""""""""""""""""""""""""""""""""""""""""""""""""""""""""""""""""""""""""""""""""""""""""""""""""""""""""""""""""""""""""""""""""""""""""""""""""""""""""""""""""""""""""""""""""""""""""""""""""""""""""""""""""""""""""""""""""""""""""""""""""""""""""""""""""""""""""""""""""""""""""""""""""""""""""""""""""""""""""""""""""""""""""""""""""""""""""""""""""""""""""""""""""""""""""""""""""""""""""""""""""""""""""""""""""""""""""""""""""""""""""""""""""""""""""""""""""""""""""""""""""""""""""""""""""""""""""""""""""""""""""""""""""""""""""""""""""""""""""""""""""""""""""""""""""""""""""""""""""""""""""""""""""""""""""""""""""""""""""""""""""""""""""""""""""""""""""""""""""""""""""""""""""""""""""""""""""""""""""""""""""""""""""""""""""""""""""""""""""""""""""""""""""""""""""""""""""""""""""""""""""""""""""""""""""""""""""""""""""""""""""""""""""""""""""""""""""""""""""""""""""""""""""""""""""""""""""""""""""""""""""""""""""""""""""""""""""""""""""""""""""""""""""""""""""""""""""""""""""""""""""""""""""""""""""""""""""""""""""""""""""""""""""""""""""""""""""""""""""""""""""""""""""""""""""""""""""""""""""""""""""""""""""""""""""""""""""""""""""""""""""""""""""""""""""""""""""""""""""""""""""""""""""""""""""""""""""""""""""""""""""""""""""""""""""""""""""""""""""""""""""""""""""""""""""""""""""""""""""""""""""""""""""""""""""""""""""""""""""""""""""""""""""""""""""""""""""""""""""""""""""""""""""""""""""""""""""""""""""""""""""""""""""""""""""""""""""""""""""""""""""""""""""""""""""""""""""""""""""""""""""""""""""""""""""""""""""""""""""""""""""""""""""""""""""""""""""""""""""""""""""""""""""""""""""""""""""""""""""""""""""""""""""""""""""""""""""""""""""""""""""""""""""""""""""""""""""""""""""""""""""""""""""""""""""""""""""""""""""""""""""""""""""""""""""""""""""""""""""""""""""""""""""""""""""""""""""""""""""""""""""""""""""""""""""""""""""""""""""""""""""""""""""""""""""""""""""""""""""""""""""""""""""""""""""""""""""""""""""""""""""""""""""""""""""""""""""""""""""""""""""""""""""""""""""""""""""""""""""""""""""""""""""""""""""""""""""""""""""""""""""""""""""""""""""""""""""""""""""""""""""""""""""""""""""""""""""""""""""""""""""""""""""""""""""""""""""""""""""""""""""""""""""""""""""""""""""""""""""""""""""""""""""""""""""""""""""""""""""""""""""""""""""""""""""""""""""""""""""""""""""""""""""""""""""""""""""""""""""""""""""""""""""""""""""""""""""""""""""""""""""""""""""""""""""""""""""""""""""""""""""""""""""""""""""""""""""""""""""""""""""""""""""""""""""""""""""""""""""""""""""""""""""""""""""""""""""""""""""""""""""""""""""""""""""""""""""""""""""""""""""""""""""""""""""""""""""""""""""""""""""""""""""""""""""""""""""""""""""""""""""""""""""""""""""""""""""""""""""""""""""""""""""""""""""""""""""""""""""""""""""""""""""""""""""""""""""""""""""""""""""""""""""""""""""""""""""""""""""""""""""""""""""""""""""""""""""""""""""""""""""""""""""""""""""""""""""""""""""""""""""""""""""""""""""""""""""""""""""""""""""""""""""""""""""""""""""""""""""""""""""""""""""""""""""""""""""""""""""""""""""""""""""""""""""""""""""""""""""""""""""""""""""""""""""""""""""""""""""""""""""""""""""""""""""""""""""""""""""""""""""""""""""""""""""""""""""""""""""""""""""""""""""""""""""""""""""""""""""""""""""""""""""""""""""""""""""""""""""""""""""""""""""""""""""""""""""""""""""""""""""""""""""""""""""""""""""""""""""""""""""""""""""""""""""""""""""""""""""""""""""""""""""""""""""""""""""""""""""""""""""""""""""""""""""""""""""""""""""""""""""""""""""""""""""""""""""""""""""""""""""""""""""""""""""""""""""""""""""""""""""""""""""""""""""""""""""""""""""""""""""""""""""""""""""""""""""""""""""""""""""""""""""""""""""""""""""""""""""""""""""""""""""""""""""""""""""""""""""""""""""""""""""""""""""""""""""""""""""""""""""""""""""""""""""""""""""""""""""""""""""""""""""""""""""""""""""""""""""""""""""""""""""""""""""""""""""""""""""""""""""""""""""""""""""""""""""""""""""""""""""""""""""""""""""""""""""""""""""""""""""""""""""""""""""""""""""""""""""""""""""""""""""""""""""""""""""""""""""""""""""""""""""""""""""""""""""""""""""""""""""""""""""""""""""""""""""""""""""""""""""""""""""""""""""""""""""""""""""""""""""""""""""""""""""""""""""""""""""""""""""""""""""""""""""""""""""""""""""""""""""""""""""""""""""""""""""""""""""""""""""""""""""""""""""""""""""""""""""""""""""""""""""""""""""""""""""""""""""""""""""""""""""""""""""""""""""""""""""""""""""""""""""""""""""""""""""""""""""""""""""""""""""""""""""""""""""""""""""""""""""""""""""""""""""""""""""""""""""""""""""""""""""""""""""""""""""""""""""""""""""""""""""""""""""""""""""""""""""""""""""""""""""""""""""""""""""""""""""""""""""""""""""""""""""""""""""""""""""""""""""""""""""""""""""""""""""""""""""""""""""""""""""""""""""""""""""""""""""""""""""""""""""""""""""""""""""""""""""""""""""""""""""""""""""""""""""""""""""""""""""""""""""""""""""""""""""""""""""""""""""""""""""""""""""""""""""""""""""""""""""""""""""""""""""""""""""""""""""""""""""""""""""""""""""""""""""""""""""""""""""""""""""""""""""""""""""""""""""""""""""""""""""""""""""""""""""""""""""""""""""""""""""""""""""""""""""""""""""""""""""""""""""""""""""""""""""""""""""""""""""""""""""""""""""""""""""""""""""""""""""""""""""""""""""""""""""""""""""""""""""""""""""""""""""""""""""""""""""""""""""""""""""""""""""""""""""""""""""""""""""""""""""""""""""""""""""""""""""""""""""""""""""""""""""""""""""""""""""""""""""""""""""""""""""""""""""""""""""""""""""""""""""""""""""""""""""""""""""""""""""""""""""""""""""""""""""""""""""""""""""""""""""""""""""""""""""""""""""""""""""""""""""""""""""""""""""""""""""""""""""""""""""""""""""""""""""""""""""""""""""""""""""""""""""""""""""""""""""""""""""""""""""""""""""""""""""""""""""""""""""""""""""""""""""""""""""""""""""""""""""""""""""""""""""""""""""""""""""""""""""""""""""""""""""""""""""""""""""""""""""""""""""""""""""""""""""""""""""""""""""""""""""""""""""""""""""""""""""""""""""""""""""""""""""""""""""""""""""""""""""""""""""""""""""""""""""""""""""""""""""""""""""""""""""""""""""""""""""""""""""""""""""""""""""""""""""""""""""""""""""""""""""""""""""""""""""""""""""""""""""""""""""""""""""""""""""""""""""""""""""""""""""""""""""""""""""""""""""""""""""""""""""""""""""""""""""""""""""""""""""""""""""""""""""""""""""""""""""""""""""""""""""""""""""""""""""""""""""""""""""""""""""""""""""""""""""""""""""""""""""""""""""""""""""""""""""""""""""""""""""""""""""""""""""""""""""""""""""""""""""""""""""""""""""""""""""""""""""""""""""""""""""""""""""""""""""""""""""""""""""""""""""""""""""""""""""""""""""""""""""""""""""""""""""""""""""""""""""""""""""""""""""""""""""""""""""""""""""""""""""""""""""""""""""""""""""""""""""""""""""""""""""""""""""""""""""""""""""""""""""""""""""""""""""""""""""""""""""""""""""""""""""""""""""""""""""""""""""""""""""""""""""""""""""""""""""""""""""""""""""""""""""""""""""""""""""""""""""""""""""""""""""""""""""""""""""""""""""""""""""""""""""""""""""""""""""""""""""""""""""""""""""""""""""""""""""""""""""""""""""""""""""""""""""""""""""""""""""""""""""""""""""""""""""""""""""""""""""""""""""""""""""""""""""""""""""""""""""""""""""""""""""""""""""""""""""""""""""""""""""""""""""""""""""""""""""""""""""""""""""""""""""""""""""""""""""""""""""""""""""""""""""""""""""""""""""""""""""""""""""""""""""""""""""""""""""""""""""""""""""""""""""""""""""""""""""""""""""""""""""""""""""""""""""""""""""""""""""""""""""""""""""""""""""""""""""""""""""""""""""""""""""""""""""""""""""""""""""""""""""""""""""""""""""""""""""""""""""""""""""""""""""""""""""""""""""""""""""""""""""""""""""""""""""""""""""""""""""""""""""""""""""""""""""""""""""""""""""""""""""""""""""""""""""""""""""""""""""""""""""""""""""""""""""""""""""""""""""""""""""""""""""""""""""""""""""""""""""""""""""""""""""""""""""""""""""""""""""""""""""""""""""""""""""""""""""""""""""""""""""""""""""""""""""""""""""""""""""""""""""""""""""""""""""""""""""""""""""""""""""""""""""""""""""""""""""""""""""""""""""""""""""""""""""""""""""""""""""""""""""""""""""""""""""""""""""""""""""""""""""""""""""""""""""""""""""""""""""""""""""""""""""""""""""""""""""""""""""""""""""""""""""""""""""""""""""""""""""""""""""""""""""""""""""""""""""""""""""""""""""""""""""""""""""""""""""""""""""""""""""""""""""""""""""""""""""""""""""""""""""""""""""""""""""""""""""""""""""""""""""""""""""""""""""""""""""""""""""""""""""""""""""""""""""""""""""""""""""""""""""""""""""""""""""""""""""""""""""""""""""""""""""""""""""""""""""""""""""""""""""""""""""""""""""""""""""""""""""""""""""""""""""""""""""""""""""""""""""""""""""""""""""""""""""""""""""""""""""""""""""""""""""""""""""""""""""""""""""""""""""""""""""""""""""""""""""""""""""""""""""""""""""""""""""""""""""""""""""""""""""""""""""""""""""""""""""""""""""""""""""""""""""""""""""""""""""""""""""""""""""""""""""""""""""""""""""""""""""""""""""""""""""""""""""""""""""""""""""""""""""""""""""""""""""""""""""""""""""""""""""""""""""""""""""""""""""""""""""""""""""""""""""""""""""""""""""""""""""""""""""""""""""""""""""""""""""""""""""""""""""""""""""""""""""""""""""""""""""""""""""""""""""""""""""""""""""""""""""""""""""""""""""""""""""""""""""""""""""""""""""""""""""""""""""""""""""""""""""""""""""""""""""""""""""""""""""""""""""""""""""""""""""""""""""""""""""""""""""""""""""""""""""""""""""""""""""""""""""""""""""""""""""""""""""""""""""""""""""""""""""""""""""""""""""""""""""""""""""""""""""""""""""""""""""""""""""""""""""""""""""""""""""""""""""""""""""""""""""""""""""""""""""""""""""""""""""""""""""""""""""""""""""""""""""""""""""""""""""""""""""""""""""""""""""""""""""""""""""""""""""""""""""""""""""""""""""""""""""""""""""""""""""""""""""""""""""""""""""""""""""""""""""""""""""""""""""""""""""""""""""""""""""""""""""""""""""""""""""""""""""""""""""""""""""""""""""""""""""""""""""""""""""""""""""""""""""""""""""""""""""""""""""""""""""""""""""""""""""""""""""""""""""""""""""""""""""""""""""""""""""""""""""""""""""""""""""""""""""""""""""""""""""""""""""""""""""""""""""""""""""""""""""""""""""""""""""""""""""""""""""""""""""""""""""""""""""""""""""""""""""""""""""""""""""""""""""""""""""""""""""""""""""""""""""""""""""""""""""""""""""""""""""""""""""""""""""""""""""""""""""""""""""""""""""""""""""""""""""""""""""""""""""""""""""""""""""""""""""""""""""""""""""""""""""""""""""""""""""""""""""""""""""""""""""""""""""""""""""""""""""""""""""""""""""""""""""""""""""""""""""""""""""""""""""""""""""""""""""""""""""""""""""""""""""""""""""""""""""""""""""""""""""""""""""""""""""""""""""""""""""""""""""""""""""""""""""""""""""""""""""""""""""""""""""""""""""""""""""""""""""""""""""""""""""""""""""""""""""""""""""""""""""""""""""""""""""""""""""""""""""""""""""""""""""""""""""""""""""""""""""""""""""""""""""""""""""""""""""""""""""""""""""""""""""""""""""""""""""""""""""""""""""""""""""""""""""""""""""""""""""""""""""""""""""""""""""""""""""""""""""""""""""""""""""""""""""""""""""""""""""""""""""""""""""""""""""""""""""""""""""""""""""""""""""""""""""""""""""""""""""""""""""""""""""""""""""""""""""""""""""""""""""""""""""""""""""""""""""""""""""""""""""""""""""""""""""""""""""""""""""""""""""""""""""""""""""""""""""""""""""""""""""""""""""""""""""""""""""""""""""""""""""""""""""""""""""""""""""""""""""""""""""""""""""""""""""""""""""""""""""""""""""""""""""""""""""""""""""""""""""""""""""""""""""""""""""""""""""""""""""""""""""""""""""""""""""""""""""""""""""""""""""""""""""""""""""""""""""""""""""""""""""""""""""""""""""""""""""""""""""""""""""""""""""""""""""""""""""""""""""""""""""""""""""""""""""""""""""""""""""""""""""""""""""""""""""""""""""""""""""""""""""""""""""""""""""""""""""""""""""""""""""""""""""""""""""""""""""""""""""""""""""""""""""""""""""""""""""""""""""""""""""""""""""""""""""""""""""""""""""""""""""""""""""""""""""""""""""""""""""""""""""""""""""""""""""""""""""""""""""""""""""""""""""""""""""""""""""""""""""""""""""""""""""""""""""""""""""""""""""""""""""""""""""""""""""""""""""""""""""""""""""""""""""""""""""""""""""""""""""""""""""""""""""""""""""""""""""""""""""""""""""""""""""""""""""""""""""""""""""""""""""""""""""""""""""""""""""""""""""""""""""""""""""""""""""""""""""""""""""""""""""""""""""""""""""""""""""""""""""""""""""""""""""""""""""""""""""""""""""""""""""""""""""""""""""""""""""""""""""""""""""""""""""""""""""""""""""""""""""""""""""""""""""""""""""""""""""""""""""""""""""""""""""""""""""""""""""""""""""""""""""""""""""""""""""""""""""""""""""""""""""""""""""""""""""""""""""""""""""""""""""""""""""""""""""""""""""""""""""""""""""""""""""""""""""""""""""""""""""""""""""""""""""""""""""""""""""""""""""""""""""""""""""""""""""""""""""""""""""""""""""""""""""""""""""""""""""""""""""""""""""""""""""""""""""""""""""""""""""""""""""""""""""""""""""""""""""""""""""""""""""""""""""""""""""""""""""""""""""""""""""""""""""""""""""""""""""""""""""""""""""""""""""""""""""""""""""""""""""""""""""""""""""""""""""""""""""""""""""""""""""""""""""""""""""""""""""""""""""""""""""""""""""""""""""""""""""""""""""""""""""""""""""""""""""""""""""""""""""""""""""""""""""""""""""""""""""""""""""""""""""""""""""""""""""""""""""""""""""""""""""""""""""""""""""""""""""""""""""""""""""""""""""""""""""""""""""""""""""""""""""""""""""""""""""""""""""""""""""""""""""""""""""""""""""""""""""""""""""""""""""""""""""""""""""""""""""""""""""""""""""""""""""""""""""""""""""""""""""""""""""""""""""""""""""""""""""""""""""""""""""""""""""""""""""""""""""""""""""""""""""""""""""""""""""""""""""""""""""""""""""""""""""""""""""""""""""""""""""""""""""""""""""""""""""""""""""""""""""""""""""""""""""""""""""""""""""""""""""""""""""""""""""""""""""""""""""""""""""""""""""""""""""""""""""""""""""""""""""""""""""""""""""""""""""""""""""""""""""""""""""""""""""""""""""""""""""""""""""""""""""""""""""""""""""""""""""""""""""""""""""""""""""""""""""""""""""""""""""""""""""""""""""""""""""""""""""""""""""""""""""""""""""""""""""""""""""""""""""""""""""""""""""""""""""""""""""""""""""""""""""""""""""""""""""""""""""""""""""""""""""""""""""""""""""""""""""""""""""""""""""""""""""""""""""""""""""""""""""""""""""""""""""""""""""""""""""""""""""""""""""""""""""""""""""""""""""""""""""""""""""""""""""""""""""""""""""""""""""""""""""""""""""""""""""""""""""""""""""""""""""""""""""""""""""""""""""""""""""""""""""""""""""""""""""""""""""""""""""""""""""""""""""""""""""""""""""""""""""""""""""""""""""""""""""""""""""""""""""""""""""""""""""""""""""""""""""""""""""""""""""""""""""""""""""""""""""""""""""""""""""""""""""""""""""""""""""""""""""""""""""""""""""""""""""""""""""""""""""""""""""""""""""""""""""""""""""""""""""""""""""""""""""""""""""""""""""""""""""""""""""""""""""""""""""""""""""""""""""""""""""""""""""""""""""""""""""""""""""""""""""""""""""""""""""""""""""""""""""""""""""""""""""""""""""""""""""""""""""""""""""""""""""""""""""""""""""""""""""""""""""""""""""""""""""""""""""""""""""""""""""""""""""""""""""""""""""""""""""""""""""""""""""""""""""""""""""""""""""""""""""""""""""""""""""""""""""""""""""""""""""""""""""""""""""""""""""""""""""""""""""""""""""""""""""""""""""""""""""""""""""""""""""""""""""""""""""""""""""""""""""""""""""""""""""""""""""""""""""""""""""""""""""""""""""""""""""""""""""""""""""""""""""""""""""""""""""""""""""""""""""""""""""""""""""""""""""""""""""""""""""""""""""""""""""""""""""""""""""""""""""""""""""""""""""""""""""""""""""""""""""""""""""""""""""""""""""""""""""""""""""""""""""""""""""""""""""""""""""""""""""""""""""""""""""""""""""""""""""""""""""""""""""""""""""""""""""""""""""""""""""""""""""""""""""""""""""""""""""""""""""""""""""""""""""""""""""""""""""""""""""""""""""""""""""""""""""""""""""""""""""""""""""""""""""""""""""""""""""""""""""""""""""""""""""""""""""""""""""""""""""""""""""""""""""""""""""""""""""""""""""""""""""""""""""""""""""""""""""""""""""""""""""""""""""""""""""""""""""""""""""""""""""""""""""""""""""""""""""""""""""""""""""""""""""""""""""""""""""""""""""""""""""""""""""""""""""""""""""""""""""""""""""""""""""""""""""""""""""""""""""""""""""""""""""""""""""""""""""""""""""""""""""""""""""""""""""""""""""""""""""""""""""""""""""""""""""""""""""""""""""""""""""""""""""""""""""""""""""""""""""""""""""""""""""""""""""""""""""""""""""""""""""""""""""""""""""""""""""""""""""""""""""""""""""""""""""""""""""""""""""""""""""""""""""""""""""""""""""""""""""""""""""""""""""""""""""""""""""""""""""""""""""""""""""""""""""""""""""""""""""""""""""""""""""""""""""""""""""""""""""""""""""""""""""""""""""""""""""""""""""""""""""""""""""""""""""""""""""""""""""""""""""""""""""""""""""""""""""""""""""""""""""""""""""""""""""""""""""""""""""""""""""""""""""""""""""""""""""""""""""""""""""""""""""""""""""""""""""""""""""""""""""""""""""""""""""""""""""""""""""""""""""""""""""""""""""""""""""""""""""""""""""""""""""""""""""""""""""""""""""""""""""""""""""""""""""""""""""""""""""""""""""""""""""""""""""""""""""""""""""""""""""""""""""""""""""""""""""""""""""""""""""""""""""""""""""""""""""""""""""""""""""""""""""""""""""""""""""""""""""""""""""""""""""""""""""""""""""""""""""""""""""""""""""""""""""""""""""""""""""""""""""""""""""""""""""""""""""""""""""""""""""""""""""""""""""""""""""""""""""""""""""""""""""""""""""""""""""""""""""""""""""""""""""""""""""""""""""""""""""""""""""""""""""""""""""""""""""""""""""""""""""""""""""""""""""""""""""""""""""""""""""""""""""""""""""""""""""""""""""""""""""""""""""""""""""""""""""""""""""""""""""""""""""""""""""""""""""""""""""""""""""""""""""""""""""""""""""""""""""""""""""""""""""""""""""""""""""""""""""""""""""""""""""""""""""""""""""""""""""""""""""""""""""""""""""""""""""""""""""""""""""""""""""""""""""""""""""""""""""""""""""""""""""""""""""""""""""""""""""""""""""""""""""""""""""""""""""""""""""""""""""""""""""""""""""""""""""""""""""""""""""""""""""""""""""""""""""""""""""""""""""""""""""""""""""""""""""""""""""""""""""""""""""""""""""""""""""""""""""""""""""""""""""""""""""""""""""""""""""""""""""""""""""""""""""""""""""""""""""""""""""""""""""""""""""""""""""""""""""""""""""""""""""""""""""""""""""""""""""""""""""""""""""""""""""""""""""""""""""""""""""""""""""""""""""""""""""""""""""""""""""""""""""""""""""""""""""""""""""""""""""""""""""""""""""""""""""""""""""""""""""""""""""""""""""""""""""""""""""""""""""""""""""""""""""""""""""""""""""""""""""""""""""""""""""""""""""""""""""""""""""""""""""""""""""""""""""""""""""""""""""""""""""""""""""""""""""""""""""""""""""""""""""""""""""""""""""""""""""""""""""""""""""""""""""""""""""""""""""""""""""""""""""""""""""""""""""""""""""""""""""""""""""""""""""""""""""""""""""""""""""""""""""""""""""""""""""""""""""""""""""""""""""""""""""""""""""""""""""""""""""""""""""""""""""""""""""""""""""""""""""""""""""""""""""""""""""""""""""""""""""""""""""""""""""""""""""""""""""""""""""""""""""""""""""""""""""""""""""""""""""""""""""""""""""""""""""""""""""""""""""""""""""""""""""""""""""""""""""""""""""""""""""""""""""""""""""""""""""""""""""""""""""""""""""""""""""""""""""""""""""""""""""""""""""""""""""""""""""""""""""""""""""""""""""""""""""""""""""""""""""""""""""""""""""""""""""""""""""""""""""""""""""""""""""""""""""""""""""""""""""""""""""""""""""""""""""""""""""""""""""""""""""""""""""""""""""""""""""""""""""""""""""""""""""""""""""""""""""""""""""""""""""""""""""""""""""""""""""""""""""""""""""""""""""""""""""""""""""""""""""""""""""""""""""""""""""""""""""""""""""""""""""""""""""""""""""""""""""""""""""""""""""""""""""""""""""""""""""""""""""""""""""""""""""""""""""""""""""""""""""""""""""""""""""""""""""""""""""""""""""""""""""""""""""""""""""""""""""""""""""""""""""""""""""""""""""""""""""""""""""""""""""""""""""""""""""""""""""""""""""""""""""""""""""""""""""""""""""""""""""""""""""""""""""""""""""""""""""""""""""""""""""""""""""""""""""""""""""""""""""""""""""""""""""""""""""""""""""""""""""""""""""""""""""""""""""""""""""""""""""""""""""""""""""""""""""""""""""""""""""""""""""""""""""""""""""""""""""""""""""""""""""""""""""""""""""""""""""""""""""""""""""""""""""""""""""""""""""""""""""""""""""""""""""""""""""""""""""""""""""""""""""""""""""""""""""""""""""""""""""""""""""""""""""""""""""""""""""""""""""""""""""""""""""""""""""""""""""""""""""""""""""""""""""""""""""""""""""""""""""""""""""""""""""""""""""""""""""""""""""""""""""""""""""""""""""""""""""""""""""""""""""""""""""""""""""""""""""""""""""""""""""""""""""""""""""""""""""""""""""""""""""""""""""""""""""""""""""""""""""""""""""""""""""""""""""""""""""""""""""""""""""""""""""""""""""""""""""""""""""""""""""""""""""""""""""""""""""""""""""""""""""""""""""""""""""""""""""""""""""""""""""""""""""""""""""""""""""""""""""""""""""""""""""""""""""""""""""""""""""""""""""""""""""""""""""""""""""""""""""""""""""""""""""""""""""""""""""""""""""""""""""""""""""""""""""""""""""""""""""""""""""""""""""""""""""""""""""""""""""""""""""""""""""""""""""""""""""""""""""""""""""""""""""""""""""""""""""""""""""""""""""""""""""""""""""""""""""""""""""""""""""""""""""""""""""""""""""""""""""""""""""""""""""""""""""""""""""""""""""""""""""""""""""""""""""""""""""""""""""""""""""""""""""""""""""""""""""""""""""""""""""""""""""""""""""""""""""""""""""""""""""""""""""""""""""""""""""""""""""""""""""""""""""""""""""""""""""""""""""""""""""""""""""""""""""""""""""""""""""""""""""""""""""""""""""""""""""""""""""""""""""""""""""""""""""""""""""""""""""""""""""""""""""""""""""""""""""""""""""""""""""""""""""""""""""""""""""""""""""""""""""""""""""""""""""""""""""""""""""""""""""""""""""""""""""""""""""""""""""""""""""""""""""""""""""""""""""""""""""""""""""""""""""""""""""""""""""""""""""""""""""""""""""""""""""""""""""""""""""""""""""""""""""""""""""""""""""""""""""""""""""""""""""""""""""""""""""""""""""""""""""""""""""""""""""""""""""""""""""""""""""""""""""""""""""""""""""""""""""""""""""""""""""""""""""""""""""""""""""""""""""""""""""""""""""""""""""""""""""""""""""""""""""""""""""""""""""""""""""""""""""""""""""""""""""""""""""""""""""""""""""""""""""""""""""""""""""""""""""""""""""""""""""""""""""""""""""""""""""""""""""""""""""""""""""""""""""""""""""""""""""""""""""""""""""""""""""""""""""""""""""""""""""""""""""""""""""""""""""""""""""""""""""""""""""""""""""""""""""""""""""""""""""""""""""""""""""""""""""""""""""""""""""""""""""""""""""""""""""""""""""""""""""""""""""""""""""""""""""""""""""""""""""""""""""""""""""""""""""""""""""""""""""""""""""""""""""""""""""""""""""""""""""""""""""""""""""""""""""""""""""""""""""""""""""""""""""""""""""""""""""""""""""""""""""""""""""""""""""""""""""""""""""""""""""""""""""""""""""""""""""""""""""""""""""""""""""""""""""""""""""""""""""""""""""""""""""""""""""""""""""""""""""""""""""""""""""""""""""""""""""""""""""""""""""""""""""""""""""""""""""""""""""""""""""""""""""""""""""""""""""""""""""""""""""""""""""""""""""""""""""""""""""""""""""""""""""""""""""""""""""""""""""""""""""""""""""""""""""""""""""""""""""""""""""""""""""""""""""""""""""""""""""""""""""""""""""""""""""""""""""""""""""""""""""""""""""""""""""""""""""""""""""""""""""""""""""""""""""""""""""""""""""""""""""""""""""""""""""""""""""""""""""""""""""""""""""""""""""""""""""""""""""""""""""""""""""""""""""""""""""""""""""""""""""""""""""""""""""""""""""""""""""""""""""""""""""""""""""""""""""""""""""""""""""""""""""""""""""""""""""""""""""""""""""""""""""""""""""""""""""""""""""""""""""""""""""""""""""""""""""""""""""""""""""""""""""""""""""""""""""""""""""""""""""""""""""""""""""""""""""""""""""""""""""""""""""""""""""""""""""""""""""""""""""""""""""""""""""""""""""""""""""""""""""""""""""""""""""""""""""""""""""""""""""""""""""""""""""""""""""""""""""""""""""""""""""""""""""""""""""""""""""""""""""""""""""""""""""""""""""""""""""""""""""""""""""""""""""""""""""""""""""""""""""""""""""""""""""""""""""""""""""""""""""""""""""""""""""""""""""""""""""""""""""""""""""""""""""""""""""""""""""""""""""""""""""""""""""""""""""""""""""""""""""""""""""""""""""""""""""""""""""""""""""""""""""""""""""""""""""""""""""""""""""""""""""""""""""""""""""""""""""""""""""""""""""""""""""""""""""""""""""""""""""""""""""""""""""""""""""""""""""""""""""""""""""""""""""""""""""""""""""""""""""""""""""""""""""""""""""""""""""""""""""""""""""""""""""""""""""""""""""""""""""""""""""""""""""""""""""""""""""""""""""""""""""""""""""""""""""""""""""""""""""""""""""""""""""""""""""""""""""""""""""""""""""""""""""""""""""""""""""""""""""""""""""""""""""""""""""""""""""""""""""""""""""""""""""""""""""""""""""""""""""""""""""""""""""""""""""""""""""""""""""""""""""""""""""""""""""""""""""""""""""""""""""""""""""""""""""""""""""""""""""""""""""""""""""""""""""""""""""""""""""""""""""""""""""""""""""""""""""""""""""""""""""""""""""""""""""""""""""""""""""""""""""""""""""""""""""""""""""""""""""""""""""""""""""""""""""""""""""""""""""""""""""""""""""""""""""""""""""""""""""""""""""""""""""""""""""""""""""""""""""""""""""""""""""""""""""""""""""""""""""""""""""""""""""""""""""""""""""""""""""""""""""""""""""""""""""""""""""""""""""""""""""""""""""""""""""""""""""""""""""""""""""""""""""""""""""""""""""""""""""""""""""""""""""""""""""""""""""""""""""""""""""""""""""""""""""""""""""""""""""""""""""""""""""""""""""""""""""""""""""""""""""""""""""""""""""""""""""""""""""""""""""""""""""""""""""""""""""""""""""""""""""""""""""""""""""""""""""""""""""""""""""""""""""""""""""""""""""""""""""""""""""""""""""""""""""""""""""""""""""""""""""""""""""""""""""""""""""""""""""""""""""""""""""""""""""""""""""""""""""""""""""""""""""""""""""""""""""""""""""""""""""""""""""""""""""""""""""""""""""""""""""""""""""""""""""""""""""""""""""""""""""""""""""""""""""""""""""""""""""""""""""""""""""""""""""""""""""""""""""""""""""""""""""""""""""""""""""""""""""""""""""""""""""""""""""""""""""""""""""""""""""""""""""""""""""""""""""""""""""""""""""""""""""""""""""""""""""""""""""""""""""""""""""""""""""""""""""""""""""""""""""""""""""""""""""""""""""""""""""""""""""""""""""""""""""""""""""""""""""""""""""""""""""""""""""""""""""""""""""""""""""""""""""""""""""""""""""""""""""""""""""""""""""""""""""""""""""""""""""""""""""""""""""""""""""""""""""""""""""""""""""""""""""""""""""""""""""""""""""""""""""""""""""""""""""""""""""""""""""""""""""""""""""""""""""""""""""""""""""""""""""""""""""""""""""""""""""""""""""""""""""""""""""""""""""""""""""""""""""""""""""""""""""""""""""""""""""""""""""""""""""""""""""""""""""""""""""""""""""""""""""""""""""""""""""""""""""""""""""""""""""""""""""""""""""""""""""""""""""""""""""""""""""""""""""""""""""""""""""""""""""""""""""""""""""""""""""""""""""""""""""""""""""""""""""""""""""""""""""""""""""""""""""""""""""""""""""""""""""""""""""""""""""""""""""""""""""""""""""""""""""""""""""""""""""""""""""""""""""""""""""""""""""""""""""""""""""""""""""""""""""""""""""""""""""""""""""""""""""""""""""""""""""""""""""""""""""""""""""""""""""""""""""""""""""""""""""""""""""""""""""""""""""""""""""""""""""""""""""""""""""""""""""""""""""""""""""""""""""""""""""""""""""""""""""""""""""""""""""""""""""""""""""""""""""""""""""""""""""""""""""""""""""""""""""""""""""""""""""""""""""""""""""""""""""""""""""""""""""""""""""""""""""""""""""""""""""""""""""""""""""""""""""""""""""""""""""""""""""""""""""""""""""""""""""""""""""""""""""""""""""""""""""""""""""""""""""""""""""""""""""""""""""""""""""""""""""""""""""""""""""""""""""""""""""""""""""""""""""""""""""""""""""""""""""""""""""""""""""""""""""""""""""""""""""""""""""""""""""""""""""""""""""""""""""""""""""""""""""""""""""""""""""""""""""""""""""""""""""""""""""""""""""""""""""""""""""""""""""""""""""""""""""""""""""""""""""""""""""""""""""""""""""""""""""""""""""""""""""""""""""""""""""""""""""""""""""""""""""""""""""""""""""""""""""""""""""""""""""""""""""""""""""""""""""""""""""""""""""""""""""""""""""""""""""""""""""""""""""""""""""""""""""""""""""""""""""""""""""""""""""""""""""""""""""""""""""""""""""""""""""""""""""""""""""""""""""""""""""""""""""""""""""""""""""""""""""""""""""""""""""""""""""""""""""""""""""""""""""""""""""""""""""""""""""""""""""""""""""""""""""""""""""""""""""""""""""""""""""""""""""""""""""""""""""""""""""""""""""""""""""""""""""""""""""""""""""""""""""""""""""""""""""""""""""""""""""""""""""""""""""""""""""""""""""""""""""""""""""""""""""""""""""""""""""""""""""""""""""""""""""""""""""""""""""""""""""""""""""""""""""""""""""""""""""""""""""""""""""""""""""""""""""""""""""""""""""""""""""""""""""""""""""""""""""""""""""""""""""""""""""""""""""""""""""""""""""""""""""""""""""""""""""""""""""""""""""""""""""""""""""""""""""""""""""""""""""""""""""""""""""""""""""""""""""""""""""""""""""""""""""""""""""""""""""""""""""""""""""""""""""""""""""""""""""""""""""""""""""""""""""""""""""""""""""""""""""""""""""""""""""""""""""""""""""""""""""""""""""""""""""""""""""""""""""""""""""""""""""""""""""""""""""""""""""""""""""""""""""""""""""""""""""""""""""""""""""""""""""""""""""""""""""""""""""""""""""""""""""""""""""""""""""""""""""""""""""""""""""""""""""""""""""""""""""""""""""""""""""""""""""""""""""""""""""""""""""""""""""""""""""""""""""""""""""""""""""""""""""""""""""""""""""""""""""""""""""""""""""""""""""""""""""""""""""""""""""""""""""""""""""""""""""""""""""""""""""""""""""""""""""""""""""""""""""""""""""""""""""""""""""""""""""""""""""""""""""""""""""""""""""""""""""""""""""""""""""""""""""""""""""""""""""""""""""""""""""""""""""""""""""""""""""""""""""""""""""""""""""""""""""""""""""""""""""""""""""""""""""""""""""""""""""""""""""""""""""""""""""""""""""""""""""""""""""""""""""""""""""""""""""""""""""""""""""""""""""""""""""""""""""""""""""""""""""""""""""""""""""""""""""""""""""""""""""""""""""""""""""""""""""""""""""""""""""""""""""""""""""""""""""""""""""""""""""""""""""""""""""""""""""""""""""""""""""""""""""""""""""""""""""""""""""""""""""""""""""""""""""""""""""""""""""""""""""""""""""""""""""""""""""""""""""""""""""""""""""""""""""""""""""""""""""""""""""""""""""""""""""""""""""""""""""""""""""""""""""""""""""""""""""""""""""""""""""""""""""""""""""""""""""""""""""""""""""""""""""""""""""""""""""""""""""""""""""""""""""""""""""""""""""""""""""""""""""""""""""""""""""""""""""""""""""""""""""""""""""""""""""""""""""""""""""""""""""""""""""""""""""""""""""""""""""""""""""""""""""""""""""""""""""""""""""""""""""""""""""""""""""""""""""""""""""""""""""""""""""""""""""""""""""""""""""""""""""""""""""""""""""""""""""""""""""""""""""""""""""""""""""""""""""""""""""""""""""""""""""""""""""""""""""""""""""""""""""""""""""""""""""""""""""""""""""""""""""""""""""""""""""""""""""""""""""""""""""""""""""""""""""""""""""""""""""""""""""""""""""""""""""""""""""""""""""""""""""""""""""""""""""""""""""""""""""""""""""""""""""""""""""""""""""""""""""""""""""""""""""""""""""""""""""""""""""""""""""""""""""""""""""""""""""""""""""""""""""""""""""""""""""""""""""""""""""""""""""""""""""""""""""""""""""""""""""""""""""""""""""""""""""""""""""""""""""""""""""""""""""""""""""""""""""""""""""""""""""""""""""""""""""""""""""""""""""""""""""""""""""""""""""""""""""""""""""""""""""""""""""""""""""""""""""""""""""""""""""""""""""""""""""""""""""""""""""""""""""""""""""""""""""""""""""""""""""""""""""""""""""""""""""""""""""""""""""""""""""""""""""""""""""""""""""""""""""""""""""""""""""""""""""""""""""""""""""""""""""""""""""""""""""""""""""""""""""""""""""""""""""""""""""""""""""""""""""""""""""""""""""""""""""""""""""""""""""""""""""""""""""""""""""""""""""""""""""""""""""""""""""""""""""""""""""""""""""""""""""""""""""""""""""""""""""""""""""""""""""""""""""""""""""""""""""""""""""""""""""""""""""""""""""""""""""""""""""""""""""""""""""""""""""""""""""""""""""""""""""""""""""""""""""""""""""""""""""""""""""""""""""""""""""""""""""""""""""""""""""""""""""""""""""""""""""""""""""""""""""""""""""""""""""""""""""""""""""""""""""""""""""""""""""""""""""""""""""""""""""""""""""""""""""""""""""""""""""""""""""""""""""""""""""""""""""""""""""""""""""""""""""""""""""""""""""""""""""""""""""""""""""""""""""""""""""""""""""""""""""""""""""""""""""""""""""""""""""""""""""""""""""""""""""""""""""""""""""""""""""""""""""""""""""""""""""""""""""""""""""""""""""""""""""""""""""""""""""""""""""""""""""""""""""""""""""""""""""""""""""""""""""""""""""""""""""""""""""""""""""""""""""""""""""""""""""""""""""""""""""""""""""""""""""""""""""""""""""""""""""""""""""""""""""""""""""""""""""""""""""""""""""""""""""""""""""""""""""""""""""""""""""""""""""""""""""""""""""""""""""""""""""""""""""""""""""""""""""""""""""""""""""""""""""""""""""""""""""""""""""""""""""""""""""""""""""""""""""""""""""""""""""""""""""""""""""""""""""""""""""""""""""""""""""""""""""""""""""""""""""""""""""""""""""""""""""""""""""""""""""""""""""""""""""""""""""""""""""""""""""""""""""""""""""""""""""""""""""""""""""""""""""""""""""""""""""""""""""""""""""""""""""""""""""""""""""""""""""""""""""""""""""""""""""""""""""""""""""""""""""""""""""""""""""""""""""""""""""""""""""""""""""""""""""""""""""""""""""""""""""""""""""""""""""""""""""""""""""""""""""""""""""""""""""""""""""""""""""""""""""""""""""""""""""""""""""""""""""""""""""""""""""""""""""""""""""""""""""""""""""""""""""""""""""""""""""""""""""""""""""""""""""""""""""""""""""""""""""""""""""""""""""""""""""""""""""""""""""""""""""""""""""""""""""""""""""""""""""""""""""""""""""""""""""""""""""""""""""""""""""""""""""""""""""""""""""""""""""""""""""""""""""""""""""""""""""""""""""""""""""""""""""""""""""""""""""""""""""""""""""""""""""""""""""""""""""""""""""""""""""""""""""""""""""""""""""""""""""""""""""""""""""""""""""""""""""""""""""""""""""""""""""""""""""""""""""""""""""""""""""""""""""""""""""""""""""""""""""""""""""""""""""""""""""""""""""""""""""""""""""""""""""""""""""""""""""""""""""""""""""""""""""""""""""""""""""""""""""""""""""""""""""""""""""""""""""""""""""""""""""""""""""""""""""""""""""""""""""""""""""""""""""""""""""""""""""""""""""""""""""""""""""""""""""""""""""""""""""""""""""""""""""""""""""""""""""""""""""""""""""""""""""""""""""""""""""""""""""""""""""""""""""""""""""""""""""""""""""""""""""""""""""""""""""""""""""""""""""""""""""""""""""""""""""""""""""""""""""""""""""""""""""""""""""""""""""""""""""""""""""""""""""""""""""""""""""""""""""""""""""""""""""""""""""""""""""""""""""""""""""""""""""""""""""""""""""""""""""""""""""""""""""""""""""""""""""""""""""""""""""""""""""""""""""""""""""""""""""""""""""""""""""""""""""""""""""""""""""""""""""""""""""""""""""""""""""""""""""""""""""""""""""""""""""""""""""""""""""""""""""""""""""""""""""""""""""""""""""""""""""""""""""""""""""""""""""""""""""""""""""""""""""""""""""""""""""""""""""""""""""""""""""""""""""""""""""""""""""""""""""""""""""""""""""""""""""""""""""""""""""""""""""""""""""""""""""""""""""""""""""""""""""""""""""""""""""""""""""""""""""""""""""""""""""""""""""""""""""""""""""""""""""""""""""""""""""""""""""""""""""""""""""""""""""""""""""""""""""""""""""""""""""""""""""""""""""""""""""""""""""""""""""""""""""""""""""""""""""""""""""""""""""""""""""""""""""""""""""""""""""""""""""""""""""""""""""""""""""""""""""""""""""""""""""""""""""""""""""""""""""""""""""""""""""""""""""""""""""""""""""""""""""""""""""""""""""""""""""""""""""""""""""""""""""""""""""""""""""""""""""""""""""""""""""""""""""""""""""""""""""""""""""""""""""""""""""""""""""""""""""""""""""""""""""""""""""""""""""""""""""""""""""""""""""""""""""""""""""""""""""""""""""""""""""""""""""""""""""""""""""""""""""""""""""""""""""""""""""""""""""""""""""""""""""""""""""""""""""""""""""""""""""""""""""""""""""""""""""""""""""""""""""""""""""""""""""""""""""""""""""""""""""""""""""""""""""""""""""""""""""""""""""""""""""""""""""""""""""""""""""""""""""""""""""""""""""""""""""""""""""""""""""""""""""""""""""""""""""""""""""""""""""""""""""""""""""""""""""""""""""""""""""""""""""""""""""""""""""""""""""""""""""""""""""""""""""""""""""""""""""""""""""""""""""""""""""""""""""""""""""""""""""""""""""""""""""""""""""""""""""""""""""""""""""""""""""""""""""""""""""""""""""""""""""""""""""""""""""""""""""""""""""""""""""""""""""""""""""""""""""""""""""""""""""""""""""""""""""""""""""""""""""""""""""""""""""""""""""""""""""""""""""""""""""""""""""""""""""""""""""""""""""""""""""""""""""""""""""""""""""""""""""""""""""""""""""""""""""""""""""""""""""""""""""""""""""""""""""""""""""""""""""""""""""""""""""""""""""""""""""""""""""""""""""""""""""""""""""""""""""""""""""""""""""""""""""""""""""""""""""""""""""""""""""""""""""""""""""""""""""""""""""""""""""""""""""""""""""""""""""""""""""""""""""""""""""""""""""""""""""""""""""""""""""""""""""""""""""""""""""""""""""""""""""""""""""""""""""""""""""""""""""""""""""""""""""""""""""""""""""""""""""""""""""""""""""""""""""""""""""""""""""""""""""""""""""""""""""""""""""""""""""""""""""""""""""""""""""""""""""""""""""""""""""""""""""""""""""""""""""""""""""""""""""""""""""""""""""""""""""""""""""""""""""""""""""""""""""""""""""""""""""""""""""""""""""""""""""""""""""""""""""""""""""""""""""""""""""""""""""""""""""""""""""""""""""""""""""""""""""""""""""""""""""""""""""""""""""""""""""""""""""""""""""""""""""""""""""""""""""""""""""""""""""""""""""""""""""""""""""""""""""""""""""""""""""""""""""""""""""""""""""""""""""""""""""""""""""""""""""""""""""""""""""""""""""""""""""""""""""""""""""""""""""""""""""""""""""""""""""""""""""""""""""""""""""""""""""""""""""""""""""""""""""""""""""""""""""""""""""""""""""""""""""""""""""""""""""""""""""""""""""""""""""""""""""""""""""""""""""""""""""""""""""""""""""""""""""""""""""""""""""""""""""""""""""""""""""""""""""""""""""""""""""""""""""""""""""""""""""""""""""""""""""""""""""""""""""""""""""""""""""""""""""""""""""""""""""""""""""""""""""""""""""""""""""""""""""""""""""""""""""""""""""""""""""""""""""""""""""""""""""""""""""""""""""""""""""""""""""""""""""""""""""""""""""""""""""""""""""""""""""""""""""""""""""""""""""""""""""""""""""""""""""""""""""""""""""""""""""""""""""""""""""""""""""""""""""""""""""""""""""""""""""""""""""""""""""""""""""""""""""""""""""""""""""""""""""""""""""""""""""""""""""""""""""""""""""""""""""""""""""""""""""""""""""""""""""""""""""""""""""""""""""""""""""""""""""""""""""""""""""""""""""""""""""""""""""""""""""""""""""""""""""""""""""""""""""""""""""""""""""""""""""""""""""""""""""""""""""""""""""""""""""""""""""""""""""""""""""""""""""""""""""""""""""""""""""""""""""""""""""""""""""""""""""""""""""""""""""""""""""""""""""""""""""""""""""""""""""""""""""""""""""""""""""""""""""""""""""""""""""""""""""""""""""""""""""""""""""""""""""""""""""""""""""""""""""""""""""""""""""""""""""""""""""""""""""""""""""""""""""""""""""""""""""""""""""""""""""""""""""""""""""""""""""""""""""""""""""""""""""""""""""""""""""""""""""""""""""""""""""""""""""""""""""""""""""""""""""""""""""""""""""""""""""""""""""""""""""""""""""""""""""""""""""""""""""""""""""""""""""""""""""""""""""""""""""""""""""""""""""""""""""""""""""""""""""""""""""""""""""""""""""""""""""""""""""""""""""""""""""""""""""""""""""""""""""""""""""""""""""""""""""""""""""""""""""""""""""""""""""""""""""""""""""""""""""""""""""""""""""""""""""""""""""""""""""""""""""""""""""""""""""""""""""""""""""""""""""""""""""""""""""""""""""""""""""""""""""""""""""""""""""""""""""""""""""""""""""""""""""""""""""""""""""""""""""""""""""""""""""""""""""""""""""""""""""""""""""""""""""""""""""""""""""""""""""""""""""""""""""""""""""""""""""""""""""""""""""""""""""""""""""""""""""""""""""""""""""""""""""""""""""""""""""""""""""""""""""""""""""""""""""""""""""""""""""""""""""""""""""""""""""""""""""""""""""""""""""""""""""""""""""""""""""""""""""""""""""""""""""""""""""""""""""""""""""""""""""""""""""""""""""""""""""""""""""""""""""""""""""""""""""""""""""""""""""""""""""""""""""""""""""""""""""""""""""""""""""""""""""""""""""""""""""""""""""""""""""""""""""""""""""""""""""""""""""""""""""""""""""""""""""""""""""""""""""""""""""""""""""""""""""""""""""""""""""""""""""""""""""""""""""""""""""""""""""""""""""""""""""""""""""""""""""""""""""""""""""""""""""""""""""""""""""""""""""""""""""""""""""""""""""""""""""""""""""""""""""""""""""""""""""""""""""""""""""""""""""""""""""""""""""""""""""""""""""""""""""""""""""""""""""""""""""""""""""""""""""""""""""""""""""""""""""""""""""""""""""""""""""""""""""""""""""""""""""""""""""""""""""""""""""""""""""""""""""""""""""""""""""""""""""""""""""""""""""""""""""""""""""""""""""""""""""""""""""""""""""""""""""""""""""""""""""""""""""""""""""""""""""""""""""""""""""""""""""""""""""""""""""""""""""""""""""""""""""""""""""""""""""""""""""""""""""""""""""""""""""""""""""""""""""""""""""""""""""""""""""""""""""""""""""""""""""""""""""""""""""""""""""""""""""""""""""""""""""""""""""""""""""""""""""""""""""""""""""""""""""""""""""""""""""""""""""""""""""""""""""""""""""""""""""""""""""""""""""""""""""""""""""""""""""""""""""""""""""""""""""""""""""""""""""""""""""""""""""""""""""""""""""""""""""""""""""""""""""""""""""""""""""""""""""""""""""""""""""""""""""""""""""""""""""""""""""""""""""""""""""""""""""""""""""""""""""""""""""""""""""""""""""""""""""""""""""""""""""""""""""""""""""""""""""""""""""""""""""""""""""""""""""""""""""""""""""""""""""""""""""""""""""""""""""""""""""""""""""""""""""""""""""""""""""""""""""""""""""""""""""""""""""""""""""""""""""""""""""""""""""""""""""""""""""""""""""""""""""""""""""""""""""""""""""""""""""""""""""""""""""""""""""""""""""""""""""""""""""""""""""""""""""""""""""""""""""""""""""""""""""""""""""""""""""""""""""""""""""""""""""""""""""""""""""""""""""""""""""""""""""""""""""""""""""""""""""""""""""""""""""""""""""""""""""""""""""""""""""""""""""""""""""""""""""""""""""""""""""""""""""""""""""""""""""""""""""""""""""""""""""""""""""""""""""""""""""""""""""""""""""""""""""""""""""""""""""""""""""""""""""""""""""""""""""""""""""""""""""""""""""""""""""""""""""""""""""""""""""""""""""""""""""""""""""""""""""""""""""""""""""""""""""""""""""""""""""""""""""""""""""""""""""""""""""""""""""""""""""""""""""""""""""""""""""""""""""""""""""""""""""""""""""""""""""""""""""""""""""""""""""""""""""""""""""""""""""""""""""""""""""""""""""""""""""""""""""""""""""""""""""""""""""""""""""""""""""""""""""""""""""""""""""""""""""""""""""""""""""""""""""""""""""""""""""""""""""""""""""""""""""""""""""""""""""""""""""""""""""""""""""""""""""""""""""""""""""""""""""""""""""""""""""""""""""""""""""""""""""""""""""""""""""""""""""""""""""""""""""""""""""""""""""""""""""""""""""""""""""""""""""""""""""""""""""""""""""""""""""""""""""""""""""""""""""""""""""""""""""""""""""""""""""""""""""""""""""""""""""""""""""""""""""""""""""""""""""""""""""""""""""""""""""""""""""""""""""""""""""""""""""""""""""""""""""""""""""""""""""""""""""""""""""""""""""""""""""""""""""""""""""""""""""""""""""""""""""""""""""""""""""""""""""""""""""""""""""""""""""""""""""""""""""""""""""""""""""""""""""""""""""""""""""""""""""""""""""""""""""""""""""""""""""""""""""""""""""""""""""""""""""""""""""""""""""""""""""""""""""""""""""""""""""""""""""""""""""""""""""""""""""""""""""""""""""""""""""""""""""""""""""""""""""""""""""""""""""""""""""""""""""""""""""""""""""""""""""""""""""""""""""""""""""""""""""""""""""""""""""""""""""""""""""""""""""""""""""""""""""""""""""""""""""""""""""""""""""""""""""""""""""""""""""""""""""""""""""""""""""""""""""""""""""""""""""""""""""""""""""""""""""""""""""""""""""""""""""""""""""""""""""""""""""""""""""""""""""""""""""""""""""""""""""""""""""""""""""""""""""""""""""""""""""""""""""""""""""""""""""""""""""""""""""""""""""""""""""""""""""""""""""""""""""""""""""""""""""""""""""""""""""""""""""""""""""""""""""""""""""""""""""""""""""""""""""""""""""""""""""""""""""""""""""""""""""""""""""""""""""""""""""""""""""""""""""""""""""""""""""""""""""""""""""""""""""""""""""""""""""""""""""""""""""""""""""""""""""""""""""""""""""""""""""""""""""""""""""""""""""""""""""""""""""""""""""""""""""""""""""""""""""""""""""""""""""""""""""""""""""""""""""""""""""""""""""""""""""""""""""""""""""""""""""""""""""""""""""""""""""""""""""""""""""""""""""""""""""""""""""""""""""""""""""""""""""""""""""""""""""""""""""""""""""""""""""""""""""""""""""""""""""""""""""""""""""""""""""""""""""""""""""""""""""""""""""""""""""""""""""""""""""""""""""""""""""""""""""""""""""""""""""""""""""""""""""""""""""""""""""""""""""""""""""""""""""""""""""""""""""""""""""""""""""""""""""""""""""""""""""""""""""""""""""""""""""""""""""""""""""""""""""""""""""""""""""""""""""""""""""""""""""""""""""""""""""""""""""""""""""""""""""""""""""""""""""""""""""""""""""""""""""""""""""""""""""""""""""""""""""""""""""""""""""""""""""""""""""""""""""""""""""""""""""""""""""""""""""""""""""""""""""""""""""""""""""""""""""""""""""""""""""""""""""""""""""""""""""""""""""""""""""""""""""""""""""""""""""""""""""""""""""""""""""""""""""""""""""""""""""""""""""""""""""""""""""""""""""""""""""""""""""""""""""""""""""""""""""""""""""""""""""""""""""""""""""""""""""""""""""""""""""""""""""""""""""""""""""""""""""""""""""""""""""""""""""""""""""""""""""""""""""""""""""""""""""""""""""""""""""""""""""""""""""""""""""""""""""""""""""""""""""""""""""""""""""""""""""""""""""""""""""""""""""""""""""""""""""""""""""""""""""""""""""""""""""""""""""""""""""""""""""""""""""""""""""""""""""""""""""""""""""""""""""""""""""""""""""""""""""""""""""""""""""""""""""""""""""""""""""""""""""""""""""""""""""""""""""""""""""""""""""""""""""""""""""""""""""""""""""""""""""""""""""""""""""""""""""""""""""""""""""""""""""""""""""""""""""""""""""""""""""""""""""""""""""""""""""""""""""""""""""""""""""""""""""""""""""""""""""""""""""""""""""""""""""""""""""""""""""""""""""""""""""""""""""""""""""""""""""""""""""""""""""""""""""""""""""""""""""""""""""""""""""""""""""""""""""""""""""""""""""""""""""""""""""""""""""""""""""""""""""""""""""""""""""""""""""""""""""""""""""""""""""""""""""""""""""""""""""""""""""""""""""""""""""""""""""""""""""""""""""""""""""""""""""""""""""""""""""""""""""""""""""""""""""""""""""""""""""""""""""""""""""""""""""""""""""""""""""""""""""""""""""""""""""""""""""""""""""""""""""""""""""""""""""""""""""""""""""""""""""""""""""""""""""""""""""""""""""""""""""""""""""""""""""""""""""""""""""""""""""""""""""""""""""""""""""""""""""""""""""""""""""""""""""""""""""""""""""""""""""""""""""""""""""""""""""""""""""""""""""""""""""""""""""""""""""""""""""""""""""""""""""""""""""""""""""""""""""""""""""""""""""""""""""""""""""""""""""""""""""""""""""""""""""""""""""""""""""""""""""""""""""""""""""""""""""""""""""""""""""""""""""""""""""""""""""""""""""""""""""""""""""""""""""""""""""""""""""""""""""""""""""""""""""""""""""""""""""""""""""""""""""""""""""""""""""""""""""""""""""""""""""""""""""""""""""""""""""""""""""""""""""""""""""""""""""""""""""""""""""""""""""""""""""""""""""""""""""""""""""""""""""""""""""""""""""""""""""""""""""""""""""""""""""""""""""""""""""""""""""""""""""""""""""""""""""""""""""""""""""""""""""""""""""""""""""""""""""""""""""""""""""""""""""""""""""""""""""""""""""""""""""""""""""""""""""""""""""""""""""""""""""""""""""""""""""""""""""""""""""""""""""""""""""""""""""""""""""""""""""""""""""""""""""""""""""""""""""""""""""""""""""""""""""""""""""""""""""""""""""""""""""""""""""""""""""""""""""""""""""""""""""""""""""""""""""""""""""""""""""""""""""""""""""""""""""""""""""""""""""""""""""""""""""""""""""""""""""""""""""""""""""""""""""""""""""""""""""""""""""""""""""""""""""""""""""""""""""""""""""""""""""""""""""""""""""""""""""""""""""""""""""""""""""""""""""""""""""""""""""""""""""""""""""""""""""""""""""""""""""""""""""""""""""""""""""""""""""""""""""""""""""""""""""""""""""""""""""""""""""""""""""""""""""""""""""""""""""""""""""""""""""""""""""""""""""""""""""""""""""""""""""""""""""""""""""""""""""""""""""""""""""""""""""""""""""""""""""""""""""""""""""""""""""""""""""""""""""""""""""""""""""""""""""""""""""""""""""""""""""""""""""""""""""""""""""""""""""""""""""""""""""""""""""""""""""""""""""""""""""""""""""""""""""""""""""""""""""""""""""""""""""""""""""""""""""""""""""""""""""""""""""""""""""""""""""""""""""""""""""""""""""""""""""""""""""""""""""""""""""""""""""""""""""""""""""""""""""""""""""""""""""""""""""""""""""""""""""""""""""""""""""""""""""""""""""""""""""""""""""""""""""""""""""""""""""""""""""""""""""""""""""""""""""""""""""""""""""""""""""""""""""""""""""""""""""""""""""""""""""""""""""""""""""""""""""""""""""""""""""""""""""""""""""""""""""""""""""""""""""""""""""""""""""""""""""""""""""""""""""""""""""""""""""""""""""""""""""""""""""""""""""""""""""""""""""""""""""""""""""""""""""""""""""""""""""""""""""""""""""""""""""""""""""""""""""""""""""""""""""""""""""""""""""""""""""""""""""""""""""""""""""""""""""""""""""""""""""""""""""""""""""""""""""""""""""""""""""""""""""""""""""""""""""""""""""""""""""""""""""""""""""""""""""""""""""""""""""""""""""""""""""""""""""""""""""""""""""""""""""""""""""""""""""""""""""""""""""""""""""""""""""""""""""""""""""""""""""""""""""""""""""""""""""""""""""""""""""""""""""""""""""""""""""""""""""""""""""""""""""""""""""""""""""""""""""""""""""""""""""""""""""""""""""""""""""""""""""""""""""""""""""""""""""""""""""""""""""""""""""""""""""""""""""""""""""""""""""""""""""""""""""""""""""""""""""""""""""""""""""""""""""""""""""""""""""""""""""""""""""""""""""""""""""""""""""""""""""""""""""""""""""""""""""""""""""""""""""""""""""""""""""""""""""""""""""""""""""""""""""""""""""""""""""""""""""""""""""""""""""""""""""""""""""""""""""""""""""""""""""""""""""""""""""""""""""""""""""""""""""""""""""""""""""""""""""""""""""""""""""""""""""""""""""""""""""""""""""""""""""""""""""""""""""""""""""""""""""""""""""""""""""""""""""""""""""""""""""""""""""""""""""""""""""""""""""""""""""""""""""""""""""""""""""""""""""""""""""""""""""""""""""""""""""""""""""""""""""""""""""""""""""""""""""""""""""""""""""""""""""""""""""""""""""""""""""""""""""""""""""""""""""""""""""""""""""""""""""""""""""""""""""""""""""""""""""""""""""""""""""""""""""""""""""""""""""""""""""""""""""""""""""""""""""""""""""""""""""""""""""""""""""""""""""""""""""""""""""""""""""""""""""""""""""""""""""""""""""""""""""""""""""""""""""""""""""""""""""""""""""""""""""""""""""""""""""""""""""""""""""""""""""""""""""""""""""""""""""""""""""""""""""""""""""""""""""""""""""""""""""""""""""""""""""""""""""""""""""""""""""""""""""""""""""""""""""""""""""""""""""""""""""""""""""""""""""""""""""""""""""""""""""""""""""""""""""""""""""""""""""""""""""""""""""""""""""""""""""""""""""""""""""""""""""""""""""""""""""""""""""""""""""""""""""""""""""""""""""""""""""""""""""""""""""""""""""""""""""""""""""""""""""""""""""""""""""""""""""""""""""""""""""""""""""""""""""""""""""""""""""""""""""""""""""""""""""""""""""""""""""""""""""""""""""""""""""""""""""""""""""""""""""""""""""""""""""""""""""""""""""""""""""""""""""""""""""""""""""""""""""""""""""""""""""""""""""""""""""""""""""""""""""""""""""""""""""""""""""""""""""""""""""""""""""""""""""""""""""""""""""""""""""""""""""""""""""""""""""""""""""""""""""""""""""""""""""""""""""""""""""""""""""""""""""""""""""""""""""""""""""""""""""""""""""""""""""""""""""""""""""""""""""""""""""""""""""""""""""""""""""""""""""""""""""""""""""""""""""""""""""""""""""""""""""""""""""""""""""""""""""""""""""""""""""""""""""""""""""""""""""""""""""""""""""""""""""""""""""""""""""""""""""""""""""""""""""""""""""""""""""""""""""""""""""""""""""""""""""""""""""""""""""""""""""""""""""""""""""""""""""""""""""""""""""""""""""""""""""""""""""""""""""""""""""""""""""""""""""""""""""""""""""""""""""""""""""""""""""""""""""""""""""""""""""""""""""""""""""""""""""""""""""""""""""""""""""""""""""""""""""""""""""""""""""""""""""""""""""""""""""""""""""""""""""""""""""""""""""""""""""""""""""""""""""""""""""""""""""""""""""""""""""""""""""""""""""""""""""""""""""""""""""""""""""""""""""""""""""""""""""""""""""""""""""""""""""""""""""""""""""""""""""""""""""""""""""""""""""""""""""""""""""""""""""""""""""""""""""""""""""""""""""""""""""""""""""""""""""""""""""""""""""""""""""""""""""""""""""""""""""""""""""""""""""""""""""""""""""""""""""""""""""""""""""""""""""""""""""""""""""""""""""""""""""""""""""""""""""""""""""""""""""""""""""""""""""""""""""""""""""""""""""""""""""""""""""""""""""""""""""""""""""""""""""""""""""""""""""""""""""""""""""""""""""""""""""""""""""""""""""""""""""""""""""""""""""""""""""""""""""""""""""""""""""""""""""""""""""""""""""""""""""""""""""""""""""""""""""""""""""""""""""""""""""""""""""""""""""""""""""""""""""""""""""""""""""""""""""""""""""""""""""""""""""""""""""""""""""""""""""""""""""""""""""""""""""""""""""""""""""""""""""""""""""""""""""""""""""""""""""""""""""""""""""""""""""""""""""""""""""""""""""""""""""""""""""""""""""""""""""""""""""""""""""""""""""""""""""""""""""""""""""""""""""""""""""""""""""""""""""""""""""""""""""""""""""""""""""""""""""""""""""""""""""""""""""""""""""""""""""""""""""""""""""""""""""""""""""""""""""""""""""""""""""""""""""""""""""""""""""""""""""""""""""""""""""""""""""""""""""""""""""""""""""""""""""""""""""""""""""""""""""""""""""""""""""""""""""""""""""""""""""""""""""""""""""""""""""""""""""""""""""""""""""""""""""""""""""""""""""""""""""""""""""""""""""""""""""""""""""""""""""""""""""""""""""""""""""""""""""""""""""""""""""""""""""""""""""""""""""""""""""""""""""""""""""""""""""""""""""""""""""""""""""""""""""""""""""""""""""""""""""""""""""""""""""""""""""""""""""""""""""""""""""""""""""""""""""""""""""""""""""""""""""""""""""""""""""""""""""""""""""""""""""""""""""""""""""""""""""""""""""""""""""""""""""""""""""""""""""""""""""""""""""""""""""""""""""""""""""""""""""""""""""""""""""""""""""""""""""""""""""""""""""""""""""""""""""""""""""""""""""""""""""""""""""""""""""""""""""""""""""""""""""""""""""""""""""""""""""""""""""""""""""""""""""""""""""""""""""""""""""""""""""""""""""""""""""""""""""""""""""""""""""""""""""""""""""""""""""""""""""""""""""""""""""""""""""""""""""""""""""""""""""""""""""""""""""""""""""""""""""""""""""""""""""""""""""""""""""""""""""""""""""""""""""""""""""""""""""""""""""""""""""""""""""""""""""""""""""""""""""""""""""""""""""""""""""""""""""""""""""""""""""""""""""""""""""""""""""""""""""""""""""""""""""""""""""""""""""""""""""""""""""""""""""""""""""""""""""""""""""""""""""""""""""""""""""""""""""""""""""""""""""""""""""""""""""""""""""""""""""""""""""""""""""""""""""""""""""""""""""""""""""""""""""""""""""""""""""""""""""""""""""""""""""""""""""""""""""""""""""""""""""""""""""""""""""""""""""""""""""""""""""""""""""""""""""""""""""""""""""""""""""""""""""""""""""""""""""""""""""""""""""""""""""""""""""""""""""""""""""""""""""""""""""""""""""""""""""""""""""""""""""""""""""""""""""""""""""""""""""""""""""""""""""""""""""""""""""""""""""""""""""""""""""""""""""""""""""""""""""""""""""""""""""""""""""""""""""""""""""""""""""""""""""""""""""""""""""""""""""""""""""""""""""""""""""""""""""""""""""""""""""""""""""""""""""""""""""""""""""""""""""""""""""""""""""""""""""""""""""""""""""""""""""""""""""""""""""""""""""""""""""""""""""""""""""""""""""""""""""""""""""""""""""""""""""""""""""""""""""""""""""""""""""""""""""""""""""""""""""""""""""""""""""""""""""""""""""""""""""""""""""""""""""""""""""""""""""""""""""""""""""""""""""""""""""""""""""""""""""""""""""""""""""""""""""""""""""""""""""""""""""""""""""""""""""""""""""""""""""""""""""""""""""""""""""""""""""""""""""""""""""""""""""""""""""""""""""""""""""""""""""""""""""""""""""""""""""""""""""""""""""""""""""""""""""""""""""""""""""""""""""""""""""""""""""""""""""""""""""""""""""""""""""""""""""""""""""""""""""""""""""""""""""""""""""""""""""""""""""""""""""""""""""""""""""""""""""""""""""""""""""""""""""""""""""""""""""""""""""""""""""""""""""""""""""""""""""""""""""""""""""""""""""""""""""""""""""""""""""""""""""""""""""""""""""""""""""""""""""""""""""""""""""""""""""""""""""""""""""""""""""""""""""""""""""""""""""""""""""""""""""""""""""""""""""""""""""""""""""""""""""""""""""""""""""""""""""""""""""""""""""""""""""""""""""""""""""""""""""""""""""""""""""""""""""""""""""""""""""""""""""""""""""""""""""""""""""""""""""""""""""""""""""""""""""""""""""""""""""""""""""""""""""""""""""""""""""""""""""""""""""""""""""""""""""""""""""""""""""""""""""""""""""""""""""""""""""""""""""""""""""""""""""""""""""""""""""""""""""""""""""""""""""""""""""""""""""""""""""""""""""""""""""""""""""""""""""""""""""""""""""""""""""""""""""""""""""""""""""""""""""""""""""""""""""""""""""""""""""""""""""""""""""""""""""""""""""""""""""""""""""""""""""""""""""""""""""""""""""""""""""""""""""""""""""""""""""""""""""""""""""""""""""""""""""""""""""""""""""""""""""""""""""""""""""""""""""""""""""""""""""""""""""""""""""""""""""""""""""""""""""""""""""""""""""""""""""""""""""""""""""""""""""""""""""""""""""""""""""""""""""""""""""""""""""""""""""""""""""""""""""""""""""""""""""""""""""""""""""""""""""""""""""""""""""""""""""""""""""""""""""""""""""""""""""""""""""""""""""""""""""""""""""""""""""""""""""""""""""""""""""""""""""""""""""""""""""""""""""""""""""""""""""""""""""""""""""""""""""""""""""""""""""""""""""""""""""""""""""""""""""""""""""""""""""""""""""""""""""""""""""""""""""""""""""""""""""""""""""""""""""""""""""""""""""""""""""""""""""""""""""""""""""""""""""""""""""""""""""""""""""""""""""""""""""""""""""""""""""""""""""""""""""""""""""""""""""""""""""""""""""""""""""""""""""""""""""""""""""""""""""""""""""""""""""""""""""""""""""""""""""""""""""""""""""""""""""""""""""""""""""""""""""""""""""""""""""""""""""""""""""""""""""""""""""""""""""""""""""""""""""""""""""""""""""""""""""""""""""""""""""""""""""""""""""""""""""""""""""""""""""""""""""""""""""""""""""""""""""""""""""""""""""""""""""""""""""""""""""""""""""""""""""""""""""""""""""""""""""""""""""""""""""""""""""""""""""""""""""""""""""""""""""""""""""""""""""""""""""""""""""""""""""""""""""""""""""""""""""""""""""""""""""""""""""""""""""""""""""""""""""""""""""""""""""""""""""""""""""""""""""""""""""""""""""""""""""""""""""""""""""""""""""""""""""""""""""""""""""""""""""""""""""""""""""""""""""""""""""""""""""""""""""""""""""""""""""""""""""""""""""""""""""""""""""""""""""""""""""""""""""""""""""""""""""""""""""""""""""""""""""""""""""""""""""""""""""""""""""""""""""""""""""""""""""""""""""""""""""""""""""""""""""""""""""""""""""""""""""""""""""""""""""""""""""""""""""""""""""""""""""""""""""""""""""""""""""""""""""""""""""""""""""""""""""""""""""""""""""""""""""""""""""""""""""""""""""""""""""""""""""""""""""""""""""""""""""""""""""""""""""""""""""""""""""""""""""""""""""""""""""""""""""""""""""""""""""""""""""""""""""""""""""""""""""""""""""""""""""""""""""""""""""""""""""""""""""""""""""""""""""""""""""""""""""""""""""""""""""""""""""""""""""""""""""""""""""""""""""""""""""""""""""""""""""""""""""""""""""""""""""""""""""""""""""""""""""""""""""""""""""""""""""""""""""""""""""""""""""""""""""""""""""""""""""""""""""""""""""""""""""""""""""""""""""""""""""""""""""""""""""""""""""""""""""""""""""""""""""""""""""""""""""""""""""""""""""""""""""""""""""""""""""""""""""""""""""""""""""""""""""""""""""""""""""""""""""""""""""""""""""""""""""""""""""""""""""""""""""""""""""""""""""""""""""""""""""""""""""""""""""""""""""""""""""""""""""""""""""""""""""""""""""""""""""""""""""""""""""""""""""""""""""""""""""""""""""""""""""""""""""""""""""""""""""""""""""""""""""""""""""""""""""""""""""""""""""""""""""""""""""""""""""""""""""""""""""""""""""""""""""""""""""""""""""""""""""""""""""""""""""""""""""""""""""""""""""""""""""""""""""""""""""""""""""""""""""""""""""""""""""""""""""""""""""""""""""""""""""""""""""""""""""""""""""""""""""""""""""""""""""""""""""""""""""""""""""""""""""""""""""""""""""""""""""""""""""""""""""""""""""""""""""""""""""""""""""""""""""""""""""""""""""""""""""""""""""""""""""""""""""""""""""""""""""""""""""""""""""""""""""""""""""""""""""""""""""""""""""""""""""""""""""""""""""""""""""""""""""""""""""""""""""""""""""""""""""""""""""""""""""""""""""""""""""""""""""""""""""""""""""""""""""""""""""""""""""""""""""""""""""""""""""""""""""""""""""""""""""""""""""""""""""""""""""""""""""""""""""""""""""""""""""""""""""""""""""""""""""""""""""""""""""""""""""""""""""""""""""""""""""""""""""""""""""""""""""""""""""""""""""""""""""""""""""""""""""""""""""""""""""""""""""""""""""""""""""""""""""""""""""""""""""""""""""""""""""""""""""""""""""""""""""""""""""""""""""""""""""""""""""""""""""""""""""""""""""""""""""""""""""""""""""""""""""""""""""""""""""""""""""""""""""""""""""""""""""""""""""""""""""""""""""""""""""""""""""""""""""""""""""""""""""""""""""""""""""""""""""""""""""""""""""""""""""""""""""""""""""""""""""""""""""""""""""""""""""""""""""""""""""""""""""""""""""""""""""""""""""""""""""""""""""""""""""""""""""""""""""""""""""""""""""""""""""""""""""""""""""""""""""""""""""""""""""""""""""""""""""""""""""""""""""""""""""""""""""""""""""""""""""""""""""""""""""""""""""""""""""""""""""""""""""""""""""""""""""""""""""""""""""""""""""""""""""""""""""""""""""""""""""""""""""""""""""""""""""""""""""""""""""""""""""""""""""""""""""""""""""""""""""""""""""""""""""""""""""""""""""""""""""""""""""""""""""""""""""""""""""""""""""""""""""""""""""""""""""""""""""""""""""""""""""""""""""""""""""""""""""""""""""""""""""""""""""""""""""""""""""""""""""""""""""""""""""""""""""""""""""""""""""""""""""""""""""""""""""""""""""""""""""""""""""""""""""""""""""""""""""""""""""""""""""""""""""""""""""""""""""""""""""""""""""""""""""""""""""""""""""""""""""""""""""""""""""""""""""""""""""""""""""""""""""""""""""""""""""""""""""""""""""""""""""""""""""""""""""""""""""""""""""""""""""""""""""""""""""""""""""""""""""""""""""""""""""""""""""""""""""""""""""""""""""""""""""""""""""""""""""""""""""""""""""""""""""""""""""""""""""""""""""""""""""""""""""""""""""""""""""""""""""""""""""""""""""""""""""""""""""""""""""""""""""""""""""""""""""""""""""""""""""""""""""""""""""""""""""""""""""""""""""""""""""""""""""""""""""""""""""""""""""""""""""""""""""""""""""""""""""""""""""""""""""""""""""""""""""""""""""""""""""""""""""""""""""""""""""""""""""""""""""""""""""""""""""""""""""""""""""""""""""""""""""""""""""""""""""""""""""""""""""""""""""""""""""""""""""""""""""""""""""""""""""""""""""""""""""""""""""""""""""""""""""""""""""""""""""""""""""""""""""""""""""""""""""""""""""""""""""""""""""""""""""""""""""""""""""""""""""""""""""""""""""""""""""""""""""""""""""""""""""""""""""""""""""""""""""""""""""""""""""""""""""""""""""""""""""""""""""""""""""""""""""""""""""""""""""""""""""""""""""""""""""""""""""""""""""""""""""""""""""""""""""""""""""""""""""""""""""""""""""""""""""""""""""""""""""""""""""""""""""""""""""""""""""""""""""""""""""""""""""""""""""""""""""""""""""""""""""""""""""""""""""""""""""""""""""""""""""""""""""""""""""""""""""""""""""""""""""""""""""""""""""""""""""""""""""""""""""""""""""""""""""""""""""""""""""""""""""""""""""""""""""""""""""""""""""""""""""""""""""""""""""""""""""""""""""""""""""""""""""""""""""""""""""""""""""""""""""""""""""""""""""""""""""""""""""""""""""""""""""""""""""""""""""""""""""""""""""""""""""""""""""""""""""""""""""""""""""""""""""""""""""""""""""""""""""""""""""""""""""""""""""""""""""""""""""""""""""""""""""""""""""""""""""""""""""""""""""""""""""""""""""""""""""""""""""""""""""""""""""""""""""""""""""""""""""""""""""""""""""""""""""""""""""""""""""""""""""""""""""""""""""""""""""""""""""""""""""""""""""""""""""""""""""""""""""""""""""""""""""""""""""""""""""""""""""""""""""""""""""""""""""""""""""""""""""""""""""""""""""""""""""""""""""""""""""""""""""""""""""""""""""""""""""""""""""""""""""""""""""""""""""""""""""""""""""""""""""""""""""""""""""""""""""""""""""""""""""""""""""""""""""""""""""""""""""""""""""""""""""""""""""""""""""""""""""""""""""""""""""""""""""""""""""""""""""""""""""""""""""""""""""""""""""""""""""""""""""""""""""""""""""""""""""""""""""""""""""""""""""""""""""""""""""""""""""""""""""""""""""""""""""""""""""""""""""""""""""""""""""""""""""""""""""""""""""""""""""""""""""""""""""""""""""""""""""""""""""""""""""""""""""""""""""""""""""""""""""""""""""""""""""""""""""""""""""""""""""""""""""""""""""""""""""""""""""""""""""""""""""""""""""""""""""""""""""""""""""""""""""""""""""""""""""""""""""""""""""""""""""""""""""""""""""""""""""""""""""""""""""""""""""""""""""""""""""""""""""""""""""""""""""""""""""""""""""""""""""""""""""""""""""""""""""""""""""""""""""""""""""""""""""""""""""""""""""""""""""""""""""""""""""""""""""""""""""""""""""""""""""""""""""""""""""""""""""""""""""""""""""""""""""""""""""""""""""""""""""""""""""""""""""""""""""""""""""""""""""""""""""""""""""""""""""""""""""""""""""""""""""""""""""""""""""""""""""""""""""""""""""""""""""""""""""""""""""""""""""""""""""""""""""""""""""""""""""""""""""""""""""""""""""""""""""""""""""""""""""""""""""""""""""""""""""""""""""""""""""""""""""""""""""""""""""""""""""""""""""""""""""""""""""""""""""""""""""""""""""""""""""""""""""""""""""""""""""""""""""""""""""""""""""""""""""""""""""""""""""""""""""""""""""""""""""""""""""""""""""""""""""""""""""""""""""""""""""""""""""""""""""""""""""""""""""""""""""""""""""""""""""""""""""""""""""""""""""""""""""""""""""""""""""""""""""""""""""""""""""""""""""""""""""""""""""""""""""""""""""""""""""""""""""""""""""""""""""""""""""""""""""""""""""""""""""""""""""""""""""""""""""""""""""""""""""""""""""""""""""""""""""""""""""""""""""""""""""""""""""""""""""""""""""""""""""""""""""""""""""""""""""""""""""""""""""""""""""""""""""""""""""""""""""""""""""""""""""""""""""""""""""""""""""""""""""""""""""""""""""""""""""""""""""""""""""""""""""""""""""""""""""""""""""""""""""""""""""""""""""""""""""""""""""""""""""""""""""""""""""""""""""""""""""""""""""""""""""""""""""""""""""""""""""""""""""""""""""""""""""""""""""""""""""""""""""""""""""""""""""""""""""""""""""""""""""""""""""""""""""""""""""""""""""""""""""""""""""""""""""""""""""""""""""""""""""""""""""""""""""""""""""""""""""""""""""""""""""""""""""""""""""""""""""""""""""""""""""""""""""""""""""""""""""""""""""""""""""""""""""""""""""""""""""""""""""""""""""""""""""""""""""""""""""""""""""""""""""""""""""""""""""""""""""""""""""""""""""""""""""""""""""""""""""""""""""""""""""""""""""""""""""""""""""""""""""""""""""""""""""""""""""""""""""""""""""""""""""""""""""""""""""""""""""""""""""""""""""""""""""""""""""""""""""""""""""""""""""""""""""""""""""""""""""""""""""""""""""""""""""""""""""""""""""""""""""""""""""""""""""""""""""""""""""""""""""""""""""""""""""""""""""""""""""""""""""""""""""""""""""""""""""""""""""""""""""""""""""""""""""""""""""""""""""""""""""""""""""""""""""""""""""""""""""""""""""""""""""""""""""""""""""""""""""""""""""""""""""""""""""""""""""""""""""""""""""""""""""""""""""""""""""""""""""""""""""""""""""""""""""""""""""""""""""""""""""""""""""""""""""""""""""""""""""""""""""""""""""""""""""""""""""""""""""""""""""""""""""""""""""""""""""""""""""""""""""""""""""""""""""""""""""""""""""""""""""""""""""""""""""""""""""""""""""""""""""""""""""""""""""""""""""""""""""""""""""""""""""""""""""""""""""""""""""""""""""""""""""""""""""""""""""""""""""""""""""""""""""""""""""""""""""""""""""""""""""""""""""""""""""""""""""""""""""""""""""""""""""""""""""""""""""""""""""""""""""""""""""""""""""""""""""""""""""""""""""""""""""""""""""""""""""""""""""""""""""""""""""""""""""""""""""""""""""""""""""""""""""""""""""""""""""""""""""""""""""""""""""""""""""""""""""""""""""""""""""""""""""""""""""""""""""""""""""""""""""""""""""""""""""""""""""""""""""""""""""""""""""""""""""""""""""""""""""""""""""""""""""""""""""""""""""""""""""""""""""""""""""""""""""""""""""""""""""""""""""""""""""""""""""""""""""""""""""""""""""""""""""""""""""""""""""""""""""""""""""""""""""""""""""""""""""""""""""""""""""""""""""""""""""""""""""""""""""""""""""""""""""""""""""""""""""""""""""""""""""""""""""""""""""""""""""""""""""""""""""""""""""""""""&
[truncated: 149,315,422 more chars]
