# Supplementary material for: Creatinine assay interferences compromises MELD accuracy and may bias liver allocation
Source: Nat Commun. 2026 Jul 23;17:7111. doi: 10.1038/s41467-026-75011-x (PMC13396164; doi:10.1038/s41467-026-75011-x)
Supplement: Supplementary file 4 — Source Data [file 41467_2026_75011_MOESM4_ESM.zip › figshare_package_FINAL_PUBLIC_DEPOSIT_V1_20260503_002637/02_workflows/F2_workflow_v01/submission_ready/public/figures/slco_F2_heatmap_public.pdf]

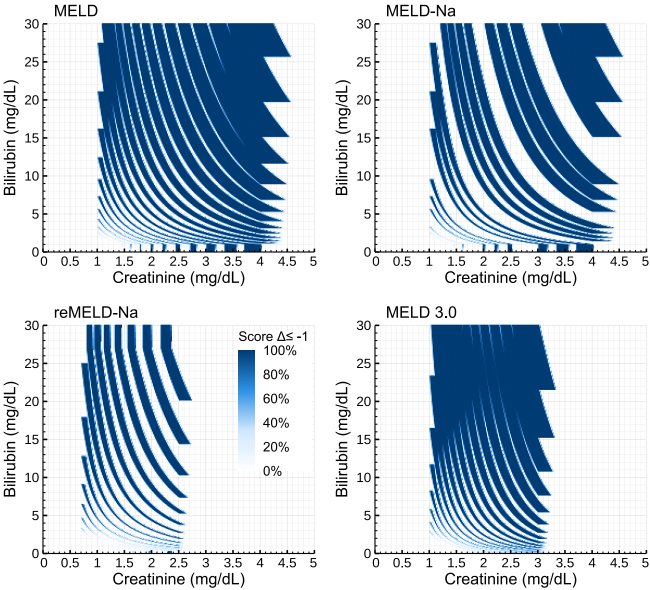
MELD
MELD-Na
reMELD-Na
MELD 3.0
Score Δ≤ -1
100%
80%
60%
40%
20%
0%
Bilirubin (mg/dL)
Creatinine (mg/dL)
0
5
10
15
20
25
30
0.5
1
1.5
2
2.5
3
3.5
4
4.5
