# Supplementary material for: Creatinine assay interferences compromises MELD accuracy and may bias liver allocation
Source: Nat Commun. 2026 Jul 23;17:7111. doi: 10.1038/s41467-026-75011-x (PMC13396164; doi:10.1038/s41467-026-75011-x)
Supplement: Supplementary file 4 — Source Data [file 41467_2026_75011_MOESM4_ESM.zip › figshare_package_FINAL_PUBLIC_DEPOSIT_V1_20260503_002637/02_workflows/F6_workflow_v01/submission_ready/public/figures/F6_SRTR_public.pdf]

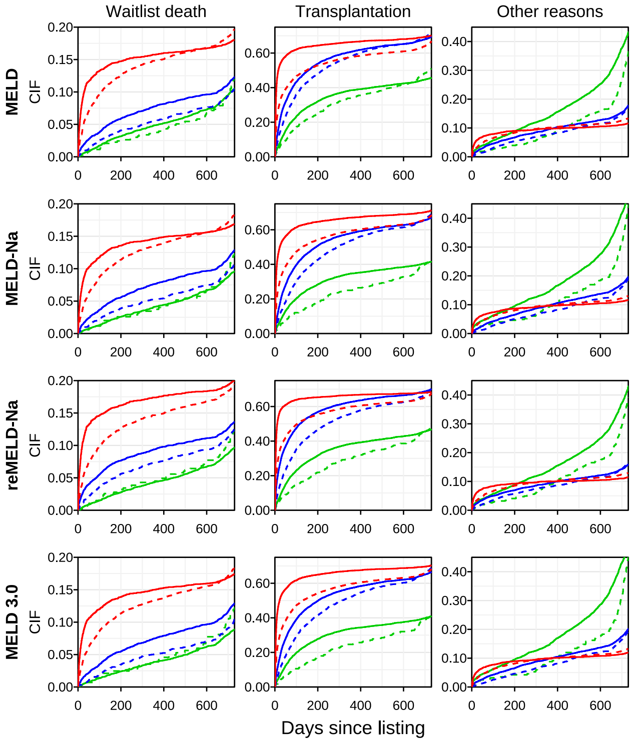
Waitlist death
Transplantation
Other reasons
MELD
MELD-Na
reMELD-Na
MELD 3.0
CIF
0.00
0.05
0.10
0.15
0.20
0.40
0.60
0.30
0
200
400
600
Days since listing
